# Catalogue of Geadephaga (Coleoptera, Adephaga) of America, north of Mexico

**DOI:** 10.3897/zookeys.245.3416

**Published:** 2012-11-28

**Authors:** Yves Bousquet

**Affiliations:** 1 Agriculture and Agri-Food Canada, Central Experimental Farm, Ottawa, Ontario, Canada

**Keywords:** Ground beetles, Trachypachidae, Rhysodidae, Carabidae, North America

## Abstract

All scientific names of Trachypachidae, Rhysodidae, and Carabidae (including cicindelines) recorded from America north of Mexico are catalogued. Available species-group names are listed in their original combinations with the author(s), year of publication, page citation, type locality, location of the name-bearing type, and etymology for many patronymic names. In addition, the reference in which a given species-group name is first synonymized is recorded for invalid taxa. Genus-group names are listed with the author(s), year of publication, page citation, type species with way of fixation, and etymology for most. The reference in which a given genus-group name is first synonymized is recorded for many invalid taxa. Family-group names are listed with the author(s), year of publication, page citation, and type genus. The geographical distribution of all species-group taxa is briefly summarized and their state and province records are indicated.

One new genus-group taxon, *Randallius* new subgenus (type species: *Chlaenius purpuricollis* Randall, 1838), one new replacement name, *Pterostichus amadeus* new name for *Pterostichus vexatus* Bousquet, 1985, and three changes in precedence, *Ellipsoptera rubicunda* (Harris, 1911) for *Ellipsoptera marutha* (Dow, 1911), *Badister micans* LeConte, 1844 for *Badister ocularis* Casey, 1920, and *Agonum deplanatum* Ménétriés, 1843 for *Agonum fallianum* (Leng, 1919), are proposed. Five new genus-group synonymies and 65 new species-group synonymies, one new species-group status, and 12 new combinations (see Appendix 5) are established.

The work also includes a discussion of the notable private North American carabid collections, a synopsis of all extant world geadephagan tribes and subfamilies, a brief faunistic assessment of the fauna, a list of valid species-group taxa, a list of North American fossil Geadephaga (Appendix 1), a list of North American Geadephaga larvae described or illustrated (Appendix 2), a list of Geadephaga species described from specimens mislabeled as from North America (Appendix 3), a list of unavailable Geadephaga names listed from North America (Appendix 4), a list of nomenclatural acts included in this catalogue (Appendix 5), a complete bibliography with indication of the dates of publication in addition to the year, and indices of personal names, supraspecific names, and species-group names.

## Introduction

The Adephaga, a name coined by the Swiss entomologist and botanist Joseph Philippe de Clairville [1742-1830] in 1806, represents the second largest suborder of Coleoptera with an estimated 39,300 species described to 2005. The group is undisputedly natural, based on the presence of several synapomorphies in the adult and immature stages (Beutel and Ribera 2005: 53; Beutel et al. 2008; Lawrence et al. 2011). The term Adephaga comes from the Greek word *adephagos* meaning gluttonous, greedy, in reference to the predaceous habits of adults and larvae of the vast majority of the species. Conventionally the Adephaga are divided into two groups, the Geadephaga for the terrestrial families and the Hydradephaga for the aquatic families.

The extant hydradephagan families include the Gyrinidae (about 875 species), Haliplidae (about 220 species), Noteridae (about 250 species), Amphizoidae (five species), Hygrobiidae (six species), Dytiscidae (about 3,700 species), Aspidytidae (two species), and Meruidae (one species). Some studies, based on structural features of the adult (Burmeister 1976; Baehr 1979) and larva (Ruhnau 1986) as well as molecular data (Shull et al. 2001; Ribera et al. 2002; Hunt et al. 2007), suggest that the Hydradephaga is monophyletic. Other studies, including recent DNA sequence analyses (Maddison et al. 2009), indicate a polyphyletic origin for the complex.

The extant geadephagan groups include the trachypachids (six species), rhysodids (about 355 species), cicindelids (about 2,415 species), and carabids (about 31,490 species). The monophyletic origin of the Geadephaga was supported in some structural and molecular studies but rejected in others (see Maddison et al. 2009 for an overview). While the taxonomic concept of the hydradephagan families is stable, that of the geadephagan families is not. Several authors consider either the trachypachids, rhysodids, or cicindelids as Carabidae.

This work catalogues all geadephagan taxa of America, north of Mexico. The last catalogue covering the Geadephaga of the region is that of Bousquet and Larochelle in 1993. Since then relatively few taxonomic studies have been published on the North American fauna. The increased interest toward the inadequately known but amazingly rich Neotropical Region is probably one of the reasons behind the situation. So, is there a need for this catalogue? For one, it is more informative than the previous one. It includes, besides the usual information on nomenclature, the type locality of each available species, locations of the primary type specimens, references to the original synonymies of invalid names, and a short description of the geographical distribution of each species. Furthermore, a number of errors were discovered in the previous catalogue and needed to be corrected.

## Brief history

The first checklist / catalogue covering the North American Geadephaga was the checklist of beetles of the United States by Friedrich Ernst Melsheimer [1784-1873] published in July 1853. The interest for this work originated with the establishment in 1842 of the first entomological society in America, The Entomological Society of Pennsylvania. The compilation of this list was one of the main objects of the Society (Sorensen 1995: 17) and it prevailed upon Melsheimer, the first and only President of the Society, to complete the task. The manuscript was delivered in 1848 to the Smithsonian Institution in Washington. Its secretary, Joseph Henry, asked Samuel S. Haldeman and John L. LeConte to advise on its publication. The two gentlemen volunteered to update the manuscript, which delayed its release considerably. The work was a straightforward list of valid species, with abridged references and synonyms but without distributional data, arranged under the valid generic names. Although limited to the United States, it included more than 90% of the species known from North America at the time. Melsheimer, a physician by profession, was the son of Frederick Valentine Melsheimer [1749-1814] who in 1806 published the first book on American entomology, a 60-page booklet entitled “*Catalogue of insects of Pennsylvania. Part first*.” It enumerates 111 genera and 1,363 species of Coleoptera (Meisel 1929: 367), though almost all of them are *nomina nuda*.

In April 1866, John Lawrence LeConte [1825-1883] published the first part of a checklist of the Coleoptera of North America (north of Mexico) for the Smithsonian Institution. It covered the Adephaga and a large section of the Polyphaga. The first 49 pages, which included the Adephaga, were reprinted with minor modifications from a list already issued in March 1863. The list included synonyms but no geographical information. The second part of the checklist, covering the Chrysomeloidea and Curculionoidea, was never published. Two additional checklists of North American beetles would be published in the United States during the xix Century, both straightforward lists without geographical data. The first one, issued in 1874, was authored by George Robert Crotch [1842-1874], a British coleopterist who at the time was assistant to Hermann Hagen at the Museum of Comparative Zoology. A supplement to Crotch’s checklist was authored in 1880 by Edward Payson Austin, an amateur coleopterist and member of the Cambridge Entomological Club in its early years. The second checklist was published in 1885 by Samuel Henshaw [1852-1941], then assistant to Professor Hyatt at Lowell Technological Institute. Three supplements, in 1887, 1889, and 1895, were later issued by Henshaw.

In Europe, the German Max Gemminger [1820-1887] and Freiherr Edgar von Harold [1830-1886] published, between 1868 and 1876, a checklist of beetles of the world in 12 volumes, compiling 77,008 species over 3,800 pages. The Geadephaga were included in the first (Carabidae including cicindelids and trachypachids), second (paussids on pages 700-706), and third volumes (rhysodids on pages 867-868), all issued in 1868. Along with each specific name the authors listed the publication year as well as the original reference and region(s) of capture. This work spurred a large number of additions and corrections by many coleopterists. It stood alone in its class until the publication of the *Coleopterorum Catalogus* under the editorship of Walther Junk and Sigmund Schenkling. Published between 1909 and 1940, this catalogue was issued in 170 parts forming 30 volumes and involved the participation of more than 60 entomologists. A list by parts and another by families can be found in Blackwelder (1957: 1022-1034). The Geadephaga were covered in parts 1 (Rhysodidae by Raffaello Gestro in 1910), 5 (Paussinae by R. Gestro in 1910), 86 (Cicindelinae by Walther Horn in 1926), 91, 92, 97, 98, 104, 112, 115, 121, 124, 126, and 127 (Carabidae, including trachypachids, by Ernst Csiki between 1927 and 1933). Second editions of the Rhysodidae, by Walter D. Hincks in 1950, and Paussinae, by Emile Janssens in 1953, were issued much later.

While the *Coleopterorum Catalogus* was being published in Berlin, Charles William Leng [1859-1941], then director of the museum at the Staten Island Institute of Arts and Sciences, released in 1920 his catalogue of the Coleoptera of America, north of Mexico, still known as the “Leng catalogue.” His goal was “to enumerate systematically all the species of Coleoptera described prior to January 1, 1919 ... with consecutive numbers, synonyms, citation of original description, and an indication of distribution.” Leng and Andrew J. Mutchler in 1927 (covering the years 1919-1924) and 1933 (for 1925-1932), Richard E. Blackwelder in 1939 (for 1933-1938), and Blackwelder and his wife, Ruth M. Blackwelder, in 1948 (for 1939-1947) published supplements to Leng’s catalogue.

In 1972, Ross H. Arnett, Jr. [1919-1999], the catalyst behind the birth of the Coleopterist’s Society and its journal *The Coleopterists Bulletin*, initiated the “North American beetle fauna project” (NABF) with the help of a small group of coleopterists. The main goal of this cooperative adventure was to “produce a series of manuals for the identification of the species of beetles of the United States and adjacent Canada, and adjacent Mexico.” Although no such book was ever published, a preliminary checklist of North American beetles, known as the “Red Version,” was compiled by 1976 by Richard E. Blackwelder and Arnett. This version was used as a “working copy” for the next one, the “Yellow Version” defined as the “definitive checklist and the one which will be kept up-to-date.” Of this version, only two families would be compiled and published (July 1977), the Cupedidae by Arnett and the Carabidae (including trachypachids but excluding cicindelids) by Terry L. Erwin, Donald R. Whitehead, and George E. Ball. The “Red Version” was reissued with modifications in 1983 under the editorship of Arnett.

In November 1978, the Science and Educational Administration, USDA, released its first fascicle, covering the family Heteroceridae, of “A catalogue of the Coleoptera of America north of Mexico.” The goal was to “supplant the Leng catalogue and supply additional essential information.” A total of 34 fascicles, treating various family-group taxa, would be published up to February 1997. Among the fascicles, one only, the Rhysodidae by Ross T. Bell in 1985, deals with Geadephaga.

In 1993, Bousquet and Larochelle published the first catalogue specifically devoted to the geadephagan beetles of North America. They listed, for the first time, the original combination of every available species-group taxon and provided a general idea of the distribution of each species by listing state and province records. One of the goals behind their work was to stimulate interest toward publication of distributional records as done regularly in Europe.

In 1998, Wolfgang Lorenz issued the first edition of his “Systematic list of extant ground beetles of the world” compiling 32,567 species (in 1861 genera) of Geadephaga. Despite being limited to scientific names with their authors and publication years, the list soon became a useful tool to those interested in carabids. A second edition was released in 2005, compiling the same information for 34,281 extant species, placed in 1929 genera.

The first catalogue of the world Coleoptera published is that of Schönherr issued in four parts, 1806, 1808, 1817 and 1826. The Carabidae were grouped in the following genera: *Scarites* (23 species), *Cychrus* (seven species), *Manticora* (two species), *Carabus* (340 species), *Calosoma* (12 species), *Galerita* (nine species), *Brachinus* (16 species), *Anthia* (27 species), *Agra* (three species), *Collyris* (four species), *Odocantha* [sic!] (seven species), *Drypta* (four species), *Cicindela* (67 species), *Elaphrus* (11 species), *Scolytes* [sic!] (three species), all included in the first volume (1806), and *Paussus* (ten species) and *Cerapterus* (two species) included in the third volume (1817). Overall 547 species of Geadephaga were listed along with references and synonyms. By comparison, the number of Carabidae (including Cicindelinae) listed in the four catalogue editions of the Dejean collection amounted to 104 (first edition, 1802), 908 (second edition, 1821), 2494 (third edition, 1833), and 2791 (fourth edition, 1836).

A comparison of the number of valid species and genera between this and previous checklists / catalogues is presented in Table 1.

**Table 1.** North American Geadephaga species/genera counts in checklists.

**Publications**	**Trachyp**	**Rhysod**	**Cicindel**	**Carabid**	**Total**
Melsheimer 1853	0	3/1	64/4	935/112	1002/117
LeConte 1866	2/1	2/2	65/4	1090/107	1159/117
Gemminger & Harold 1868	2/1	2/1	61/5	1167/124	1232/131
Crotch 1874a	2/1	2/2	67/4	1097/118	1168/125
Henshaw 1885	2/1	4/2	70/4	1179/114	1255/121
Leng 1920	2/1	4/2	114/4	2207/207	2327/214
*Coleopterorum Catalogus* 1926-33	6/1	4/2	70/4	2916/144	2996/151
Erwin et al. 1977	3/1	9/2^1^	109/4^2^	2308/169	2429/176
Bousquet & Larochelle 1993	3/1	8/2	107/4	2230/183	2348/190
Present catalogue	3/1	8/2	112/12	2316/193	2439/208

^1^ Species count from Bell (1985b) ^2^ Species count from Boyd (1982)

## Nomenclatural and distributional information

The information on species-group taxa comprises a nomenclatural and a distributional component. The nomenclatural component consists of the scientific name with its author, date and page of publication, the type locality (see section *Type locality* under “Nomenclature” below), and the repository of the name-bearing type of each valid and invalid taxon. In addition, the reference in which a given scientific name is first synonymized is listed. Such references were difficult to find for several names, simply because they were never compiled before. Taxa listed as varieties subsequently to their original descriptions were not considered as listed in synonymy but those listed as aberrations or as “simple varieties” were. Codens used for collection repositories are given in the next section. When available, the accession numbers of name-bearing types for each institution are recorded.

This catalogue deals with extant available taxa. Fossil taxa are listed in Appendix 1. Unavailable names found in the literature are listed in Appendix 4 without comment. Listings of valid species-group names are alphabetic but listings of invalid names are chronologic. Synonyms of adventive and Holarctic species found in North America are selective. Misidentifications by subsequent authors are not listed. All species-group names are given in their original combinations.

The distributional component consists of a list of state and province records, using the same two-letter postal service style abbreviations used in the 1993 catalogue (Table 2), and a short description of the distribution, usually referring to the northeasternmost, northwesternmost, southwesternmost, and southeasternmost states or provinces. In addition, records for Cape Breton Island, the Queen Charlotte Islands, Vancouver Island, and the Channel Islands are indicated in parentheses after their respective provinces or states. Western Hemisphere countries are listed for species found south of the area covered. States and provinces placed in quotation marks in the descriptive section indicate that only the state or province was given without further precision in the reference cited. The starting point for the distributional records used in this work is Bousquet and Larochelle’s (1993) catalogue. However, many of their records were undocumented or came from old lists and were not always reliable. State and province records undocumented or considered doubtful are shown in square brackets following the accepted records. Except for the *Amara* records which come from identifications generally made by Fritz Hieke, almost all records from CMNH specimens are based on identifications made by Robert L. Davidson, those from LSAM specimens on identifications made by Igor Sokolov, and those from CNC, MCZ, and USNM specimens from identifications or confirmations made by myself. The records provided by Ken Karns and Brian Raber are based on identifications made by Robert L. Davidson.

**Table 2.** Two-letter abbreviations for political regions covered by this catalogue.

AB	Alberta	MA	Massachusetts	OH	Ohio
AK	Alaska	MB	Manitoba	OK	Oklahoma
AL	Alabama	MD	Maryland	ON	Ontario
AR	Arkansas	ME	Maine	OR	Oregon
AZ	Arizona	MI	Michigan	PA	Pennsylvania
BC	British Columbia	MN	Minnesota	PE	Prince Edward Island
CA	California	MO	Missouri	PM	St.Pierre and Miquelon
CO	Colorado	MS	Mississippi	QC	Quebec
CT	Connecticut	MT	Montana	RI	Rhode Island
DC	District of Columbia	NB	New Brunswick	SC	South Carolina
DE	Delaware	NC	North Carolina	SD	South Dakota
FL	Florida	ND	North Dakota	SK	Saskatchewan
GA	Georgia	NE	Nebraska	TN	Tennessee
GL	Greenland	NF	Newfoundland	TX	Texas
IA	Iowa	NH	New Hampshire	UT	Utah
ID	Idaho	NJ	New Jersey	VA	Virginia
IL	Illinois	NM	New Mexico	VT	Vermont
IN	Indiana	NS	Nova Scotia	WA	Washington
KS	Kansas	NT	Northwest Territories	WI	Wisconsin
KY	Kentucky	NU	Nunavut	WV	West Virginia
LA	Louisiana	NV	Nevada	WY	Wyoming
LB	Labrador	NY	New York	YT	Yukon Territory

The information on supraspecific taxa consists of the scientific name with its author and date and page of publication. Type species of genus-group taxa are also given, in their original combinations, followed by the valid names in parentheses when applicable, and type genera are listed for family-group taxa. Etymology is given for all valid generic names and for some of the invalid names; the works of Brown (1956) and Cailleux and Komorn (1981) have been particularly useful.

The listing of valid supraspecific taxa is “phylogenetic,” starting with taxa putatively branching off early along the evolutionary path of the group. Synonyms of supraspecific taxa are listed chronologically. If readily available, the first reference in which a given genus-group name is synonymized is included.

In the references section, titles of journals are cited in full. Titles of papers and books using alphabets other than Latin have been translated into English and the original language listed in square brackets after the title. An improvised title is given in square brackets, in the language used by the author(s), to papers without formal title. Unless otherwise noted, all references listed were seen. Except when only the year was found, the date of publication [DP] is given in square brackets at the end of each citation.

## Institution / collection acronyms and abbreviations

Collections cited in the catalogue are referred to by the abbreviations listed below.

**ALM** Alabama Museum of Natural History, Tuscaloosa, Alabama, USA

**AMNH** American Museum of Natural History, New York, New York, USA

**ANSP** Academy of Natural Sciences, Philadelphia, Pennsylvania, USA

**BMNH** The Natural History Museum, London, United Kingdom

**BYUC** Brigham Young University, Provo, Utah, USA

**CAS** California Academy of Sciences, San Francisco, California, USA

**CMC** Cincinnati Museum of Natural History, Cincinnati, Ohio, USA

**CMN** Canadian Museum of Nature, Gatineau, Quebec, Canada

**CMNH** Carnegie Museum of Natural History, Pittsburgh, Pennsylvania, USA

**CNC** Canadian National Collection of Insects, Arachnids and Nematodes, Ottawa, Ontario, Canada

**CUIC** Cornell University Insect Collection, Cornell University, Ithaca, New York, USA

**DAPC** Darren A. Pollock collection, Eastern New Mexico University, Portales, New Mexico, USA

**DEI** Institute für Pfanzenschutzforschung (formerly Deutsches Entomologisches Institut), Kleinmachnow, Eberswalde, Germany

**EMEC** Essig Museum of Entomology Collection, University of California, Berkeley, California, USA

**ETHZ** Entomologisches Institut, Eidgenössische Technische Hochschule, Zürich, Switzerland

**FFPC** Foster Forbes Purrington collection, The Ohio State University, Columbus, Ohio, USA

**FMNH** Field Museum of Natural History, Chicago, Illinois, USA

**FSCA** Florida State Collection of Arthropods, Gainesville, Florida, USA

**GNM** Göteborgs Naturhistoriska Museum, Göteborg, Sweden

**HMUG** Hunterian Museum, University of Glasgow, Glasgow, United Kingdom

**INHS** Illinois Natural History Survey, Champaign (Urbana), Illinois, USA

**IRSN** Institut Royal des Sciences Naturelles, Brussels, Belgium

**IZWP** Museum and Institute of Zoology of the Polish Academy of Sciences, Warszawa, Poland

**KSUC** Kansas State University, Manhattan, Kansas, USA

**LACM** Los Angeles County Museum of Natural History, Los Angeles, California, USA

**LMMC** Lyman Entomological Museum, McGill University, Macdonald Campus, Sainte-Anne-de-Bellevue, Quebec, Canada

**LSAM** Louisiana State Arthropod Museum, Baton Rouge, Louisiana, USA

**LSL** Linnean Society, London, United Kingdom

**MCZ** Museum of Comparative Zoology, Harvard University, Cambridge, Massachusetts, USA

**MHNG** Muséum d’Histoire Naturelle, Geneva, Switzerland

**MHNP** Muséum National d’Histoire Naturelle, Paris, France

**MSB** Museum of Southwestern Biology, University of New Mexico, Albuquerque, New Mexico, USA

**MSNG** Museo Civico di Storia Naturale, Genoa, Italy

**MSNM** Museo Civico di Storia Naturale, Milano, Italy

**MSNT** Museo Civico di Storia Naturale, Trieste, Italy

**MSUE** Michigan State University, East Lansing, Michigan, USA

**MVM** Museum Victoria, Melbourne, Australia

**NCSU** North Carolina State University, Raleigh, North Carolina, USA

**NHMW** Naturhistorisches Museum Wien, Wien, Austria

**NIAS** National Institute for Agro-environmental Sciences, Tsukuba, Japan [formerly National Institute of Agricultural Sciences, Tokyo]

**NMNS** National Museum of Nature and Science, Tokyo, Japan

**NMP** National Museum, Prague, Czech Republic

**NRSS** Naturhistoriska Riksmuseet, Stockholm, Sweden

**NSNH** Nova Scotia Museum of Natural History, Halifax, Nova Scotia, Canada

**ODAC** Oregon Department of Agriculture, Plant Division, Salem, Oregon, USA

**ORUM** Collection Ouellet-Robert, Université de Montréal, Montreal, Quebec, Canada

**OSAC** Oregon State Arthropod Collection, Oregon State University, Corvallis, Oregon, USA

**OSUO** Ohio State University, Columbus, Ohio, USA

**PMNH** Peabody Museum of Natural History, Yale University, New Haven, Connecticut, USA

**PURC** Purdue State University, West Lafayette, Indiana, USA

**SIM** Staten Island Museum, Staten Island, New York, USA

**SMEK** Snow Museum of Entomology, University of Kansas, Lawrence, Kansas, USA

**SMTD** Staatliches Museum für Tierkunde, Dresden, Germany

**TAMU** Texas A&M University, College Station, Texas, USA

**TMB** Magyar Természettudományi Múzeum, Budapest, Hungary

**TME** Texas Museum of Entomology, Pipe Creek, Texas, USA

**UAIC** University of Arkansas, Fayetteville, Arkansas, USA

**UASM** Strickland Museum, University of Alberta, Edmonton, Alberta, Canada

**UBC** Spencer Entomological Museum, University of British Columbia, Vancouver, British Columbia, Canada

**UCD** University of California, Davis, California, USA

**UCM** University of Colorado Museum, Boulder, Colorado, USA

**UICU** University of Illinois, Urbana, Illinois, USA

**UMAA** University of Michigan, Ann Arbor, Michigan, USA

**UMM** Philipps-Universität Marburg, Zoologische Sammlung, Marburg, Germany

**UMO** The University Museum, University of Oxford, Oxford, United Kingdom

**UMSP** University of Minnesota, Saint Paul, Minnesota, USA

**USMT** Ueno Science Museum, Tokyo, Japan

**USNM** National Museum of Natural History, Smithsonian Institute, Washington, DC, USA

**USS** University of Sydney, Sydney, Australia

**UZIU** Uppsala Universitet, Zoologiska Museum, Uppsala, Sweden

**VMNH** Virginia Museum of Natural History, Martinsville, Virginia, USA

**WSU** Washington State University, Pullman, Washington, USA

**ZILR** Zoological Institute, Academy of Sciences, Saint Petersburg, Russia

**ZMH** Zoologiska Museum, University of Helsinki, Helsinki, Finland

**ZMHB** Zoologisches Museum, Humboldt Universität, Berlin, Germany

**ZMLS** Zoological Museum, Lund University, Lund, Sweden

**ZMMU** Zoological Museum, Moscow University, Moscow, Russia

**ZMUA** Zoologisch Museum, Universiteit van Amsterdam, Amsterdam, The Netherlands

**ZMUC** Zoologisk Museum, Universitets Copenhagen, Copenhagen, Denmark

**ZMUO** Zoological Museum, University of Oslo, Oslo, Norway

**ZMUT** Zoological Museum, University of Turku, Turku (= Åbo), Finland

Besides those used for provinces and states (see Table 2), the following abbreviations are used in the text:

**B.P.** Before Present

**CAN** Canada

**CBI** Cape Breton Island

**CHI** Channel Islands (Santa Barbara Islands)

**DEN** Denmark

**DP** Date of publication

**FRA** France

**ICZN** International Commission on Zoological Nomenclature

**QCI** Queen Charlotte Islands

**USA** United States of America

**VCI** Vancouver Island

In addition, the International Commission on Zoological Nomenclature is sometimes abridged to “Commission” and United States of America to “United States.”

## Geographical terms

For simplicity, North America, north of Mexico, is referred to simply as North America in the text. Middle America refers to Mexico and the republics of Central America taken collectively. The West Indies refers to the Greater and Lesser Antilles and the Bahamas. The North American continent proper is referred to as North and Middle America.

For practical reasons, the zoogeographical regions of the world are defined following national boundaries as much as possible. The Nearctic Region corresponds to Canada, the continental United States, Saint-Pierre and Miquelon, and Greenland. Although the region extends into Mexico, its southern limit is difficult to define and often varies depending on the group under study. This concept implies that North America and the Nearctic Region are equivalent in this work. The Neotropical Region comprises Middle America and South America. The Afrotropical Region consists of Africa, including Madagascar and a number of smaller islands of the Indian Ocean, such as the Comoros, the Mascarene Islands, and the Seychelles, and of the Atlantic Ocean, such as Cape Verde Islands and São Tomé, but excludes the northern countries of Morocco (including Western Sahara), Algeria, Tunisia, Libya, and Egypt west of the Suez Canal, and the Canary and Madeira Islands. The limits of the Palaearctic Region are similar to those used in the *Catalogue of Palaearctic Coleoptera* (Löbl and Smetana 2003: 8). The region thus comprises Europe, Africa north of the Sahara, and Asia as far south as the Arabian Peninsula, Pakistan, Jammu and Kashmir, Himachal Pradesh, Uttar Pradesh, Nepal, Sikkim, Bhutan, Arunachal Pradesh, China, and Taiwan. The Oriental Region is Asia south of the regions used to define the southern limit of the Palaearctic Region. It includes all the Malay Archipelago (except New Guinea). The Australian Region comprises Australia, New Zealand, New Guinea, and some smaller islands of the Pacific, such as Fiji, New Britain, New Caledonia, and Solomon Islands.

The New World consists of the Nearctic, Neotropical, and Australian Regions combined and the Old World of the Oriental, Palaearctic, and Afrotropical Regions grouped. The Northern Hemisphere is the Nearctic and Palaearctic Regions combined and the Southern Hemisphere is the Afrotropical, Oriental, Australian, and Neotropical Regions united. The Western Hemisphere consists of the Nearctic and Neotropical Regions and the Eastern Hemisphere of the Palaearctic, Afrotropical, Oriental, and Australian Regions. Far East used in reference to the Palaearctic Region includes the Russian Far Eastern Region, the Korean Peninsula, Japan, Taiwan, and China excluding the Autonomous Regions of Inner Mongolia, Sinkian Uighur, and Tibet. Middle East is used for the southwestern Asian countries, including Egypt, Turkey, Syria, Lebanon, Israel, Jordan, Saudi Arabia, Yemen, Oman, Iraq, Iran, Afghanistan, and Pakistan.

The adjective “Holarctic” is used to denote a taxon that occurs naturally in both the Nearctic and Palaearctic Regions. The adjective “Australian” (as in “Australian species”) refers to the zoogeographical region, not to the country itself. The adjective “worldwide” is used to denote a genus-group or family-group taxon represented by at least one native species in all six zoogeographical regions as defined above including both the European and Asian parts of the Palaearctic Region. The adjective “endemic” indicates that the taxon is found only in the region listed.

Names of geographical places are given in their current English forms based on *Merriam-Webster’s Geographical Dictionary*, third edition (1997).

## Nomenclature

The rules outlined in the fourth edition of the International Code of Zoological Nomenclatural, published in 1999, have been followed throughout this catalogue. The following are comments about some nomenclatural issues.

*Principle of priority*. Priority for identical taxa made available the same year, whether under the same name or not, is determined by the date, other than the year, of publication. If not specified in the work itself, the publication date is the earliest day or month on which the work is demonstrated to be in existence (ICZN 1999: Article 21.3). When both works are published or assumed to be published the same day, precedence is determined by the First Reviser (Article 24.2). Unless listed in the work itself, dates of publication besides the year can be demonstrated only for some works. Those without specific dates are listed as published the last day of the year (Article 21.3.2) and priority goes to the work with a “demonstrated” date of publication. However, the situation is subject to change with new bibliographic discoveries, which could challenge the validity of synonyms (as well as relative precedence of homonyms and validity of nomenclatural acts) and bring nomenclatural instability. In this catalogue, priority was given to the publication “in prevailing usage” when the dates of publication were determined from external sources.

*New taxa*. In the xviii and first half of the xix Century it was common practice for authors not to indicate the attribution of the new species-group taxa. Instead, some authors added the word *mihi* after the specific name, usually to indicate a taxon that the author, himself, was describing. Several collectors provided names for their specimens, even for undescribed ones, and these specimens often circulated among European coleopterists through exchange, gift, or sale. Many undescribed species were subsequently described or illustrated under the collector’s names by different authors. For these, citations are provided in this catalogue only to the first description or illustration of each species unless the term “new species” or an equivalent expression (such as an asterisk preceding the specific epithet as in Say 1823a^1^) was included with the species-group name subsequently described or illustrated. Sometimes a species was described / illustrated by different authors the same year under the same names. One example concerned several species (i.e., *Patrobus foveocollis*, *Patrobus fossifrons*, *Pterostichus adstrictus*, *Pterostichus ventricosus*, and *Pterostichus pinguedineus*)^2^ described by Eschscholtz in 1823 in the *Mémoires de la Société Impériale des Naturalistes de Moscou* (volume 6) and illustrated by Fischer von Waldheim on plates available the same year (Sherborn 1922: liii), but included in his *Entomographie de la Russie* (volume 2) issued in 1824. In such cases, citations are given for the oldest description / illustration (for exceptions see previous entry, *Principle of priority*) but references to subsequent descriptions / illustrations are noted after the entry of the valid name.

*New taxa first published as synonyms*. The International Commission on Zoological Nomenclature admits the availability of taxa first published in an available work as junior synonyms and adopted before 1961 as valid taxa or treated as senior homonyms (ICZN 1999: Article 11.6.1). In such cases the taxa date from their first publication as synonyms. Even though this ruling has existed since the publication of the ICZN first edition in 1960, it has rarely been enforced in the carabid literature. A few cases were found during the preparation of this catalogue. For example, *Notiophilus sylvaticus* has been credited in the past to Eschscholtz (1833: 24) but the name was first proposed as a junior synonym of *Notiophilus biguttatus* Fabricius by Dejean (1831: 589). The name is credited to Dejean (1831) in this catalogue. It is possible that other cases like this one will eventually be found.

*Lectotype*. Prior to 2000, a lectotype could be selected by using the term “the type” instead of “lectotype” (ICZN 1999: Article 74.5). The words “type” and “holotype” are also acceptable if the author unambiguously selects a particular syntype to act as the unique name-bearing type of the taxon. This is the case for almost all designations using the word “type” or “holotype” relating to North American Carabidae published after 1950, in particular by George E. Ball and his students. In this catalogue the expression “lectotype [as type]” or “lectotype [as holotype]” applies to such cases. Unfortunately the Commission does not mandate the addition of “lectotype” labels to selected specimens, which often creates ambiguity when authors fail to do so.

*Type locality*. According to the ICZN (1999: Article 76.1), the type locality is the geographical place of capture of the primary type (holotype or lectotype). In the absence of a primary type, the type locality encompasses the localities of all the syntypes (Article 73.2.3). This information can be obtained from labels attached to primary types or to syntypes or from the original publication (referred to as “original citation” in the text) whichever is more inclusive, or inferred from the title of the publication or even from the name of the species. When a neotype is designated, its place of capture becomes the type locality (Article 76.3) even if the specimen was collected outside the original area. In this catalogue, type localities taken from labels or from original publications are listed as indicated although the order of the elements is sometimes changed; any additional information is placed in square brackets. Many species described in the xviii and xix Centuries had but little informative place of origin, such as a country, state, province, or large geographical area (e.g., Rocky Mountains or Lake Superior). Lindroth (1961-1969) restricted the type locality of several of these North American species by selecting a specific locality or a county within the original region specified. This practice is followed in this catalogue and specific type localities are selected for several species-group taxa. Of course, only localities where a given species was actually collected can be selected.

## Notable private carabid collections

Many North American species of carabids described in the xix and beginning of the xx Centuries were from specimens held in private collections. The whereabouts of these collections are important to taxonomists. Some of the more significant ones are discussed.

### Pierre François Marie Auguste Dejean (1780-1845) Collection

Dejean, a French military officer by profession, certainly held the largest private beetle collection of his time, which he built through exchanges, purchases, gifts, and his own collecting in various parts of Europe. He described a total of 289 new carabid species-group taxa from North America, of which 182 (63%) had not been described earlier according to the present catalogue. At the sale of his collection in 1840, the carabid section (which also included the agyrtid genus *Pteroloma*) was the most significant, not only because it contained 3,014 species and 17,914 specimens, but because it was the only one to include name-bearing types. Dejean did not describe a new species-group taxon during his lifetime that he did not consider a carabid. Dejean’s carabid collection (including tiger beetles) was purchased for 7,000 francs by Marquis F. Thibault de LaFerté-Sénectère who sold it, along with his own carabids, to Baron Maximilien de Chaudoir [*q.v*.] in 1859. Dejean’s carabid specimens are at MHNP today. Lindroth (1955b) discussed the name-bearing types and status of almost all North American species described by Dejean.

### Thomas Say (1787-1834) Collection

Say was the first naturalist born in North America to describe new species of beetles from this continent. In the course of 17 years (1817-1834), he described 164 carabid species from North American material which he believed were new to science. Based on their current status, 142 (87%) had effectively not been previously described. Say left his collection by verbal bequest through his wife to the Academy of Natural Sciences in Philadelphia in 1834 (Weiss 1936: 277). After his death, which occurred in October of the same year, the collection was shipped from New Harmony, Indiana, to Philadelphia through New Orleans. In 1836, Charles Pickering sent Say’s insects to Thaddeus W. Harris in Cambridge, Massachusetts, in order to “put them in good order, and return them in a condition to be preserved” (Harris to D.H. Storer, 2 November 1836). In the same letter Harris reported “They [Say’s specimens] arrived about the middle of July; but on examination were found to be in a deplorable condition, most of the pins having become loose, the labels detached, and the insects themselves without heads, antennae and legs, or devoured by destructive larvae, and ground to powder by the perilous shakings which they had received in their transportation from New Harmony.” In a letter to C.J. Ward, dated 8 March 1837, Harris wrote “I assure you that Mr. Say’s cabinet does not contain one half of the species which he has described; of the insects in it, many are without names, and all more or less mutilated, and so badly preserved that most of them are now absolutely worthless.” On July 16, 1838, Harris indicated in a letter to S.G. Morton (see Fox 1902: 11) that he had “been obliged to bake a considerable part of the insects lately belonging to Mr. Say twice, and some of them three times, in order to destroy the vermin with which they are infested.” Say’s collection was returned to the Academy of Natural Sciences in Philadelphia in March 1842 “in such a state of ruin and dilapidation as to be almost useless” (Ruschenberger 1852: 25).

During his life, Say sent some of his specimens abroad including many to Dejean in Paris (see Dejean 1826: vi). Fortunately Dejean’s carabid collection has remained intact and in good condition to this day. In their attempt to bring taxonomic stability to Say’s names, Lindroth and Freitag (1969) selected lectotypes for eight carabid species described by Say for which Say’s authentic specimens could be located in Dejean’s collection. They also designated neotypes from the MCZ material for 131 of the remaining 156 of Say’s species leaving the tiger beetles (14 species) and a few taxa, all currently considered invalid, without type specimens. Say’s species were interpreted by Lindroth and Freitag from LeConte’s concept according to his collection. LeConte never saw Say’s collection and his interpretation of Say’s species came exclusively from the original descriptions which he considered adequate: “The entire destruction of his [Say’s] original specimens would be the subject of much greater regret, were it not for the fact that his descriptions are so clear as to leave scarcely a doubt regarding the object designated. I am thus enabled to assign to nearly all of his Coleoptera their proper place in the modern system” (LeConte 1859d: vi).

### Thaddeus William Harris (1795-1856) Collection

Harris, well known for his work in economic entomology (his profile having appeared on every cover of the *Journal of Economic Entomology* for more than 35 years), described 28 new carabid species from North America. Ten (36%) are considered valid in this catalogue. To his defence, several of his species were made available by the posthumous publication of some of his letters several decades after they were written. At Say’s suggestion, Harris sent his entire collection to Thomas Say in Philadelphia, in 1825, who labeled the specimens as well as he could. Harris’ collection, which included “4,838 specimens in 2,241 species of Coleoptera,” contained “many typical specimens described by Harris, Say, and others” (Scudder 1860: 72). It was bought by friends in 1858 and presented to the Boston Society of Natural History. Harris’ collection was transferred to the Museum of Comparative Zoology at Cambridge in April 1941 (Darlington 1941b: 273) where it stands separately from the general collection in two standard 25 drawer cabinets.

### Gustav Graf von Mannerheim (1797-1854) Collection

Mannerheim, a Finnish noble by birth and wealthy by inheritance, described 72 new North American carabid species, all from Alaska and California. Of these, 23 (32%) had not been described previously. Mannerheim never visited the New World and his descriptions were based on specimens brought back chiefly by Russian collectors such as Johann F. Eschscholtz, Eduard L. Blaschke, Egor L. Tschernikh, and Il’ia G. Vosnesensky. His library and personal collection, which consisted, at the end, of 18,000 species and nearly 100,000 specimens, were sold for the sum of 8,000 silver rubles by his widow, Countess Eva Mannerheim, in 1855 to the University of Helsinki. The money used to buy the collection came from a loan made by the Emperor to the University with the understanding that the University will pay back annually the sum of 500 rubles to the Imperial Bank of Finland which will use it for poor- and workhouses in the country (Rein 1857). Mannerheim’s collection is kept separately at the University of Helsinki (Silfverberg 1995: 43).

### Jules Antoine Adolphe Henri Putzeys (1809-1882) Collection

Putzeys described 38 new North American species of carabids; 15 (39%) are listed as valid in this catalogue. He worked in close collaboration with Chaudoir, the leading carabidologist of the time, and described several new species from specimens in Chaudoir’s collection. These specimens are now in MHNP. He also gave many of his own types to Chaudoir. His personal collection was bequeathed in 1885 to the *Société Royale Belge d’Entomologie* under the care of the *Musée Royal d’Histoire Naturelle* in Brussels. Putzeys’ collection consisted of 26,429 specimens of carabids (including cicindelids) and 6,123 species (Preudhomme de Borre 1885: clx) as well as many other beetles and various insects.

### Victor de Motschulsky (1810-1871) Collection

Motschulsky, a Russian Imperial Army Colonel, described 121 new geadephagan species from North America; 27 (22%) were undescribed at the time based on current practice. A large part of this material came from a 10-month trip he made in 1853-54 to the United States and Panama. He collected at several locations including New York, Niagara Falls, Cleveland, Cincinnati, Cawington, Lexington, the Mammoth Cave, Nashville, Louisville, New Orleans, Mobile, Montgomery, Atlanta, Washington, D.C., and Philadelphia. In the last city, he visited LeConte, Haldeman, Melsheimer, and Zeigler. The first three gentlemen gave Motschulsky several specimens from their collections including “types” (Motschulsky 1856: 16). LeConte also identified part of the beetles Motschulsky collected in Louisiana, Alabama, Georgia, and Carolina. Motschulsky’s main collection, which included almost 60,000 specimens and about 4,000 types of beetles, was bequeathed to the *Société Impériale des Naturalistes de Moscou*. It was stored in poor condition and suffered considerable damage before it was acquired in 1911 by the Zoological Museum, Moscow Lomonosov State University (Antonova 1991: 72). Keleinikova (1976) catalogued the carabid syntypes of Motschulsky’s collection at ZMMU.

### Samuel Stehman Haldeman (1812-1880) Collection

Haldeman described 45 new carabid species from North America; 22 (49%) had not been described previously. In 1869 Haldeman, who had purchased Hentz’s collection, sold his collection of beetles to Simon Snyder Rathvon of Lancaster, Pennsylvania, “for about what the cases cost” (Rathvon in Geist 1881: 125). Rathvon’s collection and library were purchased for $1,000 by Henry Bobb of East Greenville, Pennsylvania, and presented to the Franklin and Marshall College in Lancaster, Pennsylvania, as a memorial of his son (Dubbs 1903: 369). In a letter dated April 1875 and addressed to Alexander Agassiz (see below), John L. LeConte stated that he owned “all the unique types” of Haldeman. This leads one to speculate that Haldeman, a close friend of LeConte, gave his name-bearing specimens to LeConte prior to selling his collection to Rathvon.

### Maximilien Stanislavovitch Baron de Chaudoir (1816-1881) Collection

Russian aristocrat of French origin, Chaudoir was not the typical insect collector. He made a single extensive collecting trip in his life, a 40 day-journey to the Caucasus in company of M.H. Hochhuth in 1845. His collection was mostly built through purchases and gifts. The single most significant purchase was LaFerté-Sénectère’s carabid collection in 1859 which included Dejean’s original specimens. In January 1874 Chaudoir gave his tiger beetle specimens, representing 713 species, to MHNP. After his death in May 1881 his collection passed into the hands of René Oberthür in Rennes as agreed upon between Chaudoir and the Oberthür brothers. Over nearly five decades, Chaudoir described 126 new carabid species based on specimens collected in North America; 58 (46%) had not been described earlier based on this catalogue.

René Oberthür died in April 1944 and his collection, certainly one of the two largest private beetle collections ever built, was classified as “monument historique” in January 1948 by the French government. The collection, which included at least five million specimens, was acquired for the sum of 32 million francs by the Muséum d’Histoire Naturelle in Paris (MHNP) in 1951 (Cambefort 2006: 249).

### Henry Ulke (1821-1910) Collection

Although Ulke described only two North American carabids in his life, *Bembidion nevadense* in 1875 and *Pterostichus johnsoni* in 1889, his collection, which he sold in 1900 to the Carnegie Museum in Pittsburgh, was used extensively by LeConte and Horn and contains numerous syntypes of new species described by the two coleopterists. However, recognition of many of these syntypes can be difficult. Sometimes all syntypes were retained by LeConte and Horn while on other occasions all or some of them were returned to Ulke. Furthermore, syntypes returned to Ulke were often reincorporated in his collection with others of the same species from the same place. Usually these were marked with a number or colored square, but since many syntypes were left unmarked at the time, it is sometimes impossible to recognize them at the Carnegie Museum (Robert L. Davidson pers. comm. 2008).

### John Lawrence LeConte (1825-1883) Collection

LeConte is without doubt the most outstanding North American coleopterist of the xix Century, not only because he described 514 new genus-group and about 4,730 new species-group taxa of beetles (Henshaw 1882: 270), but because he was the first to work seriously on the classification of the North American fauna. During his scientific activity, which lasted almost 40 years, he described 724 new species-group taxa of Geadephaga from North America, 439 (61%) of which were not previously described. LeConte built his collection through his own collecting but also from gifts he received and identifications he provided to many persons from whom he usually retained all or some of the specimens. There is also little doubt that his father, Major John Eatton LeConte^3^, left his collection to his son. Evidence supporting this can be found in LeConte (1856a: 49) when he indicated that his second specimen of *Cicindela blanda* “came from the old collection of my father.” LeConte was a generous man and often offered some of his specimens to visitors (such as Motschulsky [*q.v*.]) or sent some to acquaintances (such as Chaudoir [*q.v*.] and Putzeys [*q.v*.]) though it seems that he retained at least one specimen of each species. Unfortunately in the xix Century the type concept for species-group taxa was not developed and LeConte sometimes gave the only syntype he had in his collection and retained one or more specimens that he acquired after the original descriptions. Therefore, syntypes of some of LeConte’s species are not in his collection. Moreover, syntypes of some of his species are difficult to find in his collection. LeConte had the habit of mixing the specimens of the new species he considered later as synonyms with those of the valid species. Since many of his specimens only bear a colored disc for label, syntypes of several of his species are not readily ascertainable.

In April 1875, LeConte wrote to his friend Alexander Agassiz, director of the Museum of Comparative Zoology in Cambridge, and expressed the wish that his collection be deposited at the museum after his death^4^. His collection was packed and transported by his longtime friend George Horn. It now stands separate from the general collection along with that of Horn.

LeConte used small colored paper disks to indicate the provenance of his specimens. The color system used is as follows:

**Pale blue** Lake Superior, Canada

**Pink** Middle states, i.e., Maryland, Delaware, New York, New Jersey, Pennsylvania, and possibly also Connecticut and Rhode Island

**Pale pink** Vermont, New Hampshire, Massachusetts

**White** Northern and eastern states, Canada, and possibly also Alaska

**Orange (brick red)** Southern and Gulf states, i.e., Virginia, North Carolina, South Carolina, Georgia, Florida, Alabama, Mississippi, Louisiana, and possibly also eastern Tennessee and Arkansas

**Dark red** Texas

**Yellow** Ohio, Illinois, Indiana, Missouri, western Tennessee, Kentucky, and possibly Iowa and the southern edge of the Great Lakes

**Pale green** Nebraska, Kansas, North Dakota, South Dakota, Oklahoma, Colorado, Wyoming, Montana

**Dark green** New Mexico

**Black** Utah

**Silver** Arizona and Valley of Gila (so including also southwestern New Mexico)

**Silver with edge cut** Baja California, Mexico

**Gold** California

**Dark blue** Oregon, Washington

**Brown** Russian America, i.e., probably the region around Colony Ross, a farming community about 75 miles north of San Francisco along the coast in California, and Alaska

### George Henry Horn (1840-1897) Collection

A physician by profession, Horn authored or coauthored more than 250 papers, in which he described 154 new genera and more than 1,600 new species of beetles, including 103 North American Geadephaga. Based on the current classification, 75 (73%) of his new geadephagan species had not been described previously. His collection and library were bequeathed to the American Entomological Society, which deposited them at the Academy of Natural Sciences in Philadelphia. In October 1974, the Horn and William G. Dietz collections were delivered to the Museum of Comparative Zoology in return for the Scudder and Morse orthopteroid insects of the MCZ (Philip D. Perkins pers. comm. 2004; see Lawrence 1973: 151). Horn’s collection is preserved along with that of LeConte apart from the general collection.

### Thomas Lincoln Casey (1857-1925) Collection

From 1884 to the end of his life, Casey described 1,864 new species-group taxa of North American Geadephaga; only 307 (16%) had not been described previously based on current concepts. Still many of his remaining “valid species” have not been subsequently studied, particularly those belonging to small species of the tribe Harpalini, and a substantial proportion will certainly end up in synonymy. Furthermore, several of Casey’s species are valid simply by chance as he did not recognize or study the proper characters (such as the male genitalia) that distinguished them from their closely related taxa known at the time. His collection, consisting of almost 117,000 specimens, including name-bearing types for more than 9,200 species-group taxa (Buchanan 1935: 7; Blackwelder 1950: 65), was built through Casey’s own collecting and by purchases. It was bequeathed to the United States National Museum in Washington, D.C. Casey (1918: 291) stated that “about a dozen” of his types “disappeared from ... [his] collection while temporarily at the Cambridge Museum.” The syntypes of some of these species (e.g., *Bembidion militare*, *Tachys occultator*, *Amara pallida*, *Amara ferruginea*, and *Amara marylandica* among Carabidae) are at the MCZ. Casey did not designate holotypes as such and therefore, unless he expressly indicated in the original description that he had but a single specimen or that a lectotype had been designated, all type specimens in his collection are syntypes.

### Willis Stanley Blatchley (1859-1940) Collection

Blatchley described 12 new North American carabid species; only two (17%) are considered valid in this work. His library and large insect collection, which included 470 name-bearing specimens, were given to Purdue University. Blatchley did not select type specimens in his publications but subsequently designated lectotypes [as types] for all the new species he had described (Blatchley 1930: 33-50).

### Charles Frederic August Schaeffer (1860-1934) Collection

Schaeffer, curator of the insect collection at the Museum of the Brooklyn Institute of Arts and Sciences, described 30 new carabid species; 22 (73%) are still valid today. In 1929, the Brooklyn Museum transferred 37,100 insect specimens, including many of Schaeffer’s carabid types, to the USNM (Debbie Feher pers. comm. 2008). Currently the type material of 25 (possibly 26) of Schaeffer’s species-group taxa are in the USNM. It is clear in his 1910 paper that Schaeffer was selecting one of the specimens from his series as “the type.” However he may not have labeled them as such because lectotypes have been designated for several of his new species by various authors.

### Henry Clinton Fall (1862-1939) Collection

A teacher by profession, Fall owned one of the largest private collections of North American beetles toward the end of his life, with an estimated 250,000 specimens (including those of Charles Liebeck which came to Fall in the 1930s) representing between 14,000 and 15,000 species or about 90% of the fauna of the time (Darlington 1940a: 46) if one excludes the “species” described by Casey. Over a period of about 40 years, Fall described 47 new North American carabid species-group taxa; 31 (66%) are still considered valid today. He left his collection, together with his correspondence, notebooks, and reprints, to the Museum of Comparative Zoology at Harvard University where his specimens are kept separately at the end of each genus in the general collection. In one of his 1910 papers, Fall designated holotypes (as “the type”) for the first time. From this publication, “type” specimens labeled as such in his collection are considered holotypes. All original specimens of his new species described prior to 1910 should be considered syntypes. Type labels on some of these specimens were probably added after the publication of the original descriptions.

### Roland Hayward (1865-1906) Collection

Hayward, a member of the Boston Stock Exchange and of the Boston Society of Natural History, described 42 new species of carabids from North America, all in the tribe Bembidiini and the genus *Amara*. Currently 32 (76%) are considered valid. His collection, which he built through purchases, gifts, exchanges, and his own collecting in New England as well as in Colorado, Manitoba, and New Brunswick, was bequeathed to the Museum of Comparative Zoology in Cambridge. Hayward did not designate type specimens for his new species.

### Edwin Cooper Van Dyke (1869-1952) Collection

Professor Van Dyke described 73 new carabid and one new trachypachid species from North America; 54 (73%) of which had not been described previously based on their current status. His collection, consisting of about 200,000 specimens (Essig 1953: 88), was presented to the California Academy of Sciences in 1924 where the holotypes of all but three of his 74 new species of Geadephaga are currently stored.

### Howard Notman (1881-1966) Collection

Notman described 38 new carabid species from North America between 1919 and 1929; 21 (55%) had not been described previously based on their current status. In 1948 he donated his entire collection to the Staten Island Institute of Arts and Sciences, where it is still today (Smetana and Herman 2001: 118). Based on Hennessey’s (1990) type catalogue of that institution, type specimens of all new species Notman collected himself, most from the Adirondacks where he owned a summer home, are in his collection in SIM (18 in total). He also described several new species from material owned by institutions, such as the USNM. Notman did not designate type specimens in his papers of 1919 and 1920 but did so after.

## Classification of Geadephaga

Unfortunately, there is no consensus among coleopterists concerning the classification of Geadephaga even at the family level. Some authors rank the cicindelids, rhysodids, and trachypachids as Carabidae while others consider one, two, or all three groups as distinct families. Even the paussines are sometimes raised to family level by modern authors. At this time, I prefer to classify the Geadephaga into three families, i.e., Trachypachidae, Rhysodidae, and Carabidae.

Following Jeannel’s (1941b-1942) classification of the carabids of France, a number of authors, mostly French and Spanish taxonomists, still recognized several families of “ground beetles.” Such an approach does not add anything to the understanding of carabid evolution. It simply adds another level to the Linnaean classification. If Jeannel’s approach is followed, it could and should have an impact on the classification of the other adephagan groups, particularly the dytiscids. Since I have been under the influence of Lindroth’s work on the carabids of Canada and Alaska, Jeannel’s approach seems to me unjustified.

Following is a discussion of the family-group taxa of Geadephaga.

### Family Trachypachidae.

Monophyly of this family is well supported by larval and adult apomorphies (Arndt and Beutel 1995; Beutel 1994; Beutel 1998). The systematic position of this group, however, is contentious. Bell (1966b, 1967), Bils (1976), Evans (1977a, 1985), Hammond (1979), Ward (1979), Burmeister (1980), Roughley (1981), Nichols (1985c), Beutel and Belkaceme (1986), Ruhnau (1986), Beutel and Roughley (1988), Acorn and Ball (1991), Arndt (1993), Deuve (1993), Arndt and Beutel (1995), Arndt (1998), and Beutel (1998) provided or discussed elements suggesting that trachypachids are more closely related to hydradephagans or part of Hydradephaga (i.e., Dytiscoidea) than to carabids. While most authors have regarded the Hydradephaga and Carabidae as distinct phyletic lineages, Bils (1976) and Nichols (1985c) argued that the hydradephagan-trachypachid lineage may have arisen within the Carabidae. Kavanaugh (1986) reevaluated the evidence supporting relationships of Trachypachidae with Hydradephaga. He concluded that trachypachids could be the sister-group of carabids and ranked the group as a subfamily within the Carabidae. Ponomarenko (1977) also postulated, from fossil evidence, that trachypachids and carabids are sister-groups that evolved from a common eodromeid ancestor. Beutel and Haas (1996), Kavanaugh (1998: 337), Fedorenko (2009), Dressler and Beutel (2010), and Martínez-Navarro et al. (2011) found support for monophyly of a clade including trachypachids and carabids. Recent molecular studies also suggested that trachypachids are more closely related to Geadephaga than to Hydradephaga (Shull et al. 2001; Maddison et al. 2009). In addition, pygidial gland compounds in trachypachids are more similar to those known from Carabidae than from Hydradephaga (Attygalle et al. 2004: 586). In this catalogue, trachypachids are included in the Geadephaga and given family rank.

The Trachypachidae includes two extant genera: *Systolosoma* Solier with two species in Chile and Argentina and *Trachypachus* Motschulsky with four species, one in Eurasia and three in western North America.

Many putative trachypachid fossils were found in Mesozoic deposits of Asia. Ponomarenko (1977), who studied the material, included all seven genera of trachypachid fossils in a distinct subfamily, Eodromeinae. Beutel (1998: 83) pointed out that the affinities between trachypachids and eodromeines are unclear because there are no apparent synapomorphic character states between the two groups.

### Family Rhysodidae.

Traditionally ranked as a distinct family, rhysodids (also known as wrinkled bark beetles) have been included within the family Carabidae in recent years by several authors following evidence or discussion provided by Bell and Bell (1962), Bell (1970), Forsyth (1972), Reichardt (1977), Baehr (1979), Beutel (1990, 1992c), Yahiro (1996), Bell (1998), Liebherr and Will (1998), and others. Some authors have treated the group as a tribe related to Scaritini or Clivinini. Reichardt (1977: 393) stated that rhysodids were “closest” to salcediines and Bell (1998: 268) even suggested that the genus *Solenogenys* Westwood, traditionally included within the Salcediini, is the sister-group to rhysodids. Erwin (1991a: 10) on the other hand included rhysodids within his subfamily Psydrinae along with gehringiines, psydrines, moriomorphines, patrobines, trechines, zolines, pogonines, and bembidiines. Molecular data published by Maddison et al. (1999: 125) suggest that rhysodids could be the sister-group to cicindelids and that both could be closely related to the subfamily Harpalinae. Others taxonomists, however, have continued to treat the rhysodids as a distinct family. Regenfuss (1975) and Nagel (1979) suggested that the Rhysodidae could be the sister-group of the remaining Geadephaga; Deuve (1993: 100) the sister-group to the other Adephaga (with the possible exception of Gehringiinae); Beutel and Roughley (1988) the sister-group of the remaining Adephaga excluding Gyrinidae; Beutel (1992a, 1993, 1998) the sister-group to Carabidae (without trachypachids). Recently Makarov (2008) found no evidence from the larval morphology suggesting that rhysodids are specialized Carabidae. Instead rhysodid larvae share several features with those of the suborder Archostemata. At this time, I prefer to rank rhysodids as a distinct family based on tradition but also on the fact that there is no solid morphological or molecular evidence presented to date pointing out that the Carabidae (with or without trachypachids) are paraphyletic in regard to rhysodids.

About 355 species of rhysodids are currently known and are placed into seven family-group taxa, namely Leoglymmiini, Medisorini, Rhysodini, Dhysorini, Sloanoglymmiini, Omoglymmiini, and Clinidiini. These taxa are usually ranked as subtribes when rhysodids are included in the carabids. I have followed Bousquet and Larochelle (1993) in listing them as tribes. Only the last two-mentioned tribes are represented in North America.

Tribe Leoglymmiini. This tribe contains a single species, *Leoglymmius lignarius* (Olliff), from Australia. Contrary to other rhysodids, the minor setae on antennomeres 5-10 are arranged in broad bands encircling the distal third of the segment and the mentum is separated from the ventral lobe of the gena by a distinct suture in its anterior half.

Tribe Medisorini. A single species, *Medisores abditus* Bell and Bell, belongs to this tribe. The few known specimens have been found in Cape Province in the Republic of South Africa.

Tribe Rhysodini. This tribe is confined to the Eastern Hemisphere and includes about 25 species in three genera: *Rhysodes* Germar (two Palaearctic species), *Kupeus* Bell and Bell (one New Zealand species), and *Kaveinga* Bell and Bell (23 Australian species).

Tribe Dhysorini. This tribe includes ten species placed in three genera, *Dhysores* Grouvelle in Africa, *Tangarona* Bell and Bell in New Zealand, and *Neodhysores* Bell and Bell in South America.

Tribe Sloanoglymmiini. This tribe has been proposed for one species, *Sloanoglymmius planatus* (Lea), endemic to southeastern Australia. The genus is taxonomically isolated and its relationship to other rhysodid genera is obscure.

Tribe Omoglymmiini. This tribe includes 180 species placed in eight genera. The group is represented in all zoogeographical regions but less so in Australia, Africa, and South America (Bell and Bell 1978: 66). The two North American species belong to the subgenus *Boreoglymmius* Bell and Bell, of the genus *Omoglymmius* Ganglbauer, along with one Japanese species. According to Bell and Bell (1983: 141), the two North American species are probably more closely related to each other than either is to the Japanese species.

Tribe Clinidiini. This tribe contains about 135 species placed in the genera *Clinidium* Kirby, *Rhyzodiastes* Fairmaire, and *Grouvellina* Bell and Bell. The species are found in all zoogeographical regions, including Madagascar, but are absent from the African continent. The North American fauna has only six species, five in the east and one in the west, included in the subgenus *Arctoclinidium* Bell of the genus *Clinidium*. This subgenus also contains three Palaearctic species, one in Japan and two in Europe. According to Bell and Bell (1985: 77), the North American species and the Japanese one form a clade and the European species another clade. These authors also placed the Japanese species, *Clinidium veneficum* Lewis, as the sister-group to *Clinidium valentinei* Bell of eastern North America.

### Family Carabidae.

Monophyly of the Carabidae, as defined here, is not evident. The layout of the prehypopharyngeal setae in the larvae (Beutel 1993) and the development of antennal pubescence in the adults (Beutel 1995) have been suggested as synapomorphies for the family. However, Arndt et al. (2005: 138) considered these character states not very convincing given the variation involved in the structures. Recent molecular sequence analyses conducted by Maddison et al. (2009) found little support for monophyly of the group no matter if the trachypachids, rhysodids, and/or cicindelids were included or excluded unless the Carabidae was considered equivalent to the Geadephaga. Therefore, the Carabidae, as defined here, could be paraphyletic in regard to rhysodids, trachypachids, and possibly even to Hydradephaga.

Carabids are found on all continents, except Antarctica, and on most islands. They range from well above the arctic circle to Tierra del Fuego and South Georgia in the Southern Hemisphere. Based on Lorenz’s (2005) checklist, 33,920 valid species are recognized.

The current classification of the Carabidae is based mainly on morphological data of adults although molecular sequence data have been used recently to discuss various aspects of carabid phylogeny. Despite several attempts there is no consensus on the classification of several subfamilies or tribes. This is particularly evident among ‘basal grade’ carabids.^5^

Fossils belonging to the family Carabidae are known from the early Jurassic (Ponomarenko 1977) which suggests that the family emergence dates back to the beginning of the Jurassic or the end of the Triassic (Kryzhanovskij 1983). Ponomarenko (1977) proposed two family-group taxa of Carabidae among Mesozoic fossils, the subfamily Protorabinae for five genera and the tribe Conjunctiini for two genera.

The world classification of family-group taxa, which has been adopted for the North American fauna in this catalogue, is outlined in Table 3.

**Table 3.** Classification of world family-group taxa of Carabidae. Taxa represented in North America are followed by a dot.

Subfamily Nebriinae	
	Tribe Pelophilini •
	Tribe Opisthiini •
	Tribe Nebriini •
	Tribe Notiokasiini
	Tribe Notiophilini •
Subfamily Cicindinae	
	Tribe Cicindini
Subfamily Carabinae	
	Tribe Cychrini •
	Tribe Pamborini
	Tribe Ceroglossini
	Tribe Carabini •
Subfamily Cicindelinae	
	Tribe Amblycheilini •
	Tribe Manticorini
	Tribe Megacephalini •
	Tribe Cicindelini •
	Tribe Ctenostomatini
	Tribe Collyridini
Subfamily Loricerinae	
	Tribe Loricerini •
Subfamily Elaphrinae	
	Tribe Elaphrini •
Subfamily Omophroninae	
	Tribe Omophronini •
Subfamily Migadopinae	
	Tribe Amarotypini
	Tribe Migadopini
Subfamily Hiletinae	
	Tribe Hiletini
Subfamily Scaritinae	
	Tribe Pasimachini •
	Tribe Carenini
	Tribe Scaritini •
	Tribe Clivinini •
	Tribe Salcediini
	Tribe Dyschiriini •
	Tribe Promecognathini •
	Tribe Dalyatini
Subfamily Broscinae	
	Tribe Broscini •
Subfamily Apotominae	
	Tribe Apotomini
Subfamily Siagoninae	
	Tribe Enceladini
	Tribe Siagonini
	Tribe Lupercini
Subfamily Melaeninae	
	Tribe Melaenini
Subfamily Gehringiinae	
	Tribe Gehringiini •
Subfamily Trechinae	
	Tribe Trechini •
	Tribe Zolini
	Tribe Bembidiini •
	Tribe Pogonini •
Subfamily Patrobinae	
	Tribe Lissopogonini
	Tribe Patrobini •
Subfamily Psydrinae	
	Tribe Psydrini •
Subfamily Moriomorphinae	
	Tribe Moriomorphini
	Tribe Amblytelini
Subfamily Nototylinae	
	Tribe Nototylini
Subfamily Paussinae	
	Tribe Metriini •
	Tribe Mystopomini
	Tribe Ozaenini •
	Tribe Protopaussini
	Tribe Paussini
Subfamily Brachininae	
	Tribe Crepidogastrini
	Tribe Brachinini •
Subfamily Harpalinae	
Supertribe Pterostichitae	
	Tribe Morionini •
	Tribe Cnemalobini
	Tribe Microcheilini
	Tribe Chaetodactylini
	Tribe Cratocerini
	Tribe Abacetini •
	Tribe Pterostichini •
	Tribe Zabrini •
	Tribe Metiini
	Tribe Drimostomatini
	Tribe Chaetogenyini
	Tribe Dercylini
	Tribe Melanchitonini
	Tribe Oodini •
	Tribe Peleciini
	Tribe Brachygnathini
	Tribe Bascanini
	Tribe Panagaeini •
	Tribe Chlaeniini •
	Tribe Cuneipectini
	Tribe Orthogoniini
	Tribe Idiomorphini
	Tribe Glyptini
	Tribe Amorphomerini
Supertribe Harpalitae	
	Tribe Licinini •
	Tribe Harpalini •
	Tribe Geobaenini
	Tribe Omphreini
	Tribe Sphodrini •
	Tribe Platynini •
	Tribe Perigonini •
	Tribe Ginemini
	Tribe Enoicini
	Tribe Atranini •
	Tribe Catapieseini
	Tribe Lachnophorini •
	Tribe Pentagonicini •
	Tribe Odacanthini •
	Tribe Calophaenini
	Tribe Ctenodactylini •
	Tribe Hexagoniini
	Tribe Cyclosomini •
	Tribe Somoplatini
	Tribe Masoreini
	Tribe Corsyrini
	Tribe Sarothrocrepidini
	Tribe Graphipterini
	Tribe Lebiini •
	Tribe Dryptini
	Tribe Galeritini •
	Tribe Zuphiini •
	Tribe Physocrotaphini
	Tribe Anthiini
	Tribe Helluonini •
	Tribe Xenaroswellianini
	Tribe Pseudomorphini •

### Subfamily Nebriinae.

This subfamily includes the tribes Nebriini, Notiokasiini, Notiophilini, Opisthiini, and Pelophilini. All but notiokasiines are Northern Hemisphere elements and represented in North America. Evidence supporting monophyly of Nebriinae is not overwhelming. The only known synapomorphy in the adult stage is the asetose parameres (Kavanaugh and Nègre 1983), a character state found in other, clearly unrelated carabid lineages. Arndt (1993: 21) listed three putative synapomorphies upon examination of the larval morphology. The molecular data analyses by Maddison et al. (1999: 125) provided only moderate support for monophyly of the subfamily and Kavanaugh’s (1998) phylogenetic analysis suggested that this subfamily represents a grade rather than a clade.

The subfamilies Nebriinae and Carabinae could be closely related as pointed out by Jeannel (1940: 7), Bell (1967: 105), Beutel (1992c: 57), and Su et al. (2004: 49). Both groups have open procoxal cavities, contrary to the remaining carabids. In addition, the external lamella of the metepimeron is completely covered and functionally replaced by an extension of the hind margin of the anepisternum (Beutel 1992c: 57). Some authors (e.g., Lorenz 2005: 125) also include the cicindines within the subfamily suggesting a close relationship between these groups. Based on similarities in the genitalia, Deuve (1993: 125) raised the possibility that the Hydradephaga, trachypachids, omophronines, and nebriines form a clade.

Tribe Pelophilini. This tribe includes a single genus, *Pelophila* Dejean, which has been retained in the tribe Nebriini until recently. Two species are known, both living in the boreal and subarctic regions: one is circumpolar, the other restricted to Canada and Alaska. Kavanaugh (1996: 34) suggested that the genus represents the sister-group to the remaining Nebriinae. One of Kavanaugh’s (1998: Fig. 2) cladograms suggested that *Pelophila* is more closely related to the tribe Nebriini than are the Opisthiini, Notiophilini, and Notiokasiini.

Tribe Opisthiini. This tribe includes two genera with five species and is doubtless monophyletic. Kavanaugh and Nègre (1983: 564) argued that opisthiines could be the sister-group to the remaining Nebriinae. On the other hand, Kavanaugh’s (1996: Fig. 1A) most parsimonious tree suggested that this tribe is the sister-group to Notiophilini and that these two tribes, along with Notiokasiini, form a clade which represents the sister-group to Nebriini.

Tribe Nebriini. This tribe contains about 600 species in the Palaearctic, Nearctic, and northern parts of the Oriental Regions. However, the group is clearly more diverse in the Palaearctic. The main genera of the tribe are *Leistus* Frölich, *Archastes* Jedlička, and particularly *Nebria* Latreille with more than 60% of the species. The limits of the genus *Nebria* are not quite settled. Kavanaugh (1995, 1996) regarded *Nippononebria* Uéno (including *Vancouveria* Kavanaugh) as the sister-group to *Leistus* while Ledoux and Roux (2005) listed *Nippononebria* and *Vancouveria* as subgenera of *Nebria* and suggested they form the sister-group to *Eonebria* Semenov and Znojko and *Sadonebria* Ledoux and Roux, a complex of 60 Palaearctic species.

Tribe Notiokasiini. This tribe contains a single species, *Notiokasis chaudoiri* Kavanaugh and Nègre, found in South America. Although the relationships of the tribe are obscure (Kavanaugh and Nègre 1983), Kavanaugh (1996: 33) found 12 synapomorphies supporting monophyly of a clade including notiokasiines, notiophilines, and opisthiines.

Tribe Notiophilini. The tribe includes a single genus, *Notiophilus* Duméril, very characteristic in the adult stage. The larvae, however, are similar in most structural features to those of Nebriini as pointed out by van Emden (1942). Jeannel (1941b: 175) included Notiophilini, Nebriini (with *Pelophila*), and Opisthiini in his family Nebriidae, suggesting implicitly a close relationship between the three groups. Kavanaugh’s (1996: Fig. 1A) most parsimonious cladogram suggested a sister-group relationship between Notiophilini and Opisthiini based on adult and larval morphological data. Based on confluent procoxal cavities, Nichols (1985c: Fig. 5) considered the tribe to be the sister-group to {Omophronini + Trachypachini + Hydradephaga}. Erwin (1991a: 11) noted that notiophilines, along with omophronines, hiletines, and trachypachids, have the first mesotarsomere slightly dilated and with squamate setae underneath. However, it remains to ascertain whether this character state is synapomorphic or convergent. Based on female reproductive tracts, Liebherr and Will (1998: 146) suggested that the tribe Notiophilini represents the sister-group to {Opisthiini + Nebriini (with *Pelophila*) + Omophronini}.

Notiophilines, with about 55 species described to date, live in the Nearctic and Palaearctic Regions and at higher altitudes in the northern parts of the Neotropical and Oriental Regions. They are more speciose in Asia than anywhere else. The phylogenetic relationships of the species have not been studied yet.

### Subfamily Cicindinae.

This subfamily includes two species, *Archaeocindis johnbeckeri* (Bänninger) from the Persian Gulf (Kuwait and Iran) and *Cicindis horni* Bruch from the Córdoba Province of Argentina. Very little can be said at this time about the relationships of the subfamily except that it represents a basal grade carabid taxon. Kryzhanovskij (1976a: 87) associated cicindines with paussines (excluding metriines) and nototylines; Nagel (1979, 1987) and Roig-Juñent et al. (2011) viewed them as the sister-group to paussines. Ball (1979: 100), however, doubted such proposed affinities between cicindines and paussines. Erwin (1985, 1991a), followed by Lorenz (2005: 125), included the Cicindini in the Nebriitae. Kavanaugh and Erwin (1991) studied the structural features and reviewed the relationships of the group. They concluded that cicindines are best placed in a distinct supertribe near the Nebriitae and Elaphritae (*sensu* Kryzhanovskij 1976a: 88). Kavanaugh’s (1998: Fig. 3) phylogenetic analysis using 153 characters of adult external and male genitalic structures suggested that cicindines may be closely related to omophronines, carabines, cychrines, and cicindelines. Aspects of the behaviour and life history of the Argentine species have been published recently (Erwin and Aschero 2004).

### Subfamily Carabinae.

This subfamily contains about 1,300 species (Lorenz 2005: [i]) placed in four tribes: Cychrini, Pamborini, Ceroglossini, and Carabini. Most authors agree that the subfamily is monophyletic. According to Deuve (2004: 32), adults of this group are characterized by two significant autopomorphies: the presence of two strip-like apodemes flanking the basal orifice of the median lobe of the aedeagus and the presence of an alveolus on the epipleurite of the abdominal segment IX at the opening of the defensive gland. Arndt (1998: 179) noted several autopomorphies in larvae of Carabinae: an extensive decrease of number of setae on the tergites and sternites with an increase in the number of pores, size reduction of the sensorial appendage on antennomere III, and a markedly sclerotized body.

Tribe Cychrini. This well-defined and likely monophyletic group of about 200 species is restricted to the Northern Hemisphere. Osawa et al. (2004: 31) and Su et al. (2004: 49), based on molecular data, argued that the tribe is probably the sister-group to the remaining clades of the subfamily. Moore (1966b), Prüser and Mossakowski (1998: 316), and Arndt (1998: 180), based on morphological data, suggested that pamborines are the closest relatives to cychrines. Jeannel (1941b: 167) indicated that cychrines are more closely related to pamborines and ceroglossines than to carabines (*sensu stricto*) and calosomatines based on the shape of the parameres.

Relationships among the four genera have not been investigated. Whether or not *Sphaeroderus* Dejean and *Scaphinotus* Dejean, the two endemic North American genera, are sister-groups, as suggested by Erwin (2007a: 139), remains to be investigated. For example, Prüser and Mossakowski (1998: 316) listed several putative synapomorphies suggesting that *Cychrus* Fabricius and *Sphaeroderus* are sister-groups (*Cychropsis* Boileau was not included in their analysis). The phylogenetic tree by Osawa et al. (2004: Fig. 5.2) based on molecular sequence data suggested that *Scaphinotus* is the sister-group to the remaining Cychrini and *Sphaeroderus* the sister-group to *Cychropsis*.

Tribe Pamborini. The 13 species currently included in this tribe are placed in two genera: *Pamborus* Latreille from Australia and the monospecific *Maoripamborus* Brookes from New Zealand. Jeannel (1941b: 94) stated that pamborines are more closely related to ceroglossines than to any other Carabinae.

Tribe Ceroglossini. This tribe comprises only the genus *Ceroglossus* Solier (eight species and 46 subspecies) which is restricted to Chile and western Argentina. The genus has traditionally been included within the Carabini but recent analyses based on molecular sequence data suggest that ceroglossines are more closely related to pamborines than to carabines (Prüser and Mossakowski 1998: 297; Su et al. 2004: 50) or form the sister-group to the remaining members of Carabinae (Osawa et al. 2004: Fig. 5.2). Arndt (1998: 179) found evidence from the larval morphology to support the latter hypothesis.

Tribe Carabini. Carabines rank among the most popular groups for beetle collectors. Adults of many species are elegant, colorful, and large (often exceeding 15 mm). Such interest has generated a market for these beetles, particularly in Europe, and unfortunately also a race to describe new varieties, morphs, and aberrations. More than 1,080 species of Carabini are recognized today worldwide. They inhabit all zoogeographical regions but are much more diverse in the Palaearctic Region than anywhere else.

The supraspecific classification of Carabini is debated. Some authors recognize only two genera, *Carabus* Linnaeus and *Calosoma* Weber, while others admit many, more or less clearly defined genera which are often grouped in two subtribes: Carabina and Calosomatina. I have followed the first approach and list all North American species in the genera *Carabus* (15 species) and *Calosoma* (41 species). The main difference between the two genera is the regression (or complete disappearance) of the ostial ligula of the aedeagus in members of *Carabus* (Deuve 2004: 33).

Based on morphological (larvae and endophallus of adults) and molecular sequence data, Deuve (2004) recognized eight major lineages within the genus *Carabus*: Spinulati, Digitulati, Lipastrimorphi, Archicarabomorphi, Tachypogenici, Metacarabi, Arcifera, and Neocarabi. The North American species are arrayed in nine subgenera: *Carabus* *s.str*. belongs to the Digitulati, *Archicarabus* Seidlitz to Archicarabomorphi, *Tachypus* Weber to Tachypogenici, *Megodontus* Solier to Neocarabi, and *Diocarabus* Reitter, *Homoeocarabus* Reitter, *Aulonocarabus* Reitter, *Hemicarabus* Géhin, and *Tanaocarabus* Reitter to Metacarabi.

Jeannel (1940: 68) recognized two major lineages within the calosomatines: the *Calosomes lobés* with a membranous ligula at the proximal opening of the male median lobe and the *Calosomes ongulés* with a chitinized ligula. The first lineage is represented in the Australian and Palaearctic Regions, and by six species belonging to the genus-group taxa *Calosoma* *s.str*. and *Calodrepa* Motschulsky in the Nearctic Region, the West Indies, and Mexico. The second lineage was divided by Jeannel (1940: 69-71) into three clades, the phyletic series of *Castrida-Caminara* represented in North America by a single species belonging to the genus-group taxon *Castrida* Motschulsky, the phyletic series of *Callisthenes* represented in the Nearctic Region by 23 species of the taxa *Chrysostigma* Kirby and *Callistenia* Lapouge, and the phyletic series of *Callitropa* represented in North America by 11 species of *Blaptosoma* Géhin, *Carabosoma* Géhin, *Camegonia* Lapouge, and *Callitropa* Motschulsky.

The systematic position of *Aplothorax burchelli* Waterhouse, a species endemic to the island of Saint Helena off the west coast of Africa, is controversial. Jeannel (1940) included the taxon within his *Calosomes ongulés* but Basilewsky (1972: 18-22) was convinced that the species is a relict of an old clade that evolved before the splitting of the *Carabus* and *Calosoma* lineages. He advocated placing the species in a distinct tribe which, in his opinion, was as justified as those of Pamborini and Ceroglossini.

### Subfamily Cicindelinae.

This group, referred to as the tiger beetles, has been regarded traditionally as a distinct family, but more and more coleopterists include it within the carabids. There is little doubt, based on characters of adults and larvae, that cicindelines form a monophyletic lineage. Relationships of the group, however, remain uncertain. It has been regarded as the sister-group to the remaining Carabidae by Nichols (1985c) and as the sister-group to Carabidae (minus paussines) by Regenfuss (1975). A close affinity between this subfamily and the Carabinae has been suggested by Erwin and Sims (1984: 366), Deuve (1993: 160; 2004: 32), Kavanaugh (1998: 338), and Liebherr and Will (1998: 151), although Liebherr and Will also emphasized that the Cicindelinae could instead be closely related to Promecognathini and Amarotypini. Maddison et al. (1999) indicated that most of their phylogenetic analyses of 18 rDNA place the Cicindelinae and Rhysodidae as sister-groups, near the Harpalinae. They also pointed out that the alternative placement of the cicindelines outside the Carabidae was more parsimonious than placing them among the basal-grade carabids. Deuve (2004: 32) noted two exceptional and primitive character states shared between cicindelines and carabines: presence of the abdominal tergite X in the male and presence of a phallobase on the aedeagus. He also pointed out numerous similarities between the two groups: the ectodermal genital ducts of the females are almost identical with a vagina differentiated in a bursa copulatrix, the presence of a sclerotized ligular apophysis, the presence of a filiform spermatheca and absence of an accessory gland, the presence of rod-shaped apophyses on the female abdominal epipleurites VIII allowing the formation of a telescopic egg-laying tube, the parameres of the aedeagus are glabrous and symmetrical, the endophallus often shows comparable dentiform sclerites, the digestion is extra-oral, and the ventral surface of the adult body often shows metallic coloration, an exceptional character state in the Adephaga.

This subfamily currently includes more than 2,500 species distributed worldwide, except Tasmania, Antarctica, and remote oceanic islands, with the greatest diversity in the tropics (Pearson 1988). Tiger beetles are classified by most authors, following Horn (1926), into two major lineages, *Alocosternales* with a very narrow and deeply longitudinally-furrowed metepisternum in the adult and *Platysternales* with a wider metepisternum that has either no longitudinal furrow or a horseshoe-shaped furrow posteriorly. These groups are often listed as supertribes Collyriditae and Cicindelitae respectively. However, based on a combined analysis of molecular and morphological data, Vogler and Barraclough (1998: 254) noted that collyridites nested within the cicindelites, rendering the latter paraphyletic. Arndt (1998: 178) also noted that, based on larval character states, Cicindelitae and the tribe Megacephalini are not monophyletic. Based on the above information, the species of Cicindelidae are simply placed here in six tribes without further grouping.

Tribe Amblycheilini. This tribe includes the genera *Omus* Eschscholtz and *Amblycheila* Say represented in North America and Mexico, and the genus *Pycnochila* Motschulsky with one species in the Strait of Magellan. Amblycheilines have been classified in the past within the megacephalines but larval characters (Arndt and Putchkov 1997) and mitochondrial and nuclear RNA gene sequences (Vogler and Barraclough 1998: 251) suggest a basal position for amblycheilines, well removed from the true megacephalines. The tribe, however, may well be a grade rather than a clade. For example, Arndt (1998: 178) placed *Omus* as the sister-group to the remaining Cicindelinae based on larval characters.

Tribe Manticorini. This tribe includes 14 species, arrayed in the genera *Mantica* Kolbe (one rarely collected species from southern Namibia) and *Manticora* Fabricius (13 Afrotropical species). Contrary to other tiger beetles, males of this tribe have asymmetric mandibles and unexpanded protarsomeres (Werner 2000: 22).

Tribe Megacephalini. This tribe includes about 200 species arrayed in 11 genera (see Naviaux 2007: 15). Even without the amblycheilines, monophyly of the tribe is doubtful. For example, the genus *Oxycheila* Dejean, traditionally considered a member of Megacephalini, nested with rather strong support within the basal groups of Cicindelini in Vogler and Barraclough’s (1998: 254) cladistic analysis based on molecular and morphological data.

Tribe Cicindelini. This tribe is by far the most diversified clade of tiger beetles. The number of recognized genera varies to a great extent among taxonomists. In this work the 98 North American species (202 species-group taxa) are placed in nine genera. All but two (*Cylindera* and *Cicindela*) of these genera are New World endemics. Arndt (1998: 178) stated that Cicindelini forms the sister-group to {Ctenostomatini + Collyridini}.

Tribe Ctenostomatini. This tribe includes two genera: *Ctenostoma* Klug with about 115 Neotropical species, and *Pogonostoma* Klug with about 110 Madagascan species. Members of this tribe are synapomorphic in lacking the articulated hook at the extremity of the inner lobe of the maxilla (Jeannel 1946: 104).

Tribe Collyridini. This tribe contains about 335 species in Asia, of which one extends into the Australian Region. The species are arrayed in two subtribes: Tricondylina for the genera *Derocrania* Chaudoir (16 species) and *Tricondyla* Latreille (about 45 species), and Collyridina for the genera *Protocollyris* Mandl (16 species), *Neocollyris* Horn (about 250 species), and *Collyris* Fabricius (ten species). Naviaux (1994: 149) indicated the structural differences between the two subtribes.

### Subfamily Loricerinae.

This subfamily contains a single genus, *Loricera* Latreille, although some authors have treated *Elliptosoma* Wollaston, with one species from Madeira, as a distinct genus. The group is restricted to the Nearctic and Palaearctic Regions with some taxa found on mountains in the northern parts of the Neotropical and Oriental Regions. Loricerini is a basal grade taxon with obscure affinity. Jeannel (1941b: 80) associated loricerines with the Carabinae, Nebriinae, Cicindelinae, Elaphrinae, Omophroninae, and Siagoninae (including *Promecognathus*) in his *Caraboidea Simplicia*, characterized by the absence of metepimeric lobes (Jeannel 1941b: 93). Bell (1967: 105) included loricerines within his *Anisochaeta Isopleuri* along with elaphrines, scaritines, and cicindelines. Arndt (1993: 22) found several common derived larval features in larvae of Loricerinae and Cicindelinae to suggest a sister-group relationship between the two taxa. Maddison et al. (1999: 126) pointed out that placement of *Loricera* within {Migadopini + Amarotypini} received relatively strong support in their 18S rDNA analyses. These three taxa were also recovered as a monophyletic unit in analyses of the same gene by Ribera et al. (2005: 290). Vigna Taglianti and Rossi (1998: 515) indicated that loricerines could be closely related to elaphrines based on the presence of the same parasitic laboulbeniales species found on these groups. Erwin (2007a: 69) listed Elaphrini as the sister-group to Loricerini.

Recently Sciaky and Facchini (1999) described a new subgenus (*Plesioloricera*) for the new Chinese species *Loricera balli* which has eight, instead of 12 or more, striae. This species could possibly be the most basal taxon of the genus.

Klausnitzer (2003) described a new species, *Loricera electrica*, based on a larva found in Baltic amber. He believes the species probably belongs to the *pilicornis* group as defined by Ball and Erwin (1969).

### Subfamily Elaphrinae.

This subfamily includes a single tribe with obscure relationships. Bell (1967) listed the Elaphrini within his *Isopleuri* along with Loricerini, Scaritini, and Cicindelini. Following Jeannel’s (1941b: 214) intuition, both Kryzhanovskij (1976a: 88) and Erwin (1985: 469) considered the elaphrines as the sister-group to Migadopini (with and without *Amarotypus* respectively) and this hypothesis was supported by Deuve’s (1993: 160) study of the female genitalia and Roig-Juñent’s (1998: Figs 9-10) parsimony analysis using 33 adult and larval characters. On the other hand, Goulet (1983: 445) regarded Melaenini and the subtribe Broscina as the taxa most closely related to Elaphrini. This possibility was found most parsimonious by Maddison et al. (1999) based on their molecular sequence analysis which did not, however, include melaenine exemplars. In addition, Yahiro (1990: 42) reported a similar type of alimentary canal in elaphrines and broscines. Roig-Juñent’s (1998) parsimony analysis shows that elaphrines are not related to broscines. Parsimony analysis based on the female reproductive tract characters placed Elaphrini as the sister-group to {Opisthiini + Nebriini + Notiophilini + Omophronini} or near the Promecognathini and Amarotypini (Liebherr and Will 1998: 146).

The tribe is represented only in the Northern Hemisphere and includes three genera, all represented in the Nearctic Region.

### Subfamily Omophroninae.

This subfamily includes a single genus, *Omophron* Latreille. Some authors have suggested that Omophronini, Trachypachidae, and the Hydradephaga form a monophyletic group. Putative synapomorphies proposed for the complex include the presence, in the larvae, of an undivided cardo and a dorsal insertion of the femoro-tibial extensor (Ruhnau 1986) and, in the adults, the atrophy of intertergal muscle M24 (Bils 1976), partial housing of the procoxae by the mesosternum, and the prominent prosternal process contacting the metasternum (Nichols 1985c). Deuve (1993: 160) noted the presence of a peculiar sclerotized structure (named “*sclerite helminthoïde*”) in the female genitalia of omophronines, nebriines (*sensu lato*), trachypachids, and the Hydradephaga which raises the possibility of close phylogenetic affinities between these groups. Jeannel (1941b: 219) suggested that cicindelines were most closely related to omophronines. Bell (1967: 106) indicated that Omophronini might be aberrant *Hemipleuri*, a group including nebriines (*sensu lato*) and carabines (*sensu lato*). Kavanaugh (1998: 338), based on a parsimonious analysis of adult characters, suggested a close affinity between Omophroninae, Carabinae, and Cicindelinae. Liebherr and Will (1998: 156) listed several “potentially synapomorphic characters” supporting placement of omophronines with nebriines (*sensu lato*) and their preferred cladogram, based on 20 characters of female ovipositors and reproductive tracts, placed them as the sister-group to notiophilines. Many authors, however, have treated the Omophronini as a basal-grade taxon with unclear affinity. Based on larval character states, Landry and Bousquet (1984) found no evidence to indicate a sister-group to Omophronini. Such conclusions were also reached by Beutel (1991) from his study of the larval head and adult thoracic structures. Erwin (2007a: 63) indicated the possibility that cicindines were closely related to omophronines.

The genus *Omophron* includes about 70 species and is represented in all zoogeographical regions except the Australian one. There is no evidence yet known to suggest that the Nearctic or the Western Hemisphere species form a clade within the genus.

### Subfamily Migadopinae.

This subfamily currently consists of two tribes: Amarotypini and Migadopini. One of the main character states of the group is the presence of a long scutellar stria reaching the apical declivity of the elytra (Jeannel 1938b: 4) as in members of *Pelophila*. This characteristic, however, is absent in members of the genus *Aquilex* Moret, which have a short scutellar stria (Moret 2005: 30). Jeannel (1938b) revised the species of migadopines (as Migadopidae) and classified them into two groups: Monolobinae for the Chilean genus *Monolobus* Solier (two species) and Migadopinae for the remaining genera which are represented in South America and in the Australian Region. He also postulated that elaphrines were without doubt the group most closely related to migadopines in the Northern Hemisphere (Jeannel 1938b: 10). Moore et al. (1987: 65) included the migadopines from Australia within the supertribe Elaphritae.

Tribe Amarotypini. This tribe includes a single species, *Amarotypus edwardsii* Bates, from New Zealand. Until recently the species was placed in the tribe Migadopini but it differs by having a setiform unguitractor plate between the tarsal claws which is missing in migadopines. Erwin (1985: 469) postulated that Amarotypini could be the sister-group to {Migadopini + Elaphrini}. In Liebherr and Will’s (1998: Fig. 57) preferred cladogram, based on 20 characters of the female ovipositors and reproductive tract, amarotypines were positioned as the sister-group to promecognathines. In Roig-Juñent (2004: Fig. 1) phylogenetic analysis, based on 57 characters of the adult morphology, the genus *Amarotypus* Bates nested inside the remaining migadopine genera, as the sister-group to {*Calathosoma* + *Stichonotus*}.

Tribe Migadopini. This group of about 30 species in 15 genera is restricted to the temperate areas of the Neotropical and Australian Regions. Moret (2005: 30) recently proposed the subtribe Aquilicina for the genera *Aquilex* Moret (one species in Ecuador) and *Rhytidognathus* Chaudoir (one species in Uruguay). He also pointed out the close relationship between the South American genus *Lissopterus* Waterhouse (two species from the Tierra del Fuego Archipelago and Falkland Islands) and the New Zealand genera *Loxomerus* Chaudoir (five species) and *Calathosoma* Jeannel (one species). The phylogenetic analysis performed by Roig-Juñent (2004) do not support Moret’s conclusions although *Aquilex* was recovered as the sister-group to the other migadopine genera. Liebherr and Will (1998: 147) alluded to the possibility that the tribe is not monophyletic.

### Subfamily Hiletinae.

This subfamily includes two genera, *Hiletus* Schiødte (= *Camaragnathus* Bocandé) with six species in tropical Africa and *Eucamaragnathus* Jeannel with 15 species in the Afrotropical (six species), Oriental (five species), and Neotropical (four species) Regions. Jeannel (1941b: 80; 1946: 209) postulated that hiletines were closely related to scaritines (*sensu lato*) based mainly on the fact that these two taxa were the only *disjuncti* (i.e., with disjunct mesocoxae) with the metepimera lobed as in the *conjuncti*. Erwin and Stork (1985: 445) believed that hiletines were related to cnemalobines (as Cnemacanthini), elaphrines, migadopines, promecognathines, pseudomorphines, scaritines, and siagonines based on some tarsal character states and suggested that this complex forms the sister-group to the paussine-brachinine clade based on the presence of distinct epimera, brushy non-styliform parameres, long empodial unguitractor plates, and non-conjunct mesocoxae. They also concluded that hiletines represent the sister-group to {scaritines + cnemalobines (as Cnemacanthini) + pseudomorphines} and that this clade was characterized by having a single long guard seta at the apex of the fifth tarsomere which projects between the two tarsal claws.

### Subfamily Scaritinae.

This subfamily is inadequately defined and possibly polyphyletic. The species possess a mesothoracic peduncle which frees the prothorax from the elytra and allows greater mobility (Basilewsky 1973: 9). It includes about 1,870 species worldwide which are grouped in this work into eight tribes: Pasimachini, Carenini, Scaritini, Clivinini, Dyschiriini, Salcediini, Promecognathini, and Dalyatini. Until the subfamily is better defined, it is difficult to comment on its relationships. Jeannel (1938a: 206) underlined a number of morphological features in the adults suggesting that scaritines and hiletines shared a common ancestor. Lindroth (1969b: xxiii) hypothesized that the similarities in “habitus and general organization” between scaritines (including promecognathines, clivinines, and dyschiriines) and broscines are probably an indication of close affinity.

Tribe Pasimachini. This tribe is represented by the genera *Pasimachus* Bonelli, with 32 species ranging collectively from southern Canada to Panama, and *Mouhotia* Laporte, with three species in eastern Asia. Monophyly of this group is doubtful. Bänninger (1950: 484) noted that if pasimachines and carenines are retained as distinct subtribes, *Mouhotia* cannot be placed in either of them and a separate subtribe would need to be established. To avoid proposing a new family-group name, *Mouhotia* is included here in the Pasimachini. Lorenz (2005: 132) included it with the carenines.

Relationships of the tribe have been little discussed. Sloane (1905b: 103) retained pasimachines and carenines under one family-group name implying a close relationship between the two groups. Nichols (1988a: 214) argued that Pasimachini is the sister-group to the tribe Carenini.

Tribe Carenini. This clade, which is endemic to Australia, includes about 195 species placed in ten genera. The genus *Scaraphites* Westwood (seven Australian species), usually listed as a member of this tribe (e.g., Lorenz 2005: 133), has been removed from it and placed in the tribe Scaritini by Moore and Lawrence (1994: 512). According to Moore and Lawrence (1994: 503), carenines represent the sister-group to the remaining Scaritini (*sensu lato*, i.e., pasimachines, scaritines, clivinines, dyschiriines).

Tribe Scaritini. This tribe, with about 495 species in 42 genera, is represented in all zoogeographical regions but is predominantly tropical. Four subtribes are currently recognized: Acanthoscelitina (one Afrotropical species in *Acanthoscelis* Dejean), Oxylobina (about 30 Oriental species in *Oxylobus* Chaudoir), Scapterina (about 25 Australian-Oriental-Afrotropical species in *Passalidius* Chaudoir, *Scapterus* Dejean, *Parathlibops* Basilewsky, *Thlibops* Putzeys, and *Steganomma* Macleay), and Scaritina (including the Madagascan storthodontines and dyscherines and the Afrotropical ochyropines [one species] and corintascarines [one species]), with the bulk of the species. Jeannel (1946: 220) assigned scapterines to his Clivinitae.

The North American fauna is represented by seven species of *Scarites* Fabricius.

Tribe Clivinini. This tribe is the most diversified group of the subfamily with about 60 genera. The inclusion of the clivinines within the Scaritinae has not been challenged often but parsimony analysis based on the female reproductive tract by Liebherr and Will (1998) suggests that clivinines could be more closely related to rhysodids than to scaritines. They also emphasized that the defensive secretions of the pygidial glands differ drastically between the two groups: clivinines use ketones or quinones while scaritines eject aliphatic acids.

At least three putative clades are recognized within the tribe and are usually ranked as subtribes. The forcipatorines, exclusively Neotropical, include the genera *Camptidius* Putzeys (one species), *Camptodontus* Dejean (14 species), *Forcipator* Maindron (four species), *Kultianella* Perrault (two species), *Obadius* Burmeister (two species), and *Stratiotes* Putzeys (two species). According to Perrault (1994: 686), members of this clade differ from those of remaining clivinines in having the ligula truncate and glabrous instead of prolonged and with at least one apical seta, the gula either vanishing posteriorly or very narrow instead of wide, the first antennomere asetose instead of having an apical seta (except in a few genera), the penultimate labial palpomere glabrous (except in one genus) instead of having two setae (except in some genera), and the clypeus glabrous (except in two species) instead of having a seta on each side. Another clade, the ardistomines, is restricted to the Western Hemisphere. Bousquet (2006c) restricted the group to the genera *Ardistomis* Putzeys, *Semiardistomis* Kult, and *Aspidoglossa* Putzeys whose members have a projection on pleurite VII. Kult (1950b) also included the genus *Neoreicheia* Kult. Whitehead (in Reichardt 1977: 386, 391) remarked that *Oxydrepanus* Putzeys was “doubtless related to *Neoreicheia*” and probably belonged to the ardistomine radiation along with such Old World genera as *Reicheia* Saulcy, *Syleter* Andrewes, and allies. Basilewsky (1973: 276) indicated that ardistomines are relatively closely related to dyschiriines. The third clade, the reicheiines, is represented only in the Old World and contains many genera including *Reicheia* Saulcy, *Trilophus* Andrewes, *Typhloreicheia* Holdhaus, *Trilophidius* Jeannel, and *Leleuporella* Basilewsky.

Iablokoff-Khnzorian (1960: 93) described a new genus, *Dyschiriomimus*, from Baltic amber which he viewed as an intermediate taxon between *Dyschirius* and *Clivina*. However, Fedorenko (1996: 37) believed the taxon is a typical clivinine more closely related to *Trilophus* and *Oxydrepanus* than to *Clivina*.

Tribe Salcediini. This tribe includes four genera placed in three subtribes following Bell (1998): *Salcedia* Fairmaire (nine Indo-African species) in Salcediina, *Holoprizus* Putzeys (one Amazonian species) and *Solenogenys* Westwood (three Brazilian species) in Solenogenyina, and *Androzelma* Dostal (one Vietnamese species) in Androzelmina. According to Bell (1998: 264), salcediines, forcipatorines, and clivinines form a well-defined clade supported by the following synapomorphies: the metepimeron is lobate and overlaps the anterior corners of abdominal sternum 2; the elytron possesses a ventral carina in form of a projected lobe which engages the dorsal angles of abdominal sternum VII; the labial pits on the mentum each have a posterior duct opening into the submental suture contrary to other scaritines in which the ducts open anteriad the suture and distant from it. Bell (1998: 265) also indicated that rhysodids shared three synapomorphies with salcediines (excluding *Androzelma*): a kind of coating on the exoskeleton, minute and retractile palpi, and a distinct lobe on which the eye is located. Furthermore among salcediines, members of *Solenogenys* share two synapomorphies with rhysodids: the medial margins of the ventral groove of the head are oblique, nearly straight, and meet near the “neck” condyle and the mandible has a dorsolateral lobe. Based on the above evidence, Bell (1998: 269) concluded that *Solenogenys* is the sister-group to rhysodids.

Tribe Dyschiriini. Relationships of this tribe within the Scaritinae are not documented. Fedorenko (1996: 37) suggested that dyschiriines share a common ancestor with clivinines but failed to disclose any characteristics that would support this claim. Jeannel (1946: 214) combined the ardistomines and dyschiriines in his Dyschiriitae.

Tribe Promecognathini. This small and well-defined tribe includes one genus with two species in western North America and four genera with six species in Cape Province in South Africa. Jeannel (1941b: 244; 1946: 206) postulated that promecognathines were closely related to siagonines without, however, offering any evidence. Lindroth (1961a: 125) and Kryzhanovskij (1976a: 88) associated promecognathines with scaritines (*sensu lato*) implying a close relationship between the two groups. Several apomorphic features, including details of the chaetotaxy, structure of the mouthparts and thorax, and marked similarity in their specialized way of attacking millipedes suggest that promecognathines could be closely related to peleciines. However, Straneo and Ball (1989) regarded the similarities between the two groups as evolutionary convergence.

McKay (1991) described a fossil from Cretaceous crater lake deposits at Orapa, Botswana, under the name *Palaeoaxinidium orapensis*, which he believed represents the sister-group to the Promecognathini.

Tribe Dalyatini. Molecular (Ribera et al. 2005) and morphological (Mateu and Bellés 2004) data suggest that the single, highly modified cave species of this tribe, *Dalyat mirabilis* Mateu from southeastern Spain, could be the sister-group to promecognathines.

### Subfamily Broscinae.

This subfamily includes a single tribe with about 290 species in 34 genera, arrayed in five subtribes (see Roig-Juñent 2000): Axonyina (five species), Broscina (about 75 species), Nothobroscina (about 90 species), Barypodina (about 25 species), and Creobiina (about 95 species). Broscines are represented in all major regions of the world, except the Afrotropical Region, but are more diverse in the Australian Region than anywhere else. They live almost exclusively in temperate areas, with only a few groups extending to the edges of the tropics. Several authors have suggested explicitly or implicitly that broscines are closely related to apotomines but Liebherr and Will’s (1998) morphological data on the female reproductive tract and the molecular sequence data provided by Maddison et al. (1999) did not support such an association. Erwin (1991a: 10) included broscines, apotomines, melaenines, and cymbionotines in his subfamily Broscinae.

Roig-Juñent’s (2000) parsimony analysis based on morphological characters of adults suggested that the three native North American genera (*Broscosoma*, *Zacotus*, and *Miscodera*) form a clade.

### Subfamily Apotominae.

This subfamily includes a single genus, *Apotomus* Illiger, with about 15 species in warm temperate and tropical regions of the Eastern Hemisphere and one species in Brazil. Kryzhanovskij (1976a: 88) and Moore et al. (1987: 122) associated apotomines with broscines and in several classifications these two groups are placed sequentially in the text. However, Roig-Juñent’s (1998: Fig. 10) parsimony analysis using 33 characters placed apotomines as the sister-group to melaenines (no *Cymbionotum* exemplars were included). Liebherr and Will (1998: 150) noted that apotomines do not have conjunct mesocoxae as in broscines and the other members of Jeannel’s *Stylifera* and that the placement of apotomines within the *Stylifera* should be rejected. They placed apotomines as a basal grade with clivinines and rhysodids but noted they could be closely related to scaritines and hiletines. Molecular data analyzed by Maddison et al. (1999: 128) did not provide support for a close relationship between apotomines and broscines.

### Subfamily Siagoninae.

This subfamily includes three genera, each arrayed in its own tribe: *Enceladus* Bonelli, *Luperca* Laporte, and *Siagona* Latreille. Relationships of the subfamily are obscure. Jeannel (1941b: 244; 1946: 206) associated siagonines with promecognathines. Erwin (1985: 467; 1991a: 9-10) listed siagonines with amarotypines, migadopines, elaphrines, promecognathines, hiletines, pseudomorphines, and scaritines (including cnemalobines, as Cnemacanthini) in his subfamily Scaritinae. The preferred cladogram of Liebherr and Will (1998: Fig. 57), based on the female reproductive tract, placed siagonines as the sister-group to the subfamily Carabinae. Some of the analyses on 18 rDNA performed by Maddison et al. (1999: 127) suggested that *Siagona* could be closely related to {*Gehringia* + *Cymbionotum*}. Based on a morphological study of larvae of *Siagona* and *Enceladus*, Grebennikov (1999: 9) did not find evidence to suggest a close relationship for siagonines.

Tribe Enceladini. Only the genus *Enceladus* belongs to this tribe, with one species found in the Guyana-Venezuelan area, possibly also in Amazonia (Reichardt 1977: 384).

Tribe Siagonini. This tribe contains only the genus *Siagona* with about 80 species in the Old World. Erwin (1978: 105) listed several apomorphic states shared by *Siagona* and *Cymbionotum* and stated that the two were undoubtedly closely related.

Tribe Lupercini. Two species of the genus *Luperca* are included in this tribe, one is found in tropical Africa, the other on the Indian subcontinent. Erwin (1978: 105) combined the genus with *Siagona* and *Cymbionotum* in his tribe Siagonini which he included in his subfamily Siagoninae along with the tribe Enceladini.

### Subfamily Melaeninae.

This subfamily includes one tribe with two genera: *Melaenus* Dejean with two species confined to the Oriental Region, Egypt, and the Afrotropical Region, excluding the Congo Basin and southern parts (Ball and Shpeley 2005: 37), and *Cymbionotum* Baudi di Selve with 20 species arrayed in two subgenera, *Procoscinia* Ball and Shpeley with two species in northern South America and *Cymbionotum* *s.str.* with 18 species confined to the warmer parts of the Old World. According to Ball and Shpeley (2005: 22), monophyly of this subfamily is indicated by the very long diverticulum of the spermathecal gland. Prior to these authors, the two genera had been variously classified. Several authors placed them in separate tribes though suggesting implicitly or explicitly that they were closely related (e.g., Erwin 1985: 469; Liebherr and Will 1998: 137). Others have separated the two rather widely. For example van Emden (1936a: 46) listed *Melaenus* in his *Harpalinae piliferae* from which the cymbionotines were excluded. Jeannel (1941b: 291-292) placed the genus *Melaenus* in his Psydritae along with psydrines, melisoderines (= moriomorphines), and meonines (= moriomorphines) and included *Cymbionotum* in a family-group taxon of its own which he considered closely related to siagonines (Jeannel 1946: 206).

Relationships of the subfamily are unclear. Liebherr and Will (1998: 150) suggested that Melaeninae could be closely related to Clivinini. Roig-Juñent’s (1998) parsimony analysis using 33 characters showed *Melaenus* to be the sister-group to apotomines; *Cymbionotum* exemplars were not included in his analysis.

This group has been reported in publications of the xix and early xx Centuries under the name Granigerini, because *Graniger algirinus* Motschulsky, the sole species included by Motschulsky in his new genus *Graniger*, was listed in synonymy with *Coscinia semelederi* Chaudoir (Chaudoir 1876d: 63). Because *Coscinia* Dejean was a junior homonym of *Coscinia* Hübner, *Graniger* Motschulsky became the valid name for this genus. However, Andrewes (1933: 3) showed that Motschulsky’s species was in fact identical with the ditomine *Carterophonus femoralis* Coquerel. *Cymbionotum* Baudi di Selve was the next available name for the species of *Coscinia* Dejean.

### Subfamily Gehringiinae.

This subfamily includes three genera placed in two subtribes: Gehringiina Darlington with a single western North American species, *Gehringia olympica* Darlington, and Helenaeina Deuve with four rarely collected species from Egypt, Turkey, Yemen, and Namibia placed in the genera *Helenaea* Schatzmayr and Koch and *Afrogehringia* Baehr, Schüle and Lorenz. The taxonomic position of the group is debated. Jeannel (1941b, 1946: 46) combined gehringiines with trachypachids and paussines (as *Caraboidea Isochaeta*) and both Lindroth (1969b) and Kryzhanovskij (1976a: 87) associated gehringiines with trachypachids. Bell (1967: 106) indicated that the form of the palpi and the anterior tibia suggest that gehringiines could be derived from the genus *Tachys* but he also raised the possibility that gehringiines could belong to his *Hemipleuri*, a group comprising the nebriines and carabines. In Nagel’s (1987: Fig. 2) cladistic analysis, gehringiines were positioned as the sister-group to {cicindines + paussines}. Beutel (1992c) indicated that the isochaetous protibia of gehringiines suggests that the group could be “an early offshoot of the metriine-paussine lineage.” In listing the tribe in his supertribe Psydritae, Erwin (1985: 468) suggested implicitly that gehringiines could be closely related to psydrines and patrobines and Lorenz (2005: 243) placed the tribe Gehringiini within the Psydrinae. Deuve (2005) made a detailed analysis of the morphology of gehringiine adults and concluded that several character states suggest “a very basal position in the phylogeny of adephagan Coleoptera” for gehringiines. However he also noted that the peculiar “abdominal type” found in the group is similar to that of the genus *Cymbionotum*. A close affiliation between *Gehringia* and *Cymbionotum* received support from the molecular analysis of Maddison et al. (1999). Arndt et al. (2005: 140) pointed out that the condition of the protibial spurs in gehringiines could not be unambiguously assigned to either of the two types found in other carabids. They noted that if the protibial spurs of gehringiines are considered to be of the isochaete type, then gehringiines could be the sister-group to Paussinae and if considered to be of the anisochaete type, they could be the sister-group to Nebriinae.

### Subfamily Trechinae.

Several authors agree that the tribes Trechini, Zolini, Bembidiini, and Pogonini are closely related and probably constitute a clade. Monophyly is supported by characteristic features of the adult morphology (Roig-Juñent and Cicchino 2001), larval morphology (Grebennikov and Maddison 2000: 226; 2005: 44), and 18S ribosomal sequence data (Maddison et al. 1999). In addition, males of Trechinae studied lack chiasmata in meiosis (Serrano 1981) contrary to most other Carabidae, a notable synapomorphy (Maddison and Ober 2011: 243). Jeannel (1941b: 299) also included mecyclothoracines in the subfamily (as Trechidae) but most recent authors place them within the Moriomorphinae. Deuve (1993: 156) included patrobines within the Trechinae.

As discussed under Patrobinae, this subfamily is probably the sister-group to patrobines.

Tribe Trechini. A relatively well-defined and very diverse group with more than 2,500 species currently arranged in 170 genera or so. Although represented in all zoogeographical regions, the tribe is more diverse in the temperate zones than in the tropics. Many species are endogean or troglodytic and flightless. Casale and Laneyrie (1982: 7) classified the Trechini into six groups placed in two major complexes, one including cnidines, trechodines, and plocamotrechines characterized by the median lobe of the aedeagus being wide open dorsally, the basal orifice lying between two symmetric lobes, and one comprising the perileptines, aepines, and trechines with the median lobe partly closed dorsally, the basal orifice opening on the ventral surface of the basal bulb. However, this classification has been challenged in recent times. Uéno (1989: 12-13) presented arguments to combine cnidines with perileptines and Grebennikov and Maddison (2005: 46-47) found evidences in the larval characters that perileptines were closely related to trechodines. In this work, the Trechini are grouped into two subtribes: Trechodina (including perileptines, cnidines, and plocamotrechines) and Trechina (including aepines).

Based on larval character states, Grebennikov and Maddison (2005) suggested that Trechini is the sister-group to {Zolini + Bembidiini + Pogonini}. Arndt’s (1993: 33) analysis of larval characters suggested that trechines are closely related to tachyines.

The North American fauna comprises about 225 species grouped into nine genera, all belonging to the subtribe Trechina. Barr (1985a: 351) recognized four series among the North American genera, the *Trechus* series with *Trechus*, the *Trechoblemus* series with *Trechoblemus*, *Blemus*, *Pseudanophthalmus*, *Neaphaenops*, and *Nelsonites*, the *Darlingtonea* series with *Darlingtonea* and *Ameroduvalius*, and the *Aphaenops* series with *Xenotrechus*. The genus *Pseudanophthalmus*, whose members are cave inhabitants except for a few rare occurrences in forest floor humus and in abandoned coal mines, is closely related to *Duvaliopsis* Jeannel which includes six edaphic species in the Carpathian and Transylvanian Alps of eastern Europe. In fact, Barr (2004: 7) listed *Duvaliopsis* as a junior synonym of *Pseudanophthalmus* since both genera are not readily separable on purely morphological grounds. *Xenotrechus*, with two species in southeastern Missouri caves, is apparently the sister-group to the monospecific genus *Chaetoduvalius* Jeannel (Barr 2004: 10) of the Apuseni Mountains in the western Carpathians, Romania.

Tribe Zolini. The 57 species of this tribe are currently arrayed in ten genera and three subtribes: Zolina with 50 species in South America (genus *Merizodus* Solier) and the Australian Region (genera *Oopterus* Guérin-Méneville, *Zolus* Sharp, *Synteratus* Broun, *Percodermus* Sloane, *Idacarabus* Lea, *Sloaneana* Csiki, and *Pterocyrtus* Sloane), Sinozolina for the genus *Sinozolus* Deuve (six Chinese species), and Chalteniina for *Chaltenia patagonica* Roig-Juñent and Cicchino of Argentina. Jeannel (1962) recognized two lineages within Zolina based on structural features of the male genitalia.

Tribe Bembidiini. This relatively well-defined tribe is represented in all zoogeographical regions of the world. Adults possess characteristic subulate apical palpomeres (except in *Horologion*), a condition found otherwise only in gehringiines and a few trechines. Bembidiines are grouped into six subtribes: Bembidiina, with about 1,350 species, is distributed worldwide but is more diverse in the temperate regions than in the tropics; Xystosomina is represented in the New World and tropical Australia (Erwin 1994: 560) by about 125 species with only one (*Mioptachys flavicauda*) found in North America; Tachyina (including lymnastines) with nearly 800 species is also worldwide but, contrary to Bembidiina, is more diverse in the tropics; Anillina with about 375 minute, apterous, and blind species is distributed in all zoogeographical regions; Horologionina with a single cave-inhabiting species, known only from the holotype collected in West Virginia; and Lovriciina represented by four cavernicolous species, placed in three genera (see Giachino et al. 2011), found in the Balkans. Erwin (1982b: 459) postulated that anillines and horologionines represent a grade of several lineages derived from *Paratachys* Casey and allies, a hypothesis refuted by Maddison and Ober (2011: 249). Arndt (1993: 33) found a number of putative synapomorphies in larvae of Tachyina and Trechini and suggested that the two taxa are sister-groups. Grebennikov (2002) and Grebennikov and Maddison (2005), working with larvae, found evidence suggesting that Anillina is the sister-group to {Tachyina + Xystosomina} and that xystosomines are probably nested within the tachyines. Van Emden (1936a) suggested that *Horologion* Valentine was closely related to psydrines and Jeannel (1949b: 93) believed it could be related to patrobines.

Tribe Pogonini. This tribe is found in all zoogeographical regions of the world but is more diverse, both in terms of species and lineages, in the Palaearctic Region. All 83 species currently recognized are more or less halobiont and live along sea coasts or near salt lakes. Jeannel (1941b: 552) stated that this group is related to mecyclothoracines (currently placed in the subfamily Moriomorphinae) of the Hawaiian islands and the Australian Region. Müller (1975) postulated that Pogonini is the sister-group to Bembidiini. Based on karyotypic data, Serrano and Galián (1998: 196) suggested that pogonines are closely related to Bembidiina. Arndt (1993: 33), working on larval characters, suggested a close relationship between pogonines and Bembidiini (excluding tachyines).

### Subfamily Patrobinae.

This subfamily, which includes the tribes Lissopogonini and Patrobini, is considered to be the sister-taxon to Trechinae by several authors based on male tarsal structure (Müller 1975), larval characteristics (Arndt 1993: 32), and similar abdominal morphology (Deuve 1993). This association is also supported by molecular sequence data (Maddison et al. 1999: 128; Maddison and Ober 2011: 243). Erwin (1985: 469) and Baehr (1998: 363) suggested that patrobines may be closely related to Moriomorphinae. Jeannel (1941b: 80-81) placed patrobines in his *Limbata Stylifera* along with apotomines, broscines, psydrines, moriomorphines, melaenines, trechines, bembidiines, pogonines, and zolines but indicated that some character states, particularly of the larvae, suggest that they may belong to the *Limbata Conchifera*.

This subfamily is found in the Northern Hemisphere and Oriental Region. Only the tribe Patrobini is represented in North America.

Tribe Lissopogonini. This tribe includes a single genus, *Lissopogonus* Andrewes, with eight species in Asia. The genus was originally described in the tribe Pogonini and subsequently transferred to the tribe Patrobini by Zamotajlov and Sciaky (1996: 40). Bousquet and Grebennikov (1999: 11) alluded to the possibility that *Lissopogonus* could be a highly derived taxon related to *Patrobus* and *Platypatrobus* based on the shared apomorphic condition of the median sulcus of the pronotum being wide and deep in the basal fifth and reaching the basal edge. Deuve and Tian (2002: 30) suggested that the genus could belong at the base of the Trechinae and Patrobinae lineages (their Trechidae).

Tribe Patrobini. The 215 species or so listed in this tribe are currently arrayed in four subtribes: Deltomerina with the genus *Deltomerus* Motschulsky only, Deltomerodina with the genus *Deltomerodes* Deuve, Patrobina with 18 genera, and Platidiolina with *Platidiolus* Chaudoir. In a cladistic analysis conducted by Roig-Juñent and Cicchino (2001: Fig. 1), this tribe is positioned as the sister-group to Amblytelini (currently included in the Moriomorphinae).

### Subfamily Psydrinae.

Following Maddison and Ober (2011: 237), this subfamily is restricted to the tribe Psydrini and includes only six species. Two (*Laccocenus ambiguus* Sloane and *Laccocenus vicinus* Moore) lives in southeastern Australia, another one (*Psydrus piceus* LeConte) ranges widely across the northern parts of North America, extending southwards to the mountains of northern California, Arizona, and New Mexico, and the other three, all members of the genus *Nomius* Laporte, are restricted to central Africa and Madagascar (two species) or to the Northern Hemisphere although apparently extinct in Asia (*Nomius pygmaeus* Dejean). Baehr’s (1998: Fig. 1) preliminary cladistic analysis using 19 characters of adults suggested that Psydrini could be the sister-group to {Patrobinae + the remaining Psydrinae [= Moriomorphinae]}. Relationships among the three genera of Psydrini have not been investigated.

### Subfamily Moriomorphinae.

Members of this subfamily were traditionally included in the Psydrinae but recent morphological (Baehr 1998) and molecular data (Maddison and Ober 2011: 237) studies suggest that the Moriomorphinae form a clade and that the group is not closely related to the true Psydrinae. Baehr (1998: 363) argued that Patrobinae could be the sister-group to Moriomorphinae. Many moriomorphines are similar to pterostichines in body form but the presence of a scrobal seta and setose parameres in almost all moriomorphines, unlike pterostichines, suggest that they are probably not closely related. Ober’s (2002) phylogenetic analysis based on molecular sequence data suggested that the subfamily Moriomorphinae, termed “austral psydrines,” could be the sister-group to {Brachininae + Harpalinae}.

This subfamily, which includes about 470 species, is represented only in the Southern Hemisphere and is particularly diverse in the Australian Region. Five tribes were traditionally recognized (see Baehr 2004): Mecyclothoracini with about 285 species placed in the genera *Neonomius* Moore and *Mecyclothorax* Sharp; Meonini with about 20 species in the genera *Raphetis* Moore, *Meonis* Laporte, *Selenochilus* Chaudoir, and *Meonochilus* Liebherr and Marris; Moriomorphini with six species in five genera, all endemic to southeastern Australia; Tropopterini with about 50 species in seven genera; and Amblytelini with six genera and about 95 species endemic to Australia, including Tasmania. Recently, Liebherr (2011) proposed an entirely new classification, dividing the moriomorphines into two groups based on characters of the parameres. His classification is adopted here.

The genus *Bembidiomorphum* Champion (two species in Chile), included in this group since van Emden (1936a: 51), belongs to the Broscini (Roig-Juñent et al. 2008: 212).

Tribe Moriomorphini. This group includes about 55 species, all endemic to the Australian Region, placed in 13 genera: *Celanida* Laporte (one species), *Melisodera* Westwood (three species), *Molopsida* White (28 species), *Moriodema* Laporte (two species), *Moriomorpha* Laporte (one species), *Neonomius* Moore (three species), *Pterogmus* Sloane (one species), *Rhaebolestes* Sloane (two species), *Rossjoycea* Liebherr (one species), *Sitaphe* Moore (eight species), *Teraphis* Laporte (six species), *Theprisa* Moore (three species), and *Trephisa* Moore (one species). These species are characterized by having elongate, parallel-sided parameres that are glabrous or sparsely clothed with very short setae.

Tribe Amblytelini. This tribe contains about 415 species in 12 genera: *Amblytelus* Erichson (43 species), *Dystrichothorax* Blackburn (48 species), *Epelyx* Blackburn (five species), *Mecyclothorax* Sharp (about 280 species), *Meonis* Laporte (16 species), *Meonochilus* Liebherr and Marris (six species), *Paratrichothorax* Baehr (one species), *Pseudamblytelus* Baehr (one species), *Raphetis* Moore (three species), *Selenochilus* Chaudoir (six species), *Trichamblytelus* Baehr (one species), and *Tropopterus* Solier (four species). These species are restricted to Australia and New Zealand except those of *Mecyclothorax* which occur also in New Guinea, Borneo, Java, and the Polynesian islands in the Pacific Ocean and *Tropopterus* which are found in Chile and Peru. Amblytelines differ from members of Moriomorphini by having more setose parameres that are either shorter, basally broader and narrowly rounded apically or elongate with whiplike apex.

### Subfamily Nototylinae.

This subfamily includes a single species, *Nototylus fryi* (Schaum), known only from the female holotype collected in the state of Espírito Santo, Brazil, in the xix Century. The species is aberrant structurally: it lacks the grooming structures of the protibiae present in all other Geadephaga except Paussini and lacks the pubescence on antennomeres 5-10 which is present in other Geadephaga except Trachypachidae, Rhysodidae, and Gehringiinae (Deuve 1994b: 141). Bänninger (1927) suggested that *Nototylus* Gemminger and Harold was related to Ozaenini, Kryzhanovskij (1976a: 87) associated it with paussines (excluding metriines) and cicindines, and Erwin (1979: 591) postulated that the species was an independently adapted myrmecophile from an ozaenine stock. However, Ball (1979: 100) doubted the possibility of a close affinity between nototylines and paussines as suggested by the above-mentioned authors. Deuve (1994b) published a detailed description of the structural character states of the species and suggested, but with some doubt, a sister-group relationship between nototylines and paussines. He noted several synapomorphies between the two groups including the compressed protibia, the tergite IX which is differentiated into a thin transverse arch, the reduced and lateral position of the laterotergite IX, and the diffuse dorsal pubescence.

### Subfamily Paussinae.

There is little doubt that this subfamily constitutes a monophyletic lineage. The known larvae share a unique transformation of the abdomen in which the epipleurites of the 9th segment are greatly enlarged and fused with the tergum of the 8th segment to form a plate, displacing the urogomphi and the 10th segment in a vertical plane (Bousquet 1986). The relationship of the subfamily is highly debated but it could be closely related to brachinines. Adults of both groups possess a two-chambered pygidial gland which produces a quinonoid secretion by mixing hydroquinones and hydrogen peroxide from the inner chamber with enzymes produced in the outer chamber (Schildknecht and Holoubek 1961); the secretion is discharged at temperatures of 55-100°C (Aneshansley et al. 1969; Aneshansley et al. 1983). The structure of the pygidial glands and the chemistry of the secretions are unique among beetles. However, based on structural dissimilarities, several authors, including Ball and McCleve (1990), Beutel (1992b), and Geiselhardt et al. (2007), believed that the similarities in the pygidial gland structures and secretions between the two groups are convergent.

Erwin and Stork (1985: 445) concluded that paussines and brachinines are closely related and form the sister-group to a large clade comprising {Elaphrini + Migadopini + Siagonini + Promecognathini + Hiletini + Pseudomorphini + Cnemacanthini (= Cnemalobini) + Scaritini} based on a suite of character states associated with tarsal claws. Deuve (1988), working on the structures of the last abdominal segments of adults, supported the view of a close relationship between paussines and brachinines. However, alternate placements of the paussines have been proposed. Jeannel (1941b: 89) placed trachypachids, gehringiines, and paussines in his *Isochaeta* based on the apical position of both protibial spurs. Kryzhanovskij (1976a: 87), followed by Lawrence and Newton (1995), included the Cicindini and Nototylini within the Paussinae, implying a close relationship between these three elements. Beutel (1995) suggested a close affinity between paussines and gehringiines. Liebherr and Will’s (1998) preferred cladogram based on 20 characters of the female ovipositors and reproductive tract placed paussines as the sister-group to the remaining Geadephaga (excluding trachypachids). An interesting observation is that of Vigna Taglianti and Rossi (1998: 516) who noted the similarity between the laboulbeniale parasitic species found on the brachinine *Pheropsophus* Solier and paussine *Pachyteles* Perty. They added that paussines and brachinines “might be more closely related than suggested by morphological data, thus supporting the result of recent biochemical studies on explosive secretions of members of these groups.”

Members of this subfamily are currently arrayed in five family-group taxa which have been ranked differently during the past few decades. In this catalogue, they are ranked as tribes. All five are probably monophyletic except for the Ozaenini which is likely paraphyletic. The phylogenetic relationships among extinct and extant genera have been expressed in a cladogram based on adult and larval characters by Geiselhardt et al. (2007: Fig. 1).

Tribe Metriini. This group includes two genera: *Metrius* Eschscholtz, with two species in western North America, and *Sinometrius* Wrase and Schmidt with a single species recently found in Hubei province in China. This tribe is usually listed as the sister-group to the remaining paussines because of the lack of the apico-lateral fold on each elytron (flange of Coanda of Stork 1985) characteristic of the remaining paussines. This fold, located at the opening of the defence gland, is apparently used to deflect discharges of secretions from the defence glands as showed by Eisner and Aneshansley (1982) for the Neotropical genus *Goniotropis* Gray. However, Vigna Taglianti et al. (1998: 292), based on a set of 20 larval characters, considered {Metriini + Ozaenini} as the sister-group to Paussini, suggesting that the elytral fold was secondarily lost in metriines or that the fold evolved twice in the subfamily.

Tribe Mystropomini. This tribe includes only the genus *Mystropomus* Chaudoir, with two Australian species. It is probably the most primitive extant genus of the subfamily excluding metriines. Adult ozaenines, protopaussines, and paussines (*sensu stricto*) are synapomorphic in having the elytral fold short, the pterothorax and abdomen parallel-sided and the epimera and anepisterna largely covered by the elytral epipleura (Beutel 1992c: 56). In adults of *Mystropomus* the elytral fold is markedly long and extends over the apical half of the elytron (Jeannel 1946: 47).

Tribe Ozaenini. This group of about 160 species is mainly represented in the tropics; only a few species enter the southern parts of the Northern Hemisphere in Japan, China, Taiwan, and southern United States. Ozaenines differ from protopaussines and paussines by having the mouthparts not modified, and from paussines also in having all 11 antennomeres normally developed. Several authors (e.g., Ball and McCleve 1990; Nagel 1997: 356; Di Giulio and Moore 2004) believed that ozaenines are paraphyletic in regard to the remaining Paussinae (excluding mystropomines and metriines). Beutel (1992b; 1995) and Di Giulio et al. (2003) proposed that the ozaenine genus *Physea* Brullé is the sister-group to {protopaussines + paussines} while Ball (in Nagel 1997: 356) regarded *Ozaena* Olivier as the best candidate based on the enlarged first antennomere and the reduced antennal cleaner of the protibia.

Tribe Protopaussini. This tribe includes eight extant Asian species placed in the genus *Protopaussus* Gestro. Some authors (e.g., Basilewsky 1953a: 23, 1962a: 6-9; Nagel 1987: 27) associated protopaussines with ozaenines based on the presence of 11 antennomeres in both groups but most have associated them with paussines. Nagel (1997: 348, 356) did not find any derived character states shared between protopaussines and ozaenines but noted that the small lacinia lacking the dense brushlike pilosity, typical of other carabids, is a putative synapomorphy for protopaussines and paussines (*sensu stricto*). From a zoogeographic point of view, it is interesting to note that a Tertiary fossil species of *Protopaussus* has been described from Dominican amber (Nagel 1997).

Tribe Paussini. This group, also known under the vernacular name “ant nest beetles,” currently includes about 565 myrmecophilous species arrayed in this work in seven subtribes: Carabidomemnina for the genera *Eohomopterus* Wasmann (two Neotropical species) and *Carabidomemnus* Kolbe (27 African species); Arthropterina for the Australian genera *Megalopaussus* Lea (one species) and *Arthropterus* Macleay (about 65 species); Cerapterina for the genera *Mesarthropterus* Wasmann (one species in Ethiopia) and *Cerapterus* Swederus (32 species in the Afrotropical and Oriental Regions with two species extending into the Himalayas); Pentaplatarthrina for the genera *Hexaplatarthrus* Jeannel (one Madagascan species) and *Pentaplatarthrus* Westwood (eight Afrotropical species); Homopterina for the genus *Homopterus* Westwood (12 Neotropical species); Heteropaussina for the genus *Heteropaussus* Thomson (about 25 species in the Afrotropical and Oriental Regions); and Paussina for the remaining 12 genera (about 385 species). Luna de Carvalho (1989: 361) used a different approach and recognized three tribes among his extant Paussinae (Paussini in this work): Cerapterini (including carabidomemnines, homopterines, heteropaussines, and arthropterines), Pentaplatarthrini, and Paussini. Within his Paussini, he included the following subtribes: Platyrhopalina for the Asian genera *Platyrhopalopsis* Desneux (three species), *Platyrhopalus* Westwood (14 species), *Stenorhopalus* Wasmann (two species), *Lebioderus* Westwood (seven species), and *Euplatyrhopalus* Desneux (six species); Ceratoderina for the genera *Paussomorphus* Raffray (three Afrotropical species), *Melanospilus* Westwood (three Oriental species with one species extending into the Himalayas), and *Ceratoderus* Westwood (seven Asian species); Leleupaussina for the genus *Leleupaussus* Luna de Carvalho (one Afrotropical species); Hylotorina for the Afrotropical genera *Granulopaussus* Kolbe (four species), *Hylopaussus* Luna de Carvalho (two species), and *Hylotorus* Dalman (six species); and Paussina for numerous genera that some authors sink into one large genus, *Paussus* Linnaeus (about 330 species in the Old World of which only two, *Paussus favieri* Fairmaire and *Paussus turcicus* Frivaldszk von Frivald, reach Europe). Nagel (1987, 1997, as Carabidomemnitae) viewed the Carabidomemnina as the sister-group of the remaining Paussini.

### Subfamily Brachininae.

There is little doubt that this group, known under the vernacular name “bombardier beetles,” constitutes a monophyletic lineage. The adults have seven (females) or eight (males) exposed abdominal sterna instead of six as in other carabids. Such modification provides a greater abdominal mobility, allowing a more efficient alignment of the defence spray. However, brachinines do not appear monophyletic in terms of their 18S rDNA (Maddison et al. 1999: 129). The group has a worldwide distribution but is clearly more diverse in the Southern Hemisphere. Most authors recognize two main lineages, ranked here as tribes, among brachinines: Brachinini, represented in most regions of the world including North America, and Crepidogastrini, restricted to southern India and Africa.

For a long time brachinines have been associated with the “Truncatipennes,” an informal name use to group several tribes whose adults have more or less truncate elytra at the apex. Jeannel (1942, 1949a) included brachinines and pseudomorphines in his *Balteifera*, implicitly suggesting a close affinity between the two groups. Liebherr and Will (1998: 152-153) placed brachinines with the {Harpalinae + Trechinae + Moriomorphinae}in their study of the female reproductive tract. These authors also alluded to the possibility of a close relationship between brachinines and clivinines. Analysis of molecular data presented by Ribera et al. (2005: 289) indicated a close relationship between brachinines and the subfamily Harpalinae, not with the Paussinae. Maddison et al. (1999: 129) suggested, from 18S r-DNA sequence analyses, an intriguing possibility, that the paussines and brachinines are closely related and that both in turn are related to Harpalinae. In my opinion their hypothesis is credible.

Tribe Crepidogastrini. This tribe is mostly represented in the Afrotropical Region but a few species are found in the Indian subcontinent. It contains the genera *Brachynillus* Reitter (three species), *Crepidogaster* Boheman (about 100 species), *Crepidogastrillus* Basilewsky (one species), *Crepidogastrinus* Basilewsky (one species), *Crepidolomus* Basilewsky (two species), and *Crepidonellus* Basilewsky (five species).

According to Erwin (1970a: 27), adults of crepidogastrines differ from those of brachinines in having the mesepimeron absent or almost so (instead of broad), the adhesive setae on the male protarsi of the “spongy” type (instead of the “seriate” type), the terminal palpomeres swollen and usually securiform (instead of subcylindrical or wedge-shaped), and the gular suture convergent behind (instead of divergent).

Tribe Brachinini. This tribe includes about 540 species of which 50, all belonging to the genus *Brachinus* Weber, occur in North America. Erwin’s (1970a: 175) study suggested that all New World species of *Brachinus*, along with a relict species found in the Himalayas, form a clade for which he proposed the subgeneric name *Neobrachinus*. He also postulated that the subgenus *Cnecostolus* Reitter, endemic to the Palaearctic Region, was the sister-group to *Neobrachinus*. Erwin (1970a: 28) arrayed the brachinine genera into four subtribes: Aptinina, Brachinina, Mastacina, and Pheropsophina. In his cladistic analysis (Erwin 1970a: Fig. 451), masticines were positioned as the sister-group to pheropsophines and the two form the sister-group to {aptinines + brachinines}.

Unlike most carabid larvae, those of brachinines are ectoparasites and feed on carabid and water beetle pupae.

### Subfamily Harpalinae.

Harpalinae is the largest subfamily of Carabidae and the one usually placed at the end of the carabid classification. Molecular data analyses (Maddison et al. 1999; Ober 2002; Ribera et al. 2005) suggest that the subfamily is monophyletic.

In this catalogue, members of Harpalinae are arrayed conveniently in two supertribes: Pterostichitae and Harpalitae. Adults of the vast majority of Pterostichitae, which includes the tribes Morionini, Abacetini, Pterostichini, Zabrini, Oodini, Panagaeini, and Chlaeniini in North America, have crossed epipleura and most secrete something else than formic acid as major constituent of the pygidial glands. Adults of Harpalitae have non-crossed epipleura and, except in the sole species of Pentagonicini studied, secrete formic acid as major constituent of their pygidial glands as far as known. The absence of a crossed epipleuron could be an evolutionary feature providing greater flexibility to aim the powerful formic acid secretion of the pygidial glands. The presence of a transverse membranous band on the stipes of larvae prompted Arndt (1998: 184) to suggest that the tribes Licinini and Harpalini, herein included in the Harpalitae, were closely related to members of Pterostichitae.

Tribe Morionini. This relatively well-defined, likely monophyletic tribe is represented in all zoogeographical regions of the world but is more diverse in the tropics than in temperate areas. Its relationships have been debated. Indeed, some larval character states suggest that morionines could be related to scaritines while some adult character states suggest they may be related to pterostichines. Bousquet (2001) discussed the larval character states of morionines in detail and concluded that they do not yield evidence to favor one hypothesis over the other. However, when features of the adults are also taken into account, there is little doubt that morionines are more closely related to pterostichines than to scaritines. Recently Will (2004: 218), following Liebherr and Will (1998: 156), found three “unambiguously optimized and unreversed synapomorphies” suggesting that cnemalobines and morionines are sister-groups. A review and cladistic analysis of the morionine genus-group taxa have been published recently (Will 2004).

Moore (1965: 5) included the Australian genus *Catadromus* Macleay (seven species) in the tribe Morionini but his view has not been retained by subsequent authors.

Tribe Cnemalobini. This tribe includes only the Neotropical genus *Cnemalobus* Guérin-Méneville (32 species in Chile, Argentina, and Uruguay). Jeannel (1941b: 286) stated that the genus should be placed near the perigonines and Reichardt (1977: 416) followed his suggestion. Erwin (1985: 467) associated cnemalobines (as Cnemacanthini) with scaritines and clivinines. Arndt (1993: 40) suggested that the tribes Cnemalobini and Harpalini form a clade based on larval characteristics. Roig-Juñent (1993: 12) suggested, from a preliminary analysis, that cnemalobines and zabrines are sister-groups and the two groups are closely related to morionines. Other cladistic analyses (Liebherr and Will 1998: 156; Will 2004: 217) placed morionines as the sister-group to cnemalobines. Molecular data (18S rDNA) analyses (Maddison et al. 1999: 129) did not endorse placement of cnemalobines with Scaritinae but supported an association with the subfamily Harpalinae.

Tribe Microcheilini. This tribe includes a single genus, *Microcheila* Brullé, with two Madagascan species. Besides their relatively aberrant facies, adults of this group possess a number of character states unusual for pterostichines. The penultimate labial palpomere has more than two setae, each sternum possesses a transverse row of setae, the protibia has a latero-apical dentiform protuberance, all tarsomeres are densely pubescent beneath, and the first four protarsomeres of the male have adhesive setae (Jeannel 1948a: 616). The elytral plica is well developed as in members of Pterostichini. The group was included, along with morionines, chaetodactylines, and pterostichines (including sphodrines and platynines), in Jeannel’s (1948a: 380) family Pterostichidae.

Tribe Chaetodactylini. This group includes a single genus, *Chaetodactyla* Tschitschérine with 20 species endemic to Madagascar. The species superficially resemble several pterostichine taxa but the male protarsomeres are not expanded and have no adhesive setae (Jeannel 1948a: 619). The group was associated with morionines, metiines, zabrines, microcheilines, and pterostichines (including sphodrines and platynines) in Jeannel’s (1942: 734-735) family Pterostichidae.

Alluaud (1935: 28) reported that one of his colleagues rearing pupae of various insect groups for parasitic Hymenoptera discovered 14 adults of *Chaetodactyla* emerging from pupal chambers of two cetonid species. Jeannel (1948a: 620) postulated that *Chaetodactyla* females probably lay their eggs on the cetoniid larvae and that the carabid larvae remain inside the cetonid pupae, eventually feeding upon them.

Tribe Cratocerini. This tribe includes the genera *Cratocerus* Dejean with two Neotropical species and *Brachidius* Chaudoir with one australo-oriental species. Chaudoir (1873a) also listed *Basoleia* Westwood (= *Catapiesis* Solier) in this tribe and Lorenz (2005: 248) also included the genus *Oxyglychus* Straneo, with one Japanese species, previously included within the caelostomines (= drimostomatines). Cratocerines have been little studied and their taxonomic position is not well established. They are usually placed within the Pterostichini (e.g., Reichardt 1977: 407). Lorenz (2005: 248-252) combined cratocerines with catapieseines and drimostomatines in his subfamily Pterostichinae.

Tribe Abacetini. This tribe is proposed here to include the abacetines proper, the loxandrines, and the celioscheseines based on a preliminary cladistic analysis conducted by Will (2000) suggesting that these three groups are closely related. Van Emden (1949) and Arndt (1988) had already drawn attention to the fact that some putative apomorphic character states were shared by abacetines (with more than 95% of the species endemic to the Old World) and loxandrines (with more than 95% of the species restricted to the New World). As defined here, this tribe, as well as all three groups included in it, is inadequately characterized except for some abacetine genera which have an asymmetrical insertion of the second antennomere in the adults, and some loxandrine genera which have the first three protarsomeres of the males obliquely expanded. Monophyly of this tribe has not yet been demonstrated.

Tribe Pterostichini. This highly diverse tribe is represented in all continents, except Antarctica, and the species are found from the arctic regions to the tropics. There are no structural features yet discovered to suggest that the tribe, as currently conceived, forms a clade and there is little doubt, as suggested by Ball (1979: 102), that it represents a grade.

A number of putative clades have been recognized within the pterostichines and some of them have received formal scientific names. These include, among others, the **euchroines** with the genera *Bothynoproctus* Tschitschérine (one Neotropical species), *Euchroa* Brullé (38 Neotropical species), *Lobobrachus* Sharp (two Neotropical species), *Setalis* Laporte (three Australian species) and, according to Will (2000: 64), *Microcephalus* Dejean (15 Neotropical species); the Northern Hemisphere **myadines** with the genus-group taxa *Aristochroa* Tschitschérine (18 Asian species), *Myas* Sturm (with about 30 species in North America and Asia placed in the subgenus *Trigonognatha* Motschulsky and one European species), *Steropanus* Fairmaire (11 Asian species, some of them endemic to the Oriental Region), and *Xenion* Tschitschérine (one European species) to which *Stereocerus* Kirby (two Holarctic species) is probably closely related (Bousquet 1999: 85); the **trigonotomines** (including deliniines) with the genera *Delinius* Westwood (three Australian species), *Leiradira* Laporte (12 Australian species), *Lesticus* Dejean (about 100 Asio-Australian species), *Trigonotoma* Dejean (about 55 Asian species), and *Euryaptus* Bates (six Asian species), *Pareuryaptus* Dubault, Lassalle and Roux (17 Asian species); the Australian **darodiliines** (including cratogastrines) with the genera *Loxogenius* Sloane (one species), *Liopasa* Tschitschérine (one species), *Cratogaster* Blanchard (five species), and *Darodilia* Laporte (ten species); the New Caledonian **abacomorphines** with the genera *Abacoleptus* Fauvel (three species), *Abacomorphus* Chaudoir (two species), *Platysmodes* Fauvel (one species), and *Setalidius* Chaudoir (two species); the **molopines** with the North American genus *Cyclotrachelus* Chaudoir (45 species) and the western Palaearctic genera *Abax* Bonelli (18 species), *Henrotiochoromus* Busulini (one species), *Molopidius* Jeannel (one species), *Molops* Bonelli (40 species), *Oscadytes* Lagar Mascaro (one species), *Percus* Bonelli (19 species), *Speomolops* Patrizi (one species), *Stenochoromus* Miller (one species), *Styracoderus* Chaudoir (three species), *Tanythrix* Schaum (three species), *Typhlochoromus* Moczarski (two species), and *Zariquieya* Jeannel (one species) to which Jeannel (1948a: 450-451) added several Madagascan genera (*Abacodes* Jeannel, *Eucamptognathus* Chaudoir, *Eudromus* Klug, *Eurypercus* Jeannel, and *Molopinus* Jeannel); and the **poecilines** as defined by Jeannel (1942: 738) with the genera *Stomis* Clairville, *Pedius* Motschulsky, *Argutor* Dejean, *Orthomus* Chaudoir, *Poecilus* Bonelli, *Phonias* des Gozis, *Bothriopterus* Chaudoir, and *Melanius* Bonelli. Some of these groups, such as the poecilines, are probably polyphyletic.

Tribe Zabrini. Zabrines are most diversified in the Palaearctic and Nearctic Regions but are also represented in the mountains of the northern Neotropical, northern Oriental, and eastern Afrotropical Regions. Some authors have recognized several, more or less clearly defined genera in this tribe, others only two, *Amara* Bonelli and *Zabrus* Clairville, each with many subgenera. Adults of zabrines are structurally most similar to members of Pterostichini and probably represent a clade within the Pterostichini as presently conceived.

Tribe Metiini. This tribe includes about 75 species restricted to the southern part of South America, predominantly in Chile and extending north to Peru and east to southern Brazil, Uruguay, and Argentina. These species are arrayed in the following genera: *Kuschelinus* Straneo (one species), *Metius* Curtis (about 60 species), *Abropus* Waterhouse (one species), *Antarctiola* Straneo (four species) and, according to Will (2000: 60), *Feroniola* Tschitschérine (nine species). Metiines are often included within the Pterostichini.

This tribe has been known in the past under the name Antarctiini. However, because its type genus *Antarctia* Dejean is a junior homonym, the family-group name Antarctiini is permanently invalid (ICZN 1999: Article 39).

Tribe Drimostomatini (including cyrtolaines). The association of the Eastern Hemisphere drimostomatines (also known under the name caelostomines) with the Western Hemisphere cyrtolaines (*Cyrtolaus* Bates with 11 Middle American species and *Barylaus* Liebherr with two species in the West Indies) was proposed by Liebherr (1986) and supported by Will’s (2000) preliminary cladistic analysis. The main characteristic of this group is the inverted aedeagus. However, this modification is absent in some groups (e.g., *Diceromerus* Chaudoir) traditionally placed within the drimostomatines and consequently monophyly of this tribe is uncertain. The drimostomatines include about 290 species arrayed in 29 genera (Lorenz 2005: 248-252, as Drimostomatina). The most speciose genera are *Caelostomus* Macleay (about 160 species, of which one is adventive in the West Indies), *Trichillinus* Straneo (21 species), *Platyxythrius* Lorenz (20 species), and *Strigomerus* Chaudoir (18 species).

The name Caelostomini, proposed by Burgeon (1935: 194), is often used for this tribe but Drimostomatini, established by Chaudoir (1872c: 283), is older and has priority. *Drimostoma* Dejean is usually treated as a junior synonym of *Caelostomus* Macleay but the family-group name Caelostomini was not proposed because of the synonymy of the type genus. Therefore, Article 40.2 of the ICZN (1999) does not apply in this case.

Tribe Chaetogenyini. This South American tribe includes five species of the genus *Chaetogenys* van Emden arrayed in two subgenera: *Chaetogenys* *s.str*. and *Camptotoma* Reiche. The group has been ranked as a subtribe of Pterostichini by some authors, including van Emden (1958), Straneo (1977), and Reichardt (1977: 408). However, the adhesive setae on the male protarsi are of the “spongy” type (Reichardt 1977: 408), not of the “seriate” type as in other pterostichines. Erwin (1985: 468) associated chaetogenyines with cuneipectines, chlaeniines, oodines, and licinines.

Tribe Dercylini. The 35 species of this exclusively Neotropical tribe are currently arrayed in one genus (*Dercylus* Laporte) with four subgenera (Moret and Bousquet 1995: 759): *Asporina* Laporte (two species), *Dercylus* *s.str*., with *Dercylodes* Chaudoir and *Pterodercylus* Kuntzen as synonyms (12 species), *Eurydercylus* Moret and Bousquet (seven species), and *Licinodercylus* Kuntzen, with *Physomerus* Chaudoir (a junior homonym) as synonym (14 species). Chaudoir (1883), Reichardt (1977), and Ball (1979: 102) suggested that dercylines were closely related to oodines. Moret and Bousquet (1995: 759) stated that the character states of the adult and of the putative larva studied indicate that dercylines are more closely related to oodines and chlaeniines than to pterostichines. Bousquet (1996a: 449) commented that dercylines were closely related to {oodines + panagaeines + chlaeniines} but that the nature of the relationship remained to be ascertained. Jeannel (1948a: 626) related dercylines to melanchitonines and Kryzhanovskij (1976a: 89) to pterostichines, microcheilines, chaetodactylines, platynines, zabrines, and cuneipectines without mentioning any character state that would justify such grouping. The adhesive setae on the male protarsi are of the “spongy” type as in chaetogenyines, oodines, and chlaeniines.

Jeannel (1948a: 627) indicated that the genus *Dercylinus* (one North American species), of which he had seen no specimen, probably belongs to dercylines and Lorenz (2005: 327) listed the genus, along with *Evolenes* (one North American species), in the subtribe Dercylina. However, these two genera are typical oodines (see Bousquet 1996a).

Tribe Melanchitonini. This tribe currently includes three genera, *Melanchiton* Andrewes (a replacement name for *Melanodes* Chaudoir), *Melanchrous* Andrewes (a replacement name for *Patellus* Chaudoir), and *Dicaelindus* Macleay. The lineage contains about 70 Old World species. As for many other groups, relationships of melanchitonines are unclear. Chaudoir (1883) included *Melanchiton* and *Melanchrous* within the tribe Oodini, likely because of the similar adhesive setae on the male protarsi. Subsequently, the two genera have been placed by some authors within the Pterostichini. Jeannel (1948a: 626) included them with dercylines in his family Dercylidae but offered no pertinent evidence to suggest that the group is monophyletic.

Straneo (1950: 65) first included the genus *Dicaelindus*, previously placed in the Pterostichini, in this tribe. Adults of *Dicaelindus* are rather similar phenetically to those of *Melanchiton*, but the male protarsi are not dilated and lack adhesive setae. Monophyly of this tribe has not yet been demonstrated.

Tribe Oodini. Members of Oodini *sensu stricto* share several apomorphic character states in the adult stage (Bousquet 1996a: 448) suggesting the tribe is monophyletic. Several authors have included or associated oodines with chlaeniines but the pygidial gland components suggest rather that panagaeines and chlaeniines are more closely related to each other than to oodines (Bousquet 1987b). Oodines, panagaeines, and chlaeniines possibly constitute a clade since the adults (except in some chlaeniines) have the metepisterna coadunate with the elytral epipleura, a synapomorphic condition that has probably been secondarily lost in some chlaeniine lineages.

Some groups, such as dercylines, melanchitonines, and geobaenines, are sometimes included within the Oodini as distinct subtribes. However, there is little evidence that they are indeed closely related to oodines and in my opinion they should be treated as distinct tribes.

This tribe is represented in all zoogeographical regions of the world and includes about 295 species in 32 genera. Jeannel (1949a: 829) recognized three family-group taxa within the oodines: sphoerodines represented in the Afrotropical Region, oodines (*sensu stricto*) represented in all zoogeographical regions, and thryptocerines represented in the Afrotropical Region.

Tribe Peleciini. Relationships of peleciines are unclear. The group has been associated with panagaeines by Kryzhanovskij (1976a: 89), Ball (1979), and Erwin (1985: 468) and included in the superfamily Odacanthomorphi, along with odacanthines, perigonines, lachnophorines, and ctenodactylines, by Jeannel (1948a: 376). Many apomorphic features, including some details of the chaetotaxy, structure of the mouthparts and thorax, and marked similarity in their specialized way of attacking millipedes, suggest that peleciines could be closely related to promecognathines. However, Straneo and Ball (1989) regarded these similarities as evolutionary convergence, not phylogenetic affinity. Larvae of *Eripus oaxacanus* Straneo and Ball, the only peleciine species known in its larval stage, are similar in some structural features to larvae of Brachinini and Pseudomorphini but Liebherr and Ball (1990) concluded that these similarities were an example of convergence due to a similar parasitic lifestyle. Arndt (1993: 36), based on larval features, suggested that peleciines, panagaeines, licinines, oodines, and chlaeniines form a clade. Liebherr and Will (1998: 156-157) noted from their analysis of the female reproductive tract that placement of peleciines as a basal group of pterostichine stock was firmly supported.

Peleciines are restricted to the Southern Hemisphere. Straneo and Ball (1989) recognized two subtribes: Agonicina for the genera *Pseudagonica* Moore and *Agonica* Sloane of Tasmania and adjacent southeastern Australia, and Peleciina (including disphericines) for the remaining genera which are represented in the Afrotropical, Oriental, and Neotropical Regions. Vigna Taglianti and Rossi (1998: 515) noted that the laboulbeniale parasitic species found in *Agonica* and in the moriomorphine genera *Pterogmus* Sloane, *Theprisa* Moore, and *Sitaphe* Moore were very similar and alluded to the possibility of a close relationship between agonicines and moriomorphines.

Tribe Brachygnathini. This tribe contains only the Neotropical genus *Brachygnathus* Perty (seven species). Relationships of the genus are uncertain. Jeannel (1949a: 849) associated it with the genus *Microcephalus* Dejean (as *Tichonia* Semenov), under the subfamily name Tichoniitae, and placed it in his family Panagaeidae. Reichardt (1977: 404) noted that inclusion of *Brachygnathus* in the tribe Panagaeini was doubtful and that the adults show some similarities to those of peleciines.

Tribe Bascanini. This tribe contains a single genus, *Bascanus* Péringuey (including *Bascanidius* Péringuey), with a few species in eastern and southern Africa. Van Emden (1936a), Basilewsky (1953a: 164-165), and Erwin (1979) suggested that bascanines are closely related to panagaeines. Csiki (1933a: 1651) associated the genus with *Melaenus* Dejean.

Tribe Panagaeini. This moderately diverse group occurs in all continents except Antarctica but is much more diverse in the tropics than in temperate regions. Panagaeines, at least those that have been analysed, secrete phenol through their pygidial glands (see Schildknecht et al. 1968; Kanehisa and Murase 1977; Moore 1979). This compound is also found, as far as known, only in some chlaeniines, which suggests that panagaeines are probably most closely related to chlaeniines. On the other hand, several authors, including Kryzhanovskij (1976a: 89), consider peleciines as the group most closely related to panagaeines.

Jeannel (1949a: 849) associated the genus *Microcephala* Dejean (as *Tichonia* Semenov) with Panagaeini but most authors, including Reichardt (1977: 407), regard it as a member of Pterostichini.

Tribe Chlaeniini. Chlaeniines are found in all zoogeographical regions of the world but are more diverse, both in terms of lineages and species, in the Afrotropical and Oriental Regions than anywhere else. Jeannel (1949a: 776) recognized six tribes among chlaeniines and Basilewsky and Grundmann (1955) ten tribes and two subfamilies. However, following Ball (1960b) and Lindroth (1969a), all the species are grouped in a single tribe in this catalogue. Several authors have suggested a close relationship between chlaeniines, panagaeines, and oodines.

Two major groups among *Chlaenius* species could be distinguished based on defensive secretions of the pygidial glands (see Schildknecht et al. 1968; Kanehisa and Murase 1977; Moore 1979; Balestrazzi et al. 1985): one secretes phenol, like panagaeines; the other one quinone. In the first group, the secretory lobes of the pygidial glands are elongate, in the second one they are shorter and thicker (Kanehisa and Shiraga 1978). I believe these two groups should be recognized either as genera or subtribes. However, owing to the lack of information on the pygidial glands and their secretions for many chlaeniine lineages, such action is futile at this time.

This tribe includes almost a thousand species worldwide arranged in 18 genera and two subtribes. The 51 North American species are assigned to the genus *Chlaenius* Bonelli and arrayed in ten subgenera of which five, *Pseudanomoglossus* Bell (one species), *Anomoglossus* Chaudoir (three species), *Callistometus* Grundmann (one species), *Brachylobus* Chaudoir (one species), and *Randallius* n.subg. (one species), are North American endemics.

Tribe Cuneipectini. This tribe includes one genus, *Cuneipectus* Sloane, with two flightless species in western Australia. Members of this group have rarely been collected and very little is known about their way of life. Kryzhanovskij (1976a: 89) listed cuneipectines in his supertribe Pterostichitae along with dercylines, zabrines, platynines, chaetodactylines, microcheilines, and pterostichines. Erwin (1985: 468) associated them with chaetogenyines, chlaeniines, oodines, and licinines in his supertribe Callistitae (= Chlaeniitae). Moore et al. (1987: 215) included them with morionines, pterostichines, abacetines, geobaenines, drimostomatines, and platynines in their Pterostichitae.

Tribe Orthogoniini. This group includes six genera represented in Asia and Africa only: *Orthogonius* Macleay (about 240 species), *Neoorthogonius* Tian and Deuve (one species), *Hexachaetus* Chaudoir (nine species), *Actenoncus* Chaudoir (four species), *Anoncopeucus* Chaudoir (two species), and *Nepalorthogonius* Habu (one species). Relationships of the tribe remain unresolved and problematic. Jeannel (1948a: 377) indicated that orthogoniines and licinines are closely related based on the shape of the frontale on the cephalic capsule of the larvae. Basilewsky (1953a: 180) associated them with glyptines, Kryzhanovskij (1976a: 90) with lebiines, anthiines, helluonines, physocrotaphines, zuphiines, galeritines, and dryptines, and Erwin (1985: 468) with idiomorphines, catapieseines, and amorphomerines. Ober and Maddison (2008: 18) found strong support in their phylogenetic analyses based on molecular data sequences for a clade comprising orthogoniines, graphipterines, and pseudomorphines. The genus *Glyptus* Brullé has been included by some authors within the tribe Orthogoniini, but both Jeannel (1948a) and Erwin (1985) believe that *Glyptus* and Orthogoniini are not closely related. Members of this tribe are termitophilous.

Tribe Idiomorphini. This tribe currently includes the genera *Idiomorphus* Chaudoir (three Indian species), *Perochnoristhus* Basilewsky (one species in Namibia), *Rathymus* Dejean (three Afrotropical species), and *Strigia* Brullé (three Oriental species) arrayed in two subtribes, Perochnoristhina for the genus *Perochnoristhus* and Idiomorphina for the remaining genera (Lorenz 2005: 391). Erwin (1984b: 378) also included the genus *Glyptus* in this tribe. Crowson (1980) stated that the genus *Perochnoristhus* could be closely related to broscines and apotomines.

Tribe Glyptini. Glyptini consists of two Afrotropical genera: *Neoglyptus* Basilewsky with six species and *Glyptus* Brullé with two species. Few authors agree on the systematic position of the group. Jeannel (1948a: 377) associated them with chlaeniines, Basilewsky (1953a: 180) with orthogoniines, and Erwin (1984b: 378) with idiomorphines. Both Chaudoir (1850a) and Lacordaire (1854) stated that glyptines were closely related to the genus *Idiomorphus* Chaudoir.

Tribe Amorphomerini. This group includes a single genus, *Amorphomerus* Sloane, represented by a few species in eastern Africa and Madagascar. Jeannel (1948a: 376) associated amorphomerines with pterostichines (*sensu lato*, including platynines), dercylines, and harpalines in his superfamily Harpalomorphi, characterized by having the mesotibiae spinose and the median lobes of the aedeagi more or less bent, with the basal bulbs well developed. He also stated (Jeannel 1948a: 731) that the tribe was more closely related to harpalines than to any other *Conchifera* groups. Kryzhanovskij (1976a: 89) associated amorphomerines with harpalines, cnemalobines (as Cnemacanthini), and agonicines (currently included in the Peleciini) and Erwin (1985: 468) associated them with idiomorphines, orthogoniines, and catapieseines. The tribe was listed as part of the tribe Lebiini by Erwin (1979).

Tribe Licinini. A clearly defined, likely monophyletic group with representatives in all zoogeographical regions of the world. Jeannel (1948a: 377) associated licinines with pentagonicines, orthogoniines, panagaeines, chlaeniines (including oodines), and glyptines, Kryzhanovskij (1976a: 89) with oodines and chlaeniines, and Erwin (1991a: 10) with oodines, chaetogenyines, chlaeniines, and cuneipectines. Ball (1992a) considered the tribe to be the sister-group to {Oodini + Chlaeniini + Panagaeini} and Ball and Bousquet (2000: 100) noted that members of the four tribes show similarities in structure of the male protarsi, genitalia, and larvae. Beutel (1992d) reported several putative synapomorphies in larval head structures between Licinini and Panagaeini, and Arndt (1993: 37) noted several synapomorphies in larvae of licinines, panagaeines, and peleciines. However, contrary to the oodine-chlaeniine-panagaeine complex, licinines have simple (i.e., non-crossed) epipleura and secrete formic acid as the major constituent of their defensive glands like harpalines and *Truncatipennes* members. Also Ober and Maddison (2008: 19) found no close relationship between licinines and the chlaeniine-oodine-panagaeine complex based on their analyses derived from molecular data sequences. Recently Liebherr and Will (1998: 144) suggested that licinines, orthogonines, panagaeines, melanchitonines, graphipterines, and loxandrines form a clade based on the presence of a villous canal extended forward on the common oviduct.

The 235 or so species are arrayed in 23 genera distributed among four subtribes following Ball (1992a).

Tribe Harpalini. This is one of the largest and most diversified carabid tribes. Although its limits are fairly stable, there is as yet no strong evidence to substantiate that the tribe is monophyletic. Based on a study of the world fauna, Noonan (1976) recognized four subtribes among harpalines: Anisodactylina, Pelmatellina, Stenolophina (including polpochilines and pachytrachelines), and Harpalina which he divided into eight genus-groups, namely Harpali, Selenophori, Bradybaeni, Acinopi, Bleusei, Dapti, Amblystomi, and Ditomi. Based on a parsimony analysis of molecular sequence data, Martínez-Navarro et al. (2005) concluded that the subtribe Harpalina was polyphyletic, that daptines were related to stenolophines, not to Harpalina, that the Selenophori group was polyphyletic and not related to Harpalina but perhaps to anisodactylines, that the Amblystomi group may be related to stenolophines instead of Harpalina, and that the subtribe Pelmatellina was related to stenolophines (see also Martínez-Navarro et al. 2003) and that the latter could be paraphyletic in regard to the former. They also advocated raising selenophorines, ditomines, and amblystomines to subtribe level.

Relationships of harpalines to other carabid groups are not well established. In the course of his work on the French fauna, Jeannel (1942: 575) associated harpalines with perigonines, anchonoderines, lachnophorines, omphreines, pterostichines (including platynines), zabrines, chaetodactylines, morionines, and metiines in his superfamily Harpalomorphi. Later, working on the Madagascan fauna, Jeannel (1948a: 376) united the harpalines with amorphomerines, dercylines, melanchitonines, pterostichines (including platynines), morionines, microcheilines, and chaetodactylines. Kryzhanovskij (1976a: 89) listed harpalines with amorphomerines, cnemalobines, and agonicines (currently included in Peleciini) in his supertribe Harpalitae. Based on the presence of a membranous transverse band on the stipes lateroventrally in larvae, Arndt (1998: 184) associated harpalines with morionines, pterostichines, zabrines, panagaeines, peleciines, chlaeniines, oodines, licinines, and cnemalobines. In a cladistic analysis conducted by Roig-Juñent and Cicchino (2001: Fig. 1), this tribe was positioned as the sister-group to {Platynini + Sphodrini}. Ruiz et al. (2008) indicated that, based on their molecular data sequence analyses, the tribe Harpalini was the sister-group to {Sphodrini + Platynini + Pterostichini + Zabrini}.

Tribe Geobaenini. The Geobaenini includes a single genus, *Geobaenus* Dejean, with four flightless species: three occur in South Africa, one in Australia. The group was first included within the tribe Harpalini and associated subsequently with pterostichines. Basilewsky (1949), because of similarity in the adhesive setae on the male protarsi, suggested that geobaenines could be closely related to melanchitonines, although later (1950, 1953, 1985) he associated the genus with platynines (as Anchomeninae or Platyninae). Liebherr and Will (1998: 144) in their study of the female reproductive tract found an “uncontested synapomorphy” uniting geobaenines with lachnophorines, odacanthines (including pentagonicines), and pseudomorphines. In these taxa, the spermathecal duct is joined to the common oviduct by an elongate sclerite.

Tribe Omphreini. This tribe includes a single genus, *Omphreus* Dejean (18 species), which is endemic to the Balkan Peninsula and Asia Minor. Omphreines have been included within the tribe Platynini by most authors but Jeannel (1942: 577), followed by Kryzhanovskij (1976a: 89), associated them with perigonines, anchonoderines (including atranines), and lachnophorines.

Tribe Sphodrini. Members of this group have been traditionally included within the Platynini. However, in recent decades numerous taxonomists dealing with the Palaearctic fauna, where this group is by far more diversified than anywhere else, rank this complex as a distinct tribe. Based on morphological characters, there seems to be little doubt that the two groups are closely related. However, from molecular data sequence analyses conducted by Ruiz et al. (2008), this relationship did not receive “the expected strong support, though it can not be completely dismissed.” The Sphodrini include about 825 species, arranged in about 40 genera, and are grouped into the following six subtribes: Atranopsina (about 100 species), Calathina (about 185 species), Dolichina (17 species), Pristosiina (about 65 species), Synuchina (almost 100 species), and Sphodrina (about 360 species). Based on Casale’s (1988: 130) cladogram, Dolichina and Synuchina are sister-groups, and Sphodrina, Calathina, and Pristosiina form a clade with Pristosiina the sister-group to the other two; the position of Atranopsina is ambiguous. From the molecular data sequence analyses conducted by Ruiz et al. (2008), only the position of the subtribe Atranopsina as the sister-group to all other subtribes was well supported.

Tribe Platynini. This is a large, complex, and worldwide group which is more diverse in the tropics than in temperate regions. There are no synapomorphies, in either adult or larval structures, yet discovered to suggest that the tribe represents a monophyletic lineage. Platynines are combined by various authors with pterostichines based on phenetic similarity between the two groups. I believe the two groups are not closely related because of the differences in elytral epipleuron configurations and pygidial gland structures and secretions. Basilewsky (1985, as Platyninae) gave an excellent introduction to the systematics of the group.

Relationships among the North American genus-group taxa have been addressed but are still inadequately understood. According to Liebherr (1991b: 5), *Tetraleucus*, *Anchomenus*, *Sericoda*, and *Elliptoleus* form a clade characterized by the synapomorphic condition of the female spermatheca having a basal reservoir and a long apical filament. Within this clade *Tetraleucus* is the sister-group to the remaining taxa. Liebherr and Schmidt’s (2004: 168) parsimony-based cladistic analysis led to the recognition of four subgenera within the genus *Agonum* forming two clades, {*Platynomicrus* Casey + *Europhilus* Chaudoir} and {*Agonum* *s.str*. + *Agonothorax* Motschulsky (= *Olisares* Motschulsky)}. Liebherr and Schmidt (2004: 153) suggested a sister-group relationship between the genus *Agonum* and the African taxa described in combination with *Agonidium* Jeannel and *Neobatenus* Jeannel as well as several others described under *Megalonychus* Chaudoir.

Tribe Perigonini. This small tribe is represented by about 115 species arranged in five genera. The place of the tribe within the carabids is unsettled. LeConte and Horn (1883: 35) and Sloane (1923: 248) included it as a separate group within the Platynini; Jeannel (1942: 577) as a distinct subfamily within his family Perigonidae along with anchonoderines, omphreines, and lachnophorines and later (Jeannel 1948a: 376) as a distinct family within his superfamily Odacanthomorphi along with lachnophorines, odacanthines, ctenodactylines, and peleciines. Kryzhanovskij (1976a: 89) followed Jeannel (1942) and combined the tribes Perigonini, Lachnophorini (including anchonoderines), and Omphreini in his supertribe Perigonitae. Erwin (1984b: 375) placed this tribe in his supertribe Lebiitae along with amorphomerines, catapieseines, graphipterines, tetragonoderines, masoreines, pentagonicines, odacanthines, and lebiines. Later (Erwin 1991a: 10) the amorphomerines and catapieseines were excluded from the Lebiitae.

The North American fauna includes two species of the genus *Perigona* Laporte which contains about 100 species worldwide. One of our species is adventive and the second one is endemic to the eastern part of the continent.

Tribe Ginemini. This tribe includes a single species, *Ginema thomasi* Ball and Shpeley, known from a single female specimen collected in the departament of Santa Cruz in Bolivia. Ball and Shpeley (2002a: 96) noted some marked similarities between this genus and members of Cyclosomini but still postulated a rather isolated position in the rank of the more derived Harpalinae lineages.

Tribe Enoicini. This tribe includes two South African genera: *Enoicus* Péringuey with one species and *Abacetodes* Straneo (= *Phimus* Péringuey, a preoccupied name) with four species. Basilewsky (1985: 15-16) associated enoicines with platynines, geobaenines, and sphodrines while earlier (Basilewsky 1953a: 61) he included them within the platynines (as Anchomenini).

Tribe Atranini. This tribe contains only two species, both included in the genus *Atranus* LeConte: one lives in Europe and the Caucasus, the other one in eastern North America. The systematic position of the genus has been debated. Dejean (1828: 122) described the North American species in the genus *Anchomenus* Bonelli, currently placed within the Platynini. LeConte (1847: 438; 1861a: 28), Seidlitz (1887: 10), and Sloane (1923: 250) associated the genus with chlaeniines, LeConte and Horn (1883: 37), Fauvel (1888: 15), and Jeannel (1942: 582) with anchonoderines, and Ball (1960b: 136), Lindroth (1966: 648), Liebherr (1986: 20), Kryzhanovskij et al. (1995: 118), and several others with platynines. Adults and larvae of *Atranus* possess several structural features not exhibited in other Platynini. In my opinion the morphological evidence relating *Atranus* to platynines is weak and for that reason the genus is retained here in its own tribe. Analysis of the pygidial secretions could be useful to indicate if the genus is more closely related to chlaeniines or to the platynine-anchonoderine lineage. Basilewsky (1962b: 155) believed the genus was more closely related to platynines than to any other group suggested to date, but because the adhesive setae on the male protarsi are of the “spongy” type rather than the “seriate” type, he advocated placing it in a distinct subfamily. Phylogenetic relationships as inferred from 28S ribosomal DNA and the wingless gene conducted by Ober and Maddison (2008) placed *Atranus* as the sister-group to the Platynini.

Tribe Catapieseini. This small Neotropical tribe includes two genera, *Catapiesis* Brullé with eight species and *Homalomorpha* Brullé with one species, ranging collectively from southern Mexico to northeastern Argentina. Catapieseines have been placed by some authors (e.g., Reichardt 1977) in the vicinity of the Morionini and Pterostichini. Lorenz (2005: 248) included them within the tribe Cratocerini, in his subfamily Pterostichinae, along with drimostomatines. Ober and Maddison (2008: 16), following Erwin (1984b: 375), placed them in their lebiomorph assemblage pointing out that catapieseines have truncate elytra and specialized eighth abdominal tergite turrets like the remaining lebiomorph taxa. Horn (1881: 163) believed that *Catapiesis* (as *Basoleia*) has a close relationship with the Helluonini. Erwin (1985: 468) listed the group within his supertribe Orthogoniitae along with idiomorphines, amorphomerines, and orthogoniines.

Tribe Lachnophorini (including anchonoderines). This small tribe includes about 120 species in ten genera. There is no solid structural or molecular evidence that would suggest this group is monophyletic. Its relationships have been discussed by several authors. Jeannel (1942: 578), followed by Kryzhanovskij (1976a: 89), associated lachnophorines with perigonines, anchonoderines (including *Atranus*), and omphreines. Liebherr (1988) suggested that lachnophorines derived from a platynine-like ancestor. He also included calophaenines within lachnophorines but Ball and Bousquet (2000: 107), following Erwin (1991b: 44), placed them with ctenodactylines.

The Lachnophorini are represented in the New World, and by one Indo-African species, *Selina westermanni* Motschulsky. Jeannel (1948a: 744) also considered the genus *Amoebea* Péringuey (one Afrotropical species) as lachnophorine. However, the name is a junior synonym of *Smeringocera* Chaudoir (six species) which belongs to the tribe Odacanthini (see Lorenz 2005: 444).

In Liebherr’s (1988) parsimony-based cladistic analysis of the West Indies lachnophorines, the taxa with setose body and setose maxillary palpomeres, represented by the genera *Euphorticus*, *Calybe*, and *Lachnophorus*, constituted a clade.

Tribe Pentagonicini. Few characteristics hold the pentagonicines together and this tribe is possibly polyphyletic. Jeannel (1949a: 767) included pentagonicines in his superfamily Callistomorphi, along with licinines, orthogoniines, panagaeines, chlaeniines, and glyptines, and indicated that pentagonicines are best placed near licinines. Moore (1966a: 162) wrote that larval characters of the pentagonicine *Scopodes* “suggest a rather close affinity with the Odacanthinae.” Liebherr (1988) included pentagonicines within the Odacanthini based mainly on the structure of the spermatheca, and Ober and Maddison (2008: 17) found support from their molecular data for such a relationship. Erwin (1984b: 375) placed this tribe in his supertribe Lebiitae along with amorphomerines, perigonines, catapieseines, graphipterines, tetragonoderines, masoreines, odacanthines, and lebiines. The author later adopted a similar arrangement (Erwin 1985: 468), with the exclusion of amorphomerines and catapieseines and the inclusion of lachnophorines. It is of interest to note that the sole species of pentagonicines studied (*Scopodes boops* Erichson) produces a saturated acid as major component (Moore 1979) of the pygidial glands, while members of the so-called *Truncatipennes*, with which this tribe is usually associated, produce formic acid (see Schildknecht et al. 1968; Moore and Wallbank 1968; Kanehisa and Murase 1977; Moore 1979).

This tribe includes close to 170 species in the world. Only six, all in the genus *Pentagonica* Schmidt-Göbel (86 species worldwide), are found in North America.

Tribe Odacanthini. The Odacanthini, with about 300 species in 30 genera, constitutes a moderately diverse group represented in all zoogeographical regions. They are more diverse in the tropics than in temperate regions. Several authors believed the group to be closely related to ctenodactylines, and both groups have been combined in a single tribe by some (Csiki 1932b, Liebke 1938, van Emden 1942). Jeannel (1948a: 376) associated odacanthines with perigonines, lachnophorines, ctenodactylines, hexagoniines, and peleciines, and Basilewsky (1962b: 154) with lachnophorines. Liebherr (1988) concluded that odacanthines (including pentagonicines) have a sister-group relationship with lachnophorines (including calophaenines) based on the presence of a bipartite spermatheca. However, phylogenetic analyses based on molecular data sequences presented by Ober and Maddison (2008: 5) did not support odacanthines and lachnophorines as a clade but did support a close relationship between pentagonicines and odacanthines and between calophaenines and lachnophorines. Erwin (1985: 468) placed the tribe Odacanthini within his Lebiitae along with perigonines, lachnophorines, graphipterines, tetragonoderines, cyclosomines (as Masoreini), pentagonicines, and lebiines.

The tribe is represented in North America by six species belonging to the New World genus *Colliuris* DeGeer, which currently includes about 80 species.

Tribe Calophaenini. This tribe includes two Neotropical genera: *Calophaena* Klug (48 species) and *Calophaenoidea* Liebke (one species). Calophaenines have been placed in the tribe Odacanthini (Horn 1881: 147; Bates 1883a: 163; Reichardt 1977: 435; Lorenz 2005: 439), Lachnophorini (Liebherr 1988: 18), or Ctenodactylini (Erwin 1991b: 44; Ball and Bousquet 2000: 107). Liebherr’s (1988) assumption of a relationship with lachnophorines received support from most molecular analyses conducted by Ober and Maddison (2008: 17). The association of calophaenines with ctenodactylines is based on similarity of the adhesive setae under the tarsomeres (Stork in Ball and Bousquet 2000: 107). Until the relationship of calophaenines is better established I prefer to place them in a distinct tribe.

Tribe Ctenodactylini. This tribe is represented only in the New World and includes about 115 species in 18 genera. Most recent authors agree that this group is closely related to the tribe Hexagoniini of the Eastern Hemisphere. Only three species are found north of Mexico and they belong to the genus *Leptotrachelus* Latreille along with about 30 more species in the tropics.

The main characteristic of the Ctenodactylini and Hexagoniini is the inverted median lobe of the aedeagus as in the drimostomatines.

Tribe Hexagoniini. This tribe contains 65 species in three genera: *Hexagonia* Kirby (47 species in Asia, Africa, New Guinea [one species], and Australia [one undescribed species, *cf*. Darlington 1968: 202]), *Dinopelma* Bates (13 species in the Oriental Region), and *Omphreoides* Fairmaire (five Madagascan species). Vigna Taglianti and Rossi (1998: 515) indicated that hexagoniines could be related to odacanthines based on similar parasitic laboulbeniales.

Tribe Cyclosomini. This tribe is used here in a restricted sense (see Ball and Bousquet 2000: 109). It includes about 120 species, predominantly tropical, placed in four genera: *Mnuphorus* Chaudoir with 11 species in the Palaearctic Region; *Cyclosomus* Latreille with 13 species in the Afrotropical and Oriental Regions; *Cyclicus* Jeannel with 22 species in the Afrotropical and Oriental Regions; and *Tetragonoderus* Dejean represented by about 80 species in the Afrotropical, Oriental, Neotropical, and Nearctic Regions, and on the southern fringe of the Palaearctic Region. Several authors, including Jeannel (1949a: 860) and Basilewsky (1984: 527), have considered the New World genus *Nemotarsus* LeConte as related to cyclosomines, but following Ball (1960b: 157) and Lindroth (1969a: 1014) the genus is listed here in the tribe Lebiini.

Cyclosomines are associated with somoplatines, graphipterines, corsyrines, masoreines, and sarothrocrepidines by most authors based on the presence of long tibial spurs in adults. However, Ball and Bousquet (2000: 109) remarked that the complex as a whole is probably not monophyletic. Molecular analyses published by Ober and Maddison (2008: 17) did not support a close relationships between cyclosomines (*sensu lato*, i.e., including somoplatines, corsyrines, masoreines, and sarothrocrepidines) and graphipterines but suggested they may be associated with members of dromiine and / or cymindidine lebiines.

Tribe Somoplatini. Ball and Bousquet (2000: 109) restricted this tribe to the genera *Somoplatus* Dejean (14 Indo-African species), *Somoplatodes* Basilewsky (two Afrotropical species), and *Lophidius* Dejean (one Afrotropical species), with *Paralophidius* Basilewsky recently placed in synonymy with *Somoplatus* (Schüle 2009: 461). Basilewsky (1986) listed these genera as part of his tribe Masoreini.

Tribe Masoreini. This tribe, as restricted by Ball and Bousquet (2000: 109), comprises the genera *Masoreus* Dejean (seven Palaearctic species), *Atlantomasoreus* Mateu (two Moroccan species), *Anaulacus* Macleay (38 species), and *Leuropus* Andrewes (one Oriental species). *Odontomasoreus* Darlington (one species from New Guinea), listed as a distinct genus by Lorenz (2005: 451), has been considered a subgenus of *Anaulacus* by Ball and Shpeley (2002b: 279). Jeannel (1949a: 860) associated masoreines with cyclosomines and nemotarsines.

Tribe Corsyrini. This tribe comprises the Palaearctic Asian genera *Corsyra* Dejean (one species) and *Discoptera* Semenov (five species). Jeannel (1949a: 860) included them with masoreines and Ball and Bousquet (2000: 109) with graphipterines. These authors did not offer evidence to support their groupings.

Tribe Sarothrocrepidini. This tribe is represented by a single genus, *Sarothrocrepis* Chaudoir, with 26 Indo-Australian species. Jeannel (1949a: 860) associated the genus with graphipterines, masoreines, cyclosomines, and nemotarsines.

Tribe Graphipterini. This tribe, represented in Africa and the Middle East, includes the genera *Graphipterus* Latreille (about 145 species), *Piezia* Brullé (18 species), and *Trichopiezia* Nègre (one species). Jeannel (1949a: 860) associated graphipterines with sarothrocrepidines, masoreines, cyclosomines, and nemotarsines and Kryzhanovskij (1976a: 90) with the same groups with the exception of the nemotarsines. Ober and Maddison (2008: 17) found no support from their molecular analyses for a close relationship between graphipterines and cyclosomines (*sensu lato*). Instead they found graphipterines to be closely related to pseudomorphines and orthogoniines.

Tribe Lebiini. A markedly complex, worldwide tribe undoubtedly more diverse both in terms of species and lineages in the tropics than in temperate regions. No synapomorphy is known to suggest that this tribe constitutes a monophyletic lineage and Ober and Maddison (2008: 18) did not recover a monophyletic Lebiini in their analyses based on molecular data sequences. The supraspecific classification is not established clearly since most modern studies on lebiines have focussed on regional faunas. The systematic position of some groups within the Lebiini is still debated. For example, the genus *Celaenephes* Schmidt-Göbel has been considered the most ancestral group of extant lebiines by Shpeley et al. (1985) but as a platynine by Basilewsky (1984). In phylogenetic analyses derived from molecular sequence data by Ober and Maddison (2008: 18), *Celaenephes* was not clearly associated with members of Lebiini.

Relationships of the tribe are not clearly understood. Jeannel (1948a: 378) included lebiines with anthiines, helluonines, dryptines, galeritines, physocrotaphines, zuphiines, and calophaenines in his superfamily Lebiomorphi. Kryzhanovskij (1976a: 90) used a similar grouping with the exception that he also included orthogoniines, a group that Jeannel (1948a: 377) and Basilewsky (1984: 528) included within the Callistomorphi (i.e., pentagonicines, licinines, panagaeines, chlaeniines, and glyptines). Erwin and Sims (1984: 357) and Erwin (1985: 468) associated lebiines with perigonines, lachnophorines, graphipterines, cyclosomines, masoreines, pentagonicines, and odacanthines.

The 220 genera currently recognized within this tribe are arrayed in the following 16 subtribes (see Ball and Bousquet 2000: 110): Celaenephina, Pericalina (including coptoderines and eucheilines), Sugimotoina, Actenonycina, Apenina, Cymindidina, Dromiusina, Lebiina, Physoderina, Metallicina, Agrina, Calleidina, Gallerucidiina, Peliocypadina, Demetriadina, and Nemotarsina. In the phylogenetic analyses from molecular data published by Ober and Maddison (2008), a small number of clades within the Lebiini were supported but none of these corresponded to the current subtribes, and the subtribes were not recovered as monophyletic.

Tribe Dryptini. Dryptines have been included within the galeritines by several authors (including Darlington 1971: 198). The two lineages are now placed in different tribes but most authors agree that they are closely related. Dryptines and galeritines share some character states with zuphiines and these three groups, referred to as supertribe Zuphiitae by Erwin and Sims (1984: 356) and Erwin (1985: 468), probably constitute a clade. Basilewsky (1960) recognized six dryptine genera in the world, only one of them, the monobasic Amazonian *Neodrypta* Basilewsky, is found in the New World. Most species are tropically-adapted in the Afrotropical, Australian, and Oriental Regions, with a few species in the southern parts of the Palaearctic Region.

Tribe Galeritini. This tribe is represented in all zoogeographical regions but is more diverse in the tropics than in the temperate zones. Basilewsky (1963b: 7) and Ball (1985) have recognized two lineages within galeritines, treated as subtribes by Ball (1985): Planetina for the genus *Planetes* Macleay (27 species in the Eastern Hemisphere) and Galeritina for the remaining genera. Lorenz (2005: 507), however, included planetines in zuphiines and Ober and Maddison (2008: Fig.5) found support for such a grouping in some of their analyses and reported that the tribe Galeritini was not monophyletic, based on their molecular data analyses. As indicated previously, this tribe is probably closely related to the Dryptini and Zuphiini.

Tribe Zuphiini. This group is represented in all zoogeographical regions of the world but is more diverse in the tropics than in temperate regions. Zuphiines are grouped by some authors (e.g., Basilewsky 1962a: 100-101) into three subtribes: Leleupidiina, Patriziina, and Zuphiina. Baehr (1985) briefly discussed the status of each of these subtribes. On the other hand, Lorenz (2005: 505-507) included patriziines within the subtribe Zuphiina. Most authors agree that Zuphiini are closely related to Galeritini. For example, LeConte and Horn (1883: 41) and Erwin (1991a: 10) combined zuphiines with galeritines and dryptines. Habu (1967) included zuphiines and galeritines in a single tribe and recognized three subtribes: Zuphiina, Galeritina, and Planetina. Moore (1998: 369) suggested a close relationship between zuphiines and physocrotaphines based on characters of adults and Jeannel (1949a: 1047) enlisted zuphiines, galeritines, dryptines, and physocrotaphines in his family Dryptidae. Ober and Maddison (2008: 18) found a well-supported “Zuphiitae” clade including zuphiines, anthiines, dryptines, galeritines, helluonines, and physocrotaphines in their phylogenetic analyses. However, the tribe Zuphiini was not found to be monophyletic.

The hypogean and monospecific genus *Ildobates* Español from the Iberian Mountain range was originally described in the Dryptini and was subsequently transferred to the Galeritini by Jeanne (1972) and finally to the Zuphiini by Ortuño et al. (2005).

Tribe Physocrotaphini. The Physocrotaphini includes the following genera: *Helluodes* Westwood (three species in southwestern India and Sri Lanka), *Physocrotaphus* Parry (one species from Sri Lanka), *Pogonoglossus* Chaudoir (35 species), and *Schuelea* Baehr (three species in New Guinea). The monobasic genus *Holoponerus* Fairmaire from New Britain was originally described as a lebiine but Darlington (1968) and Moore (1998: 370) agreed that the genus probably belongs to the physocrotaphines. Unfortunately the sole known specimen of *Holoponerus godeffroyi* Fairmaire was destroyed in 1943 during the bombing of Hamburg in World War II (Moore 1998: 370). All but two species of physocrotaphines are very rarely collected and little is known about their way of life (Sabu et al. 2008: 30). Members of *Helluodes* are probably termitophilous and those of *Pogonoglossus* are predominantly subcorticolous though some species could be litter-dwelling forms (Sabu et al. 2008: 41-42).

Jeannel (1949a: 1047) associated physocrotaphines with zuphiines, galeritines, and dryptines and most authors currently agree that these lineages are probably closely related. Jeannel (1949a: 1047) claimed that the genus *Pogonoglossus* belongs to the Zuphiini near the genus *Eunostus* Laporte but both Darlington (1968) and Moore (1998: 375) retained the genus within the Physocrotaphini. In his work on the French fauna, Jeannel (1942: 1017) associated physocrotaphines with anthiines and helluonines.

Tribe Anthiini. Anthiines are large, apterous beetles which live in the steppes and subdesert areas of Africa and southwestern Asia. The species are classified into eight or nine genera. The group is clearly defined and probably closely related to the tribe Helluonini (van Emden 1937; Jeannel 1949a: 1040; Bousquet 1987c: 928; Arndt 1993: 44). Basilewsky (1962a: 93) even suggested that anthiines could be derived from a helluonine genus close to *Triaenogenius* Chaudoir of the Afrotropical Region. Based on larval character states, Bousquet (1987c: 928) suggested that pseudomorphines and/or galeritines could be closely related to the anthiine-helluonine lineage. Erwin and Sims (1984: 356) and Erwin (1985: 468) combined the anthiines with the helluonines and physocrotaphines in their supertribe Anthiitae.

Tribe Helluonini. This is a moderately diverse group with representatives in all continents except Europe and Antarctica. Despite the fact that the group appears homogeneous from the morphology of the adults, Ober and Maddison (2008: 18) did not recover it as monophyletic in their analyses derived from molecular data sequences. Several larval features suggest that helluonines are closely related to anthiines (Bousquet 1987c; Arndt 1998: 186). However, Reichardt (1974, 1977) suggested, based on the structures of the adult mouthparts, that helluonines could be closely related to eucheilines (currently placed within the Lebiini). Liebherr and Will (1998: 145) concluded that Helluonini and Galeritini might be sister-groups based on their possession of a secondary spermathecal gland.

Sloane (1914) and Reichardt (1974) recognized two subtribes among helluonines: Helluonina with representatives in Australia and New Guinea and Helluomorphina (= Omphrina) with representatives in Asia, Africa (including Madagascar), Australia, and the Western Hemisphere. About 165 species, placed in 25 genera, are known worldwide but only eight, all belonging to the New World genus *Helluomorphoides* Ball, are found in North America.

Tribe Xenaroswellianini. Erwin (2007b: 567) suggested that this recently described tribe, which includes a single species known only from the holotype collected in the Brazilian state of Goiás, could have “a possible relationship with the enigmatic Pseudomorphini.”

Tribe Pseudomorphini. Members of Pseudomorphini are structurally aberrant possibly in response to the group’s evolution into myrmecophily. Nevertheless placement of the group within the subfamily Harpalinae has been confirmed in almost all recent analyses (Arndt et al. 2005: 141). Relationships of pseudomorphines to other harpaline tribes are unsettled as stated by Deuve (1993: 98). Erwin (1981a: 66) remarked that the male genitalia, tarsi, and adult chemical defences suggest that pseudomorphines could be “related somehow to a basal Pterostichitae stock” and that the “paramere vestiture also suggests a connection with Psydritae.” Liebherr and Will (1998: 144) indicated that pseudomorphines, geobaenines, lachnophorines, and odacanthines (including pentagonicines) may be closely related based on the presence of an elongate sclerite joining the spermathecal duct to the common oviduct. Erwin and Stork (1985: 445) concluded that {pseudomorphines + cnemacanthines (= cnemalobines) + scaritines} form a clade that could be the sister-group to Hiletini. Jeannel (1942: 1102; 1949a: 1079) associated pseudomorphines with brachinines under the name *Balteifera* but almost all authors now agree that the two lineages are not closely related. Ober and Maddison (2008: 18-19) reported strong molecular support for a clade including pseudomorphines, orthogoniines, and graphipterines. They indicated that while morphology does not support such a close relationship, all or some of the members of each tribe have obligate relationships with social insects.

## Brief faunistic assessment

The North American fauna currently consists of 2,676 valid species-group taxa (2,439 species) of Geadephaga. Of this number, 64 are adventive on this continent, leaving 2,612 (97.6%) native species-group taxa (2,375 species).

### Adventive species.

Table 4 lists the adventive species found on this continent. All but two are accidental introductions (i.e., immigrants). Several carabids were intentionally introduced in New England during the first half of the xx Century for gypsy moth (*Lymantria dispar* Linnaeus) control, including *Calosoma chinense* Kirby, *Calosoma inquisitor* Linnaeus, *Calosoma reticulatum* Fabricius, *Carabus arvensis* Herbst, *Carabus violaceus* Linnaeus, *Carabus glabratus* Paykull, and *Carabus coriaceus* Linnaeus (see Smith 1959), but only *Calosoma sycophanta* and *Carabus auratus auratus* have become established.

Among the 62 species accidentally introduced, *Laemostenus complanatus*, *Laemostenus terricola terricola*, *Somotrichus unifasciatus*, *Plochionus pallens*, and *Perigona nigriceps* are commonly found in cellars or associated with stored products and are now considered subcosmopolitan (Hinton 1945: 21, 27-34). These species have probably been introduced in food and household goods in North America and many other places in the world.

**Figure 1.**
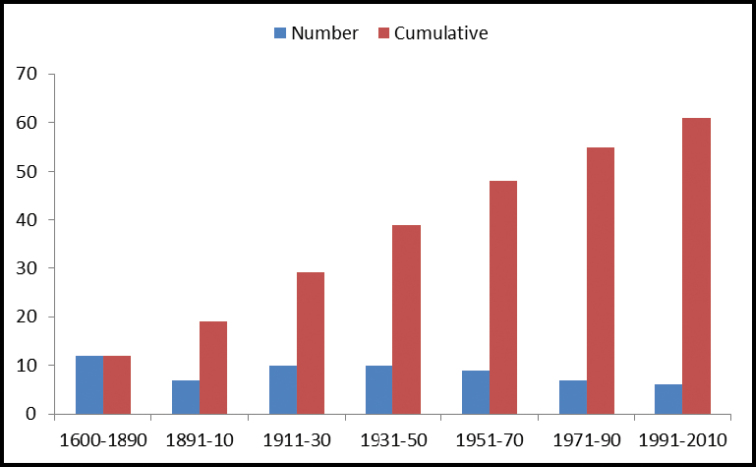
Number of adventive species of Geadephaga established in North America through time.

Most of the remaining 57 adventive species found on this continent prior to the xx Century have probably been introduced with bulk rock, bricks, mortar and no doubt soil taken aboard sailing vessels as ballast and dump ashore on this continent (Brown 1940a; Lindroth 1957c). In the xx Century, species have been introduced into North America likely through shipments of nursery stock, within the soil bagged around the roots, as suggested by Kavanaugh and Erwin (1985), Spence and Spence (1988: 166), and Bousquet (1992a). Despite that quarantine regulations were established in the 1960s for the importation of nursery stock, additional species still continue to land and become established on this continent (Fig. 1). The most recent one is *Nebria brevicollis* in the Willamette Valley in northwestern Oregon (Kavanaugh and LaBonte 2008).

All but four of the adventive species, other than those introduced with stored food products, occur naturally in Europe and their North American populations likely originated from that continent and particularly from southwestern England (Lindroth 1957c: 172). *Clivina vespertina* and *Tetragonoderus laevigatus* are native to South America (Nichols 1985b: 380; Shpeley and Ball 2008) while *Metacolpodes buchanani* (see Habu 1978: 125) and *Mochtherus tetraspilotus* (see Habu 1982: 87) are native to Asia.

As far as known, none of the adventive species of carabids found in North America are invasive in that they threaten ecosystems, habitats, or even native species.

Figure 1 illustrates the arrival of adventive species in North America through time based on the collecting date of the first inventoried specimen. The rate of arrival has been steady in the xx Century.

**Table 4.** List of species-group taxa adventive and established in North America.

**Species**	**Introduced in**			**YFC**
	**East**	**West**	**South**	
**Nebriini**				
*Leistus ferrugineus* (Linnaeus)	•			1977
*Nebria brevicollis* (Fabricius)		•		2007
**Notiophilini**				
*Notiophilus biguttatus* (Fabricius)	•	•		1923
*Notiophilus palustris* (Duftschmid)	•			1968
**Carabini**				
*Calosoma sycophanta* (Linnaeus)*	•			N/A
*Carabus granulatus granulatus* Linnaeus	•	•		1890
*Carabus nemoralis nemoralis* O.F. Müller	•	•		1890
*Carabus auratus auratus* Linnaeus*	•			N/A
**Clivinini**				
*Clivina vespertina* Putzeys			•	1948
*Clivina collaris* (Herbst)	•	•		<1838
*Clivina fossor fossor* (Linnaeus)	•	•		1915
**Dyschiriini**				
*Dyschirius globosus* Herbst		•		1978
**Broscini**				
*Broscus cephalotes* (Linnaeus)	•			1987
**Trechini**				
*Blemus discus discus* (Fabricius)	•			1933
*Trechus obtusus* Erichson		•		1927
*Trechus quadristriatus* (Schrank)	•			1965
*Trechus rubens* (Fabricius)	•			<1863
**Bembidiini**				
*Asaphidion curtum curtum* (Heyden)	•			1930
*Bembidion lampros* (Herbst)	•	•		1947
*Bembidion properans* (Stephens)	•			1942
*Bembidion obtusum* Audinet-Serville	•			1956
*Bembidion nigropiceum* (Marsham)	•			<1897
*Bembidion stephensii* Crotch	•			1891
*Bembidion bruxellense* Wesmael	•			1907
*Bembidion femoratum femoratum* Sturm	•			1967
*Bembidion tetracolum tetracolum* Say	•	•		<1823
*Elaphropus parvulus* (Dejean)		•		1940
*Porotachys bisulcatus* (Nicolai)	•	•		<1900
**Pterostichini**				
*Stomis pumicatus* (Panzer)	•			1984
*Pterostichus vernalis* (Panzer)	•			1997
*Pterostichus strenuus* (Panzer)	•	•		1937
*Pterostichus melanarius melanarius* (Illiger)	•	•		1926
*Abax parallelepipedus* (Piller & Mitterpacher)	•			1965
**Zabrini**				
*Amara aulica* (Panzer)	•			1929
*Amara apricaria* (Paykull)	•	?		<1865
*Amara fulva* (O.F. Müller)	•			1905
*Amara bifrons* (Gyllenhal)	•			1929
*Amara ovata* (Fabricius)	•	•		1925
*Amara aenea* (DeGeer)	•	•		1904
*Amara anthobia* Villa & Villa	•	•		1929
*Amara communis* (Panzer)	•			1988
*Amara eurynota* (Panzer)	•			1971
*Amara familiaris* (Duftschmid)	•	•		1901
**Harpalini**				
*Anisodactylus binotatus* (Fabricius)		•		1911
*Bradycellus harpalinus* (Audinet-Serville)		•		1951
*Acupalpus meridianus* (Linnaeus)	•	•		1931
*Ophonus puncticeps* Stephens	•			1954
*Ophonus rufibarbis* (Fabricius)	•			1953
*Harpalus rufipes* (DeGeer)	•			1937
*Harpalus affinis* (Schrank)	•	•		<1798
*Harpalus rubripes* (Duftschmid)	•			1981
**Platynini**				
*Calathus fuscipes* (Goeze)		•		1928
*Laemostenus complanatus* (Dejean)		•		<1874
*Laemostenus terricola terricola* (Herbst)	•	?		<1894
*Paranchus albipes* (Fabricius)	•			<1835
*Agonum muelleri* (Herbst)	•	•		1840
*Metacolpodes buchanani* (Hope)		•		1931
**Perigonini**				
*Perigona nigriceps* (Dejean)	•	•		<1853
**Cyclosomini**				
*Tetragonoderus laevigatus* Chaudoir			•	2007
**Lebiini**				
*Mochtherus tetraspilotus* (Macleay)			•	1992
*Somotrichus unifasciatus* (Dejean)			•	?
*Dromius fenestratus* (Fabricius)	•			1952
*Philorhizus melanocephalus* (Dejean)		•		1996
*Plochionus pallens* (Fabricius)	•	?	•	<1833

YFC: Year of first collection

### Native species.

The known North American fauna contains 2,612 native species-group taxa (2,375 species), of which 2,071 are endemic (79.3%). Among the 541 non-endemic species-group taxa, 98 (3.7% of the entire native fauna) are Holarctic and 446 (17.1%) extend south of North America as defined in this catalogue.

The number of 2,612 native species-group taxa obviously does not give an accurate account of the size of the North American carabid fauna. Several factors are involved. First, 65 species-group taxa described by Casey have not been treated subsequently. Considering that only 13.1% of Casey’s North American species-group taxa subsequently treated are currently valid, I estimate that about 50 of the remaining Casey taxa will eventually be synonymized. This would bring the number of valid Nearctic species-group taxa to about 2,560 (2,325 species). Second, several genus-group taxa have not been revised in “modern times” and obviously the number of valid species-group taxa will change. The following generic taxa are those that need revisions: *Clivina* Latreille (except *Antroforceps*), *Paraclivina* Kult, *Elaphropus* Motschulsky, *Tachys* Dejean, *Poecilus* Bonelli, *Hypherpes* Chaudoir, *Stenocellus* Casey, *Selenophorus* Dejean, *Discoderus* LeConte, *Olisthopus* Dejean, *Pinacodera* Schaum, *Apristus* Chaudoir, *Axinopalpus* LeConte, *Zuphium* Latreille, *Pseudaptinus* Laporte, and *Pseudomorpha* Kirby. Third, many species-group taxa are known but still undescribed. For example, Barr (2004: 1) reported that he was aware of 80 undescribed species of *Pseudanophthalmus*. Fourth, through modern techniques, such as DNA sequences, several “species” will probably be found to be a complex of two or more species. For example, Maddison (2008) found out that Lindroth’s (1963b) concept of *Bembidion chalceum* and *Bembidion honestum* was a complex of seven species. Finally, there is no doubt that many species remain to be discovered in North America, especially in peculiar habitats, such as caves, burrows, and deep litter.

**Figure 2.**
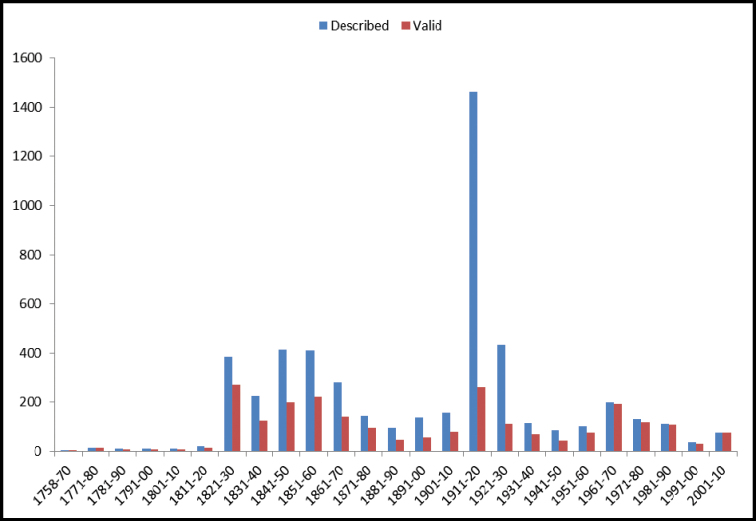
Number of North American species-group taxa of Geadephaga described and currently valid, by decade.

Figure 2 illustrates through decades the number of North American species-group taxa described and the number of those regarded as valid today based on the present catalogue. It shows that the periods between 1821-1880, 1911-1930, and 1961-1990 have been the most prolific. The first one, 1821-1880, can be associated with the works of John L. LeConte (724 geadephagan species-group taxa described), Count Dejean (289 taxa), Thomas Say (164 taxa), Baron de Chaudoir (126 taxa), and Victor Motschulsky (121 taxa). The second period, 1911-1930, relates of course to the work of Colonel Casey. By the time of his last publication in 1924, Casey had described 1,864 species-group taxa of carabids based on North American specimens while the number of taxa proposed by all authors combined since Linnaeus amounted to 2,288. The third one, 1961-1990, is connected to the magnificent work of Carl H. Lindroth (76 taxa) on the Carabidae of Canada and Alaska, the work of Thomas Barr (147 taxa) on the cave fauna, and the leadership of George Ball who, besides his own research, directed many students.

**Figure 3.**
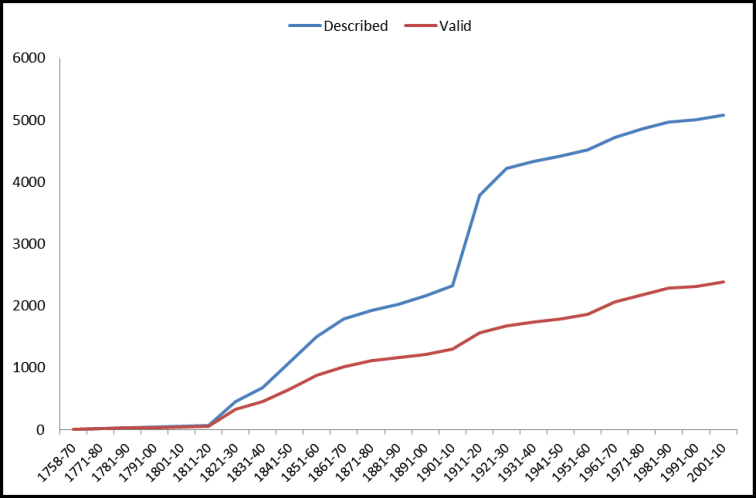
Cumulative number of species-group taxa of Geadephaga described and currently valid, by decade.

Figure 3 shows the growth, through time, of the number of species-group taxa described from North American specimens and those still considered valid. Taking into consideration the trend of description of species-group taxa still valid, the number of native species-group taxa should be slightly over 3,000 by the year 2060.

### Political regions.

Table 5 gives statistics regarding the number of geadephagan species-group taxa for each political region covered in this catalogue.

Texas (693 native species-group taxa), California (646 taxa), North Carolina (536 taxa), New York (528 taxa), and Virginia (520 taxa) have the highest number of native taxa recorded and Greenland (4 native taxa), Nunavut (34 taxa), Saint Pierre and Miquelon (50 taxa), Labrador (91 taxa), and Prince Edward Island (144 taxa) the lowest. Are these numbers representative of the relative sizes of the regions? Only to some extent. Some regions, for example Nunavut and Labrador, have been much less surveyed than others like Newfoundland proper. The geadephagan fauna of most states are still inadequately known. In the past 50 years, only Maine (Majka et al. 2011; Bousquet 2010a), New Hampshire and Vermont (Bousquet 2010a), Wisconsin (Messer 2010), Rhode Island (Sikes 2004), Connecticut (Krinsky and Oliver 2001), South Carolina (Ciegler 2000), Florida (Peck and Thomas 1998), South Dakota (Kirk and Balsbaugh 1975), and Alaska (Lindroth 1961-1969) have been the subject of faunistic assessments (see Table 3). Several states never had a checklist of their geadephagan fauna published.

**Table 5.** Species-group taxon statistics for political regions.

**Political regions**	**NbNT**	**NbAd**	**%NF**	**Rank**
Alabama	439	4	16.8	22
Alaska	248	2	09.5	56
Alberta	405	11	15.5	32
Arizona	502	3	19.2	9
Arkansas	403	4	15.5	33
British Columbia	479	28	18.3	12
California	646	12	24.7	2
Colorado	450	3	17.2	20
Connecticut	383	23	14.7	39
Delaware	201	5	07.7	59
District of Columbia	337	6	12.9	48
Florida	383	8	14.7	39
Georgia	478	3	18.3	14
Greenland	4	0	0.2	66
Idaho	338	10	12.9	47
Illinois	504	9	19.3	8
Indiana	479	9	18.3	12
Iowa	431	9	16.5	25
Kansas	408	4	15.6	30
Kentucky	371	2	14.2	42
Labrador	91	4	03.5	63
Louisiana	399	4	15.3	34
Maine	383	27	14.7	39
Manitoba	370	6	14.1	43
Maryland	408	9	15.5	30
Massachusetts	426	27	16.3	27
Michigan	474	15	18.2	16
Minnesota	419	10	16.0	28
Mississippi	439	5	16.8	22
Missouri	396	5	15.1	36
Montana	358	6	13.6	45
Nebraska	358	4	13.7	45
Nevada	239	3	09.2	57
New Brunswick	302	30	11.6	52
Newfoundland	151	27	05.8	61
New Hampshire	429	25	16.4	26
New Jersey	456	10	17.5	18
New Mexico	454	0	17.3	19
New York	528	21	20.2	4
North Carolina	536	4	20.4	3
North Dakota	275	4	10.5	54
Northwest Territories	218	1	08.3	58
Nova Scotia	258	35	09.8	55
Nunavut	34	0	01.3	65
Ohio	499	16	19.1	11
Oklahoma	387	2	14.8	37
Ontario	510	21	19.5	6
Oregon	478	22	18.2	14
Pennsylvania	500	19	19.1	10
Prince Edward Island	144	29	05.5	62
Quebec	446	32	17.1	21
Rhode Island	300	17	11.5	53
Saint Pierre and Miquelon	50	15	01.9	64
Saskatchewan	336	6	12.9	49
South Carolina	414	4	15.8	29
South Dakota	384	4	14.7	38
Tennessee	506	2	19.3	7
Texas	693	1	26.5	1
Utah	323	5	12.4	50
Vermont	397	21	15.2	35
Virginia	520	11	19.9	5
Washington	440	24	16.8	22
West Virginia	367	7	14.1	44
Wisconsin	463	16	17.7	17
Wyoming	309	4	11.8	51
Yukon Territory	201	0	07.7	59

NbNT = Number of native species-group taxa NbAd = Number of adventive species-group taxa %NF = % of the native North American fauna

**Table 6.** Checklists and taxonomic treatments on Geadephaga for North American political regions.

**Political regions**	**References**
Alabama	Löding (1945)
Alaska	Hamilton (1894a); Lindroth (1961-1969); Bousquet (1991b)
Alberta	Lindroth (1961-1969); Bousquet (1991b)
British Columbia	Hatch (1953); Lindroth (1961-1969); Bousquet (1991b)
Colorado	Wickham (1902)
Connecticut	Britton (1920); Krinsky and Oliver (2001)
District of Columbia	Ulke (1902)
Florida	Schwarz (1878); Leng (1915); Choate (1990); Peck and Thomas (1998)
Georgia	Fattig (1949)
Greenland	Henriksen (1939); Böcher (1988)
Idaho	Hatch (1953)
Indiana	Blatchley (1910)
Iowa	Wickham (1911b); Jaques and Redlinger (1946)
Kansas	Popenoe (1877)
Labrador	Sherman (1910); Lindroth (1961-1969); Bousquet (1991b); Bousquet (2010a)
Maine	Bousquet (2010a); Majka et al. (2011)
Manitoba	Lindroth (1961-1969); Bousquet (1991b)
Massachusetts	Harris (1833); Harris (1835)
New Brunswick	Lindroth (1961-1969); Bousquet (1991b); Majka et al. (2007); Bousquet (2010a)
Newfoundland	Lindroth (1955a); Lindroth (1961-1969); Bousquet (1991b); Bousquet (2010a)
New Hampshire	Bousquet (2010a)
New Jersey	Smith (1890); Smith (1900); Smith (1910)
New Mexico	Fall and Cockerell (1907)
New York	Notman (1928)
North Carolina	Brimley (1938)
Northwest Territories	Lindroth (1961-1969); Bousquet (1991b)
Nova Scotia	Lindroth (1961-1969); Lindroth (1954c); Bousquet (1991b); Majka et al. (2007); Bousquet (2010a)
Ontario	Lindroth (1961-1969); Bousquet (1991b)
Oregon	Hatch (1953)
Prince Edward Island	Lindroth (1961-1969); Bousquet (1991b); Majka et al. (2007); Majka et al. (2008); Bousquet (2010a)
Quebec	Chagnon (1917); Lindroth (1961-1969); Larochelle (1975); Larochelle (1976); Larochelle (1979); Bousquet (1991b); Laplante et al. (1991); Bousquet (2010a)
Rhode Island	Davis (1904); Sikes (2004)
Saint Pierre and Miquelon	Lindroth (1955a); Roux (1984); Bousquet (2010a)
Saskatchewan	Lindroth (1961-1969); Bousquet (1991b)
South Carolina	Kirk (1969 and 1970); Ciegler (2000)
South Dakota	Kirk and Balsbaugh (1975)
Vermont	Bousquet (2010a)
Washington	Hatch (1939b); Hatch (1953)
Wisconsin	Messer (2010)
Yukon Territory	Lindroth (1961-1969); Bousquet (1991b); Ball and Currie (1997)

## List of North American valid species-group taxa^6^

### Trachypachini

*Trachypachus gibbsii* LeConte, 1861

*Trachypachus inermis* Motschulsky, 1850

*Trachypachus slevini* Van Dyke, 1925

### Clinidiini

*Clinidium (Arctoclinidium) apertum allegheniense* Bell & Bell, 1975

*Clinidium (Arctoclinidium) apertum apertum* Reitter, 1880

*Clinidium (Arctoclinidium) baldufi* Bell, 1970

*Clinidium (Arctoclinidium) calcaratum* LeConte, 1875

*Clinidium (Arctoclinidium) rosenbergi* Bell, 1970

*Clinidium (Arctoclinidium) sculptile* (Newman, 1838)

*Clinidium (Arctoclinidium) valentinei* Bell, 1970

### Omoglymmiini

*Omoglymmius (Boreoglymmius) americanus* (Laporte, 1836)

*Omoglymmius (Boreoglymmius) hamatus* (LeConte, 1875)

### Pelophilini

*Pelophila borealis* (Paykull, 1790)**‡**

*Pelophila rudis* (LeConte, 1863)

### Opisthiini

*Opisthius richardsoni* Kirby, 1837

### Nebriini

*Leistus (Leistus) ferrugineus* (Linnaeus, 1758)**†**

*Leistus (Neoleistus) ferruginosus* Mannerheim, 1843

*Leistus (Neoleistus) longipennis* Casey, 1920

*Leistus (Neoleistus) madmeridianus* Erwin, 1970

*Nippononebria (Vancouveria) altisierrae* (Kavanaugh, 1984)

*Nippononebria (Vancouveria) campbelli* (Kavanaugh, 1984)

*Nippononebria (Vancouveria) virescens* (Horn, 1870)

*Nebria (Boreonebria) bellorum* Kavanaugh, 1979

*Nebria (Boreonebria) crassicornis crassicornis* Van Dyke, 1925

*Nebria (Boreonebria) crassicornis intermedia* Van Dyke, 1949

*Nebria (Boreonebria) frigida* Sahlberg, 1844**‡**

*Nebria (Boreonebria) gouleti* Kavanaugh, 1979

*Nebria (Boreonebria) gyllenhali castanipes* (Kirby, 1837)

*Nebria (Boreonebria) gyllenhali lassenensis* Kavanaugh, 1979

*Nebria (Boreonebria) gyllenhali lindrothi* Kavanaugh, 1979

*Nebria (Boreonebria) hudsonica* LeConte, 1863

*Nebria (Boreonebria) lacustris* Casey, 1913

*Nebria (Boreonebria) nivalis gaspesiana* Kavanaugh, 1979

*Nebria (Boreonebria) nivalis nivalis* (Paykull, 1790)**‡**

*Nebria (Nakanebria) paradisi* Darlington, 1931

*Nebria (Nakanebria) turmaduodecima* Kavanaugh, 1981

*Nebria (Reductonebria) acuta acuta* Lindroth, 1961

*Nebria (Reductonebria) acuta quileute* Kavanaugh, 1979

*Nebria (Reductonebria) acuta sonorae* Kavanaugh, 1981

*Nebria (Reductonebria) appalachia* Darlington, 1932

*Nebria (Reductonebria) arkansana arkansana* Casey, 1913

*Nebria (Reductonebria) arkansana edwardsi* Kavanaugh, 1979

*Nebria (Reductonebria) arkansana fragilis* Casey, 1924

*Nebria (Reductonebria) arkansana oowah* Kavanaugh, 1979

*Nebria (Reductonebria) charlottae* Lindroth, 1961

*Nebria (Reductonebria) danmanni* Kavanaugh, 1981

*Nebria (Reductonebria) darlingtoni* Kavanaugh, 1979

*Nebria (Reductonebria) desolata* Kavanaugh, 1971

*Nebria (Reductonebria) diversa* LeConte, 1863

*Nebria (Reductonebria) eschscholtzii* Ménétriés, 1843

*Nebria (Reductonebria) georgei* Kavanaugh, 2008

*Nebria (Reductonebria) gregaria* Fischer von Waldheim, 1820

*Nebria (Reductonebria) haida* Kavanaugh, 1984

*Nebria (Reductonebria) jeffreyi* Kavanaugh, 1984

*Nebria (Reductonebria) lituyae* Kavanaugh, 1979

*Nebria (Reductonebria) louiseae* Kavanaugh, 1984

*Nebria (Reductonebria) lyelli* Van Dyke, 1925

*Nebria (Reductonebria) mannerheimii* Fischer von Waldheim, 1828

*Nebria (Reductonebria) navajo* Kavanaugh, 1979

*Nebria (Reductonebria) obliqua chuskae* Kavanaugh, 1979

*Nebria (Reductonebria) obliqua obliqua* LeConte, 1867

*Nebria (Reductonebria) pallipes* Say, 1823

*Nebria (Reductonebria) sahlbergii modoc* Kavanaugh, 1979

*Nebria (Reductonebria) sahlbergii sahlbergii* Fischer von Waldheim, 1828

*Nebria (Reductonebria) sahlbergii triad* Kavanaugh, 1979

*Nebria (Reductonebria) suturalis* LeConte, 1850

*Nebria (Reductonebria) wallowae* Kavanaugh, 1984

*Nebria (Reductonebria) zioni oasis* Kavanaugh, 1979

*Nebria (Reductonebria) zioni zioni* Van Dyke, 1943

*Nebria (Catonebria) calva* Kavanaugh, 1984

*Nebria (Catonebria) carri* Kavanaugh, 1979

*Nebria (Catonebria) catenata* Casey, 1913

*Nebria (Catonebria) coloradensis* Van Dyke, 1943

*Nebria (Catonebria) gebleri albimontis* Kavanaugh, 1984

*Nebria (Catonebria) gebleri cascadensis* Kavanaugh, 1979

*Nebria (Catonebria) gebleri fragariae* Kavanaugh, 1979

*Nebria (Catonebria) gebleri gebleri* Dejean, 1831

*Nebria (Catonebria) gebleri rathvoni* LeConte, 1853

*Nebria (Catonebria) gebleri siskiyouensis* Kavanaugh, 1979

*Nebria (Catonebria) ingens ingens* Horn, 1870

*Nebria (Catonebria) ingens riversi* Van Dyke, 1925

*Nebria (Catonebria) kincaidi balli* Kavanaugh, 1979

*Nebria (Catonebria) kincaidi kincaidi* Schwarz, 1900

*Nebria (Catonebria) labontei* Kavanaugh, 1984

*Nebria (Catonebria) meanyi giulianii* Kavanaugh, 1981

*Nebria (Catonebria) meanyi lamarckensis* Kavanaugh, 1979

*Nebria (Catonebria) meanyi meanyi* Van Dyke, 1925

*Nebria (Catonebria) meanyi sylvatica* Kavanaugh, 1979

*Nebria (Catonebria) metallica* Fischer von Waldheim, 1820

*Nebria (Catonebria) ovipennis* LeConte, 1878

*Nebria (Catonebria) piperi* Van Dyke, 1925

*Nebria (Catonebria) piute piute* Erwin & Ball, 1972

*Nebria (Catonebria) piute sevieri* Kavanaugh, 1984

*Nebria (Catonebria) piute utahensis* Kavanaugh, 1979

*Nebria (Catonebria) praedicta* Kavanaugh & Schoville, 2009

*Nebria (Catonebria) purpurata* LeConte, 1878

*Nebria (Catonebria) schwarzi beverlianna* Kavanaugh, 1979

*Nebria (Catonebria) schwarzi schwarzi* Van Dyke, 1925

*Nebria (Catonebria) sierrablancae* Kavanaugh, 1984

*Nebria (Catonebria) spatulata sierrae* Kavanaugh, 1979

*Nebria (Catonebria) spatulata spatulata* Van Dyke, 1925

*Nebria (Catonebria) steensensis* Kavanaugh, 1984

*Nebria (Catonebria) trifaria pasquineli* Kavanaugh, 1984

*Nebria (Catonebria) trifaria trifaria* LeConte, 1878

*Nebria (Catonebria) vandykei vandykei* Bänninger, 1928

*Nebria (Catonebria) vandykei wyeast* Kavanaugh, 1979

*Nebria (Nebria) brevicollis* (Fabricius, 1792)**†**

### Notiophilini

*Notiophilus aeneus* (Herbst, 1806)

*Notiophilus aquaticus* (Linnaeus, 1758)**‡**

*Notiophilus biguttatus* (Fabricius, 1779)**†**

*Notiophilus borealis* Harris, 1869**‡**

*Notiophilus directus* Casey, 1920

*Notiophilus intermedius* Lindroth, 1955

*Notiophilus nemoralis* Fall, 1906

*Notiophilus nitens* LeConte, 1857

*Notiophilus novemstriatus* LeConte, 1847

*Notiophilus palustris* (Duftschmid, 1812)**†**

*Notiophilus semiopacus* Eschscholtz, 1833

*Notiophilus semistriatus* Say, 1823**‡**

*Notiophilus sierranus* Casey, 1920

*Notiophilus simulator* Fall, 1906

*Notiophilus sylvaticus* Dejean, 1831

### Cychrini

*Sphaeroderus bicarinatus* (LeConte, 1853)

*Sphaeroderus canadensis canadensis* Chaudoir, 1861

*Sphaeroderus canadensis lengi* Darlington, 1933

*Sphaeroderus indianae* (Blatchley, 1910)

*Sphaeroderus nitidicollis* Guérin-Méneville, 1829

*Sphaeroderus schaumii* Chaudoir, 1861

*Sphaeroderus stenostomus lecontei* Dejean, 1826

*Sphaeroderus stenostomus stenostomus* (Weber, 1801)

*Cychrus hemphillii hemphillii* Horn, 1879

*Cychrus hemphillii rickseckeri* LeConte, 1884

*Cychrus tuberculatus* Harris, 1839

*Scaphinotus (Scaphinotus) elevatus coloradensis* Van Dyke, 1907

*Scaphinotus (Scaphinotus) elevatus elevatus* (Fabricius, 1787)

*Scaphinotus (Scaphinotus) elevatus flammeus* Haldeman, 1844

*Scaphinotus (Scaphinotus) elevatus lengi* Van Dyke, 1938

*Scaphinotus (Scaphinotus) elevatus neomexicanus* Van Dyke, 1924

*Scaphinotus (Scaphinotus) elevatus tenebricosus* Roeschke, 1907

*Scaphinotus (Scaphinotus) kelloggi* (Dury, 1912)

*Scaphinotus (Scaphinotus) parisiana* Allen & Carlton, 1988

*Scaphinotus (Scaphinotus) petersi biedermani* Roeschke, 1907

*Scaphinotus (Scaphinotus) petersi catalinae* Van Dyke, 1924

*Scaphinotus (Scaphinotus) petersi corvus* (Fall, 1910)

*Scaphinotus (Scaphinotus) petersi grahami* Van Dyke, 1938

*Scaphinotus (Scaphinotus) petersi kathleenae* Ball, 1966

*Scaphinotus (Scaphinotus) petersi petersi* Roeschke, 1907

*Scaphinotus (Scaphinotus) snowi roeschkei* Van Dyke, 1907

*Scaphinotus (Scaphinotus) snowi snowi* (LeConte, 1881)

*Scaphinotus (Scaphinotus) unicolor* (Fabricius, 1787)

*Scaphinotus (Scaphinotus) vandykei* Roeschke, 1907

*Scaphinotus (Irichroa) irregularis* (Beutenmüller, 1903)

*Scaphinotus (Irichroa) viduus* (Dejean, 1826)

*Scaphinotus (Irichroa) webbi* Bell, 1959

*Scaphinotus (Nomaretus) bilobus* (Say, 1823)

*Scaphinotus (Nomaretus) cavicollis* (LeConte, 1859)

*Scaphinotus (Nomaretus) fissicollis* (LeConte, 1853)

*Scaphinotus (Nomaretus) infletus* Allen & Carlton, 1988

*Scaphinotus (Nomaretus) liebecki* Van Dyke, 1936

*Scaphinotus (Maronetus) debilis alpinus* (Beutenmüller, 1903)

*Scaphinotus (Maronetus) debilis debilis* (LeConte, 1853)

*Scaphinotus (Maronetus) hoffmani* (Barr, 2009)

*Scaphinotus (Maronetus) hubbardi* (Schwarz, 1895)

*Scaphinotus (Maronetus) imperfectus* (Horn, 1861)

*Scaphinotus (Maronetus) incompletus* (Schwarz, 1895)

*Scaphinotus (Maronetus) reichlei* (Barr, 2009)

*Scaphinotus (Maronetus) schwarzi* (Beutenmüller, 1913)

*Scaphinotus (Maronetus) tenuis* (Casey, 1914)

*Scaphinotus (Maronetus) unistriatus* (Darlington, 1932)

*Scaphinotus (Steniridia) aeneicollis* (Beutenmüller, 1903)

*Scaphinotus (Steniridia) andrewsii amplicollis* (Casey, 1920)

*Scaphinotus (Steniridia) andrewsii andrewsii* (Harris, 1839)

*Scaphinotus (Steniridia) andrewsii darlingtoni* (Valentine, 1935)

*Scaphinotus (Steniridia) andrewsii germari* (Chaudoir, 1861)

*Scaphinotus (Steniridia) andrewsii mutabilis* (Casey, 1920)

*Scaphinotus (Steniridia) andrewsii parvitarsalis* (Valentine, 1935)

*Scaphinotus (Steniridia) andrewsii waldensius* (Valentine, 1935)

*Scaphinotus (Steniridia) guyotii* (LeConte, 1863)

*Scaphinotus (Steniridia) lodingi lodingi* (Valentine, 1935)

*Scaphinotus (Steniridia) lodingi obscurus* (Valentine, 1935)

*Scaphinotus (Steniridia) ridingsii monongahelae* Leng, 1917

*Scaphinotus (Steniridia) ridingsii ridingsii* (Bland, 1863)

*Scaphinotus (Steniridia) tricarinatus* (Casey, 1914)

*Scaphinotus (Steniridia) violaceus* (LeConte, 1863)

*Scaphinotus (Pseudonomaretus) mannii* Wickham, 1919

*Scaphinotus (Pseudonomaretus) merkelii* (Horn, 1890)

*Scaphinotus (Pseudonomaretus) regularis* (LeConte, 1884)

*Scaphinotus (Pseudonomaretus) relictus* (Horn, 1881)

*Scaphinotus (Stenocantharus) angusticollis* (Mannerheim, 1823)

*Scaphinotus (Stenocantharus) hatchi* Beer, 1971

*Scaphinotus (Stenocantharus) johnsoni* Van Dyke, 1924

*Scaphinotus (Stenocantharus) velutinus* (Ménétriés, 1843)

*Scaphinotus (Brennus) bullatus* Van Dyke, 1924

*Scaphinotus (Brennus) cordatus* (LeConte, 1853)

*Scaphinotus (Brennus) crenatus* (Motschulsky, 1859)

*Scaphinotus (Brennus) cristatus* (Harris, 1839)

*Scaphinotus (Brennus) interruptus* (Ménétriés, 1843)

*Scaphinotus (Brennus) marginatus* (Fischer von Waldheim, 1820)

*Scaphinotus (Brennus) obliquus* (LeConte, 1868)

*Scaphinotus (Brennus) oreophilus* (Rivers, 1890)

*Scaphinotus (Brennus) punctatus* (LeConte, 1859)

*Scaphinotus (Brennus) riversi* (Roeschke, 1907)

*Scaphinotus (Brennus) rugiceps incipiens* (Casey, 1897)

*Scaphinotus (Brennus) rugiceps rugiceps* (Horn, 1872)

*Scaphinotus (Brennus) striatopunctatus* (Chaudoir, 1844)

*Scaphinotus (Brennus) subtilis* (Schaum, 1863)

*Scaphinotus (Brennus) ventricosus* (Dejean, 1831)

*Scaphinotus (Neocychrus) angulatus* (Harris, 1839)

*Scaphinotus (Neocychrus) behrensi* (Roeschke, 1907)

*Scaphinotus (Neocychrus) longiceps* Van Dyke, 1924

### Carabini

*Calosoma (Castrida) sayi* Dejean, 1826

*Calosoma (Calosoma) frigidum* Kirby, 1837

*Calosoma (Calosoma) sycophanta* (Linnaeus, 1758)**†**

*Calosoma (Calodrepa) aurocinctum* Chaudoir, 1850

*Calosoma (Calodrepa) scrutator* (Fabricius, 1775)

*Calosoma (Calodrepa) splendidum* Dejean, 1831

*Calosoma (Calodrepa) wilcoxi* LeConte, 1847

*Calosoma (Camegonia) marginale* Casey, 1897

*Calosoma (Camegonia) parvicolle* Fall, 1910

*Calosoma (Camegonia) prominens* LeConte, 1853

*Calosoma (Carabosoma) angulatum* Chevrolat, 1834

*Calosoma (Carabosoma) eremicola* Fall, 1910

*Calosoma (Carabosoma) peregrinator* Guérin-Méneville, 1844

*Calosoma (Carabosoma) sponsa* Casey, 1897

*Calosoma (Callitropa) externum* (Say, 1823)

*Calosoma (Callitropa) macrum* LeConte, 1853

*Calosoma (Callitropa) protractum* LeConte, 1862

*Calosoma (Blaptosoma) haydeni haydeni* Horn, 1870

*Calosoma (Blaptosoma) haydeni punctulicolle* Bates, 1891

*Calosoma (Chrysostigma) affine* Chaudoir, 1843

*Calosoma (Chrysostigma) calidum* (Fabricius, 1775)

*Calosoma (Chrysostigma) cancellatum* Eschscholtz, 1833

*Calosoma (Chrysostigma) lepidum* LeConte, 1845

*Calosoma (Chrysostigma) morrisonii* Horn, 1885

*Calosoma (Chrysostigma) obsoletum* Say, 1823

*Calosoma (Chrysostigma) semilaeve* LeConte, 1852

*Calosoma (Chrysostigma) simplex* LeConte, 1878

*Calosoma (Chrysostigma) tepidum* LeConte, 1852

*Calosoma (Callistenia) dawsoni* (Dajoz, 1997)

*Calosoma (Callistenia) dietzii* Schaeffer, 1904

*Calosoma (Callistenia) discors* LeConte, 1857

*Calosoma (Callistenia) lariversi* Van Dyke, 1943

*Calosoma (Callistenia) latipenne* Horn, 1870

*Calosoma (Callistenia) luxatum* Say, 1823

*Calosoma (Callistenia) moniliatum* (LeConte, 1852)

*Calosoma (Callistenia) monticola* Casey, 1897

*Calosoma (Callistenia) oregonum* (Gidaspow, 1959)

*Calosoma (Callistenia) placerum* (Gidaspow, 1959)

*Calosoma (Callistenia) schaefferi* Breuning, 1928

*Calosoma (Callistenia) subaeneum* Chaudoir, 1869

*Calosoma (Callistenia) subasperatum* Schaeffer, 1915

*Calosoma (Callistenia) wilkesii* (LeConte, 1852)

*Carabus (Carabus) goryi* Dejean, 1831

*Carabus (Carabus) granulatus granulatus* Linnaeus, 1758**†**

*Carabus (Carabus) vinctus* (Weber, 1801)

*Carabus (Diocarabus) chamissonis* Fischer von Waldheim, 1820

*Carabus (Aulonocarabus) truncaticollis truncaticollis* Eschscholtz, 1833**‡**

*Carabus (Homoeocarabus) maeander maeander* Fischer von Waldheim, 1820**‡**

*Carabus (Hemicarabus) serratus* Say, 1823

*Carabus (Archicarabus) nemoralis nemoralis* Müller, 1764**†**

*Carabus (Tachypus) auratus auratus* Linnaeus, 1760**†**

*Carabus (Tanaocarabus) finitimus* Haldeman, 1852

*Carabus (Tanaocarabus) forreri forreri* Bates, 1882

*Carabus (Tanaocarabus) sylvosus* Say, 1823

*Carabus (Tanaocarabus) taedatus agassii* LeConte, 1850

*Carabus (Tanaocarabus) taedatus bicanaliceps* Casey, 1920

*Carabus (Tanaocarabus) taedatus rainieri* Van Dyke, 1945

*Carabus (Tanaocarabus) taedatus taedatus* Fabricius, 1787

*Carabus (Megodontus) vietinghoffii vietinghoffii* Adams, 1812**‡**

### Amblycheilini

*Amblycheila baroni* Rivers, 1890

*Amblycheila cylindriformis* (Say, 1823)

*Amblycheila hoversoni* Gage, 1991

*Amblycheila picolominii* Reiche, 1840

*Amblycheila schwarzi* Horn, 1904

*Omus audouini* Reiche, 1838

*Omus californicus angustocylindricus* Horn, 1913

*Omus californicus californicus* Eschscholtz, 1829

*Omus californicus intermedius* Leng, 1902

*Omus californicus subcylindricus* Nunenmacher, 1940

*Omus cazieri* van den Berghe, 1994

*Omus dejeanii* Reiche, 1838

*Omus submetallicus* Horn, 1869

### Megacephalini

*Tetracha (Tetracha) carolina carolina* (Linnaeus, 1763)

*Tetracha (Tetracha) floridana* Leng & Mutchler, 1916

*Tetracha (Tetracha) virginica* (Linnaeus, 1767)

*Tetracha (Neotetracha) impressa* (Chevrolat, 1841)

### Cicindelini

*Cylindera (Cylindera) celeripes* (LeConte, 1846)

*Cylindera (Cylindera) cursitans* (LeConte, 1856)

*Cylindera (Cylindera) debilis* (Bates, 1890)

*Cylindera (Cylindera) lemniscata lemniscata* (LeConte, 1854)

*Cylindera (Cylindera) lemniscata rebaptisata* (Vaurie, 1951)

*Cylindera (Cylindera) lunalonga* (Schaupp, 1884)

*Cylindera (Cylindera) terricola cinctipennis* (LeConte, 1846)

*Cylindera (Cylindera) terricola continua* (Knaus, 1923)

*Cylindera (Cylindera) terricola imperfecta* (LeConte, 1851)

*Cylindera (Cylindera) terricola kaibabensis* (Johnson, 1990)

*Cylindera (Cylindera) terricola susanagreae* (Kippenhan, 2007)

*Cylindera (Cylindera) terricola terricola* (Say, 1824)

*Cylindera (Cylindera) unipunctata* (Fabricius, 1775)

*Cylindera (Cylindera) viridisticta arizonensis* (Bates, 1884)

*Ellipsoptera blanda* (Dejean, 1831)

*Ellipsoptera cuprascens* (LeConte, 1852)

*Ellipsoptera gratiosa* (Guérin-Méneville, 1840)

*Ellipsoptera hamata lacerata* (Chaudoir, 1854)

*Ellipsoptera hamata monti* (Vaurie, 1951)

*Ellipsoptera hirtilabris* (LeConte, 1875)

*Ellipsoptera lepida* (Dejean, 1831)

*Ellipsoptera macra ampliata* (Vaurie, 1951)

*Ellipsoptera macra fluviatilis* (Vaurie, 1951)

*Ellipsoptera macra macra* (LeConte, 1856)

*Ellipsoptera marginata* (Fabricius, 1775)

*Ellipsoptera nevadica citata* (Rumpp, 1977)

*Ellipsoptera nevadica knausii* (Leng, 1902)

*Ellipsoptera nevadica lincolniana* (Casey, 1916)

*Ellipsoptera nevadica makosika* (Spomer, 2004)

*Ellipsoptera nevadica nevadica* (LeConte, 1875)

*Ellipsoptera nevadica olmosa* (Vaurie, 1951)

*Ellipsoptera nevadica tubensis* (Cazier, 1939)

*Ellipsoptera puritana* (Horn, 1871)

*Ellipsoptera rubicunda* (Harris, 1911)

*Ellipsoptera sperata inquisitor* (Casey, 1897)

*Ellipsoptera sperata sperata* (LeConte, 1856)

*Ellipsoptera wapleri* (LeConte, 1875)

*Microthylax olivaceus* (Chaudoir, 1854)

*Opilidia chlorocephala smythi* (Harris, 1913)

*Brasiella wickhami* (Horn, 1903)

*Dromochorus belfragei* Sallé, 1877

*Dromochorus pilatei* Guérin-Méneville, 1849

*Dromochorus pruininus* Casey, 1897

*Dromochorus velutinigrens* Johnson, 1992

*Habroscelimorpha californica mojavi* (Cazier, 1937)

*Habroscelimorpha californica pseudoerronea* (Rumpp, 1958)

*Habroscelimorpha circumpicta circumpicta* (LaFerté-Sénectère, 1841)

*Habroscelimorpha circumpicta johnsonii* (Fitch, 1857)

*Habroscelimorpha circumpicta pembina* (Johnson, 1993)

*Habroscelimorpha dorsalis dorsalis* (Say, 1817)

*Habroscelimorpha dorsalis media* (LeConte, 1856)

*Habroscelimorpha dorsalis saulcyi* (Guérin-Méneville, 1840)

*Habroscelimorpha dorsalis venusta* (LaFerté-Sénectère, 1841)

*Habroscelimorpha fulgoris albilata* (Acciavatti, 1981)

*Habroscelimorpha fulgoris erronea* (Vaurie, 1951)

*Habroscelimorpha fulgoris fulgoris* (Casey, 1913)

*Habroscelimorpha gabbii* (Horn, 1867)

*Habroscelimorpha pamphila* (LeConte, 1873)

*Habroscelimorpha praetextata pallidofemora* (Acciavatti, 1981)

*Habroscelimorpha praetextata praetextata* (LeConte, 1854)

*Habroscelimorpha severa* (LaFerté-Sénectère, 1841)

*Habroscelimorpha striga* (LeConte, 1875)

*Eunota togata fascinans* (Casey, 1914)

*Eunota togata globicollis* (Casey, 1913)

*Eunota togata togata* (LaFerté-Sénectère, 1841)

*Cicindela (Cicindelidia) abdominalis* Fabricius, 1801

*Cicindela (Cicindelidia) amargosae amargosae* Dahl, 1939

*Cicindela (Cicindelidia) amargosae nyensis* Rumpp, 1956

*Cicindela (Cicindelidia) cazieri* Vogt, 1949

*Cicindela (Cicindelidia) floridana* Cartwright, 1939

*Cicindela (Cicindelidia) hemorrhagica arizonae* Wickham, 1899

*Cicindela (Cicindelidia) hemorrhagica hemorrhagica* LeConte, 1851

*Cicindela (Cicindelidia) highlandensis* Choate, 1984

*Cicindela (Cicindelidia) hornii hornii* Schaupp, 1883

*Cicindela (Cicindelidia) marginipennis* Dejean, 1831

*Cicindela (Cicindelidia) nigrocoerulea bowditchi* Leng, 1902

*Cicindela (Cicindelidia) nigrocoerulea nigrocoerulea* LeConte, 1846

*Cicindela (Cicindelidia) nigrocoerulea subtropica* Vogt, 1949

*Cicindela (Cicindelidia) obsoleta neojuvenilis* Vogt, 1949

*Cicindela (Cicindelidia) obsoleta obsoleta* Say, 1823

*Cicindela (Cicindelidia) obsoleta santaclarae* Bates, 1890

*Cicindela (Cicindelidia) obsoleta vulturina* LeConte, 1853

*Cicindela (Cicindelidia) ocellata ocellata* Klug, 1834

*Cicindela (Cicindelidia) ocellata rectilatera* Chaudoir, 1843

*Cicindela (Cicindelidia) politula barbaraannae* Sumlin, 1976

*Cicindela (Cicindelidia) politula petrophila* Sumlin, 1985

*Cicindela (Cicindelidia) politula politula* LeConte, 1875

*Cicindela (Cicindelidia) politula viridimonticola* Gage, 1988

*Cicindela (Cicindelidia) punctulata chihuahuae* Bates, 1890

*Cicindela (Cicindelidia) punctulata punctulata* Olivier, 1790

*Cicindela (Cicindelidia) roseiventris tascosaensis* Davis, 1918

*Cicindela (Cicindelidia) rufiventris cumatilis* LeConte, 1851

*Cicindela (Cicindelidia) rufiventris hentzii* Dejean, 1831

*Cicindela (Cicindelidia) rufiventris rufiventris* Dejean, 1825

*Cicindela (Cicindelidia) scabrosa* Schaupp, 1884

*Cicindela (Cicindelidia) schauppii* Horn, 1876

*Cicindela (Cicindelidia) sedecimpunctata sedecimpunctata* Klug, 1834

*Cicindela (Cicindelidia) senilis* Horn, 1867

*Cicindela (Cicindelidia) tenuisignata* LeConte, 1851

*Cicindela (Cicindelidia) trifasciata ascendens* LeConte, 1851

*Cicindela (Cicindelidia) trifasciata sigmoidea* LeConte, 1851

*Cicindela (Cicindelidia) willistoni echo* Casey, 1897

*Cicindela (Cicindelidia) willistoni estancia* Rumpp, 1962

*Cicindela (Cicindelidia) willistoni funaroi* Rotger, 1972

*Cicindela (Cicindelidia) willistoni hirtifrons* Willis, 1967

*Cicindela (Cicindelidia) willistoni praedicta* Rumpp, 1956

*Cicindela (Cicindelidia) willistoni pseudosenilis* Horn, 1900

*Cicindela (Cicindelidia) willistoni sulfontis* Rumpp, 1977

*Cicindela (Cicindelidia) willistoni willistoni* LeConte, 1879

*Cicindela (Cicindela) albissima* Rumpp, 1962

*Cicindela (Cicindela) ancocisconensis* Harris, 1852

*Cicindela (Cicindela) arenicola* Rumpp, 1967

*Cicindela (Cicindela) arida* Davis, 1928

*Cicindela (Cicindela) bellissima bellissima* Leng, 1902

*Cicindela (Cicindela) bellissima frechini* Leffler, 1979

*Cicindela (Cicindela) columbica* Hatch, 1938

*Cicindela (Cicindela) decemnotata bonnevillensis* Knisley & Kippenhan, 2012

*Cicindela (Cicindela) decemnotata decemnotata* Say, 1817

*Cicindela (Cicindela) decemnotata meriwetheri* Knisley & Kippenhan, 2012

*Cicindela (Cicindela) decemnotata montevolans* Knisley & Kippenhan, 2012

*Cicindela (Cicindela) denikei* Brown, 1934

*Cicindela (Cicindela) denverensis* Casey, 1897

*Cicindela (Cicindela) depressula depressula* Casey, 1897

*Cicindela (Cicindela) depressula eureka* Fall, 1901

*Cicindela (Cicindela) duodecimguttata* Dejean, 1825

*Cicindela (Cicindela) formosa formosa* Say, 1817

*Cicindela (Cicindela) formosa generosa* Dejean, 1831

*Cicindela (Cicindela) formosa gibsoni* Brown, 1940

*Cicindela (Cicindela) formosa pigmentosignata* Horn, 1930

*Cicindela (Cicindela) formosa rutilovirescens* Rumpp, 1986

*Cicindela (Cicindela) fulgida fulgida* Say, 1823

*Cicindela (Cicindela) fulgida pseudowillistoni* Horn, 1938

*Cicindela (Cicindela) fulgida westbournei* Calder, 1922

*Cicindela (Cicindela) hirticollis abrupta* Casey, 1913

*Cicindela (Cicindela) hirticollis athabascensis* Graves, 1988

*Cicindela (Cicindela) hirticollis coloradula* Graves, 1988

*Cicindela (Cicindela) hirticollis corpuscula* Rumpp, 1962

*Cicindela (Cicindela) hirticollis couleensis* Graves, 1988

*Cicindela (Cicindela) hirticollis gravida* LeConte, 1851

*Cicindela (Cicindela) hirticollis hirticollis* Say, 1817

*Cicindela (Cicindela) hirticollis rhodensis* Calder, 1916

*Cicindela (Cicindela) hirticollis shelfordi* Graves, 1988

*Cicindela (Cicindela) hirticollis siuslawensis* Graves, 1988

*Cicindela (Cicindela) latesignata latesignata* LeConte, 1851

*Cicindela (Cicindela) lengi jordai* Rotger, 1974

*Cicindela (Cicindela) lengi lengi* Horn, 1908

*Cicindela (Cicindela) lengi versuta* Casey, 1913

*Cicindela (Cicindela) limbalis* Klug, 1834

*Cicindela (Cicindela) limbata hyperborea* LeConte, 1863

*Cicindela (Cicindela) limbata labradorensis* Johnson, 1991

*Cicindela (Cicindela) limbata limbata* Say, 1823

*Cicindela (Cicindela) limbata nogahabarensis* Knisley, 2008

*Cicindela (Cicindela) limbata nympha* Casey, 1913

*Cicindela (Cicindela) longilabris laurentii* Schaupp, 1884

*Cicindela (Cicindela) longilabris longilabris* Say, 1824

*Cicindela (Cicindela) longilabris perviridis* Schaupp, 1884

*Cicindela (Cicindela) nebraskana* Casey, 1909

*Cicindela (Cicindela) nigrior* Schaupp, 1884

*Cicindela (Cicindela) ohlone* Freitag & Kavanaugh, 1993

*Cicindela (Cicindela) oregona guttifera* LeConte, 1856

*Cicindela (Cicindela) oregona maricopa* Leng, 1902

*Cicindela (Cicindela) oregona navajoensis* Van Dyke, 1947

*Cicindela (Cicindela) oregona oregona* LeConte, 1856

*Cicindela (Cicindela) parowana parowana* Wickham, 1905

*Cicindela (Cicindela) parowana platti* Cazier, 1937

*Cicindela (Cicindela) parowana wallisi* Calder, 1922

*Cicindela (Cicindela) patruela consentanea* Dejean, 1825

*Cicindela (Cicindela) patruela patruela* Dejean, 1825

*Cicindela (Cicindela) pimeriana* LeConte, 1867

*Cicindela (Cicindela) plutonica* Casey, 1897

*Cicindela (Cicindela) pugetana* Casey, 1914

*Cicindela (Cicindela) pulchra dorothea* Rumpp, 1977

*Cicindela (Cicindela) pulchra pulchra* Say, 1823

*Cicindela (Cicindela) purpurea audubonii* LeConte, 1845

*Cicindela (Cicindela) purpurea cimarrona* LeConte, 1868

*Cicindela (Cicindela) purpurea hatchi* Leffler, 1980

*Cicindela (Cicindela) purpurea lauta* Casey, 1897

*Cicindela (Cicindela) purpurea purpurea* Olivier, 1790

*Cicindela (Cicindela) repanda novascotiae* Vaurie, 1951

*Cicindela (Cicindela) repanda repanda* Dejean, 1825

*Cicindela (Cicindela) repanda tanneri* Knaus, 1929

*Cicindela (Cicindela) scutellaris flavoviridis* Vaurie, 1950

*Cicindela (Cicindela) scutellaris lecontei* Haldeman, 1853

*Cicindela (Cicindela) scutellaris rugata* Vaurie, 1950

*Cicindela (Cicindela) scutellaris rugifrons* Dejean, 1825

*Cicindela (Cicindela) scutellaris scutellaris* Say, 1823

*Cicindela (Cicindela) scutellaris unicolor* Dejean, 1825

*Cicindela (Cicindela) scutellaris yampae* Rumpp, 1986

*Cicindela (Cicindela) sexguttata* Fabricius, 1775

*Cicindela (Cicindela) splendida* Hentz, 1830

*Cicindela (Cicindela) tenuicincta* Schaupp, 1884

*Cicindela (Cicindela) theatina* Rotger, 1944

*Cicindela (Cicindela) tranquebarica cibecuei* Duncan, 1958

*Cicindela (Cicindela) tranquebarica diffracta* Casey, 1909

*Cicindela (Cicindela) tranquebarica joaquinensis* Knisley & Haines, 2007

*Cicindela (Cicindela) tranquebarica kirbyi* LeConte, 1867

*Cicindela (Cicindela) tranquebarica parallelonota* Casey, 1914

*Cicindela (Cicindela) tranquebarica sierra* Leng, 1902

*Cicindela (Cicindela) tranquebarica tranquebarica* Herbst, 1806

*Cicindela (Cicindela) tranquebarica vibex* Horn, 1867

*Cicindela (Cicindela) tranquebarica viridissima* Fall, 1910

*Cicindela (Cicindela) waynei* Leffler, 2001

### Loricerini

*Loricera (Loricera) decempunctata* Eschscholtz, 1833

*Loricera (Loricera) foveata* LeConte, 1851

*Loricera (Loricera) pilicornis congesta* Mannerheim, 1853 **‡**

*Loricera (Loricera) pilicornis pilicornis* (Fabricius, 1775)**‡**

### Elaphrini

*Diacheila arctica amoena* (Faldermann, 1835)**‡**

*Diacheila polita* (Faldermann, 1835)**‡**

*Blethisa catenaria* Brown, 1944**‡**

*Blethisa hudsonica* Casey, 1924

*Blethisa julii* LeConte, 1863

*Blethisa multipunctata aurata* Fischer von Waldheim, 1828**‡**

*Blethisa oregonensis* LeConte, 1853

*Blethisa quadricollis* Haldeman, 1847

*Elaphrus (Arctelaphrus) lapponicus lapponicus* Gyllenhal, 1810**‡**

*Elaphrus (Arctelaphrus) lapponicus obliteratus* Mannerheim, 1853

*Elaphrus (Neoelaphrus) cicatricosus* LeConte, 1847

*Elaphrus (Neoelaphrus) clairvillei* Kirby, 1837

*Elaphrus (Neoelaphrus) fuliginosus* Say, 1830

*Elaphrus (Neoelaphrus) laevigatus* LeConte, 1852

*Elaphrus (Neoelaphrus) lindrothi* Goulet, 1983

*Elaphrus (Neoelaphrus) olivaceus* LeConte, 1863

*Elaphrus (Elaphrus) americanus americanus* Dejean, 1831

*Elaphrus (Elaphrus) americanus sylvanus* Goulet, 1982

*Elaphrus (Elaphrus) californicus* Mannerheim, 1843

*Elaphrus (Elaphrus) finitimus* Casey, 1920

*Elaphrus (Elaphrus) lecontei* Crotch, 1876

*Elaphrus (Elaphrus) marginicollis* Goulet, 1983

*Elaphrus (Elaphrus) mimus* Goulet, 1983

*Elaphrus (Elaphrus) ruscarius* Say, 1830

*Elaphrus (Elaphrus) trossulus* Semenov, 1904**‡**

*Elaphrus (Elaphrus) tuberculatus* Mäklin, 1878**‡**

*Elaphrus (Elaphrus) viridis* Horn, 1878

*Elaphrus (Elaphroterus) angusticollis angusticollis* Sahlberg, 1844**‡**

*Elaphrus (Elaphroterus) purpurans* Hausen, 1891

### Omophronini

*Omophron (Omophron) americanum* Dejean, 1831

*Omophron (Omophron) dentatum* LeConte, 1852

*Omophron (Omophron) gilae* LeConte, 1852

*Omophron (Omophron) grossum* Casey, 1909

*Omophron (Omophron) labiatum* (Fabricius, 1801)

*Omophron (Omophron) nitidum* LeConte, 1847

*Omophron (Omophron) obliteratum* Horn, 1870

*Omophron (Omophron) ovale* Horn, 1870

*Omophron (Omophron) robustum* Horn, 1870

*Omophron (Omophron) solidum* Casey, 1897

*Omophron (Omophron) tessellatum* Say, 1823

### Pasimachini

*Pasimachus (Pasimachus) californicus* Chaudoir, 1850

*Pasimachus (Pasimachus) depressus* (Fabricius, 1787)

*Pasimachus (Pasimachus) duplicatus* LeConte, 1853

*Pasimachus (Pasimachus) elongatus* LeConte, 1846

*Pasimachus (Pasimachus) marginatus* (Fabricius, 1787)

*Pasimachus (Pasimachus) obsoletus* LeConte, 1846

*Pasimachus (Pasimachus) punctulatus* Haldeman, 1843

*Pasimachus (Pasimachus) strenuus* LeConte, 1874

*Pasimachus (Pasimachus) sublaevis* (Palisot de Beauvois, 1811)

*Pasimachus (Pasimachus) subsulcatus* Say, 1823

*Pasimachus (Pasimachus) viridans* LeConte, 1858

### Scaritini

*Scarites (Scarites) lissopterus* Chaudoir, 1881

*Scarites (Scarites) marinus* Nichols, 1986

*Scarites (Scarites) ocalensis* Nichols, 1986

*Scarites (Scarites) quadriceps* Chaudoir, 1843

*Scarites (Scarites) stenops* Bousquet & Skelley, 2010

*Scarites (Scarites) subterraneus* Fabricius, 1775

*Scarites (Scarites) vicinus* Chaudoir, 1843

### Clivinini

*Clivina (Semiclivina) dentipes* Dejean, 1825

*Clivina (Semiclivina) vespertina* Putzeys, 1867**†**

*Clivina (Clivina) choatei* Bousquet & Skelley, 2012

*Clivina (Clivina) collaris* (Herbst, 1784)**†**

*Clivina (Clivina) fossor fossor* (Linnaeus, 1758)**†**

*Clivina (Clivina) impressefrons* LeConte, 1844

*Clivina (Clivina) myops* Bousquet, 1997

*Clivina (Clivina) oregona* Fall, 1922

*Clivina (Clivina) pallida* Say, 1823

*Clivina (Clivina) planicollis* LeConte, 1857

*Clivina (Clivina) punctigera* LeConte, 1857

*Clivina (Clivina) punctulata* LeConte, 1852

*Clivina (Antroforceps) alabama* Bousquet, 2012

*Clivina (Antroforceps) rubicunda* LeConte, 1857

*Clivina (Antroforceps) sasajii* Ball, 2001

*Clivina (Leucocara) acuducta* Haldeman, 1843

*Clivina (Leucocara) americana* Dejean, 1831

*Clivina (Leucocara) californica* Van Dyke, 1925

*Clivina (Leucocara) morio* Dejean, 1831

*Clivina (Leucocara) rufa* LeConte, 1857

*Paraclivina bipustulata* (Fabricius, 1798)

*Paraclivina convexa* (LeConte, 1844)

*Paraclivina fasciata* (Putzeys, 1846)

*Paraclivina ferrea* (LeConte, 1857)

*Paraclivina marginipennis* (Putzeys, 1846)

*Paraclivina postica* (LeConte, 1846)

*Paraclivina stigmula* (Putzeys, 1846)

*Paraclivina striatopunctata* (Dejean, 1831)

*Paraclivina sulcipennis* (Putzeys, 1867)

*Schizogenius (Genioschizus) crenulatus crenulatus* LeConte, 1852

*Schizogenius (Schizogenius) amphibius* (Haldeman, 1843)

*Schizogenius (Schizogenius) auripennis* Bates, 1881

*Schizogenius (Schizogenius) brevisetosus* Whitehead, 1972

*Schizogenius (Schizogenius) chiricahuanus* Whitehead, 1972

*Schizogenius (Schizogenius) depressus* LeConte, 1852

*Schizogenius (Schizogenius) falli* Whitehead, 1972

*Schizogenius (Schizogenius) ferrugineus* Putzeys, 1846

*Schizogenius (Schizogenius) lindrothi* Whitehead, 1972

*Schizogenius (Schizogenius) lineolatus* (Say, 1823)

*Schizogenius (Schizogenius) litigiosus* Fall, 1901

*Schizogenius (Schizogenius) longipennis* Putzeys, 1867

*Schizogenius (Schizogenius) neovalidus* Whitehead, 1972

*Schizogenius (Schizogenius) ochthocephalus* Whitehead, 1972

*Schizogenius (Schizogenius) ozarkensis* Whitehead, 1972

*Schizogenius (Schizogenius) planulatus* LeConte, 1863

*Schizogenius (Schizogenius) planuloides* Whitehead, 1972

*Schizogenius (Schizogenius) pluripunctatus* LeConte, 1852

*Schizogenius (Schizogenius) pygmaeus* Van Dyke, 1925

*Schizogenius (Schizogenius) sallei* Putzeys, 1867

*Schizogenius (Schizogenius) scopaeus* Whitehead, 1972

*Schizogenius (Schizogenius) seticollis seticollis* Fall, 1901

*Schizogenius (Schizogenius) sulcifrons* Putzeys, 1846

*Schizogenius (Schizogenius) tibialis* Whitehead, 1972

*Halocoryza arenaria* (Darlington, 1939)

*Oxydrepanus rufus* (Putzeys, 1846)

*Ardistomis obliquata* Putzeys, 1846

*Ardistomis schaumii* LeConte, 1857

*Semiardistomis puncticollis* (Dejean, 1831)

*Semiardistomis viridis* (Say, 1823)

*Aspidoglossa subangulata* (Chaudoir, 1843)

### Dyschiriini

*Akephorus marinus* LeConte, 1852

*Akephorus obesus* (LeConte, 1863)

*Dyschirius abbreviatus* Putzeys, 1846

*Dyschirius aeneolus* LeConte, 1850

*Dyschirius affinis* Fall, 1901

*Dyschirius alticola* Lindroth, 1961

*Dyschirius analis* LeConte, 1852

*Dyschirius aratus* LeConte, 1852

*Dyschirius brevispinus* LeConte, 1878

*Dyschirius campicola* Lindroth, 1961

*Dyschirius carrorum* Bousquet, 1997

*Dyschirius cerberus* Larson, 1968

*Dyschirius chiricahuae* (Dajoz, 2004)

*Dyschirius comatus* Bousquet, 1988

*Dyschirius compactus* Lindroth, 1961

*Dyschirius consobrinus* LeConte, 1852

*Dyschirius criddlei* Fall, 1925

*Dyschirius curvispinus* Putzeys, 1846

*Dyschirius dejeanii* Putzeys, 1846

*Dyschirius edentulus* Putzeys, 1846

*Dyschirius erythrocerus* LeConte, 1857

*Dyschirius exochus* Whitehead, 1970

*Dyschirius ferrugineus* Bousquet, 1988

*Dyschirius gibbipennis* LeConte, 1857

*Dyschirius globosus* (Herbst, 1784)**†**

*Dyschirius globulosus* (Say, 1823)

*Dyschirius haemorrhoidalis* (Dejean, 1831)

*Dyschirius hiemalis* Bousquet, 1987

*Dyschirius interior* Fall, 1922

*Dyschirius laevifasciatus* Horn, 1878

*Dyschirius larochellei* Bousquet, 1988

*Dyschirius longulus* LeConte, 1850

*Dyschirius melancholicus* Putzeys, 1867**‡**

*Dyschirius montanus* LeConte, 1879

*Dyschirius owen* (Dajoz, 2004)

*Dyschirius pacificus* Lindroth, 1961

*Dyschirius pallipennis* (Say, 1823)

*Dyschirius patruelis* LeConte, 1852

*Dyschirius perversus* Fall, 1922

*Dyschirius pilosus* LeConte, 1857

*Dyschirius planatus* Lindroth, 1961

*Dyschirius politus politus* (Dejean, 1825)**‡**

*Dyschirius pumilus* (Dejean, 1825)

*Dyschirius quadrimaculatus* Lindroth, 1961

*Dyschirius salivagans* LeConte, 1875

*Dyschirius sculptus* Bousquet, 1988

*Dyschirius sellatus* LeConte, 1857

*Dyschirius setosus* LeConte, 1857

*Dyschirius sextoni* Bousquet, 1987

*Dyschirius soda* (Dajoz, 2004)

*Dyschirius sphaericollis* (Say, 1823)

*Dyschirius subarcticus subarcticus* Lindroth, 1961

*Dyschirius sublaevis* Putzeys, 1846

*Dyschirius tenuispinus* Lindroth, 1961

*Dyschirius terminatus* LeConte, 1846

*Dyschirius timidus* Lindroth, 1961

*Dyschirius tridentatus* LeConte, 1852

*Dyschirius truncatus* LeConte, 1857

*Dyschirius unipunctatus* Fall, 1901

*Dyschirius varidens* Fall, 1910

*Dyschirius wayah* (Dajoz, 2005)

### Promecognathini

*Promecognathus crassus* LeConte, 1868

*Promecognathus laevissimus* (Dejean, 1829)

### Broscini

*Miscodera arctica* (Paykull, 1798)**‡**

*Broscodera (Broscodera) insignis* (Mannerheim, 1852)

*Zacotus matthewsii* LeConte, 1869

*Broscus cephalotes* (Linnaeus, 1758)**†**

### Gehringiini

*Gehringia olympica* Darlington, 1933

### Trechini

*Trechoblemus westcotti* Barr, 1972

*Pseudanophthalmus abditus* Krekeler, 1973

*Pseudanophthalmus acherontis* Barr, 1959

*Pseudanophthalmus alabamae* Valentine, 1932

*Pseudanophthalmus aladdini* Valentine, 1945

*Pseudanophthalmus assimilis* Barr, 1981

*Pseudanophthalmus audax* (Horn, 1883)

*Pseudanophthalmus avernus* Valentine, 1945

*Pseudanophthalmus barberi* Jeannel, 1928

*Pseudanophthalmus barri* Krekeler, 1973

*Pseudanophthalmus beakleyi* Valentine, 1937

*Pseudanophthalmus bendermani* Barr, 1959

*Pseudanophthalmus caecus* Krekeler, 1973

*Pseudanophthalmus calcareus* Barr, 1981

*Pseudanophthalmus catherinae* Barr, 1959

*Pseudanophthalmus catoryctos* Krekeler, 1973

*Pseudanophthalmus cerberus cerberus* Barr, 1985

*Pseudanophthalmus cerberus completus* Barr, 1985

*Pseudanophthalmus chthonius* Krekeler, 1973

*Pseudanophthalmus ciliaris ciliaris* Valentine, 1937

*Pseudanophthalmus ciliaris orlindae* Barr, 1959

*Pseudanophthalmus cnephosus* Krekeler, 1973

*Pseudanophthalmus colemanensis* Barr, 1959

*Pseudanophthalmus conditus* Krekeler, 1973

*Pseudanophthalmus cordicollis* Barr, 1981

*Pseudanophthalmus cumberlandus* Valentine, 1937

*Pseudanophthalmus darlingtoni darlingtoni* Barr, 1985

*Pseudanophthalmus darlingtoni persimilis* Barr, 1985

*Pseudanophthalmus deceptivus* Barr, 1981

*Pseudanophthalmus delicatus* Valentine, 1932

*Pseudanophthalmus desertus* Krekeler, 1973

*Pseudanophthalmus digitus* Valentine, 1932

*Pseudanophthalmus distinguens* Valentine, 1948

*Pseudanophthalmus egberti* Barr, 1965

*Pseudanophthalmus elongatus* Krekeler, 1973

*Pseudanophthalmus emersoni* Krekeler, 1958

*Pseudanophthalmus engelhardti* (Barber, 1928)

*Pseudanophthalmus eremita* (Horn, 1871)

*Pseudanophthalmus exiguus* Krekeler, 1973

*Pseudanophthalmus exoticus* Krekeler, 1973

*Pseudanophthalmus farrelli* Barr, 1959

*Pseudanophthalmus fastigatus* Barr, 1981

*Pseudanophthalmus fluviatilis* Valentine, 1948

*Pseudanophthalmus fowlerae* Barr, 1980

*Pseudanophthalmus frigidus* Barr, 1981

*Pseudanophthalmus fulleri* Valentine, 1932

*Pseudanophthalmus fuscus constrictus* Valentine, 1932

*Pseudanophthalmus fuscus fuscus* Valentine, 1931

*Pseudanophthalmus georgiae* Barr, 1981

*Pseudanophthalmus globiceps* Barr, 1985

*Pseudanophthalmus gracilis* Valentine, 1931

*Pseudanophthalmus grandis elevatus* Valentine, 1932

*Pseudanophthalmus grandis grandis* Valentine, 1931

*Pseudanophthalmus hadenoecus* Barr, 1965

*Pseudanophthalmus henroti* Jeannel, 1949

*Pseudanophthalmus hesperus* Barr, 1959

*Pseudanophthalmus higginbothami* Valentine, 1931

*Pseudanophthalmus hirsutus* Valentine, 1931

*Pseudanophthalmus hoffmani* Barr, 1965

*Pseudanophthalmus holsingeri* Barr, 1965

*Pseudanophthalmus horni* (Garman, 1892)

*Pseudanophthalmus hortulanus* Barr, 1965

*Pseudanophthalmus hubbardi* (Barber, 1928)

*Pseudanophthalmus hubrichti* Valentine, 1948

*Pseudanophthalmus humeralis* Valentine, 1931

*Pseudanophthalmus hypertrichosis* Valentine, 1932

*Pseudanophthalmus hypolithos* Barr, 1981

*Pseudanophthalmus illinoisensis* Barr & Peck, 1966

*Pseudanophthalmus inexpectatus* Barr, 1959

*Pseudanophthalmus inquisitor* Barr, 1980

*Pseudanophthalmus insularis* Barr, 1959

*Pseudanophthalmus intermedius* (Valentine, 1931)

*Pseudanophthalmus intersectus* Barr, 1965

*Pseudanophthalmus jonesi* Valentine, 1945

*Pseudanophthalmus krameri* Krekeler, 1973

*Pseudanophthalmus krekeleri* Barr, 1965

*Pseudanophthalmus lallemanti* Jeannel, 1949

*Pseudanophthalmus leonae* Barr, 1960

*Pseudanophthalmus limicola* Jeannel, 1931

*Pseudanophthalmus lodingi* Valentine, 1931

*Pseudanophthalmus loganensis* Barr, 1959

*Pseudanophthalmus longiceps* Barr, 1981

*Pseudanophthalmus macradyi* Valentine, 1948

*Pseudanophthalmus major* Krekeler, 1973

*Pseudanophthalmus menetriesii campestris* Barr, 1985

*Pseudanophthalmus menetriesii menetriesii* (Motschulsky, 1862)

*Pseudanophthalmus meridionalis* Valentine, 1945

*Pseudanophthalmus montanus* Barr, 1965

*Pseudanophthalmus nelsoni* Barr, 1965

*Pseudanophthalmus nickajackensis* Barr, 1981

*Pseudanophthalmus nortoni* Barr, 1981

*Pseudanophthalmus occidentalis* Barr, 1959

*Pseudanophthalmus ohioensis* Krekeler, 1973

*Pseudanophthalmus orientalis* Krekeler, 1973

*Pseudanophthalmus orthosulcatus* Valentine, 1932

*Pseudanophthalmus packardi* Barr, 1959

*Pseudanophthalmus pallidus* Barr, 1981

*Pseudanophthalmus paradoxus* Barr, 1981

*Pseudanophthalmus parvicollis* Jeannel, 1931

*Pseudanophthalmus parvus* Krekeler, 1973

*Pseudanophthalmus paulus* Barr, 1981

*Pseudanophthalmus paynei* Barr, 1981

*Pseudanophthalmus petrunkevitchi* Valentine, 1945

*Pseudanophthalmus pholeter* Krekeler, 1973

*Pseudanophthalmus pilosus* Barr, 1985

*Pseudanophthalmus pontis* Barr, 1965

*Pseudanophthalmus potomaca* Valentine, 1932

*Pseudanophthalmus praetermissus* Barr, 1981

*Pseudanophthalmus princeps* Barr, 1979

*Pseudanophthalmus productus* Barr, 1980

*Pseudanophthalmus profundus* Valentine, 1945

*Pseudanophthalmus pubescens intrepidus* Barr, 1985

*Pseudanophthalmus pubescens pubescens* (Horn, 1869)

*Pseudanophthalmus punctatus* Valentine, 1931

*Pseudanophthalmus pusillus* Barr, 1981

*Pseudanophthalmus pusio* (Horn, 1869)

*Pseudanophthalmus puteanus* Krekeler, 1973

*Pseudanophthalmus quadratus* Barr, 1965

*Pseudanophthalmus rittmani* Krekeler, 1973

*Pseudanophthalmus robustus* Valentine, 1931

*Pseudanophthalmus rogersae* Barr, 1981

*Pseudanophthalmus rotundatus* Valentine, 1932

*Pseudanophthalmus sanctipauli* Barr, 1981

*Pseudanophthalmus scholasticus* Barr, 1981

*Pseudanophthalmus scutilus* Barr, 1981

*Pseudanophthalmus seclusus* Barr, 1981

*Pseudanophthalmus senecae* Valentine, 1932

*Pseudanophthalmus sequoyah* Barr, 1981

*Pseudanophthalmus sericus* Barr, 1981

*Pseudanophthalmus shilohensis mayfieldensis* Krekeler, 1958

*Pseudanophthalmus shilohensis shilohensis* Krekeler, 1958

*Pseudanophthalmus sidus* Barr, 1965

*Pseudanophthalmus simplex* Barr, 1980

*Pseudanophthalmus simulans* Barr, 1985

*Pseudanophthalmus solivagus* Krekeler, 1973

*Pseudanophthalmus steevesi* Barr, 1981

*Pseudanophthalmus striatus* (Motschulsky, 1862)

*Pseudanophthalmus stricticollis* Jeannel, 1931

*Pseudanophthalmus sylvaticus* Barr, 1967

*Pseudanophthalmus templetoni* Valentine, 1948

*Pseudanophthalmus tenebrosus* Krekeler, 1973

*Pseudanophthalmus tennesseensis* Valentine, 1937

*Pseudanophthalmus tenuis* (Horn, 1871)

*Pseudanophthalmus thomasi* Barr, 1981

*Pseudanophthalmus tiresias* Barr, 1959

*Pseudanophthalmus transfluvialis* Barr, 1985

*Pseudanophthalmus troglodytes* Krekeler, 1973

*Pseudanophthalmus tullahoma* Barr, 1959

*Pseudanophthalmus umbratilis* Krekeler, 1973

*Pseudanophthalmus unionis* Barr, 1981

*Pseudanophthalmus valentinei* Jeannel, 1949

*Pseudanophthalmus vanburenensis* Barr, 1959

*Pseudanophthalmus ventus* Barr, 1981

*Pseudanophthalmus vicarius* Barr, 1965

*Pseudanophthalmus virginicus* (Barr, 1960)

*Pseudanophthalmus wallacei* Barr, 1981

*Pseudanophthalmus youngi* Krekeler, 1958

*Nelsonites jonesei* Valentine, 1952

*Nelsonites walteri* Valentine, 1952

*Neaphaenops tellkampfii henroti* Jeannel, 1949

*Neaphaenops tellkampfii meridionalis* Barr, 1959

*Neaphaenops tellkampfii tellkampfii* (Erichson, 1844)

*Neaphaenops tellkampfii viator* Barr, 1979

*Blemus discus discus* (Fabricius, 1792)**†**

*Xenotrechus condei* Barr & Krekeler, 1967

*Xenotrechus denticollis* Barr & Krekeler, 1967

*Darlingtonea kentuckensis* Valentine, 1952

*Ameroduvalius jeanneli jeanneli* Valentine, 1952

*Ameroduvalius jeanneli rockcastlei* Valentine, 1952

*Trechus (Trechus) alinae* Dajoz, 1990

*Trechus (Trechus) apache* Dajoz, 1990

*Trechus (Trechus) apicalis* Motschulsky, 1845**‡**

*Trechus (Trechus) arizonae* Casey, 1918

*Trechus (Trechus) caliginis* Barr, 1985

*Trechus (Trechus) carolinae* Schaeffer, 1901

*Trechus (Trechus) chalybeus* Dejean, 1831

*Trechus (Trechus) coloradensis* Schaeffer, 1915

*Trechus (Trechus) conformis* Jeannel, 1927

*Trechus (Trechus) crassiscapus* Lindroth, 1955

*Trechus (Trechus) cumberlandus* Barr, 1962

*Trechus (Trechus) humboldti* Van Dyke, 1945

*Trechus (Trechus) hydropicus avus* Barr, 1962

*Trechus (Trechus) hydropicus beutenmuelleri* Jeannel, 1931

*Trechus (Trechus) hydropicus canus* Barr, 1962

*Trechus (Trechus) hydropicus hydropicus* Horn, 1883

*Trechus (Trechus) mitchellensis* Barr, 1962

*Trechus (Trechus) obtusus* Erichson, 1837**†**

*Trechus (Trechus) oregonensis* Hatch, 1951

*Trechus (Trechus) ovipennis* Motschulsky, 1845

*Trechus (Trechus) pomonae* Fall, 1901

*Trechus (Trechus) quadristriatus* (Schrank, 1781)**†**

*Trechus (Trechus) roanicus* Barr, 1962

*Trechus (Trechus) rubens* (Fabricius, 1792)**†**

*Trechus (Trechus) schwarzi saludae* Barr, 1979

*Trechus (Trechus) schwarzi schwarzi* Jeannel, 1931

*Trechus (Trechus) schwarzi scopulosus* Barr, 1979

*Trechus (Trechus) tenuiscapus* Lindroth, 1961

*Trechus (Trechus) yvesbousqueti* Donabauer, 2010

*Trechus (Microtrechus) aduncus* Barr, 1962

*Trechus (Microtrechus) balsamensis* Barr, 1962

*Trechus (Microtrechus) barberi* (Jeannel, 1931)

*Trechus (Microtrechus) bowlingi* Barr, 1962

*Trechus (Microtrechus) cheoahensis* Donabauer, 2005

*Trechus (Microtrechus) clingmanensis* Donabauer, 2005

*Trechus (Microtrechus) coweensis* Barr, 1979

*Trechus (Microtrechus) haoe* Barr, 1979

*Trechus (Microtrechus) haoeleadensis* Donabauer, 2005

*Trechus (Microtrechus) howellae* Barr, 1979

*Trechus (Microtrechus) inexpectatus* Barr, 1985

*Trechus (Microtrechus) luculentus cheoahbaldensis* Donabauer, 2005

*Trechus (Microtrechus) luculentus joannabaldensis* Donabauer, 2005

*Trechus (Microtrechus) luculentus luculentus* Barr, 1962

*Trechus (Microtrechus) luculentus wayahensis* Barr, 1979

*Trechus (Microtrechus) nantahalae* Barr, 1979

*Trechus (Microtrechus) nebulosus* Barr, 1962

*Trechus (Microtrechus) novaculosus* Barr, 1962

*Trechus (Microtrechus) plottbalsamensis* Donabauer, 2005

*Trechus (Microtrechus) pseudobarberi* Donabauer, 2009

*Trechus (Microtrechus) pseudonovaculosus* Donabauer, 2005

*Trechus (Microtrechus) pseudosubtilis* Donabauer, 2009

*Trechus (Microtrechus) ramseyensis* Donabauer, 2005

*Trechus (Microtrechus) rivulis* Dajoz, 2005

*Trechus (Microtrechus) rosenbergi* Barr, 1962

*Trechus (Microtrechus) satanicus* Barr, 1962

*Trechus (Microtrechus) snowbirdensis* Donabauer, 2005

*Trechus (Microtrechus) stefanschoedli* Donabauer, 2005

*Trechus (Microtrechus) stupkai* Barr, 1979

*Trechus (Microtrechus) subtilis* Barr, 1962

*Trechus (Microtrechus) talequah* Barr, 1962

*Trechus (Microtrechus) tennesseensis tauricus* Barr, 1962

*Trechus (Microtrechus) tennesseensis tennesseensis* Barr, 1962

*Trechus (Microtrechus) thomasbarri* Donabauer, 2005

*Trechus (Microtrechus) thunderheadensis* Donabauer, 2005

*Trechus (Microtrechus) tobiasi* Donabauer, 2005

*Trechus (Microtrechus) tonitru* Barr, 1962

*Trechus (Microtrechus) toxawayi* Barr, 1979

*Trechus (Microtrechus) tuckaleechee* Barr, 1962

*Trechus (Microtrechus) tusquitee* Barr, 1979

*Trechus (Microtrechus) tusquitensis* Donabauer, 2005

*Trechus (Microtrechus) uncifer* Barr, 1962

*Trechus (Microtrechus) unicoi* Barr, 1979

*Trechus (Microtrechus) valentinei* Barr, 1979

*Trechus (Microtrechus) vandykei pisgahensis* Barr, 1979

*Trechus (Microtrechus) vandykei vandykei* (Jeannel, 1927)

*Trechus (Microtrechus) verus* Barr, 1962

*Trechus (Microtrechus) wayahbaldensis* Donabauer, 2005

### Bembidiini

*Amerizus (Amerizus) oblonguloides* (Lindroth, 1963)

*Amerizus (Amerizus) oblongulus* (Mannerheim, 1852)

*Amerizus (Amerizus) spectabilis* (Mannerheim, 1852)

*Amerizus (Amerizus) utahensis* (Van Dyke, 1926)

*Amerizus (Amerizus) wingatei* (Bland, 1864)

*Lionepha casta* (Casey, 1918)

*Lionepha chintimini* (Erwin & Kavanaugh, 1981)

*Lionepha disjuncta* (Lindroth, 1963)

*Lionepha erasa* (LeConte, 1859)

*Lionepha lindrothellus* (Erwin & Kavanaugh, 1981)

*Lionepha lummi* (Erwin & Kavanaugh, 1981)

*Lionepha osculans* (Casey, 1918)

*Lionepha pseudoerasa* (Lindroth, 1963)

*Lionepha sequoiae* (Lindroth, 1963)

*Asaphidion alaskanum* Wickham, 1919

*Asaphidion curtum curtum* (Heyden, 1870)**†**

*Asaphidion yukonense* Wickham, 1919

*Bembidion (Hirmoplataphus) alpineanum* Casey, 1924

*Bembidion (Hirmoplataphus) avidum* Casey, 1918

*Bembidion (Hirmoplataphus) concolor* (Kirby, 1837)

*Bembidion (Hirmoplataphus) humboldtense* Blaisdell, 1902

*Bembidion (Hirmoplataphus) nigrum* Say, 1823

*Bembidion (Hirmoplataphus) quadrulum* LeConte, 1861

*Bembidion (Hirmoplataphus) recticolle* LeConte, 1863

*Bembidion (Hirmoplataphus) salebratum* (LeConte, 1847)

*Bembidion (Hirmoplataphus) subaerarium* Casey, 1924

*Bembidion (Hydriomicrus) brevistriatum* Hayward, 1897

*Bembidion (Hydriomicrus) californicum* Hayward, 1897

*Bembidion (Hydriomicrus) innocuum* Casey, 1918

*Bembidion (Hydriomicrus) quadratulum* Notman, 1920

*Bembidion (Hydriomicrus) semistriatum* (Haldeman, 1843)

*Bembidion (Odontium) aenulum* Hayward, 1901

*Bembidion (Odontium) bowditchii* LeConte, 1878

*Bembidion (Odontium) carinatum* (LeConte, 1852)

*Bembidion (Odontium) confusum* Hayward, 1897

*Bembidion (Odontium) coxendix* Say, 1823

*Bembidion (Odontium) durangoense* Bates, 1891

*Bembidion (Odontium) gilae* Lindroth, 1963

*Bembidion (Odontium) paraenulum* Maddison, 2009

*Bembidion (Odontium) robusticolle* Hayward, 1897

*Bembidion (Odontium) sculpturatum* (Motschulsky, 1859)

*Bembidion (Bracteon) alaskense* Lindroth, 1962**‡**

*Bembidion (Bracteon) balli* Lindroth, 1962

*Bembidion (Bracteon) carinula* Chaudoir, 1868

*Bembidion (Bracteon) foveum* Motschulsky, 1844**‡**

*Bembidion (Bracteon) hesperium* Fall, 1910

*Bembidion (Bracteon) inaequale* Say, 1823

*Bembidion (Bracteon) lapponicum* Zetterstedt, 1828**‡**

*Bembidion (Bracteon) levettei carrianum* Casey, 1924

*Bembidion (Bracteon) levettei levettei* Casey, 1918

*Bembidion (Bracteon) lorquinii* Chaudoir, 1868

*Bembidion (Bracteon) punctatostriatum* Say, 1823

*Bembidion (Bracteon) zephyrum* Fall, 1910

*Bembidion (Ochthedromus) americanum* Dejean, 1831

*Bembidion (Ochthedromus) bifossulatum* (LeConte, 1852)

*Bembidion (Ochthedromus) cheyennense* Casey, 1918

*Bembidion (Pseudoperyphus) antiquum* Dejean, 1831

*Bembidion (Pseudoperyphus) arenobile* Maddison, 2008

*Bembidion (Pseudoperyphus) bellorum* Maddison, 2008

*Bembidion (Pseudoperyphus) chalceum* Dejean, 1831

*Bembidion (Pseudoperyphus) honestum* Say, 1823

*Bembidion (Pseudoperyphus) integrum* Casey, 1918

*Bembidion (Pseudoperyphus) louisella* Maddison, 2008

*Bembidion (Pseudoperyphus) rothfelsi* Maddison, 2008

*Bembidion (Pseudoperyphus) rufotinctum* Chaudoir, 1868

*Bembidion (Cillenus) palosverdes* Kavanaugh & Erwin, 1992

*Bembidion (Actedium) lachnophoroides* Darlington, 1926

*Bembidion (Ocydromus) scopulinum* (Kirby, 1837)**‡**

*Bembidion (Peryphus) actuosum* Casey, 1918

*Bembidion (Peryphus) bruxellense* Wesmael, 1835**†**

*Bembidion (Peryphus) consanguineum* Hayward, 1897

*Bembidion (Peryphus) dauricum* (Motschulsky, 1844)**‡**

*Bembidion (Peryphus) femoratum femoratum* Sturm, 1825**†**

*Bembidion (Peryphus) lugubre* LeConte, 1857

*Bembidion (Peryphus) mexicanum* Dejean, 1831

*Bembidion (Peryphus) nevadense* Ulke, 1875

*Bembidion (Peryphus) obscurellum obscurellum* (Motschulsky, 1845)**‡**

*Bembidion (Peryphus) pernotum* Casey, 1918

*Bembidion (Peryphus) perspicuum* (LeConte, 1848)

*Bembidion (Peryphus) petrosum attuense* Lindroth, 1963

*Bembidion (Peryphus) petrosum petrosum* Gebler, 1833**‡**

*Bembidion (Peryphus) plagiatum* (Zimmermann, 1869)

*Bembidion (Peryphus) poppii schalleri* Lindroth, 1963

*Bembidion (Peryphus) rupicola* (Kirby, 1837)

*Bembidion (Peryphus) sarpedon* Casey, 1918

*Bembidion (Peryphus) satelles* Casey, 1918

*Bembidion (Peryphus) sejunctum sejunctum* Casey, 1918

*Bembidion (Peryphus) sejunctum semiaureum* Fall, 1922

*Bembidion (Peryphus) striola* (LeConte, 1852)

*Bembidion (Peryphus) tetracolum tetracolum* Say, 1823**†**

*Bembidion (Peryphus) transversale* Dejean, 1831

*Bembidion (Terminophanes) mckinleyi carneum* Lindroth, 1963

*Bembidion (Terminophanes) mckinleyi mckinleyi* Fall, 1926

*Bembidion (Asioperyphus) bimaculatum* (Kirby, 1837)

*Bembidion (Asioperyphus) lenae* Csiki, 1928**‡**

*Bembidion (Asioperyphus) postremum* Say, 1830

*Bembidion (Asioperyphus) renoanum* Casey, 1918

*Bembidion (Asioperyphus) sordidum* (Kirby, 1837)

*Bembidion (Asioperyphus) umiatense* Lindroth, 1963**‡**

*Bembidion (Peryphanes) grapii* Gyllenhal, 1827**‡**

*Bembidion (Peryphanes) lacunarium* (Zimmermann, 1869)

*Bembidion (Peryphanes) platynoides* Hayward, 1897

*Bembidion (Peryphanes) stephensii* Crotch, 1866**†**

*Bembidion (Peryphanes) subangustatum* Hayward, 1897

*Bembidion (Peryphanes) texanum* Chaudoir, 1868

*Bembidion (Peryphanes) yukonum* Fall, 1926**‡**

*Bembidion (Testediolum) commotum* Casey, 1918

*Bembidion (Testediolum) modocianum* Casey, 1924

*Bembidion (Testediolum) nebraskense* LeConte, 1863

*Bembidion (Testediolum) obscuripenne* Blaisdell, 1902

*Bembidion (Testediolum) perbrevicolle* Casey, 1924

*Bembidion (Testediolum) ulkei* Lindroth, 1963

*Bembidion (Leuchydrium) tigrinum* LeConte, 1879

*Bembidion (Bembidion) adductum* Casey, 1918

*Bembidion (Bembidion) mutatum* Gemminger & Harold, 1868

*Bembidion (Bembidion) oregonense* Hatch, 1953

*Bembidion (Bembidion) pedicellatum* LeConte, 1857

*Bembidion (Bembidion) praecinctum* LeConte, 1879

*Bembidion (Bembidion) quadrimaculatum dubitans* (LeConte, 1852)

*Bembidion (Bembidion) quadrimaculatum oppositum* Say, 1823

*Bembidion (Cyclolopha) jucundum* Horn, 1895

*Bembidion (Cyclolopha) poculare* Bates, 1884

*Bembidion (Cyclolopha) sphaeroderum* Bates, 1882

*Bembidion (Furcacampa) affine* Say, 1823

*Bembidion (Furcacampa) egens* Casey, 1918

*Bembidion (Furcacampa) fuchsii* Blaisdell, 1902

*Bembidion (Furcacampa) impotens* Casey, 1918

*Bembidion (Furcacampa) mimus* Hayward, 1897

*Bembidion (Furcacampa) nogalesium* Casey, 1924

*Bembidion (Furcacampa) timidum* (LeConte, 1847)

*Bembidion (Furcacampa) triviale* Casey, 1918

*Bembidion (Furcacampa) versicolor* (LeConte, 1847)

*Bembidion (Neobembidion) constricticolle* Hayward, 1897

*Bembidion (Neobembidion) nitidicolle* Bousquet, 2006

*Bembidion (Neobembidion) nudipenne* Lindroth, 1963

*Bembidion (Neobembidion) tencenti* Hatch, 1951

*Bembidion (Diplocampa) transparens transparens* (Gebler, 1830)**‡**

*Bembidion (Semicampa) convexulum* Hayward, 1897

*Bembidion (Semicampa) morulum* LeConte, 1863

*Bembidion (Semicampa) muscicola* Hayward, 1897

*Bembidion (Semicampa) nigrivestis* Bousquet, 2006

*Bembidion (Semicampa) praticola* Lindroth, 1963

*Bembidion (Semicampa) roosevelti* Pic, 1902

*Bembidion (Semicampa) rubiginosum* LeConte, 1879

*Bembidion (Semicampa) semicinctum* Notman, 1919

*Bembidion (Notaphus) acticola* Casey, 1884

*Bembidion (Notaphus) aeneicolle* (LeConte, 1847)

*Bembidion (Notaphus) approximatum* (LeConte, 1852)

*Bembidion (Notaphus) aratum* (LeConte, 1852)

*Bembidion (Notaphus) auxiliator* Casey, 1924

*Bembidion (Notaphus) callens* Casey, 1918

*Bembidion (Notaphus) castor* Lindroth, 1963

*Bembidion (Notaphus) coloradense* Hayward, 1897

*Bembidion (Notaphus) consimile* Hayward, 1897

*Bembidion (Notaphus) conspersum* Chaudoir, 1868

*Bembidion (Notaphus) constrictum* (Leconte, 1847)

*Bembidion (Notaphus) consuetum* Casey, 1918

*Bembidion (Notaphus) contractum* Say, 1823

*Bembidion (Notaphus) cordatum* (LeConte, 1847)

*Bembidion (Notaphus) debiliceps* Casey, 1918

*Bembidion (Notaphus) dejectum* Casey, 1884

*Bembidion (Notaphus) dorsale* Say, 1823

*Bembidion (Notaphus) evidens* Casey, 1918

*Bembidion (Notaphus) flohri* Bates, 1878

*Bembidion (Notaphus) graphicum* Casey, 1918

*Bembidion (Notaphus) hageni* Hayward, 1897

*Bembidion (Notaphus) idoneum* Casey, 1918

*Bembidion (Notaphus) indistinctum* Dejean, 1831

*Bembidion (Notaphus) insulatum* (LeConte, 1852)

*Bembidion (Notaphus) intermedium* (Kirby, 1837)

*Bembidion (Notaphus) jacobianum* Casey, 1918

*Bembidion (Notaphus) latebricola* Casey, 1918

*Bembidion (Notaphus) lecontei* Csiki, 1928

*Bembidion (Notaphus) luculentum* Casey, 1918

*Bembidion (Notaphus) mormon* Hayward, 1897

*Bembidion (Notaphus) nigripes* (Kirby, 1837)**‡**

*Bembidion (Notaphus) nubiculosum* Chaudoir, 1868

*Bembidion (Notaphus) oberthueri* Hayward, 1901

*Bembidion (Notaphus) obscuromaculatum* (Motschulsky, 1859)

*Bembidion (Notaphus) obtusangulum* LeConte, 1863

*Bembidion (Notaphus) obtusidens* Fall, 1922

*Bembidion (Notaphus) operosum* Casey, 1918

*Bembidion (Notaphus) patruele* Dejean, 1831

*Bembidion (Notaphus) pilatei* Chaudoir, 1868

*Bembidion (Notaphus) pimanum* Casey, 1918

*Bembidion (Notaphus) rapidum* (LeConte, 1847)

*Bembidion (Notaphus) scintillans* Bates, 1882

*Bembidion (Notaphus) scudderi* LeConte, 1878

*Bembidion (Notaphus) semiopacum* Casey, 1924

*Bembidion (Notaphus) semipunctatum* (Donovan, 1806)**‡**

*Bembidion (Notaphus) umbratum* (LeConte, 1847)

*Bembidion (Notaphus) versutum* LeConte, 1878

*Bembidion (Notaphus) viridicolle* (LaFerté-Sénectère, 1841)

*Bembidion (Notaphus) vividum* Casey, 1884

*Bembidion (Notaphus) vulpecula* Casey, 1918

*Bembidion (Trepanedoris) acutifrons* LeConte, 1879

*Bembidion (Trepanedoris) ampliceps* Casey, 1918

*Bembidion (Trepanedoris) anguliferum* (LeConte, 1852)

*Bembidion (Trepanedoris) canadianum* Casey, 1924

*Bembidion (Trepanedoris) clemens* Casey, 1918

*Bembidion (Trepanedoris) concretum* Casey, 1918

*Bembidion (Trepanedoris) connivens* (LeConte, 1852)

*Bembidion (Trepanedoris) elizabethae* Hatch, 1950

*Bembidion (Trepanedoris) fortestriatum* (Motschulsky, 1845)

*Bembidion (Trepanedoris) frontale* (LeConte, 1847)

*Bembidion (Trepanedoris) pseudocautum* Lindroth, 1963

*Bembidion (Trepanedoris) scenicum* Casey, 1918

*Bembidion (Trepanedoris) siticum* Casey, 1918

*Bembidion (Peryphodes) ephippigerum* (LeConte, 1852)

*Bembidion (Peryphodes) salinarium* Casey, 1918

*Bembidion (Emphanes) diligens* Casey, 1918

*Bembidion (Emphanes) vile* (LeConte, 1852)

*Bembidion (Blepharoplataphus) hastii* Sahlberg, 1827**‡**

*Bembidion (Plataphus) arcticum* Lindroth, 1963**‡**

*Bembidion (Plataphus) basicorne* Notman, 1920

*Bembidion (Plataphus) brachythorax* Lindroth, 1963**‡**

*Bembidion (Plataphus) breve* (Motschulsky, 1845)

*Bembidion (Plataphus) carolinense* Casey, 1924

*Bembidion (Plataphus) complanulum* (Mannerheim, 1853)

*Bembidion (Plataphus) compressum* Lindroth, 1963**‡**

*Bembidion (Plataphus) curtulatum* Casey, 1918

*Bembidion (Plataphus) falsum* Blaisdell, 1902

*Bembidion (Plataphus) farrarae* Hatch, 1950

*Bembidion (Plataphus) gebleri turbatum* Casey, 1918

*Bembidion (Plataphus) gordoni* Lindroth, 1963

*Bembidion (Plataphus) gratiosum* Casey, 1918

*Bembidion (Plataphus) haruspex* Casey, 1918

*Bembidion (Plataphus) hyperboraeorum* Munster, 1923**‡**

*Bembidion (Plataphus) improvidens* Casey, 1924

*Bembidion (Plataphus) kalumae* Lindroth, 1963

*Bembidion (Plataphus) kuprianovii* Mannerheim, 1843

*Bembidion (Plataphus) laxatum* Casey, 1918

*Bembidion (Plataphus) manningense* Lindroth, 1969

*Bembidion (Plataphus) neocoerulescens* Bousquet, 1993

*Bembidion (Plataphus) nigrocoeruleum* Hayward, 1897

*Bembidion (Plataphus) occultator* Notman, 1920

*Bembidion (Plataphus) oppressum* Casey, 1918

*Bembidion (Plataphus) placeranum* Casey, 1924

*Bembidion (Plataphus) planiusculum* Mannerheim, 1843

*Bembidion (Plataphus) quadrifoveolatum* Mannerheim, 1843

*Bembidion (Plataphus) rosslandicum* Lindroth, 1963

*Bembidion (Plataphus) rufinum* Lindroth, 1963

*Bembidion (Plataphus) rusticum lenensoides* Lindroth, 1963

*Bembidion (Plataphus) rusticum rusticum* Casey, 1918

*Bembidion (Plataphus) sierricola* Casey, 1924

*Bembidion (Plataphus) simplex* Hayward, 1897

*Bembidion (Plataphus) stillaguamish* Hatch, 1950

*Bembidion (Plataphus) sulcipenne hyperboroides* Lindroth, 1963

*Bembidion (Plataphus) sulcipenne prasinoides* Lindroth, 1963

*Bembidion (Plataphus) vandykei* Blaisdell, 1902

*Bembidion (Plataphus) viator* Casey, 1918

*Bembidion (Hydrium) interventor* Lindroth, 1963

*Bembidion (Hydrium) levigatum* Say, 1823

*Bembidion (Hydrium) nitidum* (Kirby, 1837)

*Bembidion (Hydrium) obliquulum* LeConte, 1859

*Bembidion (Metallina) dyschirinum* LeConte, 1861

*Bembidion (Metallina) lampros* (Herbst, 1784)**†**

*Bembidion (Metallina) properans* (Stephens, 1828)**†**

*Bembidion (Lindrochthus) wickhami* Hayward, 1897

*Bembidion (Eupetedromus) graciliforme* Hayward, 1897

*Bembidion (Eupetedromus) immaturum* Lindroth, 1954

*Bembidion (Eupetedromus) incrematum* LeConte, 1860**‡**

*Bembidion (Eupetedromus) iridipenne* Bousquet & Webster, 2006

*Bembidion (Eupetedromus) variegatum* Say, 1823

*Bembidion (Trechonepha) iridescens* (LeConte, 1852)

*Bembidion (Trechonepha) trechiforme* (LeConte, 1852)

*Bembidion (Liocosmius) festivum* Casey, 1918

*Bembidion (Liocosmius) horni* Hayward, 1897

*Bembidion (Liocosmius) mundum* (LeConte, 1852)

*Bembidion (Melomalus) planatum* (LeConte, 1847)

*Bembidion (Trichoplataphus) fugax* (LeConte, 1848)

*Bembidion (Trichoplataphus) grandiceps* Hayward, 1897

*Bembidion (Trichoplataphus) ozarkense* Maddison & Hildebrandt, 2011

*Bembidion (Trichoplataphus) planum* (Haldeman, 1843)

*Bembidion (Trichoplataphus) rolandi* Fall, 1922

*Bembidion (Phyla) obtusum* Audinet-Serville, 1821**†**

*Bembidion (Lymnaeum) laticeps* (LeConte, 1858)

*Bembidion (Lymnaeum) nigropiceum* (Marsham, 1802)**†**

*Phrypeus rickseckeri* (Hayward, 1897)

*Mioptachys flavicauda* (Say, 1823)

*Tachyta (Tachyta) angulata* Casey, 1918

*Tachyta (Tachyta) falli* (Hayward, 1900)

*Tachyta (Tachyta) inornata* (Say, 1823)

*Tachyta (Tachyta) kirbyi* Casey, 1918

*Tachyta (Tachyta) parvicornis* Notman, 1922

*Elaphropus (Barytachys) anceps* (LeConte, 1848)

*Elaphropus (Barytachys) anthrax* (LeConte, 1852)

*Elaphropus (Barytachys) brevis* (Casey, 1918)

*Elaphropus (Barytachys) brunnicollis* (Motschulsky, 1862)

*Elaphropus (Barytachys) capax* (LeConte, 1863)

*Elaphropus (Barytachys) cockerelli* (Fall, 1907)

*Elaphropus (Barytachys) congener* (Casey, 1918)

*Elaphropus (Barytachys) conjugens* (Notman, 1919)

*Elaphropus (Barytachys) dolosus* (LeConte, 1848)

*Elaphropus (Barytachys) fatuus* (Casey, 1918)

*Elaphropus (Barytachys) ferrugineus* (Dejean, 1831)

*Elaphropus (Barytachys) fuscicornis* (Chaudoir, 1868)

*Elaphropus (Barytachys) granarius* (Dejean, 1831)

*Elaphropus (Barytachys) incurvus* (Say, 1830)

*Elaphropus (Barytachys) liebecki* (Hayward, 1900)

*Elaphropus (Barytachys) monticola* (Casey, 1918)

*Elaphropus (Barytachys) nebulosus* (Chaudoir, 1868)

*Elaphropus (Barytachys) obesulus* (LeConte, 1852)

*Elaphropus (Barytachys) rapax* (LeConte, 1852)

*Elaphropus (Barytachys) renoicus* (Casey, 1918)

*Elaphropus (Barytachys) rubricauda* (Casey, 1918)

*Elaphropus (Barytachys) saturatus* (Casey, 1918)

*Elaphropus (Barytachys) sectator* (Casey, 1918)

*Elaphropus (Barytachys) sedulus* (Casey, 1918)

*Elaphropus (Barytachys) tahoensis* (Casey, 1918)

*Elaphropus (Barytachys) tripunctatus* (Say, 1830)

*Elaphropus (Barytachys) vernicatus* (Casey, 1918)

*Elaphropus (Barytachys) vivax* (LeConte, 1848)

*Elaphropus (Barytachys) xanthopus* (Dejean, 1831)

*Elaphropus (Tachyura) parvulus* (Dejean, 1831)**†**

*Micratopus aenescens* (LeConte, 1848)

*Pericompsus (Pericompsus) ephippiatus* (Say, 1830)

*Pericompsus (Pericompsus) laetulus* LeConte, 1852

*Pericompsus (Pericompsus) sellatus* LeConte, 1852

*Porotachys bisulcatus* (Nicolai, 1822)**†**

*Polyderis diaphana* (Casey, 1918)

*Polyderis laeva* (Say, 1823)

*Polyderis rufotestacea* (Hayward, 1900)

*Tachys (Tachys) bradycellinus* Hayward, 1900

*Tachys (Tachys) bryanti* Lindroth, 1966

*Tachys (Tachys) corax* LeConte, 1852

*Tachys (Tachys) halophilus* Lindroth, 1966

*Tachys (Tachys) litoralis* Casey, 1884

*Tachys (Tachys) misellus* LaFerté-Sénectère, 1841

*Tachys (Tachys) mordax* LeConte, 1852

*Tachys (Tachys) pallidus* Chaudoir, 1868

*Tachys (Tachys) pulchellus* LaFerté-Sénectère, 1841

*Tachys (Tachys) translucens* Darlington, 1937

*Tachys (Tachys) virgo* LeConte, 1852

*Tachys (Tachys) vittiger* LeConte, 1852

*Tachys (Paratachys) aeneipennis* Motschulsky, 1862

*Tachys (Paratachys) albipes* LeConte, 1863

*Tachys (Paratachys) austinicus* (Casey, 1918)

*Tachys (Paratachys) columbiensis* Hayward, 1900

*Tachys (Paratachys) edax* LeConte, 1852

*Tachys (Paratachys) hyalinus* Casey, 1918

*Tachys (Paratachys) oblitus* Casey, 1918

*Tachys (Paratachys) potomaca* (Erwin, 1981)

*Tachys (Paratachys) proximus* (Say, 1823)

*Tachys (Paratachys) pumilus* (Dejean, 1831)

*Tachys (Paratachys) rectangulus* Notman, 1919

*Tachys (Paratachys) rhodeanus* Casey, 1918

*Tachys (Paratachys) sagax* Casey, 1918

*Tachys (Paratachys) scitulus* LeConte, 1848

*Tachys (Paratachys) sequax* LeConte, 1848

*Tachys (Paratachys) spadix* Casey, 1918

*Tachys (Paratachys) umbripennis* Chaudoir, 1868

*Tachys (Paratachys) ventricosus* LeConte, 1863

*Tachys (Paratachys) vernilis* Casey, 1918

*Tachys (Paratachys) vorax* LeConte, 1852

*Anillodes debilis* (LeConte, 1853)

*Anillodes minutus* Jeannel, 1963

*Anillodes walkeri* Jeannel, 1963

*Anillinus affabilis* (Brues, 1902)

*Anillinus aleyae* Sokolov & Watrous, 2008

*Anillinus balli* Sokolov & Carlton, 2004

*Anillinus barberi* Jeannel, 1963

*Anillinus barri* Sokolov & Carlton, 2004

*Anillinus campbelli* Giachino, 2011

*Anillinus carltoni* Sokolov, 2011

*Anillinus chandleri* Sokolov, 2011

*Anillinus cherokee* Sokolov & Carlton, 2008

*Anillinus chilhowee* Sokolov, 2011

*Anillinus cieglerae* Sokolov & Carlton, 2007

*Anillinus cornelli* Sokolov & Carlton, 2004

*Anillinus daggyi* Sokolov & Carlton, 2004

*Anillinus depressus* (Jeannel, 1963)

*Anillinus docwatsoni* Sokolov & Carlton, 2004

*Anillinus dohrni* (Ehlers, 1884)

*Anillinus elongatus* Jeannel, 1963

*Anillinus erwini* Sokolov & Carlton, 2004

*Anillinus folkertsi* Sokolov & Carlton, 2004

*Anillinus fortis* (Horn, 1869)

*Anillinus gimmeli* Sokolov & Carlton, 2010

*Anillinus indianae* Jeannel, 1963

*Anillinus juliae* Sokolov & Carlton, 2010

*Anillinus kovariki* Sokolov & Carlton, 2004

*Anillinus langdoni* Sokolov & Carlton, 2004

*Anillinus lescheni* Sokolov & Carlton, 2004

*Anillinus longiceps* Jeannel, 1963

*Anillinus loweae* Sokolov & Carlton, 2004

*Anillinus magazinensis* Sokolov & Carlton, 2004

*Anillinus merritti* Sokolov & Carlton, 2010

*Anillinus moseleyae* Sokolov & Carlton, 2004

*Anillinus murrayae* Sokolov & Carlton, 2004

*Anillinus nantahala* Dajoz, 2005

*Anillinus pecki* Giachino, 2011

*Anillinus pusillus* Sokolov & Carlton, 2007

*Anillinus robisoni* Sokolov & Carlton, 2004

*Anillinus sinuaticollis* Jeannel, 1963

*Anillinus sinuatus* Jeannel, 1963

*Anillinus smokiensis* Sokolov, 2011

*Anillinus steevesi* Barr, 1995

*Anillinus stephani* Sokolov & Carlton, 2004

*Anillinus tishechkini* Sokolov & Carlton, 2004

*Anillinus turneri* Jeannel, 1963

*Anillinus unicoi* Sokolov, 2011

*Anillinus valentinei* (Jeannel, 1963)

*Anillinus virginiae* Jeannel, 1963

*Serranillus dunavani* (Jeannel, 1963)

*Serranillus jeanneli* Barr, 1995

*Serranillus septentrionis* Sokolov & Carlton, 2008

*Anillaspis caseyi* Jeannel, 1963

*Anillaspis explanata* (Horn, 1888)

*Horologion speokoites* Valentine, 1932

### Pogonini

*Thalassotrechus barbarae* (Horn, 1892)

*Diplochaetus emaciatus* (Bates, 1891)

*Diplochaetus megacephalus* Bousquet & Laplante, 1997

*Diplochaetus planatus* (Horn, 1876)

*Diplochaetus rutilus* (Chevrolat, 1863)

*Pogonus texanus* Chaudoir, 1868

### Patrobini

*Diplous (Platidius) aterrimus* (Dejean, 1828)

*Diplous (Platidius) californicus* (Motschulsky, 1844)

*Diplous (Platidius) filicornis* (Casey, 1918)

*Diplous (Platidius) rugicollis* (Randall, 1838)

*Patrobus cinctus* Motschulsky, 1860**‡**

*Patrobus fossifrons* (Eschscholtz, 1823)

*Patrobus foveocollis* (Eschscholtz, 1823)**‡**

*Patrobus lecontei* Chaudoir, 1872

*Patrobus longicornis* (Say, 1823)

*Patrobus septentrionis septentrionis* Dejean, 1828**‡**

*Patrobus stygicus* Chaudoir, 1872**‡**

*Platypatrobus lacustris* Darlington, 1938

*Platidiolus vandykei* Kurnakov, 1960

### Psydrini

*Nomius pygmaeus* (Dejean, 1831)

*Psydrus piceus* LeConte, 1846

### Metriini

*Metrius contractus contractus* Eschscholtz, 1829

*Metrius contractus planatus* Van Dyke, 1925

*Metrius contractus sericeus* Rivers, 1900

*Metrius explodens* Bousquet & Goulet, 1990

### Paussini

*Pachyteles gyllenhalii* (Dejean, 1825)

*Goniotropis kuntzeni kuntzeni* Bänninger, 1927

*Goniotropis parca* (LeConte, 1884)

*Physea hirta* LeConte, 1853

*Ozaena lemoulti* Bänninger, 1932

### Brachinini

*Brachinus (Neobrachinus) aabaaba* Erwin, 1970

*Brachinus (Neobrachinus) adustipennis* Erwin, 1969

*Brachinus (Neobrachinus) aeger* Chaudoir, 1876

*Brachinus (Neobrachinus) alexiguus* Erwin, 1970

*Brachinus (Neobrachinus) alternans* Dejean, 1825

*Brachinus (Neobrachinus) americanus* (LeConte, 1844)

*Brachinus (Neobrachinus) azureipennis* Chaudoir, 1876

*Brachinus (Neobrachinus) capnicus* Erwin, 1970

*Brachinus (Neobrachinus) cibolensis* Erwin, 1970

*Brachinus (Neobrachinus) conformis* Dejean, 1831

*Brachinus (Neobrachinus) cordicollis* Dejean, 1826

*Brachinus (Neobrachinus) costipennis* Motschulsky, 1859

*Brachinus (Neobrachinus) cyanipennis* Say, 1823

*Brachinus (Neobrachinus) cyanochroaticus* Erwin, 1969

*Brachinus (Neobrachinus) elongatulus* Chaudoir, 1876

*Brachinus (Neobrachinus) explosus* Erwin, 1970

*Brachinus (Neobrachinus) favicollis* Erwin, 1965

*Brachinus (Neobrachinus) fulminatus* Erwin, 1969

*Brachinus (Neobrachinus) fumans* (Fabricius, 1781)

*Brachinus (Neobrachinus) gebhardis* Erwin, 1965

*Brachinus (Neobrachinus) geniculatus* Dejean, 1831

*Brachinus (Neobrachinus) hirsutus* Bates, 1884

*Brachinus (Neobrachinus) ichabodopsis* Erwin, 1970

*Brachinus (Neobrachinus) imperialensis* Erwin, 1965

*Brachinus (Neobrachinus) imporcitis* Erwin, 1970

*Brachinus (Neobrachinus) janthinipennis* (Dejean, 1831)

*Brachinus (Neobrachinus) javalinopsis* Erwin, 1970

*Brachinus (Neobrachinus) kansanus* LeConte, 1863

*Brachinus (Neobrachinus) kavanaughi* Erwin, 1969

*Brachinus (Neobrachinus) lateralis* Dejean, 1831

*Brachinus (Neobrachinus) medius* Harris, 1828

*Brachinus (Neobrachinus) mexicanus* Dejean, 1831

*Brachinus (Neobrachinus) microamericanus* Erwin, 1969

*Brachinus (Neobrachinus) mobilis* Erwin, 1970

*Brachinus (Neobrachinus) neglectus* LeConte, 1844

*Brachinus (Neobrachinus) ovipennis* LeConte, 1863

*Brachinus (Neobrachinus) oxygonus* Chaudoir, 1843

*Brachinus (Neobrachinus) pallidus* Erwin, 1965

*Brachinus (Neobrachinus) patruelis* LeConte, 1844

*Brachinus (Neobrachinus) perplexus* Dejean, 1831

*Brachinus (Neobrachinus) phaeocerus* Chaudoir, 1868

*Brachinus (Neobrachinus) puberulus* Chaudoir, 1868

*Brachinus (Neobrachinus) quadripennis* Dejean, 1825

*Brachinus (Neobrachinus) rugipennis* Chaudoir, 1868

*Brachinus (Neobrachinus) sublaevis* Chaudoir, 1868

*Brachinus (Neobrachinus) tenuicollis* LeConte, 1844

*Brachinus (Neobrachinus) texanus* Chaudoir, 1868

*Brachinus (Neobrachinus) velutinus* Erwin, 1965

*Brachinus (Neobrachinus) viridipennis* Dejean, 1831

*Brachinus (Neobrachinus) vulcanoides* Erwin, 1969

### Morionini

*Morion aridus* Allen, 1969

*Morion monilicornis* (Latreille, 1805)

### Abacetini

*Loxandrus accelerans* Casey, 1918

*Loxandrus agilis* (Dejean, 1828)

*Loxandrus algidus* Allen, 1972

*Loxandrus brevicollis* (LeConte, 1846)

*Loxandrus celer* (Dejean, 1828)

*Loxandrus cervicalis* Casey, 1918

*Loxandrus cincinnati* Casey, 1924

*Loxandrus circulus* Allen, 1972

*Loxandrus collucens* Casey, 1918

*Loxandrus crenatus* LeConte, 1853

*Loxandrus duryi* Wright, 1939

*Loxandrus erraticus* (Dejean, 1828)

*Loxandrus extendus* Allen, 1972

*Loxandrus floridanus* LeConte, 1878

*Loxandrus gibbus* Allen, 1972

*Loxandrus icarus* Will & Liebherr, 1998

*Loxandrus infimus* Bates, 1882

*Loxandrus lucens* Chaudoir, 1868

*Loxandrus micans* Chaudoir, 1868

*Loxandrus minor* (Chaudoir, 1843)

*Loxandrus nitidulus* (LeConte, 1846)

*Loxandrus pactinullus* Allen, 1972

*Loxandrus parallelus* Casey, 1918

*Loxandrus parvulus* Chaudoir, 1868

*Loxandrus piceolus* Chaudoir, 1868

*Loxandrus piciventris* (LeConte, 1846)

*Loxandrus pravitubus* Allen, 1972

*Loxandrus proximus* Chaudoir, 1868

*Loxandrus pusillus* LeConte, 1853

*Loxandrus rectangulus* LeConte, 1878

*Loxandrus rectus* (Say, 1823)

*Loxandrus robustus* Allen, 1972

*Loxandrus rossi* Allen, 1972

*Loxandrus saccisecundaris* Allen, 1972

*Loxandrus saphyrinus* (Chaudoir, 1843)

*Loxandrus sculptilis* Bates, 1884

*Loxandrus spinilunatus* Allen, 1972

*Loxandrus straneoi* Will & Liebherr, 1998

*Loxandrus taeniatus* LeConte, 1853

*Loxandrus uniformis* Allen, 1972

*Loxandrus unilobus* Allen, 1972

*Loxandrus velocipes* Casey, 1918

*Loxandrus velox* (Dejean, 1828)

*Loxandrus vulneratus* Casey, 1918

*Stolonis intercepta* Chaudoir, 1874

### Pterostichini

*Abaris (Abaridius) splendidula* (LeConte, 1863)

*Hybothecus flohri* (Bates, 1882)

*Poecilus (Poecilus) chalcites* (Say, 1823)

*Poecilus (Poecilus) coloradensis* (Csiki, 1930)

*Poecilus (Poecilus) corvus* (LeConte, 1873)

*Poecilus (Poecilus) cursitor* LeConte, 1853

*Poecilus (Poecilus) cyanicolor* Chaudoir, 1876

*Poecilus (Poecilus) diplophryus* Chaudoir, 1876

*Poecilus (Poecilus) laetulus* (LeConte, 1863)

*Poecilus (Poecilus) lucublandus* (Say, 1823)

*Poecilus (Poecilus) mexicanus* Chaudoir, 1876

*Poecilus (Poecilus) occidentalis* (Dejean, 1828)

*Poecilus (Poecilus) scitulus* LeConte, 1846

*Poecilus (Poecilus) texanus* (LeConte, 1863)

*Poecilus (Derus) nearcticus* (Lindroth, 1966)**‡**

*Lophoglossus gravis* LeConte, 1873

*Lophoglossus haldemanni* (LeConte, 1846)

*Lophoglossus scrutator* (LeConte, 1846)

*Lophoglossus substrenuus* (Csiki, 1930)

*Lophoglossus tartaricus* (Say, 1823)

*Lophoglossus vernix* Casey, 1913

*Piesmus submarginatus* (Say, 1823)

*Gastrellarius blanchardi* (Horn, 1891)

*Gastrellarius honestus* (Say, 1823)

*Gastrellarius unicarum* (Darlington, 1932)

*Stomis (Neostomis) termitiformis* (Van Dyke, 1926)

*Stomis (Stomis) pumicatus* (Panzer, 1795)**†**

*Stereocerus haematopus* (Dejean, 1831)**‡**

*Stereocerus rubripes* (Motschulsky, 1860)**‡**

*Myas (Trigonognatha) coracinus* (Say, 1823)

*Myas (Trigonognatha) cyanescens* Dejean, 1828

*Pterostichus (Argutor) commutabilis* (Motschulsky, 1866)

*Pterostichus (Argutor) praetermissus* (Chaudoir, 1868)

*Pterostichus (Argutor) vernalis* (Panzer, 1795)**†**

*Pterostichus (Phonias) corrusculus* LeConte, 1873

*Pterostichus (Phonias) femoralis* (Kirby, 1837)

*Pterostichus (Phonias) patruelis* (Dejean, 1831)

*Pterostichus (Phonias) strenuus* (Panzer, 1796)**†**

*Pterostichus (Bothriopterus) adstrictus* Eschscholtz, 1823**‡**

*Pterostichus (Bothriopterus) lustrans* LeConte, 1851

*Pterostichus (Bothriopterus) mutus* (Say, 1823)

*Pterostichus (Bothriopterus) oregonus* LeConte, 1861

*Pterostichus (Bothriopterus) pensylvanicus* LeConte, 1873

*Pterostichus (Bothriopterus) trinarius* (Casey, 1918)

*Pterostichus (Melanius) castor* Goulet & Bousquet, 1983

*Pterostichus (Melanius) corvinus* (Dejean, 1828)

*Pterostichus (Melanius) ebeninus* (Dejean, 1828)

*Pterostichus (Pseudomaseus) luctuosus* (Dejean, 1828)

*Pterostichus (Pseudomaseus) tenuis* (Casey, 1924)

*Pterostichus (Feronina) barri* Bousquet, 2006

*Pterostichus (Feronina) palmi* Schaeffer, 1910

*Pterostichus (Paraferonia) lubricus* LeConte, 1853

*Pterostichus (Pseudoferonina) amadeus* Bousquet, nomen novum

*Pterostichus (Pseudoferonina) bousqueti* Bergdahl, 2011

*Pterostichus (Pseudoferonina) campbelli* Bousquet, 1985

*Pterostichus (Pseudoferonina) humidulus* (Van Dyke, 1943)

*Pterostichus (Pseudoferonina) lanei* Van Dyke, 1926

*Pterostichus (Pseudoferonina) lolo* Bergdahl, 2011

*Pterostichus (Pseudoferonina) shulli* (Hatch, 1949)

*Pterostichus (Pseudoferonina) smetanai* Bousquet, 1985

*Pterostichus (Pseudoferonina) spathifer* Bousquet, 1992

*Pterostichus (Gastrosticta) enodis* Bousquet, 1992

*Pterostichus (Gastrosticta) mutoides* Bousquet, 1992

*Pterostichus (Gastrosticta) obesulus* LeConte, 1873

*Pterostichus (Gastrosticta) ophryoderus* (Chaudoir, 1878)

*Pterostichus (Gastrosticta) punctiventris* (Chaudoir, 1878)

*Pterostichus (Gastrosticta) putus* Casey, 1913

*Pterostichus (Gastrosticta) sayanus* Csiki, 1930

*Pterostichus (Gastrosticta) subacutus* (Casey, 1918)

*Pterostichus (Gastrosticta) tumescens* LeConte, 1863

*Pterostichus (Gastrosticta) ventralis* (Say, 1823)

*Pterostichus (Morphnosoma) melanarius melanarius* (Illiger, 1798)**†**

*Pterostichus (Euferonia) coracinus* (Newman, 1838)

*Pterostichus (Euferonia) ingens* (Casey, 1918)

*Pterostichus (Euferonia) lachrymosus* (Newman, 1838)

*Pterostichus (Euferonia) novus* Straneo, 1944

*Pterostichus (Euferonia) relictus* (Newman, 1838)

*Pterostichus (Euferonia) stygicus* (Say, 1823)

*Pterostichus (Lenapterus) agonus* Horn, 1880**‡**

*Pterostichus (Lenapterus) costatus* (Ménétriés, 1851)**‡**

*Pterostichus (Lenapterus) punctatissimus* (Randall, 1838)

*Pterostichus (Lenapterus) vermiculosus* (Ménétriés, 1851)**‡**

*Pterostichus (Metallophilus) sublaevis* (Sahlberg, 1880)**‡**

*Pterostichus (Abacidus) atratus* (Newman, 1838)

*Pterostichus (Abacidus) fallax* (Dejean, 1828)

*Pterostichus (Abacidus) hamiltoni* Horn, 1880

*Pterostichus (Abacidus) permundus* (Say, 1830)

*Pterostichus (Abacidus) sculptus* LeConte, 1853

*Pterostichus (Orsonjohnsonus) johnsoni* Ulke, 1889

*Pterostichus (Lamenius) caudicalis* (Say, 1823)

*Pterostichus (Eosteropus) circulosus* Lindroth, 1966

*Pterostichus (Eosteropus) moestus* (Say, 1823)

*Pterostichus (Eosteropus) superciliosus* (Say, 1823)

*Pterostichus (Monoferonia) carolinus carolinus* Darlington, 1932

*Pterostichus (Monoferonia) carolinus fumorum* Darlington, 1932

*Pterostichus (Monoferonia) diligendus* (Chaudoir, 1868)

*Pterostichus (Monoferonia) mancus* (LeConte, 1853)

*Pterostichus (Monoferonia) primus* Darlington, 1932

*Pterostichus (Cylindrocharis) acutipes acutipes* Barr, 1971

*Pterostichus (Cylindrocharis) acutipes kentuckensis* Barr, 1971

*Pterostichus (Cylindrocharis) hypogeus* Barr, 1971

*Pterostichus (Cylindrocharis) rostratus* (Newman, 1838)

*Pterostichus (Leptoferonia) angustus* (Dejean, 1828)

*Pterostichus (Leptoferonia) beyeri* Van Dyke, 1926

*Pterostichus (Leptoferonia) blodgettensis* Will, 2007

*Pterostichus (Leptoferonia) caligans* Horn, 1891

*Pterostichus (Leptoferonia) cochlearis* Hacker, 1968

*Pterostichus (Leptoferonia) deino* Will, 2007

*Pterostichus (Leptoferonia) enyo* Will, 2007

*Pterostichus (Leptoferonia) falli* Van Dyke, 1926

*Pterostichus (Leptoferonia) fenyesi fenderi* Hacker, 1968

*Pterostichus (Leptoferonia) fenyesi fenyesi* Csiki, 1930

*Pterostichus (Leptoferonia) fuchsi* Schaeffer, 1910

*Pterostichus (Leptoferonia) hatchi* Hacker, 1968

*Pterostichus (Leptoferonia) humilis* Casey, 1913

*Pterostichus (Leptoferonia) idahoae* Csiki, 1930

*Pterostichus (Leptoferonia) inanis* Horn, 1891

*Pterostichus (Leptoferonia) infernalis* Hatch, 1936

*Pterostichus (Leptoferonia) inopinus* (Casey, 1918)

*Pterostichus (Leptoferonia) lobatus* Hacker, 1968

*Pterostichus (Leptoferonia) marinensis* Hacker, 1968

*Pterostichus (Leptoferonia) mattolensis* Hacker, 1968

*Pterostichus (Leptoferonia) pemphredo* Will, 2007

*Pterostichus (Leptoferonia) pumilus pumilus* Casey, 1913

*Pterostichus (Leptoferonia) pumilus willamettensis* Hacker, 1968

*Pterostichus (Leptoferonia) rothi* (Hatch, 1951)

*Pterostichus (Leptoferonia) sphodrinus* LeConte, 1863

*Pterostichus (Leptoferonia) stapedius* Hacker, 1968

*Pterostichus (Leptoferonia) trinitensis* Hacker, 1968

*Pterostichus (Leptoferonia) yosemitensis* Hacker, 1968

*Pterostichus (Anilloferonia) lanei* (Hatch, 1935)

*Pterostichus (Anilloferonia) malkini* (Hatch, 1953)

*Pterostichus (Anilloferonia) testaceus* (Van Dyke, 1926)

*Pterostichus (Hypherpes) adoxus* (Say, 1823)

*Pterostichus (Hypherpes) algidus* LeConte, 1853

*Pterostichus (Hypherpes) amethystinus* Mannerheim, 1843

*Pterostichus (Hypherpes) annosus* Casey, 1913

*Pterostichus (Hypherpes) arcanus* Casey, 1913

*Pterostichus (Hypherpes) baldwini* (Casey, 1924)

*Pterostichus (Hypherpes) barbarinus* Casey, 1913

*Pterostichus (Hypherpes) brachylobus* Kavanaugh & LaBonte, 2006

*Pterostichus (Hypherpes) californicus* (Dejean, 1828)

*Pterostichus (Hypherpes) canallatus* Casey, 1913

*Pterostichus (Hypherpes) castaneus* (Dejean, 1828)

*Pterostichus (Hypherpes) castanipes* (Ménétriés, 1843)

*Pterostichus (Hypherpes) congestus* (Ménétriés, 1843)

*Pterostichus (Hypherpes) craterensis* (Hatch, 1949)

*Pterostichus (Hypherpes) crenicollis* LeConte, 1873

*Pterostichus (Hypherpes) ecarinatus* Hatch, 1936

*Pterostichus (Hypherpes) esuriens* Casey, 1913

*Pterostichus (Hypherpes) gliscans* Casey, 1913

*Pterostichus (Hypherpes) gracilior* LeConte, 1873

*Pterostichus (Hypherpes) herculaneus* Mannerheim, 1843

*Pterostichus (Hypherpes) hornii* LeConte, 1873

*Pterostichus (Hypherpes) illustris* LeConte, 1851

*Pterostichus (Hypherpes) inermis* Fall, 1901

*Pterostichus (Hypherpes) isabellae* LeConte, 1851

*Pterostichus (Hypherpes) jacobinus* Casey, 1913

*Pterostichus (Hypherpes) laborans* Casey, 1913

*Pterostichus (Hypherpes) lacertus* Casey, 1913

*Pterostichus (Hypherpes) lama* (Ménétriés, 1843)

*Pterostichus (Hypherpes) lassulus* (Casey, 1920)

*Pterostichus (Hypherpes) lattini* LaBonte, 2006

*Pterostichus (Hypherpes) luscus* (Casey, 1918)

*Pterostichus (Hypherpes) menetriesii* LeConte, 1873

*Pterostichus (Hypherpes) mercedianus* (Casey, 1918)

*Pterostichus (Hypherpes) miscellus* Casey, 1913

*Pterostichus (Hypherpes) morionides* (Chaudoir, 1868)

*Pterostichus (Hypherpes) neobrunneus* Lindroth, 1966

*Pterostichus (Hypherpes) nigrocaeruleus* Van Dyke, 1926

*Pterostichus (Hypherpes) obsidianus* Casey, 1913

*Pterostichus (Hypherpes) occultus* Casey, 1913

*Pterostichus (Hypherpes) ordinarius* Casey, 1913

*Pterostichus (Hypherpes) ovalipennis* Casey, 1913

*Pterostichus (Hypherpes) panticulatus* Casey, 1913

*Pterostichus (Hypherpes) pergracilis* (Casey, 1920)

*Pterostichus (Hypherpes) planctus* LeConte, 1853

*Pterostichus (Hypherpes) protensiformis* (Casey, 1924)

*Pterostichus (Hypherpes) protractus* LeConte, 1860

*Pterostichus (Hypherpes) restrictus* (Casey, 1918)

*Pterostichus (Hypherpes) scutellaris* LeConte, 1873

*Pterostichus (Hypherpes) serripes* (LeConte, 1875)

*Pterostichus (Hypherpes) setosus* Hatch, 1951

*Pterostichus (Hypherpes) sierranus* Casey, 1913

*Pterostichus (Hypherpes) sponsor* Casey, 1913

*Pterostichus (Hypherpes) spraguei* LeConte, 1873

*Pterostichus (Hypherpes) suffusus* Casey, 1913

*Pterostichus (Hypherpes) tarsalis* LeConte, 1873

*Pterostichus (Hypherpes) tristis* (Dejean, 1828)

*Pterostichus (Hypherpes) tuberculofemoratus* Hatch, 1936

*Pterostichus (Hypherpes) vandykei* Schaeffer, 1910

*Pterostichus (Hypherpes) vicinus* Mannerheim, 1843

*Pterostichus (Hypherpes) ybousqueti* Berlov, 1999

*Pterostichus (Cryobius) arcticola* (Chaudoir, 1868)

*Pterostichus (Cryobius) auriga* Ball, 1962

*Pterostichus (Cryobius) barryorum* Ball, 1962

*Pterostichus (Cryobius) brevicornis brevicornis* (Kirby, 1837)**‡**

*Pterostichus (Cryobius) bryanti biocryus* Ball, 1962

*Pterostichus (Cryobius) bryanti bryanti* (Van Dyke, 1951)

*Pterostichus (Cryobius) bryanti bryantoides* Ball, 1962

*Pterostichus (Cryobius) bryanti cacumenis* Ball, 1966

*Pterostichus (Cryobius) bryanti stantonensis* Ball, 1966

*Pterostichus (Cryobius) bryanti tiliaceoradix* Ball, 1962

*Pterostichus (Cryobius) caribou* Ball, 1962

*Pterostichus (Cryobius) chipewyan* Ball, 1962

*Pterostichus (Cryobius) empetricola* (Dejean, 1828)**‡**

*Pterostichus (Cryobius) gerstlensis* Ball, 1962

*Pterostichus (Cryobius) hudsonicus* LeConte, 1863

*Pterostichus (Cryobius) kotzebuei* Ball, 1962

*Pterostichus (Cryobius) mandibularoides* Ball, 1966

*Pterostichus (Cryobius) nivalis* (Sahlberg, 1844)**‡**

*Pterostichus (Cryobius) parasimilis* Ball, 1962**‡**

*Pterostichus (Cryobius) pinguedineus* (Eschscholtz, 1823)**‡**

*Pterostichus (Cryobius) planus* (Sahlberg, 1885)

*Pterostichus (Cryobius) riparius* (Dejean, 1828)

*Pterostichus (Cryobius) similis* Mannerheim, 1852**‡**

*Pterostichus (Cryobius) soperi* Ball, 1966

*Pterostichus (Cryobius) surgens* LeConte, 1878

*Pterostichus (Cryobius) tareumiut* Ball, 1962**‡**

*Pterostichus (Cryobius) ventricosus ventricosus* (Eschscholtz, 1823)**‡**

*Pterostichus (Cryobius) woodi* Ball & Currie, 1997

*Cyclotrachelus (Cyclotrachelus) alabamensis* (Casey, 1920)

*Cyclotrachelus (Cyclotrachelus) approximatus* (LeConte, 1846)

*Cyclotrachelus (Cyclotrachelus) brevoorti* (LeConte, 1846)

*Cyclotrachelus (Cyclotrachelus) dejeanellus* (Csiki, 1930)

*Cyclotrachelus (Cyclotrachelus) faber* (Germar, 1824)

*Cyclotrachelus (Cyclotrachelus) freitagi* Bousquet, 1993

*Cyclotrachelus (Cyclotrachelus) fucatus* (Freitag, 1969)

*Cyclotrachelus (Cyclotrachelus) hernandensis* (Van Dyke, 1943)

*Cyclotrachelus (Cyclotrachelus) iuvenis* (Freitag, 1969)

*Cyclotrachelus (Cyclotrachelus) laevipennis* (LeConte, 1846)

*Cyclotrachelus (Cyclotrachelus) levifaber* (Freitag, 1969)

*Cyclotrachelus (Cyclotrachelus) macrovulum* (Freitag, 1969)

*Cyclotrachelus (Cyclotrachelus) ovulum* (Chaudoir, 1868)

*Cyclotrachelus (Cyclotrachelus) parafaber* (Freitag, 1969)

*Cyclotrachelus (Cyclotrachelus) spoliatus* (Newman, 1838)

*Cyclotrachelus (Cyclotrachelus) texensis* (Freitag, 1969)

*Cyclotrachelus (Cyclotrachelus) unicolor* (Say, 1823)

*Cyclotrachelus (Cyclotrachelus) vinctus* (LeConte, 1853)

*Cyclotrachelus (Evarthrus) alabamae* (Van Dyke, 1926)

*Cyclotrachelus (Evarthrus) alternans* (Casey, 1920)

*Cyclotrachelus (Evarthrus) blatchleyi* (Casey, 1918)

*Cyclotrachelus (Evarthrus) constrictus* (Say, 1823)

*Cyclotrachelus (Evarthrus) convivus* (LeConte, 1853)

*Cyclotrachelus (Evarthrus) deceptus* (Casey, 1918)

*Cyclotrachelus (Evarthrus) engelmani* (LeConte, 1853)

*Cyclotrachelus (Evarthrus) floridensis* (Freitag, 1969)

*Cyclotrachelus (Evarthrus) furtivus* (LeConte, 1853)

*Cyclotrachelus (Evarthrus) gigas* (Casey, 1918)

*Cyclotrachelus (Evarthrus) gravesi* (Freitag, 1969)

*Cyclotrachelus (Evarthrus) gravidus* (Haldeman, 1853)

*Cyclotrachelus (Evarthrus) heros* (Say, 1823)

*Cyclotrachelus (Evarthrus) hypherpiformis* (Freitag, 1969)

*Cyclotrachelus (Evarthrus) incisus* (LeConte, 1846)

*Cyclotrachelus (Evarthrus) iowensis* (Freitag, 1969)

*Cyclotrachelus (Evarthrus) lodingi* (Van Dyke, 1926)

*Cyclotrachelus (Evarthrus) nonnitens* (LeConte, 1873)

*Cyclotrachelus (Evarthrus) parasodalis* (Freitag, 1969)

*Cyclotrachelus (Evarthrus) sallei* (LeConte, 1873)

*Cyclotrachelus (Evarthrus) seximpressus* (LeConte, 1846)

*Cyclotrachelus (Evarthrus) sigillatus* (Say, 1823)

*Cyclotrachelus (Evarthrus) sinus* (Freitag, 1969)

*Cyclotrachelus (Evarthrus) sodalis colossus* (LeConte, 1846)

*Cyclotrachelus (Evarthrus) sodalis sodalis* (LeConte, 1846)

*Cyclotrachelus (Evarthrus) substriatus* (LeConte, 1846)

*Cyclotrachelus (Evarthrus) torvus* (LeConte, 1863)

*Cyclotrachelus (Evarthrus) whitcombi* (Freitag, 1969)

*Abax (Abax) parallelepipedus* (Piller & Mitterpacher, 1783)**†**

### Zabrini

*Amara (Curtonotus) alpina* (Paykull, l790)**‡**

*Amara (Curtonotus) aulica* (Panzer, 1796)**†**

*Amara (Curtonotus) blanchardi* Hayward, 1908

*Amara (Curtonotus) bokori* Csiki, 1929**‡**

*Amara (Curtonotus) carinata* (LeConte, 1847)

*Amara (Curtonotus) daurica* (Motschulsky, 1844)**‡**

*Amara (Curtonotus) deparca* (Say, 1830)

*Amara (Curtonotus) hyperborea* Dejean, 1831**‡**

*Amara (Curtonotus) jacobina* LeConte, 1855

*Amara (Curtonotus) kurnakowi* Hieke, 1994**‡**

*Amara (Curtonotus) lacustris* LeConte, 1855

*Amara (Curtonotus) pennsylvanica* Hayward, 1908

*Amara (Curtonotus) pterostichina* Hayward, 1908

*Amara (Curtonotus) thoracica* Hayward, 1908

*Amara (Curtonotus) torrida* (Panzer, 1796)**‡**

*Amara (Bradytus) apricaria* (Paykull, 1790)**†**

*Amara (Bradytus) avida* (Say, 1823)

*Amara (Bradytus) browni* Lindroth, 1968

*Amara (Bradytus) exarata* Dejean, 1828

*Amara (Bradytus) fulva* (Müller, 1776)**†**

*Amara (Bradytus) glacialis* (Mannerheim, 1853)**‡**

*Amara (Bradytus) insignis* Dejean, 1831

*Amara (Bradytus) insularis* Horn, 1875

*Amara (Bradytus) latior* (Kirby, 1837)

*Amara (Bradytus) lindrothi* Hieke, 1990

*Amara (Bradytus) neomexicana* (Casey, 1924)

*Amara (Bradytus) schwarzi* Hayward, 1908

*Amara (Neopercosia) fortis* LeConte, 1880

*Amara (Percosia) obesa* (Say, 1823)

*Amara (Xenocelia) apachensis* Casey, 1884

*Amara (Xenocelia) bradytonota* Hieke, 2001

*Amara (Xenocelia) chalcea* Dejean, 1828

*Amara (Xenocelia) discors* Kirby, 1837

*Amara (Xenocelia) gibba* (LeConte, 1847)

*Amara (Xenocelia) harpalonota* Hieke, 2001

*Amara (Xenocelia) hicksi* Lindroth, 1968**‡**

*Amara (Xenocelia) lugubris* (Casey, 1918)

*Amara (Xenocelia) merula* (Casey, 1918)

*Amara (Xenocelia) rectangula ciudadensis* (Bates, 1891)

*Amara (Xenocelia) rectangula rectangula* LeConte, 1855

*Amara (Xenocelia) spuria* Lindroth, 1968

*Amara (Reductocelia) colvillensis* Lindroth, 1968**‡**

*Amara (Celia) bifrons* (Gyllenhal, 1810)**†**

*Amara (Celia) brunnea* (Gyllenhal, 1810)**‡**

*Amara (Celia) californica californica* Dejean, 1828

*Amara (Celia) exlineae* Minsk & Hatch, 1939

*Amara (Celia) harpalina* LeConte, 1855

*Amara (Celia) idahoana* (Casey, 1924)

*Amara (Celia) musculis* (Say, 1823)

*Amara (Celia) pseudobrunnea* Lindroth, 1968

*Amara (Celia) rubrica* Haldeman, 1843

*Amara (Celia) sinuosa* (Casey, 1918)

*Amara (Celia) texana* (Putzeys, 1866)

*Amara (Celia) volatilis* (Casey, 1918)

*Amara (Amarocelia) ellipsis* (Casey, 1918)

*Amara (Amarocelia) erratica* (Duftschmid, 1812)**‡**

*Amara (Amarocelia) farcta* LeConte, 1855

*Amara (Amarocelia) interstitialis* Dejean, 1828**‡**

*Amara (Amarocelia) laevipennis* Kirby, 1837

*Amara (Amarocelia) lugens* Zimmermann, 1832

*Amara (Amarocelia) nexa* (Casey, 1918)

*Amara (Amarocelia) patruelis* Dejean, 1831

*Amara (Amarocelia) rugulifera* Hieke, 2002

*Amara (Amarocelia) sodalicia* Casey, 1924

*Amara (Amarocelia) tenebrionella* (Bates, 1882)

*Amara (Amarocelia) transberingiensis* Hieke, 2002**‡**

*Amara (Amara) aenea* (DeGeer, 1774)**†**

*Amara (Amara) aeneopolita* Casey, 1918

*Amara (Amara) anthobia* Villa & Villa, 1833**†**

*Amara (Amara) aurata* Dejean, 1828

*Amara (Amara) basillaris* (Say, 1823)

*Amara (Amara) coelebs* Hayward, 1908

*Amara (Amara) communis* (Panzer, 1797)**†**

*Amara (Amara) conflata* LeConte, 1855

*Amara (Amara) confusa* LeConte, 1847

*Amara (Amara) convexa* LeConte, 1847

*Amara (Amara) crassispina* LeConte, 1855

*Amara (Amara) cupreolata* Putzeys, 1866

*Amara (Amara) emancipata* Lindroth, 1968

*Amara (Amara) eurynota* (Panzer, 1796)**†**

*Amara (Amara) externefoveata* Hieke, 2002

*Amara (Amara) familiaris* (Duftschmid, 1812)**†**

*Amara (Amara) haywardi* Csiki, 1929

*Amara (Amara) impuncticollis* (Say, 1823)

*Amara (Amara) littoralis* Dejean, 1828**‡**

*Amara (Amara) lunicollis* Schiødte, 1837**‡**

*Amara (Amara) neoscotica* Casey, 1924

*Amara (Amara) occidentalis* Hieke, 2002

*Amara (Amara) otiosa* Casey, 1918

*Amara (Amara) ovata* (Fabricius, 1792)**†**

*Amara (Amara) pomona* Casey, 1918

*Amara (Amara) sanjuanensis* Hatch, 1949

*Amara (Amara) sera* Say, 1830

*Amara (Amara) tenax* Casey, 1918

*Amara (Amara) turbata* Casey, 1918

*Amara (Paracelia) quenseli quenseli* (Schönherr, 1806)**‡**

*Amara (Zezea) angustata* (Say, 1823)

*Amara (Zezea) angustatoides* Hieke, 2000

*Amara (Zezea) belfragei* Horn, 1892

*Amara (Zezea) flebilis* (Casey, 1918)

*Amara (Zezea) inexspectata* Hieke, 1990

*Amara (Zezea) kavanaughi* Hieke, 1990

*Amara (Zezea) longula* LeConte, 1855

*Amara (Zezea) pallipes* Kirby, 1837

*Amara (Zezea) scitula* Zimmermann, 1832

### Oodini

*Dercylinus impressus* (LeConte, 1853)

*Evolenes exarata* (Dejean, 1831)

*Anatrichis minuta* (Dejean, 1831)

*Anatrichis oblonga* Horn, 1891

*Oodinus alutaceus* (Bates, 1882)

*Oodinus pseudopiceus* Bousquet, 1996

*Lachnocrepis parallela* (Say, 1830)

*Oodes amaroides* Dejean, 1831

*Oodes americanus* Dejean, 1826

*Oodes brevis* Lindroth, 1957

*Oodes fluvialis* LeConte, 1863

*Stenocrepis (Stenocrepis) insulana* (Jacquelin du Val, 1857)

*Stenocrepis (Stenous) cuprea* (Chaudoir, 1843)

*Stenocrepis (Stenous) duodecimstriata* (Chevrolat, 1836)

*Stenocrepis (Stenous) elegans* (LeConte, 1851)

*Stenocrepis (Stenous) mexicana* (Chevrolat, 1835)

*Stenocrepis (Stenous) tibialis* (Chevrolat, 1834)

### Panagaeini

*Panagaeus (Hologaeus) cruciger* Say, 1823

*Panagaeus (Hologaeus) fasciatus* Say, 1823

*Panagaeus (Hologaeus) sallei* Chaudoir, 1862

*Micrixys distincta* (Haldeman, 1852)

### Chlaeniini

*Chlaenius (Pseudanomoglossus) maxillosus* Horn, 1876

*Chlaenius (Eurydactylus) pimalicus* Casey, 1914

*Chlaenius (Eurydactylus) tomentosus* (Say, 1823)

*Chlaenius (Anomoglossus) amoenus* Dejean, 1831

*Chlaenius (Anomoglossus) emarginatus* Say, 1823

*Chlaenius (Anomoglossus) pusillus* Say, 1823

*Chlaenius (Chlaenius) aestivus* Say, 1823

*Chlaenius (Chlaenius) augustus* Newman, 1838

*Chlaenius (Chlaenius) azurescens* Chaudoir, 1876

*Chlaenius (Chlaenius) chaudoiri* Horn, 1876

*Chlaenius (Chlaenius) cumatilis* LeConte, 1851

*Chlaenius (Chlaenius) erythropus* Germar, 1824

*Chlaenius (Chlaenius) fuscicornis* Dejean, 1831

*Chlaenius (Chlaenius) laticollis* Say, 1823

*Chlaenius (Chlaenius) orbus* Horn, 1871

*Chlaenius (Chlaenius) patruelis* LeConte, 1844

*Chlaenius (Chlaenius) platyderus* Chaudoir, 1856

*Chlaenius (Chlaenius) sericeus* (Forster, 1771)

*Chlaenius (Chlaenius) sparsus* LeConte, 1863

*Chlaenius (Chlaenius) viduus* Horn, 1871

*Chlaenius (Lithochlaenius) cordicollis* Kirby, 1837

*Chlaenius (Lithochlaenius) leucoscelis monachus* LeConte, 1851

*Chlaenius (Lithochlaenius) leucoscelis sanantonialis* Casey, 1914

*Chlaenius (Lithochlaenius) leucoscelis sonomae* Casey, 1920

*Chlaenius (Lithochlaenius) prasinus* Dejean, 1826

*Chlaenius (Lithochlaenius) purpureus* Chaudoir, 1876

*Chlaenius (Lithochlaenius) solitarius* Say, 1823

*Chlaenius (Chlaeniellus) brevilabris* LeConte, 1847

*Chlaenius (Chlaeniellus) circumcinctus* Say, 1830

*Chlaenius (Chlaeniellus) flaccidus* Horn, 1876

*Chlaenius (Chlaeniellus) floridanus* Horn, 1876

*Chlaenius (Chlaeniellus) glaucus* LeConte, 1856

*Chlaenius (Chlaeniellus) impunctifrons* Say, 1823

*Chlaenius (Chlaeniellus) nebraskensis* LeConte, 1856

*Chlaenius (Chlaeniellus) nemoralis* Say, 1823

*Chlaenius (Chlaeniellus) obsoletus* LeConte, 1851

*Chlaenius (Chlaeniellus) oxygonus* Chaudoir, 1843

*Chlaenius (Chlaeniellus) pennsylvanicus blanditus* Casey, 1920

*Chlaenius (Chlaeniellus) pennsylvanicus pennsylvanicus* Say, 1823

*Chlaenius (Chlaeniellus) pertinax* Casey, 1920

*Chlaenius (Chlaeniellus) simillimus* Chaudoir, 1856

*Chlaenius (Chlaeniellus) texanus* Horn, 1876

*Chlaenius (Chlaeniellus) tricolor tricolor* Dejean, 1826

*Chlaenius (Chlaeniellus) tricolor vigilans* Say, 1830

*Chlaenius (Chlaeniellus) vafer* LeConte, 1852

*Chlaenius (Chlaeniellus) variabilipes* Eschscholtz, 1833

*Chlaenius (Callistometus) ruficauda* Chaudoir, 1856

*Chlaenius (Brachylobus) caurinus* (Horn, 1885)

*Chlaenius (Brachylobus) lithophilus* Say, 1823

*Chlaenius (Agostenus) alternatus* Horn, 1871

*Chlaenius (Agostenus) caeruleicollis* Chaudoir, 1876

*Chlaenius (Agostenus) harpalinus* Eschscholtz, 1833

*Chlaenius (Agostenus) interruptus* Horn, 1876

*Chlaenius (Agostenus) niger* Randall, 1838

*Chlaenius (Randallius) purpuricollis* Randall, 1838

### Licinini

*Diplocheila (Isorembus) assimilis* (LeConte, 1844)

*Diplocheila (Isorembus) crossi* Will, 1998

*Diplocheila (Isorembus) impressicollis* (Dejean, 1831)

*Diplocheila (Isorembus) major major* (LeConte, 1847)

*Diplocheila (Isorembus) major melissisa* Ball, 1959

*Diplocheila (Isorembus) nupera* Casey, 1897

*Diplocheila (Isorembus) obtusa* (LeConte, 1847)

*Diplocheila (Isorembus) oregona* (Hatch, 1951)

*Diplocheila (Isorembus) striatopunctata* (LeConte, 1844)

*Diplocheila (Isorembus) undulata* Carr, 1920

*Dicaelus (Paradicaelus) ambiguus* LaFerté-Sénectère, 1841

*Dicaelus (Paradicaelus) dilatatus dilatatus* Say, 1823

*Dicaelus (Paradicaelus) dilatatus sinuatus* Ball, 1959

*Dicaelus (Paradicaelus) elongatus* Bonelli, 1813

*Dicaelus (Paradicaelus) furvus carinatus* Dejean, 1831

*Dicaelus (Paradicaelus) furvus furvus* Dejean, 1826

*Dicaelus (Paradicaelus) politus* Dejean, 1826

*Dicaelus (Paradicaelus) sculptilis intricatus* LeConte, 1873

*Dicaelus (Paradicaelus) sculptilis sculptilis* Say, 1823

*Dicaelus (Paradicaelus) sculptilis upioides* Ball, 1959

*Dicaelus (Paradicaelus) teter* Bonelli, 1813

*Dicaelus (Dicaelus) alternans* Dejean, 1826

*Dicaelus (Dicaelus) costatus* LeConte, 1853

*Dicaelus (Dicaelus) crenatus* LeConte, 1853

*Dicaelus (Dicaelus) purpuratus purpuratus* Bonelli, 1813

*Dicaelus (Dicaelus) purpuratus splendidus* Say, 1823

*Dicaelus (Dicaelus) quadratus* LeConte, 1847

*Dicaelus (Dicaelus) subtropicus* Casey, 1913

*Dicaelus (Liodicaelus) chermocki* Ball, 1959

*Dicaelus (Liodicaelus) laevipennis laevipennis* LeConte, 1847

*Dicaelus (Liodicaelus) suffusus* (Casey, 1913)

*Badister (Badister) elegans* LeConte, 1880

*Badister (Badister) ferrugineus* Dejean, 1831

*Badister (Badister) flavipes flavipes* LeConte, 1853

*Badister (Badister) maculatus* LeConte, 1853

*Badister (Badister) neopulchellus* Lindroth, 1954

*Badister (Badister) notatus* Haldeman, 1843

*Badister (Badister) obtusus* LeConte, 1878

*Badister (Badister) pulchellus* LeConte, 1847

*Badister (Baudia) grandiceps* Casey, 1920

*Badister (Baudia) micans* LeConte, 1844

*Badister (Baudia) parviceps* Ball, 1959

*Badister (Baudia) reflexus* LeConte, 1880

*Badister (Baudia) submarinus* Motschulsky, 1859

*Badister (Baudia) transversus* Casey, 1920

### Harpalini

*Notiobia (Anisotarsus) brevicollis* (Chaudoir, 1837)

*Notiobia (Anisotarsus) cephala* (Casey, 1914)

*Notiobia (Anisotarsus) maculicornis* (Chaudoir, 1843)

*Notiobia (Anisotarsus) mexicana* (Dejean, 1829)

*Notiobia (Anisotarsus) nitidipennis* (LeConte, 1847)

*Notiobia (Anisotarsus) purpurascens* (Bates, 1882)

*Notiobia (Anisotarsus) sayi* (Blatchley, 1910)

*Notiobia (Anisotarsus) terminata* (Say, 1823)

*Xestonotus lugubris* (Dejean, 1829)

*Anisodactylus (Anisodactylus) agricola* (Say, 1823)

*Anisodactylus (Anisodactylus) binotatus* (Fabricius, 1787)**†**

*Anisodactylus (Anisodactylus) californicus* Dejean, 1829

*Anisodactylus (Anisodactylus) carbonarius* (Say, 1823)

*Anisodactylus (Anisodactylus) consobrinus* LeConte, 1851

*Anisodactylus (Anisodactylus) furvus* LeConte, 1863

*Anisodactylus (Anisodactylus) harrisii* LeConte, 1863

*Anisodactylus (Anisodactylus) kirbyi* Lindroth, 1953

*Anisodactylus (Anisodactylus) lodingi* Schaeffer, 1911

*Anisodactylus (Anisodactylus) melanopus* (Haldeman, 1843)

*Anisodactylus (Anisodactylus) nigerrimus* (Dejean, 1831)

*Anisodactylus (Anisodactylus) nigrita* Dejean, 1829

*Anisodactylus (Anisodactylus) pseudagricola* Noonan, 1996

*Anisodactylus (Anisodactylus) similis* LeConte, 1851

*Anisodactylus (Gynandrotarsus) anthracinus* (Dejean, 1829)

*Anisodactylus (Gynandrotarsus) dulcicollis* (LaFerté-Sénectère, 1841)

*Anisodactylus (Gynandrotarsus) haplomus* Chaudoir, 1868

*Anisodactylus (Gynandrotarsus) harpaloides* (LaFerté-Sénectère, 1841)

*Anisodactylus (Gynandrotarsus) merula* (Germar, 1824)

*Anisodactylus (Gynandrotarsus) opaculus* (LeConte, 1863)

*Anisodactylus (Gynandrotarsus) ovularis* (Casey, 1914)

*Anisodactylus (Gynandrotarsus) rusticus* (Say, 1823)

*Anisodactylus (Gynandrotarsus) texanus* Schaeffer, 1910

*Anisodactylus (Anadaptus) alternans* (Motschulsky, 1845)

*Anisodactylus (Anadaptus) discoideus* Dejean, 1831

*Anisodactylus (Anadaptus) pitychrous* LeConte, 1861

*Anisodactylus (Anadaptus) porosus* (Motschulsky, 1845)

*Anisodactylus (Anadaptus) rudis* LeConte, 1863

*Anisodactylus (Anadaptus) sanctaecrucis* (Fabricius, 1798)

*Anisodactylus (Spongopus) verticalis* (LeConte, 1847)

*Anisodactylus (Aplocentrus) amaroides* LeConte, 1851

*Anisodactylus (Aplocentrus) caenus* (Say, 1823)

*Anisodactylus (Pseudaplocentrus) laetus* Dejean, 1829

*Geopinus incrassatus* (Dejean, 1829)

*Amphasia (Pseudamphasia) sericea* (Harris, 1828)

*Amphasia (Amphasia) interstitialis* (Say, 1823)

*Dicheirus brunneus* (Dejean, 1829)

*Dicheirus dilatatus angulatus* Casey, 1914

*Dicheirus dilatatus dilatatus* (Dejean, 1829)

*Dicheirus obtusus* LeConte, 1852

*Dicheirus piceus* (Ménétriés, 1843)

*Dicheirus strenuus* (Horn, 1869)

*Pelmatellus (Pelmatellus) obtusus* Bates, 1882

*Pelmatellus (Pelmatellus) stenolophoides parallelus* Goulet, 1974

*Stenolophus (Stenolophus) anceps* LeConte, 1857

*Stenolophus (Stenolophus) carbo* Bousquet, 1993

*Stenolophus (Stenolophus) cincticollis* LeConte, 1858

*Stenolophus (Stenolophus) dissimilis* Dejean, 1829

*Stenolophus (Stenolophus) flavipes* LeConte, 1858

*Stenolophus (Stenolophus) fuliginosus* Dejean, 1829

*Stenolophus (Stenolophus) fuscatus* Dejean, 1829

*Stenolophus (Stenolophus) humidus* Hamilton, 1893

*Stenolophus (Stenolophus) incultus* Casey, 1914

*Stenolophus (Stenolophus) limbalis* LeConte, 1857

*Stenolophus (Stenolophus) megacephalus* Lindroth, 1968

*Stenolophus (Stenolophus) ochropezus* (Say, 1823)

*Stenolophus (Stenolophus) plebejus* Dejean, 1829

*Stenolophus (Stenolophus) splendidulus* Motschulsky, 1864

*Stenolophus (Stenolophus) spretus* Dejean, 1831

*Stenolophus (Agonoderus) binotatus* (Casey, 1914)

*Stenolophus (Agonoderus) comma* (Fabricius, 1775)

*Stenolophus (Agonoderus) infuscatus* (Dejean, 1829)

*Stenolophus (Agonoderus) lecontei* (Chaudoir, 1868)

*Stenolophus (Agonoderus) lineola* (Fabricius, 1775)

*Stenolophus (Agonoderus) maculatus* (LeConte, 1869)

*Stenolophus (Agonoderus) rugicollis* (LeConte, 1859)

*Agonoleptus conjunctus* (Say, 1823)

*Agonoleptus dolosus* (Casey, 1914)

*Agonoleptus parviceps* Casey, 1914

*Agonoleptus rotundatus* (LeConte, 1863)

*Agonoleptus rotundicollis* (Haldeman, 1843)

*Agonoleptus thoracicus* (Casey, 1914)

*Agonoleptus unicolor* (Dejean, 1829)

*Bradycellus (Liocellus) curticollis* (Casey, 1924)

*Bradycellus (Liocellus) intermedius* (Fall, 1905)

*Bradycellus (Liocellus) laticollis* (Casey, 1924)

*Bradycellus (Liocellus) nitidus* (Dejean, 1829)

*Bradycellus (Liocellus) obtusus* (Fall, 1905)

*Bradycellus (Liocellus) politus* (Fall, 1905)

*Bradycellus (Liocellus) tahoensis* (Casey, 1924)

*Bradycellus (Bradycellus) fenderi* Hatch, 1951

*Bradycellus (Bradycellus) harpalinus* (Audinet-Serville, 1821)**†**

*Bradycellus (Catharellus) lecontei* Csiki, 1932

*Bradycellus (Stenocellus) ardelio* (Casey, 1914)

*Bradycellus (Stenocellus) aridus* (Casey, 1914)

*Bradycellus (Stenocellus) californicus* (LeConte, 1857)

*Bradycellus (Stenocellus) carolinensis* (Casey, 1924)

*Bradycellus (Stenocellus) congener* (LeConte, 1847)

*Bradycellus (Stenocellus) decorus* (Casey, 1914)

*Bradycellus (Stenocellus) discipulus* (Casey, 1914)

*Bradycellus (Stenocellus) exstans* (Casey, 1914)

*Bradycellus (Stenocellus) festinans* (Casey, 1914)

*Bradycellus (Stenocellus) humboldtianus* (Casey, 1924)

*Bradycellus (Stenocellus) insulsus* (Casey, 1914)

*Bradycellus (Stenocellus) larvatus* (Casey, 1914)

*Bradycellus (Stenocellus) lineatus* (Casey, 1914)

*Bradycellus (Stenocellus) lustrellus* (Casey, 1914)

*Bradycellus (Stenocellus) montanus* (Casey, 1914)

*Bradycellus (Stenocellus) nebulosus* LeConte, 1853

*Bradycellus (Stenocellus) neglectus* (LeConte, 1847)

*Bradycellus (Stenocellus) nigerrimus* Lindroth, 1968

*Bradycellus (Stenocellus) nigriceps* LeConte, 1869

*Bradycellus (Stenocellus) nubifer* LeConte, 1858

*Bradycellus (Stenocellus) picipes* (Casey, 1914)

*Bradycellus (Stenocellus) provoensis* (Casey, 1914)

*Bradycellus (Stenocellus) puncticollis* (Casey, 1914)

*Bradycellus (Stenocellus) purgatus* (Casey, 1914)

*Bradycellus (Stenocellus) rivalis* LeConte, 1858

*Bradycellus (Stenocellus) rupestris* (Say, 1823)

*Bradycellus (Stenocellus) sejunctus* (Casey, 1914)

*Bradycellus (Stenocellus) suavis* (Casey, 1914)

*Bradycellus (Stenocellus) subcordatus* Chaudoir, 1868

*Bradycellus (Stenocellus) supplex* (Casey, 1914)

*Bradycellus (Stenocellus) symetricus* (Motschulsky, 1850)

*Bradycellus (Stenocellus) tantillus* (Dejean, 1829)

*Bradycellus (Stenocellus) veronianus* (Casey, 1924)

*Bradycellus (Lipalocellus) nigrinus* (Dejean, 1829)

*Bradycellus (Lipalocellus) semipubescens* Lindroth, 1968

*Bradycellus (Triliarthrus) atrimedeus* (Say, 1823)

*Bradycellus (Triliarthrus) badipennis* (Haldeman, 1843)

*Bradycellus (Triliarthrus) conformis* (Fall, 1905)

*Bradycellus (Triliarthrus) georgei* Lindroth, 1968

*Bradycellus (Triliarthrus) kirbyi* (Horn, 1883)

*Bradycellus (Triliarthrus) lugubris* (LeConte, 1847)

*Amerinus linearis* (LeConte, 1863)

*Dicheirotrichus (Oreoxenus) mannerheimii mannerheimii* (Sahlberg, 1844)**‡**

*Dicheirotrichus (Trichocellus) cognatus* (Gyllenhal, 1827)**‡**

*Acupalpus (Acupalpus) canadensis* Casey, 1924

*Acupalpus (Acupalpus) carus* (LeConte, 1863)

*Acupalpus (Acupalpus) hydropicus* (LeConte, 1863)

*Acupalpus (Acupalpus) meridianus* (Linnaeus, 1760)**†**

*Acupalpus (Acupalpus) nanellus* Casey, 1914

*Acupalpus (Acupalpus) pumilus* Lindroth, 1968

*Acupalpus (Tachistodes) indistinctus* Dejean, 1831

*Acupalpus (Tachistodes) partiarius* (Say, 1823)

*Acupalpus (Tachistodes) pauperculus* Dejean, 1829

*Acupalpus (Tachistodes) testaceus* Dejean, 1829

*Acupalpus (Anthracus) punctulatus* Hatch, 1953

*Acupalpus (Anthracus) tener* (LeConte, 1857)

*Philodes (Philodes) alternans* (LeConte, 1853)

*Philodes (Goniolophus) flavilimbus* (LeConte, 1869)

*Philodes (Goniolophus) longulus* (Dejean, 1829)

*Philodes (Goniolophus) rectangulus* (Chaudoir, 1868)

*Pogonodaptus mexicanus* (Bates, 1878)

*Polpochila (Phymatocephalus) capitata* (Chaudoir, 1852)

*Polpochila (Phymatocephalus) erro* (LeConte, 1854)

*Polpochila (Polpochila) rotundicollis* Bates, 1882

*Piosoma setosum* LeConte, 1847

*Euryderus grossus* (Say, 1830)

*Ophonus (Metophonus) puncticeps* Stephens, 1828 **†**

*Ophonus (Metophonus) rufibarbis* (Fabricius, 1792)**†**

*Harpalus (Pseudoophonus) actiosus* Casey, 1914

*Harpalus (Pseudoophonus) compar* LeConte, 1847

*Harpalus (Pseudoophonus) erythropus* Dejean, 1829

*Harpalus (Pseudoophonus) faunus* Say, 1823

*Harpalus (Pseudoophonus) hatchi* Ball & Anderson, 1962

*Harpalus (Pseudoophonus) liobasis* Chaudoir, 1868

*Harpalus (Pseudoophonus) paratus* Casey, 1924

*Harpalus (Pseudoophonus) pensylvanicus* (DeGeer, 1774)

*Harpalus (Pseudoophonus) poncei* Will, 2002

*Harpalus (Pseudoophonus) protractus* Casey, 1914

*Harpalus (Pseudoophonus) rufipes* (DeGeer, 1774)**†**

*Harpalus (Pseudoophonus) texanus* Casey, 1914

*Harpalus (Pseudoophonus) vagans* LeConte, 1865

*Harpalus (Megapangus) caliginosus* (Fabricius, 1775)

*Harpalus (Megapangus) katiae* Battoni, 1985

*Harpalus (Plectralidus) erraticus* Say, 1823

*Harpalus (Plectralidus) retractus* LeConte, 1863

*Harpalus (Opadius) animosus* Casey, 1924

*Harpalus (Opadius) apache* Kataev, 2010

*Harpalus (Opadius) cordatus* (LeConte, 1853)

*Harpalus (Opadius) cordifer* Notman, 1919

*Harpalus (Opadius) desertus* LeConte, 1859

*Harpalus (Opadius) fraternus* LeConte, 1852

*Harpalus (Opadius) fulvilabris* Mannerheim, 1853

*Harpalus (Opadius) gravis* LeConte, 1858

*Harpalus (Opadius) indianus* Csiki, 1932

*Harpalus (Opadius) indigens* Casey, 1924

*Harpalus (Opadius) laevipes* Zetterstedt, 1828**‡**

*Harpalus (Opadius) laticeps* LeConte, 1850

*Harpalus (Opadius) lewisii* LeConte, 1865

*Harpalus (Opadius) megacephalus* LeConte, 1847

*Harpalus (Opadius) nigritarsis* Sahlberg, 1827**‡**

*Harpalus (Opadius) providens* Casey, 1914

*Harpalus (Opadius) reversus* Casey, 1924

*Harpalus (Opadius) spadiceus* Dejean, 1829

*Harpalus (Opadius) ventralis* LeConte, 1847

*Harpalus (Harpalus) affinis* (Schrank, 1781)**†**

*Harpalus (Harpalus) amputatus amputatus* Say, 1830

*Harpalus (Harpalus) atrichatus* Hatch, 1949

*Harpalus (Harpalus) balli* Noonan, 1991

*Harpalus (Harpalus) cautus* Dejean, 1829

*Harpalus (Harpalus) ellipsis* LeConte, 1847

*Harpalus (Harpalus) herbivagus* Say, 1823

*Harpalus (Harpalus) innocuus* LeConte, 1863

*Harpalus (Harpalus) martini* Van Dyke, 1926

*Harpalus (Harpalus) obnixus* Casey, 1924

*Harpalus (Harpalus) ochropus* Kirby, 1837

*Harpalus (Harpalus) opacipennis* (Haldeman, 1843)

*Harpalus (Harpalus) plenalis* Casey, 1914

*Harpalus (Harpalus) rubripes* (Duftschmid, 1812)**†**

*Harpalus (Harpalus) solitaris* Dejean, 1829**‡**

*Harpalus (Harpalus) somnulentus* Dejean, 1829

*Harpalus (Harpalus) vittatus alaskensis* Lindroth, 1968**‡**

*Harpalus (Glanodes) cohni* Ball, 1972

*Harpalus (Glanodes) corpulentus* (Casey, 1914)

*Harpalus (Glanodes) huachuca* Ball, 1972

*Harpalus (Glanodes) obliquus* Horn, 1880

*Harpalus (Glanodes) puncticeps* (Casey, 1914)

*Harpalus (Glanodes) stephani* Ball, 1972

*Harpalus (Harpalobius) fuscipalpis* Sturm, 1818**‡**

*Harpalobrachys leiroides* (Motschulsky, 1844)**‡**

*Hartonymus alternatus* (LeConte, 1863)

*Hartonymus hoodi* Casey, 1914

*Amblygnathus evansi* Ball & Maddison, 1987

*Amblygnathus iripennis* (Say, 1823)

*Amblygnathus mexicanus* Bates, 1882

*Amblygnathus subtinctus* (LeConte, 1867)

*Athrostictus punctatulus* (Putzeys, 1878)

*Selenophorus (Celiamorphus) adjunctus* (Casey, 1914)

*Selenophorus (Celiamorphus) contractus* (Casey, 1914)

*Selenophorus (Celiamorphus) discopunctatus* Dejean, 1829

*Selenophorus (Celiamorphus) ellipticus* Dejean, 1829

*Selenophorus (Celiamorphus) fossulatus* Dejean, 1829

*Selenophorus (Celiamorphus) granarius* Dejean, 1829

*Selenophorus (Celiamorphus) municeps* (Casey, 1924)

*Selenophorus (Celiamorphus) nanulus* (Casey, 1924)

*Selenophorus (Celiamorphus) subtropicus* (Casey, 1924)

*Selenophorus (Selenophorus) aeneopiceus* Casey, 1884

*Selenophorus (Selenophorus) blanchardi* Manee, 1915

*Selenophorus (Selenophorus) chaparralus* Purrington, 2000

*Selenophorus (Selenophorus) concinnus* Schaeffer, 1910

*Selenophorus (Selenophorus) cupreolus* Casey, 1914

*Selenophorus (Selenophorus) discoderoides* Schaeffer, 1910

*Selenophorus (Selenophorus) elongatus* (LeConte, 1847)

*Selenophorus (Selenophorus) famulus* Casey, 1914

*Selenophorus (Selenophorus) fatuus* LeConte, 1863

*Selenophorus (Selenophorus) gagatinus* Dejean, 1829

*Selenophorus (Selenophorus) houstoni* Casey, 1914

*Selenophorus (Selenophorus) hylacis* (Say, 1823)

*Selenophorus (Selenophorus) implicans* Casey, 1914

*Selenophorus (Selenophorus) integer* (Fabricius, 1798)

*Selenophorus (Selenophorus) laesus* (LeConte, 1858)

*Selenophorus (Selenophorus) maritimus* Casey, 1914

*Selenophorus (Selenophorus) opalinus* (LeConte, 1863)

*Selenophorus (Selenophorus) otiosus* Casey, 1914

*Selenophorus (Selenophorus) palliatus* (Fabricius, 1798)

*Selenophorus (Selenophorus) parumpunctatus* Dejean, 1829

*Selenophorus (Selenophorus) pedicularius* Dejean, 1829

*Selenophorus (Selenophorus) planipennis* LeConte, 1847

*Selenophorus (Selenophorus) riparius* Casey, 1914

*Selenophorus (Selenophorus) schaefferi* Csiki, 1932

*Selenophorus (Selenophorus) scolopaceus* Casey, 1914

*Selenophorus (Selenophorus) sinuaticollis* Notman, 1922

*Selenophorus (Selenophorus) striatopunctatus* Putzeys, 1878

*Selenophorus (Selenophorus) trepidus* (Casey, 1924)

*Selenophorus breviusculus* Horn, 1880

*Discoderus aequalis* Casey, 1914

*Discoderus amoenus* LeConte, 1863

*Discoderus congruens* Casey, 1914

*Discoderus cordicollis* Horn, 1891

*Discoderus crassicollis* Horn, 1891

*Discoderus dallasensis* Casey, 1924

*Discoderus impotens* (LeConte, 1858)

*Discoderus longicollis* Casey, 1914

*Discoderus obsidianus* Casey, 1914

*Discoderus papagonis* Casey, 1924

*Discoderus parallelus* (Haldeman, 1843)

*Discoderus parilis* (Casey, 1914)

*Discoderus peregrinus* Casey, 1924

*Discoderus pinguis* Casey, 1884

*Discoderus robustus piceus* Casey, 1914

*Discoderus robustus robustus* Horn, 1883

*Discoderus subviolaceus* Casey, 1914

*Discoderus symbolicus* Casey, 1914

*Discoderus tenebrosus* (LeConte, 1847)

*Discoderus texanus* Casey, 1924

*Stenomorphus californicus californicus* (Ménétriés, 1843)

*Stenomorphus californicus rufipes* LeConte, 1858

*Stenomorphus convexior* Notman, 1922

*Stenomorphus sinaloae* Darlington, 1936

*Trichotichnus (Trichotichnus) dichrous* (Dejean, 1829)

*Trichotichnus (Trichotichnus) vulpeculus* (Say, 1823)

*Trichotichnus (Iridessus) autumnalis* (Say, 1823)

*Trichotichnus (Iridessus) fulgens* (Csiki, 1932)

*Aztecarpalus schaefferi* Ball, 1970

*Cratacanthus dubius* (Palisot de Beauvois, 1811)

### Sphodrini

*Pseudamara arenaria* (LeConte, 1847)

*Calathus (Calathus) fuscipes* (Goeze, 1777)**†**

*Calathus (Neocalathus) calceus* Ball & Nègre, 1972

*Calathus (Neocalathus) gregarius* (Say, 1823)

*Calathus (Neocalathus) ingratus* Dejean, 1828

*Calathus (Neocalathus) opaculus* LeConte, 1854

*Calathus (Neocalathus) peropacus* Casey, 1920

*Calathus (Neocalathus) ruficollis grandicollis* Casey, 1920

*Calathus (Neocalathus) ruficollis ignicollis* Casey, 1920

*Calathus (Neocalathus) ruficollis ruficollis* Dejean, 1828

*Calathus (Acalathus) advena* (LeConte, 1846)

*Synuchus dubius* (LeConte, 1854)

*Synuchus impunctatus* (Say, 1823)

*Laemostenus (Laemostenus) complanatus* (Dejean, 1828)**†**

*Laemostenus (Pristonychus) terricola terricola* (Herbst, 1784)**†**

### Platynini

*Olisthopus brevicornis* Casey, 1913

*Olisthopus filicornis* Casey, 1913

*Olisthopus innuens* Casey, 1913

*Olisthopus iterans* Casey, 1913

*Olisthopus micans* LeConte, 1846

*Olisthopus parmatus* (Say, 1823)

*Olisthopus pusio* Casey, 1913

*Elliptoleus acutesculptus* Bates, 1882

*Sericoda bembidioides* Kirby, 1837

*Sericoda bogemannii* (Gyllenhal, 1813)**‡**

*Sericoda obsoleta* (Say, 1823)

*Sericoda quadripunctata* (DeGeer, 1774)**‡**

*Tetraleucus picticornis* (Newman, 1844)

*Anchomenus (Anchomenus) aeneolus* (LeConte, 1854)

*Anchomenus (Anchomenus) funebris* (LeConte, 1854)

*Anchomenus (Anchomenus) quadratus* (LeConte, 1854)

*Rhadine albamontana* Dajoz, 1998

*Rhadine anthicoides* Casey, 1913

*Rhadine austinica* Barr, 1974

*Rhadine babcocki* (Barr, 1960)

*Rhadine balesi* (Gray, 1937)

*Rhadine bullis* Reddell & Cokendolpher, 2004

*Rhadine caudata* (LeConte, 1863)

*Rhadine constricta* Casey, 1913

*Rhadine dissecta* (LeConte, 1863)

*Rhadine exilis* (Barr & Lawrence, 1960)

*Rhadine grubbsi* Reddell & Dupérré, 2009

*Rhadine howdeni* (Barr & Lawrence, 1960)

*Rhadine infernalis ewersi* (Barr, 1960)

*Rhadine infernalis infernalis* (Barr & Lawrence, 1960)

*Rhadine insolita* Barr, 1974

*Rhadine ivyi* Reddell & Cokendolpher, 2004

*Rhadine jejuna* (LeConte, 1878)

*Rhadine koepkei koepkei* (Barr, 1960)

*Rhadine koepkei privata* Barr, 1974

*Rhadine lanei* (Gray, 1937)

*Rhadine larvalis* LeConte, 1846

*Rhadine lindrothi* Barr, 1965

*Rhadine longiceps* Van Dyke, 1949

*Rhadine longicollis* Benedict, 1927

*Rhadine longipes* Casey, 1913

*Rhadine myrmecodes* (Horn, 1892)

*Rhadine nivalis* (Horn, 1881)

*Rhadine noctivaga* Barr, 1974

*Rhadine ozarkensis* Sanderson & Miller, 1941

*Rhadine perlevis* Casey, 1913

*Rhadine persephone* Barr, 1974

*Rhadine pertenuis* Casey, 1920

*Rhadine reyesi* Reddell & Cokendolpher, 2001

*Rhadine rossi* Van Dyke, 1949

*Rhadine rubra* (Barr, 1960)

*Rhadine russelli* Barr, 1974

*Rhadine specum crinicollis* Barr, 1974

*Rhadine specum gentilis* Barr, 1974

*Rhadine specum specum* (Barr, 1960)

*Rhadine sprousei* Reddell & Cokendolpher, 2004

*Rhadine sublustris* Casey, 1913

*Rhadine subterranea mitchelli* Barr, 1974

*Rhadine subterranea subterranea* (Van Dyke, 1919)

*Rhadine tenebrosa mckenziei* Barr, 1974

*Rhadine tenebrosa tenebrosa* (Barr, 1960)

*Rhadine testacea* Casey, 1920

*Rhadine umbra* Casey, 1913

*Mexisphodrus valverdensis* Barr, 1982

*Tanystoma cuyama* Liebherr, 1985

*Tanystoma maculicolle* (Dejean, 1828)

*Tanystoma striatum* (Dejean, 1828)

*Tanystoma sulcatum* (Dejean, 1828)

*Paranchus albipes* (Fabricius, 1794)**†**

*Oxypselaphus pusillus* (LeConte, 1854)

*Agonum (Platynomicrus) ferruginosum* (Dejean, 1828)

*Agonum (Platynomicrus) nigriceps* LeConte, 1846**‡**

*Agonum (Europhilus) anchomenoides* Randall, 1838

*Agonum (Europhilus) canadense* Goulet, 1969

*Agonum (Europhilus) consimile* (Gyllenhal, 1810)**‡**

*Agonum (Europhilus) darlingtoni* Lindroth, 1954

*Agonum (Europhilus) exaratum* (Mannerheim, 1853)**‡**

*Agonum (Europhilus) galvestonicum* (Casey, 1920)

*Agonum (Europhilus) gratiosum* (Mannerheim, 1853)**‡**

*Agonum (Europhilus) limbatum* Motschulsky, 1845

*Agonum (Europhilus) lutulentum* (LeConte, 1854)

*Agonum (Europhilus) palustre* Goulet, 1969

*Agonum (Europhilus) picicornoides* Lindroth, 1966

*Agonum (Europhilus) retractum* LeConte, 1846

*Agonum (Europhilus) simile* Kirby, 1837

*Agonum (Europhilus) sordens* Kirby, 1837

*Agonum (Europhilus) superioris* Lindroth, 1966

*Agonum (Europhilus) thoreyi* Dejean, 1828**‡**

*Agonum (Agonum) bicolor* (Dejean, 1828)**‡**

*Agonum (Agonum) muelleri* (Herbst, 1784)**†**

*Agonum (Agonum) piceolum* (LeConte, 1879)

*Agonum (Agonum) placidum* (Say, 1823)

*Agonum (Olisares) aeruginosum* Dejean, 1828

*Agonum (Olisares) affine* Kirby, 1837

*Agonum (Olisares) albicrus* Dejean, 1828

*Agonum (Olisares) anthracinum* Dejean, 1831

*Agonum (Olisares) basale* LeConte, 1846

*Agonum (Olisares) belleri* (Hatch, 1933)

*Agonum (Olisares) brevicolle* Dejean, 1828

*Agonum (Olisares) collare* (Say, 1830)

*Agonum (Olisares) corvus* (LeConte, 1860)

*Agonum (Olisares) crenistriatum* (LeConte, 1863)

*Agonum (Olisares) crenulatum* (LeConte, 1854)

*Agonum (Olisares) cupreum* Dejean, 1831

*Agonum (Olisares) cupripenne* (Say, 1823)

*Agonum (Olisares) cyanopis* (Bates, 1882)

*Agonum (Olisares) cyclifer* (Bates, 1884)

*Agonum (Olisares) deceptivum* (LeConte, 1879)

*Agonum (Olisares) decorum* (Say, 1823)

*Agonum (Olisares) deplanatum* Ménétriés, 1843

*Agonum (Olisares) elongatulum* (Dejean, 1828)

*Agonum (Olisares) errans* (Say, 1823)

*Agonum (Olisares) excavatum* Dejean, 1828

*Agonum (Olisares) extensicolle* (Say, 1823)

*Agonum (Olisares) extimum* Liebherr, 1986

*Agonum (Olisares) ferreum* Haldeman, 1843

*Agonum (Olisares) fidele* Casey, 1920

*Agonum (Olisares) fossiger* Dejean, 1828

*Agonum (Olisares) harrisii* LeConte, 1846

*Agonum (Olisares) imitans* (Notman, 1919)

*Agonum (Olisares) melanarium* Dejean, 1828

*Agonum (Olisares) metallescens* (LeConte, 1854)

*Agonum (Olisares) moerens* Dejean, 1828

*Agonum (Olisares) muiri* Liebherr, 1984

*Agonum (Olisares) mutatum* (Gemminger & Harold, 1868)

*Agonum (Olisares) nutans* (Say, 1823)

*Agonum (Olisares) octopunctatum* (Fabricius, 1798)

*Agonum (Olisares) pacificum* Casey, 1920

*Agonum (Olisares) pallipes* (Fabricius, 1787)

*Agonum (Olisares) parextimum* Liebherr, 1986

*Agonum (Olisares) propinquum* (Gemminger & Harold, 1868)

*Agonum (Olisares) punctiforme* (Say, 1823)

*Agonum (Olisares) quadrimaculatum* (Horn, 1885)

*Agonum (Olisares) quinquepunctatum* Motschulsky, 1844**‡**

*Agonum (Olisares) rigidulum* (Casey, 1920)

*Agonum (Olisares) rufipes* Dejean, 1828

*Agonum (Olisares) striatopunctatum* Dejean, 1828

*Agonum (Olisares) sulcipenne* (Horn, 1881)

*Agonum (Olisares) suturale* Say, 1830

*Agonum (Olisares) tenue* (LeConte, 1854)

*Agonum (Olisares) texanum* (LeConte, 1878)

*Agonum (Olisares) trigeminum* Lindroth, 1954

*Platynus (Microplatynus) agilis* LeConte, 1863

*Platynus (Microplatynus) pecki* Barr, 1982

*Platynus (Platynus) brunneomarginatus* (Mannerheim, 1843)

*Platynus (Platynus) daviesi* Bousquet, 2012

*Platynus (Platynus) decentis* (Say, 1823)

*Platynus (Platynus) indecentis* Liebherr & Will, 1996

*Platynus (Platynus) opaculus* LeConte, 1863

*Platynus (Platynus) ovipennis* (Mannerheim, 1843)

*Platynus (Platynus) parmarginatus* Hamilton, 1893

*Platynus (Platynus) tenuicollis* (LeConte, 1846)

*Platynus (Platynus) trifoveolatus* Beutenmüller, 1903

*Platynus (Batenus) angustatus* Dejean, 1828

*Platynus (Batenus) cincticollis* (Say, 1823)

*Platynus (Batenus) hypolithos* (Say, 1823)

*Platynus (Batenus) mannerheimii* (Dejean, 1828)**‡**

*Platynus (Batenus) prognathus* Van Dyke, 1926

*Platynus (Glyptolenopsis) ovatulus* (Bates, 1884)

*Platynus (Trapezodera) cohni* Liebherr & Will, 1996

*Platynus (Dyscolus) cazieri* Liebherr & Will, 1996

*Platynus (Dyscolus) falli* (Darlington, 1936)

*Platynus (Dyscolus) lyratus* (Chaudoir, 1879)

*Platynus (Dyscolus) megalops* (Bates, 1882)

*Platynus (Dyscolus) rufiventris* (Van Dyke, 1926)

*Metacolpodes buchanani* (Hope, 1831)**†**

### Perigonini

*Perigona (Trechicus) nigriceps* (Dejean, 1831)**†**

*Perigona (Trechicus) pallipennis* (LeConte, 1853)

### Atranini

*Atranus pubescens* (Dejean, 1828)

### Lachnophorini

*Anchonoderus quadrinotatus* Horn, 1878

*Anchonoderus schaefferi* Liebke, 1928

*Lachnophorus elegantulus* Mannerheim, 1843

*Euphorticus occidentalis* Horn, 1891

*Euphorticus pubescens* (Dejean, 1831)

*Calybe (Ega) laetula* (LeConte, 1851)

*Calybe (Ega) sallei* (Chevrolat, 1839)

*Eucaerus (Eucaerus) varicornis* LeConte, 1853

### Pentagonicini

*Pentagonica bicolor* (LeConte, 1863)

*Pentagonica felix* Bell, 1987

*Pentagonica flavipes flavipes* (LeConte, 1853)

*Pentagonica marshalli* Mateu, 1995

*Pentagonica nigricornis* Darlington, 1934

*Pentagonica picticornis* Bates, 1883

### Odacanthini

*Colliuris (Mimocasnonia) pilatei* (Chaudoir, 1848)

*Colliuris (Cosnania) lengi* (Schaeffer, 1910)

*Colliuris (Cosnania) pensylvanica* (Linnaeus, 1758)

*Colliuris (Calocolliuris) caymanensis* Darlington, 1947

*Colliuris (Calocolliuris) lioptera* (Bates, 1891)

*Colliuris (Calocolliuris) ludoviciana* (Sallé, 1849)

### Ctenodactylini

*Leptotrachelus depressus* Blatchley, 1923

*Leptotrachelus dorsalis* (Fabricius, 1801)

*Leptotrachelus pallidulus* Motschulsky, 1864

### Cyclosomini

*Tetragonoderus (Crossonychus) fasciatus* (Haldeman, 1843)

*Tetragonoderus (Crossonychus) intersectus* (Germar, 1824)

*Tetragonoderus (Crossonychus) laevigatus* Chaudoir, 1876**†**

*Tetragonoderus (Crossonychus) latipennis* LeConte, 1874

*Tetragonoderus (Crossonychus) pallidus* Horn, 1869

### Lebiini

*Mochtherus tetraspilotus* (Macleay, 1825)**†**

*Phloeoxena (Oenaphelox) signata* (Dejean, 1825)

*Eucheila (Inna) boyeri* (Solier, 1835)

*Somotrichus unifasciatus* (Dejean, 1831)**†**

*Coptodera (Coptodera) aerata* Dejean, 1825

*Coptodera (Coptodera) brunnea* Shpeley & Ball, 1994

*Coptodera (Coptodera) festiva* Dejean, 1825

*Coptodera (Coptodera) nitidula* (Buquet, 1835)

*Coptodera (Coptodera) picea* Dejean, 1826

*Cymindis (Tarulus) americana* Dejean, 1826

*Cymindis (Tarulus) arizonensis* Schaeffer, 1910

*Cymindis (Tarulus) borealis* LeConte, 1863

*Cymindis (Tarulus) californica* Horn, 1895

*Cymindis (Tarulus) cribricollis* Dejean, 1831

*Cymindis (Tarulus) elegans* LeConte, 1846

*Cymindis (Tarulus) evanescens* Casey, 1913

*Cymindis (Tarulus) interior* Lindroth, 1969

*Cymindis (Tarulus) laticollis* Say, 1830

*Cymindis (Tarulus) neglecta* Haldeman, 1843

*Cymindis (Tarulus) pilosa* Say, 1823

*Cymindis (Tarulus) planipennis* LeConte, 1863

*Cymindis (Tarulus) seriata* Hatch, 1953

*Cymindis (Tarulus) unicolor* Kirby, 1837

*Cymindis (Tarulus) uniseriata* Bates, 1884

*Cymindis (Tarulus) vaporariorum* (Linnaeus, 1758)**‡**

*Cymindis (Pinacodera) abbreviata* (Casey, 1920)

*Cymindis (Pinacodera) ampliata* (Casey, 1920)

*Cymindis (Pinacodera) atripennis* (Casey, 1920)

*Cymindis (Pinacodera) blanda* Casey, 1913

*Cymindis (Pinacodera) complanata* Dejean, 1826

*Cymindis (Pinacodera) limbata* Dejean, 1831

*Cymindis (Pinacodera) obscura* (Casey, 1920)

*Cymindis (Pinacodera) platicollis* (Say, 1823)

*Cymindis (Pinacodera) punctifera* (LeConte, 1884)

*Cymindis (Pinacodera) punctigera* LeConte, 1851

*Cymindis (Pinacodera) subcarinata* (Casey, 1920)

*Apenes (Apenes) angustata* Schwarz, 1878

*Apenes (Apenes) coriacea* (Chevrolat, 1863)

*Apenes (Apenes) hilariola* Bates, 1891

*Apenes (Apenes) lucidula lucidula* (Dejean, 1831)

*Apenes (Apenes) nebulosa* LeConte, 1867

*Apenes (Apenes) opaca* LeConte, 1851

*Apenes (Apenes) pallidipes* (Chevrolat, 1836)

*Apenes (Apenes) parallela parallela* (Dejean, 1825)

*Apenes (Apenes) sinuata* (Say, 1823)

*Dromius (Dromius) fenestratus* (Fabricius, 1794)**†**

*Dromius (Dromius) piceus* Dejean, 1831

*Philorhizus atriceps* (LeConte, 1880)

*Philorhizus melanocephalus* (Dejean, 1825)**†**

*Microlestes brevilobus brevilobus* Lindroth, 1969

*Microlestes curtipennis* (Casey, 1920)

*Microlestes lindrothi* Mateu, 1995

*Microlestes linearis* (LeConte, 1851)

*Microlestes lucidus lucidus* (LeConte, 1851)

*Microlestes major* Lindroth, 1969

*Microlestes nigrinus* (Mannerheim, 1843)

*Microlestes pusio* (LeConte, 1863)

*Apristus actuosus* Casey, 1920

*Apristus agitatus* Casey, 1920

*Apristus cephalus* Casey, 1920

*Apristus constrictus* Casey, 1920

*Apristus latens* (LeConte, 1846)

*Apristus laticollis* LeConte, 1851

*Apristus liratus* Casey, 1920

*Apristus nevadensis* Casey, 1920

*Apristus pugetanus* Casey, 1920

*Apristus subdeletus* Casey, 1920

*Apristus subsulcatus* (Dejean, 1826)

*Apristus thoracicus* Casey, 1920

*Apristus tuckeri* Casey, 1920

*Syntomus americanus* (Dejean, 1831)

*Axinopalpus biplagiatus* (Dejean, 1825)

*Axinopalpus denticulatus* Hatch, 1949

*Axinopalpus fusciceps* LeConte, 1851

*Axinopalpus illectus* Casey, 1920

*Axinopalpus pratti* Hatch, 1949

*Axinopalpus utahensis* Tanner, 1928

*Axinopalpus vittatus* Hatch, 1949

*Lebia (Loxopeza) atriceps* LeConte, 1863

*Lebia (Loxopeza) atriventris* Say, 1823

*Lebia (Loxopeza) deceptrix* Madge, 1967

*Lebia (Loxopeza) grandis* Hentz, 1830

*Lebia (Loxopeza) pimalis* (Casey, 1920)

*Lebia (Loxopeza) subdola* Madge, 1967

*Lebia (Loxopeza) subgrandis* Madge, 1967

*Lebia (Loxopeza) tricolor* Say, 1823

*Lebia (Polycheloma) lecontei* Madge, 1967

*Lebia (Lamprias) divisa* LeConte, 1850

*Lebia (Lebia) abdita* Madge, 1967

*Lebia (Lebia) abdominalis* Chaudoir, 1843

*Lebia (Lebia) analis* Dejean, 1825

*Lebia (Lebia) arizonica* Schaeffer, 1910

*Lebia (Lebia) bilineata* Motschulsky, 1859

*Lebia (Lebia) bitaeniata* Chevrolat, 1834

*Lebia (Lebia) bivittata* (Fabricius, 1798)

*Lebia (Lebia) bumeliae* Schaeffer, 1910

*Lebia (Lebia) calliope* Bates, 1883

*Lebia (Lebia) collaris* Dejean, 1826

*Lebia (Lebia) cyanipennis* Dejean, 1831

*Lebia (Lebia) esurialis* Casey, 1920

*Lebia (Lebia) fuscata* Dejean, 1825

*Lebia (Lebia) guttula* LeConte, 1851

*Lebia (Lebia) histrionica* Bates, 1883

*Lebia (Lebia) insulata* Madge, 1967

*Lebia (Lebia) lecta* Horn, 1885

*Lebia (Lebia) lobulata* LeConte, 1863

*Lebia (Lebia) marginicollis* Dejean, 1825

*Lebia (Lebia) miranda* (Horn, 1872)

*Lebia (Lebia) moesta* LeConte, 1850

*Lebia (Lebia) nigricapitata* Madge, 1967

*Lebia (Lebia) ornata* Say, 1823

*Lebia (Lebia) pectita* Horn, 1885

*Lebia (Lebia) perita* Casey, 1920

*Lebia (Lebia) perpallida* Madge, 1967

*Lebia (Lebia) pleuritica* LeConte, 1846

*Lebia (Lebia) pulchella* Dejean, 1826

*Lebia (Lebia) pumila* Dejean, 1831

*Lebia (Lebia) rufopleura* Schaeffer, 1910

*Lebia (Lebia) scalpta* Bates, 1883

*Lebia (Lebia) scapula* Horn, 1885

*Lebia (Lebia) solea* Hentz, 1830

*Lebia (Lebia) subrugosa* Chaudoir, 1871

*Lebia (Lebia) tuckeri* (Casey, 1920)

*Lebia (Lebia) viridipennis* Dejean, 1826

*Lebia (Lebia) viridis* Say, 1823

*Lebia (Lebia) vittata* (Fabricius, 1777)

*Hyboptera auxiliadora* Erwin, 2004

*Plochionus (Menidius) amandus* Newman, 1840

*Plochionus (Menidius) bicolor* Notman, 1919

*Plochionus (Menidius) discoideus* LeConte, 1880

*Plochionus (Menidius) timidus* Haldeman, 1843

*Plochionus (Plochionus) pallens* (Fabricius, 1775)**†**

*Tecnophilus croceicollis croceicollis* (Ménétriés, 1843)

*Tecnophilus croceicollis peigani* Larson, 1969

*Tecnophilus pilatei* Chaudoir, 1877

*Calleida (Calleida) circumcincta* Bates, 1883

*Calleida (Calleida) decora* (Fabricius, 1801)

*Calleida (Calleida) fimbriata* Bates, 1883

*Calleida (Calleida) fulgida* Dejean, 1831

*Calleida (Calleida) obrieni* Mateu, 1995

*Calleida (Calleida) planulata* LeConte, 1858

*Calleida (Calleida) platynoides* Horn, 1882

*Calleida (Calleida) punctata* LeConte, 1846

*Calleida (Calleida) punctulata* Chaudoir, 1848

*Calleida (Calleida) purpurea* (Say, 1823)

*Calleida (Calleida) viridipennis* (Say, 1823)

*Philophuga caerulea* Casey, 1913

*Philophuga viridicollis* (LeConte, 1846)

*Philophuga viridis amoena* (LeConte, 1846)

*Philophuga viridis horni* Chaudoir, 1877

*Philophuga viridis klamathea* Larson, 1969

*Philophuga viridis viridis* (Dejean, 1831)

*Infernophilus castaneus* (Horn, 1882)

*Onota angulicollis* (Reiche, 1842)

*Onota floridana* Horn, 1881

*Cylindronotum aeneum* Putzeys, 1845

*Agra oblongopunctata oblongopunctata* Chevrolat, 1836

*Euproctinus (Neoeuproctus) abjectus* (Bates, 1883)

*Euproctinus (Neoeuproctus) balli* Shpeley, 1986

*Euproctinus (Neoeuproctus) trivittatus* (LeConte, 1878)

*Nemotarsus elegans* LeConte, 1853

*Nemotarsus rhombifer* Bates, 1883

### Zuphiini

*Zuphium americanum* Dejean, 1831

*Zuphium delectum* Liebke, 1933

*Zuphium longicolle* LeConte, 1879

*Zuphium magnum* Schaeffer, 1910

*Zuphium mexicanum* Chaudoir, 1863

*Zuphium pseudamericanum* Mateu, 1981

*Pseudaptinus (Pseudaptinus) lecontei* (Dejean, 1831)

*Pseudaptinus (Pseudaptinus) oviceps* Van Dyke, 1926

*Pseudaptinus (Pseudaptinus) tenuicollis* (LeConte, 1851)

*Pseudaptinus (Thalpius) cubanus* (Chaudoir, 1877)

*Pseudaptinus (Thalpius) deceptor* Darlington, 1934

*Pseudaptinus (Thalpius) dorsalis* (Brullé, 1834)

*Pseudaptinus (Thalpius) hoegei* (Bates, 1883)

*Pseudaptinus (Thalpius) horni* (Chaudoir, 1872)

*Pseudaptinus (Thalpius) microcephalus* (Van Dyke, 1926)

*Pseudaptinus (Thalpius) nobilis* Liebke, 1934

*Pseudaptinus (Thalpius) pygmaeus* (Dejean, 1826)

*Pseudaptinus (Thalpius) rufulus* (LeConte, 1851)

### Galeritini

*Galerita (Progaleritina) atripes* LeConte, 1858

*Galerita (Progaleritina) bicolor* (Drury, 1773)

*Galerita (Progaleritina) forreri* Bates, 1883

*Galerita (Progaleritina) janus* (Fabricius, 1792)

*Galerita (Progaleritina) lecontei lecontei* Dejean, 1831

*Galerita (Progaleritina) mexicana* Chaudoir, 1872

*Galerita (Progaleritina) reichardti* Ball & Nimmo, 1983

*Galerita (Galerita) aequinoctialis* Chaudoir, 1852

### Helluonini

*Helluomorphoides clairvillei* (Dejean, 1831)

*Helluomorphoides ferrugineus* (LeConte, 1853)

*Helluomorphoides latitarsis* (Casey, 1913)

*Helluomorphoides nigripennis* (Dejean, 1831)

*Helluomorphoides papago* (Casey, 1913)

*Helluomorphoides praeustus bicolor* (Harris, 1828)

*Helluomorphoides praeustus floridanus* Ball, 1956

*Helluomorphoides praeustus praeustus* (Dejean, 1825)

*Helluomorphoides texanus* (LeConte, 1853)

### Pseudomorphini

*Pseudomorpha (Pseudomorpha) alleni* Van Dyke, 1953

*Pseudomorpha (Pseudomorpha) alutacea* Notman, 1925

*Pseudomorpha (Pseudomorpha) augustata* Horn, 1883

*Pseudomorpha (Pseudomorpha) behrensi* Horn, 1870

*Pseudomorpha (Pseudomorpha) castanea* Casey, 1909

*Pseudomorpha (Pseudomorpha) champlaini* Notman, 1925

*Pseudomorpha (Pseudomorpha) consanguinea* Notman, 1925

*Pseudomorpha (Pseudomorpha) cronkhitei* Horn, 1867

*Pseudomorpha (Pseudomorpha) cylindrica* Casey, 1889

*Pseudomorpha (Pseudomorpha) excrucians* Kirby, 1823

*Pseudomorpha (Pseudomorpha) falli* Notman, 1925

*Pseudomorpha (Pseudomorpha) hubbardi* Notman, 1925

*Pseudomorpha (Pseudomorpha) parallela* Van Dyke, 1943

*Pseudomorpha (Pseudomorpha) schwarzi* Notman, 1925

*Pseudomorpha (Pseudomorpha) tenebroides* Notman, 1925

*Pseudomorpha (Pseudomorpha) vandykei* Notman, 1925

*Pseudomorpha (Pseudomorpha) vicina* Notman, 1925

*Pseudomorpha (Pseudomorpha) vindicata* Notman, 1925

## Catalogue of North American Geadephaga taxa

### Family TRACHYPACHIDAE

Thomson, 1857

Trachypachini C.G. Thomson, 1857: 5. Type genus: *Trachypachus* Motschulsky, 1844.

#### Diversity.

Six species in western North America (three species), South America (two species), and northern Eurasia (one species). The species are arrayed in two genera: *Systolosoma* Solier (two South American species) and *Trachypachus* (four species).

### Genus Trachypachus

Motschulsky, 1844

Trachypachus Motschulsky, 1844: 86. Type species: *Blethisa zetterstedtii* Gyllenhal, 1827 designated by Thomson (1859: 3). Etymology. Uncertain, possibly from the Greek *trachelos* (neck, by extension pronotum) contracted and *pachys* (thick), alluding to the convex pronotum (“*corselet convexe, large*”) or from the Greek *trachys* (uneven, rough) and *pachys* [masculine].
Trachypachys Gemminger and Harold, 1868a: 46. Unjustified emendation of *Trachypachus* Motschulsky, 1844.

#### Diversity.

Northern Hemisphere, with four species in the Nearctic (three species) and Palaearctic (one species) Regions.

#### Identification.

Lindroth (1961a: 1-4) reviewed the North American species and discussed the structural differences between the three taxa.

### Trachypachus gibbsii

LeConte, 1861

Trachypachys gibbsii LeConte, 1861b: 339. Type locality: «east of Fort Colville [Washington]» (original citation). Syntype(s) in MCZ [# 85]. Etymology. The specific name honors the American geologist and ethnologist George Gibbs [1815-1873] who gathered zoological specimens for the Smithsonian while working for the Northwest Boundary Commission.
Trachypachus californicus Motschulsky, 1864: 194. Type locality: «Calif[ornie]» (original citation). Two syntypes, one listed as “corruptum,” in ZMMU (Keleinikova 1976: 190). Synonymy established by Horn (1870a: 71).
Trachypachus alticola Casey, 1920: 144. Type locality: «Lake Tahoe [Placer County], California» (original citation). Lectotype (♂), designated by Lindroth (1975: 111), in USNM [# 46830]. Synonymy established by Van Dyke (1925: 112), confirmed by Lindroth (1961a: 3).

#### Distribution.

This species ranges from southern British Columbia (Lindroth 1961a: 4) to northwestern Montana (Russell 1968: 42; Edwards 1975: 48), south to the southern part of the Sierra Nevada (Lindroth 1961a: 4) and to the Coast Ranges (Fall 1901a: 39) in California.

#### Records.

**CAN**: BC **USA**: CA, ID, MT, OR, WA

**Figure 4.**
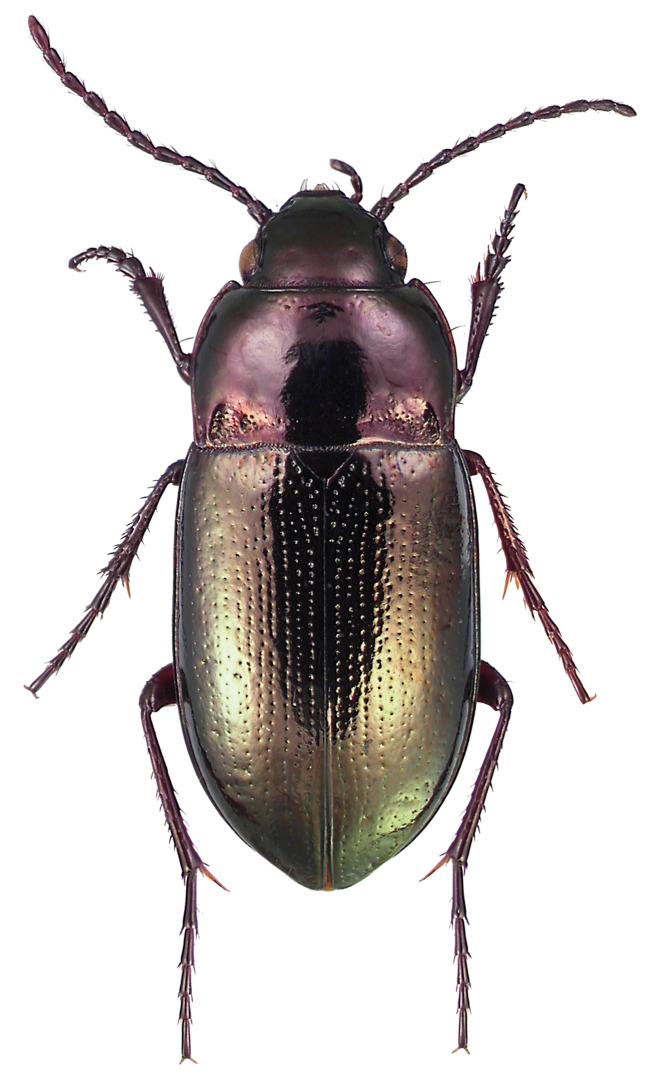
*Trachypachus gibbsii* LeConte. This species is a typical western element as are the other two North American trachypachids. Although superficially similar to some large *Bembidion* or small *Amara* and having the same ecological preferences, we now believe that these beetles, along with members of the related genus *Systolosoma* of South America, are not closely related to any groups of carabids.

### Trachypachus inermis

Motschulsky, 1850

Trachypachus inermis Motschulsky, 1850a: 16. Type locality: «California?» (original citation), herein restricted to Lake Tahoe, Placer County (see Casey 1920: 146, as *Trachypachus specularis*). Four syntypes in ZMMU (Keleinikova 1976: 201).
Trachypachus holmbergi Mannerheim, 1853: 119. Type locality: «ad ostia fl[umen] Kaktnu [= Kenai River] peninsulae Kenai [Alaska]» (original citation). Holotype [by monotypy] location unknown (possibly in ZMH). Synonymy established by LeConte (1857c: 31). Etymology. The specific name was proposed for the Finnish naturalist, geologist, and ethnographer Heinrich [Henrik] Johan Holmberg [1818-1864], who visited Russian America in 1850 and 1851 and collected insects.
Trachypachus oregonus Casey, 1920: 145. Type locality: «Oregon» (original citation). Lectotype (♀), designated by Lindroth (1975: 111), in USNM [# 46831]. Synonymy established by Van Dyke (1925: 112), confirmed by Lindroth (1961a: 1).
Trachypachus specularis Casey, 1920: 146. Type locality: «Lake Tahoe [Placer County], California» (original citation for the lectotype). Lectotype (♂), designated by Lindroth (1975: 111), in USNM [# 46832]. Synonymy established by Van Dyke (1925: 112), confirmed by Lindroth (1961a: 1).

#### Distribution.

The range of this species extends from the Kenai Peninsula in Alaska (Lindroth 1961a: 2) to northwestern Saskatchewan (Hooper 1980: 65), south to southern Colorado (Wickham 1902: 230; LeConte 1878a: 464; Bell 1971: 58), southern Utah (Garfield County, Foster F. Purrington pers. comm. 2011), and the Sierra Nevada and Coast Ranges in California (Fall 1901a: 39). One old specimen, simply labeled “Ks” is known (MCZ, collection LeConte).

#### Records.

**CAN**: AB, BC (VCI), NT, SK, YT **USA**: AK, CA, CO, ID, MT, NV, OR, UT, WA, WY [KS]

### Trachypachus slevini

Van Dyke, 1925

Trachypachus slevini Van Dyke, 1925: 111. Type locality: «Olney [Clatsop County], near Astoria, Oregon» (original citation). Holotype (♀) in CAS [# 1616]. Etymology. The specific name honors Joseph Richard Slevin [1881-1957], curator of herpetology at the California Academy of Sciences from 1928 to 1957.

#### Distribution.

This species is known only from the western regions of Washington and Oregon (Lindroth 1961a: 4).

#### Records.

**USA**: OR, WA

### Family RHYSODIDAE

Laporte, 1840

Rhysodites Laporte, 1840: 291. Type genus: *Rhysodes* Germar, 1822.

#### Diversity.

Worldwide, with about 355 species arrayed in six tribes: Clinidiini (about 135 species), Dhysorini (ten species), Leoglymmiini (one species), Medisorini (one species), Omoglymmiini (about 180 species), and Rhysodini (about 25 species). Over 90% of the species are found in the Southern Hemisphere.

#### Identification.

Bell (1970) revised the North American, Middle American, and West Indies species and provided keys for their identification.

### Tribe Clinidiini

Bell and Bell, 1978

Clinidiina R.T. Bell and J.R. Bell, 1978: 59. Type genus: *Clinidium* Kirby, 1830.

#### Diversity.

Worldwide, with about 135 species arrayed in three genera: *Clinidium* (about 75 species), *Grouvellina* Bell and Bell (17 Madagascan species), and *Rhyzodiastes* Fairmaire (about 45 species). The vast majority of species are found in the Southern Hemisphere, with only 11 species (about 8% of the world fauna) occurring in the Northern Hemisphere.

### Genus Clinidium

Kirby, 1830

Clinidium Kirby, 1830: 6. Type species: *Clinidium guildingii* Kirby, 1830 by monotypy. Etymology (original). From the Greek *clinidion* (small couch), alluding to the body shape of the adult [neuter].

#### Diversity.

About 75 species in the Nearctic (six species), Neotropical (about 65 species), and Palaearctic (three species) Regions arrayed in four subgenera: *Arctoclinidium* (nine species), *Clinidium* s.str. (about 50 Neotropical species), *Mexiclinidium* Bell and Bell (11 Middle American species), and *Tainoa* Bell and Bell (four West Indian species).

#### Identification.

Bell and Bell (1985) revised the species of the world and provided keys for their identification.

### Subgenus Arctoclinidium

Bell, 1970

Arctoclinidium R.T. Bell, 1970: 308. Type species: *Rhysodes sculptilis* Newman, 1838 by original designation. Etymology. From the Greek *arctos* (north) and the generic name *Clinidium* [*q.v*.] [neuter].

#### Diversity.

Northern Hemisphere, with nine species in North America (six species), Japan (one species), Caucasian region (one species), and southern Europe (one species).

### Clinidium apertum allegheniense

Bell and Bell, 1975

Clinidium allegheniense R.T. Bell and J.R. Bell, 1975: 65. Type locality: «Pittsburgh [Allegheny County], Pennsylvania» (original citation). Holotype (♂) in SMEK.

#### Distribution.

This subspecies is known only from southwestern Pennsylvania and the Black Mountains in western North Carolina (Bell and Bell 1985: 91). The record from “Ohio” (Bousquet and Larochelle 1993: 42) needs confirmation.

#### Records.

**USA**: NC, PA [OH]

### Clinidium apertum apertum

Reitter, 1880

Clinidium apertum Reitter, 1880: 29. Type locality: «Himalaya» (original citation), which is incorrect (Bell and Bell 1985: 90); Cartersville, Bartow County, Georgia (see Bell and Bell 1975: 66, as *Clinidium allegheniense georgicum*), herein selected. Syntype(s) in NHMW (Bell and Bell 1985: 90).
Clinidium allegheniense georgicum R.T. Bell and J.R. Bell, 1975: 66. Type locality: «Cartersville [Bartow County], Georgia» (original citation). Holotype (♂) in USNM [# 73195]. Synonymy established by Bell and Bell (1978: 65).

#### Distribution.

This subspecies is known only from the type locality in northern Georgia (Bell and Bell 1985: 90).

#### Records.

**USA**: GA

### Clinidium baldufi

Bell, 1970

Clinidium baldufi R.T. Bell, 1970: 313. Type locality: «Dayton [La Salle County], Ill[inois]» (original citation). Holotype (♂) in MCZ [# 31748]. Etymology. The specific name was proposed for Walter Valentine Balduf [1889-1969], professor of entomology at the University of Illinois.

#### Distribution.

This species ranges from New Jersey to central Iowa, including southwestern Wisconsin (Messer 2010: 33), south to southern Mississippi (Bell and Bell 1985: 89) and northern Florida (Bell 1970: 313). Old specimens simply labeled from Nebraska, Kansas, Missouri, and Texas are known (Bell 1970: 313). The records from “Arkansas” and “Louisiana” (Bousquet and Larochelle 1993: 43) need confirmation.

#### Records.

**USA**: AL, FL, GA, IA, IL, IN, KY, MD, MS, NC, NJ, OH, PA, SC, TN, VA, WI, WV [AR, KS, LA, MO, NE, TX]

### Clinidium calcaratum

LeConte, 1875

Clinidium calcaratum LeConte, 1875b: 164. Type locality: «Vancouver Island; Oregon» (original citation). Syntype(s) [3 originally cited] in MCZ [# 6831].

#### Distribution.

This species ranges from southern British Columbia, including Vancouver Island, south to Mendocino County in the Coast Ranges of California and Tuolumne County in the Sierra Nevada (Bell and Bell 1985: 84).

#### Records.

**CAN**: BC (VCI) **USA**: CA, OR, WA

### Clinidium rosenbergi

Bell, 1970

Clinidium rosenbergi R.T. Bell, 1970: 315. Type locality: «Turkey Run State Park, Parke County, Indiana» (original citation). Holotype (♂) in MCZ [# 31749].

#### Distribution.

This species ranges from northwestern Pennsylvania to eastern Missouri, south to east-central Louisiana (West Feliciana Parish, Igor M. Sokolov pers. comm. 2009) and northern Alabama (Madison County, CMNH).

#### Records.

**USA**: AL, IL, IN, KY, LA, MO, NC, OH, PA, TN, VA

### Clinidium sculptile

(Newman, 1838)

Rhysodes sculptilis Newman, 1838b: 666. Type locality: «Wheeling [Ohio County], [West] Virginia» (lectotype label). Lectotype (♂), designated by Bell and Bell (1985: 92), in BMNH.
Rhysodes conjungens Germar, 1840a: 351 [*nomen dubium*]. Type locality: «Staaten Nordamerika’s» (original citation). Holotype [by monotypy] location unknown (possibly in ZMHB). Synonymy established by LeConte (1875b: 164).

#### Distribution.

The range of this species extends from the Catskills in southern New York to west-central Indiana (Bell and Bell 1985: 92), south to east-central Louisiana (West Feliciana Parish, Igor M. Sokolov pers. comm. 2009), northern Alabama (Bell and Bell 1985: 92), and the Florida Panhandle (Peck and Thomas 1998: 15). Specimens simply labeled from Texas are known (Bell and Bell 1985: 92). The record from central Illinois (Wolcott 1896: 235) needs confirmation.

#### Records.

**USA**: AL, DC, DE, FL, GA, IN, KY, LA, MD, MO, NC, NJ, NY, OH, PA, SC, TN, VA, WV [IL, TX]

### Clinidium valentinei

Bell, 1970

Clinidium valentinei R.T. Bell, 1970: 313. Type locality: «Gorgas, Walker County, Ala[bama]» (original citation). Holotype (♂) in OSUO.

#### Distribution.

This species seems to be confined to the Appalachian Mountains from southwestern Pennsylvania to north-central Alabama and northeastern Georgia (Bell and Bell 1985: 85).

#### Records.

**USA**: AL, GA, NC, PA, SC, TN

### Tribe Omoglymmiini

Bell and Bell, 1978

Omoglymmiina R.T. Bell and J.R. Bell, 1978: 66. Type genus: *Omoglymmius* Ganglbauer, 1891.

#### Diversity.

Worldwide, with about 180 species arrayed in eight genera. The tribe is much more diverse, both in term of species and lineages, in Asia than anywhere else.

### Genus Omoglymmius

Ganglbauer, 1891

Omoglymmius Ganglbauer, 1891a: 533. Type species: *Rhysodes germari* Ganglbauer, 1891 by monotypy. Etymology. From the Greek *omos* (rough) and *glymma* (an engraved figure) [masculine].

#### Diversity.

About 150 species (Lorenz 2005: 158-159) in the Nearctic (two species), Australian, Oriental, Palaearctic (ten species, only one of them present in Europe), and Afrotropical (one species) Regions arrayed in 11 subgenera. More than 90% of the species are found in Asia.

#### Identification.

Bell and Bell (1983) revised the species of the world and provided keys for their identification.

### Subgenus Boreoglymmius

Bell and Bell, 1983

Boreoglymmius R.T. Bell and J.R. Bell, 1983: 140. Type species: *Rhysodes americanus* Laporte, 1836 by original designation. Etymology. From the Greek *bore* (north) and the last two syllables of the generic name *Omoglymmius* [masculine].

#### Diversity.

Three species in North American (two species) and Japan (one species).

### Omoglymmius americanus

(Laporte, 1836)

Rhysodes exaratus Lepeletier and Audinet-Serville [in Latreille et al.], 1825: 308 [primary homonym of *Rhysodes exaratus* Dalman, 1823]. Type locality: «Amérique septentrionale» (original citation), herein restricted to Florence, Florence County, South Carolina (see Bell and Bell 1983: 145). Syntype(s) location unknown (possibly in MHNP).
Rhysodes americanus Laporte, 1836: 58. Replacement name for *Rhysodes exaratus* Lepeletier and Audinet-Serville, 1825.
Rhysodes aratus Newman, 1838b: 664. Type locality. «Alabama» (original citation). Syntype(s) location unknown (possibly in BMNH). Synonymy established by LeConte (1875b: 162).

#### Distribution.

This species ranges from central New York to eastern Minnesota, south to east-central Texas (Bell and Bell 1983: 145) and northern Florida (Peck and Thomas 1998: 15).

#### Records.

**CAN**: ON **USA**: AL, AR, DE, FL, IA, IL, IN, KS, KY, LA, MD, MI, MN, MO, MS, NC, NE, NJ, NY, OH, OK, PA, SC, TN, TX, VA, WI

**Figure 5.**
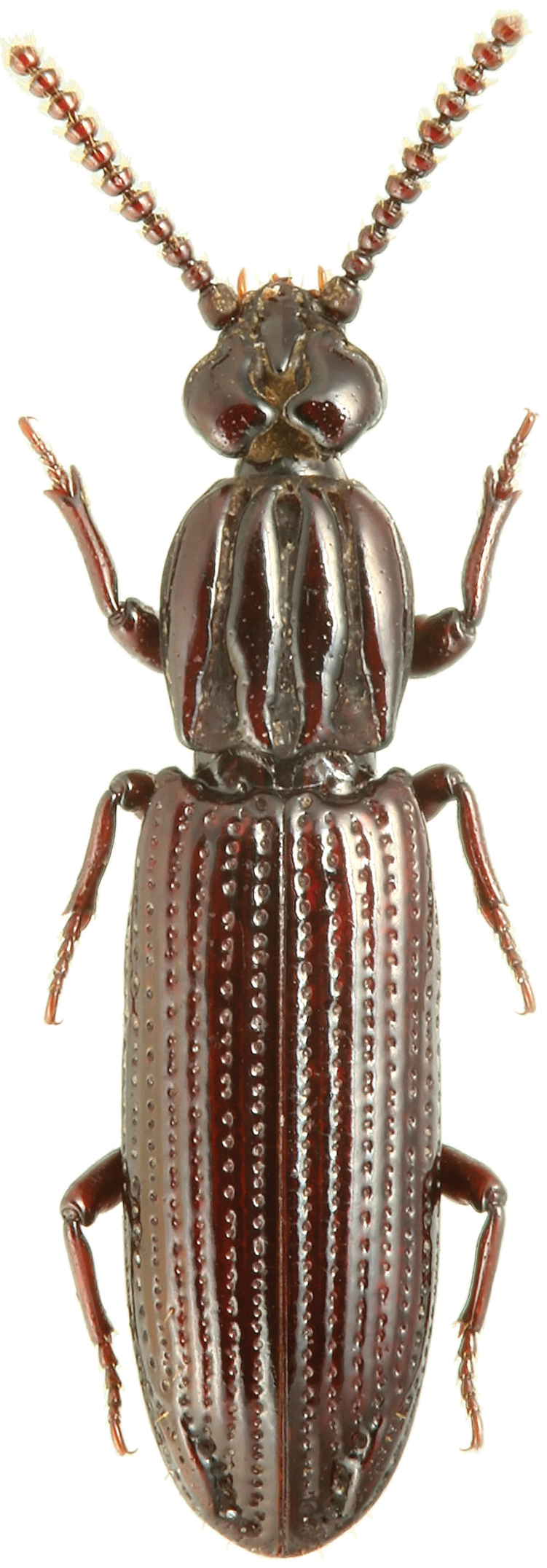
*Omoglymmius americanus* (Laporte). This species is one of the seven rhysodid species-group taxa found in eastern North America. These species live in decaying wood, such as logs, stumps or roots, where they feed on slime molds and fungi. The carabids, on the other hand, are carnivorous, herbivorous, or omnivorous feeding on both animal and plant matters.

### Omoglymmius hamatus

(LeConte, 1875)

Rhysodes hamatus LeConte, 1875b: 163. Type locality: «California» (original citation), herein restricted to Big Trees, Calaveras County (see Bell and Bell 1983: 144). Syntype(s) in MCZ [# 6830].

#### Distribution.

This species ranges from northern Idaho and southeastern Washington south to southern California, including the Sierra Nevada, and southeastern Arizona (Bell and Bell 1983: 143-144). Old specimens without specific localities from British Columbia and Texas (Bell and Bell 1983: 144) are known.

#### Records.

**USA**: AZ, CA, ID, NV, OR, WA [BC, TX]

### Family CARABIDAE

Latreille, 1802

Carabici Latreille, 1802: 80. Type genus: *Carabus* Linnaeus, 1758.

### Subfamily NEBRIINAE

Laporte, 1834

Nebriidae Laporte, 1834: 90. Type genus: *Nebria* Latreille, 1802.

#### Diversity.

About 665 species in the Nearctic (about 75 species), Neotropical (one South American species), and Palaearctic and northern parts of the Oriental (about 595 species) Regions. The species are arrayed in five tribes: Nebriini (about 600 species), Notiokasiini (one South American species), Notiophilini (about 55 species), Opisthiini (five species), and Pelophilini (two species).

### Tribe Pelophilini

Kavanaugh, 1996

Pelophilini Kavanaugh, 1996: 35. Type genus: *Pelophila* Dejean, 1821.

#### Diversity.

This tribe contains a single genus.

### Genus Pelophila

Dejean, 1821

Pelophila Dejean, 1821: 7. Type species: *Carabus borealis* Paykull, 1790 by monotypy. Etymology (see Dejean 1826: 263). From the Greek *pelos* (mud) and *philos* (beloved), alluding to the habitat of *Pelophila borealis* [feminine].

#### Diversity.

Northern Hemisphere, with one Holarctic species and one species endemic to northern North America.

#### Identification.

Lindroth (1961a: 57-60) covered both species and discussed the structural differences between them.

### Pelophila borealis

(Paykull, 1790)

Carabus borealis Paykull, 1790: 51. Type locality: «Karungi Botniae occidentalis [Norrbotten County, Sweden]» (original citation). Syntype(s) probably in NRSS.
Pelophila gebleri Mannerheim, 1823: 38. Type locality: «Sibiria ad Barnaul [Altai Kray, Russia]» (original citation). One syntype in ZMH (Silfverberg 1987: 17). Synonymy established by Dejean (1833: 22).
Pelophila marginata Mannerheim, 1823: 39. Type locality: «peninsula Kamschatka ad portum St. Petri et Pauli [apparently the town of Petropawlowsk, see Lindroth (1961a: 74)]» (original citation). One syntype in ZMH (Silfverberg 1987: 20). Synonymy established by Dejean (1833: 22).
Pelophila eschscholtzii Mannerheim, 1823: 40. Type locality: «insula Unalaschka [Aleutian Islands, Alaska]» (original citation). Lectotype (♂), designated by Lindroth (1961a: 57), in ZMH. Synonymy established by Dejean (1833: 22), confirmed by Lindroth (1961a: 57).
Pelophila elongata Mannerheim, 1823: 41. Type locality: «peninsula Kamschatka ad portum St. Petri et Pauli [apparently the town of Petropawlowsk, see Lindroth (1961a: 74)]» (original citation). One syntype in ZMH (Silfverberg 1987: 15). Synonymy established by Dejean (1833: 22).
Pelophila borealis var. *arctica* Dejean, 1826: 265. Type locality not stated. Syntype(s) location unknown (possibly in MHNP). Synonymy established by Dejean (1833: 22).
Pelophila borealis var. *dejeanii* Dejean, 1826: 265. Type locality: «environs de Barnaoul, Sibérie [Altai Kray, Russia]» (original citation). Syntype(s) probably in MHNP. Synonymy established by Dejean (1833: 22).
Pelophila laevigata Motschulsky, 1844: 92. Type locality: «près de la redoute Yamyschevo non loin du fleuve Irtych [Siberia, Russia]» (original citation). Holotype [by monotypy] in ZMMU (Keleinikova 1976: 202). Synonymy established by Bänninger (1930: 101).
Pelophila californica Motschulsky, 1844: 93. Type locality: «Californie» (original citation), which is incorrect (Lindroth 1961a: 57). Syntype(s) location unknown (possibly in ZMMU though not listed in Keleinikova 1976). Synonymy established by Bänninger (1930: 101).
Pelophila ochotica R.F. Sahlberg, 1844: 17. Type locality: «in monte Morikan [Okhotsk, Khabarovsk Kray, Siberia, Russia]» (original citation). Holotype [by monotypy] location unknown (possibly in ZMUT). Synonymy established by Bänninger (1930: 101).
Pelophila angusticollis Motschulsky, 1860: 98. Type locality: «Kamtschatka» (original citation). Five syntypes in ZMMU (Keleinikova 1976: 186). Synonymy established by Shilenkov (1994: 9).
Pelophila ulkei G.H. Horn, 1870b: 105. Type locality: «Hudson’s Bay Territory» (original citation), restricted to «Gillam, Manit[oba]» by Lindroth (1961a: 58). Holotype [by monotypy] (♂) in MCZ (collection LeConte). Synonymy established by Lindroth (1961a: 57).
Pelophila shermani Casey, 1913: 45. Type locality: «West S[ain]t Modest[e], Labrador» (original citation). Five syntypes [5 originally cited] in USNM [# 46844]. Synonymy established, under the name *Pelophila borealis ulkei* Horn, by Bänninger (1930: 102), confirmed by Lindroth (1961a: 57). Etymology. The specific name was proposed for John Dempster Sherman [1872-1960], dealer of entomological and related books and periodicals.

#### Distribution.

This species is found from northern Europe to the Bering Sea coast (Farkač 2003: 98) and from Alaska, including the Aleutian and Kodiak Islands, to Newfoundland, south to the Abitibi region in western Quebec (Larochelle 1975: 98) [see Lindroth 1963a: Fig. 59]. Fossil remnants of this species, dated between about 14,000 and 18,100 years B.P., have been unearthed in southeastern Iowa (Baker et al. 1986: 96; Schwert 1992: 76) and southern Ontario (Morgan and Morgan 1981: 1107).

#### Records.

**CAN**: AB, BC, LB, MB, NF, NT, NU, ON, QC, SK, YT **USA**: AK – **Holarctic**

**Figure 6.**
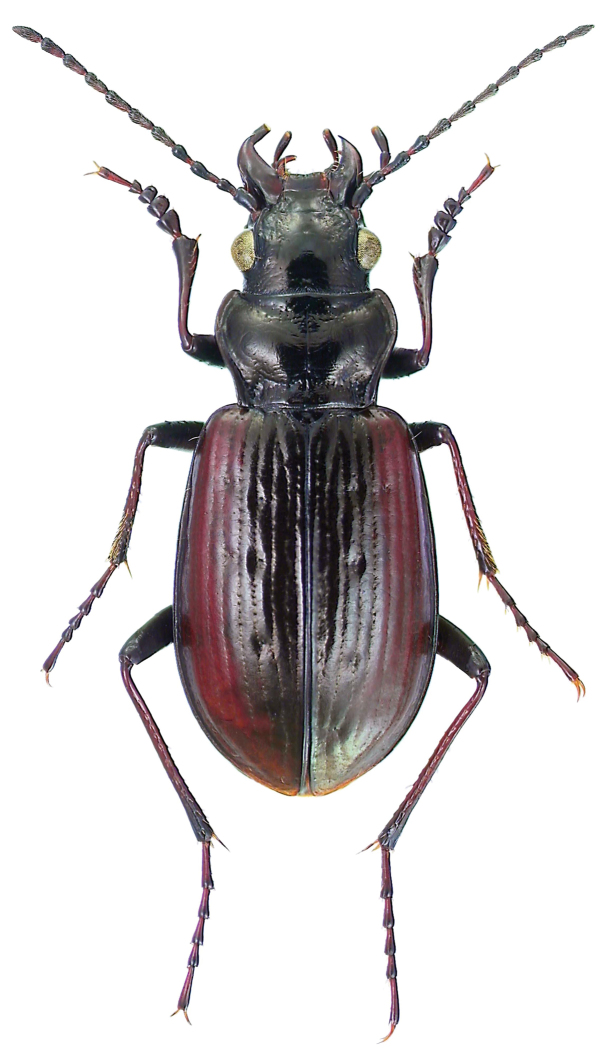
*Pelophila borealis* (Paykull). This pelophiline is one of the 97 species-group taxa which are Holarctic and found naturally in both the Palaearctic and Nearctic Regions. Most of these species are confined to the arctic, subarctic or boreal regions but a few are found mainly in the temperate regions such as *Dyschirius politus*. Most biogeographers agree that these species have spread between the two continents through Beringia during the Quaternary period.

### Pelophila rudis

(LeConte, 1863)

Nebria rudis LeConte, 1863c: 3. Type locality: «Methy [Portage] [= Portage La Loche, northern Saskatchewan]» (original citation). Holotype [by monotypy] (♂) in MCZ [# 653]. Note. In the original description, LeConte indicated that he received “one specimen” from “Mr. Kennicott” collected at “Methy.” Lindroth (1961a: 59) placed the type locality in eastern Alberta. However, I was unable to find any such locality in Alberta, although there is a locality named “Metis” in northwestern Alberta. In the same paper, LeConte (1863c: 1) described his *Cicindela hyperborea* from specimen(s) received from “Mr. R. Kennicott” collected at “Methy Portage, Hudson’s Bay Territory.” I believe LeConte meant “Methy Portage” as the collection site for *Pelophila rudis*. Located in northern Saskatchewan, Methy(e) Portage is currently called Portage La Loche.

#### Distribution.

This species is known from scattered localities from western Newfoundland (Lindroth 1955a: 39) to the Fairbanks area in Alaska (David H. Kavanaugh pers. comm. 2009), south to central British Columbia (Lindroth 1961a: 59) [see Lindroth 1963a: Fig. 65]. Fossil remnants, dated between 14,000 and 15,500 years B.P., have been unearthed in central Iowa (Schwert 1992: 76).

#### Records.

**CAN**: AB, BC, MB, NF, NT, ON, SK, YT **USA**: AK

### Tribe Opisthiini

Dupuis, 1912

Opisthiinae Dupuis, 1912: 1. Type genus: *Opisthius* Kirby, 1837.

#### Diversity.

Northern Hemisphere, with five species in North America (one species) and the Himalayas and China, including Taiwan (four species). The species are arrayed in two genera: *Opisthius* (one species) and *Paropisthius* Casey (four species).

#### Identification.

Bousquet and Smetana (1996) reviewed the species and provided a key for their identification.

### Genus Opisthius

Kirby, 1837

Opisthius Kirby, 1837: 60. Type species: *Opisthius richardsoni* Kirby, 1837 by monotypy. Etymology. Uncertain, possibly from the Greek *opisthen* (behind) or *opisthios* (hinder) [masculine]. Bousquet and Smetana (1996: 218) suggested that the name possibly reflect Kirby’s assignment of *Opisthius* to follow *Elaphrus* in his paper.

#### Diversity.

One North American species.

#### Identification.

The species was treated in Lindroth’s (1961a: 88-90) monograph on the Carabidae of Canada and Alaska.

### Opisthius richardsoni

Kirby, 1837

Opisthius richardsoni Kirby, 1837: 61. Type locality: «[probably] on an island of Lake Winnipeg» (original citation), which is incorrect (Lindroth 1961a: 89); «Medicine Hat, Al[ber]ta» selected by Lindroth (1961a: 89). Lectotype (♂), designated by Bousquet and Smetana (1996: 220), in BMNH. Etymology. The specific name was proposed for John Richardson [1787-1865], surgeon and naturalist to Sir John Franklin on two Arctic expeditions, 1819-1822 and 1825-1827.

#### Distribution.

The range of this species extends from central Saskatchewan to the Arctic Circle in central Alaska (Lindroth 1961a: 89-90), south to Tuolumne County in the Sierra Nevada of California (Dajoz 2007: 17) and north-central New Mexico (Taos County, CNC). The record from “Iowa” (Jaques and Redlinger 1946: 295) is probably based on a mislabeled specimen or a stray. Fossil remnants of this species from the late Wisconsinan age have been found in northeastern Illinois, north-central Iowa, south-central Minnesota, and northwestern Ontario (see Ashworth and Schwert 1991: 511); others from a Plio-Pleistocene sequence have been found in northwestern Greenland and Meighen Island (Böcher 1995: 18).

#### Records.

**CAN**: AB, BC (VCI), NT, SK, YT **USA**: AK, CA, CO, ID, MT, NM, NV, OR, UT, WA, WY

### Tribe Nebriini

Laporte, 1834

Nebriidae Laporte, 1834: 90. Type genus: *Nebria* Latreille, 1802.

#### Diversity.

About 600 species (Lorenz 2005: 116-124) in the Nearctic (about 60 species), Palaearctic, and northern parts of the Oriental Regions. The species are arrayed in four genera: *Archastes* Jedlička (27 Chinese species), *Leistus* (about 180 species), *Nebria* (including *Oreonebria* Daniel) (about 380 species), and *Nippononebria* (seven species).

### Genus Leistus

Frölich, 1799

Leistus Frölich, 1799: 9. Type species: *Leistus testaceus* Frölich, 1799 (= *Carabus ferrugineus* Linnaeus, 1758) designated by Daniel (1903: 171). Etymology. From the Greek *leistos* (to be carried off as booty, to be won by force) [masculine]. Note. Daniel (1903: 171) designated *Carabus ferrugineus* Linnaeus, 1758 as type species of *Leistus* Frölich, 1799, a species not originally included; however, since he listed the name in synonymy with *Leistus testaceus* Frölich, 1799, a species originally included, he is deemed to have designated the latter taxon as type species (ICZN 1999: Article 69.2.2).

#### Diversity.

Northern Hemisphere, with about 180 species (Lorenz 2005: 116-118) in the Nearctic (four species, one of them adventive) and Palaearctic (about 180 species) Regions. The species are arrayed in six subgenera: *Evanoleistus* Jedlička (about 95 Asian species), *Leistus* s.str. (about 40 species), *Nebrileistus* Bänninger (two species on Madeira and Canary Islands), *Neoleistus* (three species), *Sardoleistus* Perrault (one Mediterranean species), and *Pogonophorus* Latreille (about 35 Palaearctic species).

### Subgenus Leistus

Frölich, 1799

Leistus Frölich, 1799: 9. Type species: *Leistus testaceus* Frölich, 1799 (= *Carabus ferrugineus* Linnaeus, 1758) designated by Daniel (1903: 171).

#### Diversity.

About 40 Palaearctic species of which one is adventive in North America.

#### Identification.

Larson (1978: 307-308) discussed the structural differences between the adventive species in North America and the three native species of the subgenus *Neoleistus*.

### Leistus ferrugineus

(Linnaeus, 1758)

Carabus ferrugineus Linnaeus, 1758: 415. Type locality: «Europa» (original citation). One possible syntype in LSL (Lindroth 1957b: 331).

#### Distribution.

This European species is adventive in North America where it is known only from near Saint John’s, Newfoundland (Larson 1978: 307). The first inventoried specimen collected on this continent was caught in 1977.

#### Records.

**CAN**: NF – **Adventive**

### Subgenus Neoleistus

Erwin, 1970

Neoleistus Erwin, 1970b: 112. Type species: *Leistus ferruginosus* Mannerheim, 1843 by original designation. Etymology. From the Greek prefix *neo*- (new) and the generic name *Leistus* [*q.v*.], probably alluding to the fact that these *Leistus* species inhabit the New World [masculine].

#### Diversity.

Three western North American species.

#### Identification.

Erwin (1970b) revised the species and provided a key for their identification.

#### Taxonomic Note.

Perrault (1991a) added three species from the Far East (*Leistus angulicollis* Fairmaire, *Leistus niger* Gebler, and *Leistus shenseensis* Perrault) to this subgenus but Shilenkov (1999: 76) rejected this association and the Asian species are listed in the nominotypical subgenus by Farkač and Janata (2003: 81-82).

### Leistus ferruginosus

Mannerheim, 1843

Leistus ferrugineus Dejean, 1831: 569 [secondary homonym of *Leistus ferrugineus* (Linnaeus, 1758)]. Type locality: «détroit de Norfolk [= Sitka Sound, Baranof Island, Alaska], sur la côte nord-ouest de l’Amérique septentrionale» (original citation). Holotype [by monotypy] (♀) location unknown (possibly lost).
Leistus ferruginosus Mannerheim, 1843: 187. Type locality: «insula Sitkha [= Baranof Island, Alaska]» (original citation). Lectotype, designated by Lindroth (1961a: 56), in ZMH. Synonymy established by Mannerheim (1843: 188).
Leistus nigropiceus Casey, 1913: 45. Type locality: «Metlakatla, British Columbia» (original citation). Lectotype (♀), designated by Lindroth (1975: 111), in USNM [# 46843]. Synonymy established by Hatch (1949b: 115), confirmed by Lindroth (1954b: 121).

#### Distribution.

This species ranges from the Gulf of Alaska coast south to west-central Oregon, east to western Montana (Russell 1968: 44) [see Erwin 1970b: Fig. 7]. At least one specimen simply labeled from California is known (Erwin 1970b: 115).

#### Records.

**CAN**: AB, BC (QCI, VCI) **USA**: AK, MT, OR, WA [CA]

### Leistus longipennis

Casey, 1920

Leistus longipennis Casey, 1920: 148. Type locality: «Humboldt Co[unty], California» (original citation). Holotype [by monotypy] (♂) in USNM [# 46842].

#### Distribution.

The range of this species is restricted to the extreme northwestern tip of Humboldt County in northern California (Erwin 1970b: 117) and southwestern Oregon (Curry County, James R. LaBonte pers. comm. 1992).

#### Records.

**USA**: CA, OR

### Leistus madmeridianus

Erwin, 1970

Leistus madmeridianus Erwin, 1970b: 117. Type locality: «Jacoby Creek, 5.0 miles southeast of Arcata, Humboldt County, California» (original citation). Holotype (♂) in CAS [# 11312].

#### Distribution.

This species is known only from a few localities along the Pacific Coast in northern California [see Erwin 1970b: Fig. 7].

#### Records.

**USA**: CA

### Genus Nippononebria

Uéno, 1955

Nippononebria Uéno, 1955: 49. Type species: *Nebria pusilla* Uéno, 1955 by original designation. Etymology. From the English *nippon* (a Japanese name for Japan) and the generic name *Nebria* [*q.v*.], alluding to the country where these *Nebria*-like species known to Uéno lived [feminine].

#### Diversity.

Seven species in western North America (three species), Japan (three species), and Jilin Province in China (one species) arrayed in two subgenera: *Nippononebria* s.str. for the Japanese and Chinese species and *Vancouveria* for the Nearctic ones.

#### Taxonomic Note.

Kavanaugh (1995, 1996) regarded *Nippononebria* as the sister-group to *Leistus* while Ledoux and Roux (2005) listed *Nippononebria* and *Vancouveria* as subgenera of *Nebria* and suggested they form the sister-group to {*Eonebria* Semenov and Znojko + *Sadonebria* Ledoux and Roux}, a complex of 60 Palaearctic species. Because the North American students are used to Kavanaugh’s approach, the taxon is retained here as a distinct genus.

### Subgenus Vancouveria

Kavanaugh, 1995

Vancouveria Kavanaugh, 1995: 159. Type species: *Nebria virescens* Horn, 1870 by original designation. Etymology. From the geographic name Vancouver [feminine].

#### Diversity.

Three species in western North America.

#### Identification.

Ledoux and Roux (2005: 712) reviewed the species and provided a key for their identification.

### Nippononebria altisierrae

(Kavanaugh, 1984)

Nebria altisierrae Kavanaugh, 1984: 160. Type locality: «Olmsted Point (2560 m), Yosemite National Park [Mariposa County], Sierra Nevada, California» (original citation). Holotype (♂) in CAS [# 14338].

#### Distribution.

This species occurs at high elevations in the Sierra Nevada of California, from El Dorado County south to Sequoia National Park [see Kavanaugh 1984: Fig. 31].

#### Records.

**USA**: CA

### Nippononebria campbelli

(Kavanaugh, 1984)

Nebria campbelli Kavanaugh, 1984: 161. Type locality: «Mount Baker (1460-1520 m), Cascade Range, Whatcom County, Washington» (original citation). Holotype (♂) in CAS [# 14339].

#### Distribution.

This species ranges from the Three Brothers Mountain in southern British Columbia south to Mount Baker in Washington [see Kavanaugh 1984: Fig. 31].

#### Records.

**CAN**: BC **USA**: WA

### Nippononebria virescens

(Horn, 1870)

Nebria virescens G.H. Horn, 1870b: 100. Type locality: «Vancouver [British Columbia]» (original citation). Holotype [by monotypy] in MCZ [# 652].
Nebria brevis Casey, 1913: 55. Type locality: «Corvallis [Benton County], Oregon» (original citation for the lectotype). Lectotype (♂), designated by Lindroth (1975: 112), in USNM [# 46862]. Synonymy established with doubt by Bänninger (1925: 261), confirmed by Lindroth (1961a: 76).

#### Distribution.

This species ranges from southwestern British Columbia, including Vancouver Island, south to the northern Sierra Nevada of California in Plumas County, east to the western edge of the Rockies in west-central Idaho (Kavanaugh 1978: 349).

#### Records.

**CAN**: BC (VCI) **USA**: CA, ID, OR, WA

### Genus Nebria

Latreille, 1802

Nebria Latreille, 1802: 89. Type species: *Carabus brevicollis* Fabricius, 1792 designated by Latreille (1810: 426). Etymology. According to Ledoux and Roux (2005: 29), the name came from the Greek *nebrios* (fawn), possibly alluding to the coloration of *Nebria complanata*, the first species cited by Latreille in the genus. However Latreille (1804: 275) stated that the name derived from *nebrias* which is part of the list of unknown fishes mentioned by the elders. According to Dalby (2003: 121), *nebrias*, cited in Aristotle and others, is perhaps the dogfish *Scyliorhinus canicula* [feminine].

#### Distribution.

About 380 species (535 species-group taxa) in the arctic, subarctic, boreal, and temperate areas of the Nearctic and Palaearctic (including northern Africa and the Canary Islands) Regions arrayed in 25 subgenera (Ledoux and Roux 2005: 76 excluding *Nippononebria* and *Vancouveria*). The North American fauna has 52 species (82 species-group taxa) placed in four subgenera.

#### Identification.

Ledoux and Roux (2005) reviewed the species of the world and provided keys for the identification of the species. Lindroth’s (1961a) key included all North American species then known but many species-group taxa have been described subsequently by Kavanaugh (1979a, 1981b, 1984, 2008).

#### Taxonomic Note.

The species of *Nebria* (including *Nippononebria*) have been segregated in two main lineages by Ledoux and Roux (2005: 71-75), one (named *Vetanebri*) represented in the Palaearctic Region by 90 species and in the Nearctic Region by the three species of *Vancouveria*, the other one (*Notanebri*) containing about 290 species, 52 in the Nearctic and almost 240 in the Palaearctic.

### Subgenus Boreonebria

Jeannel, 1937

Boreonebria Jeannel, 1937b: 2. Type species: *Carabus rufescens* Strøm, 1768 (= *Carabus gyllenhali* Schönherr, 1806) by original designation. Etymology. From the Greek *bore* (north) and the generic name *Nebria* [*q.v*.], probably alluding to the northern ranges of the species of this taxon [feminine].

#### Diversity.

Thirty-one species (Ledoux and Roux 2005: 82) in North America (seven species) and Eurasia (26 species). Two species are Holarctic (*Nebria frigida* and *Nebria nivalis*).

### [gyllenhali group]

### Nebria crassicornis crassicornis

Van Dyke, 1925

Nebria crassicornis Van Dyke, 1925: 121. Type locality: «Paradise Park, [Mount] Rainier National Park [Pierce County], Washington» (original citation). Holotype (♂) in CAS [# 1627].

#### Distribution.

This subspecies is confined to a small area of the Coast Ranges and Cascade Range in southwestern British Columbia and western Washington [see Kavanaugh 1988: Fig. 15].

#### Records.

**CAN**: BC **USA**: WA

### Nebria crassicornis intermedia

Van Dyke, 1949

Nebria intermedia Van Dyke, 1949a: 49. Type locality: «Logan Pass, Glacier National Park [Flathead County], Montana» (original citation). Holotype (♂) in CAS [# 6008].

#### Distribution.

This subspecies ranges from northern British Columbia south to northeastern Oregon and southern Utah, east to northwestern Wyoming and central Utah [see Kavanaugh 1988: Fig. 15].

#### Records.

**CAN**: AB, BC **USA**: ID, MT, OR, UT, WA, WY

### Nebria frigida

Sahlberg, 1844

Nebria frigida R.F. Sahlberg, 1844: 11. Type locality: «monte Morikan cepi [Okhotsk, Khabarovsk Kray, Siberia, Russia]» (original citation). Lectotype (♀), designated by Lindroth (1961a: 81), in ZMUT.
Nebria viridis G.H. Horn, 1870b: 101. Type locality: «St. Michaels [= Saint Michael on south coast of Norton Sound], Alaska» (original citation). Lectotype (♂), designated by Kavanaugh (1979a: 116), in MCZ [# 34044]. Synonymy established by Lindroth (1961a: 81).
Nebria parvula J.R. Sahlberg, 1885b: 47. Type locality: Port Clarence, Alaska (inferred from title of the paper). Holotype [by monotypy; designated lectotype by Lindroth (1961a: 81)] (♂) in NRSS. Synonymy established, under the name *Nebria viridis* Horn, by Van Dyke (1924a: 5), confirmed by Lindroth (1961a: 81).
Nebria reducta Casey, 1920: 150. Type locality: «S[ain]t Paul Island, Alaska» (original citation), which is incorrect according to Lindroth (1961a: 24, 81). Lectotype (♀), designated by Lindroth (1975: 112), in USNM [# 46866]. Synonymy established, under the name *Nebria viridis* Horn, by Van Dyke (1924a: 5), confirmed by Lindroth (1961a: 81).

#### Distribution.

This Holarctic species is found in eastern Siberia (Farkač and Janata 2003: 88) and from the Alaskan Coast Range to the Anderson River Delta in northern Northwest Territories, south to northern British Columbia (Kavanaugh 1978: 714-715).

#### Records.

**CAN**: BC, NT, YT **USA**: AK – **Holarctic**

#### Note.

This species is placed in its own group by Ledoux and Roux (2005: 82).

### Nebria gyllenhali castanipes

(Kirby, 1837)

Helobia castanipes Kirby, 1837: 20. Type locality: «Lat. 65° [= apparently region of Great Bear Lake, Northwest Territories]» (original citation), restricted to «Nipigon, Ont[ario]» by Lindroth (1961a: 78). Lectotype (♀), designated by Kavanaugh (1979a: 111), in BMNH.
Nebria moesta LeConte, 1850: 209. Type locality: Lake Superior (inferred from title of the paper). Lectotype (♂), designated by Kavanaugh (1979a: 114), in MCZ [# 645]. Synonymy established by LeConte (1873b: 322), confirmed by Lindroth (1954b: 121).
Nebria elias Motschulsky, 1866: 276 [*nomen dubium*]. Type locality: «Amer[ique] rus[se]» (original citation). Syntype(s) lost (Keleinikova 1976: 196; Kavanaugh 1979a: 112). Synonymy established with doubt by Lindroth (1961a: 78).
Nebria labradorica Casey, 1920: 151. Type locality: «West S[ain]t Modest[e], Labrador» (original citation). Lectotype (♀), designated by Lindroth (1975: 112), in USNM [# 46855]. Synonymy established by Lindroth (1954b: 122).
Nebria prominens Casey, 1920: 151. Type locality: «M[oun]t Washington [Coos County], New Hampshire» (original citation). Lectotype (♀), designated by Lindroth (1975: 112), in USNM [# 46867]. Synonymy established by Lindroth (1954b: 122).
Nebria curtulata Casey, 1924: 20. Type locality: «W[est] S[ain]t Modest[e], Labrador» (original citation). Lectotype (♂), designated by Lindroth (1975: 112), in USNM [# 46856]. Synonymy established by Lindroth (1954b: 122).

#### Distribution.

This subspecies ranges from Greenland (Böcher 1988: 5) to Alaska (Lindroth 1961a: 79), south to northeastern Oregon, northeastern Nevada, western South Dakota, and northeastern New York (Kavanaugh 1978: 731-741).

#### Records.

**DEN**: GL **CAN**: AB, BC (VCI), LB, MB, NF, NT, ON, QC, SK, YT **USA**: AK, ID, ME, MI, MN, MT, NH, NV, NY, OR, SD, WA, WI, WY

#### Note.

The name *Nebria rufescens* (Strøm, 1768) is used by several authors instead of *Nebria gyllenhali* (Schönherr, 1806). I concur with Kavanaugh (1979a: 111) and Ledoux and Roux (2005: 107) that *Carabus rufescens* Strøm should be considered a *nomen dubium*.

### Nebria gyllenhali lassenensis

Kavanaugh, 1979

Nebria gyllenhali lassenensis Kavanaugh, 1979a: 96. Type locality: «Emerald Lake, Mount Lassen (south slope), Lassen Volcanic National Park [Shasta County], California» (original citation). Holotype (♂) in CAS [# 12511].

#### Distribution.

This subspecies is restricted to mountains in the southern part of the Cascade Range, south of the lower Columbia River valley, and the northern part of the Sierra Nevada, south to Sonora Pass [see Kavanaugh 1979a: Fig. 54].

#### Records.

**USA**: CA, OR

### Nebria gyllenhali lindrothi

Kavanaugh, 1979

Nebria gyllenhali lindrothi Kavanaugh, 1979a: 97. Type locality: «Brooklyn Lake (3,200 m), Albany County, Wyoming» (original citation). Holotype (♂) in CAS [# 12512].

#### Distribution.

This subspecies is found in the southern Rocky Mountains from the Medicine Bow Mountains and Sierra Madre of southern Wyoming south to northern New Mexico, west to the Uinta Mountains in eastern Utah and the Chuska Mountains in northeastern Arizona (David H. Kavanaugh pers. comm. 2008) [see Kavanaugh 1979a: Fig. 54].

#### Records.

**USA**: AZ, CO, NM, UT, WY

### Nebria nivalis gaspesiana

Kavanaugh, 1979

Nebria nivalis gaspesiana Kavanaugh, 1979a: 96. Type locality: «Ruisseau du Diable (980-1,070 m), Mont Albert, Gaspé-Ouest, Québec» (original citation). Holotype (♂) in CAS [# 12510].

#### Distribution.

This subspecies is known from western Newfoundland, coastal Labrador (Lindroth 1961a: 82), and the east edge of the Ungava Bay in northern Quebec; isolated on high mountains in the Gaspé Peninsula in Quebec and on Mount Katahdin in Maine [see Kavanaugh 1979a: Fig. 53].

#### Records.

**CAN**: LB, NF, QC **USA**: ME

### Nebria nivalis nivalis

(Paykull, 1790)

Carabus nivalis Paykull, 1790: 52. Type locality: «Lapponiae Lulensis [= Luleå, Norrbotten County, Sweden]» (original citation). Lectotype (♂), designated by Kavanaugh (1979a: 111), in NRSS.
Nebria bifaria Mannerheim, 1853: 120. Type locality: «insula St. Pauli [Alaska]» (original citation). Lectotype (♂), designated by Kavanaugh (1979a: 112), in ZILR. Synonymy established by Lindroth (1961a: 81).
Nebria femoralis Motschulsky, 1859b: 541 [primary homonym of *Nebria femoralis* Chaudoir, 1843]. Type locality: region of Yakutsk, east-central Siberia, Russia (inferred from title of the paper). Lectotype, designated by Shilenkov (1975: 839), in ZILR. Synonymy established by Jeannel (1937b: 4).
Nebria molbis Motschulsky, 1866: 274. Type locality: «Amérique russe» (original citation). Lectotype, designated by Kavanaugh (1979a: 114), in ZMMU. Synonymy established by Lindroth (1961a: 82).
Nebria femorata Motschulsky, 1866: 275. Type locality: «Sib[eria] bor[eali] Jakutzk [= Yakutsk, Yakutia, Siberia, Russia]» (original citation). Syntype(s) location unknown (possibly in ZILR). Synonymy established by Bänninger (1949: 144). Note. The name *Nebria femorata* Motschulsky has been interpreted as a replacement name for *Nebria femoralis* Motschulsky, 1859 by some authors (e.g., Ledoux and Roux 2005: 112). However, there is no indication in Motschulsky (1866) that he proposed the name as a replacement name. The footnote on the same page as the description of *femorata* indicates that Motschulsky (1866: 275) considered *Nebria femoralis* Chaudoir as belonging to a different genus, *Alpaeus*. Moreover in the catalogue of his new genera and species described, Motschulsky (1869: 26) listed his *Nebria femoralis* and *Nebria femorata* as different taxa.

#### Distribution.

This Holarctic subspecies is known from northern Europe to the Bering Sea Coast (Farkač and Janata 2003: 88), and from the Arctic Plains in Alaska to Baffin Island and the western edge of the Ungava Bay in northern Quebec (Kavanaugh 1978: 779-782).

#### Records.

**CAN**: BC, NT, QC, YT **USA**: AK – **Holarctic**

#### Note.

Ledoux and Roux (2005: 112) retained *Nebria bifaria* Mannerheim, 1853 as a valid subspecies of *Nebria nivalis*.

### [hudsonica group]

### Nebria bellorum

Kavanaugh, 1979

Nebria lacustris bellorum Kavanaugh, 1979a: 95. Type locality: «West Prong Little Pigeon River (at Chimneys Picnic Area; 3000’) [Sevier County], Great Smoky Mountains National Park, Tennessee» (original citation). Holotype (♂) in CAS [# 12506].

#### Distribution.

This species is restricted to the Great Smoky Mountains National Park and adjacent mountain ranges in the southern Appalachians [see Kavanaugh 1979a: Fig. 52]. Two specimens labeled from Jefferson County in Colorado and Saint Tammany Parish in Louisiana seen by Kavanaugh (1979a: 96) are likely mislabeled.

#### Records.

**USA**: NC, TN

#### Note.

This taxon, originally described as a subspecies of *Nebria lacustris* Casey, has been raised to species status by Kavanaugh et al. (2011).

### Nebria gouleti

Kavanaugh, 1979

Nebria gouleti Kavanaugh, 1979a: 94. Type locality: «Rattlesnake Creek (3000’), 10 miles s[outh]w[est] of Antone, Asotin County, Washington» (original citation). Holotype (♂) in CAS [# 12504].

#### Distribution.

This species is restricted to Washington, northern Oregon, and Idaho [see Kavanaugh 1979a: Fig. 51]. Seven specimens labeled from Longview (Highwood River) in southwestern Alberta seen by Kavanaugh (1979a: 95) are listed as doubtful by him; two specimens simply labeled from California are likely mislabeled.

#### Records.

**USA**: ID, OR, WA [AB]

### Nebria hudsonica

LeConte, 1863

Nebria hudsonica LeConte, 1863c: 3. Type locality: «Saskatchewan, Hudson’s Bay Territory» (original citation), restricted to «North Saskatchewan River at Rocky Mountain House, Alberta» by Kavanaugh (1979a: 113). Lectotype (♂), designated by Kavanaugh (1979a: 112), in MCZ [# 643].

#### Distribution.

This species ranges from the north shore of Lake Superior in western Ontario to southern Yukon Territory and southeastern Alaska (Skagway, David H. Kavanaugh pers. comm. 2008), south to the Columbia River drainage in northern Oregon and to north-central Utah and central Colorado along the Rocky Mountains (Kavanaugh 1978: 745-753). The records from New Mexico (Snow 1885: 66; Fall and Cockerell 1907: 156) are probably in error.

#### Records.

**CAN**: AB, BC, MB, NT, ON, SK, YT **USA**: AK, CO, ID, MT, OR, UT, WA, WY

### Nebria lacustris

Casey, 1913

Nebria lacustris Casey, 1913: 56. Type locality: «Bayf[ie]ld [Bayfield County], Wis[consin]» (lectotype label). Lectotype (♀), designated by Lindroth (1975: 112), in USNM [# 46865].
Nebria expansa Casey, 1913: 56. Type locality: «Indiana» (original citation for the lectotype), restricted to «Turkey Run State Park, Parke County» by Kavanaugh (1979a: 112). Lectotype (♀), designated by Lindroth (1975: 147), in USNM [# 46864]. Synonymy established by Lindroth (1961a: 77).

#### Distribution.

This species is found from northern New Brunswick (Restigouche County, CNC) to northern Minnesota, north to southeastern Manitoba, south to east-central Iowa, southern Indiana, and western North Carolina along the Appalachians [see Kavanaugh 1979a: Fig. 52]. The record from “Texas” (Casey 1913: 56) is in error (Kavanaugh 1979a: 112).

#### Records.

**CAN**: MB, NB, ON, QC **USA**: CT, DC, IA, IL, IN, KY, MA, MD, ME, MI, MN, NC, NH, NJ, NY, OH, PA, TN, VA, VT, WI, WV

### Subgenus Nakanebria

Ledoux and Roux, 2005

Nakanebria Ledoux and Roux, 2005: 183. Type species: *Nebria kurosawai* Nakane, 1960 by original designation. Etymology. From the surname of the Japanese coleopterist Takehiko Nakane [1920-1999] and the generic name *Nebria* [*q.v*.] [feminine].

#### Diversity.

Six species in western North America (two species) and the Far East (four species).

#### Taxonomic Note.

According to Ledoux and Roux (2005: 75), *Nakanebria* is the sister-group to *Reductonebria* and the two form the sister-group to *Catonebria*.

### Nebria paradisi

Darlington, 1931

Nebria vandykei Darlington, 1930: 104 [primary homonym of *Nebria vandykei* Bänninger, 1928]. Type locality: «near Paradise Valley (about 6000 feet), Mount Rainier [Pierce County], Washington» (original citation). Holotype (♂) in MCZ [# 35405].
Nebria paradisi Darlington, 1931: 24. Replacement name for *Nebria vandykei* Darlington, 1930.

#### Distribution.

This species ranges in the Cascade Range from northwestern Washington to northwestern Oregon [see Kavanaugh 1988: Fig. 19].

#### Records.

**USA**: OR, WA

### Nebria turmaduodecima

Kavanaugh, 1981

Nebria turmaduodecima Kavanaugh, 1981b: 436. Type locality: «Caribou Basin (2290 m), Trinity Alps, Siskiyou County, California» (original citation). Holotype (♂) in CAS [# 13729].

#### Distribution.

This species is endemic to the Trinity Alps in northwestern California [see Kavanaugh 1981b: Fig. 21].

#### Records.

**USA**: CA

### Subgenus Reductonebria

Shilenkov, 1975

Reductonebria Shilenkov, 1975: 834. Type species: *Nebria ochotica* Sahlberg, 1844 by original designation. Etymology. From the Latin *reducto* (bring back, reduce) and the generic name *Nebria* [*q.v*.], possibly alluding to the absence of mid-lateral setae on the pronotum of the species [feminine].

#### Diversity.

Twenty-eight species in North America (24 species) and Siberia and the Far East (four species).

#### Faunistic Note.

*Nebria carbonaria* Eschscholtz was reported from Alaska by Horn (1870b: 104) and LeConte (1878a: 479) and there is a specimen of this species in the LeConte collection labeled “Ins. S. Pauli,” one of the Pribilof Islands in Alaska. Lindroth (1961a: 74) believes the specimen is probably mislabeled since no other specimens of the species have been found on the island despite extensive search. The species was described from specimens collected “in Kamtschatka, bei St. Peter und Paul” which, according to Lindroth (1961a: 74), refers to the town of Petropavlovsk in Kamtschatka. Until recent specimens are collected on this continent, the species is not listed as a North American resident.

### [gregaria group]

### Nebria arkansana arkansana

Casey, 1913

Nebria arkansana Casey, 1913: 52. Type locality: «Indiana» (original citation for the lectotype), which according to Lindroth (1961a: 70) and Kavanaugh (1979a: 112) is incorrect; «Valley [of the] Upper San Juan [River], [Archuleta County], Color[ado]» selected by Lindroth (1961a: 70). Lectotype (♂), designated by Lindroth (1975: 111), in USNM [# 46858].

#### Distribution.

This subspecies is found in southern Wyoming, Colorado, northern New Mexico, and southeastern Utah (Kavanaugh 1978: 675-678). One specimen labeled from Nez Perce County in western Idaho is considered doubtful by Kavanaugh (1978: 678).

#### Records.

**USA**: CO, NM, UT, WY [ID]

#### Note.

This species is placed with the species of the *lyelli* group by Ledoux and Roux (2005: 195).

### Nebria arkansana edwardsi

Kavanaugh, 1979

Nebria arkansana edwardsi Kavanaugh, 1979a: 100. Type locality: «Logan Pass (7100’), Glacier National Park [Flathead County], Montana» (original citation). Holotype (♂) in CAS [# 12495]. Etymology. The subspecific name was proposed for J. Gordon Edwards [1919-2004], teacher, coleopterist, and mountaineer.

#### Distribution.

This subspecies is widely distributed in the Rocky Mountain region from southern Yukon Territory south to northeastern Nevada, southern Idaho, and northern Wyoming [see Kavanaugh 1979a: Fig. 58].

#### Records.

**CAN**: AB, BC, YT **USA**: ID, MT, NV, OR, WA, WY

### Nebria arkansana fragilis

Casey, 1924

Nebria fragilis Casey, 1924: 21. Type locality: «North Fork, Provo Cañon [Utah County], Utah» (original citation). Lectotype (♂), designated by Lindroth (1975: 112), in USNM [# 46857].
Nebria arkansana uinta Kavanaugh, 1979a: 102. Type locality: «Lost Lake (9800’), Summit County, Utah» (original citation). Holotype (♂) in CAS [# 12497]. Synonymy established by Kavanaugh (1984: 167).
Nebria fragilis teewinot Kavanaugh, 1979a: 103. Type locality: «Mount Teewinot (southeast slope; 7100-9000’), Grand Teton National Park [Teton County], Wyoming» (original citation). Holotype (♂) in CAS [# 12500]. Synonymy established by Kavanaugh (1984: 167).

#### Distribution.

This subspecies is restricted to mountains in western Wyoming and northern and central Utah (Kavanaugh 1979a: Figs 58–59).

#### Records.

**USA**: UT, WY

### Nebria arkansana oowah

Kavanaugh, 1979

Nebria arkansana oowah Kavanaugh, 1979a: 102. Type locality: «Mill Creek (at Oowah Lake; 8800’), Grand County, Utah» (original citation). Holotype (♂) in CAS [# 12496].

#### Distribution.

This subspecies is endemic to the La Sal Mountains in eastern Utah [see Kavanaugh 1979a: Fig. 58].

#### Records.

**USA**: UT

### Nebria charlottae

Lindroth, 1961

Nebria charlottae Lindroth, 1961a: 67. Type locality: «Queen Charlotte Islands [British Columbia]» (original citation), restricted to «Masset, Graham Island» by Kavanaugh (1992: 55). Holotype (♂) in CNC [# 7611].

#### Distribution.

This species is restricted to the Queen Charlotte Archipelago (Kavanaugh 1992: 55).

#### Records.

**CAN**: BC (QCI)

### Nebria gregaria

Fischer von Waldheim, 1820

Nebria gregaria Fischer von Waldheim, 1820: plate 6. Type locality: «insula Unalaschka [Alaska]» (Fischer von Waldheim 1822: 73). Lectotype (♂), designated by Kavanaugh (1979a: 113), in ZMMU.
Nebria cuneata Casey, 1913: 50. Type locality: «Alaska» (original citation). Lectotype (♂), designated by Lindroth (1975: 111), in USNM [# 46851]. Synonymy established by Lindroth (1961a: 66).

#### Distribution.

This species is endemic to the Aleutian Islands [see Kavanaugh 1981a: Fig. 8].

#### Records.

**USA**: AK

#### Note.

*Nebria macrocephala* Motschulsky was described from specimens originating «probablement de Sitka ou d’Ounalachka [Alaska]» (Motschulsky 1844: 128). The name is listed as a junior synonym of *Nebria gregaria* Fischer von Waldheim, 1820 in Ledoux and Roux (2005: 222) but according to Bänninger (1923: 131) only the specimens reported subsequently by Motschulsky (1860: 97; 1866: 273) as *Nebria macrocephala* belong to *Nebria gregaria*. The syntypes are conspecific with specimens of *Nebria stigmula* Dejean, 1826 (= *Nebria hellwigii* Panzer, 1803) as noted by Mannerheim (1853: 111). The provenance given by Motschulsky is probably incorrect.

### Nebria haida

Kavanaugh, 1984

Nebria haida Kavanaugh, 1984: 162. Type locality: «1.8 km N[orth] of Mount Needham (700-780 m), Graham Island, Queen Charlotte Islands, British Columbia» (original citation). Holotype (♂) in CAS [# 14341]. Note. Kavanaugh (1992: 56) pointed out that the peak located 1.8 km south of the collecting site is not Mount Needham as originally reported but is unnamed. The collecting site is located at the summit and surrounding slopes of another unnamed peak which Kavanaugh called «Nebria Peak».

#### Distribution.

This species is endemic to high elevations in the Queen Charlotte Islands [see Kavanaugh 1984: Fig. 31] and adjacent mainland on Mount McNeil (Kavanaugh 1992: 56) in British Columbia.

#### Records.

**CAN**: BC (QCI)

#### Note.

Clarke et al. (2001: 1416) concluded that this taxon may be more appropriately treated as a subspecies of *Nebria charlottae* given the minor differences in morphology and biology.

### Nebria jeffreyi

Kavanaugh, 1984

Nebria jeffreyi Kavanaugh, 1984: 162. Type locality: «South Fork McCoy Creek (2390-2560 m), Steens Mountains, Harney County, Oregon» (original citation). Holotype (♂) in CAS [# 14342].

#### Distribution.

This species is known only from the Steens Mountains in south-central Oregon [see Kavanaugh 1984: Fig. 31].

#### Records.

**USA**: OR

#### Note.

This species is placed with the species of the *lyelli* group by Ledoux and Roux (2005: 195).

### Nebria lituyae

Kavanaugh, 1979

Nebria lituyae Kavanaugh, 1979a: 100. Type locality: «M[oun]t Blunt (3356’), 2 miles s[outh] of Lituya Bay, Alaska» (original citation). Holotype (♂) in CAS [# 13460].

#### Distribution.

This species is known only from a small area in the Alexander Archipelago and northwestern British Columbia [see Kavanaugh 1988: Fig. 24].

#### Records.

**CAN**: BC **USA**: AK

### Nebria louiseae

Kavanaugh, 1984

Nebria louiseae Kavanaugh, 1984: 162. Type locality: «Skedans, Louise Island, Queen Charlotte Islands, British Columbia» (original citation). Holotype (♂) in CAS [# 15005].

#### Distribution.

This species is known from several islands in the Queen Charlotte Archipelago, British Columbia (Kavanaugh 1992: 55).

#### Records.

**CAN**: BC (QCI)

#### Note.

Based on DNA sequence analyses, Clarke et al. (2001: 1416) concluded that this taxon may not represent a distinct taxonomic unit but rather a variant of *Nebria charlottae*.

### Nebria sahlbergii modoc

Kavanaugh, 1979

Nebria sahlbergii modoc Kavanaugh, 1979a: 99. Type locality: «Pine Creek (4 miles e[ast] of New Pine Creek; 5700’), Modoc County, California» (original citation). Holotype (♂) in CAS [# 12513].

#### Distribution.

This subspecies is known only from the Warner Mountains in northeastern California [see Kavanaugh 1979a: Fig. 56].

#### Records.

**USA**: CA

### Nebria sahlbergii sahlbergii

Fischer von Waldheim, 1828

Nebria sahlbergii Fischer von Waldheim, 1828: 254. Type locality: «Sitcha [= Sitka, Baranof Island, Alaska]» (original citation). Lectotype (♂), designated by Kavanaugh (1979a: 115), in ZMH. Etymology. The specific name honors the Finnish naturalist Carl Reinhold Sahlberg [1779-1860] who worked mainly on beetles. Sahlberg was professor of economic and natural history at the Academy of Åbo and, after the destruction of the city and university by fire, at the University of Helsinki.
Nebria violacea Motschulsky, 1850a: 73. Type locality: «Sitka [Baranof Island, Alaska]» (original citation for *Nebria sahlbergii* var. in Mannerheim, 1843). Lectotype (♂), designated by Kavanaugh (1979a: 115), in ZMH. Synonymy established by Lindroth (1961a: 68). Note. *Nebria violacea* was proposed for Mannerheim’s (1843: 189) var. b of *Nebria sahlbergii* Fischer von Waldheim; therefore the description is by indication. The lectotype designated by Kavanaugh (1979a: 115) is the same specimen he designated as lectotype of *Nebria sahlbergii* Fischer von Waldheim, 1828.
Nebria aleuta Van Dyke, 1924a: 5. Type locality: «Mount Makushin, Unalaska Island, Alaska» (original citation). Holotype (♂) in CAS [# 3342]. Synonymy established by Lindroth (1961a: 68).

#### Distribution.

This subspecies ranges from the Aleutian Islands in Alaska (Lindroth 1961a: 70) to southwestern Northwest Territories (Tungsten, David H. Kavanaugh pers. comm. 2008), south to northwestern Montana (Edwards 1975: 50) and southern Oregon [see Kavanaugh 1988: Fig. 20]. The records from New Mexico (Snow 1885: 66; Fall and Cockerell 1907: 156), Colorado (Wickham 1902: 232; Armin 1963: 94), and Wyoming (Lavigne 1977: 46) are probably in error.

#### Records.

**CAN**: AB, BC (QCI, VCI), NT, YT **USA**: AK, MT, OR, WA

#### Note.

This species is placed in the *lyelli* group by Ledoux and Roux (2005: 195).

### Nebria sahlbergii triad

Kavanaugh, 1979

Nebria sahlbergii triad Kavanaugh, 1979a: 99. Type locality: «South Fork Salmon River (at Big Flat Campground; 1490 m), Trinity County, California» (original citation). Holotype (♂) in CAS [# 12514].

#### Distribution.

This subspecies is yet recorded only from the Klamath Mountains system in northwestern California [see Kavanaugh 1979a: Fig. 56].

#### Records.

**USA**: CA

### Nebria zioni oasis

Kavanaugh, 1979

Nebria zioni oasis Kavanaugh, 1979a: 103. Type locality: «Leeds Creek at Oak Grove Campground (6300-6500’), Washington County, Utah» (original citation). Holotype (♂) in CAS [# 12518].

#### Distribution.

This subspecies is restricted to the Pine Valley Mountains in southwestern Utah [see Kavanaugh 1979a: Fig. 60].

#### Records.

**USA**: UT

### Nebria zioni zioni

Van Dyke, 1943

Nebria zioni Van Dyke, 1943: 20. Type locality: «canyon of Zion National Park, Utah» (original citation). Holotype (♂) in CAS [# 5299].

#### Distribution.

This subspecies is known only from mountains in southwestern Utah [see Kavanaugh 1979a: Fig. 60].

#### Records.

**USA**: UT

#### Note.

This species is placed in the *lyelli* group by Ledoux and Roux (2005: 195).

### [lyelli group]

### Nebria acuta acuta

Lindroth, 1961

Nebria acuta Lindroth, 1961a: 71. Type locality: «Snowslide Gulch, 16 mi[les] E[ast] Valdez, Alaska» (original citation). Holotype (♂) in MCZ [# 30428].

#### Distribution.

This subspecies ranges from the Kenai Peninsula in Alaska (Kavanaugh 1978: 671) and southwestern Yukon Territory (Kluane Lake, Sydney G. Cannings pers. comm. 2009) south to the southern part of the Cascade Range and the Sierra Nevada in central California (Kavanaugh 1978: 671).

#### Records.

**CAN**: BC, YT **USA**: AK, CA, OR, WA

### Nebria acuta quileute

Kavanaugh, 1979

Nebria acuta quileute Kavanaugh, 1979a: 98. Type locality: «Boulder Creek (at Olympic Hot Springs; 2000’), Olympic National Park [Clallam County], Washington» (original citation). Holotype (♂) in CAS [# 12494].

#### Distribution.

This subspecies is endemic to the Olympic Peninsula in Washington [see Kavanaugh 1979a: Fig. 55].

#### Records.

**USA**: WA

### Nebria acuta sonorae

Kavanaugh, 1981

Nebria sonorae Kavanaugh, 1981b: 438. Type locality: «Chipmunk Flat, Tuolumne County, California» (original citation). Holotype (♂) in CAS [# 13731].

#### Distribution.

As far as known, this subspecies is restricted to the Sierra Nevada, between Sonora Pass and Buckeye Pass, in California [see Kavanaugh 1981b: Fig. 21].

#### Records.

**USA**: CA

### Nebria danmanni

Kavanaugh, 1981

Nebria danmanni Kavanaugh, 1981b: 437. Type locality: «Deception Basin (1830 m), Olympic National Park [Clallam County], Washington» (original citation). Holotype (♂) in CAS [# 13730].

#### Distribution.

This species is endemic to high elevations in the Olympic Mountains in northwestern Washington [see Kavanaugh 1984: Fig. 21].

#### Records.

**USA**: WA

### Nebria lyelli

Van Dyke, 1925

Nebria lyelli Van Dyke, 1925: 120. Type locality: «M[oun]t Lyell (about 11,000 feet), Yosemite National Park, California» (original citation). Holotype (♂) in CAS [# 1626].

#### Distribution.

This species is known only from Mount Lyell at the edge of the Yosemite National Park in the Sierra Nevada, California (Kavanaugh 1978: 766).

#### Records.

**USA**: CA

### Nebria wallowae

Kavanaugh, 1984

Nebria wallowae Kavanaugh, 1984: 161. Type locality: «West Fork Wallowa River (2070-2130 m), Wallowa Mountains, Wallowa County, Oregon» (original citation). Holotype (♂) in CAS [# 14347].

#### Distribution.

This species is known only from the Wallowa Mountains in northeastern Oregon [see Kavanaugh 1984: Fig. 31].

#### Records.

**USA**: OR

### [mannerheimii group]

### Nebria darlingtoni

Kavanaugh, 1979

Nebria darlingtoni Kavanaugh, 1979a: 104. Type locality: «South Fork American River, (3 miles w[est] of Riverton; 910 m), El Dorado County, California» (original citation). Holotype (♂) in CAS [# 12499].

#### Distribution.

This species is known only from the canyon of the South Fork of the American River in the Sierra Nevada, California [see Kavanaugh 1979a: Fig. 62].

#### Records.

**USA**: CA

### Nebria desolata

Kavanaugh, 1971

Nebria desolata Kavanaugh, 1971: 41. Type locality: «The Gulch (5600’), 11 mi[les] S[outh] W[est] Boulder, Garfield Co[unty], Utah» (original citation). Holotype (♂) in CAS [# 11388].

#### Distribution.

This species is known only from the type locality in south-central Utah.

#### Records.

**USA**: UT

### Nebria diversa

LeConte, 1863

Nebria livida LeConte, 1859a: 84 [secondary homonym of *Nebria livida* (Linnaeus, 1758)]. Type locality: «Cape Flattery [Clallam County, Washington]» (original citation). Lectotype (♂), designated by Kavanaugh (1979a: 113), in MCZ [# 642].
Nebria diversa LeConte, 1863b: 2. Replacement name for *Nebria livida* LeConte, 1859.
Nebria townsendi Casey, 1924: 19. Type locality: «Port Townsend [Jefferson County], Washington» (original citation). Lectotype (♀), designated by Lindroth (1975: 112), in USNM [# 46846]. Synonymy established by Hatch (1953: 59), confirmed by Lindroth (1961a: 75).

#### Distribution.

This species ranges along the Pacific Coast from the Queen Charlotte Islands to northern California (Kavanaugh 1992: 57).

#### Records.

**CAN**: BC (QCI, VCI) **USA**: CA, OR, WA

#### Note.

This species is placed in a separate group (*pallipes* group) along with *Nebria appalachia* and *Nebria pallipes* by Ledoux and Roux (2005: 195).

### Nebria eschscholtzii

Ménétriés, 1843

Nebria eschscholtzii Ménétriés, 1843: 55. Type locality: «Californie» (original citation), restricted to «South Fork of American River, 3 miles w[est] of Riverton, El Dorado County» by Kavanaugh (1979a: 112). Lectotype (♀), designated by Kavanaugh (1979a: 112), in ZILR. Etymology. The specific name honors the Estonian naturalist and explorer Johann Friedrich Gustav von Eschscholtz [1793-1831], physician and naturalist on the two Russian circumnavigational expeditions under the command of Otto Evstaf’evich von Kotzebue in 1815-1818 on the *Rurik* and in 1823-1826 on the *Predpriiatie*. Eschscholtz made substantial collections of insects in Alaska and California during these trips. He was professor of medicine and zoology and director of the Zoological Museum of the University of Dorpat in the last years of his life. Eschscholtz Bay on Kotzebue Sound, a large inlet in northwestern Alaska near Bering Strait, was named in his honor.
Nebria tenuipes Casey, 1913: 51. Type locality: «Alameda [Alameda County], California» (original citation for the lectotype). Lectotype (♀), designated by Lindroth (1975: 112), in USNM [# 46852]. Synonymy established by Hatch (1939a: 121), confirmed by Lindroth (1961a: 75).
Nebria transversa Casey, 1920: 152. Type locality: «Corvallis [Benton County], Oregon» (original citation). Holotype [by monotypy] (♀) in USNM [# 46869]. Synonymy established by Hatch (1953: 58), confirmed by Lindroth (1961a: 75).
Nebria formalis Casey, 1920: 153. Type locality: «Wawawai [Whitman County], Washington» (original citation). Lectotype (♀), designated by Lindroth (1975: 112), in USNM [# 46870]. Synonymy established by Hatch (1953: 58), confirmed by Lindroth (1961a: 75).
Nebria pallidissima Casey, 1924: 19. Type locality: «Wawawai [Whitman County], Washington» (original citation). Lectotype (♂), designated by Lindroth (1975: 112), in USNM [# 46845]. Synonymy established (as aberration) by Hatch (1953: 58), confirmed by Lindroth (1961a: 75).
Nebria pugetana Casey, 1924: 19. Type locality: «Wawawai [Whitman County], Washington» (original citation). Lectotype (♀), designated by Lindroth (1975: 112), in USNM [# 46847]. Synonymy established by Hatch (1953: 58), confirmed by Lindroth (1961a: 75).

#### Distribution.

The range of this species extends from northwestern Washington to northeastern Idaho, south to southern California [see Kavanaugh 1979b: Fig. 2]. The records from Colorado (Wickham 1902: 232; Armin 1963: 89) and Montana (Hatch 1933a: 7) must be in error.

#### Records.

**USA**: CA, ID, NV, OR, WA

### Nebria georgei

Kavanaugh, 2008

Nebria georgei Kavanaugh, 2008: 2. Type locality: «Colorado River, Grand Canyon National Park, Coconino County, Arizona» (original citation). Holotype (♂) in MSB.

#### Distribution.

This species is known only from the original two specimens collected 141 kilometers apart in the Grand Canyon National Park (Kavanaugh 2008: 5).

#### Records.

**USA**: AZ

### Nebria mannerheimii

Fischer von Waldheim, 1828

Nebria mannerheimii Fischer von Waldheim, 1828: 253. Type locality: «Sitcha sur l’île de Norfolk [= Sitka, Baranof Island, Alaska]» (original citation). Lectotype (♂), designated by Kavanaugh (1979a: 114), in ZMMU. Etymology. The specific name honors Gustav Graf von Mannerheim [1797-1854], a Finnish politician who rose from clerk to Governor of Läne Vaasa and Viipuri regions of Finland and president of the Imperial *Hofgericht* in Wiborg (currently Wyborg in Russia). Early in his life Mannerheim became interested in natural history and particularly entomology.
Nebria oregona Casey, 1913: 52. Type locality: «Clackamas Co[unty], Oregon» (original citation), restricted to «Zigzag River at Rhododendron» by Kavanaugh (1979a: 115). Lectotype (♂), designated by Lindroth (1975: 112), in USNM [# 46853]. Synonymy established by Hatch (1953: 58), confirmed by Lindroth (1961a: 74).
Nebria corvallis Casey, 1924: 20. Type locality: «Corvallis [Benton County], Oregon» (original citation). Lectotype (♂), designated by Lindroth (1975: 112), in USNM [# 46854]. Synonymy established by Hatch (1953: 58), confirmed by Lindroth (1961a: 74).
Nebria hippisleyi Casey, 1924: 21. Type locality: «Terrace, British Columbia» (original citation). Holotype [by monotypy] (♂) in USNM [# 46868]. Synonymy established by Hatch (1953: 58), confirmed by Lindroth (1961a: 74). Etymology. The specific name was proposed for Mrs. W.W. Hippisley [1880-1962] who collected beetles at or near Terrace in British Columbia. Born Marianne E. Parker, she was interested also in shells, minerals, and mosses but after a gun accident in 1911 where she lost her right arm completely she confined herself into collecting beetles. She eventually became Mrs. M.E. Clark and left her collection to the University of British Columbia.

#### Distribution.

This species ranges from the Kenai Peninsula in southern Alaska (Lindroth 1961a: 75) south to western Idaho and central Oregon (Kavanaugh 1978: 766-772). According to Kavanaugh (1978: 772), a number of specimens labeled from “California,” Nevada, and Yukon Territory are doubtful. The records from the Absaroka Range in south-central Montana (Hatch 1933a: 7) and western San Juan Mountains in Colorado (Wickham 1902: 232) are also doubtful.

#### Records.

**CAN**: BC (QCI, VCI) **USA**: AK, ID, OR, WA [CA, CO, MT, NV, YT]

### Nebria navajo

Kavanaugh, 1979

Nebria navajo Kavanaugh, 1979a: 104. Type locality: «19 miles s[outh]w[est] Kayenta (6500’), Navajo County, Arizona» (original citation). Holotype (♂) in CAS [# 12509].

#### Distribution.

This species is known only from the vicinity of the type locality in northeastern Arizona [see Kavanaugh 1979a: Fig. 63].

#### Records.

**USA**: AZ

### [obliqua group]

### Nebria appalachia

Darlington, 1932

Nebria appalachia Darlington, 1932: 153. Type locality: «below Newfound Gap (near 5,000 feet), Smoky Mountains, Tennessee» (original citation). Holotype (♂) in MCZ [# 16433].

#### Distribution.

This species is known from the southern Appalachian Mountains in western North Carolina and eastern Tennessee (Kavanaugh 1978: 674).

#### Records.

**USA**: NC, TN

#### Note.

This species is placed in a distinct group (*pallipes* group) along with *Nebria diversa* and *Nebria pallipes* by Ledoux and Roux (2005: 195).

### Nebria obliqua chuskae

Kavanaugh, 1979

Nebria obliqua chuskae Kavanaugh, 1979a: 104. Type locality: «Lukachukai Creek (at Wagon Wheel Campground; 2260 m), Apache County, Arizona» (original citation). Holotype (♂) in CAS [# 13461].

#### Distribution.

This subspecies, as far as known, is endemic to the Chuska Mountains in northeastern Arizona [see Kavanaugh 1979a: Fig. 61].

#### Records.

**USA**: AZ

### Nebria obliqua obliqua

LeConte, 1867

Nebria obliqua LeConte, 1867b: 363. Type locality: «Colorado» (original citation), restricted to «North Fork of South Platte Canyon at Santa Maria, Park County» by Kavanaugh (1979a: 114). Lectotype (♂), designated by Kavanaugh (1979a: 114), in MCZ [# 646].
Nebria obtusa LeConte, 1878a: 478. Type locality: «Green River City (6,000-7,000 feet) [Sweetwater County], Wyo[ming]» (original citation). Holotype [by monotypy] (♂) in MCZ [# 647]. Synonymy established by Kavanaugh (1979a: 114). Note. I concur with Kavanaugh (1979a: 114) that LeConte’s statement “Last ventral segment rufo-piceous (from the immaturity of the specimen)” in the description is a clear indication that LeConte had but a single specimen.
Nebria incerta Casey, 1913: 53. Type locality: «Colorado» (original citation), restricted to «North Fork of South Platte Canyon at Santa Maria, Park County» by Kavanaugh (1979a: 113). Lectotype, designated by Lindroth (1975: 112), in USNM [# 46859]. Synonymy established by Lindroth (1961a: 73).
Nebria testaceipes Casey, 1913: 54. Type locality: «Glenora, British Columbia» (original citation). Holotype [by monotypy] (♂) in USNM [# 46861]. Synonymy established, under the name *Nebria obtusa* LeConte, by Hatch (1953: 59), confirmed by Lindroth (1961a: 73).
Nebria texana Casey, 1913: 54. Type locality: «Texas» (original citation), which according to Kavanaugh (1979a: 116) is highly improbable. Holotype [by monotypy] (♂) in USNM [# 46863]. Synonymy established by Lindroth (1961a: 73).

#### Distribution.

This subspecies ranges from the west edge of the Wrangell-St. Elias National Park in southeastern Alaska to the Great Slake Lake in Northwest Territories, south to northern New Mexico, northern Arizona, central Nevada, and the lower eastern slope of the Sierra Nevada in California, east to western South Dakota and western Nebraska [see Kavanaugh 1979b: Fig. 4].

#### Records.

**CAN**: AB, BC, NT, SK, YT **USA**: AK, AZ, CA, CO, ID, MT, NE, NM, NV, OR, SD, UT, WA, WY

### Nebria pallipes

Say, 1823

Nebria pallipes Say, 1823a: 78. Type locality: «Monterey [Berkshire County], Mass[achusetts]» (neotype label). Neotype (♂), designated by Lindroth and Freitag (1969: 333), in MCZ [# 33082].

#### Distribution.

This species is found east of the Mississippi River from Nova Scotia (Lindroth 1961a: 77) to north-central Illinois (Kavanaugh 1978: 800), south to northern Alabama (Löding 1945: 12), northeastern Georgia (Leng 1910: 73; Fattig 1949: 12), and east-central South Carolina (Ciegler 2000: 20) along the Appalachian Mountains. The record from east-central Missouri (Summers 1873: 133) needs confirmation; that from Colorado (Elias 1987: 632) is likely based on a mislabeled specimen; that from northern Wisconsin along Lake Superior (Wickham 1896c: 131) probably refers to *Nebria lacustris*.

#### Records.

**CAN**: NB, NS, ON, PE, QC **USA**: AL, CT, DC, DE, GA, IL, IN, KY, MA, MD, ME, MI, NC, NH, NJ, NY, OH, PA, RI, SC, TN, VA, VT, WV [MO]

#### Note.

This species is placed in a special group (*pallipes* group) along with *Nebria appalachia* and *Nebria diversa* by Ledoux and Roux (2005: 195).

### Nebria suturalis

LeConte, 1850

Nebria suturalis LeConte, 1850: 209. Type locality: «islands at the mouth of Black Bay [Minnesota]» (original citation). Lectotype (♂), designated by Kavanaugh (1979a: 115), in MCZ [# 650]. Note. Lindroth (1961a: 73) placed the type locality in Ontario but it seems more likely that it is located in northern Minnesota near the International Border.
Nebria longula LeConte, 1878a: 478. Type locality: «Colorado» (original citation), restricted to «Longs Peak, Rocky Mountain National Park» by Kavanaugh (1979a: 113). Holotype [by monotypy] (♂) in MCZ [# 644]. Synonymy established by Kavanaugh (1979a: 113).
Nebria nimbosa Casey, 1920: 150. Type locality: «Lake of the Clouds, M[oun]t Washington [Coos County], New Hampshire» (original citation). Holotype [by monotypy] (♀) in USNM [# 46860]. Synonymy established by Bänninger (1925: 259), confirmed by Lindroth (1954b: 122).

#### Distribution.

This species ranges from the coast of Labrador and the Ungava Bay region in northern Quebec to the Rocky Mountains in western Alberta, south to central Colorado, the Adirondack Mountains in northeastern New York, and New England [see Kavanaugh 1979b: Fig. 3].

#### Records.

**CAN**: AB, LB, ON, QC **USA**: CO, ME, NH, NY, VT, WY

### Subgenus Catonebria

Shilenkov, 1975

Catonebria Shilenkov, 1975: 836. Type species: *Carabus nitidulus* Fabricius, 1787 (= *Nebria banksii* Crotch, 1871) by original designation. Etymology. From the Latin *catena* (chain), shortened to *cato*, and the generic name *Nebria* [*q.v*.], alluding to the chain-like row of tubercles on certain elytral striae in most species (David H. Kavanaugh pers. comm. 2012) [feminine].

#### Diversity.

Thirty-one species (Ledoux and Roux 2005: 240) in North America (20 species) and Siberia and the Far East (11 species).

### [metallica group]

### Nebria labontei

Kavanaugh, 1984

Nebria labontei Kavanaugh, 1984: 163. Type locality: «West Fork Wallowa River (2040-2190 m), Wallowa Mountains, Wallowa County, Oregon» (original citation). Holotype (♂) in CAS [# 14343].

#### Distribution.

As far as known, this species is endemic to the Wallowa Mountains in northeastern Oregon [see Kavanaugh 1984: Fig. 31].

#### Records.

**USA**: OR

### Nebria meanyi giulianii

Kavanaugh, 1981

Nebria meanyi giulianii Kavanaugh, 1981b: 441. Type locality: «Montgomery Creek (2380 m), Mono County, California» (original citation). Holotype (♂) in CAS [# 13732].

#### Distribution.

This subspecies is known only from the western slope of the White Mountains in eastern California [see Kavanaugh 1984: Fig. 21].

#### Records.

**USA**: CA

### Nebria meanyi lamarckensis

Kavanaugh, 1979

Nebria meanyi lamarckensis Kavanaugh, 1979a: 109. Type locality: «Lamarck Creek (above Upper Lamarck Lake; 10700-11000’), Inyo County, California» (original citation). Holotype (♂) in CAS [# 12507].

#### Distribution.

This subspecies has been found only on the eastern slope of the southern Sierra Nevada in California [see Kavanaugh 1979a: Fig. 67].

#### Records.

**USA**: CA

### Nebria meanyi meanyi

Van Dyke, 1925

Nebria meanyi Van Dyke, 1925: 118. Type locality: «close to the Nesqually River, M[oun]t Rainier National Park [Pierce County], Washington» (original citation). Holotype (♂) in CAS [# 1623]. Etymology. The specific name was proposed for Professor Edmond S. Meany [1862-1935], mountaineer, state legislator, and teacher of botany and history at the University of Washington. Mount Meany in the Olympic Mountains is named after him.

#### Distribution.

This subspecies is known from the Skagway area in southeastern Alaska and northwestern British Columbia south along the Cascade Range to Mount Shasta in north-central California [see Kavanaugh 1979a: Fig. 67]. The record from “Whitehorse Pass,” Yukon Territory (Kavanaugh 1978: 773), refers to the Skagway area in Alaska (Sydney G. Cannings pers. comm. 2009).

#### Records.

**CAN**: BC **USA**: AK, CA, OR, WA

### Nebria meanyi sylvatica

Kavanaugh, 1979

Nebria meanyi sylvatica Kavanaugh, 1979a: 109. Type locality: «Boulder Creek (at Olympic Hot Springs; 610 m), Olympic National Park [Clallam County], Washington» (original citation). Holotype (♂) in CAS [# 12508].

#### Distribution.

This subspecies in known from Vancouver Island and the Olympic Peninsula in northwestern Washington [see Kavanaugh 1979a: Fig. 67].

#### Records.

**CAN**: BC (VCI) **USA**: WA

### Nebria metallica

Fischer von Waldheim, 1820

Nebria metallica Fischer von Waldheim, 1820: plate 6. Type locality: «insula Unalaschka [Alaska]» (Fischer von Waldheim 1822: 72). Lectotype (♂), designated by Kavanaugh (1979a: 112), in ZMMU.
Nebria pacifica Chaudoir, 1850a: 424. Type locality: «Otahiti [= Tahiti]» (original citation), which is incorrect. Lectotype [as holotype], designated by Perrault (1980: 29), in MHNP. Synonymy established by Bänninger (1932: 178).

#### Distribution.

This species ranges from the Aleutian Islands south to western Montana (Hatch 1939a: 118) and southern Washington (Kavanaugh 1978: 775-778). A few specimens labeled from Arizona, California, and Oregon are known (Kavanaugh 1978: 778) but considered doubtful. The record from Colorado (Elias 1987: 632) is in error (David H. Kavanaugh pers. comm. 2012).

#### Records.

**CAN**: AB, BC (VCI) **USA**: AK, ID, MT, WA [AZ, CA, OR]

### [ovipennis group]

### Nebria carri

Kavanaugh, 1979

Nebria carri Kavanaugh, 1979a: 107. Type locality: «Dollarhide Summit (7700-7900’), Blaine County, Idaho» (original citation). Holotype (♂) in CAS [# 22918].

#### Distribution.

This species is found in the mountains of south-central and western Idaho [see Kavanaugh 1979a: Fig. 65] from the Seven Devils Mountains in the north to the Sawtooth Range in the south.

#### Records.

**USA**: ID

### Nebria gebleri albimontis

Kavanaugh, 1984

Nebria gebleri albimontis Kavanaugh, 1984: 163. Type locality: «Birch Creek (3290-3410 m), White Mountains, Mono County, California» (original citation). Holotype (♂) in CAS [# 14340].

#### Distribution.

This subspecies is known only from the type locality in the White Mountains of eastern California.

#### Records.

**USA**: CA

### Nebria gebleri cascadensis

Kavanaugh, 1979

Nebria gebleri cascadensis Kavanaugh, 1979a: 105. Type locality: «Paradise Rive (above Narada Falls; 4580-4800’), Mount Rainier National Park [Pierce County], Washington» (original citation). Holotype (♂) in CAS [# 12502].

#### Distribution.

This subspecies is found from southern British Columbia, including southern Vancouver Island, south along the Cascade Range to central Oregon [see Kavanaugh 1979a: Fig. 64]. One specimen labeled from Leavenworth Valley in Colorado seen by Kavanaugh (1979a: 106) is likely mislabeled.

#### Records.

**CAN**: BC (VCI) **USA**: OR, WA

### Nebria gebleri fragariae

Kavanaugh, 1979

Nebria gebleri fragariae Kavanaugh, 1979a: 106. Type locality: «Strawberry Creek (1,770 m), Grant County, Oregon» (original citation). Holotype (♂) in CAS [# 12501].

#### Distribution.

This subspecies is yet recorded only from the type locality in the Strawberry Mountains of eastern Oregon [see Kavanaugh 1979a: Fig. 64].

#### Records.

**USA**: OR

### Nebria gebleri gebleri

Dejean, 1831

Nebria gebleri Dejean, 1831: 573. Type locality: «détroit de Norfolk [= Sitka Sound, Baranof Island, Alaska], sur la côte nord-ouest de l’Amérique septentrionale» (original citation). Holotype [by monotypy] (♀) in MHNP (Ledoux and Roux 1992: 37). Etymology. The specific name honors Frédéric Auguste Gebler [1782-1850], physician and amateur coleopterist. Born in Germany, Gebler settled in Barnaul in Siberia at the age of 27. As physician of the vast district of Kolywano-Woskresensk, he had the opportunity to collect in several places in Siberia. After his death, Gebler’s collection was sold to Count Georges de Mniszech [1824-1881] for 1,057 silver roubles. Mniszech’s collection was bought by René Oberthür in 1885.
Nebria melanaria Hatch, 1949b: 115. Type locality: «Going-to-the-Sun Chalet, Glacier National Park [Flathead County], Montana» (original citation). Holotype (♂) in USNM. Synonymy established by Lindroth (1961a: 83).

#### Distribution.

This subspecies occurs along the Cordilleras from the Alexander Archipelago to southernmost Yukon Territory (Lindroth 1961a: 84), south to southwestern Montana, south-central Idaho, and northeastern Oregon [see Kavanaugh 1979a: Fig. 64]. The record from northern Colorado (Armin 1963: 89) is probably in error.

#### Records.

**CAN**: AB, BC, YT **USA**: AK, ID, MT, OR, WA

#### Note.

This species is placed in its own group by Ledoux and Roux (2005: 240).

### Nebria gebleri rathvoni

LeConte, 1853

Nebria rathvoni LeConte, 1853c: 400. Type locality: «Sacramento [Sacramento County], California» (original citation). Holotype [by monotypy] location unknown. Etymology. The specific name honors Simon Snyder Rathvon [1812-1891], an entomologist interested chiefly in the economic aspect of insects who resided most of his life in Lancaster County, Pennsylvania. Rathvon bought Haldeman’s collection which included that of Hentz. Note. The specimen in MCZ labeled as holotype [# 7403] is not the specimen upon which LeConte (1853c: 400) based his description since it is also labeled “6465 ft. Lake Tahoe, Cal. May 24, 1879.”

#### Distribution.

This subspecies is endemic to the Sierra Nevada and adjacent mountains in western Nevada [see Kavanaugh 1979a: Fig. 64].

#### Records.

**USA**: CA, NV

### Nebria gebleri siskiyouensis

Kavanaugh, 1979

Nebria gebleri siskiyouensis Kavanaugh, 1979a: 107. Type locality: «South Fork Salmon River (at Big Flat Campground; 1490 m), Trinity County, California» (original citation). Holotype (♂) in CAS [# 12503].

#### Distribution.

This subspecies is restricted to the Klamath Mountains system of the Coast Ranges in southwestern Oregon and northwestern California [see Kavanaugh 1979a: Fig. 64].

#### Records.

**USA**: CA, OR

### Nebria kincaidi balli

Kavanaugh, 1979

Nebria kincaidi balli Kavanaugh, 1979a: 107. Type locality: «Paradise River (above Narada Falls; 4580-4800’), Mount Rainier National Park [Pierce County], Washington» (original citation). Holotype (♂) in CAS [# 12505].

#### Distribution.

This subspecies is restricted to the Cascade Range of central Washington and northern Oregon [see Kavanaugh 1979a: Fig. 65].

#### Records.

**USA**: OR, WA

### Nebria kincaidi kincaidi

Schwarz, 1900

Nebria kincaidi Schwarz, 1900: 525. Type locality: «Farragut Bay [Alaska]» (original citation). Holotype (♂) in USNM [# 5258]. Etymology. The species name honors Trevor Kincaid [1872-1968], naturalist and professor at the University of Washington. Early in his career Kincaid worked mainly on insects and taxonomy but later became involved with oysters and was largely responsible for bringing the Japanese oyster to Washington. He served as entomologist on the Harriman Alaska Expedition in 1899 and was selected by L.O. Howard in 1908 and 1909 to go to Japan and then to southwestern Russia in search of parasites of the gypsy moth.
Nebria columbiana Casey, 1913: 48. Type locality: «Inverness [probably Inverness Passage], British Columbia» (original citation). Lectotype (♂), designated by Lindroth (1975: 113), in USNM [# 46848]. Synonymy established by Darlington (1930: 104), confirmed by Lindroth (1961a: 88).

#### Distribution.

This subspecies ranges along the mountains of the Pacific Coast from the Alexander Archipelago to northern Washington [see Kavanaugh 1979a: Fig. 65].

#### Records.

**CAN**: BC (VCI) **USA**: AK, WA

### Nebria ovipennis

LeConte, 1878

Nebria ovipennis LeConte, 1878a: 477. Type locality: «Sierra Nevada, Cal[ifornia]» (original citation), restricted to «Chipmunk Flat, Tuolumne County» by Erwin and Ball (1972: 81). Holotype [by monotypy] (♂) in MCZ [# 648].

#### Distribution.

This species is known from the Sierra Nevada in California (Erwin and Ball 1972: 81) and adjacent mountains in Washoe County, western Nevada (Kavanaugh 1978: 798).

#### Records.

**USA**: CA, NV

### Nebria spatulata sierrae

Kavanaugh, 1979

Nebria spatulata sierrae Kavanaugh, 1979a: 108. Type locality: «White Mountain (east slope, above Big Horn Lake; 3290-3480 m), Mono County, California» (original citation). Holotype (♂) in CAS [# 12516].

#### Distribution.

This subspecies is restricted to the main chain and Eastern Divide of the Sierra Nevada of California [see Kavanaugh 1979a: Fig. 66; David H. Kavanaugh pers. comm. 2012].

#### Records.

**USA**: CA

### Nebria spatulata spatulata

Van Dyke, 1925

Nebria spatulata Van Dyke, 1925: 119. Type locality: «Franklin Lake, Tulare County, California» (original citation). Holotype (♀) in CAS [# 1625].

#### Distribution.

This subspecies is endemic to the Western Divide of the southern Sierra Nevada of California [see Kavanaugh 1979a: Fig. 66; David H. Kavanaugh pers. comm. 2012].

#### Records.

**USA**: CA

### [trifaria group]

### Nebria calva

Kavanaugh, 1984

Nebria calva Kavanaugh, 1984: 164. Type locality: «Mount Baldy (3050-3350 m), s[outh]w[est] of Springerville, Apache County, Arizona» (original citation). Holotype (♂) in CNC [# 22917].

#### Distribution.

This species is yet known only from the type locality in northeastern Arizona [see Kavanaugh 1984: Fig. 32].

#### Records.

**USA**: AZ

### Nebria catenata

Casey, 1913

Nebria catenata Casey, 1913: 49. Type locality: «Colorado» (original citation), restricted to «San Juan Mountains» by Erwin and Ball (1972: 97), further to «Wolf Creek Pass, Mineral County» by Kavanaugh (1979a: 112). Lectotype (♀), designated by Lindroth (1975: 112), in USNM [# 46849].

#### Distribution.

This species is restricted to the San Juan Mountains in southwestern Colorado and the Abajo Mountains in southeastern Utah (Erwin and Ball 1972: 97).

#### Records.

**USA**: CO, UT

### Nebria coloradensis

Van Dyke, 1943

Nebria coloradensis Van Dyke, 1943: 19. Type locality: «near the Twin Lakes, Lake County, Colorado» (original citation). Holotype (♂) in CAS [# 5298].

#### Distribution.

This species is found in the Rocky Mountains in southern Wyoming and Colorado (Erwin and Ball 1972: 97).

#### Records.

**USA**: CO, WY

#### Note.

This taxon has been considered a synonym of *Nebria catenata* by Lindroth (1961a: 84), a subspecies of *Nebria trifaria* by Erwin and Ball (1972: 96) and Ledoux and Roux (2005: 285), and a distinct species by Kavanaugh (1985: 421, 423).

### Nebria ingens ingens

Horn, 1870

Nebria ingens G.H. Horn, 1870b: 98. Type locality: «high Sierra Nevada Mountains east of Visalia, California» (original citation), restricted to «Franklin Lakes, Tulare County» by Kavanaugh (1979a: 113). Lectotype (♀), designated by Kavanaugh (1979a: 113), in MCZ [# 8127].
Nebria raveni Van Dyke, 1953b: 102. Type locality: «slopes of M[oun]t Darwin (13,600 feet), Fresno County, California» (original citation). Holotype (♀) in CAS [# 8163]. Synonymy established by Lindroth (1961a: 87).

#### Distribution.

This subspecies is restricted to the Sierra Nevada in Fresno, Inyo, and Tulare Counties, California (Kavanaugh 1978: 753-754).

#### Records.

**USA**: CA

### Nebria ingens riversi

Van Dyke, 1925

Nebria riversi Van Dyke, 1925: 115. Type locality: «base of Lyell Glacier (about 11,500 feet), M[oun]t Lyell, California» (original citation). Holotype (♂) in CAS [# 1619]. Etymology. The species name honors James John Rivers [1824-1913], a naturalist born in England who studied medicine at the University of London and came under the influence of Thomas Henry Huxley. In his 40s he moved to the United States and eventually settled in California. Curator of Organic Natural History at the University of California (Berkeley), Rivers published on many subjects, including Coleoptera, Lepidoptera, spiders, and reptiles.

#### Distribution.

This subspecies is known only from Mono and Tuolumne Counties in the Sierra Nevada of California (Kavanaugh 1978: 754; David H. Kavanaugh pers. comm. 2012).

#### Records.

**USA**: CA

### Nebria piperi

Van Dyke, 1925

Nebria piperi Van Dyke, 1925: 117. Type locality: «along the margins of the Nesqually River, just below the foot of the Nesqually Glacier, M[oun]t Rainier National Park [Pierce County], Washington» (original citation). Holotype (♂) in CAS [# 1621]. Etymology. The specific name honors the American botanist Charles Vancouver Piper [1867-1926].

#### Distribution.

This species ranges along the mountains paralleling the Pacific Coast from southeastern Alaska along the British Columbia border (Lindroth 1961a: 86) and Kluane National Park in southwestern Yukon Territory (David H. Kavanaugh pers. comm. 2010) to west-central Oregon (Kavanaugh 1978: 811).

#### Records.

**CAN**: BC, YT **USA**: AK, OR, WA

#### Note.

This species is placed in the *metallica* group by Ledoux and Roux (2005: 240).

### Nebria piute piute

Erwin and Ball, 1972

Nebria trifaria piute Erwin and Ball, 1972: 95. Type locality: «La Baron Lake (9,700’), Circleville Mountain, 15.9 miles west of Junction, Beaver County, Utah» (original citation). Holotype (♂) in USNM [# 71976].

#### Distribution.

This subspecies is known only from Beaver and Piute Counties in southern Utah (Kavanaugh 1978: 813).

#### Records.

**USA**: UT

### Nebria piute sevieri

Kavanaugh, 1984

Nebria piute sevieri Kavanaugh, 1984: 164. Type locality: «Parowan Creek (2800 m), 13.5 miles S[outh] of Parowan, Markagunt Plateau, Iron County, Utah» (original citation). Holotype (♂) in CAS [# 14344].

#### Distribution.

This subspecies is known only from southern Utah [see Kavanaugh 1984: Fig. 32]. One specimen labeled from Navajo County in Arizona seen by Kavanaugh (1984: 165) is likely mislabeled.

#### Records.

**USA**: UT

### Nebria piute utahensis

Kavanaugh, 1979

Nebria trifaria utahensis Kavanaugh, 1979a: 110. Type locality: «Lonesome Beaver (7500’), Henry Mountains, Garfield County, Utah» (original citation). Holotype (♂) in CNC [# 20758].

#### Distribution.

This subspecies is restricted to the Henry Mountains in south-central Utah [see Kavanaugh 1979a: Fig. 70].

#### Records.

**USA**: UT

### Nebria praedicta

Kavanaugh and Schoville, 2009

Nebria praedicta Kavanaugh and Schoville, 2009: 74. Type locality: «north by northwest slope of Thompson Peak in upper Grizzly Lake Basin (2411-2470 m), Trinity Alps, Trinity County, California» (original citation). Holotype (♂) in CAS [# 18447].

#### Distribution.

This species is endemic to the Trinity Alps of northwestern California.

#### Records.

**USA**: CA

### Nebria purpurata

LeConte, 1878

Nebria purpurata LeConte, 1878a: 477. Type locality: «Laevenworth Valley (9,000 to 10,000 feet), above Georgetown [Clear Creek County], Colo[rado]» (original citation). Holotype [by monotypy] (♂) in MCZ [# 649].
Nebria mobilis Casey, 1913: 50. Type locality: «Colorado» (original citation). Lectotype (♀), designated by Lindroth (1975: 112), in USNM [# 46850]. Synonymy established by Lindroth (1961a: 86).

#### Distribution.

This species is found along the Rocky Mountains in Colorado and New Mexico (Kavanaugh 1978: 813-815). The record from Idaho (LeConte 1879d: 500) is probably in error.

#### Records.

**USA**: CO, NM

### Nebria schwarzi beverlianna

Kavanaugh, 1979

Nebria schwarzi beverlianna Kavanaugh, 1979a: 110. Type locality: «Hoback River (8 miles n[orth]w[est] of Bondurant; 6900’), Sublette County, Wyoming» (original citation). Holotype (♂) in CAS [# 12515].

#### Distribution.

This beautiful subspecies is recorded only from the type locality in Gros Ventre Mountains, western Wyoming [see Kavanaugh 1979a: Fig. 69].

#### Records.

**USA**: WY

### Nebria schwarzi schwarzi

Van Dyke, 1925

Nebria schwarzi Van Dyke, 1925: 116. Type locality: «Banff, Alberta» (original citation). Holotype (♂) in USNM [# 28174].

#### Distribution.

This subspecies is restricted to the Rocky Mountains in western Alberta and southeastern British Columbia [see Kavanaugh 1979a: Fig. 69].

#### Records.

**CAN**: AB, BC

#### Note.

This species is placed in the *metallica* group by Ledoux and Roux (2005: 240).

### Nebria sierrablancae

Kavanaugh, 1984

Nebria sierrablancae Kavanaugh, 1984: 164. Type locality: «Sierra Blanca (3200 m), Lincoln County, New Mexico» (original citation). Holotype (♂) in CNC [# 22919].

#### Distribution.

This species is known only from the Sierra Blanca and Capitan Mountains in central New Mexico [see Kavanaugh 1984: Fig. 32].

#### Records.

**USA**: NM

### Nebria steensensis

Kavanaugh, 1984

Nebria steensensis Kavanaugh, 1984: 165. Type locality: «South Fork McCoy Creek (2390-2560 m), Steens Mountains, Harney County, Oregon» (original citation). Holotype (♂) in CAS [# 14345].

#### Distribution.

This species is endemic to the Steens Mountains in south-central Oregon [see Kavanaugh 1984: Fig. 32].

#### Records.

**USA**: OR

### Nebria trifaria pasquineli

Kavanaugh, 1984

Nebria trifaria pasquineli Kavanaugh, 1984: 165. Type locality: «Lefthand Creek, 5 miles E[ast] of Ward, Front Range, Boulder County, Colorado» (original citation). Holotype (♂) in CAS [# 14346].

#### Distribution.

This subspecies ranges from Medicine Bow and Sierra Madre Ranges in southern Wyoming south to the Rampart Range in central Colorado [see Kavanaugh 1984: Fig. 32].

#### Records.

**USA**: CO, WY

### Nebria trifaria trifaria

LeConte, 1878

Nebria trifaria LeConte, 1878a: 478. Type locality: «American Fork Cañon (9,500 feet) [Utah County], Utah» (original citation). Lectotype (♀), designated by Kavanaugh (1979a: 116), in MCZ [# 651].
Nebria trifaria tetonensis Erwin and Ball, 1972: 95. Type locality: «South Fork of Cascade Canyon (10,000’), Teton National Park [Teton County], Wyoming» (original citation). Holotype (♂) in USNM [# 71975]. Synonymy established by Kavanaugh (1984: 167).

#### Distribution.

This subspecies ranges along the Rocky Mountains from southern Montana and southeastern Idaho south to northeastern Nevada, southern Utah, and west-central Wyoming [see Erwin and Ball 1972: Fig. 42)].

#### Records.

**USA**: ID, MT, NV, UT, WY

#### Note.

The form *tetonensis* is retained as a valid subspecies by Ledoux and Roux (2005: 285).

### Nebria vandykei vandykei

Bänninger, 1928

Nebria vandykei Bänninger, 1928: 5. Type locality: «Paradise Val[ley], M[oun]t Rainier [Pierce County], Wash[ington]» (lectotype label). Lectotype (♂), designated by Kavanaugh (1979a: 116), in ETHZ. Etymology. The species name was proposed for Edwin Cooper Van Dyke [1869-1952], an outstanding coleopterist and professor of entomology at the University of California in Berkeley.

#### Distribution.

This subspecies is known only from the Olympic Mountains and the Cascade Range of Washington [see Kavanaugh and Schoville 2009: Fig. 13].

#### Records.

**USA**: WA

### Nebria vandykei wyeast

Kavanaugh, 1979

Nebria vandykei wyeast Kavanaugh, 1979a: 109. Type locality: «Salmon River headwaters (1830-1950 m), Mount Hood, Clackamas County, Oregon» (original citation). Holotype (♂) in CAS [# 12517].

#### Distribution.

This subspecies is restricted to the Cascade Range in Oregon from Mount Hood south to the Three Sisters area [see Kavanaugh and Schoville 2009: Fig. 13].

#### Records.

**USA**: OR

### Subgenus Nebria

Latreille, 1802

Nebria Latreille, 1802: 89. Type species: *Carabus brevicollis* Fabricius, 1792 designated by Latreille (1810: 426).
Helobia Curtis, 1826: plate 103. Type species: *Carabus brevicollis* Fabricius, 1792 by original designation. Etymology. From the Greek *helos* (marsh, meadow) and *bios* (life) [feminine].
Harpazobia Gistel, 1856: 356. Type species: *Carabus brevicollis* Fabricius, 1792 by monotypy.

#### Diversity.

Sixty-five species in the Palaearctic Region, one of them adventive in North America.

### Nebria brevicollis

(Fabricius, 1792)

Carabus cursor O.F. Müller, 1776: 78 [potential *nomen oblitum*]. Type locality: environs de Paris (title of Geoffroy’s book). Syntype(s) probably in MHNP. Note. This taxon was first described by Geoffroy (1762: 146) under the name “*Bupreste noir à pattes rougeâtres*” as mentioned by Müller (1776: 78). Müller (1776: 78) reproduced Geoffroy’s original description in Latin and provided a scientific name. I consider that Müller’s name was made available by a bibliographic reference to a description and as such the type series consists of Geoffroy’s specimens (ICZN 1999: Article 72.4.4).
Carabus rufipes Goeze, 1777: 662 [primary homonym of *Carabus rufipes* DeGeer, 1774]. Type locality: environs de Paris (title of Geoffroy’s book). Syntype(s) possibly in MHNP. Synonymy established with the name *Carabus cursor* Müller by Goeze (1777: 662). Note. This taxon was first described by Geoffroy (1762: 146) under the name “*Bupreste noir à pattes rougeâtres*” as mentioned by Goeze (1777: 662) and the comment made under *Carabus cursor* also applies here. Goeze (1777: 662) also listed *Carabus ruficornis* Fabricius, 1775, currently considered a synonym of *Harpalus rufipes* (DeGeer, 1774), and *Carabus cursor* Müller, 1776 as synonyms of this taxon.
Carabus brevicollis Fabricius, 1792 [22 December]: 150 [potential *nomen protectum*]. Type locality: «Germania» (original citation). Lectotype (♂), designated by Lindroth (1961a: 78), in ZMUC. Synonymy established by Bedel (1880: 133). Note. Bedel (1881: vii) reported that he had access to the collection of Geoffroy, at the time in the hands of Maurice de Laplanche, which still contained many “authentic types.” Therefore it is likely that the synonymy proposed by Bedel (1880: 133) was based upon an examination of the type specimen(s) of the “*Bupreste noir à pattes rougeâtres*” of Geoffroy (1762) upon which *Carabus cursor* Müller, 1776 and *Carabus rufipes* Goeze, 1777 were based.
Carabus infidus Rossi, 1792 [“31 December”]: 88. Type locality: Etruria, Italy (inferred from title of the book). Syntype(s) location unknown (possibly in ZMHB). Synonymy established by Illiger (1798: 190).

#### Distribution.

This Palaearctic species is adventive in North America where it is known from the Willamette Valley in northwestern Oregon and southwestern Washington [see LaBonte 2011: Fig. 4]. Since over 3,000 specimens have been collected, the species is clearly established in the region. The first inventoried specimen found in the area was caught in late 2007 (Kavanaugh and LaBonte 2008: 482). The species has also been collected twice in eastern North America, in Quebec in 1930 and Saint Pierre and Miquelon in 1937 (Lindroth 1961a: 78) but it did not become established in this part of the continent.

#### Records.

**USA**: OR, WA – **Adventive**

### Tribe Notiophilini

Motschulsky, 1850

Notiophili Motschulsky, 1850a: iv, 16. Type genus: *Notiophilus* Duméril, 1805.

#### Diversity.

This tribe includes a single genus.

### Genus Notiophilus

Duméril, 1805

Notiophilus Duméril, 1805: 194 (as *Nothiophilus*). Type species: *Cicindela aquatica* Linnaeus, 1758 designated by Curtis (1829: plate 254). Etymology. From the Greek adjective *notios* (wet, moist, damp) and *philos* (beloved), alluding, incorrectly though, to the habitat requirements of the species known to Duméril at the time [masculine]. Note. *Notiophilus* is an incorrect subsequent spelling of *Nothiophilus* in prevailing usage and so deemed to be the correct original spelling (ICZN 1999: Article 33.3.1).
Latviaphilus Barševskis, 1994: 1. Type species: *Elaphrus biguttatus* Fabricius, 1779 by original designation. Etymology. From the geographic name Latvia and the Greek *philos* (beloved) [masculine].
Makarovius Barševskis, 1994: 1. Type species: *Notiophilus rufipes* Curtis, 1829 by original designation.

#### Diversity.

About 55 species in the arctic, subarctic, boreal, and temperate areas of the Nearctic (15 species, of which two are adventive), Neotropical (two species in mountains of Middle America), and Palaearctic (about 40 species) Regions, including northern Africa (see Barševskis 2007). Two species, *Notiophilus aquaticus* and *Notiophilus borealis*, are Holarctic.

#### Identification.

Lindroth (1961a) reviewed the North American species and provided a key for their identification. One adventive species (*Notiophilus palustris*) has been discovered subsequently in eastern Canada.

#### Faunistic Note.

*Notiophilus rufipes* Curtis is known in North America from one specimen collected in Ware County, Georgia (Barševskis 2004). In my opinion, the specimen could be mislabeled and therefore the species is not considered as a North American entity.

### Notiophilus aeneus

(Herbst, 1806)

Elaphrus aeneus Herbst, 1806: 235. Type locality: «Nordamerica» (original citation), restricted to «Boston [Suffolk County], Mass[achusetts]» by Lindroth (1961a: 93). Syntype(s) location unknown (possibly in ZMHB).
Nothiophilus porrectus Say, 1830b: (3) [3]. Type locality: «Penn[sylvania]» (neotype label). Neotype (♂), designated by Lindroth and Freitag (1969: 332), in MCZ [# 33084]. Synonymy established with doubt by Melsheimer (1853: 12).

#### Distribution.

This species ranges from Nova Scotia (Lindroth 1961a: 94) to southeastern Minnesota (Donald P. Schwert pers. comm. 1989), south at least to southeastern Nebraska (Richardson County, Foster F. Purrington pers. comm. 2009), central Missouri (Boone County, CMNH), northeastern Georgia (Leng 1910: 73; Fattig 1949: 11), and southwestern South Carolina (Ciegler 2000: 20). Two specimens labeled from the Santa Catalina Mountains in Arizona (MCZ, collection Fall) are known and so the species probably ranges farther west in southern United States.

#### Records.

**CAN**: NB, NS, ON, PE, QC **USA**: AZ, CT, DC, DE, GA, IA, IL, IN, MA, MD, ME, MI, MN, MO, NC, NE, NH, NJ, NY, OH, PA, RI, SC, TN, VA, VT, WI, WV

### Notiophilus aquaticus

(Linnaeus, 1758)

Cicindela aquatica Linnaeus, 1758: 408. Type locality: «Europa» (original citation), restricted to «Sweden» by Lindroth (1961a: 95). Syntype(s) location unknown. Note. Three Linnean specimens are present in LSL under this name, but none belong to the present species (Lindroth 1957b: 336).
Cicindela pusilla Schreber, 1759: 10. Type locality: «prope Halam [= Halle, Germany]» (original citation). Syntype(s) probably lost. Synonymy established by Fabricius (1775: 227).
Elaphrus semipunctatus Fabricius, 1775: 227. Type locality: «Halae Saxonum [= Halle, Germany]» (original citation). Three syntypes in ZMUC (Zimsen 1964: 66). Synonymy established by Fauvel (1883: 90).
Notiophilus metallicus G.R. Waterhouse, 1833: 203. Type locality: England (inferred from title of the paper). Holotype [by monotypy] location unknown (possibly in BMNH). Synonymy established by Dawson (1854: 54).
Notiophilus newmanni G.R. Waterhouse, 1833: 205. Type locality: «Snowdon [Great Britain]» (original citation). Syntype(s) [3 originally cited] location unknown (possibly in BMNH). Synonymy established by Dawson (1854: 54). Etymology. The specific name was proposed for Edward Newman [1801-1876], British naturalist and printer. Newman co-founded *The Entomological Magazine* and founded *The Entomologist*. His publications dealt mainly with entomology but he also wrote on several other natural history subjects including botany, especially ferns.
Notiophilus dauricus Chaudoir, 1850b: 164. Type locality not stated. Syntype(s) probably in MHNP (collection Chaudoir). Synonymy re-established by Reitter (1897: 361). Note. Motschulsky (1859b: 539) first described this taxon under the name *Notiophilus aquaticus* var. *dauricus* in a paper about the beetles of the government of Yakutsk, Siberia, and the name has been attributed to him since. However, Chaudoir (1850b: 164) published the name earlier as a junior synonym of *Notiophilus aquaticus* (“M. de Motschoulski m’a envoyé l’*Aquaticus* sous le nom de *Dauricus*”) and therefore the name is available from its first publication as a synonym (ICZN 1999: Article 11.6.1).
Notiophilus hardyi Putzeys, 1866a: 165. Type locality: «Terre-Neuve» (original citation). Syntype(s) in MHNP (collection Chaudoir). Synonymy established by Fall (1906: 84), confirmed by Lindroth (1954b: 121).

#### Distribution.

The range of this circumpolar species extends from Iceland to the Bering Sea Coast (Bousquet and Barševskis 2003: 96) and from Alaska (Lindroth 1961a: 95) to Newfoundland (Lindroth 1955a: 34), south to Pennsylvania (Capogreco 1989b: 4) and to New Mexico and Arizona (Fall 1906: 85; Lindroth 1961a: 95) along the Rocky Mountains. Fossil remnants from a Plio-Pleistocene sequence have been found in northwestern Greenland (Böcher 1995: 18).

#### Records.

**FRA**: PM **CAN**: AB, BC, LB, MB, NB, NF, NS (CBI), ON, QC, SK, YT **USA**: AK, AZ, CO, ID, IL, MA, ME, MI, MN, MT, NH, NM, NY, OH, PA, UT, VT, WI, WY – **Holarctic**

**Figure 7.**
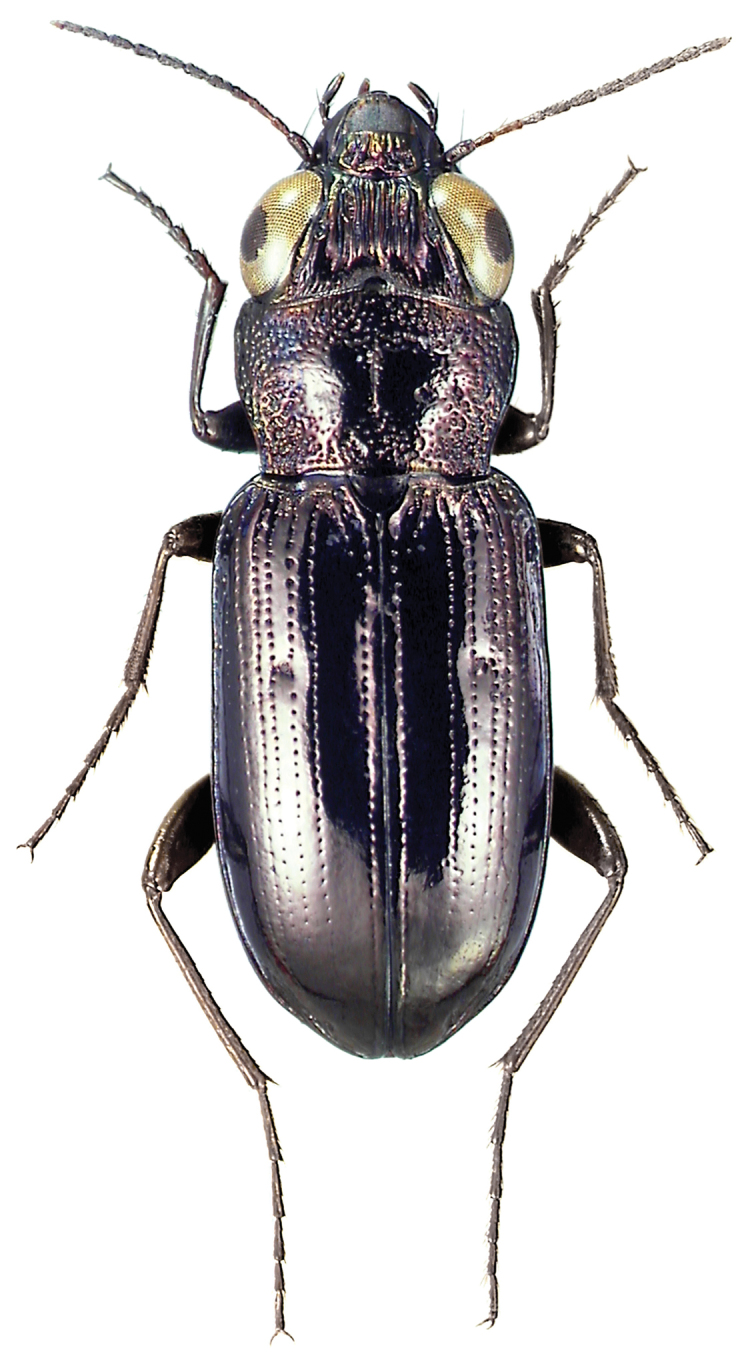
*Notiophilus aquaticus* (Linnaeus). This species is an example of a circumboreal taxon; it is found in the Northern Hemisphere without major gaps north of latitude 45. Linnaeus gave this species the epithet *aquaticus* in the 10th edition of his *Systema Naturae*, published in 1758, on the assumption that the species lived close to water. We know today that this is not the case and the species is found in relatively dry, open habitats. *Notiophilus* larvae and adults prey on collembolans. The adults are challenging to catch by hand because of their small size and swiftness.

### Notiophilus biguttatus

(Fabricius, 1779)

Elaphrus biguttatus Fabricius, 1779: 231. Type locality: «Bye [Norway]» (original citation) which according to Lindroth (1961a: 100) is located in Guldal (= Gauldal), a valley of the Gaula River, south of Trondheim. Syntype(s) destroyed (Lindroth 1961a: 100).

#### Distribution.

This Palaearctic species is adventive in North America where it is known from Newfoundland and Saint Pierre and Miquelon (Lindroth 1955a: 37) to the Gaspé Peninsula in Quebec (LeSage 1996: 23), south to Connecticut (Krinsky and Oliver 2004: 396) and “Rhode Island” (Sikes 2003: 7), and from southwestern British Columbia (Lindroth 1961a: 101). The first inventoried specimen found on the east side of this continent was caught in Newfoundland in 1923 (Brown 1950b: 197) and on the west coast in Vancouver in 1957 (Lindroth 1961a: 101). Dejean’s (1831: 589) record of this species from «Amérique septentrionale» almost certainly refers to *Notiophilus novemstriatus* LeConte.

#### Records.

**FRA**: PM **CAN**: BC, NB, NF, NS, PE, QC **USA**: CT, ME, NH, RI – **Adventive**

### Notiophilus borealis

Harris, 1869

Notiophilus borealis T.W. Harris [in Scudder], 1869: 213. Type locality: «White Mountains [Coos County, New Hampshire]» (original citation). One syntype in MCZ [# 26409].

#### Distribution.

This species is known from the Far East (Bousquet and Barševskis 2003: 96) and from Alaska (Lindroth 1961a: 97) to Newfoundland (Lindroth 1955a: 36); isolated on some mountains of New England (Lindroth 1961a: 96-97) and New York (Essex County, CNC, MCZ), and also known from northern Wyoming (Johnson County, CMNH). Fossil remnants of this species, dated between about 16,700 and 18,100 years B.P., have been unearthed in southeastern Iowa (Baker et al. 1986: 96).

#### Records.

**CAN**: AB, BC, LB, MB, NF, NT, NU, ON, QC, SK, YT **USA**: AK, ME, NH, NY, VT, WY – **Holarctic**

### Notiophilus directus

Casey, 1920

Notiophilus directus Casey, 1920: 142. Type locality: «Indiana; Keokuk, Iowa» (original citation), which according to Lindroth (1961a: 98) are unlikely; «Jasper, Al[ber]ta» selected by Lindroth (1961a: 98). Lectotype (♀), designated by Lindroth (1975: 113), in USNM [# 46841].
Notiophilus lanei Hatch, 1949b: 114. Type locality: «Pierce [Clearwater County], Idaho» (original citation). Holotype (♂) in USNM. Synonymy established by Lindroth (1954b: 121).

#### Distribution.

This species occurs in the western mountain ranges, from southwestern Alberta and southern British Columbia, south to northeastern California (Lindroth 1961a: 98), northeastern Arizona, and northern New Mexico [see Morgan and Morgan 1979: Fig. 5]. Fossil remnants, dated about 11,800 years B.P., have been unearthed in northeastern Wisconsin (Morgan and Morgan 1979: 232).

#### Records.

**CAN**: AB, BC **USA**: AZ, CA, CO, ID, MT, NM, OR, UT, WA, WY

### Notiophilus intermedius

Lindroth, 1955

Notiophilus intermedius Lindroth, 1955a: 36. Type locality: «Pinware River, Labrador» (original citation). Holotype (♂) in CNC [# 6569]. Note. Lindroth (1954b: 157) proposed the name earlier but he did not meet the requirements of availability (ICZN, Article 13.1) at the time.

#### Distribution.

This species is known from scattered localities from Newfoundland (Lindroth 1955a: 37) to the Gulf of Alaska coast (Lindroth 1961a: 98); also known from northwestern Minnesota (Clearwater County, CNC).

#### Records.

**CAN**: BC, LB, MB, NF, QC **USA**: AK, MN

### Notiophilus nemoralis

Fall, 1906

Notiophilus nemoralis Fall, 1906: 88. Type locality: «White M[oun]t[ain]s, N[ew] H[ampshire]; Moosilauke, N[ew] H[ampshire]; Rangely, M[ain]e; Camels Hump, V[ermon]t» (original citation). Syntype(s) in MCZ [# 23845].

#### Distribution.

This species is restricted to some mountains in New England (Lindroth 1961a: 100) and the Adirondacks in northeastern New York (Notman 1928: 211).

#### Records.

**USA**: MA, ME, NH, NY, VT

### Notiophilus nitens

LeConte, 1857

Notiophilus nitens LeConte, 1857c: 31. Type locality: «Prairie Paso [= possibly Bear Prairie Pass, Lewis County, Washington], Oregon [Territory]» (original citation). Holotype [by monotypy] (♂) in MCZ [# 5454].

#### Distribution.

This species is found from Vancouver Island (Lindroth 1961a: 99) to northwestern Montana (Russell 1968: 46), south to southern Oregon (Harney County, CMNH). The record from Texas (Fall 1906: 90) is probably in error.

#### Records.

**CAN**: BC (VCI) **USA**: ID, MT, OR, WA

### Notiophilus novemstriatus

LeConte, 1847

Notiophilus 9-striatus LeConte, 1847: 450. Type locality: «provinciis australibus, et mediis» (original citation), restricted to «Boston [Suffolk County], Mass[achusetts]» by Lindroth (1961a: 101). Syntype(s) in MCZ [# 655].
Notiophilus cribrilaterus Motschulsky, 1864: 193. Type locality: «Am[érique] bor[éale]» (original citation). Three syntypes in ZMMU, one labeled “Am. bor. Atlanta” (Keleinikova 1976: 193). Synonymy established by Bousquet and Larochelle (1993: 16).
Notiophilus quadrifoveatus T.W. Harris [in Scudder], 1869: 213. Type locality: «New Hampshire, Vermont, Massachusetts, New York, Pennsylvania, North Carolina, and Alabama» (original citation). One probable syntype, labeled “quadrifoveatus Harris. [handwritten] / 709 [handwritten] / 50,” in MCZ (collection Harris). Synonymy established by Fall (1906: 92), herein confirmed.
Notiophilus parvus Casey, 1920: 142. Type locality: «Catskill M[oun]t[ain]s, New York» (original citation). Lectotype (♀), designated by Lindroth (1975: 113), in USNM [# 46840]. Synonymy established by Lindroth (1954b: 121).

#### Distribution.

The range of this species extends from western Maine (Oxford County, André Larochelle pers. comm. 1990) to “South Dakota” (Kirk and Balsbaugh 1975: 15), south to east-central Texas (Riley 2011) and northern Florida (Peck and Thomas 1998: 15), west along the southwest to “Arizona” (Lindroth 1961a: 101) including northwestern Colorado (Barševskis 2009: 138). The record from Cape Breton Island in Nova Scotia (McCorquodale 2000: 339) is based on a misidentified specimen of *Notiophilus palustris*.

#### Records.

**USA**: AL, AR, AZ, CT, DC, DE, FL, GA, IA, IL, IN, KS, KY, LA, MA, MD, ME, MI, MO, MS, NC, NE, NH, NJ, NM, NY, OH, OK, PA, RI, SC, SD, TN, TX, VA, VT

### Notiophilus palustris

(Duftschmid, 1812)

Elaphrus palustris Duftschmid, 1812: 192. Type locality: «Um Linz [Austria]» (original citation). Syntype(s) probably lost. Note. Ledoux and Roux (2005: 682) reported that Duftschmid’s collection, supposedly located at the Oberösterreichisches Landesmuseum in Linz (Horn et al. 1990a: 101), is non-existent.

#### Distribution.

This Palaearctic species is adventive in North America where it is known only from Nova Scotia, including Cape Breton Island (McCorquodale 2000: 339, as *Notiophilus novemstriatus*), and Prince Edward Island (Larochelle and Larivière 1990b: 211). The first inventoried specimen found on this continent was caught in Halifax, Nova Scotia in 1968 (CNC).

#### Records.

**CAN**: NS (CBI), PE – **Adventive**

### Notiophilus semiopacus

Eschscholtz, 1833

Notiophilus semiopacus Eschscholtz, 1833: 25. Type locality: «bei St. Franzisco [San Francisco County], Californien» (original citation). Syntype(s) location unknown (possibly in ZMMU).

#### Distribution.

This species is known from California (Fall 1906: 91; Lindroth 1961a: 99), as far north as the San Francisco Bay area (Alameda County, MCZ), Arizona (Wickham 1898: 300; Lindroth 1961a: 99), and the states of Sonora and Chihuahua in Mexico (Erwin 2007a: 59). The record from “Oregon” (Fall 1906: 91) needs confirmation.

#### Records.

**USA**: AZ, CA [OR] – Mexico

### Notiophilus semistriatus

Say, 1823

Nothiophilus semistriatus Say, 1823a: 81. Type locality: «Fairfax Co[unty], V[irgini]a» (neotype label). Neotype (♂), designated by Lindroth and Freitag (1969: 332), in MCZ [# 33083].
Notiophilus confusus LeConte, 1847: 449. Type locality: United States east of the Rocky Mountains (inferred from title of the paper). Syntype(s) in MCZ [# 654]. Synonymy established by LeConte (1850: 210), confirmed by Lindroth (1961a: 94).
Notiophilus punctatus LeConte, 1850: 210. Type locality: Lake Superior (inferred from title of the paper). Four syntypes in MCZ [# 656]. Synonymy established by Lindroth (1961a: 94).
Notiophilus americanus T.W. Harris [in Scudder], 1869: 213. Type locality not stated. Syntype(s) probably lost. Synonymy established by Fall (1906: 91).
Notiophilus coloradensis Casey, 1920: 141. Type locality: «Boulder Co[unty], Colorado» (original citation). Holotype [by monotypy] (♂) in USNM [# 46837]. Synonymy established by Lindroth (1954b: 121).
Notiophilus solodovnikovi Barševskis, 2001: 38. Type locality: «Chehova M[ou]nt[ain], Sahalin isl[and], S[outh], Far East, Russia» (original citation). Holotype (♂) in Baltic Institute of Coleopterology (Daugavpils, Latvia). Synonymy established by Barševskis (2006: 66).

#### Distribution.

This species is found from Labrador to central Alaska, south to northern British Columbia (Lindroth 1961a: 94-95), New Mexico (Fall 1906: 84; Lindroth 1961a: 94) along the Rocky Mountains, Oklahoma (Latimer County, UASM), southwestern Arkansas (Columbia County, INHS), east-central Alabama (Lee County, CNC), central Georgia (Fattig 1949: 11), and southern South Carolina (Ciegler 2000: 20); also recorded from Sakhalin Island (Barševskis 2001: 38).

#### Records.

**CAN**: AB, BC, LB, MB, NB, NS (CBI), ON, QC, YT **USA**: AK, AL, AR, CO, CT, DC, DE, GA, IA, IL, IN, KS, KY, MA, MD, MI, MN, MO, NC, ND, NE, NH, NJ, NM, NY, OH, OK, PA, SC, SD, TN, VA, WI, WV – **Holarctic**

### Notiophilus sierranus

Casey, 1920

Notiophilus obscurus Fall, 1901a: 207 [primary homonym of *Notiophilus aquaticus obscurus* Dalla Torre, 1877]. Type locality: «San Bernardino Mountains (6,000 to 7,000 feet) [California]» (original citation). Syntype(s) in MCZ [# 23846].
Notiophilus sierranus Casey, 1920: 140. Type locality: «Lake Tahoe [Placer County], California» (original citation). Holotype [by monotypy] (♀) in USNM [# 46838]. Synonymy established by Lindroth (1961a: 98).
Notiophilus obscuratus Fall, 1926b: 125. Replacement name for *Notiophilus obscurus* Fall, 1901.

#### Distribution.

As far as known, this species is restricted to the Sierra Nevada in California (Fall 1906: 90; Lindroth 1961a: 99).

#### Records.

**USA**: CA

### Notiophilus simulator

Fall, 1906

Notiophilus simulator Fall, 1906: 86. Type locality: «Mullan, Montana; Leavenworth Valley and Silver Plume, 9000-11000 feet, Colorado; Houston, Texas; Coeur d’Alene, Idaho» (original citation), restricted to «Silver Plume [Clear Creek County], Color[ado]» by Lindroth (1961a: 97). Syntype(s) [5 ♀ originally cited] in MCZ [# 23847].
Notiophilus evanescens Casey, 1913: 47. Type locality: «Boulder Co[unty], Colorado» (original citation). Lectotype (♀), designated by Lindroth (1975: 113), in USNM [# 46836]. Synonymy established by Casey (1914: 356), confirmed by Lindroth (1954b: 121).

#### Distribution.

This species is found in the western mountain ranges from western Alberta and British Columbia (Lindroth 1961a: 97) south at least to central Oregon (Crook County, CNC) and southwestern Colorado (Elias 1987: 632). The record from Houston, Texas (Fall 1906: 87) needs confirmation; that from Alaska (Fall 1926a: 129) refers to *Notiophilus intermedius* (Lindroth 1961a: 97).

#### Records.

**CAN**: AB, BC **USA**: CO, ID, MT, OR, UT, WA, WY [TX]

### Notiophilus sylvaticus

Dejean, 1831

Notiophilus sylvaticus Dejean, 1831: 589. Type locality: «détroit de Norfolk [= Sitka Sound, Baranof Island, Alaska], sur la côte nord-ouest de l’Amérique septentrionale» (original citation). Holotype [by monotypy] probably in MHNP. Note. Eschscholtz (1833: 24) first described this species and the name has been attributed to him since. However, Dejean (1831: 589) published the name earlier as a junior synonym of *Notiophilus biguttatus* and the name therefore is available from its first publication as a synonym (ICZN 1999: Article 11.6.1). The date of 1829 attributed by Lindroth (1961a: 99) to this name, under the authorship of Eschscholtz, is in error.

#### Distribution.

This species ranges from southern Alaska (Lindroth 1961a: 100) to northern California, at least as far south as Mendocino County (Fall 1906: 89), east to northern Idaho (Hatch 1953: 61). Fossil remnants, dated from the Late Pleistocene, have been unearthed in northeastern Siberia (Kiselev 1981: 12).

#### Records.

**CAN**: BC (QCI, VCI) **USA**: AK, CA, ID, OR, WA

### Subfamily CARABINAE

Latreille, 1802

Carabici Latreille, 1802: 80. Type genus: *Carabus* Linnaeus, 1758.

#### Diversity.

Worldwide, with about 1,300 species (Lorenz 2005: [i]) arrayed in four tribes: Carabini (about 1,080 species), Ceroglossini (eight South American species), Cychrini (about 200 species), and Pamborini (13 species).

### Tribe Cychrini

Perty, 1830

Cychridae Perty, 1830: 6. Type genus: *Cychrus* Fabricius, 1794.

#### Diversity.

Northern Hemisphere and mountains in Mexico, with about 200 species arrayed by most authors in four genera: *Cychropsis* Boileau (about 25 Asian species), *Cychrus* (about 115 species), *Scaphinotus* (55 species), and *Sphaeroderus* (six species).

### Genus Sphaeroderus

Dejean, 1826

Sphaeroderus Dejean, 1826: 14. Type species: *Sphaeroderus lecontei* Dejean, 1826 designated by Hope (1838: 65). Etymology (original). From the Greek *sphaera* (sphere) and *dere* (neck, by extension pronotum), alluding to the round or oval shape of the pronota (“*corselet *... *arrondi, ovale ou orbiculé*”) of the species then known to Dejean [masculine].

#### Diversity.

Six species restricted to the boreal and temperate regions of eastern North America.

#### Identification.

Lindroth’s (1961a) key covers four of the species currently recognized; *Sphaeroderus indianae* was not included and *Sphaeroderus schaumii* was considered a junior synonym of *Sphaeroderus nitidicollis*. A taxonomic revision of the genus is needed.

### Sphaeroderus bicarinatus

(LeConte, 1853)

Cychrus bicarinatus LeConte, 1853c: 399. Type locality: «Habersham Co[unty], Georgia» (original citation). Holotype [by monotypy] (♂) in MCZ [# 616].
Sphaeroderus multicarinatus Darlington, 1932: 151. Type locality: «Newfound Gap (near 5,200 feet), Smoky Mountains, on the North Carolina-Tennessee state line» (original citation). Holotype (♂) in MCZ [# 16432]. Synonymy established by Bousquet and Larochelle (1993: 78) based on Barr (1974a) unpublished manuscript.

#### Distribution.

This species is found along the southern parts of the Appalachians from North Carolina and Tennessee (Darlington 1932: 152) to northern Alabama (Löding 1945: 11) and northeastern Georgia (LeConte 1853c: 399; Leng 1910: 73).

#### Records.

**USA**: AL, GA, NC, SC, TN

#### Note.

Roeschke (1907a: 263) and Lindroth (1961a: 29) regarded this form as a subspecies of *Sphaeroderus stenostomus* (Weber).

### Sphaeroderus canadensis canadensis

Chaudoir, 1861

Sphaeroderus canadensis Chaudoir, 1861b: 498. Type locality: «environs de la rivière Ottawa, Canada» (original citation). Syntype(s) in MHNP.
Sphaeroderus palpalis Motschulsky, 1866: 312. Type locality: «Hudson-Bay» (original citation), which is incorrect. Lectotype [as holotype] (♂), designated by Kryzhanovskij (1968: 186), in ZMMU. Synonymy established by Roeschke (1907a: 262), confirmed by Kryzhanovskij (1968: 186).
Sphaeroderus canadensis blanchardi Leng, 1916: 41. Type locality: «Randolph [Coos County], N[ew] H[ampshire]» (original citation). Holotype (♂) in CAS [# 4375]. Synonymy established by Darlington (1933a: 62).

#### Distribution.

The range of this subspecies extends from Cape Breton Island to southern Ontario (Lindroth 1961a: 28), south to the Black Mountains in North Carolina (Darlington 1933a: 64). The record from “South Carolina” (Bousquet and Larochelle 1993: 78) refers to *Sphaeroderus canadensis lengi*; that from “Michigan” (Bousquet and Larochelle 1993: 78) needs confirmation.

#### Records.

**CAN**: NB, NS (CBI), ON, QC **USA**: CT, KY, MA, MD, ME, NC, NH, NY, OH, PA, TN, VA, VT, WV [MI]

### Sphaeroderus canadensis lengi

Darlington, 1933

Sphaeroderus canadensis lengi Darlington, 1933a: 63. Type locality: «between Newfound Gap and Clingman’s Dome (5,000-6,600 ft.), on the North Carolina-Tennessee state line» (original citation). Holotype (♂) in MCZ [# 17238].

#### Distribution.

This subspecies occurs in the mountains south of the French Broad River (Barr 1969: 76) from southwestern North Carolina and adjacent parts of Tennessee (Darlington 1933a: 64) to northeastern Georgia (Fattig 1949: 9) and northwestern South Carolina (Ciegler 2000: 30).

#### Records.

**USA**: GA, NC, SC, TN

### Sphaeroderus indianae

(Blatchley, 1910)

Cychrus stenostomus indianae Blatchley, 1910: 42. Type locality: «Crawford County [Indiana]» (original citation for the lectotype). Lectotype (♀), designated by Blatchley (1930: 33), in PURC.

#### Distribution.

This species ranges from northwestern Ohio (Barr 1974a) to central Indiana (Blatchley 1910: 42), south to north-central Mississippi (Grenada County, Drew A. Hildebrandt pers. comm. 2009) and southwestern Virginia (Barr 1974a).

#### Records.

**USA**: IN, KY, MS, OH, TN, VA, WV

### Sphaeroderus nitidicollis

Guérin-Méneville, 1829

Sphaeroderus nitidicollis Guérin-Méneville, 1829: plate 7. Type locality: «Terre-Neuve» (Guérin-Méneville 1844a: 24). Holotype [by monotypy] in UMO (Lindroth 1969a: 1108). Note. This name has been credited to Chevrolat by almost all authors. The validation of the specific name is by association with an illustration drawn by Guérin-Méneville and published in Guérin-Méneville’s *Iconographie du Règne Animal* under the name “*Sphaeroderus nitidicollis* Chevr.” The nomenclature act was done by Guérin-Méneville and he is to be credited with the species name.
Sphaeroderus brevoorti LeConte, 1847: 443. Type locality: «Maine» (original citation). Syntype(s) in MCZ [# 617]. Synonymy established by Roeschke (1907a: 266).
Sphaeroderus granulosus Chaudoir, 1861b: 497. Type locality: «Terre Rupert, près de la baie d’Hudson» (original citation). Syntype(s) [2 ♂ originally cited] in MHNP. Synonymy established by LeConte (1866: 78).

#### Distribution.

This species ranges from Newfoundland (Lindroth 1955a: 23) to east-central Saskatchewan (Hooper 1978: 19), south to northeastern Minnesota (Gandhi et al. 2005: 923), northern Wisconsin along Lake Superior (Wickham 1896c: 131), the Adirondack Mountains in northeastern New York (Notman 1928: 208), and New England [see Lindroth 1963a: Fig. 68]. The record from southwestern Ohio (Dury 1906: 257) is probably in error. Roeschke (1907a: 268) statement that this species is found “Küstengebiet von Maine bis Washington D.C.” is in error. Fossil remnants, dated between 22,200 and 25,200 years B.P., have been unearthed in central Illinois (Schwert 1992: 76).

#### Records.

**CAN**: MB, NB, NF, NS (CBI), ON, QC, SK **USA**: ME, MN, NH, NY, VT, WI

#### Note.

Lindroth (1961a: 29) retained *brevoorti* as “a weak subspecies, at most.” I studied several specimens from Newfoundland, the Maritimes, and Quebec, including Anticosti Island, and found no structural differences between populations of the forms *nitidicollis* and *brevoorti* except for the fact that adults of the *nitidicollis* form are on average slightly larger than those of the *brevoorti* form.

### Sphaeroderus schaumii

Chaudoir, 1861

Sphaeroderus schaumii Chaudoir, 1861b: 500. Type locality: «Ohio» (original citation), herein restricted to South Bass Island in western Lake Erie (see Will et al. 1995: 68). Holotype [by monotypy] (♂) in MHNP.

#### Distribution.

This species is known from a small area from Michigan to northern Virginia (Barr 1974a) and western Maryland (Bailey et al. 1994: 320). The record from “Illinois” (Bousquet and Larochelle 1993: 78) is probably in error.

#### Records.

**USA**: DC, MD, MI, OH, VA, WV

#### Note.

Roeschke (1907a: 266), Lindroth (1961a: 29), Lorenz (2005: 64), and Erwin (2007a: 170) listed this taxon as a subspecies of *Sphaeroderus nitidicollis* Guérin-Méneville. Thomas C. Barr, Jr. (pers. comm. 1977) believes it represents a distinct species, though closely related to *Sphaeroderus nitidicollis*.

### Sphaeroderus stenostomus lecontei

Dejean, 1826

Cychrus stenostomus Say, 1823a: 72 [primary homonym of *Cychrus stenostomus* Weber, 1801]. Type locality: North America (inferred from title of the work). Syntype(s) lost. Note. Say (1823a: 72) described this taxon as a new species as indicated by the presence of an asterisk preceding the specific epithet. Say did not originally indicate the area where his specimen(s) came from but later (Say 1828: [101]) noted that the species “is not uncommon in Pennsylvania” and that he received a specimen “taken in Massachusetts.”
Sphaeroderus lecontei Dejean, 1826: 15. Type locality: «Amérique septentrionale» (original citation), restricted to «Rumney [Grafton County], New Hampsh[ire]» by Lindroth (1961a: 29). One possible syntype in MHNP (Lindroth 1955b: 12). Synonymy established by Roeschke (1907a: 263).
Sphaeroderus niagarensis Laporte, 1833: 390. Type locality: «île que forme la chute du Niagara» (original citation). Syntype(s) location unknown. Synonymy established by Chaudoir (1861b: 496).
Sphaeroderus lecontei diffractus Casey, 1914: 25. Type locality: «New Brunswick» (original citation). One syntype in USNM [# 46002]. Synonymy established by Lapouge (1933: 706), confirmed by Lindroth (1961a: 29).

#### Distribution.

This subspecies is found from Newfoundland (Lindroth 1955a: 21) to southeastern Manitoba (Lindroth 1961a: 29), south to east-central Iowa (Wickham 1888: 81, as *Cychrus stenostomus*; Lindroth 1961a: 29), northeastern Mississippi (Tishomingo County, CNC), northern Alabama (Löding 1945: 11; Madison County, CNC), northern Georgia (Fattig 1949: 9), and southern South Carolina (Ciegler 2000: 30). The records from east-central Missouri (Summers 1873: 133) and southeastern Louisiana (Summers 1874a: 79) need confirmation; that from “Saskatchewan” (Bousquet and Larochelle 1993: 78) is in error.

#### Records.

**CAN**: MB, NB, NF, NS (CBI), ON, PE, QC **USA**: AL, CT, GA, IA, IL, IN, KY, MA, MD, ME, MI, MN, MS, NC, NH, NJ, NY, OH, PA, RI, SC, TN, VA, VT, WI, WV [LA, MO]

### Sphaeroderus stenostomus stenostomus

(Weber, 1801)

Cychrus stenostomus Weber, 1801: 43. Type locality: «America» (original citation), herein restricted to Chestertown, Kent County, Maryland (CMNH). Syntype(s) location unknown. Note. This species was described the same year under the same name by Knoch (1801: 190). I have not found a date of publication other than the year for Knoch’s book. According to Evenhuis (1997b: 809), Weber’s book was published before March 1801. Weber is in “prevailing usage” as the author of this taxon.
Sphaeroderus stenostomus aequalis Casey, 1920: 172. Type locality: «Pennsylvania» (original citation). One syntype in USNM [# 46003]. Synonymy established by Erwin (2007a: 171).

#### Distribution.

This mainly coastal subspecies ranges from southeastern New York (Barr 1974a) and southeastern Pennsylvania (York County, CMNH) south to Wake County in east-central North Carolina (Brimley 1938: 115). The record from northern Georgia (Fattig 1949: 9) probably refers to *Sphaeroderus stenostomus lecontei*.

#### Records.

**USA**: DC, DE, MD, NC, NJ, NY, PA, VA, WV

### Genus Cychrus

Fabricius, 1794

Cychrus Fabricius, 1794a: 440. Type species: *Carabus rostratus* Linnaeus *sensu* Fabricius, 1775 (= *Tenebrio caraboides* Linnaeus, 1758) designated by Latreille (1810: 426). Etymology. Uncertain, possibly from *Cychreus*, son of Poseidon and Salamis, or from *Cychros*, a city in Thrace near a lake whose water was unhealthy [masculine]. Duméril (1823: 167) stated that *Cychros* was the name of a bird (probably in Pliny the Elder) but I have not been able to confirm this. Note. Fabricius (1794b: 70) used the spelling *Cychrys* for this genus in another publication issued the same year. Dates of publication of both works are unknown besides the year. Bousquet (2002b: 16) acted as First Reviser and opted for *Cychrus* as the valid name.

#### Diversity.

Northern Hemisphere, with about 115 species (Lorenz 2005: 64-66) in the Nearctic (two western species) and Palaearctic Regions.

#### Identification.

Gidaspow (1973) revised the North American species. Lindroth (1961a: 25-26) covered the species in his monograph of the Canadian and Alaskan Carabidae.

### Cychrus hemphillii hemphillii

Horn, 1879

Cychrus hemphillii G.H. Horn, 1879: 184. Type locality: «near Ogden [Weber County], Wahsatch M[oun]t[ain]s, Utah» (original citation). Syntype(s) in MCZ [# 35315]. Etymology. This species was named after Henry Hemphill [1830-1914], mason, bricklayer, and amateur malacologist in San Diego, California. Hemphill collected extensively in western United States.

#### Distribution.

This subspecies is known from southwestern Idaho, western Wyoming, and northern Utah [see Gidaspow 1973: Fig. 7].

#### Records.

**USA**: ID, UT, WY

### Cychrus hemphillii rickseckeri

LeConte, 1884

Cychrus rickseckeri LeConte, 1884: 2. Type locality: «Montana» (original citation). Holotype [by monotypy] in MCZ [# 606].

#### Distribution.

This subspecies is found from southeastern British Columbia south to northeastern Oregon (LaBonte 1988: 264), northern Idaho, and the Bitter Root Mountains in western Montana (Roeschke 1907a: 227) [see Gidaspow 1973: Fig. 7]. One specimen labeled from Logan Canyon in northern Utah is also known (Gidaspow 1973: 98).

#### Records.

**CAN**: BC **USA**: ID, MT, OR, WA [UT]

#### Note.

Lindroth (1961a: 26) treated this form as a valid species but both Roeschke (1907a: 227) and Gidaspow (1973: 96) regarded it as a subspecies of *Cychrus hemphillii* Horn.

### Cychrus tuberculatus

Harris, 1839

Cychrus tuberculatus T.W. Harris, 1839: 200. Type locality: «Oregon» (original citation, see page 199), restricted to «Portland [Multnomah County]» by Lindroth (1961a: 25). Syntype(s) apparently lost (Lindroth 1969a: 1108).
Cychrus pustulosus Casey, 1905: 160. Type locality: «Washington State» (original citation). Holotype [by monotypy] (♀) in USNM [# 46001]. Synonymy established by Roeschke (1907a: 228), confirmed by Lindroth (1961a: 25).

#### Distribution.

This species ranges from British Columbia, including the Queen Charlotte Islands and Vancouver Island, south at least to southern Oregon [see Gidaspow 1973: Fig. 7], possibly even Mendocino County in northern California (Roeschke 1907a: 229). The record from “Alaska” (Roeschke 1907a: 229) is likely in error.

#### Records.

**CAN**: BC (QCI, VCI) **USA**: OR, WA [CA]

**Figure 8.**
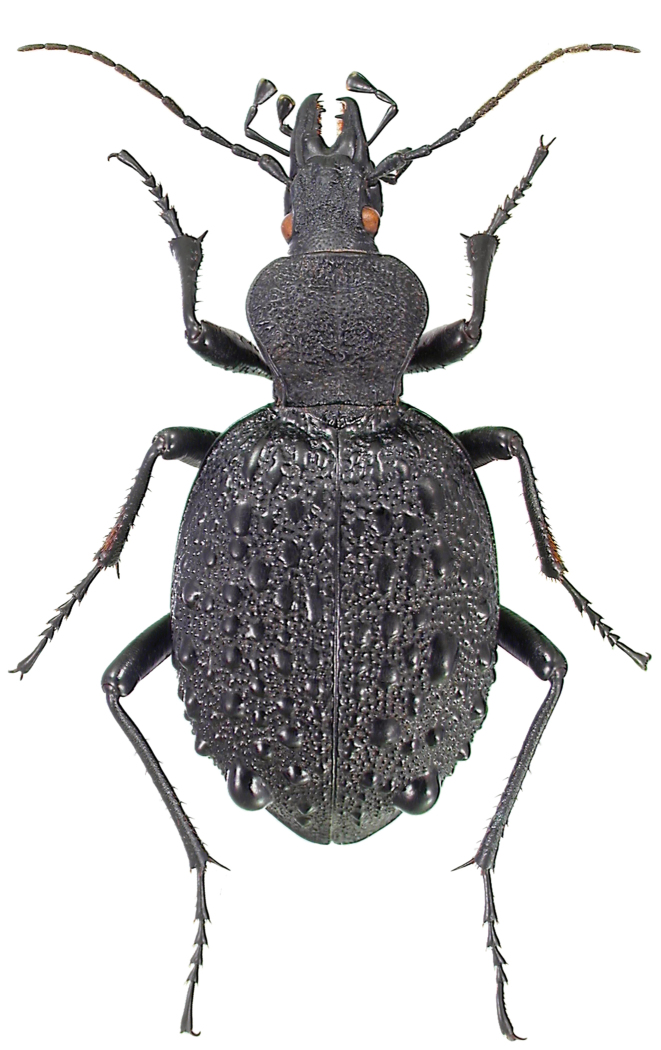
*Cychrus tuberculatus* (Harris). This species belongs to a genus that contains numerous species in the Palaearctic Region but only two in North America, both west coastal elements. The two species are morphologically very similar and likely sister-species, suggesting that a single ancestral stock crossed Beringia. *Stomis* and *Trechoblemus* are other carabid genera well represented in the Palaearctic with a single species each on the West Coast of North America.

### Genus Scaphinotus

Dejean, 1826

Scaphinotus Dejean, 1826: 17. Type species: *Carabus elevatus* Fabricius, 1787 by monotypy. Etymology. From the Greek *scaphos* (barque, nacelle, skiff) and *notos* (back, upper surface), presumably alluding to the shape of the pronotum of the sole species known to Dejean which has the sides of the pronotum markedly turned out (“*bords latéraux du corselet très-déprimés, relevés*”) giving the impression of a small boat [masculine].
Scaphonotus Agassiz, 1846: 332. Unjustified emendation of *Scaphinotus* Dejean, 1826.

#### Diversity.

Fifty-five species in the boreal and temperate regions of North America and the Sierra Madre Occidental in Mexico.

#### Identification.

Roeschke (1907a) reviewed the species known at the time.

### Subgenus Scaphinotus

Dejean, 1826

Scaphinotus Dejean, 1826: 17. Type species: *Carabus elevatus* Fabricius, 1787 by monotypy.

#### Diversity.

Nine species in North America (seven species) and the Sierra Madre Occidental of Mexico (two species).

#### Identification.

Van Dyke (1938) reviewed the species and provided a key for their identification. One new species was described subsequently by Allen and Carlton in 1988.

### [elevatus group]

### Scaphinotus elevatus coloradensis

Van Dyke, 1907

Scaphinotus elevatus coloradensis Van Dyke [in Roeschke], 1907a: 141. Type locality: Colorado (inferred from name of the species), «eastern Colorado» reported by Van Dyke (1938: 123). Holotype (♂) in AMNH [# 402] (Van Dyke 1938: 123).

#### Distribution.

The range of this subspecies extends from southern Manitoba south to “Iowa” (Lindroth 1961a: 18; Esau and Peters 1975: 510), northwestern Nebraska, and north-central New Mexico (Van Dyke 1938: 123-124). The record from Wisconsin (Rauterberg 1885: 12, as *Cychrus elevatus*) needs confirmation.

#### Records.

**CAN**: MB **USA**: CO, IA, MN, ND, NE, NM, SD [WI]

### Scaphinotus elevatus elevatus

(Fabricius, 1787)

Carabus elevatus Fabricius, 1787: 198. Type locality: «America meridionali» (original citation), which is incorrect; «Asheville [Buncombe County], North Carolina» selected by Lindroth (1961a: 17). Lectotype [as co-type], designated by Staig (1931: 14), in HMUG.

#### Distribution.

This subspecies ranges from southern New Hampshire (Merrimack and Strafford Counties, Donald S. Chandler pers. comm. 2008) south to the Florida Panhandle (Peck and Thomas 1998: 16), west to eastern Texas (Lamar and Fort Bend Counties, Brian Raber pers. comm. 2010; Riley 2011). The records from Maine (Bousquet and Larochelle 1993: 79) and Lawrence in Kansas (Van Dyke 1938: 120) need confirmation.

#### Records.

**USA**: AL, CT, DC, DE, FL, LA, MA, MD, MS, NC, NH, NJ, NY, PA, RI, SC, TX [KS, ME]

### Scaphinotus elevatus flammeus

Haldeman, 1844

Scaphinotus flammeus Haldeman, 1844: 54. Type locality: «Marietta [Washington County], Ohio» (original citation). Holotype [by monotypy] presumably lost.
Cychrus dilatatus LeConte, 1853c: 398. Type locality: «S[ain]t Louis [Missouri]» (original citation for *Cychrus flammeus* (Haldeman) *sensu* LeConte, 1847). Syntype(s) in MCZ [# 615]. Synonymy established by Roeschke (1907a: 140). Note. This name was proposed for *Scaphinotus flammeus* Haldeman, 1844 *sensu* LeConte (1847: 440).

#### Distribution.

This subspecies ranges from Nebraska to Ohio, south to Tennessee, Arkansas (Van Dyke 1938: 121), and southwestern Oklahoma (Kondratieff et al. 2005: 173). The record from “Louisiana” (Roeschke 1907a: 142) needs confirmation.

#### Records.

**USA**: AR, IA, IL, IN, KS, MO, NE, OH, OK, TN [LA]

#### Note.

Van Dyke (1938: 121) noted that “the subspecies grades gradually into typical *elevatus* along its eastern boundary and into *coloradensis* along its western boundary.”

### Scaphinotus elevatus lengi

Van Dyke, 1938

Scaphinotus elevatus lengi Van Dyke, 1938: 122. Type locality: «Dismal Swamp of Virginia» (original citation). Holotype (♂) in CAS [# 4681].

#### Distribution.

This subspecies is known so far only from the holotype collected in southeastern Virginia.

#### Records.

**USA**: VA

### Scaphinotus elevatus neomexicanus

Van Dyke, 1924

Scaphinotus elevatus neomexicanus Van Dyke, 1924b: 1. Type locality: «Cloudcroft (8000 feet) [Otero County], Sacramento Mountains, New Mexico» (original citation). Holotype (♀) in CAS [# 3016].

#### Distribution.

This subspecies is known so far only from the holotype and several specimens collected at the type locality (Eric van den Berghe and Robert L. Davidson pers. comm.) in southern New Mexico.

#### Records.

**USA**: NM

### Scaphinotus elevatus tenebricosus

Roeschke, 1907

Scaphinotus elevatus tenebricosus Roeschke, 1907a: 141. Type locality: «Eigentliche Küstenzone von New-Yersey bis Süd-Carolina» (original citation). Three syntypes [4 originally cited] in ZMUA (Boer 2002: 113, 114).

#### Distribution.

According to Roeschke (1907a: 141), this subspecies is found along the coast from New Jersey to South Carolina. Van Dyke (1938: 122) saw specimens from Virginia and North Carolina and noted that “it is apparently confined to the more maritime or coastal portion of the South Atlantic states, replacing almost entirely the [nomino]typical phase in its area of distribution.”

#### Records.

**USA**: NJ, NC, SC, VA

### Scaphinotus kelloggi

(Dury, 1912)

Cychrus kelloggi Dury, 1912: 104. Type locality: «Box Canyon [Grant County], on the Upper Gila, New Mexico» (original citation). Holotype (♂) in CMC (Vulinec and Davis 1984: 233). Etymology. The specific name was proposed for Ralph Todd Kellogg [1876-1940], an excellent collector of natural history objects, particularly birds and insects.

#### Distribution.

This species is known only from mountains in southwestern New Mexico in Grant County and other places in the neighborhood of Silver City (Van Dyke 1938: 104).

#### Records.

**USA**: NM

### Scaphinotus petersi biedermani

Roeschke, 1907

Scaphinotus biedermani Roeschke, 1907b: 571. Type locality: «Cochise Co[unty], Süd-Arizona» (original citation). Two syntypes [2 originally cited] in ZMUA (Boer 2002: 33).

#### Distribution.

This subspecies is confined to the Rincon and Huachuca Mountains of southern Arizona (Ball 1966c: 711-712).

#### Records.

**USA**: AZ

### Scaphinotus petersi catalinae

Van Dyke, 1924

Scaphinotus catalinae Van Dyke, 1924b: 2. Type locality: «near the top of M[oun]t Lemon [Pima County], Catalina Mountains, Arizona» (original citation). Holotype (♂) in CAS [# 3334].

#### Distribution.

This subspecies is known from the Santa Catalina Mountains in Pima County (Ball 1966c: 711) and Aravaipa Canyon in Pinal County (McCleve 1979: 452), southern Arizona.

#### Records.

**USA**: AZ

### Scaphinotus petersi corvus

(Fall, 1910)

Cychrus corvus Fall, 1910: 89. Type locality: «Chiricahua Mountains [Cochise County], Arizona» (original citation). Holotype [by monotypy] (♂) in MCZ [# 23841].

#### Distribution.

This subspecies is confined to the Chiricahua Mountains in Cochise County, southeastern Arizona (Ball 1966c: 714).

#### Records.

**USA**: AZ

### Scaphinotus petersi grahami

Van Dyke, 1938

Scaphinotus grahami Van Dyke, 1938: 107. Type locality: «Graham M[oun]t[ain] [Graham County], Arizona» (original citation). Holotype (♂) in CAS [# 4680].

#### Distribution.

This subspecies is found in the Pinaleño and White Mountains of southeastern Arizona (Ball 1966c: 713-714).

#### Records.

**USA**: AZ

### Scaphinotus petersi kathleenae

Ball, 1966

Scaphinotus petersi kathleenae Ball, 1966c: 714. Type locality: «east slope M[oun]t Wrightson, to west of trail between Baldy and Florida Spring, about one mile north of Baldy Spring (8400-8600’), Santa Rita Mountains, Santa Cruz County, Arizona» (original citation). Holotype (♂) in MCZ [# 31183].

#### Distribution.

This subspecies is known only from Mount Wrightson in the Santa Rita Mountains, southern Arizona (Ball 1966c: 715).

#### Records.

**USA**: AZ

### Scaphinotus petersi petersi

Roeschke, 1907

Scaphinotus petersi Roeschke, 1907a: 137. Type locality: «Pinal M[oun]t[ain]s [Gila County], circa 8 englische Meilen von Tucson, Arizona» (original citation). Holotype (♂) in ZMUA (Boer 2002: 90).

#### Distribution.

This subspecies is confined to the Pinal and Sierra Ancha Mountains in Gila County, eastern Arizona (Ball 1966c: 709).

#### Records.

**USA**: AZ

### Scaphinotus snowi roeschkei

Van Dyke, 1907

Scaphinotus roeschkei Van Dyke [in Roeschke], 1907a: 135. Type locality: «Humphrey’s Peak [Coconino County], Arizonas in 9500’ Höhe» (original citation). Holotype (♂) in ZMUA (Boer 2002: 100). Etymology. The specific name was proposed for Hans Friedrich Roeschke [1867-1934], a German physician by profession and carabidologist by avocation. Roeschke left his carabid collection (mostly members of the subfamily Carabinae) to the German neurologist, brain researcher, and entomologist Oskar Vogt [1870-1959]. Vogt’s collection was acquired by the Zoölogisch Museum of Amsterdam.

#### Distribution.

This subspecies is restricted to high mountains in Coconino County, northern Arizona (Van Dyke 1938: 103).

#### Records.

**USA**: AZ

#### Note.

Van Dyke (1938: 102) treated this form as a species but Ball (1966c: 693) retained it as a subspecies of *Scaphinotus snowi*.

### Scaphinotus snowi snowi

(LeConte, 1881)

Cychrus snowi LeConte [in LeConte and Horn], 1881: 74. Type locality: «Santa Fe cañon (7,000 feet), New Mexico» (original citation). Holotype [by monotypy] (♂) in MCZ [# 613]. Etymology. The species name was proposed for Francis Huntington Snow [1840-1908], a teacher, minister, field naturalist, mineralogist, and eventually chancellor of the University of Kansas. In one expedition to Wallace County in Kansas, Snow collected several hundred specimens of *Amblycheila cylindriformis*, which at the time was extremely rare in collections, and sold many of them for as much as $25.00 each.
Scaphinotus snowi var. *parkeri* Van Dyke, 1938: 101. Type locality: «White M[oun]t[ain]s [Navajo County], Arizona» (original citation). Holotype (♂) in CAS [# 4679]. Synonymy established by Erwin et al. (1977: 4.6). Etymology. The subspecific name was proposed for Frank Henry Parker [1910-1984], an enthusiastic beetle collector, particularly of meloids and buprestids, in Arizona. Note. This taxon was listed as a junior synonym of the nominotypical subspecies of *Scaphinotus snowi* by Erwin et al. (1977: 4.6) and as a valid subspecies by Erwin (2007a: 162). Two of the three original specimens of this taxon were taken “in company with numerous more typical specimens” (Van Dyke 1938: 101) suggesting that they are probably simply aberrant specimens.

#### Distribution.

This subspecies is found in western New Mexico, eastern Arizona, southwestern Colorado, and southeastern Utah (Van Dyke 1938: 100).

#### Records.

**USA**: AZ, CO, NM, UT

### Scaphinotus vandykei

Roeschke, 1907

Scaphinotus van dykei Roeschke, 1907a: 136. Type locality: «S[an]t[a] Maria River und Oak Creek Cañon, Arizona» (original citation). Two syntypes [2 originally cited] in ZMUA (Boer 2002: 118).
Scaphinotus fuchsi Roeschke, 1907b: 570. Type locality: «Cash Mine, M[oun]t Union [Yavapai County], Arizona» (original citation). Syntype(s) [2 originally cited] location unknown (possibly in ZMUA in collection Vogt though not listed by Boer 2002). Synonymy established by Erwin et al. (1977: 4.6).

#### Distribution.

This species is confined to southern Coconino, Yavapai, and Gila Counties in Arizona (Van Dyke 1938: 106).

#### Records.

**USA**: AZ

#### Note.

Van Dyke (1938: 106) retained *Scaphinotus fuchsi* Roeschke as a valid subspecies of *Scaphinotus vandykei* Roeschke.

### [unicolor group]

### Scaphinotus parisiana

Allen and Carlton, 1988

Scaphinotus parisiana Allen and Carlton, 1988: 130. Type locality: «northwest slope of Magazine Mountain, 13 mi[les] southwest of Paris, Logan Co[unty], Arkansas» (original citation). Holotype (♂) in UAIC.

#### Distribution.

This species is known only from the type locality in western Arkansas.

#### Records.

**USA**: AR

### Scaphinotus unicolor

(Fabricius, 1787)

Carabus unicolor Fabricius, 1787: 198 [primary homonym of *Carabus unicolor* Herbst, 1784]. Type locality: «America meridionali» (original citation), which is incorrect; Calvert, Mobile County, Alabama herein selected (see Löding 1945: 10). Lectotype [as type], designated by Staig (1931: 16), in HMUG. Note. Fabricius’ name should be permanently invalid because it is a primary homonym. However, to my knowledge *Carabus unicolor* Herbst has never been interpreted since its original description and the name is a *nomen dubium*.
Scaphinotus heros T.W. Harris, 1839: 196. Type locality: «Ohio and Indiana» (original citation). Syntype(s) lost (Van Dyke 1938: 128). Synonymy established by Erwin (2007a: 164).
Scaphinotus grandis Gistel, 1857: 52 [*nomen dubium*]. Type locality not stated. Syntype(s) lost. Synonymy established with doubt by Bousquet and Larochelle (1993: 17).
Scaphonotus hunteri Crotch, 1871: 5. Replacement name for *Scaphinotus unicolor* (Fabricius, 1787).
Scaphinotus shoemakeri Leng, 1914: 143. Type locality: «Washington, D.C. and its vicinity in Virginia and Maryland» (original citation). Holotype in CAS [# 4374]. Synonymy established by Erwin (2007a: 164).
Scaphinotus elevatus var. *floridanus* Leng, 1915: 564. Type locality: Florida (inferred from title of the paper). Holotype [by monotypy] in CAS [# 4372]. Synonymy established by Erwin (2007a: 164).

#### Distribution.

This species ranges from New Jersey (Smith 1910: 199) and Maryland (Van Dyke 1938: 128) to southwestern Illinois (Jackson County, Robert L. Davidson pers. comm. 2012), south to east-central Louisiana (West Feliciana Parish, Igor M. Sokolov pers. comm. 2009), northeastern Mississippi (Snodgrass and Cross 1983: 15), and the Florida Panhandle (Peck and Thomas 1998: 16).

#### Records.

**USA**: AL, AR, DC, FL, GA, IL, IN, KY, LA, MD, MO, MS, NC, NJ, OH, SC, TN, VA

### Subgenus Irichroa

Newman, 1838

Irichroa Newman, 1838a: 385. Type species: *Cychrus viduus* Dejean, 1826 by monotypy. Etymology. From the Latin *iris* (rainbow) and the Greek *chroa* (surface of the body), probably alluding to the lustre present on the dorsal surface of adults of the sole species in the hands of Newman [feminine].
Anabolus Gistel, 1857: 90. Type species: *Cychrus viduus* Dejean, 1826 by original designation.
Megaliridia Casey, 1920: 175. Type species: *Cychrus viduus* Dejean, 1826 designated by Bousquet and Larochelle (1993: 79). Etymology. From the Greek *mega* (large) and *iridos* (rainbow) [feminine].

#### Diversity.

Three eastern North American species.

#### Identification.

There is no published key for the identification of these species.

### Scaphinotus irregularis

(Beutenmüller, 1903)

Cychrus viduus var. *irregularis* Beutenmüller, 1903: 513. Type locality: «summit of Black Mountains, North Carolina» (original citation). Three syntypes [7 originally cited] in AMNH [# 3] (Grossbeck 1912: 360).

#### Distribution.

This species ranges along the Appalachian Mountains from southwestern Virginia (Hoffman et al. 2006: 18) to northern Georgia (Towns County, CMNH).

#### Records.

**USA**: GA, NC, TN, VA

#### Note.

This taxon has been treated as an aberration of *Scaphinotus viduus* (Dejean) by Roeschke (1907a: 144) but according to Barr (1969: 73) and Hoffman et al. (2006: 18) it represents a distinct species.

### Scaphinotus viduus

(Dejean, 1826)

Cychrus viduus Dejean, 1826: 12. Type locality: «Amérique septentrionale» (original citation), restricted to «Susquahanna [= Susquehanna] R[iver], Penns[ylvania]» by Lindroth (1961a: 18). One syntype in MHNP (Lindroth 1955b: 11).
Cychrus leonardii T.W. Harris, 1839: 193. Type locality «northern and western parts of Massachusetts and New Hampshire [page 193] and Vermont [page 194]» (original citation). One syntype [5 originally cited] in MCZ [# 26410]. Synonymy established by Darlington (1932: 146). Etymology. The specific name was proposed for Levi Washburn Leonard [1790?-1864], a Unitarian minister who settled in Dublin, New Hampshire. Leonard, a close friend and classmate of Thaddeus Harris, was an enthusiastic collector of insects and provided Harris with many specimens, including at one occasion 600 beetles, probably over 200 species, caught in the shadow of Mount Monadnock (Dow 1913: 109). Leonard is credited with opening the first library in the United States that was free to all the inhabitants of a town or city.

#### Distribution.

This species ranges from Nova Scotia (CNC) to northern Indiana (Barr 1969: 74), south to northeastern Georgia (Fattig 1949: 8) along the Appalachian Mountains. The records from northern Florida (Harris 1839: 192) and “Louisiana” (Roeschke 1907a: 145) are probably in error.

#### Records.

**CAN**: NB, NS, QC **USA**: CT, DC, DE, GA, IN, MA, ME, MD, NC, NH, NJ, NY, OH, PA, VA, VT, WV

### Scaphinotus webbi

Bell, 1959

Scaphinotus webbi R.T. Bell, 1959: 11. Type locality: «ten miles southwest of Lynchburg, Campbell County, Virginia» (original citation). Holotype (♂) in USNM [# 73630].

#### Distribution.

This species is known from a few localities in Virginia (Bell 1959: 11), northeastern West Virginia (Hampshire and Randolph Counties, CMNH), the Allegheny Plateau in southwestern Pennsylvania (Allegheny and Fayette Counties, Robert L. Davidson pers. comm. 2008), and the Big Black Mountain in southeastern Kentucky (Davis and Barbour 1978: 139).

#### Records.

**USA**: KY, PA, VA, WV

#### Note.

Hoffman et al. (2006: 18) reported that distinction between this taxon and *Scaphinotus viduus* is controversial at best and that adults of the two may be conspecific.

### Subgenus Nomaretus

LeConte, 1853

Nomaretus LeConte, 1853c: 399. Type species: *Cychrus bilobus* Say, 1823 designated by Géhin (1876b: 114). Etymology. Probably from the Greek *nomas* (roving) and *aretos* (pleasing) [masculine].

#### Diversity.

Five eastern North American species.

#### Identification.

Van Dyke (1936) reviewed the species. Gidaspow (1973: 78-89) revised the species and provided a key for their identification. One new species was subsequently described by Allen and Carlton in 1988.

### Scaphinotus bilobus

(Say, 1823)

Cychrus bilobus Say, 1823a: 73. Type locality: «Nipigon, W[estern] Ont[ario]» (neotype label). Neotype (♂), designated by Lindroth and Freitag (1969: 330), in MCZ [# 33093]. Note. Say (1823a) did not indicate the area where his specimen(s) came from but later (Say 1828: [102]) noted that his “first specimen was obtained in Missouri, and I caught another in the North-Western Territory.”

#### Distribution.

This species ranges from Cape Breton Island to southeastern Manitoba, south to eastern Kansas (Popenoe 1877: 22), southeastern Missouri, and Massachusetts [see Gidaspow 1973: Fig. 6]. The record from southeastern Pennsylvania (Rathvon 1869: 524) is likely in error.

#### Records.

**CAN**: MB, NB, NS (CBI), ON, QC **USA**: IA, IL, KS, MA, ME, MI, MN, MO, NE, NH, NY, OH, VT, WI

### Scaphinotus cavicollis

(LeConte, 1859)

Nomaretus cavicollis LeConte, 1859c: 3. Type locality: «Fort Riley [junction of Republican and Smoky Hill Rivers, Kansas]» (original citation). Holotype [by monotypy] in MCZ [# 602].

#### Distribution.

This species is known from eastern Kansas, western Missouri, western Arkansas (Allen and Thompson 1977: 32; Allen and Carlton 1988: 136), and central Oklahoma [see Gidaspow 1973: Fig. 6]. The records from “Iowa” and “Texas” (Leng and Beutenmüller 1894: 138) are likely in error; that from Buffalo, New York (Leng and Beutenmüller 1894: 138), is in error.

#### Records.

**USA**: AR, KS, MO, OK

### Scaphinotus fissicollis

(LeConte, 1853)

Nomaretus fissicollis LeConte, 1853c: 399. Type locality: «Illinois» (original citation). Holotype [by monotypy] (♀) in MCZ [# 603].

#### Distribution.

This species ranges from southeastern Minnesota (Gandhi et al. 2005: 923) south to northern Arkansas (Allen and Thompson 1977: 32; Allen and Carlton 1988: 136), west to eastern Kansas [see Gidaspow 1973: Fig. 6]. The record from “Texas” (Schwarz 1895: 270) is probably in error.

#### Records.

**USA**: AR, IA, IL, KS, MN, MO

### Scaphinotus infletus

Allen and Carlton, 1988

Scaphinotus infletus Allen and Carlton, 1988: 132. Type locality: «Alum Cove Scenic Area, Newton Co[unty], Arkansas» (original citation). Holotype (♂) in UAIC.

#### Distribution.

This species is known only from Newton County in northwestern Arkansas.

#### Records.

**USA**: AR

### Scaphinotus liebecki

Van Dyke, 1936

Scaphinotus liebecki Van Dyke, 1936: 40. Type locality: «Tex[as]» (original citation), herein restricted to New Braunfels, Comal County (see Gidaspow 1973: 82). Holotype (♂) in CAS [# 4110]. Etymology. The species name was proposed for Charles Liebeck [1863-1947], a paper scorer in Philadelphia by profession and a beetle collector in his spare time. Over the years Liebeck built up a fine collection of North American beetles, represented by more than 100,000 specimens, which he gave to Henry C. Fall with whom he had been in correspondence for more than 40 years.

#### Distribution.

This species, as far as known, is restricted to Arkansas (Stone County, CNC), western Louisiana, and eastern Texas [see Gidaspow 1973: Fig. 6].

#### Records.

**USA**: AR, LA, TX

### Subgenus Maronetus

Casey, 1914

Maronetus Casey, 1914: 30. Type species: *Maronetus tenuis* Casey, 1914 by original designation. Etymology. Anagram of the generic name *Nomaretus* [*q.v*.] [masculine].

#### Diversity.

This subgenus includes ten species-group taxa found along the Appalachians. Several undescribed taxa are known (Thomas C. Barr, Jr. pers. comm. 2009).

#### Identification.

There is no recent systematic treatment of the species of *Maronetus* and such work is much needed. A key to the species of the *debilis* group has been published (Barr 2009).

### [debilis group]

### Scaphinotus debilis alpinus

(Beutenmüller, 1903)

Nomaretus debilis var. *alpinus* Beutenmüller, 1903: 512. Type locality: «in the valley and on the summit of the Black Mountains» (original citation). Three syntypes [5 originally cited] in AMNH [# 453] (Grossbeck 1912: 360).

#### Distribution.

This subspecies has been recorded so far only from the Black Mountains in southwestern North Carolina. Roeschke (1907a: 160) reported that this form occurs above 5000 feet of altitude.

#### Records.

**USA**: NC

### Scaphinotus debilis debilis

(LeConte, 1853)

Nomaretus debilis LeConte, 1853c: 399. Type locality: «Habersham Co[unty], Georgia» (original citation). Holotype [by monotypy] in MCZ [# 604].

#### Distribution.

This subspecies is known along the Appalachian Mountains from western North Carolina (Leng and Beutenmüller 1894: 138), northeastern Georgia (Fattig 1949: 9), and northwestern South Carolina (Ciegler 2000: 32). It is found from 1800 to 5000 feet (Roeschke 1907a: 160).

#### Records.

**USA**: GA, NC, SC

### Scaphinotus hoffmani

(Barr, 2009), new combination

Maronetus hoffmani Barr, 2009: 316. Type locality: «5 km N[orth]E[ast] Dungannon, Scott County, Virginia» (original citation). Holotype (♂) in CMNH.

#### Distribution.

This species is known only from the holotype collected in southwestern Virginia.

#### Records.

**USA**: VA

### Scaphinotus incompletus

(Schwarz, 1895)

Nomaretus incompletus Schwarz, 1895: 271. Type locality: «Stone Creek, Cumberland M[oun]t[ain]s, Lee Co[unty], V[irgini]a» (original citation). Holotype [by monotypy] (♀) in USNM [# 4571].

#### Distribution.

This species is known from the Cumberland Plateau in southeastern Kentucky and the Appalachian Valley in southwestern Virginia (Barr 1969: 76; Barr 2009: 314).

#### Records.

**USA**: KY, VA

### Scaphinotus reichlei

(Barr, 2009), new combination

Maronetus reichlei Barr, 2009: 315. Type locality: «“Cesium Forest” in the Oak Ridge National Laboratory ecology study area, Anderson County, Tennessee» (original citation). Holotype (♂) in CMNH.

#### Distribution.

This species is known from two specimens collected in Cumberland and Anderson Counties, eastern Tennessee (Barr 2009: 315).

#### Records.

**USA**: TN

### [hubbardi group]

### Scaphinotus hubbardi

(Schwarz, 1895)

Nomaretus hubbardi Schwarz, 1895: 272. Type locality: «near Retreat (about 3000’), Haywood Co[unty], N[orth] C[arolina]» (original citation). One syntype in USNM [# 4572]. Etymology. The species name was proposed for Henry Guernsey Hubbard [1850-1899], a field and applied entomologist of great ability. Despite being afflicted with tuberculosis for a long time Hubbard made extensive collecting trips with his lifelong friend Eugene Schwarz.

#### Distribution.

This species is known along the Appalachian Mountains in eastern Tennessee and western North Carolina (Barr 1969: 76).

#### Records.

**USA**: NC, TN

### Scaphinotus schwarzi

(Beutenmüller, 1913)

Nomaretus schwarzi Beutenmüller, 1913: 139. Type locality: «Mount Mitchell (6,710 feet) [Yancey County], North Carolina» (original citation). Holotype [by monotypy] (♀) location unknown.

#### Distribution.

This species is known from southwestern Virginia (Hoffman et al. 2006: 18) and the Black Mountains in western North Carolina (Beutenmüller, 1913: 139).

#### Records.

**USA**: NC, VA

### [imperfectus group]

### Scaphinotus imperfectus

(Horn, 1861)

Nomaretus imperfectus G.H. Horn, 1861: 569. Type locality: «Hampshire County, Virginia» (original citation). Syntype(s) in MCZ [# 35317].

#### Distribution.

This species ranges from southeastern Ohio and Pennsylvania (Barr 1969: 76) south to western North Carolina (Thomas C. Barr, Jr. pers. comm. 2009; Leng and Beutenmüller 1894: 138).

#### Records.

**USA**: MD, NC, OH, PA, VA, WV

### Scaphinotus tenuis

(Casey, 1914)

Maronetus tenuis Casey, 1914: 31. Type locality: «Black M[oun]t[ain]s, North Carolina» (original citation). One syntype in USNM [# 46049].

#### Distribution.

This species is endemic, as far as known, to the Black Mountains (Barr 1969: 75) in southwestern North Carolina.

#### Records.

**USA**: NC

#### Note.

Barr (1969: 75) listed this taxon as a subspecies of *Scaphinotus imperfectus*.

### Scaphinotus unistriatus

(Darlington, 1932)

Nomaretus unistriatus Darlington, 1932: 149. Type locality: «Highlands [Macon County], N[orth] C[arolina]» (original citation). Holotype (♀) in MCZ [# 16431].

#### Distribution.

This species is found in the Blue Ridge Mountains (Great Balsams and Highlands Plateau) and the Nantahala Mountains (Barr 1969: 75) in southwestern North Carolina and northeastern Georgia.

#### Records.

**USA**: GA, NC

### Subgenus Steniridia

Casey, 1924

Steniridia Casey, 1924: 336. Type species: *Cychrus andrewsii* Harris, 1839 by monotypy. Etymology. From the Greek *stenos* (narrow) and *iridos* (rainbow) [feminine].

#### Diversity.

Seven species restricted to the Appalachian region.

#### Identification.

Valentine (1935) reviewed the species and provided a key for their identification.

### Scaphinotus aeneicollis

(Beutenmüller, 1903)

Cychrus aeneicollis Beutenmüller, 1903: 515. Type locality: «summit of the Black Mountains» (original citation), restricted to «M[oun]t Mitchell, North Carolina» by Valentine (1935: 359). Syntype(s) [70 originally cited] in AMNH (Grossbeck 1912: 360), MCZ [# 1793], CUIC, and FMNH.
Cychrus aeneicollis form *purpuratus* Beutenmüller, 1918: 89. Type locality: «Black Mountains in western North Carolina» (original citation). Syntype(s) in AMNH [# 1068] and ANSP. Synonymy established by Lapouge (1933: 690).

#### Distribution.

This species is endemic to the Black Mountains in western North Carolina (Barr 1969: 74). The record from the mountains of Tennessee (Roeschke 1907a: 147) needs confirmation.

#### Records.

**USA**: NC [TN]

### Scaphinotus andrewsii amplicollis

(Casey, 1920)

Irichroa violacea amplicollis Casey, 1920: 174. Type locality: «Black M[oun]t[ain]s, North Carolina» (original citation). Two syntypes in USNM [# 46008].
Irichroa andrewsi reflexa Casey, 1924: 22. Type locality: «Black M[oun]t[ain]s, North Carolina» (original citation). One syntype in USNM [# 46009]. Synonymy established by Valentine (1935: 352).
Steniridia andrewsi montana Valentine, 1935: 350. Type locality: «Beech Mountain, Avery Co[unty], North Carolina» (original citation). Holotype (♂) in USNM [# 56128]. **New synonymy** (Robert L. Davidson pers. comm. 2008).

#### Distribution.

This subspecies is found in the mountains of southwestern Virginia, western North Carolina (Valentine 1936: 227), and northeastern Tennessee (Carter County, CMNH).

#### Records.

**USA**: NC, TN, VA

### Scaphinotus andrewsii andrewsii

(Harris, 1839)

Cychrus andrewsii T.W. Harris, 1839: 195. Type locality: Chapel Hill, Orange County, North Carolina (neotype label). Neotype (♀), designated by Valentine (1935: 349), in USNM [# 56127]. Etymology. The specific name was proposed for the son of the American educator Ethan Allen Andrews [1787-1858] who taught ancient languages at the University of North Carolina and later taught at New Haven and Boston. Note. «North Carolina» was the area originally cited by Harris (1839: 195).

#### Distribution.

This subspecies is known only from Orange and Guilford Counties in northern North Carolina (Valentine 1936: 226). The record from “Tennessee” (Roeschke 1907a: 147) needs confirmation.

#### Records.

**USA**: NC [TN]

### Scaphinotus andrewsii darlingtoni

(Valentine, 1935)

Steniridia andrewsi darlingtoni Valentine, 1935: 356. Type locality: «Newfound Gap, Sevier Co[unty], Tenn[essee]» (original citation). Holotype (♂) in MCZ [# 22987].
Steniridia andrewsi nantahalae Valentine, 1936: 228. Type locality: «Cashier’s (3500 ft.), Jackson Co[unty], North Carolina» (original citation). Holotype (♂) in USNM [# 56130]. **New synonymy** (Robert L. Davidson pers. comm. 2008; see Barr 1970: 4).
Steniridia andrewsi saludae Valentine, 1936: 229. Type locality: «Melrose, Polk Co[unty], North Carolina» (original citation). Holotype (♀) in USNM [# 56132]. **New synonymy** (Robert L. Davidson pers. comm. 2008; see Barr 1970: 4).
Steniridia andrewsi barksdalei Valentine, 1936: 230. Type locality: «M[oun]t Guyot (about 3500 ft.), Swain and Haywood Co[untie]s, North Carolina» (original citation). Holotype (♂) in USNM [# 56131]. **New synonymy** (Robert L. Davidson pers. comm. 2008; see Barr 1970: 4).

#### Distribution.

This subspecies ranges along the Appalachian Mountains from the Great Smokies in eastern Tennessee and western North Carolina eastwards to the Saluda Mountains (Barr 1970: 4) in northwestern South Carolina.

#### Records.

**USA**: NC, SC, TN

### Scaphinotus andrewsii germari

(Chaudoir, 1861)

Cychrus germari Chaudoir, 1861b: 495. Type locality: «Tennessee» (original citation). Holotype [by monotypy] (♀) in MHNP. Etymology. The specific name honors the German entomologist, paleontologist, and mineralogist Ernst Friedrich Germar [1786-1853].

#### Distribution.

This subspecies is found along the Appalachians in southern West Virginia (Fayette and Webster Counties, CMNH), eastern Kentucky, southwestern Virginia, and northeastern and southeastern Tennessee (Valentine 1936: 231; Davis and Barbour 1978: 139). The records from Pennsylvania (Roeschke 1907a: 148), southwestern Ohio (Wright and Whitehouse 1941: 70), and southern Indiana (Blatchley 1910: 43) probably refer to other subspecies of the species.

#### Records.

**USA**: KY, TN, VA, WV

### Scaphinotus andrewsii mutabilis

(Casey, 1920)

Irichroa mutabilis Casey, 1920: 173. Type locality: «Uniontown [Fayette County], Pennsylvania» (original citation). Five syntypes in USNM [# 46005].
Irichroa mutabilis longicollis Casey, 1920: 173. Type locality: «Pennsylvania» (original citation). Nine syntypes in USNM [# 46006]. Synonymy established by Valentine (1935: 354).
Irichroa mutabilis modulata Casey, 1920: 174. Type locality: «Pennsylvania» (original citation). One syntype in USNM [# 46007]. Synonymy established by Valentine (1935: 354).

#### Distribution.

This subspecies ranges from southwestern Pennsylvania and southern Ohio to north-central Kentucky (Valentine 1936: 232) and northwestern Maryland (Glaser 1996: 4).

#### Records.

**USA**: KY, MD, OH, PA, WV

### Scaphinotus andrewsii parvitarsalis

(Valentine, 1935)

Steniridia andrewsi parvitarsalis Valentine, 1935: 354. Type locality: «Clayton, Rabun Co[unty], Georgia» (original citation). Holotype (♂) in USNM [# 56129].

#### Distribution.

This subspecies is known in the Appalachians from the Nantahala Mountains, adjacent Blue Ridge, and the Unicoi Mountains in southwestern North Carolina, southeastern Tennessee, and northeastern Georgia (Valentine 1936: 227; Barr 1970: 4).

#### Records.

**USA**: GA, NC, TN

### Scaphinotus andrewsii waldensius

(Valentine, 1935)

Steniridia andrewsi waldensia Valentine, 1935: 357. Type locality: «Sawyer’s Springs [Hamilton County], Tennessee» (original citation). Holotype (♂) in ANSP [# 8191].

#### Distribution.

This subspecies is known only from southeastern Kentucky (Pulaski County, CMNH) and the Walden Ridge in southeastern Tennessee (Valentine 1935: 357).

#### Records.

**USA**: KY, TN

### Scaphinotus guyotii

(LeConte, 1863)

Cychrus guyotii LeConte, 1863b: 50. Type locality: «North Carolina, near M[oun]t Le Conte» (original citation). Holotype [by monotypy] (♀) in MCZ [# 612]. Etymology. The specific name was proposed for Arnold Henri Guyot [1807-1884], geologist involved mainly with hypsometric measurements of eastern mountains, meteorology, and the reform of geographic teaching in colleges and secondary schools. Born in Neuchatel in Switzerland, Guyot emigrated to the United States at the suggestion of his friend Louis Agassiz. In 1856, he founded what is now the Princeton Museum of Natural History. Three mountains are named after him in the White Mountains, the Great Smoky Mountains, and Colorado Rockies as well as a glacier in southeastern Alaska and a crater on the moon. Note. 1. Mount LeConte is located in Sevier County, Tennessee, so LeConte (1863b: 50) probably made an error in the name of the state. 2. This species was redescribed by LeConte (1867b: 363).
Cychrus guyoti form *angelli* Beutenmüller, 1918: 89. Type locality: «Black Mountains in western North Carolina» (original citation). Syntype(s) in AMNH [# 1069] and ANSP. Synonymy established by Darlington (1932: 149).
Scaphinotus confusus Darlington, 1932: 146. Type locality: «M[oun]t Mitchell (about 6,000’), Black Mountains, North Carolina» (original citation). Holotype (♂) in MCZ [# 16430]. Synonymy established, under the name *Scaphinotus guyoti* var. *angelli* (Beutenmüller), by Valentine (1935: 363).

#### Distribution.

This species is found along the Appalachians from south-central West Virginia (Fayette County, CMNH) and Virginia (Valentine 1935: 363) to northern Georgia (Rabun, Towns, and Union Counties, CMNH) and northwestern South Carolina (Ciegler 2000: 31).

#### Records.

**USA**: GA, NC, SC, TN, VA, WV

### Scaphinotus lodingi lodingi

(Valentine, 1935)

Steniridia lodingi Valentine, 1935: 364. Type locality: «Monte Sano, Madison Co[unty], Alabama» (original citation). Holotype (♂) in USNM [# 56134].

#### Distribution.

This subspecies is found from central Tennessee (Barr 1969: 74) to west-central Alabama (Löding 1945: 11).

#### Records.

**USA**: AL, TN

### Scaphinotus lodingi obscurus

(Valentine, 1935)

Steniridia lodingi obscura Valentine, 1935: 366. Type locality: «Wadley, Randolph Co[unty], Alabama» (original citation). Holotype (♂) in USNM [# 56135].

#### Distribution.

This subspecies is known only from eastern Alabama (Löding 1945: 11).

#### Records.

**USA**: AL

### Scaphinotus ridingsii monongahelae

Leng, 1917

Scaphinotus ridingsii monongahelae Leng, 1917: 36. Type locality: «Uniontown [Fayette County], P[ennsylvani]a» (original citation). Holotype (♂) in CAS [# 4373].
Irichroa tenuiceps Casey, 1920: 172. Type locality: «Uniontown [Fayette County], Pennsylvania» (original citation). Four syntypes in USNM [# 46010]. Synonymy established by Lapouge (1933: 690).

#### Distribution.

This subspecies is known from the Appalachian Plateau in southwestern Pennsylvania and the Allegheny Mountains in West Virginia (Barr 1969: 75), western Virginia (CMNH), and northwestern Maryland (Bailey et al. 1994: 320).

#### Records.

**USA**: MD, PA, VA, WV

### Scaphinotus ridingsii ridingsii

(Bland, 1863)

Cychrus ridingsii Bland, 1863: 353. Type locality: «Hampshire Co[unty], [West] Virginia» (original citation). One syntype in ANSP [# 1017]. Etymology. The specific name was proposed for James Ridings [1803-1880]. Born in England, Ridings moved to Philadelphia in his 20s and became associated with the Entomological Society of Philadelphia, now the American Entomological Society. He collected intensively in the Philadelphia region and in Virginia but also made trips to Georgia, Kansas, and Colorado.
Steniridia ridingsi intermedia Valentine, 1935: 368. Type locality: «Natural Bridge, Rockbridge Co[unty], Virginia» (original citation). Holotype (♂) in MCZ [# 22989]. **New synonymy**. Note. Based on the information provided by Valentine (1935: 369), I do not believe that the form *intermedia*, which was based on a single specimen, is subspecifically distinct from the nominotypical form.

#### Distribution.

This subspecies is known from northwestern West Virginia and a few counties in northern and western Virginia (Valentine 1935: 367). The record from “Tennessee” (Roeschke 1907a: 150) needs confirmation.

#### Records.

**USA**: VA, WV [TN]

### Scaphinotus tricarinatus

(Casey, 1914)

Irichroa aeneicollis tricarinata Casey, 1914: 25. Type locality: «Blue Ridge M[oun]t[ain]s, North Carolina» (original citation), restricted to the «[Great] Smoky Mountains» by Valentine (1935: 362). One syntype in USNM [# 46004].

#### Distribution.

This species is found at high altitudes in the Great Balsam, Plott Balsam, Great Smoky (Barr 1969: 74), and Nantahala Mountains (Barr 1970: 5) in eastern Tennessee, western North Carolina, and northeastern South Carolina.

#### Records.

**USA**: NC, SC, TN

### Scaphinotus violaceus

(LeConte, 1863)

Cychrus violaceus LeConte, 1863c: 4. Type locality: «Mountains of Georgia» (original citation), herein restricted to Tray Mountain (4400 feet), Towns County (see Valentine 1935: 358). Syntype(s) in MCZ [# 614].
Steniridia violacea carolinae Valentine, 1935: 358. Type locality: «Blue Ridge M[oun]-t[ain]s, N[orth] C[arolina]» (original citation). Holotype (♂) in USNM [# 56133]. **New synonymy** (Robert L. Davidson pers. comm. 2008; see Barr 1970: 3).

#### Distribution.

This species has been reported from the Appalachians in western North Carolina, northern Georgia (Valentine 1935: 358), and northwestern South Carolina (Ciegler 2000: 32).

#### Records.

**USA**: GA, NC, SC

### Subgenus Pseudonomaretus

Roeschke, 1907

Pseudonomaretus Roeschke, 1907a: 154. Type species: *Cychrus relictus* Horn, 1881 designated by Casey (1914: 30). Etymology. From Greek *pseudos* (fallacy, lie) and the generic name *Nomaretus* [*q.v*.] [masculine].

#### Diversity.

Four western North American species.

#### Identification.

Gidaspow (1973: 73-78) revised the species and provided a key for their identification.

### Scaphinotus mannii

Wickham, 1919

Scaphinotus mannii Wickham, 1919a: 170. Type locality: «Wawawai [Whitman County], Wash[ington]» (original citation). Holotype (♂) in USNM [# 22545]. Etymology. This species was proposed for William M. Mann [1886-1960] who worked for the United States Bureau of Entomology early in his career and then became director of the National Zoological Park in Washington DC. Mann worked mainly on ants and termites but also with myrmecophilous insects, such as staphylinids.

#### Distribution.

This species is restricted to southeastern Washington (Gidaspow 1973: Fig. 4) and northeastern Oregon (Hatch 1953: 46; Westcott et al. 2006: 9).

#### Records.

**USA**: OR, WA

### Scaphinotus merkelii

(Horn, 1890)

Cychrus merkelii G.H. Horn, 1890: 71. Type locality: «northern Idaho» (original citation), restricted to «Coeur d’Alene [Kootenai County]» by Lindroth (1961a: 20). Holotype [by monotypy] (♀) in MCZ [# 34934]. Etymology. The specific name honors August Merkel [1837-1897], a collector of beetles. Merkel was born in Einbeck, near Hanover, Germany.
Cychrus idahoensis Webb, 1901: 133. Type locality: «Cedar Mountain, Latah County and Collins, [both] Idaho» (original citation). Syntype(s) [14 originally cited] in WSU and ZMUA (Boer 2002: 57). Synonymy established by Gidaspow (1973: 74).

#### Distribution.

This species is known from western Montana (Russell 1968: 42), northern Idaho, Whitman County in southeastern Washington (CMNH), and the Creston area (Lindroth 1961a: 20) in southeastern British Columbia [see Gidaspow 1973: Fig. 4].

#### Records.

**CAN**: BC **USA**: ID, MT, WA

### Scaphinotus regularis

(LeConte, 1884)

Cychrus regularis LeConte, 1884: 2. Type locality: «Coeur d’Aléne Mountains [Sanders County], Idaho» (original citation). Syntype(s) [2 originally cited] in MCZ [# 611].

#### Distribution.

This species is known from southeastern British Columbia, Idaho, and eastern Washington [see Gidaspow 1973: Fig. 5].

#### Records.

**CAN**: BC **USA**: ID, WA

#### Note.

This form was listed as a synonym of *Scaphinotus relictus* (Horn) by Lindroth (1961a: 20), regarded as a variety of *Scaphinotus relictus* by Roeschke (1907a: 163) and Hatch (1953: 45), and treated as a valid species by Gidaspow (1973: 77).

### Scaphinotus relictus

(Horn, 1881)

Cychrus relictus G.H. Horn, 1881: 188. Type locality: «Spokane [Spokane County], Wash[ington] Terr[itory]» (original citation). Holotype [by monotypy] (♂) in MCZ [# 33480].

#### Distribution.

This species is found in southern British Columbia, western Alberta, western Montana (Russell 1968: 42), northern Idaho, eastern Washington, and from one isolated locality in southwestern Oregon [see Gidaspow 1973: Fig. 5]. Old specimens simply labeled from California are also known (Gidaspow 1973: 77).

#### Records.

**CAN**: AB, BC **USA**: ID, MT, OR, WA [CA]

### Subgenus Stenocantharus

Gistel, 1834

Stenocantharus Gistel, 1834: 1. Type species: *Cychrus angusticollis* Mannerheim, 1823 by monotypy. Etymology (see Gistel 1829: 1068). From the Greek *stenos* (narrow) and *cantharos* (scarab) [masculine]. Note. The name *Stenocantharus* was first introduced by Gistel (1829: 1068) for “*Cychrus debilis* Dejean,” a species not available at the time.
Pemphus Motschulsky, 1866: 312. Type species: *Cychrus velutinus* Ménétriés, 1843 designated by Géhin (1876b: 114). Synonymy established by Csiki (1927: 322).

#### Diversity.

Four species restricted to the Pacific coastal and western montane regions of North America.

#### Identification.

Van Dyke (1944) and Gidaspow (1973) revised the species then placed in this subgenus. Gidaspow (1973) provided a key for their identification.

### Scaphinotus angusticollis

(Mannerheim, 1823)

Cychrus angusticollis Mannerheim [in Fischer von Waldheim], 1823: plate 46. Type locality: «insula Unalaschka [Alaska]» (Fischer von Waldheim 1824: 47), which according to Van Dyke (1944: 4) and Lindroth (1961a: 21) is likely incorrect; «Sitka [Baranof Island, Alaska]» selected by Van Dyke (1944: 4). Syntype(s) in ZILR, ZMH (Lindroth 1961a: 21), and SMTD (Grämer 1960: 101). Note. This name is credited to Fischer von Waldheim by some authors (e.g., Bousquet and Larochelle 1993: 83). However, Fischer von Waldheim’s statement (1824: 47) “le Cychrus d’Ounalaschka [i.e. *Cychrus angusticollis*] dont je dois la description exacte et un dessin élégant et fidèle à Mr. Le Comte Mannerheim” clearly points to Mannerheim as the author.
Pemphus angusticollis var. *nigripennis* Roeschke, 1907a: 167. Type locality: «Gualala, Mendocino Co[unty], Cal[ifornien]» (holotype label). Holotype (♂) in ZMUA (Boer 2002: 84). Synonymy established by Gidaspow (1973: 66).
Scaphinotus angusticollis olympiae Van Dyke, 1944: 5. Type locality: «Sol Duc Hot Springs [Clallam County], Olympic Peninsula, Wash[ington]» (original citation). Holotype (♂) in CAS [# 5343]. Synonymy established by Lindroth (1961a: 21).

#### Distribution.

This species ranges from Kodiak Island in the Gulf of Alaska to northwestern California, east at least to eastern British Columbia [see Gidaspow 1973: Fig. 2]. The record from Torrington, Alberta (Gidaspow 1973: 68) is probably based on a mislabeled specimen.

#### Records.

**CAN**: BC (QCI, VCI) **USA**: AK, CA, OR, WA

### Scaphinotus hatchi

Beer, 1971

Scaphinotus hatchi Beer, 1971: 257. Type locality: «two miles east of Islet Campground at Waldo Lake, Lane County, Oregon» (original citation). Holotype (♂) in CAS [# 11605]. Etymology. The species name honors Melville Harrison Hatch [1898-1988], coleopterist and professor at the University of Washington for more than 40 years. Hatch is best known for the five-volume series “Beetles of the Pacific Northwest” published from 1953 to 1971. His collection was transferred in 1978 to the Oregon State University and his types were subsequently moved to the USNM.

#### Distribution.

This species is known only from Waldo Lake area in western Oregon (Gidaspow 1973: 66; CNC, CMNH).

#### Records.

**USA**: OR

### Scaphinotus johnsoni

Van Dyke, 1924

Scaphinotus johnsoni Van Dyke, 1924b: 3. Type locality: «Olympic Mountains, Washington» (original citation). Holotype (♀) in CAS [# 3335]. Etymology. This specific name was proposed for Orson Bennett Johnson [1849-1917], a pioneer Pacific Northwest entomologist and first professor of natural science at the University of Washington.
Scaphinotus klahowyae Perrault, 1973b: 47. Type locality: «Klahowya, near Sappho, Clallam Co[unty], Olympic Peninsula, Washington» (original citation). Holotype (♂) location unknown (probably in MHNP). Synonymy established by Greene (1976: 326).

#### Distribution.

This rarely collected species is restricted to the southern part of Vancouver Island (Lindroth 1961a: 22) and the Olympic Mountains in northwestern Washington (Van Dyke 1924b: 3).

#### Records.

**CAN**: BC (VCI) **USA**: WA

#### Note.

This species has been placed in the subgenus *Brennus* by Lindroth (1961a: 22) and Gidaspow (1968: 155) but van den Berghe and Davidson (Robert L. Davidson pers. comm. 2008) agreed that this small species is a member of the subgenus *Stenocantharus*.

### Scaphinotus velutinus

(Ménétriés, 1843)

Cychrus velutinus Ménétriés, 1843: 53. Type locality: «Californie» (original citation), herein restricted to Fort Ross, Sonoma County (see Gidaspow 1973: 72). Syntype(s) in ZILR.
Pemphus longipes Casey, 1897: 339. Type locality: «Humboldt Co[unty], California» (original citation). Holotype [by monotypy] (♂) in USNM [# 46012]. Synonymy established by Gidaspow (1973: 70).
Pemphus opacus Casey, 1899: 97. Type locality: «Sonoma Co[unty], California» (original citation). Two syntypes [2 originally cited] in USNM [# 46011]. Synonymy established by Roeschke (1907a: 169).

#### Distribution.

This species ranges from northwestern Oregon to the San Francisco Bay area in California [see Gidaspow 1973: Fig. 3]. The record from Vancouver Island (LeConte 1869c: 370) is probably in error.

#### Records.

**USA**: CA, OR

### Subgenus Brennus

Motschulsky, 1866

Brennus Motschulsky, 1866: 311. Type species: *Cychrus ventricosus* Dejean, 1831 designated by Géhin (1876b: 114). Etymology. Unknown [masculine].

#### Diversity.

Fifteen western North American species of which two extend into the Baja California Peninsula.

#### Identification.

Gidaspow (1968) revised the species and provided a key for their identification.

### Scaphinotus bullatus

Van Dyke, 1924

Scaphinotus subtilis bullatus Van Dyke, 1924b: 3. Type locality: «at the mouth of Roaring River (5000 feet), South Fork of Kings River Cañon, Fresno County, California» (original citation). Holotype (♂) in CAS [# 3336].
Scaphinotus subtilis grandis Van Dyke, 1924b: 4. Type locality: «Cedar Creek, Tulare County, California» (original citation). Holotype (♂) in CAS [# 3338]. Synonymy established by Gidaspow (1968: 163).

#### Distribution.

This species is found within the Sierra Nevada in central California, from El Dorado County to Sequoia National Park [see Gidaspow 1968: Fig. 9].

#### Records.

**USA**: CA

### Scaphinotus cordatus

(LeConte, 1853)

Cychrus cordatus LeConte, 1853c: 399. Type locality: «San Jose [Santa Clara County], California» (original citation). Holotype [by monotypy] (♂) in MCZ [# 610].
Brennus cordatus vernicatus Casey, 1920: 183. Type locality: «near San Francisco Bay, California» (original citation). One syntype in USNM [# 46047]. Synonymy established by Gidaspow (1968: 160).
Brennus cordatus rufitarsis Casey, 1920: 184. Type locality: «S[an]ta Cruz M[oun]t[ain]s, California» (original citation). One syntype in USNM [# 46048]. Synonymy established by Gidaspow (1968: 161).

#### Distribution.

This species is restricted to central California [see Gidaspow 1968: Fig. 7].

#### Records.

**USA**: CA

### Scaphinotus crenatus

(Motschulsky, 1859)

Cychrus crenatus Motschulsky, 1859a: 161. Type locality: «Californie» (original citation), herein restricted to Fort Tejon, Kern County (see LeConte 1859a: 69, as *Cychrus striatus*). Lectotype [as holotype] (♂), designated by Kryzhanovskij (1968: 186), in ZMMU.
Cychrus striatus LeConte, 1859a: 69. Type locality: «Fort Tejon [Kern County, California]» (original citation). Holotype [by monotypy] (♂) in MCZ [# 607]. Synonymy established by LeConte (1863b: 3).
Brennus gentilis Casey, 1897: 322. Type locality: «near Monterey [Monterey County], California» (original citation). Seven syntypes [10 originally cited] in USNM [# 46021]. Synonymy established by Roeschke (1907a: 184), confirmed by Gidaspow (1968: 173).
Brennus productus Casey, 1914: 29. Type locality: «California» (original citation). One syntype in USNM [# 46023]. Synonymy established, under the name *Scaphinotus ventricosus* var. *striatus* (LeConte), by Lapouge (1933: 696), confirmed by Gidaspow (1968: 173).
Brennus montereyensis Casey, 1920: 177. Type locality: «Monterey [Monterey County], California» (original citation). One syntype in USNM [# 46022]. Synonymy established by Gidaspow (1968: 173).

#### Distribution.

This species ranges from Sonoma County in California to the Pacific Coast of Baja California Norte, east to the San Bernardino Mountains [see Gidaspow 1968: Fig. 10]. One specimen from Hidalgo state in Mexico is probably mislabeled, as pointed out by Gidaspow (1968: 176).

#### Records.

**USA**: CA – Mexico

### Scaphinotus cristatus

(Harris, 1839)

Cychrus cristatus T.W. Harris, 1839: 200. Type locality: «Oregon» (original citation, see page 199), herein restricted to Pistol River, Curry County (see Gidaspow 1968: 144). Syntype(s) lost (Roeschke 1907a: 194).
Cychrus reticulatus Motschulsky, 1850a: 90. Type locality: «California?; Unalaschka?» (original citation), listed from «Calif[ornie]» by Motschulsky (1869: 29). Lectotype [as holotype] (♂), designated by Kryzhanovskij (1968: 187), in ZMMU. Synonymy established with doubt by LeConte (1857c: 10), confirmed by Kryzhanovskij (1968: 187).
Brennus basalis Casey, 1897: 311. Type locality: «S[an]ta Cruz Co[unty], California» (original citation). Five syntypes in USNM [# 46013] and at least one in AMNH [# 441]. Synonymy established, under the name *Scaphinotus cristatus reticulatus* (Motschulsky), by Roeschke (1907a: 194).
Brennus duplicatus Casey, 1897: 312. Type locality: «Lake Co[unty], California» (original citation). Three syntypes [3 originally cited] in USNM [# 46014]. Synonymy established, under the name *Scaphinotus cristatus reticulatus* (Motschulsky), by Roeschke (1907a: 194).

#### Distribution.

This species ranges from southwestern Oregon south to the Los Angeles region in southern California, east to the northern part of the Sierra Nevada [see Gidaspow 1968: Fig. 2].

#### Records.

**USA**: CA, OR

### Scaphinotus interruptus

(Ménétriés, 1843)

Cychrus interruptus Ménétriés, 1843: 54. Type locality: «Californie» (original citation), herein restricted to Hoopa Valley, Humboldt County (see Gidaspow 1968: 159). Two syntypes in ZILR (Roeschke 1907a: 175).
Cychrus constrictus LeConte, 1853c: 398. Type locality: «San Jose [Santa Clara County], California» (original citation). Syntype(s) [2 originally cited] in MCZ [# 609]. Synonymy established by LeConte (1873b: 322).
Cychrus dissolutus Schaum, 1863: 72. Type locality: «Sacramento [Sacramento County, California]» (original citation). One syntype in ZMHB (Roeschke 1907a: 175). Synonymy established, under the name *Scaphinotus interruptus* var. *constrictus* (LeConte), by Lapouge (1933: 695).
Brennus sinuatus Casey, 1897: 330. Type locality: «California» (original citation). Syntypes [3 originally cited] location unknown. Synonymy established by Roeschke (1907a: 175), confirmed by Gidaspow (1968: 160).
Brennus politus Casey, 1897: 330. Type locality: «Hoopa Valley, Humboldt Co[unty], California» (original citation). Holotype [by monotypy] (♂) in USNM [# 46025]. Synonymy established by Gidaspow (1968: 158).
Brennus corpulentus Casey, 1897: 331. Type locality: «Oakland, Alameda Co[unty], California» (original citation). Two syntypes in USNM [# 46029]. Synonymy established, under the name *Scaphinotus interruptus* var. *constrictus* (LeConte), by Roeschke (1907a: 175), confirmed by Gidaspow (1968: 160).
Brennus integer Casey, 1914: 29. Type locality: «S[an]ta Cruz [Santa Cruz County], California» (original citation). One syntype in USNM [# 46026]. Synonymy established by Gidaspow (1968: 158).
Brennus parvulicollis Casey, 1920: 176. Type locality: «S[an]ta Cruz [Santa Cruz County], California» (original citation for *Brennus interruptus* (Ménétriés) *sensu* Casey, 1897). One syntype in USNM [# 46028]. Synonymy established, under the name *Brennus interruptus* var. *constrictus* (LeConte), by Lapouge (1933: 695), confirmed by Gidaspow (1968: 160). Note. This name was proposed for *Cychrus interruptus* Ménétriés, 1843 *sensu* Casey (1897: 333).
Brennus beringi Casey, 1920: 179. Type locality: «S[ain]t Paul Island, Alaska» (original citation) which is incorrect. One syntype in USNM [# 46024]. Synonymy established by Lindroth (1961a: 24). Etymology. The specific name honors the Danish navigator and explorer Vitus Béring [1681-1741] who is credited for being the first European to discover Alaska and the Aleutian Islands. The Bering Strait, the Bering Sea, Bering Island, and Bering Glacier are named for him.
Brennus procerus Casey, 1920: 179. Type locality: «Piedmont, Alameda Co[unty], California» (original citation). One syntype in USNM [# 46031]. Synonymy established by Gidaspow (1968: 158).

#### Distribution.

The range of this species extends over much of California, including the Coast Ranges and the Sierra Nevada, as far south as Riverside County [see Gidaspow 1968: Fig. 6]. Some specimens simply labeled from Oregon are known.

#### Records.

**USA**: CA [OR]

### Scaphinotus marginatus

(Fischer von Waldheim, 1820)

Cychrus marginatus Fischer von Waldheim, 1820: plate 7. Type locality: «insula Unalaschka [Alaska]» (Fischer von Waldheim 1822: 79). Syntype(s) in SMTD (Grämer 1960: 101; Gidaspow 1968: 149) and probably also in MHNP (collection Dejean).
Cychrus marginatus var. *fulleri* G.H. Horn, 1879: 179. Type locality: «Oregon» (original citation). Syntype(s) in MCZ [# 34835]. Synonymy established by Hatch (1953: 47), confirmed by Gidaspow (1968: 154).
Cychrus marginatus var. *gracilis* Géhin, 1885: 76. Type locality: «Mexique» (original citation), which is incorrect (Roeschke 1907a: 173). Syntype(s) in MHNP (collection Oberthür). Synonymy established by Roeschke (1907a: 171).
Brennus cupripennis Casey, 1897: 334. Type locality: «Washington State» (original citation). Two syntypes [2 ♂ originally cited] in USNM [# 46040]. Synonymy established by Hatch (1953: 47), confirmed by Lindroth (1961a: 22).
Brennus insularis Casey, 1897: 334. Type locality: «Queen Charlotte Islands [British Columbia]» (original citation). Holotype [by monotypy] (♀) in USNM [# 46041]. Synonymy established by Roeschke (1907a: 171), confirmed by Lindroth (1961a: 22).
Brennus confusus Casey, 1897: 336. Type locality: «undoubtedly on the coast between northern California and Alaska» (original citation). Two syntypes [2 originally cited] in USNM [# 46043]. Synonymy established by Hatch (1953: 47), confirmed by Lindroth (1961a: 23).
Brennus marginatus var. *fallax* Roeschke, 1907a: 174. Type locality: «Oregon, auch in Idaho und Montana, in den Bitter Root Mountains» (original citation). Three syntypes in ZMUA (Boer 2002: 49). Synonymy established by Lindroth (1961a: 23).
Brennus columbianus Casey, 1920: 180. Type locality: «Victoria, British Columbia» (original citation). Two syntypes in USNM [# 46042]. Synonymy established by Hatch (1953: 47), confirmed by Lindroth (1961a: 23).
Brennus gracilis wrangelli Casey, 1920: 182. Type locality: «Fort Wrangell, Alaska» (original citation). Holotype [by monotypy] (♀) in USNM [# 46045]. Synonymy established by Lindroth (1961a: 23).
Brennus gracilis montanicus Casey, 1920: 182. Type locality: «Helena [Lewis and Clark County], Montana» (original citation). Holotype [by monotypy] (♂) in USNM [# 46046]. Synonymy established by Lindroth (1961a: 23).
Brennus oregonus Casey, 1920: 182. Type locality: «Oregon» (original citation). One syntype in USNM [# 46044]. Synonymy established by Hatch (1953: 47), confirmed by Lindroth (1961a: 23).

#### Distribution.

The range of this species extends from the Aleutians Islands and the Gulf Coast of Alaska south to northern California, northern Arizona (Coconino County, Eric van den Berghe pers. comm. 2009), and northwestern Wyoming [see Gidaspow 1968: Fig. 4]. Gidaspow (1968: 149) considered the possibility that the species ranges further east toward the Hudson Bay because of the presence in collections of several specimens labeled from “Hudson Bay Territory.”

#### Records.

**CAN**: AB, BC (QCI, VCI) **USA**: AK, AZ, CA, ID, MT, OR, WA, WY

### Scaphinotus obliquus

(LeConte, 1868)

Cychrus obliquus LeConte, 1868b: 61. Type locality: «near Sacramento [Sacramento County], California» (original citation). Holotype [by monotypy] (♂) in MCZ [# 608].
Brennus convergens Casey, 1897: 326. Type locality: «Siskiyou Co[unty], California» (original citation). Three syntypes [4 originally cited] in USNM [# 46035]. Synonymy established by Lapouge (1933: 695), confirmed by Gidaspow (1968: 156).
Brennus opacicollis Casey, 1897: 327. Type locality: «Oregon» (original citation). Two syntypes [2 originally cited] in USNM [# 46033]. Synonymy established, under the name *Scaphinotus obliquus convergens* (Casey), by Roeschke (1907a: 180), confirmed by Gidaspow (1968: 156).
Brennus sculptipennis Casey, 1897: 327. Type locality: «California» (original citation). One syntype [3 originally cited] in USNM [# 46034]. Synonymy established, under the name *Scaphinotus obliquus convergens* (Casey), by Roeschke (1907a: 180), confirmed by Gidaspow (1968: 156).

#### Distribution.

This species is known from the northern part of California, as far south as Madera County [see Gidaspow 1968: Fig. 5], and from Washoe County in northwestern Nevada (La Rivers 1947: 133, as *Scaphinotus obliquus convergens*). Some specimens simply labeled from Oregon are known.

#### Records.

**USA**: CA, NV [OR]

### Scaphinotus oreophilus

(Rivers, 1890)

Cychrus oreophilus Rivers, 1890b: 111. Type locality: «Shingle Springs, Eldorado County, California» (original citation). Two syntypes probably in ZMUA (collection Vogt) though not listed by Boer (2002).
Brennus oreophilus hoppingi Roeschke, 1907a: 183. Type locality: «Südlicher Arm des King River (4500-5500’), Fresno Co[unty] [California]» (original citation). Five syntypes in ZMUA (Boer 2002: 56). Synonymy established by Gidaspow (1968: 164). Etymology. The specific name was proposed for Ralph Hopping [1868-1941], forest entomologist in California and later in Vernon, British Columbia. Hopping built up a large collection of western beetles which was sold by his widow to the California Academy of Sciences in 1948.
Brennus oreophilus humeralis Casey, 1914: 30. Type locality: «Mokelumne Hill, Calaveras Co[unty], California» (original citation). One syntype in USNM [# 46039]. Synonymy established by Gidaspow (1968: 164).

#### Distribution.

This species is known from central California [see Gidaspow 1968: Fig. 3] and from Grand County in eastern Utah (Gidaspow 1968: 165). The record from “Nevada” (Bousquet and Larochelle 1993: 84) needs confirmation.

#### Records.

**USA**: CA, UT [NV]

### Scaphinotus punctatus

(LeConte, 1859)

Cychrus punctatus LeConte, 1859a: 69. Type locality: «Fort Tejon [Kern County, California]» (original citation). Syntype(s) in MCZ [# 605].
Cychrus mimus G.H. Horn, 1874: 20. Type locality: «along the Santa Ana River, at San Bernardino [San Bernardino County], California» (original citation). Syntype(s) in MCZ [# 618]. Synonymy established by Gidaspow (1968: 167).
Brennus gravidus Casey, 1897: 317. Type locality: «southern California» (original citation). Syntype(s) location unknown. Synonymy established by Roeschke (1907a: 191). Note. The specimen in Casey’s collection under the name *gravidus* is the female from Monterey that Casey stated in his remarks as being probably distinct from the male type.
Brennus catenulatus Casey, 1897: 324. Type locality: «southern California» (original citation). Two syntypes in USNM [# 46038]. Synonymy established by Gidaspow (1968: 167).

#### Distribution.

This species is found in the southern third of California, including Santa Catalina Island, and in “Baja California” [see Gidaspow 1968: Fig. 7].

#### Records.

**USA**: CA (CHI) – Mexico

### Scaphinotus riversi

(Roeschke, 1907)

Brennus oreophilus riversi Roeschke, 1907a: 183. Type locality: «Hochgebirge der Sierra Nevada der Tulare und Kern Cos., in einer Höhe von etwa 5000 bis 8000 Fuss: Mt. Whitney, Round Meadow, Giant Forest [California]» (original citation). Four syntypes in ZMUA (Boer 2002: 99) and one possible syntype in SIM (Hennessey 1990: 466).

#### Distribution.

This species is found in the southern half of California, in Tulare (Sequoia National Park), Fresno, Kern, and Los Angeles Counties [see Gidaspow 1968: Fig. 5].

#### Records.

**USA**: CA

### Scaphinotus rugiceps incipiens

(Casey, 1897)

Brennus incipiens Casey, 1897: 313. Type locality: «northern California?» (original citation), herein restricted to Green Point, Humboldt County (see Gidaspow 1968: 148). Holotype [by monotypy] (♀) in USNM [# 46015].

#### Distribution.

This subspecies is found in southwestern Oregon (Westcott et al. 2006: 9) and northwestern California as far south as Colusa County [see Gidaspow 1968: Fig. 3].

#### Records.

**USA**: CA, OR

### Scaphinotus rugiceps rugiceps

(Horn, 1872)

Cychrus rugiceps G.H. Horn, 1872b: 143. Type locality: «Oregon» (original citation), herein restricted to Diamond Lake, Douglas County (see Gidaspow 1968: 148). Syntype(s) in MCZ [# 35352].
Brennus porcatus Casey, 1897: 328. Type locality: «California» (original citation). Holotype [by monotypy] (♂) in USNM [# 46036]. Synonymy established by Gidaspow (1968: 147). Note. This form was listed as a synonym of *Scaphinotus interruptus dissolutus* by Roeschke (1907a: 175).
Brennus compositus Casey, 1897: 332. Type locality: «California» (original citation). Holotype [by monotypy] (♂) in USNM [# 46030]. Synonymy established by Gidaspow (1968: 147). Note. This form was listed as a synonym of *Scaphinotus interruptus* by Roeschke (1907a: 175).
Brennus rugiceps congener Casey, 1914: 28. Type locality: «Josephine Co[unty], Oregon» (original citation). Two syntypes [2 originally cited] in USNM [# 46016]. Synonymy established by Hatch (1953: 48).

#### Distribution.

This subspecies is known for sure only from western Oregon as far south as the border with California [see Gidaspow 1968: Fig. 3]. The records from “California” (Casey 1897: 328, 332, as *Brennus porcatus* and *Brennus compositus*) need confirmation.

#### Records.

**USA**: OR [CA]

### Scaphinotus striatopunctatus

(Chaudoir, 1844)

Cychrus striatopunctatus Chaudoir, 1844: 476. Type locality: «Californie» (original citation), herein restricted to Boonville, Mendocino County (see Gidaspow 1968: 169). Holotype [by monotypy] in MHNP.
Cychrus ovalis Motschulsky, 1859a: 162. Type locality: «Californie» (original citation). Lectotype (♀), designated by Kryzhanovskij (1968: 186), in ZMMU. Synonymy established (as aberration) by Roeschke (1907a: 188), confirmed by Kryzhanovskij (1968: 186).
Brennus decipiens Casey, 1897: 316. Type locality: «near Monterey [Monterey County], California» (original citation). Eight syntypes [8 originally cited] in USNM [# 46017]. Synonymy established by Roeschke (1907a: 188).
Brennus subdepressus Casey, 1920: 177. Type locality: «Monterey [Monterey County], California» (original citation). Holotype [by monotypy] (♂) in USNM [# 46018]. Synonymy established by Lapouge (1933: 696), confirmed by Gidaspow (1968: 169).

#### Distribution.

This species is found along western California from Mendocino County south to Los Angeles County [see Gidaspow 1968: Fig. 9].

#### Records.

**USA**: CA

### Scaphinotus subtilis

(Schaum, 1863)

Cychrus subtilis Schaum, 1863: 72. Type locality: «Sacramento [Sacramento County, California]» (original citation). Syntype(s) in ZMHB.

#### Distribution.

This species is known only from central California, mostly along the west side of the Sierra Nevada from Calaveras County to Tulare and Kern Counties [see Gidaspow 1968: Fig. 8].

#### Records.

**USA**: CA

### Scaphinotus ventricosus

(Dejean, 1831)

Cychrus ventricosus Dejean, 1831: 527. Type locality: «Californie» (original citation), herein restricted to San Francisco, San Francisco County (see Eschscholtz 1833: 21). Holotype [by monotypy] (♂) in MHNP (Lindroth 1955b: 11).
Cychrus lativentris Motschulsky, 1850b: 358. Type locality not stated. Syntype(s) lost (Roeschke 1907a: 184). Synonymy established by Gidaspow (1968: 170).
Cychrus alternatus Motschulsky, 1859a: 163. Type locality: «Californie» (original citation). Syntype(s) lost (Roeschke 1907a: 188). Synonymy established by Gidaspow (1968: 170). Note. Roeschke (1907a: 188) listed this name as an aberration of *Scaphinotus striatopunctatus* (Chaudoir).
Cychrus fuchsianus Rivers, 1890a: 71. Type locality: «Eldorado and Sonoma Counties, Cal[ifornia]» (original citation). One possible syntype in ZMUA (collection Vogt) but not listed by Boer (2002). Synonymy established (as aberration) by Roeschke (1907a: 184).
Brennus symmetricus Casey, 1897: 319. Type locality: «California» (original citation). Holotype [by monotypy] (♂) in USNM [# 46032]. Synonymy established by Gidaspow (1968: 170).
Brennus strictus Casey, 1897: 322. Type locality: «California» (original citation). One syntype in USNM [# 46019]. Synonymy established, under the name *Scaphinotus ventricosus lativentris* (Motschulsky), by Roeschke (1907a: 184), confirmed by Gidaspow (1968: 173).
Brennus brevicollis Casey, 1920: 178. Type locality: «Mokelumne Hill, Calaveras Co[unty], California» (original citation). One syntype in USNM [# 46020]. Synonymy established by Gidaspow (1968: 170).

#### Distribution.

This species ranges from southern Oregon to San Luis Obispo County along the coast and to Yosemite National Park along the Sierra Nevada; also known from the Santa Catalina Island [see Gidaspow 1968: Fig. 10].

#### Records.

**USA**: CA, OR

### Subgenus Neocychrus

Roeschke, 1907

Neocychrus Roeschke, 1907a: 197. Type species: *Cychrus angulatus* Harris, 1839 designated by Lindroth (1961a: 24). Etymology. From the Greek prefix *neo*- (new) and the generic name *Cychrus* [*q.v*.] [masculine].

#### Diversity.

Three species restricted to the Pacific coastal region of North America.

#### Identification.

Van Dyke (1944) first reviewed the species. Subsequently, Gidaspow (1973) revised them and provided a key for their identification.

### Scaphinotus angulatus

(Harris, 1839)

Cychrus angulatus T.W. Harris, 1839: 200. Type locality: «Oregon» (original citation, see page 199), restricted to «Portland [Multnomah County]» by Lindroth (1961a: 24). Holotype [by monotypy] (♀) apparently lost (LeConte 1869c: 372).
Scaphinotus angulatus maritimus Van Dyke, 1924b: 5. Type locality: «near Port Angeles [Clallam County], Washington» (original citation). Holotype (♀) in CAS [# 3341]. Synonymy established by Lindroth (1961a: 25).

#### Distribution.

This species ranges from southwestern British Columbia, including Vancouver Island, to southwestern Oregon [see Gidaspow 1973: Fig. 1].

#### Records.

**CAN**: BC (VCI) **USA**: OR, WA

### Scaphinotus behrensi

(Roeschke, 1907)

Neocychrus behrensi Roeschke, 1907a: 199. Type locality: «Riesennadelholzwaldungen (= redwood cañon) im nördlichen Sonoma Co[unty], Californien» (original citation). Holotype [by monotypy] (♂) in ZMUA (Boer 2002: 32).
Scaphinotus behrensi malkini Van Dyke, 1944: 13. Type locality: «Spenser Butte, near Eugene [Lane County], Oregon» (original citation). Holotype (♂) in CAS [# 5345]. Synonymy established by Gidaspow (1973: 61).

#### Distribution.

The range of this species extends near the Pacific Coast from Lincoln County in Oregon (Gidaspow 1973: 61) to Humboldt County in northern California [see Gidaspow 1973: Fig. 1].

#### Records.

**USA**: CA, OR

### Scaphinotus longiceps

Van Dyke, 1924

Scaphinotus longiceps Van Dyke, 1924b: 5. Type locality: «interior of Humboldt County, California» (original citation). Holotype (♂) in CAS [# 3340].

#### Distribution.

This species is known only from a few specimens collected in Humboldt and Mendocino Counties (Weber and Kavanaugh 1992: 394), northern California.

#### Records.

**USA**: CA

### Tribe Carabini

Latreille, 1802

Carabici Latreille, 1802: 80. Type genus: *Carabus* Linnaeus, 1758.
Calosomii Bonelli, 1810: Tabula Synoptica. Type genus: *Calosoma* Weber, 1801.

#### Diversity.

Worldwide, with about 1,080 species arrayed in three genera: *Aplothorax* Waterhouse (one species on the island of Saint Helena in South Atlantic Ocean), *Calosoma* (about 170 species), and *Carabus* (about 910 species).

### Genus Calosoma

Weber, 1801

Calosoma Weber, 1801: 20. Type species: *Carabus sycophanta* Linnaeus, 1758 designated by Latreille (1810: 426). Etymology (original). From the Greek *calos* (beautiful) and *soma* (body), alluding to the beautiful body coloration of adults of *Calosoma sycophanta* and *Calosoma inquisitor*, the two species included by Weber in the genus [neuter].
Callisoma Agassiz, 1846: 60, 61. Unjustified emendation of *Calosoma* Weber, 1801.

#### Diversity.

Worldwide, with about 170 species in the Nearctic (41 species, of which one is adventive), Neotropical (about 55 species, many shared with North America), Australian (three species), Oriental (six species), Palaearctic (about 45 species), and Afrotropical (about 35 species) Regions. The species are arrayed in about 25 genus-group taxa.

#### Identification.

Gidaspow (1959) revised the North American species and provided a key for their identification. Subsequently Lindroth (1961a: 50, 55) listed in synonymy some of the species that were considered valid by Gidaspow (e.g., *Calosoma concretum*, *Calosoma pimelioides*, *Calosoma zimmermani*) and Dajoz (1997a) described a new species (*Calosoma dawsoni*).

#### Taxonomic Note.

The genus *Calosoma* is retained here in its wide sense following several authors, including Culot (1988). Others used different arrangements. For example, Lorenz (2005) and Erwin (2007a) listed *Callisthenes* Fischer von Waldheim as a valid genus with *Chrysostigma* Kirby and *Callistenia* Lapouge as subgenera.

#### Faunistic Note.

Burgess and Collins (1917: 86) reported that *Calosoma palmeri* Horn “occurs in California and Mexico.” The record for California is doubtful since the species, as far as known, is endemic to Guadalupe Island (Gidaspow 1959: 276).

### Subgenus Castrida

Motschulsky, 1866

Castrida Motschulsky, 1866: 300. Type species: *Calosoma sayi* Dejean, 1826 by monotypy. Etymology. Unknown [feminine].
Camedula Motschulsky, 1866: 304. Type species: *Calosoma rufipenne* Dejean, 1831 designated by Géhin (1885: xxxi). Note. The first type species designation for *Camedula* Motschulsky is that of *Calosoma glabratum* Dejean, 1831 as selected by Géhin (1876b: 114). This species has been accepted as the type species by Jeannel (1940: 199) and Gidaspow (1959: 256). However, Breuning (1928a: 93) accepted *Calosoma rufipenne* Dejean, 1831 as type species following Géhin (1885: xxxi) and this species is currently recognized as the type species (e.g., Lorenz 1998: 59, Lorenz 2005: 57). As discussed by Bousquet (2002b: 11-12), the best solution to preserve nomenclatural stability is to refer the case to the Commission in order to maintain *Calosoma rufipenne* Dejean as type species.
Callistriga Motschulsky, 1866: 307. Type species: *Carabus alternans* Fabricius, 1792 designated by Géhin (1876b: 114). Synonymy established by Csiki (1927: 11).
Calamata Motschulsky, 1866: 307. Type species: *Calamata rugata* Motschulsky, 1866 (= *Calosoma alternans granulatum* Perty, 1830) by monotypy. Synonymy established by Csiki (1927: 11).
Microcalosoma Breuning, 1927: 146. Type species: *Calosoma linelli* Mutchler, 1925 by monotypy. Etymology. From the Greek *micros* (small) and the generic name *Calosoma* [*q.v*.] [neuter].
Acampalita Lapouge, 1929b: 9. Type species: *Calosoma vagans* Dejean, 1831 by subsequent monotypy in Lapouge (1931: 418). Synonymy established by Jeannel (1940: 89). Etymology. From the Greek prefix *a*- (privative) and the generic name *Campalita* [feminine].
Catastriga Lapouge, 1929b: 9. Type species: *Calosoma trapezipenne* Chaudoir, 1869 by subsequent monotypy in Lapouge (1931: 418). Synonymy established by Gidaspow (1959: 240).
Caludema Jeannel, 1940: 89, 91. Type species: *Calosoma rufipenne* Dejean, 1831 by original designation. Synonymy established by Gidaspow (1963: 289). Etymology. Anagram of the generic name *Camedula* [*q.v*.] [neuter].

#### Diversity.

Western Hemisphere, with 14 species in the Nearctic (one species) and Neotropical (14 species) Regions.

### Calosoma sayi

Dejean, 1826

Calosoma sayi Dejean, 1826: 198. Type locality: «Amérique septentrionale» (original citation), herein restricted to Norfolk, Virginia (see Casey 1897: 344, as *Calosoma sayi virginica*). Lectotype (♀), designated by Deuve (1978: 246), in MHNP. Etymology. The specific name was proposed in honor of Thomas Say [1787-1834], American naturalist and one of the founders of the Academy of Natural Sciences in Philadelphia. Say participated in the geological expedition to the off-shore islands of Georgia and Florida (then a Spanish colony) in 1818, in Major Long’s expedition to the Rocky Mountains and the tributaries of the Missouri River in 1819 and 1820, and in Long’s expedition to the headwaters of the Mississippi River in 1823. He lived the last eight years of his life in New Harmony, Indiana, in Robert Owen’s utopian society experiment where he secretly married Lucy Way Sistare [1801-1886], an artist who illustrated some of her husband’s works.
Calosoma armata Laporte, 1835: 156. Type locality: «Mexique» (original citation). Lectotype (♂), designated by Erwin (1991a: 20), in MHNP. Synonymy established by Breuning (1927: 192).
Calosoma sayi var. *abdominale* Géhin, 1885: 58. Type locality: «Mexique» (original citation). Lectotype (♂), designated by Erwin (1991a: 20), in MHNP. Synonymy established, under the name *Calosoma alternans* var. *armatum* Laporte, by Roeschke (1900: 71).
Calosoma sayi virginica Casey, 1897: 344. Type locality: «Norfolk, V[irgini]a» (original citation). Lectotype (♂), designated by Erwin (1991a: 20), in USNM [# 37092]. Synonymy established by Roeschke (1900: 71).
Calosoma alternans var. *cuprascens* Roeschke, 1900: 71. Type locality not stated. Holotype [by monotypy] (♂) location unknown. Synonymy established by Jeannel (1940: 94).

#### Distribution.

This species ranges from Long Island, New York (Notman 1928: 209) to “Iowa” (Burgess and Collins 1917: 62), south to Guatemala (Gidaspow 1963: 301) and southern Florida except for the Keys (Peck and Thomas 1998: 15), west along southern United States to “California” (Burgess and Collins 1917: 62); also known from the Greater Antilles as far south as Puerto Rico (Gidaspow 1963: 301). The records from “Minnesota,” “North Dakota,” and “Wisconsin” (Bousquet and Larochelle 1993: 70) are probably in error or based on strays.

#### Records.

**USA**: AL, AR, AZ, CA, DC, FL, GA, IA, IL, IN, KS, KY, LA, MD, MO, MS, NC, NJ, NY, OH, OK, PA, SC, TN, TX, VA – Cuba, Dominican Republic, Guatemala, Haiti, Jamaica, Mexico, Puerto Rico

### Subgenus Calosoma

Weber, 1801

Calosoma Weber, 1801: 20. Type-species: *Carabus sycophanta* Linnaeus, 1758 designated by Latreille (1810: 426).
Callipara Motschulsky, 1866: 309. Type species: *Carabus sycophanta* Linnaeus, 1758 designated by Géhin (1876b: 114).
Syncalosoma Breuning, 1927: 179. Type species: *Calosoma frigidum* Kirby, 1837 by original designation. Synonymy established by Lapouge (1931: 400). Etymology. From the Greek prefix *syn*- (together, with) and the generic name *Calosoma* [*q.v*.] [neuter].
Acalosoma Lafer, 1989: 106. Type species: *Carabus inquisitor* Linnaeus, 1758 by original designation.

#### Diversity.

Northern Hemisphere, with six species in the Nearctic (two species, one of them adventive) and Palaearctic (five species) Regions.

#### Taxonomic Note.

This subgenus is retained in its narrow sense and excludes members of *Australodrepa* Lapouge and *Calodrepa* Motschulsky.

### Calosoma frigidum

Kirby, 1837

Calosoma frigidum Kirby, 1837: 19. Type locality: «Drummond’s Island [Chippewa County, Michigan], Canada» (original citation). One syntype in BMNH (Lindroth 1953b: 169).
Calosoma frigida levettei Casey, 1897: 344. Type locality: «Indiana» (original citation). Holotype [by monotypy] (♀) in USNM [# 37093]. Synonymy established by Breuning (1927: 180).

#### Distribution.

This species occurs from Cape Breton Island to the Skeena River drainage in west-central British Columbia (Lindroth 1961a: 47), south to northeastern Nevada (La Rivers 1947: 134), central Utah (La Rivers 1947: 135), southeastern Texas in the Galveston area (Snow 1906a: 140; Gidaspow 1959: 245), and northern Georgia (Fattig 1949: 11).

#### Records.

**CAN**: AB, BC, MB, NB, NS (CBI), ON, PE, QC, SK **USA**: CO, CT, GA, IA, IL, IN, LA, MA, MD, ME, MI, MN, MO, NC, ND, NE, NH, NJ, NV, NY, OH, OK, PA, RI, SD, TN, TX, UT, VA, VT, WI, WV

### Calosoma sycophanta

(Linnaeus, 1758)

Carabus sycophanta Linnaeus, 1758: 414. Type locality: «Europa» (original citation), restricted to «Sweden» by Lindroth (1961a: 47). One possible syntype in LSL (Lindroth 1957b: 334).

#### Distribution.

This European species was introduced as early as 1906 in many places in Canada and United States for the biological control of two introduced lymantriids: the gypsy moth, *Lymantria dispar* (Linnaeus), and the browntail moth, *Euproctis chrysorrhoea* (Linnaeus). Based on the extensive survey of Schaefer et al. (1999), the species is now established in eastern United States from southern Maine to Maryland and West Virginia, west to western Pennsylvania. The record from “Michigan” (Bousquet and Larochelle 1993: 71) must be in error. There is no confirmation that the species is established on the west coast and the record from “Washington” (Bousquet and Larochelle 1993) should be deleted.

#### Records.

**USA**: CT, DE, MA, MD, ME, NH, NJ, NY, PA, RI, VA, VT, WV – **Adventive**

**Figure 9.**
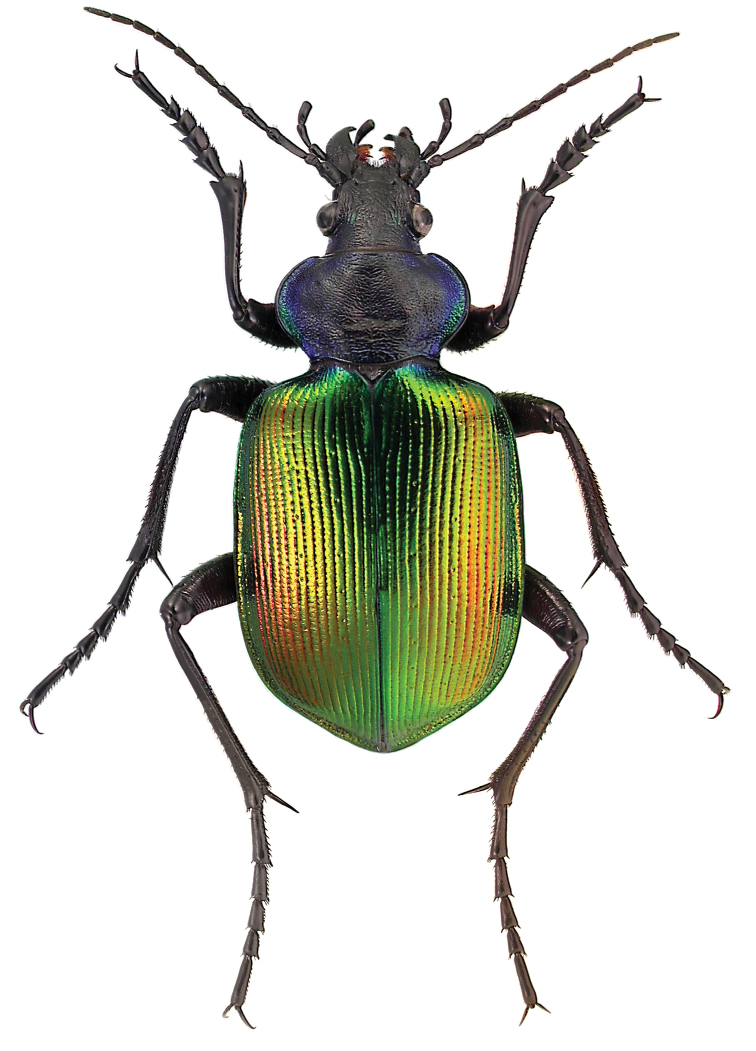
*Calosoma sycophanta* (Linnaeus). This species is one of only two intentionally introduced carabids in North America that became established on this continent, the other one being *Carabus auratus*. These species were introduced for the biological control of the gypsy moth, *Lymantria dispar*, and the browntail moth, *Euproctis chrysorrhoea*. Since its release in the vicinity of Boston in 1906-1907, *Calosoma sycophanta* has spread to southern Maine and to West Virginia.

### Subgenus Calodrepa

Motschulsky, 1866

Calodrepa Motschulsky, 1866: 310. Type species: *Carabus scrutator* Fabricius, 1775 designated by Géhin (1876b: 114). Etymology. Unknown [feminine].

#### Diversity.

Western Hemisphere, with four species in the Nearctic (four species, one of them endemic) and Neotropical (three species) Regions.

#### Taxonomic Note.

This subgenus is listed in synonymy with the nominotypical subgenus by some authors (e.g., Breuning 1927: 155; Lorenz 2005: 68).

### Calosoma aurocinctum

Chaudoir, 1850

Calosoma aurocinctum Chaudoir, 1850a: 420. Type locality: «Mexique» (original citation), herein restricted to Merida, Yucatán (see Gidaspow 1959: 248). Lectotype (♀), designated by Deuve (1978: 248), in MHNP. Note. This name is often (e.g., Gidaspow 1963: 283; Erwin 1991a: 24) cited as if it had been proposed by Chaudoir (1850a: 420) as a replacement for *Calosoma splendidum* Perbosc, 1839 (nec Dejean 1831). However, Perbosc (1839: 261) attributed his *Calosoma splendidum* to Dejean and so did not propose a new species.

#### Distribution.

This species is found from the Rio Grande in southeastern Texas (Wickham 1897: 102; Burgess and Collins 1917: 122) south to Nicaragua (Gidaspow 1963: 283) [see Gidaspow 1959: Fig. 2].

#### Records.

**USA**: TX – Mexico, Nicaragua

### Calosoma scrutator

(Fabricius, 1775)

Carabus scrutator Fabricius, 1775: 239. Type locality: «Virginia» (original citation). Two syntypes in BMNH (collection Banks) and ZMUC (Zimsen 1964: 60).

#### Distribution.

This widely distributed species ranges from western Maine (Majka et al. 2011: 45) to northeastern North Dakota (Tinerella 2003: 635), including southern Quebec (only as strays) and Ontario (Lindroth 1961a: 46), south to Venezuela (Gidaspow 1963: 283) and southern Florida (Peck and Thomas 1998: 15), west along the southwest to “California” (Gidaspow 1959: 250) and Baja California (Leng 1915: 565). Two specimens, likely strays, have been collected in New Brunswick (Webster and Bousquet 2008: 16) and on Sable Island, Nova Scotia (Majka et al. 2007: 6). The species is not known from the West Indies.

#### Records.

**CAN**: NB, NS, ON, QC **USA**: AL, AR, AZ, CA, CO, CT, DC, DE, FL, GA, IA, IL, IN, KS, KY, LA, MA, MD, ME, MI, MN, MO, MS, NC, ND, NE, NH, NJ, NM, NY, OH, OK, PA, RI, SC, SD, TN, TX, VA, WI, WV – Guatemala, Mexico, Venezuela

### Calosoma splendidum

Dejean, 1831

Calosoma splendidum Dejean, 1831: 558. Type locality: «S[ain]t-Domingue [Dominican Republic or Hispaniola]» (original citation). Holotype [by monotypy] (♀) in MHNP (Lindroth 1955b: 12).

#### Distribution.

This species is known from northeastern Georgia and southern Florida but only from a few century-old specimens (Gidaspow 1959: 248), and from the Bahamas (Turnbow and Thomas 2008: 12), Cuba, and Hispaniola (Gidaspow 1963: 283). Erwin (2007a: 104) listed this species also from Yucatán, Mexico.

#### Records.

**USA**: FL, GA – Bahamas, Cuba, Haiti, Dominican Republic, Mexico

### Calosoma wilcoxi

LeConte, 1847

Calosoma wilcoxi LeConte, 1847: 446. Type locality: «NovEboraco [= New York] ad Texas» (original citation), restricted to «New York state» by Lindroth (1961a: 46). Syntype(s) in MCZ [# 623].

#### Distribution.

This species ranges from southeastern New Hampshire (Rockingham County [probably only as strays], Donald S. Chandler pers. comm. 1992) to southeastern Minnesota (Donald P. Schwert pers. comm. 1989), including southern Quebec (only as strays) and southern Ontario (Lindroth 1961a: 47), south to southeastern Texas (Brazoria County, Brian Raber pers. comm. 2010), southeastern Louisiana (Saint John the Baptist and Saint Tammany Parishes, Igor M. Sokolov pers. comm. 2009), and central Georgia (Fattig 1949: 11). The record from “California” (Burgess and Collins 1917: 38) is likely in error or based on a stray.

#### Records.

**CAN**: ON, QC **USA**: AR, CT, DC, DE, GA, IA, IL, IN, KS, KY, LA, MA, MD, MI, MN, MO, MS, NC, NE, NH, NJ, NY, OH, OK, PA, RI, SC, TN, TX, VA, WI, WV

### Subgenus Camegonia

Lapouge, 1924

Camegonia Lapouge, 1924: 38. Type species: *Calosoma carbonatum* LeConte *sensu* Lapouge, 1924 (= *Calosoma prominens* LeConte, 1853) designated by Breuning (1928a: 95). Etymology. Unknown [feminine]. Note. Lapouge (1924: 38-39) associated two taxa with *Camegonia*, *Calosoma carbonatum* LeConte, 1862 and *Calosoma lugubre* LeConte, 1853. Breuning (1928a: 95) noted that Lapouge misidentified *Calosoma prominens* LeConte, 1853 for *Camegonia carbonata* and designated *Calosoma prominens* LeConte as type species of *Camegonia* Lapouge, 1924. That species was not originally included but since Breuning (1928a: 95) listed the species in synonymy with *Camegonia carbonata sensu* Lapouge, 1924, one of the two species originally included in *Camegonia*, he is deemed to have designated the latter species as type species (ICZN 1999: Article 69.2.2). Therefore Breuning (1928a: 95) designated as type species a species originally included as an expressly stated misidentification and the species so designated is the nominal species denoted by the name of the taxonomic species actually involved (ICZN 1999: Article 69.2.4), that is *Calosoma prominens* LeConte, 1853.

#### Diversity.

Three North American species, all of them extending into Mexico.

#### Taxonomic Note.

This subgenus is listed in synonymy with *Carabosoma* Géhin by some authors (e.g., Lorenz 2005: 70).

### Calosoma marginale

Casey, 1897

Calosoma lugubre LeConte, 1853c: 400 [primary homonym of *Calosoma lugubre* Motschulsky, 1844]. Type locality: «New Braunfels [Comal County], Texas» (original citation). Holotype [by monotypy] (♂) in MCZ [# 626].
Calosoma marginalis Casey, 1897: 340. Type locality: «Arizona?» (original citation). Holotype [by monotypy] (♂) in USNM [# 37109]. Synonymy established by Breuning (1928a: 97).
Calosoma lecontei Csiki, 1927: 21. Replacement name for *Calosoma lugubre* LeConte, 1853.

#### Distribution.

This species ranges from “Iowa” (Jaques and Redlinger 1946: 295, as *Calosoma lugubre*) to southeastern Colorado (Michels et al. 2008), south to southern Mexico (Gidaspow 1959: 254-255) and eastern Arkansas (Arkansas County, Ken Karns pers. comm. 2009). Gidaspow (1963: 282) reported the presence of two specimens from Costa Rica. The records from “Illinois” (Bousquet and Larochelle 1993: 71) and northern Arizona (Wickham 1896a: 156) need confirmation. The specimen labeled from Duparquet in Quebec (Lindroth 1961a: 49) is almost certainly mislabeled.

#### Records.

**USA**: AR, CO, IA, KS, MO, NE, NM, OK, TX [AZ, IL] – Costa Rica, Mexico

### Calosoma parvicolle

Fall, 1910

Calosoma parvicollis Fall, 1910: 90. Type locality: «San Bernardino, Riverside and Pasadena, southern California» (original citation), restricted to «Pasadena [Los Angeles County]» by Gidaspow (1959: 256). Syntype(s) in MCZ [# 23843].
Calosoma clemens Casey, 1914: 32. Type locality: «Las Vegas [Clark County], Nevada» (original citation). One syntype in USNM [# 37111]. Synonymy established by Breuning (1928a: 95).
Calosoma pertinax Casey, 1920: 163. Type locality: «Albuquerque [Bernalillo County], New Mexico» (original citation). One syntype in USNM [# 37110]. Synonymy established by Breuning (1928a: 95).

#### Distribution.

This species is found from central California to eastern Utah, south to southwestern New Mexico, Sonora in Mexico, and Baja California (Gidaspow 1959: 256).

#### Records.

**USA**: AZ, CA, NM, NV, UT – Mexico

### Calosoma prominens

LeConte, 1853

Calosoma angulatum LeConte, 1852a: 199 [primary homonym of *Calosoma angulatum* Chevrolat, 1834]. Type locality: «circa Pimas [Graham County, Arizona]» (original citation). Holotype [by monotypy] (♂) in MCZ [# 624].
Calosoma prominens LeConte, 1853c: 400. Replacement name for *Calosoma angulatum* LeConte, 1852.

#### Distribution.

This species is found from Inyo County in eastern California (Riley 1893: 239; Fall 1901a: 40) to central New Mexico, south to Sonora and the Baja California Peninsula (Gidaspow 1959: 255).

#### Records.

**USA**: AZ, CA, NM – Mexico

### Subgenus Carabosoma

Géhin, 1885

Carabosoma Géhin, 1885: xxxii. Type species: *Calosoma glabratum* Dejean, 1831 designated by Breuning (1928a: 100). Etymology. From the generic name *Carabus* [*q.v*.] and the Greek *soma* (body), alluding to the resemblance of adults to those of some *Carabus* (“*forme rappelant celles de certains carabes*”) [neuter].
Acamegonia Lapouge, 1924: 38. Type species: *Acamegonia peregrinatrix incerta* Lapouge, 1924 (= *Calosoma eremicola* Fall, 1910) by monotypy. Synonymy established by Bousquet and Larochelle (1993: 72).

#### Diversity.

Western Hemisphere, with five species in the Nearctic (four species, one of them endemic) and Neotropical (four species, only one, *Calosoma glabratum*, endemic) Regions.

### [angulatum group]

### Calosoma angulatum

Chevrolat, 1834

Calosoma angulatum Chevrolat, 1834: [no. 44]. Type locality: «Bocadelmonte [Veracruz, Mexico]» (original citation). Holotype [by monotypy] in MHNP (Erwin 1991a: 26).
Calosoma angulicolle Chaudoir, 1869a: 377. Type locality: Santa Marta, Colombia (lectotype label). Lectotype (♀), designated by Erwin (1991a: 26), in MHNP. Synonymy established by Erwin (1991a: 26).
Calosoma angulicolle var. *uniforme* Géhin, 1885: 63. Type locality: «Mazatlan [Sinaloa, Mexico]» (original citation). Syntype(s) probably in MHNP (collection Oberthür). Synonymy established by Breuning (1928a: 101).
Calosoma forreri Géhin, 1885: 64. Type locality: «Arizona» (original citation). Syntype(s) in MHNP (Deuve 1978: 253). Synonymy established by Jeannel (1940: 203).

#### Distribution.

This species is found in Mexico south at least to Colombia and Venezuela (Erwin 1991a: 26). It is also occasionally found in southwestern United States from California to Texas (Gidaspow 1959: 253).

#### Records.

**USA**: AZ, CA, NM, TX – Colombia, Costa Rica, Guatemala, Honduras, Mexico, Nicaragua, Venezuela

### [peregrinator group]

### Calosoma eremicola

Fall, 1910

Calosoma eremicola Fall, 1910: 91. Type locality: «San Clemente Island [Los Angeles County], southern California» (original citation). Syntype(s) [2 originally cited] in MCZ [# 23842].
Calosoma rugosipennis Schaeffer, 1911: 113. Type locality: «California» (original citation). Holotype [by monotypy] (♂) location unknown. Synonymy established by Gidaspow (1959: 259).
Calosoma hospes Casey, 1913: 63. Type locality: «Coronado [San Diego County], near San Diego, California» (original citation). Two syntypes in USNM [# 37114]. Synonymy established by Jeannel (1940: 206).
Acamegonia peregrinatrix incerta Lapouge, 1924: 38. Type locality: «Basse Californie [= Baja California]» (original citation). Syntype(s) location unknown. Synonymy established by Gidaspow (1959: 259).

#### Distribution.

This species is found in southern California and northern Baja California (Gidaspow 1959: 259); it is also known from one locality in southwestern New Mexico (Gidaspow 1959: 259), from Montezuma County in Colorado (FFPC), and has been reported from Nevada by Erwin (2007a: 91).

#### Records.

**USA**: CA (CHI), CO, NM, NV – Mexico

### Calosoma peregrinator

Guérin-Méneville, 1844

Calosoma peregrinator Guérin-Méneville, 1844c: 255. Type locality: intérieur du Mexique (inferred from title of the paper), herein restricted to Guadalajara, Jalisco (see Gidaspow 1959: 258). Syntype(s) probably in MHNP.
Calosoma carbonatum LeConte, 1862: 53. Type locality: «New Mexico and upper Texas» (original citation), restricted to «New Mexico» by Gidaspow (1959: 257). Syntype(s) in MCZ [# 625]. Synonymy established by Horn (1883b: 270).
Calosoma peregrinator ingens Casey, 1913: 62. Type locality: «San Diego [San Diego County], California» (original citation). Two syntypes [2 originally cited] in USNM [# 37105]. Synonymy established by Breuning (1928a: 103), confirmed by Gidaspow (1959: 258).
Calosoma peregrinator amplipennis Casey, 1913: 62. Type locality: «probably New Mexico or southern Colorado» (original citation). Holotype [by monotypy] (♂) in USNM [# 37106]. Synonymy established by Breuning (1928a: 103), confirmed by Gidaspow (1959: 258).
Calosoma subgracilis Casey, 1913: 63. Type locality not stated. Holotype [by monotypy] (♂) in USNM [# 37107]. Synonymy established by Jeannel (1940: 204), confirmed by Gidaspow (1959: 259).
Calosoma apacheana Casey, 1913: 63. Type locality: «Arizona» (original citation). Holotype [by monotypy] (♂) in USNM [# 37108]. Synonymy established by Breuning (1928a: 103), confirmed by Gidaspow (1959: 258).

#### Distribution.

The range of this species extends from Sacramento County in central California (Clark 1999: 202) to western Kansas (Popenoe 1877: 22), south to the Federal District in Mexico and Baja California (Gidaspow 1959: 258).

#### Records.

**USA**: AZ, CA, CO, KS, NM, OK, TX, UT – Mexico

### Calosoma sponsa

Casey, 1897

Calosoma sponsa Casey, 1897: 340. Type locality: «Utah» (original citation). Holotype [by monotypy] (♂) in USNM [# 37113]. Note. It is obvious that Casey (1897) incorrectly treated *Calosoma* as a feminine name. Therefore the specific name could be a noun in apposition, from the Latin *sponsa*, -*ae* (betrothed woman, bride), or a Latin adjective, from *sponsus*, -*a*, -*um* (promised, engaged, betrothed). In such case, the name is to be treated as a noun in apposition (ICZN 1999: Article 31.2.2) unless the author indicated that he or she regarded the name as an adjective or the evidence of usage is decisive. Casey (1897: 340) did not indicate that the name was an adjective and the evidence of usage is not decisive since the name has been treated as an adjective (e.g., *Calosoma sponsum*) by some authors (e.g., Erwin et al. 1977: 4.4; Bousquet and Larochelle 1993: 72; Lorenz 2005: 70; Erwin 2007: 104) and as a noun in apposition (e.g., *Calosoma sponsa*) by others (e.g., Gidaspow 1959: 260; Culot 1988: 13). Consequently the name is to be treated as a noun in apposition.
Calosoma parviceps Casey, 1897: 341. Type locality: «Arizona» (original citation). One syntype in USNM [# 37112]. Synonymy established by Breuning (1928a: 103), confirmed by Gidaspow (1959: 260).

#### Distribution.

This species is known from western Utah (Millard County, Ken Karns pers. comm. 2009), western Nevada, southern California, and “Arizona” (Gidaspow 1959: 260).

#### Records.

**USA**: AZ, CA, NV, UT

### Subgenus Callitropa

Motschulsky, 1866

Callitropa Motschulsky, 1866: 300. Type species: *Carabus externus* Say, 1823 by monotypy. Etymology. Uncertain, possibly from the Greek prefix *callo*- (beautiful) and *tropos* (manner) [feminine].
Paratropa Lapouge, 1929b: 3. Type species: *Calosoma macrum* LeConte, 1853 designated by Jeannel (1940: 209). Synonymy established by Jeannel (1940: 209).

#### Diversity.

Three North American species, two of them extending into Mexico.

### Calosoma externum

(Say, 1823)

Carabus externus Say, 1823b: 150. Type locality: «Little Rock [Pulaski County], Ark[ansas]» (neotype label). Neotype (♀), designated by Lindroth and Freitag (1969: 331), in MCZ [# 33089]. Note. «Arkansa» was the area originally cited by Say (1823b: 150).
Calosoma longipenne Dejean, 1831: 568. Type locality: «Amérique septentrionale» (original citation). One syntype in MHNP (Lindroth 1955b: 12; Deuve 1978: 247). Synonymy established by LeConte (1847: 445), confirmed by Lindroth (1955b: 12).

#### Distribution.

This species ranges from “Vermont” (Ross T. Bell pers. comm. 1989; probably only as strays) to “Nebraska,” including southernmost Ontario (only as strays), south to “Texas” (Gidaspow 1959: 274) and southern Georgia (Fattig 1949: 10).

#### Records.

**CAN**: ON **USA**: AL, AR, CT, DC, DE, GA, IA, IL, IN, KS, KY, LA, MA, MD, MI, MN, MO, MS, NC, NE, NJ, NY, OH, OK, PA, SC, TN, TX, VA, VT, WI

### Calosoma macrum

LeConte, 1853

Calosoma macrum LeConte, 1853c: 400. Type locality: «Texas» (original citation), herein restricted to San Antonio, Bexar County (see Breuning 1928a: 118). Syntype(s) in MCZ [# 621].

#### Distribution.

This species is known from “Arkansas” (Jeannel 1940: 214) to “New Mexico” (Erwin 2007a: 98), south to northeastern Mexico (Gidaspow 1959: 274) and “Louisiana” (Burgess and Collins 1917: 25).

#### Records.

**USA**: AR, LA, NM, OK, TX – Mexico

### Calosoma protractum

LeConte, 1862

Calosoma protractum LeConte, 1862: 52. Type locality: «Arizona» (original citation). Syntype(s) in MCZ [# 622].
Calosoma dolens Chaudoir, 1869a: 376. Type locality: «près d’Oaxaca, Mexique» (original citation). Lectotype (♂), designated by Deuve (1978: 250), in MHNP. Synonymy established by Breuning (1928a: 118).
Calosoma truncatum Géhin, 1885: 64. Type locality: «Mexico» (original citation). Syntype(s) probably in MHNP (collection Oberthür). Synonymy established by Breuning (1928a: 118).

#### Distribution.

This species is known from “Colorado” (Gidaspow 1959: 275) and southeastern Arizona (Snow 1906b: 161) south at least to the state of Guerrero in Mexico (Gidaspow 1959: 275). The records from southern Kansas (Snow 1903: 192; Knaus 1905a: 218; Burgess and Collins 1917: 26) are doubtful (see Gidaspow 1959: 275).

#### Records.

**USA**: AZ, CO – Mexico

### Subgenus Blaptosoma

Géhin, 1876

Blaptosoma Géhin, 1876a: 45. Type species: *Calosoma laeve* Dejean, 1826 designated by Breuning (1928b: 43). Etymology. From the Greek *blapto* (hurt) and *soma* (body) [neuter].
Aulacopterum Géhin, 1885: xxxiv. Type species: *Calosoma viridisulcatum* Chaudoir, 1863 by original designation. Synonymy established by Breuning (1928b: 43).

#### Diversity.

Seven Mexican species, one of them extending into North America.

### Calosoma haydeni haydeni

Horn, 1870

Calosoma haydeni G.H. Horn, 1870a: 69. Type locality: «southern Colorado» (original citation). Syntype(s) in MCZ [# 34552]. Etymology. This species was proposed for Ferdinand Vandeveer Hayden [1828-1887], American geologist, explorer, teacher, and physician. Hayden played the leading role in 1871 and 1872 for the establishment of Yellowstone National Park, the first national “public park or pleasuring-ground for the benefit and enjoyment of the people.”

#### Distribution.

This subspecies is known from southern Colorado (Wickham 1902: 231), northwestern Arizona, “New Mexico,” Brewster County in western Texas (Gidaspow 1959: 281), and Chihuahua in Mexico (Erwin 2007a: 96).

#### Records.

**USA**: AZ, CO, NM, TX – Mexico

### Calosoma haydeni punctulicolle

Bates, 1891

Calosoma laeve var. *punctulicolle* Bates, 1891a: 225. Type locality: «Santa Clara, in Chihuahua; Durango city; Monterey, in Nuevo Leon» (original citation). Syntype(s) probably in BMNH.

#### Distribution.

This subspecies is known from Mexico as far south as the Federal District and from Brewster County in western Texas (Gidaspow 1959: 281).

#### Records.

**USA**: TX – Mexico

### Subgenus Chrysostigma

Kirby, 1837

Chrysostigma Kirby, 1837: 19. Type species: *Carabus calidus* Fabricius, 1775 designated by Hope (1838: 47). Etymology. From the Greek *chrysos* (gold) and *stigma* (mark, spot), alluding to the conspicuous gilded elytral punctures (“elytra obscure with gilded punctiform impressions”) of the adult [neuter].
Tapinosthenes Kolbe, 1895: 56. Type species: *Calosoma cancellatum* Eschscholtz, 1833 by monotypy. Synonymy established by Jeannel (1940: 161). Etymology. From the Greek *tapeinos* (low, humble) and *sthenos* (strength) [masculine].
Lyperostenia Lapouge, 1929b: 3. Type species: *Calosoma triste* LeConte, 1845 (= *Calosoma affine* Chaudoir, 1843) by subsequent monotypy in Lapouge (1931: 382). Synonymy established by Gidaspow (1959: 260). Etymology. From the Greek *lyperos* (painful, by extension sad) and the Greek *stenos* (narrow) [feminine].

#### Diversity.

Western Hemisphere, with ten species in North America (nine species) and Middle America (four species, only one, *Chrysostigma ampliator* Bates, being endemic).

### Calosoma affine

Chaudoir, 1843

Calosoma affine Chaudoir, 1843b: 746. Type locality: «Mexique» (original citation), herein restricted to Villa Lerdo, Durango (see Gidaspow 1959: 269). Syntype(s) in MHNP (Deuve 1978: 250).
Calosoma triste LeConte, 1845a: 201. Type locality: «Missouri [Territory]» (original citation). Syntype(s) in MCZ [# 627]. Synonymy established by Jeannel (1940: 169).
Calosoma tristoides Fall, 1910: 92. Type locality: «at or near San Diego [San Diego County], California» (original citation). Syntype(s) in MCZ [# 23844]. Synonymy established by Jeannel (1940: 169).

#### Distribution.

This species ranges from southern Minnesota (Gandhi et al. 2005: 922) to the Pacific Coast in southern California, south to the state of Oaxaca in Mexico (Gidaspow 1959: 269). The records from Missouri (probably based on the type locality of *Calosoma triste*) and “Arkansas” (Burgess and Collins 1917: 87), possibly based on a stray, need confirmation.

#### Records.

**USA**: AZ, CA, CO, KS, MN, NE, NM, NV, OK, TX, UT [AR, MO] – Mexico

### Calosoma calidum

(Fabricius, 1775)

Carabus calidus Fabricius, 1775: 237. Type locality: «America» (original citation), restricted to «Rumney [Grafton County], N[ew] H[ampshire]» by Lindroth (1961a: 50). Lectotype (♂), designated by Lindroth (1961a: 50), in ZMUC.
Calosoma calida stellata Casey, 1897: 344. Type locality: «Lake Superior» (original citation). One syntype in USNM [# 37088]. Synonymy established by Gidaspow (1959: 265).
Calosoma calida expansa Casey, 1897: 344. Type locality: «Keokuk [Lee County], Iowa» (original citation). One syntype in USNM [# 37087]. Synonymy established by Casey (1913: 60), confirmed by Lindroth (1961a: 50).
Calosoma calida laticollis Casey, 1897: 344. Type locality: «Las Vegas [San Miguel County], New Mexico» (original citation). One syntype in USNM [# 37089]. Synonymy established by Breuning (1928a: 84), confirmed by Lindroth (1961a: 50).
Calosoma comes Casey, 1920: 156. Type locality: «Northwest Territory» (original citation). Holotype [by monotypy] (♂) in USNM [# 37090]. Synonymy established by Breuning (1928a: 84), confirmed by Lindroth (1961a: 50).
Calosoma concreta Casey, 1920: 157. Type locality: «apparently north of Lake Superior» (original citation). Holotype [by monotypy] (♂) in USNM [# 37091]. Synonymy established, under the name *Calosoma calidum stellatum* Casey, by Breuning (1928a: 84), confirmed by Lindroth (1961a: 50).
Chrysostigma lepidum ocellatum Lapouge, 1931: 381. Type locality: «Canada méridional, Etats-Unis» (original citation). Syntype(s) probably in MCZ, BMNH, and MHNP. Synonymy established with the name *Calosoma expansum* Casey by Lapouge (1931: 381). Note. This name was proposed for *Calosoma calidum* (Fabricius, 1775) *sensu* Dejean (1826: 197), Kirby (1837: 19), LeConte (1878e: 65), Burgess and Collins (1917: 98) and Breuning (1928a: 84).

#### Distribution.

The range of this species extends from Newfoundland (Larson and Langor 1982: 592) and Saint Pierre and Miquelon (Lindroth 1955a: 28) to eastern British Columbia (Lindroth 1961a: 51), south to “Oregon” (Hatch 1953: 52), northeastern New Mexico (Casey 1897: 344), “Kansas” (Horn 1872c: 384), east-central Missouri (Summers 1873: 133), and northern Georgia (Fattig 1949: 11); several specimens have also been caught along the Slave and Mackenzie Rivers, up to 65°30'N, in Northwest Territories (White 1851: 357; Lindroth 1961a: 51). The records from southeastern Louisiana (Summers 1874a: 79), “Alabama,” “Arkansas,” and “Mississippi” (Bousquet and Larochelle 1993: 72) are probably in error.

#### Records.

**FRA**: PM **CAN**: AB, BC, MB, NB, NS (CBI), NT, ON, QC, SK **USA**: CO, CT, DC, DE, GA, IA, IL, IN, KS, KY, MA, MD, ME, MI, MN, MO, MT, NC, ND, NE, NH, NJ, NM, NY, OH, OR, PA, RI, SD, TN, UT, VA, VT, WA, WI, WV, WY

#### Note.

Gidaspow (1959: 266) treated *Calosoma concretum* Casey as a valid species. However, both Lindroth (1961a: 50) and Obydov (2003: 534) retained the name as synonym of *Calosoma calidum* (Fabricius).

### Calosoma cancellatum

Eschscholtz, 1833

Calosoma cancellatum Eschscholtz, 1833: 23. Type locality: «bei St. Franzisco [San Francisco County], Californien» (original citation). Syntype(s) location unknown (possibly in ZMMU).
Calosoma aenescens LeConte, 1854a: 16. Type locality: «Fort Vancouver» (original citation). Syntype(s) in MCZ [# 632]. Synonymy established by LeConte (1857c: 30). Note. Fort Vancouver was a massive British outpost on the north bank of the Columbia River, slightly upstream from the mouth of the Willamette River, in Washington.
Calosoma esuriens Casey, 1913: 64. Type locality: «near San Diego [San Diego County], California» (original citation). One syntype in USNM [# 37094]. Synonymy established by Breuning (1928a: 90).
Calosoma transversa Casey, 1913: 65. Type locality: «near San Diego [San Diego County], California» (original citation). One syntype in USNM [# 37095]. Synonymy established by Breuning (1928a: 90).
Calosoma sagax Casey, 1920: 158. Type locality: «Lassen Co[unty], California» (original citation). One syntype in USNM [# 37096]. Synonymy established by Breuning (1928a: 90).
Calosoma rectilatera Casey, 1920: 158. Type locality: «Palm Spring [Riverside County], California» (original citation). One syntype in USNM [# 37097]. Synonymy established by Breuning (1928a: 91).
Calosoma praestans Casey, 1920: 159. Type locality: «Butte Co[unty], California» (original citation). One syntype in USNM [# 37098]. Synonymy established by Breuning (1928a: 91).

#### Distribution.

This species ranges from south-central British Columbia (Lindroth 1961a: 53) to north-central North Dakota (McHenry County, Foster F. Purrington pers. comm. 2009), south to “Arizona” (Gidaspow 1959: 263) and southern California along the coast (Casey 1913: 64-65, as *Calosoma esuriens* and *Calosoma transversa*). The record from “Indian Territory” (= Oklahoma) (Burgess and Collins 1917: 111) is probably in error.

#### Records.

**CAN**: BC **USA**: AZ, CA, ID, MT, ND, NV, OR, UT, WA

### Calosoma lepidum

LeConte, 1845

Calosoma lepidum LeConte, 1845a: 201. Type locality: «Missouri [Territory]» (original citation), cited from «ad flumen Yellow-stone» by LeConte (1847: 446). Syntype(s) in MCZ [# 630].

#### Distribution.

This species inhabits the Great Plains ranging from the southern parts of the Prairie Provinces south to Montana (Gidaspow 1959: 265; Lindroth 1961a: 51) and north-central South Dakota (Walworth County, CNC). The record from “Wyoming” (Bousquet and Larochelle 1993: 73) needs confirmation.

#### Records.

**CAN**: AB, MB, SK **USA**: MT, ND, SD [WY]

### Calosoma morrisonii

Horn, 1885

Calosoma morrisonii G.H. Horn, 1885a: 128. Type locality: «Colorado» (original citation). Syntype(s) in MCZ [# 35318] and ZMUA (Boer 2002: 79). Etymology. The specific name honors Herbert Knowles Morrison [1854-1885] who became a professional insect collector in the 1870s. Morrison travelled across the United States and sometimes walked 40 miles a day in pursuit of insects (Sorensen 1995: 37).
Calosoma mexicanum Géhin, 1885: 67. Type locality: «Mexique» (original citation). Syntype(s) in MHNP (Deuve 1978: 252). Synonymy established by Gidaspow (1959: 270). Note. Bruschi (2010), who saw a syntype of *Calosoma mexicanum* Géhin, believed the specimen is very similar to, and probably conspecific with, those of *Calosoma calidum* (Fabricius). If this is correct, then the provenance indicated by Géhin (1885: 67) is incorrect since *Calosoma calidum* is not found in Mexico.

#### Distribution.

This species is known from southern California to “Colorado,” south to Durango in western Mexico (Gidaspow 1959: 270).

#### Records.

**USA**: CA, CO, NM, NV – Mexico

### Calosoma obsoletum

Say, 1823

Calosoma obsoleta Say, 1823b: 149. Type locality: «F[or]t Reynolds [Pueblo County], Colo[rado]» (neotype label). Neotype (♀), designated by Lindroth and Freitag (1969: 331), in MCZ [# 33088]. Note. «Arkansa» was the area originally cited by Say (1823b: 150).
Calosoma indistinctum LeConte, 1845b: 208. Type locality: United States of America (inferred from title of the paper). Syntype(s) probably in MCZ. Synonymy established by Breuning (1928a: 110). Note. LeConte’s collection contains six specimens under the name *Calosoma obsoletum*. Any or all of them could be syntypes.
Calosoma obsoleta microsticta Casey, 1897: 345. Type locality: «Fort Wingate [McKinley County], New Mexico; Kansas» (original citation). Two syntypes [2 ♂ originally cited] in USNM [# 37104]. Synonymy established by Breuning (1928a: 110). Note. Gidaspow (1959: 267) pointed out that of the two specimens in Casey’s collection (USNM) under this name, one belongs to *Calosoma lepidum* LeConte, the other to *Calosoma obsoletum* Say. Until a lectotype is designated, the name *Calosoma microsticta* Casey is listed as a junior synonym of *Calosoma obsoletum* Say following Breuning (1928a: 110).

#### Distribution.

This species ranges from Saskatchewan (Lindroth 1961a: 50) to eastern Oregon, south to northeastern Nevada (La Rivers 1947: 136), northwestern New Mexico (Casey 1897: 345, as *Calosoma obsoleta microsticta*; McKinley County, UASM) and southwestern Oklahoma (Kondratieff et al. 2005: 171), east to eastern Minnesota (Gandhi et al. 2005: 922). The record from “Texas” (Burgess and Collins 1917: 87) needs confirmation.

#### Records.

**CAN**: AB, MB, SK **USA**: CO, IA, ID, KS, MN, MT, ND, NE, NM, NV, OK, OR, SD, UT, WY [TX]

### Calosoma semilaeve

LeConte, 1852

Calosoma semilaeve LeConte, 1852a: 199. Type locality: «San Jose; San Diego [California]» (original citation), restricted to «San Diego [San Diego County]» by Gidaspow (1959: 271). Syntype(s) in MCZ [# 628].
Calosoma semilaevis davidsoni Casey, 1914: 33. Type locality: «Alameda Co[unty], California» (original citation). Two syntypes in USNM [# 37116]. Synonymy established by Breuning (1928a: 111). Etymology. The subspecific name was proposed for George Davidson [1825-1911], surveyor, geodesist, and astronomer. Born in England, Davidson was put in charge of the survey on the west coast in 1850 soon after the United States took over California from Mexico. The Davidson Seamount off the coast of California and Mount Davidson and Davidson Street in San Francisco are named for him.
Calosoma semilaevis adjutor Casey, 1920: 162. Type locality: «Alameda [Alameda County], California» (original citation). One syntype in USNM [# 37115]. Synonymy established by Breuning (1928a: 111).

#### Distribution.

This species ranges from eastern Oregon and “Idaho” (Gidaspow 1959: 271) south to southern Arizona (Snow 1907: 141) and southern California along the coast (Fall 1901a: 40; Moore 1937: 4); also found on Guadalupe Island, Mexico (Gidaspow 1959: 271). The record from northeastern Kansas (Popenoe 1877: 22) is likely in error.

#### Records.

**USA**: AZ, CA (CHI), ID, OR, UT – Mexico

### Calosoma simplex

LeConte, 1878

Calosoma simplex LeConte, 1878d: 61. Type locality: «middle California» (original citation), herein restricted to Pinoche Hill, Merced County (see Gidaspow 1959: 272). Holotype [by monotypy] (♂) in MCZ [# 629].

#### Distribution.

This species is confined to California where it is known from Yolo County to Riverside (Gidaspow 1959: 272) and San Diego Counties (Moore 1937: 4). The records from “Arizona,” “Colorado,” “Texas,” and “Mexico” (Burgess and Collins 1917: 93) are probably in error.

#### Records.

**USA**: CA

### Calosoma tepidum

LeConte, 1852

Calosoma tepidum LeConte, 1852a: 199. Type locality: «Oregon» (original citation), herein restricted to Oregon City, Clackamas County (see Breuning 1928a: 89). Syntype(s) in MCZ [# 631].
Calosoma irregulare Walker, 1866: 312. Type locality: British Columbia (inferred from title of the book). Syntype(s) location unknown (possibly in BMNH). Synonymy established by Horn (1870a: 70).
Calosoma tepida caelator Casey, 1913: 61. Type locality: «Coeur d’Alene [Kootenai County], Idaho» (original citation). Holotype [by monotypy] (♂) in USNM [# 37101]. Synonymy established by Breuning (1928a: 88), confirmed by Lindroth (1961a: 51).
Calosoma tepida indigens Casey, 1913: 61. Type locality: «Oregon» (original citation). Two syntypes in USNM [# 37099]. Synonymy established by Breuning (1928a: 88), confirmed by Lindroth (1961a: 51).
Calosoma pellax Casey, 1920: 160. Type locality: «probably Oregon or adjacent region» (original citation). Holotype [by monotypy] (♂) in USNM [# 37100]. Synonymy established by Breuning (1928a: 88), confirmed by Lindroth (1961a: 51).
Calosoma semicuprea Casey, 1920: 161. Type locality: «probably northern Rocky Mountain region» (original citation). Holotype [by monotypy] (♂) in USNM [# 37102]. Synonymy established by Breuning (1928a: 88), confirmed by Lindroth (1961a: 51).
Calosoma cogitans Casey, 1920: 161. Type locality: «Stockton [Tooele County], Utah» (original citation). Lectotype (♀), designated by Lindroth (1975: 111), in USNM [# 37103]. Synonymy established by Breuning (1928a: 88), confirmed by Lindroth (1961a: 52).

#### Distribution.

This species is found from Vancouver Island (Lindroth 1961a: 52) to “Nebraska,” south to “Colorado” (Gidaspow 1959: 264) and southern California (La Rivers 1947: 136). The records from “North Dakota” (Bousquet and Larochelle 1993: 73) and “Alberta” (Burgess and Collins 1917: 107) need confirmation.

#### Records.

**CAN**: BC (VCI) **USA**: AZ, CA, CO, ID, MT, NE, NV, OR, UT, WA, WY [AB, ND]

#### Note.

Obydov (2003: 536), based on a study of two specimens only, concluded that *Calosoma cogitans* Casey represents a valid subspecies of *Calosoma tepidum* LeConte. I agree with Gidaspow (1959: 263) and Lindroth (1961a: 52) that the name does not apply to a distinct taxon.

### Subgenus Callistenia

Lapouge, 1929

Callistenia Lapouge, 1929b: 2. Type species: *Calosoma moniliatum* LeConte, 1852 designated by Jeannel (1940: 170). Etymology. Uncertain, possibly from the Greek *callos* (beauty) and *stenos* (narrow) [feminine].
Isostenia Lapouge, 1929b: 2. Type species: *Calosoma wilkesii* LeConte, 1852 by subsequent monotypy in Lapouge (1931: 380). Synonymy established by Jeannel (1940: 170).

#### Diversity.

Fourteen North American species in the western half of the continent.

### Calosoma dawsoni

(Dajoz, 1997), new combination

Callisthenes dawsoni Dajoz, 1997a: 70. Type locality: «proximité du Big Alkali Lake (2100 mètres) au voisinage de la route de Mammoth Lakes à Benton, Mono County, Californie» (original citation). Holotype probably in Dajoz’s collection (Paris, France).

#### Distribution.

This species is known only from the original 16 specimens collected at the type locality in the Sierra Nevada.

#### Records.

**USA**: CA

### Calosoma dietzii

Schaeffer, 1904

Calosoma dietzii Schaeffer, 1904: 197. Type locality: «Tulare Co[unty], California» (original citation). Syntype(s) [4 originally cited] location unknown. Etymology. The specific name was proposed for Ottomar Dietz [1854-1901], an enthusiastic beetle collector. Born in Germany, Dietz moved to America in his 20s, living in Milwaukee and Cincinnati before settling in New York where he was engaged in the newspaper advertising business. He was a founding member of the New York Entomological Society.
Callisthenes gravidulus Casey, 1913: 69. Type locality: «Sequoia National Park (4600’), Tulare Co[unty], California» (original citation). Holotype [by monotypy] (♂) in USNM [# 37120]. Synonymy established by Breuning (1928b: 79).

#### Distribution.

This species has been recorded so far from the Sierra Nevada in Tulare County and “South Fork” in Humboldt County, California (Gidaspow 1959: 308).

#### Records.

**USA**: CA

### Calosoma discors

LeConte, 1857

Calosoma discors LeConte, 1857c: 31. Type locality: «San Francisco; Sacramento [California]» (original citation), restricted to «Sacramento [Sacramento County]» by Gidaspow (1959: 308). Syntype(s) in MCZ [# 634].
Callisthenes discors inversus Casey, 1913: 67. Type locality: «San Francisco [San Francisco County], California» (original citation). Holotype [by monotypy] (♀) in USNM [# 37117]. Synonymy established by Breuning (1928b: 79).

#### Distribution.

This species is found in California from El Dorado and Sacramento Counties to Santa Cruz and Madera Counties (Gidaspow 1959: 308); it was cited also from Washington (Burgess and Collins 1917: 119) but the record is probably in error or based on a stray.

#### Records.

**USA**: CA

### Calosoma lariversi

Van Dyke, 1943

Calosoma lariversi Van Dyke, 1943: 17. Type locality: «near Lamoille [Elko County], Nevada» (original citation). Holotype (♂) in CAS [# 5294]. Etymology. The specific name honors Ira John La Rivers, II [1915-1977], professor of biology at the University of Nevada in Reno. La Rivers was a naturalist and published on many living groups including beetles, true bugs, ants, fishes, and algae.

#### Distribution.

According to La Rivers (1947: 137), this species is “apparently the predominant *Calosoma* of eastern Nevada, and a marked montane isolate.”

#### Records.

**USA**: NV

### Calosoma latipenne

Horn, 1870

Calosoma latipenne G.H. Horn, 1870a: 70. Type locality: «elevated regions of the South Sierras of California» (original citation). Syntype(s) in MCZ [# 8125].
Calosoma arcuata Casey, 1897: 343. Type locality: «Arizona» (original citation), which may be in error (Gidaspow 1959: 307). One syntype in USNM [# 37121]. Synonymy established with doubt, under the name *Calosoma subaeneum opimum* (Casey), by Breuning (1928b: 81), confirmed by Gidaspow (1959: 306).
Callisthenes tularensis Casey, 1913: 68. Type locality: «Tulare Co[unty], California» (original citation). One syntype in USNM [# 37119]. Synonymy established by Gidaspow (1959: 306).
Callisthenes opimus Casey, 1913: 69. Type locality: «Kern Co[unty], California» (original citation). Two syntypes in USNM [# 37118]. Synonymy established by Gidaspow (1959: 306).

#### Distribution.

This species is found in California from Sacramento County to Los Angeles and San Bernardino Counties (Gidaspow 1959: 307); according to Burgess and Collins (1917: 122), it was also collected in Reno, western Nevada.

#### Records.

**USA**: CA, NV

### Calosoma luxatum

Say, 1823

Calosoma luxata Say, 1823b: 149. Type locality: «Douglas Spring, Routt Co[unty], Colo[rado]» (neotype label). Neotype (♂), designated by Lindroth and Freitag (1969: 331), in MCZ [# 33087]. Note. «Arkansa [probably the Arkansas River since Say added “found near the Rocky mountains”]» was the area originally cited by Say (1823b: 150).
Carabus zimmermani LeConte, 1847: 445. Type locality: «Rocky Mountains» (original citation). Holotype [by monotypy] in MCZ [# 638]. Synonymy established by Jeannel (1940: 175). Etymology. The species name honors Christian Zimmermann [1800-1867], an accomplished entomologist. Born in Germany, Zimmermann immigrated to the United States at the age of 31 and eventually settled in South Carolina where he divided his time between collecting trips along the East Coast and working on his collection at home. His collection was bought by Dr. Lewis of Philadelphia and from him by George Robert Crotch who sold it to the MCZ. According to Hagen (1889: 57), “a great part [of Zimmermann’s specimens] is in Leconte’s collection, and can be recognized at once by the number on the pins in Zimmermann’s hand-writing.”
Calosoma striatulum LeConte, 1859c: 4 [primary homonym of *Calosoma striatulum* Chevrolat, 1835]. Type locality: «Milk river [probably in Montana]; Utah» (original citation). Syntype(s) in MCZ [# 639]. Synonymy established by Burgess and Collins (1917: 120), confirmed by Lindroth (1961a: 54).
Callisthenes pimelioides Walker, 1866: 312. Type locality: British Columbia (inferred from title of the book), restricted to «Oliver» by Lindroth (1961a: 54). At least one syntype in BMNH (Lindroth 1961a: 55). Synonymy established, under the name *Callisthenes zimmermani* LeConte, by LeConte (1870: 399) and Horn (1870a: 70), confirmed by Lindroth (1961a: 54).
Callisthenes luxatus var. *opacus* Géhin, 1885: 70. Type locality: «Orégon» (original citation). Syntype(s) in MHNP (Deuve 1978: 252). Synonymy established, under the name *Callisthenes luxatum zimmermanni* LeConte, by Breuning (1928b: 83).
Callisthenes exaratus Casey, 1913: 72. Type locality: «Placer Co[unty], California» (original citation). Three syntypes [3 originally cited] in USNM [# 37132]. Synonymy established, under the name *Callisthenes luxatum zimmermanni* LeConte, by Breuning (1928b: 84).
Callisthenes tegulatus Casey, 1913: 72. Type locality: «California» (original citation). One syntype in USNM [# 37128]. Synonymy established, under the name *Callisthenes luxatum zimmermanni* LeConte, by Breuning (1928b: 83).
Callisthenes tegulatus viator Casey, 1913: 72. Type locality: «California» (original citation). One syntype in USNM [# 37129]. Synonymy established, under the name *Callisthenes luxatum zimmermanni* LeConte, by Breuning (1928b: 83).
Callisthenes pustulosus Casey, 1913: 73. Type locality: «Yreka [Siskiyou County], California» (original citation). One syntype in USNM [# 37127]. Synonymy established, under the name *Callisthenes luxatum zimmermanni* LeConte, by Breuning (1928b: 84).
Callisthenes diffractus Casey, 1913: 75. Type locality: «Coolidge [McKinley County], New Mexico» (original citation). One syntype in USNM [# 37126]. Synonymy established, under the name *Callisthenes luxatum zimmermanni* LeConte, by Breuning (1928b: 84).
Callisthenes reflexus Casey, 1920: 164. Type locality: «northern Rocky Mountain region» (original citation). One syntype in USNM [# 37130]. Synonymy established, under the name *Callisthenes luxatum zimmermanni* LeConte, by Breuning (1928b: 84).
Callisthenes utensis Casey, 1920: 165. Type locality: «Stockton [Tooele County], Utah» (original citation). One syntype in USNM [# 37131]. Synonymy established, under the name *Callisthenes luxatum zimmermanni* LeConte, by Breuning (1928b: 84).
Callisthenes semotus Casey, 1920: 166. Type locality: «Stockton [Tooele County], Utah» (original citation). One syntype in USNM [# 37133]. Synonymy established, under the name *Callisthenes luxatum zimmermanni* LeConte, by Breuning (1928b: 84).
Callisthenes debilis Casey, 1920: 167. Type locality: «Oregon» (original citation). One syntype in USNM [# 37134] and one in SIM (Hennessey 1990: 466). Synonymy established, under the name *Callisthenes luxatum zimmermanni* LeConte, by Breuning (1928b: 84).
Callisthenes parowanus Casey, 1920: 167. Type locality: «Parowan [Iron County], Utah» (original citation). One syntype in USNM [# 37136]. Synonymy established, under the name *Callisthenes luxatum zimmermanni* LeConte, by Breuning (1928b: 84).
Calosoma striata Breuning, 1928b: 86. Replacement name for *Calosoma striatulum* LeConte, 1859.
Calosoma zimmermanni tahoensis Van Dyke, 1943: 18. Type locality: «slopes of M[oun]t Tallac, near Lake Tahoe [Placer County], California» (original citation). Holotype (♂) in CAS [# 5296]. Synonymy established, under the name *Calosoma zimmermanni* LeConte, by Gidaspow (1959: 318).

#### Distribution.

This species ranges from southern Saskatchewan to the Okanagan Valley in south-central British Columbia (Lindroth 1961a: 56), south to southeastern California, northwestern New Mexico (Casey, 1913: 75, as *Callisthenes diffractus*), and northern Kansas [see Gidaspow 1959: Fig. 11, as *Callisthenes luxatus*, *Callisthenes pimelioides*, and *Callisthenes zimmermanni*]. At least one specimen simply labeled from New Mexico is known (Gidaspow 1959: 311). The records from “Oklahoma” and “Texas” (Burgess and Collins 1917: 121, as *Callisthenes zimmermani*) need confirmation.

#### Records.

**CAN**: AB, BC, SK **USA**: AZ, CA, CO, ID, KS, MT, NE, NM, NV, OR, SD, UT, WA, WY [OK, TX]

#### Note.

Gidaspow (1959) considered *Callisthenes pimeloides* Walker [synonyms: *tegulatus* Casey, *viator* Casey, *pustulosus* Casey, *reflexus* Casey, and *parowanus* Casey] and *Carabus zimmermanni* LeConte [synonyms: *opacus* Géhin, *exaratus* Casey, *debilis* Casey, and *tahoensis* Van Dyke] as distinct species but Lindroth (1961a: 55) argued that the structural differences noted between these forms are no more than intraspecific variation.

### Calosoma moniliatum

(LeConte, 1852)

Callisthenes moniliatus LeConte, 1852a: 200. Type locality: «Oregon» (original citation). Syntype(s) in MCZ [# 633].
Calosoma laqueatum LeConte, 1860: 318. Type locality: «Saskatchewan» (original citation). Syntype(s) in MCZ [# 637]. Synonymy established by LeConte (1878e: 66).
Carabus bicolor Walker, 1866: 313 [primary homonym of *Carabus bicolor* Drury, 1773]. Type locality: British Columbia (inferred from title of the book). Syntype(s) in BMNH. Synonymy established, under the name *Carabus laqueatum* LeConte, by LeConte (1870: 399), confirmed by Lindroth (1961a: 53).
Callisthenes concinnus Casey, 1913: 66. Type locality: «Priest Lake [Bonner County], Idaho» (original citation). Lectotype (♂), designated by Lindroth (1975: 111), in USNM [# 37135]. Synonymy established by Jeannel (1940: 177).
Carabus taedatus var. *vancouvericus* Csiki, 1927: 286. Replacement name for *Carabus taedatus* var. *bicolor* Walker, 1866.

#### Distribution.

This species ranges from Saskatchewan to southeastern British Columbia (Lindroth 1961a: 53-54), south to “California,” “Arizona,” and “Nebraska” (Gidaspow 1959: 305). The records from “Vancouver Island” (Gidaspow 1959: 305) and “Northwest Territory, Canada” (Burgess and Collins 1917: 114) are probably in error.

#### Records.

**CAN**: AB, BC, SK **USA**: AZ, CA, ID, MT, NE, OR, WA

### Calosoma monticola

Casey, 1897

Calosoma monticola Casey, 1897: 342. Type locality: «Reno [Washoe County], Nevada» (original citation). One syntype in USNM [# 37124].
Callisthenes nevadensis Casey, 1913: 74. Type locality: «near Reno [Washoe County], Nevada» (original citation). One syntype in USNM [# 37125]. Synonymy established by Gidaspow (1959: 314).

#### Distribution.

The range of this species extends from central Washington south at least to El Dorado County in California and western Nevada (Gidaspow 1959: 315); also recorded from “Utah” (Erwin 2007a: 79). One specimen simply labeled from Wyoming is known (Gidaspow 1959: 315). Notwithstanding Gidaspow (1959: 315), Hatch (1953: 54) did not record this species from Washington, Idaho, Oregon, and British Columbia and so the records from “Oregon” and “Idaho” (Bousquet and Larochelle 1993: 74) need confirmation.

#### Records.

**USA**: CA, NV, UT, WA [ID, OR, WY]

### Calosoma oregonum

(Gidaspow, 1959)

Callisthenes oregonus Gidaspow, 1959: 317. Type species: «Oregon» (original citation). Holotype (♂) in SIM.

#### Distribution.

This species is known from four specimens without locality data from “Oregon” (Gidaspow 1959: 317).

#### Records.

**USA**: OR

### Calosoma placerum

(Gidaspow, 1959)

Callisthenes placerus Gidaspow, 1959: 309. Type locality: «Forest Hill, Placer County, California» (original citation). Holotype (♂) in CAS [# 8516].

#### Distribution.

This species is confined to California where it is found as far south as San Diego County along the coast and Tuolumne County in the Sierra Nevada (Gidaspow 1959: 310).

#### Records.

**USA**: CA

### Calosoma schaefferi

Breuning, 1928

Calosoma irregulare Schaeffer, 1915b: 235 [primary homonym of *Calosoma irregulare* Walker, 1866 and *Calosoma irregulare* Reitter, 1902]. Type locality: «Castella [Siskiyou County], California» (original citation). Holotype (♂) in USNM [# 42495].
Calosoma discors schaefferi Breuning, 1928b: 79. Replacement name for *Calosoma discors irregulare* Schaeffer, 1915.
Calosoma striatius Hatch, 1953: 54. Type locality: «Spencer’s Butte, Eugene [Lane County], Oregon» (original citation). Holotype (♂) in USNM. Synonymy established by Erwin (2007a: 81).

#### Distribution.

This species ranges from western Oregon (Hatch 1953: 54, as *Calosoma striatus*) to Santa Cruz County in California (Gidaspow 1959: 309) along the Coast Ranges.

#### Records.

**USA**: CA, OR

### Calosoma subaeneum

Chaudoir, 1869

Calosoma subaeneum Chaudoir, 1869b: 28. Type locality: «Californie» (original citation). Holotype [by monotypy] (♂) in MHNP (Deuve 1978: 250).

#### Distribution.

This species is known from “Washington,” “Idaho,” and “California” (Burgess and Collins 1917: 114; Gidaspow 1959: 305). The records from British Columbia (Hatch 1953: 53; Gidaspow 1959: 305) probably refer to *Calosoma cancellatum* Eschscholtz as pointed out by Lindroth (1961a: 53).

#### Records.

**USA**: CA, ID, WA

### Calosoma subasperatum

Schaeffer, 1915

Calosoma subasperatum Schaeffer, 1915b: 235. Type locality: «California» (original citation), herein restricted to Dorris, Siskiyou County (see Gidaspow 1959: 316). Holotype location unknown.
Callisthenes klamathensis Casey, 1920: 169. Type locality: «Klamath Co[unty], Oregon» (original citation). One syntype in USNM [# 37123]. Synonymy established by Gidaspow (1959: 315).

#### Distribution.

This rarely collected species is known from Klamath and Harney Counties in southern Oregon and Siskiyou County in northern California (Gidaspow 1959: 316); also recorded from “Nevada” (Erwin 2007a: 82).

#### Records.

**USA**: CA, NV, OR

### Calosoma wilkesii

(LeConte, 1852)

Callisthenes wilkesii LeConte, 1852a: 200. Type locality: «Oregon» (original citation). Syntype(s) in MCZ [# 635]. Etymology. The specific name honors Charles Wilkes [1798-1877], American naval officer and explorer who commanded the United States Exploring Expedition (1838-1842), commonly known as the Wilkes Expedition. The expedition included naturalists who brought back entomological specimens.

#### Distribution.

This species is found west of the Rocky Mountains from south-central British Columbia south to “California” (Lindroth 1961a: 54).

#### Records.

**CAN**: BC **USA**: CA, ID, OR, WA

### Genus Carabus

Linnaeus, 1758

Carabus Linnaeus, 1758: 413. Type species: *Carabus granulatus* Linnaeus, 1758 (ICZN 1954). Etymology. From the Greek *carabos* (an animal in Aristotle) [masculine]. The animal in question, named *Locusta* in Latin, is uncertain. According to Camus (1783: 259), *carabos* was a grasshopper, to Cuvier (1803: 369, 370) a crawfish, and to Latreille (1812: 142) a Cerambycidae. Olivier (1795: [35] 1) reported that the name *Carabus* came from the scientific name *Scarabaeus* slightly modified.

#### Diversity.

About 910 species (Lorenz 2005: 72-114) in North America (14 species), Mexico (two species), and the Palaearctic (about 895 species) and Oriental (about five species) Regions.

#### Identification.

Van Dyke (1944) reviewed the North American species and provided a key for the identification of all species except *Carabus auratus*. Lindroth (1961a: 30-42) covered all but two species (*Carabus finitimus* and *Carabus forreri*).

### Subgenus Carabus

Linnaeus, 1758

Carabus Linnaeus, 1758: 413. Type species: *Carabus granulatus* Linnaeus, 1758 (ICZN 1954).
Lichnocarabus Reitter, 1896: 161. Type species: *Tachypus vinctus* Weber, 1801 designated by Deuve (1991: 29). Etymology. From the Greek *lichnos* (greedy, dainty) and the generic name *Carabus* [*q.v*.] [masculine].
Paracarabus Lapouge, 1930: 263 [junior homonym of *Paracarabus* Reitter, 1896]. Type species: *Carabus granulatus* Linnaeus, 1758 designated by Nakane (1962: 39). Etymology. From the Greek *para* (beside, near) and the generic name *Carabus* [*q.v*.] [masculine].
Neocarabus Hatch, 1953: 50 [junior homonym of *Neocarabus* Bengtsson, 1927 and *Neocarabus* Lapouge, 1930]. Type species: *Carabus granulatus* Linnaeus, 1758 by monotypy. Note. Hatch (1949c: 144) proposed this name earlier but failed to provide at the time a description of the taxon. Therefore the name is unavailable from that date.

#### Diversity.

Twenty-six species (Deuve 2004: 109-119) in the Nearctic (three species, one of them adventive) and Palaearctic (24 species) Regions.

#### Taxonomic Note.

Members of *Archaeocarabus* Semenov (38 Chinese species) are included in this subgenus by some authors (e.g., Lorenz 2005: 72-73).

### [granulatus group]

### Carabus granulatus granulatus

Linnaeus, 1758

Carabus granulatus Linnaeus, 1758: 413. Type locality not stated; «Suecia» selected by Lindroth (1957b: 339). Four possible syntypes, only one belonging to the present species, in LSL (Lindroth 1957b: 331).
Carabus granulatus hibernicus Lindroth, 1956a: 7. Type locality: «Killarney, Kerry Co[unty], Ireland» (original citation). Holotype (♂) in BMNH. Synonymy established by Deuve (1994a: 90).

#### Distribution.

This European subspecies is adventive in North America where it is known from Newfoundland (Larson and Langor 1982: 592) to southeastern Manitoba (Roughley et al. 2010: 230; CMNH), and from east-central Minnesota (Gandhi et al. 2011: 673), Massachusetts (Van Dyke 1945a: 129), and Connecticut (Middlesex County, William L. Krinsky pers. comm. 2012) in the east, and from western British Columbia (Lindroth 1961a: 37), including the Queen Charlotte Islands (Kavanaugh 2010: 385), western Washington (Hatch 1953: 51), and Edmonton, Alberta (UASM), in the west. The first inventoried specimen collected in the east was found in New Brunswick in 1890 (Lindroth 1961a: 37) and in the west in Seattle, Washington, in 1924 (Hatch 1933c: 117).

#### Records.

**FRA**: PM **CAN**: AB, BC (QCI, VCI), MB, NB, NF, NS, ON, PE, QC **USA**: CT, MA, MN, WA – **Adventive**

### [vinctus group]

### Carabus goryi

Dejean, 1831

Carabus limbatus Say, 1823a: 77 [primary homonym of *Carabus limbatus* Fabricius, 1777]. Type locality: «Charles Co[unty], Maryland» (neotype label). Neotype (♂), designated by Lindroth and Freitag (1969: 331), in MCZ [# 33091]. Note. «Maryland» was the area originally cited by Say (1823a: 77).
Carabus goryi Dejean, 1831: 544. Type locality: «Amérique septentrionale» (original citation). Holotype [by monotypy] (♀) in MHNP (Lindroth 1955b: 12; Toulgoët 1975: 17). Synonymy established by LeConte (1847: 444), confirmed by Lindroth (1955b: 12). Etymology. The specific name was proposed for Hippolyte Louis Gory [1800-1852], captain of cavalry and coleopterist in France. Gory was a founding member of the *Société Entomologique de France*. His collection, which contained over 18,000 species, was scattered after his death.
Carabus limbatus clarkei Blumenthal, 1958: 64. Type locality: «Klingman’s Dome [= Clingmans Dome] (etwa 1900 m), Alleghenies, an der Grenze von North Carolina» (original citation). Syntype(s) [2 specimens (left elytra only) stated] location unknown. Synonymy established by Lindroth (1961a: 35). Etymology. The subspecific name was proposed for the American lepidopterist John Frederick Gates Clarke [1905-1990] who worked at the USNM.

#### Distribution.

The range of this species extends from southern Maine (Dearborn and Donahue 1993: 2) to southeastern Minnesota (Donald P. Schwert pers. comm. 1989), including southernmost Ontario (Lindroth 1961a: 35), south to northeastern Mississippi (Snodgrass and Cross 1983: 14), northern Alabama (Löding 1945: 11), and northern Georgia (Fattig 1949: 10; CMNH). The record from Idaho (LeConte 1878a: 471; Hatch 1953: 51) is in error (Lindroth 1961a: 35); that from southeastern Louisiana (Summers 1874a: 79) needs confirmation.

#### Records.

**CAN**: ON **USA**: AL, CT, DC, DE, GA, IA, IL, IN, KY, MA, MD, ME, MI, MN, MS, NC, NH, NJ, NY, OH, PA, RI, SC, TN, VA, VT, WI, WV [LA]

### Carabus vinctus

(Weber, 1801)

Tachypus vinctus Weber, 1801: 42. Type locality: «America septentrionali» (original citation), herein restricted to Newport News, Virginia (CNC). Syntype(s) location unknown.
Carabus interruptus Say, 1823a: 76 [primary homonym of *Carabus interruptus* Herbst, 1784]. Type locality: «Germantown [probably the neighborhood of Philadelphia], P[ennsylvani]a» (neotype label). Neotype (♂), designated by Lindroth and Freitag (1969: 331), in MCZ [# 33090]. Synonymy established by Dejean (1826: 79).
Carabus ligatus Germar, 1824: 6. Type locality: «America septentrionali» (original citation). Syntype(s) probably lost. Synonymy established by Horn (1876e: 127).
Carabus carinatus Dejean, 1826: 80 [primary homonym of *Carabus carinatus* Duftschmid, 1812]. Type locality: «Géorgie; environs de Philadelphie [Pennsylvania]» (original citation). Lectotype (♂), designated by Toulgoët (1975: 17), in MHNP. Synonymy established doubtfully with *Carabus ligatus* Germar by Dejean (1826: 80), confirmed by Lindroth (1955b: 12).
Carabus vinctus var. *georgiae* Csiki, 1927: 185. Replacement name for *Carabus vinctus* var. *carinatus* Dejean, 1826.

#### Distribution.

This species ranges from Massachusetts (Harris 1833: 567; Frost 1910: 86) to southeastern Minnesota (Gandhi et al. 2005: 923), including southern Ontario (Lindroth 1961a: 34), south to southeastern Mississippi (Stone County, UASM), southwestern Alabama (Van Dyke 1945a: 126), and the Florida Panhandle (Peck and Thomas 1998: 16). The record from “Louisiana” (Summers 1874a: 79) is probably in error.

#### Records.

**CAN**: ON **USA**: AL, CT, DC, DE, FL, GA, IA, IL, IN, MA, MD, MI, MN, MS, NC, NJ, NY, OH, PA, RI, SC, TN, VA, WV

### Subgenus Diocarabus

Reitter, 1896

Diocarabus Reitter, 1896: 185. Type species: *Carabus loschnikovii* Fischer von Waldheim, 1823 by monotypy. Etymology. Uncertain, possibly from the Greek *dis* (twice) or *dios* (Zeus, chief of the Greek gods) and the generic name *Carabus* [*q.v*.] [masculine].
Cryocarabus Lapouge, 1931: 575. Type species: *Carabus chamissonis* Fischer von Waldheim, 1820 by monotypy. Etymology. From the Greek *cryos* (cold) and the generic name *Carabus* [*q.v*.] [masculine].

#### Diversity.

Eleven species in North America (one northern species) and Asia (ten species, one of them extending into northern European Russia).

#### Taxonomic Note.

Deuve (2004: 186) included members of this taxon in the subgenus *Tomocarabus* Reitter, 1896.‌‌

### Carabus chamissonis

Fischer von Waldheim, 1820

Carabus chamissonis Fischer von Waldheim, 1820: plate 7. Type locality: «île d’Ounalachka [Alaska]» (Fischer von Waldheim 1822: 89). Syntype(s) in ZMH (collection Mannerheim) (Silfverberg 1987: 14), SMTD (Grämer 1960: 98), and probably also in ZMMU (collection Eschscholtz). Etymology. This species was named after the German poet Adelbert Loginovich von Chamisso [1781-1838], who served as botanist on the first scientific voyage around the world, 1815-1818, under the command of Otto Evstaf’evich von Kotzebue on the Russian ship *Rurik*.
Carabus brachyderus Wiedemann [in Wiedemann and Germar], 1821: 110. Type locality: «Unalaschka [Alaska]» (original citation). Syntype(s) location unknown (possibly in ZMUC). Synonymy established by Mannerheim (1843: 186).
Carabus groenlandicus Dejean, 1831: 554. Type locality: «côtes du Groenland» (original citation), which is incorrect (Lindroth 1955b: 12). Lectotype (♂), designated by Toulgoët (1975: 16), in MHNP. Synonymy established by LeConte (1863b: 3), confirmed by Lindroth (1955b: 12).
Carabus groenlandicus washingtoni Casey, 1920: 155. Type locality: «M[oun]t Washington [Coos County], New Hampshire» (original citation). Three syntypes [3 originally cited] in USNM [# 46058]. Synonymy established by Breuning (1932: 300).
Carabus rugosostrigatus Mandl, 1955: 237. Type locality: «Juldus [= Kaidu He, Xinjiang Uygur Autonomous Region, China]» (original citation), which is incorrect (Deuve 1991: 51). Holotype (♀) in NHMW. Synonymy established by Deuve (1991: 51).

#### Distribution.

This species occurs from the Labrador coast to the Seward Peninsula in Alaska (Lindroth 1961a: 33-34), including the Aleutians and Kodiak Island, south to northern British Columbia (CNC); isolated on high mountains in Gaspé Peninsula (Quebec), New Hampshire (Lindroth 1961a: 34), and Maine (Mount Katahdin, CNC). Fossil remnants of this species, dated between about 16,700 and 21,500 years B.P., have been unearthed in southeastern Iowa (Baker et al. 1986: 96) and northeastern Illinois (Garry et al. 1990: 394).

#### Records.

**CAN**: AB, BC, LB, MB, NT, NU, ON, QC, SK, YT **USA**: AK, ME, NH

### Subgenus Aulonocarabus

Reitter, 1896

Aulonocarabus Reitter, 1896: 192. Type species: *Carabus canaliculatus* Adams, 1812 designated by Nakane (1962‌‌: 39). Etymology. From the Greek *aulonos* (channel) and the generic name *Carabus* [*q.v*.], probably alluding to one of the two species included, *Carabus canaliculatus* which is the Latin name for channel [masculine].
Baptaulonocarabus Imura, 2002: 130. Type species: *Carabus truncaticollis* Eschscholtz, 1833 by original designation. Synonymy established by Deuve (2004: 197). Etymology. From the Greek *baptos* (dyed) and the generic name *Aulonocarabus* [*q.v*.] [masculine].

#### Diversity.

Thirteen species (Deuve 2004: 197-203) in North America (one Holarctic species) and Asia (13 species, one of them extending into northern European Russia).

### Carabus truncaticollis truncaticollis

Eschscholtz, 1833

Carabus truncaticollis Eschscholtz, 1833: 22. Type locality: «Auf den Inseln des Kamtschatkischen Meeres, St. Georg und St. Paul [Pribilof Islands, Alaska]» (original citation). Syntype(s) [2 originally cited] location unknown (possibly in ZMMU).
Carabus lutshnikianus Basilewsky, 1937: 63. Type locality: «Ile Saint-Paul, Alaska» (original citation). Holotype (♀) location unknown. Synonymy established by Van Dyke (1945a: 97).
Carabus truncaticollis alaskensis Basilewsky, 1937: 63. Type locality: «Ile Saint-Paul, Alaska» (original citation). Holotype location unknown. Synonymy established by Van Dyke (1945a: 97).

#### Distribution.

This Holarctic subspecies is known from a few old specimens collected on the Kamchatka Peninsula (Shilenkov in Kryzhanovskij et al. 1995: 43) and from Alaska, including the Pribilof Islands, to the Mackenzie River delta in northern Northwest Territories (Lindroth 1961a: 40).

#### Records.

**CAN**: NT, YT **USA**: AK – **Holarctic**

#### Note.

1. This species is listed in the subgenus *Aulonocarabus* Reitter by Kryzhanovskij et al. (1995: 43), Bousquet et al. (2003: 134), and Deuve (2004: 197) and in the subgenus *Diocarabus* by Casale and Kryzhanovskij (2003: 93) and Lorenz (2005: 85). 2. Two other subspecies of *Carabus truncaticollis* occur in Asia (Deuve 2004: 197-198).

### Subgenus Homoeocarabus

Reitter, 1896

Homoeocarabus Reitter, 1896: 144. Type species: *Carabus maeander* Fischer von Waldheim, 1820 by monotypy. Etymology. From the Greek *homoios* (like, resembling) and the generic name *Carabus* [*q.v*.] [masculine].

#### Diversity.

One Holarctic species.

### Carabus maeander maeander

Fischer von Waldheim, 1820

Carabus maeander Fischer von Waldheim, 1820: plate 10. Type locality: «Sibiria, propre Nertschinsk» (Fischer von Waldheim 1822: 104). Syntype(s) in SMTD (Grämer 1960: 96).
Carabus incompletus Fischer von Waldheim, 1828: 303. Type locality: «Kamtschatka [Russia]» (original citation). Syntype(s) location unknown. Synonymy established by Breuning (1932: 411).
Carabus ehrenbergii Fischer von Waldheim, 1829b: 368. Type locality: «Kamtschatkae [Russia]» (original citation). Syntype(s) location unknown. Synonymy established, under the name *Carabus incompletus* Fischer von Waldheim, by Heyden (1879: 166).
Carabus lapilayi Laporte, 1834: 89. Type locality: «Terre-Neuve [= Newfoundland]» (original citation). Syntype(s) location unknown. Synonymy established by LeConte (1866: 78). Etymology. The specific name was proposed for Auguste Jean Marie Bachelot de la Pylaie (also spelled de Lapilaye) [1786-1856], French botanist, explorer, and archaeologist. Bachelot de la Pylaie made two trips to Newfoundland and Saint-Pierre and Miquelon and published a flora (unfinished) of the region.
Carabus hudsonicus Motschulsky, 1866: 293. Type locality: «Hudson-Bay» (original citation). Lectotype [as holotype], designated by Kryzhanovskij (1968: 178), in ZMMU. Synonymy established with doubt by Horn (1870a: 70), confirmed by Kryzhanovskij (1968: 178).
Carabus maeander var. *simoni* Heyden, 1879: 166. Type locality: «Huds[on Bay]» (original citation). Syntype(s) location unknown. Synonymy established by Roeschke (1900: 70).
Carabus maeander var. *excostatus* Kraatz, 1880: 338. Type locality not stated. Holotype [by monotypy] location unknown (possibly in DEI). Synonymy established by Breuning (1932: 412).
Carabus lecontei Géhin, 1885: 26. Type locality: «Detroit [Wayne County, Michigan]» (original citation for *Carabus* [no] 3 of LeConte 1847: 444). Syntype(s) presumably lost. Synonymy established by Breuning (1932: 412). Note. This taxon was described by a bibliographic reference to a previously published description, that of *Carabus* [no] 3 of LeConte (1847: 444).
Carabus maeander atlanticus Lapouge, 1925: 191. Type locality: «Saint-Pierre-Miquelon» (original citation). Syntype(s) location unknown (most probably destroyed). Synonymy established, under the name *Carabus maeander* n[atio] *lapilayi* Laporte, by Breuning (1932: 412).

#### Distribution.

This Holarctic subspecies ranges from western Siberia (Bousquet et al. 2003: 154) to the Bering Sea Coast and from Alaska (Lindroth 1961a: 36) to Newfoundland (Lindroth 1955a: 23), south at least to northeastern New York (Powell 1977: 148), northern Iowa (Larsen et al. 2003: 292), and northern New Mexico (Brantley et al. 2003: 382; Sandoval County, UASM). The record from “Pennsylvania” (Bousquet and Larochelle 1993: 76) needs confirmation.

#### Records.

**FRA**: PM **CAN**: AB, BC, LB, MB, NB, NF, NS (CBI), NT, ON, PE, QC, SK **USA**: AK, CO, IA, IL, IN, ME, MI, MN, MT, ND, NM, NY, OH, SD, VT, WI [PA] – **Holarctic**

#### Note.

Two other subspecies of this species, *Carabus maeander chejuensis* Deuve from South Korea and *Carabus maeander paludis* Géhin from the Far East, Japan, and China, are found in Asia. Deuve (2004: 183) also retained *Carabus lapilayi* Laporte as a valid subspecies.

### Subgenus Hemicarabus

Géhin, 1876

Hemicarabus Géhin, 1876a: 25. Type species: *Carabus nitens* Linnaeus, 1758 designated by Géhin (1885: xix). Etymology. From the Greek prefix *hemi*- (half) and the generic name *Carabus* [*q.v*.] [masculine].

#### Diversity.

Four species in North America (one species), Asia (two species), and Europe (one species).

### Carabus serratus

Say, 1823

Carabus serratus Say, 1823a: 77. Type locality: «Asheville [Buncombe County], N[orth] C[arolina]» (neotype label). Neotype (♂), designated by Lindroth and Freitag (1969: 331), in MCZ [# 33092].
Carabus lineatopunctatus Dejean, 1826: 77. Type locality: «Amérique septentrionale» (original citation). Three possible syntypes in MHNP (Lindroth 1955b: 12). Synonymy established by Dejean (1826: 77), confirmed by Lindroth (1955b: 12).
Carabus canadensis LeConte [in Melsheimer], 1853: 10. Type locality: «Canada» (original citation for *Carabus ligatus* Germar *sensu* Kirby, 1837), restricted to «Nipigon, W[estern] Ont[ario]» by Lindroth (1961a: 40). Syntype(s) probably in BMNH. Synonymy established by Henshaw (1882: 207). Note. This name was proposed for *Carabus ligatus* Germar, 1824 *sensu* Kirby (1837: 18).
Carabus tatumi Motschulsky, 1866: 293. Type locality: «Hudson-Bay dans l’Amérique arctique» (original citation). Lectotype [as holotype] (♀), designated by Kryzhanovskij (1968: 184), in ZMMU. Synonymy established by Horn (1870a: 70), confirmed by Kryzhanovskij (1968: 184).
Carabus serratus vegasensis Casey, 1913: 59. Type locality: «Las Vegas [San Miguel County], New Mexico; probably Colorado» (original citation). Three syntypes in USNM [# 46055]. Synonymy established by Breuning (1933b: 858).

#### Distribution.

This species ranges from Newfoundland (Lindroth 1955a: 26) to the Okanagan Valley in south-central British Columbia (Lindroth 1961a: 41), south to eastern Oregon (Hatch 1953: 50), central New Mexico (Fall and Cockerell 1907: 156), eastern Kansas (Popenoe 1877: 22; Horn 1872c: 384), and northwestern South Carolina (Ciegler 2000: 29).

#### Records.

**CAN**: AB, BC, MB, NB, NF, NS (CBI), ON, PE, QC, SK **USA**: CO, CT, DC, DE, GA, IA, ID, IL, IN, KS, MA, MD, ME, MI, MN, MT, NC, ND, NE, NH, NJ, NM, NY, OH, OR, PA, RI, SC, SD, VA, VT, WA, WI, WV, WY

### Subgenus Archicarabus

Seidlitz, 1887

Archicarabus Seidlitz, 1887: 6 [Gattung]. Type species: *Carabus nemoralis* Müller, 1764 by monotypy. Etymology. From the Greek *arche* (beginning) and the generic name *Carabus* [*q.v*.] [masculine].
Archeocarabus Bengtsson, 1927: 83. Type species: *Carabus nemoralis* Müller, 1764 by original designation. Etymology. From the Greek *arche* (beginning) and the generic name *Carabus* [*q.v*.] [masculine].

#### Diversity.

Ten species in Europe and the Middle East (Deuve 2004: 167-173), one of them adventive in North America.

### Carabus nemoralis nemoralis

Müller, 1764

Carabus nemoralis O.F. Müller, 1764: 21. Type locality: Frederiksdal [Sjaelland, Denmark] (inferred from title of the book). Syntype(s) lost.
Carabus nemoralis var. *canadensis* Lapouge, 1908a: 19 [primary homonym of *Carabus canadensis* LeConte, 1853]. Type locality: «Terre-Neuve et New Brunswick» (original citation). Syntype(s) location unknown. Synonymy established by Breuning (1933a: 667). Note. The specimen from “France: Ardèche” in MHNP designated as lectotype of this taxon by Toulgoët (1976: 32) is certainly not a syntype since the taxon was described from specimens collected in Newfoundland and New Brunswick.

#### Distribution.

This European subspecies is adventive in North America where it is found from Newfoundland (Lindroth 1955a: 27) to central Minnesota (Crow Wing County, CNC), south to northeastern Virginia (Falls Church, UASM) in the east, and from the Queen Charlotte Islands (Kavanaugh 1992: 51) to central Alberta (Lindroth 1961a: 37), south to southeastern Wyoming (Burne 1989: 290), northern Utah (Salt Lake County, CMNH), and central California (Lindroth 1961a: 37); seemingly isolated in the Saskatoon area, Saskatchewan (Ronald R. Hooper pers. comm. 1990). The first inventoried specimens collected on this continent was found in New Brunswick in 1890 (Horn 1892d: 61) and on the west coast around 1909 in Seattle, Washington (Hatch 1933c: 117). The date of 1870 listed by Lindroth (1961a: 37) for the first occurrence of this species on the continent is probably a lapsus for 1890. The species was also intentionally introduced in New England as predators of gypsy moths prior to 1911 (Smith 1959: 9).

#### Records.

**CAN**: AB, BC (QCI, VCI), NB, NF, NS, ON, PE, QC, SK **USA**: CA, CT, DE, ID, IL, IN, MA, ME, MI, MN, MT, NH, NJ, NV, NY, OH, OR, PA, RI, SD, UT, VA, VT, WA, WI, WY – **Adventive**

**Figure 10.**
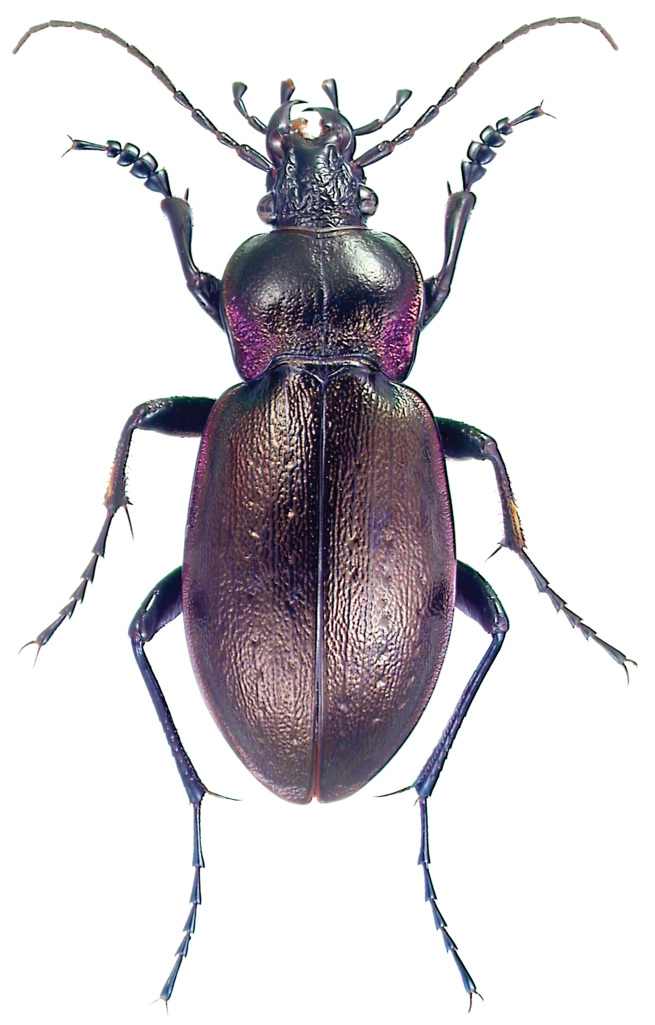
*Carabus nemoralis* Müller. This European species was first discovered on this continent in New Brunswick around 1890 and was recovered on the west side of Lake Ontario in 1919 and in southeastern Wisconsin by 1934. Unless the species went undetected for a long period of time, these dates would suggest that the species spread westwards for an average of 38 km per year. This is highly improbable for a wingless species and therefore its spread on this continent was undoubtedly enhanced by human transport unless separate introductions occurred.

### Subgenus Tachypus

Weber, 1801

Tachypus Weber, 1801: 19. Type species: *Carabus auratus* Linnaeus, 1760 designated by Ádám (1996: 11). Etymology. From the Greek *tachys* (swift, quick, fast) and *pous* (foot) [masculine].
Autocarabus Seidlitz, 1887: 9 [Gattung]. Type species: *Carabus auratus* Linnaeus, 1760 by monotypy. Etymology. From the Greek *autos* (self) and the generic name *Carabus* [*q.v*.] [masculine].
Goniocarabus Reitter, 1896: 150 [junior homonym of *Goniocarabus* Géhin, 1885]. Type species: *Carabus cancellatus* Illiger, 1798 designated by Jeannel (1941b: 109). Etymology. From the Greek *gonios* (angle) and the generic name *Carabus* [*q.v*.] [masculine].
Cancellocarabus Lutshnik, 1924: 38, 49. Replacement name for *Goniocarabus* Reitter, 1896. Etymology. From the Latin *cancellus* (lattice) and the generic name *Carabus* [*q.v*.] [masculine].

#### Diversity.

Four European species, one of them extending into Siberia. One species is adventive in eastern North America.

#### Faunistic Note.

The European *Carabus cancellatus* Illiger has been recorded from North Carolina (Horn 1883b: 270), Georgia (Fattig 1949: 10), and Wisconsin (Leng 1920: 44) but no specimens have been collected in recent decades. The species is probably not established on this continent.

### Carabus auratus auratus

Linnaeus, 1760

Carabus auratus Linnaeus, 1760: 219. Type locality: «Suecia» (original citation), which is probably incorrect (Lindroth 1957b: 328). One possible syntype in LSL (Lindroth 1957b: 328).

#### Distribution.

This European subspecies was intentionally introduced into Massachusetts in 1908 and 1910 (Smith 1959: 7) for gypsy moth control. It is now established in New England (Lindroth 1961a: 37). The first inventoried specimen collected subsequently to its release was found in June 1920 in Winchester, Massachusetts (Smith 1959: 7).

#### Records.

**USA**: CT, MA, ME, NH, VT – **Adventive**

#### Note.

In a cladistic analysis conducted by Arndt et al. (2003: 312, Fig. 7.16), this species turned out as the sister-group to all remaining species of *Carabus*.

### Subgenus Tanaocarabus

Reitter, 1896

Tanaocarabus Reitter, 1896: 135. Type species: *Carabus sylvosus* Say, 1823 designated by Breuning (1933b: 895). Etymology. From the Greek *tanaos* (outstretched, long) and the generic name *Carabus* [*q.v*.], probably alluding to the elongate shape of the adults [masculine].
Neocarabus Lapouge, 1931: 569 [junior homonym of *Neocarabus* Bengtsson, 1927]. Type species: *Carabus taedatus* Fabricius, 1787 by monotypy.
Durangocarabus Imura, 2002: 141. Type species: *Carabus forreri* Bates, 1882 by original designation. Synonymy established by Deuve (2004: 195). Etymology. From the name of the state Durango in Mexico and the generic name *Carabus* [*q.v*.] [masculine].

#### Diversity.

Five species in North America (four species) and Mexico (two species). One of Mexican species, *Carabus hendrichsi* Bolivar, Rotger, and Coronado, is endemic to the Sierra Madre Oriental.

### Carabus finitimus

Haldeman, 1852

Carabus finitimus Haldeman, 1852: 373. Type locality: «Fort Gates [Coryell County, Texas]» (original citation). Three possible syntypes, each with a dark red disc, in MCZ (collection LeConte).
Carabus lecontei Casey, 1913: 57 [primary homonym of *Carabus lecontei* Géhin, 1885]. Type locality: «Texas» (original citation). Holotype [by monotypy] (♀) in USNM [# 46052]. Synonymy established by Breuning (1933b: 896).
Carabus caseyi Angell, 1914: 75. Replacement name for *Carabus lecontei* Casey, 1913.

#### Distribution.

This species is known from Kansas (Chautauqua County, CMNH; Erwin 2007a: 110), Wichita National Forest in Oklahoma, and eastern Texas (Van Dyke 1945a: 117).

#### Records.

**USA**: KS, OK, TX

### Carabus forreri forreri

Bates, 1882

Carabus forreri Bates, 1882b: 320. Type locality: «Ciudad, Durango, Mexico» (original citation). Syntype(s) in MHNP (Toulgoët 1975: 232). Etymology. The specific name was proposed for Alfonse Forrer [1836-1899], a collector of natural history objects. Born in London, Forrer moved to the United States while a young man and, at the close of the civil war, accepted a commission from the British Museum to collect zoological material along the west coast of the United States and Mexico. He supplied many other European museums with his material and also collected for Salvin and Godman. In July 1900, his personal collection, consisting mainly of fishes, birds, and insects, was offered for sale for $500 by his widow.
Carabus townsendi Casey, 1905: 160. Type locality: «Meadow Valley (7,300 feet), six miles south of Colonia Garcia, Chihuahua, Mexico, in the Sierra Madre Mountains» (original citation). Holotype [by monotypy] in USNM. Synonymy established by Breuning (1933b: 897). Etymology. This taxon was named after the American entomologist Charles Henry Tyler Townsend [1863-1944] who published on many subjects but is primarily known for his studies on Sarcophagidae and Tachinidae. Townsend worked for many years in South America and made significant contributions in the fields of agricultural and medical entomology. At the time of his death he was living in a suburb of São Paulo where he founded his own publishing company.

#### Distribution.

This species is known from southeastern Arizona and the state of Durango in Mexico (Van Dyke 1945a: 118-119).

#### Records.

**USA**: AZ – Mexico

#### Note.

*Carabus forreri willi* Deuve is known from Chihuahua in Mexico.

### Carabus sylvosus

Say, 1823

Carabus sylvosus Say, 1823a: 75. Type locality: North America (inferred from title of the book), restricted to «Asheville [Buncombe County], N[orth] C[arolina]» by Lindroth (1961a: 41). Lectotype (♂), designated by Lindroth and Freitag (1969: 330), in MHNP.
Carabus lherminieri Dejean, 1826: 152. Type locality: «Amérique septentrionale» (original citation). One syntype in MHNP (Lindroth 1955b: 12; Toulgoët 1975: 20). Synonymy established by LeConte (1863b: 3), confirmed by Lindroth (1955b: 12). Etymology. The specific name was proposed for Félix Louis L’Herminier [1779-1833], a French pharmacist and naturalist who lived for over 30 years in Guadeloupe. L’Herminier also spent times in South Carolina where he gathered insects; some of them were sent to Dejean.

#### Distribution.

This species ranges from “Maine” (Larochelle and Larivière 1990a: 27) to northern Minnesota (Gandhi et al. 2005: 923), north to north-central Ontario (Spires 1985: 79), south to “Texas” (Van Dyke 1945a: 116, as *Carabus sylvosus lherminieri*) and central Florida (Peck and Thomas 1998: 16). The record from “Utah” (Bousquet and Larochelle 1993: 77) is in error.

#### Records.

**CAN**: ON, QC **USA**: AL, AR, CT, DC, FL, GA, IA, IL, IN, KS, KY, LA, MA, MD, ME, MI, MN, MO, MS, NC, NH, NJ, NY, OH, OK, PA, RI, SC, TN, TX, VA, VT, WI, WV

### Carabus taedatus agassii

LeConte, 1850

Carabus agassii LeConte, 1850: 209. Type locality: «Kakàbeka [Ontario]» (original citation). Syntype(s) in MCZ [# 619]. Etymology. The specific was proposed in honor of the Swiss zoologist, geologist, paleontologist, and nomenclatorist Jean Louis Rodolphe Agassiz [1807-1873], one of the best-known scientists of his time. In his late 30s, Agassiz moved to United States and became professor of zoology and geology at Harvard University where he founded the Museum of Comparative Zoology in 1859 and served as the museum’s first director until his death. Agassiz is remembered today for his theories on ice ages and other scientific accomplishments but also for his scientific racism and his resistance to Darwin’s theory of evolution.
Carabus oregonensis LeConte, 1854a: 16. Type locality: «Prairie Paso [= possibly Bear Prairie Pass, Lewis County, Washington]» (original citation). Holotype [by monotypy] (♂) in MCZ [# 620]. Synonymy established by Breuning (1933b: 719).
Carabus taedatus var. *canadicus* Roeschke, 1900: 69. Type locality: «Canada, wenigstens über den östlichen» (original citation). Syntype(s) in DEI (Edelbrock 1986: 64). Synonymy established by Breuning (1933b: 719).
Carabus patulicollis Casey, 1913: 57. Type locality: «probably Colorado» (original citation). Holotype [by monotypy] (♂) in USNM [# 46053]. Synonymy established by Lindroth (1961a: 39)
Carabus taedatus montanicus Casey, 1913: 58. Type locality: «Coeur d’Alene [Kootenai County], Idaho» (original citation). Holotype [by monotypy] (♂) in USNM [# 46051]. Synonymy established by Breuning (1933b: 719).
Carabus franciscanus Casey, 1913: 58. Type locality: «coast region near San Francisco, California» (original citation), which is probably incorrect (Lindroth 1961a: 38). Holotype [by monotypy] (♂) in USNM [# 46054]. **New synonymy**.
Carabus stocktonensis Casey, 1920: 155. Type locality: «Eureka and Stockton, Utah» (original citation). Two syntypes in USNM [# 46056]. Synonymy established by Lindroth (1961a: 39).
Carabus taedatus coloradensis Breuning, 1933b: 719. Type locality: «Longs Peak [Boulder County], Colorado» (original citation). Holotype (♂) in ZMUA (Boer 2002: 41). Synonymy established, under the name *Carabus taedatus patulicollis* Casey, by Van Dyke (1945a: 113).

#### Distribution.

This subspecies ranges from Newfoundland (Lindroth 1955a: 25) to Yukon Territory, south to the Sierra Nevada in east-central California, southern Arizona, southern New Mexico, and southwestern South Dakota (Purrington et al. 2002: 202) [see Edelbrock 1986: Fig. 69].

#### Records.

**CAN**: AB, BC (QCI), MB, NF, NT, ON, QC, SK, YT **USA**: AZ, CA, CO, ID, MT, ND, NM, NV, OR, SD, UT, WA, WY

### Carabus taedatus bicanaliceps

Casey, 1920

Carabus bicanaliceps Casey, 1920: 154. Type locality: «Olympia [Thurston County], Washington» (original citation). Holotype [by monotypy] (♀) in USNM [# 46057].

#### Distribution.

This subspecies is known from Vancouver Island and the Puget Sound area in Washington south to central Oregon between the coast and the Cascade Range (Edelbrock 1986: 49, Fig. 69).

#### Records.

**CAN**: BC (VCI) **USA**: OR, WA

### Carabus taedatus rainieri

Van Dyke, 1945

Carabus taedatus rainieri Van Dyke, 1945a: 108. Type locality: «Paradise Park (6000 ft.), M[oun]t Rainier [Pierce County], Wash[ington]» (original citation). Holotype (♂) in CAS [# 5442].

#### Distribution.

This subspecies is known from high altitude along the northern Cascades and from coastal mountains of Oregon (Edelbrock 1986: 49).

#### Records.

**CAN**: BC **USA**: OR, WA

### Carabus taedatus taedatus

Fabricius, 1787

Carabus taedatus Fabricius, 1787: 196. Type locality: «America boreali» (original citation), restricted to «Unalaska, Aleut[ian] Isl[ands] [Alaska]» by Lindroth (1961a: 38). Two syntypes in BMNH (collection Banks) and ZMUC (Zimsen 1964: 52).
Carabus baccivorus Fischer von Waldheim, 1820: plate 7. Type locality: «insula Unalaschka [Alaska]» (Fischer von Waldheim 1822: 87). Syntype(s) in ZMH (collection Mannerheim) (Silfverberg 1987: 13), SMTD (Grämer 1960: 100), and probably also in ZMMU (collection Eschscholtz). Synonymy established by LeConte (1857c: 30), confirmed by Edelbrock (1986: 43).
Carabus seriatus Wiedemann [in Wiedemann and Germar], 1821: 109. Type locality: «Unalaschka [Alaska]» (original citation). Syntype(s) location unknown (possibly in ZMUC). Synonymy established, under the name *Carabus baccivorus* Fischer von Waldheim, by Mannerheim (1843: 187).
Carabus gladiator Motschulsky, 1866: 285. Type locality: «Hudson-Bay» (original citation), which is likely incorrect. Syntype(s) in ZMMU (Edelbrock 1986: 64) though not listed by Keleinikova (1976). Synonymy established, under the name *Carabus taedatus* var. *baccivorus* Fischer von Waldheim, by Horn (1870a: 70), confirmed by Edelbrock (1986: 43).

#### Distribution.

This subspecies is restricted to southeastern Alaska including the Aleutian Islands (Edelbrock 1986: 49, Fig. 69).

#### Records.

**USA**: AK

#### Note.

1. Edelbrock (1986) studied the geographical variation in *Carabus taedatus* and recognized four species, and one of them (*Carabus agassii*) was segregated into two subspecies (*agassii* and *franciscanus*). I have followed Deuve (1994a: 144) in listing Edelbrock’s species as subspecies. Furthermore I do not recognize the form *Carabus agassii franciscanus* as subspecifically distinct. 2. This species is placed in the subgenus *Oreocarabus* Géhin by some authors (e.g., Breuning 1933b: 719; Lorenz 2005: 91).

### Subgenus Megodontus

Solier, 1848

Megodontus Solier, 1848: 58. Type species: *Carabus caelatus* Fabricius, 1801 by original designation. Etymology. From the Greek *megas* (large) and *odontos* (tooth), probably alluding to the large mentum tooth of the adult (“*dent du sinus du mentum grande*”) [masculine].
Megalodontus Jacobson, 1905: 217. Unjustified emendation of *Megodontus* Solier, 1848.
Nabicarabus Kwon and Lee, 1984: 102. Type species: *Carabus vietinghoffii* Adams, 1812 by original designation. Synonymy established by Deuve (1994a: 153). Etymology. From the Greek *nabis* (giraffe) and the generic name *Carabus* [*q.v*.] [masculine].

#### Diversity.

Twenty-two species (Deuve 2004: 363-375) in North America (one Holarctic species) and Eurasia (22 species).

### Carabus vietinghoffii vietinghoffii

Adams, 1812

Carabus vietinghoffii Adams, 1812: 170. Type locality: «ubi ducentis fere milliaribus ultra urbem Jakutzk, ad ripas fluvii Lenae [= about 200 “miles” pass the city of Yakutsk, on the banks of the Lena River], Sibiriae orientalis [Russia]» (original citation). Syntypes in SMTD (Grämer 1960: 95). Etymology. The specific name was proposed for Baron de Vietinghoff [1767-1829], Latvian naturalist also named Christoph Burchard Scheel.
Carabus vietinghovi var. *schtschegolewi* Poppius, 1906a: 15. Type locality: «oberen Lena [Siberia, Russia]» (original citation). Holotype [by monotypy] (♀) location unknown. Synonymy established by Breuning (1926: 70).
Carabus vietinghoffi alaskanus Obydov, 1996: 85. Type locality: «Umiat, Alaska» (original citation). Holotype (♂) in Obydov’s collection (Moscow, Russia). Synonymy established by Lorenz (2005: 108).

#### Distribution.

This subspecies is found in eastern Siberia and in North America from the Seward Peninsula in Alaska to Bathurst Inlet on the arctic coast of Nunavut (Lindroth 1961a: 42).

#### Records.

**CAN**: NT, NU, YT **USA**: AK – **Holarctic**

#### Note.

Two other subspecies of this species occur in eastern Asia.

### Subfamily CICINDELINAE

Latreille, 1802

Cicindeletae Latreille, 1802: 77. Type genus: *Cicindela* Linnaeus, 1758.

#### Diversity.

Worldwide, with more than 2,500 species (Lorenz 2005: 22-62) arrayed in five tribes: Amblycheilini (13 species), Cicindelini (more than 1,710 species), Collyridini (about 335 Asian species), Ctenostomatini (about 225 species in the Neotropical and Afrotropical Regions), Manticorini (14 Afrotropical species), and Megacephalini (about 200 species).

### Tribe Amblycheilini

Csiki, 1903

Amblychilinae Csiki, 1903: 124. Type genus: *Amblychila* Agassiz, 1846 (unjustified emendation of *Amblycheila* Say, 1830 not in prevailing usage) (= *Amblycheila* Say, 1830).
Omites W. Horn, 1907b: 466. Type genus: *Omus* Eschscholtz, 1829.

#### Diversity.

Western Hemisphere, with 13 species in North America (ten species), Mexico (two species), and South America (one species) arrayed in three genera: *Amblycheila* (seven species), *Omus* (five species), and *Picnochile* Motschulsky (one species in Chile and Argentina).

### Genus Amblycheila

Say, 1830

Amblycheila Say, 1830a: 67. Type species: *Manticora cylindriformis* Say, 1823 by original designation. Etymology. From the Greek *amblys* (blunt, obtuse) and *cheila* (lip, by extension labrum [upper lip]), probably alluding to absence of teeth on the apical edge of the labrum of adults of this genus contrary to those of the genus *Cicindela* [feminine].
Amblychila Agassiz, 1846: 16. Unjustified emendation of *Amblycheila* Say, 1830.
Amblyprosopa Gistel, 1850: 75. Unnecessary replacement name for *Amblycheila* Say, 1830.
Chaleposomus Chaudoir, 1861a: 337. Unnecessary replacement name for *Amblycheila* Say, 1830. Etymology. From the Greek *chalepos* (difficult, severe, harsh) and *soma* (body) [masculine].

#### Diversity.

Seven species (Pearson et al. 2006: 48) in western North America (five species) and northern Mexico (two species: *Amblycheila nyx* Sumlin from west-central Coahuila and *Amblycheila halffteri* Mateu from San Luis Potosí).

#### Identification.

Vaurie (1955) reviewed the species and offered a key for their identification. Subsequently one new North American species, *Amblycheila hoversoni* Gage, was described in 1991. Pearson et al. (2006: 22) field guide includes a key to the North American species.

#### Faunistic Note.

The record of *Amblycheila halffteri* from “Texas” (Bousquet and Larochelle 1993: 52) is in error. The species is known so far only from San Luis Potosí in Mexico.

### Amblycheila baroni

Rivers, 1890

Amblychila baroni Rivers, 1890b: 111. Type locality: «Pantano [= Pima] County, Arizona» (original citation). Holotype [by monotypy] (♀) probably in DEI. Etymology. The specific name was proposed in honor of Oscar Theodor Baron [1847-1926]. Born in Germany, Baron spent about 30 years in California where he collected extensively Lepidoptera but also Coleoptera and other insects in Mendocino and Fresno Counties. Moreover he travelled to Arizona, Mexico, Central America, and South America. He returned to Germany where he lived his last 30 years. Note. Leng (1902: 98) reported that the holotype was “found dead in a canyon ... at an elevation of 5000 feet.”
Amblychila baroni longipes Casey, 1909: 253. Type locality: «Baboquivari M[oun]t[ain]s [Pima County], Arizona» (original citation). One syntype in USNM [# 45890]. Synonymy established by Horn (1910a: 123).
Amblycheila baroni enodis Casey, 1916: 5. Type locality: «Garces, Huachuca M[oun]t[ain]s, Cochise Co[unty], Arizona» (original citation). One syntype in USNM [# 45892]. Synonymy established by Horn (1926: 52).
Amblycheila ventricosa Casey, 1924: 1. Type locality: «Huachuca M[oun]t[ain]s [Cochise County], Arizona» (original citation). Holotype [by monotypy] (♀) in USNM [# 45891]. Synonymy established by Horn (1926: 52).

#### Distribution.

This species, also known as the “Montane Giant Tiger Beetle^7^,” is restricted to mountains in southeastern Arizona and a small area in western Texas near Big Bend National Park (Pearson et al. 2006: 48).

#### Records.

**USA**: AZ, TX

### Amblycheila cylindriformis

(Say, 1823)

Manticora cylindriformis Say, 1823b: 139. Type locality: «Arkansa[s] [River] ... found at the base of the Rocky Mountains» (original citation). Syntype(s) lost. Note. Say’s specimen(s) were probably collected in Colorado.

#### Distribution.

This species, also known as the “Great Plains Giant Tiger Beetle”, ranges from southwestern South Dakota (Howden 1970: 8) and eastern Wyoming (Huber 1978: 75) south to western Texas [see Pearson et al. 2006: Map 3]. The record from “Arizona” (Horn 1910a: 123) is in error; those from “Arkansas” (Leng 1902: 97; Erwin and Pearson 2008: 20) are probably based on a misinterpretation of the type locality.

#### Records.

**USA**: CO, KS, NE, NM, OK, SD, TX, WY

### Amblycheila hoversoni

Gage, 1991

Amblycheila hoversoni Gage, 1991: 2. Type locality: «16 mi[les] south of George West, Live Oak County, Texas» (original citation). Holotype (♂) in MCZ [# 33379].

#### Distribution.

This species, also known as the “South Texas Giant Tiger Beetle”, is found only in south and west-central Texas (Pearson et al. 2006: 51).

#### Records.

**USA**: TX

### Amblycheila picolominii

Reiche, 1840

Amblycheila picolominii Reiche, 1840: 560. Type locality: «port ou baie de Saint-Francisco, dans la Nouvelle-Californie, sous le 48e degré environ de latitude septentrionale» (original citation), which is incorrect. Holotype [by monotypy] (♀) in MHNP (Horn 1904: 97). Etymology. The specific name was proposed for the collector of the type specimen, count Enea Silvio Vincenzo [Vincent] Piccolomini who spent ten years, “engaged in scientific pursuits,” in Mexico and United States. Note. 1. Reiche originally used two different spellings for this species, *picolominii* (page 560) and *piccolominii* (plate 19). As far as I know, Reiche did not subsequently use the species name and nobody acted as “First Reviser.” Therefore, I select “*picolominii*” as the correct original spelling since it is in prevailing usage. 2. George Horn (1893: 281) remarked that the specimens collected by “Picolomini” and sold by Richard Henri Dupont (born Richard Henry Puech) as found in California, which included the holotype of this species, were in fact collected “anywhere from western Texas to central Arizona.”

#### Distribution.

This species, also known as the “Plateau Giant Tiger Beetle”, occurs from the northern half of Arizona to western Texas, north to southwestern Colorado (Kippenhan 1994: 21) and southeastern Utah (Krell and Brookhart 2012: 110). The record from “Oklahoma” (Bousquet and Larochelle 1993: 52) is in error.

#### Records.

**USA**: AZ, CO, NM, TX, UT

### Amblycheila schwarzi

Horn, 1904

Amblychila schwarzi W. Horn, 1904: 98. Type locality not stated; «Truxton Valley, Peach Springs (5,000 ft.), Arizona» selected by Freitag (1999: 8). Syntype(s) [2 originally cited] probably in ZMHB. Note. This name is usually credited to Horn (1903a: 196) who first proposed it. Horn (1903a: 196) reported that “*Amblycheila piccolomini* G. Horn, Leconte, Leng ex parte” is different from “*Amblycheila piccolomini* Rivers” and gave the name *Amblycheila schwarzi* to the form not identical with “*Amblycheila piccolomini* Reiche.” In my opinion, Horn’s statement does not constitute a description by indication. The next year Horn published a description of *Amblycheila schwarzi* and stated, after seeing the type of *Amblycheila picolominii* Reiche in Paris, that his new species was the “*Amblycheila piccolomini* Rivers and Leng, ex parte.”
Amblycheila utahensis Tanner, 1951: 47. Type locality: «Diamond Valley, 15 miles north of S[ain]t George, Washington County, Utah» (original citation). Holotype (♂) in BYUC (Shawn M. Clark pers. comm. 2007). Synonymy established by Vaurie (1955: 20).

#### Distribution.

This species, also known as the “Mojave Giant Tiger Beetle”, is found from northwestern Arizona and southwestern Utah west to the desert mountains of southeastern California east of the Sierra Nevada [see Vaurie 1955: Fig. 1 and Pearson et al. 2006: Map 2].

#### Records.

**USA**: AZ, CA, NV, UT

### Genus Omus

Eschscholtz, 1829

Omus Eschscholtz, 1829: 3. Type species: *Omus californicus* Eschscholtz, 1829 by monotypy. Etymology. From the Greek *omos* (cruel), possibly alluding to the apparent ferocious habits of the species in the eyes of Eschscholtz [masculine].
Leptomus Casey, 1914: 1. Type species: *Omus submetallicus* Horn, 1869 by original designation. Synonymy established by Horn (1915: 443). Etymology. From the Greek *leptos* (fine, small, thin, delicate) and the generic name *Omus* [*q.v*.] [masculine].
Megomus Casey, 1914: 1. Type species: *Omus dejeanii* Reiche, 1838 by original designation. Synonymy established by Horn (1915: 443). Etymology. From the Greek *megas* (large) and the generic name *Omus* [*q.v*.] [masculine].

#### Diversity.

Five species in western North America inhabiting the Pacific coastal lowlands and the mountain slopes, including those of the Cascade and Sierra Nevada ranges.

#### Identification.

Casey, between 1897 and 1924, described so many forms in this genus (90 species-group taxa) that at the time of his death in 1925 it was virtually impossible to identify members of *Omus*. Cazier (1942) wrote a detailed monographic revision of the genus and recognized three species (*Anisodactylus californicus*, *Anisodactylus dejeanii*, and *Anisodactylus submetallicus*) with four subspecies for *Anisodactylus californicus*. He also provided keys for the identification of the taxa. Unfortunately his thesis was not published. In his thesis on the cicindelids of the Pacific Northwest, Leffler (1979a) followed Cazier’s (1942) conclusions concerning the taxonomy of *Omus* except that he recognized a fourth species, according specific rank to *Omus audouini* considered a synonym of *Omus californicus californicus* by Cazier. Leffler (1979a) provided a key for the separation of the species but his work also remained unpublished. Subsequently, a new species was described by van den Berghe in 1994. The field guide of Pearson et al. (2006: 23) includes a key to all five species.

### Omus audouini

Reiche, 1838

Omus audouini Reiche, 1838: 300. Type locality: «versant occidental des montagnes rocheuses, dans le district d’Oregon, aux États-Unis de l’Amérique du Nord» (original citation, see page 297). Syntype(s) probably in MHNP. Etymology. The specific name was proposed for the French zoologist Jean-Victor Audouin [1797-1841], co-founder of the *Annales des Sciences Naturelles* and a founding member of the *Société Entomologique de France*. In 1823 Audouin became assistant librarian at the Muséum d’Histoire Naturelle in Paris and obtained his doctorate in 1826. He succeeded Latreille as assistant naturalist at the chair of Crustacean and Insects at the Museum in 1830 and three years later as professor of the same chair. He died of apoplexy. He had no insect collection but held a large entomological library which he willingly made available to scholars.
Omus ambiguus Schaupp, 1884b: 121. Type locality: «M[oun]t Shasta District [Siskiyou County], Cal[ifornia]» (original citation). Holotype [by monotypy] (♂) in USNM (collection Casey). Synonymy established by Bousquet and Larochelle (1993: 52) based on Leffler (1979a: 192) unpublished thesis. Note. Leng (1902: 108) reported that the holotype was probably collected at “Upper Soda Springs” in Siskiyou County, at 3000-4000 feet.
Omus van dykei W. Horn, 1903a: 185. Type locality: «Mittel-Oregon» (original citation). Holotype [by monotypy, *cf*. Horn 1903a: 197] (♂) location unknown. Synonymy established by Boyd (1982: 2).
Omus borealis Casey, 1909: 256. Type locality: «Oregon» (original citation). One syntype in USNM [# 45857]. Synonymy established with doubt by Horn (1910a: 125), confirmed by Leffler (1979a: 198).
Omus californicus humeroplanatus W. Horn, 1910b: 293. Type locality: «Provincia Del Norte, Calif[ornia]» (original citation). Syntype(s) in MHNP. Synonymy established, under the name *Omus californicus borealis* Casey, by Horn (1915: 431).
Omus audouini parvulus Casey, 1913: 2. Type locality: «Oregon» (original citation). One syntype in USNM [# 45809]. Synonymy established by Horn (1915: 431), confirmed by Leffler (1979a: 199).
Omus oregonensis Casey, 1913: 2. Type locality: «Josephine Co[unty], Oregon» (original citation). One syntype in USNM [# 45832]. Synonymy established, under the name *Omus californicus borealis* Casey, by Hatch (1953: 36), confirmed by Leffler (1979a: 199) and van den Berghe (1994: 33). Note. According to Leffler (1979a: 199), the type series was collected at Selma and Waldo (abandoned townsite 5 km ESE of O’Brien), Josephine County, by F.W. Nunenmacher.
Omus rugipennis Casey, 1914: 3. Type locality: «northern California» (original citation). One syntype in USNM [# 45803]. Synonymy established, under the name *Omus californicus ambiguus* Schaupp, by Horn (1915: 443), confirmed by Leffler (1979a: 200). Note. According to Leffler (1979a: 200), the type series was collected at Mud Lake, 7 miles NW Cottage Grove, in Siskiyou County, by F.W. Nunenmacher.
Omus solidulus Casey, 1914: 3. Type locality: «Shasta Retreat [Bucks Lake], Siskiyou Co[unty], California» (original citation). One syntype in USNM [# 45812]. Synonymy established, under the name *Omus californicus ambiguus* Schaupp, by Horn (1915: 443), confirmed by Leffler (1979a: 200).
Omus audouini brevicornis Casey, 1916: 8. Type locality: «Humboldt Co[unty], California» (original citation). One syntype in USNM [# 45806]. Synonymy established, under the name *Omus californicus humeroplanatus* Horn, by Horn (1926: 55), confirmed by Leffler (1979a: 200). Note. According to Leffler (1979a: 200), the type series was not collected in Humboldt County as indicated by Casey (1916: 8) but at Mud Lake, 7 miles North West Cottage Grove, Siskiyou County, by F.W. Nunenmacher.
Omus audouini aequicornis Casey, 1916: 9. Type locality: «Josephine Co[unty], Oregon» (original citation). Two syntypes in USNM [# 45807]. Synonymy established, under the name *Omus californicus humeroplanatus* Horn, by Horn (1926: 55), confirmed by Leffler (1979a: 201) and van den Berghe (1994: 33). Note. According to Leffler (1979a: 201), the two original specimens were collected at Selma and Waldo in Josephine County.
Omus audouini tacomae Casey, 1916: 9. Type locality: «Tacoma [Pierce County], Washington» (original citation). One syntype in USNM [# 45804]. Synonymy established by Horn (1926: 55), confirmed by Leffler (1979a: 201).
Omus audouini delicatulus Casey, 1916: 9. Type locality: «Oregon» (original citation). One syntype in USNM [# 45814]. Synonymy established by Horn (1926: 55), confirmed by Leffler (1979a: 201).
Omus audouini distans Casey, 1916: 10. Type locality: «Seattle [King County], Washington» (original citation). One syntype in USNM [# 45813]. Synonymy established by Horn (1926: 55), confirmed by Leffler (1979a: 201).
Omus ambiguus humeralis Casey, 1916: 10. Type locality: «Humboldt Co[unty], California» (original citation). One syntype in USNM [# 45811]. Synonymy established, under the name *Omus californicus humeroplanatus* Horn, by Horn (1926: 55), confirmed by Leffler (1979a: 201). Note. According to Cazier (1942: 104), the type series was collected at 10 miles east of Orick, in Humboldt County, by F.W. Nunenmacher.
Omus thoracicus Casey, 1916: 11. Type locality: «Klamath Co[unty], Oregon» (original citation). One syntype in USNM [# 45816]. Synonymy established, under the name *Omus californicus oregonensis* Casey, by Horn (1926: 55), confirmed by Leffler (1979a: 202) and van den Berghe (1994: 33). Note. According to Leffler (1979a: 202), the type series was collected at Upper Klamath Lake, Klamath County, by F.W. Nunenmacher.
Omus [*cephalicus*] *audens* Casey, 1924: 3. Type locality: «Seattle [King County], Washington» (original citation). One syntype in USNM [# 45805]. Synonymy established by Horn (1926: 55), confirmed by Leffler (1979a: 202).
Omus [*ambiguus*] *socius* Casey, 1924: 4. Type locality: «Shasta Co[unty], California» (original citation). Holotype [by monotypy] (♂) in USNM [# 45810]. Synonymy established, under the name *Omus californicus ambiguus* Schaupp, by Horn (1926: 56), confirmed by Leffler (1979a: 203). Note. According to Leffler (1979a: 203), the holotype was collected by F.W. Nunenmacher at Round Mountain, Shasta County.

#### Distribution.

The range of this species, also known as the “Audouin’s Night-stalking Tiger Beetle”, extends from southwestern British Columbia, including southern Vancouver Island, south to northwestern California [see Pearson et al. 2006: Map 9].

#### Records.

**CAN**: BC (VCI) **USA**: CA, OR, WA

### Omus californicus angustocylindricus

Horn, 1913

Omus californicus angusto-cylindricus W. Horn, 1913: 348. Type locality: «Lassen Co[unty] borealis, Calif[ornia]» (original citation). Syntype(s) in MHNP and MCZ [# 25599].
Omus cylindricus Casey, 1914: 4. Unjustified emendation of *Omus angustocylindricus* Horn, 1913.

#### Distribution.

This subspecies, also known as the “Narrow Night-stalking Tiger Beetle”, is found above 1500 m of elevation in Plumas and Lassen Counties in northeastern California (Leffler 1979a: 218).

#### Records.

**USA**: CA

### Omus californicus californicus

Eschscholtz, 1829

Omus californicus Eschscholtz, 1829: 3. Type locality: «Cabo de los Reyes [Marin County], Californien» (original citation). Syntype(s) location unknown (possibly in ZMMU).
Omus xanti LeConte, 1859a: 69. Type locality: «Fort Tejon [Kern County, California]» (original citation). Holotype [by monotypy] (♂) in MCZ [# 23592]. Synonymy established by Horn (1903a: 196). Etymology. The species name was proposed for John Xantus de Vesey [1825-1894], a Hungarian exile who worked in the United States as a bookseller, druggist, teacher, and hospital steward. He became over the years a gifted collector of natural history specimens. He eventually returned to his native Hungary and served as Director of the Zoological Garden of Budapest and as curator of ethnography at the Hungarian National Museum.
Omus laevis G.H. Horn, 1867a: 394. Type locality: «high Sierras near the head waters of King’s and Tulé rivers [California]» (original citation), restricted to «southeast Fresno County and East Tulare County» by Ward (1982: 59). Lectotype (♂), designated by Ward (1982: 59), in MCZ [# 35314]. Synonymy established by Bousquet and Larochelle (1993: 52) based on Leffler (1979a: 209) unpublished thesis.
Omus lecontei G.H. Horn, 1872b: 143. Type locality: «near Monterey [Monterey County], California» (original citation). Lectotype (♂), designated by Ward (1982: 59), in MCZ [# 58]. Synonymy established by Bousquet and Larochelle (1993: 52) based on Leffler (1979a: 209) unpublished thesis. Note. This form is considered a valid subspecies of *Omus californicus* Eschscholtz by Knisley and Haines (2010).
Omus sequoiarum Crotch, 1874b: 73. Type locality: «Calaveras in the Sierra Nevada» (original citation). Syntype(s) [20 originally cited] in MCZ [# 57] and AMNH [# 444]. Synonymy established by Bousquet and Larochelle (1993: 52) based on Leffler (1979a: 209) unpublished thesis.
Omus edwardsii Crotch, 1874b: 73. Type locality: «Lake Tahoe [Placer County, California]» (original citation). Syntype(s) [6 originally cited] in MCZ [# 56] and AMNH [# 445] (Grossbeck 1912: 360). Synonymy established by Bousquet and Larochelle (1993: 52) based on Leffler (1979a: 209) unpublished thesis. Etymology. The specific name honors Henry Edwards [1830-1891], a stage actor by profession and enthusiastic collector of Lepidoptera and other insects. Born in England, Edwards travelled to Australia, South and Central America, Mexico, and from 1865 to 1877 resided in San Francisco before moving to eastern United States. In 1891, his widow put Edwards’ collection, which consisted of about 300,000 specimens of all orders, for sale. The American Museum of Natural History acquired the collection as well as his correspondence and notes.
Omus hornii LeConte, 1875a: 157. Type locality: «Yosemite, California» (original citation). Holotype [by monotypy] (♀) in MCZ [# 46]. Synonymy established, under the name *Omus californicus sequoiarum* Crotch, by Horn (1930: 78).
Omus hornianus W. Horn, 1892a: 91. Type locality: «California?» (original citation). Holotype [by monotypy] (♂) in DEI (Döbler 1973: 384). Synonymy established by Horn (1902b: 388).
Omus montanus Casey, 1897: 290. Type locality: «Placer Co[unty], California» (original citation). Two syntypes in USNM [# 45842]. Synonymy established by, under the name *Omus californicus edwardsii* Crotch, by Horn (1905: 54).
Omus lugubris Casey, 1897: 290. Type locality: «California» (original citation). One syntype [2 ♂ originally cited] in USNM [# 45859]. Synonymy established, under the name *Omus edwardsii* Crotch, by Leng (1902: 102).
Omus punctifrons Casey, 1897: 291. Type locality: «California» (original citation). Holotype [by monotypy] (♀) in USNM [# 45861]. Synonymy established, under the name *Omus sequoiarum* Crotch, by Leng (1902: 102).
Omus confluens Casey, 1897: 291. Type locality: «California» (original citation). Holotype [by monotypy] (♀) in USNM [# 45863]. Synonymy established, under the name *Omus sequoiarum* Crotch, by Leng (1902: 102).
Omus sculptilis Casey, 1897: 292. Type locality: «coast regions north of San Francisco, California» (original citation). Holotype [by monotypy] (♂) in USNM [# 45824]. Synonymy established by Horn (1902b: 388).
Omus elongatus Casey, 1897: 293. Type locality: «near San Francisco, California» (original citation). One syntype in USNM [# 45852]. Synonymy established, under the name *Omus lecontei* Horn, by Horn (1903a: 188, 198).
Omus californicus fuchsi W. Horn, 1903a: 188. Type locality: «Küste von Californien, südlich von San Francisco gefangen: wahrscheinlich in oder in der Nähe von Monterey Co. [= coast of California, south of San Francisco, probably in or close to Monterey County]» (original citation). One syntype in CMNH. Synonymy established, under the name *Omus californicus lecontei* Horn, by Boyd (1982: 2).
Omus mimus Casey, 1909: 256. Type locality: «probably northern California» (original citation). One syntype in USNM [# 45819]. Synonymy established with doubt by Horn (1910a: 125).
Omus dunni Casey, 1909: 258. Type locality: «vicinity of San Francisco [California]» (original citation). Two syntypes in USNM [# 45853]. Synonymy established, under the name *Omus californicus lecontei* Horn, by Horn (1910a: 126).
Omus dunni regularis Casey, 1909: 258. Type locality: «Carmel, Monterey Co[unty] [California]» (original citation). Two syntypes in USNM [# 45854]. Synonymy established, under the name *Omus californicus lecontei* Horn, by Horn (1910a: 126).
Omus dunni maritimus Casey, 1909: 259. Type locality: «Monterey Co[unty] [California]» (original citation). One syntype in USNM [# 45855]. Synonymy established, under the name *Omus californicus lecontei* Horn, by Horn (1910a: 126).
Omus cribripennis Casey, 1909: 261. Type locality: «Placerville, El Dorado Co[unty] [California]» (original citation). One syntype in USNM [# 45846]. Synonymy established by Bousquet and Larochelle (1993: 53) based on Leffler (1979a: 210) unpublished thesis.
Omus edwardsi lobatus Casey, 1909: 261. Type locality: «Placer Co[unty] [California]» (original citation). One syntype in USNM [# 45840]. Synonymy established, under the name *Omus californicus edwardsii* Crotch, by Horn (1910a: 126).
Omus montanus lucidicollis Casey, 1909: 262. Type locality: «Placer Co[unty] [California]» (original citation). Two syntypes in USNM [# 45841]. Synonymy established, under the name *Omus californicus edwardsii* Crotch, by Horn (1910a: 126).
Omus montanus brunnescens Casey, 1909: 262. Type locality: «Placer Co[unty] [California]» (original citation). Two syntypes in USNM [# 45843]. Synonymy established, under the name *Omus californicus edwardsii* Crotch, by Horn (1910a: 126).
Omus punctifrons degener Casey, 1909: 263. Type locality: «Sierra Co[unty] [California]» (original citation). One syntype in USNM [# 45865]. Synonymy established, under the name *Omus californicus punctifrons* Casey, by Horn (1910a: 125).
Omus fraterculus Casey, 1909: 263. Type locality: «Placer Co[unty] [California]» (original citation). Syntype(s) in USNM [# 45862]. Synonymy established, under the name *Omus californicus punctifrons* Casey, by Horn (1930: 78).
Omus collaris Casey, 1909: 265. Type locality: «Wawona, Mariposa Co[unty] [California]» (original citation). One syntype in USNM [# 45881]. Synonymy established, under the name *Omus hornii* LeConte, by Horn (1910a: 126).
Omus compositus Casey, 1909: 265. Type locality: «Wawona, Mariposa Co[unty] [California]» (original citation). One syntype in USNM [# 45882]. Synonymy established, under the name *Omus californicus hornii* LeConte, by Horn (1910a: 126).
Omus tularensis Casey, 1909: 265. Type locality: «Davenport (6,400 feet), Soldiers’ Camp (5,800 feet), and Colony Mill (5,415 feet), Tulare Co[unty] [California]» (original citation). Six syntypes in USNM [# 45885]. Synonymy established, under the name *Omus californicus laevis* LeConte, by Horn (1910a: 126).
Omus tularensis gracilior Casey, 1909: 266. Type locality: «Tulare Co[unty] [California]» (original citation). Holotype [by monotypy] (♂) in USNM [# 45886]. Synonymy established, under the name *Omus californicus laevis* LeConte, by Horn (1910a: 126).
Omus lugubris sierricola Casey, 1913: 3. Type locality: «California» (original citation). Holotype [by monotypy] (♂) in USNM [# 45860]. Synonymy established, under the name *Omus californicus sequoiarum* Crotch, by Horn (1915: 432).
Omus californicus intermedio-pronotalis W. Horn, 1913: 346. Type locality: «Plumas Co[unty] orientalis, Calif[ornia]» (original citation). Two syntypes in MCZ [# 25598] and IRSN. Synonymy established, under the name *Omus californicus edwardsii* Crotch, by Horn (1930: 79).
Omus californicus nunenmacheri W. Horn, 1913: 347. Type locality: «Lassen Co[unty] centralis, Calif[ornia]» (original citation). Syntype(s) [6 originally cited] in MHNP and MCZ [# 25597]. Synonymy established by Bousquet and Larochelle (1993: 53) based on Leffler’s (1979a: 210) unpublished thesis. Etymology. The subspecific name was proposed for Frederick William Nunenmacher [1870-1946], a successful collector of beetles in western United States and a specialist of Coccinellidae. His coccinellid collection went to the California Academy of Sciences and his general collection to the Field Museum of Natural History.
Omus californicus vermiculatus Casey, 1914: 5. Type locality: «probably near San Francisco, California» (original citation). Three syntypes [3 originally cited] in USNM [# 45823]. Synonymy established by Horn (1915: 443).
Omus pronotalis Casey, 1914: 10. Unjustified emendation of *Omus intermediopronotalis* Horn, 1913.
Omus sequoiarum longitarsis Casey, 1914: 12. Type locality: «Big Trees, Calaveras Co[unty], California» (original citation). One syntype in USNM [# 45858]. Synonymy established, under the name *Omus californicus sequoiarum* Crotch, by Horn (1915: 443).
Omus horni temperatus Casey, 1914: 15. Type locality: «Giant Forest, Tulare Co[unty], California» (original citation). One syntype in USNM [# 45866]. Synonymy established, under the name *Omus californicus laevis* LeConte, by Horn (1915: 443).
Omus tularensis opacellus Casey, 1914: 16. Type locality: «Tulare Co[unty], California» (original citation). One syntype in USNM [# 45889]. Synonymy established, under the name *Omus californicus laevis* LeConte, by Horn (1915: 443).
Omus levis W. Horn, 1915: 443. Unjustified emendation of *Omus laevis* Horn, 1867.
Omus shastanicus Casey, 1916: 11. Type locality: «Shasta Co[unty], California» (original citation). Two syntypes in USNM [# 45808]. Synonymy established by Bousquet and Larochelle (1993: 53) based on Leffler (1979a: 211) unpublished thesis.
Omus shastanicus cephalicus Casey, 1916: 11. Type locality: «Shasta Co[unty], California» (original citation). One syntype in USNM [# 45802]. Synonymy established, under the name *Omus californicus shastanicus* Casey, by Horn (1926: 55).
Omus shastanicus tenuiculus Casey, 1916: 12. Type locality: «Shasta Co[unty], California» (original citation). One syntype in USNM [# 45815]. Synonymy established, under the name *Omus californicus shastanicus* Casey, by Horn (1926: 55).
Omus semilucens Casey, 1916: 12. Type locality: «San Francisco Co[unty], California» (original citation). One syntype in USNM [# 45834]. Synonymy established by Horn (1926: 54).
Omus semilucens diminuens Casey, 1916: 13. Type locality: «Leona Heights, Alameda Co[unty], California» (original citation). One syntype in USNM [# 45833]. Synonymy established by Horn (1926: 55).
Omus californicus latipennis Casey, 1916: 13. Type locality: «Leona Heights, Alameda Co[unty], California» (original citation). One syntype in USNM [# 45822]. Synonymy established by Horn (1926: 55).
Omus sculptilis opacipennis Casey, 1916: 13. Type locality: «S[ain]t Helena, Napa Co[unty], California» (original citation). One syntype in USNM [# 45817]. Synonymy established by Horn (1926: 55).
Omus lacertus Casey, 1916: 15. Type locality: «Carmel, Monterey Co[unty], California» (original citation). One syntype in USNM [# 45856]. Synonymy established, under the name *Omus californicus lecontei* Horn, by Horn (1926: 59).
Omus laticollis Casey, 1916: 16. Type locality: «Tuolumne Co[unty], California» (original citation). Two syntypes in USNM [# 45864]. Synonymy established, under the name *Omus californicus fuchsi* Horn, by Horn (1930: 78).
Omus temperatus difficilis Casey, 1916: 17. Type locality: «Mariposa Co[unty], California» (original citation). One syntype in USNM [# 45867]. Synonymy established, under the name *Omus californicus hornii* LeConte, by Horn (1926: 57).
Omus temperatus mariposae Casey, 1916: 17. Type locality: «Mariposa Co[unty], California» (original citation). One syntype in USNM [# 45869]. Synonymy established, under the name *Omus californicus hornii* LeConte, by Horn (1926: 57).
Omus temperatus sparsellus Casey, 1916: 18. Type locality: «Wawona, Mariposa Co[unty], California» (original citation). One syntype in USNM [# 45873]. Synonymy established, under the name *Omus californicus hornii* LeConte, by Horn (1926: 57).
Omus subsericeus Casey, 1916: 18. Type locality: «Kaweah [Tulare County], California» (original citation). One syntype in USNM [# 45874]. Synonymy established, under the name *Omus californicus laevis* Horn, by Horn (1926: 57).
Omus collaris antennalis Casey, 1916: 19. Type locality: «Mariposa Co[unty], California» (original citation). Two syntypes in USNM [# 45875]. Synonymy established, under the name *Omus californicus laevis* Horn, by Horn (1926: 57).
Omus collaris trapezicollis Casey, 1916: 19. Type locality: «Mariposa Co[unty], California» (original citation). One syntype in USNM [# 45872]. Synonymy established, under the name *Omus californicus laevis* Horn, by Horn (1926: 57).
Omus collaris erraticus Casey, 1916: 20. Type locality: «Tuolumne Co[unty], California» (original citation). One syntype [2 originally cited] in USNM [# 45868]. Synonymy established, under the name *Omus californicus laevis* Horn, by Horn (1926: 57).
Omus horni brevis Casey, 1916: 21. Type locality: «Tuolumne Co[unty], California» (original citation). One syntype in USNM [# 45883]. Synonymy established, under the name *Omus californicus hornii* LeConte, by Horn (1926: 57).
Omus horni propinquus Casey, 1916: 21. Type locality: «Mariposa Co[unty], California» (original citation). One syntype in USNM [# 45871]. Synonymy established, under the name *Omus californicus hornii* LeConte, by Horn (1926: 57).
Omus horni asperatus Casey, 1916: 22. Type locality: «Tuolumne Co[unty], California» (original citation). One syntype in USNM [# 45879]. Synonymy established, under the name *Omus californicus hornii* LeConte, by Horn (1926: 57).
Omus horni granosus Casey, 1916: 22. Type locality: «Mariposa Co[unty], California» (original citation). One syntype in USNM [# 45880]. Synonymy established, under the name *Omus californicus hornii* LeConte, by Horn (1926: 57).
Omus horni sinuosus Casey, 1916: 23. Type locality: «Wawona, Mariposa Co[unty], California» (original citation). One syntype in USNM [# 45877]. Synonymy established, under the name *Omus californicus hornii* LeConte, by Horn (1926: 57).
Omus horni punctatus Casey, 1916: 23. Type locality: «Tuolumne Co[unty], California» (original citation). Four syntypes [4 originally cited] in USNM [# 45878]. Synonymy established, under the name *Omus californicus hornii* LeConte, by Horn (1926: 57). Note. Hennessey (1990: 467) reported the presence of a syntype of *Omus punctatus* Casey in SIM.
Omus horni farctus Casey, 1916: 23. Type locality: «Mariposa Co[unty], California» (original citation). One syntype in USNM [# 45884]. Synonymy established, under the name *Omus californicus hornii* LeConte, by Horn (1926: 57).
Omus marginalis Casey, 1916: 24. Type locality: «Tuolumne Co[unty], California» (original citation). One syntype in USNM [# 45870]. Synonymy established, under the name *Omus californicus hornii* LeConte, by Horn (1926: 57).
Omus tularensis remissus Casey, 1916: 25. Type locality: «Colony Mill (5400 ft.), Tulare Co[unty], California» (original citation). One syntype in USNM [# 45887]. Synonymy established, under the name *Omus californicus laevis* Horn, by Horn (1926: 57).
Omus laevis peropacus Casey, 1916: 25. Type locality: «Tulare Co[unty], California» (original citation). One syntype [3 originally cited] in USNM [# 45888]. Synonymy established, under the name *Omus californicus laevis* Horn, by Horn (1926: 57).
Omus cupreonitens Blaisdell and Reynolds, 1917: 49. Type locality: «shore of Humboldt Bay near Arcata, Humboldt County, California» (original citation). Holotype (♂) in USNM [# 21355]. Synonymy established by Bousquet and Larochelle (1993: 53) based on Leffler (1979a: 212) unpublished thesis.
Omus [*cupreonitens*] *reynoldsi* Casey, 1924: 5. Type locality: «Arcata, Humboldt Co[unty], California» (original citation). Holotype [by monotypy] (♂) in USNM [# 45839]. Synonymy established, under the name *Omus cupreonitens* Blaisdell and Reynolds, by Blaisdell (1925: 80).
Omus [*sculptilis*] *densicollis* Casey, 1924: 5. Type locality: «Mendocino Co[unty], California» (original citation). One syntype in USNM [# 45825]. Synonymy established by Horn (1926: 55).
Omus [*sculptilis*] *argutus* Casey, 1924: 6. Type locality: «Alameda Co[unty], California» (original citation). One syntype in USNM [# 45826]. Synonymy established by Horn (1930: 78).
Omus [*mimus*] *debiliceps* Casey, 1924: 6. Type locality: «San Francisco [San Francisco County], California» (original citation). One syntype in USNM [# 45818]. Synonymy established by Horn (1926: 54).
Omus [*mimus*] *insulsus* Casey, 1924: 6. Type locality: «S[an]ta Cruz Co[unty], California» (original citation). Two syntypes in USNM [# 45828]. Synonymy established by Horn (1926: 55).
Omus [*mimus*] *modicus* Casey, 1924: 7. Type locality: «Mendocino Co[unty], California» (original citation). Two syntypes [2 ♂ originally cited] in USNM [# 45827]. Synonymy established by Horn (1926: 55).
Omus [*mimus*] *stolidus* Casey, 1924: 7. Type locality: «Mendocino Co[unty], California» (original citation). Two syntypes in USNM [# 45829]. Synonymy established by Horn (1926: 54).
Omus [*mimus*] *subparallelus* Casey, 1924: 7. Type locality: «S[an]ta Cruz Co[unty], California» (original citation). Holotype [by monotypy] (♂) in USNM [# 45830]. Synonymy established by Horn (1926: 55).
Omus ventricosus Casey, 1924: 8. Type locality: «Mendocino Co[unty], California» (original citation). One syntype in USNM [# 45831]. Synonymy established by Horn (1926: 54).
Omus leachi Casey, 1924: 8. Type locality: «Trinity Co[unty], California» (original citation). Four syntypes [4 originally cited] in USNM [# 45838]. Synonymy established by Horn (1926: 55).
Omus pullatus Casey, 1924: 9. Type locality: «Sonoma Co[unty], California» (original citation). One syntype in USNM [# 45820]. Synonymy established by Horn (1926: 55).
Omus [*vermiculatus*] *pollens* Casey, 1924: 9. Type locality: «Marin Co[unty], California» (original citation). One syntype in USNM [# 45821]. Synonymy established by Horn (1926: 55).
Omus [*californicus*] *turbulentus* Casey, 1924: 10. Type locality: «Sonoma Co[unty], California» (original citation). One syntype in USNM [# 45835]. Synonymy established by Horn (1926: 55).
Omus [*californicus*] *aethiops* Casey, 1924: 10. Type locality: «Shasta Co[unty], California» (original citation). One syntype in USNM [# 45836]. Synonymy established by Horn (1926: 55).
Omus [*californicus*] *sparsus* Casey, 1924: 10. Type locality: «S[an]ta Cruz, California» (original citation). One syntype in USNM [# 45837]. Synonymy established by Horn (1926: 55).
Omus [*horni*] *callosus* Casey, 1924: 12. Type locality: «Tuolumne Co[unty], California» (original citation). One syntype in USNM [# 45876]. Synonymy established, under the name *Omus californicus hornii* LeConte, by Horn (1926: 57).
Omus vanlooi Nunenmacher, 1940: 144. Type locality: «Butte County, California» (original citation). Holotype (♂) in CAS [# 8164]. Synonymy established by Bousquet and Larochelle (1993: 53) based on Leffler (1979a: 212) unpublished thesis.

#### Distribution.

This subspecies, also known as the “California Night-stalking Tiger Beetle”, ranges from southwestern Oregon to southern California along the coast and through the Sierra Nevada, at elevation below 900 m near the range of *Omus californicus intermedius* (Leffler 1979a: 218; Fig. 18).

#### Records.

**USA**: CA, OR

### Omus californicus intermedius

Leng, 1902

Omus intermedius Leng, 1902: 104. Type locality: «Coulterville, Mariposa Co[unty], Cal[ifornia]» (original citation). Syntype(s) location unknown. Note. The specimen labeled “CoType 25595” in MCZ is not a syntype (see Dahl 1941: 169) but was determined by Leng; the specimen is labeled “Harris Collection - Colony Mill Ro[ad] n[orth] Kaweah California.”
Omus procerus Casey, 1909: 259. Type locality: «Tulare Co[unty] [California]» (original citation). One syntype in USNM [# 45850]. Synonymy established by Horn (1910a: 126).
Omus procerus parvicollis Casey, 1909: 260. Type locality: «Redwood and Mabel Creeks and Watson Springs, Tulare Co[unty] [California]» (original citation). Five syntypes in USNM [# 45848]. Synonymy established by Horn (1910a: 126).
Omus blaisdelli Casey, 1909: 260. Type locality: «Mokalumne Hill, Calaveras Co[unty] [California]» (original citation). Five syntypes in USNM [# 45844]. Synonymy established by Horn (1910a: 126).
Omus spissipes Casey, 1913: 3. Type locality: «Tulare Co[unty], California» (original citation). One syntype in USNM [# 45849]. Synonymy established by Horn (1915: 432).
Omus parvicollis ovipennis Casey, 1916: 14. Type locality: «Mokelumne Hill, Calaveras Co[unty], California» (original citation). One syntype in USNM [# 45851]. Synonymy established, under the name *Omus blaisdelli* Casey, by Blaisdell (1925: 80).
Omus [*blaisdelli*] *torvus* Casey, 1924: 11. Type locality: «Lampson’s Flat (1800 feet), California» (original citation). One syntype in USNM [# 45845]. Synonymy established, under the name *Omus blaisdelli* Casey, by Blaisdell (1925: 80).
Omus [*cribripennis*] *maurus* Casey, 1924: 11. Type locality: «El Dorado Co[unty], California» (original citation). Three syntypes [3 originally cited] in USNM [# 45847]. Synonymy established by Horn (1926: 58).

#### Distribution.

This subspecies, also known as the “Intermediate Night-stalking Tiger Beetle”, is found above 900 meters in the Sierra Nevada between El Dorado and Tulare Counties, California (Leffler 1979a: 222).

#### Records.

**USA**: CA

### Omus californicus subcylindricus

Nunenmacher, 1940

Omus subcylindricus Nunenmacher, 1940: 143. Type locality: «Santa Clara County, California» (original citation). Holotype (♂) in CAS [# 8165].

#### Distribution.

This subspecies, also known as the “Subcylindrical Night-stalking Tiger Beetle”, is restricted to San Martin, Santa Clara County, in western California (Leffler 1979a: 217).

#### Records.

**USA**: CA

### Omus cazieri

van den Berghe, 1994

Omus cazieri van den Berghe, 1994: 33. Type locality: «north side M[oun]t Ashland (1080-1200 m), Jackson Co[unty], Oregon» (original citation). Holotype (♂) in CMNH. Etymology. The specific name was proposed in honor of Mont Adelbert Cazier [1911-1995], curator at the American Museum of Natural History and later professor of zoology at Arizona State University. Cazier published on the behaviour, ecology, and systematics of various arthropod groups, including tiger beetles.

#### Distribution.

This taxon, also known as the “Mount Ashland Night-stalking Tiger Beetle”, is known only from Jackson County in southwestern Oregon.

#### Records.

**USA**: OR

### Omus dejeanii

Reiche, 1838

Omus dejeanii Reiche, 1838: 299. Type locality: «versant occidental des montagnes rocheuses, dans le district d’Oregon, aux États-Unis de l’Amérique du Nord» (original citation, see page 297), herein restricted to Bull Run, Clackamas County, Oregon (see Casey 1916: 8, as *Omus dejeani foveatus*). Syntype(s) probably in MHNP. Etymology. The specific name was proposed in honor of Pierre François Marie Auguste Dejean [1780-1845], a French military officer who held the title of aide-de-camp to Napoleon Bonaparte from 1813 to 1815. Forced to leave his country in January 1816, he was able to come back in 1818 thanks to the relations of his father. At the death of this one in 1824, Dejean inherited his double title of count and pair-de-France. In 1844, Dejean was awarded the rank of “Grand Croix” (the highest) of the prestigious order Légion d’Honneur.
Omus dejeani robustus Casey, 1916: 7. Type locality: «Seattle [King County], Washington» (original citation). Two syntypes in USNM [# 45800]. Synonymy established by Horn (1926: 54).
Omus dejeani foveatus Casey, 1916: 8. Type locality: «Bull Run, Clackamas Co[unty], Oregon» (original citation). Two syntypes in USNM [# 45801]. Synonymy established by Horn (1926: 54).

#### Distribution.

This species, also known as the “Greater Night-stalking Tiger Beetle”, is found from southwestern British Columbia, including Vancouver Island, to southwestern Oregon [see Pearson et al. 2006: Map 6]. The record from northern California (Leng 1902: 105) needs confirmation; that from “Montana” (Leng 1902: 105) is in error.

#### Records.

**CAN**: BC (VCI) **USA**: OR, WA [CA]

**Figure 11.**
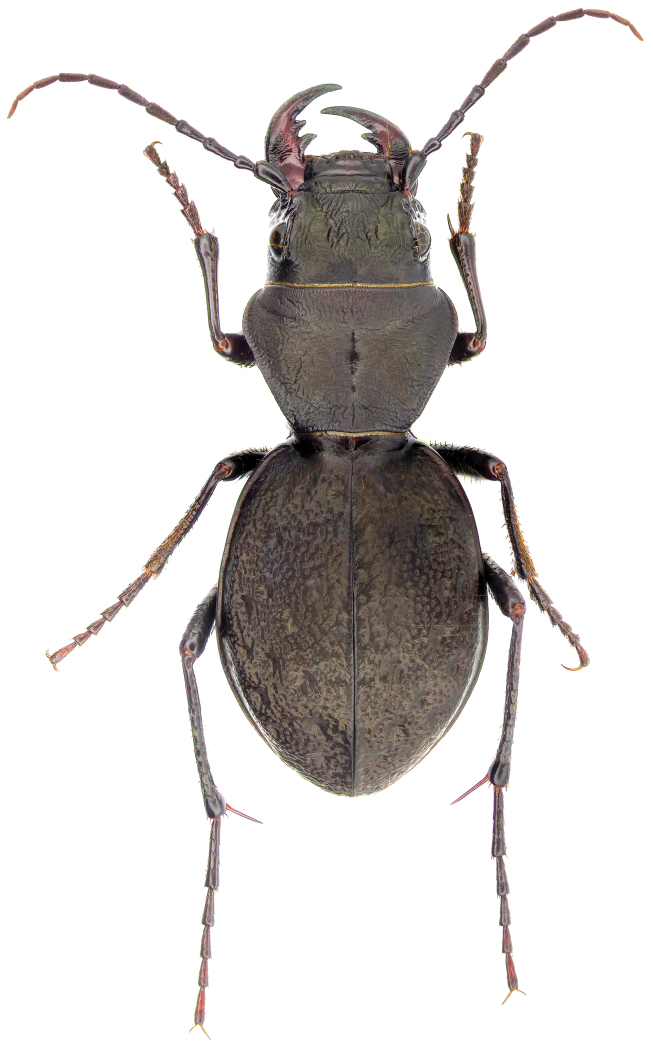
*Omus dejeanii* Reiche. The Greater Night-stalking Tiger Beetle is one of the few species-group taxa currently recognized in the genus *Omus* although over one hundred taxa have been described, particularly by Thomas Casey. Even today, taxonomists do not agree on the number of valid taxa that should be recognized in *Omus*. The difficulty of defining the species-group taxa is not unusual among old, apterous carabid lineages because they tend to form small, local populations.

### Omus submetallicus

Horn, 1869

Omus submetallicus G.H. Horn, 1869b: 129. Type locality: «California» (original citation), restricted, incorrectly so, to «mountains near Alleghany City, Sierra Co[unty]» by Ward (1982: 59), based on information provided by Fuchs to Leng (1902: 109), and subsequently to «Warthan Canyon, mouth of Mulch Canyon, 601 m, 43.6 km (by road) E[ast] San Lucas, St[ate] H[y]w[a]y 198, Fresno Co[unty]» by Leffler (1986a: 38). Holotype [by monotypy] (♂) in MCZ [# 33469].
Omus submetallicus niger Cazier, 1937b: 94. Type locality: «Wartham Canyon, Fresno Co[unty], California» (original citation). Holotype (♂) in CAS [# 4478]. Synonymy established by Bousquet and Larochelle (1993: 52) based on Leffler’s statement (1979a: 181).

#### Distribution.

This species, also known as the “Lustrous Night-stalking Tiger Beetle”, is restricted to a small area east of Warthan Canyon in western Fresno County, central California (Knisley and Haines 2010: 245).

#### Records.

**USA**: CA

### Tribe Megacephalini

Laporte, 1834

Megacephalidae Laporte, 1834: 33. Type genus: *Megacephala* Latreille, 1802.
Megalocephalidae Gistel, 1850: 75. Type genus: *Megalocephala* Gistel, 1850 (= *Megacephala* Latreille, 1802).
Oxycheilites J. Thomson, 1857: 17, 53. Type genus: *Oxycheila* Dejean, 1825.
Tetrachae Leng and Mutchler, 1916: 683. Type genus: *Tetracha* Hope, 1838.

#### Diversity.

About 200 species in the Nearctic (four species), Neotropical (about 165 species), Australian (25 species), Palaearctic (one species), and Afrotropical (12 species) Regions. The species are arrayed in 11 genera (see Naviaux 2007: 10-11): *Aniara* Hope (one Neotropical species), *Australicapitona* Sumlin (eight Australian species), *Cheiloxia* Guérin-Méneville (two Neotropical species), *Grammognatha* Motschulsky (one Mediterranean species), *Megacephala* Latreille (12 Afrotropical species), *Metriocheila* Thomson (one Neotropical species), *Oxycheila* Dejean (about 30 Neotropical species), *Phaeoxantha* Chaudoir (12 Neotropical species), *Pseudotetracha* Fleutiaux (17 Australian species), *Pseudoxycheila* Guérin-Méneville (about 20 Neotropical species), and *Tetracha* (about 95 species).

### Genus Tetracha

Hope, 1838

Gnatho Illiger, 1807: 348 [potential *nomen oblitum*, see Bousquet (2002b: 23)]. Type species: *Cicindela carolina* Linnaeus, 1763 designated by Bousquet (2002b: 24). Etymology. Uncertain, either from the Latin *Gnatho* (name of a parasite in *Eunuchus*, title of a comedy by Terence; a parasite in general) or the Greek *gnathos* (jaw).
Tetracha Hope, 1838: 6 [potential *nomen protectum*]. Type species: *Cicindela carolina* Linnaeus, 1763 by monotypy. Etymology. From the Greek *tetra* (four) and perhaps *chaite* (long hair) contracted, possibly alluding to the presence of four setae on the labrum (Naviaux 2007: 16) [feminine].

#### Diversity.

Western Hemisphere, with about 95 species (110 species-group taxa) (Naviaux 2007: 3) in the Nearctic (four species, only one endemic) and Neotropical (about 95 species) Regions. The species are arrayed in seven subgenera: *Apterotetracha* Naviaux (three Brazilian species), *Microtetracha* Naviaux (one Argentine species), *Neotetracha* (about 55 species), *Oblongotetracha* Naviaux (one Venezuelan species), *Paratetracha* Naviaux (five South American species), *Prototetracha* Naviaux (one Mexican species), and *Tetracha* s.str. (29 species).

#### Identification.

Naviaux (2007) recently revised the species and provided keys for the subgenera and the species groups but not for all species. Pearson et al. (2006: 24) field guide to the tiger beetles of North America included a key to all North American species (*Tetracha impressa* under the name *Tetracha affinis*) except *Tetracha floridana*. Naviaux (2007: 42-43) redescribed *Tetracha floridana* and pointed out the structural differences between the species and *Tetracha carolina*.

#### Taxonomic Note.

In their phylogenetic analysis based on the nuclear 18S and the mitochondrial 16S and cytochrome oxidase III genes, Zerm et al. (2007) found out that the genus *Tetracha* was paraphyletic in regard to the monospecific genus *Aniara* Hope and that the two genera and *Phaeoxantha* form a clade.

#### Faunistic Note.

From a zoogeographical point of view it is of interest to note that a specimen identical to present day *Tetracha carolina carolina* has been found in amber from the east coast of Germany (Horn 1906).

### Subgenus Tetracha

Hope, 1838

Tetracha Hope, 1838: 6. Type species: *Cicindela carolina* Linnaeus, 1763 by original designation.

#### Diversity.

Twenty-nine species in North America (three species, one of them endemic) and the Neotropical Region (28 species), including the West Indies.

### [carolina group]

### Tetracha carolina carolina

(Linnaeus, 1763)

Cicindela carolina Linnaeus, 1763: 395. Type locality: «Carolina» (original citation). Two possible syntypes in LSL (Lindroth 1957b: 336).
Megacephala carolinensis Latreille, 1805: 175. Unjustified emendation of *Megacephala carolina* (Linnaeus, 1763).
Megacephala mexicana Gray, 1831: 263. Type locality: «Mexico» (original citation). Syntype(s) location unknown. Synonymy established by Cresson (1861: 8).
Megacephala boisduvalii Gistel, 1837: 7. Type locality: «Mexico» (original citation). Syntype(s) lost. Synonymy established by Horn (1903b: 220). Etymology. The specific name honors the French naturalist and physician Jean Baptiste Antoine Déchauffour de Boisduval [1801-1879] who for some time was employed by count Dejean as curator of his collection. Boisduval is primarily known for his work on Lepidoptera. Among other things he published with John Eatton LeConte an iconography of the Lepidoptera and caterpillars of North America between 1829 and 1834.

#### Distribution.

The range of this subspecies, also known as the “Pan-American Big-headed Tiger Beetle” (Pearson et al. 2006: 57) or the “Carolina Metallic Tiger Beetle” (Erwin and Pearson 2008: 70), extends from Maryland to southeastern California, south to Nicaragua (Naviaux 2010: 70) and the Florida Keys [see Pearson et al. 1997: Fig. 3]; two specimens from San Diego County in California are also known (Moore 1937: 3). The records from the Bahamas, Cuba, Cayman Islands, and Jamaica refer to *Tetracha carolina occidentalis*. The records from “Colorado” (Wickham 1902: 228), “Connecticut,” “Indiana,” “New Jersey,” and “New York” (see Bousquet and Larochelle 1993: 54) are probably in error or based on strays; that from Nebraska (Bruner 1901: 97) needs confirmation (see Spomer et al. 2008a: 11).

#### Records.

**USA**: AL, AR, AZ, CA, DC, FL, GA, IA, IL, KS, KY, LA, MD, MO, MS, NC, NM, NV, OK, SC, TN, TX, VA [NE] – Guatemala, Mexico, Nicaragua

#### Note.

Three more subspecies are recognized by Naviaux (2007: 40): *Tetracha carolina chevrolatii* Chaudoir from the Yucatán Peninsula and Belize, *Tetracha carolina moraveci* Naviaux from the state of Mexico, and *Tetracha carolina occidentalis* (Klug) from the West Indies.

### Tetracha floridana

Leng and Mutchler, 1916

Tetracha carolina var. *floridana* Leng and Mutchler, 1916: 688. Type locality: «[The] Everglade[s], Fl[orid]a» (original citation). Holotype in AMNH [# 129] (Dahl 1941: 170).

#### Distribution.

This species, also known as the “Florida Metallic Tiger Beetle”, is found only in southern Florida, from Dixie County to the Keys (Choate 2003: 63).

#### Records.

**USA**: FL

### [virginica group]

### Tetracha virginica

(Linnaeus, 1767)

Cicindela virginica Linnaeus, 1767: 657. Type locality: «Carolina» (original citation). Syntype(s) probably lost. Note. *Cicindela virginata*, usually attributed to Linnaeus, is an incorrect subsequent spelling introduced by Gmelin (1790: 1922).
Tetracha virginica var. *melaena* Cartwright, 1935: 70. Type locality: «Clemson College [Anderson and Pickens Counties], S[outh] C[arolina]» (original citation). Holotype (♂) in USNM [# 50765]. Synonymy established by Boyd (1982: 5).

#### Distribution.

The range of this species, also known as the “Virginia Big-headed Tiger Beetle” (Pearson et al. 2006: 59) or the “Virginia Metallic Tiger Beetle” (Erwin and Pearson 2008: 100), extends from southwestern Connecticut (Dunn 1985b: 21) to southwestern Nebraska (Spomer et al. 2008a: 54), south to southwestern Texas, northeastern Mexico (Pearson et al. 2006: 60), and the Florida Keys [see Pearson et al. 2006: Map 13]; apparently isolated in southeastern Arizona (Dajoz 2004: 116) and south-central Wisconsin (Lawton 1971: 57). The records from “Colorado” (Boyd 1982: 5) and “Tabasco” (Erwin and Pearson 2008: 100) need confirmation.

#### Records.

**USA**: AL, AR, AZ, CT, DC, DE, FL, GA, IA, IL, IN, KS, KY, LA, MD, MO, MS, NC, NE, NJ, NY, OH, OK, PA, SC, TN, TX, VA, WI, WV [CO] – Mexico

### Subgenus Neotetracha

Naviaux, 2007

Neotetracha Naviaux, 2007: 29, 79. Type species: *Megacephala distinguenda* Dejean, 1831 by original designation. Etymology. From Greek prefix *neo*- (new) and the generic name *Tetracha* [*q.v*.] [feminine].

#### Diversity.

About 55 Neotropical species, one of them extending into southeastern Texas.

### Tetracha impressa

(Chevrolat, 1841)

Megacephala impressa Chevrolat, 1841: [plate 55] 3. Type locality: «en allant de la Vera-Cruz à Mexico» (original citation). Holotype [by monotypy] (♀) in MHNP (Naviaux 2007: 143).

#### Distribution.

This species, also known as the “Upland Metallic Tiger Beetle”, ranges from southeastern Texas (Darlington 1935b: 161, as *Tetracha angustata*) to Veracruz, Mexico (Naviaux 2007: 144).

#### Records.

**USA**: TX – Mexico

#### Note.

This species has passed under the name *Tetracha angustata* (Chevrolat, 1841) or *Tetracha affinis angustata* in the North American literature until recently.

### Tribe Cicindelini

Latreille, 1802

Cicindeletae Latreille, 1802: 77. Type genus: *Cicindela* Linnaeus, 1758.

#### Diversity.

Worldwide, with more than 1,710 species arrayed in five subtribes: Apteroessina (one species from south India known only from three partial specimens collected in the early xix Century), Cicindelina (more than 1,090 species), Iresina (about 45 species, most in the Neotropical and Australian Regions), Dromicina [= Prothymina] (about 475 species in the Neotropical, Australian, Oriental, Asian Palaearctic, and Afrotropical Regions), and Theratina (about 100 Asian species in the genus *Therates* Latreille).

### Subtribe Cicindelina

Latreille, 1802

Cicindeletae Latreille, 1802: 77. Type genus: *Cicindela* Linnaeus, 1758.

#### Diversity.

Worldwide, with about 1,090 species. The North American fauna is represented by 98 species (about 9% of the world fauna). The number of genera recognized varies greatly depending on the approach used (lumper versus splitter).

#### Identification.

Willis (1968) published a simplified key based on external characters for 91 North American forms, representing all the current recognized species except for the following 11: *Cicindela albissima*, *Cicindela arida*, *Cicindela cazieri*, *Cicindela highlandensis*, *Cicindela nigrior*, *Cicindela ohlone*, *Cicindela scabrosa*, *Cicindela waynei*, *Habroscelimorpha fulgoris*, *Cylindera lunalonga*, and *Dromochorus velutinigrens*. Pearson et al. (2006: 24-42) field guide to the North American tiger beetles included a key to all currently recognized species, except *Cylindera lunalonga* which has been raised to species level very recently, and a few subspecies. The key is based on external characters and distribution ranges.

### Genus Cylindera

Westwood, 1831

Cylindera Westwood, 1831: 300. Type species: *Cicindela germanica* Linnaeus, 1758 by monotypy. Etymology. From the Greek *cylindros* (cylinder), probably alluding to the more cylindrical shape of the sole species known to Westwood [feminine].

#### Diversity.

About 210 species in all zoogeographical regions. The North American fauna includes eight species (about 4% of the world fauna) placed in the nominotypical subgenus.

#### Taxonomic Note.

Lorenz (2005: 54-58) listed the following taxa as subgenera of *Cylindera*: *Apterodela* Rivalier (four species), *Conidera* Rivalier (two species), *Eriodera* Rivalier (one species), *Eugrapha* Rivalier (32 species), *Gaymara* Freitag and Barnes (five Neotropical species), *Glomera* Acciavatti and Pearson (two species), *Ifasina* Jeannel (67 species), *Leptinomera* Rivalier (25 species), *Oligoma* Rivalier (two species), *Plectographa* Rivalier (19 species), *Setinteridenta* Acciavatti (one species), and *Verticina* Rivalier (six species). *Cicindelina* Jeannel (one Madagascan species) was transferred from the genus *Cicindela* to *Cylindera* by Moravec (2010: 198).

### Subgenus Cylindera

Westwood, 1831

Cylindera Westwood, 1831: 300. Type species: *Cicindela germanica* Linnaeus, 1758 by monotypy.
Eumecus Motschulsky, 1850a: 4. Type species: *Cicindela germanica* Linnaeus, 1758 designated by Motschulsky (1862b: 22).
Cicindosa Motschulsky, 1864: 173. Type species: *Cicindosa obliquealba* Motschulsky, 1864 (= *Cicindela morio* Klug, 1834) designated by Horn (1915: 236). Synonymy established by Freitag and Barnes (1989: 317).
Cylindrodera Bedel, 1879: 6. Unjustified emendation of *Cylindera* Westwood, 1831. Note. Bedel (1879) used the spelling *Cylindera* in the key (page 3) and *Cylindrodera* in the text (page 6), both as valid.
Cylindella Jacobson, 1924: 238. Unnecessary replacement name for *Cylindera* Westwood, 1831.

#### Diversity.

About 45 species (Lorenz 2005: 55-56) in the Nearctic (eight species, 14 species-group taxa), Neotropical (about 15 species), Oriental (four species), Palaearctic (14 species), and Afrotropical (six species) Regions.

### Cylindera celeripes

(LeConte, 1846)

Cicindela celeripes LeConte, 1846b: 183. Type locality: «ad fluminis Kansas Republican Fork [Kansas]» (original citation). Syntype(s) in MCZ [# 4]. Note. According to MacRae and Brown (2011b: 231), the type locality is the area occupied by present day Fort Riley Military Reservation in the Flint Hills near Junction City, Riley County, Kansas, at the confluence of the Kansas and Republican Rivers.

#### Distribution.

This species, also known as the “Swift Tiger Beetle”, has been recorded from Nebraska and western Iowa south to north-central Texas and north-central Arkansas [see MacRae and Brown 2011b: Fig. 8]. The record from Indiana (Montgomery and Montgomery 1931: 359) was based on misidentified *Cylindera cursitans* (Knisley et al. 1990: 279); those from “Illinois,” and “Dakota” (Leng 1902: 117) are in error or based on strays. According to MacRae and Brown (2011b: 230), once abundant in Nebraska, western Iowa, and eastern Kansas, the species has declined below detectable levels in much of this area during the past century.

#### Records.

**USA**: AR, IA, KS, MO, NE, OK, TX

### Cylindera cursitans

(LeConte, 1856)

Cicindela cursitans LeConte, 1856a: 60. Type locality: «Fort Riley [junction of Republican and Smoky Hill Rivers, Kansas]» (original citation). Holotype [by monotypy] (♀) in MCZ [# 10].
Cicindela alata Liljeblad, 1932: 215. Type locality: «Chicago [Cook County], Illinois» (original citation). Holotype (♂) in FMNH (Kippenhan 1996b: 52). Synonymy established by Horn (1935: 66). Note. Kippenhan (1996b: 53) reported that the original specimens of Liljeblad were “introduced” into Chicago since *Cylindera cursitans* is unknown from the vicinity of the city.

#### Distribution.

The range of this species, also known as the “Ant-like Tiger Beetle”, extends from North Dakota and west-central Minnesota (Tinerella and Rider 2000: 367) to the Ohio River Valley in West Virginia (Kirchner and Kondratieff 1999: 84), south to western Alabama (Löding 1945: 10), Louisiana, and northern Kansas (Popenoe 1877: 22) [see Pearson et al. 2006: Map 90]; also recorded from “Montana” (Erwin and Pearson 2008: 207).

#### Records.

**USA**: AL, AR, IA, IL, IN, KS, KY, LA, MN, MO, MS, ND, NE, OH, SD, TN, WV [MT]

### Cylindera debilis

(Bates, 1890)

Cicindela debilis Bates, 1890: 509. Type locality: «Ciudad in Durango [Mexico]» (original citation). Three syntypes in DEI (Döbler 1973: 372).
Cicindela debilis var. *segnis* E.D. Harris, 1913: 69. Type locality: «Sonoita [Santa Cruz County], Ariz[ona]» (original citation). Syntype(s) in MCZ [# 23553]. Synonymy established by Cazier (1954: 287).

#### Distribution.

This species, also known as the “Grass-runner Tiger Beetle”, ranges from southeastern Arizona to southwestern Texas [see Pearson et al. 2006: Map 87], south to Durango (Cazier 1954: 287).

#### Records.

**USA**: AZ, NM, TX – Mexico

### Cylindera lemniscata lemniscata

(LeConte, 1854)

Cicindela lemniscata LeConte, 1854d: 220. Type locality not stated; cited from «probably the valley of the Gila [River]» by LeConte (1856a: 59). Holotype [by monotypy] (♂) in MCZ [# 19].

#### Distribution.

This subspecies, also known as the “White-striped Tiger Beetle”, ranges from southern California to southwestern New Mexico, as far north as southwestern Utah (Tanner 1929a: 86), south to Nayarit (Cazier 1960: 12) [see Shook 1989: Fig. 1].

#### Records.

**USA**: AZ, CA, NM, NV, UT – Mexico

### Cylindera lemniscata rebaptisata

(Vaurie, 1951)

Cicindela lemniscata rufipes Vaurie, 1950: 5 [primary homonym of *Cicindela rufipes* Klug, 1825]. Type locality: «Van Horn, Culberson County, Texas» (original citation). Holotype (♂) in AMNH [# 1209].
Cicindela lemniscata rebaptisata Vaurie, 1951: 12. Replacement name for *Cicindela lemniscata rufipes* Vaurie, 1950.

#### Distribution.

This subspecies, the “Rouged Tiger Beetle”, is known from northeastern New Mexico south to southern Durango and Tamaulipas [see Shook 1989: Fig. 1].

#### Records.

**USA**: NM, TX – Mexico

#### Note.

Intergrades between the two subspecies of *Cylindera lemniscata* occur over a small area in south-central New Mexico and northern Chihuahua [see Shook 1989: Fig. 1]. On the Baja California Peninsula, this species is represented by *Cylindera lemniscata bajacalifornica* (Shook).

### Cylindera lunalonga

(Schaupp, 1884)

Cicindela lunalonga Schaupp, 1884b: 122. Type locality: «Sierra Nevada, Cal[ifornia]» (original citation), herein restricted to near Westwood, Lassen County (see Kippenhan and Knisley 2009: 30). Holotype [by monotypy] (♀) apparently destroyed.
Cicindela lunalonga var. *tuolumnae* Leng, 1902: 157. Type locality: «Hetch Hetchy Valley, Tuolumne Co[unty], Cal[ifornia]» (original citation). Lectotype (♀), designated by Dahl (1941: 191), in AMNH [# 1228]. Synonymy established (as aberration) by Horn (1905: 23), confirmed by Kippenhan and Knisley (2009: 30).
Cicindela tularensis Casey, 1914: 19. Type locality: «Tulare Co[unty], California» (original citation). Four syntypes [4 originally cited] in USNM [# 45919]. Synonymy established by Horn (1915: 444), confirmed by Kippenhan and Knisley (2009: 31).
Cicindela pusilla wagneri Cazier, 1937c: 117. Type locality: «Friant, Freno Co[unty], Cal[ifornia]» (original citation). Holotype (♀) in AMNH [# 1203]. Synonymy established by Cazier (1948: 17), confirmed by Kippenhan and Knisley (2009: 31).

#### Distribution.

This species, also known as the “Meadow Tiger Beetle”, is restricted to the western and northern slopes of the Sierra Nevada and adjacent eastern portion of the San Joaquin Valley of California (Woodcock et al. 2006: 869). Specimens labeled from Riverside in California, Prescott in Arizona (see Cazier 1939: 28), and Olmito and San Benito in Texas are doubtful or in error (Woodcock et al. 2006: 869); the records from San Pedro Mártir Mountains in Baja California (Cazier 1948: 18) and “Nevada” (Boyd 1982: 16) need confirmation. Extensive surveys in the past 20 years have produce a sole extant population, near Westwood in Lassen County leading Woodcock et al. (2006: 875) to suggest that the species should be considered as a candidate for listing as a Threatened and Endangered species by the U.S. Fish and Wildlife Service.

#### Records.

**USA**: CA [NV]

#### Note.

This form has been considered a subspecies of *Cylindera terricola* (Say) or a full species depending on the author until mitochondrial DNA analysis indicates that it is a distinct species (Woodcock et al. 2006).

### Cylindera terricola cinctipennis

(LeConte, 1846)

Cicindela cinctipennis LeConte, 1846b: 182. Type locality: «apud Rocky Mountains» (original citation), cited from «Platte and Arkansas River» by LeConte (1856a: 46). Syntype(s) in MCZ [# 6].
Cicindela cyanella LeConte, 1856a: 46. Type locality: «Yellowstone River, Upper Missouri [probably in northeastern Montana]» (original citation). Holotype [by monotypy] in MCZ [# 11]. Synonymy established (as aberration) by Horn (1905: 23).

#### Distribution.

The range of the “Belted-winged Tiger Beetle” is disputed. According to Johnson (1990b: Fig. 1), it ranges from west-central Alberta to southeastern Saskatchewan, south to central New Mexico and central Arizona; isolated in southwestern Utah and southwestern Yukon Territory. According to Pearson et al. (2006: 153), it is found in “lower elevations of Colorado, New Mexico, and Arizona.” Erwin and Pearson (2008: 222) recorded the subspecies from the same states as Pearson et al. (2006: 153) and added Kansas (also cited by Leng 1902: 155).

#### Records.

**CAN**: AB, SK, YT **USA**: AZ, CO, MT, ND, NE, NM, SD, UT, WY

### Cylindera terricola continua

(Knaus, 1923)

Cicindela pusilla imperfecta form *continua* Knaus, 1923: 195. Type locality: «Baldwin Lake near Pine Knot (8,500 feet), San Bernardino Mountains [San Bernardino County], California» (original citation). Holotype probably in KSUC. Note. This taxon was clearly proposed as an infrasubspecific entity by Knaus (1923: 195) but since it was adopted as the valid name of a subspecies before 1985 (e.g., Boyd 1982: 16), it is deemed to be subspecific from its original publication (ICZN 1999: Article 45.6.4.1). The subspecies is credited to Pearson, Knisley and Kazilek (2006: 153) by some authors (e.g., Kippenhan 2007: 7; Erwin and Pearson 2008: 222) but since these authors failed to indicate explicitly that the taxon was intentionally new, a mandatory requirement (ICZN 1999: Article 16.1), the name could not be credited to them even if Knaus’ name was unavailable.

#### Distribution.

This subspecies, the “Interior Tiger Beetle”, is known from Nye County in western Nevada and from the Traverse Mountain Range in western Ventura County eastwards to the San Bernardino Mountains in San Bernardino County and the southern tip of the Sierra Nevada in Kern County, California (Kippenhan 2007: 14, Fig. 8). The record from “NE” (Erwin and Pearson 2008: 222) is probably an error for “NV.”

#### Records.

**USA**: CA, NV

#### Note.

Freitag (1999: 87) listed this taxon as a junior synonym of *Cylindera terricola imperfecta* (LeConte) but Pearson et al. (2006: 153) and Kippenhan (2007: 7) retained it as a valid subspecies.

### Cylindera terricola imperfecta

(LeConte, 1851)

Cicindela imperfecta LeConte, 1851: 171. Type locality: «California borealis» (original citation), cited from «Sacramento» by LeConte (1856a: 45). One syntype in MCZ [# 17].

#### Distribution.

This subspecies, the “Imperfect Tiger Beetle”, ranges from westernmost Alberta and British Columbia, as far north as Fort Saint John along the Peace River (Catling 2007: 19), south to southern Utah, southern Nevada, and Mono County (Kippenhan 2007: 13) in west-central California [see Johnson 1990b: Fig. 1]; also recorded from “Arizona” and “Wyoming” by Erwin and Pearson (2008: 222). The record from New Mexico (Fall and Cockerell 1907: 155) must be in error.

#### Records.

**CAN**: AB, BC **USA**: CA, ID, MT, NV, OR, UT, WA [AZ, WY]

### Cylindera terricola kaibabensis

(Johnson, 1990)

Cicindela pusilla kaibabensis Johnson, 1990b: 4. Type locality: «8 miles north of Kaibab Lodge, Coconino Co[unty], Arizona» (original citation). Holotype (♂) in AMNH [# 1551].

#### Distribution.

This subspecies, the “Kaibab Tiger Beetle”, is known only from the Kaibab Plateau of northern Arizona (Johnson 1990b: 1).

#### Records.

**USA**: AZ

### Cylindera terricola susanagreae

(Kippenhan, 2007)

Cicindela terricola susanagreae Kippenhan, 2007: 15. Type locality: «4 mi[les] N[orth] of Big Pine, Inyo Co[unty], Calif[ornia]» (original citation). Holotype (♂) in CAS.

#### Distribution.

This subspecies, the “Susan’s Tiger Beetle”, is restricted to the valleys east of the Sierra Nevada Mountains in Mono and Inyo Counties, California [see Kippenhan 2007: Fig. 8].

#### Records.

**USA**: CA

### Cylindera terricola terricola

(Say, 1824)

Cicindela pusilla Say, 1817b: 21 [primary homonym of *Cicindela pusilla* Schreber, 1759]. Type locality: «the Missouri, above the confluence of the river Platte» (original citation, see page 19 for *Cicindela formosa*). Syntype(s) lost.
Cicindela terricola Say, 1824: 269. Type locality: «North-west Territory» (original citation). Syntype(s) lost. Synonymy established by Horn (1915: 390). Note. In the late 1810s and early 1820s, the Northwest Territory consisted of Ohio and parts of Michigan and Minnesota. Say’s specimen(s) was collected during the expedition to Saint Peter’s River which extended also into southeastern Manitoba and western Ontario. Say’s specimen(s) of *Cylindera terricola* was probably collected in Manitoba.
Cicindela pusilla sayanella Casey, 1914: 19. Type locality: «Monroe Cañon, Sioux Co[unty], Nebraska» (original citation). One syntype in USNM [# 45920]. Synonymy established by Horn (1915: 444).

#### Distribution.

This subspecies, also known as the “Variable Tiger Beetle”, ranges from northwestern Ontario (Lawton 2008: 73) to southeastern Saskatchewan, south to northwestern Nebraska [see Johnson 1990b: Fig. 1]. The record from Minnesota (Horn 1928: 12) needs confirmation.

#### Records.

**CAN**: MB, ON, SK **USA**: ND, NE, SD [MN]

### Cylindera unipunctata

(Fabricius, 1775)

Cicindela unipunctata Fabricius, 1775: 225. Type locality: «America» (original citation), herein restricted to “Brown’s Woods”, near Pittsburgh, Allegheny County, Pennsylvania (see Leng 1902: 119). Lectotype [as type], designated by Staig (1931: 8), in HMUG.

#### Distribution.

The range of this species, also known as the “One-spotted Tiger Beetle”, extends from northeastern New York to central Georgia and southern Mississippi, west to western Missouri [see Pearson et al. 1997: Fig. 19]. The record from “Texas” (Horn 1915: 389) is probably in error or based on a stray.

#### Records.

**USA**: AL, AR, DC, GA, IA, IL, IN, KY, MD, MN, MO, MS, NC, NJ, NY, OH, PA, SC, TN, VA, WV

### Cylindera viridisticta arizonensis

(Bates, 1884)

Cicindela viridisticta var. *arizonensis* Bates, 1884: 260. Type locality: «Arizona; northern Sonora, Mexico» (original citation). Syntype(s) in BMNH.

#### Distribution.

This subspecies, the “Pygmy Tiger Beetle”, ranges from central Arizona (Pearson et al. 2006: 156) south to Durango (Cazier 1954: 285).

#### Records.

**USA**: AZ – Mexico

#### Note.

The subspecies *Cylindera viridisticta viridisticta* (Bates) and *Cylindera viridisticta interjecta* (Horn) are Mexican endemics.

### Genus Ellipsoptera

Dokhtouroff, 1883

Ellipsoptera Dokhtouroff, 1883b: 70. Type species: *Cicindela hirtilabris* LeConte, 1875 designated by Horn (1915: 236). Etymology. From the Latin *ellipsis* (lack, defect) and the Greek *pteron* (wing) [feminine].

#### Diversity.

Thirteen North American species (27 species-group taxa), of which five extend into Mexico, as far south as the state of Yucatan, and one into the Bahamas and Cuba.

### [marginata group]

### Ellipsoptera blanda

(Dejean, 1831)

Cicindela blanda Dejean, 1831: 238. Type locality: «Amérique septentrionale» (original citation), herein restricted to Lynches River State Park, Florence County, South Carolina (see Cartwright 1935: 75). Syntype(s) in MHNP.
Cicindela tarsalis LeConte, 1852b: 66. Type locality: «Canootche river, Georgia» (original citation). Holotype [by monotypy] (♂) in MCZ [# 39]. Synonymy established by LeConte (1856a: 49).

#### Distribution.

This species, also known as the “Sandbar Tiger Beetle”, is endemic to the Coastal Plain ranging from southeastern North Carolina to the Florida Panhandle west to southeastern Mississippi and southwestern Louisiana [see Pearson et al. 2006: Map 97].

#### Records.

**USA**: AL, FL, GA, LA, MS, NC, SC

### Ellipsoptera cuprascens

(LeConte, 1852)

Cicindela cuprascens LeConte, 1852b: 65. Type locality: «Arkansas river» (original citation), cited from «Missouri and Kansas» by LeConte (1856a: 49). Syntype(s) in MCZ [# 9].
Cicindela cuprascens amnicola Casey, 1913: 37. Type locality: «Kentucky, Illinois and Missouri» (original citation). Four syntypes in USNM [# 45956]. Synonymy established by Horn (1915: 395).
Cicindela mundula Casey, 1913: 37. Type locality: «Vicksburg [Warren County], Mississippi» (original citation). One syntype in USNM [# 45957]. Synonymy established by Horn (1915: 395).

#### Distribution.

This species, also known as the “Coppery Tiger Beetle”, ranges from southern Ohio to southwestern Manitoba and central Montana, south to east-central New Mexico, eastern Texas, and southern Alabama [see Pearson et al. 2006: Map 100]; also known from one locality in northern Georgia. The record from “Wisconsin” (Bousquet and Larochelle 1993: 68) is likely in error (Peter W. Messer pers. comm. 2007).

#### Records.

**CAN**: MB **USA**: AL, AR, CO, GA, IA, IL, IN, KS, KY, LA, MN, MO, MS, MT, ND, NE, NM, OH, OK, SD, TN, TX, WV, WY

### Ellipsoptera gratiosa

(Guérin-Méneville, 1840)

Cicindela gratiosa Guérin-Méneville, 1840: 37. Type locality: Pensacola, Escambia County, Florida (inferred from title of the paper). One syntype in MCZ (collection LeConte).

#### Distribution.

This species, also known as the “Whitish Tiger Beetle”, inhabits the Coastal Plain ranging from Virginia (Hoffman et al. 2006: 17) to the Florida Panhandle, west to southwestern Alabama (Löding 1945: 10) [see Pearson et al. 2006: Map 106]; also recorded from “Mississippi” (Erwin and Pearson 2008: 229). The record from Louisiana (Boyd 1982: 17) is probably in error or based on a stray.

#### Records.

**USA**: AL, FL, GA, NC, SC, VA [MS]

### Ellipsoptera hamata lacerata

(Chaudoir, 1854)

Cicindela lacerata Chaudoir, 1854: 115. Type locality: «Floride» (original citation), herein restricted to Cedar Key, Levy County (see Leng 1915: 561, as *Cicindela hamata*). Syntype(s) in MHNP.

#### Distribution.

This subspecies, the “Gulf Beach Tiger Beetle”, is found along the Gulf coast of Florida, including the Keys (Choate 2003: Map 61), and Alabama (Löding 1945: 10). The record from Horn Island, Mississippi (Richmond 1968: 234), is in error for *Ellipsoptera hamata monti* (Graves and Pearson 1973: 187); those from “North Carolina,” “South Carolina,” “Georgia,” and “Louisiana” (Erwin and Pearson 2008: 229) are probably in error or based on strays.

#### Records.

**USA**: AL, FL

#### Note.

The nominotypical subspecies is known from the states of Tabasco and Veracruz and *Ellipsoptera hamata pallifera* (Chaudoir) from the states of Quintana Roo and Yucatán (Erwin and Pearson 2008: 229-230).

### Ellipsoptera hamata monti

(Vaurie, 1951)

Cicindela hamata monti Vaurie, 1951: 4. Type locality: «Ten miles southwest of Sabine, Jefferson County, Texas» (original citation). Holotype (♂) in AMNH [# 1213].

#### Distribution.

This subspecies, the “Coastal Tiger Beetle”, is found along the Gulf Coast from Mississippi (Graves and Pearson 1973: 187) to the state of Veracruz in Mexico (Erwin and Pearson 2008: 230).

#### Records.

**USA**: LA, MS, TX – Mexico

### Ellipsoptera hirtilabris

(LeConte, 1875)

Cicindela hirtilabris LeConte, 1875a: 161. Type locality: «near Hogarth’s landing, and near Spring Cove, Florida» (original citation), herein restricted to Hogarths Landing, Saint Johns County. Syntype(s) in MCZ [# 15].

#### Distribution.

This species, also known as the “Moustached Tiger Beetle”, ranges from east-central Georgia (Beaton 2008: 42) to southern Florida (Choate 2003: Map 63; Pearson et al. 2006: 174). The record from “North Carolina” (Erwin and Pearson 2008: 230) is probably in error or based on a stray.

#### Records.

**USA**: FL, GA

### Ellipsoptera lepida

(Dejean, 1831)

Cicindela lepida Dejean, 1831: 255. Type locality: «Amérique septentrionale» (original citation), herein restricted to Trenton, Mercer County, New Jersey (see LeConte 1846b: 181). Syntype(s) in MHNP.
Cicindela lepida insomnis Casey, 1913: 35. Type locality: «Seward Co[unty], Kansas» (original citation). One syntype in USNM [# 46000]. Synonymy established by Horn (1915: 395).

#### Distribution.

This species, also known as the “Ghost Tiger Beetle”, ranges from southern Quebec to southeastern Alberta (Hilchie 1985: 333), south to Chihuahua (Cazier 1954: 297), central Texas, southern Louisiana, and eastern North Carolina, west to western Arizona and eastern Nevada [see Pearson et al. 1997: Fig. 17]. The record from South Carolina, based on a specimen in CMNH, is probably in error (Knisley and Schultz 1997: 113). According to Erwin and Pearson (2008: 231), the Ghost Tiger Beetle has been extirpated over much of its former range due to habitat loss.

#### Records.

**CAN**: AB, MB, ON, QC, SK **USA**: AL, AR, AZ, CO, CT, DE, IA, IL, IN, KS, KY, LA, MA, MD, MI, MN, MO, MS, MT, NC, ND, NE, NJ, NM, NV, NY, OH, OK, PA, SD, TN, TX, UT, VA, WI, WY – Mexico

### Ellipsoptera macra ampliata

(Vaurie, 1951)

Cicindela macra ampliata Vaurie, 1951: 10. Type locality: «Denton County, Texas» (original citation). Holotype (♂) in AMNH [# 1214].

#### Distribution.

This subspecies, the “Denton Tiger Beetle”, is known from Dallas, Denton, and Kaufman Counties in northern Texas (Vaurie 1951: 10) [see Willis 1967: Fig. 137].

#### Records.

**USA**: TX

### Ellipsoptera macra fluviatilis

(Vaurie, 1951)

Cicindela macra fluviatilis Vaurie, 1951: 8. Type locality: «Red River, north of Quanah, Hardeman County, Texas» (original citation). Holotype (♂) in AMNH [# 1215].

#### Distribution.

This subspecies, the “Panhandle Tiger Beetle”, is found in Oklahoma, northern Texas, and northeastern New Mexico [see Pearson et al. 2006: Map 101]. The records from “Arkansas” and “Kansas” (Boyd 1982: 17) need confirmation.

#### Records.

**USA**: NM, OK, TX [AR, KS]

#### Note.

Willis (1967) indicated the presence of narrow zones of intergradation between this subspecies and the other two.

### Ellipsoptera macra macra

(LeConte, 1856)

Cicindela macra LeConte, 1856a: 50. Type locality: «Wisconsin and Minnesota» (original citation), herein restricted to Jordan, Scott County, Minnesota (see Horn 1928: 13). Syntype(s) in MCZ [# 21].
Cicindela macra mercurialis Casey, 1913: 36. Type locality: «Iowa» (original citation). One syntype in USNM [# 45955]. Synonymy established by Horn (1915: 395).
Cicindela macra topekana Casey, 1916: 31. Type locality: «M[oun]t Hope, Kansas» (original citation). Three syntypes [3 originally cited] in USNM [# 45954]. Synonymy established by Horn (1926: 299).

#### Distribution.

This subspecies, also known as the “Sandy Stream Tiger Beetle”, ranges from Beaver Islands in northern Mic﻿higan (Dunn 1987: 11) to southern Wyoming, south to north-central Colorado (Kippenhan 1990: 314), Arkansas, and Tennessee [see Pearson et al. 2006: Map 101]. The record from “Ohio” (Willis 1967: 269) needs confirmation (see Graves and Brzoska 1991: 28); that from “Texas” (Freitag 1999: 94) probably refers to the *fluviatilis* form; those from central and southeastern New Mexico (Fall and Cockerell 1907: 155) are probably in error.

#### Records.

**USA**: AR, CO, IA, IL, IN, KS, KY, LA, MI, MN, MO, NE, OK, SD, TN, WI, WY [OH]

#### Note.

Intergrades between this and the *fluviatilis* forms occur in southern Kansas and eastern Oklahoma (Pearson et al. 2006: 169).

### Ellipsoptera marginata

(Fabricius, 1775)

Cicindela marginata Fabricius, 1775: 226. Type locality: «Virginia» (original citation), herein restricted to Cobbs Island, Chesterfield County (see Harris 1911: 57). One syntype in ZMUC (Zimsen 1964: 65).
Cicindela variegata Dejean, 1825: 84. Type locality: «Amérique septentrionale» (original citation). Holotype [by monotypy] (♀) in MHNP. Synonymy established by Say (1830a: 68).

#### Distribution.

This species, also known as the “Margined Tiger Beetle”, is found along the Atlantic Coast from Kings County in Nova Scotia (Neil and Majka 2008: 4) to the Florida Keys and along the Gulf Coast in Florida [see Pearson et al. 2006: Map 96]; it is also recorded from the Bahamas and Cuba (Peck 2005: 27). According to Dunn (1983: 4), the species has declined significantly along the New Hampshire coast.

#### Records.

**CAN**: NS **USA**: CT, DC, DE, FL, GA, MA, MD, ME, NC, NH, NJ, NY, RI, SC, VA – Bahamas, Cuba

**Figure 12.**
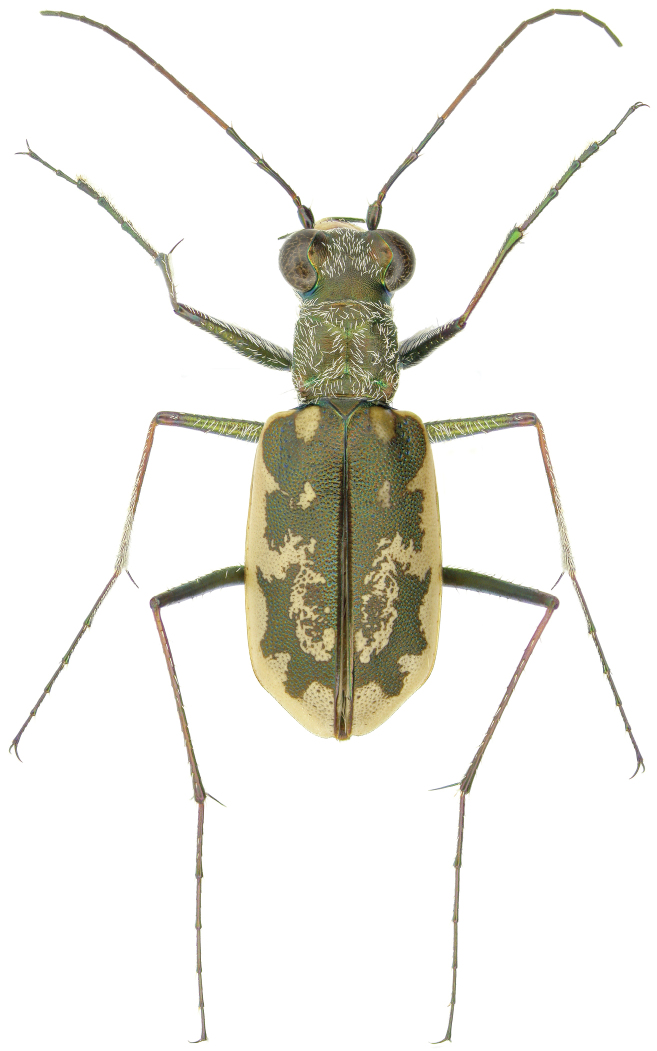
*Cicindela marginata* Fabricius. This species is a typical coastal species inhabiting mudflats and salt marshes from New Brunswick to southern Florida and along the Florida Gulf coast; it is also known from the Bahamas and the coast of Cuba. The breeding populations in New Brunswick were discovered only recently. Whether the species had been in the area for some time but went unnoticed, or extended its range in recent years, is the subject of speculation.

### Ellipsoptera puritana

(Horn, 1871)

Cicindela puritana G.H. Horn, 1871: 325. Type locality: «N[ew] H[ampshire]» (lectotype label). Lectotype (♂), designated by Ward (1982: 61), in MCZ [# 16273].

#### Distribution.

This species, also known as the “Puritan Tiger Beetle”, is now restricted to two areas, one along the Connecticut River in southern Massachusetts and Connecticut, the other one along the Chesapeake Bay in Maryland (Pearson et al. 2006: 170, 193). It formerly occurred along the Connecticut River as far north as central New Hampshire.

#### Records.

**USA**: CT, MA, MD, NH, NJ, NY, VA, VT

#### Note.

This species has been listed as threatened by the U.S. Fish and Wildlife Service in August 1990.

### Ellipsoptera wapleri

(LeConte, 1875)

Cicindela wapleri LeConte, 1875a: 158. Type locality: «Mississippi» (original citation). Holotype [by monotypy] (♂) in MCZ [# 44]. Etymology. The specific name was proposed for Émile Wapler, a French from Mulhouse, who collected insects in southern United States.

#### Distribution.

This species, also known as the “White-sand Tiger Beetle”, is found along a small area within the Coastal Plain from south-central Georgia to eastern Louisiana [see Pearson et al. 2006: Map 98]. Beaton (2008: 42) did not find this species at any historical sites in Georgia during his survey of the tiger beetles of the state.

#### Records.

**USA**: AL, FL, GA, LA, MS

### [sperata group]

### Ellipsoptera nevadica citata

(Rumpp, 1977)

Cicindela nevadica citata Rumpp, 1977: 175. Type locality: «8 kilometers west-southwest of Willcox [Cochise County, Arizona]» (original citation). Holotype (♂) in CAS [# 12528].

#### Distribution.

This subspecies, the “Chiracahua Tiger Beetle”, is known from southeastern Arizona and Sonora, Mexico (Spomer 2004: 409); also recorded from “New Mexico” (Erwin and Pearson 2008: 233).

#### Records.

**USA**: AZ [NM] – Mexico

### Ellipsoptera nevadica knausii

(Leng, 1902)

Cicindela knausii Leng, 1902: 166. Type locality: «near Kackley, Belvidere, and Great Spirit Springs, Kansas» (original citation). Syntype(s) location unknown. Etymology. This taxon was proposed in honor of Warren Knaus [1858-1937], amateur coleopterist and founder, owner, and editor of *The Democrat* in McPherson, Kansas which became, after the purchase of *The Opinion* in 1912, the *Democratic Opinion* until the time of his death. Knaus made several collecting trips to western United States and assembled a collection of nearly 90,000 specimens which he donated to the Kansas State University (at the time the Kansas State College) in Manhattan, March 1917. The collection was transferred to the university only in July 1937, shortly after Knaus’ death.

#### Distribution.

This subspecies, the “Knaus’s Tiger Beetle”, ranges from southern Manitoba to southeastern Alberta, south to central New Mexico and central Texas [see Willis 1967: Fig. 143].

#### Records.

**CAN**: AB, MB, SK **USA**: CO, KS, MT, ND, NE, NM, OK, SD, TX, WY

### Ellipsoptera nevadica lincolniana

(Casey, 1916)

Cicindela lincolniana Casey, 1916: 32. Type locality: «Lincoln [Lancaster County], Nebraska» (original citation). Two syntypes in USNM [# 45959].

#### Distribution.

This subspecies, also known as the “Salt Creek Nevada Tiger Beetle”, has been reported yet only from around the type locality in eastern Nebraska [see Willis 1967: Fig. 143]. Population estimates vary yearly from a few hundred to under a thousand specimens (Spomer et al. 2008a: 43).

#### Records.

**USA**: NE

#### Note.

This species has been listed as endangered by the U.S. Fish and Wildlife Service in October 2005.

### Ellipsoptera nevadica makosika

(Spomer, 2004)

Cicindela nevadica makosika Spomer, 2004: 410. Type locality: «Indian Creek, Pennington Co[unty], S[outh]D[akota]» (original citation). Holotype (♂) in USNM.

#### Distribution.

This subspecies, the “Indian Creek Tiger Beetle”, is known only from the type locality in the South Dakota Badlands (Spomer 2004: 410).

#### Records.

**USA**: SD

### Ellipsoptera nevadica nevadica

(LeConte, 1875)

Cicindela nevadica LeConte, 1875a: 159. Type locality: «Nevada» (original citation), herein restricted to Ash Meadows, Nye County (see Willis 1967: 280). Syntype(s) in MCZ [# 26].

#### Distribution.

This subspecies, also known as the “Nevada Tiger Beetle”, is found in the Great Basin region of Nevada and California south to northern Sonora [see Willis 1967: Fig. 143].

#### Records.

**USA**: CA, NV – Mexico

#### Note.

Another subspecies, *Ellipsoptera nevadica metallica* (Sumlin), is known from the state of Coahuila.

### Ellipsoptera nevadica olmosa

(Vaurie, 1951)

Cicindela nevadica olmosa Vaurie, 1951: 6. Type locality: «Los Olmos, Kenedy County, Texas» (original citation). Holotype (♂) in AMNH [# 1216].

#### Distribution.

This subspecies, the “Olmos Creek Tiger Beetle”, ranges from southeastern Arizona to southeastern Texas, including northern Coahuila [see Willis 1967: Fig. 143].

#### Records.

**USA**: AZ, NM, TX – Mexico

### Ellipsoptera nevadica tubensis

(Cazier, 1939)

Cicindela nevadica tubensis Cazier, 1939: 25. Type locality: «Tuba City, Coconino Co[unty], Arizona» (original citation). Holotype (♀) probably in AMNH.

#### Distribution.

This subspecies, the “Tube Tiger Beetle”, ranges from northeastern Utah to central Arizona and northwestern New Mexico [see Willis 1967: Fig. 143].

#### Records.

**USA**: AZ, CO, NM, UT

### Ellipsoptera rubicunda

(Harris, 1911)

Cicindela cuprascens sperata var. *rubicunda* E.D. Harris, 1911 [before 31 May]: 55. Type locality: «Albuquerque and Deming, New Mex[ico]» (original citation). Syntype(s) in MCZ [# 25613]. Note. 1. Even if Harris (1911: 55) expressly gave this name an infrasubspecific rank, it is subspecific because it was adopted as the valid name of a subspecies before 1985 (e.g., Rumpp 1977: 176) (ICZN 1999: Article 45.6.4.1). 2. This name has been listed as a junior synonym of *Ellipsoptera marutha* (Dow) since Cazier (1954: 296). However, Harris’ name is older than Dow’s name. Indeed *Ellipsoptera marutha* was made available by Dow in the June 1911 (page 272) issue of volume 22 of the *Entomological News* mailed on 31 May. In the same issue (page 283), Henry Skinner reviewed Harris’ catalogue where the name *rubicunda* was proposed. Furthermore, Dow (1911: 271) reported “I have a good share of the color forms recognized in the E.D. Harris catalogue” indicating that he had Harris’ catalogue at the time. The statement of Harris (1911: 55) that “*marutha* is the brilliant green form lately described by Dow” was certainly in error. There is no precise date on Harris’ catalogue except that the “introduction” is dated February 1911. 3. *Cicindela cuprascens sperata* var. *marutha* was first described by Harris (1911: 55) in the same catalogue. However, his name was not adopted as the valid name of a subspecies since, to my knowledge, *marutha* has always been subsequently attributed to Dow.
Cicindela sperata var. *marutha* Dow, 1911 [31 May]: 272. Type locality: «[possibly] F[or]t Wingate [McKinley County], New Mexico» (original citation). Syntype(s) [2 originally cited] in AMNH [# 1204]. Synonymy established by Cazier (1954: 296). Note. Dow (1911: 271) did not formerly state a locality for his syntypes but simply wrote that he received the material “from Mr. John Woodgate, Ft. Wingate, New Mexico.”

#### Distribution.

This species, also known as the “Aridland Tiger Beetle”, is found from northern Utah to southern Colorado, south to Chihuahua (Cazier 1954: 297) [see Pearson et al. 1997: Fig. 29]. The record from “Arkansas” (Boyd 1982: 17) is in error or based on a stray.

#### Records.

**USA**: AZ, CO, NM, TX, UT – Mexico

#### Note.

Rumpp (1977: 176) recognized *marutha* and *rubicunda* as distinct subspecies under the specific name *Ellipsoptera marutha*. I have followed Freitag (1999: 95) in considering the two taxa as synonyms.

### Ellipsoptera sperata inquisitor

(Casey, 1897)

Cicindela inquisitor Casey, 1897: 298. Type locality: «Austin [Travis County], Texas» (original citation). One syntype in USNM [# 45958].

#### Distribution.

This subspecies, the “Inquisitor Tiger Beetle”, is found in central Texas (Pearson et al. 2006: 171).

#### Records.

**USA**: TX

### Ellipsoptera sperata sperata

(LeConte, 1856)

Cicindela sperata LeConte, 1856a: 50. Type locality: «Rio Grande, at various places» (original citation), herein restricted to El Paso, El Paso County, Texas (see Harris 1911: 55). Syntype(s) in MCZ [# 36].

#### Distribution.

This subspecies, also known as the “Rio Grande Tiger Beetle”, ranges from northern Utah to northeastern New Mexico [see Pearson et al. 2006: Map 103], south to Durango and Tamaulipas (Cazier 1954: 296). The records from western Kansas (Popenoe 1877: 22; Snow 1878: 63) and “Oklahoma” (Boyd 1982: 17) need confirmation; that from Yuma, California (Leng 1902: 167) is likely in error.

#### Records.

**USA**: AZ, CO, NM, TX, UT [KS, OK] – Mexico

#### Note.

Another subspecies, *Ellipsoptera sperata vauriei* (Cazier), is known from the state of Sonora in Mexico.

### Genus Microthylax

Rivalier, 1954

Microthylax Rivalier, 1954: 260. Type species: *Cicindela digueti* Horn, 1897 by original designation. Etymology. From Greek *micros* (small, little) and *thylax* (bag, sack, pouch), alluding to the small size of the endophallus (“*pénis *... *avec un sac interne très peu développé et occupant seulement la portion subterminale du pénis*”) [masculine].

#### Diversity.

Three species (five species-group taxa) in United States and Cuba (one species) and Mexico (two species), including Baja California.

### Microthylax olivaceus

(Chaudoir, 1854)

Cicindela olivacea Chaudoir, 1854: 118. Type locality: «Cuba» (original citation). Syntype(s) [2 originally cited] in MHNP.

#### Distribution.

This species, also known as the “Olive Tiger Beetle”, is found in Cuba and southern Florida, including the Keys [see Woodruff and Graves 1963: Fig. 3]. Apparently the species has not been sighted in Florida since the 1980s (Pearson et al. 2006: 149).

#### Records.

**USA**: FL – Cuba

### Genus Opilidia

Rivalier, 1954

Opilidia Rivalier, 1954: 261. Type species: *Cicindela macrocnema* Chaudoir, 1852 by original designation. Etymology. Unknown [feminine].

#### Diversity.

Six Neotropical species, of which one is represented by a distinct subspecies in southeastern Texas.

### Opilidia chlorocephala smythi

(Harris, 1913)

Cicindela smythi E.D. Harris, 1913: 67. Type locality: «ocean side of Padre Island [Kenedy County], Texas» (original citation). Syntype(s) in MCZ [# 23551]. Etymology. This species was named after Eugene Graywood Smyth [1886-1975], economic entomologist with the United States Department of Agriculture. Smyth had an interest in tiger beetles.

#### Distribution.

This subspecies, the “Smyth’s Beach Tiger Beetle”, is known only from the type locality in southeastern Texas. No specimens have been collected since the original ones (over 80 specimens) in June 1912.

#### Records.

**USA**: TX

#### Note.

The nominotypical subspecies is found along the Gulf Coast of Mexico, as far south as Veracruz, and from Honduras (Erwin and Pearson 2008: 290).

### Genus Brasiella

Rivalier, 1954

Brasiella Rivalier, 1954: 261. Type species: *Cicindela argentata* Fabricius, 1801 by original designation. Etymology. Unknown [feminine].

#### Diversity.

Thirty-eight Neotropical species (Lorenz 2005: 58), of which one extends into southwestern North America.

#### Faunistic Note.

The Cuban *Brasiella viridicollis* (Dejean) is known in North America from a single specimen, probably a stray, collected in the Florida Keys in 1983 (Schiefer 2005: 551; Pearson et al. 2006: 151). The species is not considered here as a North American entity.

### Brasiella wickhami

(Horn, 1903)

Cicindela wickhami W. Horn, 1903a: 182. Type locality: «Tucson, S[üd] Arizona» (original citation). Three syntypes in DEI (Döbler 1973: 418).

#### Distribution.

This species, also known as the “Sonoran Tiger Beetle”, ranges from southern Arizona south to Sinaloa; it is also found in southern Baja California Peninsula (Cazier 1954: 286). The record from “California” (Erwin and Pearson 2008: 118) needs confirmation.

#### Records.

**USA**: AZ [CA] – Mexico

### Genus Dromochorus

Guérin-Méneville, 1849

Dromochorus Guérin-Méneville, 1849: [plate 162] 1. Type species: *Dromochorus pilatei* Guérin-Méneville, 1849 by monotypy. Etymology (original). From the Greek *dromos* (running) and *choros* (land, country, by extension field), probably alluding to the circumstance upon which the holotype of *Colliuris pilatei* was found [masculine].
Dromeochora Gistel, 1850: 75. Unnecessary replacement name for *Dromochorus* Guérin-Méneville, 1849.

#### Diversity.

Four North American species, one of them extending into northern Mexico.

### Dromochorus belfragei

Sallé, 1877

Dromochorus belfragei Sallé, 1877: 6. Type locality: «Dallas, Wasco, sur les bords de la Trinity-River, Texas» (original citation). Syntype(s) probably in MHNP. Etymology. The specific name honors Gustav Wilhelm Belfrage [1834-1882], a professional insect collector in Texas. Belfrage was born in Stockholm, Sweden, and moved to the United States in his 20s. During Belfrage’s time, the standard rate was five cents per specimen (Sorensen 1995: 37).
Dromochorus bellefragei Heyne, 1893: 3. Unjustified emendation for *Dromochorus belfragei* Sallé, 1877.
Dromochorus sericeus Casey, 1897: 294. Type locality: «Texas» (original citation). Two syntypes [3 originally cited] in USNM [# 45894] and one in DEI (Döbler 1973: 409). Synonymy established by Leng (1902: 110).

#### Distribution.

This species, also known as the “Loamy-ground Dromo Tiger Beetle”, ranges from central and coastal Texas (Pearson et al. 2006: 160) south to Tamaulipas (Cazier 1954: 297); it was also recorded from southeastern Colorado (Michels et al. 2008).

#### Records.

**USA**: CO, TX – Mexico

### Dromochorus pilatei

Guérin-Méneville, 1849

Dromochorus pilatei Guérin-Méneville, 1849: [plate 162] 2. Type locality: «Velasco [Brazoria County], Texas» (original citation). Holotype [by monotypy] (♂) probably in MHNP (collection J. Thomson, see Schaupp 1884a: 85). Etymology. The specific name honors Louis Pilate [1816-1852], a French naturalist traveller who collected in Alabama, Louisiana, Texas, and the state of Yucatán in Mexico. Pilate died at the age of 36 of hypertrophy of the heart.
Cicindela maga LeConte, 1875a: 161. Type locality: «near Lake Ponchartrain [Jefferson Parish], Louisiana» (original citation). Syntype(s) [3 originally cited] in MCZ [# 22]. Synonymy established by Sallé (1877: 5).

#### Distribution.

This species, also known as the “Cajun Dromo Tiger Beetle”, is found along and near the Gulf Coast in southern Louisiana (Schaupp 1884a: 85) and northeastern Texas [see Pearson et al. 2006: Map 92].

#### Records.

**USA**: LA, TX

### Dromochorus pruininus

Casey, 1897

Dromochorus pruininus Casey, 1897: 294. Type locality: «Kansas» (original citation), herein restricted to Onaga, Pottawatomie County (see Harris 1911: 51, as *Cicindela belfragei*). Three syntypes [3 originally cited] in USNM [# 45893].

#### Distribution.

This species, also known as the “Frosted Dromo Tiger Beetle”, ranges from northern Kansas and central Missouri (MacRae and Brown 2011a) south to Nueces and Webb Counties in southern Texas and west into the Texas Panhandle (Pearson et al. 2006: 160). There is also one record from east-central Nebraska (Pearson et al. 2006: 160).

#### Records.

**USA**: KS, MO, OK, TX [NE]

### Dromochorus velutinigrens

Johnson, 1992

Dromochorus velutinigrens Johnson, 1992 [5 February]: 50. Type locality: «10 km east of Riviera, Kleberg Co[unty], Texas» (original citation). Holotype (♂) in CMN.
Dromochorus venetavelutinus Gage, 1992 [“31 December”]: 4. Type locality: «Port Mansfield, Willacy County, Texas» (original citation). Holotype (♀) in TME. Synonymy established by Pearson et al. (1997: 37).

#### Distribution.

This species, also known as the “Velvet Dromo Tiger Beetle”, is found only in southern Texas, primarily along the coast [see Pearson et al. 2006: Map 94].

#### Records.

**USA**: TX

### Genus Habroscelimorpha

Dokhtouroff, 1883

Habroscelimorpha Dokhtouroff, 1883b: 69. Type species: *Cicindela dorsalis* Say, 1817 designated by Horn (1915: 236). Etymology. From the generic name *Habroscelis* (an unjustified emendation of *Abroscelis*) and the Greek *morphe* (form), probably alluding to the resemblance of the adults to those of *Abroscelis* [feminine].
Habroscelidomorpha Bertkau, 1884: 266. Unjustified emendation of *Habroscelimorpha* Dokhtouroff, 1883.

#### Diversity.

Western Hemisphere, with 18 species (33 species-group taxa) in the Nearctic (nine species, 18 species-group taxa) and Neotropical (14 species) Regions.

### Habroscelimorpha californica mojavi

(Cazier, 1937)

Cicindela californica mojavi Cazier, 1937c: 116. Type locality: «Mojave, Cal[ifornia]» (holotype label). Holotype (♂) in AMNH [# 1200].

#### Distribution.

This subspecies, the “California Tiger Beetle”, ranges from the Mojave Desert of southern California (Pearson et al. 2006: 138) south to northeastern Baja California Peninsula and northwestern Sonora (Cazier 1954: 289).

#### Records.

**USA**: CA – Mexico

#### Note.

This form intergrades with the *pseudoerronea* form at Soda Lake in northeastern San Bernardino County, California (Pearson et al. 2006: 139).

### Habroscelimorpha californica pseudoerronea

(Rumpp, 1958)

Cicindela californica pseudoerronea Rumpp, 1958: 150. Type locality: «seven miles north of Furnace Creek (260 feet), Death Valley, Inyo County, California» (original citation). Holotype (♂) in CAS [# 17194].

#### Distribution.

This subspecies, the “Inland Tiger Beetle”, is found only in the Death Valley in Inyo and San Bernardino Counties, California (Rumpp 1958: 151).

#### Records.

**USA**: CA

#### Note.

The nominotypical subspecies is found in the Baja California Peninsula and *Habroscelimorpha californica brevihamata* (Horn) occurs in the states of Sinaloa and Sonora in Mexico.

### Habroscelimorpha circumpicta circumpicta

(LaFerté-Sénectère, 1841)

Cicindela circumpicta LaFerté-Sénectère, 1841a: 39. Type locality: Texas (inferred from title of the paper), herein restricted to Brownsville, Cameron County (see Willis 1967: 243). Syntype(s) probably in MHNP (collection Chaudoir).
Cicindela circumpicta inspiciens Casey, 1913: 33. Type locality: «Point Isabel [Cameron County], Texas» (original citation). One syntype in USNM [# 45997]. Synonymy established by Horn (1915: 390).

#### Distribution.

This subspecies, also known as the “Cream-edged Tiger Beetle”, is found from southeastern Oklahoma to northeastern Mexico [see Johnson 1993b: Fig. 1].

#### Records.

**USA**: OK, TX – Mexico

#### Note.

This form intergrades with the *johnsonii* form along the Oklahoma-Texas border (Pearson et al. 2006: 140).

### Habroscelimorpha circumpicta johnsonii

(Fitch, 1857)

Cicindela johnsonii Fitch, 1857: 487. Type locality: «prairies west of Arkansas» (original citation). Syntype(s) location unknown (no original specimens have been located by Barnes 1988: 107). Etymology. The specific name was proposed for Benjamin P. Johnson [1793-1869], lawyer, politician, office holder, and for more than 20 years Secretary of the New York State Agricultural Society.
Cicindela circumpicta ambiens Casey, 1913: 33. Type locality: «Kansas» (original citation). One syntype in USNM [# 45996]. Synonymy established by Drew and Van Cleave (1962: 108).
Cicindela circumpicta salinae Vaurie, 1951: 3. Type locality: «Lincoln (Salt Basin), Lancaster County, Nebraska» (original citation). Holotype (♂) in AMNH [# 1212]. Synonymy established by Willis (1967: 250).

#### Distribution.

This subspecies, the “Johnson’s Tiger Beetle”, ranges from central Missouri to southeastern Colorado, north to west-central Nebraska (Spomer et al. 2008a: 58), south to New Mexico and southwestern Texas [see Johnson 1993b: Fig. 1]. The taxon is also found in Coahuila in northern Mexico (Murray 1979: 55). The records from “North Dakota” (Boyd 1982: 14; Freitag 1999: 77) probably refer to the *pembina* form.

#### Records.

**USA**: CO, KS, MO, NE, NM, OK, TX – Mexico

### Habroscelimorpha circumpicta pembina

(Johnson, 1993)

Cicindela circumspicta pembina Johnson, 1993b: 55. Type locality: «near Pembina, Pembina County, North Dakota» (original citation). Holotype in AMNH [# 1550].

#### Distribution.

This subspecies, the “Pembina Tiger Beetle”, is known only from a small area in northeastern North Dakota [see Johnson 1993b: Fig. 1].

#### Records.

**USA**: ND

### Habroscelimorpha dorsalis dorsalis

(Say, 1817)

Cicindela dorsalis Say, 1817b: 20. Type locality: «New Jersey» (original citation), herein restricted to Ocean City, Cape May County (see Boyd 1978: 231). Syntype(s) lost.
Cicindela signata Dejean, 1825: 124. Type locality: «Amérique septentrionale» (original citation). Syntype(s) in MHNP. Synonymy established by Dejean (1826: 414).
Cicindela dorsalis semipicta Casey, 1897: 299 [primary homonym of *Cicindela semipicta* Fairmaire, 1871]. Type locality not stated. One syntype in USNM [# 45994]. Synonymy established by Leng (1902: 161).
Cicindela munifica Casey, 1913: 31. Type locality: «Rhode Island» (original citation). Two syntypes in USNM [# 45993]. Synonymy established by Horn (1915: 392).
Cicindela dorsalis lineoscripta Casey, 1924: 16. Replacement name for *Cicindela dorsalis semipicta* Casey, 1897.

#### Distribution.

This subspecies, also known as the “Eastern Beach Tiger Beetle”, once occurred along the Atlantic Coast from Cape Cod, Massachusetts to the Chesapeake Bay. It is now found at two isolated sites on the coast of Massachusetts and along both shores of the Chesapeake Bay in Maryland and Virginia (Boyd and Rust 1982: 234; Pearson et al. 2006: 144). The subspecies was successfully reintroduced at Sandy Hook, New Jersey (Pearson et al. 2006: 192). The records from “Delaware” (Bousquet and Larochelle 1993: 65) and from near Lancaster, Pennsylvania (Cresson 1861: 12) are probably based on strays.

#### Records.

**USA**: CT, MA, MD, NJ, NY, RI, VA

#### Note.

This subspecies is listed as threatened under the Endangered Species Act by the U.S. Fish and Wildlife in 1990 (Pearson et al. 2006: 191).

### Habroscelimorpha dorsalis media

(LeConte, 1856)

Cicindela media LeConte, 1856a: 47. Type locality: «sea coast of Georgia and South Carolina» (original citation), herein restricted to Hilton Head Island, Beaufort County, South Carolina (see Cartwright 1935: 75). Syntype(s) in MCZ [# 23].

#### Distribution.

This subspecies, the “Eastern Beach Tiger Beetle”, is found along the Atlantic Coast from Ocean County in New Jersey to southern Florida (Boyd and Rust 1982: 234).

#### Records.

**USA**: DE, FL, GA, MD, NC, NJ, SC, VA

#### Note.

According to Knisley and Schultz (1997: 103), the ranges of this form and of the *dorsalis* form are contiguous in southern New Jersey, the southern tip of coastal Virginia, and near the mouth of the Chesapeake Bay on the Virginia side and little intergradation can be observed on those sites.

### Habroscelimorpha dorsalis saulcyi

(Guérin-Méneville, 1840)

Cicindela saulcyi Guérin-Méneville, 1840: 37. Type locality: Pensacola, Escambia County, Florida (inferred from title of the paper). Two syntypes in IRSN. Etymology. The specific name honors Ernest de Saulcy [1803-1899], a French naval officer who sailed to many places including America. Saulcy was interested in natural history and collected insects. He was the older brother of the archaeologist and numismatist Louis-Félicien-Joseph [Félix] de Saulcy [1807-1880] who also collected beetles and uncle of Félicien de Saulcy [1832-1912], who worked on the systematics of Coleoptera, particularly those of small size.
Cicindela castissima Bates, 1884: 260. Type locality: «Arcas Islets [off the coast of Campeche], Gulf of Mexico» (original citation). Syntype(s) in BMNH. Synonymy established by Horn (1905: 23).
Cicindela apricoidea Casey, 1913: 32. Type locality: «seabeaches of Louisiana and Mississippi» (original citation). Four syntypes [4 originally cited] in USNM [# 45995]. Synonymy established by Horn (1915: 393).

#### Distribution.

This subspecies, the “Saulcy’s Beach Tiger Beetle”, ranges from the Gulf Coast of Florida to the Mississippi River delta in Louisiana (Pearson et al. 2006: 144); it is also known from the Isla Arcas in Campeche (Bates 1884: 260). The records from Cuba (Leng and Mutchler 1916: 697, as *Cicindela dorsalis* var. *venusta*; Cazier 1954: 294; Erwin and Pearson 2008: 247) are based on mislabeled specimens (Valdés 1999: 13).

#### Records.

**USA**: AL, FL, LA, MS – Mexico

### Habroscelimorpha dorsalis venusta

(LaFerté-Sénectère, 1841)

Cicindela venusta LaFerté-Sénectère, 1841a: 37. Type locality: Texas (inferred from title of the paper). Syntype(s) probably in MHNP (collection Chaudoir).

#### Distribution.

This subspecies, the “Gulf Beach Tiger Beetle”, is found from coastal Mississippi (Lago et al. 2002: 201) south along the Gulf to the state of Tamaulipas (Cazier 1954: 294). The record from east-central Colorado (Snow 1877: 16) is probably in error.

#### Records.

**USA**: LA, MS, TX – Mexico

### Habroscelimorpha fulgoris albilata

(Acciavatti, 1981)

Cicindela fulgoris albilata Acciavatti, 1981: 238. Type locality: «2 miles east at playa lakes, Salt Flat, Hudspeth County, Texas» (original citation). Holotype (♂) in AMNH [# 1473].

#### Distribution.

This subspecies, the “Pale Tiger Beetle”, is known from the Salt Basin of western Texas and adjacent New Mexico and from a single site in Dawson County, Texas (Acciavatti 1981: 239).

#### Records.

**USA**: NM, TX

### Habroscelimorpha fulgoris erronea

(Vaurie, 1951)

Cicindela californica viridicyanea Vaurie, 1950: 1 [primary homonym of *Cicindela viridicyanea* Audouin and Brullé, 1839]. Type locality: «Wil[l]cox, Cochise County, Arizona» (original citation). Holotype (♂) in AMNH [# 1208].
Cicindela californica erronea Vaurie, 1951: 12. Replacement name for *Cicindela californica viridicyanea* Vaurie, 1950.

#### Distribution.

This subspecies, the “Willcox Tiger Beetle”, is known only from the type locality in southeastern Arizona (Acciavatti 1981: 238).

#### Records.

**USA**: AZ

### Habroscelimorpha fulgoris fulgoris

(Casey, 1913)

Cicindela praetextata fulgoris Casey, 1913: 34. Type locality: «El Paso [El Paso County], Texas» (original citation). Lectotype (♂), designated by Acciavatti (1981: 237), in USNM [# 45998].
Cicindela praetextata stringens Casey, 1913: 34. Type locality: «El Paso [El Paso County], Texas» (original citation). One syntype in USNM [# 45999]. Synonymy established by Acciavatti (1981: 237)

#### Distribution.

This subspecies, also known as the “Glittering Tiger Beetle”, is found from east-central Arizona to north-central New Mexico, south to the Rio Grande area in westernmost Texas and southeastern New Mexico [see Acciavatti 1981: Fig. 1]; also recorded from Chihuahua in Mexico (Murray 1979: 55).

#### Records.

**USA**: AZ, NM, TX – Mexico

#### Note.

This subspecies intergrades with the *albilata* form in southeastern New Mexico (Pearson et al. 2006: 143).

### Habroscelimorpha gabbii

(Horn, 1867)

Cicindela gabbii G.H. Horn, 1867a: 395. Type locality: «near Wilmington (San Pedro) [Los Angeles County], California» (original citation). Lectotype (♂), designated by Ward (1982: 62), in MCZ [# 35316]. Etymology. The specific name honors the American paleontologist William More Gabb [1839-1878] who worked for the California Geological Survey.

#### Distribution.

This species, also known as the “Western Tidal Flat Tiger Beetle”, occurs along the Pacific Coast from southern California to central Baja California Peninsula, and along the Gulf of California Coast from northern Sonora to Sinaloa (Cazier 1954: 291) [see Pearson et al. 1997: Fig. 28]. According to Pearson et al. (2006: 139), this species is now found in the United States only in three or four protected areas in Ventura, Orange, and San Diego Counties.

#### Records.

**USA**: CA – Mexico

### Habroscelimorpha pamphila

(LeConte, 1873)

Cicindela pamphila LeConte, 1873b: 321. Type locality: «Texas» (original citation), herein restricted to Corpus Christi, Nueces County (see LeConte 1881: xxxvi). Syntype(s) in MCZ [# 29].

#### Distribution.

This species, also known as the “Gulfshore Tiger Beetle”, is found along the Gulf Coast from eastern Mississippi (Grammer 2009) to northern Tamaulipas in Mexico (Pearson et al. 2006: 145).

#### Records.

**USA**: LA, MS, TX – Mexico

### Habroscelimorpha praetextata pallidofemora

(Acciavatti, 1981)

Cicindela praetextata pallidofemora Acciavatti, 1981: 236. Type locality: «Virgin River, S[ain]t George, Washington County, Utah» (original citation). Holotype (♂) in AMNH [# 1474].

#### Distribution.

This subspecies, the “Virgin River Tiger Beetle”, is found only along the Virgin River in southwestern Utah and southeastern Nevada [see Acciavatti 1981: Fig. 1].

#### Records.

**USA**: NV, UT

### Habroscelimorpha praetextata praetextata

(LeConte, 1854)

Cicindela praetextata LeConte, 1854d: 220. Type locality: «San Diego to El Paso» (original citation); «probably found in the valley of the Gila» (LeConte 1856a: 58), herein restricted to Phoenix, Maricopa County, Arizona (see Harris 1911: 53). Lectotype (♂), designated by Acciavatti (1981: 233), in MCZ [# 32].

#### Distribution.

This subspecies, also known as the “Riparian Tiger Beetle”, is found from the Gila River Basin in eastern Arizona westwards to the Salton Sea Basin in California, north to southern Nevada [see Pearson et al. 2006: Map 74]. The record from New Mexico (Fall and Cockerell 1907: 155) needs confirmation. According to Erwin and Pearson (2008: 251), the species has been extirpated from many of its historic sites.

#### Records.

**USA**: AZ, CA, NV [NM]

### Habroscelimorpha severa

(LaFerté-Sénectère, 1841)

Cicindela severa LaFerté-Sénectère, 1841a: 41. Type locality: Texas (inferred from title of the paper), herein restricted to Port Isabel, Cameron County (see Leng 1902: 173, as “Point Isabel”). Syntype(s) probably in MHNP (collection Chaudoir).
Cicindela yucatana W. Horn, 1897a: 354 (as *yukatana*). Type locality: «Yucatan mer.» (syntype label). One syntype in MHNP (Cassola 1994: 2). Synonymy established by Horn (1897a: 354), confirmed by Cassola (1994: 2). Note. This taxon was briefly described by Horn (1897a: 354) but treated as a junior synonym of *Cicindela severa*. It was redescribed and treated as a valid taxon by Horn (1903b: 219), Cazier (1954: 261), and Johnson (1993a: 42), the first and last authors based on misidentified specimens of *Cicindela wellingi* Cassola and Sawada (see Cassola 1994). Therefore the name was first published as a junior synonym but treated as an available name before 1961. In such case, the name is available but dates from its first publication as a synonym (ICZN 1999: Article 50.7).
Cicindela severa alabamae Casey, 1920: 134. Type locality: «Coden [Mobile County], Alabama» (original citation). Three syntypes in USNM [# 45963]. Synonymy established by Horn (1926: 284).

#### Distribution.

This subspecies, also known as the “Saltmarsh Tiger Beetle”, is found along the Gulf Coast from the Florida Keys to Tamaulipas in Mexico (Cazier 1954: 261) [see Pearson et al. 1997: Fig. 4]; also recorded from Yucatán (Horn 1897a: 354).

#### Records.

**USA**: AL, FL, LA, MS, TX – Mexico

#### Note.

*Habroscelimorpha yucatana* (Horn) from Yucatán is considered a subspecies of *Habroscelimorpha severa* by some authors, including Erwin and Pearson (2008: 253).

### Habroscelimorpha striga

(LeConte, 1875)

Cicindela striga LeConte, 1875a: 160. Type locality: «Lake Harvey [Hillsborough County], Florida» (original citation). Syntype(s) in MCZ [# 38].

#### Distribution.

This species, also known as the “Elusive Tiger Beetle”, is found along the Atlantic Coast from southern South Carolina (Cartwright 1935: 73; Ciegler 1997: 191) to central Florida, and along the Gulf Coast of Florida [see Pearson et al. 1997: Fig. 10].

#### Records.

**USA**: FL, GA, SC

### Genus Eunota

Rivalier, 1954

Eunota Rivalier, 1954: 259. Type species: *Cicindela togata* LaFerté-Sénectère, 1841 by original designation. Etymology. From the Greek *eu* (good, beautiful) and *notos* (back, dorsum), probably alluding to the nice coloration of the adults [feminine].

#### Diversity.

One North American species which extends into northern Mexico.

### Eunotatogata fascinans

(Casey, 1914)

Cicindela fascinans Casey, 1914: 23. Type locality: «Santa Rosa [Guadalupe County], New Mexico» (original citation). One syntype in USNM [# 45953].

#### Distribution.

This subspecies, the “Salt Flat Tiger Beetle”, is known only from Torrance and Guadalupe Counties in central New Mexico and Hudspeth County in western Texas (Pearson et al. 2006: 148).

#### Records.

**USA**: NM, TX

### Eunotatogata globicollis

(Casey, 1913)

Cicindela togata var. *apicalis* W. Horn, 1897b: 17 [primary homonym of *Cicindela apicalis* Chaudoir, 1843]. Type locality: «Nebraska; Ka[c]kley, Kansas» (original citation). Three syntypes in DEI (Döbler 1973: 358).
Cicindela globicollis Casey, 1913: 35. Type locality: «Clark Co[unty], Kansas» (original citation). Three syntypes [3 originally cited] in USNM [# 45952]. Synonymy established by Horn (1915: 396).
Cicindela togata latilabris Willis, 1967: 286. Replacement name for *Cicindela togata apicalis* Horn, 1897.

#### Distribution.

This subspecies, the “Alkali Tiger Beetle”, ranges from eastern Nebraska (Carter 1989: 15) and central Colorado (Kippenhan 1990: 312) south to northern Texas (Gaumer and Murray 1971: 10) and southeastern New Mexico (Acciavatti et al. 1980: 31) [see Pearson et al. 2006: Map 80]. The record from north-central Utah (Tanner 1929a: 87) is probably in error.

#### Records.

**USA**: CO, KS, NE, NM, OK, TX

### Eunotatogata togata

(LaFerté-Sénectère, 1841)

Cicindela togata LaFerté-Sénectère, 1841a: 40. Type locality: Texas (inferred from title of the paper), herein restricted to Port Isabel, Cameron County (see Harris 1911: 57). Syntype(s) probably in MHNP (collection Chaudoir).

#### Distribution.

This subspecies, also known as the “White-cloaked Tiger Beetle”, is known from scattered localities from southern South Carolina (Cartwright 1935: 75) to northeastern Texas, south to northern Florida (Choate 2003: Map 48) and Tamaulipas (Cazier 1954: 297) [see Pearson et al. 2006: Map 80]. Ciegler (1997: 191) reported that the last specimen seen from South Carolina was collected in 1935 and that the species may be extinct in the state.

#### Records.

**USA**: AL, FL, LA, MS, SC, TX – Mexico

### Genus Cicindela

Linnaeus, 1758

Cicindela Linnaeus, 1758: 407. Type species: *Cicindela campestris* Linnaeus, 1758 designated by Latreille (1810: 425). Etymology. From the Latin *cicindela* (glow-worm in Pliny the Elder) [feminine].
Cicindella Gistel, 1850: 75. Unnecessary replacement name for *Cicindela* Linnaeus, 1758.

#### Diversity.

Worldwide, with about 340 species described by 2005 assigned to 24 subgenera (Lorenz 2005: 41-51). The North American fauna is represented by 60 species (about 18% of the world fauna) placed in two subgenera.

#### Taxonomic Note.

The genus is employed here in a restricted sense as used by most taxonomists working on the Palaearctic and African faunas and recently by Erwin and Pearson (2008) for the North American fauna.

### Subgenus Cicindelidia

Rivalier, 1954

Cicindelidia Rivalier, 1954: 255. Type species: *Cicindela carthagena* Dejean, 1831 by original designation. Etymology. From the generic name *Cicindela* [*q.v*.] and the Latin suffix -*idia* (little, small), probably alluding to the small size (“*toutes les espèces sont de taille petite ou moyenne*”) of adults of these tiger beetles [feminine].

#### Diversity.

Western Hemisphere, with about 65 species (Lorenz 2005: 48-49) in the Nearctic (21 species, 42 species-group taxa) and Neotropical (about 55 species).

#### Faunistic Note.

1. *Cicindela fera* Chevrolat is known north of Mexico from a single specimen collected in the 1950s at the southern border of Arizona and New Mexico (Pearson et al. 2006: 125). The specimen was probably a stray and the species is not listed here as a North American entity. 2. *Cicindela sommeri* Mannerheim is known from the Sierra Madre Occidental of western Mexico and from several specimens labeled from San Diego County, California (Leng 1902: 181; Pearson et al. 2006: 136). However, there is serious doubt about the origin of the California specimens and the species is not included here as a North American entity.

### Cicindela abdominalis

Fabricius, 1801

Cicindela abdominalis Fabricius, 1801: 237. Type locality: «Carolina» (original citation), herein restricted to McClellanville, Charleston County, South Carolina (see Cartwright 1935: 74). One syntype in ZMUC (Zimsen 1964: 64).
Cicindela ventralis Newman [in Doubleday], 1838: 414 [primary homonym of *Cicindela ventralis* Dejean, 1825]. Type locality: «S[ain]t John’s Bluff [Duval County], Florida» (original citation). Syntype(s) [9 originally cited] location unknown. Synonymy established by Harris and Leng (1916: 18).
Cicindela abdominalis faceta Casey, 1913: 38. Type locality not stated. Holotype [by monotypy] (♀) in USNM [# 45969]. Synonymy established by Horn (1915: 385), confirmed by Choate (1984: 75).

#### Distribution.

This species, also known as the “Eastern Pinebarrens Tiger Beetle”, is found mainly along the Coastal Plain and Piedmont Plateau from Long Island in southeastern New York (Leng 1928: 206) to central Florida, west to southeastern Louisiana [see Pearson et al. 1997: Fig. 11].

#### Records.

**USA**: AL, DE, FL, GA, LA, MD, MS, NC, NJ, NY, PA, SC, VA

### Cicindela amargosae amargosae

Dahl, 1939

Cicindela willistoni amargosae Dahl, 1939: 221. Type locality: «four miles north of Furnace Creek, Death Valley, Inyo County, California» (original citation). Holotype (♂) in CAS [# 8152].

#### Distribution.

This subspecies, also known as the “Great Basin Tiger Beetle”, is found in the Death Valley area in eastern California (Leffler 1987: 8).

#### Records.

**USA**: CA

#### Note.

1. Rumpp (1956: 141) reported the presence of intergrade populations between this subspecies and the *nyensis* form at places located between the type localities of *amargosae* and *nyensis*. 2. *Cicindela amargosae* has been regarded as a subspecies of *Cicindela senilis* LeConte by some authors (e.g., Willis 1968) or *Cicindela willistoni* LeConte (e.g., Kippenhan 1996b: 56) but treated as a closely related but distinct species by Leffler (1987: 8) and Pearson et al. (2006: 117).

### Cicindela amargosae nyensis

Rumpp, 1956

Cicindela amargosae nyensis Rumpp, 1956: 140. Type locality: «1.6 miles south of Springdale, Nye County, Nevada» (original citation). Holotype (♂) in CAS [# 17193].

#### Distribution.

This subspecies, also known as the “Nye Tiger Beetle”, is found in southeastern Oregon (Leffler 1979a: Fig. 60) and western Nevada (Rumpp 1956: 140).

#### Records.

**USA**: NV, OR

#### Note.

Kippenhan (2005) indicated from an analysis of populations that the variation in the dorsal coloration in *Cicindela amargosae* did not coincide with the accepted subspecific criteria. Probably the form *nyensis* should not be recognized as a valid entity. Kippenhan (2005: Fig. 1) provided a detailed map of the known populations of *Cicindela amargosae*.

### Cicindela cazieri

Vogt, 1949

Cicindela cazieri Vogt, 1949: 6. Type locality: «ten miles north of Rio Grande City, Starr County, Texas» (original citation). Holotype (♂) in USNM [# 59057].

#### Distribution.

This species, also known as the “Cazier’s Tiger Beetle”, is found along a small area in Jim Hogg and Starr Counties, southeastern Texas [see Pearson et al. 2006: Map 63], and in Tamaulipas, Mexico (Erwin and Pearson 2008: 127).

#### Records.

**USA**: TX – Mexico

#### Note.

This taxon is listed as a subspecies of *Cicindela politula* LeConte by some authors (e.g., Murray and Acciavatti 1976).

### Cicindela floridana

Cartwright, 1939

Cicindela abdominalis var. *floridana* Cartwright, 1939: 364. Type locality: «Miami [Dade County], Florida» (original citation). Holotype (♂) in USNM [# 53417].

#### Distribution.

This species is known only from a few sites in the Richmond Heights area of Miami (Brzoska et al. 2011: 5).

#### Records.

**USA**: FL

### Cicindela hemorrhagica arizonae

Wickham, 1899

Cicindela rufiventris var. *arizonae* Wickham, 1899: 226. Type locality: «Cañon of the Colorado River [Arizona]» (original citation). One syntype in USNM [# 56137].

#### Distribution.

This subspecies, also known as the “Grand Canyon Tiger Beetle”, is restricted to the Colorado River at the bottom of the Grand Canyon in northern Arizona and along the Virgin River in adjacent Utah and Nevada (Pearson et al. 2006: 135).

#### Records.

**USA**: AZ, NV, UT

### Cicindela hemorrhagica hemorrhagica

LeConte, 1851

Cicindela hemorrhagica LeConte, 1851: 171. Type locality: «San Diego [San Diego County, California]» (original citation). Syntype(s) in MCZ [# 14].
Cicindela bisignata Dokhtouroff, 1883a: 12. Type locality: «Californie» (original citation). Syntype(s) location unknown (possibly in DEI). Synonymy established by Horn (1905: 22).
Cicindela haemorrhagica var. *pacifica* Schaupp, 1884a: 106. Type locality: «San Diego [San Diego County], Cal[ifornia]» (original citation). Syntype(s) apparently destroyed. Synonymy established (as aberration) by Horn (1905: 22).
Cicindela woodgatei Casey, 1913: 40. Type locality: «Jemez Springs [Sandoval County], New Mexico» (original citation). Sixteen syntypes in USNM [# 45965]. Synonymy established by Cazier (1948: 11).
Cicindela pacifica nevadiana Casey, 1924: 16. Type locality: «Las Vegas [Clark County], Nevada» (original citation). Two syntypes [2 originally cited] in USNM [# 45964]. Synonymy established by Horn (1926: 290).

#### Distribution.

The range of this subspecies, also known as the “Wetsalts Tiger Beetle”, extends from central Washington to northwestern Wyoming, south to western Texas and along the Pacific Coast to the northern parts of the Baja California Peninsula (Cazier 1948: 11) [see Pearson et al. 1997: Fig. 35].

#### Records.

**USA**: AZ, CA (CHI), CO, ID, NM, NV, OR, TX, UT, WA, WY – Mexico

#### Note.

1. Some authors (e.g., Nagano 1982: 39) have treated *Cicindela pacifica* Schaupp as a valid subspecies of *Cicindela hemorrhagica* LeConte. 2. Freitag (1999: 62), Pearson et al. (2006: 135), and Erwin and Pearson (2008: 141) considered *Cicindela woodgatei* Casey as a valid subspecies of *Cicindela hemorrhagica* LeConte despite the fact that there seem to be no consistent characters to separate the adults from those of the nominate form. 3. Another subspecies, *Cicindela hemorrhagica hentziana* Leng, is found in Baja California; its record from “Utah” (Leng 1920: 42) is in error.

### Cicindela highlandensis

Choate, 1984

Cicindela highlandensis Choate, 1984: 74. Type locality: «0.25 mi[les] south of Josephine Creek, 4.3 mi[les] north of junction of Rt. S-17 and 621, Highlands Co[unty], Florida» (original citation). Holotype (♀) in FSCA.

#### Distribution.

This species, also known as the “Highlands Tiger Beetle”, is found in Highlands and Polk Counties, central Florida (Choate 2003: 84; Pearson et al. 2006: 126).

#### Records.

**USA**: FL

### Cicindela hornii hornii

Schaupp, 1883

Cicindela anthracina G.H. Horn, 1880a: 139 [primary homonym of *Cicindela anthracina* Klug, 1834]. Type locality: «Fort Bayard [Grant County], New Mexico» (original citation). Lectotype (♀), designated by Ward (1982: 60), in MCZ [# 33470].
Cicindela hornii Schaupp, 1883d: 80. Replacement name for *Cicindela anthracina* Horn, 1880. Note. There is no indication on page 80 that Schaupp proposed this name as a replacement name but this is evident on page 88 published in 1884.
Cicindela ritteri Bates, 1890: 496. Type locality: «Villa Lerdo in Durango [Mexico]» (original citation). Holotype [by monotypy] (♀) in BMNH. Synonymy established (as aberration) by Horn (1905: 21).

#### Distribution.

This species, also known as the “Horn’s Tiger Beetle”, ranges from southern Arizona to southwestern Texas, south to Durango (Cazier 1954: 248) [see Pearson et al. 1997: Fig. 25].

#### Records.

**USA**: AZ, NM, TX – Mexico

#### Note.

The subspecies *Cicindela hornii scotina* Bates is known from the states of Chihuahua, Durango, and Zacatecas in Mexico (Erwin and Pearson 2008: 146).

### Cicindela marginipennis

Dejean, 1831

Cicindela marginipennis Dejean, 1831: 260. Type locality: «Amérique septentrionale» (original citation), herein restricted to the banks of the Susquehanna below the bridge at Harrisburg, Dauphin County, Pennsylvania (see Leng 1902: 179). Syntype(s) in MHNP.

#### Distribution.

The range of this species, also known as the “Cobblestone Tiger Beetle”, is disjunct: one population is known from New Brunswick (Sabine 2005: 53) south to central New Jersey (Boyd 1978: Fig. 28), northwestern West Virginia (Allen and Acciavatti 2002: 26), southeastern Kentucky (Laudermilk et al. 2010: 28), and southeastern Indiana; the second is found in northeastern Mississippi and western Alabama [see Pearson et al. 2006: Map 67]. The record from “South Carolina” (Choate 2003: Map 30) needs confirmation.

#### Records.

**CAN**: NB **USA**: AL, IN, KY, ME, MS, NH, NJ, NY, OH, PA, VT, WV [SC]

#### Note.

This species is listed on the IUCN Red List of Threatened Species (IUCN 2007) and has been extirpated from many historical sites (Erwin and Pearson 2008: 155).

### Cicindela nigrocoerulea bowditchi

Leng, 1902

Cicindela bowditchi Leng, 1902: 124. Type locality: «vicinity of Durango [La Plata County], Colo[rado]» (original citation). Lectotype (♀), designated by Dahl (1941: 190), in MCZ [# 16272]. Etymology. The specific name was proposed for Frederick Channing Bowditch [*c*. 1853-1925], a conveyancer by profession and amateur coleopterist. Bowditch accompanied Samuel Hubbard Scudder in Colorado and Wyoming to collect fossils from Florissant shales.

#### Distribution.

This subspecies, the “Bowditch’s Tiger Beetle”, is known from southwestern Colorado (Kippenhan 1994: 65) and northwestern New Mexico (Rumpp 1962: 172). The record from “Arizona” (Boyd 1982: 11) is in error or based on a stray.

#### Records.

**USA**: CO, NM

#### Note.

This subspecies intergrades with the nominate form in north-central New Mexico (Pearson et al. 2006: 119).

### Cicindela nigrocoerulea nigrocoerulea

LeConte, 1846

Cicindela nigrocoerulea LeConte, 1846b: 181. Type locality: «ad flumen Arkansas» (original citation); cited from «near Bent’s Fort [Colorado] on the Arkansas River» by LeConte (1856a: 35). Syntype(s) in MCZ [# 27]. Note. According to Leng (1902: 124), LeConte’s original specimens consisted of “one pair found near Bent’s Fort on the Arkansas River, about 100 miles east of Pueblo, Col., and between Upper Dry Creek and Lower Dry Creek.”
Cicindela robusta Leng, 1902: 124. Type locality: «Alpine [Brewster County], Tex[as]» (original citation for the lectotype). Lectotype (♀), designated by Dahl (1941: 190), in AMNH [# 1231]. Synonymy established (as aberration) by Horn (1905: 21).
Cicindela [*nigrocoerulea*] *feminalis* Casey, 1909: 269. Type locality: «Las Animas [Bent County], Colorado» (original citation). Three syntypes in USNM [# 45906]. Synonymy established by Horn (1915: 381).
Cicindela snowi Casey, 1909: 269. Type locality: «Congress Junction [Yavapai County], Arizona» (original citation). One syntype in USNM [# 45908]. Synonymy established by Horn (1915: 381).
Cicindela [*snowi*] *triplicans* Casey, 1909: 270. Type locality: «Robinson [probably Robinson Place, Moffat County], Colorado» (original citation). One syntype in USNM [# 45907]. Synonymy established by Horn (1915: 381).
Cicindela [*snowi*] *velutoidea* Casey, 1909: 270. Type locality: «probably Colorado» (original citation). Holotype [by monotypy] (♂) in USNM [# 45909]. Synonymy established by Horn (1915: 381).

#### Distribution.

This subspecies, also known as the “Black Sky Tiger Beetle”, occurs from Salton Sea in southern California (LaRue 1991: 49) and southernmost Nevada (Kippenhan 200﻿2: 381) to western Texas (Gaumer and Murray 1971: 10), north to southwestern Utah (Tanner 1929a: 85) and northeastern Colorado (Kippenhan 1990: 311), south to Aguascalientes and San Luis Potosí in Mexico (Cazier 1960: 8).

#### Records.

**USA**: AZ, CA, CO, KS, NM, NV, OK, TX, UT – Mexico

### Cicindela nigrocoerulea subtropica

Vogt, 1949

Cicindela nigrocoerulea subtropica Vogt, 1949: 2. Type locality: «five miles southwest of Mission, S[outh]W[est] Hidalgo Co[unty], Texas» (original citation for the holotype, see Bellamy 1991: 736). Holotype (♀) in USNM [# 59055].

#### Distribution.

This subspecies, the “Subtropic Tiger Beetle”, is confined to Hidalgo and Cameron Counties in southern Texas (Pearson et al. 2006: 119).

#### Records.

**USA**: TX

### Cicindela obsoleta neojuvenilis

Vogt, 1949

Cicindela obsoleta neojuvenilis Vogt, 1949: 4. Type locality: «five miles southwest of Mission, S[outh]W[est] Hidalgo County, Texas» (original citation). Holotype (♂) in USNM [# 59056].

#### Distribution.

This subspecies, the “Rio Grande Grassland Tiger Beetle”, is known from the lower Rio Grande Valley in southern Texas from Maverick County to Hidalgo County (Pearson et al. 2006: 122), north to Kimble County in central Texas (Mawdsley 2009: 9).

#### Records.

**USA**: TX

### Cicindela obsoleta obsoleta

Say, 1823

Cicindela obsoleta Say, 1823b: 143. Type locality: «banks of the Arkansa river, near the mountains, Missouri Territory [= probably Colorado]» (original citation). Syntype(s) lost.
Cicindela prasina LeConte, 1856a: 31. Type locality: «Arkansas River below Bent’s Fort [Colorado]» (original citation). Holotype [by monotypy] (♂) in MCZ [# 33]. Synonymy established by Cresson (1861: 15), confirmed by Mawdsley (2009: 5).

#### Distribution.

This subspecies, also known as the “Large Grassland Tiger Beetle”, ranges from western Kansas to central Arizona, north to northern Colorado (Kippenhan 1994: 66), south to southern New Mexico and southwestern Texas; also known from one locality in eastern Kansas [see Mawdsley 2009: Fig. 18]. The record from “Utah” (Boyd 1982: 11) is likely in error.

#### Records.

**USA**: AZ, CO, KS, NM, OK, TX

#### Note.

This subspecies intergrades with the *santaclarae* form in New Mexico and western Texas (Mawdsley 2009: 6). Two other subspecies of this species are found in Mexico, *Cicindela obsoleta juvenilis* Horn from the states of Jalisco, Nayarit, Sonora, and Sinaloa and *Cicindela obsoleta latemaculata* Becker from the state of Durango (Erwin and Pearson 2008: 159, 160).

### Cicindela obsoleta santaclarae

Bates, 1890

Cicindela obsoleta var. or race *santaclarae* Bates, 1890: 493. Type locality: «Santa Clara in Chihuahua [Mexico]» (original citation). One syntype in BMNH (Mawdsley 2009: 6) and one in SIM (Hennessey 1990: 467).
Cicindela santaclarae var. *anita* Dow, 1911: 271. Type locality: «F[or]t Wingate, N[ew] Mex[ico]» (syntype label). One syntype in AMNH [# 1205] (Mawdsley 2009: 6) and one in CUIC. Synonymy established by Horn (1915: 382), confirmed by Mawdsley (2009: 6).

#### Distribution.

This subspecies, the “Santa Clara Grassland Tiger Beetle”, ranges from southern Colorado (Kippenhan 1994: 67) south to northern Durango (Cazier 1954: 251), including southwestern Texas and western Arizona [see Mawdsley 2009: Fig. 18].

#### Records.

**USA**: AZ, CO, NM, TX – Mexico

### Cicindela obsoleta vulturina

LeConte, 1853

Cicindela vulturina LeConte, 1853b: 439. Type locality: «Eagle Pass [Maverick County, Texas]» (original citation). One syntype in MCZ [# 43].

#### Distribution.

This subspecies, the “Prairie Tiger Beetle”, ranges from southern Missouri and north-central Arkansas to north-central Texas, south to southeastern Texas [see Mawdsley 2009: Fig. 18] and Coahuila in Mexico (Cazier 1954: 250). The record from central New Mexico (Fall and Cockerell 1907: 154) is suspect (Mawdsley 2009: 8); that from “Colorado” (Wickham 1902: 228) is probably in error.

#### Records.

**USA**: AR, LA, MO, OK, TX [NM] – Mexico

### Cicindela ocellata ocellata

Klug, 1834

Cicindela flavo-punctata Chevrolat, 1834 [8 March]: [no. 28] [primary homonym of *Cicindela flavopunctata* Audouin, 1832]. Type locality: Mexico (inferred from title of the book). Syntype(s) location unknown (possibly in UMO).
Cicindela ocellata Klug, 1834 [19 November]: 33. Type locality: «Jalapa [=Jalapa Enríquez, Veracruz, Mexico]» (original citation). Holotype [by monotypy] (♂) location unknown. Synonymy established by Gemminger and Harold (1868a: 15).
Cicindela incerta Chevrolat, 1835c: [no. 127]. Type locality: «Tutepec, Véra-Cruz? [Mexico]» (original citation). Holotype [by monotypy] location unknown (possibly in UMO). Synonymy established, under the name *Cicindela flavopunctata* Chevrolat, by Gemminger and Harold (1868a: 15).
Cicindela humeralis Chevrolat, 1841: [plate 59] 13. Type locality: Mexico (inferred from title of the paper). Syntype(s) location unknown (possibly in UMO). Synonymy established, under the name *Cicindela flavopunctata* Chevrolat, by Gemminger and Harold (1868a: 15).
Cicindela flavopunctata var. *chiapana* Bates, 1890: 505. Type locality: «Tapachula in Chiapas; La Noria in Sinaloa; Guatemala, near the city» (original citation), restricted to «Tapachula, Chiapas» by Freitag (1999: 66). Two syntypes in DEI (Döbler 1973: 368). Synonymy established, under the name *Cicindela flavopunctata humeralis* Chevrolat, by Horn (1915: 387).

#### Distribution.

This subspecies, also known as the “Ocellated Tiger Beetle”, occurs from southeastern Arizona and adjacent New Mexico (Pearson et al. 2006: 133) south to Costa Rica (Blackwelder 1944: 18); also recorded from “Texas” (Erwin and Pearson 2008: 162).

#### Records.

**USA**: AZ, NM [TX] – Belize, Costa Rica, El Salvador, Guatemala, Honduras, Mexico, Nicaragua

### Cicindela ocellata rectilatera

Chaudoir, 1843

Cicindela rectilatera Chaudoir, 1843b: 693. Type locality: «Mexique» (original citation). Syntype(s) in MHNP.
Cicindela texana LeConte, 1863b: 1. Type locality: «Fredericksburg, Texas; Tampico, Mexico; Rio Bravo» (original citation for *Cicindela decostigma* Chevrolat *sensu* LeConte, 1856). Syntype(s) probably in MCZ. Synonymy established by LeConte (1867b: 363). Note. This name was proposed for *Cicindela decostigma* Chevrolat, 1835 *sensu* LeConte (1856a: 54).

#### Distribution.

This subspecies, the “Dark-abdomened Tiger Beetle”, ranges from western Louisiana (Graves and Pearson 1973: 180) to northern New Mexico [see Pearson et al. 2006: Map 66], including southern Oklahoma (Schmidt 2004: 5), south to southern Tamaulipas (Cazier 1954: 278).

#### Records.

**USA**: LA, NM, OK, TX – Mexico

### Cicindela politula barbaraannae

Sumlin, 1976

Cicindela politula barbaraannae Sumlin, 1976b: 523. Type locality: «Hueco Mountains, 18.6 mi[les] E[ast] El Paso, Hudspeth Co[unty], Texas» (original citation). Holotype (♂) in CAS [# 13147].

#### Distribution.

This subspecies, the “Barbaraann’s Tiger Beetle”, occurs in the Hueco, Sierra Diablo, and Apache mountains in western Texas (Gage 1988: 146-147) and in the Sacramento Mountains of southern New Mexico (Pearson et al. 2006: 129) where it is found above 1500 m.

#### Records.

**USA**: NM, TX

### Cicindela politula petrophila

Sumlin, 1985

Cicindela politula petrophila Sumlin, 1985: 223. Type locality: «Guadalupe Mountains National Park, Culberson Co[unty], Texas» (original citation). Holotype (♂) in SMEK.

#### Distribution.

This subspecies, the “Rock-loving Tiger Beetle”, is known only from above 1670 m in the Guadalupe Mountains in western Texas and southeastern New Mexico (Gage 1988: 146).

#### Records.

**USA**: NM, TX

### Cicindela politula politula

LeConte, 1875

Cicindela politula LeConte, 1875a: 159. Type locality: «Texas» (original citation), herein restricted to Signal Mountains, Howard County (see Cazier 1939: 24 as *Cicindela alleni*). Syntype(s) in MCZ [# 31].
Cicindela politula cribrum Casey, 1913: 39. Type locality: «Texas» (original citation). One syntype in USNM [# 45968]. Synonymy established by Horn (1915: 385).
Cicindela alleni Cazier, 1939: 24 [primary homonym of *Cicindela alleni* Horn, 1908]. Type locality: «Signal M[oun]t[ain]s, Howard Co[unty], Texas» (original citation). Holotype (♀) in AMNH [# 1199]. Synonymy established by Sumlin (1985: 221).
Cicindela alleniana Mandl, 1961: 25. Replacement name for *Cicindela alleni* Cazier, 1939.

#### Distribution.

This subspecies, also known as the “Limestone Tiger Beetle”, occurs from Carter and Murray Counties in southern Oklahoma (Pearson et al. 2006: 128) south to Coahuila and Nuevo León (Sumlin 1985: Fig. 9).

#### Records.

**USA**: OK, TX – Mexico

#### Note.

Another subspecies, *Cicindela politula laetipennis* Horn, is known from the state of Coahuila in Mexico.

### Cicindela politula viridimonticola

Gage, 1988

Cicindela politula viridimonticola Gage, 1988: 143. Type locality: «129.16 kilometers south of Artesia (above 2192.8 m), Eddy County, New Mexico» (original citation). Holotype (♂) in FSCA.

#### Distribution.

This subspecies, the “Green Mountain Tiger Beetle”, is known only from the type locality in southeastern New Mexico.

#### Records.

**USA**: NM

### Cicindela punctulata chihuahuae

Bates, 1890

Cicindela punctulata var. *chihuahuae* Bates, 1890: 500. Type locality: «Arizona; Mexico: Santa Clara in Chihuahua, and Chihuahua City» (original citation). Syntype(s) in BMNH.
Cicindela fontinaria Casey, 1916: 33. Type locality: «Jemez Springs [Sandoval County], New Mexico» (original citation). One syntype in USNM [# 45960]. Synonymy established by Boyd (1982: 11).

#### Distribution.

This subspecies, the “Chihuahua Tiger Beetle”, is known from northeastern Colorado to west-central Nevada [see Pearson et al. 2006: Map 56], south to Chihuahua (Cazier 1954: 253). The records from Oklahoma (Drew and Van Cleave 1962: 113), “Nebraska,” “Kansas,” and “Texas” (Freitag 1999: 68) apparently refer to intergrades and these records are registered under the nominotypical subspecies.

#### Records.

**USA**: AZ, CO, NM, NV, UT – Mexico

#### Note.

Bertholf (1983: 21) listed this form in synonymy with the nominotypical subspecies.

### Cicindela punctulata punctulata

Olivier, 1790

Cicindela punctulata Olivier, 1790a: [No. 33] 27. Type locality: «Nouvelle-Jersey» (original citation), herein restricted to Bay Head, Ocean County (see Harris 1911: 39). Syntype(s) location unknown (possibly in MHNP).
Cicindela micans Fabricius, 1798: 61. Type locality: «America boreali» (original citation). One syntype in ZMUC (Zimsen 1964: 64). Synonymy established by Schönherr (1806: 245).
Cicindela punctulata var. *jenisonii* Gistel, 1837: 55. Type locality: «America septentrionali» (original citation). Syntype(s) lost. Synonymy established by Horn (1905: 22).
Cicindela boulderensis Casey, 1909: 271. Type locality: «Boulder Co[unty], Colorado» (original citation). One syntype in USNM [# 45961]. Synonymy established by Horn (1915: 383).
Cicindela prolixa Casey, 1916: 33. Type locality: «Akron [Washington County], Colorado» (original citation). One syntype in USNM [# 45962]. Synonymy established by Leng (1920: 41).

#### Distribution.

This subspecies, also known as the “Punctured Tiger Beetle”, ranges from New Brunswick to southern Alberta, south to southern Texas and southern Florida [see Pearson et al. 2006: Map 56].

#### Records.

**CAN**: AB, MB, NB, ON, QC, SK **USA**: AL, AR, CO, CT, DC, DE, FL, GA, IA, ID, IL, IN, KS, KY, LA, MA, MD, ME, MI, MN, MO, MS, MT, NC, ND, NE, NH, NJ, NM, NY, OH, OK, PA, RI, SC, SD, TN, TX, UT, VA, VT, WI, WV, WY

#### Note.

The two subspecies of *Cicindela punctulata* intergrade over a large area in southwestern United States (Pearson et al. 2006: 123) and northern Mexico (Murray 1979: 51). Another subspecies, *Cicindela punctulata catharinae* Chevrolat, is endemic to Mexico.

### Cicindela roseiventris tascosaensis

Davis, 1918

Cicindela roseiventris linearis W. Horn, 1905: 22 [primary homonym of *Cicindela linearis* Chaudoir, 1843]. Type locality: «San Carlos [= Cuidad Quesada], Costa Rica» (original citation). Five syntypes in DEI (Döbler 1973: 406).
Cicindela tascosaensis W.T. Davis, 1918: 34. Type locality: «Tascosa [Oldham County], Texas» (original citation). Holotype (♂) location unknown. Synonymy established by Horn (in Davis 1922: 130). Note. The holotype was in W.T. Davis’ collection but is not at AMNH (Lee Herman pers. comm. 2009) or SIM (Hennessey 1990). One syntype exists in DEI (Döbler 1973: 414).

#### Distribution.

This subspecies, also known as the “Tascoa Tiger Beetle”, has been recorded from Oldham County in northwestern Texas (Davis 1918: 34), Alajuela province in Costa Rica (Horn 1905: 22), and Panama (Erwin and Pearson 2008: 178).

#### Records.

**USA**: TX – Costa Rica, Panama

#### Note.

1. No specimens of this subspecies have been collected in United States since the original ones in 1917 and the subspecies has never been found in Mexico. Cazier (1954: 279) believed that the US specimens of *Cicindela tascosaensis* were probably mislabeled. However, Davis (1922: 130) wrote to the collector of the Texan specimens, Miss Mildred McGill, who replied on December 1920 that she remembered well collecting the tiger beetles “on the sandy, grassy spots of the ground, and on the wide floors of white sand rocks” about “a mile or a little more” of the house she lived in. 2. Two other subspecies, *Cicindela roseiventris mexicana* Klug and *Cicindela roseiventris roseiventris* Chevrolat, are found in Mexico and in Central America.

### Cicindela rufiventris cumatilis

LeConte, 1851

Cicindela cumatilis LeConte, 1851: 173. Type locality: «Louisiana» (original citation), herein restricted to Shreveport, Caddo Parish (see Chevrolat, 1852: 419 as *Cicindela guexiana*). Syntype(s) in MCZ [# 8].
Cicindela guexiana Chevrolat, 1852: 419. Type locality: «Shreveport [Caddo Parish], Louisiane» (original citation). Syntype(s) location unknown (possibly in UMO). Synonymy established by Melsheimer (1853: 2). Etymology. The specific name was proposed for John A. Guex [?-1858]. Born in Geneva in Switzerland, Guex came to America as a young man and settled in New York. He was interested in Coleoptera and provided many European correspondents with specimens from America. His collection of beetles, containing over 17,000 species, was presented to the Academy of Natural Sciences in Philadelphia in 1854.

#### Distribution.

This subspecies, the “Mexican Red-bellied Tiger Beetle”, ranges from Louisiana (Pearson et al. 2006: 131) south through Texas to Queretaro and Veracruz (Murray 1979: 53). The records from “Georgia” (Boyd 1982: 12) and western Alabama (Löding 1945: 9) probably refer to the nominotypical subspecies; those from “Mississippi,” “Arkansas” (Boyd 1982: 12), and Oklahoma (Drew and Van Cleave 1962: 114) are probably based on intergrades and these records are listed under the nominotypical subspecies.

#### Records.

**USA**: LA, TX – Mexico

### Cicindela rufiventris hentzii

Dejean, 1831

Cicindela haemorrhoidalis T.W. Harris, 1828a: 91 [primary homonym of *Cicindela haemorrhoidalis* Wiedemann, 1823]. Type locality not stated. Two possible syntypes in MCZ (collection Harris). Note. Gould (1834: 53) reported that this species was first discovered by Harris on the “summit of Blue Hill, in Milton [Norfolk County, Massachusetts].”
Cicindela haemorrhoidalis Hentz, 1830: 254 [primary homonym of *Cicindela haemorrhoidalis* Wiedemann, 1823]. Type locality: «Massachusetts» (original citation). Syntype(s) lost. Synonymy established with *Cicindela hentzii* Dejean by Dejean (1833: 4).
Cicindela hentzii Dejean, 1831: 248 (as *heutzii*). Type locality: «Amérique septentrionale» (original citation), herein restricted to Stoneham, Middlesex County, Massachusetts (see Frost, 1920: 230 as *Cicindela hentzi* var. *niveihamata*). Syntype(s) probably in MHNP. Synonymy established by Gould (1834: 52). Etymology. The specific name honors Nicholas Marcellus Hentz [1797-1856], the first authority on spiders in the United States. In his early years, Hentz published on beetles and described new species from Massachusetts and Pennsylvania. His collection, consisting of about 1,500 species, most of them Coleoptera from all parts of the United States, was purchased for $550 by friends and presented to the Boston Society of Natural History in 1836 (Weiss 1936: 280); little was left of the collection by 1861 (Wilson 1973: 71). Note. This name was originally proposed under the spelling *heutzii* because Dejean believed the name of the collector was Heutz. LeConte (1856a: 55) emended Dejean’s name to *hentzii* since the name of the collector was Hentz. This is an unjustified emendation. However since the emendation is in prevailing usage and attributed to the original author and date, it is deemed to be a justified emendation (ICZN 1999: Article 33.2.3.1) and the spelling becomes the correct original spelling (ICZN 1999: Article 32.2.2).
Cicindela erythrogaster T.W. Harris [in Scudder], 1891: 138. Type locality not stated. Holotype [by monotypy] lost. Synonymy established by Horn (1915: 386).
Cicindela hentzi var. *niveihamata* Frost, 1920: 230. Type locality: «Middlesex Fells Reservation near the shore of Spot Pond in the town of Stoneham [Middlesex County], Mass[achusetts]» (original citation). Holotype (♂) in MCZ [# 34727]. Synonymy established by Horn (1926: 287).

#### Distribution.

This subspecies, the “Hentz’s Tiger Beetle”, is found only along eastern Massachusetts [see Leonard and Bell 1999: Fig. 111]. The record from “Rhode Island” (Bousquet and Larochelle 1993: 64) is in error or based on a stray.

#### Records.

**USA**: MA

### Cicindela rufiventris rufiventris

Dejean, 1825

Cicindela rufiventris Dejean, 1825: 102. Type locality: «Saint-Domingue [= Dominican Republic or Hispaniola]» (original citation), which is incorrect; East Plains, a desert tract of stunted pines and oaks, near Brookville, about ten miles inland from Barnegat, Ocean County, New Jersey (see Leng 1902: 177) herein selected. Syntype(s) in MHNP.
Cicindela rufiventris collusor Casey, 1913: 39. Type locality not stated. Holotype [by monotypy] (♀) in USNM [# 45967]. Synonymy established by Horn (1915: 386).

#### Distribution.

This subspecies, also known as the “Eastern Red-bellied Tiger Beetle”, ranges from southwestern Vermont (Leonard and Bell 1999: 104) to southwestern Missouri, south to east-central Texas and the Florida Panhandle [see Pearson et al. 2006: Map 64].

#### Records.

**USA**: AL, AR, CT, DC, DE, FL, GA, IL, IN, KY, LA, MA, MD, MO, MS, NC, NJ, NY, OH, OK, PA, RI, SC, TN, TX, VA, VT, WV

#### Note.

This subspecies intergrades with the *cumatilis* form through southern Missouri, Arkansas, and Louisiana (Pearson et al. 2006: 131). Another subspecies, *Cicindela rufiventris reducens* Horn, is known from the states of Jalisco and Colima in Mexico.

### Cicindela scabrosa

Schaupp, 1884

Cicindela abdominalis var. *scabrosa* Schaupp, 1884a: 108. Type locality: «Fl[orid]a» (original citation), herein restricted to Crescent City, Putnam County (see Casey 1913: 38, as *Cicindela extenuata*). Syntype(s) apparently destroyed.
Cicindela extenuata Casey, 1913: 38. Type locality: «Crescent City [Putnam County], Florida» (original citation). Two syntypes in USNM [# 45970]. Synonymy established by Leng (1915: 563) and Horn (1915: 385), confirmed by Choate (1984: 76).

#### Distribution.

This species, also known as the “Scabrous Tiger Beetle”, is found from southeastern Georgia to southern Florida [see Pearson et al. 2006: Map 61].

#### Records.

**USA**: FL, GA

### Cicindela schauppii

Horn, 1876

Cicindela schauppii G.H. Horn, 1876a: 240. Type locality: «Corsicana [Navarro County], eastern Texas» (original citation). Lectotype (♂), designated by Ward (1982: 62), in MCZ [# 10042]. Etymology. This species was named after Franz G. Schaupp [1840?-1904], a German immigrant who settled in New York City but spent his last twenty years in Texas. Schaupp sustained himself mostly by teaching languages and as a hobby collected and studied beetles. He was instrumental in the establishment of the Brooklyn Entomological Society.

#### Distribution.

This species, also known as “Schaupp’s Tiger Beetle”, occurs from southeastern Kansas and northwestern Arkansas [see Pearson et al. 2006: Map 69] south to Nuevo León (Cazier 1954: 287). The record from “Missouri” (Erwin and Pearson 2008: 181) needs confirmation.

#### Records.

**USA**: AR, KS, OK, TX [MO] – Mexico

### Cicindela sedecimpunctata sedecimpunctata

Klug, 1834

Cicindela 16-punctata Klug, 1834: 32. Type locality: «Mexico» (original citation). Holotype [by monotypy] (♂) location unknown.
Cicindela rufiventris var. *ventanasa* Bates, 1890: 503. Type locality: «Ventanas in Durango, and La Noria in Sinaloa [Mexico]» (original citation). One syntype in DEI (Döbler 1973: 416) and two in IRSN. Synonymy established by Horn (1905: 21).
Cicindela sedecimpunctata sonorana Casey, 1913: 40. Type locality: «Arizona, New Mexico and southward to Durango» (original citation). Twenty-five syntypes in USNM [# 45966]. Synonymy established by Horn (1915: 386).

#### Distribution.

This subspecies, also known as the “Western Red-bellied Tiger Beetle”, ranges from northeastern New Mexico to central Arizona [see Pearson et al. 2006: Map 65], south to Guanajuato in Mexico (Cazier 1954: 271).

#### Records.

**USA**: AZ, NM, TX – Mexico

#### Note.

Besides the nominotypical subspecies, four other subspecies are known, ranging collectively from Mexico to Costa Rica (see Pearson et al. 2006: 132; Erwin and Pearson 2008: 185-186).

### Cicindela senilis

Horn, 1867

Cicindela senilis G.H. Horn, 1867a: 395. Type locality: «California» (original citation), herein restricted to San Rafael, Alameda County (see Leng 1902: 142). Lectotype (♂), designated by Ward (1982: 60), in MCZ [# 33471].
Cicindela senilis exoleta Casey, 1909: 272. Type locality: «Oakland [Alameda County], California» (original citation). One syntype in USNM [# 45927]. Synonymy established by Harris (1911: 22).
Cicindela senilis frosti Varas Arangua, 1928: 174. Type locality: «Manhattan, Los Angeles Co[unty], California» (original citation). Syntype(s) [2 ♂ originally cited] in CAS [# 8149]. Synonymy established by Cazier (1937a: 159). Etymology. The subspecific name was proposed in honor of Charles Albert Frost [1872-1962], a civil engineer with the Waterworks Division of the Metropolitan District Commission in Massachusetts and amateur coleopterist. Frost left his collection of more than 50,000 specimens to the Museum of Comparative Zoology.

#### Distribution.

This species, also known as the “Senile Tiger Beetle”, is found along western California, as far north as Sonoma and Lake Counties, and the northern part of the Baja California Peninsula. According to Pearson et al. (2006: 116), it is now known in the United States only from a few protected coastal populations and two interior populations, one near Lake Elsinore in western Riverside County and one near Jacumba in San Diego County.

#### Records.

**USA**: CA (CHI) – Mexico

### Cicindela tenuisignata

LeConte, 1851

Cicindela tenuisignata LeConte, 1851: 171. Type locality: «ad flumen Novum [= New River, Imperial County], in desertis fluminis Colorado [California]» (original citation). Syntype(s) in MCZ [# 40].
Cicindela psilogramma Bates, 1890: 507. Type locality: «Villa Lerdo in Durango [Mexico]» (original citation). Two syntypes in DEI (Döbler 1973: 403) and four in IRSN. Synonymy established by Horn (1892b: 97).

#### Distribution.

This species, also known as the “Thin-lined Tiger Beetle”, ranges from western Nebraska (Brust 2007: 9) to southern California [see Pearson et al. 1997: Fig. 38], south to northern Sinaloa and southern Tamaulipas (Cazier 1954: 257).

#### Records.

**USA**: AZ, CA, CO, KS, NE, NM, NV, OK, TX, UT – Mexico

### Cicindela trifasciata ascendens

LeConte, 1851

Cicindela ascendens LeConte, 1851: 172. Type locality: «Georgia» (original citation), herein restricted to Saint Simons Island, Glynn County (see Beaton 2008: 41). Holotype [by monotypy] in MCZ [# 2].
Cicindela serpens LeConte, 1851: 173. Type locality: «Key West [Monroe County], Florida» (original citation). Syntype(s) in MCZ [# 34]. Synonymy established by Leng (1902: 160).

#### Distribution.

This highly vagile subspecies, also known as the “Ascendant Tiger Beetle”, is found primarily along the Atlantic and Gulf Coasts from Virginia to southern Florida, west to eastern Texas [see Pearson et al. 2006: Map 70], south to Panama (Erwin and Pearson 2008: 197). This form has also been found inland as far north as north-central Kansas (Charlton and Kopper 2000: 266) and along the Atlantic Coast as far north as Massachusetts (Comboni and Schultz 1989: 151); however there is no known established populations inland anywhere in North America (Pearson and Vogler 2001: 105). The record from the “West Indies” (LeConte 1856a: 51) needs confirmation.

#### Records.

**USA**: AL, AR, FL, GA, KS, LA, MA, MD, MO, MS, NC, NJ, OK, SC, TN, TX, VA – Belize, Costa Rica, El Salvador, Guatemala, Honduras, Mexico, Nicaragua, Panama.

#### Note.

Besides the two subspecies found in North America, six other subspecies are recognized among this polymorphic species in Middle and South America and the West Indies.

### Cicindela trifasciata sigmoidea

LeConte, 1851

Cicindela sigmoidea LeConte, 1851: 172. Type locality: «San Diego [San Diego County, California]» (original citation). Syntype(s) in MCZ [# 35] and MHNP (collection Chaudoir).

#### Distribution.

This subspecies, the “Sigmoid Tiger Beetle”, is found along the Pacific Coast from southern California (Nagano 1982: 38) to the Baja California Peninsula (Cazier 1954: 291); it is occasionally found inland, as far as the Salton Sea in Imperial County (see LaRue 1991). The record from “Arizona” (Freitag 1999: 74) needs confirmation.

#### Records.

**USA**: CA (CHI) [AZ] – Mexico

### Cicindela willistoni echo

Casey, 1897

Cicindela echo Casey, 1897: 298. Type locality: «Great Salt Lake, Utah» (original citation). Four syntypes in USNM [# 45923].
Cicindela echo amedeensis Casey, 1909: 272 (as *amadeensis*). Type locality: «Amedee, Cal[ifornia]» (original citation). Two syntypes in USNM [# 45924]. Synonymy established by Harris (1911: 23).
Cicindela spaldingi Casey, 1924: 14. Type locality: «Callao [Juab County], Utah» (original citation). One syntype in USNM [# 45922]. Synonymy established by Boyd (1982: 10). Etymology. The specific name was proposed in honor of Thomas Utting Spalding [1866-1929]. Born in England, Spalding came to America and headed west in search of gold. He eventually settled in Utah and became an excellent collector of Lepidoptera and Coleoptera which he sold to students in the east. In 1918 alone, his Lepidoptera sales amounted to $1,150 (Tanner 1929b: 344). At that time he started selling beetles to Thomas Casey who bought in all 820 specimens from him.

#### Distribution.

This subspecies, the “Echo Tiger Beetle”, is found mainly within the Great Basin from Wyoming to southern Oregon, south to east-central California, southern Nevada, and southern Utah (Pearson et al. 2006: 114).

#### Records.

**USA**: CA, ID, NV, OR, UT, WY

### Cicindela willistoni estancia

Rumpp, 1962

Cicindela willistoni estancia Rumpp, 1962: 166. Type locality: «7.0 miles east of Willard, Torrance County, New Mexico» (original citation). Holotype (♂) in CAS [# 17199].

#### Distribution.

This subspecies, the “Torrance Tiger Beetle”, is known only from Torrance County in central New Mexico (Pearson et al. 2006: 114).

#### Records.

**USA**: NM

### Cicindela willistoni funaroi

Rotger, 1972

Cicindela willistoni funaroi Rotger, 1972: 25. Type locality: «4.2 miles from the Catholic Church building of San Ysidro, Sandoval County, New Mexico» (original citation). Holotype (♂) in Ronald L. Huber collection (Bloomington, Minnesota).

#### Distribution.

This subspecies, the “Funaro’s Tiger Beetle”, is known only from the type locality in northwestern New Mexico.

#### Records.

**USA**: NM

### Cicindela willistoni hirtifrons

Willis, 1967

Cicindela willistoni hirtifrons Willis, 1967: 301. Type locality: «Big Salt Marsh, 11 mi[les] N[orth]E[ast] of Hudson, Stafford Co[unty], Kansas» (original citation). Holotype (♂) in SMEK.

#### Distribution.

This subspecies, the “Hairy-fronted Tiger Beetle”, is found in central Kansas, western Oklahoma, west-central Texas, and east-central New Mexico (Willis 1967: 302); also recorded from “Arizona” (Erwin and Pearson 2008: 202).

#### Records.

**USA**: KS, NM, OK, TX [AZ]

### Cicindela willistoni praedicta

Rumpp, 1956

Cicindela willistoni praedicta Rumpp, 1956: 135. Type locality: «3.5 miles south of Shoshone, Inyo County, California» (original citation). Holotype (♂) in CAS [# 17200].

#### Distribution.

This subspecies, the “Augured Tiger Beetle”, is known from Inyo County in eastern California and Nye County in Nevada (Rumpp 1956: 135).

#### Records.

**USA**: CA, NV

### Cicindela willistoni pseudosenilis

Horn, 1900

Cicindela pseudosenilis W. Horn, 1900: 117. Type locality: «Owen’s lake, Inyo Co[unty], California» (original citation). Syntype(s) in DEI (Döbler 1973: 418) and MCZ [# 23808].

#### Distribution.

This subspecies, the “Owens Lake Tiger Beetle”, is restricted to Owens and adjacent Panamint Valley of east-central California (Pearson et al. 2006: 115).

#### Records.

**USA**: CA

### Cicindela willistoni sulfontis

Rumpp, 1977

Cicindela willistoni sulfontis Rumpp, 1977: 170. Type locality: «5.6 kilometers west-southwest of Willcox [Cochise County, Arizona]» (original citation). Holotype (♂) in CAS [# 12530].

#### Distribution.

This subspecies, the “Sulphur Valley Tiger Beetle”, is endemic to the Sulphur Springs Valley in southeastern Arizona.

#### Records.

**USA**: AZ

### Cicindela willistoni willistoni

LeConte, 1879

Cicindela willistoni LeConte, 1879d: 507. Type locality: «Lake Como [Carbon County], Wyoming Territory» (original citation). Syntype(s) in MCZ [# 45]. Etymology. The specific name honors Samuel Wendell Williston [1852-1918], well known American paleontologist, dipterist, and teacher.

#### Distribution.

This subspecies, also known as the “Williston’s Tiger Beetle”, is endemic to the Laramie Plain of Wyoming (Rumpp 1962: 168).

#### Records.

**USA**: WY

#### Note.

This subspecies intergrades with the *echo* form to the west (Pearson et al. 2006: 114). In a cladistic analysis based on molecular data by Vogler and Welsh (1997), *Cicindela willistoni* clearly embedded within the subgenus *Cicindela* while morphological characters suggest that it belongs to the subgenus *Cicindelidia*.

### Subgenus Cicindela

Linnaeus, 1758

Cicindela Linnaeus, 1758: 407. Type species: *Cicindela campestris* Linnaeus, 1758 designated by Latreille (1810: 425).
Pachydela Rivalier, 1954: 253. Type species: *Cicindela scutellaris* Say, 1823 by original designation. Synonymy established by Boyd (1982: 6).
Tribonia Rivalier, 1954: 254. Type species: *Cicindela tranquebarica* Herbst, 1806 by original designation. Synonymy established by Boyd (1982: 6).

#### Diversity.

Northern Hemisphere, with about 75 species (Lorenz 2005: 43-48) in the Nearctic (38 species, of which four extend into northern Mexico; 92 species-group taxa) and Palaearctic (36 species) Regions.

### [decemnotata group]

### Cicindela ancocisconensis

Harris, 1852

Cicindela ancocisconensis T.W. Harris, 1852: 305. Type locality: «mountain streams near the White M[oun]t[ain]s, N[ew] H[ampshire]» (original citation), herein restricted to Conway, Carroll County (see Wilson and Larochelle 1980: 33). Syntypes in MCZ [# 24] (collection LeConte, see LeConte 1856a: 38). Note. According to his son, Edward, Thaddeus William Harris originally collected this species at Conway, on an island in the river (see Wilson and Larochelle 1980: 33).
Cicindela catharina T.W. Harris [in Scudder], 1869: 229. Unnecessary replacement name for *Cicindela ancocisconensis* Harris, 1852.
Cicindela ancocisconensis dowiana Casey, 1914: 23. Type locality: «De Bruce [Sullivan County], New York» (original citation). Two syntypes in USNM [# 45975]. Synonymy established by Horn (1915: 444).
Cicindela ancocisconensis carolinae Casey, 1916: 28. Type locality: «North Carolina» (original citation). One syntype in USNM [# 45974]. Synonymy established by Horn (1926: 267).
Cicindela ancocisconensis eriensis Casey, 1916: 29. Type locality: «Buffalo [Erie County], New York» (original citation). Two syntypes [2 originally cited] in USNM [# 45976]. Synonymy established by Horn (1926: 267).

#### Distribution.

This eastern species, also known as the “Appalachian Tiger Beetle”, ranges from southwestern New Brunswick (Webster and Bousquet 2008: 16) south to northeastern Georgia, west at least to eastern Kentucky (Laudermilk et al. 2010: 28) [see Pearson et al. 1997: Fig. 15]. Old records from Indiana, northern Illinois, and western Missouri (see Wilson and Larochelle 1980: 37-38) suggest that the species was more widely distributed at one time. Beaton (2008: 40) did not find the species in Georgia during his intensive survey of tiger beetles in the state.

#### Records.

**CAN**: NB, QC **USA**: GA, KY, MA, MD, ME, NC, NH, NJ, NY, OH, PA, TN, VA, VT, WV [IL, IN, MO]

### Cicindela arida

Davis, 1928

Cicindela denverensis var. *propinqua* Knaus, 1923: 194 [primary homonym of *Cicindela propinqua* Chaudoir, 1835]. Type locality: «Ash Meadow (2,050 feet), Nye County, Nevada» (original citation). Holotype (♂) location unknown (possibly in KSUC).
Cicindela arida A.C. Davis, 1928: 65. Type locality: «Death Valley Junction [Inyo County], California» (original citation). Holotype (♂) in USNM [# 56263]. Synonymy established by Nicolay and Weiss (1932: 352).

#### Distribution.

This species, the “Death Valley Tiger Beetle”, is known from the Death Valley region (Kritsky and Horner 1998: 17) in Inyo County, California, and Nye County, Nevada.

#### Records.

**USA**: CA, NV

#### Note.

This form has been listed as a subspecies of *Cicindela tranquebarica* Herbst by most authors, including Erwin and Pearson (2008: 191), but Kritsky and Horner (1998: 17) found enough structural differences to substantiate that it represents a distinct species.

### Cicindela decemnotata bonnevillensis

Knisley and Kippenhan, 2012

Cicindela decemnotata bonnevillensis Knisley and Kippenhan [in Knisley et al.], 2012: 19. Type locality: «playa south of Delle, Tooele Co[unty], Utah» (original citation). Holotype (♂) in MCZ.

#### Distribution.

This subspecies is restricted to the area of ancient Lake Bonneville in north-central Utah [see Knisley et al. 2012: Fig. 23].

#### Records.

**USA**: UT

### Cicindela decemnotata decemnotata

Say, 1817

Cicindela decemnotata Say, 1817a: [25]. Type locality: «sandy alluvions of the Missouri, above the confluence of the river Platte» (original citation). Holotype [by monotypy] (♀) lost.
Cicindela decemnotata albertina Casey, 1913: 24. Type locality: «Lethbridge, Alberta» (original citation). Two syntypes [2 originally cited] in USNM [# 45937]. Synonymy established by Horn (1915: 374).
Cicindela lantzi E.D. Harris, 1913: 68. Type locality: «Jefferson [Park County], Col[orado]» (original citation). Syntype(s) in MCZ [# 23552]. Synonymy established by Horn (1915: 374). Etymology. The specific name honors David Ernest Lantz [1855-1918], a naturalist and teacher whose main field of study was economic mammology.

#### Distribution.

This subspecies, also known as the “Badlands Tiger Beetle”, ranges in patchy colonies from eastern Alaska south through the Rocky Mountains to northeastern New Mexico and southern Utah, east to western North Dakota and western Nebraska [see Knisley et al. 2012: Figs 23, 24]. The records from “Kansas” (Leng 1902: 134) and “Manitoba” (Knisley et al. 2012: 14) need confirmation.

#### Records.

**CAN**: AB, SK, YT **USA**: AK, CO, ID, MT, ND, NE, NM, UT, WY [KS, MB]

### Cicindela decemnotata meriwetheri

Knisley and Kippenhan, 2012

Cicindela decemnotata meriwetheri Knisley and Kippenhan [in Knisley et al.], 2012: 15. Type locality: «Grand Coulee Dam Airport, Grant Co[unty], Washington» (original citation). Holotype (♂) in MCZ. Etymology. The subspecific name was proposed for Meriwether Lewis [1774-1809], American explorer, soldier, and public administrator, well-known for his role as leader of the Lewis and Clark Expedition, 1804-06, the first American expedition to the Pacific Coast.

#### Distribution.

This subspecies ranges from south-central British Columbia to southeastern Washington [see Knisley et al. 2012: Fig. 23].

#### Records.

**CAN**: BC **USA**: WA

### Cicindela decemnotata montevolans

Knisley and Kippenhan, 2012

Cicindela decemnotata montevolans Knisley and Kippenhan [in Knisley et al.], 2012: 22. Type locality: «1.2 mi S[outh] H[igh]w[a]y 89 @ Cache-Rich Co[unty] line, Cache Co[unty], Utah» (original citation). Holotype (♂) in MCZ.

#### Distribution.

This subspecies is restricted to high elevations of the Bear River Mountains of southeastern Idaho and northeastern Utah [see Knisley et al. 2012: Fig. 23].

#### Records.

**USA**: ID, UT

### Cicindela denverensis

Casey, 1897

Cicindela denverensis Casey, 1897: 297. Type locality: «Denver [Denver County], Colorado» (original citation). One syntype in USNM [# 45939].
Cicindela purpurea var. *ludoviciana* Leng, 1902: 132. Type locality: «Vowell’s Mill, Natchitoches Parish, in the northwestern part of Louisiana» (original citation). Lectotype (♂), designated by Dahl (1941: 171), in AMNH [# 1222]. Synonymy established by Schincariol and Freitag (1991: 1347).
Cicindela denverensis conquisita Casey, 1914: 357. Type locality: «Sioux Co[unty], Nebraska» (original citation). One syntype in USNM [# 45940]. Synonymy established by Horn (1915: 444).
Cicindela denverensis oreada Casey, 1914: 358. Type locality: «Benkelman [Dundy County], Nebraska» (original citation). One syntype in USNM [# 45941]. Synonymy established by Horn (1915: 444).
Cicindela plattensis Smyth, 1933: 202. Type locality: «valley of the South Platte» (original citation). Syntype(s) location unknown. Synonymy established, under the name *Cicindela denverensis conquisita* Casey, by Nicolay (1934: 154).

#### Distribution.

This species, also known as the “Green Claybank Tiger Beetle”, inhabits the Great Plains from eastern Montana and North Dakota south to northern Louisiana, northern Texas, and northeastern New Mexico [see Schincariol and Freitag 1991: Fig. 13].

#### Records.

**USA**: AR, CO, KS, LA, MT, ND, NE, NM, OK, SD, TX, WY

#### Note.

According to Pearson et al. (2006: 92), individuals with green elytra and blue head and thorax from northwestern Louisiana and southwestern Arkansas (originally described under the name *ludoviciana*) may be either an isolated population of this species or a local green morph of *Cicindela splendida*. They also added that based on the ecology, behavior, and distribution, the greenish population is more likely a local variant of *Cicindela splendida*.

### Cicindela fulgida fulgida

Say, 1823

Cicindela fulgida Say, 1823b: 141. Type locality: «near the mountains on the Nebraska (Platte) and Arkansa rivers, Missouri Territory» (original citation). Syntype(s) lost.
Cicindela fulgida subnitens Calder, 1922a: 62. Type locality: «Lincoln [Lancaster County], Nebr[aska]» (original citation). Holotype location unknown (possibly in UMAA). Synonymy established by Horn (1926: 275).
Cicindela fulgida williamlarsi Knudsen, 1985: 182. Type locality: «San Ysidro, Sandoval Co[unty], N[ew]M[exico]» (original citation). Holotype (♂) in CAS [# 17196]. Synonymy established implicitly by Kippenhan (1994: 52).
Cicindela fulgida winonae Knudsen, 1985: 184. Type locality: «Grants, Valencia Co[unty], N[ew]M[exico]» (original citation). Holotype (♂) in CAS [# 15836]. Synonymy established implicitly by Kippenhan (1994: 52).
Cicindela fulgida rumppi Knudsen, 1985: 185. Type locality: «Laguna del Perro, 7.2 mi[les] E[ast] of Willard, Torrance Co[unty], New Mexico» (original citation). Holotype (♂) in CAS [# 17195]. Synonymy established implicitly by Kippenhan (1994: 52). Etymology. The subspecific name was proposed for Norman L. Rumpp [1913-1991], an engineer for the Navy Department at the U.S. Naval Weapons Center in China Lake, California, by profession and a cicindelophile by avocation.

#### Distribution.

This subspecies, also known as the “Crimson Saltflat Tiger Beetle”, ranges from Minnesota to southern Alberta (Hilchie 1985: 330), south to northeastern Arizona and northern Texas [see Pearson et al. 1997: Map 11]. The record from “Saskatchewan” (Freitag 1999: 27) needs confirmation.

#### Records.

**CAN**: AB **USA**: AZ, CO, KS, MN, MT, ND, NE, NM, OK, SD, TX, UT, WY [SK]

#### Note.

Pearson et al. (2006: 99), followed by Erwin and Pearson (2008: 138), considered *Cicindela fulgida williamlarsi* Knudsen and *Cicindela fulgida winonae* Knudsen as synonyms of *Cicindela fulgida pseudowillistoni* Horn. They also listed *Cicindela fulgida rumppi* Knudsen as a valid subspecies restricted to the Laguna del Perro area in Torrance County, central New Mexico.

### Cicindela fulgida pseudowillistoni

Horn, 1938

Cicindela fulgida pseudo-willistoni W. Horn, 1938: 13. Type locality: «Como-See (8000 Fuß hoch) [Carbon County], Süd-Wyoming» (original citation). Lectotype (♀), designated by Kippenhan (1996a: 38), in DEI.

#### Distribution.

This subspecies, also known as the “Alkaline Tiger Beetle”, is found in southern Wyoming and northwestern Colorado (Kippenhan 1996a: 42).

#### Records.

**USA**: CO, WY

#### Note.

Pearson et al. (2006: 99) recorded this subspecies from a much larger area, throughout the western Great Plains and intermontane southern Rocky Mountains.

### Cicindela fulgida westbournei

Calder, 1922

Cicindela fulgida elegans Calder, 1922a: 62 [primary homonym of *Cicindela elegans* Fischer von Waldheim, 1823]. Type locality: «Westbourne, Man[itoba]» (original citation). Holotype location unknown (possibly in UMAA).
Cicindela westbournei Calder, 1922b: 191. Replacement name for *Cicindela elegans* Calder, 1922.

#### Distribution.

This subspecies, also known as the “Westbourne’s Tiger Beetle”, is found in southern Manitoba, southern Saskatchewan (Wallis 1961: 51), north-central North Dakota, and northwestern Minnesota (Knudsen 1985: 186); also recorded from “Montana” (Erwin and Pearson 2008: 139).

#### Records.

**CAN**: MB, SK **USA**: MN, ND, UT [MT]

### Cicindela latesignata latesignata

LeConte, 1851

Cicindela latesignata LeConte, 1851: 172. Type locality: «San Diego [San Diego County, California]» (original citation). Syntype(s) in MCZ [# 18].
Cicindela latesignata obliviosa Casey, 1913: 20. Type locality: «San Diego [San Diego County], California» (original citation). Three syntypes in USNM [# 45925]. Synonymy established by Horn (1915: 376).

#### Distribution.

This subspecies, also known as the “Western Beach Tiger Beetle”, is found along the Pacific Coast from southern California to the central parts of the Baja California Peninsula (Cazier 1948: 14) and also along the Gulf of California coast in northern Sonora [see Pearson et al. 1997: Fig. 27]. According to Pearson et al. (2006: 195), this taxon is now gone from most of its former sites in southern California.

#### Records.

**USA**: CA – Mexico

#### Note.

Some authors (e.g., Nagano 1982: 37) consider *Cicindela obliviosa* Casey as a valid subspecies. *Cicindela latesignata parkeri* Cazier is found in Sonora and Baja California in Mexico.

### Cicindela lengi jordai

Rotger, 1974

Cicindela lengi jordai Rotger, 1974: 9. Type locality: «Heart Canyon, four miles north of Aztec, San Juan Co[unty], New Mexico» (original citation). Holotype (♂) in Ronald L. Huber collection (Bloomington, Minnesota).

#### Distribution.

This subspecies, the “Jorda’s Tiger Beetle”, is known from northeastern Arizona (Bertholf 1983: 12) and northern New Mexico (Acciavatti et al. 1980: 30). Based on Pearson et al. (2006: Map 47), it is also found in southern Utah and southwestern Colorado. The record from “Wyoming” (Boyd 1982: 9) probably refers to the nominotypical subspecies.

#### Records.

**USA**: AZ, CO, NM, UT

### Cicindela lengi lengi

Horn, 1908

Cicindela venusta LeConte, 1846b: 179 [primary homonym of *Cicindela venusta* LaFerté-Sénectère, 1841]. Type locality: «apud flumen Platte» (original citation); cited from «near the Forks of Platte River» by LeConte (1856a: 39). Syntype(s) in MCZ [# 42].
Cicindela lengi W. Horn, 1908b: 738. Replacement name for *Cicindela venusta* LeConte, 1846.

#### Distribution.

This subspecies, also known as the “Blowout Tiger Beetle”, ranges from western South Dakota (Spomer et al. 2008a: 57) and southern Wyoming, south to northern New Mexico, extreme northwestern Texas, and southern Oklahoma [see Pearson et al. 2006: Map 47]. The records from “Montana” (Horn 1915: 372) and “Iowa” (Boyd 1982: 9) need confirmation.

#### Records.

**USA**: CO, KS, MO, NE, NM, OK, SD, TX, WY [IA, MT]

#### Note.

This subspecies intergrades with the *jordai* form in the southwestern part of its range and with the *versuta* form in the northern part of its range.

### Cicindela lengi versuta

Casey, 1913

Cicindela venusta versuta Casey, 1913: 24. Type locality: «Aweme, Manitoba» (original citation). Four syntypes in USNM [# 45973].
Cicindela venusta gracilenta Casey, 1913: 25. Type locality: «Montana» (original citation). One syntype in USNM [# 45972]. Synonymy established by Bousquet and Larochelle (1993: 57).

#### Distribution.

This subspecies, the “Adroit Tiger Beetle”, ranges from southern Manitoba to northern Alberta (Wallis 1961: 56), south to Wyoming and northwestern South Dakota (Spomer et al. 2008a: 34).

#### Records.

**CAN**: AB, MB, SK **USA**: MT, ND, SD, WY

### Cicindela limbalis

Klug, 1834

Cicindela limbalis Klug, 1834: 29. Type locality: «Nord-Amerika» (original citation), herein restricted to Eastport, Washington County, Maine (see LeConte, 1846b: 177, as *Cicindela spreta*). Holotype [by monotypy] (♀) location unknown.
Cicindela amoena LeConte, 1846b: 177. Type locality: «prope provinciae Missouri terminum occidentalem» (original citation). Holotype [by monotypy] (♀) in MCZ [# 1]. Synonymy established by LeConte (1863b: 1).
Cicindela spreta LeConte, 1846b: 177. Type locality: «Eastport [Washington County], Maine» (original citation). Syntype(s) in MCZ. Synonymy established by LeConte (1863b: 1). Note. According to Frost (1920: 229), there is one syntype of this taxon in the LeConte collection and another one in the Harris collection labeled “Eastport, Me” and the manuscript number “1502.” The syntype in the LeConte collection is probably mixed with LeConte’s specimens of *Cicindela limbalis*.
Cicindela purpurea var. *transversa* Leng, 1902: 131. Type locality: «Ill[inois]» (original citation for the lectotype). Lectotype (♂), designated by Dahl (1941: 171), in AMNH [# 1221]. Synonymy established by Schincariol and Freitag (1991: 1346).
Cicindela limbalis awemeana Casey, 1913: 23. Type locality: «Aweme, Manitoba» (original citation). Four syntypes in USNM [# 45932]. Synonymy established by Horn (1915: 374).
Cicindela limbalis eldorensis Casey, 1913: 23. Type locality: «Eldora [Boulder County], Colorado» (original citation). One syntype in USNM [# 45933]. Synonymy established by Horn (1915: 374).
Cicindela purpurea limbalis f. *militaris* Varas Arangua, 1929: 242. Type locality: «West Point, Ramsey y Peekskill [New York], Hartford, Conn[ecticut], Warwick, R[hode] I[sland], Rock City, N[ew] Y[ork]» (original citation). Syntype(s) location unknown. Synonymy established by Nicolay and Weiss (1932: 347). Note. Even if this taxon was originally proposed at an infrasubspecific rank, it is deemed to be subspecific from its original publication because it was adopted as the valid name of a subspecies before 1985 (e.g., Leng and Mutchler 1933: 9) (see ICZN 1999: Article 45.6.4.1).
Cicindela sedalia Smyth, 1933: 201. Type locality: «Sedalia [Douglas County, Colorado]» (original citation). Syntype(s) location unknown. Synonymy established by Kippenhan (1994: 47).

#### Distribution.

This species, also known as the “Common Claybank Tiger Beetle”, ranges from Newfoundland to eastern British Columbia, north to northern Yukon Territory (Eagle River, Sydney G. Cannings pers. comm. 2009), south to eastern Utah, northern New Mexico, central Missouri, southern Pennsylvania, and New Jersey [see Schincariol and Freitag 1991: Fig. 13; Pearson et al. 1997: Fig. 3]. According to Knisley and Schultz (1997: 114), the literature records from Virginia, western North Carolina, and northwestern Georgia could refer instead to *Cicindela splendida*. The record from the District of Columbia (Boyd 1982: 8) needs confirmation.

#### Records.

**CAN**: AB, BC, MB, NB, NF, NS (CBI), NT, ON, QC, SK, YT **USA**: CO, CT, IA, IL, IN, KS, KY, MA, ME, MI, MN, MO, MT, ND, NE, NH, NJ, NM, NY, OH, PA, RI, SD, UT, VT, WI, WY [DC, GA, NC, VA]

#### Note.

Spomer et al. (2008a: 25) noted that this species intergrades occasionally with *Cicindela denverensis*, rarely with *Cicindela splendida*. Based on results from a limited mitochondrial DNA analysis, Woodcock and Knisley (2010) concluded that *Cicindela limbalis*, *Cicindela splendida*, and *Cicindela denverensis* may represent a single species.

### Cicindela nigrior

Schaupp, 1884

Cicindela scutellaris var. *nigrior* Schaupp, 1884a: 87. Type locality: «G[eorgi]a» (original citation). Syntype(s) apparently destroyed in the San Francisco earthquake of 1906 (Horn et al. 1990b: 345).

#### Distribution.

This species, also known as the “Autumn Tiger Beetle”, is confined to the Coastal Plain and Piedmont Plateau ranging from “North Carolina” (Knisley and Schultz 1997: 116) to the Florida Panhandle (Choate 2003: Map 13), west to southeastern Mississippi (George County, Drew A. Hildebrandt pers. comm. 2009).

#### Records.

**USA**: AL, FL, GA, MS, NC, SC

### Cicindela ohlone

Freitag and Kavanaugh, 1993

Cicindela ohlone Freitag and Kavanaugh [in Freitag et al.], 1993: 114. Type locality: «Soquel, Santa Cruz Co[unty], Calif[ornia]» (original citation). Holotype (♂) in CAS [# 17109].

#### Distribution.

This species, the “Ohlone Tiger Beetle”, is known only from remnant stands of native grassland on coastal terraces in Santa Cruz County, California (Freitag et al. 1993: 117). According to Pearson et al. (2006: 193), it is known from only nine sites where populations range from less than 100 to several hundreds.

#### Records.

**USA**: CA

#### Note.

This species has been listed as endangered under the Endangered Species Act by the U.S. Fish and Wildlife Service in October 2001. Collection of specimens is illegal (Pearson et al. 2006: 88).

### Cicindela parowana parowana

Wickham, 1905

Cicindela parowana Wickham, 1905: 165. Type locality: «beaches of Little Salt Lake, near Parowan [Iron County], Utah» (original citation). Syntype(s) in DEI (Döbler 1973: 379), MCZ [# 23809], and USNM [# 56136].
Cicindela parowana remittens Casey, 1924: 14. Type locality: «Callao [Juab County], Utah» (original citation). Holotype [by monotypy] (♀) in USNM [# 56136]. Synonymy established by Horn (1926: 275).

#### Distribution.

This subspecies, also known as the “Dark Saltflat Tiger Beetle”, occurs from southeastern Oregon and southwestern Idaho to southwestern Utah (Leffler 1987: 7). The record from “Washington” (Freitag 1999: 39) probably refers to the *wallisi* form.

#### Records.

**USA**: ID, NV, OR, UT

#### Note.

Some authors, including Freitag (1999: 39), consider *Cicindela remittens* Casey as a valid subspecies of *Cicindela parowana* Wickham. According to Pearson et al. (2006: 100), all three subspecies of *Cicindela parowana* intergrade in a narrow zone in southeastern Oregon.

### Cicindela parowana platti

Cazier, 1937

Cicindela parowana platti Cazier, 1937a: 161. Type locality: «Benton’s Crossing, Mono Co[unty], Calif[ornia]» (original citation). Holotype (♂) in AMNH [# 1202].

#### Distribution.

This subspecies, the “Platt Tiger Beetle”, occurs in southeastern Oregon, east-central California, and western Nevada [see Pearson et al. 2006: Map 42].

#### Records.

**USA**: CA, NV, OR

### Cicindela parowana wallisi

Calder, 1922

Cicindela azurea Calder, 1922a: 62 [primary homonym of *Cicindela azurea* Krausse, 1910]. Type locality: «Penticton, B[ritish]C[olumbia]» (original citation). Holotype (♂) in CNC [# 7315].
Cicindela wallisi Calder, 1922b: 191. Replacement name for *Cicindela azurea* Calder, 1922. Etymology. Although not indicated, the specific name was likely proposed for John Braithwaite Wallis [1877-1962], assistant superintendent of the Winnipeg Public Schools. Wallis wrote “The Cicindelidae of Canada” published in 1961.

#### Distribution.

This subspecies, the “Wallis’ Tiger Beetle”, ranges from south-central British Columbia south to southeastern Oregon (Leffler 1987: 7), including southwestern Idaho (Shook 1984: 159).

#### Records.

**CAN**: BC **USA**: OR, WA

### Cicindela pimeriana

LeConte, 1867

Cicindela pimeriana LeConte, 1867b: 363. Type locality: «Sonora» (original citation for *Cicindela viatica* Chevrolat *sensu* LeConte, 1856). Holotype [by monotypy] (♀) in MCZ [# 30]. Note. This name was proposed for *Cicindela viatica* Chevrolat, 1835 *sensu* LeConte (1856a: 62).
Cicindela cochisensis Casey, 1909: 274. Type locality: «Douglas [Cochise County], Arizona» (original citation). Six syntypes in USNM [# 45921]. Synonymy established by Harris (1911: 24).

#### Distribution.

This species, also known as the “Cochise Tiger Beetle”, is restricted to southeastern Arizona, southwestern New Mexico [see Pearson et al. 1997: Fig. 31] and adjacent regions in Sonora, Mexico (LeConte 1856a: 62, as *Cicindela viatica* Chevrolat).

#### Records.

**USA**: AZ, NM – Mexico

### Cicindela plutonica

Casey, 1897

Cicindela [*purpurea*] *plutonica* Casey, 1897: 296. Type locality: «Placer Co[unty], California» (original citation). One syntype in USNM [# 45948].
Cicindela plutonica leachi Cazier, 1936: 124. Type locality: «Warner M[oun]t[ain]s (9,000 to 10,000 feet), Modoc Co[unty], Calif[ornia]» (original citation). Holotype (♀) in AMNH [# 1523]. Synonymy established by Bousquet and Larochelle (1993: 59) based on Leffler (1979a: 367) unpublished thesis. Etymology. The subspecific name was proposed in honor of Edwin R. Leach [1878-1971], an amateur coleopterist living in California who was chiefly interested in scarabaeids. Leach donated his collection, estimated at 40-50,000 specimens, to the California Academy of Sciences.

#### Distribution.

This rare species, also known as the “Alpine Tiger Beetle”, ranges from southern Idaho to south-central Oregon, south to east-central California and west-central Utah [see Pearson et al. 2006: Map 31]. The record from one locality in northern Montana (see Pearson et al. 2006: Map 31) is possibly based on a stray.

#### Records.

**USA**: CA, ID, NV, OR, UT [MT]

### Cicindela pugetana

Casey, 1914

Cicindela pugetana Casey, 1914: 20. Type locality: «British Columbia» (original citation). Holotype [by monotypy] (♂) in USNM [# 45942].

#### Distribution.

This species, also known as the “Sagebrush Tiger Beetle”, is found from southern British Columbia south to north-central Oregon [see Pearson et al. 1997: Fig. 32].

#### Records.

**CAN**: BC **USA**: OR, WA

#### Note.

According to Pearson et al. (2006: 89), further studies may show that this taxon is rather a subspecies of *Cicindela plutonica* Casey.

### Cicindela purpurea audubonii

LeConte, 1845

Cicindela audubonii LeConte, 1845a: 201. Type locality: «flum[inis] Yellow-Stone» (original citation), cited from «ad ripas fluminis Yellowstone, apud Fort Union [Roosevelt County, northeastern Montana]» by LeConte (1845b: 207). Syntype(s) in MCZ [# 3]. Etymology. The species name honors John James Audubon [1785-1851], celebrated American ornithologist, naturalist, hunter, and painter.
Cicindela purpurea var. *graminea* Schaupp, 1884a: 89. Type locality: «Kans[as], Cal[ifornia]» (original citation). Syntype(s) apparently destroyed. Synonymy established by Nicolay and Weiss (1932: 346).
Cicindela purpurea auguralis Casey, 1913: 21. Type locality: «Colorado» (original citation). Three syntypes [3 originally cited] in USNM [# 45929]. Synonymy established, under the name *Cicindela purpurea graminea* Schaupp, by Horn (1915: 373).
Cicindela purpurea inducta Casey, 1913: 22. Type locality: «Colorado» (original citation). One syntype in USNM [# 45931]. Synonymy established, under the name *Cicindela purpurea graminea* Schaupp, by Horn (1915: 373).
Cicindela purpurea var. *nigerrima* Leng, 1919a: 139. Type locality: «at the West» (original citation for *Cicindela purpurea audubonii* LeConte *sensu* LeConte, 1856), restricted to «Chimney Gulch, Golden, Colorado» by Dahl (1941: 170). Syntype(s) probably in MCZ. Synonymy established by Hatch (1953: 37). Note. This name was proposed for *Cicindela purpurea* var. *audubonii* LeConte, 1845 *sensu* LeConte (1856a: 37). Therefore the type series consists of the specimen(s) which had been misidentified (ICZN 1999: Article 72.4.2). The specimen in AMNH [# 1220] designated as lectotype by Dahl (1941: 170) is not a syntype.

#### Distribution.

This subspecies, the “Audubon’s Tiger Beetle”, ranges from southern Manitoba to central British Columbia, south to east-central California, Arizona, and northern Texas [see Pearson et al. 2006: Map 28]. The records from “Wisconsin,” “Illinois,” “Kentucky,” “Tennessee” and “Arkansas” (Boyd 1982: 7) apparently refer to the nominate form.

#### Records.

**CAN**: AB, BC, MB, SK **USA**: AZ, CA, CO, IA, ID, KS, MN, MT, ND, NE, NM, NV, OK, OR, SD, TX, UT, WA, WY

### Cicindela purpurea cimarrona

LeConte, 1868

Cicindela cimarrona LeConte, 1868a: 49. Type locality: «south of Raton Mountain [= Barela Mesa, Colfax County, New Mexico]» (original citation). Syntype(s) [6 originally cited] in MCZ [# 5].
Cicindela purpurea ardelio Casey, 1913: 21. Type locality: «New Mexico» (original citation). Holotype [by monotypy] (♂) in USNM [# 45930]. Synonymy established by Horn (1915: 374).

#### Distribution.

This subspecies, the “Cimarron Tiger Beetle”, is known from north and central Colorado (Kippenhan 1994: 44-45), much of New Mexico (Acciavatti et al. 1980: 30), and southeastern Arizona (Bertholf 1983: 22) [see Pearson et al. 2006: Map 28]. The records from northern (Tanner 1929a: 79) and southwestern (Horn 1926: 266) Utah need confirmation.

#### Records.

**USA**: AZ, CO, NM [UT]

### Cicindela purpurea hatchi

Leffler, 1980

Cicindela mirabilis Casey, 1914: 358 [primary homonym of *Cicindela mirabilis* Laporte, 1835]. Type locality: «Dutch Flat, Placer Co[unty], California» (original citation). Holotype [by monotypy] (♀) in USNM [# 45936].
Cicindela purpurea hatchi Leffler, 1980: 128. Replacement name for *Cicindela purpurea mirabilis* Casey, 1914.

#### Distribution.

This subspecies, also known as the “Hatch’s Tiger Beetle”, ranges from Vancouver Island to the central region of the Sierra Nevada in California (Leffler 1987: 4).

#### Records.

**CAN**: BC (VCI) **USA**: CA, OR, WA

#### Note.

This subspecies intergrades with the *lauta* form in the Willamette Valley of Oregon south to Shasta and Modoc Counties in northwestern California (Pearson et al. 2006: 87).

### Cicindela purpurea lauta

Casey, 1897

Cicindela [*purpurea*] *lauta* Casey, 1897: 296. Type locality: «Siskiyou Co[unty], California» (original citation), restricted to «Dunsmuir, Shasta Co[unty]» by Leffler (1987: 5). Two syntypes in USNM [# 45734].
Cicindela lauta franciscana Casey, 1913: 23. Type locality: «California» (original citation). One syntype in USNM [# 45935]. Synonymy established by Horn (1915: 373), confirmed by Leffler (1980: 128).

#### Distribution.

This subspecies, the “Elegant Tiger Beetle”, ranges from the Columbia River in southern Washington to northwestern California (Leffler 1987: 4).

#### Records.

**USA**: CA, OR, WA

### Cicindela purpurea purpurea

Olivier, 1790

Cicindela purpurea Olivier, 1790a: [No. 33] 14. Type locality: «Géorgie» (original citation). Syntype(s) location unknown (possibly in MHNP).
Cicindela marginalis Fabricius, 1801: 240. Type locality: «Canada» (original citation). One syntype in ZMUC (Zimsen 1964: 64). Synonymy established with doubt by Say (1818: 419).
Cicindela purpurea var. *ramosa* Gistel, 1837: 31. Type locality: «America septentrionali, Canada» (original citation). Syntype(s) lost. Synonymy established by Horn (1915: 373).

#### Distribution.

This subspecies, also known as the “Cow Path Tiger Beetle”, ranges from southern Quebec to Minnesota, south to central Arkansas and northern Georgia [see Pearson et al. 2006: Map 28]. Beaton (2008: 39) indicated that despite extensive search he was unable to find any population of this species in Georgia, including at all known historical sites, and Ciegler (1997: 189) noted that it has not been collected in South Carolina since 1936. The record from “Nova Scotia” (Bousquet and Larochelle 1993: 59) was based on a misidentified specimen (see Majka et al. 2007: 6); that from “New Brunswick” (Erwin and Pearson 2008: 175) needs confirmation.

#### Records.

**CAN**: ON, QC **USA**: AL, AR, CT, DC, DE, GA, IA, IL, IN, KS, KY, MA, MD, ME, MI, MN, MO, MS, NC, ND, NE, NH, NJ, NY, OH, OK, PA, RI, SC, SD, TN, VA, VT, WI, WV [NB]

#### Note.

This subspecies intergrades with the *audubonii* form over a wide area from North Dakota to Oklahoma (Pearson et al. 2006: 86).

### Cicindela scutellaris flavoviridis

Vaurie, 1950

Cicindela scutellaris flavoviridis Vaurie, 1950: 2. Type locality: «Forestburg, Montague County, Texas» (original citation). Holotype (♂) in AMNH [# 1210].

#### Distribution.

This subspecies, the “Chartreuse Tiger Beetle”, is known only from north-central Texas (Pearson et al. 2006: 102).

#### Records.

**USA**: TX

### Cicindela scutellaris lecontei

Haldeman, 1853

Cicindela lecontei Haldeman, 1853: 361. Type locality: «Wisconsin» (original citation). One possible syntype, a ♀ labeled “[yellow disc] / C. Lecontei Hald. [handwritten],” in MCZ (collection LeConte).
Cicindela criddlei Casey, 1913: 19. Type locality: «Aweme, Manitoba» (original citation). Four syntypes [4 originally cited] in USNM [# 45916]. Synonymy established by Wallis (1961: 35).

#### Distribution.

This subspecies, the “LeConte’s Tiger Beetle”, ranges from southern Quebec to southern Manitoba (Wallis 1961: 35-36), south to Kansas, northern Mississippi, West Virginia, and Connecticut [see Pearson et al. 2006: Map 43). The records from “Saskatchewan” and “Alberta” (Bousquet and Larochelle 1993: 60) refer to the nominotypical subspecies.

#### Records.

**CAN**: MB, ON, QC **USA**: CT, IA, IL, IN, KS, MA, ME, MI, MN, MO, MS, ND, NE, NH, NY, OH, PA, SD, TN, VT, WI, WV

#### Note.

This subspecies intergrades with the nominate form over a broad area in the Great Plains and with the *unicolor* form in northern Missouri and Tennessee (Pearson et al. 2006: 102, 103).

### Cicindela scutellaris rugata

Vaurie, 1950

Cicindela varians Ljungh, 1799: 147 [*nomen oblitum*, see Boyd (2000)]. Type locality: «extra Europam» (original citation). Lectotype (♀), designated by Cassola (1999: 76), in ZMLS.
Cicindela scutellaris rugata Vaurie, 1950: 3 [*nomen protectum*]. Type locality: «Vowell’s Mill, Natchitoches [Parish] County, Louisiana» (original citation). Holotype (♂) in AMNH [# 1211]. Synonymy established by Cassola (1999: 76).

#### Distribution.

This subspecies, the “Rugate Tiger Beetle”, is known from southwestern Arkansas (Ward 1972: 70), western Louisiana (Graves and Pearson 1973: 175), and eastern Texas (Vaurie 1950: 4). The record from “Oklahoma” (Erwin and Pearson 2008: 183) needs confirmation.

#### Records.

**USA**: AR, LA, TX [OK]

#### Note.

This subspecies intergrades with the nominate form in the northwestern part of its range and with the *lecontei* form in the northeastern part of its range (Pearson et al. 2006: 103).

### Cicindela scutellaris rugifrons

Dejean, 1825

Cicindela rugifrons Dejean, 1825: 51. Type locality: «Amérique septentrionale» (original citation), herein restricted to Cambridge, Middlesex County, Massachusetts (see Harris 1828a: 90, as *Cicindela denticulata*). Holotype [by monotypy] (♀) probably in MHNP.
Cicindela modesta Dejean, 1825: 52. Type locality: «Saint-Domingue» (original citation), which is incorrect (see Dejean 1831: 210). Syntype(s) in MHNP. Synonymy established by LeConte (1856a: 35). Note. This name is listed in synonymy with *Cicindela scutellaris lecontei* Haldeman by Horn (1928: 12).
Cicindela denticulata T.W. Harris, 1828a: 90. Type locality: «near Sweet Auburn in Cambridge [Middlesex County, Massachusetts]» (original citation). Syntype(s) presumably lost. Synonymy established by LeConte (1846b: 175).
Cicindela denticulata Hentz, 1830: 253 [primary homonym of *Cicindela denticulata* Harris, 1828]. Type locality: «Massachusetts» (original citation). Syntype(s) lost. Synonymy established by Gould (1834: 46).
Cicindela denticulata var. *oberleitneri* Gistel, 1837: 55. Type locality: «America boreali (Massachusetts)» (original citation). Syntype(s) lost. Synonymy established by Horn (1907a: 22).
Cicindela scutellaris var. *carolina* E.D. Harris, 1911: 28. Type locality: «neighborhood of Raleigh [Wake County], N[orth] C[arolina]» (original citation). Syntype(s) in MCZ [# 25609]. Synonymy established by Horn (1915: 379). Note. Harris (1911: 28) stated that *carolina* is “a slightly differentiated race of *rugifrons* existing in the neighborhood of Raleigh, N.C.” but at the same time listed the variety from Surry and Newport News in Virginia as well as Raleigh, Southern Pines, Hamlet, Montague, and Manly in North Carolina.

#### Distribution.

This subspecies, also known as the “Wrinkle-fronted Tiger Beetle”, ranges east of the Appalachians from Massachusetts (Leonard and Bell 1999: 133) to North Carolina (Harris 1911: 28, as *Cicindela scutellaris* var. *carolina*). The records from South Carolina (Cartwright 1935: 72) and “Georgia” (J.E. LeConte 1849: 25) probably refer to the *unicolor* form.

#### Records.

**USA**: CT, DC, DE, MA, MD, NC, NJ, NY, PA, RI, VA

#### Note.

This form intergrades with the *lecontei* form in Massachusetts and Connecticut and with the *unicolor* form in North Carolina (Pearson et al. 2006: 103).

### Cicindela scutellaris scutellaris

Say, 1823

Cicindela scutellaris Say, 1823b: 140 (as *scutelaris*). Type locality: «the Arkansa [River]» (original citation). Syntype(s) lost. Note. The incorrect subsequent spelling *scutellaris* is in prevailing usage and attributed to the publication of the original spelling; therefore it is deemed to be the correct original spelling (ICZN 1999: Article 33.3.1).
Cicindela scutellaris billingsi Casey, 1924: 14. Type locality: «Billings [Yellowstone County], Montana» (original citation). Holotype [by monotypy] (♂) in USNM [# 45915]. Synonymy established by Horn (1926: 276).
Cicindela shantzi Casey, 1924: 14. Type locality: «Colorado» (original citation). Holotype [by monotypy] (♂) in USNM [# 45914]. Synonymy established by Horn (1926: 276).

#### Distribution.

This subspecies, also known as the “Festive Tiger Beetle”, ranges from southwestern Saskatchewan and southeastern Alberta (Wallis 1961: 35) south to east-central New Mexico (Acciavatti et al. 1980: 30), northern Texas (Gaumer and Murray 1971: 10), Arkansas (Graves and Pearson 1973: 175), and northwestern Mississippi (Bolivar County, Drew A. Hildebrandt pers. comm. 2009). The record from “Iowa” (Freitag 1999: 49) probably refers to the *lecontei* form.

#### Records.

**CAN**: AB, SK **USA**: AR, CO, KS, MS, MT, ND, NE, NM, OK, SD, TX, WY

#### Note.

According to Pearson et al. (2006: 102), this subspecies intergrades with the *lecontei* form over a broad zone in eastern South Dakota, Nebraska, and Kansas.

### Cicindela scutellaris unicolor

Dejean, 1825

Cicindela unicolor Dejean, 1825: 52. Type locality: «Amérique septentrionale» (original citation), herein restricted to Wilmington, New Hanover County, North Carolina (see Leng 1902: 125). Syntype(s) in MHNP.

#### Distribution.

This subspecies, the “Unicolored Tiger Beetle”, is known from eastern Tennessee and North Carolina (Pearson et al. 2006: 103) south to central Florida (Peck and Thomas 1998: 16; Choate 2003: Map 74), southern Alabama (Löding 1945: 9), and southern Mississippi (Graves and Pearson 1973: 174); also recorded from “Virginia” (Erwin and Pearson 2008: 184). The records from Texas (Tucker 1906: 85), “Louisiana,” “Colorado” (Leng 1902: 125), New Mexico (Fall and Cockerell 1907: 155), and Oklahoma (Drew and Van Cleave 1962: 115) could be based on strays, on intergrades, or be in error.

#### Records.

**USA**: AL, FL, GA, MS, NC, SC, TN [VA]

#### Note.

According to Pearson et al. (2006: 103), this form intergrades with the *rugifrons* form in North Carolina and with the *lecontei* form in northern Missouri and Tennessee.

### Cicindela scutellaris yampae

Rumpp, 1986

Cicindela scutellaris yampae Rumpp, 1986: 140. Type locality: «Maybell Sand Hills (between two and 6 km east of Maybell), Moffat County, Colorado» (original citation). Holotype (♂) in AMNH [# 1526].

#### Distribution.

This subspecies, the “Yampa Tiger Beetle”, is known only from the Maybell Sand Hills area in northwestern Colorado (Kippenhan 1994: 55).

#### Records.

**USA**: CO

### Cicindela splendida

Hentz, 1830

Cicindela splendida Hentz, 1830: 254. Type locality: «North Carolina» (original citation), herein restricted to Asheville, Buncombe County (see Harris 1911: 7). One possible syntype in MCZ [# 20].
Cicindela purpurea splendida f. *cyanocephala* Varas Arangua, 1929: 239 [primary homonym of *Cicindela cyanocephala* Fabricius, 1798]. Type locality: «Kansas, Nebraska» (original citation). Syntype(s) location unknown. Synonymy established by Schincariol and Freitag (1991: 1345). Note. Even if this taxon was originally proposed at an infrasubspecific rank, it is deemed to be subspecific from its original publication because it was adopted before 1985 as the valid name of a subspecies (e.g., Leng and Mutchler 1933: 9) (see ICZN 1999: Article 45.6.4.1).
Cicindela splendida var. *cyanocephalata* Eckhoff, 1939: 211. Replacement name for *Cicindela splendida* var. *cyanocephala* Varas Arangua, 1929.
Cicindela splendida var. *cyanocephalonota* Eckhoff, 1970: 32. Unnecessary replacement name for *Cicindela splendida* var. *cyanocephalata* Eckhoff, 1939.

#### Distribution.

This species, also known as the “Splendid Tiger Beetle”, occurs from southern Pennsylvania to eastern Wyoming, north to southern Wisconsin, south to central Texas, northeastern Georgia, and northern South Carolina [see Schincariol and Freitag 1991: Fig. 13; Pearson et al. 1997: Fig. 20]; The records from “New York” (Schaupp 1884a: 90), “Minnesota” (Horn 1928: 10), and “New Mexico” (Freitag 1999: 50, 51) need confirmation.

#### Records.

**USA**: AL, AR, CO, DC, GA, IA, IL, IN, KS, KY, LA, MD, MO, MS, NC, NE, OH, OK, PA, SC, SD, TN, TX, VA, WI, WV, WY [MN, NM, NY]

#### Note.

1. The name *Cicindela discus*, credited to Klug (1834: 23), is often listed as a synonym of *Cicindela splendida* Hentz but Klug did not apply the name to a new species. 2. According to Pearson et al. (2006: 91), recent DNA studies on this species, *Cicindela denverensis* and *Cicindela limbalis* suggest that members of all three could be conspecific. Spomer et al. (2008a: 23) noted that this species hybridizes with *Cicindela denverensis* in a narrow zone in central Nebraska and possibly also with *Cicindela limbalis*.

### Cicindela tenuicincta

Schaupp, 1884

Cicindela latesignata var. *tenuicincta* Schaupp, 1884b: 122. Type locality: «Colorado» (original citation), which is probably incorrect (Freitag 1999: 55); Saltair, Salt Lake County, Utah (see Leng 1902: 138) herein selected. Holotype [by monotypy] (♀) in USNM [# 1204].

#### Distribution.

This species, also known as the “Short-legged Tiger Beetle”, is found from southern Oregon to east-central California, east to southeastern Utah [see Pearson et al. 2006: Map 48]. The records from “Arizona” and “New Mexico” (Bousquet and Larochelle 1993: 61) are in error or based on strays.

#### Records.

**USA**: CA, NV, OR, UT

### Cicindela tranquebarica cibecuei

Duncan, 1958

Cicindela tranquebarica cibecuei Duncan, 1958: 43. Type locality: «Cibecue Creek, near Cibecue, Gila County, Arizona» (original citation). Holotype (♂) in AMNH [# 1206].

#### Distribution.

This subspecies is known only from east-central Arizona [see Kritsky and Horner 1998: Fig. 5].

#### Records.

**USA**: AZ

#### Note.

Bertholf (1983: 26) considered this form as a synonym of the *lassenica* form (= *Cicindela tranquebarica parallelonota* Casey).

### Cicindela tranquebarica diffracta

Casey, 1909

Cicindela diffracta Casey, 1909: 273. Type locality: «Las Vegas [San Miguel County], New Mexico» (original citation). One syntype in USNM [# 45950].
Cicindela admiscens Casey, 1913: 25. Type locality: «Jemez Springs [Sandoval County], New Mexico» (original citation). Thirteen syntypes in USNM [# 45951]. Synonymy established by Horn (1915: 376).

#### Distribution.

This subspecies, the “Diffracted Tiger Beetle”, is found from southern Wyoming to New Mexico and eastern Arizona (Kritsky and Horner 1998: 25, Fig. 5). According to Pearson et al. (2006: 107), it occurs in southern Nevada, northern Arizona, and New Mexico; also recorded from Utah (Tanner 1929a: 80) and “Nebraska” (Erwin and Pearson 2008: 192).

#### Records.

**USA**: AZ, CO, NM, NV, WY [NE, UT]

### Cicindela tranquebarica joaquinensis

Knisley and Haines, 2007

Cicindela tranquebarica joaquinensis Knisley and Haines, 2007: 112. Type locality: «near Guernsey, Kings Co[unty], Ca[lifornia]» (original citation). Holotype (♂) in CAS.

#### Distribution.

This subspecies, the “Joaquin Tiger Beetle”, is known only from the San Joaquin Valley of California.

#### Records.

**USA**: CA

#### Note.

According to Knisley and Haines (2007: 109), this subspecies intergrades with the *vibex* form along the margins of the San Joaquin Valley.

### Cicindela tranquebarica kirbyi

LeConte, 1867

Cicindela kirbyi LeConte, 1867b: 362. Type locality: northern parts of North America (inferred from title of Kirby’s 1837 book). Syntype(s) in BMNH. Note. This subspecies was described by the inclusion of a drawing of the left elytron as well as by indication to *Cicindela obliquata* Dejean, 1825 *sensu* Kirby (1837: 10).

#### Distribution.

This subspecies, the “Kirby’s Tiger Beetle”, ranges from Manitoba to Alberta, north to southern Northwest Territories; it southern limit is debated. Kritsky and Horner (1998: Fig. 5) placed it at southern Wyoming and northern Nebraska while Pearson et al. (2006: Map 45) placed it at southeastern New Mexico and northern Texas. I have accepted the latest range for the records. The subspecies is also recorded from “British Columbia,” “Ontario,” “Arkansas,” “Iowa,” “Minnesota,” “Missouri,” “Utah,” and “Washington” by Erwin and Pearson (2008: 193).

#### Records.

**CAN**: AB, MB, NT, SK **USA**: CO, KS, MT, ND, NE, NM, OK, SD, TX, WY [AR, BC, IA, MN, MO, ON, UT, WA]

#### Note.

This subspecies intergrades with the nominotypical form over a large area in the Midwest (Pearson et al. 2006: 106).

### Cicindela tranquebarica parallelonota

Casey, 1914

Cicindela parallelonota Casey, 1914: 21. Type locality: «Las Vegas [Clark County], Nevada» (original citation). One syntype in USNM [# 45943].
Cicindela lassenica Casey, 1914: 22. Type locality: «California» (original citation). One syntype in USNM [# 45946]. Synonymy established by Kritsky and Horner (1998: 25).
Cicindela moapana Casey, 1914: 22. Type locality: «McGill (6500 feet), White Pine Co[unty], Nevada» (original citation). One syntype in USNM [# 45949]. Synonymy established by Kritsky and Horner (1998: 25).
Cicindela tranquebarica var. *inyo* Fall, 1917: 106. Type locality: «Olancha [Inyo County], California» (original citation). Holotype (♀) in MCZ [# 23838]. Synonymy established by Kritsky and Horner (1998: 25).
Cicindela tranquebarica var. *owena* Fall, 1917: 106. Type locality: «Olancha [Inyo County], California» (original citation). Holotype (♂) in MCZ [# 23839]. Synonymy established implicitly with the name *Cicindela tranquebarica inyo* Fall by Cazier (1939: 27).
Cicindela kirbyi uintana Casey, 1924: 15. Type locality: «Zion Cañon, Utah» (original citation). Holotype [by monotypy] (♀) in USNM [# 45938]. Synonymy established, under the name *Cicindela tranquebarica owena* Fall, by Horn (1926: 272).

#### Distribution.

This subspecies, also known as the “Opal Tiger Beetle”, occurs in Utah, Nevada, and eastern California (Kritsky and Horner 1998: 25).

#### Records.

**USA**: CA, NV, UT

#### Note.

Some authors, including Pearson et al. (2006: 107, 108), consider the forms *inyo* and *moapana* as distinct subspecies: the first one is confined to the Owens Valley of interior central California and adjacent Nevada and the second one is found in east-central Nevada and adjacent Utah. Freitag (1999: 57) and Erwin and Pearson (2008: 194) listed the *lassenica* form as a distinct subspecies and recorded it from “California,” “Nevada,” “Utah,” and “Arizona.”

### Cicindela tranquebarica sierra

Leng, 1902

Cicindela vulgaris var. *sierra* Leng, 1902: 146. Type locality: «Sierra Co[unty], Cal[ifornia]» (original citation). Lectotype (♀), designated by Dahl (1941: 172), in AMNH [# 1224].

#### Distribution.

This subspecies, the “Sierra Tiger Beetle”, is restricted to the Sierra Nevada in eastern California [see Kritsky and Horner 1998: Fig. 5].

#### Records.

**USA**: CA

### Cicindela tranquebarica tranquebarica

Herbst, 1806

Cicindela tranquebarica Herbst, 1806: 178. Type locality: «Trankenbar [= Tranquebar, Tamil Nadu, India]» (original citation), which is incorrect; herein restricted to Charlotte, Mecklenburg County, North Carolina (see Harris 1911: 18, as *Cicindela tranquebarica* var. *vulgarisminor*). Syntype(s) location unknown (possibly in ZMHB).
Cicindela vulgaris Say, 1818: 409. Type locality: «North America» (original citation). Syntype(s) lost. Synonymy established by LeConte (1863b: 1).
Cicindela obliquata Dejean, 1825: 72. Type locality: «Amérique septentrionale» (original citation). Syntype(s) in MHNP. Synonymy established, under the name *Cicindela vulgaris* Say, by Dejean (1826: 414).
Cicindela vulgaris var. *horiconensis* Leng, 1902: 145. Type locality: «Lake George [Warren County], N[ew] Y[ork] (original citation for the lectotype). Lectotype (♂), designated by Dahl (1941: 172), in AMNH [# 1223]. Synonymy established by Horn (1905: 20).
Cicindela tranquebarica form *minor* Leng, 1910: 80. Type locality: «Louisiana; Georgia» (original citation). Syntype(s) location unknown. Synonymy established by Horn (1930: 81).
Cicindela tranquebarica var. *vulgaris-minor* E.D. Harris, 1911: 18. Type locality: «Charlotte, Goldsboro, High Point, Montague, Southern Pines, Jamestown, Manly [all in] No[rth] Car[olina]; Vowell’s Mill, Louisiana» (original citation). Syntype(s) in MCZ [# 25603]. Synonymy established, under the name *Cicindela tranquebarica minor* Leng, by Horn (1915: 376).
Cicindela tranquebarica turbulenta Casey, 1913: 25. Type locality: «Vicksburg [Warren County], Mississippi» (original citation). One syntype in USNM [# 45944]. Synonymy established by Horn (1915: 376).
Cicindela crinifrons Casey, 1913: 26. Type locality: «Asheville and Southern Pines, North Carolina» (original citation). Nine syntypes in USNM [# 45945]. Synonymy established, under the name *Cicindela tranquebarica minor* Leng, by Horn (1915: 376).
Cicindela wichitana Casey, 1914: 21. Type locality: «Kansas» (original citation). Four syntypes [4 originally cited] in USNM [# 45947]. Synonymy established by Horn (1915: 444).
Cicindela tranquebarica var. *viridula* Varas Arangua, 1928: 173. Type locality: «Concord, Massachusetts; Rhode Island; Connecticut; Long Island, N[ew] Y[ork]» (original citation). Syntype(s) in CAS [# 8151]. Synonymy established by Horn (1930: 81).

#### Distribution.

This subspecies, also known as the “Oblique-lined Tiger Beetle”, ranges over much of eastern North America, from Newfoundland to Nebraska, south to northern Texas and northern Florida (Choate 2003: Map 26) [see Kritsky and Horner 1998: Fig. 5; Pearson et al. 2006: Map 45]. Several state and province records (e.g., AB, CA, ID, MB, MT, ND, NT, NV, OR, SK, UT, WY) in Boyd (1982: 9), Bousquet and Larochelle (1993: 61), and Freitag (1999: 57) apparently refer to other subspecies of *Cicindela tranquebarica* Herbst. Choate (2003: 81) reported that there are no recent collection records of this species in Florida.

#### Records.

**CAN**: LB, NB, NF, NS (CBI), ON, PE, QC **USA**: AR, CO, CT, DC, DE, FL, GA, IA, IL, IN, KS, KY, LA, MA, MD, ME, MI, MN, MO, MS, NC, NE, NH, NJ, NY, OH, OK, PA, RI, SC, SD, TN, TX, VA, VT, WI, WV

#### Note.

*Cicindela tranquebarica* is extremely variable in color and maculation which lead to the recognition of several subspecies. However, it appears that the species varies seasonally. As remarked by Sumlin (1976a: 103) “if one were to sample this population [at Owen’s Lake, Inyo County, California] from early April to the middle of June one would have approximately 5 subspecific phenotypes represented; i.e., *inyo* Fall (green in color with narrow to thick lunules), *kirbyi* LeConte (brown or black in color with very wide lunules), *cibecuei* Duncan (blue in color with wide lunules), *borealis* E.D. Harris (brown or black in color with very narrow lunules), and *parallelonota* Casey (light to dark green in color with thickened lunules).” A thorough study of the variation in this species would probably lead to a reduction in the number of subspecies.

### Cicindela tranquebarica vibex

Horn, 1867

Cicindela vibex G.H. Horn, 1867a: 395. Type locality: «Fort Klamath [Klamath County], Oregon» (original citation). Lectotype (♂), designated by Ward (1982: 61), in MCZ [# 33472].
Cicindela vulgaris var. *roguensis* E.D. Harris, 1901: 226. Type locality: «basin of the Rogue River, S[outh]-W[est] Oregon» (original citation). Syntype(s) [20 originally cited] in MCZ [# 25605]. Synonymy established by Harris (1911: 19).
Cicindela tranquebarica var. *borealis* E.D. Harris, 1911: 19. Type locality: «Kootenay region of British Columbia» (original citation). Syntype(s) in MCZ [# 25604]. Synonymy established by Bousquet and Larochelle (1993: 61) based on Leffler (1979a: 578) unpublished thesis. Note. Harris (1911: 19) stated that the taxon is “a race indigenous to the Kootenay region of British Columbia” but listed it from Spokane in Washington, Provo in Utah, as well as Kaslo, Lardo River, Ainsworth, and Bear Foot Mountains in British Columbia.

#### Distribution.

This subspecies, the “Wealed Tiger Beetle”, ranges from British Columbia to western Montana, south to northern Utah and northern California [see Kritsky and Horner 1998: Fig. 5]. The record from “Northwest Territories” (Boyd 1982: 10) is probably in error.

#### Records.

**CAN**: BC **USA**: CA, ID, MT, NV, OR, UT, WA, WY

#### Note.

Freitag (1999: 55) and Pearson et al. (2006: 107) considered the form *borealis* as a valid subspecies although Pearson et al. stated that “perhaps the entire population represents a zone of intergradation and not a distinct subspecies.” Erwin and Pearson (2008: 195) also retained the *roguensis* form as a distinct subspecies listing it from several western states (i.e., ID, OR, MT, NV, WA, WY).

### Cicindela tranquebarica viridissima

Fall, 1910

Cicindela vulgaris var. *viridissima* Fall, 1910: 89. Type locality: «near San Bernardino and Colton; Tulare County [in] southern California» (original citation). Syntype(s) in MCZ [# 23840].

#### Distribution.

This taxon, also known as the “Santa Ana Tiger Beetle”, is found in southern California, primarily in Orange and western San Bernardino Counties (Pearson et al. 2006: 108).

#### Records.

**USA**: CA

### [formosa group]

### Cicindela denikei

Brown, 1934

Cicindela sexguttata denikei Brown, 1934: 22. Type locality: «Ingolf, Ont[ario]» (original citation). Holotype (♂) in CNC [# 3529].

#### Distribution.

This species, also known as the “Laurentian Tiger Beetle”, is restricted to a small area in southeastern Manitoba, northwestern (Lawton 2008: 73) and southwestern Ontario, and adjacent parts of Minnesota (Kaulbars and Freitag 1993a: 307; Pearson et al. 2006: 95); isolated at Manitoulin Island, Ontario (Bouchard et al. 2006: 21).

#### Records.

**CAN**: MB, ON **USA**: MN

### Cicindela formosa formosa

Say, 1817

Cicindela formosa Say, 1817a: [23]. Type locality: «sandy alluvions of the Missouri, above the confluence of the river Platte» (original citation). Syntype(s) lost.
Cicindela formosa luxuriosa Casey, 1913: 24. Type locality: «near Denver [Denver County], Colorado» (original citation). Two syntypes in USNM [# 45971]. Synonymy established by Horn (1915: 371).
Cicindela formosa fletcheri Criddle, 1925: 127. Type locality: «Sunshine Road, Marias River [Chouteau County], Montana» (original citation). Holotype (♂) in CNC [# 1418]. Synonymy established by Horn (1926: 262). Etymology. The subspecific name was proposed for James Fletcher [1852-1908], the first entomologist and botanist in the Dominion Department of Agriculture in Ottawa. Born in England, Fletcher worked mainly on economic insects and particularly Lepidoptera larvae.

#### Distribution.

This subspecies, also known as the “Big Sand Tiger Beetle”, ranges from southern Saskatchewan and southern Alberta south to New Mexico and southern Texas (Gaumer 1977: 188-189); also recorded from “Manitoba” and “Minnesota” (Erwin and Pearson 2008: 135).

#### Records.

**CAN**: AB, SK **USA**: CO, KS, MT, ND, NE, NM, OK, SD, TX, WY [MB, MN]

#### Note.

Gaumer (1977: 194-195) reported the presence of intergrade populations between this subspecies and the *generosa* form in central United States and with the *pigmentosignata* form in north-central and central Texas.

### Cicindela formosa generosa

Dejean, 1831

Cicindela generosa Dejean, 1831: 231. Type locality: «Amérique septentrionale» (original citation), herein restricted to Ballardvale, Essex County, Massachusetts (see Harris 1911: 2). Syntype(s) in MHNP.
Cicindela formosa var. *manitoba* Leng, 1902: 137. Type locality: «Aweme, Manitoba» (original citation). Lectotype, designated by Dahl (1941: 170), in AMNH [# 1219]. Synonymy established by Horn (1926: 263). Note. Rumpp (1986: 145) listed this name in synonymy with the nominotypical subspecies.

#### Distribution.

This subspecies, also known as the “Eastern Sand Tiger Beetle”, ranges from Kings County in Nova Scotia (Neil and Majka 2008: 4) to southern Manitoba, south to northeastern South Dakota (Spomer et al. 2008a: 21), central Louisiana, southern Mississippi, and southern Virginia (Gaumer 1977: 202-203). The records from “Saskatchewan,” “Montana,” “Nebraska” (Erwin and Pearson 2008: 136), “Colorado” (Leng 1902: 136), and “Alabama” (Freitag 1999: 25) need confirmation.

#### Records.

**CAN**: MB, NS, ON, QC **USA**: AR, CT, DE, IA, IL, IN, KY, LA, MA, MD, ME, MI, MN, MO, MS, ND, NH, NJ, NY, OH, PA, RI, SD, TN, VA, VT, WI, WV [AL, CO, MT, NE, SK]

### Cicindela formosa gibsoni

Brown, 1940

Cicindela formosa gibsoni Brown, 1940b: 182. Type locality: «Great Sand Hills, west of Swift Current, Sask[atchewan]» (original citation). Holotype (♂) in CNC [# 4885]. Etymology. This subspecies was named after Arthur Gibson [1875-1959], Dominion entomologist with a special interest in Lepidoptera. Gibson had no formal training but learned under James Fletcher and Charles Gordon Hewitt, both with the Department of Agriculture in Ottawa.

#### Distribution.

This subspecies, also known as the “Gibson’s Sand Tiger Beetle”, is known from southwestern Saskatchewan (Wallis 1961: 38; Gaumer 1977: 216) and northwestern Colorado (Kippenhan 1994: 41). The record from “Alberta” (Bousquet and Larochelle 1993: 55) is in error; that from “North Dakota” (Freitag 1999: 26) needs confirmation; that from “Utah” (Erwin and Pearson 2008: 136) is probably based on intergrades found along the Green River (see Pearson et al. 2006: 84).

#### Records.

**CAN**: SK **USA**: CO [SD, UT]

#### Note.

In his unpublished thesis, Gaumer (1977: 219) treated the Colorado population of this subspecies as a distinct subspecies of *Cicindela formosa*. This subspecies intergrades narrowly with the *formosa* form on all sides of its small range in southern Saskatchewan and along the Green River in northeastern Utah (Pearson et al. 2006: 84).

### Cicindela formosa pigmentosignata

Horn, 1930

Cicindela formosa pigmento-signata W. Horn, 1930: 76. Type locality: «Mineola [and] Rosser, Texas» (original citation). Syntype(s) [2 originally cited] in USNM [# 41843] and DEI (Döbler 1973: 378).

#### Distribution.

This subspecies, also known as the “Reddish-green Sand Tiger Beetle”, is found in southwestern Arkansas, northern Louisiana, and eastern Texas (Gaumer 1977: 210).

#### Records.

**USA**: AR, LA, TX

### Cicindela formosa rutilovirescens

Rumpp, 1986

Cicindela formosa rutilovirescens Rumpp, 1986: 142. Type locality: «Mescalero Sands (1300 m), 55 to 65 km due east of Roswell, Chaves County, New Mexico» (original citation). Holotype (♂) in CAS [# 12984].

#### Distribution.

This subspecies, also known as the “Mescalero Sand Tiger Beetle”, is found in Terry and Yoakum Counties in northwestern Texas and in Chaves, Eddy, and Roosevelt Counties in eastern New Mexico (Rumpp 1986: 143).

#### Records.

**USA**: NM, TX

#### Note.

Rumpp (1986: 144) reported the presence of intergrade populations between this subspecies and the nominotypical form in Bailey and Lamb Counties, northwestern Texas, and Quay County, eastern New Mexico.

### Cicindela longilabris laurentii

Schaupp, 1884

Cicindela longilabris var. *laurentii* Schaupp, 1884a: 87. Type locality: «Col[orado]» (original citation), herein restricted to Golden, Jefferson County (see Leng 1902: 121). Syntype(s) apparently destroyed.
Cicindela longilabris var. *oslari* Leng, 1902: 121. Type locality: «southwest slope of M[oun]t Wilson of the San Miguel Range (12,000 feet) [Dolores County], Colorado» (original citation for the lectotype). Lectotype (♀), designated by Dahl (1941: 189), in AMNH [# 1226]. Synonymy established by Spanton (1988: 123). Etymology. The subspecific name was proposed in honor of Ernest J. Oslar [1858-1944], a resident of Denver who had an interest in Colorado Coleoptera. Oslar was born in England.
Cicindela longilabris var. *vestalia* Leng, 1902: 121. Type locality: «Maiden [Fergus County], Montana» (original citation). Lectotype (♀), designated by Dahl (1941: 188), in AMNH [# 1227]. Synonymy established by Spanton (1988: 123).
Cicindela oslari densissima Casey, 1924: 12. Type locality: «probably Colorado» (original citation). One syntype in USNM [# 45895]. Synonymy established, under the name *Cicindela longilabris oslari* Leng, by Horn (1926: 273), confirmed by Spanton (1988: 123).
Cicindela oslari estesiana Casey, 1924: 13. Type locality: «Colorado» (original citation). Three syntypes [3 originally cited] in USNM [# 45896]. Synonymy established by Horn (1930: 82), confirmed by Spanton (1988: 123).

#### Distribution.

This subspecies, the “Laurent’s Long-lipped Tiger Beetle”, ranges from north-central Montana to western South Dakota, south to central New Mexico, central Arizona, and southern Nevada [see Spanton 1988: Fig. 39].

#### Records.

**USA**: AZ, CO, ID, MT, NM, NV, SD, UT, WY

### Cicindela longilabris longilabris

Say, 1824

Cicindela longilabris Say, 1824: 268. Type locality: «1 km W[est] of Silver Islet on Perry Bay, Sibley Prov[incial] P[ar]k, Ont[ario]» (neotype label). Neotype (♂), designated by Spanton (1988: 123), in MCZ [# 32908]. Note. «North-west Territory» was the area originally cited by Say (1824: 268).
Cicindela albilabris Kirby, 1837: 12. Type locality: «Lat. 64° and also Canada» (original citation). One syntype in BMNH (Lindroth 1953b: 169). Synonymy established by LeConte (1846b: 178).
Cicindela longilabris novaterrae Leng, 1919a: 140. Type locality: «near Bay S[ain]t George, Newfoundland» (original citation). Holotype (♀) in AMNH [# 1225]. Synonymy established by Spanton (1988: 123).
Cicindela oslari terracensis Casey, 1924: 13. Type locality: «Terrace, British Columbia» (original citation). Holotype [by monotypy] (♂) in USNM [# 45897]. Synonymy established by Horn (1926: 273), confirmed by Spanton (1988: 123).

#### Distribution.

The range of this subspecies, also known as the “Boreal Long-lipped Tiger Beetle”, extends from Newfoundland to eastern Alaska, south to southern Alberta, central Minnesota, central Wisconsin, and southern New York [see Spanton 1988: Fig. 39]. Intergrade populations between the three subspecies of *Cicindela longilabris* are found in southeastern British Columbia, southwestern Alberta, western Montana, Idaho, southeastern Washington, and northeastern Oregon [see Spanton 1988: Fig. 39]; these records are listed under this subspecies.

#### Records.

**CAN**: AB, BC, LB, MB, NB, NF, NS (CBI), NT, ON, PE, QC, SK, YT **USA**: AK, ID, ME, MI, MN, MT, NH, NY, OR, VT, WA, WI

### Cicindela longilabris perviridis

Schaupp, 1884

Cicindela longilabris var. *perviridis* Schaupp, 1884a: 87. Type locality: «Cal[ifornia], Oregon, Utah and Newf[oun]dl[an]d» (original citation), restricted to «Sierra» and «Placer» Counties in California by Leng (1902: 122). Syntype(s) location unknown. Note. Leng (1902: 123) reported that the “type of this species” was in Charles Fuchs collection. Much of Fuchs’ collection was destroyed in the San Francisco earthquake and fire of 1906.
Cicindela ostenta Casey, 1913: 17. Type locality: «California» (original citation). One syntype in USNM [# 45898]. Synonymy established by Horn (1915: 377).
Cicindela perviridis placerensis Casey, 1913: 18. Type locality: «Placer Co[unty], California» (original citation). Two syntypes in USNM [# 45905]. Synonymy established by Horn (1915: 377).
Cicindela ostenta columbiana Casey, 1924: 13. Type locality: «British Columbia» (original citation). Holotype [by monotypy] (♂) in USNM [# 45899]. Synonymy established by Wallis (1961: 50).

#### Distribution.

The range of this subspecies, the “Green Long-lipped Tiger Beetle”, extends along the Cascade Range and Sierra Nevada from southwestern British Columbia to east-central California (Spanton 1988: 129, Fig. 39). The records from Colorado (Wickham 1902: 228), “Idaho,” and “Montana” (Boyd 1982: 6) probably refer to the *laurentii* form; that from “Nevada” (Bousquet and Larochelle 1993: 58) needs confirmation.

#### Records.

**CAN**: BC **USA**: CA, OR, WA [NV]

### Cicindela nebraskana

Casey, 1909

Cicindela montana LeConte, 1861b: 338 [primary homonym of *Cicindela montana* Charpentier, 1825]. Type locality: «valleys of the Rocky Mountains» (original citation), restricted to «valleys of the Bitter Root Mountains of eastern Idaho and western Montana» by Spanton (1988: 131). Syntype(s) in MCZ [# 25].
Cicindela [*longilabris*] *nebraskana* Casey, 1909: 268. Type locality: «Nebraska» (original citation). One syntype in USNM [# 45902]. Synonymy established by Boyd (1982: 6).
Cicindela montana canadensis Casey, 1913: 17. Type locality: «Calgary, Alberta» (original citation). Three syntypes in USNM [# 45903]. Synonymy established by Horn (1915: 377).
Cicindela spissitarsis Casey, 1913: 18. Type locality: «Aweme, Manitoba» (original citation). One syntype in USNM [# 45904]. Synonymy established by Horn (1926: 273).
Cicindela calgaryana Casey, 1914: 18. Type locality: «Lethbridge, Alberta» (original citation). Three syntypes in USNM [# 45901]. Synonymy established by Horn (1915: 444).
Cicindela montana uteana Casey, 1924: 12. Type locality: «Provo [Utah County], Utah» (original citation). Holotype [by monotypy] (♂) in USNM [# 45900]. Synonymy established by Horn (1926: 273).
Cicindela longilabris chamberlaini Knaus, 1925: 182. Type locality: «Stein Mountains, Harney County, southeast Oregon» (original citation). Holotype (♂) location unknown (possibly in KSUC). Synonymy established by Spanton (1988: 131). Etymology. The subspecific name was proposed for Willard Joseph Chamberlin [1890-1971], professor and forest entomologist at the Oregon State University. Chamberlin (not Chamberlain as thought by Knaus) worked mainly on Buprestidae and Scolytinae. He sold his collection in 1950 to the California Academy of Sciences.

#### Distribution.

This species, also known as the “Prairie Long-lipped Tiger Beetle”, ranges from the Fraser River in British Columbia to northwestern Ontario (Lawton 2008: 72), south to west-central Nebraska (Spomer et al. 2008a: 54), northern Colorado, southern Utah, and east-central California [see Spanton 1988: Fig. 40]. The records from “New Mexico” (Freitag 1999: 35) and Minnesota (Horn 1928: 11) need confirmation.

#### Records.

**CAN**: AB, BC, MB, ON, SK **USA**: CA, CO, ID, MT, ND, NE, NV, OR, SD, UT, WA, WY [MN, NM]

### Cicindela patruela consentanea

Dejean, 1825

Cicindela consentanea Dejean, 1825: 63. Type locality: «Amérique septentrionale» (original citation), herein restricted to Lakehurst, Ocean County, New Jersey (see Leng 1902: 130). Syntype(s) in MHNP.

#### Distribution.

This subspecies, also known as the “Consenta’s Tiger Beetle”, was once found in Long Island and New Jersey but is now restricted to the Pine Barrens region of New Jersey (Mawdsley 2007: 17). Single specimens, possibly strays, are known from Delaware, “Maryland,” and “Pennsylvania” (Mawdsley 2007: 17). The taxon is also recorded from “North Carolina” and “Virginia” by Erwin and Pearson (2008: 166).

#### Records.

**USA**: NJ, NY [DE, MD, NC, PA, VA]

### Cicindela patruela patruela

Dejean, 1825

Cicindela patruela Dejean, 1825: 62. Type locality: «Amérique septentrionale» (original citation), herein restricted to Chickies Rock, Lancaster County, Pennsylvania (see Leng 1902: 129). Syntype(s) in MHNP.
Cicindela montana Hentz [in Scudder], 1869: 53 [primary homonym of *Cicindela montana* LeConte, 1861]. Type locality: «near the Pilot Mountain [North Carolina]» (original citation). Syntype(s) lost. Synonymy established by Horn (1915: 381).
Cicindela patruela huberi Johnson, 1990a: 27. Type locality: «2.4 miles southwest of Mather, Monroe Co[unty], Wisconsin» (original citation). Holotype (♂) in FSCA. Synonymy established by Kaulbars and Freitag (1993a: 308).

#### Distribution.

This subspecies, also known as the “Northern Barrens Tiger Beetle”, ranges from New Hampshire (Leonard and Bell 1999: 47) to Minnesota, south to northern Alabama (Löding 1945: 9) northeastern Georgia, and northwestern South Carolina [see Kaulbars and Freitag 1993a: Fig. 40]. The species has been collected also at two sites in the Outaouais region in western Quebec and eastern Ontario (Leonard and Bell 1999: 47). The record from New Jersey (Smith 1910: 197) is questionable (see Boyd 1978: 215). Beaton (2008: 39) indicated that he was unable to find any extant populations of this species in Georgia despite intensive searching at historical sites and other areas of suitable habitat.

#### Records.

**CAN**: ON, QC **USA**: AL, CT, DC, DE, GA, IN, KY, MA, MD, MI, MN, NC, NH, NY, OH, PA, RI, SC, TN, VA, VT, WI, WV [NJ]

### Cicindela pulchra dorothea

Rumpp, 1977

Cicindela pulchra dorothea Rumpp, 1977: 172. Type locality: «5.2 to 6.2 kilometers southeast of Willcox [Cochise County, Arizona]» (original citation). Holotype (♂) in CAS [# 12529].

#### Distribution.

This subspecies, the “Dorothy’s Tiger Beetle”, in known from southeastern Arizona (Bertholf 1983: 21), New Mexico (Acciavatti et al. 1980: 30), and a small area in western Texas [see Pearson et al. 2006: Map 39].

#### Records.

**USA**: AZ, NM, TX

### Cicindela pulchra pulchra

Say, 1823

Cicindela pulchra Say, 1823b: 142. Type locality: «in the country bordering the Platte and Arkansa rivers near the mountains, Missouri Territory» (original citation). Syntype(s) lost.

#### Distribution.

This subspecies, also known as the “Beautiful Tiger Beetle”, ranges from western South Dakota (Larsen and Willis 2008: 480; Brust 2010: 1) and northeastern Wyoming (Brust 2011: 78) south to northern Texas, New Mexico, and Arizona (Bertholf 1983: 20) [see Pearson et al. 2006: Map 39]; also recorded from Nuevo León (Erwin and Pearson 2008: 172).

#### Records.

**USA**: AZ, CO, KS, NE, NM, OK, SD,TX, WY

### Cicindela sexguttata

Fabricius, 1775

Cicindela sex guttata Fabricius, 1775: 226. Type locality: «Virginia» (original citation). Four syntypes in ZMUC (Zimsen 1964: 65).
Cicindela violacea Fabricius, 1801: 232. Type locality: «Carolina» (original citation). Syntype(s) probably lost. Synonymy established by Dejean (1833: 2).
Cicindela sexguttata var. *harrisii* Leng, 1902: 128. Type locality: «Lake Memphremagog [Quebec], Canada» (original citation for the lectotype). Lectotype (♂), designated by Dahl (1941: 190), in AMNH [# 1230]. Synonymy established by Horn (1905: 21). Etymology. The subspecific name was proposed in honor of Edward Doubleday Harris [1839-1919], one of the sons of Thaddeus Harris (see *Agonum harrisii* LeConte). Edward Harris, an architect by profession, was interested in genealogy, history, and entomology. He specialized on tiger beetles and his collection is now at the Museum of Comparative Zoology.
Cicindela sexguttata var. *4-guttata* C.A. Davis, 1903: 271. Type locality: «from northern Rhode Island into Massachusetts» (original citation). Syntype(s) location unknown. Synonymy established by Horn (1905: 21).
Cicindela levettei Casey, 1909: 270. Type locality: «Iowa» (original citation). Five syntypes in USNM [# 45918]. Synonymy established by Harris (1911: 29).
Cicindela levettei tridens Casey, 1909: 271. Type locality: «Vowell’s Mill, northwestern Louisiana, to Onaga, Kansas» (original citation). Two syntypes in USNM [# 45917]. Synonymy established by Harris (1911: 29).
Cicindela illinoensis Mares, 1921: 310. Type locality: «Riverside [Cook County], Illinois» (original citation). Holotype (♂) in INHS (Webb 1980: 78). Synonymy established by Horn (1926: 279).
Cicindela kansanus Knaus, 1928: 24. Type locality: «near Onaga, Pottawatomie County, Kansas; Bourbon County, Kansas; West Point, Cuming County, South Bend, Cass County, Lawrence, Nuckolls County, Omaha, Douglas County [all Nebraska]; near Iowa City [Iowa]» (original citation). Syntype(s) [10 originally cited] location unknown (possibly in KSUC). Synonymy established, under the name *Cicindela sexguttata violacea* Fabricius, by Horn (1930: 83).

#### Distribution.

This species, also known as the “Six-spotted Tiger Beetle”, ranges from Nova Scotia to the Black Hills in southwestern South Dakota, south to east-central Texas and northern Florida [see Kaulbars and Freitag 1993a: Fig. 39; Pearson et al. 2006: Map 36]. The record from “North Dakota” (Freitag 1999: 50) needs confirmation.

#### Records.

**CAN**: NB, NS, ON, QC **USA**: AL, AR, CT, DC, DE, FL, GA, IA, IL, IN, KS, KY, LA, MA, MD, ME, MI, MN, MO, MS, NC, NE, NH, NJ, NY, OH, OK, PA, RI, SC, SD, TN, TX, VA, VT, WI, WV [ND]

### [hirticollis group]

### Cicindela albissima

Rumpp, 1962

Cicindela limbata albissima Rumpp, 1962: 181. Type locality: «Coral Pink Sand Dunes (6,300 feet), 14 miles south of M[oun]t Carmel Junction, Kane County, Utah» (original citation). Holotype (♂) in CAS [# 17198].

#### Distribution.

This species, also known as the “Coral Pink Sand Dune Tiger Beetle”, is confined to a small area of less than 400 hectares in the Coral Pink Sand Dunes formation in southwestern Utah [see Johnson 1991: Fig. 22] and is considered an endangered species.

#### Records.

**USA**: UT

#### Note.

This taxon was listed as a subspecies of *Cicindela limbata* Say by Johnson (1991) and Freitag (1999: 32). Molecular studies have shown that it represents a distinct species (Morgan et al. 2000).

### Cicindela arenicola

Rumpp, 1967

Cicindela arenicola Rumpp, 1967: 130. Type locality: «S[ain]t Anthony Sand Dunes, 11 km northeast of S[ain]t Anthony, Fremont County, Idaho» (original citation). Holotype (♂) in CAS [# 9374].

#### Distribution.

This species, also known as the “St. Anthony Dune Tiger Beetle”, is found in the Snake River Valley of southeastern Idaho [see Shook and Clark 1988: Fig. 1; Pearson et al. 1997: Fig. 22] and in southwestern Montana (Winton et al. 2010: 43).

#### Records.

**USA**: ID, MT

### Cicindela bellissima bellissima

Leng, 1902

Cicindela bellissima Leng, 1902: 142. Type locality: «Yaquina Bay [Lincoln County], on the seacoast of Oregon» (original citation). Lectotype (♂), designated by Dahl (1941: 188), in AMNH [# 1218].

#### Distribution.

This subspecies, also known as the “Pacific Coast Tiger Beetle”, ranges along the seacoast from Grays Harbor County in northern Washington to Del Norte County in northern California [see Leffler 1979a: Fig. 30; Pearson et al. 2006: Map 26].

#### Records.

**USA**: CA, OR, WA

### Cicindela bellissima frechini

Leffler, 1979

Cicindela bellissima frechini Leffler, 1979b: 466. Type locality: «Mukkah Bay, Clallam Co[unty], Washington» (original citation). Holotype (♂) in AMNH [# 1487].

#### Distribution.

This subspecies, also known as the “Frechin’s Tiger Beetle”, is known only from a small area around Neah Bay in the extreme northwestern Olympic Peninsula, Washington (Pearson et al. 2006: 82).

#### Records.

**USA**: WA

### Cicindela columbica

Hatch, 1938

Cicindela bellissima columbica Hatch, 1938: 234. Type locality: «Perry [Franklin County], Wash[ington]» (original citation). Holotype (♀) in USNM. Note. According to Leffler and Pearson (1976: 41), the type locality is at the junction of the Palouse and Snake Rivers.

#### Distribution.

This species, also known as the “Columbia River Tiger Beetle”, was once found along the Columbia, Salmon, and Snake Rivers in north-central Oregon, south-central Washington, and western Idaho [see Leffler 1979a: Fig. 29; Pearson et al. 1997: Fig. 27]. According to Pearson et al. (2006: 81), it is now known only from a few sites along the Salmon River in Idaho.

#### Records.

**USA**: ID, OR, WA

#### Note.

This species is listed on the IUCN Red List of Threatened Species (IUCN 2007).

### Cicindela depressula depressula

Casey, 1897

Cicindela depressula Casey, 1897: 297. Type locality: «Placer Co[unty], California» (original citation). Four syntypes in USNM [# 45987].

#### Distribution.

This montane subspecies, also known as the “Dispirited Tiger Beetle”, ranges from southern Alaska south to the Sierra Nevada in east-central California and western Nevada; also found in the Rocky Mountains in southeastern British Columbia, northern Idaho, and western Montana [see Freitag 1965: Fig. 33; Pearson et al. 2006: Map 19].

#### Records.

**CAN**: BC **USA**: AK, CA, ID, MT, NV, OR, WA

#### Note.

Intergrade populations are known between the two subspecies of *Cicindela depressula* in western Washington at intermediate altitudes (Pearson et al. 2006: 70).

### Cicindela depressula eureka

Fall, 1901

Cicindela eureka Fall, 1901b: 307. Type locality: «Humboldt County, California» (original citation). Syntype(s) [8 originally cited] in MCZ [# 23837].

#### Distribution.

This subspecies, also known as the “Eureka Tiger Beetle”, inhabits a narrow area along or near the Pacific Coast from northern Washington to northern California [see Freitag 1965: Fig. 33].

#### Records.

**USA**: CA, OR, WA

### Cicindela duodecimguttata

Dejean, 1825

Cicindela duodecimguttata Dejean, 1825: 73. Type locality: «Amérique septentrionale» (original citation), herein restricted to Framingham, Middlesex County, Massachusetts (see Harris 1911: 11). Syntype(s) in MHNP.
Cicindela proteus Kirby, 1837: 9. Type locality: «Canada» (original citation). Two syntypes in BMNH (Lindroth 1953b: 169). Synonymy established by LeConte (1846b: 181).
Cicindela bucolica Casey, 1913: 28. Type locality: «Aweme, Manitoba» (original citation). Four syntypes in USNM [# 45978]. Synonymy established by Horn (1915: 374).
Cicindela hudsonica Casey, 1916: 29. Type locality: «Hudson Bay Territory» (original citation). One syntype in USNM [# 45979]. Synonymy established by Horn (1926: 268).
Cicindela repanda edmontonensis Carr, 1920: 218. Type locality: «Edmonton, Al[ber]ta» (original citation). Holotype (♀) in CNC [# 407]. Synonymy established, under the name *Cicindela bucolica* Casey, by Casey (1924: 16), confirmed by Freitag (1965: 103).

#### Distribution.

This species, also known as the “Twelve-spotted Tiger Beetle”, is found from Northwest Territories and the eastern front of the Rocky Mountains in Alberta to Newfoundland (Lindroth 1955a: 16), south to Alabama, central Texas, and Colorado (Kippenhan 1990: 309) [see Freitag 1965: Fig.17]. The record from Vancouver, British Columbia (Wallis 1961: 22) is possibly based on a mislabeled specimen.

#### Records.

**FRA**: PM **CAN**: AB, LB, MB, NB, NF, NS (CBI), NT, ON, PE, QC, SK **USA**: AL, AR, CO, CT, DC, DE, GA, IA, IL, IN, KS, KY, LA, MA, MD, ME, MI, MN, MO, MS, MT, NC, ND, NE, NH, NJ, NY, OH, OK, PA, RI, SC, SD, TN, TX, VA, VT, WI, WV, WY

### Cicindela hirticollis abrupta

Casey, 1913

Cicindela gravida abrupta Casey, 1913: 31. Type locality: «Sacramento [Sacramento County], California» (original citation). Two syntypes in USNM [# 45992].

#### Distribution.

This subspecies, also known as the “Sacramento Valley Hairy-necked Tiger Beetle”, is endemic to a small area within the Sacramento Valley of California (Graves et al. 1988: 660). An extensive survey in 2001-2004 within the known distributional range of the subspecies yield no specimens and Knisley and Fenster (2006) concluded that the subspecies has been extirpated in the late 1980s to early 1990s possibly from the construction of the Oroville Dam on the Feather River in the 1960s (see also Fenster and Knisley 2006).

#### Records.

**USA**: CA

### Cicindela hirticollis athabascensis

Graves, 1988

Cicindela hirticollis athabascensis Graves [in Graves et al.], 1988: 666. Type locality: «Lake Athabasca, Thompson Bay Dunes, Saskatchewan» (original citation). Holotype (♂) in CNC [# 20586].

#### Distribution.

This subspecies, the “Athabascan Tiger Beetle”, is known only from the Lake Athabasca Sand Dunes of northern Alberta and Saskatchewan (Graves et al. 1988: 667). This is the northernmost and most isolated known population of *Cicindela hirticollis*. The record from “Northwest Territories” (Erwin and Pearson 2008: 142) needs confirmation.

#### Records.

**CAN**: AB, SK [NT]

### Cicindela hirticollis coloradula

Graves, 1988

Cicindela hirticollis coloradula Graves [in Graves et al.], 1988: 668. Type locality: «I-40 & Little Co[lorado] R[iver], Navajo Co[unty], A[ri]z[ona]» (original citation). Holotype (♂) in USNM [# 105096].

#### Distribution.

This subspecies, also known as the “Colorado River Tiger Beetle”, is restricted to the valley of the Little Colorado River in Navajo County, northeastern Arizona (Graves et al. 1988: 669).

#### Records.

**USA**: AZ

### Cicindela hirticollis corpuscula

Rumpp, 1962

Cicindela hirticollis corpuscula Rumpp, 1962: 174. Type locality: «Potholes, Imperial County, California» (original citation). Holotype (♂) in CAS [# 17197].

#### Distribution.

This subspecies, the “Southwest Hairy-necked Tiger Beetle”, ranges from western Colorado and western New Mexico westwards to southeastern California and the northern parts of the Baja California Peninsula [see Graves et al. 1988: Fig. 6]. The record from “Wyoming” (Erwin and Pearson 2008: 143) needs confirmation. According to Pearson et al. (2006: 73), the subspecies is now probably extirpated from many former sites along the Gila River in central and western Arizona.

#### Records.

**USA**: AZ, CA, CO, NM, NV, UT [WY] – Mexico

### Cicindela hirticollis couleensis

Graves, 1988

Cicindela hirticollis couleensis Graves [in Graves et al.], 1988: 669. Type locality: «Vantage [Kittitas County], Wash[ington]» (original citation). Holotype (♂) in USNM [# 105097].

#### Distribution.

This subspecies, the “Coulee Tiger Beetle”, inhabits mainly the Columbia-Snake river system (Graves et al. 1988: 671) ranging from southern British Columbia to southern Oregon and Idaho. Its western limit is in Cowlitz County, within 100 km of the coast (Pearson et al. 2006: 73). The record from “Montana” (Erwin and Pearson 2008: 143) needs confirmation.

#### Records.

**CAN**: BC **USA**: ID, OR, WA [MT]

### Cicindela hirticollis gravida

LeConte, 1851

Cicindela gravida LeConte, 1851: 170. Type locality: «San Diego [San Diego County, California]» (original citation). Syntype(s) in MCZ [# 12].

#### Distribution.

This subspecies, The “Pacific Hairy-necked Tiger Beetle”, is restricted to the Pacific Coast of northern Baja California and southern California (Graves et al. 1988: 659), as far north as Santa Cruz County (Pearson et al. 2006: 73). According to Erwin and Pearson (2008: 143), this taxon is threatened and has been eliminated over most of its former range.

#### Records.

**USA**: CA (CHI) – Mexico

### Cicindela hirticollis hirticollis

Say, 1817

Cicindela hirticollis Say, 1817b: 20. Type locality: «Pennsylvania» (original citation). Syntype(s) lost.
Cicindela albohirta Dejean, 1826: 425. Type locality: «Amérique septentrionale» (original citation). Syntype(s) [2 ♂ originally cited] in MHNP. Synonymy established by LeConte (1846b: 180).
Cicindela unita Kollar, 1836: 330. Type locality: «America boreali» (original citation). Syntype(s) location unknown. Synonymy established by Melsheimer (1853: 2).

#### Distribution.

This subspecies, also known as the “Hairy-necked Tiger Beetle”, ranges east of the Mississippi River from New Jersey to southern Florida (Peck and Thomas 1998: 16), west to the Mississippi River Valley (Graves et al. 1988: 658, Fig. 6) then north to the southern region of the Great Lakes. According to Allen and Acciavatti (2002: 12), this subspecies is considered rare and even endangered throughout the Ohio River drainage and other regions. The records from Oklahoma (Drew and Van Cleave 1962: 110), “Kansas,” and “Nebraska” (Erwin and Pearson 2008: 144) are based on intergrades and are recorded here under the *shelfordi* form; that from “Utah” (Erwin and Pearson 2008: 144) is probably in error or based on a stray.

#### Records.

**USA**: AL, AR, CT, DC, DE, FL, GA, IA, IL, IN, KY, LA, MD, MI, MN, MO, MS, NC, NJ, NY, OH, PA, SC, TN, TX, VA, WI, WV

#### Note.

This subspecies intergrades with the *shelfordi* form along the Mississippi River and with the *rhodensis* form in the southern Great Lakes region and at Long Island, New York (Graves et al. 1988: 658, 661, 663).

### Cicindela hirticollis rhodensis

Calder, 1916

Cicindela hirticollis var. *nigrita* C.A. Davis, 1903: 273 [primary homonym of *Cicindela nigrita* Dejean, 1825]. Type locality: «Warwick [Kent County], R[hode] I[sland]» (original citation). Syntype(s) location unknown.
Cicindela hirticollis var. *rhodensis* Calder, 1916 [12 April]: 93. Replacement name for *Cicindela hirticollis* var. *nigrita* Davis, 1903.
Cicindela shermani Casey, 1916 [29 November]: 30. Type locality: «Marquette [Marquette County], Michigan» (original citation). One syntype in USNM [# 45990]. Synonymy established by Horn (1930: 81).

#### Distribution.

This subspecies, the “Rhode’s Tiger Beetle”, ranges from Newfoundland to the Lake Superior region in Ontario, south to southeastern Wisconsin (Messer 2010: 33), Michigan, Connecticut, and Rhode Island [see Graves et al. 1988: Fig. 6]. Numerous state and province records (e.g., MB, IA, IL, IN, MN, ND, NE, SD) listed by Erwin and Pearson (2008: 145) are reported here under other subspecies of *Cicindela hirticollis*.

#### Records.

**FRA**: PM **CAN**: LB, NB, NF, NS (CBI), ON, PE, QC **USA**: CT, MA, ME, MI, NH, NY, RI, VT

### Cicindela hirticollis shelfordi

Graves, 1988

Cicindela hirticollis shelfordi Graves [in Graves et al.], 1988: 664. Type locality: «banks of Cimarron R[iver] near Guthrie [Logan County], Okla[homa]» (original citation). Holotype (♂) in USNM [# 105095]. Etymology. The subspecific name was proposed in honor of Professor Victor Ernest Shelford [1877-1968], animal ecologist and pioneer of North American tiger beetle biology.

#### Distribution.

This subspecies, the “Shelford’s Tiger Beetle”, inhabits the Great Plains from southern Manitoba to southern Alberta, south to New Mexico and eastern Texas (Graves et al. 1988: 665, Fig. 6). Numerous state records (e.g., AR, AZ, LA, MN, UT) listed by Erwin and Pearson (2008: 145) are reported here under other subspecies of *Cicindela hirticollis*.

#### Records.

**CAN**: AB, MB, SK **USA**: CO, IA, KS, MN, MO, MT, ND, NE, NM, OK, SD, TX, WY

#### Note.

This subspecies intergrades with the nominate form over a large area west of the Mississippi river, from southern Manitoba to Louisiana (Graves et al. 1988: Fig. 6), and with the *corpuscula* form along the Green River in eastern Utah (Graves et al. 1988: 666).

### Cicindela hirticollis siuslawensis

Graves, 1988

Cicindela hirticollis siuslawensis Graves [in Graves et al.], 1988: 671. Type locality: «3 mi[les] n[orth] Florence, Lane Co[unty], Ore[gon]» (original citation). Holotype (♂) in AMNH [# 1542].

#### Distribution.

This subspecies, the “Northwest Hairy-necked Tiger Beetle”, is found along the Pacific Coast from northern Washington to northern California (Graves et al. 1988: 672). According to Pearson et al. (2006: 74), it is now extirpated from most historic sites.

#### Records.

**USA**: CA, OR, WA

### Cicindela limbata hyperborea

LeConte, 1863

Cicindela hyperborea LeConte, 1863c: 1. Type locality: «Methy Portage [= Portage La Loche, northern Saskatchewan], Hudson’s Bay Territory» (original citation). Syntype(s) in MCZ [# 16]. Note. Methy(e) Portage was the longest portage in the regular fur trade. It lies between Lac La Loche (the top of the Churchill River system) on the southeast and the Clearwater River, which flows into the Athabasca River, on the northwest.

#### Distribution.

This subspecies, the “Hyperboreal Tiger Beetle”, ranges from southern Northwest Territories to east-central Alberta, central Saskatchewan, west-central Manitoba [see Johnson 1991: Fig. 22] and northeastern Manitoba (Woodcock et al. 2011: 118).

#### Records.

**CAN**: AB, MB, NT, SK

#### Note.

This subspecies intergrades with the *nympha* form along a narrow zone in central Alberta (Pearson et al. 2006: 76).

### Cicindela limbata labradorensis

Johnson, 1991

Cicindela limbata labradorensis Johnson, 1991: 261. Type locality: «Goose Bay, Labrador, Newfoundland» (original citation). Holotype (♂) in CNC [# 20225].

#### Distribution.

This subspecies, the “Labrador Tiger Beetle”, is known only from southern Labrador (Johnson 1991: Fig. 22; Brzoska and Stamatov 2008: 50-51).

#### Records.

**CAN**: LB

### Cicindela limbata limbata

Say, 1823

Cicindela limbata Say, 1823b: 141. Type locality: «on the Nebraska (Platte) and Arkansa Rivers» (original citation). Syntype(s) lost.
Cicindela limbigera Gemminger and Harold, 1868a: 20. Unnecessary replacement name for *Cicindela limbata* Say, 1823.

#### Distribution.

This subspecies, also known as the “Sandy Tiger Beetle”, is restricted to a small area enclosing southeastern Wyoming, southern South Dakota, Nebraska, and northeastern Colorado [see Johnson 1991: Fig. 22]. The record from “Kansas” (Boyd 1982: 7) needs confirmation.

#### Records.

**USA**: CO, NE, SD, WY [KS]

### Cicindela limbata nogahabarensis

Knisley, 2008

Cicindela limbata nogahabarensis Knisley [in Knisley et al.], 2008: 280. Type locality: «Nogahabara Sand Dunes, Koyukuk National Wildlife Refuge, Alaska» (original citation). Holotype (♂) in CAS.

#### Distribution.

This subspecies, the “Nogahabar Tiger Beetle”, is known only from the type locality.

#### Records.

**USA**: AK

### Cicindela limbata nympha

Casey, 1913

Cicindela limbigera nympha Casey, 1913: 20. Type locality: «Aweme, Manitoba» (original citation). Five syntypes in USNM [# 45928].

#### Distribution.

This subspecies, also known as the “Nymphal Tiger Beetle”, ranges from central Alberta to northwestern Minnesota, south to northern South Dakota (Spomer et al. 2008a: 19) and northern Montana [see Johnson 1991: Fig. 22; Pearson et al. 2006: Map 21].

#### Records.

**CAN**: AB, MB, SK **USA**: MN, MT, ND, SD

### Cicindela oregona guttifera

LeConte, 1856

Cicindela guttifera LeConte, 1856a: 42. Type locality: «Santa Fe [Santa Fe County], New Mexico» (original citation). Syntype(s) in MCZ [# 13].
Cicindela sterope Casey, 1913: 28. Type locality: «Kansas» (original citation). One syntype in USNM [# 45991]. Synonymy established by Horn (1915: 378).
Cicindela audax Casey, 1913: 29. Type locality: «Colorado» (original citation). One syntype in USNM [# 45980]. Synonymy established by Horn (1915: 378).
Cicindela oregona oregonella Casey, 1924: 16. Type locality: «Deer Creek, Provo Cañon, Utah» (original citation). Holotype [by monotypy] (♂) in USNM [# 45983]. Synonymy established by Freitag (1965: 111).

#### Distribution.

This subspecies, the “Dappled Tiger Beetle”, ranges from eastern Alaska to western Northwest Territories, north to above the Arctic Circle (Brzoska 2008: 65), south to the Queen Charlotte Islands and, along the Rocky Mountains, to central New Mexico [see Freitag 1965: Fig. 18]; also recorded from “Arizona” (Erwin and Pearson 2008: 163) and “Idaho” (Boyd 1982: 7).

#### Records.

**CAN**: AB, BC (QCI), NT, YT **USA**: AK, CO, KS, MT, NM, UT, WY [AZ, ID]

#### Note.

This subspecies intergrades with the nominate form in southern British Columbia south, along the crest of the Rocky Mountains, to central Utah, with the *navajoensis* form in western Colorado and New Mexico, and with the *maricopa* form in western Utah (Pearson et al. 2006: 68).

### Cicindela oregona maricopa

Leng, 1902

Cicindela oregona var. *maricopa* Leng, 1902: 150. Type locality: «Phoenix [Maricopa County], Arizona» (original citation). Lectotype (♂), designated by Dahl (1941: 189), in AMNH [# 1229].
Cicindela provensis Casey, 1924: 15. Type locality: «Parowan and Provo Cañons, Utah» (original citation). Five syntypes [5 originally cited] in USNM [# 45981]. Synonymy established by Horn (1926: 274).
Cicindela provensis mormonella Casey, 1924: 15. Type locality: «Eureka, Provo Cañon, Parowan and Vineyard, Utah» (original citation). Six syntypes [6 originally cited] in USNM [# 45982]. Synonymy established by Horn (1926: 274).
Cicindela provensis nephiana Casey, 1924: 16. Type locality: «Parowan [Iron County], Utah» (original citation). One syntype in USNM [# 45989]. Synonymy established by Horn (1926: 274).

#### Distribution.

This subspecies, the “Maricopa Tiger Beetle”, ranges from southern California to south-central New Mexico, north to southern Nevada [see Freitag 1965: Fig. 18]. According to Pearson et al. (2006: 68), distinct individuals of this form are confined to southeastern and central Arizona. The record from “Texas” (Bousquet and Larochelle 1993: 58) is in error; that from “Utah” is apparently based on intergrades.

#### Records.

**USA**: AZ, CA, NM, NV

#### Note.

1. According to Freitag (1965: 111), the type series of *Cicindela provensis* Casey, *Cicindela mormonella* Casey, and *Cicindela nephiana* Casey are hybrid specimens of *Cicindela oregona guttifera* x *Cicindela oregona maricopa*. Boyd (1982: 7) and Freitag (1999: 37) listed these names as synonyms of *Cicindela oregona maricopa* Leng and I am following them. 2. Intergrade populations between this subspecies and the *navajoensis* form are known in southwestern New Mexico (Pearson et al. 2006: 69).

### Cicindela oregona navajoensis

Van Dyke, 1947

Cicindela oregona navajoensis Van Dyke, 1947: 155. Type locality: «15 miles W[est] N[orth] W[est] Kayenta [Navajo County], Arizona» (original citation). Holotype (♂) in CAS [# 5864].

#### Distribution.

This subspecies, the “Navajo Tiger Beetle”, inhabits a small area in the southern parts of the Rocky Mountains in Utah, southwestern Colorado, northern Arizona, and New Mexico [see Freitag 1965: Fig. 18; Pearson et al. 2006: Map 18].

#### Records.

**USA**: AZ, CO, NM, UT

### Cicindela oregona oregona

LeConte, 1856

Cicindela oregona LeConte, 1856a: 41. Type locality: «Oregon Territory and northern California, as far as San Francisco» (original citation). Syntype(s) in MCZ [# 28].
Cicindela depressula scapularis Casey, 1909: 272. Type locality: «California» (original citation). One syntype in USNM [# 45988]. Synonymy established by Hatch (1953: 41).
Cicindela guttifera sonoma Casey, 1913: 29. Type locality: «maritime regions north of San Francisco, California» (original citation). Two syntypes in USNM [# 45984]. Synonymy established by Horn (1915: 378).
Cicindela quadripennis Casey, 1913: 30. Type locality: «Hawthorne [Mineral County], Nevada» (original citation). One syntype in USNM [# 45986]. Synonymy established by Horn (1915: 378).
Cicindela ovalipennis Casey, 1913: 30. Type locality: «Hawthorne [Mineral County], Nevada» (original citation). One syntype in USNM [# 45985]. Synonymy established by Horn (1915: 378).

#### Distribution.

This subspecies, also known as the “Western Tiger Beetle”, ranges from Vancouver Island to western Alberta, south to central Utah and southern California along the Mexican border [see Freitag 1965: Fig. 18]; also recorded from Baja California (Murray 1979: 50) and northern Sonora (Cazier 1954: 242). The records from New Mexico (Fall and Cockerell 1907: 155), “Arizona,” and “New Mexico” (Boyd 1982: 6) probably refer to the *guttifera* form.

#### Records.

**CAN**: AB, BC (VCI) **USA**: CA (CHI), ID, MT, NV, OR, UT, WA, WY – Mexico

### Cicindela repanda novascotiae

Vaurie, 1951

Cicindela repanda novascotiae Vaurie, 1951: 1. Type locality: «Truro, Nova Scotia» (original citation). Holotype (♂) in AMNH [# 1217].

#### Distribution.

This subspecies, the “Nova Scotia Tiger Beetle”, is known from Nova Scotia, including Cape Breton Island, Prince Edward Island, and the Magdalen Islands in Quebec (Leonard and Bell 1999: 101). The record from “New Brunswick” (Boyd 1982: 6) needs confirmation.

#### Records.

**CAN**: NS (CBI), PE, QC [NB]

### Cicindela repanda repanda

Dejean, 1825

Cicindela repanda Dejean, 1825: 74. Type locality: «Amérique septentrionale» (original citation), herein restricted to Framingham, Middlesex County, Massachusetts (see Harris 1911: 9). Syntype(s) in MHNP.
Cicindela repanda unijuncta Casey, 1897: 299. Type locality: «El Paso [El Paso County], Texas» (original citation). Two syntypes in USNM [# 45977]. Synonymy established (as aberration) by Horn (1902a: 234).
Cicindela repanda hoosieri Mares, 1921: 310. Type locality: «Cedar Lake, Lake County, Indiana» (original citation). Holotype (♀) in INHS (Webb 1980: 78). Synonymy established by Horn (1926: 269).
Cicindela repanda-unijuncta form *duncani* Knaus, 1924: 126. Type locality: «near Phoenix, Ariz[ona]» (original citation). Holotype [by monotypy] probably in KSUC. Synonymy established, under the name *Cicindela duodecimguttata repanda hoosieri* Mares, by Horn (1930: 81). Note. Even if this taxon was originally proposed at infrasubspecific rank, it is deemed to be subspecific from its original publication because it was adopted as the valid name of a subspecies (e.g., Leng and Mutchler 1927: 7) before 1985 (see ICZN 1999: Article 45.6.4.1).
Cicindela repanda var. *maehleri* Robinson, 1948: 27. Type locality: «Brazos County, Texas» (original citation). Holotype (♂) in AMNH [# 1207]. Synonymy established by Boyd (1982: 6). Etymology. The subspecific name was proposed for Kenneth Leforest Maehler [1912-1991] who worked as plant quarantine entomologist with the United States Department of Agriculture.

#### Distribution.

This subspecies, also known as the “Bronze Tiger Beetle”, ranges from Newfoundland to southwestern British Columbia, south to southern Oregon, northeastern Arizona, central Texas, and northern Florida (Choate 2003: Map 18) [see Pearson et al. 2006: Map 16]. The record from “Northwest Territories” (Boyd 1982: 6) needs confirmation.

#### Records.

**CAN**: AB, BC, LB, MB, NB, NF, NS, ON, PE, QC, SK **USA**: AL, AR, AZ, CO, CT, DC, DE, FL, GA, IA, ID, IL, IN, KS, KY, LA, MA, MD, ME, MI, MN, MO, MS, MT, NC, ND, NE, NH, NJ, NM, NY, OH, OK, OR, PA, RI, SC, SD, TN, TX, UT, VA, VT, WA, WI, WV, WY [NT]

#### Note.

Intergrade populations between this subspecies and the *tanneri* form are known from northwestern Colorado (Kippenhan 1994: 29).

### Cicindela repanda tanneri

Knaus, 1929

Cicindela tanneri Knaus, 1929: 47. Type locality: «Green River [Emery County], Utah» (original citation). Holotype (♂) location unknown (possibly in KSUC). Etymology. The specific name honors Vasco Myron Tanner [1892-1989], naturalist and entomologist at Brigham Young University in Provo, Utah.

#### Distribution.

This subspecies, the “Tanner’s Tiger Beetle”, is known only from the Green River Valley in eastern Utah (Pearson et al. 2006: 65).

#### Records.

**USA**: UT

### Cicindela theatina

Rotger, 1944

Cicindela theatina Rotger, 1944: 76. Type locality: «edge of the Great Sand Dunes in the San Luis Valley (about 8,200 feet) [Alamosa County], Colorado» (original citation). Holotype (♂) in AMNH (Robert Acciavatti pers. comm. 2008).

#### Distribution.

This species, also known as the “Colorado Dune Tiger Beetle”, is restricted to the Great Sand Dunes National Monument and adjacent areas in Alamosa, Costilla, and Saguache Counties in south-central Colorado (Kippenhan 1990: 310; Pearson et al. 2006: 79). The record from “New Mexico” (Boyd 1982: 7) is in error.

#### Records.

**USA**: CO

### Cicindela waynei

Leffler, 2001

Cicindela waynei Leffler, 2001: 20. Type locality: «Bruneau Dunes State Park (910m), Owyhee Co[unty], Idaho» (original citation). Holotype (♂) in CAS [# 12266].

#### Distribution.

This species, also known as the “Bruneau Dune Tiger Beetle”, has yet been found only at the type locality in southwestern Idaho [see Leffler 2001: Fig. 1].

#### Records.

**USA**: ID

### Subfamily LORICERINAE

Bonelli, 1810

Loricerides Bonelli, 1810: Tabula Synoptica. Type genus: *Loricera* Latreille, 1802.

#### Diversity.

This subfamily contains a single tribe.

### Tribe Loricerini

Bonelli, 1810

Loricerides Bonelli, 1810: Tabula Synoptica. Type genus: *Loricera* Latreille, 1802.

#### Diversity.

This tribe includes a single genus.

### Genus Loricera

Latreille, 1802

Loricera Latreille, 1802: 88. Type species: *Carabus pilicornis* Fabricius, 1775 by monotypy. Etymology. Probably from the Greek *loron* (thong) and *ceras* (horn, by extension antenna) [feminine].
Lorocera Agassiz, 1846: 216. Unjustified emendation of *Loricera* Latreille, 1802.

#### Diversity.

Thirteen species in the Nearctic (three species, one of them Holarctic), Neotropical (two species in mountains of Middle America), and Palaearctic (nine species) Regions arrayed in three subgenera: *Elliptosoma* Wollaston (one species from Madeira*)*, *Loricera* s.str. (11 species), and *Plesioloricera* Sciaky and Facchini (one species from Szechwan, China).

#### Identification.

Ball and Erwin (1969: 883) published a key to all species then known, including the three found in North America. Lindroth (1961a: 121-125) covered the Nearctic species.

### Subgenus Loricera

Latreille, 1802

Loricera Latreille, 1802: 88. Type species: *Carabus pilicornis* Fabricius, 1775 by monotypy.

#### Diversity.

Eleven species in North America (three species), Middle America (two species), Asia (seven species), and Europe (one species which is also found in Asia and North America).

### Loricera decempunctata

Eschscholtz, 1833

Loricera decempunctata Eschscholtz, 1833: 25. Type locality: «Norfolksund [= Sitka Sound], Insel Sitcha [= Baranof Island, Alaska]» (original citation). Syntype(s) location unknown (possibly in ZMMU and collection LeConte in MCZ).

#### Distribution.

This species ranges from Kodiak Island and the Kenai Peninsula in southern Alaska south to northern California (Lindroth 1961a: 122; Humboldt County, MCZ).

#### Records.

**CAN**: BC (QCI, VCI) **USA**: AK, CA, OR, WA

### Loricera foveata

LeConte, 1851

Loricera foveata LeConte, 1851: 180. Type locality: «San Francisco [San Francisco County, California]» (original citation). Holotype [by monotypy] (♀) in MCZ [# 77].

#### Distribution.

This species is found along the coastal region from Washington (Hatch 1953: 64) to at least the San Francisco Bay area (LeConte 1851: 180).

#### Records.

**USA**: CA, OR, WA

### Loricera pilicornis congesta

Mannerheim, 1853

Loricera congesta Mannerheim, 1853: 121. Type locality: «peninsula Kenai [Alaska]» (original citation). Holotype [by monotypy] probably in ZILR.

#### Distribution.

This Holarctic subspecies is found on the Kuril Islands and on the Aleutian Islands and Kenai Peninsula in Alaska (Lindroth 1961a: 125).

#### Records.

**USA**: AK – **Holarctic**

**Figure 13.**
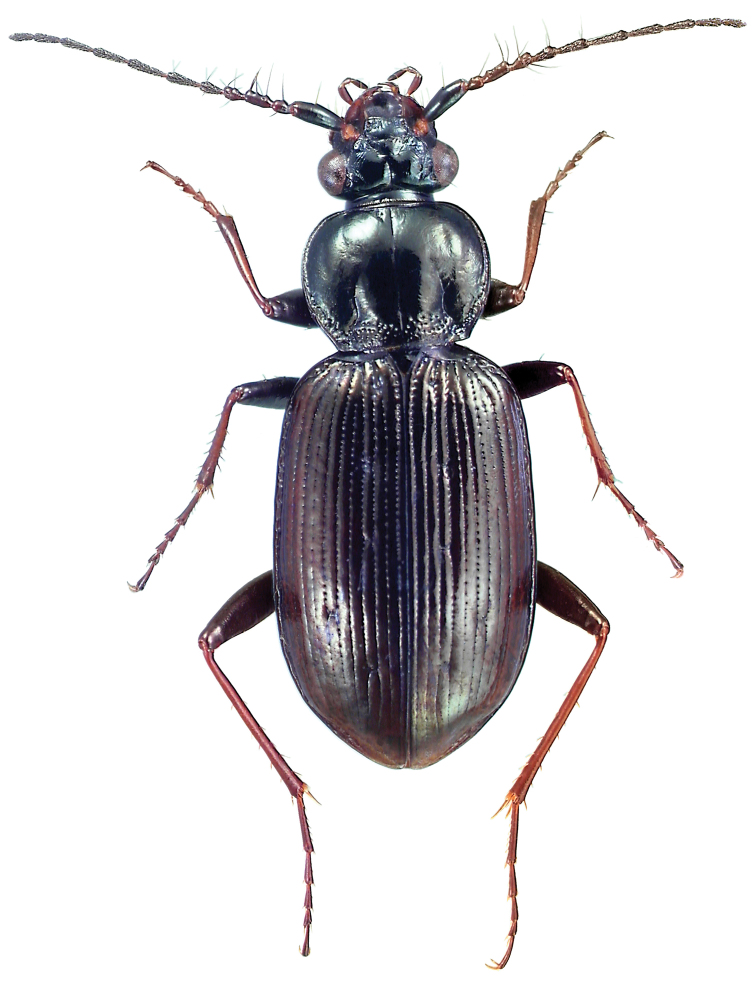
*Loricera pilicornis* (Fabricius). This is a widely distributed Holarctic species, occurring in Europe as far south as northern Spain and continental Italy, over Asia as far south as Kazakhstan and Sichuan, and in North America as far south as the San Bernardino Mountains in southern California and northern West Virginia. The specific name derives from the Latin *pili* (hairs) and *cornus* (horn) in reference to the presence of conspicuous stiff setae on the basal antennomeres of the adults. These setae, oriented in three main directions, act as a trap for the capture of collembolan prey.

### Loricera pilicornis pilicornis

(Fabricius, 1775)

Carabus pilicornis Fabricius, 1775: 243. Type locality: «Anglia [= England]» (original citation). One syntype in BMNH (collection Banks).
Carabus seticornis O.F. Müller, 1776: 79. Type locality: Denmark and Norway (inferred from title of the book). Syntype(s) probably lost. Synonymy established by Illiger (1798: 193).
Loricera semipunctata Eschscholtz, 1833: 25. Type locality: «bei St. Franzisco [San Francisco County], Californien» (original citation). Syntype(s) location unknown (possibly in ZMMU). Synonymy established, under the name *Loricera caerulescens* Linnaeus *sensu auctorum* (= *Carabus pilicornis* Fabricius), by Horn (1878c: 29).
Loricera rufilabris Motschulsky, 1845b: 340. Type locality: «Kamtschatka [Russia]» (original citation). Nine syntypes in ZMMU (Keleinikova 1976: 215). Synonymy established by Mannerheim (1846: 246).
Loricera californica LeConte, 1863c: 3. Type locality: «San Francisco [San Francisco County], California» (original citation). One syntype in MCZ [# 657]. Synonymy established by Lindroth (1961a: 123).
Loricera neoscotica LeConte, 1863c: 3. Type locality: «Nova Scotia» (original citation). Two syntypes in MCZ [# 5453]. Synonymy established, under the name *Loricera caerulescens* Linnaeus *sensu auctorum* (= *Carabus pilicornis* Fabricius), by Horn (1878c: 29), confirmed by Lindroth (1961a: 123).
Loricera uteana Casey, 1920: 147. Type locality: «Provo [Utah County], Utah» (original citation). Lectotype (♀), designated by Lindroth (1975: 113), in USNM [# 46835]. Synonymy established by Lindroth (1961a: 123).
Loricera pilicornis sierrae Van Dyke, 1925: 113. Type locality: «near Tallac, Lake Tahoe [Placer County], California» (original citation). Holotype (♂) in CAS [# 1618]. Synonymy established by Lindroth (1961a: 123).

#### Distribution.

This Holarctic subspecies ranges over most of Europe and a large part of Asia (see Bousquet 2003a: 98) and from the west coast of Alaska above the arctic circle (Lindroth 1961a: 125) to Newfoundland (Lindroth 1955a: 33), south to northern West Virginia (Tucker and Preston Counties, CMNH), western Nebraska (Kimball County, USNM), northern New Mexico (Sandoval and San Miguel Counties, UASM), southern Arizona (Greenlee County, UASM), and the San Bernardino Mountains in southern California (Fall 1901a: 40, as *Loricera californica*).

#### Records.

**FRA**: PM **CAN**: AB, BC, LB, MB, NB, NF, NS (CBI), NT, ON, PE, QC, SK, YT **USA**: AK, AZ, CA, CO, CT, IA, ID, IN, MA, ME, MI, MN, MT, ND, NE, NH, NJ, NM, NY, OH, OR, PA, SD, UT, VT, WA, WI, WV, WY – **Holarctic**

### Subfamily ELAPHRINAE

Latreille, 1802

Elaphrii Latreille, 1802: 81. Type genus: *Elaphrus* Fabricius, 1775.

#### Diversity.

This subfamily includes a single tribe.

### Tribe Elaphrini

Latreille, 1802

Elaphrii Latreille, 1802: 81. Type genus: *Elaphrus* Fabricius, 1775.

#### Diversity.

Northern Hemisphere, with about 50 species arrayed in three genera, all represented in North America.

### Genus Diacheila

Motschulsky, 1844

Diacheila Motschulsky, 1844: 74 (as *Diaheila*). Type species: *Harpalus arcticus* Gyllenhal, 1810 designated by Lacordaire (1854: 47). Etymology. Probably from the Greek *dia* (through, between, during) and *cheilos* (lip, by extension either labrum [upper lip] or labium [lower lip]) [feminine]. Note. Motschulsky (1844) originally used two different spellings for the name of this genus: *Diacheila* (pages 13, 76, v) and *Diaheila* (pages 15, 74). He subsequently used *Diachila* (Motschulsky 1850a: vi, 17; 1864: 195) and *Diaheila* (Motschulsky 1869: 10). Following article 24.2.4 (ICZN 1999), *Diaheila* should be the correct original spelling. *Diacheila* is an incorrect spelling but since it is in prevailing usage and attributed to the publication of the original spelling, it is deemed to be the correct original spelling (ICZN 1999: Article 33.3.1).
Arctobia C.G. Thomson, 1857: 16. Type species: *Harpalus arcticus* Gyllenhal, 1810 by monotypy. Etymology. From the Greek *arctos* (north) and *bios* (life) [feminine].

#### Diversity.

Three species in the arctic and subarctic areas of the Nearctic (two Holarctic species) and Palaearctic (three species) Regions.

#### Identification.

Lindroth (1954a) reviewed the species and provided a key for their identification. The two species found in North America were also covered in his monograph of the Carabidae of Canada and Alaska (Lindroth 1961a: 102-104).

#### Taxonomic Note.

Goulet (1983: 447) regarded *Diacheila* as the sister-group to {*Blethisa* + *Elaphrus*}.

### Diacheila arctica amoena

(Faldermann, 1835)

Blethisa amoena Faldermann, 1835: 358. Type locality: «montibus Altaicis [Mongolia]» (original citation). Syntype(s) probably in ZILR.
Diachila subpolaris LeConte, 1863c: 2. Type locality: «Hudson’s Bay» (original citation). One syntype in CMNH (Lindroth 1954b: 121). Synonymy established by Lindroth (1954b: 122).
Diachila americana Motschulsky, 1864: 195. Type locality: «Amér[ique] arctique. Hudson Bay» (original citation). Syntype(s) location unknown (possibly in ZMMU though not listed in Keleinikova 1976). Synonymy established with the name *Diachila subpolaris* LeConte by Horn (1870a: 70).

#### Distribution.

This Holarctic subspecies is known in Asia from Kazakhstan, Mongolia, and eastern Siberia (Goulet 2003: 206) and in the Nearctic Region from a few localities in Alaska, Northwest Territories (Lindroth 1961a: 102), northern Alberta (Bourassa and Wood 2011: 144; Fort McMurray area, Gerald J. Hilchie pers. comm. 2009), and Labrador (Lindroth 1961a: 102). Fossil remnants, dated between 10,400 and 17,000 years B.P., have been unearthed in Cape Breton Island, Nova Scotia (Miller 1997: 250) and Iowa (Schwert 1992: 76).

#### Records.

**CAN**: AB, LB, NT **USA**: AK – **Holarctic**

#### Note.

The nominotypical subspecies is found in northern Europe and eastern Siberia.

**Figure 14.**
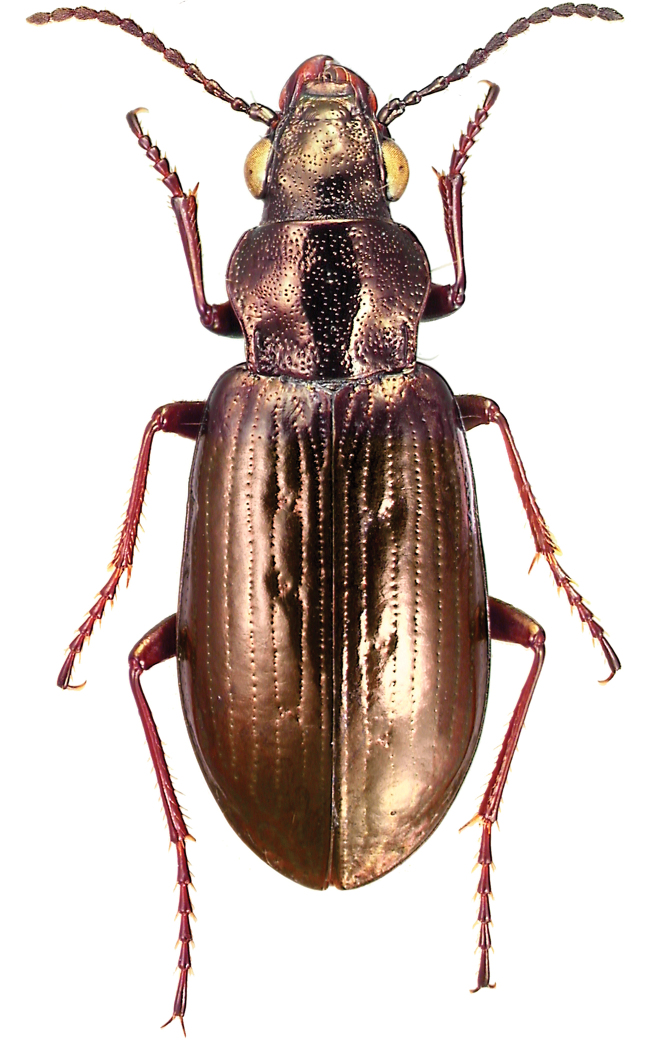
*Diacheila arctica amoena* (Faldermann). This subspecies is widely distributed over the arctic and subarctic regions of the Northern Hemisphere, ranging from Labrador to Kazakhstan, but populations seem to be highly localized. The German coleopterist Franz Faldermann found the adults of this taxon “pleasant” hence his scientific name *amoena*. The nominotypical subspecies ranges from the Nordic regions of Scandinavia to the Komi Republic in northern European Russia.

### Diacheila polita

(Faldermann, 1835)

Blethisa polita Faldermann, 1835: 359. Type locality: «montes Altaici [Mongolia]» (original citation). Holotype [by monotypy] probably in ZILR.
Nebria xiaoxinganensis Li and Liang [in Li], 1992: 28. Type locality: «M[oun]t Xiaoxinganlieng, Yichun, Heilongjiang Province [China]» (original citation). Holotype (♂) location unknown. Synonymy established by Ledoux et al. (2003: 80).

#### Distribution.

This Holarctic species is known from scattered localities in Norway, European Russia, Siberia, Mongolia (Goulet 2003: 206), and northeastern China (Li 1992: 30, as *Nebria xiaoxinganensis*) in the Palaearctic Region, and from Alaska to northwestern Northwest Territories [see Morgan and Morgan 1981: map 3] in the Nearctic Region. Fossil remnants of this species, believed to be 2.0-2.5 million years old, have been found in Greenland (Bennike and Böcher 1990: 336; Böcher 1995: 20); others, dated between about 12,000 and 21,500 years B.P., have been unearthed in Iowa and north-central Illinois (Baker et al. 1986: 96; Garry et al. 1990: 394; Schwert 1992: 76; Woodman et al. 1996: 17), northeastern Pennsylvania (Barnosky et al. 1988: 178), southern Ontario and southern Quebec (Morgan and Morgan 1981: 1107).

#### Records.

**CAN**: NT, YT **USA**: AK – **Holarctic**

### Genus Blethisa

Bonelli, 1810

Blethisa Bonelli, 1810: 48, Tabula Synoptica. Type species: *Carabus multipunctatus* Linnaeus, 1758 designated by Dejean (1826: 266). Etymology. Unknown [feminine].
Helobium Leach, 1815: 83. Type species: *Carabus multipunctatus* Linnaeus, 1758 by monotypy. Etymology. From the Greek *helos* (marsh, meadow) and *bios* (life) [neuter].
Rhaphiona Fischer von Waldheim [in Zoubkoff], 1829: 155. Type species: *Blethisa eschscholtzii* Zoubkoff, 1829 by monotypy. Synonymy established by Lindroth (1954a: 13). Etymology (original). From the Greek *rhaphion* (awl), alluding to the shape of the palpi (“*palpes subuliformes*”) of the adults [feminine].

#### Diversity.

Eight species in the arctic, subarctic, boreal, and temperate areas of North America (six species) and Eurasia (four species). Two species-group taxa are Holarctic (*Blethisa catenaria* and *Blethisa multipunctata aurata*).

#### Identification.

Lindroth (1954a) reviewed the species and subsequently treated all the North American species in his monograph of the Carabidae of Canada and Alaska (Lindroth 1961a: 104-108). Goulet and Smetana (1983) provided a key to all known species.

#### Faunistic Note.

*Blethisa eschscholtzii* Zoubkoff has been recorded from North American on the basis of a single specimen in USNM labeled “5 mi. E Sanderson, Texas” (Lindroth 1954b: 157). Obviously the specimen is mislabeled. The species is restricted to the region north of the Caspian Sea and the area around Lake Balkhash in southeastern Kazakhstan.

### Blethisa catenaria

Brown, 1944

Blethisa catenaria Brown, 1944: 4. Type locality: «near Fort Prince of Wales, Churchill, Man[itoba]» (original citation). Holotype (♀) in CNC [# 5412].

#### Distribution.

This species is found in northern European Russia and eastern Siberia in the Palaearctic Region (Goulet 2003: 206) and from western Alaska to the western shore of the Hudson Bay in northern Manitoba [see Nielsen et al. 1987: Fig. 17b; Morgan et al. 1986: Fig. 1]. Fossil remnants from the Pliocene or early Pleistocene have been unearthed in northwestern Greenland, Meighen Island, Ellesmere Island, and eastern Siberia (Böcher 1995: 20).

#### Records.

**CAN**: MB, NT, NU, YT **USA**: AK – **Holarctic**

### Blethisa hudsonica

Casey, 1924

Blethisa hudsonica Casey, 1924: 18. Type locality: «Edmonton, Alberta» (original citation). Lectotype (♂), designated by Lindroth (1975: 113), in USNM [# 46834].

#### Distribution.

This Nearctic species ranges from Newfoundland (Lindroth 1955a: 31) to westernmost Yukon Territory (Goulet et al. 2009: 33), south to southern British Columbia (Lindroth 1961a: 106), northwestern Montana (Russell 1968: 46; LaBonte and Johnson 1989: 170), northern Illinois (Blatchley 1910: 50), and Massachusetts (Middlesex County, MCZ, USNM) [see Morgan et al. 1986: Fig. 4]. The record from “Pennsylvania” (Bousquet and Larochelle 1993: 86, as *Blethisa multipunctata aurata*), based on several specimens labeled from Erie County in CMNH, is doubtful (Robert L. Davidson pers. comm. 2008); that from Circle in Alaska (Lindroth 1961a: 106) needs confirmation.

#### Records.

**FRA**: PM **CAN**: AB, BC, LB, MB, NB, NF, NS (CBI), NT, ON, PE, QC, SK, YT **USA**: IL, MA, ME, MI, MT, ND, NH, NY, VT, WI [AK, PA]

#### Note.

This species has passed under the name *Blethisa multipunctata* (Linnaeus, 1758) or *Blethisa multipunctata aurata* Fischer von Waldheim, 1828 in the North American literature.

### Blethisa julii

LeConte, 1863

Blethisa julii LeConte, 1863c: 2. Type locality: «Nova Scotia» (original citation), herein restricted to Cheticamp, Cape Breton Island (see Lindroth 1954c: 300). Syntype(s) in MCZ [# 5452]. Etymology. This species was named after Julius Ulke [1833-1910], brother of Henry Ulke (see *Bembidion ulkei*), a photographer and also a beetle collector. In the 1860s, Julius and Henry lived on Tenth Street in Washington across the Ford’s Theater and had a portrait studio on Pennsylvania Avenue. Julius took the historic photograph of the room in which Abraham Lincoln died on 15 April 1865 a few minutes after the president’s body was removed.

#### Distribution.

The range of this species extends from Newfoundland (Lindroth 1955a: 30) to the foothills of the Rocky Mountains in Alberta (Lindroth 1961a: 107-108), south to northern New York and New England (Lindroth 1961a: 107) [see Morgan et al. 1986: Fig. 3]. The record from Michigan (Bousquet and Larochelle 1993: 86), based on a specimen labeled from Lake Superior in CMNH, needs confirmation.

#### Records.

**CAN**: AB, MB, NB, NF, NS (CBI), NT, ON, QC, SK **USA**: ME, NH, NY, VT [MI]

### Blethisa multipunctata aurata

Fischer von Waldheim, 1828

Blethisa aurata Fischer von Waldheim, 1828: 262. Type locality: «Kamtschatka [Russia]» (original citation). Syntype(s) in ZMH (Lindroth 1961a: 106), MHNP (collection Dejean), and SMTD (Grämer 1960: 102).
Blethisa inexspectata Goulet and Smetana, 1983: 551. Type locality: «Prudhoe Bay R[oa]d, Bonanza Cr[eek] (900’), Alaska» (original citation). Holotype (♂) in CNC [# 15404]. Synonymy established by Goulet et al. (2009: 28).

#### Distribution.

This Holarctic subspecies is known from Hokkaidō in Japan and the Russian Far Eastern Region, including Sakhalin and Kamchatka, in the Palaearctic Region and from Alaska, as far south as Anchorage, and northwestern Northwest Territories in the Nearctic Region (Goulet et al. 2009: 33).

#### Records.

**CAN**: NT **USA**: AK – **Holarctic**

#### Note.

The nominotypical subspecies ranges from the Atlantic Coast in Europe to the Lake Baikal region in Siberia (Goulet et al. 2009: 33).

### Blethisa oregonensis

LeConte, 1853

Blethisa oregonensis LeConte, 1853c: 401. Type locality: «Oregon» (original citation), herein restricted to 7.5 km NNW of Monroe, Finley National Wildlife Refuge, Benton County (see LaBonte and Johnson 1989: 171). Holotype [by monotypy] (♀) in MCZ [# 641].
Blethisa acutangula Chaudoir, 1861b: 524. Type locality: «Orégon» (original citation). Holotype [by monotypy] (♂) in MHNP. Synonymy established by LeConte (1866: 78).
Blethisa columbica Casey, 1909: 277. Type locality: «British Columbia» (original citation). Lectotype (♂), designated by Lindroth (1975: 113), in USNM [# 46833]. Synonymy established by Hatch (1949b: 114), confirmed by Lindroth (1954b: 121).

#### Distribution.

This species is found mainly west of the Cascade Range from southwestern British Columbia, including Vancouver Island, to south-central Oregon (LaBonte and Johnson 1989: 171).

#### Records.

**CAN**: BC (VCI) **USA**: OR, WA

### Blethisa quadricollis

Haldeman, 1847

Blethisa quadricollis Haldeman, 1847: 149. Type locality: «southern shore of Lake Superior» (original citation), herein restricted to Marquette, Marquette County, Michigan (see Hubbard and Schwarz 1878: 627). Syntype(s) presumably lost.
Blethisa americana T.W. Harris [in Scudder], 1869: 211. Type locality not stated. Holotype [by monotypy] probably lost. Synonymy established by LeConte (in Scudder 1869: 212).

#### Distribution.

The range of this species extends from Newfoundland (Lindroth 1955a: 31-32) to southern Alaska, south to central British Columbia (Lindroth 1961a: 106), northwestern Montana (LaBonte and Johnson 1989: 170), northern Indiana (Blatchley 1910: 50), and New Jersey (Smith 1890: 73) [see Morgan et al. 1986: Fig. 2; Ball and Currie 1997: Fig. 3].

#### Records.

**CAN**: AB, BC, LB, MB, NB, NF, NS (CBI), NT, ON, PE, QC, SK, YT **USA**: AK, IL, IN, MA, ME, MI, MN, MT, NH, NJ, NY, OH, PA, RI, VT, WI

### Genus Elaphrus

Fabricius, 1775

Elaphrus Fabricius, 1775: 227. Type species: *Cicindela riparia* Linnaeus, 1758 designated by Latreille (1810: 425). Etymology. From the Greek *elaphros* (nimble), probably alluding to the agility of the adults in the field [masculine].

#### Diversity.

Thirty-nine species in the arctic, subarctic, boreal, and temperate areas of North America (19 species) and Eurasia (24 species). These species are arrayed in five subgenera, all but *Sinoelaphrus* Shi and Liang (one species in northern China) represented in North America. Four species are Holarctic.

#### Identification.

Goulet (1983) revised the species then known and provided a key for their identification.

### Subgenus Arctelaphrus

Semenov, 1926

Arctelaphrus Semenov, 1926: 39. Type species: *Elaphrus lapponicus* Gyllenhal, 1810 by original designation. Etymology. From the Greek *arctos* (north) and the generic name *Elaphrus* [*q.v*.] [masculine].

#### Diversity.

One species in the subarctic regions of North America, Asia, and Europe.

#### Taxonomic Note.

Goulet (1983) concluded from his phylogenetic analysis using adult and larval characters that this subgenus was the most basal lineage of *Elaphrus*.

### Elaphrus lapponicus lapponicus

Gyllenhal, 1810

Elaphrus lapponicus Gyllenhal, 1810: 8. Type locality: «Lapponia» (original citation), restricted to «Abisko, Swed[en]» by Lindroth (1961a: 111). Syntype(s) in GNM (Lindroth 1961a: 111).
Elaphrus elongatus Fischer von Waldheim, 1828: 266. Type locality: «Kamtschatka [Russia]» (original citation). Syntype(s) in ZMH (Lindroth 1961a: 111) and SMTD (Grämer 1960: 102). Synonymy established by Dejean (1831: 587), confirmed by Lindroth (1961a: 111).
Elaphrus obscurior Kirby, 1837: 63. Type locality: «Lat. 65° [= apparently region of Great Bear Lake, Northwest Territories]» (original citation). Holotype [by monotypy] in BMNH (Lindroth 1953b: 177). Synonymy established by Lindroth (1953b: 177).

#### Distribution.

This Holarctic subspecies is widely distributed in the subarctic areas. In the Palaearctic Region, it ranges from the British Isles to the Kamchatka Peninsula, and in the Nearctic Region from Alaska to Labrador [see Goulet 1983: Fig. 157]. Fossil remnants of this species, dated between 10,100 and 20,700 years B.P., have been unearthed in southern Quebec (Mott et al. 1981: 146), southern Ontario (Morgan and Morgan 1981: 1107), Illinois (Schwert 1992: 76), and southeastern Iowa (Baker et al. 1986: 96); others, believed to be 2.0-2.5 million years old, have also been found in Greenland and Meighen Island (Bennike and Böcher 1990: 336; Böcher 1995: 22).

#### Records.

**CAN**: AB, BC, LB, MB, NT, NU, ON, QC, SK, YT **USA**: AK – **Holarctic**

### Elaphrus lapponicus obliteratus

Mannerheim, 1853

Elaphrus obliteratus Mannerheim, 1853: 117. Type locality: «ad portum Pauli insulae Kadjak [= Port Harbour, Kodiak Island, Alaska]» (original citation). Lectotype (♂), designated by Lindroth (1961a: 111), in ZMH.

#### Distribution.

This subspecies is known only from a few localities on Kodiak Island, Alaska (Goulet 1983: 244).

#### Records.

**USA**: AK

### Subgenus Neoelaphrus

Hatch, 1951

Neoelaphrus Hatch, 1951: 113. Type species: *Elaphrus uliginosus* Fabricius, 1792 by original designation. Etymology. From the Greek prefix *neo*- (new) and the generic name *Elaphrus* [*q.v*.] [masculine].

#### Diversity.

Fourteen species in the Nearctic (six species) and Palaearctic (eight species) Regions.

#### Taxonomic Note.

Goulet’s (1983) phylogenetic analysis based on adult and larval characters suggests that *Neoelaphrus* is the sister-group to {*Elaphrus* s.str. + *Elaphroterus*}.

#### Faunistic Note.

Fossil remnants of *Elaphrus sibiricus* Motschulsky, probably about 2.0-2.5 million years B.P., have been unearthed from northwestern Yukon Territory (Elias and Matthews 2002: 914) and northwestern Greenland (Böcher 1995: 22). The species is currently found in Siberia, Japan, Mongolia, and Inner Mongolia in China (Goulet 2003: 207).

### [clairvillei group]

### Elaphrus clairvillei

Kirby, 1837

Elaphrus clairvillei Kirby, 1837: 61 (as *clairvillii*). Type locality: «from New York to Cumberland-house» (original citation), restricted to «Nipigon, Ont[ario]» by Lindroth (1961a: 112). Holotype [by monotypy] in BMNH (Lindroth 1953b: 176). Etymology. The specific name honors the Swiss naturalist Joseph Phillippe de Clairville [1742-1830]. Born in France, Clairville studied medicine and natural history at Montpellier and in 1782 moved to Winterthur, near Zurich. During the French occupation he was forced to leave Switzerland and fled to Erlangen, Germany. His entomological interests were mainly in Odonata, Diptera, and Coleoptera and his collection of beetles is now at the Natural History Museum in Basel. Note. The incorrect subsequent spelling *clairvillei* is in prevailing usage and attributed to the publication of the original spelling; therefore it is deemed to be the correct original spelling (ICZN 1999: Article 33.3.1).
Elaphrus politus LeConte, 1850: 209. Type locality: «Maple Island [Ontario]» (original citation). Holotype [by monotypy] (♀) in MCZ [# 171]. Synonymy established by LeConte (1870: 396), confirmed by Goulet (1983: 271).
Elaphrus clairvillei frosti Hippisley, 1922: 64. Type locality: «some three and one-half miles southwest of Terrace as the crow flies, or six miles by the road [British Columbia]» (original citation). Holotype probably in MCZ (collection Frost). Synonymy established by Lindroth (1961a: 112).
Elaphrus torreyensis Tanner, 1942: 137. Type locality: «Torrey, Wayne County, Utah» (original citation). Holotype (♀) in BYUC (Shawn M. Clark pers. comm. 2007). Synonymy established by Lindroth (1961a: 112). Note. Tanner (1942: 138) listed the type locality as “Torrey, Wayne County, Utah” but mistakenly wrote a few lines below that the holotype and a paratype [of *Elaphrus torreyensis*] were “taken at Escalante, Garfield Co[unty].”

#### Distribution.

This species extends throughout the temperate and boreal regions of North America from Newfoundland to Alaska, south to northern California along the west coast, to the White Mountains in eastern Arizona and to central New Mexico along the Rocky Mountains, to west-central Nebraska (Arthur County, Foster F. Purrington pers. comm. 2010), and to New Jersey along the east coast [see Goulet 1983: Fig. 160]. The record from east-central Missouri (Summers 1873: 132) needs confirmation.

#### Records.

**CAN**: AB, BC (QCI, VCI), LB, MB, NB, NF, NS (CBI), NT, ON, PE, QC, SK, YT **USA**: AK, AZ, CA, CO, CT, ID, MA, ME, MI, MN, MT, NE, NH, NJ, NM, NV, NY, OH, OR, PA, RI, SD, UT, VT, WA, WI, WY [MO]

### Elaphrus laevigatus

LeConte, 1852

Elaphrus laevigatus LeConte, 1852a: 200. Type locality: «San Francisco [San Francisco County, California]» (original citation). Three syntypes in MCZ [# 660].
Elaphrus politus Casey, 1897: 345 [primary homonym of *Elaphrus politus* LeConte, 1850]. Type locality: «San Francisco [San Francisco County], California» (original citation). Lectotype (♀), designated by Lindroth (1975: 113), in USNM [# 46822]. Synonymy established by Van Dyke (1925: 113), confirmed by Goulet (1983: 280).
Elaphrus caseyi Leng, 1919b: 203. Replacement name for *Elaphrus politus* Casey, 1897.

#### Distribution.

This species is known from western Nevada and California, from the Oregon border to the Los Angeles area [see Goulet 1983: Fig. 161].

#### Records.

**USA**: CA, NV

### Elaphrus olivaceus

LeConte, 1863

Elaphrus olivaceus LeConte, 1863c: 1. Type locality: «Catskill Mountains, New York» (original citation). One syntype in CMNH (collection Ulke).

#### Distribution.

This species ranges from the west coast of Newfoundland to southern British Columbia, south to southeastern Utah, central Colorado, central Illinois, and New Jersey [see Goulet 1983: Fig. 161]. The species is known from only one locality west of the Rocky Mountains, in southern British Columbia.

#### Records.

**CAN**: AB, BC, MB, NB, NF, NS (CBI), NT, ON, PE, QC, SK **USA**: CO, CT, IL, IN, MA, ME, MI, MN, MT, ND, NE, NH, NJ, NY, RI, SD, UT, VT, WI, WY

### [fuliginosus group]

### Elaphrus cicatricosus

LeConte, 1847

Elaphrus cicatricosus LeConte, 1847: 448. Type locality: «NovEboraci [= New York]» (original citation). Holotype [by monotypy] (♀) in MCZ [# 658].
Elaphrus rhodeanus Casey, 1924: 17. Type locality: «Boston Neck [Washington County], Rhode Island» (original citation). Lectotype (♀), designated by Lindroth (1975: 113), in USNM [# 46825]. Synonymy established by Lindroth (1961a: 114).

#### Distribution.

This species ranges from Maine, southern Quebec, and Michigan south to northern Mississippi (Pontotoc County, Drew A. Hildebrandt pers. comm. 2009) and North Carolina (Northampton County, CNC) [see Goulet 1983: Fig. 159]. The record from Georgia (Fattig 1949: 11) needs confirmation; those from eastern Iowa (Wickham 1911b: 5; King 1914: 320) are probably in error.

#### Records.

**CAN**: QC **USA**: CT, DE, MA, MD, ME, MI, MS, NC, NH, NJ, NY, OH, PA, RI, TN, VA, VT, WV [GA]

### Elaphrus fuliginosus

Say, 1830

Elaphrus fuliginosus Say, 1830b: (1) [3]. Type locality: «Rumney [Grafton County], N[ew] H[ampshire]» (neotype label). Neotype (♂), designated by Lindroth and Freitag (1969: 332), in MCZ [# 33086]. Note. «Pennsylvania» was the area originally cited by Say (1830b: (1) [3]).

#### Distribution.

This species ranges from Quebec to southern Manitoba, south to Nebraska and Maryland [see Goulet 1983: Fig. 158]. It is also known from one unexpected locality in central Alberta (Bousquet 1987a: 112), which suggests that the species is more widely distributed at least in the northern part of its range.

#### Records.

**CAN**: AB, MB, ON, QC **USA**: CT, IA, IL, IN, MA, MD, ME, MI, MN, ND, NE, NH, NJ, NY, PA, RI, SD, VT, WI

**Figure 15.**
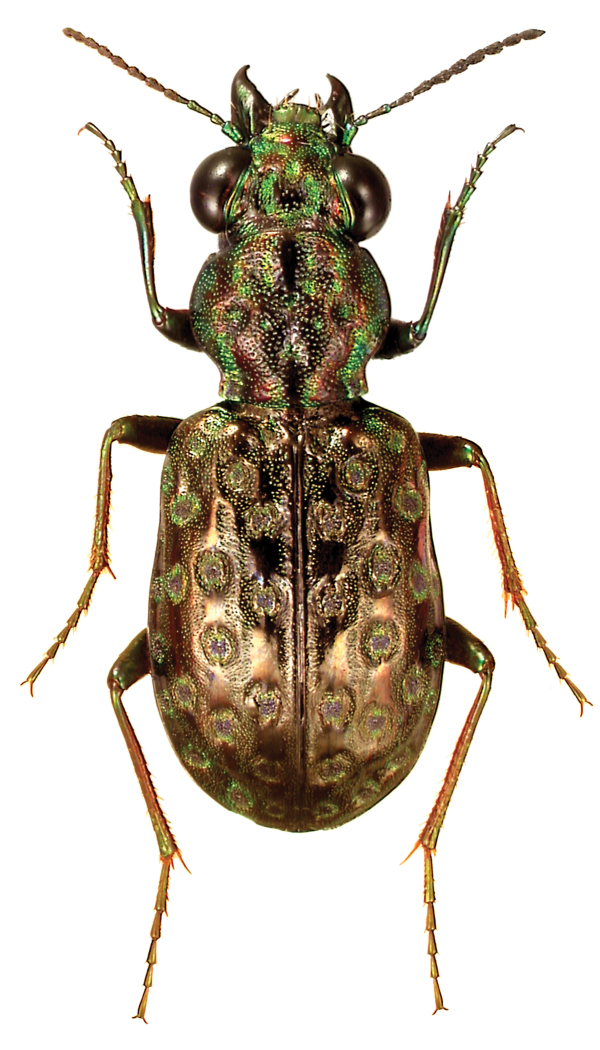
*Elaphrus fuliginosus* Say. Why the great naturalist Thomas Say gave this eastern species the name *fuliginosus* (sooty) is not evident. It may refer to the mirrors on the elytra which gives the impression that the animals are dirty. Members of *Elaphrus* have the ability to produce stridulating chirps by rubbing rows of bristles on the dorsal surface of the abdomen against two areas of parallel ridges on the ventral surface of the elytra. The sound is produced when the beetle is under stress.

### Elaphrus lindrothi

Goulet, 1983

Elaphrus lindrothi Goulet, 1983: 264. Type locality: «3 mi[les] n[orth] Pomona, Jackson Co[unty], Ill[inois]» (original citation). Holotype (♂) in CNC [# 18010].

#### Distribution.

This species is known from two areas, Maryland and southeastern Virginia (Surry County, CNC), and southern Indiana, southern Illinois, and southern Tennessee (Marion County, CMNH) [see Goulet 1983: Fig. 159]. The apparent gap is probably due to inadequate samplings.

#### Records.

**USA**: IL, IN, MD, TN, VA

### Subgenus Elaphrus

Fabricius, 1775

Elaphrus Fabricius, 1775: 227. Type species: *Cicindela riparia* Linnaeus, 1758 designated by Latreille (1810: 425).
Trichelaphrus Semenov, 1926: 39. Type species: *Cicindela riparia* Linnaeus, 1758 by original designation. Etymology. From the Greek *trichos* (hair) and the generic name *Elaphrus* [*q.v*.], alluding to the accessory setae on the abdominal sterna (“*sterno semper plus minusve piloso*”) of the adult [masculine].

#### Diversity.

Eighteen species in North America (ten species) and Eurasia (ten species). Two species (*Elaphrus trossulus* and *Elaphrus tuberculatus*) are Holarctic.

### Elaphrus americanus americanus

Dejean, 1831

Elaphrus americanus Dejean, 1831: 588. Type locality: «Amérique septentrionale» (original citation), restricted to «Great Bear Lake, N[orth] W[est] Terr[itories]» by Lindroth (1961a: 115). Holotype [by monotypy] (♀) in MHNP (Lindroth 1955b: 12).
Elaphrus intermedius Kirby, 1837: 62. Type locality: «Gr[eat] Bear L[ake] [Northwest Territories]» (syntype label). Three syntypes in BMNH (Lindroth 1953b: 176). Synonymy established by Lindroth (1961a: 115).
Elaphrus punctatissimus LeConte, 1850: 210. Type locality: «Sault [= Sault Sainte Marie, Michigan according to Lindroth (1961a: 115)]» (original citation). Nine syntypes in MCZ [# 661]. Synonymy established by LeConte (1873b: 321), confirmed by Goulet (1983: 307).
Elaphrus sinuatus LeConte, 1850: 210. Type locality: «Pic [north shore of Lake Superior, Ontario]» (original citation). Two syntypes [2 originally cited] in MCZ [# 663]. Synonymy established, under the name *Elaphrus punctatissimus* LeConte, by LeConte (1853c: 402), confirmed by Goulet (1983: 307).
Elaphrus gratiosus Mannerheim, 1853: 118. Type locality: «as ostia fl[umen] Kaktnu [= Kenai River] peninsulae Kenai [Alaska]» (original citation). Lectotype (♂), designated by Lindroth (1961a: 115), in ZMH. Synonymy established, under the name *Elaphrus punctatissimus* LeConte, by Motschulsky (1855b: 79), confirmed by Lindroth (1961a: 115).
Elaphrus bituberosus Casey, 1924: 17. Type locality: «Terrace, British Columbia» (original citation). Lectotype (♂), designated by Lindroth (1975: 113), in USNM [# 46823]. Synonymy established by Lindroth (1961a: 115).

#### Distribution.

This subspecies ranges from Newfoundland to southwestern Alaska, south to central British Columbia, southeastern Alberta, northern Minnesota, north-central Ohio (Purrington and Stanton 1996: 44), northern New York, and Maine [see Goulet and Baum 1982: Fig. 1]. The records from “California,” “Wyoming,” “South Dakota,” “Iowa” (see Bousquet and Larochelle 1993: 87), and Missouri (Anonymous 2007) are likely based on mislabeled specimens or are in error.

#### Records.

**CAN**: AB, BC, LB, MB, NB, NF, NS (CBI), NT, ON, PE, QC, SK, YT **USA**: AK, ME, MI, MN, NH, NY, OH, WI

### Elaphrus americanus sylvanus

Goulet, 1982

Elaphrus americanus sylvanus Goulet [in Goulet and Baum], 1982: 2271. Type locality: «16 mi[les] N[orth] of Powers, Coos Co[unty], Oregon» (original citation). Holotype (♂) in CNC [# 18011].

#### Distribution.

This subspecies ranges from the Queen Charlotte Islands (Kavanaugh 1992: 58) to southwestern Alberta, south to central Colorado, central Idaho, and southern Oregon (Goulet and Baum 1982: 2272; Fig. 1).

#### Records.

**CAN**: AB, BC (QCI, VCI) **USA**: CO, ID, OR, WA

### Elaphrus californicus

Mannerheim, 1843

Elaphrus californicus Mannerheim, 1843: 190. Type locality: «California» (original citation), herein restricted to Quincy, Plumas County (see Goulet 1983: 302). Lectotype (♀), designated by Lindroth (1961a: 118), in ZMH.
Elaphrus similis LeConte, 1847: 449. Type locality: «Long’s Peak [Boulder County, Colorado]» (original citation). One syntype in MCZ [# 662]. Synonymy established by LeConte (1863b: 2), confirmed by Goulet (1983: 299).
Elaphrus hesperius Casey, 1920: 138. Type locality: «Cal. [with a red dot on the “a”] [= Gualala, Mendocino County, California]» (lectotype label). Lectotype (♀), designated by Lindroth (1975: 113), in USNM [# 46829]. Synonymy established by Lindroth (1961a: 118).

#### Distribution.

This species is widely distributed from central Alaska to Nova Scotia, south to northern Virginia, central Missouri, northern New Mexico, and southern California near the Mexican border [see Goulet 1983: Fig. 172]. The records from “North Carolina,” northeastern Florida, “Louisiana,” and “Texas” [see Goulet 1983: Fig. 172] need confirmation.

#### Records.

**CAN**: AB, BC (VCI), MB, NB, NS, NT, ON, PE, QC, SK **USA**: AK, CA, CO, CT, DC, IA, ID, IL, IN, KS, KY, MA, MD, ME, MI, MN, MO, MT, ND, NE, NH, NJ, NM, NV, NY, OH, OR, PA, SD, UT, VA, VT, WA, WI, WV, WY [FL, LA, NC, TX]

### Elaphrus finitimus

Casey, 1920

Elaphrus finitimus Casey, 1920: 137. Type locality: «California» (original citation). One syntype in USNM [# 46827].

#### Distribution.

This species extends from western Montana to southern Oregon, south to southernmost California, central Arizona, and southern Colorado [see Goulet 1983: Fig. 170].

#### Records.

**USA**: AZ, CA, CO, ID, MT, NV, OR, UT

### Elaphrus lecontei

Crotch, 1876

Elaphrus lecontei Crotch [in Horn], 1876c: 246. Type locality: «Long’s Peak [Boulder County, Colorado]» (original citation for *Elaphrus intermedius* Kirby *sensu* LeConte, 1847). Two syntypes in MCZ [# 170]. Note. This name was proposed for *Elaphrus intermedius* Kirby, 1837 *sensu* LeConte (1847: 449).
Elaphrus devinctus Casey, 1920: 139. Type locality: «Wray [Yuma County], Colorado» (original citation). Lectotype (♀), designated by Lindroth (1975: 113), in USNM [# 46826]. Synonymy established by Lindroth (1961a: 114).
Elaphrus spissicornis Casey, 1924: 18. Type locality: «Parowan (6000 ft.) [Iron County], Utah» (original citation). Lectotype (♀), designated by Lindroth (1975: 113), in USNM [# 46824]. Synonymy established by Lindroth (1961a: 114).

#### Distribution.

This species ranges from northwestern Quebec along the James Bay (Chisasibi, Serge Laplante pers. comm. 2011) to central British Columbia, north to the Great Slave Lake in Northwest Territories, south to southern California, southern Arizona, and central Kansas [see Goulet 1983: Fig. 169]. Fossil remnants from a Plio-Pleistocene sequence have been unearthed in northwestern Greenland (Böcher 1995: 23).

#### Records.

**CAN**: AB, BC, MB, NT, ON, QC, SK **USA**: AZ, CA, CO, ID, KS, MN, MT, ND, NE, NV, OR, SD, UT, WA, WY

### Elaphrus marginicollis

Goulet, 1983

Elaphrus marginicollis Goulet, 1983: 288. Type locality: «Jack’s Gulch, Roosevelt N[ational] F[orest], Colorado» (original citation). Holotype (♂) in USNM.

#### Distribution.

This species is known from a few localities in southeastern Washington, northern California, southeastern Wyoming, and Colorado [see Goulet 1983: Fig. 168].

#### Records.

**USA**: CA, CO, WA, WY

### Elaphrus mimus

Goulet, 1983

Elaphrus mimus Goulet, 1983: 290. Type locality: «Angwin [Napa County], Cal[ifornia]» (original citation). Holotype (♂) in CAS [# 16493].

#### Distribution.

This species is known only from the original two specimens collected at the type locality.

#### Records.

**USA**: CA

### Elaphrus ruscarius

Say, 1830

Elaphrus ruscarius Say, 1830b: (1) [3]. Type locality: «Columbia [Lancaster County], P[ennsylvani]a» (neotype label). Neotype (♂), designated by Lindroth and Freitag (1969: 332), in MCZ [# 33085]. Note. «Pennsylvania, Mississippi, Arkansas, Missouri and the Rocky Mountains» were the areas originally cited by Say (1830b: (1) [3]).
Elaphrus texanus Casey, 1924: 17. Type locality: «Galveston [Galveston County], Texas» (original citation). Lectotype (♀), designated by Lindroth (1975: 113), in USNM [# 46828]. Synonymy established by Lindroth (1961a: 119).

#### Distribution.

This species ranges from southern Quebec to northern Minnesota, south to eastern Texas (Casey, 1924: 17, as *Elaphrus texanus*), east-central Louisiana (Allen 1965: 61), southwestern Alabama (Löding 1945: 11), and northern Florida (Peck and Thomas 1998: 16) [see Goulet 1983: Fig. 168].

#### Records.

**CAN**: ON, QC **USA**: AL, AR, CT, DC, DE, FL, GA, IA, IL, IN, KS, KY, LA, MA, MD, ME, MI, MN, MO, MS, NC, NE, NH, NJ, NY, OH, OK, PA, RI, SC, TN, TX, VA, VT, WI, WV

### Elaphrus trossulus

Semenov, 1904

Elaphrus trossulus Semenov, 1904: 21. Type locality: «Mongoliâ occid[entalis]: inter Ulan-daban et opp. Kobdo; syst. fl. Kobdo; syst. fl. Sansai; ad lac. Shar-nur» (original citation). Syntype(s) probably in ZILR.
Elaphrus parviceps Van Dyke, 1925: 112. Type locality: «Teller, Seward Peninsula, Alaska» (original citation). Holotype (♀) in CAS [# 1617]. Synonymy established by Goulet and Smetana (1997: 204).

#### Distribution.

This species is found in the montane regions of northern Mongolia and neighbouring Russia west and south of Lake Baikal, in northeastern Siberia, and in the arctic regions from the Commander Islands in the Bering Sea to the eastern shore of James Bay in Quebec (Morgan and Pilny 1997: 146) [see Goulet 1983: Fig. 171]. Fossil remnants of this species, dated between 10,100 and 11,050 years old, have been found in southern Quebec (Mott et al. 1981: 146); others, older than 33,000 years B.P., has been unearthed in southwestern Ontario (Warner et al. 1988: 37).

#### Records.

**CAN**: MB, NT, NU, ON, QC, YT **USA**: AK – **Holarctic**

#### Note.

According to Shilenkov (in Kryzhanovskij et al. 1995: 61), the type material of *Elaphrus trossulus* Semenov is conspecific with members of *Elaphrus tuberculatus* Mäklin. However, Goulet and Smetana (1997: 203) concluded that adults of *Elaphrus trossulus* and *Elaphrus parviceps* Van Dyke “cannot be separated.” Lindroth (1961a: 116) regarded *Elaphrus parviceps* as a junior synonym of *Elaphrus riparius* (Linnaeus), a species restricted to the Palaearctic Region according to Goulet (1983: 313).

### Elaphrus tuberculatus

Mäklin, 1878

Elaphrus tuberculatus Mäklin, 1878: 16. Type locality: «Briochowska öarne (70°39' n. br.) [= Brochowsky Island] inom Jenisej floden [Russia]» (original citation). Holotype [by monotypy] location unknown (possibly in ZMH).
Elaphrus latipennis J.R. Sahlberg, 1880: 10. Type locality: «prope vicum Dudinka [Taimyr Autonomous Okrug, Russia]» (original citation). Holotype [by monotypy] location unknown (possibly in ZMH). Synonymy established by Semenov (1910: 433).
Elaphrus latipennis var. *orientalis* Semenov, 1904: 20. Type locality: «Bulun [Yakutia, Russia]» (original citation). Syntype(s) [2 originally cited] location unknown (possibly in ZILR). Synonymy established by Goulet (1983: 316).
Elaphrus tumidiceps Munster, 1924: 288. Type locality: «Lakselv in Porsanger Finmarkiae [Norway]» (original citation). Holotype (♂) in ZMUO (see Lindroth 1939a: 62). Synonymy established by Bänninger (1932: 184).

#### Distribution.

This Holarctic species ranges from northern Scandinavia to eastern Siberia, and from Alaska to the Mackenzie River in the Northwest Territories [see Goulet 1983: Fig. 171]. Fossil remnants of this species, believed to be 2.0-2.5 million years old, have been found in Greenland (Bennike and Böcher 1990: 336; Böcher 1995: 23).

#### Records.

**CAN**: NT, YT **USA**: AK – **Holarctic**

### Elaphrus viridis

Horn, 1878

Elaphrus viridis G.H. Horn, 1878b: 52 [primary homonym of *Elaphrus riparius viridis* Letzner, 1849] [potential *nomen protectum*]. Type locality: «California» (original citation), herein restricted to 9.5 miles south of Dixon, Solano County (see Goulet 1983: 292). Holotype [by monotypy] (♀) in MCZ [# 34043]. Note. Although it is obvious that infrasubspecific rank was meant for *Elaphrus riparius viridis* and many other names in Letzner’s work (1849), the fact that Csiki (1927: 420) treated it as a senior homonym makes the name subspecific from the date of its establishment (ICZN 1999: Article 45.6.4.1). However since the reversal of precedence (ICZN 1999: Article 23.9) could probably be applied to this case, I believe it is essential to preserve the current name of this endangered species.
Elaphrus horni Csiki, 1927: 420. Replacement name for *Elaphrus viridis* Horn, 1878.

#### Distribution.

This species is known from a small area in Solano County, California [see Goulet 1983: Fig. 168].

#### Records.

**USA**: CA

#### Note.

This species, also known under the vernacular name “Delta Green Ground Beetle”, is considered an endangered species by the World Wildlife Fund and listed on the IUCN Red List of Threatened Species.

### Subgenus Elaphroterus

Semenov, 1896

Elaphroterus Semenov, 1896: 309. Type species: *Elaphrus aureus* Müller, 1821 designated by Semenov (1926: 39). Etymology. Unknown [masculine].
Elaphrotatus Semenov, 1896: 308. Type species: *Elaphrus punctatus* Motschulsky, 1844 designated by Semenov (1926: 39). Synonymy established by Goulet (1983: 322).

#### Diversity.

Five species in northern North America (two species) and Eurasia (four species). One species (*Elaphroterus angusticollis*) is Holarctic.

### Elaphrus angusticollis angusticollis

Sahlberg, 1844

Elaphrus angusticollis R.F. Sahlberg, 1844: 20. Type locality: «fluminis Ochotae [= River Ochota, near Okhotsk, Khabarovsk Kray, Siberia, Russia]» (original citation). One syntype in ZMH (Silfverberg 1987: 12).
Elaphrus angustus Chaudoir, 1850b: 161. Type locality: «Sibérie orientale» (original citation). Syntype(s) in MHNP. Synonymy established by Palmén (1944: 24).

#### Distribution.

This Holarctic subspecies ranges from the Lena River in eastern Siberia to the Bering Sea Coast, and from Alaska to the Mackenzie River in Northwest Territories [see Goulet 1983: Fig. 173]. Fossil remnants from a Plio-Pleistocene sequence have been unearthed in northwestern Greenland (Böcher 1995: 23).

#### Records.

**CAN**: NT, YT **USA**: AK – **Holarctic**

#### Note.

The subspecies *Elaphrus angusticollis longicollis* Sahlberg occurs in the Palaearctic Region.

### Elaphrus purpurans

Hausen, 1891

Elaphrus pallipes G.H. Horn, 1878b: 51 [primary homonym of *Elaphrus pallipes* Duftschmid, 1812]. Type locality: «Oregon and British Columbia» (original citation), restricted to «Oregon» by Lindroth (1961a: 119). Syntype(s) in MCZ [# 8126]. Note. The specimen marked as type in MCZ is labeled «B[ritish] Col[umbia]».
Elaphrus pallipes var. *purpurans* Hausen, 1891a: 251. Type locality: «British Columbia» (original citation). Holotype in LMMC (Goulet and Smetana 1997: 218). Synonymy established (as aberration) by Csiki (1927: 424).

#### Distribution.

This species ranges from central Alaska to the Mackenzie River in Northwest Territories, south to southeastern Alberta, central Idaho, and central California along the west coast [see Goulet 1983: Fig. 173].

#### Records.

**CAN**: AB, BC, NT, YT **USA**: AK, CA, ID, MT, OR, WA

### Subfamily OMOPHRONINAE

Bonelli, 1810

Omophronii Bonelli, 1810: Tabula Synoptica. Type genus: *Omophron* Latreille, 1802.

#### Diversity.

This subfamily includes a single tribe.

### Tribe Omophronini

Bonelli, 1810

Omophronii Bonelli, 1810: Tabula Synoptica. Type genus: *Omophron* Latreille, 1802.
Scolyti Motschulsky, 1850a: 91. Type genus: *Scolytus* Fabricius, 1790 (= *Omophron* Latreille, 1802).
Epactiini Fauvel, 1888: 1. Type genus: *Epactius* Schneider, 1791 (= *Omophron* Latreille, 1802).

#### Diversity.

This tribe includes a single genus.

### Genus Omophron

Latreille, 1802

Scolytus Fabricius, 1790: 221 [junior homonym of *Scolytus* Geoffroy, 1762]. Type species: *Carabus limbatus* Fabricius, 1777 designated by Latreille (1810: 426). Etymology. Unknown [masculine]. Note. Latreille’s designation was intended for *Omophron* Latreille but since *Omophron* is a replacement name for *Scolytus* Fabricius, both have the same type species and the type fixation for either applies also to the other (ICZN 1999: Article 67.8).
Omophron Latreille, 1802: 89. Replacement name for *Scolytus* Fabricius, 1790. Etymology. From the Greek *omophron* (merciless, savage) [neuter, see Allen and Duff (1992: 85)].

#### Diversity.

About 70 species in the Nearctic (11 species), Neotropical (six species in Middle America), Oriental (16 species), Palaearctic (16 species), and Afrotropical (20 species) Regions. The species are arrayed in two subgenera: *Omophron* s.str. (about 60 species) and *Phrator* Semenov (eight species in the Mediterranean region and Africa).

### Subgenus Omophron

Latreille, 1802

Scolytus Fabricius, 1790: 221 [junior homonym of *Scolytus* Geoffroy, 1762]. Type species: *Carabus limbatus* Fabricius, 1777 designated by Latreille (1810: 426).
Epactius Schneider [in Fabricius], 1791: 23. Replacement name for *Scolytus* Fabricius, 1790. Etymology. From the Greek *epactios* (on the shore) [masculine].
Lithophilus Schneider [in Fabricius], 1791: 23. Replacement name for *Scolytus* Fabricius, 1790. Etymology. From the Greek *lithos* (stone) and *philos* (beloved) [masculine].
Omophron Latreille, 1802: 89. Replacement name for *Scolytus* Fabricius, 1790. Note. See Bousquet and Larochelle (1993: 89, footnote) for priority of *Omophron* over both Schneider’s names.
Scolyttus Billberg, 1820: 24. Unjustified emendation of *Scolytus* Fabricius, 1790.
Homophron Fischer von Waldheim, 1828: 255. Unjustified emendation of *Omophron* Latreille, 1802.
Homophron Semenov, 1922: 41 [junior homonym of *Homophron* Fischer von Waldheim, 1828]. Type species: *Omophron tessellatum* Say, 1823 by original designation. Synonymy established by Csiki (1927: 405).
Istor Semenov, 1922: 43. Type species: *Omophron robustum* Horn, 1870 by original designation. Synonymy established by Csiki (1927: 405).
Paromophron Semenov, 1922: 40. Type species: *Omophron ovale* Horn, 1870 by original designation. Synonymy established by Csiki (1927: 405). Etymology. From the Greek *para* (near, next to) and the generic name *Omophron* [*q.v*.] [neuter].
Prosecon Semenov, 1922: 44. Type species: *Omophron gilae* LeConte, 1852 by original designation. Synonymy established by Csiki (1927: 406).
Stenomophron Semenov, 1922: 42. Type species: *Omophron baenningeri* Dupuis, 1912 by original designation. Synonymy established by Csiki (1927: 406). Etymology. From the Greek *stenos* (narrow) and the generic name *Omophron* [*q.v*.] [neuter].
Phromoon Lutshnik, 1933a: 132. Replacement name for *Homophron* Semenov, 1922. Etymology. Anagram of the generic name *Omophron* [*q.v*.] [neuter].

#### Diversity.

About 60 species in the Nearctic (11 species), Neotropical (six species in Middle America), Oriental (16 species), Palaearctic (15 species), and Afrotropical (13 species) Regions.

#### Identification.

Benschoter and Cook (1956) revised the North American species and provided a key for their identification. Lindroth’s (1961a: 10) key covered seven species.

### Omophron americanum

Dejean, 1831

Omophron americanum Dejean, 1831: 583. Type locality: «Amérique septentrionale» (original citation), restricted to «Montreal area, Queb[ec]» by Lindroth (1961a: 12). One syntype in MHNP (Lindroth 1955a: 31).
Omophron saii Kirby, 1837: 65. Type locality: «Canada» (original citation). One syntype in BMNH (Lindroth 1953b: 177). Synonymy established by LeConte (1847: 447), confirmed by Lindroth (1953b: 177).
Omophron lacustre Casey, 1897: 301. Type locality: «Bayfield [Bayfield County, Wisconsin], Lake Superior» (original citation). One syntype in USNM [# 48086]. Synonymy established by Benschoter and Cook (1956: 426).
Omophron texanum Casey, 1897: 302. Type locality: «Austin [Travis County], Texas» (original citation). Holotype [by monotypy] (♀) in USNM [# 48088]. Synonymy established, under the name *Omophron lacustre* Casey, by Bänninger (1921: 119).
Omophron fontinale Casey, 1913: 41. Type locality: «Jemez Springs [Sandoval County], New Mexico» (original citation). Two syntypes in USNM [# 48090]. Synonymy established, under the name *Omophron texanum* Casey, by Bänninger (1921: 118).
Omophron iridescens Casey, 1913: 41. Type locality: «Vicksburg [Warren County], Mississippi» (original citation). One syntype in USNM [# 48079]. Synonymy established by Benschoter and Cook (1956: 426).
Omophron lengi Casey, 1920: 135. Type locality: «South Carolina» (original citation). One syntype in USNM [# 48085]. Synonymy established by Benschoter and Cook (1956: 426).
Omophron illustre Casey, 1920: 136. Type locality: «Vineyard [Utah County], Utah» (original citation). One syntype in USNM [# 48094]. Synonymy established by Benschoter and Cook (1956: 426).
Homophron tanneri Chandler, 1941: 100. Type locality: «Moab, San Juan Co[unty], Utah» (original citation). Holotype (♀) in BYUC (Shawn M. Clark pers. comm. 2007). Synonymy established by Benschoter and Cook (1956: 426).
Homophron tanneri proximum Chandler, 1941: 102. Type locality: «Box Canyon near the junction of Calf Creek and the Escalante River in Garfield Co[unty], Utah» (original citation). Holotype in BYUC (Shawn M. Clark pers. comm. 2007). Synonymy established by Benschoter and Cook (1956: 426).

#### Distribution.

This species ranges from Newfoundland (Lindroth 1955a: 150) to the foothills of the Rocky Mountains in Alberta (Lindroth 1961a: 12), south to northeastern Arizona, the state of Coahuila in Mexico (Benschoter and Cook 1956: 427), and the Florida Panhandle (Peck and Thomas 1998: 15). The record from “Vera Cruz” (Benschoter and Cook 1956: 428) needs confirmation.

#### Records.

**CAN**: AB, MB, NB, NF, NS (CBI), ON, PE, QC, SK **USA**: AL, AR, AZ, CO, CT, DC, DE, FL, GA, IA, ID, IL, IN, KS, KY, LA, MA, MD, ME, MI, MN, MO, MS, MT, NC, ND, NE, NH, NJ, NM, NY, OH, OK, PA, RI, SC, SD, TN, TX, UT, VA, VT, WI, WV, WY – Mexico

### Omophron dentatum

LeConte, 1852

Omophron dentatum LeConte, 1852a: 200. Type locality: «San Diego [San Diego County, California]» (original citation). Syntype(s) in MCZ [# 130].

#### Distribution.

This species seems to be confined to the southern half of California (Benschoter and Cook 1956: 422) and the Baja California Peninsula (Erwin 2007a: 64). Old specimens simply labeled from Arizona and Texas are known (Benschoter and Cook 1956: 422) but are probably mislabeled.

#### Records.

**USA**: CA (CHI) – Mexico

### Omophron gilae

LeConte, 1852

Omophron gilae LeConte, 1852a: 201. Type locality: «ad fluminis Gilae ripas» (original citation). Syntype(s) in MCZ [# 129].
Omophron pallidum Casey, 1897: 305. Type locality: «southwestern Utah» (original citation). Two syntypes [2 originally cited] in USNM [# 48093]. Synonymy established by Benschoter and Cook (1956: 416).
Omophron gilae pimalis Casey, 1913: 44. Type locality: «Arizona» (original citation). One syntype in USNM [# 48092]. Synonymy established by Benschoter and Cook (1956: 416).

#### Distribution.

This species ranges from southern California to western Colorado (Benschoter and Cook 1956: 416) and northern New Mexico (Taos County, UASM), south to Sonora, Mexico (Erwin 2007a: 65). Old specimens simply labeled from Texas are known (Benschoter and Cook 1956: 416).

#### Records.

**USA**: AZ, CA, CO, NM, UT [TX] – Mexico

### Omophron grossum

Casey, 1909

Omophron grossum Casey, 1909: 275. Type locality: «Texas» (original citation). One syntype in USNM [# 48089].

#### Distribution.

This species ranges from western Wisconsin (Messer 2010: 34) to southern Nebraska (Adams County, Foster F. Purrington pers. comm. 2010), south at least to northeastern Texas (Benschoter and Cook 1956: 425), east-central Louisiana (West Feliciana Parish, Igor M. Sokolov pers. comm. 2009), and southwestern Mississippi (Lago and Zucarro 1984: 118; Wilkinson County, UASM).

#### Records.

**USA**: AR, IA, KS, LA, MO, MS, NE, OK, TX, WI

### Omophron labiatum

(Fabricius, 1801)

Scolytus labiatus Fabricius, 1801: 248. Type locality: «Carolina» (original citation). Lectotype, designated by Lindroth (1969a: 1108), in ZMUC.

#### Distribution.

This species is found along the Atlantic and Gulf of Mexico coasts, from Sable Island off the coast of Nova Scotia (Lindroth 1969a: 1108) to southern Florida (Peck and Thomas 1998: 15), west to southeastern Texas (Benschoter and Cook 1956: 420).

#### Records.

**CAN**: NS **USA**: AL, CT, DC, FL, GA, LA, MA, MD, ME, MS, NC, NJ, NY, PA, SC, TX, VA

### Omophron nitidum

LeConte, 1847

Omophron nitidum LeConte, 1847: 447. Type locality: «Territorio Missouriensi et provinciis occidentalibus» (original citation), herein restricted to Kansas City, Missouri (see Benschoter and Cook 1956: 420). Syntype(s) in MCZ [# 128].
Omophron nitens Chaudoir, 1868a: 60. Type locality: «Texas» (original citation). Syntype(s) probably in MHNP. Synonymy established by Horn (1870a: 72).

#### Distribution.

This species ranges from northern Nebraska to northwestern Indiana, north to the Minneapolis region in western Minnesota, south to Alabama and southern Texas (Benschoter and Cook 1956: 420, 422). The records from Wisconsin (Rauterberg 1885: 11) and Charity Island in Michigan (Andrews 1916: 72) are probably based on misidentified *Omophron americanum*.

#### Records.

**USA**: AL, AR, IA, IL, IN, KS, LA, MN, MO, MS, NE, OK, TN, TX

### Omophron obliteratum

Horn, 1870

Omophron obliteratum G.H. Horn, 1870a: 73. Type locality: «Camp Grant [Pinal County] on the San Pedro River, a tributary of the Gila, Arizona» (original citation). Holotype [by monotypy] (♀) in MCZ [# 33479].
Omophron sonorae Casey, 1897: 304. Type locality: «Sonora, probably near Hermosillo, Mexico» (original citation). Four syntypes [5 originally cited] in USNM [# 48091]. Synonymy established by Bänninger (1921: 116).
Omophron obliteratum utense Casey, 1913: 43. Type locality: «Leeds and S[ain]t George, Utah» (original citation). Three syntypes in USNM [# 48095]. Synonymy established by Benschoter and Cook (1956: 414).
Omophron obliteratum subimpressum Casey, 1913: 43. Type locality: «New Mexico» (original citation). One syntype in USNM [# 48096]. Synonymy established by Benschoter and Cook (1956: 414).

#### Distribution.

This species ranges from southern California to western Texas, north to southern Utah (Benschoter and Cook 1956: 416), south to Zacatecas in Mexico (Erwin 2007a: 67). The record from “Montana” (Bousquet and Larochelle 1993: 90) is likely in error.

#### Records.

**USA**: AZ, CA, NM, TX, UT – Mexico

### Omophron ovale

Horn, 1870

Omophron ovale G.H. Horn, 1870a: 75. Type locality: «Fort Crook [Shasta County], California» (original citation). Syntype(s) [2 originally cited] in MCZ [# 33478].
Omophron concinnum Casey, 1897: 302. Type locality: «Siskiyou Co[unty], California» (original citation). Holotype [by monotypy] (♂) in USNM [# 48084]. Synonymy established by Bänninger (1921: 114).
Omophron gemma Casey, 1897: 304. Type locality: «Eel river, near its entrance into Humboldt Bay, Humboldt Co[unty], California» (original citation). Six syntypes in USNM [# 48081]. Synonymy established by Bänninger (1921: 114).
Omophron frater Casey, 1913: 41. Type locality: «California» (original citation). One syntype in USNM [# 48082]. Synonymy established by Bänninger (1921: 114).

#### Distribution.

The range of this species extends from southwestern Saskatchewan (Ronald R. Hooper pers. comm. 1990) to Vancouver Island (Lindroth 1961a: 13), south to northern California and southern Wyoming (Benschoter and Cook 1956: 426).

#### Records.

**CAN**: AB, BC (VCI), SK **USA**: CA, ID, MT, NV, OR, UT, WA, WY

### Omophron robustum

Horn, 1870

Omophron robustum G.H. Horn, 1870a: 73. Type locality: «Nova Scotia region» (original citation), which is incorrect (Fall 1920: 211; Lindroth 1961a: 10); «Toronto [Ontario]» selected by Lindroth (1961a: 10). Holotype [by monotypy] (♂) in MCZ [# 131].
Omophron brevipenne Casey, 1909: 276. Type locality: «Ohio» (original citation). One syntype in USNM [# 48087]. Synonymy established by Fall (1920: 211).
Omophron decoloratum Fall, 1920: 211. Type locality: «Gray Co[unty], Kansas» (original citation). Holotype (♀) in MCZ [# 23883]. Synonymy established by Benschoter and Cook 1956: 416).

#### Distribution.

This species ranges from the southern part of the Ontario Peninsula to south-central North Dakota (Benschoter and Cook 1956: 418), south to northwestern Texas (Hutchinson County, Robert L. Davidson pers. comm. 2012) and northwestern Tennessee (Lake County, CMNH); seemingly isolated in southeastern Alberta (Lindroth 1961a: 11).

#### Records.

**CAN**: AB, ON **USA**: IA, IL, IN, KS, MI, MN, ND, NE, OH, OK, SD, TN, TX, WI

### Omophron solidum

Casey, 1897

Omophron solidum Casey, 1897: 303. Type locality: «Marin to Humboldt Co[untie]s, California» (original citation). Ten syntypes in USNM [# 48083].
Omophron lawrencei Hatch, 1953: 69. Type locality: «Medford [Jackson County], Or[egon]» (original citation). Holotype (♀) in USNM. Synonymy established by Benschoter and Cook (1956: 425).

#### Distribution.

This species is known from southwestern Oregon and northern California (Benschoter and Cook 1956: 425).

#### Records.

**USA**: CA, OR

### Omophron tessellatum

Say, 1823

Omophron tessellatus Say, 1823b: 152. Type locality: «K[ansa]s» (neotype label). Neotype (♂), designated by Lindroth and Freitag (1969: 356), in MCZ [# 32964]. Note. «Elk-horn Creek, Missouri [Territory]» was the area originally cited by Say (1823b: 150). *Omophron tesselatum* is an incorrect subsequent spelling, introduced by LeConte (1847: 447), not currently in prevailing usage.
Omophron lecontei Dejean, 1831: 582. Type locality: «Amérique septentrionale» (original citation). One syntype in MHNP (Lindroth 1955b: 31). Synonymy established by LeConte (1847: 447), confirmed by Lindroth (1955b: 31).
Omophron [*tessellatum*] *ellipticum* Casey, 1909: 276. Type locality: «Rhode Island» (original citation). One syntype in USNM [# 48080]. Synonymy established by Bänninger (1921: 118).

#### Distribution.

This species ranges from Cape Breton Island to southern Alberta, south to southwestern Arizona, northwestern Oklahoma (Cimarron County, CNC), southwestern Arkansas (Hempstead County, MCZ), and Virginia (Benschoter and Cook 1956: 424). One specimen labeled from Alameda County in western California, seen by Benschoter and Cook (1956: 424), is possibly mislabeled. The record from “Texas” (Bousquet and Larochelle 1993: 90) needs confirmation.

#### Records.

**CAN**: AB, MB, NB, NS (CBI), ON, PE, QC, SK **USA**: AR, AZ, CO, CT, IA, IL, IN, KS, KY, MA, MD, ME, MI, MN, MO, MT, ND, NE, NH, NJ, NM, NY, OH, OK, PA, RI, SD, VA, VT, WI [CA, TX]

### Subfamily SCARITINAE

Bonelli, 1810

Scaritides Bonelli, 1810: Tabula Synoptica. Type genus: *Scarites* Fabricius, 1775.

#### Diversity.

Worldwide, with about 1,870 species arrayed in eight tribes: Carenini (about 195 species), Clivinini (about 820 species), Dalyatini (one species), Dyschiriini (about 300 species), Pasimachini (35 species), Promecognathini (eight species), Salcediini (about 15 species), and Scaritini (about 495 species).

### Tribe Pasimachini

Putzeys, 1867

Pasimachides Putzeys, 1867b: 3. Type genus: *Pasimachus* Bonelli, 1813.

#### Diversity.

Thirty-five species arrayed in two genera: *Mouhotia* Laporte (three Oriental species) and *Pasimachus*.

### Genus Pasimachus

Bonelli, 1813

Pasimachus Bonelli, 1813: 476. Type species: *Scarites depressus* Fabricius, 1787 designated by Hope (1838: 94). Etymology. Probably from the Greek *pasi* (the whole, all, very, by extension universal) and *machetes* (warrior, fighter) contracted [masculine].

#### Diversity.

Western Hemisphere, with 32 species arrayed in two subgenera: *Emydopterus* Lacordaire (13 Middle American species) and *Pasimachus* s.str. (19 species).

#### Identification.

Bänninger (1950) reviewed all the species and provided a key for their identification. Purrington and Drake (2005: 254-255) published a key to the North American species. A modern taxonomic revision of the genus is needed as challenging problems remain to be resolved (Ball and Bousquet 2000: 76).

### Subgenus Pasimachus

Bonelli, 1813

Pasimachus Bonelli, 1813: 476. Type species: *Scarites depressus* Fabricius, 1787 designated by Hope (1838: 94).

#### Diversity.

Nineteen species in the temperate, subtropical, and tropical areas of North America (11 species) and Middle America (13 species).

#### Faunistic Note.

Snow (1907: 141) recorded *Pasimachus mexicanus* Gray from Pima County in Arizona. Because the presence of this species in United States has not been confirmed subsequently, *Pasimachus mexicanus* is not retained as a North American entity in this catalogue.

### [depressus group]

### Pasimachus californicus

Chaudoir, 1850

Pasimachus californicus Chaudoir, 1850a: 437. Type locality: «Californie» (original citation), which is incorrect; El Paso, El Paso County, Texas, herein selected (see Casey 1913: 86, as *Pasimachus californicus transversus*). Syntype(s) in MHNP.
Pasimachus validus LeConte, 1858a: 14. Type locality: «Kansas, Texas, Arizona» (original citation). Syntype(s) in MCZ [# 5464]. Synonymy established by LeConte (1874a: 273).
Pasimachus corpulentus LeConte, 1858a: 15. Type locality: «Laredo to Ringgold Barracks, Texas; Sonora» (original citation). Syntype(s) in MCZ [# 5465]. Synonymy established by LeConte (1874a: 273).
Pasimachus californicus transversus Casey, 1913: 86. Type locality: «El Paso [El Paso County], Texas» (original citation). One syntype in USNM [# 46893]. Synonymy established by Leng (1920: 47).
Pasimachus cephalotes Casey, 1913: 87. Type locality: «Texas» (original citation). One syntype in USNM [# 46894]. Synonymy established by Leng (1920: 47).
Pasimachus acomanus Casey, 1913: 87. Type locality: «Jemez Springs [Sandoval County], New Mexico» (original citation). Holotype [by monotypy] (♀) in USNM [# 46895]. Synonymy established by Leng (1920: 47).
Pasimachus obesus Casey, 1913: 88. Type locality: «Arizona» (original citation). One syntype in USNM [# 46896]. Synonymy established by Leng (1920: 47).

#### Distribution.

This species ranges from “Utah” (LeConte 1874a: 273) to southeastern Nebraska (Pawnee County, Peter W. Messer pers. comm. 2008), south at least to eastern Arkansas (Cook and Holt 2006: 2313) and Michoacán in Mexico (Ball and Shpeley 1992a: 46).

#### Records.

**USA**: AR, AZ, CO, KS, NE, NM, OK, TX, UT – Mexico

### Pasimachus depressus

(Fabricius, 1787)

Scarites depressus Fabricius, 1787: 206. Type locality: «Cajennae [= Cayenne, French Guiana]» (original citation), which is incorrect; Southern Pines, Moore County, North Carolina, herein selected (see Casey, 1913: 82, as *Pasimachus depressus carolinensis*). Lectotype [as type], designated by Staig (1931: 21), in HMUG.
Tenebrio complanatus Gmelin, 1790: 1993. Type locality: «Cayenna [= Cayenne, French Guiana]» (original citation), which is incorrect. Syntype(s) location unknown. Synonymy established by Schönherr (1806: 126).
Pasimachus morio LeConte, 1846a: 145. Type locality: «Carolina» (original citation). Syntype(s) in MCZ [# 5458]. Synonymy established by Bänninger (1950: 510).
Pasimachus laevis LeConte, 1846a: 146. Type locality: «New Jersey» (original citation). Syntype(s) in MCZ [# 5459]. Synonymy established by Melsheimer (1853: 7).
Pasimachus limbatus Zimmermann [in LeConte], 1874a: 271. Type locality not stated. Syntype(s) probably lost. Synonymy established by Leng (1920: 47).
Pasimachus depressus carolinensis Casey, 1913: 82. Type locality: «Southern Pines [Moore County], North Carolina» (original citation). Four syntypes in USNM [# 46882]. Synonymy established by Leng (1920: 47).
Pasimachus champlaini Casey, 1913: 82. Type locality: «Carlisle Junction [Cumberland County], Pennsylvania» (original citation). Holotype [by monotypy] in USNM [# 46883]. Synonymy established by Leng (1920: 47).

#### Distribution.

This species ranges from southeastern New York (Notman 1928: 211) and New Jersey (Bänninger 1950: 491) to Wisconsin (Messer 2010: 34), south to southeastern Texas (Galveston County, MCZ; Bänninger 1950: 491) and central Florida (Lake County, MCZ).

#### Records.

**USA**: AL, AR, DC, DE, FL, GA, IA, IL, IN, KS, KY, LA, MD, MO, MS, NC, NJ, NY, OH, OK, PA, SC, TN, TX, VA, WI, WV

### Pasimachus duplicatus

LeConte, 1853

Pasimachus duplicatus LeConte, 1853c: 395. Type locality: «Creek boundary [= boundary of the Creek Indian Reservation at that time, located near or in Oklahoma], Missouri Territory» (original citation). Two syntypes in MCZ [# 5460].
Pasimachus costifer LeConte, 1854c: 79. Type locality: «Laredo to Ringgold Barracks [Texas]» (original citation). Five syntypes in MCZ [# 5461]. Synonymy established by Erwin et al. (1977: 4.11).

#### Distribution.

The range of this species extends from northern Arizona to “Missouri” (Bänninger 1950: 492), north to northern Nebraska (Cherry and Sheridan Counties, Peter W. Messer pers. comm. 2004), south to southern Texas (Johnson 1978: 67) and northeastern Mexico (Bänninger 1950: 492).

#### Records.

**USA**: AZ, CO, KS, MO, NE, NM, OK, TX – Mexico

#### Note.

Bänninger (1950: 510) retained *Pasimachus costifer* LeConte as a questionable subspecies of *Pasimachus duplicatus*.

### Pasimachus elongatus

LeConte, 1846

Pasimachus elongatus LeConte, 1846a: 147. Type locality: «Territorio Missouriensi» (original citation). Syntype(s) in MCZ [# 5463].
Pasimachus pimalis Casey, 1913: 84. Type locality: «Arizona» (original citation). Holotype [by monotypy] (♀) in USNM [# 46885]. Synonymy established with doubt by Bänninger (1950: 501).
Pasimachus angustulus Casey, 1913: 84. Type locality: «Nebraska» (original citation). Holotype [by monotypy] (♂) in USNM [# 46890]. Synonymy established, under the name *Pasimachus pimalis* Casey, by Leng (1920: 47).
Pasimachus angustulus evanescens Casey, 1913: 84. Type locality: «probably Colorado» (original citation). Holotype [by monotypy] (♂) in USNM [# 46886]. Synonymy established, under the name *Pasimachus pimalis* Casey, by Leng (1920: 47).
Pasimachus vegasensis Casey, 1913: 85. Type locality: «Las Vegas [San Miguel County], New Mexico» (original citation). Holotype [by monotypy] (♂) in USNM [# 46887]. Synonymy established, under the name *Pasimachus pimalis* Casey, by Leng (1920: 47).
Pasimachus vernicatus Casey, 1913: 85. Type locality: «Kansas» (original citation). Holotype [by monotypy] (♂) in USNM [# 46888]. Synonymy established, under the name *Pasimachus pimalis* Casey, by Leng (1920: 47).

#### Distribution.

This species ranges from the southern part of the Prairie Provinces (Lindroth 1961a: 131) south to northern Sonora (Bates 1884: 264), western and northern Texas (Wheeler, Grayson, Presidio, and Brewster Counties, MCZ, CMNH; Bänninger 1950: 490), and southeastern Louisiana (Summers 1874a: 79), east to Indiana (Blatchley 1910: 55; Bänninger 1950: 490) and southwestern Michigan (Dunn 1982a: 206). The record from southwestern Virginia (Horn 1869a: 123) is probably in error.

#### Records.

**CAN**: AB, MB, SK **USA**: AR, AZ, CO, IA, ID, IL, IN, KS, LA, MI, MN, MO, MT, ND, NE, NM, OH, OK, SD, TX, WI, WY – Mexico

**Figure 16.**
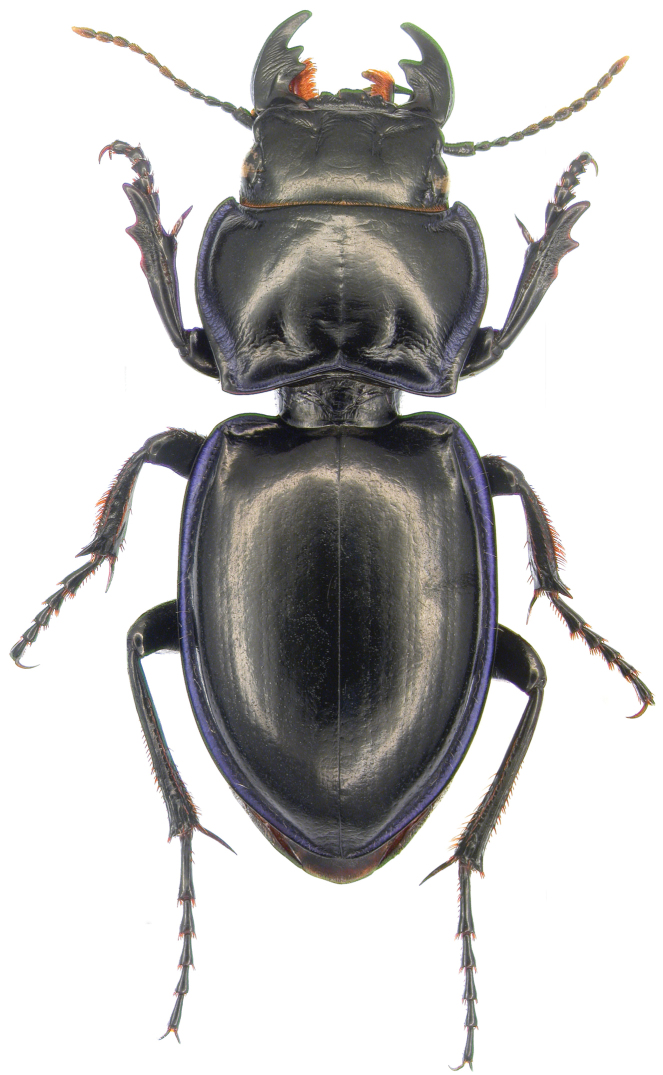
*Pasimachus elongatus* LeConte. Because of its large size and robust shape, this species is one of the most conspicuous carabid elements of the grassland regions of North America. It belongs to a genus which is endemic to North and Middle America. Although not firmly established, the sister taxon to *Pasimachus* could be *Mouhotia*, a genus of massive and handsome species endemic to southeast Asia.

### Pasimachus obsoletus

LeConte, 1846

Pasimachus obsoletus LeConte, 1846a: 148. Type locality: «ad flumen Platte, prope Rocky Mountains» (original citation). Three syntypes in MCZ [# 5462].
Pasimachus acuminatus Casey, 1913: 88. Type locality: «probably Colorado» (original citation). Holotype [by monotypy] in USNM [# 46889]. Synonymy established by Leng (1920: 47).
Pasimachus vestigialis Casey, 1913: 89. Type locality: «New Mexico; El Paso and Marfa, Texas» (original citation). Three syntypes in USNM [# 46891]. Synonymy established by Leng (1920: 47).
Pasimachus atronitens Casey, 1913: 89. Type locality: «San Bernardino Ranch and Douglas, Cochise Co[unty], Arizona» (original citation). Six syntypes [6 originally cited] in USNM [# 46892]. Synonymy established by Leng (1920: 47).

#### Distribution.

This species is found from “Iowa” (Bänninger 1950: 492) to southeastern Wyoming (Laramie County, CMNH), south to southeastern Arizona (Casey 1913: 89, as *Pasimachus atronitens*) and Chihuahua in northern Mexico (Bänninger 1950: 492).

#### Records.

**USA**: AZ, CO, IA, KS, NE, NM, OK, SD, TX, WY – Mexico

#### Note.

Bänninger (1950: 510) retained *Pasimachus atronitens* and *Pasimachus acuminatus* as questionable subspecies of *Pasimachus obsoletus*. Erwin (2011b: 51) treated *Pasimachus atronitens* as a valid species.

### Pasimachus punctulatus

Haldeman, 1843

Pasimachus punctulatus Haldeman, 1843b: 298. Type locality: «Alabama» (original citation). One possible syntype, labeled “[orange disc] / P. punctulatus !Hald. [handwritten],” in MCZ (collection LeConte).
Pasimachus missuricus Gistel, 1857: 27. Type locality: «Missuri» (original citation). Syntype(s) lost. Synonymy established by Bänninger (1950: 501).
Pasimachus sinuatus Casey, 1913: 83. Type locality: «S[ain]t Louis, Missouri» (original citation). Holotype [by monotypy] in USNM [# 46884]. Synonymy established by Leng (1920: 47).

#### Distribution.

This species ranges from New Jersey (Smith 1890: 74; Smith 1910: 201) and north-central Virginia (Carrington 2002: 107) to west-central Kansas (Snow 1878: 63; Knaus 1907: 233), south to central Texas (LeConte 1846a: 146; Lee County, MCZ) and the Florida Panhandle (Peck and Thomas 1998: 16); also recorded from Durango in Mexico (García 2004: 289, as *Pasimachus punctatus* Haldeman). The record from Cochise County, Arizona (Snow 1906b: 161) is probably in error.

#### Records.

**USA**: AL, AR, FL, IL, IN, KS, KY, LA, MO, MS, NC, NJ, OH, OK, PA, SC, TN, TX, VA, WV – Mexico

### Pasimachus viridans

LeConte, 1858

Pasimachus viridans LeConte, 1858b: 61. Type locality: «Sonora [Mexico]» (original citation). Holotype [by monotypy] in MCZ [# 5466].
Pasimachus ignicinctus Bates, 1891a: 230. Type locality: «Canelas, Sierra Madre of Durango [Mexico]» (original citation). Syntype(s) probably in BMNH. Synonymy established by Erwin et al. (1977: 4.11).
Pasimachus viridans ambiens Casey, 1913: 90. Type locality: «Arizona» (original citation). Four syntypes [4 originally cited] in USNM [# 46898]. Synonymy established by Leng (1920: 47).

#### Distribution.

This species ranges from southern Arizona (Schaeffer 1905: 142) south at least to Durango in Mexico (Bänninger 1950: 493).

#### Records.

**USA**: AZ – Mexico

#### Note.

Bänninger (1950: 510) retained *Pasimachus ignicinctus* Bates as a valid subspecies of *Pasimachus viridans*.

### [marginatus group]

### Pasimachus marginatus

(Fabricius, 1787)

Scarites marginatus Fabricius, 1787: 206. Type locality: «Cajennae [= Cayenne, French Guiana]» (original citation), which is incorrect. Lectotype [as type], designated by Staig (1931: 23), in HMUG.
Pasimachus crassus Casey, 1913: 81. Type locality: «Southern Pines [Moore County], North Carolina» (original citation). Four syntypes in USNM [# 46881]. Synonymy established by Erwin et al. (1977: 4.11).

#### Distribution.

This species ranges from southern Maryland (Peter W. Messer pers. comm. 2010) to the Florida Keys (Nichols 1988b: Fig. 5-4; Peck and Thomas 1998: 16), west to “Texas” (Leng 1915: 565; Bänninger 1950: 488), north along the Mississippi River drainage to southwestern Kentucky (Mammoth Cave National Park, CMNH).

#### Records.

**USA**: AL, FL, GA, KY, LA, MD, MS, NC, SC, TN, TX

#### Note.

Bänninger (1950: 509) retained *Pasimachus crassus* Casey as a questionable subspecies of *Pasimachus marginatus*.

### Pasimachus subsulcatus

Say, 1823

Pasimachus subsulcatus Say, 1823a: 19. Type locality: «Georgia and Florida» (original citation), restricted to «Florida» by Lindroth and Freitag (1969: 333). Lectotype, designated by Lindroth and Freitag (1969: 333), in MHNP (collection Dejean).
Pasimachus floridanus Casey, 1913: 79. Type locality: «Palm Beach [Palm Beach County], Florida» (original citation). Six syntypes [6 originally cited] in USNM [# 46879]. Synonymy established by Erwin et al. (1977: 4.11).
Pasimachus subsulcatus subnitens Casey, 1913: 79. Type locality: «Florida» (original citation). Holotype [by monotypy] in USNM [# 46878]. Synonymy established by Leng (1915: 567).
Pasimachus opacipennis Casey, 1913: 80. Type locality: «Florida» (original citation). Holotype [by monotypy] in USNM [# 46880]. Synonymy established with doubt by Bänninger (1950: 509).

#### Distribution.

This species is probably restricted to the Coastal Plain ranging from South Carolina (Kirk 1970: 9; Ciegler 2000: 38) to southern Florida including the Keys (Peck and Thomas 1998: 17), west to southeastern Louisiana (Allen 1965: 62).

#### Records.

**USA**: AL, FL, GA, LA, SC

#### Note.

Bänninger (1950: 510) retained *Pasimachus floridanus* Casey and *Pasimachus subnitens* Casey as questionable subspecies of *Pasimachus subsulcatus*. Nichols (1988a: 217) retained *Pasimachus floridanus* as a valid species but added that “further study is needed to determine ... whether it is a taxon worthy of distinction from *Pasimachus subsulcatus* Say.”

### [strenuus group]

### Pasimachus strenuus

LeConte, 1874

Pasimachus strenuus LeConte, 1874a: 267. Type locality: «Florida» (original citation). Syntype(s) [2 originally cited] in MCZ [# 5455].
Pasimachus strenuus robustus Casey, 1913: 78. Type locality: «Florida» (original citation). Holotype [by monotypy] in USNM [# 46877]. Synonymy established by Leng (1915: 566).

#### Distribution.

This species is known from southeastern Georgia (Fattig 1949: 12), the Florida Peninsula and Panhandle (Peck and Thomas 1998: 17), and southwestern Alabama (Löding 1945: 12).

#### Records.

**USA**: AL, FL, GA

### Pasimachus sublaevis

(Palisot de Beauvois, 1811)

Scarites sublaevis Palisot de Beauvois, 1811: 107. Type locality: «Caroline du sud» (original citation). Syntype(s) probably lost.
Pasimachus substriatus Haldeman, 1843c: 313. Type locality: «Long Island [New York]» (original citation). One possible syntype, labeled “[pink disc] / P. substriatus Lec. [handwritten] / sublaevis 15 [handwritten],” in MCZ (collection LeConte). Synonymy established by LeConte (1874a: 268).
Pasimachus assimilis LeConte, 1846a: 148. Type locality: «Georgia» (original citation). Syntype(s) in MCZ [# 5457]. Synonymy established by LeConte (1853c: 395).
Pasimachus rugosus LeConte, 1846a: 149. Type locality: «Nova Caesarea» [= New Jersey] (original citation). Syntype(s) in MCZ [# 5456]. Synonymy established by LeConte (1853c: 395).
Pasimachus brevitarsis Casey, 1913: 76. Type locality: «Pass Christian [Harrison County], Mississippi» (original citation). Two syntypes [2 originally cited] in USNM [# 46875]. Synonymy established with doubt by Bänninger (1950: 509).
Pasimachus oblongus Casey, 1913: 77. Type locality: «Southern Pines [Moore County], North Carolina» (original citation). Four syntypes [4 originally cited] in USNM [# 46876]. Synonymy established with doubt, under the name *Pasimachus substriatus* Haldeman, by Bänninger (1950: 509).

#### Distribution.

The range of this species extends from Massachusetts (Miliotis 1974: 114) to central Iowa (Purrington and Drake 2005: 256), south to southeastern Mississippi (Casey 1913: 76, as *Pasimachus brevitarsis*) and southern Florida including the Keys (Nichols 1988b: Fig. 5-4; Peck and Thomas 1998: 17). The records from South Dakota (Kirk and Balsbaugh 1975: 15, as *Scarites substriatus*), “Kansas” (Bousquet and Larochelle 1993: 96), and southeastern Louisiana (Summers 1874a: 79) need confirmation.

#### Records.

**USA**: AL, DC, FL, GA, IA, IL, IN, MA, MS, NC, NJ, NY, OH, SC, TN, VA [KS, LA, SD]

#### Note.

Bänninger (1950: 510) retained *Pasimachus substriatus* Haldeman as a questionable subspecies of *Pasimachus sublaevis*. Nichols (1988a: 224-225) treated it as a junior synonym of *Pasimachus sublaevis*.

### Tribe Scaritini

Bonelli, 1810

Scaritides Bonelli, 1810: Tabula Synoptica. Type genus: *Scarites* Fabricius, 1775.

#### Diversity.

Worldwide, with about 495 species (Lorenz 2005: 135-141 and the genus *Scaraphites* Westwood) placed in 42 genera arrayed in four subtribes: Acanthoscelitina (one species on the southwest coast of Africa), Oxylobina (29 Asian species in the genus *Oxylobus* Chaudoir), Scapterina (22 species in the Eastern Hemisphere except Europe), and Scaritina (about 445 species).

### Subtribe Scaritina

Bonelli, 1810

Scaritides Bonelli, 1810: Tabula Synoptica. Type genus: *Scarites* Fabricius, 1775.

#### Diversity.

Worldwide, with about 445 species arrayed in 37 genera. The tribe is much more diversified in term of species (about 51% of the world fauna) in the Afrotropical (particularly on Madagascar) than anywhere else. The Western Hemisphere has only four endemic genus-group taxa: *Antilliscaris* Bänninger (four West Indian species), *Baenningeria* Reichardt (two species on the Galápagos), *Glyptogrus* Bates (seven Neotropical species), and *Taeniolobus* Chaudoir (about 40 Neotropical species). The Nearctic (with seven species) and Australian (with 12 species) Regions are underrepresented.

### Genus Scarites

Fabricius, 1775

Scarites Fabricius, 1775: 249. Type species: *Scarites subterraneus* Fabricius, 1775 designated by Andrewes (1929: 225). Etymology. From the Greek *scaritis* (gem of the color of the fish named *scaros*, probably a wrasse, in Pliny the Elder) [masculine].

#### Diversity.

About 190 species (Lorenz 2005: 137-140) arrayed in four subgenera: *Orientolobus* Dostal (eight Indo-African species), *Parallelomorphus* Motschulsky (15 Old World species), *Scarites* s.str. (about 130 species), and *Taeniolobus* Chaudoir (about 40 Neotropical species). The genus is more diversified in term of species in the tropics of the Old World (about 60.5% of the world fauna) than anywhere else.

#### Identification.

Bänninger (1938) reviewed the species and provided keys for their identification. Three new North American species were subsequently described. Bousquet and Skelley (2010) published a key to all Nearctic species except *Scarites lissopterus* Chaudoir.

### Subgenus Scarites

Fabricius, 1775

Scarites Fabricius, 1775: 249. Type species: *Scarites subterraneus* Fabricius, 1775 designated by Andrewes (1929: 225).
Scallophorites Motschulsky, 1857b: 95. Type species: *Scarites striatus* Dejean, 1825 by original designation. Synonymy established by Jeannel (1946: 240).
Scaritolius Fairmaire, 1905: 115. Type species: *Scaritolius politus* Fairmaire, 1905 (= *Scarites fairmairei* Bänninger, 1933) by monotypy. Synonymy established by Bänninger (1933: 104).

#### Diversity.

About 130 species in the Nearctic (seven species), Neotropical (about 25 species), Oriental (about 45 species), Palaearctic (about 25 species, most of them in Asia), and Afrotropical (about 45 species) Regions. The bulk of the species (about 77% of the world fauna) are found in the tropics of the Old World.

#### Taxonomic Note.

The taxonomy of the species of the *quadriceps* group is based on Stephen W. Nichols’ unpublished manuscript entitled “A provisional key to the North American species of the *Scarites subterraneus* group” as well as his thesis (Nichols 1988a).

### [quadriceps group]

### Scarites lissopterus

Chaudoir, 1881

Scarites quadriceps var. *lissopterus* Chaudoir, 1881: 93. Type locality: «Dallas [Dallas County], Texas» (original citation). Syntype(s) probably in MHNP.

#### Distribution.

This species inhabits the Great Plains from south-central Kansas (Sedgwick County, MCZ) to the Rio Grande in Texas (Hidalgo and Cameron Counties, MCZ, USNM), west to western Texas (El Paso County, USNM), including southeastern and central New Mexico (Chaves County, CMNH; Ellis et al. 2001: 16), east to northeastern Louisiana (Franklin and Tensas Parishes, Igor M. Sokolov pers. comm. 2009). The records from “Wisconsin” and “Iowa” (Bousquet and Larochelle 1993: 96) are likely in error.

#### Records.

**USA**: KS, LA, NM, OK, TX

#### Note.

Bänninger (1938: 152) regarded this taxon as a subspecies of *Scarites subterraneus* Fabricius.

### Scarites quadriceps

Chaudoir, 1843

Scarites quadriceps Chaudoir, 1843b: 729. Type locality: «Amérique septentrionale» (original citation). Syntype(s) in MHNP.
Scarites substriatus Haldeman, 1844: 54. Type locality not stated. Syntype(s) possibly in MCZ. Synonymy established by LeConte (1846b: 210). Note. Two specimens in collection LeConte (MCZ), labeled “[orange disc] / Scarites substriatus Hald. quadriceps Chaud. distinctus Hald. [handwritten]” and “[orange disc] / substriatus 2 [handwritten],” could be syntypes of *Scarites substriatus* and / or *Scarites distinctus*.
Scarites distinctus Haldeman, 1844: 54. Type locality: «Georgia?» (original citation). Syntype(s) possibly in MCZ. Synonymy established by LeConte (1846b: 210).
Scarites intermedius LeConte, 1845a: 201. Type locality: «provinciis occidentalibus» (original citation). Syntype(s) in MCZ [# 675]. Synonymy established, under the name *Scarites distinctus* Haldeman, by LeConte (1863b: 3).
Scarites ephialtus LeConte, 1845a: 201. Type locality: «provinciis Australibus» (original citation). Syntype(s) in MCZ [# 676]. Synonymy established, under the name *Scarites intermedius* LeConte, by Melsheimer (1853: 7).
Scarites affinis LeConte, 1845a: 201. Type locality: United States of America (inferred from title of the paper). Syntype(s) in MCZ [# 674]. Synonymy established, under the name *Scarites vicinus* Chaudoir, by LeConte (1846b: 211).

#### Distribution.

This species ranges along the Coastal Plain from New Jersey (Smith 1890: 74, as *Scarites subterraneus* var. *substriatus*; CNC) to southern Florida (Nichols 1988b: Fig. 5-7; Peck and Thomas 1998: 17), west to southeastern Texas (Jefferson County, USNM). Several records (i.e., AR, IA, IL, IN, KS, KY, MI, MN, MO, OH, OK, ON, PA, SD, TN, WI) listed in Bousquet and Larochelle (1993: 97) refer to other species, particularly *Scarites vicinus* Chaudoir.

#### Records.

**USA**: AL, FL, GA, LA, MD, MS, NC, NJ, SC, TX

### Scarites vicinus

Chaudoir, 1843

Scarites vicinus Chaudoir, 1843b: 728. Type locality: «environs de la Nouvelle Orléans [Louisiana]» (original citation). Syntype(s) in MHNP.

#### Distribution.

This species ranges from southern Ontario (CNC) to eastern North Dakota (Tinerella 2003: 635 as *Scarites quadriceps*), south to northeastern Texas (Cass County, USNM) and northern Alabama (Madison County, USNM).

#### Records.

**CAN**: ON **USA**: AL, AR, IA, IL, IN, KS, KY, LA, MN, MO, MS, ND, NE, OH, OK, TN, TX, WI

### [subterraneus group]

### Scarites marinus

Nichols, 1986

Scarites marinus Nichols, 1986: 258. Type locality: «Big Pine Key, Monroe Co[unty], Florida» (original citation). Holotype (♀) in CUIC [# 6891].

#### Distribution.

This species is known from coastal Florida, including the Keys (Peck and Thomas 1998: 17), the Bahamas, Cuba, and the Yucatán Peninsula in southern Mexico [see Nichols 1986: Fig. 9]. One old specimen simply labeled from Louisiana is known (Nichols 1986: 261).

#### Records.

**USA**: FL [LA] – Bahamas, Cuba, Mexico

### Scarites ocalensis

Nichols, 1986

Scarites ocalensis Nichols, 1986: 261. Type locality: «Jacksonville, Duval Co[unty], Florida» (original citation). Holotype (♂) in CUIC [# 6890].

#### Distribution.

This species is endemic to the Florida Peninsula north of Lake Okeechobee [see Nichols 1986: Fig. 10].

#### Records.

**USA**: FL

### Scarites stenops

Bousquet and Skelley, 2010

Scarites stenops Bousquet and Skelley, 2010: 46. Type locality: «4.0 mi[les] S[outh]W[est] Archer on Rt-24, Levy Co[unty], Florida» (original citation). Holotype (♂) in FSCA.

#### Distribution.

This species is known only from the holotype.

#### Records.

**USA**: FL

### Scarites subterraneus

Fabricius, 1775

Scarites subterraneus Fabricius, 1775: 249. Type locality: «P[oin]t Pelee, Ont[ario]» (neotype label). Neotype (♂), designated by Nichols (1985a: 1214), in CNC [# 20654]. Note. “America” was the area originally listed by Fabricius (1775: 249).
Carabus interruptus Herbst, 1784: 133. Type locality: «Ostindien» (original citation), which is incorrect. Syntype(s) location unknown (possibly in ZMHB). Synonymy established by Fabricius (1801: 124).
Scarites subterreus Bonelli, 1813: 466. Type locality: «Amérique septentrionale» (original citation). Syntype(s) location unknown. Synonymy established by Chaudoir (1881: 94).
Scarites beckwithii Stephens, 1827: 37. Type locality: «near Dover [and] Yorkshire coast [United Kingdom]» (original citation). Syntype(s) [3 originally cited] in BMNH. Synonymy established with doubt by Chaudoir (1855: 104), confirmed by Nichols (1988a: 48).
Scarites denticollis Chaudoir, 1843b: 729. Type locality: «Nouvelle Orléans [Orleans Parish, Louisiana]» (original citation). Syntype(s) in MHNP. Synonymy established by Chaudoir (1881: 94), confirmed by Nichols (1988a: 48).
Scarites patruelis LeConte, 1845a: 201. Type locality: «Georgia» (original citation). Syntype(s) in MCZ [# 673]. Synonymy established with doubt, under the name *Scarites denticollis* Chaudoir, by Chaudoir (1855: 104).
Scarites californicus LeConte, 1852a: 198. Type locality: «ad San Diego [San Diego County, California]» (original citation). Syntype(s) [2 originally cited] in MCZ [# 672]. Synonymy established by Bousquet and Larochelle (1993: 97) based on Nichols (1988a: 49) unpublished thesis.
Scarites texanus Chaudoir, 1881: 94. Type locality: «Texas et dans le Yucatan (?)» (original citation). Syntype(s) in MHNP (Nichols 1988a: 51). Synonymy established by Nichols (in Bousquet and Skelley 2010: 47).
Scarites durangoensis Bates, 1891a: 232. Type locality: «Villa Lerdo in Durango [Mexico]» (original citation). Syntype(s) probably in BMNH. Synonymy established, under the name *Scarites californicus* LeConte, by Bänninger (1933: 119).

#### Distribution.

This species is found from southeastern New Hampshire (Rockingham and Strafford Counties, Donald S. Chandler pers. comm. 2008) to eastern North Dakota (Cass County, Donald P. Schwert pers. comm. 1989), including southern Ontario (Lindroth 1961a: 129), south to the Yucatán Peninsula (Nichols 1988a: 52), the Florida Keys (Peck and Thomas 1998: 17), and Cuba (Darlington 1934: 67; Nichols 1988b: Fig. 5-8), west along the southwest to southwestern California (LeConte, 1852a: 198, as *Scarites californicus*) and Baja California (Horn 1894: 307).

#### Records.

**CAN**: ON **USA**: AL, AR, AZ, CA, CT, CO, DC, DE, FL, GA, IA, IL, IN, KS, KY, LA, MA, MD, MI, MN, MO, MS, NC, ND, NE, NH, NJ, NM, NY, OH, OK, PA, RI, SC, SD, TN, TX, VA, VT, WI, WV – Cuba, Mexico

#### Note.

Bänninger (1938: 151) retained *Scarites patruelis* LeConte, *Scarites californicus* LeConte, and *Scarites texanus* Chaudoir as valid subspecies of *Scarites subterraneus*. All three names are listed in synonymy with *Scarites subterraneus* by Nichols (1988a: 49).

### Tribe Clivinini

Rafinesque, 1815

Clivinidia Rafinesque, 1815: 109. Type genus: *Clivina* Latreille, 1802.

#### Diversity.

Worldwide, with about 820 species (Lorenz 2005: 141-150) arrayed provisionally in three subtribes, Forcipatorina (25 Neotropical species), Ardistomina (about 90 species), and Clivinina (about 705 species). The last two subtribes are represented in North America. The Nearctic fauna includes 57 species (about 7% of the world fauna).

### Subtribe Clivinina

Rafinesque, 1815

Clivinidia Rafinesque, 1815: 109. Type genus: *Clivina* Latreille, 1802.

#### Diversity.

Worldwide, with about 705 species. The Northern Hemisphere is represented by about 170 species (roughly 24% of the world fauna) and North America by 52 species (about 7% of the world fauna).

### Genus Clivina

Latreille, 1802

Clivina Latreille, 1802: 96. Type species: *Scarites arenarius* Fabricius, 1775 (= *Tenebrio fossor* Linnaeus, 1758) by monotypy. Etymology. From the Greek *clivina* (kind of bird in Pliny the Elder) [feminine].

#### Diversity.

Worldwide, with about 375 species described (Lorenz 2005: 141-145) arrayed in nine subgenera: *Antroforceps*, *Clivina* s.str., *Cliviniella* Kult (four Afrotropical species), *Dacca* Putzeys (one Oriental species), *Eoclivina* Kult (eight Indo-African species), *Physoclivina* Kult (one Afrotropical species), *Reichardtula* Whitehead, *Leucocara*, and *Semiclivina*. Seventeen species, three of them adventive, are found in the boreal (marginal), temperate, and subtropical areas of North America.

### Subgenus Semiclivina

Kult, 1947

Semiclivina Kult, 1947: 31. Type species: *Clivina dentipes* Dejean, 1825 by original designation. Etymology. From the Latin prefix *semi*- (half) and the generic name *Clivina* [*q.v*.] [feminine].

#### Diversity.

Western Hemisphere, with at least 30 species in the Nearctic (two species, one of them adventive) and Neotropical (at least 30 species) Regions. One species, possibly adventive, is known from the suburbs of Sydney, Australia (Baehr 2008: 23-25).

#### Identification.

Nichols (1985b: 380) discussed the structural differences between the two species found in North America.

#### Taxonomic Note.

This subgenus as defined by Kult (1947: 31) includes the species of groups 19 (mistakenly reported as group 29 by Kult) and 24 of Putzeys (1867b: 145, 166-178). Recently Dostal (2011) listed this taxon as a distinct genus and described a new subgenus, *Uroclivina* Dostal, for the species of *Semiclivina* with a peculiar tubercle behind the posterior edge of the eye.

### Clivina dentipes

Dejean, 1825

Clivina dentipes Dejean, 1825: 415. Type locality: «île de Cuba» (original citation). Holotype [by monotypy] location unknown (possibly lost according to Lindroth 1955b: 13 and Nichols 1988a: 160).
Clivina fissipes Putzeys, 1846: 89. Type locality: «Texas» (original citation). Holotype [by monotypy] in UMO (Nichols 1988a: 160). Synonymy established, under the name *Clivina corvina* Putzeys, by Melsheimer (1853: 8), confirmed by Nichols (1988a: 160).
Clivina corvina Putzeys, 1846: 92. Type locality: «Nouvelle Orléans [Orleans Parish, Louisiana]» (original citation). Syntype(s) [2 originally cited] probably in MHNP (collection Chaudoir). Synonymy established by LeConte (1879a: 33).
Clivina confusa LeConte, 1852a: 198. Type locality: «ad fluminis Colorado ripas» (original citation). Three syntypes in MCZ [# 5468]. Synonymy established, under the name *Clivina corvina* Putzeys, by Melsheimer (1853: 8).
Clivina georgiana LeConte, 1857b: 81. Type locality: Georgia (inferred from the species name). Syntype(s) location unknown (probably in MCZ). Synonymy established by Putzeys (1867b: 173).

#### Distribution.

The range of this species extends from Connecticut (Krinsky and Oliver 2001: 44) to eastern South Dakota (Kirk and Balsbaugh 1975: 16), including southernmost Ontario (Bousquet 1987a: 119), south to southern Texas (Zapeta, Kleberg, and Gonzales Counties, CMNH; Leng 1915: 570) and southern Florida (Peck and Thomas 1998: 17), west along the south to the Colorado River drainage in San Bernardino County, California (Fall 1901a: 41); also recorded from Cuba (Dejean 1825: 415; Jacquelin du Val 1857: 15), Jamaica (Nichols 1988b: Fig. 5-14), and Mexico as far south as Oaxaca (Erwin 2011b: 169). One old specimen labeled “Mass” is known (MCZ).

#### Records.

**CAN**: ON **USA**: AL, AR, AZ, CA, CO, CT, DC, DE, FL, GA, IA, IL, IN, KS, KY, LA, MD, MI, MO, MS, NC, NE, NJ, NM, NY, OH, OK, PA, SC, SD, TN, TX, VA, WI, WV [MA] – Cuba, Jamaica, Mexico

### Clivina vespertina

Putzeys, 1867

Clivina vespertina Putzeys, 1867b: 176. Type locality: «Montevideo [Uruguay]» (original citation). Lectotype, designated by Nichols (1985b: 380), in MHNP.

#### Distribution.

This species is native to South America and is adventive in North America where it is known from southeastern United States (Nichols 1985b: 380). The first inventoried specimen collected on this continent was found in Mobile, Alabama in 1948 (Nichols 1985b: 380).

#### Records.

**USA**: AL, LA, MS – **Adventive**

### Subgenus Clivina

Latreille, 1802

Clivina Latreille, 1802: 96. Type species: *Scarites arenarius* Fabricius, 1775 (= *Tenebrio fossor* Linnaeus, 1758) by monotypy.

#### Diversity.

Worldwide, with over 250 species described. The number of species cannot be assessed at this time since many species included by Lorenz (2005: 141-145) in this subgenus belong to *Semiclivina*, *Reichardtula*, and *Leucocara*. The Nearctic Region has only ten described species and two of them are adventive.

#### Identification.

Bousquet (1997c: 347-348) published a key to all North American species and two unnamed ones. One species (*Clivina choatei*) was described subsequently.

### Clivina choatei

Bousquet and Skelley, 2012

Clivina choatei Bousquet and Skelley, 2012: 44. Type locality: «4.0 mi SW Archer, Levy Co[unty], Florida» (original citation). Holotype (♂) in FSCA.

#### Distribution.

This species is known from Levy and Gilchrist Counties in northern Florida.

#### Records.

**USA**: FL

### Clivina collaris

(Herbst, 1784)

Carabus collaris Herbst, 1784: 141. Type locality: «Berlin [Germany]» (original citation). Syntype(s) location unknown (possibly in ZMHB).
Clivina elongata Randall, 1838b: 34 [primary homonym of *Clivina elongata* Ahrens, 1830]. Type locality: «Boston [Suffolk County], Mass[achusetts]» (original citation). Syntype(s) lost. Synonymy established by LeConte (1879a: 34).
Clivina randalli LeConte, 1857b: 82. Replacement name for *Clivina elongata* Randall, 1838.

#### Distribution.

This European species is adventive in North America where is it known from southern Quebec (Larochelle 1975: 78) to southwestern Ohio (Dury 1879: 162) and Connecticut (Krinsky and Oliver 2001: 44), from southern Manitoba (Pollock 1991b: 298), from British Columbia (Lindroth 1961a: 162), and from Washington (Hatch 1953: 66). The first inventoried specimen collected on this continent was found prior to 1838 (Randall 1838b: 34, as *Clivina elongata*) in Massachusetts. The record from northeastern Kansas (Popenoe 1877: 22, as *Clivina elongata*) is probably in error.

#### Records.

**CAN**: BC, MB, ON, QC **USA**: CT, MA, ME, NH, OH, WA – **Adventive**

### Clivina fossor fossor

(Linnaeus, 1758)

Tenebrio fossor Linnaeus, 1758: 417. Type locality: «Upsalia [= Uppsala, Sweden]» (original citation). One possible syntype in LSL (Lindroth 1957b: 338).

#### Distribution.

This Palaearctic subspecies is adventive in North America where it is found in the east from Newfoundland (Lindroth 1955a: 45) to northern Minnesota (Petrice et al. 2002: 9), south to northern Pennsylvania (Bradford County, CMNH) and in the west from southwestern British Columbia (Bousquet 1987a: 119) to south-central Saskatchewan (Ronald R. Hooper pers. comm. 2002), south to northwestern Wyoming (Teton County, Foster F. Purrington pers. comm. 2010) and southern Oregon (Nelson and Reynolds 1987: 12). The first inventoried specimen collected in the east was found in 1915 in the Montreal region (Lindroth 1961a: 161) and in the west in 1937 in western Washington (Hatch 1949b: 118). The records from Georgia (Fattig 1949: 14) and Alabama (Löding 1945: 12) are probably in error; those from “Ohio” (Hamilton 1889b: 93), “Illinois,” “Indiana,” and “Idaho” (Bousquet and Larochelle 1993: 102) need confirmation.

#### Records.

**FRA**: PM **CAN**: AB, BC, NB, NF, NS (CBI), ON, PE, QC, SK **USA**: CT, MA, ME, MI, MN, NH, NY, OR, PA, VT, WA, WI, WY [ID, IL, IN, OH] – **Adventive**

#### Note.

The subspecies *Clivina fossor sachalinica* Nakane is found in the Far East and Japan.

**Figure 17.**
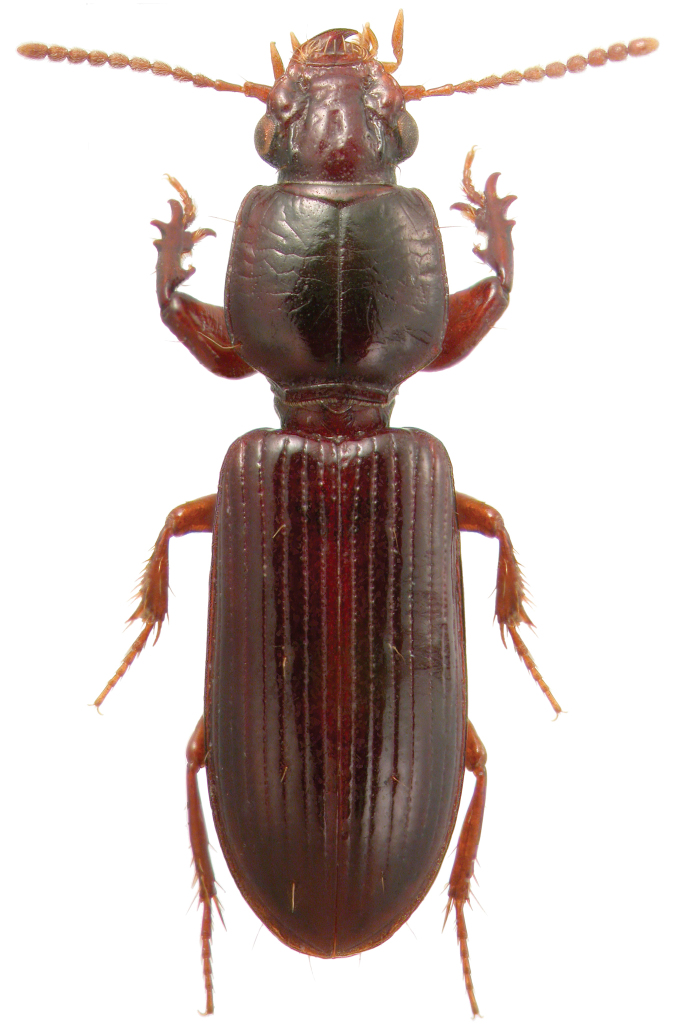
*Clivina fossor* (Linnaeus). This *Clivina* is one of the 62 carabid species accidentally introduced and established in North America and one of 18 that have been introduced independently on the Atlantic and Pacific Coasts. Many of the adventive species reached our borders in ballast used by Europeans to improve the stability of their trade ships on their way to America. Ballast consisted of stones, broken slates, mortar, bricks, rubbish, soil, and sand that were usually dumped along the shore at ports of entry and certainly constituted a good environment for the survival of carabids during ocean voyages.

### Clivina impressefrons

LeConte, 1844

Clivina impressefrons LeConte, 1844: 50. Type locality: «New York» (original citation), herein restricted to Olivebridge, Ulster County (CMNH). Two syntypes in MCZ [# 5469]. Note. The specimen with the type label in the LeConte collection is not a syntype because it bears an orange disc (= southern states). Two specimens with pink discs (= middle states) are possible syntypes. The spelling *impressifrons* is an incorrect subsequent spelling, introduced by LeConte (1846b: 213), not currently in prevailing usage.

#### Distribution.

The range of this species, also known under the vernacular name “slender seed-corn Beetle”, extends from the Saint Lawrence Plain in southern Quebec (Lindroth 1961a: 164) to northern Utah (Knowlton and Wood 1947: 94; Davis and Utah Counties, USNM), south to southern Texas (Hlavac 1967: 31; Johnson 1978: 67) and northern Georgia (Fattig 1949: 14; Hlavac 1967: 30). At least one specimen simply labeled from Florida is known (Hlavac 1967: 30). The record from Idaho (Anonymous 1960: 642) needs confirmation.

#### Records.

**CAN**: ON, QC **USA**: AL, AR, CO, CT, DC, DE, GA, IA, IL, IN, KS, KY, LA, MA, MD, ME, MI, MN, MO, MS, NC, NE, NH, NJ, NM, NY, OH, OK, PA, RI, SC, SD, TN, TX, UT, VA, VT, WI, WV [FL, ID]

### Clivina myops

Bousquet, 1997

Clivina myops Bousquet, 1997c: 343. Type locality: «Raleigh [Wake County], N[orth]C[arolina]» (original citation). Holotype (♂) in CNC [# 22215].

#### Distribution.

This species is known from the holotype collected in east-central North Carolina and six old specimens labeled “D C,” “Md,” and “Ill.” (CMNH).

#### Records.

**USA**: DC, IL, MD, NC

### Clivina oregona

Fall, 1922

Clivina oregona Fall, 1922b: 164. Type locality: «Corvallis [Benton County], Oregon» (original citation). Holotype in MCZ [# 23857].

#### Distribution.

This species occurs from southern British Columbia (Lindroth 1961a: 163) and northern Idaho (Hatch 1953: 66) south to central Oregon (Fall 1922b: 164; Benton County, MCZ).

#### Records.

**CAN**: BC **USA**: ID, OR, WA

#### Note.

Hlavac (1967: 27) believed this species was morphologically distinct enough to propose a new subgenus, *Betaclivina*, for it. His thesis being unpublished, the name is unavailable.

### Clivina pallida

Say, 1823

Clivina pallida Say, 1823a: 22. Type locality: «S[outh] C[arolina]» (neotype label). Neotype (♂), designated by Lindroth and Freitag (1969: 334), in MCZ [# 33076]. Note. «Chinquoteage island, coast of Virginia» was the area originally cited by Say (1823a: 22).
Clivina rufescens Dejean, 1831: 504. Type locality: «Amérique septentrionale» (original citation). Syntype(s) location unknown (see Lindroth 1955b: 13 and Lindroth and Freitag 1969: 334). Synonymy established by LeConte (1846b: 214).

#### Distribution.

This species ranges from southern Maine (Nelson 1995: 71) to “Illinois” (Hlavac 1967: 23), including southeastern Michigan (Saint Clair and Wayne Counties, CMNH) and west-central Indiana (Montgomery County, R. Michael Brattain collection), south to north-central Texas (Knaus 1905b: 348), southeastern Louisiana (Tangipahoa Parish, USNM) and the Florida Panhandle (Peck and Thomas 1998: 17).

#### Records.

**USA**: AL, AR, DC, FL, GA, IL, IN, LA, MD, ME, MI, MS, NC, NJ, NY, OH, PA, SC, TN, TX, VA

### Clivina planicollis

LeConte, 1857

Clivina planicollis LeConte, 1857b: 81. Type locality: «South Carolina» (original citation). Lectotype (♂), designated by Whitehead (1974: 454), in MCZ [# 5471]. Note. LeConte (1857b: 81, 82) originally used two spellings for the name of this species, *planicollis* (page 81) and *planicolis* (page 82). Because LeConte (1863b: 4) subsequently used the spelling *planicollis*, this spelling is the correct original spelling (ICZN 1999: Article 24.2.4).
Clivina texana LeConte, 1863c: 4. Type locality: «Texas» (original citation), restricted to «Bentsen State Park, Mission, Hidalgo County» by Whitehead (1974: 454). Lectotype (♂), designated by Whitehead (1974: 454), in MCZ [# 5470]. Synonymy established by Whitehead (1974: 454).

#### Distribution.

This species ranges from South Carolina (LeConte 1857b: 82; Ciegler 2000: 42) and northern Georgia (Fattig 1949: 14; Floyd County, MCZ) west to southwestern Texas (Dajoz 2004: 117; El Paso County, MCZ), north along the Mississippi River drainage to west-central Indiana (Tippecanoe County, CMNH) and east-central Kansas (Dickinson and Douglas Counties, MCZ); also recorded from Jalisco, Nayarit, Tamaulipas (Erwin 2011b: 183) and Veracruz in Mexico (Bates 1881: 32). The record from the District of Columbia (Ulke 1902: 6) needs confirmation.

#### Records.

**USA**: AL, AR, GA, IN, KS, LA, MS, OK, SC, TX [DC] – Mexico

#### Note.

Hlavac (1967: 19) believed this species was morphologically distinct enough to propose a new subgenus, *Alphaclivina*, for it. His thesis being unpublished, the name is unavailable.

### Clivina punctigera

LeConte, 1857

Clivina punctigera LeConte, 1857b: 81. Type locality: «South Carolina» (original citation). One syntype in MCZ [# 5473].

#### Distribution.

This species ranges from southern Missouri (Hlavac 1967: 32; Ripley County, CMNH) to central Virginia (Nelson County, USNM) and the District of Columbia (Ulke 1902: 37), including southern Ohio (Washington County, UASM; Wright and Whitehouse 1941: 70), south to the Florida Panhandle (Jackson County, CNC), southwestern Mississippi (Copiah County, MCZ), and southern Texas (Zapata, San Patricio and Dimmit Counties, CMNH, UASM).

#### Records.

**USA**: AL, AR, DC, IL, IN, FL, GA, LA, MO, MS, OH, OK, SC, TN, TX, VA, WV

### Clivina punctulata

LeConte, 1852

Clivina punctulata LeConte, 1852a: 198. Type locality: «San Jose [Santa Clara County, California]» (original citation). Two syntypes in MCZ [# 5472].

#### Distribution.

This species is found in California from Siskiyou County (CAS) to San Diego County (Moore 1937: 5).

#### Records.

**USA**: CA

### Subgenus Antroforceps

Barr, 1967

Antroforceps Barr, 1967a: 66. Type species: *Antroforceps bolivari* Barr, 1967 by original designation. Etymology. Probably from the Greek *antron* (cave) and part of the name Forcipatorina, a scaritine subtribe in which Barr placed his new taxon [masculine].

#### Diversity.

Four species in the temperate regions of eastern North America (three species) and caves in the Sierra de Guatemala mountains in northeastern Mexico (one species, *Clivina bolivari* Barr).

#### Identification.

Ball (2001) revised the species and provided a key for their identification. One species (*Clivina alabama*) was described subsequently.

#### Taxonomic Note.

In a cladistic analysis of the Western Hemisphere subgenera proposed by Ball (2001: Fig. 9), this taxon turn out as the sister-group to {*Semiclivina* + *Leucocara* (under the name *Reichardtula*)}.

### Clivina alabama

Bousquet, 2012

Clivina alabama Bousquet [in Bousquet and Skelley], 2012: 47. Type locality: «0.5 mi S[outh] Highland Lake, Blount Co[unty], Al[abama]» (original citation). Holotype in CNC [# 24034].

#### Distribution.

This species is known only from two specimens collected in Blount County, north-central Alabama.

#### Records.

**USA**: AL

### Clivina rubicunda

LeConte, 1857

Clivina rubicunda LeConte, 1857b: 81. Type locality: «Louisiana» (original citation). Two syntypes in MCZ [# 5474].

#### Distribution.

This species is confined to eastern United States ranging from Connecticut (Krinsky and Oliver 2001: 46) to southeastern Kansas (Knaus 1885: 57), including eastern Iowa (Ball 2001: 147), south to “Louisiana” (LeConte 1857b: 81) and central Florida (Peck and Thomas 1998: 17).

#### Records.

**USA**: AL, CT, DC, FL, GA, IA, IL, IN, KS, LA, MD, MI, MS, NJ, NY, OH, PA, SC, VA

### Clivina sasajii

Ball, 2001

Clivina sasajii Ball, 2001: 147. Type locality: «Latimer County, Oklahoma» (original citation). Holotype (♂) in TAMU.

#### Distribution.

This species is known only from eastern Oklahoma (Ball 2001: 147).

#### Records.

**USA**: OK

### Subgenus Leucocara

Bousquet, 2009

Leucocara Bousquet, 2009: 43. Type species: *Clivina americana* Dejean, 1831 by original designation. Etymology (original). From the Greek *leukos* (white) and *kara* (head) [feminine]. The name was proposed in honor of Donald Robert Whitehead [1938-1990], taxonomist at the United States Department of Agriculture who had an interest in Clivinini in general.

#### Diversity.

This subgenus includes 72 species in the Nearctic (5 species), Palaearctic (6 species), Oriental (18 species) and Afrotropical (43 species) Regions (see Bousquet 2009: Table 2).

#### Identification.

There is no key for the identification of the North American species of this subgenus. Nichols (1988a: 147-153) covered two species (*Clivina acuducta* and *Clivina americana*) in his thesis. A revision of the group is needed.

#### Taxonomic Note.

Until recently, the species of this subgenus were included in the subgenus *Reichardtula* Whitehead, a replacement name for *Eupalamus* Schmidt-Göbel.

### Clivina acuducta

Haldeman, 1843

Clivina acuducta Haldeman, 1843b: 299. Type locality: «P[ennsylvani]a and Ala[bama]» (original citation), restricted to «S[outh]E[astern] Penns[ylvania]» by Lindroth (1961a: 160). One possible syntype, labeled “[orange disc] / C. americana Dej. acuducta Hald. [handwritten],” in MCZ (collection LeConte).
Clivina cordata Putzeys, 1846: 86. Type locality: «Nouvelle Orléans [Orleans Parish, Louisiana]» (original citation). Holotype [by monotypy] in MHNP (collection Chaudoir). **New synonymy** based on Nichols (1988a: 148) unpublished thesis.
Clivina ludoviciana Putzeys, 1867b: 138. Type locality: «Louisiane» (original citation). Syntype(s) [5 originally cited] in MHNP (collection Chaudoir). Synonymy established by LeConte (1879a: 34).

#### Distribution.

This species ranges from Massachusetts (Norfolk County, CMNH) to southwestern Wisconsin (Grant County, CMNH), south to eastern Texas (Riley 2011; San Augustine County, CMNH) and southern Florida (Nichols 1988a: 150).

#### Records.

**USA**: AL, AR, FL, GA, IL, IN, KY, LA, MA, MD, MO, MS, NC, NJ, NY, OH, PA, SC, TN, TX, VA, WI, WV

#### Note.

*Clivina acuducta* has been listed in synonymy with *Clivina americana* in most catalogues (Leng 1920: 48; Erwin et al. 1977: 4.12; Bousquet and Larochelle 1993: 103) but Nichols (1988a: 148) treated it as a valid species.

### Clivina americana

Dejean, 1831

Clivina americana Dejean, 1831: 503. Type locality: «Amérique septentrionale» (original citation), restricted to «Boston [Suffolk County], Mass[achusetts]» by Lindroth (1961a: 160). Seven possible syntypes in MHNP (Lindroth 1955b: 13).
Clivina analis Putzeys, 1846: 81. Type locality: «Texas (original citation). Holotype [by monotypy] in MHNP (collection Chaudoir). Synonymy established by Putzeys (1867b: 138), confirmed by Nichols (1988a: 151).
Clivina morula LeConte, 1857b: 81. Type locality: United States of America (inferred from title of the paper). Syntype(s) in MCZ [# 5476]. Synonymy established by Nichols (in Bousquet 2009: 44). Note. This name has been recorded by mistake as *Clivina merula* LeConte by Putzeys (1867b: 192).

#### Distribution.

This species ranges from Nova Scotia (Lindroth 1954c: 301) to southeastern North Dakota (Tinerella 2003: 635), south to western (Dajoz 2007: 23) and southern (Gonzales County, MCZ) Texas, southern Louisiana (Hine 1906: 76, as *Clivina analis*; Calcasieu and Orleans Parishes, MCZ), and southern Florida including the Keys (Peck and Thomas 1998: 17); also recorded from the Bahamas (Nichols 1988a: 153).

#### Records.

**CAN**: NB, NS, ON, QC **USA**: AL, AR, CT, DC, DE, FL, GA, IA, IL, IN, KS, KY, LA, MA, MD, ME, MI, MN, MO, MS, NC, ND, NE, NH, NJ, NY, OH, OK, PA, RI, SC, SD, TN, TX, VA, VT, WI, WV – Bahamas

### Clivina californica

Van Dyke, 1925

Clivina californica Van Dyke, 1925: 124. Type locality: «shores of Clear Lake, Lake County, California» (original citation). Holotype in CAS [# 1631].

#### Distribution.

This species is known only from the type locality.

#### Records.

**USA**: CA

### Clivina morio

Dejean, 1831

Clivina morio Dejean, 1831: 506. Type locality: «Amérique septentrionale» (original citation), herein restricted to 4.2 miles northeast of Abita Springs, Saint Tammany Parish, Louisiana (LSAM). Holotype [by monotypy] in MHNP.

#### Distribution.

This species is known from Harrison County in southeastern Mississippi (Drew A. Hildebrandt pers. comm. 2010), Saint Tammany Parish in southeastern Louisiana, Trinity County in eastern Texas (Bousquet 2009: 44), and LeFlore County in eastern Oklahoma (Matthew Gimmel collection). The records from southeastern New York (Leng and Beutenmüller 1893: 135), Lancaster County in Pennsylvania (Rathvon 1869: 524), northwestern Georgia (Fattig 1949: 15), and southwestern Florida (Leng 1915: 571) are in error (see Bousquet 2006c: 3).

#### Records.

**USA**: LA, MS, OK, TX

### Clivina rufa

LeConte, 1857

Clivina rufa LeConte, 1857b: 81. Type locality: «Illinois» (original citation). One syntype in MCZ [# 5475].

#### Distribution.

This species ranges from southeastern New York (Notman 1928: 213) to eastern South Dakota (Kirk and Balsbaugh 1975: 17), south to eastern Oklahoma (Latimer County, UASM), “Louisiana” (LeConte 1879a: 34), and southern Florida (Peck and Thomas 1998: 17). The record from the lower peninsula of Michigan (Hubbard and Schwarz 1878: 644) needs confirmation.

#### Records.

**USA**: AL, AR, DC, DE, FL, GA, IA, IL, IN, KS, LA, NC, NJ, NY, OK, SD [MI]

### Genus Paraclivina

Kult, 1947

Paraclivina Kult, 1947: 31. Type species: *Clivina burmeisteri* Putzeys, 1866 by original designation. Etymology. From the Greek *para* (near, next to) and the generic name *Clivina* [*q.v*.] [feminine].

#### Diversity.

At least 33 species (see Bousquet 2009: table 1) in temperate, subtropical, and tropical areas of the Nearctic (nine species) and Neotropical (29 species) Regions. Kult’s (1947: 31) statement that *Paraclivina* is represented by two species in Australia is apparently erroneous (see Baehr 2008: 23).

#### Identification.

There is no revision of the North American species of this genus. The last key published, that of LeConte (1879a), included seven of the eight species recorded at the time (*Paraclivina sulcipennis* was omitted). Since then, one tropical species (*Paraclivina fasciata*) has been recovered in Florida but it is unclear if it is established in the area. Putzeys (1846 and 1867b) included descriptions of all the North American species currently recognized. In his 1867 work, these species were listed in groups 21 (most species) and 22 (*Paraclivina fasciata* and *Paraclivina ferrea*) of the genus *Clivina*. A revision of the genus is needed.

#### Taxonomic Note.

This taxon is very likely monophyletic, characterized by the synapomorphic condition of the lateral bead of the pronotum uninterrupted and removed from the base. The genus *Clivina* is markedly speciose and inadequately understood both taxonomically and phylogenetically. In these circumstances, I believe it is more convenient to isolate this taxon as a distinct genus even if eventually it may prove to be nested within *Clivina*. In a cladistic analysis of the five Western Hemisphere subgenera of *Clivina* (*sensu auctorum*) conducted by Ball (2001: Fig. 9), this taxon turn out as the sister-group to the remaining subgenera.

### Paraclivina bipustulata

(Fabricius, 1798)

Scarites 2pustulatus Fabricius, 1798: 44. Type locality: «America boreali» (original citation). Three syntypes in ZMUC (Zimsen 1964: 41).
Scarites quadrimaculatus Palisot de Beauvois, 1811: 107. Type locality: «Caroline du sud» (original citation). Syntype(s) probably lost. Synonymy established by Say (1823a: 21).

#### Distribution.

The range of this species extends from Massachusetts (Purrington 1997: 96) to eastern South Dakota (Kirk and Balsbaugh 1975: 17; French et al. 2004: 557), south to southeastern Texas (Snow 1906a: 141; Cameron County, MCZ) and southern Florida including the Keys (Peck and Thomas 1998: 17), west along the southwest to southern Arizona (Dajoz 2004: 116; Cochise and Pima Counties, UASM); also recorded from several islands of the West Indies, Mexico, Honduras, and Nicaragua (Erwin 2011b: 162). The record from “New Hampshire” (Bousquet and Larochelle 1993: 103) needs confirmation.

#### Records.

**CAN**: ON **USA**: AL, AR, AZ, CT, DC, DE, FL, GA, IA, IL, IN, KS, KY, LA, MA, MD, MI, MN, MO, MS, NC, NE, NJ, NM, NY, OH, OK, PA, RI, SC, SD, TN, TX, VA, WI, WV [NH] – Cayman Islands, Cuba, Dominican Republic, Haiti, Jamaica, Mexico

### Paraclivina convexa

(LeConte, 1844)

Clivina convexus LeConte, 1844: 50. Type locality: «Georgia» (original citation). Two syntypes in MCZ [# 5478].
Clivina bisignata Putzeys, 1846: 102. Type locality: «Amérique boréale» (original citation). Holotype [by monotypy] probably in MHNP (collection Chaudoir). Synonymy established with doubt by LeConte (1857b: 82), accepted by Putzeys (1867b: 156).

#### Distribution.

This species is known from Long Island, New York (Cooper 1935: 144) to east-central South Carolina (Ciegler 2000: 41) and “Georgia” (LeConte 1844: 50) and from “Louisiana” (LeConte 1879a: 34) and Cuba (Chevrolat 1863: 193, as *Clivina bisignata*). The records from “Arkansas” (Bousquet and Larochelle 1993: 103) and “Texas” (Leng and Beutenmüller 1893: 96) need confirmation.

#### Records.

**USA**: GA, LA, NC, NJ, NY, SC, VA [AR, TX] – Cuba

### Paraclivina fasciata

(Putzeys, 1846)

Clivina fasciata Putzeys, 1846: 106. Type locality: «Merida, Yucatan» (original citation). Syntype(s) [5 originally cited] in UMO (Nichols 1988a: 135) and probably also MHNP.
Clivina klugii Putzeys, 1846: 106. Type locality: «Colombie; Cumana [Venezuela]» (original citation). Syntype(s) [2 originally cited] in MHNP (Nichols 1988a: 136). Synonymy established by Nichols (in Bousquet 2009: 38). Etymology. The specific name honors the German entomologist Johann Christoph Friedrich Klug [1775-1856], professor and eventually director of the Zoological Museum at the University in Berlin.
Clivina sculptifrons Putzeys, 1846: 107. Type locality: «Colombie» (original citation). Syntype(s) [3 originally cited] in MHNP (Nichols 1988a: 136). Synonymy established by Nichols (in Bousquet 2009: 38).
Dyschirius insularis Jacquelin du Val, 1857: 13. Type locality: Cuba (inferred from title of the book). Holotype [by monotypy] in MHNP. Synonymy established by Nichols (in Bousquet 2009: 38).
Clivina dilutipennis Putzeys, 1867b: 162. Type locality: «San Andres Tuxtla, Mexique» (original citation). Syntype(s) [4 originally cited] in MHNP (collection Chaudoir). Synonymy established by Nichols (in Bousquet 2009: 38).
Clivina dissimilis Blatchley, 1923: 15 [primary homonym of *Clivina dissimilis* Putzeys, 1846]. Type locality: «Dunedin [Pinellas County, Florida]» (original citation). Holotype [by monotypy] (♂) in PURC. Synonymy established by Erwin (2011b: 171).
Clivina floridae Csiki, 1927: 503. Replacement name for *Clivina dissimilis* Blatchley, 1923.

#### Distribution.

This species has been reported from several islands of the West Indies and several countries from southern Mexico to South America (Erwin 2011b: 171). It is also known from one specimen, the holotype of *Clivina dissimilis* Blatchley, collected in Pinellas County, central Florida. The species is apparently adventive in the Philippines and the Marianas (Darlington 1970: 12).

#### Records.

**USA**: FL – Bahamas, Brazil, Cayman Islands, Colombia, Costa Rica, Cuba, Dominica, Dominican Republic, Grenada, Guadeloupe, Haiti, Honduras, Jamaica, Mexico, Panama, Puerto Rico, Saint Croix, Saint Thomas, Venezuela, Virgin Islands

### Paraclivina ferrea

(LeConte, 1857)

Clivina ferrea LeConte, 1857b: 81. Type locality: «Illinois; Catskill [New York]» (original citation). Two syntypes in MCZ [# 5477].

#### Distribution.

This species occurs from southeastern New York (LeConte 1857b: 82) to southeastern South Dakota (Kirk and Balsbaugh 1975: 17), south to southern Texas (Wickham 1897: 103; Johnson 1978: 67) and northern Florida (Leon County, USNM), west along southern United States to southern California (Imperial, Riverside, and Kern Counties, CAS, USNM) and the Baja California Peninsula (Horn 1894: 307).

#### Records.

**USA**: AL, AR, AZ, CA, CO, DC, FL, GA, IA, IL, IN, KS, LA, MD, MO, MS, NC, NJ, NM, NY, OH, OK, PA, SC, SD, TN, TX, VA, WI – Mexico

#### Note.

This species has been recorded by mistake as *Clivina ferruginea* LeConte by Horn (1872c: 384).

### Paraclivina marginipennis

(Putzeys, 1846)

Clivina marginipennis Putzeys, 1846: 101. Type locality: «Nouvelle Orléans [Orleans Parish, Louisiana] et Yucatan [Mexico]; Guadeloupe» (original citation), restricted to «Louisiane» by Putzeys (1867b: 149). Syntype(s) [4 originally cited but restricted to one by Putzeys (1867b: 149)] in MHNP (collection Chaudoir).

#### Distribution.

This species is known from northeastern Mississippi (Tishomingo County, Drew A. Hildebrandt pers. comm. 2009), southeastern Louisiana (Putzeys 1867b: 149), and some islands of the West Indies (Nichols 1988a: 143; Peck 2009b: 5).

#### Records.

**USA**: LA, MS – Dominica, Dominican Republic, Guadeloupe, Puerto Rico, Saint Lucia

### Paraclivina postica

(LeConte, 1846)

Clivina postica LeConte, 1846b: 213. Type locality: «ad Rocky Mountains» (original citation). Syntype(s) in MCZ [# 5479].

#### Distribution.

This species ranges from southwestern Ohio (Dury 1882: 218) to southeastern South Dakota (Kirk and Balsbaugh 1975: 17), including southern Wisconsin (Messer 2010: 35), south to east-central Texas (Riley 2011), southeastern Louisiana (Jefferson Parish, MCZ; Allen 1965: 62; LeConte 1879a: 34), southern Mississippi (Hancock County, Drew A. Hildebrandt pers. comm. 2008), central Alabama (Shelby County, CMNH), and southwestern South Carolina (Ciegler 2000: 42).

#### Records.

**USA**: AR, AL, GA, IA, IL, IN, KS, LA, MO, MS, NE, OH, OK, SC, SD, TN, TX, WI

### Paraclivina stigmula

(Putzeys, 1846)

Clivina stigmula Putzeys, 1846: 104. Type locality: «Texas» (original citation). Syntype(s) [2 originally cited] in MHNP (collection Chaudoir).

#### Distribution.

This species is known for sure only from “Texas” (Putzeys 1846: 104; MCZ). The record from “Kansas” (Bousquet and Larochelle 1993: 104) needs confirmation.

#### Records.

**USA**: TX [KS]

### Paraclivina striatopunctata

(Dejean, 1831)

Clivina striatopunctata Dejean, 1831: 505. Type locality: «Amérique septentrionale» (original citation). Four possible syntypes in MHNP (Lindroth 1955b: 13).
Clivina picea Putzeys, 1846: 103. Type locality: «Louisiane» (original citation). Holotype [by monotypy] location unknown (possibly in UMO, collection Chevrolat). Synonymy established by Putzeys (1867b: 155).

#### Distribution.

This species may be restricted to the Coastal Plain and Piedmont Plateau. It is known from Staten Island, New York (Leng 1915: 569) and New Jersey (Hamilton 1889a: 30; Leng and Beutenmüller 1893: 96) south to southern Florida (Peck and Thomas 1998: 17), west to south-central Texas (Bexar County, CMNH, USNM). The record from eastern Iowa (Wickham 1911b: 5) is probably in error. One specimen simply labeled from Tennessee (CMNH) is also known.

#### Records.

**USA**: AL, DE, FL, GA, LA, MS, NJ, NY, SC, TX [TN]

### Paraclivina sulcipennis

(Putzeys, 1867)

Clivina sulcipennis Putzeys, 1867b: 156. Type locality: «États-Unis du Sud» (original citation). Holotype [by monotypy] probably in IRSN.

#### Distribution.

This species is known only from the Atlantic shore of Virginia (Hoffman et al. 2006: 18), southern North Carolina (Brunswick County, Ken Karns pers. comm. 2009), southeastern South Carolina (Charleston County, USNM), Florida as far south as Collier County (USNM), and southwestern Alabama (Van Dyke 1925: 125; Baldwin County, MCZ).

#### Records.

**USA**: AL, FL, NC, SC, VA

### Genus Schizogenius

Putzeys, 1846

Schizogenius Putzeys, 1846: 131. Type species: *Schizogenius strigicollis* Putzeys, 1846 designated by Desmarest (1851: 102). Etymology. From the Greek *schizo* (cleave, split) and *geneion* (chin, by extension mentum), alluding to the indented shape of the lateral lobes of the mentum (“*lobes lateraux* [du menton] *profondément échancrés, tellement que chacun d’eux semble partagé en deux ailes*”) of the adult [masculine].

#### Diversity.

Western Hemisphere, with 75 species in temperate, subtropical, and tropical areas of the Nearctic (24 species) and Neotropical (about 60 species) Regions arrayed in three subgenera: *Genioschizus* (10 species), *Listropus* Putzeys (eight Neotropical species), and *Schizogenius* s.str. (57 species). One species has been described by Baehr (1983) from the Fiji Islands in the Central Pacific Ocean based upon a single specimen. In my opinion, the specimen could be mislabeled and confirmation is needed.

#### Identification.

Whitehead (1972) revised all the North American and some of the Neotropical species.

### Subgenus Genioschizus

Whitehead, 1972

Genioschizus Whitehead, 1972: 144. Type species: *Schizogenius crenulatus* LeConte, 1849 by original designation. Etymology (original). From the Greek *geneion* (chin, by extension mentum) and *schizo* (cleave, split); also an anagram of *Schizogenius* [*q.v*.] [masculine].

#### Diversity.

Ten species in North America (one species) and Middle and South America (ten species).

### Schizogenius crenulatus crenulatus

LeConte, 1852

Schizogenius crenulatus LeConte, 1852a: 197. Type locality: «ad flumen Colorado» (original citation), restricted to «Colorado River opposite Yuma, Yuma County, Arizona» by Whitehead (1972: 150). Lectotype, designated by Whitehead (1972: 150), in MCZ [# 5480].

#### Distribution.

This subspecies is found in southeastern California and Arizona south to southern Sinaloa and northern Nayarit [see Whitehead 1972: Fig. 74].

#### Records.

**USA**: AZ, CA – Mexico

#### Note.

The subspecies *Schizogenius crenulatus chiapatecus* Whitehead is found in Mexico and Honduras.

### Subgenus Schizogenius

Putzeys, 1846

Schizogenius Putzeys, 1846: 131. Type species: *Schizogenius strigicollis* Putzeys, 1846 designated by Desmarest (1851: 102).

#### Diversity.

Fifty-seven species in the Nearctic (23 species) and Neotropical (45 species) Regions.

### [brevisetosus group]

### Schizogenius brevisetosus

Whitehead, 1972

Schizogenius brevisetosus Whitehead, 1972: 206. Type locality: «Sanderson [Terrell County], Tex[as]» (original citation). Holotype (♂) in CNC [# 12868].

#### Distribution.

This species is found along the Colorado River in eastern New Mexico to the Rio Grande drainage system in central Texas and Coahuila [see Whitehead 1972: Fig. 146]; it is also recorded from southwestern Oklahoma (Kondratieff et al. 2005: 173).

#### Records.

**USA**: NM, OK, TX – Mexico

### [depressus group]

### Schizogenius depressus

LeConte, 1852

Schizogenius depressus LeConte, 1852a: 197. Type locality: «ad flumen Colorado» (original citation). Lectotype (♂), designated by Whitehead (1972: 287), in MCZ [# 5843].

#### Distribution.

This species ranges from the Okanagan Valley in southern British Columbia to northwestern South Dakota, south to the state of Mexico and southern California [see Whitehead 1972: Fig. 241].

#### Records.

**CAN**: BC **USA**: AZ, CA (CHI), CO, ID, MT, NM, OR, SD, TX, UT, WA, WY – Mexico

### Schizogenius falli

Whitehead, 1972

Schizogenius falli Whitehead, 1972: 281. Type locality: «4.8 mi[les] e[ast] Sabinas Hidalgo (800’), Rio Sabinas Hidalgo, Nuevo Leon, Mex[ico]» (original citation). Holotype (♂) in MCZ [# 31981]. Etymology. The specific name honors Henry Clinton Fall [1862-1939], a high school teacher in Chicago, Pomona, and Pasadena, and reputed coleopterist.

#### Distribution.

The known range of this species extends from southern Illinois to southern California, south to Baja California Sur and Colima in Mexico [see Whitehead 1972: Fig. 240].

#### Records.

**USA**: AR, AZ, CA, CO, IL, MO, NE, NM, OK, TX, UT – Mexico

### Schizogenius litigiosus

Fall, 1901

Schizogenius litigiosus Fall, 1901a: 210. Type locality: «middle and northern California» (original citation), restricted to «Sylvania [Sonoma County]» by Whitehead (1972: 268). Syntype(s) in MCZ [# 23858].

#### Distribution.

This species ranges west of the Rocky Mountains from Vancouver Island to western Idaho, south to southern California [see Whitehead 1972: Fig. 237].

#### Records.

**CAN**: BC (VCI) **USA**: CA, ID, NV, OR, WA

### Schizogenius ochthocephalus

Whitehead, 1972

Schizogenius ochthocephalus Whitehead, 1972: 285. Type locality: «Davis [Yolo County], Cal[ifornia]» (original citation). Holotype (♂) in UCD.

#### Distribution.

This species is known only from a few localities in northern and central California [see Whitehead 1972: Fig. 240] and from San Bernardino County in the southeast (Dajoz 2007: 20).

#### Records.

**USA**: CA

### Schizogenius pygmaeus

Van Dyke, 1925

Schizogenius pygmaeus Van Dyke, 1925: 125. Type locality: «shores of Clear Lake, Lake County, California» (original citation). Holotype in CAS [# 1632].
Schizogenius championi Kult, 1950a: 142. Type locality: «Pantaleon (700 ft.), Guatemala» (original citation). Holotype in BMNH. Synonymy established by Whitehead (1972: 270). Etymology. The specific name was proposed in honor of the British coleopterist George Charles Champion [1851-1927] who is best known for his contribution to the *Biologia Centrali-Americana*. Engaged by Godman and Salvin, the editors and publishers of the series, Champion collected in Guatemala, Panama, and Colombia between 1879 and 1883. He served as Secretary and Chief Assistant to the editors and contributed major sections to nine volumes of the series on the Heteroptera and Coleoptera.

#### Distribution.

This widely distributed species ranges from northern California south through Arizona and western New Mexico to Colombia, including Baja California [see Whitehead 1972: Fig. 239].

#### Records.

**USA**: AZ, CA, NM – Colombia, Costa Rica, El Salvador, Guatemala, Honduras, Mexico, Nicaragua, Panama

### Schizogenius scopaeus

Whitehead, 1972

Schizogenius scopaeus Whitehead, 1972: 278. Type locality: «Limpia Canyon, 2 mi[les] n[orth] w[est] Fort Davis [Jeff Davis County], Texas» (original citation). Holotype (♂) in MCZ [# 31980].

#### Distribution.

This species ranges from southern Missouri to southeastern Colorado south to Nuevo León and Tamaulipas [see Whitehead 1972: Fig. 238].

#### Records.

**USA**: AR, CO, MO, OK, TX – Mexico

### Schizogenius sulcifrons

Putzeys, 1846

Schizogenius sulcifrons Putzeys, 1846: 134. Type locality: «Amérique boréale» (original citation), restricted to «Rumney [Grafton County], N[ew] H[ampshire]» by Lindroth (1961a: 167). Lectotype (♀), designated by Lindroth (1961a: 167), in UMO.

#### Distribution.

This species is widely distributed east of the Mississippi River from New Brunswick (Lindroth 1961a: 167) to eastern Illinois, south to east-central Louisiana (West Feliciana Parish, Igor M. Sokolov pers. comm. 2009), southern Mississippi (Clairborne, Covington, Pearl River, Stone, and Wilkinson Counties, Drew A. Hildebrandt pers. comm. 2008), and western North Carolina (Whitehead 1972: 267, Fig. 236). Old specimens simply labeled from “Georgia,” “South Carolina,” and “Wisconsin” are known (Whitehead 1972: 267). The record from “Alabama” (Bousquet and Larochelle 1993: 106) needs confirmation.

#### Records.

**CAN**: NB, ON, QC **USA**: DC, IL, IN, KY, LA, MA, MD, ME, MS, NC, NH, NJ, NY, OH, PA, TN, VA, VT, WV [AL, GA, SC, WI]

### [ferrugineus group]

### Schizogenius auripennis

Bates, 1881

Schizogenius auripennis Bates, 1881: 38. Type locality: «Teleman, Guatemala» (original citation). Lectotype, designated by Whitehead (1972: 182), in BMNH.
Schizogenius peninsularis Van Dyke, 1949a: 50. Type locality: «5 miles south of Miraflores, Lower California» (original citation). Holotype in CAS [# 6010]. Synonymy established by Whitehead (1972: 182).

#### Distribution.

This species ranges from southern Arizona south to Costa Rica in Pacific drainage areas [see Whitehead 1972: Fig. 101].

#### Records.

**USA**: AZ – Belize, Costa Rica, Guatemala, Honduras, Mexico

### Schizogenius ferrugineus

Putzeys, 1846

Schizogenius ferrugineus Putzeys, 1846 [January]: 135. Type locality: «Galveston [Galveston County], Texas» (original citation). Holotype [by monotypy] probably in MHNP (collection Chaudoir).
Clivina sulcata LeConte, 1846b [August]: 214. Type locality: «NovEboraci [= New York]» (original citation). Two syntypes in MCZ [# 31157]. Synonymy established by LeConte (1853c: 396), confirmed by Lindroth (1961a: 168).

#### Distribution.

This species ranges from southern Quebec (Larochelle 1975: 106) to South Dakota (Kirk and Balsbaugh 1975: 16), north to southwestern Saskatchewan (CNC), south to central Texas and northern Florida, west along southern United States to southeastern Arizona [see Whitehead 1972: Fig. 100]. Also recorded from the Bahamas (Erwin 2011b: 215).

#### Records.

**CAN**: ON, QC, SK **USA**: AL, AR, AZ, CO, CT, DC, DE, FL, GA, IA, IL, IN, KS, KY, LA, MA, ME, MD, MI, MN, MO, MS, NC, NE, NH, NJ, NM, NY, OH, OK, PA, SC, SD, TX, VA, VT, WI – Bahamas

### [lindrothi group]

### Schizogenius lindrothi

Whitehead, 1972

Schizogenius lindrothi Whitehead, 1972: 199. Type locality: «7 mi[les] n[orth] Southport, Bay Co[unty], Florida» (original citation). Holotype (♂) in MCZ [# 31970]. Etymology. The specific name honors Carl Hildebrand Lindroth [1905-1979], Swedish naturalist and carabid taxonomist. His systematic treatment of the carabid fauna of Canada and Alaska, achieved after 20 years, was the main catalyzer behind the interest for carabid taxonomy in North America that arose in the 1960s.

#### Distribution.

This species is currently known only from a few specimens collected in Florida, Guatemala, and Costa Rica (Whitehead 1972: 201). Also recorded from Honduras and Panama (Erwin 2011b: 218).

#### Records.

**USA**: FL – Costa Rica, Guatemala, Honduras, Panama

#### Note.

Whitehead (1972: 201) stated that he was uncertain if the two Central American specimens he saw were conclusively conspecific with those from Florida.

### [lineolatus group]

### Schizogenius lineolatus

(Say, 1823)

Clivina lineolata Say, 1823a: 22. Type locality: «Allegheny [Allegheny County], P[ennsylvani]a» (neotype label). Neotype (♂), designated by Lindroth and Freitag (1969: 334), in MCZ [# 33078].

#### Distribution.

This species is distributed from New Brunswick (Webster and Bousquet 2008: 16) to northeastern Montana, south to the Rio Grande Valley in south-central Texas, Tamaulipas in Mexico, and central Alabama [see Whitehead 1972: Fig. 206]. One old specimen simply labeled from “South Carolina” is known (Whitehead 1972: 251). Except for four apparently mislabeled “Florida” specimens, the species is unknown south of New Jersey along the Atlantic Coast.

#### Records.

**CAN**: NB, ON, QC **USA**: AL, AR, CT, DC, IA, IL, IN, KS, KY, MA, MD, ME, MN, MO, MS, MT, NC, NE, NH, NJ, NY, OH, OK, PA, RI, SD, TN, TX, VA, VT, WI, WV, WY [SC] – Mexico

### [longipennis group]

### Schizogenius chiricahuanus

Whitehead, 1972

Schizogenius chiricahuanus Whitehead, 1972: 257. Type locality: «Cave C[ree]k (ca. 6000’), Chiricahua M[oun]t[ain]s, Cochise Co[unty], Ariz[ona]» (original citation). Holotype (♂) in MCZ [# 31968].

#### Distribution.

This species is known only from a few localities in southern Arizona [see Whitehead 1972: Fig. 209].

#### Records.

**USA**: AZ

### Schizogenius longipennis

Putzeys, 1867

Schizogenius tristriatus var. *longipennis* Putzeys, 1867b: 227. Type locality: «Mexique» (original citation), restricted to «Fortin de las Flores, Veracruz» by Whitehead (1972: 254). Lectotype (♀), designated by Whitehead (1972: 254), in IRSN.
Schizogenius validus Fall, 1901a: 210. Type locality: «Rio Verdi [= Verde River] in central Arizona» (original citation). Lectotype (♂), designated by Whitehead (1972: 254), in MCZ [# 23860]. Synonymy established by Whitehead (1972: 254).

#### Distribution.

The range of this species extends from southern Arizona to Tamaulipas, south to Costa Rica [see Whitehead 1972: Fig. 208].

#### Records.

**USA**: AZ – Belize, Costa Rica, Guatemala, Honduras, Mexico

### Schizogenius neovalidus

Whitehead, 1972

Schizogenius neovalidus Whitehead, 1972: 252. Type locality: «Gila River, n[ea]r Cliff, Grant Co[unty], New Mexico» (original citation). Holotype (♂) in MCZ [# 31967].

#### Distribution.

This species is known from eastern Arizona and southwestern New Mexico [see Whitehead 1972: Fig. 207].

#### Records.

**USA**: AZ, NM

### [pluripunctatus group]

### Schizogenius pluripunctatus

LeConte, 1852

Schizogenius pluripunctatus LeConte, 1852a: 197. Type locality: «ad flumen Colorado» (original citation). Lectotype, designated by Whitehead (1972: 221), in MCZ [# 5484].
Schizogenius simplex LeConte, 1852a: 197. Type locality: «Colorado [River]» (original citation). Lectotype, designated by Whitehead (1972: 221), in MCZ [# 5485]. Synonymy established by LeConte (1857b: 83), confirmed by Whitehead (1972: 221).

#### Distribution.

This species ranges from Arizona and western New Mexico, south to Nayarit [see Whitehead 1972: Fig. 147].

#### Records.

**USA**: AZ, NM – Mexico

### Schizogenius seticollis seticollis

Fall, 1901

Schizogenius seticollis Fall, 1901a: 209. Type locality: «Pomona [Los Angeles County], Cal[ifornia]» (lectotype label). Lectotype, designated by Whitehead (1972: 209), in MCZ [# 23859].

#### Distribution.

This subspecies is restricted to central and western California, from Shasta County in the north to San Diego County in the south [see Whitehead 1972: Fig. 147].

#### Records.

**USA**: CA

#### Note.

The subspecies *Schizogenius seticollis vandykei* Whitehead is known only from a few localities in Baja California Sur (Whitehead 1972: 213).

### [sallei group]

### Schizogenius sallei

Putzeys, 1867

Schizogenius sallei Putzeys, 1867b: 228. Type locality: «Texas» (original citation), herein restricted to Garner State Park, Uvalde County (see Whitehead 1972: 230). Lectotype (♂), designated by Whitehead (1972: 229), in IRSN. Etymology. The specific name was proposed for Auguste Sallé [1820-1896], a naturalist traveller and later insect dealer in Paris. Sallé, accompanied by his mother, lived most of the time between 1832 and 1860 in Mexico and Venezuela and travelled also to southern United States, the West Indies, and Central America where he collected insects. Most of these specimens were sent to Louis Alexandre August Chevrolat who divided them in lots and sold them. In 1839, these lots were offered at 30 francs for 100 specimens plus 3 francs 40 centimes for the transport from America to Paris. His Central American collections were purchased by Godman and Salvin for the *Biologia Centrali-Americana* (Papavero 1971: 178-179).

#### Distribution.

This species ranges from Kansas south to the Rio Grande Valley in southern Texas [see Whitehead 1972: Fig. 185]. One specimen simply labeled from “Ohio” (Whitehead 1972: 229) could be mislabeled. The record from south-central Colorado (Wickham 1902: 232) is probably in error.

#### Records.

**USA**: KS, OK, TX [OH]

### [tristriatus group]

### Schizogenius amphibius

(Haldeman, 1843)

Clivina amphibia Haldeman, 1843b: 299. Type locality: southeastern Pennsylvania (Haldeman 1843a: 295). Lectotype, designated by Whitehead (1972: 236), in MCZ (collection LeConte).
Clivina frontalis LeConte, 1846b: 215. Type locality: «Westchester Co[unty], N[ew] Y[ork]» (original citation). Lectotype (♂), designated by Whitehead (1972: 236), in MCZ [# 5482]. Synonymy established by Melsheimer (1853: 8), confirmed by Whitehead (1972: 236).

#### Distribution.

This species ranges from Maine, southern Quebec, and Michigan south to Tennessee and North Carolina [see Whitehead 1972: Fig. 190]; also seen from eastern Oklahoma (Le Flore County, FFPC). The records from eastern Iowa (Wickham 1911b: 6) and Missouri (Summers 1873: 133) need confirmation; that from southwestern Colorado (Wickham 1902: 232) must be in error.

#### Records.

**CAN**: QC **USA**: CT, DC, IL, IN, KY, MA, MD, ME, MI, NC, NH, NJ, NY, OH, OK, PA, RI, TN, VA, VT, WV [IA, MO, TX]

#### Note.

Whitehead (1972: 237) noted that four specimens from Texas, without definite locality data, may represent an isolated form of this species.

**Figure 18.**
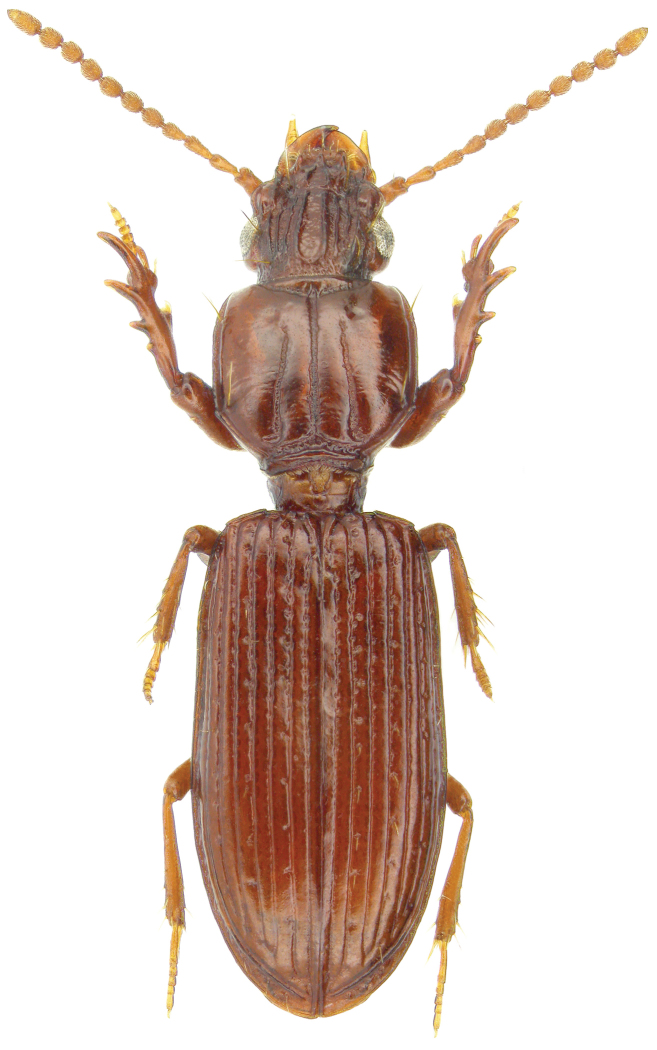
*Schizogenius amphibius* (Haldeman). This small clivinine belongs to a genus endemic to the Western Hemisphere and is closely related to *Halocoryza* which is represented, besides North America, along the east and west coast of Africa and on many islands in the eastern parts of the Indian Ocean. The adults usually live along river banks near the water and this ecological preference was hinted at by Samuel Haldeman through the species’ epithet.

### Schizogenius ozarkensis

Whitehead, 1972

Schizogenius ozarkensis Whitehead, 1972: 240. Type locality: «5 mi[les] n[orth] Stringtown, Atoka Co[unty], Oklahoma» (original citation). Holotype (♂) in MCZ [# 31978].

#### Distribution.

This species is known only from a few specimens collected in or near the Ozark Mountains in southern Missouri, western Arkansas, and eastern Oklahoma [see Whitehead 1972: Fig. 189].

#### Records.

**USA**: AR, MO, OK

### Schizogenius planulatus

LeConte, 1863

Schizogenius planulatus LeConte, 1863c: 5. Type locality: «New York» (original citation), herein restricted to Ithaca, Tompkins County (see Whitehead 1972: 240). Lectotype (♀), designated by Whitehead (1972: 238), in MCZ [# 5481].

#### Distribution.

This species is known for sure only from a few specimens collected in New York, West Virginia, and Kentucky [see Whitehead 1972: Fig. 189]; it was also reported from southwestern Ohio (Dury 1910: 66) and northeastern Georgia (Fattig 1949: 15).

#### Records.

**USA**: KY, NY, WV [GA, OH]

### Schizogenius planuloides

Whitehead, 1972

Schizogenius planuloides Whitehead, 1972: 241. Type locality: «Cypress Mills [Blanco County], Texas» (original citation). Holotype (♂) in USNM [# 74164].

#### Distribution.

This species is known from a few localities throughout much of Texas [see Whitehead 1972: Fig. 189].

#### Records.

**USA**: TX

### Schizogenius tibialis

Whitehead, 1972

Schizogenius tibialis Whitehead, 1972: 234. Type locality: «19.3 mi[les] n[orth]w[est] Tamazunchale (500’), S[an] L[uis] P[otosí], Mexico» (original citation). Holotype (♂) in MCZ [# 31979].

#### Distribution.

This species occurs from southern Texas south to southeastern Oaxaca and northern Chiapas [see Whitehead 1972: Fig. 188].

#### Records.

**USA**: TX – Mexico

### Genus Halocoryza

Alluaud, 1919

Halocoryza Alluaud, 1919: 100. Type species: *Halocoryza maindroni* Alluaud, 1919 by monotypy. Etymology. From the Greek *halos* (sea) and generic name *Coryza*, alluding to the presence along the sea (under “*de grosses pierres le long de la jetée *... *ces pierres sont vraisemblablement submergées à marée haute*”) of adults of these *Coryza*-like species [feminine].

#### Diversity.

Four species are placed in this genus: one (*Halocoryza acapulcana* Whitehead) is found on the Pacific Coast of Mexico and on the Galápagos Islands, one (*Halocoryza arenaria*) in southeastern North America, the West Indies, Brazil, and the west coast of Africa, another one (*Halocoryza maindroni* Alluaud) on the east coast of Africa and several islands along the western part of the Indian Ocean, including Madagascar, and the southern part of the Red Sea, and the last one (*Halocoryza whiteheadiana* Erwin) on Baja California Sur.

#### Identification.

Whitehead (1967) and Erwin (2011a) reviewed the species and provided keys for their identification.

### Halocoryza arenaria

(Darlington, 1939)

Schizogenius arenarius Darlington, 1939: 84. Type locality: «near Barahona, Dominican Republic» (original citation). Holotype in MCZ [# 23505].

#### Distribution.

This species is known from southern Florida, the Yucatán Peninsula, Jamaica (Nichols 1988b: Fig. 5-12), Puerto Rico, the Dominican Republic (Whitehead 1969: 36), the Bahamas, Cuba, Cayman Islands (Peck 2005: 29) and several islands of the Lesser Antilles (Peck 2009a: 12). The species has been recorded also from the Gulf of Biafra in Africa (Bruneau de Miré 1979) and the state of Pernambuco, Brazil (Nichols 1988b: 89).

#### Records.

**USA**: FL – Bahamas, Barbados, Brazil, Cayman Islands, Cuba, Dominica, Dominican Republic, Jamaica, Martinique, Mexico, Puerto Rico, Saint Lucia, Saint Vincent, Virgin Islands

### Genus Oxydrepanus

Putzeys, 1867

Oxydrepanus Putzeys, 1867b: 103. Type species: *Dyschirius rufus* Putzeys, 1846 designated by Bousquet and Larochelle (1993: 107). Etymology. From the Greek *oxys* (acute) and *drepanos* (sickle), probably alluding to the acute, sickle-shaped mandibles (“*mandibules *... *très arquées, très aigües à l’extrémité*”) of the adult [masculine].

#### Diversity.

Thirteen species in the subtropical and tropical regions of the Western Hemisphere, including the West Indies, with one species reaching southeastern North America.

#### Identification.

Nichols (1988a: 172-182) covered the four species found in the West Indies in his thesis and provided a key for their identification.

### Oxydrepanus rufus

(Putzeys, 1846)

Dyschirius rufus Putzeys, 1846: 44. Type locality: «Havane, Cuba» (original citation). Holotype [by monotypy] in UMO (Nichols 1988a: 180).
Dyschirius brevicarinatus Putzeys, 1846: 53. Type locality: «Cuba» (original citation). Syntype(s) [2 originally cited] probably in IRSN. Synonymy established by Putzeys (1861: 70).

#### Distribution.

This species has been reported from southeastern Louisiana (Colby 2002: 37), southern Florida (Darlington 1935a: 161), the Bahamas (Peck and Thomas 1998: 18), Cuba (Darlington 1934: 70), Cayman Islands and Puerto Rico (Peck 2005: 29), Dominican Republic (Erwin 2011b: 204), Jamaica, and eastern Mexico in Veracruz and Campeche (Nichols 1988a: 181).

#### Records.

**USA**: FL, LA – Bahamas, Cayman Islands, Cuba, Dominican Republic, Jamaica, Mexico, Puerto Rico

### Subtribe Ardistomina

Putzeys, 1867

Ardistomides Putzeys, 1867b: 4, 200. Type genus: *Ardistomis* Putzeys, 1846.

#### Diversity.

Western Hemisphere, with about 90 species, arrayed in three genera. All but five species are found in the Neotropical Region. One of the North American species has been collected in Japan.

#### Identification.

Bousquet (2006c) revised the North American species and provided a key for their identification.

### Genus Ardistomis

Putzeys, 1846

Ardistomis Putzeys, 1846: 118. Type species: *Ardistomis fasciolata* Putzeys, 1846 designated by Desmarest (1851: 102). Etymology. From the Greek *ardis* (point of an arrow) and *stoma* (mouth), probably alluding to the apex of the glossal sclerite which is membranous and markedly projected forward (“*la languette *... *se rétrécit de la base à l’apex qui se termine en une longue pointe membraneuse*”), one of the main characteristics of adults of this genus according to Putzeys [feminine]. Note. Lorenz (1998: 48) treated *Ardistomis* as masculine. However, the ending -*is* in Latin is of variable gender (masculine or feminine) and in such case the Commission (ICZN 1999: Article 30.1.4.2) rules that the name is to be treated as masculine unless its author, when establishing the name treated it as feminine in combination with an adjectival species-group name. Putzeys (1846) treated *Ardistomis* as feminine and so the name is feminine.
Ardistomus Csiki, 1927: 547. Unjustified emendation of *Ardistomis* Putzeys, 1846.

#### Diversity.

This genus contains 44 species (Valdés 2009: 70) restricted to the temperate, subtropical, and tropical regions of the Nearctic (two species) and Neotropical (42 species) Regions, including the West Indies.

### Ardistomis obliquata

Putzeys, 1846

Ardistomis obliquata Putzeys, 1846: 120. Type locality: «Amérique boréale» (original citation), restricted to «Saint Catherines Island [Liberty County], Georgia» by Bousquet (2006c: 19). Holotype [by monotypy] in MHNP (Nichols 1988a: 94).

#### Distribution.

This species ranges along the Coastal Plain and Piedmont Plateau from New Jersey (Smith 1890: 76; Smith 1910: 202) and southeastern Pennsylvania (Rathvon 1969: 524) to southern Florida, west to central Louisiana [see Bousquet 2006c: Fig. 35].

#### Records.

**USA**: AL, DC, FL, GA, LA, MS, NC, NJ, NY, PA, SC, TN, VA

### Ardistomis schaumii

LeConte, 1857

Ardistomis schaumii LeConte, 1857b: 80. Type locality: «Louisiana» (original citation), restricted to «Alexandria, Rapides Parish» by Bousquet (2006c: 18). Lectotype, designated by Bousquet (2006c: 12), in MCZ [# 5486]. Etymology. The specific name honors Hermann Rudolphe Schaum [1819-1865], a German physician who eventually turned entomologist and worked primarily on Coleoptera. Schaum made a trip to eastern United States from 1847 to 1849 and stayed for some time with LeConte. In 1857 he became professor at the Royal University in Berlin.

#### Distribution.

This species is found from South Carolina (Ciegler 2000: 43) to southern Florida, west to the Rio Grande in south-central Texas, north along the Mississippi River drainage to southern Illinois [see Bousquet 2006c: Fig. 34].

#### Records.

**USA**: AL, AR, FL, GA, IL, LA, MS, OK, SC, TN, TX, VA

### Genus Semiardistomis

Kult, 1950

Semiardistomis Kult, 1950b: 301. Type species: *Clivina labialis* Chaudoir, 1837 by original designation. Etymology. From the Latin prefix *semi*- (half) and the generic name *Ardistomis* [*q.v*.] [masculine]. Note. As for *Ardistomis*, this name could be masculine or feminine because of its ending -*is*. In such case, the Commission (ICZN 1999: Article 30.1.4.2) rules that the name is to be treated as masculine unless its author, when establishing the name treated it as feminine in combination with an adjectival species-group name. Kult (1950: 301) proposed *Semiardistomis* as a subgenus and so did not combine it with a species-group name. However, he treated *Ardistomis* as masculine and *Semiardistomis*, contrary to *Ardistomis*, is masculine.
Ardistomiellus Kult, 1950b: 303. Type species: *Clivina viridis* Say, 1823 by original designation. Synonymy established by Whitehead (in Reichardt 1977: 392). Etymology. From the generic name *Ardistomis* [*q.v*.] and the suffix -*ellus* (small, little) [masculine].

#### Diversity.

Twenty species (Valdés 2012) restricted to the temperate, subtropical, and tropical regions of the Western Hemisphere, including the West Indies.

### Semiardistomis puncticollis

(Dejean, 1831)

Clivina puncticollis Dejean, 1831: 508. Type locality: «Amérique septentrionale» (original citation), restricted to «Highlands Hammock State Park, Hardee Co[unty], Florida» by Bousquet (2006c: 12). Lectotype (♀), designated by Bousquet (2006c: 15), in MHNP.

#### Distribution.

This species ranges from southeastern Iowa (Wickham 1911b: 6; King 1914: 321) to western Kentucky, south to the Rio Grande in southeastern Texas, east to southern Florida, north along the coast to southern Virginia [see Bousquet 2006c: Fig. 33]. The record from southeastern Pennsylvania (Rathvon 1869: 524) is probably in error.

#### Records.

**USA**: AL, AR, FL, GA, IA, IL, IN, KS, KY, LA, MO, MS, NC, OK, SC, TX, VA

### Semiardistomis viridis

(Say, 1823)

Clivina viridis Say, 1823a: 21. Type locality: «Phila[delphia] Neck, P[ennsylvani]a» (neotype label). Neotype (♂), designated by Lindroth and Freitag (1969: 334), in MCZ [# 33077].
Clivina rostrata Dejean, 1825: 419. Type locality: «Géorgie» (original citation). Lectotype, designated by Bousquet (2006c: 12), in MHNP. Synonymy established by Dejean (1826: 478), confirmed by Nichols (1988a: 195).
Ardistomis vicina Putzeys, 1846: 129. Type locality: «Amérique boréale» (original citation). Lectotype, designated by Bousquet (2006c: 12), in MHNP. Synonymy established by LeConte (1857b: 80), confirmed by Nichols (1988a: 195).

#### Distribution.

The range of this species extends from Long Island, New York to southwestern Wisconsin (Messer 2010: 35), south to the Rio Grande in south-central Texas and southern Florida [see Bousquet 2006c: Fig. 32]; also recorded from the Bahamas (Turnbow and Thomas 2008: 15).

#### Records.

**USA**: AL, AR, CT, DC, DE, FL, GA, IL, IN, KS, KY, LA, MD, MO, MS, NC, NJ, NY, OH, OK, PA, SC, TN, TX, VA, WI, WV – Bahamas

### Genus Aspidoglossa

Putzeys, 1846

Aspidoglossa Putzeys, 1846: 108. Type species: *Aspidoglossa submetallica* Putzeys, 1846 designated by Desmarest (1851: 102). Etymology. From the Greek *aspidos* (shield) and *glossa* (tongue), probably alluding to the shape of the glossal sclerite [feminine].

#### Diversity.

Twenty-six species (Lorenz 2005: 146-147) in the temperate, subtropical, and tropical regions of the Western Hemisphere, including the West Indies. One species only is found in North America.

### Aspidoglossa subangulata

(Chaudoir, 1843)

Dyschirius subangulatus Chaudoir, 1843b: 738. Type locality: «Nouvelle-Orléans [Orleans Parish, Louisiana]» (original citation). Lectotype, designated by Bousquet (2006c: 10), in MHNP.
Dyschirius humeralis Chaudoir, 1843b: 737. Type locality: «Nouvelle-Orléans [Orleans Parish, Louisiana]» (original citation). Syntype(s) in MHNP. Synonymy established by Melsheimer (1853: 9).
Aspidoglossa fraterna Putzeys, 1846: 114. Type locality: «Amérique boréale» (original citation). Syntype(s) [7 originally cited] in MHNP (collection Chaudoir) and UMO. Synonymy established by Melsheimer (1853: 9).
Aspidoglossa vicina Putzeys, 1846: 114. Type locality: «Caroline» (original citation). Syntype(s) [2 ♂ originally cited] location unknown (possibly in UMO in collection Chevrolat). Synonymy established by Melsheimer (1853: 9).

#### Distribution.

This species ranges over eastern United States from Washington D.C. to northeastern Kansas, including southeastern Iowa (Wickham 1911b: 6; King 1914: 323), south to the big bend along the Rio Grande in Texas and northeastern Mexico (Bousquet 2006c: 9) and southern Florida; also known from southeastern Arizona [see Bousquet 2006c: Fig. 31]. Two specimens labeled from Klamath County, Oregon (AMNH) and Gallatin County, Montana (CAS) are known (Bousquet 2006c: 9). The species has been recorded also from Japan (Habu 1963: 19).

#### Records.

**USA**: AL, AR, AZ, DC, FL, GA, IA, IL, IN, KS, KY, LA, MD, MI, MO, MS, NC, NM, OH, OK, PA, SC, TN, TX, VA [MT, OR] – Mexico

### Tribe Dyschiriini

Kolbe, 1880

Dyschiriini Kolbe, 1880: 266. Type genus: *Dyschirius* Bonelli, 1810.

#### Diversity.

Worldwide, with about 300 species arrayed in nine genera: *Akephorus* (two species), *Clivinopsis* Bedel (three species in northern Africa, Kazakhstan, and Turkmenistan), *Cribrodyschirius* Bruneau de Miré (seven species in Africa, Madagascar, and Asia), *Dyschirius* (about 255 species), *Neodyschirius* Kult (one Afrotropical species), *Reicheiodes* Ganglbauer (16 Palaearctic species, including the Himalayas), *Setodyschirius* Fedorenko (13 species from Australia), *Torretassoa* Schatzmayr (one species in Egypt and Yemen), and *Caledyschirius* Bulirsch (five species in New Caledonia). The Northern Hemisphere is well represented with about 220 species (73.5% of the world fauna).

### Genus Akephorus

LeConte, 1852

Akephorus LeConte, 1852a: 194. Type species: *Akephorus marinus* LeConte, 1852 by monotypy. Etymology. From the Greek *ake* (point) and *phoro* (to bear, carry), possibly alluding to the acute apical spur of the protibia of the adult (“*tibiae anticae spinis terminalibus longissimis*”) [masculine]. Note. *Acephorus* is an incorrect subsequent spelling for *Akephorus*, first used by LeConte (1853c: 396), not in prevailing usage.

#### Diversity.

Two species restricted to the seashore of the Pacific in North America.

#### Identification.

The two species can be differentiated using Lindroth’s (1961a) and Bousquet’s (1988a) keys to *Dyschirius*.

#### Taxonomic Note.

This taxon has been considered a subgenus of *Dyschirius* by Lindroth (1961a) and a distinct genus by Fedorenko (1996). The phylogenetic analysis of Fedorenko (1996) did not yield clear evidence as to the position of the group. Furthermore there are little structural characteristics for members of *Akephorus*. The presence of conspicuous microsculpture on the body is also found in a few *Dyschirius* groups, such as the *exochus* group, phenetically similar to the remaining *Dyschirius*. The body shape, however, is distinctive. With the current phylogenetic knowledge of the tribe, I see no harm in retaining this taxon as a distinct genus.

### Akephorus marinus

LeConte, 1852

Akephorus marinus LeConte, 1852a: 195. Type locality: «circa San Diego [San Diego County, California]» (original citation). Three syntypes in MCZ [# 685].

#### Distribution.

This species is confined to the seashore of the Pacific Coast of California, as far north as San Mateo County (CAS), and of the Baja California Peninsula (CNC).

#### Records.

**USA**: CA (CHI) – Mexico

### Akephorus obesus

(LeConte, 1863)

Dyschirius obesus LeConte, 1863b: 50. Type locality: «San Francisco [San Francisco County], Cal[ifornia]» (original citation). One syntype in MCZ [# 679].

#### Distribution.

This species ranges along the seashore of the Pacific from Kunghit Island in the Queen Charlotte Islands (James C. Bergdahl pers. comm. 2009) south at least to central California (LeConte 1867b: 363; San Mateo County, CAS).

#### Records.

**CAN**: BC (QCI, VCI) **USA**: CA, OR, WA

### Genus Dyschirius

Bonelli, 1810

Dyschirius Bonelli, 1810: Tabula Synoptica. Type species: *Scarites thoracicus* Rossi, 1790 designated by Curtis (1831: plate 354). Etymology. Possibly from the Greek *dis* (twice, double) and *cheiros* (hand), alluding to the presence of a curved spine laterally and a movable spur medially on the protibia of the adult [masculine].
Dischirius Duponchel, 1844: 151. Unjustified emendation of *Dyschirius* Bonelli, 1810.
Dyschiridius Jeannel, 1941b: 260. Type species: *Dyschirius arenosus* Stephens, 1827 (= *Scarites thoracicus* Rossi, 1790) by original designation.

#### Diversity.

About 245 species (Lorenz 2005: 151-154) in the Nearctic (about 60 species, one of them adventive), Neotropical (about 20 species, five shared with North America), Oriental (about 25 species), Palaearctic (about 140 species, many shared with the Oriental and Afrotropical Regions), and Afrotropical (about 30 species) Regions.

#### Identification.

The North American species have never been revised. Bousquet (1988a) published a key to all species found in the area and briefly discussed each species-group. Subsequently five new species have been described by Bousquet (1997a) and Dajoz (2004), an adventive species has been detected (*Dyschirius globosus*) in North America, two species have been treated as junior synonyms (*Dyschirius filiformis* with *Dyschirius sublaevis* and *Dyschirius integer* with *Dyschirius dejeanii*), and two species had their names changed (*Dyschirius nigricornis* for *Dyschirius melancholicus* and *Dyschirius integer* for *Dyschirius dejeanii*).

#### Taxonomic Note.

Fedorenko (1996) recognized two genera within *Dyschirius* (minus *Akephorus*): *Dyschirius* with about 20 Palaearctic species and *Dyschiriodes* for the remaining species. However, there seems to be no consistent character state that separates the two “genera” except possibly for the basal sclerite of the endophallus which is “strongly sclerotized” in *Dyschirius* and “poorly sclerotized” in *Dyschiriodes*. In addition there is no solid evidence presented suggesting that the genus *Dyschirius* of authors is polyphyletic or that *Dyschirius sensu* Fedorenko and *Dyschiriodes* are sister-taxa. The genus *Dyschirius sensu auctorum* (prior to Fedorenko 1996) is a well-defined taxon and I see no reason to recognize *Dyschiriodes* as generically distinct. A similar approach was adopted by Balkenohl (2003: 223-230) in the *Catalogue of Palaearctic Coleoptera*, Lorenz (2005: 151-154), and Erwin (2011b: 97-128).

Fedorenko (1996) recognized five subgenera within “*Dyschiriodes*:” *Antidyschirius* Fedorenko, 1996 [type species: *Dyschirius laevifasciatus* Horn, 1878], *Eudyschirius* Fedorenko, 1996 [type species: *Dyschirius lafertei* Putzeys, 1846], *Chiridysus* Fedorenko, 1996 [type species: *Dyschirius strumosus* Erichson, 1837], *Dyschiriodes* Jeannel, 1941 [type species: *Clivina punctata* Dejean *sensu* Jeannel, 1941 (= *Clivina minuta* Dejean, 1825)], and *Paradyschirius* Fedorenko, 1996 [type species: *Dyschirius parallelus* Motschulsky, 1844]. In my opinion there are several other species groups actually recognized within *Dyschirius* that deserve a subgeneric name. For that reason I have listed the North American species under species-group names and did not retain Fedorenko’s subgenera until further studies are done on the North American fauna. For information, the species of the *laevifasciatus* group belong to *Antidyschirius*; those of the *tridentatus*, *ferrugineus*, and *brevispinus* groups, as well as *Dyschirius globosus* (classified here in the *dejeanii* group), *Dyschirius comatus* (placed here in the *pilosus* group), *Dyschirius criddlei* and *Dyschirius edentulus* (both included here in the *politus* group) belong to *Eudyschirius*; those of the *analis* and *quadrimaculatus* groups to *Paradyschirius*; and those of all remaining North American groups to *Dyschiriodes sensu stricto*.

### [analis group]

### Dyschirius affinis

Fall, 1901

Dyschirius affinis Fall, 1901a: 209. Type locality: «Massachusetts, eastern New York» (original citation). Syntype(s) in MCZ [# 23848].
Dyschirius duplicatus Fall, 1901a: 209. Type locality: «Luling and El Paso, Texas» (original citation). Syntype(s) in MCZ [# 23851]. Synonymy established by Bousquet (1988a: 372).

#### Distribution.

This species ranges from Maine (Larochelle and Larivière 1990a: 28; Androscoggin County, CNC) to southeastern Montana (Herman 1986: 62), south to westernmost (Fall 1901a: 209, as *Dyschirius duplicatus*) and southern Texas (Cameron, San Patricio, and Zapata Counties, CMNH, MCZ) and Maryland (Erwin 1981b: 140). The record from “Arizona” (Bousquet and Larochelle 1993: 98) needs confirmation.

#### Records.

**CAN**: ON, QC **USA**: AR, CO, CT, DC, IA, IL, IN, KS, MA, MD, ME, MT, ND, NE, NH, NM, NY, OH, OK, PA, TN, TX, VA, VT, WI, WY [AZ]

### Dyschirius analis

LeConte, 1852

Dyschirius analis LeConte, 1852a: 196. Type locality: «ad fluminis Colorado ripas» (original citation). Four syntypes in MCZ [# 684].
Dyschirius hintoni Kult, 1950a: 137. Type locality: «Jalapa, Veracruz, Mexico» (original citation). Holotype location unknown (originally in Kult’s collection). Synonymy established by Fedorenko (1996: 200). Etymology. The specific name was proposed for the British entomologist Howard Everest Hinton [1912-1977] who worked at the British Museum of Natural History and later at Bristol University. Hinton published mainly on insect anatomy, physiology, biochemistry, and behavior.

#### Distribution.

The range of this species extends from eastern Washington (Hatch 1953: 67) to southern California (Fall 1901a: 41; Moore 1937: 5), east to southwestern Louisiana (Cameron Parish, LSAM), south to Veracruz in Mexico (Kult 1950a: 138, as *Dyschirius hintoni*). The records from southeastern British Columbia (Hatch 1953: 67) and “Idaho” (Bousquet and Larochelle 1993: 98) need confirmation; that from “Nebraska” (Bousquet and Larochelle 1993: 98) is probably in error.

#### Records.

**USA**: AZ, CA, LA, NM, NV, OR, TX, WA [BC, ID] – Mexico

### Dyschirius haemorrhoidalis

(Dejean, 1831)

Clivina haemorrhoidalis Dejean, 1831: 511. Type locality: «Amérique septentrionale» (original citation), herein restricted to Bent Creek, Appomattox County, Virginia (CNC). Four possible syntypes in MHNP (Lindroth 1955b: 13).

#### Distribution.

This species is found from southern New Hampshire (Hillsborough County, Ross T. Bell pers. comm. 2008) to “North Dakota” (Donald P. Schwert pers. comm. 1989), south to eastern Texas (San Augustine and Wood Counties, CMNH; Bousquet 1988a: 373; Riley 2011) and southern Florida (Peck and Thomas 1998: 17). The record from “New Mexico” (Bousquet and Larochelle 1993: 99) needs confirmation.

#### Records.

**CAN**: ON **USA**: AL, AR, CT, DC, FL, GA, IA, IL, IN, KS, KY, LA, MA, MD, MI, MN, MO, MS, NC, ND, NE, NH, NJ, NY, OH, OK, PA, RI, SC, SD, TN, TX, VA, WI, WV [NM]

### Dyschirius terminatus

LeConte, 1846

Dyschirius terminatus LeConte, 1846b: 212. Type locality: «NovEboraci [= New York]» (original citation). Two syntypes in MCZ [# 5467].

#### Distribution.

This species ranges from Massachusetts (Hampshire County, CNC) to northeastern Kansas (Douglas County, MCZ), including central Wisconsin (Messer 2010: 34) and southeastern Iowa (Des Moines County, MCZ), south to San Luis Potosí in Mexico (Bousquet 1988a: 373) and the Florida Panhandle (Peck and Thomas 1998: 17), west along southern United States to southeastern California (Imperial County, CAS; Dajoz 2007: 16). One old specimen simply labeled “Neb” is known (MCZ).

#### Records.

**USA**: AL, AR, AZ, CA, CT, DE, FL, IA, IL, IN, KS, LA, MA, MD, MI, MO, MS, NC, NJ, NM, NY, OH, OK, PA, SC, TN, TX, VA, WI [NE] – Mexico

### [brevispinus group]

### Dyschirius brevispinus

LeConte, 1878

Dyschirius brevispinus LeConte, 1878c: 593. Type locality: «Detroit [Wayne County, Michigan]» (original citation). Holotype [by monotypy] (♀) in MCZ [# 678].

#### Distribution.

This species is known from scattered localities from the Saint Lawrence Plain in southern Quebec (Larochelle 1975: 81; Bousquet 1987a: 112) to southeastern Michigan (LeConte 1878c: 593), south to southeastern Pennsylvania (Dauphin County, MCZ; Lindroth 1961a: 139); also known from northeastern Nebraska (Clopton 1991: 61).

#### Records.

**CAN**: ON, QC **USA**: MI, NE, OH, PA, VT

### Dyschirius tenuispinus

Lindroth, 1961

Dyschirius tenuispinus Lindroth, 1961a: 139. Type locality: «Medicine Hat, Al[ber]ta» (original citation). Holotype (♂) in CNC [# 7753].

#### Distribution.

This species is known only from a few specimens collected in southern Alberta (Lindroth 1961a: 139; Bousquet 1987a: 113), northeastern Colorado (Bell 1971: 56; Lavigne 1978: 104), and northeastern Arizona (Apache County, UASM).

#### Records.

**CAN**: AB **USA**: AZ, CO

### [carrorum group]

### Dyschirius carrorum

Bousquet, 1997

Dyschirius carrorum Bousquet, 1997a: 92 (as *carri*). Type locality: «Tp. 8 Rge. 14 W.1 Mer. [= about 8 km north of Glenboro], Manitoba» (original citation). Holotype (♂) in CNC [# 22220]. Etymology. This specific name was proposed for John Lawrence Carr [1922-2006] and Bertha Carr of Calgary, Alberta, who collected the holotype. Over several decades of collecting in western North America, the couple assembled a large and extremely valuable collection of beetles which they gave to the Canadian National Collection of Insects in 2000. Note. By a lapsus, *carri* was the original spelling of the specific name despite having been proposed for a man and a woman together. Because there is a clear evidence of an inadvertent error in the original publication, the spelling must be corrected (ICZN 1999: Article 32.5.1) and the original spelling is deemed to be *carrorum* (see ICZN 1999: Article 31.1.2).

#### Distribution.

This species is known only from the holotype collected in south-central Manitoba.

#### Records.

**CAN**: MB

### [exochus group]

### Dyschirius exochus

Whitehead, 1970

Dyschirius exochus Whitehead, 1970: 183. Type locality: «Cedar Lane [Matagorda County], Tex[as]» (original citation). Holotype (♂) in SMEK.

#### Distribution.

This species is known from the type locality in southeastern Texas, from Jackson County in southeastern Mississippi (CMNH), and from two localities in the Florida Panhandle (Frank 1985: 481; Herman 1986: 63).

#### Records.

**USA**: FL, MS, TX

### Dyschirius sculptus

Bousquet, 1988

Dyschirius sculptus Bousquet, 1988a: 370. Type locality: «Pass-a-Grille Beach [Pinellas County], Fl[orida]» (original citation). Holotype (♂) in CUIC [# 6456].

#### Distribution.

This species is known only from the holotype collected along the Gulf Coast of Florida.

#### Records.

**USA**: FL

### [ferrugineus group]

### Dyschirius ferrugineus

Bousquet, 1988

Dyschirius ferrugineus Bousquet, 1988a: 371. Type locality: «Goose Isl[and] St[ate] P[ar]k, Aransas County, Texas» (original citation). Holotype (♂) in USNM.

#### Distribution.

This species is known only from a few localities along the Gulf Coast in southern Texas (Bousquet 1988a: 371; FMNH).

#### Records.

**USA**: TX

### [globulosus group]

### Dyschirius aeneolus

LeConte, 1850

Dyschirius aeneolus LeConte, 1850: 204. Type locality: Lake Superior (inferred from title of the paper), herein restricted to Marquette, Marquette County, Michigan (see Hubbard and Schwarz 1878: 627). Two syntypes [2 originally cited] in MCZ [# 692].
Dyschirius frigidus Mannerheim, 1853: 123. Type locality: «ad fl. Tchunitén peninsulae Kenai [Alaska]» (original citation). Holotype [by monotypy] location unknown. Synonymy established by Lindroth (1961a: 152).

#### Distribution.

This species ranges from southern Labrador to the Kenai Peninsula in Alaska (Lindroth 1961a: 152, as *Dyschirius frigidus*), south to northern Idaho (Hatch 1953: 67), northeastern Minnesota (Gandhi et al. 2005: 923), northeastern Illinois (Peter W. Messer pers. comm. 2008), and the upper and lower peninsulas of Michigan (Hubbard and Schwarz 1878: 627, 644). The records from Colorado (LeConte 1879d: 500; Wickham 1902: 232), Washington and southwestern Oregon (Herman 1986: 60, as *Dyschirius frigidus*; Hatch 1953: 67), and “California” (Leng and Beutenmüller 1894: 184) need confirmation; that from Iowa (Wickham 1911b: 5) is probably in error.

#### Records.

**CAN**: AB, BC, LB, NT, ON, QC, SK, YT **USA**: AK, ID, IL, MI, MN, WI [CA, CO, OR, WA]

### Dyschirius alticola

Lindroth, 1961

Dyschirius alticola Lindroth, 1961a: 152. Type locality: «Rossland Trail, B[ritish] C[olumbia]» (original citation). Holotype in CNC [#7607].

#### Distribution.

This species is known from a few montane locations in south-central British Columbia (Lindroth 1961a: 152; Bousquet 1987a: 116), Idaho (Bear Lake County, CNC), and the Sierra Nevada of California (Papp 1978: 165; Mono and Placer Counties, USNM). The record from “Wyoming” (Bousquet and Larochelle 1993: 101) needs confirmation.

#### Records.

**CAN**: BC **USA**: CA, ID [WY]

### Dyschirius chiricahuae

(Dajoz, 2004)

Dyschiriodes chiricahuae Dajoz, 2004: 117. Type locality: «en haut du Pinery Canyon (vers 2000 mètres), monts Chiricahua, à 20 km environ à l’ouest de la localité de Portal, Cochise County, Arizona» (original citation). Holotype in Dajoz’s collection (Paris, France).

#### Distribution.

This species is known only from two specimens collected at the type locality in southeastern Arizona.

#### Records.

**USA**: AZ

### Dyschirius compactus

Lindroth, 1961

Dyschirius compactus Lindroth, 1961a: 152. Type locality: «Stanley, B[ritish] C[olumbia]» (original citation). Holotype (♂) in CAS [# 8161].

#### Distribution.

This species is known from a few montane locations from southwestern Alberta to south-central British Columbia (Lindroth 1961a: 152; Bousquet 1987a: 116).

#### Records.

**CAN**: AB, BC

### Dyschirius consobrinus

LeConte, 1852

Dyschirius consobrinus LeConte, 1852a: 196. Type locality: «San Francisco [San Francisco County, California]» (original citation). Holotype [by monotypy] (♂) in MCZ [# 690].

#### Distribution.

This species ranges from southwestern Washington (Herman 1986: 60) to Riverside County (CAS) in southeastern California and “the middle Sierras” (Fall 1901a: 41). The records from southeastern British Columbia and northern Idaho (Hatch 1953: 67) are probably in error.

#### Records.

**USA**: CA, OR, WA

### Dyschirius dejeanii

Putzeys, 1846

Dyschirius dejeanii Putzeys, 1846: 25. Type locality: «Amérique boréale» (original citation). Holotype [by monotypy] in MHNP (collection Chaudoir).
Dyschirius apicalis LeConte, 1850: 204 [primary homonym of *Dyschirius apicalis* Putzeys, 1846]. Type locality: Lake Superior (inferred from title of the paper). Syntype(s) in MCZ [# 691]. Synonymy established implicitly by Bousquet (2008a: 517).
Dyschirius integer LeConte, 1852a: 196. Type locality: «ad flumen Colorado» (original citation). Three syntypes in MCZ [# 689]. Synonymy established with the name *Dyschirius apicalis* LeConte by Lindroth (1961a: 149), confirmed by Bousquet (2008a: 517).
Dyschirius nigripes LeConte, 1853c: 396. Replacement name for *Dyschirius apicalis* LeConte, 1850.
Dyschirius transmarinus Mannerheim, 1853: 122 [*nomen dubium*]. Type locality: «insula Sitkha [= Baranof Island, Alaska]» (original citation). Syntype(s) location unknown (possibly in ZILR). Synonymy established with doubt, under the name *Dyschirius integer* LeConte, by Bousquet (1988a: 379).
Dyschirius basalis LeConte, 1857b: 77. Type locality: «Fort Yuma, Colorado River [Imperial County], California» (original citation). Four syntypes in MCZ [# 7401]. Synonymy established, under the name *Dyschirius integer* LeConte, by Bousquet (1988a: 379). Note. Fort Yuma was located on the California side of the Colorado River, opposite Yuma, on a bluff one hundred feet above the river and at an altitude of 260 feet.
Dyschirius sulcatus LeConte, 1859c: 34. Replacement name for *Dyschirius apicalis* LeConte, 1850.

#### Distribution.

This species ranges from Newfoundland (Lindroth 1955a: 42, as *Dyschirius nigripes*) to east-central Alaska (Lindroth 1961a: 150), south to southern California (Fall 1901a: 41, as *Dyschirius basalis*), central New Mexico (Socorro County, CNC), Kansas (Popenoe 1877: 22; Horn 1872c: 384, as *Dyschirius sulcatus*), central Pennsylvania (Northumberland County, CMNH), and northeastern New Jersey (Smith 1910: 201, as *Dyschirius nigripes*; Bergen County, MCZ).

#### Records.

**CAN**: AB, BC, MB, NB, NF, NS (CBI), NT, ON, PE, QC, SK, YT **USA**: AK, AZ, CA, CO, CT, IA, ID, IL, IN, KS, MA, ME, MI, MN, MT, ND, NE, NH, NJ, NM, NV, NY, OH, OR, PA, RI, SD, UT, VT, WA, WI, WY

### Dyschirius gibbipennis

LeConte, 1857

Dyschirius gibbipennis LeConte, 1857b: 77. Type locality: «San Diego [San Diego County], California» (original citation). Holotype [by monotypy] (♀) in MCZ [# 682].

#### Distribution.

This species ranges from southwestern Oregon (Herman 1986: 60) to southern California (Fall 1901a: 41; San Diego County, CAS). The record from “Arizona” (Bousquet and Larochelle 1993: 101) needs confirmation.

#### Records.

**USA**: CA (CHI), OR [AZ]

### Dyschirius globosus

(Herbst, 1784)

Carabus globosus Herbst, 1784: 142. Type locality: «Berlin [Germany]» (original citation). Syntype(s) location unknown (possibly in ZMHB).
Scarites gibbus Fabricius, 1792: 96. Type locality: «Germania» (original citation). Two syntypes in ZMUC (Zimsen 1964: 42). Synonymy established by Fuessly (1794: 160).
Dyschirius glomerosus Bousquet, 1997a: 94. Type locality: «Lulu Island, B[ritish] C[olumbia]» (original citation). Holotype (♂) in CNC [# 22221]. Synonymy established by Bousquet (2002a: 84).

#### Distribution.

This European species is adventive in North America where it is known only from the Vancouver area in southwestern British Columbia (Bousquet 1997a: 94, as *Dyschirius glomerosus*). The first inventoried specimen collected on this continent was found in 1978.

#### Records.

**CAN**: BC – **Adventive**

### Dyschirius globulosus

(Say, 1823)

Clivina gl *obulosa* Say, 1823a: 23. Type locality: «Arlington [Middlesex County], Mass[achusetts]» (neotype label). Neotype (♀), designated by Lindroth and Freitag (1969: 333), in MCZ [# 33081].
Dyschirius parvus LeConte, 1850: 204. Type locality: Lake Superior (inferred from title of the paper). Syntype(s) in MCZ [# 683]. Synonymy established by LeConte (1879a: 31), confirmed by Lindroth (1961a: 154).

#### Distribution.

The range of this species extends from Newfoundland to central Alaska, south to southern British Columbia (Lindroth 1961a: 155), northern Arizona (Coconino County, CMNH), southern New Mexico (Otero County, CNC), east-central Texas (Riley 2011), southeastern Louisiana (Summers 1874a: 79), northern Mississippi (Drew A. Hildebrandt pers. comm. 2007), and southern Florida (Peck and Thomas 1998: 17).

#### Records.

**CAN**: AB, BC, MB, NB, NF, NS (CBI), ON, PE, QC, SK, YT **USA**: AK, AL, AZ, CO, CT, DC, DE, FL, GA, IA, ID, IL, IN, KS, KY, LA, MA, MD, ME, MI, MN, MO, MS, NC, ND, NE, NH, NJ, NM, NY, OH, OK, PA, RI, SC, SD, TX, VA, VT, WI, WV, WY

### Dyschirius hiemalis

Bousquet, 1987

Dyschirius hiemalis Bousquet, 1987a: 116. Type locality: «R.B. Miller Res[earch] St[atio]n, 17 mi[les] w[est] Turner Valley (4,900’), Alberta» (original citation). Holotype (♂) in CNC [# 19238].

#### Distribution.

This species ranges from Labrador and the Ungava Bay area to Alaska, south to south-central British Columbia; isolated on the Shickshock Mountains in Gaspé Peninsula, Quebec [see Bousquet 1987a: map 1]. Fossil remnants, dated between 10,400 and 12,600 years B.P., have been unearthed in Cape Breton Island, Nova Scotia (Miller 1997: 250).

#### Records.

**CAN**: AB, BC, LB, MB, NT, ON, QC, SK, YT **USA**: AK

#### Note.

This taxon has been treated as a subspecies of *Dyschirius melancholicus* Putzeys by Fedorenko (1996: 149) but retained as a distinct species by Bousquet (1987a: 116) and Balkenohl and Lompe (2003: 99).

### Dyschirius longulus

LeConte, 1850

Dyschirius longulus LeConte, 1850: 204. Type locality: Lake Superior (inferred from title of the paper). Three syntypes in MCZ [# 693].

#### Distribution.

This species is found from Newfoundland (Lindroth 1955a: 43) to south-central British Columbia (Lindroth 1961a: 156), south to northwestern Minnesota (Gandhi et al. 2005: 924). The records from South Dakota (Kirk and Balsbaugh 1975: 16), Indiana (Blatchley 1910: 57), the upper and lower peninsulas of Michigan (Hubbard and Schwarz 1878: 627, 644; Hatch 1925: 548; Silvey 1936: 657), Ohio (Leng and Beutenmüller 1893: 91; Dury 1902: 110), and “Pennsylvania” (Bousquet and Larochelle 1993: 101) are in error.

#### Records.

**CAN**: AB, BC, MB, NF, QC, SK **USA**: MN

### Dyschirius melancholicus

Putzeys, 1867

Dyschirius melancholicus Putzeys, 1867b: 41. Type locality: «Daourie» (original citation). Holotype [by monotypy] probably in IRSN.
Dyschirius helleni J. Müller, 1923: 78. Type locality: «Dudinka [Taimyr Autonomous Okrug, Russia]» (original citation). Lectotype, designated by Fedorenko (1996: 148), in MSNT. Synonymy established by Fedorenko (1990: 37). Etymology. The specific name was proposed for Wolter Hellén [1890-1979], curator at the Zoological Museum of the University of Helsinki, excellent collector, and specialist on parasitic Hymenoptera of the Finnish fauna.
Dyschirius norvegicus Munster, 1923b: 249. Type locality: «Sørum i Våge (antagelig i Tromsdalen) ved Tromsø og Gorzzejok i Karasjok» (original citation), restricted to «Sörum, Vågå, Norway» by Lindroth (1961a: 153). Syntype(s) [4 originally cited] location unknown (possibly in ZMUO). Synonymy established, under the name *Dyschirius helleni* Müller, by Hellén (1934: 52), confirmed by Fedorenko (1990: 37).
Dyschirius secretus Fall, 1926a: 130. Type locality: «Anchorage, Alaska» (original citation). Holotype (♀) in MCZ [# 23854]. Synonymy established, under the name *Dyschirius helleni* Müller, by Lindroth (1954b: 122).

#### Distribution.

This Holarctic species ranges from Scandinavia (Balkenohl 2003: 226) to the Hudson Bay in northeastern Manitoba (Garry 1993: 94, as *Dyschirius nigricornis*). Fossil remnants, dated between 10,800 and 20,530 years B.P., have been unearthed in central North Dakota (Ashworth and Schwert 1992: 260), northeastern Iowa (Schwert 1992: 76; Woodman et al. 1996: 17), and southeastern Iowa (Baker et al. 1986: 96).

#### Records.

**CAN**: MB, NT, NU, YT **USA**: AK – **Holarctic**

#### Note.

This species has long been known under the name *Dyschirius nigricornis* Motschulsky, 1844 in the North American literature.

### Dyschirius planatus

Lindroth, 1961

Dyschirius planatus Lindroth, 1961a: 155. Type locality: «Waterton Park, Al[ber]ta» (original citation). Holotype (♂) in CNC [# 7603].

#### Distribution.

This species is known from the southern part of the Prairie Provinces (Lindroth 1961a: 155; Bousquet 1987a: 118) and from northwestern Montana (Teton County, CNC). The records from British Columbia (Jarrett and Scudder 2001: 382), “Wyoming,” and “Minnesota” (Bousquet and Larochelle 1993: 101) need confirmation.

#### Records.

**CAN**: AB, MB, SK **USA**: MT [BC, MN, WY]

### Dyschirius subarcticus subarcticus

Lindroth, 1961

Dyschirius subarcticus Lindroth, 1961a: 151. Type locality: «Circle, Alaska» (original citation). Holotype in MCZ [# 30423].

#### Distribution.

This subspecies is found from Alaska to the Great Slave Lake area in Northwest Territories (Lindroth 1961a: 151).

#### Records.

**CAN**: NT, YT **USA**: AK

#### Note.

The subspecies *Dyschirius subarcticus altaicus* Fedorenko occurs in the eastern part of the Palaearctic Region.

### Dyschirius timidus

Lindroth, 1961

Dyschirius timidus Lindroth, 1961a: 154. Type locality: «Onah, Manit[oba]» (original citation). Holotype (♂) in CNC [# 7605].

#### Distribution.

This species is known only from southern Manitoba (Lindroth 1961a: 154) and Alberta (Langor et al. 2006: 13).

#### Records.

**CAN**: AB, MB

### Dyschirius wayah

(Dajoz, 2005)

Dyschiriodes wayah Dajoz, 2005: 208. Type locality: «Wayah Bald, dans la Nantahala National Forest, Macon County, Caroline du Nord» (original citation). Holotype in Dajoz’s collection (Paris, France).

#### Distribution.

This species is known only from the type locality in southwestern North Carolina.

#### Records.

**USA**: NC

### [laevifasciatus group]

### Dyschirius laevifasciatus

Horn, 1878

Dyschirius laevifasciatus G.H. Horn, 1878b: 52. Type locality: «Oregon» (original citation), herein restricted to Blodgett, Benton County (CNC). Syntype(s) [3 originally cited] in MCZ [# 8181] and CMNH (collection Ulke).

#### Distribution.

This species is found from the foothills of the Rocky Mountains in southern Alberta to south-central British Columbia (Lindroth 1961a: 140), south at least to central Oregon (Benton and Lincoln Counties, CNC, MCZ, USNM). One old specimen labeled “Cal” is known (MCZ).

#### Records.

**CAN**: AB, BC **USA**: OR, WA [CA]

### [pilosus group]

### Dyschirius comatus

Bousquet, 1988

Dyschirius comatus Bousquet, 1988a: 378. Type locality: «Highland Hammock, Highland[s] Co[unty], Fl[orid]a» (original citation). Holotype (♂) in USNM.

#### Distribution.

This species is confined to the Coastal Plain ranging from North Carolina to central Florida, west to southeastern Louisiana (Tangipahoa and Saint Tammany Parishes, LSAM) [see Bousquet 1988a: Fig. 38]. One specimen simply labeled from Texas (CMNH) is known.

#### Records.

**USA**: AL, FL, LA, MS, NC, SC [TX]

### Dyschirius pilosus

LeConte, 1857

Dyschirius pilosus LeConte, 1857b: 80. Type locality: «New Orleans [Orleans Parish, Louisiana]» (original citation). Lectotype, designated by Bousquet (1988a: 377), in MCZ [# 697].
Dyschirius hispidus LeConte, 1863c: 4. Type locality: «western states» (original citation). Lectotype (♀), designated by Bousquet (1988a: 377), in MCZ [# 34045]. Synonymy established by Lindroth (1961a: 156).

#### Distribution.

This species ranges from southern New Brunswick (Webster and Bousquet 2008: 16) to southern Manitoba, south to eastern Texas, southeastern Louisiana (LeConte 1857b: 80; East Baton Rouge Parish, LSAM), Mississippi (Bolivar and Issaquena Counties, Drew A. Hildebrandt pers. comm. 2009), and northeastern North Carolina [see Bousquet 1988a: Fig. 38]. The records from southwestern Georgia (Fattig 1949: 13) and southwestern Alabama (Löding 1945: 12) probably refer to *Dyschirius comatus*; that from “Montana” (Bousquet and Larochelle 1993: 100) is probably in error.

#### Records.

**CAN**: MB, NB, ON, QC **USA**: CT, DC, IA, IL, IN, KS, KY, LA, MA, MD, ME, MI, MN, MO, MS, NC, ND, NE, NH, NJ, NY, OH, OK, PA, TN, TX, VA, VT, WI, WV

### Dyschirius setosus

LeConte, 1857

Dyschirius setosus LeConte, 1857b: 79. Type locality: «Massachusetts and New York» (original citation), restricted to «Mass[achusetts]» by Lindroth (1961a: 157). Three syntypes in MCZ [# 696].
Dyschirius alternatus Hatch, 1949b: 117. Type locality: «Grand Coulee [Grant County], Washington» (original citation). Holotype in USNM. Synonymy established by Lindroth (1961a: 157).

#### Distribution.

This species is found from Prince Edward Island (Larochelle and Larivière 1990a: 27) to south-central British Columbia (Lindroth 1961a: 157), south to eastern Washington (Hatch 1953: 68, as *Dyschirius alternatus*), northern Utah (Utah County, USNM), Oklahoma (Custer and Latimer Counties, CMNH, UASM), and Long Island in New York (MCZ). Seemingly isolated at Fairbanks, Alaska (Lindroth 1961a: 157). The records from “New Jersey,” “Maryland” (Bousquet and Larochelle 1993: 100), and “Colorado” (Wickham 1902: 232) need confirmation.

#### Records.

**CAN**: AB, BC, MB, NB, NS, ON, PE, QC, SK **USA**: AK, IA, ID, IL, MA, ME, MI, MN, MT, ND, NE, NH, NY, OK, PA, RI, SD, UT, VT, WA, WI, WY [CO, MD, NJ]

### [politus group]

### Dyschirius cerberus

Larson, 1968

Dyschirius cerberus Larson, 1968: 1108. Type locality: «Atchison [Atchison County], Kansas» (original citation). Holotype (♀) in USNM [# 69973].

#### Distribution.

This species is known only from the holotype collected in northeastern Kansas.

#### Records.

**USA**: KS

### Dyschirius criddlei

Fall, 1925

Dyschirius criddlei Fall, 1925: 309. Type locality: «Baldur, Manitoba» (original citation). Holotype (♂) in MCZ [# 23849]. Etymology. This specific name honors Norman Criddle [1875-1933], a naturalist and entomologist established in Manitoba who collected extensively in his province.

#### Distribution.

This species is known from southern Manitoba (Lindroth 1961a: 148), North Dakota (Grand Forks County, CNC, UASM), southeastern Nebraska (Foster F. Purrington pers. comm. 2010), Kansas (Stafford County, CNC, UASM), New Mexico (Chaves County, CNC, UASM), northwestern Texas (Hutchinson County, Darren A. Pollock pers. comm. 2011), and southern Florida (Monroe County, FFPC, UASM).

#### Records.

**CAN**: MB **USA**: FL, KS, ND, NE, NM, TX

### Dyschirius edentulus

Putzeys, 1846

Dyschirius edentulus Putzeys, 1846: 51. Type locality: «Galveston [Galveston County], Texas» (original citation). Holotype [by monotypy] in MHNP (collection Chaudoir).
Dyschirius colossus Larson, 1968: 1110. Type locality: «Goose Island State Park, 9 mi[les] north of Rockport [Aransas County], Texas» (original citation). Holotype (♂) in USNM [# 69974]. Synonymy established by Whitehead (1970: 182).

#### Distribution.

This species is known only from north-central Oklahoma (Herman 1986: 61), southeastern Texas (Putzeys 1846: 52; Larson 1968: 1110, as *Dyschirius colossus*; Cameron and Aransas Counties, MCZ, UASM), and Florida (Dixie County, CMNH; Monroe County, FFPC). The record from the lower peninsula of Michigan (Hubbard and Schwarz 1878: 644) is in error.

#### Records.

**USA**: FL, OK, TX

### Dyschirius erythrocerus

LeConte, 1857

Dyschirius erythrocerus LeConte, 1857b: 78. Type locality: «Ohio; Pennsylvania» (original citation). Lectotype (♀), designated by Bousquet (1988a: 375), in MCZ [# 680].

#### Distribution.

This species ranges from southern New Brunswick (Webster and Bousquet 2008: 16) to southeastern South Dakota (Kirk and Balsbaugh 1975: 16), south to southwestern Oklahoma (Kondratieff et al. 2005: 172), southern Louisiana (Saint Martin Parish, LSAM), northern Mississippi (Bolivar, Marshall, and Warren Counties, Peter W. Messer pers. comm. 2009), and Delaware [see Bousquet 1988a: Fig. 37]. The records from the Bahamas (Turnbow and Thomas 2008: 12) and Horn Island, Mississippi (Richmond 1968: 233) refer to *Dyschirius larochellei* Bousquet; that from Colorado (Wickham 1902: 232) is in error.

#### Records.

**CAN**: NB, ON, QC **USA**: AR, CT, DE, IA, IL, IN, KS, KY, LA, MA, ME, MI, MN, MS, NE, NH, NJ, NY, OH, OK, PA, SD, TN, VT, WI

#### Note.

Bulirsch (2009: 19) reported that *Dyschirius weyrauchi* Kult, described from one specimen collected in Peru in 1900, is a junior synonym of *Dyschirius erythrocerus*. If this synonymy is correct, then the specimen from Peru is probably mislabeled.

### Dyschirius larochellei

Bousquet, 1988

Dyschirius larochellei Bousquet, 1988a: 374. Type locality: «6 mi[les] s[outh] L[ake] Placid, Archb[old] B[iological] St[ation], Florida» (original citation). Holotype (♂) in CNC [# 19659].

#### Distribution.

This species is found along the Atlantic Coast from western Newfoundland (Lindroth 1955a: 45, as *Dyschirius erythrocerus*) to southern Florida, including the Keys (Peck and Thomas 1998: 17), the Bahamas, Cuba, and Hispaniola (Nichols 1988b: Fig. 5-6, as *D*. nr. *erythrocerus*), west along the Gulf Coast to southern Texas; also known from Yucatán in southern Mexico (Whitehead 1970: 181, as *Dyschirius erythrocerus*) [see Bousquet 1988a: Fig. 37].

#### Records.

**CAN**: NB, NF, NS **USA**: CT, FL, LA, MA, MS, NH, NJ, NY, SC, VA, TX – Bahamas, Cuba, Hispaniola, Mexico

### Dyschirius pacificus

Lindroth, 1961

Dyschirius pacificus Lindroth, 1961a: 144. Type locality: «Tofino, Vanc[ouver] Isl[and], B[ritish] C[olumbia]» (original citation). Holotype in CNC [# 7609].

#### Distribution.

This species is confined to the Pacific Coast ranging from the Queen Charlotte Islands (Kavanaugh 1992: 59) south at least to northern California (Mendocino County, CNC).

#### Records.

**CAN**: BC (QCI, VCI) **USA**: CA, OR, WA

### Dyschirius perversus

Fall, 1922

Dyschirius perversus Fall, 1922c: 172. Type locality: «Miami, Manitoba» (original citation). Holotype in MCZ [# 23853].
Dyschirius desertus Fall, 1925: 310. Type locality: «Olancha (Owen’s Lake) [Inyo County], California» (original citation). Holotype (♀) in MCZ [# 23850]. Synonymy established by Bousquet (1988a: 374).

#### Distribution.

This rarely collected species is known from scattered localities in the southern parts of the Prairie Provinces (Lindroth 1961a: 147; Bousquet 1988a: 373), southeastern Oregon (Harney County, CNC), northwestern (Pershing County, MCZ) and west-central (Bechtel et al. 1983: 474) Nevada, north-central Utah (Utah County, USNM), and eastern California (Fall 1925: 310, as *Dyschirius desertus*; Inyo and Plumas Counties, CAS).

#### Records.

**CAN**: AB, MB **USA**: CA, NV, OR, UT

### Dyschirius politus politus

(Dejean, 1825)

Clivina polita Dejean, 1825: 422. Type locality: «environs de Paris; aussi en Allemagne» (original citation). Syntype(s) in MHNP.
Dyschirius irkutensis Fleischer, 1899: 11, 23. Type locality: «Quellgebiet des Irkut [= Irkutsk, Russia]» (original citation). At least one syntype in NMP (Fedorenko 1996: 160). Synonymy established by Ganglbauer (1906 : 266).
Dyschirius aureolus Notman, 1920b: 26. Type locality: «Schoharie [Schoharie County], N[ew] Y[ork]» (original citation). Two syntypes [2 ♂ originally cited] in SIM (Hennessey 1990: 466). Synonymy established by Lindroth (1954b: 122).
Dyschirius politus jenisseiensis G. Müller, 1924: 68. Type locality: «Dudinka, nella regione del fiume Jenissei [Taymyr Autonomous Okrug, northern Russia]» (original citation). Holotype [by monotypy] location unknown. Synonymy established by Fedorenko (1992: 102).

#### Distribution.

This Holarctic subspecies is known from the British Islands to eastern Siberia, as far south as Italy, Bulgaria, Iran, Turkmenistan, Kyrgyzstan, and Mongolia (Balkenohl 2003: 226), and in the Nearctic Region from Alaska (Lindroth 1961a: 146) to Newfoundland (Lindroth 1955a: 36), south to southwestern Pennsylvania (Allegheny County, CMNH), eastern South Dakota (Kirk and Balsbaugh 1975: 16), northern New Mexico (Rio Arriba County, USNM), northeastern Nevada (Elko County, MCZ), and southwestern California (Los Angeles County, CAS).

#### Records.

**CAN**: AB, BC, MB, NB, NF, NT, ON, QC, SK, YT **USA**: AK, CA, CO, ID, MA, ME, MI, MT, NH, NM, NV, NY, OH, OR, PA, SD, UT, VT, WA, WI, WY – **Holarctic**

#### Note.

The subspecies *Dyschirius politus chamunensis* Fedorenko and *Dyschirius politus meridianus* Fedorenko occur in Asia.

### Dyschirius sphaericollis

(Say, 1823)

Clivina sphaericollis Say, 1823a: 23. Type locality: «Rumney [Grafton County], N[ew] H[ampshire]» (neotype label). Neotype (♀), designated by Lindroth and Freitag (1969: 334), in MCZ [# 33080].
Dyschirius subpunctatus Hatch, 1949b: 116. Type locality: «Vantage [Kittitas County], Washington» (original citation). Holotype (♂) in USNM. Synonymy established by Bousquet (1988a: 374).

#### Distribution.

The range of this species extends from Newfoundland (Lindroth 1955a: 44) to south-central British Columbia, south to eastern Oregon (Baker County, MCZ), southeastern Arizona (Greenlee and Graham Counties, CMNH), central New Mexico (Socorro County, CNC), southern Texas (Herman 1986: 61; Dajoz 2004: 117), eastern Tennessee (Knox County, MCZ), and North Carolina (Herman 1986: 63). The records from “Georgia” (J.E. LeConte 1849: 25), Florida (Leng 1915: 568), and San Bernardino County in southwestern California (Riley 1893: 239) need confirmation.

#### Records.

**CAN**: AB, BC, MB, NB, NF, NS (CBI), ON, PE, QC, SK **USA**: AR, AZ, CO, CT, DC, IA, ID, IL, IN, KS, KY, LA, MA, MD, ME, MI, MN, MO, MS, MT, NC, ND, NE, NH, NJ, NM, NY, OH, OK, OR, PA, RI, SD, TN, TX, UT, VA, VT, WA, WI, WV, WY [CA, FL, GA]

### Dyschirius truncatus

LeConte, 1857

Dyschirius truncatus LeConte, 1857b: 78. Type locality: «Illinois» (original citation). Holotype [by monotypy] (♀) in MCZ [# 681].

#### Distribution.

This species ranges from southern Manitoba to south-central British Columbia, north to east-central Alaska (Lindroth 1961a: 147), south to southern California (Fall 1901a: 41), central Arizona (Griffith 1900: 565), and southern Colorado (LeConte 1879d: 500; Wickham 1902: 232; Douglas County, CNC); also recorded from Baja California Sur (Horn 1895: 225). One old specimen labeled “Pittsburg VI Pa” (CMNH) is known.

#### Records.

**CAN**: AB, BC, MB, NT, SK **USA**: AK, AZ, CA, CO, IA, ID, IL, IN, MO, ND, NE, NV, SD, UT, WA, WI [PA] – Mexico

### [pumilus group]

### Dyschirius abbreviatus

Putzeys, 1846

Dyschirius abbreviatus Putzeys, 1846 [January]: 12. Type locality: «Texas» (Putzeys 1861: 71), restricted to «Galveston [Galveston County]» by Putzeys (1867b: 51). Holotype [by monotypy] in MHNP (collection Chaudoir). Note. Putzeys (1846: 13) originally gave «Yucatan» as type locality but changed it to «Texas» later (Putzeys 1861: 71). This species is a senior primary homonym of *Dyschirius abbreviatus* Chaudoir, 1846 [June].

#### Distribution.

This species inhabits the Coastal Plain ranging from the coast of southern North Carolina (Brunswick County, Ken Karns pers. comm. 2009) to southern Florida (Nichols 1988b: Fig. 5-5; Peck and Thomas 1998: 17), west to southeastern Texas (Putzeys 1867b: 51).

#### Records.

**USA**: AL, FL, GA, LA, MS, NC, TX

#### Note.

According to Whitehead (1970: 185), members of *Dyschirius darlingtoni* Kult, 1950 from Mexico are probably conspecific with those of *Dyschirius abbreviatus*.

### Dyschirius aratus

LeConte, 1852

Dyschirius aratus LeConte, 1852a: 196. Type locality: «ad flumis Gilae ripas» (original citation). Two syntypes in MCZ [# 701].

#### Distribution.

This species ranges from west-central Wisconsin (Messer 2010: 34) and southern Manitoba (Lindroth 1961a: 142, as *Dyschirius dentiger*) to the Okanagan Valley in south-central British Columbia (Bousquet 1987a: 115), south to southeastern California (Whitehead 1970: 186), Sonora (CNC), Chihuahua (CNC), southeastern Texas (Galveston County, CNC), and southeastern Mississippi (Jackson County, Drew A. Hildebrandt pers. comm. 2007).

#### Records.

**CAN**: AB, BC, MB **USA**: AZ, CA, CO, ID, KS, MS, MT, NE, NM, NV, OK, OR, TX, UT, WA, WI, WY – Mexico

### Dyschirius curvispinus

Putzeys, 1846

Dyschirius curvispinus Putzeys, 1846: 41. Type locality: «Galveston [Galveston County], Texas» (original citation). Holotype [by monotypy] in MHNP (collection Chaudoir).

#### Distribution.

This species is known from southern Maine (Kennebec County, Robert E. Nelson pers. comm. 1989), Connecticut (Litchfield County, CMNH) and “Rhode Island” (Sikes 2003: 7) in the northeast and from the Florida Panhandle (Peter W. Messer pers. comm. 2008) to southeastern Texas (Putzeys 1846: 42; Whitehead 1970: 186; Herman 1986: 61) in the southeast.

#### Records.

**USA**: CT, FL, LA, ME, MS, RI, TX

### Dyschirius montanus

LeConte, 1879

Dyschirius montanus LeConte, 1879d: 507. Type locality: «[Fort] Garland [Costilla County], Colo[rado]» (original citation). Two syntypes in MCZ [# 699].
Dyschirius thompsoni Hatch, 1949b: 117. Type locality: «Condon [Gilliam County], Oregon» (original citation). Holotype (♀) in USNM. Synonymy established by Hatch (1953: 68), confirmed by Lindroth (1961a: 141).

#### Distribution.

This species ranges from southern Quebec (Larochelle 1975: 82) to south-central British Columbia (Lindroth 1961a: 141), south to the Sierra Nevada in California (Dajoz 2004: 119), southeastern Arizona (Graham County, CNC), south-central Colorado (Wickham 1902: 232), Nebraska (Clopton 1991: 61), Wisconsin (Messer 2010: 34), and “Michigan” (Garry A. Dunn pers. comm. 1986). One old specimen simply labeled from New Mexico is known (Fall and Cockerell 1907: 156).

#### Records.

**CAN**: AB, BC, MB, ON, QC, SK **USA**: AZ, CA, CO, MI, MN, NE, NV, OR, SD, WA, WI, WY [NM]

### Dyschirius owen

(Dajoz, 2004)

Dyschiriodes owen Dajoz, 2004: 118. Type locality: «Lieu dit Fish Slough, 25 km au nord de Bishop, Inyo County, Californie» (original citation). Holotype in Dajoz’s collection (Paris, France).

#### Distribution.

This species is known only from the two specimens collected at the type locality in eastern California.

#### Records.

**USA**: CA

### Dyschirius pumilus

(Dejean, 1825)

Clivina pumila Dejean, 1825: 425. Type locality: «Amérique septentrionale» (original citation). One syntype in MHNP (Nichols 1988a: 209).
Dyschirius dentiger LeConte, 1857b: 79. Type locality: «New York and Pennsylvania» (original citation). Two syntypes in MCZ [# 695]. Synonymy established by Putzeys (1867b: 55), confirmed by Bousquet (1988a: 373).
Dyschirius rufiventris LeConte, 1857b: 79. Type locality: «Louisiana» (original citation). Holotype [by monotypy] (♂) in MCZ [# 698]. Synonymy established by Leng (1920: 48), confirmed by Bousquet (1988a: 373).
Dyschirius falciger LeConte, 1878b: 373. Type locality: «Tampa and Lake Harney [Florida]» (original citation). At least two syntypes, possibly four, in MCZ. Synonymy established by LeConte (1879a: 31).

#### Distribution.

This species is known along the Coastal Plain and Piedmont Plateau from southeastern New York (Notman 1928: 212) to southern Florida including the Keys (Peck and Thomas 1998: 17), west to southern Texas (Aransas and Brooks Counties, CNC), and also from Minnesota (Gandhi et al. 2005: 924) and west-central Wisconsin (Messer 2010: 34). The record from southern Arizona (Wickham 1898: 300) is probably in error (Nichols 1988a: 209).

#### Records.

**USA**: AL, DC, FL, GA, LA, MD, MN, MS, NC, NJ, NY, PA, SC, TX, VA, WI

### Dyschirius sextoni

Bousquet, 1987

Dyschirius sextoni Bousquet, 1987a: 113. Type locality: «Belleville, Ont[ario]» (original citation). Holotype (♂) in CNC [# 19237]. Etymology. The specific name was proposed for Richard Sexton [1930-2003], a friend of the author and a beetle collector in Quebec.

#### Distribution.

This species is known only from the type locality in southern Ontario, from Monroe and Sheboygan Counties in Wisconsin (Purrington and Maxwell 1998: 190; Messer 2010: 35), and from Highlands County in central Florida (Vince Golia collection).

#### Records.

**CAN**: ON **USA**: FL, WI

### Dyschirius soda

(Dajoz, 2004)

Dyschiriodes soda Dajoz, 2004: 119. Type locality: «Soda Lake près de Baker, San Bernardino County, Californie» (original citation). Holotype in Dajoz’s collection (Paris, France).

#### Distribution.

This species is known only from eight specimens collected at the type locality in southeastern California.

#### Records.

**USA**: CA

### Dyschirius sublaevis

Putzeys, 1846

Dyschirius sublaevis Putzeys, 1846: 42. Type locality: «Galveston [Galveston County], Texas» (original citation). Syntype(s) [2 originally cited] in MHNP (collection Chaudoir).
Dyschirius filiformis LeConte, 1857b: 78. Type locality: «Coney Island [Kings County], near New York [New York]» (original citation). Three syntypes in MCZ [# 694]. Synonymy established by Erwin (2011b: 124) based on Nichols’ (1988) unpublished thesis.

#### Distribution.

This species is found along the Atlantic and Gulf of Mexico coasts from Maine (Larochelle and Larivière 1990a: 27, as *Dyschirius filiformis*; York County, CNC) and New Hampshire (Rockingham County, CNC) to southern Florida including the Keys (Nichols 1988b: Fig. 5-6; Peck and Thomas 1998: 17), west to southeastern Texas (Putzeys 1846: 43; Wickham 1897: 103); also recorded from the Bahamas (Turnbow and Thomas 2008: 12), Cuba (Nichols 1988b: Fig. 5-4), Cayman Islands (Erwin 2011b: 124), and Yucatán, Mexico (Peck 2005: 28). The record from northeastern Kansas (Popenoe 1878: 78, as *Dyschirius filiformis*) needs confirmation; one old specimen labeled “Milwaukee WIS” is also known (MCZ).

#### Records.

**USA**: AL, CT, DE, FL, GA, LA, MA, MD, ME, MS, NC, NH, NJ, NY, RI, TX, VA [KS, WI] – Bahamas, Cayman Islands, Cuba, Mexico

### [quadrimaculatus group]

### Dyschirius quadrimaculatus

Lindroth, 1961

Dyschirius quadrimaculatus Lindroth, 1961a: 148. Type locality: «Irvine, Al[ber]ta» (original citation). Holotype (♂) in CNC [# 7606].

#### Distribution.

This species is known from the Prairie Provinces (Lindroth 1961a: 148), “Montana” (Bousquet 1988a: 372), Wyoming (Park County, CNC), and “North Dakota” (Bousquet 1988a: 372). The record from “Idaho” (Bousquet and Larochelle 1993: 98) needs confirmation.

#### Records.

**CAN**: AB, MB, SK **USA**: MT, ND, WY [ID]

### [sellatus group]

### Dyschirius campicola

Lindroth, 1961

Dyschirius campicola Lindroth, 1961a: 143. Type locality: «Del Rio [Val Verde County], Tex[as]» (original citation). Holotype (♂) in MCZ [# 30422].

#### Distribution.

This species ranges from the southern part of the Prairie Provinces (Lindroth 1961a: 143; Bousquet 1987a: 115) to southern Arizona (Bousquet 1987a: 115; Dajoz 2007: 21), southern Texas, and central Arkansas, east to northeastern Ohio [see Davidson and Lee 1990: Fig. 1)].

#### Records.

**CAN**: AB, MB **USA**: AR, AZ, CO, ID, IL, KS, ND, NE, NM, OH, OK, SD, TX, UT, WY

### Dyschirius pallipennis

(Say, 1823)

Clivina pallipennis Say, 1823a: 24. Type locality: «Angl[e]sea [Cape May County], N[ew] J[ersey]» (neotype label). Neotype (♀), designated by Lindroth and Freitag (1969: 334), in MCZ [# 33079]. Note. «Egg-harbour [New Jersey], coast of Virginia and Florida» were the areas originally cited by Say (1823a: 24).

#### Distribution.

The range of this species extends from southern Quebec (Larochelle 1975: 82) to southern Alberta (Bousquet 1987a: 115), south to northern Texas (Bowie and Winkler Counties, CMNH, USNM), southeastern Louisiana (West Baton Rouge Parish, LSAM), and southern Florida (Herman 1986: 63).

#### Records.

**CAN**: AB, ON, QC, SK **USA**: AR, FL, GA, IL, KS, LA, MI, MO, MS, MT, ND, NE, NJ, NY, OK, PA, SD, TX, VA

### Dyschirius salivagans

LeConte, 1875

Dyschirius salivagans LeConte, 1875c: 169. Type locality: «Great Salt Lake [Davis County], Utah» (original citation). Holotype [by monotypy] (♀) in MCZ [# 700].

#### Distribution.

This species has been reported from southern Oregon (Herman 1986: 60), western Nevada (Bousquet 1988a: 376), and Utah (Knowlton 1939: 2; Lindroth 1961a: 144; Herman 1986: 61). The records from South Dakota (Kirk and Balsbaugh 1975: 16) and Albuquerque in New Mexico (Fall and Cockerell 1907: 156) need confirmation.

#### Records.

**USA**: NV, OR, UT [NM, SD]

### Dyschirius sellatus

LeConte, 1857

Dyschirius sellatus LeConte, 1857b: 78. Type locality: «Atlantic City [Atlantic County], on the coast of New Jersey» (original citation). Three syntypes in MCZ [# 677].

#### Distribution.

This species ranges from Newfoundland (Lindroth 1955a: 45) to central Florida (Peck and Thomas 1998: 17), west to northeastern Texas (Lindroth 1961a: 143), including southeastern Mississippi (George and Greene Counties, Drew A. Hildebrandt pers. comm. 2008), and north to southern North Dakota (Burleigh County, Donald P. Schwert pers. comm. 1989) and eastern Minnesota (Gandhi et al. 2005: 924). The record from “Pennsylvania” (Bousquet and Larochelle 1993: 100) needs confirmation.

#### Records.

**CAN**: NB, NF, NS, PE, QC **USA**: FL, GA, IL, LA, MD, MN, MO, MS, NC, ND, NE, NJ, NY, OK, SD, TX [PA]

### [tridentatus group]

### Dyschirius interior

Fall, 1922

Dyschirius interior Fall, 1922c: 172. Type locality: «Baldur, Manitoba» (original citation). Holotype in MCZ [# 23852].
Dyschirius arizonicus Van Dyke, 1943: 22. Type locality: «Holbrook [Navajo County], Arizona» (original citation). Holotype in CAS [# 5303]. Synonymy established by Bousquet (1988a: 371).

#### Distribution.

This species ranges from the southern part of the Prairie Provinces (Lindroth 1961a: 139) south to east-central California (Dajoz 2004: 119), northern Arizona (Van Dyke 1943: 22, as *Dyschirius arizonicus*; Mojave and Navajo Counties, MCZ, USNM), and western Texas (Randall and Ward Counties, CMNH, USNM); also known from southwestern Oregon (Curry County, DAPC).

#### Records.

**CAN**: AB, MB, SK **USA**: AZ, CA, CO, ID, NE, NM, NV, OR, TX, UT, WY

### Dyschirius patruelis

LeConte, 1852

Dyschirius patruelis LeConte, 1852a: 196. Type locality: «San Diego [San Diego County, California]» (original citation). Three syntypes in MCZ [# 686].

#### Distribution.

This species is found along the Pacific Coast in southern California (LeConte 1852a: 196). The record from “Oregon” (Leng 1920: 47) needs confirmation; that from southwestern British Columbia (Hatch 1953: 67) is in error.

#### Records.

**USA**: CA [OR]

### Dyschirius tridentatus

LeConte, 1852

Dyschirius tridentatus LeConte, 1852a: 195. Type locality: «ad San Diego [San Diego County, California]» (original citation). Holotype [by monotypy] (♀) in MCZ [# 688].
Dyschirius convexus LeConte, 1852a: 195. Type locality: «San Diego [San Diego County, California]» (original citation). Syntype(s) in MCZ [# 687]. Synonymy established by LeConte (1858a: 29), confirmed by Lindroth (1961a: 138).
Dyschirius quadridens Motschulsky, 1859a: 133. Type locality: California (inferred from title of the paper). One syntype, listed as “corruptum,” in ZMMU (Keleinikova 1976: 213). Synonymy established by LeConte (1863b: 3).

#### Distribution.

This species is found from northern Washington (Lindroth 1961a: 138) to western Montana (Russell 1968: 47), south to southwestern New Mexico (Sierra County, CMNH) and southwestern California (LeConte 1852a: 195; Moore 1937: 5). The records from “Nebraska” (Bousquet and Larochelle 1993: 98) and northwestern British Columbia (Hatch 1953: 67) are probably in error. One old specimen simply labeled “Van” is known (MCZ).

#### Records.

**USA**: AZ, CA, ID, MT, NM, NV, OR, WA [BC]

### Dyschirius unipunctatus

Fall, 1901

Dyschirius unipunctatus Fall, 1901a: 207. «Pomona, San Bernardino, Riverside [California]» (original citation). Syntype(s) in MCZ [# 23855].

#### Distribution.

This species is found in southern California (Fall 1901a: 207).

#### Records.

**USA**: CA

### Dyschirius varidens

Fall, 1910

Dyschirius varidens Fall, 1910: 93. Type locality: «Los Angeles [Los Angeles County], California» (original citation). Holotype in MCZ [# 23856].

#### Distribution.

This species ranges from “Washington” (Hatch 1953: 67) to western Montana (Jefferson County, CNC), south to northeastern Nevada (Elko County, CNC) and southern California (Fall 1910: 93; San Diego County, CAS, UASM). The record from “Wyoming” (Bousquet and Larochelle 1993: 98) needs confirmation.

#### Records.

**USA**: CA, ID, MT, NV, OR, WA [WY]

### Tribe Promecognathini

LeConte, 1853

Promecognathi LeConte, 1853c: 371, 394. Type genus: *Promecognathus* Chaudoir, 1846.
Axinidiini Basilewsky, 1963a: 307. Type genus: *Axinidium* Sturm, 1843.

#### Diversity.

Eight species in North America (two species) and South Africa (six species) arrayed in five genera: *Axinidium* Sturm (two species), *Holaxinidium* Basilewsky (one species), *Metaxinidium* Basilewsky (two species), *Paraxinidium* Basilewsky (one species), and *Promecognathus* (two species).

### Genus Promecognathus

Chaudoir, 1846

Promecognathus Chaudoir, 1846: 524. Type species: *Eripus laevissimus* Dejean, 1829 by monotypy. Etymology (original). From the Greek *promeces* (advanced, in front of, by extension elongate) and *gnathos* (jaw), alluding to the elongate mandibles (“*mandibulae longissimae*”) of the adults [masculine].

#### Diversity.

Two species restricted to western North America.

#### Identification.

Lindroth (1961a: 125-128) commented on the structural differences between the two species.

#### Taxonomic Note.

The status of the two forms as distinct species is questionable in my opinion. Van Dyke (1925: 123) considered the two forms as conspecific.

### Promecognathus crassus

LeConte, 1868

Promecognathus crassus LeConte, 1868b: 62. Type locality: «California» (original citation), herein restricted to Monterey, Monterey County (see Casey 1913: 94, as *Promecognathus corpulentus*). Three syntypes in MCZ [# 640].
Promecognathus contractus Casey, 1913: 94. Type locality: «Napa Co[unty], Cal[ifornia]» (syntype label). One syntype in USNM [# 46872]. Synonymy established by Lindroth (1961a: 127). Note. Casey (1913: 94) cited, probably by error, the type locality as “Lake Co[unty], California.”
Promecognathus corpulentus Casey, 1913: 94. Type locality: «Monterey [Monterey County], California» (original citation). Lectotype, designated by Lindroth (1975: 113), in USNM [# 46873]. Synonymy established by Lindroth (1961a: 127).
Promecognathus grandiceps Casey, 1913: 94. Type locality: «California» (original citation). Lectotype, designated by Lindroth (1975: 113), in USNM [# 46874]. Synonymy established by Lindroth (1961a: 127).

#### Distribution.

This species is found along the Pacific Coast from southwestern British Columbia, including Vancouver Island (Lindroth 1961a: 128), to central California (Casey 1913: 94, as *Promecognathus corpulentus*).

#### Records.

**CAN**: BC (VCI) **USA**: CA, OR, WA

**Figure 19.**
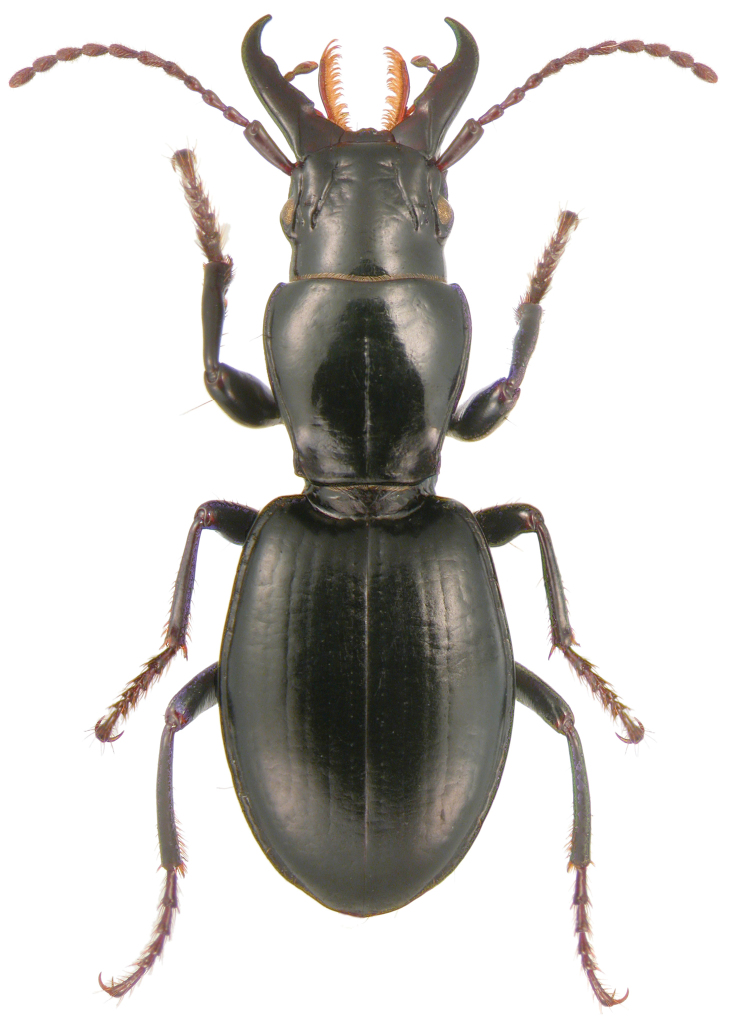
*Promecognathus crassus* LeConte. This species, along with its close relative *Promecognathus laevissimus*, are western coastal elements that feed on polydesmid millipedes. The adults straddle their prey, moving quickly toward the head to pierce the neck and sever the ventral nerve cord with their long mandibles, thereby preventing the millipedes from using their cyanide defense spray. Promecognathines represent a very old, relic lineage, likely originating from Pangea, represented today only in western North America and the Cape Province in South Africa.

### Promecognathus laevissimus

(Dejean, 1829)

Eripus laevissimus Dejean, 1829: 11. Type locality: «Californie» (original citation), restricted to «San Francisco [San Francisco County]» by Lindroth (1961a: 127). Holotype [by monotypy (see Dejean 1829: 9)] in MHNP (Lindroth 1955b: 13).
Promecognathus debilis Casey, 1897: 346. Type locality: «S[an]ta Cruz [Santa Cruz County], California» (original citation). Holotype [by monotypy] in USNM [# 46871]. Synonymy established by Lindroth (1961a: 127).

#### Distribution.

This species is known from “Oregon” south at least to central California (Lindroth 1961a: 127; Fresno County, CAS), east to Washoe County in northwestern Nevada (La Rivers 1947: 139; Lindroth 1961a: 127).

#### Records.

**USA**: CA, NV, OR

### Subfamily BROSCINAE

Hope, 1838

Broschidae Hope, 1838: 80. Type genus: *Broscus* Panzer, 1813.

#### Diversity.

One tribe is included in this subfamily.

### Tribe Broscini

Hope, 1838

Broschidae Hope, 1838: 80. Type genus: *Broscus* Panzer, 1813.

#### Diversity.

About 290 species (Häckel et al. 2010) in the Nearctic (four species, one of them adventive), Neotropical (about 30 species, one in Mexico, the other ones in South America), Australian (about 180 species), Oriental (five species), and Palaearctic (about 70 species) Regions placed in 34 genera. The genera are arrayed in five subtribes (Roig-Juñent 2000): Broscina (about 75 species), Creobiina (about 95 species in South America and the Australian Region), Anoxyina (five species in Mexico, Iran, and the Oriental Region), Baripodina (about 25 South American species), and Nothobroscina (about 90 species in the Australian Region and one in Chile). The group is better represented in the New World with about 215 species (74% of the world fauna) than in the Old World.

### Subtribe Broscina

Hope, 1838

Broschidae Hope, 1838: 80. Type genus: *Broscus* Panzer, 1813.
Cephalotida Heer, 1838: 7. Type genus: *Cephalotes* Bonelli, 1810 (= *Broscus* Panzer, 1813). Note. This family-group name is permanently invalid, being based on a preoccupied type genus (ICZN 1999: Article 39).
Zacotini G.H. Horn, 1881: 165, 169. Type genus: *Zacotus* LeConte, 1869.

#### Diversity.

About 75 species in the Nearctic (four species), Oriental (four species), and Palaearctic (about 65 species) Regions. One species (*Miscodera arctica*) is Holarctic and one is adventive (*Broscus cephalotes*) in North America.

### Genus Miscodera

Eschscholtz, 1830

Miscodera Eschscholtz, 1830: 63. Type species: *Scarites arcticus* Paykull, 1798 by monotypy. Etymology (original). From the Greek *mischos* (peduncle) and *dere* (neck, by extension pronotum), alluding to the pedunculate body shape of the adults [feminine].
Leiochiton Curtis, 1831: plate 346. Type species: *Scarites arcticus* Paykull, 1798 by original designation. Etymology. From the Greek *leios* (smooth) and *chiton* (tunic, by extension cuticle), alluding to the smooth body (“very smooth and highly polished”) of the adults [masculine].
Liochiton Agassiz, 1846: 203, 212. Unjustified emendation of *Leiochiton* Curtis, 1831.

#### Diversity.

One Holarctic species in the subarctic and boreal regions.

#### Identification.

Lindroth (1961a: 170-171) covered the species.

#### Taxonomic Note.

Cladistic analysis of broscine genera performed by Roig-Juñent (2000) placed this genus as the sister-group of the Holarctic genus *Broscodera* Lindroth.

### Miscodera arctica

(Paykull, 1798)

Scarites arcticus Paykull, 1798: 85. Type locality: «Botnia occidentali & orientali [= Gulf of Bothnia, Sweden]» (original citation). Syntype(s) probably in NRSS.
Leiochiton readii Curtis, 1831: plate 346. Type locality: «on Cold-edge [Road], the moor due north of Halifax [West Yorkshire, United Kingdom]» (original citation). Syntype(s) location unknown. Synonymy established by Ganglbauer (1891a: 145).
Miscodera erythropus Motschulsky, 1844: 76. Type locality: «montagnes du Hamar-Daban près de la station Chybet [Irkutsk Oblast, Russia]» (original citation). Three syntypes in ZMMU (Keleinikova 1976: 196). Synonymy established by Horn (1881: 168).
Miscodera americana Mannerheim, 1853: 134. Type locality: «ad fl[umen] Skeljanktnu peninsulae Kenai [Alaska]» (original citation). Holotype [by monotypy] location unknown. Synonymy established by Horn (1881: 168).
Miscodera hardyi Chaudoir, 1861b: 525. Type locality: «S[ain]t Pierre [et] Miquelon» (original citation). Syntype(s) [2 originally cited] in MHNP. Synonymy established by Horn (1881: 168).

#### Distribution.

This circumpolar species ranges from Ireland to the Bering Sea coast (Bousquet 2003b: 237) and from Alaska (Lindroth 1961a: 171) to Newfoundland (Lindroth 1955a: 133), south to northeastern New York (Notman 1928: 241), the upper peninsula of Michigan (Chippewa County, MCZ), northern Wisconsin (Bayfield County, MCZ), northern Colorado (Dajoz 1989: 337; Gilpin County, CMNH), southern Montana, and northern Washington (Hatch 1933b: 7). Fossil remnants of this species, believed to be 2.0-2.5 million years old, have been found in Greenland (Bennike and Böcher 1990: 336; Böcher 1995: 23); others, about 20,530 years B.P., have been unearthen in northeastern Iowa (Woodman et al. 1996: 17).

#### Records.

**FRA**: PM **CAN**: AB, BC, LB, MB, NB, NF, NS (CBI), NT, ON, QC, SK, YT **USA**: AK, CO, ME, MI, MT, NH, NY, VT, WA, WI, WY – **Holarctic**

### Genus Broscodera

Lindroth, 1961

Broscodera Lindroth, 1961b: 150. Type species: *Miscodera insignis* Mannerheim, 1852 by original designation. Etymology. From the generic name *Broscus* [*q.v*.] and the Greek *dere* (neck, by extension pronotum) [feminine].

#### Diversity.

Northern Hemisphere, with four species in the Nearctic (one species) and Palaearctic (three species) Regions arrayed in two subgenera: *Broscodera* s.str. (one species) and *Sinobrosculus* Deuve (three species in Nepal, Gansu, and Sichuan).

#### Taxonomic Note.

Cladistic analysis of broscine genera performed by Roig-Juñent (2000) placed this genus as the sister-group of the Holarctic genus *Miscodera* Eschscholtz.

### Subgenus Broscodera

Lindroth, 1961

Broscodera Lindroth, 1961b: 150. Type species: *Miscodera insignis* Mannerheim, 1852 by original designation.

#### Diversity.

One species in the temperate regions of western North America.

#### Identification.

Lindroth (1961a: 171-172) treated the species.

### Broscodera insignis

(Mannerheim, 1852)

Miscodera insignis Mannerheim, 1852: 296. Type locality: «insula Sitkha [= Baranof Island, Alaska]» (original citation). Holotype [by monotypy; designated lectotype by Lindroth (1969a: 1111)] (♂) in ZMH.

#### Distribution.

This species is confined to the Pacific Coast and adjacent Coast Ranges, ranging from the Alexander Archipelago in southeastern Alaska to western Oregon (Lindroth 1961a: 172). The record from southeastern Wyoming (Lavigne 1977: 45) must be based on a mislabeled specimen.

#### Records.

**CAN**: BC (QCI) **USA**: AK, OR, WA

### Genus Zacotus

LeConte, 1869

Zacotus LeConte, 1869c: 373. Type species: *Zacotus matthewsii* LeConte, 1869 by monotypy. Etymology. From the Greek *zacotos* (very angry) [masculine].

#### Diversity.

One species in temperate western North America.

#### Identification.

The species is covered in Lindroth’s (1961a: 172-173) monograph.

#### Taxonomic Note.

Cladistic analysis of broscine genera performed by Roig-Juñent (2000) placed this genus as the sister-group of the Asian genus *Eobroscus* Kryzhanovskij.

### Zacotus matthewsii

LeConte, 1869

Zacotus matthewsii LeConte, 1869c: 373. Type locality: Vancouver Island, British Columbia (inferred from title of the paper), herein restricted to Tofino (see Lindroth 1961a: 173). Holotype [by monotypy] (♀) in MCZ [# 5854]. Etymology. The specific name was proposed for Henry and Joseph Matthews, brothers of the British Coleopterist Reverend Andrew Matthews [1815-1897] who specialized in small beetles. Henry and Joseph Matthews collected in British Columbia, including Vancouver Island. Note. There is a clear evidence of an inadvertent error in the original publication of the name *matthewsii*. It should have been spelled *matthewsiorum* since the species was proposed for two men together (see ICZN 1999: Article 31.1.2). However, the spelling *matthewsii* has been in used since LeConte proposed the name and I believe it should be preserved. The case should be submitted to the Commission for a ruling.
Zacotus angustus Casey, 1920: 290. Type locality: «Josephine Co[unty], Oregon» (original citation). Lectotype (♂), designated by Lindroth (1975: 113), in USNM [47697]. Synonymy established by Hatch and Fender (1944: 188).
Zacotus subopacus Hopping, 1925: 206. Type locality: «Princeton, B[ritish] C[olumbia]» (original citation). Holotype (♀) in CNC [# 1380]. Synonymy established by Hatch and Fender (1944: 188).
Zacotus fredericki Nunenmacher, 1944: 12. Type locality: «Lincoln County, Oregon» (original citation). Holotype (♂) in FMNH (Goldman 2006). Synonymy established by Hatch and Fender (1944: 188).

#### Distribution.

This species ranges from the southern part of the Alexander Archipelago to northwestern California, east to the Bitter Root Mountains in southwestern Montana (Ball 1956b: 34, Fig. 1).

#### Records.

**CAN**: BC (QCI, VCI) **USA**: AK, CA, ID, MT, OR, WA

#### Note.

Based on variation in color, lustre, and surface sculpture in adults, Ball (1956b) recognized a western and eastern “races” for this species.

### Genus Broscus

Panzer, 1813

Cephalotes Bonelli, 1810: Tabula Synoptica [junior homonym of *Cephalotes* Latreille, 1802]. Type species: *Carabus cephalotes* Linnaeus, 1758 by subsequent monotypy in Panzer (1813: 62). Etymology. From the Greek *cephalotos* (headed) [masculine].
Broscus Panzer, 1813: 62. Replacement name for *Cephalotes* Bonelli, 1810. Etymology. Uncertain, possibly a contracted form of the Greek *bibrosco* (eat, gnaw, consume) [masculine].

#### Diversity.

Twenty-three species in the Palaearctic Region, one of them adventive in eastern North America.

#### Identification.

Larochelle and Larivière (1989a) provided a description of the external structures as well as the male and female genitalia of the species found in North America.

#### Taxonomic Note.

Cladistic analysis of broscine genera performed by Roig-Juñent (2000) placed this genus as the sister-group of the temperate Asian genus *Craspedonotus* Schaum.

### Broscus cephalotes

(Linnaeus, 1758)

Carabus cephalotes Linnaeus, 1758: 414. Type locality: «Europa» (original citation), restricted to «Suecia [Sweden]» by Lindroth (1957b: 339). Three possible syntypes in LSL (Lindroth 1957b: 330).

#### Distribution.

This Palaearctic species is adventive in North America where it is known from Cape Breton Island and eastern Prince Edward Island (Larochelle and Larivière 1989a: Fig. 4). The first inventoried specimen found on this continent was caught in 1987.

#### Records.

**CAN**: NS (CBI), PE – **Adventive**

**Figure 20.**
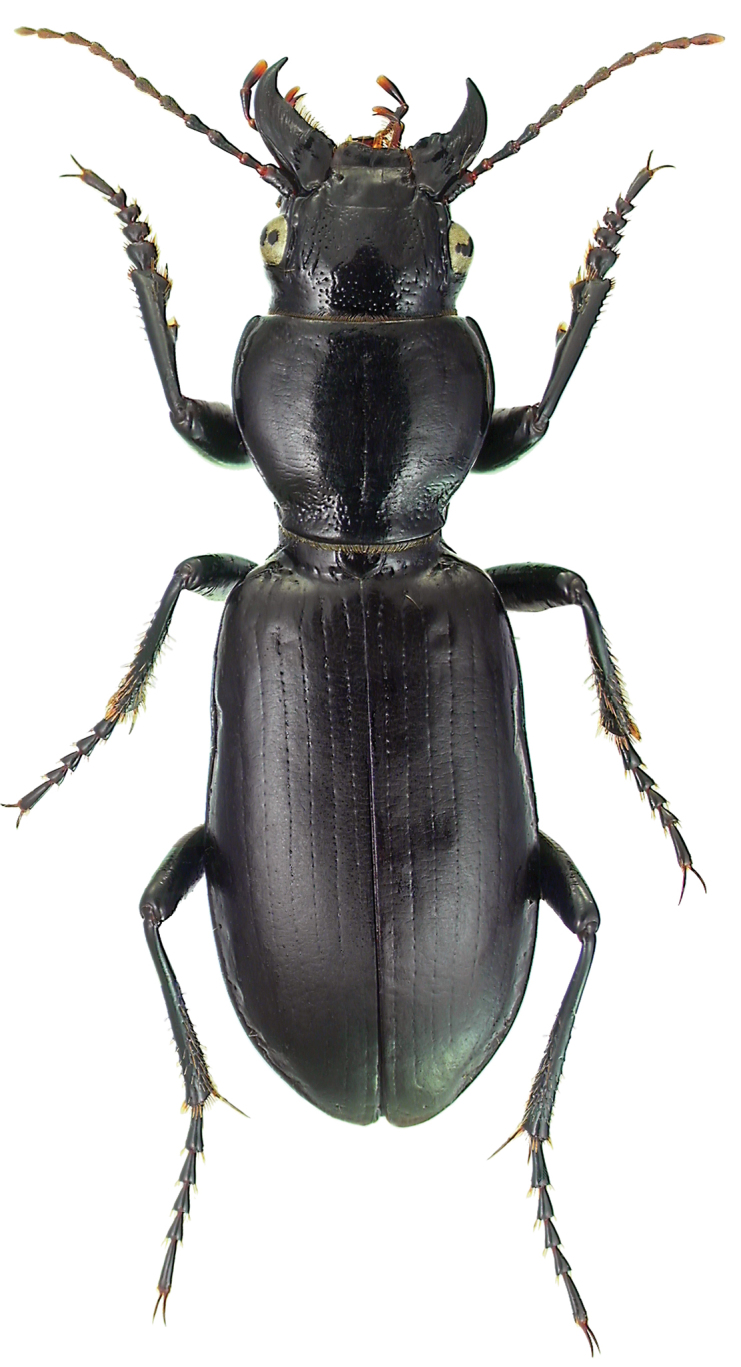
*Broscus cephalotes* (Linnaeus). This relatively large Palaearctic carabid is one of the most recently established species in North America. The first recorded specimen was found in the late 1980s and, considering the species’ size, it is doubtful that it would have escaped notice for a long period. On this continent, the species has been found only on bare, fine sand along coastal beaches in Prince Edward Island and Cape Breton in company with amphipods of the genus *Gammarus* which probably make up part of their diet. The pathway of introduction of this broscine on American soil is uncertain.

### Subfamily GEHRINGIINAE

Darlington, 1933

Gehringiini Darlington, 1933b: 110. Type genus: *Gehringia* Darlington, 1933.

#### Diversity.

Five species placed in two subtribes: Gehringiina (one species) and Helenaeina (three rarely collected species in the genus *Helenaea* Schatzmayr and Koch from Egypt, Turkey, and Yemen and one species in the genus *Afrogehringia* Baehr, Schüle and Lorenz from Namibia).

#### Identification.

Baehr et al. (2009: 106) provided a key to all species of this subfamily.

### Tribe Gehringiini

Darlington, 1933

Gehringiini Darlington, 1933b: 110. Type genus: *Gehringia* Darlington, 1933.

#### Diversity.

Three genera and five species are placed in this tribe.

### Subtribe Gehringiina

Darlington, 1933

Gehringiini Darlington, 1933b: 110. Type genus: *Gehringia* Darlington, 1933.

#### Diversity.

One North American species belongs to this subtribe.

### Genus Gehringia

Darlington, 1933

Gehringia Darlington, 1933b: 110. Type species: *Gehringia olympica* Darlington, 1933 by original designation. Etymology. From the surname of Dr. John George Gehring [1857-1932] of Bethel, Maine, from whom Darlington was collecting “on shares” when he secured his specimens on Olympic Mountains. Born in Cleveland, Ohio, Gehring came to Bethel at 30 and eventually opened a clinic for the treatment of persons with nervous disorders. Dr. Gehring and his “inn” were the prototype and scene of Novelist Robert Herrick’s The Master of the Inn. The name is feminine.

#### Diversity.

One species in western North America.

#### Identification.

The species was covered in Lindroth’s (1961a: 4-5) monograph on the carabids of Canada and Alaska.

### Gehringia olympica

Darlington, 1933

Gehringia olympica Darlington, 1933b: 111. Type locality: «near Sol Duc Hot Springs [Clallam County], Olympic M[oun]t[ain]s, Washington» (original citation). Holotype (♀) in MCZ [# 17243].

#### Distribution.

This minute species ranges from central Alaska (66.79376°N, 150.73164°W, Derek S. Sikes pers. comm. 2008) to southern Northwest Territories (CNC), south to northwestern Montana (Edwards 1975: 48), southeastern Idaho (Caribou County, CNC), and northeastern Oregon (Lindroth 1961a: 5).

#### Records.

**CAN**: AB, BC, NT, YT **USA**: AK, ID, MT, OR, WA

### Subfamily TRECHINAE

Bonelli, 1810

Trechii Bonelli, 1810: Tabula Synoptica. Type genus: *Trechus* Clairville, 1806.

#### Diversity.

Worldwide, with about 5,410 species arrayed in four tribes: Bembidiini (about 2,630 species), Pogonini (about 80 species), Trechini (about 2,650 species), and Zolini (about 50 species). The North American fauna is represented by about 615 species (roughly 11.3% of the world fauna).

### Tribe Trechini

Bonelli, 1810

Trechii Bonelli, 1810: Tabula Synoptica. Type genus: *Trechus* Clairville, 1806.

#### Diversity.

Worldwide, with about 2,650 species arrayed in two subtribes: Trechina (about 2,470 species) and Trechodina (about 180 species). Only the subtribe Trechina is represented in North America.

### Subtribe Trechina

Bonelli, 1810

Trechii Bonelli, 1810: Tabula Synoptica. Type genus: *Trechus* Clairville, 1806.

#### Diversity.

Worldwide, with about 2,470 species (Lorenz 2005: 168-200). The North American fauna is represented by about 225 species (roughly 9% of the world fauna) arrayed in nine genera.

### Genus Trechoblemus

Ganglbauer, 1891

Trechoblemus Ganglbauer, 1891a: 187. Type species: *Carabus micros* Herbst, 1784 by monotypy. Etymology. From the generic names *Trechus* [*q.v*.] and *Blemus* [*q.v*.] [masculine].

#### Diversity.

Northern Hemisphere, with six species in temperate areas of the Nearctic (one western species) and Palaearctic (five species) Regions.

#### Identification.

Barr (1972) provided a description of the external structures and male genitalia of the North American species.

### Trechoblemus westcotti

Barr, 1972

Trechoblemus westcotti Barr, 1972: 142. Type locality: «Hillsboro, Washington County, Oregon» (original citation). Holotype (♂) in ODAC.

#### Distribution.

This species is known only from a few localities in the Willamette Valley in northwestern Oregon.

#### Records.

**USA**: OR

### Genus Pseudanophthalmus

Jeannel, 1920

Pseudanophthalmus Jeannel, 1920b: 154. Type species: *Anophthalmus menetriesi* Motschulsky, 1862 by original designation. Etymology. From the Greek *pseudos* (fallacy, lie) and the generic name *Anophthalmus* [masculine].
Aphanotrechus Barr, 1960c: 65. Type species: *Aphanotrechus virginicus* Barr, 1960 by original designation. Synonymy established by Barr and Krekeler (1967: 1322). Etymology. From the Greek *aphanes* (invisible, obscure) and the generic name *Trechus* [*q.v*.] [masculine].

#### Diversity.

About 145 described species, though more than 220 are known (Barr 2004: 1), restricted to eastern North America south of the last glaciation.

#### Identification.

Barr (2004: 11-16) provided a key for the identification of the 26 species groups currently recognized. Barr (1959) revised the species of the *robustus* group (four species). Krekeler (1973) revised the species of the *barri* (two species), *horni* (13 species), *inexpectatus* (six species), and *rittmani* (three species) groups and provided keys for the identification of the species of the *horni* and *inexpectatus* groups. Barr (1981) revised the species of the *alabamae* (two species), *hirsutus* (seven species), *hubrichti* (six species), *hypolithos* (five species), *jonesi* (eight species), and *tennesseensis* (four species) groups but did not provide keys for their identification. He also treated the *engelhardti* group but many species were added subsequently.

#### Taxonomic Note.

*Duvaliopsis* Jeannel, with six species in Carpathian and Transylvanian Alps of eastern Europe, is listed as a synonym of *Pseudanophthalmus* by Barr (2004: 7). Most other trechine students treat *Duvaliopsis* as a distinct genus.

*Tennessarius* Valentine (1952: 15), listed as a junior synonym of *Pseudanophthalmus* Jeannel by Barr (1962b: 111), is an unavailable name since the original description was not accompanied by the fixation of a type species (ICZN 1999: Article 13.3). Barr (2004: 7) provided a type species for *Tennessarius* along with a bibliographic reference to a previously published description but since he listed *Tennessarius* in synonymy with *Pseudanophthalmus*, the name is still unavailable (ICZN 1999: Article 11.6).

### [alabamae group]

### Pseudanophthalmus alabamae

Valentine, 1932

Pseudanophthalmus alabamae Valentine, 1932a: 273. Type locality: «Manitou Cave, 1.5 miles southwest of F[or]t Payne, at the foot of Lookout M[oun]t[ain] [DeKalb County], Ala[bama]» (original citation). Holotype (♂) in USNM [# 44279].

#### Distribution.

This species is known from a number of caves in DeKalb County, northeastern Alabama (Barr 2004: 40).

#### Records.

**USA**: AL

### Pseudanophthalmus georgiae

Barr, 1981

Pseudanophthalmus georgiae Barr, 1981: 90. Type locality: «Blowing Spring Cave, 4 km N[orth]E[ast] Cloudland and 1.6 km N[orth]W[est] Chelsea at the east base of Lookout Mountain, Chattooga Co[unty], Georgia» (original citation). Holotype (♂) in AMNH.

#### Distribution.

This species is known only from a few caves in Chattooga and Walker Counties, northwestern Georgia (Barr 2004: 40).

#### Records.

**USA**: GA

### [audax group]

### Pseudanophthalmus audax

(Horn, 1883)

Anophthalmus audax G.H. Horn, 1883b: 272. Type locality: «Ronald’s cave [Hart County, Kentucky]» (original citation). Holotype [by monotypy] (♂) in MCZ [# 8229].

#### Distribution.

This species is known only from two caves in Hart and Edmonson Counties, Kentucky (Barr 2004: 24).

#### Records.

**USA**: KY

### Pseudanophthalmus emersoni

Krekeler, 1958

Pseudanophthalmus emersoni Krekeler, 1958: 176. Type locality: «Donnehue’s Cave, one mile southwest of Bedford, Lawrence Co[unty], Ind[iana]» (original citation). Holotype (♂) in FMNH. Etymology. The specific name honors Professor Alfred Edward Emerson [1896-1976] of the University of Chicago who worked on the systematics, phylogeny, distribution, and natural history of termites. His collection of more than one million specimens was given to the American Museum of Natural History.

#### Distribution.

This species is known only from two caves in Lawrence County, southern Indiana (Barr 2004: 24).

#### Records.

**USA**: IN

### Pseudanophthalmus packardi

Barr, 1959

Pseudanophthalmus packardi Barr, 1959: 22. Type locality: «Bat Cave, Carter Caves State Park, Carter Co[unty], Kentucky» (original citation). Holotype (♂) in AMNH. Etymology. The specific name was proposed for Alpheus Spring Packard, Jr. [1839-1905], American geologist and entomologist, professor of zoology and geology at Brown University, who collected the first specimen of this species.

#### Distribution.

This species is known from several caves in Carter and Elliott Counties, northeastern Kentucky (Barr 2004: 24).

#### Records.

**USA**: KY

### [barri group]

### Pseudanophthalmus barri

Krekeler, 1973

Pseudanophthalmus barri Krekeler, 1973: 64. Type locality: «Indian Cave, 0.5 mile southwest of Charlestown, Clark Co[unty], Ind[iana]» (original citation). Holotype (♂) in FMNH.

#### Distribution.

This species is known from several caves in southern Clark County, southern Indiana (Barr 2004: 24).

#### Records.

**USA**: IN

### Pseudanophthalmus troglodytes

Krekeler, 1973

Pseudanophthalmus troglodytes Krekeler, 1973: 65. Type locality: «Highbaugh Cave, 4.5 miles northwest of Jeffersontown, Jefferson Co[unty], K[entuck]y» (original citation). Holotype (♂) in FMNH.

#### Distribution.

This species is known only from two nearby caves in Jefferson County, north-central Kentucky (Barr 2004: 24).

#### Records.

**USA**: KY

### [cumberlandus group]

### Pseudanophthalmus acherontis

Barr, 1959

Pseudanophthalmus tiresias acherontis Barr, 1959: 20. Type locality: «Echo Cave, 2 miles northeast of Rockvale, Rutherford Co[unty], Tennessee» (original citation). Holotype (♂) in AMNH.

#### Distribution.

This species is known from several caves in Rutherford and Wilson Counties, Tennessee (Barr 2004: 33).

#### Records.

**USA**: TN

### Pseudanophthalmus bendermani

Barr, 1959

Pseudanophthalmus tiresias bendermani Barr, 1959: 21. Type locality: «Benderman Cave, Maury Co[unty], Tennessee» (original citation). Holotype (♂) in AMNH.

#### Distribution.

This species is known only from the type-locality cave located two miles west of Southport in west-central Tennessee (Barr 2004: 34).

#### Records.

**USA**: TN

### Pseudanophthalmus catherinae

Barr, 1959

Pseudanophthalmus tiresias catherinae Barr, 1959: 17. Type locality: «Petty Cave, Marshall Co[unty], Tennessee» (original citation). Holotype (♂) in AMNH.

#### Distribution.

This species is known only from the type-locality cave in south-central Tennessee (Barr 2004: 33).

#### Records.

**USA**: TN

### Pseudanophthalmus cumberlandus

Valentine, 1937

Pseudanophthalmus cumberlandus Valentine, 1937: 96. Type locality: «Piper Cave, Monoville [Smith County], Tennessee» (original citation). Holotype (♂) in USNM [# 56124].

#### Distribution.

This species is known from a few caves in Smith and Macon Counties, northern Tennessee (Barr 1980: 91; Barr 2004: 33).

#### Records.

**USA**: TN

### Pseudanophthalmus inquisitor

Barr, 1980

Pseudanophthalmus inquisitor Barr, 1980: 94. Type locality: «Sheals Cave, Clay Co[unty], Tennessee» (original citation). Holotype (♂) in AMNH.

#### Distribution.

This species is known only from the type-locality cave in northern Tennessee (Barr 2004: 34).

#### Records.

**USA**: TN

### Pseudanophthalmus insularis

Barr, 1959

Pseudanophthalmus tiresias insularis Barr, 1959: 18. Type locality: «Baker Station Cave, in northern Davidson Co[unty], Tennessee» (original citation). Holotype (♂) in AMNH.

#### Distribution.

This species is known only from the type-locality cave in north-central Tennessee (Barr 2004: 33).

#### Records.

**USA**: TN

### Pseudanophthalmus occidentalis

Barr, 1959

Pseudanophthalmus tiresias occidentalis Barr, 1959: 18. Type locality: «De Priest Branch Cave, Lewis Co[unty], Tennessee» (original citation). Holotype (♂) in AMNH.

#### Distribution.

This species is known from two nearby caves in Lewis and Hickman Counties, western Tennessee (Barr 1980: 93; Barr 2004: 33).

#### Records.

**USA**: TN

### Pseudanophthalmus productus

Barr, 1980

Pseudanophthalmus productus Barr, 1980: 91. Type locality: «Neil Fisher Cave (= Rip Van Winkle Cave), Smith Co[unty], Tennessee» (original citation). Holotype (♂) in AMNH.

#### Distribution.

This species is known from several caves in Smith, Putnam, and Jackson Counties, northern Tennessee (Barr 1980: 92; Barr 2004: 33).

#### Records.

**USA**: TN

### Pseudanophthalmus tiresias

Barr, 1959

Pseudanophthalmus tiresias tiresias Barr, 1959: 16. Type locality: «Indian Grave Point Cave, 6 miles southwest of Smithville, DeKalb Co[unty], Tennessee» (original citation). Holotype (♂) in AMNH.

#### Distribution.

This species is still known only from two nearby caves in central Tennessee (Barr 1980: 92; Barr 2004: 33).

#### Records.

**USA**: TN

### Pseudanophthalmus tullahoma

Barr, 1959

Pseudanophthalmus tiresias tullahoma Barr, 1959: 20. Type locality: «Carroll Cave, Coffee Co[unty], Tennessee» (original citation). Holotype (♂) in AMNH.

#### Distribution.

This species is known only from Carroll and Riley Creek caves, Coffee County, south-central Tennessee (Barr 1980: 93; Barr 2004: 33).

#### Records.

**USA**: TN

### [engelhardti group]

### Pseudanophthalmus aladdini

Valentine, 1945

Pseudanophthalmus lodingi aladdini Valentine, 1945: 637. Type locality: «Aladdin Cave, upper end of Sharp Cove, Madison Co[unty], Ala[bama]» (original citation). Holotype (♂) in USNM [# 57049].

#### Distribution.

This species is known from a few caves in Madison County, northern Alabama (Barr 2004: 35).

#### Records.

**USA**: AL

### Pseudanophthalmus deceptivus

Barr, 1981

Pseudanophthalmus deceptivus Barr, 1981: 43. Type locality: «Fisher Cave, near the top of Newmans Ridge, between Blackwater and Kyles Ford, Lee Co[unty], Virginia» (original citation). Holotype (♀) in AMNH.

#### Distribution.

This species is known only from the type-locality cave in southwestern Virginia (Barr 2004: 34).

#### Records.

**USA**: VA

### Pseudanophthalmus distinguens

Valentine, 1948

Pseudanophthalmus lodingi distinguens Valentine, 1948: 12. Type locality: «Inge Cave, 5½ miles south of Trinity, Morgan County, Ala[bama]» (original citation). Holotype (♂) probably in ALM.

#### Distribution.

This species is known from a few caves in Morgan County, northern Alabama (Barr 2004: 36).

#### Records.

**USA**: AL

### Pseudanophthalmus engelhardti

(Barber, 1928)

Anophthalmus engelhardti Barber, 1928: 195. Type locality: «English Cave [Claiborne County], Powell River, six miles south of Cumberland Gap, Tennessee» (original citation). Holotype (♂) in USNM [# 40824]. Etymology. The specific name honors George Paul Engelhardt [1871-1942], curator of natural history at the Brooklyn Museum and an authority on clear-wing moths.

#### Distribution.

This species is known only from the type-locality cave in northeastern Tennessee (Barr 2004: 34).

#### Records.

**USA**: TN

### Pseudanophthalmus fastigatus

Barr, 1981

Pseudanophthalmus fastigatus Barr, 1981: 50. Type locality: «Horseshoe Cave, 7 km S[outh]W[est] Chickamauga, Walker Co[unty], Georgia» (original citation). Holotype (♂) in AMNH.

#### Distribution.

This species is known only from the type-locality cave in northwestern Georgia (Barr 2004: 35).

#### Records.

**USA**: GA

### Pseudanophthalmus fluviatilis

Valentine, 1948

Pseudanophthalmus lodingi fluviatilis Valentine, 1948: 12. Type locality: «Rock House Cave, 1 mile south of Oleander, Marshall County, Ala[bama]» (original citation). Holotype (♂) probably in ALM.

#### Distribution.

This species is known from several caves in Marshall and Morgan Counties, northern Alabama (Barr 2004: 36).

#### Records.

**USA**: AL

### Pseudanophthalmus fulleri

Valentine, 1932

Pseudanophthalmus fulleri Valentine, 1932a: 272. Type locality: «Tennessee Cave [= 41 Crystal Caves], [near] Chattanooga [Hamilton County], Tenn[essee]» (original citation). Holotype (♀) in USNM [# 44277].

#### Distribution.

This species is known only from a number of caves in Hamilton County, southeastern Tennessee, and Dade County, northwestern Georgia (Barr 1981: 49; Barr 2004: 35).

#### Records.

**USA**: GA, TN

### Pseudanophthalmus hesperus

Barr, 1959

Pseudanophthalmus hesperus Barr, 1959: 15. Type locality: «Bethel Cave, Perry Co[unty], Tennessee» (original citation). Holotype (♂) in AMNH.

#### Distribution.

This species is known from a few caves in Perry and Wayne Counties, Tennessee (Barr 2004: 36).

#### Records.

**USA**: TN

### Pseudanophthalmus holsingeri

Barr, 1965

Pseudanophthalmus holsingeri Barr, 1965a: 63. Type locality: «Fugates Cave, at Gibson Station, Lee Co[unty], Virginia» (original citation). Holotype (♂) in USNM [# 75261].

#### Distribution.

This species has been found yet only at the type-locality cave in southwestern Virginia (Barr 2004: 35).

#### Records.

**USA**: VA

### Pseudanophthalmus humeralis

Valentine, 1931

Pseudanophthalmus humeralis Valentine, 1931: 253. Type locality: «Crystal Cave, Monteagle [Grundy County], Tenn[essee]» (original citation). Holotype (♂) in USNM [# 44262].
Pseudanophthalmus humeralis brevis Valentine, 1932a: 273. Type locality: «Wonder Cave, Monteagle [Grundy County], Tenn[essee]» (original citation). Holotype (♂) in USNM [# 44278]. Synonymy established by Jeannel (1949b: 84).

#### Distribution.

This species is known from a few caves in Grundy and Franklin Counties, southern Tennessee (Barr 2004: 35).

#### Records.

**USA**: TN

### Pseudanophthalmus lodingi

Valentine, 1931

Pseudanophthalmus lödingi Valentine, 1931: 252. Type locality: «Shelta Cave, Huntsville [Madison County], Ala[bama]» (original citation). Holotype (♂) in USNM [# 44261].

#### Distribution.

This species is known from several caves in Madison County, northern Alabama (Barr 2004: 35).

#### Records.

**USA**: AL

### Pseudanophthalmus meridionalis

Valentine, 1945

Pseudanophthalmus lodingi meridionalis Valentine, 1945: 639. Type locality: «Saltpeter or Nyman Cave, ten miles southwest of Guntersville, Marshall Co[unty], Ala[bama]» (original citation). Holotype (♂) in USNM [# 57051].

#### Distribution.

This species is known from a few caves in northern Alabama (Barr 2004: 36).

#### Records.

**USA**: AL

### Pseudanophthalmus nickajackensis

Barr, 1981

Pseudanophthalmus nickajackensis Barr, 1981: 51. Type locality: «Nickajack Cave, 1.0 km S[outh] Shellmound Station near the mouth of Nickajack Cove, Marion Co[unty], Tennessee» (original citation). Holotype (♂) in AMNH.

#### Distribution.

This species is known only from the type-locality cave in southern Tennessee (Barr 2004: 35).

#### Records.

**USA**: TN

### Pseudanophthalmus nortoni

Barr, 1981

Pseudanophthalmus nortoni Barr, 1981: 48. Type locality: «Grassy Creek Cave, 0.7 km S[outh] Washington, Rhea Co[unty], Tennessee» (original citation). Holotype (♂) in AMNH.

#### Distribution.

This species is known only from the type-locality cave in east-central Tennessee (Barr 2004: 35).

#### Records.

**USA**: TN

### Pseudanophthalmus profundus

Valentine, 1945

Pseudanophthalmus lodingi profundus Valentine, 1945: 637. Type locality: «Natural Well, Monte Sano, Madison Co[unty], Ala[bama]» (original citation). Holotype (♂) in USNM [# 57048].
Pseudanophthalmus lodingi aquaticus Valentine, 1945: 638. Type locality: «Cave Spring Cave, between Troy and Keels M[oun]t[ain]s, near Berkley, Madison Co[unty], Ala[bama]» (original citation). Holotype (♂) in USNM [# 57050]. Synonymy established by Barr (2004: 35).

#### Distribution.

This species is known from a few caves in northern Alabama (Barr 2004: 35).

#### Records.

**USA**: AL

### Pseudanophthalmus rotundatus

Valentine, 1932

Pseudanophthalmus rotundatus Valentine, 1932a: 271. Type locality: «English Cave [Claiborne County], Tenn[essee]» (original citation). Holotype (♂) in USNM [# 44276].

#### Distribution.

This species has been reported from a number of caves in Claiborne and Hancock Counties in northeastern Tennessee and Lee County in southwestern Virginia (Barr 2004: 35).

#### Records.

**USA**: TN, VA

### Pseudanophthalmus sequoyah

Barr, 1981

Pseudanophthalmus sequoyah Barr, 1981: 52. Type locality: «Ellis (= Sequoyah) Cave, 3.3 km S[outh]W[est] Sulphur Springs, Dekalb Co[unty], Alabama» (original citation). Holotype (♂) in AMNH.

#### Distribution.

This species is known only from the type-locality cave in northeastern Alabama (Barr 2004: 35).

#### Records.

**USA**: AL

### Pseudanophthalmus sidus

Barr, 1965

Pseudanophthalmus sidus Barr, 1965a: 64. Type locality: «Meredith Saltpeter Cave, Shanghai Boat Dock, 6 miles southeast of LaFollette, Campbell Co[unty], Tennessee» (original citation). Holotype (♂) in AMNH (Barr 1981: 48).

#### Distribution.

This species is known only from the type-locality cave in northern Tennessee (Barr 2004: 35).

#### Records.

**USA**: TN

### Pseudanophthalmus steevesi

Barr, 1981

Pseudanophthalmus steevesi Barr, 1981: 53. Type locality: «Randolph Cave, 1.7 km S[outh]W[est] Blount Springs, Blount Co[unty], Alabama» (original citation). Holotype (♂) in AMNH.

#### Distribution.

This species is known from a few caves in Blount County, north-central Alabama (Barr 2004: 35).

#### Records.

**USA**: AL

### Pseudanophthalmus wallacei

Barr, 1981

Pseudanophthalmus wallacei Barr, 1981: 46. Type locality: «Weaver Cave, 3.0 km N[orth] Clinton, Anderson Co[unty], Tennessee on the southeast (left) side of Clinch River» (original citation). Holotype (♀) in AMNH.

#### Distribution.

This species is known only from the type-locality cave in eastern Tennessee (Barr 2004: 34).

#### Records.

**USA**: TN

### [eremita group]

### Pseudanophthalmus conditus

Krekeler, 1973

Pseudanophthalmus conditus Krekeler, 1973: 73. Type locality: «Lawrence Cave, 0.5 mile south-southwest of Perryville, Boyle Co[unty], K[entuck]y» (original citation). Holotype (♂) in FMNH.

#### Distribution.

This species is known only from two caves in central Kentucky (Barr 2004: 27).

#### Records.

**USA**: KY

### Pseudanophthalmus eremita

(Horn, 1871)

Anophthalmus eremita G.H. Horn, 1871: 328. Type locality: «Wyandotte Cave [Crawford County, Indiana]» (original citation). Holotype [by monotypy] in MCZ [# 34553].

#### Distribution.

This species is known from two caves in Crawford and Harrison Counties, southern Indiana (Barr 2004: 27).

#### Records.

**USA**: IN

### [gracilis group]

### Pseudanophthalmus gracilis

Valentine, 1931

Pseudanophthalmus gracilis Valentine, 1931: 253. Type locality: «Tommie’s Cave [= Tawneys Cave], Newport [Giles County], V[irgini]a» (original citation). Holotype (♀) in USNM [# 44263].

#### Distribution.

This species is known from a few caves in Giles and Craig Counties, western Virginia (Barr 2004: 21).

#### Records.

**USA**: VA

### Pseudanophthalmus hadenoecus

Barr, 1965

Pseudanophthalmus hadenoecus Barr, 1965a: 53. Type locality: «Mystic Cave, Pendleton Co[unty], West Virginia» (original citation). Holotype (♂) in USNM [# 75266].

#### Distribution.

This species is known only from the type-locality cave, near Onego, in eastern West Virginia (Barr 2004: 21).

#### Records.

**USA**: WV

### [grandis group]

### Pseudanophthalmus fuscus constrictus

Valentine, 1932

Pseudanophthalmus fuscus constrictus Valentine, 1932a: 267. Type locality: «Organ Cave, 2 miles southeast of Ronceverte, east of the Greenbrier River [Greenbrier County], W[est] V[irgini]a» (original citation). Holotype (♂) in USNM [# 44272].

#### Distribution.

This subspecies is known only from a few caves in Greenbrier and Monroe Counties, southeastern West Virginia (Barr 2004: 17).

#### Records.

**USA**: WV

### Pseudanophthalmus fuscus fuscus

Valentine, 1931

Pseudanophthalmus fuscus Valentine, 1931: 254. Type locality: «Kaufman’s Cave [= Coffman Cave], Frankford [Greenbrier County], W[est] V[irgini]a» (original citation). Holotype (♀) in USNM [# 44266].
Pseudanophthalmus subaequalis Valentine, 1931: 255. Type locality: «Kaufman’s Cave [= Coffman Cave], Frankford [Greenbrier County], W[est] V[irginia]» (original citation). Holotype (♂) in USNM [# 44265]. Synonymy established by Valentine (1932a: 267).

#### Distribution.

This subspecies is known from several caves in Greenbrier County, southeastern West Virginia (Barr 2004: 17).

#### Records.

**USA**: WV

### Pseudanophthalmus grandis elevatus

Valentine, 1932

Pseudanophthalmus grandis elevatus Valentine, 1932a: 265. Type locality: «Organ Cave, 2 miles southeast of Ronceverte, east of the Greenbrier River [Greenbrier County], W[est] V[irgini]a» (original citation). Holotype (♂) in USNM [# 44269].

#### Distribution.

This subspecies is known only from a few caves in Greenbrier and Monroe Counties, southeastern West Virginia (Barr 2004: 17).

#### Records.

**USA**: WV

### Pseudanophthalmus grandis grandis

Valentine, 1931

Pseudanophthalmus grandis Valentine, 1931: 254. Type locality: «Higginbotham’s large cave, Frankford [Greenbrier County], W[est] V[irgini]a» (original citation). Holotype (♂) in USNM [# 44264].

#### Distribution.

This subspecies is known from several caves in Greenbrier County, southeastern West Virginia (Barr 2004: 17).

#### Records.

**USA**: WV

### Pseudanophthalmus henroti

Jeannel, 1949

Pseudanophthalmus henroti Jeannel, 1949b: 69. Type locality: «Arbuckle’s cave, près de Lewisburg, rive droite de Greenbrier river, Greenbrier County, West Virginia» (original citation). Holotype (♂) probably in MHNP. Etymology. The specific name was proposed for Henri Henrot [1913-1973], physician in Paris and specialist of the cave fauna, particularly the catopids. His collection was offered to the Muséum d’Histoire Naturelle, Paris, in 1973.

#### Distribution.

This species is known from a few caves in southern Greenbrier County, West Virginia (Barr 2004: 17).

#### Records.

**USA**: WV

### Pseudanophthalmus hypertrichosis

Valentine, 1932

Pseudanophthalmus hypertrichosis Valentine, 1932a: 266. Type locality: «Martha Clarke Cave, 2 miles southwest of Hillsboro [Pocahontas County], W[est] V[irgini]a» (original citation). Holotype (♂) in USNM [# 44271].

#### Distribution.

This species is known only from a few caves in southern Pocahontas County, east-central West Virginia (Barr 2004: 17).

#### Records.

**USA**: WV

### Pseudanophthalmus krekeleri

Barr, 1965

Pseudanophthalmus krekeleri Barr, 1965a: 52. Type locality: «Rich Mountain Cave, Randolph Co[unty], West Virginia» (original citation). Holotype (♂) in USNM [# 75264]. Etymology. This species is named after Carl Herman Krekeler [1920-2012], professor of biology at Valparaiso University in Indiana. Krekeler published three papers on cave carabids.

#### Distribution.

This species is known only from a few specimens collected at the type-locality cave in northeastern West Virginia (Barr 2004: 18).

#### Records.

**USA**: WV

### Pseudanophthalmus montanus

Barr, 1965

Pseudanophthalmus montanus Barr, 1965a: 52. Type locality: «Bennett Cave, Tucker Co[unty], West Virginia» (original citation). Holotype (♂) in USNM [# 75265].

#### Distribution.

This species is known only from two caves in Tucker County, northeastern West Virginia (Barr 2004: 18).

#### Records.

**USA**: WV

### Pseudanophthalmus orthosulcatus

Valentine, 1932

Pseudanophthalmus grandis orthosulcatus Valentine, 1932a: 265. Type locality: «cave opening about 800 ft. north of the village Greenville [= Greenville Saltpeter Cave] [Monroe County], W[est] V[irgini]a» (original citation). Holotype (♀) in USNM [# 44270].

#### Distribution.

This species is known only from a few caves in Monroe County, southeastern West Virginia (Barr 2004: 17).

#### Records.

**USA**: WV

### Pseudanophthalmus sylvaticus

Barr, 1967

Pseudanophthalmus sylvaticus Barr, 1967b: 167. Type locality: «east base of Kennison Mountain near the Cranberry Glades (1000 m), Pocahontas Co[unty], West Virginia» (original citation). Holotype (♂) in USNM [# 75271].

#### Distribution.

This species is known only from two nearby places in the Yew Mountains, east-central West Virginia (Barr 2004: 17).

#### Records.

**USA**: WV

### Pseudanophthalmus virginicus

(Barr, 1960)

Aphanotrechus virginicus Barr, 1960c: 66. Type locality: «Hugh Young Cave, 0.5 mile southeast of Maiden Spring, Tazewell Co[unty], Virginia» (original citation). Holotype (♀) in AMNH [# 1046].

#### Distribution.

This species is still known only from the type-locality cave in southwestern Virginia (Barr 2004: 18).

#### Records.

**USA**: VA

### [hirsutus group]

### Pseudanophthalmus assimilis

Barr, 1981

Pseudanophthalmus assimilis Barr, 1981: 65. Type locality: «Ellis (= Sequoyah) Cave, 3.3 km S[outh]W[est] Sulphur Springs, DeKalb Co[unty], Alabama» (original citation). Holotype (♂) in AMNH.

#### Distribution.

This species is known only from two caves in DeKalb County, northeastern Alabama (Barr 2004: 37).

#### Records.

**USA**: AL

### Pseudanophthalmus delicatus

Valentine, 1932

Pseudanophthalmus hirsutus delicatus Valentine, 1932a: 270. Type locality: «Gilly’s cave, 1 mile south of Pennington Gap [Lee County], V[irgini]a» (original citation). Holotype (♂) in USNM [# 44274].

#### Distribution.

This species is known from several caves in Lee County, southwestern Virginia (Barr 1981: 60; Barr 2004: 37).

#### Records.

**USA**: VA

### Pseudanophthalmus digitus

Valentine, 1932

Pseudanophthalmus digitus Valentine, 1932a: 270. Type locality: «[Tennessee Caverns] on the south side of the Tennessee River about 5 miles west of Chattanoogan [Hamilton County], Raccoon M[oun]t[ain], Tenn[essee]» (original citation). Holotype (♀) in USNM [# 44275].

#### Distribution.

This species is known from caves in Hamilton County, Tennessee, and Dade County, Georgia (Barr 1981: 64; Barr 2004: 37).

#### Records.

**USA**: GA, TN

### Pseudanophthalmus hirsutus

Valentine, 1931

Pseudanophthalmus hirsutus Valentine, 1931: 252. Type locality: «King Solomon’s Cave [= Cudjos Cave], Cumberland Gap [Lee County, Virginia], Tenn[essee]» (original citation). Holotype (♂) in USNM [# 44260].

#### Distribution.

This species is known from two nearby caves in Cumberland Gap National Park, Lee County, southwestern Virginia (Barr 1981: 60; Barr 2004: 37).

#### Records.

**USA**: VA

### Pseudanophthalmus paulus

Barr, 1981

Pseudanophthalmus paulus Barr, 1981: 63. Type locality: «Nobletts Cave, 4.8 km W[est] Sweetwater on the west side of Watson Ridge, Monroe Co[unty], Tennessee» (original citation). Holotype (♀) in AMNH.

#### Distribution.

This species is known only from the type-locality cave in southeastern Tennessee (Barr 2004: 37).

#### Records.

**USA**: TN

### Pseudanophthalmus sericus

Barr, 1981

Pseudanophthalmus sericus Barr, 1981: 62. Type locality: «Lane Cave, in the valley of Moccasin Creek, Scott Co[unty], Virginia» (original citation). Holotype (♂) in AMNH.

#### Distribution.

This species is known only from the type-locality cave in southwestern Virginia (Barr 2004: 37).

#### Records.

**USA**: VA

### Pseudanophthalmus ventus

Barr, 1981

Pseudanophthalmus ventus Barr, 1981: 64. Type locality: «Blowing Cave, in town of Sequatchie, Marion Co[unty], Tennessee» (original citation). Holotype (♂) in AMNH.

#### Distribution.

This species is known only from the type-locality cave in southern Tennessee (Barr 2004: 37).

#### Records.

**USA**: TN

### [horni group]

### Pseudanophthalmus abditus

Krekeler, 1973

Pseudanophthalmus horni abditus Krekeler, 1973: 44. Type locality: «Swope Cave, 4.5 miles north of Versailles, Woodford Co[unty], K[entuck]y» (original citation). Holotype (♂) in FMNH.

#### Distribution.

This species is known from a few caves in Woodford and Jessamine Counties, Kentucky (Barr 2004: 22).

#### Records.

**USA**: KY

### Pseudanophthalmus caecus

Krekeler, 1973

Pseudanophthalmus horni caecus Krekeler, 1973: 43. Type locality: «Clifton Cave, 0.6 mile east-southeast of Clifton, Woodford Co[unty], K[entuck]y» (original citation). Holotype (♂) in FMNH.

#### Distribution.

This species is known only from the type-locality cave in east-central Kentucky (Barr 2004: 22).

#### Records.

**USA**: KY

### Pseudanophthalmus chthonius

Krekeler, 1973

Pseudanophthalmus chthonius Krekeler, 1973: 50. Type locality: «Wilson Cave, 1.5 miles northwest of Kent, Jefferson Co[unty], Ind[iana]» (original citation). Holotype (♂) in FMNH.

#### Distribution.

This species is known from a few caves in Jefferson, Jennings, and Clark Counties, southern Indiana (Krekeler 1973: 51; Barr 2004: 23).

#### Records.

**USA**: IN

### Pseudanophthalmus desertus

Krekeler, 1973

Pseudanophthalmus desertus desertus Krekeler, 1973: 49. Type locality: «Jones Cave, 0.8 mile northeast of Locust Grove, Clark Co[unty], K[entuck]y» (original citation). Holotype (♂) in FMNH.

#### Distribution.

This species is known from a few widely scattered caves in Clark, Henry, Scott, and Owen Counties, Kentucky (Krekeler 1973: 49; Barr 2004: 22).

#### Records.

**USA**: KY

#### Note.

According to Barr (2004: 22), the Henry and Owen County populations possibly represent a distinct species.

### Pseudanophthalmus elongatus

Krekeler, 1973

Pseudanophthalmus elongatus Krekeler, 1973: 46. Type locality: «Old Fort Cave, 1.2 miles northeast of Harrodsburg, Mercer Co[unty], K[entuck]y» (original citation). Holotype (♂) in FMNH.

#### Distribution.

This species is known from a few caves in Mercer and Garrard Counties, central Kentucky (Barr 2004: 22).

#### Records.

**USA**: KY

### Pseudanophthalmus exoticus

Krekeler, 1973

Pseudanophthalmus exoticus Krekeler, 1973: 53. Type locality: «Townsend Cave, 4 miles west-northwest of Zachariah, [Lee County] Estill Co[unty], K[entuck]y» (original citation). Holotype (♂) in FMNH.

#### Distribution.

This species is known only from the holotype collected in Lee County, not Estill County as reported by Krekeler (1973: 53), in eastern Kentucky (Barr 2004: 23).

#### Records.

**USA**: KY

### Pseudanophthalmus horni

(Garman, 1892)

Anophthalmus horni Garman, 1892: 241. Type locality: «within the corporate limits of Lexington [Fayette County, Kentucky]» (original citation), restricted to «Reid (= Picadome) Cave, at Picadome School» by Barr (2004: 22). Syntype(s) location unknown (probably in USNM). Note. Barber (1928: 196) stated that Garman sent six specimens of this species, labeled “Lexington, Ky. 10.9.92,” to the USNM. These specimens are probably syntypes.
Pseudanophthalmus horni garmani Jeannel, 1949b: 49. Type locality: «Reid’s cave, à 2 km W[est] de Lexington, Fayette County, Kentucky» (original citation). Holotype in MHNP. Synonymy established by Krekeler (1973: 41). Etymology. The subspecific name was proposed for Harrison Garman [1856-1944], professor at the University of Illinois, later at the University of Kentucky, and state entomologist at the Kentucky Agricultural Experiment Station. Garman was an outstanding entomologist but not a specialist on any groups or subjects.
Pseudanophthalmus horni minor Jeannel, 1949b: 49. Type locality: «Phelp’s cave, à 5 miles S[outh]-W[est] de Lexington, Fayette County, Kentucky» (original citation). Holotype probably in MHNP. Synonymy established by Krekeler (1973: 42).

#### Distribution.

This species is known from several caves in Fayette County, north-central Kentucky (Barr 2004: 22).

#### Records.

**USA**: KY

### Pseudanophthalmus krameri

Krekeler, 1973

Pseudanophthalmus krameri Krekeler, 1973: 54. Type locality: «Cave Hill Cave, 5 miles northwest of West Union, Adams Co[unty], Ohio» (original citation). Holotype (♂) in FMNH.

#### Distribution.

This species is known only from the type-locality cave in southern Ohio (Barr 2004: 23).

#### Records.

**USA**: OH

### Pseudanophthalmus major

Krekeler, 1973

Pseudanophthalmus desertus major Krekeler, 1973: 50. Type locality: «Beaver Cave, 3 miles northeast of Oddville, Harrison Co[unty], K[entuck]y» (original citation). Holotype (♂) in FMNH.

#### Distribution.

This species is known only from the type-locality cave, 35 miles northeast of Lexington, northern Kentucky (Barr 2004: 22).

#### Records.

**USA**: KY

### Pseudanophthalmus ohioensis

Krekeler, 1973

Pseudanophthalmus ohioensis Krekeler, 1973: 52. Type locality: «Freeland Cave, 6 miles southeast of Peebles, Adams Co[unty], Ohio» (original citation). Holotype (♂) in FMNH.

#### Distribution.

This species is known only from the type-locality cave in southern Ohio (Barr 2004: 23).

#### Records.

**USA**: OH

### Pseudanophthalmus pholeter

Krekeler, 1973

Pseudanophthalmus pholeter Krekeler, 1973: 55. Type locality: «Adams Cave, 5 miles south-southwest of Richmond, Madison Co[unty], K[entuck]y» (original citation). Holotype (♂) in FMNH.

#### Distribution.

This species is known only from the type-locality cave in east-central Kentucky (Barr 2004: 23).

#### Records.

**USA**: KY

### Pseudanophthalmus solivagus

Krekeler, 1973

Pseudanophthalmus solivagus Krekeler, 1973: 44. Type locality: «Weber Cave, 2 miles northwest of Nonesuch, Woodford Co[unty], K[entuck]y» (original citation). Holotype (♂) in FMNH.

#### Distribution.

This species is known from a few caves in Woodford and Jessamine Counties, Kentucky (Barr 2004: 22).

#### Records.

**USA**: KY

### Pseudanophthalmus tenebrosus

Krekeler, 1973

Pseudanophthalmus tenebrosus Krekeler, 1973: 48. Type locality: «Stevens Creek Cave, 0.85 mile east-southeast of Orville, Henry Co[unty], K[entuck]y» (original citation). Holotype (♂) in FMNH.

#### Distribution.

This species is known only from the type-locality cave in northern Kentucky (Barr 2004: 22).

#### Records.

**USA**: KY

### [hubbardi group]

### Pseudanophthalmus avernus

Valentine, 1945

Pseudanophthalmus hubbardi avernus Valentine, 1945: 648. Type locality: «Endless Caverns, Rockingham Co[unty], V[irgini]a» (original citation). Holotype (♂) in USNM [# 57053].

#### Distribution.

This species is known only from the type-locality cave in west-central Virginia (Barr 2004: 18).

#### Records.

**USA**: VA

### Pseudanophthalmus hubbardi

(Barber, 1928)

Anophthalmus hubbardi Barber, 1928: 196. Type locality: «Luray Cave, Page County, Virginia» (original citation). Holotype (♀) in USNM [# 40823].

#### Distribution.

This species is known only from the type-locality cave in northern Virginia (Barr 2004: 18).

#### Records.

**USA**: VA

### Pseudanophthalmus intersectus

Barr, 1965

Pseudanophthalmus intersectus Barr, 1965a: 57. Type locality: «Crossroads Cave, near Millboro Springs, Bath Co[unty], Virginia» (original citation). Holotype (♂) location unknown (originally in T.C. Barr’s collection).

#### Distribution.

This species is known only from the type-locality cave in western Virginia (Barr 2004: 18).

#### Records.

**USA**: VA

### Pseudanophthalmus limicola

Jeannel, 1931

Pseudanophthalmus hubbardi limicola Jeannel, 1931: 450. Type locality: «Maddens cave, à 4 milles de New-Market, Shenandoah Co[unty], Virginia» (original citation). Holotype in MHNP.

#### Distribution.

This species is known from a few nearby caves in Shenandoah County, northern Virginia (Barr 2004: 18).

#### Records.

**USA**: VA

### Pseudanophthalmus parvicollis

Jeannel, 1931

Pseudanophthalmus hubbardi parvicollis Jeannel, 1931: 450. Type locality: «Battlefield Crystal cave, située à un mille au nord de Strasburg [Rockingham County], Virginia» (original citation). Holotype (♀) in USNM [# 43665].

#### Distribution.

This species is known only from the type-locality cave in northern Virginia (Barr 2004: 18).

#### Records.

**USA**: VA

### Pseudanophthalmus potomaca

Valentine, 1932

Pseudanophthalmus potomaca Valentine, 1932a: 262. Type locality: «[Kenny] Simmons’ Cave, on South Branch Potomac River, 10 miles south of Franklin [Pendleton County], W[est] V[irgini]a» (original citation). Holotype (♂) in USNM [# 44267].

#### Distribution.

This species is known from two nearby caves in Pendleton County, eastern West Virginia, and Highland County, western Virginia (Barr 2004: 18).

#### Records.

**USA**: VA, WV

### Pseudanophthalmus senecae

Valentine, 1932

Pseudanophthalmus potomaca senecae Valentine, 1932a: 263. Type locality: «Seneca Caves [= Stratosphere Balloon Cave; see Barr 1965a: 58], on the North Fork Potomac River, 4 miles northeast of Riverton [Pendleton County], W[est] V[irgini]a» (original citation). Holotype (♂) in USNM [# 44268].

#### Distribution.

This species is known from a few nearby caves in eastern West Virginia (Barr 2004: 19).

#### Records.

**USA**: WV

### [hubrichti group]

### Pseudanophthalmus egberti

Barr, 1965

Pseudanophthalmus egberti Barr, 1965a: 49. Type locality: «Starnes Cave, Giles Co[unty], Virginia» (original citation). Holotype (♂) in USNM [# 75268].

#### Distribution.

This species is known from two caves in Giles County, western Virginia (Barr 1981: 69; Barr 2004: 38).

#### Records.

**USA**: VA

### Pseudanophthalmus hubrichti

Valentine, 1948

Pseudanophthalmus hubrichti Valentine, 1948: 13. Type locality: «Dougherty’s Cave, 4½ miles north of Lebanon, Russell Co[unty], V[irgini]a» (original citation). Holotype (♂) probably in ALM. Etymology. The specific name was given in honor of Leslie Raymond Hubricht [1908-2005], world authority on the land snails of eastern United States, author of numerous articles on North American land and freshwater mollusks, and prolific collector.

#### Distribution.

This species is known only from the type-locality cave in southwestern Virginia (Barr 2004: 38).

#### Records.

**USA**: VA

### Pseudanophthalmus paradoxus

Barr, 1981

Pseudanophthalmus paradoxus Barr, 1981: 70. Type locality: «Sensabaugh Saltpeter Cave, about 8 km W[est] Kingsport, Hawkins Co[unty], Tennessee» (original citation). Holotype (♂) in AMNH.

#### Distribution.

This species is known only from the type-locality cave in northeastern Tennessee (Barr 2004: 38).

#### Records.

**USA**: TN

### Pseudanophthalmus quadratus

Barr, 1965

Pseudanophthalmus quadratus Barr, 1965a: 60. Type locality: «Straleys Cave, Giles Co[unty], Virginia» (original citation). Holotype (♂) in USNM [# 75262].

#### Distribution.

This species is known only from two nearby caves near Eggleston, western Virginia (Barr 2004: 38).

#### Records.

**USA**: VA

### Pseudanophthalmus sanctipauli

Barr, 1981

Pseudanophthalmus sanctipauli Barr, 1981: 67. Type locality: «Banners Corner Cave, near S[ain]t Paul, Russell Co[unty], Virginia» (original citation). Holotype (♂) in AMNH.

#### Distribution.

This species is known only from two caves in Russell and Scott Counties, southwestern Virginia (Barr 2004: 38).

#### Records.

**USA**: VA

### Pseudanophthalmus vicarius

Barr, 1965

Pseudanophthalmus vicarius Barr, 1965a: 48. Type locality: «Hugh Young Cave, near Maiden Spring, Tazewell Co[unty], Virginia» (original citation). Holotype (♂) in USNM [# 75267].

#### Distribution.

This species is known from a number of caves in Tazewell County, southwestern Virginia (Barr 1981: 70; Barr 2004: 38).

#### Records.

**USA**: VA

### [hypolithos group]

### Pseudanophthalmus calcareus

Barr, 1981

Pseudanophthalmus calcareus Barr, 1981: 85. Type locality: «Limestone Cave, on the northwest slope of Pine Mountain, 2.5 km N[orth]N[orth]W[est] of the common corner of Whitley Co[unty], Kentucky, and Campbell and Claiborne Co[untie]s, Tennessee, in Whitley Co[unty], Kentucky» (original citation). Holotype (♂) in AMNH.

#### Distribution.

This species is known only from the type-locality cave in Whitley County, southeastern Kentucky (Barr 2004: 40).

#### Records.

**USA**: KY

### Pseudanophthalmus frigidus

Barr, 1981

Pseudanophthalmus frigidus Barr, 1981: 86. Type locality: «Icebox Cave, 25 m above L & N railroad tracks on north side of Cumberland River in the town of Pineville, 1.0 km S[outh]E[ast] of the courthouse, Bell Co[unty], Kentucky» (original citation). Holotype (♂) in AMNH.

#### Distribution.

This species is known only from the type-locality cave in southeastern Kentucky (Barr 2004: 40).

#### Records.

**USA**: KY

### Pseudanophthalmus hypolithos

Barr, 1981

Pseudanophthalmus hypolithos Barr, 1981: 83. Type locality: «Old Quarry Cave, 1.8 km S[outh]S[outh]E[ast] Ashcamp, Pike Co[unty], Kentucky» (original citation). Holotype (♂) in AMNH.

#### Distribution.

This species is known from the type-locality cave in Pike County and two abandoned coal mines in Floyd County, eastern Kentucky (Barr 2004: 40).

#### Records.

**USA**: KY

### Pseudanophthalmus praetermissus

Barr, 1981

Pseudanophthalmus praetermissus Barr, 1981: 87. Type locality: «Kern’s Cave No. 1, Scott Co[unty], Virginia» (original citation). Holotype (♂) in AMNH.

#### Distribution.

This species is known only from the type-locality cave in southwestern Virginia (Barr 2004: 40).

#### Records.

**USA**: VA

### Pseudanophthalmus scholasticus

Barr, 1981

Pseudanophthalmus scholasticus Barr, 1981: 84. Type locality: «Sawmill Hollow Cave, 2 km N[orth]N[orth]W[est] Nolansburg and 600 m E[ast]S[outh]E[ast] Pine Mountain Settlement School on the northwest side of Pine Mountain, Harlan Co[unty], Kentucky» (original citation). Holotype (♂) in AMNH.

#### Distribution.

This species is known only from the type-locality cave in southeastern Kentucky (Barr 2004: 40).

#### Records.

**USA**: KY

### [inexpectatus group]

### Pseudanophthalmus cnephosus

Krekeler, 1973

Pseudanophthalmus cnephosus Krekeler, 1973: 61. Type locality: «Eli Reed Cave, 6.5 miles east-southeast of Hodgenville, Larue Co[unty], K[entuck]y» (original citation). Holotype (♂) in FMNH.

#### Distribution.

This species is known only from two caves in Larue and Nelson Counties, central Kentucky (Barr 2004: 21).

#### Records.

**USA**: KY

### Pseudanophthalmus inexpectatus

Barr, 1959

Pseudanophthalmus inexpectatus Barr, 1959: 10. Type locality: «White Cave, Mammoth Cave National Park, Edmonson Co[unty], Kentucky» (original citation). Holotype (♂) in AMNH.

#### Distribution.

This species is known from a few caves located in Mammoth Cave National Park, Kentucky (Barr 2004: 21).

#### Records.

**USA**: KY

### Pseudanophthalmus orientalis

Krekeler, 1973

Pseudanophthalmus inexpectatus orientalis Krekeler, 1973: 59. Type locality: «Wilson Cave, 1 mile southeast of Black Gnat, Green Co[unty], K[entuck]y» (original citation). Holotype (♂) in FMNH.

#### Distribution.

This species is known from several caves in Green, Hart, and Taylor Counties, central Kentucky (Barr 2004: 21).

#### Records.

**USA**: KY

### Pseudanophthalmus parvus

Krekeler, 1973

Pseudanophthalmus parvus Krekeler, 1973: 62. Type locality: «Tatum Cave, 1.8 miles west-southwest of Riley, Marion Co[unty], K[entuck]y» (original citation). Holotype (♂) in FMNH.

#### Distribution.

This species is known only from the type-locality cave in central Kentucky (Barr 2004: 21).

#### Records.

**USA**: KY

### Pseudanophthalmus puteanus

Krekeler, 1973

Pseudanophthalmus puteanus Krekeler, 1973: 60. Type locality: «Old Well Cave, 0.6 mile southeast of Nevada, Mercer Co[unty], K[entuck]y» (original citation). Holotype (♂) in FMNH.

#### Distribution.

This species is known only from two nearby caves in Mercer and Boyle Counties, Kentucky (Barr 2004: 21).

#### Records.

**USA**: KY

### Pseudanophthalmus umbratilis

Krekeler, 1973

Pseudanophthalmus umbratilis Krekeler, 1973: 62. Type locality: «Robinson Cave, 4.5 miles west-northwest of Lancaster, Garrard Co[unty], K[entuck]y» (original citation). Holotype (♂) in FMNH.

#### Distribution.

This species is known from several caves in Garrard, Fayette, Woodford, and Owen Counties, Kentucky (Barr 2004: 21).

#### Records.

**USA**: KY

### [intermedius group]

### Pseudanophthalmus intermedius

(Valentine, 1931)

Neaphaenops intermedius Valentine, 1931: 249. Type locality: «Wonder Cave, Monteagle [Grundy County], Tenn[essee]» (original citation). Holotype [designated lectotype by Erwin and House (1978: 244)] (♂) in USNM [# 44256].

#### Distribution.

This species is known from several caves in Grundy and Franklin Counties, southern Tennessee (Barr 2004: 29).

#### Records.

**USA**: TN

### Pseudanophthalmus macradyi

Valentine, 1948

Pseudanophthalmus macradyi Valentine, 1948: 9 (as *macradei*). Type locality: «Higginbotham’s Cave, McMinnville, Warren Co[unty], Tenn[essee]» (original citation). Holotype (♂) probably in ALM. Etymology. The specific name was proposed for Edward McCrady [1906-1981], American teacher, research scientist, practicing portrait painter, and popular speaker. McCrady also played the violin, composed one symphony, published translations of Greek and Latin classics, explored caves in Tennessee, was president of the University of the South in Sewanee, Tennessee, and was *de facto* mayor of the city. Note. The spelling *macradyi*, introduced by Barr (2004: 29), is a justified emendation of *macradei* in my opinion, and the original name must be corrected (see ICZN 1999: Article 32.5.1).

#### Distribution.

This species is known only from several caves in Warren and Grundy Counties, Tennessee (Barr 2004: 29).

#### Records.

**USA**: TN

### Pseudanophthalmus templetoni

Valentine, 1948

Pseudanophthalmus intermedius templetoni Valentine, 1948: 7. Type locality: «Higginbotham’s Cave, McMinnville, Warren Co[unty], Tenn[essee]» (original citation). Holotype (♂) probably in ALM.

#### Distribution.

This species is known from several caves in Warren and Grundy Counties, Tennessee (Barr 2004: 29).

#### Records.

**USA**: TN

### Pseudanophthalmus vanburenensis

Barr, 1959

Pseudanophthalmus templetoni vanburenensis Barr, 1959: 15. Type locality: «McElroy Cave, 1.5 miles northeast of Bone Cave P.O., Van Buren Co[unty], Tennessee» (original citation). Holotype (♂) in AMNH.

#### Distribution.

This species is known only from caves in Van Buren County, central Tennessee (Barr 2004: 29).

#### Records.

**USA**: TN

### [jonesi group]

### Pseudanophthalmus cordicollis

Barr, 1981

Pseudanophthalmus cordicollis Barr, 1981: 82. Type locality: «Little Kennedy Cave, Wise Co[unty], Virginia» (original citation). Holotype (♂) in AMNH.

#### Distribution.

This species is known only from a few caves in Wise County, southwestern Virginia (Barr 2004: 39).

#### Records.

**USA**: VA

### Pseudanophthalmus jonesi

Valentine, 1945

Pseudanophthalmus jonesi Valentine, 1945: 645. Type locality: «Saltpeter or Brady Cave, southeast slope of Walden Ridge, Grassy Cove, Cumberland Co[unty], Tenn[essee]» (original citation). Holotype (♂) in USNM [# 57052]. Etymology. This species was proposed for the American geologist and archaeologist Walter Bryan Jones [1895-1977] of the University of Alabama. Jones was the founder and director of the Alabama Museum of Natural History.

#### Distribution.

This species is known only from three caves in Grassy Cove, Cumberland County, in east-central Tennessee (Barr 1981: 73; Barr 2004: 39).

#### Records.

**USA**: TN

### Pseudanophthalmus longiceps

Barr, 1981

Pseudanophthalmus longiceps Barr, 1981: 79. Type locality: «Fisher Cave, near the top of Newmans Ridge, between Blackwater and Kyles Ford, Lee Co[unty], Virginia» (original citation). Holotype (♂) in AMNH.

#### Distribution.

This species is known from two caves in Lee County, southwestern Virginia, and Hancock County, northeastern Tennessee (Barr 1981: 80; Barr 2004: 39).

#### Records.

**USA**: TN, VA

### Pseudanophthalmus pallidus

Barr, 1981

Pseudanophthalmus pallidus Barr, 1981: 78. Type locality: «Chadwell Cave, 6 km N[orth]E[ast] Tazewell, 425 m S[outh] of Cedar Fork Road, and 1000 m N[orth] of Henderson Knob (400 m) [Claiborne County, Tennessee]» (original citation). Holotype (♂) in AMNH.

#### Distribution.

This species is known from a few caves in Claiborne County, northeastern Tennessee (Barr 2004: 39). The record from “Virginia” (Bousquet and Larochelle 1993: 114) is in error (see Hoffman et al. 2006: 19).

#### Records.

**USA**: TN

### Pseudanophthalmus rogersae

Barr, 1981

Pseudanophthalmus rogersae Barr, 1981: 75. Type locality: «Sawmill Hollow Cave, 2.0 km N[oth]N[orth]W[est] Nolansburg and 600 m E[ast]S[outh]E[ast] Pine Mountain Settlement School on the northwest slope of Pine Mountain (700 m) [Harlan County, Kentucky]» (original citation). Holotype (♀) in AMNH.

#### Distribution.

This species is known only from the type-locality cave in southeastern Kentucky (Barr 2004: 39). The record from “Virginia” (Bousquet and Larochelle 1993: 115) is in error (see Hoffman et al. 2006: 19).

#### Records.

**USA**: KY

### Pseudanophthalmus scutilus

Barr, 1981

Pseudanophthalmus scutilus Barr, 1981: 73. Type locality: «New Mammoth Cave, 1.5 km E[ast] Elk Valley on the north side of Pine Mountain, Campbell Co[unty], Tennessee» (original citation). Holotype (♂) in AMNH.

#### Distribution.

This species is known only from the type-locality cave in northern Tennessee (Barr 2004: 39).

#### Records.

**USA**: TN

### Pseudanophthalmus seclusus

Barr, 1981

Pseudanophthalmus seclusus Barr, 1981: 76. Type locality: «Flannery Cave, Scott Co[unty], Virginia» (original citation). Holotype (♂) in AMNH.

#### Distribution.

This species is known from several caves in the Rye Cove karst near Clinchport in southwestern Virginia (Barr 1981: 77).

#### Records.

**USA**: VA

### Pseudanophthalmus thomasi

Barr, 1981

Pseudanophthalmus thomasi Barr, 1981: 80. Type locality: «Blair-Collins Cave (490 m), Scott Co[unty], Virginia» (original citation). Holotype (♂) in AMNH.

#### Distribution.

This species is known only from two caves in Scott County, southwestern Virginia (Barr 2004: 39).

#### Records.

**USA**: VA

### [leonae group]

### Pseudanophthalmus leonae

Barr, 1960

Pseudanophthalmus leonae Barr, 1960a: 310. Type locality: «Hert Hollow Cave, two miles S[outh]W[est] of Springville, Lawrence Co[unty], Indiana» (original citation). Holotype (♂) in AMNH [# 1047].

#### Distribution.

This species is known only from the type-locality cave, located two miles southwest of Springville, southern Indiana (Barr 2004: 27).

#### Records.

**USA**: IN

### [menetriesii group]

### Pseudanophthalmus cerberus cerberus

Barr, 1985

Pseudanophthalmus cerberus cerberus Barr, 1985b: 123. Type locality: «Rhoton Cave, 3.3 km S[outh]W[est] Hestand on N[orth] side valley of Sweetwater Creek, Monroe Co[unty], Kentucky» (original citation). Holotype (♂) in AMNH.

#### Distribution.

This subspecies is known from several caves in Monroe, Barren, Metcalfe, Adair, Cumberland, and Clay Counties in southern Kentucky and Jackson County in northern Tennessee (Barr 1985b: 124).

#### Records.

**USA**: KY, TN

### Pseudanophthalmus cerberus completus

Barr, 1985

Pseudanophthalmus cerberus completus Barr, 1985b: 124. Type locality: «Cole Cave, 1.8 km N Austin, Barren Co[unty], Kentucky» (original citation). Holotype (♂) in AMNH.

#### Distribution.

This subspecies is known from a number of caves in central Barren County, southern Kentucky (Barr 2004: 32).

#### Records.

**USA**: KY

#### Note.

According to Barr (2004: 32), intergrades between this subspecies and the nominotypical subspecies occur in Bowles Branch Cave in Barren County.

### Pseudanophthalmus darlingtoni darlingtoni

Barr, 1985

Pseudanophthalmus darlingtoni darlingtoni Barr, 1985b: 125. Type locality: «Jones Cave, 4.3 km N[orth]N[orth]E[ast] Columbia on E[ast] side valley of Butler Branch, Adair Co[unty], Kentucky» (original citation). Holotype (♂) in AMNH.

#### Distribution.

This subspecies is known from several caves in northeastern Metcalfe, northern Adair, and southern Green Counties, southern Kentucky (Barr 1985b: 126; Barr 2004: 32).

#### Records.

**USA**: KY

### Pseudanophthalmus darlingtoni persimilis

Barr, 1985

Pseudanophthalmus darlingtoni persimilis Barr, 1985b: 126. Type locality: «Woodard Cave, 5.0 km N[orth]W[est] Donansburg near Little Barren River, Green Co[unty], Kentucky» (original citation). Holotype (♂) in AMNH.

#### Distribution.

This subspecies is known from several caves in central Green and eastern Hart Counties, Kentucky (Barr 2004: 32).

#### Records.

**USA**: KY

### Pseudanophthalmus globiceps

Barr, 1985

Pseudanophthalmus globiceps Barr, 1985b: 122. Type locality: «Barnes Smith Cave, 5.7 km N[orth] Hinesdale, Hart Co[unty], Kentucky» (original citation). Holotype (♂) in AMNH.

#### Distribution.

This species is known only from the type-locality cave in central Kentucky (Barr 2004: 31).

#### Records.

**USA**: KY

### Pseudanophthalmus menetriesii campestris

Barr, 1985

Pseudanophthalmus menetriesi campestris Barr, 1985b: 119. Type locality: «Walnut Hill Cave, 3.3 km S[outh] Park City, Barren Co[unty], Kentucky» (original citation). Holotype (♂) in AMNH.

#### Distribution.

This subspecies is found in several caves in Barren and Warren Counties, southern Kentucky (Barr 2004: 31).

#### Records.

**USA**: KY

#### Note.

According to Barr (2004: 31), intergrades between this subspecies and the nominotypical subspecies occur in caves in Warren County.

### Pseudanophthalmus menetriesii menetriesii

(Motschulsky, 1862)

Anophthalmus ménétriesii Motschulsky, 1862b: 41. Type locality: «caverne des Mammouths [Edmonson County, Kentucky]» (original citation). One syntype in ZMMU (Keleinikova 1976: 205).
Anophthalmus ventricosus Motschulsky, 1862b: 42. Type locality: «caverne des Mammouths [Edmonson County, Kentucky]» (original citation). One syntype in ZMMU (Keleinikova 1976: 222). Synonymy established by Jeannel (1928: 121).
Anophthalmus angulatus LeConte, 1863c: 18. Type locality: «Mammoth Cave [Edmonson County], Kentucky» (original citation). One syntype in MCZ [# 5598]. Synonymy established by Horn (1869a: 127).

#### Distribution.

This subspecies is known from several caves in Hart, Edmonson, Barren, and Warren Counties in Kentucky (Barr 2004: 31).

#### Records.

**USA**: KY

### Pseudanophthalmus pilosus

Barr, 1985

Pseudanophthalmus pilosus Barr, 1985b: 120. Type locality: «Bland Cave, 1.8 km N[orth]W[est] Spurrier on N[orth] side Akers Valley, Hardin Co[unty], Kentucky» (original citation). Holotype (♂) in AMNH.

#### Distribution.

This species is known from caves in Hardin and Hart Counties, central Kentucky (Barr 2004: 31).

#### Records.

**USA**: KY

### Pseudanophthalmus simulans

Barr, 1985

Pseudanophthalmus simulans Barr, 1985b: 120. Type locality: «Cub Run Cave, at Cub Run, Hart Co[unty], Kentucky» (original citation). Holotype (♂) in AMNH.

#### Distribution.

This species is known only from the type-locality cave in central Kentucky (Barr 2004: 31).

#### Records.

**USA**: KY

### Pseudanophthalmus striatus

(Motschulsky, 1862)

Anophthalmus striatus Motschulsky, 1862b: 41. Type locality: «caverne des Mammouths [Edmonson County, Kentucky]» (original citation). One syntype in ZMMU (Keleinikova 1976: 218).
Anophthalmus interstitialis Hubbard, 1880: 52. Type locality: «Washington’s Hall in the Mammoth Cave [Edmonson County, Kentucky]» (original citation). Holotype [by monotypy] (♀) in USNM [# 23860]. Synonymy established with doubt by Horn (1883b: 272), confirmed by Jeannel (1928: 124).

#### Distribution.

This species is known from several caves in Hart, Edmonson, Metcalf, and Warren Counties, southern Kentucky (Barr 2004: 32).

#### Records.

**USA**: KY

#### Note.

The MCZ holds a specimen [# 7397], incorrectly labeled lectotype, of *Promecognathus interstitialis* Hubbard from Cave City, Kentucky.

### Pseudanophthalmus transfluvialis

Barr, 1985

Pseudanophthalmus transfluvialis Barr, 1985b: 123. Type locality: «McGinnis Cave, 4.2 km S[outh]W[est] Bowling Green, Warren Co[unty], Kentucky» (original citation). Holotype (♂) in AMNH.

#### Distribution.

This species is known from a few caves in Warren and Logan Counties, southern Kentucky (Barr 1985b: 123; Barr 2004: 31).

#### Records.

**USA**: KY

### [petrunkevitchi group]

### Pseudanophthalmus hoffmani

Barr, 1965

Pseudanophthalmus hoffmani Barr, 1965a: 58. Type locality: «Buchanan Saltpeter Cave, at Ellendale, 6 miles northeast of Saltville, Smyth Co[unty], Virginia» (original citation). Holotype (♂) in USNM [# 75260].

#### Distribution.

This species is known from a few caves in Smyth and Bland Counties, Virginia (Barr 2004: 19).

#### Records.

**USA**: VA

### Pseudanophthalmus hortulanus

Barr, 1965

Pseudanophthalmus hortulanus Barr, 1965a: 59. Type locality: «Cassells Cave, at the northwest end of Burkes Garden, Tazewell Co[unty], Virginia» (original citation). Holotype (♂) in USNM [# 75263].

#### Distribution.

This species is known only from the type-locality cave in southwestern Virginia (Barr 2004: 19).

#### Records.

**USA**: VA

### Pseudanophthalmus petrunkevitchi

Valentine, 1945

Pseudanophthalmus petrunkevitchi Valentine, 1945: 652. Type locality: «Skyline Cavern, two miles southwest of Front Royal, Warren Co[unty], V[irgini]a» (original citation). Holotype (♂) in USNM [# 57054]. Etymology. The specific name was proposed in honor of the eminent arachnologist Alexander Ivanovitch Petrunkevitch [1875-1964]. Born in Ukraine, Petrunkevitch settled in New Haven, Connecticut, where he taught from 1910 to 1944 at Yale University. He published extensively on spiders and scorpions and wrote two volumes of poetry under the pseudonym of Alexandr Jan-Ruban.

#### Distribution.

This species, the only one of the genus *Pseudanophthalmus* with pigmented eyespots, is known from a few specimens collected at two nearby caves in Warren and Pages Counties, northern Virginia (Barr 2004: 19).

#### Records.

**USA**: VA

### [pubescens group]

### Pseudanophthalmus ciliaris ciliaris

Valentine, 1937

Pseudanophthalmus ciliaris Valentine, 1937: 95. Type locality: «Dunbar’s Cave, Clarksville [Montgomery County], Tennessee» (original citation). Holotype (♂) in USNM [# 56125].

#### Distribution.

This subspecies occurs in several caves in Montgomery, Cheatham, and Robertson Counties in northern Tennessee and Christian and Logan Counties in southern Kentucky (Barr 2004: 30).

#### Records.

**USA**: KY, TN

### Pseudanophthalmus ciliaris orlindae

Barr, 1959

Pseudanophthalmus orlindae Barr, 1959: 7. Type locality: «Jesse James Cave, 1.5 miles southeast of Orlinda, Robertson Co[unty], Tennessee» (original citation). Holotype (♂) in AMNH.

#### Distribution.

This subspecies is known from a few caves in Logan and Simpson Counties, southern Kentucky, and Robertson County, northern Tennessee (Barr 2004: 30).

#### Records.

**USA**: KY, TN

#### Note.

According to Barr (2004: 30), intergrades between this subspecies and the nominotypical subspecies occur in one cave in Robertson County, Tennessee.

### Pseudanophthalmus colemanensis

Barr, 1959

Pseudanophthalmus ciliaris colemanensis Barr, 1959: 6. Type locality: «Coleman Cave, 8 miles west of Clarksville, Montgomery Co[unty], Tennessee» (original citation). Holotype (♂) in AMNH.

#### Distribution.

This species is known from several caves in Montgomery County, northern Tennessee (Barr 2004: 31).

#### Records.

**USA**: TN

### Pseudanophthalmus loganensis

Barr, 1959

Pseudanophthalmus ciliaris loganensis Barr, 1959: 7. Type locality: «Cook Cave, 1 mile east of Adairville, Logan Co[unty], Kentucky» (original citation). Holotype (♂) in AMNH.

#### Distribution.

This species is known from several caves in Warren, Simpson, and Logan Counties, southern Kentucky, and Robertson and Sumner Counties, northern Tennessee (Barr 2004: 30).

#### Records.

**USA**: KY, TN

### Pseudanophthalmus princeps

Barr, 1979

Pseudanophthalmus princeps Barr, 1979a: 17. Type locality: «Hoy Cave, 1.9 miles north of county courthouse in Franklin under US 31W, Simpson County, Kentucky» (original citation). Holotype (♂) in AMNH [# 1418].

#### Distribution.

This species is known from a few caves in Simpson and Warren Counties in southern Kentucky and Sumner County in northern Tennessee (Barr 2004: 30).

#### Records.

**USA**: KY, TN

### Pseudanophthalmus pubescens intrepidus

Barr, 1985

Pseudanophthalmus pubescens intrepidus Barr, 1985b: 127. Type locality: «Buchanan Cave, 1.3 km W[est] Gainesville and 30 m E[ast] KY Rt. 101, at head of hollow tributary to Difficult Creek, Allen Co[unty], Kentucky» (original citation). Holotype (♂) in AMNH.

#### Distribution.

This subspecies is known two caves in Allen and Barren Counties, southern Kentucky (Barr 1985b: 128).

#### Records.

**USA**: KY

### Pseudanophthalmus pubescens pubescens

(Horn, 1869)

Anophthalmus pubescens G.H. Horn, 1869a: 126. Type locality: «Cave City cave [Barren County, Kentucky]» (original citation). Holotype [by monotypy] in MCZ [# 8230].

#### Distribution.

This subspecies is known from several caves from Hart and Metcalfe Counties westwards to eastern Warren County, Kentucky (Barr 2004: 30).

#### Records.

**USA**: KY

#### Note.

According to Barr (2004: 30), intergrades between the two subspecies of *Promecognathus pubescens* are found in Beckton Cave, Barren County.

### [pusio group]

### Pseudanophthalmus higginbothami

Valentine, 1931

Pseudanophthalmus higginbothami Valentine, 1931: 251. Type locality: «Higginbotham’s large cave, within the radius of a mile at Frankford [Greenbrier County], W[est] V[irgini]a» (original citation). Holotype (♂) in USNM [# 44259].

#### Distribution.

This species is known only from a few caves in Greenbrier and Pocahontas Counties, eastern West Virginia (Jeannel 1949b: 75; Barr 2004: 20).

#### Records.

**USA**: WV

### Pseudanophthalmus lallemanti

Jeannel, 1949

Pseudanophthalmus lallemanti Jeannel, 1949b: 74. Type locality: «Davis cave [= General Davis Cave], à 5 à 6 miles W[est] de Ronceverte et à 10 miles S[outh]W[est] de Lewisburg, Greenbrier County, West Virginia» (original citation). Holotype probably in MHNP.

#### Distribution.

This species is known only from the type-locality cave in southeastern West Virginia (Barr 2004: 20).

#### Records.

**USA**: WV

### Pseudanophthalmus nelsoni

Barr, 1965

Pseudanophthalmus nelsoni Barr, 1965a: 44. Type locality: «Old Tunnel Cave, Alleghany Co[unty], Virginia» (original citation). Holotype (♂) in USNM [# 75270]. Etymology. The specific name honors Gayle H. Nelson [1926-2005], professor of human anatomy for almost 60 years and entomologist with an interest in Buprestidae and Schizopodiae (Coleoptera) and Pentatomidae (Hemiptera).

#### Distribution.

This species is known only from two caves in Allegheny County, western Virginia (Barr 2004: 20).

#### Records.

**USA**: VA

### Pseudanophthalmus pontis

Barr, 1965

Pseudanophthalmus pontis Barr, 1965a: 45. Type locality: «Buck Hill Cave, at Natural Bridge, Rockbridge Co[unty], Virginia» (original citation). Holotype (♂) in USNM [# 75269].

#### Distribution.

This species is still known only from the type-locality cave in northern Virginia (Barr 2004: 20).

#### Records.

**USA**: VA

### Pseudanophthalmus punctatus

Valentine, 1931

Pseudanophthalmus pusio var. *punctatus* Valentine, 1931: 250. Type locality: «Tommie’s Cave [= Tawneys Cave], Newport [Giles County], V[irgini]a» (original citation). Holotype (♂) in USNM [# 44258].

#### Distribution.

This species is known from a few caves in Giles County, western Virginia (Barr 2004: 20).

#### Records.

**USA**: VA

### Pseudanophthalmus pusio

(Horn, 1869)

Anophthalmus pusio G.H. Horn, 1869a: 125. Type locality: «Erhart’s cave, Montgomery County, Virginia» (original citation). One syntype in MCZ.
Pseudanophthalmus pusio bathycola Valentine, 1932a: 268. Type locality: «Aunt Nellie’s Cave, on tributary of the North Fork Roanoke River, 3 miles southeast of Blacksburg [Montgomery County], V[irgini]a» (original citation). Holotype (♂) in USNM [# 44273]. Synonymy established by Barr (1965a: 43).

#### Distribution.

This species is found in several caves in Montgomery and Roanoke Counties, western Virginia (Barr 2004: 20).

#### Records.

**USA**: VA

### [rittmani group]

### Pseudanophthalmus catoryctos

Krekeler, 1973

Pseudanophthalmus catoryctos Krekeler, 1973: 72. Type locality: «Adams Cave, 5 miles south-southwest of Richmond, Madison Co[unty], K[entuck]y» (original citation). Holotype (♂) in FMNH.

#### Distribution.

This species is known only from the type-locality cave in east-central Kentucky (Barr 2004: 23).

#### Records.

**USA**: KY

### Pseudanophthalmus exiguus

Krekeler, 1973

Pseudanophthalmus exiguus exiguus Krekeler, 1973: 70. Type locality: «Watson Cave, 0.8 mile north-northeast of Cobhill, Estill Co[unty], K[entuck]y» (original citation). Holotype (♂) in FMNH.
Pseudanophthalmus exiguus furtivus Krekeler, 1973: 71. Type locality: «California Cave, 1.5 miles north-northeast of Ravenna, Estill Co[unty], K[entuck]y» (original citation). Holotype (♂) in FMNH. Synonymy established by Barr (2004: 23).

#### Distribution.

This species is known from several caves in Estill, Powell, and Lee Counties, eastern Kentucky (Barr 2004: 23).

#### Records.

**USA**: KY

### Pseudanophthalmus rittmani

Krekeler, 1973

Pseudanophthalmus rittmani Krekeler, 1973: 68. Type locality: «Baker Cave, 1.7 miles east of Cobhill, Estill Co[unty], K[entuck]y» (original citation). Holotype (♂) in FMNH.

#### Distribution.

This species is known from several caves in Estill and Powell Counties, eastern Kentucky (Barr 2004: 23).

#### Records.

**USA**: KY

### [robustus group]

### Pseudanophthalmus beakleyi

Valentine, 1937

Pseudanophthalmus robustus beakleyi Valentine, 1937: 97 (as *beaklei*). Type locality: «Bunkum Cave, Byrdstown [Pickett County], Tennessee» (original citation). Holotype (♂) in USNM [# 56123]. Note. This species was named after John C. Beakley. The spelling *beakleyi*, introduced by Barr (2004: 28), is a justified emendation of *beaklei* in my opinion, and the original name must be corrected (see ICZN 1999: Article 32.5.1).
Pseudanophthalmus robustus lupus Barr, 1959: 14. Type locality: «Wolf River Cave, Fentress Co[unty], Tennessee» (original citation). Holotype (♂) in AMNH. Synonymy established by Barr (1962b: 114).

#### Distribution.

This species is known from several caves in Fentress, Overton, and Pickett Counties, northern Tennessee, and in McCreary and Wayne Counties, southern Kentucky (Barr 2004: 28).

#### Records.

**USA**: KY, TN

### Pseudanophthalmus farrelli

Barr, 1959

Pseudanophthalmus robustus farrelli Barr, 1959: 12. Type locality: «Indian Grave Point Cave, in Dry Creek Valley, DeKalb Co[unty], Tennessee» (original citation). Holotype (♂) in AMNH.

#### Distribution.

This species occurs in several caves in DeKalb and Smith Counties, Tennessee (Barr 2004: 28).

#### Records.

**USA**: TN

### Pseudanophthalmus robustus

Valentine, 1931

Pseudanophthalmus robustus Valentine, 1931: 250. Type locality: «Johnson’s Cave, Monterey [Putnam County], Tenn[essee]» (original citation). Holotype (♂) in USNM [# 44257].
Pseudanophthalmus robustus neglectus Jeannel, 1949b: 50. Type locality: «Higginbotham’s caves, à 6 miles S[outh]E[ast] de Mc Minville, Warren County, Tennessee» (original citation). Holotype in MHNP. Synonymy established by Barr (1962b: 112).
Pseudanophthalmus robustus megosteus Barr, 1959: 12. Type locality: «Big Bone Cave, Van Buren Co[unty], Tennessee» (original citation). Holotype (♂) in AMNH. Synonymy established by Barr (1962b: 112).

#### Distribution.

This species is known from a several caves in DeKalb, Overtone, Putnam, Warren, White, Van Buren, and Grundy Counties, Tennessee (Barr 2004: 28).

#### Records.

**USA**: TN

### Pseudanophthalmus valentinei

Jeannel, 1949

Pseudanophthalmus valentinei Jeannel, 1949b: 51. Type locality: «Johnson’s cave, à 7 miles S[outh]W[est] de Monterey, sur la route de Sparta, Putnam County, Tennessee» (original citation). Holotype probably in MHNP. Etymology. The specific name was proposed for Joseph Manson Valentine [1902-1994] who was associated for a time with the University of Alabama at Tuscaloosa and had an interest in cave carabids. In the 1950s Valentine became interested in archaeology, particularly the association between caves and the Mayas (Stuart B. Peck pers. comm. 2008).

#### Distribution.

This species is found in caves in Putnam and Overton Counties, northern Tennessee (Barr 2004: 28).

#### Records.

**USA**: TN

### [simplex group]

### Pseudanophthalmus fowlerae

Barr, 1980

Pseudanophthalmus fowlerae Barr, 1980: 88. Type locality: «Sheals Cave, 0.8 km e[ast] of Celina, Clay Co[unty], Tennessee» (original citation). Holotype (♂) in AMNH [# 1505].

#### Distribution.

This species is known only from the type-locality cave in northern Tennessee (Barr 2004: 32).

#### Records.

**USA**: TN

### Pseudanophthalmus simplex

Barr, 1980

Pseudanophthalmus simplex Barr, 1980: 86. Type locality: «Carter Cave, 5.5 km s[outh]s[outh]w[est] of Flynns Lick, Jackson Co[unty], Tennessee» (original citation). Holotype (♂) in AMNH [# 1506].

#### Distribution.

This species is known from a few caves in Jackson County, northern Tennessee (Barr 2004: 32).

#### Records.

**USA**: TN

### [tennesseensis group]

### Pseudanophthalmus paynei

Barr, 1981

Pseudanophthalmus paynei Barr, 1981: 56. Type locality: «Moores Bridge Cave, 1.3 km N[orth] Clinton on east (left) side of Clinch River, Anderson Co[unty], Tennessee» (original citation). Holotype (♂) in AMNH.

#### Distribution.

This species is known only from a few caves in Anderson County, eastern Tennessee (Barr 2004: 37).

#### Records.

**USA**: TN

### Pseudanophthalmus pusillus

Barr, 1981

Pseudanophthalmus pusillus Barr, 1981: 56. Type locality: «Martin Cave, 7.2 km S[outh]W[est] Clinton beside Southern Railroad tracks, Anderson Co[unty], Tennessee» (original citation). Holotype (♂) in AMNH.

#### Distribution.

This species is known only from the type-locality cave in eastern Tennessee (Barr 2004: 36).

#### Records.

**USA**: TN

### Pseudanophthalmus tennesseensis

Valentine, 1937

Pseudanophthalmus tennesseensis Valentine, 1937: 98 (as *tenesensis*). Type locality: «Grand Caverns [= Atomic Caverns], Byington [Knox County], Tennessee» (original citation). Holotype (♂) in USNM [# 56126]. Note. The spelling *tennesseensis* is an incorrect subsequent spelling, introduced by Barr (1981: 55), in prevailing usage and attributed to the publication of the original spelling; therefore it is deemed to be the correct original spelling (ICZN 1999: Article 33.3.1).

#### Distribution.

This species is known from a few caves in Knox and Roane Counties, eastern Tennessee (Barr 1981: 55; Barr 2004: 36).

#### Records.

**USA**: TN

### Pseudanophthalmus unionis

Barr, 1981

Pseudanophthalmus unionis Barr, 1981: 57. Type locality: «Wright Cave, Union Co[unty], Tennessee» (original citation). Holotype (♂) in AMNH.

#### Distribution.

This species is known from two caves in Union County, northeastern Tennessee (Barr 2004: 37).

#### Records.

**USA**: TN

### [tenuis group]

### Pseudanophthalmus barberi

Jeannel, 1928

Pseudanophthalmus barberi Jeannel, 1928: 133. Type locality: «Rockhaven cave, aux environs de Brandenburg, Meade Co[unty], Kentucky» (original citation). Holotype (♀) in USNM [# 75689]. Etymology. The specific name was proposed for Herbert Spencer Barber [1882-1950], protégé of Eugene Schwarz, who worked at the United State Department of Agriculture and at the USNM. Barber published mainly on chrysomelids, bruchids, and lampyrids.

#### Distribution.

This species is known from several caves in Meade, Breckinridge, Hardin, Hart, and Larue Counties, Kentucky (Barr 2004: 26).

#### Records.

**USA**: KY

### Pseudanophthalmus illinoisensis

Barr and Peck, 1966

Pseudanophthalmus illinoisensis Barr and Peck, 1966: 520. Type locality: «Cave Spring Cave, Hardin Co[unty], Illinois» (original citation). Holotype (♂) in USNM [# 75259].

#### Distribution.

This species is known only from the type-locality cave (Barr 2004: 26).

#### Records.

**USA**: IL

### Pseudanophthalmus shilohensis mayfieldensis

Krekeler, 1958

Pseudanophthalmus mayfieldensis Krekeler, 1958: 178. Type locality: «Mayfield’s Cave, five miles west-by-northwest of Bloomington, Monroe Co[unty], Ind[iana]» (original citation). Holotype (♂) in FMNH.
Pseudanophthalmus boonensis Krekeler, 1958: 180. Type locality: «Boone Cave, one mile north of Freeman, Owen Co[unty], Ind[iana]» (original citation). Holotype (♂) in FMNH. Synonymy established by Barr (2004: 26).

#### Distribution.

This subspecies is known from several caves in Monroe, Lawrence, and Owen Counties, southern Indiana (Barr 2004: 26).

#### Records.

**USA**: IN

#### Note.

Barr (2004: 26) noted that intergrades between this subspecies and *Promecognathus shilohensis shilohensis* occur in some caves in Lawrence County.

### Pseudanophthalmus shilohensis shilohensis

Krekeler, 1958

Pseudanophthalmus shilohensis Krekeler, 1958: 178. Type locality: «Shiloh Cave, six miles west-by-northwest of Bedford, Lawrence Co[unty], Ind[iana]» (original citation). Holotype (♂) in FMNH.

#### Distribution.

This subspecies is known from a few caves in central Lawrence County, southern Indiana (Barr 2004: 26).

#### Records.

**USA**: IN

### Pseudanophthalmus stricticollis

Jeannel, 1931

Pseudanophthalmus eremita stricticollis Jeannel, 1931: 451. Type locality: «Marengo cave, Crawford Co[unty], Indiana» (original citation). Holotype in MHNP.
Pseudanophthalmus eremita morrisoni Jeannel, 1931: 451. Type locality: «Donnelson’s [= Donaldson] cave, à Mitchell, près de Bedford, Lawrence Co[unty], Indiana» (original citation). Holotype in MHNP. Synonymy established by Barr (2004: 25).
Pseudanophthalmus jeanneli Krekeler, 1958: 171. Type locality: «Elrod’s Cave, two miles east of Orangeville, Orange Co[unty], Ind[iana]» (original citation). Holotype (♂) in FMNH. Synonymy established by Barr (2004: 25).
Pseudanophthalmus tenuis blatchleyi Barr, 1960a: 316. Type locality: «Truitt’s Cave, near Bloomington, Monroe Co[unty], Indiana» (original citation). Holotype (♂) in USNM [# 75258]. Synonymy established by Barr (2004: 26).

#### Distribution.

This species is known from several caves in Crawford, Washington, Orange, Lawrence, and Monroe Counties, southern Indiana (Barr 2004: 25).

#### Records.

**USA**: IN

### Pseudanophthalmus tenuis

(Horn, 1871)

Anophthalmus tenuis G.H. Horn, 1871: 327. Type locality: «Wyandotte Cave [Crawford County], southern Indiana» (original citation). Syntype(s) [3 originally cited] in MCZ [# 34325].
Pseudanophthalmus eremita longicollis Jeannel, 1949b: 57. Type locality: «Bradford cave, à 5 à 6 miles au N[orth]E[ast] de Salisbury, et à 16 miles de New Albany [Harrison County], Crawford County, Indiana» (original citation). Holotype in MHNP. Synonymy established by Barr (1960a: 312).
Pseudanophthalmus bloomi Krekeler, 1958: 172. Type locality: «Langdon Cave, three miles southwest of White Cloud, Harrison Co[unty], Ind[iana]» (original citation). Holotype (♂) in FMNH. Synonymy established by Barr (1960a: 312).

#### Distribution.

This species is known from several caves in Crawford, Harrison, and Washington Counties, southern Indiana (Barr 2004: 25).

#### Records.

**USA**: IN

### Pseudanophthalmus youngi

Krekeler, 1958

Pseudanophthalmus youngi Krekeler, 1958: 175. Type locality: «Clifty Caves, four miles north of Campbellsburg, Washington Co[unty], Ind[iana]» (original citation). Holotype (♂) in FMNH. Etymology. The specific name was proposed in honor of Frank Nelson Young, Jr. [1915-1998], professor at the University of Indiana. Young published on many subjects, including herpetology, malacology, and medical entomology, but he is best known for his work on water beetles and periodical cicadas.
Pseudanophthalmus donaldsoni Krekeler, 1958: 175. Type locality: «Donaldson Cave complex (Donaldson’s Cave, Twin Caves, Bronson’s Cave), four miles east of Mitchell, Lawrence Co[unty], Ind[iana]» (original citation). Holotype (♂) in FMNH. Synonymy established by Barr (2004: 26).

#### Distribution.

This species is known from several caves in Crawford, Orange, Lawrence, and Washington Counties, southern Indiana (Barr 2004: 26).

#### Records.

**USA**: IN

### Genus Nelsonites

Valentine, 1952

Nelsonites Valentine, 1952: 13. Type species: *Nelsonites jonesei* Valentine, 1952 by original designation. Etymology (original). From the first name of Nelson Bolling Jones, a cave explorer, kill in action in Germany on April 2, 1945 [masculine].

#### Diversity.

Two cave-inhabiting Appalachian species.

#### Identification.

Valentine (1952) discussed the structural differences between the two species.

### Nelsonites jonesei

Valentine, 1952

Nelsonites jonesei Valentine, 1952: 16. Type locality: «Richardson’s Cave, 2.5 miles east of Somerset, Pulaski County, K[entuck]y» (original citation). Holotype (♂) probably in ALM.

#### Distribution.

This species is known from a few caves in Pulaski County, southeastern Kentucky.

#### Records.

**USA**: KY

### Nelsonites walteri

Valentine, 1952

Nelsonites walteri Valentine, 1952: 18. Type locality: «Johnson’s Cave, 7 miles southwest of Monterey, Putnam County, Tenn[essee]» (original citation). Holotype (♂) probably in ALM. Etymology. The specific name is based on the first name of the American geologist and archaeologist Walter Bryan Jones [1895-1977] of the University of Alabama (see *Pseudanophthalmus jonesi*).

#### Distribution.

This species is known from two nearby caves in Putnam and Van Buren Counties, Tennessee.

#### Records.

**USA**: TN

### Genus Neaphaenops

Jeannel, 1920

Neaphaenops Jeannel, 1920b: 154. Type species: *Anophthalmus tellkampfii* Erichson, 1844 by original designation. Etymology. From the Greek *neo* (new) and the generic name *Aphaenops* [masculine].

#### Diversity.

One polymorphic cave-inhabiting species in the Appalachians.

#### Identification.

Barr (1979a) revised the species and provided a key for the identification of its subspecies.

### Neaphaenops tellkampfii henroti

Jeannel, 1949

Neaphaenops tellkampfi henroti Jeannel, 1949b: 90. Type locality: «Sig Shacklett’s cave, à 5 miles S[outh]W[est] de Garrett, sur le territoire de Guston, Meade County, Kentucky» (original citation). Holotype in MHNP.

#### Distribution.

This subspecies is known from several caves in Breckinridge, Hardin, Hart, and Meade Counties in northwest and central Kentucky (Barr 1979a: 8).

#### Records.

**USA**: KY

### Neaphaenops tellkampfii meridionalis

Barr, 1959

Neaphaenops tellkampfi meridionalis Barr, 1959: 23. Type locality: «Hoy Cave, 2 miles north of Franklin, Simpson Co[unty], Kentucky» (original citation). Holotype (♂) in AMNH.

#### Distribution.

This subspecies has been found in several caves in Allen, Simpson, Warren, and Logan Counties in southern Kentucky (Barr 1979a: 9).

#### Records.

**USA**: KY

### Neaphaenops tellkampfii tellkampfii

(Erichson, 1844)

Anophthalmus tellkampfii Erichson [in Tellkampf], 1844: 384. Type locality: Mammoth Cave, Edmonson County, Kentucky (inferred from title of the paper). One syntype (with prothorax missing) in ZMHB (Bernd Jaeger pers. comm. 2007). Etymology. The specific name honors August Otto Theodor Tellkampf [1812-1883]. Born in German, Tellkampf immigrated to the United States at the age of 27 where he practiced medicine in Cleveland and New York. He was interested in speleology and visited Mammoth Cave in October 1842. He was a member of the Lyceum of Natural History of New York.

#### Distribution.

This subspecies has been collected in several caves in Allen, Barren, Edmonson, Hart, and Warren Counties in central and southern Kentucky (Barr 1979a: 6).

#### Records.

**USA**: KY

#### Note.

Intergrades between this subspecies and the *meridionalis* form occur in a narrow zone in Warren and Allen Counties (Barr 1979a: 10) and between this subspecies and the *viator* form in eastern Hart County (Barr 1979a: 8).

### Neaphaenops tellkampfii viator

Barr, 1979

Neaphaenops tellkampfi viator Barr, 1979a: 7. Type locality: «Brush Creek Cave, 0.8 mile east and slightly north of Lobb on the west side of Brush Creek, in western Green County, Kentucky» (original citation). Holotype (♂) in AMNH [# 1417].

#### Distribution.

This subspecies is known from several caves in Green, Hart, and Metcalfe Counties in central and southern Kentucky (Barr 1979a: 8).

#### Records.

**USA**: KY

### Genus Blemus

Dejean, 1821

Blemus Dejean, 1821: 16. Type species: *Carabus discus* Fabricius, 1792 designated by Westwood (1838: 5). Etymology. Uncertain, possibly from the Greek *blema* (a throw, cast; a shot, wound; a coverlet) [masculine]. The name was proposed by Franz Anton Ziegler and made available by Dejean.
Lasiotrechus Ganglbauer, 1891a: 187, 191. Type species: *Carabus discus* Fabricius, 1792 by monotypy. Etymology. From the Greek *lasios* (hairy) and the generic name *Trechus* [*q.v*.], alluding to the densely pubescent elytra (“*Nur die Flügeldecken pubescent*”) of the adult [masculine].

#### Diversity.

Two Palaearctic species, one of them adventive in North America.

#### Identification.

The species found in North America was covered in Lindroth’s (1961a: 194) monograph.

### Blemus discus discus

(Fabricius, 1792)

Carabus discus Fabricius, 1792: 164. Type locality: «Germania» (original citation). Lectotype (♀), designated by Uéno (1974: 269), in ZMUC.
Carabus unifasciatus Panzer, 1796b: no 7. Type locality: Germany (inferred from title of the book). Syntype(s) location unknown (possibly in ZMHB). Synonymy established by Illiger (1798: 187).

#### Distribution.

This Palaearctic subspecies is adventive in North America where it is known from Nova Scotia (NSMC) to Wisconsin (Messer 2010: 35), south to southern Pennsylvania (Bradford and Allegheny Counties, CMNH). The first inventoried specimen collected on this continent was found in the Montreal area in 1933 (Brown 1940a: 69).

#### Records.

**CAN**: NB, NS, ON, PE, QC **USA**: CT, MA, ME, MI, NH, NY, OH, PA, VT, WI – **Adventive**

#### Note.

The subspecies *Blemus discus orientalis* Jeannel is known from Yunnan, China.

### Genus Xenotrechus

Barr and Krekeler, 1967

Xenotrechus Barr and Krekeler, 1967: 1322. Type species: *Xenotrechus denticollis* Barr and Krekeler, 1967 by original designation. Etymology. From the Greek *xenos* (stranger, guest) and the generic name *Trechus* [*q.v*.] [masculine].

#### Diversity.

Two troglobitic species restricted to southeastern Missouri.

#### Identification.

Barr and Krekeler (1967) provided a description of the external structures and male genitalia of both species.

### Xenotrechus condei

Barr and Krekeler, 1967

Xenotrechus condei Barr and Krekeler, 1967: 1323. Type locality: «Friedman’s Cave, 5 miles west-southwest of Imperial, Jefferson Co[unty], Missouri» (original citation). Holotype (♂) in USNM.

#### Distribution.

This species is known from two nearby caves, Friedman’s and Pleasant Valley Caves, in eastern Missouri.

#### Records.

**USA**: MO

### Xenotrechus denticollis

Barr and Krekeler, 1967

Xenotrechus denticollis Barr and Krekeler, 1967: 1323. Type locality: «Kohm’s Cave, 1.9 miles south-south-west of (the cathedral in) S[ain]te Genevieve, S[ain]te Genevieve Co[unty], Missouri» (original citation). Holotype (♂) in USNM.

#### Distribution.

This species is known from two nearby caves, Kohm’s and Sims Caves, in eastern Missouri.

#### Records.

**USA**: MO

### Genus Darlingtonea

Valentine, 1952

Darlingtonea Valentine, 1952: 19. Type species: *Darlingtonea kentuckensis* Valentine, 1952 by monotypy. Etymology (original). From the surname of Philip J. Darlington, Jr. (see *Agonum darlingtoni* Lindroth). The name is feminine.

#### Diversity.

One cave-inhabiting species in Kentucky.

#### Identification.

Valentine (1952) lengthy described, neatly illustrated, and discussed the structural differences between members of *Darlingtonea* and those of the other troglobitic genera of the Appalachian region.

### Darlingtonea kentuckensis

Valentine, 1952

Darlingtonea kentuckensis Valentine, 1952: 22. Type locality: «Richardson’s Cave, 2.5 miles east of Somerset, Pulaski County, K[entuck]y» (original citation). Holotype (♂) probably in ALM.
Darlingtonea kentuckensis lexingtoni Valentine, 1952: 24. Type locality: «Big Saltpeter Cave, 8 miles north of Livingston, Rockcastle County, K[entuck]y» (original citation). Holotype (♂) probably in ALM. **New synonymy**. Note. This synonymy is based on the following unpublished comment (dated March 1969) by Thomas Barr: “specimens [of *Darlingtonea kentuckensis*] from the southwestern part of the range are darker and more robust than those from the northeastern part of the range. The variation is probably clinal, however; there are no distinct separations that would warrant the naming of geographical races.”

#### Distribution.

This species is known from several caves in Kentucky located as far north as Estill County and as far south as Fentress County [see Marsh 1969: Fig. 5].

#### Records.

**USA**: KY

### Genus Ameroduvalius

Valentine, 1952

Ameroduvalius Valentine, 1952: 24. Type species: *Ameroduvalius jeanneli* Valentine, 1952 by original designation. Etymology (original). From the English adjective American (of America) and the generic name *Duvalius*, alluding to the presence of members of the *Duvalius* line in the New World [masculine].

#### Diversity.

One cave-inhabiting species in Kentucky.

#### Identification.

Valentine (1952: 24-29) described, illustrated, and discussed the status of the species.

### Ameroduvalius jeanneli jeanneli

Valentine, 1952

Ameroduvalius jeanneli Valentine, 1952: 27. Type locality: «Sloan’s Valley (Cassidy) Cave, 6 miles southeast of Burnside, Pulaski County, K[entuck]y» (original citation). Holotype (♂) probably in ALM. Etymology. The specific name honors René Jeannel [1879-1965], a French biogeographer and taxonomist of worldwide reputation. Jeannel, who held the entomology chair at the Muséum d’Histoire Naturelle in Paris from 1932 to 1950, published on many groups of insects but is better known for his work on cave beetles and Carabidae. Cambefort (2006: 198) qualified Jeannel of the most important French entomologist of the xx Century.

#### Distribution.

This subspecies is known from several caves in the Somerset area, southeastern Kentucky.

#### Records.

**USA**: KY

### Ameroduvalius jeanneli rockcastlei

Valentine, 1952

Ameroduvalius jeanneli rockcastlei Valentine, 1952: 29. Type locality: «Big Saltpeter Cave, Rockcastle County [Kentucky]» (original citation). Holotype (♂) probably in ALM.

#### Distribution.

This subspecies is known only from the type-locality cave in southeastern Kentucky.

#### Records.

**USA**: KY

### Genus Trechus

Clairville, 1806

Trechus Clairville, 1806: 22. Type species: *Carabus rubens* Fabricius, 1801 designated by Blanchard [in Audouin et al. 1841: plate 25]. Etymology. From the Greek *trechis* (runner), probably alluding to the quickness of the adults in the field [masculine]. Note. As stated by Andrewes (1939: 157), the first valid type species designation for *Trechus* Clairville, 1806 is that of Latreille (1810: 426) who designated *Carabus meridianus* Linnaeus, 1760. This species is also the type species of *Acupalpus* Latreille, 1829. Acceptance of Latreille’s designation would require nomenclatural changes for two extensive, well-known genera. A request should be addressed to the International Commission on Zoological Nomenclature to suppress Latreille’s designation. A first request was postponed (ICZN 1950).

#### Diversity.

About 870 species arrayed in eight subgenera: *Arabotrechus* Mateu (one Yemenite species), *Atlantotrechus* Lompe (one species in the Madeira Islands), *Elgonophyes* Jeannel (one Afrotropical species), *Elgonotrechus* Jeannel (14 Afrotropical species), *Meruitrechus* Jeannel (two Afrotropical species), *Microtrechus* (41 species), *Minitrechus* Vigna Taglianti and Magrini (one species in Ethiopia), and *Trechus* s.str. (about 775 species). More than 92% of the species are found in the Palaearctic Region.

### Subgenus Trechus

Clairville, 1806

Trechus Clairville, 1806: 22. Type species: *Carabus rubens* Fabricius, 1801 designated by Blanchard [in Audouin et al. 1841: plate 25].
Calotrechus Wollaston, 1854: 64. Type species: *Trechus nigrocruciatus* Wollaston, 1854 designated by Jeannel (1927: 114). Synonymy established by Casale and Laneyrie (1982: 124). Etymology. From the Greek *calos* (beautiful) and the generic name *Trechus* [*q.v*.] [masculine].
Antoinella Jeannel, 1937c: 83. Type species: *Duvalius groubei* Antoine, 1935 by original designation. Synonymy established by Casale (2011: 14). Etymology. This name was proposed in honor of Maurice Antoine (1886-1962). Born in Caen in Normandy, France, Antoine moved to Morocco in his 30s where he taught natural science at a secondary school in Casablanca from 1919 to 1948. He is well-known for his monographic treatment (1955-1962) of the carabid fauna of Morocco.
Altaiotrechus Iablokoff-Khnzorian, 1971: 155. Type species: *Altaiotrechus alticola* Iablokoff-Khnzorian, 1971 (= *Trechus kuraicus* Shilenkov, 1995) by monotypy. Synonymy established by Shilenkov (in Kryzhanovskij et al. 1995: 69). Etymology. From the geographical name *altai* (mountain system in Asia) and the generic name *Trechus* [*q.v*.] [masculine].
Hydrotrechus Carabajal, García and Rodríguez, 2000: 123. Type species: *Hydrotrechus cantabricus* Carabajal, García and Rodríguez, 2000 by original designation. Synonymy established by Ortuño and Jiménez-Valverde (2011: 28).

#### Diversity.

About 775 species in North America (23 species, of which three are adventive), mountains in Mexico (about five species), Oriental (two species in the Philippines), Palaearctic (about 725 species), and Afrotropical (about 25 species on Mont Elgon, Mont Meru, and Ethiopia) Regions. More than 95% of the species inhabit the Northern Hemisphere.

#### Identification.

There is no published key for the identification of the species of this subgenus.

### [chalybeus group]

### Trechus apicalis

Motschulsky, 1845

Trechus apicalis Motschulsky, 1845b: 347. Type locality: «Kamtschatka [Siberia, Russia]» (original citation). Six syntypes in ZMMU (Keleinikova 1976: 187).
Trechus kamtschatkensis Putzeys, 1847: 308. Type locality: «Kamtschatka [Siberia, Russia]» (original citation). Syntype(s) location unknown (possibly in IRSN). Synonymy established by Putzeys (1870: 166).
Epaphius micans LeConte, 1847: 414. Type locality: «Lapointe [Madeline Island, Wisconsin], Lacus Superioris» (original citation). Syntype(s) in MCZ [# 5596]. Synonymy established by Lindroth (1963b: 202).
Epaphius fulvus LeConte, 1847: 415 [secondary homonym of *Trechus fulvus* Dejean, 1831]. Type locality: «Lapointe [Madeline Island, Wisconsin], Lacus Superioris» (original citation). Syntype(s) in MCZ. Synonymy established, under the name *Trechus apicalis micans* (LeConte), by Jeannel (1927: 172), confirmed by Lindroth (1963b: 202).
Trechus canadensis Putzeys, 1870: 160. Type locality: «Terre neuve (S[ain]t Pierre [et] Miquelon); Toronto» (original citation). Syntype(s) [5 originally cited] in MHNP (collection Chaudoir). Synonymy established by Jeannel (1931: 428).
Trechus borealis Schaeffer, 1915a: 47. Type locality: «Battle Harbor, Labrador; Bay S[ain]t George, Newfoundland; New Jersey; Bellport, L[on]g Island [New York]» (original citation), restricted to «Battle Harbor, Labr[ador]» by Lindroth (1963b: 202). Syntype(s) in USNM (Lindroth 1963b: 202, though not listed by Erwin and House 1978). Synonymy established, under the name *Trechus apicalis micans* (LeConte), by Jeannel (1927: 172), confirmed by Lindroth (1963b: 202).
Trechus pallescens Casey, 1918: 407. Replacement name for *Trechus fulvus* (LeConte 1847).
Trechus puritanus Casey, 1918: 407. Type locality: «Fall River [Bristol County], Massachusetts» (original citation). Lectotype (♂), designated by Lindroth (1975: 114), in USNM [# 46077]. Synonymy established, under the name *Trechus apicalis micans* (LeConte), by Jeannel (1927: 172), confirmed by Lindroth (1963b: 202).
Trechus rhodensis Casey, 1918: 408. Type locality: «Boston Neck [Washington County], Rhode Island» (original citation). Lectotype (♂), designated by Lindroth (1975: 114), in USNM [# 46078]. Synonymy established, under the name *Trechus apicalis micans* (LeConte), by Jeannel (1927: 172), confirmed by Lindroth (1963b: 202).
Trechus brumalis Casey, 1918: 408. Type locality: «W[est] S[ain]t Modest[e], Labrador» (original citation). Lectotype (♂), designated by Lindroth (1975: 114), in USNM [# 46079]. Synonymy established, under the name *Trechus apicalis micans* (LeConte), by Jeannel (1927: 172), confirmed by Lindroth (1963b: 202).

#### Distribution.

This species ranges from Newfoundland (Lindroth 1955a: 78-79, as *Trechus apicalis micans*) to Alaska, south to southeastern British Columbia (Lindroth 1963b: 202), southern Colorado (Elias 1987: 632; Mineral County, UASM) along the Rocky Mountains, and northeastern West Virginia (Tucker County, CMNH). Also found in the Far East and on Hokkaidō, Japan (Moravec et al. 2003: 326).

#### Records.

**FRA**: PM **CAN**: AB, BC, LB, MB, NB, NF, NS (CBI), NT, ON, PE, QC, SK, YT **USA**: AK, CO, CT, IN, MA, ME, MI, MN, MT, NH, NJ, NY, OH, PA, RI, VT, WI, WV – **Holarctic**

**Figure 21.**
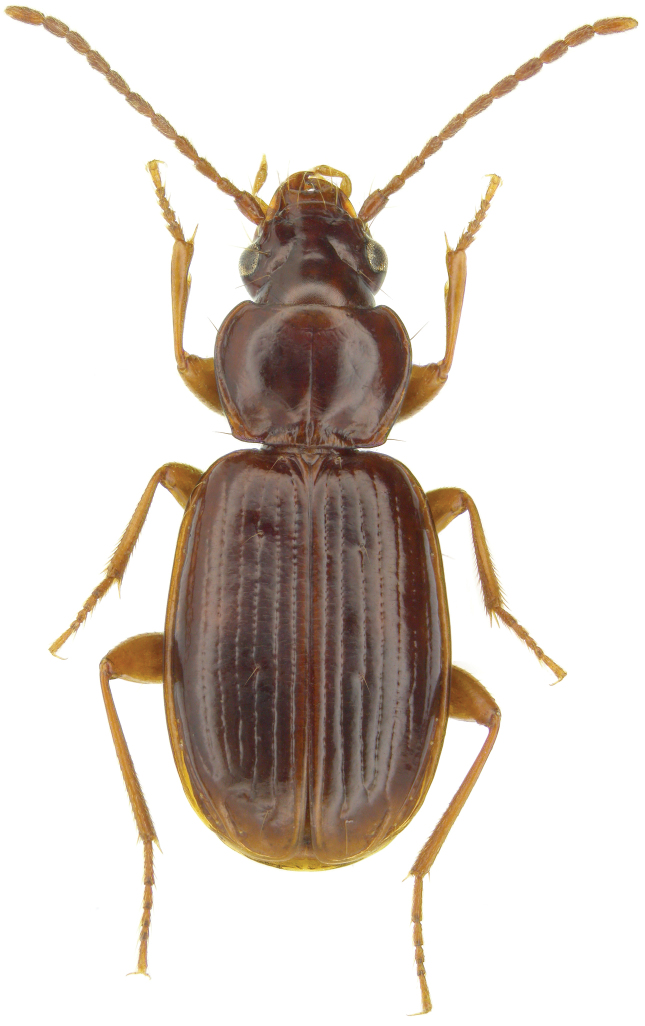
*Trechus apicalis* Motschulsky. This trechine species is “wing dimorphic” with the vast majority of individuals being micropterous (with short wing vestiges) and a few macropterous (with long wings). Usually macropterous individuals of dimorphic species are able to fly but this is not always the case as flight muscles could be atrophied. Carl Lindroth argued that in stable periods, when the species’ habitat is not subject to drastic changes, the brachypterous form normally predominates but in unstable periods, the situation is reversed.

### Trechus chalybeus

Dejean, 1831

Trechus chalybeus Dejean, 1831: 17. Type locality: «île d’Ounalaschka, l’une des îles Aleutiennes [Alaska]» (original citation). One syntype in MHNP (Lindroth 1955b: 14).
Trechus californicus Motschulsky, 1845b: 347. Type locality: «île Sitka [= Baranof Island, Alaska]» (original citation). Four syntypes in ZMMU (Keleinikova 1976: 190). Synonymy established by Horn (1875: 131).
Trechus tahoensis Casey, 1918: 407. Type locality: «Lake Tahoe [Placer County], California» (original citation). Lectotype (♀), designated by Lindroth (1975: 114), in USNM [# 46076]. Synonymy established by Jeannel (1927: 169), confirmed by Lindroth (1961a: 197).
Trechus chalybaeus brachyderus Jeannel, 1931: 422. Type locality: «Bears Paw Mountains, Montana» (original citation). Holotype (♂) in USNM [# 43660]. Synonymy established by Lindroth (1961a: 197).

#### Distribution.

This species ranges from the Aleutian Islands in Alaska (Lindroth 1961a: 198) south to the Sierra Nevada in eastern California (Inyo and Tulare Counties, CAS; Casey 1918: 407, as *Trechus tahoensis*; Dajoz 1990: 158) and to New Mexico along the Rocky Mountains (Snow 1885: 67; Fall and Cockerell 1907: 158).

#### Records.

**CAN**: AB, BC (QCI, VCI) **USA**: AK, CA, CO, ID, MT, NM, NV, OR, WA, WY

### Trechus coloradensis

Schaeffer, 1915

Trechus chalybaeus var. *coloradensis* Schaeffer, 1915a: 48. Type locality: «Colorado» (original citation). Syntype(s) in USNM [# 42515].
Trechus chalybaeus var. *utahensis* Schaeffer, 1915a: 48 [*nomen dubium*]. Type locality: «southwest Utah» (original citation). Lectotype (♂), designated by Lindroth (1963b: 201) and described as “severely mutilated,” in USNM [# 75690]. Synonymy established with doubt by Lindroth (1963b: 201).
Trechus saxatilis Casey, 1918: 408. Type locality: «Colorado» (original citation). Lectotype (♀), designated by Lindroth (1975: 114), in USNM [# 46080]. Synonymy established with doubt by Jeannel (1927: 171), confirmed by Lindroth (1963b: 201).
Trechus coloradensis arcticollis Jeannel, 1931: 419. Type locality: « Moscow, Cedar mountains, Idaho [see page 430]» (original citation). Holotype (♂) in USNM [# 43662]. Synonymy established by Lindroth (1963b: 201). Note. The “Cedar Mountain” referred to is probably that in Latah County, which is also the type locality of *Scaphinotus merkelii*.
Trechus coloradensis gravidulus Jeannel, 1931: 419. Type locality: «New Mexico» (original citation). Holotype (♂) in USNM [# 43661]. Synonymy established by Lindroth (1963b: 201).
Trechus pugetensis Hatch, 1951: 113. Type locality: «Seattle [King County], Wash[ington]» (original citation). Holotype (♂) in USNM. Synonymy established by Lindroth (1963b: 201).

#### Distribution.

This species ranges from northwestern Washington (Hatch 1951: 113, as *Trechus pugetensis*) and northern Idaho (Lindroth 1963b: 201) south to northern New Mexico (Dajoz 1990: 158; Santa Fe County, CMNH), northeastern Arizona (Donabauer 2010a: 41), and northwestern California (Humboldt County, James R. LaBonte pers. comm. 2008).

#### Records.

**USA**: AZ, CA, CO, ID, NM, UT, WA

### Trechus crassiscapus

Lindroth, 1955

Trechus chalybaeus crassiscapus Lindroth, 1955a: 80. Type locality: «Cow Head, N[ew]f[ound]l[an]d» (original citation). Holotype (♂) in CNC [# 6571].

#### Distribution.

This species ranges from Newfoundland and the coast of Labrador to northeastern Minnesota (Gandhi et al. 2005: 924), south to mountains in northern New York and New England [see Lindroth 1963a: Fig. 69]. The record from “Massachusetts” (Bousquet and Larochelle 1993: 120) is in error.

#### Records.

**CAN**: LB, NB, NF, NS (CBI), ON, QC **USA**: ME, MN, NH, NY, VT, WI

### Trechus oregonensis

Hatch, 1951

Trechus oregonensis Hatch, 1951: 114. Type locality: «Hood R[i]v[er] Rapids, Parkdale [Hood River County], Ore[gon]» (original citation). Holotype (♂) in USNM.

#### Distribution.

This species inhabits the North American Cordilleras ranging from British Columbia and southwestern Alberta (Lindroth 1963b: 201) south to western Montana (Russell 1968: 48) and the Sierra Nevada in California (Lindroth 1963b: 201). The record from “Colorado” (Bousquet and Larochelle 1993: 121) needs confirmation.

#### Records.

**CAN**: AB, BC **USA**: CA, ID, MT, OR, WA [CO]

### Trechus tenuiscapus

Lindroth, 1961

Trechus tenuiscapus Lindroth, 1961a: 198. Type locality: «Cameron L[ake], Waterton Park, Al[ber]ta» (original citation). Holotype (♂) in CNC [# 11722].

#### Distribution.

This species ranges from southern Yukon Territory (Lindroth 1961a: 200) south to mountains in northwestern Montana (Edwards 1975: 51), west-central Idaho (Boise County, CMNH), and western Oregon (Lane County, Foster F. Purrington pers. comm. 2009). The record from “Northwestern Territories” (Bousquet and Larochelle 1993: 121) needs confirmation.

#### Records.

**CAN**: AB, BC (VCI), YT **USA**: ID, MT, OR, WA [NT]

### Trechus yvesbousqueti

Donabauer, 2010

Trechus yvesbousqueti Donabauer, 2010a: 41. Type locality: «Escudilla M[oun]t[ain] (2730 m), Apache N[ational] F[orest], 8 mi[les] N[orth]E[ast] Alpine [Apache County], Ariz[ona]» (original citation). Holotype (♂) in CNC [# 23928].

#### Distribution.

This species is known only from Apache County (Donabauer 2010a: 41) in northeastern Arizona.

#### Records.

**USA**: AZ

### [hydropicus group]

### Trechus caliginis

Barr, 1985

Trechus caliginis Barr, 1985b: 128. Type locality: «Camp Creek Bald, just below summit (about 1460 m), Greene Co[unty], Tennessee/Madison Co[unty], North Carolina» (original citation). Holotype (♂) in AMNH.

#### Distribution.

This species is known only from the type locality in the Bald Mountains between Greeneville, Tennessee and Asheville, North Carolina (Barr 1985b: 128).

#### Records.

**USA**: TN/NC

### Trechus carolinae

Schaeffer, 1901

Trechus carolinae Schaeffer, 1901: 212. Type locality: «M[oun]t Mitchell [Yancey County], North Carolina» (original citation). Holotype [by monotypy] in AMNH [# 404] (Grossbeck 1912: 361).

#### Distribution.

This species is known only from the type locality, at the summit of Mount Mitchell where it is found in deep spruce and fir needle duff.

#### Records.

**USA**: NC

### Trechus cumberlandus

Barr, 1962

Trechus cumberlandus Barr, 1962a: 76. Type locality: «Elisha Steele Cave, 3 miles east of Monticello, Wayne Co[unty], Kentucky» (original citation). Holotype (♂) in USNM [# 65975].

#### Distribution.

This species is found in caves in the Cumberland Plateau, from Rockcastle County in southeastern Kentucky southwest to Grundy County in southern Tennessee (Barr 1979b: 37).

#### Records.

**USA**: KY, TN

### Trechus hydropicus avus

Barr, 1962

Trechus beutenmulleri avus Barr, 1962a: 72. Type locality: «Grandfather Mountain, Avery Co[unty], North Carolina» (original citation). Holotype (♂) in USNM [# 65973].

#### Distribution.

This subspecies is known from Grandfather Mountain in Avery County and Three Top Mountain in Ashe County in western North Carolina (Barr 1979b: 43).

#### Records.

**USA**: NC

#### Note.

This subspecies intergrades with the *beutenmuelleri* form on Beech Mountain, Avery County, North Carolina (Barr 1979b: 43).

### Trechus hydropicus beutenmuelleri

Jeannel, 1931

Trechus beutenmülleri Jeannel, 1931: 436. Type locality: «mount Mitchell [Yancey County], North Carolina» (original citation). Holotype probably in MHNP. Etymology. The species was proposed for William Beutenmüller [1864-1934], curator of insects at the American Museum of Natural History. Beutenmüller collected extensively in the vicinity of New York and at Black Mountain in North Carolina and published mainly on Lepidoptera.

#### Distribution.

This subspecies ranges southwestwards from the Roan Mountain in Tennessee to the Black and Great Craggy Mountains at the edge of the Blue Ridge in western North Carolina (Barr 1979b: 44).

#### Records.

**USA**: NC, TN

### Trechus hydropicus canus

Barr, 1962

Trechus beutenmulleri canus Barr, 1962a: 73. Type locality: «White Top Mountain, Grayson Co[unty], Virginia» (original citation). Holotype (♂) in USNM [# 65974].

#### Distribution.

This subspecies is known from Grayson, Washington, and Lee Counties, southwestern Virginia, and Harlan and Letcher Counties, southeastern Kentucky (Barr 1979b: 36).

#### Records.

**USA**: KY, VA

### Trechus hydropicus hydropicus

Horn, 1883

Trechus hydropicus G.H. Horn, 1883b: 273. Type locality: «Virginia» (original citation), restricted to «Bald Knob, Mountain Lake, Giles County» by Barr (1979b: 42). Syntype(s) in MCZ [# 8231].

#### Distribution.

This subspecies is known from western Maryland, Virginia, and eastern West Virginia (Barr 1979b: 36). The record from Sassafras Mountain in northwestern South Carolina (Ciegler 2000: 44) is apparently in error (see Ciegler 2003: [1]) as well as the state record of “South Carolina” by Bousquet and Larochelle (1993: 121).

#### Records.

**USA**: MD, VA, WV

### Trechus mitchellensis

Barr, 1962

Trechus mitchellensis Barr, 1962a: 75. Type locality: «Celo Mountain, Yancey Co[unty], North Carolina» (original citation). Holotype (♂) in USNM [# 65976].

#### Distribution.

This species is found in the Black Mountains in Yancey, Buncombe, and McDowell Counties, North Carolina (Barr 1979b: 37).

#### Records.

**USA**: NC

### Trechus roanicus

Barr, 1962

Trechus roanicus Barr, 1962a: 73. Type locality: «Roan Mountain, Carter Co[unty], Tennessee» (original citation). Holotype (♂) in USNM [# 65977].

#### Distribution.

This species is known from Carter County in northeastern Tennessee and from Mitchell County in western North Carolina (Barr 1979b: 37).

#### Records.

**USA**: NC, TN

### Trechus schwarzi saludae

Barr, 1979

Trechus schwarzi saludae Barr, 1979b: 46. Type locality: «one mile east of Melrose, on the south side of the gorge of the North Pacolet River (1300 feet), Polk County, North Carolina» (original citation). Holotype (♂) in AMNH [# 1503].

#### Distribution.

This subspecies is known from several specimens collected at the type locality in southwestern North Carolina.

#### Records.

**USA**: NC

### Trechus schwarzi schwarzi

Jeannel, 1931

Trechus schwarzi Jeannel, 1931: 437. Type locality: «Roan High Knob [Carter County], North Carolina» (original citation), which is probably incorrect (Barr 1962a: 75); «Retreat, Haywood Co[unty], N[orth] C[arolina]» selected by Barr (1962a: 74). Holotype (♀) in USNM [# 43663].

#### Distribution.

This subspecies is restricted to the Pishah Ledge, which is the eastern arm of the Great Balsam Mountains, in western North Carolina (Barr 1979b: 45).

#### Records.

**USA**: NC

### Trechus schwarzi scopulosus

Barr, 1979

Trechus schwarzi scopulosus Barr, 1979b: 46. Type locality: «below summit of Craggy Dome (5600 feet), Buncombe County, North Carolina» (original citation). Holotype (♂) in AMNH [# 1504].

#### Distribution.

This subspecies is known from the Black and Great Craggy mountains and adjacent Blue Ridge, in Buncombe, McDowell, and Yancey Counties, western North Carolina (Barr 1979b: 46).

#### Records.

**USA**: NC

### [ovipennis group]

### Trechus alinae

Dajoz, 1990

Trechus alinae Dajoz, 1990: 156. Type locality: «Mont San Jacinto State Park (2600 m), Riverside County, Californie» (original citation). Holotype (♂) in Dajoz’s collection (Paris, France).

#### Distribution.

This species is known only from the San Jacinto Mountains in southeastern California.

#### Records.

**USA**: CA

### Trechus apache

Dajoz, 1990

Trechus apache Dajoz, 1990: 153. Type locality: «Apache au lieu-dit Skeleton Canyon, Cochise County, Arizona» (original citation). Holotype (♂) in Dajoz’s collection (Paris, France).

#### Distribution.

This species is known only from the holotype.

#### Records.

**USA**: AZ

### Trechus arizonae

Casey, 1918

Trechus arizonae Casey, 1918: 409. Type locality: «Arizona» (original citation). One syntype in USNM [# 46081].

#### Distribution.

This species is endemic to the Pinaleno Mountains in Graham County, southeastern Arizona (Donabauer 2010a: 39).

#### Records.

**USA**: AZ

### Trechus conformis

Jeannel, 1927

Trechus conformis Jeannel, 1927: 188. Type locality: «Lagunitas, sur la côte de Tomales bay, Marin County, Californie» (original citation). Holotype (♂) in MHNP.

#### Distribution.

This species is known only from the holotype.

#### Records.

**USA**: CA

### Trechus humboldti

Van Dyke, 1945

Trechus humboldti Van Dyke, 1945b: 101. Type locality: «near Orick, Humboldt County, California» (original citation). Holotype (♂) in CAS [# 5434].

#### Distribution.

This species is known from northwestern California (Van Dyke 1945b: 101) and southern Oregon (Lane County, CMNH).

#### Records.

**USA**: CA, OR

### Trechus ovipennis

Motschulsky, 1845

Trechus ovipennis Motschulsky, 1845b: 348. Type locality: «Californie» (original citation), which was regarded as incorrect by Jeannel (1931: 433); «Sithka [Baranof Island], Alaska» selected by Lindroth (1961a: 195). Syntype(s) in MCZ and probably also in ZMMU. Note. Keleinikova (1976: 210) reported the presence of two syntypes in ZMMU, one labeled “Am.b.occ. Sitka,” the second “California.”

#### Distribution.

This species ranges along the Pacific Coast from southeastern Alaska (Lindroth 1961a: 196) to at least Marin County, central California (Kavanaugh and Erwin 1985: 177).

#### Records.

**CAN**: BC (QCI, VCI) **USA**: AK, CA, OR, WA

### Trechus pomonae

Fall, 1901

Trechus pomonae Fall, 1901a: 211. Type locality: «Pomona and Pasadena [California]» (original citation). Syntype(s) [3 originally cited] in MCZ [# 23872].

#### Distribution.

This species is known only from southwestern California (Jeannel 1927: 189; Donabauer 2010a: 39).

#### Records.

**USA**: CA

### [quadristriatus group]

### Trechus obtusus

Erichson, 1837

Trechus laevis Stephens, 1835: 384 [potential *nomen oblitum*]. Type locality: «near London [United Kingdom]» (original citation). Syntype(s) location unknown (possibly in BMNH).
Trechus obtusus Erichson, 1837 [15 September]: 122 [potential *nomen protectum*]. Type locality: Mark Brandenburg [Prussia] (inferred from title of the book). Syntype(s) location unknown (possibly in ZMHB). Synonymy established by Waterhouse (1863: 148).
Trechus castanopterus Heer, 1837 [after 10 December]: 46 [second section]. Type locality: Matt; Andermatt [Switzerland] (Heer 1837: 73 [first section]). Syntype(s) location unknown (possibly in ETHZ). Synonymy established by Schaum (1860: 641).

#### Distribution.

This European species is adventive in North America where it is known from the Queen Charlotte Islands (Kavanaugh 1992: 61) to north-central Idaho (LaBonte 1989: 17; Hatten et al. 2007: 359), south to northern Utah (Davis and Salt Lake Counties, CMNH) and west-central California [see Kavanaugh and Erwin 1985: Fig. 1]. The first inventoried specimen collected on this continent was found in North Creek, King County, Washington in 1925 (Kavanaugh and Erwin 1985: 171). The species is also adventive in Hawaii since 1998 (Liebherr and Takumi 2003).

#### Records.

**CAN**: BC (QCI, VCI) **USA**: CA, ID, OR, UT, WA – **Adventive**

### Trechus quadristriatus

(Schrank, 1781)

Carabus quadristriatus Schrank, 1781: 218. Type locality: Austria (inferred from title of the book). Syntype(s) probably lost.
Carabus minutus Fabricius, 1792: 167 [primary homonym of *Carabus minutus* Rossi, 1790]. Type locality: «Germania» (original citation). One syntype in ZMUC (Zimsen 1964: 60). Synonymy established by Dawson (1854: 169).
Carabus tempestivus Panzer, 1799: no 6. Type locality: «Dresdae [Germany]» (original citation). Syntype(s) location unknown (possibly in ZMHB). Synonymy established, under the name *Trechus minutus* (Fabricius), by Erichson (1837: 121).

#### Distribution.

This European species is adventive in North America where it is known from Nova Scotia (Majka et al. 2006: 603) to northern Wisconsin (Iron County, CMNH; Messer 2010: 35), as far north as the Abitibi region in Quebec (Paquin and Dupérré 2002: 87), south to northeastern West Virginia (Hampshire County, CMNH) and eastern Maryland (Queen Annes County, Foster F. Purrington pers. comm. 2009). The first inventoried specimen collected on this continent was found at Port Credit, southern Ontario, in 1965 (Bousquet et al. 1984: 215).

#### Records.

**CAN**: NS, ON, QC **USA**: MD, MI, NY, PA, WI, WV – **Adventive**

### [rubens group]

### Trechus rubens

(Fabricius, 1792)

Carabus rubens Fabricius, 1792: 140. Type locality: «Kiliae [= Kiel, Germany]» (original citation). Syntype(s) location unknown.
Bembidium paludosum Gyllenhal, 1810: 34. Type locality: Sweden (inferred from title of the book). Syntype(s) location unknown (possibly in UZIU). Synonymy established by Schiødte (1841: 327).
Carabus palpalis Duftschmid, 1812: 183. Type locality: «um Linz [Austria]» (original citation). Holotype [by monotypy] probably lost. Synonymy established, under the name *Trechus paludosus* (Gyllenhal), by Redtenbacher (1856: 68).

#### Distribution.

This European species is adventive in North America where it is known from Newfoundland (Lindroth 1955a: 82; Larson and Langor 1982: 593) to western Quebec (Larochelle 1975: 112), and from the New England area (Bousquet and Larochelle 1993: 121). The first inventoried specimen collected on this continent was found prior to 1863 (LeConte 1863b: 14) probably in Nova Scotia as recorded by Horn (1875: 131). The record from eastern Ontario (Hamilton 1889b: 94) is probably in error.

#### Records.

**FRA**: PM **CAN**: NB, NF, NS (CBI), PE, QC **USA**: ME, NH, VT – **Adventive**

### Subgenus Microtrechus

Jeannel, 1927

Microtrechus Jeannel, 1927: 585. Type species: *Microtrechus vandykei* Jeannel, 1927 by original designation. Etymology. From the Greek *micros* (small, little) and the generic name *Trechus* [*q.v*.], alluding to the small size of adults of these *Trechus* species [masculine].

#### Diversity.

Forty-one species (46 species-group taxa) restricted to the Appalachian Mountains in North Carolina, Tennessee, South Carolina, and Georgia and currently placed in three species groups.

#### Identification.

Barr (1979b) revised and provided a key to the species of this subgenus (22 species, 29 species-group taxa). Subsequently, 15 new species and two subspecies have been described by Barr (1985b), Dajoz (2005), and Donabauer (2005a, b).

### [nebulosus group]

### Trechus balsamensis

Barr, 1962

Trechus balsamensis Barr, 1962a: 87. Type locality: «Water Rock Knob, Haywood-Jackson Counties, North Carolina» (original citation). Holotype (♂) in USNM [# 65979].

#### Distribution.

This species is known only from the Plott Balsam Mountains, western North Carolina.

#### Records.

**USA**: NC

### Trechus cheoahensis

Donabauer, 2005

Trechus cheoahensis Donabauer, 2005b: 90. Type locality: «Cheoah Bald, Graham/Swain Co[unty], N[orth]C[arolina]» (original citation). Holotype (♂) in Donabauer’s collection (Vienna, Austria).

#### Distribution.

This species is known only from the type locality.

#### Records.

**USA**: NC

### Trechus clingmanensis

Donabauer, 2005

Trechus clingmanensis Donabauer, 2005b: 72. Type locality: «Clingmans Dome, G[reat]S[moky]M[ountains], Servier/Swain Co[unty], T[en]n[essee]/N[orth]C[arolina]» (original citation). Holotype (♂) in Donabauer’s collection (Vienna, Austria).

#### Distribution.

This species is known only from the type locality.

#### Records.

**USA**: NC/TN

### Trechus haoeleadensis

Donabauer, 2005

Trechus haoeleadensis Donabauer, 2005b: 85. Type locality: «Haoe Lead, Unicoi M[oun]t[ai]ns, Graham/Monroe Co[unty], N[orth]C[arolina]/T[en]n[essee]» (original citation). Holotype (♂) in Donabauer’s collection (Vienna, Austria).

#### Distribution.

This species is known only from the type locality.

#### Records.

**USA**: NC/TN

### Trechus luculentus cheoahbaldensis

Donabauer, 2005

Trechus luculentus cheoahbaldensis Donabauer, 2005b: 89. Type locality: «Cheoah Bald, Graham/Swain Co[unty], N[orth]C[arolina]» (original citation). Holotype (♂) in Donabauer’s collection (Vienna, Austria).

#### Distribution.

This subspecies is known only from the type locality.

#### Records.

**USA**: NC

### Trechus luculentus joannabaldensis

Donabauer, 2005

Trechus luculentus joannabaldensis Donabauer, 2005b: 89. Type locality: «Joanna Bald, Snowbird Mountains, Graham/Cherokee Co[unty], N[orth]C[arolina]/T[en]n[essee]» (original citation). Holotype (♂) in Donabauer’s collection (Vienna, Austria).

#### Distribution.

This subspecies is known only from the type locality.

#### Records.

**USA**: NC/TN

### Trechus luculentus luculentus

Barr, 1962

Trechus luculentus Barr, 1962a: 88. Type locality: «Clingmans Dome, Swain Co[unty], North Carolina» (original citation). Holotype (♂) in USNM [# 65981].

#### Distribution.

This subspecies is known from the central Great Smoky Mountains in North Carolina and Tennessee (Barr 1979b: 40).

#### Records.

**USA**: NC, TN

### Trechus luculentus wayahensis

Barr, 1979

Trechus luculentus wayahensis Barr, 1979b: 70. Type locality: «head of Dirty John Creek, southwest slope of Winespring Bald (near Wayah Bald) (4900 feet), Macon County, North Carolina» (original citation). Holotype (♂) in AMNH [# 1497].

#### Distribution.

This subspecies is endemic to the Nantahala Mountains in southwestern North Carolina (Donabauer 2005b: 88).

#### Records.

**USA**: NC

### Trechus nantahalae

Barr, 1979

Trechus nantahalae Barr, 1979b: 72. Type locality: «0.4 mile northwest of Burningtown Gap on the southwest slope of Burningtown Bald, at a seep along the Appalachian Trail (4300 feet), Macon County, North Carolina» (original citation). Holotype (♂) in AMNH [# 1498].

#### Distribution.

This species is known only from the type locality in the northern Nantahala Mountains, southwestern North Carolina.

#### Records.

**USA**: NC

### Trechus nebulosus

Barr, 1962

Trechus nebulosus Barr, 1962a: 86. Type locality: «M[oun]t Kephart, Sevier Co[unty], Tennessee» (original citation). Holotype (♂) in USNM [# 65982].

#### Distribution.

This species is found in central and eastern Great Smoky Mountains in North Carolina and Tennessee (Barr 1979b: 40).

#### Records.

**USA**: NC, TN

### Trechus novaculosus

Barr, 1962

Trechus novaculosus Barr, 1962a: 89. Type locality: «Clingmans Dome, Swain Co[unty], North Carolina» (original citation). Holotype (♂) in USNM [# 65983].

#### Distribution.

This species is found in central Great Smoky Mountains in North Carolina and Tennessee (Barr 1979b: 40).

#### Records.

**USA**: NC, TN

### Trechus pseudonovaculosus

Donabauer, 2005

Trechus pseudonovaculosus Donabauer, 2005b: 80. Type locality: «Clingmans Dome, G[reat]S[moky]M[ountains], Servier [sic!]/Swain Co[unty], T[en]n[essee]/N[orth]C[arolina]» (original citation). Holotype (♂) in Donabauer’s collection (Vienna, Austria).

#### Distribution.

This species is known only from the type locality.

#### Records.

**USA**: NC/TN

### Trechus ramseyensis

Donabauer, 2005

Trechus ramseyensis Donabauer, 2005b: 74. Type locality: «Ramsey Cascade, G[reat]S[moky]M[ountains], Servier [sic!] Co[unty], T[en]n[essee]» (original citation). Holotype (♂) in Donabauer’s collection (Vienna, Austria).

#### Distribution.

This species is known only from the type locality.

#### Records.

**USA**: TN

### Trechus rosenbergi

Barr, 1962

Trechus rosenbergi Barr, 1962a: 89. Type locality: «Water Rock Knob, Haywood-Jackson Counties, North Carolina» (original citation). Holotype (♂) in USNM [# 65984].

#### Distribution.

This species is known from the Plott Balsam Mountains and Great Balsam Mountains in western North Carolina (Barr 1979b: 40).

#### Records.

**USA**: NC

### Trechus snowbirdensis

Donabauer, 2005

Trechus snowbirdensis Donabauer, 2005b: 78. Type locality: «Joanna Bald, Snowbird M[oun]t[ain]s, Graham Co[unty], N[orth]C[arolina]» (original citation). Holotype (♂) in Donabauer’s collection (Vienna, Austria).

#### Distribution.

This species is known only from the type locality.

#### Records.

**USA**: NC

### Trechus stefanschoedli

Donabauer, 2005

Trechus stefanschoedli Donabauer, 2005b: 87. Type locality: «Thunderhead M[oun]t[ai]n, G[reat]S[moky]M[ountains], Blount/Swain Co[unty], T[en]n[essee]/N[orth]C[arolina]» (original citation). Holotype (♂) in Donabauer’s collection (Vienna, Austria).

#### Distribution.

This species is known only from the type locality.

#### Records.

**USA**: NC/TN

### Trechus stupkai

Barr, 1979

Trechus stupkai Barr, 1979b: 65. Type locality: «edge of Ramsay Prong, about 150 yards above Ramsay Cascades (4600 feet), Great Smoky Mountains National Park, Sevier County, Tennessee» (original citation). Holotype (♂) in AMNH [# 1499]. Etymology. The specific name was proposed in honor of Arthur Stupka [1907-1999], head naturalist of the Great Smoky Mountains National Park for 30 years. He also worked before as naturalist and ranger in Yosemite and Acadia National Parks.

#### Distribution.

This species is known only from the holotype collected in eastern Tennessee.

#### Records.

**USA**: TN

### Trechus tennesseensis tauricus

Barr, 1962

Trechus tennesseensis tauricus Barr, 1962a: 87. Type locality: «Bull Cave Sinkhole, Blount Co[unty], Tennessee» (original citation). Holotype (♂) in USNM [# 65988].

#### Distribution.

This subspecies is known only from Bull Cave in the Cades Cove Mountains, Great Smoky Mountains, in eastern Tennessee (Barr 1979b: 40).

#### Records.

**USA**: TN

### Trechus tennesseensis tennesseensis

Barr, 1962

Trechus tennesseensis tennesseensis Barr, 1962a: 87. Type locality: «Berry Cave, Roane Co[unty], Tennessee» (original citation). Holotype (♂) in USNM [# 65989].

#### Distribution.

This subspecies is known only from the original 28 specimens collected in a cave in the Appalachian Valley, 8 miles south of Kingston and ¼ mile west of the Tennessee River on the southeast side of a valley east of Huckleberry Ridge (Barr 1962a: 87).

#### Records.

**USA**: TN

### Trechus thomasbarri

Donabauer, 2005

Trechus thomasbarri Donabauer, 2005b: 75. Type locality: «Haoe Lead, Unicoi M[oun]t[ain]s, Graham/Monroe Co[unty], N[orth]C[arolina]/T[en]n[essee]» (original citation). Holotype (♂) in Donabauer’s collection (Vienna, Austria). Etymology. This species was proposed for Thomas Calhoun Barr, Jr. [1931-2011], an expert on North American cave Carabidae. Barr taught at the Zoology Department, University of Kentucky, in Lexington.

#### Distribution.

This species is known only from the holotype.

#### Records.

**USA**: NC/TN

### Trechus tobiasi

Donabauer, 2005

Trechus tobiasi Donabauer, 2005b: 84. Type locality: «Tusquitee Bald, Macon/Clay Co[unty], N[orth]C[arolina]» (original citation). Holotype (♂) in Donabauer’s collection (Vienna, Austria).

#### Distribution.

This species is known only from the type locality in the Nantahala Mountains.

#### Records.

**USA**: NC

### Trechus tuckaleechee

Barr, 1962

Trechus tuckaleechee Barr, 1962a: 86. Type locality: «Tuckaleechee Caverns, Blount Co[unty], Tennessee» (original citation). Holotype (♂) in USNM [# 65991].

#### Distribution.

This species is known only from the original specimens collected in a large stream cavern at the north side of the Great Smoky Mountains.

#### Records.

**USA**: TN

### Trechus unicoi

Barr, 1979

Trechus luculentus unicoi Barr, 1979b: 68. Type locality: «Stratton Meadows (4900 feet), Monroe County, Tennessee, and Graham County, North Carolina» (original citation). Holotype (♂) in AMNH [# 1496].

#### Distribution.

This species is endemic to the Unicoi Mountains in southeastern Tennessee and western North Carolina (Barr 1979b: 40).

#### Records.

**USA**: NC, TN

#### Note.

This taxon was first described as a subspecies but raised to species by Donabauer (2005b: 87).

### Trechus valentinei

Barr, 1979

Trechus valentinei Barr, 1979b: 62. Type locality: «Appalachian Trail near summit of M[oun]t Kephart (approximately 6000 feet), Great Smoky Mountains National Park, Sevier County, Tennessee» (original citation). Holotype (♂) in AMNH [# 1501].

#### Distribution.

This species is found in the central Great Smoky Mountains in Tennessee and North Carolina (Barr 1979b: 40).

#### Records.

**USA**: NC, TN

### Trechus verus

Barr, 1962

Trechus verus Barr, 1962a: 81. Type locality: «M[oun]t Sterling, Haywood Co[unty], North Carolina» (original citation). Holotype (♂) in USNM [# 65993].

#### Distribution.

This species is known only from the conifer forests in the eastern end of the Great Smoky Mountains in Haywood County, western North Carolina, and Cocke County, eastern Tennessee.

#### Records.

**USA**: NC, TN

### Trechus wayahbaldensis

Donabauer, 2005

Trechus wayahbaldensis Donabauer, 2005b: 69. Type locality: «Wayah Bald, Macon Co[unty], N[orth]C[arolina]» (original citation). Holotype (♂) in Donabauer’s collection (Vienna, Austria).

#### Distribution.

This species is known only from the type locality.

#### Records.

**USA**: NC

### [uncifer group]

### Trechus aduncus

Barr, 1962

Trechus aduncus Barr, 1962a: 82. Type locality: «M[oun]t Pisgah, Haywood Co[unty], North Carolina» (original citation). Holotype (♂) in USNM [# 65978].

#### Distribution.

This species occurs in the Great Balsam Mountains in western North Carolina (Barr 1979b: 39).

#### Records.

**USA**: NC

### Trechus coweensis

Barr, 1979

Trechus aduncus coweensis Barr, 1979b: 59. Type locality: «summit of Yellow Mountain (5000 feet), 4 miles southwest of Glenville, Macon-Jackson Counties, North Carolina» (original citation). Holotype (♂) in AMNH [# 1492].

#### Distribution.

This species is known only from the Cowee Mountains in southwestern North Carolina (Donabauer 2005a: 56).

#### Records.

**USA**: NC

### Trechus howellae

Barr, 1979

Trechus aduncus howellae Barr, 1979b: 60. Type locality: «east face of Big Butt (4800 feet), Coweeta Hydrologic Laboratory, Macon County, North Carolina» (original citation). Holotype (♂) in AMNH [# 1493]. Etymology. The subspecific name was proposed for Thelma Howell [1901-1979], executive director of the Highlands Biological Station for more than 25 years. The Station established the *Thelma Howell Memorial Scholarship* for investigators.

#### Distribution.

This species is known only from Big Butt in the Nantahala Mountains, southwestern North Carolina (Donabauer 2005a: 57).

#### Records.

**USA**: NC

### Trechus inexpectatus

Barr, 1985

Trechus inexpectatus Barr, 1985b: 129. Type locality. «Camp Creek Bald, just below summit (1460 m), Greene Co[unty], Tennessee/Madison Co[unty], North Carolina» (original citation). Holotype (♂) in AMNH.

#### Distribution.

This species is known only from the type locality.

#### Records.

**USA**: TN/NC

### Trechus plottbalsamensis

Donabauer, 2005

Trechus plottbalsamensis Donabauer, 2005a: 55. Type locality: «Waterrock Knob, Haywood/Jackson Co[unty], N[orth]C[arolina]» (original citation). Holotype (♂) in Donabauer’s collection (Vienna, Austria).

#### Distribution.

This species is known only from the type locality in the Plott Balsam Mountains, southwestern North Carolina.

#### Records.

**USA**: NC

### Trechus satanicus

Barr, 1962

Trechus satanicus Barr, 1962a: 81. Type locality: «west end of Graveyard Fields near Devils Courthouse, Haywood Co[unty], North Carolina» (original citation). Holotype (♂) in USNM [# 65985].

#### Distribution.

This species is endemic to the western Pisgah Ridge (Donabauer 2005a: 57) in the Great Balsam Mountains.

#### Records.

**USA**: NC

### Trechus talequah

Barr, 1962

Trechus talequah Barr, 1962a: 82. Type locality: «Haw Knob, Monroe Co[unty], Tennessee» (original citation). Holotype (♂) in USNM [# 65987].

#### Distribution.

This species has been found yet only in the Unicoi Mountains, between 4800-5000 feet, in North Carolina and Tennessee (Barr 1979b: 40).

#### Records.

**USA**: NC, TN

### Trechus thunderheadensis

Donabauer, 2005

Trechus thunderheadensis Donabauer, 2005a: 54. Type locality: «Thunderhead Mountain, G[reat]S[moky]M[ountains], Blount/Swain Co[unty], N[orth]C[arolina]/T[en]n[essee]» (original citation). Holotype (♂) in Donabauer’s collection (Vienna, Austria).

#### Distribution.

This species is known only from Thunderhead Mountain at the junction of Swain County in North Carolina and Blount County in Tennessee.

#### Records.

**USA**: NC/TN

### Trechus toxawayi

Barr, 1979

Trechus aduncus toxawayi Barr, 1979b: 59. Type locality: «0.25 mile west of the summit of Toxaway Mountain (4600 feet), Jackson County, North Carolina» (original citation). Holotype (♂) in AMNH [# 1494].

#### Distribution.

This species is known only from the Toxaway Mountain in southwestern North Carolina.

#### Records.

**USA**: NC

### Trechus tusquitensis

Donabauer, 2005

Trechus tusquitensis Donabauer, 2005a: 57. Type locality: «Tusquitee Bald, Macon/Clay Co[unty], N[orth]C[arolina]» (original citation). Holotype (♂) in Donabauer’s collection (Vienna, Austria).

#### Distribution.

This species is known only from the type locality in southwestern North Carolina.

#### Records.

**USA**: NC

### Trechus uncifer

Barr, 1962

Trechus uncifer Barr, 1962a: 80. Type locality: «Clingmans Dome, Sevier Co[unty], Tennessee - Swain Co[unty], North Carolina» (original citation). Holotype (♂) in USNM [# 65992].

#### Distribution.

This species is known from the central Great Smoky Mountains east to Plott Balsam Mountains in Sevier and Cocke Counties, eastern Tennessee, and in Haywood and Jackson Counties, western North Carolina (Barr 1979b: 38).

#### Records.

**USA**: NC, TN

### [vandykei group]

### Trechus barberi

(Jeannel, 1931)

Microtrechus barberi Jeannel, 1931: 444. Type locality: «Retreat [Haywood County], North Carolina» (original citation). Holotype (♂) in USNM [# 43664].

#### Distribution.

The range of this common species extends from the vicinity of Asheville, western North Carolina, to the mountains of northeast Georgia (Barr 1979b: 38) and northwestern South Carolina (Ciegler 2000: 44).

#### Records.

**USA**: GA, NC, SC, TN

### Trechus bowlingi

Barr, 1962

Trechus bowlingi Barr, 1962a: 78. Type locality: «M[oun]t Kephart, Sevier Co[unty], Tennessee - Swain Co[unty], North Carolina» (original citation). Holotype (♂) in USNM [# 65980].

#### Distribution.

This species is found in the spruce-fir forests, between 3000-6500 feet, in the Great Smoky Mountains of North Carolina and Tennessee (Barr 1979b: 38; Donabauer 2009: 137).

#### Records.

**USA**: NC, TN

### Trechus haoe

Barr, 1979

Trechus haoe Barr, 1979b: 51. Type locality: «Haoe Lead (4800 feet) above Joyce Kilmer Memorial Forest, Graham County, North Carolina» (original citation). Holotype (♂) in AMNH [# 1495].

#### Distribution.

This species is known only from the Unicoi Mountains, along the Tennessee-North Carolina border (Donabauer 2009: 138).

#### Records.

**USA**: NC

### Trechus pseudobarberi

Donabauer, 2009

Trechus pseudobarberi Donabauer, 2009: 136. Type locality: «Waterrock Knob, Haywood/Jackson Co[unty], N[orth]C[arolina]» (original citation). Holotype (♂) in Donabauer’s collection (Wien, Austria).

#### Distribution.

This species is known only from two localities in western North Carolina (Donabauer 2009: 136).

#### Records.

**USA**: NC

### Trechus pseudosubtilis

Donabauer, 2009

Trechus pseudosubtilis Donabauer, 2009: 133. Type locality: «Cataloochee Balsam (summit), G[reat]S[moky]M[ountains], Swain/Haywood Co[unty], T[en]n[essee]/N[orth]C[arolina]» (original citation). Holotype (♂) in Donabauer’s collection (Wien, Austria).

#### Distribution.

This species is known only from the type locality.

#### Records.

**USA**: NC/TN

### Trechus rivulis

Dajoz, 2005

Trechus rivulis Dajoz, 2005: 208. Type locality: «Buck Creek [Nantahala Forest, Clay County, North Carolina]» (original citation). Holotype in Dajoz’s collection (Paris, France).

#### Distribution.

This species is known only from the type locality in southwestern North Carolina.

#### Records.

**USA**: NC

### Trechus subtilis

Barr, 1962

Trechus subtilis Barr, 1962a: 80. Type locality: «M[oun]t Sterling, Haywood Co[unty], North Carolina» (original citation). Holotype (♂) in USNM [# 65986].

#### Distribution.

This species has been found at several locations in the Great Smoky Mountains and the Plott Balsams in western North Carolina (Barr 1979b: 38; Donabauer 2009: 131).

#### Records.

**USA**: NC

### Trechus tonitru

Barr, 1962

Trechus tonitru Barr, 1962a: 79. Type locality: «Thunderhead, Great Smoky Mountains National Park, Blount Co[unty], Tennessee» (original citation). Holotype (♂) in USNM [# 65990].

#### Distribution.

This species is known only from the type locality in the western Great Smoky Mountains on the border between Blount County, Tennessee, and Swain County, North Carolina.

#### Records.

**USA**: TN

### Trechus tusquitee

Barr, 1979

Trechus tusquitee Barr, 1979b: 52. Type locality: «Tusquitee Bald, Clay-Macon Counties, North Carolina» (original citation). Holotype (♂) in AMNH [# 1500].

#### Distribution.

This species is known at present from small mountain ranges in southwestern North Carolina (Barr 1979b: 37).

#### Records.

**USA**: NC

### Trechus vandykei pisgahensis

Barr, 1979

Trechus vandykei pisgahensis Barr, 1979b: 50. Type locality: «M[oun]t Pisgah (5000 feet), Haywood-Buncombe Counties, North Carolina» (original citation). Holotype (♂) in AMNH [# 1502].

#### Distribution.

This subspecies is found in the Great Balsam and Cowee Mountains in western North Carolina (Barr 1979b: 37).

#### Records.

**USA**: NC

### Trechus vandykei vandykei

(Jeannel, 1927)

Microtrechus vandykei Jeannel, 1927: 587. Type locality: «Black Mountains, Monts Alleghany, Virginia» (original citation). Holotype in MHNP.

#### Distribution.

This subspecies is found in the Bald and Unaka Mountains of Tennessee and in the Black and Great Craggy Mountains of North Carolina (Barr 1979b: 37; Donabauer 2009: 138). Populations very similar to those of *Trechus vandykei* have been reported by Donabauer (2009: 138) in the Great Smoky Mountains.

#### Records.

**USA**: NC, TN

### Tribe Bembidiini

Stephens, 1827

Bembidiidae Stephens, 1827: 5. Type genus: *Bembidium* Gyllenhal, 1810 (unjustified emendation of *Bembidion* Latreille, 1802, not in prevailing usage) (= *Bembidion* Latreille, 1802).

#### Diversity.

Worldwide, with about 2,630 species arrayed in six subtribes: Anillina (about 375 species), Bembidiina (about 1,340 species), Horologionina (one species), Lovriciina (four species), Tachyina (about 790 species), and Xystosomina (about 125 species). The Northern Hemisphere contains roughly 55.5% of the world fauna and North America alone 14.8% (about 390 species).

### Subtribe Bembidiina

Stephens, 1827

Bembidiidae Stephens, 1827: 5. Type genus: *Bembidium* Gyllenhal, 1810 (unjustified emendation of *Bembidion* Latreille, 1802) (= *Bembidion* Latreille, 1802).
Peryphidae Kirby, 1837: 52. Type genus: *Peryphus* Dejean, 1821.

#### Diversity.

Worldwide, with about 1,350 species. The number of genera admitted varies greatly depending on the authors. In this work, the species are arrayed in nine genera following Maddison (2012: 570): *Amerizus* Chaudoir, *Asaphidion* des Gozis, *Bembidion* Latreille, *Caecidium* Uéno, *Lionepha* Casey, *Ocys* Gistel, *Orzolina* Machado, *Sakagutia* Uéno, and *Sinechostictus* Motschulsky (including *Pseudolimnaeum* Kraatz). The majority of the species are found in the Northern Hemisphere (roughly 75.5% of the world fauna).

### Genus Amerizus

Chaudoir, 1868

Amerizus Chaudoir, 1868b: 216. Type species: *Trechus spectabilis* Mannerheim, 1852 by monotypy. Etymology. From the Greek *a* (without) and *merizo* (to divide, split), probably alluding to the apparent undivided outer lobe of the maxilla (“*maxillae tenues, elongatae, mala externa *... *haud biarticulata*”) of adults [masculine].

#### Diversity.

About 50 species in North America (five species) and Asia (about 45 species) placed in two subgenera: *Tiruka* Andrewes for most Asian species and *Amerizus* for the Nearctic species and one Asian species.

#### Taxonomic Note.

Toledano (2011) noted that *Amerizus* is closely related to the genus *Caecidium* Uéno (two species in Japan). Lindroth (1980: 203) pointed out that *Bembidion (Gnatholymnaeum) blackburni* (Sharp) from Hawaii is structurally similar to the species of this genus and indeed listed the species within the subgenus *Amerizus*. However, Liebherr (2008: 36) argued against such association. Both *Caecidium* and *Bembidion blackburni* were not sequenced by Maddison (2012).

### Subgenus Amerizus

Chaudoir, 1868

Amerizus Chaudoir, 1868b: 216. Type species: *Trechus spectabilis* Mannerheim, 1852 by monotypy.

#### Diversity.

Six species in North America (five species) and the Altai Mountains in Russia (*Amerizus teles* Belousov and Dudko, 2010).

#### Identification.

Lindroth (1963b: 403-406) treated four of the five North American species, leaving *Amerizus utahensis*.

### Amerizus oblonguloides

(Lindroth, 1963)

Bembidion oblonguloides Lindroth, 1963b: 404. Type locality: «Prince Rupert, B[ritish] C[olumbia]» (original citation). Holotype (♂) in CNC [# 8383].

#### Distribution.

This species is restricted to the Queen Charlotte Islands and adjacent mainland (Kavanaugh 1992: 69).

#### Records.

**CAN**: BC (QCI)

### Amerizus oblongulus

(Mannerheim, 1852)

Trechus oblongulus Mannerheim, 1852: 299. Type locality: «insula Sitkha [= Baranof Island, Alaska]» (original citation). Lectotype (♂), designated by Lindroth (1963b: 404), in MCZ [# 5560].
Amerizus crassicornis Casey, 1918: 165. Type locality: «Inverness [probably Inverness Passage], British Columbia» (original citation). Lectotype (♂), designated by Lindroth (1975: 122), in USNM [# 46899]. Synonymy established by Lindroth (1963b: 404).
Amerizus keeni Casey, 1918: 166. Type locality: «Metlakatla, British Columbia» (original citation). Lectotype (♂), designated by Lindroth (1975: 122), in USNM [# 46901]. Synonymy established by Lindroth (1963b: 404). Etymology. The specific name was proposed for the English clergyman John Henry Keen [1851 (or early 1852)-1950]. Keen was sent as missionary to the Haida Indian on the Queen Charlotte Islands for eight years (1890-1898) and a year later to Metlakatla, an Indian village a few miles northwest of Prince Rupert in British Columbia, for fourteen years. During those years, Keen collected extensively in the province and sent specimens to many museums and individuals, including Casey.

#### Distribution.

This species ranges from the Kenai Peninsula in Alaska (Lindroth 1963b: 405) south to “California” (Hayward 1897: 131) and to the Sangre de Cristo Mountains in northeastern New Mexico (Ball 1966b: 30). The record from “Mexico” (Hayward 1897: 131) is probably in error.

#### Records.

**CAN**: BC (QCI) **USA**: AK, CA, ID, NM, OR, WA

### Amerizus spectabilis

(Mannerheim, 1852)

Trechus spectabilis Mannerheim, 1852: 298. Type locality: «insula Sitkha [= Baranof Island, Alaska]» (original citation). Lectotype (♂), designated by Lindroth (1963b: 403), in ZMH.
Amerizus longicornis Casey, 1918: 166. Type locality: «S[an]ta Cruz M[oun]t[ain]s [Santa Clara County], California» (original citation). Lectotype (♂), designated by Lindroth (1975: 122), in USNM [# 46900]. Synonymy established by Lindroth (1963b: 403).

#### Distribution.

The range of this species extends from the Alexander Archipelago (Mannerheim 1852: 298; Lindroth 1963b: 404) south at least to the Santa Cruz Mountains of the Coast Ranges (Casey 1918: 166, as *Amerizus longicornis*) and the Sierra Nevada in California (Kavanaugh 1992: 68).

#### Records.

**CAN**: BC (QCI, VCI) **USA**: AK, CA, OR, WA

### Amerizus utahensis

(Van Dyke, 1926)

Bembidium utahensis Van Dyke, 1926a: 66. Type locality: «near Salt Lake City [Salt Lake County], Utah» (original citation). Holotype (♂) in CAS [# 1819].

#### Distribution.

This species is known only from Utah.

#### Records.

**USA**: UT

#### Note.

This species was originally placed in the subgenus *Lymneops* Casey but subsequently transferred to *Amerizus* Chaudoir by Van Dyke (1949b: 56).

### Amerizus wingatei

(Bland, 1864)

Bembidium wingatei Bland, 1864: 319. Type locality: «near Bellefonte [Centre County], Pennsylvania» (original citation). One syntype in ANSP [# 1034]. Etymology. The specific name was proposed for John D. Wingate, a dentist in Bellefonte, Pennsylvania who had an interest in natural history. Wingate presented over 1,500 beetles to the Academy of Natural Sciences in Philadelphia in 1861.

#### Distribution.

This eastern species occurs from Newfoundland (Lindroth 1955a: 78, as *Bembidium oblongulum*) to northern Minnesota (Petrice et al. 2002: 9; Gandhi et al. 2005: 925), south to Tennessee (Carter County, CMNH) and North Carolina (Lindroth 1963b: 406; Mitchell and Yancey Counties, CMNH) along the Appalachian Mountains.

#### Records.

**FRA**: PM **CAN**: LB, NB, NF, NS (CBI), ON, PE, QC **USA**: IL, IN, KY, MA, ME, MI, MN, NC, NH, NY, OH, PA, TN, VA, VT, WI, WV

#### Note.

This species has long been confused with *Amerizus oblongulus* Mannerheim, under that name, following Horn (1875: 131).

### Genus Lionepha

Casey, 1918

Lionepha Casey, 1918: 18. Type species: *Bembidium erasum* LeConte, 1859 by original designation. Etymology. From Greek *leios* (smooth) and the generic name *Nepha*, probably alluding to the polish body (“body ... polished in great part”) of the adult [feminine].

#### Diversity.

Nine species in western North America.

#### Identification.

Erwin and Kavanaugh (1981) revised the species and provided a key for their identification.

### [casta group]

### Lionepha casta

(Casey, 1918)

Bembidion castum Casey, 1918: 20. Type locality: «S[an]ta Cruz M[oun]t[ain]s [Santa Clara County], California» (original citation). Lectotype (♂), designated by Lindroth (1975: 116), in USNM [# 36818].
Bembidion serenum Casey, 1918: 21. Type locality: «Arcata, Humboldt Co[unty], California» (original citation for the lectotype). Lectotype (♀), designated by Lindroth (1975: 116), in USNM [# 36819]. Synonymy established by Lindroth (1963b: 261), confirmed by Erwin and Kavanaugh (1981: 52).
Bembidion brumale Casey, 1918: 22. Type locality: «Metlakatla, British Columbia» (original citation). Lectotype (♀), designated by Lindroth (1975: 116), in USNM [# 36823]. Synonymy established by Erwin and Kavanaugh (1981: 52).
Bembidion vacivum Casey, 1918: 22. Type locality: «Skeena River at Terrace, British Columbia» (original citation). Lectotype (♀), designated by Lindroth (1975: 116), in USNM [# 36822]. Synonymy established, under the name *Bembidion brumale* Casey, by Lindroth (1963b: 262), confirmed by Erwin and Kavanaugh (1981: 52).
Bembidion nescium Casey, 1918: 30. Type locality: «Metlakatla, British Columbia» (original citation). Lectotype (♂), designated by Lindroth (1975: 116), in USNM [# 36845]. Synonymy established by Lindroth (1963b: 261), confirmed by Erwin and Kavanaugh (1981: 52).

#### Distribution.

This species ranges from the southern part of the Alexander Archipelago, Alaska (Lindroth 1963b: 261) and west-central British Columbia south to central California along the coast mountain systems [see Erwin and Kavanaugh 1981: Fig. 23].

#### Records.

**CAN**: BC (QCI, VCI) **USA**: AK, CA, OR, WA

### [erasa group]

### Lionepha chintimini

(Erwin and Kavanaugh, 1981)

Bembidion chintimini Erwin and Kavanaugh, 1981: 63. Type locality: «Mary’s Peak, 8 miles W[est] of Philomath (1220 m), Benton County, Oregon» (original citation). Holotype (♀) in CNC [# 16452].

#### Distribution.

This species is known from several mountains in the Coast Ranges of Oregon (David R. Maddison pers. comm. 2012).

#### Records.

**USA**: OR

### Lionepha disjuncta

(Lindroth, 1963)

Bembidion disjunctum Lindroth, 1963b: 264. «Sonora Pass [Tuolumne County], Sierra Nevada, Calif[ornia]» (original citation). Holotype (♂) in MCZ [# 32533].

#### Distribution.

This species is known from four localities, the northernmost in southern British Columbia and the southernmost in the mid-Sierra Nevada [see Erwin and Kavanaugh 1981: Fig. 24].

#### Records.

**CAN**: BC **USA**: CA, OR

### Lionepha erasa

(LeConte, 1859)

Bembidium erasum LeConte, 1859a: 83. Type locality: «Oregon» (original citation), restricted to «Fort Klamath, Klamath Co[unty]» by Erwin and Kavanaugh (1981: 59). Lectotype (♀), designated by Erwin and Kavanaugh (1981: 59), in MCZ [# 5490].
Bembidion lascivum Casey, 1918: 21. Type locality: «Lake Tahoe [Placer County], California» (original citation for the lectotype). Lectotype (♂), designated by Lindroth (1975: 116), in USNM [# 36821]. Synonymy established by Lindroth (1963b: 261).
Bembidion lubricum Casey, 1918: 21. Type locality: «Truckee [Nevada County], California» (original citation for the lectotype). Lectotype (♂), designated by Lindroth (1975: 116), in USNM [# 36820]. Synonymy established by Lindroth (1963b: 261).
Bembidion probatum Casey, 1918: 22. Type locality: «Boulder Co[unty], Colorado» (original citation). Lectotype (♀), designated by Lindroth (1975: 116), in USNM [# 36817]. Synonymy established by Lindroth (1963b: 261).

#### Distribution.

This species ranges from southwestern Alberta and southeastern British Columbia south to the San Jacinto Mountains in southern California, including the Sierra Nevada and Cascade Range, and northern Colorado along the Rocky Mountains [see Erwin and Kavanaugh 1981: Fig. 25].

#### Records.

**CAN**: AB, BC **USA**: CA, CO, ID, MT, NV, OR, WA, WY

### Lionepha lindrothellus

(Erwin and Kavanaugh, 1981)

Bembidion lindrothellus Erwin and Kavanaugh, 1981: 61. Type locality: «Haines Highway Mile 31.5, Little Boulder Creek, Alaska» (original citation). Holotype (♂) in MCZ [# 32549].

#### Distribution.

This species is known from a few localities along the Pacific Coast from southern Alaska near the Yukon Territory border to northern Washington [see Erwin and Kavanaugh 1981: Fig. 26].

#### Records.

**CAN**: BC (VCI) **USA**: AK, WA

#### Note.

This species corresponds to *Bembidion brumale* Casey *sensu* Lindroth (1963b: 262).

### Lionepha lummi

(Erwin and Kavanaugh, 1981)

Bembidion lummi Erwin and Kavanaugh, 1981: 62. Type locality: «Friday Harbour, San Juan Island, San Juan County, Washington» (original citation). Holotype (♀) in CAS [# 13652].

#### Distribution.

This species is known from two localities, one on the eastern edge of the Central Plateau in eastern British Columbia, the other on an island on Puget Sound, Washington [see Erwin and Kavanaugh 1981: Fig. 27].

#### Records.

**CAN**: BC **USA**: WA

### [osculans group]

### Lionepha osculans

(Casey, 1918)

Bembidion osculans Casey, 1918: 20. Type locality: «probably the coast regions south of San Francisco, California» (original citation). Lectotype (♀), designated by Lindroth (1975: 116), in USNM [# 36816].
Bembidion speculum Casey, 1918: 20. Type locality: «Marin Co[unty], California» (original citation). Lectotype (♀), designated by Lindroth (1975: 116), in USNM [# 36815]. Synonymy established by Lindroth (1963b: 259).

#### Distribution.

This species ranges from the lower Columbia River drainage in northwestern Idaho and southern Washington south to the Sierra Nevada and Coast Ranges in central California [see Erwin and Kavanaugh 1981: Fig. 20].

#### Records.

**USA**: CA, ID, OR, WA

### Lionepha pseudoerasa

(Lindroth, 1963)

Bembidion pseudoerasum Lindroth, 1963b: 260. Type locality: «Truckee [Nevada County], Calif[ornia]» (original citation). Holotype (♂) in USNM [# 76638].

#### Distribution.

This species is endemic to the Sierra Nevada in California from Nevada County to Sequoia National Park [see Erwin and Kavanaugh 1981: Fig. 21].

#### Records.

**USA**: CA

### Lionepha sequoiae

(Lindroth, 1963)

Bembidion sequoiae Lindroth, 1963b: 260. Type locality: «Sequoia [National] Park, Calif[ornia]» (original citation). Holotype (♂) in MCZ [# 32532].

#### Distribution.

This species ranges along the Cascade Range and Sierra Nevada from southern British Columbia to central California [see Erwin and Kavanaugh 1981: Fig. 22].

#### Records.

**CAN**: BC **USA**: CA, OR, WA

### Genus Asaphidion

des Gozis, 1886

Tachypus Dejean, 1821: 18 [junior homonym of *Tachypus* Weber, 1801]. Type species: *Elaphrus picipes* Duftschmid, 1812 (= *Cicindela caraboides* Schrank, 1781) designated by Duponchel (1842: 542). Etymology. From the Greek *tachys* (swift, quick, fast) and *pous* (foot) [masculine].
Asaphidion des Gozis, 1886: 6. Replacement name for *Tachypus* Dejean, 1821. Etymology. From the Greek *asaphes* (indistinct, obscure) and the suffix -*idion* (small, little) [neuter].
Pseudelaphrus Acloque, 1896: 81. Type species: *Cicindela flavipes* Linnaeus, 1760 designated by Bousquet (2002b: 42). Synonymy established by Antoine (1955: 126). Etymology. From the Greek *pseudos* (fallacy, lie) and the generic name *Elaphrus* [*q.v*.] [masculine].
Asaphidium Jacobson, 1906: 277. Unjustified emendation of *Asaphidion* des Gozis, 1886.
Basaphidion Netolitzky, 1935c: 168. Type species: *Cicindela caraboides* Schrank, 1781 by original designation.

#### Diversity.

Northern Hemisphere, with 39 species in the arctic, subarctic, boreal, and temperate areas of the Nearctic (three species, one of them adventive) and Palaearctic (37 species) Regions.

#### Identification.

Lindroth (1963b: 203-206) covered all three species found in North America, the adventive *Asaphidion curtum* under the name *Asaphidion flavipes* (Linnaeus).

### Asaphidion alaskanum

Wickham, 1919

Asaphidion alaskanum Wickham, 1919b: 178. Type locality: «15 miles below New Rampart House, on the Porcupine River, Alaska» (original citation). Holotype (♀) in USNM [# 22562].

#### Distribution.

This species is found from Alaska to northwestern Northwest Territories (Lindroth 1963b: 204). Fossil remnants of this species, believed to be 2.0-2.5 million years old, have been found in Greenland and Meighen Island (Bennike and Böcher 1990: 336; Böcher 1995: 23).

#### Records.

**CAN**: NT, YT **USA**: AK

### Asaphidion curtum curtum

(Heyden, 1870)

Tachypus curtus Heyden, 1870: 65. Type locality: «Valenzia, Albufera [Spain]» (original citation). Syntype(s) [2 originally cited] location unknown (possibly in DEI).

#### Distribution.

This European and North African subspecies is adventive in North America where it is known from Maine (Larochelle and Larivière 1990a: 28, 33, as *Asaphidion flavipes*) and southeastern New Hampshire (Bell 1989b: 204, as *Asaphidion flavipes*) to Long Island, New York (Davidson and Langworthy 1981: 280, as *Asaphidion flavipes*). The first inventoried specimens collected on this continent were found in the late 1920s in Long Island (Cooper 1930: 21, as *Asaphidion flavipes*), the next ones were found in 1976 also on Long Island (Davidson and Langworthy 1981: 280).

#### Records.

**USA**: CT, MA, ME, NH, NY, RI – **Adventive**

#### Note.

Two other subspecies, one from Morocco (*Asaphidion curtum moroccanum* Antoine) and the other from the Canary Islands (*Asaphidion curtum delatorrei* Uyttenboogaart), are recognized.

**Figure 22.**
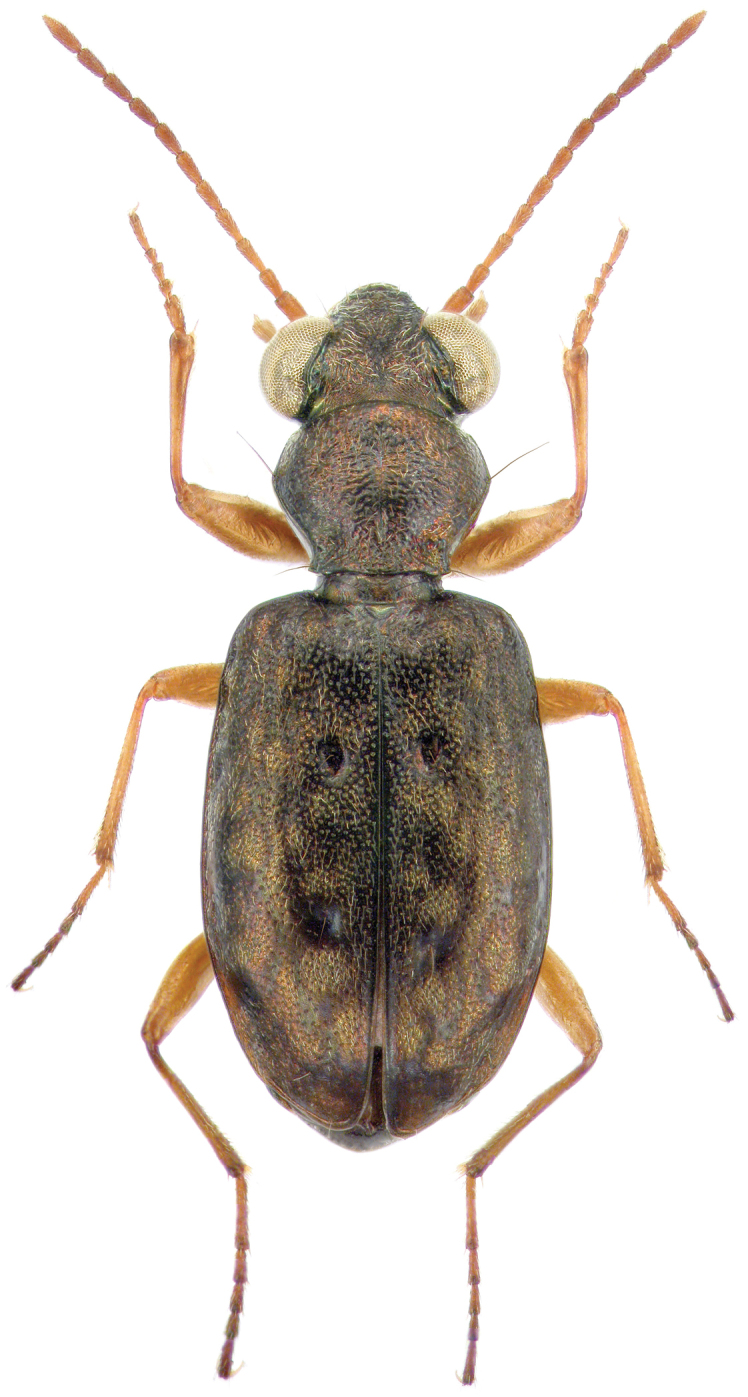
*Asaphidion curtum curtum* (Heyden). This adventive bembidiine was reported in the North American literature under the name *Asaphidion flavipes* (Linnaeus) until a study of the male genitalia showed that the specimens were in fact conspecific with the morphologically similar *Asaphidion curtum*. *Asaphidion* species are odd-looking bembidiines and Linnaeus originally associated them with tiger beetles probably because of their large eyes and absence of elytral striae.

### Asaphidion yukonense

Wickham, 1919

Asaphidion yukonense Wickham, 1919b: 180. Type locality: «Yukon Crossing, Yukon Territory» (original citation). Holotype (♀) in USNM [# 22563].

#### Distribution.

This species is known from Alaska, Yukon Territory, northeastern British Columbia, and west-central Alberta [see Morgan and Morgan 1979: Fig. 4; Morgan and Morgan 1981: map 4]. Fossil remnants of this species from the Pleistocene and early Holocene have been found in west-central Illinois, northeastern Wisconsin, Vermont, southern Ontario, and northwestern Ontario (see Ashworth and Schwert 1991: 512; Bajc et al. 1997: 691).

#### Records.

**CAN**: AB, BC, YT **USA**: AK

### Genus Bembidion

Latreille, 1802

Bembidion Latreille, 1802: 82. Type species: *Carabus quadriguttatus* Fabricius, 1775 (= *Cicindela quadrimaculata* Linnaeus, 1760) designated by Andrewes (1935: 17). Etymology. From the Greek *bembix* (whirl, top) and the suffix -*idion* (little), probably alluding to the erratic movements of adults of these small species in the field [neuter]. Note. Notwithstanding Maddison’s (1993: 151) remarks and Toledano’s (1999: 197-199) lengthy discussion, the first valid type species designation for *Bembidion* Latreille, 1802 is that of Andrewes (1935: 17) who designated *Cicindela quadrimaculata* Linnaeus, 1760. Even if this species was not an originally included species, the fact that Andrewes (1935: 17) listed it in synonymy with *Carabus quadriguttatus* Fabricius, 1775, a species originally included, he is deemed to have designated the latter species as type species (ICZN 1999: Article 69.2.2).
Bembidium Gyllenhal, 1810: 12. Unjustified emendation of *Bembidion* Latreille, 1802.
Bembecidium Agassiz, 1846: 45. Unjustified emendation of *Bembidium* Gyllenhal, 1810.
Bembicidium Gemminger and Harold, 1868a: 405. Unjustified emendation of *Bembidion* Latreille, 1802.

#### Diversity.

Worldwide, with about 1,250 species (Lorenz 2005: 215-236, as Bembidiina excluding *Phrypeus*, *Amerizus*, *Lionepha*,and *Asaphidion*) arrayed in about 105 subgenera. The Northern Hemisphere includes roughly 75% of the species and the Westhern Hemisphere approximately 30% of the species. The North American fauna alone is represented by 253 species (about 20% of the world fauna) placed in 37 subgenera. Eight of the North American species are adventive and 20 species are Holarctic.

#### Identification.

Hayward (1897) reviewed the North American species but his work is now outdated. Lindroth (1963b) covered 191 (six of them in the key alone) of the 253 species found in North America (about 75% of the fauna).

#### Taxonomic Note.

Maddison (2012: 570) presented an entirely new classification of this genus based on molecular data analyses. He found support for monophyly of *Bembidion* exclusive of the subgenus *Phyla*; however, he retained the taxon in the genus pending further study. He recognized three series, the *Odontium*, *Ocydromus*, and *Bembidion* Series with the following subgenera represented in North America left unplaced: *Blepharoplataphus*, *Plataphus* (including *Plataphodes*), *Hydrium* (including *Eurytrachelus*), *Metallina*, *Lindrochthus*, *Eupetedromus*, *Trechonepha*, *Liocosmius*, *Melomalus*, *Trichoplataphus*, *Phyla*, and *Lymnaeum*. The North American taxa in the *Odontium* Series were placed in two complexes, the *Hydriomicrus* (*Hirmoplataphus* and *Hydriomicrus*) and *Odontium* (*Odontium*, *Bracteon*, *Ochthedromus*, and *Pseudoperyphus*) Complexes. The North American taxa in the *Ocydromus* Series were placed in two complexes, the *Princidium* (*Cillenus* and *Actedium*) and *Ocydromus* (*Ocydromus*, *Peryphus*, *Terminophanes*, *Asioperyphus*, *Peryphanes*, *Testediolum*, and *Leuchydrium*) Complexes. The North American taxa in the *Bembidion* Series were placed in three complexes, the *Bembidion* (*Bembidion* s.str. and *Cyclolopha*), *Furcacampa* (*Furcacampa* and *Neobembidion*) and *Diplocampa* (*Diplocampa* and *Semicampa*) Complexes with *Notaphus*, *Trepanedoris*, *Peryphodes*, and *Emphanes* left unplaced.

#### Faunistic Note.

Edwards (1975: 53) recorded a “*Bembidion nigricornis* Hayward” from Glacier National Park in Montana. I was unable to find any *Bembidion* of that name or with a similar name described by Hayward or any other North American authors.

### Subgenus Hirmoplataphus

Lindroth, 1963

Hirmoplataphus Lindroth, 1963b: 300. Type species: *Bembidium hirmocaelum* Chaudoir, 1850 by original designation. Etymology. From the Greek *eirmos* (series, string) or the first two syllables of one of the included species, *Bembidium hirmocaelum*, and the generic name *Plataphus* [*q.v*.] [masculine]. Note. The name *Hirmoplataphus* was first proposed by Netolitzky (1942: 46). However, because he failed to designate a type species, the name cannot be attributed from this publication (ICZN 1999: Article 13.3).

#### Diversity.

Northern Hemisphere, with 11 species in the Nearctic (nine species) and Palaearctic (two species: *Bembidium friebi* Netolitzky and *Bembidium hirmocaelum* Chaudoir) Regions.

#### Identification.

There is no taxonomic revision of the North American species and such study is needed. Lindroth (1963b: 301-305) covered all but three (*Bembidium alpineanum*, *Bembidium avidum*, and *Bembidium subaerarium*) species described by Casey.

### Bembidion alpineanum

Casey, 1924

Bembidion alpineanum Casey, 1924: 30. Type locality: «Nevada Co[unty], California» (original citation). Holotype [by monotypy] (♀) location unknown (missing in USNM as of September 2006).

#### Distribution.

This species is known only from the holotype collected in eastern California.

#### Records.

**USA**: CA

### Bembidion avidum

Casey, 1918

Bembidion avidum Casey, 1918: 53. Type locality: «Reno [Washoe County], Nevada» (original citation). Lectotype (♂), designated by Erwin (1984a: 167), in USNM [# 36886].

#### Distribution.

According to Erwin (1984a: 167), this species “is found in the Basin and Range Province of the western United States.”

#### Records.

**USA**: NV

### Bembidion concolor

(Kirby, 1837)

Peryphus concolor Kirby, 1837: 54. Type locality: northern parts of British America (inferred from title of the book), restricted to «Edmonton, Al[ber]ta» by Lindroth (1963b: 304). One syntype in BMNH (Lindroth 1953b: 176).
Ochthedromus longulus LeConte, 1847: 456. Type locality: «Aquilae Portum [= Eagle Harbor, Keweenaw County, Michigan] Lacus Superioris» (original citation). Syntype(s) in MCZ [# 5496]. Synonymy established by Lindroth (1953b: 176).
Ochthedromus subaeneus LeConte, 1847: 457. Type locality: «Lacum Superiorem» (original citation). Syntype(s) in MCZ [# 5507]. Synonymy established, under the name *Bembidion longulum* (LeConte), by Hayward (1897: 133), confirmed by Lindroth (1963b: 304).

#### Distribution.

This species ranges from Newfoundland (Lindroth 1955a: 56, as *Bembidion longulum*) to eastern Alaska (Lindroth 1963b: 304), south to northern Oregon (Gilliam and Morrow Counties, CMNH), central Nevada (Eureka County, Ken Karns pers. comm. 2009), northern Arizona (Coconino County, Ken Karns pers. comm. 2009), central New Mexico (Fall and Cockerell 1907: 157; Casey 1918: 54, as *Bembidion longulum*), eastern South Dakota (Kirk and Balsbaugh 1975: 17), the upper peninsula of Michigan (LeConte 1847: 456, as *Bembidion longulum*), and northeastern New York (Casey 1918: 51). The record from “California” (Lindroth 1955a: 56) needs confirmation (see Lindroth 1963b: 304); those from “New Hampshire” and “Maine” (Bousquet and Larochelle 1993: 133) are probably in error (Ross T. Bell pers. comm. 2008).

#### Records.

**CAN**: AB, BC (VCI), MB, NB, NF, NS, NT, ON, QC, SK, YT **USA**: AK, AZ, CO, ID, MI, MT, ND, NM, NV, NY, OR, SD, UT, WA, WI, WY [CA]

### Bembidion humboldtense

Blaisdell, 1902

Bembidium humboldtensis Blaisdell, 1902: 74. Type locality: «Humboldt County, Cal[ifornia]» (original citation). Syntype(s) [12 originally cited] in CAS [# 2658 and 2659] and MCZ [# 2010].
Bembidion chetcoens Hatch, 1953: 88. Type locality: «Myrtle Grove, Chetco R[iver] [Coos County], Ore[gon]» (original citation). Holotype (♂) in USNM. Synonymy established by Lindroth (1963b: 305).

#### Distribution.

As far as known, this species is confined to the west coast of southern Oregon (Hatch 1953: 88, as *Bembidion chetcoens*) and northern California (Blaisdell 1902: 74; Casey 1918: 55). The record from the central Sierra Nevada (Papp 1978: 164) needs confirmation.

#### Records.

**USA**: CA, OR

### Bembidion nigrum

Say, 1823

Bembidium niger Say, 1823a: 85. Type locality: «Rumney [Grafton County], N[ew] H[ampshire]» (neotype label). Neotype (♂), designated by Lindroth and Freitag (1969: 336), in MCZ [# 33070].
Bembidion nigrum facile Casey, 1918: 48. Type locality: «Illinois» (original citation). Lectotype (♀), designated by Lindroth (1975: 118), in USNM [# 36875]. Synonymy established by Lindroth (1963b: 301).
Bembidion morosum Casey, 1918: 49. Type locality: «Virginia» (original citation). Lectotype (♂), designated by Lindroth (1975: 118), in USNM [# 36876]. Synonymy established by Lindroth (1963b: 301).

#### Distribution.

The range of this eastern species extends from Cape Breton Island (Lindroth 1954c: 302) to eastern South Dakota (Kirk and Balsbaugh 1975: 17), south to east-central Louisiana (West Feliciana Parish, Igor M. Sokolov pers. comm. 2009), southwestern Mississippi (Drew A. Hildebrandt pers. comm. 2007), and northern Georgia (Fattig 1949: 17). The record from “Kansas” (Bousquet and Larochelle 1993: 134) needs confirmation.

#### Records.

**CAN**: NB, NS (CBI), ON, PE, QC **USA**: AL, AR, CT, DC, DE, GA, IA, IL, IN, KY, LA, MA, MD, ME, MI, MO, MS, NC, NH, NJ, NY, OH, PA, SD, TN, VA, VT, WI, WV [KS]

### Bembidion quadrulum

LeConte, 1861

Bembidium quadrulum LeConte, 1861b: 340. Type locality: «east of Fort Colville [Washington]» (original citation). Lectotype (♂), designated by Erwin (1984a: 180), in MCZ [# 5498].
Bembidion tartareum Casey, 1918: 49. Type locality: «Coeur d’Alene [Kootenai County], Idaho» (original citation). Lectotype (♀), designated by Lindroth (1975: 118), in USNM [# 36877]. Synonymy established by Hatch (1953: 87), confirmed by Lindroth (1963b: 303).
Bembidion callidum Casey, 1918: 50. Type locality: «Truckee [Nevada County], California» (original citation). Lectotype (♂), designated by Erwin (1984a: 180), in USNM [# 36878]. Synonymy established by Erwin (1984a: 180).
Bembidion tritum Casey, 1918: 50. Type locality: «Boulder Co[unty], Colorado» (original citation). Lectotype (♀), designated by Erwin (1984a: 180), in USNM [# 36879]. Synonymy established by Erwin (1984a: 180).
Bembidion aegrotum Casey, 1918: 51. Type locality: «Colorado» (original citation). Lectotype (♂), designated by Erwin (1984a: 180), in USNM [# 36880]. Synonymy established by Erwin (1984a: 180).
Bembidion porrectum Casey, 1918: 55. Type locality: «Las Vegas [San Miguel County], New Mexico» (original citation). Lectotype (♀), designated by Lindroth (1975: 118), in USNM [# 36891]. Synonymy established by Lindroth (1963b: 303).
Bembidion viridinigrum Casey, 1924: 31. Type locality: «Govan [Lincoln County], Washington» (original citation). Lectotype (♂), designated by Lindroth (1975: 118), in USNM [# 36881]. Synonymy established by Hatch (1953: 87), confirmed by Lindroth (1963b: 303).

#### Distribution.

The range of this western species extends from southern Yukon Territory (Lindroth 1963b: 304) south to the Sierra Nevada in California (Casey 1918: 50, as *Bembidion callidum*), northern Arizona (Navajo County, CNC), and northern New Mexico (Casey 1918: 55, as *Bembidion porrectum*) along the Rocky Mountains.

#### Records.

**CAN**: AB, BC (VCI), YT **USA**: AZ, CA, CO, ID, MT, NM, OR, UT, WA, WY

### Bembidion recticolle

LeConte, 1863

Bembidium recticolle LeConte, 1863c: 19. Type locality: «New Mexico» (original citation). One possible syntype in CMNH (collection Ulke). Note. The specimen of *Bembidion recticolle* in MCZ, labeled “6-7,000 ft. Green River City Wyo. July 20-27, 1877” and “type 5499,” is not a syntype.
Bembidium tetragonoderum Chaudoir, 1868b: 240. Type locality: «Californie» (original citation). Lectotype (♀), designated by Lindroth (1963b: 304), in MHNP. Synonymy established by Hayward (1897: 61), confirmed by Lindroth (1963b: 304).
Bembidion oblatum Casey, 1918: 52. Type locality: «Nevada» (original citation). Lectotype (♀), designated by Lindroth (1975: 118), in USNM [# 36888]. Synonymy established by Lindroth (1963b: 304).
Bembidion pertinax Casey, 1918: 53. Type locality: «Nevada» (original citation). Lectotype (♀), designated by Lindroth (1975: 118), in USNM [# 36887]. Synonymy established by Lindroth (1963b: 304).
Bembidion umbraticola Casey, 1918: 54. Type locality: «Provo [Utah County], Utah» (original citation). Lectotype (♀), designated by Lindroth (1975: 118), in USNM [# 36889]. Synonymy established by Lindroth (1963b: 305).

#### Distribution.

This western species is found from south-central Alberta to Vancouver Island, north to the Skeena River drainage in central British Columbia (Lindroth 1963b: 305), south at least to central California in the Coast Ranges (Casey 1918: 53, as *Bembidion tetragonoderum*) and “New Mexico” (Hayward 1897: 61). The record from southeastern Wisconsin (Messer 2010: 36) needs confirmation since the specimen could be mislabeled.

#### Records.

**CAN**: AB, BC (VCI) **USA**: AZ, CA, CO, ID, MT, NM, NV, OR, UT, WA, WY [WI]

### Bembidion salebratum

(LeConte, 1847)

Ochthedromus salebratus LeConte, 1847: 453. Type locality: «LaPointe [Ashland County, Wisconsin], Lacus Superioris» (original citation). Six syntypes in MCZ [# 5495].
Ochthedromus purpurascens LeConte, 1847: 454. Type locality: «Lacum Sabulosam [= probably Sandy Lake, northern Minnesota] prope Mississippi scaturigines» (original citation). One syntype in MCZ [# 5497]. Synonymy established by Lindroth (1963b: 302).
Bembidion inopinum Casey, 1918: 51. Type locality: «Maine» (original citation). Lectotype (♂), designated by Lindroth (1975: 118), in USNM [# 36890]. Synonymy established by Lindroth (1963b: 302).
Bembidion consessor Casey, 1918: 52. Type locality: «Bluff Point, Lake Champlain, New York» (original citation). Lectotype (♀), designated by Lindroth (1975: 118), in USNM [# 36885]. Synonymy established by Lindroth (1963b: 302).
Bembidion mackinacensis Hatch, 1929: 135. Type locality: «Douglas Lake, Mich[igan]» (original citation). Holotype (♂) in USNM. Synonymy established by Lindroth (1963b: 302).

#### Distribution.

This species ranges from Newfoundland (Lindroth 1955a: 56) to the foothills of the Rockies in Alberta and northeastern British Columbia (Lindroth 1963b: 302), south to southeastern Wyoming (Hayward 1897: 60, as *Bembidion concolor*), central Minnesota (Crow Wing and Sherburne Counties, CNC), Vermont (Addison County, CNC), and Maine (Aroostook County, CNC). The record from southern South Dakota (Kirk and Balsbaugh 1975: 17) needs confirmation; those from “Idaho,” “Iowa,” and “Pennsylvania” (Bousquet and Larochelle 1993: 134) are probably in error. Fossil remnants from a Plio-Pleistocene sequence have been unearthed in northwestern Greenland (Böcher 1995: 25).

#### Records.

**CAN**: AB, BC, MB, NB, NF, NS (CBI), NT, ON, QC, SK **USA**: ME, MI, MN, MT, ND, NH, NY, VT, WI, WY [SD]

#### Note.

This species has passed under the name *Bembidion concolor* (Kirby, 1837) until Lindroth (1963b).

### Bembidion subaerarium

Casey, 1924

Bembidion subaerarium Casey, 1924: 31. Type locality: «Blue Lakes, Alpine Co[unty], California» (original citation). Lectotype (♂), designated by Erwin (1984a: 183), in USNM [# 36896].

#### Distribution.

This species is known only from the type locality in the Sierra Nevada in California.

#### Records.

**USA**: CA

### Subgenus Hydriomicrus

Casey, 1918

Hydriomicrus Casey, 1918: 87. Type species: *Leja semistriata* Haldeman, 1843 designated by Lindroth (1963b: 305). Etymology. From the Greek *hydrias* (from water) and *micros* (small, little), possibly alluding to the small size of the adult and the habitat requirement of the species [masculine].

#### Diversity.

Five North American species, two in the east and three in the west.

#### Identification.

Lindroth’s (1963b: 212-229) key to *Bembidion* included all but one species (*Bembidion innocuum*); the two species found in Canada were treated in detail (Lindroth 1963b: 306).

#### Taxonomic Note.

Based on molecular data analyses, Maddison (2012: 568) concluded that this subgenus is closely related to *Hirmoplataphus*.

### Bembidion brevistriatum

Hayward, 1897

Bembidium brevistriatum Hayward, 1897: 58. Type locality: «Dunsmuir, Pomona Mountains, Santa Rosa [in] California» (original citation). Three syntypes in MCZ [# 2009].

#### Distribution.

This species is known from southwestern Oregon (Hatch 1953: 88) to southern California “in the higher mountain cañons or valleys between the ranges” (Fall 1901a: 42).

#### Records.

**USA**: CA, OR

### Bembidion californicum

Hayward, 1897

Bembidium californicum Hayward, 1897: 84. Type locality: «Pom[ona] [Los Angeles County], Cal[ifornia]» (lectotype label). Lectotype (♀), designated by Erwin (1984a: 168), in MCZ [# 16290].

#### Distribution.

This species is known from southwestern Oregon (Josephine County, James R. LaBonte pers. comm. 1992) to the southern part of California where it is “widely distributed” (Fall 1901a: 42). The record from northwestern Montana (Edwards 1975: 51) needs confirmation.

#### Records.

**USA**: CA, OR [MT]

### Bembidion innocuum

Casey, 1918

Bembidion innocuum Casey, 1918: 63. Type locality: «Hoopa Valley, Humboldt Co[unty], California» (original citation). Lectotype (♂), designated by Erwin (1984a: 168), in USNM [# 36894]. Note. This name has been listed in synonymy with *Bembidion californicum* Hayward by Erwin (1984a: 168) but according to Maddison (2012: 535) it is a senior synonym of *Bembidion marinianum* Casey.
Bembidion marinianum Casey, 1924: 29. Type locality: «Marin Co[unty], California» (original citation). Lectotype (♀), designated by Erwin (1984a: 176), in USNM [# 36893]. Synonymy established by Maddison (2012: 535).

#### Distribution.

This species is known only from Del Norte (Maddison 2012: Supplementary content Table S1), Marin (Casey 1924: 29, as *Bembidion marinianum*) and Humboldt (Casey 1918: 63) Counties in coastal California.

#### Records.

**USA**: CA

### Bembidion quadratulum

Notman, 1920

Bembidium quadratulum Notman, 1920a: 296. Type locality: «Moss Pond, M[ount] Redfield, Essex Co[unty], N[ew] Y[ork]» (original citation). Holotype [by monotypy] (♀) in SIM (Hennessey 1990: 466).
Bembidium proximum Notman, 1920a: 297. Type locality: «Moss Pond, M[ount] Redfield, Essex Co[unty], N[ew] Y[ork]» (original citation). Holotype [by monotypy] (♂) in SIM (Hennessey 1990: 466). Synonymy established by Lindroth (1963b: 306).

#### Distribution.

This species ranges from Newfoundland (Lindroth 1955a: 50) to the Laurentides region in Quebec (Larochelle 1975: 60), south to southwestern (Hardin County, CMNH) and eastern Tennessee (Johnson, Morgan, Roane, and Union Counties, CMNH).

#### Records.

**CAN**: NB, NF, NS (CBI), QC **USA**: CT, ME, NH, NY, TN, VT

### Bembidion semistriatum

(Haldeman, 1843)

Leja semistriata Haldeman, 1843b: 303. Type locality: southeastern Pennsylvania (Haldeman 1843a: 298). Syntype(s) presumably lost.

#### Distribution.

The range of this eastern species extends from Nova Scotia (Lindroth 1954c: 302) to west-central Indiana (Blatchley 1910: 79), including southeastern Michigan (Wayne County, CMNH), south to northwestern Arkansas (Newton County, CNC, UASM), northeastern Mississippi (Tishomingo County, Drew A. Hildebrandt pers. comm. 2010), and southern Georgia (Torres and Ruberson 2006: 31).

#### Records.

**CAN**: NB, NS, ON, QC **USA**: AR, CT, DC, GA, IN, KY, MA, MD, ME, MI, MS, NC, NH, NJ, NY, OH, PA, RI, TN, VA, VT, WV

### Subgenus Odontium

LeConte, 1847

Odontium LeConte, 1847: 452. Type species: *Bembidium coxendix* Say, 1823 designated by Jeannel (1941b: 542). Etymology. From the Greek *odontos* (tooth), alluding to the mentum tooth (“*mentum dente longissimo, convexo, subobtuso*”) of the adult [neuter].
Ocys Gistel, 1848a: xi [junior homonym of *Ocys* Stephens, 1828]. Type species: *Elaphrus striatus* Fabricius, 1792 by monotypy. Synonymy established by Bousquet (2002b: 35). Etymology. From the Geek *ocys* (swift, quick) [masculine].
Cylindrobracteon Netolitzky, 1939: 7, 16. Type species: *Bembidion fusiforme* Netolitzky, 1914 by original designation. Synonymy established by Maddison (1993: 160). Etymology. From the Greek *cylindros* (roller, cylinder) and the generic name *Bracteon* [*q.v*.] [neuter].

#### Diversity.

Twenty-three species in the Nearctic (ten species), Neotropical (one species also present in southern Arizona), Palaearctic (12 species), and Oriental (one species, *Bembidion subfusum* Darlington, in the Philippines) Regions.

#### Identification.

Lindroth (1963b: 241-246, as *bowditchi* and *coxendix* groups) treated all the species found in North America, *Bembidion durangoense* under the name *Bembidion arizonae*. One species, *Bembidion paraenulum*, was subsequently described.

### Bembidion aenulum

Hayward, 1901

Bembidium aenulum Hayward, 1901: 156. Type locality: «Cedar Co[unty], Iowa» (original citation). Holotype [by monotypy] (♀) in MCZ [# 2006].

#### Distribution.

This species is known from eastern South Dakota to southwestern Nebraska, east to eastern Iowa [see Maddison and Arnold 2009: Fig. 9]. Two specimens simply labeled “Missouri” and “Wisconsin” (Maddison and Arnold 2009: 60) are known.

#### Records.

**USA**: IA, NE, SD [MO, WI]

### Bembidion bowditchii

LeConte, 1878

Bembidium bowditchii LeConte, 1878a: 451. Type locality: «Green River City (6,000 to 7,000 feet) [Sweetwater County], Wyoming» (original citation). Three syntypes in MCZ [# 5492].

#### Distribution.

This species is known from a few localities in southern British Columbia (Lindroth 1963b: 242), northwestern Washington (Maddison 2012: Supplementary content Table S1), “Idaho” (Maddison 1993: 161), western Montana (Russell 1968: 49), and southwestern Wyoming (Hayward 1897: 51).

#### Records.

**CAN**: BC **USA**: ID, MT, WA, WY

### Bembidion carinatum

(LeConte, 1852)

Odontium carinatum LeConte, 1852a: 186. Type locality: «ad flumen Colorado» (original citation). Two syntypes in MCZ [# 97].

#### Distribution.

This species is known from a few localities in southeastern California and southern Arizona (Lindroth 1963b: 245; Dajoz 2007: 19).

#### Records.

**USA**: AZ, CA

### Bembidion confusum

Hayward, 1897

Bembidium nitidulum Dejean, 1831: 84 [secondary homonym of *Bembidion nitidulum* (Marshall, 1802)]. Type locality: «Amérique septentrionale» (original citation), restricted to «Grand Bend, Ont[ario]» by Lindroth (1963b: 245). Holotype [by monotypy] (♀) in MHNP (Lindroth 1955b: 13).
Bembidium confusum Hayward, 1897: 52. Replacement name for *Bembidium nitidulum* Dejean, 1831.
Bembidion confusum aeneorubrum Casey, 1918: 11. Type locality: «Ohio» (original citation). Lectotype (♀), designated by Lindroth (1975: 115), in USNM [# 36804]. Synonymy established (as aberration) by Csiki (1928: 38), confirmed by Lindroth (1963b: 245).
Bembidion confusum marquettense Casey, 1918: 11. Type locality: «Marquette [Marquette County], Michigan» (original citation). Lectotype (♀), designated by Lindroth (1975: 115), in USNM [# 36805]. Synonymy established (as aberration) by Csiki (1928: 38), confirmed by Lindroth (1963b: 245).

#### Distribution.

This species is found east of the Rocky Mountains from New Brunswick (Queens County, Reginald P. Webster pers. comm. 2008) to southeastern Alberta (Lindroth 1963b: 245), south to southeastern Colorado (Maddison 1985: 111), northwestern (Jones County, CNC) and east-central (Riley 2011) Texas, and central Florida (Peck and Thomas 1998: 18).

#### Records.

**CAN**: AB, MB, NB, ON, QC, SK **USA**: AL, AR, CO, CT, DC, DE, FL, GA, IA, IL, IN, KS, KY, LA, MA, MD, ME, MI, MN, MO, MS, ND, NE, NH, NJ, NY, OH, OK, PA, RI, SC, SD, TN, TX, VA, VT, WI

### Bembidion coxendix

Say, 1823

Bembidium coxendix Say, 1823b: 151. Type locality: «F[or]t Pierre [Stanley County], [South] Dakota» (neotype label). Neotype (♂), designated by Lindroth and Freitag (1969: 335), in MCZ [# 33071].
Bembidion snowi Casey, 1918: 12. Type locality: «Kansas» (original citation). Lectotype (♂), designated by Lindroth (1975: 115), in USNM [# 36803]. Synonymy established by Lindroth (1963b: 243).
Bembidion unicum Casey, 1918: 12. Type locality: «Laredo [Webb County], Texas» (original citation). Lectotype (♀), designated by Lindroth (1975: 115), in USNM [# 36798]. Synonymy established by Lindroth (1963b: 243).
Bembidion venator Casey, 1918: 12. Type locality: «El Paso [El Paso County], Texas» (original citation). Lectotype (♀), designated by Lindroth (1975: 115), in USNM [# 36801]. Synonymy established by Lindroth (1963b: 243).
Bembidion vigilans Casey, 1918: 13. Type locality: «probably Indiana» (original citation). Lectotype (♂), designated by Lindroth (1975: 115), in USNM [# 36802]. Synonymy established by Lindroth (1963b: 243).
Bembidion weesi Hatch and Ortenburger, 1930: 11. Type locality: «Little River, Cleveland Co[unty], Okla[homa]» (original citation). Holotype (♂) in USNM. Synonymy established by Lindroth (1963b: 243). Etymology. The specific name was proposed for Asa Orrin Weese [1885-1955], professor of zoology at the University of Oklahoma. Weese was mainly interested in community ecology and succession.

#### Distribution.

The range of this species extends from southwestern and north-central Pennsylvania (Westmoreland and Lycoming Counties, Robert L. Davidson pers. comm. 2008) to Alberta (Lindroth 1963b: 244), south to central New Mexico (Fall and Cockerell 1907: 157; Socorro County, UASM), southern Texas (Snow 1906a: 141; Casey 1918: 12, as *Bembidion unicum*; Cameron and Victoria Counties, MCZ, UASM), northeastern Louisiana (Franklin Parish, Igor M. Sokolov pers. comm. 2009), northwestern Mississippi (Bolivar County, Foster F. Purrington pers. comm. 2012), and southwestern Georgia (Fattig 1949: 16). The records from “New Hampshire,” “Vermont,” “Idaho,” and “Northwest Territories” (Bousquet and Larochelle 1993: 126) are in error; that from Connecticut (Krinsky and Oliver 2001: 70) was based on misidentified specimens of *Bembidion confusum* (William L. Krinsky pers. comm. 2009).

#### Records.

**CAN**: AB, MB, SK **USA**: AR, CO, DC, GA, IA, IL, IN, KS, LA, MI, MN, MO, MS, MT, NC, ND, NE, NM, OH, OK, PA, SC, SD, TN, TX, WI, WY

### Bembidion durangoense

Bates, 1891

Bembidium durangoense Bates, 1891a: 263. Type locality: «Villa Lerdo in Durango» (original citation). Lectotype (♀), designated by Erwin (1984a: 171), in BMNH.
Bembidion arizonae Lindroth, 1963b: 246. Type locality: «San Carlos, Gila R[iver] Valley, Ariz[ona]» (original citation). Holotype (♂) in MCZ [# 34747]. **New synonymy** (David R. Maddison pers. comm. 2007).

#### Distribution.

This species is known from southern Arizona (Lindroth 1963b: 246; Dajoz 2007: 21) and northern Mexico (Bates 1891a: 263).

#### Records.

**USA**: AZ – Mexico

### Bembidion gilae

Lindroth, 1963

Bembidion gilae Lindroth, 1963b: 246. Type locality: «San Carlos, Gila R[iver] Valley, Ariz[ona]» (original citation). Holotype (♂) in MCZ [# 32536].

#### Distribution.

This species is known from the Gila River drainage in southern Arizona.

#### Records.

**USA**: AZ

#### Note.

This taxon has been listed in synonymy with *Bembidion durangoense* Bates by Erwin (1984a: 171) but according to David R. Maddison (pers. comm. 2007) it represents a distinct species.

### Bembidion paraenulum

Maddison, 2009

Bembidion paraenulum Maddison [in Maddison and Arnold], 2009: 56. Type locality: «Big Otter River at route 24, Bedford Co[unty], Virginia» (original citation). Holotype (♂) in USNM.

#### Distribution.

This species ranges from southern New Hampshire south to northern Florida [see Maddison and Arnold 2009: Fig. 9], west to eastern Texas (Hardin County, Robert L. Davidson pers. comm. 2012), including eastern Louisiana (East Feliciana Parish, Igor M. Sokolov pers. comm. 2009).

#### Records.

**USA**: AL, FL, GA, LA, MS, NC, NH, TX, VA

### Bembidion robusticolle

Hayward, 1897

Bembidium robusticolle Hayward, 1897: 50. Type locality: «Iowa; Michigan; Kansas» (original citation). Two syntypes in MCZ [# 2007].

#### Distribution.

This species is known from a few scattered localities from Connecticut (Lindroth 1963b: 243; Krinsky and Oliver 2001: 82) and northern Vermont (Lamoille County, Ross T. Bell pers. comm. 2008) to western North Dakota (McKenzie County, Donald P. Schwert pers. comm. 1989), south at least to “Kansas” (Hayward 1897: 50) and “Kentucky” (Lindroth 1963b: 243).

#### Records.

**USA**: CT, IA, IL, KS, KY, MI, MO, ND, VT, WI

### Bembidion sculpturatum

(Motschulsky, 1859)

Odontium sculpturatum Motschulsky, 1859a: 132. Type locality: «Col[onie] Ross [farming community about 75 miles north of San Francisco along the coast, California]» (original citation). One syntype in MCZ (Lindroth 1963b: 245).

#### Distribution.

This species is known from eastern Washington (Hatch 1953: 80) to central California (Lindroth 1963b: 245).

#### Records.

**USA**: CA, OR, WA

### Subgenus Bracteon

Bedel, 1879

Bracteon Bedel, 1879: 27. Type species: *Elaphrus litoralis* Olivier, 1790 designated by Bedel (1881: xxiii). Etymology. From the Latin *bractea* (bract, i.e., a specialized leaf very different from the other ones), possibly alluding to the distinctness of adults of this taxon compared to other members of the genus *Bembidion* [neuter].
Chrysobracteon Netolitzky, 1914: 166. Type species: *Carabus velox* Linnaeus, 1760 designated by Netolitzky (1939: 7). Synonymy established by Maddison (1993: 161). Etymology. From the Greek *chrysos* (gold) and the generic name *Bracteon* [*q.v*.] [neuter].
Parabracteon Notman, 1929a: 157. Type species: *Bembidion tuberculatum* Notman, 1929 (= *Bembidium carinula* Chaudoir, 1868) by monotypy. Synonymy established with the name *Chrysobracteon* Netolitzky by Lindroth (1963b: 230). Etymology. From the Greek *para* (near, next to) and the generic name *Bracteon* [*q.v*.] [neuter].
Litoreobracteon Netolitzky, 1939: 7, 17. Type species: *Elaphrus litoralis* Olivier, 1790 by original designation. Synonymy established with the name *Chrysobracteon* Netolitzky by Lindroth (1962: 1). Etymology. From the Latin *litoreus* (of the seashore, littoral) and the generic name *Bracteon* [*q.v*.] [neuter].
Argyrobracteon Netolitzky, 1939: 7, 19. Type species: *Bembidion argenteolum* Ahrens, 1812 by original designation. Synonymy established with the name *Chrysobracteon* Netolitzky by Lindroth (1962: 1). Etymology. From the Greek *argyros* (silver) and the generic name *Bracteon* [*q.v*.] [neuter].
Conicibracteon Netolitzky, 1939: 7, 19. Type species: *Bembidium stenoderum* Bates, 1873 by original designation. Synonymy established with the name *Chrysobracteon* Netolitzky by Lindroth (1962: 1). Etymology. From the Greek *conicos* (conelike) and the generic name *Bracteon* [*q.v*.] [neuter].
Stylobracteon Netolitzky, 1939: 8, 20. Type species: *Bembidion baikaloussuricum* Netolitzky, 1939 (= *Bembidium conicolle* Motschulsky, 1845) by original designation. Synonymy established with the name *Chrysobracteon* Netolitzky by Lindroth (1962: 1). Etymology. From the Greek *stylos* (pillar, column) and the generic name *Bracteon* [*q.v*.] [neuter].
Foveobracteon Netolitzky, 1939: 21. Type species: *Bembidium foveum* Motschulsky, 1844 by original designation. Synonymy established with the name *Chrysobracteon* Netolitzky by Lindroth (1962: 1). Etymology. From the Latin *fovea* (pit, foveole) and the generic name *Bracteon* [*q.v*.] [neuter].

#### Diversity.

Northern Hemisphere, with 17 species in the Nearctic (11 species) and Palaearctic (nine species) Regions. Three species are Holarctic.

#### Identification.

Maddison (1993) revised the species of this subgenus and provided a key for their identification.

### Bembidion alaskense

Lindroth, 1962

Bembidion alaskense Lindroth, 1962: 9. Type locality: «Grants Cabin, Toms L[ake] [= Lake Brooks], Alaska» (original citation). Holotype (♂) in CAS [# 9307].
Bembidion colvillense Lindroth, 1965: 126. Type locality: «Umiat, Alaska» (original citation). Holotype (♂) in MCZ [# 32753]. Synonymy established by Maddison (1993: 177).

#### Distribution.

This Holarctic species is known from the region of Lake Baikal in Siberia and Alaska, from the Brooks Range south to the Alaska Peninsula [see Maddison 1993: Fig. 264]. Fossil remnants from a Plio-Pleistocene sequence have been found in northwestern Greenland (Böcher 1995: 24).

#### Records.

**USA**: AK – **Holarctic**

### Bembidion balli

Lindroth, 1962

Bembidion balli Lindroth, 1962: 15. Type locality: «[Fort] McMurray, Alberta» (original citation). Holotype (♂) in CNC [# 8375].

#### Distribution.

This species is known only from west-central Saskatchewan and eastern Alberta [see Maddison 1993: Fig. 261]. The record from Churchill, Manitoba (Elias 1984: 142) is based on misidentified *Bembidion carinula* (Maddison 1993: 174). Fossil remnants, dated about 13,200 years B.P., have been unearthed in southern Ontario (Karrow et al. 2007: 290).

#### Records.

**CAN**: AB, SK

### Bembidion carinula

Chaudoir, 1868

Bembidium carinula Chaudoir, 1868b: 239. Type locality: «Terre de Rupert [former name for the entire drainage basin of Hudson’s Bay, thus including most of northern Quebec, Ontario, Manitoba, etc. (Lamb 1971)]»; herein restricted to Fort Rupert (formerly Rupert House), Quebec (CNC). Lectotype (♂), designated by Lindroth (1963b: 237), in MHNP.
Bembidion tuberculatum Notman, 1929a: 157. Type locality: «Marquette [Marquette County], Mich[igan]» (original citation). Holotype (♀) in SIM (Hennessey 1990: 466). Synonymy established by Lindroth (1962: 12).

#### Distribution.

A common species ranging from Newfoundland to southwestern British Columbia, north to central Yukon Territory and the Ungava Bay in Quebec, south to northern Iowa and southwestern Pennsylvania [see Maddison 1993: Fig. 265]. The records from “Georgia,” “Arkansas,” “Oregon” and Vancouver Island (Hayward 1897: 46) are probably in error; that from “Kentucky” (Maddison 1993: Fig. 265) needs confirmation. Lindroth’s (1963b: 237) record from Washington is based on a misidentified *Bembidion lapponicum* (Maddison 1993: 182). Three specimens labeled “Or.,” “Tex,” and from Pena Blanca in Arizona seen by Maddison (1993: 182) are probably mislabeled.

#### Records.

**CAN**: AB, BC, LB, MB, NB, NF, NT, NU, ON, QC, SK, YT **USA**: CT, IA, IL, IN, MA, ME, MI, MN, NH, NJ, NY, OH, PA, VT, WI [KY]

### Bembidion foveum

Motschulsky, 1844

Bembidium foveum Motschulsky, 1844: 271. Type locality: «bords du Lac Baïcal [Irkutsk Oblast, Russia]» (original citation). Lectotype (♂), designated by Lafer (1975: 59), in ZMMU.
Bembidion bryanti Carr, 1932: 191 [primary homonym of *Bembidion bryanti* Andrewes, 1921]. Type locality: «above Sans Sault rapids, 70 miles below Fort Norman [Northwest Territories]» (original citation). Holotype (♂) in CNC [# 3435]. Synonymy established by Lindroth (1962: 16).
Bembidion beringi Netolitzky, 1939: 21. Type locality: «Petropawlowsk, Kamtschatka [Russia]» (original citation). Holotype (♂) in NRSS. Synonymy established by Lindroth (1962: 16).
Bembidion grahami Hatch, 1951: 114. Type locality: «Finlay Forks, B[ritish] C[olumbia]» (original citation). Holotype (♂) in USNM. Synonymy established by Lindroth (1962: 16). Etymology. The specific name was proposed in honor of Roy Graham [1908-1939], paleobotanist and assistant geologist in British Columbia.

#### Distribution.

This Holarctic species ranges in the Palaearctic Region from northeastern Europe to Kamchatka, and in the Nearctic Region, from Alaska east to Churchill, Manitoba, and south to central Saskatchewan [see Maddison 1993: Fig. 262a].

#### Records.

**CAN**: BC, MB, NT, SK, YT **USA**: AK – **Holarctic**

#### Note.

*Bembidion glabriusculum* Motschulsky, 1844, listed as a questionable synonym of this species by Lindroth (1962: 16), is a junior synonym of *Bembidion argenteolum* Ahrens (Maddison 1993: 176).

### Bembidion hesperium

Fall, 1910

Bembidium hesperium Fall, 1910: 95 (as *hesperum*). Type locality: «Vancouver Island [British Columbia]» (original citation), herein restricted to Duncan (CNC). Holotype (♂) in MCZ [# 23862]. Note. The name *Bembidion hesperium* has been credited to Casey (1918: 9) by some authors on the basis that Casey proposed a replacement name for *Bembidium hesperum* Fall, 1910, supposedly a junior primary homonym of *Bembidion hesperus* Crotch, 1867. In fact, Casey (1918) emended Fall’s name to *Bembidion hesperium* since the name *Bembidion hesperum* is an incorrect latinization for *Bembidion hesperium* (adjective) or *Bembidion hesperus* (noun in apposition). Casey’s emendation is currently in prevailing usage and so deemed to be a justified emendation (ICZN 1999: Article 33.2.3.1).
Bembidion binarium Casey, 1918: 9. Type locality: «California» (original citation). Lectotype (♂), designated by Lindroth (1975: 115), in USNM [# 36796]. Synonymy established by Lindroth (1962: 8).

#### Distribution.

This western species ranges from southeastern British Columbia to Vancouver Island, south to east-central California [see Maddison 1993: Fig. 269].

#### Records.

**CAN**: BC (VCI) **USA**: CA, OR, WA

### Bembidion inaequale

Say, 1823

Bembidium inaequalis Say, 1823b: 151. Type locality: «M[oun]t Pleasant [Henry County], I[ow]a» (neotype label). Neotype (♂), designated by Lindroth and Freitag (1969: 335), in MCZ [# 33073]. Note. «near Engineer Cantonment [winter quarter along the west bank of the Missouri River north of modern Omaha, Nebraska]» was the area originally cited by Say (1823b: 152).
Bembidium arenarium Dejean, 1831: 80. Type locality: «Amérique septentrionale» (original citation). One syntype in MHNP (Lindroth 1955b: 13). Synonymy established by LeConte (1847: 452), confirmed by Lindroth (1955b: 13).
Bembidium lacustre LeConte, 1847: 451. Type locality: «Lacum Superiorem» (original citation). Lectotype [as holotype] (♀), designated by Maddison (1993: 191), in MCZ [# 5491]. Synonymy established by Hatch (1953: 81), confirmed by Lindroth (1954b: 124).
Bembidion opaciceps Casey, 1918: 8. Type locality: «California» (original citation). Lectotype (♀), designated by Lindroth (1975: 115), in USNM [# 36794]. Synonymy established by Lindroth (1962: 7).

#### Distribution.

This species ranges from Cape Breton Island to western Alaska, south to northwestern California, south-central Colorado, northern Kansas, southeastern Louisiana (East Baton Rouge Parish, Igor M. Sokolov pers. comm. 2009), northern Alabama, and northern Georgia [see Maddison 1993: Fig. 273]. The records from “Florida,” “Arkansas,” and “Texas” (Hayward 1897: 44) are probably in error.

#### Records.

**CAN**: AB, BC (QCI, VCI), MB, NB, NS (CBI), ON, PE, QC, SK, YT **USA**: AK, AL, CA, CO, CT, DC, GA, IA, ID, IL, IN, KS, KY, LA, MA, MD, ME, MI, MN, MO, MS, MT, NC, ND, NE, NH, NJ, NV, NY, OH, OR, PA, SD, TN, UT, VA, VT, WA, WI, WV, WY

### Bembidion lapponicum

Zetterstedt, 1828

Bembidium impressum var. *lapponicum* Zetterstedt, 1828: 6. Type locality: «flumen Tornense [= Torne-älve] prope Wittangi [Sweden]» (original citation). Lectotype (♂), designated by Lindroth (1963b: 239), in ZMLS.
Bembidium latiusculum Motschulsky, 1844: 272. Type locality: «Daurie et Kamtchatka» (original citation), restricted to «Dauria [= Transbaikalia, Siberia, Russia]» by Lindroth (1962: 13). Syntype(s) location unknown (possibly in ZMMU though not listed in Keleinikova 1976). Synonymy established by Lindroth (1962: 13).
Bembidium lapponicum C.G. Thomson, 1857: 18 [primary homonym of *Bembidium impressum* var. *lapponicum* Zetterstedt, 1828]. Type locality: «Torneå-elf nära Vittangi [Sweden]» (original citation). Syntype(s) in ZMLS (Charpentier 1972: 290). **New synonymy**. Note. Thomson (1857: 18) proposed this name for a new species referring to var.b of *Bembidium impressum* cited byZetterstedt (1837: 23), a transcription of Zetterstedt (1828: 6). Apparently he did not consider the italicized word “*lapponicum*” that followed “Var. b.” as a scientific name for the variety.
Bembidium jenisseense J.R. Sahlberg, 1880: 14. Type locality: «[Verkhne-]Imbatsk [Lower Yenisei, Russia]» (original citation for the lectotype). Lectotype (♀), designated by Lindroth (1962: 13), in ZMUT. Synonymy established by Lindroth (1939b: 71).
Bembidium pugetanum Fall, 1916: 13. Type locality: «Seattle [King County], Washington» (original citation). Holotype (♂) in MCZ [# 23865]. Synonymy established by Lindroth (1962: 13).

#### Distribution.

This Holarctic species ranges in the Palaearctic Region from northernmost Scandinavia to Kamchatka, south to Mongolian Peoples’ Republic. In North America, it occurs commonly in the northwest from Alaska to the Anderson River; it is also found at scattered localities in Washington, northern Oregon, Idaho, and Wyoming, and in western Canada east to central Saskatchewan [see Maddison 1993: Fig. 267a].

#### Records.

**CAN**: AB, BC, NT, SK, YT **USA**: AK, ID, OR, WA, WY – **Holarctic**

### Bembidion levettei carrianum

Casey, 1924

Bembidion carrianum Casey, 1924: 23. Type locality: «Edmonton, Alberta» (original citation). Lectotype (♀), designated by Lindroth (1975: 115), in USNM [# 36800].

#### Distribution.

This transamerican subspecies ranges from western Newfoundland (Lindroth 1955a: 47) and Labrador to western Alaska, south to west-central British Columbia, southeastern New Mexico, southern Wisconsin, southwestern Michigan, central New York, and southeastern New Brunswick [see Maddison 1993: Fig. 272].

#### Records.

**CAN**: AB, BC, LB, MB, NB, NF, NS (CBI), NT, NU, ON, QC, SK, YT **USA**: AK, CO, ME, MI, MN, ND, NH, NM, NY, VT, WI

### Bembidion levettei levettei

Casey, 1918

Bembidion levettei Casey, 1918: 9. Type locality: «Colorado» (original citation). Lectotype (♂), designated by Lindroth (1975: 114), in USNM [# 36793]. Etymology. The specific name was proposed for Dr. Gilbert M. Levette who worked as assistant for the Indiana Geological Survey in Indianapolis. Levette collected natural history specimens, particularly shells, beetles, reptiles and amphibians. His collection of Coleoptera was purchased by Thomas Casey in 1890.

#### Distribution.

The range of this subspecies extends from central Washington to southwestern Alberta, south to northern New Mexico [see Maddison 1993: Fig. 272]. One specimen labeled “Cal.” is known (Maddison 1993: 191). Fossil remnants from a Plio-Pleistocene sequence have been unearthed in northwestern Greenland and Banks Island (Böcher 1995: 23).

#### Records.

**CAN**: AB, BC **USA**: CO, ID, MT, NM, WA, WY

#### Note.

This species has long been known in the North American literature under the name *Bembidion lit[t]orale* (Olivier).

### Bembidion lorquinii

Chaudoir, 1868

Bembidium lorquinii Chaudoir, 1868b: 239. Type locality: «Californie» (original citation), herein restricted to Klamath River at Orleans, Humboldt County (CNC). Lectotype (♂), designated by Lindroth (1963b: 235), in MHNP. Etymology. This name was proposed for Pierre Lorquin [1797-1873], a French naturalist traveller who visited several places in the world to collect insects. He was one of the earliest to collect intensively in California where he arrived during the gold excitement in 1850. He stayed in the state until 1859 when he left for China.
Bembidion tacomae Casey, 1924: 22. Type locality: «Wawawai [Whitman County], Washington» (original citation). Lectotype (♂), designated by Lindroth (1975: 115), in USNM [# 36797]. Synonymy established by Lindroth (1962: 9).

#### Distribution.

This western species is found from southwestern British Columbia, including Vancouver Island, to western Idaho, south to southwestern California [see Maddison 1993: Fig. 270]. The records from Wyoming (LeConte 1878a: 465), Colorado (Wickham 1902: 232; Lindroth 1963b: 236), and “Montana” (Hayward 1897: 46) are probably in error.

#### Records.

**CAN**: BC (VCI) **USA**: CA, ID, NV, OR, WA

### Bembidion punctatostriatum

Say, 1823

Bembidium punctato-striatum Say, 1823a: 83. Type locality: «Rumney [Grafton County], N[ew] H[ampshire]» (neotype label). Neotype (♂), designated by Lindroth and Freitag (1969: 335), in MCZ [# 33072].
Bembidium sigillare Say, 1830c: 24 [*nomen dubium*]. Type locality: «Missouri [Territory]» (original citation). Syntype(s) lost. Synonymy established with doubt by LeConte (1857a: 4).
Bembidium stigmaticum Dejean, 1831: 83. Type locality: «Amérique septentrionale» (original citation). Holotype [by monotypy] in MHNP (Lindroth 1955b: 13). Synonymy established, under the name *Bembidium sigillare* Say, by LeConte (1847: 451), confirmed by Lindroth (1955b: 13).

#### Distribution.

This species shows a disjunct distribution. In the east, it ranges from Nova Scotia (Majka et al. 2007: 8), Maine, and southern Quebec south to the District of Columbia and Kentucky; in the west it occurs from western North Dakota and Saskatchewan to southwestern British Columbia, north to northern Northwest Territories and southwestern Alaska (Elias 1988: 41) [see Maddison 1993: Fig. 268]. I have also seen one specimen from northwestern Colorado (Eagle County, CMNH). The records from “Missouri,” “Arkansas” (Hayward 1897: 47), southwestern Iowa (Wickham 1911b: 6), “Washington” and “Oregon” (Hatch 1953: 81) need confirmation; that from “California” (Hayward 1897: 47) is probably in error.

#### Records.

**CAN**: AB, BC, NB, NS, NT, ON, QC, SK, YT **USA**: AK, CO, CT, DC, IN, KY, MA, ME, MI, MT, ND, NH, NJ, NY, OH, PA, VT, WV [AR, IA, MO, OR, WA]

### Bembidion zephyrum

Fall, 1910

Bembidium zephyrum Fall, 1910: 96. Type locality: «Humboldt County, California» (original citation). Holotype (♂) in MCZ [# 23870].
Bembidion zephyrum tristiculum Casey, 1924: 22. Type locality: «southern Oregon» (original citation). Lectotype (♂), designated by Lindroth (1975: 115), in USNM [# 36795]. Synonymy established by Hatch (1953: 81), confirmed by Lindroth (1962: 6).
Bembidion marginosum Casey, 1924: 23. Type locality: «Del Norte Co[unty], California» (original citation). Lectotype (♂), designated by Lindroth (1975: 115), in USNM [# 36799]. Synonymy established by Lindroth (1962: 6).
Bembidion zephyrium Bousquet and Larochelle, 1993: 125. Unjustified emendation of *Bembidion zephyrum* Fall, 1910.

#### Distribution.

This species ranges from the Queen Charlotte Islands (Kavanaugh 1992: 62) south to San Francisco, California; it is mainly found on the Pacific Coast though a few inland localities are also known [see Maddison 1993: Fig. 271]. The record from Mono County in eastern California (Dajoz 2007: 17) needs confirmation.

#### Records.

**CAN**: BC (QCI, VCI) **USA**: CA, OR, WA

### Subgenus Ochthedromus

LeConte, 1847

Ochthedromus LeConte, 1847: 453. Type species: *Bembidium americanum* Dejean, 1831 designated by Lindroth (1963b: 249). Etymology. From the Greek *ochthos* (bank) and *dromos* (running), alluding to the habitat where the species of this taxon are found [masculine].

#### Diversity.

Three North American species.

#### Identification.

Lindroth (1963b: 249-250) recognized two species, *Bembidium americanum* and *Bembidium bifossulatum*, in this subgenus and treated both in his monograph. Maddison (1993: 160) showed that one of the species, *Bembidium bifossulatum*, consisted of two distinct forms which he treated as subspecies. Subsequently (Maddison 2012: 541) he listed them as distinct species.

### Bembidion americanum

Dejean, 1831

Bembidium americanum Dejean, 1831: 87. Type locality: «Amérique septentrionale» (original citation), restricted to «W[est] Roxbury [Suffolk County], Mass[achusetts]» by Lindroth (1963b: 249). One syntype in MHNP (Lindroth 1955b: 13).
Bembidion illini Casey, 1918: 15. Type locality: «Keokuk [Lee County], Iowa» (original citation). Lectotype (♀), designated by Lindroth (1975: 115), in USNM [# 36811]. Synonymy established by Lindroth (1963b: 249).

#### Distribution.

This species ranges from southern New Brunswick (Webster and Bousquet 2008: 16) to southwestern Wisconsin (Richland County, Peter W. Messer pers. comm. 2008), south to eastern Texas (Sabine County, Brian Raber pers. comm. 2010; Riley 2011) and central Florida (Peck and Thomas 1998: 18). The record from “Minnesota” (Bousquet and Larochelle 1993: 126) needs confirmation.

#### Records.

**CAN**: NB, ON, QC **USA**: AL, AR, CT, DC, DE, FL, GA, IA, IL, IN, KS, KY, LA, MA, MD, ME, MI, MO, MS, NC, NH, NJ, NY, OH, OK, PA, RI, SC, TN, TX, VA, VT, WI, WV [MN]

### Bembidion bifossulatum

(LeConte, 1852)

Ochthedromus bifossulatus LeConte, 1852a: 186. Type locality: «San Diego [San Diego County, California]» (original citation). Two syntypes in MCZ [# 98].
Bembidion regestum Casey, 1918: 16. Type locality: «probably Kansas» (original citation). Lectotype (♂), designated by Lindroth (1975: 115), in USNM [# 36809]. Synonymy established by Lindroth (1963b: 250), confirmed by Maddison (1993: 160).
Bembidion ferreum Casey, 1924: 24. Type locality: «Iron Co[unty], Utah» (original citation). Lectotype (♀), designated by Lindroth (1975: 115), in USNM [# 36806]. Synonymy established by Lindroth (1963b: 250), confirmed by Maddison (1993: 160).

#### Distribution.

This species ranges from southern British Columbia, including Vancouver Island (Lindroth 1963b: 250), south to southern California (LeConte 1852a: 186) and southwestern Utah (Casey 1918: 14, as *Bembidion ferreum*). The previous records from other states and provinces refer to *Bembidion cheyennense*.

#### Records.

**CAN**: BC (VCI) **USA**: CA, ID, NV, OR, UT, WA

#### Note.

If the lectotypes currently associated with the labels for *Bembidion regestum* and *Bembidion sufflatum* are the original ones, then both type localities are in error. However, both lectotypes have been dissected and remounted in the last decade and possibly the specimens were not properly reassociated with the labels.

### Bembidion cheyennense

Casey, 1918

Bembidion cheyennense Casey, 1918: 15. Type locality: «Cheyenne [Laramie County], Wyoming» (original citation). Lectotype (♂), designated by Lindroth (1975: 115), in USNM [# 36808].
Bembidion sufflatum Casey, 1918: 15. Type locality: «California» (original citation). Lectotype (♂), designated by Lindroth (1975: 115), in USNM [# 36807]. Synonymy established by Maddison (1993: 160). Note. See “Note” section under the previous species.
Bembidion nuperum Casey, 1918: 16. Type locality: «undoubtedly Colorado» (original citation). Holotype [by monotypy] (♂) in USNM [# 36810]. Synonymy established by Maddison (1993: 160).

#### Distribution.

The range of this species extends from Nova Scotia (Majka et al. 2007: 7) to the Rocky Mountains in Alberta (Lindroth 1963b: 250, as *Bembidion bifossulatum*), south to southern Arizona (Pima and Graham Counties, CMNH), Durango in Mexico (CNC), southeastern Missouri (Wayne County, CNC), northern Ohio (Lee 1994: 58, as *Bembidion bifossulatum*), and northwestern Pennsylvania (Erie County, CMNH). The record from “Arkansas” (Bousquet and Larochelle 1993: 127, as *Bembidion bifossulatum*) needs confirmation.

#### Records.

**CAN**: AB, MB, NS, ON, QC, SK **USA**: AZ, CO, IA, KS, MI, MN, MO, MT, ND, NE, NH, NM, OH, OK, PA, SD, TX, VT, WI, WY [AR] – Mexico

### Subgenus Pseudoperyphus

Hatch, 1950

Pseudoperyphus Hatch, 1950: 100. Type species: *Bembidium chalceum* Dejean, 1831 by original designation. Etymology. From the Greek *pseudos* (fallacy, lie) and the generic name *Peryphus* [*q.v*.] [masculine].
Bracteomimus Lindroth, 1955a: 49. Type species: *Bembidium chalceum* Dejean, 1831 by original designation. Etymology. From the generic name *Bracteon* [*q.v*.] and the Latin *mimus* or the Greek *mimos* (actor) [masculine].

#### Diversity.

Nine North American species, one of them extending into northern Mexico.

#### Identification.

Maddison (2008) revised the species and provided a key for their identification. Some of the species are very difficult to recognize on external characters and the male genitalia must be examined.

### [chalceum group]

### Bembidion antiquum

Dejean, 1831

Bembidium antiquum Dejean, 1831: 88. Type locality: «Amérique septentrionale» (original citation), herein restricted to McCance, Westmoreland County, Pennsylvania (see Maddison 2008: 188). Holotype [by monotypy] (♂) in MHNP (Lindroth 1955b: 13).
Ochthedromus dilatatus LeConte, 1847: 455. Type locality: «Columbiam [= Columbia, Lancaster County], P[ennsylvani]a» (original citation). One syntype in MCZ [# 5493]. Synonymy established by Maddison (2008: 175).

#### Distribution.

This species ranges from Nova Scotia to northwestern Wisconsin, south to northern Arkansas, central Mississippi (Madison County, Drew A. Hildebrandt pers. comm. 2008), northern Alabama, and central North Carolina [see Maddison 2008: Fig. 21].

#### Records.

**CAN**: NB, NS, ON, QC **USA**: AL, AR, DC, IA, IL, IN, KY, MA, MD, ME, MO, MS, NC, NH, NJ, NY, OH, PA, TN, VA, VT, WI, WV

### Bembidion bellorum

Maddison, 2008

Bembidion bellorum Maddison, 2008: 170. Type locality: «Tygart Valley River near Valley Head (690 m), Randolph Co[unty], W[est]V[irginia]» (original citation). Holotype (♂) in USNM.

#### Distribution.

This species ranges from New England and northeastern New York south to northeastern Kentucky [see Maddison 2008: Fig. 20A].

#### Records.

**USA**: KY, ME, NH, NY, OH, PA, VT, WV

### Bembidion chalceum

Dejean, 1831

Bembidium chalceum Dejean, 1831: 89. Type locality: «Amérique septentrionale» (original citation), restricted to «Rumney [Grafton County], N[ew] H[ampshire]» by Lindroth (1963b: 247). Lectotype (♂), designated by Maddison (2008: 166), in MHNP.

#### Distribution.

The range of this species extends from Newfoundland to southwestern Northwest Territories, south to northeastern California, northern Colorado, north-central Arkansas, and South Carolina [see Maddison 2008: Fig. 18]. The record from “Mississippi” (Bousquet and Larochelle 1993: 126) probably refers to another species of the subgenus.

#### Records.

**CAN**: AB, BC, MB, NB, NF, NS (CBI), NT, ON, QC, SK **USA**: AR, CA, CO, CT, DC, IA, IL, IN, KS, KY, MA, MD, ME, MI, MN, MO, MT, NC, NE, NH, NJ, NY, OH, OK, OR, PA, RI, SC, SD, VA, VT, WA, WI, WV, WY

### Bembidion louisella

Maddison, 2008

Bembidion louisella Maddison, 2008: 177. Type locality: «North Aspy River near the town of Cape North, Cape Breton Island, Nova Scotia» (original citation). Holotype (♂) in CNC [# 23575].

#### Distribution.

This species ranges from Newfoundland to southeastern Quebec, south to central New Hampshire [see Maddison 2008: Fig. 22]

#### Records.

**CAN**: NB, NF, NS (CBI), QC **USA**: ME, NH, VT

### Bembidion rothfelsi

Maddison, 2008

Bembidion rothfelsi Maddison, 2008: 167. Type locality: «Ottauquechee River, Bridgewater (250 m), Windsor Co[unty], Vermont» (original citation). Holotype (♂) in USNM. Etymology. The specific name was proposed in honor of Klaus Rothfels [1919-1987], one of Canada’s outstanding cytogeneticists. Working at the University of Toronto, Rothfels specialized on the evolutionary analysis of speciation in black flies.

#### Distribution.

This species ranges from Cape Breton Island to east-central Missouri, south to southwestern Mississippi and southern North Carolina along the Appalachian Mountains [see Maddison 2008: Fig. 19].

#### Records.

**CAN**: NB, NS (CBI), ON, QC **USA**: CT, DE, IL, MD, ME, MO, MS, NC, NH, NY, OH, PA, TN, VA, VT, WV

### [honestum group]

### Bembidion arenobile

Maddison, 2008

Bembidion arenobilis Maddison, 2008: 181. Type locality: «Dan River in Danville, at Robertson Bridge, Virginia» (original citation). Holotype (♂) in USNM.

#### Distribution.

This species ranges from the Roanoke River drainage in Virginia south to southeastern Mississippi in the Piedmont, east to eastern North Carolina [see Maddison 2008: Fig. 23]

#### Records.

**USA**: AL, MS, NC, SC, VA

### Bembidion honestum

Say, 1823

Bembidium honestum Say, 1823a: 82. Type locality: «Water Gap, N[ew] J[ersey]-P[ennsylvani]a [possibly Delaware Water Gap in Monroe County, New Jersey]» (neotype label). Neotype (♂), designated by Lindroth and Freitag (1969: 336), in MCZ [# 33067].
Ochthedromus basalis LeConte, 1847: 454. Type locality: «provinciis mediis» (original citation). Four syntypes in MCZ [# 5494]. Synonymy established by Melsheimer (1853: 26), confirmed by Maddison (2008: 179).
Bembidium platyderum Chaudoir, 1868b: 242. Type locality: «New-York» (original citation). Lectotype (♀), designated by Lindroth (1963b: 248), in MHNP. Synonymy established by Lindroth (1963b: 248), confirmed by Maddison (2008: 179).

#### Distribution.

This species ranges from Cape Breton Island to eastern Illinois, south to North Carolina along the Appalachian Mountains [see Maddison 2008: Fig. 23]. One old specimen simply labeled from Wisconsin is known (Messer 2010: 35). The records from “Iowa,” “Kansas” (Hayward 1897: 56), and “Michigan” (Bousquet and Larochelle 1993: 126) need confirmation; that from Churchill in northern Manitoba (Elias 1984: 142) is in error (Maddison 2008: 181); those from “Wyoming,” “Texas” (Hayward 1897: 56), “Arkansas,” and “Mississippi” (Bousquet and Larochelle 1993: 126) are probably in error.

#### Records.

**CAN**: NB (CBI), NS, ON, QC **USA**: CT, DC, DE, IL, IN, KY, MA, MD, ME, NC, NH, NJ, NY, OH, PA, VA, VT, WV [IA, KS, MI, WI]

### Bembidion integrum

Casey, 1918

Bembidion integrum Casey, 1918: 79. Type locality: «Texas» (original citation), restricted to «Colorado River near Utley, Bastrop County» by Maddison (2008: 182). Lectotype (♂), designated by Lindroth (1975: 115), in USNM [# 36904].

#### Distribution.

This species ranges from west-central Wisconsin to south-central Montana, south to north-central New Mexico, southern Texas, northwestern Tamaulipas in Mexico, eastern Louisiana (East Feliciana Parish, Igor M. Sokolov pers. comm. 2009), and southern Mississippi, east to west-central Indiana [see Maddison 2008: Fig. 24].

#### Records.

**USA**: AR, CO, IA, IL, IN, KS, LA, MN, MO, MS, MT, NE, NM, OK, SD, TX, WI – Mexico

### Bembidion rufotinctum

Chaudoir, 1868

Bembidium rufotinctum Chaudoir, 1868b: 241. Type locality: Amérique septentrionale (inferred from title of the paper), restricted to «Lowell [Middlesex County], Mass[achusetts]» by Lindroth (1963b: 249). Lectotype (♀), designated by Lindroth (1963b: 249), in MHNP.
Bembidium blanchardi Hayward, 1897: 56. Type locality: «Lowell [Middlesex County], Mass[achusetts]» (original citation). Two syntypes in MCZ [# 2008]. Synonymy established by Hayward (1901: 157), confirmed by Maddison (2008: 179).

#### Distribution.

This species ranges from New Brunswick (Webster and DeMerchant 2012: 5) and southern Quebec (André Larochelle and Reginald P. Webster pers. comm. 1997, 2009) south to northern Georgia (Choate and Choate 1995: 371) and northwestern South Carolina (Ciegler 2003: [1]) along the Appalachian Mountains, east to northeastern Massachusetts [see Maddison 2008: Fig. 20B).

#### Records.

**CAN**: NB, QC **USA**: GA, KY, MA, NC, NH, NY, PA, SC, TN, VA, VT

### Subgenus Cillenus

Samouelle, 1819

Cillenus Samouelle, 1819: 148. Type species: *Cillenus lateralis* Samouelle, 1819 by monotypy. Etymology. Unknown [masculine].
Cillenum Curtis, 1828: plate 200. Unjustified emendation of *Cillenus* Samouelle, 1819.

#### Diversity.

Two species, one along the Pacific Coast of North America, the other (*Bembidion laterale* Samouelle) along the coasts of Europe and northern Africa. The three other species listed in this subgenus by Lorenz (2005: 216) belong to the subgenus *Desarmatocillenus* Netolitzky (see Lindroth 1980).

#### Identification.

Kavanaugh and Erwin (1992: 317) emended Lindroth’s (1963b) key to *Bembidion* to accommodate the new species.

#### Taxonomic Note.

This taxon is ranked as a distinct genus by many authors, with *Armatocillenus* Dupuis (three species in Taiwan and New Guinea), *Desarmatocillenus* Netolitzky (14 species in southeastern Asia and the Australian Region), *Chinocillenus* Netolitzky (one Chinese species), *Corallicillenus* Uéno (two Japanese species), and *Novicillenus* Uéno and Habu (two Japanese species) listed as subgenera (see Lorenz 2005: 216). Recently Sasakawa (2007) synonymized *Novicillenus* and *Corallicillenus* with *Desarmatocillenus*. Recent molecular data analyses conducted by Maddison and Ober (2011: 251) and Maddison (2012: 568) strongly support *Cillenus* as a member of *Bembidion*.

### Bembidion palosverdes

Kavanaugh and Erwin, 1992

Bembidion palosverdes Kavanaugh and Erwin, 1992: 312. Type locality: «Point Vicente, Los Angeles County, California» (original citation). Holotype (♂) in CAS [# 16806].

#### Distribution.

This species is known only from two localities on the Palos Verdes Peninsula along the coast of southern California (Kavanaugh and Erwin 1992: 315).

#### Records.

**USA**: CA

### Subgenus Actedium

Motschulsky, 1864

Actedium Motschulsky, 1864: 182. Type species: *Bembidium kuesteri* Schaum, 1845 designated by Dallas (1866: 426). Etymology. From the Greek *acte* (seashore, coastal) and the suffix -*idion* (little), alluding to the habitat where the two small carabid species in the hands of Motschulsky were found (“*les espèces vivent sur le bord de la mer*”) [neuter].

#### Diversity.

Northern Hemisphere, with five species in the Nearctic (one species) and Palaearctic (four European and northern African species, one of them endemic to the Canary Islands) Regions.

#### Identification.

Lindroth (1963b: 265) treated the North American species.

### Bembidion lachnophoroides

Darlington, 1926

Bembidion lachnophoroides Darlington, 1926: 34. Type locality: «Medicine Hat, Alberta» (original citation). Holotype (♂) in CNC [# 2300].

#### Distribution.

This species is known from the Whitefox River drainage in east-central Saskatchewan and several localities in Alberta, as far north as the Fort Vermilion area (Bousquet 1987a: 120; CNC).

#### Records.

**CAN**: AB, SK

### Subgenus Ocydromus

Clairville, 1806

Ocydromus Clairville, 1806: 20. Type species: *Carabus modestus* Fabricius, 1801 designated by Jeannel (1941b: 481). Etymology. From the Greek *ocys* (swift, quick) and *dromos* (running), probably alluding to the fast movement of the adults in the field [masculine].
Protoperyphus Alluaud, 1926: 11, 12. Type species: *Bembidion derelictum* Alluaud, 1926 by monotypy. Etymology. From the Greek *protos* (first) and the generic name *Peryphus* [*q.v*.] [masculine].
Synechoperyphus Netolitzky, 1942: 48. Type species: *Bembidium transsylvanicum* Bielz, 1852 by original designation. Synonymy established by Schuler (1962: 2), confirmed by Belousov (in Kryzhanovskij et al. 1995: 87). Etymology. From the Greek *synechos* (continuous) and the generic name *Peryphus* [*q.v*.] [masculine].

#### Diversity.

About 40 species in the Nearctic (one Holarctic species), Palaearctic (about 30 species), and Afrotropical (nine species) Regions.

#### Identification.

Lindroth (1963b: 342-343) covered the species found in North America.

### Bembidion scopulinum

(Kirby, 1837)

Peryphus scopulinus Kirby, 1837: 53. Type locality: «Lat. 54° [= along North Saskatchewan River]» (original citation), restricted to «Edmonton, Al[ber]ta» by Lindroth (1963b: 342). Two syntypes [2 originally cited] in BMNH (Lindroth 1953b: 176).
Peryphus oblique-lunatus Motschulsky, 1844: 244. Type locality: «environs de Kiakhta [northern Mongolia]» (original citation). One syntype in ZMMU (Keleinikova 1976: 208). Synonymy established, under the name *Bembidion thermarum* (Motschulsky), by Netolitzky (1935a: 35).
Omala thermarum Motschulsky, 1844: 255. Type locality: «Alpes du Hamar-Daban [Irkutsk Oblast], Sibérie orientale [Russia]» (original citation). Four syntypes in ZMMU (Keleinikova 1976: 224). Synonymy established by Lindroth (1963b: 342).
Ochthedromus gelidus LeConte, 1847: 464. Type locality: «Lacum Superiorem» (original citation). Four syntypes in MCZ [# 5517]. Synonymy established by LeConte (1873b: 325), confirmed by Lindroth (1963b: 342).
Bembidion scopulinum bellulum Casey, 1918: 71. Type locality: «Las Vegas [San Miguel County], New Mexico» (original citation). Holotype [by monotypy] (♀) in USNM [# 36902]. Synonymy established (as aberration) by Csiki (1928: 111), confirmed by Erwin (1984a: 181).

#### Distribution.

This Holarctic species is found in Kazakhstan, Siberia, Mongolia, Liaoning in China, the Korean Peninsula, and Japan (Marggi et al. 2003: 255), and from mainland Alaska (Lindroth 1963b: 343) to Newfoundland (Lindroth 1955a: 58-59), south to Connecticut (Litchfield County, William L. Krinsky pers. comm. 2008; Lindroth 1963b: 343), north-central Ohio (Lee 1994: 59), southeastern South Dakota (Kirk and Balsbaugh 1975: 18), and northern New Mexico (Casey 1918: 71, as *Bembidion scopulinum bellulum*; Taos County, UASM) along the Rocky Mountains. The record from northeastern Georgia (Fattig 1949: 17) is probably in error; that from “Pennsylvania” (Bousquet and Larochelle 1993: 138) needs confirmation.

#### Records.

**FRA**: PM **CAN**: AB, BC, LB, MB, NB, NF, NS (CBI), NT, ON, PE, QC, SK, YT **USA**: AK, CO, CT, IL, IN, MA, ME, MI, MN, MT, ND, NH, NM, NY, OH, RI, SD, VT, WI, WY – **Holarctic**

### Subgenus Peryphus

Dejean, 1821

Peryphus Dejean, 1821: 17. Type species: *Carabus littoralis* Olivier, 1795 (= *Bembidium tetracolum* Say, 1823) designated by Westwood (1838: 7). Etymology. Possibly from the Greek *peri* (very) and *phos* (light, by extension bright, clear) [masculine]. The name was proposed by Johann Karl Megerle von Mühlfeld and made available by Dejean.

#### Diversity.

About 65 species in the Nearctic (21 species, three of them adventive), Neotropical (four species in Middle America shared with North America), Palaearctic (about 50 species), and Afrotropical (one species in Ethiopia, *Bembidion scottustulatum* Netolitzky) Regions. Three species (*Bembidion dauricum*, *Bembidion obscurellum* and *Bembidion petrosum*) are Holarctic.

#### Identification.

Lindroth (1963b: 330-342, as *tetracolum*, *transversale*, *striola*, and in part *grapei* groups) covered all but six (*Bembidion lugubre*, *Bembidion pernotum*, *Bembidion mexicanum*, *Bembidion perspicuum*, *Bembidion sarpedon*, and *Bembidion femoratum*) of the species found in North America. Maddison and Swanson (2010: 26-29) briefly discussed the status of each species of the *transversale* group but did not include a key to separate all species.

### [dauricum group]

### Bembidion dauricum

(Motschulsky, 1844)

Leja daurica Motschulsky, 1844: 256. Type locality: «au-delà du [Lac] Baïcal [Siberia, Russia]» (original citation). Ten syntypes in ZMMU (Keleinikova 1976: 194) and one in ZILR (Netolitzky 1935a: 29).
Bembidion armeniacum pseudoproperans Netolitzky, 1920a: 69. Type locality: «Tunkun-Sajan im Baikalgebiete [Siberia, Russia]» (original citation). Syntypes in NHMW (Lindroth 1963b: 323). Synonymy established by Netolitzky (1935a: 29).
Bembidion lysholmi Munster, 1930: 353. Type locality: «ad Melbo [Lofoten] Norvegiae septentrionalis» (original citation). Holotype (♀) in ZMUO (Lindroth 1963b: 323). Synonymy established by Lindroth (1939b: 80). Etymology. The specific name was proposed for Bjarne Lysholm [1861-1939], a Norwegian physician who turned company manager. Lysholm devoted the last 25 years of his life to natural history and particularly to beetles. He was a founding member of the Norwegian Entomological Society in 1904, served as president of The Royal Norwegian Society of Sciences, and was awarded the Knight’s Cross of the Order of St. Olav for scientific accomplishments.

#### Distribution.

This Holarctic species ranges from Scandinavia to eastern Siberia and Mongolia (Marggi et al. 2003: 261) in the Palaearctic Region and from the Chukchi coast in Alaska to the Hudson Bay coast in northern Manitoba (Lindroth 1963b: 325); it also occurs quite isolated in the southern parts of the Rocky Mountains in Colorado and New Mexico (Lindroth 1969a: 1114). Fossil remnants of this species, dated between about 16,700 and 18,100 years B.P., have been unearthed in southeastern Iowa (Baker et al. 1986: 96); others, older than 33,000 years B.P., in southwestern Ontario (Warner et al. 1988: 35).

#### Records.

**CAN**: MB, NT, YT **USA**: AK, CO, NM – **Holarctic**

### [striola group]

### Bembidion actuosum

Casey, 1918

Bembidion actuosum Casey, 1918: 65. Type locality: «Boulder Co[unty], Colorado» (original citation). Lectotype (♀), designated by Erwin (1984a: 166), in USNM [# 36899].
Bembidion debilicolle Casey, 1924: 34. Type locality: «Lake Co[unty], California» (original citation). Lectotype (♀), designated by Erwin (1984a: 166), in USNM [# 36935]. Synonymy established by Erwin (1984a: 166).
Bembidion urgens Casey, 1924: 35. Type locality: «Modoc Co[unty], California» (original citation). Lectotype (♀), designated by Lindroth (1975: 118), in USNM [# 36939]. Synonymy established, under the name *Bembidion debilicolle* Casey, by Lindroth (1963b: 318).

#### Distribution.

This species inhabits the North American Cordilleras from southwestern Alberta and southern British Columbia (Lindroth 1963b: 318, as *Bembidion debilicolle*) south at least to Lake County in the Coast Ranges of California (Casey 1924: 34, as *Bembidion debilicolle*), northern Utah (Salt Lake and Utah Counties, CMNH), and north-central Colorado (Casey 1918: 65; Armin 1963: 169, as *Bembidion debilicolle*) along the Rocky Mountains.

#### Records.

**CAN**: AB, BC **USA**: CA, CO, ID, MT, NV, OR, UT, WY

### Bembidion consanguineum

Hayward, 1897

Bembidium consanguineum Hayward, 1897: 76. Type locality: «California, Arizona, Utah, Colorado and Guadaloupe Island» (original citation), restricted to «Ariz[ona]» by Lindroth (1963b: 316). Four syntypes in MCZ [# 16285].

#### Distribution.

This species is known from southeastern Wyoming (Lavigne 1977: 44), south-central Colorado (Wickham 1902: 233; Lindroth 1963b: 316), and “New Mexico” (Casey 1918: 74) west to Guadaloupe Island off the Pacific Coast of Baja California (Hayward 1897: 76). The record from “Montana” (Bousquet and Larochelle 1993: 135) needs confirmation; that from “Oregon” (Notman 1919b: 227) is in error according to Lindroth (1963b: 316).

#### Records.

**USA**: AZ, CA, CO, NM, NV, UT, WY [MT] – Mexico

### Bembidion nevadense

Ulke, 1875

Bembidium nevadense Ulke, 1875: 811. Type locality: «Nevada» (original citation). Lectotype (♀), designated by Lindroth (1963b: 316), in CMNH.
Bembidion viaticum Casey, 1918: 65. Type locality: «New Mexico» (original citation). Lectotype (♀), designated by Erwin (1984a: 177), in USNM [# 36884]. Synonymy established by Erwin (1984a: 177).

#### Distribution.

This species is widely distributed in the North American Cordilleras from southern British Columbia south to central California along the Sierra Nevada (Lindroth 1963b: 317), southeastern Arizona (Cochise County, UASM, Ken Karns pers. comm. 2009), and “New Mexico” (Casey 1918: 65, as *Bembidion viaticum*) along the Rocky Mountains.

#### Records.

**CAN**: BC **USA**: AZ, CA, ID, MT, NM, NV, OR, UT, WA, WY

### Bembidion plagiatum

(Zimmermann, 1869)

Ochthedromus plagiatus Zimmermann [in LeConte], 1869b: 247. Type locality: «Maryland» (original citation). Lectotype (♂), designated by Lindroth (1963b: 319), in MCZ [# 32620].

#### Distribution.

This species, the only one of the group occurring in eastern North America, is known from southernmost Ontario (Lindroth 1963b: 319) to “New Jersey,” south to south-central North Carolina (Hoffman 1982: 146), northeastern Georgia (Horn and Ulyshen 2009: 121), eastern Alabama (Tallapoosa County, UASM, Drew A. Hildebrandt pers. comm. 2009), and east-central Mississippi (Lowndes County, Drew A. Hildebrandt pers. comm. 2008).

#### Records.

**CAN**: ON **USA**: AL, GA, KY, MD, MS, NC, NJ, OH, PA, TN, VA, WV

### Bembidion satelles

Casey, 1918

Bembidion satelles Casey, 1918: 71. Type locality: «Truckee [Nevada County], California» (original citation). Holotype [by monotypy] (♀) in USNM [# 36903].

#### Distribution.

This species is known from a few localities in the Sierra Nevada north of Yosemite National Park (Lindroth 1963b: 318; Maddison 1985: 113).

#### Records.

**USA**: CA

### Bembidion striola

(LeConte, 1852)

Ochthedromus striola LeConte, 1852a: 190. Type locality: «San Diego [San Diego County, California]» (original citation). Three syntypes in MCZ [# 5513].
Bembidion vancouveri Casey, 1918: 73. Type locality: «Victoria, British Columbia» (original citation). Lectotype (♀), designated by Lindroth (1975: 118), in USNM [# 36933]. Synonymy established by Hatch (1953: 90), confirmed by Lindroth (1963b: 315).
Bembidion shastanicum Casey, 1918: 74. Type locality: «Dunsmuir, Siskiyou Co[unty], California» (original citation). Lectotype (♀), designated by Lindroth (1975: 118), in USNM [# 36936]. Synonymy established by Lindroth (1963b: 315).
Bembidion angustior Casey, 1924: 33. Type locality: «Del Norte Co[unty], California» (original citation). Lectotype (♂), designated by Lindroth (1975: 118), in USNM [# 36934]. Synonymy established by Lindroth (1963b: 315).
Bembidion modulatum Casey, 1924: 34. Type locality: «Josephine Co[unty], Oregon» (original citation). Holotype [by monotypy] (♂) in USNM [# 36938]. Synonymy established by Hatch (1953: 90), confirmed by Lindroth (1963b: 315).

#### Distribution.

This species ranges from Vancouver Island (Lindroth 1963b: 315) south to southern California (LeConte 1852a: 190) where it is “widely distributed” (Fall 1901a: 42). The records from Colorado (Wickham 1902: 233), New Mexico (Fall and Cockerell 1907: 157), central Arizona (Griffith 1900: 565), Utah (Knowlton 1939: 2), northern Idaho (Hatch 1953: 90), and northeastern Kansas (Popenoe 1878: 79) are probably in error.

#### Records.

**CAN**: BC (VCI) **USA**: CA (CHI), OR, WA

### [tetracolum group]

### Bembidion bruxellense

Wesmael, 1835

Bembidium femoratum Gyllenhal, 1827: 406 [primary homonym of *Bembidium femoratum* Sturm, 1825]. Type locality: Sweden (inferred from title of the book). Syntype(s) location unknown (possibly in UZIU).
Peryphus elegans Stephens, 1835 [31 March]: 386 [secondary homonym of *Bembidion elegans* Germar, 1824]. Type locality: «near London [United Kingdom]» (original citation). One syntype in BMNH (Netolitzky 1935: 133). Synonymy established by Netolitzky (1935: 133).
Bembidion bruxellense Wesmael, 1835 [“31 December”]: 47. Type locality: «environs de Bruxelles [Belgium]» (original citation). Syntype(s) location unknown (possibly in IRSN). Synonymy established by Putzeys (1845b: 140).

#### Distribution.

This European species is adventive in North America where it is known from Newfoundland (Lindroth 1955a: 61, as *Bembidion rupestre*) to western Quebec (Larochelle 1975: 53), and from Maine (Larochelle and Larivière 1990a: 28). The record from “New Hampshire” (Bousquet and Larochelle 1993: 137) needs confirmation. The first inventoried specimen collected on this continent was found in Newfoundland in 1907 (Lindroth 1957c: 151).

#### Records.

**FRA**: PM **CAN**: NB, NF, NS (CBI), PE, QC **USA**: ME [NH] – **Adventive**

### Bembidion femoratum femoratum

Sturm, 1825

Bembidium femoratum Sturm, 1825: 117. Type locality: «Würzburg, Preußen [Germany]» (original citation). Syntype(s) location unknown.

#### Distribution.

This European subspecies is adventive in North America where it is known from New Brunswick and Nova Scotia (Bousquet 1992a: 504; Majka 2005: 536) and from one specimen discovered in 2003 in Grant County, Washington (Robert L. Davidson pers. comm. 2008). The first inventoried specimen collected on this continent was found near Halifax, Nova Scotia in 1967 (Bousquet 1992a: 504).

#### Records.

**CAN**: NB, NS (CBI) **USA**: WA – **Adventive**

### Bembidion obscurellum obscurellum

(Motschulsky, 1845)

Peryphus obscurellus Motschulsky, 1845a: 27. Type locality: «Kamtschatka [Russia]» (original citation). One syntype in ZMMU (Keleinikova 1976: 208).
Peryphus maritimus Motschulsky, 1850a: 11 [primary homonym of *Peryphus maritimus* Stephens, 1839]. Type locality: «Kamtschatka [Russia]» (original citation). Syntype(s) location unknown (possibly in ZMMU though not listed in Keleinikova 1976). Synonymy established by Netolitzky (1935a: 32).
Peryphus fuscicrus Motschulsky, 1855b: 79. Type locality: «ad castellum Nicolajevsk peninsulae Kenai [Alaska]» (original citation for *Peryphus lucidus* (LeConte) *sensu* Mannerheim, 1853). Syntype(s) in ZMMU (Lindroth 1963b: 338). Synonymy established by Netolitzky (1918: 25). Note. This taxon was described by indication for *Bembidion lucidum* (LeConte, 1847) *sensu* Mannerheim (1853: 150).
Bembidium mixtum LeConte, 1863b: 14. Type locality: «castellum Nicolajevsk peninsulae Kenai [Alaska]» (original citation for *Bembidium lucidus* (LeConte) var. *b* of Mannerheim, 1853). Two possible syntypes in MCZ [# 5516]. Synonymy established by Hayward (1897: 134). Note. This taxon was described by indication for *Bembidium lucidum* (LeConte, 1847) var. *b* of Mannerheim (1853: 114) and therefore the type series consists of the specimen(s) denoted by the reference (ICZN 1999: Article 72.4.4). The two specimens labeled “R[ussian] A[merica]” in MCZ are possibly syntypes received by LeConte from Mannerheim.
Bembidium repandum J.R. Sahlberg, 1875: 78. Type locality: «af floden Varsuga invid hafstranden i ryska Lappmarken (66°20’) [= Varsuga River in Kola Peninsula, Russia]» (original citation). Two syntypes [8 originally cited] in ZMH (Silfverberg 1987: 23). Synonymy established by Lindroth (1963b: 338).
Bembidion cribrulum Netolitzky, 1910: 217. Replacement name for *Bembidion maritimum* (Motschulsky, 1850).
Bembidion caducum Casey, 1918: 80. Type locality: «Cheyenne [Laramie County], Wyoming» (original citation). Lectotype (♂), designated by Lindroth (1975: 119), in USNM [# 36906]. Synonymy established, under the name *Bembidion fuscicrus* (Motschulsky), by Fall (1922c: 172), confirmed by Lindroth (1963b: 338).
Bembidion albidipenne Casey, 1918: 80. Type locality: «Montrose (6000 ft.) [Montrose County], Colorado» (original citation). Lectotype (♀), designated by Lindroth (1975: 119), in USNM [# 36907]. Synonymy established, under the name *Bembidion fuscicrus* (Motschulsky), by Fall (1922c: 172), confirmed by Lindroth (1963b: 338).
Bembidion parowanum Casey, 1918: 80. Type locality: «Little Salt Lake, Parowan [Iron County], Utah» (original citation). Lectotype (♂), designated by Lindroth (1975: 119), in USNM [# 36909]. Synonymy established by Lindroth (1963b: 338).
Bembidion petulans Casey, 1918: 81. Type locality: «apparently Colorado» (original citation). Holotype [by monotypy] (♂) in USNM [36908]. Synonymy established, under the name *Bembidion caducum* Casey, by Nicolay and Weiss (1934: 197), confirmed by Lindroth (1963b: 338).

#### Distribution.

This Holarctic subspecies ranges from Scandinavia to northeastern Siberia (Motschulsky 1845a: 27) and Mongolia (Marggi et al. 2003: 262) and in North America from Alaska (Lindroth 1963b: 339) to Newfoundland (Bousquet 1987a: 120), south to Virginia (Hoffman et al. 2006: 20), east-central Iowa (Iowa County, MCZ), southern New Mexico (Fall and Cockerell 1907: 157), southwestern Nevada (Esmeralda County, CMNH), and “California” (Cooper 1976: 163).

#### Records.

**CAN**: AB, BC, MB, NB, NF, NS, NT, ON, PE, QC, SK, YT **USA**: AK, CA, CO, IA, ID, IL, IN, ME, MI, MN, MT, ND, NH, NM, NV, NY, OH, OR, PA, RI, SD, UT, VA, VT, WA, WI, WY – **Holarctic**

#### Note.

Five more subspecies are recognized within the Palaearctic fauna.

### Bembidion petrosum attuense

Lindroth, 1963

Bembidion petrosum attuense Lindroth, 1963b: 334. Type locality: «Peaceful R[iver], Attu, Aleutian Islands, Alaska» (original citation). Holotype (♂) in MCZ [# 35407].

#### Distribution.

This subspecies is known only from numerous specimens collected at the type locality.

#### Records.

**USA**: AK

### Bembidion petrosum petrosum

Gebler, 1833

Bembidium petrosum Gebler, 1833: 275. Type locality: western Siberia (inferred from title of the paper); «Smjeinogorsk, S[outh of] Barnaul, Ob R[iver], Siberia, Russia» selected by Lindroth (1963b: 332). Lectotype (♂), designated by Lindroth (1963b: 332), in ZMH.
Ochthedromus substrictus LeConte, 1848: 465. Type locality: «Lacum Superiorem» (original citation). Syntype(s) in MCZ [# 5515]. Synonymy established, under the name *Bembidion lucidum* (LeConte), by LeConte (1863b: 14), confirmed by Lindroth (1954b: 126).
Ochthedromus lucidus LeConte, 1848: 466 [secondary homonym of *Bembidion lucidum* (Faldermann, 1835)]. Type locality: «Lacum Superiorem» (original citation). Syntype(s) in MCZ [# 5514]. Synonymy established by Lindroth (1953b: 176).
Peryphus subinflatus Motschulsky, 1859a: 126. Type locality: «colonie Ross [farming community about 75 miles north of San Francisco along the coast, California]» (original citation). Lectotype, designated by Bousquet and Larochelle (1993: 16), in ZMMU. Synonymy established by Bousquet and Larochelle (1993: 16).
Bembidium wagneri Tschitschérine, 1893: 377. Type locality: «Krasnojarsk [Siberia, Russia]» (original citation). Syntype(s) [3 originally cited] in ZILR. Synonymy established by Netolitzky (1934: 73).
Bembidion distinguendum siebkei J. Müller, 1918: 94. Type locality: «Scandinavien» (original citation), restricted to «Mo[e]n [Troms County], R[iver] Målselv, N[orthern] Norway» by Lindroth (1963b: 333). Syntype(s) in ZMUT (Lindroth 1963b: 333). Synonymy established by Munster (1921: 60), confirmed by Lindroth (1963b: 333). Note. This name is credited to Sparre Schneider (1910: 72) by several authors (e.g., Lorenz 2005: 232; Marggi et al. 2003: 262; Kryzhanovskij et al. 1995: 86). The species’ name is included and discussed by Sparre Schneider (1910: 72) but no character states are mentioned that would make the name available (see also Netolitzky 1934: 73).
Bembidion lepusculus Casey, 1918: 75. Type locality: «Colorado» (original citation). Holotype [by monotypy] (♂) in USNM [# 36937]. Synonymy established by Lindroth (1954b: 125).
Bembidion castalium Casey, 1918: 75. Type locality: «Las Vegas [San Miguel County], New Mexico» (original citation). Lectotype (♀), designated by Lindroth (1975: 119), in USNM [# 36940]. Synonymy established by Lindroth (1954b: 126).
Bembidion exiguiceps Casey, 1924: 34. Type locality: «Terrace, British Columbia» (original citation). Lectotype (♀), designated by Lindroth (1975: 119), in USNM [# 36942]. Synonymy established by Lindroth (1954b: 128).
Bembidion wenatchee Hatch, 1950: 101. Type locality: «Wenatchee [Chelan County], Washington» (original citation). Holotype (♀) in USNM. Synonymy established by Hatch (1953: 90), confirmed by Lindroth (1954b: 130).
Bembidion petrosum carlhlindrothi Kangas, 1980: 364. Type locality: «Osnatsennaja, am Jenissej, Sibirien» (original citation). Holotype (♂) in ZMH. Synonymy established by Lindroth (1985: 195).

#### Distribution.

This Holarctic subspecies is known from Scandinavia to eastern Siberia (Marggiet al. 2003: 262) and from Alaska (Lindroth 1963b: 334) to Newfoundland (Lindroth 1955a: 60-61), south to “Rhode Island” (Sikes 2003: 7), southwestern Pennsylvania (Allegheny County, CMNH), “Nebraska” (Hayward 1897: 81, as *Bembidion lucidum*), northeastern New Mexico (Casey, 1918: 75, as *Bembidion castalium*), southern Arizona (Wickham 1898: 300, as *Bembidion lucidum*), and southeastern California (San Bernardino County, MCZ).

#### Records.

**FRA**: PM **CAN**: AB, BC, LB, MB, NB, NF, NS (CBI), NT, ON, PE, QC, SK, YT **USA**: AK, AZ, CA, CO, ID, IL, MA, ME, MI, MN, MT, ND, NE, NH, NM, NV, NY, OH, OR, PA, RI, UT, VT, WA, WI, WY – **Holarctic**

### Bembidion poppii schalleri

Lindroth, 1963

Bembidion poppii schalleri Lindroth, 1963b: 335. Type locality: «Colville R[iver], Umiat, Alaska» (original citation). Holotype (♂) in MCZ [# 34571].

#### Distribution.

This subspecies is known only from a few localities on mainland Alaska.

#### Records.

**USA**: AK

#### Note.

Besides the nominotypical subspecies found in eastern Siberia, two more subspecies have been described from Japan and Szechwan, China.

### Bembidion rupicola

(Kirby, 1837)

Peryphus rupicola Kirby, 1837: 53. Type locality: «Lat. 54° [= along North Saskatchewan River] and 65° [= apparently region of Great Bear Lake, Northwest Territories]» (original citation), restricted to «Edmonton, Al[ber]ta» by Lindroth (1963b: 336). Two syntypes in BMNH (Lindroth 1953b: 176).
Bembidion aversans Casey, 1924: 35. Type locality: «Mexico» (original citation). Lectotype (♀), designated by Erwin (1984a: 181), in USNM [# 36941]. Synonymy established by Erwin (1984a: 181).

#### Distribution.

The range of this species extends from the Ontario Peninsula (Bousquet 1987a: 120) to southern British Columbia, north to western Alaska (Lindroth 1963b: 337-338), south to northern Oregon (Gilliam and Morrow Counties, CMNH; Hatch 1953: 90; Lindroth 1963b: 337), southern Arizona (Dajoz 2007: 21), western Texas (Jeff Davis County, MCZ), and northwestern Pennsylvania (Warren and Forest Counties, CMNH). The species has been reported also from “Mexico” (Casey 1924: 35, as *Bembidion aversans*). The record from “California” (Bousquet and Larochelle 1993: 138) was based on a misidentified specimen of *Bembidion petrosum* in MCZ.

#### Records.

**CAN**: AB, BC, MB, NT, ON, SK **USA**: AK, AZ, CO, IA, ID, MI, MN, MT, ND, NE, NM, NV, OH, OR, PA, SD, TX, UT, WA, WI, WY – Mexico

### Bembidion sejunctum sejunctum

Casey, 1918

Bembidion sejunctum Casey, 1918: 79. Type locality: «somewhere between Fort Wingate and Jemez Springs, New Mexico» (original citation). Lectotype (♂), designated by Lindroth (1975: 119), in USNM [# 36905].

#### Distribution.

The range of this subspecies is disjunct. It occurs in the east along the coast of Newfoundland, Saint Pierre and Miquelon (Lindroth 1955a: 63), the Maritimes, Magdalen Islands (Lindroth 1963b: 340), and the Saint Lawrence Estuary (Larochelle 1975: Fig. 169) and in the west from the Mackenzie River in central Northwest Territories south to northern New Mexico (Taos County, UASM), including southwestern North Dakota (Tinerella 2003: 635) [see Lindroth 1963a: Fig. 66].

#### Records.

**FRA**: PM **CAN**: AB, LB, NB, NF, NS (CBI), NT, PE, QC, SK **USA**: CO, ND, NM, UT, WY

### Bembidion sejunctum semiaureum

Fall, 1922

Bembidion semiaureum Fall, 1922c: 171. Type locality: «Humboldt Co[unty], California» (original citation). Holotype in MCZ [# 23867].
Bembidion fenderi Hatch, 1950: 102. Type locality: «Seaview [Pacific County], Washington» (original citation). Holotype (♂) in USNM. Synonymy established by Lindroth (1954b: 130).

#### Distribution.

This subspecies is confined to the west coast ranging from the Queen Charlotte Islands (Kavanaugh 1992: 66) south at least to northern California (Fall 1922c: 171; Hatch 1953: 91) [see Lindroth 1963a: Fig. 66].

#### Records.

**CAN**: BC (QCI, VCI) **USA**: CA, OR, WA

### Bembidion tetracolum tetracolum

Say, 1823

Carabus littoralis Olivier, 1795: [35] 110 [secondary homonym of *Bembidion litoralis* (Olivier, 1790)]. Type locality: «environs de Paris, sur les bords de la Seine [France]» (original citation). Syntype(s) location unknown (possibly in MHNP). Note. The use of a single or double consonant in the spellings of species-group names is deemed to be identical (ICZN 1999: Article 58.7); therefore the two Olivier’s names are homonyms.
Bembidium tetracolum Say, 1823a: 89. Type locality: «Arlington [Middlesex County], Mass[achusetts]» (neotype label). Neotype (♂), designated by Lindroth and Freitag (1969: 336), in MCZ [# 33068]. Synonymy established, under the name *Bembidion ustulatum* (Linnaeus) *sensu auctorum* (= *Bembidion tetracolum* Say), by Fassati (1950: 42).
Bembidion rupicola nactum Casey, 1918: 77. Type locality: «lower Hudson Valley, New York» (original citation). Lectotype (♀), designated by Lindroth (1975: 119), in USNM [# 36943]. Synonymy established by Lindroth (1954b: 126).

#### Distribution.

This Palaearctic subspecies is adventive in North America where it is known from Newfoundland (Lindroth 1955a: 62, as *Bembidion ustulatum*) to western Montana (Russell 1968: 54), including southern Quebec and the Ontario Peninsula (Lindroth 1963b: 332), south to northern Utah (Salt Lake County, CMNH), west-central Nebraska (Keith County, Foster F. Purrington pers. comm. 2010), and central Virginia (Davidson 1995: 16); also in the Pacific Northwest from southwestern British Columbia (Lindroth 1963b: 332) to northern California (Notman 1929b: 222; Tehama County, Foster F. Purrington pers. comm. 2010). The records from Colorado (Wickham 1902: 233, as *Bembidion ustulatum*), “Tennessee,” and “North Carolina” (Bousquet and Larochelle 1993: 138) need confirmation; that from “Texas” (Lindroth 1955a: 62) could be based on a mislabeled specimen. The first inventoried specimen collected on this continent was found prior to 1823, likely along the east coast. The species has been unearthed from a xvii Century colonial site in Boston, Massachusetts (Bain 1998: 39), suggesting that the species was established on this continent long before Thomas Say collected his specimens. The species is also adventive in southeastern Australia (Moore et al. 1987: 136).

#### Records.

**FRA**: PM **CAN**: BC, NB, NF, NS, ON, PE, QC **USA**: CA, CT, DC, IA, IL, IN, MA, ME, MI, MN, MT, ND, NE, NH, NJ, NY, OH, OR, PA, RI, SD, UT, VA, VT, WA, WI [CO, NC, TN, TX] – **Adventive**

#### Note.

This species has been known for a long time under the name *Bembidion ustulatum* (Linnaeus, 1758). Lindroth (1957b: 334-335) suggested to drop the name to promote stability. Four other subspecies of *Bembidion tetracolum* are found in the Palaearctic Region.

### [transversale group]

### Bembidion lugubre

LeConte, 1857

Bembidium lugubre LeConte, 1857a: 6. Type locality: «valley of the Rio Grande» (original citation). Holotype [by monotypy] (♀) in MCZ [# 5511].
Bembidium mexicanum var. *sallaei* Bates, 1882a: 148. Type locality: «Puebla, Toluca, Etla, Guanajuato, near the capital, Oaxaca [in] Mexico; river Sarstoon, British Honduras; near the city, Totonicapam, Panajachel [in] Guatemala» (original citation). Lectotype (♂) from «Mexico», designated by Erwin (1982b: 469), in MHNP (collection Chaudoir). Synonymy established by Maddison and Swanson (2010: 28).
Bembidion canonicum Casey, 1918: 61. Type locality: «Arizona» (original citation). Holotype [by monotypy] (♂) in USNM [# 36920]. Synonymy established by Maddison and Swanson (2010: 28).
Bembidion retectum Casey, 1918: 61. Type locality: «S[ain]t George [Washington County], Utah» (original citation). Lectotype (♂), designated by Erwin (1984a: 177), in USNM [# 36923]. Synonymy established by Maddison and Swanson (2010: 28).
Bembidion mexicanum var. *sallei* Csiki, 1928: 102. Unjustified emendation of *Bembidion mexicanum* var. *sallaei* Bates, 1882.

#### Distribution.

This species ranges from “California” to “Texas,” south at least to Oaxaca, Mexico (Maddison and Swanson 2010: 29).

#### Records.

**USA**: AZ, CA, NM, TX, UT – Mexico

### Bembidion mexicanum

Dejean, 1831

Bembidium mexicanum Dejean, 1831: 126. Type locality: «Mexique» (original citation). Lectotype (♂), designated by Erwin (1982b: 469), in MHNP.
Bembidium stabile LeConte, 1879d: 508. Type locality: «La Veta [Huerfano County], Colo[rado] (original citation for the lectotype). Lectotype (♀), designated by Erwin (1984a: 176), in MCZ [# 5512]. Synonymy established by Erwin (1984a: 176), confirmed by Maddison and Swanson (2010: 28).
Bembidion badiipenne Casey, 1918: 60. Type locality: «somewhere on the road between F[or]t Wingate and Jemez Springs, New Mexico» (original citation). Lectotype (♀), designated by Erwin (1984a: 176), in USNM [# 36913]. Synonymy established by Erwin (1984a: 176), confirmed by Maddison and Swanson (2010: 28).
Bembidion lugubre vafrum Casey, 1918: 60. Type locality: «Arizona» (original citation). Lectotype (♂), designated by Erwin (1984a: 176), in USNM [# 36921]. Synonymy established by Erwin (1984a: 176), confirmed by Maddison and Swanson (2010: 28).

#### Distribution.

This species ranges from South Dakota (Maddison and Swanson 2010: 28) and eastern Wyoming (Lavigne 1977: 44, as *Bembidion lugubre*) south to Costa Rica (Erwin 1982b: 469, Fig. 65).

#### Records.

**USA**: AZ, CO, NE, NM, SD, WY – Belize, Costa Rica, Guatemala, Mexico

### Bembidion pernotum

Casey, 1918

Bembidion pernotum Casey, 1918: 62. Type locality: «Jemez Springs [Sandoval County], New Mexico» (original citation for the lectotype). Lectotype (♀), designated by Erwin (1984a: 178), in USNM [# 36922].

#### Distribution.

This species is known from southern Colorado and northern New Mexico (Maddison and Swanson 2010: 29).

#### Records.

**USA**: CO, NM

### Bembidion perspicuum

(LeConte, 1848)

Ochthedromus perspicuus LeConte, 1848: 466. Type locality: «ad Rocky Mountains» (original citation). One syntype in MCZ [# 5510].
Bembidion acomanum Casey, 1918: 59. Type locality: «Jemez Springs [Sandoval County], New Mexico» (original citation for the lectotype). Lectotype (♀), designated by Lindroth (1975: 119), in USNM [# 36916]. Synonymy established by Maddison and Swanson (2010: 27).
Bembidion excursum Casey, 1918: 59. Type locality: «Tuçson [Pima County], Arizona» (original citation). Lectotype (♀), designated by Lindroth (1975: 119), in USNM [# 36915]. Synonymy established by Maddison and Swanson (2010: 27).

#### Distribution.

This species ranges from “Oregon” (Maddison and Swanson 2010: 27) to western Nebraska (Keith County, Robert L. Davidson pers. comm. 2012), south to western Texas (Jeff Davis and Presidio Counties, CNC) and southern California (Maddison and Swanson 2010: 27).

#### Records.

**USA**: AZ, CA, CO, KS, NE, NM, NV, OK, TX, UT

### Bembidion sarpedon

Casey, 1918

Bembidion sarpedon Casey, 1918: 58. Type locality: «Cañon City [Fremont County], Colorado» (original citation). Lectotype (♂), designated by Lindroth (1975: 119), in USNM [# 36914].
Bembidion animatum Casey, 1918: 62. Type locality: «Jemez Springs [Sandoval County], New Mexico» (original citation for the lectotype). Lectotype (♀), designated by Lindroth (1975: 119), in USNM [# 36918]. Synonymy established by Maddison and Swanson (2010: 27).

#### Distribution.

This species ranges from “Wyoming” south to northern New Mexico and northeastern Arizona (Maddison and Swanson 2010: 28).

#### Records.

**USA**: AZ, CO, NM, UT, WY

### Bembidion transversale

Dejean, 1831

Bembidium transversale Dejean, 1831: 110. Type locality: «territoire du nord-ouest, Amérique septentrionale» (original citation), restricted to «Nipigon, Ont[ario]» by Lindroth (1963b: 341). Holotype [by monotypy] (♀) in MHNP (Lindroth 1955b: 13).
Peryphus erosus Motschulsky, 1850a: 10. Type locality: «California» (original citation), listed from «St. Francisco [San Francisco County]» by Motschulsky (1859a: 128). Lectotype, designated by Bousquet and Larochelle (1993: 16), in ZMMU. Synonymy established by Hayward (1897: 134).
Ochthedromus mannerheimii LeConte, 1852a: 190 [secondary homonym of *Bembidium mannerheimii* Sahlberg, 1827]. Type locality: «San Diego [San Diego County, California]» (original citation). Lectotype (♀), designated by Maddison and Swanson (2010: 26), in MCZ [# 35571]. Synonymy established with the name *Peryphus erosus* Motschulsky by LeConte (1863b: 14), confirmed by Maddison and Swanson (2010: 26).
Bembidium haplogonum Chaudoir, 1868b: 241. Type locality: «Californie» (original citation). Syntype(s) location unknown (not in MHNP according to Lindroth 1963b: 341). Synonymy established by Hayward (1897: 134).
Bembidion marinicum Casey, 1918: 57. Type locality: «Marin Co[unty], California» (original citation). Holotype [by monotypy] (♀) in USNM [# 36919]. Synonymy established by Lindroth (1963b: 341).
Bembidion tuolumne Casey, 1924: 30. Type locality: «Tuolumne Co[unty], California» (original citation). Lectotype (♂), designated by Lindroth (1975: 119), in USNM [# 36917]. Synonymy established by Lindroth (1963b: 341).

#### Distribution.

This species ranges from Newfoundland (Lindroth 1955a: 59) to Alaska (Maddison and Swanson 2010: 27), south to southern California (LeConte 1852a: 190; Maddison 1985: 114), “Utah,” “Colorado” (Maddison and Swanson 2010: 27), northern Michigan (Hubbard and Schwarz 1878: 629; Dunn 1985a: 10; CNC), and central New York (Notman 1928: 215). The record from South Dakota (Kirk and Balsbaugh 1975: 17) refers to *Bembidion mexicanum* (USNM); thatfrom “Illinois” (Bousquet and Larochelle 1993: 138) needs confirmation.

#### Records.

**CAN**: AB, BC (QCI, VCI), LB, MB, NB, NF, NS (CBI), NT, ON, QC, SK, YT **USA**: AK, CA, CO, ID, MI, MN, MT, ND, NV, NY, OR, UT, WA, WI, WY [IL]

#### Note.

Maddison and Swanson (2010: 27) stated that specimens of this species from western British Columbia, western Oregon, and California represent another form, with no consistent differences in COI or 28S from the typical form but with clearly different mentum shape. They added that *Bembidion transversale* as currently understood could be a complex of multiple species.

### Subgenus Terminophanes

Müller-Motzfeld, 1998

Terminophanes Müller-Motzfeld, 1998: 73. Type species: *Bembidium terminale* Heer, 1841 by original designation. Etymology. Probably from the specific name of the type species and the last two syllables of the generic name *Peryphanes* [feminine].

#### Diversity.

About 15 species in the Nearctic (one northwestern species), Oriental (one species, *Bembidion vitalisi* Andrewes from Laos), and Palaearctic (14 species) Regions. Some of the species included by Lorenz (2005: 229) in the subgenus *Ocydromus* belong to this subgenus according to Toledano (2000: 66).

#### Identification.

Lindroth (1963b: 307-310) covered the species found in North America.

### Bembidion mckinleyi carneum

Lindroth, 1963

Bembidion mckinleyi carneum Lindroth, 1963b: 309. Type locality: «Racing R[iver], Brit[ish] Col[umbia]» (original citation). Holotype (♂) in CNC [# 8391].

#### Distribution.

This subspecies is known only from the Rocky Mountains and foothills in British Columbia and Alberta, as far north as northern British Columbia near the Yukon Territory border (Lindroth 1963b: 309). The record from southwestern Alaska (Elias 1988: 41) possibly refers to the nominotypical subspecies and needs confirmation.

#### Records.

**CAN**: AB, BC [AK]

### Bembidion mckinleyi mckinleyi

Fall, 1926

Bembidion mckinleyi Fall, 1926a: 132. Type locality: «McKinley Park, Alaska» (original citation). Holotype in MCZ [# 23863].

#### Distribution.

This subspecies is known only from southwestern Yukon Territory and Alaska from the Gulf Coast to beyond the arctic circle (Lindroth 1963b: 308). Fossil remnants of this species, dated between about 10,000 and 11,000 years old, have been found in southern Quebec (Mott et al. 1981: 146) and southern Ontario (Pilny et al. 1987: 620).

#### Records.

**CAN**: YT **USA**: AK

#### Note.

Another subspecies, *Bembidion mckinleyi scandicum* Lindroth, is found along the northern parts of the Palaearctic Region from Sweden to the Far East and Mongolia (Marggi et al. 2003: 268).

### Subgenus Asioperyphus

Vysoký, 1986

Asioperyphus Vysoký, 1986: 94. Type species: *Bembidium infuscatum* Dejean, 1831 by original designation. Etymology. From the Latin *Asia* (Asia) and the generic name *Peryphus* [*q.v*.], alluding to the region where the species known to Vysoký lived [masculine].
Chinoperyphus Vysoký, 1986: 96. Type species: *Bembidion obenbergeri* Lutshnik, 1928 by original designation. Synonymy established by Müller-Motzfeld (1998: 73). Etymology. From the Latin *China* (China) and the generic name *Peryphus* [*q.v*.], alluding to the country where the type species occurs [masculine].

#### Diversity.

Northern Hemisphere, with about 30 species in the Nearctic (six species) and Palaearctic (27 species) Regions. Two species (*Bembidion umiatense* and *Bembidion lenae*) are Holarctic.

#### Identification.

Lindroth (1963b: 326-330, as *bimaculatum* and *lenae* groups) covered all but one (*Bembidion renoanum*) of the species found in North America.

### Bembidion bimaculatum

(Kirby, 1837)

Peryphus bimaculatus Kirby, 1837: 52. Type locality: «Lat. 65° [= apparently region of Great Bear Lake, Northwest Territories]» (original citation). Two syntypes in BMNH (Lindroth 1953b: 175).

#### Distribution.

This species ranges from north-central Ontario (CNC) to the Arctic Plains of Alaska (Lindroth 1963b: 326), south to San Bernardino County in southeastern California (Dajoz 2007: 20), northeastern Arizona (Apache County, UASM; Wickham 1896a: 157), and southern New Mexico (Fall and Cockerell 1907: 157). The record from “Kansas” (Hamilton 1894a: 7) is probably in error.

#### Records.

**CAN**: AB, BC (VCI), MB, NT, ON, SK, YT **USA**: AK, AZ, CA, CO, ID, MT, NM, NV, OR, UT, WA, WY

### Bembidion lenae

Csiki, 1928

Bembidium intermedium Poppius, 1906a: 32 [secondary homonym of *Bembidion intermedium* (Kirby, 1837)]. Type locality: «Ytyk-haja, Lena [River, Russia]» (lectotype label). Lectotype (♂), designated by Fassati (1952: 32), in ZMH.
Bembidion poppii var. *lenae* Csiki, 1928: 108. Replacement name for *Bembidion poppii* var. *intermedium* Poppius, 1906.

#### Distribution.

This Holarctic species is found from eastern Siberia (Marggi et al. 2003: 243) to the Anderson River delta in northern Northwest Territories (Lindroth 1963b: 311).

#### Records.

**CAN**: NT, YT **USA**: AK – **Holarctic**

### Bembidion postremum

Say, 1830

Bembidium postremum Say, 1830c: 23. Type locality: «Allegheny [Allegheny County], P[ennsylvani]a» (neotype label). Neotype (♂), designated by Lindroth and Freitag (1969: 336), in MCZ [# 33069]. Note. «Pennsylvania» was the area originally cited by Say (1830c: 23).

#### Distribution.

This eastern species is found from “New Brunswick” (Larochelle and Larivière 1990a: 28) to northern Minnesota (Gandhi et al. 2005: 925), south to northeastern Iowa (Purrington and Larsen 1997: 50), southwestern Ohio (Dury 1910: 66), and southwestern Pennsylvania (Hayward 1897: 80; Lindroth and Freitag 1969: 336). The record from northeastern Georgia (Fattig 1949: 17) is probably in error.

#### Records.

**CAN**: NB, QC **USA**: IA, IL, IN, MA, ME, MN, NH, NY, OH, PA, VT, WI

### Bembidion renoanum

Casey, 1918

Bembidion renoanum Casey, 1918: 72. Type locality: «Reno [Washoe County], Nevada» (original citation). Lectotype (♂), designated by Erwin (1984a: 181), in USNM [# 36932].

#### Distribution.

This species is known only from the type locality in western Nevada.

#### Records.

**USA**: NV

### Bembidion sordidum

(Kirby, 1837)

Peryphus sordidus Kirby, 1837: 52. Type locality: «Lat. 54° [= along North Saskatchewan River]» (original citation), restricted to «Edmonton, Al[ber]ta» by Lindroth (1963b: 328). Holotype [by monotypy] in BMNH (Lindroth 1953b: 176).

#### Distribution.

The range of this species extends from the Ungava Bay area (Larochelle 1975: Fig. 173) in northern Quebec to the Arctic Plains of Alaska (Lindroth 1963b: 329), south to east-central Utah (Grand County, UASM), southwestern Colorado (Elias 1987: 632), southern South Dakota (Kirk and Balsbaugh 1975: 18), and the Saint Lawrence River drainage in southern Quebec (Larochelle 1975: Fig. 173). The record from “Michigan” (Bousquet and Larochelle 1993: 137) needs confirmation. Fossil remnants, dated from about 10,400 and 21,500 years B.P., have been unearthed in eastern Minnesota, northeastern Wisconsin, Iowa, northern Illinois (Schwert 1992: 77), northeastern Pennsylvania (Barnosky et al. 1988: 178), and Cape Breton Island in Nova Scotia (Miller 1997: 250).

#### Records.

**CAN**: AB, BC, MB, NT, ON, QC, SK, YT **USA**: AK, CO, MT, ND, SD, UT, WY [MI]

### Bembidion umiatense

Lindroth, 1963

Bembidion umiatense Lindroth, 1963b: 329. Type locality: «Umiat, Alaska» (original citation). Holotype (♂) in CNC [# 8380].

#### Distribution.

This Holarctic species is found from the northern regions of European Russia to eastern Siberia (Marggi et al. 2003: 244) and in North America from the Arctic Plains in Alaska (Lindroth 1963b: 330) to Contwoyto Lake in western Nunavut (Shpeley and Pilny 1995: 233).

#### Records.

**CAN**: NU, YT **USA**: AK – **Holarctic**

### Subgenus Peryphanes

Jeannel, 1941

Peryphanes Jeannel, 1941b: 484. Type species: *Peryphus dalmatinus* Dejean, 1831 by original designation. Etymology. Uncertain, possibly from the generic name *Peryphus* [*q.v*.] contracted and the Greek *phanos* (light, bright) [feminine].

#### Diversity.

Northern Hemisphere, with about 35 species in the Nearctic (seven species, one of them adventive) and Palaearctic (about 30 species) Regions. Two species (*Bembidion grapii* and *Bembidion yukonum*), placed in this subgenus for convenience, are Holarctic.

#### Identification.

Lindroth (1963b) covered all but one (*Bembidion subangustatum*) of the species found in North America.

### Bembidion grapii

Gyllenhal, 1827

Bembidium brunnipes C.R. Sahlberg, 1827a [19 May]: 191 [primary homonym of *Bembidium brunnipes* Sturm, 1825]. Type locality: «Fennia Australi» (original citation). Syntype(s) location unknown (possibly in ZMH).
Bembidium grapii Gyllenhal, 1827 [July-October]: 403. Type locality: «Lapponia boreali» (original citation), restricted to «Abisko, Sweden» by Lindroth (1963b: 319). Syntype(s) location unknown (possibly in UZIU). Synonymy established with the name *Bembidion sahlbergii* Dejean by Schaum (1861: 407). Note. The spelling *grapei* is an incorrect subsequent spelling, first introduced by Zetterstedt (1837: 25), not currently in prevailing usage.
Bembidium sahlbergii Dejean, 1831: 144. Type locality: «Finlande» (original citation). Syntype(s) probably in MHNP. Synonymy established with the name *Bembidion brunnipes* Sahlberg by Dejean (1831: 144).
Peryphus picipes Kirby, 1837: 54. Type locality: «Lat. 65° [= apparently region of Great Bear Lake, Northwest Territories]» (original citation). Two syntypes [2 originally cited] in BMNH (Lindroth 1953b: 176). Synonymy established by Fall (1926a: 133), confirmed by Lindroth (1953b: 176).
Bembidium aereum Jacquelin du Val, 1851: 508. Type locality: «Laponie méridionale» (original citation). Holotype [by monotypy] probably in MHNP. Synonymy established by Piochard de La Brûlerie (1876: 447).
Ochthedromus nitens LeConte, 1857c: 10. Type locality: «ora orientali insulae Kadjak» (original citation for *Ochthedromus picipes* (Kirby) *sensu* Mannerheim, 1853). Syntype(s) location unknown (possibly in ZMH). Synonymy established by Henshaw (1885: 6). Note. This name was proposed for *Peryphus picipes* Kirby, 1837 *sensu* Mannerheim (1853: 151). Therefore the type series consists of the specimen(s) which had been misidentified (ICZN 1999: Article 72.4.2). The specimen labeled “Type 5519” in MCZ is not a syntype since it bears also a pale blue disc (= north shore of Lake Superior).
Metallina planicollis Motschulsky, 1860: 91. Type locality: «Kamtschatka [Russia]» (original citation). Three syntypes in ZMMU (Keleinikova 1976: 211). Synonymy established, under the name *Bembidion picipes* (Kirby), by Netolitzky (1935a: 29).
Bembidium islandicum Sharp, 1900: 254. Type locality: «Reykjavik [Iceland]» (original citation). Syntypes location unknown. Synonymy established by Lindroth (1931: 169).
Bembidion scrutatum Casey, 1918: 64. Type locality: «Eldora [Boulder County], Colorado» (original citation). Lectotype (♂), designated by Erwin (1984a: 173), in USNM [# 36987]. Synonymy established by Erwin (1984a: 173).
Bembidion nitens seductum Casey, 1918: 66. Type locality: «Eldora [Boulder County], Colorado» (original citation). Lectotype (♀), designated by Lindroth (1975: 118), in USNM [# 36900]. Synonymy established by Lindroth (1963b: 319).
Bembidion grapei v. *nitiduloides* Munster, 1930: 354. Type locality: «Kaafjord i Alten og desuten ved Jennestad [Norway]» (original citation). Holotype location unknown (possibly in ZMUO). Synonymy established by Munster (1932: 82).

#### Distribution.

This Holarctic species ranges in North America from the Seward Peninsula in Alaska (Lindroth 1963b: 320) to Newfoundland (Lindroth 1955a: 57) and Greenland (Böcher 1988: 12), including the Aleutian and Kodiak Islands, south to mountains in New England (Lindroth 1963b: 320), southeastern upper peninsula of Michigan (Mackinac County, UMAA), northern Wisconsin (Bayfield County, MCZ), northeastern Minnesota (Kamal J.K. Gandhi pers. comm. 2008), southern New Mexico (Fall and Cockerell 1907: 157), and southeastern Arizona (Dajoz 2007: 21; Lindroth 1969a: 1114). The records from “Massachusetts” and “Pennsylvania” (Bousquet and Larochelle 1993: 136) are in error.

#### Records.

**DEN**: GL **FRA**: PM **CAN**: AB, BC, LB, MB, NB, NF, NS (CBI), NT, ON, QC, SK, YT **USA**: AK, AZ, CO, ME, MI, MN, MT, NH, NM, NV, NY, UT, VT, WI, WY – **Holarctic**

#### Note.

This species is listed as *incertae sedis* by Marggi et al. (2003: 271) and Maddison (2012: 542). It belongs to the *Ocydromus* Complex. For convenience, the species is listed in the subgenus *Peryphanes* where Lindroth (1963b: 319, as *grapei* group) placed it.

### Bembidion lacunarium

(Zimmermann, 1869)

Ochthedromus lacunarius Zimmermann [in LeConte], 1869b: 248. Type locality: «middle states» (original citation), restricted to «White Sulphur Springs [Greenbrier County], W[est] V[irgini]a» by Lindroth (1963b: 325). Syntype(s) probably lost.
Bembidion militare Casey, 1884c: 65. Type locality: «Willets Point [Queens County], Long Island [New York]» (original citation). Holotype [by monotypy] in MCZ [# 5518]. Synonymy established, under the name *Bembidion picipes* (Kirby) *sensu* Hayward (= *Bembidion lacunarium* Zimmermann), by Hayward (1897: 83), confirmed by Lindroth (1954b: 125).
Bembidion histricum Casey, 1918: 68. Type locality: «probably Indiana» (original citation). Holotype [by monotypy] (♂) in USNM [# 36927]. Synonymy established by Lindroth (1963b: 325).

#### Distribution.

This eastern species is found from New Brunswick (Webster and Bousquet 2008: 17) to “North Dakota” (Donald P. Schwert pers. comm. 1989), south to Big Bend National Park in western Texas (Dajoz 2007: 23) and Tennessee (Cannon, Clay, Fayette, Macon, Madison, Overton, Smith, Wayne, and Wilson Counties, CMNH), including northeastern New Mexico (Fall and Cockerell 1907: 157, as *Bembidion picipes*). The record from British Columbia (Jarrett and Scudder 2001: 381) was based on misidentified specimens of *Bembidion platynoides* Hayward (UBC).

#### Records.

**CAN**: NB, ON, QC **USA**: CT, IA, IN, KS, KY, MA, MD, ME, MI, MN, MO, ND, NH, NJ, NM, NY, OH, PA, SD, TN, TX, VA, VT, WI, WV

#### Note.

This species has been known for a long time under the name *Bembidion picipes* (Kirby). Lindroth (1953b: 176) showed that Kirby’s syntypes of *Bembidion picipes* were conspecific with those of *Bembidion grapii* Gyllenhal.

### Bembidion platynoides

Hayward, 1897

Peryphus concolor Motschulsky, 1850a: 9 [primary homonym of *Peryphus concolor* Kirby, 1837]. Type locality: «California» (original citation), cited from «St. Francisco» by Motschulsky (1859a: 129). Lectotype, designated by Bousquet and Larochelle (1993: 16), in ZMMU.
Bembidium platynoides Hayward, 1897: 78. Type locality: «Pomona [Los Angeles County], California» (original citation for the lectotype). Lectotype (♂), designated by Erwin (1984a: 179), in MCZ [# 16287]. Synonymy established by Bousquet and Larochelle (1993: 16).
Bembidion insopitans Casey, 1918: 68. Type locality: «Victoria, British Columbia» (original citation). Lectotype (♀), designated by Lindroth (1975: 118), in USNM [# 36926]. Synonymy established by Lindroth (1963b: 322).
Bembidion optatum Casey, 1918: 69. Type locality: «Redwood Creek, Humboldt Co[unty], California» (original citation). Lectotype (♀), designated by Lindroth (1975: 118), in USNM [# 36929]. Synonymy established by Lindroth (1963b: 322).
Bembidion merens Casey, 1918: 70. Type locality: Gualala, Mendocino County, California (lectotype label according to Lindroth 1975: 119). Lectotype (♂), designated by Lindroth (1975: 119), in USNM [# 36930]. Synonymy established by Lindroth (1963b: 322).
Bembidion sedulum Casey, 1918: 70. Type locality: «southern California» (original citation). Lectotype (♂), designated by Erwin (1984a: 179), in USNM [# 36931]. Synonymy established by Erwin (1984a: 179).

#### Distribution.

The range of this western species extends from Vancouver Island (Lindroth 1963b: 323) to western Montana (Russell 1968: 53; Edwards 1975: 53), north to central British Columbia (Lindroth 1963b: 323), south to southern California (Hayward 1897: 79; Fall 1901a: 42; Moore 1937: 6). The record from “New Mexico” (Bousquet and Larochelle 1993: 136) is probably in error.

#### Records.

**CAN**: BC (VCI) **USA**: AZ, CA (CHI), ID, MT, OR, WA

### Bembidion stephensii

Crotch, 1866

Peryphus affinis Stephens, 1835: 386 [secondary homonym of *Bembidion affine* (Say, 1823)]. Type locality: «near Marton, Yorkshire [United Kingdom]» (original citation). Syntype(s) location unknown.
Bembidium stephensii Crotch, 1866: 110. Replacement name for *Bembidium affine* (Stephens, 1835). Etymology. The specific name honors the British entomologist James Francis Stephens [1792-1852] who worked as a clerk in the admiralty at Somerset House in London and later at the British Museum. Stephens devoted a large part of his life to study British insects. His collection was purchased by the British Museum and his fine library was acquired by Henry Tibbats Stainton who continued Stephens’ practice of allowing fellow entomologists to use his library on Wednesday evenings. Stainton produced a catalogue of Stephens’ books in 1853, under the title *Bibliotheca Stephensiana*.
Bembidium canadense Hayward, 1897: 77. Type locality: «near Ottawa [Ontario], Canada» (original citation). One syntype [2 originally cited] in MCZ [# 16286]. Synonymy established by Lindroth (1954b: 125).

#### Distribution.

This Palaearctic species is adventive in North America where it is known from Newfoundland (Lindroth 1955a: 58) to northeastern Wisconsin (Forest County, CMNH; Messer 2010: 36), south to northern Ohio (Lee 1994: 58) and Massachusetts (Lindroth 1955a: 58). The records from “Rhode Island” and “Virginia” (Bousquet and Larochelle 1993: 136) need confirmation. The first inventoried specimen collected on this continent was found in Ottawa, Ontario in 1891 (Lindroth 1963b: 323).

#### Records.

**FRA**: PM **CAN**: NB, NF, NS (CBI), ON, PE, QC **USA**: MA, ME, NH, NY, OH, VT, WI [RI, VA] – **Adventive**

### Bembidion subangustatum

Hayward, 1897

Bembidium subangustatum Hayward, 1897: 83. Type locality: «New Mexico and Arizona» (original citation). Two syntypes [“about a dozen” originally cited] in MCZ [# 16289].

#### Distribution.

This species is known from western Texas (Jeff Davis County, Ken Karns pers. comm. 2009), southern New Mexico (Fall and Cockerell 1907: 157), southern Arizona (Casey 1918: 67), southeastern California (Dajoz 2007: 20), and “Mexico” (Casey 1918: 67).

#### Records.

**USA**: AZ, CA, NM, TX – Mexico

### Bembidion texanum

Chaudoir, 1868

Bembidium texanum Chaudoir, 1868b: 240. Type locality: «Texas» (original citation), herein restricted to Kerrville, Kerr County (CNC). Lectotype (♂), designated by Lindroth (1963b: 325), in MHNP.
Bembidion inquietum Casey, 1918: 67. Type locality: «Jemez Springs [Sandoval County], New Mexico» (original citation). Lectotype (♀), designated by Erwin (1984a: 184), in USNM [# 36924]. Synonymy established by Erwin (1984a: 184).
Bembidion cogitans Casey, 1918: 69. Type locality: «probably Indiana» (original citation). Lectotype (♂), designated by Erwin (1984a: 184), in USNM [# 36928]. Synonymy established by Erwin (1984a: 184).

#### Distribution.

This species ranges from northwestern Ohio (Holeski and Graves 1982: 216) to southern Manitoba (Lindroth 1963b: 326), south to southeastern Arizona (Maddison 1985: 114; Dajoz 2007: 21), northwestern New Mexico (Casey 1918: 67 as *Bembidion inquietum*), west-central and central Texas (Blanco, Crockett, Edwards, Travis, and Williamson Counties, CMNH), southeastern Louisiana (Summers 1874a: 81), and northern Mississippi (Grenada and Pontotoc Counties, Drew A. Hildebrandt pers. comm. 2010).

#### Records.

**CAN**: MB **USA**: AR, AZ, CO, IA, IL, IN, KS, KY, LA, MN, MO, MS, NE, NM, OH, OK, SD, TN, TX, WI

### Bembidion yukonum

Fall, 1926

Bembidion yukonum Fall, 1926a: 131. Type locality: «Dawson, Yukon Territory» (original citation). Holotype (♂) in MCZ [# 23869].
Bembidion nitidulum var. *grapeioides* Munster, 1930: 354. Type locality: «Neiden i Syd-Varanger [Norway]» (original citation). Holotype location unknown (possibly in ZMUO). Synonymy established by Lindroth (1954b: 129).
Bembidion nitidulum var. *sahlbergioides* Munster, 1932: 80. Unnecessary replacement name for *Bembidion nitidulum* var. *grapeioides* Munster, 1930.

#### Distribution.

This Holarctic species is found from Scandinavia to eastern Siberia (Marggi et al. 2003: 272) and in North America from the Arctic Plains in northern Alaska (Lindroth 1963b: 321) to the north shore of the Saint Lawrence Estuary (Larochelle 1975: Fig. 183), south to east-central British Columbia (Lindroth 1963b: 321).

#### Records.

**CAN**: BC, MB, NT, QC, YT **USA**: AK – **Holarctic**

#### Note.

This species is listed as *incertae sedis* by Marggi et al. (2003: 272). It was not sequenced by Maddison (2012). For convenience, the species is listed in the subgenus *Peryphanes* where Lindroth (1963b: 321, as *grapei* group) placed it.

### Subgenus Testediolum

Ganglbauer, 1891

Testediolum Ganglbauer, 1891a: 153. Type species: *Bembidium glaciale* Heer, 1837 designated by Jeannel (1941a: 97). Etymology. From the generic name *Testedium* and the Latin suffix -*olum* (small, little) [neuter].
Peryphidium Tschitschérine, 1895: 233. Type species: *Bembidium tjanschanicum* Tschitschérine, 1895 (= *Bembidion kokandicum* Solsky, 1874) by monotypy. Synonymy established by Netolitzky (1921: 210). Etymology. From the generic name *Peryphus* [*q.v*.] and the Latin suffix -*idium* (small, little) [neuter].

#### Diversity.

Northern Hemisphere, with 24 species in the Nearctic (6 species) and Palaearctic (18 species) Regions arrayed in three species groups, two of these groups being exclusively Eurasian. One species from India, *Bembidion braminum* Andrewes, is usually also included in this subgenus (see Lorenz 2005: 234) but its taxonomic position should be reevaluated.

#### Identification.

Lindroth (1963b: 312-314, as *nebraskense* group) covered all but two species (*Bembidion modocianum* and *Bembidion perbrevicolle*) described by Casey.

### Bembidion commotum

Casey, 1918

Bembidion commotum Casey, 1918: 23. Type locality: «Lake Tahoe [Placer County], California» (original citation). Lectotype (♀), designated by Lindroth (1975: 118), in USNM [# 36824].
Bembidion seclusum Casey, 1918: 23. Type locality: «Placer Co[unty], California» (original citation). Holotype [by monotypy] (♂) in USNM [# 36825]. Synonymy established by Lindroth (1963b: 313).

#### Distribution.

This species is found in the North American Cordilleras from southeastern Alberta and southern British Columbia south to the Sierra Nevada in California (Lindroth 1963b: 313) and to central Colorado (Wickham 1902: 233, as *Bembidion breve*) along the Rocky Mountains.

#### Records.

**CAN**: AB, BC **USA**: CA, CO, ID, MT, NV, OR, WA, WY

#### Note.

This species has been known until the 1960s under the name *Bembidion breve* (Motschulsky).

### Bembidion modocianum

Casey, 1924

Bembidion modocianum Casey, 1924: 29. Type locality: «Modoc Co[unty], California» (original citation). Lectotype (♂), designated by Erwin (1984a: 177), in USNM [# 36882].

#### Distribution.

This species is known so far only from the type series collected in northeastern California.

#### Records.

**USA**: CA

### Bembidion nebraskense

LeConte, 1863

Bembidium nebraskense LeConte, 1863c: 19. Type locality: «Nebraska [Territory], near the Rocky Mountains [probably in present day Colorado]» (original citation). Holotype [by monotypy] (♀) in MCZ [# 5506].
Bembidion denveranum Casey, 1918: 64. Type locality: «Boulder Co[unty], Colorado» (original citation). Lectotype (♂), designated by Erwin (1984a: 177), in USNM [# 36898]. Synonymy established by Erwin (1984a: 177).
Bembidion tractabile Casey, 1918: 64. Type locality: «Utah» (original citation). Lectotype (♀), designated by Erwin (1984a: 177), in USNM [# 36901]. Synonymy established by Erwin (1984a: 177).
Bembidion govanicum Casey, 1924: 31. Type locality: «Govan [Lincoln County], Washington» (original citation). Lectotype (♂), designated by Lindroth (1975: 118), in USNM [# 36895]. Synonymy established by Hatch (1953: 89), confirmed by Lindroth (1963b: 312).

#### Distribution.

This western species is known from southwestern Alberta and southern British Columbia (Lindroth 1963b: 312) south at least to northeastern California (Modoc County, CNC; Hayward 1897: 69), Nevada (White Pine County, CMNH), northern Utah (Rich County, UASM), and northern New Mexico (Taos County, UASM; Hayward 1897: 69; Maddison 2012: Supplementary content Table S1). The record from “Nebraska” (Hayward 1897: 69) probably originated from the original statement about the type locality.

#### Records.

**CAN**: AB, BC **USA**: CA, CO, ID, MT, NM, NV, OR, UT, WA, WY

### Bembidion obscuripenne

Blaisdell, 1902

Bembidium obscuripenne Blaisdell, 1902: 74. Type locality: «Oregon» (original citation), restricted to «Dallas [Polk County]» by Lindroth (1963b: 314). Syntype(s) [2 originally cited] in CAS [# 2657].
Bembidium whitneyi Fall, 1910: 96. Type locality: «M[oun]t Whitney (8,000 to 11,000 feet) [Tulare-Inyo Counties], California» (original citation). Syntype(s) in MCZ [# 23868]. Synonymy established by Lindroth (1963b: 314).
Bembidium micans Notman, 1919b: 227. Type locality: «Dallas [Polk County], Ore[gon]» (original citation). Syntype(s) [2 originally cited] location unknown (originally in collection C.W. Leng). Synonymy established by Lindroth (1963b: 314).
Bembidion immaculosum Hatch, 1950: 101. Type locality: «Spokane [Spokane County], Washington» (original citation). Holotype (♂) in USNM. Synonymy established by Lindroth (1963b: 314).

#### Distribution.

This species is found along the North American Cordilleras from northern Washington (Hatch 1950: 101, as *Bembidion immaculosum*) to Sequoia National Park in southeastern California along the Sierra Nevada (Fall 1910: 96, as *Bembidion whitneyi*; Dajoz 2007: 16). The records from southern Idaho (Horning and Barr 1970: 24; Stafford et al. 1986: 288, as *Bembidion immaculosum*) and British Columbia (Hatch 1953: 89, as *Bembidion nevadense* sensu Hatch) need confirmation.

#### Records.

**USA**: CA, OR, WA [BC, ID]

### Bembidion perbrevicolle

Casey, 1924

Bembidion perbrevicolle Casey, 1924: 25. Type locality: «Placer Co[unty], California» (original citation). Lectotype (♀), designated by Erwin (1984a: 178), in USNM [# 36826].

#### Distribution.

According to Erwin (1984a: 178), this species occurs in the foothills of the Sierra Nevada of California.

#### Records.

**USA**: CA

### Bembidion ulkei

Lindroth, 1963

Bembidion ulkei Lindroth, 1963b: 314. Type locality: «Nevada» (original citation). Holotype (♂) in CMNH. Etymology. The specific name honors Henry Ulke [1821-1910], a German by birth, who emigrated with his two brothers in 1852 to the United States and settled first in New York, then Philadelphia and finally in Washington DC. He was a portrait painter by profession and painted presidents (hence his soubriquet of “painter of presidents”), cabinet officers, and worthy individuals. Following the death of his wife in 1893 he lost interest in entomology and sold his collection in November 1900 to the Carnegie Museum in Pittsburgh.

#### Distribution.

This species is known only from the original six specimens collected in “Nevada.”

#### Records.

**USA**: NV

### Subgenus Leuchydrium

Casey, 1918

Leuchydrium Casey, 1918: 46. Type species: *Bembidium tigrinum* LeConte, 1879 by monotypy. Etymology. From Greek *leukos* (white) and the generic name *Hydrium* [*q.v*.], alluding to the pale coloration of adults of the sole species (“pallid coloration”) [neuter].

#### Diversity.

One North American species along the Pacific Coast.

#### Identification.

The species is included in Lindroth’s (1963b: 224) key to the Canadian *Bembidion*.

### Bembidion tigrinum

LeConte, 1879

Bembidium tigrinum LeConte, 1879d: 509. Type locality: «southern part of California» (original citation), herein restricted to Santa Barbara, Santa Barbara County (Fall 1901a: 43). Holotype [by monotypy] (♀) in MCZ [# 5521].

#### Distribution.

This species ranges along the Pacific Coast from the southern tip of Vancouver Island (Bousquet 1987a: 121) to southern California (LeConte 1879d: 509; Fall 1901a: 9).

#### Records.

**CAN**: BC **USA**: CA, OR, WA

### Subgenus Bembidion

Latreille, 1802

Bembidion Latreille, 1802: 82. Type species: *Carabus quadriguttatus* Fabricius, 1775 (= *Cicindela quadrimaculata* Linnaeus, 1760) designated by Andrewes (1935: 17).
Lopha Dejean, 1821: 17. Type species: *Cicindela quadrimaculata* Linnaeus, 1760 designated by Westwood (1838: 7).
Taractus Gistel, 1856: 359. Type species: *Cicindela quadrimaculata* Linnaeus, 1760 designated by Bousquet (2002b: 50). Etymology. Possibly from the Greek *taractes* (disturbed) [masculine].

#### Diversity.

Northern Hemisphere, with 11 species in the Nearctic (six species) and Palaearctic (six species) Regions. One species (*Bembidion quadrimaculatum*) is Holarctic but with different subspecies in the Nearctic and Palaearctic Regions.

#### Identification.

Lindroth (1963b: 382-387) covered four of the North American species in his monograph. A revision of the Nearctic species is needed.

### Bembidion adductum

Casey, 1918

Bembidion adductum Casey, 1918: 149. Type locality: «Paraiso Hot Springs, Monterey Co[unty], California» (original citation). Lectotype (♂), designated by Erwin (1984a: 166), in USNM [# 37062].
Bembidion relictum Casey, 1918: 153 [primary homonym of *Bembidion relictum* Apfelbeck, 1904]. Type locality: «Truckee [Nevada County], California» (original citation). Holotype [by monotypy] (♂) in USNM [# 37067]. Synonymy established by Erwin (1984a: 166).
Bembidion reliquum Csiki, 1928: 145. Replacement name for *Bembidion relictum* Casey, 1918.

#### Distribution.

This species is known from the Sierra Nevada (Casey 1918: 153, as *Bembidion relictum*; Dajoz 2007: 17) and the Coast Ranges in west-central California. The record from eastern Oregon (Hatch 1953: 98, as *Bembidion relictum*) needs confirmation.

#### Records.

**USA**: CA [OR]

### Bembidion mutatum

Gemminger and Harold, 1868

Ochthedromus axillaris LeConte, 1850: 211 [secondary homonym of *Bembidion axillare* (Motschulsky, 1844)]. Type locality: «Sault [= Sault Sainte Marie; according to Lindroth (1963b: 386) probably the locality in Ontario]» (original citation). Two syntypes in MCZ [# 5550]. Note. Lindroth (1963b: 386) pointed out that the syntype, labeled as type, in MCZ has the forebody of this species glued to the hindbody of *Bembidion quadrimaculatum* (Linnaeus). The description of *Bembidion axillare* points to the forebody as being part of the original specimen.
Bembicidium mutatum Gemminger and Harold, 1868a: 416. Replacement name for *Bembicidiun axillare* (LeConte, 1850).
Bembidion vegetum Casey, 1918: 151. Type locality: «Boulder Co[unty], Colorado» (original citation). Lectotype (♀), designated by Lindroth (1975: 121), in USNM [# 37064]. Synonymy established by Lindroth (1963b: 386).

#### Distribution.

The range of this species extends from Newfoundland (Lindroth 1955a: 73) to east-central Alaska (Lindroth 1963b: 387), south to Nechako River in central British Columbia, northern New Mexico (Fall and Cockerell 1907: 157), west-central Minnesota (Gandhi et al. 2005: 924), the Adirondack Mountains in northeastern New York (Notman 1928: 218), and New England (Lindroth 1963b: 387). The record from “Washington” (Bousquet and Larochelle 1993: 146) is probably in error.

#### Records.

**CAN**: AB, BC, MB, NB, NF, NS, NT, ON, QC, SK, YT **USA**: AK, CO, ID, ME, MI, MN, NH, NM, NY, UT, VT, WI, WY

### Bembidion oregonense

Hatch, 1953

Bembidion oregonense Hatch, 1953: 98. Type locality: «Clear Lake [Marion County], Ore[gon]» (original citation). Holotype (♀) in USNM.

#### Distribution.

As far as known, this species has been recorded from the type locality and Frenchglen, Harney County, in Oregon (Hatch 1953: 98).

#### Records.

**USA**: OR

### Bembidion pedicellatum

LeConte, 1857

Bembidium pedicellatum LeConte, 1857a: 6. Type locality: «Lancaster Co[unty], Pennsylvania» (original citation). Holotype [by monotypy] (♂) in MCZ [# 5551].
Bembidion strigulosum Casey, 1918: 150. Type locality: «District of Columbia» (original citation). Lectotype (♂), designated by Erwin (1984a: 178), in USNM [# 37068]. Synonymy established by Lindroth (1963b: 383), confirmed by Erwin (1984a: 178).
Bembidion fastidiosum Casey, 1918: 150. Type locality: «S[ain]t Louis, Missouri» (original citation for the lectotype). Lectotype (♂), designated by Erwin (1984a: 178), in USNM [# 37069]. Synonymy established by Lindroth (1963b: 383), confirmed by Erwin (1984a: 178).

#### Distribution.

This species ranges from southeastern Pennsylvania (Delaware and Lebanon Counties, Robert L. Davidson pers. comm. 2008) to southeastern Minnesota (Gandhi et al. 2005: 925), south to central Texas (Bosque County, CMNH) and eastern Tennessee (Sevier County, CNC). The records from “New Jersey” and “North Carolina” (Bousquet and Larochelle 1993: 146) need confirmation.

#### Records.

**USA**: DC, DE, IA, IL, IN, KY, KS, MD, MI, MN, MO, NE, OH, PA, TN, TX, VA, WI, WV [NC, NJ]

### Bembidion praecinctum

LeConte, 1879

Bembidium praecinctum LeConte, 1879d: 509. Type locality: «Alamosa [Alamosa County], Colo[rado]» (original citation). Holotype [by monotypy] (♀) in MCZ [# 5547].
Bembidion veridicum Casey, 1918: 152. Type locality: «Elko [Elko County], Nevada» (original citation). Lectotype (♂), designated by Erwin (1984a: 179), in USNM [# 37066]. Synonymy established by Erwin (1984a: 179).
Bembidion alutaceum Hatch, 1950: 103. Type locality: «Mottet Meadow, Blue M[oun]t[ain]s [Union County], Oregon» (original citation). Holotype (♂) in USNM. Synonymy established by Lindroth (1963b: 385).

#### Distribution.

This species is found from southern Manitoba (Lindroth 1963b: 386) to southeastern Washington (Hatch 1953: 98), including southern Saskatchewan and Alberta (Lindroth 1963b: 386), south at least to Santa Barbara County in southwestern California (Maddison 1985: 113), southern Arizona (Dajoz 2007: 21), and southern Colorado (LeConte 1879d: 509; Wickham 1902: 234).

#### Records.

**CAN**: AB, MB, SK **USA**: AZ, CA, CO, ID, MT, NV, OR, UT, WA, WY

### Bembidion quadrimaculatum dubitans

(LeConte, 1852)

Ochthedromus dubitans LeConte, 1852a: 189. Type locality: «San Francisco [San Francisco County, California]» (original citation). Syntype(s) in MCZ [# 5548].
Ochthedromus cruralis LeConte, 1852a: 189. Type locality: «San Jose [Santa Clara County, California]» (original citation). Syntype(s) in MCZ [# 5549]. Synonymy established by Hayward (1897: 136), confirmed by Lindroth (1963b: 384).
Bembidion gregale Casey, 1918: 148. Type locality: «Agassiz, British Columbia» (original citation). Lectotype (♀), designated by Lindroth (1975: 121), in USNM [# 37060]. Synonymy established by Hatch (1953: 98), confirmed by Lindroth (1963b: 384).
Bembidion pugetanum Casey, 1918: 148 [primary homonym of *Bembidion pugetanum* Fall, 1916]. Type locality: «Spokane [Spokane County], Washington» (original citation). Lectotype (♂), designated by Lindroth (1975: 121), in USNM [# 37061]. Synonymy established by Hatch (1953: 98), confirmed by Lindroth (1963b: 384).
Bembidion sapphicum Casey, 1918: 149. Type locality: «Reno [Washoe County], Nevada» (original citation). Lectotype (♂), designated by Lindroth (1975: 121), in USNM [# 37063]. Synonymy established by Lindroth (1963b: 384).
Bembidion tenax Casey, 1918: 152. Type locality: «Fort Wingate [McKinley County], New Mexico» (original citation). Lectotype (♂), designated by Erwin (1984a: 180), in USNM [# 37065]. Synonymy established by Erwin (1984a: 180).
Bembidion caseyi Leng, 1919b: 202. Replacement name for *Bembidion pugetanum* Casey, 1918.

#### Distribution.

This subspecies ranges from the southwestern edge of Lac Winnipeg in southern Manitoba to Vancouver Island (Hayward 1897: 119), north to the Brook Range in Alaska (Lindroth 1963b: 385), south to the Baja California Peninsula (Horn 1894: 308) and southern New Mexico (Fall and Cockerell 1907: 157). The records from Kansas (Snow 1903: 193; Knaus 1905a: 218) need confirmation.

#### Records.

**CAN**: AB, BC (VCI), MB, NT, SK, YT **USA**: AK, AZ, CA, CO, ID, MT, NM, NV, OR, WA, WY [KS] – Mexico

#### Note.

Three other subspecies, including the nominotypical one, are known from the Palaearctic Region (Toledano 1999: 205-210).

### Bembidion quadrimaculatum oppositum

Say, 1823

Bembidium oppositum Say, 1823a: 86. Type locality: «Rumney [Grafton County], N[ew] H[ampshire]» (neotype label). Neotype (♂), designated by Lindroth and Freitag (1969: 338), in MCZ [# 33062].

#### Distribution.

This subspecies ranges from Newfoundland (Lindroth 1955a: 72) to the Rocky Mountains in Alberta (Lindroth 1963b: 384), south to central Colorado (Wickham 1902: 235), “Texas” (Lindroth 1963b: 384), northern Georgia (Fattig 1949: 18), and east-central South Carolina (Ciegler 2000: 48).

#### Records.

**CAN**: AB, MB, NB, NF, NS (CBI), ON, PE, QC, SK **USA**: CO, CT, DC, GA, IA, IL, IN, KS, MA, MD, ME, MI, MN, MO, NC, ND, NE, NH, NJ, NY, OH, OK, PA, RI, SC, SD, TN, TX, VA, VT, WI, WV

### Subgenus Cyclolopha

Casey, 1918

Cyclolopha Casey, 1918: 144. Type species: *Bembidium sphaeroderum* Bates, 1882 designated by Erwin (1982b: 483). Etymology. From the Greek *cyclos* (circle) and the generic name *Lopha* [*q.v*.], alluding to the sphaerical body shape (“oblong-oval, convex body”) of adults of these *Lopha*-like species [feminine].

#### Diversity.

Six Neotropical species of which three extend into southwestern North America.

#### Identification.

Perrault (1982a) revised the species except *Bembidion jucundum* Horn.

### Bembidion jucundum

Horn, 1895

Bembidium jucundum G.H. Horn, 1895: 230. Type locality: «San José del Cabo [Baja California, Mexico]» (original citation for the lectotype). Lectotype (♂), designated by Erwin (1984a: 175), in CAS [# 1].

#### Distribution.

This species is known from the Baja California Peninsula and “Arizona” (Hayward 1897: 121).

#### Records.

**USA**: AZ – Mexico

### Bembidion poculare

Bates, 1884

Bembidium poculare Bates, 1884: 291. Type locality: «Mexico» (original citation). One syntype in BMNH (Perrault 1982a: 98).
Bembidium dilaticolle Notman, 1919b: 227. Type locality: «Huachuca M[oun]t[ain]s [Cochise County], Ariz[ona]» (original citation). Holotype [by monotypy] (♀) in SIM (Hennessey 1990: 466). Synonymy established by Perrault (1982a: 98).

#### Distribution.

This species ranges from southeastern Arizona and southwestern New Mexico (Hidalgo County, CMNH) south to the Isthmus of Tehuantepec in southern Mexico [see Perrault 1982a: Fig. 64].

#### Records.

**USA**: AZ, NM – Mexico

### Bembidion sphaeroderum

Bates, 1882

Bembidium sphaeroderum Bates, 1882a: 147. Type locality: «Jalapa [Veracruz], Mexico» (original citation). Lectotype (♂), designated by Perrault (1982a: 100), in BMNH.
Bembidion occultum Casey, 1918: 144. Type locality: «Grand Cañon of the Colorado, Arizona» (original citation). One syntype in USNM [# 37056]. Synonymy established by Perrault (1982a: 100).
Bembidion minax Casey, 1918: 146. Type locality: «Jemez Springs [Sandoval County], New Mexico» (original citation). One syntype in USNM [# 37059]. Synonymy established by Perrault (1982a: 100).

#### Distribution.

This species is found from Arizona and New Mexico south to Oaxaca in Mexico [see Perrault 1982a: Fig. 64].

#### Records.

**USA**: AZ, NM – Mexico

### Subgenus Furcacampa

Netolitzky, 1931

Furcacampa Netolitzky, 1931: 158. Type species: *Bembidium affinis* Say, 1823 by original designation. Etymology. From the Latin *furca* (fork) and the generic name *Campa* [feminine].

#### Diversity.

Western Hemisphere, with nine species in North America (nine species) and northern Neotropical (three species in Cuba and Mexico, all shared with North America) Regions.

#### Identification.

Lindroth (1963b: 376-381, as *affine* and *versicolor* groups) covered six of the nine species, leaving three species described by Casey. A taxonomic revision of the subgenus is needed.

### [affine group]

### Bembidion affine

Say, 1823

Bembidium affinis Say, 1823a: 86. Type locality: «Mobile [Mobile County], Ala[bama]» (neotype label). Neotype (♂), designated by Lindroth and Freitag (1969: 338), in MCZ [# 33063].
Bembidium decipiens Dejean, 1831: 159. Type locality: «Amérique septentrionale» (original citation). One syntype in MHNP (Lindroth 1955b: 14). Synonymy established by LeConte (1847: 462), confirmed by Lindroth (1955b: 14).
Bembidium fallax Dejean, 1831: 189. Type locality: «Amérique septentrionale» (original citation). One syntype in MHNP (Lindroth 1955b: 14). Synonymy established by LeConte (1850: 211), confirmed by Lindroth (1955b: 14).
Bembidion thespis Casey, 1918: 128. Type locality: «Marquette [Marquette County], Michigan» (original citation). Lectotype (♂), designated by Lindroth (1975: 121), in USNM [# 37001]. Synonymy established by Lindroth (1963b: 376).

#### Distribution.

This species ranges from southwestern Quebec (CNC) to “North Dakota” (Donald P. Schwert pers. comm. 1989), south to east-central Texas (Casey 1918: 128; Riley 2011) and the Florida Panhandle (Peck and Thomas 1998: 18); also recorded from Cuba (Jacquelin du Val 1857: 23). The records from Arizona (Wickham 1896a: 157; Hayward 1897: 122) and “Colorado” (Wickham 1902: 235) need confirmation.

#### Records.

**CAN**: ON, QC **USA**: AL, AR, CT, DC, DE, FL, GA, IA, IL, IN, KS, KY, LA, MA, MD, MI, MO, MS, NC, ND, NH, NJ, NY, OH, OK, PA, RI, SC, SD, TN, TX, VA, VT, WI, WV [AZ, CO] – Cuba

### [versicolor group]

### Bembidion egens

Casey, 1918

Bembidion egens Casey, 1918: 132. Type locality: «Jemez Springs [Sandoval County], New Mexico» (original citation). Lectotype (♂), designated by Erwin (1984a: 171), in USNM [# 37016].
Bembidion demissum Casey, 1918: 133. Type locality: «near Benson [Cochise County], Arizona» (original citation). Lectotype (♀), designated by Erwin (1984a: 171), in USNM [# 37019]. Synonymy established by Erwin (1984a: 171).

#### Distribution.

This species is known from central Wyoming (Natrona County, CMNH) south to northwestern New Mexico (Casey 1918: 132) and southern Arizona (Casey 1918: 133, as *Bembidion demissum*; Cochise and Pima Counties, CMNH). The record from northeastern California (Notman 1929b: 223, as *Bembidion demissum*) needs confirmation.

#### Records.

**USA**: AZ, CO, NM, WY [CA]

### Bembidion fuchsii

Blaisdell, 1902

Bembidium fuchsii Blaisdell, 1902: 77. Type locality: «Blue Lakes, Alpine County, Cal[ifornia]» (original citation). Holotype (♂) in CAS [# 2664]. Etymology. The specific name was proposed for Charles Fuchs [1839-1914], an enthusiastic coleopterist. Born in Germany, Fusch immigrated to the United States at the age of 25 and settled first in New York and later in California. A large part of his collection was lost in the San Francisco earthquake and fire in 1906. He was instrumental in the establishment of the Brooklyn Entomological Society in 1872 and the California Entomological Club in 1901 which became a year later the Pacific Coast Entomological Society.

#### Distribution.

This species is known from eastern Washington (Hatch 1953: 97) to central Wyoming (Natrona County, CMNH), south at least to the Sierra Nevada in east-central California (Blaisdell 1902: 77).

#### Records.

**USA**: CA, ID, OR, WA, WY

### Bembidion impotens

Casey, 1918

Ochthedromus pictus LeConte, 1847: 461 [secondary homonym of *Bembidion pictum* (Duftschmid, 1812)]. Type locality: «Rocky Mountains» (original citation). Syntype(s) in MCZ [# 5537].
Emphanes flavopictus Motschulsky, 1858: 153. Replacement name for *Emphanes pictus* (LeConte, 1847). Note. This name was first introduced by Motschulsky (1850a: 13) in a catalogue but the taxon was not described. Subsequently Motschulsky (1858: 153) listed the name in synonymy with *Ochthedromus pictus* LeConte and pointed out that since LeConte’s name was preoccupied, his name could be preserved. In my opinion such statement should be interpreted as the introduction of a replacement name. Casey (1918: 135) eventually described *Bembidion flavopictum* Motschulsky from the “coast region, from S[an]ta Cruz to Humboldt, California.” This name should have precedence over *Bembidion impotens* Casey, 1918 but since Casey’s name has been used as valid since the 1920s, I believe it should be preserved and the case eventually submitted to the Commission for a ruling. The reversal of precedence (ICZN 1999: Article 23.9.1) could not be applied since Motschulsky’s name has been used as a valid name after 1899 by Casey (1918: 135).
Bembidion impotens Casey, 1918: 129. Replacement name for *Bembidion pictum* (LeConte, 1847).
Bembidion aestivum Casey, 1918: 129. Type locality: «Highland Park [Lake County], northern Illinois» (original citation). Lectotype (♂), designated by Lindroth (1975: 121), in USNM [# 37005]. Synonymy established by Lindroth (1963b: 380).
Bembidion frugale Casey, 1918: 130. Type locality: «Yuma Reservation, California» (original citation). Lectotype (♂), designated by Erwin (1984a: 174), in USNM [# 37006]. Synonymy established by Erwin (1984a: 174).
Bembidion imbelle Casey, 1918: 130. Type locality: «Dallas [Dallas County], Texas» (original citation for the lectotype). Lectotype (♂), designated by Erwin (1984a: 174), in USNM [# 37007]. Synonymy established by Erwin (1984a: 174).
Bembidion gratuitum Casey, 1918: 130. Type locality: «Arizona» (original citation). Lectotype (♂), designated by Erwin (1984a: 174), in USNM [# 37004]. Synonymy established by Erwin (1984a: 174).
Bembidion virgatulum Casey, 1918: 131. Type locality: «Reno [Washoe County], Nevada» (original citation for the lectotype). Lectotype (♂), designated by Erwin (1984a: 174), in USNM [# 37003]. Synonymy established by Erwin (1984a: 174).
Bembidion indigens Casey, 1918: 133. Type locality: «Federal District, Mexico» (original citation). Lectotype (♂), designated by Erwin (1984a: 174), in USNM [# 37017]. Synonymy established by Erwin (1984a: 174).
Bembidion pullulum Casey, 1918: 133. Type locality: «Amecameca, Mexico» (original citation). Lectotype (♂), designated by Erwin (1984a: 174), in USNM [# 37018]. Synonymy established by Erwin (1984a: 174).

#### Distribution.

This species occurs from southwestern New Brunswick (Larochelle and Larivière 1990a: 29, 34) to southern British Columbia (Lindroth 1963b: 381), south to southeastern California (Casey 1918: 130, as *Bembidion frugale*; Andrews et al. 1979: 28), the Federal District in Mexico (Casey 1918: 133, as *Bembidion indigens*), and southern Florida (Peck and Thomas 1998: 18).

#### Records.

**CAN**: BC, MB, NB, ON, QC, SK **USA**: AR, AZ, CA, CO, CT, FL, GA, IA, ID, IL, IN, KS, KY, LA, MA, MD, ME, MI, MN, MO, MS, MT, NC, NE, NJ, NM, NV, OH, OK, OR, PA, SC, SD, TN, TX, UT, VA, VT, WA, WI, WV, WY – Mexico

### Bembidion mimus

Hayward, 1897

Bembidium mimus Hayward, 1897: 108. Type locality not stated; «Nipigon, Ont[ario]» selected by Lindroth (1963b: 380). One possible syntype in MCZ [# 5536] labeled “L[ake] Sup[erior].”
Bembidion rotundiceps Casey, 1918: 132. Type locality: «Long Island, New York» (original citation). Lectotype (♀), designated by Lindroth (1975: 121), in USNM [# 37012]. Synonymy established by Lindroth (1963b: 380).
Bembidion pellax Casey, 1918: 136. Type locality: «Boston Neck [Washington County], Rhode Island» (original citation). Lectotype (♂), designated by Lindroth (1975: 121), in USNM [# 37015]. Synonymy established by Lindroth (1954b: 127).

#### Distribution.

The range of this species extends from southern Newfoundland (Lindroth 1955a: 72) to southern Saskatchewan (Ronald R. Hooper pers. comm. 1990), south to northern Nebraska (Cherry County, Foster F. Purrington pers. comm. 2010), “Illinois” (Lindroth 1955a: 72), and northeastern West Virginia (Tucker County, CMNH). The record from the Atlantic Coast in South Carolina (Kirk 1969: 10; Ciegler 2000: 48) needs confirmation.

#### Records.

**CAN**: MB, NB, NF, NS (CBI), ON, PE, QC, SK **USA**: CT, IA, IL, MA, ME, MI, MN, NE, NH, NJ, NY, OH, PA, RI, SD, VT, WI, WV [SC]

### Bembidion nogalesium

Casey, 1924

Bembidion nogalesium Casey, 1924: 42. Type locality: «Nogales, S[an]ta Cruz Co[unty], Arizona» (original citation). Lectotype (♀), designated by Erwin (1984a: 177), in USNM [# 37020].

#### Distribution.

This species has been collected in western Texas (Brewster County, CMNH), southern Arizona (Cochise, Pima, and Pinal Counties, CMNH; Casey, 1924: 42), and in Baja California Sur (CMNH).

#### Records.

**USA**: AZ, TX – Mexico

### Bembidion timidum

(LeConte, 1847)

Ochthedromus timidus LeConte, 1847: 460. Type locality: «Rocky Mountains» (original citation), restricted to «San Luis Valley [Alamosa County], Color[ado]» by Lindroth (1963b: 378). Holotype [by monotypy] (♀) in MCZ [# 5534].
Bembidium sordidulum Chaudoir, 1868b: 244. Type locality: «Californie» (original citation). Holotype [by monotypy; designated lectotype by Lindroth (1963b: 378)] (♀) in MHNP. Synonymy established by Hayward (1901: 158), confirmed by Lindroth (1963b: 378).
Bembidion oregonum Casey, 1924: 42. Type locality: «Josephine Co[unty], Oregon» (original citation). Lectotype (♂), designated by Lindroth (1975: 121), in USNM [# 37010]. Synonymy established by Hatch (1953: 97), confirmed by Lindroth (1963b: 378).

#### Distribution.

This species is found from western Ontario to Vancouver Island (Lindroth 1963b: 379), north to northern Yukon Territory (Maddison 1985: 114) and southern Northwest Territories (Lindroth 1963b: 379), south to southern California (Fall 1901a: 42; Andrews et al. 1979: 28), southern Arizona (Wickham 1898: 300; Griffith 1900: 565; Snow 1907: 142), central New Mexico (Ellis et al. 2001: 16), southern South Dakota (Kirk and Balsbaugh 1975: 19), and northwestern Minnesota (Polk County, CMNH; Kittson and Roseau Counties, Peter W. Messer pers. comm. 2009). The record from “Michigan” (Bousquet and Larochelle 1993: 145) needs confirmation.

#### Records.

**CAN**: AB, BC (VCI), MB, NT, ON, SK, YT **USA**: AZ, CA, CO, ID, MN, MT, ND, NM, NV, OR, SD, UT, WA, WY [MI]

### Bembidion triviale

Casey, 1918

Bembidion triviale Casey, 1918: 134. Type locality: «Lake Co[unty], California» (original citation). Lectotype (♂), designated by Erwin (1984a: 184), in USNM [# 37008].
Bembidion temperans Casey, 1918: 135. Type locality: «Lake Co[unty], California» (original citation). Lectotype (♀), designated by Erwin (1984a: 184), in USNM [# 37009]. Synonymy established by Erwin (1984a: 184).

#### Distribution.

This species is known only from Lake County in the California Coast Ranges.

#### Records.

**USA**: CA

### Bembidion versicolor

(LeConte, 1847)

Notaphus variegatus Kirby, 1837: 58 [secondary homonym of *Bembidion variegatum* Say, 1823]. Type locality: northern parts of British America (inferred from title of the book), restricted to «Nipigon, Ont[ario]» by Lindroth (1963b: 377). Two syntypes in BMNH labeled «S[outh] of L[ake] Winnipeg» (Lindroth 1953b: 176).
Ochthedromus versicolor LeConte, 1847: 462. Replacement name for *Ochthedromus variegatus* (Kirby, 1837). Note. Because this name was proposed as a replacement name, the specimen labeled as type [# 5535] of *Bembidion versicolor* in MCZ has no status.
Bembidion tolerans Casey, 1918: 132. Type locality: «Metlakatla, British Columbia» (original citation). Lectotype (♀), designated by Lindroth (1975: 121), in USNM [# 37014]. Synonymy established by Lindroth (1954b: 127).
Bembidion terracense Casey, 1924: 41. Type locality: «Terrace, British Columbia» (original citation). Lectotype (♂), designated by Lindroth (1975: 121), in USNM [# 37011]. Synonymy established by Lindroth (1954b: 128).
Bembidion wisconsinium Casey, 1924: 41. Type locality: «Bayfield [Bayfield County], Wisconsin» (original citation). Lectotype (♀), designated by Lindroth (1975: 121), in USNM [# 37013]. Synonymy established by Lindroth (1963b: 377).

#### Distribution.

This species is found from Newfoundland (Lindroth 1955a: 70-71) to central Alaska (Lindroth 1963b: 378), south to northwestern Oregon (Westcott et al. 2006: 7), southern Colorado (LeConte 1879d: 501; Wickham 1902: 234; Elias 1987: 632), southwestern Nebraska (Keith County, CMNH), and southern South Carolina (Ciegler 2000: 49). The records from “Kansas” (Popenoe 1878: 79) and Arkansas (Wickham 1897: 104) need confirmation; those from Texas (Wickham 1897: 104), New Mexico (Fall and Cockerell 1907: 157) and Arizona (Wickham 1897: 104; Griffith 1900: 565) possibly refer to *Bembidion impotens*.

#### Records.

**FRA**: PM **CAN**: AB, BC (QCI, VCI), LB, MB, NB, NF, NS (CBI), NT, ON, PE, QC, SK **USA**: AK, CO, CT, DE, IA, ID, IL, IN, KY, MA, ME, MI, MN, MT, NC, ND, NE, NH, NJ, NY, OH, OR, PA, RI, SC, SD, VA, VT, WA, WI, WV, WY [AR, KS]

### Subgenus Neobembidion

Bousquet, 2006

Neobembidion Bousquet [in Bousquet and Webster], 2006: 34. Type species: *Bembidion constricticolle* Hayward, 1897 by original designation. Etymology. From the Greek prefix *neo*- (new) and the generic name *Bembidion* [*q.v*.], alluding to the fact that these *Bembidion* inhabit the New World [neuter].

#### Diversity.

Four North American species.

#### Identification.

Bousquet and Webster (2006) provided a key for the identification of three species. Subsequently, a species described by Casey and incorrectly placed in another subgenus, as a synonym, was found to belong to this subgenus (see below under *Bembidion tencenti*).

### Bembidion constricticolle

Hayward, 1897

Bembidium constricticolle Hayward, 1897: 112. Type locality: «valley of the San Juan River, N[ew] Mex[ico]; Winslow, Ariz[ona]; Colorado Springs, Colo[rado]» (original citation), restricted to «San Juan Valley [San Juan County]» by Lindroth (1963b: 346). Four syntypes in MCZ [# 16300].

#### Distribution.

This species is found from southern Alberta (Lindroth 1969a: 1115) to southeastern Arizona (Cochise County, CMNH), southern New Mexico (Hidalgo County, CMNH; Fall and Cockerell 1907: 157), and western Texas (Brewster and Culberson Counties, CMNH, MCZ).

#### Records.

**CAN**: AB **USA**: AZ, CO, NM, TX, UT, WY

### Bembidion nitidicolle

Bousquet, 2006

Bembidion nitidicolle Bousquet [in Bousquet and Webster], 2006: 32. Type locality: «Rosefield (s[outh] e[ast] of Val Marie), Sask[atchewan]» (original citation). Holotype (♂) in CNC [# 23457].

#### Distribution.

This species is known from southern Saskatchewan, central South Dakota (Bousquet and Webster 2006: 32), and southeastern Colorado (Pueblo County, Foster F. Purrington pers. comm. 2009).

#### Records.

**CAN**: SK **USA**: CO, SD

#### Note.

Members of this species are probably conspecific with *Bembidion tencenti* Hatch (see “Note” section under *Bembidion tencenti*).

### Bembidion nudipenne

Lindroth, 1963

Bembidion nudipenne Lindroth, 1963b: 347. Type locality: «Brandon, Manit[oba]» (original citation). Holotype (♂) in CNC [# 8377].

#### Distribution.

This species ranges from southern Manitoba to southern Alberta (Lindroth 1963b: 347), south to southern Texas (Kenedy County, CNC).

#### Records.

**CAN**: AB, MB, SK **USA**: CO, ND, TX

### Bembidion tencenti

Hatch, 1951

Bembidion tencenti Hatch, 1951: 115. Type locality: «Tencent L[ake], Harney Co[unty], Ore[gon]» (original citation). Holotype (♂) in USNM.

#### Distribution.

This species is known only from the holotype collected in southeastern Oregon.

#### Records.

**USA**: OR

#### Note.

This taxon has been listed as a junior synonym of *Bembidion dejectum* Casey by Lindroth (1963b: 356) but the holotype is not conspecific with members of *Bembidion dejectum*. The specimen belongs to the subgenus *Neobembidion* and is externally very similar and probably conspecific with members of *Bembidion nitidicolle* Bousquet.

### Subgenus Diplocampa

Bedel, 1896

Diplocampa Bedel, 1896: 56, 70. Type species: *Bembidium assimile* Gyllenhal, 1810 designated by Jeannel (1941b: 462). Etymology. From the Greek prefix *diplo*- (double) and the generic name *Campa*, alluding to the frontal sulcus being double (“*les bourrelets frontaux sont dédoublés*”) in the adult [feminine]. Note. Bedel (1896: 56) originally included two species in his new genus-group taxon: *Bembidion assimile* Gyllenhal and *Bembidion fumigatum* (Duftschmid).
Paralopha Casey, 1918: 153 [junior homonym of *Paralopha* Bethune-Baker, 1908]. Type species: *Ochthedromus sulcatus* LeConte, 1847 (= *Peryphus transparens* Gebler, 1830) by original designation. Synonymy established by Netolitzky (1931: 164). Etymology. From the Greek *para* (near, next to) and the generic name *Lopha* [*q.v*.] [feminine].

#### Diversity.

Northern Hemisphere, with eight species in the Nearctic (one Holarctic species) and Palaearctic (eight species) Regions.

#### Identification.

Lindroth (1963b: 393-395) covered the North American species in his monograph of the Canadian and Alaskan Carabidae.

#### Taxonomic Note.

One species (*Bembidion longipenne* Putzeys) from Venezuela is currently included in this subgenus but is probably not consubgeneric with members of *Diplocampa*. *Bembidion hesperus* Crotch, described from the Azores and listed in this subgenus by Lorenz (2005: 223), is a junior synonym of *Bembidion ambiguum* Dejean (Lindroth 1960b: 6), a species of the subgenus *Neja* Motschulsky.

### Bembidion transparens transparens

(Gebler, 1830)

Peryphus transparens Gebler, 1830: 61. Type locality: «prope Barnaul [Altai Kray, southwestern Siberia, Russia]» (original citation). Syntype(s) probably in MHNP (Lindroth 1963b: 393).
Ochthedromus sulcatus LeConte, 1847: 463. Type locality: «Lacum Superiorem» (original citation). Three syntypes in MCZ [# 5552]. Synonymy established by Hatch (1953: 99), confirmed by Lindroth (1954b: 127).
Ochthedromus trepidus LeConte, 1847: 463. Type locality: «Lacum Superiorem» (original citation). Holotype [by monotypy] in MCZ [# 5557]. Synonymy established, under the name *Bembidion sulcatum* (LeConte), by LeConte (1857a: 5), confirmed by Lindroth (1954b: 128).
Bembidiun contaminatum J.R. Sahlberg, 1875: 83. Type locality: «Åbo [= Turku, Finland]» (original citation for the lectotype). Lectotype (♀), designated by Lindroth (1963b: 393), in ZMH. Synonymy established (as aberration) by Csiki (1928: 136), confirmed by Lindroth (1963b: 393).
Bembidion edmontonense Casey, 1924: 37. Type locality: «Edmonton, Alberta» (original citation). Lectotype (♀), designated by Lindroth (1975: 121), in USNM [# 36953]. Synonymy established by Lindroth (1954b: 129).

#### Distribution.

This Holarctic subspecies is found from northern Europe (Marggi et al. 2003: 248) to eastern Siberia (Kryzhanovskij et al. 1995: 82) and from the Chukchi Sea coast in Alaska (Lindroth 1963b: 394) to Newfoundland (Lindroth 1955a: 75-76), south to northern Pennsylvania (Bradford County, CMNH), northeastern Illinois (Lake County, CNC), east-central South Dakota (Kirk and Balsbaugh 1975: 20), and “Oregon” (Hatch 1953: 99).

#### Records.

**FRA**: PM **CAN**: AB, BC (VCI), LB, MB, NB, NF, NS (CBI), NT, ON, PE, QC, SK, YT **USA**: AK, IA, ID, IL, IN, MA, ME, MI, MN, MT, NH, NY, OH, OR, PA, SD, VT, WA, WI – **Holarctic**

#### Note.

The subspecies *Bembidion transparens prostratum* (Motschulsky) occurs in the eastern part of Asia.

### Subgenus Semicampa

Netolitzky, 1910

Semicampa Netolitzky, 1910: 217. Type species: *Bembidium schuppelii* Dejean, 1831 designated by Jeannel (1941b: 462). Etymology. From the Latin *semi*- (half) and the generic name *Campa* [feminine].

#### Diversity.

Northern Hemisphere, with 26 species in the Nearctic (eight species) and Palaearctic (18 species) Regions.

#### Identification.

Bousquet and Webster (2006) provided a key for the identification of all North American species except *Bembidion rubiginosum*. Lindroth (1963b: 387-393) covered six of the eight North American species.

### Bembidion convexulum

Hayward, 1897

Bem ﻿*bidium convexulum* Hayward, 1897: 106. Type locality: «British Columbia; Laggan, Alberta» (original citation), restricted to «Creston, B[ritish] C[olumbia]» by Lindroth (1963b: 392). Syntype(s) [5 originally cited] in MCZ [# 16297].
Bembidion novellum Casey, 1918: 113. Type locality: «Truckee [Nevada County], California» (original citation). Holotype [by monotypy] (♂) in USNM [# 37021]. Synonymy established by Lindroth (1963b: 392).

#### Distribution.

This species inhabits the North American Cordilleras from the arctic circle in eastern Alaska and west-central Yukon Territory (Lindroth 1963b: 392) south to San Bernardino County in southeastern California (Dajoz 2007: 20, as *Bembidion convexiuscula*), northern Idaho (Hatch 1953: 94), northwestern Wyoming (Teton County, UASM), and east-central Montana (Russell 1968: 57).

#### Records.

**CAN**: AB, BC, YT **USA**: AK, CA, ID, MT, OR, WA, WY

### Bembidion morulum

LeConte, 1863

Bembidium morulum LeConte, 1863c: 19. Type locality: «Hudson’s Bay Territory» (original citation), restricted to «Churchill, Manit[oba]» by Lindroth (1963b: 389). Two syntypes in CMNH (collection Ulke).
Bembidion browni Lindroth, 1955a: 73. Type locality: «Churchill, Manitoba» (original citation). Holotype (♂) in CNC [# 6572]. Synonymy established by Lindroth (1963b: 389). Note. Lindroth (1954b: 158) proposed the name earlier but he did not meet the requirements of availability (ICZN, Article 13.1) at the time.

#### Distribution.

This species is known from Newfoundland to central Alaska, south to northeastern British Columbia and southern Alberta in the Rocky Mountains (Lindroth 1963b: 389). Fossil remnants of this species, dated between about 10,400 and 28,000 years B.P., have been unearthed in eastern Minnesota, northeastern Wisconsin, Illinois (Schwert 1992: 77), Iowa (Baker et al. 1986: 96; Schwert 1992: 77), central North Dakota (Ashworth and Schwert 1992: 260), and Cape Breton Island in Nova Scotia (Miller 1997: 250).

#### Records.

**CAN**: AB, BC, MB, NF, NT, ON, SK, YT **USA**: AK

### Bembidion muscicola

Hayward, 1897

Bembidium muscicola Hayward, 1897: 122. Type locality: «Cambridge [Middlesex County], Massachusetts» (original citation for the lectotype). Lectotype (♂), designated by Lindroth (1963b: 387), in MCZ [# 16303].

#### Distribution.

This eastern species ranges from Cape Breton Island to southern Saskatchewan (Lindroth 1963b: 388), south to eastern South Dakota (Larsen and Purrington 2010: 571), northern Illinois (Hayward 1897: 123), and northeastern West Virginia (Tucker County, CMNH).

#### Records.

**CAN**: MB, NB, NS (CBI), ON, PE, QC, SK **USA**: IL, MA, ME, MI, MN, NH, NJ, NY, PA, RI, SD, VT, WI, WV

### Bembidion nigrivestis

Bousquet, 2006

Bembidion nigrivestis Bousquet [in Bousquet and Webster], 2006: 24. Type locality: «Sainte-Catherine-de-Hatley (Stanstead), Quebec» (original citation). Holotype (♂) in CNC [# 23455].

#### Distribution.

This species ranges from New Brunswick and Maine west to eastern Minnesota and southeastern Manitoba (Bousquet and Webster 2006: 24).

#### Records.

**CAN**: MB, NB, ON, QC **USA**: MA, ME, MI, MN, NH, WI

### Bembidion praticola

Lindroth, 1963

Bembidion praticola Lindroth, 1963b: 388. Type locality: «Amberley, S[outh] Kincardine, L[ake] Huron, Ont[ario]» (original citation). Holotype (♂) in CNC [# 8385].

#### Distribution.

This species is found from New Brunswick (Bousquet 1987a: 121) to the Osoyoos Valley in southern British Columbia, south to central Washington (Lindroth 1963b: 389), northern Iowa (Purrington et al. 2002: 201), northeastern Illinois (Lake County, CNC), and New York (Liebherr and Song 2002: 132; Saint Lawrence County, CMNH). The species seems to be local.

#### Records.

**CAN**: BC, NB, ON, QC, SK **USA**: IA, IL, ME, MI, MN, NH, NY, WA, WI

### Bembidion roosevelti

Pic, 1902

Bembidium concinnum Blaisdell, 1902: 78 [secondary homonym of *Bembidion concinnum* (Stephens, 1828)]. Type locality: «Eldorado County, Cal[ifornia]» (original citation). Syntype(s) in CAS [# 2665].
Bembidium roosevelti Pic, 1902: 71. Replacement name for *Bembidium concinnum* Blaisdell, 1902. Note. The spelling *roosvelti* used by some authors, including Lindroth (1963b: 392), is an incorrect subsequent spelling that I consider not to be in prevailing usage. Pic (1902: 71) did not give the etymology of the specific name.
Bembidium perconcinnum Blaisdell, 1904: 349. Replacement name for *Bembidium concinnum* Blaisdell, 1902.
Bembidion blaisdelli Casey, 1918: 222. Replacement name for *Bembidion concinnum* Blaisdell, 1902.

#### Distribution.

This species is found from the southern part of the Prairie Provinces (Lindroth 1963b: 393) to eastern Oregon (Baker County, CNC), south to Riverside County in southern California (Dajoz 2007: 19), central Nevada (Lander County, CNC), central Utah (Sevier County, CNC), southern Wyoming (Lindroth 1963b: 393), and southwestern North Dakota (Bowman County, CNC).

#### Records.

**CAN**: AB, MB, SK **USA**: CA, ID, MT, ND, NV, OR, UT, WY

### Bembidion rubiginosum

LeConte, 1879

Bembidium rubiginosum LeConte, 1879d: 508. Type locality: «[Fort] Garland [Costilla County], Colo[rado]» (original citation). Holotype [by monotypy] (♀) in MCZ [# 5541].

#### Distribution.

This species is known from Saskatchewan and Alberta (David R. Maddison pers. comm. 2012), southwestern Idaho (Owyhee County, CNC), southeastern Wyoming (Lavigne 1977: 44), and Costilla County in south-central Colorado (LeConte 1879d: 508; Wickham 1902: 234).

#### Records.

**CAN**: AB, SK **USA**: CO, ID, WY

### Bembidion semicinctum

Notman, 1919

Bembidium semicinctum Notman, 1919c: 129. Type locality: «Mooers, Clinton Co[unty], N[ew] Y[ork]» (original citation). Holotype [by monotypy] (♀) in SIM (Hennessey 1990: 466).

#### Distribution.

This eastern species ranges from Cape Breton Island (CNC) to “Michigan” (Garry A. Dunn pers. comm. 1986), south to northeastern West Virginia (Randolph County, CMNH).

#### Records.

**CAN**: NB, NS (CBI), ON, QC **USA**: CT, MA, ME, MI, NH, NJ, NY, PA, RI, VT, WV

### Subgenus Notaphus

Dejean, 1821

Notaphus Dejean, 1821: 16. Type species: *Carabus ustulatus* Linnaeus *sensu* Illiger, 1798 (= *Carabus varius* Olivier, 1795) designated by Westwood (1838: 7). Etymology. Probably from the Latin *nota* (mark) and the Greek *phos* (light, by extension bright, clear), alluding to the elytral spots of the adult rather than from the Greek *notos* (dorsum) and *phos* (light, by extension bright, clear) [masculine]. The name was proposed by Johann Karl Megerle von Mühlfeld and made available by Dejean.
Austronotaphus Jeannel, 1962: 620. Type species: *Poecilus luridus* Blanchard, 1843 by original designation. Synonymy established by Lorenz (1998: 204), confirmed by Toledano (2002: 5). Etymology. From the Greek *auster*, -*tri* (south) and the generic name *Notaphus* [*q.v*.] [masculine].
Notaphidius Jeannel, 1962: 620, 622. Type species: *Notaphus aricensis* Jeannel, 1962 by original designation. Synonymy established by Lorenz (1998: 204), confirmed by Toledano (2002: 5).
Notaphiellus Jeannel, 1962: 618, 631. Type species: *Bembicidium solieri* Gemminger & Harold, 1868 by original designation. Synonymy established by Maddison (2012: 564).

#### Diversity.

About 90 species in the Nearctic (50 species), Neotropical (about 35 species), and Palaearctic (seven species) Regions. One species from the Philippines (*Bembidion igorot* Darlington) and one from Myanmar (*Bembidion serpentinum* Landin) are also listed in this subgenus by Lorenz (2005: 220). Two species (*Bembidion nigripes* and *Bembidion semipunctatum*) are Holarctic. One South American species (*Bembidion brullei* Gemminger and Harold) has been introduced in Australia and New Zealand (Lindroth 1976a: 197-198). The North American species are arrayed here in six species groups for convenience.

#### Identification.

Lindroth (1963b: 357-375, as *scudderi*, *obtusangulum*, *dorsale*, *contractum*, and *oberthueri* groups) covered 28 of the 50 North American species. A taxonomic revision of the North American species is needed.

### [contractum group]

### Bembidion acticola

Casey, 1884

Bembidion acticola Casey, 1884c: 63. Type locality: «N[ew] J[ersey]» (lectotype label). Lectotype (♂), designated by Erwin (1984a: 166), in USNM [# 37042]. Note. According to Casey (1884c: 63), the type specimens from New Jersey came from Atlantic City and Cape May.
Bembidion argutum Casey, 1918: 123. Type locality: «Boston Neck [Washington County], Rhode Island» (original citation). Lectotype (♂), designated by Erwin (1984a: 166), in USNM [# 37045]. Synonymy established by Erwin (1984a: 166).
Bembidion assensum Casey, 1924: 41. Type locality: «near Brooklyn [Kings County], L[ong] I[sland], New York» (original citation). Lectotype (♀), designated by Erwin (1984a: 166), in USNM [# 37046]. Synonymy established by Erwin (1984a: 166).

#### Distribution.

This species is found along the Atlantic Coast from Long Island (Casey 1924: 41, as *Bembidion assensum*) to Maryland (Erwin 1984a: 166).

#### Records.

**USA**: DE, MD, NJ, NY, RI

### Bembidion constrictum

(LeConte, 1847)

Ochthedromus constrictus LeConte, 1847: 462. Type locality: United States east of the Rocky Mountains (inferred from title of the paper), restricted to «Ipswich [Essex County], Mass[achusetts]» by Lindroth (1963b: 373). Four syntypes in MCZ [# 5538].
Bembidion vernula Casey, 1884c: 62. Type locality: «Cap May [Cap May County], New Jersey» (original citation). Holotype [by monotypy] (♀) in USNM [# 37038]. Synonymy established by Horn (1885b: 108), confirmed by Erwin (1984a: 169).
Bembidion festinans Casey, 1918: 121. Type locality: «Texas» (original citation). Lectotype (♀), designated by Erwin (1984a: 169), in USNM [# 37043]. Synonymy established by Erwin (1984a: 169).
Bembidion constrictum civile Casey, 1918: 124. Type locality: «Brownsville [Cameron County], Texas» (original citation). Lectotype (♀), designated by Erwin (1984a: 170), in USNM [# 37044]. Synonymy established (as aberration) by Csiki (1928: 59), confirmed by Erwin (1984a: 170).

#### Distribution.

This species is found along the Atlantic and Gulf of Mexico coasts from the Maritime Provinces (Lindroth 1963b: 373; Bousquet 1987a: 121) to southern Florida including the Keys (Leng 1915: 572), west to southeastern Texas (Wickham 1897: 104; Casey 1918: 124, as *Bembidion constrictum civile*). The records from Ohio (Everly 1938: 141), Indiana (Wolcott and Montgomery 1933: 125), South Dakota (Kirk and Balsbaugh 1975: 19), “Nebraska” (Hayward 1897: 108), Kansas (Knaus 1887: 87; Snow 1903: 193), and “New Mexico” (Hayward 1897: 108) probably refer to *Bembidion viridicolle* LaFerté-Sénectère.

#### Records.

**CAN**: NB, NS (CBI), PE **USA**: AL, CT, DC, DE, FL, GA, LA, MA, MD, ME, MS, NC, NH, NJ, NY, RI, VA, TX

### Bembidion contractum

Say, 1823

Bembidium contractum Say, 1823a: 85. Type locality: «Ipswich [Essex County], Mass[achusetts]» (neotype label). Neotype (♂), designated by Lindroth and Freitag (1969: 337), in MCZ [# 33064].

#### Distribution.

This species is found along the Atlantic Coast from Newfoundland (Lindroth 1955a: 70), the Maritime Provinces (Lindroth 1963b: 373), and the Magdalen Islands (Larochelle 1975: 55) to southern Florida (Peck and Thomas 1998: 18) and along the Gulf Coast to southern Louisiana (Summers 1874a: 81; Hine 1906: 76; Allen 1965: 65). The records from “Ohio,” “Tennessee” (Hayward 1897: 109), eastern Kansas (Popenoe 1877: 24), and “Pennsylvania” (Bousquet and Larochelle 1993: 144) need confirmation.

#### Records.

**FRA**: PM **CAN**: NB, NF, NS (CBI), PE, QC **USA**: AL, CT, DC, DE, FL, GA, LA, MA, MD, ME, NH, NJ, NY, RI, SC [KS, OH, PA, TN]

### Bembidion luculentum

Casey, 1918

Bembidion luculentum Casey, 1918: 122. Type locality: «Indian River [Brevard County], Florida» (original citation). Lectotype (♂), designated by Erwin (1984a: 176), in USNM [# 37050].
Bembidion prosperum Casey, 1918: 122. Type locality: «Lake Worth [Palm Beach County], Florida» (original citation). Lectotype (♀), designated by Erwin (1984a: 176), in USNM [# 37049]. Synonymy established by Erwin (1984a: 176).

#### Distribution.

This species is known so far only from the Florida Peninsula (Peck and Thomas 1998: 18).

#### Records.

**USA**: FL

### Bembidion pilatei

Chaudoir, 1868

Bembidium pilatei Chaudoir, 1868b: 243. Type locality: «Texas» (original citation). Holotype [by monotypy] (♀) in MHNP (Lindroth 1963b: 373).

#### Distribution.

This species is known from central Texas (Lee County, CMNH), southwestern Louisiana (Cameron Parish, Igor M. Sokolov pers. comm. 2009), and southwestern Florida (Monroe County, CMNH).

#### Records.

**USA**: FL, LA, TX

#### Note.

Hayward (1901: 158) listed *Bembidion pilatei* Chaudoir as a synonym of *Bembidion constrictum* LeConte but Lindroth (1963b: 373) regarded the two forms as distinct species.

### Bembidion viridicolle

(LaFerté-Sénectère, 1841)

Notaphus viridicollis LaFerté-Sénectère, 1841a: 48. Type locality: Texas (inferred from title of the paper). Lectotype (♂), designated by Lindroth (1963b: 374), in MHNP.
Bembidium apicale Jacquelin du Val, 1857: 23 [primary homonym of *Bembidium apicale* Ménétriés, 1832]. Type locality: Cuba (inferred from title of the book). Syntype(s) location unknown. Synonymy established by Darlington (1934: 77).
Bembidium hamiferum Chaudoir, 1868b: 244. Type locality: «Texas» (original citation). Lectotype (♂), designated by Lindroth (1963b: 374), in MHNP. Synonymy established by Hayward (1901: 157), confirmed by Lindroth (1963b: 374).
Bembicidium chevrolati Gemminger and Harold, 1868a: 409. Replacement name for *Bembicidium apicale* Jacquelin du Val, 1857. Etymology. The specific name was proposed to honor Louis Alexandre Auguste Chevrolat [1799-1884], one of the leading coleopterist of his time. Born in Paris, Chevrolat was an employee at the Minister of Finances and despite his obligations wrote extensively on the taxonomy of beetles (particularly Curculionidae). Chevrolat gathered an impressive collection which was dispersed during his life time and by his heirs. Nevertheless, most of his specimens are now in MHNP (Cambefort 2006: 145).
Bembidion particeps Casey, 1918: 124. Type locality: «Arizona» (original citation). Lectotype (♀), designated by Erwin (1984a: 185), in USNM [# 37039]. Synonymy established by Erwin (1984a: 185).

#### Distribution.

This species ranges from Connecticut (Krinsky and Oliver 2001: 88) to southeastern Alberta (Lindroth 1963b: 375), including southernmost Ontario (Bousquet 1987a: 121), north to Fort Smith in southern Northwest Territories (Lindroth 1963b: 374-375), south to northeastern Arizona (Apache County, CMNH), west-central New Mexico (Cibola County, CMNH), southern Texas (Johnson 1978: 67), and southern Florida, including the Keys (Peck and Thomas 1998: 18). The species is also known from the Bahamas (Darlington 1953: 5, as *Bembidion chevrolati*), several islands of the West Indies (Peck and Thomas 1998: 18), and “Mexico” (Erwin et al. 1977: 4.21).

#### Records.

**CAN**: AB, MB, NT, ON, SK **USA**: AZ, CO, CT, FL, GA, IA, IN, IL, KS, LA, MS, ND, NE, NJ, NM, OH, OK, PA, SC, SD, TX, VA, WI, WY – Bahamas, Cuba, Dominica Republic, Haiti, Jamaica, Mexico, Puerto Rico

### Bembidion vividum

Casey, 1884

Bembidion vividum Casey, 1884c: 66. Type locality: «Cap May [Cape May County], New Jersey» (original citation). Lectotype (♀), designated by Erwin (1984a: 185), in USNM [# 37048].

#### Distribution.

This species is known only from the type locality, from Atlantic County in New Jersey (CMNH), and from one specimen simply labeled “Md” (CMNH).

#### Records.

**USA**: MD, NJ

#### Note.

This form was listed in synonymy with *Bembidion contractum* Say by Horn (1885b: 108) but considered a valid species by Erwin (1984a: 185).

### [dorsale group]

### Bembidion dorsale

Say, 1823

Bembidium dorsalis Say, 1823a: 84. Type locality: «M[iss]o[uri]» (neotype label). Neotype (♀), designated by Lindroth and Freitag (1969: 337), in MCZ [# 33066]. Note. «Missouri [Territory]» was the area originally cited by Say (1823a: 85).

#### Distribution.

This species is found from southeastern Manitoba to the foothills of the Rocky Mountains in southern Alberta, north to northeastern British Columbia (Lindroth 1963b: 359), south to northeastern Wyoming (Crook County, CNC), Kansas (Popenoe 1877: 24; Snow 1903: 193), Missouri (Summers 1873: 147; Lindroth and Freitag 1969: 337), southern Illinois (Hayward 1897: 101), and southwestern Ohio (Dury 1902: 111). The record from “Colorado” (Bousquet and Larochelle 1993: 141) needs confirmation.

#### Records.

**CAN**: AB, BC, MB, SK **USA**: IA, IL, IN, KS, MI, MN, MO, MT, ND, NE, OH, SD, WI, WY [CO]

### Bembidion oberthueri

Hayward, 1901

Bembidium oberthüri Hayward, 1901: 158. Type locality: «Winnipeg, Man[itoba]» (original citation for the lectotype). Lectotype (♀), designated by Lindroth (1963b: 375), in MCZ. Etymology. The specific name honors René Oberthür [1852-1944], a wealthy amateur coleopterist. His father, François-Charles Oberthür, himself an amateur lepidopterist, owned the largest printing house in France at one time, with about 1,000 employees, and became extremely rich through his creativity. At Rennes he built a specially-made house just to hold the Lepidoptera collection of his oldest son, Charles, and the Coleoptera collection of René. The Oberthür collection was built almost entirely through purchases, by financing collecting expeditions abroad, and by trade. Indeed, the Oberthürs agreed to provide several missionary congregations with their printed material free of charge in exchange for an obligation for the missionaries to collect all insects they saw. During World War II, Georg Frey, himself a beetle collector who eventually gathered one of the two largest personal collections ever built (the other was the Oberthür collection), was an officer in the German army; he made sure that the Oberthür building housing the collection was properly heated and maintained (Cambefort 2006: 244-250). Note. This name was proposed for *Notaphus viridicollis* LaFerté-Sénectère, 1841 *sensu* Hayward (1897: 103) and the description was by indication (see ICZN 1999: Article 12.2). Three specimens in MCZ are labeled as “Type” [# 16296] but the lectotype is the one labeled “Winnipeg Man July 7-12, 1887 / Roland Hayward Coll.”

#### Distribution.

The range of this species extends from New Brunswick to the foothills of the Rocky Mountains in Alberta (Lindroth 1963b: 375), south to south-central Montana (Carbon County, UASM), “Nebraska,” “Illinois” (Hayward 1897: 103, as *Bembidion viridicolle*), and southeastern West Virginia (Greenbrier County, David R. Maddison pers. comm. 2011). The record from San Bernardino County, California (Cooper 1976: 163) is probably based on a mislabeled specimen; that from “Indiana” (Bousquet and Larochelle 1993: 144) needs confirmation. One specimen from Kamloops, British Columbia, was regarded as possibly mislabeled by Lindroth (1963b: 375).

#### Records.

**CAN**: AB, MB, NB, ON, QC, SK **USA**: CT, IA, IL, MA, ME, MI, MN, MT, ND, NE, NH, NY, OH, PA, SD, VT, WI, WV [BC, IN]

### Bembidion umbratum

(LeConte, 1847)

Ochthedromus umbratus LeConte, 1847: 458. Type locality: «ad Rocky Mountains» (original citation), restricted to «Green R[iver] [Sweetwater County], Wyom[ing]» by Lindroth (1963b: 360). Two syntypes in MCZ [# 5529].
Notaphus variolosus Motschulsky, 1859a: 131. Type locality: «environs de Ross [farming community about 75 miles north of San Francisco along the coast, California]» (original citation). Lectotype, designated by Bousquet (1997b: 332), in ZMMU. Synonymy established by Lindroth (1963b: 360), confirmed by Bousquet (1997b: 332).

#### Distribution.

This species is found from Saskatchewan to western British Columbia, north to southern Northwest Territories (Lindroth 1963b: 361), south to central California along the coast (Motschulsky, 1859a: 131, as *Bembidion variolosum*) and southern Colorado (LeConte 1879d: 501; Wickham 1902: 234). The record from “Alaska” (Hayward 1897: 102, as *Bembidion variolosum*) needs confirmation.

#### Records.

**CAN**: AB, BC, NT, SK **USA**: CA, CO, ID, MT, NV, OR, WA, WY [AK]

### Bembidion versutum

LeConte, 1878

Bembidium versutum LeConte, 1878c: 594. Type locality: «Marquette [Marquette County, Michigan], Lake Superior» (original citation). Six syntypes in MCZ [# 5528].

#### Distribution.

This species ranges from New Brunswick (Larochelle and Larivière 1990a: 29) to northwestern Wisconsin (Hayward 1897: 100; Messer 2010: 36; Barron County, CMNH), south to northeastern Ohio (Lake and Trumbull Counties, CMNH, Harry J. Lee pers. comm. 2008) and northeastern West Virginia (Hampshire and Tucker Counties, CMNH). The record from southwestern Iowa (Wickham 1911b: 6) is probably in error.

#### Records.

**CAN**: NB, ON, QC **USA**: CT, MA, ME, MI, NH, NJ, NY, OH, PA, RI, VT, WI, WV

### [obtusangulum group]

### Bembidion callens

Casey, 1918

Bembidion callens Casey, 1918: 112. Type locality: «Tuçson [Pima County], Arizona» (original citation). Lectotype (♀), designated by Erwin (1984a: 168), in USNM [# 37033].

#### Distribution.

This species has been recorded yet only from the type locality in southern Arizona.

#### Records.

**USA**: AZ

### Bembidion dejectum

Casey, 1884

Bembidion dejectum Casey, 1884c: 67. Type locality: «Arizona» (original citation). Lectotype (♂), designated by Lindroth (1975: 120), in USNM [# 37054].
Bembidion fidele Casey, 1918: 113. Type locality: «Provo [Utah County], Utah» (original citation). Lectotype (♂), designated by Lindroth (1975: 120), in USNM [# 37032]. Synonymy established by Lindroth (1963b: 356).
Bembidion aberti Hatch, 1950: 102. Type locality: «L[ake] Abert [Lake County], Oregon» (original citation). Holotype (♂) in USNM. Synonymy established by Lindroth (1963b: 356).

#### Distribution.

The range of this species extends from southern Manitoba (Lindroth 1963b: 357) to eastern Washington (Hatch 1953: 96, as *Bembidion aberti*), including southern Alberta (Lindroth 1963b: 357), south to Inyo County in eastern California (UASM), southeastern Arizona (Snow 1906b: 161; Casey 1884c: 67), east-central Colorado (Wickham 1902: 234), and west-central Kansas (Knaus 1905a: 218).

#### Records.

**CAN**: AB, MB, SK **USA**: AZ, CA, CO, KS, ND, NE, NV, OR, SD, UT, WA, WY

### Bembidion hageni

Hayward, 1897

Ochthedromus sexpunctatus LeConte, 1852a: 186 [secondary homonym of *Bembidion sexpunctatum* Heer, 1841]. Type locality: «ad Colorado [River, California]» (original citation). Holotype [by monotypy] (♀) in MCZ [# 99].
Bembidium hageni Hayward, 1897: 89. Replacement name for *Bembidium sexpunctatum* (LeConte, 1852). Etymology. The specific name honors Herman August Hagen [1817-1893], physician, entomologist, and bibliographer. Born in East Prussia, Hagen moved to Cambridge in Massachusetts at the age of 50 on the insistence of Louis Agassiz and worked at the Museum of Comparative Zoology. He eventually became professor of entomology at the University. His impressive library was bought by the MCZ.

#### Distribution.

This rarely collected species is known only from the Colorado River valley in southeastern California to western New Mexico (Cibola County, CMNH).

#### Records.

**USA**: AZ, CA, NM

### Bembidion lecontei

Csiki, 1928

Ochthedromus grandicollis LeConte, 1852a: 189 [secondary homonym of *Bembidion grandicolle* Motschulsky, 1850]. Type locality: «San Diego [San Diego County, California]» (original citation). Holotype [by monotypy] (♀) in MCZ [# 5539].
Bembidion lecontei Csiki, 1928: 62. Replacement name for *Bembidion grandicolle* (LeConte, 1852).

#### Distribution.

This species is known from southwestern California (Fall 1901a: 43; Moore 1937: 8).

#### Records.

**USA**: CA

### Bembidion mormon

Hayward, 1897

Bembidium mormon Hayward, 1897: 110. Type locality: «vicinity of Salt Lake, Utah; Sherman, Wyo[ming]; California» (original citation), restricted to «Salt L[ake City] [Salt Lake County]» by Lindroth (1963b: 356). At least two syntypes [9 originally cited] in MCZ [# 16298].

#### Distribution.

This species ranges from the Okanagan Valley in southern British Columbia (Lindroth 1963b: 356) south to Inyo County in the Sierra Nevada of California (CMNH, MCZ, UASM) and southern Colorado (Elias 1987: 632). The record from southeastern South Dakota (Kirk and Balsbaugh 1975: 19) needs confirmation.

#### Records.

**CAN**: BC **USA**: CA, CO, ID, NV, OR, UT, WA, WY [SD]

### Bembidion obtusangulum

LeConte, 1863

Bembidium obtusangulum LeConte, 1863c: 19. Type locality: «Nebraska [Territory], near the Rocky Mountains [probably in present day Colorado]» (original citation). One syntype in CMNH (collection Ulke). Note. The specimen of *Bembidion obtusangulum* in the LeConte’s collection (MCZ), labeled “8-10,000 ft. South Park, Col. Aug. 11-16,1877 / Type 5540 / B. obtusangulum Lec. [handwritten],” is not a syntype as indicated by Lindroth (1963b: 355).
Bembidion cornix Casey, 1918: 111. Type locality: «Washington State» (original citation). Lectotype (♂), designated by Lindroth (1975: 120), in USNM [# 37031]. Synonymy established by Hatch (1953: 96), confirmed by Lindroth (1963b: 355).

#### Distribution.

The range of this species extends from southwestern Manitoba to the Okanagan Valley in British Columbia (Lindroth 1963b: 356), south to central California along the Sierra Nevada (Dajoz 2007: 18), central Colorado (LeConte 1878a: 465; Wickham 1902: 234) along the Rocky Mountains, and northwestern Nebraska (Sheridan County, CNC).

#### Records.

**CAN**: AB, BC, MB, SK **USA**: CA, CO, ID, MT, ND, NE, OR, UT, WA, WY

### [patruele group]

### Bembidion aeneicolle

(LeConte, 1847)

Ochthedromus aeneicollis LeConte, 1847: 459. Type locality: «Lacum Superiorem» (original citation). One syntype in MCZ [# 5531].

#### Distribution.

This species is known from southeastern Manitoba to southwestern British Columbia (Lindroth 1963b: 365), south to eastern Washington (Hatch 1953: 96), northwestern Utah (Toole County, CMNH), south-central Colorado (Wickham 1902: 234; LeConte 1879d: 501; Lindroth 1963b: 365; Armin 1963: 165), central Nebraska (Blaine County, Foster F. Purrington pers. comm. 2010), and southeastern Wisconsin (Milwaukee County, Peter W. Messer pers. comm. 2009). The record from “Northwest Territories” (Bousquet and Larochelle 1993: 141) was based on four old specimens in the CNC labeled “N.W.T.” which does not refer to present day Northwest Territories or Nunavut.

#### Records.

**CAN**: AB, BC, MB, SK **USA**: CO, ID, MT, ND, NE, SD, UT, WA, WI, WY

### Bembidion approximatum

(LeConte, 1852)

Ochthedromus approximatus LeConte, 1852a: 187. Type locality: «San Diego [San Diego County, California]» (original citation for the lectotype). Lectotype (♂), designated by Erwin (1984a: 167), in MCZ [# 5526].
Notaphus flammulipennis Motschulsky, 1859a: 129. Type locality: «Col[onie] Ross [farming community about 75 miles north of San Francisco along the coast, California]» (original citation). One syntype in ZMMU (Keleinikova 1976: 197) and a possible one in MCZ (collection LeConte). Synonymy established by LeConte (1863b: 14).
Notaphus laterimaculatus Motschulsky, 1859a: 130. Type locality: «Califor[nie]» (lectotype label). Lectotype, designated by Bousquet and Larochelle (1993: 15), in ZMMU. Synonymy established by Bousquet and Larochelle (1993: 15).
Bembidium suspectum Blaisdell, 1902: 76. Type locality: «Oregon City [Clackamas County], Ore[gon], on the shore of the Willamette river» (original citation). Holotype (♀) in CAS [# 2663]. Synonymy established by Hatch (1953: 93), confirmed by Lindroth (1963b: 359).
Bembidion simulator Casey, 1918: 93. Type locality: Santa Rosa, Sonoma County, California (lectotype label according to Lindroth 1975: 120). Lectotype (♀), designated by Lindroth (1975: 120), in USNM [# 36958]. Synonymy established by Lindroth (1963b: 359).
Bembidion cernens Casey, 1918: 100. Type locality: «San Diego [San Diego County], California» (original citation). Lectotype (♀), designated by Erwin (1984a: 167), in USNM [# 36997]. Synonymy established by Erwin (1984a: 167).
Bembidion haustum Casey, 1924: 38. Type locality: «Alameda Co[unty], California» (original citation). Holotype [by monotypy] (♀) in USNM [# 37000]. Synonymy established by Erwin (1984a: 167).
Bembidion stevensoni Hatch, 1953: 93. Type locality: «Stevenson [Skamania County], Wash[ington]» (original citation). Holotype (♀) in USNM. Synonymy established by Lindroth (1963b: 359).

#### Distribution.

This species is found from Skamania County in southern Washington (Hatch 1953: 93, as *Bembidion stevensoni*) to the San Diego area in southwestern California (LeConte 1852a: 187; Moore 1937: 7). The records from Nevada (Bechtel et al. 1983: 474), “British Columbia,” “Arizona,” “Dacota” (Hayward 1897: 99), and northern Idaho (Hatch 1953: 93) are likely based on misidentified specimens (see Lindroth 1963b: 359).

#### Records.

**USA**: CA, OR, WA

### Bembidion auxiliator

Casey, 1924

Bembidion auxiliator Casey, 1924: 38. Type locality: «San Joaquin Co[unty], California» (original citation). Lectotype (♂), designated by Erwin (1984a: 167), in USNM [# 36964].

#### Distribution.

This species is known only from the type series collected in central California.

#### Records.

**USA**: CA

### Bembidion castor

Lindroth, 1963

Bembidion castor Lindroth, 1963b: 366. Type locality: «Walsingham n[ea]r Simcoe, S[outh] Ont[ario]» (original citation). Holotype (♂) in CNC [# 8390].

#### Distribution.

The range of this species extends from western Newfoundland to the Rocky Mountains in Alberta (Lindroth 1963b: 367), south to north-central Colorado (Motz and Morgan 2001: Fig. 4d), northern Nebraska (Cherry County, Foster F. Purrington pers. comm. 2010), central Iowa (Story County, CNC), western West Virginia (Cabell County, CMNH), and Maryland (Erwin 1981b: 147). The records from northern Oklahoma (French et al. 2001: 228; Elliott et al. 2006: 125) need confirmation.

#### Records.

**CAN**: AB, MB, NB, NF, NS, ON, QC, SK **USA**: CO, CT, IA, MA, MD, ME, MI, MT, ND, NE, NH, NY, OH, PA, SD, VA, VT, WI, WV [OK]

### Bembidion coloradense

Hayward, 1897

Bembidium coloradense Hayward, 1897: 98. Type locality: «Vicinity of Rico (85-10000 ft.), Dolores Co[unty], Col[orado]» (lectotype label). Lectotype (♀), designated by Erwin (1984a: 169), in MCZ [# 16295].
Bembidion imperitum Casey, 1918: 91. Type locality: «Victoria, Vancouver Island [British Columbia]» (original citation for the lectotype). Lectotype (♂), designated by Lindroth (1975: 120), in USNM [# 36950]. Synonymy established by Lindroth (1963b: 357).
Bembidion prociduum Casey, 1918: 91. Type locality: «Washington State» (original citation). Lectotype (♂), designated by Lindroth (1975: 120), in USNM [# 36951]. Synonymy established, under the name *Bembidion imperitum* Casey, by Nicolay and Weiss (1934: 197), confirmed by Lindroth (1963b: 357).
Bembidion amplipenne Casey, 1924: 36. Type locality: «Trout Creek, Juab Co[unty], Utah» (original citation). Lectotype (♀), designated by Lindroth (1975: 120), in USNM [# 36952]. Synonymy established by Lindroth (1963b: 357).
Bembidion albertanum Casey, 1924: 40. Type locality: «Edmonton, Alberta» (original citation). Lectotype (♀), designated by Lindroth (1975: 120), in USNM [# 36984]. Synonymy established by Lindroth (1963b: 357).

#### Distribution.

This species ranges from southern Manitoba to Vancouver Island (Lindroth 1963b: 358), north to the Great Slave Lake area in Northwest Territories (Bousquet 1987a: 121), south at least to Sierra County in the Sierra Nevada of California (MCZ; Lindroth 1963b: 358), the Grand Canyon in northern Arizona (CMNH), the Sacramento Mountains in southern New Mexico (CMNH), west-central Minnesota (Gandhi et al. 2005: 924), and central Wisconsin (Messer 2010: 36).

#### Records.

**CAN**: AB, BC (VCI), MB, NT, SK **USA**: AZ, CA, CO, ID, MN, MT, ND, NM, NV, OR, UT, WA, WI

### Bembidion conspersum

Chaudoir, 1868

Ochthedromus tesselatus LeConte, 1852a: 188 [secondary homonym of *Bembidion tesselatum* Brullé, 1843]. Type locality: «San Diego [San Diego County, California]» (original citation). Lectotype (♀), designated by Erwin (1984a: 169), in MCZ [# 5532].
Bembidium conspersum Chaudoir, 1868b [July]: 244. Replacement name for *Bembidium tesselatum* (LeConte, 1852).
Bembicidium xanthostictum Gemminger and Harold, 1868a [August]: 424. Replacement name for *Bembicidium tesselatum* (LeConte, 1852).

#### Distribution.

According to Erwin (1984a: 169), this species is found throughout California.

#### Records.

**USA**: CA

### Bembidion consuetum

Casey, 1918

Bembidion consuetum Casey, 1918: 93. Type locality: «Gualala River, Mendocino Co[unty], California» (original citation). Lectotype (♀), designated by Erwin (1984a: 170), in USNM [# 36955].
Bembidion augurale Casey, 1918: 92. Type locality: «San Francisco [San Francisco County], California» (original citation). Lectotype (♂), designated by Erwin (1984a: 170), in USNM [# 36954]. Synonymy established by Erwin (1984a: 170).

#### Distribution.

This species, as far as known, is restricted to the San Francisco Bay area in California (Erwin 1984a: 170).

#### Records.

**USA**: CA

### Bembidion cordatum

(LeConte, 1847)

Ochthedromus cordatus LeConte, 1847: 457. Type locality: «NovEboraci [= New York]» (original citation). Lectotype (♀), designated by Erwin (1984a: 170), in MCZ [# 5522].
Bembidion placabile Casey, 1918: 119. Type locality: «Big Springs [Howard County], Texas» (original citation). Lectotype (♂), designated by Erwin (1984a: 170), in USNM [# 37034]. Synonymy established by Erwin (1984a: 170).

#### Distribution.

This species is found from western New Hampshire (Cooper 1976: 164) to eastern North Dakota (Grand Forks County, CNC), north to southern Manitoba (Lindroth 1963b: 362) and southern Saskatchewan (Ronald R. Hooper pers. comm. 2002), south to southern Arizona (Snow 1906b: 161; Coconino County, CMNH), including north-central Utah (Cache County, CMNH), the Big Bend National Park in western Texas (Dajoz 2007: 23), west-central Arkansas (Garland County, Robert L. Davidson pers. comm. 2012), northern Tennessee (Montgomery County, Foster F. Purrington pers. comm. 2010), and west-central West Virginia (Roane County, CMNH).

#### Records.

**CAN**: MB, ON, SK **USA**: AR, AZ, CO, IA, IL, IN, KS, MI, MO, ND, NE, NH, NM, NY, OH, OK, PA, SD, TN, TX, UT, VA, VT, WI, WV, WY

### Bembidion debiliceps

Casey, 1918

Bembidion debiliceps Casey, 1918: 104. Type locality: «Corvallis [Benton County], Oregon» (original citation). Lectotype (♀), designated by Lindroth (1975: 120), in USNM [# 36961].

#### Distribution.

This species is found along the Pacific Coast from southwestern British Columbia, including Vancouver Island (Lindroth 1963b: 369), south at least to west-central Oregon (Casey 1918: 104).

#### Records.

**CAN**: BC (VCI) **USA**: OR, WA

### Bembidion evidens

Casey, 1918

Bembidion evidens Casey, 1918: 93. Type locality: «Milford [Beaver County], Utah» (original citation). Lectotype (♂), designated by Erwin (1984a: 171), in USNM [# 36956].

#### Distribution.

This species is known only from the type locality in southwestern Utah.

#### Records.

**USA**: UT

### Bembidion graphicum

Casey, 1918

Bembidion graphicum Casey, 1918: 108. Type locality: «Bayfield [Bayfield County], Wisconsin» (original citation). Lectotype (♂), designated by Lindroth (1975: 120), in USNM [# 36972].
Bembidion lassulum Casey, 1918: 118. Type locality: «Tuçson [Pima County], Arizona» (original citation). Holotype [by monotypy] (♀) in USNM [# 37040]. Synonymy established by Erwin (1984a: 173).

#### Distribution.

The range of this species extends from western Ontario (Lindroth 1963b: 369) to central Yukon Territory (Dawson, CNC), south to south-central Oregon (Lake County, UASM), southern Arizona (Casey 1918: 118, as *Bembidion lassulum*), southern Colorado (Elias 1987: 632), and northeastern Wisconsin (Casey 1918: 108). The records from “Nebraska” and “South Dakota” (Bousquet and Larochelle 1993: 142) need confirmation.

#### Records.

**CAN**: AB, BC, MB, NT, ON, SK, YT **USA**: AZ, CO, MN, MT, OR, UT, WA, WI, WY [NE, SD]

### Bembidion idoneum

Casey, 1918

Bembidion idoneum Casey, 1918: 90. Type locality: «Mendocino Co[unty], California» (original citation for the lectotype). Lectotype (♂), designated by Erwin (1984a: 173), in USNM [# 36946].
Bembidion idoneum obsequens Casey, 1918: 90. Type locality: «Lake Co[unty], California» (original citation). Lectotype (♂), designated by Erwin (1984a: 173), in USNM [# 36947]. Synonymy established (as aberration) by Csiki (1928: 61), confirmed by Erwin (1984a: 173).
Bembidion efficiens Casey, 1918: 90. Type locality: «Mokelumne Hill, Calaveras Co[unty], California» (original citation). Lectotype (♀), designated by Erwin (1984a: 173), in USNM [# 36949]. Synonymy established by Erwin (1984a: 173).
Bembidion continens Casey, 1918: 91. Type locality: «Siskiyou Co[unty], California» (original citation). Lectotype (♀), designated by Erwin (1984a: 173), in USNM [# 36948]. Synonymy established by Erwin (1984a: 173).

#### Distribution.

According to Erwin (1984a: 174), the range of this species “extends throughout the western United States in the mountainous regions;” the only documented records are those of the type localities in California and that from Valley County in central Idaho (Maddison 2012: Supplementary content Table S1). David R. Maddison (pers. comm. 2012) caught the species in Steens Mountains, southeastern Oregon.

#### Records.

**USA**: CA, ID, OR

### Bembidion indistinctum

Dejean, 1831

Bembidium indistinctum Dejean, 1831: 67. Type locality: «Californie» (original citation), restricted to «San Diego [San Diego County]» by Lindroth (1963b: 361). One syntype in MHNP (Lindroth 1955b: 14).
Ochthedromus consentaneus LeConte, 1852a: 187. Type locality: «San Diego [San Diego County, California]» (original citation). Syntype(s) in MCZ [# 5527]. Synonymy established by Lindroth (1963b: 361).
Bembidion curiosum Casey, 1918: 95. Type locality: «San Francisco [San Francisco County], California» (original citation). Lectotype (♂), designated by Lindroth (1975: 120), in USNM [# 36989]. Synonymy established by Lindroth (1963b: 361).
Bembidion devinctum Casey, 1918: 97. Type locality: «Alameda [Alameda County], California» (original citation). Lectotype (♀), designated by Lindroth (1975: 120), in USNM [# 36988]. Synonymy established by Lindroth (1963b: 361).
Bembidion extricatum Casey, 1918: 98. Type locality: «San Diego [San Diego County], California» (original citation). Lectotype (♀), designated by Lindroth (1975: 120), in USNM [# 36987]. Synonymy established by Lindroth (1963b: 361).
Bembidion caudex Casey, 1918: 98. Type locality: «Tehatchapi [= Tehachapi] Pass [Kern County], California» (original citation). Lectotype (♀), designated by Lindroth (1975: 120), in USNM [# 36983]. Synonymy established by Lindroth (1963b: 361).
Bembidion consentaneum barbarae Casey, 1918: 99. Type locality: «S[an]ta Barbara [Santa Barbara County], California» (original citation). Lectotype (♀), designated by Lindroth (1975: 120), in USNM [# 36985]. Synonymy established (as aberration), under the name *Bembidion consentaneum* (LeConte), by Csiki (1928: 59), confirmed by Lindroth (1963b: 361).
Bembidion derisor Casey, 1918: 99. Type locality: «eastern shore of San Francisco Bay, California» (original citation). Lectotype (♀), designated by Erwin (1984a: 174), in USNM [# 36986]. Synonymy established by Erwin (1984a: 174).
Bembidion expositum Casey, 1918: 101. Type locality: «Tehatchapi [= Tehachapi] Pass [Kern County], California» (original citation). Lectotype (♂), designated by Erwin (1984a: 174), in USNM [# 36999]. Synonymy established by Erwin (1984a: 174).
Bembidion formale Casey, 1918: 101. Type locality: «San Francisco [San Francisco County], California» (original citation). Lectotype (♂), designated by Erwin (1984a: 174), in USNM [# 36995]. Synonymy established by Erwin (1984a: 174).
Bembidion formale reconditum Casey, 1918: 102. Type locality: «San Diego [San Diego County], California» (original citation). Lectotype (♂), designated by Erwin (1984a: 174), in USNM [# 36996]. Synonymy established (as aberration), under the name *Bembidion formale* Casey, by Csiki (1928: 60), confirmed by Erwin (1984a: 174).
Bembidion franciscanum Casey, 1918: 102. Type locality: «Alameda [Alameda County], California» (original citation). Lectotype (♂), designated by Erwin (1984a: 174), in USNM [# 36991]. Synonymy established by Erwin (1984a: 174).
Bembidion ornatellum Casey, 1918: 102. Type locality: «Alameda [Alameda County], California» (original citation). Lectotype (♂), designated by Erwin (1984a: 174), in USNM [# 36992]. Synonymy established by Erwin (1984a: 174).

#### Distribution.

This species is found from Kupreanof Island in the Alexander Archipelago to southern California (Moore 1937: 7; Kavanaugh 1992: 67).

#### Records.

**CAN**: BC (QCI, VCI) **USA**: AK, CA (CHI), OR, WA

### Bembidion insulatum

(LeConte, 1852)

Ochthedromus insulatus LeConte, 1852a: 186. Type locality: «San Diego [San Diego County, California]» (original citation). Three syntypes in MCZ [# 100].
Bembidion caliginosum Casey, 1918: 119. Type locality: «Douglas [Cochise County], Arizona» (original citation). Lectotype (♂), designated by Erwin (1984a: 175), in USNM [# 37035]. Synonymy established by Erwin (1984a: 175).

#### Distribution.

This species ranges from southern Manitoba to southern British Columbia though not reaching the coast, north to Fort Smith in southern Northwest Territories (Lindroth 1963b: 363), south to northern Baja California (CNC), southern Arizona (Casey 1918: 119, as *Bembidion caliginosum*; Maddison 2012: Supplementary content Table S1), southwestern New Mexico (Hidalgo County, CNC), and southeastern South Dakota (Kirk and Balsbaugh 1975: 19). The record from northwestern Texas (Merickel and Wangberg 1981: 157) needs confirmation.

#### Records.

**CAN**: AB, BC, MB, NT, SK **USA**: AZ, CA, MT, ND, NM, NV, OR, SD, UT, WA, WY [TX] – Mexico

### Bembidion intermedium

(Kirby, 1837)

Notaphus intermedius Kirby, 1837: 58. Type locality: «Lat. 54° [= along North Saskatchewan River]» (original citation), restricted to «Edmonton, Al[ber]ta» by Lindroth (1963b: 365). Holotype [by monotypy] (♀) in BMNH (Lindroth 1953b: 176).

#### Distribution.

This species occurs from the Saint Lawrence Plain in southern Quebec (Larochelle 1975: 57) to central British Columbia, north to east-central Alaska (Lindroth 1963b: 365), south to southern Colorado (Wickham 1902: 234; Bell 1971: 28), southern South Dakota (Kirk and Balsbaugh 1975: 18), southern Wisconsin (Messer 2010: 36), and southwestern Pennsylvania (Allegheny County, Robert L. Davidson pers. comm. 2008). The records from Missouri (Casey 1918: 109), southern Oklahoma (Hatch and Ortenburger (1930: 11), Texas (Wickham 1897: 104), Kansas (Popenoe 1877: 24; Snow 1878: 63), New Mexico (Fall and Cockerell 1907: 157), southern Arizona (Wickham 1897: 104), and “Delaware” (Bousquet and Larochelle 1993: 142) are probably in error.

#### Records.

**CAN**: AB, BC, MB, ON, QC, SK **USA**: AK, CO, MI, MN, MT, NY, OH, PA, SD, VT, WI, WY

### Bembidion jacobianum

Casey, 1918

Bembidion jacobianum Casey, 1918: 101. Type locality: «San Diego [San Diego County], California» (original citation). Lectotype (♀), designated by Erwin (1984a: 175), in USNM [# 36993].
Bembidion procax Casey, 1918: 103. Type locality: «San Diego [San Diego County], California» (original citation). Lectotype (♂), designated by Erwin (1984a: 175), in USNM [# 36994]. Synonymy established by Erwin (1984a: 175).

#### Distribution.

This species is known only from the type locality in southwestern California.

#### Records.

**USA**: CA

### Bembidion latebricola

Casey, 1918

Bembidion latebricola Casey, 1918: 100. Type locality: «Arizona» (original citation). Lectotype (♂), designated by Erwin (1984a: 175), in USNM [# 36998].

#### Distribution.

This species is known only from the type series.

#### Records.

**USA**: AZ

### Bembidion nigripes

(Kirby, 1837)

Notaphus nigripes Kirby, 1837: 57. Type locality: «Lat. 54° [= along North Saskatchewan River]» (original citation), restricted to «Edmonton, Al[ber]ta» by Lindroth (1963b: 369). Two syntypes [3 originally cited] in BMNH (Lindroth 1953b: 176).
Notaphus quadraticollis Mannerheim, 1853: 148. Type locality: «ad ostia fl[umen] Kaktnu [= Kenai River] peninsulae Kenai [Alaska]» (original citation). Syntype(s) location unknown (Lindroth 1963b: 370). Synonymy established with doubt by LeConte (1860: 316).
Bembidion retractum Casey, 1918: 109. Type locality: «Boulder Co[unty], Colorado» (original citation). Lectotype (♂), designated by Lindroth (1975: 120), in USNM [# 36977]. Synonymy established by Lindroth (1963b: 370).
Bembidion concitatum Casey, 1924: 35. Type locality: «Marquette [Marquette County], Michigan» (original citation for *Bembidion nigripes* (Kirby) *sensu* Casey, 1918). Lectotype (♂), designated by Lindroth (1975: 120), in USNM [# 36959]. Synonymy established by Hatch (1953: 93), confirmed by Lindroth (1963b: 370). Note. This name was proposed for *Notaphus nigripes* Kirby, 1837 *sensu* Casey (1918: 92).

#### Distribution.

This species ranges from Newfoundland (Lindroth 1955a: 67) to Vancouver Island, north to central Alaska and the Mackenzie River delta in northern Northwest Territories (Lindroth 1963b: 370-371), south to El Dorado County in eastern California (Maddison 1985: 113), “Utah” (Hayward 1897: 105), northern New Mexico (Taos County, UASM), eastern North Dakota (Tinerella 2003: 635), northeastern Wisconsin (Messer 2010: 36), and the upper peninsula of Michigan (Casey 1924: 35, as *Bembidion concitatum*); also found in eastern Siberia (Lindroth 1963b: 371) and Japan (Marggi et al. 2003: 254). The records from western Maine (Harvey and Knight (1897: 79), northeastern New York (Notman 1928: 216), “Pennsylvania” (Leng and Beutenmüller 1893: 136), “Illinois” (Bousquet and Larochelle 1993: 142), southern South Dakota (Kirk and Balsbaugh 1975: 18), and southern Oklahoma (Elliott et al. 2006: 125) are likely in error. Fossil remnants, dated between 15,100 and 15,300 years B.P., have been unearthed in central Iowa (Schwert 1992: 77).

#### Records.

**FRA**: PM **CAN**: AB, BC (VCI), LB, MB, NB, NF, NS, NT, ON, PE, QC, SK, YT **USA**: AK, CA, CO, ID, MI, MN, MT, ND, NM, NV, OR, UT, WA, WI, WY – **Holarctic**

### Bembidion obscuromaculatum

(Motschulsky, 1859)

Notaphus obscuromaculatus Motschulsky, 1859a: 130. Type locality: «Calif[ornia] bor[ealis]» (lectotype label). Lectotype (♂), designated by Bousquet (1997b: 332), in ZMMU.

#### Distribution.

This species is yet known only from the lectotype.

#### Records.

**USA**: CA

#### Note.

This species is distinct from all other North American *Bembidion* (see Bousquet 1997b: 332) but only the original specimen is known. It is possible that the lectotype is mislabeled. Motschulsky (1859a: 130) added at the end of this species description “Je crois que cette espèce se rencontre aussi dans les possessions russes, notamment sur l’île Kenoi [Alaska]”.

### Bembidion operosum

Casey, 1918

Bembidion operosum Casey, 1918: 103. Type locality: «S[an]ta Cruz [Santa Cruz County], California» (original citation). Lectotype (♀), designated by Erwin (1984a: 178), in USNM [# 36960].

#### Distribution.

This species is yet known only from the type locality and Santa Barbara County (Maddison 1985: 113) in western California.

#### Records.

**USA**: CA

### Bembidion patruele

Dejean, 1831

Bembidium patruele Dejean, 1831: 69. Type locality: «Amérique septentrionale» (original citation), restricted to «Rumney [Grafton County], N[ew] H[ampshire]» by Lindroth (1963b: 371). One syntype in MHNP (Lindroth 1955b: 14).
Notaphus posticum Haldeman, 1843b: 303. Type locality: southeastern Pennsylvania (Haldeman 1843a: 297). Lectotype [as type] (♂), designated by Lindroth (1955a: 68), in MCZ [# 28694]. Synonymy established by Lindroth (1955b: 14).
Bembidium fraternum LeConte, 1857a: 6. Type locality: «Habersham County, Georgia» (original citation). Holotype [by monotypy] (♀) in MCZ [# 5530]. Synonymy established, under the name *Bembidion posticum* (Haldeman), by Lindroth (1954b: 127).
Bembidion imitator Casey, 1918: 105. Type locality: «Kamloops, British Columbia» (original citation). Lectotype (♂), designated by Lindroth (1975: 120), in USNM [# 36962]. Synonymy established by Hatch (1953: 94), confirmed by Lindroth (1963b: 371).
Bembidion monstratum Casey, 1918: 106. Type locality: «Highland Park [Lake County], Illinois» (original citation). Lectotype (♂), designated by Lindroth (1975: 120), in USNM [# 36971]. Synonymy established, under the name *Bembidion posticum* (Haldeman), by Lindroth (1954b: 126).
Bembidion fenisex Casey, 1918: 106. Type locality: «Indiana» (original citation). Lectotype (♂), designated by Lindroth (1975: 120), in USNM [# 36969]. Synonymy established, under the name *Bembidion posticum* (Haldeman), by Lindroth (1954b: 126).
Bembidion plectile Casey, 1918: 107. Type locality: «Indiana» (original citation for the lectotype). Lectotype (♂), designated by Lindroth (1975: 120), in USNM [# 36968]. Synonymy established, under the name *Bembidion posticum* (Haldeman), by Lindroth (1954b: 126).
Bembidion mediocre Casey, 1918: 107. Type locality: «undoubtedly from somewhere in the Atlantic regions» (original citation). Holotype [by monotypy] (♂) in USNM [# 36967]. Synonymy established by Erwin (1984a: 178).
Bembidion exclusum Casey, 1918: 109. Type locality: «Highland Park [Lake County], Illinois» (original citation). Lectotype (♂), designated by Lindroth (1975: 120), in USNM [# 38970]. Synonymy established, under the name *Bembidion posticum* (Haldeman), by Lindroth (1954b: 127).
Bembidion marcidum Casey, 1918: 110. Type locality: «lower Hudson Valley, New York» (original citation for the lectotype). Lectotype (♀), designated by Lindroth (1975: 121), in USNM [# 36973]. Synonymy established, under the name *Bembidion posticum* (Haldeman), by Lindroth (1954b: 127).
Bembidion editum Casey, 1918: 125. Type locality: «near the Delaware River, New Jersey» (original citation). Lectotype (♂), designated by Erwin (1984a: 178), in USNM [# 37041]. Synonymy established by Erwin (1984a: 178).
Bembidion subexiguum Casey, 1924: 37. Type locality: «Terrace, British Columbia» (original citation). Lectotype (♂), designated by Lindroth (1975: 121), in USNM [# 36957]. Synonymy established by Lindroth (1963b: 371).
Bembidion contristans Casey, 1924: 37. Type locality: «Rhode Island» (original citation). Lectotype (♂), designated by Lindroth (1975: 121), in USNM [# 36974]. Synonymy established, under the name *Bembidion posticum* (Haldeman), by Lindroth (1954b: 127).

#### Distribution.

This widely distributed species ranges from Newfoundland (Lindroth 1955a: 68) to western British Columbia, north to southern Northwest Territories (Lindroth 1963b: 372), south to Oregon (Hatch 1953: 94), southern Colorado (Elias 1987: 632), southern Texas (Kenedy County, CNC), northwestern Florida (Holmes County, UASM), and so﻿uthern South Carolina (Ciegler 2000: 48).

#### Records.

**CAN**: AB, BC, MB, NB, NF, NS (CBI), NT, ON, PE, QC, SK **USA**: AL, AR, CO, CT, DC, DE, FL, GA, IA, ID, IL, IN, KY, LA, MA, MD, ME, MI, MN, MO, MS, MT, NC, ND, NH, NJ, NY, OH, OK, OR, PA, RI, SC, SD, TN, TX, VA, VT, WA, WI, WV, WY

### Bembidion pimanum

Casey, 1918

Bembidion pimanum Casey, 1918: 98. Type locality: «near Benson [Cochise County], Arizona» (original citation). Lectotype (♀), designated by Erwin (1984a: 179), in USNM [# 36982].

#### Distribution.

This species is known only from the type locality in southeastern Arizona.

#### Records.

**USA**: AZ

### Bembidion semiopacum

Casey, 1924

Bembidion semiopacum Casey, 1924: 39. Type locality: «San Joaquin Co[unty], California» (original citation). Holotype [by monotypy] (♀) in USNM [# 36965].

#### Distribution.

This species is known only from the California central valley (Erwin 1984a: 182).

#### Records.

**USA**: CA

### Bembidion semipunctatum

(Donovan, 1806)

Carabus semipunctatus Donovan, 1806: 22. Type locality: «shore of the Severn sea, near the village of Newton, Glamorganshire [Wales, Great Britain]» (original citation). Holotype [by monotypy] probably lost (Lindroth 1963b: 367).
Bembidium elegantulum R.F. Sahlberg, 1844: 56. Type locality: «fluminis Ochotae [River Ochota, near Okhotsk, Khabarovsk Kray, Siberia, Russia]» (original citation). Lectotype (♀), designated by Lindroth (1963b: 367), in ZMUT. Synonymy established by Lindroth (1963b: 367).
Notaphus alternans Motschulsky, 1845b: 349. Type locality: «Kamtschatka [Russia]» (original citation). One syntype in ZMMU (Keleinikova 1976: 185). Synonymy established by Shilenkov (1994: 20).
Bembidion provoanum Casey, 1918: 105. Type locality: «Provo [Utah County], Utah» (original citation). Lectotype (♀), designated by Erwin (1984a: 182), in USNM [# 36966]. Synonymy established by Erwin (1984a: 182).
Bembidion accuratum Casey, 1924: 39. Type locality: «Edmonton, Alberta» (original citation). Lectotype (♂), designated by Lindroth (1975: 120), in USNM [# 36963]. Synonymy established by Lindroth (1963b: 367).

#### Distribution.

This Holarctic species is found from Great Britain to eastern Siberia and Japan (Marggi et al. 2003: 254), and from the Arctic Plains in northern Alaska to the Hudson Bay shore in northwestern Ontario, south to southeastern Minnesota (Olmsted Country, CMNH), north-central Utah (Casey 1918: 105, as *Bembidion provoanum*), and northeastern California (Notman 1929b: 223, as *Bembidion provoanum*).

#### Records.

**CAN**: AB, BC, MB, NT, ON, SK, YT **USA**: AK, CA, MN, MT, OR, UT, WA – **Holarctic**

#### Note.

*Elaphrus sturmii*, credited to Duftschmid (1812: 203), is usually listed as junior synonym of this species (e.g., Marggi et al. 2003: 254). However, it is quite evident that Duftschmid (1812: 203) referred to *Carabus sturmii* Panzer listed on the preceding page (page 202).

### Bembidion vulpecula

Casey, 1918

Bembidion vulpecula Casey, 1918: 126. Type locality: «Brownsville [Cameron County], Texas» (original citation). Lectotype (♀), designated by Erwin (1984a: 185), in USNM [# 37047].

#### Distribution.

This species is known from southern Texas (Johnson 1978: 67) and “Mexico” (Erwin 1984a: 185).

#### Records.

**USA**: TX – Mexico

### [rapidum group]

### Bembidion aratum

(LeConte, 1852)

Ochthedromus aratus LeConte, 1852a: 189. Type locality: «ad flumen Gila» (original citation). Lectotype (♀), designated by Erwin (1982b: 479), in MCZ [# 3343].
Bembidion definitum Casey, 1918: 116. Type locality: «Tuçson [Pima County], Arizona» (original citation). Holotype [by monotypy] (♂) in USNM [# 37030]. Synonymy established by Erwin (1982b: 479).

#### Distribution.

This species is known from the Gila River drainage in southwestern New Mexico (David R. Maddison pers. comm. 2007) and southern Arizona (Casey 1918: 116, as *Bembidion definitum*; Cochise and Santa Cruz Counties, CMNH), south to San Luis Potosí in the Sierra Madre Oriental (Ball and Shpeley 1992a: 47, as *Bembidion definitum*). The record from Inyo County in eastern California (Riley 1893: 239) needs confirmation.

#### Records.

**USA**: AZ, NM [CA] – Mexico

### Bembidion nubiculosum

Chaudoir, 1868

Ochthedromus laticollis LeConte, 1852a: 187 [secondary homonym of *Bembidion laticolle* (Duftschmid, 1812)]. Type locality: «Colorado [River, California]» (original citation). Lectotype (♂), designated by Erwin (1984a: 177), in MCZ [# 5523].
Bembidium nubiculosum Chaudoir, 1868b [July]: 244. Replacement name for *Bembidium laticolle* (LeConte, 1852).
Bembicidium platyderum Gemminger and Harold, 1868a [August]: 418 [primary homonym of *Bembidium platyderum* Chaudoir, 1868]. Replacement name for *Bembicidium laticolle* (LeConte, 1852).
Ochthedromus pardalis Zimmermann [in LeConte], 1869b: 247. Replacement name for *Ochthedromus laticollis* LeConte, 1852.
Bembidion nubiculosum daphnis Casey, 1918: 120. Type locality: «El Paso [El Paso County], Texas» (original citation). Lectotype (♂), designated by Erwin (1984a: 177), in USNM [# 37036]. Synonymy established by Erwin (1984a: 177).
Bembidion amnicum Casey, 1918: 121. Type locality: «Brownsville [Cameron County], Texas» (original citation). Lectotype (♂), designated by Erwin (1984a: 178), in USNM [# 37037]. Synonymy established by Erwin (1984a: 178).

#### Distribution.

This species is found from the Baja California Peninsula (Horn 1894: 308) and southeastern California (Casey 1918: 120; Andrews et al. 1979: 28) to southeastern Texas (Snow 1906a: 141; Casey, 1918: 121, as *Bembidion amnicum*) and northern Mexico (Erwin 1984a: 178). The record from “Utah” (Hayward 1897: 96) needs confirmation.

#### Records.

**USA**: AZ, CA, NM, TX [UT] – Mexico

### Bembidion rapidum

(LeConte, 1847)

Ochthedromus rapidus LeConte, 1847: 460. Type locality: «ad Rocky Mountains» (original citation), restricted to «Colorado Spring[s] [El Paso County], Color[ado]» by Lindroth (1963b: 363). Lectotype (♀), designated by Erwin (1984a: 180), in MCZ [# 5533].
Bembidion docile Casey, 1918: 126. Type locality: «Arizona» (original citation). Lectotype (♀), designated by Erwin (1984a: 180), in USNM [# 37055]. Synonymy established by Erwin (1984a: 180).
Bembidion sociale Casey, 1918: 127. Type locality: «Marquette [Marquette County], Michigan» (original citation for the lectotype). Lectotype (♀), designated by Lindroth (1975: 120), in USNM [# 37051]. Synonymy established by Lindroth (1963b: 363).
Bembidion negligens Casey, 1918: 127. Type locality: «El Paso [El Paso County], Texas» (original citation). Holotype [by monotypy] (♂) in USNM [# 37052]. Synonymy established by Erwin (1984a: 180).
Bembidion fugitans Casey, 1918: 127. Type locality: «Arizona» (original citation). Lectotype (♂), designated by Erwin (1984a: 180), in USNM [# 37053]. Synonymy established by Erwin (1984a: 180).

#### Distribution.

The range of this species extends from Nova Scotia (Majka et al. 2007: 8) to the Rocky Mountains in Alberta (Lindroth 1963b: 364), south to southeastern Arizona (Dajoz 2007: 21; Cochise County, UASM), southern Texas (Johnson 1978: 67), northern Alabama (Madison County, CMNH), and eastern South Carolina (Ciegler 2000: 48), west along the southwest to southern California (Lindroth 1963b: 364; Los Angeles County, CMNH).

#### Records.

**CAN**: AB, MB, NB, NS, ON, QC, SK **USA**: AL, AR, AZ, CA, CO, CT, GA, IA, IL, IN, KS, KY, LA, MA, MD, ME, MI, MN, MO, MS, MT, NC, ND, NE, NH, NM, NY, OH, OK, PA, RI, SC, SD, TN, TX, UT, VA, VT, WI, WV, WY

### Bembidion scintillans

Bates, 1882

Bembidium scintillans Bates, 1882a: 150. Type locality: «Capulalpam [= Calpulalpam, Oaxaca], Mexico» (original citation for the lectotype). Lectotype (♀), designated by Erwin (1982b: 479), in BMNH.
Bembidium vinnulum Casey, 1884b: 15. Type locality: «Arizona» (original citation). Lectotype (♀), designated by Erwin (1982b: 479), in USNM [# 37029]. Synonymy established by Horn (1886b: xii).

#### Distribution.

This species is known from the Gila River drainage in southwestern New Mexico (David R. Maddison pers. comm. 2007) and southeastern Arizona (Graham and Greenlee Counties, CMNH; Casey 1884b: 15, as *Bembidion vinnulum*), and from southeastern Mexico (Bates 1882a: 150).

#### Records.

**USA**: AZ, NM – Mexico

#### Note.

This taxon has been listed in synonymy with *Bembidion aratum* (LeConte) by Erwin (1982b: 479) but according to Maddison (2012: 535) it represents a distinct species.

### [scudderi group]

### Bembidion consimile

Hayward, 1897

Bembidium consimile Hayward, 1897: 88. Type locality: «Lincoln, Nebraska; Salt Lake, Utah; Colorado Springs, Colo[rado]» (original citation), restricted to «Lincoln [Lancaster County], Nebraska» by Lindroth (1963b: 353). Two syntypes [“about a dozen” originally cited] in MCZ [# 16293].

#### Distribution.

This species is found from southern Manitoba to southern Alberta (Lindroth 1963b: 353), south to Mono County in east-central California (Dajoz 2007: 17), west-central Nevada (Bechtel et al. 1983: 474), central Colorado, and Nebraska (Hayward 1897: 88).

#### Records.

**CAN**: AB, MB, SK **USA**: CA, CO, ND, NE, NV, SD, UT, WY

### Bembidion flohri

Bates, 1878

Bembidium flohri Bates, 1878a: 602. Type locality: «near the capital, Mexico» (original citation). Lectotype (♂), designated by Erwin (1984a: 172), in BMNH.
Bembidium henshawi Hayward, 1897: 87. Type locality: «Salt Lake [Millard County], Utah» (original citation for the lectotype). Lectotype (♂), designated by Erwin (1984a: 172), in MCZ [# 16292]. Synonymy established by Erwin (1984a: 172). Etymology. The specific name was proposed for Samuel Henshaw [1852-1941], American entomologist, librarian, and biographer. Henshaw was assistant to Professor Alpheus Hyatt at Lowell Institute from 1876 to 1891 and worked at the Museum of Comparative Zoology from 1891 to 1927, first as assistant in entomology and librarian, then as curator, and finally as director.

#### Distribution.

This species ranges from southern Manitoba to central British Columbia (Lindroth 1963b: 354, as *Bembidion henshawi*), south to southern California (Hayward 1897: 88) and to the “Mexican transverse volcanic belt” (Erwin 1984a: 172).

#### Records.

**CAN**: AB, BC, MB, SK **USA**: CA, CO, MT, ND, NV, OR, UT, WA, WY – Mexico

### Bembidion obtusidens

Fall, 1922

Bembidion obtusidens Fall, 1922c: 170. Type locality: «Baldur, Manitoba» (original citation). Holotype (♂) in MCZ [# 23864].

#### Distribution.

This species occurs from northwestern Ontario (CNC) to central Alberta (Lindroth 1963b: 355), south to central Utah (Fall 1922c: 171), southeastern Wyoming (Albany County, MCZ), and east-central South Dakota (Kirk and Balsbaugh 1975: 20). The record from “Colorado” (Bousquet and Larochelle 1993: 140) needs confirmation.

#### Records.

**CAN**: AB, MB, ON, SK **USA**: MT, ND, SD, UT, WY [CO]

### Bembidion scudderi

LeConte, 1878

Bembidium scudderi LeConte, 1878a: 451. Type locality: «Salt Lake Valley (4,300 feet) [Salt Lake County, Utah]» (original citation). Holotype [by monotypy] (♂) in MCZ [# 5520]. Etymology. The specific name honors the celebrated American paleontologist, entomologist, and nomenclatorist Samuel Hubbard Scudder [1837-1911] who worked primarily on Orthoptera and Lepidoptera at Harvard University.
Bembidion spissicorne Casey, 1924: 40. Type locality: «Bellevue (3400 ft.) [Weber County], Utah» (original citation). Lectotype (♀), designated by Lindroth (1975: 120), in USNM [# 36990]. Synonymy established by Lindroth (1963b: 351).

#### Distribution.

This species is found from southern Manitoba to the Okanagan Valley in British Columbia (Lindroth 1963b: 352), south to Kern County in southern California (Fall 1901a: 43), northern Utah (LeConte 1878a: 451; Casey, 1924: 40, as *Bembidion spissicorne*; Box Elder and Utah Counties, MCZ), and Yellowstone National Park in Wyoming (MCZ). The record from “Colorado” (Bousquet and Larochelle 1993: 140) needs confirmation.

#### Records.

**CAN**: AB, BC, MB, SK **USA**: CA, MT, ND, NV, OR, UT, WA, WY [CO]

### Subgenus Trepanedoris

Netolitzky, 1918

Trepanedoris Netolitzky, 1918: 24. Type species: *Carabus doris* Panzer, 1796 by original designation. Etymology. From the generic name *Trepanes* and the specific name of the type species *doris* [masculine].

#### Diversity.

Northern Hemisphere, with 16 species in the Nearctic (13 species) and Palaearctic (three species) Regions. The North American species are placed in two groups following Maddison (2012: 544).

#### Identification.

Lindroth (1963b: 395-402) covered all North American species except for three (*Bembidion scenicum*, *Bembidion clemens* and *Bembidion elizabethae*).

### [connivens group]

### Bembidion acutifrons

LeConte, 1879

Bembidium acutifrons LeConte, 1879d: 509. Type locality: «Alamosa [Alamosa County], Colo[rado]» (original citation). Holotype [by monotypy] (♀) in MCZ [# 5553].
Bembidion microreticulatum Hatch, 1950: 105. Type locality: «Stickney L[ake] [Snohomish County], Washington» (original citation). Holotype (♂) in USNM. Synonymy established by Lindroth (1963b: 400).

#### Distribution.

The range of this species extends from southern Manitoba to Vancouver Island, north to east-central Alaska (Lindroth 1963b: 400), south to western Oregon (Hatch 1953: 100, as *Bembidion microreticulatum*), southern Colorado (LeConte 1879d: 509; Wickham 1902: 235), and east-central South Dakota (Kirk and Balsbaugh 1975: 20).

#### Records.

**CAN**: AB, BC (VCI), MB, NT, SK **USA**: AK, CO, MT, ND, OR, SD, UT, WA, WY

### Bembidion ampliceps

Casey, 1918

Bembidion ampliceps Casey, 1918: 161. Type locality: «Gilroy Hot Springs, S[an]ta Clara Co[unty], California» (original citation). Holotype [by monotypy] (♂) in USNM [# 37084].

#### Distribution.

This species is known from east-central Oregon (Union County, CMNH; Lindroth 1963b: 401) to southeastern California (Dajoz 2007: 20). The records from southwestern British Columbia and eastern Washington (Hatch 1953: 100) are probably in error (see Lindroth 1963b: 401).

#### Records.

**USA**: CA, OR

### Bembidion canadianum

Casey, 1924

Bembidion canadianum Casey, 1924: 43. Type locality: «Edmonton, Alberta» (original citation). Lectotype (♀), designated by Lindroth (1975: 122), in USNM [# 37083].

#### Distribution.

This species is found from the Saint Lawrence Estuary in Quebec (Larochelle 1975: 53) to south-central British Columbia (Jarrett and Scudder 2001: 381) and central Washington (Grant County, Foster F. Purrington pers. comm. 2011), north to south-central Northwest Territories (near Hay River, CNC), south to southern Utah (Garfield County, FFPC).

#### Records.

**CAN**: AB, BC, MB, NT, ON, QC, SK **USA**: MT, UT, WA

### Bembidion clemens

Casey, 1918

Bembidion clemens Casey, 1918: 159. Type locality: «Provo [Utah County], Utah» (original citation). Lectotype (♀), designated by Erwin (1984a: 168), in USNM [# 37080].
Bembidion vapidum Casey, 1918: 160. Type locality: «M[oun]t Diablo [Contra Costa County], California» (original citation). Lectotype (♂), designated by Erwin (1984a: 169), in USNM [# 37073]. Synonymy established by Erwin (1984a: 169).
Bembidion disparile Casey, 1918: 161. Type locality: «S[an]ta Barbara [Santa Barbara County], California» (original citation). Lectotype (♂), designated by Erwin (1984a: 169), in USNM [# 37074]. Synonymy established by Erwin (1984a: 169).
Bembidion invidiosum Casey, 1918: 162. Type locality: «road between Fort Wingate and Jemez Springs [Sandoval County], New Mexico» (original citation). Lectotype (♂), designated by Erwin (1984a: 169), in USNM [# 37081]. Synonymy established by Erwin (1984a: 169).
Bembidion remotum Casey, 1918: 163. Type locality: «Paraiso Hot Springs, Monterey Co[unty], California» (original citation). Lectotype (♂), designated by Erwin (1984a: 169), in USNM [# 37086]. Synonymy established by Erwin (1984a: 169).

#### Distribution.

This species is known from western California (Casey 1918: 160, 161, 163 as *Bembidion vapidum*, *Bembidion disparile*, and *Bembidion remotum*), north-central Utah (Casey 1918: 159), central Arizona (Maddison 2012: Supplementary content Table S1), and northwestern New Mexico (Casey 1918: 162, as *Bembidion invidiosum*).

#### Records.

**USA**: AZ, CA, NM, UT

### Bembidion connivens

(LeConte, 1852)

Ochthedromus connivens LeConte, 1852a: 188. Type locality: «San Francisco [San Francisco County, California]» (original citation). Two syntypes in MCZ [# 5556].
Bembidion digressum Casey, 1918: 155. Type locality: «S[ain]t Helena, Napa Co[unty], California» (original citation). Lectotype (♀), designated by Lindroth (1975: 122), in USNM [# 37075]. Synonymy established by Fall (1926a: 134), confirmed by Lindroth (1963b: 401).

#### Distribution.

This species is found from Vancouver Island (Lindroth 1963b: 401) to northwestern Montana (Russell 1968: 58), south to central New Mexico (Fall and Cockerell 1907: 157) and southwestern California (Moore 1937: 8).

#### Records.

**CAN**: BC (VCI) **USA**: CA, CO, ID, MT, NM, OR, WA

### Bembidion elizabethae

Hatch, 1950

Bembidion elizabethae Hatch, 1950: 104. Type locality: «Licten Springs, King Co[unty], Washington» (original citation). Holotype in USNM. Etymology. The specific name is based on the first name of Elizabeth Farrar (see *Bembidion farrarae*).

#### Distribution.

This species is known from the type locality and Lincoln County in western Oregon (Maddison 2012: Supplementary content Table S1).

#### Records.

**USA**: OR, WA

#### Note.

This name has been listed in synonymy with *Bembidion connivens* (LeConte) by Lindroth (1963b: 401) but according to Maddison (2012: 535) it applies to a valid species.

### Bembidion frontale

(LeConte, 1847)

Ochthedromus frontalis LeConte, 1847: 462. Type locality: «Detroit [Wayne County, Michigan]» (original citation). Syntype(s) in MCZ [# 26893].

#### Distribution.

This species is found from Cape Breton Island (Bousquet 1987d: 105) to south-central British Columbia, south to northern Idaho (Kootenai County, CNC), Missouri (Summers 1873: 147), and West Virginia (Pocahontas and Randolph Counties, CMNH). The records from “Washington,” “Oregon,” and “California” (Hayward 1897: 127, as *Bembidion assimile*) probably refer to *Bembidion siticum*; those from Colorado (Wickham 1902: 235) and New Mexico (Fall and Cockerell 1907: 157, as *Bembidion assimile*) need confirmation as they could also refer to *Bembidion siticum*.

#### Records.

**CAN**: BC, MB, NB, NS (CBI), ON, PE, QC, SK **USA**: CT, IA, ID, IL, IN, KS, MA, ME, MI, MN, MO, ND, NH, NJ, NY, OH, PA, RI, SD, VA, VT, WI, WV [CO, NM]

#### Note.

This species has passed under the name *Bembidion assimile* Gyllenhal, 1810 until the 1950s.

### Bembidion scenicum

Casey, 1918

Bembidion scenicum Casey, 1918: 159. Type locality: «Lake Tahoe [Placer County], California» (original citation). Lectotype (♂), designated by Erwin (1984a: 181), in USNM [# 37079].

#### Distribution.

This species is known only from the type locality in the Sierra Nevada.

#### Records.

**USA**: CA

### Bembidion siticum

Casey, 1918

Bembidion siticum Casey, 1918: 157. Type locality: «Gualala River, Mendocino Co[unty], California» (original citation). Lectotype (♂), designated by Lindroth (1975: 122), in USNM [# 36803].
Bembidion adolescens Casey, 1918: 158. Type locality: «Booneville, Mendocino Co[unty], California» (original citation). Lectotype (♀), designated by Lindroth (1975: 122), in USNM [# 37071]. Synonymy established by Lindroth (1963b: 402).

#### Distribution.

This species is known from Vancouver Island (Lindroth 1963b: 402) to northwestern Montana (Russell 1968: 58), south to “Nevada” (Lindroth 1963b: 402) and Mendocino County in western California (Casey 1918: 157).

#### Records.

**CAN**: BC (VCI) **USA**: CA, ID, MT, NV, OR, WA

### [fortestriatum group]

### Bembidion anguliferum

(LeConte, 1852)

Omala polita Motschulsky, 1845a: 29 [*nomen dubium*]. Type locality: «Californie» (original citation). Syntype(s) location unknown (possibly in ZMMU though not listed in Keleinikova 1976).
Ochthedromus angulifer LeConte, 1852a: 188. Type locality: «San Francisco [San Francisco County, California]» (original citation). Holotype [by monotypy] (♂) in MCZ [# 5554]. Synonymy established with doubt by LeConte (1863b: 15). Note. As for *Bembidion ephippigerum* (see “Note” following the species name), the evidence of usage indicates that the name has been treated as an adjective (e.g., *Bembidion anguliferum*) since Hayward (1897: 125) and not as a noun in apposition (e.g., *Bembidion angulifer*).
Bembidion tersum Casey, 1918: 162. Type locality: «Lake Tahoe [Placer County], California» (original citation). Lectotype (♂), designated by Lindroth (1975: 121), in USNM [# 37082]. Synonymy established by Lindroth (1963b: 396).

#### Distribution.

According to Lindroth (1963b: 396), this species is “probably restricted to C[entral] Calif[ornia].” It has been recorded so far from Placer (Casey 1918: 162, as *Bembidion tersum*), Mono (Dajoz 2007: 17), Nevada (Lindroth 1963b: 396), and San Francisco Counties (LeConte 1852a: 188).

#### Records.

**USA**: CA

### Bembidion concretum

Casey, 1918

Bembidion concretum Casey, 1918: 156. Type locality: «Marquette [Marquette County], Michigan» (original citation). Lectotype (♀), designated by Lindroth (1975: 121), in USNM [# 37076].
Bembidion congruens Casey, 1918: 156. Type locality: «Boston Neck [Washington County], Rhode Island» (original citation). Lectotype (♀), designated by Lindroth (1975: 121), in USNM [# 37077]. Synonymy established by Lindroth (1963b: 395).
Bembidion habile Casey, 1918: 162. Type locality: «Highland Park [Lake County], Illinois» (original citation). Lectotype (♂), designated by Lindroth (1975: 121), in USNM [# 37085]. Synonymy established by Lindroth (1963b: 395).

#### Distribution.

This species ranges from Newfoundland (Lindroth 1955a: 77, as *Bembidion anguliferum*) to east-central Alaska, south to eastern Oregon (Harney County, UASM), southern South Dakota (Kirk and Balsbaugh 1975: 20), “Indiana” (Schrock 1985: 346), and western Virginia (Richard L. Hoffman pers. comm. 1992).

#### Records.

**CAN**: AB, BC, MB, NB, NF, NS (CBI), NT, ON, PE, QC, SK, YT **USA**: AK, IL, IN, MA, MD, ME, MI, MN, MT, ND, NH, NY, OH, OR, PA, RI, SD, VA, VT, WA, WI, WV

#### Note.

This species has passed under the name *Bembidion anguliferum* (LeConte, 1852) until the 1960s.

### Bembidion fortestriatum

(Motschulsky, 1845)

Omala fortestriata Motschulsky, 1845b: 352. Type locality: «île Sitka [= Baranof Island, Alaska]» (original citation). Lectotype (♂), designated by Bousquet (1997b: 330), in ZMMU.
Ochthedromus cautus LeConte, 1847: 464. Type locality: «Rocky Mountains» (original citation). Holotype [by monotypy] (♀) in MCZ [# 5555]. Synonymy established by Lindroth (1963b: 396).
Bembidium fortistriatum Mannerheim, 1852: 302. Unjustified emendation of *Bembidium fortestriatum* (Motschulsky, 1845).
Bembidion umbraticum Casey, 1918: 158. Type locality: «Marquette [Marquette County], Michigan» (original citation). Lectotype (♂), designated by Lindroth (1975: 122), in USNM [# 37078]. Synonymy established by Lindroth (1963b: 397).
Bembidion peregrinum Casey, 1918: 159. Type locality: «Massett, Queen Charlotte Islands [British Columbia]» (original citation). Holotype [by monotypy] (♀) in USNM [# 37072]. Synonymy established by Lindroth (1963b: 397).
Bembidion concurrens Fall, 1926a: 134. Type locality: «Scow Bay (north of Wrangel), Alaska» (original citation). Holotype (♂) in MCZ [# 23861]. Synonymy established by Lindroth (1963b: 397).

#### Distribution.

This species occurs from Newfoundland (Lindroth 1963b: 397) to central Alaska (Lindroth 1963b: 397), south to northeastern Oregon (Umatilla County, MCZ), western Montana (Russell 1968: 57), western Nebraska (Keith County, CMNH), and northeastern West Virginia (Tucker County, CMNH). The records from central New Mexico (Fall and Cockerell 1907: 157, as *Bembidion cautum*) and south-central Colorado (Wickham 1902: 235, as *Bembidion cautum*) probably refer to *Bembidion connivens*.

#### Records.

**CAN**: AB, BC (QCI, VCI), MB, NB, NF, NS (CBI), NT, ON, PE, QC, SK, YT **USA**: AK, CT, ID, IL, IN, MA, ME, MI, MN, MT, NE, NH, NY, OR, PA, RI, VT, WA, WI, WV

### Bembidion pseudocautum

Lindroth, 1963

Bembidion pseudocautum Lindroth, 1963b: 397. Type locality: «Grand Bend, L[ake] Huron, Ont[ario]» (original citation). Holotype (♂) in CNC [# 8387].

#### Distribution.

This species is found from Nova Scotia (Maddison 2012: Supplementary content Table S1) to the Okanagan Valley in British Columbia (Lindroth 1963b: 399), north to southern Northwest Territories (CNC), south to northern Illinois (Purrington et al. 2002: 200; McHenry County, MCZ), southwestern Pennsylvania (Allegheny County, CMNH), and Connecticut (Lindroth 1963b: 398).

#### Records.

**CAN**: AB, BC, MB, NB, NS, NT, ON, QC, SK **USA**: CT, IL, MA, ME, MI, MN, NH, NY, OH, PA, VT, WI

### Subgenus Peryphodes

Casey, 1918

Peryphodes Casey, 1918: 85. Type species: *Ochthedromus ephippiger* LeConte, 1852 by original designation (see Casey 1918: 89). Etymology. From the generic name *Peryphus* and the Greek suffix -*odes* (likeness), alluding to the resemblance of the adults to those of *Peryphus* [masculine].

#### Diversity.

Two North American species in the temperate regions.

#### Identification.

Lindroth (1963b: 345-346) treated both species.

### Bembidion ephippigerum

(LeConte, 1852)

Ochthedromus ephippiger LeConte, 1852a: 188. Type locality: «San Diego [San Diego County, California]» (original citation). Two syntypes in MCZ [# 5542]. Note. According to ICZN (1999: example for Article 31.2.2), names ending in -*fer* and -*ger* may be nouns in apposition (e.g., *Bembidion ephippiger*) or adjectives in the masculine gender (e.g., *Bembidion ephippigerum*). In such case the name is to be treated as a noun in apposition unless the author indicated that the name was an adjective or the evidence of usage is decisive. LeConte (1852a: 188) did not specify that the name was an adjective but the evidence of usage is decisive because the name has been treated as an adjective since Leng (1920: 51).

#### Distribution.

This species is confined to the west coast ranging from Vancouver Island (Lindroth 1963b: 345) to southern California (LeConte 1852a: 188; Fall 1901a: 42). The records from “Montana,” “Wyoming,” “Utah,” “Arizona” (Hayward 1897: 111), and “Colorado” (Csiki 1928: 118) almost certainly refer to *Bembidion salinarium* Casey.

#### Records.

**CAN**: BC (VCI) **USA**: CA (CHI), OR, WA

### Bembidion salinarium

Casey, 1918

Bembidion salinarium Casey, 1918: 86. Type locality: «Parowan [Iron County], Utah» (original citation for the lectotype). Lectotype (♀), designated by Lindroth (1975: 119), in USNM [# 36944].

#### Distribution.

The range of this species extends from southern Manitoba (Lindroth 1963b: 346) to the Okanagan Valley in south-central British Columbia (Bousquet 1987a: 121), south at least to Inyo County in California (MCZ), “Arizona” (Lindroth 1963b: 346), southern Colorado (Alamosa County, CNC), and northeastern North Dakota (Ramsay County, MCZ).

#### Records.

**CAN**: AB, BC, MB, SK **USA**: AZ, CA, CO, MT, ND, NV, OR, UT, WA, WY

### Subgenus Emphanes

Motschulsky, 1850

Omala Motschulsky, 1844: 238 [junior homonym of *Omala* Schumacher, 1817]. Type species: *Bembidium normannum* Dejean, 1831 designated by Jeannel (1941b: 457). Etymology. Uncertain, possibly from the Greek *homalos* (even, equal) [feminine].
Emphanes Motschulsky, 1850a: 12. Replacement name for *Omala* Motschulsky, 1844. Etymology. From the Greek *emphanes* (clear, evident) [feminine].

#### Diversity.

Northern Hemisphere, with 21 species in the Nearctic (two species) and Palaearctic (19 species) Regions.

#### Identification.

Lindroth (1963b: 381-382) treated both species found in North America.

### Bembidion diligens

Casey, 1918

Bembidion diligens Casey, 1918: 114. Type locality: «Provo [Utah County], Utah» (original citation). Lectotype (♂), designated by Lindroth (1975: 121), in USNM [# 37022].
Bembidion parabile Casey, 1918: 114. Type locality: «Reno [Washoe County], Nevada» (original citation). Lectotype (♀), designated by Lindroth (1975: 121), in USNM [# 37023]. Synonymy established by Lindroth (1963b: 382).
Bembidion vilescans Casey, 1924: 43. Type locality: «Bellevue (3400 ft.) [Weber County], Utah» (original citation). Holotype [by monotypy] (♀) in USNM [# 37024]. Synonymy established by Lindroth (1963b: 382).

#### Distribution.

This species ranges from central British Columbia to southwestern Manitoba (Lindroth 1963b: 382), south to northern Nebraska (Cherry County, Robert L. Davidson pers. comm. 2012), southwestern Colorado (Elias 1987: 632), and Mono County in east-central California (Dajoz 2007: 17).

#### Records.

**CAN**: AB, BC, MB, SK **USA**: CA, CO, MT, NE, NV, OR, SD, UT, WY

### Bembidion vile

(LeConte, 1852)

Ochthedromus vilis LeConte, 1852a: 189. Type locality: «San Diego [San Diego County, California]» (original citation). Holotype [by monotypy] in MCZ [# 5545].

#### Distribution.

This species is confined to the Pacific Coast from southern Vancouver Island (Lindroth 1963b: 382) to southern California (LeConte 1852a: 189; Fall 1901a: 43). The record from northern Idaho (Hatch 1953: 95) is probably in error.

#### Records.

**CAN**: BC (VCI) **USA**: CA, OR, WA

### Subgenus Blepharoplataphus

Netolitzky, 1920

Blepharoplataphus Netolitzky, 1920b: 96. Type species: *Bembidium virens* Gyllenhal, 1827 by original designation. Etymology. From the Greek *blepharis* (eyelash) and the generic name *Plataphus* [*q.v*.], alluding to the transverse row of small setae on the abdominal sternites (“*wimperartiger Härchen vor dem Hinterrande der Abdominal sternite*”) of the adult [masculine].
Parataphus Jedlička, 1932: 38. Type species: *Bembidion heyrovskyi* Jedlička, 1932 by monotypy. Synonymy established by Toledano (2008: 10, 23). Etymology. From the Greek *para* (near, next to) and the last two syllables of the genus-group name *Notaphus* [*q.v*.] [masculine].

#### Diversity.

Northern Hemisphere, with five species in the Nearctic (one Holarctic species) and Palaearctic (five species) Regions.

#### Identification.

Lindroth (1963b: 297-298) covered the species found in North America.

#### Taxonomic Note.

This taxon is listed as a junior synonym of *Trichoplataphus* Netolitzky by some authors (e.g., Kryzhanovskij et al. 1995: 84). Toledano (2000: 36-37) discussed the taxonomic status of both taxa. According to Maddison (2012: 569), *Blepharoplataphus* is closely related to the subgenus *Plataphus*.

### Bembidion hastii

Sahlberg, 1827

Bembidium hastii C.R. Sahlberg, 1827a: 195. Type locality: «Lapponia» (original citation), restricted to «Enontekis [Finland]» by Lindroth (1963b: 297). Lectotype (♂), designated by Lindroth (1963b: 297), in ZMH.
Peryphus litigiosus Motschulsky, 1844: 246. Type locality: «Sibérie orientale» (original citation), restricted to «Irkutsk [Russia]» by Netolitzky (1943a: 109). Seven syntypes in ZMMU (Keleinikova 1976: 203). Synonymy established by Lindroth (1963b: 297).
Peryphus cupreus Motschulsky, 1844: 247 [secondary homonym of *Bembidion cupreum* Gory, 1833]. Type locality: «bords du fleuve Selenga près de Verkhne-Oudinsk [Siberia, Russia]» (original citation). One syntype in ZMMU (Keleinikova 1976: 194). Synonymy established, under the name *Bembidion litigiosum* (Motschulsky), by Netolitzky (1935a: 21).
Peryphus ventricosus Motschulsky, 1860: 89 [*nomen dubium*]. Type locality: «rivages du fl[euve] Kodogorek, Kamtschatka [Russia]» (original citation). One badly damage syntype in ZMMU (Netolitzky 1935a: 20; Keleinikova 1976: 222). Synonymy established with doubt by Lindroth (1963b: 297).
Bembicidium cupripenne Gemminger and Harold, 1868a: 410. Replacement name for *Bembicidium cupreum* (Motschulsky, 1844).

#### Distribution.

The range of this Holarctic species extends from Norway to the Far East (Marggi et al. 2003: 246) in the Palaearctic Region and from the Seward Peninsula in Alaska east to northern Labrador (Lindroth 1963b: 298).

#### Records.

**CAN**: BC, LB, MB, NT, ON, QC, YT **USA**: AK – **Holarctic**

### Subgenus Plataphus

Motschulsky, 1864

Plataphus Motschulsky, 1864: 184. Type species: *Elaphrus prasinus* Duftschmid, 1812 designated by Jeannel (1941b: 532). Etymology. From the Greek *platys* (flat) and *phos* (light, by extension appearance), alluding to the flat body (“*corps très déprimé*”) of the adult [masculine]. Note. Concerning the type species designation, see Bousquet (2002b: 40).
Plataphodes Ganglbauer, 1891a: 152. Type species: *Peryphus fellmanni* Mannerheim, 1823 by monotypy. Synonymy established by Maddison (2012: 569). Etymology. From the generic name *Plataphus* [*q.v*.] and the Greek suffix -*odes* (likeness), alluding to the resemblance of the species to those of *Plataphus* [masculine].
Micromelomalus Casey, 1918: 37. Type species: *Bembidium planiusculum* Mannerheim, 1843 designated by Netolitzky (1943b: 61). Synonymy established by Netolitzky (1943b: 61). Etymology. From the Greek *micros* (small, little) and the generic name *Melomalus* [*q.v*.] [masculine].
Trachelonepha Casey, 1918: 37. Type species: *Bembidium falsum* Blaisdell, 1902 designated by Lindroth (1963b: 283). Synonymy established by Lindroth (1963b: 283). Etymology. From the Greek *trachelos* (neck, by extension pronotum) and the generic name *Nepha* [feminine].

#### Diversity.

Northern Hemisphere, with about 75 species in the Nearctic (36 species) and Palaearctic (45 species) Regions. Only two species (*Bembidion hyperboraeorum* and *Bembidion prasinum* Duftschmid) occur in Europe. Seven species are Holarctic, some of them represented by different subspecies in the Nearctic and Palaearctic Regions.

#### Identification.

Lindroth (1963b: 268-297, as *simplex*, *kuprianovii*, *incertum*, and *planiusculum* groups) covered all the species found in North America except *Bembidion falsum*, *Bembidion oppressum*, *Bembidion vandykei*, and *Bembidion placeranum*. Subsequently to Lindroth’s (1963b) work, the names of five species were changed because of homonymy or synonymy: *Bembidion flebile* for *Bembidion curtulatum*, *Bembidion lenense* for *Bembidion sulcipenne*, *Bembidion coerulescens* for *Bembidion neocoerulescens*, *Bembidion incertum* for *Bembidion breve*, and *Bembidion ochropus* for *Bembidion manningense*. Species identifications are difficult and examination of the male genitalia is usually required for confirmation.

### [planiusculum group]

### Bembidion basicorne

Notman, 1920

Bembidium basicorne Notman, 1920c: 185. Type locality: «Windsor, Broome Co[unty], N[ew] Y[ork]» (original citation). Three syntypes [3 originally cited] in SIM (Hennessey 1990: 466).

#### Distribution.

The range of this species extends from “Nova Scotia” (Larochelle and Larivière 1990a: 28) to northeastern Ohio (Lee 1994: 58), south to western North Carolina (Swain County, CMNH) and south-central Tennessee (Grundy County, CMNH) along the Appalachian Mountains.

#### Records.

**CAN**: NS, QC **USA**: MA, ME, NC, NH, NY, OH, PA, TN, VA, VT, WV

### Bembidion carolinense

Casey, 1924

Bembidion carolinense Casey, 1924: 27. Type locality: «Blacks M[oun]t[ain]s, North Carolina» (original citation). Holotype [by monotypy] (♂) in USNM [# 36862].
Bembidion keeneanum Casey, 1924: 28. Type locality: «Keene Heights, Essex Co[unty], New York» (original citation). Lectotype (♀), designated by Lindroth (1975: 117), in USNM [# 36863]. Synonymy established by Lindroth (1954b: 128).

#### Distribution.

This species ranges from Newfoundland (Lindroth 1955a: 55) to southern Quebec (Larochelle 1975: 54), south to northeastern Tennessee (Greene and Unicoi Counties, CMNH) and northeastern Georgia (Fattig 1949: 18) along the Appalachian Mountains.

#### Records.

**FRA**: PM **CAN**: NB, NF, NS (CBI), QC **USA**: GA, MA, ME, NC, NH, NY, TN, VA, VT

### Bembidion curtulatum

Casey, 1918

Bembidion curtulatum Casey, 1918: 39. Type locality: «Hoopa Valley, Humboldt Co[unty], California» (original citation). Lectotype (♂), designated by Lindroth (1975: 117), in USNM [# 36859].
Bembidion effetum Casey, 1918: 40. Type locality: «Soda Springs, Anderson Valley, in or near Mendocino Co[unty], California» (original citation). Holotype [by monotypy, see page 43; designated lectotype by Erwin (1984a: 170)] (♀) in USNM [# 36861]. Synonymy established by Erwin (1984a: 170).
Bembidion flebile Casey, 1918: 41. Type locality: Santa Rosa, Sonoma County, California (lectotype label according to Lindroth 1975: 117). Lectotype (♀), designated by Lindroth (1975: 117), in USNM [# 36867]. Synonymy established with doubt by Lindroth (1963b: 295), confirmed by Erwin (1984a: 170).
Bembidion timefactum Casey, 1918: 41. Type locality: Soda Springs, Anderson Valley, Mendocino County, California (lectotype label according to Lindroth 1975: 117). Lectotype (♀), designated by Lindroth (1975: 117), in USNM [# 36864]. Synonymy established, under the name *Bembidion flebile* Casey, by Lindroth (1954b: 124).
Bembidion decrepitum Casey, 1918: 41. Type locality: «Colorado» (original citation). Lectotype (♂), designated by Lindroth (1975: 117), in USNM [# 36868]. Synonymy established, under the name *Bembidion flebile* Casey, by Lindroth (1954b: 124).

#### Distribution.

The range of this species extends from central Alaska to west-central Northwest Territories (Maddison 1985: 111, as *Bembidion flebile*), south at least to west-central California along the Coast Ranges (Casey 1918: 41, as *Bembidion flebile*) and to southwestern Colorado (Hinsdale County, CMNH; Casey 1918: 41, as *Bembidion decrepitum*) along the Rocky Mountains.

#### Records.

**CAN**: AB, BC (VCI), NT, YT **USA**: AK, CA, CO, ID, MT, OR, WA

#### Note.

Maddison (2012: 535) noted that there at least two species within the current concept of this species.

### Bembidion falsum

Blaisdell, 1902

Bembidium falsum Blaisdell, 1902: 76. Type locality: «Mendocino County, Cal[ifornia]» (original citation). Lectotype (♂), designated by Erwin (1984a: 171), in CAS [# 2661].
Bembidion extensum Casey, 1918: 42. Type locality: «Soda Springs, Anderson Valley, Mendocino Co[unty], California» (original citation). Lectotype (♀), designated by Erwin (1984a: 171), in USNM [# 36870]. Synonymy established by Erwin (1984a: 171).
Bembidion kincaidi Hatch, 1950: 100. Type locality: «M[oun]t Baker [Whatcom County], Washington» (original citation). Holotype (♂) in USNM [# 75671]. Synonymy established by Hatch (1953: 86), confirmed by Erwin (1984a: 172).

#### Distribution.

This species is found along the Cascade Range and Coastal Ranges (Erwin 1984a: 172) from northwestern Washington to central California.

#### Records.

**USA**: CA, OR, WA

### Bembidion gebleri turbatum

Casey, 1918

Bembidion turbatum Casey, 1918: 32. Type locality: «Boulder Co[unty], Colorado» (original citation). Lectotype (♀), designated by Lindroth (1975: 117), in USNM [# 36847].
Bembidion conflictum Casey, 1918: 32. Type locality: «Red Cliff [Eagle County], Colorado» (original citation). Lectotype (♂), designated by Lindroth (1975: 117), in USNM [# 36848]. Synonymy established by Lindroth (1954b: 124).

#### Distribution.

This subspecies ranges from southern Yukon Territory (Lindroth 1963b: 291) to northern Colorado along the Rocky Mountains (Casey 1918: 32) and to “California” (Lindroth 1963b: 291).

#### Records.

**CAN**: AB, BC (VCI), YT **USA**: CA, CO, ID, MT, UT, WA, WY

#### Note.

Three other subspecies of *Bembidion gebleri* Gebler live in eastern Asia.

### Bembidion gordoni

Lindroth, 1963

Bembidion gordoni Lindroth, 1963b: 284. Type locality: «Kitchener, B[ritish] C[olumbia]» (original citation). Holotype (♂) in UBC. Etymology. The specific name was proposed for Gordon Stace Smith [1866-1962], a dedicated beetle collector in British Columbia.

#### Distribution.

This mountain species is known from southern British Columbia south at least to central Oregon along the Cascade Range (Lindroth 1963b: 286), west to Gallatin County in southern Montana (Maddison 2012: Supplementary content Table S1).

#### Records.

**CAN**: BC **USA**: MT, OR, WA

### Bembidion gratiosum

Casey, 1918

Bembidion gratiosum Casey, 1918: 34. Type locality: «Colorado» (original citation). Lectotype (♂), designated by Lindroth (1975: 117), in USNM [# 36851].

#### Distribution.

The range of this species extends from the Alaska Peninsula to southern Yukon Territory (Lindroth 1963b: 290), south at least to Ouray County in southwestern Colorado (CNC) along the Rocky Mountains (Lindroth 1963b: 289) and to the Sierra Nevada in eastern California (Dajoz 2007: 16).

#### Records.

**CAN**: AB, BC, YT **USA**: AK, CA, CO, MT, OR, UT, WA

### Bembidion hyperboraeorum

Munster, 1923

Bembidion hyperboraeorum Munster, 1923a: 238. Type locality: «Staburselven ad Bojobaeski [northern Norway]» (original citation for the holotype according to Lindroth 1963b: 294). Holotype in ZMUO (Lindroth 1969a: 1114).

#### Distribution.

This Holarctic species ranges from Scandinavia to eastern Siberia and the Far East (Marggi et al. 2003: 265) and from the Seward Peninsula in Alaska (Lindroth 1963b: 295) to the Hudson Bay in northeastern Manitoba (Garry 1993: 95).

#### Records.

**CAN**: MB, NT, NU, YT **USA**: AK – **Holarctic**

### Bembidion kalumae

Lindroth, 1963

Bembidion kalumae Lindroth, 1963b: 286. Type locality: «Kalum L[ake] N[orth] Terrace, B[ritish] C[olumbia]» (original citation). Holotype (♂) in CNC [# 8395].

#### Distribution.

This species is known only from a few localities in central and west-central British Columbia (Lindroth 1963b: 286).

#### Records.

**CAN**: BC

### Bembidion oppressum

Casey, 1918

Bembidion oppressum Casey, 1918: 40. Type locality: «Duncan’s Mills, Sonoma Co[unty], California» (original citation). Lectotype (♂), designated by Erwin (1984a: 178), in USNM [# 36866].

#### Distribution.

This species is known from the Coast Ranges from Humboldt County (Notman 1929b: 222) to central California (Erwin 1984a: 178).

#### Records.

**USA**: CA

### Bembidion planiusculum

Mannerheim, 1843

Bembidium planiusculum Mannerheim, 1843: 216. Type locality: «insula Sitkha [= Baranof Island, Alaska]» (original citation). Two syntypes in ZMH (Silfverberg 1987: 22) and MHNG (Marggi 2010: 186); a possible syntype in MCZ [# 5502]. Note. Lindroth (1963b: 287) designated a specimen in ZMH as lectotype but, according to Silfverberg (1987: 22), the specimen is not a syntype. In such case, the specimen loses its status of lectotype (ICZN 1999: Article 74.2).

#### Distribution.

This species is known from the Kenai Peninsula in Alaska (Lindroth 1963b: 288) south to northern Washington (Glesne et al. 2000: 89) and central Alberta (Maddison 1985: 113). All records prior to Lindroth (1963b) listed by Bousquet and Larochelle (1993: 132) need confirmation.

#### Records.

**CAN**: AB, BC (QCI, VCI) **USA**: AK, WA [CO, MT, NM, OR, UT, WY]

### Bembidion rufinum

Lindroth, 1963

Bembidion rufinum Lindroth, 1963b: 287. Type locality: «Big Boulder Creek, E[ast] Pine Pass, B[ritish] C[olumbia]» (original citation). Holotype (♂) in CNC [# 8382].

#### Distribution.

The range of this species extends from the Haines district in southeastern Alaska to southeastern Yukon Territory (Lindroth 1963b: 287), south to western Montana (Russell 1968: 51) along the Rocky Mountains and foothills and to “California” (Lindroth 1963b: 287). The record from “Idaho” (Bousquet and Larochelle 1993: 132) needs confirmation.

#### Records.

**CAN**: AB, BC, YT **USA**: AK, CA, MT [ID]

### Bembidion rusticum lenensoides

Lindroth, 1963

Bembidion rusticum lenensoides Lindroth, 1963b: 293. Type locality: «Elmendorf, Anchorage, Alaska» (original citation). Holotype (♂) in MCZ [# 35345].

#### Distribution.

This subspecies occurs from northern Alaska to southeastern Yukon Territory (Lindroth 1963b: 293), south to northwestern Montana (Edwards 1975: 54, as *Bembidion rusticum*) and central British Columbia (Lindroth 1963b: 293).

#### Records.

**CAN**: AB, BC, YT **USA**: AK, MT

### Bembidion rusticum rusticum

Casey, 1918

Bembidion rusticum Casey, 1918: 33. Type locality: «Catskill M[oun]t[ain]s, New York» (original citation). Holotype [by monotypy] (♂) in USNM [# 36854].
Bembidion notmani Casey, 1924: 27. Type locality: «Keene Heights, Essex Co[unty], New York» (original citation). Holotype [by monotypy] (♂) in USNM [# 36844]. Synonymy established by Lindroth (1954b: 128). Etymology. The specific name was proposed for Howard Notman [1881-1966], painter and amateur entomologist interested in Lepidoptera and Coleoptera.

#### Distribution.

This subspecies is found from Newfoundland (Lindroth 1955a: 54) to the Lake Superior area in western Ontario (Lindroth 1963b: 293), south to north-central Pennsylvania (Lycoming County, Robert L. Davidson pers. comm. 2008; Lindroth 1955a: 54).

#### Records.

**FRA**: PM **CAN**: LB, NB, NF, NS (CBI), ON, QC **USA**: MA, ME, NH, NY, PA, VT

#### Note.

This subspecies is reported from eastern Siberia and the Far East (Marggi et al. 2003: 266) but the specimens probably belong to a distinct (still undescribed) subspecies as pointed out by Lindroth (1963b: 293).

### Bembidion sierricola

Casey, 1924

Bembidion sierricola Casey, 1924: 28. Type locality: «Nevada Co[unty], California» (original citation). Lectotype (♀), designated by Lindroth (1975: 117), in USNM [# 36871].

#### Distribution.

This species is known from a few mountain locations in the Similkameen district in southern British Columbia (Lindroth 1963b: 286) and from the Sierra Nevada in California (Casey 1924: 28; Maddison 1985: 114).

#### Records.

**CAN**: BC **USA**: CA

### Bembidion simplex

Hayward, 1897

Bembidium simplex Hayward, 1897: 63. Type locality: «Labrador, Hudson Bay Territory, New Hampshire, Vermont, Massachusetts, Lake Superior region, Missouri, Highlands [in] N[orth] C[arolina]» (original citation). At least five syntypes in MCZ [# 16282].
Bembidion essexense Casey, 1924: 26. Type locality: «Keene Heights, Essex Co[unty], New York» (original citation). Holotype [by monotypy] (♂) in USNM [# 36843]. Synonymy established by Lindroth (1954b: 128).

#### Distribution.

The range of this species extends from western Maine (Oxford County, MCZ; Larochelle and Larivière 1990a: 28) to southeastern Wisconsin (Rauterberg 1885: 22; Messer 2010: 36), including the Appalachian region in southern Quebec (Larochelle 1975: 62), south to Tennessee (Sevier County, CMNH) and North Carolina (Hayward 1897: 64; Brimley 1938: 117; Swain County, CMNH) along the Appalachian Mountains. The records from “Labrador,” “Hudson Bay Territory,” and “Missouri” (Hayward 1897: 64) refer to other species.

#### Records.

**CAN**: QC **USA**: CT, MA, ME, MI, NC, NH, NY, OH, PA, TN, VA, VT, WI, WV

#### Note.

Lindroth (1963b: 268) included this species in its own group. However, I believe the species is closely related to some of the species included by Lindroth (1963b) in his *planiusculum* group.

### Bembidion stillaguamish

Hatch, 1950

Bembidion stillaguamish Hatch, 1950: 98. Type locality: «Snoqualmie Falls, Snoqualmie R[iver], King Co[unty], Washington» (original citation). Holotype (♀) in USNM.

#### Distribution.

This species ranges from southwestern British Columbia, including Vancouver Island (Lindroth 1963b: 290), to southwestern Oregon (Hatch 1950: 98).

#### Records.

**CAN**: BC (VCI) **USA**: OR, WA

### Bembidion sulcipenne hyperboroides

Lindroth, 1963

Bembidion lenense hyperboroides Lindroth, 1963b: 294. Type locality: «Colville R[iver], Umiat, Alaska» (original citation). Holotype (♂) in MCZ [# 34750].

#### Distribution.

This subspecies is known from Alaska, including the arctic zone and Kodiak Island, southeastern Yukon Territory, and adjacent northern British Columbia (Lindroth 1963b: 294).

#### Records.

**CAN**: BC, YT **USA**: AK

### Bembidion sulcipenne prasinoides

Lindroth, 1963

Bembidion lenense prasinoides Lindroth, 1963b: 294. Type locality: «Millertown, C[entral] N[ew]f[ound]l[an]d» (original citation). Holotype (♂) in CNC [# 8376].

#### Distribution.

This subspecies ranges from Newfoundland (Lindroth 1955a: 52, as *Bembidion lenense*) to southwestern Alaska (Elias 1988: 41), south to southern British Columbia (Lindroth 1963b: 294) and the Saint Lawrence Estuary near Quebec City (Larochelle 1975: Fig. 175).

#### Records.

**CAN**: AB, BC, LB, MB, NB, NF, NS, QC **USA:** AK

### Bembidion vandykei

Blaisdell, 1902

Bembidium vandykei Blaisdell, 1902: 75. Type locality: «Mendocino County, Cal[ifornia]» (original citation). Syntype(s) in CAS [# 2660].
Bembidion vespertinum Casey, 1918: 40. Type locality: «coast regions just north of San Francisco, California» (original citation). Four syntypes in USNM [# 36860]. Synonymy established by Lindroth (1963b: 283).
Bembidion electum Casey, 1918: 42. Type locality: «Hydesville, Eel River Valley, Humboldt Co[unty], California» (original citation). Holotype [by monotypy] (♀) in USNM [# 36869]. Synonymy established by Lindroth (1963b: 283).

#### Distribution.

This species is known from the California west coast region as far south as the San Francisco area (Casey 1918: 40, as *Bembidion vespertinum*).

#### Records.

**USA**: CA

### [complanulum group]

### Bembidion arcticum

Lindroth, 1963

Bembidion arcticum Lindroth, 1963b: 274. Type locality: «Unalaska, Aleut[ian] Isl[ands], Alaska» (original citation). Holotype (♂) in MCZ [# 34746].

#### Distribution.

This Holarctic species ranges from western Siberia (Marggi et al. 2003: 265) to the Dempster Highway in Yukon Territory (Maddison 1985: 111; UASM), including the Aleutian Islands and Alaska Peninsula (Lindroth 1963b: 275), south to Saint Elias Mountains in northwestern British Columbia (Jarrett and Scudder 2001: 380).

#### Records.

**CAN**: BC, YT **USA**: AK – **Holarctic**

### Bembidion brachythorax

Lindroth, 1963

Bembidion brachythorax Lindroth, 1963b: 279. Type locality: «Upper Chatanika R[iver], Alaska» (original citation). Holotype (♂) in MCZ [# 34748].

#### Distribution.

This species is found from eastern Siberia (Marggi et al. 2003: 265) to the Hudson Bay coast in northern Manitoba (Holliday 1982: 116), including the Great Slave Lake area in Northwest Territories (Lindroth 1963b: 279).

#### Records.

**CAN**: MB, NT, YT **USA**: AK – **Holarctic**

### Bembidion breve

(Motschulsky, 1845)

Peryphus brevis Motschulsky, 1845a: 28. Type locality: «Sitka [Baranof Island, Alaska]» (original citation). Lectotype (♂), designated by Bousquet and Larochelle (1993: 16), in ZMMU.
Notaphus incertus Motschulsky, 1845b: 350. Type locality: «île Sitka [= Baranof Island, Alaska]» (original citation). One possible syntype in MCZ (Lindroth 1963b: 272). Synonymy established by Netolitzky (1935a: 23), confirmed by Bousquet and Larochelle (1993: 16).
Peryphus tetraglyptus Mannerheim, 1853: 151. Type locality: «insula Kadjak [Alaska]» (original citation for the lectotype). Lectotype (♂), designated by Lindroth (1963b: 273), in ZMH. Synonymy established, under the name *Bembidion incertum* (Motschulsky), by Hayward (1897: 133), confirmed by Lindroth (1963b: 273).
Bembidion blanditum Casey, 1918: 23. Type locality: «Metlakatla, British Columbia» (original citation). Lectotype (♀), designated by Lindroth (1975: 116), in USNM [# 36829]. Synonymy established, under the name *Bembidion incertum* (Motschulsky), by Hatch (1953: 84), confirmed by Lindroth (1963b: 273).
Bembidion saturatum Casey, 1918: 24. Type locality: «Placer Co[unty], California» (original citation). Lectotype (♀), designated by Lindroth (1975: 117), in USNM [# 36831]. Synonymy established, under the name *Bembidion incertum* (Motschulsky), by Lindroth (1963b: 273).
Bembidion ampliatum Casey, 1918: 24. Type locality: «Colorado» (original citation). Lectotype (♂), designated by Lindroth (1975: 117), in USNM [# 36828]. Synonymy established, under the name *Bembidion incertum* (Motschulsky), by Lindroth (1963b: 273).
Bembidion lividulum Casey, 1918: 25. Type locality: «Placer Co[unty], California» (original citation). Lectotype (♀), designated by Lindroth (1975: 117), in USNM [# 36830]. Synonymy established, under the name *Bembidion incertum* (Motschulsky), by Lindroth (1963b: 273).
Bembidion improvisum Casey, 1918: 25. Type locality: «Colorado» (original citation). Lectotype (♂), designated by Lindroth (1975: 117), in USNM [# 36832]. Synonymy established, under the name *Bembidion incertum* (Motschulsky), by Lindroth (1963b: 273).
Bembidion testatum Casey, 1918: 30. Type locality: «Lake Tahoe [Nevada County], California» (original citation). Lectotype (♂), designated by Erwin (1984a: 174), in USNM [# 36842]. Synonymy established, under the name *Bembidion incertum* (Motschulsky), by Erwin (1984a: 174).

#### Distribution.

The range of this species extends from the Aleutian Islands and the Gulf of Alaska coast to southwestern Yukon Territory (Lindroth 1963b: 273, as *Bembidion incertum*), south to north-central New Mexico (Fall and Cockerell 1907: 157; Hayward 1897: 68, as *Bembidion incertum*) along the Rocky Mountains and to the Sierra Nevada in eastern California (Casey 1918: 30, as *Bembidion testatum*).

#### Records.

**CAN**: AB, BC (QCI), YT **USA**: AK, CA, CO, ID, MT, NM, NV, OR, WA, WY

### Bembidion complanulum

(Mannerheim, 1853)

Peryphus complanulus Mannerheim, 1853: 152. Type locality: «in ora orientali insulae Kadjak [Alaska]» (original citation). Lectotype (♂), designated by Lindroth (1963b: 282), in ZMH.
Bembidion parvulum Notman, 1922b: 99 [primary homonym of *Bembidion parvulum* Dejean, 1831]. Type locality: «Paradise Park (6,000 ft.), M[oun]t Rainier [Pierce County], Wash[ington]» (original citation). Holotype (♀) in USNM [# 26594]. Synonymy established by Hatch (1953: 84), confirmed by Lindroth (1954b: 128).

#### Distribution.

The range of this species extends from the Aleutian Islands and the Alaska Peninsula (Lindroth 1963b: 283) south along the Rocky Mountains to northwestern Montana (Russell 1968: 51; Edwards 1975: 51) and to the central Sierra Nevada in California (Papp 1978: 164). The records from “Northwest Territories” (Bousquet and Larochelle 1993: 130) and from the San Juan River drainage in southwestern Colorado (Wickham 1902: 232) are probably in error.

#### Records.

**CAN**: AB, BC (QCI) **USA**: AK, CA, MT, OR, WA

### Bembidion compressum

Lindroth, 1963

Bembidion compressum Lindroth, 1963b: 276. Type locality: «Umiat, arctic Alaska» (original citation). Holotype (♂) in MCZ [# 34749].

#### Distribution.

This Holarctic species is found from eastern Siberia (Marggi et al. 2003: 265) to the Hudson Bay in northeastern Manitoba (Garry 1993: 95), including northern Alberta (Birch Mountains, Gerald J. Hilchie pers. comm. 2009). Fossil remnants of this species, dated between about 16,700 and 18,100 years B.P., have been unearthed in southeastern Iowa (Baker et al. 1986: 96).

#### Records.

**CAN**: AB, MB, NT, NU, YT **USA**: AK – **Holarctic**

### Bembidion farrarae

Hatch, 1950

Bembidion farrarae Hatch, 1950: 99. Type locality: «Sluskin Falls, M[oun]t Rainier [Pierce County], Washington» (original citation). Holotype (♀) in USNM. Etymology. The specific name was proposed for Elizabeth Farrar (later Mrs. Thomas G. Kinney) who did a master thesis, under the direction of Melville H. Hatch in 1936, at the University of Washington on the Bembidiini of the state of Washington.

#### Distribution.

This species is known from the Kenai Peninsula in Alaska (Lindroth 1963b: 278) south to “Oregon” (Hatch 1953: 84) and from north-central Colorado (Lindroth 1963b: 278).

#### Records.

**CAN**: BC (QCI) **USA**: AK, CO, OR, WA

### Bembidion haruspex

Casey, 1918

Bembidion haruspex Casey, 1918: 31. Type locality: «Inverness [probably Inverness Passage], British Columbia» (original citation for the lectotype). Lectotype (♀), designated by Lindroth (1975: 117), in USNM [# 36846].

#### Distribution.

This species ranges from British Columbia, as far north as the Prince Rupert area (Lindroth 1963b: 274), to Mendocino County in western California (Maddison 1985: 112) and to southern Colorado along the Rocky Mountains (Elias 1987: 632).

#### Records.

**CAN**: BC (VCI) **USA**: CA, CO, ID, MT, OR, WA

### Bembidion improvidens

Casey, 1924

Bembidion improvidens Casey, 1924: 25. Type locality: «Placer Co[unty], California» (original citation). Lectotype (♂), designated by Lindroth (1975: 117), in USNM [# 36841].

#### Distribution.

This species is known from Mount Rainier in Washington (Lindroth 1963b: 283) and the Sierra Nevada in California (Casey 1924: 25; Papp 1978: 164).

#### Records.

**USA**: CA, WA

### Bembidion kuprianovii

Mannerheim, 1843

Bembidium kuprianovii Mannerheim, 1843: 217. Type locality: «insula Sitkha [= Baranof Island, Alaska]» (original citation). Lectotype (♀), designated by Lindroth (1963b: 269), in ZMH.
Bembidium biimpressum Mannerheim, 1843: 217. Type locality: «insula Sitkha [= Baranof Island, Alaska]» (original citation). Syntype(s) location unknown. Synonymy established by Lindroth (1963b: 269).
Peryphus ovipennis Motschulsky, 1845b: 352. Type locality: «île Sitka [= Baranof Island, Alaska]» (original citation). Syntype(s) in ZMMU (Netolitzky 1935a: 23). Synonymy established by Mannerheim (1852: 376), confirmed by Netolitzky (1935a: 23).
Bembidium funereum LeConte, 1860: 320. Type locality: «Saskatchewan River [probably in Alberta according to Lindroth (1963b: 269)]» (original citation). One syntype in MCZ [# 5504]. Synonymy established by Netolitzky (1942: 37), confirmed by Lindroth (1963b: 269).
Bembidium maeklini Hayward, 1897: 66. Type locality: «Woskresensk [= Resurrection Bay, Kenai Peninsula, Alaska]» (holotype label). Holotype in MCZ [# 34087]. Synonymy established by Lindroth (1963b: 269). Note. This name was first proposed, but not made available, by LeConte (1863b: 14). In his original description, Hayward stated “The only specimens [of this species] known to me are in the LeConte collection. The type is probably from Alaska. The other three are from British Columbia.” Three specimens are labeled as type of this species at MCZ. Two of them, numbered “type 16284,” are from California and are not syntypes.
Bembidion adultum Casey, 1918: 33. Type locality: «Truckee [Nevada County], California» (original citation). Lectotype (♀), designated by Lindroth (1975: 116), in USNM [# 36853]. Synonymy established by Lindroth (1963b: 269).
Bembidion dilutum Casey, 1918: 33. Type locality: «Truckee [Nevada County], California» (original citation). Lectotype (♀), designated by Lindroth (1975: 116), in USNM [# 36855]. Synonymy established by Lindroth (1963b: 269).
Bembidion bucolicum Casey, 1918: 34. Type locality: «Stikine River Cañon, British Columbia» (original citation). Lectotype (♂), designated by Lindroth (1975: 116), in USNM [# 36852]. Synonymy established by Netolitzky (1931: 161), confirmed by Lindroth (1963b: 269).

#### Distribution.

This species ranges from central Alaska including the Alaska Peninsula and Kodiak Island (Lindroth 1963b: 270) to western Northwest Territories (Tungsten, UASM), south at least to northern Utah (LeConte 1878a: 465, as *Bembidion maeklini*), including western Montana (Russell 1968: 50; Edwards 1975: 52) and north-central Wyoming (Big Horn County, Ken Karns pers. comm. 2009; Hamilton 1894a: 7, as *Bembidion funerum* [sic]), and the Sierra Nevada in California (Casey 1918: 33, as *Bembidion adultum* and *Bembidion dilutum*). The record from southwestern Colorado (Wickham 1902: 233, as *Bembidion maeklini*) needs confirmation.

#### Records.

**CAN**: AB, BC, NT, YT **USA**: AK, CA, ID, MT, NV, OR, UT, WA, WY [CO]

### Bembidion laxatum

Casey, 1918

Bembidion laxatum Casey, 1918: 24. Type locality: «Placer Co[unty], California» (original citation). Lectotype (♂), designated by Lindroth (1975: 117), in USNM [# 36833].
Bembidion adumbratum Casey, 1918: 26. Type locality: «Placer Co[unty], California» (original citation). Lectotype (♂), designated by Lindroth (1975: 117), in USNM [#36827]. Synonymy established by Lindroth (1963b: 274).
Bembidion rainieri Hatch, 1950: 97. Type locality: «Sunrise Park, M[oun]t Rainier [Pierce County], Washington» (original citation). Holotype (♀) in USNM. Synonymy established by Lindroth (1963b: 274).

#### Distribution.

This species is known from Mount Rainier in Washington and the Sierra Nevada in California (Lindroth 1963b: 274). The record from British Columbia (Jarrett and Scudder 2001: 381) needs confirmation.

#### Records.

**USA**: CA, WA [BC]

### Bembidion manningense

Lindroth, 1969

Bembidion ochropus Lindroth, 1963b: 278 [primary homonym of *Bembidion ochropus* Andrewes, 1935]. Type locality: «Pasayten R[iver] E[ast] Manning Park, B[ritish] C[olumbia]» (original citation). Holotype (♂) in CNC [# 8393].
Bembidion manningense Lindroth, 1969a: 1114. Replacement name for *Bembidion ochropus* Lindroth, 1963.

#### Distribution.

This species ranges from the Yukon River in western Alaska to the Mackenzie River delta in northwestern Northwest Territories (Lindroth 1963b: 278-279, as *Bembidion ochropus*), south through the Rocky Mountains to western Montana (Russell 1968: 51) and mountains in southern British Columbia (Lindroth 1963b: 279, as *Bembidion ochropus*).

#### Records.

**CAN**: AB, BC, NT, YT **USA**: AK, MT

### Bembidion neocoerulescens

Bousquet, 1993

Bembidium coerulescens Van Dyke, 1926a: 65 [primary homonym of *Bembidion andreae coerulescens* Dalla Torre, 1877]. Type locality: «Niles Cañon, Alameda County, California» (original citation). Holotype (♂) in CAS [# 1818]. Note. Even if it is obvious that infrasubspecific rank was meant when Dalla Torre (1877: 56) proposed his *Bembidion andreae coerulescens* (see Lindroth 1963b: 271, footnote), the fact that Csiki (1928: 163) and Van Dyke (1949b: 56) treated Dalla Torre’s name as a senior homonym makes the name subspecific from the date of its establishment (ICZN 1999: Article 45.6.4.1).
Bembidion van dykei Csiki, 1928: 163 [primary homonym of *Bembidion vandykei* Blaisdell, 1902]. Replacement name for *Bembidion coerulescens* Van Dyke, 1926.
Bembidion umbraticola Van Dyke, 1949b: 56 [primary homonym of *Bembidion umbraticola* Casey, 1918]. Replacement name for *Bembidion coerulescens* Van Dyke, 1926.
Bembidion neocoerulescens Bousquet [in Bousquet and Larochelle], 1993: 9. Replacement name for *Bembidion coerulescens* Van Dyke, 1926.

#### Distribution.

This species is known yet only from the San Francisco Bay area in California.

#### Records.

**USA**: CA

### Bembidion nigrocoeruleum

Hayward, 1897

Bembidium nigrocoeruleum Hayward, 1897: 66. Type locality: «Washington» (original citation). One syntype [7 originally cited] in MCZ [# 16283].
Bembidion expansipenne Casey, 1924: 26. Type locality: «Siskiyou Co[unty], California» (original citation). Lectotype (♀), designated by Lindroth (1975: 116), in USNM [# 36840]. Synonymy established by Lindroth (1963b: 271).

#### Distribution.

This species is known from southeastern British Columbia (Syringa Provincial Park, James C. Bergdahl pers. comm. 2008) south at least to northern California (Casey 1924: 26, as *Bembidion expansipenne*) and northwestern Montana (Edwards 1975: 53).

#### Records.

**CAN**: BC **USA**: CA, MT, OR, WA

### Bembidion occultator

Notman, 1920

Bembidium occultator Notman, 1920a: 295. Type locality: «Meadow Pond, M[oun]t Redfield, Essex Co[unty], N[ew] Y[ork]» (original citation). Holotype [by monotypy] (♂) in SIM (Hennessey 1990: 466).

#### Distribution.

The range of this species extends from Newfoundland (Lindroth 1955a: 51) to southeastern Alberta (Lindroth 1963b: 282), south to east-central Minnesota (Aitkin County, CMNH) and mountains in New York and New England (Lindroth 1963b: 282). The four specimens labeled from the Anchorage area in southern Alaska (Lindroth 1963b: 282) could be mislabeled in my opinion. The record from “Pennsylvania” (Bousquet and Larochelle 1993: 131) needs confirmation.

#### Records.

**CAN**: AB, LB, NB, NF, NS (CBI), ON, QC, SK **USA**: ME, MI, MN, NH, NY, VT [AK, PA]

### Bembidion placeranum

Casey, 1924

Bembidion placeranum Casey, 1924: 28. Type locality: «Placer Co[unty], California» (original citation). Holotype [by monotypy] (♀) in USNM [# 36865].

#### Distribution.

According to Erwin (1984a: 179), this species is found in the foothills of the Sierra Nevada in California.

#### Records.

**USA**: CA

### Bembidion quadrifoveolatum

Mannerheim, 1843

Bembidium quadrifoveolatum Mannerheim, 1843: 218. Type locality: «insula Sitkha [= Baranof Island, Alaska]» (original citation). One syntype in ZMH (Silfverberg 1987: 23). Note. Lindroth (1963b: 275) designated a lectotype in ZMH but, according to Silfverberg (1987: 23), the specimen is not a syntype. In such case, the specimen loses its status of lectotype (ICZN 1999: Article 74.2).
Bembidion illex Casey, 1918: 31. Type locality: «Metlakatla, British Columbia» (original citation). Lectotype (♂), designated by Lindroth (1975: 117), in USNM [# 36850]. Synonymy established by Hatch (1953: 85), confirmed by Lindroth (1963b: 275).

#### Distribution.

This species ranges from the Aleutian Islands and Alaska Peninsula (Lindroth 1963b: 276) to south-central Yukon Territory, south to the Sierra Nevada in California (Papp 1978: 164; Dajoz 2007: 16) and at least to northwestern Montana along the Rocky Mountains (Russell 1968: 50; Edwards 1975: 53).

#### Records.

**CAN**: AB, BC (QCI), YT **USA**: AK, CA, MT, OR, WA

### Bembidion rosslandicum

Lindroth, 1963

Bembidion rosslandicum Lindroth, 1963b: 277. Type locality: «Rossland Trail, S[outh] Brit[ish] Col[umbia]» (original citation). Holotype (♂) in CNC [# 8392].

#### Distribution.

This species is known from a few localities in northwestern Montana (Russell 1968: 51), southwestern Alberta, and southeastern British Columbia (Lindroth 1963b: 277).

#### Records.

**CAN**: AB, BC **USA**: MT

### Bembidion viator

Casey, 1918

Bembidion viator Casey, 1918: 31. Type locality: «Massett, Queen Charlotte Islands [British Columbia]» (original citation). Lectotype (♂), designated by Lindroth (1975: 117), in USNM [# 36849].

#### Distribution.

This species is known from the Queen Charlotte Archipelago and adjacent mainland in British Columbia (Kavanaugh 1992: 65) and from northwestern Washington (Skagit County, James C. Bergdahl pers. comm. 2008).

#### Records.

**CAN**: BC (QCI) **USA**: WA

### Subgenus Hydrium

LeConte, 1847

Hydrium LeConte, 1847: 453. Type species: *Bembidium levigatum* Say, 1823 by monotypy. Etymology. From the Greek *hydrias* (from water), probably alluding to the habitat requirement of the species [neuter].
Eudromus Kirby, 1837: 55 [junior homonym of *Eudromus* Klug, 1835]. Type species: *Peryphus nitidus* Kirby, 1837 by monotypy. Synonymy established, under the name *Platytrachelus* Motschulsky, by Ganglbauer (1891b: 17). Etymology. From the Greek *eu* (well) and *dromos* (running) [masculine].
Platytrachelus Motschulsky, 1844: xi [junior homonym of *Platytrachelus* Schönherr, 1843]. Type species: *Platytrachelus sibiricus* Motschulsky, 1844 (= *Bembicidium vitiosum* Gemminger and Harold, 1868) designated by Netolitzky (1939: 9). Etymology. From the Greek *platys* (broad, wide) and *trachelos* (neck, by extension pronotum), alluding to the wide pronotum (“*corselet presqu’aussi large que les élytres*”) of the adult of *Bembidion sibiricum* [masculine].
Eurytrachelus Motschulsky, 1850a: 15. Replacement name for *Platytrachelus* Motschulsky, 1844. Synonymy established by Maddison (2012: 569). Etymology. From the Greek *eurys* (broad, wide) and *trachelos* (neck, by extension pronotum) [masculine].
Pogonidium Ganglbauer, 1891a: 151. Type species: *Elaphrus laticollis* Duftschmid, 1812 by monotypy. Synonymy established with the name *Eudromus* Kirby by Csiki (1906: 37). Etymology. From the generic name *Pogonus* [*q.v*.] and the Latin suffix -*idium* (little) [neuter].

#### Diversity.

Northern Hemisphere, with ten species in the Nearctic (four species) and Palaearctic (six species) Regions.

#### Identification.

Lindroth (1963b: 250-254, as *nitidum* and *levigatum* groups) covered all four North American species.

#### Taxonomic Note.

Habu and Uéno (1955: 45) described the subgenus *Aptenidium* (one Japanese species) in the genus *Hydrium* implying (though not discussed by the authors) a close relationship between the two genus-group taxa. Based on the original description, *Aptenidium* is likely not closely related to *Hydrium*.

### [levigatum group]

### Bembidion levigatum

Say, 1823

Bembidium levigatum Say, 1823a: 84. Type locality: «Missouri [Territory]» (original citation). Lectotype (♀), designated by Lindroth and Freitag (1969: 335), in MHNP (collection Dejean). Note. The spelling *laevigatum* is an incorrect subsequent spelling, introduced by Dejean (1831: 150), not in prevailing usage.
Bembidion laevigatum delawarense Casey, 1924: 24. Type locality: «Pennsylvania» (original citation). Holotype [by monotypy] (♂) in USNM [# 36814]. Synonymy established by Lindroth (1963b: 254).

#### Distribution.

This widely distributed species ranges from east-central Maine (Majka et al. 2011: 45) to “Montana” (Hayward 1897: 40) and southeastern Alberta (CNC), south to eastern Utah (Grand County, CNC), the Rio Grande in western Texas (Brewster County, CNC) and northern Chihuahua (Bates 1891a: 263), and the Florida Panhandle (Peck and Thomas 1998: 18).

#### Records.

**CAN**: AB **USA**: AL, AR, CO, DC, DE, FL, GA, IA, IL, IN, KS, KY, LA, MD, ME, MI, MN, MO, MS, MT, NC, ND, NE, NH, NM, NY, OH, OK, PA, SC, SD, TN, TX, UT, VA, WI, WV – Mexico

### [nitidum group]

### Bembidion interventor

Lindroth, 1963

Bembidion interventor Lindroth, 1963b: 252. Type locality: «Creston, B[ritish] C[olumbia]» (original citation). Holotype (♂) in CNC [# 8394].

#### Distribution.

This species occurs from the Laurentides region in western Quebec (Larochelle 1975: 57) to central Alaska (Lindroth 1963b: 253), south at least to northern Oregon (Morrow County, CMNH), northern Colorado (Eagle County, CMNH) along the Rocky Mountains, and southwestern North Dakota (Morton County, UASM).

#### Records.

**CAN**: AB, BC, MB, NT, ON, QC, SK, YT **USA**: AK, CO, ID, MT, ND, OR, WA, WY

### Bembidion nitidum

(Kirby, 1837)

Peryphus nitidus Kirby, 1837: 55. Type locality: «Lat. 54° [= along North Saskatchewan River]» (original citation), restricted to «Edmonton, Al[ber]ta» by Lindroth (1963b: 251). One syntype [2 originally cited] in BMNH (Lindroth 1953b: 176).
Bembidion edolatum Casey, 1924: 24. Type locality: «Duluth [Saint Louis County], Minnesota» (original citation). Lectotype (♂), designated by Lindroth (1975: 115), in USNM [# 36813]. Synonymy established by Lindroth (1963b: 251).

#### Distribution.

The range of this species extends from Prince Edward Island (Majka et al. 2008: 130) and New Brunswick (Webster and Bousquet 2008: 16) to eastern Alaska (Lindroth 1969a: 1114), south to eastern Idaho (Clark County, UASM), southern Colorado (Wickham 1896c: 132; Maddison 1985: 95) along the Rocky Mountains, “Kansas” (Wickham 1896c: 132), Missouri (Summers 1873: 147), northwestern Indiana (Blatchley 1910: 69), and New York (Notman 1928: 214). One old specimen simply labeled “N.M.” is known (MCZ). The record from “New Jersey” (Wickham 1896c: 132) needs confirmation.

#### Records.

**CAN**: AB, BC, MB, NB, NT, ON, PE, QC, SK, YT **USA**: AK, CO, IA, ID, IN, KS, MA, ME, MI, MN, MO, MT, ND, NE, NH, NY, OH, SD, VT, WI, WY [NJ, NM]

### Bembidion obliquulum

LeConte, 1859

Bembidium obliquulum LeConte, 1859a: 83. Type locality: «California» (original citation), herein restricted to Dog Island Park, Red Bluff, Tehama County (CNC). One syntype in MCZ [# 7399].
Bembidium aptum LeConte, 1859b: 281. Type locality: «Oregon» (original citation). One syntype in MCZ [# 7400]. Synonymy established by LeConte (1859b: 287), confirmed by Lindroth (1963b: 254).
Bembidion obliqulum Casey, 1918: 17. Unjustified emendation of *Bembidion obliquulum* LeConte, 1859.
Bembidion nitidum var. *josephineum* Casey, 1924: 25. Type locality: «Josephine Co[unty], Oregon» (original citation). Holotype [by monotypy] in USNM [# 36812]. Synonymy established by Hatch (1953: 82), confirmed by Lindroth (1963b: 254).

#### Distribution.

This species is known from scattered localities from northern Idaho (Bonner County, MCZ) to west-central Washington (Grays Harbor County, CMNH), south to southern California (Ventura County, CMNH). The record from British Columbia (Jarrett and Scudder 2001: 381) needs confirmation.

#### Records.

**USA**: CA, ID, OR, WA [BC]

### Subgenus Metallina

Motschulsky, 1850

Leja Dejean, 1821: 17. Type species: *Carabus celer* Fabricius, 1792 (= *Carabus lampros* Herbst, 1784) designated by Desmarest (1851: 192). Etymology. From the Greek *leia* (smooth) [feminine]. The name was proposed by Johann Karl Megerle von Mühlfeld and made available by Dejean. Note. Regarding the type species designation, see Bousquet (2002b: 31).
Metallina Motschulsky, 1850a: 13. Unnecessary replacement name for *Leja* Dejean, 1821.

#### Diversity.

Northern Hemisphere, with seven species in the Nearctic (three species, of which two are adventive) and Palaearctic (six species) Regions.

#### Identification.

Lindroth (1963b: 254-258, as *lampros* group) covered all three species found in North America.

#### Nomenclatural Note.

All recent authors seen (e.g., Marggi et al. 2003: 251; Lorenz 2005: 218; Ortuño and Toribio 2005: 278; Maddison 2012: 545) used *Metallina* as the valid name for this subgenus despite that *Leja* is the oldest available name and is not a junior homonym of any genus-group names. I am following them but the case needs to be submitted to the Commission for a ruling.

#### Taxonomic Note.

According to Maddison (2012: 569), this group is closely related to subgenus *Hydrium*.

### Bembidion dyschirinum

LeConte, 1861

Bembidium dyschirinum LeConte, 1861b: 340. Type locality: «east of Fort Colville [Washington]» (original citation). Holotype [by monotypy] (♀) in MCZ [# 5544].
Bembidion aleneanum Casey, 1918: 114. Type locality: «Coeur d’Alene [Kootenai County], Idaho» (original citation). Lectotype (♂), designated by Lindroth (1975: 115), in USNM [# 37028]. Synonymy established by Lindroth (1963b: 255).
Bembidion perturbatum Casey, 1918: 115. Type locality: «Boulder Co[unty], Colorado» (original citation). Lectotype (♀), designated by Lindroth (1975: 115), in USNM [# 37025]. Synonymy established by Lindroth (1963b: 255).
Bembidion agitabile Casey, 1918: 115. Type locality: «Coeur d’Alene [Kootenai County], Idaho» (original citation). Lectotype (♀), designated by Lindroth (1975: 115), in USNM [# 37026]. Synonymy established by Hatch (1950: 97), confirmed by Lindroth (1963b: 255).
Bembidion atrolucens Casey, 1918: 115. Type locality: «Bull Run, Clackamas Co[unty], Oregon» (original citation). Lectotype (♂), designated by Lindroth (1975: 116), in USNM [# 37027]. Synonymy established by Lindroth (1963b: 255).
Bembidion speculinum Casey, 1924: 32. Type locality: «Terrace, British Columbia» (original citation). Holotype [by monotypy] (♀) in USNM [# 36892]. Synonymy established by Lindroth (1963b: 255).
Bembidion keechelus Hatch, 1950: 97. Type locality: «L[ake] Keechelus [Kittitas County], Washington» (original citation). Holotype (♂) in USNM. Synonymy established by Lindroth (1963b: 255).

#### Distribution.

This species ranges from southeastern Alaska to southern Yukon Territory (Lindroth 1963b: 256), south along the Rocky Mountains to central Colorado (Wickham 1902: 234; Hayward 1897: 115; Casey 1918: 115, as *Bembidion perturbatum*) and to the Sierra Nevada in California (Dajoz 2007: 17). Fossil remnants from a Plio-Pleistocene sequence have been found in northwestern Greenland (Böcher 1995: 24).

#### Records.

**CAN**: AB, BC (QCI, VCI), YT **USA**: AK, CA, CO, ID, MT, OR, UT, WA, WY

### Bembidion lampros

(Herbst, 1784)

Carabus lampros Herbst, 1784: 143. Type locality: «Berlin [Germany]» (original citation). Syntype(s) location unknown (possibly in ZMHB).
Carabus rufipes Paykull, 1790: 101 [primary homonym of *Carabus rufipes* DeGeer, 1774 and *Carabus rufipes* Goeze, 1777]. Type locality: «Svecia australi» (original citation). Syntype(s) possibly in NRSS. Synonymy established, under the name *Carabus celer* Fabricius, by Schönherr (1806: 223).
Carabus celer Fabricius, 1792: 167 [primary homonym of *Carabus celer* Gmelin, 1790]. Type locality: «Europa» (original citation). One syntype in ZMUC (Zimsen 1964: 60). Synonymy established by Schönherr (1806: 223).
Carabus tristis Fabricius, 1792: 167 [primary homonym of *Carabus tristis* Schaller, 1783 and *Carabus tristis* Herbst, 1786]. Type locality: «Germania» (original citation). One syntype in ZMUC (Zimsen 1964: 60). Synonymy established by Schaum (1860: 717).
Carabus pulchellus Marsham, 1802: 454 [primary homonym of *Carabus pulchellus* Razoumowsky, 1789 and *Carabus pulchellus* Panzer, 1796]. Type locality: Great Britain (inferred from title of the book). One syntype probably in BMNH (Stephens’ collection, see Netolitzky 1935: 134). Synonymy established by Jacquelin du Val (1851: 504).
Carabus acutus Marsham, 1802: 461. Type locality: Great Britain (inferred from title of the book). Two syntypes probably in BMNH (Stephens’ collection, see Stephens 1828b: 27). Synonymy established by Jacquelin du Val (1851: 504).
Bembidium felixianum Heer, 1837: 52 [second section]. Type locality: «Rheinwald [southeastern Switzerland]» (original citation). Syntype(s) location unknown (possibly in ETHZ). Synonymy established by Jacquelin du Val (1851: 504).

#### Distribution.

This Palaearctic species is adventive in North America where it is known from southern British Columbia (Hatch 1953: 83; Smith et al. 2004: 96) to northwestern Oregon (Westcott et al. 2006: 7) in the west and from Newfoundland (Lindroth 1955a: 49; Larson and Langor 1982: 592) in the east. The first inventoried specimen collected in the eastern part of this continent was found in 1949 (see Lindroth 1955a: 49) and in the western part in 1947 (Hatch 1949c: 145). Fossil remnants from a Plio-Pleistocene sequence have been unearthed in northwestern Greenland (Böcher 1995: 24).

#### Records.

**CAN**: BC, NF **USA**: OR, WA – **Adventive**

#### Note.

*Carabus pygmaeus* attributed to Paykull (1798: 148) is often listed as a junior synonym of this species. However, Paykull did not describe a new species under that name since he referred to *Carabus pygmaeus* Fabricius, 1792.

### Bembidion properans

(Stephens, 1828)

Tachypus properans Stephens, 1828b: 26. Type locality: «near London; Spitchweek, Devon; Netley, Salop [United Kingdom]» (original citation), restricted to «London, Engl[and]» by Lindroth (1963b: 256). Five syntypes in BMNH (Netolitzky 1935b: 135).
Tachypus chalceus Stephens, 1828b: 27. Type locality: «near London [United Kingdom]» (original citation). Syntype(s) in BMNH. Synonymy established by Jacquelin du Val (1851: 504).
Bembidium velox Erichson, 1837: 134 [secondary homonym of *Bembidion velox* (Linnaeus, 1761)]. Type locality: Mark Brandenburg [Prussia] (inferred from title of the book). Syntype(s) probably in ZMHB. Synonymy established by Jacquelin du Val (1851: 505).
Bembidium 14-striatum C.G. Thomson, 1871: 361. Replacement name for *Bembidium velox* Erichson, 1837. Note. Because Thomson proposed this name as a replacement name, the lectotype of *Bembidion quatuordecimstriatum* designated by Lindroth (1963b: 256) in ZMLS has no status.

#### Distribution.

This Palaearctic species is adventive in North America where it is known from Newfoundland (Larson and Langor 1982: 592) to the coastal area of New Brunswick (Bousquet 1987a: 120), including the Magdalen Islands in the Gulf of Saint Lawrence (Larochelle 1975: 60), and from southeastern Maine (Larochelle and Larivière 1990a: 28, 33). One specimen has been collected recently in a campground in Kittitas County, central Washington (Robert L. Davidson pers. comm. 2008). The first inventoried specimen collected on this continent was found in Nova Scotia in 1942 (Majka et al. 2007: 7).

#### Records.

**CAN**: NB, NF, NS (CBI), PE, QC **USA**: ME, WA – **Adventive**

### Subgenus Lindrochthus

Maddison, 2012

Lindrochthus Maddison, 2012: 570. Type species: *Bembidium wickhami* Hayward, 1897 by original designation. Etymology (original). From the surname of Carl Lindroth and the ending “chthus” to suggest its similarity to *Philochthus* [masculine].

#### Diversity.

One species in western North America.

#### Identification.

The species is described in detail, under the name *Bembidion carlhi*, by Erwin and Kavanaugh (1981: 37, 39).

### Bembidion wickhami

Hayward, 1897

Bembidium wickhami Hayward, 1897: 112. Type locality: «Dunsmuir [Siskiyou County], Cal[ifornia]» (original citation for the lectotype). Lectotype (♂), designated by Erwin (1984a: 185), in MCZ [# 16299]. Etymology. The specific name was proposed for Henry Frederick Wickham [1866-1933], professor of zoology and entomology at the University of Iowa. For many years, Wickham collected fossil insects at Florissant in Colorado.
Bembidion delectum Casey, 1918: 44. Type locality: «Gilroy Hot Springs, S[an]ta Clara Co[unty], California» (original citation). Lectotype (♂), designated by Erwin (1984a: 185), in USNM [# 36872]. Synonymy established by Erwin (1984a: 185).
Bembidion carlhi Erwin and Kavanaugh, 1981: 37. Type locality: «Steamboat Falls, Douglas County, Oregon» (original citation). Holotype (♀) in CAS [# 13657]. Synonymy established by Erwin (1984a: 185). Etymology. The species name is based on the first name and middle initial of Carl Hildebrand Lindroth (see *Schizogenius lindrothi*).

#### Distribution.

This species is known from the Cascade Range in southwestern Oregon south to central California along the Coast Ranges (Casey, 1918: 44, as *Bembidion delectum*), including the Klamath Mountains, and along the Sierra Nevada [see Erwin and Kavanaugh 1981: Fig. 18, as *Bembidion carlhi*]; also recorded from “Nevada” (Hayward 1897: 112).

#### Records.

**USA**: CA, NV, OR

### Subgenus Eupetedromus

Netolitzky, 1911

Eupetedromus Netolitzky, 1911: 190. Type species: *Carabus dentellus* Thunberg, 1787 designated by Jeannel (1941b: 451). Etymology. From the Greek *eupetes* (easy) and *dromos* (running) [masculine].

#### Diversity.

Eleven species in the Nearctic (five species) and Palaearctic (seven species) Regions. One species (*Bembidion incrematum*) is Holarctic.

#### Identification.

Bousquet and Webster (2006) provided a key for the identification of all North American species. Lindroth (1963b: 348-351, 360) covered four species, three he placed in the *incrematum* group, the other (*Bembidion variegatum*) in the *dorsale* group.

### Bembidion graciliforme

Hayward, 1897

Bembidium graciliforme Hayward, 1897: 97. Type locality: «Massachusetts, Pennsylvania, Michigan, Illinois and Iowa» (original citation), restricted to «Iowa» by Lindroth (1963b: 350). Syntype(s) in MCZ [# 16294].

#### Distribution.

The range of this species extends from Nova Scotia (CNC) to eastern Minnesota (Epstein and Kulman 1990: 214), south to east-central Iowa (Johnson County, USNM), west-central Indiana (Blatchley 1910: 76), and southeastern West Virginia (Greenbrier County, MCZ). The record from southern South Dakota (Kirk and Balsbaugh 1975: 19) needs confirmation.

#### Records.

**CAN**: NB, NS, ON, QC **USA**: CT, IA, IL, IN, MA, ME, MD, MI, MN, NH, NJ, NY, OH, PA, RI, VA, VT, WI, WV [SD]

### Bembidion immaturum

Lindroth, 1954

Bembidion immaturum Lindroth, 1954b: 158. Type locality: «Steady Brook, Newfoundland» (holotype label). Holotype (♂) in CNC [# 6570]. Note. Although Lindroth’s description is very short, I believe he met the requirements of availability for a species-group name published after 1930 (ICZN 1999: Article 13.1).

#### Distribution.

This species occurs from Newfoundland (Lindroth 1955a: 66) to southeastern Ontario (Ottawa, CNC), south to New England and the Adirondack Mountains in northeastern New York (Lindroth 1963b: 349). The record from eastern Iowa (Cooper 1976: 163) needs confirmation.

#### Records.

**CAN**: NB, NF, NS (CBI), ON, QC **USA**: ME, NH, NY, VT [IA]

### Bembidion incrematum

LeConte, 1860

Bembidium incrematum LeConte, 1860: 316. Type locality: «Sitka [Baranof Island, Alaska]» (syntype label). Syntype(s) in MCZ [# 5524]. Note. LeConte (1860: 316) stated that *Bembidion incrematum* was the species described by Mannerheim (1852: 300) under the name *Bembidion nigripes* (Kirby).
Bembidium arcuatum LeConte, 1878c: 594. Type locality: «Marquette [Marquette County, Michigan], Lake Superior» (original citation). Three syntypes in MCZ [# 5525]. Synonymy established by Lindroth (1954b: 127).
Bembidion mobile Casey, 1918: 95. Type locality: «Metlakatla, British Columbia» (original citation). Lectotype (♀), designated by Lindroth (1975: 119), in USNM [# 36979]. Synonymy established by Hatch (1953: 92), confirmed by Lindroth (1954b: 126).
Bembidion semotum Casey, 1918: 96. Type locality: «Truckee [Nevada County], California» (original citation). Lectotype (♂), designated by Lindroth (1975: 119), in USNM [# 36978]. Synonymy established by Lindroth (1954b: 126).
Bembidion nubiferum Casey, 1918: 96. Type locality: «Duncan’s Mills, Sonoma Co[unty], California» (original citation). Lectotype (♂), designated by Lindroth (1975: 119), in USNM [# 36980]. Synonymy established by Lindroth (1954b: 126).
Bembidion gulosum Casey, 1918: 96. Type locality: «Coeur d’Alene [Kootenai County], Idaho» (original citation). Lectotype (♀), designated by Lindroth (1975: 119), in USNM [# 36981]. Synonymy established by Hatch (1953: 92), confirmed by Lindroth (1954b: 126).
Bembidium lengi Notman, 1919a: 98. Type locality: «Cochrane, Ont[ario]; Ausable Lakes, Essex Co[unty], N[ew] Y[ork]» (original citation). One syntype in SIM (Hennessey 1990: 466). Synonymy established by Lindroth (1954b: 129).
Bembidion oblectans Casey, 1924: 36. Type locality: «Edmonton, Alberta» (original citation). Holotype [by monotypy] (♀) in USNM [# 36975]. Synonymy established by Lindroth (1954b: 128).
Bembidion fortunatum Casey, 1924: 36 [primary homonym of *Bembidium fortunatum* Wollaston, 1871]. Type locality: «Edmonton, Alberta» (original citation). Lectotype (♀), designated by Lindroth (1975: 119), in USNM [# 36976]. Synonymy established by Lindroth (1954b: 128).

#### Distribution.

This species ranges from Newfoundland (Lindroth 1955a: 64) to east-central Alaska (Lindroth 1963b: 349), south at least to Sonoma County in western California (Casey, 1918: 96, as *Bembidion nubiferum*), northeastern New Mexico (Mora County, Foster F. Purrington pers. comm. 2012), east-central Ohio (Usis and MacLean 1998: 67), the Adirondack Mountains in New York (Notman 1928: 217, as *Bembidion arcuatum*), and southeastern Maine (Washington County, CNC); also known from eastern Siberia (Marggi et al. 2003: 250). The records from “Massachusetts” (Bousquet and Larochelle 1993: 139), “Pennsylvania,” and “West Virginia” (Hamilton 1894a: 8, as *Bembidion dentellum*) need confirmation.

#### Records.

**FRA**: PM **CAN**: AB, BC (QCI, VCI), LB, MB, NB, NF, NS (CBI), NT, ON, PE, QC, SK, YT **USA**: AK, CA, CO, ID, ME, MI, MT, ND, NH, NY, OH, OR, SD, VT, WA, WI [MA, PA, WV] – **Holarctic**

#### Note.

This spec﻿ies has passed for a long time under the name *Bembidion dentellum* (Thunberg, 1787).

### Bembidion iridipenne

Bousquet and Webster, 2006

Bembidion iridipenne Bousquet and Webster, 2006: 29. Type locality: «Lincoln, Sunbury Co[unty], New Brunswick» (original citation). Holotype (♂) in CNC [# 23456].

#### Distribution.

This species ranges from New Brunswick to southwestern Quebec, south to Virginia (Bousquet and Webster 2006: 29).

#### Records.

**CAN**: NB, QC **USA**: NH, PA, VA, VT

### Bembidion variegatum

Say, 1823

Bembidium variegatum Say, 1823a: 89. Type locality: «Rivervale [Bergen County], N[ew] J[ersey]» (neotype label). Neotype (♂), designated by Lindroth and Freitag (1969: 337), in MCZ [# 33065].
Bembidium postfasciatum Hamilton, 1893: 305. Type locality: near Allegheny, Allegheny County, Pennsylvania (inferred from title of the paper). Four syntypes [8 originally cited] in CMNH (collection Ulke). Synonymy established by Casey (1918: 111).

#### Distribution.

The range of this species extends from New Brunswick (CNC) to eastern South Dakota (Kirk and Balsbaugh 1975: 19), south to western (Dajoz 2007: 23) and east-central (Tucker 1906: 85) Texas, southwestern Alabama (Baldwin County, CMNH), northeastern Georgia (Leng 1910: 73; Fattig 1949: 17), and eastern South Carolina (Ciegler 2000: 49). The record from Colorado (Wickham 1902: 234) needs confirmation; that from British Columbia (Jarrett and Scudder 2001: 382) was based on misidentified specimens of *Bembidion patruele*, *Bembidion graphicum*, and *Bembidion nigripes* (UBC).

#### Records.

**CAN**: NB, ON, QC **USA**: AL, AR, CT, DC, DE, GA, IA, IL, IN, KS, KY, LA, MA, MD, ME, MI, MO, MS, NC, NE, NH, NJ, NY, OH, OK, PA, RI, SC, SD, TN, TX, VA, VT, WI, WV [CO]

#### Note.

This species has been listed in the subgenus *Notaphus* by Lindroth (1963b: 360) but placed in the subgenus *Eupetedromus* by Bousquet and Webster (2006: 30).

### Subgenus Trechonepha

Casey, 1918

Trechonepha Casey, 1918: 19. Type species: *Ochthedromus iridescens* LeConte, 1852 designated by Lindroth (1963b: 265). Etymology. From the generic names *Trechus* [*q.v*.] and *Nepha*, probably alluding to the resemblance of these *Nepha*-like species to those of *Trechus* (“habitus somewhat as in *Trechus*”) [feminine].

#### Diversity.

Western Nearctic Region, with two species.

#### Identification.

Both species are included in Lindroth’s (1963b: 266-267) monograph.

### Bembidion iridescens

(LeConte, 1852)

Ochthedromus iridescens LeConte, 1852a: 191. Type locality: «San Jose [Santa Clara County, California]» (original citation). Holotype [by monotypy] (♀) in MCZ [# 5559].
Peryphus parallelocollis Motschulsky, 1859a: 125. Type locality: «St. Francisco [San Francisco County, California]» (original citation). Lectotype, designated by Bousquet (1997b: 331), in ZMMU. Synonymy established by Lindroth (1963b: 266), confirmed by Bousquet (1997b: 331).
Bembidion fabrum Casey, 1918: 27. Type locality: «Redwood Creek, Humboldt Co[unty], California» (original citation). Holotype [by monotypy] (♀) in USNM [# 36839]. Synonymy established by Lindroth (1963b: 266).
Bembidion obliviosum Casey, 1918: 27. Type locality: «Spokane [Spokane County], Washington» (original citation). Lectotype (♀), designated by Lindroth (1975: 116), in USNM [# 36835]. Synonymy established by Lindroth (1963b: 266).
Bembidion volatile Casey, 1918: 28. Type locality: «Gilroy Hot Springs, S[an]ta Clara Co[unty], California» (original citation). Lectotype (♂), designated by Lindroth (1975: 116), in USNM [# 36836]. Synonymy established by Lindroth (1963b: 266).
Bembidion impium Casey, 1918: 28. Type locality: «Agassiz, British Columbia» (original citation). Lectotype (♀), designated by Lindroth (1975: 116), in USNM [# 36837]. Synonymy established by Hatch (1953: 84), confirmed by Lindroth (1963b: 267).
Bembidion parallelocolle amicum Casey, 1918: 29. Type locality: «Hoopa Valley, Humboldt Co[unty], California» (original citation). Lectotype (♂), designated by Lindroth (1975: 116), in USNM [# 36834]. Synonymy established (as aberration), under the name *Bembidion parallelecolle* (Motschulsky), by Csiki (1928: 72), confirmed by Lindroth (1963b: 267).
Bembidion deceptor Casey, 1918: 29. Type locality: «Metlakatla, British Columbia» (original citation). Lectotype (♂), designated by Lindroth (1975: 116), in USNM [# 36838]. Synonymy established, under the name *Bembidion obliviosum* Casey, by Hatch (1953: 84), confirmed by Lindroth (1963b: 267).
Bembidion repens Casey, 1918: 35. Type locality: «Booneville, Mendocino Co[unty], California» (original citation). Lectotype (♀), designated by Lindroth (1975: 116), in USNM [# 36856]. Synonymy established by Lindroth (1963b: 267).

#### Distribution.

This species ranges from central Alberta (Lindroth 1963b: 267) to the Queen Charlotte Islands (Kavanaugh 1992: 64), south to southern California (Fall 1901a: 10; Moore 1937: 6) and northern Utah (Knowlton 1939: 2; Davis and Salt Lake Counties, CMNH).

#### Records.

**CAN**: AB, BC (QCI, VCI) **USA**: CA (CHI), ID, MT, NV, OR, UT, WA

### Bembidion trechiforme

(LeConte, 1852)

Ochthedromus trechiformis LeConte, 1852a: 190. Type locality: «ad montes, circa S[an]ta Isabel [San Bernardino County, California]» (original citation). Two syntypes in MCZ [# 5558].

#### Distribution.

This species is known from southern California (Fall 1901a: 43; Moore 1937: 6; Maddison 2012: Supplementary content Table S1). The record from “Washington” (Hayward 1897: 128) was based on a misidentified *Bembidion iridescens* (Lindroth 1963b: 267).

#### Records.

**USA**: CA

### Subgenus Liocosmius

Casey, 1918

Liocosmius Casey, 1918: 43. Type species: *Ochthedromus mundus* LeConte, 1852 designated by Lindroth (1963b: 343). Etymology. From the Greek *leios* (smooth) and *cosmos* (ornament, decoration), probably alluding to the polished surface (“highly polished throughout”) of the adults [masculine].

#### Diversity.

Three western North American species.

#### Identification.

Lindroth (1963b: 343-345) treated two species (*Bembidion mundum* and *Bembidion horni*) and listed the other one, based on the original description alone, as synonym of *Bembidion mundum*. Later he rectified the synonymy (Lindroth 1969a: 1115).

### Bembidion festivum

Casey, 1918

Bembidion festivum Casey, 1918: 45. Type locality: «S[an]ta Barbara [Santa Barbara County], California» (original citation). Lectotype (♀), designated by Erwin (1984a: 172), in USNM [# 36874].

#### Distribution.

This species has been recorded from Benton and Jackson Counties in western Oregon (Westcott et al. 2006: 7), from Humboldt and Yolo Counties in northern California (Maddison 2012: Supplementary content Table S1), and from southwestern California (Casey 1918: 45; Moore 1937: 6).

#### Records.

**USA**: CA, OR

### Bembidion horni

Hayward, 1897

Bembidium horni Hayward, 1897: 116. Type locality: «Arizona; California» (original citation), restricted to «Tehachapi [Kern County], Calif[ornia]» by Lindroth (1963b: 344). Four syntypes [“about a dozen” originally cited] in MCZ [# 16301].

#### Distribution.

This species is known from southern California (Fall 1901a: 43; Moore 1937: 6), northern Arizona (Navajo and Apache Counties, UASM; Hayward 1897: 117), southern Utah (San Juan and Kane Counties, MCZ, UASM), and western New Mexico (Maddison 2012: Supplementary content Table S1). The records from “Washington” and “Oregon” (Bousquet and Larochelle 1993: 139) are likely in error.

#### Records.

**USA**: AZ, CA, NM, UT

### Bembidion mundum

(LeConte, 1852)

Lopha bifasciata Motschulsky, 1850a: 12 [secondary homonym of *Bembidion bifasciatum* (Stephens, 1828)]. Type locality: «California?» (original citation). Lectotype (♂), designated by Bousquet (1997b: 331), in ZMMU.
Ochthedromus mundus LeConte, 1852a: 190. Type locality: «San Jose [Santa Clara County, California]» (original citation). Two syntypes in MCZ [# 35335]. Synonymy established with doubt by LeConte (1857c: 10), confirmed by Bousquet (1997b: 331).
Bembidion hilare Casey, 1918: 44. Type locality: «Cloverdale, Sonoma Co[unty], California» (original citation). Lectotype (♂), designated by Erwin (1984a: 173), in USNM [# 36873]. Synonymy established by Lindroth (1963b: 344).

#### Distribution.

This species is found from southwestern British Columbia, including Vancouver Island (Lindroth 1963b: 344), south to southern California (Fall 1901a: 43, as *Bembidion bifasciatum*) and northern Arizona (Snow 1906b: 161; Coconino County, CMNH), including southwestern (Tanner 1928: 269, as *Bembidion bifasciatum*) and eastern Utah (Grand County, Foster F. Purrington pers. comm. 2010).

#### Records.

**CAN**: BC (VCI) **USA**: AZ, CA, NV, OR, UT, WA

### Subgenus Melomalus

Casey, 1918

Melomalus Casey, 1918: 37. Type species: *Ochthedromus planatus* LeConte, 1847 designated by Netolitzky (1943b: 61). Etymology. Unknown [masculine].

#### Diversity.

Northern Hemisphere, with two species in the Nearctic (*Bembidion planatum*) and Palaearctic (*Bembidion altaicum* Gebler in Asia) Regions.

#### Identification.

The North American species is treated in Lindroth’s (1963b: 284) monograph.

#### Taxonomic Note.

This subgenus was placed in synonymy with *Plataphus* Motschulsky by Netolitzky (1943b: 61) and Lindroth (1963b: 283) but considered a valid taxon by Toledano (2008: 12). According to Maddison (2012: 569), *Melomalus* is not closely related to the subgenus *Plataphus*.

### Bembidion planatum

(LeConte, 1847)

Ochthedromus planatus LeConte, 1847: 456. Type locality: «Lacum Superiorem» (original citation), restricted to «Isle Royal[e] [Keweenaw County], Mich[igan]» by Lindroth (1963b: 284). Four syntypes in MCZ [# 5500].
Peryphus aequalis Walker, 1866: 316. Type locality: British Columbia (inferred from title of the book). Syntype(s) location unknown (possibly in BMNH). Synonymy established by LeConte (1870: 400).
Bembidion solutum Casey, 1918: 38. Type locality: «near San Francisco [San Francisco County], California» (original citation). Lectotype (♀), designated by Lindroth (1975: 117), in USNM [# 36857]. Synonymy established by Lindroth (1954b: 124).
Bembidion adjutor Casey, 1918: 39. Type locality: Duncan’s Mills, Sonoma County, California (lectotype label according to Lindroth 1975: 117). Lectotype [as type] (♀), designated by Lindroth (1975: 117), in USNM [# 36858]. Synonymy established by Lindroth (1954b: 124).

#### Distribution.

This species ranges from Newfoundland (Lindroth 1955a: 52) to the Norton Sound area in Alaska (Lindroth 1963b: 284), south to central California along the Coast Ranges, southern Colorado along the Rocky Mountains, Isle Royale in northernmost Michigan (Hubbard and Schwarz 1878: 629), and Cape Breton Island [see Lindroth 1963a: Fig. 63]. Fossil remnants from a Plio-Pleistocene sequence have been unearthed in northwestern Greenland (Böcher 1995: 24).

#### Records.

**CAN**: AB, BC (VCI), NB, NF, NS (CBI), NT, ON, QC, YT **USA**: AK, CA, CO, ID, MI, MT, NV, OR, UT, WA, WY

### Subgenus Trichoplataphus

Netolitzky, 1914

Trichoplataphus Netolitzky, 1914: 51. Type species: *Bembidium lissonotum* Bates, 1873 designated by Netolitzky (1943a: 109). Etymology. From the Greek *trichos* (hair) and the generic name *Plataphus* [*q.v*.], alluding to the presence of sparse setae on the metasternum, metacoxae, and abdominal sterna (“*die schüttere Behaarung des Metasternums, der Hinterhüften und aller sichtbaren Ventralsegmente*”) on the adults of these *Plataphus-*related species [masculine].
Triporus Andrewes, 1921: 251. Type species: *Bembidium kara* Andrewes, 1921 by monotypy. Synonymy established by Netolitzky (1943a: 109). Etymology. From the Latin prefix *tri*- (three) and *porus* (holes), alluding to the presence of three discal setigerous punctures on the third elytral interval (“elytra ... stria ... 3 with three well-marked pores”) of the adult [masculine].

#### Diversity.

Northern Hemisphere, with 22 species in the Nearctic (five eastern species) and Palaearctic (17 Asian species) Regions (see Toledano and Schmidt 2010).

#### Identification.

Four of the North American species are included in Lindroth’s (1963b: 212-229) key to species of *Bembidion*; three of these were treated in detail (Lindroth 1963b: 298-300). One new species was described subsequently by Hildebrandt and Maddison (2011) who provided a modified version of Lindroth’s key to include the new species.

### Bembidion fugax

(LeConte, 1848)

Ochthedromus fugax LeConte, 1848: 467. Type locality: «Illinois» (original citation). Lectotype [as type], designated by Lindroth (1963b: 300), in MCZ [# 5508].
Bembidion champlaini Casey, 1918: 56. Type locality: «N[ew] Cumberland [Cumberland County], Pennsylvania» (original citation). Lectotype (♂), designated by Lindroth (1975: 118), in USNM [# 36912]. Synonymy established by Nicolay and Weiss (1934: 196), confirmed by Lindroth (1963b: 299).

#### Distribution.

This eastern species ranges from “Vermont” (Hayward 1897: 72) and southwestern Massachusetts (Hampden County, MCZ) to “Illinois” (Hayward 1897: 72), south to northern and eastern Tennessee (Cheatham, Fentress, and Sevier Counties, CMNH, MCZ). The record from southern Wisconsin (Rauterberg 1885: 22) needs confirmation; those from Missouri (Summers 1873: 147) and the Rocky Mountains in northern Colorado (Wickham 1902: 232) must be in error.

#### Records.

**USA**: DC, IL, IN, MA, MD, MI, NJ, NY, OH, PA, TN, VA, VT [WI]

### Bembidion grandiceps

Hayward, 1897

Bembidium grandiceps Hayward, 1897: 70. Type locality: «Lowell [in] Mass[achusetts], New Jersey, Pennsylvania, the District of Columbia and Texas» (original citation). One syntype (labeled “Tex.”) in ANSP [# 1030]. Note. Six specimens, labeled “Pen” (1), “D.C.” (3), and “Tex.” (2), in LeConte’s collection (MCZ) are probably also syntypes.

#### Distribution.

This species is known from central Iowa (Maddison 2012: Supplementary content Table S1), northeastern Kansas (Pottawatomie County, CMNH; Lindroth 1963b: 218; Knaus 1903: 188), eastern Oklahoma (Latimer County, UASM) and central Texas (Blanco County, UASM; Hayward 1897: 70). According to Hildebrandt and Maddison (2011: 273), the records from Massachusetts, “New Jersey,” “Pennsylvania,” the District of Columbia (Hayward 1897: 70), and New York (Notman 1928: 216) are likely incorrect and may be based on *Bembidion fugax*.

#### Records.

**USA**: IA, KS, OK, TX

### Bembidion ozarkense

Maddison and Hildebrandt, 2011

Bembidion ozarkense Maddison and Hildebrandt [in Hildebrandt and Maddison], 2011: 268. Type locality: «Current River at Van Buren (135 m), Carter Co[unty], Missouri» (original citation). Holotype (♂) in OSAC.

#### Distribution.

This species is known from the Ozark Plateau of Missouri and Arkansas [see Hildebrandt and Maddison 2011: Fig. 6].

#### Records.

**USA**: AR, MO

### Bembidion planum

(Haldeman, 1843)

Peryphus planus Haldeman, 1843b: 303. Type locality: southeastern Pennsylvania (Haldeman 1843a: 298). One possible syntype, a ♂ labeled “[pale green disc] / O. planus (Hald) Lec. [handwritten] / B. guexii Chaud [handwritten],” in MCZ (collection LeConte).
Ochthedromus planipennis LeConte, 1850: 211 [*nomen dubium*]. Type locality: «Kaministiquia River below Kakàbeka Falls [= near Thunder Bay, Ontario]» (original citation). Syntype(s) location unknown. Synonymy established with doubt by Lindroth (1963b: 300). Note. This name has been listed in synonymy with *Bembidion fugax* by LeConte (1857a: 5) himself. As expressed by Lindroth (1963b: 300), the type locality given suggests that the type specimen(s) was conspecific with *Bembidion planum* rather than *Bembidion fugax*.
Bembidium guexii Chaudoir, 1868b: 242. Unnecessary replacement name for *Bembidium planum* (Haldeman, 1843).
Bembidion vulsum Casey, 1918: 55. Type locality: «Catskill M[oun]t[ain]s, New York» (original citation). Lectotype (♀), designated by Lindroth (1975: 117), in USNM [# 36911]. Synonymy established by Nicolay and Weiss (1934: 196), confirmed by Lindroth (1963b: 298).
Bembidion filicorne Casey, 1918: 56. Type locality: «Oak Ridge [Ocean County], New Jersey» (original citation). Lectotype (♂), designated by Lindroth (1975: 118), in USNM [# 36910]. Synonymy established by Nicolay and Weiss (1934: 196), confirmed by Lindroth (1963b: 298).

#### Distribution.

The range of this eastern species extends from Nova Scotia (Lindroth 1954c: 302) to “Minnesota” (Hildebrandt and Maddison 2011: 274), south to eastern Oklahoma (Le Flore County, FFPC ), northern Arkansas (Lindroth 1969a: 1114; Pope, Searcy, and Washington Counties, CMNH), northern Mississippi (Drew A. Hildebrandt pers. comm. 2010), central Alabama (Jefferson County, Drew A. Hildebrandt pers. comm. 2009), northern Georgia (Fattig 1949: 17), and northwestern South Carolina (Ciegler 2000: 48).

#### Records.

**CAN**: NB, NS, ON, QC **USA**: AL, AR, CT, DC, GA, IA, IL, IN, KY, MA, MD, ME, MI, MN, MO, MS, NC, NH, NJ, NY, OH, OK, PA, RI, SC, TN, VA, VT, WI, WV

### Bembidion rolandi

Fall, 1922

Bembidion rolandi Fall, 1922c: 171. Type locality: «E[ast] Park, a suburb of Phila[delphia], Penn[sylvania]» (original citation). Holotype (♀) in MCZ [# 23866]. Etymology. Although not indicated, the species name was probably proposed for Roland Hayward [1865-1906], who as an amateur studied the taxonomy of the North American Bembidiini and *Amara*.

#### Distribution.

This eastern species ranges from Nova Scotia (Larochelle and Larivière 1990a: 28; Maddison 2012: Supplementary content Table S1) to the Ontario Peninsula (Lindroth 1963b: 300) and northern Ohio (Ashtabula, Cuyahoga, Erie, and Lorain Counties, Harry J. Lee, Jr. pers. comm. 2008), south to west-central Tennessee (Hickman County, CMNH) and northeastern Virginia (Richard L. Hoffman pers. comm. 1992).

#### Records.

**CAN**: NB, NS, ON, QC **USA**: DC, KY, MA, MD, ME, NJ, NY, OH, PA, TN, VA, VT, WV

### Subgenus Phyla

Motschulsky, 1844

Phyla Motschulsky, 1844: 238 (as *Phayla*). Type species: *Bembidion obtusum* Audinet-Serville, 1821 designated by Netolitzky (1939: 10). Etymology. Uncertain, possibly from the Greek *phylax* (guard) or *phyle* (tribe, race), or even *philia* (love) since Motschulsky used the spelling *Phila* later (Motschulsky 1850a: 14) [feminine]. Note. Motschulsky (1844) used two different original spellings for this name, *Phayla* (page 238) and *Phyla* (pages 260-263). Since he subsequently used the spelling *Phyla* (e.g., Motschulsky 1869: 8), this spelling becomes the correct original spelling (ICZN 1999: Article 24.2.4). *Phaula* used by Bedel (1879: 26, 34) is an incorrect subsequent spelling, not an unjustified emendation since there is no demonstrably intentional change (see ICZN 1999: Article 33.2).
Microcys J.R. Sahlberg, 1908: 11. Type species: *Microcys liliputanus* Sahlberg, 1908 by monotypy. Synonymy established by Müller (1918: 68). Etymology. From the Greek *mikros* (small) and the generic name *Ocys* [masculine].

#### Diversity.

West Palaearctic Region, with nine species of which one is adventive in eastern North America.

#### Identification.

The species found in North America was covered in Lindroth’s (1963b: 258) monograph.

#### Taxonomic Note.

Maddison (2012: 561) noted that this taxon may be the sister-group of the remaining *Bembidion*.

### Bembidion obtusum

Audinet-Serville, 1821

Bembidion obtusum Audinet-Serville, 1821: 83. Type locality: «Paris [France]» (original citation). Syntype(s) probably lost.

#### Distribution.

This Palaearctic species is adventive in North America where it is known from Prince Edward Island (Majka et al. 2008: 130), and from western Quebec, as far north as the Abitibi region (Paquin and Dupérré 2002: 86), to the Algoma District in northern Ontario (Pearce et al. 2003), south to southern Michigan (Purrington et al. 2002: 200), northeastern Ohio, and Long Island, New York [see Hoebeke et al. 1991: Fig. 1]. The first inventoried specimen collected on this continent was found in the Great Lake region in Ontario in 1956 (Lindroth 1963b: 258).

#### Records.

**CAN**: ON, PE, QC **USA**: OH, MI, NY, PA, VT – **Adventive**

### Subgenus Lymnaeum

Stephens, 1828

Lymnaeum Stephens, 1828b: 2. Type species: *Carabus nigropiceus* Marsham, 1802 designated by Westwood (1838: 6). Etymology (original). From the Greek *limne* (marsh, pond, pool) [neuter].
Limnaeum Agassiz, 1846: 210, 218. Unjustified emendation of *Lymnaeum* Stephens, 1828.
Lymneops Casey, 1918: 168. Type species: *Lymneops angusticeps* Casey, 1918 (= *Lymnaeum laticeps* LeConte, 1858) by original designation. Synonymy established by Fall (1922a: 84). Etymology. From the generic name *Lymnaeum* and the Greek suffix -*ops* (having the appearance of) [masculine].
Limneops Csiki, 1928: 164. Unjustified emendation of *Lymneops* Casey, 1918.

#### Diversity.

Northern Hemisphere, with four coastal species in North America (two species, one endemic along the Pacific and one adventive species along the Atlantic) and Europe (three species: *Bembidion abeillei* Bedel, *Bembidion eichleri* Marggi, Wrase and Huber, and *Bembidion nigropiceum* Marsham).

#### Identification.

Both species found in North America are included in Lindroth’s (1963b: 213) key to the Canadian and Alaskan *Bembidion*.

### Bembidion laticeps

(LeConte, 1858)

Lymnaeum laticeps LeConte, 1858b: 61. Type locality: «San Diego [San Diego County], California» (original citation). Holotype [by monotypy] (♀) in MCZ [# 5562].
Lymneops angusticeps Casey, 1918: 169. Type locality: «San Pedro [Los Angeles County], California» (original citation). Lectotype (♂), designated by Erwin (1984a: 176), in USNM [# 46903]. Synonymy established by Fall (1922a: 84), confirmed by Erwin (1984a: 176).

#### Distribution.

This species occurs on the seashore along the coast of California.

#### Records.

**USA**: CA

### Bembidion nigropiceum

(Marsham, 1802)

Carabus nigropiceus Marsham, 1802: 468. Type locality: Great Britain (inferred from title of the book). Syntype(s) probably in BMNH (collection Stephens).
Bembidium sulcatulum Chaudoir [in Chaudoir and Hochhuth], 1846: 233. Type locality: «Kertch [= Kerch, eastern Crimea, Ukraine]» (original citation). Syntype(s) probably in MHNP. Synonymy established by Chaudoir (1850b: 182).
Bembidium puritanum Hayward, 1897: 129. Type locality: «Massachusetts» (original citation). Syntype(s) [4 originally cited] in MCZ [# 16304] and ANSP. Synonymy established by Erwin and Kavanaugh (1980: 241).

#### Distribution.

This adventive species was known on this continent until recently only from the four specimens upon which Hayward (1897: 129) described his *Bembidion puritanum*. Erwin and Kavanaugh (1980: 241) stated that the species was probably “accidentally introduced into Massachusetts from England through shipping in the late 1800s, that a population was sustained for a short time, but that it is not likely to have persisted to the present.” However, more than 80 specimens were collected between 2007 and 2010 in Boston Harbor, Suffolk County, Massachusetts (Davidson and Rykken 2011: 491).

#### Records.

**USA**: MA – **Adventive**

### Genus Phrypeus

Casey, 1924

Phrypeus Casey, 1924: 43. Type species: *Bembidium rickseckeri* Hayward, 1897 designated by Lindroth (1963b: 407). Etymology. Anagram of the generic name *Peryphus* [*q.v*.] [masculine].

#### Diversity.

One species in western North America.

#### Identification.

Lindroth (1963b: 407-408) covered the species in his monograph of the Canadian and Alaskan Carabidae.

#### Taxonomic Note.

Molecular data analyses presented by Maddison and Ober (2011) and Maddison (2012) strongly suggest that this genus is not closely related to the other members of Bembidiina. However, its relationship is not established. Because there are no family-group name proposed for *Phrypeus*, the genus is left for lack of anything better in the subtribe Bembidiina.

### Phrypeus rickseckeri

(Hayward, 1897)

Bembidium rickseckeri Hayward, 1897: 85. Type locality: «California, Oregon and Washington» (original citation), restricted to «Sylvania [= Camp Meeker, Sonoma County], Cal[ifornia]» by Lindroth (1963b: 408). Syntype(s) [8 originally cited] in MCZ [# 16291]. Etymology. The specific name honors Lucius Edgar Ricksecker [1841-1913], an enthusiastic naturalist and collector of natural history objects, especially beetles. Ricksecker collected mainly in southern California and particularly along the west coast. His collection and library, at the time in Santa Rosa, were totally destroyed by the earthquake of 18 April 1906.
Phrypeus rutilinus Casey, 1924: 44. Type locality: «Josephine Co[unty], Oregon» (original citation). Lectotype (♀), designated by Lindroth (1975: 122), in USNM [# 36945]. Synonymy established by Hatch (1953: 92), confirmed by Lindroth (1963b: 408).

#### Distribution.

This species ranges from Vancouver Island (Lindroth 1963b: 408) to western Montana (Russell 1968: 58), south to Nevada County in the Sierra Nevada (Dajoz 2007: 16) and to Sonoma County along the Pacific Coast (Lindroth 1963b: 408).

#### Records.

**CAN**: BC (VCI) **USA**: CA, MT, OR, WA

**Figure 23.**
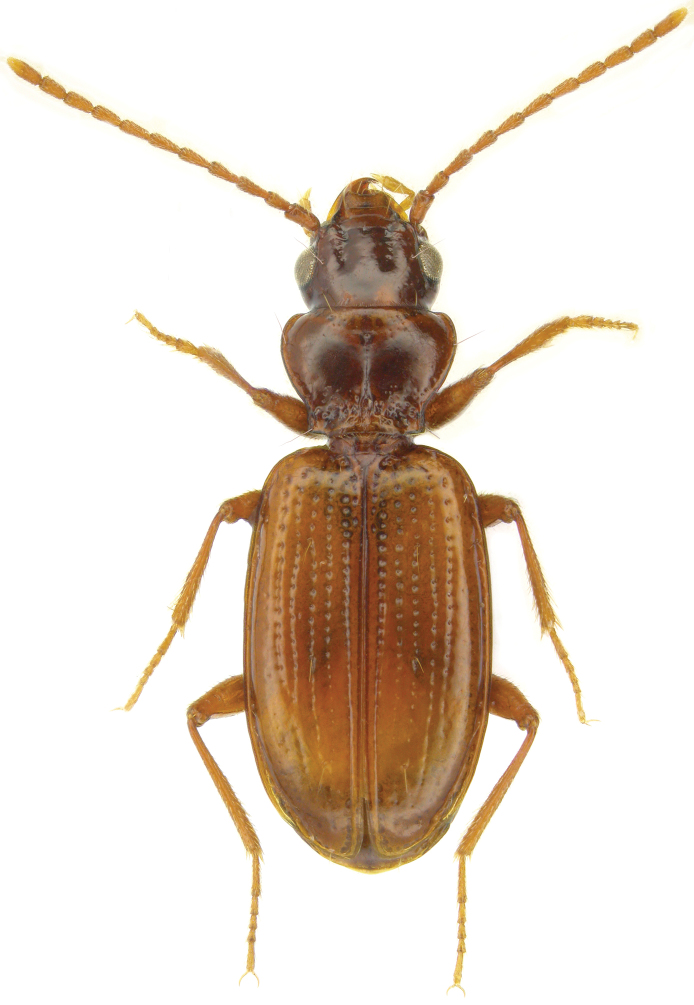
*Phrypeus rickseckeri* (Hayward). Thomas Casey is well known to North American coleopterists for the numerous species he proposed that are considered invalid today. His extremely rigid species concept allowed almost no morphological variation. He was, however, more successful at the generic level and many of his taxa are still recognized as valid today. One of these is *Phrypeus* which he proposed for a small, peculiar bembidiine species living along river banks in the coastal region of western North America. Associated with the subtribe Bembidiina for a long time, recent molecular analyses have revealed that the species is not closely related to that group.

### Subtribe Xystosomina

Erwin, 1994

Xystosomina Erwin, 1994: 560. Type genus: *Xystosomus* Schaum, 1863.

#### Diversity.

New World, with about 125 species arrayed in seven genera: *Erwiniana* Paulsen and Smith (about 55 Neotropical species), *Geballusa* Erwin (five Neotropical species), *Gouleta* Erwin (four Neotropical species), *Inpa* Erwin (one South American species), *Mioptachys* (13 species), *Philipis* Erwin (38 Australian species), and *Xystosomus* Schaum (nine Neotropical species). The North American fauna is represented by a single species.

### Genus Mioptachys

Bates, 1882

Tachymenis Motschulsky, 1862b: 27 [junior homonym of *Tachymenis* Weigmann, 1835]. Type species: *Bembidium flavicaudus* Say, 1823 designated by Casey (1918: 220). Etymology. From the generic name *Tachys* [*q.v*.] and the Greek *menis* (wrath) [masculine].
Mioptachys Bates, 1882a: 144. Type species: *Mioptachys trechoides* Bates, 1882 designated by Erwin (1974a: 145). Synonymy established by Erwin (1974a: 145). Etymology. Uncertain, possibly from the Greek prefix *mio*- (less) and the generic name *Tachys* [*q.v*.] [masculine].

#### Diversity.

Western Hemisphere, with 13 species in temperate, subtropical, and tropical areas of the Nearctic (one species) and Neotropical (12 species) Regions.

#### Identification.

The North American species is treated in Lindroth’s (1966: 441) monograph under the genus *Tachys*.

### Mioptachys flavicauda

(Say, 1823)

Bembidium flavicaudus Say, 1823a: 87. Type locality: «W[hite] S[ulphur] Springs [Greenbrier County], W[est] V[irgini]a» (neotype label). Neotype (♂), designated by Lindroth and Freitag (1969: 339), in MCZ [# 33055].
Tachymenis reflexicollis Motschulsky, 1862b: 31. Type locality: «environs de New-York» (original citation). Two syntypes in ZMMU (Keleinikova 1976: 214). Synonymy established by Lindroth (1966: 441).
Tachymenis marginicollis Motschulsky, 1862b: 32. Type locality: «environs de la Nouvelle Orléans [Orleans Parish], Louisiane» (original citation). Lectotype, designated by Erwin (1974a: 141), in ZMMU. Synonymy established by Casey (1918: 221), confirmed by Erwin (1974a: 145).

#### Distribution.

This species ranges from the Nova Scotia Peninsula (Christopher G. Majka pers. comm. 2007) to western Washington (Hatch 1953: 104), north to southern British Columbia (Lindroth 1966: 441) and southern Alberta (CNC), south to southwestern California (Los Angeles County, CAS), southeastern Arizona (Dajoz 2007: 21), south-central Texas (Bastrop County, CNC), and southern Florida (Peck and Thomas 1998: 18).

#### Records.

**CAN**: AB, BC, NB, NS, ON, QC **USA**: AL, AR, AZ, CA, CT, DC, FL, GA, IA, IL, IN, KS, KY, LA, MA, MD, ME, MI, MN, MO, MS, NC, ND, NE, NH, NJ, NM, NY, OH, OK, OR, PA, RI, SC, SD, TN, TX, VA, VT, WA, WI, WV

**Figure 24.**
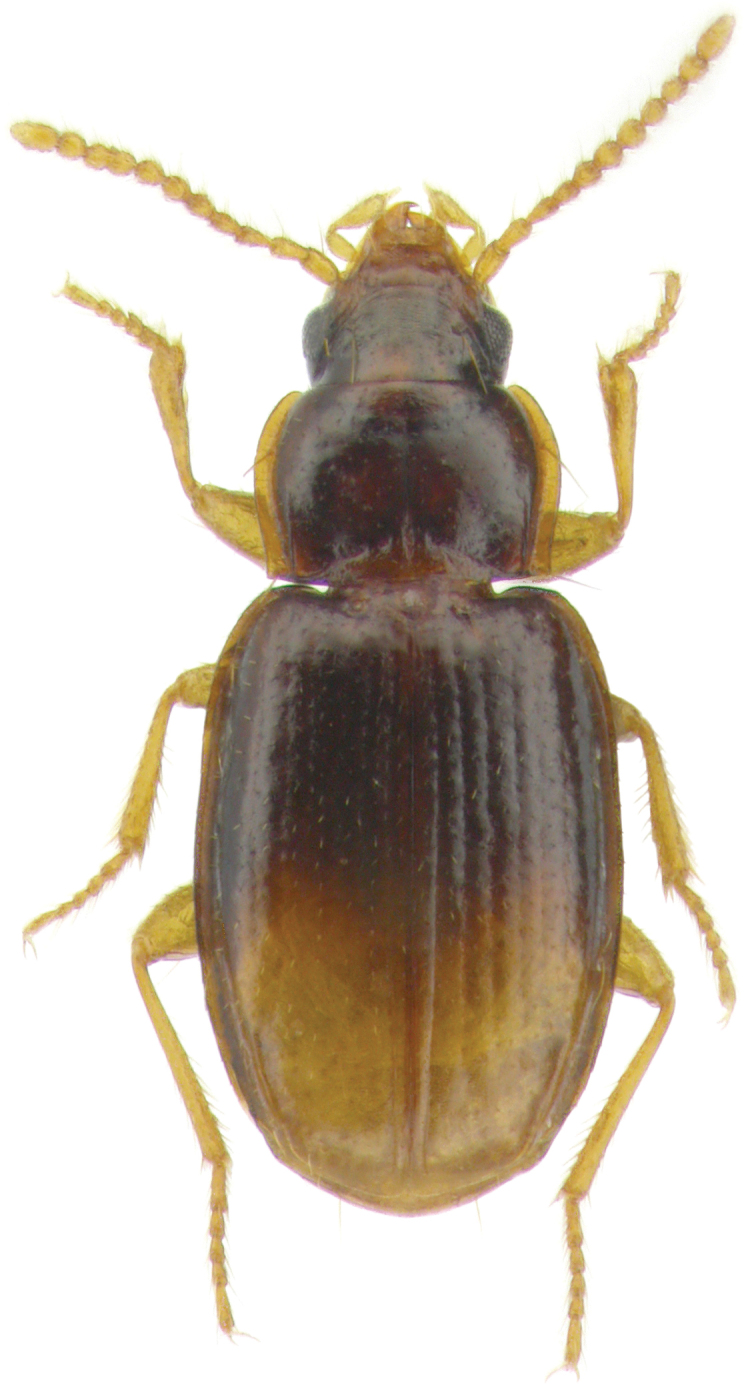
*Mioptachys flavicauda* (Say). This small species is a characteristic element of the fauna that lives under the bark of dead deciduous trees. Despite its size, it is easily recognized in the field by its conspicuous yellow apical third of the elytra. Thomas Say, the first entomologist born on this continent worthy of the name, was struck by the coloration and for that reason proposed the name *flavicauda*, derived from the Latin *flavus* (yellow) and *cauda* (tail), for the species.

### Subtribe Tachyina

Motschulsky, 1862

Tachyaires Motschulsky, 1862b: 24. Type genus: *Tachys* Dejean, 1821. Note. The stem of *Tachys* is *Tachy*- (Madge 1989 : 468).
Micratopini Casey, 1914: 42. Type genus: *Micratopus* Casey, 1914.
Limnastini Jeannel, 1937a: 245. Type genus: *Limnastis* Ganglbauer, 1891 (unjustified emendation of *Lymnastis* Motschulsky, 1862, not in prevailing usage) (= *Lymnastis* Motschulsky, 1862).

#### Diversity.

Worldwide, with about 790 species (Lorenz 2005: 207-215). The Northern Hemisphere is represented by about 230 species (roughly 29% of the world fauna) and North America by 73 species (9.2% of the world fauna). Two species are adventive (*Porotachys bisulcatus* and *Elaphropus parvulus*) in the Nearctic Region. No species are Holarctic.

#### Note.

The genitive form of *tachys* is *tachéos* (see Alonso-Zarazaga 2007) and the family-group name based on the genus *Tachys* should be spelled Tacheina. However the spelling Tachyina being in prevailing usage, it is maintained (ICZN 1999: Article 29.5).

### Genus Tachyta

Kirby, 1837

Tachyta Kirby, 1837: 56. Type species: *Tachyta picipes* Kirby, 1837 (= *Bembidium inornatum* Say, 1823) by monotypy. Etymology. From the Greek *tachytes* (swiftness) [feminine].

#### Diversity.

Worldwide, with 24 species in boreal, temperate, subtropical, and tropical areas. The species are arrayed in two subgenera: *Paratachyta* Erwin (five species in the Eastern Hemisphere) and *Tachyta* s.str. (19 species).

#### Identification.

Erwin (1975) revised the species and provided a key for their identification.

### Subgenus Tachyta

Kirby, 1837

Tachyta Kirby, 1837: 56. Type species: *Tachyta picipes* Kirby, 1837 (= *Bembidium inornatum* Say, 1823) by monotypy.

#### Diversity.

Worldwide, with 19 species in the Nearctic (five species), Neotropical (three species, only one of them endemic), Australian (five species), Oriental (four species), Palaearctic (two species), and Afrotropical (four species) Regions.

### [falli group]

### Tachyta angulata

Casey, 1918

Tachyta angulata Casey, 1918: 216. Type locality: «Bayfield [Bayfield County], Wisconsin» (original citation). Lectotype (♂), designated by Erwin (1974a: 150), in USNM [# 46966].

#### Distribution.

This species ranges from Cape Breton Island (Lindroth 1954c: 303) to west-central Yukon Territory, south to Veracruz in Mexico, northeastern Mississippi (Oktibbeha County, Drew A. Hildebrandt pers. comm. 2009), and northern Georgia [see Erwin 1975: Fig. 121]. The record from “Oregon” (Bousquet and Larochelle 1993: 149) needs confirmation.

#### Records.

**CAN**: AB, BC, MB, NB, NS (CBI), ON, QC, SK, YT **USA**: AZ, CT, DC, GA, IN, MA, MD, ME, MI, MN, MO, MS, MT, NC, NH, NJ, NM, NY, PA, RI, SC, VA, VT, WI, WV [OR] – Mexico

### Tachyta falli

(Hayward, 1900)

Tachys falli Hayward, 1900: 199. Type locality: «Siskiyou Co[unty], Cal[ifornia]» (lectotype label). Lectotype (♂), designated by Erwin (1974a: 150), in MCZ [# 7048].

#### Distribution.

This western species ranges from southwestern British Columbia, including Vancouver Island, to western Idaho, south to Fresno County in California along the Sierra Nevada and to the San Francisco Bay area along the Coast Ranges [see Erwin 1975: Fig. 111].

#### Records.

**CAN**: BC (VCI) **USA**: CA, ID, OR, WA

### [inornata group]

### Tachyta inornata

(Say, 1823)

Bembidium inornatum Say, 1823a: 87. Type locality: «Asheville [Buncombe County], N[orth] C[arolina]» (neotype label). Neotype (♂), designated by Lindroth and Freitag (1969: 339), in MCZ [# 33056].
Tachyta picipes Kirby, 1837: 56. Type locality: «Lat. 54° [= along North Saskatchewan River]» (original citation). Lectotype (♀), designated by Erwin (1974a: 151), in BMNH. Synonymy established by LeConte (1848: 471), confirmed by Lindroth (1953b: 176).
Tachys rivularis Motschulsky, 1850a: 8. Type locality: «C[alifornia]» (lectotype label). Lectotype (♀), designated by Erwin (1974a: 151), in ZMMU. Synonymy established by Hayward (1900: 233), confirmed by Erwin (1975: 45).
Tachyta collaris Casey, 1918: 218. Type locality: «Washington State» (original citation). Lectotype (♂), designated by Erwin (1974a: 151), in USNM [# 46968]. Synonymy established by Hatch (1953: 103), confirmed by Erwin (1975: 45).
Tachyta arizonica Casey, 1918: 219. Type locality: «Arizona» (original citation). Lectotype (♂), designated by Erwin (1974a: 151), in USNM [# 46969]. Synonymy established by Erwin (1975: 45).
Tachyta debilicollis Casey, 1918: 219. Type locality: «Catskill M[oun]t[ain]s [Greene County], New York» (original citation). Lectotype (♂), designated by Erwin (1974a: 151), in USNM [# 46971]. Synonymy established by Erwin (1975: 45).
Tachyta californica Casey, 1918: 219. Type locality: «Humboldt Co[unty], California» (original citation for the lectotype). Lectotype (♀), designated by Erwin (1974a: 151), in USNM [# 46970]. Synonymy established by Hatch (1953: 103), confirmed by Erwin (1975: 45).

#### Distribution.

This species ranges from Maine and southern Quebec to south-central British Columbia, south to southwestern California, Belize and Guatemala, and southern Florida, including the Keys; also known from Cuba [see Erwin 1975: Fig. 135].

#### Records.

**CAN**: BC, ON, QC, SK **USA**: AL, AR, AZ, CA, CO, CT, DC, DE, FL, GA, IA, ID, IL, IN, KS, KY, LA, MA, MD, ME, MI, MO, MS, MT, NC, ND, NH, NJ, NM, NV, NY, OH, OK, OR, PA, RI, SC, SD, TN, TX, VA, VT, WA, WI, WV – Belize, Cuba, Guatemala, Mexico

### Tachyta kirbyi

Casey, 1918

Tachyta kirbyi Casey, 1918: 216. Type locality: «Duluth [Saint Louis County], Minnesota» (original citation). Lectotype (♂), designated by Erwin (1974a: 151), in USNM [# 46967].

#### Distribution.

The range of this species extends from the Gaspé Peninsula in eastern Quebec to west-central British Columbia, south to northwestern Oregon, south-central New Mexico through the Rocky Mountains, the Black Hills in southwestern South Dakota, east-central Minnesota, western New York near the Great Lakes, and Massachusetts (Erwin 1975: 65) along the east coast [see Erwin 1975: Fig. 135]. The record of *Tachys rivularis* from “Sitka” in the Alexander Archipelago (Motschulsky 1850a: 8) probably refers to this species.

#### Records.

**CAN**: AB, BC, ON, QC, SK **USA**: CO, ID, MA, ME, MN, MT, ND, NH, NM, NY, OR, SD, VT, WA, WI [AK]

### Tachyta parvicornis

Notman, 1922

Tachyta parvicornis Notman, 1922b: 100. Type locality: «S[ain]t Petersburg [Pinellas County], Fl[orid]a» (original citation). Holotype (♂) in FSCA.

#### Distribution.

This species ranges from Massachusetts to the Black Hills in southwestern South Dakota, south to southern Arizona, southern Texas, southern Mississippi (Harrison and Lamar Counties, Drew A. Hildebrandt pers. comm. 2008), and southern Florida, including the Keys [see Erwin 1975: Fig. 156].

#### Records.

**USA**: AL, AR, AZ, CO, DC, FL, GA, IN, LA, MA, MD, MS, NC, NJ, NM, NY, PA, RI, SC, SD, TN, TX, VA, WI, WV

### Genus Elaphropus

Motschulsky, 1839

Elaphropus Motschulsky, 1839: 73. Type species: *Elaphropus caraboides* Motschulsky, 1839 by monotypy. Etymology. From the Greek *elaphros* (nimble) and *pous* (foot), probably alluding to the agility of the adults in the field [masculine].

#### Diversity.

Worldwide, with about 340 species (Lorenz 2005: 207-211, as *Elaphropus* and *Nototachys*) arrayed in seven subgenera following Sciaky and Vigna Taglianti (2003, as phyletic line of *Elaphropus*): *Amaurotachys* Jeannel (Old World), *Barytachys*, *Elaphropus* s.str. (about 40 species in the Eastern Hemisphere), *Sphaerotachys* Müller (Eastern Hemisphere) with *Nototachys* Alluaud, *Tachyphanes* Jeannel, and *Trepanotachys* Alluaud as synonyms, *Tachylopha* Motschulsky (40 species in the Eastern Hemisphere), *Tachyura*, and *Tachyuropsis* Shilenkov (one species in the Far East).

#### Taxonomic Note.

Several authors have discussed the taxonomic status of *Elaphropus* and related taxa and most have different opinions as to the statuses they should be assigned. In view of this, I prefer to include all taxa of the phyletic line of *Elaphropus* (sensu Sciaky and Vigna Taglianti 2003) into a single genus, as done by Erwin (1974a) and Shilenkov (2002), and recognize several subgenera.

### Subgenus Barytachys

Chaudoir, 1868

Barytachys Chaudoir, 1868b: 213. Type species: *Bembidium incurvum* Say, 1830 designated by Jeannel (1941b: 434). Etymology. From the Greek *barys* (heavy) and the generic name *Tachys* [*q.v*.], probably alluding to the convex (and thus heavy compared to the other species of *Tachys*) body of adults of these species of *Tachys* [masculine].

#### Diversity.

Western Hemisphere, with 37 species in North America (28 species), Middle America (ten species), and the West Indies (three species, two of them endemic).

#### Identification.

Hayward (1900: 201-212, as *incurvus* group) reviewed the North American species then known (15 species). Lindroth (1966: 414-424, as *incurvus* group) treated 12 species (one in the key only) found in North America. A taxonomic revision of the subgenus is needed.

#### Taxonomic Note.

Sciaky and Vigna Taglianti (2003) considered *Elaphropus* s.str. and *Barytachys* as two distinct genera, arguing that “*Barytachys* could possibly be considered a subgenus of *Elaphropus*, but from a biogeographical point of view, this classification seems highly implausible.” However, there seem to be no significant character states to differentiate the two taxa. In their key, members of *Elaphropus* are characterized by their “body almost always depigmented, nearly uniform in colour, almost round and convex” and those of *Barytachys* by their “body generally blackish, sometimes with lighter markings, oval and relatively flat.” *Elaphropus* is said to be represented only in the Eastern Hemisphere while *Barytachys* is represented in the Western Hemisphere. Based on the evidence presented I am not convince that these two taxa should be considered distinct. However, for the time being, *Barytachys* is retained as a valid subgenus pending further study.

### Elaphropus anceps

(LeConte, 1848)

Tachys anceps LeConte, 1848: 470. Type locality: «Rocky Mountains» (original citation), restricted to «Nebraska» by Erwin (1974a: 133). Lectotype (♀), designated by Erwin (1974a: 133) in MCZ [# 5583].

#### Distribution.

The range of this species extends from Cape Breton Island to southeastern Alberta, north to southern Northwest Territories (Lindroth 1966: 418-419), south to southeastern Arizona (Dajoz 2007: 21), northwestern Texas (Nolan County, CMNH), northwestern Louisiana (Caddo Parish, Igor M. Sokolov pers. comm. 2009), and Maryland (Clark et al. 2006: 1306). The records from “British Columbia,” “Washington,” “Oregon,” “Idaho,” “Utah” (Bousquet and Larochelle 1993: 150), based on specimens identified under this name in USNM, need confirmation.

#### Records.

**CAN**: AB, MB, NB, NS (CBI), NT, ON, QC, SK **USA**: AR, AZ, CO, CT, IA, IL, IN, KS, KY, LA, MA, MD, ME, MI, MO, MT, ND, NE, NH, NJ, NM, NY, OH, OK, PA, RI, SD, TN, TX, VA, VT, WI, WV, WY [BC, ID, OR, UT, WA]

#### Note.

Lindroth (1966: 417) considered *Elaphropus nebulosus* (Chaudoir), *Elaphropus vernicatus* (Casey), and *Elaphropus congener* (Casey) as synonyms of this species. Erwin (1974a) treated *Elaphropus nebulosus* and *Elaphropus congener* as valid species and *Elaphropus vernicatus* as a synonym of *Elaphropus unionis* (Csiki).

### Elaphropus anthrax

(LeConte, 1852)

Tachys anthrax LeConte, 1852a: 192. Type locality: «San Diego [San Diego County, California]» (original citation). Holotype [by monotypy; designated lectotype by Erwin (1974a: 133)] (♂) in MCZ [# 5580].

#### Distribution.

This species ranges from southern Vancouver Island (CNC) to northern Idaho (Hatch 1953: 103), south to southern California (LeConte 1852a: 192; Fall 1901a: 44).

#### Records.

**CAN**: BC (VCI) **USA**: CA, ID, OR, WA

### Elaphropus brevis

(Casey, 1918)

Tachyura brevis Casey, 1918: 182. Type locality: «Fortress Monroe [= Hampton], Virginia» (original citation). Lectotype (♀), designated by Erwin (1974a: 133), in USNM [# 46918].

#### Distribution.

This species is known only from the type locality in southeastern Virginia.

#### Records.

**USA**: VA

#### Note.

This form was considered a synonym of *Elaphropus granarius* (Dejean) by Lindroth (1966: 414) but listed as a valid species by Erwin (1974a: 133).

### Elaphropus brunnicollis

(Motschulsky, 1862)

Tachyura brunnicollis Motschulsky, 1862b: 28. Type locality: «environs de Mobile [Mobile County, Alabama]» (original citation). Lectotype, designated by Erwin (1974a: 133), in ZMMU.
Barytachys gemellus Casey, 1884c: 71. Type locality: «Cap May [Cape May County], New Jersey» (original citation). Lectotype (♀), designated by Erwin (1974a: 133), in USNM [# 46916]. Synonymy established by Erwin (1974a: 133). Note. Hayward (1900: 204) and Lindroth (1966: 411) treated *Elaphropus gemellus* (Casey) as a synonym of *Elaphropus fuscicornis* (Chaudoir).
Tachys subpunctatus Blatchley, 1924: l64. Type locality: «near Dunedin [Pinellas County, Florida]» (original citation). Holotype [by monotypy] (♂) in PURC. Synonymy established by Erwin (1974a: 133).

#### Distribution.

This species is currently known only from the Coastal Plain ranging from New Jersey (Casey 1884c: 71, as *Barytachys gemellus*) to at least central Florida (Blatchley 1924: 164, as *Tachys subpunctatus*), west to southwestern Alabama (Motschulsky 1862b: 28).

#### Records.

**USA**: AL, FL, GA, NJ, SC, VA

### Elaphropus capax

(LeConte, 1863)

Tachys capax LeConte, 1863c: 20. Type locality: «Washington, District of Columbia» (original citation). Lectotype (♀), designated by Erwin (1974a: 137), in MCZ [# 5579].

#### Distribution.

The range of this species extends from western Vermont (Addison and Chittenden Counties, CMNH) to eastern Iowa (Hayward 1900: 209), including southernmost Ontario (Bousquet 1987a: 122), south to southeastern Louisiana (East Baton Rouge and Saint Tammany Parishes, Igor M. Sokolov pers. comm. 2009), southern Mississippi (Peter W. Messer pers. comm. 2008), and southern Florida (Peck and Thomas 1998: 18).

#### Records.

**CAN**: ON **USA**: DC, FL, GA, IA, IL, IN, LA, MA, MD, MO, MS, NJ, NY, OH, PA, TN, VA, VT

#### Note.

This form was regarded as a valid species by Hayward (1900: 209), Casey (1918: 187), Lindroth (1966: 422), and Bousquet (1987a: 122) but listed as a synonymy of *Elaphropus vivax* (LeConte) by Erwin (1974a: 137).

### Elaphropus cockerelli

(Fall, 1907)

Tachys cockerelli Fall, 1907: 218. Type locality: «Romeroville [San Miguel County], New Mexico» (original citation). Lectotype (♀), designated by Erwin (1974a: 133), in MCZ [# 23871]. Etymology. The specific name honors Theodore Dru Alison Cockerell [1866-1948], professor at the University of Colorado and accomplished naturalist. Cockerell wrote on many subjects including scale insects, fossil plants, fossil insects, biogeography, geology, and particularly wild bees, his special interest.

#### Distribution.

This species is known from northeastern New Mexico (Fall 1907: 218) and southeastern Arizona (Cochise County, USNM).

#### Records.

**USA**: AZ, NM

### Elaphropus congener

(Casey, 1918)

Tachyura congener Casey, 1918: 181. Type locality: «Austin [Travis County], Texas» (original citation for the lectotype). Lectotype (♀), designated by Erwin (1974a: 133), in USNM [# 46914].

#### Distribution.

This species has been reported from localities in South Dakota (Kirk and Balsbaugh 1975: 21), west-central Mississippi, and eastern and central Texas (Casey 1918: 181).

#### Records.

**USA**: MS, SD, TX

#### Note.

This form was considered a synonym of *Elaphropus anceps* (LeConte) by Lindroth (1966: 417) but listed as a valid species by Erwin (1974a: 133).

### Elaphropus conjugens

(Notman, 1919)

Tachys trechiformis Hayward, 1900: 216 [primary homonym of *Tachys trechiformis* Jordan, 1894]. Type locality: «Verde River [Maricopa County], Arizona» (original citation). Holotype [by monotypy] (♂) in MCZ [# 7051].
Tachys conjugens Notman, 1919b: 229. Type locality: «Rincon M[oun]t[ain]s [Pima County], Ariz[ona]» (original citation). One syntype [2 ♀ originally cited] in SIM (Hennessey 1990: 466). Synonymy established by Erwin (1974a: 133).
Tachys trechoides Csiki, 1928: 201. Replacement name for *Tachys trechiformis* Hayward, 1900.

#### Distribution.

This species is known only from southern Arizona.

#### Records.

**USA**: AZ

### Elaphropus dolosus

(LeConte, 1848)

Tachys dolosus LeConte, 1848: 470. Type locality: «Rocky Mountains» (original citation). Lectotype (♀), designated by Erwin (1974a: 133), in MCZ [# 5584].
Tachys audax LeConte, 1852a: 193. Type locality: «ad Colorado et Gilae ripas» (original citation), restricted to «Colorado River, Arizona» by Erwin (1974a: 134). Lectotype (♀), designated by Erwin (1974a: 134), in MCZ [# 5585]. Synonymy established by Erwin (1974a: 134). Note. This form has been considered a valid species by Hayward (1900: 206) and Casey (1918: 177).
Tachyura apacheana Casey, 1918: 184. Type locality: «Riverside [Pinal County], Arizona» (original citation). Lectotype (♂), designated by Erwin (1974a: 134), in USNM [# 46922]. Synonymy established by Erwin (1974a: 134).

#### Distribution.

This species ranges from southern New Hampshire (Hillsborough County, CMNH) to south-central South Dakota (Kirk and Balsbaugh 1975: 21), including southern Ontario (Lindroth 1966: 419), south to southern Texas (Casey 1918: 177, as *Tachyura audax*; Hayward 1900: 205), southwestern Alabama (Baldwin County, CMNH), and the District of Columbia (Beutenmüller 1897: 40), west along southern United States to southern California (Fall 1901a: 44, as *Tachys audax*) and the Baja California Peninsula (Horn 1894: 308, as *Tachys audax*). The records from “Alberta,” “Idaho,” “Montana,” “Wyoming” (Bousquet and Larochelle 1993: 151) and northern California (Notman 1929b: 223) are probably in error.

#### Records.

**CAN**: ON **USA**: AL, AR, AZ, CA, CO, DC, IA, IL, IN, KS, MA, MO, NE, NH, NM, NY, OH, OK, PA, SD, TN, TX, VT, WI, WV – Mexico

### Elaphropus fatuus

(Casey, 1918)

Tachyura fatua Casey, 1918: 187. Type locality: «Lake Worth [Palm Beach County], Florida» (original citation). Lectotype (♂), designated by Erwin (1974a: 134), in USNM [# 46930].

#### Distribution.

This species is known only from the type locality in southern Florida.

#### Records.

**USA**: FL

### Elaphropus ferrugineus

(Dejean, 1831)

Bembidium ferrugineum Dejean, 1831: 59. Type locality: «Amérique septentrionale» (original citation), restricted to «Iowa City [Johnson County], Iowa» by Lindroth (1966: 423). Holotype [by monotypy] apparently lost (Lindroth 1955b: 14). Note. The sole specimen of *Elaphropus ferrugineus* Dejean in Dejean’s collection is a specimen of *Elaphropus tripunctatus* (Lindroth 1966: 424) and does not agree with the original description of *Bembidion ferrugineum* as provided by Dejean.
Tachys ovipennis Chaudoir, 1868b: 215. Type locality: Amérique septentrionale (inferred from title of the paper). Lectotype, designated by Erwin (1974a: 134), in MHNP. Synonymy established by Hayward (1900: 234), confirmed by Lindroth (1966: 423). Note. It is possible that the lectotype of *Tachys ovipennis* Chaudoir is the holotype of *Bembidion ferrugineum* Dejean. Chaudoir (1868b: 215) wrote after the description of his *Tachys ovipennis* “Dejean, chose difficile à comprendre, l’avait confondu avec son *ferrugineus*, dont il est pourtant tellement distinct.”

#### Distribution.

This species ranges from “Massachusetts” (Hayward 1900: 212) and Connecticut (Krinsky and Oliver 2001: 90) to northeastern Kansas (Popenoe 1878: 79), including eastern Iowa (Lindroth 1966: 423), south to northwestern and central Texas (Barr 1964: 3), southern Alabama (Conecuh County, CMN), and the Florida Panhandle (Washington County, CNC). The records from “Colorado” (Beutenmüller 1897: 39) and “New Hampshire” (Bousquet and Larochelle 1993: 151) need confirmation.

#### Records.

**USA**: AL, AR, CT, DC, FL, GA, IA, IL, IN, KS, KY, MA, MD, MO, NC, NJ, NY, OH, OK, PA, SC, TN, TX [CO, NH]

#### Note.

Under this name, LeConte (1863b: 15) listed “*truncorum* Hald.” as synonym. I have not found any species described by that name in Haldeman’s publications.

### Elaphropus fuscicornis

(Chaudoir, 1868)

Tachys fuscicornis Chaudoir, 1868b: 214. Type locality: «Louisiane» (original citation). Lectotype (♀), designated by Erwin (1974a: 134), in MHNP.

#### Distribution.

This species seems restricted to the Coastal Plain ranging from New Jersey (Smith 1890: 79; Smith 1910: 203) to southern Florida (Leng 1915: 573), west to “Louisiana” (Chaudoir 1868b: 214) including southwestern Alabama (Löding 1945: 14).

#### Records.

**USA**: AL, FL, GA, LA, NJ

### Elaphropus granarius

(Dejean, 1831)

Bembidium granarium Dejean, 1831: 61. Type locality: «Amérique septentrionale» (original citation), restricted to «M[oun]t Washington [Coos County], N[ew] H[ampshire]» by Lindroth (1966: 414). Holotype [by monotypy] (♀) in MHNP.
Tachys occultus LeConte, 1848: 470. Type locality: «Georgia» (original citation). Lectotype (♂), designated by Erwin (1974a: 135), in MCZ [#5582]. Synonymy established by Horn (1875: 132), confirmed by Lindroth (1966: 414). Note. This form is listed as a valid species by Erwin (1974a: 135).
Barytachys glossema Casey, 1884c: 70. Type locality: «near Philadelphia [Philadelphia County], Pennsylvania» (original citation). Lectotype (♂), designated by Erwin (1974a: 134), in USNM [# 46917]. Synonymy established by Horn (1885b: 108), confirmed by Lindroth (1966: 414).

#### Distribution.

The range of this species extends from Nova Scotia (Lindroth 1966: 416) to “Nebraska” (Hayward 1900: 204), south to northern Oklahoma (French et al. 2001: 228), southeastern Louisiana (East Baton Rouge, Tangipahoa, and Saint Tammany Parishes, Igor M. Sokolov pers. comm. 2009), and northern Florida (Peck and Thomas 1998: 18). The records from southern Colorado (Elias 1987: 632) and “Minnesota” (Bousquet and Larochelle 1993: 151) need confirmation.

#### Records.

**CAN**: NB, NS, ON, QC **USA**: AL, AR, CT, DC, FL, GA, IA, IL, IN, KS, LA, MA, MD, ME, MI, MO, MS, NC, NE, NH, NJ, NY, OH, OK, PA, RI, SC, VA, VT, WI [CO, MN]

### Elaphropus incurvus

(Say, 1830)

Bembidium incurvum Say, 1830c: 26. Type locality: «N[orth] Ill[inois]» (neotype label). Neotype (♂), designated by Lindroth and Freitag (1969: 338), in MCZ [# 33060]. Note. «Indiana» was the area originally cited by Say (1830c: 26).
Tachyura parallela Casey, 1918: 182 [secondary homonym of *Tachys parallela* Motschulsky, 1850]. Type locality: «Boston Neck [Washington County], Rhode Island» (original citation). Lectotype (♀), designated by Erwin (1974a: 134), in USNM [# 46915]. Synonymy established by Lindroth (1966: 416).
Tachys rhodensis Csiki, 1928: 195. Replacement name for *Tachys parallelus* (Casey, 1918).

#### Distribution.

This species ranges from Cape Breton Island (Bousquet 1987a: 121) to the Okanagan Valley in south-central British Columbia (Lindroth 1966: 416), south to “Oregon” (Hayward 1900: 205; Hatch 1953: 104), central New Mexico (Fall and Cockerell 1907: 157), and southeastern Tennessee (Sequatchie and Bledsoe Counties, Robert L. Davidson pers. comm. 2009). Several state records listed in Bousquet and Larochelle (1993: 151) need confirmation.

#### Records.

**CAN**: BC, MB, NB, NS (CBI), ON, PE, QC, SK **USA**: CO, CT, DC, DE, IA, ID, IL, IN, KS, KY, MA, MD, ME, MI, MN, MO, MS, MT, ND, NE, NH, NJ, NM, NY, OH, OR, PA, RI, SD, TN, VT, WA, WI, WV [AR, FL, LA, NC, SC, TX]

### Elaphropus liebecki

(Hayward, 1900)

Tachys liebecki Hayward, 1900: 207. Type locality: «Texas» (original citation). Lectotype (♂), designated by Erwin (1974a: 134), in MCZ [# 7049].

#### Distribution.

This species is known only from the type series.

#### Records.

**USA**: TX

### Elaphropus monticola

(Casey, 1918)

Tachyura monticola Casey, 1918: 179. Type locality: «Jemez Springs [Sandoval County], New Mexico» (original citation). Lectotype (♂), designated by Erwin (1974a: 135), in USNM [# 46910].

#### Distribution.

This species is known only from the type locality in northwestern New Mexico.

#### Records.

**USA**: NM

### Elaphropus nebulosus

(Chaudoir, 1868)

Tachys nebulosus Chaudoir, 1868b: 214. Type locality: «Louisiane» (original citation). Lectotype (♀), designated by Erwin (1974a: 135), in MHNP.
Tachyura solita Casey, 1918: 178. Type locality: «Galveston [Galveston County], Texas» (original citation). Lectotype (♀), designated by Erwin (1974a: 135), in USNM [# 46908]. Synonymy established by Erwin (1974a: 135).
Tachyura soror Casey, 1918: 179. Type locality: «Austin [Travis County], Texas» (original citation for the lectotype). Lectotype (♀), designated by Erwin (1974a: 135), in USNM [# 46909]. Synonymy established by Erwin (1974a: 135).

#### Distribution.

This species is known from North Carolina (Brimley 1938: 118) to “Utah” (Hayward 1900: 207), south to southeastern Arizona (Cochise County, USNM), southeastern Texas (Casey 1918: 178, as *Tachuyra solita*), southwestern Mississippi (Casey 1918: 179), and northern Georgia (Fattig 1949: 18); also recorded from the state of Veracruz in Mexico and Guatemala (Bates 1882a: 141). The records from eastern Washington, eastern Oregon, southwestern Idaho (Hatch 1953: 103), “Nevada” (Bousquet and Larochelle 1993: 151), and Pennsylvania (Bates 1882a: 141) are probably in error.

#### Records.

**USA**: AZ, CO, GA, KS, LA, MS, NC, NM, SC, TX, UT – Guatemala, Mexico

#### Note.

This form was listed as a synonym of *Elaphropus anceps* (LeConte) by Lindroth (1966: 417) but regarded as a valid species by Hayward (1900: 207), Casey (1918: 179), and Erwin (1974a: 135).

### Elaphropus obesulus

(LeConte, 1852)

Tachys obesulus LeConte, 1852a: 192. Type locality: «in vallem fluminis Gila [Arizona]» (original citation). Lectotype (♀), designated by Erwin (1974a: 135), in MCZ [# 5581].

#### Distribution.

This species is known from Sonora in Mexico (USNM), southern Arizona (Pima and Santa Cruz Counties, CMNH, USNM), and “California” (Hayward 1900: 211). The record from southwestern Utah (Tanner 1928: 270) needs confirmation.

#### Records.

**USA**: AZ, CA [UT] – Mexico

### Elaphropus rapax

(LeConte, 1852)

Tachys rapax LeConte, 1852a: 192. Type locality: «ad fluminis Gilae ripas [Arizona]» (original citation). Lectotype (♂), designated by Erwin (1974a: 134), in MCZ [# 5586].

#### Distribution.

This species is known from southeastern Washington (Walla Walla County, MCZ) and northern Idaho (Hatch 1953: 103) south to southern California (Fall 1901a: 43) and central Arizona (Griffith 1900: 565; Hayward 1900: 206; Casey 1918: 177), east along the south to western Texas (Casey 1918: 177; El Paso County, MCZ).

#### Records.

**USA**: AZ, CA, ID, NM, NV, OR, TX, WA

#### Note.

This form has been listed in synonymy with *Elaphropus dolosus* (LeConte) by Erwin (1974a: 134) but treated as a valid species by Hayward (1900: 205), Casey (1918: 177), and Lindroth (1966: 419). Upon examination of the type specimens of both taxa, I agree with the last-mentioned authors.

### Elaphropus renoicus

(Casey, 1918)

Tachyura renoica Casey, 1918: 183. Type locality: «Reno [Washoe County], Nevada» (original citation for the lectotype). Lectotype (♀), designated by Erwin (1974a: 135), in USNM [# 46920].

#### Distribution.

This species is known from east-central Oregon (Grant County, James R. LaBonte pers. comm. 1992), Humboldt County in northwestern California, and Washoe County in northwestern Nevada (Casey 1918: 183).

#### Records.

**USA**: CA, NV, OR

### Elaphropus rubricauda

(Casey, 1918)

Tachyura rubricauda Casey, 1918: 186. Type locality: «Galveston [Galveston County], Texas» (original citation for the lectotype). Lectotype (♀), designated by Erwin (1974a: 135), in USNM [# 46928].
Tachyura gaudens Casey, 1918: 188. Type locality: «Valley of the Rio Grande from Brownsville to El Paso» (original citation). Lectotype (♂), designated by Erwin (1974a: 136), in USNM [# 46933]. Synonymy established by Erwin (1974a: 135).
Tachyura fracta Casey, 1918: 188. Type locality: «Waco [McLennan County], Texas» (original citation for the lectotype). Lectotype (♂), designated by Erwin (1974a: 136), in USNM [# 46932]. Synonymy established by Erwin (1974a: 135).

#### Distribution.

This species is known from “Indiana” to southeastern Iowa (Casey 1918: 186), south to the Rio Grande in southern Texas (Casey 1918: 188, as *Tachyura gaudens*), central Louisiana, and west-central Mississippi (Casey 1918: 186).

#### Records.

**USA**: IA, IN, LA, MO, MS, TX

### Elaphropus saturatus

(Casey, 1918)

Tachyura saturata Casey, 1918: 187. Type locality: «Asheville [Buncombe County], North Carolina» (original citation for the lectotype). Lectotype (♀), designated by Erwin (1974a: 136), in USNM [# 46931].

#### Distribution.

The range of this species extends from Nova Scotia (Lindroth 1966: 422) to southwestern Wisconsin (Messer 2010: 37), south to central Louisiana (Grant Parish, CMNH), southwestern Alabama (Clarke County, CMNH), and northern Georgia (Fattig 1949: 19). The records from “Minnesota” and “Kansas” (Bousquet and Larochelle 1993: 152) need confirmation.

#### Records.

**CAN**: NB, NS, ON, QC **USA**: AL, AR, CT, GA, IA, LA, MD, ME, NC, NH, NY, OH, PA, TN, VA, VT, WI, WV [KS, MN]

### Elaphropus sectator

(Casey, 1918)

Tachyura sectator Casey, 1918: 180. Type locality: «Provo [Utah County], Utah» (original citation). Lectotype (♂), designated by Erwin (1974a: 136), in USNM [# 46911].
Tachyura sectator sospes Casey, 1918: 180. Type locality: «Arizona» (original citation). Lectotype (♀), designated by Erwin (1974a: 136), in USNM [# 46912]. Synonymy established by Erwin (1974a: 136).

#### Distribution.

This species is known from “Arizona” and northern Utah (Casey 1918: 180).

#### Records.

**USA**: AZ, UT

### Elaphropus sedulus

(Casey, 1918)

Tachyura sedula Casey, 1918: 184. Type locality: «S[an]ta Cruz [Santa Cruz County], California» (original citation for the lectotype). Lectotype (♀), designated by Erwin (1974a: 136), in USNM [# 46923].
Tachyura profuga Casey, 1918: 185. Type locality: «Reno [Washoe County], Nevada» (original citation). Lectotype (♀), designated by Erwin (1974a: 136), in USNM [# 46924]. Synonymy established by Erwin (1974a: 136).

#### Distribution.

This species has been reported from northwestern Nevada (Casey 1918: 185, as *Tachyura profuga*), Santa Cruz County in western California (Casey 1918: 184-185; Notman 1929b: 223; Dajoz 2007: 19), and southwestern Oregon (Westcott et al. 2006: 8).

#### Records.

**USA**: CA, NV, OR

### Elaphropus tahoensis

(Casey, 1918)

Tachyura tahoensis Casey, 1918: 183. Type locality: «Lake Tahoe [Placer County], California» (original citation). Holotype [by monotypy] (♀) in USNM [# 46921].

#### Distribution.

This species is known only from the type locality in the Sierra Nevada.

#### Records.

**USA**: CA

### Elaphropus tripunctatus

(Say, 1830)

Bembidium tripunctatum Say, 1830c: 26. Type locality: «N[ew] J[ersey]» (neotype label). Neotype (♂), designated by Lindroth and Freitag (1969: 338), in MCZ [# 33059]. Note. «Indiana» was the area originally cited by Say (1830c: 26).
Tachyura laredoana Casey, 1918: 189. Type locality: «Laredo [Webb County], Texas» (original citation). Holotype [by monotypy] (♀) in USNM [# 46934]. Synonymy established by Erwin (1974a: 136).
Tachyura serva Casey, 1918: 189. Type locality: «Bluff Point, Lake Champlain, New York» (original citation). Holotype [by monotypy] (♀) in USNM [# 46935]. Synonymy established by Erwin (1974a: 136).
Tachyura barnesi Stehr, 1947: 284. Type locality: «along the Muskingum River in Muskingum Township, Washington County, Ohio» (original citation). Holotype (♀) probably in OSUO (Erwin 1974a: 136). Synonymy established by Erwin (1974a: 136).

#### Distribution.

This species ranges from Cape Breton Island (Lindroth 1966: 423) to “Iowa” (Hayward 1900: 210), south to southern Texas (Casey 1918: 189, as *Tachyura laredoana*) and northern Georgia (Fattig 1949: 19). The records from “South Dakota” and “Wyoming” (Bousquet and Larochelle 1993: 152) are probably in error.

#### Records.

**CAN**: NB, NS (CBI), ON, QC **USA**: AL, AR, DC, DE, GA, IA, IL, IN, KS, KY, LA, MA, MD, ME, MI, MO, NC, NH, NJ, NY, OH, OK, PA, TN, TX, VA, VT, WV

### Elaphropus vernicatus

(Casey, 1918)

Tachyura vernicata Casey, 1918: 181. Type locality: «probably Indiana» (original citation). Lectotype (♀), designated by Erwin (1974a: 136), in USNM [# 46913]. Note. This name was listed as synonym of *Elaphropus anceps* (LeConte) by Lindroth (1966: 417).
Tachyura laetifica Casey, 1918: 183 [secondary homonym of *Tachys laetificus* Bates, 1873]. Type locality: «S[ain]t Louis, Missouri» (original citation for the lectotype). Lectotype (♀), designated by Erwin (1974a: 136), in USNM [# 46919]. Synonymy established by Erwin (1974a: 136).
Tachys unionis Csiki, 1928: 202. Replacement name for *Tachys laetificus* (Casey, 1918).

#### Distribution.

This species is known from Nova Scotia (Majka et al. 2007: 8) to southern Saskatchewan (Ronald R. Hooper pers. comm. 2008), south to Arkansas (Franklin County, CNC) and North Carolina (Casey 1918: 183, as *Tachyura laetifica*).

#### Records.

**CAN**: MB, NB, NS, ON, QC, SK **USA**: AR, CT, DC, IA, IL, IN, KY, MA, MD, ME, MO, NC, NH, NJ, NY, OH, PA, RI, TN, VA, VT, WV

### Elaphropus vivax

(LeConte, 1848)

Tachys vivax LeConte, 1848: 468. Type locality: «ubique usque ad Rocky Mountains» (original citation), restricted to «N[orth] Carol[ina]» by Lindroth (1966: 422). Lectotype (♂), designated by Erwin (1974a: 137), in MCZ [# 5578].
Tachys mendax LeConte, 1848: 469. Type locality: «NovEboraci [= New York] et ad Rocky Mountains» (original citation), restricted incorrectly to «New England» by Erwin (1974a: 137). Lectotype (♀), designated by Erwin (1974a: 137), in MCZ [# 5589]. Synonymy established by Erwin (1974a: 137). Note. Hayward (1900: 233) and Casey (1918: 185) listed this name in synonymy with *Elaphropus xanthopus* (Dejean).

#### Distribution.

The range of this species extends from Nova Scotia (Majka and Vickery 2008) to “Wisconsin” (Rauterberg 1885: 23), including southern Quebec and southern Ontario (Lindroth 1966: 423), south to northwestern Texas (Nolan County, CMNH), southwestern Alabama (Clarke County, CMNH), and northern Florida (Suwanee County, CMNH). The record from southern Arizona (Snow 1907: 142) is probably in error.

#### Records.

**CAN**: NS, ON, QC **USA**: AL, AR, CT, DC, DE, FL, GA, IA, IL, IN, KS, KY, LA, MA, MD, ME, MI, MO, MS, NC, NH, NJ, NY, OH, OK, PA, SC, TN, TX, VA, VT, WI, WV

### Elaphropus xanthopus

(Dejean, 1831)

Bembidium xanthopus Dejean, 1831: 60. Type locality: «Amérique septentrionale» (original citation), restricted to «Long Isl[and], N[ew] Y[ork]» by Lindroth (1966: 420). Lectotype (♂), designated by Erwin (1974a: 137), in MHNP.
Tachyura xanthopus laxipennis Casey, 1918: 185. Type locality: «Boston Neck [Washington County], Rhode Island» (original citation). Holotype [by monotypy] (♀) in USNM [# 46925]. Synonymy established by Erwin (1974a: 137).
Tachyura xanthopus famelica Casey, 1918: 185. Type locality: «Boston Neck [Washington County], Rhode Island» (original citation). Holotype [by monotypy] (♂) in USNM [# 46926]. Synonymy established by Erwin (1974a: 137).
Tachyura ancilla Casey, 1918: 186. Type locality: «Southern Pines [Moore County], North Carolina» (original citation). Lectotype (♀), designated by Erwin (1974a: 137), in USNM [# 46929]. Synonymy established by Erwin (1974a: 137).
Tachyura levipes Casey, 1918: 186. Type locality: «Long Island, New York» (original citation for the lectotype). Lectotype (♂), designated by Erwin (1974a: 134), in USNM [# 46927]. Synonymy established by Lindroth (1966: 420). Note. This taxon is listed as a valid species by Erwin (1974a: 134).

#### Distribution.

This species ranges from Nova Scotia (Lindroth 1966: 422) to western Kansas (Trego County, CNC), south to southeastern Texas (Wickham 1897: 104; Cameron County, CNC) and central Florida (Peck and Thomas 1998: 18). The species was also recorded from the Bahamas (Darlington 1953: 5) and Cuba (Darlington 1934: 78).

#### Records.

**CAN**: NS, ON, QC **USA**: AL, AR, CT, DC, DE, FL, GA, IL, IN, KS, KY, LA, MA, MD, ME, MI, MO, MS, NC, NH, NJ, NY, OH, OK, PA, RI, SC, TN, TX, VA, VT, WI, WV – Bahamas, Cuba

### Subgenus Tachyura

Motschulsky, 1862

Tachyura Motschulsky, 1862b: 27. Type species: *Elaphrus quadrisignatus* Duftschmid, 1812 designated by Jeannel (1941b: 434). Etymology. Uncertain, possibly from the generic name *Tachys* [*q.v*.] and the Greek *oura* (tail) [feminine]. Note. The first valid type species designation for *Tachyura* Motschulsky, 1862 is that of Schatzmayr and Koch (1934: 21) who designated *Bembidion focki* Hummel, 1822 (= *Trechus bisulcatus* Nicolai, 1822). Acceptance of that species as type species would make *Tachyura* a senior subjective synonym of *Porotachys* Netolitzky, 1914. I suggest conserving the type species designation of Jeannel to preserve stability (see Bousquet 2002b: 49).

#### Diversity.

According to Sciaky and Vigna Taglianti (2003: 90), members of *Tachyura* occur in the Old World though a few species extends into the Australian Region in New Guinea. The number of species is difficult to assess. Kopecký (2003: 278-280) listed 47 species for the Palaearctic Region. One species is adventive in North America.

#### Identification.

The species was included in Lindroth’s (1966: 410) key to the species of *Tachys*.

### Elaphropus parvulus

(Dejean, 1831)

Bembidium parvulum Dejean, 1831: 57. Type locality: «Espagne; midi de la France; Dalmatie» (original citation), restricted to «Spain» by Erwin (1974a: 135). Lectotype (♂), designated by Erwin (1974a: 135), in MHNP.
Bembidium pulicarium Dejean, 1831: 62. Type locality: «Saxe [= Saxony, Germany]» (original citation). Syntype(s) [4 originally cited] probably in MHNP. Synonymy established by Jacquelin du Val (1852: 201).

#### Distribution.

This European species is adventive in North America where it is known from southwestern British Columbia, Washington, Oregon, and Idaho [see LaBonte and Nelson 1998: Fig. 1]. The first inventoried specimen reported from this continent was collected in Seattle in 1940 (Hatch 1950: 105).

#### Records.

**CAN**: BC **USA**: ID, OR, WA – **Adventive**

### Genus Micratopus

Casey, 1914

Micratopus Casey, 1914: 42. Type species: *Micratopus fusciceps* Casey, 1914 (= *Blemus aenescens* LeConte, 1848) by monotypy. Etymology. From the Greek *micros* (small, little) and *topos* (place), possibly alluding to the small size (“body very small”) of the adult [masculine].

#### Diversity.

Western Hemisphere, with five species in the Nearctic (one species) and Neotropical (four species) Regions, including the West Indies. Erwin (1974a: 125) stated that he was aware of 35 undescribed species in this genus.

#### Identification.

Barr (1971b) redescribed the North American species and illustrated its male genitalia.

#### Taxonomic Note.

This taxon is possibly the sister-group to *Lymnastis* Motschulsky (Erwin 1974a: 125), a genus of about 40 species in the Eastern Hemisphere with one endemic species, *Lymnastis americanus* Darlington, in Cuba. According to Robert L. Davidson (pers. comm. 2008), there is at least two species of *Micratopus*, besides *Micratopus aenescens*, that occur in southern United States. One of them, found in Florida, is probably the Cuban species.

### Micratopus aenescens

(LeConte, 1848)

Blemus aenescens LeConte, 1848: 473. Type locality: «Georgia» (original citation). Holotype [by monotypy; designated lectotype by Erwin (1974a: 138)] (♀) in MCZ [# 5577].
Micratopus fusciceps Casey, 1914: 43. Type locality: «Vicksburg [Warren County], Mississippi» (original citation). Lectotype, designated by Erwin (1974a: 138), in USNM [# 46973]. Synonymy established by Barr (1971b: 34).

#### Distribution.

This species occurs from Connecticut (Krinsky and Oliver 2001: 96) to “Indiana” (Schrock 1985: 351), south to southeastern Texas (San Patricio County, UASM) and southern Florida including the Keys (Peck and Thomas 1998: 18), west along the southwest to southwestern California (Riverside County, CAS); one specimen, possibly mislabeled, was seen from western Oregon (Benton County, CNC). The record from “Illinois” (Bousquet and Larochelle 1993: 152) needs confirmation.

#### Records.

**USA**: AL, AR, AZ, CA, CT, FL, GA, IN, KY, LA, MD, MI, MS, NC, NM, OH, OK, PA, SC, TN, TX, VA [IL, OR]

### Genus Pericompsus

LeConte, 1852

Pericompsus LeConte, 1852a: 191. Type species: *Bembidium ephippiatum* Say, 1830 designated by LeConte (1859e: 553). Etymology. From the Greek *peri* (very) and *compsos* (elegant), probably alluding to the nice color of the body of adults of the three species LeConte had before him [masculine].

#### Diversity.

New World, with about 70 species arrayed in three subgenera: *Eidocompsus* Erwin (13 Neotropical species), *Pericompsus* s.str. (about 45 species), and *Upocompsus* Erwin (nine Australian species).

#### Identification.

Erwin (1974b) revised the species and provided a key for their identification.

### Subgenus Pericompsus

LeConte, 1852

Pericompsus LeConte, 1852a: 191. Type species: *Bembidium ephippiatum* Say, 1830 designated by LeConte (1859e: 553).
Tachysops Casey, 1918: 171. Type species: *Bembidium ephippiatum* Say, 1830 designated by Jeannel (1941b: 423). Etymology. From the generic name *Tachys* [*q.v*.] and the Greek suffix -*ops* (having the appearance of) [masculine].
Tachysalia Casey, 1918: 173. Type species: *Pericompsus laetulus* LeConte, 1852 by original designation. Synonymy established by Jeannel (1941b: 423).
Leiotachys Jeannel, 1962: 611, 614. Type species: *Bembidium circuliforme* Solier, 1849 by original designation. Synonymy established by Erwin (1974b: 5). Etymology. From the Greek *leios* (smooth) and the generic name *Tachys* [*q.v*.] [masculine].
Leptotachys Jeannel, 1962: 611, 615. Type species: *Leptotachys pallidus* Jeannel, 1962 (= *Tachys univittatus* Jensen-Haarup, 1910) by original designation. Synonymy established by Reichardt (1977: 399). Etymology. From the Greek *leptos* (thin, slender) and the generic name *Tachys* [*q.v*.] [masculine].

#### Diversity.

Western Hemisphere, with about 45 species in the Nearctic (three species) and Neotropical (about 45 species) Regions.

### [ephippiatus group]

### Pericompsus ephippiatus

(Say, 1830)

Bembidium ephippiatum Say, 1830c: 25. Type locality: «Ind[iana]» (neotype label). Neotype (♂), designated by Lindroth and Freitag (1969: 338), in MCZ [# 33061]. Note. «Indiana» was the area originally cited by Say (1830c: 25).

#### Distribution.

This species ranges from southwestern New Hampshire (Choate 1977: 115) to northern Kansas, south to Honduras and central Florida [see Erwin 1974b: Fig. 73]. The record from southern Wisconsin (Rauterberg 1885: 23) needs confirmation.

#### Records.

**USA**: AL, AR, CT, DC, FL, GA, IA, IL, IN, KS, KY, LA, MA, MD, MO, MS, NC, NH, NJ, NY, OH, OK, PA, SC, SD, TN, TX, VA, VT, WV [WI] – Honduras, Mexico

### Pericompsus laetulus

LeConte, 1852

Pericompsus laetulus LeConte, 1852a: 192. Type locality: «in valle fluminis Gila, circa Pimas [Arizona]» (original citation). Lectotype (♀), designated by Erwin (1974b: 50), in MCZ [# 5588].

#### Distribution.

This species is known from central Oklahoma (Grady County, CMNH), southwestern New Mexico, and southeastern Arizona, south to Durango in Mexico. The species is apparently absent from the desert region of northern Mexico [see Erwin 1974b: Fig. 75].

#### Records.

**USA**: AZ, NM, OK – Mexico

### [sellatus group]

### Pericompsus sellatus

LeConte, 1852

Pericompsus sellatus LeConte, 1852a: 191. Type locality: «ad flumen Colorado» (original citation), herein restricted to Yuma, Yuma County, Arizona (see Erwin 1974b: 79). Lectotype (♀), designated by Erwin (1974b: 78), in MCZ [# 5587].

#### Distribution.

This species is known only from “California” and southern Arizona [see Erwin 1974b: Fig. 134].

#### Records.

**USA**: AZ, CA

### Genus Porotachys

Netolitzky, 1914

Porotachys Netolitzky, 1914: 174. Type species: *Trechus bisulcatus* Nicolai, 1822 by monotypy. Etymology. From the Greek *poros* (hole) and the generic name *Tachys* [*q.v*.], alluding to the presence of the pair of labial pits on the mentum (“*mentum biporosum*”) of the adult [masculine].
Macrotachys Uéno, 1953: 39, 42. Type species: *Tachys recurvicollis* Andrewes, 1925 by monotypy. Synonymy established by Shilenkov (2002: 35). Etymology. From the Greek *macros* (long) and the generic name *Tachys* [*q.v*.] [masculine].

#### Diversity.

Five species in the Palaearctic (three species) and Oriental (two species) Regions. Among the Palaearctic species, one is endemic to Japan (*Porotachys recurvicollis* Andrewes) and one to Turkey (*Porotachys ottomanus* Schweiger). The three species from the Sinai (*Porotachys efflatouani*, *Porotachys shahinei*, and *Porotachys zulficari*, all described by Schatzmayr and Koch) included in this genus by Kopecký (2003: 274-275) belong to *Sphaerotachys* (see Sciaky and Vigna Taglianti 2003: 93). One species is adventive in North America.

#### Identification.

The species found in North America was covered in Lindroth’s (1966: 424) monograph.

### Porotachys bisulcatus

(Nicolai, 1822)

Trechus bisulcatus Nicolai, 1822 [10 September]: 26. Type locality: Halle, Germany (inferred from title of the book). Syntype(s) probably lost (Lindroth 1966: 424).
Bembidion fockii Hummel, 1822 [November]: 27. Type locality: «insula Pharmacopolarum» (original citation). Holotype [by monotypy] location unknown. Synonymy established by Gaubil (1849: 265). Note. Despite extensive search, I have been unable to find any precision about the type locality.
Tachys frontalis Hayward, 1900: 212. Type locality: «Peekskill [Westchester County], N[ew] Y[ork]» (lectotype label). Lectotype (♂), designated by Erwin (1974a: 144), in MCZ [# 7050]. Synonymy established by Lindroth (1966: 424).

#### Distribution.

This Palaearctic species is adventive in North America where it ranges from Nova Scotia (Majka et al. 2006: 605) to eastern North Dakota (Cass County, Donald P. Schwert pers. comm. 1989), south along the east coast to New Jersey (Smith 1910: 203, as *Tachys frontalis*); also recorded from Missouri (Anonymous 2007). The species is also known from the Vancouver area in southwestern British Columbia (CNC), western Washington (Nelson 1987: 394), and northern Oregon (Westcott et al. 2006: 8). The first inventoried specimen collected on this continent was found in the east prior to 1900 (Hayward 1900: 212, as *Tachys frontalis*).

#### Records.

**CAN**: BC, NB, NS, ON, QC **USA**: CT, MA, ME, MI, MN, MO, ND, NH, NJ, NY, OH, OR, PA, RI, VT, WA, WI – **Adventive**

### Genus Polyderis

Motschulsky, 1862

Polyderis Motschulsky, 1862b: 27. Type species: *Tachys brevicornis* Chaudoir, 1846 designated by Jeannel (1941b: 424). Etymology. Possibly from the Greek *polys* (many) and *deris* (fight) [feminine].
Microtachys Casey, 1918: 210. Type species: *Bembidium laevum* Say, 1823 designated by Jeannel (1941b: 424). Synonymy established by Antoine (1955: 107). Etymology. From the Greek *micros* (small, little) and the generic name *Tachys* [*q.v*.] [masculine].
Brachytachys Basilewsky, 1953a: 51. Type species: *Brachytachys curtulus* Basilewsky, 1953 by original designation. Synonymy established by Bruneau de Miré (1964: 71). Etymology. From the Greek *brachys* (short) and the generic name *Tachys* [*q.v*.] [masculine].
Neotachys Kult, 1961: 2. Type species: *Bembidium algiricum* Lucas, 1846 by original designation. Synonymy established by Lindroth (1966: 424). Etymology. From the Greek *neo* (new) and the generic name *Tachys* [*q.v*.] [masculine].
Polyderidius Jeannel, 1962: 611. Type species: *Polyderidius rapoporti* Jeannel, 1962 by original designation. Synonymy established by Erwin (1974a: 143).

#### Diversity.

Worldwide, with about 40 species in the Nearctic (three species), Neotropical (ten species), Australian (nine species), Oriental (eight species), Palaearctic (nine species), and Afrotropical (ten species) Regions. One species is also known from the Hawaiian Islands.

#### Identification.

Lindroth (1966: 424-427) treated two (*Polyderis laeva* and *Polyderis rufotestacea*) of the three species found in North America.

#### Taxonomic Note.

This taxon is considered a subgenus of *Tachys* by some authors (e.g., Lorenz 2005: 211).

### Polyderis diaphana

(Casey, 1918)

Tachys diaphanus Casey, 1918: 214. Type locality: «Austin [Travis County], Texas» (original citation). Lectotype (♂), designated by Erwin (1974a: 144), in USNM [# 46965].

#### Distribution.

This species is known only from the type locality in central Texas.

#### Records.

**USA**: TX

### Polyderis laeva

(Say, 1823)

Bembidium laevum Say, 1823a: 88. Type locality: «Arlington [Middlesex County], Mass[achusetts]» (neotype label). Neotype (♀), designated by Lindroth and Freitag (1969: 339), in MCZ [# 33057]. Note. Because Say (1823a: 88) used the epithet *laevum* with the generic name *Bembidium*, the specific name derives from the Latin adjective *laevus*, -*a*, -*um* (left, clumsy [figurative]), not from the Latin adjective *laevis*, -*e* (smooth). Therefore, used with the feminine generic name *Polyderis*, the specific name is *laeva*, not *laevis* as used by modern authors.
Bembidium troglodytes Dejean, 1831: 44. Type locality: «Amérique septentrionale» (original citation). Holotype [by monotypy] in MHNP (Lindroth 1955b: 14). Synonymy established by LeConte (1848: 472), confirmed by Lindroth (1955b: 14).
Tachys congestus Casey, 1918: 212. Type locality: «Norfolk, Virginia» (original citation). Lectotype (♀), designated by Erwin (1974a: 144), in USNM [# 46962]. Synonymy established by Erwin (1974a: 144).
Tachys unistriatus Casey, 1918: 212 [primary homonym of *Tachys unistriatus* Putzeys, 1876]. Type locality: «Pennsylvania» (original citation). Lectotype (♂), designated by Erwin (1974a: 144), in USNM [# 46963]. Synonymy established by Erwin (1974a: 144).
Tachys rectus Casey, 1918: 213. Type locality: «Alexandria [Rapides Parish], Louisiana» (original citation). Lectotype (♀), designated by Erwin (1974a: 144), in USNM [# 46961]. Synonymy established by Erwin (1974a: 144).
Tachys flumenalis Casey, 1918: 213. Type locality: «Vicksburg [Warren County], Mississippi» (original citation). Lectotype (♀), designated by Erwin (1974a: 144), in USNM [# 46964]. Synonymy established by Erwin (1974a: 144).
Tachys unistriolatus Csiki, 1928: 202. Replacement name for *Tachys unistriatus* Casey, 1918.

#### Distribution.

This species ranges from Nova Scotia (Bousquet 1987a: 123) to southwestern North Dakota (Smith et al. 1979: 92), south to southeastern Texas (Casey 1918: 213) and southern Florida including the Keys (Peck and Thomas 1998: 18).

#### Records.

**CAN**: NB, NS, ON, QC **USA**: AR, CT, DC, FL, GA, IA, IL, IN, KS, KY, LA, MA, MD, ME, MI, MO, MS, NC, ND, NE, NH, NJ, NY, OH, OK, PA, RI, SC, SD, TN, TX, VA, VT, WI

### Polyderis rufotestacea

(Hayward, 1900)

Tachys rufotestaceus Hayward, 1900: 217. Type locality: «Pom[ona] [Los Angeles County], Cal[ifornia]» (lectotype label). Lectotype (♂), designated by Erwin (1974a: 144), in MCZ [# 7052].

#### Distribution.

This species is known from central Idaho (Custer County, Ken Karns pers. comm. 2009), southeastern Oregon (Malheur County, James R. LaBonte pers. comm. 1992), California, from Lake County (CAS) to Riverside County (CAS) east to Mono and Inyo Counties (CAS; Dajoz 2007: 18), southern Arizona (Cochise and Maricopa Counties, CMNH; Hayward 1900: 217), southern Texas (Zapata County, CMNH; Casey 1918: 211), northwestern Arkansas (Newton County, Peter W. Messer pers. comm. 2008), and eastern South Dakota (Kirk and Balsbaugh 1975: 21; French et al. 2004: 557). The record from Aklavik in northern Northwest Territories (Lindroth 1966: 427) is quite obviously based on a mislabeled specimen.

#### Records.

**USA**: AR, AZ, CA, ID, OR, SD, TX

### Genus Tachys

Dejean, 1821

Tachys Dejean, 1821: 16. Type species: *Tachys scutellaris* Stephens, 1828 designated by Hope (1838: 61) (see ICZN 1990). Etymology. From the Greek *tachys* (swift, quick, fast), probably alluding to the quickness of the adults in the field [masculine]. The name was proposed by Franz Anton Ziegler and made available by Dejean.

#### Diversity.

Worldwide, with about 180 species (Lorenz 2005: 212-214, as *Paratachys* and *Tachys*) arrayed in two subgenera, both represented in North America.

#### Nomenclatural Note.

The first use of the name *Tachys* is by Schönherr (1806: 221) who included 23 species-group taxa under it. Ádám (1996: 11) selected *Carabus quadriguttatus* Fabricius, 1775 (= *Cicindela quadrimaculata* Linnaeus, 1760), one of the species originally included, as type species of *Tachys* Schönherr, 1806. This action implies that *Tachys* Dejean, 1821 is a junior homonym of *Tachys* Schönherr, 1806 which is a junior objective synonym of *Bembidion* Latreille, 1802. Obviously, to preserve nomenclatural stability, the name *Tachys* Schönherr, 1806 should be rejected.

#### Taxonomic Note.

Shilenkov (2002) treated *Tachys* and *Paratachys* as distinct genera, pointing out four major structural differences between the two taxa. Sciaky and Vigna Taglianti (2003) took the same approach. In view of the large concept used here for the genus *Elaphropus*, I believe it is more appropriate to retain the two taxa as subgenera of *Tachys*, following in this sense Kopecký (2003: 275-277) and Lorenz (2005: 212-214). Sciaky and Vigna Taglianti (2003: 82) suggested that *Polyderis* is closely related to *Tachys* and *Paratachys* based on the presence of labial pits.

### Subgenus Tachys

Dejean, 1821

Tachys Dejean, 1821: 16. Type species: *Tachys scutellaris* Stephens, 1828 designated by Hope (1838: 61) (see ICZN 1990).
Tachymantes Gistel, 1856: 359. Type species: *Tachys scutellaris* Stephens, 1828 designated by Bousquet (2002b: 49).
Isotachys Casey, 1918: 204. Type species: *Tachys vittiger* LeConte, 1852 designated by Lindroth (1966: 427). Synonymy established by Ball (1960b: 119). Etymology. From the Greek *isos* (equal) and the generic name *Tachys* [*q.v*.] [masculine].

#### Diversity.

About 35 species in the Nearctic (11 species), Neotropical (seven species), Australian (one non-endemic species), Oriental (five species), Palaearctic (12 species, none in the Far East), and Afrotropical (seven species, three shared with Eurasia) Regions. One species, *Tachys oahuensis* Blackburn, is endemic to the Hawaiian Islands.

#### Identification.

Lindroth (1966: 427-431) treated five (one of them, *Tachys corax*, in the key only) of the species found in North America and Hayward (1900: 218-224) eight species. A taxonomic revision of the species of this subgenus is needed.

### Tachys bradycellinus

Hayward, 1900

Tachys bradycellinus Hayward, 1900: 224. Type locality: «Louisiana» (original citation). Holotype [by monotypy] (♂) in MCZ [# 7053].

#### Distribution.

This species has been reported from a few islands in the West Indies (Peck and Thomas 1998: 18; Steiner 2008: 131), “Louisiana” (Hayward 1900: 224), and southeastern Texas (Casey 1918: 208); it is also known from southern Mississippi (Hancock and Jackson Counties, Drew A. Hildebrandt pers. comm. 2009) and Grady County in central Oklahoma (Robert L. Davidson pers. comm. 2012). The record from “Florida” (Bousquet and Larochelle 1993: 153) needs confirmation.

#### Records.

**USA**: LA, MS, OK, TX [FL] – Cuba, Hispaniola, Jamaica, Navassa

### Tachys bryanti

Lindroth, 1966

Tachys bryanti Lindroth, 1966: 430. Type locality: «Edmonton, Al[ber]ta» (original citation). Holotype (♂) in CAS [# 10007]. Etymology. The species was named after Owen Bryant [1882-1958], president of the Calgary-based Bryant Oil Company and insect collector in his spare time. Bryant left his entire estate, including his insect collection, to the California Academy of Sciences.

#### Distribution.

This species is still known only from the holotype.

#### Records.

**CAN**: AB

### Tachys corax

LeConte, 1852

Tachys corax LeConte, 1852a: 194. Type locality: «ad flumen Novum [= New River, Imperial County], desertorum Colorado [California]» (original citation). Holotype [by monotypy] (♀) in MCZ [# 5572].
Tachys funebris Casey, 1918: 205. Type locality: «Provo [Utah County], Utah» (original citation). Lectotype (♀), designated by Erwin (1974a: 147), in USNM [# 46954]. Synonymy established by Erwin (1974a: 147).
Tachys esurialis Casey, 1918: 209. Type locality: «San Diego [San Diego County], California» (original citation). Lectotype (♀), designated by Erwin (1974a: 147), in USNM [# 46953]. Synonymy established by Erwin (1974a: 147).

#### Distribution.

This species ranges from eastern Washington (Hatch 1953: 104) to “Montana” (Hayward 1900: 223), south to western Texas (Dajoz 2007: 23), southeastern New Mexico (Chaves County, CNC), and the Baja California Peninsula (Horn 1894: 308). The records from “Nebraska” and “Texas” (Hayward 1900: 223) probably refer to *Tachys halophilus* Lindroth.

#### Records.

**USA**: AZ, CA, CO, MT, NM, NV, OR, TX, UT, WA – Mexico

### Tachys halophilus

Lindroth, 1966

Tachys halophilus Lindroth, 1966: 428. Type locality: «Woodside, N[orth]W[est] Portage-la-Prairie, Manit[oba]» (original citation). Holotype (♂) in CNC [# 9233].

#### Distribution.

This species is known from the southern parts of the Prairie Provinces (Lindroth 1966: 429; Bousquet 1987a: 123) and eastern North Dakota (Grand Forks County, UASM, USNM). The records of *Tachys corax* from “Nebraska” and “Texas” (Hayward 1900: 223) probably refer to this species.

#### Records.

**CAN**: AB, MB, SK **USA**: ND [NE, TX]

### Tachys litoralis

Casey, 1884

Tachys litoralis Casey, 1884b: 15. Type locality: «Atlantic City [Atlantic County], New Jersey» (original citation). Holotype [by monotypy] (♀) in USNM [# 46955]. Note. Hayward (1900: 234) listed this name in synonymy with *Tachys pallidus* Chaudoir.
Tachys occultator Casey, 1884c: 69. Type locality: «Cap May [Cape May County], New Jersey» (original citation). Holotype [by monotypy] (♀) in MCZ [# 5576]. Synonymy established by Erwin (1974a: 148).
Tachys omissus Casey, 1918: 206. Type locality: «Fortress Monroe [= Hampton], Virginia» (original citation). Lectotype (♀), designated by Erwin (1974a: 148), in USNM [# 46956]. Synonymy established by Erwin (1974a: 148).
Tachys luridicollis Casey, 1918: 207. Type locality: «Galveston [Galveston County], Texas» (original citation). Lectotype (♂), designated by Erwin (1974a: 148), in USNM [# 46957]. Synonymy established by Erwin (1974a: 148).
Tachys torrescans Casey, 1918: 207. Type locality: «Pass Christian [Harrison County], Mississippi» (original citation). Lectotype (♀), designated by Erwin (1974a: 148), in USNM [# 46958]. Synonymy established by Erwin (1974a: 148).

#### Distribution.

This species is found along the Coastal Plain from Long Island, New York (Cooper 1935: 144) to southern Florida, including the Keys (Peck and Thomas 1998: 18), west to southeastern Texas (Casey 1918: 207, as *Tachys luridicollis*); also known from some islands in the West Indies (Darlington 1934: 78; Darlington 1941a: 11, as *Tachys occultator* Casey).

#### Records.

**USA**: AL, DE, FL, GA, LA, MS, NJ, NY, SC, TX, VA – Bahamas, Cuba, Jamaica

### Tachys misellus

LaFerté-Sénectère, 1841

Tachys misellus LaFerté-Sénectère, 1841a: 48. Type locality: Texas (inferred from title of the paper). Lectotype (♂), designated by Erwin (1974a: 148), in MHNP.
Tachys lymnaeoides Bates, 1882a: 139. Type locality: «Champerico, Guatemala» (original citation). Lectotype (♂), designated by Erwin (1974a: 148), in BMNH. Synonymy established by Erwin (1974a: 148).

#### Distribution.

This species has been reported from southwestern Alabama (Löding 1945: 14), “Texas” (LaFerté-Sénectère 1841a: 48), and Guatemala (Bates 1882a: 139, as *Tachys lymnaeoides*). The record from “Arizona” (Bousquet and Larochelle 1993: 154) needs confirmation.

#### Records.

**USA**: AL, TX [AZ] – Guatemala

### Tachys mordax

LeConte, 1852

Tachys mordax LeConte, 1852a: 193. Type locality: «ad Colorado [River]» (original citation). Holotype [by monotypy; designated lectotype by Erwin (1974a: 148)] (♀) in MCZ [# 5564].

#### Distribution.

This species has been recorded from “California,” “Arizona,” and Salt Lake Valley in Utah (Hayward 1900: 220). The record from “Colorado” (LeConte 1858a: 28) probably refers to the Colorado River.

#### Records.

**USA**: AZ, CA, UT

### Tachys pallidus

Chaudoir, 1868

Tachys pallidus Chaudoir, 1868b: 212. Type locality: «Texas» (original citation). Lectotype (♂), designated by Erwin (1974a: 149), in MHNP.

#### Distribution.

This species is known from New Jersey (Beutenmüller 1897: 37; Hayward 1900: 221; Smith 1910: 203), the Florida Peninsula (Leng 1915: 574), Big Pine Key, one of the Florida Keys (Foster F. Purrington pers. comm. 2012), Claiborne County in southwestern Mississippi (Robert L. Davidson pers. comm. 2012), Cameron Parish in southwestern Louisiana (Hine 1906: 76), “several localities” in Texas (Hayward 1900: 221), and Chaves County in southeastern New Mexico (Robert L. Davidson pers. comm. 2012); it has been recorded also from Guana Island (Valentine and Ivie 2005: 275) in the British Virgin Islands.

#### Records.

**USA**: FL, LA, MS, NJ, NM, TX – Guana Island

### Tachys pulchellus

LaFerté-Sénectère, 1841

Tachys pulchellus LaFerté-Sénectère, 1841a: 45. Type locality: Texas (inferred from title of the paper). Lectotype, designated by Erwin (1974a: 149), in MHNP.
Tachys pugnax Casey, 1918: 207. Type locality: «Galveston [Galveston County], Texas» (original citation). Lectotype (♂), designated by Erwin (1974a: 149), in USNM [# 46959]. Synonymy established by Erwin (1974a: 149).
Tachys subtropicus Casey, 1918: 208. Type locality: «Brownsville [Cameron County], Texas» (original citation). Lectotype (♀), designated by Erwin (1974a: 149), in USNM [# 46960]. Synonymy established by Erwin (1974a: 149).

#### Distribution.

This species is known from southeastern Georgia (Glynn County, CMNH) and the Florida Peninsula (Monroe and Pinellas Counties, CMNH; Hayward 1900: 221, as *Tachys occultator* Casey *sensu* Hayward) west to southeastern New Mexico (Chaves County, CNC). The records from the District of Columbia (Ulke 1902: 6), Missouri (Summers 1873: 147), and “Yucatan” (Chaudoir 1868: 212) need confirmation.

#### Records.

**USA**: AL, FL, GA, LA, MS, NM, TX [DC, MO]

### Tachys translucens

Darlington, 1937

Tachys translucens Darlington, 1937: 123. Type locality: «Boquerón (mouth of Guantánamo Bay), Oriente Prov[ince] [=Santiago de Cuba], Cuba» (original citation). Holotype (♂) in MCZ [# 22484].

#### Distribution.

This species is known from Cuba and from Big Pine Key, Monroe County, in southern Florida (Foster F. Purrington and Robert L. Davidson pers. comm. 2009). This is a **new record** for America north of Mexico.

#### Records.

**USA**: FL – Cuba

### Tachys virgo

LeConte, 1852

Tachys virgo LeConte, 1852a: 194. Type locality: «San Diego [San Diego County, California]» (original citation). Holotype [by monotypy; designated lectotype by Erwin (1974a: 150)] (♀) in MCZ [# 5567].

#### Distribution.

This species is found along the seacoast from Santa Barbara County (Fall 1901a: 43) to San Diego County (LeConte 1852a: 194; Moore 1937: 8) in southwestern California.

#### Records.

**USA**: CA

### Tachys vittiger

LeConte, 1852

Tachys vittiger LeConte, 1852a: 193. Type locality: «San Diego [San Diego County, California]» (original citation). Lectotype (♀), designated by Erwin (1974a: 150), in MCZ [# 5565].
Tachys marginellus LeConte, 1852a: 193. Type locality: «ad flumen Colorado, circa millia xxx a mare» (original citation), according to Hayward (1900: 222) taken at the «Valley of the Colorado River». Lectotype (♀), designated by Erwin (1974a: 150), in MCZ [# 5566]. Synonymy established by Hayward (1900: 234), confirmed by Erwin (1974a: 150).
Tachys picturatus Putzeys, 1874: 119. Type locality: «Guana Island, au nord-est d’Antigoa» (original citation, see page 120). Lectotype (♂), designated by Erwin (1974a: 150), in IRSN. Synonymy established by Erwin (1974a: 150).
Tachys beebei Mutchler, 1925: 223. Type locality: «South Seymour [Galapagos Islands, Ecuador]» (original citation). Holotype (♂) in AMNH [# 258]. Synonymy established by Erwin (1973a: 125). Etymology. The specific name honors the American naturalist, explorer, and author Charles William Beebe [1877-1962] who worked as curator of ornithology for the New York Zoological Society from 1899 to 1952.
Tachys ensenadae Mutchler, 1934: 3. Type locality: «Ensenada, Puerto Rico» (original citation). Holotype (♂) in AMNH [# 1054]. Synonymy established by Erwin (1973a: 125).

#### Distribution.

This species is known from Vancouver Island (Lindroth 1966: 428, listed as “doubtful”), along the seacoast in southern California, including Santa Catalina Island (LeConte 1852a: 193; Fall 1901a: 43), Baja California Norte (Moore and Legner 1974: 289), Guatemala (Bates 1882a: 139), the Galápagos Islands (Mutchler 1925: 223, as *Tachys beebei*), Antigua (Putzeys 1874: 119, as *Tachys picturatus*), Montserrat (Ivie et al. 2008: 238, as *Tachys ensenada*), Guana Island (Valentine and Ivie 2005: 275, as *Tachys ensenada*), and Puerto Rico (Mutchler 1934: 3, as *Tachys ensenadae*). The records from Colorado (Wickham 1902: 235; LeConte 1858a: 28, as *Tachys marginellus*) must be in error.

#### Records.

**CAN**: BC (VCI) **USA**: CA (CHI) – Antigua and Barbuda, British Virgin Islands, Dominican Republic, Ecuador, Guana Island, Guatemala, Mexico, Montserrat, Puerto Rico

### Subgenus Paratachys

Casey, 1918

Paratachys Casey, 1918: 174. Type species: *Paratachys austinicus* Casey, 1918 by original designation. Etymology. From the Greek *para* (near, next to) and the generic name *Tachys* [*q.v*.] [masculine].
Eotachys Jeannel, 1941b: 424, 426. Type species: *Elaphrus bistriatus* Duftschmid, 1812 by original designation. Synonymy established by Erwin (1971: 236). Etymology. From the Greek *eos* (dawn, east) and the generic name *Tachys* [*q.v*.] [masculine].
Macrotachys Kult, 1961: 2 [junior homonym of *Macrotachys* Uéno, 1953]. Type species: *Bembidium fulvicolle* Dejean, 1831 by original designation. Synonymy established, under the name *Eotachys* Jeannel, by Lindroth (1966: 431). Etymology. From the Greek *macros* (long) and the generic name *Tachys* [*q.v*.] [masculine].

#### Diversity.

Worldwide, with about 145 species in the Nearctic (20 species), Neotropical (about 35 species), Australian (about 15 species), Oriental, Palaearctic (about 30 species), and Afrotropical Regions. Erwin (1974a: 126) stated that the number of New World species known to him is over 300.

#### Identification.

Lindroth (1966: 431-436) treated six and Hayward (1900: 224-232) 11 (ten in the key) of the 20 species found in North America. A revision of the group is needed.

### Tachys aeneipennis

Motschulsky, 1862

Tachys aeneipennis Motschulsky, 1862b: 29. Type locality: «environs de Mobile [Mobile County, Alabama]» (original citation). Lectotype (♂), designated by Erwin (1974a: 139), in ZMMU.

#### Distribution.

This species has been reported from southern Alabama (Motschulsky 1862b: 29) and western Mississippi (Casey 1918: 201).

#### Records.

**USA**: AL, MS

### Tachys albipes

LeConte, 1863

Tachys albipes LeConte, 1863c: 20. Type locality: «Louisiana» (original citation). Lectotype (♂), designated by Erwin (1974a: 137), in MCZ [# 5575].
Tachys putzeysi Fleutiaux and Sallé, 1890: 368. Type locality: «Camp-Jacob [Guadeloupe]» (original citation). Lectotype (♀), designated by Erwin (1974a: 137), in MHNP. Synonymy established by Erwin (1974a: 139).

#### Distribution.

This species is known from the Coastal Plain ranging from North Carolina (Brimley 1938: 118) to southern Florida (Peck and Thomas 1998: 18), west to “Louisiana” (LeConte 1863c: 20), and from Cuba (Darlington 1934: 80) and Guadeloupe (Fleutiaux and Sallé, 1890: 368, as *Tachys putzeysi*) in the West Indies.

#### Records.

**USA**: FL, GA, LA, NC, SC – Cuba, Guadeloupe

### Tachys austinicus

(Casey, 1918)

Paratachys austinicus Casey, 1918: 174. Type locality: «Austin [Travis County], Texas» (original citation). Lectotype (♀), designated by Erwin (1974a: 139), in USNM [# 46905].

#### Distribution.

This species is known from southwestern Pennsylvania (Allegheny County, CMNH), Virginia (Hoffman et al. 2006: 21), South Carolina (Ciegler 2000: 54), east-central Georgia (Emanuel County, Robert L. Davidson pers. comm. 2012), central Florida (Pinellas County, CMNH), southwestern Alabama (Baldwin County, Robert L. Davidson pers. comm. 2012), northeastern Mississippi (Oktibbeha County, Drew A. Hildebrandt pers. comm. 2009), Texas (Cameron, Nolan, Travis, and Zapata Counties, CMNH; Riley 2011), and central Arkansas (Pulaski County, CMNH). The record from South Dakota (Kirk and Balsbaugh 1975: 21) needs confirmation.

#### Records.

**USA**: AL, AR, FL, GA, MS, PA, SC, TX, VA [SD]

### Tachys columbiensis

Hayward, 1900

Tachys columbiensis Hayward, 1900: 231. Type locality: «Charlotte H. [= Charlotte Harbour, an inlet in the Gulf of Mexico, in Charlotte and Lee Counties], Fl[orid]a» (lectotype label). Lectotype (♀), designated by Erwin (1974a: 140), in MCZ [# 7054].

#### Distribution.

This species seems confined to the Coastal Plain and Piedmont Plateau ranging from southeastern Pennsylvania (Lebanon and Dauphin Counties, CMNH) to southern Florida, including the Keys (Peck and Thomas 1998: 18), west to Arkansas (Pulaski and Garland Counties, CMNH) and eastern Texas (Sabine County, Robert L. Davidson pers. comm. 2012), including Alabama (Löding 1945: 14) and Winston County in east-central Mississippi (Drew A. Hildebrandt pers. comm. 2009).

#### Records.

**USA**: AL, AR, FL, GA, MS, NC, PA, SC, TX, VA

### Tachys edax

LeConte, 1852

Tachys edax LeConte, 1852a: 194. Type locality: California (inferred from title of the paper), restricted to «Gilroy Hot Springs, Santa Clara Co[unty]» by Erwin (1974a: 140). Lectotype (♂), designated by Erwin (1974a: 140), in MCZ [# 5573].

#### Distribution.

This species ranges from south-central British Columbia (Lindroth 1966: 436) south to “Utah” (Hayward 1900: 231) and at least Santa Clara in west-central California (Erwin 1974a: 140).

#### Records.

**CAN**: BC **USA**: CA, ID, NV, OR, UT, WA

### Tachys hyalinus

Casey, 1918

Tachys hyalinus Casey, 1918: 200. Type locality: «Austin [Travis County], Texas» (original citation). Lectotype (♀), designated by Erwin (1974a: 141), in USNM [# 46946].
Tachys temporalis Casey, 1918: 200. Type locality: «near the city [of New York], New York» (original citation). Lectotype (♀), designated by Erwin (1974a: 141), in USNM [# 46947]. Synonymy established by Erwin (1974a: 141).

#### Distribution.

This species has been reported only from central Texas (Casey 1918: 200) and southeastern New York (Casey 1918: 200, as *Tachys temporalis*).

#### Records.

**USA**: NY, TX

### Tachys oblitus

Casey, 1918

Tachys oblitus Casey, 1918: 195. Type locality: «New Jersey» (original citation). Lectotype (♀), designated by Erwin (1974a: 141), in USNM [# 46937].
Tachys cuneatus Casey, 1918: 195. Type locality: «near the city [of New York], New York» (original citation). Lectotype (♂), designated by Erwin (1974a: 141), in USNM [# 46938]. Synonymy established by Erwin (1974a: 141).
Tachys cuneatus appalachius Casey, 1918: 195. Type locality: «Asheville [Buncombe County], North Carolina» (original citation). Lectotype (♀), designated by Erwin (1974a: 141), in USNM [# 46939]. Synonymy established by Erwin (1974a: 141).
Tachys iowensis Casey, 1918: 195. Type locality: «Cedar Rapids [Linn County], Iowa» (original citation). Lectotype (♂), designated by Erwin (1974a: 141), in USNM [# 46940]. Synonymy established by Erwin (1974a: 141).
Tachys gentilis Casey, 1918: 197. Type locality: «probably Indiana» (original citation). Lectotype (♂), designated by Erwin (1974a: 141), in USNM [# 46943]. Synonymy established by Erwin (1974a: 141).
Tachys obliquus Casey, 1918: 201. Type locality: «Willets Point [Queens County], Long Island, New York» (original citation). Lectotype (♀), designated by Erwin (1974a: 141), in USNM [# 46949]. Synonymy established by Erwin (1974a: 141).

#### Distribution.

This species ranges from southern Quebec (Larochelle 1975: 111, as *Tachys obliquus*) to eastern Iowa (Casey 1918: 195, as *Tachys iowensis*), south to eastern Texas (Polk County, CMNH), central Louisiana (Grant Parish, CMNH), northern Alabama (De Kalb and Madison Counties, CMNH), and central Georgia (Butts County, CMNH). The record from central Florida (Frost 1975: 37, as *Tachys obliquus*) needs confirmation.

#### Records.

**CAN**: ON, QC **USA**: AL, AR, CT, GA, IA, IL, IN, KY, LA, MA, MD, NC, NH, NJ, NY, OH, PA, RI, SC, TN, TX, VA, VT, WV [FL]

### Tachys potomaca

(Erwin, 1981)

Paratachys potomaca Erwin, 1981b: 152. Type locality: «Plummers Island, Montgomery County, Maryland» (original citation). Holotype (♂) in USNM [# 76906].

#### Distribution.

This species is known from south-central Ohio (Fairfield County, CMNH) to “Massachusetts,” south to “North Carolina” (Erwin 1981b: 152).

#### Records.

**USA**: MA, MD, NC, OH, PA, VA

### Tachys proximus

(Say, 1823)

Bembidium proximus Say, 1823a: 88. Type locality: «Brookline [Norfolk County], Mass[achusetts]» (neotype label). Neotype (♀), designated by Lindroth and Freitag (1969: 335), in MCZ [# 33058].
Tachys nubifer Casey, 1918: 200. Type locality: «Vicksburg [Warren County], Mississippi» (original citation). Lectotype (♀), designated by Erwin (1974a: 142), in USNM [# 46948]. Synonymy established by Erwin (1974a: 142).

#### Distribution.

This species ranges from southeastern Maine (Majka et al. 2011: 46) and the Saint Lawrence Plain in southern Quebec (Larochelle 1975: 111) to eastern South Dakota (Kirk and Balsbaugh 1975: 21), south to southeastern New Mexico, northern Coahuila in Mexico (Barr and Reddell 1967: 269), and central Florida (Seminole County, CMNH). The record from Jamaica (Darlington 1941a: 11) needs confirmation.

#### Records.

**CAN**: ON, QC **USA**: AL, AR, CT, DC, DE, FL, GA, IA, IL, IN, KS, KY, LA, MA, MD, ME, MO, MS, NC, NE, NJ, NM, NY, OH, OK, PA, SC, SD, TN, TX, VA, VT, WI, WV – Mexico

### Tachys pumilus

(Dejean, 1831)

Bembidium pumilum Dejean, 1831: 43. Type locality: «Amérique septentrionale» (original citation), restricted to «New York» by Erwin (1974a: 142). Lectotype (♀), designated by Erwin (1974a: 142), in MHNP.
Tachys corruscus LeConte, 1848: 472. Type locality: «NovEboraci [= New York] et ad Rocky Mountains» (original citation), restricted to «New York» by Lindroth (1966: 435). Lectotype (♀), designated by Erwin (1974a: 142), in MCZ [# 5571]. Synonymy established by Erwin (1974a: 142).
Tachys coruscus Bates, 1882a: 139. Unjustified emendation of *Tachys corruscus* LeConte, 1848.

#### Distribution.

The range of this species extends from southwestern Quebec (LeSage 1996: 23) to southern Wisconsin (Messer 2010: 37), south to southeastern Texas (Kleberg County, CMNH; Hayward 1900: 230) and southern Florida (Peck and Thomas 1998: 18); also recorded from the Bahamas (Darlington 1953: 6, as *Tachys corruscus*), Cuba (Darlington 1934: 83), Jamaica (Darlington 1941a: 11), Puerto Rico (Wolcott 1941: 81), Mexico, and Guatemala (Bates 1882a: 139). The records from Yuma, California (Hayward 1900: 230), Sacramento Mountains in New Mexico (Fall and Cockerell 1907: 158), and “Colorado” (Leng 1920: 54) probably refer to other species.

#### Records.

**CAN**: QC **USA**: AL, AR, CT, DC, DE, FL, GA, IA, IL, IN, KS, KY, LA, MA, MD, MO, MS, NC, NH, NJ, NY, OH, OK, PA, RI, SC, TN, TX, VA, WI – Bahamas, Cuba, Guatemala, Jamaica, Mexico, Puerto Rico

### Tachys rectangulus

Notman, 1919

Tachys rectangulus Notman, 1919b: 229. Type locality: «North America» (original citation). Holotype [by monotypy] (♂) location unknown (originally in collection C.W. Leng).

#### Distribution.

This species is known only from the holotype.

#### Records.

None.

### Tachys rhodeanus

Casey, 1918

Tachys rhodeanus Casey, 1918: 198. Type locality: «Boston Neck [Washington County], Rhode Island» (original citation). Lectotype (♀), designated by Erwin (1974a: 142), in USNM [# 46944].

#### Distribution.

This species is known from scattered localities from New Brunswick (Webster and Bousquet 2008: 17) to northern Ohio (Cuyahoga County, Harry J. Lee, Jr. pers. comm. 2008), south to southern South Carolina (Ciegler 2003: [2]) and southwestern Alabama (Baldwin County, CMNH).

#### Records.

**CAN**: NB, ON, QC **USA**: AL, CT, DC, MA, NH, OH, PA, RI, SC, VA, VT, WV

### Tachys sagax

Casey, 1918

Tachys sagax Casey, 1918: 197. Type locality: «Highland Park [Lake County], Illinois» (original citation). Lectotype (♂), designated by Erwin (1974a: 142), in USNM [# 46942].

#### Distribution.

Besides the type locality, this species is known from several counties in Virginia (Hoffman et al. 2006: 21), Maryland (Lepping 2009: 65), Montgomery County in northern Tennessee (Foster F. Purrington pers. comm. 2011), Oktibbeha County in northeastern Mississippi (Drew A. Hildebrandt pers. comm. 2009), and Desha County in southeastern Arkansas (Foster F. Purrington pers. comm. 2010).

#### Records.

**USA**: AR, IL, MD, MS, TN, VA

### Tachys scitulus

LeConte, 1848

Tachys scitulus LeConte, 1848: 471. Type locality: «Columbiam [= Columbia, Lancaster County] Pennsylvaniae» (original citation). Lectotype (♀), designated by Erwin (1974a: 142), in MCZ [# 5568].
Tachys pallescens Casey, 1918: 199 [primary homonym of *Tachys pallescens* Bates, 1873]. Type locality: «Keokuk [Lee County], Iowa» (original citation for the lectotype). Lectotype (♂), designated by Erwin (1974a: 142), in USNM [# 46945]. Synonymy established by Erwin (1974a: 142).
Tachys pallidiusculus Csiki, 1928: 191. Replacement name for *Tachys pallescens* Casey, 1918.

#### Distribution.

This species ranges from New Brunswick (Bousquet 1987a: 124) to central South Dakota (Kirk and Balsbaugh 1975: 21), south to central Texas (Blanco and Travis Counties, CMNH; Hayward 1900: 229; Riley 2011) and southern Florida (Peck and Thomas 1998: 18); also recorded from Cuba (Darlington 1934: 81) and Jamaica (Darlington 1941a: 12).

#### Records.

**CAN**: NB, ON, QC **USA**: AL, AR, CT, DC, DE, FL, GA, IA, IL, IN, KS, KY, LA, MA, MD, ME, MI, MN, MO, MS, NC, NH, NJ, NY, OH, OK, PA, SC, SD, TN, TX, VA, VT, WI, WV – Cuba, Jamaica

### Tachys sequax

LeConte, 1848

Tachys sequax LeConte, 1848: 472. Type locality: «ad Rocky Mountains» (original citation). Lectotype (♀), designated by Erwin (1974a: 142), in MCZ [# 5570].

#### Distribution.

This species is known only from the lectotype. The record from “Colorado” (Leng 1920: 54) needs confirmation.

#### Records.

**USA**: [CO]

### Tachys spadix

Casey, 1918

Tachys spadix Casey, 1918: 202. Type locality: «El Paso [El Paso County], Texas» (original citation). Holotype [by monotypy] (♂) in USNM [# 46950].
Tachys laxicollis Casey, 1918: 202. Type locality: «Houston [Harris County], Texas» (original citation). Holotype [by monotypy] (♀) in USNM [# 46951]. Synonymy established by Erwin (1974a: 142).

#### Distribution.

This species is known only from east-central and westernmost Texas (Casey 1918: 202).

#### Records.

**USA**: TX

### Tachys umbripennis

Chaudoir, 1868

Tachys umbripennis Chaudoir, 1868b: 213. Type locality: «Louisiane» (lectotype label). Lectotype (♀), designated by Erwin (1974a: 143), in MHNP.

#### Distribution.

This species has been reported from “Arkansas” (Casey 1918: 193), “Louisiana” (Chaudoir 1868b: 213), and southeastern Texas along the Gulf Coast (Casey 1918: 193).

#### Records.

**USA**: AR, LA, TX

#### Note.

This form has been listed as a synonym of *Tachys pumilus* (Dejean) by Hayward (1900: 235) but regarded as a valid species by Casey (1918: 193) and Erwin (1974a: 143).

### Tachys ventricosus

LeConte, 1863

Tachys ventricosus LeConte, 1863c: 20. Type locality: «Louisiana» (original citation). Lectotype (♂), designated by Erwin (1974a: 143), in MCZ [# 5574].
Tachys oopterus Chaudoir, 1868b: 212. Type locality: «Louisiane» (original citation). Lectotype (♂), designated by Erwin (1974a: 143), in MHNP. Synonymy established by Horn (1875: 132), confirmed by Erwin (1974a: 143).

#### Distribution.

This species is known along the Coastal Plain from southwestern Georgia (Fattig 1949: 19) to southern Florida (Peck and Thomas 1998: 18), west to southeastern Texas (Casey 1918: 197).

#### Records.

**USA**: AL, FL, GA, LA, TX

### Tachys vernilis

Casey, 1918

Tachys vernilis Casey, 1918: 202. Type locality: «Brownsville [Cameron County], Texas» (original citation). Lectotype (♂), designated by Erwin (1974a: 143), in USNM [# 46952].

#### Distribution.

This species is known only from the type locality in southeastern Texas.

#### Records.

**USA**: TX

### Tachys vorax

LeConte, 1852

Tachys vorax LeConte, 1852a: 194. Type locality: «ad fluminis Gilae et Colorado ripas» (original citation), restricted to «Gila River, Arizona» by Erwin (1974a: 143). Lectotype (♀), designated by Erwin (1974a: 143), in MCZ [# 5569].

#### Distribution.

This species is found from southeastern Texas (Wickham 1897: 104) to southwestern California (Fall 1901a: 43; Moore 1937: 8) and the Baja California Peninsula (Horn 1894: 308), north to southwestern Utah (Tanner 1928: 270). The records from the Bahamas (Darlington 1953: 6), Cuba (Darlington 1934: 82; Mateu 1977: 378), Jamaica (Darlington 1941a: 11), and Puerto Rico (Wolcott 1936: 188) need confirmation; that from eastern Washington (Hatch 1953: 105) is probably in error; that from “Colorado” (LeConte 1858a: 28) probably refers to the Colorado River.

#### Records.

**USA**: AZ, CA, NM, TX, UT – Mexico

### Subtribe Anillina

Jeannel, 1937

Anillini Jeannel, 1937a: 241, 243. Type genus: *Anillus* Jacquelin du Val, 1851.
Scotodipnina Jeannel, 1937a: 241, 265. Type genus: *Scotodipnus* Schaum, 1860.
Typhlocharina Jeanne, 1973: 95, 98. Type genus: *Typhlocharis* Dieck, 1869. Note. The stem of *Typhlocharis* is *Typhlocharit*- (Madge 1989: 469).

#### Diversity.

Worldwide, with about 375 species in the Nearctic (50 species), Neotropical (24 species), Australian (about 45 species), Oriental (about 22 species), Palaearctic (about 175 species), and Afrotropical (about 60 species) Regions. There is no genus-group taxa shared between the Western Hemisphere and the Eastern Hemisphere.

### Genus Anillodes

Jeannel, 1963

Anillodes Jeannel, 1963a: 54. Type species: *Anillus debilis* LeConte, 1853 by original designation. Etymology. From the generic name *Anillus* and the Greek suffix -*odes* (likeness), alluding to the resemblance of the adults to those of *Anillus* [masculine].

#### Diversity.

The genus contains three species found in California.

#### Identification.

Jeannel (1963a: 54-58) revised the species. Soon after, Jeannel (1963b) published a supplement to his monograph and pointed out that his *Anillodes debilis* consisted of two very similar species, one of which being undescribed (*Anillodes minutus*). A revision of the genus is needed.

#### Taxonomic Note.

A study of the type specimens of *Anillodes affabilis* (Brues), *Anillodes sinuatus* Jeannel, and *Anillodes debilis* (LeConte) showed that the first two species, described from Texas, are morphologically more similar to members of *Anillinus* Casey than to *Anillodes debilis*, the type species of *Anillodes* described from California. In fact, *Anillodes affabilis* and *Anillodes sinuatus* are externally indistinguishable from many *Anillinus* species and examination of the male genitalia is required for positive identifications. Based on this fact, the Texas species of *Anillodes* are transferred to *Anillinus* and the California species are retained in the genus *Anillodes*.

### Anillodes debilis

(LeConte, 1853)

Anillus debilis LeConte, 1853c: 397. Type locality: «San Jose [Santa Clara County], California» (original citation). Two syntypes [3 originally cited] in MCZ [# 5563].

#### Distribution.

This species is known from a few specimens collected in Tulare and Santa Clara Counties in California (Jeannel 1963a: 56).

#### Records.

**USA**: CA

### Anillodes minutus

Jeannel, 1963

Anillodes minutus Jeannel, 1963b: 147. Type locality: «Bulkaap camp ground, aux environs de Springville [Tulare County], Californie» (original citation). Holotype in MHNP.

#### Distribution.

This species is known only from the two original specimens collected at the type locality in south-central California.

#### Records.

**USA**: CA

### Anillodes walkeri

Jeannel, 1963

Anillodes walkeri Jeannel, 1963a: 56. Type locality: «Deer Creek Grove [Tulare County, California]» (original citation). Holotype in MHNP.

#### Distribution.

This species is known from a few specimens collected near Springville in south-central California.

#### Records.

**USA**: CA

### Genus Anillinus

Casey, 1918

Anillinus Casey, 1918: 167. Type species: *Anillinus carolinae* Casey, 1918 by original designation. Etymology. From the generic name *Anillus* and the Latin suffix -*inus* (pertaining to), alluding to the close affinity of the species of this genus to those of *Anillus* [masculine].
Micranillodes Jeannel, 1963a: 57. Type species: *Micranillodes depressus* Jeannel, 1963 by original designation. Etymology. From the Greek *micros* (small, little) and the generic name *Anillodes* [*q.v*.] [masculine]. **New synonymy**. Note. A study of the holotype of *Micranillodes depressus* Jeannel revealed that the species is externally similar to members of *Anillinus*.
Troglanillus Jeannel, 1963b: 147. Type species: *Troglanillus valentinei* Jeannel, 1963 by original designation. Synonymy established by Barr (1995: 240). Etymology. From the Greek *trogle* (hole) and the generic name *Anillus* [masculine].

#### Diversity.

Western Hemisphere, with 44 species in eastern North America (42 species), most of them in the Appalachian region, and the state of Santa Catarina in Brazil (two species).

#### Identification.

Sokolov et al. (2004) reviewed the North American species and provided a key for the identification of all but four species (*Anillinus daggyi*, *Anillinus dohrni*, *Anillinus longiceps*, and *Anillinus valentinei*). Subsequently 16 new species were described by Dajoz (2005), Sokolov et al. (2007), Sokolov and Watrous (2008), Sokolov and Carlton (2008, 2010), Sokolov (2011), and Giachino (2011) and three species are transferred to the genus in this work.

### Anillinus affabilis

(Brues, 1902), new combination

Anillus affabilis Brues, 1902: 366. Type locality: «Austin [Travis County], Texas» (original citation). Lectotype (♂), designated by Erwin and House (1978: 233), in USNM [# 75691].

#### Distribution.

This species is known only from the three original specimens collected at the type locality in central Texas.

#### Records.

**USA**: TX

### Anillinus aleyae

Sokolov and Watrous, 2008

Anillinus aleyae Sokolov and Watrous, 2008: 538. Type locality: «Ozark Underground Lab[oratory] (800 m), 36.5585°N, 92.8134°W, Taney Co[unty], M[iss]o[uri]» (original citation). Holotype (♂) in USNM.

#### Distribution.

This species is known only from Taney and Barry Counties in southwestern Missouri (Sokolov and Watrous 2008: 541).

#### Records.

**USA**: MO

### Anillinus balli

Sokolov and Carlton, 2004

Anillinus balli Sokolov and Carlton [in Sokolov et al.], 2004: 208. Type locality: «Bald Rock Picnic Area, Laurel Co[unty], K[entuck]y» (original citation). Holotype (♂) in USNM.

#### Distribution.

This species is known from the original two specimens collected at the type locality in southeastern Kentucky (Sokolov et al. 2004: 208).

#### Records.

**USA**: KY

### Anillinus barberi

Jeannel, 1963

Anillinus barberi Jeannel, 1963b: 150. Type locality: «Plummers island [Montgomery County, Maryland]» (original citation). Holotype (♀) in USNM [# 69545].

#### Distribution.

This species is known from a few specimens collected at the type locality on the Potomac River in Maryland and northern Virginia (Sokolov et al. 2004: 198).

#### Records.

**USA**: MD, VA

### Anillinus barri

Sokolov and Carlton, 2004

Anillinus barri Sokolov and Carlton [in Sokolov et al.], 2004: 208. Type locality: «Indian Bound[a]ry Campground, Monroe Co[unty], T[en]n[essee]» (original citation). Holotype (♂) in USNM.

#### Distribution.

This species is known from Monroe County (Sokolov et al. 2004: 209) and Polk County (Giachino 2011: 113) in southeastern Tennessee.

#### Records.

**USA**: TN

### Anillinus campbelli

Giachino, 2011

Anillinus campbelli Giachino, 2011: 111. Type locality: «Van Hook Glade, 4 mi[les] W[est] Highlands (3500 f) [Macon County], N[orth] Car[olina]» (original citation). Holotype (♂) in CNC [# 23998].

#### Distribution.

This species is known only from the type locality in southwestern North Carolina.

#### Records.

**USA**: NC

### Anillinus carltoni

Sokolov, 2011

Anillinus carltoni Sokolov, 2011: 6. Type locality: «ca. 0.5 km E[ast] Thunderhead Mountain at 35°34'3"N, 83°42'4"W (1,510m), Great Smoky Mountain[s] National Park, Blount County, Tennessee» (original citation). Holotype (♂) in USNM.

#### Distribution.

This species is known only from the type locality along the state line between Tennessee and North Carolina.

#### Records.

**USA**: TN

### Anillinus chandleri

Sokolov, 2011

Anillinus chandleri Sokolov, 2011: 11. Type locality: «Sumter National Forest, 33°37.20'N, 82°5.55'W, Edgefield County, South Carolina» (original citation). Holotype (♂) in USNM.

#### Distribution.

This species is known only from the holotype collected in western South Carolina near the Georgia border.

#### Records.

**USA**: SC

### Anillinus cherokee

Sokolov and Carlton, 2008

Anillinus cherokee Sokolov and Carlton, 2008: 40. Type locality: «Joyce Kilmer Memorial, Spirit Ridge, Nantahala National Forest, Graham Co[unty], N[orth]C[arolina]» (original citation). Holotype (♂) in USNM.

#### Distribution.

This species is known from the southeastern parts of Blount County in Tennessee and adjacent parts of Nantahala National Forest in Graham County, North Carolina (Sokolov and Carlton 2008: 42).

#### Records.

**USA**: NC, TN

### Anillinus chilhowee

Sokolov, 2011

Anillinus chilhowee Sokolov, 2011: 8. Type locality: «Chilhowee Mountain at 35°7.0'N, 84°37.44'W (715 m), Cherokee National Forest, Polk County, Tennessee» (original citation). Holotype (♂) in USNM.

#### Distribution.

This species is known only from the holotype.

#### Records.

**USA**: TN

### Anillinus cieglerae

Sokolov and Carlton, 2007

Anillinus cieglerae Sokolov and Carlton [in Sokolov et al.], 2007: 5. Type locality: «West Prong Campground, Great Smoky Mountain[s] National Park, Blount Co[unty], T[en]n[essee]» (original citation). Holotype (♂) in USNM.

#### Distribution.

This species is known only from central and northeastern Blount County in Tennessee and adjacent parts of Swain County in North Carolina (Sokolov et al. 2007: 7).

#### Records.

**USA**: NC, TN

### Anillinus cornelli

Sokolov and Carlton, 2004

Anillinus cornelli Sokolov and Carlton [in Sokolov et al.], 2004: 209. Type locality: «Crowders M[oun]t[ain] St[ate] P[ar]k, Gaston Co[unty], N[orth]C[arolina]» (original citation). Holotype (♂) in USNM.

#### Distribution.

This species is known only from two nearby localities in Gaston County, North Carolina, and York County, South Carolina (Sokolov et al. 2004: 210).

#### Records.

**USA**: NC, SC

### Anillinus daggyi

Sokolov and Carlton, 2004

Anillinus daggyi Sokolov and Carlton [in Sokolov et al.], 2004: 210. Type locality: «Glen Alpine Springs, Burke Co[unty], N[orth]C[arolina]» (original citation). Holotype (♂) in NCSU.

#### Distribution.

This species is known from two nearby localities in Burke County, western North Carolina (Sokolov et al. 2004: 211).

#### Records.

**USA**: NC

### Anillinus depressus

(Jeannel, 1963), new combination

Micranillodes depressus Jeannel, 1963a: 58. Type locality: «Travis Co[unty], Texas» (original citation). Holotype (♀) in USNM [# 69551].

#### Distribution.

This species is known only from the holotype collected in central Texas.

#### Records.

**USA**: TX

### Anillinus docwatsoni

Sokolov and Carlton, 2004

Anillinus docwatsoni Sokolov and Carlton [in Sokolov et al.], 2004: 212. Type locality: «Chimney Rock, Rutherford Co[unty], N[orth]C[arolina]» (original citation). Holotype (♂) in NCSU. Etymology. The specific name was proposed for Arthel Lane “Doc” Watson [1923-2012], American guitar player and singer of bluegrass, folk, country, blues, and gospel music.

#### Distribution.

This species is known only from the type locality in southwestern North Carolina (Sokolov et al. 2004: 212).

#### Records.

**USA**: NC

### Anillinus dohrni

(Ehlers, 1884)

Anillus dohrni Ehlers, 1884: 36. Type locality: «Florida» (original citation). Holotype [by monotypy] (♀) in ANSP [# 8165]. Etymology. The species was proposed in honor of the German entomologist Carl August Dohrn [1806-1892]. Wealthy by inheritance, Dohrn specialized in Coleoptera.

#### Distribution.

This species is known only from the holotype.

#### Records.

**USA**: FL

#### Note.

Jeannel (1963a: 76-77) reported that “Horn (1888: 27)” doubted the state provenance of the holotype and that the specimens in the USNM from Clayton, Georgia belong to *Anillinus dohrni*. I have not found any statement of that sort made by Horn. On the other hand, Schwarz (1891: 24) had “some doubt regarding the correctness of the [type] locality.”

### Anillinus elongatus

Jeannel, 1963

Anillinus elongatus Jeannel, 1963b: 151. Type locality: «Morgan Cr[eek], Chapel Hill, Orange Co[unty], N[orth] C[arolina]» (original citation for the neotype). Neotype (♂), designated by Sokolov et al. (2004: 199), in NCSU. Note. Remnants of the holotype are in the USNM [# 69544] (Sokolov et al. 2004: 199).

#### Distribution.

This species is known from several localities in Orange, Mecklenburg, and Cabarrus Counties in North Carolina (Sokolov et al. 2004: 200).

#### Records.

**USA**: NC

### Anillinus erwini

Sokolov and Carlton, 2004

Anillinus erwini Sokolov and Carlton [in Sokolov et al.], 2004: 213. Type locality: «Rough Ridge Trailhead, 8 mi[les] S[outh]W[est] Blowing Rock (4,200’), Watauga Co[unty], N[orth]C[arolina]» (original citation). Holotype (♂) in USNM.

#### Distribution.

This species is known from southwestern Virginia and North Carolina, north of the French Broad River (Sokolov et al. 2004: 213).

#### Records.

**USA**: NC, VA

### Anillinus folkertsi

Sokolov and Carlton, 2004

Anillinus folkertsi Sokolov and Carlton [in Sokolov et al.], 2004: 214. Type locality: «1 mi[le] S[outh] Claiborne Dam, Monroe County, Ala[bama]» (original citation). Holotype (♂) in USNM. Etymology. The specific name was proposed in honor of the naturalist and conservationist George W. Folkerts [1938-2007], professor in the Department of Biological Sciences at Auburn University for 38 years. Folkerts was a well-known expert on the ecology of disappearing habitat types and declining species.

#### Distribution.

This species is known only from the type locality in southwestern Alabama (Sokolov et al. 2004: 214).

#### Records.

**USA**: AL

### Anillinus fortis

(Horn, 1869)

Anillus fortis G.H. Horn, 1869a: 127. Type locality: «mountainous regions of eastern Tennessee» (original citation). Holotype [by monotypy] (♀) in MCZ [# 35575].
Anillinus carolinae Casey, 1918: 168. Type locality: «Blacks M[oun]t[ain]s, North Carolina» (original citation). Four syntypes [4 originally cited] in USNM [# 46902]. Synonymy established by Jeannel (1937a: 352), confirmed by Barr (1995: 241).

#### Distribution.

This species is known from North Carolina, north of the French Broad River (Sokolov et al. 2004: 201), and presumably eastern Tennessee (Horn 1869a: 127). The records from the District of Columbia (Ulke 1902: 6), Virginia (Schwarz 1891: 24), South Carolina (Ciegler 2000: 55), and northeastern Georgia (Leng 1910: 73; Fattig 1949: 18) are probably in error.

#### Records.

**USA**: NC, TN

### Anillinus gimmeli

Sokolov and Carlton, 2010

Anillinus gimmeli Sokolov and Carlton, 2010: 11. Type locality: «White Oak Sink, 35°38.1'N, 83°45.3'W, Great Smoky Mountain[s] National Park, Blount Co[unty], T[en]n[essee]» (original citation). Holotype in USNM.

#### Distribution.

This species is known only from the eastern part of Rich Mountain ridge, Blount County, eastern Tennessee (Sokolov and Carlton 2010: 12).

#### Records.

**USA**: TN

### Anillinus indianae

Jeannel, 1963

Anillinus indianae Jeannel, 1963b: 152. Type locality: «Mitchell, Lawrence Co[unty], Indiana» (original citation). Holotype in USNM [# 69548].

#### Distribution.

This species is known only from the original four specimens collected in Lawrence and Crawford Counties in southern Indiana (Sokolov et al. 2004: 202).

#### Records.

**USA**: IN

### Anillinus juliae

Sokolov and Carlton, 2010

Anillinus juliae Sokolov and Carlton, 2010: 7. Type locality: «north-eastern part of Starr Mountain, 35°20.067'N, 84°24.514'W, McMinn Co[unty], T[en]n[essee]» (original citation). Holotype (♂) in USNM.

#### Distribution.

This species is known only from the type locality in southeastern Tennessee.

#### Records.

**USA**: TN

### Anillinus kovariki

Sokolov and Carlton, 2004

Anillinus kovariki Sokolov and Carlton [in Sokolov et al.], 2004: 214. Type locality: «Mays Pond, 3.5 mi[les] N[orth]W[est] Monticello, Jefferson Co[unty], Florida» (original citation). Holotype (♂) in FSCA.

#### Distribution.

This species is known only from the type locality in northern Florida (Sokolov et al. 2004: 215).

#### Records.

**USA**: FL

### Anillinus langdoni

Sokolov and Carlton, 2004

Anillinus langdoni Sokolov and Carlton [in Sokolov et al.], 2004: 215. Type locality: «Laurel Falls Trail (747 m), G[reat]S[moky]M[ountains]N[ational]P[ark], Sevier Co[unty], T[en]n[essee]» (original citation). Holotype (♂) in USNM.

#### Distribution.

This species is known only from the Great Smoky Mountains in Cocke, Monroe, and Sevier Counties, Tennessee (Sokolov et al. 2004: 216; Giachino 2011: 111).

#### Records.

**USA**: TN

### Anillinus lescheni

Sokolov and Carlton, 2004

Anillinus lescheni Sokolov and Carlton [in Sokolov et al.], 2004: 217. Type locality: «Latimer Co[unty], Oklahoma» (original citation). Holotype (♂) in USNM.

#### Distribution.

This species is known only from Latimer County in eastern Oklahoma (Sokolov et al. 2004: 217).

#### Records.

**USA**: OK

### Anillinus longiceps

Jeannel, 1963

Anillinus longiceps Jeannel, 1963b: 149. Type locality: «Crystal Cave, Monteagle [Grundy County], Tennessee» (original citation). Holotype (♂) in USNM [# 69547].

#### Distribution.

This species is known only from the holotype collected in southern Tennessee.

#### Records.

**USA**: TN

### Anillinus loweae

Sokolov and Carlton, 2004

Anillinus loweae Sokolov and Carlton [in Sokolov et al.], 2004: 218. Type locality: «Cataloochee Divide Trail near Purchase, G[reat]S[moky]M[ountains]N[ational]P[ark], Haywood Co[unty], N[orth]C[arolina]» (original citation). Holotype (♂) in USNM.

#### Distribution.

This species is known in the Appalachians from western North Carolina, eastern Tennessee, and northwestern South Carolina (Sokolov et al. 2004: 219; Giachino 2011: 110).

#### Records.

**USA**: NC, SC, TN

### Anillinus magazinensis

Sokolov and Carlton, 2004

Anillinus magazinensis Sokolov and Carlton [in Sokolov et al.], 2004: 220. Type locality: «M[oun]t Magazine, 0.5 mi[le] S[outh] Greenfield Picnic Area, Logan Co[unty], Ar[kansas]» (original citation). Holotype (♂) in USNM.

#### Distribution.

This species is known only from Mount Magazine in western Arkansas (Sokolov et al. 2004: 221).

#### Records.

**USA**: AR

### Anillinus merritti

Sokolov and Carlton, 2010

Anillinus merritti Sokolov and Carlton, 2010: 9. Type locality: «Twentymile Creek valley, 35°28.8'N, 83°50.7'W, Great Smoky Mountains National Park, Swain Co[unty], N[orth]C[arolina]» (original citation). Holotype (♂) in USNM.

#### Distribution.

This species is known from two localities in Swain and Macon Counties in western North Carolina (Sokolov and Carlton 2010: 11).

#### Records.

**USA**: NC

### Anillinus moseleyae

Sokolov and Carlton, 2004

Anillinus moseleyae Sokolov and Carlton [in Sokolov et al.], 2004: 221. Type locality: «Appalachian Trail, 1.5 mi[les] N[orth]E[ast] Newfound Gap, G[reat]S[moky]M[ountains]N[ational]P[ark], Swain Co[unty], N[orth]C[arolina]» (original citation). Holotype (♂) in USNM.

#### Distribution.

This species is known only from high elevations in central Great Smoky Mountains National Park in eastern Tennessee and western North Carolina (Sokolov et al. 2004: 222).

#### Records.

**USA**: NC, TN

### Anillinus murrayae

Sokolov and Carlton, 2004

Anillinus murrayae Sokolov and Carlton [in Sokolov et al.], 2004: 222. Type locality: «Collins Picnic Area, G[reat]S[moky]M[ountains]N[ational]P[ark], Swain Co[unty], N[orth]C[arolina]» (original citation). Holotype (♂) in USNM.

#### Distribution.

This species is known only from Swain and Jackson Counties in North Carolina (Sokolov et al. 2004: 223).

#### Records.

**USA**: NC

### Anillinus nantahala

Dajoz, 2005

Anillinus nantahala Dajoz, 2005: 210. Type locality: «Wayah Bald [Nantahala National Forest, Macon County, North Carolina]» (original citation). Holotype in Dajoz’s collection (Paris, France).

#### Distribution.

This species is known only from the type locality in southwestern North Carolina.

#### Records.

**USA**: NC

### Anillinus pecki

Giachino, 2011

Anillinus pecki Giachino, 2011: 114. Type locality: «Linville Falls (3500 f), Avery Co[unty], N[orth] Car[olina]» (original citation). Holotype (♂) in CNC [# 23999].

#### Distribution.

This species is known from Avery and Wilkes Counties along the Blue Ridge Parkway in western North Carolina (Giachino 2011: 115).

#### Records.

**USA**: NC

### Anillinus pusillus

Sokolov and Carlton, 2007

Anillinus pusillus Sokolov and Carlton [in Sokolov et al.], 2007: 8. Type locality: «Twentymile Trail, Great Smoky Mountains National Park, Swain Co[unty], N[orth]C[arolina]» (original citation). Holotype (♂) in USNM.

#### Distribution.

This species is known from southwestern Blount County in Tennessee and Swain County in North Carolina in the Great Smoky Mountains National Park (Sokolov et al. 2007: 10).

#### Records.

**USA**: NC, TN

### Anillinus robisoni

Sokolov and Carlton, 2004

Anillinus robisoni Sokolov and Carlton [in Sokolov et al.], 2004: 223. Type locality: «5.0 mi[les] S[outh]W[est] Big Fork, Polk Co[unty], Ar[kansas]» (original citation). Holotype (♂) in USNM.

#### Distribution.

This species is known only from the southern parts of the Ouachita National Forest in western Arkansas (Sokolov et al. 2004: 224).

#### Records.

**USA**: AR

### Anillinus sinuaticollis

Jeannel, 1963

Anillinus sinuaticollis Jeannel, 1963b: 152. Type locality: «Roane Co[unty], Tennessee» (original citation). Holotype (♀) in USNM [# 69541].

#### Distribution.

This species is known only from the holotype collected in eastern Tennessee (Sokolov et al. 2004: 204).

#### Records.

**USA**: TN

### Anillinus sinuatus

(Jeannel, 1963), new combination

Anillodes sinuatus Jeannel, 1963a: 57. Type locality: «Bexar Co[unty], Texas» (original citation). Holotype (♀) in USNM [# 69549].

#### Distribution.

This species is known only from the holotype collected in south-central Texas.

#### Records.

**USA**: TX

### Anillinus smokiensis

Sokolov, 2011

Anillinus smokiensis Sokolov, 2011: 9. Type locality: «Gregory Cave at 35°36.59'N, 83°48.35'W (605 m), southern slope of Rich Mountain ridge, Great Smoky Mountain[s] National Park, Blount County, Tennessee» (original citation). Holotype (♂) in USNM.

#### Distribution.

This species is known only from eight specimens collected at the type-locality cave.

#### Records.

**USA**: TN

### Anillinus steevesi

Barr, 1995

Anillinus steevesi Barr, 1995: 243. Type locality: «Cloudland Canyon State Park, Dade County, Georgia» (original citation). Holotype (♂) in CMNH.

#### Distribution.

This species is known from Tishomingo County in northeastern Mississippi, northern Alabama (Sokolov and Carlton 2010: 13; Giachino 2011: 111), Blount County in eastern Tennessee, Swain County in western North Carolina, and Dade County in northwestern Georgia (Sokolov et al. 2004: 205).

#### Records.

**USA**: AL, GA, MS, NC, TN

### Anillinus stephani

Sokolov and Carlton, 2004

Anillinus stephani Sokolov and Carlton [in Sokolov et al.], 2004: 224. Type locality: «Latimer Co[unty], Oklahoma» (original citation). Holotype (♂) in FSCA.

#### Distribution.

This species is known only from Latimer County in eastern Oklahoma (Sokolov et al. 2004: 225).

#### Records.

**USA**: OK

### Anillinus tishechkini

Sokolov and Carlton, 2004

Anillinus tishechkini Sokolov and Carlton [in Sokolov et al.], 2004: 226. Type locality: «Winona Forest Dr., 10 mi[les] W[est] L[ake] Sylvia, Perry Co[unty], Ar[kansas]» (original citation). Holotype (♂) in USNM.

#### Distribution.

This species is known only from the type locality in central Arkansas (Sokolov et al. 2004: 226).

#### Records.

**USA**: AR

### Anillinus turneri

Jeannel, 1963

Anillinus turneri Jeannel, 1963a: 77. Type locality: «Peach Co[unty], Georgia» (original citation). Holotype (♀) in USNM [# 69543].

#### Distribution.

This species is known for sure only from the two original specimens collected in central Georgia. The records from York and Lancaster Counties in northern South Carolina (Ciegler 2000: 55) need confirmation.

#### Records.

**USA**: GA [SC]

### Anillinus unicoi

Sokolov, 2011

Anillinus unicoi Sokolov, 2011: 3. Type locality: «Stratton Meadows at 35°20.229'N, 84°1.862'W (1300m), Unicoi Mountains, Graham County, North Carolina» (original citation). Holotype (♂) in USNM.

#### Distribution.

This species is known only from the holotype collected in the central part of the Unicoi Mountains in North Carolina.

#### Records.

**USA**: NC

### Anillinus valentinei

(Jeannel, 1963)

Troglanillus valentinei Jeannel, 1963b: 148. Type locality: «Fort-Payne Cave [DeKalb County], Alabama» (original citation). Holotype (♂) in USNM [# 69550].

#### Distribution.

This true troglobite species is confined to caves in northeastern Alabama east of the Wills Creek anticline (Barr 1995: 243).

#### Records.

**USA**: AL

### Anillinus virginiae

Jeannel, 1963

Anillinus virginiae Jeannel, 1963a: 76. Type locality: «Skyland [Page County], Virginia» (original citation). Holotype (♀) in USNM [# 69546].

#### Distribution.

This species is known for sure only from the type locality in northern Virginia (Sokolov et al. 2004: 207). The record from “West Virginia” (Bousquet and Larochelle 1993: 157) needs confirmation.

#### Records.

**USA**: VA [WV]

### Genus Serranillus

Barr, 1995

Serranillus Barr, 1995: 246. Type species: *Serranillus jeanneli* Barr, 1995 by original designation. Etymology. From the Latin noun *serra* (saw) and the generic name *Anillus*, alluding to the serrulate lateral margins of the elytra (“subhumeral margin strongly crenelate”) [masculine].

#### Diversity.

Three species in eastern North America.

#### Identification.

Sokolov and Carlton (2008: 40) provided a key for the identification of all species.

### Serranillus dunavani

(Jeannel, 1963)

Anillinus dunavani Jeannel, 1963a: 76. Type locality: «Rocky Bottom (1500 m), Sassafras mountains, Pickens Co[unty], South Carolina» (original citation). Holotype (♂) in USNM [# 69542].

#### Distribution.

This species is known only from the holotype collected in northwestern South Carolina. The records from North Carolina and northeastern Georgia (Barr 1995: 245) refer to *Anillinus loweae* Sokolov and Carlton (Sokolov et al. 2004: 189).

#### Records.

**USA**: SC

### Serranillus jeanneli

Barr, 1995

Serranillus jeanneli Barr, 1995: 247. Type locality: «along Ball Creek (approximately 950 m), Coweeta Hydrologic Laboratory, Macon Co[unty], North Carolina» (original citation). Holotype (♂) in CMNH.

#### Distribution.

This species is known only from the type locality in southwestern North Carolina.

#### Records.

**USA**: NC

### Serranillus septentrionis

Sokolov and Carlton, 2008

Serranillus septentrionis Sokolov and Carlton, 2008: 38. Type locality: «Black Horse Gap, Blue Ridge P[ark]w[a]y, Botetourt Co[unty], V[irgini]a» (original citation). Holotype (♂) in USNM.

#### Distribution.

This species is known from Giles and Botetourt Counties in Virginia (Sokolov and Carlton 2008: 40).

#### Records.

**USA**: VA

### Genus Anillaspis

Casey, 1918

Anillaspis Casey, 1918: 168. Type species: *Anillus explanatus* Horn, 1888 by original designation. Etymology. Uncertain, possibly from the generic name *Anillus* and the Greek *aspis* (shield) [feminine].

#### Diversity.

Two species in the Sierra Nevada of California.

#### Identification.

Jeannel (1963a: 79) provided a key for the identification of the two species.

### Anillaspis caseyi

Jeannel, 1963

Anillaspis caseyi Jeannel, 1963a: 80. Type locality: «Placer Co[unty], California» (original citation). Holotype (♀) location unknown (not in USNM). Etymology. The species name was proposed in honor of Thomas Lincoln Casey [1857-1925], American military officer, engineer, coleopterist, conchologist, and astronomer. Casey is well known for his controversial concept of species where little room was left for variation. His taxonomic approach was heavily criticized by his peers but nevertheless he always believed he was right. He once replied to a friend about these criticisms “I am just a generation ahead of the rest of you.” Note. Jeannel (1963a: 80) reported that the holotype of this species, which came from Casey’s collection, is labeled “Alabaster Cave, Cal.” but since Casey (1918: 168) noted that the sole specimen of *Anillinus explanatus* he had, upon which *Anillinus caseyi* was described, came from Placer County, the label is apocryphal.

#### Distribution.

This species is known only from the holotype collected in eastern California.

#### Records.

**USA**: CA

### Anillaspis explanata

(Horn, 1888)

Anillus explanatus G.H. Horn, 1888: 26. Type locality: «Alabaster Cave [Eldorado County], California» (original citation). Holotype [by monotypy] (♀) in MCZ [# 35574].

#### Distribution.

This species is known only from the holotype collected in eastern California.

#### Records.

**USA**: CA

### Subtribe Horologionina

Jeannel, 1949

Horologionidae Jeannel, 1949b: 91. Type genus: *Horologion* Valentine, 1932.

#### Diversity.

One troglobitic species in eastern North America.

### Genus Horologion

Valentine, 1932

Horologion Valentine, 1932b: 2. Type species: *Horologion speokoites* Valentine, 1932 by monotypy. Etymology (original). From the Greek *horologion* (hourglass), probably alluding to the shape of the body of the adult which, because of the markedly constricted pronotum posteriorly, resembles an hourglass [neuter].

#### Diversity.

One species in eastern United States.

#### Identification.

Valentine (1932b) published a detailed description of the species enhanced by several accurate drawings.

### Horologion speokoites

Valentine, 1932

Horologion speokoites Valentine, 1932b: 3. Type locality: «Arbuckle’s Cave, Maxwelton [Greenbrier County], W[est] V[irgini]a» (original citation). Holotype (♂) in USNM [# 44255].

#### Distribution.

This species is known only from the holotype collected in a cave three miles north of Lewisburg, near Maxwelton, in southeastern West Virginia. The cave has two rather small rooms connected by a narrow, descending, and tortuous passage. The specimen was found in the lower room, which was wet, muddy, and quite dark (Valentine 1932b: 1-2).

#### Records.

**USA**: WV

### Tribe Pogonini

Laporte, 1834

Pogonidae Laporte, 1834: 70. Type genus: *Pogonus* Dejean, 1821.
Pogonopsini Bedel, 1900a: 20. Type genus: *Pogonopsis* Bedel, 1898.

#### Diversity.

About 85 species (Lorenz 2005: 237-238) in the Nearctic (six species), Neotropical (five species), Australian (about 15 species), Palaearctic (about 50 species), and Afrotropical (five species) Regions arrayed in 12 genera: *Bedeliolus* Semenov (three Palaearctic species), *Cardiaderus* Dejean (one Palaearctic species), *Diodercarus* Lutshnik (one Palaearctic species), *Diplochaetus* (four species), *Ochtozetus* Chaudoir (two South American species), *Olegius* Komarov (one species from Turkmenistan), *Pogonistes* Chaudoir (eight Palaearctic species), *Pogonopsis* Bedel (one north African species), *Pogonus* (about 55 species), *Sirdenus* Dejean (five Palaearctic species, one of them extending into the Afrotropical Region), *Syrdenoidius* Baehr and Hudson (one species from south Australia), and *Thalassotrechus* (one species).

#### Identification.

Bousquet and Laplante (1997) revised the Western Hemisphere species and provided a key for their identification.

### Genus Thalassotrechus

Van Dyke, 1918

Thalassotrechus Van Dyke, 1918 [4 October]: 303. Type species: *Trechus barbarae* Horn, 1892 by original designation. Etymology. From the Greek *thalassa* (sea) and the generic name *Trechus* [*q.v*.], alluding to the habitat (“in crevices of those rocks situated just below the high tide mark”) where adults of these *Trechus*-like species (“it superficially resembles ... the typical genus *Trechus*”) are found [masculine].
Anatrechus Casey, 1918 [12 November]: 411. Type species: *Trechus barbarae* Horn, 1892 by original designation. Etymology. From the Greek *ana* (up, back) and the generic name *Trechus* [*q.v*.] [masculine].

#### Diversity.

One species confined to the Pacific Coast of California and Baja California.

### Thalassotrechus barbarae

(Horn, 1892)

Trechus barbarae G.H. Horn, 1892c: 41. Type locality: «Santa Barbara [Santa Barbara County], Cal[ifornia]» (original citation). Lectotype (♂), designated by Bousquet and Laplante (1997: 705), in MCZ [# 34324].
Thalassotrechus nigripennis Van Dyke, 1918: 304. Type locality: «Moss Beach, San Mateo County, California» (original citation). Holotype (♂) in CAS [# 3286]. Synonymy established by Evans (1977b: 86).

#### Distribution.

This species is found along the Pacific Coast from northern California to southern Baja California [see Bousquet and Laplante 1997: map 1].

#### Records.

**USA**: CA – Mexico

### Genus Diplochaetus

Chaudoir, 1872

Diplochaetus Chaudoir, 1872a: 36. Type species: *Pogonus rutilus* Chevrolat, 1863 by monotypy. Etymology (original). From the Greek *diplos* (double) and *chaete* (hair), alluding to the presence of two setae at the apex of the glossal sclerite (“*ligula medio apice setis binis approximatis instructa*”) of the adult [masculine].

#### Diversity.

Western Hemisphere, with four species in the Nearctic (four species, two of them endemic) and Neotropical (two species) Regions, including the West Indies.

### Diplochaetus emaciatus

(Bates, 1891)

Pogonus emaciatus Bates, 1891a: 260. Type locality: «Mazatlan [Sinaloa, Mexico]» (original citation). Lectotype (♀), designated by Bousquet and Laplante (1997: 712), in BMNH.
Diplochaetus desertus Van Dyke, 1953a: 98. Type locality: «on the margins of Salton Sea, Riverside Co[unty], California» (original citation). Holotype (♂) in CAS [# 8160]. Synonymy established by Bousquet and Laplante (1997: 712).

#### Distribution.

This species is known from a few localities around Salton Sea in southern California, from the northern part of the Baja California Peninsula, and from the state of Sinaloa along the Pacific Coast in Mexico [see Bousquet and Laplante 1997: map 2].

#### Records.

**USA**: CA – Mexico

### Diplochaetus megacephalus

Bousquet and Laplante, 1997

Diplochaetus megacephalus Bousquet and Laplante, 1997: 711. Type locality: «Bottomless Lakes S[tate] P[ark], Chaves Co[unty], N[ew]M[exico]» (original citation). Holotype (♂) in CNC [# 22161].

#### Distribution.

This species is presently known from numerous specimens collected in two localities in Chaves County, New Mexico [see Bousquet and Laplante 1997: map 2].

#### Records.

**USA**: NM

### Diplochaetus planatus

(Horn, 1876)

Pogonus depressus LeConte, 1874b: 44 [primary homonym of *Pogonus depressus* Motschulsky, 1844]. Type locality: «San Diego [San Diego County], California» (original citation). Lectotype, designated by Bousquet and Laplante (1997: 713), in MCZ [# 5595].
Pogonus planatus G.H. Horn, 1876c: 250. Replacement name for *Pogonus depressus* LeConte, 1874.

#### Distribution.

This species ranges from south-central Oregon to south-central Kansas, south to western Texas near the Rio Grande, southeastern Arizona [see Bousquet and Laplante 1997: map 3], and southwestern California (LeConte 1874b: 44). One specimen without specific locality is known from Nebraska (Bousquet and Laplante 1997: 714).

#### Records.

**USA**: AZ, CA, KS, NM, NV, OK, OR, TX, UT [NE]

### Diplochaetus rutilus

(Chevrolat, 1863)

Pogonus rutilus Chevrolat, 1863: 197. Type locality: «Cuba» (original citation). Syntype(s) location unknown (possibly in UMO in collection Chevrolat and MHNP in collection Chaudoir).
Pogonus parallelus LeConte, 1874b: 44 [primary homonym of *Pogonus parallelus* Chaudoir, 1872]. Type locality: «Texas» (original citation). Holotype [by monotypy] (♂) in MCZ [# 34824]. Synonymy established by Bousquet and Laplante (1997: 708). Note. LeConte (1874b: 44) noted that the sole specimen he had was a female.
Pogonus lecontei G.H. Horn, 1876c: 250. Replacement name for *Pogonus parallelus* LeConte, 1874.

#### Distribution.

This species is known from New Jersey, southern South Carolina (Ciegler 2000: 56), the Florida Peninsula including the Keys, some islands in the Greater Antilles, southeastern Texas, Chiapas in Mexico, southern New Mexico (Otero County, CMNH), and southeastern Arizona [see Bousquet and Laplante 1997: map 2]. The species is also found in Colombia (Bousquet and Laplante 1997: 710) and Venezuela (Chaudoir 1872a: 36).

#### Records.

**USA**: AZ, FL, NJ, NM, SC, TX – Colombia, Cuba, Cayman Islands, Dominican Republic, Mexico, Venezuela

### Genus Pogonus

Dejean, 1821

Pogonus Dejean, 1821: 2, 9. Type species: *Carabus littoralis* Duftschmid, 1812 by monotypy. Etymology. Uncertain, possibly from the Greek *pogonos* (beard) [masculine]. The name was proposed by Franz Anton Ziegler and made available by Dejean.

#### Diversity.

About 55 species in two subgenera: *Pogonoidius* Carret (six Palaearctic species) and *Pogonus* s.str. (about 50 species).

### Subgenus Pogonus

Dejean, 1821

Pogonus Dejean, 1821: 2, 9. Type species: *Carabus littoralis* Duftschmid, 1812 by monotypy.

#### Diversity.

About 50 species in the Nearctic (one species), Australian (17 species), Palaearctic (about 30 species), and Afrotropical (four species) Regions.

### Pogonus texanus

Chaudoir, 1868

Pogonus texanus Chaudoir, 1868b: 344. Type locality: «Texas» (original citation), herein restricted to Goose Island State Park, Aransas County (see Bousquet and Laplante 1997: 716). One syntype [2 ♀ originally cited] in MHNP and one in MCZ (collection LeConte, see LeConte 1874b: 45).

#### Distribution.

This species is known only from a few localities in southwestern Louisiana and eastern Texas south to the Rio Grande [see Bousquet and Laplante 1997: map 1]; it was also collected at least twice at Atlantic City, New Jersey (G.E. Horn in Smith 1890: 79) in the xix Century.

#### Records.

**USA**: LA, NJ, TX

### Subfamily PATROBINAE

Kirby, 1837

Patrobidae Kirby, 1837: 50. Type genus: *Patrobus* Dejean, 1821.

#### Diversity.

About 220 species in the Nearctic, Oriental, and Palaearctic Regions arrayed in two tribes: Lissopogonini (six species in the Oriental and Palaearctic Regions) and Patrobini (about 215 species).

### Tribe Patrobini

Kirby, 1837

Patrobidae Kirby, 1837: 50. Type genus: *Patrobus* Dejean, 1821.

#### Diversity.

Northern Hemisphere, with about 215 species arrayed in four subtribes following Zamotajlov (2002): Deltomerina (about 115 species), Deltomerodina (13 species in the genus *Deltomerodes* Deuve), Patrobina (about 80 species), and Platidiolina (five species). The North American fauna is represented by 13 species (roughly 6.2 % of the world fauna).

#### Identification.

Darlington (1938) and Lindroth (1961a) reviewed the North American species and provided keys for their identification. Subsequently to Lindroth’s work, one species (*Patrobus septentrionis*) was shown to consist of two distinct forms by Zamotajlov (2003c).

### Subtribe Deltomerina

Chaudoir, 1872

Deltomeridae Chaudoir, 1872a: 51. Type genus: *Deltomerus* Motschulsky, 1850.

#### Diversity.

Northern Hemisphere, with about 115 species in the Nearctic (four species) and Palaearctic (about 105 species) Regions arrayed in nine genera: *Deltomerus* (about 70 species in eastern Europe, northern Africa, and the Middle East), *Diplous* (23 species), *Himalopenetretus* Zamotajlov (one species in Uttar Pradesh), *Ledouxius* Zamotajlov (seven species in Kashmir and Pakistan), *Minipenetetrus* Zamotajlov (one Chinese species), *Naxipenetetrus* Zamotajlov (two Chinese species), *Patanitretus* Zamotajlov (one species in Pakistan), *Penetretus* Motschulsky (five south European species), and *Qiangopatrobus* Zamotajlov (three Chinese species).

### Genus Diplous

Motschulsky, 1850

Diplous Motschulsky, 1850a: x. Type species: *Patrobus sibiricus* Motschulsky, 1844 by monotypy. Etymology. From the Greek *diplous* (double), possibly alluding to the emarginate mentum tooth (“*mentum dente medio bicuspi*”) of the adult [masculine].

#### Distribution.

Twenty-three species (Lorenz 2005: 241-242) in the Nearctic (four species) and Palaearctic (19 species in Asia only) Regions arrayed in two subgenera: *Diplous* s.str. (17 species) and *Platidius* (six species).

### Subgenus Platidius

Chaudoir, 1872

Platidius Chaudoir, 1872a: 51. Type species: *Patrobus aterrimus* Dejean, 1828 designated by Darlington (1938: 147). Etymology. Probably from the Greek *platys* (flat), referring to the flat body (“*forme aplatie*”) of the adult [masculine].

#### Diversity.

Six species in the Nearctic (four species) and Palaearctic (*Diplous depressus* Gebler and *Diplous dolini* Zamotajlov in eastern Asia) Regions.

#### Identification.

Marek and Kavanaugh (2005) revised the North American species and provided a key for their identification.

### Diplous aterrimus

(Dejean, 1828)

Patrobus aterrimus Dejean, 1828: 32. Type locality: «détroit de Norfolk [= Sitka Sound, Baranof Island, Alaska], sur la côte nord-ouest de l’Amérique septentrionale» (original citation). Syntype(s) in MHNP and possibly also in MCZ (collection LeConte).
Patrobus fulcratus LeConte, 1869c: 374. Type locality: Vancouver Island, British Columbia (inferred from title of the paper). Syntype(s) [2 originally cited] in MCZ [# 5594]. Synonymy established by Horn (1875: 130), confirmed by Darlington (1938: 150).
Platidius breviceps Casey, 1918: 402. Type locality: «Boulder Co[unty], Colorado» (original citation). Lectotype (♂), designated by Lindroth (1975: 114), in USNM [# 46074]. Synonymy established by Darlington (1938: 150).
Platidius tenuitarsis Casey, 1918: 403. Type locality: «Colorado» (original citation). Lectotype (♂), designated by Lindroth (1975: 114), in USNM [# 46071]. Synonymy established (as aberration) by Csiki (1928: 342), confirmed by Darlington (1938: 150).
Platidius coloradensis Casey, 1918: 403. Type locality: «Red Cliff [Eagle County], Colorado» (original citation). Lectotype (♂), designated by Lindroth (1975: 114), in USNM [# 46072]. Synonymy established (as aberration) by Csiki (1928: 342), confirmed by Darlington (1938: 150).
Platidius reflexus Casey, 1918: 403. Type locality: «Colorado» (original citation). Lectotype (♀), designated by Lindroth (1975: 114), in USNM [# 46073]. Synonymy established (as aberration) by Csiki (1928: 341), confirmed by Darlington (1938: 150).

#### Distribution.

The range of this species extends from northern Northwest Territories and northern Yukon Territory south to central Oregon (Darlington 1938: 150), north-central Utah, and southern Colorado [see Marek and Kavanaugh 2005: Fig. 10].

#### Records.

**CAN**: AB, BC (QCI, VCI), NT, YT **USA**: AK, CO, ID, MT, OR, UT, WA, WY

### Diplous californicus

(Motschulsky, 1844)

Patrobus californicus Motschulsky, 1844: 131. Type locality: «Californie» (original citation), cited from «environs de St. Francisco [San Francisco County]» by Motschulsky (1859a: 123). Lectotype, designated by Marek and Kavanaugh (2005: 159), in MCZ [# 8232].
Patrobus trochantericus LeConte, 1869c: 375. Type locality: «Fort Crook [Shasta County], northern California» (original citation). Two syntypes in MCZ [# 5593]. Synonymy established by Horn (1875: 131), confirmed by Darlington (1938: 149).
Platidius latipennis Casey, 1918: 399. Type locality: Gualala, Mendocino County, California (lectotype label according to Lindroth 1975: 114). Lectotype (♂), designated by Lindroth (1975: 114), in USNM [# 46067]. Synonymy established (as aberration) by Csiki (1928: 342), confirmed by Darlington (1938: 149).
Platidius incisus Casey, 1918: 399. Type locality: «south of San Francisco, California» (original citation). Lectotype (♀), designated by Lindroth (1975: 114), in USNM [# 46065]. Synonymy established (as aberration) by Csiki (1928: 342), confirmed by Darlington (1938: 149).
Platidius strenuus Casey, 1918: 400. Type locality: «Washington State» (original citation). Lectotype (♂), designated by Lindroth (1975: 114), in USNM [# 46069]. Synonymy established (as aberration) by Csiki (1928: 342), confirmed by Darlington (1938: 149).
Platidius rectus Casey, 1918: 400. Type locality: «Reno [Washoe County], Nevada» (original citation). Lectotype (♂), designated by Lindroth (1975: 114), in USNM [# 46066]. Synonymy established (as aberration) by Csiki (1928: 342), confirmed by Darlington (1938: 149).
Platidius sierranus Casey, 1918: 401. Type locality: «Mokelumne Hill, Calaveras Co[unty], California» (original citation for the lectotype). Lectotype (♂), designated by Lindroth (1975: 114), in USNM [# 46068]. Synonymy established (as aberration) by Csiki (1928: 342), confirmed by Darlington (1938: 149).
Platidius breviusculus Casey, 1918: 401. Type locality: «Reno [Washoe County], Nevada» (original citation). Holotype [by monotypy] (♂) in USNM [# 46070]. Synonymy established by Darlington (1938: 149).

#### Distribution.

This species ranges from western Montana (Russell 1968: 47) to Vancouver Island (Lindroth 1961a: 188), south to the northern part of the Coast Ranges in California and the Sierra Nevada (Darlington 1938: 149; Marek and Kavanaugh 2005: 160).

#### Records.

**CAN**: BC (VCI) **USA**: CA, ID, MT, NV, OR, WA

### Diplous filicornis

(Casey, 1918)

Platidius filicornis Casey, 1918: 404. Type locality: «Redwood Creek, Humboldt Co[unty], California» (original citation). Lectotype (♀), designated by Lindroth (1975: 114), in USNM [# 46075].

#### Distribution.

This species ranges from the Skeena River drainage in central British Columbia (Lindroth 1961a: 189) south to the Coast Ranges and the Sierra Nevada in central California (Darlington 1938: 151; Marek and Kavanaugh 2005: 164).

#### Records.

**CAN**: BC **USA**: CA, OR, WA

### Diplous rugicollis

(Randall, 1838)

Patrobus rugicollis Randall, 1838a: 1 (as *angicollis*). Type locality: «Grafton Notch, [near] Bethel [Oxford County], M[ain]e» (neotype label). Neotype (♂), designated by Darlington (1938: 152), in MCZ [# 23357]. Note. «Hallowell» in Maine was the place originally cited by Randall (1838a: 2). The spelling *angicollis* was an error for *rugicollis* as indicated in the “Errata” inserted in the *Boston Journal of Natural History* (volume 2, page 560). The spelling *rugicollis* is in prevailing usage and attributed to the publication of the original spelling; therefore it is deemed to be the correct original spelling (ICZN 1999: Article 33.3.1).
Patrobus longipalpus Notman, 1919b: 231. Type locality: «Keene Heights, Essex Co[unty], N[ew] Y[ork]» (original citation). Holotype [by monotypy] (♀) in SIM (Hennessey 1990: 466). Synonymy established by Darlington (1938: 152).

#### Distribution.

This eastern species is found from Cape Breton Island (Lindroth 1961a: 189) to the Saint Lawrence Valley in southern Quebec (Larochelle 1975: 81), south to northeastern West Virginia (Randolph County, CMNH). Fossil remnants from the Early Wisconsinan have been unearthed in southern Ontario (Morgan and Morgan 1981: 1107).

#### Records.

**CAN**: NB, NS (CBI), QC **USA**: MA, ME, NH, NY, PA, VT, WV

### Subtribe Patrobina

Kirby, 1837

Patrobidae Kirby, 1837: 50. Type genus: *Patrobus* Dejean, 1821.

#### Diversity.

Northern Hemisphere, with about 80 species in the Nearctic (eight species) and Palaearctic (about 75 species, only six of them occurring in Europe) Regions arrayed in ten genera: *Apatrobus* Habu and Baba (28 species), *Archipatrobus* Zamotajlov (three species), *Chaetapatrobus* Lafer (one species), *Chinapenetetrus* Kurnakov (11 species), *Dimorphopatrobus* Casale and Sciaky (one species), *Minypatrobus* Uéno (four species), *Parapenetretus* Kurnakov (14 species), *Patrobus* (16 species), *Platypatrobus* (one species), and *Quasipenetretus* Zamotajlov (one species).

### Genus Patrobus

Dejean, 1821

Patrobus Dejean, 1821: 10. Type species: *Carabus rufipes* Fabricius *sensu* Duftschmid, 1812 (= *Carabus atrorufus* Strøm, 1768) designated by Curtis (1827: plate 192). Etymology. Uncertain, possibly from the Greek *patros* (father, by extension homeland) and the Latin suffix -*bus* (having the quality of) rather than the Greek *bos* (ox, cow, cattle) [masculine]. According to Desmarest (1851: 132), the name derived from the Greek *petros* (stone) and *bios* (life). The name was proposed by Johann Karl Megerle von Mühlfeld and made available by Dejean.
Neopatrobus Darlington, 1938: 155. Type species: *Feronia longicornis* Say, 1823 by original designation. Synonymy established by Lorenz (1998: 226). Etymology. From the Greek prefix *neo*- (new) and the generic name *Patrobus* [*q.v*.] [masculine].
Geopatrobus Darlington, 1938: 157. Type species: *Platysma foveocollis* Eschscholtz, 1823 by original designation. Synonymy established by Bousquet and Larochelle (1993: 159). Etymology. From the Greek prefix *geo*- (earth) and the generic name *Patrobus* [*q.v*.] [masculine].

#### Diversity.

Sixteen species in the arctic, subarctic, boreal, and temperate areas of the Nearctic (seven species) and Palaearctic (13 species) Regions. Four species are Holarctic.

### [fossifrons group]

### Patrobus fossifrons

(Eschscholtz, 1823)

Platysma fossifrons Eschscholtz, 1823: 104. Type locality: «Kamtschatka [Russia] et Unalaschka [Alaska]» (original citation), restricted to «Unalaska» by Darlington (1938: 162). Syntype(s) in ZMH (Silfverberg 1987: 16) and probably also in ZMMU (Lindroth 1961a: 181). Note. This species was also made available the same year by an illustration in Fischer von Waldheim (1823: plate 19, figure 4).
Patrobus longiventris Mannerheim, 1853: 145. Type locality: «in ora orientali insulae Kadjak [Alaska]» (original citation). Lectotype (♀), designated by Lindroth (1961a: 181), in ZMH. Synonymy established by Darlington (1938: 161), confirmed by Lindroth (1961a: 181).
Patrobus fulvus Mannerheim, 1853: 145. Type locality: «insula Kadjak [Alaska]» (original citation). Lectotype (♂), designated by Lindroth (1961a: 181), in ZMH. Synonymy established, under the name *Patrobus latiusculus* Chaudoir, by Chaudoir (1872a: 46), confirmed by Lindroth (1961a: 181).
Patrobus latiusculus Chaudoir, 1872a: 46. Type locality: «côte orientale de l’île Kadjak près de la côte nord-ouest de l’Amérique [Alaska]; Orégon» (original citation), restricted to «Kodiak Island [Alaska]» by Darlington (1938: 162). Syntype(s) in MHNP. Synonymy established by Darlington (1938: 161).
Patrobus fossifrons dimorphicus Darlington, 1938: 161. Type locality: «near Victoria, Vancouver Island, British Columbia» (original citation). Holotype (♂) in MCZ [# 22983]. Synonymy established by Lindroth (1961a: 181).

#### Distribution.

This western species ranges from southeastern Alberta to Vancouver Island, north to the Gulf Coast of Alaska, including the Aleutian Islands (Lindroth 1961a: 181), south to Mono County in the Sierra Nevada (CAS) and mountains in southwestern Colorado (Darlington 1938: 162-163). The record from Kamchatka (Eschscholtz 1823: 104) is probably in error since the species is not listed from the Palaearctic Region by Zamotajlov (2003b: 284-285).

#### Records.

**CAN**: AB, BC (VCI) **USA**: AK, CA, CO, ID, MT, OR, UT, WA, WY

#### Note.

Pohl (1998) reported that members of this species can be segregated into a “coastal” and “inland” morphs and that hybridization occurs between the coastal form of *Patrobus fossifrons* and *Patrobus stygicus*.

### Patrobus lecontei

Chaudoir, 1872

Patrobus rufipes LeConte, 1863c: 18 [secondary homonym of *Patrobus rufipes* (Duftschmid, 1812)]. Type locality: «North Red River [in southern Manitoba or on Minnesota-Dakota line]» (original citation). Syntype(s) in MCZ [# 5592].
Patrobus lecontei Chaudoir, 1872a: 47. Replacement name for *Patrobus rufipes* LeConte, 1863.
Patrobus canadensis Casey, 1924: 67. Type locality: «Edmonton, Alberta» (original citation). Lectotype (♂), designated by Lindroth (1975: 113), in USNM [# 46064]. Synonymy established by Darlington (1938: 159).
Patrobus lecontei gravidus Darlington, 1938: 160. Type locality: «Little River, southwest Newfoundland» (original citation). Holotype (♂) in MCZ [# 21782]. Synonymy established by Lindroth (1955a: 82).

#### Distribution.

This species is found from Newfoundland (Lindroth 1955a: 83) to the Okanagan River in south-central British Columbia, north to the Peace River in northeastern British Columbia (Lindroth 1961a: 181), south to eastern South Dakota (Kirk and Balsbaugh 1975: 21), northern Minnesota (Lindroth 1963a: Fig. 61), and the Pontiac region in southwestern Quebec (Lindroth 1961a: 181); isolated in high mountains in Colorado (Darlington 1938: 160).

#### Records.

**FRA**: PM **CAN**: AB, BC, MB, NB, NF, QC, SK **USA**: CO, MN, ND, SD

### Patrobus stygicus

Chaudoir, 1872

Patrobus stygicus Chaudoir, 1872a: 46. Type locality: «côte méridionale de Terre-Neuve» (original citation). Holotype [by monotypy] (♂) in MHNP.

#### Distribution.

This species ranges from Newfoundland (Lindroth 1955a: 83) to the Chukchi Sea coast in northwestern Alaska (Lindroth 1961a: 183), south to north-central Washington (Pohl 1998: 690), northern Minnesota (Clearwater and Lake Counties, CNC), the upper peninsula of Michigan (Escanaba, MCZ), and north-central Maine (Larochelle and Larivière 1990a: 28; Piscataquis County, CNC). The species is also known from the Far East (Zamotajlov 2003b: 285). Fossil remnants, dated between 10,400 and 15,400 years B.P., have been unearthed in Cape Breton Island in Nova Scotia (Miller 1997: 250) and central Iowa (Schwert 1992: 76); others from a Plio-Pleistocene sequence have been found in northwestern Greenland (Böcher 1995: 23).

#### Records.

**CAN**: AB, BC, LB, MB, NF, NT, ON, QC, SK, YT **USA**: AK, ME, MI, MN, WA – **Holarctic**

### [foveocollis group]

### Patrobus foveocollis

(Eschscholtz, 1823)

Platysma foveocollis Eschscholtz, 1823: 105. Type locality: «Unalaschka [Aleutian Islands, Alaska]» (original citation). Syntype(s) in ZMH (Silfverberg 1987: 16) and possibly also in ZMMU (Lindroth 1961a: 185). Note. This species was also made available the same year by an illustration in Fischer von Waldheim (1823: plate 19, figure 5).
Pterostichus tenuis LeConte, 1850: 207. Type locality: Lake Superior (inferred from title of the paper). Syntype(s) in MCZ [# 5590]. Synonymy established by Lindroth (1955a: 85).
Patrobus angusticollis Mannerheim, 1853: 146. Type locality: «ad sinum Woskresensk [= Resurrection Bay] peninsulae Kenai [Alaska]» (original citation). Syntype(s) in MHNP (collection Chaudoir, see Chaudoir 1872a: 45). Synonymy established, under the name *Patrobus foveocollis tenuis* (LeConte), by Darlington (1938: 167).
Patrobus obtusiusculus Chaudoir, 1872a: 43. Type locality: «Terre de Rupert près de la baie d’Hudson» (original citation). Holotype [by monotypy] (♀) probably in MHNP. Synonymy established with doubt, under the name *Patrobus foveocollis tenuis* (LeConte), by Darlington (1938: 167).
Patrobus laeviceps Casey, 1918: 397. Type locality: «W[est] S[ain]t Modest[e], Labrador» (original citation). Lectotype (♂), designated by Lindroth (1975: 114), in USNM [# 46060]. Synonymy established, under the name *Patrobus foveocollis tenuis* (LeConte), by Darlington (1938: 167), confirmed by Lindroth (1961a: 185).
Patrobus insularis Casey, 1918: 397. Type locality: «S[ain]t Paul Island, Alaska» (original citation), which is doubtful (Lindroth 1961a: 24, 185-186). Lectotype (♀), designated by Lindroth (1975: 114), in USNM [# 46059]. Synonymy established, under the name *Patrobus foveocollis tenuis* (LeConte), by Darlington (1938: 167), confirmed by Lindroth (1961a: 185).

#### Distribution.

This Holarctic species is found in easternmost Siberia (Zamotajlov 2003b: 285) and on this continent from Alaska, south of the Arctic Circle and including the Aleutian and Kodiak Islands (Lindroth 1961a: 186), to Newfoundland (Lindroth 1955a: 85), south to Mont Katahdin in Maine, the White Mountains in New Hampshire (Darlington 1938: 169), the Adirondack Mountains in northeastern New York (Notman 1928: 221, as *Patrobus septentrionis* var. *tenuis*), the upper peninsula of Michigan along Lake Superior (Hubbard and Schwarz 1878: 629, as *Patrobus tenuis*), northeastern Minnesota (Gandhi et al. 2005: 925), northern Wyoming (Johnson County, CMNH), and northern British Columbia (Lindroth 1961a: 186); isolated in high mountains in Colorado (Darlington 1938: 169; Armin 1963: 191).

#### Records.

**CAN**: AB, BC, LB, MB, NB, NF, NT, ON, QC, SK, YT **USA**: AK, CO, ME, MI, MN, NH, NY, VT, WY – **Holarctic**

### [longicornis group]

### Patrobus longicornis

(Say, 1823)

Feronia longicornis Say, 1823a: 40. Type locality: «Arlington [Middlesex County], Mass[achusetts]» (neotype label). Neotype (♂), designated by Darlington (1938: 158), in MCZ [# 22982].
Patrobus americanus Dejean, 1828: 34. Type locality: «Amérique septentrionale» (original citation). Syntype(s) probably in MHNP. Synonymy established by Dejean (1828: 34).

#### Distribution.

This widely distributed species ranges from Newfoundland (Lindroth 1955a: 82) to at least the Okanagan Valley in British Columbia, north to southern Northwest Territories (Lindroth 1961a: 180), south to southern Arizona (Darlington 1938: 158), the Sacramento Mountains in New Mexico (Fall and Cockerell 1907: 158), northern Oklahoma (Tulsa and Cimarron Counties, CNC), and northeastern Florida (Darlington 1938: 158). The record from “Texas” (Wickham 1896c: 132) needs confirmation.

#### Records.

**FRA**: PM **CAN**: AB, BC, MB, NB, NF, NS (CBI), NT, ON, PE, QC, SK **USA**: AL, AR, AZ, CO, CT, DC, FL, GA, IA, ID, IL, IN, KS, KY, LA, MA, MD, ME, MI, MN, MO, MS, MT, NC, ND, NE, NH, NJ, NM, NY, OH, OK, OR, PA, RI, SC, SD, TN, UT, VA, VT, WA, WI, WV, WY [TX]

### [septentrionis group]

### Patrobus cinctus

Motschulsky, 1860

Patrobus cinctus Motschulsky, 1860: 91. Type locality: «îles Kourilles» (original citation for the lectotype). Lectotype (♂), designated by Zamotajlov (2003c: 241), in ZMMU.
Patrobus fuscipennis Motschulsky, 1860: 91. Type locality: «Kamtschatka [Russia]» (original citation). Lectotype (♀), designated by Zamotajlov (2003c: 241), in ZMMU. Synonymy established by Zamotajlov (2003a: 22).
Patrobus tritus Casey, 1920: 186. Type locality: «Marquette [Marquette County], Michigan» (original citation for *Patrobus tenuis* LeConte *sensu* Casey, 1918). Lectotype (♀), designated by Lindroth (1975: 114), in USNM [# 46063]. Synonymy established by Zamotajlov (2003a: 22). Note. This name was proposed for *Patrobus tenuis* (LeConte, 1850) *sensu* Casey (1918: 396).

#### Distribution.

The range of this species remains largely undocumented. It was reported by Zamotajlov (2003c: 242) from eastern Siberia, including the Kurils and Commander Islands, southern Alaska, including the Aleutian Islands, the upper peninsula of Michigan along Lake Superior (Casey 1920: 186, as *Patrobus tritus*), southern Quebec, and “Newfoundland.”

#### Records.

**CAN**: NF, QC **USA**: AK, MI – **Holarctic**

### Patrobus septentrionis septentrionis

Dejean, 1828

Patrobus alpinus Curtis, 1827: plate 192 [*nomen oblitum*, see Zamotajlov (2003c: 240)]. Type locality: «near the summit of Craig-calloch, one of the Dochart Hills [Scotland, United Kingdom]» (original citation). Syntype(s) location unknown (possibly in MVM).
Patrobus septentrionis Dejean, 1828 [29 November]: 29 [*nomen protectum*]. Type locality: «Laponie et dans les parties septentrionales de la Suède et de la Finlande» (original citation), restricted to «Lapland» by Darlington (1938: 166). Lectotype (♂), designated by Zamotajlov (2003c: 240), in MHNP. Synonymy established by Dawson (1854: 72).
Patrobus hyperboreus Dejean, 1828 [29 November]: 30. Type locality: «Groenland» (original citation). Lectotype (♀), designated by Zamotajlov (2003c: 240), in MHNP. Synonymy established by Schaum (1858: 377), confirmed by Zamotajlov (2003c: 240).
Harpalus picicornis Zetterstedt, 1828 [“31 December”]: 32. Type locality: «Tornensi [Torne Lappmark, Sweden]; Wittangi, Juckasjervi et Karesuando [Sweden]; Norvegia» (original citation). Twenty-one syntypes in ZMLS (Lindroth 1938: 19). Synonymy established by Zetterstedt (1837: 40). Note. According to Lindroth (1938: 19), of the 21 syntypes in ZMLS 19 are conspecific with this species and two with *Patrobus atrorufus*.
Patrobus lapponicus Chaudoir, 1844: 440. Type locality not stated. Holotype [by monotypy] (♂) probably in MHNP. Synonymy established by Schaum (1861: 202).
Patrobus lacustris Motschulsky, 1844: 130. Type locality: «[nearby] fleuve Ichim dans les Steppes des Kirguises [Siberia, Russia]» (original citation). Two syntypes in ZMMU (Keleinikova 1976: 202). Synonymy established, under the name *Patrobus lapponicus* Chaudoir, by Mäklin (1855: 32).
Patrobus rubripennis C.G. Thomson, 1857: 26. Type locality: «Lappland; Dovre [Norway]» (original citation). Syntype(s) in ZMLS (Charpentier 1972: 291). Synonymy established by Chaudoir (1872a: 44).
Patrobus labradorinus Casey, 1918: 395. Type locality: «W[est] S[ain]t Modest[e], Labrador» (original citation). Lectotype (♂), designated by Lindroth (1975: 114), in USNM [# 46062]. Synonymy established (as aberration) by Csiki (1928: 341), confirmed by Darlington (1938: 165).
Patrobus minuens Casey, 1918: 396. Type locality: «W[est] S[ain]t Modest[e], Labrador» (original citation). Lectotype (♂), designated by Lindroth (1975: 114), in USNM [# 46061]. Synonymy established (as aberration) by Csiki (1928: 341), confirmed by Darlington (1938: 165).

#### Distribution.

This circumpolar species ranges from Greenland (Böcher 1988: 8) west to Iceland (Zamotajlov 2003c: 241), south on this continent to northern Maine (Darlington 1938: 166; Dearborn and Donahue 1993: 5), northern New Hampshire (Coos County, Ross T. Bell pers. comm. 2008; Leng and Beutenmüller 1895: 75), northern Michigan (Lindroth 1961a: 184), northern Minnesota (Petrice et al. 2002: 9; Gandhi et al. 2005: 926), and southern Montana (Hatch 1933a: 7); isolated on high mountains in Colorado (Wickham 1902: 235; Zamotajlov 2003c: 241). Three specimens labeled “W[ashington]T[erritory]” are known (MCZ). Some of the records listed here may in fact refer to *Patrobus cinctus*.

#### Records.

**DEN**: GL **CAN**: AB, BC, LB, MB, NB, NF, NT, ON, QC, SK, YT **USA**: AK, CO, ME, MI, MN, MT, NH, WA – **Holarctic**

#### Note.

According to Zamotajlov and Isaev (2006), this species is represented by four subspecies in the Palaearctic Region: the nominotypical subspecies, *Patrobus septentrionis australis* Sahlberg, *Patrobus septentrionis volgensis* Zamotajlov and Isaev, and *Patrobus septentrionis sajanus* Zamotajlov.

### Genus Platypatrobus

Darlington, 1938

Platypatrobus Darlington, 1938: 146. Type species: *Platypatrobus lacustris* Darlington, 1938 by original designation. Etymology. From the Greek *platys* (broad, wide, flat) and the generic name *Patrobus* [*q.v*.], alluding to the body shape of adults (“form ... rather broad, depressed”) of the *Patrobus*-like species [masculine].

#### Diversity.

One species in boreal and northern temperate regions of North America.

#### Identification.

Bousquet and Grebennikov (1999) redescribed the species and illustrated the male and female genitalia.

#### Taxonomic Note.

With the exception of accessory setae on the pronotum, elytra, and mesosternum, this species is structurally very similar to those of *Patrobus* and possibly simply represents an offspring of *Patrobus*. The larva does not differ significantly from those of *Patrobus* (Bousquet and Grebennikov 1999).

### Platypatrobus lacustris

Darlington, 1938

Platypatrobus lacustris Darlington, 1938: 146. Type locality: «Ba[t]ch[a]w[a]n[a](g) B[ay] [north of Sault Sainte Marie, Ontario], Lake Superior» (original citation). Holotype (♀) in MCZ [# 21781].

#### Distribution.

This species ranges from New Brunswick to Great Slave Lake in Northwest Territories, south to the foothills of the Rocky Mountains in southern Alberta, northeastern Minnesota (Gandhi et al. 2005: 926), northeastern Ohio (Ashtabula and Trumbull Counties, Harry J. Lee, Jr. pers. comm. 2008), northeastern Pennsylvania (Pike County, CMNH), and Connecticut (Krinsky and Oliver 2004: 396) [see Bousquet 1987a: map 2].

#### Records.

**CAN**: AB, MB, NB, NT, ON, QC, SK **USA**: CT, ME, MN, NH, OH, PA, VT

**Figure 25.**
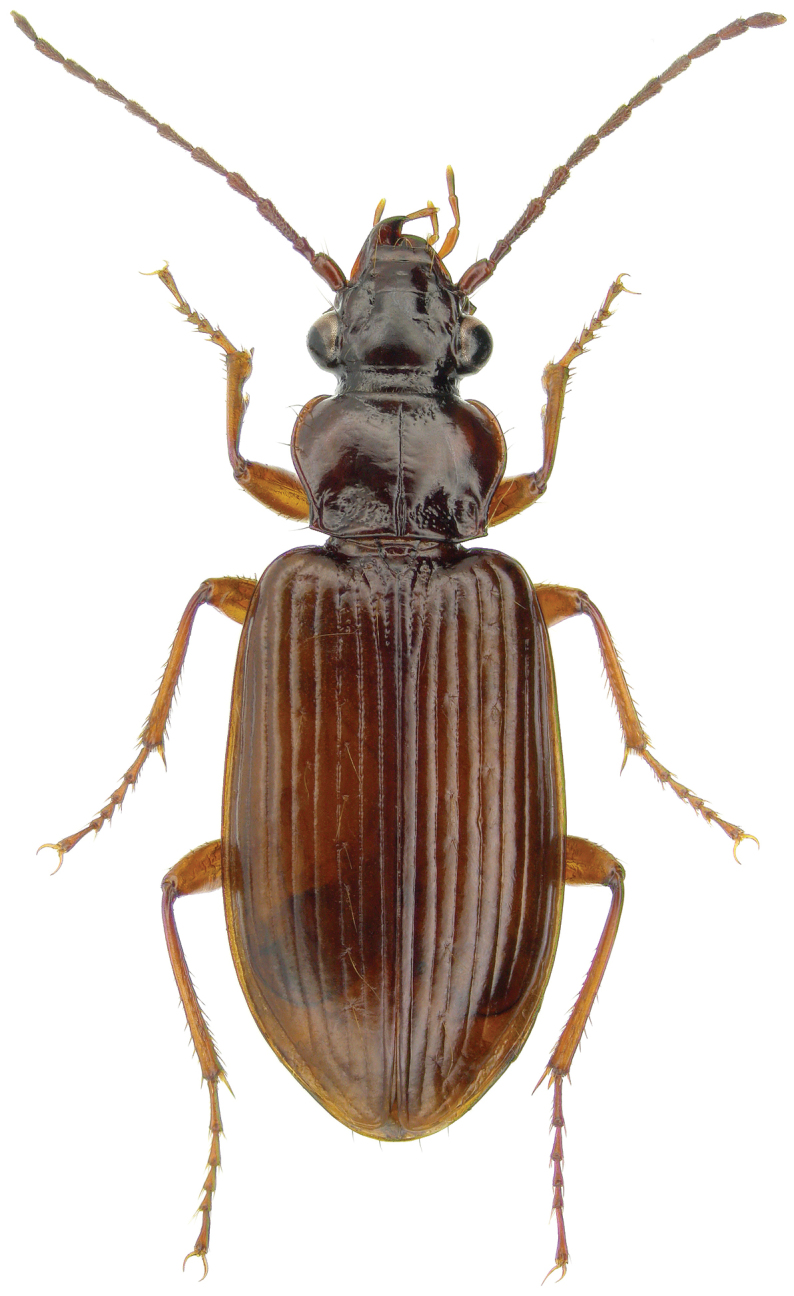
*Platypatrobus lacustris* Darlington. Described in 1938, this species remained a great rarity for more than 20 years. The few specimens known had all been captured at light. Henri Goulet, then a summer student working at the Biosystematics Research Centre in Ottawa (now part of the Eastern Cereal and Oilseed Research Centre), found out that a mite of the genus *Protodinychus*, members of which occurred commonly in beaver houses, had been found on one of the specimens of *Patrobus lacustris*. Armed with this information, Goulet visited an abandoned beaver house in Gatineau Park in southwestern Quebec and found more than 50 specimens of *Platypatrobus*. The habitat requirements of the species were discovered: *Platypatrobus lacustris* lives in the walls of active or recently deserted beaver houses.

### Subtribe Platidiolina

Zamotajlov and Lafer, 2001

Platidiolina Zamotajlov and Lafer, 2001: 411. Type genus: *Platidiolus* Chaudoir, 1878.

#### Diversity.

Five species placed in one genus.

### Genus Platidiolus

Chaudoir, 1878

Platidiolus Chaudoir, 1878: 77 (as *Piatidiolus*). Type species: *Platidiolus rufus* Chaudoir, 1878 by monotypy. Etymology. From the generic name *Platidius* and the Latin suffix -*olus* (little, small), alluding to the smaller size of adults of the *Platidius*-like species (“*infiniment plus petit que le Platidius depressus*”) [masculine]. Note. *Platidiolus* is an incorrect subsequent spelling of *Piatidiolus* in prevailing usage and attributed to the publication of the original spelling; therefore, it is deemed to be the correct original spelling (ICZN 1999: Article 33.3.1).
Patroboidea Van Dyke, 1926a: 67. Type species: *Patroboidea rufa* Van Dyke, 1925 (= *Platidiolus vandykei* Kurnakov, 1960) by monotypy. Synonymy established by Kurnakov (1960: 275). Etymology. From the generic name *Patrobus* [*q.v*.] and the Greek suffix -*oidea* (like, resembling), alluding to the resemblance of the adults to those of some species of *Patrobus* (“it belongs near the genus *Patrobus*, and in general has much the appearance of the flatter members of that genus”) [feminine].

#### Diversity.

Five species in Siberia (four species) and western North America (one species).

### Platidiolus vandykei

Kurnakov, 1960

Patroboidea rufa Van Dyke, 1926a: 69 [secondary homonym of *Platidiolus rufus* Chaudoir, 1878]. Type locality: «along the Snohomish River near Monroe [Snohomish County], Washington» (original citation). Holotype (♂) in CAS [# 1820].
Platydiolus van dykei Kurnakov, 1960: 276. Replacement name for *Platidiolus rufus* (Van Dyke, 1926).

#### Distribution.

This rarely collected species is known from Yukon Territory (CNC), northeastern Alaska (Lindroth 1961a: 191), and the Kodiak Island (Dave H. Kavanaugh pers. comm. 2009) south to northwestern Oregon (Westcott et al. 2006: 8) and northwestern Montana (Edwards 1975: 50).

#### Records.

**CAN**: AB, BC, YT **USA**: AK, MT, OR, WA

### Subfamily PSYDRINAE

LeConte, 1853

Psydri LeConte, 1853c: 371, 393. Type genus: *Psydrus* LeConte, 1846.

#### Diversity.

This subfamily includes a single tribe.

### Tribe Psydrini

LeConte, 1853

Psydri LeConte, 1853c: 371, 393. Type genus: *Psydrus* LeConte, 1846.
Nomiidae des Gozis, 1875: 3. Type genus: *Nomius* Laporte, 1835. Note. The spelling of the family-group name based on *Nomius* was emended to Nomiusidae by the International Commission on Zoological Nomenclature (ICZN 2011) to remove the homonymy with the hymenopteran family-group name Nomiidae Robertson, 1904.

#### Diversity.

Six species arrayed in three genera: *Laccocenus* Sloane (two species in southeastern Australia), *Nomius* (three species), and *Psydrus* (one species).

### Genus Nomius

Laporte, 1835

Nomius Laporte, 1835: 144. Type species: *Nomius graecus* Laporte, 1835 (= *Morio pygmaeus* Dejean, 1831) by monotypy. Etymology. From the Greek *nomas* (roving), alluding to the presence in Greece of a species which Laporte believed was closely related to members of the tropical genus *Ozaena* (“*cet insecte est très-voisin des Ozaena et *... *la découverte en Europe d’un insecte qui rentre dans un groupe naturel d’insectes jusqu’ici propres aux parties les plus chaudes du globe*”) [masculine].
Aplochile LeConte, 1846b: 208. Type species: *Morio pygmaeus* Dejean, 1831 by monotypy. Etymology (original). From the Greek *haplos* (simple) and *cheilos* (lip, by extension labrum) [feminine].
Haplochile LeConte, 1850: 204. Unjustified emendation of *Aplochile* LeConte, 1846.

#### Diversity.

Three species, two in tropical Africa and one in North America, Mexico, southern Europe, and the Middle East.

#### Identification.

The species found in North America was treated in Lindroth’s (1961a: 175) monograph.

### Nomius pygmaeus

(Dejean, 1831)

Morio pygmaeus Dejean, 1831: 512. Type locality: «Amérique septentrionale» (original citation), restricted to «Minaki, n[orth]w[est] of Kenora, Ont[ario]» by Lindroth (1961a: 175). Holotype [by monotypy] in MHNP (Lindroth 1955b: 13).
Nomius graecus Laporte, 1835: 145. Type locality: «Grèce» (original citation). Syntype(s) in MHNP (collection Oberthür *via* James Thomson). Synonymy established by Schaum (1857b: lxxviii).

#### Distribution.

The range of this species, also known as the “stinking Beetle”, extends from southern Quebec, as far north as Mistassini (Larochelle 1975: 95), to the Queen Charlotte and Vancouver Islands (Lindroth 1961a: 175), south at least to central California (Sequoia National Park, CAS), southern Arizona (Ober and Maddison 2008: 24; Cochise County, CNC), “Oklahoma” (David R. Maddison pers. comm. 2008), Alabama (LeConte 1846b: 209), northern Georgia (Fattig 1949: 16), and southern South Carolina (Ciegler 2000: 56); one specimen was collected in the highlands of Chiapas in Mexico (Reichardt 1977: 394). In the Old World, this species has been found in southern Europe, Cyprus (Fauvel 1889b: 96), Morocco, and Iran (Talysh) (Hůrka 2003: 346).

#### Records.

**CAN**: AB, BC (QCI, VCI), MB, ON, QC, SK **USA**: AL, AR, AZ, CA, CO, DE, GA, ID, IL, IN, ME, MI, MN, MT, NC, NJ, NY, OH, OK, OR, PA, SC, TN, UT, WA, WI, WV – Mexico

**Figure 26.**
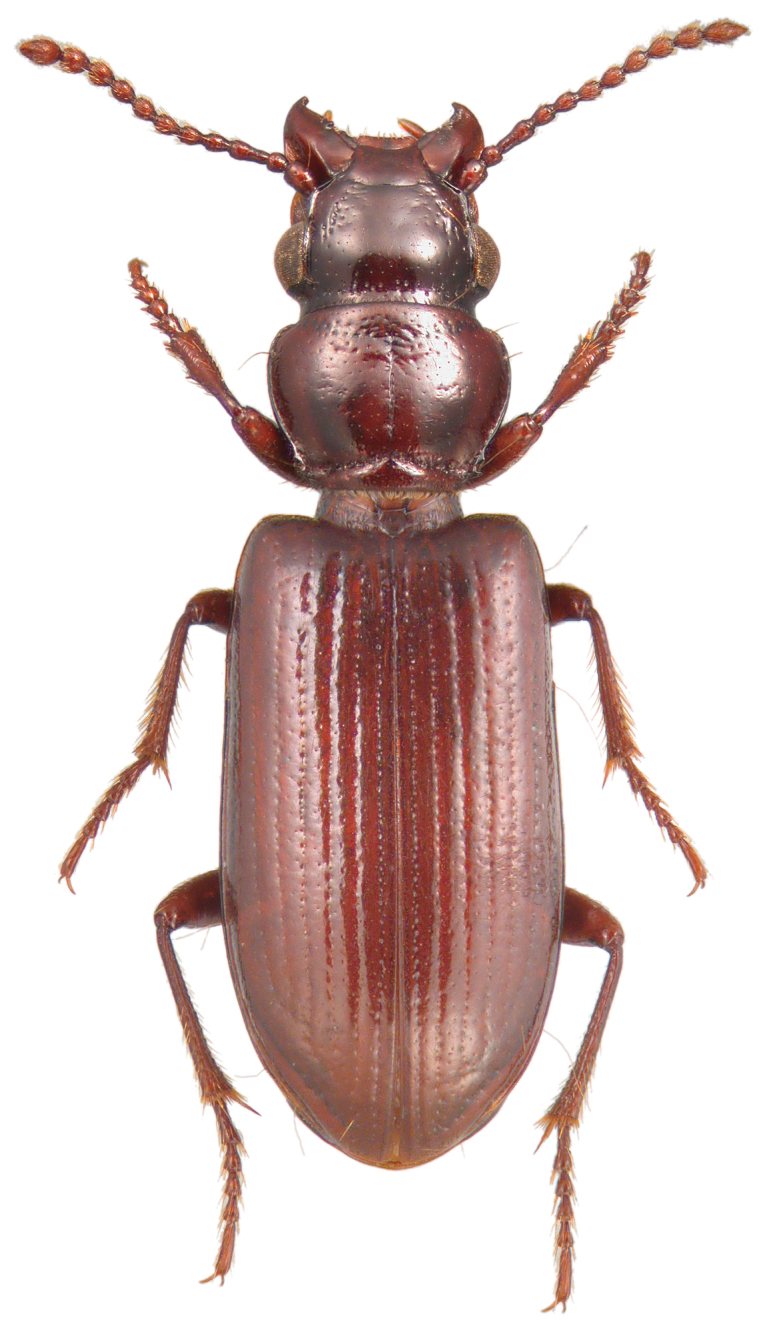
*Nomius pygmaeus* (Dejean). This species is known under the vernacular name “stinking beetle” because of the strong fetid smell that the adults produce. They are attracted to lights and sometimes find their way into houses. It was reported in the literature that at one occasion an entire village had to be evacuated because of the odor produced by these small beetles. The species was often listed as very common at light in the xix Century but is rare today. The species has an unusual range being found in North America and Europe and there is no evidence that it was transported by man from one continent to the other.

### Genus Psydrus

LeConte, 1846

Psydrus LeConte, 1846a: 153. Type species: *Psydrus piceus* LeConte, 1846 by monotypy. Etymology. From the Greek *psydros* (lying) [masculine].
Monillipatrobus Hatch, 1933c: 118. Type species: *Monillipatrobus punctatus* Hatch, 1933 (= *Psydrus piceus* LeConte, 1846) by original designation. Synonymy established by Hatch (1935: 118). Etymology. From the Latin *monile* (necklace, string of beads, by extension moniliform) and the generic name *Patrobus* [*q.v*.], alluding to the shape of the antennae (“antenna ... with the segments ... submoniliform”) of adults of the species which Hatch placed in the tribe Patrobini [masculine].

#### Diversity.

One North American species.

#### Identification.

The species was treated in Lindroth’s (1961a: 175-176) monograph.

### Psydrus piceus

LeConte, 1846

Psydrus piceus LeConte, 1846a: 154. Type locality: «Eagle River [Keweenaw County, Michigan], lacus Superioris» (original citation). One syntype in MCZ [# 5489].
Monillipatrobus punctatus Hatch, 1933c: 118. Type locality: «Seattle [King County], Wash[ington]» (original citation). Holotype (♂) in USNM. Synonymy established by Hatch (1935: 118).

#### Distribution.

This species is known from scattered localities from the Lac Saint-Jean area in Quebec (Larochelle 1975: 99) to Vancouver Island, north to the Skeena River in northwestern British Columbia (Lindroth 1961a: 176), south to the San Gabriel and San Bernardino Mountains in southern California (Noonan 1967: 92), southern Arizona (McCleve 1975: 176), southwestern New Mexico (Grant County, USNM), and the upper peninsula of Michigan along Lake Superior (LeConte 1846a: 154). The record from “Illinois” and “South Dakota” (Bousquet and Larochelle 1993: 158) need confirmation.

#### Records.

**CAN**: AB, BC (VCI), MB, ON, QC, SK **USA**: AZ, CA, CO, ID, MI, MT, NM, NV, OR, WA [IL, SD]

**Figure 27.**
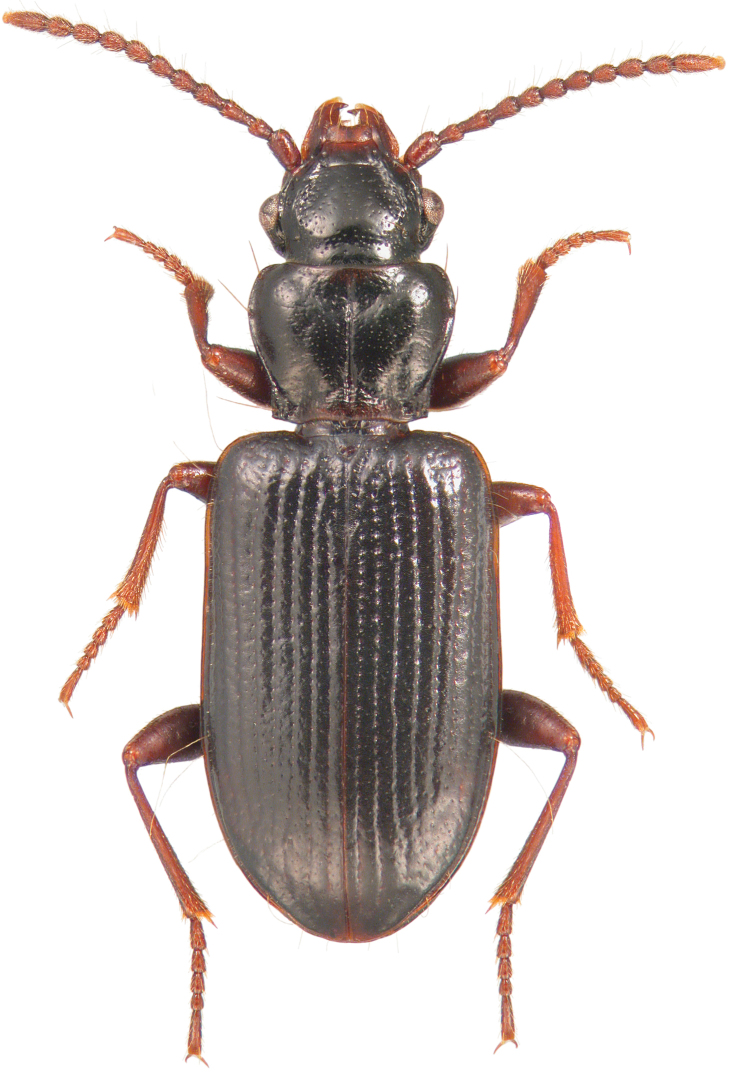
*Psydrus piceus* LeConte. This species is widely distributed west of the Rocky Mountains where it is usually found under the bark of relatively large coniferous trees. It is also found within the boreal regions east of the Rockies but is very rarely collected there. This species belongs to the same tribe as *Nomius pygmaeus* but unlike that species, the adults do not emit an unpleasant smell.

### Subfamily PAUSSINAE

Latreille, 1806

Paussili Latreille, 1806: 234. Type genus: *Paussus* Linnaeus, 1775.

#### Diversity.

Worldwide, with about 735 species in 47 genera. These genera are placed in five tribes: Metriini (three species), Mystropomini (two species in the genus *Mystropomus*), Ozaenini (about 180 species), Paussini (about 560 species), and Protopaussini (eight species in the genus *Protopaussus* Gestro). The Northern Hemisphere fauna is represented by about 70 species (roughly 9.5% of the world fauna) and North America by only seven species (less than 1% of the world fauna).

### Tribe Metriini

LeConte, 1853

Metrii LeConte, 1853c: 371, 394. Type genus: *Metrius* Eschscholtz, 1829.

#### Diversity.

Three species in the genera *Metrius* (two species) and *Sinometrius* Wrase and Schmidt (one Chinese species).

### Genus Metrius

Eschscholtz, 1829

Metrius Eschscholtz, 1829: 7. Type species: *Metrius contractus* Eschscholtz, 1829 by monotypy. Etymology. From the Greek *metrios* (moderate, modest) [masculine].

#### Diversity.

Two species in western North America.

#### Identification.

Bousquet and Goulet (1990) commented on the structural differences between the two species and on the three subspecies of *Metrius contractus*.

### Metrius contractus contractus

Eschscholtz, 1829

Metrius contractus Eschscholtz, 1829: 7. Type locality: «Californien» (original citation), restricted to «San Francisco [San Francisco County]» by Lindroth (1961a: 6). One syntype probably in MHNP (collection Dejean), others possibly in ZMMU.

#### Distribution.

This subspecies ranges from southwestern British Columbia (Lindroth 1961a: 8) to San Luis Obispo County in California along the Coast Ranges and, usually at low elevation, to Tulare County in the Sierra Nevada (Bousquet and Goulet 1990: 17, Fig. 6).

#### Records.

**CAN**: BC **USA**: CA, OR, WA

### Metrius contractus planatus

Van Dyke, 1925

Metrius contractus planatus Van Dyke, 1925: 122. Type locality: «above Fallen Leaf Lake, Lake Tahoe region [Placer County], California» (original citation). Holotype (♂) in CAS [# 1629].

#### Distribution.

This subspecies is found in the Sierra Nevada near Lake Tahoe and in Yosemite National Park (Bousquet and Goulet 1990: 17, Fig. 6).

#### Records.

**USA**: CA

### Metrius contractus sericeus

Rivers, 1900

Metrius sericeus Rivers, 1900: 389. Type locality: «slopes of Mount Whitney (7,000 feet) [California]» (original citation). One syntype [2 originally cited] in LACM and ETHZ.

#### Distribution.

This subspecies occurs in the southern part of the Sierra Nevada from Kings Canyon and Sequoia National Park to northern Kern County (Bousquet and Goulet 1990: 17, Fig. 6).

#### Records.

**USA**: CA

### Metrius explodens

Bousquet and Goulet, 1990

Metrius explodens Bousquet and Goulet, 1990: 13. Type locality: «39 mi[les] N[orth]E[ast] Lowell (2860’), Idaho Co[unty], Idaho» (original citation). Holotype (♂) in CAS [# 16494].

#### Distribution.

This species is known from three localities in Idaho County in north-central Idaho (James C. Bergdahl pers. comm. 2008) and in Ravalli County in western Montana (Moore 2008: 81).

#### Records.

**USA**: ID, MT

### Tribe Ozaenini

Hope, 1838

Ozaenidae Hope, 1838: 107. Type genus: *Ozaena* Olivier, 1811.
Pseudozaenini Sloane, 1905a: 705. Type genus: *Pseudozaena* Laporte, 1834.
Physeitae Jeannel, 1946: 47. Type genus: *Physea* Brullé, 1835.
Pachytelini Jeannel, 1946: 47, 48. Type genus: *Pachyteles* Perty, 1830.
Eustrini Jeannel, 1946: 47, 48. Type genus: *Eustra* Schmidt-Göbel, 1846.

#### Diversity.

About 180 species arrayed in 21 genera in the Neotropical Region (about 120 species of which five extend into southwestern United States), Asia (about 40 species in five endemic genera, most species in the Oriental Region, a few in New Guinea, Japan, Taiwan, and Nepal), and Afrotropical Region (about 20 species in two endemic genera).

#### Identification.

All North American species have been covered in the revision of the ozaenine species of southwestern United States and of selected ones from Mexico by Ball and McCleve (1990). The work includes a key for the identification of ten species.

### Genus Pachyteles

Perty, 1830

Pachyteles Perty, 1830: 3. Type species: *Pachyteles striola* Perty, 1830 designated by Hope (1838: 99). Etymology. From the Greek *pachys* (thick) and *telos* (end), alluding to the incrassate last antennomere of the adult (“*nomen datum ob antennas versus apicem incrassatas*”) [masculine].

#### Diversity.

About 50 species ranging from southwestern North America to Chile and Uruguay.

#### Taxonomic Note.

Recent molecular data analyses (Moore 2008) suggest that *Pachyteles *s.str., *Goniotropis*, and *Tropopsis* Solier (two South American species), which have been treated as subgenera of *Pachyteles* (Ball and McCleve 1990: 90), are not so closely related as suspected from the morphological data. These groups are recognized here as distinct genera.

### Pachyteles gyllenhalii

(Dejean, 1825)

Ozaena gyllenhalii Dejean, 1825: 436. Type locality: «îles de l’Amérique» (original citation), herein restricted to Cayamas, Cuba (see Ball and McCleve 1990: 95). Lectotype [as holotype] (♀), designated by Ball and McCleve (1990: 94), in MHNP. Etymology. The specific name honors Leonhard Gyllenhal [1752-1840], Swedish military officer and entomologist. His main publication, *Insecta Suecica*, for which he was awarded the gold medal of the Swedish Academy of Sciences, took him about 30 years to complete. Gyllenhal left his collection to the Royal Swedish Society of Sciences in Uppsala; it was eventually transferred to the Uppsala University Museum of Zoology.
Ozaena verticalis Chaudoir, 1848: 104. Type locality: «Colombie» (original citation). Lectotype (♀), designated by Ball and McCleve (1990: 94), in MHNP. Synonymy established by Ball and McCleve (1990: 94).
Goniotropis pallida Chevrolat, 1863: 190. Type locality: «Havana, Cuba» (original citation). Holotype [by monotypy] location unknown (possibly in UMO). Synonymy established by Darlington (1934: 67).
Pachyteles testaceus G.H. Horn, 1869b: 129. Type locality: «Fort Grant [= Camp Grant, Pinal County], Arizona» (original citation). Lectotype (♀), designated by Ball and McCleve (1990: 94), in MCZ [# 34891]. Synonymy established by Ball and McCleve (1990: 94).

#### Distribution.

This species ranges from southeastern California (San Bernardino County, CMNH) to southern Texas (Cameron County, CMNH), south to Brazil; it is also known from Cuba and from the Tres Marias Islands off the Pacific Coast of Mexico (Ball and McCleve 1990: 95).

#### Records.

**USA**: AZ, CA, TX – Brazil, Colombia, Costa Rica, Cuba, Mexico, Surinam

### Genus Goniotropis

Gray, 1831

Goniotropis Gray, 1831: 273. Type species: *Goniotropis brasiliensis* Gray, 1831 by monotypy. Etymology. From the Greek *gonia* (angle, corner) and *tropis* (keel, ridge), probably alluding to the flange of Coanda of the adult which is keel-like and located on the posterolateral angle of the elytron (Ball and McCleve 1990: 90) [feminine].
Scythropasus Chaudoir, 1854: 293. Type species: *Scythropasus elongatus* Chaudoir, 1854 by monotypy. Synonymy established by Bänninger (1927: 207). Etymology (original). From the Greek *scythropazo* (to look sullen) [masculine]. Note. Chaudoir (1854: 295-296) originally included four species in this genus-group name but stated that, besides *Scythropasus elongatus*, the three other species were placed in the genus for the time being (“*provisoirement*”). This statement is interpreted here as if the three species were conditionally included in the genus and thus are deemed not to be originally included (ICZN 1999: Article 67.2.5).

#### Diversity.

About 40 species extending collectively from southwestern United States to Brazil and Paraguay.

### Goniotropis kuntzeni kuntzeni

Bänninger, 1927

Goniotropis kuntzeni Bänninger, 1927: 204. Type locality: «Canelas Durango, Mexico» (original citation). Holotype (♀) in ETHZ.

#### Distribution.

This subspecies is known from Arizona and the states of Durango and Sonora in northwestern Mexico (Ball and McCleve 1990: 92).

#### Records.

**USA**: AZ – Mexico

#### Note.

*Pachyteles kuntzeni maracayensis* (Deuve) is known from Venezuela.

### Goniotropis parca

(LeConte, 1884)

Pacheteles parca LeConte, 1884: 2. Type locality: «Arizona» (original citation), herein restricted to Molino Basin, Santa Catalina Mountains, Pima County (CNC). Holotype [by monotypy] (♀) in MCZ [# 5487].
Pachyteles beyeri Notman, 1919b: 225. Type locality: «San Felipe, Low[er] Cal[ifornia]» (original citation). Holotype [by monotypy] (♂) in SIM (Hennessey 1990: 466). Synonymy established by Ball and McCleve (1990: 91).

#### Distribution.

This species is known from southern Arizona, western Durango, northern Sonora, and Baja California (Ball and McCleve 1990: 91).

#### Records.

**USA**: AZ – Mexico

### Genus Physea

Brullé, 1835

Trachelizus Solier [in Brullé], 1835a: 258 [junior homonym of *Trachelizus* Dejean, 1834]. Type species: *Trachelizus rufus* Brullé, 1835 (= *Ozaena testudinea* Klug, 1834) by monotypy. Etymology. From the Greek verb *trachelizo* (bent back the neck, to lay bare or expose or uncover) [masculine]. Note. This name has been credited to Brullé since he was the author of the book. However, the remark made by Brullé (1835a: 258) “Les trachélizes.* - Trachelizus*. Solier. Qui ont été formés tout récemment dans un mémoire manuscrit que M. Solier a confié à M. Audouin afin qu’il fût inséré dans notre travail” leaves little doubt that the name should be credited to Solier.
Physea Brullé, 1835a: 473. Replacement name for *Trachelizus* Solier, 1835. Etymology (original). From the Greek *physeo* (to blow, inflate), probably alluding to the bulging elytra (“*les élytres sont renflées*” see page 259) of the adults [feminine].

#### Diversity.

Six species in the Neotropical Region, of which one extends into southern United States.

### Physea hirta

LeConte, 1853

Physea hirta LeConte, 1853c: 393. Type locality: «Mexico ... a few miles south of our boundary» (original citation). Lectotype [as holotype] (♂), designated by Ball and McCleve (1990: 87), in MCZ [# 5488]. Note. The lectotype is labeled “Physea hirta Lec. Tampico Hald.” Whether or not the specimen is really a syntype is open to question. Tampico, located in the southern part of the Tamaulipas state, is not “a few miles south of” the US boundary.

#### Distribution.

This species ranges from southeastern Texas (Ball and McCleve 1990: 87) to Guatemala (Bates 1881: 27).

#### Records.

**USA**: TX – Guatemala, Mexico

### Genus Ozaena

Olivier, 1811

Ozaena Olivier, 1811: 617. Type species: *Ozaena dentipes* Olivier, 1811 by monotypy. Etymology. From the Latin *ozaena* (a fetid polyp in the nose), alluding to the distasteful odor produced by the liquid expulsed from the mouth of the carabid adults when disturbed or from their body in general (“*exprime la puanteur de la liqueur que la plupart des Carabes font sortir de leur bouche lorsqu’on les inquiète, ou celle de leur corps en général*”) [feminine].
Ictinus Laporte, 1834: 53. Type species: *Ictinus tenebrioides* Laporte, 1834 (= *Ozaena dentipes* Olivier, 1811) by monotypy. Synonymy established by Laporte (1835: 144). Etymology. Uncertain, possibly from the Greek *ictinos* (kite) or *Ictinos* (the name of the Greek architect who built the Parthenon between 444-439) [masculine].

#### Diversity.

Twelve Neotropical species, of which one extends into southwestern United States.

### Ozaena lemoulti

Bänninger, 1932

Ozaena lemoulti Bänninger, 1932: 184. Type locality: «Saint Jean du Maroni, Franz[ösisch] Guayana» (original citation). Holotype (♂) in ETHZ. Etymology. The specific name was proposed for Eugène Henri Le Moult [1882-1965], collector of insects in Guyana and later insect dealer in Paris. His father was the director of the penitentiary at Cayenne and Le Moult used convicts to collect *Morpho* in large number. In 1913 he published his first sale catalogue of insects and a pair of the large cerambycid *Titanus giganteus* was offered for 500 francs (Moret 1995: 404).
Ozaena halffteri Ogueta, 1965: 83. Type locality: «Tlapacoyan, Veracruz, México» (original citation). Holotype (♀) location unknown. Synonymy established by Ball and McCleve (1990: 100).

#### Distribution.

This species ranges from southern Arizona to northern Argentina, including the Cayman Islands in the West Indies (Ball and Shpeley 1990: 814-815).

#### Records.

**USA**: AZ – Argentina, Belize, Bolivia, Brazil, Costa Rica, Cayman Islands, Colombia, Ecuador, Guatemala, Guyana, Mexico, Paraguay, Panama, Venezuela.

### Subfamily BRACHININAE

Bonelli, 1810

Brachinii Bonelli, 1810: Tabula Synoptica. Type genus: *Brachinus* Weber, 1801.

#### Diversity.

About 650 species arrayed in two tribes: Brachinini (about 535 species) and Crepidogastrini (about 115 species, most from the Afrotropical Region, a few from the Oriental Region). The Northern Hemisphere is represented by about 230 species (roughly 35% of the world fauna) and North America by 48 species (roughly 7.3% of the world fauna).

### Tribe Brachinini

Bonelli, 1810

Brachinii Bonelli, 1810: Tabula Synoptica. Type genus: *Brachinus* Weber, 1801.

#### Diversity.

About 535 species arrayed in nine genera. The genera are classified in four subtribes following Erwin (1970a: 27-38): Aptinina (about 55 Old World species in three genera), Brachinina (about 305 species), Mastacina (about 50 Old World species in the genus *Mastax* Fischer von Waldheim), and Pheropsophina Jeannel (about 125 species in the Eastern Hemisphere genus *Pheropsophus* Dejean and the Western Hemisphere genus *Pheropsophidius* Hubenthal).

### Subtribe Brachinina

Bonelli, 1810

Brachinii Bonelli, 1810: Tabula Synoptica. Type genus: *Brachinus* Weber, 1801.

#### Diversity.

About 305 species arrayed in three genera: *Aptinoderus* Hubenthal (five species in southern Africa), *Brachinus* (about 300 species), and *Brachinulus* Basilewsky (one species on Príncipe Island in the Gulf of Guinea).

### Genus Brachinus

Weber, 1801

Brachinus Weber, 1801: 22. Type species: *Carabus crepitans* Linnaeus, 1758 designated by Latreille (1810: 426). Etymology (original). From the Greek *brachyno* (shorten), probably alluding to the short elytra (“*elytra rigida abbreuiata truncata*”) of the adult [masculine].
Brachynus Agassiz, 1846: 50, 51. Unjustified emendation of *Brachinus* Weber, 1801.

#### Distribution.

About 300 species (Lorenz 2005: 17-20) in the Nearctic (50 species), Neotropical (about 50 species, several shared with North America), Australian (one species in New Guinea), Oriental, Palaearctic (about 120 species), and Afrotropical Regions. These species are arrayed in nine subgenera: *Brachinus* s.str. (ten species), *Brachynolomus* Reitter (43 species), *Metabrachinus* Jeannel (38 Afrotropical species), *Aploa* Hope (three species), *Brachinoaptinus* Lutshnik (17 species), *Aptinomimus* Alluaud (seven Madagascan species), *Dysbrachinus* Schuler (two species), *Cnecostolus* Reitter (11 species), and *Neobrachinus* (about 85 species), with 89 species not assigned to subgenera.

#### Identification.

Erwin (1970a) revised the North and Middle American species. No new species from the region have been described subsequently.

### Subgenus Neobrachinus

Erwin, 1970

Neobrachinus Erwin, 1970a: 47. Type species: *Carabus fumans* Fabricius, 1781 by original designation. Etymology. From the Greek prefix *neo*- (new) and the generic name *Brachinus* [*q.v*.], probably alluding to the fact that all but one of these *Brachinus* species inhabit the New World [masculine].

#### Distribution.

Eighty-three species in the temperate, subtropical, and tropical regions of the Western Hemisphere and one species (*Brachinus dryas* Andrewes) in the Himalayas. Forty-eight species inhabit North America.

#### Taxonomic Note.

In a cladistic analysis based on adult characters carried out by Erwin (1970a: 170), *Neobrachinus* was positioned as the sister-group to *Cnecostolus* whose members inhabit Eurasia.

#### Faunistic Note.

The records of *Brachinus cinctipennis* Chevrolat from Albuquerque in New Mexico and Nogales in Arizona (Schaeffer 1910: 401) are probably in error since the species is recorded only from Mexico, as far north as San Luis Potosí, by Erwin (1970a: 98).

### [aabaaba group]

### Brachinus aabaaba

Erwin, 1970

Brachinus aabaaba Erwin, 1970a: 161. Type locality: «Presa de Guadalupe, 53.3 miles west of Ciudad del Maiz, San Luis Potosi, Mexico» (original citation). Holotype (♂) in MCZ [# 34679].

#### Distribution.

This species is found from northeastern Kansas south to southeastern New Mexico and San Luis Potosí in Mexico [see Erwin 1970a: Fig. 450].

#### Records.

**USA**: KS, NM, TX – Mexico

### [alternans group]

### Brachinus alternans

Dejean, 1825

Brachinus alternans Dejean, 1825: 316. Type locality: «Géorgie» (original citation), herein restricted to Thomasville, Thomas County (see Erwin 1970a: 90). Lectotype (♀), designated by Erwin (1970a: 88), in MHNP.
Brachinus librator Dejean, 1831: 425. Type locality: «Amérique septentrionale» (original citation). Holotype [by monotypy; designated lectotype by Erwin (1970a: 88)] (♂) in MHNP. Synonymy established by Lindroth (1969a: 1099) based on Erwin’s (1969c) thesis.
Brachinus deyrollii LaFerté-Sénectère, 1841a: 42. Type locality: «Missouri» (lectotype label). Lectotype (♂), designated by Erwin (1970a: 88), in MHNP. Synonymy established by Lindroth (1969a: 1099) based on Erwin’s (1969c) thesis. Etymology. The specific name was proposed for the French amateur coleopterist Achille Deyrolle [1818-1865], a dealer of natural history specimens in Paris.
Brachinus strenuus LeConte, 1844: 48. Type locality: «Georgia» (original citation). Lectotype (♀), designated by Erwin (1970a: 88), in MCZ [# 5844]. Synonymy established with doubt, under the name *Brachinus deyrollii* LaFerté-Sénectère, by LeConte (1858a: 28), confirmed by Erwin (1970a: 88).
Brachinus tormentarius LeConte, 1846b: 200. Type locality: «provinciis occidentalibus» (original citation). Lectotype (♀), designated by Erwin (1970a: 88), in MCZ [# 5845]. Synonymy established by Lindroth (1969a: 1099) based on Erwin’s (1969c) thesis.
Brachinus distinguendus Chaudoir, 1868b: 287. Type locality: Amérique septentrionale (inferred from title of the paper). Lectotype (♂), designated by Erwin (1970a: 88), in MHNP. Synonymy established by Lindroth (1969a: 1099) based on Erwin’s (1969c) thesis.

#### Distribution.

This species extends from Connecticut and “Rhode Island” (Sikes 2003: 8) to southern Minnesota and northeastern Nebraska, south to the Rio Grande in south-central New Mexico and southeastern Texas, and the Florida Keys [see Erwin 1970a: Fig. 201].

#### Records.

**USA**: AL, AR, CT, DC, FL, GA, IA, IL, IN, KS, KY, LA, MD, MI, MN, MO, MS, NC, NE, NJ, NM, NY, OH, OK, PA, RI, SC, TN, TX, VA, WV

### Brachinus rugipennis

Chaudoir, 1868

Brachinus rugipennis Chaudoir, 1868b: 297. Type locality: Amérique septentrionale (inferred from title of the paper), restricted to «Texas» by Erwin (1970a: 91), herein to Elkhart, Anderson County (see Erwin 1970a: 92). Holotype [by monotypy; designated lectotype by Erwin (1970a: 91)] (♀) in MHNP.

#### Distribution.

This species ranges from Massachusetts to western Wyoming and western Colorado, south to central New Mexico, southeastern Texas, southeastern Mississippi (George County, Drew A. Hildebrandt pers. comm. 2008), and the Florida Keys [see Erwin 1970a: Fig. 200]. The populations in the northeast are isolated from the remaining ones but this is probably the result of inadequate collecting.

#### Records.

**USA**: AL, AR, CO, FL, GA, KS, MA, MS, NE, NJ, NM, OK, PA, TN, TX, VA, WY

### Brachinus viridipennis

Dejean, 1831

Brachinus viridipennis Dejean, 1831: 426. Type locality: «Amérique septentrionale» (original citation), restricted to «Mobile [Mobile County], Alabama» by Erwin (1970a: 90). Holotype [by monotypy; designated lectotype by Erwin (1970a: 90)] (♀) in MHNP.
Brachinus viridis LeConte, 1844: 49. Type locality: «Georgia» (original citation). Lectotype (♂), designated by Erwin (1970a: 90), in MCZ [# 5840]. Synonymy established by Chaudoir (1868b: 289), confirmed by Erwin (1970a: 144).

#### Distribution.

This species is found only in the southern parts of the United States from southern South Carolina to southern Florida, west to southern Arkansas and eastern Texas [see Erwin 1970a: Fig. 199]. The record from New Jersey (Smith 1910: 212) needs confirmation.

#### Records.

**USA**: AL, AR, FL, GA, SC, TX [NJ]

### [americanus group]

### Brachinus alexiguus

Erwin, 1970

Brachinus alexiguus Erwin, 1970a: 57. Type locality: «College Station [Brazos County], Texas» (original citation). Holotype (♂) in MCZ [# 34684].

#### Distribution.

This species is known from east-central Texas, Latimer County in Oklahoma [see Erwin 1970a: Fig. 89], and from southwestern Mississippi (Adams County, Drew A. Hildebrandt pers. comm. 2008).

#### Records.

**USA**: MS, OK, TX

### Brachinus americanus

(LeConte, 1844)

Aptinus americanus LeConte, 1844: 48. Type locality: «Georgia» (original citation). Lectotype (♀), designated by Erwin (1970a: 55), in MCZ [# 5839].

#### Distribution.

This species ranges from western New York to southeastern Minnesota, south to southeastern Texas, east-central Louisiana (West Feliciana Parish, Igor M. Sokolov pers. comm. 2009), and northern Florida [see Erwin 1970a: Fig. 88].

#### Records.

**USA**: AL, AR, FL, GA, IA, IL, IN, KS, KY, LA, MI, MN, MO, MS, NC, NY, OH, OK, PA, TN, TX, WI, WV

### Brachinus capnicus

Erwin, 1970

Brachinus capnicus Erwin, 1970a: 60. Type locality: «Smokemont [Swain County], Great Smoky Mountains National Park, North Carolina» (original citation). Holotype (♀) in CUIC [# 4566].

#### Distribution.

This species is known only from the holotype.

#### Records.

**USA**: NC

### Brachinus microamericanus

Erwin, 1969

Brachinus microamericanus Erwin, 1969b: 287. Type locality: «Dundee [Tunica County], Mississippi» (original citation). Holotype (♂) in UMAA.

#### Distribution.

This species is known only from a few specimens collected in eastern Michigan, Missouri, northwestern Louisiana (Bossier Parish, Igor M. Sokolov pers. comm. 2009), and northwestern Mississippi [see Erwin 1970a: Fig. 90].

#### Records.

**USA**: LA, MI, MO, MS

### [cordicollis group]

### Brachinus cordicollis

Dejean, 1826

Brachinus cordicollis Dejean, 1826: 466. Type locality: «Amérique septentrionale» (original citation), restricted to «Fairfax Co[unty], V[irgini]a» by Lindroth (1969a: 1105). Lectotype (♂), designated by Erwin (1970a: 144), in MHNP.
Brachinus velox LeConte, 1846b: 206. Type locality: «NovEboraci [= New York]» (original citation). Lectotype (♂), designated by Erwin (1970a: 144), in MCZ [# 5850]. Synonymy established by LeConte (1863a: 524), confirmed by Erwin (1970a: 144).
Brachinus leptocerus Chaudoir, 1868b: 296. Type locality: Amérique septentrionale (inferred from title of the paper), restricted to «New York» by Lindroth (1969a: 1105). Lectotype (♀), designated by Erwin (1970a: 144), in MHNP. Synonymy established with doubt, under the name *Brachinus velox* LeConte, by Chaudoir (1868b: 296), confirmed by Erwin (1970a: 144).
Brachynus gracilis Blatchley, 1910: 160. Type locality: «Marshall County [Indiana]» (original citation). Lectotype (♂), designated by Blatchley (1930: 33), in PURC. Synonymy established by Lindroth (1969a: 1105) based on Erwin’s (1969c) thesis.

#### Distribution.

The range of this species extends from New Brunswick to east-central South Dakota (Kirk and Balsbaugh 1975: 40), south to northwestern Arkansas, northwestern Tennessee (Cheatham County, CMNH), and Virginia. The species is also known from a number of localities in the southern part of the Rocky Mountains, in Colorado and New Mexico [see Erwin 1970a: Fig. 391]. The records from Georgia (J.E. LeConte 1849: 25; Fattig 1949: 42), southwestern Alabama (Löding 1945: 23), and “Utah” (Horn 1872c: 384; Erwin 1970a: 147) need confirmation.

#### Records.

**CAN**: NB, ON, QC **USA**: AR, CO, CT, IL, IN, KS, KY, MA, MD, ME, MI, MN, MO, NE, NH, NJ, NM, NY, OH, PA, RI, SD, TN, VA, VT, WI, WV [AL, GA, UT]

### Brachinus cyanochroaticus

Erwin, 1969

Brachinus cyanochroaticus Erwin, 1969a: 283. Type locality: «Eleven miles west of York, Benson County, North Dakota» (original citation). Holotype (♂) in MCZ [# 34687].

#### Distribution.

This species ranges from southwestern New Brunswick (Webster and Bousquet 2008: 23) to south-central British Columbia, south to southwestern Idaho, central Colorado, Missouri, and New Jersey along the Atlantic Coast [see Erwin 1970a: Fig. 392].

#### Records.

**CAN**: BC, MB, NB, ON, QC, SK **USA**: CO, CT, IA, ID, IL, IN, KS, MA, ME, MI, MN, MO, MT, ND, NE, NH, NJ, NY, OH, PA, SD, VT, WI, WY

### Brachinus fulminatus

Erwin, 1969

Brachinus fulminatus Erwin, 1969b: 288. Type locality: «Wayland, Middlesex County, Massachusetts» (original citation). Holotype (♂) in MCZ [# 34690].

#### Distribution.

This species is known from western New York and southern New Hampshire (Hillsborough, Rockingham, and Strafford Counties, Ross T. Bell pers. comm. 2008) south to North Carolina, from several localities in southern Indiana [see Erwin 1970a: Fig. 414], and from Waukesha County in southeastern Wisconsin (Messer 2010: 34).

#### Records.

**USA**: CT, DE, IN, MA, MD, NC, NH, NJ, NY, PA, RI, VA, WI

### Brachinus ichabodopsis

Erwin, 1970

Brachinus ichabodopsis Erwin, 1970a: 150. Type locality: «Saint John’s River, Hardkinsville, Florida» (original citation). Holotype (♂) in MCZ [# 33509]. Note. I have not been able to find a place with the name “Hardkinsville,” or alphabetically close to, in Florida. I have seen the holotype label and the locality is clearly written “Hardkinsville.”

#### Distribution.

This species is known only from the original two specimens, one collected at the type locality and the other at an unspecified locality in Florida (Erwin 1970a: 150).

#### Records.

**USA**: FL

### Brachinus janthinipennis

(Dejean, 1831)

Aptinus janthinipennis Dejean, 1831: 412. Type locality: «Amérique septentrionale» (original citation), restricted to «New York» by Lindroth (1969a: 1106), herein to Ithaca, Tompkins County (see Erwin 1970a: 158). Holotype [by monotypy; designated lectotype by Erwin (1970a: 156)] (♀) in MHNP.
Brachinus pumilio LeConte, 1846b: 208. Type locality: «NovEboraci, ad lacum Onondaga [Onondaga County, New York]» (original citation). Lectotype (♂), designated by Erwin (1970a: 156), in MCZ [# 5841]. Synonymy established by Lindroth (1969a: 1106) based on Erwin’s (1969c) thesis.

#### Distribution.

The range of this species extends from Maine (Larochelle and Larivière 1990a: 33) and southern Quebec to western South Dakota, south to central Texas and central Georgia (Butts County, CMNH; Horn and Ulyshen 2009: 121) [see Erwin 1970a: Fig. 417].

#### Records.

**CAN**: ON, QC **USA**: CO, CT, GA, IA, IL, IN, KS, MA, MD, ME, MI, MN, MO, MS, ND, NE, NH, NJ, NY, OH, OK, PA, RI, SD, TX, VA, VT, WI

### Brachinus mobilis

Erwin, 1970

Brachinus mobilis Erwin, 1970a: 159. Type locality: «Mobile [Mobile County], Alabama» (original citation). Holotype (♂) in CUIC [# 4568].

#### Distribution.

This species is known only from the original five specimens collected at the type locality in southwestern Alabama.

#### Records.

**USA**: AL

### Brachinus oxygonus

Chaudoir, 1843

Brachinus oxygonus Chaudoir, 1843b: 714. Type locality: «Amérique septentrionale» (original citation), restricted to «Highlands County, Florida» by Erwin (1970a: 151). Lectotype (♂), designated by Erwin (1970a: 151), in MHNP.
Brachinus stenomus Chaudoir, 1868b: 291. Type locality: Amérique septentrionale (inferred from title of the paper), restricted to «Highlands County, Florida» by Erwin (1970a: 151). Holotype [by monotypy; designated lectotype by Erwin (1970a: 151)] (♂) in MHNP. Synonymy established by Erwin (1970a: 151).

#### Distribution.

This species ranges from North Carolina to the Florida Keys, west to southern Alabama [see Erwin 1970a: Fig. 416]; also known from one locality in central Missouri (Erwin 1970a: 153).

#### Records.

**USA**: AL, FL, GA, MO, NC, SC

### Brachinus sublaevis

Chaudoir, 1868

Brachinus sublaevis Chaudoir, 1868b: 293. Type locality: Amérique septentrionale (inferred from title of the paper), restricted to «Florida» by Erwin (1970a: 149), herein to Brooksville, Hernando County (see Erwin 1970a: 150). Holotype [by monotypy; designated lectotype by Erwin (1970a: 149)] (♀) in MHNP.
Brachynus pulchellus Blatchley, 1910: 161. Type locality: «Posey County [Indiana]» (original citation for the lectotype). Lectotype (♀), designated by Blatchley (1930: 33), in PURC. Synonymy established by Erwin (1970a: 149).

#### Distribution.

This species is known from southeastern Michigan southeastwards to southern Florida, including the Keys, and southwestwards to east-central and southwestern Texas [see Erwin 1970a: Fig. 393]. The records from southeastern South Dakota (Kirk and Balsbaugh 1975: 39) and southwestern Ohio (Wright and Whitehouse 1941: 70, as *Brachinus pulchellus*) need confirmation.

#### Records.

**USA**: AL, AR, FL, GA, IN, KY, LA, MI, MO, MS, OK, SC, TN, TX, VA [OH, SD]

### Brachinus vulcanoides

Erwin, 1969

Brachinus vulcanoides Erwin, 1969b: 287. Type locality: «Baychester [Bronx County], New York» (original citation). Holotype (♂) in MCZ [# 34688].

#### Distribution.

This species is known from a small area along the Atlantic Coast from New Hampshire to New Jersey [see Erwin 1970a: Fig. 415]. One specimen labeled from Crescent City, Florida is probably mislabeled (Erwin 1970a: 155).

#### Records.

**USA**: CT, MA, NH, NJ, NY, RI

### [costipennis group]

### Brachinus costipennis

Motschulsky, 1859

Brachynus costipennis Motschulsky, 1859a: 138. Type locality: «Californie» (original citation), herein restricted to Dry Creek, 9.0 miles northwest of Healdsburg, Sonoma County (see Erwin 1970a: 88). Lectotype (♀), designated by Erwin (1970a: 85), in MCZ [# 8329].
Brachynus carinulatus Motschulsky, 1859a: 139. Type locality: Californie (inferred from title of the paper). Lectotype (♂), designated by Erwin (1970a: 85), in ZMMU. Synonymy established by Erwin (1965: 4).
Brachynus cognatus Chaudoir, 1876a: 74. Type locality: «Orizaba [Veracruz, Mexico]» (lectotype label). Lectotype (♀), designated by Erwin (1970a: 85), in MHNP. Synonymy established by Erwin (1970a: 85).
Brachinus cognatus var. *cancellatus* Bates, 1891a: 269. Type locality: «Chihuahua City, Mexico» (lectotype label). Lectotype (♂), designated by Erwin (1970a: 85), in BMNH. Synonymy established by Erwin (1970a: 85).

#### Distribution.

This species ranges from northern California to eastern Utah, south to Guatemala (Erwin 1973b: 82) and northern Baja California [see Erwin 1970a: Fig. 197]. Specimens labeled from “Arkansas” and “Kansas” are known but could be mislabeled (Erwin 1970a: 87).

#### Records.

**USA**: AZ, CA, NM, TX, UT – Guatemala, Mexico

### [explosus group]

### Brachinus explosus

Erwin, 1970

Brachinus explosus Erwin, 1970: 161. Type locality: «Tamazunchale, San Luis Potosi, Mexico» (original citation). Holotype (♀) in MCZ [34689].

#### Distribution.

This species is known from the type locality and “Arizona” (Erwin 2011b: 285).

#### Records.

**USA**: AZ – Mexico

### [fumans group]

### Brachinus azureipennis

Chaudoir, 1876

Brachynus azureipennis Chaudoir, 1876a: 75. Type locality: «Matamoros, Etat de Puebla [Mexico]» (original citation). Lectotype (♂), designated by Erwin (1970a: 115), in MHNP.

#### Distribution.

This species ranges from southeastern Arizona south to Guerrero, Mexico [see Erwin 1970a: Fig. 276].

#### Records.

**USA**: AZ – Mexico

### Brachinus cibolensis

Erwin, 1970

Brachinus cibolensis Erwin, 1970a: 98. Type locality: «Southwest Research Station (5,400 feet), five miles west of Portal, Cochise County, Arizona» (original citation). Holotype (♂) in AMNH [# 1232].

#### Distribution.

This species ranges from northern Arizona and central New Mexico south to Durango City, Mexico [see Erwin 1970a: Fig. 219].

#### Records.

**USA**: AZ, NM – Mexico

### Brachinus conformis

Dejean, 1831

Brachinus conformis Dejean, 1831: 427. Type locality: «Amérique septentrionale» (original citation), restricted to «Florida» by Erwin (1970a: 119), herein to Highlands Hammock State Park, Highlands County (see Erwin 1970a: 121). Lectotype (♂), designated by Erwin (1970a: 119), in MHNP.

#### Distribution.

This species is confined to northern and central Florida [see Erwin 1970a: Fig. 302].

#### Records.

**USA**: FL

### Brachinus cyanipennis

Say, 1823

Brachinus cyanipennis Say, 1823b: 143. Type locality: «Ames [Story County], Iowa» (neotype label). Neotype (♂), designated by Lindroth and Freitag (1969: 350), in MCZ [# 32998]. Note. «Near Engineer Cantonment [winter quarter along the west bank of the Missouri River north of modern Omaha, Nebraska], Missouri [Territory]» was the area originally cited by Say (1823b: 144).
Brachinus cephalotes Dejean, 1825: 317. Type locality: «Amérique septentrionale» (original citation). Lectotype (♂), designated by Erwin (1970a: 127), in MHNP. Synonymy established by Chaudoir (1868b: 297), confirmed by Erwin (1970a: 127).
Brachinus rejectus LeConte, 1863a: 525. Type locality: «middle and western states; Kansas» (original citation), restricted to «Kansas» by Lindroth (1969a: 1103). Lectotype (♀), designated by Erwin (1970a: 127), in MCZ [# 5843]. Synonymy established by Chaudoir (1868b: 297), confirmed by Erwin (1970a: 127).

#### Distribution.

This species ranges from Cumberland County in Nova Scotia (Majka and Gilhen 2008: 1) to southern Manitoba, south to eastern Texas, northwestern Louisiana (Natchitoches Parish, Igor M. Sokolov pers. comm. 2009), southern Alabama, northwestern South Carolina (Ciegler 2000: 35), and Virginia along the east coast [see Erwin 1970a: Fig. 330]. The species is also recorded from an unspecified locality in New Mexico (Erwin 1970a: 128). The records from “Georgia” (J.E. LeConte 1849: 25, as *Brachinus cephalotes*) and Enterprise in Florida (Castle and Laurent 1896: 303) need confirmation.

#### Records.

**CAN**: MB, NB, NS, ON, QC **USA**: AL, AR, CT, DC, IA, IL, IN, KS, KY, LA, MA, MD, ME, MI, MN, MO, MS, ND, NE, NH, NJ, NY, OH, OK, PA, RI, SC, SD, TN, TX, VA, VT, WI [FL, GA, NM]

### Brachinus favicollis

Erwin, 1965

Brachinus favicollis Erwin, 1965: 11. Type locality: «Jamul, San Diego County, California» (original citation). Holotype (♂) in CAS [# 9132].

#### Distribution.

The range of this species extends from eastern Arizona to the Pacific Coast in southern California and Baja California Norte [see Erwin 1970a: Fig. 365].

#### Records.

**USA**: AZ, CA – Mexico

### Brachinus fumans

(Fabricius, 1781)

Carabus fumans Fabricius, 1781: 307. Type locality: «America» (original citation), restricted to «Springfield [Hampden County], Mass[achusetts]» by Lindroth (1969a: 1105). Lectotype (♂), designated by Lindroth (1969a: 1105), in ZMUC.
Brachinus cyanopterus LeConte, 1844: 49. Type locality: «New York» (original citation). Lectotype (♀), designated by Erwin (1970a: 134), in MCZ [# 5847]. Synonymy established by LeConte (1846b: 203), confirmed by Erwin (1970a: 134).
Brachinus sufflans LeConte, 1846b: 204. Type locality: «NovEboraci [= New York]» (original citation). Lectotype (♀), designated by Erwin (1970a: 134), in MCZ [# 5648]. Synonymy established by Chaudoir (1868b: 292), confirmed by Erwin (1970a: 134).
Brachinus affinis LeConte, 1846b: 204. Type locality: «Indiana ad flumen Ohio» (original citation). Lectotype (♂), designated by Erwin (1970a: 134), in MCZ [# 31881]. Synonymy established with doubt by Chaudoir (1868b: 292), confirmed by Erwin (1970a: 134).
Brachinus amplipennis Bates, 1891a: 268. Type locality: «Paso del Norte, Chihuahua [Mexico]» (lectotype label). Lectotype (♀), designated by Erwin (1970a: 136), in BMNH. Synonymy established by Lindroth (1969a: 1105) based on Erwin’s (1969c) thesis.
Brachinus tabasconus Bates, 1891a: 268. Type locality: «San Juan Bautista, Tabasco [Mexico]» (lectotype label). Lectotype (♂), designated by Erwin (1970a: 136), in BMNH. Synonymy established by Lindroth (1969a: 1105) based on Erwin’s (1969c) thesis.
Brachinus atbarae Stehr, 1950: 102. Type locality: «Atbara, B[ritish] C[olumbia]» (original citation). Holotype (♂) in OSUO. Synonymy established by Lindroth (1969a: 1105) based on Erwin’s (1969c) thesis.

#### Distribution.

This widely distributed species ranges from southwestern New Brunswick (Webster and Bousquet 2008: 23) to south-central British Columbia, south to southwestern California, the state of Tabasco in Mexico, and central Florida [see Erwin 1970a: Fig. 369].

#### Records.

**CAN**: AB, BC, MB, NB, ON, QC **USA**: AL, AR, AZ, CA, CO, CT, DC, DE, FL, GA, IA, ID, IL, IN, KS, KY, LA, MA, MD, ME, MI, MN, MO, MS, MT, NC, ND, NE, NH, NJ, NM, NV, NY, OH, OK, OR, PA, RI, SC, SD, TN, TX, UT, VA, VT, WA, WI, WV, WY – Mexico

### Brachinus gebhardis

Erwin, 1965

Brachinus gebhardis Erwin, 1965: 6. Type locality: «Uvas Creek, 5 miles west of Morgan Hill, Santa Clara County, California» (original citation). Holotype (♂) in CAS [# 9047].

#### Distribution.

This species ranges from northern California to southern Baja California Sur and southeastern Arizona [see Erwin 1970a: Fig. 332].

#### Records.

**USA**: AZ, CA – Mexico

### Brachinus imperialensis

Erwin, 1965

Brachinus imperialensis Erwin, 1965: 17. Type locality: «Potholes, Imperial County, California» (original citation). Holotype (♂) in CAS [# 9133].

#### Distribution.

The range of this species extends from the Pacific Coast in southern California to northeastern Texas, north to northeastern Colorado, south to San Luis Potosí in central Mexico [see Erwin 1970a: Fig. 364].

#### Records.

**USA**: AZ, CA, CO, NM, NV, TX – Mexico

### Brachinus imporcitis

Erwin, 1970

Brachinus imporcitis Erwin, 1970a: 114. Type locality: «Pinal Creek, Globe, Gila County, Arizona» (original citation). Holotype (♂) in CUIC [# 4567].

#### Distribution.

This species is known only from Arizona [see Erwin 1970a: Fig. 278].

#### Records.

**USA**: AZ

### Brachinus javalinopsis

Erwin, 1970

Brachinus javalinopsis Erwin, 1970a: 109. Type locality: «Willcox, Cochise County, Arizona» (original citation). Holotype (♂) in AMNH [# 1233].

#### Distribution.

This species ranges from western Arizona to the Texas Panhandle and southeastern Texas [see Erwin 1970a: Fig. 254]; also known from northwestern Mississippi (Coahoma County, Drew A. Hildebrandt pers. comm. 2008).

#### Records.

**USA**: AZ, MS, NM, TX

### Brachinus kavanaughi

Erwin, 1969

Brachinus kavanaughi Erwin, 1969b: 287. Type locality: «along Coal Creek, Superior, Boulder County, Colorado» (original citation). Holotype (♂) in CAS [# 10361].

#### Distribution.

This species is found west of the Appalachian Mountains from western New York to eastern Wyoming, including southwestern Wisconsin (Messer 2010: 34) and est-central Minnesota (Gandhi et al. 2011: 673), south to northeastern Mexico [see Erwin 1970a: Fig. 253].

#### Records.

**USA**: CO, IL, KS, MN, MO, NE, NY, OH, OK, SD, TX, WI, WY – Mexico

### Brachinus medius

Harris, 1828

Brachinus medius T.W. Harris, 1828b: 117. Type locality not stated; «Boston [Suffolk County], Massachusetts» selected by Erwin (1970a: 129). Lectotype (♂), designated by Erwin (1970a: 129), in MCZ [# 26411].
Brachinus minutus T.W. Harris, 1828b: 117. Type locality not stated; «Boston [Suffolk County], Massachusetts» selected by Erwin (1970a: 129). Lectotype (♂), designated by Erwin (1970a: 129), in MCZ [# 26412]. Synonymy established by Lindroth (1969a: 1104) based on Erwin’s (1969c) thesis.

#### Distribution.

This species ranges from the Atlantic Coast in New England to south-central British Columbia, south to southern California, northeastern Mexico, and central Florida [see Erwin 1970a: Fig. 331].

#### Records.

**CAN**: BC, ON, QC **USA**: AL, AR, CA, CO, CT, DC, FL, GA, IA, ID, IL, IN, KS, LA, MA, ME, MI, MN, MO, MS, MT, ND, NE, NH, NJ, NY, OH, OK, OR, PA, RI, SC, SD, TX, UT, VT, WA, WI – Mexico

### Brachinus mexicanus

Dejean, 1831

Brachinus mexicanus Dejean, 1831: 428. Type locality: «Mexique» (original citation), restricted to «Baja California» by Erwin (1970a: 104), herein to Cataviña (see Erwin 1970a: 106). Lectotype (♂), designated by Erwin (1970a: 104), in MHNP.
Brachinus convexus Chaudoir, 1837a: 7. Type locality: «Mexique» (original citation). Lectotype (♀), designated by Erwin (1970a: 104), in MHNP. Synonymy established by Chaudoir (1876a: 73), confirmed by Erwin (1970a: 104).
Brachynus le contei Motschulsky, 1859a: 139 [primary homonym of *Brachinus lecontei* LeConte, 1844]. Type locality: California (inferred from title of the paper). Two syntypes in ZMMU (Erwin 1965: 10). Synonymy established by Lindroth (1969a: 1099) based on Erwin’s (1969c) thesis.
Brachinus fidelis LeConte, 1863a: 524. Type locality: «Kern [Kern County, California]» (lectotype label). Lectotype (♀), designated by Erwin (1970a: 104), in MCZ [# 5852]. Synonymy established, under the name *Brachinus lecontei* Motschulsky, by Erwin (1965: 9). Note. LeConte (1863a: 524) reported this species from “New Mexico, Arizona and Lower California” suggesting that the specimen selected as lectotype may not be a syntype.

#### Distribution.

This species occurs from eastern Washington to northern Illinois, south to Guatemala and the southern part of the Baja California Peninsula [see Erwin 1970a: Fig. 252].

#### Records.

**USA**: AR, AZ, CA, IL, NM, NV, OR, SD, TX, UT, WA – Guatemala, Mexico

### Brachinus neglectus

LeConte, 1844

Brachinus neglectus LeConte, 1844: 49. Type locality: «Georgia» (original citation), herein restricted to Thomasville, Thomas County (see Erwin 1970a: 111). Lectotype (♂), designated by Erwin (1970a: 110), in MCZ [# 31775].

#### Distribution.

This species is restricted to the Coastal Plain where it ranges from North Carolina to central Florida, west to “Mississippi” (Drew A. Hildebrandt pers. comm. 2007) [see Erwin 1970a: Fig. 255].

#### Records.

**USA**: AL, FL, GA, MS, NC, SC

### Brachinus ovipennis

LeConte, 1863

Brachinus ovipennis LeConte, 1863a: 525. Type locality: «middle and southern states» (original citation), herein restricted to Conneaut Lake, Crawford County, Pennsylvania (see Erwin 1970a: 122). Lectotype (♂), designated by Erwin (1970a: 121), in MCZ [# 31774]. Note. Erwin (1970a: 121) restricted the type locality to «Vermont» but that state is not part of LeConte’s concept of the middle states. The lectotype bears a pink label indicating middle states.

#### Distribution.

This species ranges from southeastern Maine (Majka et al. 2011: 47) and southern Quebec to southwestern South Dakota, south to northern Texas, “Mississippi” (Drew A. Hildebrandt pers. comm. 2007) and, east of the Appalachian Mountains, to Long Island, New York [see Erwin 1970a: Fig. 303].

#### Records.

**CAN**: ON, QC **USA**: CT, IA, IL, IN, KS, MA, ME, MI, MN, MO, MS, NY, OH, OK, PA, RI, SD, TX, VT, WI

### Brachinus patruelis

LeConte, 1844

Brachinus patruelis LeConte, 1844: 50. Type locality: «New York» (original citation), herein restricted to Southold, Suffolk County (see Erwin 1970a: 119). Lectotype (♀), designated by Erwin (1970a: 117), in MCZ [# 5842].

#### Distribution.

This species ranges from southwestern Maine (Majka et al. 2011: 48) to Wisconsin (Messer 2010: 34), including northeastern Ohio (Ashtabula County, Foster F. Purrington pers. comm. 2009), south to Maryland (Kent County, CMNH) [see Erwin 1970a: Fig. 301]. The records from “Georgia” (J.E. LeConte 1849: 25) and Missouri (Summers 1873: 133) are likely in error.

#### Records.

**USA**: CT, IL, MA, MD, ME, MI, NH, NJ, NY, OH, RI, WI

### Brachinus perplexus

Dejean, 1831

Brachinus perplexus Dejean, 1831: 426. Type locality: «Amérique septentrionale» (original citation), restricted to «Florida» by Erwin (1970a: 141), herein to Royal Palm State Park, Dade County (see Erwin 1970a: 142). Lectotype (♂), designated by Erwin (1970a: 141), in MHNP.
Brachinus le contei LeConte, 1844: 49. Type locality: «Georgia» (original citation). One possible syntype, with an orange label (= southern states), in MCZ (collection LeConte under the name *Brachinus perplexus*). Synonymy established by LeConte (1863a: 524).

#### Distribution.

This species ranges from southeastern Virginia (Norfolk, Robert L. Davidson pers. comm. 2012) to eastern Oklahoma, south to southeastern Texas and southwestern Florida [see Erwin 1970a: Fig. 366]. The records from New York (LeConte 1846b: 203), New Jersey (Smith 1910: 212), southeastern Iowa (King 1914: 322; Hendrickson 1930: 94), and Missouri (Summers 1873: 133) need confirmation.

#### Records.

**USA**: AL, AR, FL, GA, LA, MS, NC, OK, SC, TN, TX, VA [IA, MO, NJ, NY]

### Brachinus phaeocerus

Chaudoir, 1868

Brachinus phaeocerus Chaudoir, 1868b: 300. Type locality: «Texas» (original citation, see page 299), herein restricted to Big Bend National Park, Brewster County (see Erwin 1970a: 114). Lectotype (♂), designated by Erwin (1970a: 111), in MHNP.

#### Distribution.

This species is restricted to the Great Plains and ranges from northeastern Iowa (Black Hawk County, Doug A. Veal pers. comm. 2009) to north-central Colorado, south to Chihuahua in northern Mexico and southeastern Texas [see Erwin 1970a: Fig. 279]. The specimens collected near Ithaca, New York (Erwin 1970a: 114), are possibly strays or mislabeled.

#### Records.

**USA**: AZ, CO, IA, KS, NE, NM, OK, TX [NY] – Mexico

### Brachinus puberulus

Chaudoir, 1868

Brachinus puberulus Chaudoir, 1868b: 294. Type locality: «Texas» (original citation), herein restricted to Brownsville, Cameron County (see Erwin 1970a: 140). Lectotype (♂), designated by Erwin (1970a: 140), in MHNP.

#### Distribution.

This species is known from a few specimens collected in the Gulf Plain in eastern Texas [see Erwin 1970a: Fig. 368].

#### Records.

**USA**: TX

### Brachinus quadripennis

Dejean, 1825

Brachinus quadripennis Dejean, 1825: 316. Type locality: «Amérique septentrionale» (original citation), restricted to «Florida» by Lindroth (1969a: 1101), herein to Gainesville, Alachua County (see Erwin 1970a: 103). Lectotype (♀), designated by Erwin (1970a: 99), in MHNP.
Brachinus stygicornis Say, 1830b: (1) [3]. Type locality: «South Bend [Cass County], Neb[raska]» (neotype label). Neotype (♂), designated by Lindroth and Freitag (1969: 350), in MCZ [# 32997]. Synonymy established by Lindroth and Freitag (1969: 350). Note. «Engineer Cantonment [= winter quarter along the west bank of the Missouri River north of modern Omaha, Nebraska] near Council Bluff, Missouri [Territory]» was the area originally cited by Say (1830b: (1) [3]).
Brachinus tschernikhii Mannerheim, 1843: 184. Type locality: «California» (original citation). Syntype(s) probably lost (Erwin 1965: 13). Synonymy established by Lindroth (1969a: 1102) based on Erwin’s (1969c) thesis. Etymology. The specific name was proposed for Egor [Georgii] Leont’evich Tschernikh [Chernykh] [*ca*. 1813-1843], agriculturist at Fort Ross and also a successful beetle collector in Alaska and California. Tschernikh was in charge of one of the ranch, the Tschernikh Ranch, located five miles north of Bodega Bay (near present-day Freestone). Along with Vosnesensky, naturalist and curator at the Zoological Museum of the Academy of Natural Sciences in Saint Petersburg, he was the first to climb Mount Saint Helena in Napa County in June 1841.

#### Distribution.

The range of this species extends from Massachusetts to south-central British Columbia, south to southern California along the Pacific Coast, north-central Utah, central Kansas, southeastern Texas, southern Mississippi (George and Wilkinson Counties, Drew A. Hildebrandt pers. comm. 2008), and southern Florida [see Erwin 1970a: Fig. 251]. At least one specimen simply labeled from Arizona is known (Erwin 1970a: 101); the record from southwestern New Mexico (Fall and Cockerell 1907: 160, as *Brachinus tschernikhii*) needs confirmation.

#### Records.

**CAN**: AB, BC, MB, ON, SK **USA**: AL, AR, CA, FL, GA, IA, ID, IL, IN, KS, LA, MA, MI, MN, MO, MS, MT, ND, NE, NH, NJ, NY, OH, OR, PA, RI, SC, SD, TN, TX, UT, WA, WI [AZ, NM]

### Brachinus tenuicollis

LeConte, 1844

Brachinus tenuicollis LeConte, 1844: 49. Type locality: North America (inferred from title of the paper), restricted to «New York» by Erwin (1970a: 123), herein to Oswego, Oswego County (see Erwin 1970a: 125). Syntype location unknown. Note. LeConte (1846b: 199) subsequently stated that he had but a single specimen of this species. The specimen selected as lectotype by Erwin (1970a: 123) is probably not the syntype (see “Note” under *Brachinus similis*).
Brachinus ballistarius LeConte, 1846b: 199. Type locality: «NovEboraci [= New York]» (original citation). Holotype [by monotypy; designated lectotype by Erwin (1970a: 123)] (♂) in MCZ [# 5846]. Synonymy established by Lindroth (1969a: 1103) based on Erwin’s (1969c) thesis.
Brachinus similis LeConte, 1846b: 199. Type locality: «NovEboraci [= New York]» (original citation). Lectotype (♀), designated by Erwin (1970a: 123), in MCZ [# 5849]. Synonymy established by Lindroth (1969a: 1103) based on Erwin’s (1969c) thesis. Note. Erwin (1970a: 123) selected the same specimen as lectotype for both *Brachinus tenuicollis* LeConte and *Brachinus similis* LeConte. Based on the labels attached to the specimen, the lectotype is likely a syntype of *Brachinus similis* and consequently not of *Brachinus tenuicollis*.

#### Distribution.

This species ranges from southern Quebec to southeastern Wyoming (Lavigne 1977: 44), south to southwestern Texas and northern Florida [see Erwin 1970a: Fig. 329].

#### Records.

**CAN**: ON, QC **USA**: AR, CO, CT, DC, DE, FL, IA, IL, IN, KS, KY, LA, MA, MD, MI, MN, MO, MS, NE, NH, NJ, NM, NY, OH, OK, SC, TN, TX, VT, WI, WY

### Brachinus velutinus

Erwin, 1965

Brachinus velutinus Erwin, 1965: 17. Type locality: «Davis, Yolo County, California» (original citation). Holotype (♂) in UCD.

#### Distribution.

This species is known so far only from the Central Valley in California between the Sierra Nevada and the Coast Ranges [see Erwin 1970a: Fig. 367].

#### Records.

**USA**: CA

### [hirsutus group]

### Brachinus hirsutus

Bates, 1884

Brachinus hirsutus Bates, 1884: 295. Type locality: «Pinos Altos in Chihuahua, Mexico» (original citation). Lectotype (♂), designated by Erwin (1970a: 93), in BMNH.

#### Distribution.

This species ranges from southern Utah to the Rio Grande in southwestern Texas, south to the Mexican High Plateau in Jalisco [see Erwin 1970a: Fig. 220].

#### Records.

**USA**: AZ, NM, TX, UT – Mexico

### Brachinus pallidus

Erwin, 1965

Brachinus pallidus Erwin, 1965: 8. Type locality: «Mad River, 5 miles east of Mad River Post Office, Trinity County, California» (original citation). Holotype (♂) in CAS [# 9134].

#### Distribution.

This species ranges from northeastern Washington to southern California [see Erwin 1970a: Fig. 218].

#### Records.

**USA**: CA, OR, WA

### [kansanus group]

### Brachinus kansanus

LeConte, 1863

Brachinus kansanus LeConte, 1863a: 524. Type locality: «Kansas» (original citation). Lectotype (♂), designated by Erwin (1970a: 83), in MCZ [# 5851].

#### Distribution.

This species is mainly restricted to the Great Plains and ranges from southwestern Ohio to western Kansas south to southern Oklahoma and western Arkansas [see Erwin 1970a: Fig. 198].

#### Records.

**USA**: AR, IA, IL, KS, MN, MO, NE, OH, OK

### [lateralis group]

### Brachinus adustipennis

Erwin, 1969

Brachinus adustipennis Erwin, 1969b: 287. Type locality: «Myakka River, Myakka River State Park, Sarasota County, Florida» (original citation). Holotype (♂) in MCZ [# 34683].

#### Distribution.

This widely distributed species ranges from southeastern New York to southern Nebraska (Franklin County, Foster F. Purrington pers. comm. 2010), north to southwestern Wisconsin (Messer 2010: 34) and Michigan, south to Panama, southern Florida, and Cuba, west to the coast of the Gulf of California [see Erwin 1970a: Fig. 166].

#### Records.

**USA**: AL, AR, FL, GA, IA, IL, IN, KS, KY, LA, MA, MI, MO, MS, NE, NM, NY, OK, SC, TN, TX, VA, WI – Costa Rica, Cuba, Mexico, Panama

### Brachinus aeger

Chaudoir, 1876

Brachynus aeger Chaudoir, 1876: 82. Type locality: «Nouvelle-Grenade» (original citation). Lectotype (♀), designated by Erwin (1970: 77), in MHNP.

#### Distribution.

This species ranges from southern Sonora in Mexico to Colombia [see Erwin 1970: Fig. 168]. It was also recorded from “Texas” (Erwin 2011b: 277).

#### Records.

**USA**: TX – Belize, Colombia, Costa Rica, Guatemala, Guyana, Honduras, Mexico, Panama, Venezuela

### Brachinus lateralis

Dejean, 1831

Brachinus lateralis Dejean, 1831: 424. Type locality: «Amérique septentrionale» (original citation), restricted to «Imperial County, California» by Erwin (1970a: 73). Lectotype (♂), designated by Erwin (1970a: 73), in MHNP.
Brachinus leucoloma Chaudoir, 1868b: 301. Type locality: «Rio-Gila, en Californie» (original citation). Holotype [by monotypy; designated lectotype by Erwin (1970a: 73)] (♂) in MHNP. Synonymy established by Erwin (1965: 7).

#### Distribution.

This species extends from northern Arizona, southern Nevada, and southern California south to the southern part of the Baja California Peninsula and Chiapas in Mexico [see Erwin 1970a: Fig. 169]; also recorded from Nicaragua (Erwin 2011b: 292). The record from Cuba (Jacquelin du Val 1857: 12) must be in error.

#### Records.

**USA**: AZ, CA, NV – Mexico, Nicaragua

### [texanus group]

### Brachinus elongatulus

Chaudoir, 1876

Brachynus elongatulus Chaudoir, 1876a: 75. Type locality: «Mexique (Terres froides, Mexico, Orizaba, Guanaxuato)» (original citation), restricted to «Orizaba [Veracruz]» by Erwin (1970a: 65). Lectotype (♀), designated by Erwin (1970a: 65), in MHNP.
Brachynus elongatulus var. *brevior* Chaudoir, 1876a: 75. Type locality: «Etat d’Oaxaca, Mexique» (original citation). Lectotype (♀), designated by Erwin (1970a: 65), in MHNP. Synonymy established by Erwin (1970a: 65).

#### Distribution.

This species extends along the Pacific Coast from central Oregon to the southern extremity of the Baja California Peninsula, and from western Arizona to southwestern Oklahoma (Kondratieff et al. 2005: 171), south to Oaxaca, Mexico [see Erwin 1970a: Fig. 109].

#### Records.

**USA**: AZ, CA, NM, OK, OR, TX – Mexico

### Brachinus geniculatus

Dejean, 1831

Brachinus geniculatus Dejean, 1831: 428. Type locality: «environs de Carthagène [Colombia]» (original citation). Syntype(s) probably in MHNP.
Brachinus ventralis Mannerheim, 1837: 40. Type locality: «Columbia ad Maracay» (original citation). Syntype(s) location unknown. Synonymy established by Chaudoir (1876a: 78).
Brachynus rhytiderus Chaudoir, 1876a: 76. Type locality: «Mexique» (original citation), restricted to «San Luis Potosi» by Erwin (1970a: 63). Lectotype (♂), designated by Erwin (1970a: 63), in MHNP. Synonymy established by Erwin (1973b: 79).

#### Distribution.

This species ranges from east-central Texas south at least to northern Colombia (Dejean 1831: 428) [see Erwin 1970a: Fig. 110].

#### Records.

**USA**: TX – Belize, Colombia, Costa Rica, El Salvador, Guatemala, Honduras, Mexico, Nicaragua, Panama.

### Brachinus texanus

Chaudoir, 1868

Brachinus texanus Chaudoir, 1868b: 299. Type locality: «Texas» (original citation), herein restricted to Wolfe City, Hunt County (see Erwin 1970a: 63). Lectotype (♂), designated by Erwin (1970a: 60), in MHNP.

#### Distribution.

This species ranges from Massachusetts to “Minnesota,” south to the Rio Grande River in southern Texas and west-central Florida [see Erwin 1970a: Fig. 108]. The species is quite common west of the Mississippi Basin and south of latitude 40°, but known only from a few isolated localities outside this area. The record from southeastern Alberta (Erwin 1970a: 62) is possibly based on a mislabeled specimen or a stray; that from Riverside County, California (Dajoz 2007: 19) is probably in error.

#### Records.

**USA**: AL, AR, FL, KS, LA, MA, MI, MN, MO, MS, ND, NE, NY, OK, TN, TX, VA, WI [AB]

### Subfamily HARPALINAE

Bonelli, 1810

Harpalii Bonelli, 1810: Tabula Synoptica. Type genus: *Harpalus* Latreille, 1802. Note. The oldest available name for this taxon is Graphipterinae Latreille, 1802. I agree with Bouchard et al. (2011: 122) that the name Harpalinae should be maintained.

#### Diversity.

Worldwide, with about 19,600 species arrayed here in two supertribes for convenience: Pterostichitae and Harpalitae.

### Supertribe PTEROSTICHITAE

Bonelli, 1810

Pterostichii Bonelli, 1810: Tabula Synoptica. Type genus: *Pterostichus* Bonelli, 1810.

#### Diversity.

Worldwide, with about 6,400 species arrayed in the following 24 tribes: Abacetini (about 810 species), Amorphomerini (two species in the genus *Amorphomerus* Sloane), Bascanini (ten species in the genus *Bascanus* Péringuey), Brachygnathini (seven species in the genus *Brachygnathus* Perty), Chaetodactylini (about 20 species in the genus *Chaetodactyla* Tschitschérine), Chaetogenyini (five species), Chlaeniini (about 980 species), Cnemalobini (about 30 species), Cratocerini (four species), Cuneipectini (two species in the genus *Cuneipectus* Sloane), Dercylini (35 species in the genus *Dercylus* Laporte), Drimostomatini [= Caelostomini] (about 290 species), Glyptini (nine species), Idiomorphini (ten species), Melanchitonini (about 70 species), Metiini (about 75 species), Microcheilini (two species in the genus *Microcheila* Brullé), Morionini (about 85 species), Oodini (about 300 species), Orthogoniini (about 120 species), Panagaeini (about 270 species), Peleciini (about 75 species), Pterostichini (about 2,500 species), and Zabrini (about 700 species).

### Tribe Morionini

Brullé, 1835

Morioniens Brullé, 1835b: 36. Type genus: *Morion* Latreille, 1810. Note. The stem of *Morion* is *Morion*- (Madge 1989 : 465).

#### Diversity.

About 85 species (Lorenz 2005: 247-248) in the Nearctic (two species, none endemic), Neotropical (11 species), Australian (16 species), Oriental (11 species), Palaearctic (six species), and Afrotropical (40 species) Regions. These species are arrayed in nine genera: *Buderes* Murray (one Afrotropical species), *Hyperectenus* Alluaud (two Afrotropical species), *Hyperion* Laporte (one Australian species), *Megamorio* Chaudoir (six Afrotropical species), *Morion* (about 40 species), *Morionidius* Chaudoir (five Asian species), *Moriosomus* Motschulsky (two Neotropical species), *Platynodes* Westwood (one Afrotropical species), and *Stereostoma* Kirby (28 Afrotropical species).

### Genus Morion

Latreille, 1810

Morion Latreille, 1810: 159. Type species: *Harpalus monilicornis* Latreille, 1805 by monotypy. Etymology. Uncertain, possibly from the Greek *morion* (piece, part, portion, section) or from the Latin *morio*, -*onis* (fool, simpleton); morio or morion was also the name of a gem in Pliny the Elder [masculine]. Note. *Morio* is an incorrect subsequent spelling not in prevailing usage, introduced by Lamarck (1817: 510) and accepted by Latreille (1818b: 385) himself as the proper Latin name for the French name Morion.

#### Diversity.

About 40 species (Lorenz 2005: 247) in temperate, subtropical, and tropical areas of the Nearctic (two species), Neotropical (9 species), Australian (15 species), Oriental (eight species), Palaearctic (four species), and Afrotropical (three species) Regions.

#### Identification.

Allen (1969) reviewed the Western Hemisphere species.

### Morion aridus

Allen, 1969

Morion aridus Allen, 1969: 146. Type locality: «Organ Pipe Cactus National Monument, Pima [Graham County], Arizona» (original citation). Holotype (♂) in MCZ [# 31609].

#### Distribution.

This species is known from southern Arizona, the Baja California Peninsula (Allen 1969: 146), and the state of Sonora (CAS).

#### Records.

**USA**: AZ – Mexico

### Morion monilicornis

(Latreille, 1805)

Harpalus monilicornis Latreille, 1805: 206. Type locality: «Americae insulis» (original citation), which is likely incorrect; Savannah, Chatham County, Georgia (see Palisot de Beauvois 1811: 107, as *Scarites georgiae*) herein selected. Syntype(s) location unknown (possibly in MHNP).
Scarites georgiae Palisot de Beauvois, 1811: 107. Type locality: «Savan[n]ah [Chatham County], Géorgie» (original citation). Syntype(s) probably lost. Synonymy established by Dejean (1825: 430).

#### Distribution.

This species ranges from east-central South Dakota to central New York, south to southern Florida and southeastern Texas (Allen 1969: 147). The record from Cuba (Jacquelin du Val 1857: 18) needs confirmation.

#### Records.

**USA**: AL, AR, FL, GA, IA, IL, KY, LA, MD, MO, MS, NC, NY, OH, OK, SC, SD, TX, VA

### Tribe Abacetini

Chaudoir, 1873

Abacétides Chaudoir, 1873a: 5. Type genus: *Abacetus* Dejean, 1828.
Celioschesini Jeannel, 1948a: 381, 442. Type genus: *Celioschesis* Tschitschérine, 1898 (= *Aristopus* LaFerté-Sénectère, 1853). Note. The stem of *Celioschesis* is *Celioschese*- (Madge 1989 : 461).
Loxandrina Erwin and Sims, 1984: 383. Type genus: *Loxandrus* LeConte, 1853.

#### Diversity.

Worldwide, with approximately 810 species in about 45 genera. The tribe is underrepresented in the Northern Hemisphere with around 75 species (less than 10% of the world fauna). Only five species, all in the genus *Abacetus*, are known from Europe and northern Africa. The Western Hemisphere is represented by about 235 species (29% of the world fauna) in the genera *Adrimus* Bates (22 Neotropical species), *Loxandrus* (about 190 species), *Metoncidus* Bates (one Neotropical species), *Oxycrepis* Reiche (four Neotropical species), and *Stolonis* (19 species). All these genera belong to the loxandrine complex.

#### Taxonomic Note.

Members of this tribe are often included in the tribe Pterostichini.

### Genus Loxandrus

LeConte, 1853

Megalostylus Chaudoir, 1842: 855 [junior homonym of *Megalostylus* Schönherr, 1840]. Type species: *Feronia recta* Say, 1823 designated by Casey (1918: 325). Etymology (original). From the Greek *megale* (large) and *stylos* (pillar, by extension base), alluding to the robust first antennomere (“*antennes *... *1er article plus gros que tous les autres *... *fort long*”) of the adult [masculine]. Note. Casey’s designation was intended for *Loxandrus* LeConte but since *Loxandrus* is a replacement name for *Megalostylus* Chaudoir, both have the same type species and the type fixation for either applies also to the other (ICZN 1999: Article 67.8).
Loxandrus LeConte, 1853a: 250. Replacement name for *Megalostylus* Chaudoir, 1842. Etymology. From the Greek *loxos* (slanting, by extension oblique) and *andros* (male), alluding to the obliquely truncate male protarsomeres 1-3 (“*antici maris articulis *... *valde obliquis*”) of the species [masculine].

#### Diversity.

About 210 species in the Nearctic (44 species), Neotropical (about 150 species), and Australian (about 20 species) Regions.

#### Identification.

Allen (1972) revised the North American species though he left out more than 20 species because their type series were not located or included only females. These species were studied by Bousquet (2006a) leading to several new synonymies.

### [agilis group]

### Loxandrus accelerans

Casey, 1918

Loxandrus accelerans Casey, 1918: 386. Type locality: «Galveston [Galveston County], Texas» (original citation). Lectotype (♀), designated by Allen (1977: 286), in USNM [# 47338].

#### Distribution.

This species is known only from the type locality in southeastern Texas.

#### Records.

**USA**: TX

### Loxandrus agilis

(Dejean, 1828)

Feronia agilis Dejean, 1828: 244. Type locality: «Amérique septentrionale» (original citation), herein restricted to Archbold Biological Station, Highlands County, Florida (CNC). Lectotype (♂), designated by Allen (1972: 91), in MHNP.
Loxandrus calathinus LeConte, 1878b: 376. Type locality: «Tampa [Hillsborough County], Florida» (original citation). Lectotype (♀), designated by Bousquet (2006a: 146), in MCZ [# 5693]. Synonymy established by Bousquet (2006a: 146).
Loxandrus flavilimbus Blatchley, 1918: 418. Type locality: «Dunedin [Pinellas County, Florida]» (original citation). Lectotype (♀), designated by Blatchley (1930: 43), in PURC. Synonymy established by Allen (1972: 92).
Loxandrus cursitans Casey, 1918: 387. Type locality: «Dunedin [Pinellas County], Florida» (original citation). Lectotype (♀), designated by Allen (1977: 286), in USNM [# 47348]. Synonymy established by Bousquet (2006a: 148).

#### Distribution.

This species has been recorded from southeastern South Carolina (Ciegler 2003: [2]), northern Georgia (J.E. LeConte 1849: 26; Fattig 1949: 28), all over Florida except the Keys (Peck and Thomas 1998: 19), southwestern Alabama (Löding 1945: 17), Mississippi (Snodgrass and Cross 1983: 18), one locality (probably Mittie in Cherokee County) in eastern Texas (see Allen 1972: 94), and the Bahamas (Peck and Thomas 1998: 19). The record from “Arkansas” (Wickham 1896b: 43) needs confirmation; those from the District of Columbia (Ulke 1902: 7) and southwestern Ohio (Wright and Whitehouse 1941: 70) are probably in error.

#### Records.

**USA**: AL, FL, GA, MS, SC, TX [AR] – Bahamas

### Loxandrus algidus

Allen, 1972

Loxandrus algidus Allen, 1972: 108. Type locality: «Maryland» (original citation). Holotype (♂) in UMSP.

#### Distribution.

This species is known from a few specimens collected in Maryland (Allen 1972: 110; CNC), eastern Alabama, and southeastern Oklahoma (Bousquet 2006a: 151-152).

#### Records.

**USA**: AL, MD, OK

### Loxandrus cervicalis

Casey, 1918

Loxandrus cervicalis Casey, 1918 [12 November]: 385. Type locality: «Sarasota [Sarasota County], Florida» (original citation). Lectotype (♂), designated by Allen (1977: 286), in USNM [# 47340].
Loxandrus mundus Casey, 1918 [12 November]: 385. Type locality: «LaBelle [Hendry County], Florida» (original citation). Lectotype (♀), designated by Allen (1977: 286), in USNM [# 47341]. Synonymy established, under the name *Loxandrus brunneus* Blatchley, by Casey (1924: 79).
Loxandrus suturalis Casey, 1918 [12 November]: 384. Type locality: «Dunedin [Pinellas County], Florida» (original citation). Lectotype (♀), designated by Allen (1977: 286), in USNM [# 47339]. Synonymy established by Bousquet (2006a: 151).
Loxandrus brunneus Blatchley, 1918 [31 December]: 417 [primary homonym of *Loxandrus brunneus* Sloane, 1903]. Type locality: «shore of Lake Okeechobee, four miles southeast of Moore Haven [Glades County, Florida]» (original citation for the lectotype). Lectotype (♂), designated by Blatchley (1930: 43), in PURC. Synonymy established by Allen (1972: 98).
Loxandrus blatchleyi Csiki, 1930: 569. Replacement name for *Loxandrus brunneus* Blatchley, 1918.

#### Distribution.

This species is known from several localities in northern and central Florida and one locality in central Arkansas [see Allen 1972: Fig. 176]. The records from South Carolina (Kirk 1969: 11) and Georgia (Fattig 1949: 28, also as *Loxandrus mundus*) need confirmation.

#### Records.

**USA**: AR, FL [GA, SC]

### Loxandrus extendus

Allen, 1972

Loxandrus extendus Allen, 1972: 82. Type locality: «Arkansas» (original citation). Holotype (♂) in MCZ [# 31941].

#### Distribution.

This species is known from Dubois and Vigo Counties in western Indiana, from an unspecified locality in Arkansas (Allen 1972: 84), and from Noxubee County in eastern Mississippi (CMNH).

#### Records.

**USA**: AR, IN, MS

### Loxandrus floridanus

LeConte, 1878

Loxandrus floridanus LeConte, 1878b: 376. Type locality: «[Fort] Capron and Enterprise [Florida]» (original citation). Seven syntypes in MCZ [# 5694].
Loxandrus comptus Casey, 1918: 387. Type locality: «Dunedin [Pinellas County], Florida» (original citation). Lectotype (♀), designated by Allen (1977: 286), in USNM [# 47349]. Synonymy established by Bousquet (2006a: 147).
Loxandrus scitus Casey, 1918: 388. Type locality: «Dunedin [Pinellas County], Florida» (original citation). Lectotype (♂), designated by Allen (1977: 286), in USNM [# 47351]. Synonymy established by Allen (1972: 89).
Loxandrus contumax Casey, 1918: 388. Type locality: «Indian River [Brevard County], Florida» (original citation). Lectotype (♀), designated by Allen (1977: 286), in USNM [# 47352]. Synonymy established by Bousquet (2006a: 147).
Loxandrus breviusculus Casey, 1924: 80. Type locality: «Dunedin [Pinellas County], Florida» (original citation). Holotype [by monotypy] (♀) in USNM [# 47350]. Synonymy established by Bousquet (2006a: 146).

#### Distribution.

This species is known from the Bahamas and along the Coastal Plain from southern Florida west to eastern Texas, north to northeastern Louisiana [see Allen 1972: Fig. 171]. The record from southern Georgia (Fattig 1949: 27, as *Loxandrus comptus*) needs confirmation.

#### Records.

**USA**: AL, FL, LA, MS, TX [GA] – Bahamas

### Loxandrus icarus

Will and Liebherr, 1998

Loxandrus icarus Will and Liebherr, 1998: 234. Type locality: «Eliz. Furnace Cpgd., G[eorge] Washington N[ational] F[orest], Shenandoah Co[unty], V[irgini]a» (original citation). Holotype (♂) in CUIC [# 7038].

#### Distribution.

This species is known only from the original 13 specimens collected at the type locality.

#### Records.

**USA**: VA

### Loxandrus parallelus

Casey, 1918

Loxandrus parallelus Casey, 1918: 386. Type locality: «Monroe [probably in Ouachita Parish], Louisiana» (original citation). Holotype [by monotypy] (♂) in USNM [# 47342].

#### Distribution.

This species is known from northeastern Arkansas south to southeastern Louisiana, east to eastern Mississippi (Noxubee County, CMNH) and west to east-central Texas [see Allen 1972: Fig. 170]; also recorded from South Carolina (Kirk 1969: 11; Ciegler 2000: 59). One specimen simply labeled from Kentucky is known (Allen 1972: 98)

#### Records.

**USA**: AR, LA, MS, SC, TX [KY]

### Loxandrus parvulus

Chaudoir, 1868

Loxandrus parvulus Chaudoir, 1868b: 342. Type locality: «Caroline» (original citation). Holotype [by monotypy] (♀) in MHNP (Bousquet 2006a: 149).
Loxandrus aduncus Allen, 1972: 96. Type locality: «Winter Park [Orange County], Florida» (original citation). Holotype (♂) in MCZ [# 31955]. Synonymy established by Bousquet (2006a: 149).

#### Distribution.

This species is known from a few localities in central Florida and southern Alabama [see Allen 1972: Fig. 174, as *Loxandrus aduncus*]. It probably occurs also farther north as implied by the type locality given by Chaudoir (1868b: 342).

#### Records.

**USA**: AL, FL

### Loxandrus piceolus

Chaudoir, 1868

Loxandrus piceolus Chaudoir, 1868b: 343. Type locality: «Texas» (original citation). Holotype [by monotypy] (♀) in MHNP (Bousquet 2006a: 149).

#### Distribution.

This species is known only from the holotype (Bousquet 2006a: 149).

#### Records.

**USA**: TX

### Loxandrus piciventris

(LeConte, 1846)

Argutor piciventris LeConte, 1846b: 337. Type locality: «Georgia» (original citation). Lectotype (♀), designated by Bousquet (2006a: 150), in MCZ [# 5698].

#### Distribution.

This species is known only from the type series.

#### Records.

**USA**: GA

### Loxandrus pusillus

LeConte, 1853

Loxandrus pusillus LeConte, 1853a: 252. Type locality: «Georgia» (original citation), herein restricted to Little Satilla River at junction with US 301, Pierce County (see Allen 1972: 136). Holotype [by monotypy] (♀) in MCZ [# 5699].
Loxandrus minutus Allen, 1972: 134. Type locality: «Myakka River State Park, Sarasota Co[unty], Florida» (original citation). Holotype (♂) in MCZ [# 31952]. Synonymy established by Bousquet (2006a: 151).

#### Distribution.

This species is known from southeastern Georgia to central Florida including the Panhandle (Peck and Thomas 1998: 19) [see Allen 1972: Fig. 175, as *Loxandrus minutus*].

#### Records.

**USA**: FL, GA

### Loxandrus rossi

Allen, 1972

Loxandrus rossi Allen, 1972: 106. Type locality: «Paragould, Greene County, Arkansas» (original citation). Holotype (♂) in MSUE.

#### Distribution.

This species is known from a few localities in central Georgia, northern Florida (Peck and Thomas 1998: 19), northeastern Alabama (Allen 1972: 108), and northeastern Arkansas [see Allen 1972: Fig. 174].

#### Records.

**USA**: AL, AR, FL, GA

### Loxandrus saccisecundaris

Allen, 1972

Loxandrus saccisecundaris Allen, 1972: 78. Type locality: «Georgia» (original citation). Holotype (♂) in MCZ [# 31949].

#### Distribution.

This species is known only from the holotype collected in Georgia from an unspecified locality.

#### Records.

**USA**: GA

### Loxandrus spinilunatus

Allen, 1972

Loxandrus spinilunatus Allen, 1972: 100. Type locality: «Arkansas» (original citation). Holotype (♂) in MCZ [# 31942].

#### Distribution.

This species is known from two unspecified localities in Arkansas and Louisiana (Allen 1972: 102).

#### Records.

**USA**: AR, LA

### Loxandrus taeniatus

LeConte, 1853

Loxandrus taeniatus LeConte, 1853a: 252. Type locality: «Louisiana» (original citation), herein restricted to Lake Chicot State Park, Evangeline Parish (see Allen 1972: 87). Holotype [by monotypy] (♂) in MCZ [# 5697].

#### Distribution.

This species is known along the Coastal Plain from North Carolina and South Carolina, west to southern Louisiana and eastern Arkansas [see Allen 1972: Fig. 175]. Specimens simply labeled from Georgia are known (Allen 1972: 87). The records from Missouri (Summers 1873: 134), central Kansas (Knaus 1885: 58), and southeastern Texas (Casey 1918: 384) need confirmation.

#### Records.

**USA**: AL, AR, LA, MS, NC, SC [GA, KS, MO, TX]

### Loxandrus unilobus

Allen, 1972

Loxandrus unilobus Allen, 1972: 76. Type locality: «Carlisle, Lonoke Co[unty], Arkansas» (original citation). Holotype (♂) in MCZ [# 31945].

#### Distribution.

This species is found along the Coastal Plain from northeastern Florida to central Louisiana and east-central Arkansas [see Allen 1972: Fig. 172]. At least one specimen simply labeled from Texas is known (Allen 1972: 78).

#### Records.

**USA**: AR, FL, LA [TX]

### Loxandrus velox

(Dejean, 1828)

Feronia velox Dejean, 1828: 245. Type locality: «Amérique septentrionale» (original citation). Lectotype (♂), designated by Allen (1972: 158), in MHNP.

#### Distribution.

This species is known for sure only from the four original specimens. The records from “Pennsylvania” (LeConte 1853a: 252), South Carolina (Kirk 1969: 11), Georgia (J.E. LeConte 1849: 26; Leng 1910: 73; Fattig 1949: 28), and southwestern Alabama (Löding 1945: 17) need confirmation.

#### Records.

[AL, GA, PA, SC]

#### Note.

Dejean’s name was listed as synonym of *Loxandrus rectus* (Say) by LeConte (1846b: 338) but later considered a valid species (e.g., LeConte 1853a: 252; LeConte 1878b: 376).

### [celer group]

### Loxandrus celer

(Dejean, 1828)

Feronia celeris Dejean, 1828: 246. Type locality: «Amérique septentrionale» (original citation), herein restricted to Saint Simons Island, Glynn County, Georgia (see Allen 1972: 45). Lectotype (♂), designated by Allen (1972: 44), in MHNP.
Loxandrus rapidus Chaudoir, 1868b: 344. Type locality: «Louisiane» (original citation). Lectotype (♂), designated by Allen (1972: 44), in MHNP. Synonymy established by Allen (1972: 44).
Loxandrus parvicollis Casey, 1918: 389. Type locality: «Galveston [Galveston County], Texas» (original citation). Holotype [by monotypy] (♀) in USNM [# 47343]. Synonymy established by Bousquet (2006a: 149).
Loxandrus concinnus Casey, 1918: 391. Type locality: «Dunedin [Pinellas County], Florida» (original citation). Lectotype (♀), designated by Allen (1977: 286), in USNM [# 47353]. Synonymy established by Bousquet (2006a: 147).

#### Distribution.

This species ranges from Virginia (Virginia Beach County, VMNH) to southern Florida, west to southeastern Texas (Casey 1918: 389, as *Loxandrus parvicollis*) and central Oklahoma (Grady County, Robert L. Davidson pers. comm. 2012), south to Chiapas in southern Mexico; also known from the Bahamas, Cuba, Cayman Islands, and Puerto Rico (Peck 2005: 31, as *Loxandrus celeris*) [see Allen 1972: Fig. 147]. The records from “Pennsylvania” (LeConte 1853a: 252) and southwestern Ohio (Wright and Whitehouse 1941: 70) need confirmation.

#### Records.

**USA**: AL, FL, GA, LA, MS, OK, SC, TX, VA [OH, PA] – Bahamas, Cayman Islands, Cuba, Mexico, Puerto Rico

### [crenatus group]

### Loxandrus crenatus

LeConte, 1853

Loxandrus crenatus LeConte, 1853a: 253. Type locality: «Georgia» (original citation), herein restricted to Brawbridge, Decatur County (see Allen 1972: 112). Holotype [by monotypy] (♂) in MCZ [# 5701].
Loxandrus crenulatus Chaudoir, 1868b: 343. Type locality: «Texas» (original citation). Lectotype (♀), designated by Bousquet (2006a: 148), in MHNP. Synonymy established by Bousquet (2006a: 148).

#### Distribution.

This species inhabits the Coastal Plain from southeastern Virginia (Norfolk, Robert L. Davidson pers. comm. 2008) to central Florida, west to southeastern Texas (Brazoria County, Brian Raber pers. comm. 2010) [see Allen 1972: Fig. 162], including western Arkansas (Polk and Garland Counties, Robert L. Davidson pers. comm. 2012); also recorded from Cuba (Darlington 1934: 91).

#### Records.

**USA**: AL, AR, FL, GA, LA, MS, NC, SC, TX, VA – Cuba

### Loxandrus proximus

Chaudoir, 1868

Loxandrus proximus Chaudoir, 1868b: 344. Type locality: «je crois qu’il est originaire du Texas» (original citation). Holotype [by monotypy] (♀) in MHNP.

#### Distribution.

This species is at present known only from the holotype which Chaudoir (1868b: 344) believed had been collected in Texas.

#### Records.

**USA**: TX

### [erraticus group]

### Loxandrus brevicollis

(LeConte, 1846)

Argutor brevicollis LeConte, 1846b: 338. Type locality: «provinciis australibus, et NovEboraci [= New York]» (original citation), restricted to «Enterprise [Volusia County], Florida» by Allen (1972: 142). Three syntypes in MCZ [# 5695].
Loxandrus fulgens Casey, 1918: 388. Type locality: «Vicksburg [Warren County], Mississippi» (original citation). Lectotype (♀), designated by Allen (1977: 286), in USNM [# 47344]. Synonymy established by Bousquet (2006a: 148).

#### Distribution.

This species is found from southeastern Pennsylvania to Iowa (Wickham 1888: 82; King 1914: 323; Jaques and Redlinger 1946: 297), south to southern Louisiana and central Florida including the Panhandle (Peck and Thomas 1998: 19) [see Allen 1972: Fig. 160]. Old specimens simply labeled from Massachusetts and Oklahoma are known (Allen 1972: 144).

#### Records.

**USA**: AL, AR, DC, DE, FL, GA, IA, IL, IN, LA, MO, MS, NC, PA, SC, TN, VA [MA, OK]

### Loxandrus cincinnati

Casey, 1924

Loxandrus cincinnati Casey, 1924: 80. Type locality: «Cincinnati [Hamilton County], Ohio» (original citation). Lectotype (♂), designated by Allen (1977: 286), in USNM [# 47355].
Loxandrus cincinnatiensis Csiki, 1930: 570. Unjustified emendation of *Loxandrus cincinnati* Casey, 1924.

#### Distribution.

This species is known from southwestern Ohio and northeastern Kentucky to western Illinois, south to north-central Texas, southern Alabama, and the Florida Panhandle (Peck and Thomas 1998: 19) [see Allen 1972: Fig. 168]. One specimen simply labeled from “South Carolina” is known (Allen 1972: 156).

#### Records.

**USA**: AL, AR, FL, IL, IN, KY, LA, MO, MS, OH, TN, TX [SC]

### Loxandrus circulus

Allen, 1972

Loxandrus circulus Allen, 1972: 157. Type locality: «District of Columbia» (original citation). Holotype (♂) in MCZ [# 31950].

#### Distribution.

This species is known from a few localities in Maryland, the District of Columbia, northern Ohio, Mississippi (Drew A. Hildebrandt pers. comm. 2007), and Alabama [see Allen 1972: Fig. 159]. The record from “Illinois” (Bousquet and Larochelle 1993: 161) needs confirmation.

#### Records.

**USA**: AL, DC, MD, MS, OH [IL]

### Loxandrus erraticus

(Dejean, 1828)

Feronia erratica Dejean, 1828: 240. Type locality: «Amérique septentrionale» (original citation), restricted to «Mobile [Mobile County], Alab[ama]» by Lindroth (1966: 538). One syntype in MHNP (Lindroth 1955b: 15).

#### Distribution.

This species ranges along the Coastal Plain from Rhode Island and Connecticut (William L. Krinsky pers. comm. 2008) to central Florida, west to southern Louisiana and north along the Mississippi River drainage to east-central Illinois [see Allen 1972: Fig. 155]. The record from southwestern Ohio (Dury 1902: 113) needs confirmation.

#### Records.

**USA**: AL, AR, CT, DC, DE, FL, GA, IL, IN, KY, LA, MO, MS, NC, NJ, PA, RI, SC [OH]

### Loxandrus gibbus

Allen, 1972

Loxandrus gibbus Allen, 1972: 146. Type locality: «Tuscaloosa [Tuscaloosa County], Alabama» (original citation). Holotype (♂) in MCZ [# 31953].

#### Distribution.

This species is known along the Coastal Plain from southern North Carolina to central Florida, west to northeastern Louisiana and southern Arkansas, north along the Mississippi River drainage to southwestern Indiana and southwestern Illinois (Allen 1972: 147-148, Fig. 156).

#### Records.

**USA**: AL, AR, FL, IL, IN, LA, MO, MS, NC, TN, SC

### Loxandrus minor

(Chaudoir, 1843)

Megalostylus minor Chaudoir, 1843b: 766. Type locality: «Nouvelle Orléans [Orleans Parish, Louisiana]» (original citation). Holotype [by monotypy] (♀) in MHNP.
Loxandrus inquietus Casey, 1918: 389. Type locality: «Indiana» (original citation). Lectotype (♀), designated by Lindroth (1975: 125), in USNM [# 47345]. Synonymy established by Lindroth (1966: 540).

#### Distribution.

This species ranges from southern Ontario (Lindroth 1966: 540) south to southeastern Louisiana (Chaudoir 1843b: 766), west to eastern Kansas [see Allen 1972: Fig. 167]. The records from New Jersey (Smith 1910: 206), District of Columbia (Ulke 1902: 7), South Carolina (Kirk 1969: 11; Ciegler 2000: 59), “Georgia” (J.E. LeConte 1849: 26), Florida (Frost 1964: 138), and southwestern Alabama (Löding 1945: 17) need confirmation.

#### Records.

**CAN**: ON **USA**: AR, IA, IL, IN, KS, LA, MO [AL, DC, FL, GA, NJ, SC]

### Loxandrus nitidulus

(LeConte, 1846)

Argutor nitidulus LeConte, 1846b: 339. Type locality: «provinciis australibus» (original citation), herein restricted to Lucedale, George County, Mississippi (see Allen 1972: 153). Four syntypes in MCZ [# 5696].

#### Distribution.

This species ranges from northern Indiana to eastern Iowa, south to northern Louisiana and southwestern Alabama [see Allen 1972: Fig. 161). One specimen simply labeled from Florida is known (Allen 1972: 153).

#### Records.

**USA**: AL, AR, IA, IL, IN, LA, MO, MS, TN [FL]

### Loxandrus robustus

Allen, 1972

Loxandrus robustus Allen, 1972: 153. Type locality: «Carlisle, Lonoke Co[unty], Arkansas» (original citation). Holotype (♂) in MCZ [# 31939].

#### Distribution.

This species is known from one locality in southwestern Indiana, several localities in Arkansas, and one locality in Noxubee County in eastern Mississippi (CMNH) [see Allen 1972: Fig. 169].

#### Records.

**USA**: AR, IN, MS

### Loxandrus uniformis

Allen, 1972

Loxandrus uniformis Allen, 1972: 130. Type locality: «Mobile [Mobile County], Alabama» (original citation). Holotype (♂) in CAS [# 11543].

#### Distribution.

This species is known from a few localities in northwestern Ohio, South Carolina, and southwestern Alabama [see Allen 1972: Fig. 165]. Specimens simply labeled from Virginia and Arkansas are known (Allen 1972: 132).

#### Records.

**USA**: AL, OH, SC [AR, VA]

### Loxandrus velocipes

Casey, 1918

Loxandrus velocipes Casey, 1918: 390. Type locality: «District of Columbia» (original citation). Lectotype (♂), designated by Lindroth (1975: 125), in USNM [# 47346].
Loxandrus inferus Allen, 1972: 137. Type locality: «Rossyln, Nelson Co[unty], Virginia» (original citation). Holotype (♂) in MCZ [# 31940]. Synonymy established by Will and Liebherr (1998: 237).

#### Distribution.

This species ranges from Maryland (Erwin 1981b: 160) to southeastern Minnesota (Allen 1972: 137), including north-central Ohio (Lee 1994: 61) and southern Ontario (Lindroth 1966: 539), south to southeastern Louisiana and southern Georgia (Torres and Ruberson 2006: 32) [see Allen 1972: Fig. 158]. The record from “Connecticut” (Bousquet and Larochelle 1993: ) was based on a misidentified specimen of *Loxandrus vitiosus* (Krinsky and Oliver 2001: 102).

#### Records.

**CAN**: ON **USA**: AL, AR, DC, GA, IA, IL, IN, LA, MD, MN, MO, MS, NC, OH, SC, TN, VA, WI, WV

### [infimus group]

### Loxandrus infimus

Bates, 1882

Loxandrus infimus Bates, 1882a: 87. Type locality: «Cubilguitz [Alta Verapaz], Guatemala» (original citation for the lectotype). Lectotype (♂), designated by Allen (1972: 30), in BMNH.
Loxandrus mutans Darlington, 1936c: 180. Type locality: «Etang Lachaux, Haiti» (original citation). Holotype (♂) in MCZ [# 22022]. Synonymy established by Allen (1972: 30).

#### Distribution.

This species is found from northern Sonora and southeastern Texas south to Brazil (Allen 1972: 34); also known from Haiti [see Allen 1972: Fig. 144].

#### Records.

**USA**: TX – Brazil, Guatemala, Haiti, Mexico

### Loxandrus pactinullus

Allen, 1972

Loxandrus pactinullus Allen, 1972: 34. Type locality: «8.1 mi[les] E[ast] Villamar (5500 ft), Michoacan, Mexico» (original citation). Holotype (♂) in MCZ [# 31943].

#### Distribution.

This species ranges from southeastern Arizona (Allen and Ball 1980: 500) south to Morelos in Mexico [see Allen 1972: Fig. 154].

#### Records.

**USA**: AZ – Mexico

### Loxandrus sculptilis

Bates, 1884

Loxandrus sculptilis Bates, 1884: 278. Type locality: «Tolé [Chiriquí], Panama» (original citation for the lectotype). Lectotype (♂), designated by Allen (1972: 38), in BMNH.

#### Distribution.

This species ranges from southern Texas (Allen and Ball 1980: 501) and central Sinaloa south to Panama [see Allen 1972: Fig. 153].

#### Records.

**USA**: TX – Mexico, Panama

### [micans group]

### Loxandrus duryi

Wright, 1939

Loxandrus duryi Wright, 1939: 257. Type locality: «near Goshen, Clermont County, Ohio» (original citation). Holotype (♂) location unknown. Etymology. The specific name honors Ralph Dury [1899-1984], naturalist, director of the Cincinnati Museum of Natural History, and authority on freshwater bivalves. Ralph was the son of Charles Dury [1847-1931], collector of natural history objects in Ohio who published new species of Coleoptera and lists of beetles found in the vicinity of Cincinnati, Ohio.

#### Distribution.

The range of this species extends from central New York (Bousquet 2006a: 152) to east-central Iowa (Johnson County, CMNH), including southern Ontario (CNC), south to southeastern Texas (Cameron County, CMNH), central Louisiana, and northeastern Alabama (DeKalb County, CMNH) [see Allen 1972: Fig. 166].

#### Records.

**CAN**: ON **USA**: AL, IA, IL, IN, LA, MO, MS, NY, OH, PA, TX, VA

### Loxandrus micans

Chaudoir, 1868

Loxandrus micans Chaudoir, 1868b: 342. «Opelousas [Saint Landry Parish], Louisiane» (original citation). Holotype [by monotypy] (♂) in MHNP.

#### Distribution.

This species is known from three localities in southwestern Tennessee, eastern Arkansas (Allen 1972: 114, Fig. 165), and southern Louisiana (Chaudoir 1868b: 342).

#### Records.

**USA**: AR, LA, TN

### Loxandrus straneoi

Will and Liebherr, 1998

Loxandrus straneoi Will and Liebherr, 1998: 231. Type locality: «30°51'23"N, 88°44'45"W, Rt26 at Pascagoula R[iver], George Co[unty], Mississippi» (original citation). Holotype (♂) in CUIC [# 7037]. Etymology. This species was named after Stefano Ludovico Straneo [1902-1997], a teacher, administrator, and author. Straneo was also deeply involved as an amateur in the study of Carabidae and particularly the tribe Pterostichini. He described 64 new genera and almost 1,200 new species.

#### Distribution.

This species is known only from George and Noxubee Counties in eastern Mississippi (Will and Liebherr 1998: 231).

#### Records.

**USA**: MS

### Loxandrus vulneratus

Casey, 1918

Loxandrus vulneratus Casey, 1918: 390. Type locality: «Indiana» (original citation). Lectotype (♀), designated by Allen (1977: 286), in USNM [# 47354].
Loxandrus vitiosus Allen, 1972: 115. Type locality: «4 mi[les] w[est] Mineral Springs, Howard Co[unty], Arkansas» (original citation). Holotype (♂) in UAIC. Synonymy established by Bousquet (2006a: 151).

#### Distribution.

This species ranges from Connecticut (Krinsky and Oliver 2001: 102) and southeastern Pennsylvania to southwestern Illinois, south to southern Louisiana, southwestern Alabama, and central South Carolina (Ciegler 2000: 60) [see Allen 1972: Fig. 167]. One specimen simply labeled from Florida is known (Allen 1972: 116).

#### Records.

**USA**: AL, AR, CT, DC, IL, IN, LA, MD, MO, MS, NC, OH, PA, SC, TN, VA [FL]

### [rectangulus group]

### Loxandrus rectangulus

LeConte, 1878

Loxandrus rectangulus LeConte, 1878b: 377. Type locality: «Enterprise [Volusia County, Florida]» (original citation). Two syntypes [2 originally cited] in MCZ [# 5700].

#### Distribution.

This species is known from central and southern Florida (Peck and Thomas 1998: 19), the Bahamas (Turnbow and Thomas 2008: 13), Grand Cayman Island, and southern Mexico [see Allen 1972: Fig. 146]. One specimen simply labeled from Texas is known (Allen 1972: 42).

#### Records.

**USA**: FL [TX] – Bahamas, Cayman Islands, Mexico

### [rectus group]

### Loxandrus collucens

Casey, 1918

Loxandrus collucens Casey, 1918: 382. Type locality: «Houston [Harris County], Texas» (original citation). Lectotype (♀), designated by Allen (1977: 286), in USNM [# 47336].
Loxandrus ludovicianus Casey, 1918: 383. Type locality: «Cane River [Natchitoches Parish], Louisiana» (original citation). Holotype [by monotypy] (♀) in USNM [# 47337]. Synonymy established by Bousquet (2006a: 149).

#### Distribution.

This species is known from a few localities in northwestern Louisiana (Casey 1918: 383, as *Loxandrus ludovicianus*) and eastern and southern Texas (Casey, 1918: 382; Brazoria, Fort Bend, Lasalle, and Live Oak Counties, CMNH).

#### Records.

**USA**: LA, TX

### Loxandrus lucens

Chaudoir, 1868

Loxandrus lucens Chaudoir, 1868b: 342. Type locality: «Havre [Hill County], Texas» (original citation). Lectotype (♂), designated by Allen (1972: 129), in MHNP.

#### Distribution.

This species is found west of the Appalachians from central Kentucky south to eastern Texas and southern Alabama (Allen 1972: 130, Fig. 163). The record from northern Georgia (Fattig 1949: 27) is probably in error.

#### Records.

**USA**: AL, AR, KY, LA, TN, TX

### Loxandrus pravitubus

Allen, 1972

Loxandrus pravitubus Allen, 1972: 72. Type locality: «Brownsville [Cameron County], Texas» (original citation). Holotype (♂) in MCZ [# 31957].

#### Distribution.

This species is found along the Coastal Plain from southern North Carolina to the Rio Grande in southeastern Texas [see Allen 1972: Fig. 159].

#### Records.

**USA**: AL, LA, MS, NC, TX

### Loxandrus rectus

(Say, 1823)

Feronia recta Say, 1823a: 58. Type locality: «S[outh] C[arolina]» (neotype label). Neotype (♂), designated by Lindroth and Freitag (1969: 343), in MCZ [# 33034].
Feronia lucidula Dejean, 1828: 239. Type locality: «Amérique septentrionale» (original citation). Lectotype (♂), designated by Allen (1972: 68), in MHNP. Synonymy established by LeConte (1853a: 251), confirmed by Allen (1972: 68).
Megalostylus laticollis Chaudoir, 1843b: 766. Type locality: «Nouvelle Orléans [Orleans Parish, Louisiana]» (original citation). Lectotype (♂), designated by Allen (1972: 68), in MHNP. Synonymy established by LeConte (1853a: 251), confirmed by Allen (1972: 68).
Loxandrus iris Motschulsky, 1866: 242. Type locality: «Nouv[elle] Orléans [Orleans Parish, Louisiana]» (original citation). Lectotype (♂), designated by Bousquet and Larochelle (1993: 15), in ZMMU. Synonymy established by Bousquet and Larochelle (1993: 15).
Loxandrus rectus mandibularis Casey, 1918: 382. Type locality: «District of Columbia» (original citation). Lectotype (♂), designated by Allen (1977: 286), in USNM [# 47334]. Synonymy established by Allen (1972: 68).
Loxandrus limatus Casey, 1918: 382. Type locality: «District of Columbia» (original citation). Lectotype (♀), designated by Allen (1977: 286), in USNM [# 47335]. Synonymy established by Bousquet (2006a: 149).

#### Distribution.

This species ranges from the District of Columbia to southern Illinois, north to northern Ohio (Lee 1994: 61), south to southeastern Texas and northern Florida including the Panhandle (Peck and Thomas 1998: 19) [see Allen 1972: Fig. 157]. The record from southeastern Pennsylvania (Rathvon 1869: 525) needs confirmation.

#### Records.

**USA**: AL, AR, DC, FL, GA, IL, IN, KY, LA, MD, MO, MS, NC, OH, SC, TN, TX, VA [PA]

### [saphyrinus group]

### Loxandrus saphyrinus

(Chaudoir, 1843)

Megalostylus saphyrinus Chaudoir, 1843b: 766. Type locality: «Nouvelle Orléans [Orleans Parish, Louisiana]» (original citation). Lectotype (♂), designated by Allen (1972: 126), in MHNP.
Loxandrus reflexus LeConte, 1878b: 376. Type locality: «Tampa [Hillsborough County, Florida]» (original citation). Lectotype (♀), designated Bousquet (2006a: 151), in MCZ [# 5692]. Synonymy established by Bousquet (2006a: 151).
Loxandrus lateralis Casey, 1918: 381. Type locality: «Sarasota [Sarasota County], Florida» (original citation). Lectotype (♀), designated by Allen (1977: 286), in USNM [# 47347]. Synonymy established by Bousquet (2006a: 148).
Loxandrus sapphirinus Csiki, 1930: 573. Unjustified emendation of *Loxandrus saphyrinus* (Chaudoir, 1843).

#### Distribution.

This species is found from North Carolina to northwestern Arkansas, including south-central Kentucky (Metcalfe County, Peter W. Messer pers. comm. 2012), south to eastern Texas and southern Florida [see Allen 1972: Fig. 164].

#### Records.

**USA**: AL, AR, FL, GA, KY, LA, MS, NC, SC, TN, TX

### Genus Stolonis

Motschulsky, 1866

Stolonis Motschulsky, 1866: 230. Type species: *Stolonis notula* Motschulsky, 1866 by monotypy. Etymology. Uncertain, possibly from the Greek *stolonis* (shoot) [feminine].
Prostolonis Mateu, 1976: 69. Type species: *Prostolonis martinezi* Mateu, 1976 by original designation. Synonymy established by Allen and Ball (1980: 529). Etymology. From the Latin prefix *pro*- (before, in front of) and the generic name *Stolonis* [*q.v*.] [feminine].

#### Diversity.

Twenty species in the Neotropical Region, one of them extending into southern United States.

#### Identification.

Will (2005) provided a key for the identification of the species of this genus.

#### Taxonomic Note.

This genus-group taxon has been ranked as a subgenus of *Oxycrepis* Reiche by Allen and Ball (1980) but as a distinct genus by Will (2005).

### Stolonis intercepta

Chaudoir, 1874

Stolonis intercepta Chaudoir, 1874a: 87. Type locality: «Jucatan [= Yucatán, Mexico]» (original citation). Lectotype (♂), designated by Allen and Ball (1980: 530), in MHNP.
Stolonis ulkei G.H. Horn, 1885a: 129. Type locality: «Texas» (original citation). One syntype [2 originally cited] in MCZ [# 34452] and one in CMNH (collection Ulke). Synonymy established by Allen and Ball (1980: 530).

#### Distribution.

This species ranges from southern Arizona and southern Texas along the Gulf of Mexico coast south to southern Mexico (Allen and Ball 1980: 532). The record from southwestern Ohio (Dury 1906: 257) must be in error or based on a stray.

#### Records.

**USA**: AZ, TX – Mexico

### Tribe Pterostichini

Bonelli, 1810

Pterostichii Bonelli, 1810: Tabula Synoptica. Type genus: *Pterostichus* Bonelli, 1810.
Poecilii Bonelli, 1810: Tabula Synoptica. Type genus: *Poecilus* Bonelli, 1810.
Molopides Bonelli, 1810: Tabula Synoptica. Type genus: *Molops* Bonelli, 1810.
Féroniens Dejean, 1825: 3. Type genus: *Feronia* Latreille, 1816 (= *Poecilus* Bonelli, 1810).
Trigonotomidae Laporte, 1834: 75 (as Trogonotomidae). Type genus: *Trigonotoma* Dejean, 1828.
Catadromiens Brullé, 1835a: 277, 328. Type genus: *Catadromus* Macleay, 1825.
Stomides Gené, 1839: 50. Type genus: *Stomis* Clairville, 1806.
Euchroides Chaudoir, 1874b: 16. Type genus: *Euchroa* Brullé, 1835.
Platysmatini Tschitschérine, 1899a: 84. Type genus: *Platysma* Bonelli, 1810. Note. The stem of *Platysma* is *Platysmat*- (Madge 1989: 467).
Myadina Jacobson, 1907: 334. Type genus: *Myas* Sturm, 1826. Note. The stem of *Myas* is *Myad*- (Madge 1989: 465).
Abaxini Schuler, 1970: 115. Type genus: *Abax* Bonelli, 1810. Note. The stem of *Abax* is *Abac*- (Madge 1989: 460).
Aristochroodini Sciaky, 1996: 437. Type genus: *Aristochroodes* Marcilhac, 1993.

#### Diversity.

Worldwide, with about 2,620 species. The North American fauna is represented by about 255 species (9.7% of the world fauna), of which six are adventive, arrayed in 38 genus-group taxa including the eastern endemic *Abacidus* (five species), *Cyclotrachelus* (43 species), *Cylindrocharis* (three species), *Feronina* (two species), *Gastrellarius* (three species), *Gastrosticta* (ten species), *Lophoglossus* (six species), *Monoferonia* (four species), *Paraferonia* (one species), and *Piesmus* (one species), the western endemic *Anilloferonia* (three species), *Leptoferonia* (26 species), *Orsonjohnsonus* (one species), and *Pseudoferonina* (nine species), as well as *Hypherpes* (60 species) and *Lamenius* (one species).

### Genus Abaris

Dejean, 1831

Abaris Dejean, 1831: 780. Type species: *Abaris aenea* Dejean, 1831 by monotypy. Etymology (original). From the Greek *a* (absence) and *barys* (heavy), probably alluding to the small size (and thus light) for pterostichines of adults of the sole species in the hands of Dejean [feminine].
Abarys Agassiz, 1846: 1. Unjustified emendation of *Abaris* Dejean, 1831.

#### Diversity.

Twenty-seven species in the Neotropical Region, one of them reaching southwestern United States. The species are arrayed in two subgenera: *Abaris* s.str. (13 species) and *Abaridius* (14 species).

#### Identification.

Will (2002b) published a revision of all known species.

#### Taxonomic Note.

A cladistic analysis placed the genus *Neotalus* Will, with one South American species, as the sister-group to *Abaris* (Will 2002b).

### Subgenus Abaridius

Chaudoir, 1874

Abaridius Chaudoir, 1874a: 97. Type species: *Abaris tachypoides* Bates, 1871 by monotypy. Etymology. From the generic name *Abaris* [*q.v*.] and the Latin suffix -*ius* (pertaining to), alluding to the close affinity of the taxon to *Abaris* (“*pour indiquer son affinité avec les vrais Abarys*”) [masculine].
Abareoidius Rye, 1875: 240. Unjustified emendation of *Abaridius* Chaudoir, 1874.

#### Diversity.

Fourteen species in the Neotropical Region, one of them reaching southwestern United States.

### Abaris splendidula

(LeConte, 1863)

Pterostichus splendidulus LeConte, 1863c: 10. Type locality: «Fort Yuma, California» (original citation). Holotype [by monotypy] (♀) in MCZ [# 5648].

#### Distribution.

This species is known from southern Arizona, possibly also southeastern California as suggested by the type locality, Sonora in northwestern Mexico, and the southern part of the Baja California Peninsula [see Will 2002b: Fig. 17].

#### Records.

**USA**: AZ [CA] – Mexico

### Genus Hybothecus

Chaudoir, 1874

Hybothecus Chaudoir, 1874b: 3. Type species: *Hybothecus incrassatus* Chaudoir, 1874 by monotypy. Etymology. From the Greek *hybos* (hump) and *thece* (case, container), possibly alluding to the markedly convex elytra (“*remarquable par la forte convexité des élytres*”) of the adult [masculine].
Ophryogaster Chaudoir, 1878: 59. Type species: *Feronia anomala* Chaudoir, 1878 designated by Straneo (1977: 115). Synonymy established by Lorenz (2005: 261) based on a communication of Will (Kiplin W. Will pers. comm. 2007). Etymology. From the Greek *ophryos* (brow) and *gaster* (stomach, by extension abdomen), probably alluding to the transverse furrow on the last three visible abdominal sterna (“*profond sillon transversal, qui longe le bord antérieur des 3 derniers segments de l’abdomen*”) of the adult [feminine].
Incastichus Moret, 1997: 304. Type species: *Incastichus aequidianus* Moret, 1997 by original designation. Synonymy established by Lorenz (2005: 261). Etymology. From *Inca* (the South American Indian peoples) and the Greek *stichos* (line, row) [masculine].

#### Diversity.

Seven species in the Neotropical Region, one of them reaching southwestern United States.

#### Identification.

There is no taxonomic revision or available key for the identification of the species.

### Hybothecus flohri

(Bates, 1882)

Ophryogaster flohri Bates, 1882a: 84. Type locality: «probably near Mexico city, Mexico» (original citation). Syntype(s) location unknown (possibly in ZMHB; not in BMNH according to Stuart Hine pers. comm. 1991). Etymology. The specific name was proposed for Julius Flohr [1837-1896], a physician born in Germany who left for Mexico City in 1859. Flohr was a collector of natural history specimens.
Pterostichus arizonicus Schaeffer, 1910: 393. Type locality: «Tucson [Pima County], Arizona» (original citation). Holotype (♀) in USNM [# 42500]. Synonymy established by Bousquet and Larochelle (1993: 17).

#### Distribution.

This species is known from southern Arizona (Schaeffer 1910: 393, as *Pterostichus arizonicus*; Graham, Pima and Santa Cruz Counties, UASM) south to Chiapas (UASM).

#### Records.

**USA**: AZ – Mexico

### Genus Poecilus

Bonelli, 1810

Poecilus Bonelli, 1810: Tabula Synoptica. Type species: *Carabus cupreus* Linnaeus, 1758 designated by Curtis (1827: plate 187). Etymology. From the Greek *poecilos* (varicolored), possibly alluding to the variously colored species Bonelli had in his hands [masculine].
Feronia Latreille, 1816: 191. Type species: *Carabus cupreus* Linnaeus, 1758 designated by Blanchard [in Audouin et al. 1842: plate 22]. Etymology. Divinity of the ancient Italians, guardian of the plants and the emancipated (Theil 1882: 1071) [feminine].
Thalia Hope, 1838: 70 [junior homonym of *Thalia* Bruguière, 1791]. Unnecessary replacement name for *Feronia* Latreille, 1816. Etymology. Thalia was one of the Muse [feminine].
Enchores Gistel, 1848a: x, xi. Unnecessary replacement name for *Poecilus* Bonelli, 1810.
Feronius Wencker and Silbermann, 1866: 7. Unnecessary replacement name for *Feronia* Latreille, 1816.

#### Diversity.

About 140 species (Lorenz 2005: 266-269) in the Nearctic (13 species) and Palaearctic (128 species) Regions; five of the North American species extend into northern Mexico and Cuba. The species of *Poecilus* are arrayed in seven subgenera: *Poecilus* s.str. (about 70 species), *Angoleus* Villa and Villa (23 species), *Parapedius* Seidlitz (two species in the Mediterranean region), *Carenostylus* Chaudoir (one Mediterranean species), *Derus* (23 species), *Metapedius* Fiori (one species from Italy), and *Pseudoderus* Seidlitz (18 species in western Asia).

#### Identification.

No review or identification key is available for the North American species and a revision of the genus is needed.

#### Taxonomic Note.

*Lyropedius* Seidlitz (two eastern European species) is treated as a subgenus of *Poecilus* by some authors, including Kryzhanovskij and Abdurakhmanov (1983). Bousquet (1999: 58) pointed out that the taxon, based on character of the male genitalia, is probably not closely related to *Poecilus*. The taxon is listed as a subgenus of *Pterostichus* by Bousquet (2003d: 498).

### Subgenus Poecilus

Bonelli, 1810

Poecilus Bonelli, 1810: Tabula Synoptica. Type species: *Carabus cupreus* Linnaeus, 1758 designated by Curtis (1827: plate 187).
Leconteus Lutshnik, 1915a: 414. Type species: *Feronia chalcites* Say, 1823 by original designation. Synonymy established by Csiki (1930: 589). Etymology. From the surname of John Lawrence LeConte [masculine].
Americobius Lutshnik, 1915a: 414. Type species: *Feronia azteca* Tschitschérine, 1897 by original designation. Synonymy established by Csiki (1930: 589). Etymology. From the English prefix americo- (relating to America) and the Greek *bios* (life) [masculine].
Parapoecilus Jeannel, 1942: 746, 751. Type species: *Carabus dimidiatus* Olivier, 1795 (= *Carabus kugelanni* Panzer, 1797) by original designation. Synonymy established by Lindroth (1966: 478). Etymology. From the Greek *para* (near, beside) and the generic name *Poecilus* [*q.v*.] [masculine].

#### Diversity.

About 70 species (Lorenz 2005: 266-268) in the Nearctic (12 species, of which five extend into northern Mexico or Cuba) and Palaearctic (about 60 species) Regions.

#### Identification.

Chaudoir (1876c) reviewed the species but his work is outdated. Lindroth’s (1966) monograph covers seven of the 12 North American species.

### [chalcites group]

### Poecilus chalcites

(Say, 1823)

Feronia chalcites Say, 1823a: 56. Type locality: «Washington, D[istrict of] C[olumbia]» (neotype label). Neotype (♂), designated by Lindroth and Freitag (1969: 342), in MCZ [# 33038]. Note. «the Missouri» was the area originally cited by Say (1823a: 56).
Feronia sayi Brullé, 1835c: 277. Unnecessary replacement name for *Feronia chalcites* Say, 1823.
Poecilus micans Chaudoir, 1843b: 767. Type locality: «Nouvelle Orléans [Orleans Parish, Louisiana]» (original citation). Syntype(s) in MHNP. Synonymy established, under the name *Poecilus sayi* (Brullé), by LeConte (1853a: 254).

#### Distribution.

This species ranges from southwestern New Brunswick (Majka et al. 2007: 9) to the Black Hills in western South Dakota (Kirk and Balsbaugh 1975: 21), south to northern Chihuahua (Bates 1891a: 249), southern Texas (Johnson 1978: 67), and the Florida Panhandle (Okaloosa County, CNC); also recorded from Cuba (Darlington 1934: 91). The records from Arizona (Snow 1906b: 162, as *Poecilus sayi*) and “North Dakota” (Bousquet and Larochelle 1993: 165) need confirmation.

#### Records.

**CAN**: NB, ON, QC **USA**: AL, AR, CO, CT, DC, DE, FL, GA, IA, IL, IN, KS, KY, LA, MA, MD, ME, MI, MN, MO, MS, NC, NE, NH, NJ, NM, NY, OH, OK, PA, RI, SC, SD, TN, TX, VA, VT, WI, WV [AZ, ND] – Cuba, Mexico

### Poecilus diplophryus

Chaudoir, 1876

Poecilus subcordatus LeConte, 1851: 181 [secondary homonym of *Poecilus subcordatus* (Chaudoir, 1842)]. Type locality: «ad flumina Colorado et Gila» (original citation). Lectotype (♀), designated by Bousquet (1999: 62), in MCZ [# 79].
Poecilus diplophryus Chaudoir, 1876c: 42. Replacement name for *Poecilus subcordatus* LeConte, 1851.
Poecilus pimalis Casey, 1913: 138. Type locality: «Tuçson [Pima County], Arizona» (original citation). Holotype [by monotypy] (♀) in USNM [# 47087]. Synonymy established by Bousquet and Larochelle (1993: 17).

#### Distribution.

This species ranges from “Colorado” (LeConte 1858a: 28) to west-central Nevada (Bechtel et al. 1983: 474), south to the Baja California Peninsula (Horn 1894: 309) and “New Mexico” (Schaupp 1882c: 41). The record from est-central South Dakota (Kirk and Balsbaugh 1975: 21) needs confirmation.

#### Records.

**USA**: AZ, CA, CO, NM, NV, UT [SD] – Mexico

### Poecilus scitulus

LeConte, 1846

Poecilus scitulus LeConte, 1846b: 334. Type locality: «fluminis Platte furcationem [= Platte River, Nebraska]» (original citation). Lectotype (♀), designated by Bousquet (1999: 64), in MCZ [# 5634].

#### Distribution.

The range of this species extends from southern Manitoba to the Fraser River in south-central British Columbia (Lindroth 1966: 481), south to southern California (Fall 1901a: 44) and the Mexico City environs on the Mexican Plateau (Ball and Shpeley 1992a: 52).

#### Records.

**CAN**: AB, BC, MB, SK **USA**: AZ, CA, CO, ID, KS, MN, MT, ND, NE, NM, NV, OK, OR, SD, TX, UT, WA, WY – Mexico

### [lucublandus group]

### Poecilus coloradensis

(Csiki, 1930)

Poecilus bicolor LeConte, 1846b: 232 [secondary homonym of *Pterostichus bicolor* (Aragona, 1830)]. Type locality: «ad Rocky Mountains» (original citation). One syntype in MCZ [# 5640]. Note. LeConte (1853a: 255) stated that he had a single specimen of this species.
Pterostichus coloradensis Csiki, 1930: 591. Replacement name for *Pterostichus bicolor* (LeConte, 1846).

#### Distribution.

This species is known for sure only from the syntype. The records from northeastern Kansas (Horn 1872c: 385; Popenoe 1878: 78) are suspect.

#### Records.

**USA**: [CO, KS]

#### Note.

Adults of this species are structurally similar to, and possibly conspecific with, those of *Poecilus lucublandus* Say as defined by Lindroth (1966: 482-483).

### Poecilus corvus

(LeConte, 1873)

Pterostichus corvus LeConte, 1873a: 307. Type locality: «Dacota» (original citation), herein restricted to Newell, Butte County, South Dakota (see Kirk and Balsbaugh 1975: 21). Lectotype (♂), designated by Bousquet (1999: 61), in MCZ [# 5636].

#### Distribution.

This species is found from western Ontario (CNC) to southeastern Alberta (Lindroth 1966: 484), south to southeastern Oregon (Harney County, James R. LaBonte pers. comm. 2009; Hatch 1953: 115), central Wyoming (Lavigne 1977: 47), western South Dakota (Kirk and Balsbaugh 1975: 21), and western Minnesota (Tinerella and Rider 2001: 320; Gandhi et al. 2005: 926). The record from northern British Columbia (Jarrett and Scudder 2001: 382) needs confirmation.

#### Records.

**CAN**: AB, MB, ON, SK **USA**: MN, MT, ND, OR, SD, WY [BC]

### Poecilus cursitor

LeConte, 1853

Poecilus cursorius LeConte, 1851: 181 [secondary homonym of *Poecilus cursorius* (Dejean, 1828)]. Type locality: «S[an]ta Isabel [San Bernardino County, California]» (lectotype label). Lectotype (♂), designated by Bousquet (1999: 61), in MCZ [# 78]. Note. LeConte (1851: 181) originally cited this species from «San Diego, ad montes» but later (1853a: 255) listed it from «Santa Isabel, California». Because the lectotype label indicates that the specimen was collected at Santa Isabel, this is the type locality. Santa Isabel is a Mohave settlement near Needles in San Bernardino County along the Colorado River.
Poecilus cursitor LeConte, 1853a: 254. Replacement name for *Poecilus cursorius* LeConte, 1851.

#### Distribution.

This species is known from western Oregon (Hatch 1953: 115, as *Pterostichus occidentalis*) to southern California (Fall 1901a: 45, as *Pterostichus occidentalis*).

#### Records.

**USA**: CA, OR

#### Note.

This species has been known for a long time under the name *Poecilus occidentalis* (Dejean, 1828) but Lindroth (1966: 484) showed that *Poecilus cursitor* and *Poecilus occidentalis* are different species.

### Poecilus laetulus

(LeConte, 1863)

Pterostichus laetulus LeConte, 1863c: 10. Type locality: «San Jose [Santa Clara County, California]» (original citation for *Poecilus occidentalis* Dejean *sensu* LeConte, 1853). Lectotype (♂), designated by Bousquet (1999: 62), in MCZ [# 5635]. Note. This name was proposed for *Pterostichus occidentalis* (Dejean, 1828) *sensu* LeConte (1853a: 253). By an unfortunate error, LeConte (1863c: 10) used the specific name *californicus* instead of *occidentalis*.

#### Distribution.

This species ranges from southeastern Washington (Hatch 1953: 115) to southern California (Fall 1901a: 44; Moore 1937: 9) and southern Arizona (Wickham 1898: 300). The records from New Mexico (Wickham 1896a: 157; Fall and Cockerell 1907: 158) and northeastern Kansas (Popenoe 1878: 78) are probably based on misidentified specimens.

#### Records.

**USA**: AZ, CA (CHI), OR, WA

### Poecilus lucublandus

(Say, 1823)

Feronia lucublanda Say, 1823a: 55. Type locality: «Ithaca [Tompkins County], N[ew] Y[ork]» (neotype label). Neotype (♂), designated by Lindroth and Freitag (1969: 342), in MCZ [# 33036].
Feronia convexicollis Say, 1823a: 50. Type locality: «Devil’s Lake [Ramsey County], N[orth] Dak[ota]» (neotype label). Neotype (♂), designated by Lindroth and Freitag (1969: 342), in MCZ [# 33035]. Synonymy established by Lindroth (1966: 482). Note. «Missouri [Territory]» was the area originally cited by Say (1823a: 51).
Poecilus fraternus Say, 1824: 270. Type locality: «North-west Territory» (original citation). Syntype(s) lost. Synonymy established by LeConte (1853a: 255). Note. This name has been listed as a *nomen dubium* by Lindroth and Freitag (1969: 342).
Poecilus castanipes Kirby, 1837: 37. Type locality: northern parts of British America (inferred from title of the book). Holotype [by monotypy] (♀) in BMNH. Synonymy established by LeConte (1853a: 255), confirmed by Lindroth (1953b: 172).
Poecilus dilatatus LeConte, 1846b: 232. Type locality: «NovEboraci [= New York]» (original citation). Lectotype (♀), designated by Bousquet (1999: 63), in MCZ [# 5639]. Synonymy established by LeConte (1853a: 255), confirmed by Bousquet (1999: 63).
Pterostichus manhattanis Casey, 1884c: 72. Type locality: «Willets Point [Queens County], New York Harbor» (original citation). Holotype [by monotypy] (♂) in USNM [# 47083]. Synonymy established by Horn (1885b: 108), confirmed by Lindroth (1966: 482).
Poecilus elucens Casey, 1924: 76. Type locality: «Edmonton, Alberta» (original citation). Lectotype (♂), designated by Lindroth (1975: 123), in USNM [# 47084]. Synonymy established by Lindroth (1966: 482).
Poecilus planifer Casey, 1924: 76. Type locality: «Agassiz, British Columbia» (original citation). Lectotype (♀), designated by Lindroth (1975: 124), in USNM [# 47088]. Synonymy established by Hatch (1953: 116), confirmed by Lindroth (1966: 482).
Poecilus lucublandus acomanus Casey, 1924: 76. Type locality: «Jemez Springs [Sandoval County], New Mexico» (original citation). Lectotype (♂), designated by Bousquet (1999: 62), in USNM [# 47086]. **New synonymy**.
Poecilus lucublandus louisinus Casey, 1924: 76. Type locality: «S[ain]t Louis, Missouri» (original citation). Holotype [by monotypy] (♂) in USNM [# 47082]. **New synonymy**.

#### Distribution.

This widely distributed species ranges from Cape Breton Island (Lindroth 1954c: 303) to Vancouver Island, north at least to Fort Smith in southern Northwest Territories (Lindroth 1966: 483), south to “Oregon” (Hatch 1953: 116), northern New Mexico (Fall and Cockerell 1907: 158), the Texas Panhandle (Michels et al. 2010: 743), southwestern Oklahoma (Kondratieff et al. 2005: 173), northern Georgia (Fattig 1949: 26), and central South Carolina (Ciegler 2000: 61).

#### Records.

**CAN**: AB, BC (VCI), MB, NB, NS (CBI), NT, ON, PE, QC, SK **USA**: CO, CT, DC, DE, GA, IA, ID, IL, IN, KS, KY, MA, MD, ME, MI, MN, MO, MT, NC, ND, NE, NH, NJ, NM, NY, OH, OK, OR, PA, RI, SC, SD, TN, TX, UT, VA, VT, WA, WI, WV, WY

#### Note.

The lectotypes of *Poecilus lucublandus acomanus* Casey and *Poecilus lucublandus louisinus* Casey are conspecific with members of *Poecilus lucublandus* as defined by Lindroth (1966) and these names are here placed in synonymy. However, the species exhibits considerable structural variation within its range and it is possible, even likely, that more than one species are included within the actual concept of *Poecilus lucublandus*.

### Poecilus occidentalis

(Dejean, 1828)

Feronia occidentalis Dejean, 1828: 231. Type locality: «Californie» (original citation). One syntype in MHNP (Lindroth 1966: 484).

#### Distribution.

This species is known from southern California (Fall 1901a: 45).

#### Records.

**USA**: CA

### [incertae sedis]

### Poecilus cyanicolor

Chaudoir, 1876

Poecilus cyaneus LeConte, 1846b: 231 [primary homonym of *Poecilus cyaneus* Gory, 1833]. Type locality: «ad Rocky Mountains» (original citation), cited from «near Long’s Peak [Boulder County, Colorado], Missouri Territory» by LeConte (1853a: 254). Lectotype (♀), designated by Bousquet (1999: 62), in MCZ [# 5637].
Poecilus cyanicolor Chaudoir, 1876c: 43. Replacement name for *Poecilus cyaneus* LeConte, 1846.
Poecilus cyanicolor var. *connexus* Chaudoir, 1876c: 44. Type locality: «Montagnes-Rocheuses; Colorado» (original citation). Syntype(s) [3 originally cited] probably in MHNP. Synonymy established by Csiki (1930: 595).

#### Distribution.

This species is known yet only from north-central (LeConte 1853a: 254) and southeastern Colorado (Michels et al. 2008), “Kansas” (Horn 1872c: 385), and a few localities in central Mexico (Bates 1882a: 84).

#### Records.

**USA**: CO, KS – Mexico

### Poecilus mexicanus

Chaudoir, 1876

Poecilus mexicanus Chaudoir, 1876c: 44. Type locality: «Mexique» (original citation). Syntype(s) probably in MHNP and MHNG (collection Reiche).
Poecilus snowi Casey, 1913: 137. Type locality: «San Bernardino Ranch, Cochise Co[unty], Arizona» (original citation). Lectotype (♂), designated by Allen (1977: 286), in USNM [# 47085]. Synonymy established with doubt by Casey (1918: 376).

#### Distribution.

This species ranges from southeastern Arizona (Casey 1913: 137, as *Poecilus snowi*) south at least to the state of Oaxaca in Mexico (Bates 1882a: 84).

#### Records.

**USA**: AZ – Mexico

### Poecilus texanus

(LeConte, 1863)

Pterostichus texanus LeConte, 1863c: 10. Type locality: «Texas» (original citation). Lectotype (♂), designated by Bousquet (1999: 64), in MCZ [# 5638].

#### Distribution.

This species is known from southeastern Arizona (Snow 1906b: 162) and southern Texas (Snow 1906a: 141; Duval County, CMNH). The records from Kansas (Knaus 1901: 110; Snow 1903: 193) need confirmation.

#### Records.

**USA**: AZ, TX [KS]

### Subgenus Derus

Motschulsky, 1844

Derus Motschulsky, 1844: xi. Type species: *Argutor politus* Motschulsky, 1844 (= *Platysma ravum* Lutshnik, 1922) by monotypy. Etymology. From the Greek *dere* (neck, by extension pronotum) [masculine].
Derulus Tschitschérine, 1896a: 112. Type species: *Feronia samojedora* Sahlberg, 1880 designated by Lindroth (1966: 476). Synonymy established by Lindroth (1966: 476).

#### Diversity.

Twenty-three species (Lorenz 2005: 269), mostly in the arctic and subarctic areas, in North America (one species), Asia (21 species), and eastern Europe (three species).

#### Identification.

No revision or identification key is available for the species. Lindroth (1966: 476-478) covered the North American species.

### Poecilus nearcticus

(Lindroth, 1966)

Pterostichus nearcticus Lindroth, 1966: 477. Type locality: «Anderson R[iver] delta, N[orth]W[est] Terr[itories]» (original citation). Holotype (♂) in CNC [# 9727].

#### Distribution.

This species ranges from the Anderson River delta in northern Northwest Territories (Lindroth 1966: 478) to the Titaluk River (North Slope County) in northern Alaska (CNC). The species is also known from a few localities in eastern Siberia (Alfimov and Berman 2009: Fig. 1). Fossil remnants of this species, from the early Pleistocene and Late Wisconsin, have been found in the lower Kolymian Basin in northeastern Siberia (Matthews 1974a: 208) and western Alaska (Matthews 1974b: 1365) respectively.

#### Records.

**CAN**: NT, YT **USA**: AK – **Holarctic**

### Genus Lophoglossus

LeConte, 1853

Lophoglossus LeConte, 1853a: 248. Type species: *Feronia tartarica* Say, 1823 designated by Casey (1918: 324). Etymology. From the Greek *lophos* (crest) and *glossa* (tongue), alluding to the carinate glossal sclerite (“*ligula carinata*”) of the adult [masculine].

#### Diversity.

Six species in the temperate regions of eastern North America.

#### Identification.

Will (1999) reviewed the species and provided a key for their identification.

### Lophoglossus gravis

LeConte, 1873

Lophoglossus gravis LeConte, 1873a: 316. Type locality: «probably Pennsylvania» (original citation). Holotype [by monotypy] (♂) in MCZ [# 34935].

#### Distribution.

This species is known from a few localities along the Atlantic Coastal Plain and Piedmont Plateau from central New Jersey (Smith 1910: 205) to central South Carolina (Ceigler 2000: 62). The record from “Pennsylvania” (LeConte 1873a: 316) needs confirmation.

#### Records.

**USA**: DC, GA, MD, NC, NJ, SC, VA [PA]

### Lophoglossus haldemanni

(LeConte, 1846)

Lyperus haldemanni LeConte, 1846b: 341. Type locality: «Alabama» (original citation), restricted to «Mobile, Mobile Co[unty]» by Will (1999: 272). Syntype(s) in MCZ [# 5642]. Etymology. The specific name honors Samuel Stehman Haldeman [1812-1880], American naturalist, linguist, and archaeologist. Haldeman spent most of his adult life teaching zoology, geology, philology, and chemistry at various colleges and Universities. As a naturalist, he was mainly interested in mollusks and insects, particularly beetles.
Lophoglossus haldemani LeConte, 1853a: 248. Unjustified emendation of *Lophoglossus haldemanni* (LeConte, 1846).

#### Distribution.

This species ranges from southwestern Indiana (Blatchley 1910: 97; Schrock 1985: 354) to southeastern Iowa (Wickham 1911b: 6; Henry County, CNC), south to eastern Texas and central Florida (Will 1999: 272, Fig. 35). The record from North Carolina (Brimley 1938: 121) needs confirmation; that from “New York” (Wickham 1895a: 187) is likely in error.

#### Records.

**USA**: AL, AR, FL, GA, IA, IL, IN, KS, KY, LA, MO, MS, OK, TN, TX [NC]

### Lophoglossus scrutator

(LeConte, 1846)

Lyperus scrutator LeConte, 1846b: 342. Type locality: «provinciis australibus» (original citation), cited from «Cleveland [Cuyahoga County], Ohio» by LeConte (1853a: 249). Lectotype (♀), designated by Bousquet (1999: 69), in MCZ [# 5644].
Feronia canadensis Chaudoir, 1868b: 331. Type locality: «Toronto [Ontario], dans le Canada occidental» (original citation). Holotype [by monotypy] (♀) location unknown (possibly in MHNP). Synonymy established by LeConte (1873a: 316).
Lophoglossus illini Casey, 1913: 145. Type locality: «Illinois» (original citation). Lectotype (♂), designated by Allen (1977: 286), in USNM [# 47096]. Synonymy established by Bousquet and Larochelle (1993: 15).
Lophoglossus bispiculatus Casey, 1913: 146. Type locality: «Indiana» (original citation). Lectotype (♂), designated by Allen (1977: 286), in USNM [# 47097]. Synonymy established by Bousquet and Larochelle (1993: 15).

#### Distribution.

This species ranges from southern Quebec (Larochelle 1975: 105) to southeastern Minnesota (Gandhi et al. 2005: 926), south to Missouri (Summers 1873: 145) and West Virginia (Will 1999: 270). The records from North Carolina (Brimley 1938: 121), South Carolina (Kirk 1970: 11), and Georgia (J.E. LeConte 1849: 26; Fattig 1949: 26) need confirmation.

#### Records.

**CAN**: ON, QC **USA**: CT, IA, IL, IN, MA, MI, MN, MO, NJ, NY, OH, PA, VT, WI, WV [GA, NC, SC]

### Lophoglossus substrenuus

(Csiki, 1930)

Pterostichus strenuus LeConte, 1853a: 249 [secondary homonym of *Pterostichus strenuus* (Panzer, 1796)]. Type locality: «New York» (original citation). Holotype [by monotypy] (♂) in MCZ [# 5643]. Note. Will (1999: 267) selected «Odenten, Ann Arundel Co[unty], M[arylan]d» as type locality but this selection should be rejected since LeConte (1853a: 249) originally specified that his specimen came from New York.
Pterostichus substrenuus Csiki, 1930: 627. Replacement name for *Pterostichus strenuus* LeConte, 1853.

#### Distribution.

This species ranges from Long Island in New York (Schaupp 1883b: 31) and New Jersey to central Missouri, north to northeastern Illinois, south to southeastern Louisiana (Saint Tammany Parish, Igor M. Sokolov pers. comm. 2009) and central Florida [see Will 1999: Fig. 34].

#### Records.

**USA**: AL, AR, FL, GA, IL, IN, KY, LA, MO, MS, NC, NJ, NY, PA, SC, TN, VA

### Lophoglossus tartaricus

(Say, 1823)

Feronia tartarica Say, 1823a: 44. Type locality: «Mobile [Mobile County], Ala[bama]» (neotype label). Neotype (♂), designated by Lindroth and Freitag (1969: 343), in MCZ [# 32963].
Feronia complanata Dejean, 1828: 281. Type locality: «Amérique septentrionale» (original citation). Syntype(s) probably in MHNP. Synonymy established by LeConte (1846b: 341).

#### Distribution.

As far as known, this species ranges along the Coastal Plain from the coast of South Carolina to central Florida, west to east-central Texas, north along the Mississippi Valley to southern Indiana (Will 1999: 271). The records from “Iowa” (Jaques and Redlinger 1946: 297), “Illinois” (Wolcott 1900: 469), “New York” (Wickham 1895a: 187; Casey 1913: 144), New Jersey (Smith 1910: 205), and southeastern Pennsylvania (Rathvon 1869: 526, as *Pterostichus complanata*) need confirmation.

#### Records.

**USA**: AL, AR, FL, GA, IN, LA, MS, SC, TX [IA, IL, NJ, NY, PA]

### Lophoglossus vernix

Casey, 1913

Lophoglossus vernix Casey, 1913: 146. Type locality: «Lyme [New London County], Connecticut» (original citation). Lectotype (♂), designated by Lindroth (1975: 124), in USNM [# 47098].

#### Distribution.

This species is known from a few localities along or near the Atlantic Coast from New Hampshire to Virginia (Will 1999: 268).

#### Records.

**USA**: CT, MA, MD, NH, RI, VA

### Genus Piesmus

LeConte, 1846

Piesmus LeConte, 1846b: 340. Type species: *Feronia submarginata* Say, 1823 by monotypy. Etymology (original). From the Greek *pieszo* (to press), alluding to the depressed shape of the adults (“*habitus depressus*”) [masculine].

#### Diversity.

One species in the temperate regions of eastern North America.

### Piesmus submarginatus

(Say, 1823)

Feronia submarginata Say, 1823a: 45. Type locality: «Hope [Hempstead County], Ark[ansas]» (neotype label). Neotype (♂), designated by Lindroth and Freitag (1969: 340), in MCZ [# 33041].
Poecilus monedula Germar, 1824: 18. Type locality: «America boreali» (original citation). Syntype(s) probably lost. Synonymy established by LeConte (1853a: 246).

#### Distribution.

This species is found from southern Ohio (Purrington and Stanton 1996: 43; Silverman et al. 2008: 733) south to “Texas” (LeConte 1858a: 28), southern Mississippi (Hancock County, CNC), and central Florida (Peck and Thomas 1998: 19). Bates (1882a: 83) recorded it from “Mexico.”

#### Records.

**USA**: AL, AR, FL, GA, IL, KY, LA, MS, NC, OH, OK, SC, TN, TX, VA, WV – Mexico

### Genus Gastrellarius

Casey, 1918

Gastrellarius Casey, 1918: 321, 338. Type species: *Feronia honesta* Say, 1823 by original designation. Etymology. Unknown [masculine].

#### Diversity.

Three species in the temperate regions of eastern North America.

#### Identification.

Darlington (1932) provided a key for the identification of the species.

### Gastrellarius blanchardi

(Horn, 1891)

Pterostichus blanchardi G.H. Horn, 1891: 33. Type locality: «Highland[s] [Macon County], North Carolina» (original citation). Holotype [by monotypy] (♂) in MCZ [# 34426]. Etymology. The specific name honors Frederick Blanchard [1843-1912] who worked in the banking business at Lowell, Massachusetts. Blanchard was an ardent beetle collector, essentially New England species, and worked regularly with LeConte’s collection. He bequeathed his collection to the Museum of Comparative Zoology.

#### Distribution.

This species is found along the Appalachian Mountains from southern West Virginia (Hoffman 1998: 36) to northern Georgia (Leng 1910: 73; Fattig 1949: 21) and northwestern South Carolina (Ciegler 2000: 63).

#### Records.

**USA**: GA, NC, SC, VA, WV

### Gastrellarius honestus

(Say, 1823)

Feronia honesta Say, 1823a: 51. Type locality: «Rumney [Grafton County] N[ew] H[ampshire]» (neotype label). Neotype (♂), designated by Lindroth and Freitag (1969: 340), in MCZ [# 33051].
Feronia fastidita Dejean, 1828: 323. Type locality: «Amérique septentrionale» (original citation). One syntype in MHNP (Lindroth 1955b: 14). Synonymy established by LeConte (1863b: 9), confirmed by Lindroth (1955b: 14).
Stomis americana Laporte, 1834: 72. Type locality: «Amérique du Nord» (original citation). Syntype(s) probably in MHNP (collection Chaudoir). Synonymy established, under the name *Feronia fastidita* Dejean, by Chaudoir (1847: 13).
Gastrellarius atronitens Casey, 1918: 339. Type locality: «Indiana» (original citation). Lectotype (♂), designated by Lindroth (1975: 123), in USNM [# 47044]. Synonymy established by Lindroth (1966: 472).
Gastrellarius scolopaceus Casey, 1918: 340. Type locality: «probably Indiana» (original citation). Holotype [by monotypy] (♂) in USNM [# 47046]. Synonymy established by Lindroth (1966: 472).
Gastrellarius deficiens Casey, 1918: 340. Type locality: «Indiana» (original citation). Lectotype (♀), designated by Lindroth (1975: 123), in USNM [# 47045]. Synonymy established by Lindroth (1966: 472).

#### Distribution.

This species ranges from the Nova Scotia Peninsula (Schaupp 1882c: 41; Chritopher G. Majka pers. comm. 2008) to western Minnesota (Gandhi et al. 2005: 926), south along the Appalachian Mountains to northern Alabama (Löding 1945: 15), northern Georgia (Leng 1910: 73; Fattig 1949: 21), and northwestern South Carolina (Ciegler 2000: 63).

#### Records.

**CAN**: NB, NS, ON, QC **USA**: AL, CT, DC, GA, IL, IN, KY, MA, MD, ME, MI, MN, NC, NH, NJ, NY, OH, PA, SC, TN, VA, VT, WI, WV

### Gastrellarius unicarum

(Darlington, 1932)

Pterostichus unicarum Darlington, 1932: 155. Type locality: «Newfound Gap (5,000-5,200 feet), Smoky Mountains, North Carolina-Tennessee state line» (original citation). Holotype (♂) in MCZ [# 16434].

#### Distribution.

This species is known from the Great Smokies along the Tennessee and North Carolina border (Darlington 1932: 155; Barr 1969: 81) and Rabun Bald in northeastern Georgia (Fattig 1949: 22).

#### Records.

**USA**: GA, NC, TN

### Genus Stomis

Clairville, 1806

Stomis Clairville, 1806: 46. Type species: *Carabus pumicatus* Panzer, 1795 by monotypy. Etymology. Probably from the Greek *stoma* (mouth) [masculine].

#### Diversity.

About 40 species in the temperate areas of the Nearctic (two species, one of them adventive) and Palaearctic Regions, arrayed in two subgenera, both represented in North America.

### Subgenus Neostomis

Bousquet, 1983

Neostomis Bousquet, 1983: 1599. Type species: *Pterostichus termitiformis* Van Dyke, 1926 by original designation. Etymology. From the Greek prefix *neo*- (new) and the generic name *Stomis* [*q.v*.], alluding to the fact that the sole species of *Stomis* placed in this taxon inhabits the New World [masculine].

#### Diversity.

One North American species along the Pacific Coast.

#### Identification.

Hacker (1968) redescribed the species.

### Stomis termitiformis

(Van Dyke, 1926)

Pterostichus termitiformis Van Dyke, 1926a: 74. Type locality: «Marshfield [= Coos Bay, Coos County], Oregon» (original citation). Holotype (♂) in CAS [# 1826].

#### Distribution.

This rarely collected species is known from one specimen collected along the west coast of Vancouver Island (Bousquet 2000: 81) and a few others collected along or near the coast of Oregon (Hacker 1968: 42).

#### Records.

**CAN**: BC **USA**: OR

### Subgenus Stomis

Clairville, 1806

Stomis Clairville, 1806: 46. Type species: *Carabus pumicatus* Panzer, 1795 by monotypy.
Stomatomius Gistel, 1856: 358. Type species: *Carabus pumicatus* Panzer, 1795 designated by Bousquet (2002b: 48).
Stobeus Fairmaire, 1889: 8. Type species: *Stobeus collucens* Fairmaire, 1889 by original designation. Synonymy established by Bousquet (1988b: 230). Etymology. Unknown [masculine].
Eustomis Semenov, 1889a: 378. Type species: *Stomis formosus* Semenov, 1889 by monotypy. Synonymy established by Bousquet (1983: 1597). Etymology. From the Greek *eu* (agreeable, original, primitive) and the generic name *Stomis* [*q.v*.] [masculine].

#### Diversity.

Thirty-six species in Asia (29 species) and Europe (seven species), one of them adventive in eastern North America.

#### Identification.

There is no taxonomic revision of the subgenus. The sole species in North America was included in Lindroth’s (1966: 443) monograph.

### Stomis pumicatus

(Panzer, 1795)

Carabus pumicatus Panzer, 1795: 60. Type locality: «Mannhemii [Baden-Wûrttemberg, Germany]» (original citation). Syntype(s) possibly in ZMHB.
Carabus tenuis Marsham, 1802: 468. Type locality: Great Britain (inferred from title of the book). Syntype(s) possibly in BMNH. Synonymy established by Samouelle (1819: 153).

#### Distribution.

This European species is adventive in North America where it is known from a few localities in Cape Breton Island (Bousquet 1987a: 124), Prince Edward Island (CNC), and New Brunswick (Kent County, Christopher G. Majka pers. comm. 2011). The first inventoried specimen collected on this continent was found in Cape Breton Highland National Park in 1984 (Bousquet 1987a: 124). The previous North American record from Hemmingford, Quebec (Darlington 1940b: 252) was based on a mislabeled specimen (see Bousquet 1987a: 124).

#### Records.

**CAN**: NB, NS (CBI), PE – **Adventive**

### Genus Stereocerus

Kirby, 1837

Stereocerus Kirby, 1837: 34. Type species: *Stereocerus similis* Kirby, 1837 (= *Feronia haematopus* Dejean, 1831) by monotypy. Etymology. From the Greek *stereos* (solid, firm, hard) and *ceros* (horn, by extension antenna), alluding to the stout antennae (“antennae robust”) of the adults [masculine].
Boreobia Tschitschérine, 1896b: 375. Type species: *Feronia imitatrix* Tschitschérine, 1896 (= *Feronia haematopus* Dejean, 1831) designated by Bousquet (1984a: 4). Etymology. From the Greek *boreas* (north) and *bios* (life) [feminine].

#### Diversity.

Two Holarctic species in the arctic, subarctic, and boreal regions.

#### Identification.

Lindroth (1966) redescribed both species and provided a mean for their identification through his key to Canadian species of *Pterostichus*.

### Stereocerus haematopus

(Dejean, 1831)

Feronia haematopus Dejean, 1831: 769. Type locality: «côtes du Labrador» (original citation), herein restricted to Hopedale (CNC). One syntype [3 originally cited] in MHNP (Lindroth 1955b: 15).
Stereocerus similis Kirby, 1837: 34. Type locality: «Lat. 54° [= along North Saskatchewan River]» (original citation). Lectotype (♂), designated by Bousquet (1999: 86), in BMNH. Synonymy established by LeConte (1865a: xxxvii), confirmed by Bousquet (1999: 86).
Feronia strigicollis R.F. Sahlberg, 1844: 42. Type locality: circa Okhotsk, Khabarovsk Kray, Siberia, Russia (inferred from introduction and title of the paper). Lectotype (♂), designated by Lindroth (1966: 531), in ZMUT. Synonymy established by Lindroth (1954b: 135).
Feronia imitatrix Tschitschérine, 1896b: 373. Type locality: «Novaja Zemlja [= Novaya Zemlya, Russia]» (original citation). Syntype(s) [2 originally cited] in ZILR. Synonymy established by Lindroth (1966: 532).
Pterostichus lapponicus Jedlička, 1937: 46. Type locality: «Lappon» (original citation). Holotype (♀) in NMP. Synonymy established by Bousquet (1999: 86).

#### Distribution.

This Holarctic species ranges in the Palaearctic Region from northern European Russia to the Far East, south to Manchuria (Bousquet 2003d: 517), and in the Nearctic Region from Alaska (Lindroth 1966: 533) to Newfoundland (Lindroth 1955a: 88), south to high mountains in New England (Lindroth 1966: 533), the Black Hills in southwestern South Dakota (Robert L. Davidson pers. comm. 2008), and Wyoming (Lindroth 1966: 533). The record from “New York” (Notman 1928: 226) needs confirmation; that from Ohio (Everly 1927: 155) is in error. Fossil remnants of this species, dated between about 10,400 and 18,100 years B.P., have been unearthed in southeastern Iowa (Baker et al. 1986: 96) and Cape Breton Island in Nova Scotia (Miller 1997: 250); others, believed to be 2.0-2.5 million years old, have been found in Greenland (Bennike and Böcher 1990: 336; Böcher 1995: 28).

#### Records.

**CAN**: AB, BC, LB, MB, NF, NT, NU, ON, QC, SK, YT **USA**: AK, ME, MT, NH, SD, VT, WY [NY] – **Holarctic**

### Stereocerus rubripes

(Motschulsky, 1860)

Steroderus rubripes Motschulsky, 1860: 94. Type locality: «Sibérie arctique» (original citation), restricted to «Anadyr [Russia]» by Lindroth (1969a: 1119). One syntype in ZMMU (Lindroth 1969a: 1119).
Pterostichus kryzhanovskii Lindroth, 1966: 533. Type locality: «Atkasuk [= Atqasuk], Meade R[iver], Alaska» (original citation). Holotype (♂) in MCZ [# 33502]. Synonymy established by Lindroth (1969a: 1119). Etymology. The specific name was proposed in honor of Oleg Leonidovich Kryzhanovskij [1918-1997], one of the leading Russian entomologists of his time. A veteran of World War II, Kryzhanovskij worked at the Zoological Institute of Saint Petersburg on taxonomy of various groups of beetles with a special interest in Carabidae and Histeridae. He was also known for his work on biogeography.

#### Distribution.

This Holarctic species ranges from the northern part of European Russia (Bousquet 2003d: 517) to the Arctic Slope in northern Alaska. The record from “Yukon Territory” (Ball and Currie 1997: 452) could not be confirmed.

#### Records.

**USA**: AK [YT] – **Holarctic**

### Genus Myas

Sturm, 1826

Myas Sturm, 1826: 6, 171. Type species: *Abax chalybaeus* Palliardi, 1825 by monotypy. Etymology. Uncertain, possibly from the Latin *myax*, -*acis* (kind of pearl mussels in Pliny the Elder) or the Greek *mys*, *myos* (mouse), or *myia* (fly) [masculine]. The name was proposed by Franz Anton Ziegler and first described by Dejean (1828: 423-424).

#### Diversity.

Thirty-two species (Lorenz 2005: 265-266) in the temperate regions of North America (two species), Asia (29 species), and Europe (one species), arrayed in two subgenera (Bousquet 1999: 90): *Myas* s.str. for the European species and *Trigonognatha* for the other ones.

### Subgenus Trigonognatha

Motschulsky, 1857

Trigonognatha Motschulsky, 1857a: 25. Type species: *Trigonognatha cuprescens* Motschulsky, 1857 by monotypy. Etymology. From the Greek *trigonos* (triangular) and *gnathos* (jaw), alluding to the triangular mandibles (“*mandibules triangulaires*”) of the adult [feminine].
Aurisma Fairmaire, 1889: 9. Type species: *Aurisma delavayi* Fairmaire, 1889 by monotypy. Synonymy established by Tschitschérine (1898a: 77).
Neomyas Allen, 1980: 17. Type species: *Myas cyanescens* Dejean, 1828 by original designation. Synonymy established by Bousquet (1999: 90). Etymology. From the Greek prefix *neo*- (new) and the generic name *Myas* [*q.v*.] [masculine].

#### Diversity.

Thirty-one species (Lorenz 2005: 266) in eastern North America (two species) and eastern Asia (29 species).

#### Identification.

Allen (1980) revised the North American under the generic name *Neomyas*. Lindroth (1966) redescribed both species.

### Myas coracinus

(Say, 1823)

Abax coracinus Say, 1823a: 59. Type locality: «Rock(e)ville, P[ennsylvani]a» (neotype label). Neotype (♂), designated by Lindroth and Freitag (1969: 339), in MCZ [# 33053].

#### Distribution.

This species ranges from southwestern Connecticut (Krinsky and Oliver 2001: 104) to northeastern Illinois, north to the northwestern region of the lower peninsula of Michigan (Allen 1980: 20; Hatch 1925: 549), south to northern Mississippi (Union County, Drew A. Hildebrandt pers. comm. 2010), northern Alabama (Löding 1945: 15), and northwestern Georgia (Fattig 1949: 21). The records from southern Wisconsin (Rauterberg 1885: 16) and “Massachusetts” (Bousquet and Larochelle 1993: 168) need confirmation.

#### Records.

**CAN**: ON **USA**: AL, CT, DC, DE, GA, DE, IL, IN, KY, MD, MI, MS, NC, NJ, NY, OH, PA, TN, VA, VT, WV [MA, WI]

### Myas cyanescens

Dejean, 1828

Myas cyanescens Dejean, 1828: 425. Type locality: «Amérique septentrionale» (original citation), restricted to «M[oun]t Toby [Franklin County], Mass[achusetts]» by Lindroth (1966: 445). One syntype in MHNP (Lindroth 1955b: 14).
Myas foveatus LeConte, 1847: 355. Type locality: «NovEboraci [= New York]» (original citation). Lectotype (♀), designated by Bousquet (1999: 91), in MCZ [# 5599]. Synonymy established by LeConte (1869b: 249), confirmed by Lindroth (1955b: 14).
Neomyas lindrothi Allen, 1980: 20. Type locality: «Graybeard Mountain, North Carolina» (original citation). Holotype (♂) location unknown (missing in AMNH, Richard L. Hoffman pers. comm. 2003). **New synonymy**. Note. Allen (1980) established *Neomyas lindrothi* exclusively on the presence of a distinct scale group on the right lateral surface of the endophallus; this group of scales being absent in *Myas cyanescens*. In my opinion, this character alone is probably not of specific nature considering among others that the endophallic scales are extremely variable in members of *Myas cyanescens* as noted by Allen (1980: 24) himself.

#### Distribution.

This species is found from “Nova Scotia” (Larochelle and Larivière 1990a: 29) to northwestern Minnesota (Allen 1980: 24), south to northern Alabama (Löding 1945: 15) and Georgia (Fattig 1949: 21, as *Myas foveatus*). A specimen simply labeled from Texas is known (Allen 1980: 26).

#### Records.

**CAN**: NB, NS, ON, QC **USA**: AL, CT, GA, IA, IL, MA, MD, ME, MI, MN, NC, NH, NJ, NY, OH, PA, RI, TN, VA, VT, WI, WV [TX]

### Genus Pterostichus

Bonelli, 1810

Pterostichus Bonelli, 1810: Tabula Synoptica. Type species: *Carabus fasciatopunctatus* Creutzer, 1799 designated by Curtis (1828: plate 196). Etymology. From the Greek *pteron* (wing, by extension elytron) and *stichos* (row, line), possibly alluding to the striae on the elytra of the adults [masculine].

#### Diversity.

About 1,055 species (Lorenz 2005: 269-287, 289, as *Pterostichus*, *Lyropedius*, and *Abacidus*) in the Nearctic (about 180 species, of which four are adventive) and Palaearctic (about 875 species) Regions. *Percolaus* Bates with two Middle American species, *Mayaferonia* Ball and Roughley with two Middle American species, and *Allotriopus* Bates with eight Mexican species are also includes in the genus *Pterostichus* by some authors (e.g., Ball and Roughley 1982).

#### Faunistic Note.

*Pterostichus (Steropus) madidus* (Fabricius), a European species, is known in North America from two specimens, one collected in 1913 in Quebec (Lindroth 1966: 476) and the other one in 1984 in Somerset County, Pennsylvania (Cameron and Reeves 1990: 127). Until more specimens are collected on this continent, the species is not considered as a North American resident.

#### Taxonomic Note.

Based on phylogenetic analyses from two nuclear gene sequences (wingless and 28SrDNA), Sasakawa and Kubota (2007) concluded that the genus *Pterostichus sensu* Bousquet (1999) was monophyletic with the exception of *Bothriopterus* which forms a clade with *Stomis* and *Myas*.

### Subgenus Argutor

Dejean, 1821

Argutor Dejean, 1821: 11. Type species: *Carabus vernalis* Panzer, 1795 designated by Curtis (1837: plate 666). Etymology. Uncertain, possibly from the Latin *argutor* (to stand about, to trample on) [masculine]. The name was proposed by Johann Karl Megerle von Mühlfeld and made available by Dejean.
Lagarus Chaudoir, 1838: 10. Type species: *Carabus vernalis* Panzer, 1795 by original designation. Etymology (original). From the Greek *lagaros* (loose, thin, by extension spindly), alluding to the body shape of adults of the two species Chaudoir included in this taxon [masculine].
Gluptodactylus Gautier des Cottes, 1869: 147. Type species: *Carabus vernalis* Panzer, 1795 by monotypy. Etymology (original). From the Greek *glyptos* (carved) and *dactylos* (finger), alluding to the presence of a sulcus on the male first tarsomeres (“*♂ les premiers articles des tarses *... *sillonnés en dessus*”) [masculine].
Aulacotarsus Reiche [in Reiche and Lallemant], 1872: 16. Unnecessary replacement name for *Gluptodactylus* Gautier des Cottes, 1869. Etymology. From the Greek *aulacos* (furrow) and *tarsos* (tarsus) [masculine].
Eolagarus Tschitschérine, 1899d: 287. Type species: *Lagarus dulcis* Bates, 1883 by monotypy. Synonymy established, under the name *Lagarus* Chaudoir, implicitly by Lindroth (1966: 501), explicitly by Lafer (1984: 19). Etymology. From the Greek *eos* (east) and the generic name *Lagarus* [*q.v*.] [masculine].
Pseudargutor Casey, 1918: 324. Type species: *Feronia erythropus* Dejean, 1828 (= *Loxandrus commutabilis* Motschulsky, 1866) by original designation. Synonymy established, under the name *Lagarus* Chaudoir, by Lindroth (1966: 501). Etymology (original). From the Greek *pseudos* (fallacy, lie) and the generic name *Argutor* [*q.v*.] [masculine].
Pseudolagarus Lutshnik, 1922: 70. Type species: *Platysma leconteianum* Lutshnik, 1922 (= *Loxandrus commutabilis* Motschulsky, 1866) by original designation. Synonymy established, under the name *Lagarus* Chaudoir, by Lindroth (1966: 501). Etymology (original). From the Greek *pseudos* (fallacy, lie) and the generic name *Lagarus* [*q.v*.] [masculine].
Paralagarus Lutshnik, 1922: 71. Type species: *Platysma chunghusorum* Lutshnik, 1922 (= *Pterostichus sulcitarsis* Morawitz, 1863) by original designation. Synonymy established, under the name *Lagarus* Chaudoir, by Lafer (1984: 19). Etymology. From the Greek *para* (near, beside) and the generic name *Lagarus* [*q.v*.] [masculine].

#### Diversity.

Thirteen species in the boreal and temperate areas of the Nearctic (three species, one of them adventive) and Palaearctic (11 species) Regions.

#### Identification.

Bousquet and Webster (2004) reviewed the Nearctic species.

#### Taxonomic Note.

This subgenus was known until the late 1980s under the name *Lagarus* Chaudoir.

### [commutabilis group]

### Pterostichus commutabilis

(Motschulsky, 1866)

Feronia erythropus Dejean, 1828: 243 [secondary homonym of *Pterostichus erythropus* (Marsham, 1802)]. Type locality: «Amérique septentrionale» (original citation), restricted to «Rumney [Grafton County], N[ew] H[ampshire]» by Lindroth (1966: 502). One syntype in MHNP (Lindroth 1966: 502).
Platyderus nitidus Kirby, 1837: 29 [secondary homonym of *Pterostichus nitidus* (Dejean, 1828)]. Type locality: «Lat. 54° [= along North Saskatchewan River]» (original citation). Lectotype (♀), designated by Bousquet (1999: 99), in BMNH. Synonymy established by LeConte (1846b: 231), confirmed by Bousquet (1999: 99).
Loxandrus commutabilis Motschulsky, 1866: 243. Type locality: «Am[érique] b[oréale]» (lectotype label). Lectotype (♀), designated by Bousquet and Larochelle (1993: 15), in ZMMU. Synonymy established by Bousquet and Larochelle (1993: 15).
Platysma leconteianum Lutshnik, 1922: 70. Replacement name for *Platysma erythropus* (Dejean, 1828) and *Platysma nitida* (Kirby, 1837).

#### Distribution.

This species ranges from Cape Breton Island and Prince Edward Island to south-central British Columbia, south to the Lake Tahoe region in the Sierra Nevada, southern Colorado along the Rocky Mountains, northeastern Kansas (Popenoe 1877: 23, as *Pterostichus erythropus*), southern Indiana, and Delaware along the Atlantic Coast [see Bousquet and Webster 2004: Fig. 5].

#### Records.

**CAN**: BC, MB, NB, NS (CBI), ON, PE, QC, SK **USA**: CA, CO, CT, DE, IA, IL, IN, KS, MA, ME, MI, MN, ND, NE, NH, NJ, NY, OH, PA, RI, SD, VT, WA, WI

### Pterostichus praetermissus

(Chaudoir, 1868)

Feronia praetermissa Chaudoir, 1868b: 331. Type locality: «Louisiane» (lectotype label). Lectotype (♀), designated by Bousquet and Larochelle (1993: 14), in MCZ [# 34423].

#### Distribution.

This species ranges from Nova Scotia (Christopher G. Majka pers. comm. 2004) to the Missouri River in southeastern South Dakota and eastern Nebraska, south to southwestern Texas (Dajoz 2007: 23, as *Pterostichus commutabilis*), northern Alabama, and northern Florida [see Bousquet and Webster 2004: Fig. 6].

#### Records.

**CAN**: NS, ON, QC **USA**: AL, CT, FL, GA, IA, IL, IN, LA, MA, MD, ME, MI, MN, NE, NH, NJ, NY, OH, PA, RI, SC, SD, TX, VA, VT, WI

### [vernalis group]

### Pterostichus vernalis

(Panzer, 1795)

Carabus vernalis Panzer, 1795: 60 [primary homonym of *Carabus vernalis* Müller, 1776]. Type locality: Germany (inferred from title of the book). Syntype(s) possibly in ZMHB. Note. Panzer’s name should be permanently invalid because it is a primary homonym. However, *Carabus vernalis* Müller has never been interpreted since its original description to my knowledge and the name is a *nomen dubium*.
Carabus crenatus Duftschmid, 1812: 92. Type locality: «um Linz [Austria]» (original citation). Syntype(s) probably lost. Synonymy established by Dejean (1821: 11).

#### Distribution.

This European species is adventive in North America where it is known from southern Quebec, south of the Saint Lawrence River, eastern Ontario (near Avonmore, CNC), and northern Vermont (Bousquet and Webster 2004: 658). The first inventoried specimen collected on this continent was found in 1997 (Byers et al. 2001: 84). The previous record from Quebec (Lindroth 1966: 503) was based on a mislabeled specimen.

#### Records.

**CAN**: ON, QC **USA**: VT – **Adventive**

### Subgenus Phonias

des Gozis, 1886

Phonias des Gozis, 1886: 8. Type species: *Platysma interstincta* Sturm, 1824 (= *Platysma ovoidea* Sturm, 1824) by original designation. Etymology. Uncertain, possibly from the Greek *phonos* (slaughter, murder) and suffix -*ios* (pertaining to) [masculine].
Micromaseus Casey, 1918: 324 [junior homonym of *Micromaseus* Desbrochers des Loges, 1906]. Type species: *Feronia patruelis* Dejean, 1831 by original designation. Etymology. From the Greek *micros* (small, little) and the generic name *Omaseus* [*q.v*.] [masculine].
Omaseulus Lutshnik, 1929: 5. Replacement name for *Micromaseus* Casey, 1918. Etymology. From the generic name *Omaseus* [*q.v*.] and the Latin suffix -*ulus* (small, little) [masculine].
Americomaseus Csiki, 1930: 644. Replacement name for *Micromaseus* Casey, 1918. Etymology. From the English prefix americo- (relating to America) and the generic name *Omaseus* [*q.v*.] [masculine].
Biphonias Jeanne, 1988: 74. Type species: *Pterostichus longinquus* Bates, 1873 by original designation. Synonymy established by Kryzhanovskij et al. (1995: 99). Etymology. From the Latin prefix *bi*- (two) and the generic name *Phonias* [*q.v*.] [masculine].

#### Diversity.

Twenty-eight species (Lorenz 2005: 275) in the boreal and temperate areas of the Nearctic (four species, one of them adventive) and Palaearctic (25 species) Regions.

#### Identification.

Lindroth (1966) reviewed the Nearctic species.

### Pterostichus corrusculus

LeConte, 1873

Pterostichus corrusculus LeConte, 1873a: 314. Type locality: «Massachusetts» (original citation), herein restricted to Medford, Middlesex County (CNC). Lectotype (♂), designated by Bousquet (1999: 103), in MCZ [# 5650].
Pterostichus corusculus Csiki, 1930: 644. Unjustified emendation of *Pterostichus corrusculus* LeConte, 1873.

#### Distribution.

This small *Pterostichus* is known from New Brunswick (Webster and Bousquet 2008: 18) to northern Wisconsin (Marquette County, CMNH; Rauterberg 1885: 16; Messer 2010: 37), south to central Pennsylvania (Clinton County, CMNH) and northeastern New Jersey (Bergen County, CNC).

#### Records.

**CAN**: NB, ON, QC **USA**: CT, MA, ME, NH, NJ, NY, PA, RI, WI

### Pterostichus femoralis

(Kirby, 1837)

Argutor femoralis Kirby, 1837: 30. Type locality: «Lat. 54° [= along North Saskatchewan River]» (original citation), restricted to «Edmonton, Al[ber]ta» by Lindroth (1966: 504). Lectotype (♂), designated by Bousquet (1999: 103), in BMNH.
Pterostichus desidiosus LeConte, 1863c: 11. Type locality: «western states» (original citation). Lectotype (♀), designated by Bousquet (1999: 104), in MCZ [# 5649]. Synonymy established by LeConte (1873a: 309), confirmed by Bousquet (1999: 104).
Micromaseus aequicollis Casey, 1918: 379. Type locality: «S[ain]t Louis, Missouri» (original citation). Holotype [by monotypy] (♂) in USNM [# 47074]. Synonymy established by Lindroth (1966: 504).

#### Distribution.

The range of this species extends from the Saguenay River in Quebec (Larochelle 1975: 101) to east-central British Columbia (Jarrett and Scudder 2001: 383), south to northeastern New Mexico (Fall and Cockerell 1907: 158; San Miguel County, MCZ), the Texas Panhandle (Robert L. Davidson pers. comm. 2008), and the District of Columbia (Ulke 1902: 6). The records from “Georgia” (J.E. LeConte 1849: 26) and southern Louisiana (Summers 1874a: 80) need confirmation.

#### Records.

**CAN**: AB, BC, MB, NB, ON, QC, SK **USA**: AR, CO, CT, DC, IA, IL, IN, KS, MA, MI, MN, MO, MT, ND, NE, NH, NJ, NM, NY, OH, OK, PA, SD, TX, VT, WI [GA, LA]

### Pterostichus patruelis

(Dejean, 1831)

Feronia patruelis Dejean, 1831: 759. Type locality: «Amérique septentrionale» (original citation), restricted to «W[est] Roxbury [Suffolk County], Mass[achusetts]» by Lindroth (1966: 505). Holotype [by monotypy] (♀) in MHNP.
Argutor bicolor Kirby, 1837: 30. Type locality: «Lat. 54° [= along North Saskatchewan River]» (original citation). Lectotype (♂), designated by Bousquet (1999: 104), in BMNH. Synonymy established by LeConte (1870: 396), confirmed by Bousquet (1999: 104).
Argutor linearis Mannerheim, 1853: 126. Type locality: «ad ostia fl[umen] Kaktnu [= Kenai River] peninsulae Kenai [Alaska]» (original citation). Syntype(s) location unknown (Lindroth 1966: 505). Synonymy established by Hamilton (1894a: 9).

#### Distribution.

This species occurs from Newfoundland (Lindroth 1955a: 91) to the Gulf Coast of Alaska (Lindroth 1966: 506), south at least to northeastern Washington (Pend Oreille County, CNC), eastern Kansas (Popenoe 1877: 23; Lindroth 1966: 505), and southwestern Virginia (Hoffman 1998: 39).

#### Records.

**FRA**: PM **CAN**: AB, BC, LB, MB, NB, NF, NS (CBI), ON, PE, QC, SK, YT **USA**: AK, CT, IA, IL, IN, KS, MA, MD, ME, MI, MN, MO, MT, ND, NE, NH, NJ, NY, OH, PA, RI, SD, VA, VT, WA, WI, WV

### Pterostichus strenuus

(Panzer, 1796)

Carabus strenuus Panzer, 1796b: no 6. Type locality: «Brunsvigiae [= Brunswick, Germany]» (original citation). Syntype(s) location unknown (possibly in ZMHB).

#### Distribution.

This European species is adventive in North America on both coasts where it is found in southeastern Newfoundland (Lindroth 1955a: 91; Larson and Langor 1982: 593) and the Vancouver area in British Columbia (Spence and Spence 1988: 158, Fig. 8). The first inventoried specimen collected on this continent was found in Newfoundland in 1937 (see Lindroth 1955a: 91).

#### Records.

**CAN**: BC, NF – **Adventive**

### Subgenus Bothriopterus

Chaudoir, 1835

Tilodes Fischer von Waldheim, 1829a: 19 [potential *nomen oblitum*, see Bousquet (2002c: 179)]. Type species: *Carabus oblongopunctatus* Fabricius, 1787 designated by Bousquet (1999: 104). Etymology. Unknown [masculine].
Bothriopterus Chaudoir, 1835: 447 [potential *nomen protectum*]. Type species: *Carabus oblongopunctatus* Fabricius, 1787 designated by Chaudoir (1838: 9). Etymology (see Chaudoir 1838: 9). From the Greek *bothros* (pit, dimple) and *pteron* (wing, by extension elytron), probably alluding to the foveolate discal setigerous punctures on the third elytral interval in adults of *Pterostichus oblongopunctatus* and related species [masculine].
Dysidius Chaudoir, 1838: 8. Type species: *Feronia morosa* Dejean, 1828 (= *Feronia muta* Say, 1823) by original designation. Synonymy established by Goulet (1974a: 27). Etymology (original). From the Greek *dys*- (bad, with difficulty; referring to by Chaudoir as deplorable, sad) and *eidos* (form, appearance), alluding to the specific name of the sole species (*Feronia morosa*) Chaudoir attributed to this genus-group taxon [masculine].
Geopezus Gistel, 1856: 358. Type species: *Carabus oblongopunctatus* Fabricius, 1787 by monotypy.
Parargutor Casey, 1918: 324. Type species: *Pterostichus lustrans* LeConte, 1851 by original designation. Synonymy established, under the name *Dysidius* Chaudoir, by Lindroth (1966: 489). Etymology. From the Greek *para* (near, beside) and the generic name *Argutor* [*q.v*.] [masculine].

#### Diversity.

Eighteen species (Lorenz 2005: 273) in the arctic (marginal), subarctic, boreal, and temperate areas of the Nearctic (six species, one of them circumboreal) and Palaearctic (12 species, including two in the Himalayas) Regions, with one species (*Pterostichus tropicalis* Bates) in the mountains of northern Mexico.

#### Identification.

Tschitschérine (1900d) published a key to the species but his work is outdated. Lindroth (1966: 484-491) reviewed the North American species under his *adstrictus* and *mutus* groups.

### [adstrictus group]

### Pterostichus adstrictus

Eschscholtz, 1823

Pterostichus adstrictus Eschscholtz, 1823: 103. Type locality: «Unalaschka [Aleutian Islands, Alaska]» (original citation). Three syntypes in ZMH (Silfverberg 1987: 11). Note. This species was also made available the same year by an illustration in Fischer von Waldheim (1823: plate 19, figure 1).
Feronia vitrea Dejean, 1828: 320. Type locality: «Kamtschatka [Russia]; île de Sitka sur la côte nord-ouest de l’Amérique septentrionale [Alaska]» (original citation). Syntype(s) probably in MHNP. Synonymy established by Tschitschérine (1900d: 609).
Feronia luczotii Dejean, 1828: 321. Type locality: «île de Terre-Neuve [= Newfoundland]» (original citation). Syntype(s) probably in MHNP. Synonymy established by Tschitschérine (1900d: 609). Etymology. The specific name was proposed for François-Marie-Julien Luczot de La Thébaudais [1769-1844], Chief engineer of Morbihan in France and amateur entomologist with an interest in Lepidoptera and Coleoptera. It is under his chairmanship that 18 entomologists formed the *Société Entomologique de France* on January 31, 1832 at the headquarters of the *Société philomathique* (Peyerimhoff 1932: 4). His son, ship lieutenant Charles Luczot, brought him insects from Newfoundland, Chile, and Madagascar and some of them found their way into the hands of Dejean.
Harpalus borealis Zetterstedt, 1828: 32. Type locality: «Ofver-Tornea, Wittangi et Juckasjervi [Sweden]» (original citation), restricted to «Vittangi [Norrbotten], Lapl[and]» by Lindroth (1966: 485). Lectotype (♂), designated by Lindroth (1966: 485), in ZMLS. Synonymy established by Mannerheim (1852: 295), confirmed by Lindroth (1966: 485).
Omaseus bulwerii Stephens, 1828a: 113. Type locality: «near Dublin [Ireland]» (original citation). Syntype(s) probably in BMNH. Synonymy established, under the name *Pterostichus orinomus* (Stephens), by Wilson and Duncan (1834: 83).
Omaseus orinomum Stephens, 1828a: 114. Type locality: «base of Ben Lomond, in Scotland; Westmoreland, Cumberland, and Yorkshire [United Kingdom]» (original citation). Syntype(s) probably in BMNH. Synonymy established with the name *Pterostichus vitreus* (Dejean) by LeConte (in Bell 1859: 248).
Feronia oblongo-notata Say, 1830b: (6) [3]. «Aweme, Man[itoba]» (neotype label). Neotype (♂), designated by Lindroth and Freitag (1969: 343), in MCZ [# 33037]. Synonymy established, under the name *Pterostichus luczotii* (Dejean), by LeConte (1850: 206). Note. «N[orth] W[est] Territory» was the area originally cited by Say (1830b: (6) [3]).
Pterostichus seriepunctatus Mannerheim, 1843: 204. Type locality: «insula Sitkha [= Baranof Island, Alaska]» (original citation). Lectotype (♂), designated by Lindroth (1966: 485), in ZMH. Synonymy established by Tschitschérine (1900d: 609), confirmed by Lindroth (1966: 485).
Feronia commixta Chaudoir, 1850b: 135. Type locality: «Sitka [Alaska]» (original citation). Syntype(s) probably in MHNP. Synonymy established by Lindroth (1966: 485).
Bothriopterus sexpunctatus Mannerheim, 1853: 127. Type locality: «insula Kadjak [Alaska]» (original citation for the lectotype). Lectotype (♂), designated by Lindroth (1966: 485), in ZMH. Synonymy established by Tschitschérine (1900d: 609), confirmed by Lindroth (1954b: 133).
Pterostichus motschulskyi Mäklin, 1855: 39. Type locality: «continenti Americes borealis ad fontes Kaknu [= Skilak Lake or Kenai River, both on Kenai Peninsula, Alaska]» (original citation for *Pterostichus orinomus* (Stephens) *sensu* Mannerheim, 1852). Lectotype (♂), designated by Bousquet (1999: 108), in ZMH. Synonymy established, under the name *Pterostichus luczotii* (Dejean), by LeConte (1863b: 9), confirmed by Bousquet (1999: 108). Note. This name was proposed for *Pterostichus orinomus* (Stephens, 1828) *sensu* Mannerheim (1852: 295).
Platysma arctica Reiche, 1857: viii. Type locality: «Islandia, etiam in Lapponia» (original citation). Syntype(s) location unknown (possibly in MHNG). Synonymy established by Tschitschérine (1900d: 609).
Platysma obtusangula Motschulsky, 1859a: 150. Type locality: California (inferred from title of the paper). One syntype in ZMMU (Keleinikova 1976: 208), possibly another one in MCZ (Lindroth 1966: 485). Synonymy established, under the name *Platysma sexpunctatus* (Mannerheim), by LeConte (1863b: 9), confirmed by Lindroth (1954b: 133).
Platysma oblongiuscula Motschulsky, 1859a: 151. Type locality: California (inferred from title of the paper). One syntype, listed as “corruptum,” in ZMMU (Keleinikova 1976: 208). Synonymy established by LeConte (1863b: 9).
Bothriopterus latescans Casey, 1913: 139. Type locality: «Lake Tahoe [Placer County], California» (original citation). Lectotype (♀), designated by Lindroth (1975: 124), in USNM [# 47089]. Synonymy established by Lindroth (1954b: 133).
Bothriopterus sericeus Casey, 1913: 140. Type locality: «Clackamas Co[unty], Oregon» (original citation). Lectotype (♀), designated by Lindroth (1975: 124), in USNM [# 47090]. Synonymy established by Hatch (1953: 118), confirmed by Lindroth (1954b: 133).
Bothriopterus latebricola Casey, 1913: 141. Type locality: «Truckee [Nevada County], California» (original citation). Lectotype (♀), designated by Lindroth (1975: 124), in USNM [# 47092]. Synonymy established by Lindroth (1954b: 133).
Bothriopterus shastanus Casey, 1913: 141. Type locality: «Siskiyou Co[unty], California» (original citation). Lectotype (♂), designated by Lindroth (1975: 124), in USNM [# 47093]. Synonymy established by Hatch (1953: 118), confirmed by Lindroth (1954b: 133).
Bothriopterus saxatilis Casey, 1913: 142. Type locality: «Boulder Co[unty], Colorado» (original citation for the lectotype). Lectotype (♂), designated by Lindroth (1975: 124), in USNM [# 47095]. Synonymy established by Hatch (1953: 118), confirmed by Lindroth (1954b: 133).
Bothriopterus laxicollis Casey, 1913: 142. Type locality: «Colorado» (original citation). Lectotype (♂), designated by Lindroth (1975: 124), in USNM [# 47094]. Synonymy established by Lindroth (1954b: 133).
Bothriopterus angusticollis Casey, 1924: 77. Type locality: «Provo Cañon [Utah County], Utah» (original citation). Lectotype (♂), designated by Lindroth (1975: 124), in USNM [# 47091]. Synonymy established by Lindroth (1954b: 133).

#### Distribution.

This circumpolar species ranges from Iceland to the coast of Bering Sea (Bousquet 2003d: 487) and from Alaska, including the Aleutian, Kodiak, and possibly Pribilof Islands (Lindroth 1966: 487), to Newfoundland (Lindroth 1955a: 94), south to north-central Pennsylvania (LeConte 1867a: 346, as *Platysma luczotii*), northeastern Indiana (Blatchley 1910: 98, as *Platysma luczotii*; Schrock 1985: 353), the Black Hills in western South Dakota (Kirk and Balsbaugh 1975: 22), the Black Range in west-central New Mexico (Fall and Cockerell 1907: 158, as *Platysma luczotii*), and the Sierra Nevada in California (Dajoz 2007: 16). The record from Virginia (Goulet 1974a: 19) is apparently based on misidentified specimens of *Platysma pensylvanicus* (Hoffman 1998: 39); that from “Nebraska” (Schaupp 1882c: 42, as *Platysma luczotii*) probably refers to Colorado.

#### Records.

**FRA**: PM **CAN**: AB, BC (QCI, VCI), LB, MB, NB, NF, NS (CBI), NT, ON, PE, QC, SK, YT **USA**: AK, AZ, CA, CO, CT, IA, ID, IN, MA, ME, MI, MN, MT, ND, NH, NM, NY, OR, PA, RI, SD, UT, VT, WA, WI, WY – **Holarctic**

### Pterostichus oregonus

LeConte, 1861

Pterostichus oregonus LeConte, 1861b: 339. Type locality: «east of Fort Colville [Washington]» (original citation). Lectotype (♂), designated by Bousquet (1999: 110), in MCZ [# 5647].
Omaseus colligatus Walker, 1866: 314. Type locality: British Columbia (inferred from title of the book). Lectotype (♀), designated by Bousquet (1999: 111), in BMNH. Synonymy established by LeConte (1870: 399), confirmed by Bousquet (1999: 111).

#### Distribution.

This species ranges from southern British Columbia (Lindroth 1966: 489) south to northern Utah (Cache, Davis and Utah Counties, Robert L. Davidson pers. comm. 2012) and eastern Oregon (Hatch 1953: 118).

#### Records.

**CAN**: BC **USA**: ID, OR, UT, WA

### Pterostichus pensylvanicus

LeConte, 1873

Pterostichus pensylvanicus LeConte, 1873a: 314. Type locality: «mountains of Pennsylvania» (original citation). Lectotype (♂), designated by Bousquet (1999: 111), in MCZ [# 5646].

#### Distribution.

The range of this species extends from Newfoundland (Lindroth 1955a: 93) to central British Columbia, north to Fort Smith in southern Northwest Territories (Lindroth 1966: 487), south to the Black Hills in western South Dakota (Kirk and Balsbaugh 1975: 22), southeastern Iowa (Wickham 1911b: 6), and west-central Virginia (Hoffman 1998: 39).

#### Records.

**CAN**: AB, BC, MB, NB, NF, NS (CBI), NT, ON, PE, QC, SK **USA**: CT, IA, IL, IN, MA, MD, ME, MI, MN, ND, NH, NJ, NY, OH, PA, RI, SD, VA, VT, WI, WV

### [mutus group]

### Pterostichus lustrans

LeConte, 1851

Pterostichus lustrans LeConte, 1851: 181. Type locality: «San Francisco, San Jose, et S[an]ta Isabel [California]» (original citation), herein restricted to San Francisco [San Francisco County]. Lectotype (♂), designated by Bousquet (1999: 109), in MCZ [# 80].
Platysma puncticollis Motschulsky, 1859a: 149. Type locality: «Col[onie] Ross [farming community about 75 miles north of San Francisco along the coast, California]» (original citation). Lectotype (probably ♂), designated by Bousquet (1997b: 334), in ZMMU. Synonymy established by Leng (1920: 58), confirmed by Bousquet (1997b: 335).
Parargutor atrolucens Casey, 1918: 378. Type locality: «Clackamas Co[unty], Oregon» (original citation). Lectotype (♂), designated by Lindroth (1975: 124), in USNM [# 47072]. Synonymy established by Lindroth (1966: 490).

#### Distribution.

This species ranges from Vancouver Island (Lindroth 1966: 491) and northern Idaho (Idaho County, CNC) south to central New Mexico (Bernalillo County, CMNH), southern Arizona (Wickham 1898: 300; Snow 1906b: 162), and southern California (Fall 1901a: 45; Moore 1937: 9).

#### Records.

**CAN**: BC (VCI) **USA**: AZ, CA (CHI), CO, ID, NM, NV, OR, UT, WA

### Pterostichus mutus

(Say, 1823)

Feronia muta Say, 1823a: 44. Type locality: «Black M[oun]t[ain]s, N[o]rth C[arolina]» (neotype label). Neotype (♂), designated by Lindroth and Freitag (1969: 343), in MCZ [# 33039].
Omaseus politus T.W. Harris, 1828c: 123. Type locality: «N[ew] Y[ork]» (lectotype label). Lectotype (♂), designated by Bousquet (1999: 110), in MCZ [# 34571]. Synonymy established, under the name *Pterostichus morosus* (Dejean), by Harris (1833: 567), confirmed by Bousquet (1999: 110).
Feronia morosa Dejean, 1828: 282. Type locality: «Amérique septentrionale» (original citation). Syntype(s) probably in MHNP. Synonymy established by LeConte (1853a: 242).
Feronia carbonaria Dejean, 1828: 283. Type locality: «Amérique septentrionale» (original citation). Holotype [by monotypy] (♂) probably in MHNP. Synonymy established by LeConte (1846b: 335).
Omaseus picicornis Kirby, 1837: 33. Type locality: northern parts of British America (inferred from title of the book). Lectotype (♂), designated by Bousquet (1999: 110), in BMNH. Synonymy established by LeConte (1846b: 335), confirmed by Bousquet (1999: 110).
Pterostichus stenopus Hausen, 1891a: 253. Type locality: «S[ain]t[e] Rose [= present day Laval], P[rovince of] Q[uebec]» (original citation). Holotype [by monotypy] (♂) location unknown. Synonymy established by Horn (1892c: 41).
Pterostichus pulvinatus Hausen, 1891b: 159. Type locality: «nord du Vermont» (original citation). Holotype [by monotypy] location unknown. Synonymy established by Horn (1892c: 41).
Dysidius egens Casey, 1924: 74. Type locality: «New Jersey» (original citation). Lectotype (♀), designated by Lindroth (1975: 124), in USNM [# 47071]. Synonymy established by Lindroth (1954b: 132).

#### Distribution.

This species ranges from western Newfoundland (Lindroth 1955a: 92) to “Montana” (Lindroth 1966: 490), south to northeastern New Mexico (San Miguel County, CNC), northeastern Kansas (Popenoe 1877: 23), and northeastern Georgia (Leng 1910: 73; Fattig 1949: 27); isolated in southwestern British Columbia, including Vancouver Island (Lindroth 1966: 490). The record from “Idaho” (Bousquet and Larochelle 1993: 170) needs confirmation. Lindroth (1966: 490) postulated that the best explanation for the presence of this species in southwestern British Columbia is that it was accidentally introduced into the area.

#### Records.

**CAN**: BC (VCI), MB, NB, NF, NS (CBI), ON, PE, QC **USA**: CO, CT, DC, DE, GA, IA, IL, IN, KS, MA, MD, ME, MI, MN, MT, NC, ND, NE, NH, NJ, NM, NY, OH, PA, RI, SD, TN, VA, VT, WI, WV [ID]

### Pterostichus trinarius

(Casey, 1918)

Pterostichus purpuratus LeConte, 1853a: 242 [primary homonym of *Pterostichus purpuratus* Heer, 1841]. Type locality: «Ohio» (original citation). Lectotype (♂), designated by Bousquet (1999: 111), in MCZ [# 5645].
Dysidius purpuratus trinarius Casey, 1918: 377. Type locality: «Camphill [Cumberland County], Pennsylvania» (original citation). Lectotype (♀), designated by Lindroth (1975: 124), in USNM [# 47070]. Synonymy established by Nicolay and Weiss (1934: 210), confirmed by Lindroth (1966: 489).
Pterostichus ohionis Csiki, 1930: 638. Replacement name for *Pterostichus purpuratus* LeConte, 1853.

#### Distribution.

This species ranges from southeastern South Dakota (Kirk and Balsbaugh 1975: 22) to southwestern New York (Smith 1910: 205), south to Virginia (Nicolay and Weiss 1934: 211; Carrington 2002: 108), central West Virginia (Carrington 2002: 108), and central Missouri (Boone County, CMNH).

#### Records.

**USA**: IA, IL, IN, KY, MD, MI, MO, NJ, NY, OH, PA, SD, VA, WI, WV

### Subgenus Melanius

Bonelli, 1810

Melanius Bonelli, 1810: Tabula Synoptica. Type species: *Carabus aterrimus* Herbst, 1784 by subsequent monotypy in Latreille (1816: 194). Etymology. Uncertain, possibly from the Greek *melanos* (black), alluding to the black coloration of adults of the species in the hands of Bonelli [masculine].
Omaseus Dejean, 1821: 12. Type species: *Carabus aterrimus* Herbst, 1784 designated by Curtis (1824: plate 15). Etymology. Unknown [masculine]. The name was proposed by Franz Anton Ziegler and made available by Dejean.
Lyperus Chaudoir, 1838: 12. Type species: *Carabus aterrimus* Herbst, 1784 by original designation. Etymology (original). From Greek *lyperos* (painful, by extension sad) [masculine].
Lyperosomus Motschulsky, 1850a: 47. Unnecessary replacement name for *Lyperus* Chaudoir, 1838. Etymology. From Greek *lyperos* (painful, by extension sad) and *soma* (body) [masculine].
Metamelanius Tschitschérine, 1900b: 395. Type species: *Feronia ebenina* Dejean, 1828 by monotypy. Synonymy established by Lindroth (1966: 498). Etymology. From the Greek *meta* (near, among) and the generic name *Melanius* [*q.v*.] [masculine].

#### Diversity.

Eight species in the boreal and temperate regions of the Nearctic (three eastern species) and Palaearctic (five species, of which three occur in the Far East) Regions.

#### Identification.

Lindroth (1966) reviewed the North American species. One new species was described subsequently by Goulet and Bousquet (1983).

### [corvinus group]

### Pterostichus corvinus

(Dejean, 1828)

Feronia corvina Dejean, 1828 [29 November]: 281. Type locality: «Amérique septentrionale» (original citation), restricted to «Rumney [Grafton County], N[ew] H[ampshire]» by Lindroth (1966: 499). One syntype [2 originally cited] in MHNP (Lindroth 1966: 499).
Omaseus subpunctatus T.W. Harris, 1828c [7 November]: 123. Type locality: «V[ermon]t» (lectotype label). Lectotype (♂), designated by Bousquet (1999: 115), in MCZ [# 34570]. Synonymy established with doubt by Harris (1833: 567), confirmed by Bousquet (1999: 115). Note. This name has always been listed as a junior synonym of Dejean’s name and for that reason the name is retained here as invalid. The publication date of Dejean’s work is from an external source (see Bousquet 2004a: 46) and the actual publication date is probably at least two to three weeks earlier.
Omascus tenebrosus Chaudoir, 1837b: 30. Type locality: «Amérique septentrionale» (original citation). Syntype(s) probably in MHNP. Synonymy established by LeConte (1863b: 9).
Omaseus aequalis Casey, 1924: 72. Type locality: «New Jersey» (original citation). Lectotype (♀), designated by Lindroth (1975: 125), in USNM [# 47065]. Synonymy established by Nicolay and Weiss (1934: 210), confirmed by Lindroth (1954b: 132).

#### Distribution.

The range of this species extends from Nova Scotia (Majka et al. 2007: 9) to south-central British Columbia, as far north as northeastern British Columbia (Lindroth 1966: 499-500) and southern Northwest Territories (CNC), south to eastern Washington (Hatch 1953: 117), northern Colorado (Haubold 1951: 704; Armin 1963: 224), southern Iowa (Union County, Foster F. Purrington pers. comm. 2011), and northeastern South Carolina (Ciegler 2000: 66). The record from southwestern California (Moore 1937: 9) is probably in error.

#### Records.

**CAN**: AB, BC, MB, NB, NS, NT, ON, PE, QC, SK **USA**: CO, CT, DC, GA, IA, IL, IN, MA, MD, ME, MI, MN, ND, NE, NH, NJ, NY, OH, PA, RI, SC, SD, VA, VT, WA, WI, WV

### [ebeninus group]

### Pterostichus castor

Goulet and Bousquet, 1983

Pterostichus castor Goulet and Bousquet, 1983: 281. Type locality: «Limbour [= Gatineau], Qué[bec]» (original citation). Holotype (♂) in CNC [# 17028].

#### Distribution.

This species is restricted to a small area from New Brunswick (Webster and DeMerchant 2012: 6) to western Ontario, south to northern Wisconsin (Messer 2010: 37), northern Michigan (Dunn 1985a: 12), and northeastern Pennsylvania (Pike County, CMNH) [see Goulet and Bousquet 1983: map 1].

#### Records.

**CAN**: NB, ON, QC **USA**: MI, NH, PA, VT, WI

### Pterostichus ebeninus

(Dejean, 1828)

Feronia ebenina Dejean, 1828: 307. Type locality: «Amérique septentrionale» (original citation), restricted to «New Orleans [Orleans Parish], Louis[iana]» by Lindroth (1966: 498). Syntype(s) location unknown (possibly in MHNP).
Lyperus acutangulus Chaudoir, 1843b: 771. Type locality: «Nouvelle-Orléans [Orleans Parish, Louisiana]» (original citation). Syntype(s) [2 originally cited] probably in MHNP. Synonymy established by Schaupp (1882c: 42).

#### Distribution.

This species ranges from “Rhode Island” (Sikes 2003: 7) to “Minnesota” (Gandhi et al. 2005: 926), including southernmost Ontario (Lindroth 1966: 499, possibly only as strays), south to “Texas” (Schaupp 1882c: 42; Nicolay and Weiss 1934: 209), southeastern Louisiana (Lindroth 1966: 498), and southern Florida (Peck and Thomas 1998: 20).

#### Records.

**CAN**: ON **USA**: AL, AR, DC, FL, GA, IA, IL, IN, LA, MD, MI, MN, MO, MS, NC, NJ, NY, OH, PA, RI, SC, TN, TX, VA, WI

### Subgenus Pseudomaseus

Chaudoir, 1838

Pseudomaseus Chaudoir, 1838: 10. Type species: *Carabus nigrita* Paykull, 1792 by original designation. Etymology (original). From the Greek *pseudos* (fallacy, lie) and the generic name *Omaseus* [*q.v*.] [masculine].

#### Diversity.

Eighteen species (Lorenz 2005: 274-275) in the boreal and temperate regions of the Nearctic (two species) and Palaearctic (16 species) Regions.

#### Identification.

Bousquet and Pilon (1984) commented on the structural differences between the two North American species.

### Pterostichus luctuosus

(Dejean, 1828)

Feronia luctuosa Dejean, 1828 [29 November]: 284. Type locality: «Amérique septentrionale» (original citation), restricted to «Arlington [Middlesex County], Mass[achusetts]» by Lindroth (1966: 501). One syntype in MHNP (Lindroth 1966: 501).
Omaseus hamatus T.W. Harris, 1828c [7 November]: 123. Type locality not stated. Lectotype (♂), designated by Bousquet (1999: 119), in MCZ [# 34568]. Synonymy established with doubt by Harris (1833: 567), confirmed by Bousquet (1999: 119). Note. See “Note” under *Omaseus subpunctatus*.
Pterostichus abjectus LeConte, 1853a: 243. Type locality: «middle states and Lake Superior» (original citation). Lectotype (♂), designated by Bousquet (1999: 119), in MCZ [# 5641]. Synonymy established by LeConte (1863b: 9), confirmed by Bousquet (1999: 119).
Omaseus brevibasis Casey, 1924: 73. Type locality: «near the city [of New York], New York» (original citation). Lectotype (♀), designated by Lindroth (1975: 125), in USNM [# 47066]. Synonymy established by Bousquet and Larochelle (1993: 16).
Omaseus confluens Casey, 1924: 73. Type locality: «Boston Neck [Washington County], Rhode Island» (original citation). Lectotype (♂), designated by Lindroth (1975: 125), in USNM [# 47069]. Synonymy established by Nicolay and Weiss (1934: 209), confirmed by Lindroth (1954b: 132).

#### Distribution.

This species ranges from Newfoundland to Vancouver Island, south to west-central Washington, central Colorado (Jefferson County, Robert L. Davidson pers. comm. 2008), east-central South Dakota, northern Illinois, and eastern Virginia [see Bousquet and Pilon 1984: Fig. 8].

#### Records.

**FRA**: PM **CAN**: AB, BC (VCI), MB, NB, NF, NS (CBI), ON, PE, QC, SK **USA**: CO, CT, DC, IA, ID, IL, IN, MA, MD, ME, MI, MN, ND, NE, NH, NJ, NY, OH, PA, RI, SD, VA, VT, WA, WI, WV

### Pterostichus tenuis

(Casey, 1924)

Omaseus tenuis Casey, 1924: 73. Type locality: «New Jersey» (original citation). Lectotype (♀), designated by Lindroth (1975: 125), in USNM [# 47068].
Omaseus testaceus Casey, 1924: 74. Type locality: «Boston Neck [Washington County], Rhode Island» (original citation). Holotype [by monotypy] (♀) in USNM [# 47067]. Synonymy established by Bousquet and Pilon (1984: 389).

#### Distribution.

This species ranges from eastern Newfoundland to northeastern Alberta, south to northern Colorado, northern Illinois, and southwestern North Carolina (Macon and Jackson Counties, CNC) [see Bousquet and Pilon 1984: Fig. 7].

#### Records.

**CAN**: AB, MB, NB, NF, NS (CBI), ON, PE, QC **USA**: CO, CT, IL, MA, MD, ME, MI, MN, NC, NH, NJ, NY, OH, PA, RI, VT, WI, WV

### Subgenus Feronina

Casey, 1918

Feronina Casey, 1918: 322. Type species: *Pterostichus palmi* Schaeffer, 1910 by original designation. Etymology. Probably a slight modification of the generic name *Feronia* [*q.v*.] [feminine].

#### Diversity.

Two species in the Appalachian region of eastern North America.

#### Identification.

Bousquet (2006b) commented on the structural differences between the two species.

### Pterostichus barri

Bousquet, 2006

Pterostichus barri Bousquet, 2006b: 158. Type locality: «4 mi[les] w[est] Pennington Gap, U.S. 421, Lee Co[unty], V[irgini]a» (original citation). Holotype (♂) in CNC [# 23463].

#### Distribution.

This species is known from northeastern Kentucky, eastern and central West Virginia, and southwestern Virginia (Bousquet 2006b: 159).

#### Records.

**USA**: KY, VA, WV

### Pterostichus palmi

Schaeffer, 1910

Pterostichus palmi Schaeffer, 1910: 393. Type locality: «North Carolina» (original citation), restricted to «Mount Mitchell State Park, Yancey Co[unty]» by Bousquet (1999: 125). Holotype (♂) in USNM [# 42499]. Etymology. The specific name honors Charles Palm [1836-1917], founder of the firm Palm, Fechteler & Co. in New York City which introduced the decalcomania industry in the United States. Born in Germany, Palm collected butterflies and beetles. His Coleoptera collection went to the American Museum of Natural History.

#### Distribution.

This species is found at high elevation in the Appalachian Mountains in North Carolina, Tennessee (Barr 1969: 72), Kentucky (Jackson County, Foster F. Purrington pers. comm. 2009), and northern Georgia (Fattig 1949: 24), including the Black, Bald, Great Balsam, and Great Smoky Mountains. The record from “Virginia” (Bousquet and Larochelle 1993: 172) needs confirmation.

#### Records.

**USA**: GA, KY, NC, TN [VA]

### Subgenus Paraferonia

Casey, 1918

Paraferonia Casey, 1918: 323. Type species: *Pterostichus lubricus* LeConte, 1853 by original designation. Etymology. From the Greek *para* (near) and the generic name *Feronia* [*q.v*.] [feminine].

#### Diversity.

One species in the Appalachian Mountains of eastern North America.

### Pterostichus lubricus

LeConte, 1853

Pterostichus lubricus LeConte, 1853a: 240. Type locality: «upper part of Georgia» (original citation). Lectotype (♂), designated by Bousquet (1999: 126), in MCZ [# 5629].

#### Distribution.

This species is endemic to the Appalachian Mountains from southwestern Virginia (Hoffman 1998: 38) to northern Georgia (Fattig 1949: 26) and northwestern South Carolina (Ciegler 2000: 67), including the Great Balsam and Great Smoky Mountains (Barr 1969: 80).

#### Records.

**USA**: GA, NC, SC, TN, VA

### Subgenus Pseudoferonina

Ball, 1965

Pseudoferonina Ball, 1965: 107. Type species: *Pterostichus lanei* Van Dyke, 1926 by original designation. Etymology. From the Greek *pseudos* (fallacy, lie) and the generic name *Feronina* [*q.v*.] [feminine].
Melvilleus Ball, 1965: 110. Type species: *Platysma shulli* Hatch, 1949 by original designation. Synonymy established by Bousquet (1985a: 254). Etymology. From the first name of Melville Hatch (see *Scaphinotus hatchi*) [masculine].

#### Diversity.

Nine species in the Pacific Northwest.

#### Identification.

Bousquet (1985a) wrote a key for the identification of the species. Three new species were described subsequently (Bousquet 1992b; Bergdahl and Kavanaugh 2011).

### [humidulus group]

### Pterostichus campbelli

Bousquet, 1985

Pterostichus campbelli Bousquet, 1985a: 257. Type locality: «1 mi[le] S[outh] Hebo, Tillamook Co[unty], Ore[gon]» (original citation). Holotype (♂) in CNC [# 18401].

#### Distribution.

This species is known from several localities in western Oregon. Two specimens simply labeled “Was” are also known (Bousquet 1985a: 258).

#### Records.

**USA**: OR [WA]

### Pterostichus humidulus

(Van Dyke, 1943)

Pterostichus pacificus Van Dyke, 1926a: 114 [secondary homonym of *Pterostichus pacificus* (Poppius, 1906)]. Type locality: «near Hoquiam [Grays Harbor County], Washington» (original citation). Holotype (♂) in CAS [# 1854].
Feronia humidula Van Dyke, 1943: 23. Replacement name for *Feronia pacifica* (Van Dyke, 1926).

#### Distribution.

This species is known from western Washington (Bousquet 1985a: 259). The record from northwestern Oregon (Hatch 1953: 114) probably refers to *Pterostichus campbelli*.

#### Records.

**USA**: WA

### Pterostichus smetanai

Bousquet, 1985

Pterostichus smetanai Bousquet, 1985a: 254. Type locality: «Bear Cr[ee]k (3200’), Spirit L[a]k[e], M[oun]t S[ain]t Helens [Skamania County], Wash[ington]» (original citation). Holotype (♂) in CNC [# 18400].

#### Distribution.

This species is known from the type locality in southern Washington and one locality in northwestern Oregon (Westcott et al. 2006: 8).

#### Records.

**USA**: OR, WA

### [lanei group]

### Pterostichus amadeus

Bousquet, new replacement name

Pterostichus vexatus Bousquet, 1985a: 253 [primary homonym of *Pterostichus vexatus* Broun, 1908]. Type locality: «Harvard [Latah County], Ida[ho]» (original citation). Holotype (♂) in CAS [# 15227].
Pterostichus amadeus Bousquet. New replacement name for *Pterostichus vexatus* Bousquet, 1985. Note. *Pterostichus vexatus* Bousquet, 1985 is a primary homonym of *Pterostichus vexatus* Broun, 1908, a valid species from New Zealand currently placed in the genus *Holcaspis* Chaudoir (Butcher 1984: 85; Larochelle and Larivière 2001: 98). The International Commission on Zoological Nomenclature (1999: 59) rules that the “junior name [of a primary homonym] is permanently invalid” (Article 57.2). However, the commission moderates this ruling in Article 23.9.5 which states that “when an author discovers that a species-group name in use is a junior primary homonym ... of another species-group name also in use, but the names apply to taxa not considered congeneric after 1899, the author must not automatically replace the junior homonym; the case should be referred to the Commission for a ruling under the plenary power and meanwhile prevailing usage of both names is to be maintained.” Since Broun described his new species in 1908, both taxa described under the name *Pterostichus vexatus* were considered congeneric after 1899 and therefore the name *Pterostichus vexatus* Bousquet, 1985 must be changed.

#### Distribution.

This species is known from Latah and Idaho Counties (CNC) in northern Idaho and Mineral County in west-central Montana (James C. Bergdahl pers. comm. 2008).

#### Records.

**USA**: ID, MT

### Pterostichus bousqueti

Bergdahl, 2011

Pterostichus bousqueti Bergdahl [in Bergdahl and Kavanaugh], 2011: 80. Type locality: «small tributaries of the South Fork of the Payette River near Lowman (ca. 1200 m), Boise County, Idaho» (original citation). Holotype (♂) in CAS.

#### Distribution.

This species is known from two creeks near their confluences with the South Fork of the Payette River near Lowman in west-central Idaho (Bergdahl and Kavanaugh 2011: 81).

#### Records.

**USA**: ID

### Pterostichus lanei

Van Dyke, 1926

Pterostichus lanei Van Dyke, 1926a: 76. Type locality: «Wawawai [Whitman County], Washington» (original citation). Holotype (♂) in CAS [# 1828]. Etymology. The specific name was proposed for Merton C. Lane [1893-1965] who worked as an entomologist with the United States Department of Agriculture in the Walla Walla field office in Washington. Heading the USDA’s Pacific Northwest Wireworm Project, Lane became an authority on the biology and taxonomy of the Pacific Northwest elaterids.

#### Distribution.

This species is known from a few localities in southeastern Washington and west-central Idaho (Bousquet 1985a: 259).

#### Records.

**USA**: ID, WA

### Pterostichus lolo

Bergdahl, 2011

Pterostichus lolo Bergdahl [in Bergdahl and Kavanaugh], 2011: 85. Type locality: «Cottonwood Creek near the confluence of Orogrande Creek and the North Fork of the Clearwater River (ca. 870 m), Clearwater County, Idaho» (original citation). Holotype (♂) in CAS.

#### Distribution.

This species is known only from the type locality.

#### Records.

**USA**: ID

### Pterostichus shulli

(Hatch, 1949)

Platysma shulli Hatch, 1949a: 80. Type locality: «Pierce (3200 ft.) [Clearwater County], Idaho» (original citation). Holotype (♂) in USNM. Etymology. The specific name was proposed for Wesley Earl Shull [1899-?] who worked on economic entomology in Idaho. Shull was head of the Entomology Department at University of Idaho between 1938 and 1946.

#### Distribution.

This species is known only from the type locality in northeastern Idaho.

#### Records.

**USA**: ID

### Pterostichus spathifer

Bousquet, 1992

Pterostichus spathifer Bousquet, 1992b: 519. Type locality: «Isabella Creek (1,700’), Clearwater Co[unty], Idaho» (original citation). Holotype (♂) in CAS [# 16024].

#### Distribution.

This species is known only from several specimens collected at the type locality in northeastern Idaho.

#### Records.

**USA**: ID

### Subgenus Gastrosticta

Casey, 1918

Gastrosticta Casey, 1918: 323, 371. Type species: *Feronia ventralis* Say, 1823 by original designation. Etymology. From the Greek *gastros* (stomach, by extension abdomen) and *stictos* (punctured), alluding to the coarse punctures on the sides of the abdominal sterna (“conspicuous coarse punctuation of the lateral parts of the abdomen”) of the adult [feminine].

#### Diversity.

Ten species in the temperate regions of North America, east of the Rocky Mountains.

#### Identification.

Bousquet (1992b) published a preliminary key to the species.

### Pterostichus enodis

Bousquet, 1992

Pterostichus enodis Bousquet, 1992b: 516. Type locality: «Tex[as]» (original citation). Holotype (♂) in CMNH.

#### Distribution.

This species is known only from the holotype.

#### Records.

**USA**: TX

### Pterostichus mutoides

Bousquet, 1992

Pterostichus mutoides Bousquet, 1992b: 517. Type locality: «G[eorgi]a» (original citation). Holotype (♂) in CMNH.

#### Distribution.

This species is known only from two specimens collected in “Georgia.”

#### Records.

**USA**: GA

### Pterostichus obesulus

LeConte, 1873

Pterostichus obesulus LeConte, 1873a: 314. Type locality: «Georgia» (original citation). Holotype [by monotypy] (♀) in MCZ [# 32326].

#### Distribution.

This species is known only from a few localities in west-central Georgia (Fattig 1949: 25), northern Florida (Bousquet 1992b: 511, 513) including the Panhandle (Wakulla County, CMNH), and “Louisiana” (Bousquet 1992b: 513).

#### Records.

**USA**: FL, GA, LA

### Pterostichus ophryoderus

(Chaudoir, 1878)

Feronia ophryodera Chaudoir, 1878: 64. Type locality: «Mississippi» (original citation), herein restricted to 5 miles south of Toomsuba, Lauderdale County (CNC). Lectotype (♀), designated by Bousquet (1992b: 515), in MHNP.

#### Distribution.

This species is known only from several localities in Mississippi (Bousquet 1992b: 515; Drew A. Hildebrandt pers. comm. 2007) and western Alabama (Greene County, Foster F. Purrington pers. comm. 2010).

#### Records.

**USA**: AL, MS

### Pterostichus punctiventris

(Chaudoir, 1878)

Feronia punctiventris Chaudoir, 1878: 66. Type locality: «Texas» (original citation). Lectotype (♀), designated by Bousquet (1992b: 516), in MHNP.
Gastrosticta amnicola Casey, 1918: 372. Type locality: «S[ain]t Louis, Missouri» (original citation). Lectotype (♂), designated by Allen (1977: 286), in USNM [# 47116]. Synonymy established by Bousquet (1992b: 515).

#### Distribution.

This species is known from a few localities from northern Georgia (Fattig 1949: 26, as *Gastrosticta amnicola*) to eastern Oklahoma (Latimer and Pushmataha Counties, CNC; Cherokee County, CMNH), north to east-central Missouri (Bousquet 1992b: 516), south to “Texas” (Chaudoir 1878: 66) and southwestern Alabama (Löding 1945: 16, as *Gastrosticta amnicola*).

#### Records.

**USA**: AL, AR, GA, MO, MS, OK, TX

### Pterostichus putus

Casey, 1913

Pterostichus putus Casey, 1913: 135. Type locality: «Texas» (original citation). Lectotype (♂), designated by Allen (1977: 286), in USNM [# 47117].

#### Distribution.

This species is known only from a few localities in Grayson County (CNC) in Texas.

#### Records.

**USA**: TX

### Pterostichus sayanus

Csiki, 1930

Feronia obscura Say, 1830b: (5) [3] [primary homonym of *Feronia obscura* Dejean, 1828]. Type locality: «All[eghen]y, P[ennsylvani]a» (neotype label). Neotype (♂), designated by Lindroth and Freitag (1969: 342), in MCZ [# 32962]. Note. «Indiana» was the area originally cited by Say (1830b: (5) [3]).
Pterostichus sayanus Csiki, 1930: 676. Replacement name for *Pterostichus obscurus* (Say, 1830).

#### Distribution.

This species ranges from southwestern Pennsylvania (Lindroth and Freitag 1969: 342) to “Iowa” (Jaques and Redlinger 1946: 297), south to east-central Louisiana (West Feliciana Parish, LSAM), northern Alabama (Löding 1945: 16; Madison County, CMNH), and northern Georgia (Fattig 1949: 25).

#### Records.

**USA**: AL, AR, GA, IA, IL, IN, KY, LA, MO, NC, OH, PA, TN, VA, WV

### Pterostichus subacutus

(Casey, 1918)

Gastrosticta subacuta Casey, 1918: 373. Type locality: «Texas» (original citation), restricted to «7.9 mi[les] S[outh] of Cuero, De Witt Co[unty]» by Bousquet (1999: 132). Lectotype (♀), designated by Allen (1977: 286), in USNM [# 47118].

#### Distribution.

This species is known from a few localities in Oklahoma (Latimer and Marshall Counties, CMNH, CNC), eastern, central, and southern Texas (De Witt, Madison, Anderson, Lee, and Houston Counties, CMNH, CNC, MCZ; Riley 2011), and “Louisiana” (CNC).

#### Records.

**USA**: LA, OK, TX

### Pterostichus tumescens

LeConte, 1863

Pterostichus tumescens LeConte, 1863c: 11. Type locality: «Louisiana» (original citation), herein restricted to Lake Claiborne, Claiborne Parish (CNC). Lectotype (♂), designated by Bousquet (1999: 132), in MCZ [# 5630].

#### Distribution.

This species is known from southwestern Florida (Collier County, Foster F. Purrington pers. comm. 2011) and from central and southern Mississippi (Will and Gill 2008: 121; Hancock and Leake Counties, CMNH, CNC) to southeastern Oklahoma (Pushmataha County, CNC), south at least to east-central Texas (Harris County, CNC).

#### Records.

**USA**: AR, FL, LA, MS, OK, TX

### Pterostichus ventralis

(Say, 1823)

Feronia ventralis Say, 1823a: 46. Type locality: «Douglas Co[unty], Kan[sas]» (neotype label), herein restricted to Lawrence (CNC). Neotype (♂), designated by Lindroth and Freitag (1969: 341), in MCZ [# 32961]. Note. «Missouri [Territory]» was the area originally cited by Say (1823a: 46).

#### Distribution.

This species is known from eastern Kansas (Lindroth and Freitag 1969: 341; Douglas County, CNC, UASM) to “Iowa” (Jaques and Redlinger 1946: 297) and southwestern Illinois (Saint Clair County, MCZ; Wickham 1895a: 185), south to northwestern Louisiana (Bossier Parish, LSAM; LeConte 1853a: 241) and south-central Texas (Comal County, CMNH). The record from “Florida” (Wickham 1895a: 185) needs confirmation (see Leng 1915: 576).

#### Records.

**USA**: AR, IA, IL, KS, LA, MO, OK, TX [FL]

### Subgenus Morphnosoma

Lutshnik, 1915

Morphnosoma Lutshnik, 1915b: 424. Type species: *Carabus vulgaris* Linnaeus *sensu auctorum* (= *Carabus melanarius* Illiger, 1798) by original designation. Etymology. From the Greek *morphnos* (kind of eagle in Pliny the Elder) and *soma* (body) [neuter].
Omaseidius Jeannel, 1942: 781. Type species: *Carabus vulgaris* Linnaeus *sensu auctorum* (= *Carabus melanarius* Illiger, 1798) by original designation.

#### Diversity.

Two Palaearctic species (*Pterostichus alexeji* Zamotajlov and Kryzhanovskij and *Pterostichus melanarius* with three subspecies), one of them adventive in North America. The status of *Pterostichus brevistylis* Jeannel, described from Switzerland, remains unclear.

#### Taxonomic Note.

Sasakawa and Kubota (2006) combined members of *Petrophilus* Chaudoir (type species: *Feronia findelii* Dejean, 1828), *Euryperis* Motschulsky (type species: *Euryperis uralensis* Motschulsky, 1850), *Morphnosoma*, *Euferonia*, *Feroperis* Lafer (type species: *Feronia jungens* Tschitschérine, 1893), and *Moritapterus* Berlov (type species: *Pterostichus thunbergi* Morawitz, 1862) into a single subgeneric taxon, *Petrophilus* Chaudoir, 1838.

### Pterostichus melanarius melanarius

(Illiger, 1798)

Carabus melanarius Illiger, 1798: 163. Type locality: Prussia (inferred from title of the book). Syntype(s) probably lost (Lindroth 1966: 491).

#### Distribution.

This European species is adventive in North America on both coasts and is now found from Newfoundland (Lindroth 1955a: 87; Larson and Langor 1982: 592) to Vancouver Island (Lindroth 1966: 492), south to northwestern California (Del Norte County, James R. LaBonte pers. comm. 1992), northern Utah (Davis County, CMNH), northern Colorado (Miller and Peairs 2008: 34), South Dakota (Ellsbury et al. 1998: 621; French et al. 2004: 557; Larsen and Purrington 2010: 571), northeastern Iowa (Purrington and Larsen 1997: 50), east-central Ohio (Usis and MacLean 1998: 67), and central Pennsylvania (Capogreco 1989b: 4; Byers et al. 2001: 85). The first inventoried specimen collected on this continent was found in Nova Scotia in 1926 (Lindroth 1957c: 153) and in the west in Seattle in 1927 (Hatch 1933c: 120).

#### Records.

**CAN**: AB, BC (VCI), MB, NB, NF, NS (CBI), ON, PE, QC, SK **USA**: CA, CO, CT, IA, ID, IL, IN, MA, ME, MI, MN, MT, ND, NH, NY, OH, OR, PA, RI, SD, UT, VT, WA, WI, WY – **Adventive**

#### Note.

Two other subspecies of *Pterostichus melanarius* are found in Europe, *Pterostichus melanarius bulgaricus* Lutshnik and *Pterostichus melanarius cardioderus* Chaudoir (Bousquet 2003d: 499).

### Subgenus Euferonia

Casey, 1918

Euferonia Casey, 1918: 322, 365. Type species: *Feronia stygica* Say, 1823 by original designation. Etymology. From the Greek *eu* (well, by extension large) and the generic name *Feronia* [*q.v*.], alluding to the large size (“usually rather large in size”) of adults of these *Feronia* (= *Pterostichus*) species [feminine].
Moritapterus O. Berlov, 2000: 4. Type species: *Pterostichus thunbergi* Morawitz, 1862 by original designation. **New synonymy**. Etymology. From the surname of the Japanese carabidologist Seiji Morita and the Greek *pteron* (wing, by extension elytron) [masculine]. Note. Sasakawa and Kubota (2006) listed this name in synonymy with *Morphnosoma* Lutshnik along with *Euferonia* Casey.

#### Diversity.

Eleven species in the boreal and temperate regions of eastern North America (six species) and Japan (five species: *Pterostichus robustistylis* Sasakawa and Kubota, *Pterostichus sejunctus* Bates, *Pterostichus thunbergi* Morawitz, *Pterostichus tschitscherianus* Jacobson, and *Pterostichus tuberifer* Sasakawa).

#### Identification.

Nicolay and Weiss (1934) and Lindroth (1966) summarily reviewed the North American species. A thorough revision of this group is needed.

#### Taxonomic Note.

This subgenus is often combined with *Morphnosoma* Lutshnik (e.g., Sasakawa and Kubota 2005; Lorenz 2005). The Japanese species are phenetically most similar to the North American *Pterostichus lachrymosus* (see Bousquet 1999: 137).

### [lachrymosus group]

### Pterostichus lachrymosus

(Newman, 1838)

Feronia lachrymosa Newman, 1838a: 387. Type locality: «northern states of America» (original citation, see page 388), restricted to «M[oun]t Mitchell, Black M[oun]t[ain]s, N[orth] C[arolina]» by Lindroth (1966: 495). Lectotype (♀), designated by Lindroth (1966: 495), in BMNH.

#### Distribution.

This species occurs from New Brunswick (Bousquet 1987a: 124) to the Ontario Peninsula (Lindroth 1966: 495), south along the Appalachian Mountains to the Great Smokies in eastern Tennessee (Stockton 1954: Fig. 19; CNC) and to northern Georgia (Fattig 1949: 24). The record from southwestern Arkansas (Stockton 1954: 16) is probably in error.

#### Records.

**CAN**: NB, ON, QC **USA**: CT, DC, GA, KY, MA, MD, ME, NC, NH, NJ, NY, OH, PA, SC, TN, VA, VT, WV

### [stygicus group]

### Pterostichus coracinus

(Newman, 1838)

Feronia coracina Newman, 1838a: 386. Type locality: «northern states of America» (original citation, see page 388), restricted to «Rumney [Grafton County], N[ew] H[ampshire]» by Lindroth (1966: 494). Syntype(s) lost (Lindroth 1966: 494).
Feronia monedula Newman, 1838a: 386 [*nomen dubium*]. Type locality: «northern states of America» (original citation, see page 388). Syntype(s) probably lost (Lindroth 1966: 494). Synonymy established with doubt by LeConte (1873a: 317).
Feronia moerens Newman, 1838a: 387. Type locality: «northern states of America» (original citation, see page 388). Lectotype (♂), designated by Lindroth (1966: 494), in BMNH. Synonymy established by LeConte (1870: 399), confirmed by Lindroth (1966: 494).
Pterostichus adiunctus LeConte, 1853a: 245. Type locality: «Lake Superior» (original citation). Lectotype (♀), designated by Bousquet (1999: 138), in MCZ [# 5631]. Synonymy established by LeConte (1870: 399), confirmed by Lindroth (1966: 494).
Pterostichus flebilis LeConte, 1853a: 245. Type locality: «Lake Superior» (original citation). Holotype [by monotypy] (♂) in MCZ [# 5632]. Synonymy established by LeConte (1870: 399), confirmed by Lindroth (1966: 494).
Pterostichus erebeus Casey, 1913: 134. Type locality: «Bayfield [Bayfield County], Wisconsin» (original citation). Lectotype (♀), designated by Lindroth (1975: 124), in USNM [# 47063]. Synonymy established by Lindroth (1966: 494).
Euferonia roanica Casey, 1920: 188. Type locality: «Roan Mountain, North Carolina» (original citation). Lectotype (♂), designated by Lindroth (1975: 124), in USNM [# 47059]. Synonymy established by Lindroth (1966: 494).
Euferonia coracina venator Casey, 1920: 189. Type locality: «Danville [Montour County], Pennsylvania» (original citation). Lectotype (♂), designated by Lindroth (1975: 124), in USNM [# 47060]. Synonymy established by Nicolay and Weiss (1934: 204), confirmed by Lindroth (1966: 494).
Euferonia coracina ludibunda Casey, 1920: 189. Type locality: «Buena Vista Spring, Franklin Co[unty], Pennsylvania» (original citation). Lectotype (♀), designated by Lindroth (1975: 124), in USNM [# 47061]. Synonymy established, under the name *Pterostichus coracinus erebeus* Casey, by Nicolay and Weiss (1934: 206), confirmed by Lindroth (1966: 494).
Euferonia lacustris Casey, 1924: 71. Type locality: «near Chicago, Illinois» (original citation). Lectotype (♂), designated by Lindroth (1975: 124), in USNM [# 47062]. Synonymy established by Nicolay and Weiss (1934: 204), confirmed by Lindroth (1966: 494).
Euferonia strigosula Casey, 1924: 72. Type locality: «Hagerstown [Washington County], Maryland» (original citation). Lectotype (♂), designated by Lindroth (1975: 124), in USNM [# 47056]. Synonymy established, under the name *Pterostichus coracinus roanicus* (Casey), by Nicolay and Weiss (1934: 205), confirmed by Lindroth (1966: 494).
Euferonia washingtonensis Nicolay and Weiss, 1934: 203. Type locality: «slopes of M[oun]t Madison [Coos County], New Hampshire» (original citation). Holotype (♂) in MCZ [# 22985]. Synonymy established by Lindroth (1966: 494).
Euferonia washingtonensis var. *rufitarsis* Nicolay and Weiss, 1934: 204. Type locality: «Black Mountain, North Carolina» (original citation). Holotype (♂) in MCZ [# 22986]. Synonymy established by Lindroth (1966: 494).

#### Distribution.

This species is found from Newfoundland (Lindroth 1955a: 86) to eastern South Dakota (French et al. 2004: 557), north to the James Bay area in Quebec (Larochelle 1975: 86), south along the Appalachian Mountains to eastern Tennessee (Nicolay and Weiss 1934: 206, as *Euferonia coracina roanica*) and northern Georgia (Fattig 1949: 24). The record from the Black Hills in southwestern South Dakota (Stockton 1954: 15) is doubtful.

#### Records.

**FRA**: PM **CAN**: LB, NB, NF, NS (CBI), ON, PE, QC **USA**: CT, DC, GA, IA, IL, IN, KY, MA, MD, ME, MI, MN, NC, NH, NJ, NY, OH, PA, RI, SC, SD, TN, VA, VT, WI, WV

### Pterostichus ingens

(Casey, 1918)

Euferonia ingens Casey, 1918: 367. Type locality: «Willow Spring [Cook County], Ill[inois]» (lectotype label). Lectotype (♀), designated by Lindroth (1975: 124), in USNM [# 47055].
Euferonia iripennis Nicolay and Weiss, 1934: 200 [secondary homonym of *Pterostichus iripennis* (Chaudoir, 1868)]. Type locality: «Camden [Kershaw County], South Carolina» (original citation). Holotype (♂) in MCZ [# 22984]. Synonymy established by Lindroth (1975: 124).

#### Distribution.

Until the status of this taxon is better defined, its distribution remains inadequately documented. It has been recorded from Indiana, Illinois, Iowa, Missouri (Casey 1918: 367), and South Carolina (Nicolay and Weiss 1934: 200, as *Euferonia iripennis*).

#### Records.

**USA**: IA, IL, IN, MO, SC

#### Note.

Lindroth (1966: 493) discussed the status of this taxon and at the time regarded it as an extreme, southern form of *Pterostichus stygicus*. Lindroth (1975: 124) later listed it as specifically distinct from *Pterostichus stygicus* and listed *Pterostichus iripennis* (Nicolay and Weiss) as synonym.

### Pterostichus novus

Straneo, 1944

Pterostichus novus Straneo, 1944: 127. Type locality: «Detroit [Wayne County, Michigan]» (original citation). Holotype probably in MSNM (collection Straneo).

#### Distribution.

This species is known from scattered localities from southwestern Quebec (Bousquet 1998: 105) to eastern North Dakota (Cass and Ransom Counties, CNC), north to southern Manitoba (Lindroth 1966: 495), south to southern South Dakota including the Black Hills (Kirk and Balsbaugh 1975: 23), northeastern Illinois (Purrington et al. 2002: 201), and northeastern New Jersey (FSCA). The record from Maryland (Erwin 1981b: 162), based on a single old specimen, needs confirmation.

#### Records.

**CAN**: MB, ON, QC **USA**: IL, MI, MN, ND, NJ, NY, OH, PA, SD, WI [MD]

### Pterostichus relictus

(Newman, 1838)

Feronia relicta Newman, 1838a: 387. Type locality: «northern states of America» (original citation, see page 388), restricted to «Allegheny, Penns[ylvania]» by Lindroth (1966: 495). Lectotype, designated by Lindroth (1966: 495), in BMNH.
Pterostichus protensus LeConte, 1863c: 12. Type locality: «Pennsylvania» (original citation). Lectotype (♂), designated by Bousquet (1999: 139), in MCZ [# 5633]. Synonymy established by LeConte (1873a: 306), confirmed by Lindroth (1966: 495).

#### Distribution.

The range of this species extends from northeastern New York (Notman 1928: 222) to southern Michigan (Eaton and Washtenaw Counties, UMAA), including southernmost Ontario (Lindroth 1966: 496), south to southwestern Indiana (Blatchley 1910: 93) and along the Appalachian Mountains to northern South Carolina (Ciegler 2000: 67) and central Alabama (Löding 1945: 16; Stockton 1954: Fig. 18). The records from eastern Kansas (Snow 1880: 78, as *Pterostichus protensus*; Knaus 1907: 233) are likely in error.

#### Records.

**CAN**: ON **USA**: AL, GA, IN, KY, MI, NC, NY, OH, PA, SC, TN, VA

### Pterostichus stygicus

(Say, 1823)

Feronia stygica Say, 1823a: 41. Type locality: «Rumney [Grafton County], N[ew] H[ampshire]» (neotype label). Neotype (♂), designated by Lindroth and Freitag (1969: 341), in MCZ [# 33045].
Omaseus bisigillatus T.W. Harris, 1828c: 123. Type locality: «Maine» (lectotype label). Lectotype (♀), designated by Bousquet (1999: 139), in MCZ [# 34569]. Synonymy established by Harris (1833: 567), confirmed by Bousquet (1999: 139).
Feronia picipes Newman, 1838a: 377. Type locality: «Trenton Falls [Oneida County, New York]» (lectotype label). Lectotype (♂), designated by Bousquet (1999: 139), in BMNH. Synonymy established by LeConte (1870: 399), confirmed by Bousquet (1999: 139). Note. LeConte (1873a: 306) noted that “the type of *Feronia picipes* Newm. in the British Museum belongs to this [*Pterostichus stygicus* Say] species, but the description does not agree, and seems to refer rather to *Pterostichus submarginatus*; there seems to have been some confusion of labels.”
Omaseus rugicollis Haldeman, 1843b: 300. Type locality: «western Penn[sylvania]» (original citation). One possible syntype, a ♂ labeled “[yellow disc] / P. stygicus (Say) Lec. rugicollis Hald. [handwritten],” in MCZ (collection LeConte). Synonymy established by LeConte (1853a: 246).
Pterostichus probus Casey, 1913: 133. Type locality: «Asheville [Buncombe County], North Carolina» (original citation). Lectotype (♀), designated by Lindroth (1975: 124), in USNM [# 47053]. Synonymy established by Nicolay and Weiss (1934: 201), confirmed by Lindroth (1966: 492).
Pterostichus vapidus Casey, 1913: 134. Type locality: «Adirondack M[oun]t[ain]s, New York» (original citation). Holotype [by monotypy] (♂) in USNM [# 47058]. Synonymy established by Lindroth (1966: 492).
Euferonia quadrifera Casey, 1918: 366. Type locality: «Ontario» (original citation). Lectotype (♂), designated by Lindroth (1975: 124), in USNM [# 47064]. Synonymy established by Nicolay and Weiss (1934: 201), confirmed by Lindroth (1966: 493).
Euferonia umbonata Casey, 1918: 368. Type locality: «Boston Neck [Washington County], Rhode Island» (original citation). Lectotype (♂), designated by Lindroth (1975: 124), in USNM [# 47057]. Synonymy established by Nicolay and Weiss (1934: 201), confirmed by Lindroth (1966: 493).
Euferonia subaequalis Casey, 1918: 368. Type locality: «Indiana» (original citation). Lectotype (♀), designated by Lindroth (1975: 124), in USNM [# 47054]. Synonymy established by Nicolay and Weiss (1934: 201), confirmed by Lindroth (1966: 493).

#### Distribution.

This species ranges from Maine (Dearborn and Donahue 1993: 5) to central Iowa (O’Rourke et al. 2008: 126), south to “Louisiana” (Nicolay and Weiss 1934: 201) and southwestern Georgia (Fattig 1949: 24), west to northeastern Kansas (Popenoe 1877: 23). The record from “Minnesota” (Bousquet and Larochelle 1993: 174) needs confirmation. Fossil remnants from a Plio-Pleistocene sequence have been unearthed in northwestern Greenland (Böcher 1995: 26).

#### Records.

**CAN**: ON, QC **USA**: CT, DC, DE, GA, IA, IL, IN, KS, KY, LA, MA, MD, ME, MI, MO, MS, NC, NH, NJ, NY, OH, PA, RI, SC, TN, VA, VT, WI, WV [MN]

### Subgenus Lenapterus

Berlov, 1996

Lenapterus O. Berlov, 1996: 11. Type species: *Lyperopherus vermiculosus* Ménétriés, 1851 by original designation. Etymology (original). From the name of the river Lena and the Greek *pteron* (wing, by extension elytron) or the first two syllables of the generic name *Pterostichus* [*q.v*.] [masculine].
Galapterus O. Berlov and Plutenko, 1997: 47. Type species: *Pterostichus galae* Farkač and Plutenko, 1996 by original designation. Synonymy established by Sundukov (2005: 803). Etymology. From the first name of Plutenko’s wife and the Greek *pteron* (wing, by extension elytron) or the first two syllables of the generic name *Pterostichus* [*q.v*.] [masculine].

#### Diversity.

Eleven species in the arctic, subarctic, and boreal regions of North America (four species) and Asia (ten species, one of them extending to European Russia). Three species are Holarctic.

#### Identification.

Lindroth (1966) reviewed the North American species. Budarin (1976) reviewed the world fauna, including two species (*Pterostichus mirus* Tschitschérine and *Pterostichus rugosus* Gebler) that belong, however, to the subgenus *Metallophilus* Chaudoir. Recently Sundukov (2005) revised all known species and provided a key for their identification.

#### Taxonomic Note.

This subgenus has been known for sometimes under the name *Lyperopherus* Motschulsky (e.g., Ball 1960b: 127; Lindroth 1966: 525).

### Pterostichus agonus

Horn, 1880

Pterostichus agonus G.H. Horn, 1880a: 140. Type locality: «Yukon River, Alaska» (original citation). Holotype [by monotypy] in MCZ [# 34414].
Feronia tschuchtschorum J.R. Sahlberg, 1885a: 12. Type locality: «Jinretlen; Rirajtinop» (original citation), restricted to «Rirajtinop n[ea]r Pitlekaj, Chuk[o]tchi Penins[ula] [Russia]» by Lindroth (1966: 528). Syntype(s) in NRSS (Lindroth 1966: 528). Synonymy established by Lindroth (1966: 528).
Pterostichus corallipes Jedlička, 1937: 44. Type locality: «Mandschurei: Chingan-Mont Buchalu» (original citation). Holotype (♂) in NMP. Synonymy established by Bousquet (1999: 143).
Pterostichus agonus averenskii O. Berlov and E. Berlov, 1997: 50. Type locality: «upper part of Kele River at the junction with Kakchin River (1200 m), Verkhoyanskiy Khrebet, Yakutia [= Sakha Republic, Siberia, Russia]» (original citation). Holotype (♀) in Berlov’s collection. Synonymy established by Sundulov (2005: 810).

#### Distribution.

This Holarctic species is found in the arctic regions of eastern Siberia and northeastern China (Bousquet 2003d: 497) and on this continent from the Seward Peninsula in Alaska to northwestern Northwest Territories (Lindroth 1966: 528).

#### Records.

**CAN**: NT, YT **USA**: AK – **Holarctic**

### Pterostichus costatus

(Ménétriés, 1851)

Lyperopherus costatus Ménétriés, 1851: 49. Type locality: «Taimyrflusse unter 73¼ ° n. Br. [= Taimura River, Siberia, Russia]» (original citation). Lectotype (♀), designated by Lindroth (1966: 529), in ZMH.

#### Distribution.

This Holarctic species ranges from the Yenisei River in Russia (Lindroth 1966: 530) to the Melville Peninsula in northern Nunavut, Canada [see Nielsen et al. 1987: Fig. 18c].

#### Records.

**CAN**: NT, NU, YT **USA**: AK – **Holarctic**

### Pterostichus punctatissimus

(Randall, 1838)

Feronia punctatissima Randall, 1838a: 3. Type locality: «near the summit of the Blue Mountains [Franklin County, Maine]» (original citation). Holotype [by monotypy] lost.

#### Distribution.

This species is found from Newfoundland (Lindroth 1955a: 87) to southeastern Yukon Territory (Lindroth 1966: 527), including northeastern British Columbia (CNC), north to northwestern Northwest Territories, south to central Alberta (Lindroth 1966: 527), northeastern Minnesota (Cook County, CNC), some mountains in New York (Notman 1928: 223), and “Massachusetts” (Wickham 1895a: 186). The record from Cuyahoga County in northern Ohio (Bubna 1902: 193) needs confirmation. Fossil remnants of this species, dated between about 11,700 and 18,100 years B.P., have been unearthed in northeastern Wisconsin, central Minnesota (Schwert 1992: 77), and central and southeastern Iowa (Schwert 1992: 77; Baker et al. 1986: 96).

#### Records.

**CAN**: AB, BC, LB, MB, NB, NF, NS (CBI), NT, ON, QC, SK, YT **USA**: MA, ME, MI, MN, NH, NY, VT [OH]

**Figure 28.**
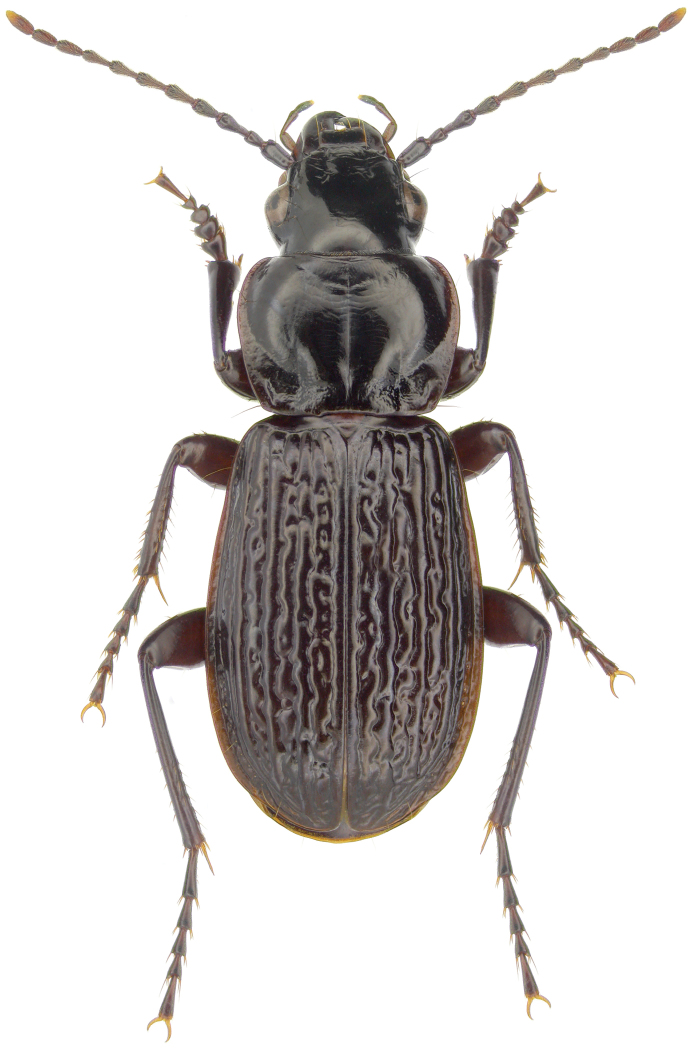
*Pterostichus punctatissimus* Randall. This species is one of the most attractive *Pterostichus* in North America. The sculpture of the elytra is irregular giving the impression to the naked eye that the striae bear large punctures. For that reason Randall named the species *punctatissimus* meaning the most punctured. Although not as common as *Pterostichus adstrictus*, this species is also a characteristic element of boreal forests in the Nearctic Region.

### Pterostichus vermiculosus

(Ménétriés, 1851)

Lyperopherus vermiculosus Ménétriés, 1851: 48. Type locality: «Indega-Bucht [= Indiga Bay, on the Barents Sea coast] des Europäisch-Russischen Eismeeres unter 67°40’N. Br.» (original citation). Lectotype (♀), designated by Lindroth (1966: 528), in ZMH.
Lyperopherus intricatus Ménétriés, 1851: 49. Type locality: «Boganida [River, Siberia, Russia]» (original citation). Lectotype (♂), designated by Lindroth (1966: 528), in ZMH. Synonymy established by Lindroth (1966: 528).
Lyperopherus innuitorum Brown, 1950a: 231. Type locality: «Kidluit Bay, Richards Island, N[orth]W[est]T[erritories]» (original citation). Holotype (♂) in CNC [# 5775]. Synonymy established by Lindroth (1954b: 131).

#### Distribution.

This Holarctic species is found in the arctic regions from European Russia to the Melville Peninsula and Southampton Island in eastern Nunavut, Canada (Lindroth 1966: 528). Fossil remnants of this species, dated between about 16,700 and 18,100 years B.P., have been unearthed in southeastern Iowa (Baker et al. 1986: 96); others from a Plio-Pleistocene sequence have been found in northwestern Greenland (Böcher 1995: 28).

#### Records.

**CAN**: NT, NU, YT **USA**: AK – **Holarctic**

### Subgenus Metallophilus

Chaudoir, 1838

Metallophilus Chaudoir, 1838: 9. Type species: *Feronia interrupta* Dejean, 1828 by original designation. Etymology (original). From the Greek *metallon* (mine) and *philos* (beloved) [masculine].
Lyperopherus Motschulsky, 1844: 156. Type species: *Poecilus rugosus* Gebler, 1823 designated by Lindroth (1966: 525). Synonymy established by Bousquet (1999: 144). Etymology. From the Greek *lyperos* (painful, by extension sad) and *phero* (to bear, carry) [masculine].
Tundraphilus O. Berlov, 1996: 12. Type species: *Feronia sublaevis* Sahlberg, 1880 by original designation. Synonymy established by Bousquet (1999: 144). Etymology. From the English name tundra (treeless ecosystem of the arctic regions) and the Greek *philos* (beloved) [masculine].

#### Diversity.

Eight species in North America (one Holarctic species) and Palaearctic Asia (eight species, one extending to European Russia).

#### Taxonomic Note.

According to Bousquet (1999: 145), this group is closely related and possibly paraphyletic in regard to *Myosodus* Fischer von Waldheim, a subgenus of 22 species centered in the Caucasus Mountains. Brinev and Shilenkov (2001) retained *Tundraphilus* as a valid subgenus, listing *Pterostichus sublaevis* Sahlberg, *Pterostichus orion* Tschitschérine, *Pterostichus pfitzenmayeri* Poppius, and *Pterostichus kamtschaticus* Motschulsky in it.

### Pterostichus sublaevis

(Sahlberg, 1880)

Feronia sublaevis J.R. Sahlberg, 1880: 24. Type locality: «Tolstoinos, in littore arenoso fluminis Jenissej [= Tolstyy Nos, Taymyr Autonomous Okrug, Russia]» (original citation). Holotype [by monotypy] (♂) in NRSS.
Pterostichus sublaevis var. *unicoloripes* Poppius, 1910: 335. Type locality: «Chara-Ulach-Gebirgen [= Kharaulakhskiy Khrebet] an der Lena-Mündung, unweit der Insel Tit-ary [northern Siberia]» (original citation). Syntype(s) location unknown (apparently not at ZMH according to Silfverberg’s (1987) list of insect types). Synonymy established (as aberration) by Csiki (1930: 678).
Pterostichus rufofemoralis Van Dyke, 1926a: 113. Type locality: «Iron Creek, sixty miles north of Nome, Alaska» (original citation). Holotype (♀) in CAS [# 1853]. Synonymy established by Lindroth (1966: 530).

#### Distribution.

This Holarctic tundra species is found from the northern part of European Russia (Brinev and Shilenkov 2001) to northwestern Northwest Territories (Lindroth 1966: 531). Fossil remnants of this species, dated between about 16,700 and 18,100 years B.P., have been unearthed in southeastern Iowa (Baker et al. 1986: 96).

#### Records.

**CAN**: NT, YT **USA**: AK – **Holarctic**

### Subgenus Abacidus

LeConte, 1863

Albux J.E. LeConte, 1849: 26 [*nomen oblitum*, see Bousquet (2008b: 328)]. Type species: *Feronia striata* Dejean, 1828 (= *Pterostichus sculptus* LeConte, 1853) by monotypy. Etymology. Unknown. Note. Hardy et al. (1986: 472) argued that the paper in which this name was proposed was written by John Eatton LeConte and not his son, John Lawrence LeConte.
Abacidus LeConte, 1863b: 9 [*nomen protectum*]. Type species: *Feronia fallax* Dejean, 1828 designated by Casey (1913: 136). Etymology. From the generic name *Abax* [*q.v*.] and the Latin suffix -*idus* (having the nature of), alluding to the superficial resemblance of the adults to those of *Abax* in which Dejean originally placed the two species known to LeConte [masculine].
Peristethus LeConte, 1863b: 9 [junior homonym of *Peristethus* Kaup, 1858]. Type species: *Feronia permunda* Say, 1830 by monotypy. Synonymy established by Casey (1913: 136). Etymology. From the Greek *peri* (around, near) and *stethos* (breast, chest) [masculine].

#### Diversity.

Five species in the temperate regions of eastern North America.

#### Identification.

Lindroth (1966: 535) provided a key to all species, including also *Pterostichus obesulus* LeConte, a species that actually belongs to the subgenus *Gastrosticta*. Sadek (1982) wrote a M.Sc. thesis on the taxonomy of this group.

#### Taxonomic Note.

Lindroth (1966: 534), followed recently by Lorenz (2005: 289), treated this group as a distinct genus. Structural characters of the adults and larvae clearly suggest that members of *Abacidus* are congeneric with those of *Pterostichus*. In fact, the group is probably closely related to *Metallophilus* and *Myosodus* (see Bousquet 1999: 148).

### [fallax group]

### Pterostichus fallax

(Dejean, 1828)

Feronia fallax Dejean, 1828: 391. Type locality: «Amérique septentrionale» (original citation), restricted to «Faison, Duplin Co[unty], North Carolina» by Bousquet (1999: 149). Syntype(s) probably in MHNP.

#### Distribution.

This species ranges from east-central Iowa to North Carolina, including southern Michigan (Sadek 1982: 23), south to northern Florida (Peck and Thomas 1998: 20) and “Texas” (Schaupp 1882c: 41).

#### Records.

**USA**: AL, AR, FL, GA, IA, IL, MI, MO, MS, NC, SC, TN, TX

### [hamiltoni group]

### Pterostichus hamiltoni

Horn, 1880

Pterostichus hamiltoni G.H. Horn, 1880a: 139. Type locality: «near Allegheny City [Allegheny County], Pennsylvania» (original citation). Lectotype (♂), designated by Bousquet (1999: 149), in MCZ [# 8234]. Etymology. The specific name honors John Hamilton [1827-1897], a physician by profession who had an interest in entomology and ornithology.

#### Distribution.

This species is known from scattered localities from Ohio (Purrington et al. 1989: 107; Usis and MacLean 1998: 67) and southwestern Pennsylvania (Horn 1880a: 139; Allegheny, Fayette, and Westmoreland Counties, CMNH) south to northern Georgia (Fattig 1949: 25).

#### Records.

**USA**: GA, MD, OH, PA, WV

#### Note.

In the *Zoological Record* for the year 1880, this species is registered (page 23) as “*Pterostichus (Peristethus) maximiliani*.”

### [permundus group]

### Pterostichus atratus

(Newman, 1838)

Feronia atrata Newman, 1838a: 386. Type locality: «northern states of America» (original citation, see page 388), restricted to «Cleveland [Cuyahoga County], Ohio» by Lindroth (1966: 536). Lectotype (♂), designated by Bousquet (1999: 149), in BMNH.

#### Distribution.

This species is found from eastern Maryland (Queen Annes County, Foster F. Purrington pers. comm. 2009) to southern Wisconsin (Sadek 1982: 34), including Pelee Island in southernmost Ontario (Lindroth 1966: 537), south to southern Louisiana (East Baton Rouge Parish, CNC) and the Florida Panhandle (Peck and Thomas 1998: 19).

#### Records.

**CAN**: ON **USA**: AL, FL, GA, IL, IN, KY, LA, MD, MO, MS, NC, OH, PA, SC, TN, VA, WI, WV

### Pterostichus permundus

(Say, 1830)

Feronia permunda Say, 1830b: (6) [3]. Type locality: «Wabash Vall[ey], Richland & Lawrence Co[unties], Ill[inois]» (neotype label). Neotype (♂), designated by Lindroth and Freitag (1969: 341), in MCZ [# 33042]. Note. «Indiana» was the area originally cited by Say (1830b: (6) [3]).
Abacidus planifer Casey, 1913: 136. Type locality: «Indiana» (original citation). Holotype [by monotypy] (♀) in USNM [# 47081]. Synonymy established by Lindroth (1966: 535).

#### Distribution.

This species occurs from central New York (Hajek et al. 2007: 880) to southeastern South Dakota (Kirk and Balsbaugh 1975: 23; Ellsbury et al. 1998: 621), north to southernmost Ontario (Bousquet 1987a: 125) and northeastern Michigan, south to northeastern Texas and northeastern Florida (Sadek 1982: 25, Fig. 16). The records from Quebec (Larochelle 1975: 31) and “New Mexico” (Bousquet and Larochelle 1993: 174) are based on mislabeled specimens or are in error.

#### Records.

**CAN**: ON **USA**: AL, AR, FL, GA, IA, IL, IN, KS, LA, MI, MN, MO, MS, NE, NY, OH, OK, PA, SC, SD, TN, TX, WI

### Pterostichus sculptus

LeConte, 1853

Feronia striata Dejean, 1828: 390 [secondary homonym of *Pterostichus striatus* (Rossi, 1792)]. Type locality: «Amérique septentrionale» (original citation), restricted to «Highlands, Macon Co[unty], North Carolina» by Bousquet (1999: 149). Syntype(s) probably in MHNP.
Pterostichus sculptus LeConte, 1853a: 248. Replacement name for *Pterostichus striatus* (Dejean, 1828).

#### Distribution.

The range of this species extends from “New York” (Wickham 1895a: 186) to Iowa (Wickham 1888: 82; Jaques and Redlinger 1946: 297), south to eastern Arkansas (Sadek 1982: 31), Alabama (Löding 1945: 16), Georgia (Fattig 1949: 25; House and All 1981: 195; CMNH, MCZ), and South Carolina (Ciegler 2000: 68).

#### Records.

**USA**: AL, AR, GA, IA, MD, NC, NJ, NY, OH, PA, SC, TN

### Subgenus Orsonjohnsonus

Hatch, 1933

Orsonjohnsonus Hatch, 1933c: 119. Type species: *Pterostichus johnsoni* Ulke, 1889 by original designation. Etymology. From the name and surname of Orson Bennett Johnson (see *Scaphinotus johnsoni*) [masculine].

#### Diversity.

One species in the Pacific Northwest.

#### Identification.

The species was included in Lindroth’s (1966: 473) monograph.

### Pterostichus johnsoni

Ulke, 1889

Pterostichus johnsoni Ulke, 1889: 59. Type locality: «Oregon» (original citation), herein restricted to Camp Creek, 3.5 miles southeast of Rhododendron, Clackamas County (CNC). Lectotype (♂), designated by Bousquet (1999: 151), in CMNH.

#### Distribution.

This species is known from northern Washington to central Oregon (Foltz 2011).

#### Records.

**USA**: OR, WA

### Subgenus Lamenius

Bousquet, 1999

Lamenius Bousquet, 1999: 151. Type species: *Feronia caudicalis* Say, 1823 by original designation. Etymology (original). Anagram of the generic name *Melanius* [*q.v*.] [masculine].

#### Diversity.

One species in the boreal and temperate regions of North America.

#### Identification.

The species was included in Lindroth’s (1966: 500) monograph.

#### Taxonomic Note.

This species has been included for a long time with the species of *Melanius* Bonelli.

### Pterostichus caudicalis

(Say, 1823)

Feronia caudicalis Say, 1823a: 56. Type locality: «Arlington [Middlesex County], Mass[achusetts]» (neotype label). Neotype (♂), designated by Lindroth and Freitag (1969: 343), in MCZ [# 33031].
Pterostichus agrestis Bland, 1865: 381. Type locality: «Colorado Territory» (original citation). Lectotype (♂), designated by Bousquet (1999: 153), in ANSP [# 2702]. Synonymy established by LeConte (1873a: 308), confirmed by Bousquet (1999: 153).

#### Distribution.

This species is found from Newfoundland (Lindroth 1955a: 89-90) to the Okanagan Valley in south-central British Columbia, north to Fort Smith in southern Northwest Territories (Lindroth 1966: 500), south to northeastern Nevada (Elko County, CNC), central Colorado (Elias 1987: 632; Wickham 1902: 236), Missouri (Summers 1873: 134), and northern Georgia (Fattig 1949: 27).

#### Records.

**CAN**: AB, BC, MB, NF, NT, ON, QC, SK **USA**: CO, CT, DC, GA, IA, ID, IL, IN, KS, MA, MD, ME, MI, MN, MO, MT, NC, ND, NE, NH, NJ, NV, NY, OH, OR, PA, RI, SD, UT, VA, VT, WA, WI, WV, WY

### Subgenus Eosteropus

Tschitschérine, 1902

Eosteropus Tschitschérine, 1902a: 500. Type species: *Platysma creperum* Tschitschérine, 1902 designated by Bousquet (1984b: 1612). Etymology. From the Greek *eos* (east) and the generic name *Steropus* [masculine].
Refonia Casey, 1918: 323. Type species: *Feronia moesta* Say, 1823 by original designation. Synonymy established by Bousquet (1984b: 1612). Etymology. Anagram of the generic name *Feronia* [*q.v*.] [feminine].

#### Diversity.

Forty-two species in the arctic, subarctic, boreal, and temperate regions of North America (three species), Palaearctic Asia (34 species), and Europe (five species).

#### Identification.

Lindroth (1966: 474-475, 406-497, as *circulosus* and *moestus* groups) treated all three North American species in his review of the Canadian *Pterostichus*.

### [circulosus group]

### Pterostichus circulosus

Lindroth, 1966

Pterostichus circulosus Lindroth, 1966: 474. Type locality: «Circle, Alaska» (original citation). Holotype (♂) in MCZ [# 33503].

#### Distribution.

This species is known from a few localities in Yukon Territory (Ball and Currie 1997: 451; CNC) and Alaska (Lindroth 1966: 475). The species is also cited, with a question mark, from the Magadan region in eastern Siberia (Budarin 1985: 15).

#### Records.

**CAN**: YT **USA**: AK

### [moestus group]

### Pterostichus moestus

(Say, 1823)

Feronia moesta Say, 1823a: 42. Type locality: «Asheville [Buncombe County], N[orth] C[arolina]» (neotype label). Neotype (♂), designated by Lindroth and Freitag (1969: 341), in MCZ [# 33044].
Evarthrus perseverus Motschulsky, 1866: 260. Type locality: «Am[érique] bor[éale]» (original citation). Lectotype (♀), designated by Bousquet (1984a: 2), in ZMMU. Synonymy established by Bousquet (1984a: 2).

#### Distribution.

This species occurs from the neighborhood of New York City (Schaupp 1883b: 31; Ulster County, CMNH) to northeastern Ohio (Lee 1994: 60), south along the Appalachian Mountains to northern Georgia (Fattig 1949: 25; CNC) and northwestern South Carolina (Ciegler 2000: 67). The record from “Indiana” (Schrock 1985: 354) needs confirmation. The old records from Ontario and Quebec (see Lindroth 1966: 497) and Lake Superior (LeConte 1853a: 247) were based on misidentified or mislabeled specimens.

#### Records.

**USA**: DC, DE, GA, KY, MD, NC, NJ, NY, OH, PA, SC, VA, WV [IN]

### Pterostichus superciliosus

(Say, 1823)

Feronia superciliosa Say, 1823b: 144. «P[ennsylvani]a» (neotype label). Neotype (♂), designated by Lindroth and Freitag (1969: 341), in MCZ [# 34659].

#### Distribution.

This species is known from scattered localities from Staten Island, New York (Smith 1910: 205) to northwestern Pennsylvania (Forest County, CMNH), south along the Appalachian Mountains to northeastern Georgia (Fattig 1949: 25). The record from “Michigan” (Wickham 1895a: 185) is in error.

#### Records.

**USA**: GA, MD, NC, NJ, NY, PA, VA, WV

### Subgenus Monoferonia

Casey, 1918

Monoferonia Casey, 1918: 322, 363. Type species: *Evarthrus mancus* LeConte, 1853 by original designation. Etymology. From the Greek *monos* (one) and the generic name *Feronia* [*q.v*.], probably alluding to the presence of a single discal setigerous puncture on the third elytral interval in these *Feronia* (= *Pterostichus*) species [feminine].

#### Diversity.

Four species in the Appalachian region of eastern North America.

#### Identification.

Darlington (1932) reviewed the species and provided a key for their identification.

### Pterostichus carolinus carolinus

Darlington, 1932

Pterostichus carolinus carolinus Darlington, 1932: 162. Type locality: «Little Switzerland (about 3,400 feet) [Mitchell County], Black Mountains, N[orth] C[arolina]» (original citation). Holotype (♂) in MCZ [# 16437].

#### Distribution.

This subspecies is known from the Black Mountains in western North Carolina (Darlington 1932: 162; Barr 1969: 72) and from northeastern Georgia near the border with South Carolina (Fattig 1949: 21). The record from “Tennessee” (Bousquet and Larochelle 1993: 171) needs confirmation.

#### Records.

**USA**: GA, NC [TN]

### Pterostichus carolinus fumorum

Darlington, 1932

Pterostichus carolinus fumorum Darlington, 1932: 163. Type locality: «between Newfound Gap and Clingman’s Dome (5,000-6,642 feet), Smoky Mountains, along the North Carolina-Tennessee state line» (original citation). Holotype (♂) in MCZ [# 16438].

#### Distribution.

This subspecies is endemic to the Great Smoky Mountains along the North Carolina-Tennessee border (Darlington 1932: 163).

#### Records.

**USA**: NC, TN

### Pterostichus diligendus

(Chaudoir, 1868)

Feronia diligenda Chaudoir, 1868b: 334. Type locality: Amérique septentrionale (inferred from title of the paper), restricted to «M[oun]t Holyoke [Hampshire County], Mass[achusetts]» by Lindroth (1966: 469). Holotype [by monotypy] (♂) in MHNP (Lindroth 1966: 469).
Pterostichus osculans Casey, 1884b: 2. Type locality not stated. Lectotype (♀), designated by Lindroth (1975: 123), in USNM [# 47115]. Synonymy established by Horn (1885b: 108), confirmed by Lindroth (1966: 469).
Pterostichus apalachius G.H. Horn, 1892c: 41. Type locality: «Pen[nsylvania]» (syntype labels). Six syntypes in MCZ (collection LeConte). Synonymy established, under the name *Pterostichus osculans* Casey, by Casey (1913: 133). Note. This name was proposed for *Pterostichus diligendus* (Chaudoir, 1868) *sensu* LeConte (1873a: 305) and credited to LeConte by Horn (1892c). LeConte’s collection includes six syntypes, all labeled “Pen.”

#### Distribution.

This species is found along the Appalachian region from southern Quebec (Larochelle 1975: 101) to Kentucky (Powell County, CNC) and northeastern Georgia (Fattig 1949: 23). The records from northwestern Indiana (Wolcott and Montgomery 1933: 126, as *Monoferonia osculans*) and “Illinois” (Schaupp 1882c: 41) need confirmation.

#### Records.

**CAN**: QC **USA**: CT, DC, GA, KY, MA, MD, ME, NC, NH, NJ, NY, OH, PA, VA, VT, WV [IL, IN]

### Pterostichus mancus

(LeConte, 1853)

Evarthrus mancus LeConte, 1853a: 234. Type locality: «Nakutshi Valley, Habersham County, Georgia» (original citation). Lectotype [as type] (♂), designated by Darlington (1932: 161), in MCZ [# 16439].
Pterostichus mancus plethorus Darlington, 1932: 161. Type locality: «M[oun]t Mitchell (5,000-6,711 feet), Black Mountains, North Carolina» (original citation). Holotype (♂) in MCZ [# 16436]. Synonymy established by Bousquet (1999: 122).

#### Distribution.

This species is known from the Roan, Grandfather, and Black Mountains in North Carolina (Barr 1969: 72) and northern Georgia (Fattig 1949: 23).

#### Records.

**USA**: GA, NC

### Pterostichus primus

Darlington, 1932

Pterostichus primus Darlington, 1932: 159. Type locality: «Deep Creek (Bryson City) (near 2,000 feet) [Swain County], N[orth] C[arolina]» (original citation). Holotype (♂) in MCZ [# 16435].

#### Distribution.

This species is apparently endemic to the Great Smoky and Unicoi Mountains along the North Carolina - Tennessee border (Barr 1969: 80).

#### Records.

**USA**: NC, TN

### Subgenus Cylindrocharis

Casey, 1918

Cylindrocharis Casey, 1918: 326. Type species: *Feronia rostrata* Newman *sensu* Casey, 1918 (= *Pterostichus acutipes acutipes* Barr, 1971) by original designation. Etymology. From the Greek *cylindros* (roller, cylinder) and *charis* (grace, beauty), probably alluding to the more or less cylindrical appearance of adults of these nice carabids [feminine].

#### Diversity.

Three species in the Appalachian region.

#### Identification.

Barr (1971a) revised the species.

### Pterostichus acutipes acutipes

Barr, 1971

Pterostichus acutipes acutipes Barr, 1971a: 7. Type locality: «Round Mountain (2500 feet), easternmost Buncombe County, North Carolina» (original citation). Holotype (♂) in AMNH [# 1341].

#### Distribution.

This subspecies occurs in the Appalachians from the Black Mountains in North Carolina to northern Georgia (Barr 1971a: 9). The record from “Kentucky” (Bousquet and Larochelle 1993: 176) refers to *Pterostichus acutipes kentuckensis*.

#### Records.

**USA**: GA, NC, SC, TN

### Pterostichus acutipes kentuckensis

Barr, 1971

Pterostichus acutipes kentuckensis Barr, 1971a: 9. Type locality: «Jessamine Creek gorge, 3 miles south of Wilmore, Jessamine County, Kentucky» (original citation). Holotype (♂) in AMNH [# 1342].

#### Distribution.

This subspecies is known from a few localities in central Kentucky (Barr 1971a: 11) and southwestern Virginia (Hoffman 1998: 37).

#### Records.

**USA**: KY, VA

### Pterostichus hypogeus

Barr, 1971

Pterostichus hypogeus Barr, 1971a: 11. Type locality: «west side of Big Butt (mountain) in the upper Bearpen Creek basin (4800 feet), Coweeta Hydrologic Laboratory, Macon County, North Carolina» (original citation). Holotype (♂) in AMNH [# 1343].

#### Distribution.

This species is known from the Nantahalas to the Snowbird Mountains in Mason and Graham Counties, North Carolina (Barr 1971a: 12).

#### Records.

**USA**: NC

### Pterostichus rostratus

(Newman, 1838)

Feronia rostrata Newman, 1838a: 387. Type locality: «Trenton Falls [Oneida County, New York]» (lectotype label). Lectotype (♂), designated by Lindroth (1966: 457), in BMNH.
Stereocerus grandiceps LeConte, 1846b: 336. Type locality: «NovEboraci [= New York]» (original citation). One syntype in MCZ [# 5615] (see LeConte 1853a: 236). Synonymy established by LeConte (1863b: 8), confirmed by Lindroth (1966: 457).
Cylindrocharis sulcatula Casey, 1918: 327. Type locality: «probably Indiana» (original citation). Lectotype (♂), designated by Lindroth (1975: 122), in USNM [# 47043]. Synonymy established by Van Dyke (1926a: 75), confirmed by Lindroth (1966: 457).
Cylindrocharis piceata Casey, 1918: 327. Type locality: «New York» (original citation for the lectotype). Lectotype (♂), designated by Lindroth (1975: 122), in USNM [# 47042]. Synonymy established by Van Dyke (1926a: 75), confirmed by Lindroth (1966: 457).

#### Distribution.

This species ranges from New Brunswick to southern Ontario (Lindroth 1966: 458), south along the Appalachian Mountains to northeastern Alabama (Löding 1945: 15) and northern Georgia (Fattig 1949: 21), including the higher mountains of Tennessee and North Carolina (Barr 1971a: 7). The records from “Iowa” (Bousquet and Larochelle 1993: 176) and southwestern Alabama (Löding 1945: 15) are likely in error.

#### Records.

**CAN**: NB, ON, QC **USA**: AL, CT, DC, GA, KY, MA, MD, ME, NC, NH, NJ, NY, PA, RI, SC, TN, VA, VT, WV

### Subgenus Leptoferonia

Casey, 1918

Leptoferonia Casey, 1918: 321, 336. Type species: *Feronia angusta* Dejean, 1828 by original designation. Etymology. From the Greek *leptos* (thin, slender) and the generic name *Feronia* [*q.v*.], alluding to the slender shape of adults of these *Feronia* (= *Pterostichus*) species [feminine].

#### Diversity.

Twenty-six species in western North America.

#### Identification.

Hacker (1968) revised the species. Since then, four new species have been described by Will (2007), one species (*Pterostichus rothi*) has been transferred from *Anilloferonia* to this subgenus (Will 2007), and one subspecies (*Pterostichus stapedius yosemitensis*) has been raised to species level by Bousquet (1999: 164).

### [fenyesi group]

### Pterostichus cochlearis

Hacker, 1968

Pterostichus cochlearis Hacker, 1968: 19. Type locality: «Weitchpec-Orick Road (1400 ft.), Humboldt County, Calif[ornia]» (original citation). Holotype (♂) in USNM [# 69601].

#### Distribution.

This species is restricted to the Coast Ranges in southwestern Oregon and northwestern California [see Hacker 1968: Fig. 35].

#### Records.

**USA**: CA, OR

### Pterostichus fenyesi fenderi

Hacker, 1968

Pterostichus fenyesi fenderi Hacker, 1968: 18. Type locality: «2 miles northwest of Petrolia (200 ft.), Humboldt County, Calif[ornia]» (original citation). Holotype (♂) in USNM [# 69600].

#### Distribution.

This subspecies is found from the Eel River in Humboldt County southwards to Mendocino County, California [see Hacker 1968: Fig. 35].

#### Records.

**USA**: CA

### Pterostichus fenyesi fenyesi

Csiki, 1930

Pterostichus ovicollis Schaeffer, 1910: 393 [secondary homonym of *Pterostichus ovicollis* (Reitter, 1884)]. Type locality: «California» (original citation), herein restricted to 9 miles northwest of Blocksburg, Humboldt County (see Hacker 1968: 18). Holotype (♀) in USNM [# 42498].
Pterostichus fényesi Csiki, 1930: 582. Replacement name for *Pterostichus ovicollis* Schaeffer, 1910.

#### Distribution.

This subspecies is known only from Humboldt County, north of Eel River, in northwestern California [see Hacker 1968: Fig. 35] and from southwestern Oregon (Curry County, James R. LaBonte pers. comm. 2009).

#### Records.

**USA**: CA, OR

### [fuchsi group]

### Pterostichus angustus

(Dejean, 1828)

Feronia angusta Dejean, 1828: 328. Type locality: «Californie» (original citation), herein restricted to Montgomery Woods State Park, Mendocino County (see Hacker 1968: 29). Holotype [by monotypy] (♂) probably in MHNP.
Pterostichus linearis LeConte, 1853a: 239. Type locality: «San Francisco [California]» (original citation). Holotype [by monotypy] (♀) in MCZ [# 5606]. Synonymy established by LeConte (1857c: 8), confirmed by Hacker (1968: 28).
Pterostichus crucialis Casey, 1913: 126. Type locality: «S[an]ta Cruz M[oun]t[ain]s, California» (original citation). Lectotype (♂), designated by Bousquet (1999: 165), in USNM [# 47047]. Synonymy established by Van Dyke (1926a: 75), confirmed by Hacker (1968: 28).

#### Distribution.

This species is found in western California from near Mount Shasta in Siskiyou County south to the Monterey area in Monterey County [see Hacker 1968: Fig. 38].

#### Records.

**USA**: CA

### Pterostichus enyo

Will, 2007

Pterostichus enyo Will, 2007: 55. Type locality: «12 mi[les] W[est] Weaverville, 2.3-6.8 mi[les] N[orth] on FR 421 (1700-2000 ft.), Trinity Co[unty], California» (original citation). Holotype (♀) in AMNH.

#### Distribution.

This species is known only from the holotype collected in northwestern California.

#### Records.

**USA**: CA

### Pterostichus fuchsi

Schaeffer, 1910

Pterostichus fuchsi Schaeffer, 1910: 392. Type locality: «California» (original citation), herein restricted to 8 miles west of Willits, Mendocino County (see Hacker 1968: 22). Holotype (♀) in USNM [# 42501].
Leptoferonia fugax Casey, 1918: 337 [secondary homonym of *Pterostichus fugax* (Morawitz, 1862)]. Type locality: «California» (original citation). Lectotype (♂), designated by Bousquet (1999: 170), in USNM [# 47050]. Synonymy established by Van Dyke (1926a: 75), confirmed by Hacker (1968: 21).
Pterostichus fugiens Csiki, 1930: 582. Replacement name for *Pterostichus fugax* (Casey, 1918).

#### Distribution.

This species is restricted to the Coast Ranges in Mendocino and Sonoma Counties, California [see Hacker 1968: Fig. 36].

#### Records.

**USA**: CA

### Pterostichus humilis

Casey, 1913

Pterostichus humilis Casey, 1913: 128. Type locality: «Hoopa Valley of the Trinity River, Humboldt Co[unty], California» (original citation). Lectotype (♀), designated by Bousquet (1999: 171), in USNM [# 47051].
Leptoferonia larvalis Casey, 1918: 337. Type locality: «Hoopa Valley, Humboldt Co[unty], California» (original citation). Lectotype (♀), designated by Bousquet (1999: 171), in USNM [# 47052]. Synonymy established by Hacker (1968: 27).

#### Distribution.

This species ranges along the Coast Ranges from southern Oregon southwards to Humboldt County in northern California [see Hacker 1968: Fig. 37].

#### Records.

**USA**: CA, OR

### Pterostichus lobatus

Hacker, 1968

Pterostichus lobatus Hacker, 1968: 23. Type locality: «3 miles south of Rockport (800 ft.), Mendocino County, Calif[ornia]» (original citation). Holotype (♂) in USNM [# 69603].

#### Distribution.

This species is found along the Pacific Coast of California south of the Eel River in Humboldt County to the mouth of the Navarro River in Mendocino County [see Hacker 1968: Fig. 37].

#### Records.

**USA**: CA

### Pterostichus marinensis

Hacker, 1968

Pterostichus marinensis Hacker, 1968: 22. Type locality: «2 miles northwest of Pan Toll Camp, west slope of Mount Tamalpais (1700 ft.), Marin County, Calif[ornia]» (original citation). Holotype (♂) in USNM [# 69602].

#### Distribution.

This species is, as far as known, restricted to a small area along the Pacific Coast from the Tomales Bay to the north side of the Pacific entrance of the San Francisco Bay [see Hacker 1968: Fig. 36].

#### Records.

**USA**: CA

### Pterostichus mattolensis

Hacker, 1968

Pterostichus mattolensis Hacker, 1968: 24. Type locality: «4 miles south of Honeydew (1500 ft.), Humboldt County, Calif[ornia]» (original citation). Holotype (♂) in USNM [# 69604].

#### Distribution.

This species is restricted to the Coast Ranges in southern Humboldt County and northern Mendocino County, California [see Hacker 1968: Fig. 37].

#### Records.

**USA**: CA

### Pterostichus trinitensis

Hacker, 1968

Pterostichus trinitensis Hacker, 1968: 26. Type locality: «8 miles northeast of Zenia (3500 ft.), Trinity County, Calif[ornia]» (original citation). Holotype (♂) in USNM [# 69605].

#### Distribution.

This species is found in southwestern Humboldt, southwestern Trinity, and northern Mendocino Counties in the Coast Ranges, northwestern California [see Hacker 1968: Fig. 37].

#### Records.

**USA**: CA

### [hatchi group]

### Pterostichus blodgettensis

Will, 2007

Pterostichus blodgettensis Will, 2007: 50. Type locality: «Bacon Cr[ee]k n[ear] Loop R[oa]d (1250 m), Blodgett Experimental Forest, El Dorado Co[unty], California» (original citation). Holotype (♂) in EMEC.

#### Distribution.

This species is known only from the holotype.

#### Records.

**USA**: CA

### Pterostichus hatchi

Hacker, 1968

Pterostichus hatchi Hacker, 1968: 31. Type locality: «2 miles southwest of Ganns (6500 ft.), Calaveras County, Calif[ornia]» (original citation). Holotype (♂) in USNM [# 69606].

#### Distribution.

This species is restricted to a small area of the Sierra Nevada in Calaveras, El Dorado, and Tuolumne Counties, east-central California [see Hacker 1968: Fig. 39].

#### Records.

**USA**: CA

### Pterostichus stapedius

Hacker, 1968

Pterostichus stapedius stapedius Hacker, 1968: 32. Type locality: «Beasore Meadows (6700 ft.), Madera County, Calif[ornia]» (original citation). Holotype (♂) in USNM [# 69607].

#### Distribution.

This species is restricted to the Sierra Nevada from the Yosemite National Park to near King Canyon National Park, California [see Hacker 1968: Fig. 39].

#### Records.

**USA**: CA

### Pterostichus yosemitensis

Hacker, 1968

Pterostichus stapedius yosemitensis Hacker, 1968: 34. Type locality: «½ mile north of Crane Flat Ranger Station (6100 ft.), Tuolumne County, Calif[ornia]» (original citation). Holotype (♂) in USNM [# 69608].

#### Distribution.

This species is restricted to a small area of the Sierra Nevada in Tuolumne County, California [see Hacker 1968: Fig. 39].

#### Records.

**USA**: CA

#### Note.

This taxon has been considered a subspecies of *Pterostichus stapedius* by Hacker (1968: 34) but regarded as a valid species by Bousquet (1999: 164).

### [inanis group]

### Pterostichus caligans

Horn, 1891

Pterostichus caligans G.H. Horn, 1891: 32. Type locality: «Sylvania [= Camp Meeker, Sonoma County], Cal[ifornia]» (original citation). Lectotype (♀), designated by Bousquet (1999: 167), in MCZ [# 34425].

#### Distribution.

This species is restricted to the Coast Ranges of California from Mendocino County to southern Sonoma County, east to western Napa County [see Hacker 1968: Fig. 38].

#### Records.

**USA**: CA

### Pterostichus deino

Will, 2007

Pterostichus deino Will, 2007: 54. Type locality: «Deer C[ree]k Meadow [Tehama County], Cal[ifornia]» (original citation). Holotype (♀) in CAS.

#### Distribution.

This species is known from two localities in Tehama and Butte Counties in northern California.

#### Records.

**USA**: CA

### Pterostichus idahoae

Csiki, 1930

Pterostichus elongatus Schaeffer, 1910: 391 [secondary homonym of *Pterostichus elongatus* (Duftschmid, 1812)]. Type locality: «Moscou M[oun]t[ain]s [Latah County], Idaho» (original citation). Holotype (♀) in USNM [# 42496].
Pterostichus idahoae Csiki, 1930: 582. Replacement name for *Pterostichus elongatus* Schaeffer, 1910.

#### Distribution.

This species is found in northern Idaho and western Montana (Hacker 1968: 39).

#### Records.

**USA**: ID, MT

### Pterostichus inanis

Horn, 1891

Pterostichus inanis G.H. Horn, 1891: 32. Type locality: «Nev[ada]» (lectotype label), herein restricted to Reno, Washoe County (see Hacker 1968: 41). Lectotype (♂), designated by Bousquet (1999: 172), in MCZ [# 34424].

#### Distribution.

This species is known from “Washington” to northern California along the Coast Ranges, from the Sierra Nevada, and from Reno in northwestern Nevada (Hacker 1968: 41). Hacker (1968: 41) implied that the species occurs in British Columbia but the record needs confirmation.

#### Records.

**USA**: CA, NV, OR, WA [BC]

### Pterostichus pemphredo

Will, 2007

Pterostichus pemphredo Will, 2007: 52. Type locality: «Bacon Cr[ee]k n[ear] Loop R[oa]d (1250 m), Blodgett Experimental Forest, El Dorado Co[unty], California» (original citation). Holotype (♀) in EMEC.

#### Distribution.

This species is known only from the type locality.

#### Records.

**USA**: CA

### [inopinus group]

### Pterostichus infernalis

Hatch, 1936

Pterostichus infernalis Hatch, 1936: 705. Type locality: «Devils L[a]k[e] [Lincoln County], Ore[gon]» (original citation). Holotype (♂) in USNM.

#### Distribution.

This species ranges from northwestern Oregon, as far east as the eastern slopes of the Coast Ranges, southwards to northwestern California [see Hacker 1968: Fig. 34].

#### Records.

**USA**: CA, OR

### Pterostichus inopinus

(Casey, 1918)

Leptoferonia inopina Casey, 1918: 338. Type locality: «Josephine Co[unty], Oregon» (original citation), restricted to «Golden» by Hacker (1968: 11). Lectotype (♂), designated by Bousquet (1999: 172), in USNM [# 47048].

#### Distribution.

This species is restricted to western Oregon, as far north as Mount Hood National Forest, and northwestern California [see Hacker 1968: Fig. 32]. The record from “Washington” in Bousquet and Larochelle (1993: 179) is based on two specimens from Yakima County which Hacker (1968: 11) regarded as mislabeled.

#### Records.

**USA**: CA, OR

### Pterostichus pumilus pumilus

Casey, 1913

Pterostichus longicollis LeConte, 1853a: 239 [secondary homonym of *Pterostichus longicollis* (Duftschmid, 1812)]. Type locality: «Oregon» (original citation), herein restricted to Bull Run, Clackamas County (see Casey 1913: 127). Lectotype (♀), designated by Bousquet (1999: 178), in MCZ [# 5613].
Feronia oregona Chaudoir, 1868b: 335 [secondary homonym of *Pterostichus oregonus* LeConte, 1861]. Type locality: «Orégon» (original citation). Holotype [by monotypy] (♀) location unknown (possibly in MHNP). Synonymy established by LeConte (1873a: 304).
Pterostichus pumilus Casey, 1913: 127 (as *pumilis*). Type locality: «Clackamas Co[unty], Oregon» (original citation). Lectotype (♂), designated by Lindroth (1975: 123), in USNM [# 47049]. Synonymy established by Van Dyke (1926a: 75), confirmed by Lindroth (1966: 471). Note. The incorrect subsequent spelling *pumilus*, introduced by Casey (1914: 356), is in prevailing usage and attributed to the publication of the original spelling; therefore it is deemed to be the correct original spelling (ICZN 1999: Article 33.3.1).
Micromaseus longicollis Casey, 1924: 75 [secondary homonym of *Pterostichus longicollis* (Duftschmid, 1812) and *Pterostichus longicollis* LeConte, 1853]. Type locality: «Seattle [King County], Washington» (original citation). Holotype [by monotypy] (♂) in USNM [# 47073]. Synonymy established by Hatch (1953: 112), confirmed by Lindroth (1966: 471).
Pterostichus oregonis Csiki, 1930: 582. Replacement name for *Pterostichus longicollis* LeConte, 1853.

#### Distribution.

This subspecies ranges from southwestern British Columbia, including Vancouver Island (Lindroth 1966: 471), southwards to the Green Peter Mountain (Hacker 1968: 13) in the Oregon Cascades [see Hacker 1968: Fig. 33].

#### Records.

**CAN**: BC (VCI) **USA**: OR, WA

### Pterostichus pumilus willamettensis

Hacker, 1968

Pterostichus pumilus willamettensis Hacker, 1968: 13. Type locality: «hills east of the Willamette Valley 5 miles north of Mabel (700 ft.), Linn County, Oreg[on]» (original citation). Holotype (♂) in USNM [# 69599].

#### Distribution.

This subspecies is endemic to the Willamette Valley and surrounding foothills in western Oregon [see Hacker 1968: Fig. 33].

#### Records.

**USA**: OR

### [rothi group]

### Pterostichus rothi

(Hatch, 1951)

Anilloferonia rothi Hatch, 1951: 117. Type locality: «Mary’s Peak, Benton Co[unty], Ore[gon]» (original citation). Holotype (♀) in CAS [# 8158]. Etymology. The specific name was proposed for Vincent Daniel Roth [1924-1997] who run for a long period the South Western Research Station of the American Museum of Natural History in the Chiricahua Mountains, Arizona. Roth was interested in spiders.

#### Distribution.

This species is known from four sites in Lincoln and Benton Counties in western Oregon (Brenner 2005).

#### Records.

**USA**: OR

### [sphodrinus group]

### Pterostichus beyeri

Van Dyke, 1926

Pterostichus beyeri Van Dyke, 1926a: 71. Type locality: «Bitter Root Mountains, Montana» (original citation). Holotype (♂) in CAS [# 1823]. Etymology. The specific name honors Gustav Beyer [1840-1924], a fur manufacturer but also a naturalist and insect collector. Beyer was a founding member of the New York Entomological Society.
Pterostichus idahoensis Hatch, 1936: 706. Type locality: «Pierce [Clearwater County], Id[aho]» (original citation). Holotype (♀) in USNM. Synonymy established by Hatch (1953: 112).

#### Distribution.

This species is known from a few specimens collected in northern Idaho and along the western edge of Montana (Hacker 1968: 36; see Will and Gill 2008: Fig. 15).

#### Records.

**USA**: ID, MT

### Pterostichus sphodrinus

LeConte, 1863

Pterostichus sphodrinus LeConte, 1863c: 10. Type locality: «Nebraska [Territory]» (original citation). Lectotype (♂), designated by Bousquet (1999: 180), in CMNH (collection Ulke).
Monoferonia idahoanus Casey, 1924: 78. Type locality: «Moscow M[oun]t[ain] [Latah County], Idaho» (original citation). Holotype [by monotypy] (♂) in USNM [# 47114]. Synonymy established by Darlington (1932: 158), confirmed by Lindroth (1966: 470).

#### Distribution.

This species is found in the Rocky Mountains in southern Alberta, southeastern British Columbia (Lindroth 1966: 470-471), northeastern Washington, northern Idaho (Hatch 1953: 112), and western Montana (Russell 1968: 59; Edwards 1975: 55; Hansen et al. 2009: 353). The record from “Alaska” (Erwin et al. 1977: 4.33; Bousquet and Larochelle 1993: 182) is probably in error.

#### Records.

**CAN**: AB, BC **USA**: ID, MT, WA

### [incertae sedis]

### Pterostichus falli

Van Dyke, 1926

Pterostichus falli Van Dyke, 1926a: 73. Type locality: «east side of the hills east of Hollywood, Los Angeles County, California» (original citation). Holotype (♂) in CAS [# 1824].

#### Distribution.

This species is actually known only from the type locality (Hacker 1968: 37) in southern California.

#### Records.

**USA**: CA

### Subgenus Anilloferonia

Van Dyke, 1926

Anilloferonia Van Dyke, 1926a: 115. Type species: *Anilloferonia testacea* Van Dyke, 1926 by original designation. Etymology. From the Greek *an* (without), *illoz* (eyes), and the generic name *Feronia*, referring to the absence of eyes (“eyes wanting”) in adults of these *Feronia* (= *Pterostichus*) species [feminine].

#### Diversity.

Three species in the Pacific Northwest are currently recognized.

#### Identification.

Hatch (1953) provided a key for the identification of the species.

#### Taxonomic Note.

*Pterostichus rothi* has been included within this subgenus by most authors but recent molecular data analyses (Will and Gill 2008: 115) suggest that the species is most closely related to members of *Leptoferonia* Casey.

### Pterostichus lanei

(Hatch, 1935)

Anilloferonia lanei Hatch, 1935: 116 [secondary homonym of *Pterostichus lanei* Van Dyke, 1926]. Type locality: «Seaside [Clatsop County], Ore[gon]» (original citation). Holotype (♂) in USNM.

#### Distribution.

This taxon is limited to the Coast Ranges in northwestern Oregon (James R. LaBonte pers. comm. 1994).

#### Records.

**USA**: OR

#### Note.

The name *Pterostichus lanei* (Hatch, 1935) is a secondary homonym of *Pterostichus (Pseudoferonina) lanei* Van Dyke, 1926. James R. LaBonte (pers. comm. 2012) is planning to propose a replacement name for *Pterostichus lanei* Hatch.

### Pterostichus malkini

(Hatch, 1953)

Anilloferonia malkini Hatch, 1953: 119. Type locality: «Charleston, Coos Co[unty], Ore[gon]» (original citation). Holotype (♀) in USNM. Etymology. The specific name was proposed for Borys Malkin [1917-2009], ethnologist, naturalist, collector, photographer, and filmmaker (a documentary). Malkin, a Polish immigrant to the United States, collected beetles in the Philippines, New Guinea, Australia, Europe, Africa, and across the United States. He sold his North American beetle collection to the Field Museum of Natural History in June 1959.

#### Distribution.

This taxon is confined to the Coast Ranges in southwestern Oregon (James R. LaBonte pers. comm. 1994).

#### Records.

**USA**: OR

### Pterostichus testaceus

(Van Dyke, 1926)

Anilloferonia testacea Van Dyke, 1926a: 116. Type locality: «Yakima Indian Forest Reserve on the east side of Mount Adams (about 4000 feet), Washington» (original citation). Holotype (♀) in CAS [# 1855].

#### Distribution.

This species is confined to the Cascade Range in Washington and Oregon (James R. LaBonte pers. comm. 1994).

#### Records.

**USA**: OR, WA

### Subgenus ^8^ Hypherpes

Chaudoir, 1838

Hypherpes Chaudoir, 1838: 8. Type species: *Feronia castanea* Dejean, 1828 designated by Bousquet (1984a: 4). Etymology. Uncertain, but more likely from the Greek *ypherpo* (to creep on secretly) rather than from the Greek *hypo* (under, beneath), *phero* (to bear, carry), and the Latin *pes* (foot) [masculine]. The name was proposed by Johann Friedrich Eschscholtz and made available by Chaudoir.
Haplocoelus Chaudoir, 1838: 8. Type species: *Feronia tristis* Dejean, 1828 by original designation. Synonymy established by Csiki (1930: 578). Etymology (original). From the Greek *haplo*- (simple, single) and *coilos* (hollow, by extension sulcus, stria, impression), possibly alluding to the single laterobasal impression on each side of the pronotum of the adult [masculine].
Brachystilus Chaudoir, 1838: 10. Type species: *Feronia californica* Dejean, 1828 by original designation. Synonymy established implicitly by LeConte (1853a: 238), explicitly by Csiki (1930: 579). Etymology (original). From the Greek *brachys* (short) and *stylos* (pillar, column, by extension support) [masculine]. Note. Chaudoir (1838) used two different spellings for this taxon: *Brachystilus* (page 10) and *Brachystylus* (page 17). Bousquet and Larochelle (1993: 176) acted as First Revisers and selected *Brachystilus* as the original spelling, so that the name *Brachystylus* Schönherr, 1845, a valid taxon in Curculionidae, would not enter in homonymy.
Holciophorus LeConte, 1853a: 249. Type species: *Feronia atra* Dejean, 1828 (= *Feronia lama* Ménétriés, 1843) by monotypy. Synonymy established by Van Dyke (1926a: 73). Etymology. Uncertain, possibly from the Greek *holcio* (rudder) and *phoro* (to bear, carry) [masculine].
Gonoderus Motschulsky, 1859a: 149. Type species: *Feronia adoxa* Say, 1823 designated by Lindroth (1966: 467). Synonymy established by Csiki (1930: 578).
Hammatomerus Chaudoir, 1868b: 337. Type species: *Feronia morionides* Chaudoir, 1868 by monotypy. Synonymy established by Van Dyke (1926a: 73). Etymology. From the Greek *hammatos* (knot, noose, cord) and *meros* (part, by extension segment) [masculine].
Pheryphes Casey, 1920: 186. Type species: *Pterostichus tarsalis* LeConte, 1873 designated by Bousquet (1984a: 4). Synonymy established by Bousquet and Larochelle (1993: 176). Etymology. Anagram of the generic name *Hypherpes* [*q.v*.] [masculine].

#### Diversity.

Sixty species in eastern (two species) and western (58 species) North America.

#### Identification.

Casey (1913) reviewed most of the species then known (under *planctus*, *californicus*, *menetriesi*, *congestus*, and *adoxus* groups) but his key is very difficult to use. A taxonomic revision of the species is much needed.

#### Taxonomic Note.

Bousquet (1999) combined members of *Leptoferonia*, *Anilloferonia*, and *Hypherpes* into a single subgeneric taxon. Based on molecular sequence data, Will and Gill (2008) recovered these groups as monophyletic and suggested to recognize them as distinct subgenera.

### [adoxus group]

### Pterostichus adoxus

(Say, 1823)

Feronia adoxa Say, 1823a: 46. Type locality: «M[oun]t Wachusett [Worcester County], Mass[achusetts]» (neotype label). Neotype (♂), designated by Lindroth and Freitag (1969: 340), in MCZ [# 33052].
Pterostichus reiectus LeConte, 1853a: 236. Type locality: «New York» (original citation). Holotype [by monotypy] (♂) in MCZ [# 5612]. Synonymy established by LeConte (1873a: 314).
Hypherpes sufflatus Casey, 1920: 187. Type locality: «probably Indiana» (original citation). Holotype [by monotypy; designated lectotype by Perrault (1973a: 39)] (♀) in USNM [# 47040]. Synonymy established by Lindroth (1966: 467).

#### Distribution.

This species ranges from Cape Breton Island (Bousquet 1987d: 105) to northern Minnesota (Petrice et al. 2002: 9), south along the Appalachian Mountains at least to eastern Tennessee (Carter and Morgan Counties, CMNH) and northwestern South Carolina (Ciegler 2000: 66). The records from Georgia (Fattig 1949: 21), northern Alabama (Löding 1945: 15), and Iowa (Wickham 1911b: 6; King 1914: 321) need confirmation since they could refer to *Pterostichus tristis*; that from “Wyoming” (Wickham 1896c: 132) is in error.

#### Records.

**CAN**: NB, NS (CBI), ON, QC **USA**: CT, DC, DE, IN, KY, MA, MD, ME, MI, MN, NC, NH, NJ, NY, OH, PA, SC, TN, VA, VT, WI, WV [AL, GA, IA]

### Pterostichus tristis

(Dejean, 1828)

Feronia tristis Dejean, 1828: 324. Type locality: «Amérique septentrionale» (original citation), herein restricted to Nakutshi Valley, Habersham County, Georgia (see LeConte, 1853a: 236, as *Pterostichus sustentus*). Lectotype, designated by Perrault (1973a: 39), in MHNP.
Feronia interfector Newman, 1838a: 387. Type locality: «northern states of America» (original citation, see page 388). Syntype(s) probably lost (Lindroth 1966: 467). Synonymy established by Perrault (1973a: 39).
Pterostichus sustentus LeConte, 1853a: 236. Type locality: «Nakutshi Valley, Habersham County, Georgia» (original citation). Lectotype (♀), designated by Perrault (1973a: 39), in MCZ [# 5611]. Synonymy established by Perrault (1973a: 39).
Pterostichus subarcuatus LeConte, 1853a: 238. Type locality: «New York» (original citation). Lectotype (♀), designated by Perrault (1973a: 39), in MCZ [# 5618]. Synonymy established by Perrault (1973a: 39).
Gonoderus cordicollis Motschulsky, 1859a: 149. Type locality: «Etats-Unis» (original citation). Lectotype (♂), designated by Bousquet and Larochelle (1993: 14), in ZMMU. Synonymy established by Bousquet and Larochelle (1993: 14).
Pterostichus zephyrus Casey, 1884b: 2. Type locality not stated. Holotype [by monotypy; designated lectotype by Perrault (1973a: 39)] (♂) in USNM [# 47041]. Synonymy established by Perrault (1973a: 39).
Pterostichus tetricula Casey, 1913: 130. Type locality: «Bayfield [Bayfield County], Wisconsin» (original citation). Lectotype (♀), designated by Perrault (1973a: 39), in USNM [# 47039]. Synonymy established by Perrault (1973a: 39).

#### Distribution.

This species has a similar distribution pattern than the preceding one ranging from Cape Breton Island (Bousquet 1987d: 105) to northern Minnesota (Petrice et al. 2002: 9), south to east-central Iowa (Iowa County, MCZ), Tennessee in the Great Smokies (CNC, MCZ), Georgia (Perrault 1973a: 37), and northwestern South Carolina (Ciegler 2000: 68). One old specimen labeled “La.” (MCZ) is probably mislabeled.

#### Records.

**CAN**: NB, NS (CBI), ON, PE, QC **USA**: CT, GA, IA, IL, IN, KY, MA, MD, ME, MI, MN, NC, NH, NJ, NY, OH, PA, RI, SC, TN, VA, VT, WI, WV

### [amethystinus group]

### Pterostichus amethystinus

Mannerheim, 1843

Pterostichus amethystinus Mannerheim, 1843: 201. Type locality: «insula Sitkha [= Baranof Island, Alaska]» (original citation). Syntype(s) location unknown (Lindroth 1966: 464).
Pterostichus amethystinus novellus Casey, 1913: 102. Type locality: Hydesville, Valley of Eel River, Humboldt County, California (lectotype label according to Lindroth 1975: 123). Lectotype (♂), designated by Lindroth (1975: 123), in USNM [# 46981]. Synonymy established by Hatch (1936: 701), confirmed by Lindroth (1966: 464).
Pterostichus amethystinus metlakatlae Casey, 1913: 102. Type locality: «Metlakatla, British Columbia» (original citation). Lectotype (♀), designated by Lindroth (1975: 123), in USNM [# 46983]. Synonymy established by Hatch (1936: 701), confirmed by Lindroth (1966: 464).
Pterostichus jejunus Casey, 1913: 104. Type locality: «probably California» (original citation). Holotype [by monotypy] (♀) in USNM [# 46986]. Synonymy established by Lindroth (1966: 464).
Hypherpes stoicus Casey, 1924: 68. Type locality: «Inverness [probably Inverness Passage], British Columbia» (original citation). Lectotype (♂), designated by Lindroth (1975: 123), in USNM [# 46980]. Synonymy established by Hatch (1936: 701), confirmed by Lindroth (1966: 464).

#### Distribution.

This species is found along the Pacific Coast from the Yakutat Bay in southeastern Alaska (Lindroth 1966: 465) to Mendocino County in California (Van Dyke 1919b: 5).

#### Records.

**CAN**: BC (QCI, VCI) **USA**: AK, CA, OR, WA

### Pterostichus obsidianus

Casey, 1913

Pterostichus obsidianus Casey, 1913: 102. Type locality: «Monterey [Monterey County], California» (original citation). Lectotype (♂), designated by Bousquet (1999: 175), in USNM [# 46982].

#### Distribution.

This species is known only from the lectotype collected in coastal California.

#### Records.

**USA**: CA

#### Note.

The lectotype of *Pterostichus obsidianus* Casey is structurally similar to and possibly conspecific with members of *Pterostichus scutellaris* LeConte.

### Pterostichus scutellaris

LeConte, 1873

Pterostichus scutellaris LeConte, 1873a: 312. Type locality: «California» (original citation). Lectotype (♂), designated by Bousquet (1999: 179), in MCZ [# 5607].

#### Distribution.

I have seen this species from Marin and Fresno Counties in California (CNC); according to Van Dyke (1919b: 5), it extends south to Monterey County in California. The syntype studied by Lindroth (1966: 462) is not conspecific with the lectotype.

#### Records.

**USA**: CA

### [arcanus group]

### Pterostichus arcanus

Casey, 1913

Pterostichus arcanus Casey, 1913: 103. Type locality: «S[an]ta Cruz M[oun]t[ain]s, California» (original citation). Lectotype (♂), designated by Bousquet (1999: 166), in USNM [# 46985].

#### Distribution.

This species is known only from the lectotype collected in the Coast Ranges of California.

#### Records.

**USA**: CA

### [castaneus group]

### Pterostichus castaneus

(Dejean, 1828)

Feronia castanea Dejean, 1828: 326. Type locality: «détroit de Norfolk [= Sitka Sound, Baranof Island, Alaska] sur la côte nord-ouest de l’Amérique septentrionale» (original citation). Syntype(s) location unknown (possibly in MHNP and MCZ, see Lindroth 1966: 465).
Feronia brunnea Dejean, 1828: 327. Type locality: «Californie» (original citation). Holotype [by monotypy] (♀) in MHNP. Synonymy established by LeConte (1873a: 312), confirmed by Lindroth (1966: 465).
Feronia sejungenda Chaudoir, 1868b: 336. Type locality: «Californie» (original citation). One syntype [2 ♀ originally cited] in MHNP. Synonymy established with doubt, under the name *Pterostichus brunneus* (Dejean), by Tschitschérine (1900c: 467), herein confirmed. Note. Tschitschérine (1900c: 467) wrote that this taxon was probably a synonym of the true *Pterostichus brunneum*. I have seen one of the two syntypes, a damage specimen in Chaudoir’s collection in MHNP missing the abdomen, the posterior legs and most of the remaining tarsi. The specimen matches those of *Pterostichus castaneus* (Dejean) in all its external characters.
Pterostichus wrangelli Casey, 1913: 131. Type locality: «Fort Wrangell, Alaska» (original citation). Holotype [by monotypy] (♂) in USNM [# 47000]. Synonymy established by Casey (1918: 325), confirmed by Lindroth (1966: 466).
Hypherpes terracensis Casey, 1924: 68. Type locality: «Terrace, British Columbia» (original citation). Lectotype (♂), designated by Lindroth (1975: 123), in USNM [# 46984]. Synonymy established by Hatch (1953: 110), confirmed by Lindroth (1966: 466).

#### Distribution.

This species ranges from the southeastern coast of Alaska (Lindroth 1966: 466) to “California” (Dejean 1828: 327, as *Feronia brunnea*; Chaudoir 1868b: 336, as *Feronia sejungenda*), east to southeastern Bri﻿tish Columbia (Lindroth 1966: 466) and “Idaho” (Van Dyke 1924a: 8).

#### Records.

**CAN**: BC (QCI, VCI) **USA**: AK, CA, ID, OR, WA

### Pterostichus tuberculofemoratus

Hatch, 1936

Pterostichus tuberculo-femoratus Hatch, 1936: 701. Type locality: «Crater L[ake] [Klamath County], Ore[gon]» (original citation). Holotype (♂) in USNM.

#### Distribution.

This species is known from a few localities in southern Oregon (Hatch 1936: 702).

#### Records.

**USA**: OR

### [castanipes group]

### Pterostichus castanipes

(Ménétriés, 1843)

Feronia castanipes Ménétriés, 1843: 59. Type locality: «Rio del Sacramento [= Sacramento River], Californie» (original citation). Lectotype (♀), designated by Bousquet (1999: 168), in ZMH.
Pterostichus contractus LeConte, 1851: 182. Type locality: «San Jose [Santa Clara County, California]» (original citation). Lectotype (♂), designated by Bousquet (1999: 168), in MCZ [# 83]. Synonymy established by LeConte (1873a: 304).
Pterostichus gregalis Casey, 1913: 121. Type locality: «Humboldt Co[unty], California» (original citation). Lectotype (♂), designated by Bousquet (1999: 171), in USNM [# 47027]. **New synonymy** (Serge Laplante pers. comm. 1998).

#### Distribution.

At present, this species is known only from the Coast Ranges in north and central California. The record from Utah (Knowlton and Wood 1947: 94) is no doubt in error.

#### Records.

**USA**: CA

### [congestus group]

### Pterostichus congestus

(Ménétriés, 1843)

Feronia congesta Ménétriés, 1843: 59. Type locality: «Californie» (original citation), herein restricted to Anderson Valley, Mendocino County (see Casey, 1913: 105, as *Pterostichus breviusculus*). Lectotype (♀), designated by Bousquet (1999: 168), in ZMH.
Brachystylus curtipennis Motschulsky, 1859a: 148. Type locality: «St. Francisco [California]» (original citation). Lectotype (♀), designated by Bousquet and Larochelle (1993: 12), in ZMMU. Synonymy established by Bousquet and Larochelle (1993: 12).
Pterostichus breviusculus Casey, 1913: 105. Type locality: «Anderson Valley, Mendocino Co[unty], California» (original citation). Lectotype (♂), designated by Bousquet (1999: 166), in USNM [# 46992]. **New synonymy** (Serge Laplante pers. comm. 1998).
Pterostichus breviusculus mimus Casey, 1913: 105. Type locality: «Sonoma Co[unty], California» (original citation). Lectotype (♀), designated by Bousquet (1999: 166), in USNM [# 46990]. **New synonymy** (Serge Laplante pers. comm. 1998).
Pterostichus plutonicus Casey, 1913: 106. Type locality: «coast regions north of San Francisco, California» (original citation). Lectotype (♂), designated by Bousquet (1999: 177), in USNM [# 46991]. **New synonymy** (Serge Laplante pers. comm. 1998).

#### Distribution.

This species is found along the Coast Ranges from Oregon (Lane County, CNC) to southern California (Fall 1901a: 44).

#### Records.

**USA**: CA, OR

### Pterostichus crenicollis

LeConte, 1873

Pterostichus crenicollis LeConte, 1873a: 311. Type locality: «Washington Territory and Vancouver» (original citation), restricted to «Wash[ington]» by Lindroth (1966: 460). Lectotype (♂), designated by Bousquet (1999: 169), in MCZ [# 5602].
Pterostichus rectilatus Casey, 1913: 106. Type locality: «Clackamas Co[unty], Oregon» (original citation). Lectotype (♂), designated by Lindroth (1975: 122), in USNM [# 46996]. Synonymy established by Hatch (1953: 111), confirmed by Lindroth (1966: 460).
Pterostichus pugetanus Casey, 1913: 107. Type locality: «Clackamas Co[unty], Oregon» (original citation). Lectotype (♀), designated by Lindroth (1975: 122), in USNM [# 46995]. Synonymy established by Casey (1918: 328), confirmed by Lindroth (1966: 460).

#### Distribution.

This species is restricted to the Coast Ranges and adjacent areas from the Gulf Coast of Alaska (Lindroth 1966: 460) to northwestern California (Notman 1929b: 222; Will and Gill 2008: 123).

#### Records.

**CAN**: BC (QCI, VCI) **USA**: AK, CA, OR, WA

### Pterostichus suffusus

Casey, 1913

Pterostichus suffusus Casey, 1913: 106. Type locality: «Humboldt Co[unty], California» (original citation). Lectotype (♂), designated by Bousquet (1999: 181), in USNM [# 46993].
Pterostichus cuneatulus Casey, 1913: 108. Type locality: «Humboldt Co[unty], California» (original citation). Lectotype (♂), designated by Bousquet (1999: 169), in USNM [# 46998]. **New synonymy** (Serge Laplante pers. comm. 1998).

#### Distribution.

As far as known, this species is found along the Coast Ranges in northern California.

#### Records.

**USA**: CA

### [craterensis group]

### Pterostichus craterensis

(Hatch, 1949)

Platysma craterense Hatch, 1949a: 80. Type locality: «Crater L[ake] [Klamath County], Ore[gon]» (original citation). Holotype (♂) in USNM.

#### Distribution.

This species is known from a few specimens collected in Klamath County, southern Oregon.

#### Records.

**USA**: OR

### [ecarinatus group]

### Pterostichus ecarinatus

Hatch, 1936

Pterostichus ecarinatus Hatch, 1936: 702. Type locality: «Waha [Nez Perce County], Id[aho]» (original citation). Holotype (♂) in USNM.

#### Distribution.

This species is found along the Rocky Mountains and their foothills in western Alberta, eastern British Columbia (Lindroth 1966: 466), northeastern Washington, northern Idaho (Hatch 1953: 109), and western Montana (Russell 1968: 58; Edwards 1975: 54; Will and Gill 2008: 123).

#### Records.

**CAN**: AB, BC **USA**: ID, MT, WA

### [gracilior group]

### Pterostichus annosus

Casey, 1913

Pterostichus annosus Casey, 1913: 122. Type locality: «California» (original citation). Holotype [by monotypy] (♂) in USNM [# 47030].

#### Distribution.

This species is known only from the holotype.

#### Records.

**USA**: CA

### Pterostichus baldwini

(Casey, 1924)

Hypherpes baldwini Casey, 1924: 70. Type locality: «Baldwin Lake (8600 ft.) [San Bernardino County], California» (original citation). Lectotype (♂), designated by Bousquet (1999: 166), in USNM [# 47035].

#### Distribution.

This species is known only from the San Bernardino Mountains in southern California.

#### Records.

**USA**: CA

### Pterostichus esuriens

Casey, 1913

Pterostichus esuriens Casey, 1913: 122. Type locality: «Hermitage and Guallala, Mendocino Co[unty], California» (original citation), restricted to «Hermitage» by Bousquet (1999: 169). Lectotype (♀), designated by Bousquet (1999: 169), in USNM [# 47033].

#### Distribution.

This species is known only from two specimens collected in Mendocino County, California.

#### Records.

**USA**: CA

### Pterostichus gracilior

LeConte, 1873

Brachystylus longicollis Motschulsky, 1859a: 147 [secondary homonym of *Pterostichus longicollis* (Duftschmid, 1812)]. Type locality: «Cap de l’Amiral Drak[e] [= Drakes Head, Marin County, California]» (original citation). Syntype(s) location unknown (possibly in ZMMU though not listed in Keleinikova 1976).
Pterostichus gracilior LeConte, 1873a: 304. Replacement name for *Pterostichus longicollis* (Motschulsky, 1859).

#### Distribution.

This species seems to be confined to the mountains of southern California (Fall 1901a: 44). The records from “Oregon,” “Nevada,” and “Nebraska” (Schaupp 1882c: 41) are probably in error.

#### Records.

**USA**: CA

### Pterostichus hornii

LeConte, 1873

Pterostichus hornii LeConte, 1873a: 313. Type locality: «S[outh]E[ast] Sierras of California» (original citation), herein restricted to near Fort Tejon, Kern County (see Van Dyke 1927a: 196). Holotype [by monotypy] (♀) in MCZ [# 5610]. Etymology. The specific name honors George Henry Horn [1840-1897], long-time friend of J.L. LeConte. An obstetrician by profession, Horn devoted his leisure to taxonomic studies of North American beetles. Along with his mentor LeConte, he coauthored in 1883 the *Classification of the Coleoptera of North America* which was a stepping-stone for the development of coleopterology on this continent.

#### Distribution.

This species is known from a few localities in the southern parts of the Sierra Nevada. The record from Baja California (Horn 1894: 308) needs confirmation.

#### Records.

**USA**: CA

### Pterostichus lacertus

Casey, 1913

Pterostichus lacertus Casey, 1913: 123. Type locality: «Hydesville, Valley of Eel River, Humboldt Co[unty], California» (original citation). Lectotype (♀), designated by Bousquet (1999: 173), in USNM [# 47038].

#### Distribution.

This species is known only from the lectotype collected in northwestern California.

#### Records.

**USA**: CA

### Pterostichus mercedianus

(Casey, 1918)

Hypherpes mercedianus Casey, 1918: 335. Type locality: «Merced Creek [Mariposa County], California» (original citation). One syntype in USNM [# 47031].
Hypherpes protensipennis Casey, 1918: 336. Type locality: «Berkeley [Alameda County], Cal[ifornia]» (lectotype label). Lectotype (♂), designated by Bousquet (1999: 177), in USNM [# 47029]. **New synonymy** (Serge Laplante pers. comm. 1998).

#### Distribution.

This species is known from the Coast Ranges and Sierra Nevada in central California.

#### Records.

**USA**: CA

### Pterostichus panticulatus

Casey, 1913

Pterostichus panticulatus Casey, 1913: 124. Type locality: «with scarcely a doubt from California» (original citation). Holotype [by monotypy] (♀) in USNM [# 47034].

#### Distribution.

This species is known only from the holotype.

#### Records.

**USA**: CA

### Pterostichus pergracilis

(Casey, 1920)

Hypherpes pergracilis Casey, 1920: 187. Type locality: «Olancha, Inyo Co[unty], California» (original citation). Lectotype (♀), designated by Bousquet (1999: 177), in USNM [# 47032].

#### Distribution.

This species is known only from the lectotype.

#### Records.

**USA**: CA

### Pterostichus protensiformis

(Casey, 1924)

Hypherpes protensiformis Casey, 1924: 70. Type locality: «Berkeley [Alameda County], California» (original citation). Lectotype (♂), designated by Bousquet (1999: 177), in USNM [# 47016].

#### Distribution.

This species is known only from the type locality in western California.

#### Records.

**USA**: CA

### Pterostichus sierranus

Casey, 1913

Pterostichus sierranus Casey, 1913: 124. Type locality: «Lake Tahoe and Merced Cr[eek], California» (original citation), restricted to «Lake Tahoe [Placer County]» by Bousquet (1999: 180). Lectotype (♀), designated by Bousquet (1999: 180), in USNM [# 47028].

#### Distribution.

This species is known only from Placer (Casey 1913: 124) and Tuolumne Counties (Dajoz 2007: 17) in the Sierra Nevada.

#### Records.

**USA**: CA

#### Note.

Casey (1913) based the description of this species on two specimens, one from Lake Tahoe, the other from Merced Creek. The specimen from Merced Creek was later (Casey 1918: 335) considered a distinct species which Casey named *Hypherpes mercedianus*.

### Pterostichus sponsor

Casey, 1913

Pterostichus sponsor Casey, 1913: 123. Type locality: «Monterey [Monterey County], California» (original citation). Lectotype (♀), designated by Bousquet (1999: 180), in USNM [# 47037].

#### Distribution.

This species is known only from the lectotype collected in west-central California.

#### Records.

**USA**: CA

### Pterostichus spraguei

LeConte, 1873

Pterostichus spraguei LeConte, 1873a: 313. Type locality: «Nevada» (original citation). Lectotype (♂), designated by Bousquet (1999: 181), in MCZ [# 5609]. Etymology. The specific name was proposed for Philip Shaw Sprague [1829-1874], an active member of the Boston Society of Natural History. Sprague started collecting beetles in his 30s and his collection went to the Boston Society.

#### Distribution.

Besides the lectotype, I have seen one specimen, probably of this species, from Mono County, California (CNC). The species was also reported from Zion National Park, Utah, by Tanner (1928: 270).

#### Records.

**USA**: CA, NV [UT]

### [herculaneus group]

### Pterostichus herculaneus

Mannerheim, 1843

Pterostichus herculaneus Mannerheim, 1843: 201. Type locality: «insula Sithka [= Baranof Island, Alaska]» (original citation), which is probably incorrect (Lindroth 1966: 463). Syntype(s) location unknown (Lindroth 1966: 463).
Pterostichus scenicus Casey, 1913: 103. Type locality: «British Columbia» (original citation). Holotype [by monotypy] (♀) in USNM [# 46987]. Synonymy established by Hatch (1953: 108), confirmed by Lindroth (1966: 464).

#### Distribution.

This species ranges from northwestern Montana (Russell 1968: 58) to Vancouver Island (Lindroth 1966: 464), south to the Coast Ranges in southwestern Oregon (Niwa and Peck 2002: 787) and Tuolumne County in the Sierra Nevada (Will and Gill 2008: 123). The record from southeastern Colorado (Wickham 1902: 235) is likely in error.

#### Records.

**CAN**: BC (VCI) **USA**: CA, ID, MT, OR, WA

### Pterostichus laborans

Casey, 1913

Pterostichus laborans Casey, 1913: 116. Type locality: «Lake Tahoe [Placer County], California» (original citation). Lectotype (♂), designated by Bousquet (1999: 173), in USNM [# 47020].
Pterostichus tahoensis Casey, 1913: 113. Type locality: «Lake Tahoe [Placer County], California» (original citation). Lectotype (♂), designated by Bousquet (1999: 181), in USNM [# 47011]. **New synonymy** (Serge Laplante pers. comm. 1998).
Hypherpes placerensis Casey, 1918: 334. Type locality: «Placer Co[unty], California» (original citation). Lectotype (♂), designated by Bousquet (1999: 177), in USNM [# 47015]. **New synonymy** (Serge Laplante pers. comm. 1998).

#### Distribution.

This species is known only from the Lake Tahoe area in the Sierra Nevada, California.

#### Records.

**USA**: CA

### Pterostichus lattini

LaBonte, 2006

Pterostichus lattini LaBonte, 2006: 204. Type locality: «Marys Peak (1092 m), Benton Co[unty], Or[egon]» (original citation). Holotype (♂) in CAS [# 18122].

#### Distribution.

This species is known from the Cascade Range and Coast Ranges of western Oregon [see LaBonte 2006: Fig. 5].

#### Records.

**USA**: OR

### Pterostichus neobrunneus

Lindroth, 1966

Pterostichus neobrunneus Lindroth, 1966: 462. Type locality: «Oliver, B[ritish] C[olumbia]» (original citation). Holotype (♂) in CNC [# 9224].

#### Distribution.

This species occurs west of the Rocky Mountains from southern British Columbia, including Vancouver Island (Lindroth 1966: 463), to northern Oregon (Hatch 1953: 109, as *Pterostichus brunneus*).

#### Records.

**CAN**: BC (VCI) **USA**: OR, WA

#### Note.

Prior to Lindroth (1966), this species was reported in the literature under the name *Pterostichus brunneus* (Dejean, 1828).

### Pterostichus occultus

Casey, 1913

Pterostichus occultus Casey, 1913: 112. Type locality: «Siskiyou Co[unty], California» (original citation). Lectotype (♀), designated by Bousquet (1999: 176), in USNM [# 47009].

#### Distribution.

This species is known only from the lectotype collected in northern California.

#### Records.

**USA**: CA

### Pterostichus planctus

LeConte, 1853

Pterostichus planctus LeConte, 1853a: 239. Type locality: «Sac[ramento] [Sacramento County, California]» (lectotype label). Lectotype (♀), designated by Bousquet (1999: 177), in MCZ [# 5603].

#### Distribution.

This species is known from “Oregon” (LeConte 1853a: 239) and a few localities in the Sierra Nevada (Fall 1901a: 45).

#### Records.

**USA**: CA, OR

### Pterostichus protractus

LeConte, 1860

Pterostichus protractus LeConte, 1860: 319. Type locality: «Jasper House [= Jasper, Alberta]» (original citation). Lectotype (♂), designated by Bousquet (1999: 178), in MCZ [# 5604].
Pterostichus inornatus Bland, 1865: 381. Type locality: «Colorado Territory» (original citation). Lectotype (♂), designated by Bousquet (1999: 178), in ANSP [# 2701]. Synonymy established by LeConte (1873a: 303), confirmed by Bousquet (1999: 178).
Pterostichus fontinalis Casey, 1913: 110. Type locality: «Yellowstone Park, Wyoming» (original citation). Lectotype (♂), designated by Lindroth (1975: 122), in USNM [# 47004]. Synonymy established by Lindroth (1966: 463).
Pterostichus zunianus Casey, 1913: 111. Type locality: «New Mexico» (original citation). Lectotype (♂), designated by Bousquet (1999: 183), in USNM [# 47005]. **New synonymy** (Serge Laplante pers. comm. 1998).
Hypherpes vivax Casey, 1918: 333. Type locality: «Yellowstone Park, Wyoming» (original citation). Lectotype (♂), designated by Lindroth (1975: 122), in USNM [# 47007]. Synonymy established by Lindroth (1966: 463).
Hypherpes intectus Casey, 1918: 333. Type locality: «Boulder Co[unty], Colorado» (original citation). Lectotype (♂), designated by Bousquet (1999: 173), in USNM [# 47006]. **New synonymy** (Serge Laplante pers. comm. 1998).
Hypherpes provensis Casey, 1924: 69. Type locality: «Provo Cañon [Utah County], Utah» (original citation for the lectotype). Lectotype (♂), designated by Lindroth (1975: 123), in USNM [# 47017]. Synonymy established by Lindroth (1966: 463).
Hypherpes utensis Casey, 1924: 69. Type locality: «Provo Cañon [Utah County], Utah» (original citation). Lectotype (♀), designated by Lindroth (1975: 123), in USNM [# 47010]. Synonymy established by Lindroth (1966: 463).

#### Distribution.

This species occurs from the foothills of the Rocky Mountains in south-central Alberta to the Kootenay River drainage in southeastern British Columbia (Lindroth 1966: 463), south to the Sierra Nevada in California (Fall 1901a: 45; Papp 1978: 167), northern Arizona (Apache and Coconino Counties, CNC), and the Sangre de Cristo Mountains in New Mexico (Fall and Cockerell 1907: 158; Casey, 1913: 111, as *Pterostichus zunianus*). The record from Baja California (Horn 1894: 308) is suspect; that from “Nebraska” (Schaupp 1882c: 40) needs confirmation.

#### Records.

**CAN**: AB, BC **USA**: AZ, CA, CO, ID, MT, NM, NV, OR, UT, WA, WY [NE]

### Pterostichus vandykei

Schaeffer, 1910

Pterostichus van dykei Schaeffer, 1910: 392. Type locality: «Moscou M[oun]t[ain]s, Idaho» (original citation). Lectotype (♀), designated by Erwin and House (1978: 247), in USNM [# 42497].

#### Distribution.

This species is known from northern Idaho and adjacent eastern Washington (Hatch 1953: 109).

#### Records.

**USA**: ID, WA

### Pterostichus ybousqueti

Berlov, 1999

Pterostichus parens Casey, 1913: 112 [secondary homonym of *Pterostichus parens* (Tschitschérine, 1897)]. Type locality: «Siskiyou Co[unty], Cal[ifornia]» (lectotype label). Lectotype (♂), designated by Bousquet (1999: 176), in USNM [# 47008].
Pterostichus ybousqueti O. Berlov, 1999: 60. Replacement name for *Pterostichus parens* Casey, 1913.

#### Distribution.

This species is known only from Siskiyou County in northern California.

#### Records.

**USA**: CA

### [illustris group]

### Pterostichus illustris

LeConte, 1851

Pterostichus illustris LeConte, 1851: 182. Type locality: «S[an] D[iego] [San Diego County, California]» (lectotype label). Lectotype (♂), designated by Bousquet (1999: 172), in MCZ [# 82].

#### Distribution.

As far as known, this species seems to be restricted to southwestern California.

#### Records.

**USA**: CA

#### Note.

This name has been listed as a junior synonym of *Pterostichus congestus* Ménétriés by LeConte (1858a: 28; 1873a: 304) but, as pointed out by Bousquet and Larochelle (1993: 18), it applies to a distinct species.

### [inermis group]

### Pterostichus inermis

Fall, 1901

Pterostichus inermis Fall, 1901a: 211. Type locality: «foot-hill cañons of the Sierras near Pomona [Los Angeles County, California]» (original citation). Lectotype (♂), designated by Bousquet (1999: 172), in MCZ [# 23873].

#### Distribution.

This species is known so far only from southwestern California.

#### Records.

**USA**: CA

### Pterostichus miscellus

Casey, 1913

Pterostichus miscellus Casey, 1913: 115. Type locality: «S[an]ta Barbara [Santa Barbara County], California» (original citation). Lectotype (♂), designated by Bousquet (1999: 175), in USNM [# 47019].

#### Distribution.

This very distinct species is still known only from the lectotype collected at Santa Barbara along the coast of California.

#### Records.

**USA**: CA

### [isabellae group]

### Pterostichus canallatus

Casey, 1913

Pterostichus canallatus Casey, 1913: 120. Type locality: «S[an]ta Barbara [Santa Barbara County], California» (original citation). Lectotype (♂), designated by Bousquet (1999: 167), in USNM [# 47026].

#### Distribution.

This species is known only from the type locality in southwestern California.

#### Records.

**USA**: CA

### Pterostichus gliscans

Casey, 1913

Pterostichus gliscans Casey, 1913: 119. Type locality: «San Clemente Island [Los Angeles County], California» (original citation). Lectotype (♂), designated by Bousquet (1999: 170), in USNM [# 47025].

#### Distribution.

This species is known only from the original specimens collected in San Clemente Island, part of the Channel Islands, in the Pacific Ocean along the coast of southern California.

#### Records.

**USA**: CA (CHI)

### Pterostichus isabellae

LeConte, 1851

Pterostichus isabellae LeConte, 1851: 182. Type locality: «S[an]ta Isabel [San Bernardino County, California]» (original citation), cited from «‘Bill Williams’ Ranch’ in the mountains east of San Diego» by LeConte (1853a: 237). Lectotype (♂), designated by Bousquet (1999: 173), in MCZ [# 84].

#### Distribution.

According to Fall (1901b: 44), this species is “common and widely diffuse” in southern California.

#### Records.

**USA**: CA (CHI)

### Pterostichus jacobinus

Casey, 1913

Pterostichus jacobinus Casey, 1913: 118. Type locality: «San Diego [San Diego County], California» (original citation). Lectotype (♀), designated by Bousquet (1999: 173), in USNM [# 47024].

#### Distribution.

This species is known yet only from San Diego County in southwestern California.

#### Records.

**USA**: CA

### Pterostichus ovalipennis

Casey, 1913

Pterostichus ovalipennis Casey, 1913: 119. Type locality: «between Indio and Colton [probably in Riverside County], California» (original citation). Lectotype (♀), designated by Bousquet (1999: 177), in USNM [# 47023].

#### Distribution.

This species is known only from the lectotype collected in southwestern California.

#### Records.

**USA**: CA

### [lama group]

### Pterostichus lama

(Ménétriés, 1843)

Feronia atra Dejean, 1828: 339 [secondary homonym of *Pterostichus ater* (Sahlberg, 1817)]. Type locality: «Californie» (original citation), herein restricted to Lake Tahoe, Placer County (see Casey, 1913: 97, as *Holciophorus pollens*). Syntype(s) probably in MHNP (Lindroth 1966: 458).
Feronia lama Ménétriés, 1843: 60. Type locality: «Californie» (original citation), herein restricted to Lake Tahoe, Placer County (see Casey, 1913: 97). Syntype(s) location unknown (Lindroth 1966: 458). Synonymy established by LeConte (1853a: 250).
Percus aterrimus Motschulsky, 1845b: 341. Type locality: «Californie» (original citation). Holotype [by monotypy] (♂) location unknown (possibly in ZMMU though not listed in Keleinikova 1976). Synonymy established by Motschulsky (1845b: 341).
Holciophorus vancouveri Casey, 1913: 97. Type locality: «Victoria, Vancouver Island [British Columbia]» (original citation). Lectotype (♂), designated by Lindroth (1975: 122), in USNM [# 46974]. Synonymy established by Hatch (1953: 107), confirmed by Lindroth (1966: 458).
Holciophorus pollens Casey, 1913: 97. Type locality: «Lake Tahoe [Placer County], California» (original citation). Lectotype (♂), designated by Lindroth (1975: 122), in USNM [# 46975]. Synonymy established by Lindroth (1966: 458).
Holciophorus cephalus Casey, 1913: 98. Type locality: «California» (original citation). Holotype [by monotypy] (♂) in USNM. Synonymy established, under the name *Pterostichus pollens* (Casey), by Casey (1924: 68).
Holciophorus domitor Casey, 1913: 98. Type locality not stated. Lectotype (♂), designated by Lindroth (1975: 122), in USNM. Synonymy established by Lindroth (1966: 458).

#### Distribution.

This large species is found from the Queen Charlotte Islands (Kavanaugh 1992: 70) south to southern California (Fall 1901a: 44, as *Pterostichus ater*; Will and Gill 2008: 124), including Washoe County in northwestern Nevada (La Rivers 1946: 102, as *Feronia ater*).

#### Records.

**CAN**: BC (QCI, VCI) **USA**: CA, NV, OR, WA

### [lassulus group]

### Pterostichus lassulus

(Casey, 1920)

Hypherpes lassulus Casey, 1920: 186. Type locality: «Olancha, Inyo Co[unty], California» (original citation). Lectotype (♀), designated by Bousquet (1999: 174), in USNM [# 46999].

#### Distribution.

This species is known only from the lectotype collected in eastern California.

#### Records.

**USA**: CA

### [menetriesii group]

### Pterostichus menetriesii

LeConte, 1873

Pterostichus menetriesii LeConte, 1873a: 304. Type locality: «Cal[ifornia]» (lectotype label). Lectotype (♀), designated by Bousquet (1999: 175), in MCZ [# 34326]. Etymology. The specific name was proposed for Edouard Ménétriés [1802-1861], the leading Russian entomologist of his time. Born in France, Ménétriés worked at the Museum of the Jardin des Plantes under Cuvier and Latreille before acting as conservator of rarities at the Zoological Museum in the Imperial Academy of Sciences in Saint Petersburg. He described many North American beetles collected in Alaska and northern California by various Russian expeditions. His collection is in the Zoological Institute in Saint Petersburg.

#### Distribution.

This species is found along the Coast Ranges in central California (Marin, San Mateo, and San Francisco Counties, Will and Gill 2008: 125, CNC). It was also reported from Santa Rosa Island in the Pacific Ocean (Fall 1901a: 44).

#### Records.

**USA**: CA (CHI)

### [morionides group]

### Pterostichus morionides

(Chaudoir, 1868)

Feronia morionides Chaudoir, 1868b: 337. Type locality: «Californie» (original citation). Syntype(s) [2 originally cited] probably in MHNP.

#### Distribution.

This species is found along the northern (Siskiyou County, CNC) and central parts of the Sierra Nevada (Will and Gill 2008: 118, 125).

#### Records.

**USA**: CA

#### Note.

In analyses using molecular data sequences, this species was consistently positioned as the sister-group to {*Pterostichus adoxus* + *Pterostichus tristis*} which occur in eastern North America (Will and Gill 2008: 118).

### [nigrocaeruleus group]

### Pterostichus brachylobus

Kavanaugh and LaBonte, 2006

Pterostichus brachylobus Kavanaugh and LaBonte, 2006: 217. Type locality: «Neskowin Creek at Neskowin Campground, Tillamook County, Oregon» (original citation). Holotype (♂) in CAS [# 18121].

#### Distribution.

This species is known only along the coast in central Oregon [see Kavanaugh and LaBonte 2006: Fig. 14].

#### Records.

**USA**: OR

### Pterostichus nigrocaeruleus

Van Dyke, 1926

Pterostichus nigrocaeruleus Van Dyke, 1926a: 70. Type locality: «back of Port Angeles [Clallam County], Washington» (original citation). Holotype (♂) in CAS [# 1821].

#### Distribution.

This species is found along the Pacific Coast from Vancouver Island to northern California (Lindroth 1966: 467).

#### Records.

**CAN**: BC (VCI) **USA**: CA, OR, WA

### [restrictus group]

### Pterostichus luscus

(Casey, 1918)

Hypherpes luscus Casey, 1918: 332. Type locality: «Colorado» (original citation). Lectotype (♂), designated by Bousquet (1999: 179), in USNM [# 47003].

#### Distribution.

This species is yet known only from the lectotype.

#### Records.

**USA**: CO

#### Note.

This name has been listed in synonymy with *Pterostichus restrictus* (Casey) by Erwin et al. (1977: 4.36), based on Armin (1963: 220) unpublished thesis. However, I believe the name probably applies to a distinct species.

### Pterostichus restrictus

(Casey, 1918)

Pterostichus longulus LeConte, 1873a: 312 [secondary homonym of *Pterostichus longulus* (Reiche and Saulcy, 1856)]. Type locality: «Colorado» (original citation). Lectotype (♂), designated by Bousquet (1999: 179), in MCZ [# 5605].
Hypherpes restrictus Casey, 1918: 331. Type locality: «Boulder Co[unty], Col[orado]» (lectotype label). Lectotype (♂), designated by Bousquet (1999: 179), in USNM [# 47001]. Synonymy established by Erwin et al. (1977: 4.36) based on Armin (1963: 220) unpublished thesis.
Hypherpes elumbis Casey, 1918: 332. Type locality: «probably Colorado» (original citation). Holotype [by monotypy] (♀) in USNM [# 47002]. Synonymy established by Erwin et al. (1977: 4.36) based on Armin (1963: 220) unpublished thesis.
Pterostichus restrictus var. *lecontellus* Csiki, 1930: 581. Replacement name for *Pterostichus restrictus* var. *longulus* LeConte, 1873.

#### Distribution.

This species is found along the Rocky Mountains from southern Montana (Hatch 1933a: 7) to northern New Mexico (Snow 1885: 67; Fall and Cockerell 1907: 157).

#### Records.

**USA**: CO, MT, NM, WY

### [setosus group]

### Pterostichus setosus

Hatch, 1951

Pterostichus setosus Hatch, 1951: 116. Type locality: «Wrangle Gap, Rogue R[iver] N[ational] F[orest] [Jackson County], Ore[gon]» (original citation). Holotype (♂) in USNM.

#### Distribution.

This very distinct species is known only from southwestern Oregon (Hatch 1951: 116; Niwa and Peck 2002: 787).

#### Records.

**USA**: OR

### [tarsalis group]

### Pterostichus serripes

(LeConte, 1875)

Holciophorus serripes LeConte, 1875c: 169. Type locality: «Yosemite Valley [Mariposa County], Cal[ifornia]» (original citation). Lectotype (♂), designated by Bousquet (1999: 180), in MCZ [# 5600].

#### Distribution.

This species is known so far only from the Yosemite Valley in the Sierra Nevada, California.

#### Records.

**USA**: CA

### Pterostichus tarsalis

LeConte, 1873

Pterostichus tarsalis LeConte, 1873a: 311. Type locality: «Lake Taho[e] Valley [Placer County], Sierra Nevada [California]» (original citation). Lectotype (♂), designated by Bousquet (1999: 181), in MCZ [# 5601].
Pterostichus sequoiarum Casey, 1913: 101. Type locality: «Big Trees [Calaveras County], California» (original citation). Lectotype (♀), designated by Bousquet and Larochelle (1993: 17), in USNM [# 46979]. Synonymy established by Bousquet and Larochelle (1993: 17).
Hypherpes spissitarsis Casey, 1918: 329. Type locality: «Lake Tahoe [Placer County], California» (original citation). Holotype [by monotypy] (♂) in USNM [# 46978]. Synonymy established by Bousquet and Larochelle (1993: 14).

#### Distribution.

This species is known for sure only from the Sierra Nevada, California. The record from “Oregon” (Schaupp 1882c: 40) needs confirmation.

#### Records.

**USA**: CA [OR]

### [vicinus group]

### Pterostichus algidus

LeConte, 1853

Feronia valida Dejean, 1828: 325 [primary homonym of *Feronia concinna* var. *valida* Dejean, 1828 (page 293)]. Type locality: «détroit de Norfolk [= Sitka Sound, Baranof Island, Alaska] sur la côte nord-ouest de l’Amérique septentrionale» (original citation). Syntype(s) in MHNP.
Pterostichus algidus LeConte, 1853a: 238. Type locality: «Oregon» (original citation). Lectotype (♀), designated by Bousquet (1999: 164), in MCZ [# 7398]. Synonymy established by LeConte (1873a: 303), confirmed by Bousquet (1999: 164).
Pterostichus humboldti Casey, 1913: 114. Type locality: «Humboldt Co[unty], California» (original citation). Lectotype (♂), designated by Bousquet (1999: 171), in USNM [# 47012]. **New synonymy** (Serge Laplante pers. comm. 1998).
Pterostichus bucolicus Casey, 1913: 115. Type locality: «Humboldt Co[unty], California» (original citation for the lectotype). Lectotype (♂), designated by Bousquet (1999: 167), in USNM [# 47018]. **New synonymy** (Serge Laplante pers. comm. 1998).
Hypherpes innatus Casey, 1918: 328. Type locality: «Canada, west of the Rocky M[oun]t[ain]s» (original citation). Lectotype (♀), designated by Lindroth (1975: 122), in USNM [# 46989]. Synonymy established by Lindroth (1966: 460).
Hypherpes responsor Casey, 1918: 330. Type locality: «Victoria, Vancouver Island [British Columbia]» (original citation). Lectotype (♀), designated by Lindroth (1975: 122), in USNM [# 46997]. Synonymy established by Hatch (1953: 108), confirmed by Lindroth (1966: 460).
Hypherpes kansanus Casey, 1918: 330. Type locality: «Kansas (probably western)» (original citation), which is incorrect. Lectotype (♂), designated by Bousquet (1999: 173), in USNM [# 46988]. **New synonymy** (Serge Laplante pers. comm. 1998).
Hypherpes anthrax Casey, 1918: 331. Type locality: «Vancouver Island [British Columbia]» (original citation). Lectotype (♀), designated by Lindroth (1975: 122), in USNM [# 46994]. Synonymy established by Lindroth (1966: 460).

#### Distribution.

This species ranges along the Pacific Coast from southern Alaska (Lindroth 1966: 462) to northern California (Fall 1901a: 45; Casey, 1913: 114, as *Pterostichus humboldti*).

#### Records.

**CAN**: BC (QCI, VCI) **USA**: AK, CA, OR, WA

### Pterostichus barbarinus

Casey, 1913

Pterostichus barbarinus Casey, 1913: 116. Type locality: «S[an]ta Barbara [Santa Barbara County], California» (original citation). Lectotype (♂), designated by Bousquet (1999: 166), in USNM [# 47022].

#### Distribution.

This species is known only from the type locality in southwestern California.

#### Records.

**USA**: CA

### Pterostichus californicus

(Dejean, 1828)

Feronia californica Dejean, 1828: 222. Type locality: «Californie» (original citation), herein restricted to Hoopa Valley, Humboldt County (see Casey 1913: 114, as *Pterostichus cupidus*). Syntype(s) probably in MHNP.
Pterostichus simplex LeConte, 1851: 181. Type locality: «San Jose [Santa Clara County, California]» (lectotype label). Lectotype (♀), designated by Bousquet (1999: 167), in MCZ [# 81]. Synonymy established by LeConte (1857c: 8).
Brachystylus amplicollis Motschulsky, 1859a: 146. Type locality: Californie (inferred from title of the paper). Lectotype (♀), designated by Bousquet (1999: 167), in MCZ [# 8233]. Synonymy established by LeConte (1873a: 304).
Brachystylus parallelus Motschulsky, 1859a: 147 [*nomen dubium*]. Type locality: «St. Francisco [San Francisco County, California]» (original citation). Lectotype (♂), designated by Bousquet and Larochelle (1993: 13), in ZMMU. Synonymy established with doubt by Bousquet and Larochelle (1993: 13).
Pterostichus cupidus Casey, 1913: 114. Type locality: «Hoopa Valley, Humboldt Co[unty], California» (original citation). Lectotype (♂), designated by Bousquet (1999: 167), in USNM [# 47014]. Synonymy established by Erwin et al. (1977: 4.36), confirmed by Bousquet (1999: 167).
Pterostichus diabolus Casey, 1913: 120. Type locality: «M[oun]t Diablo [Contra Costa County], California» (original citation). Lectotype (♂), designated by Bousquet (1999: 169), in USNM [# 47036]. **New synonymy** (Serge Laplante pers. comm. 1998).

#### Distribution.

As far as known, the range of this species extends along the Coast Ranges of California from Humboldt County (Casey 1913: 114, as *Pterostichus cupidus*) to Santa Clara County (LeConte 1851: 181, as *Pterostichus simplex*).

#### Records.

**USA**: CA

### Pterostichus ordinarius

Casey, 1913

Pterostichus ordinarius Casey, 1913: 116. Type locality: «North Fork, Madera Co[unty], California» (original citation). Lectotype (♂), designated by Bousquet (1999: 176), in USNM [# 47021].

#### Distribution.

This species is known only from the original specimens collected at the type locality in the Sierra Nevada.

#### Records.

**USA**: CA

### Pterostichus vicinus

Mannerheim, 1843

Pterostichus vicinus Mannerheim, 1843: 200. Type locality: «California» (original citation). Lectotype (♂), designated by Bousquet (1999: 183), in ZMH.
Hypherpes alamedae Casey, 1918: 334. Type locality: «Alameda [Alameda County], California» (original citation). Lectotype (♀), designated by Bousquet (1999: 164), in USNM [# 47013]. **New synonymy** (Serge Laplante pers. comm. 1998).

#### Distribution.

This species seems to be restricted to the Coast Ranges in California. Fall (1901b: 44) statement that the species is “common in the Sierras and throughout the region [i.e., southern California] to the west” needs confirmation.

#### Records.

**USA**: CA

### Subgenus Cryobius

Chaudoir, 1838

Cryobius Chaudoir, 1838: 11. Type species: *Poecilus ventricosus* Eschscholtz, 1823 by original designation. Etymology (original). From the Greek *cryos* (cold) and *bios* (life), alluding to the cold habitat where these species live [masculine].
Haptoderus Chaudoir, 1838: 10. Type species: *Feronia spadicea* Dejean, 1828 (= *Feronia pumilio* Dejean, 1828) by original designation. Synonymy established by Bousquet (1999: 183). Etymology (original). From the Greek *hapto* (fasten to, by extension to apply) and *dere* (neck, by extension pronotum) [masculine].
Pseudorthomus Chaudoir, 1838: 12, 19. Type species: *Feronia amaroides* Dejean, 1828 by original designation. Synonymy established by Bousquet (1999: 183). Etymology (original). From the Greek *pseudos* (fallacy, lie) and the generic name *Orthomus* [masculine].
Pseudocryobius Motschulsky, 1850a: ix. Type species: *Feronia nivalis* Sahlberg, 1844 designated by Bousquet (1984a: 4). Synonymy established by Poppius (1906b: 7). Etymology (original). From the Greek *pseudos* (fallacy, lie) and the generic name *Cryobius* [*q.v*.] [masculine].
Abaxodes Gistel, 1857: 31. Type species: *Feronia abaxoides* Dejean, 1828 designated by Strand (1917: 76). Etymology. From the generic name *Abax* [*q.v*.] and the Greek -*odes* (likeness) [masculine].
Orites Schaum, 1858: 442, 463. Type species: *Platysma negligens* Sturm, 1824 by monotypy. Synonymy established by Schatzmayr (1942: 77). Etymology. From the Greek *oreites* (mountaineer) [masculine].
Pyreneorites Jeannel, 1937b: 11, 16. Type species: *Feronia pusilla* Dejean, 1828 by original designation. Synonymy established by Bousquet (1999: 183). Etymology. From the geographical name Pyrenees and the generic name *Orites* [*q.v*.] [masculine].
Euhaptoderus Jeanne, 1969: 33. Type species: *Carabus unctulatus* Duftschmid, 1812 by original designation. Synonymy established by Bousquet (1999: 183). Etymology. From the Greek *eu* (agreeable, original, primitive) and the generic name *Haptoderus* [*q.v*.] [masculine].
Iberoderus Jeanne, 1969: 34. Type species: *Feronia nemoralis* Graells, 1851 by original designation. Synonymy established by Bousquet (1999: 183). Etymology. From the geographic name Iberia (the Iberian Peninsula) and the Greek *dere* (neck, by extension pronotum) [masculine].
Cryobiopterus O. Berlov, 1997: 36. Type species: *Argutor brevicornis* Kirby, 1837 by original designation. Synonymy established by Bousquet (1999: 183). Etymology. From the Greek *cryos* (cold), *bios* (life), and *pteron* (wing, by extension elytron) [masculine].

#### Diversity.

About 115 species in the arctic, subarctic, and boreal regions of North America (23 species) and the Palaearctic Region (about 100 species). Several species are Holarctic.

#### Identification.

Ball (1966a) revised the North American species which he arrayed in three groups. Since then, two new species have been described, one (*Pterostichus haftorni*) by Lindroth (1969a) which has been subsequently synonymized, the other one (*Pterostichus woodi*) by Ball and Currie (1997), and several species related to *Pterostichus planus* have been downgraded to subspecies of *Pterostichus bryanti* by Ball and Currie (1997). Species identifications are difficult in part because of the important intraspecific variation observed in most species.

#### Taxonomic Note.

Lorenz (2005: 275) listed *Parahaptoderus* Jeanne as a junior synonym of *Cryobius*. However, the taxon, which is composed of three European species of which one extends to Turkey, is probably not closely related to *Cryobius* (see Bousquet 1999: 187).

### [brevicornis group]

### Pterostichus brevicornis brevicornis

(Kirby, 1837)

Argutor brevicornis Kirby, 1837: 31. Type locality: «Lat. 65° [= apparently region of Great Bear Lake, Northwest Territories]» (original citation), restricted to «Good Hope, N[orth]W[est] T[erritories]» by Ball (1966a: 105). Lectotype (♂), designated by Ball (1966a: 105), in BMNH.
Feronia subtilis R.F. Sahlberg, 1844: 35. Type locality: circa Okhotsk, Khabarovsk Kray, Siberia, Russia (inferred from introduction and title of the paper). Lectotype [as holotype] (♀), designated by Ball (1966a: 105), in ZMH. Synonymy established by Ball (1966a: 105).
Cryobius fastidiosus Mannerheim, 1853: 131. Type locality: «ad sinum Woskresenk [= Resurrection Bay] peninsulae Kenai [Alaska]» (original citation). Lectotype (♂), designated by Silfverberg (1987: 16), in ZMH. Synonymy established by Lindroth (1954b: 131).
Pseudocryobius quinquepunctatus Motschulsky, 1860: 93. Type locality: «Kamtschatka [Russia]» (original citation). One syntype in ZMMU (Keleinikova 1976: 213). Synonymy established by Ball (1966a: 105).
Feronia fragilis Mäklin, 1878: 20. Type locality: «Sopotschnaja Korga (71°40’ n. lat.) [Siberia, Russia]» (original citation). Syntype(s) location unknown. Synonymy established by Ball (1966a: 105). Note. The lectotype selected by Ball (1966a: 105), apparently in ZMH though not listed in Silfverberg’s (1987) type list, is labeled “Waigatsch” (= Vaygach Island in Siberia) and is probably not a syntype.
Feronia infima Mäklin, 1878: 20 [primary homonym of *Feronia infima* Chaudoir, 1868]. Type locality: «Jenisej, nemligen åtminstone från Mesenkin åt söder ända nedom 61 breddgraden [Russia]» (original citation). Syntype(s) location unknown (possibly in NRSS). Synonymy established, under the name *Pterostichus fastidiosus* (Mannerheim), by Poppius (1906b: 192), confirmed by Lindroth (1954b: 132).
Feronia arctica J.R. Sahlberg, 1880: 31. Replacement name for *Feronia infima* Mäklin, 1878.
Feronia epipleuralis J.R. Sahlberg, 1885b: 49. Type locality: Port Clarence, Alaska (inferred from title of the paper). Syntype(s) [3 originally cited] in ZMH. Synonymy established, under the name *Pterostichus fastidiosus* (Mannerheim), by Poppius (1906b: 192), confirmed by Ball (1966a: 105).
Platysma aquilonium Tschitschérine, 1904: 125. Type locality: «insula Kolgujev: Bugrino-Stanovishtshe [in Barents Sea, Russia]» (original citation). Syntype(s) [2 ♂ originally cited] probably in ZILR. Synonymy established, under the name *Pterostichus fastidiosus* (Mannerheim), by Poppius (1906b: 192).
Cryobius carbo Poppius, 1906b: 182. Type locality not stated. Lectotype (♀), designated by Ball (1966a: 105), in ZMH. Synonymy established by Ball (1966a: 105).
Cryobius fastidiosus minusculus Poppius, 1906b: 199. Type locality: «Bulkur, Unterste Lena [Yakutia, Siberia, Russia]» (original citation). Lectotype (♀), designated by Ball (1966a: 105), in ZMH. Synonymy established by Ball (1966a: 105).
Cryobius delicatus Casey, 1918: 375. Type locality: «S[ain]t Paul Island, Alaska» (original citation). Lectotype [as holotype] (♂), designated by Ball (1966a: 89), in USNM [# 47079]. **New synonymy**. Note. Ball (1966a) retained this name for a subspecies of *Pterostichus brevicornis* endemic to the Bering Sea Islands but, as pointed out by himself (Ball 1966a: 121) and Lindroth (1966: 523), there are no constant structural character states between the two forms and intermediate specimens are known. Based on these facts, I prefer not to retain this form as a valid subspecies.

#### Distribution.

This Holarctic subspecies ranges from the Kola Peninsula in northern European Russia eastwards to Newfoundland (Ball 1966a: 119); the species is represented by isolated populations in the Gaspé Peninsula, Maine, New Hampshire, Vermont, New York, Michigan (Ball 1966a: 119-121), northeastern Minnesota (Cook County, CNC), and northern Wyoming (Big Horn County, FFPC) [see Ball and Currie 1997: Fig. 4]. The record from northern Colorado (Armin 1963: 217) is likely in error. Fossil remnants, older than 33,000 years B.P., have been unearthed in southwestern Ontario (Warner et al. 1988: 37); others from a Plio-Pleistocene sequence have been found in northwestern Greenland (Böcher 1995: 28).

#### Records.

**CAN**: AB, BC, LB, MB, NF, NT, NU, ON, QC, YT **USA**: AK, ME, MI, MN, NH, NY, VT, WY – **Holarctic**

#### Note.

The subspecies *Pterostichus brevicornis yasudai* Morita is endemic to the island of Hokkaidō, Japan.

### Pterostichus empetricola

(Dejean, 1828)

Feronia empetricola Dejean, 1828: 331. Type locality: «île d’Ounalaschka, l’une des îles Aleutiennes [Alaska]» (original citation for the lectotype). Lectotype (♀), designated by Ball (1966a: 122), in MHNP.
Cryobius ruficollis Mannerheim, 1853: 131. Type locality: «insula Afognak [Alaska]» (original citation). Holotype [by monotypy; designated lectotype by Ball (1966a: 122)] (♀) in MHNP. Synonymy established by Poppius (1906b: 65), confirmed by Ball (1966a: 122).
Cryobius rotundicollis Mannerheim, 1853: 132 [secondary homonym of *Pterostichus rotundicollis* (Duftschmid, 1812)]. Type locality: «insula Atkha [= Atka Island, Aleutian Islands, Alaska]» (original citation). Holotype [by monotypy] in ZILR. Synonymy established by Ball (1966a: 122).
Cryobius pacificus Poppius, 1906b: 184. Type locality: «Sibir[ia] or[ientali] [Russia]» (lectotype label). Lectotype (♀), designated by Ball (1966a: 122), in ZMH. Synonymy established by Ball (1966a: 122).
Pterostichus globicollis Csiki, 1930: 654. Replacement name for *Pterostichus rotundicollis* (Mannerheim, 1853).

#### Distribution.

This Holarctic species ranges from the Kuril and Commander Islands (Eremin 1998: 298) on the east coast of Asia to southern Yukon Territory, including Kodiak and Aleutian Islands, south to west-central British Columbia (Lemieux and Lindgren 2004: 562) [see Ball 1963: Fig. 3].

#### Records.

**CAN**: BC, YT **USA**: AK – **Holarctic**

#### Note.

Lindroth (1966: 524) stated that the taxonomic status of this taxon as a distinct species from *Pterostichus brevicornis* Kirby is questionable. Ball (1966a: 123) evoked the fact that the taxon may be regarded as a “parthenogenetic race” of *Pterostichus brevicornis*. Both authors concluded that this taxon is parthenogenetic since, as far as known, no males have been found to date that could be associated with it. This is also the case for the Asian specimens studied by Eremin (1998).

### Pterostichus mandibularoides

Ball, 1966

Pterostichus mandibularoides Ball, 1966a: 125. Type locality: «Anderson R[iver] delta, Northwest Territories» (original citation). Holotype (♂) in CNC [# 9232].

#### Distribution.

This species is found from the western shore of Hudson Bay in Nunavut westwards to the coast of the Bering Sea in Alaska, southwards to northeastern British Columbia and northwestern Alberta (UASM) [see Nielsen et al. 1987: Fig. 18b].

#### Records.

**CAN**: AB, BC, NT, NU, YT **USA**: AK

### Pterostichus nivalis

(Sahlberg, 1844)

Feronia nivalis R.F. Sahlberg, 1844: 37. Type locality: «monte Morikan [Okhotsk, Khabarovsk Kray, Russia]» (original citation). Lectotype (♀), designated by Bousquet (1999: 191), in ZMH.
Feronia thulensis J.R. Sahlberg, 1885a: 18. Type locality: «Irkajpi [Chukchi Peninsula, Russia]» (original citation for the lectotype). Lectotype (♂), designated by Ball (1966a: 129), in NRSS. Synonymy established by Ball (1966a: 128).

#### Distribution.

The range of this Holarctic species extends from eastern Siberia (Ball 1966a: 131) eastwards to east-central Yukon Territory (Dempster Highway Mi 35, CNC), south to the Alaska Peninsula [see Ball 1963: Fig. 3].

#### Records.

**CAN**: YT **USA**: AK – **Holarctic**

### [pinguedineus group]

### Pterostichus arcticola

(Chaudoir, 1868)

Feronia arcticola Chaudoir, 1868b: 339. Type locality: «Groenland» (original citation), which is incorrect (Ball 1966a: 29); «Labrador» selected by Ball (1966a: 29), herein restricted to Hopedale (see Ball 1966a: 35). Lectotype (♂), designated by Ball (1966a: 29), in MHNP.
Feronia labradorensis Chaudoir, 1868b: 340. Type locality: «Labrador» (original citation). Holotype [by monotypy] (♂) in MHNP. Synonymy established by Ball (1966a: 29).

#### Distribution.

This species ranges from the Labrador coast and Baffin Island to central Alaska, south at least to central British Columbia (Summit Lake, CNC); isolated on the Adirondack Mountains of New York, the White Mountains of New Hampshire, and the Shickshock Mountains in Gaspé Peninsula [see Ball 1963: Fig. 8; Nielsen et al. 1987: Fig. 17d; Ball and Currie 1997: Fig. 2]. Fossil remnants of this species, dated between about 16,700 and 18,100 years B.P., have been unearthed in southeastern Iowa (Baker et al. 1986: 96).

#### Records.

**CAN**: BC, LB, MB, NT, NU, QC, YT **USA**: AK, ME, NH, NY

### Pterostichus auriga

Ball, 1962

Pterostichus auriga Ball, 1962: 14. Type locality: «Crowbill Mountain, Cape Thompson, Alaska» (original citation). Holotype (♂) in MCZ [# 31311].

#### Distribution.

This species is known from two localities along the west coast of Alaska (Ball 1966a: 82).

#### Records.

**USA**: AK

### Pterostichus barryorum

Ball, 1962

Pterostichus barryorum Ball, 1962: 17. Type locality: «Delta Islands, Anderson R[iver], Northwest Territories» (original citation). Holotype (♂) in CNC [# 9230].

#### Distribution.

This species ranges from northwestern Nunavut and central Northwest Territories westwards to northeastern Alaska (Ball 1962: 19). The record from McMurray in northern Alberta (Ball 1966a: 49), based upon a single specimen, has been rejected by Ball and Currie (1997: 476). Fossil remnants of this species, dated between about 16,700 and 20,530 years B.P., have been unearthed in southeastern and northeastern Iowa (Baker et al. 1986: 96; Schwert 1992: 77; Woodman et al. 1996: 17).

#### Records.

**CAN**: NT, NU, YT **USA**: AK

### Pterostichus bryanti biocryus

Ball, 1962

Pterostichus biocryus Ball, 1962: 10. Type locality: «Crowbill Mountain, Cape Thompson, Alaska» (original citation). Holotype (♂) in MCZ [# 31309].

#### Distribution.

This subspecies is known only from the type locality in the arctic tundra and from the “Alpine interior Alaska” (Matthews 1974b: 1365).

#### Records.

**USA**: AK

### Pterostichus bryanti bryanti

(Van Dyke, 1951)

Feronia bryanti Van Dyke, 1951: 27. Type locality: «Aklavik, Mackenzie Delta, Northwest Territor[ies]y» (original citation). Holotype (♂) in CAS [# 6197].

#### Distribution.

This subspecies is known only from a few localities in the arctic from northwestern Northwest Territories (Ball 1966a: 66) to Prudhoe Bay in Alaska, including the Richardson Mountains in Yukon Territory (Ball and Currie 1997: 489).

#### Records.

**CAN**: NT, YT **USA**: AK

### Pterostichus bryanti bryantoides

Ball, 1962

Pterostichus bryantoides Ball, 1962: 11. Type locality: «141 Meridian, 69-20 [= 69°20'N, 141°W], Alaska [near the Yukon Territory border]» (original citation). Holotype (♂) in USNM.

#### Distribution.

This subspecies is known for sure only from Kotzebue on the Seward Peninsula (Matthews 1974b: 1365) and the type locality in northeastern Alaska. The record from “Yukon Territory” (Ball and Currie 1997: 452) could not be confirmed.

#### Records.

**USA**: AK [YT]

### Pterostichus bryanti cacumenis

Ball, 1966

Pterostichus cacumenis Ball, 1966a: 67. Type locality: «Eagle Summit, Mile 108.5, Steese Highway, Alaska» (original citation). Holotype (♀) in MCZ [# 31310].
Pterostichus haftorni Lindroth, 1969a: 1118. Type locality: «M[oun]t Harper, S[outh]E[ast] Fairbanks, Alaska» (original citation). Holotype (♂) in MCZ [# 35351]. Synonymy established by Ball and Currie (1997: 486). Etymology. The specific name was proposed for the Norwegian ornithologist and biologist Svein Haftorn [1925-2003] who collected the original two specimens.

#### Distribution.

This subspecies is known from a few localities between Yukon River and Fairbanks in the Yukon-Tanana Highlands in Alaska (Ball and Currie 1997: 486).

#### Records.

**USA**: AK

### Pterostichus bryanti stantonensis

Ball, 1966

Pterostichus stantonensis Ball, 1966a: 66. Type locality: «Stanton, Wood Bay, Northwest Territories» (original citation). Holotype (♀) in CNC [# 9372].

#### Distribution.

This subspecies is known only from the type locality located at the mouth of the Anderson River along the northwestern coast of Northwest Territories (Ball and Currie 1997: 486).

#### Records.

**CAN**: NT

### Pterostichus bryanti tiliaceoradix

Ball, 1962

Pterostichus tiliaceoradix Ball, 1962: 8. Type locality: «Savage River (2660 feet), M[oun]t McKinley National Park [Alaska]» (original citation). Holotype (♂) location unknown (not located in USNM as on 27 September 2006).

#### Distribution.

This subspecies is known only from a few localities in central Alaska (Lindroth 1966: 515).

#### Records.

**USA**: AK

### Pterostichus chipewyan

Ball, 1962

Pterostichus chipewyan Ball, 1962: 24. Type locality: «Churchill, Manitoba» (original citation). Holotype (♂) in CNC [# 9090].

#### Distribution.

This species is found from the Hudson Bay coast in northern Ontario to northeastern British Columbia, north to central Northwest Territories [see Ball 1963: Fig. 9].

#### Records.

**CAN**: AB, BC, MB, NT, ON

### Pterostichus gerstlensis

Ball, 1962

Pterostichus gerstlensis Ball, 1962: 22. Type locality: «Big Gerstle R[iver], mi[le] 1393, Alaska Highway, Alaska» (original citation). Holotype (♂) in MCZ [# 31305].

#### Distribution.

This species is known only from eastern and central Alaska [see Ball 1963: Fig. 8].

#### Records.

**USA**: AK

### Pterostichus hudsonicus

LeConte, 1863

Pterostichus hudsonicus LeConte, 1863c: 11. Type locality: «Hudson’s Bay Territory» (original citation), restricted to «Coppermine, Northwest Territories [= Nunavut]» by Bousquet (1999: 190). Lectotype (♂), designated by Ball (1966a: 49), in MCZ [# 5651].

#### Distribution.

The range of this species extends from the Hudson Bay area in Nunavut to Kuskokwim Bay on the western coast of Alaska, south to northeastern British Columbia [see Ball 1963: Fig. 9] and northern Alberta (Birch Mountains, Gerald J. Hilchie pers. comm. 2009, determination of George E. Ball). The records from northern Colorado (Wickham 1902: 236; Armin 1963: 218) are likely in error.

#### Records.

**CAN**: AB, BC, NT, NU, YT **USA**: AK

### Pterostichus kotzebuei

Ball, 1962

Pterostichus kotzebuei Ball, 1962: 19. Type locality: «Kotzebue, Alaska» (original citation). Holotype (♂) in MCZ [# 31304].

#### Distribution.

This species is known from the Dempster Highway in Yukon Territory (UASM) to the Seward Peninsula in western Alaska [see Ball 1963: Fig. 8].

#### Records.

**CAN**: YT **USA**: AK

### Pterostichus parasimilis

Ball, 1962

Pterostichus parasimilis Ball, 1962: 6. Type locality: «Umiat, Alaska» (original citation). Holotype (♂) in MCZ [# 31307].

#### Distribution.

This Holarctic species is known from the Kamchatka Peninsula, several islands in the Bering Sea, mainland Alaska, and the Ogilvie Mountains in western Yukon Territory [see Ball 1963: Fig. 4].

#### Records.

**CAN**: YT **USA**: AK – **Holarctic**

### Pterostichus pinguedineus

(Eschscholtz, 1823)

Poecilus pinguedineus Eschscholtz, 1823: 106. Type locality: «Unalaschka [Aleutian Islands, Alaska]» (original citation). Syntype(s) probably in ZILR. Note. This species was also made available the same year, under the name *Poecilus pinguedinis*, by an illustration in Fischer von Waldheim (1823: plate 19, figure 7).
Feronia frigida Dejean, 1828: 334. Type locality: «Kamschatka [Russia]» (original citation). Lectotype (♀), designated by Ball (1966a: 70), in MHNP. Synonymy established by Ball (1966a: 70).
Argutor mandibularis Kirby, 1837: 31. Type locality: «Lat. 54° [= along North Saskatchewan River]» (original citation). Lectotype [as type] (♂), designated by Ball (1966a: 70), in BMNH. Synonymy established by Ball (1966a: 70).
Feronia subsinuosa Chaudoir, 1868b: 339. Type locality: «Kadjak [Island, Alaska]» (original citation). Holotype [by monotypy] (♀) in MHNP. Synonymy established by Ball (1966a: 70).
Feronia diplogma Chaudoir, 1868b: 340. Type locality: «côte nord-ouest de l’Amérique du Nord» (original citation). Holotype [by monotypy] (♀) in MHNP. Synonymy established by Ball (1966a: 70).
Feronia stuxbergi Mäklin, 1878: 17. Type locality: «Krestowskoj [Taimyr Autonomous Okrug, Siberia, Russia]» (lectotype label). Lectotype (♂), designated by Ball (1966a: 70), in ZMH. Synonymy established by Ball (1966a: 70). Etymology. The specific name honors the Swedish explorer and naturalist Anton Julius Stuxberg [1849-1902] who took part to the Arctic zoological expeditions with Adolf Erika Nordenskiöld in 1875-1876 and 1878-1879. Stuxberg worked at the geological museum in Göteborg until his death.
Feronia despecta J.R. Sahlberg, 1885a: 14. Type locality: «Pitlekaj [Chukchi Peninsula, Russia]» (lectotype label). Lectotype (♀), designated by Ball (1966a: 70), in NRSS. Synonymy established by Ball (1966a: 70).
Feronia sulcipennis J.R. Sahlberg, 1885a: 15. Type locality: «Pitlekaj [Chukchi Peninsula, Russia]» (original citation). Holotype [by monotypy] (♀) in NRSS. Synonymy established by Ball (1966a: 71).
Feronia laeviuscula J.R. Sahlberg, 1885a: 16 [secondary homonym of *Pterostichus longicollis* var. *laeviusculus* Letzner, 1852]. Type locality: «Nunamo, S[ain]t Lawrence Bay [Chukchi Peninsula, Siberia, Russia]» (original citation). Holotype [by monotypy] (♀) in NRSS. Synonymy established by Ball (1966a: 71).
Feronia splendida J.R. Sahlberg, 1885b: 49. Type locality: Port Clarence, Alaska (inferred from title of the paper). Holotype [by monotypy] (♀) in NRSS. Synonymy established by Ball (1966a: 71).
Cryobius subnitidulus Poppius, 1906b: 62. Type locality: «Insel Kadjak [Alaska]» (original citation). Syntype(s) [2 originally cited] in ZMH (Poppius 1906b: 64). Synonymy established, under the name *Pterostichus subsinuosus* (Chaudoir), by Poppius (1908a: 2).
Cryobius holmbergi Poppius, 1906b: 70. Type locality: «Insel Kadjak [Alaska]» (original citation). Holotype [by monotypy] (♀) in ZMH. Synonymy established by Ball (1966a: 71).
Cryobius incognitus Poppius, 1906b: 91. Type locality: «Amer[ica] ross[ica]» (original citation). Holotype [by monotypy] (♀) in ZILR. Synonymy established by Ball (1966a: 71).
Cryobius stuxbergi var. *fortestriatus* Poppius, 1906b: 103. Type locality: «Briochowskiostroff, Fl[umen] Jenissej, arktisch Sibirien [Russia]» (original citation). Holotype [by monotypy] (♀) in ZMH. Synonymy established by Ball (1966a: 71).
Cryobius stuxbergi repandus Poppius, 1906b: 104. Type locality: «Lena infer[ior] [Yakutia, Siberia, Russia]» (lectotype label). Lectotype (♀), designated by Ball (1966a: 71), in ZMH. Synonymy established by Ball (1966a: 71).
Cryobius alaskensis Poppius, 1906b: 116. Type locality: «Insel Kadjak, Alaska» (original citation). Syntype(s) [4 originally cited] in ZILR. Synonymy established by Ball (1966a: 71).
Pterostichus montanellus Poppius, 1907: 21. Type locality: «White Mountains [New Hampshire]» (original citation). Two syntypes [2 originally cited] in ZMHB (Lindroth 1966: 518) and ZMH (Silfverberg 1987: 20). Synonymy established by Ball (1966a: 71).
Pterostichus beringensis Poppius, 1908b: 4. Type locality: «St. Lawrence Bai [Chukchi Peninsula, Russia]» (original citation). Holotype [by monotypy] location unknown. Synonymy established by Bousquet (2003d: 492).
Cryobius beringi Casey, 1918: 374. Type locality: «S[ain]t Paul Island, Alaska» (original citation). Lectotype [as holotype] (♀), designated by Ball (1966a: 71), in USNM [# 47078]. Synonymy established by Ball (1966a: 71).
Cryobius washingtoni Casey, 1920: 190. Type locality: «White M[oun]t[ain]s, New Hampshire» (original citation). Lectotype (♀), designated by Ball (1966a: 71), in USNM [# 47075]. Synonymy established by Ball (1966a: 71).
Pterostichus anadyricus Csiki, 1930: 651. Replacement name for *Pterostichus laeviusculus* (Sahlberg, 1885).

#### Distribution.

This Holarctic species ranges from the Yenisei River in Siberia eastwards to the Mackenzie River delta in western Northwest Territories; isolated on the top of some mountains in New England and on the Shickshock Mountains of the Gaspé Peninsula, Quebec [see Ball 1963: Fig. 5]. The record from Wyoming (Lavigne 1977: 47) is in error. Fossil remnants of this species, dated between about 12,000 and 18,100 years B.P., have been unearthed in central and southeastern Iowa (Schwert 1992: 77; Baker et al. 1986: 96) and northeastern Pennsylvania (Barnosky et al. 1988: 178); others, older than 33,000 years B.P., have been found in southwestern Ontario (Warner et al. 1988: 37).

#### Records.

**CAN**: NT, QC, YT **USA**: AK, ME, NH, VT – **Holarctic**

#### Note.

*Platysma poppiusianum* Jacobson, 1907 (a replacement name for *Pterostichus insulicola* Poppius, 1906) was listed as a junior synonym of this species by Ball (1966a: 71), as a junior synonym of *Pterostichus subgibbus* (Motschulsky, 1860) by Kryzhanovskij et al. (1975: 137), and treated as a valid species by Eremin (1990) and Kryzhanovskijet al. (1995: 102).

### Pterostichus planus

(Sahlberg, 1885)

Feronia plana J.R. Sahlberg, 1885b: 50. Type locality: Port Clarence, Alaska (inferred from title of the paper). Lectotype (♀), designated by Lindroth (1969a: 1118), in ZMLS.
Feronia blaisdelli Van Dyke, 1943: 24. Type locality: «Nome, Alaska» (original citation). Holotype (♂) in CAS [# 5304]. Synonymy established by Ball (1966a: 69).

#### Distribution.

This species is known only from a few localities in the Seward Peninsula, western Alaska [see Ball 1963: Fig. 6].

#### Records.

**USA**: AK

### Pterostichus similis

Mannerheim, 1852

Pterostichus similis Mannerheim, 1852: 296. Type locality: «insula St. Georgii [Pribilof Islands, Alaska]» (original citation). Lectotype (♂), designated by Ball (1966a: 53), in ZMH.
Cryobius quadricollis Mannerheim, 1853: 133. Type locality: «insula St. Georgii [Pribilof Islands, Alaska]» (original citation). Holotype [by monotypy] probably in ZILR. Synonymy established by LeConte (1873a: 310).

#### Distribution.

This species is found from eastern Siberia to the Dempster Highway in Yukon Territory (UASM), including several islands of the Bering Sea [see Ball 1963: Fig. 4].

#### Records.

**CAN**: YT **USA**: AK – **Holarctic**

#### Note.

Matthews (1974b: 1365) remarked that some specimens of this species have anomalous male genitalia. He interpreted this condition as either *Pterostichus similis* “is dimorphic in certain genital characters” or that it “includes two species” as currently defined.

### Pterostichus soperi

Ball, 1966

Pterostichus soperi Ball, 1966a: 26. Type locality: «Aklavik, N[orth]w[est] T[erritories]» (original citation). Holotype (♂) in CAS [# 9316]. Etymology. This species was named after the Canadian Joseph Dewey Soper [1893-1982], arctic explorer, zoologist, and prolific author. Soper, as biologist for the Canadian Wildlife Service, made important contributions to the knowledge of arctic birds and mammals and to arctic geography.

#### Distribution.

This species is known from the Norton Sound Inlet in western Alaska to the Coronation Gulf in northern Nunavut (Ball 1966a: 28-29).

#### Records.

**CAN**: NT, NU, YT **USA**: AK

### Pterostichus surgens

LeConte, 1878

Pterostichus surgens LeConte, 1878a: 449. Type locality: «Alma (10,000 ft.) [Park County], Col[orado]» (lectotype label). Lectotype (♂), designated by Ball (1966a: 24), in MCZ [# 5652].

#### Distribution.

This species is found along the Rocky Mountains from southern Alberta to Utah and Colorado (Ball 1966a: 26).

#### Records.

**CAN**: AB **USA**: CO, MT, UT, WY

### Pterostichus tareumiut

Ball, 1962

Pterostichus tareumiut Ball, 1962: 15. Type locality: «Point Barrow, Alaska» (original citation). Holotype (♂) in MCZ [# 31306].

#### Distribution.

This Holarctic species is known in North America along the arctic coast from western Alaska to north-central Nunavut, and from Victoria and Banks Islands; in the Palaearctic Region the species is known only from the northern Bering Sea Coast (Ball 1966a: 47).

#### Records.

**CAN**: NT, NU, YT **USA**: AK – **Holarctic**

### Pterostichus woodi

Ball and Currie, 1997

Pterostichus woodi Ball and Currie, 1997: 487. Type locality: «km. 155, Dempster H[igh]w[a]y, Yukon [Territory]» (original citation). Holotype (♂) in CNC [# 23110]. Etymology. The specific name was proposed for Donald Monty Wood [1933-], dipterist at Agriculture and Agri-Food Canada (Canadian National Collection of Insects) who collected most of the specimens of the type series.

#### Distribution.

This species is known only from the Ogilvie Mountains in Yukon Territory.

#### Records.

**CAN**: YT

### [ventricosus group]

### Pterostichus caribou

Ball, 1962

Pterostichus caribou Ball, 1962: 3. Type locality: «Eskimo Point, Northwest Territories [= Nunavut]» (original citation). Holotype (♂) in CNC [# 9094].

#### Distribution.

This species ranges from northern Alaska to the western shores of the Hudson Bay in northern Manitoba and Southampton Island in Nunavut [see Ball 1963: Fig. 2; Nielsen et al. 1987: Fig. 18a]; isolated in eastern Ohio (Usis and MacLean 1998: 67). Fossil remnants of this species, dated between about 16,700 and 21,500 years B.P., have been unearthed in north-central Illinois (Garry et al. 1990: 394) and east-central Iowa (Baker et al. 1986: 96).

#### Records.

**CAN**: MB, NT, NU, YT **USA**: AK, OH

### Pterostichus riparius

(Dejean, 1828)

Feronia riparia Dejean, 1828: 332. Type locality: «détroit de Norfolk [= Norfolk Sound, Baranof Island, Alaska] sur la côte nord-ouest de l’Amérique du Nord [Alaska]» (original citation). Holotype [by monotypy; designated lectotype by Ball (1966a: 83)] (♀) in MHNP.
Omaseus fuscoaeneus Chaudoir, 1835: 448. Type locality: «Détroit de Norfolk [= Norfolk Sound, Baranof Island, Alaska]» (original citation). Syntype(s) location unknown. Synonymy established by LeConte (1869b: 248).
Cryobius fatuus Mannerheim, 1853: 130. Type locality: «Kadjak [Alaska]» (lectotype label). Lectotype (♂), designated by Bousquet (1999: 193), in ZMH. Synonymy established by Tschitschérine (1891: 143), confirmed by Ball (1966a: 83).
Cryobius breviusculus Casey, 1918: 375 [secondary homonym of *Pterostichus breviusculus* (Sahlberg, 1844)]. Type locality: «S[ain]t Paul Island, Alaska» (original citation). Lectotype [as holotype] (♀), designated by Ball (1966a: 84), in USNM [# 47077]. Synonymy established by Ball (1966a: 84).
Pterostichus laevilatus Notman, 1919b: 231. Type locality: «Golden, B[ritish] C[olumbia]» (original citation). Holotype [by monotypy] (♀) in USNM [# 75390]. Synonymy established by Hatch (1953: 114).
Cryobius patulus Casey, 1920: 190. Type locality: «Stickine River Cañon, British Columbia» (original citation). Lectotype [as holotype], designated by Ball (1966a: 84), in USNM [# 47076]. Synonymy established by Hatch (1953: 114), confirmed by Ball (1966a: 84).
Pterostichus caseyi Csiki, 1930: 652. Replacement name for *Pterostichus breviusculus* (Casey, 1918).

#### Distribution.

This species ranges from central Alaska to eastern Alberta, including western Northwest Territories, south to west-central Montana, northern Idaho, and northern Oregon [see Ball 1963: Fig. 2]. The records from northern Colorado (Packard 1877: 811; Wickham 1902: 236; Armin 1963: 218; Elias 1987: 632, as *Pterostichus fatuus*) need confirmation. Fossil remnants from a Plio-Pleistocene sequence have been unearthed in northwestern Greenland (Böcher 1995: 28).

#### Records.

**CAN**: AB, BC (QCI), NT, YT **USA**: AK, ID, MT, OR, WA [CO]

### Pterostichus ventricosus ventricosus

(Eschscholtz, 1823)

Poecilus ventricosus Eschscholtz, 1823: 106. Type locality: «Unalaschka [Aleutian Islands, Alaska]» (original citation). Two syntypes in ZMH (Silfverberg 1987: 26). Note. This species was also made available the same year by an illustration in Fischer von Waldheim (1823: plate 19, figure 6).
Platysma borealis Ménétriés, 1851: 50 [secondary homonym of *Pterostichus borealis* (Zetterstedt, 1828)]. Type locality: «Taimyrsee bei 74½° n. Br[eite]; Boganida [Russia]» (original citation). Syntypes location unknown (possibly in ZMH). Synonymy established by Ball (1966a: 89).
Omaseus rufiscapus Mannerheim, 1853: 126. Type locality: «insula Kadjak [Alaska]» (original citation). Syntype(s) probably in MHNP (see Chaudoir 1868b: 341). Synonymy established by Ball (1966a: 89).
Cryobius hyperboreus Mannerheim, 1853: 127. Type locality: «insula St. Georgii [Pribilof Islands, Alaska]» (original citation). Holotype [by monotypy] (♀) in ZMH. Synonymy established, under the name *Pterostichus subexaratus* (Mannerheim), by Poppius (1906b: 31), confirmed by Ball (1966a: 88).
Cryobius subexaratus Mannerheim, 1853: 128. Type locality: «insula Unalaschka [Alaska]» (original citation for the lectotype). Lectotype (♂), designated by Bousquet (1999: 194), in ZMH. Synonymy established by Ball (1966a: 88).
Cryobius vindicatus Mannerheim, 1853: 129. Type locality: «oris meridionali et occidentali insulae Kadjak [Alaska]» (original citation). Lectotype (♂), designated by Bousquet (1999: 194), in ZMH. Synonymy established by Ball (1966a: 89).
Cryobius subcaudatus Mannerheim, 1853: 132. Type locality: «ad rivulos fl[umen] Tschunuktnu peninsulae Kenai [Alaska]» (original citation). Lectotype (♀), designated by Ball (1966a: 89), in ZMH. Synonymy established by Ball (1966a: 89). Note. Brown (1952: 340) noted that based on Mannerheim’s map (1853: plate 2), “Tschunuktnu” River is either Resurrection Creek or Sixmile Creek and that these flow into Turnagain Arm of Cook Inlet and Sunrise respectively.
Feronia quadrangularis J.R. Sahlberg, 1885a: 20. Type locality: «Pitlekaj [Chukchi Peninsula, Siberia]» (original citation). Holotype [by monotypy] (♂) in ZMH. Synonymy established by Ball (1966a: 88).
Feronia rugifera Tschitschérine, 1891: 141. Type locality: «île d’Ounalaschka [Aleutian Islands, Alaska] (original citation). Holotype [by monotypy] (♀) probably in ZILR. Synonymy established by Ball (1966a: 88).
Feronia ventricosa var. *brevicollis* Tschitschérine, 1891: 142 [secondary homonym of *Feronia brevicollis* (LeConte, 1846)]. Type locality not stated. Syntype(s) location unknown (possibly in ZMMU). Synonymy established by Ball (1966a: 88).
Feronia borealis var. *gracilior* Tschitschérine, 1896b: 376. Type locality: «Novaja Zemlja [= Novaya Zemlya, Russia]» (original citation). Syntype(s) [3 originally cited] in ZILR. Synonymy established, under the name *Pterostichus borealis* (Ménétriés), by Poppius (1906b: 97).
Pterostichus vegae Poppius, 1906b: 39. Type locality: «Tschuktschen-Halbinsel bei Jinretlen, NO Sibirien [Russia]» (original citation). Holotype [by monotypy] (♀) in NRSS (Ball 1966a: 88). Synonymy established by Ball (1966a: 88).
Cryobius czekanowskii Poppius, 1906b: 46. Type locality: «Fl[umen] Jenissej: Tolstoinos [=Tolstyy Nos, Taymyr Autonomous Okrug, Russia]» (original citation for the lectotype). Lectotype [as holotype] (♀), designated by Ball (1966a: 89), in ZMH. Synonymy established by Ball (1966a: 89).
Cryobius sedakowi Poppius, 1908a: 3. Type locality: «Bureja-Fluss, Ost-Sibirien [Russia]» (original citation). Holotype [by monotypy] (♂) in MHNP (collection Chaudoir). Synonymy established by Ball (1966a: 89).
Platysma ventricosum var. *aleutorum* Lutshnik, 1915c: 427. Replacement name for *Platysma ventricosum* var. *brevicolle* (Tschitschérine, 1891).
Cryobius otariidinus Casey, 1918: 374. Type locality: «S[ain]t Paul Island, Alaska» (original citation), which is probably incorrect (Ball 1966a: 89). Lectotype [as holotype] (♀), designated by Ball (1966a: 89), in USNM [# 47080]. Synonymy established by Ball (1966a: 89).
Pterostichus boreus Csiki, 1930: 652. Replacement name for *Pterostichus borealis* (Ménétriés, 1851).

#### Distribution.

This Holarctic species ranges from Novaya Zemlya in the Arctic Ocean off the coast of European Russia east to Franklin Bay on the coast of Northwest Territories, south in the Nearctic Region to central Northwest Territories and Umnak Island in the Aleutian Islands [see Ball 1963: Fig. 2]. Fossil remnants of this species, dated between about 16,700 and 20,530 years B.P., have been unearthed in northeastern and southeastern Iowa (Schwert 1992: 77; Baker et al. 1986: 96; Woodman et al. 1996: 17); others, older than 33,000 years B.P., have been found in southwestern Ontario (Warner et al. 1988: 37).

#### Records.

**CAN**: NT, YT **USA**: AK – **Holarctic**

#### Note.

The subspecies *Pterostichus ventricosus nechaevi* Lafer and Kuznetsov is endemic to Sakhalin Island and *Pterostichus ventricosus paludosus* (Sahlberg) (junior synonym: *Pterostichus tungusicus* Poppius) to southeastern Siberia (Ball 1966a: 101).

### Genus Cyclotrachelus

Chaudoir, 1838

Cyclotrachelus Chaudoir, 1838: 27. Type species: *Feronia tenebricosa* Dejean, 1828 (= *Molops faber* Germar, 1824) by monotypy. Etymology (original). From the Greek *cyclos* (circle) and *trachelos* (neck, by extension pronotum), alluding to the rounded shape of the pronotum (“*corselet arrondi*”) of the adults in the hands of Chaudoir [masculine].

#### Diversity.

Forty-five species in the temperate and subtropical regions of North America with one species (*Pterostichus substriatus*) extending into northern Mexico.

#### Identification.

Freitag (1969) revised the species. Since the publication of his revision, a replacement name has been proposed for *Cyclotrachelus obsoleta* and two subspecies are raised to species level in this publication.

#### Taxonomic Note.

This genus, certainly one of the most characteristic pterostichine elements in North America, could be closely related to *Molops* Bonelli (Freitag 1969), whose species inhabit the mountains of Europe and Turkey, or to the *Sterocorax* complex (*Sterocorax* Ortuño, *Paleocorax* Ortuño, *Iberopus* Ortuño), a clade endemic to the Iberian Peninsula and currently included within the genus *Pterostichus* Bonelli (see Bousquet 1999: 198). Based on molecular sequence data, Will and Gill (2008: 113) found that the genus *Pterostichus* was monophyletic if *Cyclotrachelus* were included in the genus and recommended to treat *Cyclotrachelus* as a subgenus of *Pterostichus*.

### Subgenus Cyclotrachelus

Chaudoir, 1838

Cyclotrachelus Chaudoir, 1838: 27. Type species: *Feronia tenebricosa* Dejean, 1828 (= *Molops faber* Germar, 1824) by monotypy.
Fortax Motschulsky, 1866: 246. Type species: *Feronia morio* Dejean, 1828 (= *Pterostichus dejeanellus* Csiki, 1930) designated by Freitag (1969: 101). Synonymy established by Bousquet (1999: 199). Etymology. From the Latin *fortax* (carrier, bearer) [masculine].
Ferestria Leng, 1915: 576. Type species: *Broscus laevipennis* LeConte, 1846 by original designation. Synonymy established, under the name *Fortax* Motschulsky, by Csiki (1930: 674). Etymology. Possibly from the Latin *fero* (to bear, carry) and *stria* (groove, stria) [feminine].

#### Diversity.

Eighteen species, most restricted to the Coastal and Gulf Plains.

### [approximatus group]

### Cyclotrachelus approximatus

(LeConte, 1846)

Broscus approximatus LeConte, 1846b: 354. Type locality: «Pennsylvania» (original citation). Lectotype (♀), designated by Freitag (1969: 106), in MCZ [# 5628].

#### Distribution.

This species is restricted to a small area from “Pennsylvania” (LeConte 1846b: 354) to North Carolina [see Freitag 1969: Fig. 126]. The record from Georgia (Fattig 1949: 24) needs confirmation.

#### Records.

**USA**: DC, MD, NC, PA, VA [GA]

### Cyclotrachelus freitagi

Bousquet, 1993

Feronia obsoleta Say, 1830b: (5) [3] [primary homonym of *Feronia obsoleta* Say, 1823]. Type locality: «Cades Cove, Blount [Blount County], Tenn[essee]» (neotype label). Neotype (♂), designated by Lindroth and Freitag (1969: 340), in MCZ [# 33046]. Note. «Indiana» was the area originally cited by Say (1830b: (5) [3]).
Cyclotrachelus freitagi Bousquet [in Bousquet and Larochelle], 1993: 10. Replacement name for *Cyclotrachelus obsoletus* (Say, 1830).

#### Distribution.

The range of this species extends from West Sister Island (Ohio) in the Western Basin of Lake Erie (Will et al. 1995: 62) to northeastern Illinois, south to southern Alabama and northern Georgia [see Freitag 1969: Fig. 126]. The record from Missouri (Summers 1873: 134) needs confirmation.

#### Records.

**USA**: AL, GA, IL, IN, KY, MI, MS, NC, OH, SC, TN, VA [MO]

### Cyclotrachelus iuvenis

(Freitag, 1969)

Evarthrus iuvenis Freitag, 1969: 107. Type locality: «24 miles north of Roanoke [Roanoke County], Virginia» (original citation). Holotype (♂) in MCZ [# 31591].

#### Distribution.

This species is known from a small area in west-central and southern West Virginia (Roane and Raleigh Counties, CMNH), western Virginia, and North Carolina [see Freitag 1969: Fig. 126].

#### Records.

**USA**: NC, VA, WV

### [faber group]

### Cyclotrachelus faber

(Germar, 1824)

Molops faber Germar, 1824: 23. Type locality: «Kentucky» (original citation), which is probably incorrect (Freitag 1969: 125); Gainesville, Alachua County, Florida (see Freitag 1969: 126) herein selected. Syntype(s) probably lost.
Feronia tenebricosa Dejean, 1828: 301. Type locality: «Amérique septentrionale» (original citation). One syntype in MHNP (Lindroth 1955b: 14). Synonymy established by LeConte (1846b: 353), confirmed by Lindroth (1955b: 14).
Cyclotrachelus roticollis Casey, 1918: 349. Type locality: «Dunedin [Pinellas County], Florida» (original citation). Lectotype [as holotype] (♂), designated by Freitag (1969: 125), in USNM [# 47108]. Synonymy established by Freitag (1969: 125).
Cyclotrachelus fallaciosus Casey, 1924: 77. Type locality: «Dunedin [Pinellas County], Florida» (original citation). Lectotype [as holotype] (♂), designated by Freitag (1969: 125), in USNM [# 47109]. Synonymy established by Freitag (1969: 125).

#### Distribution.

This species is found in southern Georgia and Florida including the Keys [see Freitag 1969: Fig. 129]. The record from northwestern South Carolina (Kirk 1970: 11) is probably in error.

#### Records.

**USA**: FL, GA

### Cyclotrachelus levifaber

(Freitag, 1969)

Evarthrus levifaber Freitag, 1969: 123. Type locality: «Camden [Kershaw County], S[outh] C[arolina]» (original citation). Holotype (♀) in MCZ [# 31592].

#### Distribution.

This species is known only from a few specimens collected in “North Carolina,” northern South Carolina, and “Georgia” [see Freitag 1969: Fig. 129].

#### Records.

**USA**: GA, NC, SC

### Cyclotrachelus parafaber

(Freitag, 1969)

Evarthrus parafaber Freitag, 1969: 122. Type locality: «Mobile [Mobile County], Ala[bama]» (original citation). Holotype (♂) in CAS [# 813].

#### Distribution.

This species is known only from several specimens collected at the type locality in southern Alabama [see Freitag 1969: Fig. 129].

#### Records.

**USA**: AL

### [laevipennis group]

### Cyclotrachelus dejeanellus

(Csiki, 1930)

Feronia morio Dejean, 1828: 302 [secondary homonym of *Pterostichus morio* (Duftschmid, 1812)]. Type locality: «Amérique septentrionale» (original citation), restricted to «Alma [Bacon County], Georgia» by Freitag (1969: 102). Lectotype [as type], designated by Freitag (1969: 102), in MHNP.
Pterostichus dejeanellus Csiki, 1930: 674. Replacement name for *Pterostichus morio* (Dejean, 1828).
Evarthrus taurus Van Dyke, 1943: 25. Type locality: «near Punta Gorda [Charlotte County], Florida» (original citation). Holotype (♂) in CAS [# 5306]. Synonymy established by Freitag (1969: 102).

#### Distribution.

This species ranges from southern Georgia to southwestern Florida [see Freitag 1969: Fig. 125]. The record from Alabama (Löding 1945: 16) needs confirmation.

#### Records.

**USA**: FL, GA [AL]

### Cyclotrachelus hernandensis

(Van Dyke, 1943)

Evarthrus hernandensis Van Dyke, 1943: 26. Type locality: «near Brooksville, Hernando County, Florida» (original citation). Holotype (♂) in CAS [# 5308].

#### Distribution.

This species is known from a few localities in the western parts of the Florida Peninsula [see Freitag 1969: Fig. 125].

#### Records.

**USA**: FL

### Cyclotrachelus laevipennis

(LeConte, 1846)

Broscus laevipennis LeConte, 1846b: 354. Type locality: «Georgia» (original citation), restricted to «Clayton, Rabun County» by Bousquet (1999: 201). Lectotype (♀), designated by Freitag (1969: 103), in MCZ [# 5627].
Evarthrus acutus LeConte, 1853a: 231. Type locality: «Louisiana» (original citation). Lectotype (♀), designated by Freitag (1969: 104), in MCZ [# 5626]. Synonymy established by Freitag (1969: 104).
Ferestria nanula Casey, 1918: 364. Type locality: «Mobile [Mobile County], Alabama» (original citation). Lectotype [as holotype] (♀), designated by Freitag (1969: 104), in USNM [# 47111]. Synonymy established by Freitag (1969: 104).
Ferestria simiola Casey, 1920: 192. Type locality: «Mobile [Mobile County], Alabama» (original citation). Holotype [by monotypy] (♀) in USNM [# 47112]. Synonymy established by Freitag (1969: 104).
Ferestria castigata Casey, 1920: 192. Type locality: «Mobile [Mobile County], Alabama» (original citation). Lectotype [as holotype] (♂), designated by Freitag (1969: 104), in USNM [# 47110]. Synonymy established by Freitag (1969: 104).
Ferestria bullata Casey, 1920: 193. Type locality: «Mobile [Mobile County], Alabama» (original citation). Holotype [by monotypy] (♀) in USNM [# 47113]. Synonymy established by Freitag (1969: 104).

#### Distribution.

This species inhabits the Gulf Plain and southern Piedmont Plateau from South Carolina to the Florida Panhandle and “Louisiana” (LeConte 1853a: 231, as *Evarthrus acutus*) [see Freitag 1969: Fig. 125].

#### Records.

**USA**: AL, FL, GA, LA, MS, SC

### [ovulum group]

### Cyclotrachelus alabamensis

(Casey, 1920)

Evarthrus constrictus Bates, 1882a: 80 [secondary homonym of *Evarthrus constrictus* (Say, 1823)]. Type locality: «Mexico» (original citation), which is likely incorrect. Holotype [by monotypy] (♀) in BMNH.
Evarthrinus alabamensis Casey, 1920: 198. Type locality: «Allen [= Allenville, Mobile County], Alabama» (original citation). Lectotype [as holotype] (♂), designated by Freitag (1969: 117), in USNM [# 47136]. Synonymy established by Freitag (1969: 117).
Evarthrinus lilliputicus Casey, 1920: 199. Type locality: «Mobile [Mobile County], Alabama» (original citation). Lectotype [as holotype] (♂), designated by Freitag (1969: 117), in USNM [# 47137]. Synonymy established by Freitag (1969: 117).
Pterostichus batesellus Csiki, 1930: 671. Replacement name for *Pterostichus constrictus* (Bates, 1882).

#### Distribution.

This species is known only from Mobile County in southwestern Alabama (Freitag 1969: 118). The record from “Mississippi” (Bousquet and Larochelle 1993: 186) was based on a misidentified specimen (Drew A. Hildebrandt pers. comm. 2007).

#### Records.

**USA**: AL

### Cyclotrachelus macrovulum

(Freitag, 1969)

Evarthrus macrovulum Freitag, 1969: 119. Type locality: «Mobile [Mobile County], Ala[bama]» (original citation). Holotype (♂) in CAS [# 9814].

#### Distribution.

This species is known only from southern Alabama, southern Mississippi (Drew A. Hildebrandt pers. comm. 2007), and east-central (West Feliciana Parish, LSAM) and southern Louisiana [see Freitag 1969: Fig. 128].

#### Records.

**USA**: AL, LA, MS

### Cyclotrachelus ovulum

(Chaudoir, 1868)

Feronia ovulum Chaudoir, 1868b: 332. Type locality: «Georgetown, Caroline du Sud» (original citation), which according to Freitag (1969: 119) is probably in error for Georgetown, Georgia. Holotype [by monotypy] (♀) in MHNP.

#### Distribution.

This species is known from southern Georgia and northern Florida [see Freitag 1969: Fig. 128].

#### Records.

**USA**: FL, GA

### Cyclotrachelus texensis

(Freitag, 1969)

Evarthrus texensis Freitag, 1969: 121. Type locality: «12 mi[les] W[est] Kirbyville, Tyler Co[unty], Texas» (original citation). Holotype (♂) in MCZ [# 35349].

#### Distribution.

This species is known from a few specimens collected in eastern Texas [see Freitag 1969: Fig. 128].

#### Records.

**USA**: TX

### Cyclotrachelus vinctus

(LeConte, 1853)

Evarthrus vinctus LeConte, 1853a: 232. Type locality: «Nakutshi valley, Habersham Co[unty], Georgia» (original citation). Lectotype (♀), designated by Freitag (1969: 115), in MCZ [# 5623].

#### Distribution.

This species is found in a small area including eastern Tennessee, southwestern North Carolina, northwestern South Carolina, and northeastern Georgia [see Freitag 1969: Fig. 128].

#### Records.

**USA**: GA, NC, SC, TN

### [spoliatus group]

### Cyclotrachelus brevoorti

(LeConte, 1846)

Feronia brevoorti LeConte, 1846b: 352. Type locality: «Alabama» (original citation), restricted to «Mobile, Mobile County» by Bousquet (1999: 200). Lectotype (♂), designated by Freitag (1969: 114), in MCZ [# 5625]. Etymology. This species was named for James Carson Brevoort [1818-1887], a wealthy American collector.

#### Distribution.

This species is found along the Coastal Plain from South Carolina to central Mississippi [see Freitag 1969: Fig. 127].

#### Records.

**USA**: AL, FL, GA, MS, SC

### Cyclotrachelus fucatus

(Freitag, 1969)

Evarthrus fucatus Freitag, 1969: 111. Type locality: «Leesburg, Cherokee Co[unty], Ala[bama]» (original citation). Holotype (♂) in UMAA.

#### Distribution.

This species ranges from southwestern Pennsylvania to southwestern Illinois (Union County, CNC), south to northern Alabama and northwestern Georgia [see Freitag 1969: Fig. 127].

#### Records.

**USA**: AL, GA, IL, KY, OH, PA, TN, WV

### Cyclotrachelus spoliatus

(Newman, 1838)

Feronia spoliata Newman, 1838a: 386. Type locality: «northern states of America» (original citation, see page 388), restricted to «Southern Pines [Moore County], N[orth] C[arolina]» by Freitag (1969: 113). Lectotype [as type] (♂), designated by Freitag (1969: 113), in BMNH.
Evarthrus rotundatus LeConte, 1853a: 230. Type locality: «Athens [Clarke County], Georgia» (original citation). Holotype [by monotypy] (♀) in MCZ [# 5624]. Synonymy established by Freitag (1969: 113). Note. The specimen labeled “Va / rotundatus 2” and designated lectotype by Freitag (1969: 113) is not a syntype.
Evarthrinus pinorum Casey, 1920: 198. Type locality: «Southern Pines [Moore County], North Carolina» (original citation). Lectotype [as holotype] (♂), designated by Freitag (1969: 113), in USNM [# 47135]. Synonymy established by Freitag (1969: 113).

#### Distribution.

This species is found in a small area from the District of Columbia south to southern South Carolina [see Freitag 1969: Fig. 127] including northeastern Georgia (LeConte 1853a: 230). The records from “New York” (Leng and Beutenmüller 1893: 138, as *Pterostichus rotundatus*) and northern Alabama (Löding 1945: 15) need confirmation.

#### Records.

**USA**: DC, GA, NC, SC, VA [AL, NY]

### Cyclotrachelus unicolor

(Say, 1823)

Feronia unicolor Say, 1823a: 40. Type locality: «Upson Co[unty], G[eorgi]a» (neotype label). Neotype (♀), designated by Lindroth and Freitag (1969: 340), in MCZ [# 33049].

#### Distribution.

This species is restricted to the Coastal Plain and southern Piedmont from South Carolina (Ciegler 2000: 71) to northeastern Alabama, south to the Florida Panhandle [see Freitag 1969: Fig. 127]. The record from North Carolina (Brimley 1938: 119) needs confirmation.

#### Records.

**USA**: AL, FL, GA, SC [NC]

### Subgenus Evarthrus

LeConte, 1853

Evarthrus LeConte, 1853a: 226, 227. Type species: *Feronia sigillata* Say, 1823 designated by Casey (1918: 322). Etymology. From the Greek *ev* (original, primitive, by extension simple) and *arthron* (joint), possibly alluding to the simple (i.e., non carinate) basal antennomeres (“*antennae articulis basalibus simplicibus*”) of the adult [masculine].
Anaferonia Casey, 1918: 341. Type species: *Feronia constricta* Say, 1823 by original designation. Synonymy established by Freitag (1969: 126). Etymology. From the Greek prefix *ana*- (up, back, again) and the generic name *Feronia* [*q.v*.] [feminine].
Megasteropus Casey, 1918: 350. Type species: *Megasteropus gigas* Casey, 1918 by original designation. Synonymy established by Freitag (1969: 126). Etymology. From the Greek *megas* (large) and the generic name *Steropus* [masculine].
Eumolops Casey, 1918: 351. Type species: *Eumolops sexualis* Casey, 1918 (= *Evarthrus torvus torvus* LeConte, 1863) by original designation. Synonymy established by Freitag (1969: 127). Etymology. From the Greek *eu* (agreeable, primitive) and the generic name *Molops* [masculine].
Evarthrinus Casey, 1918: 357. Type species: *Eumolops decepta* Casey, 1918 designated by Freitag (1969: 127). Synonymy established by Freitag (1969: 127). Etymology. From the generic name *Evarthrus* [*q.v*.] and the Latin suffix -*inus* (pertaining to) [masculine].
Evarthrops Casey, 1920: 194. Type species: *Evarthrus furtivus* LeConte, 1853 designated by Freitag (1969: 127). Synonymy established by Freitag (1969: 127). Etymology. From the generic name *Evarthrus* [*q.v*.] and the Greek suffix -*ops* (having the appearance of) [masculine].

#### Diversity.

Twenty-seven species are known.

### [blatchleyi group]

### Cyclotrachelus blatchleyi

(Casey, 1918)

Evarthrus blatchleyi Casey, 1918: 360. Type locality: «Dunedin [Pinellas County], Florida» (original citation). Lectotype [as holotype] (♂), designated by Freitag (1969: 131), in USNM [# 47122]. Etymology. The specific name honors Willis Stanley Blatchley [1859-1940], geologist and naturalist in the true sense of the word, author of several books on Coleoptera, Hemiptera, and Orthoptera.

#### Distribution.

This species ranges from southern Georgia to southwestern Florida [see Freitag 1969: Fig. 131].

#### Records.

**USA**: FL, GA

### Cyclotrachelus floridensis

(Freitag, 1969)

Evarthrus floridensis Freitag, 1969: 132. Type locality: «Winter Park [Orange County], Fl[orid]a» (original citation). Holotype (♂) in MCZ [# 31593].

#### Distribution.

This species is endemic to a small area in the eastern part of central Florida [see Freitag 1969: Fig. 131].

#### Records.

**USA**: FL

### [gigas group]

### Cyclotrachelus gigas

(Casey, 1918)

Megasteropus gigas Casey, 1918: 350. Type locality: «Texas» (original citation), restricted to «Victoria, Victoria County» by Bousquet (1999: 206). Lectotype [as holotype] (♀), designated by Freitag (1969: 165), in USNM [# 47123].

#### Distribution.

This species is known from two localities in southeastern Texas [see Freitag 1969: Fig. 136].

#### Records.

**USA**: TX

### Cyclotrachelus heros

(Say, 1823)

Feronia heros Say, 1823b: 145. Type locality: «Tex[as]» (neotype label), restricted to «Dallas, Dallas County» by Bousquet (1999: 207). Neotype (♂), designated by Lindroth and Freitag (1969: 340), in MCZ [# 33048]. Note. «the Arkansa» was the area originally cited by Say (1823b: 145).

#### Distribution.

This species is known from southeastern Oklahoma south to Comal County, Texas [see Freitag 1969: Fig. 136]. The record from “Arkansas” (Freitag 1969: 167) derives from the locality originally cited by Say (1823b: 145) which possibly refer to the Arkansas Territory. Prior to 1825, this territory included, besides Arkansas, all of the present state of Oklahoma. It could also refer to the Arkansas River which flows through Colorado, southern Kansas, northeastern Oklahoma, and Arkansas. The record from southeastern Kansas (Knaus 1907: 233) needs confirmation.

#### Records.

**USA**: OK, TX [KS]

### Cyclotrachelus sallei

(LeConte, 1873)

Evarthrus sallei LeConte, 1873a: 319. Type locality: United States of America (inferred from title of the paper); restricted to «Texas» by Freitag (1969: 165), and to «Dallas, Dallas County» by Bousquet (1999: 208). Lectotype (♂), designated by Freitag (1969: 165), in MCZ [# 5663].

#### Distribution.

This species is known only from eastern Texas [see Freitag 1969: Fig. 136].

#### Records.

**USA**: TX

### [gravesi group]

### Cyclotrachelus gravesi

(Freitag, 1969)

Evarthrus gravesi Freitag, 1969: 167. Type locality: «Pearl (Jackson), Rankin Co[unty], Miss[issippi]» (original citation). Holotype (♀) in MCZ [# 35350].

#### Distribution.

This species is known from the holotype and four specimens collected in Hinds County in southern Mississippi (Drew A. Hildebrandt pers. comm. 2009).

#### Records.

**USA**: MS

### [hypherpiformis group]

### Cyclotrachelus hypherpiformis

(Freitag, 1969)

Evarthrus hypherpiformis Freitag, 1969: 145. Type locality: «Prairies [= possibly Prairie Lakes], s[outh] Demopolis, Marengo Co[unty], Alabama» (original citation). Holotype (♂) in UMAA.

#### Distribution.

This species is known only from a few specimens collected in western Alabama and northeastern Mississippi [see Freitag 1969: Fig. 132].

#### Records.

**USA**: AL, MS

### [incisus group]

### Cyclotrachelus incisus

(LeConte, 1846)

Feronia incisa LeConte, 1846b: 345. Type locality: «ad Rocky Mountains» (original citation), restricted to «Iowa City, Johnson County, Iowa» by Bousquet (1999: 207). Lectotype (♂), designated by Freitag (1969: 127), in MCZ [# 5620].
Feronia lixa LeConte, 1846b: 346. Type locality: «ad Rocky Mountains» (original citation), cited from «near Long’s Peak [Boulder County, Colorado]» by LeConte (1853a: 232). Lectotype (♀), designated by Freitag (1969: 127), in MCZ [# 5622]. Synonymy established by LeConte (1873a: 319), confirmed by Freitag (1969: 127).
Feronia abdominalis LeConte, 1846b: 347. Type locality: «ad Rocky Mountains» (original citation), cited from «near Long’s Peak [Boulder County, Colorado]» by LeConte (1853a: 232). Lectotype (♂), designated by Freitag (1969: 128), in MCZ [# 5621]. Synonymy established by LeConte (1873a: 319), confirmed by Freitag (1969: 128).
Anaferonia distincta Casey, 1918: 342. Type locality: «Iowa» (original citation). Lectotype [as holotype] (♂), designated by Freitag (1969: 128), in USNM [# 47103]. Synonymy established by Freitag (1969: 128).
Anaferonia iowana Casey, 1918: 347. Type locality: «Iowa» (original citation). Lectotype [as holotype] (♂), designated by Freitag (1969: 128), in USNM [# 47107]. Synonymy established by Freitag (1969: 128).
Anaferonia fausta Casey, 1918: 348. Type locality: «Pennsylvania» (original citation). Lectotype [as holotype] (♂), designated by Freitag (1969: 128), in USNM [# 47104]. Synonymy established by Freitag (1969: 128).

#### Distribution.

This species is found from southwestern Pennsylvania to southeastern South Dakota, south to southern Colorado (Miller and Peairs 2008: 34; LeConte 1879d: 500; Wickham 1902: 236), Oklahoma, and central Arkansas [see Freitag 1969: Fig. 130]. The records from South Carolina (Kirk 1970: 11; Ciegler 2000: 70) are probably in error; that from “Kentucky” (Bousquet and Larochelle 1993: 188) needs confirmation.

#### Records.

**USA**: AR, CO, IA, IL, IN, KS, MO, NE, OH, OK, PA, SD, VA [KY]

### Cyclotrachelus whitcombi

(Freitag, 1969)

Evarthrus whitcombi Freitag, 1969: 129. Type locality: «Hot Springs [Garland County], Ark[ansas]» (original citation). Holotype (♂) in CAS [# 9815]. Etymology. The specific name was proposed in honor of Willard Hall Whitcomb [1915-2002], entomologist and teacher who made important contributions in the field of terrestrial arthropod biology.

#### Distribution.

This species is known from a few localities in eastern Oklahoma, southern Arkansas, and northwestern Louisiana (Natchitoches Parish, LSAM) [see Freitag 1969: Fig. 130].

#### Records.

**USA**: AR, LA, OK

### [seximpressus group]

### Cyclotrachelus alabamae

(Van Dyke, 1926)

Evarthrus vagans alabamae Van Dyke, 1926a: 118. Type locality: «near Mobile [Mobile County], Alabama» (original citation). Holotype (♂) in CAS [# 1858].

#### Distribution.

This species is restricted to the Gulf Coastal Plain from southwestern Alabama to eastern Texas, north to southern Arkansas [see Freitag 1969: Fig. 132]. Freitag (1969: 142) suggested that the specimens labeled from Clay County in northeastern Kansas and Lawrence County in northeastern Arkansas are probably mislabeled.

#### Records.

**USA**: AL, AR, LA, MS, TX [KS]

### Cyclotrachelus engelmani

(LeConte, 1853)

Evarthrus engelmani LeConte, 1853a: 228. Type locality: «Texas» (original citation), restricted to «Cuero, DeWitt County» by Bousquet (1999: 206). Lectotype (♂), designated by Freitag (1969: 142), in MCZ [# 5655]. Etymology. The species name was proposed for George Engelmann [1809-1884], physician and eminent botanist. Born in Germany, Engelmann immigrated to the United States and settled in Saint Louis. LeConte originally spelled his name “Engelman.”
Evarthrus engelmanni Freitag, 1969: 142. Unjustified emendation of *Evarthrus engelmani* LeConte, 1853.

#### Distribution.

This species is found only in eastern Texas (Freitag 1969: 143, Fig. 132).

#### Records.

**USA**: TX

### Cyclotrachelus nonnitens

(LeConte, 1873)

Evarthrus nonnitens LeConte, 1873a: 320. Type locality: «Red River, Louisiana» (original citation). Holotype [by monotypy; designated lectotype by Freitag (1969: 144)] (♀) in MCZ [# 5656].
Evarthrus enormis Casey, 1918: 361. Type locality: «Houston [Harris County], Texas» (original citation). Lectotype [as holotype] (♀), designated by Freitag (1969: 144), in USNM [# 47125]. Synonymy established by Freitag (1969: 144).

#### Distribution.

This species is found along the Gulf Coastal Plain from eastern Texas and southern Arkansas to western Mississippi [see Freitag 1969: Fig. 132]. The record from northwestern South Carolina (Kirk 1970: 11) is in error.

#### Records.

**USA**: AR, LA, MS, TX

### Cyclotrachelus seximpressus

(LeConte, 1846)

Feronia seximpressa LeConte, 1846b: 350. Type locality: «ad Rocky Mountains, prope Long’s Peak [Boulder County, Colorado]» (original citation), which is improbable, herein restricted to Lake Texoma State Park, Marshall County, Oklahoma (see Freitag 1969: 141). Lectotype (♂), designated by Freitag (1969: 139), in MCZ [# 5653].
Evarthrus rubripes Casey, 1918: 359. Type locality: «S[ain]t Louis, Missouri» (original citation for the lectotype). Lectotype [as holotype] (♂), designated by Freitag (1969: 140), in USNM [# 47121]. Synonymy established by Freitag (1969: 140).

#### Distribution.

This species is found west of the Appalachians Mountains from southwestern Pennsylvania to eastern Nebraska, north to southern Wisconsin and southeastern Minnesota, south to east-central Texas and southern Mississippi (Walthall County, Paul K. Lago pers. comm. 2009) [see Freitag 1969: Fig. 132].

#### Records.

**USA**: AR, IA, IL, IN, KS, MI, MN, MO, MS, NE, OH, OK, PA, TX, WI

### [sigillatus group]

### Cyclotrachelus convivus

(LeConte, 1853)

Evarthrus conviva LeConte, 1853a: 229. Type locality: «Alabama» (original citation), restricted to «Mobile, Mobile County» by Bousquet (1999: 206). Holotype [by monotypy; designated lectotype by Freitag (1969: 137)] (♂) in MCZ [# 5654].
Evarthrus basilaris Motschulsky, 1866: 261. Type locality: «Mobile [Mobile County, Alabama]» (original citation). Lectotype (♂), designated by Bousquet (1984a: 2), in ZMMU. Synonymy established by Bousquet (1984a: 2).
Evarthrus sigillatus parallelus Casey, 1918: 359. Type locality: «Indiana» (original citation). Lectotype [as holotype] (♂), designated by Freitag (1969: 137), in USNM [# 47119]. Synonymy established by Freitag (1969: 137).

#### Distribution.

This species ranges from southwestern Pennsylvania to the Mississippi River in western Illinois, south to southeastern Louisiana and southern Alabama [see Freitag 1969: Fig. 131]. The record from South Carolina (Kirk 1970: 11; Ciegler 2000: 70) is probably in error.

#### Records.

**USA**: AL, AR, IL, IN, KY, LA, MO, MS, OH, PA, TN, WV

### Cyclotrachelus sigillatus

(Say, 1823)

Feronia sigillata Say, 1823a: 42. Type locality: «Phila[delphia] [Philadelphia County], P[ennsylvani]a» (neotype label). Neotype (♀), designated by Lindroth and Freitag (1969: 340), in MCZ [# 33047]. Note. «Germantown [Pennsylvania]» was the area originally cited by Say (1823a: 43).
Feronia vidua Dejean, 1828: 278. Type locality: «Amérique septentrionale» (original citation). One syntype in MHNP (Lindroth 1955b: 15). Synonymy established by LeConte (1846b: 350), confirmed by Lindroth (1955b: 15).
Feronia americana Dejean, 1828: 392. Type locality: «Amérique septentrionale» (original citation). Lectotype [as type] (♂), designated by Freitag (1969: 133), in MHNP. Synonymy established by Freitag (1969: 133).
Feronia orbata Newman, 1838a: 386. Type locality: «northern states of America» (original citation, see page 388). Lectotype [as type] (♀), designated by Freitag (1969: 133), in BMNH. Synonymy established by Freitag (1969: 133).
Evarthrus nimius Motschulsky, 1866: 260. Type locality: «Ohio» (original citation), which is incorrect. Lectotype (♀), designated by Bousquet (1984a: 3), in ZMMU. Synonymy established by Bousquet (1984a: 3).
Evarthrus breviformis Casey, 1918: 360. Type locality: «Southern Pines [Moore County], North Carolina» (original citation). Lectotype [as holotype] (♀), designated by Freitag (1969: 133), in USNM [# 47120]. Synonymy established by Freitag (1969: 133).
Evarthrus montanus Van Dyke, 1926a: 116 [secondary homonym of *Pterostichus montanus* (Motschulsky, 1844)]. Type locality: «base of the Black Mountains, North Carolina» (original citation). Holotype (♂) in CAS [# 1856]. Synonymy established by Freitag (1969: 133).
Pterostichus carolinensis Csiki, 1930: 673. Replacement name for *Pterostichus montanus* (Van Dyke, 1926).

#### Distribution.

This species is found along the Appalachians, Piedmont Plateau, and Coastal Plain from Massachusetts to the Florida Panhandle and eastern Alabama [see Freitag 1969: Fig. 131]. The records from southeastern Louisiana (Summers 1874a: 80, as *Evarthrus americanus*), Missouri (Summers 1873: 134, as *Evarthrus orbatus*), “Iowa” (Wickham 1911b: 6; King 1914: 321; Jaques and Redlinger 1946: 297, as *Evarthrus orbatus*), and Ohio (Dury 1902: 113; Walker 1957: 270, as *Evarthrus americanus*) are in error.

#### Records.

**USA**: AL, DC, DE, FL, GA, MA, MD, NC, NJ, NY, PA, SC, TN, VA, WV

### Cyclotrachelus sinus

(Freitag, 1969)

Evarthrus sinus Freitag, 1969: 136. Type locality: «Alabama Port, Mobile Co[unty], Ala[bama]» (original citation). Holotype (♂) in MCZ [# 31594].

#### Distribution.

This species is known from a few localities in southern Alabama and southern Mississippi [see Freitag 1969: Fig. 131].

#### Records.

**USA**: AL, MS

### [sodalis group]

### Cyclotrachelus alternans

(Casey, 1920)

Evarthrinus alternans Casey, 1920: 196. Type locality: «Keokuk [Lee County], Iowa» (original citation). Lectotype [as holotype] (♂), designated by Freitag (1969: 153), in USNM [# 47131].

#### Distribution.

This species ranges from northwestern Wisconsin to northeastern South Dakota, south to central Missouri [see Freitag 1969: Fig. 134]. The record from “North Dakota” (Bousquet and Larochelle 1993: 187) needs confirmation.

#### Records.

**USA**: IA, IL, MN, MO, SD, WI [ND]

### Cyclotrachelus furtivus

(LeConte, 1853)

Evarthrus furtivus LeConte, 1853a: 234. Type locality: «Lake superior» (original citation), which is incorrect; «Virginia» selected by Freitag (1969: 152), restricted to «Mount Vernon, Fairfax County» by Bousquet (1999: 206). Holotype [by monotypy; designated lectotype by Freitag (1969: 152)] (♂) in MCZ [# 5662].

#### Distribution.

The range of this species extends from southern Pennsylvania and New Jersey to central Virginia [see Freitag 1969: Fig. 133]. The records from Ohio (Everly 1927: 155; Everly 1938: 141), “Michigan” (Leng 1920: 57), Indiana (Blatchley 1910: 101), North Carolina (Brimley 1938: 199), and Georgia (Fattig 1949: 22) are probably in error.

#### Records.

**USA**: DC, MD, NJ, PA, VA, WV

### Cyclotrachelus iowensis

(Freitag, 1969)

Evarthrus iowensis Freitag, 1969: 154. Type locality: «Iowa City [Johnson County], Iowa» (original citation). Holotype (♂) in USNM [# 69820].

#### Distribution.

This species is known from a few localities in southeastern Minnesota, Iowa, and eastern South Dakota [see Freitag 1969: Fig. 134].

#### Records.

**USA**: IA, MN, SD

### Cyclotrachelus lodingi

(Van Dyke, 1926)

Evarthrus lodingi Van Dyke, 1926a: 118. Type locality: «Monte Sano, Madison County, Alabama» (original citation). Holotype (♂) in CAS [# 1860].

#### Distribution.

This species is restricted to Tennessee and northern Alabama [see Freitag 1969: Fig. 133].

#### Records.

**USA**: AL, TN

#### Note.

This form has been treated as a subspecies of *Cyclotrachelus sodalis* (LeConte) by Freitag (1969) but I believe it should be regarded as a distinct species based on the character states listed by Freitag (1969: 148).

### Cyclotrachelus parasodalis

(Freitag, 1969)

Evarthrus parasodalis Freitag, 1969: 150. Type locality: «Washington Co[unty], Ark[ansas]» (original citation). Holotype (♂) in MCZ [# 31608].

#### Distribution.

This species is known only from Arkansas [see Freitag 1969: Fig. 133].

#### Records.

**USA**: AR

### Cyclotrachelus sodalis colossus

(LeConte, 1846)

Feronia colossus LeConte, 1846b: 343. Type locality: «Missouri» (original citation), cited from «near the Kansas River» by LeConte (1853a: 233), restricted to «Lathrop, Clinton County» by Bousquet (1999: 209). Lectotype (♂), designated by Freitag (1969: 146), in MCZ [# 5658].
Feronia corax LeConte, 1846b: 347. Type locality: «ad Rocky Mountains» (original citation), cited from «near Long’s Peak [Boulder County, Colorado]» by LeConte (1853a: 229). Lectotype (♂), designated by Freitag (1969: 146), in MCZ [# 5661]. Synonymy established by LeConte (1873a: 318), confirmed by Freitag (1969: 146).

#### Distribution.

This subspecies is found west of the Mississippi River from southeastern Minnesota (Gandhi et al. 2005: 926), western Iowa and eastern Nebraska south to southern Kansas and central Missouri [see Freitag 1969: Fig. 133]. At least one specimen simply labeled from Arkansas is known (Freitag 1969: 150). The records from southern Wisconsin (Rauterberg 1885: 15) and northern Colorado (LeConte 1853a: 229, as *Feronia corax*) need confirmation; that from southeastern Louisiana (Summers 1874a: 80) is probably in error.

#### Records.

**USA**: IA, KS, MN, MO, NE [AR, CO, WI]

### Cyclotrachelus sodalis sodalis

(LeConte, 1846)

Feronia sodalis LeConte, 1846b: 349. Type locality: «Pennsylvania» (original citation), restricted to «Pittsburgh, Allegheny County» by Bousquet (1999: 209). Lectotype (♂), designated by Freitag (1969: 146), in MCZ [# 5659].
Feronia vagans LeConte, 1846b: 349 [primary homonym of *Feronia vagans* Dejean, 1831]. Type locality: «provinciis occidentalibus» (original citation), cited from «Ohio» by LeConte (1853a: 229). Lectotype (♂), designated by Freitag (1969: 147), in MCZ [# 5664]. Synonymy established by Freitag (1969: 147).
Evarthrus fatuus LeConte, 1853a: 233. Type locality: «Iowa» (original citation). Holotype [by monotypy; designated lectotype by Freitag (1969: 147)] (♂) in MCZ [# 5060]. Synonymy established by LeConte (1873a: 318), confirmed by Freitag (1969: 147). Note. LeConte (1853a: 233) reported that the sole specimen he had was a female.
Evarthrus licinoides Motschulsky, 1866: 261. Type locality: «Am[érique] bor[éale]» (original citation). Lectotype (♀), designated by Bousquet (1984a: 3), in ZMMU. Synonymy established by Bousquet (1984a: 3).
Eumolops sulcata Casey, 1918: 355. Type locality: «Florida» (original citation), which is probably incorrect (Freitag 1969: 147). Lectotype [as holotype] (♂), designated by Freitag (1969: 147), in USNM [# 47134]. Synonymy established by Freitag (1969: 147).
Evarthrinus retractus Casey, 1920: 197. Type locality: «probably Indiana» (original citation). Holotype [by monotypy] (♀) in USNM [# 47132]. Synonymy established by Freitag (1969: 147).
Evarthrinus inflatipennis Casey, 1924: 78. Type locality: «near Chicago [Cook County], northern Illinois» (original citation). Lectotype [as holotype] (♀), designated by Freitag (1969: 147), in USNM [# 47133]. Synonymy established by Freitag (1969: 147).

#### Distribution.

This subspecies ranges from southwestern Vermont (Bell and Nielsen 1978: 8) to northeastern Minnesota, including southernmost Ontario, south to northeastern Mississippi and eastern Tennessee [see Freitag 1969: Fig. 133]. The record from northwestern Missouri (Freitag 1969: 149) probably refers to *Cyclotrachelus sodalis colossus*.

#### Records.

**CAN**: ON **USA**: IA, IL, IN, KY, MI, MN, MS, NJ, NY, OH, PA, TN, VA, VT, WI

### [substriatus group]

### Cyclotrachelus constrictus

(Say, 1823)

Feronia constricta Say, 1823b: 147. Type locality: «Colo[rado] Spr[ings] [El Paso County], Col[orado]» (neotype label). Neotype (♂), designated by Lindroth and Freitag (1969: 340), in MCZ [# 33050]. Note. «the Arkansa river near the rocky mountains» was the area originally cited by Say (1823b: 148).
Feronia ovipennis LeConte, 1846b: 345. Type locality: «ad Rocky Mountains» (original citation). Lectotype (♀), designated by Freitag (1969: 158), in MCZ [# 5619]. Synonymy established by LeConte (1873a: 319), confirmed by Freitag (1969: 158).
Anaferonia vernicata Casey, 1918: 344. Type locality: «Alamogordo [Otero County], New Mexico» (original citation). Lectotype [as holotype] (♂), designated by Freitag (1969: 159), in USNM [# 47105]. Synonymy established by Freitag (1969: 159).
Anaferonia pimalis Casey, 1918: 345. Type locality: «Arizona» (original citation). Lectotype [as holotype] (♀), designated by Freitag (1969: 147), in USNM [# 47106]. Synonymy established by Freitag (1969: 159).
Anaferonia pudica Casey, 1918: 346. Type locality: «Texas» (original citation). Lectotype [as holotype] (♀), designated by Freitag (1969: 147), in USNM [# 47101]. Synonymy established by Freitag (1969: 159).
Anaferonia papago Casey, 1918: 346. Type locality: «Arizona» (original citation). Lectotype [as holotype] (♀), designated by Freitag (1969: 147), in USNM [# 47102]. Synonymy established by Freitag (1969: 159).

#### Distribution.

This species ranges from southeastern South Dakota and western Iowa south to central Texas, west to central Arizona [see Freitag 1969: Fig. 134].

#### Records.

**USA**: AZ, CO, IA, KS, NE, NM, OK, SD, TX

### Cyclotrachelus substriatus

(LeConte, 1846)

Feronia substriata LeConte, 1846b: 344. Type locality: «ad Rocky Mountains» (original citation), restricted to «Sterling, Logan County, Colorado» by Bousquet (1999: 210). Lectotype (♀), designated by Freitag (1969: 156), in MCZ [# 5616].
Evarthrus latebrosus LeConte, 1853a: 233. Type locality: «Missouri Territory; Illinois» (original citation), restricted to «Missouri Territory» by Freitag (1969: 156). Lectotype (♂), designated by Freitag (1969: 156), in MCZ [# 5617]. Synonymy established by LeConte (1873a: 319), confirmed by Freitag (1969: 156).
Anaferonia evanescens Casey, 1918: 343. Type locality: «Colonia Garcia, Sierra Madre M[oun]t[ain]s, Chihuahua, Mexico» (original citation). Lectotype [as holotype] (♀), designated by Freitag (1969: 156), in USNM [# 47100]. Synonymy established by Freitag (1969: 156).
Anaferonia pantex Casey, 1918: 344. Type locality: «Texas» (original citation). Lectotype [as holotype] (♂), designated by Freitag (1969: 156), in USNM [# 47099]. Synonymy established by Freitag (1969: 156).

#### Distribution.

This species ranges from southern Minnesota to southern Wyoming, south to the state of Durango and southeastern Texas [see Freitag 1969: Fig. 134]. The record from Missouri (Summers 1873: 134) needs confirmation.

#### Records.

**USA**: AZ, CO, KS, MN, NE, NM, OK, SD, TX, WY [MO] – Mexico

### [torvus group]

### Cyclotrachelus deceptus

(Casey, 1918)

Evarthrus texanus Motschulsky, 1866: 261 [secondary homonym of *Pterostichus texanus* LeConte, 1863]. Type locality: «Texas» (original citation), restricted to «Dallas, Dallas County]» by Bousquet (1999: 210). Lectotype (♀), designated by Bousquet (1984a: 3), in ZMMU.
Eumolops decepta Casey, 1918: 357. Type locality: «Indiana» (original citation), which is incorrect (Freitag 1969: 161). Lectotype [as holotype] (♀), designated by Freitag (1969: 161), in USNM [# 47356]. Synonymy established by Bousquet (1984a: 3).
Eumolops impolita Casey, 1918: 358. Type locality: «Texas» (original citation). Lectotype [as holotype] (♂), designated by Freitag (1969: 161), in USNM [# 47130]. Synonymy established by Freitag (1969: 161).
Evarthrinus minax Casey, 1920: 194. Type locality: «Indiana» (original citation), which is probably incorrect (Freitag 1969: 161). Lectotype [as holotype] (♂), designated by Freitag (1969: 161), in USNM [# 47124]. Synonymy established by Freitag (1969: 161).
Pterostichus texicola Csiki, 1930: 659. Replacement name for *Pterostichus texanus* (Motschulsky, 1866).

#### Distribution.

This species is known from Texas, as far south as DeWitt County and west to El Paso County [see Freitag 1969: Fig. 135]. The record from “Oklahoma” (Bousquet and Larochelle 1993: 189) needs confirmation.

#### Records.

**USA**: TX [OK]

#### Note.

This taxon has been regarded as a subspecies of *Cyclotrachelus torvus* (LeConte) by Freitag (1969) but I believe it should be treated as a distinct species.

### Cyclotrachelus gravidus

(Haldeman, 1853)

Evarthrus gravidus Haldeman, 1853: 361. Type locality not stated; «Texas» selected by Freitag (1969: 163), restricted to «Kerrville, Kerr County» by Bousquet (1999: 206). One possible syntype, a ♀ labeled “[dark red disc] / E. gravidus Hald. [handwritten],” in MCZ (collection LeConte).
Eumolops ampla Casey, 1918: 353. Type locality: «Texas» (original citation). Lectotype [as holotype] (♀), designated by Freitag (1969: 163), in USNM [# 47126]. Synonymy established by Freitag (1969: 163).

#### Distribution.

This species is known from southern Oklahoma (Hatch and Ortenburger 1930: 11, as *Eumolops ampla*) and Texas, as far south as Comal County and west to El Paso County [see Freitag 1969: Fig. 135].

#### Records.

**USA**: OK, TX

### Cyclotrachelus torvus

(LeConte, 1863)

Evarthrus torvus LeConte, 1863c: 9. Type locality: «Kansas» (original citation). Syntype(s) location unknown. Note. The specimen in MCZ [# 5657] labeled “Col[orado]” and selected lectotype by Freitag (1969: 160) is not a syntype.
Feronia acuminata Chaudoir, 1868b: 333. Type locality: «Texas» (original citation). Lectotype (♂), designated by Freitag (1969: 160), in MHNP. Synonymy established by Freitag (1969: 160).
Eumolops prominens Casey, 1918: 353. Type locality: «Florida» (original citation), which is probably incorrect (Freitag 1969: 161). Lectotype [as holotype] (♀), designated by Freitag (1969: 161), in USNM [# 47128]. Synonymy established by Freitag (1969: 161).
Eumolops sexualis Casey, 1918: 354. Type locality: «Las Vegas [San Miguel County], New Mexico» (original citation). Lectotype [as holotype] (♂), designated by Freitag (1969: 161), in USNM [# 47124]. Synonymy established by Freitag (1969: 161).
Eumolops inflatula Casey, 1918: 354. Type locality: «Akron [Washington County], Colorado» (original citation). Lectotype [as holotype] (♀), designated by Freitag (1969: 161), in USNM [# 47127]. Synonymy established by Freitag (1969: 161).

#### Distribution.

This species inhabits the Great Plains from southwestern Minnesota (Gandhi et al. 2005: 926) to western South Dakota, south to southern New Mexico and southern Arkansas [see Freitag 1969: Fig. 135]. The record from “Arizona” (Bousquet and Larochelle 1993: 189) is probably in error; one specimen labeled from Hidalgo County in southeastern Texas is probably mislabeled (Freitag 1969: 163).

#### Records.

**USA**: AR, CO, IA, KS, MO, MN, NE, NM, OK, SD, WY

### Genus Abax

Bonelli, 1810

Abax Bonelli, 1810: Tabula Synoptica. Type species: *Carabus striola* Fabricius, 1792 (= *Carabus parallelepipedus* Piller and Mitterpacher, 1783) designated by Westwood (1838: 4). Etymology. From the Latin *abax* (counting-board), possibly alluding to the shape of the pronotum of the adults [masculine].

#### Diversity.

Eighteen species in Europe, with one species extending into Turkey, arrayed in two subgenera: *Abax* s.str. (16 species) and *Abacopercus* Ganglbauer (two species). One species is adventive in eastern North America.

### Subgenus Abax

Bonelli, 1810

Abax Bonelli, 1810: Tabula Synoptica. Type species: *Carabus striola* Fabricius, 1792 (= *Carabus parallelepipedus* Piller and Mitterpacher, 1783) designated by Westwood (1838: 4).
Bobsus Fischer von Waldheim, 1829a: 20. Type species: *Carabus parallelepipedus* Piller and Mitterpacher, 1783 by monotypy.

#### Diversity.

Sixteen species in Europe and Turkey, one of them adventive in the Nearctic Region.

#### Identification.

The species in North America was covered in Lindroth’s (1969a: 1116) monograph.

### Abax parallelepipedus

(Piller and Mitterpacher, 1783)

Carabus parallelepipedus Piller and Mitterpacher, 1783: 105. Type locality: Slavonia (inferred from title of the book). Syntype(s) probably lost (Lindroth 1969a: 1116).
Carabus ater Villers, 1789: 364 [primary homonym of *Carabus ater* Goeze, 1777]. Type locality: «Gallia Aust.» (original citation). Syntype(s) location unknown. Synonymy established by Csiki (1916: 15).

#### Distribution.

This European species is adventive in North America where it is known from Sydney, Nova Scotia (Brown 1967: 87) and western Newfoundland (David W. Langor pers. comm. 1990). The first inventoried specimen collected on this continent was found in 1965 in Nova Scotia.

#### Records.

**CAN**: NF, NS – **Adventive**

### Tribe Zabrini

Bonelli, 1810

Zabrides Bonelli, 1810: Tabula Synoptica. Type genus: *Zabrus* Clairville, 1806.

#### Diversity.

About 700 species arrayed in two subtribes: Zabrina (107 species of *Zabrus* in Europe and western Asia, the vast majority restricted to the Mediterranean region) and Amarina. More than 98% of the species inhabit the Northern Hemisphere.

### Subtribe Amarina

Zimmermann, 1832

Amaroiden Zimmermann, 1832: 6. Type genus: *Amara* Bonelli, 1810.
Isopleuridae Kirby, 1837: 49. Type genus: *Isopleurus* Kirby, 1837 (= *Celia* Zimmermann, 1832).
Agronomaeidae Gistel, 1848b: [2]. Type genus: *Agronoma* Gistel, 1848 (= *Amara* Bonelli, 1810).
Pangeteidae Gistel, 1856: 358. Type genus: *Pangetes* Gistel, 1856 (= *Amara* Bonelli, 1810).

#### Diversity.

About 595 species placed in one genus.

### Genus Amara

Bonelli, 1810

Amara Bonelli, 1810: Tabula Synoptica. Type species: *Carabus vulgaris* Linnaeus *sensu* Panzer, 1797 (= *Amara lunicollis* Schiødte, 1837) designated by Westwood (1838: 4). Etymology. Probably from the Greek *amara* (trench, by extension furrow, stria), alluding to the presence of laterobasal impressions on the pronotum (“*thorax latus, basi transverse impressus, utrinq. stria sesquialtera angulis rectis*”) of the adult rather than from the Greek prefix *a*- (privative) and *mairo* (to shine, by extension to be clear) alluding to the fact that the adults are dark colored as advocated by Zimmermann (1832: 30) or *a*- (with) and *mairo* (to shine) as advocated by Jacquelin du Val (1855: 31) [feminine].

#### Diversity.

About 595 species (Hieke 2007) in the Nearctic (105 species, including ten adventive species), Neotropical (13 species in Middle America, only two of them endemic), Oriental (five species), Palaearctic (about 505 species, 16 of them Holarctic), and Afrotropical (about 12 species, nine of them endemic) Regions. These species are arrayed in 47 subgenera.

#### Note.

Almost all new state and province records listed here are based on specimens determined by Fritz Hieke.

### Subgenus Curtonotus

Stephens, 1827

Curtonotus Stephens, 1827: 67. Type species: *Carabus convexiusculus* Marsham, 1802 designated by Westwood (1838: 4). Etymology (original, see page 138). From the Greek *cyrtos* (curved, by extension convex) and *notos* (back, upper surface), alluding to the convex body (“body ... very convex”) of the adult [masculine].
Leirus Dejean, 1828: 457. Type species: *Carabus aulicus* Panzer, 1796 designated by Jeannel (1942: 946). Synonymy established by Zimmermann (1832: 38). Note. This name has been credited to Zimmermann (1832: 38) by most authors and to Fischer von Waldheim (1829a: 16) by Bousquet (2002c: 176). However, Dejean (1828: 457) first described the taxon without citing any species with it. The species first associated with *Leirus* are those listed by Fischer von Waldheim (1829a: 16). The name *Leirus* was proposed by Johann Karl Megerle von Mühlfeld and first made available by Dahl (1823: 6) but his work has been suppressed by the Commission (ICZN 1964).
Cyrtonotus Agassiz, 1846: 108, 114. Unjustified emendation of *Curtonotus* Stephens, 1827.
Lirus Agassiz, 1846: 204, 213. Unjustified emendation of *Leirus* Dejean, 1828.
Leirodema Tschitschérine, 1894: 394. Type species: *Amara sifanica* Tschitschérine, 1894 designated by Hieke (1995a: 54). Synonymy established by Kryzhanovskij (1974: 177).
Feronalius Casey, 1918: 226. Type species: *Amara pterostichina* Hayward, 1908 by original designation. Synonymy established by Hieke (1993: 149).
Paracurtonotus Habu, 1942: 494. Type species: *Leirus giganteus* Motschulsky, 1844 by original designation. Synonymy established by Habu (1953: 39). Etymology. From the Greek *para* (beside, near) and the generic name *Curtonotus* [*q.v*.] [masculine].
Paracyrtonotus Baliani, 1943: 48. Type species: *Amara mixta* Baliani, 1943 by monotypy. Synonymy established by Hieke (1993: 102). Etymology. From the Greek *para* (beside, near) and the generic name *Cyrtonotus* [*q.v*.] [masculine].

#### Diversity.

Eighty-five species (Hieke 2007) in the arctic, subarctic, boreal, and temperate regions of North America (15 species of which three extend into Mexico), Asia (about 60 species), and Europe (13 species, all but possibly one shared with Asia). Six species are Holarctic and one is adventive in eastern North America.

#### Identification.

There is no modern taxonomic revision of the species and such study would be useful. Lindroth (1968, as *aulica* group) covered ten of the 15 species found in North America.

### Amara alpina

(Paykull, l790)

Carabus alpinus Paykull, l790: 119. Type locality: «fummis alpibus Lapponicis & Dalecarlicis» (original citation), restricted to «Abisko, Torne L[ap]p[mar]k, Sweden» by Lindroth (1968: 673). Lectotype (♀), designated by Lindroth (1968: 675), in NRSS.
Amara brunnipennis Dejean, 1831: 800. Type locality: «Labrador» (original citation). Holotype [by monotypy] (♂) in MHNP (Lindroth 1955b: 16). Synonymy established by Lindroth (1968: 673).
Leirus borealis Chaudoir, 1843b: 775. Type locality not stated. Syntype(s) apparently lost (Lindroth 1968: 673). Synonymy established, under the name *Amara brunnipennis* Dejean, by Chaudoir (1861c: 198).
Leirus brevicornis Ménétriés, 1851: 51. Type locality: «Boganida [River] [Taimyr Peninsula, Siberia, Russia]» (original citation). Syntype(s) probably in ZMMU (Lindroth 1968: 673). Synonymy established by Csiki (1929: 459).
Amara obtusa LeConte, 1855: 348. Type locality: «Russian America» (original citation). Syntype(s) in MCZ [# 5671]. Synonymy established, under the name *Amara brunnipennis* Dejean, by Hayward (1908: 60), confirmed by Lindroth (1954b: 134).
Curtonotus caligatus Putzeys, 1866b: 252. Type locality: «Ile S[ain]t-Georges, Kamtschatka [Russia]» (original citation). Holotype [by monotypy] (♂) in MHNP (collection Chaudoir). Synonymy established by Sahlberg (1882: 188).
Curtonotus cognatus Putzeys, 1866b: 253. Type locality: «Norwége» (original citation). Holotype [by monotypy] in MHNP (collection Chaudoir). Synonymy established with doubt (as aberration) by Jacobson (1907: 362).
Amara subsulcata J.R. Sahlberg, 1880: 33. Type locality: «Dudinka, Yenisei R[iver] [Taimyr Autonomous Okrug, Russia]» (original citation for the lectotype). Lectotype (♀), designated by Lindroth (1968: 674), in NRSS. Synonymy established by Munster (1927: 303), confirmed by Lindroth (1968: 674). Note. The specimen designated as lectotype by Silfverberg (1987: 25) is invalid since there was a prior valid designation of a lectotype (see ICZN 1999: Article 74.1.1).
Amara angustata J.R. Sahlberg, 1885b: 51 [secondary homonym of *Amara angustata* (Say, 1823)]. Type locality: Port Clarence, Alaska (inferred from title of the paper). Holotype [by monotypy] (♀) in ZMH. Synonymy established by Lindroth (1968: 674).
Amara pullula Poppius, 1906a: 55. Type locality: «Bulkur (c:a 71° 45’ n. Br.), unterste Lena [Siberia, Russia]» (original citation). Holotype [by monotypy] (♂) in ZMH (Silfverberg 1987: 23). Synonymy established by Lindroth (1968: 674).
Amara birulai Poppius, 1913: 82. Type locality: «Kasatschie, Jana-Mündung [Siberia, Russia]» (original citation). Syntype(s) [2 ♂ originally cited] location unknown. Synonymy established by Hieke (1999b: 156).
Curtonotus rubripennis Casey, 1918: 232. Type locality: «Colorado» (original citation). Lectotype (♂), designated by Lindroth (1975: 131), in USNM [# 47152]. Synonymy established, under the name *Amara alpina brunnipennis* Dejean, by Lindroth (1954b: 134).
Curtonotus deficiens Casey, 1918: 232. Type locality: «summit of M[oun]t Washington [Coos County], New Hampshire» (original citation). Lectotype (♀), designated by Lindroth (1975: 131), in USNM [# 47154]. Synonymy established, under the name *Amara alpina brunnipennis* Dejean, by Lindroth (1954b: 134).
Curtonotus argutus Casey, 1918: 233. Type locality: «M[oun]t Washington [Coos County], New Hampshire» (original citation). Lectotype (♂), designated by Lindroth (1975: 131), in USNM [# 47153]. Synonymy established, under the name *Amara alpina brunnipennis* Dejean, by Lindroth (1954b: 134).
Curtonotus inanis Casey, 1918: 233. Type locality: «summit of M[oun]t Washington [Coos County], New Hampshire» (original citation). Holotype [by monotypy] (♀) in USNM [# 47155]. Synonymy established, under the name *Amara alpina brunnipennis* Dejean, by Lindroth (1954b: 134).
Curtonotus subtilis Casey, 1924: 45. Type locality: «Stupart Bay [northern Quebec], possibly Labrador» (original citation). Holotype [by monotypy] (♂) in USNM [# 47151]. Synonymy established, under the name *Amara alpina brunnipennis* Dejean, by Lindroth (1954b: 135). Note. Stupart Bay is a bay along the coast of the Hudson Strait in northern Quebec, near Maricourt (Wakeham).
Amara alaskana Csiki, 1929: 459. Replacement name for *Amara angustata* Sahlberg, 1885.

#### Distribution.

This Holarctic species is found from Great Britain to the Pacific Coast of Siberia (Hieke 2003a: 558), and from the west coast of Alaska, including the Saint Lawrence Island, to northwestern Newfoundland, south to the James Bay area and to northern New Mexico along the Rocky Mountains [see Lindroth 1963b: Fig. 58]; isolated on the Shickshock Mountains in Gaspé Peninsula (Larochelle 1975: 42) and some mountains in New England (Lindroth 1966: 676). The record from “New York” (Notman 1928: 226) needs confirmation. According to Lindroth (1968: 676), this is the carabid occurring farthest north in North America. Fossil remnants of this species, dated between about 14,000 and 21,500 years B.P., have been unearthed in southeastern and central Iowa (Baker et al. 1986: 96; Schwert 1992: 78) and north-central Illinois (Garry et al. 1990: 394); others from interglacial deposits have been found in northwestern Greenland (Bennike 2000: 31).

#### Records.

**CAN**: AB, BC, LB, MB, NF, NT, NU, ON, QC, YT **USA**: AK, CO, ME, NH, NM, UT, VT, WY [NY] – **Holarctic**

### Amara aulica

(Panzer, 1796)

Carabus ruficornis DeGeer, 1774: 95 [*nomen dubium*]. Type locality not stated. Syntype(s) lost.
Carabus aulicus Panzer, 1796b: no 3. Type locality: «Brunsvigiae [= Brunswick, Germany]» (original citation). Syntype(s) location unknown (possibly in ZMHB). Synonymy established with doubt by Schönherr (1806: 181).

#### Distribution.

This European species is adventive in North America where it is known from Newfoundland (Larson and Langor 1982: 593) and Saint Pierre and Miquelon (Lindroth 1955a: 95) to the Saint Lawrence Valley in Quebec (Larochelle 1975: 42), south to Boston Harbor, Massachusetts (Davidson et al. 2011: 512). The first inventoried specimen collected on this continent was found in Cape Breton Island, Nova Scotia in 1929 (Fall 1934: 171). The record from the Similkameen Valley in British Columbia (Smith et al. 2004: 96) was based on a misidentified specimen of *Amara carinata* LeConte (personal observation).

#### Records.

**FRA**: PM **CAN**: NB, NF, NS (CBI), PE, QC **USA**: MA, ME, NH – **Adventive**

### Amara blanchardi

Hayward, 1908

Amara blanchardi Hayward, 1908: 22. Type locality: «Provo [Utah County], Utah» (original citation). Holotype (♂) in MCZ [# 25666].
Curtonotus sponsor Casey, 1918: 228. Type locality: «Siskiyou Co[unty], California» (original citation). Lectotype (♂), designated by Hieke (1994: 303), in USNM [# 47141]. Synonymy established by Hieke (1994: 303).
Curtonotus tartareus Casey, 1918: 229. Type locality: «probably Colorado» (original citation). Holotype [by monotypy] (♂) in USNM [# 47143]. Synonymy established by Hieke (1994: 303).

#### Distribution.

This western species occurs from southern Saskatchewan (MCZ) to south-central British Columbia (Lindroth 1968: 666), south to east-central California (Inyo County, CAS) and northwestern New Mexico (San Juan County, CNC).

#### Records.

**CAN**: AB, BC, SK **USA**: AZ, CA, CO, ID, MT, NM, NV, OR, UT, WA, WY

### Amara bokori

Csiki, 1929

Amara sahlbergi Poppius, 1906a: 54 [secondary homonym of *Amara sahlbergi* (Zetterstedt, 1838)]. Type locality: «Saostroff, fl. Jenissej [Taimyr Autonomous Okrug, Siberia, Russia]» (original citation). Lectotype (♂), designated by Lindroth (1968: 676), in ZMH. Etymology. The specific name was proposed for John Reinhold Sahlberg (see *Amara john-sahlbergi*).
Amara bokori Csiki, 1929: 461. Replacement name for *Amara sahlbergi* Poppius 1906.

#### Distribution.

This species ranges from eastern Siberia to the Hudson Bay coast in northern Manitoba (Lindroth 1968: 677).

#### Records.

**CAN**: MB, NT, NU, YT **USA**: AK – **Holarctic**

### Amara carinata

(LeConte, 1847)

Curtonotus carinatus LeConte, 1847: 368. Type locality: «ad Rocky Mountains» (original citation), cited from «Nebraska Territory, near the Rocky Mountains» by LeConte (1855: 347), restricted to «Bent Co[unty], Color[ado]» by Lindroth (1968: 668). Syntype(s) in MCZ [# 5668].
Curtonotus laticollis LeConte, 1847: 368 [secondary homonym of *Amara laticollis* Stephens, 1828]. Type locality: «ad Rocky Mountains» (original citation), cited from «Nebraska Territory, near the Rocky Mountains» by LeConte (1855: 347). One syntype in MCZ [# 5667]. Synonymy established by Lindroth (1968: 668).
Curtonotus adstrictus Putzeys, 1866b: 238. Type locality: «États-Unis» (original citation). Lectotype (♂), designated by Lindroth (1968: 668), in MHNP. Synonymy established, under the name *Amara laticollis* (LeConte), by Casey (1918: 227), confirmed by Lindroth (1968: 668).
Curtonotus concretus Casey, 1918: 228. Type locality: «California» (original citation). Holotype [by monotypy] (♀) in USNM [# 47138]. Synonymy established by Hieke (1994: 304).
Curtonotus spadiceus Casey, 1918: 229. Type locality: «Jemez Springs, Las Vegas and Fort Wingate, New Mexico» (original citation). Lectotype [as holotypus] (♂), designated by Hieke (1994: 305), in USNM [# 47144]. Synonymy established by Hieke (1994: 304).
Curtonotus catenulatus Casey, 1918: 230. Type locality: «Billings [Yellowstone County], Montana» (original citation). Lectotype (♂), designated by Lindroth (1975: 130), in USNM [# 47145]. Synonymy established by Lindroth (1968: 668).
Curtonotus gilvipes Casey, 1924: 46. Type locality: «Rosebank, Man[itoba]» (lectotype label). Lectotype [as holotype] (♂), designated by Lindroth (1975: 130), in USNM [# 47147]. Synonymy established by Lindroth (1968: 668).

#### Distribution.

This species ranges from the Gaspé Peninsula (Larochelle 1975: 43) to south-central British Columbia (Lindroth 1968: 669), south to the Sierra Nevada in California (Dajoz 2007: 16), central New Mexico (Bernalillo County, CMNH; Lindroth 1968: 668), the Texas Panhandle (Michels et al. 2010: 743), northwestern Arkansas (Carroll County, MCZ), and northeastern Ohio (Lake County, CMNH). The record from “West Virginia” (Bousquet and Larochelle 1993: 190) is in error; that from “Yukon Territory” (Ball and Currie 1997: 452) could not be confirmed.

#### Records.

**CAN**: AB, BC, MB, ON, QC, SK **USA**: AR, AZ, CA, CO, IA, ID, IL, IN, KS, MA, MN, MO, MT, ND, NE, NH, NM, OH, OR, SD, TX, UT, VT, WI, WY [YT]

### Amara daurica

(Motschulsky, 1844)

Leirus dauricus Motschulsky, 1844: 177. Type locality: «Kiachta [= Kyakhta], Baikal [Russia]» (lectotype label). Lectotype, designated by Hieke (1993: 77), in ZMMU. Note. Motschulsky (1844: 177) originally cited that he found this species “qu’au delà du Baïcal sur les bords bourbeux des fleuves Ouda et Selenga.”
Curtonotus contractus Putzeys, 1866b: 241. Type locality: «Sibér[ie]» (lectotype label). Lectotype (♂), designated by Hieke (1993: 77), in MHNP (collection Chaudoir). Synonymy established by Hieke (1993: 75).
Amara monostigma Jedlička, 1957a: 28. Type locality: «Nertschinsk, Sib. or. [Transbaikalien, Russia]» (original citation). Holotype (♂) in NMP. Synonymy established, under the name *Amara contracta* (Putzeys), by Kryzhanovskij (1975: 92).

#### Distribution.

This species is found in Kazakhstan, Mongolia, northern China, and across Siberia (Hieke 2003a: 559). In North America, it is known only from the Anderson River Delta area in northwestern Northwest Territories (Hieke 1994: 306).

#### Records.

**CAN**: NT – **Holarctic**

#### Note.

The North American specimens differ from the Siberian ones in structural details and, according to Hieke (1994: 307), it is possible that they constitute a distinct subspecies.

### Amara deparca

(Say, 1830)

Feronia deparca Say, 1830b: (6) [3]. Type locality: «Mexico» (original citation), herein restricted to 10 miles west of El Salto, Durango (CNC). Syntype(s) lost.
Curtonotus substriatus Putzeys, 1866b: 242. Type locality: «Mexique» (original citation). Syntype(s) [4 originally cited] in MHNP (collection Chaudoir). Synonymy established by Bates (1882a: 76).
Amara bowditchi Hayward, 1908: 24. Type locality: «Phoenix [Maricopa County], Ariz[ona]» (original citation). Holotype (♂) in MCZ [# 25667]. Synonymy established by Hieke (1993: 144).

#### Distribution.

This species ranges from Baja California Norte (CAS), southeastern California (Inyo and San Bernardino Counties, CAS, CMNH), and southern Nevada (Clark County, MCZ) east to northwestern Oklahoma (Texas County, CMNH), north to northern Colorado and “Utah” (Hayward 1908: 25, as *Amara bowditchi*), south at least to the state of México (Bates 1882a: 76).

#### Records.

**USA**: AZ, CA, CO, NM, NV, OK, TX, UT – Mexico

### Amara hyperborea

Dejean, 1831

Amara hyperborea Dejean, 1831: 800. Type locality: «Labrador» (original citation), herein restricted to Hebron (CNC). Holotype [by monotypy] (♀) in MHNP (Lindroth 1955b: 16).
Curtonotus elongatus LeConte, 1850: 207. Type locality: Lake Superior (inferred from title of the paper), cited as «one male found floating in Lake Superior, near the northern shore» by LeConte (1855: 348). One syntype in MCZ [# 5672]. Synonymy established by Lindroth (1954b: 134).
Lirus ovipennis Motschulsky, 1859a: 156. Type locality: «Californie» (original citation), which is incorrect. Lectotype [as holotype] (♂), designated by Hieke (1993: 148), in ZMMU. Synonymy established by Hieke (1993: 148).
Lirus longicollis Motschulsky, 1860: 95. Type locality: «Kamtschatka» (original citation for the lectotype). Lectotype [as holotype], designated by Hieke (1990: 252), in ZMMU. Synonymy established by Putzeys (1865: 338), confirmed by Hieke (1975: 296).
Amara peregrina Morawitz, 1862: 258. Type locality: «Zagan-oluj [Chita region, eastern Siberia, Russia]» (lectotype label). Lectotype (♂), designated by Hieke (1990: 252), in ZILR. Synonymy established by Lindroth (1953a: 18), confirmed by Hieke (1990: 252).
Curtonotus pedestris Putzeys, 1866b: 254. Type locality: «Udskoe Ochotsk [Khabarovsk Kray, Siberia, Russia]» (original citation). Holotype [by monotypy] (♀) in MHNP. Synonymy established by Hieke (1990: 252).
Curtonotus tristis Putzeys, 1866b: 255. Type locality: «Owho-Bay, Canada boréal» (original citation). Holotype [by monotypy] (♂) in MHNP (collection Chaudoir). Synonymy established by Lindroth (1968: 678).
Curtonotus canadensis Putzeys, 1866b: 256. Type locality: «Canada boréal» (original citation). Holotype [by monotypy] (♀) in MHNP (collection Chaudoir). Synonymy established by Lindroth (1968: 678).
Curtonotus dejeani Putzeys, 1866b: 258. Type locality: «Kamtschatka [Russia]» (original citation). Holotype [by monotypy] (♂) in MHNP. Synonymy established, under the name *Amara longicollis* (Motschulsky), by Tschitschérine (1894: 389), confirmed by Hieke (1975: 296).
Harpalus simulans J.R. Sahlberg, 1880: 44. Type locality: «insulam Tschornaja, ad flumen Jenissej [Russia]» (original citation). Syntype(s) [3 originally cited] in NRSS (Lindroth 1955a: 99). Synonymy established with doubt, under the name *Amara peregrina* Morawitz, by Hellén (1930: 6), confirmed by Lindroth (1954b: 134).
Curtonotus imperfectus Brown, 1930: 232. Type locality: «Bradore Bay, Que[bec]» (original citation). Holotype (♀) in CNC [# 3163]. Synonymy established by Lindroth (1954b: 134).

#### Distribution.

This Holarctic species ranges from northern Finland to the Pacific Coast, south to Mongolia and northeastern China (Hieke 2003a: 560) and from Alaska (Lindroth 1968: 679) to Newfoundland (Lindroth 1955a: 99), south to the upper peninsula of Michigan (Lindroth 1968: 678). The record from the White Mountains in New Hampshire (Wickham 1896b: 37) needs confirmation; those from “Vermont” (Hamilton 1894a: 10) and Colorado (Snow 1877: 17; Wickham 1902: 236; Armin 1963: 208, as *Amara elongatus*) are probably in error.

#### Records.

**CAN**: AB, BC, LB, MB, NF, NT, ON, QC, SK, YT **USA**: AK, MI [NH] – **Holarctic**

### Amara jacobina

LeConte, 1855

Amara jacobina LeConte, 1855: 346. Type locality: «San Diego [San Diego County], California» (original citation). Holotype [by monotypy; designated lectotype by Hieke (1990: 285)] (♂) in MCZ [# 5665].
Amara stupida LeConte, 1855: 347. Type locality: «Sacramento [Sacramento County], California» (original citation). Holotype [by monotypy; designated lectotype by Hieke (1990: 286)] (♀) in MCZ [# 5666]. Synonymy established by Hieke (1990: 283).

#### Distribution.

The range of this species extends from “Washington” (Hayward 1908: 23) to the Baja California Peninsula (Horn 1894: 309) and Arizona (Griffith 1900: 565; Hayward 1908: 23). The records from north-central Utah (Horn 1894: 309), “Colorado” (Wickham 1902: 236), and New Mexico (Fall and Cockerell 1907: 159) need confirmation.

#### Records.

**USA**: AZ, CA, OR, WA [CO, NM, UT] – Mexico

### Amara kurnakowi

Hieke, 1994

Amara kurnakowi Hieke, 1994: 310. Type locality: «r[iver] Omsuktschan, bas[in] Kolymy [Siberia, Russia]» (original citation). Holotype (♂) in ZMHB (Hieke 2007).
Amara kurnakovi Budarin and Kryzhanovskij [in Budarin], 1995: 27. Type locality: «Magadan area: Omsukchan River (Kolyma River basin) [Siberia, Russia]» (original citation). Holotype (♂) in ZILR. Synonymy established by Hieke (1997: 225).

#### Distribution.

This Holarctic species is known from northeastern Siberia and the Anderson River Delta in northwestern Northwest Territories (Hieke 1994: 311).

#### Records.

**CAN**: NT – **Holarctic**

### Amara lacustris

LeConte, 1855

Amara lacustris LeConte, 1855: 346. Type locality: «north shore of Lake Superior [Ontario]» (original citation). Holotype [by monotypy] (♀) in MCZ [# 5669].
Curtonotus manitobensis Casey, 1924: 46. Type locality: «Rosebank, Man[itoba]» (lectotype label). Lectotype (♀), designated by Lindroth (1975: 130), in USNM [# 47142]. Synonymy established by Lindroth (1954b: 135).

#### Distribution.

This species occurs from Nova Scotia (Larochelle and Larivière 1990a: 30, 34) to southwestern British Columbia, north to the coast of Nunavut and northern Alaska (Lindroth 1968: 671), south to northwestern Washington (Hatch 1953: 121), south-central Utah (Wayne County, CNC), southwestern Colorado (La Plata County, CNC), the Black Hills in southwestern South Dakota (Kirk and Balsbaugh 1975: 25), and “Michigan” (Garry A. Dunn pers. comm. 1986). The record from “Nebraska” (Bousquet and Larochelle 1993: 191) needs confirmation.

#### Records.

**CAN**: AB, BC, MB, NB, NS, NT, NU, ON, SK, YT **USA**: AK, CO, ID, MI, MN, MT, ND, SD, UT, WA, WI, WY [NE]

### Amara pennsylvanica

Hayward, 1908

Curtonotus fulvipes Putzeys, 1866b: 235 [secondary homonym of *Amara fulvipes* (Audinet-Serville, 1821)]. Type locality: «Missouri» (original citation). Holotype [by monotypy] (♂) in MHNP (collection Chaudoir).
Amara pennsylvanica Hayward, 1908: 34. Replacement name for *Amara fulvipes* (Putzeys, 1866). Note. Because Hayward’s name was expressly proposed as a replacement name, the syntypes of *Amara pennsylvanica* in MCZ [# 25668] have no status (see ICZN 1999: Article 72.7).

#### Distribution.

This species ranges from Nova Scotia (Lindroth 1968: 678) to “Wyoming” (Hayward 1908: 34), south to northeastern Colorado (Sedgwick County, Ken Karns pers. comm. 2009), northeastern Texas (Dallas County, MCZ), southwestern Alabama (Mobile County, USNM), and northwestern South Carolina (Ciegler 2000: 75). An old specimen simply labeled from “New Mexico” (MCZ) is known. The record from western Montana (Hatch 1933a: 8) needs confirmation.

#### Records.

**CAN**: NS, ON, QC **USA**: AL, AR, CO, CT, DC, DE, GA, IA, IL, IN, KS, KY, MA, MD, MI, MN, MO, NC, NE, NH, NJ, NY, OH, OK, PA, RI, SC, SD, TN, TX, VA, WI, WV, WY [MT, NM]

### Amara pterostichina

Hayward, 1908

Curtonotus putzeysi Bates, 1878a: 600 [secondary homonym of *Amara putzeysii* Fairmaire, 1867 and *Amara putzeysii* Horn, 1875]. Type locality: «near the capital, Mexico» (original citation). Lectotype (♂), designated by Hieke (1993: 150), in BMNH. Etymology. The specific name was given in honor of the Belgian Jules Antoine Adolphe Henri Putzeys [1809-1882] who, despite his judicial and administrative duties, published extensively on botany and entomology, particularly Odonata and Carabidae.
Amara pterostichina Hayward, 1908: 20. Type locality: «Coolidge [McKinley County], N[ew] M[exico]» (lectotype label). Lectotype (♂), designated by Hieke (1993: 149), in MCZ [# 25664]. Synonymy established by Hieke (1993: 149).
Amara batesiana Csiki, 1929: 466. Replacement name for *Amara putzeysi* Bates, 1878.

#### Distribution.

This species ranges from southern Arizona (Cochise County, CNC) to western Texas (Dajoz 2007: 23; Brewster and Jeff Davis Counties, CNC, MCZ), south to the Federal District and central Veracruz in Mexico (Bates 1882a: 76).

#### Records.

**USA**: AZ, NM, TX – Mexico

### Amara thoracica

Hayward, 1908

Amara thoracica Hayward, 1908: 21. Type locality: «Colorado Springs [El Paso County], Colorado» (original citation). Holotype (♂) in MCZ [# 25665].

#### Distribution.

The range of this species extends from southern Saskatchewan (CNC) to the Okanagan Valley in south-central British Columbia (Lindroth 1968: 668), south to northern Arizona (Coconino County, CMNH) and central New Mexico (Torrance County, CMNH; Milford et al. 2000: 21).

#### Records.

**CAN**: AB, BC, SK **USA**: AZ, CO, MT, NM, NV, WY

### Amara torrida

(Panzer, 1796)

Carabus torridus Panzer, 1796b: no 2. Type locality: Germany (inferred from title of the book), which according to Lindroth (1968: 671) is incorrect; «Jokkmokk, Swed[en] Lapl[and]» selected by Lindroth (1968: 671). Syntype(s) location unknown (possibly in ZMHB).
Amara melanogastrica Dejean, 1828: 519. Type locality: «île d’Ounalaschka, l’une des îles Aleutiennes [Alaska]» (original citation). Two syntypes in MHNP (Lindroth 1955b: 16). Synonymy established by Hatch (1953: 121), confirmed by Lindroth (1955b: 16).
Leirus eschscholtzii Chaudoir, 1837b: 36. Type locality: «Kamtchatka [Russia]» (original citation). Lectotype (♂), designated by Lindroth (1968: 671), in MHNP. Synonymy established by Lindroth (1968: 671).
Curtonotus rufimanus Kirby, 1837: 35. Type locality: «Lat. 54° [= along North Saskatchewan River]» (original citation), restricted to «Edmonton, Al[ber]ta» by Lindroth (1968: 671). One syntype in BMNH (Lindroth 1953b: 172). Synonymy established by Lindroth (1953b: 172).
Curtonotus brevilabris Kirby, 1837: 35. Type locality: «Lat. 65° [= apparently region of Great Bear Lake, Northwest Territories]» (original citation). Holotype [by monotypy] (♂) in BMNH (Lindroth 1953b: 172). Synonymy established, under the name *Amara rufimana* (Kirby), by Horn (1876e: 129), confirmed by Lindroth (1953b: 172).
Leirus rufimanus Motschulsky, 1844: 176 [secondary homonym of *Amara rufimana* (Kirby, 1837)]. Type locality: «Kamtchatka [Russia]» (original citation for the lectotype). Lectotype (♂), designated by Lindroth (1968: 671), in MHNP (collection Chaudoir). Synonymy established by Lindroth (1968: 671).
Leirus picipes Motschulsky, 1844: 176. Type locality: «sur les bords de l’Irtych à Omsk [Siberia, Russia]» (original citation). Three syntypes in ZMMU (Hieke 1995b: 324). Synonymy established by Hieke (1995b: 324).
Amara infausta LeConte, 1855: 347. Replacement name for *Amara rufimana* (Motschulsky, 1844).
Lirus californicus Motschulsky, 1859a: 155 [*nomen dubium*]. Type locality: California (inferred from title of the paper and specific epithet), which is incorrect, and «ile Kadiak [Alaska]» (original citation). Lectotype (♂), designated by Hieke (1993: 147), in ZILR. Synonymy established by Hieke (1993: 146).
Curtonotus reflexus Putzeys, 1866b: 241. Type locality: «Terre- Neuve (S[ain]t-Pierre-Miquelon)» (original citation). Lectotype (♀), designated by Lindroth (1968: 671), in IRSN. Synonymy established, under the name *Amara rufimana* (Kirby), by Hayward (1908: 60), confirmed by Lindroth (1954b: 134).
Curtonotus somnolentus Putzeys, 1866b: 243. Type locality: «Ounalaschka [Aleutian Islands, Alaska]» (original citation). Syntype(s) [2 originally cited] probably in MHNP (collection Chaudoir) and IRSN. Synonymy established by Lindroth (1968: 671).
Curtonotus holmbergi Putzeys, 1866b: 250. Type locality: «Amérique Russe» (original citation). Holotype [by monotypy] (♂) in MHNP (collection Chaudoir). Synonymy established by Lindroth (1968: 671).
Curtonotus striolatus Putzeys, 1866b: 251. Replacement name for *Curtonotus rufimanus* (Motschulsky, 1844).
Amara cylindrica LeConte, 1878a: 450. Type locality: «South Park (8,000 to 10,000 feet), Colorado; Slave Lake; Lake Winnipeg [Manitoba]» (original citation). Syntype(s) in MCZ [# 5670]. Synonymy established by Lindroth (1953b: 172).
Amara ruficornis J.R. Sahlberg, 1880: 32 [secondary homonym of *Amara ruficornis* (DeGeer, 1774)]. Type locality: «Turuchansk [= Turukhansk, Krasnoyarsk Kray, Siberia, Russia]» (original citation). Holotype [by monotypy] (♂) in NRSS. Synonymy established by Lindroth (1968: 672).
Amara hudsonica Hayward, 1908: 29. Type locality: «Ungava Bay, Hudson Bay Territory» (original citation). Syntype(s) [3 originally cited] in USNM (Lindroth 1968: 672). Synonymy established by Lindroth (1968: 672).
Curtonotus labradorensis Casey, 1918: 231. Type locality: «W[est] S[ain]t Modest[e], Labrador» (original citation). Lectotype (♂), designated by Lindroth (1975: 131), in USNM [# 47148]. Synonymy established by Lindroth (1954b: 134).
Curtonotus scrutatus Casey, 1918: 231. Type locality: «W[est] S[ain]t Modest[e], Labrador» (original citation). Lectotype (♂), designated by Lindroth (1975: 131), in USNM [# 47150]. Synonymy established by Lindroth (1954b: 134).
Curtonotus albertanus Casey, 1924: 45. Type locality: «Edmonton, Alberta» (original citation). Lectotype (♂), designated by Lindroth (1975: 131), in USNM [# 47146]. Synonymy established by Lindroth (1954b: 134).
Curtonotus brevipennis Casey, 1924: 46. Type locality: «Western Hudson Bay region» (original citation). Holotype [by monotypy] (♀) in USNM [# 47149]. Synonymy established by Lindroth (1954b: 135).
Curtonotus durus Casey, 1924: 47. Type locality: «Edmonton, Alberta» (original citation). Lectotype (♀), designated by Lindroth (1975: 131), in USNM [# 47139]. Synonymy established by Lindroth (1954b: 135).
Curtonotus biarcuatus Casey, 1924: 47. Type locality: «Edmonton, Alberta» (original citation). Lectotype (♀), designated by Lindroth (1975: 131), in USNM [# 47140]. Synonymy established by Lindroth (1954b: 135).
Amara consueta Fall, 1926a: 135. Type locality: «Skagway, Alaska» (original citation). Holotype (♂) in MCZ [# 23874]. Synonymy established by Lindroth (1968: 672).
Amara john-sahlbergi Csiki, 1929: 464. Replacement name for *Amara ruficornis* Sahlberg, 1880. Etymology. The specific name was proposed in honor of the Finnish John Reinhold Sahlberg [1845-1920], professor of entomology at the University of Helsinki and one of the leading European entomologist of his time. Sahlberg collected extensively in Scandinavia but also made several expeditions to other parts of Europe, northern Africa, the Middle East, and Siberia. He worked primarily on Hemiptera and Coleoptera. Both his father, Reinhold Ferdinand Sahlberg [1811-1874], and grandfather, Carl Reinhold Sahlberg [1779-1860], were also naturalists and involved in entomology.
Amara tenuestriata Baliani, 1943: 47. Type locality: «Ochtsk, Siberia sett. or. [Russia]» (original citation). Holotype (♂) in MSNG (collection Baliani). Synonymy established by Hieke (1999b: 188).
Amara pulla Jedlička, 1957a: 30. Type locality: «Tripoli, Baikal [Russia]» (original citation). Holotype (♂) in NMP. Synonymy established by Hieke (1995b: 324). Note. According to Hieke (1995b: 324), the type locality is incorrect and possibly refers to the village Tibelti, about 20 km west of Kultuk in Irkutsk Oblast, Russia.
Amara turanica Jedlička, 1957a: 30. Type locality: «Turan, Baikal [Buryatia, Russia]» (original citation). Holotype in NMP. Synonymy established by Hieke (1993: 101).
Amara nairica Iablokoff-Khnzorian, 1964: 283. Type locality: «Gegamgebirgskette, südöstlich vom Sevansee (etwa 3000 m) [Armenia]» (original citation). Holotype (♂) location unknown. Synonymy established by Iablokoff-Khnzorian (1975: 26) (see Hieke 1975: 320).

#### Distribution.

This circumpolar species is found from northern Finland to the Far East (Hieke 2003a: 561) and from the west coast of Alaska (Lindroth 1968: 673) to Newfoundland (Lindroth 1955a: 96-97), south to Nova Scotia (Lindroth 1968: 673), the Magdalen Islands, Gaspé Peninsula (Larochelle 1975: 48), southern Minnesota (Gandhi et al. 2005: 929), northern Colorado (LeConte, 1878a: 450, as *Amara cylindrica*; Hayward 1908: 28, as *Amara rufimana*; Armin 1963: 209, as *Amara melanogastrica*) along the Rocky Mountains, and southeastern British Columbia (Lindroth 1968: 673); isolated on Mount Washington in New Hampshire (CNC). The records from Prince Edward Island (Bousquet and Larochelle 1993: 192, see Majka et al. 2008: 131), “Wisconsin” (Wickham 1896b: 36, as *Amara rufimana*), northwestern Iowa (Wickham 1911b: 6, as *Amara rufimana*), and “South Dakota” (Hayward 1908: 28, as *Amara rufimana*) are probably in error; those from “New Mexico” (Hamilton 1894a: 10, as *Amara eschscholtzii*) and Seboomook in Maine (Dearborn and Donahue 1993: 6) need confirmation. Fossil remnants of this species, dated between about 16,700 and 18,100 years B.P., have been unearthed in southeastern Iowa (Baker et al. 1986: 96).

#### Records.

**FRA**: PM **CAN**: AB, BC, LB, MB, NB, NF, NS, NT, ON, QC, SK, YT **USA**: AK, CO, MN, MT, NH, WY [ME, NM] – **Holarctic**

### Subgenus Bradytus

Stephens, 1827

Bradytus Stephens, 1827: 67. Type species: *Carabus ferrugineus* Linnaeus *sensu* Stephens, 1828 (= *Carabus fulvus* Müller, 1776) designated by Westwood (1838: 4). Etymology (original, see page 136). From the Greek *bradytes* (slowness), probably alluding to the slow pace of the adults in nature [masculine].
Linconus Fischer von Waldheim, 1829a: 16. Type species: *Carabus apricarius* Paykull, 1790 designated by Bousquet (2002c: 176). Synonymy established by Bousquet (2002c: 176).
Omius Fischer von Waldheim, 1829a: 16. Type species: *Carabus consularis* Duftschmid, 1812 designated by Bousquet (2002c: 177). Synonymy established by Bousquet (2002c: 177).
Pseudobradytus Csiki, 1908: 353. Type species: *Amara crenata* Dejean, 1828 by monotypy. Synonymy established by Jeannel (1942: 939). Etymology. From the Greek *pseudos* (fallacy, lie) and the generic name *Bradytus* [*q.v*.] [masculine].

#### Diversity.

Thirty-five species (Hieke 2007) in the arctic, subarctic, boreal, and temperate regions of North America (12 species, of which two are adventive), Asia (26 species), and Europe (seven species, all shared with Asia). One species (*Amara glacialis*) is Holarctic.

#### Identification.

There is no modern taxonomic revision of the species. Lindroth (1968, as *apricaria* and *insignis* groups) covered all but two (*Amara neomexicana* and *Amara lindrothi*) of the North American species.

### [fulva group]

### Amara apricaria

(Paykull, 1790)

Carabus apricarius Paykull, 1790: 125. Type locality: «Dalecarlia [= Dalarna, Sweden]» (original citation). Lectotype (♂), designated by Lindroth (1968: 683), in NRSS.
Amara pygmea Couper, 1865: 60. Type locality: «Quebec» (original citation). Lectotype (♂), designated by Lindroth (1971: 111), in ORUM. Synonymy established by Lindroth (1971: 111).
Amara putzeysii G.H. Horn, 1875: 129 [primary homonym of *Amara putzeysii* Fairmaire, 1867]. Type locality: «S[ain]t Pierre [and] Miquelon» (original citation). Holotype [by monotypy] (♀) in MCZ [# 666]. Synonymy established with doubt by Horn (in Hamilton 1889b: 95), confirmed by Lindroth (1954b: 135). Note. Horn (1875: 129) noted that the sole specimen he had was a male.
Amara putzeysiana Csiki, 1929: 457. Replacement name for *Amara putzeysii* Horn, 1875.

#### Distribution.

This Palaearctic species is adventive in North America where it is known from Newfoundland (Lindroth 1955a: 100-101) and southern Labrador (Lindroth 1954d: 368) to the Kenai Peninsula in Alaska (Derek S. Sikes pers. comm. 2008), south to northern California (Trinity and Lassen Counties, CAS), west-central Nevada (Bechtel et al. 1983: 474), southern Colorado (Huerfano and Pueblo Counties, CMNH), Kansas (Trego County, CNC), and Virginia (Hoffman et al. 2006: 23; Clarke County, USNM). The first inventoried specimen collected on this continent was found in Quebec prior to 1865 (Couper 1865: 60, as *Amara pygmea*). Several records (e.g., AL, AR, GA, NT, SC, YT) listed in Bousquet and Larochelle (1993: 192) are in error or need confirmation.

#### Records.

**FRA**: PM **CAN**: AB, BC (VCI), LB, MB, NB, NF, NS (CBI), NT, ON, PE, QC, SK **USA**: AK, CA, CO, CT, DC, DE, IA, ID, IL, IN, KS, MA, MD, ME, MI, MN, MT, ND, NE, NH, NJ, NV, NY, OH, OR, PA, RI, SD, UT, VA, VT, WA, WI, WY – **Adventive**

### Amara avida

(Say, 1823)

Zabrus avidus Say, 1823b: 148. Type locality: «N[orth] Fork S[outh] Platte Can[y]on (7-8000 ft.), Col[orado]» (neotype label). Neotype (♂), designated by Lindroth and Freitag (1969: 343), in MCZ [# 33033].
Amara confinis Dejean, 1828: 510. Type locality: «Amérique septentrionale» (original citation). One syntype in MHNP (Lindroth 1955b: 16). Synonymy established by LeConte (1847: 367), confirmed by Lindroth (1955b: 16).

#### Distribution.

This species ranges from Newfoundland (Lindroth 1955a: 100) to southeastern British Columbia (Lindroth 1968: 690), south to southern Colorado (Wickham 1902: 236; Armin 1963: 200), southeastern Kansas (Knaus 1907: 233), Missouri (Summers 1873: 145), and southwestern North Carolina (Macon County, MCZ). The record from southeastern Louisiana (Summers 1874a: 80) is likely in error.

#### Records.

**FRA**: PM **CAN**: AB, BC, MB, NB, NF, NS (CBI), ON, PE, QC, SK **USA**: CO, CT, DC, IA, IL, IN, KS, MA, MD, ME, MI, MN, MO, MT, NC, ND, NE, NH, NJ, NY, OH, PA, RI, SD, VA, VT, WI

### Amara browni

Lindroth, 1968

Amara browni Lindroth, 1968: 686. Type locality: «Reindeer Depot, N[orth] W[est] Terr[itories]» (original citation). Holotype (♂) in CNC [# 10509]. Etymology. The specific name was proposed for Williamson James Brown [1902-1977], one of Canada’s leading coleopterist of his time. Brown worked at Agriculture Canada on the systematics of several families with a special interest on scarabaeids, elaterids, and chrysomelids. He was also interested by the adventive species in Canada, the problem of sibling species, and the arctic beetles.

#### Distribution.

This species is known from a few localities in Yukon Territory and the Anderson River delta in northern Northwest Territories (Lindroth 1968: 687).

#### Records.

**CAN**: NT, YT

### Amara exarata

Dejean, 1828

Amara exarata Dejean, 1828: 509. Type locality: «Amérique septentrionale» (original citation), restricted to «Norfolk [Norfolk County], Mass[achusetts]» by Lindroth (1968: 680). One syntype in MHNP (Lindroth 1955b: 16).
Amara furtiva Say, 1830b: (7) [3]. Type locality: «Wabash Vall[ey], Richland & Lawrence Co[unties], Ill[inois]» (neotype label). Neotype (♂), designated by Lindroth and Freitag (1969: 344), in MCZ [# 33032]. Synonymy established by Horn (1875: 128). Note. «Indiana» was the area originally cited by Say (1830b: (7) [3]).
Bradytus gravidus Casey, 1918: 236. Type locality: «Douglas Co[unty], Kansas» (original citation). Lectotype (♀), designated by Lindroth (1975: 131), in USNM [# 47156]. Synonymy established by Lindroth (1968: 680).
Bradytus curtus Casey, 1918: 236. Type locality: «S[ain]t Louis, Missouri» (original citation). Lectotype (♀), designated by Lindroth (1975: 131), in USNM [# 47158]. Synonymy established by Lindroth (1968: 680).
Bradytus stygialis Casey, 1918: 237. Type locality: «Pennsylvania» (original citation). Lectotype (♂), designated by Lindroth (1975: 131), in USNM [# 47157]. Synonymy established by Lindroth (1968: 680).

#### Distribution.

The range of this species extends from southern New Hampshire (Sullivan County, MCZ) to eastern South Dakota (Kirk and Balsbaugh 1975: 26; French et al. 2004: 557), south to northeastern Texas (Dallas County, MCZ), northeastern Georgia (Fattig 1949: 28; Clayton County, USNM), and northwestern South Carolina (Ciegler 2000: 74). The record from “Florida” (Bousquet and Larochelle 1993: 192) is probably in error.

#### Records.

**CAN**: ON **USA**: AL, CT, DC, GA, IA, IL, IN, KS, MA, MD, MI, MN, MO, NC, NE, NH, NJ, NY, OH, OK, PA, RI, SC, SD, TN, TX, VA, WI, WV

### Amara fulva

(Müller, 1776)

Carabus fulvus O.F. Müller, 1776: 77. Type locality: Denmark and Norway (inferred from title of the book); «Denmark» selected by Lindroth (1968: 680). Syntype(s) lost (Lindroth 1968: 680).
Carabus concolor Olivier, 1795: [35] 80 [primary homonym of *Carabus concolor* Gmelin, 1790 and *Carabus concolor* Fabricius, 1792]. Type locality: «environs de Paris» (original citation). Syntype(s) location unknown (possibly in MHNP). Synonymy established by Illiger (1801: 53).

#### Distribution.

This European species is adventive in North America where it is known only in eastern Canada from Newfoundland (Lindroth 1955a: 101) to the Gaspé Peninsula (Larochelle 1975: 44) and the north shore of the Saint Lawrence in Quebec (Brown 1932b: 200), including southern Labrador (Lindroth 1955a: 101). The first inventoried specimen collected on this continent was found in Newfoundland in 1905 (Lindroth 1955a: 101).

#### Records.

**CAN**: LB, NB, NF, NS (CBI), PE, QC – **Adventive**

### Amara glacialis

(Mannerheim, 1853)

Bradytus glacialis Mannerheim, 1853: 135. Type locality: «ad fl[umen] Tchuniten peninsulae Kenai [Alaska]» (original citation). Holotype [by monotypy] location unknown (possibly in ZILR). Note. Mannerheim (1853: 135) stated that he had a single specimen of this species from “fl. Tchuniten” but also mentioned that he received from Motschulsky specimens of this species collected “ad litora continentis Americes borealis” which he referred to his “Var. b.” Since specimens referred to distinct variants are excluded from the type series (ICZN 1999: Article 72.4.1), the description of this species is based exclusively on a single specimen which is the holotype by monotypy. The sentence “Occurrit etiam in peninsula Kamschatka” added by Mannerheim after the statement that his specimens of “Var. b” were sent to him by Motschulsky clearly refers to the variety. The specimen labeled as “Type 8326” in MCZ is not the holotype.
Amara trybomi J.R. Sahlberg, 1880: 34. Type locality: «Tolstoinos [=Tolstyy Nos, Taymyr Autonomous Okrug, Russia]» (original citation for the lectotype). Lectotype (♂), designated by Lindroth (1968: 684), in NRSS. Synonymy established by Poppius (1906a: 49), confirmed by Lindroth (1968: 684). Etymology. The specific name was proposed in honor of the Swedish zoologist Filip Trybom [1850-1913], who was interested initially in entomology and participated in a number of expeditions to northern Russia. He eventually became a fishery research commissioner.
Bradytus nainensis Casey, 1918: 238. Type locality: «Nain, Labrador» (original citation). Lectotype (♂), designated by Lindroth (1975: 131), in USNM [# 47159]. Synonymy established by Lindroth (1954b: 135).

#### Distribution.

This species is found from the Yenisei River in western Siberia to the coast of Labrador [see Alfimov and Berman 2009: Fig. 1].

#### Records.

**CAN**: BC, LB, MB, NT, NU, ON, QC, SK, YT **USA**: AK – **Holarctic**

### Amara latior

(Kirby, 1837)

Curtonotus latior Kirby, 1837: 36. Type locality: northern parts of British America (inferred from title of the book), restricted to «Swift Current, Sask[atchewan]» by Lindroth (1968: 682). Holotype [by monotypy] (♀) in BMNH (Lindroth 1953b: 172).
Amara oregona LeConte, 1855: 349. Type locality: «Fort Vancouver, Oregon [Territory]» (original citation). Holotype [by monotypy] (♂) in MCZ [# 7388]. Synonymy established by Horn (1875: 128), confirmed by Lindroth (1968: 682). Note. Fort Vancouver was a massive British outpost on the north bank of the Columbia River, slightly upstream from the mouth of the Willamette River, in Washington.
Amara libera LeConte, 1855: 349. Type locality: «Lake Superior, Illinois, Wisconsin, Nebraska» (original citation). Syntype(s) in MCZ [# 7387]. Synonymy established with doubt by LeConte (1859c: 33), confirmed by Lindroth (1968: 682).
Bradytus laevistriatus Putzeys, 1866b: 262. Type locality: «Etats-Unis» (original citation). Lectotype (♂), designated by Lindroth (1968: 682), in IRSN. Synonymy established by LeConte (1873b: 323), confirmed by Lindroth (1968: 682).
Bradytus humphreysi Casey, 1918: 240. Type locality: «Humphreys Peak [Coconino County], Arizona» (original citation). Lectotype (♂), designated by Lindroth (1975: 131), in USNM [# 47160]. Synonymy established by Lindroth (1968: 682).
Bradytus deceptus Casey, 1918: 241. Type locality: «New Mexico» (original citation). Lectotype (♀), designated by Lindroth (1975: 131), in USNM [# 47161]. Synonymy established by Lindroth (1968: 682).
Bradytus relictus Casey, 1918: 242. Type locality: «Denver [Denver County], Colorado» (original citation). Lectotype (♀), designated by Lindroth (1975: 131), in USNM [# 47164]. Synonymy established by Lindroth (1968: 682).

#### Distribution.

The range of this species extends from western Newfoundland (Lindroth 1955a: 102) to Vancouver Island (Lindroth 1968: 682), south to northwestern California (Humboldt County, USNM; Hayward 1908: 44), Arizona (Casey, 1918: 240, as *Bradytus humphreysi*; Chen et al. 2006: 171; Coconino County, USNM), New Mexico (Fall and Cockerell 1907: 158; Casey, 1918: 241, as *Bradytus deceptus*; Taos County, CMNH), “Texas” (Lindroth 1955a: 102), and northern Virginia (Page County, USNM).

#### Records.

**CAN**: AB, BC (VCI), MB, NB, NF, NS, ON, PE, QC, SK **USA**: AR, AZ, CA, CO, CT, DC, IA, ID, IL, IN, KS, KY, MA, ME, MI, MN, MT, ND, NE, NH, NJ, NM, NY, OH, OR, PA, SD, TX, UT, VA, VT, WA, WI, WY

### Amara lindrothi

Hieke, 1990

Amara lindrothi Hieke, 1990: 234. Type locality: «Kluane [= Silver City], Y[ukon] T[erritory]» (original citation). Holotype (♂) in CNC [# 23036].

#### Distribution.

This species is known from a few localities in Labrador (CNC), northern Manitoba (CNC), northeastern Alberta (Andrew Lake, UASM), and Yukon Territory as far north as Swim Lakes (CNC), south to east-central Nevada (White Pine County, CMNH) and northern New Mexico (Hieke 1990: 234).

#### Records.

**CAN**: AB, LB, MB, YT **USA**: CO, NM, NV, UT, WY

### Amara neomexicana

(Casey, 1924)

Bradytus neomexicanus Casey, 1924: 48. Type locality: «Maxwell [Colfax County], New Mexico» (original citation). Holotype [by monotypy] (♂) in USNM [# 47167].
Bradytus novellus Stehr, 1949: 207. Type locality: «F[or]t Davis [Jeff Davis County], Davis M[oun]t[ain]s, Texas» (original citation). Holotype (♂) in MSUE. Synonymy established by Hieke (1997: 240).
Bradytus vegasensis Stehr, 1949: 207. Type locality: «6 mi[les] N[orth] E[ast] of Las Vegas [San Miguel County], New Mexico» (original citation). Holotype (♂) in MSUE. Synonymy established, under the name *Amara novella* (Stehr), by Hieke (1994: 326).

#### Distribution.

This species is known from Arizona (Coconino National Forest, CNC), northern New Mexico (Casey 1924: 48; Stehr 1949: 207, as *Bradytus vegasensis*), and western Texas (Stehr 1949: 207, as *Bradytus novellus*).

#### Records.

**USA**: AZ, NM, TX

### Amara schwarzi

Hayward, 1908

Isopleurus septentrionalis LeConte, 1847: 358 [secondary homonym of *Amara septentrionalis* Curtis, 1840]. Type locality: «Lacum Superiorem» (original citation), herein restricted to Marquette, Marquette County, Michigan (USNM). Syntype(s) in MCZ [# 7386].
Amara schwarzi Hayward, 1908: 42. Replacement name for *Amara septentrionalis* (LeConte, 1847). Note. Because Hayward’s name was expressly proposed as a replacement name, the syntype of *Amara schwarzi* Hayward in MCZ [# 25669] has no status (see ICZN 1999: Article 72.7).

#### Distribution.

This species inhabits the Laurentian Highlands from the north shore of the Saint Lawrence Gulf (Larochelle 1975: 48) to the Great Slave Lake in the Northwest Territories (Lindroth 1968: 684), south to central Washington (Kittitas County, CMNH), southwestern Alberta (Indian Creek at 49º48’N - 114º07’W, CNC), northeastern Minnesota, and the upper peninsula of Michigan (Lindroth 1968: 684). The record from South Dakota (Kirk and Balsbaugh 1975: 26) was based on a small specimen of *Amara latior* in MCZ; those from Labrador (Larochelle 1975: 48) and Yukon Territory (Lindroth 1968: 684), and probably also from Colorado (Wickham 1902: 237), refer to *Amara lindrothi* (CNC). The records from Ohio (Everly 1927: 155), New Jersey (Smith 1910: 206), and “New York” (Leng and Beutenmüller 1893: 139; Wickham 1896b: 38) are likely in error; that from northern Wisconsin along Lake Superior (Wickham 1896c: 133, as *Amara septentrionalis*) needs confirmation.

#### Records.

**CAN**: AB, MB, NT, ON, QC **USA**: MI, MN, WA [WI]

### [insignis group]

### Amara insignis

Dejean, 1831

Amara insignis Dejean, 1831: 796. Type locality: «Californie» (original citation), herein restricted to San Diego, San Diego County (see Casey 1918: 295, as *Amara tarsalis*). Two syntypes in MHNP (Lindroth 1955b: 17).
Celia coerulea Motschulsky, 1859a: 153. Type locality: «St. Francisco [San Francisco County, California]» (original citation). Lectotype (♂), designated by Bousquet (1997b: 335), in ZMMU. Synonymy established by Hayward (1908: 61), confirmed by Bousquet (1997b: 335).
Amara tarsalis Casey, 1918: 295. Type locality: «San Diego [San Diego County], California» (original citation). Seven syntypes in USNM [# 47276]. Synonymy established implicitly by Lindroth (1968: 692).
Amara guadalupensis Casey, 1918: 295. Type locality: «Island of Guadalupe, [Baja] California» (original citation). One syntype in USNM [# 47277]. Synonymy established implicitly by Lindroth (1968: 692).

#### Distribution.

This species is found along the Pacific Coast from central California (Lindroth 1968: 692) south to Guadalupe Island off the coast of Baja California (Casey 1918: 295, as *Amara guadalupensis*). The record from “Oregon [Territory]” (LeConte 1857c: 9) is probably in error.

#### Records.

**USA**: CA (CHI) – Mexico

### Amara insularis

Horn, 1875

Amara insularis G.H. Horn, 1875: 128. Type locality: «island of San Clemente [Los Angeles County] on the coast of California» (original citation). Two syntypes [2 originally cited] in MCZ [# 668] (collection LeConte).

#### Distribution.

This species is known from “all the islands” (Fall 1901a: 45) off the coast of southern California.

#### Records.

**USA**: CA (CHI)

### Subgenus Neopercosia

Hieke, 1978

Neopercosia Hieke, 1978: 289. Type species: *Amara fortis* LeConte, 1880 by original designation. Etymology. From the Greek prefix *neo*- (new) and the generic name *Percosia* [feminine].

#### Diversity.

One species in southern North America.

#### Identification.

The species was redescribed in Hieke’s (1978: 290-292) revision of the subgenus *Percosia*.

### Amara fortis

LeConte, 1880

Amara fortis LeConte, 1880b: 164. Type locality: «Texas» (original citation), restricted to «Waco [McLennan County]» by Horn (1892b: 27). Syntype(s) in MCZ [# 5685].

#### Distribution.

This species is known from McLennan (Horn 1892b: 27) and Dallas (Hieke 1978: 292) Counties in Texas and from southwestern Oklahoma (Kondratieff et al. 2005: 171).

#### Records.

**USA**: OK, TX

### Subgenus Percosia

Zimmermann, 1832

Percosia Zimmermann, 1832: 18. Type species: *Amara sicula* Dejean, 1831 designated by Hope (1838: 86). Etymology. Uncertain, possibly from the Latin adjective *percosius* (relative to Percosius, king of Cyzicus an ancient city of Mysia in Asia Minor) [feminine].
Spelaeobia Gistel, 1856: 358. Type species: *Carabus patricius* Duftschmid, 1812 (= *Carabus equestris* Duftschmid, 1812) by monotypy. Synonymy established by Bousquet (2002b: 47).

#### Diversity.

Six species in the boreal and temperate regions of North America (one species), Asia (two species), Europe (three species, two shared with Asia), and northern Africa (two endemic species).

#### Identification.

Hieke (1978) revised the species of this subgenus.

### Amara obesa

(Say, 1823)

Feronia obesa Say, 1823a: 37. Type locality: «Charity Is[land] [Arenac County], Mich[igan]» (neotype label). Neotype (♂), designated by Lindroth and Freitag (1969: 344), in MCZ [# 33030]. Note. «Harrowgate», a neighborhood in the northeast section of Philadelphia, Pennsylvania, was the area originally cited by Say (1823a: 37).
Percosia diffinis LeConte, 1847: 359. Type locality: «ad Rocky Mountains» (original citation), cited from «Nebraska [Territory]» by LeConte (1855: 354). Five syntypes in MCZ [# 5686]. Synonymy established by Horn (1892b: 39), confirmed by Lindroth (1968: 690).
Percosia extensa Casey, 1918: 244. Type locality: «Clackamas Co[unty], Oregon» (original citation). Lectotype (♀), designated by Lindroth (1975: 131), in USNM [# 47172]. Synonymy established by Hatch (1953: 129), confirmed by Lindroth (1968: 690).
Percosia latissima Casey, 1918: 245. Type locality: «Highland Park [Lake County], Illinois» (original citation). Lectotype (♂), designated by Lindroth (1975: 131), in USNM [# 47170]. Synonymy established by Lindroth (1968: 690).
Percosia ventricosa Casey, 1918: 245. Type locality: «Long Island, New York» (original citation). Lectotype (♀), designated by Lindroth (1975: 131), in USNM [# 47171]. Synonymy established by Lindroth (1968: 690).
Percosia sulcatula Casey, 1920: 199. Type locality: «Mesa, Boulder Co[unty], Colorado» (original citation). Lectotype (♀), designated by Lindroth (1975: 131), in USNM [# 47173]. Synonymy established by Hatch (1953: 129), confirmed by Lindroth (1968: 694).

#### Distribution.

This species ranges from Newfoundland (Lindroth 1955a: 102) to southern Alaska (CNC), south to central California (Merced County, CAS), northern Arizona (Chen et al. 2006: 171; Apache and Coconino Counties, CMNH, CNC, UASM), northeastern Texas (Hieke 1978: 289), and northern Georgia (Fattig 1949: 28).

#### Records.

**CAN**: AB, BC (VCI), MB, NB, NF, NS (CBI), NT, ON, PE, QC, SK, YT **USA**: AK, AR, AZ, CA, CO, CT, DC, GA, IA, ID, IL, IN, KS, MA, MD, ME, MI, MN, MO, MS, MT, NC, ND, NE, NH, NJ, NM, NV, NY, OH, OK, OR, PA, RI, SC, SD, TN, TX, UT, VA, VT, WA, WI, WV, WY

### Subgenus Xenocelia

Hieke, 2001

Xenocelia Hieke, 2001: 7. Type species: *Carabus municipalis* Duftschmid, 1812 by original designation. Etymology. From the Greek *xenos* (stranger, guest) and the generic name *Celia* [*q.v*.] [feminine].

#### Diversity.

Thirty-two species (Hieke 2007) in the arctic (marginal), subarctic, boreal, and temperate regions of North America (11 species, two extending into northern Mexico), Asia (17 species), and Europe (ten species, many shared with Asia and one extending into northern Africa). One species (*Amara hicksi*) is Holarctic.

#### Identification.

Hieke (2001) revised the species. Two Asian species have been described subsequently.

### Amara apachensis

Casey, 1884

Amara apachensis Casey, 1884b: 3. Type locality: «Arizona» (original citation), herein restricted to Tucson, Pima County (see Casey 1918: 241, as *Bradytus specularis*). Lectotype (♀), designated by Hieke (1993: 115), in USNM [# 47202].
Bradytus specularis Casey, 1918: 241. Type locality: «Tuçson [Pima County], Arizona» (original citation). Lectotype [as typus] (♀), designated by Hieke (1994: 336), in USNM [# 47163]. Synonymy established by Hieke (1994: 336).
Celia amplipennis Casey, 1918: 266. Type locality: «Arizona» (original citation). Lectotype [as holotypus] (♂), designated by Hieke (1993: 116), in USNM [# 47194]. Synonymy established by Hieke (1993: 115).
Celia patula Casey, 1918: 270. Type locality: «Arizona» (original citation). Lectotype (♂), designated by Hieke (1993: 116), in USNM [# 47203]. Synonymy established by Hieke (1993: 115).
Celia decora Notman, 1922b: 101. Type locality: «Sabino Canyon [Pima County], Ariz[ona]» (original citation). Holotype (♂) in USNM [# 26592]. Synonymy established by Hieke (1993: 115).

#### Distribution.

This species ranges from northern Oregon to southern California and Sonora, east to western Texas and Chihuahua in Mexico (Hieke 2001: 128). The record from southwestern Utah (Tanner 1928: 270) needs confirmation.

#### Records.

**USA**: AZ, CA, NM, OR, TX [UT] – Mexico

### Amara bradytonota

Hieke, 2001

Amara bradytonota Hieke, 2001: 136. Type locality: «Texas» (original citation), herein restricted to Gatesville, Coryell County (see Hieke 2001: 136). Holotype (♂) in USNM.

#### Distribution.

This species is known from two localities, Manhattan in northeastern Kansas and Gatesville in central Texas (Hieke 2001: 136).

#### Records.

**USA**: KS, TX

### Amara chalcea

Dejean, 1828

Amara chalcea Dejean, 1828: 476. Type locality: «Amérique septentrionale» (original citation), restricted to «Billerica [Middlesex County], Mass[achusetts]» by Lindroth (1968: 700). One syntype in MHNP (Hieke 2001: 114).
Celia impunctata Putzeys, 1867a: 170 [*nomen dubium*; secondary homonym of *Amara fulva impunctata* Letzner, 1852]. Type locality: «Etats-Unis» (original citation). Holotype [by monotypy] (♀) location unknown. **New synonymy**. Note. Putzeys (1867a: 171) compared his new species to *Amara chalcea* Dejean and observed several but relatively minor structural differences between the two. However, all the differences noted, including the size, seem to fall within the variability of *Amara chalcea* as described by Lindroth (1968: 700). The holotype of *Celia impunctata* could not be located in MHNP (Fritz Hieke pers. comm. 2001). Obviously, the name is a *nomen dubium* but for convenience it is placed here in synonymy with *Amara chalcea* Dejean.
Celia pinorum Casey, 1918: 264. Type locality: «Southern Pines [Moore County], North Carolina» (original citation). Lectotype (♀), designated by Lindroth (1975: 132), in USNM [# 47191]. Synonymy established by Lindroth (1968: 700).
Celia sodalis Casey, 1918: 265. Type locality: «Staten Island, New York» (original citation). Lectotype (♀), designated by Lindroth (1975: 133), in USNM [# 47192]. Synonymy established by Lindroth (1968: 700).
Celia corvina Casey, 1918: 266. Type locality: «Southern Pines [Moore County], North Carolina» (original citation). Lectotype (♂), designated by Lindroth (1975: 133), in USNM [# 47193]. Synonymy established by Lindroth (1968: 700).
Celia schotti Casey, 1918: 266. Type locality: «Jamaica [Queens County], Long Island, New York» (original citation). Lectotype (♂), designated by Lindroth (1975: 133), in USNM [# 47195]. Synonymy established by Lindroth (1968: 700). Etymology. The specific name was proposed in honor of Fred M. Schott [1887-1946], long time entomologist with the New Jersey Department of Agriculture. His main interests were his entomological books as well as his collection of moths and beetles.
Celia sphaerops Casey, 1918: 267. Type locality: «Wyandanch [Suffolk County], Long Island, New York» (original citation). Lectotype (♂), designated by Lindroth (1975: 132), in USNM [# 47196]. Synonymy established by Hieke (1993: 123).
Celia maneei Casey, 1924: 55. Type locality: «Southern Pines [Moore County], North Carolina» (original citation). Lectotype (♀), designated by Lindroth (1975: 133), in USNM [# 47197]. Synonymy established by Lindroth (1968: 700). Etymology. The specific name honors the Reverend Abram Herbert Manee [1858-1927] of Southern Pines. Manee was a great collector of insects, especially Coleoptera. His collection is in the North Carolina Department of Agriculture in Raleigh.
Amara jucunda Csiki, 1929: 439. Replacement name for *Amara impunctata* (Putzeys, 1867).

#### Distribution.

This species is found from western Maine (Oxford County, Ross T. Bell pers. comm. 2008) to southeastern Wyoming (Hieke 2001: 116), including southernmost Ontario (Lindroth 1968: 700), south to the Taos Mountains in northern New Mexico (LeConte 1876: 299; McKinley County, USNM), “Texas” (Horn 1892b: 32), southwestern Alabama (Hieke 2001: 116), northern Georgia (Fattig 1949: 29), and central South Carolina (Ciegler 2000: 74).

#### Records.

**CAN**: ON **USA**: AL, AR, CO, CT, DC, GA, IA, IL, IN, KS, MA, MD, ME, MI, MN, MO, NC, NE, NH, NJ, NM, NY, OH, PA, RI, SC, TX, VT, WI, WY

### Amara discors

Kirby, 1837

Amara discors Kirby, 1837: 40. Type locality: northern parts of British America (inferred from title of the book), restricted to «Saskatoon, Sask[atchewan]» by Lindroth (1968: 697). Holotype [by monotypy] (♀) in BMNH (Lindroth 1953b: 173).
Celia nevadica Casey, 1918: 264. Type locality: «Reno [Washoe County], Nevada» (original citation). Lectotype (♂), designated by Lindroth (1975: 132), in USNM [# 47190]. Synonymy established by Lindroth (1968: 697).
Celia scolopax Casey, 1918: 269. Type locality: «Boulder Co[unty], Colorado» (original citation for the lectotype). Lectotype (♂), designated by Lindroth (1975: 132), in USNM [# 47201]. Synonymy established by Lindroth (1968: 697).
Bradytus spaldingi Casey, 1924: 48. Type locality: «Callao [Juab County], Utah» (original citation). Lectotype (♂), designated by Lindroth (1975: 132), in USNM [# 47166]. Synonymy established by Lindroth (1968: 697).
Celia parowana Casey, 1924: 57. Type locality: «summit of Parowan M[oun]t[ain]s, Utah» (original citation). Lectotype (♂), designated by Lindroth (1975: 132), in USNM [# 47205]. Synonymy established by Lindroth (1968: 697).
Amara wakelandi Hatch, 1949a: 82. Type locality: «Succor Cr[eek] [Owyhee County], Ida[ho]» (original citation). Holotype (♂) in USNM. Synonymy established by Lindroth (1968: 697). Etymology. The specific name was proposed for Claude Wakeland [1888-1960], professor of entomology at the University of Idaho.

#### Distribution.

This western species ranges from southern Manitoba to southern British Columbia, south to southeastern California (San Bernardino County, David H. Kavanaugh pers. comm. 2008), southern Utah (Garfield County, CNC), south-central New Mexico, and northwestern Nebraska (Hieke 2001: 118).

#### Records.

**CAN**: AB, BC, MB, SK **USA**: CA, CO, ID, MT, NE, NM, NV, OR, SD, UT, WA, WY

### Amara gibba

(LeConte, 1847)

Celia gibba LeConte, 1847: 360. Type locality: «Lacum Superiorem» (original citation), herein restricted to Marquette, Marquette County, Michigan (see Casey 1918: 259, as *Celia paganica*). Holotype [by monotypy] (♂) in MCZ [# 3688].
Celia paganica Casey, 1918: 259. Type locality: «Marquette [Marquette County], Michigan» (original citation). Lectotype (♀), designated by Lindroth (1975: 132), in USNM [# 47179]. Synonymy established by Hieke (1993: 135).

#### Distribution.

This species ranges from Newfoundland to Alaska including Kodiak Island; it is also known from the Lake Superior region in northern Michigan (Hieke 2001: 113) and from northeastern New York (Essex County, CNC).

#### Records.

**CAN**: AB, BC, LB, MB, NB, NF, NS (CBI), NT, ON, QC, YT **USA**: AK, MI, NY

#### Note.

This form, listed as a synonym of *Amara discors* Kirby by Lindroth (1968: 697), was regarded as a valid species by Hieke (1990: 226).

### Amara harpalonota

Hieke, 2001

Amara harpalonota Hieke, 2001: 119. Type locality: «Spok[ane] Falls [Spokane County], Wash[ington]» (original citation). Holotype (♂) in USNM.

#### Distribution.

This species is known from a few localities in eastern Washington, northern Oregon, and west-central Idaho (Hieke 2001: 119).

#### Records.

**USA**: ID, OR, WA

### Amara hicksi

Lindroth, 1968

Amara hicksi Lindroth, 1968: 699. Type locality: «Norman Wells, N[orth] W[est] Terr[itories]» (original citation). Holotype (♂) in CNC [# 10511]. Etymology. The specific name honors Stanton D. Hicks [1910-1983], an amateur coleopterist with a special interest for the fauna of southern Ontario. Hicks, a Canadian, worked at the Canadian National Collection of Insects. Note. The type locality was listed by error as “Good Hope, North West Territory” by Hieke (2001: 58).

#### Distribution.

This Holarctic species is found from western Northwest Territories to Alaska and from Kamtschatka to northern European Russia, south to Mongolia and northern China (Hieke 2001: 61).

#### Records.

**CAN**: NT **USA**: AK – **Holarctic**

### Amara lugubris

(Casey, 1918)

Celia lugubris Casey, 1918: 268. Type locality: «Reno [Washoe County], Nevada» (original citation). Lectotype (♂), designated by Lindroth (1975: 132), in USNM [# 47198].

#### Distribution.

This species ranges from northern California to north-central Colorado, south to southwestern New Mexico and southern Arizona (Hieke 2001: 126-127).

#### Records.

**USA**: AZ, CA, CO, NM, NV, UT

#### Note.

This form was considered a synonym of *Amara discors* Kirby by Lindroth (1968: 697) but treated as a valid species by Hieke (2001: 124).

### Amara merula

(Casey, 1918)

Celia merula Casey, 1918: 267. Type locality: «probably Colorado» (original citation), restricted to «Pass n[ea]r Ouray, Red M[oun]t[ain]s, Colorado» by Hieke (1993: 128). Holotype [by monotypy] (♀) in USNM [# 47204].

#### Distribution.

This species ranges from the Kodiak Island and Kenai Peninsula in Alaska south to the Sierra Nevada in east-central California, northern Arizona, and northern New Mexico along the Rocky Mountains (Hieke 2001: 122-124).

#### Records.

**CAN**: AB, BC **USA**: AK, AZ, CA, CO, ID, MT, NM, NV, OR, UT, WA, WY

#### Note.

This form was regarded as a synonym of *Amara discors* Kirby by Lindroth (1968: 697) but treated as a valid species by Hieke (1993: 128).

### Amara rectangula ciudadensis

(Bates, 1891)

Celia ciudadensis Bates, 1891a: 248. Type locality: «Ciudad, in Durango» (original citation). Two syntypes in BMNH (Hieke 1990: 229). Note. According to Selander and Vaurie (1962: 27), the type locality “is presumably the settlement of Ciudad or La Ciudad near the border of Sinaloa 148 km. west-northwest of the city of Durango.”
Amara nupera G.H. Horn, 1892b: 33. Type locality: «Salida [Chaffee County], Colo[rado]» (lectotype label). Lectotype (♂), designated by Hieke (1993: 132), in MCZ [# 34449]. Synonymy established by Hieke (2001: 130).
Bradytus obsolescens Casey, 1918: 242. Type locality: «Colorado Springs [El Paso County], Colorado» (original citation). Lectotype (♀), designated by Lindroth (1975: 132), in USNM [# 47162]. Synonymy established, under the name *Amara nupera* Horn, by Hieke (1993: 132).
Bradytus aequalis Casey, 1918: 242. Type locality: «Colonia Garcia, Sierra Madre M[oun]t[ain]s, Chihuahua, Mexico» (original citation). Lectotype [as typus] (♂), designated by Hieke (1997: 194), in USNM [# 47168]. Synonymy established, under the name *Amara nupera* Horn, by Hieke (1997: 194).
Celia greenei Casey, 1918: 269. Type locality: «S[an]ta Fé [Santa Fe County], New Mexico» (original citation). Lectotype (♂), designated by Hieke (1993: 135), in USNM [# 47199]. Synonymy established, under the name *Amara nupera* Horn, by Hieke (1993: 132).
Bradytus maxwelli Casey, 1924: 49. Type locality: «Maxwell [Colfax County], New Mexico» (original citation). Lectotype [as typus], designated by Hieke (1994: 322), in USNM [# 47165]. Synonymy established, under the name *Amara nupera* Horn, by Hieke (1994: 322).
Amara maxvellensis Csiki, 1929: 457. Unjustified emendation of *Amara maxwelli* (Casey, 1924) (cited as *maxvelli*).
Bradytus celianus Stehr, 1949: 208. Type locality: «Jemez M[oun]t[ain]s [Sandoval County], New Mexico» (original citation). Holotype (♂) in MSUE. Synonymy established, under the name *Amara nupera* Horn, by Hieke (1994: 306).

#### Distribution.

This subspecies ranges from northern California to northwestern Oklahoma (Cimarron County, CMNH), north to northern Colorado, south to Durango in Mexico (Hieke 2001: 135-136), including western Texas (Jeff Davis County, CMNH), and south-central California (Hieke 2001: 135).

#### Records.

**USA**: AZ, CA, CO, NM, OK, TX, UT – Mexico

#### Note.

Lindroth (1968: 697) listed *Amara obsolescens* Casey as a junior synonym of *Amara discors* Kirby but Hieke (1993: 132) considered it a synonym of *Amara nupera* Horn.

### Amara rectangula rectangula

LeConte, 1855

Amara rectangula LeConte, 1855: 355. Type locality: «S[an] Fr[ancisco] [San Francisco County, California]» (lectotype label). Lectotype (♂), designated by Hieke (1993: 132), in MCZ [# 5691]. Note. LeConte (1855: 355) stated that his specimen from San Francisco was a female.

#### Distribution.

This subspecies ranges from northern Idaho and eastern Washington south at least to Monterey County in western California (Hieke 2001: 134).

#### Records.

**USA**: CA, ID, OR, WA

### Amara spuria

Lindroth, 1968

Amara spuria Lindroth, 1968: 696. Type locality: «Rampart, Alaska» (original citation). Holotype (♂) in MCZ [# 31227].

#### Distribution.

This species is known from a few localities in Northwest Territories, Yukon Territory (CNC), Alaska, and from Manning Provincial Park in southern British Columbia (Hieke 2001: 110).

#### Records.

**CAN**: BC, NT, YT **USA**: AK

### Subgenus Reductocelia

Lafer, 1989

Reductocelia Lafer, 1989: 165. Type species: *Amara kuznetzovi* Lafer, 1989 (= *Amara chalcophaea sachalinica* Hieke, 1999) by original designation. Etymology. From the Latin *reducto* (reduce) and the generic name *Celia* [*q.v*.], probably alluding to the absence of the lateromedial setae on the pronotum of the adult [feminine].
Bradycelia Lafer, 1989: 166. Type species: *Amara nigromontana* Lafer, 1989 (= *Amara lucens* Baliani, 1943) by original designation. Synonymy established by Hieke (1997: 229). Etymology. Probably from the generic names *Bradytus* [*q.v*.] contracted and *Celia* [*q.v*.] [feminine].

#### Diversity.

Twenty species (Hieke 2007) in northern North America (one Holarctic species) and eastern Asia.

#### Identification.

Hieke (1999a) revised the species of this subgenus. Subsequently, ten new Chinese species were described.

### Amara colvillensis

Lindroth, 1968

Amara colvillensis Lindroth, 1968: 687. Type locality: «Colville R[iver], Umiat, Alaska» (original citation). Holotype (♂) in MCZ [# 35344].

#### Distribution.

This Holarctic species is found in Siberia east of the Lena River and in North America from Alaska to the Hudson Bay coast in Manitoba and Nunavut (Hieke 1999a: 347).

#### Records.

**CAN**: MB, NU, YT **USA**: AK – **Holarctic**

### Subgenus Celia

Zimmermann, 1832

Celia Zimmermann, 1832: 18. Type species: *Harpalus bifrons* Gyllenhal, 1810 designated by Westwood (1838: 4). Etymology (original). From the Greek *cello* (to run) [feminine].
Acrodon Zimmermann, 1832: 40. Type species *Harpalus brunneus* Gyllenhal, 1810 by monotypy. Synonymy established by Dawson (1854: 117). Etymology. From the Greek *acron* (top, summit) and *odon* (tooth), alluding to the entire apex of the mentum tooth (“*menti dente intermedio simplici*”) [masculine].
Isopleurus Kirby, 1837: 49. Type species: *Isopleurus nitidus* Kirby, 1837 (= *Celia sinuosa* Casey, 1918) by monotypy. Synonymy established implicitly by Lindroth (1954b: 136), explicitly by Lindroth (1968: 692). Etymology. From the Greek *isos* (equal) and *pleura* (side) [masculine].
Adocron Lutshnik, 1927: 58. Type species: *Amara praetermissa* Sahlberg, 1827 by original designation. Etymology. Anagram of the generic name *Acrodon* [*q.v*.] [masculine].
Embrikiella Lutshnik, 1935: 265. Type species: *Amara kachovskyi* Lutshnik, 1935 (= *Leiocnemis tartariae* Bates, 1878) by monotypy. Etymology (original). From the first name of the Norwegian zoologist Embrik Strand [1876-1947].

#### Diversity.

Forty species (Hieke 2007) in North America (12 species), Middle America (two species, one of them endemic, *Amara chihuahuae* Casey), Asia (22 species), and Europe and northern Africa (14 species, several shared with Asia). One species found in the Nearctic Region is adventive (*Amara bifrons*), another one is Holarctic (*Amara brunnea*).

#### Identification.

There is no modern revision of the species of this subgenus and such work is much needed.

### Amara bifrons

(Gyllenhal, 1810)

Harpalus bifrons Gyllenhal, 1810: 144. Type locality: «Bahusia [= Bohuslän], Hallandia [Sweden]» (original citation). Lectotype (♂), designated by Lindroth (1968: 700), in UZIU.

#### Distribution.

This European species is adventive in North America where it is known from Newfoundland (Lindroth 1955a: 105) to western Quebec (Larochelle 1975: 43), south to Massachusetts (Davidson et al. 2011: 513). The first inventoried specimen collected on this continent was found in Cape Breton Island in 1929 (Lindroth 1963a: Fig. 39).

#### Records.

**FRA**: PM **CAN**: LB, NB, NF, NS (CBI), PE, QC **USA**: MA, ME, NH – **Adventive**

### Amara brunnea

(Gyllenhal, 1810)

Harpalus brunneus Gyllenhal, 1810: 143. Type locality: Sweden (inferred from title of the book), restricted to «Göteborg» by Lindroth (1968: 704). Lectotype (♂), designated by Lindroth (1968: 704), in UZIU.
Harpalus lapponicus C.R. Sahlberg, 1827c: 250. Type locality: «Lapponica» (original citation). One syntype in ZMH (Silfverberg 1987: 19). Synonymy established by Schaum (1858: 548).
Harpalus sahlbergi Zetterstedt, 1837: 36. Unnecessary replacement name for *Harpalus lapponicus* Sahlberg, 1827.
Celia amplicollis Mannerheim, 1853: 139. Type locality: «ad rivulum fl[umen] Tschunuktnu peninsulae Kenai [Alaska]» (original citation). Holotype [by monotypy] location unknown (possibly in collection Chaudoir, MHNP, *fide* Lindroth 1968: 704). Synonymy established by Putzeys (1866b: 197), confirmed by Lindroth (1968: 704). Note. Regarding the type locality, see “Note” section for *Cryobius subcaudatus* Mannerheim.
Harpalus mongolicus Jedlička, 1966: 221. Type locality: «16 km SO von Somon Bajanzogt, Zentral aimak, Mongolei» (original citation). Holotype (♀) in TMB. Synonymy established by Hieke (1973: 116).

#### Distribution.

This Holarctic species is found from northern Manitoba (CNC) to the Kenai Peninsula in Alaska (Lindroth 1968: 706), and in the Palaearctic Region from the Far East to Norway, south to Spain, Italy, Kazakhstan, and Mongolia (Hieke 2003a: 556). The records from British Columbia (Hamilton 1894a: 11; Jarrett and Scudder 2001: 379) need confirmation; those from “Washington,” “Colorado” (Hamilton 1894a: 11; Wickham 1902: 238), and Idaho (Horning and Barr 1970: 24, as *Amara amplicollis*) are in error.

#### Records.

**CAN**: AB, MB, NT, YT **USA**: AK [BC] – **Holarctic**

### Amara californica californica

Dejean, 1828

Amara californica Dejean, 1828: 474. Type locality: «Californie» (original citation), herein restricted to San Clemente Island, Los Angeles County (see Casey, 1918: 294, as *Amara perspecta*). One syntype in MHNP (Lindroth 1955b: 17).
Amara mexicana Dejean, 1831: 792. Type locality: «Mexique» (original citation). Holotype [by monotypy] (♀) in MHNP. Synonymy established by Hieke (1975: 318).
Celia championi Bates, 1882a: 77. Type locality: «Quezaltenango, 7800 ft. [Guatemala]» (lectotype label). Lectotype (♂), designated by Hieke (1990: 219), in BMNH. Synonymy established by Hieke (1990: 219).
Amara robustula G.H. Horn, 1892b: 32. Type locality: «California» (original citation). Holotype [by monotypy] (♂) in MCZ [# 3154]. Synonymy established by Hieke (1993: 138).
Celia consors Casey, 1918: 250. Type locality: «Salazar, Mex[ico], Mexico» (original citation). Lectotype [as holotypus] (♀), designated by Hieke (1993: 122), in USNM [# 47251]. Synonymy established by Hieke (1993: 122).
Celia gnara Casey, 1918: 255. Type locality: «near the city, Mexico» (original citation). Lectotype [as typus] (♀), designated by Hieke (1993: 125), in USNM [# 47261]. Synonymy established by Hieke (1993: 125).
Amara perspecta Casey, 1918: 294. Type locality: «San Clemente Island [Los Angeles County], California» (original citation). Lectotype (♀), designated by Lindroth (1975: 131), in USNM [# 47275]. Synonymy established by Lindroth (1968: 693).
Celia pugetana Casey, 1924: 56. Type locality: «Wawawai [Whitman County], Washington» (original citation). Lectotype (♂), designated by Lindroth (1975: 131), in USNM [# 47206]. Synonymy established by Lindroth (1968: 693).

#### Distribution.

This subspecies ranges from Vancouver Island (Lindroth 1968: 694) to eastern South Dakota (Brookings County, USNM), south to Costa Rica (Hieke 1990: 221) and the Baja California Peninsula (Horn 1894: 309).

#### Records.

**CAN**: BC (VCI) **USA**: AZ, CA (CHI), CO, ID, MT, NM, NV, OR, SD, TX, UT, WA, WY – Costa Rica, Guatemala, Mexico

#### Note.

The subspecies *Amara californica costaricensis* (Bates) is found in Costa Rica (Hieke 1990: 221).

### Amara exlineae

Minsk and Hatch, 1939

Amara exlineae Minsk and Hatch, 1939: 215. Type locality: «Paradise Park, M[oun]t Rainier [Pierce County], Wash[ington]» (original citation). Holotype (♂) in USNM. Etymology. The specific name was proposed for the American arachnologist Harriet Idola Exline [1909-1968], also known as Mrs. Donald Leslie Frizzell.

#### Distribution.

This species is known from southwestern Alberta (Waterton Lakes National Park, UASM), southern British Columbia (Jarrett and Scudder 2001: 379), Washington (Minsk and Hatch 1939: 215; Pierce County, UASM), eastern Oregon (Baker County, CNC), and northwestern Montana (Flathead County, UASM). There is one specimen, identified by Hieke, from Mayo in central Yukon Territory (CNC).

#### Records.

**CAN**: AB, BC **USA**: MT, OR, WA [YT]

#### Note.

This form was considered a synonym of *Amara idahoana* Casey by Lindroth (1968: 703) but regarded as a valid species by Hieke (1993: 123).

### Amara harpalina

LeConte, 1855

Amara harpalina LeConte, 1855: 355. Type locality: «Santa Fe [Santa Fe County], New Mexico» (original citation). Two syntypes [2 ♀ originally cited] in MCZ [# 5690].
Celia acutangula Putzeys, 1866b: 194. Type locality: «États-Unis» (original citation). Holotype [by monotypy] (♂) in MHNP (collection Chaudoir). Synonymy established by Horn (1892b: 40).
Celia shantzi Casey, 1918: 285. Type locality: «Akron [Washington County], Colorado» (original citation). Lectotype [as typus] (♀), designated by Hieke (1993: 140), in USNM [# 47225]. Synonymy established by Hieke (1993: 139). Etymology. The specific name was proposed for Homer Leroy Shantz [1876-1958], American botanist, professor and president of the University of Arizona.

#### Distribution.

This species is known from eastern Oregon (Baker County, USNM) to western South Dakota (Lawrence County, USNM), north to southeastern Montana (Powder River County, USNM), south to western Texas (Culberson County, CMNH) and central Arizona (Gila County, MCZ).

#### Records.

**USA**: AZ, CO, ID, MT, NM, OR, TX, UT, WY

### Amara idahoana

(Casey, 1924)

Celia idahoana Casey, 1924: 56. Type locality: «Moscow [Latah County], Idaho» (original citation). Holotype [by monotypy] (♂) in USNM [# 47207].

#### Distribution.

This species ranges from southern Alaska (Anchorage area, UASM) to southern Northwest Territories (CNC), south to northern Oregon (Hood River County, MCZ) and southern Colorado (Elias 1987: 633).

#### Records.

**CAN**: AB, BC (VCI), NT **USA**: AK, CO, ID, MT, OR, WA, WY

### Amara musculis

(Say, 1823)

Feronia musculis Say, 1823a: 35. Type locality: «eastern shore of Virginia; Florida» (original citation), restricted to «coast of Virginia» by Lindroth (1968: 707). Lectotype (♂), designated by Lindroth (1968: 707), in MHNP (collection Dejean). Note. Say (1823a: 35) probably made an error by spelling the specific name *musculis*. He did not give the etymology of the name but the word likely derives from the Latin noun *musculus*, -*i* (little mouse) and should have been spelled in the nominative singular (e.g., *musculus*), in the genitive singular (e.g., *musculi*) or in the genitive plural (e.g., *musculorum*) (see ICZN 1999: Articles 11.9.1.2 and 11.9.1.3). All authors seen, starting with LeConte (1855: 355), have used the spelling *musculus* until Lindroth (1968: 707) returned to the original spelling *musculis*. I consider that Say used an inappropriate connecting vowel and the name is not to be corrected (ICZN 1999: Article 32.5.1).
Acrodon contempta LeConte, 1847: 367. Type locality: «NovEboraci [= New York], et ad Rocky Mountains» (original citation). Syntype(s) presumably lost (not located in MCZ). Synonymy established by LeConte (1855: 355).
Celia lyncea Casey, 1918: 280. Type locality: «Sheepshead Bay [Kings County], Long Island, New York» (original citation). Lectotype (♀), designated by Lindroth (1975: 133), in USNM [# 47214]. Synonymy established by Lindroth (1968: 707).
Celia nugator Casey, 1918: 281. Type locality: «New Jersey seashore» (original citation for the lectotype). Lectotype (♂), designated by Lindroth (1975: 133), in USNM [# 47215]. Synonymy established by Lindroth (1968: 707).
Celia curticeps Casey, 1918: 281. Type locality: «Fortress Monroe [= Hampton], Virginia» (original citation). Lectotype (♂), designated by Lindroth (1975: 133), in USNM [# 47218]. Synonymy established by Lindroth (1968: 707).
Celia pimalis Casey, 1918: 283. Type locality: «Arizona» (original citation). Lectotype (♂), designated by Hieke (1993: 137), in USNM [# 47223]. Synonymy established by Hieke (1993: 137).
Celia shoemakeri Casey, 1918: 283. Type locality: «Long Island, New York» (original citation). Lectotype (♀), designated by Lindroth (1975: 133), in USNM [# 47222]. Synonymy established by Lindroth (1968: 707). Etymology. The specific name was proposed for Ernest Shoemaker, an amateur entomologist and painter of insects from Brooklyn, New York. His collection of more than 60,000 specimens, chiefly beetles, was presented to the USNM in 1959.
Celia vegrandis Casey, 1918: 287. Type locality: «Wisconsin» (original citation). Lectotype (♀), designated by Lindroth (1975: 133), in USNM [# 47228]. Synonymy established by Lindroth (1968: 708).
Celia fluminea Casey, 1918: 287. Type locality: «Vicksburg [Warren County], Mississippi» (original citation). Lectotype (♂), designated by Lindroth (1975: 133), in USNM [# 47229]. Synonymy established by Lindroth (1968: 708).
Celia crenulata Casey, 1918: 288. Type locality: «Duluth [Saint Louis County], Minnesota» (original citation). Holotype [by monotypy] (♂) in USNM [# 47237]. Synonymy established by Lindroth (1968: 708).
Celia limbalis Casey, 1918: 289. Type locality: «Southern Pines [Moore County], North Carolina» (original citation). Lectotype (♀), designated by Lindroth (1975: 133), in USNM [# 47232]. Synonymy established by Lindroth (1968: 708).
Celia paulula Casey, 1918: 289. Type locality: «Wading River [Suffolk County], Long Island, New York» (original citation). Lectotype (♂), designated by Lindroth (1975: 133), in USNM [# 47235]. Synonymy established by Lindroth (1968: 708).
Celia scintilla Casey, 1918: 290. Type locality: «Fortress Monroe [= Hampton], Virginia» (original citation). Lectotype (♂), designated by Lindroth (1975: 133), in USNM [# 47236]. Synonymy established by Lindroth (1968: 708).
Celia brevitarsis Casey, 1918: 290. Type locality: «Southern Pines [Moore County], North Carolina» (original citation). Lectotype (♂), designated by Lindroth (1975: 133), in USNM [# 47238]. Synonymy established by Lindroth (1968: 708).
Celia minnesotana Casey, 1924: 57. Type locality: «Duluth [Saint Louis County], Minnesota» (original citation). Holotype [by monotypy] (♀) in USNM [# 47230]. Synonymy established by Lindroth (1975: 133).

#### Distribution.

This widely distributed species ranges from Nova Scotia (Sable Island, CNC) to south-central British Columbia (Lindroth 1968: 708), south to southern California (San Diego County, CNC), southern New Mexico (Grant County, USNM; Wickham 1896c: 133; Fall and Cockerell 1907: 158), northern Oklahoma (French et al. 2001: 228; Alfalfa County, CMNH), central Mississippi (Casey 1918: 287, as *Celia fluminea*), and the Florida Panhandle (Okaloosa County, CNC). One old specimen labeled from “Texas” (USNM) is known.

#### Records.

**CAN**: AB, BC, MB, NB, NS, ON, QC, SK **USA**: AL, AR, AZ, CA, CO, CT, DC, DE, FL, GA, IA, ID, IL, IN, KS, KY, LA, MA, MD, ME, MI, MN, MO, MS, MT, NC, ND, NE, NH, NJ, NM, NY, OH, OK, OR, PA, RI, SC, SD, TN, UT, VA, VT, WA, WI, WV, WY [TX]

### Amara pseudobrunnea

Lindroth, 1968

Amara pseudobrunnea Lindroth, 1968: 706. Type locality: «Swift R[iver], Yukon [Territory]» (original citation). Holotype (♂) in CNC [# 10512].

#### Distribution.

This northern species ranges from the coast of Labrador to central Alaska (Lindroth 1968: 707), south in the west to northern Idaho (Bonner County, CNC) and northwestern Montana (Edwards 1975: 56).

#### Records.

**CAN**: AB, BC, LB, MB, NU, QC, SK, YT **USA**: AK, ID, MT

### Amara rubrica

Haldeman, 1843

Amara rubrica Haldeman, 1843b: 301. Type locality: southeastern Pennsylvania (Haldeman 1843a: 297). One probable syntype, a ♀ labeled “[pink disc] / A. rubrica Hald. [handwritten],” in MCZ (collection LeConte).
Amara pallida Casey, 1884b: 5. Type locality: «Willets Point [Queens County], New York Harbor» (original citation). Holotype [by monotypy] (♂) in MCZ [# 34446]. Synonymy established by Horn (1885b: 108), confirmed by Lindroth (1968: 708).
Amara ferruginea Casey, 1884b: 5. Type locality: «Atlantic City [Atlantic County], New Jersey» (original citation). Holotype [by monotypy] (♂) in MCZ [# 34447]. Synonymy established by Horn (1885b: 108), confirmed by Lindroth (1968: 708).
Celia lucina Casey, 1918: 279. Type locality: «Delaware» (original citation). Lectotype (♀), designated by Lindroth (1975: 133), in USNM [# 47224]. Synonymy established by Lindroth (1968: 708).
Celia proditor Casey, 1918: 280. Type locality: «Cape May [Cape May County], New Jersey» (original citation). Lectotype (♀), designated by Lindroth (1975: 134), in USNM [# 47213]. Synonymy established by Lindroth (1968: 708).
Celia haldemani Casey, 1918: 281. Type locality: «Douglas Co[unty], Kan[sas]» (lectotype label). Lectotype [as holotypus] (♀), designated by Hieke (1993: 127), in USNM [# 47219]. Synonymy established by Hieke (1993: 126).
Celia liquida Casey, 1918: 282. Type locality: «Long Island, New York» (original citation). Lectotype (♀), designated by Lindroth (1975: 134), in USNM [# 47220]. Synonymy established by Lindroth (1968: 708).
Celia politissima Casey, 1918: 284. Type locality: «Long Island, New York» (original citation). Lectotype (♀), designated by Lindroth (1975: 134), in USNM [# 47217]. Synonymy established by Lindroth (1968: 709).
Celia lubrica Casey, 1918: 286. Type locality: «New Jersey» (original citation). Lectotype (♀), designated by Lindroth (1975: 134), in USNM [# 47226]. Synonymy established by Lindroth (1968: 709).
Celia nigripennis Casey, 1918: 286. Type locality: «Willets Point [Queens County], Long Island, New York» (original citation). Lectotype (♀), designated by Lindroth (1975: 134), in USNM [# 47227]. Synonymy established by Lindroth (1968: 709).
Celia piceonitens Casey, 1924: 58. Type locality: «Southern Pines [Moore County], North Carolina» (original citation). Lectotype (♀), designated by Lindroth (1975: 134), in USNM [# 47234]. Synonymy established by Lindroth (1968: 709).

#### Distribution.

This species ranges from Nova Scotia (Majka et al. 2007: 9) to southwestern North Dakota (Tinerella 2003: 636), south to central Colorado (Summit County, UASM; Horn 1892b: 37; Wickham 1902: 238), northeastern Texas (Lamar County, Brian Raber pers. comm. 2010), northern Alabama (Madison County, CMNH, USNM), and northwestern South Carolina (Ciegler 2000: 75).

#### Records.

**CAN**: NB, NS, ON, QC **USA**: AL, CO, CT, DC, DE, GA, IA, IN, KS, MA, MD, ME, MI, MN, MS, NC, NE, NH, NJ, NY, OH, OK, PA, RI, SC, SD, TN, TX, VA, VT, WI, WV

### Amara sinuosa

(Casey, 1918)

Isopleurus nitidus Kirby, 1837: 50 [secondary homonym of *Amara nitida* Sturm, 1825]. Type locality: «Rocky Mountains» (original citation). Holotype [by monotypy] (♂) in BMNH (Lindroth 1953b: 175).
Acrodon subaenea LeConte, 1850: 208 [secondary homonym of *Amara subaenea* Sturm, 1825]. Type locality: Lake Superior (inferred from title of the paper). Four syntypes in MCZ [# 5689], one in MHNP (collection Chaudoir) (Putzeys 1866b: 194). Synonymy established by LeConte (1873b: 324), confirmed by Lindroth (1968: 701).
Celia sinuosa Casey, 1918: 277. Type locality: «Aldermere, British Columbia» (original citation). Lectotype (♀), designated by Lindroth (1975: 133), in USNM [# 47211]. Synonymy established by Lindroth (1968: 701).
Celia elusa Casey, 1918: 277. Type locality: «probably Colorado» (original citation). Holotype [by monotypy] (♀) in USNM [# 47212]. Synonymy established by Lindroth (1968: 701).
Celia nupta Casey, 1918: 278. Type locality: «Lake Champlain, New York» (original citation). Lectotype (♂), designated by Lindroth (1975: 133), in USNM [# 47208]. Synonymy established by Lindroth (1968: 701).
Celia thoracica Casey, 1918: 278. Type locality: «Boulder Co[unty], Colorado» (original citation). Lectotype (♂), designated by Lindroth (1975: 133), in USNM [# 47209]. Synonymy established by Lindroth (1968: 701).
Celia hospes Casey, 1918: 279. Type locality: «Boulder Co[unty], Colorado» (original citation). Lectotype (♀), designated by Lindroth (1975: 133), in USNM [# 47210]. Synonymy established by Lindroth (1968: 701).
Amara subaenescens Csiki, 1929: 445. Replacement name for *Amara subaenea* (LeConte, 1850).

#### Distribution.

This species is found from Newfoundland (Lindroth 1955a: 104-105, as *Amara subaenescens*) to the Queen Charlotte Islands (Kavanaugh 1992: 75) and Vancouver Island, north to the Alaskan coast (Lindroth 1968: 703), south at least to east-central Utah (Grand County, CMNH, CNC), southern Colorado (Costilla and Mineral Counties, UASM), the Black Hills in western South Dakota (USNM), northwestern Indiana (Blatchley 1910: 109, as *Amara subaenea*), and northern New York (Casey 1918: 278, as *Celia nupta*). The records from southeastern New Jersey (Smith 1910: 206, as *Amara subaenea*), “Nebraska” (Bousquet and Larochelle 1993: 199), and Kansas (Knaus 1898: 19 and Snow 1903: 193, as *Amara subaenea*) need confirmation.

#### Records.

**CAN**: AB, BC (QCI, VCI), LB, MB, NB, NF, NT, ON, PE, QC, SK, YT **USA**: AK, CO, IA, ID, IL, IN, ME, MI, MN, MT, NH, NY, SD, UT, WI, WY [KS, NE, NJ]

### Amara texana

(Putzeys, 1866)

Celia texana Putzeys, 1866b: 196. Type locality: «Texas» (original citation). Syntype(s) [2 originally cited] in MHNP (collection Chaudoir).

#### Distribution.

This species is known from central and southern Texas (Horn 1892b: 38; Riley 2011; Bexar, Harris, and Lee Counties, MCZ) and central and southwestern New Mexico (Bernalillo and Luna Counties, CMNH). The record from southeastern Kansas (Knaus 1885: 58) needs confirmation.

#### Records.

**USA**: NM, TX [KS]

### Amara volatilis

(Casey, 1918)

Celia volatilis Casey, 1918: 287. Type locality: «Fortress Monroe [= Hampton], Virginia» (original citation). Lectotype [as typus] (♀), designated by Hieke (1993: 142), in USNM [# 47233].
Celia virginica Casey, 1918: 289. Type locality: «Fortress Monroe [= Hampton], Virginia» (original citation). Lectotype (♂), designated by Hieke (1993: 142), in USNM [# 47231]. Synonymy established by Hieke (1993: 141).

#### Distribution.

This species is found along the Atlantic Coast from “New York” to northern Florida (Hieke 1993: 143).

#### Records.

**USA**: FL, NC, NJ, NY, SC, VA

### Subgenus Amarocelia

Motschulsky, 1862

Amarocelia Motschulsky, 1862a: 4. Type species: *Amara punctatostriata* Motschulsky, 1860 (= *Amara interstitialis* Dejean, 1828) designated by Hieke (1995a: 17). Etymology. From the generic names *Amara* [*q.v*.] and *Celia* [*q.v*.] [feminine]. Note. Hieke (1995a: 17) listed *Amara interstitialis* Dejean, 1828 as type species of *Amarocelia* Motschulsky, a species not originally included. However, since he placed *Amara interstitialis* Dejean in synonymy with *Amara punctatostriata* Motschulsky, 1860, a species originally included, he is deemed to have designated the latter species as type species (ICZN 1999: Article 69.2.2).

#### Diversity.

Thirteen species in North America, of which two are also found in Mexico and three are Holarctic.

#### Identification.

Lindroth (1968) covered all but four (*Amara nexa*, *Amara sodalicia*, *Amara lugens*, and *Amara tenebrionella*) species known at the time. Two species were described subsequently by Hieke (2002). A revision of the subgenus is needed.

### Amara ellipsis

(Casey, 1918)

Celia ellipsis Casey, 1918: 252. Type locality: «Kansas» (original citation). Lectotype (♂), designated by Lindroth (1975: 134), in USNM [# 47259].
Celia winnipegensis Casey, 1924: 53. Type locality: «Winnipeg, Manitoba» (original citation). Holotype [by monotypy] (♂) in USNM [# 47255]. Synonymy established by Lindroth (1968: 718).

#### Distribution.

This species ranges from the Magdalen Islands in the Gulf of Saint Lawrence (CNC) to the Queen Charlotte Islands (Kavanaugh 1992: 75), south to southern California (San Bernardino County, CAS, MCZ), southern Arizona (Greenlee County, UASM), southern New Mexico (Otero County, MCZ), “Kansas” (Casey 1918: 252), central Michigan (Dunn 1982b: 37), and eastern New Brunswick (Webster and Bousquet 2008: 18).

#### Records.

**CAN**: AB, BC (QCI), MB, NB, ON, QC, SK **USA**: AZ, CA, CO, IA, ID, KS, MI, MN, MT, ND, NE, NM, NV, OR, SD, UT, WA, WI, WY

### Amara erratica

(Duftschmid, 1812)

Carabus erraticus Duftschmid, 1812: 120. Type locality: Austria (inferred from title of the book). Holotype [by monotypy] probably lost (Lindroth 1968: 716).
Amara punctulata Dejean, 1828: 472. Type locality: «Kamtschatka [Russia]; détroit de Norfolk sur la côte nord-ouest de l’Amérique du Nord [Alaska]» (original citation). One syntype in MHNP (Lindroth 1955b: 17). Synonymy established by Zimmermann (1832: 24), confirmed by Lindroth (1955b: 17).
Amara septentrionalis Schiødte, 1837: 170. Type locality: «Lapland [probably Swedish Lappland]» (original citation). Lectotype, designated by Hansen and Martin (2000: 214), in ZMUC. Synonymy established by Schiødte (1841: 187).
Amara affinis Motschulsky, 1850a: 60 [primary homonym of *Amara affinis* Dejean, 1828 and *Amara affinis* Motschulsky, 1845]. Type locality: «Kamtschatka [Russia]» (original citation). Lectotype (♀), designated by Hieke (1975: 328), in ZMMU. Synonymy established by Hieke (1975: 328).
Amara obscuricornis Motschulsky, 1859b: 540. Type locality: region of Yakutsk, east-central Siberia, Russia (inferred from title of the paper); «Kamtschatka» listed by Motschulsky (1850a: 60). Lectotype (♂), designated by Hieke (1975: 323), in ZMMU. Synonymy established by Hieke (1975: 322). Note. Motschulsky described this taxon in a paper about beetles collected by Pavlofski in the “gouvernement de Iakoutsk.” In the same publication, Motschulsky refers to his book *Die Kaefer Russlands* (1850) where the species is listed, but not described, and the provenance given is “Kamtschatka.” The lectotype, designated by Hieke (1975: 322), bears two labels: “Jakutsk” and “Amara obscuricornis Motsch. Sib. or. Kamtsch.”
Amara sibirica Csiki, 1929: 427. Replacement name for *Amara affinis* Motschulsky, 1850.

#### Distribution.

This circumpolar species is found over most of the boreal-alpine region in the Palaearctic Region and in North America from Alaska (Lindroth 1968: 717) to Newfoundland (Lindroth 1955a: 107), south to New England (Lindroth 1968: 716), northern Wisconsin (Messer 2010: 38), southern Colorado (Mineral County, UASM; Lindroth 1968: 716), northern Utah (Salt Lake County, CMNH), and Mount Rainier in western Washington (Lindroth 1968: 717). Several state records (e.g., CA, IA, MI, MN, ND, NM, OR) listed in Bousquet and Larochelle (1993: 196) and taken from earlier authors probably refer to other species or need confirmation.

#### Records.

**FRA**: PM **CAN**: AB, BC, LB, MB, NB, NF, NT, NU, ON, QC, SK, YT **USA**: AK, CO, ID, ME, MT, NH, UT, VT, WA, WI, WY – **Holarctic**

### Amara farcta

LeConte, 1855

Amara farcta LeConte, 1855: 353. Type locality: «New Mexico» (original citation). Two syntypes in MCZ [# 5684].
Celia lauta Casey, 1918: 248. Type locality: «southern Arizona» (original citation). Lectotype (♂), designated by Hieke (1993: 128), in USNM [# 47248]. Synonymy established by Hieke (1993: 127).
Celia formalis Casey, 1918: 248. Type locality: «Provo [Utah County], Utah» (original citation). Lectotype (♀), designated by Lindroth (1975: 134), in USNM [# 47241]. Synonymy established by Lindroth (1968: 712).
Celia finitima Casey, 1918: 249. Type locality: «Truckee [Nevada County], California» (original citation). Lectotype [as typus] (♂), designated by Hieke (1993: 124), in USNM [# 47256]. Synonymy established by Hieke (1993: 124).
Celia subdepressa Casey, 1918: 249. Type locality: «Reno [Washoe County], Nevada» (original citation). Lectotype (♀), designated by Lindroth (1975: 134), in USNM [# 47242]. Synonymy established by Lindroth (1968: 712).
Celia shastanica Casey, 1918: 251. Type locality: «Siskiyou Co[unty], California» (original citation). Lectotype (♂), designated by Lindroth (1975: 134), in USNM [# 47252]. Synonymy established by Lindroth (1968: 712).
Celia solita Casey, 1918: 252. Type locality: «Nebraska» (original citation). Lectotype (♂), designated by Lindroth (1975: 134), in USNM [# 47240]. Synonymy established by Lindroth (1968: 712).
Celia vancouveri Casey, 1924: 50. Type locality: «Peachland, B[ritish] C[olumbia]» (lectotype label). Lectotype (♂), designated by Lindroth (1975: 134), in USNM [# 47243]. Synonymy established by Hatch (1953: 125), confirmed by Lindroth (1968: 712).
Celia olympia Casey, 1924: 50. Type locality: «Wilbur [Lincoln County], Washington» (original citation). Holotype [by monotypy] (♂) in USNM [# 47245]. Synonymy established by Hatch (1953: 125), confirmed by Lindroth (1968: 712).
Celia subsimilis Casey, 1924: 50. Type locality: «Govan [Lincoln County], Washington» (original citation). Lectotype (♂), designated by Lindroth (1975: 134), in USNM [# 47244]. Synonymy established by Hatch (1953: 125), confirmed by Lindroth (1968: 713).
Celia marginatella Casey, 1924: 51. Type locality: «Monarch [Manitoba]» (holotype label). Holotype [by monotypy] (♀) in USNM [# 47246]. Synonymy established by Lindroth (1968: 713).
Celia parallela Casey, 1924: 51. Type locality: «L[eth]bridge, Al[ber]ta» (lectotype label). Lectotype (♂), designated by Lindroth (1975: 134), in USNM [# 47249]. Synonymy established by Lindroth (1968: 713).
Celia albertae Casey, 1924: 51. Type locality: «L[eth]bridge, Al[ber]ta» (holotype label). Holotype [by monotypy] (♀) in USNM [# 47250]. Synonymy established by Lindroth (1968: 713).
Celia funebris Casey, 1924: 52. Type locality: «Winnipeg, Man[itoba]» (holotype label). Holotype [by monotypy] (♂) in USNM [# 47254]. Synonymy established by Lindroth (1968: 713).

#### Distribution.

This species ranges from eastern Manitoba to southwestern British Columbia, north at least to the Magunday River in southern Yukon Territory (Lindroth 1968: 714), south to southeastern California (San Bernardino County, CAS), southern Arizona (Maricopa County, UASM), southern New Mexico (Grant County, USNM; LeConte 1855: 353), and Durango in Mexico (UASM). The record from Wisconsin (Rauterberg 1885: 17) is probably in error; that from “Ontario” (Bousquet and Larochelle 1993: 196), based on a specimen from Nipigon Bay (CMNH) determined by Hieke, needs confirmation as the specimen could be mislabeled or could be a stray.

#### Records.

**CAN**: AB, BC, MB, SK, YT **USA**: AZ, CA, CO, ID, MT, ND, NE, NM, NV, OR, SD, UT, WA, WY [ON] – Mexico

### Amara interstitialis

Dejean, 1828

Amara interstitialis Dejean, 1828: 472. Type locality: «Kamtschatka [Russia]» (original citation). Two syntypes [2 originally cited] in MHNP (Lindroth 1955b: 16).
Amara borealis Motschulsky, 1844: 184. Type locality: «Petrop[avlosk], Kamtsch[atka Peninsula], Sib[eria] or[iental] [Russia]» (lectotype label). Lectotype (♂), designated by Hieke (1993: 59), in ZMMU. Synonymy established by Morawitz (1862: 235), confirmed by Hieke (1993: 59). Note. Motschulsky (1844: 184) gave the provenance of the syntypes as “Tourkinsk au-delà du Baïcal ... [et] environs de St.-Pétersbourg” suggesting that the lectotype designated by Hieke (1993: 59) may not be a syntype.
Amara punctato-striata Motschulsky, 1860: 96. Type locality: «Daourie» (original citation). Two syntypes in ZMMU (Keleinikova 1976: 212). Synonymy established by Hieke (1995a: 112).
Amara interstitialis var. *puncticollis* J.R. Sahlberg, 1875: 109 [primary homonym of *Amara puncticollis* Dejean, 1828]. Type locality: «Soroka [= Belomorsk], by vid Hvita hafvet i ryska Karelen [= Republic of Karelia, Russia]» (original citation). Three syntypes in ZMH (Silfverberg 1987: 23). Synonymy established by Hieke (1995a: 113).
Amara interstitialis var. *fennica* Csiki, 1929: 439. Replacement name for *Amara interstitialis* var. *puncticollis* Sahlberg, 1875.
Amara tschitaensis Jedlička, 1957b: 101. Type locality: «Tschita, Transbaikal [Russia]» (original citation). Holotype (♂) in NMP. Synonymy established by Hieke (1973: 107).

#### Distribution.

This Holarctic species ranges from Finland to the Kamtschatka Peninsula (Hieke 2003a: 556) and from Alaska to northwestern Northwest Territories (Lindroth 1968: 716). The records from British Columbia (Jarrett and Scudder 2001: 380) and west-central Montana (Hansen et al. 2009: 353) need confirmation.

#### Records.

**CAN**: NT, YT **USA**: AK [BC, MT] – **Holarctic**

### Amara laevipennis

Kirby, 1837

Amara laevipennis Kirby, 1837: 40. Type locality: «Lat. 54° [= along North Saskatchewan River]» (original citation), restricted to «Jasper, Al[ber]ta» by Lindroth (1968: 717). Two syntypes [“three or four” originally cited] in BMNH (Lindroth 1953b: 173).
Amara aeneolucens Casey, 1918: 312. Type locality: «Duluth [Saint Louis County, Minnesota], Lake Superior» (original citation for the lectotype). Lectotype (♀), designated by Lindroth (1975: 134), in USNM [# 47324]. Synonymy established by Lindroth (1968: 717).

#### Distribution.

This boreal species is found from Newfoundland to southwestern British Columbia, north to east-central Alaska (Lindroth 1968: 717), south to southwestern California (Ventura County, MCZ), northeastern New Mexico (Snow 1885: 67; Las Vegas, CMNH) along the Rocky Mountains, the Black Hills in western South Dakota (Lawrence County, USNM), northeastern Minnesota along Lake Superior (Casey 1918: 312, as *Amara aeneolucens*), northwestern Pennsylvania (Warren County, CMNH), and “Massachusetts” (LeConte 1855: 353; MCZ).

#### Records.

**CAN**: AB, BC, MB, NB, NF, NS (CBI), NT, ON, PE, QC, SK **USA**: AK, CA, CO, ID, MA, ME, MI, MN, MT, NH, NM, NY, OR, PA, SD, UT, VT, WA, WI, WY

### Amara lugens

Zimmermann, 1832

Amara lugens Zimmermann, 1832: 25. Type locality: «Orizaba [Veracruz, Mexico]» (lectotype label). Lectotype (♀), designated by Hieke (1990: 213), in ZMHB.
Celia högei Bates, 1882a: 77. Type locality: «Las Vigas [Veracruz], Mexico» (original citation for the lectotype). Lectotype (♂), designated by Hieke (1990: 218), in BMNH. Synonymy established by Hieke (1990: 218).
Celia mora Casey, 1918: 253. Type locality: «Colonia Garcia, Sierra Madre M[oun]t[ain]s, Chihuahua, Mexico» (original citation). Lectotype (♂), designated by Hieke (1993: 132), in USNM [# 47257]. Synonymy established by Hieke (1993: 132).

#### Distribution.

This species ranges from northern Arizona (Coconino County, USNM) and New Mexico (San Miguel County, USNM) south at least to the Isthmus of Tehuantepec (Bates 1882a: 77).

#### Records.

**USA**: AZ, NM – Mexico

### Amara nexa

(Casey, 1918)

Celia nexa Casey, 1918: 253. Type locality: «Truckee [Nevada County], California» (original citation). Three syntypes [3 originally cited] in USNM [# 47260].

#### Distribution.

This species is known only from the Sierra Nevada in California (Casey 1918: 253; Alpine, Amador, Eldorado, Placer, Plumas, and Shasta Counties, CAS, MCZ, USNM).

#### Records.

**USA**: CA

### Amara patruelis

Dejean, 1831

Amara patruelis Dejean, 1831: 793. Type locality: «Amérique septentrionale» (original citation), restricted to «W[est] Roxbury [Suffolk County], Mass[achusetts]» by Lindroth (1968: 714). Holotype [by monotypy] (♂) in MHNP (Lindroth 1955b: 16).
Amara inaequalis Kirby, 1837: 39. Type locality: «Lat. 54° [= along North Saskatchewan River]» (original citation). One syntype in BMNH (Lindroth 1953b: 173). Synonymy established by Casey (1918: 248), confirmed by Lindroth (1953b: 173).
Amara splendida Haldeman, 1843b: 300. Type locality: southeastern Pennsylvania (Haldeman 1843a: 297). Syntype(s) presumably lost. Synonymy established by Casey (1918: 248).
Celia reducta Casey, 1918: 252. Type locality: «Colorado» (original citation). Lectotype (♂), designated by Lindroth (1975: 134), in USNM [# 47258]. Synonymy established by Lindroth (1968: 714).
Celia columbiana Casey, 1924: 52. Type locality: «British Columbia» (original citation). Holotype [by monotypy] (♀) in USNM [# 47247]. Synonymy established by Hatch (1953: 126), confirmed by Lindroth (1954b: 136).

#### Distribution.

This species ranges from Newfoundland (Lindroth 1955a: 107) to the Seward Peninsula in western Alaska (Lindroth 1968: 715), south to “California” (Lindroth 1968: 715), central Colorado (Elias 1987: 633; Casey 1918: 252, as *Celia reducta*), eastern South Dakota (Brookings County, USNM), and southwestern North Carolina (Jackson County, USNM) along the Appalachian Mountains. The record from Siberia (Lindroth 1968: 715) refers to *Amara transberingiensis* Hieke (Hieke 2002: 671).

#### Records.

**CAN**: AB, BC, MB, NB, NF, NS, NT, ON, PE, QC, SK, YT **USA**: AK, CA, CO, CT, DC, IA, ID, IL, IN, MA, ME, MI, MN, MT, NC, ND, NH, NJ, NV, NY, OR, PA, RI, SD, UT, VA, VT, WA, WI, WY

### Amara rugulifera

Hieke, 2002

Amara rugulifera Hieke, 2002: 660. Type locality: «S[ain]t Elmo [San Bernardino County], California» (original citation). Holotype (♂) in USNM.

#### Distribution.

This species is known from Andrew Lake in northeastern Alberta to south-central British Columbia, south to southeastern California, southeastern Arizona, and northeastern New Mexico (Hieke 2002: 660).

#### Records.

**CAN**: AB, BC **USA**: AZ, CA, CO, MT, NM, UT, WY

### Amara sodalicia

Casey, 1924

Amara sodalicia Casey, 1924: 62. Type locality: «Maxwell [Colfax County], New Mexico» (original citation). Holotype [by monotypy] (♀) in USNM [# 47311].

#### Distribution.

This species is known only from a few specimens collected in Colfax and Catron Counties, New Mexico (Hieke 1997: 250).

#### Records.

**USA**: NM

### Amara tenebrionella

(Bates, 1882)

Celia tenebrionella Bates, 1882a: 78. Type locality: «Las Vigas [Veracruz], Mexico» (original citation). Lectotype (♂), designated by Hieke (1990: 222), in BMNH.
Celia xanthognatha Bates, 1882a: 78. Type locality: «Las Vigas [Veracruz], Mexico» (original citation). Lectotype [as holotypus] (♂), designated by Hieke (1990: 224), in BMNH. Synonymy established by Hieke (1990: 223).
Celia tenebrionella var. *aeneicolor* Bates, 1891a: 248. Type locality: «Ciudad, in Durango» (original citation). Lectotype (♂), designated by Hieke (1990: 222), in BMNH. Synonymy established by Hieke (1990: 222).

#### Distribution.

This species is known from the Rocky Mountains in northern Arizona (Hieke 1990: 223) and from the states of Durango (Bates 1891a: 248, as *Celia tenebrionella* var. *aeneicolor*) and Veracruz (Bates 1882a: 78) in Mexico.

#### Records.

**USA**: AZ – Mexico

### Amara transberingiensis

Hieke, 2002

Amara transberingiensis Hieke, 2002: 668. Type locality: «Inuvik, North West Territor[ies]y» (original citation). Holotype (♂) in CNC [# 22916].

#### Distribution.

This species is known from the Hudson Bay region in northeastern Manitoba, from northern British Columbia (CNC), and from the Northwest Territories to the Seward Peninsula in Alaska. In the Palaearctic Region, the species ranges from eastern Siberia to the Taimyr Peninsula, south at least to the Yakutsk area (Hieke 2002: 668).

#### Records.

**CAN**: BC, MB, NT, YT **USA**: AK – **Holarctic**

### Subgenus Amara

Bonelli, 1810

Amara Bonelli, 1810: Tabula Synoptica. Type species: *Carabus vulgaris* Linnaeus *sensu* Panzer, 1797 (= *Amara lunicollis* Schiødte, 1837) designated by Westwood (1838: 4).
Linomus Fischer von Waldheim, 1829a: 16. Type species: *Carabus lucidus* Duftschmid, 1812 by monotypy. Synonymy established by Bousquet (2002c: 176).
Agronoma Gistel, 1848b: [2]. Type species: *Carabus familiaris* Duftschmid, 1812 designated by Bouchard et al. (2011: 145). Synonymy established by Bouchard et al. (2011: 145).
Pangetes Gistel, 1856: 358. Type species: *Carabus ovatus* Fabricius, 1792 designated by Bousquet (2002b: 37). Synonymy established by Bousquet (2002b: 37).

#### Diversity.

Eighty-seven species (Hieke 2007) in North America (29 species, of which six are adventive), Middle America (four species of which one, *Amara dolosa* Say, is endemic), Asia (about 55 species), Europe and northern Africa (about 25 species, most shared with western Asia), and the Afrotropical Region (nine species). Two species are Holarctic (*Amara littoralis* and *Amara lunicollis*).

#### Identification.

No taxonomic revision has been published for the species of this group and such study is much needed. Hieke (2000) revised the six species of the *impuncticollis* group found in North America. Lindroth (1968) covered all but three (*Amara haywardi*, *Amara pomona*, and *Amara sera*) of the currently valid North American species known at the time. Since his publication, four taxa listed by Lindroth as synonyms (*Amara neoscotica*, *Amara otiosa*, *Amara tenax*, and *Amara turbata*) have been revalidated (Hieke 1994, 2000, 2003b), two new species described (Hieke 2002), and three adventive species (*Amara communis*, *Amara eurynota*, and *Amara ovata*) discovered on this continent. Identifications of some species are difficult and require examination of the male genitalia for confirmation.

### [aurata group]

### Amara aurata

Dejean, 1828

Amara aurata Dejean, 1828: 475. Type locality: «Californie» (original citation), herein restricted to Monterey, Monterey County (see Casey 1918: 272, as *Celia rotundiceps*). One syntype in MHNP (Lindroth 1955b: 17).
Amara imitatrix G.H. Horn, 1892b: 34. Type locality: «California, Washington and Vancouver» (original citation), restricted to «Calif[ornia]» by Lindroth (1968: 710). Four syntypes in MCZ [# 34450]. Synonymy established by Lindroth (1968: 710).
Celia rotundiceps Casey, 1918: 272. Type locality: «Monterey [Monterey County], California» (original citation). Lectotype [as typus] (♂), designated by Hieke (1993: 120), in USNM [# 47264]. Synonymy established by Hieke (1993: 119).
Celia jacinto Casey, 1918: 272. Type locality: «San Diego [San Diego County], California» (original citation). Lectotype (♂), designated by Hieke (1993: 120), in USNM [# 47265]. Synonymy established by Hieke (1993: 119).
Celia farallonica Casey, 1918: 273. Type locality: «Farallone Islands, San Francisco Co[unty], California» (original citation). Lectotype (♂), designated by Hieke (1993: 121), in USNM [# 47269]. Synonymy established by Hieke (1993: 119).
Celia proba Casey, 1918: 274. Type locality: «San Francisco [San Francisco County], California» (original citation). Lectotype (♂), designated by Lindroth (1975: 134), in USNM [# 47271]. Synonymy established by Lindroth (1968: 710).
Celia clementina Casey, 1918: 274. Type locality: «San Clemente Island [Los Angeles County], California» (original citation). Lectotype (♂), designated by Hieke (1993: 121), in USNM [# 47270]. Synonymy established by Hieke (1993: 119).
Celia hilaris Casey, 1918: 275. Type locality: «Cal. [with a “x” over the a] [= Redwood Creek, Humboldt County, California]» (lectotype label). Lectotype (♂), designated by Hieke (1993: 121), in USNM [# 47272]. Synonymy established by Hieke (1993: 119).
Celia evanida Casey, 1918: 275. Type locality: «Columbia River Valley, Oregon» (original citation). Lectotype (♂), designated by Lindroth (1975: 134), in USNM [# 47273]. Synonymy established by Hatch (1953: 126), confirmed by Lindroth (1968: 710).
Celia angustior Casey, 1918: 275. Type locality: «Siskiyou Co[unty], California» (original citation). Lectotype (♂), designated by Hieke (1993: 121), in USNM [# 47274]. Synonymy established by Hieke (1993: 119).
Celia govanensis Casey, 1924: 53. Type locality: «Govan [Lincoln County], Washington» (original citation for the lectotype). Lectotype (♀), designated by Lindroth (1975: 134), in USNM [# 47266]. Synonymy established by Hatch (1953: 126), confirmed by Lindroth (1968: 710).
Celia fragilis Casey, 1924: 53. Type locality: «Govan [Lincoln County], Washington» (original citation for the lectotype). Lectotype (♂), designated by Lindroth (1975: 134), in USNM [# 47267]. Synonymy established by Hatch (1953: 126), confirmed by Lindroth (1968: 710).

#### Distribution.

The range of this western species extends from western Montana (Russell 1968: 61; Hansen et al. 2009: 353) to Vancouver Island, north to the Prince Rupert area in British Columbia (Lindroth 1968: 711), south to Baja California Norte (CAS). The records from northern Colorado (Armin 1963: 201) and South Dakota (Kirk and Balsbaugh 1975: 26) need confirmation.

#### Records.

**CAN**: BC (VCI) **USA**: CA (CHI), ID, MT, NV, OR, WA [CO, SD] – Mexico

### [cupreolata group]

### Amara crassispina

LeConte, 1855

Amara crassispina LeConte, 1855: 352. Type locality: «Lake Superior» (original citation). Holotype [by monotypy] (♀) in MCZ [# 5676].

#### Distribution.

This species ranges from “Maine” to “Montana” (Hieke 2003b: 207), south to southern Colorado (Elias 1987: 633), southern Texas (Bastrop and Kerr Counties, CNC), and the Florida Panhandle (Okaloosa County, CNC); also recorded from “Oregon” (Hieke 2003b: 207).

#### Records.

**USA**: AL, AR, CO, CT, DC, FL, GA, IL, IN, KS, KY, LA, MA, MD, ME, MI, MN, MO, MT, NC, ND, NE, NH, NJ, NY, OH, OK, OR, PA, RI, SC, SD, TN, TX, VA, WI, WV

### Amara cupreolata

Putzeys, 1866

Amara cupreolata Putzeys, 1866b: 180. Type locality: «Etats-Unis» (original citation), restricted to «New Jersey» by Lindroth (1968: 733). Lectotype (♂), designated by Lindroth (1968: 738), in MHNP (collection Chaudoir).
Amara enervis Casey, 1918: 306. Type locality: «Pennsylvania» (original citation). Lectotype (♂), designated by Lindroth (1975: 136), in USNM [# 47309]. Synonymy established by Lindroth (1968: 733), confirmed by Hieke (2003b: 204).

#### Distribution.

This species is found from Prince Edward Island (King County, CNC) and Nova Scotia (NSNH) to southwestern British Columbia (Creston area, UASM), south to northern Idaho (Hatten et al. 2007: 359), northern Colorado (Miller and Peairs 2008: 34; Larimer County, UASM), southern Oklahoma (Elliott et al. 2006: 126), southwestern Alabama (Mobile County, USNM), and North Carolina (Hieke 2003b: 205). Several state records (e.g., AR, DC, DE, GA, MS, NE, SC, UT) reported in Bousquet and Larochelle (1993: 195) refer to other species of the group or need confirmation.

#### Records.

**CAN**: AB, BC, MB, NB, NS, ON, PE, QC, SK **USA**: AL, CO, CT, IA, ID, IL, IN, KS, KY, MA, MD, ME, MI, MN, MO, MT, NC, ND, NH, NJ, NY, OH, OK, PA, RI, SD, TN, TX, VA, VT, WI, WV

### Amara haywardi

Csiki, 1929

Amara parviceps Hayward, 1908: 54 [secondary homonym of *Amara parviceps* (Putzeys, 1866)]. Type locality: «Lake Superior» (original citation). Holotype (♂) in MCZ [# 19605].
Amara haywardi Csiki, 1929: 418. Replacement name for *Amara parviceps* Hayward, 1908.
Amara convexissima Hieke, 2002: 634. Type locality: «S[outh] Ill[inois]» (holotype label). Holotype (♂) in USNM. Synonymy established by Hieke (2003b: 207).

#### Distribution.

This species is yet known only from two specimens, both holotypes.

#### Records.

**USA**: IL

#### Note.

This form was considered a synonym of *Amara cupreolata* Putzeys by Lindroth (1968: 733) but regarded as a valid species by Hieke (2003b: 207).

### Amara neoscotica

Casey, 1924

Amara neoscotica Casey, 1924: 59. Type locality: «Halifax, N[ova] S[cotia]» (lectotype label). Lectotype (♂), designated by Lindroth (1975: 136), in USNM [# 47278].

#### Distribution.

This species ranges from Nova Scotia (Hieke 2003b: 206) to the Rocky Mountains in Alberta (CNC), south to “Colorado,” “Illinois,” and “New Jersey” (Hieke 2003b: 206).

#### Records.

**CAN**: AB, MB, NB, NS, ON, QC, SK **USA**: CO, IL, MA, ME, MI, NJ, VT, WI

#### Note.

This form was considered a synonym of *Amara cupreolata* Putzeys by Lindroth (1954b: 136) but regarded as a valid species by Hieke (2003b: 205).

### Amara tenax

Casey, 1918

Amara tenax Casey, 1918: 302. Type locality: «Colorado» (original citation). Lectotype (♀), designated by Lindroth (1975: 136), in USNM [# 47300].
Amara inflaticollis Casey, 1924: 63. Type locality: «Aweme, Man[itoba]» (lectotype label). Lectotype (♂), designated by Lindroth (1975: 136), in USNM [# 47319]. Synonymy established by Hieke (2003b: 206).

#### Distribution.

This species is found from southern Manitoba (Casey 1924: 63, as *Amara inflaticollis*) to southeastern British Columbia (Robson, CNC), north to the Great Slave Lake area in Northwest Territories (CNC), south to “Nevada” (Hieke 2003b: 207), western Colorado (Montrose County, MCZ), “Illinois” (Hieke 2003b: 207), and Massachusetts (Middlesex County, MCZ).

#### Records.

**CAN**: AB, BC, MB, NT, SK **USA**: CO, IA, IL, KS, MA, MI, MN, ND, NE, NV, NY, SD, UT, VT, WI, WY

#### Note.

This form was considered a synonym of *Amara cupreolata* Putzeys by Lindroth (1968: 733) but regarded as a valid species by Hieke (2003b: 206).

### [impuncticollis group]

### Amara impuncticollis

(Say, 1823)

Feronia impuncticollis Say, 1823a: 36. Type locality: «N[orth] Cumberl[an]d, P[ennsylvani]a» (neotype label). Neotype (♂), designated by Lindroth and Freitag (1969: 344), in MCZ [# 33029]. Note. «Pennsylvania; on the Missouri» were the areas originally cited by Say (1823a: 36).
Amara anthracina Haldeman, 1843b: 300. Type locality: southeastern Pennsylvania (Haldeman 1843a: 297). Syntype(s) presumably lost. Synonymy established by LeConte (1855: 351).
Amara difficilis LeConte, 1847: 362. Type locality: «Territorio Missouriensi» (original citation). Syntype(s) location unknown. Synonymy established by LeConte (1855: 351).
Amara arcuata Casey, 1918: 296. Type locality: «Willets Point [Queens County], Long Island, New York» (original citation). Lectotype (♀), designated by Lindroth (1975: 135), in USNM [# 47283]. Synonymy established by Lindroth (1968: 728).
Amara edax Notman, 1920c: 186. Type locality: «Windsor, Broome Co[unty], N[ew] Y[ork]» (original citation). Holotype [by monotypy] (♂) in SIM (Hennessey 1990: 466). Synonymy established by Lindroth (1968: 728).
Amara wadei Casey, 1924: 63. Type locality: «Hagerstown [Washington County], Maryland» (original citation). Lectotype (♀), designated by Lindroth (1975: 135), in USNM [# 47296]. Synonymy established by Lindroth (1968: 728). Etymology. The specific name was proposed for Joseph Sanford Wade [1880-1961], scientific assistant at the U.S. Bureau of Entomology in Washington DC.

#### Distribution.

This species is found east of the Rocky Mountains from Maine to at least southern Wisconsin, including southern Quebec and the Ontario Peninsula, south to southern Texas (Hieke 2000: 69), southeastern Louisiana (East Baton Rouge Parish, Igor M. Sokolov pers. comm. 2009), and southern Georgia (Torres and Ruberson 2006: 31). One specimen is known also from Nuevo León in Mexico and four from Costa Rica (Hieke 2000: 69). Several old records listed by Bousquet and Larochelle (1993: 196) (e.g., CO, CT, DE, FL, MB, MN, MS, NB, NF, NM, NS, PE, RI, SD, SK, VT) refer to *Amara otiosa* Casey or need confirmation. The record from Montana listed by Hieke (2000: 69) refers to Missouri; that from British Columbia (Jarrett and Scudder 2001: 380) is likely in error.

#### Records.

**CAN**: ON, QC **USA**: AL, AR, DC, GA, IA, IL, IN, KS, KY, LA, MA, MD, ME, MI, MO, NC, NE, NH, NJ, NY, OH, OK, PA, SC, TN, TX, VA, WI, WV – Costa Rica, Mexico

### Amara littoralis

Dejean, 1828

Amara littoralis Dejean, 1828: 467. Type locality: «détroit de Norfolk [= Norfolk Sound, Baranof Island, Alaska] sur la côte nord-ouest de l’Amérique septentrionale» (original citation). Holotype [by monotypy] probably in MHNP. Note. Mannerheim (1843: 207) first described this species and the name has been attributed to him since. However Dejean (1828: 467) published the name as a junior synonym of *Amara plebeja* and the name is available from its publication as a synonym (ICZN 1999: Article 11.6.1). Consequently the lectotype designated by Lindroth (1968: 730) in ZMH is not part of the type series and loses its status (ICZN 1999: Article 72.4.3).
Amara fallax LeConte, 1847: 362. Type locality: «Lacum Superiorem» (original citation). Holotype [by monotypy] (♀) in MCZ [# 5678]. Synonymy established by Lindroth (1968: 730), confirmed by Hieke (1994: 318).
Amara acuminata Casey, 1918: 297 [secondary homonym of *Amara acuminata* (Paykull, 1798)]. Type locality: «S[an]ta Fé [Santa Fe County], New Mexico» (original citation). Lectotype [as holotypus] (♀), designated by Hieke (1993: 107), in USNM [# 47281]. Synonymy established by Hieke (1993: 107).
Amara mystica Casey, 1918: 298. Type locality: «San Francisco [San Francisco County], California» (original citation). Lectotype (♀), designated by Lindroth (1975: 135), in USNM [# 47286]. Synonymy established by Lindroth (1968: 730), confirmed by Hieke (1994: 318).
Amara hesperia Casey, 1918: 298. Type locality: «Cal[ifornia]» (lectotype label). Lectotype (♂), designated by Lindroth (1975: 135), in USNM [# 47285]. Synonymy established by Lindroth (1968: 730), confirmed by Hieke (1994: 319).
Amara keeni Casey, 1918: 299. Type locality: «Inverness [probably Inverness Passage], British Columbia» (original citation). Lectotype (♂), designated by Lindroth (1975: 135), in USNM [# 47289]. Synonymy established by Lindroth (1968: 730), confirmed by Hieke (1994: 319).
Amara lacustrina Casey, 1918: 299. Type locality: «Bayfield [Bayfield County, Wisconsin], Lake Superior» (original citation for the lectotype). Lectotype (♂), designated by Lindroth (1975: 135), in USNM [# 47287]. Synonymy established by Lindroth (1968: 730), confirmed by Hieke (1994: 319).
Amara laurana Casey, 1918: 300. Type locality: «Boulder [Boulder County], Colorado» (original citation). Lectotype (♂), designated by Lindroth (1975: 135), in USNM [# 47288]. Synonymy established by Lindroth (1968: 730), confirmed by Hieke (1994: 319).
Amara teres Notman, 1922a: 146. Type locality: «Westfield, Chautauqua Co[unty], N[ew] Y[ork]» (original citation). Holotype (♀) in SIM (Hennessey 1990: 466). Synonymy established with doubt by Lindroth (1968: 730).
Amara oodiformis Casey, 1924: 58. Type locality: «Ilo [= Craigmont, Lewis County], Idaho» (holotype label). Holotype [by monotypy] (♂) in USNM [# 47182]. Synonymy established by Lindroth (1968: 730), confirmed by Hieke (1994: 319).
Amara acuticauda Casey, 1924: 58. Replacement name for *Amara acuminata* Casey, 1918.
Amara convergens Casey, 1924: 59. Type locality: «Peachland, B[ritish] C[olumbia]» (lectotype label). Lectotype (♂), designated by Lindroth (1975: 136), in USNM [# 47284]. Synonymy established by Lindroth (1968: 730), confirmed by Hieke (1994: 319).
Amara pullmani Casey, 1924: 61. Type locality: «Pullman [Whitman County], Washington» (original citation). Lectotype (♂), designated by Lindroth (1975: 136), in USNM [# 47279]. Synonymy established by Lindroth (1968: 730), confirmed by Hieke (1994: 319).

#### Distribution.

This Holarctic species ranges from Newfoundland to the Gulf Coast of Alaska, including Kodiak Island (Lindroth 1968: 731), south to southern California (Dajoz 2007: 20; San Diego County, CNC), northern New Mexico (Casey 1918: 297, as *Amara acuminata*; Rio Arriba County, CMNH), western Texas (Dajoz 2007: 23; Brewster County, CMNH), east-central Alabama (Lee County, CNC; LeConte 1855: 352), and eastern South Carolina (Ciegler 2000: 74). In the Palaearctic Region, the species is known only from northwestern Siberia and the Kamchatka Peninsula (Hieke 2000: 67).

#### Records.

**CAN**: AB, BC (QCI), MB, NB, NF, NS, NT, ON, PE, QC, SK, YT **USA**: AK, AL, AR, AZ, CA, CO, CT, DC, DE, GA, IA, ID, IL, IN, KS, KY, LA, MA, MD, ME, MI, MN, MO, MT, NC, ND, NE, NH, NJ, NM, NV, NY, OH, OK, OR, PA, RI, SC, SD, TN, TX, UT, VA, VT, WA, WI, WV, WY – **Holarctic**

### Amara otiosa

Casey, 1918

Amara otiosa Casey, 1918: 300. Type locality: «Duluth [Saint Louis County], Minnesota» (original citation). Lectotype (♂), designated by Lindroth (1975: 135), in USNM [# 47290].

#### Distribution.

This species ranges from Newfoundland to southern Manitoba, south to northeastern Colorado, central Missouri, and North Carolina (Hieke 2000: 72-74). One specimen simply labeled from Texas is known (Hieke 2000: 69).

#### Records.

**CAN**: MB, NB, NF, NS (CBI), ON, PE, QC **USA**: CO, IL, MA, ME, MI, MN, MO, NC, NH, NJ, NY, OH, PA, RI, VT, WI, WV [TX]

#### Note.

Lindroth (1968: 728) regarded this form as a synonym of *Amara impuncticollis* Say but Hieke (2000: 71) considered it as a valid species.

### Amara ovata

(Fabricius, 1792)

Carabus ovatus Fabricius, 1792: 154 [primary homonym of *Carabus ovatus* Paykull, 1790]. Type locality: «Saxonia [Germany]» (original citation). Two syntypes in ZMUC (Zimsen 1964: 57). Note. *Carabus ovatus* Paykull, 1790 cannot be interpreted at this time. Paykull originally described it as the *Carabus vulgaris* Linnaeus var. β and later (Paykull 1798: 167) listed it as a synonym of *Carabus helopioides* Fabricius, 1792. Until the type specimen(s) can be study Paykull’s name should be considered a *nomen dubium*.

#### Distribution.

This European species is adventive in North America where it is known in the east from Nova Scotia (Majka et al. 2006: 605) to southern Wisconsin (Messer 2010: 38), south to Virginia (Hieke 2000: 82). The first inventoried specimen collected on the east coast was found in Massachusetts in 1925. The species is also adventive in the western part of North America where it is known from southeastern British Columbia and Alberta (Hieke 2000: 82). The first inventoried specimen collected on the west coast was found in southeastern British Columbia in 1936 (see Hieke 2000: 82). One specimen simply labeled from Iowa is known (Hieke 2000: 82). The record from “Cleveland, Or.” (Hieke 2000: 82) obviously refers to Ohio.

#### Records.

**CAN**: AB, BC, NB, NS, ON, PE, QC **USA**: CT, MA, NH, NY, OH, PA, RI, VA, WI [IA] – **Adventive**

### Amara sera

Say, 1830

Amara sera Say, 1830b: (7) [3]. Type locality: «Mexico» (original citation). Syntype(s) lost.
Amara azteca Bates, 1882a: 79. Type locality: «Oaxaca, Mexico» (lectotype label). Lectotype (♂), designated by Hieke (1993: 108), in BMNH. Synonymy established by Hieke (1993: 108).

#### Distribution.

The range of this species extends from southern Arizona and western Texas south to Guatemala (Hieke 1993: 110).

#### Records.

**USA**: AZ, TX – Guatemala, Mexico

### Amara turbata

Casey, 1918

Amara turbata Casey, 1918: 307. Type locality: «Akron [Washington County], Colorado» (original citation). Lectotype (♂), designated by Lindroth (1975: 135), in USNM [# 47310].
Amara microcephala Casey, 1924: 62. Type locality: «Southern Pines [Moore County], North Carolina» (original citation). Lectotype (♂), designated by Lindroth (1975: 136), in USNM [# 47293]. Synonymy established by Hieke (1994: 337).
Amara recticollis Casey, 1924: 62. Type locality: «Southern Pines [Moore County], North Carolina» (original citation). Holotype [by monotypy] (♂) in USNM [# 47291]. Synonymy established by Hieke (1994: 337).

#### Distribution.

This species ranges from southern Quebec to southeastern Alberta, south to southern Arizona, southern Texas, west-central Alabama, and North Carolina (Hieke 2000: 76-79).

#### Records.

**CAN**: AB, ON, QC, SK **USA**: AL, AR, AZ, CO, IA, ID, IL, IN, KS, KY, MA, MD, MI, MT, NC, NE, NH, NJ, NM, NV, NY, PA, RI, SD, TN, TX, UT, WI, WV, WY

#### Note.

Lindroth (1968: 730) regarded this form as a synonym of *Amara littoralis* Mannerheim but Hieke (1994: 337) considered it as a valid species.

### [incertae sedis]

### Amara aenea

(DeGeer, 1774)

Carabus aeneus DeGeer, 1774: 98. Type locality not stated; «Uppsala, Sweden» selected by Lindroth (1968: 732). Lectotype (♀), designated by Lindroth (1968: 732), in NRSS.
Amara devincta Casey, 1918: 307. Type locality: «New London [New London County], Connecticut» (original citation). Holotype [by monotypy] (♂) in USNM [# 47312]. Synonymy established by Darlington (1936d: 20), confirmed by Lindroth (1968: 733).

#### Distribution.

This European species is adventive in North America where it is known in the east from Newfoundland (Lindroth 1955a: 111) to southwestern Manitoba (Stjernberg 2011: 71), south to northeastern Oklahoma (Hieke 1990: 203), northern Louisiana (Morehouse and West Carroll Parishes, Igor M. Sokolov pers. comm. 2009), and the Florida Panhandle (Okaloosa County, CNC) and in the west from southern British Columbia (Jarrett and Scudder 2001: 378; CNC) to southeastern Alberta (CNC), south to northern Colorado, north-central Arizona, and the San Francisco Bay area in west-central California [see Hieke 1990: Fig. 23]. The first inventoried specimen collected in the eastern part of this continent was found in Brooklyn, New York, in 1904 and in the western part in San Francisco, California, in 1941 (see Hieke 1990: 202, 204). This species was recorded from “Amérique septentrionale” by Dejean (1828: 466), under the name *Amara trivialis* Gyllenhal, a synonym of *Amara aenea*, suggesting that it was already present in North America by that time. The identification of the specimen(s) was confirmed by Lindroth (1968: 732). However, considering that the next documented specimen from this continent was collected only at the beginning of the xx Century, there is doubt about the provenance of Dejean’s specimen(s).

#### Records.

**CAN**: AB, BC, MB, NB, NF, NS (CBI), ON, PE, QC **USA**: AL, AR, AZ, CA, CO, CT, DC, DE, FL, GA, IA, ID, IL, IN, KS, LA, MA, MD, ME, MI, MN, MO, MS, MT, NC, NE, NH, NJ, NV, NY, OH, OK, OR, PA, RI, SC, TN, VA, VT, WA, WI, WV, WY – **Adventive**

### Amara aeneopolita

Casey, 1918

Amara aeneopolita Casey, 1918: 304. Type locality: «Marquette [Marquette County], Michigan» (original citation). Lectotype (♂), designated by Lindroth (1975: 135), in USNM [# 47304].

#### Distribution.

This species ranges from Newfoundland (Lindroth 1955a: 108) to west-central Northwest Territories, south to northern Alberta (Lindroth 1968: 723), east-central South Dakota (Kirk and Balsbaugh 1975: 27), central Iowa (O’Rourke et al. 2008: 126; Larsen et al. 2003: 292), and northeastern New York (Essex and Hamilton Counties, USNM); apparently isolated in central Colorado (Elias 1987: 633). The record from “Yukon Territory” (Ball and Currie 1997: 453) could not be confirmed.

#### Records.

**CAN**: AB, LB, MB, NB, NF, NT, ON, QC, SK **USA**: CO, IA, ME, MI, ND, NH, NY, SD, VT [YT]

### Amara anthobia

Villa and Villa, 1833

Amara anthobia A. Villa and G.B. Villa, 1833: 33. Type locality not stated; «Italia» selected by Lindroth (1968: 731). Syntype(s) probably lost (Lindroth 1968: 731).

#### Distribution.

This European species is adventive in North America where it is known from southern British Columbia (Jarrett and Scudder 2001: 379) to southern California (Los Angeles County, CMNH). The first inventoried specimen collected in that area was found in southwestern Washington in 1929 (Hatch and Kincaid 1958: 5). The species has been recorded also from the Washington D.C. area since 1964 (Hieke 1990: 205) and has been recently collected in Westchester County in southeastern New York (Peter W. Messer pers. comm. 2011).

#### Records.

**CAN**: BC **USA**: CA, MD, NY, OR, VA, WA – **Adventive**

### Amara basillaris

(Say, 1823)

Feronia basillaris Say, 1823a: 35. Type locality: «Dover [Norfolk County], Mass[achusetts]» (neotype label). Neotype (♂), designated by Lindroth and Freitag (1969: 343), in MCZ [# 33028].
Amara lucidula Dejean, 1828: 477. Type locality: «Amérique septentrionale» (original citation). Two syntypes in MHNP (Lindroth 1955b: 17). Synonymy established by LeConte (1855: 351), confirmed by Lindroth (1955b: 17).
Amara marylandica Casey, 1884b: 4. Type locality: «banks of the Potomac River, below Washington, Maryland» (original citation). Holotype [by monotypy] (♀) in MCZ [# 31228]. Synonymy established by Horn (1885b: 108), confirmed by Lindroth (1968: 735).

#### Distribution.

This species ranges from “New Hampshire” (Hayward 1908: 54) to southeastern South Dakota (Kirk and Balsbaugh 1975: 27), south to Oklahoma (Alfalfa and Logan Counties, CMNH, USNM), northeastern Georgia (Clarke County, USNM), and central South Carolina (Ciegler 2000: 74). One old specimen labeled “Tex” (USNM) is known.

#### Records.

**USA**: CT, DC, DE, GA, IA, IL, IN, KS, KY, MA, MD, MN, NC, NE, NH, NJ, NY, OK, PA, RI, SC, SD, VA, WV [TX]

### Amara coelebs

Hayward, 1908

Amara coelebs Hayward, 1908: 58. Type locality: «Osoyoos, British Columbia» (original citation). Lectotype (♂), designated by Lindroth (1968: 723), in MCZ [# 25671].
Amara rustica Casey, 1918: 303. Type locality: «Southern Pines [Moore County], North Carolina» (original citation). Lectotype (♀), designated by Lindroth (1975: 135), in USNM [# 47302]. Synonymy established by Lindroth (1968: 723). Note. Since the type locality is in North Carolina, outside the species range, the lectotype of *Amara rustica* possibly belongs to another species or the specimen is mislabeled.
Amara nebraskana Casey, 1918: 304. Type locality: «Nebraska» (original citation). Lectotype (♂), designated by Lindroth (1975: 135), in USNM [# 47298]. Synonymy established by Lindroth (1968: 723).
Amara oblongula Casey, 1918: 305. Type locality: «Boulder Co[unty], Colorado» (original citation). Lectotype (♂), designated by Lindroth (1975: 135), in USNM [# 47299]. Synonymy established by Lindroth (1968: 723).
Amara leviceps Casey, 1924: 60. Type locality: «Govan [Lincoln County], Washington» (original citation for the lectotype). Lectotype (♂), designated by Lindroth (1975: 135), in USNM [# 47308]. Synonymy established by Hatch (1953: 125), confirmed by Lindroth (1968: 723).

#### Distribution.

This species ranges from southern Manitoba to south-central British Columbia (Lindroth 1968: 724-725), south to east-central Washington (Casey 1924: 60, as *Amara leviceps*), northwestern New Mexico (Sandoval County, UASM, USNM), Kansas (Cheyenne, Douglas and Riley Counties, CNC, UASM, USNM), and central Illinois (Macon County, USNM).

#### Records.

**CAN**: AB, BC, MB, SK **USA**: CO, IA, ID, IL, KS, MN, ND, NE, NM, SD, WA, WI, WY

### Amara communis

(Panzer, 1797)

Carabus communis Panzer, 1797: no 2. Type locality: Germany (inferred from title of the book). Syntype(s) location unknown (possibly in ZMHB).

#### Distribution.

This European species is adventive in North America where it is known from the Maritime Provinces (Bousquet 1992a: 504; Majka 2005: 534) and Connecticut (Majka 2005: 534) [see Majka 2005: Fig. 1]. The record from the Gaspé Peninsula, Quebec (Majka 2005: 534), is based on a misidentified specimen of *Amara aenea* (CNC). The first inventoried specimen collected on this continent was found in New Brunswick in 1988 (Bousquet 1992a: 504).

#### Records.

**CAN**: NB, NS, PE **USA**: CT – **Adventive**

### Amara conflata

LeConte, 1855

Amara conflata LeConte, 1855: 352. Type locality: «San Francisco [San Francisco County], California» (original citation). Syntype(s) in MCZ [# 5677].
Amara impressicollis Motschulsky, 1859a: 153. Type locality: «St. Francisco [San Francisco County, California]» (original citation). One syntype in ZMMU (Keleinikova 1976: 200). Synonymy established by LeConte (1863b: 10).
Celia semota Casey, 1918: 251. Type locality: «Clackamas Co[unty], Oregon» (original citation). Lectotype (♀), designated by Lindroth (1975: 135), in USNM [# 47253]. Synonymy established by Lindroth (1968: 726).
Amara diffidens Casey, 1918: 306. Type locality: «near San Francisco [San Francisco County], California» (original citation). Lectotype (♀), designated by Lindroth (1975: 135), in USNM [# 47305]. Synonymy established by Lindroth (1968: 726).
Amara leydeni Casey, 1918: 306. Type locality: «Coeur d’Alene [Kootenai County], Idaho» (original citation). Lectotype (♂), designated by Lindroth (1975: 135), in USNM [# 47307]. Synonymy established by Hatch (1953: 124), confirmed by Lindroth (1968: 727).

#### Distribution.

This species ranges from northwestern Montana (Russell 1968: 60) to Vancouver Island (Lindroth 1968: 728), south to southern California (San Diego County, CAS, CNC) and northern Utah (Davis, Salt Lake, and Utah Counties, CMNH).

#### Records.

**CAN**: BC (VCI) **USA**: CA (CHI), ID, MT, OR, UT, WA

### Amara confusa

LeConte, 1847

Amara confusa LeConte, 1847: 361. Type locality: «ad Rocky Mountains» (original citation), cited from «Nebraska [Territory], near the Rocky Mountains [probably present day Colorado]» by LeConte (1855: 352). Three syntypes in MCZ [# 5680].
Amara subpunctata LeConte, 1855: 352. Type locality: «at the Rocky Mountains» (original citation). Holotype [by monotypy] (♂) in MCZ [# 5679]. Synonymy established by Lindroth (1968: 725).
Amara protensa Putzeys, 1866b: 183. Type locality: «bords du fl[euve] Ruper [= Ruper River, Quebec], Baie d’Hudson» (original citation), which is likely incorrect. Lectotype (♀), designated by Lindroth (1968: 725), in MHNP. Synonymy established by Lindroth (1968: 725).
Celia modulata Casey, 1918: 250. Type locality: «Fort Wingate [McKinley County], New Mexico» (original citation). Lectotype (♀), designated by Hieke (1993: 131), in USNM [# 47239]. Synonymy established by Hieke (1993: 131).
Amara impedita Casey, 1918: 310. Type locality: «Wyoming» (original citation). Lectotype (♂), designated by Lindroth (1975: 135), in USNM [# 47320]. Synonymy established by Lindroth (1968: 726).
Amara ebenina Casey, 1918: 310. Type locality: «Boulder Co[unty], Colorado» (original citation). Lectotype (♀), designated by Lindroth (1975: 135), in USNM [# 47322]. Synonymy established by Lindroth (1968: 726).
Amara castalia Casey, 1918: 311. Type locality: «Jemez Springs [Sandoval County], New Mexico» (original citation). Lectotype [as holotypus] (♂), designated by Hieke (1993: 114), in USNM [# 47321]. Synonymy established by Hieke (1993: 114).
Amara viridula Casey, 1924: 60. Type locality: «Lethbridge, Alberta» (lectotype label). Lectotype (♂), designated by Lindroth (1975: 135), in USNM [# 47301]. Synonymy established by Lindroth (1968: 726).
Amara oblongiformis Casey, 1924: 60. Type locality: «Govan [Lincoln County], Washington» (original citation). Holotype [by monotypy] (♂) in USNM [# 47306]. Synonymy established by Lindroth (1968: 726).
Amara acomana Casey, 1924: 61. Type locality: «Maxwell [Colfax County], New Mexico» (original citation). Lectotype (♂), designated by Lindroth (1975: 135), in USNM [# 47313]. Synonymy established by Lindroth (1968: 726).
Amara subarctica Casey, 1924: 64. Type locality: «Boucher, Sask[atchewan]» (lectotype label). Lectotype (♂), designated by Lindroth (1975: 135), in USNM [# 47323]. Synonymy established by Lindroth (1968: 726).

#### Distribution.

This species is found from southern Manitoba to south-central British Columbia, north to central Alaska (Lindroth 1968: 726), south to the Sierra Nevada in east-central California (Tuolumne County, MCZ), central Arizona (Gila County, MCZ), central New Mexico (Fall and Cockerell 1907: 158), southern Kansas (Knaus 1905a: 218; Casey 1918: 311), and northern Indiana (Blatchley 1910: 107, as *Amara protensa*).

#### Records.

**CAN**: AB, BC, MB, SK, YT **USA**: AK, AZ, CA, CO, ID, IL, IN, KS, MT, ND, NE, NM, NV, OR, SD, UT, WA, WY

### Amara convexa

LeConte, 1847

Amara convexa LeConte, 1847: 363. Type locality: «Lacum Superiorem» (original citation), restricted to «Port Arthur, Ont[ario]» by Lindroth (1968: 734). Holotype [by monotypy] (♀) in MCZ [# 5682].
Amara polita LeConte, 1847: 364 [secondary homonym of *Amara polita* (Chaudoir, 1846)]. Type locality: «ad Rocky Mountains» (original citation). Four syntypes in MCZ [# 5681]. Synonymy established by Horn (1875: 127), confirmed by Lindroth (1968: 734).
Amara wingatei Casey, 1918: 308. Type locality: «Fort Wingate [McKinley County], New Mexico» (original citation). Lectotype (♂), designated by Lindroth (1975: 136), in USNM [# 47314]. Synonymy established by Lindroth (1968: 734).
Amara oviformis Casey, 1918: 308. Type locality: «Boulder Co[unty], Colorado» (original citation). Lectotype (♀), designated by Lindroth (1975: 136), in USNM [# 47315]. Synonymy established by Lindroth (1968: 734).
Amara piceola Casey, 1918: 309. Type locality: «Las Vegas [San Miguel County], New Mexico» (original citation). Holotype [by monotypy] (♂) in USNM [# 47316]. Synonymy established by Lindroth (1968: 734).
Celia frugalis Casey, 1924: 54. Type locality: «Aweme, Man[itoba]» (original citation). Lectotype (♂), designated by Lindroth (1975: 136), in USNM [# 47268]. Synonymy established by Lindroth (1968: 734).
Amara cockerelli Casey, 1924: 64 [primary homonym of *Amara cockerelli* Wickham, 1912]. Type locality: «Jimtown [= Jamestown, Boulder County], Colorado» (original citation). Lectotype (♀), designated by Lindroth (1975: 136), in USNM [# 47317]. Synonymy established by Lindroth (1968: 734).
Amara breviformis Casey, 1924: 65. Type locality: «Boulder [Boulder County], Colorado» (original citation). Holotype [by monotypy] (♂) in USNM [# 47318]. Synonymy established by Lindroth (1968: 734).
Amara cockerelliana Csiki, 1929: 411. Replacement name for *Amara cockerelli* Casey, 1924.
Amara lecontei Csiki, 1929: 419. Replacement name for *Amara polita* LeConte, 1847.

#### Distribution.

The range of this species extends from Nova Scotia (Majka et al. 2007: 9) to central British Columbia (Lindroth 1968: 735), south to southeastern Arizona (Greenlee County, MCZ), southern New Mexico (Grant County, USNM), western Texas (Jeff Davis County, Ken Karns pers. comm. 2009), and southern New Jersey (Cape May County, MCZ). The record from North Carolina (Brimley 1938: 122, as *Amara polita*) is probably in error. Three old specimens labeled from Brownsville in southeastern Texas (USNM) are known.

#### Records.

**CAN**: AB, BC, MB, NS, ON, QC, SK **USA**: AZ, CO, CT, IA, ID, IL, IN, KS, MA, ME, MI, MN, MT, ND, NE, NH, NJ, NM, NY, OH, OK, PA, SD, TX, UT, VT, WI, WY

### Amara emancipata

Lindroth, 1968

Amara emancipata Lindroth, 1968: 718. Type locality: «Anarchist M[oun]t[ain], Osoyoos, B[ritish] C[olumbia]» (original citation). Holotype (♀) in CNC [# 10513].

#### Distribution.

This species is found along and west of the Rocky Mountains from southwestern Alberta to south-central British Columbia, south to “Utah” and northern Colorado (Hieke 2002: 642).

#### Records.

**CAN**: AB, BC **USA**: CO, ID, MT, UT, WA, WY

### Amara eurynota

(Panzer, 1796)

Carabus eurynotus Panzer, 1796a: no 23 (as *eyrinotus*). Type locality: Germany (inferred from title of the book). Syntype(s) location unknown (possibly in ZMHB). Note. The spelling *eurynota* is an incorrect subsequent spelling, introduced by Latreille (1804: 366), in prevailing usage and attributed to Panzer (1796a); therefore it is deemed to be the correct original spelling (ICZN 1999: Article 33.3.1).
Carabus acuminatus Paykull, 1798: 166 [primary homonym of *Carabus acuminatus* Olivier, 1790]. Type locality: Sweden (inferred from title of the book). Syntype(s) possibly in NRSS. Synonymy established by Illiger (1801: 50).

#### Distribution.

This European species is adventive in North America where it is known only from a few localities in eastern Newfoundland (Bousquet 1987a: 128-129). The first inventoried specimen collected on this continent was found in 1971.

#### Records.

**CAN**: NF – **Adventive**

### Amara externefoveata

Hieke, 2002

Amara externefoveata Hieke, 2002: 642. Type locality: «Southern Pines [Moore County], North Carolina» (original citation). Holotype (♂) in USNM.

#### Distribution.

This species is known only from two specimens collected in Maryland [not Pennsylvania as stated by Hieke (2002: 643)] and North Carolina (Hieke 2002: 643).

#### Records.

**USA**: MD, NC

### Amara familiaris

(Duftschmid, 1812)

Carabus familiaris Duftschmid, 1812: 119. Type locality: Austria (inferred from title of the book). Syntype(s) probably lost (Lindroth 1968: 731).
Amara humilis Casey, 1918: 302. Type locality: «Boston Neck [Washington County], Rhode Island» (original citation for the lectotype). Lectotype (♂), designated by Lindroth (1975: 136), in USNM [# 47292]. Synonymy established by Darlington (1936d: 20), confirmed by Lindroth (1968: 733).

#### Distribution.

This European species is adventive in North America where it is known in the east from Newfoundland (Lindroth 1955a: 111) to southern Saskatchewan (Ronald R. Hooper pers. comm. 2007), south to central Oklahoma (Grady County, Robert L. Davidson pers. comm. 2012), northwestern Mississippi (Panola County, CMNH) and the Florida Panhandle (Okaloosa County, CNC) [see Hieke 1990: Fig. 25]. The first inventoried specimen collected on the east coast was found in Rhode Island in 1901 (Hieke 1990: 207). The species is also adventive in the western parts of North America where it ranges from southeastern Alaska to central California, east to western Montana [see Hieke 1990: Fig. 25]. The first inventoried specimen collected on the west coast was found in western Washington in 1913 (Hatch 1953: 26).

#### Records.

**FRA**: PM **CAN**: AB, BC (VCI), MB, NB, NF, NS (CBI), ON, PE, QC, SK **USA**: AK, AL, AR, CA, CT, DC, FL, IA, ID, IL, IN, KY, MA, MD, ME, MI, MO, MS, MT, NC, NH, NJ, NY, OH, OK, OR, PA, RI, SC, TN, VA, VT, WA, WI, WV – **Adventive**

### Amara lunicollis

Schiødte, 1837

Amara lunicollis Schiødte, 1837: 164. Type locality: «Ravneholm, nordlige Sjaelland [Denmark]» (original citation). Lectotype, designated by Hansen and Martin (2000: 213), in ZMUC.
Amara limbata Schiødte, 1837: 166. Type locality: «Amager [Denmark]» (original citation). Lectotype (♂), designated by Hansen and Martin (2000: 213), in ZMUC. Synonymy established by Schaum (1858: 529).
Amara poeciloides Heer, 1837: 40 [second section]. Type locality: «Camogaskerthal (6800 [feet]) [Switzerland]» (Heer 1837: 59 [first section]). Syntype(s) location unknown (possibly in ETHZ). Synonymy established by Schaum (1858: 528).
Amara assimilis Chaudoir, 1844: 446. Type locality: «Kieff [= Kiev, Ukraine]» (original citation). Syntype(s) in MHNP. Synonymy established by Chaudoir (1861c: 198).
Amara inepta LeConte, 1855: 351. Type locality: «Oregon [Territory]» (original citation). Holotype [by monotypy] (♀) in MCZ [# 5683]. Synonymy established by Hieke (1994: 309). Note. Lindroth (1968: 730) treated *Amara inepta* LeConte as a junior synonym of *Amara littoralis* Mannerheim but Hieke (1994: 309) considered it a junior synonym of *Amara lunicollis* Schiødte.
Amara marquettensis Casey, 1918: 304. Type locality: «Marquette [Marquette County], Michigan» (original citation). Lectotype (♀), designated by Lindroth (1975: 135), in USNM [# 47303]. Synonymy established by Lindroth (1954b: 136).
Amara carriana Casey, 1924: 65. Type locality: «Edmonton, Alberta» (original citation). Lectotype (♂), designated by Lindroth (1975: 135), in USNM [# 47325]. Synonymy established by Lindroth (1954b: 136).
Amara arsenjevi Lutshnik, 1935: 258. Type locality: «fl. Tumain, Prov. Ussuriensis, Sibirie or. [Russia]» (original citation). One syntype in ZILR (Hieke 1973: 9). Synonymy established by Hieke (1973: 9).
Amara zaisani Jedlička, 1964: 290. Type locality: «Zaisan, Central Aimak, Mongolei» (original citation). Holotype (♂) in TMB. Synonymy established by Hieke (1972: 417).

#### Distribution.

This circumpolar species ranges from eastern Siberia to Ireland, south to Spain, Italy, Mongolia, and northern China (Hieke 2003a: 550) and from Alaska (Lindroth 1968: 721) to Newfoundland (Lindroth 1955a: 109), south to northeastern West Virginia (Randolph County, CMNH), east-central Ohio (Usis and MacLean 1998: 67), southern New Mexico (Otero County, CNC), and southwestern Oregon (Niwa and Peck 2002: 787).

#### Records.

**CAN**: AB, BC, LB, MB, NB, NF, NS (CBI), NT, ON, PE, QC, SK, YT **USA**: AK, CO, CT, MA, ME, MI, MN, MT, NH, NM, NY, OH, OR, PA, RI, VT, WI, WV – **Holarctic**

### Amara occidentalis

Hieke, 2002

Amara occidentalis Hieke, 2002: 654. Type locality: «Clamath [= Klamath] Falls n[ea]r Algoma [Klamath County], Oregon» (original citation). Holotype (♂) in USNM.

#### Distribution.

This species is known from a few specimens collected in Baker and Klamath Counties in Oregon and Marin and El Dorado Counties in California (Hieke 2002: 654).

#### Records.

**USA**: CA, OR

### Amara pomona

Casey, 1918

Amara brunnipes Motschulsky, 1859a: 154 [primary homonym of *Amara similata brunnipes* Letzner, 1852]. Type locality: «St. Francisco [San Francisco County, California]» (original citation). Lectotype (♀), designated by Hieke (1993: 112), in ZMMU. Note. This name was listed as “*Amara brunnipennis*” by Motschulsky (1869: 26).
Amara pomona Casey, 1918: 301. Type locality: «California» (original citation). Lectotype [as typus] (♂), designated by Hieke (1993: 112), in USNM [# 47294]. Synonymy established by Hieke (1993: 111).
Amara vigilax Casey, 1918: 301. Type locality: «near San Francisco [San Francisco County], California» (original citation). Lectotype [as typus] (♀), designated by Hieke (1993: 112), in USNM [# 47295]. Synonymy established by Hieke (1993: 111).
Amara americana Csiki, 1929: 410. Replacement name for *Amara brunnipes* Motschulsky, 1859.

#### Distribution.

This species ranges from northern Washington south to the Mexican border in southern California, east to eastern Nevada (Hieke 1993: 114). One specimen simply labeled from Mexico is known (Hieke 1993: 114).

#### Records.

**USA**: CA, NV, WA – Mexico

### Amara sanjuanensis

Hatch, 1949

Amara san-juanensis Hatch, 1949a: 81. Type locality: «Brown’s Is[land], San Juan Is[lands] [San Juan County], Wash[ington]» (original citation). Holotype (♂) in USNM.

#### Distribution.

This species is, as far as known, restricted to southern British Columbia, including Vancouver Island (Lindroth 1968: 722), northern Washington, northern Idaho (Hatch 1953: 124), and northwestern Montana (Russell 1968: 60).

#### Records.

**CAN**: BC (VCI) **USA**: ID, MT, WA

### Subgenus Paracelia

Bedel, 1899

Paracelia Bedel, 1899: 169, 174. Type species: *Amara simplex* Dejean, 1828 by original designation. Etymology. From the Greek *para* (beside, near) and the generic name *Celia* [*q.v*.] [feminine].
Iranoleirides Hieke, 1978: 300. Type species: *Amara astrabadensis* Lutshnik, 1935 by original designation. Synonymy established by Hieke (2006: 296). Etymology. From the geographical name Iran and the generic name *Leirides* [masculine].

#### Diversity.

Twenty-one species (Hieke 2007) in the Palaearctic Region (including the Himalayas and northern Africa), one of them (*Amara quenseli*) extending into the Nearctic Region and one (*Amara simplex* Dejean) extending into the Afrotropical Region.

#### Identification.

Hieke (2006) revised the species of this subgenus.

### Amara quenseli quenseli

(Schönherr, 1806)

Carabus quenseli Schönherr, 1806: 201. Type locality: «Lapponia» (original citation), restricted to «Abisko, Torne Lappmark [Sweden]» by Lindroth (1968: 694). Lectotype (♂), designated by Lindroth (1968: 694), in NRSS. Etymology. The specific name honors the Swedish naturalist Conrad Quensel [1767-1806] who worked as professor of natural history and conservator in Stockholm.
Harpalus despectus C.R. Sahlberg, 1827b: 245. Type locality: «Lapponia» (original citation). Lectotype, designated by Silfverberg (1987: 15), in ZMH. Synonymy established by Sahlberg (1875: 106).
Amara remotestriata Dejean, 1828: 473. Type locality: «île d’Ounalaschka, l’une des îles Aleutiennes [Alaska]» (original citation). Two syntypes in MHNP (Lindroth 1955b: 17). Synonymy established by Hatch (1953: 128), confirmed by Lindroth (1954b: 135).
Amara monticola Dejean, 1831: 794. Type locality: «alpes de la Savoie [France]» (original citation). Holotype [by monotypy] (♀) in MHNP. Synonymy established by Heer (1837: 56), confirmed by Schaum (1858: 542).
Celia remota Zimmermann, 1832: 27. Type locality: «Unalaschka [Aleutian Islands, Alaska]» (original citation). Syntype(s) probably lost. Synonymy established with the name *Amara remotestriata* Dejean by Zimmermann (1832: 27).
Celia microcephala Motschulsky, 1844: 191. Type locality: «Tourkinsk [= Turka on the east bank of Lake Baikal, Russia]» (original citation). Two syntypes in ZMMU (Keleinikova 1976: 205). Synonymy established by Hieke (1995a: 102).
Isopleurus terrestris LeConte, 1847: 358. Type locality: «Fort Laramie, flumanis Platte [Nebraska]» (original citation). Syntype(s) in MCZ [# 5687]. Synonymy established, under the name *Amara remotestriata* Dejean, by Horn (1892b: 40).
Celia indistincta Mannerheim, 1853: 137. Type locality: «insula Unalaschka [Aleutian Islands, Alaska]» (original citation). Holotype [by monotypy] probably in ZMMU (Lindroth 1968: 694). Synonymy established, under the name *Amara remotestriata* Dejean, by Horn (1892b: 40).
Celia relucens Mannerheim, 1853: 138. Type locality: «Kenai, Cast[ellum] Nicol[ajevsk] [= Fort Saint Nicholas, Alaska]» (lectotype label). Lectotype (♂), designated by Lindroth (1968: 694), in ZMH. Synonymy established, under the name *Amara remotestriata* Dejean, by LeConte (1855: 354), confirmed by Lindroth (1968: 694).
Celia purpurascens Motschulsky, 1859a: 152. Type locality: California (inferred from title of the paper). Lectotype (♂), designated by Hieke (1993: 138), in ZMMU. Synonymy established by Hieke (1993: 137).
Amara femoralis G.H. Horn, 1892b: 30 [primary homonym of *Amara femoralis* Dejean, 1831]. Type locality: «M[oun]t Lincoln and at Argentine Pass (11,000 to 13,000 feet) [Colorado]» (original citation). Four syntypes in MCZ [# 667]. Synonymy established by Lindroth (1968: 694).
Celia laxicollis Casey, 1918: 258. Type locality: «Sheepshead Bay, Long Island, New York» (original citation for the lectotype). Lectotype (♂), designated by Lindroth (1975: 131), in USNM [# 47175]. Synonymy established by Lindroth (1968: 694).
Celia brumalis Casey, 1918: 259. Type locality: «Marquette [Marquette County], Michigan» (original citation). Lectotype (♂), designated by Lindroth (1975: 132), in USNM [# 47178]. Synonymy established by Lindroth (1968: 694).
Celia defecta Casey, 1918: 260. Type locality: «Jemez Springs [Sandoval County], New Mexico» (original citation). Lectotype (♀), designated by Lindroth (1975: 132), in USNM [# 47177]. Synonymy established by Lindroth (1968: 694).
Celia exposita Casey, 1918: 260. Type locality: «Columbia River Valley, Oregon» (original citation). Lectotype (♀), designated by Lindroth (1975: 132), in USNM [# 47183]. Synonymy established by Hatch (1953: 128), confirmed by Lindroth (1968: 694).
Celia mimica Casey, 1918: 260. Type locality: «Boston Neck [Washington County], Rhode Island» (original citation). Lectotype [as holotype] (♂), designated by Lindroth (1975: 132), in USNM [# 47184]. Synonymy established by Lindroth (1968: 694).
Celia fontinalis Casey, 1918: 261. Type locality: «Jemez Springs [Sandoval County], New Mexico» (original citation). Lectotype (♀), designated by Lindroth (1975: 132), in USNM [# 47185]. Synonymy established by Lindroth (1968: 694).
Celia eldorensis Casey, 1918: 261. Type locality: «Eldora [Boulder County], Colorado» (original citation). Lectotype (♀), designated by Lindroth (1975: 132), in USNM [# 47180]. Synonymy established by Lindroth (1968: 694).
Celia docilis Casey, 1918: 262. Type locality: «Colorado» (original citation). Lectotype (♂), designated by Lindroth (1975: 132), in USNM [# 47180]. Synonymy established by Lindroth (1968: 694).
Celia brunnescens Casey, 1918: 262. Type locality: «probably Colorado» (original citation). Holotype [by monotypy] (♀) in USNM [# 47189]. Synonymy established by Lindroth (1968: 694).
Celia definita Casey, 1918: 263. Type locality: «Yreka [Siskiyou County], California» (original citation). Lectotype (♀), designated by Lindroth (1975: 132), in USNM [# 47187]. Synonymy established by Lindroth (1968: 694).
Celia obligata Casey, 1918: 263. Type locality: «Utah» (original citation). Lectotype (♀), designated by Lindroth (1975: 132), in USNM [# 47188]. Synonymy established by Lindroth (1968: 694).
Celia piperi Casey, 1924: 49. Type locality: «Grayling, near Bay City [Bay County], Michigan» (original citation). Lectotype (♀), designated by Lindroth (1975: 132), in USNM [# 47174]. Synonymy established by Lindroth (1968: 694).
Celia explanatula Casey, 1924: 54. Type locality: «Similkameen R[iver], B[ritish] C[olumbia]» (lectotype label). Lectotype (♀), designated by Lindroth (1975: 132), in USNM [# 47176]. Synonymy established by Hatch (1953: 128), confirmed by Lindroth (1968: 694).
Celia tahomae Casey, 1924: 55. Type locality: «Wawawai [Whitman County], Washington» (original citation). Lectotype (♀), designated by Lindroth (1975: 132), in USNM [# 47200]. Synonymy established by Hatch (1953: 128), confirmed by Lindroth (1968: 694).
Celia washoeana Casey, 1924: 55. Type locality: «Nevada» (original citation). Lectotype (♂), designated by Lindroth (1975: 132), in USNM [# 47181]. Synonymy established by Lindroth (1968: 694).
Celia cervicalis Casey, 1924: 56. Type locality: «N[orth]W[est] T[erritories], Can[ada]» (lectotype label). Lectotype (♂), designated by Lindroth (1975: 132), in USNM [# 47182]. Synonymy established by Lindroth (1968: 694).
Amara horni Csiki, 1929: 436. Replacement name for *Amara femoralis* Horn, 1892.
Amara uenoi Habu, 1972: 33. Type locality: «M[oun]t Rishiri (1600 m), Rishiri Is[land], Japan» (original citation). Holotype (♂) in NMNS. Synonymy established by Hieke (1999b: 189).

#### Distribution.

This Holarctic species is widely distributed in the Nearctic Region from Newfoundland (Lindroth 1955a: 103) to Alaska, including the Kodiak, Aleutian and Pribilof Islands (Lindroth 1968: 696), south to southern California (Fall 1901a: 45, as *Amara remotestriata*), northern Arizona (Villa-Castillo and Wagner 2002: 246; Apache County, UASM), northern New Mexico (Fall and Cockerell 1907: 158, as *Amara remotestriata* and *Amara femoralis*; Milford et al. 2000: 21; Rio Arriba and Sandoval Counties, CMNH, UASM), Oklahoma (Hamilton 1894b: 353; Hatch and Ortenburger 1930: 8, as *Amara remotestriata*), and southwestern North Carolina (Jackson County, USNM).

#### Records.

**FRA**: PM **CAN**: AB, BC, LB, MB, NB, NF, NS (CBI), NT, ON, PE, QC, SK, YT **USA**: AK, AZ, CA, CO, CT, DE, IA, ID, IL, IN, KS, MA, MD, ME, MI, MN, MO, MT, NC, ND, NE, NH, NJ, NM, NV, NY, OH, OK, OR, PA, RI, SD, UT, VA, VT, WA, WI, WY – **Holarctic**

#### Note.

The subspecies *Amara quenseli silvicola* Zimmermann is endemic to Europe (Hieke 2003a: 565).

### Subgenus Zezea

Csiki, 1929

Triaena LeConte, 1847: 365 [junior homonym of *Triaena* Hübner, 1818]. Type species: *Feronia angustata* Say, 1823 designated by Chevrolat (1849: 643). Etymology (original). From the Greek *triaina* (trident), alluding to the trifid protibial spur of the adults (“*tibiisque anticis calcare trifido terminatis*”) [feminine].
Zezea Csiki, 1929: 402. Replacement name for *Triaena* LeConte, 1847. Etymology. Unknown [feminine].
Pseudotriaena Minsk and Hatch, 1939: 216. Type species: *Amara glabrata* Minsk and Hatch, 1939 (= *Amara longula* LeConte, 1855) by original designation. Synonymy established by Lindroth (1968: 735). Etymology. From the Greek *pseudos* (fallacy, lie) and the generic name *Triaena* [*q.v*.] [feminine].

#### Diversity.

Twenty-five species (Hieke 2007) in North America (nine species, all endemic), Asia (six species, only one endemic), Europe (13 species, six endemic), and northern Africa (four species, two endemic).

#### Identification.

Lindroth (1968: 735-741, as *pallipes* group) covered all the North American species except *Amara belfragei*. Three new species have been described subsequently (Hieke 1990, 2000) and one of Lindroth’s species (*Amara angustata*) was found to consist of two species (Hieke 2000). Hieke (2000) revised the species of the *angustata* group.

### [angustata group]

### Amara angustata

(Say, 1823)

Feronia angustata Say, 1823a: 36. Type locality: «Indep[e]nd[e]nce [Buchanan County], Iowa» (neotype label). Neotype (♀), designated by Lindroth and Freitag (1969: 345), in MCZ [# 33027]. Note. «on the Missouri» was the area originally cited by Say (1823a: 37).
Amara indistincta Haldeman, 1843b: 300. Type locality: «Easton, P[ennsylvani]a» (neotype label). Neotype (♂), designated by Hieke (2000: 49), in CNC [# 23535]. Synonymy established by LeConte (1855: 350).

#### Distribution.

This species ranges from southern Quebec to southeastern British Columbia, south to southeastern Wyoming, southeastern Texas (Fort Bend and Harris Counties, Brian Raber pers. comm. 2010), east-central Louisiana (West Feliciana Parish, Igor M. Sokolov pers. comm. 2009), southern Mississippi (Hinds County, Drew A. Hildebrandt pers. comm. 2010), northern Alabama (Lawrence County, UASM), and South Carolina (Hieke 2000: 49-51). The records from “St. Louis, Montana” and “Cleveland, Oregon” listed by Hieke (2000: 51) refers to Missouri and Ohio respectively. The records from “New Brunswick” and “Prince Edward Island” (Bousquet and Larochelle (1993: 199) refer to other species; that from west-central Colorado (Wickham 1902: 237) needs confirmation.

#### Records.

**CAN**: BC, MB, ON, QC, SK **USA**: AL, CT, DC, DE, IA, IL, IN, KS, KY, LA, MA, MD, ME, MI, MN, MO, MS, NC, ND, NE, NH, NJ, NY, OH, PA, RI, SC, SD, TN, TX, VA, VT, WI, WV, WY [CO]

### Amara angustatoides

Hieke, 2000

Amara angustatoides Hieke, 2000: 55. Type locality: «Chicago [Cook County], Ill[inois]» (original citation). Holotype (♂) in USNM.

#### Distribution.

This species ranges from New Brunswick to South Dakota, south to southern Pennsylvania (Hieke 2000: 55-57) and northeastern West Virginia (Randolph County, CMNH).

#### Records.

**CAN**: NB, ON, QC **USA**: CT, IA, IL, IN, MA, ME, MD, MI, MN, NH, NY, OH, PA, SD, VT, WI, WV

### Amara belfragei

Horn, 1892

Amara belfragei G.H. Horn, 1892a: 19. Type locality: «Waco [McLennan County], Texas» (original citation). Four syntypes in MCZ [# 34448].

#### Distribution.

This species is known from a few localities in central and southern Texas (Hieke 2000: 58).

#### Records.

**USA**: TX

### Amara flebilis

(Casey, 1918)

Triaena flebilis Casey, 1918: 316. Type locality: «Marquette [Marquette County, Michigan], Lake Superior» (original citation). Lectotype (♀), designated by Lindroth (1975: 136), in USNM [# 47326].

#### Distribution.

This species ranges from Cape Breton Island to southern Manitoba, south to northern Arizona, northeastern Colorado, northern Illinois, and northeastern Virginia (Hieke 2000: 53-55). The record “Cleveland, Oregon” (Hieke 2000: 55) refers to Ohio.

#### Records.

**CAN**: MB, NB, NS (CBI), ON, QC **USA**: AZ, CO, CT, DC, IA, IL, IN, MA, MD, ME, MI, MN, ND, NH, NJ, NY, OH, PA, VA, VT, WI, WV

#### Note.

This form has been treated as a synonym of *Amara angustata* (Say) by Lindroth (1968: 736) but considered a valid species by Hieke (2000: 53).

### [scitula group]

### Amara inexspectata

Hieke, 1990

Amara inexspectata Hieke, 1990: 196. Type locality: «San Francisco [San Francisco County], Ca[lifornia]» (original citation). Holotype (♂) in CAS [# 16389].

#### Distribution.

This species occurs along the Pacific Coast from central Oregon (Lincoln County, CNC) to Monterey County in California (Hieke 1990: 196).

#### Records.

**USA**: CA, OR

### Amara kavanaughi

Hieke, 1990

Amara kavanaughi Hieke, 1990: 198. Type locality: «10 mi[les] S[outh] of Dixon, Solano Co[unty], California» (original citation). Holotype (♂) in CAS [# 16390].

#### Distribution.

This species is known from southern Oregon (Lake County, CNC) and several localities in northern and central California (Hieke 1990: 198).

#### Records.

**USA**: CA, OR

### Amara longula

LeConte, 1855

Amara longula LeConte, 1855: 350. Type locality: «San Francisco [San Francisco County, California]» (original citation). Three syntypes in MCZ [# 5675].
Amara afoveolata Hayward, 1908: 47. Type locality: «Vancouver Island [British Columbia] and California» (original citation), restricted to «Vanc[ouver] Isl[and]» by Lindroth (1968: 740). Three syntypes [3 originally cited] in MCZ [# 25670]. Synonymy established by Lindroth (1968: 740). Note. According to Lindroth (1968: 740), the syntype from California is conspecific with members of *Amara scitula*.
Triaena profuga Casey, 1918: 318. Type locality: «Arizona» (original citation). Lectotype (♀), designated by Lindroth (1975: 137), in USNM [# 47330]. Synonymy established by Lindroth (1968: 740).
Amara alaxnoguia Minsk and Hatch, 1939: 217. Type locality: «Seattle [King County], Wash[ington]» (original citation). Holotype (♂) in USNM. Synonymy established by Hatch (1953: 129), confirmed by Lindroth (1968: 740).
Amara atrichata Minsk and Hatch, 1939: 217. Type locality: «Seattle [King County], Wash[ington]» (original citation). Holotype (♂) in USNM. Synonymy established by Lindroth (1968: 740).
Amara glabrata Minsk and Hatch, 1939: 217. Type locality: «Seattle [King County], Wash[ington]» (original citation). Holotype (♂) in USNM. Synonymy established by Hatch (1953: 129), confirmed by Lindroth (1968: 740).

#### Distribution.

This species is found from southwestern British Columbia, including Vancouver Island (Lindroth 1968: 740), to southern California (Hayward 1908: 46; Moore 1937: 10; CAS) and “Arizona” (Casey 1918: 318, as *Triaena profuga*), including eastern Nevada (White Pine County, UASM). The records from “Idaho” and “Utah” (Bousquet and Larochelle 1993: 200) are probably in error.

#### Records.

**CAN**: BC (VCI) **USA**: CA, NV, OR, WA

### Amara pallipes

Kirby, 1837

Amara pallipes Kirby, 1837: 39. Type locality: northern parts of British America (inferred from title of the book), restricted to «Edmonton, Al[ber]ta» by Lindroth (1968: 737). Holotype [by monotypy] (♂) in BMNH (Lindroth 1953b: 173).
Triaena depressa LeConte, 1847: 365. Type locality: «Lacum Superiorem» (original citation). Holotype [by monotypy] (♀) in MCZ [# 5674]. Synonymy established by LeConte (1855: 350), confirmed by Lindroth (1968: 737).
Triaena shermani Casey, 1918: 315. Type locality: «Marquette [Marquette County, Michigan], Lake Superior» (original citation). Lectotype (♀), designated by Lindroth (1975: 136), in USNM [# 47327]. Synonymy established by Lindroth (1968: 737).
Triaena lawrenceana Casey, 1924: 66. Type locality: «Ogdensburg [Saint Lawrence County], New York» (original citation). Holotype [by monotypy] (♂) in USNM [# 47331]. Synonymy established by Lindroth (1968: 737).
Triaena parallela Casey, 1924: 67. Type locality: «Lake Champlain, New York» (original citation). Lectotype (♂), designated by Lindroth (1975: 136), in USNM [# 47333]. Synonymy established by Lindroth (1968: 737).
Amara kincaidi Minsk and Hatch, 1939: 217. Type locality: «Renton [King County], Wash[ington]» (original citation). Holotype (♂) in USNM. Synonymy established by Hatch (1953: 129), confirmed by Lindroth (1968: 737).

#### Distribution.

The range of this species extends from Cape Breton Island (Lindroth 1954c: 305) to Vancouver Island (Lindroth 1968: 739), north to Fort Smith in southern Northwest Territories (Bousquet 1987a: 130), south to western Washington (Hatch 1953: 129), southwestern Colorado (Montezuma County, CNC), Iowa (Hayward 1908: 46; Wickham 1911b: 6; Dickinson County, USNM), and Virginia (Casey 1918: 314). The records from “Nevada,” “Tennessee” (Bousquet and Larochelle 1993: 200), and Georgia (J.E. LeConte 1849: 26; Fattig 1949: 30) need confirmation.

#### Records.

**CAN**: AB, BC (VCI), MB, NB, NS (CBI), NT, ON, QC, SK **USA**: CO, CT, DC, IA, ID, IL, IN, MA, ME, MI, MN, MT, ND, NH, NJ, NY, OH, PA, RI, SD, VA, VT, WA, WI, WV [GA, NV, TN]

### Amara scitula

Zimmermann, 1832

Amara scitula Zimmermann, 1832: 32. Type locality: «Neu-Californien» (original citation), restricted to «Waterman [= probably Barstow in San Bernardino County], Calif[ornia]» by Lindroth (1968: 739). Holotype [by monotypy] in ZILR (*fide* Zimmermann 1832: 33).
Bradytus immundus Casey, 1918: 243. Type locality: «Gualala, Mendocino Co[unty], California» (original citation). Lectotype [as typus] (♂), designated by Hieke (1994: 308), in USNM [# 47169]. Synonymy established by Hieke (1994: 308).
Amara provoana Casey, 1918: 300. Type locality: «Provo [Utah County], Utah» (original citation). Lectotype (♀), designated by Lindroth (1975: 136), in USNM [# 47280]. Synonymy established by Lindroth (1968: 739).
Triaena uinta Casey, 1918: 317. Type locality: «Provo [Utah County], Utah» (original citation). Lectotype (♂), designated by Lindroth (1975: 136), in USNM [# 47328]. Synonymy established by Lindroth (1968: 739).
Triaena vapida Casey, 1918: 317. Type locality: «Provo [Utah County], Utah» (original citation). Lectotype (♀), designated by Lindroth (1975: 136), in USNM [# 47329]. Synonymy established by Lindroth (1968: 739).
Amara obliqua Casey, 1924: 63. Type locality: «Victoria, B[ritish] C[olumbia]» (holotype label). Holotype [by monotypy] (♂) in USNM [# 47297]. Synonymy established by Lindroth (1968: 739).
Triaena irregularis Casey, 1924: 66. Type locality: «V[ancouver] I[sland]» (holotype label). Holotype [by monotypy] (♂) in USNM [# 47332]. Synonymy established by Lindroth (1968: 739). Note. According to Lindroth (1975: 137), the type locality originally given by Casey (1924: 66), “Washington State,” is incorrect.

#### Distribution.

This species ranges from southern Manitoba to Vancouver Island (Lindroth 1968: 740), south to southern California (Fall 1901a: 45; Moore 1937: 10) and southwestern Colorado (Wickham 1902: 237), east to northwestern South Dakota (Kirk and Balsbaugh 1975: 27).

#### Records.

**CAN**: AB, BC (VCI), MB, SK **USA**: CA, CO, ID, MT, NV, OR, SD, UT, WA, WY

### Tribe Oodini

LaFerté-Sénectère, 1851

Oodites LaFerté-Sénectère, 1851: 214, 266. Type genus: *Oodes* Bonelli, 1810.
Thryptocerini Jeannel, 1949a: 775, 829. Type genus: *Thryptocerus* Chaudoir, 1878.
Sphaerodini Jeannel, 1949a: 829. Type genus: *Sphoerodes* Chaudoir, 1883. Note. This family-group name is based on *Sphaerodes*, an incorrect subsequent spelling of *Sphoerodes* not in prevailing usage.
Simoini Basilewsky, 1953a: 153. Type genus: *Simous* Chaudoir, 1882.

#### Diversity.

Worldwide, with about 300 species in 32 genera (Lorenz 2005: 324-327). The Northern Hemisphere is represented by about 45 species (only 14.5% of the world fauna) and the Western Hemisphere by 55 species (about 18.5%). Seventeen species occur in North America.

#### Identification.

Bousquet (1996a) revised and provided a key to the Nearctic, Mexican, and West Indian species.

### Genus Dercylinus

Chaudoir, 1883

Dercylinus Chaudoir, 1883: 525. Type species: *Evolenes impressa* LeConte, 1853 by monotypy. Etymology. From the generic name *Dercylus* and the Latin suffix -*inus* (pertaining to) [masculine].

#### Diversity.

One species in temperate North America.

### Dercylinus impressus

(LeConte, 1853)

Evolenes impressa LeConte, 1853c: 392. Type locality: «N[ew] Orl[eans] [Orleans Parish, Louisiana]» (holotype label). Holotype [by monotypy] (♀) in MCZ [# 5870].

#### Distribution.

This species is known from a few scattered localities in North Carolina (Brimley 1938: 127), Rabun County in northeastern Georgia (Fattig 1949: 45), southwestern Alabama (Washington County, Paul K. Lago pers. comm. 2009), northeastern Mississippi (Tishomingo County, Drew A. Hildebrandt pers. comm. 2008), southern Louisiana (Bousquet 1996a: 453), central Arkansas (Pulaski County, CMNH), eastern Missouri (Anonymous 2007), east-central Illinois (Coles County, Robert L. Davidson pers. comm. 2012), eastern Oklahoma (Bousquet 1996a: 453), and eastern Texas (Sabine County, Brian Raber pers. comm. 2010; Riley 2011).

#### Records.

**USA**: AL, AR, GA, IL, LA, MO, MS, NC, OK, TX

### Genus Evolenes

LeConte, 1853

Evolenes LeConte, 1853c: 392. Type species: *Oodes exaratus* Dejean, 1831 designated by Bousquet and Larochelle (1993: 202). Etymology. From the Greek *ev* (well) and *olene* (elbow, arm, by extension tibia), possibly alluding to the expanded protibiae (“*tibiae anticae latiores*”) of the adult [feminine].

#### Diversity.

One species in temperate North America.

### Evolenes exarata

(Dejean, 1831)

Oodes exaratus Dejean, 1831: 678. Type locality: «Amérique septentrionale» (original citation), restricted to «Mobile, Mobile Co[unty], Alabama» by Bousquet (1996a: 454). Holotype [by monotypy] (♀) in MHNP (Lindroth 1955b: 26).

#### Distribution.

This species is restricted to the Coastal Plain ranging from the District of Columbia to the Florida Panhandle, west to southeastern Mississippi (Drew A. Hildebrandt pers. comm. 2007) [see Bousquet 1996a: map 1].

#### Records.

**USA**: AL, DC, FL, GA, MS, NC, SC

### Genus Anatrichis

LeConte, 1853

Anatrichis LeConte, 1853c: 391. Type species: *Oodes minutus* Dejean, 1831 by original designation. Etymology. Probably from the Greek *an* (without) and *trichos* (hair) and alluding to the absence of hairs underneath the metatarsomeres of the adults (“*tarsi posteriores subtus non pubescentes*”) contrary to those of *Lachnocrepis* [feminine].

#### Diversity.

Six species (Bousquet 1996a: 456), possibly eight (Lorenz 2005: 324), in the temperate and tropical areas of the Nearctic (two species), Neotropical, Australian, and Oriental Regions.

### Anatrichis minuta

(Dejean, 1831)

Oodes minutus Dejean, 1831: 677. Type locality: «Amérique septentrionale» (original citation), restricted to «Homestead [Dade County], Flor[ida]» by Lindroth (1969a: 1002). Holotype [by monotypy] (♂) in MHNP (Lindroth 1955b: 26).

#### Distribution.

This species ranges from Massachusetts to central Kansas, south to southeastern Texas and southern Florida [see Bousquet 1996a: map 2]; also recorded from the Bahamas (Turnbow and Thomas 2008: 10).

#### Records.

**USA**: AL, AR, CT, DC, FL, GA, IL, IN, KS, KY, LA, MA, MD, MO, MS, NC, NH, OH, OK, RI, SC, TN, TX, VA, WV – Bahamas

### Anatrichis oblonga

Horn, 1891

Anatrichis oblonga G.H. Horn, 1891: 37. Type locality: «near the lower Rio Grande, Texas» (original citation), restricted to «Brownsville, Cameron Co[unty]» by Bousquet (1996a: 457). Holotype [by monotypy] (♀) in MCZ [# 2945].

#### Distribution.

This species ranges from eastern Texas south to Honduras; it also occurs in the Bahamas and Cuba [see Bousquet 1996a: 459, map 3].

#### Records.

**USA**: TX – Bahamas, Belize, Cuba, Guatemala, Honduras, Mexico

### Genus Oodinus

Motschulsky, 1865

Oodinus Motschulsky, 1865: 352. Type species: *Oodinus piceus* Motschulsky, 1865 by monotypy. Etymology. From the generic name *Oodes* [*q.v*.] and the Latin suffix -*inus* (pertaining to) [masculine].
Oodiellus Chaudoir, 1882: 322. Type species: *Oodiellus mexicanus* Chaudoir, 1882 (= *Anatrichis alutaceus* Bates, 1882) designated by Bousquet and Larochelle (1993: 202). Synonymy established with doubt by Bates (1884: 269), confirmed by Spence (1983: 568). Etymology. From the generic name *Oodes* [*q.v*.] and the Latin suffix -*ellus* (small) [masculine].

#### Diversity.

Ten species in the temperate and tropical areas of the Nearctic (two species), Neotropical, and Australian (one species in the Malay Archipelago) Regions.

### Oodinus alutaceus

(Bates, 1882)

Anatrichis alutaceus Bates, 1882a [February]: 48. Type locality: «Cordova [Veracruz], Mexico» (original citation). Lectotype (♀), designated by Bousquet (1996a: 464), in BMNH.
Oodiellus mexicanus Chaudoir, 1882 [27 December]: 323. Type locality: «Mexique» (original citation). Syntype(s) [2 originally cited] probably in MHNP. Synonymy established by Sallé (in Chaudoir 1882: 323).

#### Distribution.

This species ranges from southeastern Texas (Bousquet 1996a: 465; map 5) south to Columbia (Martínez 2003: 17).

#### Records.

**USA**: TX – Colombia, Costa Rica, Honduras, Mexico

### Oodinus pseudopiceus

Bousquet, 1996

Oodinus pseudopiceus Bousquet, 1996a: 462. Type locality: «Hillsborough R[iver] S[tate] P[ark], Hillsboro[ugh] Co[unty], Fl[orid]a» (original citation). Holotype (♂) in CNC [# 21759].

#### Distribution.

This species is known from the Florida Peninsula and several islands of the West Indies [see Bousquet 1996a: map 4].

#### Records.

**USA**: FL – Bahamas, Cuba, Dominican Republic

### Genus Lachnocrepis

LeConte, 1853

Lachnocrepis LeConte, 1853c: 391. Type species: *Oodes parallelus* Say, 1830 by monotypy. Etymology. From the Greek *lachnos* (soft hair, down) and *crepis* (boot, sandal), alluding to the presence of dense hairs underneath all tarsomeres (“*tarsi omnes subtus pilosi*”) of the adult [feminine].
Eulachnocrepis Habu, 1956b: 96. Type species: *Oodes prolixus* Bates, 1873 by original designation. Synonymy established by Bousquet (1996a: 467). Etymology. From the Greek *eu* (agreeable, original, primitive) and the generic name *Lachnocrepis* [*q.v*.] [feminine].

#### Diversity.

Three species in temperate regions of North America (one species) and eastern Asia (two species).

### Lachnocrepis parallela

(Say, 1830)

Oodes parallelus Say, 1830b: (3) [3]. Type locality: «L[ouisian]a» (neotype label), restricted to «New Orleans, Orleans Parish» by Bousquet (1996a: 468). Neotype (♂), designated by Lindroth and Freitag (1969: 352), in MCZ [# 32975]. Note. «Louisiana» was the area originally cited by Say (1830b: (3) [3]).

#### Distribution.

This species has a disjunct distribution over eastern North America. In the north, it ranges from Nova Scotia (Majka et al. 2007: 11) to southeastern Manitoba, south to Nebraska, northwestern Tennessee (Obion County, CMNH), and Maryland; in the south, it occurs along the Coastal Plain from Georgia (Fattig 1949: 45) to southern Florida, west to southern Louisiana [see Bousquet 1996a: map 6]. The species could reach the Rio Grande since Summers (1874b: 135) reported seeing one specimen “from Mexico, near the Rio Grande.”

#### Records.

**CAN**: MB, NB, NS, ON, QC **USA**: AL, CT, DC, FL, GA, IA, IL, IN, LA, MA, MD, ME, MI, MN, MO, MS, NC, ND, NE, NH, NJ, NY, OH, PA, RI, SD, TN, VA, VT, WI, WV

**Figure 29.**
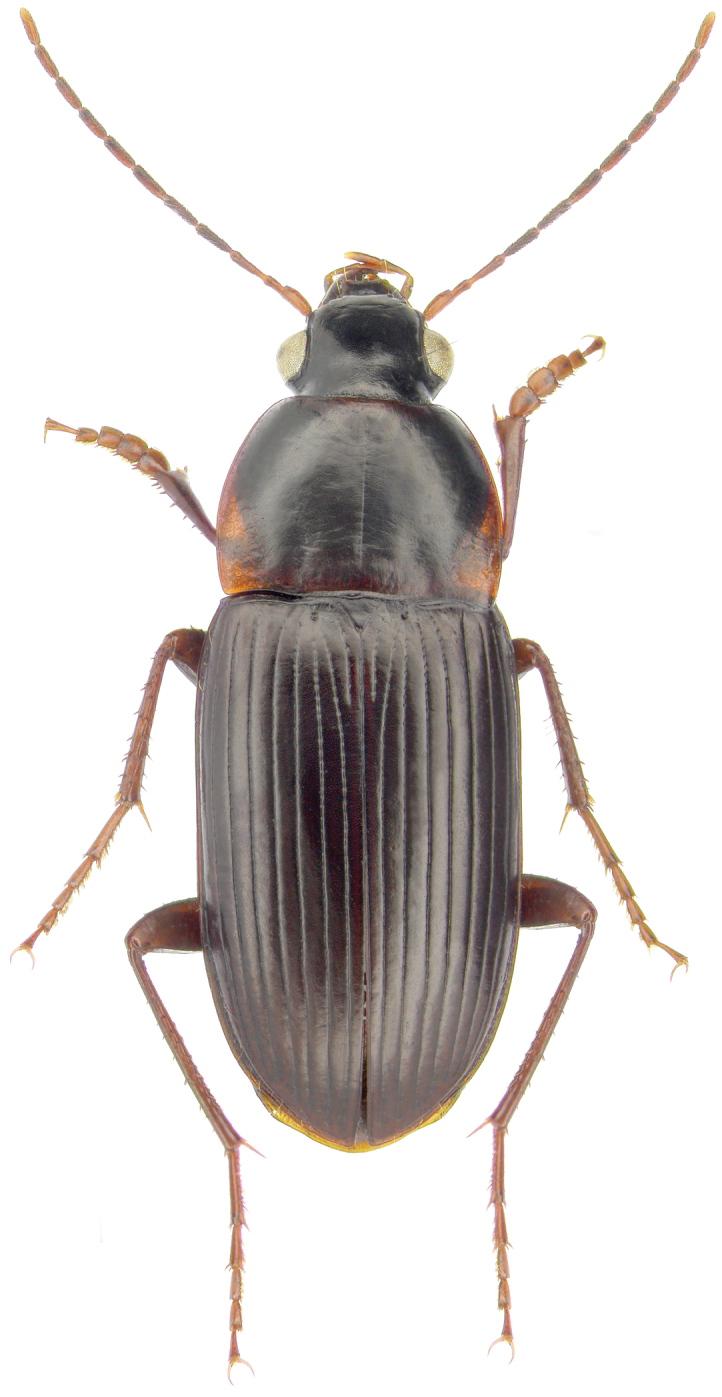
*Lachnocrepis parallela* (Say). This species is found in marshes and swamps in temperate eastern North America and is closely related to two species living under similar conditions in eastern Asia. The genus *Lachnocrepis* is another example of taxa exhibiting disjunct eastern Asia-eastern North America distributional patterns. Most current biogeographers agree that such disjunct patterns result from range restrictions of taxa associated with the mixed mesophytic forest during the mid-Tertiary.

### Genus Oodes

Bonelli, 1810

Oodes Bonelli, 1810: Tabula Synoptica. Type species: *Carabus helopioides* Fabricius, 1792 by subsequent monotypy in Panzer (1813: 51). Etymology. From the Greek *oön* (egg) and the suffix -*oides* (having the form of), alluding to the ovoid body shape (“*corp*[*us*] *parum convexum, ovale*”) of the adult [masculine].

#### Diversity.

About 50 species in temperate and tropical areas of the Nearctic (four species), Palaearctic (11 species), Oriental, Afrotropical (one species), and Australian Regions.

### Oodes amaroides

Dejean, 1831

Oodes amaroides Dejean, 1831: 674. Type locality: «Amérique septentrionale» (original citation), restricted to «Mobile [Mobile County], Alab[ama]» by Lindroth (1969a: 1000). Holotype [by monotypy] (♀) in MHNP (Lindroth 1955b: 26). Note. Chaudoir’s collection in MHNP contains two specimens, a male and a female, of this species and the male has been designated lectotype by Bousquet (1996a: 472). The designation is invalid since Dejean (1831: 675) expressly stated that he had but a single female of this species.

#### Distribution.

This species ranges from southern Maine to east-central Minnesota (Kamal J.K. Gandhi pers. comm. 2008), south to southeastern Texas and the Florida Peninsula; also known from one locality in Cuba [see Bousquet 1996a: map 7]. The record from southeastern Wyoming (Lavigne 1977: 47) is probably based on a mislabeled specimen or a stray.

#### Records.

**CAN**: ON **USA**: AL, AR, CT, DC, DE, FL, GA, IA, IL, IN, KS, KY, LA, MA, MD, ME, MI, MN, MO, MS, NC, NH, NJ, NY, OH, OK, PA, RI, SC, TN, TX, VA, WI, WV – Cuba

### Oodes americanus

Dejean, 1826

Oodes americanus Dejean, 1826: 377. Type locality: «Amérique septentrionale» (original citation), restricted to «New Orleans [Orleans Parish], Louis[iana]» by Lindroth (1969a: 997). Lectotype (♂), designated by Bousquet (1996a: 474), in MHNP.

#### Distribution.

This species is restricted to the Coastal Plain ranging from Maryland to central Florida, west to central Louisiana [see Bousquet 1996a: map 9].

#### Records.

**USA**: AL, FL, GA, LA, MD, MS, NC, SC, VA

### Oodes brevis

Lindroth, 1957

Oodes brevis Lindroth, 1957a: 63. Type locality: «Fairfax Co[unty], Virginia» (original citation). Holotype (♂) in MCZ [# 29603].

#### Distribution.

This species ranges from southern New Hampshire to northwestern Arkansas, north to southernmost Ontario and the central part of the lower peninsula of Michigan, south to eastern Texas and the Florida Panhandle [see Bousquet 1996a: map 10]. The record from “Iowa” (Bousquet and Larochelle 1993: 200) needs confirmation.

#### Records.

**CAN**: ON **USA**: AL, AR, CT, DC, FL, GA, IL, IN, LA, MA, MD, MI, MO, MS, NC, NH, NJ, NY, OH, RI, PA, SC, TN, TX, VA [IA]

### Oodes fluvialis

LeConte, 1863

Oodes fluvialis LeConte, 1863c: 13. Type locality: «Canada and western states» (original citation), restricted to «Turkey Point, Ont[ario]» by Lindroth (1969a: 997). Lectotype (♂), designated by Bousquet (1996a: 473), in MCZ [# 5867].

#### Distribution.

This species occurs from southern Maine and southern Quebec to western Minnesota, south to east-central Texas (Riley 2011), southwestern Louisiana and southern Florida [see Bousquet 1996a: map 8].

#### Records.

**CAN**: ON, QC **USA**: AL, CT, DC, FL, GA, IA, IL, IN, LA, MA, MD, ME, MI, MN, MO, MS, NC, NE, NH, NJ, NY, OH, PA, RI, SC, TN, TX, VA, VT, WI

### Genus Stenocrepis

Chaudoir, 1857

Stenocrepis Chaudoir, 1857: 45. Type species: *Oodes cayennensis* Buquet, 1835 designated by Chaudoir (1883: 488). Etymology (original). From the Greek *stenos* (narrow) and *crepis* (sole), alluding to the narrow male protarsomeres 1-3 (“*tarsi in mare articulis tribus dilatatis, angustis*”) [feminine].

#### Diversity.

About 30 species in temperate and tropical areas of the Nearctic (six species) and Neotropical Regions, including the West Indies. The species are currently arrayed in two subgenera: *Stenocrepis* s.str. and *Stenous* Chaudoir.

### Subgenus Stenocrepis

Chaudoir, 1857

Stenocrepis Chaudoir, 1857: 45. Type species: *Oodes cayennensis* Buquet, 1835 designated by Chaudoir (1883: 488).

#### Diversity.

Ten species, ranging from southern Texas to central Argentina and including the West Indies, belong to this subgenus.

### Stenocrepis insulana

(Jacquelin du Val, 1857)

Oodes insulanus Jacquelin du Val, 1857: 20. Type locality: Cuba (inferred from title of the book). Syntype(s) location unknown (possibly in MHNP).
Oodes texanus LeConte, 1863c: 13. Type locality: «Texas» (original citation). Lectotype (♀), designated by Bousquet (1996a: 481), in MCZ [# 5869]. Synonymy established by Bousquet (1996a: 481).
Stenocrepis chalcas Bates, 1882a: 47. Type locality: «Cordova [Veracruz], Mexico» (original citation). Lectotype (♂), designated by Bousquet and Larochelle (1993: 17), in BMNH. Synonymy established, under the name *Stenocrepis texanus* (LeConte), by Bousquet and Larochelle (1993: 17).
Stenocrepis chalcochrous Chaudoir, 1883: 487. Type locality: «Mexique» (original citation). Holotype [by monotypy] (♂) in MHNP. Synonymy established, under the name *Stenocrepis chalcas* Bates, by Sallé (in Chaudoir 1883: 487), confirmed by Bousquet (1996a: 481).

#### Distribution.

This species ranges from southern Texas to southern Mexico [see Bousquet 1996a: map 11]; it is also known from the Bahamas, Cuba, Cayman Islands, Trinidad and Tobago, and French Guiana (Bousquet 1996a: 482-483). The record from southeastern Louisiana (Summers 1874a: 80, as *Oodes texanus*) is suspect; that from northeastern Kansas (Knaus 1901: 110) refers to *Stenocrepis cuprea* (Knaus 1903: 187).

#### Records.

**USA**: TX [LA] – Bahamas, Cuba, Cayman Islands, French Guiana, Trinidad and Tobago

### Subgenus Stenous

Chaudoir, 1857

Stenous Chaudoir, 1857: 39. Type species: *Oodes cupreus* Chaudoir, 1843 designated by Bousquet and Larochelle (1993: 201). Etymology (original). From the Greek *stenos* (narrow) and *oön* (egg), referring to the shape of the body (“*habitus anguste ovatus*”) of the adult [masculine].
Crossocrepis Chaudoir, 1857: 50. Type species: *Oodes quatuordecimstriatus* Chaudoir, 1843 by monotypy. Synonymy established by Bousquet (1996a: 483). Etymology. From the Greek *crossos* (fringe) and *crepis* (sole) [feminine].

#### Diversity.

Twenty species in temperate and tropical areas of the Nearctic (five species) and Neotropical (19 species) Regions, including the West Indies.

### Stenocrepis cuprea

(Chaudoir, 1843)

Oodes cupreus Chaudoir, 1843b: 761. Type locality: «Nouvelle-Orléans [Orleans Parish, Louisiana]» (original citation). Lectotype (♂), designated by Bousquet (1996a: 491), in MHNP.
Oodes leucodactylus LaFerté-Sénectère, 1851: 273. Type locality: «New Orléans [Orleans Parish, Louisiana]» (original citation). Syntype(s) probably in MHNP (collection Chaudoir). Synonymy established by Chaudoir (1857: 40).

#### Distribution.

This species ranges from southeastern Wisconsin (Messer 2010: 39) and southernmost Ontario south to southern Florida and the Rio Grande in southern Texas, west along the Rio Grande to central New Mexico [see Bousquet 1996a: map 15]. The species is not yet know from Mexico but it certainly occurs at least along the states bordering Texas. The record from “Pennsylvania” (Bousquet and Larochelle 1993: 201) needs confirmation.

#### Records.

**CAN**: ON **USA**: AL, AR, FL, GA, IA, IL, IN, KS, KY, LA, MD, MI, MO, MS, NE, NM, OH, OK, SC, TN, TX, VA, WI, WV [PA]

### Stenocrepis duodecimstriata

(Chevrolat, 1836)

Oodes 12-striatus Chevrolat, 1836b: [no. 173]. Type locality: «Tuspan [Veracruz, Mexico]» (original citation). Holotype [by monotypy] (♂) in UMO.
Oodes humilis LaFerté-Sénectère, 1851: 270. Type locality: «Mexico» (original citation). Syntype(s) probably in MHNP (collection Chaudoir). Synonymy established by Bates (1882a: 46).
Oodes striatellus LaFerté-Sénectère, 1851: 272. Type locality: «Mexico» (original citation). Syntype(s) probably in MHNP (collection Chaudoir). Synonymy established by Chaudoir (1883: 494).
Stenous le contei Chaudoir, 1857: 41. Type locality: «Louisiane» (original citation). Lectotype (♂), designated by Bousquet (1996a: 485), in MHNP. Synonymy established by Chaudoir (1883: 494), confirmed by Bousquet (1996a: 485).

#### Distribution.

This species ranges from Long Island, New York, to south-central Oklahoma, south to Nicaragua (Bates 1882a: 46) and southern Florida, west to the Mexican state of Nayarit; also known from several islands in the Bahamas and the Greater Antilles [see Bousquet 1996a: map 12].

#### Records.

**USA**: AL, AR, FL, GA, IL, KY, LA, MD, MS, NC, NJ, NY, OH, OK, SC, VA, TX, WV – Bahamas, Cuba, Dominican Republic, Guatemala, Haiti, Jamaica, Mexico, Nicaragua

### Stenocrepis elegans

(LeConte, 1851)

Oodes elegans LeConte, 1851: 180. Type locality: «ad fluminis Gilae ripas, circa Pimas [Arizona]» (original citation). Lectotype (♂), designated by Bousquet (1996a: 495), in MCZ [# 76].

#### Distribution.

This species ranges from southwestern California to eastern Arizona, south along the Gulf of California to Nayarit [see Bousquet 1996a: map 17]. A few specimens simply labeled from New Mexico and Texas are known.

#### Records.

**USA**: AZ, CA [NM, TX] – Mexico

### Stenocrepis mexicana

(Chevrolat, 1835)

Oodes mexicanus Chevrolat, 1835b: [no. 102]. Type locality: Mexico (inferred from title of the book). Holotype [by monotypy] (♀) in UMO (Bousquet 1996a: 487).
Oodes 14-striatus Chaudoir, 1843b: 759. Type locality: «près de la Nouvelle-Orléans [Orleans Parish, Louisiana]» (original citation). Lectotype (♂), designated by Bousquet (1996a: 487), in MHNP. Synonymy established by Bousquet (1996a: 487).
Oodes picipes LeConte, 1844: 52. Type locality: «Georgia» (original citation). Lectotype (♀), designated by Bousquet (1996a: 487), in MCZ [# 5868]. Synonymy established, under the name *Stenocrepis quatuordecimstriata* (Chaudoir), by Chaudoir (1857: 51), confirmed by Bousquet (1996a: 487).
Oodes stenocephalus LaFerté-Sénectère, 1851: 271. Type locality: «New Orléans [Orleans Parish, Louisiana]» (original citation). Syntype(s) probably in MHNP (collection Chaudoir). Synonymy established, under the name *Stenocrepis quatuordecimstriata* (Chaudoir), by Chaudoir (1857: 51).
Stenocrepis sulcatus Chevrolat, 1863: 195. Type locality: «Cuba» (original citation). Holotype [by monotypy] (♂) location unknown. Synonymy established by Bousquet (1996a: 487).

#### Distribution.

This species ranges from New Jersey to southern Wisconsin, south through southeastern Kansas (Knaus 1903: 188) and eastern and central Texas to southern Mexico, and to the Florida Keys, west to the Pacific Coast in the Mexican state of Colima; also recorded from the Bahamas (Turnbow and Thomas 2008: 15) and Cuba [see Bousquet 1996a: map 13]. The record from “New Mexico” (Bousquet and Larochelle 1993: 201, as *Stenocrepis quatuordecimstriata*) is likely in error.

#### Records.

**USA**: AL, AR, FL, GA, IL, IN, KS, KY, LA, MS, NC, NJ, OH, OK, SC, TN, TX, VA, WI, WV – Bahamas, Cuba, Mexico

### Stenocrepis tibialis

(Chevrolat, 1834)

Amara tibialis Chevrolat, 1834: [no. 46]. Type locality: Mexico (inferred from title of the book), herein restricted to Mazatlán, Sinaloa (USNM). Lectotype (♂), designated by Bousquet (1996a: 493), in UMO.
Oodes femoralis Chaudoir, 1835: 444. Type locality: «Cuba» (original citation). Lectotype (♀), designated by Bousquet (1996a: 493), in MHNP. Synonymy established by Chaudoir (1837b: 50), confirmed by Bousquet (1996a: 493).
Oodes pallipes Brullé, 1836: plate 2, figure 6. Type locality: «sur les bords du Rio grande, province de Santa-Cruz [Argentina]» (Brullé 1837: 32). Syntype(s) location unknown (possibly in MHNP). Synonymy established, under the name *Stenocrepis femoralis* (Chaudoir), by Brullé (1837: 32).
Oodes chlorophanus Erichson, 1847: 72. Type locality: Peru (inferred from title of the publication). Syntype(s) location unknown. Synonymy established by Bates (1882a: 46). Note. Chaudoir (1883: 502) retained this name for a distinct, closely related species but the position of Bates (1882a: 46) seems more plausible (Bousquet 1996a: 493).

#### Distribution.

This species ranges from southern Texas south to Argentina (Brullé 1837: 32, as *Oodes pallipes*) and along the West Indies from Cuba to at least Puerto Rico [see Bousquet 1996a: map 16].

#### Records.

**USA**: TX – Argentina, Brazil, Cayman Islands, Colombia, Costa Rica, Cuba, Dominican Republic, Ecuador, Guatemala, Haiti, Jamaica, Mexico, Peru, Puerto Rico.

### Tribe Panagaeini

Bonelli, 1810

Panagaeides Bonelli, 1810: Tabula Synoptica. Type genus: *Panagaeus* Latreille, 1802.
Tefflini Basilewsky, 1946: 7. Type genus: *Tefflus* Leach, 1819.

#### Diversity.

Worldwide, with about 270 species in 19 genera (Lorenz 2005: 319-323, as Panagaeina). The Northern Hemisphere is represented by only about 40 species (15.5 % of the world fauna). Four species in two genera are found in North America.

### Genus Panagaeus

Latreille, 1802

Panagaeus Latreille, 1802: 91. Type species: *Carabus cruxmajor* Linnaeus, 1758 by monotypy. Etymology. Uncertain, possibly from the Greek *pan*- (all, the whole) and *agaios* (admirable, enviable), alluding to the overall beautiful coloration of the adult, or from the Greek *panagios* (very saintly, very pious), alluding to the cross-shaped color band on the elytra of the species Latreille had before him [masculine].

#### Diversity.

Fourteen species in temperate and tropical areas of the Nearctic (three species), Neotropical (four species), and Palaearctic (eight species) Regions arrayed in two subgenera: *Panagaeus* (Palaearctic Region) and *Hologaeus* (Western Hemisphere).

#### Identification.

Ogueta (1966a) reviewed the Western Hemisphere species and provided a key for their identification.

### Subgenus Hologaeus

Ogueta, 1966

Hologaeus Ogueta, 1966b: 396. Type species: *Panagaeus cruciger* Say, 1823 by original designation. Etymology. From the Greek *holo*- (whole, entire) and *agaios* (admirable, enviable), probably alluding to the overall beautiful coloration of the adults [masculine].

#### Diversity.

Western Hemisphere, with six species in the Nearctic (three species) and Neotropical (four species) Regions.

### Panagaeus cruciger

Say, 1823

Panagaeus crucigerus Say, 1823a: 69. Type locality: «Surf City [Ocean County], N[ew] J[ersey]» (neotype label). Neotype (♂), designated by Lindroth and Freitag (1969: 335), in MCZ [# 33075]. Note. «Senipuxten, eastern shore of Maryland» was the area originally cited by Say (1823a: 70).
Panagaeus lapidarius LaFerté-Sénectère, 1851: 223. Type locality: «New-Orléans [Louisiana]» (original citation). Syntype(s) probably in MHNP (collection Chaudoir). Synonymy established by Schaum (1852: 143).

#### Distribution.

This species ranges from Long Island, New York (Notman 1928: 213; MCZ), to southern Florida (Peck and Thomas 1998: 20), west to eastern Texas along the Gulf Coast (Snow 1906a: 141; Galveston County, USNM); also recorded from northern Indiana (Blatchley 1910: 65; Schrock 1985: 352) and along the Ohio River in southwestern Ohio (Dury 1902: 111). The record from “Pennsylvania” (Bousquet and Larochelle 1993: 202) needs confirmation.

#### Records.

**USA**: AL, DE, FL, IN, LA, MD, MS, NC, NJ, NY, OH, TX, VA [PA]

### Panagaeus fasciatus

Say, 1823

Panagaeus fasciatus Say, 1823a: 70. Type locality: «P[ennsylvani]a» (neotype label). Neotype (♂), designated by Lindroth and Freitag (1969: 335), in MCZ [# 33074].

#### Distribution.

This species is found from Massachusetts (Harris in Scudder 1869: 264) and Connecticut (Krinsky and Oliver 2001: 136) to western South Dakota (Larsen and Purrington 2010: 571), including southern Ontario (Lindroth 1969a: 969), south to southeastern Texas (San Patricio County, CNC) and southern Florida (Peck and Thomas 1998: 20); also recorded from northeastern New Mexico (Fall and Cockerell 1907: 157).

#### Records.

**CAN**: ON **USA**: AL, AR, CT, DC, DE, FL, GA, IA, IL, IN, KS, KY, LA, MA, MD, MI, MN, MO, MS, NC, NE, NJ, NM, NY, OH, OK, PA, SC, SD, TN, TX, VA, WI, WV

### Panagaeus sallei

Chaudoir, 1862

Panagaeus sallei Chaudoir, 1862: 353. Type locality: «Aculzingo, Etat de Vera-Cruz, Mexique» (original citation). Syntype(s) [3 originally cited] in MHNP.
Panagaeus sallaei Bates, 1882a: 41. Unjustified emendation of *Panagaeus sallei* Chaudoir, 1862.

#### Distribution.

This species ranges from central and southern Arizona (Cochise, Santa Cruz, and Yavapai Counties, CMNH, CNC; LeConte 1879c: 60) and western and south-central Texas (Presidio and Bexar Counties, CMNH, MCZ; LeConte 1879c: 60), south at least to central Veracruz and the Federal District in Mexico (Ogueta 1966a: 12); also found in the Baja California Peninsula (Horn 1894: 307).

#### Records.

**USA**: AZ, TX – Mexico

### Genus Micrixys

LeConte, 1854

Eugnathus LeConte, 1853c: 375 [junior homonym of *Eugnathus* Schönherr, 1833]. Type species: *Panagaeus distinctus* Haldeman, 1852 by monotypy. Etymology. From the Greek *eu* (well, by extension large) and *gnathos* (jaw), probably alluding to the well-developed mandibles (“*mandibulae crassae dilatatae*”) of the adult [masculine].
Micrixys LeConte, 1854d: 220. Replacement name for *Eugnathus* LeConte, 1853. Etymology. From the Greek *micros* (small, little) and *ixys* (waist), probably alluding to the abrupt basal constriction of the pronotum (“*thorax postice sensim valde angustatus sub-pedunculatus*”, see LeConte 1853c: 375) of the adult [feminine].

#### Diversity.

Two species in temperate and tropical areas of the Nearctic (one species) and Neotropical (two species in Mexico) Regions.

#### Identification.

Van Dyke (1927b: 93) discussed the structural differences between the two species.

### Micrixys distincta

(Haldeman, 1852)

Panagaeus distinctus Haldeman, 1852: 373. Type locality: «Santa Fé [Santa Fe County, New Mexico]» (original citation). One possible syntype, a ♀ labeled “[dark green disc] / distinctus 3 [handwritten],” in MCZ (collection LeConte).

#### Distribution.

This species ranges from southwestern Tennessee (Hardeman County, CMNH) to southwestern Arizona (Maricopa County, USNM), north to southeastern Colorado (Michels et al. 2008; Las Animas County, Robert L. Davidson pers. comm. 2008) and northeastern Kansas (Riley County, CNC), south to the state of Guanajuato on the Mexican Plateau (Ball and Shpeley 1992a: 60). The record from southern Wisconsin (Rauterberg 1885: 12) is probably in error.

#### Records.

**USA**: AZ, CO, KS, NM, OK, TN, TX – Mexico

### Tribe Chlaeniini

Brullé, 1834

Chlaenides Brullé, 1834b: 123. Type genus: *Chlaenius* Bonelli, 1810. Note. 1. Under Article 11.7.2 of the Code, family-group names published in vernacular forms, such as this one, can be made available only if they were published before 1900, have been latinized by later authors, and have been generally accepted as valid and as dating from the first publication in vernacular form. These conditions are met since at least 1998 when Lorenz (1998: 308) used the name Chlaeniini Brullé, 1834 for this tribe. 2. The family-group name Callistidae Laporte, 1834 has been used as the valid name for this tribe by some authors. Tome IV of the *Histoire naturelle des insectes* authored by Brullé was issued in two livraisons and the first one (pages 1-240) was recorded on August 2 by the *Bibliographie de la France*. Laporte’s *Etudes entomologiques* was also issued in two livraisons and the first one (pages 1-94 + pls 1-2), in which the name Callistidae was made available, was recorded on August 9 by the *Bibliographie de la France*. Based on this evidence alone, Brullé’s name is retained here as the valid one for the tribe.

#### Diversity.

Worldwide, with about 980 species (Lorenz 2005: 328-342) arrayed in two subtribes: Callistina (77 afro-oriental and one Palaearctic species) and Chlaeniina (about 900 species).

### Subtribe Chlaeniina

Brullé, 1834

Chlaenides Brullé, 1834b: 123. Type genus: *Chlaenius* Bonelli, 1810.
Lissaucheniidae Gistel, 1848b: [2]. Type genus: *Lissauchenius* Macleay, 1825.
Rhopalomelini Alluaud, 1930: 105. Type genus: *Rhopalomelus* Boheman, 1848.
Chlaeniodini Jeannel, 1949a: 776, 777. Type genus: *Chlaeniodus* Jeannel, 1949.
Eccoptomenini Jeannel, 1949a: 776, 821. Type genus: *Eccoptomenus* Chaudoir, 1850.
Chlaenionini Jeannel, 1949a: 776. Type genus: *Chlaenionus* Kuntzen, 1913.
Procletini Basilewsky, 1950: 49. Type genus: *Procletus* Péringuey, 1896.
Pleroticini Basilewsky, 1950: 50. Type genus: *Pleroticus* Péringuey, 1896.
Callistoidini Basilewsky, 1950: 51. Type genus: *Callistoides* Motschulsky, 1864.
Harpaglossini Basilewsky, 1950: 52. Type genus: *Harpaglossus* Motschulsky, 1858.
Chlaenioctenini Basilewsky and Grundmann, 1955: 204. Type genus: *Chlaenioctenus* Bates, 1892.
Brachylobini Basilewsky and Grundmann, 1955: 204. Type genus: *Brachylobus* Chaudoir, 1876.
Leptodinodini Basilewsky and Grundmann, 1955: 205. Type genus: *Leptodinodes* Jeannel, 1949.

#### Diversity.

Worldwide, with about 900 species (Lorenz 2005: 328-341). The vast majority of species (about 81 % of the world fauna) are found in the Oriental and Afrotropical Regions.

### Genus Chlaenius

Bonelli, 1810

Chlaenius Bonelli, 1810: Tabula Synoptica. Type species: *Carabus festivus* Panzer, 1796 designated by Madge (1975: 581). Etymology. From the Greek *chlaena* (coat, blanket), possibly alluding to the pubescence on the elytra of the species in the hands of Bonelli [masculine]. Note. Madge (1975: 581) correctly pointed out that the first valid type species of *Chlaenius* Bonelli, 1810 is *Carabus spoliatus* Rossi, 1792 as designated by Hope (1838: 75). However, acceptance of Hope’s designation would seriously affect the current nomenclature in the genus and I agree with Madge that a request should be addressed to the International Commission on Zoological Nomenclature to set aside Hope’s designation and select that of *Carabus festivus* Panzer, 1796 as type species of *Chlaenius*.

#### Diversity.

Worldwide, with about 855 species (Lorenz 2005: 328-341) arrayed in 62 subgenera. The genus is mainly represented in the Afrotropical and Oriental Regions with about 650 species (approximately 76% of the world fauna). The North American fauna has 52 species (6% of the world fauna) in the boreal, temperate, and subtropical regions.

#### Identification.

Bell (1960) revised the North American species. Since the publication of this work, one Neotropical species was discovered in southern Texas (*Chlaenius azurescens*), the name of one species (*Chlaenius perplexus*) was replaced by an older one (*Chlaenius circumcinctus*), and two of Bell’s species (*Chlaenius cursor* and *Chlaenius herbaceus*) have been shown to be a complex of two species each and the species found in North America to be *Chlaenius sparsus* and *Chlaenius patruelis* respectively. A number of subspecies recognized by Bell (1960) are placed in synonymy in this catalogue.

### Subgenus Pseudanomoglossus

Bell, 1960

Pseudanomoglossus Bell, 1960: 101. Type species: *Chlaenius maxillosus* Horn, 1876 by original designation. Etymology. From the Greek *pseudos* (fallacy, lie) and the generic name *Anomoglossus* [*q.v*.] [masculine].

#### Diversity.

One species along the Coastal Plain of eastern North America.

### Chlaenius maxillosus

Horn, 1876

Chlaenius maxillosus G.H. Horn, 1876d: 260. Type locality: «Lake Harney and Haulover, Florida» (original citation), restricted to «Lake Harney [Volusia County]» by Bell (1960: 102). One syntype [2 ♀ originally cited] in MCZ [# 8004].

#### Distribution.

This species is found from southeastern Georgia (Fattig 1949: 44) to southern Florida (Bell 1960: 102), west to southeastern Texas (Aransas County, USNM); also recorded from South Bimini Island in the Bahamas (Darlington 1953: 8).

#### Records.

**USA**: AL, FL, GA, MS, TX – Bahamas

### Subgenus Eurydactylus

LaFerté-Sénectère, 1851

Eurydactylus LaFerté-Sénectère, 1851: 255. Type species: *Epomis tomentosus* Say, 1823 by monotypy. Etymology (original). From the Greek *eurys* (broad, wide) and *dactylos* (finger), alluding to the strong dilatation of the male protarsomeres (“*tarses antérieurs du mâle présentant une dilatation tout à fait exceptionnelle*”) [masculine].
Glyptoderus LaFerté-Sénectère, 1851: 260. Type species: *Glyptoderus aurolimbatus* LaFerté-Sénectère, 1851 by original designation. Synonymy established by Bell (1960: 102). Etymology. From the Greek *glyptos* (carved) and *dere* (neck, by extension pronotum) [masculine].

#### Diversity.

Seven species in the Nearctic (two species) and Neotropical (five species) Regions.

### Chlaenius pimalicus

Casey, 1914

Chlaenius pimalicus Casey, 1914: 38. Type locality: «southern Arizona» (original citation), herein restricted to Douglas, Cochise County (see Bell 1960: 103). Three syntypes [3 originally cited] in USNM [# 47702].

#### Distribution.

To date this species has been recorded only from southeastern Arizona (Bell 1960: 103).

#### Records.

**USA**: AZ

### Chlaenius tomentosus

(Say, 1823)

Epomis tomentosus Say, 1823a: 60. Type locality: «Penn[sylvania]» (neotype label), herein restricted to Pocono Lake, Monroe County (see Bell 1960: 104). Neotype (♀), designated by Lindroth and Freitag (1969: 350), in MCZ [# 32996]. Note. «Pennsylvania; the Missouri» were the areas originally cited by Say (1823a: 61).
Amara luctuosa Germar, 1824: 10. Type locality: «Kentucky» (original citation). Syntype(s) probably lost (Lindroth 1969a: 974). Synonymy established by LeConte (1847: 438).
Chlaenius amplus LeConte, 1856b: 29. Type locality: «Georgia» (original citation). One syntype [2 originally cited] in MCZ [# 5866]. Synonymy established by Horn (1876d: 276), confirmed by Bell (1960: 103).
Chlaenius zunianus Casey, 1914: 38. Type locality: «Fort Wingate [McKinley County], New Mexico» (original citation). Lectotype (♀), designated by Lindroth (1975: 144), in USNM [# 47718]. Synonymy established by Lindroth (1969a: 974).
Chlaenius tomentosus lacustrinus Casey, 1920: 298. Type locality: «Bayfield [Bayfield County, Wisconsin], Lake Superior» (original citation). Lectotype (♂), designated by Lindroth (1975: 144), in USNM [# 47717]. Synonymy established by Bell (1960: 103).

#### Distribution.

This species ranges from Maine to southern Alberta, south to southeastern Arizona, central Texas, and central Florida (Bell 1960: 104-105).

#### Records.

**CAN**: AB, MB, ON, QC, SK **USA**: AL, AR, AZ, CO, CT, DC, DE, FL, GA, IA, IL, IN, KS, KY, LA, MA, MD, ME, MI, MN, MO, MS, NC, ND, NE, NH, NJ, NM, NY, OH, OK, PA, RI, SC, SD, TN, TX, VA, VT, WI, WV

### Subgenus Anomoglossus

Chaudoir, 1857

Anomoglossus Chaudoir, 1857: 4. Type species: *Chlaenius emarginatus* Say, 1823 designated by Bell (1960: 105). Etymology (original). From the Greek *anomos* (abnormal) and *glossa* (tongue), alluding to the unusual shape of the ligula (“*ligula omnino abnormis*”), corresponding to the glossa and paraglossae or the glossal sclerite, of the adult [masculine].

#### Diversity.

Three species in eastern North America.

#### Taxonomic Note.

The generic name *Agreuter* Lepeletier and Audinet-Serville (in Latreille et al. 1828: 633) was proposed for two species, *Chlaenius chlorodius* Dejean, 1826 and *Chlaenius elegantulus* Dejean, 1826 (= *Chlaenius pusillus* Say, 1823). Bousquet (2002b: 6) designated *Chlaenius elegantulus* as type species and so the name is listed as a synonym of *Anomoglossus* Chaudoir (e.g., Lorenz 2005: 333). However, Duponchel (1840a: 196) had selected *Chlaenius chlorodius* as the type species and so *Agreuter* is a senior synonym of *Amblygenius* LaFerté-Sénectère, 1851.

### Chlaenius amoenus

Dejean, 1831

Chlaenius amoenus Dejean, 1831: 648. Type locality: «Amérique septentrionale» (original citation), restricted to «Georgia» by Bell (1960: 106), herein to Ellijay, Gilmer County (see Fattig 1949: 44). Two syntypes in MHNP (Lindroth 1955b: 26).

#### Distribution.

This species ranges from Virginia (Robert L. Davidson pers. comm. 1992) to northeastern Iowa (Purrington et al. 2002: 201), south to eastern Texas (Sabine County, CMNH), southeastern Louisiana (Colby 2002: 37), and northern Florida (Bell 1960: 106).

#### Records.

**USA**: AL, AR, FL, GA, IA, IL, LA, MO, MS, NC, SC, TN, TX, VA

### Chlaenius emarginatus

Say, 1823

Chlaenius emarginatus Say, 1823a: 63. Type locality: North America (inferred from title of the paper); restricted to «White Sulphur Springs [Greenbrier County], W[est] V[irgini]a» by Lindroth and Freitag (1969: 351). Lectotype (♂), designated by Lindroth and Freitag (1969: 351), in MHNP (collection Dejean).
Anomoglossus delectans Casey, 1914: 39. Type locality: «central New York» (original citation). Lectotype (♀), designated by Lindroth (1975: 144), in USNM [# 47719]. Synonymy established by Bell (1960: 106).
Anomoglossus gravis Casey, 1914: 40. Type locality: «Vicksburg [Warren County], Mississippi» (original citation). One syntype in USNM [# 47721]. Synonymy established by Bell (1960: 106).
Anomoglossus semotus Casey, 1920: 299. Type locality: «Lake Worth [Palm Beach County], Florida» (original citation). One syntype in USNM [# 47720]. Synonymy established by Bell (1960: 106).

#### Distribution.

This species ranges from Nova Scotia (Lindroth 1969a: 975) to eastern South Dakota (Kirk and Balsbaugh 1975: 36; French et al. 2004: 557), south to central Oklahoma and central Florida (Bell 1960: 107). Old specimens labeled “Tex” are also known (Bell 1960: 107).

#### Records.

**CAN**: NB, NS, ON, QC **USA**: AL, AR, CT, DC, DE, FL, GA, IA, IL, IN, KS, KY, LA, MA, MD, ME, MI, MN, MO, MS, NC, NE, NH, NJ, NY, OH, OK, PA, RI, SC, SD, TN, VA, VT, WI, WV [TX]

### Chlaenius pusillus

Say, 1823

Chlaenius pusillus Say, 1823a: 63. Type locality: «Frankl[i]nv[i]lle, P[ennsylvani]a» (neotype label). Neotype (♂), designated by Lindroth and Freitag (1969: 352), in MCZ [# 32977].
Chlaenius elegantulus Dejean, 1826: 367. Type locality: «Amérique septentrionale» (original citation). One syntype in MHNP (Lindroth 1955b: 26). Synonymy established by Say (1830b: (3)[3]), confirmed by Lindroth (1955b: 26).
Anomoglossus nanulus Casey, 1914: 41. Type locality: «Alexandria [Rapides Parish], Louisiana» (original citation). One syntype in USNM [# 47722]. Synonymy established by Bell (1960: 107).

#### Distribution.

This species ranges from southern Quebec (Saint-Clet, CNC) to southeastern Minnesota (Gandhi et al. 2005: 929), south to southern Texas and central Florida (Bell 1960: 108). At least one specimen labeled “Mass” is known (Bell 1960: 108).

#### Records.

**CAN**: ON QC **USA**: AL, AR, CT, DC, DE, GA, FL, IA, IL, IN, KS, KY, LA, MD, MI, MN, MO, MS, NC, NE, NJ, NY, OH, OK, PA, RI, SC, TN, TX, VA, WI [MA]

### Subgenus Chlaenius

Bonelli, 1810

Chlaenius Bonelli, 1810: Tabula Synoptica. Type species: *Carabus festivus* Panzer, 1796 designated by Madge (1975: 581).
Chaelinus Basilewsky and Grundmann, 1954: 242. Type species: *Carabus festivus* Panzer, 1796 by original designation. Etymology. Anagram of the generic name *Chlaenius* [*q.v*.] [masculine].
Chinelaus Basilewsky and Grundmann, 1954: 242. Type species: *Epomis pallipes* Gebler, 1823 by original designation. Etymology. Anagram of the generic name *Chlaenius* [*q.v*.] [masculine].
Merochlaenius Grundmann, 1955: 280. Type species: *Chlaenius aestivus* Say, 1823 by original designation. Synonymy established by Bousquet and Larochelle (1993: 204). Etymology. From the Greek *meros* (thigh, femur) and the generic name *Chlaenius* [*q.v*.] [masculine].
Pachychlaenius Grundmann, 1955: 282 (as *Pachychleanius*). Type species: *Chlaenius erythropus* Germar, 1824 by original designation. Synonymy established by Bousquet and Larochelle (1993: 204). Etymology. From the Greek *pachys* (thick) and the generic name *Chlaenius* [*q.v*.] [masculine].
Sericochlaenius Grundmann, 1955: 286. Type species: *Chlaenius cumatilis* LeConte, 1851 by original designation. Synonymy established by Bousquet and Larochelle (1993: 204). Etymology. From the Greek *sericon* (silk) and the generic name *Chlaenius* [*q.v*.] [masculine].

#### Diversity.

About 140 species in all zoogeographical regions except the Australian Region. The Nearctic Region is represented by 14 species.

### [aestivus group]

### Chlaenius aestivus

Say, 1823

Chlaenius aestivus Say, 1823a: 62. Type locality: «Rosslyn [Arlington County], V[irgini]a» (neotype label). Neotype (♂), designated by Lindroth and Freitag (1969: 351), in MCZ [# 32979].
Chlaenius cobaltinus Dejean, 1826: 331. Type locality: «Amérique septentrionale» (original citation). Two possible syntypes in MHNP (Lindroth 1955b: 25). Synonymy established by Brullé (1835c: 284).
Chlaenius congener LeConte, 1844: 51. Type locality: «Georgia» (original citation). Four syntypes in MCZ [# 5859]. Synonymy established by LeConte (1853c: 390).

#### Distribution.

This species ranges from Massachusetts (Bell 1960: 120) to east-central Iowa (Wickham 1888: 82), including southernmost Ontario (Lindroth 1969a: 981), south to southeastern Louisiana (Allen 1965: 75) and central Florida (Bell 1960: 120). The record from eastern Kansas (Popenoe 1877: 23) needs confirmation.

#### Records.

**CAN**: ON **USA**: AL, AR, CT, DC, DE, FL, GA, IA, IL, IN, KY, LA, MA, MD, MI, MO, MS, NC, NH, NJ, NY, OH, OK, PA, SC, TN, VA, WI [KS]

### Chlaenius augustus

Newman, 1838

Chlaenius augustus Newman, 1838a: 490. Type locality: «Wilmington [New Castle County], Delaware» (original citation). Syntype(s) probably lost.
Chlaenius lecontei Haldeman, 1843b: 304. Type locality: North America (inferred from title of the paper). Two probable syntypes, a ♀ labeled “[orange disc] / C. augustus Newm. Lecontei Hald. [handwritten]” and a ♂ labeled “[orange disc] / 355. [handwritten] / angustus 2 [handwritten],” in MCZ (collection LeConte). Synonymy established by LeConte (1847: 433).

#### Distribution.

This species ranges from New Jersey (Bell 1960: 121) to east-central South Carolina (Ciegler 2000: 80) and southwestern Alabama (Löding 1945: 23; Bell 1960: 121). The record from “Mississippi” (Bousquet and Larochelle 1993: 204) needs confirmation.

#### Records.

**USA**: AL, DE, GA, NC, NJ, SC, TN, VA [MS]

### Chlaenius azurescens

Chaudoir, 1876

Chlaenius azurescens Chaudoir, 1876b: 220. Type locality: «Orizaba [Veracruz]» (original citation). Syntype(s) in MHNP.

#### Distribution.

This species ranges from southern Texas (Johnson 1978: 68) to Veracruz (Davidson 1980: 88) along the east coast of Mexico.

#### Records.

**USA**: TX – Mexico

### Chlaenius platyderus

Chaudoir, 1856

Chlaenius diffinis Chaudoir, 1856: 279 [primary homonym of *Chlaenius diffinis* LaFerté-Sénectère, 1851]. Type locality: «midi des Etats-Unis» (original citation), herein restricted to Willard, Greene County, Missouri (see Bell 1960: 122). Syntype(s) in MHNP.
Chlaenius platyderus Chaudoir, 1856: 280. Type locality: «Louisiane» (original citation). Holotype [by monotypy] in MHNP. Synonymy established by Chaudoir (1876b: 218), confirmed by Bell (1960: 122).
Chlaenius kuntzeni Csiki, 1931: 958. Replacement name for *Chlaenius diffinis* Chaudoir, 1856.

#### Distribution.

This species ranges from “Massachusetts” to central North Dakota (Bell 1960: 122), north to southeastern Manitoba (Ryan and Holliday 2006: 414), south to northeastern New Mexico, central Texas, northwestern Georgia (Fattig 1949: 44) along the Appalachians, and Long Island, New York, along the east coast (Bell 1960: 122).

#### Records.

**CAN**: MB **USA**: AR, CO, CT, GA, IA, IL, IN, KS, KY, LA, MA, MD, MI, MN, MO, MS, NC, ND, NE, NJ, NM, NY, OH, OK, PA, RI, SD, TN, TX, VA, WI, WV

### Chlaenius viduus

Horn, 1871

Chlaenius viduus G.H. Horn, 1871: 325. Type locality: «Missouri probably near S[ain]t Louis» (original citation). Holotype [by monotypy] (♀) in MCZ [# 34530].

#### Distribution.

This rarely collected species is known from Arkansas, southern Missouri (Bell 1960: 121), and eastern Oklahoma (Latimer County, UASM). Specimens labeled «S[outh] Ill[inois]» are known (Bell 1960: 121).

#### Records.

**USA**: AR, MO, OK [IL]

### [cursor group]

### Chlaenius chaudoiri

Horn, 1876

Chlaenius chaudoiri G.H. Horn, 1876d: 270. Type locality: «Texas; Tamaulipas, Mexico» (original citation), restricted to «Texas» by Bell (1960: 118), herein to Palmetto State Park, Gonzalez County (see Bell 1960: 118). Two syntypes [2 originally cited] in MCZ [# 8001]. Etymology. The specific name honors Maximilien Stanislavovitch Baron de Chaudoir [1816-1881]. Born in Ukraine and wealthy by inheritance, Chaudoir devoted his life to the study of Carabidae. His work includes many revisions of highly diverse, world-wide genera.

#### Distribution.

This species is known from southeastern Arizona to central Texas (Bell 1960: 118), south to Durango and San Luis Potosí in Mexico (Davidson 1980: 133).

#### Records.

**USA**: AZ, NM, TX – Mexico

### Chlaenius cumatilis

LeConte, 1851

Chlaenius cumatilis LeConte, 1851: 179. Type locality: «San Diego, et S[an]ta Isabel [California]» (original citation), restricted to «San Diego [San Diego County]» by Bell (1960: 117). Six syntypes in MCZ [# 72].
Chlaenius cumatilis sparsellus Casey, 1914: 37. Type locality: «Arizona» (original citation). Two syntypes in USNM [# 47703]. Synonymy established by Bell (1960: 117).

#### Distribution.

This species is known from Sonora (Davidson 1980: 129), southern Arizona, southern California (Bell 1960: 117), and the Baja California Peninsula (Horn 1894: 311).

#### Records.

**USA**: AZ, CA (CHI) – Mexico

### Chlaenius patruelis

LeConte, 1844

Chlaenius patruelis LeConte, 1844: 51. Type locality: «Georgia» (original citation), herein restricted to Saint Simons Island, Glynn County (see Fattig 1949: 44). Two syntypes in MCZ [# 5855].

#### Distribution.

This species is known from southern Alabama, southern Georgia, and throughout Florida (Bell 1960: 118). A specimen labeled “N. Car.” is known (Bell 1960: 118).

#### Records.

**USA**: AL, FL, GA [NC]

#### Note.

This species was known for a long time under the name *Chlaenius herbaceus* Chevrolat, 1834 (see Bell 1960: 117) until Bell (1966a) showed that *Chlaenius patruelis* and *Chlaenius herbaceus* were two distinct species. *Chlaenius herbaceus* is known only from Veracruz and Campeche in Mexico (Davidson 1980: 125).

### Chlaenius sparsus

LeConte, 1863

Chlaenius sparsus LeConte, 1863c: 12. Type locality: «Cape San Lucas, Lower California [Baja California Sur]» (original citation). Four syntypes in MCZ [# 5857].

#### Distribution.

This species is known from the Baja California Peninsula and from southern Arizona south, through central and western Mexico, to Central America (Robert L. Davidson pers. comm. 2007).

#### Records.

**USA**: AZ – Mexico

#### Note.

Horn (1876d: 275) listed this name as a junior synonym of *Chlaenius cursor* Chevrolat, 1835 described from “Orixaba” in Veracruz. However, both names apply to distinct species (Robert L. Davidson pers. comm. 2007).

### [laticollis group]

### Chlaenius erythropus

Germar, 1824

Chlaenius erythropus Germar, 1824: 11. Type locality: «America septentrionali» (original citation), restricted to «Mobile [Mobile County], Alab[ama]» by Lindroth (1969a: 982). Lectotype (♂), designated by Lindroth (1969a: 982), in ZMHB.
Chlaenius rufilabris Dejean, 1826: 329. Type locality: «Louisiane; Géorgie» (original citation). Syntype(s) [2 originally cited] in MHNP. Synonymy established with doubt by Dejean (1826: 329), confirmed by Chaudoir (1856: 278).
Chlaenius umbritarsis Casey, 1920: 291. Type locality: «Louisiana» (original citation). One syntype in USNM [# 47698]. Synonymy established by Bell (1960: 130).
Chlaenius oblongipennis Casey, 1920: 292. Type locality: «Lake Worth [Palm Beach County], Florida» (original citation). One syntype in USNM [# 47699]. Synonymy established by Bell (1960: 130).

#### Distribution.

This species ranges from New Jersey (Bell 1960: 131) to eastern South Dakota (Kirk and Balsbaugh 1975: 36), including southernmost Ontario (Lindroth 1969a: 982), south to northeastern New Mexico (Union County, CNC), southeastern Texas, and southern Florida (Bell 1960: 131).

#### Records.

**CAN**: ON **USA**: AL, AR, CT, DC, FL, GA, IA, IL, IN, KS, KY, LA, MI, MN, MO, MS, NC, NE, NJ, NM, OH, OK, PA, SC, SD, TN, TX, VA, WI

### Chlaenius fuscicornis

Dejean, 1831

Chlaenius fuscicornis Dejean, 1831: 647. Type locality: «Amérique septentrionale» (original citation), herein restricted to New Orleans, Orleans Parish, Louisiana (see Bell 1960: 130). One syntype in MHNP (Lindroth 1955b: 25).

#### Distribution.

This species is found in southeastern United States from South Carolina (Kirk 1970: 16; Ciegler 2000: 80) to northern Arkansas, south to southeastern Texas and southeastern Georgia (Bell 1960: 130). The records from Illinois (Leng and Beutenmüller 1893: 143; Blatchley 1910: 166), “Iowa” (Jaques and Redlinger 1946: 296), Kansas (Popenoe 1877: 23; Snow 1903: 193), “Oklahoma” (Arnold 2008), and Missouri (Summers 1873: 145) need confirmation; that from southeastern Pennsylvania (Rathvon 1869: 525) must be in error.

#### Records.

**USA**: AL, AR, GA, LA, MS, SC, TN, TX [IA, IL, KS, MO, OK]

### Chlaenius laticollis

Say, 1823

Chlaenius laticollis Say, 1823a: 64. Type locality: «Tonganoxie [Leavenworth County], Ka[nsas]» (neotype label). Neotype (♀), designated by Lindroth and Freitag (1969: 351), in MCZ [# 32978]. Note. «the Missouri» was the area originally cited by Say (1823a: 64).
Chlaenius rufipes Dejean, 1826: 331. Type locality: «Caroline» (original citation). One syntype in MHNP (Lindroth 1955b: 25). Synonymy established by LaFerté-Sénectère (1851: 252), confirmed by Lindroth (1955b: 25).
Chlaenius brevicollis LeConte, 1847: 432 [secondary homonym of *Chlaenius brevicollis* (Chaudoir, 1843)]. Type locality: «Georgia» (original citation). One syntype in MCZ [# 5858]. Synonymy established, under the name *Chlaenius rufipes* Dejean, by LeConte (1863b: 10).
Chlaenius brachyderus Chaudoir, 1856: 279. Type locality: «Louisiane» (original citation). Syntype(s) in MHNP. Synonymy established with the name *Chlaenius brevicollis* LeConte by Chaudoir (1856: 279).

#### Distribution.

This species ranges from southern Maine (Majka et al. 2011: 47) to southeastern Nebraska (Nemaha County, Foster F. Purrington pers. comm. 2012), including southernmost Ontario (Bousquet 1987a: 132), south to Durango and Nuevo León (Davidson 1980: 92) in Mexico and southern Florida (Bell 1960: 129), west along southern United States to southeastern Arizona (Dajoz 2004: 116). The record from Tabasco in Mexico (Davidson 1980: 92) is suspect; that from southern Wisconsin (Rauterberg 1885: 18) needs confirmation.

#### Records.

**CAN**: ON **USA**: AL, AR, AZ, CT, DC, DE, FL, GA, IA, IL, IN, KS, KY, LA, MA, MD, ME, MI, MO, MS, NC, NH, NJ, NM, NY, OH, OK, PA, SC, TN, TX, VA, WV [WI] – Mexico

### Chlaenius orbus

Horn, 1871

Chlaenius orbus G.H. Horn, 1871: 326. Type locality: «central and western Texas» (original citation), herein restricted to Temple, Bell County (see Bell 1960: 128). Syntype(s) in MCZ [# 34529] and IZWP [# 1319] (Mroczkowski 1960: 379).

#### Distribution.

This species ranges from southeastern Arizona to southeastern Texas, south to Oaxaca in Mexico (Bell 1960: 128); also recorded from San Bernardino County in southeastern California (Dajoz 2007: 20).

#### Records.

**USA**: AZ, CA, NM, TX – Mexico

### [sericeus group]

### Chlaenius sericeus

(Forster, 1771)

Carabus sericeus Forster, 1771: 58. Type locality: «America septentrionali» (original citation), restricted to «W[est] Roxbury [Suffolk County], Mass[achusetts]» by Lindroth (1969a: 982). Syntype(s) probably lost (Lindroth 1969a: 982).
Carabus forsteri Turton, 1802: 464. Unnecessary replacement name for *Carabus sericeus* Forster, 1771. Etymology. The specific name was proposed for Johann Reinhold Forster [1729-1798], an English Lutheran pastor who served as naturalist on James Cook’s second circumnavigation of the globe on the *Resolution*. He was the father of the naturalist Georg Adam Forster [1754-1794] who often travelled with him.
Chlaenius viridifrons Eschscholtz, 1833: 27. Type locality: «bei St. Franzisco [San Francisco County], Californien» (original citation). Syntype(s) location unknown (possibly in ZMMU). **New synonymy**.
Chlaenius perviridis LeConte, 1847: 434. Type locality: «ad Rocky Mountains» (original citation). Holotype [by monotypy] (♂) in MCZ [# 5856]. Synonymy established by LeConte (1856b: 27) and Chaudoir (1856: 286).
Chlaenius regularis LeConte, 1851: 179. Type locality: «ad flumina Colorado et Gila» (original citation). One syntype in MCZ [# 73]. Synonymy established, under the name *Chlaenius sericeus* var. *perviridis* LeConte, by Chaudoir (1876b: 218).
Chlaenius regularis apacheanus Casey, 1914: 34. Type locality: «Arizona (southwestern) and the adjacent parts of California» (original citation). Five syntypes [5 originally cited] in USNM [# 47700]. Synonymy established, under the name *Chlaenius sericeus regularis* LeConte, by Bell (1960: 126).
Chlaenius sericeus uteanus Casey, 1914: 35. Type locality: «Provo [Utah County], Utah» (original citation). Lectotype (♂), designated by Lindroth (1975: 144), in USNM [# 47701]. Synonymy established by Bell (1960: 123).
Chlaenius sierricola Casey, 1914: 36. Type locality: «Mokelumne Hill, Calaveras Co[unty], California» (original citation). One syntype in USNM [# 47711]. **New synonymy**.

#### Distribution.

This transamerican species ranges from Newfoundland (Lindroth 1955a: 135) to southwestern British Columbia (Lindroth 1969a: 983), south to southern California (Bell 1960: 125, 127), Chihuahua (Davidson 1980: 84) and northern Sonora (Bates 1884: 267, as *Chlaenius regularis*) in Mexico, and central Florida (Peck and Thomas 1998: 20).

#### Records.

**CAN**: AB, BC, MB, NB, NF, NS (CBI), ON, PE, QC, SK **USA**: AL, AR, AZ, CA, CO, CT, DC, DE, FL, GA, IA, ID, IL, IN, KS, KY, LA, MA, MD, ME, MI, MN, MO, MS, MT, NC, ND, NE, NH, NJ, NM, NV, NY, OH, OK, OR, PA, RI, SC, SD, TN, TX, UT, VA, VT, WA, WI, WV, WY – Mexico

#### Note.

Bell (1960: 123-128) recognized four subspecies within *Chlaenius sericeus*: *sericeus* s.str., *regularis*, *viridifrons*, and *sierricola*, all separated by color and relatively minor differences in the pronotum shape.

### Subgenus Lithochlaenius

Kryzhanovskij, 1976

Hemichlaenius Lutshnik, 1933b: 169 [junior homonym of *Hemichlaenius* Bates, 1892]. Type species: *Chlaenius rambouseki* Lutshnik, 1933 by monotypy. Etymology. From the Greek prefix *hemi*- (half) and the generic name *Chlaenius* [*q.v*.] [masculine].
Lithochlaenius Kryzhanovskij, 1976b: 9, 12. Replacement name for *Hemichlaenius* Lutshnik, 1933. Etymology. From the Greek *lithos* (stone) and the generic name *Chlaenius* [*q.v*.] [masculine].
Agilochlaenius Kirschenhofer, 1997: 116. Type species: *Chlaenius latro* LaFerté-Sénectère, 1851 by original designation. Synonymy established by Kirschenhofer (2000: 58). Etymology. From the Latin *agilis* (quick, nimble) and the generic name *Chlaenius* [*q.v*.] [masculine].

#### Diversity.

Nineteen species can be assigned to this subgenus: five in North America (Bell 1960: 109, as *solitarius* group), six in the Neotropical Region (*Chlaenius argentinicus* Jedlička, *Chlaenius chlorochrous* Chaudoir, *Chlaenius ecuadoricus* Jedlička, *Chlaenius leucoscelis* Chevrolat, *Chlaenius peruanus* Erichson, and *Chlaenius purpureus* Chaudoir), and ten in the Palaearctic Region (see Liu et al. 2011), of which one extends into the Oriental Region.

#### Taxonomic Note.

Liu et al. (2011: 22) argued that it is premature to place any of the Western Hemisphere species of *Chlaenius* in the subgenus *Lithochlaenius* because they differ from the Palaearctic species of *Lithochlaenius* in having the lateral edge of elytron rounded at level of the humerus, instead of being angulate. However, I agree with Robert L. Davidson (pers. comm.) that the Western Hemisphere species related to *Chlaenius prasinus* are closely related to the Palaearctic species of *Lithochlaenius* and at this time I prefer to include these species in the subgenus *Lithochlaenius*.

### Chlaenius cordicollis

Kirby, 1837

Chlaenius cordicollis Kirby, 1837: 22. Type locality: «Canada» (original citation), restricted to «S[outh] Cajuga, Ont[ario]» by Lindroth (1969a: 979). Two syntypes in BMNH (Lindroth 1953b: 169).

#### Distribution.

This species ranges from New Brunswick (Restigouche and Queens Counties, CNC) to southern Manitoba (Lindroth 1969a: 981), south to northwestern Arkansas (Bell 1960: 112), northwestern Mississippi (Coahoma County, Drew A. Hildebrandt pers. comm. 2008), and North Carolina (Robert L. Davidson pers. comm. 1992). The record from southwestern Colorado (Elias 1987: 634) needs confirmation.

#### Records.

**CAN**: MB, NB, ON, QC **USA**: AR, CT, DC, DE, IA, IL, IN, KY, MA, MD, ME, MI, MN, MO, MS, NC, ND, NH, NJ, NY, OH, PA, SD, TN, VA, VT, WI, WV [CO]

### Chlaenius leucoscelis monachus

LeConte, 1851

Chlaenius monachus LeConte, 1851: 180. Type locality: «valle fluminis Gilae» (original citation), herein restricted to Phoenix, Maricopa County, Arizona (see Bell 1960: 114). Holotype [by monotypy] (♂) in MCZ [# 75].
Chlaenius gilensis Casey, 1914: 35. Type locality: «Yuma [Yuma County], Arizona» (original citation). Two syntypes [2 originally cited] in USNM [# 47708]. Synonymy established by Bell (1960: 114).

#### Distribution.

This subspecies is found in the drainage basin of the Colorado River in southwestern Colorado, southern Utah, Arizona, and southeastern California (Bell 1960: 114).

#### Records.

**USA**: AZ, CA, CO, UT

### Chlaenius leucoscelis sanantonialis

Casey, 1914

Chlaenius sanantonialis Casey, 1914: 36. Type locality: «Texas» (original citation), herein restricted to Hot Springs (Big Bend National Park), Brewster County (see Bell 1960: 115). Two syntypes [2 ♂ originally cited] in USNM [# 47707].

#### Distribution.

This subspecies ranges from central Colorado to southernmost New Mexico and central Texas (Bell 1960: 115), including northwestern Oklahoma (Cimarron County, CMNH, CNC).

#### Records.

**USA**: CO, NM, OK, TX

### Chlaenius leucoscelis sonomae

Casey, 1920

Chlaenius sonomae Casey, 1920: 294. Type locality: «north of San Francisco, California» (original citation). Ten syntypes [10 originally cited] in USNM [# 47705].
Chlaenius recticollis Casey, 1920: 294. Type locality: «California» (original citation). One syntype in USNM [# 47706]. Synonymy established by Bell (1960: 114).

#### Distribution.

This subspecies is known from southwestern Oregon (Hatch 1953: 163, as *Chlaenius leucoscelis*) and through much of California as far south as Kern County (Bell 1960: 114).

#### Records.

**USA**: CA, OR

### Chlaenius prasinus

Dejean, 1826

Chlaenius prasinus Dejean, 1826: 345. Type locality: «Amérique septentrionale» (original citation), herein restricted to Vicksburg, Warren County, Mississippi (see Casey 1920: 292, as *Chlaenius regestus*). One syntype in MHNP (Lindroth 1955b: 25).
Chlaenius smaragdinus Chaudoir, 1843b: 755. Type locality: «près de la Nouvelle-Orléans [Orleans Parish, Louisiana]» (original citation). Syntype(s) in MHNP. Synonymy established by Melsheimer (1853: 13).
Chlaenius regestus Casey, 1920: 292. Type locality: «Vicksburg [Warren County], Mississippi» (original citation). One syntype in USNM [# 47704]. Synonymy established by Bell (1960: 110).

#### Distribution.

This species ranges from Massachusetts to “Colorado” (Horn 1876d: 267), including southern Michigan and “Minnesota” (Gandhi et al. 2005: 929), south at least to central Texas and southern Florida (Bell 1960: 110-111).

#### Records.

**USA**: AL, AR, CO, CT, DC, FL, GA, IA, IL, IN, KS, KY, LA, MA, MD, MI, MN, MO, MS, NC, NE, NJ, NY, OH, OK, PA, SC, TN, TX, VA, WI, WV

### Chlaenius purpureus

Chaudoir, 1876

Chlaenius purpureus Chaudoir, 1876b: 246. Type locality: «Orizaba [Veracruz], Mexique» (original citation). Holotype [by monotypy] (♂) in MHNP.

#### Distribution.

This species ranges from central Missouri (Morgan County, CMNH) to Arizona (Bell 1960: 110), south to Nicaragua (Bates 1882a: 45); also found along the Baja California Peninsula (Davidson 1980: 69).

#### Records.

**USA**: AZ, KS, MO, NM, TX – Guatemala, Mexico, Nicaragua

### Chlaenius solitarius

Say, 1823

Chlaenius solitarius Say, 1823a: 65. Type locality: «Opposite Dubuque [Dubuque County], Iowa» (neotype label). Neotype (♂), designated by Lindroth and Freitag (1969: 351), in MCZ [# 32991]. Note. «on the Missouri» was the area originally cited by Say (1823a: 66).
Chlaenius chlorophanus Dejean, 1831: 662. Type locality: «Amérique septentrionale» (original citation). Holotype [by monotypy] (♂) in MHNP (Lindroth 1955b: 25). Synonymy established by LeConte (1867a: 347), confirmed by Lindroth (1955b: 25).

#### Distribution.

This species inhabits the Great Plains and the Great Central Plains from southern Alberta to southwestern Pennsylvania (Allegheny and Westmoreland Counties, Robert L. Davidson pers. comm. 2008), south to northern Mississippi (Drew A. Hildebrandt pers. comm. 2007), east-central Louisiana (West Feliciana Parish, Igor M. Sokolov pers. comm. 2009), central Texas, and central New Mexico (Bell 1960: 111). The records from southwestern Ohio (Dury 1906: 257) and “Utah” (Bousquet and Larochelle 1993: 206) need confirmation. The record from “Georgia” (J.E. LeConte 1849: 26) is probably in error; that from Beaver Islands in Michigan (Hatch 1925: 553) was based on misidentified specimens of *Chlaenius cordicollis* (Dunn 1985a: 10). The specimen labeled from northeastern Florida (see Bell 1960: 111) is probably mislabeled (Robert L. Davidson pers. comm. 1992). Old specimens simply labeled from Michigan and Minnesota are known (Bell 1960: 111).

#### Records.

**CAN**: AB **USA**: AR, CO, IA, IL, IN, KS, KY, LA, MO, MS, MT, ND, NE, NM, OK, PA, SD, TN, TX, WI, WY [MI, MN, OH, UT]

### Subgenus Chlaeniellus

Reitter, 1908

Chlaeniellus Reitter, 1908: 185. Type species: *Carabus vestitus* Paykull, 1790 designated by Jeannel (1942: 971). Etymology. From the generic name *Chlaenius* [*q.v*.] and the Latin suffix -*ellus* (small, little) [masculine].
Agrochlaenius Basilewsky and Grundmann, 1954: 240. Type species: *Buprestis variegatus* Geoffroy, 1785 (= *Chlaenius olivieri* Crotch, 1870) by original designation. Etymology. From the Greek *agros* (field) and the generic name *Chlaenius* [*q.v*.] [masculine]. Note. This name was first proposed by Lutshnik (1933b: 171). However, because he failed to designate a type species, the name cannot be attributed from this publication (ICZN 1999: Article 13.3).

#### Diversity.

About 80 species in all zoogeographical regions except the Australian Region. The subgenus is best represented in the Northern Hemisphere with 58 species (about 73.5% of the world fauna) of which 17 are found in North America.

### [circumcinctus group]

### Chlaenius circumcinctus

Say, 1830

Chlaenius circumcinctus Say, 1830b: (3) [3]. Type locality: «L[ouisian]a» (neotype label), herein restricted to New Orleans, Orleans Parish (see Chaudoir, 1843b: 753, as *Chlaenius virens*). Neotype (♂), designated by Lindroth and Freitag (1969: 351), in MCZ [# 32994]. Note. «Louisiana» was the area originally cited by Say (1830b: (3) [3]).
Chlaenius perplexus Dejean, 1831: 655. Type locality: «Sénégal» (original citation), which is incorrect. Holotype [by monotypy] (♂) in MHNP (Lindroth 1955b: 25). Synonymy established by Horn (1876d: 276), confirmed by Lindroth (1955b: 25).
Chlaenius virens Chaudoir, 1843b: 753. Type locality: «Nouvelle-Orléans [Orleans Parish, Louisiana]» (original citation). Syntype(s) in MHNP. Synonymy established with doubt by LeConte (1847: 440), confirmed by Chaudoir (1856: 284).
Chlaenius poeyi Chevrolat, 1863: 194. Type locality: «Cuba» (original citation). Syntype(s) location unknown (possibly in UMO). Synonymy established, under the name *Chlaenius perplexus* Dejean, by Chaudoir (1876b: 237). Etymology. The specific name was proposed for the Cuban ichthyologist and entomologist Felipe Poey y Aloy [1799-1891]. Poey went to Paris and studied under Cuvier. In 1842 he was appointed professor and eventually director of the Zoological Museum in Havana.

#### Distribution.

This species ranges from southern Georgia (Fattig 1949: 43) and throughout Florida west along the Gulf Coast to southeastern Texas, north to north-central Oklahoma (Alfalfa County, CMNH) and central Arkansas (Pulaski County, Robert L. Davidson pers. comm. 2012), south at least to Veracruz along the Gulf Coast (Bell 1960: 146, as *Chlaenius perplexus* Dejean); also known from Cuba and Puerto Rico (Bell 1960: 146, as *Chlaenius perplexus* Dejean) and the Dominican Republic (CMNH).

#### Records.

**USA**: AL, AR, FL, GA, LA, MS, OK, TX – Cuba, Dominica Republic, Mexico, Puerto Rico

#### Note.

This species has been known for a long time under the name *Chlaenius perplexus* Dejean, 1831 but Say’s name has priority (see Bousquet 1993: 6).

### [glaucus group]

### Chlaenius glaucus

LeConte, 1856

Chlaenius glaucus LeConte, 1856b [25 March]: 28. Type locality: «Colorado river, near the junction of the Gila» (original citation). Syntype(s) in MCZ [# 5862].
Chlaenius sericinitens Chaudoir, 1856 [after 25 November]: 284. Type locality: «Rio Colorado, Californie» (original citation). Holotype [by monotypy] (♀) in MHNP. Synonymy established by LeConte (1863b: 11).

#### Distribution.

This species is found from central California (Bell 1960: 144) to western Texas (Jeff Davis, Ken Karns pers. comm. 2009), south to northern Sonora (Bates 1884: 267).

#### Records.

**USA**: AZ, CA, NM, TX – Mexico

### Chlaenius simillimus

Chaudoir, 1856

Chlaenius simillimus Chaudoir, 1856: 283. Type locality: «près de S[an] Francisco, Californie» (original citation). Syntype(s) in MHNP.

#### Distribution.

This species is known only from a few specimens collected in the lower Sacramento River Valley of northwestern California (Bell 1960: 145).

#### Records.

**USA**: CA

### [impunctifrons group]

### Chlaenius impunctifrons

Say, 1823

Chlaenius impunctifrons Say, 1823a: 64. Type locality: «Dorchester [Suffolk County], Ma[ssachusetts]» (neotype label). Neotype (♂), designated by Lindroth and Freitag (1969: 350), in MCZ [# 32995].

#### Distribution.

This species ranges from New Brunswick (Webster and Bousquet 2008: 21) to North Dakota (Tinerella 2003: 635), north to southern Manitoba (Lindroth 1969a: 984), south to eastern Texas (Houston County, CNC; Riley 2011) and southern Florida (Bell 1960: 137). The records from “Wyoming” and “Colorado” (Bousquet and Larochelle 1993: 207) are likely in error.

#### Records.

**CAN**: MB, NB, ON, QC **USA**: AL, AR, CT, DC, DE, FL, GA, IA, IL, IN, KS, KY, LA, MA, MD, ME, MI, MN, MO, MS, NC, ND, NE, NH, NJ, NY, OH, OK, PA, RI, SC, SD, TN, TX, VA, VT, WI, WV

#### Note.

The name *Chlaenius indutus* LaFerté-Sénectère, 1851 is often listed as a junior synonym of this species (e.g., Bell 1960: 136; Lindroth 1969a: 983). However LaFerté-Sénectère never provided a description of the taxon and the name is a *nomen nudum*.

### [nemoralis group]

### Chlaenius brevilabris

LeConte, 1847

Chlaenius brevilabris LeConte, 1847: 437. Type locality: «ad Insulam Longam NovEboraci [= Long Island, New York]» (original citation). Three syntypes in MCZ [# 5864].
Chlaenius consimilis LeConte, 1847: 437 [*nomen dubium*]. Type locality: «ad Rocky Mountains» (original citation). Holotype [by monotypy] (♂) in MCZ [# 5865]. Synonymy established by Horn (1876d: 276). Note. According to Bell (1960: 143), the holotype of *Chlaenius consimilis* is a deformed specimen that could be instead an abnormal *Chlaenius tricolor*.
Chlaenius texanellus Casey, 1914: 37. Type locality: «Galveston [Galveston County], Texas» (original citation). Nineteen syntypes in USNM [# 47712]. **New synonymy**. Note. Bell (1960: 143) retained this name for a subspecies of *Chlaenius brevilabris* based on different forebody metallic lustre; I believe the form does not warrant subspecific status.

#### Distribution.

This species ranges from south-central New Hampshire (Merrimack County, Ross T. Bell pers. comm. 2008) to eastern South Dakota (Kirk and Balsbaugh 1975: 35), including southern Ontario (Lindroth 1969a: 987), south to southern Texas (Bell 1960: 143) and southwestern South Carolina (Hampton County, CMNH). The records from “Utah” (Bousquet and Larochelle 1993: 207) and New Mexico (Fall and Cockerell 1907: 160) need confirmation.

#### Records.

**CAN**: ON **USA**: AL, AR, CT, DC, IA, IL, IN, KS, KY, LA, MD, MI, MN, MO, MS, NE, NH, NJ, NY, OH, OK, PA, SC, SD, TN, TX, VA, VT, WI, WV [NM, UT]

### Chlaenius nemoralis

Say, 1823

Chlaenius nemoralis Say, 1823a: 65. Type locality: «Winter Park [Orange County], Fl[orid]a» (neotype label). Neotype (♂), designated by Lindroth and Freitag (1969: 351), in MCZ [# 32992]. Note. «Pennsylvania; Georgia and Florida» were the areas originally cited by Say (1823a: 65).
Chlaenius longicollis Chaudoir, 1843b: 752. Type locality: «Nouvelle Orléans [Orleans Parish, Louisiana]» (original citation). Holotype [by monotypy] (♀) in MHNP. Synonymy established by Chaudoir (1876b: 241).

#### Distribution.

This species ranges from Massachusetts (Easton 1909: 37) to northeastern South Dakota (Kirk and Balsbaugh 1975: 36), including southern Ontario (Lindroth 1969a: 984), south to central Texas and southern Florida (Bell 1960: 140). The record from “New Hampshire” (Bousquet and Larochelle 1993: 208) needs confirmation.

#### Records.

**CAN**: ON **USA**: AL, AR, CT, DC, DE, FL, GA, IA, IL, IN, KS, KY, LA, MA, MD, MI, MN, MO, MS, NC, ND, NE, NJ, NY, OH, OK, ON, PA, RI, SC, SD, TN, TX, VA, WI, WV [NH]

### Chlaenius oxygonus

Chaudoir, 1843

Chlaenius oxygonus Chaudoir, 1843b: 753. Type locality: «Nouvelle-Orléans [Orleans Parish, Louisiana]» (original citation). Holotype [by monotypy] (♂) in MHNP.

#### Distribution.

This species occurs from northwestern Georgia and southwestern South Carolina (Hampton County, CMNH) to central Florida, west to eastern Texas, north to central Oklahoma (Grady County, CMNH) and east-central Arkansas (Bell 1960: 141).

#### Records.

**USA**: AL, AR, FL, GA, LA, MS, OK, SC, TX

### Chlaenius tricolor tricolor

Dejean, 1826

Chlaenius tricolor Dejean, 1826: 334. Type locality: «Géorgie» (original citation), herein restricted to Atlanta, Fulton County (see Bell 1960: 141). One syntype in MHNP (Lindroth 1955b: 25).
Chlaenius quadricollis Kirby, 1837: 22. Type locality: «Canada» (original citation). One syntype in BMNH (Lindroth 1953b: 169). Synonymy established with doubt by Chaudoir (1856: 283), confirmed by Lindroth (1953b: 169).
Chlaenius atripennis LeConte, 1847: 436. Type locality: «provinciis mediis» (original citation). Syntype(s) in MCZ [# 5863]. Synonymy established by LeConte (1853c: 390), confirmed by Lindroth (1969a: 986).

#### Distribution.

This subspecies ranges from Newfoundland (Lindroth 1955a: 134) to northwestern Montana (Russell 1968: 66), south to central New Mexico (Fall and Cockerell 1907: 160), central Texas, northwestern Georgia, and northern South Carolina (Bell 1960: 142).

#### Records.

**CAN**: AB, NB, NF, NS, ON, QC, SK **USA**: AL, AR, CO, CT, DC, DE, GA, IA, IL, IN, KS, KY, LA, MA, MD, ME, MI, MN, MO, MS, MT, NC, ND, NE, NH, NJ, NM, NY, OH, OK, PA, RI, SC, SD, TN, TX, VA, VT, WI, WV

#### Note.

Bell (1960: 142) noted the presence of intergrade specimens between the two forms of *Chlaenius tricolor* in the area immediately west of the Rocky Mountains.

### Chlaenius tricolor vigilans

Say, 1830

Chlaenius vigilans Say, 1830b: (3) [3]. Type locality: «Mexico» (original citation). Syntype(s) lost.
Chlaenius chalybeipennis Chevrolat, 1835a: [no. 72]. Type locality: «[apparently] environs de la Véra-Cruz [Mexico]» (original citation). Syntype(s) location unknown (possibly in UMO). Synonymy established by Chaudoir (1876b: 241).
Chlaenius jacinto Casey, 1920: 295. Type locality: «California (southern) and Arizona» (original citation). Six syntypes [6 originally cited] in USNM [# 47710]. Synonymy established by Bell (1960: 142).

#### Distribution.

This subspecies ranges from southern British Columbia, including Vancouver Island (Lindroth 1969a: 986), to southern California (Bell 1960: 142) and Guatemala (Bates 1882a: 44).

#### Records.

**CAN**: BC (VCI) **USA**: AZ, CA, ID, NV, OR, UT, WA – Guatemala, Mexico

### [pennsylvanicus group]

### Chlaenius flaccidus

Horn, 1876

Chlaenius flaccidus G.H. Horn, 1876d: 265. Type locality: «Waco County, Texas» (original citation). Holotype [by monotypy] (♀) in MCZ [# 8003].

#### Distribution.

This species is known only from a few localities in central and southern Texas (Bell 1960: 149). The record from Georgia (Fattig 1949: 43) is likely in error (Bell 1960: 149).

#### Records.

**USA**: TX

### Chlaenius floridanus

Horn, 1876

Chlaenius floridanus G.H. Horn, 1876d: 263. Type locality: «Florida» (original citation), herein restricted to Winter Park, Orange County (MCZ). Syntype(s) [3 originally cited] in MCZ [# 8002] and possibly CMNH (collection Ulke).

#### Distribution.

This species is known from northern Georgia (Fattig 1949: 43) to southern Florida (Peck and Thomas 1998: 20) and from Inagua in the Bahamas (Darlington 1953: 8).

#### Records.

**USA**: FL, GA – Bahamas

### Chlaenius nebraskensis

LeConte, 1856

Chlaenius nebraskensis LeConte, 1856b: 28. Type locality: «Yellowstone River [probably in Montana]; Santa Fe [New Mexico]» (original citation), restricted to «Yellowstone River» by Bell (1960: 148). Syntype(s) in MCZ [# 5861].
Chlaenius sedulus Casey, 1920: 296. Type locality: «Amarillo [Potter County], Texas» (original citation). One syntype in USNM [# 47714]. Synonymy established by Bell (1960: 148).

#### Distribution.

This species is found from southern Saskatchewan and southern Alberta (Lindroth 1969a: 988) south to southern Arizona and southern Texas (Bell 1960: 148).

#### Records.

**CAN**: AB, SK **USA**: AZ, CO, KS, MT, ND, NE, NM, NV, OK, SD, TX, UT

### Chlaenius pennsylvanicus blanditus

Casey, 1920

Chlaenius blanditus Casey, 1920: 297. Type locality: «Vineyard [Utah County], Utah» (original citation). Lectotype (♂), designated by Lindroth (1975: 144), in USNM [# 47713].

#### Distribution.

This subspecies ranges from northern Utah to southern Arizona, east to western Texas (Bell 1960: 147).

#### Records.

**USA**: AZ, NM, TX, UT

### Chlaenius pennsylvanicus pennsylvanicus

Say, 1823

Chlaenius pennsylvanicus Say, 1823a: 66. Type locality: «Penn[sylvania]» (neotype label). Neotype (♂), designated by Lindroth and Freitag (1969: 351), in MCZ [# 32993].
Chlaenius pubescens T.W. Harris, 1828d: 132. Type locality not stated. Syntype(s) presumably lost. Synonymy established by LeConte (1856b: 27).
Chlaenius vicinus Dejean, 1831: 659. Type locality: «Amérique septentrionale» (original citation). Two syntypes in MHNP (Lindroth 1955b: 25). Synonymy established, with *Chlaenius pubescens* Harris, by Dejean (1833: 25), confirmed by Lindroth (1955b: 25).
Chlaenius impunctifrons Kirby, 1837: 21 [primary homonym of *Chlaenius impunctifrons* Say, 1823]. Type locality: northern parts of British America (inferred from title of the paper). One syntype in BMNH (Lindroth 1953b: 169). Synonymy established by LeConte (1870: 397), confirmed by Lindroth (1953b: 169).
Chlaenius fulgiceps Newman, 1838a: 490. Type locality: «Ohio» (original citation). Syntype(s) probably lost (Lindroth 1969a: 987). Synonymy established by LeConte (1847: 436).

#### Distribution.

This subspecies ranges from Newfoundland (Lindroth 1955a: 134) to Vancouver Island (Lindroth 1969a: 987), south at least to southwestern Oregon, southern Colorado along the Rocky Mountains (Bell 1960: 147), southeastern Louisiana (Saint Tammany Parish, Igor M. Sokolov pers. comm. 2009; Summers 1874a: 80), and southern Florida (Peck and Thomas 1998: 20). The record from southwestern California (Moore 1937: 12) is probably in error.

#### Records.

**FRA**: PM **CAN**: AB, BC (VCI), MB, NB, NF, NS (CBI), ON, PE, QC, SK **USA**: AL, AR, CO, CT, DC, FL, GA, IA, ID, IL, IN, KS, KY, LA, MA, MD, ME, MI, MN, MO, MS, MT, NC, ND, NE, NH, NJ, NY, OH, OR, PA, RI, SC, SD, TN, VA, VT, WA, WI, WV, WY

### [vafer group]

### Chlaenius pertinax

Casey, 1920

Chlaenius pertinax Casey, 1920: 295. Type locality: «New Augustine [= Saint Augustine, Saint Johns County], Florida» (original citation). One syntype in USNM [# 47709].

#### Distribution.

This species is endemic to the Florida Peninsula (Bell 1960: 165; Peck and Thomas 1998: 20).

#### Records.

**USA**: FL

### Chlaenius texanus

Horn, 1876

Chlaenius texanus G.H. Horn, 1876d: 261. Type locality: «Texas» (original citation), herein restricted to Brownsville, Cameron County (MCZ). Syntype(s) [2 originally cited] in MCZ [# 34531].

#### Distribution.

This species is known from southern Mississippi (Hancock County, Drew A. Hildebrandt pers. comm. 2008), southern Texas, and northern Mexico (Bell 1960: 151).

#### Records.

**USA**: MS, TX – Mexico

### Chlaenius vafer

LeConte, 1852

Chlaenius vafer LeConte, 1852b: 66. Type locality: «Creek Boundary [= boundary of the Creek Indian Reservation at that time, located near or in Oklahoma]» (original citation). Syntype(s) in MCZ [# 5860]. Note. LeConte (1852b: 65) stated that this and most of the other species from the expedition were collected “from the boundary of the tract of land ... set apart for the Creek Indians.”

#### Distribution.

This species ranges from southeastern Nebraska (Lancaster County, Foster F. Purrington pers. comm. 2010) and central Missouri (Boone County, CMNH) south to Mississippi (CMNH), southwestern Louisiana (Hine 1906: 77), and southern Texas (Bell 1960: 151). The record from eastern South Dakota (Kirk and Balsbaugh 1975: 36) needs confirmation.

#### Records.

**USA**: AR, KS, LA, MO, MS, NE, OK, TX [SD]

### [variabilipes group]

### Chlaenius obsoletus

LeConte, 1851

Chlaenius obsoletus LeConte, 1851: 180. Type locality: «San Diego et Colorado [River] [California]» (original citation), herein restricted to San Diego, San Diego County. Syntype(s) in MCZ [# 74].
Chlaenius rogator Motschulsky, 1859a: 157. Type locality: «St. Francisco [San Francisco County, California]» (original citation). One syntype in ZMMU (Keleinikova 1976: 214). Synonymy established with doubt by Chaudoir (1876b: 244).

#### Distribution.

This species ranges from central California (Bell 1960: 150) south to “Nicaragua” (Bates 1882a: 44) and the Baja California Peninsula (Horn 1894: 311), including western New Mexico (Catron County, UASM; Snow 1885: 67; Fall and Cockerell 1907: 160) and western Texas (Jeff Davis County, UASM). The record from “Utah” (Bousquet and Larochelle 1993: 208) needs confirmation.

#### Records.

**USA**: AZ, CA (CHI), NM, TX [UT] – Guatemala, Mexico, Nicaragua

### Chlaenius variabilipes

Eschscholtz, 1833

Chlaenius variabilipes Eschscholtz, 1833: 27. Type locality: «bei St. Franzisco [San Francisco County], Californien» (original citation). Syntype(s) location unknown.
Chlaenius asperulus Ménétriés, 1843: 55. Type locality: «Californie» (original citation). One syntype in ZMH (collection Mannerheim) (Silfverberg 1987: 12). Synonymy established by LeConte (1856b: 28).
Chlaenius obscurus LeConte, 1851: 179. Type locality: «San Jose, et ad flumen Colorado» (original citation). Six syntypes in MCZ [# 70]. Synonymy established by LeConte (1856b: 28) and Chaudoir (1856: 270).

#### Distribution.

The range of this species extends from southeastern Oregon (Harney County, James R. LaBonte pers. comm. 1992) and west-central Nevada (Bell 1960: 150) south to the Baja California Peninsula (Horn 1894: 311) and at least Durango in Mexico (Ball and Shpeley 1992a: 61). The specimens labeled from Texas (Bell 1960: 150) are probably mislabeled or represent strays.

#### Records.

**USA**: AZ, CA, NV, OR, UT [TX] – Mexico

### Subgenus Callistometus

Grundmann, 1956

Callistometus Grundmann, 1956: 265. Type species: *Chlaenius ruficauda* Chaudoir, 1856 by original designation. Etymology. Uncertain, possibly from the Greek prefix *callo*- (beautiful), *stoma* (mouth), and the Latin suffix -*etus* (provided with) [masculine].

#### Diversity.

Three species in southwestern North America (one species) and Mexico (three species).

### Chlaenius ruficauda

Chaudoir, 1856

Chlaenius apicalis LeConte, 1851: 179 [secondary homonym of *Chlaenius apicalis* (Wiedemann, 1819)]. Type locality: «ad fluminis Colorado ripas» (original citation). Three syntypes in MCZ [# 71].
Chlaenius posticus LeConte, 1853c: 390 [secondary homonym of *Chlaenius posticus* (Fabricius, 1798)]. Replacement name for *Chlaenius apicalis* LeConte, 1851.
Chlaenius ruficauda Chaudoir, 1856: 194. Replacement name for *Chlaenius apicalis* LeConte, 1851 and *Chlaenius posticus* LeConte, 1853.

#### Distribution.

This species ranges from southern California to southwestern New Mexico, south to Oaxaca in Mexico (Bell 1960: 116).

#### Records.

**USA**: AZ, CA, NM – Mexico

#### Note.

The name *Chlaenius dimidiatus* Motschulsky, 1858 is often listed as a junior synonym of this species (e.g., Csiki 1931: 971; Bell 1960: 115). However, Motschulsky never provided a description of the taxon and the name is a *nomen nudum*.

### Subgenus Brachylobus

Chaudoir, 1876

Brachylobus Chaudoir, 1876b: 287. Type species: *Chlaenius lithophilus* Say, 1823 by monotypy. Etymology (original). From the Greek *brachys* (short) and *lobos* (lobes), alluding to the markedly short lateral lobes of the mentum (“*mentum vix emarginatum, lobis cum parte media confusis, nec eandem superantibus*”) of the adult [masculine].

#### Diversity.

Two species in the boreal and temperate regions of North America.

### Chlaenius caurinus

(Horn, 1885)

Brachylobus caurinus G.H. Horn, 1885a: 134. Type locality: «near Yreka [Siskiyou County], Cal[iforni]a» (original citation). Holotype [by monotypy] (♂) in MCZ [# 34536].

#### Distribution.

This species is known only from the type locality in north-central California (Bell 1960: 139).

#### Records.

**USA**: CA

#### Note.

Bell (1960: 139) stated that the holotype and still only known specimen may simply be an aberrant individual of *Chlaenius lithophilus*.

### Chlaenius lithophilus

Say, 1823

Chlaenius lithophilus Say, 1823a: 62. Type locality: «Rivervale [Morris County], N[ew] J[ersey]» (neotype label). Neotype (♂), designated by Lindroth and Freitag (1969: 352), in MCZ [# 32976].
Chlaenius viridanus Dejean, 1831: 660. Type locality: «Amérique septentrionale» (original citation). One syntype in MHNP (Lindroth 1955b: 26). Synonymy established by LeConte (1847: 434), confirmed by Lindroth (1955b: 26).
Chlaenius smaragdiger Motschulsky, 1865: 338. Type locality: «Pensylvanie» (original citation). One syntype in ZMMU (Keleinikova 1976: 217). Synonymy established by Chaudoir (1876b: 288).
Brachylobus lithophilus indigaceus Casey, 1914: 41. Type locality: «Texas» (original citation). Two syntypes [2 originally cited] in USNM [# 47723]. **New synonymy**. Note. Bell (1960: 138) retained *Chlaenius indigaceus* Casey as a subspecies but I believe, as done implicitly by Lindroth (1969a: 989), that the form, which is based exclusively on color, does not warrant a subspecific status.

#### Distribution.

This species ranges from Newfoundland (Lindroth 1955a: 135) to central British Columbia, north to Fort Smith in southern Northwest Territories (Lindroth 1969a: 989), south to northeastern Washington (Hatch 1953: 163), “New Mexico” (Robert L. Davidson pers. comm. 1992), northern Texas (Cooke County, CNC), and northeastern Georgia (Bell 1960: 138). The record from “Oregon” (Wickham 1896c: 135) needs confirmation.

#### Records.

**CAN**: AB, BC, MB, NB, NF, NS (CBI), NT, ON, PE, QC, SK **USA**: AL, AR, CO, CT, DC, GA, IA, ID, IL, IN, KS, KY, MA, MD, ME, MI, MN, MO, MS, MT, NC, ND, NE, NH, NJ, NM, NY, OH, OK, PA, SD, TN, TX, UT, VA, VT, WA, WI, WV, WY [OR]

### Subgenus Agostenus

Fischer von Waldheim, 1829

Agostenus Fischer von Waldheim, 1829a: 15. Type species: *Carabus sulcicollis* Paykull, 1798 designated by Jeannel (1942: 971). Etymology. Uncertain, possibly from the Greek *agon* (gathering) or *agos* (leader) or the incorrect transcription of *agan* (very) and *stenos* (narrow, tight) [masculine]. The name was proposed by Franz Anton Ziegler and made available by Fischer von Waldheim. Note. This name has been incorrectly credited to Motschulsky (1850a: x, 65) until Kryzhanovskij et al. (1995: 158).
Pelasnus Fischer von Waldheim, 1829a: 15. Type species: *Carabus quadrisulcatus* Paykull *sensu* Illiger, 1798 (= *Agostenus costulatus* Motschulsky, 1859) by monotypy. Note. This name has been incorrectly credited to Motschulsky (1850a: x, 65, as *Pelasmus*) until Kryzhanovskij et al. (1995: 158).
Agostenops Lutshnik, 1933b: 171. Type species: *Chlaenius alutaceus* Gebler, 1830 by monotypy. Synonymy established by Kryzhanovskij et al. (1995: 158). Etymology. From the generic name *Agostenus* [*q.v*.] and the Greek suffix -*ops* (having the appearance of) [masculine].

#### Diversity.

Nine species in the Nearctic (five species), Neotropical (one Mexican species also found in Arizona), and Palaearctic (four species) Regions.

### Chlaenius alternatus

Horn, 1871

Chlaenius alternatus G.H. Horn, 1871: 327. Type locality: «Saskatchewan region» (original citation), herein restricted to Meota, Saskatchewan (CNC). Holotype [by monotypy] (♂) in MCZ [# 34534].
Chlaenius albertanus Casey, 1924: 93. Type locality: «Edmonton, Alberta» (original citation). Lectotype (♀), designated by Lindroth (1975: 144), in USNM [# 47716]. Synonymy established by Bell (1960: 134).

#### Distribution.

This species ranges from Newfoundland (Larson and Langor 1982: 594) to central Alaska (Lindroth 1969a: 993), south to northeastern Washington (Hatch 1953: 162), central Colorado along the Rocky Mountains, western South Dakota (Meade County, Foster F. Purrington pers. comm. 2010), and eastern Minnesota (Bell 1960: 134). The species is unknown east of the Mississippi south of the Canadian border. Fossil remnants of this species, dated between about 16,700 and 25,200 years B.P., have been unearthed in southeastern Iowa (Baker et al. 1986: 96) and southeastern Illinois (Schwert 1992: 78).

#### Records.

**CAN**: AB, BC, MB, NB, NF, NS, NT, ON, QC, SK **USA**: AK, CO, MN, MT, ND, SD, WA, WY

### Chlaenius caeruleicollis

Chaudoir, 1876

Chlaenius caeruleicollis Chaudoir, 1876b: 78. Type locality: «Las Peras [Oaxaca], Mexique» (original citation). Syntype(s) in MHNP.
Chlaenius insperatus G.H. Horn, 1885a: 134. Type locality: «Arizona» (original citation). Three syntypes in MCZ [# 34532]. Synonymy established by Horn (1886b: xii).

#### Distribution.

This species is known from a few specimens simply labeled from Arizona and from Mexico as far south as Oaxaca and central Veracruz (Bell 1960: 132; Davidson 1980: 31).

#### Records.

**USA**: AZ – Mexico

### Chlaenius harpalinus

Eschscholtz, 1833

Chlaenius harpalinus Eschscholtz, 1833: 27. Type locality: «bei St. Franzisco [San Francisco County], Californien» (original citation). Syntype(s) location unknown (Lindroth 1969a: 990).
Chlaenius crestonensis Brown, 1933: 43. Type locality: «Creston, B[ritish] C[olumbia]» (original citation). Holotype (♂) in CNC [# 3401]. Synonymy established by Hatch (1953: 161), confirmed by Bell (1960: 132).

#### Distribution.

This species ranges from southeastern Alberta to Vancouver Island (Lindroth 1969a: 990-991), south at least to central California and northern Utah (Bell 1960: 133). One old specimen labeled from “Arizona” is also known (Bell 1960: 133).

#### Records.

**CAN**: AB, BC (VCI) **USA**: CA, ID, MT, NV, OR, UT, WA [AZ]

### Chlaenius interruptus

Horn, 1876

Chlaenius interruptus G.H. Horn, 1876d: 259. Type locality: «Washington County, Oregon» (original citation). One syntype [2 originally cited] in MCZ [# 34533] and one in CMNH (collection Ulke).

#### Distribution.

This species is restricted to the Pacific Coast ranging from southern British Columbia, including Vancouver Island (Lindroth 1969a: 993), south through the Coast Range to northern California (Bell 1960: 135). The record from Inyo County, California (Dajoz 2007: 19) needs confirmation. Fossil remnants from a Plio-Pleistocene sequence have been unearthed in northwestern Greenland (Böcher 1995: 25).

#### Records.

**CAN**: BC (VCI) **USA**: CA, OR, WA

### Chlaenius niger

Randall, 1838

Chlaenius niger Randall, 1838b: 34. Type locality: «vicinity of Boston [Massachusetts]» (original citation). Syntype(s) lost.
Chlaenius niger var. *ludoviciana* Leng, 1915: 592. Type locality: «Louisiana» (original citation). Holotype location unknown. Synonymy established by Bell (1960: 133).

#### Distribution.

This species ranges from Newfoundland (Lindroth 1955a: 133) to south-central British Columbia (Lindroth 1969a: 992), south to northeastern Washington (Hatch 1953: 162), “Wyoming” (Robert L. Davidson pers. comm. 1992), east-central Texas, southern Florida, and Cuba (Bell 1960: 133-134). The record from “Yukon Territory” (Ball and Currie 1997: 453) could not be confirmed.

#### Records.

**CAN**: AB, BC, MB, NB, NF, NS (CBI), NT, ON, PE, QC, SK **USA**: AL, AR, CT, DC, FL, GA, IA, IL, IN, KS, LA, MA, MD, ME, MI, MN, MO, MS, MT, NE, NH, NJ, NY, OH, OK, PA, RI, SC, TN, TX, VA, VT, WA, WI, WY, TX [YT] – Cuba

#### Note.

The name *Chlaenius exaratus* LaFerté-Sénectère, 1851 is often listed as a junior synonym of this species (e.g., Bell 1960: 133; Lindroth 1969a: 991). However, LaFerté-Sénectère never provided a description of the taxon and the name is a *nomen nudum*.

### Subgenus Randallius

Bousquet, n.subg.

Randallius Bousquet, n.subg. Type species: *Chlaenius purpuricollis* Randall, 1838. Etymology. The generic name is proposed in honor of John Witt Randall [1813-1892] who in his 20s published two descriptive papers on the beetles of Maine and Massachusetts. One of his new species is the type species of this subgenus. Randall did not publish anything else on entomology after these two papers but wrote a volume of poems in 1856 entitled “Consolations of Solitude.” His collection is lost (Sprague 1875: 374). The gender of the name is masculine.

#### Diversity.

One North American species.

#### Taxonomic Note.

In my opinion, *Chlaenius purpuricollis* is taxonomically isolated and within the actual structure of the genus *Chlaenius* deserves to be placed in its own subgenus. See Bell (1960: 135) and Lindroth (1969a: 994) as “*purpuricollis* group” for the descriptive character states of this new taxon.

### Chlaenius purpuricollis

Randall, 1838

Chlaenius purpuratus T.W. Harris, 1836: 67 [*nomen dubium*]. Type locality: North America (inferred from title of the paper). Syntype(s) lost. Note. This species was briefly described mainly by comparison with two specimens that Harris attributed with doubt to *Chlaenius aestivus*. Its identity is doubtful but for convenience the name is listed in synonymy with *Chlaenius purpuricollis*.
Chlaenius purpuricollis Randall, 1838b: 35. Type locality: Massachusetts (inferred from title of the paper). Syntype(s) lost. **New synonymy**.
Chlaenius frostii Carr, 1920: 219. Type locality: «Edmonton, Al[ber]ta» (original citation). Holotype (♂) in CNC [# 440]. **New synonymy**. Note. Bell (1960: 136) retained *Chlaenius frostii* Carr as a subspecies but I believe, as done implicitly by Lindroth (1969a: 994), that the form does not warrant subspecific status.
Chlaenius punctipennis Casey, 1920: 298. Type locality: «Wildur [= Wilbur, Lincoln County], Washington» (original citation). Lectotype (♂), designated by Lindroth (1975: 144), in USNM [# 47715]. Synonymy established by Hatch (1953: 162).

#### Distribution.

This species is found from southern Quebec (Lindroth 1969a: 994) to southeastern British Columbia (CNC), north to Fort Smith in southern Northwest Territories (Bousquet 1987a: 132), south to Oregon (Bell 1960: 136), New Mexico (Perrault 1970: 56), Kansas (Popenoe 1878: 78; Bell 1960: 136), and “New Jersey” (Smith 1890: 92; Smith 1910: 213).

#### Records.

**CAN**: AB, BC, MB, NT, ON, QC, SK **USA**: CO, CT, IA, ID, IL, IN, KS, MA, MI, MN, ND, NE, NH, NJ, NM, NY, OR, PA, RI, UT, WA, WI, WY

**Figure 30.**
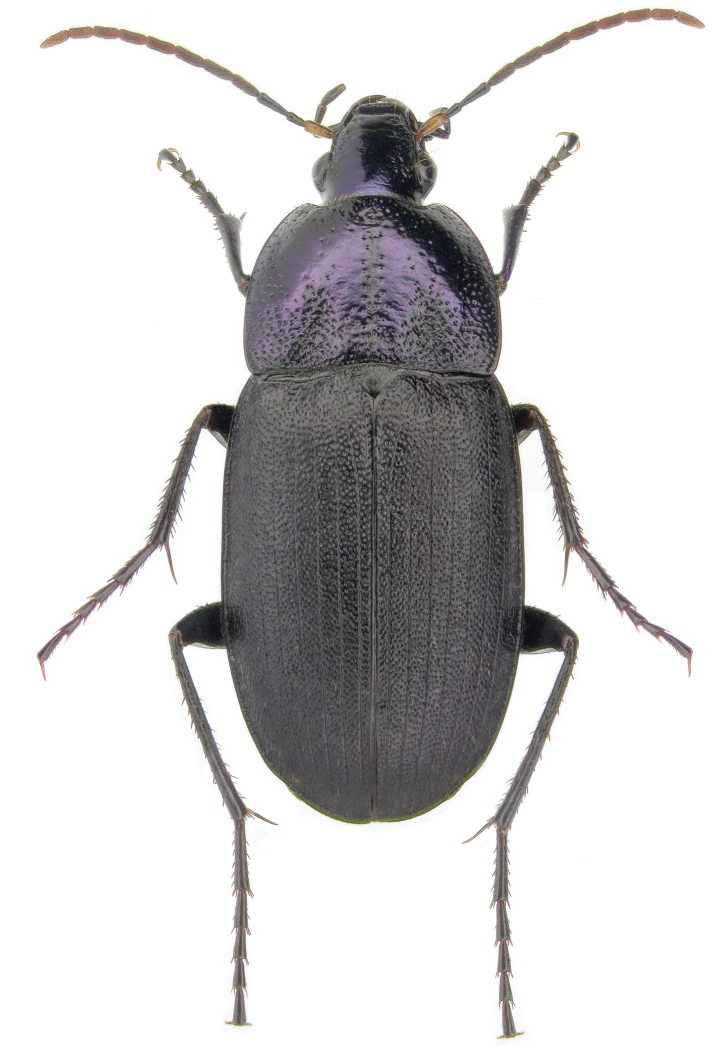
*Chlaenius purpuricollis* Randall. This relatively small chlaeniine had been rarely collected until pitfall trapping showed that it is common in alvars, which are flat, relatively open grassy areas with shallow or sporadic soil cover over calcareous bedrock. Alvars are known from Sweden, Estonia, New York, Michigan, Ohio, and particularly central Canada. The chlaeniine is also found in similar habitats in other parts of North America.

### Supertribe HARPALITAE

Bonelli, 1810

Harpalii Bonelli, 1810: Tabula Synoptica. Type genus: *Harpalus* Latreille, 1802.

#### Diversity.

Worldwide, with about 13,200 species placed in the following 32 tribes: Anthiini (about 130 species), Atranini (two species), Calophaenini (about 50 species), Catapieseini (nine species), Corsyrini (six species), Ctenodactylini (about 65 species), Cyclosomini (about 120 species), Dryptini (about 85 species), Enoicini (five South African species), Galeritini (about 135 species), Geobaenini (four species), Ginemini (one South American species), Graphipterini (about 155 species), Harpalini (about 2,725 species), Helluonini (about 180 species), Hexagoniini (about 65 species), Lachnophorini (about 115 species), Lebiini (about 4,260 species), Licinini (about 235 species), Masoreini (about 50 species), Odacanthini (about 325 species), Omphreini (14 species), Pentagonicini (about 165 species), Perigonini (about 120 species), Physocrotaphini (about 40 species), Platynini (about 2,670 species), Pseudomorphini (about 320 species), Sarothrocrepidini (about 25 species), Somoplatini (13 species), Sphodrini (about 825 species), Xenaroswellianini (one species), and Zuphiini (about 290 species).

### Tribe Licinini

Bonelli, 1810

Licinii Bonelli, 1810: Tabula Synoptica. Type genus: *Licinus* Latreille, 1802.

#### Diversity.

Worldwide, with about 235 species arrayed in four subtribes: Dicaelina (45 species), Dicrochilina (30 species), Lestignathina (about 60 species), and Licinina (about 100 species). The Northern Hemisphere is represented by about 150 species (roughly 64% of the world fauna) and North America alone by 62 species (approximately 26.5 %).

### Subtribe Dicaelina

Laporte, 1834

Dicoelidae Laporte, 1834: 83. Type genus: *Dicoelus* Agassiz, 1846 (unjustified emendation of *Dicaelus* Bonelli, 1813 not in prevailing usage) (= *Dicaelus* Bonelli, 1813).
Rembidae Gistel, 1848: [2]. Type genus: *Rembus* Macleay, 1825.

#### Diversity.

Forty-five species arrayed in two genera, both represented in the Nearctic Region.

### Genus Diplocheila

Brullé, 1835

Rembus W.S. Macleay, 1825: 16 [junior homonym of *Rembus* Germar, 1824]. Type species: *Carabus impressus* Fabricius, 1798 (= *Rembus daldorfi* Crotch, 1871) designated by Westwood (1838: 5). Etymology. Uncertain, possibly from the Greek *rhembo* (turn, revolve, roll) [masculine]. Note. This taxon was made available also by Lepeletier and Audinet-Serville [in Latreille et al.] the same year but the work of Latreille et al. was issued in October 1825 (Evenhuis 1997a: 228) while that of Macleay in July 1825 (Evenhuis 1997b: 507).
Diplocheila Brullé, 1835a: 407. Replacement name for *Rembus* Macleay, 1825. Etymology (original). From the Greek *diplo* (double) and *cheilos* (lip), alluding to the markedly emarginate labrum (“*lèvre supérieure *... *presque divisée en deux parties*”) of the adult [feminine].
Diplochila Agassiz, 1846: 125. Unjustified emendation of *Diplocheila* Brullé, 1835.
Rhembus Agassiz, 1846: 322, 323. Unjustified emendation of *Rembus* Macleay, 1825.

#### Distribution.

Twenty-nine species in the boreal, temperate, and tropical areas of the Nearctic (nine species), Oriental (12 species), Palaearctic (nine species), and Afrotropical (two species) Regions. The species are placed in three subgenera: *Diplocheila* (nine Oriental and one east Palaearctic species), *Isorembus* (18 species), and *Neorembus* Ball (one Asian species).

#### Identification.

Ball (1959) revised the species of this genus. Since this publication, one new North American species has been described by Will (1998) and a few nomenclatural changes affecting the Nearctic species have been introduced by Lindroth (1968-1969a): Ball’s *Diplocheila modesta* corresponds to *Diplocheila assimilis* and his *Diplocheila assimilis* to *Diplocheila impressicollis*. Lindroth’s (1968: 940-941) key encompasses seven North American species.

### Subgenus Isorembus

Jeannel, 1949

Isorembus Jeannel, 1949a: 771. Type species: *Rembus aegyptiacus* Dejean, 1831 by original designation. Etymology. From the Greek *isos* (equal) and the generic name *Rembus* [*q.v*.] [masculine].
Submera Habu, 1956a: 50, 58. Type species: *Rembus zeelandicus* Redtenbacher, 1868 by original designation. Synonymy established by Ball (1959: 48). Etymology (original). From the generic name *Rembus* [*q.v*.] spelled in reverse with the addition of a terminal *a* [feminine]. Note. Lafer and Kataev (2008) argued that *Submera* should be considered as a valid subgenus.
Shirahataia Habu, 1956a: 50, 63. Type species: *Submera macromandibularis* Habu and Tanaka, 1956 by original designation. Synonymy established by Ball (1959: 48). Etymology. The generic name was proposed for Kôtarô Shirahata [1914-1980], a Japanese policeman interested mainly in Odonata but also in various groups of beetles. After his retirement in 1969, Shirahata (born K. Suzuki) worked as a part-time researcher at the Yamagata Prefectural Museum (Shun-Ichi Uéno pers. comm. 2009).

#### Diversity.

Eighteen species in the Nearctic (nine species), Oriental (three species), Palaearctic (five species), and Afrotropical (two species, one shared with northern Africa and Spain) Regions. Several species listed in this subgenus by Lorenz (2005: 342-343) belong to the subgenus *Diplocheila *s.str.

### Diplocheila assimilis

(LeConte, 1844)

Rembus assimilis LeConte, 1844: 51. Type locality: «Georgia» (original citation). One syntype in MCZ [# 5703].
Diplocheila modesta Casey, 1920: 203. Type locality: «Montreal [Quebec]» (original citation). One syntype in USNM [# 47379]. Synonymy established by Lindroth (1968: 943).

#### Distribution.

This species is found from New Brunswick (Webster and Bousquet 2008: 20) to east-central South Dakota (Kirk 1971: 239), south to “Georgia” (LeConte 1844: 51).

#### Records.

**CAN**: NB, ON, QC **USA**: CT, GA, IA, IL, IN, MA, MD, ME, MI, MN, NH, NJ, NY, OH, PA, SC, SD, VT, WI

### Diplocheila crossi

Will, 1998

Diplocheila crossi Will, 1998: 96. Type locality: «Leroy Percy S[tate] P[ark], Washington Co[unty], Mississippi» (original citation). Holotype (♀) in CUIC [# 6971]. Etymology. The specific name was proposed for the American entomologist William Henley Cross [1928-1984] who is best known for his work on the ecology of the boll weevil with the USDA in Starkville, Mississippi.

#### Distribution.

This species is known from a few localities in the Florida Panhandle and along the Mississippi Basin in northwestern Mississippi, western Tennessee, and southern Illinois [see Will 1998: Fig. 10].

#### Records.

**USA**: FL, IL, MS, TN

### Diplocheila impressicollis

(Dejean, 1831)

Rembus impressicollis Dejean, 1831: 682. Type locality: “Amérique septentrionale” (original citation), restricted to «Syracuse [Onondaga County], N[ew] Y[ork]» by Lindroth (1968: 944). One syntype in MHNP (Lindroth 1955b: 17).
Rembus laticollis LeConte, 1847: 419. Type locality: «Syracusas NovEboraci [= Syracuse, Onondaga County, New York]» (original citation). Two syntypes in MCZ [# 5702]. Synonymy established by Lindroth (1955b: 17).
Diplochila cliens Casey, 1897: 348. Type locality: «Kansas» (original citation). One syntype in USNM [# 47376]. Synonymy established by Lindroth (1968: 944).
Diplochila planulata Casey, 1913: 149. Type locality: «Austin [Travis County], Texas» (original citation). One syntype in USNM [# 47377]. Synonymy established by Lindroth (1968: 944).
Diplocheila foveata Casey, 1920: 201. Type locality: «Lake Champlain, New York» (original citation). One syntype in USNM [# 47378]. Synonymy established by Lindroth (1968: 944).

#### Distribution.

This species ranges from southern Quebec (Larochelle 1975: 80) to west-central South Dakota (Kirk and Balsbaugh 1975: 34), south to central New Mexico (Ball 1959: 71, as *Diplocheila assimilis*), the Rio Grande in southeastern Texas (Ball 1959: 72, as *Diplocheila assimilis planulata*), and southern Virginia (Hoffman 2010: 22). The records from “North Dakota” (Bousquet and Larochelle 1993: 209) and Connecticut (Britton 1920: 214, as *Diplocheila laticollis*; see Krinsky and Oliver 2001: 4) need confirmation.

#### Records.

**CAN**: ON, QC **USA**: DC, IA, IL, IN, KS, MA, MD, MI, MN, MO, MS, NE, NH, NJ, NM, NY, OH, OK, PA, RI, SD, TX, VA, VT, WI [CT, ND]

### Diplocheila major major

(LeConte, 1847)

Rembus major LeConte, 1847: 418. Type locality: «urbes Detroit et NovAurelianum [= New Orleans, Louisiana]» (original citation), restricted to «Detroit [Wayne County], Mich[igan]» by Lindroth (1969a: 945). Three syntypes in MCZ [# 5704].
Diplochila expansa Casey, 1913: 148. Type locality: «Keokuk [Lee County], Iowa» (original citation). Two syntypes in USNM [# 47372]. Synonymy established by Ball (1959: 75).
Diplochila oblonga Casey, 1913: 148. Type locality: «Kansas» (original citation). Three syntypes in USNM [# 47374]. Synonymy established by Ball (1959: 75).
Diplocheila procera Casey, 1920: 200. Type locality: «Lake Superior» (original citation). One syntype in USNM [# 47373]. Synonymy established by Ball (1959: 75).

#### Distribution.

This subspecies ranges from “Rhode Island” to east-central South Dakota (Ball 1959: 78), south to east-central Kansas (Snow 1880: 78; Ball 1959: 78), “Missouri” (Ball 1960b: 78), and New Jersey (Smith 1890: 82; Smith 1910: 206). The records from Louisiana (Summers 1874a: 80; Casey 1913: 148) and Alabama (Löding 1945: 18) refer to subspecies *melissisa*; those from “North Dakota” (Bousquet and Larochelle 1993: 209) and north-central Colorado (Wickham 1902: 238) need confirmation.

#### Records.

**CAN**: ON **USA**: CT, IA, IL, IN, KS, MI, MN, MO, NE, NJ, NY, OH, PA, RI, SD, WI [CO, ND]

### Diplocheila major melissisa

Ball, 1959

Diplocheila major melissisa Ball, 1959: 78. Type locality: «Clewiston, Hendry County, Fl[orid]a» (original citation). Holotype (♀) in MCZ [# 31159].

#### Distribution.

This subspecies ranges along the Coastal Plain from eastern Virginia (Hoffman et al. 2006: 24) to southern Florida (Peck and Thomas 1998: 21), west to southeastern Texas (Ball 1959: 80); also recorded from Cuba (Ball 1959: 80).

#### Records.

**USA**: AL, FL, LA, MS, NC, SC, TX, VA – Cuba

### Diplocheila nupera

Casey, 1897

Diplochila nupera Casey, 1897: 348. Type locality: «Lake Worth [Palm Beach County], Florida» (original citation). Holotype [by monotypy] (♀) in USNM [# 47382].
Rembus angusticollis Blatchley, 1928b: 61. Type locality: «Dunedin [Pinellas County], Fl[orid]a» (original citation). Holotype [by monotypy] (♀) in PURC. Synonymy established by Ball (1959: 72).

#### Distribution.

As far as known, this species is endemic to the southern half of the Florida Peninsula (Ball 1959: 73).

#### Records.

**USA**: FL

### Diplocheila obtusa

(LeConte, 1847)

Rembus obtusus LeConte, 1847: 420. Type locality: «Longs’s Peak [Boulder County, Colorado]» (original citation). Three syntypes in MCZ [# 5706].
Diplocheila parallela Casey, 1920: 204. Type locality: «Homer [Champaign County], Illinois» (original citation). One syntype in USNM [# 47383]. Synonymy established by Lindroth (1954b: 136).

#### Distribution.

This species occurs from Cape Breton Island (Bousquet 1987d: 107) to south-central British Columbia, north to central Northwest Territories along the Mackenzie River (Lindroth 1969a: 948), south to eastern Washington, “Nevada” (Ball 1959: 82), the Sierra Blanca in south-central New Mexico (Fall and Cockerell 1907: 159), northwestern Texas (Oldham County, Foster F. Purrington pers. comm. 2010), “Missouri” (Ball 1959: 82), northwestern Tennessee (Stewart County, Foster F. Purrington pers. comm. 2010), and western Virginia (Hoffman et al. 2006: 24). The record from southern Louisiana (Summers 1874a: 80) is probably in error; that from “Arkansas” (Bousquet and Larochelle 1993: 209) needs confirmation.

#### Records.

**CAN**: AB, BC, MB, NB, NS (CBI), NT, ON, PE, QC, SK **USA**: CO, CT, IA, ID, IL, IN, KS, MA, ME, MI, MN, MO, MT, ND, NE, NH, NM, NV, NY, OH, PA, SD, TN, TX, VA, VT, WA, WI, WY [AR]

### Diplocheila oregona

(Hatch, 1951)

Rembus oregona Hatch, 1951: 119. Type locality: «McMinnville [Yamhill County], Ore[gon]» (original citation). Holotype (♂) in USNM.

#### Distribution.

This species ranges from southeastern Manitoba to the Okanagan Valley in southern British Columbia (Lindroth 1968: 943), south to northwestern Oregon (Hatch 1951: 119), “Utah” (Ball 1959: 64), northwestern Wyoming (Sikes 1994), and “North Dakota” (Donald P. Schwert pers. comm. 1989).

#### Records.

**CAN**: AB, BC, MB, SK **USA**: MT, ND, NV, OR, UT, WY

### Diplocheila striatopunctata

(LeConte, 1844)

Rembus striatopunctatus LeConte, 1844: 50. Type locality: «Carlisle [Cumberland County], Pennsylvania» (original citation). Syntype(s) in MCZ [# 2729].
Diplochila alternans Casey, 1897: 347. Type locality: «Bayfield [Bayfield County, Wisconsin]» (original citation). One syntype in USNM [# 47375]. Synonymy established by Ball (1959: 60).
Diplocheila amplipennis Casey, 1920: 202. Type locality: «Lake Superior» (original citation). One syntype in USNM [# 47380]. Synonymy established by Ball (1959: 60).
Diplocheila brevicollis Casey, 1920: 203. Type locality: «Ogdensburg [Saint Lawrence County], New York» (original citation). One syntype in USNM [# 47381]. Synonymy established by Ball (1959: 60).

#### Distribution.

This species ranges from central Nova Scotia (Majka et al. 2007: 10) to the Okanagan Valley in British Columbia, north to Fort Smith in southern Northwest Territories (Lindroth 1968: 941), south to Inyo County in California (Dajoz 2007: 19), southeastern Arizona (Dajoz 2007: 21, as *Diplochaetus striatopunctata*), “Kansas,” and northeastern New Jersey (Ball 1959: 63). Old specimens simply labeled from Texas and Georgia are known (Ball 1959: 63). The record from “Virginia” (Bousquet and Larochelle 1993: 209) needs confirmation.

#### Records.

**CAN**: AB, BC, MB, NB, NS, NT, ON, QC, SK **USA**: AZ, CA, IA, ID, IL, IN, KS, MI, MN, MO, MT, ND, NE, NH, NJ, NY, OH, OR, PA, SD, UT, VT, WA, WI [GA, TX, VA]

### Diplocheila undulata

Carr, 1920

Diplochila undulata Carr, 1920: 218. Type locality: «Edmonton, Al[ber]ta» (original citation). Holotype (♂) in CNC [# 408].

#### Distribution.

This species is known from Alberta, as far north as Edmonton, southern Manitoba (Lindroth 1969a: 946), Minnesota (Tinerella and Rider 2001: 321; Kamal J.K. Gandhi pers. comm. 2008), Wisconsin (Messer 2010: 39), “Illinois” (Ball 1959: 83), and southeastern Nebraska (Hall County, UASM).

#### Records.

**CAN**: AB, MB **USA**: IL, MN, NE, WI

### Genus Dicaelus

Bonelli, 1813

Dicaelus Bonelli, 1813: 446. Type species: *Dicaelus violaceus* Bonelli, 1813 (= *Dicaelus purpuratus* Bonelli, 1813) designated by Hope (1838: 82). Etymology. From the Greek *dis* (double) and *coilos* (hollow), alluding to the pair of impressions on the anterior part of the frons (“*la tête porte sur le devant deux enfoncements très-considérables *... *c’est de ces deux impressions que j’ai tiré le nom Dicaelus*”) of the adult [masculine].
Dicoelus Agassiz, 1846: 122, 123. Unjustified emendation of *Dicaelus* Bonelli, 1813.

#### Distribution.

Sixteen species (24 species-group taxa) restricted to the temperate and tropical zones of North America and Mexico (south to the vicinity of Mexico City). Three subgenera are recognized: *Dicaelus *s.str., *Paradicaelus*, and *Liodicaelus*.

#### Identification.

Ball (1959) revised the species of this genus. Since the publication of this work, Ball (1992a) described one new Mexican species (*Diplocheila franclemonti*), one Mexican subspecies was raised to specific level (*Diplocheila abbreviatus* Bates), and another one (*Diplocheila laevipennis dicaeloides*) was placed in synonymy. A few changes are introduced here: *Diplocheila subtropicus* and *Diplocheila quadratus* treated as subspecies are raised to specific level, and *Diplocheila purpuratus darlingtoni* is placed in synonymy with *Diplocheila quadratus*.

### Subgenus Paradicaelus

Ball, 1959

Paradicaelus Ball, 1959: 103. Type species: *Dicaelus furvus* Dejean, 1826 by original designation. Etymology. From the Greek *para* (near) and the generic name *Dicaelus* [*q.v*.] [masculine].

#### Diversity.

Seven North American species arrayed in three species groups.

### [elongatus group]

### Dicaelus elongatus

Bonelli, 1813

Dicaelus elongatus Bonelli, 1813: 448. Type locality: «Amérique septentrionale» (original citation, see page 449), restricted to «Blacks M[oun]t[ain]s, N[orth] C[arolina]» by Lindroth (1969a: 950). Syntype(s) location unknown (possibly in MHNG in collection Jurine, see Casale and Giachino 1998: 68).
Dicaelus simplex Dejean, 1826: 389. Type locality: «Amérique septentrionale» (original citation). One syntype in MHNP (Lindroth 1955b: 18). Synonymy established by Horn (1880b: xvii).
Dicaelus obscurus LeConte, 1847: 429. Type locality: «provinciis australibus» (original citation). Syntype(s) in MCZ [# 5716]. Synonymy established, under the name *Dicaelus simplex* Dejean, by LeConte (1853c: 389), confirmed by Ball (1959: 106).
Dicaelus debiliceps Casey, 1913: 151. Type locality: «Buena Vista Spring, Franklin Co[unty], Pennsylvania» (original citation). One syntype in USNM [# 47359]. Synonymy established by Ball (1959: 106).
Dicaelus ashevillensis Casey, 1920: 205. Type locality: «Asheville [Buncombe County], North Carolina» (original citation). Holotype [by monotypy] (♂) in USNM [# 47360]. Synonymy established by Ball (1959: 106).

#### Distribution.

This species ranges from Maine (Foss 2001: 14) to eastern South Dakota (Kirk and Balsbaugh 1975: 35), including southern Ontario (Lindroth 1969a: 950), south to east-central Texas (Riley 2011) and southern Florida, including the Keys (Peck and Thomas 1998: 21). The record from Montreal, Quebec (Perrault 1977: 153) needs confirmation.

#### Records.

**CAN**: ON **USA**: AL, AR, CT, DC, DE, FL, GA, IA, IL, IN, KS, KY, LA, MA, MD, ME, MI, MO, MS, NC, NE, NH, NJ, NY, OH, OK, PA, RI, SC, SD, TN, TX, VA, VT, WI [QC]

### [furvus group]

### Dicaelus dilatatus dilatatus

Say, 1823

Dicaelus dilatatus Say, 1823a: 68. Type locality: «Camp Hill[s] [Cumberland County], P[ennsylvani]a» (neotype label). Neotype (♂), designated by Lindroth and Freitag (1969: 343), in MCZ [# 33026]. Note. Say (1823a) did not indicate the area where his specimen(s) came from but later (Say 1825: [53]) noted that the species was “an inhabitant of Pennsylvania.”
Dicaelus dejeanii Dejean, 1831: 687. Type locality: «Amérique septentrionale» (original citation), restricted to «coastal plain of Georgia» by Ball (1959: 126). Holotype [by monotypy] (♀) in MHNP (Lindroth 1955b: 17). Synonymy established by Brullé (1835c: 282).
Dicaelus planicollis LeConte, 1847: 427. Type locality: «Georgia ad montes» (original citation). Holotype [by monotypy] (♀) in MCZ [# 5709]. Synonymy established by Ball (1959: 130).
Dicaelus carolinensis Casey, 1913: 150. Type locality: «Southern Pines [Moore County], North Carolina» (original citation). Holotype [by monotypy] (♂) in USNM [# 47357]. Synonymy established by Ball (1959: 130).

#### Distribution.

This subspecies ranges from “Maine” (Larochelle and Larivière 1990a: 32) to west-central New York, south to northern Florida (Ball 1959: 131-132), west to eastern Texas (Sabine County, Brian Raber pers. comm. 2010).

#### Records.

**USA**: AL, CT, DC, DE, FL, GA, MA, MD, ME, MS, NC, NH, NJ, NY, PA, RI, SC, TX, VA, VT, WV

#### Note.

Ball (1959: 132) reported the presence of intergrade populations of this and the *sinuatus* forms in Mississippi, Alabama, northern Florida, Georgia, South Carolina, and North Carolina. For practical reasons, these populations are considered to belong to the nominotypical subspecies.

### Dicaelus dilatatus sinuatus

Ball, 1959

Dicaelus dilatatus sinuatus Ball, 1959: 132. Type locality: «Mount Pleasant, Henry County, Iowa» (original citation). Holotype (♂) location unknown. Note. The holotype was originally deposited in the Iowa Natural History Survey Collection. The whereabouts of that collection is unknown to the author. Paratypes of the species are in SMEK [# 5445] (Byers and Karren 1968: 3).

#### Distribution.

This subspecies ranges from western Pennsylvania to southeastern Iowa (Ball 1959: 134) and west-central Illinois (Willand et al. 2011: 273 as *Dicaelus dilatatus*), south to southern Tennessee and southwestern North Carolina (Ball 1959: 134).

#### Records.

**USA**: IA, IL, IN, KY, NC, OH, PA, TN, VA, WV

### Dicaelus furvus carinatus

Dejean, 1831

Dicaelus carinatus Dejean, 1831: 689. Type locality: «Amérique septentrionale» (original citation), restricted to «coastal plain of Georgia» by Ball (1959: 123). Holotype [by monotypy] (♀) in MHNP (Lindroth 1955b: 18).

#### Distribution.

This subspecies ranges from eastern Minnesota (Gandhi et al. 2005: 929) south to eastern Texas (Sabine County, Brian Raber pers. comm. 2010) and southern Mississippi, east to northwestern Georgia (Ball 1959: 123).

#### Records.

**USA**: AL, AR, GA, IA, KS, MN, MO, MS, NE, OK, TX

### Dicaelus furvus furvus

Dejean, 1826

Dicaelus furvus Dejean, 1826: 388. Type locality: «Amérique septentrionale» (original citation), restricted to «Pennsylvania, east of the Susquehanna River» by Ball (1959: 121). One syntype in MHNP (Lindroth 1955b: 18).
Dicaelus ovalis LeConte, 1847: 427. Type locality: United States east of the Rocky Mountains (inferred from title of the paper). Syntype(s) in MCZ [# 5715]. Synonymy established by Ball (1959: 120).

#### Distribution.

This subspecies ranges from eastern Pennsylvania to west-central Illinois (McCravy and Willand 2008: 157), south to Tennessee (Ball 1959: 122) and South Carolina (Kirk 1969: 14; Kirk 1970: 15; Ciegler 2000: 84). The records from New York (Notman 1928: 229, as *Dicaelus ovalis*) and “Georgia” (J.E. LeConte 1849: 26) need confirmation; that from southern Louisiana (Summers 1874a: 80) is probably in error.

#### Records.

**USA**: DC, DE, IL, IN, KY, MD, NC, NJ, OH, PA, SC, TN, VA, WV [GA, NY]

#### Note.

Ball (1959: 122) reported the presence of intergrade populations between this and the *carinatus* forms in Ohio, Indiana, Illinois, Tennessee, and North Carolina. For practical reasons, these populations are considered here to belong to the nominotypical subspecies.

### Dicaelus sculptilis intricatus

LeConte, 1873

Dicaelus sculptilis intricatus LeConte, 1873b: 324. Type locality: North America (inferred from title of the paper); restricted to «eastern Pennsylvania» by Ball (1959: 143). Syntype(s) location unknown (Lindroth 1969a: 952).

#### Distribution.

This subspecies is found from southernmost Ontario and southeastern Michigan south to southern Indiana and central Maryland (Ball 1959: 143-144).

#### Records.

**CAN**: ON **USA**: IN, MD, MI, OH, PA, VA, WV

### Dicaelus sculptilis sculptilis

Say, 1823

Dicaelus sculptilis Say, 1823a: 68. Type locality: «Platte Co[unty], M[iss]o[uri]» (neotype label). Neotype (♂), designated by Lindroth and Freitag (1969: 345), in MCZ [# 33025]. Note. «Missouri [Territory]» was the area originally cited by Say (1823a: 69).
Dicaelus ocellatus Blatchley, 1912: 77. Type locality: «H[ot] Springs [Garland County], Ark[ansas]» (original citation). Lectotype (♂), designated by Blatchley (1930: 37), in INHS. Synonymy established by Ball (1959: 141).

#### Distribution.

This subspecies is found in the southern part of the Great Plains from northeastern Kansas to central Iowa, south to central Arkansas and eastern Oklahoma (Ball 1959: 142) including southwestern Illinois (Union County, CNC). The record from western Pennsylvania by LeConte (1859d: 53) is probably in error.

#### Records.

**USA**: AR, IA, IL, KS, MO, OK

#### Note.

Ball (1959: 142) reported the presence of intergrade populations between all three subspecies of *Dicaelus sculptilis* in Iowa.

### Dicaelus sculptilis upioides

Ball, 1959

Dicaelus sculptilis upioides Ball, 1959: 144. Type locality: «Winnipeg, Manitoba» (original citation). Holotype (♂) in CUIC [# 3516].

#### Distribution.

This subspecies ranges from western Ontario to Saskatoon in Saskatchewan (Lindroth 1969a: 953), south to southern New Mexico (Ball 1959: 145), southeastern Nebraska (Lancaster County, Foster F. Purrington pers. comm. 2009), and southern Illinois (Ball 1959: 145).

#### Records.

**CAN**: MB, ON, SK **USA**: CO, IA, IL, MN, ND, NE, NM, SD, WI, WY

### [politus group]

### Dicaelus ambiguus

LaFerté-Sénectère, 1841

Dicaelus ambiguus LaFerté-Sénectère, 1841a: 44. Type locality: «Amer[ica] bor[ealis]» (original citation in Dejean 1836: 31), herein restricted to Columbia, Lancaster County, Pennsylvania (see LeConte, 1847: 430, as *Dicaelus reflexus*). Syntype(s) probably in MHNP (collection Chaudoir).
Dicaelus opacus LaFerté-Sénectère, 1841a: 43. Type locality: Texas (inferred from title of the paper). Syntype(s) probably in MHNP (collection Chaudoir). Synonymy established by LeConte (1873b: 324).
Dicaelus reflexus LeConte, 1847: 430. Type locality: «Columbiam [= Columbia, Lancaster County] Pensylvaniae» (original citation). Syntype(s) in MCZ [# 5717]. Synonymy established with doubt by LeConte (1863b: 10), confirmed by Ball (1959: 112).
Dicaelus turbulentus LeConte, 1863c: 12. Type locality: «Missouri» (original citation). Four syntypes in MCZ [# 5718]. Synonymy established by Horn (1880b: xvii), confirmed by Ball (1959: 112).

#### Distribution.

This species is known from northern New Jersey (Smith 1910: 207) to southeastern Iowa, south to southeastern Texas (Ball 1959: 113) and the Florida Panhandle (Peck and Thomas 1998: 21).

#### Records.

**USA**: AL, AR, DC, FL, GA, IA, IL, IN, KY, LA, MD, MO, MS, NC, NJ, OH, OK, PA, SC, TN, TX, VA

### Dicaelus politus

Dejean, 1826

Dicaelus politus Dejean, 1826: 391. Type locality: «Amérique septentrionale» (original citation), restricted to «Uniontown, Penns[ylvania]» by Lindroth (1969a: 951). One syntype in MHNP (Lindroth 1955b: 18).
Dicaelus leonardii T.W. Harris, 1828d: 132. Type locality not stated. Possible syntype(s) in MCZ. Synonymy established by Harris (1829: 1), confirmed by Ball (1959: 110). Note. Harris’s collection contains two specimens under the name *Dicaelus politus*, both from New Hampshire. Ball (1959: 110) reported the presence of one syntype in MCZ, probably referring to the ♂ specimen in LeConte’s collection labeled “[pink disc] / D. politus Dej. Leonardii Harris. [handwritten].”
Dicaelus angustus Casey, 1913: 152. Type locality: «Tennessee» (original citation). One syntype in USNM [# 47363]. Synonymy established by Ball (1959: 110).

#### Distribution.

This species ranges from southern Quebec (Larochelle 1975: 80) to northern Iowa, south to northern Alabama (Ball 1959: 111) and South Carolina (Ciegler 2000: 84). The record from “Florida” (Horn 1880c: 52) needs confirmation.

#### Records.

**CAN**: ON, QC **USA**: AL, CT, DC, DE, GA, IA, IL, IN, KY, MA, MD, ME, MI, MO, MS, NC, NH, NJ, NY, OH, PA, RI, SC, TN, VA, VT, WI, WV [FL]

### Dicaelus teter

Bonelli, 1813

Dicaelus teter Bonelli, 1813: 449. Type locality: «Amérique septentrionale» (original citation), restricted to «Pike [Wyoming County], N[ew] Y[ork]» by Lindroth (1969a: 952). Syntype(s) probably in MHNP (Casale and Giachino 1998: 68).
Dicaelus ovipennis Casey, 1913: 152. Type locality: «North Carolina» (original citation). One syntype in USNM [# 47362]. Synonymy established by Ball (1959: 114).

#### Distribution.

This species ranges from southern Quebec (Larochelle 1975: 80) to southwestern Indiana, south to northern Alabama (Ball 1959: 115), northern Georgia (Fattig 1949: 31), and northwestern South Carolina (Ciegler 2000: 85). The record from South Dakota (Kirk and Balsbaugh 1975: 35) is likely in error.

#### Records.

**CAN**: ON, QC **USA**: AL, CT, DC, GA, IN, KY, MA, MD, MI, NC, NJ, NY, OH, PA, SC, TN, VA, VT, WV

### Subgenus Dicaelus

Bonelli, 1813

Dicaelus Bonelli, 1813: 446. Type species: *Dicaelus violaceus* Bonelli, 1813 (= *Dicaelus purpuratus* Bonelli, 1813) designated by Hope (1838: 82).

#### Diversity.

Six North American species, one of them extending into northern Mexico, arrayed in three species groups.

### [costatus group]

### Dicaelus costatus

LeConte, 1853

Dicaelus costatus LeConte, 1853c: 389. Type locality: «Texas» (original citation), herein restricted to Kingsville, Kleberg County (see Ball 1959: 168). Syntype(s) [3 originally cited] in MCZ [# 5708].
Dicaelus costatus var. *lerdoensis* Bates, 1891a: 238. Type locality: «Villa Lerdo, in Durango» (original citation). Syntype(s) [2 originally cited] probably in BMNH. Synonymy established by Ball (1959: 166).

#### Distribution.

This species is known from eastern Texas south to southern Tamaulipas and Durango (Ball 1959: 167) in Mexico. Two specimens are known from northern Florida and “Virginia” (Ball 1959: 167).

#### Records.

**USA**: TX [FL, VA] – Mexico

### [crenatus group]

### Dicaelus alternans

Dejean, 1826

Dicaelus alternans Dejean, 1826: 387. Type locality: «Amérique septentrionale» (original citation), herein restricted to Saint Simons Island, Glynn County, Georgia (see Ball 1959: 164). One syntype in MHNP (Lindroth 1955b: 18).

#### Distribution.

This species is known from southeastern coastal Georgia south to southern Florida (Ball 1959: 164).

#### Records.

**USA**: FL, GA

### Dicaelus crenatus

LeConte, 1853

Dicaelus crenatus LeConte, 1853c: 389. Type locality: «Louisiana» (original citation). Holotype [by monotypy] (♀) in MCZ [# 5714].

#### Distribution.

This species inhabits the southern part of the Great Plains from “Kansas” to southeastern Missouri (Ball 1959: 162), south to northwestern Mississippi (Coahoma County, Drew A. Hildebrandt pers. comm. 2008), “Louisiana” (LeConte 1853c: 389), and southern Texas (Ball 1959: 162). The records from Alabama (Löding 1945: 18), the Florida Panhandle (Harris and Whitcomb 1974: 99), Georgia (Fattig 1949: 31), and North Carolina (Brimley 1938: 122) need confirmation.

#### Records.

**USA**: AR, KS, LA, MO, MS, OK, TX [AL, FL, GA, NC]

### Dicaelus subtropicus

Casey, 1913

Dicaelus subtropicus Casey, 1913: 151. Type locality: «Palm Beach [Palm Beach County], Florida» (original citation). Six syntypes [6 originally cited] in USNM [# 47358].

#### Distribution.

This species is known only from a few localities in the southern third of Florida, including the Keys (Ball 1959: 165).

#### Records.

**USA**: FL

#### Note.

This form has been considered as a subspecies of *Dicaelus alternans* Dejean by Ball (1959: 164) but I believe it is more appropriately treated as a distinct species.

### [purpuratus group]

### Dicaelus purpuratus purpuratus

Bonelli, 1813

Dicaelus purpuratus Bonelli, 1813: 447. Type locality: «Caroline» (original citation), incorrectly restricted to «Kentucky» by Lindroth (1969a: 953), herein restricted to Raleigh, Wake County, North Carolina (CNC). Syntype(s) location unknown (possibly in MHNG in collection Jurine, see Casale and Giachino 1998: 68).
Dicaelus violaceus Bonelli, 1813: 447. Type locality: «Caroline» (original citation). Syntype(s) location unknown (possibly in MHNG in collection Jurine). Synonymy established by LeConte (1863b: 10).
Dicaelus chalybeus Dejean, 1826: 385. Type locality: «Louisiane» (original citation). One syntype in MHNP (Lindroth 1955b: 17). Synonymy established with doubt by Dejean (1826: 385), confirmed by Lindroth (1969a: 953).
Dicaelus cyaneus Dejean, 1831: 686. Type locality: «Amérique septentrionale» (original citation). Holotype [by monotypy] (♂) in MHNP (Lindroth 1955b: 18). Synonymy established by Horn (1880c: 52).
Dicaelus confusus LeConte, 1847: 424. Type locality: «Georgia» (original citation). Holotype [by monotypy] (♀) in MCZ [# 5711]. Synonymy established by Horn (1880c: 52), confirmed by Ball (1959: 154).
Dicaelus iricolor LeConte, 1847: 426. Type locality: «urbem S[ain]t Louis [Missouri]» (original citation). Syntype(s) in MCZ [# 5712]. Synonymy established by Horn (1880c: 52), confirmed by Ball (1959: 154).

#### Distribution.

The range of this subspecies extends from Massachusetts to western Wisconsin (Ball 1959: 156), south to southeastern Texas (Cameron County, Brian Raber pers. comm. 2010), southern Louisiana (Summers 1874a: 80; Allen 1965: 73), southwestern Alabama (Ball 1959: 156), and central Georgia (Fattig 1949: 30).

#### Records.

**CAN**: ON **USA**: AL, CT, DC, GA, IA, IL, IN, KY, LA, MA, MD, MI, MO, MS, NC, NJ, NY, OH, PA, SC, TN, TX, VA, WI, WV

### Dicaelus purpuratus splendidus

Say, 1823

Dicaelus splendidus Say, 1823a: 69. Type locality: «Platte Co[unty], M[iss]o[uri]» (neotype label). Neotype (♀), designated by Lindroth and Freitag (1969: 343), in MCZ [# 33024]. Note. «Missouri [Territory]» was the area originally cited by Say (1823a: 69).
Dicaelus decoloratus LeConte, 1847: 423. Type locality: «Texas» (original citation). Holotype [by monotypy] (♀) in MCZ [# 5710]. Synonymy established with doubt by LeConte (1853c: 388), confirmed by Ball (1959: 156).
Dicaelus speciosus Casey, 1913: 152. Type locality: «New Mexico» (original citation). Two syntypes in USNM [# 47361]. Synonymy established by Fall (1932: 20), confirmed by Ball (1959: 156).

#### Distribution.

The range of this subspecies extends from “Minnesota” (Ball 1959: 158) to southwestern North Dakota (Tinerella 2003: 636), south to southeastern Arizona, southeastern Texas (Ball 1959: 158), and southeastern Louisiana (Allen 1965: 73), including west-central Illinois (Willand et al. 2011: 273).

#### Records.

**USA**: AR, AZ, CO, IA, IL, KS, LA, MN, MO, ND, NE, NM, OK, SD, TX

### Dicaelus quadratus

LeConte, 1847

Dicaelus quadratus LeConte, 1847: 422. Type locality: «Georgia» (original citation). Two syntypes in MCZ [# 5713].
Dicaelus lecontei LaFerté-Sénectère, 1851: 277. Type locality: «Amer[ica] bor[ealis]» (original citation). Syntype(s) probably in MHNP (collection Chaudoir). Synonymy established by LeConte (1853c: 388).
Dicaelus darlingtoni Fall, 1932: 19. Type locality: «Homestead [Dade County], Florida» (original citation). Holotype (♀) in MCZ [# 23875]. **New synonymy**.

#### Distribution.

This species has been recorded from “Georgia” (LeConte 1847: 422), the Florida Panhandle and Peninsula including the Keys (Peck and Thomas 1998: 21), and southwestern Alabama (Löding 1945: 18; Ball 1959: 159).

#### Records.

**USA**: AL, FL, GA

#### Note.

Following Lindroth (1969a: 954), I believe this taxon should be regarded as a valid species, not as a subspecies of *Dicaelus purpuratus* Bonelli.

### Subgenus Liodicaelus

Casey, 1913

Liodicaelus Casey, 1913: 154. Type species: *Liodicaelus evanescens* Casey, 1913 (= *Dicaelus laevipennis* LeConte, 1847) by original designation. Etymology. From the Greek prefix *lio*- (smooth) and the generic name *Dicaelus* [*q.v*.] [masculine].

#### Diversity.

Five species in North America (three species) and Mexico (four species, two of them endemic).

### Dicaelus chermocki

Ball, 1959

Dicaelus chermocki Ball, 1959: 175. Type locality: «Carr Canyon (7,500’), Huachuca M[oun]t[ain]s, Cochise County, Arizona» (original citation). Holotype (♀) in MCZ [# 31162]. Etymology. The specific name was given in honor of Ralph Lucien Chermock [1918-1977], professor of biology at the University of Alabama and eventually director of the Alabama Museum of Natural History. His interest, shared with his wife Ottilie Diana Chermock, was directed mainly to Lepidoptera. Each year, the Ralph L. Chermock Prize is presented to the most outstanding graduate student in systematic and ecology at the University of Alabama.

#### Distribution.

This species is confined to the Huachuca and Chiricahua Mountains of southeastern Arizona [see Ball 1992a: Fig. 17].

#### Records.

**USA**: AZ

### Dicaelus laevipennis laevipennis

LeConte, 1847

Dicaelus laevipennis LeConte, 1847: 421. Type locality: «flumen Platte, prope rupem caminatam (Chimney) [Colorado]» (original citation). Two syntypes in MCZ [# 5707].
Liodicaelus evanescens Casey, 1913: 154. Type locality: «San Bernardino Ranch, Cochise Co[unty], Arizona» (original citation). One syntype in USNM [# 47364]. Synonymy established by Ball (1959: 170).
Dicaelus laevipennis dicaeloides Ball, 1959: 172. Type locality: «Cloudcroft (9,000’), Otero County, New Mexico» (original citation). Holotype (♂) in MCZ [# 31161]. Synonymy established by Ball (1992a: 365).

#### Distribution.

This subspecies ranges from southeastern Alberta to southwestern South Dakota, south to southeastern Texas, Chihuahua in Mexico, and southeastern Arizona [see Ball 1992a: Fig. 18].

#### Records.

**CAN**: AB **USA**: AZ, CO, KS, MT, NE, NM, OK, SD, TX, UT – Mexico

#### Note.

The subspecies *Dicaelus laevipennis flohri* Bates is endemic to Mexico ranging from the state of Durango to the Mexico City area (Ball 1992a: 366).

### Dicaelus suffusus

(Casey, 1913)

Liodicaelus suffusus Casey, 1913: 155. Type locality: «Sierra Madre M[oun]t[ain]s, Chihuahua» (original citation), restricted to «vicinity of Madera» by Ball (1992a: 364). Nine syntypes [9 originally cited] in USNM [# 47365].

#### Distribution.

This species is known only from the Chiricahua Mountains in southern Arizona and the Sierra Madre Occidental in Chihuahua and Sonora [see Ball 1992a: Fig. 17].

#### Records.

**USA**: AZ – Mexico

### Subtribe Licinina

Bonelli, 1810

Licinii Bonelli, 1810: Tabula Synoptica. Type genus: *Licinus* Latreille, 1802.
Badistidae Gistel, 1856: 357. Type genus: *Badistes* Agassiz, 1846 (unjustified emendation of *Badister* Clairville, 1806, not in prevailing usage) (= *Badister* Clairville, 1806).

#### Diversity.

Worldwide, with about 100 species in the Nearctic (14 species of *Badister*), Neotropical (five species, one of them belonging to the Chilean genus *Eutogeneius* Solier), Australian (11 species), Oriental (four species), Palaearctic (68 species), and Afrotropical (three species of *Badister*) Regions. Approximately 82% of the species are found in the Northern Hemisphere.

#### Faunistic Note.

Several specimens of *Licinus silphoides* (Rossi) were taken alive in Massachusetts as noted by LeConte (1873b: 324). It is likely that a population persisted for a while but since no specimen has been caught in North America in the xx Century, the species is certainly not established on this continent.

### Genus Badister

Clairville, 1806

Badister Clairville, 1806: 90. Type species: *Carabus bipustulatus* Fabricius, 1792 (= *Carabus bullatus* Schrank, 1798) by monotypy. Etymology. From the Greek *badister*, -*os* (walker) [masculine].
Badistes Agassiz, 1846: 42. Unjustified emendation of *Badister* Clairville, 1806.

#### Distribution.

Forty-eight species in the boreal, temperate, and tropical areas in the Nearctic (14 species), Neotropical (five species in Mexico, the West Indies, and Peru), Australian (two species), Oriental (one species), Palaearctic (27 species), and Afrotropical (three species) Regions. The species are arrayed in three subgenera: *Badister* (23 species), *Baudia* (22 species), and *Trimorphus* Stephens (two Palaearctic species).

#### Identification.

Ball (1959) revised the North American species. No new North American species have been described since. Lindroth’s (1969a) key encompasses all North American species but *Badister submarinus*.

### Subgenus Badister

Clairville, 1806

Badister Clairville, 1806: 90. Type species: *Carabus bipustulatus* Fabricius, 1792 (= *Carabus bullatus* Schrank, 1798) by monotypy.
Amblychus Gyllenhal, 1810: 74. Type species: *Carabus bipustulatus* Fabricius, 1792 (= *Carabus bullatus* Schrank, 1798) designated by Jeannel (1942: 1000). Etymology. From the Greek *amblys* (blunt, obtuse) [masculine].

#### Diversity.

Twenty-four species in North America (eight species, one of them extending into the Bahamas), Mexico (three species, one of them endemic), Peru (one species), and the Palaearctic Region (14 species).

#### Identification.

Erwin and Ball (2011: 407-409) published a key to the Western Hemisphere species.

### [elegans group]

### Badister elegans

LeConte, 1880

Badister elegans LeConte, 1880b: 165. Type locality: «Bosque Co[unty, Texas]» (original citation). Two syntypes [3 originally cited] in MCZ [# 5722].

#### Distribution.

This rarely collected species ranges from “Illinois” to eastern Kansas (Ball 1959: 206), south to Coahuila and Tamaulipas (Ball 1992a: 375) in northern Mexico, southeastern Louisiana (East Baton Rouge Parish, Igor M. Sokolov pers. comm. 2009), and western Alabama (Tuscaloosa County, UASM). The records from southwestern New Jersey (Smith 1910: 207), “Florida” (Leng 1915: 580), “Delaware” (Houghton 1905: 211), and “Colorado” (Csiki 1931: 903) need confirmation.

#### Records.

**USA**: AL, AR, IL, KS, LA, MO, OK, TX [CO, DE, FL, NJ] – Mexico

### Badister flavipes flavipes

LeConte, 1853

Badister flavipes LeConte, 1853c: 388. Type locality: «Louisiana» (original citation). Two syntypes [2 originally cited] in MCZ [# 5725].
Europhilus iridipennis Motschulsky, 1865: 321. Type locality: «environs de la nouvelle Orléans [Orleans Parish, Louisiana]» (original citation). Lectotype (♀), designated by Liebherr (1991b: 120), in ZMMU. Synonymy established by Liebherr (1991b: 120).
Badister laticeps Blatchley, 1910: 118. Type locality: «Perry County [Indiana]» (original citation for the lectotype). Lectotype (♀), designated by Blatchley (1930: 33), in PURC. **New synonymy**.
Badister flavicornis Casey, 1920: 208. Type locality: «Cedar Rapids [Linn County], Iowa» (original citation). One syntype in USNM [# 47368]. Synonymy established, under the name *Badister flavipes laticeps* Blatchley, by Ball (1959: 211).

#### Distribution.

This subspecies ranges from south-central New York (Ball 1959: 213) to southeastern Nebraska (Lancaster County, UASM), including southern Wisconsin (Rauterberg 1885: 17; Purrington et al. 2002: 201), south to southern Texas (Gonzales County, CMNH; Brazoria County, Robert L. Davidson pers. comm. 2008) and southern Florida (Peck and Thomas 1998: 21); also recorded from the Bahamas (Darlington 1953: 8).

#### Records.

**USA**: AR, DC, FL, GA, IA, IL, IN, KS, KY, LA, MD, MI, MO, MS, NE, NY, OH, PA, TN, TX, WI – Bahamas.

#### Note.

Ball (1959) recognized three subspecies within this taxon, two of them occurring north of Mexico. Because the differences between the two forms are largely based on color and that intermediate specimens are known from Louisiana (Ball 1959: 210), I prefer not to recognize the color forms as distinct subspecies. *Badister flavipes mexicanus* Van Dyke is endemic to Mexico.

### Badister maculatus

LeConte, 1853

Badister maculatus LeConte, 1853c: 387. Type locality: «Lancaster [Lancaster County], P[ennsylvani]a» (original citation). Holotype [by monotypy] (♀) in MCZ [# 5721].

#### Distribution.

This rarely collected species ranges from southwestern New Jersey (Smith 1910: 207) to central Kansas (Knaus 1898: 19), south to east-central Texas (Riley 2011), central Louisiana (Concordia Parish, CNC), and northern Florida (Peck and Thomas 1998: 21).

#### Records.

**USA**: AL, AR, DC, FL, GA, IA, IN, KS, KY, LA, MO, MS, NJ, OH, OK, PA, SC, VA, TX

### [notatus group]

### Badister notatus

Haldeman, 1843

Badister notatus Haldeman, 1843b: 299. Type locality: southeastern Pennsylvania (Haldeman 1843a: 296). Syntype(s) presumably lost.
Badister terminalis LeConte, 1844: 51. Type locality: «New York» (original citation). One syntype in MCZ [# 5719]. Synonymy established by LeConte (1847: 417), confirmed by Ball (1959: 215).
Badister angustus Casey, 1920: 207. Type locality: «Illinois» (original citation). One syntype in USNM [# 47367]. Synonymy established by Ball (1959: 215).
Badister gilvipes Casey, 1920: 207. Type locality: «Long Island, New York» (original citation). One syntype in USNM [# 47366]. Synonymy established by Ball (1959: 215).

#### Distribution.

This species is found from southwestern New Brunswick (Webster and Bousquet 2008: 20) to eastern South Dakota (Kirk and Balsbaugh 1975: 35; French et al. 2004: 557), south to east-central Texas (Brazos County, CNC; Riley 2011), northern Georgia (Ball 1959: 216-217), and northwestern South Carolina (Ciegler 2000: 85).

#### Records.

**CAN**: NB, ON, QC **USA**: AL, AR, CT, DC, DE, GA, IA, IL, IN, KS, KY, LA, MA, MD, ME, MI, MN, MO, MS, NC, NE, NH, NJ, NY, OH, OK, PA, RI, SC, SD, TN, TX, VA, VT, WI, WV

### [pulchellus group]

### Badister ferrugineus

Dejean, 1831

Badister ferrugineus Dejean, 1831: 690. Type locality: «Californie» (original citation), herein restricted to San Francisco, San Francisco County (see Eschscholtz 1833: 28). Holotype [by monotypy] (♂) in MHNP (Lindroth 1955b: 18).
Badister anthracinus LeConte, 1859a: 83. Type locality: «Oregon» (original citation). One syntype in MCZ [# 5724]. Synonymy established by Lindroth (1969a: 961).

#### Distribution.

This species ranges from southwestern British Columbia, including Vancouver Island (Lindroth 1969a: 962), south to southern California (Ball 1959: 200-201). One old specimen labeled “Nev.” is known (Ball 1959: 200).

#### Records.

**CAN**: BC (VCI) **USA**: CA, OR, WA [NV]

### Badister neopulchellus

Lindroth, 1954

Badister neopulchellus Lindroth, 1954b: 153. Type locality: «West Roxbury [Suffolk County], Massachusetts» (original citation). Holotype (♂) in MCZ [# 29071].

#### Distribution.

This species ranges from Cape Breton Island to southwestern British Columbia, north to the Great Slave Lake in Northwest Territories (Lindroth 1969a: 959), south to south-central California (Ball 1992a: 375), northeastern Kansas (Popenoe 1878: 78, as *Badister pulchellus*), and northeastern Georgia (Fattig 1949: 31, as *Badister pulchellus*). Two specimens simply labeled “Tex” (MCZ) are known (Ball 1959: 196).

#### Records.

**CAN**: AB, BC, MB, NB, NS (CBI), NT, ON, PE, QC, SK **USA**: CA, CO, CT, DC, GA, IA, ID, IL, IN, KS, MA, ME, MI, MN, MO, MT, NC, ND, NE, NH, NJ, NY, OH, OR, PA, RI, SD, UT, VT, WA, WI, WV, WY [TX]

**Figure 31.**
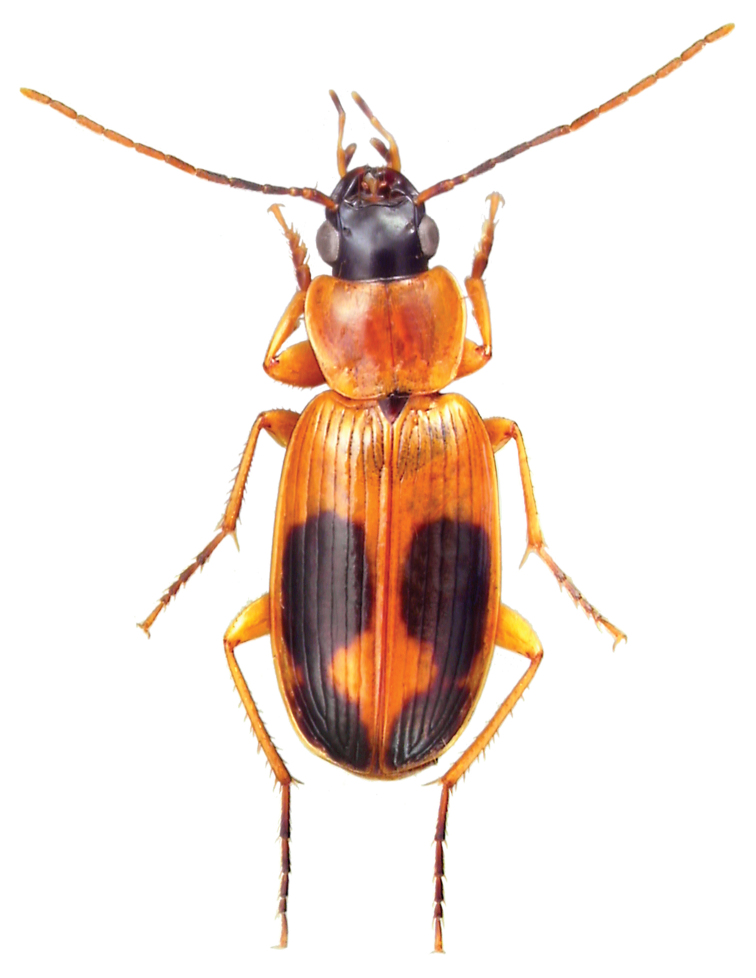
*Badister neopulchellus* Lindroth. This nicely colored licinine was first recognized by Carl Lindroth when he realized that the *Badister pulchellus* of authors included two species, this one which is transcontinental and relatively commonly collected and *Badister pulchellus*, a much rarer species restricted to the eastern parts of the continent. With the advent of molecular tools, there is little doubt that several of our carabid species will eventually be showed to be complexes of species that cannot be separately with confidence based on morphology alone.

### Badister obtusus

LeConte, 1878

Badister obtusus LeConte, 1878c: 594. Type locality: «Marquette [Marquette County, Michigan], Lake Superior» (original citation). Holotype [by monotypy] (♀) in MCZ [# 5723].

#### Distribution.

This species is found from Cape Breton Island (Bousquet 1987a: 132) to south-central British Columbia (Jarrett and Scudder 2001: 380), north to central Northwest Territories (Ball 1992a: 375), south to northeastern New Mexico (Ball 1992a: 375) along the Rocky Mountains, the Guadalupe Mountains in western Texas (Ball 1992a: 375), the upper peninsula of Michigan (LeConte 1878c: 594), southern Ontario (Lindroth 1969a: 961), and southern Maine (Majka et al. 2011: 46). The record from the Pacific Northwest (Hatch 1953: 961) refers to *Badister neopulchellus* (Lindroth 1969a: 959).

#### Records.

**CAN**: AB, BC, MB, NB, NS (CBI), NT, ON, QC, SK **USA**: CO, ME, MI, MN, MT, ND, NM, SD, TX, WI

### Badister pulchellus

LeConte, 1847

Badister pulchellus LeConte, 1847: 418. Type locality: «Evansville Indianae, et in provinciis orientalibus» (original citation), restricted to «Evanston [error for Evansville, Vanderburgh County]» by Ball (1959: 204). Three syntypes in MCZ [# 5720].

#### Distribution.

This rarely collected species is known from southern Ontario (Lindroth 1969a: 959), northern Ohio (Ashtabula County, Harry J. Lee, Jr. pers. comm. 2008), southern Michigan (Clinton County, CMNH), northern and southwestern Indiana (LeConte 1847: 418; Ball 1959: 205), western Illinois, “Tennessee” (Ball 1959: 205), southwestern Alabama (Lindroth 1954b: 153), and Connecticut (New London County, Foster F. Purrington pers. comm. 2009). Most old (pre-1950s) records of this species refer to *Badister neopulchellus*.

#### Records.

**CAN**: ON **USA**: AL, CT, IL, IN, MI, OH, TN

### Subgenus Baudia

Ragusa, 1884

Baudia Ragusa, 1884: 3. Type species: *Carabus peltatus* Panzer, 1796 by monotypy. Etymology. From the surname of the Italian entomologist Flaminio Baudi di Selve [1821-1901] [feminine].

#### Diversity.

Twenty-two species in the Nearctic (six species), Neotropical (one species shared with North America), Australian (two species, one of them endemic to New Caledonia), Oriental (one species shared with the Australian Region), Palaearctic (11 species), and Afrotropical (three endemic species) Regions.

### [micans group]

### Badister grandiceps

Casey, 1920

Badister grandiceps Casey, 1920: 209. Type locality: «District of Columbia» (original citation). Holotype [by monotypy] (♀) in USNM [# 47369].

#### Distribution.

This transamerican species ranges from Cape Breton Island to Vancouver Island, south to “Oregon” (Lindroth 1969a: 964-965), northern Utah (Ball 1959: 225), east-central South Dakota (Kirk and Balsbaugh 1975: 35), northeastern Illinois (Purrington et al. 2002: 200), and the District of Columbia (Ball 1959: 225). The record from “Nebraska” (Bousquet and Larochelle 1993: 213) needs confirmation.

#### Records.

**CAN**: AB, BC (VCI), MB, NB, NS (CBI), ON, PE, QC **USA**: CT, DC, IA, IL, IN, KY, MA, ME, MI, MN, MT, NH, NJ, NY, OH, OR, PA, SD, UT, VT, WA, WI [NE]

### Badister micans

LeConte, 1844

Badister micans LeConte, 1844: 52. Type locality: «Georgia» (original citation). One syntype, a ♂ labeled “[orange disc] / micans 3 [handwritten],” in MCZ (collection LeConte).
Badister ocularis Casey, 1920: 210. Type locality: «Illinois» (original citation). Holotype [by monotypy] (♀) in USNM [# 47370]. Synonymy established with doubt by Bousquet and Larochelle (1993: 213), herein confirmed.

#### Distribution.

This species occurs from Nova Scotia (Lindroth 1954c: 305) to western Minnesota (Gandhi et al. 2005: 929, as *Badister ocularis*), south to northwestern Tennessee (Lake County, UASM) and northern Georgia (Leng 1910: 73; Fattig 1949: 32). The records from “Florida” (Schaupp 1882b: 7), northeastern Kansas (Popenoe 1878: 78), and southern Texas (Wickham 1897: 105) need confirmation.

#### Records.

**CAN**: NB, NS, ON, PE, QC **USA**: CT, GA, IL, IN, MA, ME, MI, MN, NH, NJ, NY, OH, PA, RI, SC, TN, VA, VT, WI [FL, KS, TX]

#### Note.

Casey (1920: 206-207), followed by Ball (1959: 227) and Lindroth (1969a: 966), stated that the species described by LeConte in 1844 as *Badister micans* was “very different” from that described under the same name in 1847 (page 418). However the only difference I can see between the 1844 and 1847 descriptions is the size: 4½ lines (= 9 mm) in the 1844 description and .24 [inch] (= 6 mm) in the 1847 description. In 1844, Georgia is listed as the provenance of the specimen(s) and in 1847 he reported he had a specimen from Georgia and one from Long’s Peak. LeConte (1844: 52) added that *Badister micans* “differs very much in its general appearance from all the species of this genus” which also led Casey (1920: 206) to believe that the specimen was “probably not a *Badister*.” However, the other two species of *Badister* described by LeConte in 1844 were *Badister terminalis* (= *Badister notatus* Haldeman) and *Badister testaceus* (= *Philodes alternans* LeConte). Both species are indeed quite different from *Badister ocularis*. My interpretation is that LeConte made a simple mistake in the size of the species in his 1844 description. Blatchley (1928a: 47) also commented on this species.

### Badister parviceps

Ball, 1959

Badister parviceps Ball, 1959: 225. Type locality: «Crusoe Lake, Wayne County, New York» (original citation). Holotype (♂) in CUIC [# 3517].

#### Distribution.

This species ranges from southwestern Quebec (Bousquet 1987a: 132) to southeastern Minnesota (Ball 1959: 226), north to southern Manitoba (Lindroth 1969a: 967), south to southeastern Texas (Ball 1992a: 377) and northwestern Alabama (Colbert County, CMNH). One specimen labeled from Creston, British Columbia in UBC seen by Lindroth (1969a: 967) is probably mislabeled.

#### Records.

**CAN**: MB, ON, QC **USA**: AL, AR, CT, DC, IA, IL, IN, KS, MA, MD, MI, MN, MO, MS, NY, OH, PA, TN, TX, VA, WI, WV

### Badister reflexus

LeConte, 1880

Badister reflexus LeConte, 1880b: 166. Type locality: «New York, Michigan, Louisiana» (original citation), restricted to «New York» by Ball (1959: 222). Four syntypes in MCZ [# 5727].
Badister seclusus Blatchley, 1922: 12. Type locality: «Dunedin [Pinellas County], Fl[orid]a» (original citation). Lectotype (♀), designated by Blatchley (1930: 45), in PURC. Synonymy established by Ball (1959: 222).

#### Distribution.

This species is known from Rhode Island (Sikes and Webster 2005: 315) to southern Wisconsin (Messer 2010: 39), including southernmost Ontario (Lindroth 1969a: 966, probably only as strays), south to the Rio Grande in southeastern Texas (Ball 1992a: 376), the Florida Keys, and the Greater Antilles (Ball 1992a: 376-377); it is also known from a single specimen, possibly a stray, collected in southwestern New Brunswick (Webster and Bousquet 2008: 21) and from Quintana Roo in the Yucatán Peninsula (Ball 1992a: 376). The record from “Quebec” (Bousquet and Larochelle 1993: 213) is in error.

#### Records.

**CAN**: NB, ON **USA**: AL, AR, CT, DC, FL, GA, IA, IL, IN, LA, MA, MD, MI, MS, NC, NE, NJ, NY, OH, OK, PA, RI, SC, TN, TX, VA, WI, WV – Bahamas, Cayman Islands, Cuba, Dominican Republic, Haiti, Jamaica, Mexico

#### Note.

In the *Zoological Record* for the year 1880 this species is registered (page 22) under the name “*Badister albescens*.”

### Badister submarinus

Motschulsky, 1859

Badistes submarinus Motschulsky, 1859a: 158. Type locality: «environs de la nouvelle Helvétie [= Sacramento, Sacramento County, California]» (original citation). One syntype in ZMMU (Keleinikova 1976: 219) and one in MCZ [# 8327].

#### Distribution.

This species is known from three females collected in southern Oregon (Ball 1992a: 377) and north-central California (Motschulsky 1859a: 158).

#### Records.

**USA**: CA, OR

### [transversus group]

### Badister transversus

Casey, 1920

Badister transversus Casey, 1920: 210. Type locality: «probably Indiana» (original citation), restricted to «Elkhart [Elkhart County], Ind[iana]» by Lindroth (1969a: 962). Holotype [by monotypy] (♀) in USNM [# 47371].

#### Distribution.

This species ranges from New Brunswick (Webster and Bousquet 2008: 20) to southern Manitoba (Lindroth 1969a: 964; Roughley et al. 2010: 230), south to central Nebraska (Loup County, Foster F. Purrington pers. comm. 2010), northern Indiana, and New Jersey (Ball 1959: 219).

#### Records.

**CAN**: MB, NB, ON, QC **USA**: CT, IL, IN, MA, ME, MI, MN, NE, NH, NJ, NY, OH, PA, SD, VT, WI

#### Note.

This species has been referred to the subgenus *Trimorphus* Stephens (type species: *Trimorphus scapularis* Stephens, 1828 (= *Carabus sodalis* Duftschmid, 1812)) by Ball (1959) but Lindroth (1969a: 962) concluded that it is probably more closely related to members of *Baudia* than to those of *Trimorphus*.

### Tribe Harpalini

Bonelli, 1810

Harpalii Bonelli, 1810: Tabula Synoptica. Type genus: *Harpalus* Latreille, 1802.

#### Diversity.

Worldwide, with about 2,725 species arrayed in four subtribes: Anisodactylina (about 345 species), Harpalina (about 1,665 species), Pelmatellina (about 90 species), and Stenolophina (about 625 species).

### Subtribe Anisodactylina

Lacordaire, 1854

Eurytrichini LeConte, 1847: 376 [*nomen oblitum*, see Bouchard et al. (2011: 128, 837-839)]. Type genus: *Eurytrichus* LeConte, 1847 (= *Anisotarsus* Chaudoir, 1837). Note. Family-group name based on a type genus considered to be a junior synonym is not to be replaced (ICZN 1999: Article 40.1) unless it was replaced before 1961 and the replacement name is in prevailing usage (ICZN 1999: Article 40.2). In this case, the replacement name, Anisotarsi Csiki, 1932, is not in prevailing usage and so Eurytrichini LeConte, 1847 is the oldest available name for this subtribe.
Anisodactylides Lacordaire, 1854: 257, 268 [*nomen protectum*]. Type genus: *Anisodactylus* Dejean, 1829.
Anisotarsi Csiki, 1932a: 1039 [replacement name for Eurytrichini]. Type genus: *Anisotarsus* Chaudoir, 1837.

#### Diversity.

Worldwide, with about 345 species arrayed in 30 genera (Lorenz 2005: 348-353). The North American fauna is represented by 48 species (about 14% of the world fauna).

#### Taxonomic Note.

Ball and Bousquet (2000: 90), following Noonan (1973: 388), recognized two genus-groups among the North American taxa, the Notiobii (an unavailable name; referred to as the “Notiobioid main branch” by Noonan) for the genus *Notiobia* and Anisodactyli (“Anisodactyloid main branch” of Noonan) for all remaining genera.

### Genus Notiobia

Perty, 1830

Notiobia Perty, 1830: 13. Type species: *Notiobia nebrioides* Perty, 1830 by monotypy. Etymology (original). From the Greek adjective *notios* (wet, moist, damp) and *bios* (life), alluding to the damp habitat where the species in the hands of Perty lived (“habitatione verosimile demptum est”) [feminine].

#### Diversity.

About 95 species in the Nearctic (eight species), Neotropical (47 species), Australian (28 species), and Afrotropical (ten species) Regions arrayed in three subgenera: *Anisotarsus* (53 species), *Diatypus* Murray (ten Ethiopian species), and *Notiobia* s.str. (26 Neotropical species).

### Subgenus Anisotarsus

Chaudoir, 1837

Anisotarsus Chaudoir, 1837b: 41. Type species: *Anisotarsus brevicollis* Chaudoir, 1837 designated by Casey (1914: 209). Etymology (original). From the Greek *anisos* (unequal) and *tarsos* (tarsus), alluding to the fact that the male protarsomere 1 is distinctly smaller than the following three (“*premier article [du tarse du mâle] très petit, proportionnellement aux 2e, 3e et 4e articles, qui sont larges, cordiformes*”) in the two species that Chaudoir included in this taxon [masculine].
Diaphoromerus Chaudoir, 1843a: 402. Type species: *Diaphoromerus iridipennis* Chaudoir, 1843 by monotypy. Synonymy established by Noonan (1973: 295). Etymology (original). From the Greek *diaphoros* (different) and *meros* (part, segment) [masculine].
Eurytrichus LeConte, 1847: 387. Type species: *Feronia terminata* Say, 1823 designated by van Emden (1953b: 525). Synonymy established by LeConte (1870: 403). Etymology (original). From the Greek *eurys* (wide, extensive) and *trichos* (hair), alluding to the dense setae on the ventral surface of the male tarsi (“*tarsis ♂ subtus dense pilosis*”) [masculine].
Harpalodes Motschulsky, 1864: 208. Type species: *Harpalus fulgens* Dejean, 1829 (= *Poecilus chalcitis* Germar, 1824) by original designation. Synonymy established by Bousquet (2002b: 25). Etymology. From the generic name *Harpalus* [*q.v*.] and the Greek suffix -*odes* (likeness) [masculine].
Stilbolidus Casey, 1914: 171. Type species: *Harpalus mexicanus* Dejean, 1829 by original designation. Synonymy established by Lindroth (1968: 864).

#### Diversity.

Fifty-three species in the Nearctic (eight species, four of them endemic), Neotropical (21 species), and Australian (28 species) Regions.

#### Identification.

Noonan (1973) revised the North and Middle American species and provided a key for their identification.

### [brevicollis group]

### Notiobia brevicollis

(Chaudoir, 1837)

Anisotarsus brevicollis Chaudoir, 1837b: 42. Type locality: «Mexique» (original citation), restricted to «state of Puebla» by Noonan (1973: 300). Lectotype (♀), designated by Noonan (1973: 300), in MHNP.
Anisotarsus laeviusculus Chaudoir, 1837b: 43. Type locality: «Mexique» (original citation). Lectotype (♂), designated by Noonan (1973: 300), in MHNP. Synonymy established by Bates (1882a: 49), confirmed by Noonan (1973: 300).

#### Distribution.

This species ranges from southeastern Arizona to southeastern Texas, south to southern Mexico; it is also known from southern Baja California [see Noonan 1973: Fig. 139].

#### Records.

**USA**: AZ, NM, TX – Mexico

### [terminata group]

### Notiobia cephala

(Casey, 1914)

Anisotarsus cephalus Casey, 1914: 215. Type locality: «Florida» (original citation), restricted to «Gainesville, Alachua Co[unty]» by Shpeley (2001: 117). Lectotype [as holotype] (♀), designated by Noonan (1973: 305), in USNM [# 47978].
Anisotarsus tenuitarsis Casey, 1914: 215. Type locality: «Lake Worth [Palm Beach County], Florida» (original citation). Lectotype (♂), designated by Noonan (1973: 305), in USNM [# 47979]. Synonymy established by van Emden (1953b: 544), confirmed by Shpeley (2001: 117).

#### Distribution.

This species is endemic to the Florida Peninsula (Shpeley 2001: 118).

#### Records.

**USA**: FL

#### Note.

This form has been listed as a synonym of *Notiobia nitidipennis* (LeConte) by Noonan (1973: 305) but treated as a distinct species by Shpeley (2001).

### Notiobia maculicornis

(Chaudoir, 1843)

Harpalus maculicornis Chaudoir, 1843b: 787. Type locality: «près de la Nouvelle-Orléans [Orleans Parish, Louisiana]» (original citation). Lectotype (♂), designated by Noonan (1973: 307), in MHNP.
Harpalus patronus Casey, 1914: 89. Type locality: «Morgan City [Saint Mary Parish], Louisiana» (original citation). One syntype in USNM [# 47793]. Synonymy established by Noonan (1973: 307).
Anisodactylus depressus Notman, 1919b: 236. Type locality: «Austin [Travis County], Tex[as]» (original citation). Lectotype (♂), designated by Noonan (1973: 307), in USNM [# 75704]. Synonymy established by Noonan (1973: 307).

#### Distribution.

This species is known from Connecticut (Krinsky and Oliver 2001: 212), Long Island, New York (Noonan 1973: 307), the coast of southern North Carolina (Brunswick County, Ken Karns pers. comm. 2009), and from “Kansas” and northern Arkansas south to southern Mississippi and eastern Texas [see Noonan 1973: Fig. 134]. The records from New Mexico (Snow 1885: 67; Fall and Cockerell 1907: 162, as *Anisodactylus maculicornis*) are likely in error.

#### Records.

**USA**: AR, CT, KS, LA, MS, NC, NY, OK, TX

### Notiobia mexicana

(Dejean, 1829)

Harpalus mexicanus Dejean, 1829: 288. Type locality: «Mexique» (original citation), herein restricted to Guadalajara, Jalisco (see Casey 1914: 208, as *Stilbolidus aztecanus*). Lectotype (♂), designated by Noonan (1973: 320), in MHNP.
Anisodactylus arizonae Casey, 1884b: 6. Type locality: «Arizona» (original citation). Holotype [by monotypy] (♂) in USNM [# 47964]. Synonymy established by Horn (1886b: xii), confirmed by Noonan (1973: 320).
Stilbolidus aztecanus Casey, 1914: 208. Type locality: «Guadalajara [Jalisco], Mexico» (original citation). Lectotype (♀), designated by Noonan (1973: 320), in USNM [# 47965]. Synonymy established by van Emden (1953b: 540), confirmed by Noonan (1973: 320).

#### Distribution.

This species ranges from the mountains in Arizona and northern New Mexico south to northern Panama; also known from the Laguna Mountains in southern Baja California [see Noonan (1973: Fig. 137)].

#### Records.

**USA**: AZ, NM, TX – Costa Rica, Guatemala, Mexico, Panama

### Notiobia nitidipennis

(LeConte, 1847)

Eurytrichus nitidipennis LeConte, 1847: 388. Type locality: «Georgia» (original citation). Holotype [by monotypy] (♂) in MCZ [# 5963].
Anisotarsus delicatus Casey, 1914: 214. Type locality: «Asheville [Buncombe County], North Carolina» (original citation). Lectotype (♀), designated by Noonan (1973: 305), in USNM [# 47977]. Synonymy established by van Emden (1953b: 544), confirmed by Lindroth (1968: 869) and Noonan (1973: 305).
Harpalus conspectus Casey, 1924: 103. Type locality: «M[on]t Royal [= Montreal], Quebec» (original citation). Lectotype (♀), designated by Lindroth (1975: 142), in USNM [# 47806]. Synonymy established by Lindroth (1968: 869).
Harpalus agitabilis Casey, 1924: 104. Type locality: «District of Columbia» (original citation). Holotype [by monotypy] (♂) in USNM [# 47825]. Synonymy established by Noonan (1973: 305).

#### Distribution.

This species ranges from Maine and southern Quebec to southwestern South Dakota (Larsen and Purrington 2010: 571), south to eastern Texas and northeastern Georgia (Shpeley 2001: 119), including “Colorado” (van Emden 1953b: 544) [see Noonan 1973: Fig. 135]. The previous records from Florida (Noonan 1973) refers to *Notiobia cephala* (Casey) (see Shpeley 2001).

#### Records.

**CAN**: ON, QC **USA**: AL, AR, CO, CT, DC, GA, IA, IL, IN, MA, MD, ME, MI, MO, MS, NC, NJ, NY, OH, OK, PA, RI, SC, SD, TN, TX, VA, VT, WI, WV

### Notiobia purpurascens

(Bates, 1882)

Anisotarsus purpurascens Bates, 1882a: 50. Type locality: «Las Vigas [Veracruz], Mexico» (original citation for the lectotype). Lectotype (♂), designated by Noonan (1973: 311), in BMNH.
Anisotarsus convexulus Casey, 1914: 210. Type locality: «Galveston [Galveston County], Texas» (original citation). Lectotype (♀), designated by Noonan (1973: 311), in USNM [# 47968]. Synonymy established by Noonan (1973: 311).
Anisotarsus inaudax Casey, 1914: 211. Type locality: «Galveston and westward nearly to El Paso, Texas» (original citation), restricted to «Galveston [Galveston County]» by Noonan (1973: 311). Lectotype (♀), designated by Noonan (1973: 311), in USNM [# 47967]. Synonymy established by Noonan (1973: 311).
Anisotarsus extraneus Casey, 1914: 212. Type locality: «Los Angeles Co[unty], California» (original citation). Lectotype [as holotype] (♀), designated by Noonan (1973: 311), in USNM [# 47969]. Synonymy established by Noonan (1973: 311).
Anisotarsus calathoides Casey, 1914: 212. Type locality: «Arizona» (original citation). Lectotype (♂), designated by Noonan (1973: 311), in USNM [# 47970]. Synonymy established by Noonan (1973: 311).

#### Distribution.

This species ranges from the San Francisco Bay area to southwestern Alabama, including central Missouri (Shockley and Cline 2004: 281), south to central Mexico and southern California [see Noonan 1973: Fig. 145]. The species is adventive on several islands of Hawaii (Liebherr 2009: 403).

#### Records.

**USA**: AL, AZ, CA, LA, MO, MS, NM, TX – Mexico

### Notiobia sayi

(Blatchley, 1910)

Eurytrichus piceus LeConte, 1847: 388 [secondary homonym of *Anisodactylus piceus* (Ménétriés, 1843)]. Type locality: «NovEboraci [= New York]» (original citation). Holotype [by monotypy] (♂) in MCZ [# 5962].
Anisodactylus sayi Blatchley, 1910: 198. Replacement name for *Anisodactylus piceus* (LeConte, 1847).

#### Distribution.

This species ranges from southern Quebec (Larochelle 1975: 51, as *Anisotarsus piceus*) to northeastern South Dakota (Kirk and Balsbaugh 1975: 32, as *Anisotarsus piceus*), south to eastern Texas and northeastern Florida [see Noonan 1973: Fig. 133].

#### Records.

**CAN**: ON, QC **USA**: AL, AR, CT, DC, FL, GA, IA, IL, IN, KS, KY, LA, MA, MD, MI, MN, MO, MS, NC, NE, NH, NJ, NY, OH, OK, PA, RI, SC, SD, TN, TX, VT, WI

### Notiobia terminata

(Say, 1823)

Feronia terminata Say, 1823a: 48. Type locality: «Cleveland [Cuyahoga County], O[hio]» (neotype label). Neotype (♂), designated by Lindroth and Freitag (1969: 355), in MCZ [# 32981].
Harpalus similis Say, 1823a: 29. Type locality: «Fl[orid]a» (neotype label). Neotype (♂), designated by Lindroth and Freitag (1969: 355), in MCZ [# 32982]. Synonymy established by Noonan (1973: 313). Note. «North Carolina» was the original area cited by Say (1823a: 30).
Harpalus agilis Dejean, 1829: 357. Type locality: «Amérique septentrionale» (original citation), restricted to «Archbold Biological Station, Highland Co[unty], Florida» by Noonan (1973: 313). Lectotype (♂), designated by Noonan (1973: 313), in MHNP. Synonymy established, under the name *Notiobia similis* (Say), by Melsheimer (1853: 23), confirmed by Noonan (1973: 313).
Harpalus ocreatus Say, 1830c: 20. Type locality: «San Cristrobal las Casas (7000’), Chiapas, Mex[ico]» (neotype label). Neotype (♂), designated by Noonan (1973: 313), in MCZ [# 34663]. Synonymy established by Noonan (1973: 313). Note. «Mexico» was the area originally cited by Say (1830c: 20).
Harpalus testaceus Haldeman, 1843b: 301. Type locality: southeastern Pennsylvania (Haldeman 1843a: 297). One possible syntype, a ♀ labeled “[pink disc] / 291. [handwritten] / E. terminatus (Say) Lec. testaceus Hald. [handwritten],” in MCZ (collection LeConte). Synonymy established by LeConte (1863b: 12), confirmed by Lindroth (1968: 867).
Anisotarsus foveicollis Bates, 1884: 269. Type locality: «Volcan de Chiriqui [Chiriqui], Panama» (original citation for the lectotype). Lectotype (♂), designated by Noonan (1973: 314), in BMNH. Synonymy established by Noonan (1973: 314).
Anisotarsus subvirens Casey, 1914: 213. Type locality: «Austin [Travis County], Texas» (original citation). Lectotype (♀), designated by Noonan (1973: 314), in USNM [# 47976]. Synonymy established by Noonan (1973: 314).
Anisotarsus floridanus Casey, 1914: 214. Type locality: «Florida» (original citation), restricted to «Archbold Biological Station, Highland Co[unty]» by Noonan (1973: 314). Lectotype (♂), designated by Noonan (1973: 314), in USNM [# 47982]. Synonymy established by Noonan (1973: 314).
Anisotarsus hebes Casey, 1924: 136. Type locality: «Dallas [Dallas County], Texas» (original citation). Lectotype (♂), designated by Noonan (1973: 314), in USNM [# 47974]. Synonymy established with doubt, under the name *Notiobia terminatus subvirens* (Casey), by van Emden (1953b: 542), confirmed by Noonan (1973: 314).
Anisotarsus angusticollis Casey, 1924: 137. Type locality: «S[ain]t Louis, Missouri» (original citation). Lectotype (♂), designated by Noonan (1973: 314), in USNM [# 47975]. Synonymy established with doubt by van Emden (1953b: 542), confirmed by Lindroth (1968: 867) and Noonan (1973: 314).
Anisotarsus fuscipennis Casey, 1924: 137. Type locality: «Bayfield [Bayfield County], Wisconsin» (original citation for the lectotype). Lectotype (♂), designated by Noonan (1973: 314), in USNM [# 47971]. Synonymy established with doubt by van Emden (1953b: 542), confirmed by Lindroth (1968: 867) and Noonan (1973: 314).
Anisotarsus parallelus Casey, 1924: 138. Type locality: «S[ain]t Louis, Missouri» (original citation). Lectotype [as holotype] (♂), designated by Noonan (1973: 314), in USNM [# 47973]. Synonymy established by Noonan (1973: 314).
Anisotarsus subovalis Casey, 1924: 138. Type locality: «Charleston [Mississippi County], Missouri» (original citation). Lectotype [as holotype] (♂), designated by Noonan (1973: 314), in USNM [# 47972]. Synonymy established with doubt by van Emden (1953b: 542), confirmed by Noonan (1973: 314).
Anisotarsus inerrans Casey, 1924: 138. Type locality: «S[ain]t Louis, Missouri» (original citation). Lectotype [as holotype] (♂), designated by Noonan (1973: 314), in USNM [# 47966]. Synonymy established, with doubt under the name *Notiobia similis* (Say), by van Emden (1953b: 541), confirmed by Noonan (1973: 314).
Anisotarsus connivens Casey, 1924: 139. Type locality: «Marion Co[unty], Florida» (original citation). Lectotype [as holotype] (♂), designated by Noonan (1973: 314), in USNM [# 47981]. Synonymy established by Noonan (1973: 314).
Anisotarsus vernicatus Casey, 1924: 140. Type locality: «Everglade Co[unty], Florida» (original citation). Lectotype [as holotype] (♂), designated by Noonan (1973: 314), in USNM [# 47980]. Synonymy established by Noonan (1973: 314).

#### Distribution.

This widely distributed species ranges from western Newfoundland (CNC) to South Dakota (Kirk and Balsbaugh 1975: 32), south to Panama and southern Florida, west to southeastern Arizona [see Noonan 1973: Fig. 144]; also known from single specimens collected in southeastern Saskatchewan (Ronald R. Hooper pers. comm. 2007) and northern Colorado (Larimer County, William L. Krinsky pers. comm. 2008). The species is also recorded from Bermuda (Hilburn and Gordon 1989: 677).

#### Records.

**CAN**: NB, NF, NS, ON, PE, QC, SK **USA**: AL, AR, AZ, CO, CT, DC, DE, FL, GA, IA, IL, IN, KS, KY, LA, MA, MD, ME, MI, MN, MO, MS, NC, NE, NH, NJ, NY, OH, OK, PA, RI, SC, SD, TN, TX, VA, VT, WI, WV – Bermuda, Costa Rica, Guatemala, Honduras, Mexico, Panama

### Genus Xestonotus

LeConte, 1853

Xestonotus LeConte, 1853c: 383. Type species: *Selenophorus lugubris* Dejean, 1829 by monotypy. Etymology. From the Greek *xestos* (smoothed, polished) and *notos* (back, by extension dorsum) [masculine].

#### Diversity.

One North American species in the temperate regions.

#### Identification.

The species is included in Lindroth’s (1968: 864) monograph.

#### Taxonomic Note.

Lindroth (1968: 864) regarded this taxon as a subgenus of *Anisodactylus* while Noonan (1973: 347) treated it as a valid genus. As discussed by Bousquet and Tchang (1992: 760), larval characters support Noonan’s view.

### Xestonotus lugubris

(Dejean, 1829)

Selenophorus lugubris Dejean, 1829: 118. Type locality: «Amérique septentrionale» (original citation), restricted to «Lexington [Middlesex County], Mass[achusetts]» by Lindroth (1968: 864). One syntype in MHNP (Lindroth 1955b: 30).
Harpalus manhattanis Casey, 1884b: 9. Type locality: «Staten Island, near the city of New York [New York]» (original citation). Holotype [by monotypy] (♂) in USNM [# 47927]. Synonymy established by Horn (1885b: 109), confirmed by Lindroth (1968: 864).
Anisodactylus tioganus Casey, 1924: 123. Type locality: «Tioga Co[unty], Pennsylvania» (original citation). Lectotype (♂), designated by Lindroth (1975: 142), in USNM [# 47911]. Synonymy established by Lindroth (1968: 864).

#### Distribution.

This species ranges from Nova Scotia (Lindroth 1954c: 308) and Prince Edward Island (Majka et al. 2008: 132) to southeastern South Dakota (Kirk and Balsbaugh 1975: 31), south to southern Kansas (Snow 1903: 194), southern Arkansas (Kraim 1983: 229), and northwestern North Carolina (Watauga County, USNM). The records from “Colorado” (Snow 1877: 17; Wickham 1902: 243) need confirmation.

#### Records.

**CAN**: NB, NS, ON, PE, QC **USA**: AR, CT, DC, IA, IL, IN, KS, MA, MD, ME, MI, MN, MO, NC, NH, NJ, NY, OH, PA, RI, SD, VA, VT, WI, WV [CO]

### Genus Anisodactylus

Dejean, 1829

Anisodactylus Dejean, 1829: 4, 132. Type species: *Carabus binotatus* Fabricius, 1787 designated by Westwood (1838: 4). Etymology. From the Greek *anisos* (unequal) and *dactylos* (finger), probably referring to the fact that the male protarsomere 1 is distinctly smaller than the following three (“*premier article des 4 tarses antérieurs des mâles plus petit que les suivants*”) in the species that Dejean included in this taxon [masculine].

#### Diversity.

About 50 species in North America (33 species, one of them adventive), Mexico (seven species, two of them endemic), and the Palaearctic Region (17 species) arrayed in nine subgenera: *Anadaptus* (seven species), *Anisodactylus* s.str. (22 species), *Aplocentrus* (two species), *Gynandrotarsus* (10 species), *Hexatrichus* Tschitschérine (four west Palaearctic species), *Pseudanisodactylus* Noonan (three Palaearctic species), *Pseudaplocentrus* (one species), *Pseudodichirus* Lutshnik (one west Palaearctic species), and *Spongopus* (one species). One Palaearctic species (*Anisodactylus binotatus*) is adventive in New Zealand (Larochelle and Larivière 2005: 34).

### Subgenus Anisodactylus

Dejean, 1829

Anisodactylus Dejean, 1829: 4, 132. Type species: *Carabus binotatus* Fabricius, 1787 designated by Westwood (1838: 4).
Cephalogyna Casey, 1918: 414. Type species: *Anisodactylus lodingi* Schaeffer, 1911 by original designation. Synonymy established by Lindroth (1968: 849). Etymology. From the Greek *cephale* (head) and *gyne* (female), alluding to the head of the female which is larger than in the male (“very large head, notably larger in the female than in the male”) [feminine].
Pseudhexatrichus Noonan, 1973: 282, 352. Type species: *Anisodactylus dejeanii* Buquet, 1840 (= *Carabus heros* Fabricius, 1801) by original designation. Synonymy established by Noonan (1996: 10). Etymology. From the Greek *pseudos* (fallacy, lie) and the generic name *Hexatrichus* [masculine].

#### Diversity.

Twenty-two species in the Nearctic (14 species, of which two extends into the Baja California Peninsula and one is adventive) and Palaearctic (nine species, including the Himalayas) Regions.

#### Identification.

Noonan (1996) revised all the species. One Palaearctic species, *Anisodactylus emarginatus* Ito, has been described subsequently. Lindroth’s key (1968: 835-838) included all North American species except *Anisodactylus pseudagricola* described subsequently.

### [binotatus group]

### Anisodactylus binotatus

(Fabricius, 1787)

Carabus 2notatus Fabricius, 1787: 199. Type locality: «Kiliae [Germany]» (original citation). Lectotype (♀), designated by Lindroth (1968: 851), in ZMUC.

#### Distribution.

This Palaearctic species is adventive in North America where it is known from the Skeena River valley in central British Columbia (Spence and Spence 1988: 158) to northeastern Oregon, east to west-central Montana (Hansen et al. 2009: 353) [see Noonan 1996: Fig. 230]; the species has been recorded also from Sacramento County in California (Clark 1999: 202). The specimen simply labeled from Nevada and the one labeled from Dickenson County in Iowa (Noonan 1996: 113) are likely mislabeled. The first inventoried specimen collected on this continent was found in 1911 at Portland, Oregon (Noonan 1996: 38). The species is also adventive in New Zealand since 1938 (Larochelle and Larivière 2005: 35).

#### Records.

**CAN**: BC (VCI) **USA**: CA, MT, OR, WA – **Adventive**

### Anisodactylus consobrinus

LeConte, 1851

Anisodactylus consobrinus LeConte, 1851: 183. Type locality: «California borealis» (original citation), herein restricted to Arcata, Humboldt County (see Noonan 1996: 105). Lectotype (♂), designated by Noonan (1996: 30), in MCZ [# 88].
Anisodactylus brevicollis LeConte, 1851: 183. Type locality: «S[an]ta Isabel [= Mohave settlement near Needles in San Bernardino County along the Colorado River, California]» (original citation). Lectotype [as holotype] (♀), designated by Noonan (1996: 30), in MCZ [# 87]. Synonymy established by Horn (1880d: 177), confirmed by Noonan (1996: 30).

#### Distribution.

This species ranges from northern Idaho and northern Washington south to northern Baja California Peninsula. A few specimens are known also from central Arizona and southern New Mexico [see Noonan 1996: Fig. 225].

#### Records.

**USA**: AZ, CA (CHI), ID, NM, NV, OR, WA – Mexico

### [californicus group]

### Anisodactylus californicus

Dejean, 1829

Anisodactylus californicus Dejean, 1829: 148. Type locality: «Californie» (original citation), herein restricted to Sequoia Lake, Sequoia National Forest, Fresno County (see Noonan 1996: 123). Holotype [by monotypy] (♀) in MHNP (Lindroth 1955b: 30).
Anisodactylus confusus LeConte, 1851: 183. Type locality: «San Francisco [San Francisco County, California]» (original citation). Lectotype (♂), designated by Noonan (1996: 55), in MCZ [# 86]. Synonymy established by Horn (1875: 130), confirmed by Noonan (1996: 55).
Harpalus depressicollis Motschulsky, 1859a: 136. Type locality: California (inferred from title of the paper). Lectotype [as holotype] (♀), designated by Noonan (1996: 55), in ZMMU. Synonymy established by Noonan (1996: 55).
Anisodactylus obsolescens Casey, 1914: 188. Type locality: «San Francisco [San Francisco County], California» (original citation). Lectotype (♀), designated by Lindroth (1975: 141), in USNM [# 47922]. Synonymy established by Lindroth (1968: 854).
Anisodactylus angustus Casey, 1914: 188. Type locality: «San Diego [San Diego County], California» (original citation). Lectotype (♂), designated by Lindroth (1975: 142), in USNM [# 47920]. Synonymy established by Lindroth (1968: 854).
Anisodactylus oregonus Casey, 1914: 189. Type locality: «Clackamas Co[unty], Oregon» (original citation). Lectotype (♂), designated by Lindroth (1975: 142), in USNM [# 47919]. Synonymy established by Hatch (1953: 174), confirmed by Lindroth (1968: 854).
Anisodactylus sinuatus Casey, 1914: 190. Type locality: «Oregon» (original citation). Lectotype (♀), designated by Lindroth (1975: 142), in USNM [# 47918]. Synonymy established by Lindroth (1968: 854).
Anisodactylus paganicus Casey, 1914: 190. Type locality: «Provo [Utah County], Utah» (original citation). Lectotype (♂), designated by Lindroth (1975: 142), in USNM [# 47917]. Synonymy established by Lindroth (1968: 854).
Anisodactylus humeralis Casey, 1914: 190. Type locality: «Reno [Washoe County], Nevada» (original citation). Holotype [by monotypy] (♀) in USNM [# 47921]. Synonymy established by Lindroth (1968: 854).
Anisodactylus aleneanus Casey, 1924: 124. Type locality: «Coeur d’Alene [Kootenai County], Idaho» (original citation). Lectotype (♂), designated by Lindroth (1975: 142), in USNM [# 47923]. Synonymy established by Hatch (1953: 174), confirmed by Lindroth (1968: 854).
Anisodactylus comes Casey, 1924: 124. Type locality: «Valley of Redwood Creek, Humboldt Co[unty], California» (original citation). Lectotype (♀), designated by Lindroth (1975: 142), in USNM [# 47924]. Synonymy established by Lindroth (1968: 854).
Anisodactylus maestus Casey, 1924: 124. Type locality: «S[an]ta Cruz [Santa Cruz County], California» (original citation). Lectotype (♀), designated by Lindroth (1975: 142), in USNM [# 47925]. Synonymy established by Lindroth (1968: 854).

#### Distribution.

This western species ranges from central Alberta to Vancouver Island, south to the Baja California Peninsula, southern Arizona, and northern Colorado [see Noonan 1996: Fig. 240]. Specimens labeled from “New Mexico,” Dallas (Texas), Jalisco in west-central Mexico, “Nebraska,” Lake County in Illinois, and “New Jersey” are known.

#### Records.

**CAN**: AB, BC (VCI) **USA**: AZ, CA (CHI), CO, ID, MT, NV, OR, UT, WA, WY [IL, NE, NM, TX] – Mexico

### Anisodactylus furvus

LeConte, 1863

Anisodactylus furvus LeConte, 1863c: 14. Type locality: «upper part of Georgia» (original citation), herein restricted to Barnesville, Lamar County (see Noonan 1996: 135). Holotype [by monotypy] (♀) in MCZ [# 5952].

#### Distribution.

This eastern species ranges from New Jersey to northwestern Missouri, north to southernmost Ontario, south to east-central Texas and the Florida Panhandle [see Noonan 1996: Fig. 244]. Three specimens labeled from “Colorado,” Santa Cruz County in Arizona, and Lane County in Oregon are known.

#### Records.

**CAN**: ON **USA**: AL, AR, DE, FL, GA, IL, IN, KY, LA, MA, MI, MO, MS, NC, NJ, OH, OK, PA, SC, TN, TX, VA [AZ, CO]

### Anisodactylus similis

LeConte, 1851

Anisodactylus similis LeConte, 1851: 183. Type locality: «Oregon» (original citation), herein restricted to Ochoco National Forest, Crook County (see Noonan 1996: 133). Lectotype [as holotype] (♂), designated by Noonan (1996: 60), in MCZ [# 89].
Anisodactylus semipunctatus LeConte, 1859a: 83. Type locality: «Oregon, California» (original citation), restricted to «Oregon» by Noonan (1996: 60). Lectotype (♂), designated by Noonan (1996: 60), in MCZ [# 5954]. Synonymy established by Horn (1875: 130), confirmed by Lindroth (1968: 855).
Anisodactylus puncticollis Chaudoir, 1868b: 161. Type locality: «île de Vancouver [British Columbia]» (original citation). Holotype [by monotypy] (♂) in MHNP. Synonymy established by Horn (1875: 130), confirmed by Lindroth (1968: 855).
Anisodactylus incisus Casey, 1914: 185. Type locality: «Hoopa Valley, Humboldt Co[unty], California» (original citation). Lectotype (♂), designated by Lindroth (1975: 142), in USNM [# 47914]. Synonymy established by Lindroth (1968: 855).
Anisodactylus solidus Casey, 1914: 186. Type locality: «n[ea]r San Francisco [California]» (original citation). Lectotype (♂), designated by Lindroth (1975: 142), in USNM [# 47912]. Synonymy established by Lindroth (1968: 855).
Anisodactylus incertus Casey, 1914: 186. Type locality: «S[an]ta Clara Co[unty], California» (original citation). Lectotype (♀), designated by Noonan (1996: 60), in USNM [# 47916]. Synonymy established by Noonan (1975: 227).
Anisodactylus sericatus Casey, 1914: 187. Type locality: «San Francisco Bay, California» (original citation). Lectotype (♀), designated by Noonan (1975: 227), in USNM [# 47915]. Synonymy established by Noonan (1975: 227).

#### Distribution.

This species occurs from central British Columbia south to the Mexican border in California and the eastern edge of the Rocky Mountains in northern Colorado [see Noonan 1996: Fig. 244]. Two old specimens labeled from “Arizona” and “Texas” are known.

#### Records.

**CAN**: BC (VCI) **USA**: CA (CHI), CO, ID, MT, NV, OR, UT, WA [AZ, TX]

### [carbonarius group]

### Anisodactylus carbonarius

(Say, 1823)

Harpalus carbonarius Say, 1823a: 32. Type locality: «Camden [Kershaw County], S[outh] C[arolina]» (neotype label). Neotype (♂), designated by Lindroth and Freitag (1969: 353), in MCZ [# 32987].
Anisodactylus luctuosus Dejean, 1829: 151. Type locality: «Amérique septentrionale» (original citation). Lectotype (♂), designated by Noonan (1996: 69), in MHNP. Synonymy established by LeConte (1847: 382), confirmed by Lindroth (1955b: 29).
Anisodactylus rufipennis LeConte, 1847: 381. Type locality: «Brooklyn, insulae Longae NovEboraci [= New York]» (original citation). Holotype [by monotypy] (♂) in MCZ [# 5953]. Synonymy established by Horn (1880d: 177), confirmed by Noonan (1996: 69).
Triplectrus brevior Casey, 1924: 126. Type locality: «Pennsylvania» (original citation). Lectotype (♀), designated by Lindroth (1975: 141), in USNM [# 47928]. Synonymy established by Lindroth (1968: 848).

#### Distribution.

This species is found from southern Quebec to northeastern South Dakota (Kirk and Balsbaugh 1975: 31), south to northern Colorado (Miller and Peairs 2008: 34), northeastern Texas, and central Florida [see Noonan 1996: Fig. 247]; the species has been collected at three sites in Washington which suggest that it has been introduced into the Pacific northwest.

#### Records.

**CAN**: ON, QC **USA**: AL, AR, CO, CT, DC, DE, FL, GA, IA, IL, IN, KS, KY, MA, MD, MI, MN, MO, MS, NC, NE, NH, NJ, NY, OH, OK, PA, RI, SC, SD, TN, TX, VA, VT, WA, WI, WV

#### Taxonomic Note.

This species was included in the subgenus *Gynandrotarsus* by Lindroth (1968: 848) but in the nominotypical subgenus by Noonan (1973: 351).

### Anisodactylus harrisii

LeConte, 1863

Anisodactylus harrisii LeConte, 1863c: 14. Type locality: «middle and eastern states» (original citation), restricted to «Marion [Plymouth County], Mass[achusetts]» by Lindroth (1968: 850). Lectotype (♂), designated by Noonan (1996: 71), in MCZ [# 5968].
Anisodactylus lacertosus Casey, 1924: 123. Type locality: «probably Indiana» (original citation), restricted to «Indiana» by Noonan (1996: 71). Holotype [by monotypy] (♀) in USNM [# 47913]. Synonymy established by Lindroth (1968: 850).

#### Distribution.

This species occurs from Nova Scotia to southwestern British Columbia, south to southern California, southern Arizona, the Texas Panhandle (Michels et al. 2010: 743), east-central Louisiana (West Feliciana Parish, Igor M. Sokolov pers. comm. 2009), central Mississippi, and west-central South Carolina (Ciegler 2000: 90) [see Noonan 1996: Fig. 248].

#### Records.

**CAN**: AB, BC, MB, NB, NS, ON, PE, QC, SK **USA**: AL, AR, AZ, CA, CT, CO, DE, GA, IA, ID, IL, IN, KS, LA, MA, MD, ME, MI, MN, MO, MS, MT, NC, ND, NE, NH, NJ, NM, NY, OH, OR, PA, RI, SC, SD, TX, UT, VA, VT, WA, WI, WV

### Anisodactylus lodingi

Schaeffer, 1911

Anisodactylus lodingi Schaeffer, 1911: 114. Type locality: «Mobile [Mobile County], Alabama» (original citation). Lectotype (♂), designated by Erwin and House (1978: 234), in USNM [# 42494]. Etymology. The species name was proposed for Henry Peter Löding [1869-1942], a florist and horticulturist in Mobile, Alabama. Löding is best known in the scientific community as a collector of Coleoptera. He was awarded the honorary degree of Doctor of Science by the University of Alabama in 1932. His collection is at the same University.

#### Distribution.

This species ranges from Long Island, New York, south to northern Florida and southeastern Mississippi [see Noonan 1996: Fig. 246]. One specimen labeled from “Massachusetts” is known.

#### Records.

**USA**: AL, FL, GA, MS, NY, NC [MA]

### Anisodactylus nigerrimus

(Dejean, 1831)

Harpalus nigerrimus Dejean, 1831: 842. Type locality: «Amérique septentrionale» (original citation), restricted to «Marion [Plymouth County], Mass[achusetts]» by Lindroth (1968: 850). Holotype [by monotypy] (♂) in MHNP (Lindroth 1955b: 30; Noonan 1996: 65).
Harpalus laticollis Kirby, 1837: 43. Type locality: northern parts of British America (inferred from title of the book). Holotype [by monotypy] (♀) in BMNH. Synonymy established by LeConte (1850: 208), confirmed by Lindroth (1953b: 174).
Anisodactylus punctulatus LeConte, 1863c: 14. Type locality: «Middle States» (original citation). Lectotype [as holotype] (♂), designated by Noonan (1996: 65), in MCZ [# 5955]. Synonymy established by Horn (1880d: 177), confirmed by Lindroth (1968: 851).
Harpalus opacus Casey, 1884b: 8. Type locality: «eastern Pennsylvania» (original citation). Holotype [by monotypy] (♀) in USNM [# 47910]. Synonymy established by Horn (1885b: 109), confirmed by Lindroth (1968: 851).

#### Distribution.

This species is found from Newfoundland to western Ontario, south to northwestern Arkansas, southern Mississippi, and northern Georgia, west to eastern Kansas and Nebraska [see Noonan 1996: Fig. 245]. Seven specimens labeled from “Texas,” “South Dakota,” “California,” southwestern Saskatchewan, and southeastern Alberta are also known (Noonan 1996: 135-138). Several specimens were recently caught in the southern Similkameen Valley in British Columbia (Smith et al. 2004: 96) suggesting that the range of the species extends farther west or that the species was introduced in the area; a misidentification is also possible.

#### Records.

**CAN**: NB, NF, NS (CBI), ON, PE, QC **USA**: AL, AR, CT, DC, DE, GA, IA, IL, IN, KS, KY, LA, MA, MD, ME, MI, MN, MO, MS, NC, ND, NE, NH, NJ, NY, OH, PA, RI, SC, TN, VA, VT, WI, WV [AB, BC, CA, SD, SK, TX]

### [melanopus group]

### Anisodactylus agricola

(Say, 1823)

Harpalus agricolus Say, 1823a: 33. Type locality: «Allegheny, P[ennsylvani]a» (neotype label). Neotype (♀), designated by Lindroth and Freitag (1969: 354), in MCZ [# 32985].
Harpalus paradoxus Haldeman, 1843b: 302. Type locality: southeastern Pennsylvania (Haldeman 1843a: 297). Syntype(s) presumably lost. Synonymy established by Melsheimer (1853: 23).
Anisodactylus striatus LeConte, 1847: 380. Type locality: «Evansville, I[ow]a alterumque ad Rocky Mountains» (original citation), restricted to «Evansville, Iowa» by Noonan (1996: 44). Lectotype (♀), designated by Noonan (1996: 44), in MCZ [# 5956]. Synonymy established by Melsheimer (1853: 23), confirmed by Noonan (1996: 44).

#### Distribution.

This species ranges from Maine and southern Quebec to southeastern Minnesota, south to northern Arkansas (Kraim 1983: 176), central Alabama, and northwestern South Carolina (Ciegler 2000: 89) [see Noonan 1996: Fig. 235]. The record from “Colorado” (Leng 1920: 72) is probably in error.

#### Records.

**CAN**: ON, QC **USA**: AL, AR, CT, DC, DE, GA, IA, IL, IN, KS, KY, MA, MD, ME, MI, MN, MO, NC, NE, NH, NJ, NY, OH, PA, RI, SC, TN, VA, WI, WV

### Anisodactylus melanopus

(Haldeman, 1843)

Harpalus melanopus Haldeman, 1843b: 302. Type locality: southeastern Pennsylvania (Haldeman 1843a: 297). Neotype (♂), designated by Noonan (1996: 48), in MCZ.

#### Distribution.

This species ranges from Massachusetts to southeastern Minnesota (Gandhi et al. 2005: 930), south to southern Missouri, “Mississippi” (Drew A. Hildebrandt pers. comm. 2007), and central South Carolina (Ciegler 2000: 90) [see Noonan 1996: Fig. 237]. There are two specimens from more western sites: one from western Nebraska, the other one from Utah. Whether these specimens are strays or represent elements of permanent populations remain to be seen. The record from South Dakota (Kirk and Balsbaugh 1975: 31) needs confirmation.

#### Records.

**CAN**: ON **USA**: CT, DC, DE, GA, IA, IL, IN, MA, MD, MI, MN, MO, MS, NC, NJ, NY, OH, PA, RI, SC, VA, VT, WI, WV [NE, SD, UT]

### Anisodactylus pseudagricola

Noonan, 1996

Anisodactylus pseudagricola Noonan, 1996: 46. Type locality: «Cornwall [Litchfield County], C[onnecticu]t» (original citation). Holotype (♂) in CUIC [# 7001].

#### Distribution.

This species, known only from relatively few specimens, ranges from New Hampshire to eastern Michigan, south to northern Ohio (Ashtabula and Huron Counties, Harry J. Lee, Jr. pers. comm. 2008) and southern Pennsylvania (Somerset County, Robert L. Davidson pers. comm. 2012) [see Noonan 1996: Fig. 236]. One specimen collected in central Missouri (Boone County, CMNH) and identified by Gerald R. Noonan in 1994 is known (Robert L. Davidson pers. comm. 2012).

#### Records.

**CAN**: ON **USA**: CT, MA, MI, MO, NH, NY, OH, PA

### [nigrita group]

### Anisodactylus kirbyi

Lindroth, 1953

Anisodactylus kirbyi Lindroth, 1953b: 174. Type locality: «Cheticamp, Nova Scotia» (holotype label). Holotype (♂) in CNC [# 6574]. Etymology. The specific name honors the English clergyman, entomologist, and naturalist William Kirby [1759-1850] who is best known to the North American entomological community for his taxonomic treatment of the insects brought back by John Richardson from the second arctic expedition (1825-1827) of Sir John Franklin.

#### Distribution.

This species has a disjunct distribution. In the east, the species is found from Cape Breton Island to southeastern Manitoba south to southern Indiana, southern Pennsylvania, and New Jersey; in the west, the species ranges from southern British Columbia south to southwestern California, east to northern Idaho [see Noonan 1996: Fig. 238].

#### Records.

**CAN**: BC (VCI), MB, NB, NS (CBI), ON, PE, QC **USA**: CA, CT, ID, IL, IN, MA, MD, ME, MI, MN, ND, NH, NJ, NY, OH, OR, PA, RI, SD, VT, WA, WI

### Anisodactylus nigrita

Dejean, 1829

Anisodactylus nigrita Dejean, 1829: 149. Type locality: «Amérique septentrionale» (original citation), restricted to «Forest Hills [Suffolk County], Mass[achusetts]» by Lindroth (1968: 852). Lectotype (♂), designated by Noonan (1996: 53), in MHNP.
Harpalus interpunctatus Kirby, 1837: 42. Type locality: «Lat. 54° [= along North Saskatchewan River]» (original citation). Lectotype (♂), designated by Noonan (1996: 53), in BMNH. Synonymy established by Lindroth (1953b: 174).
Anisodactylus lecontei Chaudoir, 1868b: 161 [primary homonym of *Anisodactylus lecontei* Gemminger and Harold, 1868]. Type locality: «provinciis mediis» (original citation for *Anisodactylus nigrita* Dejean *sensu* LeConte, 1847). Two syntypes in MCZ. **New synonymy**. Note. This name was proposed for *Anisodactylus nigrita* Dejean, 1829 *sensu* LeConte (1847: 379). LeConte’s collection contains four specimens under the name *Anisodactylus nigrita*. They are labeled as follow: 1. (♂) “[white disc above, yellow under] / = type de nigrita Dej [handwritten] / A. nigrita Dej. [handwritten];” 2. (♀) “[pink under] / nigrita 2 [handwritten];” 3. (♀) “[white disc above, yellow under] / nigrita 3 [handwritten];” 4. (♀) “[pink under] / 260. [handwritten] / var. interpunctatus (? Kirby) Lec. [handwritten] / nigrita 4 [handwritten].” The two specimens with pink discs (= middle states) are probably syntypes. Lindroth (1968: 853) listed this name with doubt as a synonym of *Anisodactylus kirbyi* Lindroth. However, all four specimens in LeConte’s collection are conspecific with members of *Anisodactylus nigrita* Dejean.

#### Distribution.

This species ranges from Nova Scotia to southern British Columbia, south to southern Washington, Colorado along the Rocky Mountains, Nebraska, and eastern Tennessee and western North Carolina along the Appalachian Mountains [see Noonan 1996: Fig. 239]. A few specimens simply labeled from California, Kansas, and Mississippi are known. The record from southwestern Alabama (Löding 1945: 25) is probably in error.

#### Records.

**CAN**: AB, BC, MB, NB, NS, ON, PE, QC, SK **USA**: CO, CT, DC, IA, IL, IN, KY, MA, MD, ME, MI, MN, NC, ND, NE, NH, NJ, NY, OH, PA, RI, SD, TN, VA, VT, WA, WI, WV [CA, KS, MS]

### Subgenus Gynandrotarsus

LaFerté-Sénectère, 1841

Gynandrotarsus LaFerté-Sénectère, 1841b: 202. Type species: *Gynandrotarsus harpaloides* LaFerté-Sénectère, 1841 by monotypy. Etymology. From the Greek *gyne* (female), *andros* (male), and *tarsos* (tarsus), probably alluding to the expanded first protarsomere of the female (“*le premier article des tarses antérieurs des femelles *... *une fois et demie aussi large et deux fois aussi long que l’article correspondant des mâles*”) as in the male [masculine].
Triplectrus LeConte, 1847: 381. Type species: *Harpalus rusticus* Say, 1823 designated by Lindroth (1968: 843). Synonymy established by Casey (1914: 172). Etymology. From the Greek *treis* (three) and *plectron* (spur), alluding to the trifid apical spur of the protibia (“*tibiae anticae calcare terminale trifido*”) of the adult [masculine].

#### Diversity.

Ten species in North America (nine species) and Mexico (four species, one of them endemic, *Anisodactylus darlingtoni* Noonan).

#### Identification.

Noonan (1973) revised all species.

### [dulcicollis group]

### Anisodactylus dulcicollis

(LaFerté-Sénectère, 1841)

Harpalus dulcicollis LaFerté-Sénectère, 1841a: 44. Type locality: Texas (inferred from title of the paper). Lectotype (♂), designated by Noonan (1973: 362), in MHNP.
Anisodactylus ellipticus LeConte, 1847: 384. Type locality: «NoviAureliani [= New Orleans, Orleans Parish, Louisiana]» (original citation). Lectotype [as type] (♂), designated by Noonan (1973: 362), in MCZ [# 5967]. Synonymy established by LeConte (1866: 78), confirmed by Noonan (1973: 362).
Anisodactylus elongatus Chaudoir, 1868b: 163. Type locality: «Texas» (original citation). Holotype [by monotypy] (♂) in MHNP (Noonan 1973: 362). Synonymy established by Lindroth (1968: 847).
Triplectrus modicus Casey, 1914: 178. Type locality: «Houston [Harris County], Texas» (original citation). Lectotype (♀), designated by Lindroth (1975: 141), in USNM [# 47941]. Synonymy established by Lindroth (1968: 847).

#### Distribution.

This species ranges from extreme southern Ontario (Bousquet 1987a: 130) to eastern Nebraska, south to southeastern Texas and west-central Georgia [see Noonan 1973: Fig. 165]. Three specimens labeled from “Colorado,” Arizona (Huachuca Mountains), and California (Los Angeles County) are known. The record from New Jersey (Smith 1890: 98) needs confirmation.

#### Records.

**CAN**: ON **USA**: AL, AR, GA, IA, IL, IN, KS, KY, LA, MO, MS, NC, NE, OH, OK, SC, TN, TX, VA [AZ, CA, CO, NJ]

### Anisodactylus harpaloides

(LaFerté-Sénectère, 1841)

Gynandrotarsus harpaloides LaFerté-Sénectère, 1841b: 203. Type locality: «Texas» (original citation). Lectotype (♀), designated by Noonan (1973: 360), in MHNP.
Triplectrus beryllus Casey, 1924: 131. Type locality: «McPherson [McPherson County], Kansas» (original citation). Lectotype (♂), designated by Noonan (1973: 360), in USNM [# 47973]. Synonymy established by Noonan (1973: 360).

#### Distribution.

This species ranges from northeastern Nebraska to eastern Virginia (Hoffman et al. 2006: 26), south to northern Mississippi (Lafayette and Panola Counties, Paul K. Lago pers. comm. 2009), northwestern Louisiana, and northeastern Texas (Noonan 1973: 361, Fig. 159). The records from “North Carolina” (Leng 1920: 72), South Carolina (Kirk 1969: 13; Kirk 1970: 14; Ciegler 2000: 90), Georgia (Fattig 1949: 50), “Florida” (Noonan 1973: 361), and southwestern Louisiana (Hine 1906: 77) need confirmation.

#### Records.

**USA**: AR, IL, KS, LA, MO, MS, NE, OK, TN, TX, VA [FL, GA, NC, SC]

### Anisodactylus opaculus

(LeConte, 1863)

Gynandrotarsus opaculus LeConte, 1863c: 16. Type locality: «Texas» (original citation), herein restricted to Austin, Travis County (see Casey 1924: 130, as *Triplectrus paulus*). Holotype [by monotypy] (♀) in MCZ [# 5948].
Triplectrus paulus Casey, 1924: 130. Type locality: «Austin [Travis County], Texas» (original citation). Lectotype (♂), designated by Noonan (1973: 359), in USNM [# 47942]. Synonymy established by Noonan (1973: 359).

#### Distribution.

This species ranges from northern Kansas to northeastern Kentucky (Fayette County, Robert L. Davidson pers. comm. 2012) south to western Alabama and Nuevo León in northeastern Mexico [see Noonan 1973: Fig. 158]. The western limit of the species range is along the Rio Grande in western Texas, although it has been recorded from one locality in southwestern Arizona (Snow 1907: 142). The species is also known from south-central Wisconsin (Sauk County, Robert L. Davidson pers. comm. 2012). The records from South Carolina (Kirk 1969: 13; Kirk 1970: 14; Ciegler 2000: 90) need confirmation.

#### Records.

**USA**: AL, AR, KS, KY, LA, MO, MS, NM, OK, TN, TX, WI [AZ, SC] – Mexico

### Anisodactylus texanus

Schaeffer, 1910

Anisodactylus texanus Schaeffer, 1910: 404. Type locality: «New Braunfels [Comal County], Texas» (original citation). Lectotype (♂), designated by Noonan (1973: 361), in USNM [# 75703].

#### Distribution.

This species ranges from southeastern Louisiana to southeastern Arizona, south to northern Mexico [see Noonan 1973: Fig. 164].

#### Records.

**USA**: AZ, LA, TX – Mexico

### [rusticus group]

### Anisodactylus anthracinus

(Dejean, 1829)

Harpalus anthracinus Dejean, 1829: 369. Type locality: «Mexique» (original citation), herein restricted to Majaica (7000’), 45 miles northwest Chihuahua, Chihuahua (CNC). Holotype [by monotypy] (♀) in MHNP.
Anisodactylus dilatatus Say, 1830c: 18. Type locality: «37 mi[les] west of Durango (8400’), D[uran]go [Mexico]» (neotype label). Neotype (♂), designated by Noonan (1973: 366), in MCZ [# 34664]. Synonymy established by Bates (1882a: 52). Note. «Mexico» was the area originally cited by Say (1830c: 18).
Triplectrus convexus Casey, 1914: 176. Type locality: «Arizona» (original citation), restricted to «Madera Canyon, Pima County» by Noonan (1973: 366). Lectotype (♂), designated by Noonan (1973: 366), in USNM [# 47938]. Synonymy established by Noonan (1973: 366).

#### Distribution.

This species ranges from Tulare County in east-central California to southwestern Texas, south to the Federal District area of Mexico [see Noonan 1973: Fig. 160].

#### Records.

**USA**: AZ, CA, NM, TX – Mexico

### Anisodactylus haplomus

Chaudoir, 1868

Anisodactylus haplomus Chaudoir, 1868b: 163. Type locality: Amérique septentrionale (inferred from title of the paper), restricted to «Galveston [Galveston County], Tex[as]» by Lindroth (1968: 845). Holotype [by monotypy] (♀) in MHNP (Noonan 1973: 363).
Triplectrus peropacus Casey, 1914: 176. Type locality: «Galveston [Galveston County], Texas» (original citation for the lectotype). Lectotype (♂), designated by Lindroth (1975: 141), in USNM [# 47935]. Synonymy established by Lindroth (1968: 845).
Triplectrus breviceps Casey, 1924: 129. Type locality: «Mobile [Mobile County], Alabama» (original citation). Lectotype (♀), designated by Lindroth (1975: 141), in USNM [# 47936]. Synonymy established by Lindroth (1968: 845).
Triplectrus longicollis Casey, 1924: 129. Type locality: «District of Columbia» (original citation). Holotype [by monotypy] (♂) in USNM [# 47973]. Synonymy established by Noonan (1973: 364).

#### Distribution.

This species ranges from Long Island, New York, to northeastern Oklahoma (Foster F. Purrington pers. comm. 2010), south to east-central Texas (Riley 2011) and southern Florida [see Noonan 1973: Fig. 166].

#### Records.

**USA**: AL, AR, DC, FL, GA, IL, KY, LA, MO, MS, NC, NY, OH, OK, SC, TN, TX, VA

### Anisodactylus merula

(Germar, 1824)

Harpalus merula Germar, 1824: 24. Type locality: «Kentucky» (original citation). Lectotype (♀), designated by Lindroth (1968: 845), in MHNP.
Anisodactylus pinguis LeConte, 1847: 382. Type locality: «ad Rocky Mountains» (original citation). Lectotype (♀), designated by Noonan (1973: 368), in MCZ [# 5950]. Synonymy established by Melsheimer (1853: 23), confirmed by Lindroth (1968: 845).
Anisodactylus crassus LeConte, 1847: 382. Type locality: «NovEboraci [= New York]» (original citation). Lectotype (♀), designated by Noonan (1973: 368), in MCZ [# 5951]. Synonymy established by Melsheimer (1853: 23), confirmed by Lindroth (1968: 845).
Anisodactylus gravidus LeConte, 1847: 383. Type locality: «NovEboraci [= New York]» (original citation). Lectotype (♀), designated by Noonan (1973: 368), in MCZ [# 5949]. Synonymy established by Melsheimer (1853: 9), confirmed by Lindroth (1968: 845).
Triplectrus aethiops Casey, 1914: 175. Type locality: «Austin and Waco, Texas» (original citation), restricted to «Austin [Travis County]» by Casey (1924: 128). Lectotype (♀), designated by Noonan (1973: 369), in USNM [# 47932]. Synonymy established by Noonan (1973: 369).
Triplectrus marginatus Casey, 1924: 126. Type locality: «Grayling, near Bay City [Bay County], Michigan» (original citation). Lectotype [as holotype] (♂), designated by Noonan (1973: 369), in USNM [# 47934]. Synonymy established by Lindroth (1968: 845).
Triplectrus wolcotti Casey, 1924: 127. Type locality: «near Chicago [Cook County], Illinois» (original citation). Lectotype (♀), designated by Lindroth (1975: 141), in USNM [# 47930]. Synonymy established by Lindroth (1968: 845). Etymology. The specific name honors Albert Burk Wolcott [1869-1950] who worked for a long time as preparator of educational exhibits for the public schools of Chicago. An amateur coleopterist, Wolcott specialized in Cleridae. His clerid collection and library were deposited at the Field Museum of Natural History, Chicago in 1946.
Triplectrus sulcipennis Casey, 1924: 128. Type locality: «Waco [McLennan County], Texas» (original citation). Lectotype (♀), designated by Noonan (1973: 369), in USNM [# 47931]. Synonymy established by Noonan (1973: 369).
Triplectrus kempi Casey, 1924: 130. Type locality: «Lake George [Warren County], New York» (original citation). Lectotype (♀), designated by Lindroth (1975: 141), in USNM [# 47940]. Synonymy established by Lindroth (1968: 845). Etymology. The specific name was proposed for James Furman Kemp [1859-1926], distinguished professor of geology at Columbia University.

#### Distribution.

This eastern species ranges from southern Quebec to west-central Minnesota, north to southwestern Manitoba (Lindroth 1968: 846), south to southeastern Texas and the Florida Keys [see Noonan 1973: Fig. 161]. One specimen labeled from Anaheim, California is known. The records from “Arizona” and “North Dakota” (Bousquet and Larochelle 1993: 217) need confirmation.

#### Records.

**CAN**: MB, ON, QC **USA**: AL, AR, CO, CT, DC, FL, GA, IA, IL, IN, KS, KY, LA, MA, MD, ME, MI, MN, MO, MS, NC, NE, NH, NJ, NY, OH, OK, PA, RI, SC, SD, TN, TX, VA, VT, WI [AZ, CA, ND]

### Anisodactylus ovularis

(Casey, 1914)

Triplectrus ovularis Casey, 1914: 177. Type locality: «S[ain]t Louis, Missouri» (original citation for the lectotype). Lectotype (♂), designated by Noonan (1973: 372), in USNM [# 47939].
Triplectrus semirubidus Casey, 1924: 127. Type locality: «Highland Park [Lake County], north of Chicago, Illinois» (original citation). Lectotype (♀), designated by Lindroth (1975: 141), in USNM [# 47929]. Synonymy established by Lindroth (1968: 846).

#### Distribution.

This species ranges from Long Island, New York, to central South Dakota (Kirk and Balsbaugh 1975: 31), including southern Ontario, south to northeastern Texas, northeastern Georgia (House and All 1981: 195; Morrill 1992: 181), and west-central South Carolina (Ciegler 2000: 90) [see Noonan 1973: Fig. 163].

#### Records.

**CAN**: ON **USA**: AR, GA, IA, IL, IN, KS, LA, MD, MI, MN, MO, MS, NE, NJ, NY, OH, OK, PA, RI, SC, SD, TN, TX, VA, WI, WV

### Anisodactylus rusticus

(Say, 1823)

Harpalus rusticus Say, 1823a: 32. Type locality: «Rumney [Grafton County], N[ew] H[ampshire]» (neotype label). Neotype (♂), designated by Lindroth and Freitag (1969: 353), in MCZ [# 32986].
Anisodactylus tristis Dejean, 1829: 158. Type locality: «Amérique septentrionale» (original citation). Lectotype (♂), designated by Noonan (1973: 365), in MHNP. Synonymy established by Horn (1880d: 177), confirmed by Lindroth (1955b: 29).
Triplectrus oblongus Casey, 1924: 128. Type locality: «Nisbet [Lycoming County], Pennsylvania» (original citation). Holotype [by monotypy] (♀) in USNM [# 47933]. Synonymy established by Lindroth (1968: 843).

#### Distribution.

This species ranges from Prince Edward Island to Yellowstone National Park in Wyoming, north to southern Manitoba (Lindroth 1968: 845), south to northern Colorado (Miller and Peairs 2008: 34), eastern Texas, and northern Florida [see Noonan 1973: Fig. 162]. The records from “Arizona” (Leng 1920: 72) and New Mexico (Fall and Cockerell 1907: 162) need confirmation.

#### Records.

**CAN**: MB, NB, NS, ON, PE, QC **USA**: AL, AR, CO, CT, DC, DE, FL, GA, IA, IL, IN, KS, KY, LA, MA, MD, ME, MI, MN, MO, MS, NC, ND, NE, NH, NJ, NY, OH, OK, PA, RI, SC, SD, TN, TX, VA, VT, WI, WV, WY [AZ, NM]

### Subgenus Anadaptus

Casey, 1914

Anadaptus Casey, 1914: 203. Type species: *Anisodactylus discoideus* Dejean, 1831 designated by Lindroth (1968: 838). Etymology. From the Greek *ana* (upward, outward) and the generic name *Daptus*, alluding to the possibility of a close relationship between members of this taxon and those of *Daptus* (“this is one of the most interesting genera ... and the outward suggestion of Daptus may not be so very fanciful after all”) [masculine].

#### Diversity.

Seven species in North America (six species) and Mexico (*Anadaptus rotundangulus* Bates).

#### Identification.

Noonan (2001) revised the species and provided a key for their identification.

### Anisodactylus alternans

(Motschulsky, 1845)

Harpalus alternans Motschulsky, 1845b: 343. Type locality: «Californie» (original citation), herein restricted to San Jose, Santa Clara County (see LeConte 1851: 184, as *Anisodactylus alternans* LeConte). Holotype [by monotypy] (♀) in ZMMU (Bousquet and Larochelle 1993: 14).
Anisodactylus alternans LeConte, 1851: 184. Type locality: «San Jose [Santa Clara County, California]» (original citation). Lectotype (♀), designated by Noonan (2001: 314), in MCZ [# 90]. Synonymy established by Bousquet and Larochelle (1993: 14).
Anisodactylus nivalis G.H. Horn, 1880d: 172. Type locality: «Nev[ada]» (lectotype label). Lectotype (♀), designated by Noonan (2001: 315), in MCZ [# 2956]. Synonymy established by Noonan (2001: 315).
Anadaptus parvulus Casey, 1914: 204. Type locality: «California» (original citation). Lectotype (♂), designated by Lindroth (1975: 141), in USNM [# 47962]. Synonymy established, under the name *Anisodactylus nivalis* Horn, by Lindroth (1968: 841).

#### Distribution.

This western species is found from southeastern British Columbia south to the Mexican border in California, southwestern Utah, and central Colorado [see Noonan 2001: Fig. 51].

#### Records.

**CAN**: BC **USA**: CA, CO, ID, MT, NV, OR, UT, WA

### Anisodactylus discoideus

Dejean, 1831

Anisodactylus discoideus Dejean, 1831: 831. Type locality: «Amérique septentrionale» (original citation), restricted to «Rumney [Grafton County], N[ew] H[ampshire]» by Lindroth (1968: 839). Holotype [by monotypy] (♂) in MHNP (Lindroth 1955b: 30).

#### Distribution.

This species ranges from southern Nova Scotia to eastern Montana, north to southern Manitoba (Lindroth 1968: 839) and west-central Saskatchewan, south to southeastern Texas and northern Florida [see Noonan 2001: Fig. 49]. The species is also, quite unexpectedly, found in western Oregon, some 1200 km from the western edge of the species main distribution area (Noonan 2001: 313).

#### Records.

**CAN**: MB, NB, NS, ON, QC, SK **USA**: CT, DC, FL, GA, IA, IL, IN, KS, KY, MA, MD, ME, MI, MN, MO, MT, NC, ND, NE, NH, NJ, NY, OH, OR, PA, RI, SC, SD, TN, TX, VA, VT, WI, WV

### Anisodactylus pitychrous

LeConte, 1861

Anisodactylus pitychrous LeConte, 1861b: 339. Type locality: «California» (original citation). Holotype [by monotypy] (♀) in MCZ [# 5956]. Note. LeConte (1861b: 339) mentioned that the sole specimen he had was a male.

#### Distribution.

This species ranges from southeastern Manitoba (CNC) to northwestern Washington, south to the Los Angeles area in California and, along the Rocky Mountains, to Arizona and northern New Mexico [see Noonan 2001: Fig. 44].

#### Records.

**CAN**: AB, MB, SK **USA**: AZ, CA, CO, ID, NM, NV, OR, UT, WA

### Anisodactylus porosus

(Motschulsky, 1845)

Ophonus porosus Motschulsky, 1845b: 344. Type locality: «Californie» (original citation). Holotype [by monotypy; designated lectotype by Bousquet (1997b: 330)] (♂) in ZMMU.
Ophonus sublaevis Motschulsky, 1859a: 138. Type locality: «California» (lectotype label). Lectotype (♀), designated by Bousquet (1997b: 334), in ZMMU. Synonymy established by Horn (1880d: 178), confirmed by Bousquet (1997b: 334).
Anisodactylus chalceus LeConte, 1859c: 2 [secondary homonym of *Anisodactylus chalceus* (Brullé, 1838)]. Type locality: «Santa Fé [Santa Fe County, New Mexico]» (original citation). Lectotype (♀), designated by Noonan (2001: 316), in MCZ [# 5958]. Synonymy established by Horn (1880d: 178), confirmed by Noonan (2001: 316).
Anisodactylus viridescens LeConte, 1861b: 339. Type locality: «Calif[ornia]» (lectotype label). Lectotype (♂), designated by Noonan (2001: 316), in MCZ [# 5957]. Synonymy established by Horn (1880d: 178), confirmed by Noonan (2001: 316).
Anisodactylus lecontei Gemminger and Harold, 1868a: 256. Replacement name for *Anisodactylus chalceus* LeConte, 1859.
Anadaptus idahoensis Hatch, 1949a: 88. Type locality: «Sand Point [Bonner County], Id[aho]» (original citation). Holotype (♂) in USNM. Synonymy established, under the name *Anisodactylus viridescens* LeConte, by Hatch (1953: 177), confirmed by Noonan (2001: 317).

#### Distribution.

This species occurs from northern Idaho and eastern Washington, south to central California [see Noonan 2001: Fig. 49]. The records from New Mexico (LeConte, 1859c: 2; Fall and Cockerell 1907: 162) and “Arizona” (Leng 1920: 73) need confirmation. One specimen labeled “V[ancouver] I[sland]” is known (Lindroth 1968: 842, as *Anisodactylus viridescens*).

#### Records.

**USA**: CA, ID, OR, WA [AZ, BC, NM]

### Anisodactylus rudis

LeConte, 1863

Anisodactylus rudis LeConte, 1863c: 15. Type locality: «California» (original citation). Holotype [by monotypy] (♂) in MCZ [# 5959].

#### Distribution.

This species ranges from southwestern British Columbia to northwestern Wyoming (Park County, Ken Karns pers. comm. 2009), south to northeastern Nevada and the Mexican border in California [see Noonan 2001: Fig. 56].

#### Records.

**CAN**: BC **USA**: CA, ID, NV, OR, WA, WY

### Anisodactylus sanctaecrucis

(Fabricius, 1798)

Carabus st.crucis Fabricius, 1798: 58. Type locality: «Americae Insulis» (original citation), which is incorrect; «Washington, D.C.» selected by Lindroth (1968: 839). Syntype(s) presumably lost (Zimsen 1964: 57).
Harpalus baltimoriensis Say, 1823a: 33. Type locality: «Pen[n]ington Gap [Lee County], V[irgini]a» (neotype label). Neotype (♂), designated by Lindroth and Freitag (1969: 354), in MCZ [# 32983]. Synonymy established by Schaum (1847: 47).
Anadaptus uteanus Casey, 1924: 136. Type locality: «Stockton [Tooele County], Utah» (original citation). Lectotype (♀), designated by Lindroth (1975: 141), in USNM [# 47963]. Synonymy established by Lindroth (1968: 839).

#### Distribution.

This species ranges from the Sept-Iles region in easternmost Quebec to Vancouver Island, south to the Sierra Nevada in east-central California, southern Colorado in the Rocky Mountains, Oklahoma, southern Louisiana, and northern Florida; seemingly isolated in northwestern British Columbia [see Noonan 2001: Fig. 50].

#### Records.

**CAN**: AB, BC (VCI), MB, NB, NS, ON, PE, QC, SK **USA**: AL, AR, CA, CO, CT, DC, DE, FL, GA, IA, ID, IL, IN, KS, KY, LA, MA, MD, ME, MI, MN, MO, MS, MT, NC, ND, NE, NH, NJ, NY, OH, OK, OR, PA, RI, SC, SD, TN, UT, VA, VT, WA, WI, WV, WY

### Subgenus Spongopus

LeConte, 1847

Spongopus LeConte, 1847: 377. Type species: *Spongopus verticalis* LeConte, 1847 by monotypy. Etymology. From the Greek *spongos* (spongy) and *pous* (foot), probably alluding to the adhesive setae on the male protarsomeres giving the impression of a sponge [masculine].

#### Diversity.

One North American species in the temperate regions.

#### Identification.

Lindroth (1968: 863-864) covered the species.

#### Taxonomic Note.

Lindroth (1968: 862) and Noonan (1973: 374) treated this taxon as a subgenus of *Anisodactylus*. As discussed by Bousquet and Tchang (1992: 762), larval characters suggest that *Spongopus* is probably not closely related to *Anisodactylus* and thus may be more appropriately treated as a distinct genus as done by Ball (1960b: 144).

### Anisodactylus verticalis

(LeConte, 1847)

Spongopus verticalis LeConte, 1847: 378. Type locality: «Paterson [Passaic County], Novae Caesareae [= New Jersey]» (original citation). One syntype in MCZ [# 5960].

#### Distribution.

This species ranges from New Brunswick (Webster and Bousquet 2008: 19) to southern Saskatchewan (Roche Percee, CNC), south to east-central Texas (Riley 2011), northwestern Louisiana (Natchitoches Parish, Igor M. Sokolov pers. comm. 2009), northern Georgia (Fattig 1949: 52), and northwestern South Carolina (Ciegler 2000: 91).

#### Records.

**CAN**: MB, NB, ON, QC, SK **USA**: AR, CT, DC, GA, IA, IL, IN, KS, KY, LA, MA, MD, ME, MI, MN, MO, MS, NC, ND, NE, NH, NJ, NY, OH, OK, PA, SC, SD, TN, TX, VA, VT, WI

### Subgenus Aplocentrus

LeConte, 1847

Aplocentrus LeConte, 1847: 385. Type species: *Harpalus caenus* Say, 1823 designated by Lindroth (1968: 857). Etymology. From the Greek *haplos* (simple) and *centron* (spur), alluding to the simple (i.e., not trifid as compared to members of *Triplectrus* [= *Gynandrotarsus*]) apical spur of protibia of the adult (“*tibiae anticae calcare terminali simplice*”) [masculine].
Haplocentrus Csiki, 1932a: 1077. Unjustified emendation of *Aplocentrus* LeConte, 1847.
Aplocentroides Ball and Bousquet, 2000: 92. Unnecessary replacement name for *Aplocentrus* LeConte, 1847. Etymology. From the generic name *Aplocentrus* [*q.v*.] and the Greek suffix -*oides* (resembling, having the form of) [masculine]. Note. *Aplocentrus calliops* Rafinesque, 1819, the type species and sole species in the genus *Aplocentrus* Rafinesque, 1819 is “apparently a mythical” species of fish based on a purely imaginative drawing that Audubon prepared as a hoax (Eschmeyer 1998: 1843). Therefore, the name *Aplocentrus* Rafinesque, 1819 is a *nomen nudum* and the replacement name, *Aplocentroides* Ball and Bousquet, 2000, is unnecessary.

#### Diversity.

Two North American species in the temperate regions.

#### Identification.

Both species are included in Lindroth’s (1968: 857, 860) monograph.

### Anisodactylus amaroides

LeConte, 1851

Anisodactylus amaroides LeConte, 1851: 184. Type locality: «San Francisco [San Francisco County, California]» (original citation). Three syntypes in MCZ [# 91].

#### Distribution.

This species is found west of the Rocky Mountains, from south-central British Columbia (Lindroth 1968: 857) south to southern California (Fall 1901a: 51; Moore 1937: 13).

#### Records.

**CAN**: BC **USA**: CA, OR, WA

### Anisodactylus caenus

(Say, 1823)

Harpalus caenus Say, 1823a: 34. Type locality: «Amer[ica] Bor[ealis]» (lectotype label), restricted to «Newark [Essex County], N[ew] J[ersey]» by Lindroth (1968: 860). Lectotype (♀), designated by Lindroth and Freitag (1969: 354), in MHNP.
Anisodactylus subaeneus LeConte, 1847: 385. Type locality: «Brooklyn, NovEboraci [= New York]» (original citation). Syntype(s) in MCZ [# 5965]. Synonymy established by Horn (1880d: 178).
Anisodactylus obscurus LeConte, 1847: 386. Type locality: «Massachusetts» (original citation). Syntype(s) in MCZ [# 5964]. Synonymy established by Horn (1880d: 178).
Anisodactylus viridans Casey, 1924: 125. Type locality: «McPherson [McPherson County], Kansas» (original citation). One syntype in USNM [# 47926]. Synonymy established with doubt by Lindroth (1968: 860).

#### Distribution.

This species ranges from “Massachusetts” (LeConte, 1847: 386, as *Anisodactylus obscurus*) to central Kansas (Casey 1924: 125, as *Anisodactylus viridans*), including central Iowa (Larsen and Purrington 2010: 570), south to northern Texas (Tarrant and Dallas Counties, CNC, UASM), northeastern Louisiana (Allen 1965: 70), southwestern Alabama (Löding 1945: 25), and northwestern South Carolina (Ciegler 2000: 89).

#### Records.

**USA**: AL, AR, CT, DC, DE, GA, IA, IL, IN, KS, KY, LA, MA, MD, MI, MO, MS, NC, NJ, NY, OH, OK, PA, SC, TN, TX, VA

### Subgenus Pseudaplocentrus

Noonan, 1973

Pseudaplocentrus Noonan, 1973: 377. Type species: *Anisodactylus laetus* Dejean, 1829 by original designation. Etymology. From the Greek *pseudos* (fallacy, lie) and the generic name *Aplocentrus* [*q.v*.] [masculine].

#### Diversity.

One eastern North American species in the temperate and subtropical regions.

#### Identification.

The species is covered in Lindroth’s (1968: 860) monograph.

### Anisodactylus laetus

Dejean, 1829

Anisodactylus laetus Dejean, 1829: 154. Type locality: «Amérique septentrionale» (original citation), restricted to «Mobile [Mobile County], Alab[ama]» by Lindroth (1968: 860). One syntype in MHNP (Lindroth 1955b: 30).
Harpalus gemmeus Casey, 1914: 108. Type locality: «Urbana [Champaign County], Illinois» (original citation). Lectotype (♀), designated by Lindroth (1975: 142), in USNM [# 47817]. Synonymy established by Lindroth (1975: 142).

#### Distribution.

This species occurs from southeastern New York (Notman 1928: 248) to eastern South Dakota (Kirk and Balsbaugh 1975: 31), including southernmost Ontario (Bousquet 1987a: 131), south to east-central Texas (Riley 2011; Horn 1880d: 175) and southern Florida (Peck and Thomas 1998: 21).

#### Records.

**CAN**: ON **USA**: AL, AR, DC, FL, GA, IA, IL, KS, KY, LA, MI, MN, MO, MS, NC, NE, NJ, NY, OH, OK, PA, SC, SD, TX, VA, WI

### Genus Geopinus

LeConte, 1847

Geopinus LeConte, 1847: 371. Type species: *Daptus incrassatus* Dejean, 1829 by monotypy. Etymology. From the Greek prefix *geo-* (earth) and *pinos* (dirt, filth), probably alluding to the habitat where LeConte found his specimens (“*terram laborans, quasi fodiens*”) [masculine].

#### Diversity.

One North American species in the temperate regions.

#### Identification.

The species is covered in Lindroth’s (1968: 832-833) monograph.

#### Taxonomic Note.

This taxon has always been treated as a valid genus. In a phylogenetic analysis based on characters of the adult conducted by Noonan (1973: Fig. 241), this taxon nested within the genus *Anisodactylus*. Bousquet and Tchang’s (1992: 769) phylogenetic analysis based on larval characters suggested that *Geopinus* may be the sister-group to *Gynandrotarsus* of the genus *Anisodactylus*. Based on these two studies, *Anisodactylus* as presently recognized is probably paraphyletic. However, I prefer to leave *Geopinus*, with its highly distinctive species, as a distinct genus until more conclusive evidence is found.

### Geopinus incrassatus

(Dejean, 1829)

Daptus incrassatus Dejean, 1829: 21. Type locality: «Amérique septentrionale» (original citation), restricted to «Hope [Hempstead County], Ark[ansas]» by Lindroth (1968: 832). One syntype [2 originally cited] in MHNP (Lindroth 1955b: 26).
Geopinus incrassatus fluviaticus Casey, 1914: 52. Type locality: «Keokuk [Lee County], Iowa» (original citation). Lectotype (♀), designated by Lindroth (1975: 141), in USNM [# 47724]. Synonymy established by Lindroth (1968: 832).

#### Distribution.

This widely distributed species ranges from Maine (Robert E. Nelson pers. comm. 1989) and southern Quebec (Larochelle 1975: 85) to southwestern Idaho and northwestern Nevada, north to the southern parts of the Prairie Provinces, south to northern Arizona (Johnson and Clark 1989: 443; Fig. 1), southern Texas (Johnson 1978: 67), and southern Georgia (Fattig 1949: 46).

#### Records.

**CAN**: AB, MB, ON, QC, SK **USA**: AL, AR, AZ, CO, CT, DC, DE, GA, IA, ID, IL, IN, KS, LA, MA, MD, ME, MI, MN, MO, MS, NC, ND, NE, NH, NJ, NM, NV, NY, OH, OK, PA, RI, SC, SD, TN, TX, UT, VA, VT, WI, WY

**Figure 32.**
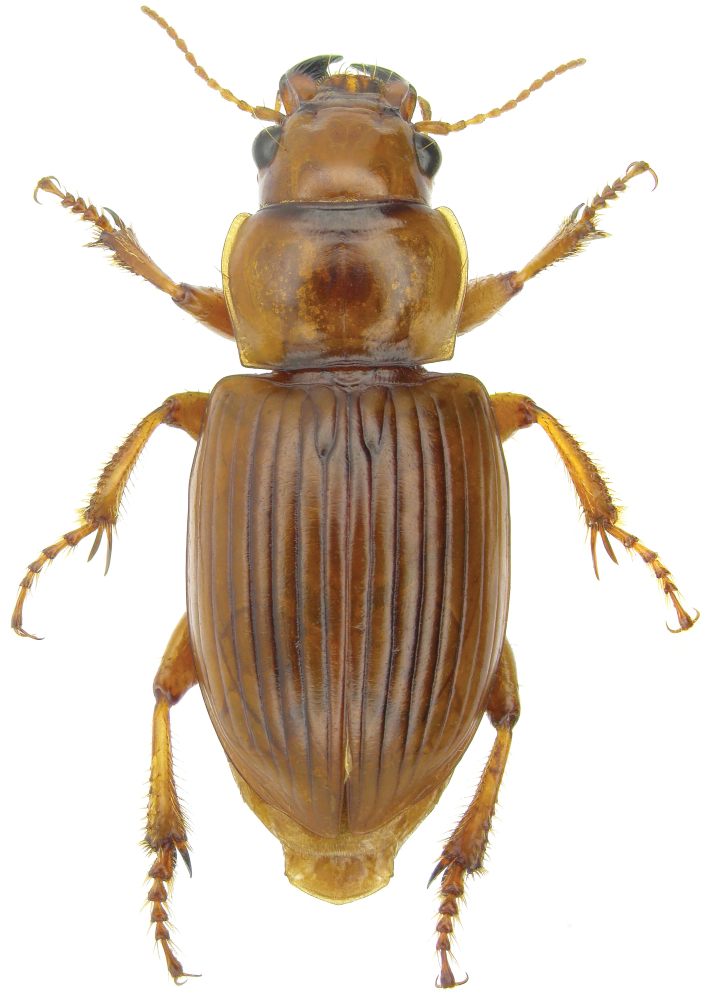
*Geopinus incrassatus* (Dejean). This species is a good illustration of the fact that overall morphological similarity is not always a good indicator of close relationship. It was described by Dejean in the genus *Daptus* which currently includes five species in the Palaearctic Region. John Lawrence LeConte recognized its distinctness and proposed a new genus for it but still most taxonomists interested in harpalines thought it was closely related to daptines. However, several small structural details of the adults and larvae suggest that the species is simply an aberrant member of the genus *Anisodactylus* and is not closely related to daptines.

### Genus Amphasia

Newman, 1838

Amphasia Newman, 1838a: 388. Type species: *Amphasia fulvicollis* Newman, 1838 (= *Feronia interstitialis* Say, 1823) by monotypy. Etymology. From the Greek *amphi* (double) and *phasis* (appearance), probably alluding to the presence in the adults of character states found in two related genera (“apparently partaking of the characters of *Harpalus* and *Mazoreus*”) [feminine]. According to Duponchel (1840b: 374), the name *Amphasia* derives from the Greek *amphi* (around, on both sides) and *asis* (slime, mud, by extension marsh); there is no evidence in Newman (1838) pointing to that interpretation.

#### Diversity.

Two North American species arrayed in two subgenera.

#### Identification.

Lindroth (1968: 860-861) covered both species in his treatment of the genus *Anisodactylus*.

#### Taxonomic Note.

Lindroth (1968: 860) regarded this taxon as a subgenus of *Anisodactylus* while Noonan (1973: 347) treated it as a valid genus. Larval characters support Noonan’s view (Bousquet and Tchang 1992: Fig. 42).

### Subgenus Pseudamphasia

Casey, 1914

Pseudamphasia Casey, 1914: 195. Type species: *Harpalus sericeus* Harris, 1828 by original designation. Etymology. From the Greek *pseudos* (fallacy, lie) and the generic name *Amphasia* [*q.v*.] [feminine].

#### Diversity.

One North American species in the temperate regions.

### Amphasia sericea

(Harris, 1828)

Harpalus sericeus T.W. Harris, 1828b: 117. Type locality not stated; «Camden [Kershaw County], S[outh] C[arolina]» selected by Lindroth (1968: 861). Three syntypes in MCZ (collection Harris).
Harpalus femoratus Dejean, 1829: 224. Type locality: «Amérique septentrionale» (original citation). One syntype in MHNP (Lindroth 1955b: 30). Synonymy established by Dejean (1831: 837), confirmed by Lindroth (1955b: 30).

#### Distribution.

This species ranges from New Brunswick (Webster and Bousquet 2008: 19) to western South Dakota (Kirk and Balsbaugh 1975: 32), north to southern Saskatchewan (Ronald R. Hooper pers. comm. 1990), south to east-central Texas (Riley 2011), south-central Louisiana (Evangeline Parish, MCZ) and the Florida Panhandle (Liberty County, CNC).

#### Records.

**CAN**: MB, NB, ON, QC, SK **USA**: AL, AR, CT, DC, DE, FL, GA, IA, IL, IN, KS, KY, LA, MA, MD, ME, MI, MN, MO, MS, NC, NE, NH, NJ, NY, OH, OK, PA, RI, SC, SD, TN, TX, VA, VT, WI, WV

### Subgenus Amphasia

Newman, 1838

Amphasia Newman, 1838a: 388. Type species: *Amphasia fulvicollis* Newman, 1838 (= *Feronia interstitialis* Say, 1823) by monotypy.

#### Diversity.

One North American species in the temperate regions.

### Amphasia interstitialis

(Say, 1823)

Feronia interstitialis Say, 1823a: 57. Type locality: «Camp Hill [Cumberland County], P[ennsylvani]a» (neotype label). Neotype (♂), designated by Lindroth and Freitag (1969: 354), in MCZ [# 32984]. Note. «Missouri [Territory]; Pennsylvania» were the areas originally cited by Say (1823a: 57).
Harpalus obscuripennis Dejean, 1829: 247. Type locality: «Amérique septentrionale» (original citation). One syntype [2 originally cited] in MHNP (Lindroth 1955b: 30). Synonymy established by Say (1830c: 19), confirmed by Lindroth (1955b: 30).
Amphasia fulvicollis Newman, 1838a: 388. Type locality: «Trenton Falls [Oneida County, New York]» (original citation). Holotype [by monotypy] in BMNH. Synonymy established by LeConte (1847: 376).
Amphasia mollis Casey, 1924: 131. Type locality: «Highland Park [Lake County], north of Chicago, Illinois» (original citation). Holotype [by monotypy] (♀) in USNM [# 47944]. Synonymy established by Lindroth (1968: 860).

#### Distribution.

This species ranges from southern Quebec (Larochelle 1975: 49) to southeastern South Dakota (Kirk and Balsbaugh 1975: 32), south to east-central Texas (Riley 2011), northern Arkansas (Kraim 1983: 221), and west-central South Carolina (Kirk 1970: 14; Ciegler 2000: 92).

#### Records.

**CAN**: ON, QC **USA**: AR, CT, DC, DE, IA, IL, IN, KS, KY, MA, MD, ME, MI, MN, MO, NC, NE, NH, NJ, NY, OH, OK, PA, RI, SC, SD, TN, TX, VA, VT, WI, WV

### Genus Dicheirus

Mannerheim, 1843

Dicheirus Mannerheim, 1843 [“31 December”, permit delivered on 28 March]: 211. Type species: *Harpalus dilatatus* Dejean, 1829 designated by Desmarest (1851: 126). Etymology. From the Greek *dis* (twice) and *cheiros* (hand), probably alluding to the trifid (mistakenly thought to be double) apical spur of the protibia (“*tibiae anticae spina apicali duplici*”) of the adult [masculine].
Diplocheirus Ménétriés, 1843 [29 July]: 62. Type species: *Harpalus dilatatus* Dejean, 1829 by monotypy. Etymology. From the Greek prefix *diplo*- (double) and *cheiros* (hand) [masculine]. Note. With the evidence available, this name should have precedence. However, the name is not in prevailing usage and for that reason it is not retained here as valid (see *Principle of priority* in the “Nomenclature” section).
Dichirus Agassiz, 1846: 122. Unjustified emendation of *Dicheirus* Mannerheim, 1843.
Dicherius Motschulsky, 1859a: 138. Unjustified emendation of *Dicheirus* Mannerheim, 1843.

#### Diversity.

Five North American species, of which two extend into northern Baja California and Guadalupe Island.

#### Identification.

Noonan (1968) revised the species and provided a key for their identification.

### Dicheirus brunneus

(Dejean, 1829)

Harpalus brunneus Dejean, 1829: 239 [primary homonym of *Harpalus brunneus* Gyllenhal, 1810]. Type locality: «Californie» (original citation), restricted to «5.5 mi[les] N[orth] W[est] Ruth Dam, Trinity County» by Noonan (1968: 294). Holotype [by monotypy] (♀) in MHNP (Lindroth 1955b: 30). Note. This name is a junior primary homonym of *Harpalus brunneus* Gyllenhal, 1810 [= *Amara brunnea* (Gyllenhal, 1810)]. Since both names apply to taxa not considered congeneric since 1899, the case is to be referred to the Commission and meanwhile prevailing usage of both names must be maintained (ICZN 1999: Article 23.9.5).
Anisodactylus pilosus G.H. Horn, 1880d: 165. Type locality: «San Joaquin Valley, California» (original citation). Lectotype (♀), designated by Noonan (1968: 295), in MCZ [# 8006]. Synonymy established by Noonan (1968: 293).
Dicheirus exiguus Casey, 1924: 133. Type locality: «Del Norte Co[unty], California» (original citation). Two syntypes in USNM [# 47951]. Synonymy established by Noonan (1968: 293).
Dicheirus gracilis Casey, 1924: 134. Type locality: «Govan [Lincoln County], Washington» (original citation). Holotype [by monotypy] (♀) in USNM [# 47949]. Synonymy established by Noonan (1968: 293).

#### Distribution.

This species is known from east-central Washington (Casey 1924: 134, as *Dicheirus gracilis*) south to Eldorado County in eastern California [see Noonan 1968: Fig. 16].

#### Records.

**USA**: CA, OR, WA

### Dicheirus dilatatus angulatus

Casey, 1914

Dicheirus angulatus Casey, 1914: 199. Type locality: «San Diego [San Diego County], California» (original citation). Six syntypes [6 originally cited] in USNM [# 47952].
Dicheirus blaisdelli Van Dyke, 1926a: 125. Type locality: «Poway, San Diego County, California» (original citation). Holotype (♀) in CAS [# 1869]. Synonymy established by Noonan (1968: 299). Etymology. The species name was proposed for Frank Elsworth Blaisdell [1862-1947], physician and professor of surgery at Stanford University School of Medicine. Blaisdell was also a naturalist and interested in beetles, particularly the Tenebrionidae and Melyridae. He gave his collection of almost 200,000 beetles to the California Academy of Sciences where he pursued his interest after his retirement. In my opinion, his taxonomic treatment of the large and difficult tenebrionid genus *Eleodes* in 1909 is exceptional and avant-gardist.

#### Distribution.

This subspecies is known from a few localities in southernmost California and northern Baja California [see Noonan 1968: Fig. 19].

#### Records.

**USA**: CA – Mexico

### Dicheirus dilatatus dilatatus

(Dejean, 1829)

Harpalus dilatatus Dejean, 1829: 241. Type locality: «Californie» (original citation), herein restricted to Sequoia National Park, Tulare County (see Noonan 1968: 299). One syntype [2 ♂ originally cited] in MHNP (Lindroth 1955b: 30).
Harpalus hirsutus Ménétriés, 1843: 61. Type locality: «Californie» (original citation). Syntype(s) in ZMH (collection Mannerheim) (Silfverberg 1987: 17) and probably also in ZILR. Synonymy established by Horn (1880d: 176).
Dicheirus firmus Casey, 1924: 132. Type locality: «Alameda and San Francisco, California» (original citation). Two syntypes in USNM [# 47946]. Synonymy established by Noonan (1968: 296).
Dicheirus multiplex Casey, 1924: 132. Type locality: «Port Harford, S[an] Luis Obispo Co[unty], California» (original citation). One syntype in USNM [# 47947]. Synonymy established by Noonan (1968: 296).
Dicheirus beniciensis Casey, 1924: 133. Type locality: «Benicia [Solano County], California» (original citation). Three syntypes [3 originally cited] in USNM [# 47945]. Synonymy established by Noonan (1968: 296).
Dicheirus beniciensis validicornis Casey, 1924: 133. Type locality: «California» (original citation). Two syntypes [2 originally cited] in USNM [# 47948]. Synonymy established by Noonan (1968: 296).

#### Distribution.

This subspecies is found over most of California, south to the Los Angeles area and San Clemente Island [see Noonan 1968: Fig. 19].

#### Records.

**USA**: CA (CHI)

### Dicheirus obtusus

LeConte, 1852

Dicheirus obtusus LeConte, 1852a: 185. Type locality: «San Jose [Santa Clara County, California]» (original citation). Lectotype (♂), designated by Noonan (1968: 287), in MCZ [# 93].
Anisodactylus immanis G.H. Horn, 1880d: 165. Type locality: «San Joaquin Valley, California» (original citation). Lectotype (♀), designated by Noonan (1968: 288), in MCZ [# 8007]. Synonymy established by Noonan (1968: 286).
Dicheirus brevisetosus Casey, 1914: 198. Type locality: «Lake Tahoe [Placer County], California» (original citation). Holotype [by monotypy, see page 202] (♂) in USNM [# 47950]. Synonymy established by Noonan (1968: 286).

#### Distribution.

This species is known from northeastern Oregon south to Los Angeles County in southern California [see Noonan 1968: Fig. 15].

#### Records.

**USA**: CA, OR

### Dicheirus piceus

(Ménétriés, 1843)

Harpalus piceus Ménétriés, 1843: 61. Type locality: «Californie» (original citation), herein restricted to Blocksburg, Humboldt County (see Noonan 1968: 290). One syntype in ZMH (collection Mannerheim) (Silfverberg 1987: 22).
Dicheirus villosus Motschulsky, 1845b: 344. Type locality: «Californie» (original citation). Holotype [by monotypy] probably in ZMMU (collection Eschscholtz). Synonymy established by Horn (1880d: 177).
Dicheirus irregularis Motschulsky, 1845b: 345. Type locality not stated; «Calif[ornia]» reported by Motschulsky (1869: 14). Holotype [by monotypy] probably in ZMMU (collection Eschscholtz). Synonymy established by Horn (1880d: 177).
Dicheirus paralellus LeConte, 1851: 184. Type locality: «S[an] D[iego] [San Diego County, California]» (lectotype label). Lectotype (♀), designated by Noonan (1968: 291), in MCZ [# 92]. Synonymy established by LeConte (1858a: 29), confirmed by Noonan (1968: 288).
Dicheirus alutaceus Casey, 1914: 200. Type locality: «Valley of Eel River, Humboldt Co[unty], California» (original citation). Lectotype (♀), designated by Lindroth (1975: 142), in USNM [# 47953]. Synonymy established by Noonan (1968: 288).
Dicheirus piceus rupimontis Casey, 1914: 200. Type locality: «Utah» (original citation). One syntype in USNM [# 47955]. Synonymy established by Noonan (1968: 288).
Dicheirus piceus angustulus Casey, 1914: 200. Type locality: «Trinity River and Redwood Creek, Humboldt Co[unty], California» (original citation). Two syntypes in USNM [# 47956]. Synonymy established by Noonan (1968: 288).
Dicheirus australinus Casey, 1914: 201. Type locality: «San Diego and San Clemente Island, California» (original citation). Fifteen syntypes [15 originally cited] in USNM [# 47960]. Synonymy established by Noonan (1968: 288).
Dicheirus australinus insularis Casey, 1914: 201. Type locality: «Guadalupe Island» (original citation). Fifteen syntypes [15 originally cited] in USNM [# 47961]. Synonymy established by Noonan (1968: 288).
Dicheirus decoloratus Casey, 1914: 201. Type locality: «Arizona» (original citation). Holotype [by monotypy] (♂) in USNM [# 47957]. Synonymy established by Noonan (1968: 288).
Dicheirus incidens Casey, 1924: 134. Type locality: «Columbia River [Clatsop County], Oregon» (original citation). Three syntypes [3 originally cited] in USNM [# 47954]. Synonymy established by Hatch (1953: 177), confirmed by Noonan (1968: 288).
Dicheirus facilis Casey, 1924: 135. Type locality: «Columbia River [Clatsop County], Oregon» (original citation). Two syntypes [2 ♀ originally cited] in USNM [# 47959]. Synonymy established by Hatch (1953: 177), confirmed by Noonan (1968: 288).
Dicheirus sodalis Casey, 1924: 135. Type locality: «Wawawai [Whitman County], Washington» (original citation). Holotype [by monotypy] (♀) in USNM [# 47958]. Synonymy established by Hatch (1953: 177), confirmed by Noonan (1968: 289).

#### Distribution.

This species ranges from Vancouver Island to northwestern Montana (Russell 1968: 67), south to northwestern Utah and the Mexican border in California [see Noonan 1968: Fig. 18]; also recorded from Guadalupe Island in the Pacific (Noonan 1968: 289).

#### Records.

**CAN**: BC (VCI) **USA**: AZ, CA (CHI), ID, MT, OR, UT, WA – Mexico

### Dicheirus strenuus

(Horn, 1869)

Anisodactylus strenuus G.H. Horn, 1869b: 130. Type locality: «Fort Tejon [Kern County], California» (original citation). Lectotype, designated by Noonan (1968: 296), in MCZ [# 34550].

#### Distribution.

This species is known only from a few localities in Kern and Tulare Counties in southern California [see Noonan 1968: Fig. 17].

#### Records.

**USA**: CA

### Subtribe Pelmatellina

Bates, 1882

Pelmatellinae Bates, 1882a: 67. Type genus: *Pelmatellus* Bates, 1882.

#### Diversity.

New World, with about 90 species arrayed in eight genera: *Hakaharpalus* Larochelle and Larivière (five New Zealand species), *Kupeharpalus* Larochelle and Larivière (three New Zealand species), *Lecanomerus* Chaudoir (35 Australian species), *Nemaglossa* Solier (one Chilean species), *Notospeophonus* Moore (three species in Australia), *Pelmatellus* (28 species), *Syllectus* Bates (three New Zealand species), and *Trachysarus* Reed (eight species from the Juan Fernández).

#### Taxonomic Note.

In the phylogenetic analysis of the tribe Harpalini using molecular sequence data conducted by Martínez-Navarro et al. (2005) members of Pelmatellina clustered within those of Stenolophina.

### Genus Pelmatellus

Bates, 1882

Pelmatellus Bates, 1882a: 68. Type species: *Pelmatellus nitescens* Bates, 1882 designated by Goulet (1974b: 84). Etymology. Possibly from the Greek *pelma* (sole of the foot) and the Latin *tellus* (earth) [masculine].

#### Diversity.

Twenty-eight species in the Nearctic (two species) and Neotropical (28 species) Regions arrayed in three subgenera: *Pelmatellopsis* Perrault (17 South American species), *Pelmatellus* s.str. (10 species), and *Thenarellus* Bates (one Middle American species).

### Subgenus Pelmatellus

Bates, 1882

Pelmatellus Bates, 1882a: 68. Type species: *Pelmatellus nitescens* Bates, 1882 designated by Goulet (1974b: 84).

#### Diversity.

Ten North and Middle American species.

#### Identification.

Goulet (1974b) revised the species and provided a key for their identification.

### Pelmatellus obtusus

Bates, 1882

Pelmatellus obtusus Bates, 1882a: 68. Type locality: «Ostuncalco, Guatemala» (original citation for the lectotype). Lectotype (♂), designated by Goulet (1974b: 90), in BMNH.
Bradycellus lucidus Casey, 1884b: 8 [primary homonym of *Bradycellus lucidus* Bates, 1878]. Type locality: «Arizona» (original citation). One syntype in USNM [# 47990]. Synonymy established by Goulet (1974b: 90).
Tachycellus turbatus Fall, 1905: 173. Type locality: «Beulah (8,000 ft.), and Cloudcroft (9,000 ft.), New Mexico» (original citation). Syntype(s) [2 originally cited] in MCZ [# 23880]. Synonymy established with the name *Pelmatellus lucidus* (Casey) by Casey (1914: 234).
Pelmatellus sinuosus Casey, 1914: 235. Type locality: «Salazar, Mex[ico state], Mexico» (original citation). Holotype [by monotypy] (♀) in USNM [# 47991]. Synonymy established by Goulet (1974b: 90).

#### Distribution.

The range of this species extends from southeastern Arizona and southwestern New Mexico south to Guatemala [see Goulet 1974b: Fig. 32].

#### Records.

**USA**: AZ, NM – Guatemala, Mexico

### Pelmatellus stenolophoides parallelus

Goulet, 1974

Pelmatellus stenolophoides parallelus Goulet, 1974b: 93. Type locality: «M[oun]t Lemmon [Pima County], Catalina M[oun]t[ain]s (9150 feet), Arizona» (original citation). Holotype (♂) in CAS [# 12171].

#### Distribution.

This subspecies is known only from the holotype collected in southern Arizona.

#### Records.

**USA**: AZ

#### Note.

The subspecies *Pelmatellus stenolophoides stenolophoides* Bates occurs from central Mexico to Guatemala. The record from “Arizona” (Bousquet and Larochelle 1993: 213) for the nominotypical subspecies is an error and refers to the subspecies *Pelmatellus stenolophoides parallelus*.

### Subtribe Stenolophina

Kirby, 1837

Stenolophidae Kirby, 1837: 46. Type genus: *Stenolophus* Dejean, 1821.
Polpochilinae Bates, 1891b: 10. Type genus: *Polpochila* Solier, 1849.
Acupalpini Tschitschérine, 1900a: 342, 351. Type genus: *Acupalpus* Latreille, 1829.
Cratocarini Casey, 1914: 48, 299. Type genus: *Cratocara* LeConte, 1863 (= *Polpochila* Solier, 1849).
Bradycellini Jeannel, 1942: 693, 700. Type genus: *Bradycellus* Erichson, 1837.
Anthracini Schuler, 1970: 113, 114. Type genus: *Anthracus* Motschulsky, 1850.

#### Diversity.

Worldwide, with about 625 species (Lorenz 2005: 353-362) arrayed in approximately 35 genera. The Northern Hemisphere is represented by about 325 species (roughly 52% of the world fauna) and North America alone by 102 species (16.3%).

#### Taxonomic Note.

Ball and Bousquet (2000: 90) recognized two genus-groups among the North American taxa, the Polpochili for the genus *Polpochila* and Stenolophi for all remaining genera.

### Genus Stenolophus

Dejean, 1821

Stenolophus Dejean, 1821: 15. Type species: *Carabus vaporariorum* Linnaeus *sensu* Fabricius, 1787 (= *Carabus teutonus* Schrank, 1781) designated by Westwood (1838: 5). Etymology. Probably from the Greek *stenos* (narrow) and *lophos* (crest, ridge) [masculine]. The name was proposed by Johann Karl Megerle von Mühlfeld and made available by Dejean.

#### Diversity.

About 170 species (Lorenz 2005: 353-355, excluding *Agonoleptus*) arrayed in four subgenera: *Agonoderus* (seven species), *Astenolophus* Habu (11 Palaearctic species in Asia, two of them extending into the Oriental Region), *Egadroma* Motschulsky (about 90 species in Africa, Asia, and the Australian Region), and *Stenolophus* s.str. (about 60 species). The North American fauna has 22 species (roughly 13% of the world fauna). *Egadroma* is often listed as a separate genus.

### Subgenus Stenolophus

Dejean, 1821

Stenolophus Dejean, 1821: 15. Type species: *Carabus vaporariorum* Linnaeus *sensu* Fabricius, 1787 (= *Carabus teutonus* Schrank, 1781) designated by Westwood (1838: 5).
Notiocharis Gistel, 1856: 359. Type species: *Carabus vaporariorum* Linnaeus *sensu* Fabricius, 1787 (= *Carabus teutonus* Schrank, 1781) designated by Bousquet (2002b: 34). Etymology. From the Greek *notios* (southern) and *charis* (grace) [feminine].

#### Diversity.

About 60 species in the Nearctic (15 species), Neotropical (ten species), Oriental (11 species), and Palaearctic (about 25 species) Regions.

#### Identification.

Lindroth (1968: 905-907) treated all currently recognized valid species in his key except for *Stenolophus splendidulus*. Bousquet (1997b: 335) commented on *Stenolophus splendidulus* and illustrated the median lobe.

### Stenolophus anceps

LeConte, 1857

Stenolophus anceps LeConte, 1857c: 28. Type locality: «San Francisco [San Francisco County, California]» (original citation). Syntype(s) in MCZ [# 5926].
Stenolophus fidelis Casey, 1914: 275. Type locality: «Reno [Washoe County], Nevada» (original citation). Lectotype (♂), designated by Lindroth (1975: 143), in USNM [# 48039]. Synonymy established by Lindroth (1968: 910).
Stenolophus peregrinus Casey, 1914: 278. Type locality: «Provo [Utah County], Utah» (original citation). Seven syntypes [7 originally cited] in USNM [# 48045]. **New synonymy** (Gerald R. Noonan pers. comm. 2008).

#### Distribution.

This species ranges from Vancouver Island (Lindroth 1968: 911) to western Montana (Russell 1968: 67), south to southern Colorado (Elias 1987: 634) and southern California (Fall 1901a: 50; Andrews et al. 1979: 28).

#### Records.

**CAN**: BC (VCI) **USA**: CA, CO, ID, MT, NV, OR, UT, WA

### Stenolophus carbo

Bousquet, 1993

Harpalus carbonarius Dejean, 1829: 398 [primary homonym of *Harpalus carbonarius* Say, 1823]. Type locality: «Amérique septentrionale» (original citation), restricted to «Hope [Hempstead County], Ark[ansas]» by Lindroth (1968: 908). Holotype [by monotypy] (♀) in MHNP (Lindroth 1955b: 31).
Stenolophus carbo Bousquet [in Bousquet and Larochelle], 1993: 11. Replacement name for *Stenolophus carbonarius* (Dejean, 1829).

#### Distribution.

The range of this species extends from northern New Hampshire (Reeves et al. 1983: 459) to eastern South Dakota (Kirk and Balsbaugh 1975: 33), including southernmost Ontario (Lindroth 1968: 908), south to southern Texas (Johnson 1978: 67) and southern Florida (Peck and Thomas 1998: 22). The record from Nova Scotia (Lindroth 1954c: 309) is considered doubtful by Majka et al. (2007: 10).

#### Records.

**CAN**: ON **USA**: AL, AR, CT, DC, FL, GA, IA, IL, IN, KY, LA, MA, MD, MI, MO, MS, NC, NH, NJ, NY, OK, RI, SC, SD, TN, TX, VA, WI [NS]

### Stenolophus cincticollis

LeConte, 1858

Stenolophus cincticollis LeConte, 1858b: 60. Type locality: «Colorado River, near the Gila [southwestern Arizona]» (original citation). Holotype [by monotypy] in MCZ [# 5927].
Stenolophus lamprotus Bates, 1891a: 246. Type locality: «Villa Lerdo, in Durango» (original citation). Syntype(s) [2 originally cited] in BMNH. **New synonymy** (Gerald R. Noonan pers. comm. 2008).
Stenolophus semitinctus Casey, 1914: 279. Type locality: «El Paso [El Paso County], Texas» (original citation). Four syntypes [4 originally cited] in USNM [# 48046]. **New synonymy** (Gerald R. Noonan pers. comm. 2008).
Stenolophus extensicollis Casey, 1924: 145. Type locality: «Tempe [Maricopa County], Arizona» (original citation). Holotype [by monotypy] (♂) in USNM [# 48044]. **New synonymy** (Gerald R. Noonan pers. comm. 2008).

#### Distribution.

This species ranges from southeastern California (Imperial County, MCZ, USNM) to westernmost Texas (Casey 1914: 279, as *Stenolophus semitinctus*), south at least to the state of Durango in Mexico. The record from “Colorado” (Csiki 1932a: 1260) needs confirmation.

#### Records.

**USA**: AZ, CA, NM, TX [CO] – Mexico

### Stenolophus dissimilis

Dejean, 1829

Stenolophus dissimilis Dejean, 1829: 410. Type locality: «Louisiane» (original citation). Two syntypes [2 originally cited] in MHNP (Lindroth 1955b: 31).

#### Distribution.

This species ranges from northeastern Ohio (Purrington et al. 1999: 47) to southern Wisconsin (Rauterberg 1885: 21; Messer 2010: 40), north to Charity Island in the Michigan waters of Lake Huron (Andrews 1916: 79), south to southern Texas (Johnson 1978: 67), northern Georgia (Fattig 1949: 54), and northwestern South Carolina (Ciegler 2000: 94). The record from New Jersey (Smith 1910: 216) needs confirmation.

#### Records.

**USA**: AL, AR, GA, IL, IN, KS, KY, LA, MI, MO, MS, NC, OH, OK, SC, TN, TX, WI [NJ]

### Stenolophus flavipes

LeConte, 1858

Stenolophus flavipes LeConte, 1858b: 60. Type locality: «San Diego [San Diego County], California» (original citation). Syntype(s) in MCZ [# 5928].

#### Distribution.

This species is known from northern California (Shasta County, USNM) to southern California (Fall 1901a: 50; Dajoz 2007: 20) and “Arizona” (Leng 1920: 74). The record from “Oregon” (Schaupp 1883a: 14) is probably in error (Hatch 1953: 184).

#### Records.

**USA**: AZ, CA

### Stenolophus fuliginosus

Dejean, 1829

Stenolophus fuliginosus Dejean, 1829: 423. Type locality: «Amérique septentrionale» (original citation), restricted to «Rumney [Grafton County], N[ew] H[ampshire]» by Lindroth (1968: 909). One syntype in MHNP (Lindroth 1955b: 31).
Stenolophus versicolor Kirby, 1837: 46. Type locality: «Lat. 54° [= along North Saskatchewan River]» (original citation). Three syntypes [3 originally cited] in BMNH (Lindroth 1953b: 175). Synonymy established by LeConte (1847: 410), confirmed by Lindroth (1953b: 175).
Stenolophus fuscipennis LeConte, 1847: 410. Type locality: «NovEboraci [= New York]» (original citation). Syntype(s) in MCZ [# 5924]. Synonymy established by LeConte (1853c: 386), confirmed by Lindroth (1968: 910).

#### Distribution.

The range of this species extends from Newfoundland (Lindroth 1955a: 149) to Vancouver Island (Lindroth 1968: 910), south to the Sierra Nevada in California (Dajoz 2007: 16), northern Colorado (Haubold 1951: 706; Armin 1963: 122), and eastern Georgia (Fattig 1949: 54). The record from southern Louisiana (Summers 1874a: 81) needs confirmation.

#### Records.

**CAN**: AB, BC (VCI), MB, NB, NF, NS (CBI), ON, PE, QC, SK **USA**: AR, CA, CO, CT, DC, GA, IA, ID, IL, IN, MA, MD, ME, MI, MN, MT, NC, ND, NH, NJ, NY, OH, OR, PA, RI, SC, SD, VA, VT, WA, WI, WY [LA]

### Stenolophus fuscatus

Dejean, 1829

Stenolophus fuscatus Dejean, 1829: 426. Type locality: «Amérique septentrionale» (original citation), restricted to «Galesburg [Knox County], Illin[ois]» by Lindroth (1968: 914). Holotype [by monotypy] (♂) in MHNP (Lindroth 1955b: 31). Note. Dejean (1829: 427) stated that the unique specimen of this species in his collection was a female.

#### Distribution.

This species occurs from southwestern New Brunswick (Webster and Bousquet 2008: 19) to eastern South Dakota (Kirk and Balsbaugh 1975: 33), south to northeastern Kansas (Popenoe 1878: 79) and south-central Maryland (Prince Georges County, USNM). The record from southern Colorado (Elias 1987: 634) needs confirmation.

#### Records.

**CAN**: NB, ON, QC **USA**: CT, IL, IN, KS, MA, MD, ME, MI, NE, NH, NJ, NY, OH, PA, RI, SD, VT, WV [CO]

### Stenolophus humidus

Hamilton, 1893

Stenolophus humidus Hamilton, 1893: 306. Type locality: near Allegheny [Pennsylvania] (inferred from title of the paper). Six syntypes [15 originally cited] in CMNH.

#### Distribution.

This species ranges from southern Nova Scotia (Christopher G. Majka pers. comm. 2007) to southwestern Michigan (Allegan County, CMNH), south to southeastern Mississippi (Stone County, Drew A. Hildebrandt pers. comm. 2008), northern Georgia (Fattig 1949: 54), and eastern South Carolina (Ciegler 2000: 94).

#### Records.

**CAN**: NS, ON, QC **USA**: CT, DC, GA, KY, MA, MD, ME, MI, MS, NC, NH, NJ, NY, OH, PA, RI, SC, VA, VT, WV

### Stenolophus incultus

Casey, 1914

Stenolophus incultus Casey, 1914: 275. Type locality: «Truckee [Nevada County], California» (original citation). Lectotype (♀), designated by Lindroth (1975: 143), in USNM [# 48040].
Stenolophus abstinens Casey, 1914: 273. Type locality: «Arizona» (original citation). Two syntypes in USNM [# 48037]. **New synonymy** (Gerald R. Noonan pers. comm. 2008).
Stenolophus remissus Casey, 1914: 274. Type locality: «southern California» (original citation). One syntype in USNM [# 48038]. **New synonymy** (Gerald R. Noonan pers. comm. 2008).
Stenolophus consors Casey, 1914: 276. Type locality: «Gualala River, Mendocino Co[unty], California» (original citation). Lectotype (♂), designated by Lindroth (1975: 143), in USNM [# 48041]. Synonymy established by Lindroth (1968: 911).
Stenolophus debiliceps Casey, 1914: 276. Type locality: «Lake Tahoe [Placer County], California» (original citation). Holotype [by monotypy] (♀) in USNM [# 48042]. Synonymy established by Lindroth (1968: 911).

#### Distribution.

This species is found from Vancouver Island (Lindroth 1968: 911) to northern Idaho (Hatch 1953: 183), south to “Arizona” (Casey 1914: 273, as *Stenolophus abstinens*) and southern California (Casey 1914: 274, as *Stenolophus remissus*). The record from “Montana” (Bousquet and Larochelle 1993: 220) needs confirmation.

#### Records.

**CAN**: BC (VCI) **USA**: AZ, CA, ID, OR, UT, WA [MT]

### Stenolophus limbalis

LeConte, 1857

Stenolophus limbalis LeConte, 1857c: 28. Type locality: «San Jose [Santa Clara County], California» (original citation). Syntype(s) in MCZ [# 5923].
Stenolophus indistinctus Motschulsky, 1859a: 134. Type locality: «St. José [Santa Clara County], California» (lectotype label). Lectotype (♂), designated by Bousquet (1997b: 333), in ZMMU. Synonymy established by LeConte (1863b: 13), confirmed by Bousquet (1997b: 333).
Stenolophus longitarsis Casey, 1914: 277. Type locality: «Oregon» (original citation). Lectotype (♂), designated by Lindroth (1975: 143), in USNM [# 48043]. Synonymy established by Hatch (1953: 184), confirmed by Lindroth (1968: 909).

#### Distribution.

This species ranges from Vancouver Island (Lindroth 1968: 909) to northwestern Montana (Russell 1968: 67), south to central Utah (Casey 1914: 276) and southern California (Fall 1901a: 50; Moore 1937: 14). The species is adventive on Oahu, Hawaii (Liebherr 2009: 403).

#### Records.

**CAN**: BC (VCI) **USA**: CA (CHI), ID, MT, NV, OR, UT, WA

### Stenolophus megacephalus

Lindroth, 1968

Stenolophus megacephalus Lindroth, 1968: 914. Type locality: «Bala, N[orth]W[est] Gravenhurst, Ont[ario]» (original citation). Holotype (♂) in CNC [# 10579].

#### Distribution.

This species is restricted to a small area from southern Quebec and the Ontario Peninsula (Bousquet 1987a: 131) south to central Pennsylvania (Clinton County, CMNH) and “Rhode Island” (Sikes 2003: 8).

#### Records.

**CAN**: ON, QC **USA**: CT, KY, MA, NH, NY, PA, RI

### Stenolophus ochropezus

(Say, 1823)

Feronia ochropeza Say, 1823a: 54. Type locality: North America (inferred from title of the paper); restricted to «Camden [Kershaw County], S[outh] C[arolina]» by Lindroth (1968: 911). Lectotype (♂), designated by Lindroth and Freitag (1969: 356), in MHNP (collection Dejean).
Stenolophus convexicollis LeConte, 1847: 409. Type locality: «Rocky Mountains» (original citation). Syntype(s) in MCZ [# 5929]. Synonymy established by LeConte (1869a: 379), confirmed by Lindroth (1968: 912).
Stenolophus rotundicollis Motschulsky, 1859a: 135. Type locality: «California» (lectotype label). Lectotype (♀), designated by Bousquet (1997b: 333), in ZMMU. Synonymy established by Bousquet (1997b: 333).
Stenolophus laticollis Motschulsky, 1864: 202. Type locality: «N[ew] Orl[eans] [Orleans Parish, Louisiana]» (original citation). Lectotype (♀), designated by Bousquet and Larochelle (1993: 18), in ZMMU. Synonymy established by Bousquet and Larochelle (1993: 18).
Stenolophus gracilis Casey, 1884b: 14. Type locality: «Arizona» (original citation). Holotype [by monotypy] presumably lost (Lindroth 1968: 912). Synonymy established by Horn (1885b: 109).
Stenolophus testaceicollis Casey, 1924: 145. Type locality: «Boston Neck [Washington County], Rhode Island» (original citation). Holotype [by monotypy] (♀) in USNM [# 48035]. Synonymy established by Lindroth (1968: 912).
Stenolophus floridanus Casey, 1924: 145. Type locality: «Florida» (original citation). Lectotype (♂), designated by Lindroth (1975: 143), in USNM [# 48036]. Synonymy established by Lindroth (1968: 912).

#### Distribution.

This species ranges from Nova Scotia (Lindroth 1954c: 309) to southeastern Saskatchewan (Ronald R. Hooper pers. comm. 2007), south to southern Texas (Johnson 1978: 67) and southern Florida (Peck and Thomas 1998: 22), west along the southwest to southern California (Horn 1894: 312; Andrews et al. 1979: 28); also found in southern Baja California (Horn 1894: 312), the Bahamas (Darlington 1953: 10), Cuba (Darlington 1934: 112), Jamaica (Darlington 1941a: 14), Dominican Republic (Robert L. Davidson pers. comm. 2012) and Puerto Rico (Wolcott 1936: 191).

#### Records.

**CAN**: MB, NB, NS (CBI), ON, QC, SK **USA**: AL, AR, AZ, CA, CO, CT, DC, DE, FL, GA, IA, IL, IN, KS, KY, LA, MA, MD, ME, MI, MN, MO, MS, NC, NE, NH, NJ, NM, NY, OH, OK, PA, RI, SC, SD, TN, TX, UT, VA, VT, WI, WV – Bahamas, Cuba, Dominican Republic, Jamaica, Mexico, Puerto Rico

### Stenolophus plebejus

Dejean, 1829

Stenolophus plebejus Dejean, 1829: 424. Type locality: «Amérique septentrionale» (original citation), restricted to «Wellesley, Mass[achusetts]» by Lindroth (1968: 913). One syntype [2 ♂ originally cited] in MHNP (Lindroth 1955b: 31).
Acupalpus lugubris Haldeman, 1843b: 302 [*nomen dubium*]. Type locality: southeastern Pennsylvania (Haldeman 1843a: 297). Syntype(s) presumably lost. Synonymy established by Casey (1914: 279).
Stenolophus humeralis Motschulsky, 1864: 202. Type locality: «Am[érique] b[oréale]» (lectotype label). Lectotype (♂), designated by Bousquet and Larochelle (1993: 18), in ZMMU. Synonymy established by Bousquet and Larochelle (1993: 18).
Stenolophus rivularis Casey, 1924: 146. Type locality: «S[ain]t Louis, Missouri» (original citation). Holotype [by monotypy] (♀) in USNM [# 48048]. Synonymy established by Lindroth (1968: 913).

#### Distribution.

The range of this species extends from southern Quebec (Larochelle 1975: 255) to eastern Iowa (Johnson County, USNM), south to southeastern Texas (Aransas County, USNM) and southern Florida (Peck and Thomas 1998: 22); also recorded from Bermuda (Hilburn and Gordon 1989: 677). The records from central Colorado (Wickham 1902: 242) and Tabasco in Mexico (Bates 1891a: 246) are probably in error.

#### Records.

**CAN**: ON, QC **USA**: AL, AR, CT, DC, FL, GA, IA, IL, IN, KY, LA, MA, MD, ME, MI, MO, MS, NC, NH, NJ, NY, OH, OK, PA, RI, SC, TN, TX, VA, VT, WV – Bermuda

### Stenolophus splendidulus

Motschulsky, 1864

Stenolophus splendidulus Motschulsky, 1864: 201. Type locality: «Am[érique] bor[éale]» (original citation). Lectotype (♂), designated by Bousquet (1997b: 335), in ZMMU.

#### Distribution.

Besides the three original specimens, I assign to this species a series of specimens collected in southernmost Ontario (Rondeau Provincial Park, CNC).

#### Records.

**CAN**: ON

### Stenolophus spretus

Dejean, 1831

Stenolophus spretus Dejean, 1831: 845. Type locality: «Amérique septentrionale» (original citation). Holotype [by monotypy] (♂) in MHNP (Lindroth 1955b: 31).

#### Distribution.

This species is known from New Jersey (Smith 1910: 215; Lindroth 1968: 905) to the Florida Keys (Peck and Thomas 1998: 22), west along the Gulf Plain to southern Texas (Johnson 1978: 67), north to western Kansas (Scott County, CMNH).

#### Records.

**USA**: AL, AR, DC, FL, GA, KS, LA, MD, MS, NJ, OK, SC, TX, VA

### Subgenus Agonoderus

Dejean, 1829

Agonoderus Dejean, 1829: 49. Type species: *Carabus lineola* Fabricius, 1775 designated by Brullé (1835b: 15). Etymology (original). From the Greek *a* (absence), *gonia* (angle), and *dere* (neck, by extension pronotum), alluding to the rounded posterior angles of the pronotum of adults (“*corselet ovalaire ou en carré dont les angles sont arrondis*”) of the three species Dejean had before him [masculine].

#### Diversity.

Seven species in North America, of which two extend into northern Mexico, Bermuda, the Bahamas, and Cuba.

#### Identification.

The subgenus has been reviewed by Heading (1964) but his work remains unpublished. Lindroth (1968: 916-920, as *comma* group) covered four species. A revision of the group would be useful.

### Stenolophus binotatus

(Casey, 1914)

Agonoderus binotatus Casey, 1914: 291. Type locality: «Alexandria [Rapides Parish], Louisiana» (original citation). One syntype in USNM [# 48060].

#### Distribution.

This species is known from southern Oklahoma (Marshall County, CMNH) to eastern Coahuila in Mexico (UASM), east to southeastern Louisiana (Heading 1964: 34).

#### Records.

**USA**: LA, OK, TX – Mexico

### Stenolophus comma

(Fabricius, 1775)

Carabus comma Fabricius, 1775: 248. Type locality: «America» (original citation), restricted to «Arlington [Middlesex County], Mass[achusetts]» by Lindroth (1968: 918). Lectotype (♂), designated by Lindroth (1968: 918), in ZMUC.
Trechus similis Kirby, 1837: 48. Type locality: «Lat. 54° [= along North Saskatchewan River]» (original citation). Two syntypes [2 originally cited] in BMNH (Lindroth 1953b: 175). Synonymy established by LeConte (1873b: 325), confirmed by Lindroth (1953b: 175).
Agonoderus dorsalis LeConte, 1847: 373. Type locality: «provinciis mediis et occidentalibus» (original citation). Syntype(s) in MCZ [# 5876]. Synonymy established by LeConte (1869a: 375). Note. Fall (1933: 104) pointed out that the specimen labeled “dorsalis” in LeConte’s collection is conspecific with those of *Stenolophus lecontei* Chaudoir and since LeConte (1869a: 375) listed his *dorsalis* in synonymy with *Stenolophus comma* (Fabricius), it seems likely that the specimen labeled *dorsalis* is not a syntype.
Agonoderus oculatus Casey, 1914: 295. Type locality: «Austin [Travis County], Texas» (original citation). Holotype [by monotypy] (♂) in USNM [# 48066]. Synonymy established by Bousquet and Larochelle (1993: 221), based on Heading’s (1964) unpublished thesis.
Agonoderus gracilitarsis Casey, 1914: 296. Type locality: «New York» (original citation for the lectotype). Lectotype, designated by Lindroth (1975: 143), in USNM [# 48068]. Synonymy established by Lindroth (1968: 918).
Agonoderus quadricollis Casey, 1914: 296. Type locality: «Oklahoma» (original citation). Holotype [by monotypy] (♂) in USNM [# 48067]. Synonymy established by Bousquet and Larochelle (1993: 221), based on Heading’s (1964) unpublished thesis.
Agonoderus obliqulus Casey, 1914: 297. Type locality: «Provo [Utah County], Utah» (original citation for the lectotype). Lectotype (♀), designated by Lindroth (1975: 143), in USNM [# 48070]. Synonymy established by Lindroth (1968: 918).
Agonoderus pallescens Casey, 1914: 297. Type locality: «Arizona» (original citation). Five syntypes [5 originally cited] in USNM [# 48071]. Synonymy established by Bousquet and Larochelle (1993: 221), based on Heading’s (1964) unpublished thesis.
Agonoderus latipennis Casey, 1914: 298. Type locality: «Utah» (original citation). Holotype [by monotypy] in USNM [# 48072]. Synonymy established by Bousquet and Larochelle (1993: 221), based on Heading’s (1964) unpublished thesis.

#### Distribution.

This species ranges from Newfoundland (Lindroth 1955a: 150) to Vancouver Island (Lindroth 1968: 919), south to southeastern California (Dajoz 2007: 20), southern Arizona, central Texas, northern Alabama, and east-central South Carolina (Ciegler 2000: 93) [see Heading 1964: Fig. 19]. The record from “Mississippi” (Bousquet and Larochelle 1993: 221) needs confirmation.

#### Records.

**FRA**: PM **CAN**: AB, BC (VCI), MB, NB, NF, NS, ON, PE, QC, SK **USA**: AL, AR, AZ, CA, CO, CT, DC, DE, GA, IA, ID, IL, IN, KS, KY, LA, MA, MD, ME, MI, MN, MO, MT, NC, ND, NE, NH, NJ, NM, NY, OH, OK, OR, PA, RI, SC, SD, TN, TX, UT, VA, VT, WA, WI, WV, WY [MS]

### Stenolophus infuscatus

(Dejean, 1829)

Agonoderus infuscatus Dejean, 1829: 54. Type locality: «Amérique septentrionale» (original citation), restricted to «Mobile [Mobile County], Alab[ama]» by Lindroth (1968: 917). Syntype(s) in MHNP.
Agonoderus suturalis LeConte, 1847: 373. Type locality: «NovEboraci [= New York]» (original citation). Syntype(s) in MCZ [# 5874]. Synonymy established by LeConte (1853c: 381).

#### Distribution.

This species ranges from “New York” (LeConte 1847: 373) and New Jersey to southern Florida including the Keys (Peck and Thomas 1998: 22), west to northern Oklahoma (Alfalfa County, Robert L. Davidson pers. comm. 2008) and southeastern Texas [see Heading 1964: Fig. 18]; also recorded from the Bahamas and Cuba (Darlington 1953: 11). The records from southeastern Minnesota (Gandhi et al. 2005: 931) and southwestern Ohio (Blatchley 1910: 177) need confirmation.

#### Records.

**USA**: AL, DE, FL, GA, LA, MD, MS, NC, NJ, NY, OK, SC, TX, VA [MN, OH] – Bahamas, Cuba.

### Stenolophus lecontei

(Chaudoir, 1868)

Agonoderus lecontei Chaudoir, 1868b: 164. Type locality: «provinciis australibus et occidentalibus» (original citation for *Agonoderus pallipes* Fabricius *sensu* LeConte, 1847), restricted to «Hope [Hempstead County], Ark[ansas]» by Lindroth (1968: 919). Lectotype (♂), designated by Lindroth (1968: 919), in MHNP. Note. This name was proposed for *Agonoderus pallipes* (Fabricius, 1801) *sensu* LeConte (1847: 373).
Agonoderus idoneus Casey, 1914: 292. Type locality: «Keokuk [Lee County], Iowa» (original citation). One syntype in USNM [# 48061]. Synonymy established by Bousquet and Larochelle (1993: 222), based on Heading’s (1964) unpublished thesis.
Agonoderus plagiatus Casey, 1914: 294. Type locality: «Wisconsin» (original citation). Lectotype (♀), designated by Lindroth (1975: 143), in USNM [# 48062]. Synonymy established by Lindroth (1968: 919).
Agonoderus tarsalis Casey, 1914: 294. Type locality: «El Paso [El Paso County], Texas» (original citation). One syntype in USNM [# 48064]. Synonymy established by Bousquet and Larochelle (1993: 222), based on Heading’s (1964) unpublished thesis.
Agonoderus vividus Casey, 1914: 294. Type locality: «Arkansas» (original citation). One syntype in USNM [# 48063]. Synonymy established by Bousquet and Larochelle (1993: 222), based on Heading’s (1964) unpublished thesis.
Agonoderus vacans Casey, 1914: 294. Type locality: «Waco [McLennan County], Texas» (original citation). Two syntypes in USNM [# 48065]. Synonymy established by Bousquet and Larochelle (1993: 222), based on Heading’s (1964) unpublished thesis.

#### Distribution.

The range of this species extends from western Maine (Kennebec and Somerset Counties, Ross T. Bell pers. comm. 2008) to eastern South Dakota (Ellsbury et al. 1998: 621), south to east-central Texas (Riley 2011) and the Florida Panhandle (Peck and Thomas 1998: 22). Also known from northern Idaho (Hatten et al. 2007: 359) and from a single, old (1926) specimen (possibly mislabeled) from western Oregon (Westcott et al. 2006: 9). The records from northern Colorado (Armin 1963: 115) and New Mexico (Fall and Cockerell 1907: 161, *Agonoderus pallipes*) need confirmation.

#### Records.

**CAN**: ON, QC **USA**: AL, AR, CT, DC, DE, FL, GA, IA, ID, IL, IN, KS, KY, LA, MA, MD, ME, MI, MN, MO, MS, NC, NE, NH, NJ, NY, OH, OK, PA, RI, SC, SD, TN, TX, VA, VT, WI, WV [CO, NM, OR]

### Stenolophus lineola

(Fabricius, 1775)

Carabus lineatus Forster, 1771: 59 [potential *nomen oblitum*]. Type locality: «Americâ septentrionali» (original citation). Syntype(s) lost.
Carabus lineola Fabricius, 1775: 244 [potential *nomen protectum*]. Type locality: «America septentrionali» (original citation), restricted to «Hope [Hempstead County], Ark[ansas]» by Lindroth (1968: 917). One syntype in BMNH (Zimsen 1964: 57; Lindroth 1968: 917). Synonymy established by Goeze (1777: 655).
Carabus furcatus Fabricius, 1792: 164. Type locality: «America» (original citation). Syntype(s) probably lost (Lindroth 1968: 917). Synonymy established with doubt by Dejean (1829: 51).
Carabus chrysomalinus Frölich, 1792: 162. Type locality: «Virginien» (original citation). Syntype(s) lost. Synonymy established by Crotch (1871: 10).

#### Distribution.

This species is found from Prince Edward Island to southern Alberta (Lindroth 1968: 918), south to “Baja California” (Leng 1920: 74), southeastern California (Andrews et al. 1979: 28), southern Arizona (Heading 1964: Fig. 18), north-central Mexico (Heading 1964: 30), and central Florida (Peck and Thomas 1998: 22); also known from one specimen collected in northern Oregon (Westcott et al. 2006: 9) and reported as “common in light traps” in Bermuda (Hilburn and Gordon 1989: 677).

#### Records.

**CAN**: AB, MB, ON, PE, QC, SK **USA**: AL, AR, AZ, CA, CO, CT, DC, DE, FL, GA, IA, ID, IL, IN, KS, KY, LA, MA, MD, ME, MI, MN, MO, MS, MT, NC, NE, NH, NJ, NM, NV, NY, OH, OK, OR, PA, RI, SC, SD, TN, TX, UT, VA, VT, WI, WV, WY – Bermuda, Mexico

### Stenolophus maculatus

(LeConte, 1869)

Agonoderus maculatus LeConte, 1869a: 374. Type locality: «California and Nevada» (original citation). Syntype(s) in MCZ [# 5873].

#### Distribution.

This species is found throughout California, in southeastern Arizona [see Heading 1964: Fig. 18], and in “Nevada” (LeConte 1869a: 374); one specimen labeled from south-central Washington is also known (Heading 1964: 32).

#### Records.

**USA**: AZ, CA, NV [WA]

### Stenolophus rugicollis

(LeConte, 1859)

Agonoderus rugicollis LeConte, 1859a [February]: 83. Type locality: «California» (original citation), herein restricted to North Fork, Madera County (see Casey 1914: 297, as *Stenolophus rectus*). Syntype(s) in MCZ [# 5875].
Dichirus pallidus Motschulsky, 1859a [after 27 November]: 137. Type locality: «Calif[ornia]» (lectotype label). Lectotype (probably ♀), designated by Bousquet (1997b: 333), in ZMMU. Synonymy established by Casey (1914: 298), confirmed by Bousquet (1997b: 333).
Agonoderus rectus Casey, 1914: 297. Type locality: «North Fork, Madera Co[unty], California» (original citation). Holotype [by monotypy] (♀) in USNM [# 48069]. Synonymy established by Bousquet and Larochelle (1993: 222), based on Heading’s (1964) unpublished thesis.

#### Distribution.

This species is known from central Washington to southwestern California [see Heading 1964: Fig. 19].

#### Records.

**USA**: CA, OR, WA

### Genus Agonoleptus

Casey, 1914

Agonoleptus Casey, 1914: 284. Type species: *Agonoleptus parviceps* Casey, 1914 by monotypy. Etymology. From the Greek *a* (without), *gonia* (angle), and *leptos* (fine, thin, delicate), probably alluding to the fact that adults of the sole, relatively small species known to Casey have the posterior angles of the pronotum rounded (“*the hind angles of the prothorax are rounded*”) [masculine].

#### Diversity.

Seven North American species in the temperate regions.

#### Identification.

There is no key for the identification of all species. Lindroth (1968: 921-924) covered four species, leaving *Agonoleptus dolosus* and *Agonoleptus parviceps*.

#### Taxonomic Note.

Adults and particularly larvae (personal observation) are structurally quite different from those of *Stenolophus* s.str. and *Agonoderus* and I am unable to find any character states that would conclusively suggest that *Agonoleptus* is closely related to the other two taxa. For that reason, I consider *Agonoleptus* as a distinct genus.

### [conjunctus group]

### Agonoleptus conjunctus

(Say, 1823), new combination

Trechus conjunctus Say, 1823a: 90. Type locality: «Wh[i]t[e] Sulphur Spr[i]ngs [Greenbrier County], W[est] V[irgini]a» (neotype label). Neotype (♂), designated by Lindroth and Freitag (1969: 356), in MCZ [# 32966].
Acupalpus humilis Dejean, 1829: 462. Type locality: «Amérique septentrionale» (original citation). Holotype [by monotypy] (♀) in MHNP (Lindroth 1955b: 31). Synonymy established by Lindroth (1955b: 31).
Acupalpus misellus Dejean, 1829: 467. Type locality: «Amérique septentrionale» (original citation). One syntype in MHNP (Lindroth 1955b: 31). Synonymy established by Say (1830c: 21), confirmed by Lindroth (1955b: 31).
Trechus immunis Kirby, 1837: 48. Type locality: «Lat. 54° [= along North Saskatchewan River]» (original citation). Syntype(s) [2 originally cited] in BMNH (Lindroth 1953b: 175). Synonymy established by LeConte (1873b: 325), confirmed by Lindroth (1953b: 175).
Acupalpus obesus Bates, 1878a: 593. Type locality: «near the capital, Mexico» (original citation). Syntype(s) probably in BMNH. Synonymy established by Bates (1882a: 71).
Stenolophus captiosus Casey, 1914: 281. Type locality: «Boulder Co[unty], Colorado» (original citation for the lectotype). Lectotype, designated by Lindroth (1975: 143), in USNM [# 48050]. Synonymy established by Lindroth (1968: 921).
Stenolophus moquinus Casey, 1914: 282. Type locality: «Arizona» (original citation). Lectotype (♀), designated by Lindroth (1975: 143), in USNM [# 48051]. Synonymy established by Lindroth (1968: 921).

#### Distribution.

This species ranges from Cape Breton Island (Lindroth 1954c: 309) to Vancouver Island (Lindroth 1968: 922), south to the Sierra Nevada in California (Dajoz 2007: 16), at least San Luis Potosí in Mexico (CNC), and central Florida (Peck and Thomas 1998: 22). The record from Guatemala (Bates 1882a: 71) needs confirmation.

#### Records.

**CAN**: AB, BC (VCI), MB, NB, NS (CBI), ON, PE, QC, SK **USA**: AL, AR, AZ, CA, CO, CT, DC, DE, FL, GA, IA, ID, IL, IN, KS, KY, LA, MA, MD, ME, MI, MN, MO, MS, MT, NC, ND, NE, NH, NJ, NM, NY, OH, OK, OR, PA, RI, SC, SD, TN, TX, UT, VA, VT, WA, WI, WV, WY – Mexico

### Agonoleptus rotundatus

(LeConte, 1863), new combination

Stenolophus rotundatus LeConte, 1863c: 17. Type locality: «Louisiana» (original citation). One syntype in MCZ [# 5925].

#### Distribution.

This species ranges from east-central Ohio (Usis and MacLean 1998: 67) to southeastern Wyoming (Lavigne 1977: 47) and northeastern Colorado (Lavigne 1978: 102), including southernmost Ontario (Lindroth 1968: 924; Bousquet 1987a: 131), south to southeastern Texas (Casey 1914: 282) and northwestern South Carolina (Kirk 1970: 15). The record from “New York” (Bousquet and Larochelle 1993: 222) needs confirmation.

#### Records.

**CAN**: ON **USA**: AL, AR, CO, IN, KS, KY, LA, MD, MO, MS, OH, OK, PA, NC, SC, TN, TX, VA, WV, WY [NY]

### Agonoleptus rotundicollis

(Haldeman, 1843), new combination

Acupalpus rotundicollis Haldeman, 1843b: 302. Type locality: southeastern Pennsylvania (Haldeman 1843a: 297). One possible syntype, a ♂ labeled “[pink disc] / var. rotundicollis Hald. [handwritten] / conjunctus 3 [handwritten],” in MCZ (collection LeConte).
Mazoreus americanus Motschulsky, 1864: 234. Type locality: «Am[érique] bor[éale]» (original citation). Lectotype, designated by Bousquet and Larochelle (1993: 15), in ZMMU. Synonymy established by Bousquet and Larochelle (1993: 15).
Stenolophus scitulus Casey, 1884c: 78. Type locality: «near Philadelphia [Philadelphia County], Pennsylvania» (original citation). Lectotype (♂), designated by Lindroth (1975: 143), in USNM [# 48053]. Synonymy established by Lindroth (1968: 922).
Stenolophus scitulus incitatus Casey, 1914: 283. Type locality: «probably New Jersey» (original citation). Holotype [by monotypy] (♂) in USNM [# 48054]. Synonymy established by Lindroth (1968: 922).

#### Distribution.

The range of this species extends from southern Quebec (Larochelle 1975: 109) to eastern Minnesota (Gandhi et al. 2005: 931), south to west-central Mississippi (Washington County, CMNH) and northwestern South Carolina (Kirk 1970: 15). The record from Colorado (Elias 1987: 634) is probably in error.

#### Records.

**CAN**: ON, QC **USA**: CT, MA, MD, ME, MI, MN, MS, IA, IL, NC, NJ, NY, OH, PA, RI, SC, TN, VA, VT, WI, WV

### Agonoleptus thoracicus

(Casey, 1914), new combination

Stenolophus thoracicus Casey, 1914: 282. Type locality: «S[ain]t Louis, Missouri» (original citation for the lectotype). Lectotype (♀), designated by Lindroth (1975: 143), in USNM [# 48052].

#### Distribution.

This species ranges from east-central Vermont to southeastern North Dakota, south to northeastern Kansas, Tennessee, and northeastern Virginia [see Bousquet and Messer 2010: Fig. 3].

#### Records.

**USA**: DC, IA, IL, IN, KS, KY, MA, MD, MI, MO, ND, NJ, NY, OH, PA, SD, TN, VA, VT, WI

#### Note.

This taxon has been listed in synonymy with *Agonoleptus conjunctus* (Say) by Lindroth (1968: 921) but considered a valid species by Bousquet and Messer (2010).

### [unicolor group]

### Agonoleptus dolosus

(Casey, 1914), new status, new combination

Stenolophus unicolor dolosus Casey, 1914: 280. Type locality: «Los Angeles Co[unty], California» (original citation). Two syntypes in USNM [# 48049].

#### Distribution.

This species is known only from the type series collected in southwestern California.

#### Records.

**USA**: CA

#### Note.

I have studied both syntypes, including the male genitalia of one of them, and I have little doubt that they belong to a distinct species, though probably closely related to *Agonoleptus unicolor* (Dejean) based on Lindroth’s (1968: 924) description of the holotype of *Agonoleptus unicolor*. The name *Stenolophus dolosus* is listed as a junior synonym of *Stenolophus unicolor* (Dejean, 1829) in Lorenz (2005: 354).

### Agonoleptus parviceps

Casey, 1914

Agonoleptus parviceps Casey, 1914: 285. Type locality: «Colorado Springs [El Paso County], Colorado» (original citation). Lectotype (♀), designated by Bousquet (1990: 203), in USNM [# 48055].

#### Distribution.

This species is known from two localities in eastern Colorado and central New Mexico (Bousquet 1990: 203).

#### Records.

**USA**: CO, NM

### Agonoleptus unicolor

(Dejean, 1829), new combination

Stenolophus unicolor Dejean, 1829: 411. Type locality: «Californie» (original citation). Holotype [by monotypy] (♂) in MHNP (Lindroth 1955b: 31).

#### Distribution.

This species is known for sure only from southern California, as far north as the San Francisco Bay (Casey 1914: 280). The records from Colorado (Wickham 1902: 242; Elias 1987: 634) need confirmation; that from the Similkameen Valley in British Columbia (Smith et al. 2004: 96) was based on misidentified specimens of *Agonoleptus conjuctus* (Say).

#### Records.

**USA**: CA [CO]

### Genus Bradycellus

Erichson, 1837

Bradycellus Erichson, 1837: 64. Type species: *Carabus collaris* Paykull, 1798 (= *Acupalpus caucasicus* Chaudoir, 1846) designated by Andrewes (1935: 20). Etymology. From the Greek *bradys* (slow) and *cello* (to run), probably alluding to the slow pace of the adults in nature [masculine]. Note. As pointed out by Andrewes (1935: 20), the first valid type species designation for *Bradycellus* Erichson is that of *Harpalus placidus* Gyllenhal, 1827 as selected by Westwood (1838: 6). This species is currently included in *Trichocellus* Ganglbauer, 1892. In order to preserve stability, an application should be submitted to the Commission to suppress Westwood’s designation.

#### Diversity.

About 130 species (Lorenz 2005: 356-358) arrayed in ten subgenera: *Atlantocellus* Wrase and Jaeger (five species from the Canary and Madeira Islands), *Bradycelloides* Habu (one east Palaearctic species), *Bradycellus* s.str. (45 species), *Catharellus* (one species), *Desbordesius* Maindron (one east Palaearctic species), *Liocellus* (eight species), *Lipalocellus* (two species), *Stenocellus* (34 species), *Tachycellus* Morawitz (27 Palaearctic species from Asia with one species extending into the Oriental Region), and *Triliarthrus* (six species). The North American fauna is represented by 51 species (roughly 39% of the world fauna).

#### Taxonomic Note.

Noonan (1976: 21) also included *Psychristus* Andrewes (six species) and *Nipponobradycellus* Habu (four species) in this genus. In Lorenz (2005: 358), *Nipponobradycellus* is listed as a subgenus of the genus *Psychristus*.

### Subgenus Liocellus

Motschulsky, 1864

Liocellus Motschulsky, 1864: 207. Type species: *Acupalpus nitidus* Dejean, 1829 designated by Lindroth (1968: 882). Etymology. From the Greek prefix *lio*- (smooth) and *cello* (to run) [masculine].
Glycerius Casey, 1884c: 79. Type species: *Acupalpus nitidus* Dejean, 1829 by monotypy. Etymology. Possibly from the Greek *glyceros* (sweet) [masculine].

#### Diversity.

Eight species in North America (seven species) and Mexico (one species).

#### Identification.

Fall (1905: 175, as *Glycerius*) published a key to the four North American species then known. Casey (1924) subsequently added three new species. The subgenus is in need of a taxonomic revision.

### Bradycellus curticollis

(Casey, 1924)

Glycerius curticollis Casey, 1924: 140. Type locality: «Monterey [Monterey County], California» (original citation). Two syntypes [2 originally cited] in USNM [# 47987].

#### Distribution.

This species is known only from the type locality in west-central California.

#### Records.

**USA**: CA

### Bradycellus intermedius

(Fall, 1905)

Glycerius intermedius Fall, 1905: 176. Type locality: «San Bernardino M[oun]t[ain]s [and] Lake Tahoe, California» (original citation). Syntype(s) [3 ♀ originally cited] in MCZ [# 23877].

#### Distribution.

This species is known from the Sierra Nevada and San Bernardino Mountains (Fall 1905: 176) and from San Diego County (Moore 1937: 14) in California.

#### Records.

**USA**: CA

### Bradycellus laticollis

(Casey, 1924)

Glycerius laticollis Casey, 1924: 141. Type locality: «Reno [Washoe County], Nevada» (original citation). Two syntypes in USNM [# 47989].

#### Distribution.

This species is known only from the type locality in northwestern Nevada.

#### Records.

**USA**: NV

### Bradycellus nitidus

(Dejean, 1829)

Acupalpus nitidus Dejean, 1829: 474. Type locality: «Californie» (original citation). One syntype in MHNP (Lindroth 1955b: 30).
Acupalpus obsoletus Say, 1830c: 22. Type locality: «Mexico» (original citation). Syntype(s) lost. Synonymy established by Horn (1894: 312).

#### Distribution.

The range of this species extends from southwestern Washington (Hatch and Kincaid 1958: 6; Pacific County, MCZ) southwards to the Baja California Peninsula (Horn 1894: 312) and Guatemala (Ball and Shpeley 1992a: 58). The record from “British Columbia” (Horn 1894: 312) is likely in error (Lindroth 1968: 883).

#### Records.

**USA**: AZ, CA (CHI), NM, OR, UT, WA – Guatemala, Mexico

### Bradycellus obtusus

(Fall, 1905)

Glycerius obtusus Fall, 1905: 176. Type locality: «Pasadena, Azusa, Claremont, [all in] southern California» (original citation). Syntype(s) in MCZ [# 23878].

#### Distribution.

This species is known so far only from southern California (Fall 1905: 176).

#### Records.

**USA**: CA

### Bradycellus politus

(Fall, 1905)

Glycerius politus Fall, 1905: 176. Type locality: «The Dalles, Oregon to southern California» (original citation), restricted to «Pomona [Los Angeles County], Calif[ornia]» by Lindroth (1968: 883). Syntype(s) in MCZ [# 23879].

#### Distribution.

This species ranges from the Okanagan Valley in south-central British Columbia (Lindroth 1968: 883) to southern California (Fall 1905: 176; Dajoz 2007: 20).

#### Records.

**CAN**: BC **USA**: CA, OR, WA

### Bradycellus tahoensis

(Casey, 1924)

Glycerius tahoensis Casey, 1924: 140. Type locality: «Lake Tahoe [Placer County], California» (original citation). Two syntypes [2 originally cited] in USNM [# 47988].

#### Distribution.

This species is known only from the type locality in the Sierra Nevada.

#### Records.

**USA**: CA

### Subgenus Bradycellus

Erichson, 1837

Bradycellus Erichson, 1837: 64. Type species: *Carabus collaris* Paykull, 1798 (= *Acupalpus caucasicus* Chaudoir, 1846) designated by Andrewes (1935: 20).
Tetraplatypus Tschitschérine, 1897: 62. Type species: *Acupalpus similis* Dejean, 1829 (= *Trechus ruficollis* Stephens, 1828) by monotypy. Synonymy established by Lindroth (1968: 883), accepted by Jaeger (2008: 1513-1514). Etymology. From the Greek *tetra* (four), *platys* (wide, broad) and *pous* (foot), possibly alluding to the expanded male mesotarsomeres (“*♂ ont les tarses intermédiaires faiblement dilatés*”) [masculine].

#### Diversity.

About 45 species in the Nearctic (two species, one of them adventive), Neotropical (25 species), and Palaearctic (17 species) Regions.

#### Identification.

The two species found in North America have been covered by Lindroth (1968: 883-884, as *harpalinus* group).

### Bradycellus fenderi

Hatch, 1951

Bradycellus fenderi Hatch, 1951: 120. Type locality: «Depoe Bay [Lincoln County], Ore[gon]» (original citation). Holotype location unknown (not in USNM). Etymology. This species was named after Kenneth M. Fender [1910-1987], a rural mail carrier at McMinnville, Oregon, and collector of Pacific Northwest beetles. Along with his wife, Dorothy McKey, Fender specialized in Lycidae and Cantharidae and the two published several taxonomic papers together.

#### Distribution.

This species is found along the Pacific Coast from northwestern Washington (Nelson 1988b: 56) to southwestern Oregon (Coos County, CNC).

#### Records.

**USA**: OR, WA

### Bradycellus harpalinus

(Audinet-Serville, 1821)

Carabus fulvus Marsham, 1802: 456 [primary homonym of *Carabus fulvus* Müller, 1776]. Type locality: Great Britain (inferred from title of the book). Syntype(s) probably in BMNH (collection Stephens).
Trechus harpalinus Audinet-Serville, 1821: 84. Type locality: «environs de Paris [France]» (original citation). Syntype(s) probably lost. Synonymy established by Dejean (1829: 472).

#### Distribution.

This Palaearctic species is adventive in North America where it is known from the Vancouver area in southwestern British Columbia (Lindroth 1968: 883) south to Oregon (Westcott et al. 2006: 7; Lincoln County, Foster F. Purrington pers. comm. 2009). The first inventoried specimen collected on this continent was found in Vancouver in 1951 (Lindroth 1968: 883).

#### Records.

**CAN**: BC **USA**: OR, WA – **Adventive**

### Subgenus Catharellus

Casey, 1914

Catharellus Casey, 1914: 242. Type species: *Geobaenus cordicollis* LeConte, 1847 (= *Bradycellus lecontei* Csiki, 1932) by monotypy. Etymology. Uncertain, possibly from the Greek *catharos* (clean, pure) and the Latin suffix -*ellus* (small, little) [masculine].

#### Diversity.

One North American species.

#### Identification.

The species was covered in Lindroth’s (1968: 885) treatment of the Canadian *Bradycellus*.

### Bradycellus lecontei

Csiki, 1932

Geobaenus cordicollis LeConte, 1847: 406 [secondary homonym of *Bradycellus cordicollis* Wesmael, 1835]. Type locality: «Lacum Superiorem» (original citation), herein restricted to Marquette, Marquette County, Michigan (see Hubbard and Schwarz 1878: 629). One syntype in MCZ [# 5938].
Bradycellus lecontei Csiki, 1932a: 1233. Replacement name for *Bradycellus cordicollis* (LeConte, 1847).

#### Distribution.

This species ranges from Newfoundland (Lindroth 1955a: 147) to eastern Alaska (Lindroth 1968: 885), south to northern Oregon (Hatch 1953: 182, as *Bradycellus cordicollis*), northern Colorado (Larimer County, UASM; Lindroth 1955a: 147), eastern South Dakota (Kirk and Balsbaugh 1975: 32), and northeastern West Virginia (Tucker County, CMNH).

#### Records.

**FRA**: PM **CAN**: AB, BC, LB, MB, NB, NF, NS (CBI), NT, ON, PE, QC, SK, YT **USA**: AK, CO, ID, IL, MA, ME, MI, MN, MT, ND, NH, NY, OR, PA, SD, VT, WA, WI, WV, WY

### Subgenus Stenocellus

Casey, 1914

Stenocellus Casey, 1914: 243. Type species: *Trechus rupestris* Say, 1823 designated by Lindroth (1968: 885). Etymology. From the Greek *stenos* (narrow) and the last two syllables of the generic name *Bradycellus*, alluding to the narrower body of these *Bradycellus*-related species (“*distinguished at once from Bradycellus by their more slender elongate parallel *... *form*”) [masculine].

#### Diversity.

Thirty-four species in North America (33 species), Mexico (three species, one endemic to Guadalupe Island), and the Bahamas (one species).

#### Identification.

Casey (1914: 243-257) published a key to the species then known except *Stenocellus nebulosus*, *Stenocellus nigriceps*, and *Stenocellus subcordatus*. He subsequently described three new species (Casey 1924). Lindroth (1968, as *rupestris*, *nigriceps*, and *tantillus* groups) covered nine species. The subgenus is in need of a revision.

#### Faunistic Note.

According to Kataev and Matalin (in Kryzhanovskij et al. 1995: 135), the sole syntype (a badly damage ♂) of *Stenolophus elongatus* Motschulsky, 1860 in ZMMU, reported from the Kuril Islands, is a specimen of this subgenus. Since no species of *Stenocellus* are known from the Far East, they concluded that the specimen is mislabeled and probably originated from northwestern North America.

### [nigriceps group]

### Bradycellus insulsus

(Casey, 1914)

Stenocellus insulsus Casey, 1914: 246. Type locality: «near the city [of New York], New York» (original citation). Lectotype (♂), designated by Lindroth (1975: 143), in USNM [# 47999].

#### Distribution.

This species, as far as known, ranges from southeastern New Hampshire (Rockingham County, Ross T. Bell pers. comm. 2008) to northwestern Minnesota (Gandhi et al. 2005: 930), including southernmost Ontario (Lindroth 1968: 892), south to South Carolina (Charleston County, CNC).

#### Records.

**CAN**: ON **USA**: CT, MD, MI, MN, NH, NJ, NY, OH, PA, SC, VA, VT, WV

### Bradycellus neglectus

(LeConte, 1847)

Geobaenus neglectus LeConte, 1847: 407. Type locality: «insulam Mackinaw [Mackinac County, Michigan]» (original citation). Holotype [by monotypy] in MCZ [# 5937].

#### Distribution.

This species ranges from Newfoundland (Lindroth 1955a: 147-148) to northeastern British Columbia (Lindroth 1968: 892), south to southwestern Oklahoma (Kondratieff et al. 2005: 171), southern Arkansas (Kraim 1983: 277), northeastern Georgia (Fattig 1949: 53), and southeastern South Carolina (Ciegler 2000: 96). The record from southwestern Alabama (Löding 1945: 26) needs confirmation; that from “Utah” (Bousquet and Larochelle 1993: 225) is probably in error.

#### Records.

**CAN**: AB, BC, MB, NB, NF, NS (CBI), ON, PE, QC, SK **USA**: AR, CT, GA, IL, MA, ME, MI, MN, NC, ND, NH, NY, OH, OK, PA, SC, SD, TN, VT, WI [AL]

### Bradycellus nigriceps

LeConte, 1869

Bradycellus nigriceps LeConte, 1869a: 381. Type locality: «New Jersey and Virginia» (original citation), restricted to «New Jersey» by Lindroth (1968: 889). One syntype [2 originally cited] in MCZ [# 5945].
Stenocellus occultus Casey, 1914: 246. Type locality: Bluff Point, Lake Champlain, New York (lectotype label according to Lindroth 1975: 143). Lectotype, designated by Lindroth (1975: 143), in USNM [# 47997]. Synonymy established by Lindroth (1968: 889).

#### Distribution.

This species ranges from New Brunswick (Lindroth 1968: 891) to North Dakota (Tinerella 2003: 636), north to southeastern Manitoba (Lindroth 1968: 891), south to east-central Arkansas (Kraim 1983: 280), northeastern Mississippi (Snodgrass and Cross 1983: 16), and southeastern South Carolina (Ciegler 2000: 96). The record from “New Mexico” (Fall and Cockerell 1907: 162) is probably in error.

#### Records.

**CAN**: MB, NB, ON, QC **USA**: AR, CT, DC, IA, IL, IN, MA, MD, ME, MI, MN, MS, ND, NH, NJ, NY, OH, PA, SC, VA, VT, WI, WV

### Bradycellus supplex

(Casey, 1914)

Stenocellus supplex Casey, 1914: 245. Type locality: «Atlantic City [Atlantic County], New Jersey» (original citation). Two syntypes in USNM [# 48000].

#### Distribution.

This species is known only from the type locality.

#### Records.

**USA**: NJ

#### Note.

The two syntypes are morphologically similar to adults of *Bradycellus insulsus* Casey and may be conspecific with them.

### [rupestris group]

### Bradycellus congener

(LeConte, 1847)

Geobaenus congener LeConte, 1847: 407. Type locality: «Rocky Mountains» (original citation). Syntype(s) in MCZ [# 5939].
Stenocellus alutaceus Casey, 1914: 250. Type locality: «Siskiyou Co[unty], California» (original citation). Lectotype (♀), designated by Lindroth (1975: 143), in USNM [# 48005]. Synonymy established by Lindroth (1968: 888).

#### Distribution.

This species ranges from Nova Scotia (Majka et al. 2007: 10) to Vancouver Island (Lindroth 1968: 888), south at least to central California (Clark 1999: 202; Dajoz 2007: 17), northern Sonora in Mexico (Bates 1884: 277), southern Texas (Johnson 1978: 67), southeastern Louisiana (East Baton Rouge Parish, Igor M. Sokolov pers. comm. 2009), Tennessee (Cumberland County, CNC), and southern West Virginia (McDowell County, Ken Karns pers. comm. 2009).

#### Records.

**CAN**: AB, BC (VCI), MB, NS, ON, QC, SK **USA**: AZ, CA, CO, IA, ID, IL, KS, LA, MA, ME, MI, MN, MT, ND, NH, NM, NV, OH, OR, SD, TN, TX, UT, VT, WA, WI, WV, WY – Mexico

### Bradycellus lustrellus

(Casey, 1914)

Stenocellus lustrellus Casey, 1914: 251. Type locality: «Redwood Creek, Humboldt Co[unty], California» (original citation). Lectotype (♀), designated by Lindroth (1975: 142), in USNM [# 48006].

#### Distribution.

This species is known only from the type locality along the Pacific Coast of California.

#### Records.

**USA**: CA

### Bradycellus nebulosus

LeConte, 1853

Acupalpus suturalis LeConte, 1847: 411 [primary homonym of *Acupalpus suturalis* Dejean, 1829]. Type locality: «Georgia» (original citation). One syntype in MCZ [# 5946].
Bradycellus nebulosus LeConte, 1853c: 385. Replacement name for *Bradycellus suturalis* (LeConte, 1847).

#### Distribution.

This species is known from northwestern Georgia (Fattig 1949: 53) and “Texas” (Horn 1883a: 52). The record from Baja California (Horn 1894: 312) needs confirmation.

#### Records.

**USA**: GA, TX

#### Note.

Fall (1905: 171) stated that *Bradycellus nebulosus* is “closely resembling and probably not distinct from *rupestris*.”

### Bradycellus nigerrimus

Lindroth, 1968

Bradycellus nigerrimus Lindroth, 1968: 889. Type locality: «Maple Creek, Sask[atchewan]» (original citation). Holotype (♂) in CNC [# 10578].

#### Distribution.

This species is known from a small area including southern Saskatchewan, southern Alberta (Lindroth 1968: 889), and southwestern North Dakota (Tinerella 2003: 636). The records from “Montana” and “Wyoming” (Bousquet and Larochelle 1993: 225) need confirmation.

#### Records.

**CAN**: AB, SK **USA**: ND [MT, WY]

### Bradycellus nubifer

LeConte, 1858

Bradycellus nubifer LeConte, 1858b: 60. Type locality: «San Diego, California and at Tuvac in northern Sonora» (original citation), restricted to «San Diego [San Diego County]» by Lindroth (1968: 887). Syntype(s) in MCZ [# 5940].
Bradycellus ventralis LeConte, 1858b: 61. Type locality: «Gila River» (original citation). Holotype [by monotypy] in MCZ [# 5941]. Synonymy established by LeConte (1869a: 382), confirmed by Lindroth (1968: 887).
Stenocellus nubicollis Casey, 1914: 253. Type locality: «Cal [with a dot within the “C”] [= San Francisco, San Francisco County, California]» (lectotype label). Lectotype (♂), designated by Lindroth (1975: 142), in USNM [# 48011]. Synonymy established by Lindroth (1968: 887).

#### Distribution.

The range of this western species extends from central Alberta to Vancouver Island (Lindroth 1968: 887), south to southern California and northern Sonora (LeConte 1858b: 60). The record from southern Texas (Johnson 1978: 67) needs confirmation.

#### Records.

**CAN**: AB, BC (VCI) **USA**: AZ, CA, ID, NV, OR, WA [TX] – Mexico

### Bradycellus rupestris

(Say, 1823)

Trechus rupestris Say, 1823a: 91. Type locality: «Arlington [Middlesex County], Mass[achusetts]» (neotype label). Neotype (♂), designated by Lindroth and Freitag (1969: 355), in MCZ [# 32967].
Acupalpus elongatulus Dejean, 1829: 457. Type locality: «Amérique septentrionale» (original citation). One syntype in MHNP (Lindroth 1955b: 30). Synonymy established by Harris (1833: 568), confirmed by Lindroth (1955b: 30).
Stenolophus cinctus Say, 1830c: 20 [*nomen dubium*]. Type locality: «Massachusetts» (original citation). Syntype(s) lost. Synonymy established by Melsheimer (1853: 25).
Acupalpus debilipes Say, 1830c: 21 [*nomen dubium*]. Type locality: «Indiana» (original citation). Syntype(s) lost. Synonymy established doubtfully with *Bradycellus parallelus* Chaudoir by LeConte (1869a: 382).
Trechus flavipes Kirby, 1837: 47. Type locality: «Lat. 54° [= along North Saskatchewan River]» (original citation). Two syntypes in BMNH (Lindroth 1953b: 175). Synonymy established by LeConte (1847: 407), confirmed by Lindroth (1953b: 175).
Bradycellus parallelus Chaudoir, 1868b: 166. Type locality: «Louisiane» (original citation). One syntype in MHNP (Lindroth 1968: 886). Synonymy established by Schaupp (1883c: 50), confirmed by Lindroth (1968: 886).
Stenocellus antennalis Casey, 1914: 245. Type locality: «Catskill M[oun]t[ain]s, New York» (original citation). Lectotype (♂), designated by Lindroth (1975: 142), in USNM [# 47998]. Synonymy established by Lindroth (1968: 886).

#### Distribution.

This species ranges from Cape Breton Island (Lindroth 1954c: 308) to southeastern Wyoming (Lavigne 1977: 44), south to southern New Mexico (Fall and Cockerell 1907: 162), southern Texas (Wickham 1897: 113), southern Louisiana (Summers 1874a: 81; Allen 1965: 72, as *Bradycellus debilipes*), northern Georgia (Fattig 1949: 53), and eastern South Carolina (Ciegler 2000: 96). One specimen simply labeled from Florida is known (Leng 1915: 599).

#### Records.

**CAN**: NB, NS (CBI), ON, QC **USA**: AL, AR, CO, CT, DC, DE, GA, IA, IL, IN, KS, KY, LA, MA, MD, ME, MI, MN, MO, MS, NC, NE, NH, NJ, NM, NY, OH, OK, PA, RI, SC, SD, TN, TX, VA, VT, WI, WV, WY [FL]

### [subcordatus group]

### Bradycellus subcordatus

Chaudoir, 1868

Bradycellus subcordatus Chaudoir, 1868b: 166. Type locality: Amérique septentrionale (inferred from title of the paper). Holotype [by monotypy] (♂) in MHNP (Lindroth 1968: 881).

#### Distribution.

This species is known only from the holotype. Casey (1914: 258) assumed that the species was “possibly a native of California” because the holotype was sent to Chaudoir by Motschulsky. Although Motschulsky described several species from the west coast, he never set foot in western United States.

#### Records.

**USA**: ?CA

### [tantillus group]

### Bradycellus californicus

(LeConte, 1857)

Stenolophus californicus LeConte, 1857c: 29. Type locality: «California» (original citation). Four syntypes in MCZ [# 5942].

#### Distribution.

This species is found from Vancouver Island (Lindroth 1968: 894) to western Montana (Russell 1968: 68), south to west-central Nevada (Lyon County, CNC) and southern California (Fall 1901a: 50).

#### Records.

**CAN**: BC (VCI) **USA**: CA (CHI), ID, MT, NV, OR, WA

### Bradycellus rivalis

LeConte, 1858

Bradycellus rivalis LeConte, 1858b: 61. Type locality: «Colorado Desert, at New River and at the Colorado» (original citation). Four syntypes in MCZ [# 5943].

#### Distribution.

This species is known from “Texas” (Horn 1894: 312), Arizona (Griffith 1900: 566; Casey 1914: 249), southeastern California (Fall 1901a: 50), and the Baja California Peninsula (Horn 1894: 312).

#### Records.

**USA**: AZ, CA, TX – Mexico

### Bradycellus tantillus

(Dejean, 1829)

Acupalpus tantillus Dejean, 1829: 465. Type locality: «Amérique septentrionale» (original citation), restricted to «Mobile [Mobile County], Alab[ama]» by Lindroth (1968: 892). One syntype in MHNP (Lindroth 1955b: 31).
Acupalpus difficilis Dejean, 1829: 467. Type locality: «Amérique septentrionale» (original citation). One syntype in MHNP (Lindroth 1955b: 31). Synonymy established by Chaudoir (1868b: 167), confirmed by Lindroth (1955b: 31).

#### Distribution.

This species ranges from southwestern Maine (Majka et al. 2011: 46) to eastern South Dakota (Kirk and Balsbaugh 1975: 33), including southern Ontario (Lindroth 1968: 893; Bousquet 1987a: 131), south to southeastern Texas (Fort Bend, Brian Raber pers. comm. 2010), northern Louisiana (Allen 1965: 72), southern Florida (Peck and Thomas 1998: 21), and the Bahamas (Darlington 1953: 10). The record from “Quebec” (Bousquet and Larochelle 1993: 226) was based on a misidentified specimen of *Bradycellus nigriceps* (CNC); those from northern Colorado (Haubold 1951: 706; Armin 1963: 118) need confirmation.

#### Records.

**CAN**: ON **USA**: AL, AR, CT, DC, FL, GA, IA, IL, IN, LA, MA, MD, MO, MS, NC, NJ, NY, OH, OK, PA, RI, SC, SD, TN, TX, VA, VT, WV [CO] – Bahamas

### [incertae sedis]

### Bradycellus ardelio

(Casey, 1914)

Stenocellus ardelio Casey, 1914: 254. Type locality: «Siskiyou Co[unty], California» (original citation). One syntype in USNM [# 48016].

#### Distribution.

This species is known only from the type locality in northern California.

#### Records.

**USA**: CA

### Bradycellus aridus

(Casey, 1914)

Stenocellus aridus Casey, 1914: 248. Type locality: «San Diego [San Diego County], California» (original citation). Holotype [by monotypy] (♂) in USNM [# 48001].

#### Distribution.

This species is known only from the type locality in southwestern California.

#### Records.

**USA**: CA

### Bradycellus carolinensis

(Casey, 1924)

Stenocellus carolinensis Casey, 1924: 142. Type locality: «Southern Pines [Moore County], North Carolina» (original citation). One syntype in USNM [# 48020].

#### Distribution.

This species is known only from the type locality.

#### Records.

**USA**: NC

### Bradycellus decorus

(Casey, 1914)

Stenocellus decorus Casey, 1914: 248. Type locality: «Tuçson [Pima County], Arizona» (original citation). Holotype [by monotypy] (♀) in USNM [# 48004].

#### Distribution.

This species is known only from the type locality in southern Arizona.

#### Records.

**USA**: AZ

### Bradycellus discipulus

(Casey, 1914)

Stenocellus discipulus Casey, 1914: 252. Type locality: «Hoopa Valley, Humboldt Co[unty], California» (original citation). One syntype in USNM [# 48008].

#### Distribution.

This species is known only from the type locality in northwestern California.

#### Records.

**USA**: CA

### Bradycellus exstans

(Casey, 1914)

Stenocellus exstans Casey, 1914: 253. Type locality: «Sacramento Co[unty], California» (original citation). Holotype [by monotypy] (♀) in USNM [# 48014].

#### Distribution.

This species is known only from the type locality in north-central California.

#### Records.

**USA**: CA

### Bradycellus festinans

(Casey, 1914)

Stenocellus festinans Casey, 1914: 257. Type locality: «Sedgwick Co[unty], Kansas and Texas» (original citation). Three syntypes [3 originally cited] in USNM [# 48022].

#### Distribution.

This species ranges from southern Montana (Hatch 1933a: 10) and South Dakota (Kirk and Balsbaugh 1975: 33) south to “Texas” (Casey 1914: 257) and southern Arkansas (Kraim 1983: 283). The record from Cuba (Darlington 1934: 110) is doubtful.

#### Records.

**USA**: AR, KS, MT, SD, TX, WY

### Bradycellus humboldtianus

(Casey, 1924)

Stenocellus humboldtianus Casey, 1924: 142. Type locality: «Hoopa Valley, Humboldt Co[unty], California» (original citation). One syntype in USNM [# 48012].

#### Distribution.

This species is known only from the type locality in northwestern California.

#### Records.

**USA**: CA

### Bradycellus larvatus

(Casey, 1914)

Stenocellus larvatus Casey, 1914: 256. Type locality: «El Paso [El Paso County], Texas» (original citation). Holotype [by monotypy] (♀) in USNM [# 48018].

#### Distribution.

This species is known only from the type locality in westernmost Texas.

#### Records.

**USA**: TX

### Bradycellus lineatus

(Casey, 1914)

Stenocellus lineatus Casey, 1914: 253. Type locality: «S[an]ta Cruz M[oun]t[ain]s, California» (original citation). One syntype in USNM [# 48013].

#### Distribution.

This species is known only from the type locality in western California.

#### Records.

**USA**: CA

### Bradycellus montanus

(Casey, 1914)

Stenocellus montanus Casey, 1914: 251. Type locality: «Truckee [Nevada County], California» (original citation). Two syntypes [2 originally cited] in USNM [# 48010].

#### Distribution.

This species is known only from the type locality in the Sierra Nevada.

#### Records.

**USA**: CA

### Bradycellus picipes

(Casey, 1914)

Stenocellus picipes Casey, 1914: 255. Type locality: «Lake Tahoe [Placer County], California» (original citation). Six syntypes [7 originally cited] in USNM [# 48015].

#### Distribution.

This species is known only from the type locality in the Sierra Nevada.

#### Records.

**USA**: CA

### Bradycellus provoensis

(Casey, 1914)

Stenocellus provoensis Casey, 1914: 256. Type locality: «Provo [Utah County], Utah» (original citation). One syntype in USNM [# 48017].

#### Distribution.

This species is known only from the type locality in north-central Utah.

#### Records.

**USA**: UT

### Bradycellus puncticollis

(Casey, 1914)

Stenocellus puncticollis Casey, 1914: 251. Type locality: «San Francisco Bay and northward in the coast regions, California» (original citation). Nine syntypes [10 originally cited] in USNM [# 48009].

#### Distribution.

This species is known from the type series and San Diego County (Moore 1937: 14) in California.

#### Records.

**USA**: CA

### Bradycellus purgatus

(Casey, 1914)

Stenocellus purgatus Casey, 1914: 249. Type locality: «near San Diego [San Diego County], California» (original citation). Two syntypes in USNM [# 48003].

#### Distribution.

This species is known only from the type locality in southwestern California.

#### Records.

**USA**: CA

### Bradycellus sejunctus

(Casey, 1914)

Stenocellus sejunctus Casey, 1914: 252. Type locality: «Alameda Co[unty] and Santa Rosa, California» (original citation). Two syntypes in USNM [# 48007].

#### Distribution.

This species is known for sure only from the type series collected in western California; it was also recorded from Humboldt County (Notman 1929b: 222).

#### Records.

**USA**: CA

### Bradycellus suavis

(Casey, 1914)

Stenocellus suavis Casey, 1914: 257. Type locality: «Austin [Travis County], Texas» (original citation). One syntype in USNM [# 48021].

#### Distribution.

This species is known for sure only from the type locality in east-central Texas. The record from South Dakota (Kirk and Balsbaugh 1975: 33) needs confirmation.

#### Records.

**USA**: TX [SD]

### Bradycellus symetricus

(Motschulsky, 1850)

Stenolophus symetricus Motschulsky, 1850a: 23. Type locality: «California» (original citation), cited from «Colonie Ross [farming community about 75 miles north of San Francisco along the coast, California]» by Motschulsky (1859a: 134). Syntype(s) location unknown (possibly in ZMMU though not listed in Keleinikova 1976).
Stenocellus symmetricus Casey, 1914: 254. Unjustified emendation of *Stenocellus symetricus* (Motschulsky, 1850).

#### Distribution.

This species is known for sure only from the type series collected on the west coast in central California. Casey (1914: 254) reported it from Saint Helena, Sonoma County, California.

#### Records.

**USA**: CA

### Bradycellus veronianus

(Casey, 1924)

Stenocellus veronianus Casey, 1924: 142. Type locality: «Vero [Beach] [Indian River County], Florida» (original citation). One syntype in USNM [# 48019].

#### Distribution.

This species is known yet only from the type locality.

#### Records.

**USA**: FL

### Subgenus Lipalocellus

Ball and Bousquet, 2000

Liocellus Tschitschérine, 1901: 247 [junior homonym of *Liocellus* Motschulsky, 1864]. Type species: *Harpalus nigrinus* Dejean, 1829 by original designation. Etymology. From the Greek prefix *lio*- (smooth) and *cello* (to run) [masculine].
Lipalocellus Ball and Bousquet, 2000: 94. Replacement name for *Liocellus* Tschitschérine, 1901. Etymology. The name was composed from an arbitrary combination of syllables [masculine].

#### Diversity.

Two North American species.

#### Identification.

Lindroth (1968: 894-898, as *nigrinus* group) reviewed the species.

### Bradycellus nigrinus

(Dejean, 1829)

Harpalus nigrinus Dejean, 1829: 399. Type locality: «détroit de Norfolk [= Norfolk Sound, Baranof Island, Alaska], sur la côte nord-ouest de l’Amérique septentrionale» (original citation). One syntype [2 ♂ originally cited] in MHNP (Lindroth 1955b: 30).
Trechus tibialis Kirby, 1837: 46. Type locality: northern parts of British America (inferred from title of the book). Holotype [by monotypy] in BMNH (Lindroth 1953b: 175). Synonymy established with doubt by Chaudoir (1868b: 167), confirmed by Lindroth (1953b: 175).
Geobaenus quadricollis LeConte, 1847: 405. Type locality: «Lacum Superiorem» (original citation). Holotype [by monotypy] (♀) in MCZ [# 5944]. Synonymy established, under the name *Bradycellus tibialis* (Kirby), by Chaudoir (1868b: 167), confirmed by LeConte (1869a: 382) and Lindroth (1968: 896).

#### Distribution.

This species extends from Newfoundland (Lindroth 1955a: 146) to southeastern Alaska, including Kodiak Island (Lindroth 1968: 896), south to mountains in southeastern California (Lindroth 1968: 896; San Bernardino County, CNC) and New Mexico (Fall and Cockerell 1907: 162), northwestern Indiana (Blatchley 1910: 193), and southeastern West Virginia (Greenbrier County, CNC). The record from “North Carolina” (Bousquet and Larochelle 1993: 226) needs confirmation.

#### Records.

**FRA**: PM **CAN**: AB, BC (QCI, VCI), MB, NB, NF, NS (CBI), ON, PE, QC, SK, YT **USA**: AK, CA, CO, CT, ID, IL, IN, MA, ME, MI, MN, MT, NH, NM, NY, OH, OR, PA, UT, VA, VT, WA, WI, WV [NC]

### Bradycellus semipubescens

Lindroth, 1968

Bradycellus semipubescens Lindroth, 1968: 896. Type locality: «Fairy Lake, Queb[ec]» (original citation). Holotype (♂) in CNC [# 10577].

#### Distribution.

This species ranges from Nova Scotia (Majka et al. 2007: 10) to central Minnesota (Gandhi et al. 2005: 930), north to southeastern Manitoba (Roughley et al. 2010: 230), south at least to east-central Missouri (Saint Louis, MCZ), southwestern Illinois (Saint Clair County, MCZ), and southeastern Pennsylvania (Dauphin County, MCZ). The single specimen from McMurray, Alberta (Lindroth 1968: 898) is possibly mislabeled or represents a stray.

#### Records.

**CAN**: MB, NB, NS, ON, QC **USA**: IA, IL, MA, ME, MI, MN, MO, NH, NY, PA, VT, WI [AB]

### Subgenus Triliarthrus

Casey, 1914

Triliarthrus Casey, 1914: 238. Type species: *Stenolophus badipennis* Haldeman, 1843 designated by Lindroth (1968: 898). Etymology. Probably from the Greek prefix *tri*- (three), *lis* (smooth), and *arthron* (joint), alluding to the glabrous antennomeres 1-3 of the adults (“the first three joints of the antennae are glabrous”) [masculine].

#### Diversity.

Six North American species in the boreal and temperate regions.

#### Identification.

Lindroth (1968: 898-903, as *badipennis* group) reviewed all species.

### Bradycellus atrimedeus

(Say, 1823)

Feronia atrimedea Say, 1823a: 39. Type locality: «Iowa City [Johnson County, Iowa]» (neotype label). Neotype (♀), designated by Lindroth and Freitag (1969: 355), in MCZ [# 32968]. Note. «the Missouri» was the area originally cited by Say (1823a: 40). Say (1823a: 39) obviously made an error in the epithet *atrimedea* which comes from the Latin adjectives *atrox*, -*ocis* (black) and *medius*, -*a*, -*um* (middle). Most authors, starting with LeConte (1847: 404), have used the spelling *atrimedius*, but this spelling is not in prevailing usage since Lindroth (1968: 899).

#### Distribution.

The range of this species extends from New Brunswick (Lindroth 1968: 899) to eastern South Dakota (Kirk and Balsbaugh 1975: 32), north to southeastern Manitoba (Lindroth 1968: 899), south to “Texas” (Fall 1905: 172), Tennessee (Sevier County, CNC), and northeastern Georgia (Fattig 1949: 53).

#### Records.

**CAN**: MB, NB, ON, QC **USA**: DC, GA, IA, IL, IN, KS, KY, MA, MD, ME, MI, MN, MO, NC, NE, NH, NJ, NY, OH, PA, RI, SD, TN, TX, VA, VT, WI, WV

### Bradycellus badipennis

(Haldeman, 1843)

Stenolophus badipennis Haldeman, 1843b: 302. Type locality: southeastern Pennsylvania (Haldeman 1843a: 297). One possible syntype, labeled “[pink disc] /510. [handwritten] / B. badiipennis (Hald.) Lec. ruficrus ‡ Lec. [handwritten],” in MCZ (Lindroth 1968: 899).
Triliarthrus badiipennis Casey, 1914: 239. Unjustified emendation of *Triliarthrus badipennis* (Haldeman, 1843).
Triliarthrus properus Casey, 1914: 240. Type locality: «West Point [Orange County], New York» (original citation). Lectotype (♂), designated by Lindroth (1975: 143), in USNM [# 47995]. Synonymy established by Fall (1930: 251), confirmed by Lindroth (1968: 898).
Stenolophus curticollis Casey, 1924: 146. Type locality: «Somerset [Montgomery County], Maryland» (original citation). Lectotype (♀), designated by Lindroth (1975: 143), in USNM [# 48047]. Synonymy established by Lindroth (1968: 898).

#### Distribution.

This species is known from southwestern New Brunswick (Webster and Bousquet 2008: 19) to northern Minnesota (Gandhi et al. 2005: 930), including southern Quebec and the Ontario Peninsula (Lindroth 1968: 899), south to northeastern Kansas (Popenoe 1877: 24), Tennessee (Knox County, CNC), northern Georgia (Fattig 1949: 53), and eastern South Carolina (Ciegler 2000: 96). The records from Colorado (Wickham 1902: 242; Armin 1963: 119) are probably in error.

#### Records.

**CAN**: NB, ON, QC **USA**: CT, DC, GA, IA, IL, IN, KS, KY, MA, MD, ME, MI, MN, MO, NC, NH, NJ, NY, OH, PA, RI, SC, TN, VA, VT, WI, WV

### Bradycellus conformis

(Fall, 1905)

Tachycellus conformis Fall, 1905: 173. Type locality: «Washington State and P[oin]t Reyes, California» (original citation), restricted to «Washington State» by Lindroth (1968: 903). Syntype(s) in MCZ [# 23881].
Triliarthrus tetricus Casey, 1914: 241. Type locality: «Hoopa Valley, Humboldt Co[unty], California» (original citation). Lectotype (♀), designated by Lindroth (1975: 143), in USNM [# 47996]. Synonymy established by Fall (1930: 251), confirmed by Lindroth (1968: 903).

#### Distribution.

This species is restricted to the Pacific Coast from southern British Columbia, including Vancouver Island (Lindroth 1968: 903), south at least to the San Francisco Bay in California (Fall 1905: 173). The record from northern Colorado (Armin 1963: 119) is likely in error.

#### Records.

**CAN**: BC (VCI) **USA**: CA, OR, WA

### Bradycellus georgei

Lindroth, 1968

Bradycellus georgei Lindroth, 1968: 899. Type locality: «McMurray, Al[ber]ta» (original citation). Holotype (♀) in CNC [# 10576].

#### Distribution.

This species is yet known only from a few localities in Alberta.

#### Records.

**CAN**: AB

### Bradycellus kirbyi

(Horn, 1883)

Tachycellus kirbyi G.H. Horn, 1883a: 51. Type locality: «near Grimsby, Ontario» (original citation). Eight syntypes in MCZ [# 34551].

#### Distribution.

This species ranges from southwestern New Brunswick (Webster and Bousquet 2008: 19) to central Minnesota (Sherburne County, CNC), including southern Quebec and the Ontario Peninsula (Lindroth 1968: 901), south to northeastern West Virginia (Randolph County, CMNH).

#### Records.

**CAN**: NB, ON, QC **USA**: CT, MA, ME, MI, MN, NH, NJ, NY, OH, PA, RI, VA, VT, WI, WV

### Bradycellus lugubris

(LeConte, 1847)

Geobaenus lugubris LeConte, 1847: 405. Type locality: «Lacum Superiorem» (original citation). Two syntypes in MCZ [# 5947].
Triliarthrus protractus Casey, 1914: 239. Type locality: «Massachusetts» (original citation). Lectotype (♂), designated by Lindroth (1975: 143), in USNM [# 47994]. Synonymy established by Lindroth (1968: 901).
Tachycellus frosti Fall, 1930: 251. Type locality: «Natick [Middlesex County], Massachusetts» (original citation). Holotype (♂) in MCZ [# 23882]. Synonymy established, under the name *Bradycellus protractus* (Casey), by Lindroth (1954b: 143).

#### Distribution.

The range of this species extends from northeastern Newfoundland (Lindroth 1955a: 146, as *Triliarthrus protractus*) to the Rocky Mountains in Alberta (Lindroth 1968: 902), south to Minnesota (Lindroth 1955a: 146; Epstein and Kulman 1990: 214), Tennessee (Lindroth 1968: 901), and southwestern North Carolina (Jackson County, CNC).

#### Records.

**CAN**: AB, MB, NB, NF, NS (CBI), ON, PE, QC, SK **USA**: CT, DC, IL, MA, ME, MI, MN, NC, NH, NJ, NY, OH, PA, TN, VA, VT, WI, WV

### Genus Amerinus

Casey, 1884

Amerinus Casey, 1884b: 7. Type species: *Bradycellus linearis* LeConte, 1863 by monotypy. Etymology. Uncertain, possibly a simple modification of the generic name *Amerizus* [*q.v*.] to which the adults of the sole species vaguely resemble or from the English adjective American and the Latin suffix -*inus* (pertaining to), alluding to the region where the species live [masculine].

#### Diversity.

One North American species in the temperate regions.

#### Identification.

The species was treated by Lindroth (1968: 877-878).

### Amerinus linearis

(LeConte, 1863)

Bradycellus linearis LeConte, 1863c: 16. Type locality: «Pennsylvania; Wisconsin» (original citation). One syntype in MCZ [# 5935].
Amerinus longipennis Casey, 1914: 259. Type locality: «Vicksburg [Warren County], Mississippi» (original citation). Holotype [by monotypy] (♀) in USNM [# 48026]. Synonymy established by Bousquet and Larochelle (1993: 12).
Amerinus fuscicornis Casey, 1914: 260. Type locality: «Fort Monroe, Virginia» (original citation). Holotype [by monotypy] (♂) in USNM [# 48025]. Synonymy established by Bousquet and Larochelle (1993: 12).

#### Distribution.

This species ranges from Massachusetts (Lindroth 1968: 878) to southeastern Wisconsin (Rauterberg 1885: 20), including southernmost Ontario (Bousquet 1987a: 131), south to southeastern Texas (Harris County, Brian Raber pers. comm. 2010), northern Louisiana (Allen 1965: 71, as *Amerinus longipennis*; Claiborne Parish, CNC), west-central Mississippi (Casey 1914, as *Amerinus longipennis*), and west-central South Carolina (Ulyshen et al. 2005: 252).

#### Records.

**CAN**: ON **USA**: AR, DC, IA, IL, IN, LA, MA, MD, MO, MS, NC, NJ, NY, OH, PA, SC, TX, VA, WI

### Genus Dicheirotrichus

Jacquelin du Val, 1855

Dicheirotrichus Jacquelin du Val, 1855: 35, 65. Type species: *Harpalus obsoletus* Dejean, 1829 by original designation. Etymology (original). From the Greek *dis* (twice), *cheiros* (hand), and *trichos* (hair) [masculine].
Dichirotrichus Gemminger and Harold, 1868a: 262. Unjustified emendation of *Dicheirotrichus* Jacquelin du Val, 1855.

#### Diversity.

Forty-six species (Lorenz 2005: 358) in the Nearctic (two Holarctic species) and Palaearctic (46 species) Regions arrayed in five subgenera: *Cardiostenus* Tschitschérine (four Palaearctic species), *Dicheirotrichus* s.str. (nine Palaearctic species), *Oreoxenus* (one Holarctic species), *Pelagophilus* Tschitschérine (one west Palaearctic species), and *Trichocellus* (31 species).

#### Identification.

Lindroth (1968: 874-877) covered both species found in North America under the generic name *Trichocellus*.

### Subgenus Oreoxenus

Tschitschérine, 1899

Oreoxenus Tschitschérine, 1899b: 445. Type species: *Bradycellus mannerheimii* Sahlberg, 1844 designated by Noonan (1976: 22). Etymology. From the Greek *oreos* (mountain) and *xenos* (stranger, foreigner, guest), probably alluding to the mountainous habits of the species (“*les espèces *... *paraissent alpines*”) in the hands of Tschitschérine [masculine].

#### Diversity.

One Holarctic species.

### Dicheirotrichus mannerheimii mannerheimii

(Sahlberg, 1844)

Bradycellus mannerheimii R.F. Sahlberg, 1844: 51. Type locality: «montis Morikan [Okhotsk, Khabarovsk Kray, Siberia, Russia]» (original citation). One syntype in ZMH (Silfverberg 1987: 20).
Trichocellus porsildi Brown, 1932a: 3. Type locality: «Baker L[ake], N[orth]W[est] T[erritories]» (original citation). Holotype (♂) in CNC [# 3249]. Synonymy established by Lindroth (1968: 877). Etymology. The specific name was proposed in honor of the botanist Alf Erling Porsild [1901-1977] who collected some of the specimens of the type series. Born in Copenhagen, Porsild grew up on the University of Copenhagen Arctic Station in Qeqertarsuaq, Greenland. He settled in Ottawa where he held the position of curator at the National Museum of Canada in Ottawa between 1936 and 1945 and eventually head of the department of botany from 1945 to 1967. Mount Porsild in the Yukon Territory is named after him.

#### Distribution.

This Holarctic subspecies ranges from eastern Siberia (Jaeger and Kataev 2003: 402) to Labrador (Lindroth 1954d: 370); isolated at high altitude in Colorado (Lindroth 1968: 877). Fossil remnants from the Early Wisconsinan have been unearthed in southern Ontario (Morgan and Morgan 1981: 1108).

#### Records.

**CAN**: AB, BC, LB, MB, NT, QC, SK, YT **USA**: AK, CO – **Holarctic**

#### Note.

Two other subspecies, *Dicheirotrichus mannerheimii oreophilus* K. Daniel and J. Daniel and *Dicheirotrichus mannerheimii ponojensis* Sahlberg (synonym: *Dicheirotrichus setiporus* Reitter) are found in the Palaearctic Region.

### Subgenus Trichocellus

Ganglbauer, 1891

Trichocellus Ganglbauer, 1891a: 366. Type species: *Harpalus placidus* Gyllenhal, 1827 designated by Andrewes (1934: 201). Etymology. From the Greek *trichos* (hair) and *cello* (to run) [masculine].

#### Diversity.

Thirty-one species in the Nearctic (one Holarctic species) and Palaearctic (31 species) Regions.

### Dicheirotrichus cognatus

(Gyllenhal, 1827)

Harpalus cognatus Gyllenhal, 1827 [July-October]: 455. Type locality: Höberg, N. Vånga, Västergötland, Sweden (lectotype label). Lectotype (♂), designated by Lindroth (1968: 875), in UZIU.
Harpalus deutschii C.R. Sahlberg, 1827d [23 June]: 261. Type locality: «Lapponia» (original citation). One syntype in ZMH (Silfverberg 1987: 15). Synonymy established by Dejean (1829: 440). Note. This name may be older than *Dicheirotrichus cognatus* but is not in “prevailing usage” (see *Principle of priority* under “Nomenclature” section).
Trechus ruficrus Kirby, 1837: 47. Type locality: northern parts of British America (inferred from title of the book). Holotype [by monotypy] in BMNH (Lindroth 1953b: 175). Synonymy established by Horn (1876e: 130), confirmed by Lindroth (1953b: 175).
Ophonus obscuritarsis Motschulsky, 1844: 228. Type locality: «Omsk [Russia]» (lectotype label). Lectotype (♂), designated by Kataev and Shilenkov (in Kryzhanovskij et al. 1995: 136), in ZMMU. Synonymy established by Kataev and Shilenkov (in Kryzhanovskij et al. 1995: 136).
Bradycellus marginicollis Motschulsky, 1845b: 345. Type locality: «Kamtschatka [Russia]» (original citation). Lectotype (♂), designated by Kataev and Shilenkov (in Kryzhanovskij et al. 1995: 136), in ZMMU. Synonymy established by Kataev and Shilenkov (in Kryzhanovskij et al. 1995: 136).
Acupalpus axillaris Mannerheim, 1853: 124. Type locality: «insula Kadjak [Alaska]» (original citation). Lectotype (♂), designated by Lindroth (1968: 875), in ZMH. Synonymy established by Schaupp (1883c: 50), confirmed by Lindroth (1968: 875).
Stenolophus quadripunctatus Mannerheim, 1853: 125. Type locality: «insula Sitkha [= Baranof Island, Alaska]» (original citation). Syntype(s) location unknown (possibly in ZMH). **New synonymy**. Note. Mannerheim (1853: 125) described a “var[iety] b” of *Acupalpus axillaris* Mannerheim, 1853 and noted that it refers to the name *Stenolophus quadripunctatus*, provided by Ménétriés, and listed but not described in Motschulsky (1850a: 22). As such he made the name available.
Acupalpus longiusculus Mannerheim, 1853: 125. Type locality: «ad sinum Woskresensk [= Resurrection Bay] peninsulae Kenai [Alaska]» (original citation). Lectotype (♂), designated by Lindroth (1968: 875), in ZMH. Synonymy established by LeConte (1863b: 12), confirmed by Lindroth (1968: 875).
Acupalpus conflagratus Mannerheim, 1853: 126. Type locality: «ad ostia fl[umen] Kaktnu [= Kenai River] peninsulae Kenai [Alaska]» (original citation). Lectotype (♂), designated by Lindroth (1968: 875), in ZMH. Synonymy established by Chaudoir (1868b: 166), confirmed by Lindroth (1968: 875).
Bradycellus nitens LeConte, 1858b: 60. Type locality: «San Diego [San Diego County], California» (original citation). Holotype [by monotypy] in MCZ [# 5936]. Synonymy established by LeConte (1869a: 382), confirmed by Lindroth (1968: 875).
Bradycellus enwaldi J.R. Sahlberg [in Reuter], 1882: 154. Type locality: «Ponoj [=Ponoy, Kola Peninsula, Russia]» (original citation). Two syntypes in ZMH (Silfverberg 1987: 16). Synonymy established by Hellén (1930: 5).
Trichocellus boreellus Casey, 1914: 229. Type locality: «Queen Charlotte Islands [British Columbia]» (original citation). Lectotype (♂), designated by Lindroth (1975: 142), in USNM [# 47983]. Synonymy established by Hatch (1953: 180), confirmed by Lindroth (1968: 875).
Trichocellus lateralis Casey, 1914: 230. Type locality: «Fort Wingate [McKinley County], New Mexico» (original citation). Lectotype (♀), designated by Lindroth (1975: 142), in USNM [# 47984]. Synonymy established by Lindroth (1968: 875).
Trichocellus monticola Casey, 1914: 230. Type locality: «Truckee [Nevada County], California» (original citation). Lectotype (♀), designated by Lindroth (1975: 142), in USNM [# 47985]. Synonymy established by Lindroth (1968: 875).
Trichocellus punctipennis Casey, 1914: 230. Type locality: «Reno [Washoe County], Nevada» (original citation). Lectotype (♀), designated by Lindroth (1975: 142), in USNM [# 47986]. Synonymy established by Lindroth (1968: 875).

#### Distribution.

This circumpolar species ranges from Iceland to eastern Siberia (Jaeger and Kataev 2003: 403) and from Alaska (Lindroth 1968: 876) to Greenland (Böcher 1988: 14), south to southeastern Massachusetts (Cape Cod, CNC), central Iowa (Wickham 1911b: 8), central South Dakota (Kirk and Balsbaugh 1975: 32), the Sacramento Mountains in New Mexico (Fall and Cockerell 1907: 162), and the Baja California Peninsula (Horn 1894: 312). The records from southeastern New York (Notman 1928: 249) and “Pennsylvania” (Bousquet and Larochelle 1993: 227) need confirmation.

#### Records.

**DEN**: GL **FRA**: PM **CAN**: AB, BC (QCI), LB, MB, NB, NF, NS (CBI), NT, NU, ON, PE, QC, SK, YT **USA**: AK, AZ, CA, CO, IA, ID, IN, MA, ME, MI, MN, MT, ND, NH, NM, NV, OR, SD, UT, VT, WA, WI, WY [NY, PA] – Mexico – **Holarctic**

### Genus Acupalpus

Latreille, 1829

Acupalpus Latreille, 1829: 391. Type species: *Carabus meridianus* Linnaeus, 1760 designated by Blanchard [in Audouin et al. 1842: plate 21]. Etymology. From the Latin nouns *acus* (needle, pin) and *palpus* (feeler, by extension palp), alluding to the acuminate last maxillary palpomere (“*les palpes extérieurs se terminent par un article pointu au bout*”) of the adult [masculine].

#### Diversity.

Worldwide, with about 125 species in the Nearctic (12 species), Neotropical (ten species), Australian (about ten species), Oriental (about 15 species), Palaearctic (about 50 species), and Afrotropical (30 species) Regions arrayed in eight subgenera: *Acupalpus* s.str. (about 75 species), *Ancylostria* Schauberger (four species), *Anthracus* (32 species), *Palcuapus* Habu (one east Palaearctic species), *Pseudanthracus* Habu (three species), *Setacupalpus* Habu (two east Palaearctic species), *Subacupalpus* Habu (two Asian species), and *Tachistodes* (four species). The North American fauna is represented by 12 species (roughly 9.6% of the world fauna), one of them is adventive.

#### Taxonomic Note.

Noonan (1976: 23) treated *Hemiaulax* Bates (two species), with *Idiomelas* Tschitchérine (two species) as synonym, as a subgenus of *Acupalpus*. In Lorenz (2005: 361), *Hemiaulax* and *Idiomelas* are listed as valid genera with *Egaploa* Alluaud (two species) considered a subgenus of *Idiomelas*.

### Subgenus Acupalpus

Latreille, 1829

Acupalpus Latreille, 1829: 391. Type species: *Carabus meridianus* Linnaeus, 1760 designated by Blanchard [in Audouin et al. 1842: plate 21].
Manicellus Motschulsky, 1864: 207. Type species: *Stenolophus elegans* Dejean, 1829 designated by Jeannel (1942: 712). Synonymy established by Tschitschérine (1900a: 365).

#### Diversity.

Worldwide with about 75 species in the Nearctic (six species, one of them adventive), Neotropical (ten species), Australian (three species), Oriental (six species), Palaearctic (28 species), and Afrotropical (22 species) Regions.

#### Identification.

Lindroth (1968: 929-934, as *meridianus* group) covered all species found in North America.

### Acupalpus canadensis

Casey, 1924

Acupalpus canadensis Casey, 1924: 144. Type locality: «M[oun]t Royal [= Montreal], Quebec» (original citation). Holotype [by monotypy] in USNM [# 48032].

#### Distribution.

This species ranges from Newfoundland (Lindroth 1955a: 149) to southern Saskatchewan (Lindroth 1968: 934), south to “South Dakota” (Lindroth 1955a: 149), northern Indiana (La Porte County, CMNH), and central Pennsylvania (Lycoming and Clinton Counties, CMNH). The record from “Delaware” (Bousquet and Larochelle 1993: 228) was based on a misidentified specimen of *Acupalpus carus* in MCZ.

#### Records.

**CAN**: MB, NB, NF, NS (CBI), ON, PE, QC, SK **USA**: IN, MA, ME, MI, MN, ND, NH, NY, OH, PA, SD, VT, WI

### Acupalpus carus

(LeConte, 1863)

Stenolophus carus LeConte, 1863c: 18. Type locality: «Hudson’s Bay Territory and Illinois» (original citation), restricted to «Illinois» by Lindroth (1968: 930). Syntype(s) in MCZ [# 5933].
Acupalpus expertus Casey, 1914: 267. Type locality: «Boston Neck [Washington County], Rhode Island» (original citation). Lectotype (♀), designated by Lindroth (1975: 143), in USNM [# 48031]. Synonymy established by Lindroth (1968: 930).
Acupalpus trivialis Casey, 1914: 268. Type locality: «Lake Champlain, New York» (original citation). Lectotype (♀), designated by Lindroth (1975: 143), in USNM [# 48034]. Synonymy established by Lindroth (1968: 930).
Acupalpus subrectus Casey, 1924: 143. Type locality: «Beverly Hills [Cook County], Illinois» (original citation). Lectotype, designated by Lindroth (1975: 144), in USNM [# 48030]. Synonymy established by Lindroth (1968: 930).
Acupalpus curtipennis Casey, 1924: 144. Type locality: «northern Illinois» (original citation). Holotype [by monotypy] (♀) in USNM [# 48029]. Synonymy established by Lindroth (1968: 930).

#### Distribution.

The range of this species extends from Saint Pierre and Miquelon and southwestern Newfoundland (Lindroth 1955a: 148, as *Acupalpus expertus*) to southern Saskatchewan (Ronald R. Hooper pers. comm. 2007), south to northern Louisiana (Allen 1965: 72) and southern North Carolina (Macon County, CNC); also known from northwestern Oregon (Westcott et al. 2006: 6), Washington (Spokane County, CMNH), northern Idaho (Hatch 1953: 185), and south-central British Columbia (Lindroth 1968: 931). The record from northern Colorado (Armin 1963: 114) needs confirmation.

#### Records.

**FRA**: PM **CAN**: BC, MB, NB, NF, NS (CBI), ON, PE, QC, SK **USA**: AR, CT, DC, DE, IA, ID, IL, IN, LA, MA, ME, MI, MN, MO, NC, NH, NJ, NY, OH, OR, PA, RI, SD, VT, WA, WI, WV [CO]

### Acupalpus hydropicus

(LeConte, 1863)

Stenolophus hydropicus LeConte, 1863c: 17. Type locality: «New York» (original citation). Five syntypes in MCZ [# 5932].

#### Distribution.

This species ranges from southeastern New Hampshire (Rockingham County, Ross T. Bell pers. comm. 2008) to at least northern Illinois (Cook County, CMNH), south to northeastern Louisiana (Allen 1965: 72) and northern Florida (Alachua County, CNC).

#### Records.

**USA**: CT, DC, DE, FL, IL, IN, KY, LA, MA, MD, MI, MO, NH, NJ, NY, OH, PA, RI, VA, VT, WV

### Acupalpus meridianus

(Linnaeus, 1760)

Carabus meridianus Linnaeus, 1760: 221. Type locality: Sweden (inferred from title of the book). Three possible syntypes, two belonging to the present species, in LSL (Lindroth 1957b: 332).

#### Distribution.

This Palaearctic species is adventive in North America where it is known from the Quebec City area (Chantal 1971: 202) in the east and from south-central Saskatchewan (Ronald R. Hooper pers. comm. 2007) to Vancouver Island (Lindroth 1968: 930), including central Alberta (Pollock 1991a: 705), south to “Oregon” (Hatch 1953: 185) and northern Idaho (Hatten et al. 2007: 359) in the west. The first inventoried specimen collected on this continent was found in Seattle in 1931 (Hatch 1946: 77).

#### Records.

**CAN**: AB, BC (VCI), QC, SK **USA**: ID, OR, WA – **Adventive**

### Acupalpus nanellus

Casey, 1914

Acupalpus nanellus Casey, 1914: 268. Type locality: «Boston Neck [Washington County], Rhode Island» (original citation). Lectotype, designated by Lindroth (1975: 144), in USNM [# 48033].

#### Distribution.

This species ranges from Nova Scotia (Lindroth 1968: 933) to northwestern Minnesota (Polk County, CMNH), north to southeastern Manitoba (Roughley et al. 2010: 230; CMNH), south at least to southwestern Pennsylvania (Allegheny County, CMNH) and Rhode Island (Casey 1914: 268). One old specimen simply labeled from Illinois is known (CMNH). The record from “Connecticut” (Bousquet and Larochelle 1993: 228) needs confirmation (see Krinsky and Oliver 2001: 4).

#### Records.

**CAN**: MB, NB, NS, ON, QC **USA**: MA, ME, MI, MN, NH, NY, OH, PA, RI, VT, WI [CT, IL]

### Acupalpus pumilus

Lindroth, 1968

Acupalpus pumilus Lindroth, 1968: 931. Type locality: «Amberley S[outh] Kincardine, Ont[ario]» (original citation). Holotype (♂) in CNC [# 10580].

#### Distribution.

The range of this species extends from Nova Scotia (Lindroth 1968: 932) to northwestern Minnesota (Polk County, CMNH), south to west-central West Virginia (Mason County, CMNH) and Delaware (Lindroth 1968: 932). The record from eastern South Dakota (Kirk and Balsbaugh 1975: 33) needs confirmation.

#### Records.

**CAN**: NS, ON, PE, QC **USA**: CT, DE, IL, MA, ME, MI, MN, NH, NY, OH, PA, VA, WI, WV [SD]

### Subgenus Tachistodes

Casey, 1914

Tachistodes Casey, 1914: 286. Type species: *Acupalpus pauperculus* Dejean, 1829 designated by Lindroth (1968: 934). Etymology. Uncertain, possibly from the generic name *Tachys* [*q.v*.] and the Greek -*odes* (likeness), alluding to the resemblance of adults of the species included by Casey in this group to those of *Tachys* [masculine].

#### Diversity.

Four North American species, one of them extending into the Bahamas.

#### Identification.

Lindroth (1968: 934-938, as *pauperculus* group) covered all species.

### Acupalpus indistinctus

Dejean, 1831

Acupalpus indistinctus Dejean, 1831: 846. Type locality: «Amérique septentrionale» (original citation), restricted to «Mobile [Mobile County], Alab[ama]» by Lindroth (1968: 935). Holotype [by monotypy] in MHNP.
Tachistodes obscurus Casey, 1924: 147. Type locality: «near the city [of New York], New York» (original citation). Lectotype, designated by Lindroth (1975: 144), in USNM [# 48057]. Synonymy established by Lindroth (1968: 935).

#### Distribution.

This species ranges from Massachusetts (Suffolk County, MCZ) to central Iowa (Wickham 1911b: 8), including southernmost Ontario (Lindroth 1968: 935; Bousquet 1987a: 132), south to southern Texas (Johnson 1978: 67) and central Florida (Peck and Thomas 1998: 21); also recorded from north-central Maine (Majka et al. 2011: 46). The records from Colorado (Elias 1987: 634) and southeastern Arizona (Dajoz 2004: 116) need confirmation.

#### Records.

**CAN**: ON **USA**: AL, AR, CT, DC, DE, FL, GA, IA, IL, IN, KS, KY, LA, MA, MD, ME, MI, MS, NC, NJ, NY, OH, OK, PA, RI, SC, TN, TX, VA, WI, WV [AZ, CO]

### Acupalpus partiarius

(Say, 1823)

Trechus partiarius Say, 1823a: 90. Type locality: «Gorham [Jackson County], Ill[inois]» (neotype label). Neotype (♀), designated by Lindroth and Freitag (1969: 356), in MCZ [# 32965].
Tachistodes lyratus Casey, 1924: 147. Type locality: «Kansas» (original citation). Lectotype, designated by Lindroth (1975: 144), in USNM [# 48058]. Synonymy established by Lindroth (1968: 937).
Tachistodes convergens Casey, 1924: 148. Type locality: «Rockaway Beach [Queens County], Long Island, New York» (original citation). Lectotype, designated by Lindroth (1975: 143), in USNM [# 48059]. Synonymy established by Lindroth (1968: 937).

#### Distribution.

This species is found from Maine (Procter 1946: 111) to South Dakota (Kirk and Balsbaugh 1975: 33), including southern Quebec (Larochelle 1975: 31) and southern Ontario (Lindroth 1968: 937), south to southeastern Colorado (Miller and Peairs 2008: 34), southeastern Texas (Casey 1914: 288), and northeastern Florida (Saint Johns County, MCZ).

#### Records.

**CAN**: ON, QC **USA**: AR, CO, CT, DE, FL, GA, IA, IL, IN, KS, KY, LA, MA, MD, ME, MI, MN, MO, MS, NC, NE, NH, NJ, NY, OH, OK, PA, RI, SC, SD, TN, TX, VA, VT, WI

### Acupalpus pauperculus

Dejean, 1829

Acupalpus pauperculus Dejean, 1829: 463. Type locality: «Amérique septentrionale» (original citation), restricted to «Sherborn [Middlesex County], Mass[achusetts]» by Lindroth (1968: 935). Syntype(s) in MHNP.
Acupalpus consimilis Dejean, 1829: 465. Type locality: «Amérique septentrionale» (original citation). Syntype(s) in MHNP. Synonymy established by Chaudoir (1868b: 165), confirmed by LeConte (1869a: 376) and Lindroth (1968: 935).

#### Distribution.

This species occurs from Nova Scotia (Lindroth 1954c: 309) to eastern South Dakota (Kirk and Balsbaugh 1975: 33; French et al. 2004: 557), south to southeastern Colorado (Miller and Peairs 2008: 34), southern Texas (Wickham 1897: 113; Harris County, Foster F. Purrington pers. comm. 2009), and southern Florida (Monroe County, Drew A. Hildebrandt pers. comm. 2007).

#### Records.

**CAN**: NB, NS (CBI), ON, PE, QC **USA**: AL, AR, CO, CT, DC, DE, FL, GA, IA, IL, IN, KS, KY, LA, MA, MD, ME, MI, MN, MO, MS, NC, NH, NJ, NY, OH, OK, PA, RI, SC, SD, TN, TX, VA, VT, WI, WV

### Acupalpus testaceus

Dejean, 1829

Acupalpus testaceus Dejean, 1829: 460. Type locality: «Amérique septentrionale» (original citation), restricted to «Camden [Kershaw County], S[outh] C[arolina]» by Lindroth (1968: 937). Syntype(s) in MHNP.
Acupalpus micros LeConte, 1847: 412. Type locality: «provinciis australibus» (original citation). Syntype(s) in MCZ [# 5878]. Synonymy established by LeConte (1853c: 386), confirmed by Lindroth (1968: 937).
Tachistodes fusciceps Casey, 1914: 288. Type locality: «Atlantic City [Atlantic County], New Jersey» (original citation). Lectotype (♀), designated by Lindroth (1975: 144), in USNM [# 48056]. Synonymy established by Lindroth (1968: 937).

#### Distribution.

This species ranges from southern Quebec (Larochelle 1975: 32) to southeastern South Dakota (Kirk and Balsbaugh 1975: 33), south to eastern Texas (Snow 1906a: 141, as *Agonoderus micros*; Nacogdoches, Orange, and San Augustine Counties, CNC, CMNH), northeastern Louisiana (Allen 1965: 72), southern Florida, and the Bahamas (Peck and Thomas 1998: 21).

#### Records.

**CAN**: ON, QC **USA**: AL, AR, CT, DC, DE, FL, GA, IA, IL, IN, KS, KY, LA, MA, MD, ME, MI, MO, MS, NC, NH, NJ, NY, OH, OK, PA, RI, SC, SD, TN, TX, VA, WI, WV – Bahamas

### Subgenus Anthracus

Motschulsky, 1850

Anthracus Motschulsky, 1850a: 21. Type species: *Carabus consputus* Duftschmid, 1812 by original designation. Etymology. From the Greek *anthrax* (coal), probably alluding to the color of adults of the sole species Motschulsky had before him [masculine].
Balius Schiødte, 1861: 184 [junior homonym of *Balius* Guérin-Méneville, 1857]. Type species: *Carabus consputus* Duftschmid, 1812 by monotypy.

#### Diversity.

Thirty-two species in the Nearctic (two western species), Australian (five species), Oriental (five species), Palaearctic (15 species), and Afrotropical (eight species) Regions.

#### Identification.

The two North American species have never been compared and the specific independence of the two not tested.

#### Taxonomic Note.

This taxon is considered a distinct genus by several authors, including Jaeger and Kataev (2003: 399). However, the main character state separating members of *Anthracus* from those of *Acupalpus* is the shape of the pronotum which is cordiform with the sides sinuate in basal half and the posterior angles well distinct, right (see Jeannel 1948a: 714; Basilewsky 1951: 232; Habu 1973a: 301-302). However this character is not constant. Antoine (1959: 453) reported that adults of *Acupalpus (Acupalpus) cantabricus cantabricus* Piochard de la Brûlerie in Morocco have the sides of the pronotum arcuate and the posterior angles rounded while those of *Acupalpus cantabricus zaerensis* Antoine (these two taxa are now considered separate species) have the sides sinuate and the posterior angles right and that some specimens greatly resemble adults of *Anthracus*. Basilewsky (1951: 237) separated *Acupalpus (Acupalpus) angulatus* Jeannel from most other species in his key to Afrotropical *Acupalpus* by the clearly sinuate sides of pronotum with the posterior angles markedly acute. Other character states usually reported, such as the mandibles and antennae relatively longer, the prosternum with small setae, and the median line of the pronotum deeper, more or less sulciform in *Anthracus* are also variable and not at all diagnostic of *Anthracus*. Until the relationships between members of the acupalpine complex are better known, I prefer to follow Ball (1960b: 147), Lindroth (1968: 925), Noonan (1976: 24), and Ball and Bousquet (2000: 94) and treat *Anthracus* as a subgenus of *Acupalpus*.

### Acupalpus punctulatus

Hatch, 1953

Acupalpus punctulatus Hatch, 1953: 185. Type locality: «Forest Grove [Washington County], Or[egon]» (original citation). Holotype (♀) in USNM.

#### Distribution.

This species is known from a few localities from northwestern Oregon (Hatch 1953: 185) to southeastern Oregon (Harney County, CNC).

#### Records.

**USA**: OR

### Acupalpus tener

(LeConte, 1857)

Stenolophus tener LeConte, 1857c: 29. Type locality: «San Jose [Santa Clara County], California» (original citation). Holotype [by monotypy] (♀) in MCZ [# 5931].

#### Distribution.

This species is known from California, from Humboldt County (CAS) to Santa Clara County (LeConte, 1857c: 29).

#### Records.

**USA**: CA

### Genus Philodes

LeConte, 1861

Philodes LeConte, 1861a: 33. Type species: *Stenolophus alternans* LeConte, 1853 designated by Lindroth (1968: 927). Etymology. Uncertain, possibly from the Greek *philos* (beloved) and the suffix -*odes* (likeness) [masculine].

#### Diversity.

Four North American species, one of them extending into the Bahamas, placed in two subgenera.

#### Identification.

Lindroth (1968: 925-926) treated all currently recognized species in his key to Canadian *Acupalpus*.

#### Taxonomic Note.

This taxon has been treated as a subgenus of *Acupalpus* by North American students since Ball (1960b: 147). However, members of the group are characteristic in having three or more discal setae on the third elytral interval and the endophallus with four large, curved spines and a basal pillow of pigmented hairs. To my knowledge, all other acupalpines have one or no discal seta on the third elytral interval. The situation regarding the sclerites in the endophallus is more complex. While most species of *Acupalpus* (including *Anthracus*) are described as having the internal sac unarmed, there are exceptions. Jeannel (1948a: 720) reported that the endophallus of the Madagascan *Anthracus madecassus* Jeannel has some large spines (*quelques grosses dents visibles par transparence*), Habu (1981: 44) described the endophallus of *Acupalpus hilaris* Tschitschérine as having “four large thorn-like copulatory pieces,” and Antoine (1959: 451) stated that the internal sac of *Acupalpus elegans* Dejean has 18 large hooks arranged in two rows (*18 grands crochets à base ovale régulièrement disposés sur deux rangs, un droit et un gauche*) reminiscent of the condition found in members of *Stenolophus*. However, in members of *Philodes* the four endophallus structures are proportionally bigger than in any of these species. At this time, I prefer to regard *Philodes* as generically distinct from *Acupalpus* even though *Acupalpus* may eventually be shown to be paraphyletic in regard to *Philodes*.

### Subgenus Philodes

LeConte, 1861

Philodes LeConte, 1861a: 33. Type species: *Stenolophus alternans* LeConte, 1853 designated by Lindroth (1968: 927).

#### Diversity.

One species occurring over eastern North America.

### Philodes alternans

(LeConte, 1853)

Badister testaceus LeConte, 1844: 52 [secondary homonym of *Acupalpus testaceus* Dejean, 1829]. Type locality: «Pennsylvania» (original citation). Three syntypes in MCZ [# 5930].
Stenolophus alternans LeConte, 1853c: 386. Replacement name for *Stenolophus testaceus* (LeConte, 1844).

#### Distribution.

This species ranges from southern Quebec (Larochelle 1975: 31) south to eastern Oklahoma (Latimer County, UASM), northwestern Arkansas (Newton County, Peter W. Messer pers. comm. 2008), southeastern Mississippi (George and Greene Counties, Drew A. Hildebrandt pers. comm. 2008), east-central Kentucky (Jessamine County, MCZ), and northern Virginia (Hoffman and Roble 2000: 38).

#### Records.

**CAN**: ON, QC **USA**: AR, DC, DE, IL, IN, KY, MS, NH, NJ, NY, OH, OK, PA, VA, VT

### Subgenus Goniolophus

Casey, 1914

Goniolophus Casey, 1914: 262. Type species: *Goniolophus lucens* Casey, 1914 (= *Stenolophus flavilimbus* LeConte, 1869) by original designation. Etymology. From the Greek *gonia* (angle, corner) and *lophos* (crest, comb, tuft) [masculine].

#### Diversity.

Three eastern North American species of which one extends into the Bahamas.

#### Taxonomic Note.

This subgenus is in need of a taxonomic revision. I have seen at least four species in eastern North America, one of them (from Baker and Ware Counties in Georgia and Highlands County in Florida) being undescribed.

### Philodes flavilimbus

(LeConte, 1869)

Stenolophus flavilimbus LeConte, 1869a: 378. Type locality: «Georgia» (original citation). Holotype [by monotypy] in MCZ [# 5934].
Goniolophus lucens Casey, 1914: 264. Type locality: «Galveston [Galveston County], Texas» (original citation). Four syntypes in USNM [# 48028]. **New synonymy**.

#### Distribution.

This species ranges from Georgia (LeConte 1869a: 378; Fattig 1949: 53) to eastern Texas along the Gulf Coast (Casey 1914: 264, as *Goniolophus lucens*), south to central Florida (Peck and Thomas 1998: 21), north to north-central Tennessee (Smith County, Robert L. Davidson pers. comm. 2012) and central Arkansas (Montgomery and Arkansas Counties, Foster F. Purrington pers. comm. 2009).

#### Records.

**USA**: AL, AR, FL, GA, LA, MS, TN, TX

### Philodes longulus

(Dejean, 1829)

Acupalpus longulus Dejean, 1829: 459. Type locality: «Amérique septentrionale» (original citation). One syntype in MHNP (Lindroth 1955b: 31).

#### Distribution.

This species ranges from southern Virginia (Hoffman and Roble 2000: 38) and Tennessee (Bradley, Crockett, Loudon, Maury, and Smith Counties, CMNH) to southern Florida (Peck and Thomas 1998: 21) and the Bahamas (Darlington 1953: 11), west at least to east-central Texas (Riley 2011), including southeastern Louisiana (Saint Tammany and Tangipahoa Parishes, CNC). The records from Rhode Island (Davis 1904: 14), Delaware (Houghton 1905: 212), and “Arkansas” (Bousquet and Larochelle 1993: 228) need confirmation.

#### Records.

**USA**: AL, FL, GA, KY, LA, MS, NC, SC, TN, TX, VA [AR, DE, RI] – Bahamas

### Philodes rectangulus

(Chaudoir, 1868)

Acupalpus rectangulus Chaudoir, 1868b: 167. Type locality: Amérique septentrionale (inferred from title of the paper), restricted to «Brookline [Norfolk County], Mass[achusetts]» by Lindroth (1968: 928). Lectotype (♂), designated by Lindroth (1968: 928), in MHNP.

#### Distribution.

The range of this species extends from southern Quebec (Larochelle 1975: 32) to eastern South Dakota (Kirk and Balsbaugh 1975: 33), south to eastern Texas (San Augustine and Hardin Counties, CMNH, UASM) and southern Florida (Peck and Thomas 1998: 21).

#### Records.

**CAN**: ON, QC **USA**: AL, AR, DC, DE, FL, GA, IA, IL, IN, LA, MA, MD, MI, MO, MS, NC, NY, OH, OK, PA, RI, SC, SD, TN, TX, VA, VT, WI

### Genus Pogonodaptus

Horn, 1881

Pogonodaptus G.H. Horn, 1881: 178. Type species: *Pogonodaptus piceus* G.H. Horn, 1881 (= *Polpochila mexicana* Bates, 1878) by monotypy. Etymology. From the generic names *Pogonus* [*q.v*.] and *Daptus*, alluding to the resemblance of the adults to those of the two genera (“a small species resembling *Daptus* and somewhat also *Pogonus*”) [masculine].

#### Diversity.

Two species in southern United States and Middle America (one species) and the West Indies (one species in Haiti).

#### Identification.

Darlington (1936c: 205) discussed the structural differences between the two species.

### Pogonodaptus mexicanus

(Bates, 1878)

Polpochila mexicana Bates, 1878a: 589. Type locality: «Vera Cruz, Mexico» (original citation). Syntype(s) probably in BMNH.
Pogonodaptus piceus G.H. Horn, 1881: 179. Type locality: «Texas» (original citation). Holotype [by monotypy] (♀) in MCZ [# 34537]. Synonymy established by Bates (1884: 277).

#### Distribution.

This species is known from central (Grady County, Robert L. Davidson pers. comm. 2008) and southwestern Oklahoma (Kondratieff et al. 2005: 173), Arkansas (Garland and Ouachita Counties, Robert L. Davidson pers. comm. 2012), western Louisiana (Sabine Parish, CMNH), and southern Texas (Wickham 1897: 113) south at least to Nicaragua (Blackwelder 1944: 48).

#### Records.

**USA**: AR, LA, OK, TX – Guatemala, Mexico, Nicaragua

### Genus Polpochila

Solier, 1849

Melanotus Dejean, 1831: 698 [junior homonym of *Melanotus* Eschscholtz, 1829]. Type species: *Melanotus flavipes* Dejean, 1831 designated by Hope (1838: 111). Etymology (original). From the Greek *melas* (black) and *notos* (back, dorsum), alluding to the coloration of the adults [masculine].
Polpochila Solier, 1849: 217. Type species: *Polpochila parallela* Solier, 1849 (= *Melanotus chilensis* Chaudoir, 1837) by monotypy. Synonymy established by LeConte (1869b: 248). Etymology. Unknown [feminine].
Cratocara LeConte, 1863b: 11. Replacement name for *Melanotus* Dejean, 1831. Etymology. From the Greek *cratos* (strength) and *cara* (head) [feminine].

#### Diversity.

About 25 species in the Western Hemisphere arrayed in two subgenera: *Phymatocephalus* (two species) and *Polpochila* s.str. (23 species).

#### Identification.

Nègre (1963) revised the species, including all three found in North America, known at the time.

### Subgenus Phymatocephalus

Schaum, 1864

Phymatocephalus Schaum, 1864: 125. Type species: *Phymatocephalus riehlii* Schaum, 1864 (= *Melanotus erro* LeConte, 1854) by monotypy. Etymology. From the Greek *phymatos* (tumor, growth) and *cephale* (head), alluding to the big head (“*caput crassum*”) of the adult [masculine].

#### Diversity.

Two species in southern North America and Mexico.

### Polpochila capitata

(Chaudoir, 1852)

Melanotus capitatus Chaudoir, 1852: 83. Type locality: «Mexique» (original citation). Syntype(s) in MHNP.

#### Distribution.

This species ranges from southern Arizona southwards through the Sonoran and Chihuahuan deserts to Oaxaca in Mexico (Ball and Shpeley 1992a: 55). The records from southwestern New Mexico (Fall and Cockerell 1907: 161) and “California” (Csiki 1932a: 1061) need confirmation.

#### Records.

**USA**: AZ [CA, NM] – Mexico

### Polpochila erro

(LeConte, 1854)

Melanotus erro LeConte, 1854d: 221. Type locality not stated; Tucson [Pima County], Arizona, herein selected (see Nègre 1963: 215). Holotype [by monotypy] in MCZ [# 5877].
Phymatocephalus riehlii Schaum, 1864: 126. Type locality: «Mexico» (original citation). Syntype(s) location unknown (possibly in UMM in collection Riehl). Synonymy established by Nègre (1963: 215). Etymology. The specific name was proposed for Friedrich Riehl [1795-1876], an auditor in Germany who collected beetles. Riehl gave his well-ordered collection, which contained many specimens from the Western Hemisphere including some received from Gundlach in Cuba, to the Zoological Institute of the University of Marburg in Germany.
Cratocara mentalis Casey, 1914: 302. Type locality: «supposed[ly] Arizona» (original citation). Holotype [by monotypy] (♂) in USNM [# 48074]. Synonymy established by Nègre (1963: 215).

#### Distribution.

This species is known from southern Arizona (Nègre 1963: 215) to western Texas (Culberson, Jeff Davis, and Presidio Counties, CMNH), south to Durango and Sinaloa (Nègre 1963: 215).

#### Records.

**USA**: AZ, NM, TX – Mexico

### Subgenus Polpochila

Solier, 1849

Polpochila Solier, 1849: 217. Type species: *Polpochila parallela* Solier, 1849 (= *Melanotus chilensis* Chaudoir, 1837) by monotypy.

#### Diversity.

Twenty-three species in the Nearctic (one species) and Neotropical (23 species) Regions.

### Polpochila rotundicollis

Bates, 1882

Polpochila rotundicollis Bates, 1882a: 74. Type locality: «Leon [Guanajuato], Mexico» (original citation). Syntype(s) probably in BMNH.
Cratocara brunnea Casey, 1914: 302. Type locality: «Willcox [Cochise County], Arizona» (original citation). Three syntypes [3 originally cited] in USNM [# 48073]. Synonymy established by Nègre (1963: 218).

#### Distribution.

This species is known from southern Arizona to the state of Guerrero in western Mexico (Nègre 1963: 218).

#### Records.

**USA**: AZ – Mexico

### Subtribe Harpalina

Bonelli, 1810

Harpalii Bonelli, 1810: Tabula Synoptica. Type genus: *Harpalus* Latreille, 1802.
Ophonidae Laporte, 1834: 68. Type genus: *Ophonus* Dejean, 1821.
Stenomorphidae Laporte, 1834: 71. Type genus: *Stenomorphus* Dejean, 1831.
Daptini LeConte, 1847: 371. Type genus: *Daptus* Fischer von Waldheim, 1823.
Amblystomini Fauvel, 1889a: 17. Type genus: *Amblystomus* Erichson, 1837.
Selenophorini Casey, 1914: 48, 134. Type genus: *Selenophorus* Dejean, 1829. Synonymy established by Jeannel (1948a: 640).
Trichotichnini Jeannel, 1942: 615, 624. Type genus: *Trichotichnus* Morawitz, 1863. Synonymy established by Jeannel (1948a: 640).
Eriotomi Antoine, 1959: 331, 354. Type genus: *Eriotomus* Piochard de la Brûlerie, 1873 (= *Oedesis* Motschulsky, 1850). Synonymy established by Noonan (1976: 29).
Granigerini Antoine, 1959: 326, 331, 357. Type genus: *Graniger* Motschulsky, 1864. Synonymy established by Noonan (1976: 29).
Bleusei Antoine, 1959: 386, 427. Type genus: *Bleusea* Bedel, 1896. Synonymy established by Noonan (1976: 29).
Cratacanthi Lindroth, 1968: 742. Type genus: *Cratacanthus* Dejean, 1829. Synonymy established by Noonan (1976: 29).

#### Diversity.

Worldwide, with about 1,665 species (Lorenz 2005: 362-387, as Harpalina, Amblystomina, and Ditomina). The Northern Hemisphere is represented by about 895 species (53.5% of the world fauna) and North America alone by 138 species (about 8%), of which five are adventive.

#### Taxonomic Note.

Noonan (1976) arrayed the genera of this subtribe into the following genus-groups: Acinopi (no North American representatives), Amblystomi (no North American representatives), Bleusei (no North American representatives), Bradybaeni (no North American representatives), Dapti (including *Cratacanthus*), Ditomi (no North American representatives), Harpali (including *Euryderus*, *Harpalobrachys*, *Harpalus*, *Hartonymus*, *Ophonus*, and *Piosoma*), and Selenophori (including *Amblygnathus*, *Athrostictus*, *Aztecarpalus*, *Discoderus*, *Selenophorus*, *Stenomorphus*, and *Trichotichnus*). Ball and Bousquet (2000: 91) followed Noonan’s classification with the exception that the Selenophori were divided into the Selenophori proper and the Trichotichni (including *Aztecarpalus* and *Trichotichnus*).

### Genus Piosoma

LeConte, 1847

Piosoma LeConte, 1847: 374. Type species: *Piosoma setosum* LeConte, 1847 by monotypy. Etymology (original). From the Greek *pio* (fat) and *soma* (body), alluding to the chubby shape of the adults (“*corpus crassum*”) [neuter].

#### Diversity.

One North American species in the temperate regions.

#### Identification.

Lindroth (1968: 745-746) treated the species.

### Piosoma setosum

LeConte, 1847

Piosoma setosum LeConte, 1847: 375. Type locality: «circiter Long’s Peak [Boulder County, Colorado], Rocky Mountains» (original citation). Syntype(s) in MCZ [# 5872].
Piosoma brevipennis Casey, 1914: 54. Type locality not stated. Holotype [by monotypy] (♂) in USNM [# 47725]. Synonymy established by Lindroth (1968: 745).

#### Distribution.

This species ranges from Saskatchewan to central British Columbia (Lindroth 1968: 745-746), south to southern Arizona (Snow 1907: 142; Pima County, CMNH), central New Mexico (Fall and Cockerell 1907: 161; Bernalillo and Socorro Counties, CMNH, UASM), and southeastern Kansas (Wilson County, MCZ). One old specimen labeled “Tex” is known (MCZ).

#### Records.

**CAN**: AB, BC, SK **USA**: AZ, CO, ID, KS, MT, ND, NE, NM, NV, SD, UT, WY [TX]

**Figure 33.**
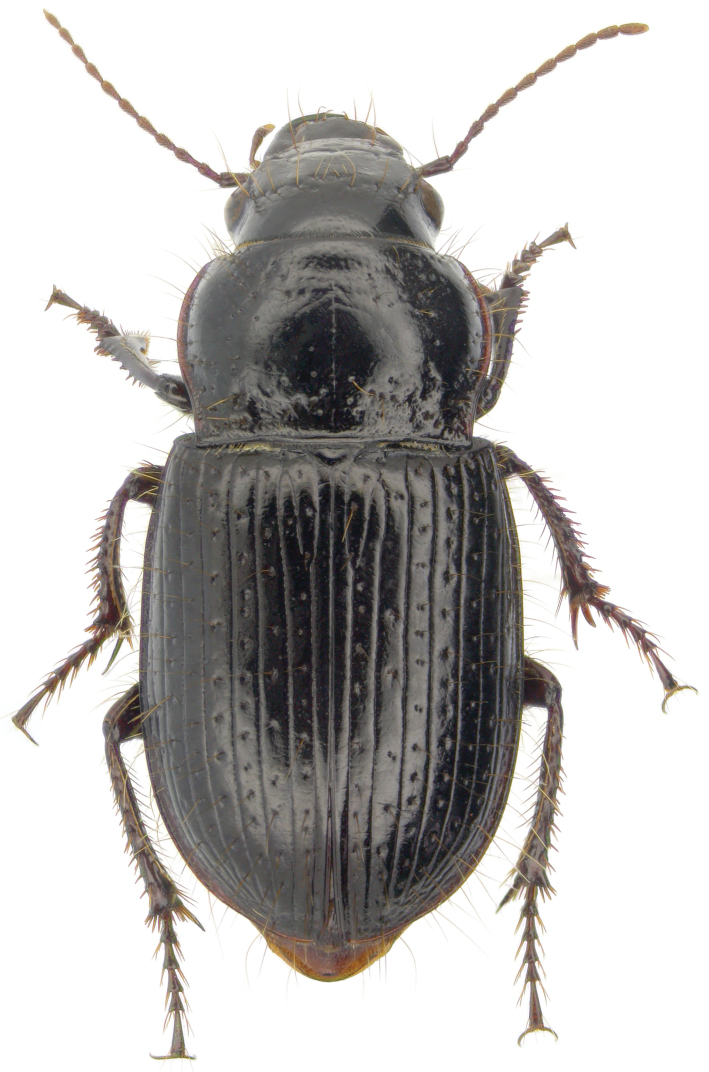
*Piosoma setosum* LeConte. Adults of this species are easily recognized by the presence of many, long and erect setae over the body, hence the specific name *setosum*. Prairie inhabitants, the first documented adults were found by John Lawrence LeConte during his expedition of 1845 which brought him along the Platte River up to Fort Laramie in Wyoming, thence to the foot of the Rocky Mountains.

### Genus Euryderus

LeConte, 1846

Euryderus LeConte, 1846a: 151. Type species: *Euryderus zabroides* LeConte, 1846 (= *Amara grossa* Say, 1830) by monotypy. Etymology. From the Greek *eurys* (broad, wide) and *dere* (neck, by extension pronotum), alluding to the broad pronotum (“*thorax *... *longitudine duplo latior*”) of the adult [masculine].
Nothopus LeConte, 1852b: 67. Unnecessary replacement name for *Euryderus* LeConte, 1846 [incorrectly supposed to be a junior homonym of *Eurydera* Laporte, 1831]. Etymology. Uncertain, possibly from the Greek *nothos* (spurious) and *pous* (foot) [masculine].

#### Diversity.

One North American species in the temperate regions.

#### Identification.

Ball (1960b) and Lindroth (1968: 747-748) treated the species.

### Euryderus grossus

(Say, 1830)

Amara grossa Say, 1830b: (7) [3]. Type locality: «Denver [Denver County], Col[orado]» (neotype label). Neotype (♀), designated by Lindroth and Freitag (1969: 352), in MCZ [# 32974]. Note. «North-West Territory» was the area originally cited by Say (1830b: (7) [3]).
Euryderus zabroides LeConte, 1846a: 152. Type locality: «apud flumen Platte supra furcationem» (original citation). Holotype [by monotypy] in MCZ [# 5871]. Synonymy established with doubt by LeConte (1863b: 11), accepted by Ball (1960a: 49).
Nothopus valens Casey, 1914: 55. Type locality: «Keokuk [Lee County], Iowa» (original citation for the lectotype). Lectotype [as type], designated by Ball (1960a: 49), in USNM [# 47727]. Synonymy established with doubt by Leng (1920: 70), confirmed by Ball (1960b: 49).
Nothopus obtusus Casey, 1914: 56. Type locality: «Colorado» (original citation). Lectotype [as type], designated by Ball (1960a: 49), in USNM [# 47729]. Synonymy established by Ball (1960a: 49).
Nothopus zabroides privatus Casey, 1914: 56. Type locality: «El Paso [El Paso County], Texas» (original citation). Lectotype [as type], designated by Ball (1960a: 50), in USNM [# 47726]. Synonymy established by Ball (1960a: 50).
Nothopus arizonicus Casey, 1914: 56. Type locality: «Arizona» (original citation). Lectotype [as type], designated by Ball (1960a: 50), in USNM [# 47728]. Synonymy established by Ball (1960a: 50).

#### Distribution.

This species ranges from western New York (Ball 1960a: 59, Fig. 3) to the Okanagan Valley in British Columbia (Lindroth 1968: 747), south to northeastern Oregon (Hatch 1953: 165), southern Arizona, southern Texas (Ball 1960a: Fig. 3), southern Louisiana (Allen 1965: 69), and northwestern Mississippi (Bolivar County, Drew A. Hildebrandt pers. comm. 2008), east to southern Georgia (Torres and Ruberson 2006: 32). The records from southeastern Pennsylvania (Rathvon 1869: 526, as *Nothopus zabroides*) and “California” (Leng 1920: 70, as *Nothopus valens*) are probably in error.

#### Records.

**CAN**: AB, BC, MB, ON, SK **USA**: AR, AZ, CO, GA, IA, ID, IL, IN, KS, LA, MI, MN, MO, MS, MT, ND, NE, NM, NY, OH, OK, OR, SD, TX, UT, WA, WI, WY

### Genus Ophonus

Dejean, 1821

Ophonus Dejean, 1821: 13. Type species: *Carabus sabulicola* Panzer, 1796 designated by Guérin-Méneville (1827: 244) (see ICZN 1990). Etymology. Uncertain, possibly from the Greek prefix *ob-* (to, toward, against, opposite) modified by the elision of the “*b*” and *phonos* (to slaughter, murder) [masculine]. According to Desmarest (1851: 125), the name derives from the mythological name *Ophioneus*. The name was proposed by Franz Anton Ziegler and made available by Dejean.

#### Diversity.

About 70 Palaearctic species arrayed in six subgenera: *Brachyophonus* Sciaky (two species), *Hesperophonus* Antoine (16 species), *Incisophonus* Sciaky (one species), *Macrophonus* Tschitschérine (three species), *Metophonus* (about 40 species), and *Ophonus* s.str. (ten species). A single species is found in the Far East and two species are adventive in the Nearctic Region.

#### Identification.

Lindroth (1968: 756-758, as *rufibarbis* group) reviewed both species found in North America and provided a mean for their identification through his key to *Harpalus*. Both species are also included in Noonan’s (1991: 20-45) key to the North American species of *Harpalus* excluding the subgenera *Pseudoophonus* and *Glanodes*.

### Subgenus Metophonus

Bedel, 1897

Metophonus Bedel, 1897: 111. Type species: *Harpalus syriacus* Dejean, 1829 by original designation. Etymology. From the Greek *meta* (between, among, beyond) and the generic name *Ophonus* [*q.v*.] [masculine].
Sulcophonus Schauberger, 1933: 130. Type species: *Harpalus sulcifer* Tschitschérine, 1902 (= *Ophonus cribrellus* Reiche and Saulcy, 1855) by monotypy. Synonymy established by Sciaky (1987: 70). Etymology. From the Latin *sulcus* (furrow, groove) and the generic name *Ophonus* [*q.v*.] [masculine].

#### Diversity.

About 40 Palaearctic species, of which a single one, *Ophonus stricticollis* Tschitschérine, is found in eastern Asia. Two species are adventive in eastern North America.

### Ophonus puncticeps

Stephens, 1828

Ophonus puncticeps Stephens, 1828a: 163. Type locality: «near Dover [United Kingdom]» (original citation). Lectotype (♂), designated by Lindroth (1968: 757), in BMNH.

#### Distribution.

This Palaearctic species is adventive in North America where it is known from Prince Edward Island and Nova Scotia (Majka et al. 2006: 606) to eastern Iowa (Linn County, Doug A. Veal pers. comm. 2009), south to southern Pennsylvania [see Larochelle and Larivière 1989b: Fig. 1] and New Jersey (Steffens and Davidson 1979: 64). The first inventoried specimen collected on this continent was found in Long Island, New York, in 1954 (Dietrich 1958: 46).

#### Records.

**CAN**: NB, NS, ON, PE, QC **USA**: CT, IA, IL, MA, ME, MI, NH, NJ, NY, OH, PA, RI, VT, WI – **Adventive**

### Ophonus rufibarbis

(Fabricius, 1792)

Carabus rufibarbis Fabricius, 1792: 159. Type locality: «Germania» (original citation). Lectotype (♂), designated by Lindroth (1968: 757), in ZMUC.

#### Distribution.

This Palaearctic species is adventive in North America where it is known from Montreal (Lindroth 1968: 758) and the Quebec City region (Landry and Rancourt 1976: 53; CNC) in Quebec. The first inventoried specimen collected on this continent was found in Montreal in 1953.

#### Records.

**CAN**: QC – **Adventive**

### Genus Harpalus

Latreille, 1802

Harpalus Latreille, 1802: 92. Type species: *Carabus proteus* Paykull, 1790 (= *Carabus affinis* Schrank, 1781) designated by Andrewes (1935: 19). Etymology (see Latreille 1804: 325). From the Greek *harpaleos* (grasping, greedy, voracious, by extension living by plunder) [masculine]. According to Mulsant (1830: 327), so named “*à cause de la guerre qu’ils font aux insectes plus faibles qu’eux*.” Note. As stated by Andrewes (1935) and Noonan (1976: 31), the first valid type species designation for *Harpalus* Latreille is *Carabus ruficornis* Fabricius, 1775 (= *Carabus rufipes* DeGeer, 1774) as designated by Latreille (1810: 426). This species is currently included in the subgenus *Pseudoophonus* Motschulsky, 1844. Acceptance of Latreille’s designation would require nomenclatural changes at the subgeneric level within the genus *Harpalus*. A request should be addressed to the Commission to suppress Latreille’s designation. A first request was postponed (ICZN 1950).
Harpaleus Billberg, 1820: 23. Unjustified emendation of *Harpalus* Latreille, 1802.

#### Diversity.

About 415 species (Lorenz 2005: 363-372) in the Nearctic, Neotropical (Mexico only), Australian (one adventive species in New Zealand), Oriental, Palaearctic, and Afrotropical Regions. The Northern Hemisphere is represented by about 360 species (roughly 87% of the world fauna) and North America alone by 59 species (approximately 14%). Five species are Holarctic and three North American species are adventive.

### Subgenus Pseudoophonus

Motschulsky, 1844

Holosus Fischer von Waldheim, 1829a: 21 [potential *nomen oblitum*, see Bousquet (2002c: 176)]. Type species: *Carabus ruficornis* Fabricius, 1775 (= *Carabus rufipes* DeGeer, 1774) designated by Bousquet (2002c: 176). Etymology. Uncertain, possibly from the Greek *holos* (whole, entire) and *sos* (sound) [masculine].
Pseudoophonus Motschulsky, 1844: 196 [potential *nomen protectum*]. Type species: *Carabus ruficornis* Fabricius, 1775 (= *Carabus rufipes* DeGeer, 1774) designated by Desmarest (1851: 124). Etymology. From the Greek *pseudos* (fallacy, lie) and the generic name *Ophonus* [*q.v*.] [masculine]. Note. *Pseudophonus* is an incorrect subsequent spelling, introduced by Ménétriés (1848: 37), not in prevailing usage.
Empeirus Motschulsky 1844: 197 (as *Empeinus*). Type species: *Harpalus pastor* Motschulsky, 1844 designated by Noonan (1976: 36). Synonymy established by Kataev (2002: 192). Note. Motschulsky originally used the spelling *Empeinus* (p. 197) but corrected it to *Empeirus* in his corrigendum (p. xi) issued simultaneously with the original work. Therefore *Empeirus* is the correct original spelling (ICZN 1999: Article 32.5.1.1) despite that Motschulsky used *Empeinus* in his subsequent publications (1848: 487; 1850a: vii, 25).
Platus Motschulsky, 1844: 197. Type species: *Harpalus calcitrapus* Motschulsky, 1844 (= *Carabus calceatus* Duftschmid, 1812) designated by Noonan (1976: 33).
Pardileus des Gozis 1882: 289. Type species: *Carabus calceatus* Duftschmid, 1812 by original designation. Synonymy established by Ball and Anderson (1962: 4).
Migadophonus Tschitschérine, 1897: 47. Type species: *Ophonus aenigma* Tschitschérine, 1897 by monotypy. Synonymy established by Habu (1968: 287). Etymology. From the Greek *migados* (mixed pell-mell) and the generic name *Ophonus* [*q.v*.] [masculine].
Neopardileus Habu, 1954b: 281. Type species: *Ophonus itoshimanus* Habu, 1954 (= *Carabus calceatus* Duftschmid, 1812) by monotypy. Etymology. From the Greek prefix *neo*- (new) and the generic name *Pardileus* [masculine].

#### Diversity.

About 60 species in North America (13 species), Mexico (four species, one of them, *Harpalus alienus* Bates, endemic), Asia (about 45 species), and Europe and northern Africa (three species, none endemic).

#### Identification.

Ball and Anderson (1962) revised the North American species and provided a key for their identification. Lindroth (1968: 758-765) covered ten species. Subsequently one new species was described by Will (2002a).

### [compar group]

### Harpalus actiosus

Casey, 1914

Harpalus actiosus Casey, 1914: 79. Type locality: «Keokuk [Lee County], Iowa» (original citation for the lectotype). Lectotype (♂), designated by Ball and Anderson (1962: 67), in USNM [# 47759].
Harpalus pubitarsis Casey, 1914: 82. Type locality: «probably Indiana» (original citation for the lectotype). Lectotype (♂), designated by Ball and Anderson (1962: 67), in USNM [# 47762]. Synonymy established by Ball and Anderson (1962: 67).

#### Distribution.

This species ranges from New Jersey and Virginia to northeastern North Dakota, south to the Gulf Coast of Texas and northwestern Mississippi (Bolivar County, Drew A. Hildebrandt pers. comm. 2007); also known from north-central Colorado [see Ball and Anderson 1962: Fig. 39].

#### Records.

**USA**: AR, CO, IA, IL, IN, KS, MD, MO, MS, ND, NE, NJ, OH, OK, PA, SD, TX, VA, WI

### Harpalus compar

LeConte, 1847

Carabus bicolor Fabricius, 1775: 241 [primary homonym of *Carabus bicolor* Drury, 1773]. Type locality: «America» (original citation), restricted to «Rumney [Grafton County], N[ew] H[ampshire]» by Lindroth (1968: 763). Lectotype (♀), designated by Lindroth (1968: 763) in ZMUC.
Harpalus compar LeConte, 1847: 395. Type locality: United States east of the Rocky Mountains (inferred from title of the paper). Lectotype (♀), designated by Ball and Anderson (1962: 70), in MCZ [# 5895]. Synonymy established by Ball and Anderson (1962: 70).
Harpalus nactus Casey, 1914: 82. Type locality: «Arizona» (original citation). Lectotype (♀), designated by Ball and Anderson (1962: 70), in USNM [# 47773]. Synonymy established by Hatch (1932: 174), confirmed by Ball and Anderson (1962: 70).
Harpalus feroculus Casey, 1924: 98. Type locality: «Boston Neck [Washington County], Rhode Island» (original citation). Holotype [by monotypy] (♀) in USNM [# 47760]. Synonymy established with doubt by Hatch (1932: 174), confirmed by Ball and Anderson (1962: 70).
Harpalus admissus Casey, 1924: 99. Type locality: «Duluth [Saint Louis County], Minnesota» (original citation for the lectotype). Lectotype (♂), designated by Ball and Anderson (1962: 70), in USNM [# 47772]. Synonymy established by Ball and Anderson (1962: 70).
Harpalus excubans Casey, 1924: 99. Type locality: «Watch Hill [Washington County], Rhode Island» (original citation). Lectotype (♂), designated by Lindroth (1975: 137), in USNM [# 47775]. Synonymy established with doubt by Lindroth (1968: 763). Note. Ball and Anderson (1962: 79) regarded the lectotype of *Harpalus excubans* Casey as an hybrid *erythropus* x *bicolor*. Lindroth (1968: 764) believed the specimen was probably a small *Harpalus bicolor*.

#### Distribution.

This species ranges from Nova Scotia to southern Manitoba, south to southeastern Arizona, southeastern Texas, and central Florida [see Ball and Anderson 1962: Fig. 39].

#### Records.

**CAN**: MB, NS, ON, QC **USA**: AL, AR, AZ, CO, CT, DC, DE, FL, GA, IA, IL, IN, KS, KY, LA, MA, ME, MI, MN, MO, MS, NC, ND, NE, NH, NJ, NM, NY, OH, OK, PA, RI, SC, SD, TN, TX, VA, VT, WI, WV, WY

### Harpalus erythropus

Dejean, 1829

Harpalus erythropus Dejean, 1829: 258. Type locality: «Amérique septentrionale» (original citation), restricted to «Dorchester [Suffolk County], Mass[achusetts]» by Lindroth (1968: 764). One syntype in MHNP (Lindroth 1955b: 27).
Harpalus rufopiceus Casey, 1914: 80. Type locality: «S[ain]t Louis, Missouri» (original citation). Lectotype (♀), designated by Ball and Anderson (1962: 76), in USNM [# 47765]. Synonymy established by Hatch (1932: 174), confirmed by Ball and Anderson (1962: 76).
Harpalus deludens Casey, 1914: 80. Type locality: «Keokuk [Lee County], Iowa» (original citation). Lectotype (♀), designated by Ball and Anderson (1962: 77), in USNM [# 47763]. Synonymy established by Hatch (1932: 174), confirmed by Ball and Anderson (1962: 76).
Harpalus effetus Casey, 1914: 81. Type locality: «Willets Point [Queens County], Long Island, New York» (original citation). Holotype [by monotypy] (♂) in USNM [# 47769]. Synonymy established by Hatch (1932: 174), confirmed by Ball and Anderson (1962: 76).
Harpalus fenisex Casey, 1914: 81. Type locality: «S[ain]t Louis, Missouri» (original citation). Holotype [by monotypy] (♂) in USNM [# 47767]. Synonymy established by Hatch (1932: 174), confirmed by Ball and Anderson (1962: 76).
Harpalus abstrusus Casey, 1914: 87. Type locality: «Boston Neck [Washington County], Rhode Island» (original citation). Lectotype (♂), designated by Ball and Anderson (1962: 79), in USNM [# 47764]. Synonymy established with doubt by Lindroth (1968: 764). Note. Ball and Anderson (1962: 76) regarded the lectotype of *Harpalus abstrusus* Casey as an hybrid *erythropus* x *bicolor*. Lindroth (1968: 764) believed the specimen was probably an abnormal *Harpalus erythropus*.
Harpalus cupiens Casey, 1924: 98. Type locality: «northern Illinois» (original citation). Holotype [by monotypy] (♀) in USNM [# 47766]. Synonymy established with doubt by Hatch (1932: 174), confirmed by Ball and Anderson (1962: 76).

#### Distribution.

This species is widely distributed east of the Rocky Mountains from Nova Scotia (Lindroth 1968: 764) to southern Manitoba, south to north-central Colorado, southern Oklahoma, and central Florida [see Ball and Anderson 1962: Fig. 39]. The record from south-central Montana (Hatch 1933a: 10) is probably in error.

#### Records.

**CAN**: MB, NS, ON, QC **USA**: AL, AR, CO, CT, DC, DE, FL, GA, IA, IL, IN, KS, KY, MA, MD, ME, MI, MN, MO, MS, NC, ND, NE, NH, NJ, NY, OH, OK, PA, RI, SC, SD, TN, VA, VT, WI, WV, WY

### Harpalus paratus

Casey, 1924

Harpalus paratus Casey, 1924: 100. Type locality: «Akron [Washington County], Colorado» (original citation for the lectotype). Lectotype (♂), designated by Ball and Anderson (1962: 73), in USNM [# 47771].
Harpalus spaldingi Casey, 1924: 103. Type locality: «Vineyard [Utah County], Utah» (original citation). Holotype [by monotypy] (♂) in USNM [# 47768]. Synonymy established by Ball and Anderson (1962: 74).

#### Distribution.

This primarily prairie species ranges from southeastern Michigan to southern Alberta, south to central Arizona and the Rio Grande Basin in south-central Texas [see Ball and Anderson 1962: Fig. 39]. According to Ball and Anderson (1962: 76), the species is also found in “Mexico.” The records from “Arkansas” and “Pennsylvania” (Bousquet and Larochelle 1993: 232) need confirmation.

#### Records.

**CAN**: AB, SK **USA**: AZ, CO, IA, ID, IN, KS, MI, MN, ND, NE, NM, OH, OK, SD, TX, UT, WI [AR, PA] – Mexico

### Harpalus vagans

LeConte, 1865

Harpalus longicollis LeConte, 1847: 396 [primary homonym of *Harpalus longicollis* Rambur, 1838]. Type locality: «NovEboraci [= New York]» (original citation). Lectotype (♂), designated by Ball and Anderson (1962: 64), in MCZ [# 5887].
Harpalus vagans LeConte, 1865b: 102. Type locality: «western states» (original citation). Lectotype (♀), designated by Ball and Anderson (1962: 64), in MCZ [# 5886]. Synonymy established by Ball and Anderson (1962: 64).
Harpalus haldemani Casey, 1914: 79. Type locality: «Pennsylvania» (original citation). Lectotype (♀), designated by Ball and Anderson (1962: 64), in USNM [# 47766]. Synonymy established by Ball and Anderson (1962: 64).
Harpalus dolosus Casey, 1914: 84. Type locality: «R[hode] I[sland]» (lectotype label). Lectotype (♀), designated by Ball and Anderson (1962: 79), in USNM [# 47778]. Synonymy established by Lindroth (1968: 762). Note. Ball and Anderson (1962: 79) regarded the lectotype of *Harpalus dolosus* Casey as an hybrid *erythropus* x *bicolor*. Lindroth (1968: 764) believed the specimen was conspecific with members of *Harpalus longicollis*.
Harpalus latescans Casey, 1924: 97. Type locality: «Pennsylvania» (original citation). Lectotype (♂), designated by Ball and Anderson (1962: 64), in USNM [# 47758]. Synonymy established by Hatch (1932: 174), confirmed by Ball and Anderson (1962: 64).

#### Distribution.

This eastern species occurs from the Saguenay River in southern Quebec to eastern South Dakota, south to central Kansas, southern Mississippi, and southwestern Georgia [see Ball and Anderson 1962: Fig. 39]. The record from “Nova Scotia” (Bousquet and Larochelle 1993: 231) was based on a misidentified specimen of *Harpalus erythropus* (Majka and Bousquet 2008: 474).

#### Records.

**CAN**: ON, QC **USA**: AL, AR, CT, DC, GA, IA, IL, IN, KS, MA, MD, MI, MO, MS, NC, NJ, NY, OH, OK, PA, RI, SC, SD, TN, VA, VT, WI, WV

### [pensylvanicus group]

### Harpalus liobasis

Chaudoir, 1868

Harpalus mexicanus Chaudoir, 1837b: 46 [primary homonym of *Harpalus mexicanus* Dejean, 1828]. Type locality: «Mexique» (original citation). Syntype(s) probably in MHNP.
Harpalus liobasis Chaudoir, 1868b: 170. Type locality: «côte occidentale de l’Amérique septentrionale» (original citation). Holotype [by monotypy; designated lectotype by Lindroth (1968: 761)] (♂) in MHNP. Synonymy established by Erwin et al. (1977: 4.48)].
Harpalus montezumae Csiki, 1932a: 1183. Replacement name for *Harpalus mexicanus* Chaudoir, 1837.

#### Distribution.

This species is known only from “Arizona” (Erwin et al. 1977: 4.48) and “Mexico” (Bates 1882a: 57). The record from “near Philadelphia,” Pennsylvania (Casey 1914: 82) is likely in error.

#### Records.

**USA**: AZ – Mexico

#### Note.

Ball and Anderson (1962: 45) listed *Harpalus liobasis* Chaudoir as a junior synonym of *Harpalus pensylvanicus* but Lindroth (1968: 761) treated it as a distinct species.

### Harpalus pensylvanicus

(DeGeer, 1774)

Carabus pensylvanicus DeGeer, 1774: 108. Type locality: «Pensylvanie» (original citation). Lectotype (♂), designated by Lindroth (1968: 760), in NRSS.
Harpalus longior Kirby, 1837: 43. Type locality: «Lat. 54° [= along North Saskatchewan River]» (original citation), which according to Lindroth (1968: 760) is probably incorrect. Two syntypes [2 originally cited] in BMNH (Lindroth 1953b: 174). Synonymy established by Lindroth (1953b: 174).
Harpalus pennsylvanicus mormonicus Casey, 1914: 86. Type locality: «Utah» (original citation). Lectotype (♂), designated by Ball and Anderson (1962: 47), in USNM [# 47779]. Synonymy established by Hatch (1932: 174), confirmed by Ball and Anderson (1962: 47).
Harpalus immixtus Casey, 1924: 97. Type locality: «Keene Valley, Adirondack M[oun]t[ain]s [Essex County], New York» (original citation). Lectotype (♂), designated by Ball and Anderson (1962: 47), in USNM [# 47761]. Synonymy established by Ball and Anderson (1962: 47).

#### Distribution.

This common species occurs over a large area in North America from Prince Edward Island (Lindroth 1968: 761) and Nova Scotia to south-central British Columbia, south to southern California, northwestern Mexico, southeastern Texas, southern Florida, and the Bahamas [see Ball and Anderson 1962: Fig. 38].

#### Records.

**CAN**: BC, MB, NB, NS, ON, PE, QC, SK **USA**: AK, AL, AR, AZ, CA, CO, CT, DC, DE, FL, GA, IA, ID, IL, IN, KS, KY, LA, MA, MD, ME, MI, MN, MO, MS, MT, NC, ND, NE, NH, NJ, NM, NV, NY, OH, OK, OR, PA, RI, SC, SD, TN, TX, UT, VA, VT, WA, WI, WV, WY – Bahamas, Mexico

### Harpalus protractus

Casey, 1914

Harpalus protractus Casey, 1914: 85. Type locality: «S[ain]t Louis, Missouri» (original citation). Holotype [by monotypy] (♂) in USNM [# 47776].
Harpalus thoracinus Casey, 1914: 85. Type locality not stated. Holotype [by monotypy] (♂) in USNM [# 47770]. Synonymy established by Ball and Anderson (1962: 57).

#### Distribution.

This species is found from Maryland to southern Nebraska (Nuckolls County, Foster F. Purrington pers. comm. 2011), south to south-central Oklahoma, northern Louisiana, and east-central Alabama [see Ball and Anderson 1962: Fig. 38].

#### Records.

**USA**: AL, GA, IL, IN, KS, KY, LA, MD, MO, MS, NC, NE, OH, OK, SC, TN, VA, WV

### Harpalus texanus

Casey, 1914

Harpalus texanus Casey, 1914: 83. Type locality: «Austin [Travis County], Texas» (original citation). Lectotype (♀), designated by Ball and Anderson (1962: 51), in USNM [# 47777].

#### Distribution.

This species is restricted to the Coastal Plain ranging from south-central North Carolina to southern Florida, west to eastern Texas [see Ball and Anderson 1962: Fig. 38].

#### Records.

**USA**: FL, GA, MS, NC, TX

### [rufipes group]

### Harpalus faunus

Say, 1823

Harpalus faunus Say, 1823a: 28. Type locality: «Penn[sylvania]» (neotype label). Neotype (♂), designated by Lindroth and Freitag (1969: 352), in MCZ [# 32970].
Harpalus badius Dejean, 1829: 254. Type locality: «Amérique septentrionale» (original citation). Holotype [by monotypy] (♂) in MHNP (Lindroth 1955b: 27). Synonymy established with doubt by Brullé (1835c: 286), confirmed by Lindroth (1955b: 27). Note. Dejean (1829: 256) noted that the unique specimen of *Harpalus badius* he had was a female.
Harpalus convivus LeConte, 1865b: 102. Type locality: «New York» (original citation). Lectotype (♀), designated by Ball and Anderson (1962: 38), in MCZ [# 5885]. Synonymy established by Ball and Anderson (1962: 38).

#### Distribution.

This species ranges from southwestern Maine (Majka et al. 2011: 46) and southern Quebec (Larochelle 1975: 87) to western North Dakota, south to western (Dajoz 2007: 23) and northeastern Texas, southeastern Louisiana, and the Florida Panhandle (Peck and Thomas 1998: 21); also known from northern (Villa-Castillo and Wagner 2002: 246) and east-central Arizona [see Ball and Anderson 1962: Fig. 37].

#### Records.

**CAN**: MB, ON, QC **USA**: AR, AZ, CO, CT, DC, FL, GA, IA, IL, IN, KS, KY, LA, MA, MD, ME, MI, MN, MO, MS, NC, ND, NE, NH, NJ, NY, OH, OK, PA, RI, SC, SD, TN, TX, VA, VT, WI, WV

### Harpalus hatchi

Ball and Anderson, 1962

Harpalus hatchi Ball and Anderson, 1962: 41. Type locality: «Harvey County, Kansas» (original citation). Holotype (♂) in SMEK [# 6478] (Byers and Karren 1968: 3).

#### Distribution.

This species is known only from three specimens collected in Kansas (Ball and Anderson 1962: 43) and five specimens from central Oklahoma (Seminole County, Robert L. Davidson pers. comm. 2008).

#### Records.

**USA**: KS, OK

### Harpalus rufipes

(DeGeer, 1774)

Carabus rufipes DeGeer, 1774: 96. Type locality not stated; «Sweden» selected by Lindroth (1968: 759). Lectotype (♂), designated by Lindroth (1968: 759), in NRSS.
Carabus ruficornis Fabricius, 1775: 241 [primary homonym of *Carabus ruficornis* DeGeer, 1774]. Type locality: «Europa» (original citation). Lectotype (♂), designated by Lindroth (1968: 759), in ZMUC. Synonymy established by Schönherr (1806: 182).

#### Distribution.

This adventive species is found from Newfoundland (Larson and Langor 1982: 593) to near Montreal, Quebec (Mercado Cárdenas and Buddle 2007: 140), south to Connecticut (Krinsky and Oliver 2001: 210) and Rhode Island (Zhang et al. 1994: 69). The record from Wayne County, New York (Hajek et al. 2007) is in error (see Hajek et al. 2009: 913). The first inventoried specimen collected on this continent was found on Prince Edward Island in 1937 (Brown 1950b: 199).

#### Records.

**FRA**: PM **CAN**: NB, NF, NS (CBI), PE, QC **USA**: CT, MA, ME, NH, RI, VT – **Adventive**

### [incertae sedis]

### Harpalus poncei

Will, 2002

Harpalus poncei Will, 2002a: 448. Type locality: «Naples [Collier County], Fl[orid]a» (original citation). Holotype (♂) in CUIC [# 7161]. Etymology. The specific name honors the Spanish explorer Juan Ponce de León [1460-1521] who died in Cuba of wounds sustained during an expedition against the Carib Indians of Guadeloupe.

#### Distribution.

This species is known only from two specimens collected in 1963 in two localities in Florida, along the coast of the Gulf of Mexico [see Will 2002a: Fig. 5].

#### Records.

**USA**: FL

### Subgenus Megapangus

Casey, 1914

Megapangus Casey, 1914: 71. Type species: *Carabus caliginosus* Fabricius, 1775 by monotypy. Etymology. From the Greek *megas* (large) and the generic name *Pangus* [masculine].

#### Diversity.

Two North American species, both extending into northern Mexico.

#### Identification.

Will (1997) reviewed the species of this subgenus and provided a key for their identification.

#### Taxonomic Note.

This taxon is listed as a synonym of *Pseudoophonus* Motschulsky by Kataev et al. (2003: 384).

### Harpalus caliginosus

(Fabricius, 1775)

Carabus caliginosus Fabricius, 1775: 240. Type locality: «America» (original citation), restricted to «Hope [Hempstead County], Arkansas» by Lindroth (1968: 765). Lectotype (♂), designated by Lindroth (1968: 765), in ZMUC.
Harpalus caliginosus dux Casey, 1924: 94. Type locality: «southern Illinois» (original citation). Lectotype (♀), designated by Lindroth (1975: 138), in USNM [# 47741]. Synonymy established by Lindroth (1968: 765).

#### Distribution.

This species ranges from Nova Scotia (King County, CNC) to Washington, north to southern Manitoba (Lindroth 1968: 765) and southern Saskatchewan (CNC), south to the northern part of the Baja California Peninsula, northeastern Mexico, and northern Florida [see Will 1997: Fig. 1A].

#### Records.

**CAN**: MB, NS, ON, QC, SK **USA**: AL, AR, AZ, CA, CO, CT, DC, DE, FL, GA, IA, ID, IL, IN, KS, KY, LA, MA, MD, ME, MI, MN, MO, MS, NC, ND, NE, NH, NJ, NM, NV, NY, OH, OK, OR, PA, RI, SC, SD, TN, TX, UT, VA, VT, WA, WI, WV, WY – Mexico

### Harpalus katiae

Battoni, 1985

Harpalus katiae Battoni, 1985: 356. Type locality: «Burkburnett at Red river, Wichita Co[unty], Texas» (original citation). Holotype (♂) location unknown.

#### Distribution.

This species ranges from northern Colorado (Will 1997: 47) to southern Virginia (Hoffman and Roble 2000: 37), north to Wisconsin (Purrington et al. 2002: 201), south to northern Florida, northeastern Mexico (Will 1997: 47, 49), southeastern New Mexico (Dunn 1986: 2), and southeastern Arizona (Cochise County, Ken Karns pers. comm. 2009) [see Will 1997: Fig. 1B].

#### Records.

**USA**: AL, AR, AZ, CO, GA, FL, IL, LA, MO, MS, NC, NE, NM, OK, SC, TN, TX, VA, WI – Mexico

### Subgenus Plectralidus

Casey, 1914

Plectralidus Casey, 1914: 72. Type species: *Harpalus erraticus* Say, 1823 designated by El-Moursy (1959: 37). Etymology. From the Greek *plectron* (spur), probably alluding to the dentiform process on the elytral edge delimiting laterally the subapical sinuation (“external dentition of the elytral apices”) [masculine].

#### Diversity.

Two North American species.

#### Identification.

El-Moursy (1959) and Noonan (1991: 131-135) revised the species. Both species were also treated by Lindroth (1968: 766-767).

### Harpalus erraticus

Say, 1823

Harpalus erraticus Say, 1823a: 27 (as *eraticus*). Type locality: «Medora [Reno County], K[ansa]s» (neotype label). Neotype (♂), designated by Lindroth and Freitag (1969: 352), in MCZ [# 32973]. Note. The incorrect subsequent spelling *erraticus*, introduced by Harris (1833: 568), is in prevailing usage and attributed to the publication of the original spelling; therefore it is deemed to be the correct original spelling (ICZN 1999: Article 33.3.1).
Harpalus caudalis Casey, 1914: 73. Type locality: «S[ain]t L[ouis], M[iss]o[uri]» (lectotype label). Lectotype (♂), designated by Lindroth (1975: 138), in USNM [# 47742]. Synonymy established by El-Moursy (1959: 41).

#### Distribution.

This species occurs east of the Rocky Mountains ranging from southern Quebec to southeastern Alberta, south to central New Mexico, southern Kansas, central Alabama [see Noonan 1991: Fig. 285], and southeastern South Carolina (Ciegler 2000: 100).

#### Records.

**CAN**: AB, MB, ON, QC, SK **USA**: AL, AR, AZ, CO, CT, DC, DE, GA, IA, IL, IN, KS, MA, MD, ME, MI, MN, MO, MS, MT, ND, NE, NH, NJ, NM, NY, OH, RI, SC, SD, TN, VT, WI

### Harpalus retractus

LeConte, 1863

Harpalus impiger LeConte, 1854c: 79 [secondary homonym of *Harpalus impiger* (Duftschmid, 1812)]. Type locality: «Santa Fe, New Mexico; Frontera [New Mexico]» (original citation), restricted to «Santa Fe [Santa Fe County]» by Lindroth (1968: 767). Lectotype (♂), designated by Noonan (1991: 134), in MCZ [# 5884].
Harpalus retractus LeConte, 1863b: 13. Replacement name for *Harpalus impiger* LeConte, 1854.
Harpalus collucens Casey, 1914: 73. Type locality: «Fort Wingate [McKinley County], New Mexico» (original citation). Lectotype (♂), designated by Noonan (1991: 134), in USNM [# 47743]. Synonymy established by El-Moursy (1959: 41).
Harpalus acomanus Casey, 1914: 73. Type locality: «Jemez Springs [Sandoval County], New Mexico» (original citation). Twelve syntypes [12 originally cited] in USNM [# 47744]. Synonymy established by El-Moursy (1959: 41).
Harpalus rectangulus Casey, 1914: 74. Type locality: «Arizona» (original citation). Holotype [by monotypy] (♂) in USNM [# 47745]. Synonymy established by El-Moursy (1959: 41).

#### Distribution.

This species is restricted to mountains in “Utah” (Noonan 1991: 135), Arizona, New Mexico, and southern Colorado [see Noonan 1991: Fig. 285]. The record from “Mexico” (Csiki 1932a: 1186) is probably in error.

#### Records.

**USA**: AZ, CO, NM, UT

### Subgenus Opadius

Casey, 1914

Opadius Casey, 1914: 66. Type species: *Cratognathus cordatus* LeConte, 1853 by original designation. Etymology. Possibly from the Greek *opados* (accompanying, attending) [masculine].
Pharalus Casey, 1914: 68. Type species: *Pangus testaceus* LeConte, 1853 (= *Harpalus indianus* Csiki, 1932) by monotypy. **New synonymy**. Etymology. Anagram of the generic name *Harpalus* [*q.v*.] [masculine].
Euharpalops Casey, 1924: 116. Type species: *Euharpalops wadei* Casey, 1924 (= *Harpalus fraternus* LeConte, 1852) by original designation. **New synonymy**. Etymology. From the Greek *eu* (good, agreeable, very), the generic name *Harpalus* [*q.v*.], and the suffix -*ops* (having the appearance of) [masculine].
Haploharpalus Schauberger, 1926: 44. Type species: *Harpalus froelichii* Sturm, 1818 designated by Habu (1973a: 68). **New synonymy**. Etymology. From the Greek prefix *haplo*- (single) and the generic name *Harpalus* [*q.v*.] [masculine].
Cordoharpalus Hatch, 1949a: 87. Type species: *Harpalus cordifer* Notman, 1919 by original designation. Synonymy established by Bousquet and Larochelle (1993: 234). Etymology. From the Latin *cordis* (heart) and the generic name *Harpalus* [*q.v*.] [masculine].

#### Diversity.

This group is represented in the Nearctic and Palaearctic Regions but the number of species cannot be assessed at this time. The North American fauna has 19 species.

#### Identification.

The species of this subgenus have been revised by Noonan (1991: 45-127). Lindroth (1968) covered all the species except *Harpalus gravis*, *Harpalus cordatus*, and *Harpalus apache*.

#### Taxonomic Note.

This taxon corresponds to the *fraternus* stock of Noonan (1991: 45). Although I have doubt about monophyly of this complex, I have followed Noonan’s (1991) conclusion since it is the last major study on these species. Kataev et al. (2003: 371) listed *Opadius* Casey and its synonyms as synonyms of the subgenus *Harpalus* Latreille. In 2010, Kataev included *Harpalus apache* and *Harpalus cordatus* (as *cordatus* group) in the subgenus *Glanodes* Casey.

### [cordatus group]

### Harpalus apache

Kataev, 2010

Harpalus apache Kataev, 2010: 829. Type locality: «near Santa Rosa, Guadalupe Co[unty], N[ew] M[exico]» (original citation). Holotype (♂) in FMNH.

#### Distribution.

This species is known only from the type locality in east-central New Mexico.

#### Records.

**USA**: NM

### Harpalus cordatus

(LeConte, 1853)

Cratognathus cordatus LeConte, 1853c: 381. Type locality: «New Mexico» (original citation), herein restricted to Albuquerque, Bernalillo County (see Noonan 1991: 99). Lectotype (♀), designated by Noonan (1991: 95), in MCZ [# 5913].
Harpalus tadorcus Ball, 1972: 185. Replacement name for *Harpalus cordatus* (LeConte, 1853).

#### Distribution.

This species is restricted to arid grasslands in eastern Arizona, southern Colorado, and New Mexico [see Noonan 1991: Fig. 276].

#### Records.

**USA**: AZ, CO, NM

#### Note.

Ball (1972: 185) proposed *Harpalus tadorcus* to replace *Harpalus cordatus* (LeConte, 1853), a junior secondary homonym of *Harpalus cordatus* (Duftschmid, 1812), both species being then placed in the genus *Harpalus*. Since Duftschmid’s species is currently included in the genus *Ophonus* and the replacement name was proposed after 1960, the original species-group name is to be reinstated (ICZN 1999: Article 59.4).

### Harpalus cordifer

Notman, 1919

Harpalus cordifer Notman, 1919b: 235. Type locality: «Br[itish] Col[umbia]» (original citation), herein restricted to Mission City (CNC). Lectotype (♂), designated by Noonan (1991: 93), in SIM.
Harpalus washingtoniensis Van Dyke, 1926a: 123. Type locality: «Port Angeles [Clallam County], Washington» (original citation). Holotype (♂) in CAS [# 1865]. Synonymy established by Hatch (1949a: 87), confirmed by Lindroth (1968: 786).

#### Distribution.

This species ranges from the Alexander Archipelago and northwestern British Columbia south to southwestern Oregon, east to eastern Washington [see Noonan 1991: Fig. 276].

#### Records.

**CAN**: BC (VCI) **USA**: AK, OR, WA

### [desertus group]

### Harpalus desertus

LeConte, 1859

Harpalus desertus LeConte, 1859c: 3. Type locality: «near Santa Fé [Santa Fe County, New Mexico]» (original citation as deduced from page vi). Holotype [by monotypy] (♀) in MCZ [# 5907].
Harpalus furtivus LeConte, 1865b: 103. Type locality: «Colorado Territory» (original citation), restricted to «Colorado» by Noonan (1991: 116). Holotype [by monotypy] (♂) in MCZ [# 5905]. Synonymy established by Noonan (1991: 116).
Harpalus lucidus LeConte, 1865b: 104 [primary homonym of *Harpalus lucidus* Morawitz, 1863]. Type locality: «Nebraska [Territory], near the Rocky Mountains [probably in present day Colorado]» (original citation). Holotype [by monotypy] (♂) in MCZ [# 5908]. Synonymy established by Lindroth (1968: 810).
Harpalus clandestinus LeConte, 1878a: 450. Type locality: «[Fort] Garland (8,000 feet) [Costilla County], Col[orado]» (original citation). Holotype [by monotypy] (♂) in MCZ [# 3904]. Synonymy established by Noonan (1991: 116).
Harpalus lustrans Casey, 1884a: 64. Replacement name for *Harpalus lucidus* LeConte, 1865.
Harpalus probatus Casey, 1914: 119. Type locality: «Jemez Springs [Sandoval County], New Mexico» (original citation for the lectotype). Lectotype (♂), designated by Noonan (1991: 117), in USNM [# 47849]. Synonymy established by Noonan (1991: 117).
Harpalus nitescans Casey, 1914: 119. Type locality: «Socorro Co[unty], New Mexico» (original citation). Lectotype (♂), designated by Noonan (1991: 116), in USNM [# 47847]. Synonymy established by Noonan (1991: 117).
Harpalus clientus Casey, 1914: 120. Type locality: «Socorro Co[unty], New Mexico» (original citation). Lectotype (♀), designated by Noonan (1991: 116), in USNM [# 47850]. Synonymy established by Noonan (1991: 116).
Harpalus malacus Casey, 1914: 121. Type locality: «New Mexico» (original citation). Holotype [by monotypy] (♂) in USNM [# 47848]. Synonymy established by Noonan (1991: 116).
Harpalus illectus Casey, 1914: 121. Type locality: «Virgin River [Clark County], Utah» (original citation). Holotype [by monotypy] (♂) in USNM [# 47852]. Synonymy established by Noonan (1991: 116).
Harpalus nugax Casey, 1914: 122. Type locality: «New Mexico» (original citation). Lectotype (♀), designated by Noonan (1991: 117), in USNM [# 47851]. Synonymy established by Noonan (1991: 117).
Harpalus vacivus Casey, 1914: 123. Type locality: «Jemez Springs [Sandoval County], New Mexico» (original citation). Lectotype (♂), designated by Lindroth (1975: 140), in USNM [# 47853]. Synonymy established by Lindroth (1968: 810).
Harpalus socors Casey, 1914: 124. Type locality: «Arizona» (original citation). Lectotype (♂), designated by Noonan (1991: 117), in USNM [# 47854]. Synonymy established by Noonan (1991: 117).
Harpalus curticornis Casey, 1914: 124. Type locality: «Salida [Chaffee County], Colorado» (original citation). Lectotype (♂), designated by Noonan (1991: 116), in USNM [# 47853]. Synonymy established by Noonan (1991: 116).
Harpalus cyrtonotoides Notman, 1919b: 234 [*nomen dubium*]. Type locality: «Col[orado]» (original citation). Syntype(s) [2 ♂ originally cited] location unknown (originally in collection C.W. Leng). **New synonymy**. Note. *Harpalus cyrtonotoides* is here considered a doubtful synonym of *Harpalus desertus* LeConte based on comment made by Ball and Bousquet (2000: 98).
Harpalus maxwelli Casey, 1924: 108. Type locality: «Maxwell [Colfax County], New Mexico» (original citation). Holotype [by monotypy] (♂) in USNM [# 47856]. Synonymy established by Noonan (1991: 117).
Harpalus dulciculus Casey, 1924: 109. Type locality: «Akron [Washington County], Colorado» (original citation). Lectotype (♂), designated by Lindroth (1975: 140), in USNM [# 47830]. Synonymy established by Lindroth (1968: 810).
Harpalus captiosus Casey, 1924: 110. Type locality: «Akron [Washington County], Colorado» (original citation). Holotype [by monotypy] (♀) in USNM [# 47831]. Synonymy established by Lindroth (1968: 810).
Harpalus metuens Casey, 1924: 110. Type locality: «Arizona» (original citation for the lectotype). Lectotype (♀), designated by Lindroth (1975: 140), in USNM [# 47829]. Synonymy established by Lindroth (1968: 810).
Harpalus maxwellensis Csiki, 1932a: 1183. Unjustified emendation of *Harpalus maxwelli* Casey, 1924.
Harpalus couleensis Hatch, 1949a: 87. Type locality: «Upper Grand Coulee [Grant County, Washington]» (original citation). Holotype (♂) in USNM. Synonymy established, under the name *Harpalus lustrans* Casey, by Hatch (1953: 171), confirmed by Lindroth (1968: 810).

#### Distribution.

This western species occurs from south-central British Columbia to western Minnesota (Gandhi et al. 2005: 930), south to east-central and westernmost Texas, southern Arizona, and northern California [see Noonan 1991: Figs 279-282].

#### Records.

**CAN**: AB, BC, SK **USA**: AZ, CA, CO, ID, KS, MN, MT, ND, NE, NM, NV, OK, OR, SD, TX, UT, WA, WY

#### Note.

Noonan (1991: 119-124) recognized two morphs within this species, the *desertus* and *furtivus* morphs, that Lindroth (1968: 809) regarded as distinct species. Noonan (1991: 124) indicated that intergradation between the two morphs occurs throughout the areas in which the *furtivus* morph occurs.

### Harpalus gravis

LeConte, 1858

Harpalus gravis LeConte, 1858b: 60. Type locality: «San Antonio [Bexar County], Texas» (original citation). Holotype [by monotypy] (♂) in MCZ [# 5911].

#### Distribution.

This species is found along the Coastal Plain and Piedmont Plateau from southeastern New York and New Jersey to northern Florida, west to southeastern Oklahoma and southeastern Texas [see Noonan 1991: Fig. 270]. The record from southwestern Ohio (Blatchley 1910: 186) is probably in error.

#### Records.

**USA**: AL, AR, FL, GA, LA, MO, MS, NC, NJ, NY, OK, SC, TX, VA

### Harpalus indianus

Csiki, 1932

Pangus testaceus LeConte, 1853c: 385 [secondary homonym of *Harpalus testaceus* Haldeman, 1843]. Type locality: «Illinois» (original citation), herein restricted to Chicago, Cook County (see Noonan 1991: 115). Lectotype (♀), designated by Noonan (1991: 113), in MCZ [# 5910]. Note. The lectotype designated by Noonan (1991: 113) is labeled “Io[w]a” and is probably not a syntype. One specimen with a yellow disc (which includes Illinois) in the LeConte collection (MCZ) may be one of the two syntypes cited originally by LeConte (1853c: 385).
Harpalus indianus Csiki, 1932a: 1186. Replacement name for *Harpalus testaceus* (LeConte, 1853).

#### Distribution.

This species ranges from southernmost Ontario (Bousquet 1987a: 130) to southeastern South Dakota (Kirk and Balsbaugh 1975: 28), south to southeastern Kansas (Noonan 1991: 115) and northern Mississippi (Drew A. Hildebrandt pers. comm. 2007). Two old specimens simply labeled from Louisiana and Florida are known (Noonan 1991: 115).

#### Records.

**CAN**: ON **USA**: AR, IA, IL, IN, KS, MI, MO, MS, NE, OH, SD, TN, WI [FL, LA]

### [laticeps group]

### Harpalus animosus

Casey, 1924

Harpalus montanus LeConte, 1865b: 102 [primary homonym of *Harpalus montanus* Sturm, 1818]. Type locality: «Colorado Territory» (original citation), restricted to «Colorado» by Noonan (1991: 46). Lectotype (♂), designated by Noonan (1991: 46), in MCZ [# 5898].
Harpalus animosus Casey, 1924: 101. Type locality: «Miner’s Peak [Iron County], Utah» (original citation). Lectotype (♀), designated by Lindroth (1975: 138), in USNM [# 47783]. Synonymy established by Lindroth (1968: 774).
Harpalus montuosus Csiki, 1932a: 1183. Replacement name for *Harpalus montanus* LeConte, 1865.

#### Distribution.

This western species occurs from northwestern British Columbia to southwestern Saskatchewan, south to north-central New Mexico and northern Arizona in the Rocky Mountains and to northern Oregon along the coast [see Noonan 1991: Fig. 267]. The record from south-central South Dakota (Kirk and Balsbaugh 1975: 29, as *Harpalus montuosus*) needs confirmation.

#### Records.

**CAN**: AB, BC (VCI), SK **USA**: AZ, CO, ID, MT, NM, NV, OR, UT, WA, WY [SD]

### Harpalus laticeps

LeConte, 1850

Harpalus laticeps LeConte, 1850: 208. Type locality: Lake Superior (inferred from title of the paper), herein restricted to Marquette, Marquette County, Michigan (see Noonan 1991: 56). Lectotype (♂), designated by Noonan (1991: 53), in MCZ [# 5900].
Harpalus solutus Casey, 1914: 90. Type locality: «New Hampshire» (original citation). Lectotype (♀), designated by Lindroth (1975: 138), in USNM [# 47784]. Synonymy established by Lindroth (1968: 774).

#### Distribution.

This species is transcontinental in the north ranging from Newfoundland (Lindroth 1955a: 139; probably only as strays) to western Yukon Territory, south to northern Oregon along the west coast, southeastern Arizona and central New Mexico in the Rocky Mountains, the Black Hills in western South Dakota, northern Illinois, and southwestern Pennsylvania (Noonan 1991: 56) in the east [see Noonan 1991: Fig. 268]. The records from Connecticut (Britton 1920: 218; see Krinsky and Oliver 2001: 5), Indiana (Blatchley 1910: 186), and southwestern Oklahoma (Kondratieff et al. 2005: 172) need confirmation.

#### Records.

**CAN**: AB, BC, LB, MB, NB, NF, NS (CBI), NT, ON, PE, QC, SK, YT **USA**: AK, AZ, CO, ID, IL, MA, ME, MI, MN, MT, ND, NH, NM, NV, NY, OR, PA, SD, VT, WA, WI, WY [CT, IN, OK]

### Harpalus providens

Casey, 1914

Harpalus viduus LeConte, 1865b: 103 [primary homonym of *Harpalus viduus* LeConte, 1859]. Type locality: «Rock Island [Rock Island County], Illinois» (original citation). Holotype [by monotypy] (♀) in MCZ [# 5901].
Harpalus providens Casey, 1914: 90. Type locality: «New Jersey» (original citation). Lectotype (♀), designated by Lindroth (1975: 138), in USNM [# 472785]. Synonymy established by Lindroth (1968: 775).

#### Distribution.

This eastern species ranges from southern Quebec to northern Minnesota, south to central Missouri and southwestern Virginia (Hoffman and Roble 2000: 38) [see Noonan 1991: Fig. 267]; also known from southwestern South Dakota (Larsen and Purrington 2010: 571). An old specimen simply labeled from Kansas and one labeled from Alabama are known (Noonan 1991: 51).

#### Records.

**CAN**: ON, QC **USA**: CT, DC, IA, IL, IN, KY, MA, MD, ME, MI, MN, MO, NE, NH, NJ, NY, OH, PA, RI, SD, VA, VT, WI, WV [AL, KS]

### [nigritarsis group]

### Harpalus megacephalus

LeConte, 1847

Harpalus megacephalus LeConte, 1847: 397. Type locality: «Lacum Superiorem» (original citation), herein restricted to Marquette, Marquette County, Michigan (see Hubbard and Schwarz 1878: 629). Lectotype (♂), designated by Noonan (1991: 108), in MCZ [# 5888].

#### Distribution.

This species ranges from Maine (Piscataquis County, CNC) and northern New Brunswick (Webster and Bousquet 2008: 20) west to Manitoba and Minnesota (Lindroth 1968: 785), south to the Black Hills in western South Dakota (CMNH). The record from the Ungava Bay in Labrador (Sherman 1910: 181) is probably in error.

#### Records.

**CAN**: MB, NB, ON, QC **USA**: ME, MI, MN, SD, WI

#### Note.

This species is externally very similar to *Harpalus fulvilabris* and Lindroth (1968: 783) included these two species in one species group. However, Noonan (1991) listed the two species in different species groups suggesting that the similarities are probably due to convergent evolution.

### Harpalus nigritarsis

Sahlberg, 1827

Harpalus nigritarsis C.R. Sahlberg, 1827b: 237. Type locality: «Lapponia» (original citation). Lectotype (♂), designated by Noonan (1991: 100), in ZMH.
Harpalus femoralis Motschulsky, 1844: 215 [primary homonym of *Harpalus femoralis* Stephens, 1828]. Type locality: «montagnes du Hamar-Daban au sud du Baïcal [Irkutsk Oblast, Russia]» (original citation). Lectotype (♂), designated by Kataev and Shilenkov (in Kryzhanovskij et al. 1995: 145), in ZMMU. Synonymy established by Kataev and Shilenkov (in Kryzhanovskij et al. 1995: 145).
Harpalus proximus LeConte, 1847: 398. Type locality: «Lacum Superiorem» (original citation). Lectotype (♀), designated by Noonan (1991: 100), in MCZ [# 5891]. Synonymy established by Lindroth (1968: 795).
Harpalus curtatus Mannerheim, 1853: 124. Type locality: «ad sinum Woskresensk [= Resurrection Bay] peninsulae Kenai [Alaska]» (original citation). Lectotype (♂), designated by Noonan (1991: 100), in MHNP. Synonymy established by Lindroth (1968: 795).
Harpalus recensus Casey, 1914: 99. Type locality: «W[est] S[ain]t Modest[e], Labrador» (original citation for the lectotype). Lectotype (♂), designated by Lindroth (1975: 139), in USNM [# 47798]. Synonymy established, under the name *Harpalus nigritarsis proximus* LeConte, by Lindroth (1954b: 141).
Harpalus mansuetus Casey, 1914: 104. Type locality: «Tallac [El Dorado County], California» (original citation). Lectotype (♂), designated by Noonan (1991: 100), in USNM [# 47810]. Synonymy established by Noonan (1991: 100).
Harpalus seclusus Casey, 1914: 106. Type locality: «Colorado» (original citation). Lectotype (♂), designated by Lindroth (1975: 139), in USNM [# 47813]. Synonymy established by Noonan (1991: 100).
Harpalus opicus Casey, 1914: 106. Type locality: «Placer Co[unty], California» (original citation). Lectotype (♀), designated by Lindroth (1975: 139), in USNM [# 47808]. Synonymy established by Noonan (1991: 100).
Harpalus fanaticus Casey, 1924: 102. Type locality: «The Mammoth, summit of Parowan M[oun]t[ain]s (10000 ft.), Utah» (original citation). Lectotype (♀), designated by Noonan (1991: 101), in USNM [# 47786]. Synonymy established by Noonan (1991: 101).
Harpalus parowanus Casey, 1924: 105. Type locality: «The Mammoth, Parowan M[oun]t[ain]s (10000 feet), Utah» (original citation). Lectotype (♂), designated by Lindroth (1975: 139), in USNM [# 47815]. Synonymy established, under the name *Harpalus seclusus* Casey, by Lindroth (1968: 797).
Harpalus sibiricus Csiki, 1932a: 1141. Replacement name for *Harpalus femoralis* Motschulsky, 1844.

#### Distribution.

This Holarctic species ranges in the Nearctic Region from Newfoundland to the Alaskan coast, south to the Sierra Nevada in California, southern Arizona, southeastern Nebraska, and New Hampshire and New York [see Noonan 1991: Figs 277 and 278]. The species is known only from a few scattered localities in the prairies. The record from southern Indiana (Wiedenmann et al. 1992: 286) needs confirmation. In the Palaearctic Region, the species is known from Sweden to the Far East and the Altai in Kazakhstan (Kataev et al. 2003: 379).

#### Records.

**FRA**: PM **CAN**: AB, BC (VCI), LB, MB, NB, NF, NS (CBI), NT, ON, QC, YT **USA**: AK, AZ, CA, CO, ID, MI, MN, MT, NE, NH, NM, NV, NY, OR, SD, UT, WA, WI, WY [IN] – **Holarctic**

#### Note.

Noonan (1991: 102-104) recognized two morphs in this species, the *nigritarsis* and *seclusus* morphs, which Lindroth (1968) regarded as distinct species. Noonan stated that a broad zone of intergradation between the two morphs occurs in the northern part of the Rocky Mountains and adjacent mountains and in lowland areas of northwestern United States, western Canada, and Alaska.

### [spadiceus group]

### Harpalus fraternus

LeConte, 1852

Harpalus fraternus LeConte, 1852a: 185. Type locality: «Oregon» (original citation), herein restricted to Moro, Sherman County (see Casey 1924: 116, as *Euharpalops wadei*). Holotype [by monotypy; designated lectotype by Noonan 1991: 70] (♂) in MCZ [# 96].
Harpalus oblitus LeConte, 1859c: 2 [primary homonym of *Harpalus oblitus* Dejean, 1829]. Type locality: «Santa Fé [Santa Fe County, New Mexico]» (original citation). Holotype [by monotypy] (♂) in MCZ [# 5903]. Synonymy established by LeConte (1861b: 339), confirmed by Noonan (1991: 70).
Harpalus occidentalis Chaudoir, 1868b: 168. Type locality: «Vancouver [British Columbia]» (original citation). Holotype [by monotypy] (♂) in MHNP. Synonymy established by Horn (1875: 126), confirmed by Noonan (1991: 70).
Harpalus lecontei Casey, 1914: 117. Replacement name for *Harpalus oblitus* LeConte, 1859.
Harpalus fraternus nimius Casey, 1924: 100. Type locality: «Columbia River [Clatsop County], Oregon» (original citation). Holotype [by monotypy] (♂) in USNM [# 47781]. Synonymy established by Hatch (1953: 172), confirmed by Noonan (1991: 71).
Harpalus praestans Casey, 1924: 101. Type locality: «Provo Cañon [Utah County], Utah» (original citation). Lectotype (♀), designated by Lindroth (1975: 138), in USNM [# 47782]. Synonymy established by Lindroth (1968: 771).
Euharpalops wadei Casey, 1924: 116. Type locality: «Moro [Sherman County], Oregon» (original citation). Lectotype (♂), designated by Lindroth (1975: 138), in USNM [# 47746]. Synonymy established by Hatch (1953: 172), confirmed by Noonan (1991: 71).
Harpalus hoppingi Lindroth, 1968: 777. Type locality: «Vernon, B[ritish] C[olumbia]» (original citation). Holotype (♂) in CAS [# 9860]. Synonymy established by Noonan (1991: 71). Etymology. The specific name was proposed for George Redstone Hopping [1899-1974], entomologist for the Federal Government in Vernon and later in Calgary. Hopping published many papers on forest entomology and management of stands subject to insect attack but also on taxonomy of various groups of beetles including Cerambycidae and Scolytinae.

#### Distribution.

This western species occurs from Vancouver Island to southeastern Manitoba (Roughley et al. 2010: 230), north to northeastern British Columbia, south to western Texas, southern Arizona, and the White Mountains in California, east to eastern Nebraska [see Noonan 1991: Fig. 271].

#### Records.

**CAN**: AB, BC (VCI), MB, SK **USA**: AZ, CA, CO, ID, KS, MT, NE, NM, NV, OR, SD, TX, UT, WA, WY

#### Note.

Noonan (1991: 75) recognized two forms within this species, a southern one, known as *Harpalus lecontei*, that Lindroth (1968) treated as a distinct species, and a more northern one which corresponds to *Harpalus fraternus* LeConte *sensu* Lindroth.

### Harpalus fulvilabris

Mannerheim, 1853

Harpalus fulvilabris Mannerheim, 1853: 123. Type locality: «in ora orientali insulae Kadjak [Alaska]» (original citation). Lectotype (♂), designated by Lindroth (1968: 783), in ZMH [# 2348].
Harpalus subaeneus Mannerheim, 1853: 123. Type locality: «in ora orientali insulae Kadjak [Alaska]» (original citation). Syntype(s) probably in ZMH. **New synonymy**. Note. This taxon was described by Mannerheim (1853: 123) as “var[iety] b” of *Harpalus fulvilabris* Mannerheim, 1853.

#### Distribution.

This species is transcontinental in the north, ranging from Newfoundland to the Aleutian Islands in Alaska, south to the Lillooet Land District in British Columbia, north-central New Mexico along the Rocky Mountains, the Black Hills in western South Dakota, and New England in the east [see Noonan 1991: Fig. 276].

#### Records.

**CAN**: AB, BC, LB, MB, NB, NF, NS (CBI), NT, ON, PE, QC, SK, YT **USA**: AK, CO, ME, MI, MN, NH, NM, NY, SD, VT, WI

### Harpalus indigens

Casey, 1924

Harpalus indigens Casey, 1924: 114. Type locality: «Monmouth [Kennebec County], Maine» (original citation). Holotype [by monotypy] (♂) in USNM [# 47863].
Opadius piperi Casey, 1924: 93. Type locality: «Grayline, near Bay City [Bay County], Michigan» (original citation). Lectotype (♀), designated by Lindroth (1975: 139), in USNM [# 47740]. Synonymy established by Lindroth (1968: 782).
Harpalus beatulus Casey, 1924: 114. Type locality: «Marquette [Marquette County], Michigan» (original citation). Holotype [by monotypy] (♂) in USNM [# 47862]. Synonymy established by Lindroth (1968: 782).

#### Distribution.

This species ranges from the Nova Scotia Peninsula (Christopher G. Majka pers. comm. 2008) to southern South Dakota (Kirk and Balsbaugh 1975: 30), north to southeastern Manitoba (Roughley et al. 2010: 230), south to Nebraska and southern Pennsylvania (Noonan 1991: 85; Fig. 272).

#### Records.

**CAN**: MB, NB, NS, ON, QC **USA**: CT, IA, IL, MA, ME, MI, NE, NH, NJ, NY, PA, SD, VT, WI

### Harpalus laevipes

Zetterstedt, 1828

Harpalus laevipes Zetterstedt, 1828: 26. Type locality: «Juckasjervi Lapponiae Tornensis [Sweden]; Bossekop [= Bossogohppi, Finnmark, Norway] prope Altengaard Finmarkiae» (original citation). Four syntypes in ZMLS (Lindroth 1938: 18).
Harpalus quadripunctatus Dejean, 1829: 326. Type locality: «Styrie [Austria]» (original citation for the lectotype). Lectotype (♂), designated by Noonan (1991: 58), in MHNP. Synonymy established by Schaum (1860: 596).
Harpalus impressipennis Motschulsky, 1844: 213 [primary homonym of *Harpalus impressipennis* Dejean, 1829]. Type locality: «Tourkinsk [= Turka] sur le bord oriental du [Lac] Baïcal» (original citation). Three syntypes in ZMMU (Keleinikova 1976: 200). Synonymy established by Kryzhanovskij et al. (1995: 142).
Harpalus rufimanus LeConte, 1847: 402 [secondary homonym of *Harpalus rufimanus* (Marsham, 1802)]. Type locality: «Lacum Superiorem» (original citation). Lectotype (♂), designated by Noonan (1991: 58), in MCZ [# 5897]. Synonymy established, under the name *Harpalus egregius* Casey, by Lindroth (1968: 776).
Harpalus alienus LeConte, 1879d: 508 [primary homonym of *Harpalus alienus* Bates, 1878]. Type locality: «[La] Veta Pass [Costilla County, Colorado]» (original citation). Holotype [by monotypy] (♂) in MCZ [# 5912]. Synonymy established, under the name *Harpalus egregius* Casey, by Lindroth (1968: 776).
Harpalus sachalinensis Matsumura, 1911: 110. Type locality: «Kusunnai [Sakhalin, Russia]» (original citation). Holotype (♀) location unknown. Synonymy established by Kataev (in Kryzhanovskij et al. 1995: 142).
Harpalus pimalicus Casey, 1914: 87. Type locality: «base of Humphrey’s Peak [Coconino County], Arizona» (original citation). Lectotype (♂), designated by Noonan (1991: 58), in USNM [# 47774]. Synonymy established, under the name *Harpalus quadripunctatus* Dejean, by Noonan (1991: 58).
Harpalus egregius Casey, 1914: 88. Replacement name for *Harpalus alienus* LeConte, 1879.
Harpalus instructus Casey, 1924: 107. Type locality: «Edmonton, Alberta» (original citation). Lectotype (♂), designated by Lindroth (1975: 138), in USNM [# 47833]. Synonymy established, under the name *Harpalus egregius* Casey, by Lindroth (1968: 776).
Harpalus motschoulskyanus Schauberger, 1928: 80. Replacement name for *Harpalus impressipennis* Motschulsky, 1844.
Harpalus baergi Csiki, 1932a: 1180. Replacement name for *Harpalus rufimanus* LeConte, 1847.
Harpalus cascadiensis Hatch, 1949a: 85. Type locality: «Van Horn, Skagit Co[unty], Wash[ington]» (original citation). Holotype (♂) in USNM. Synonymy established, under the name *Harpalus egregius* Casey, by Lindroth (1968: 776).

#### Distribution.

This Holarctic species ranges in North America from Newfoundland to southeastern Alaska (Lindroth 1968: 777), south to west-central Oregon, southern Nevada, southeastern Arizona and south-central New Mexico along the Rocky Mountains, the lower peninsula of Michigan, and southern New York [see Noonan 1991: Fig. 269]. The records from southern Indiana (Wiedenmann et al. 1992: 282, as *Harpalus egregius*) and “Iowa” (Jaques and Redlinger 1946: 297, as *Harpalus rufimanus*) need confirmation.

#### Records.

**CAN**: AB, BC (VCI), LB, MB, NB, NF, NS (CBI), NT, ON, PE, QC, SK, YT **USA**: AK, AZ, CO, ME, MI, MN, MT, NH, NM, NV, NY, OR, UT, WA, WI [IA, IN] – **Holarctic**

### Harpalus lewisii

LeConte, 1865

Harpalus lewisii LeConte, 1865b: 103. Type locality: «Marquette [Marquette County, Michigan], Lake Superior» (original citation for the lectotype). Lectotype (♂), designated by Noonan (1991: 62), in MCZ [# 5899]. Etymology. The specific name was proposed for Samuel Lewis, a physician from Philadelphia who had an interest in North American Coleoptera. Lewis provided LeConte and other coleopterists with fine specimens from many parts of the continent.
Harpalus aesopus Casey, 1914: 117. Type locality: «Plattsburg [Clinton County], New York» (original citation). Lectotype (♀), designated by Lindroth (1975: 138), in USNM [# 47780]. Synonymy established by Casey (1924: 101), confirmed by Lindroth (1968: 773).

#### Distribution.

This species ranges from Nova Scotia to central Alberta and southeastern Northwest Territories, south to southern Wisconsin and Connecticut [see Noonan 1991: Fig. 270]. All known sites in the United States are east of the Mississippi.

#### Records.

**CAN**: AB, MB, NB, NS, NT, ON, QC, SK **USA**: CT, IN, MA, ME, MI, MN, ND, NH, NY, VT, WI

### Harpalus reversus

Casey, 1924

Harpalus funestus LeConte, 1847: 402 [primary homonym of *Harpalus funestus* Audinet-Serville, 1821]. Type locality: «prope Long’s Peak [Boulder County, Colorado], Rocky Mountains» (original citation). Lectotype (♂), designated by Noonan (1991: 66), in MCZ [# 5902].
Harpalus reversus Casey, 1924: 103. Type locality: «Marquette [Marquette County], Michigan» (original citation). Lectotype (♀), designated by Lindroth (1975: 138), in USNM [# 47794]. Synonymy established by Lindroth (1968: 779).
Harpalus funerarius Csiki, 1932a: 1182. Replacement name for *Harpalus funestus* LeConte, 1847.

#### Distribution.

With the exception of two specimens labeled from “British Columbia” and “Victoria” on Vancouver Island (Noonan 1991: 70), this species occurs east of the Rocky Mountains from central Alberta to the Quebec City area (Larochelle 1975: 87, map 302), south to Massachusetts, northwestern Pennsylvania (Erie County, Robert L. Davidson pers. comm. 2012), southern Kansas, central New Mexico, and southeastern Arizona [see Noonan 1991: Fig. 271]. This species is much more abundant in the prairies of central North America and both Lindroth (1968: 779; 1971: 1456, 1457) and Noonan (1991: 69) concluded that the species was originally an inhabitant of the prairies that expanded its range eastwards after humans cut down forests in the east.

#### Records.

**CAN**: AB, MB, ON, QC, SK **USA**: AZ, CO, IA, KS, MA, ME, MI, MN, MT, ND, NE, NH, NM, NY, PA, SD, VT, WI, WY [BC]

### Harpalus spadiceus

Dejean, 1829

Harpalus spadiceus Dejean, 1829: 336. Type locality: «Amérique septentrionale» (original citation), restricted to «White Sulphur Springs [Greenbrier County], W[est] Virg[ina]» by Lindroth (1968: 780). Holotype [by monotypy] (♀) in MHNP (Lindroth 1955b: 27).
Harpalus comis Haldeman, 1843b: 301 [*nomen dubium*]. Type locality: southeastern Pennsylvania (Haldeman 1843a: 297). Syntype(s) presumably lost. Synonymy established with doubt by LeConte (1863b: 13).
Harpalus carolinae Schaeffer, 1910: 402. Type locality: «Black Mountains, North Carolina» (original citation). Lectotype (♂), designated by Erwin and House (1978: 238), in USNM [# 42509]. Synonymy established by Noonan (1991: 86).

#### Distribution.

This eastern species ranges from southern Quebec, southern Ontario, and the New England states south through the Appalachian mountains to northern Georgia [see Noonan 1991: Figs 273-275]. The record from southwestern Manitoba (Stjernberg 2011: 70) is probably in error.

#### Records.

**CAN**: ON, QC **USA**: CT, DC, DE, GA, KY, MA, NC, NH, NJ, NY, OH, PA, RI, SC, TN, VA, VT, WV

#### Note.

Noonan (1991: 87-88) recognized two morphs for this species, the *spadiceus* and *carolinae* morphs, that Lindroth (1968: 780-781) treated as distinct species. According to Noonan (1991: 88), the two morphs intergrade over an area extending southwards from Virginia to Georgia.

### Harpalus ventralis

LeConte, 1847

Harpalus ventralis LeConte, 1847: 399. Type locality: «prope Long’s Peak [Boulder County, Colorado]» (original citation). Lectotype (♀), designated by Noonan (1991: 80), in MCZ [# 5892].
Harpalus electus Casey, 1924: 115. Type locality: «Edmonton, Alberta» (original citation). Holotype [by monotypy] (♀) in USNM [# 47861]. Synonymy established by Lindroth (1968: 781).

#### Distribution.

This species ranges from central Alberta to southeastern Manitoba (Roughley et al. 2010: 230), south to western Kansas, northern New Mexico, and northeastern Utah [see Noonan 1991: Fig. 272].

#### Records.

**CAN**: AB, MB, SK **USA**: CO, KS, MN, MT, ND, NE, NM, SD, UT, WY

### Subgenus Harpalus

Latreille, 1802

Harpalus Latreille, 1802: 92. Type species: *Carabus proteus* Paykull, 1790 (= *Carabus affinis* Schrank, 1781) designated by Andrewes (1935: 19).
Proteonus Fischer von Waldheim, 1829a: 21. Type species: *Carabus distinguendus* Duftschmid, 1812 designated by Bousquet (2002c: 178). Synonymy established by Bousquet (2002c: 178).
Amblystus Motschulsky, 1864: 209. Type species: *Carabus rubripes* Duftschmid, 1812 by original designation. Synonymy established by Tschitschérine (1901: 240).
Harpalomerus Casey, 1914: 76. Type species: *Harpalus amputatus* Say, 1830 designated by Lindroth (1968: 769). Synonymy established by Kataev et al. (2003: 371). Etymology. From the generic name *Harpalus* [*q.v*.] and the Greek *meros* (part, segment) [masculine].

#### Diversity.

This subgenus includes native species in North America, Mexico, and the Palaearctic and Afrotropical Regions. The total number of species cannot be determined at this time. The North American fauna is represented by 17 species, of which two are adventive.

#### Identification.

Noonan (1991) revised all the North American species except the two adventive ones but his key (pages 20-45) included all 17 species. Lindroth (1968) covered all but three (*Harpalus balli*, *Harpalus martini*, and *Harpalus rubripes*) species in his monograph of the Canadian Carabidae.

### [affinis group]

### Harpalus affinis

(Schrank, 1781)

Carabus aeneus Fabricius, 1775: 245 [primary homonym of *Carabus aeneus* DeGeer, 1774]. Type locality: «Lipsia [= Leipzig, Saxony, Germany]» (original citation). Lectotype (♂), designated by Lindroth (1968: 768), in ZMUC.
Carabus affinis Schrank, 1781: 212. Type locality: Austria (inferred from title of the book). Syntype(s) probably lost (Lindroth 1968: 768). Synonymy established by Schönherr (1806: 204).
Carabus proteus Paykull, 1790: 115. Type locality: «Svecia australi» (original citation). Syntype(s) probably in NRSS. Synonymy established by Fabricius (1792: 156).
Harpalus viridi-aeneus Palisot de Beauvois, 1811: 108. Type locality: «Pensylvanie» (original citation). Syntype(s) probably lost (Lindroth 1968: 768). Synonymy established by Hatch (1949a: 84).
Harpalus viridis Say, 1823a: 31. Type locality: «East Boston, Mass[achusetts]» (neotype label). Neotype (♂), designated by Lindroth and Freitag (1969: 352), in MCZ [# 32972]. Synonymy established by Brullé (1835c: 287).
Harpalus assimilis Dejean, 1829: 272. Type locality: «Amérique septentrionale» (original citation). One syntype in MHNP (Lindroth 1955b: 26). Synonymy established, under the name *Harpalus viridis* Say, by Say (1830c: 19), confirmed by Lindroth (1955b: 26).
Harpalus convictor Casey, 1884b: 12. Type locality: «Willets Point [Queens County], Long Island [New York]» (original citation). One syntype in USNM [# 47747]. Synonymy established, under the name *Harpalus viridiaeneus* Palisot de Beauvois, by Horn (1885b: 109), confirmed by Lindroth (1954b: 141).
Harpalus canonicus Casey, 1884b: 12. Type locality: «Rhode Island» (original citation). One syntype in USNM [# 47748]. Synonymy established, under the name *Harpalus viridiaeneus* Palisot de Beauvois, by Horn (1885b: 109), confirmed by Lindroth (1968: 768).
Harpalus lustralis Casey, 1884b: 12. Type locality: «New York State» (original citation). One syntype in USNM [# 47749]. Synonymy established, under the name *Harpalus viridiaeneus* Palisot de Beauvois, by Horn (1885b: 109), confirmed by Lindroth (1968: 768).
Harpalus aenescens Casey, 1884b: 12. Type locality: «Rhode Island; Willets Point, Long Island» (original citation). One syntype [4 originally cited] in USNM [# 47750]. Synonymy established, under the name *Harpalus viridiaeneus* Palisot de Beauvois, by Horn (1885b: 109), confirmed by Lindroth (1968: 768).

#### Distribution.

This Palaearctic species is adventive in North America where it is known in the east from Newfoundland (Lindroth 1955a: 137) to eastern Minnesota (Ramsey County, CMNH), south to “Kansas” (Lindroth 1955a: 137) and northern (Tucker and Preston Counties, CMNH) and eastern West Virginia (Pendleton County, Foster F. Purrington pers. comm. 2009). The record from southern Florida (Peck and Thomas 1998: 21) may represent a separate introduction. In the west, the species ranges from Alberta and British Columbia, including Vancouver Island (Lindroth 1968: 769), south to western Oregon (Clackamas, Linn, and Yamhill Counties, CMNH) and southwestern Idaho (Owyhee County, Ken Karns pers. comm. 2009); the species is also found in southeastern Arizona (Cochise County, CNC). The first inventoried specimen caught in the east was collected prior to 1798 in Pennsylvania (Palisot de Beauvois, 1811: 108, as *Harpalus viridiaeneus*). The species is also adventive in New Zealand since 1975 (Larochelle and Larivière 2005: 52).

#### Records.

**FRA**: PM **CAN**: AB, BC (VCI), LB, NB, NF, NS (CBI), ON, PE, QC **USA**: AZ, CT, DE, FL, IA, ID, IL, IN, KS, MA, MD, ME, MI, MN, MO, NH, NJ, NY, OH, OR, PA, RI, VA, VT, WA, WI, WV – **Adventive**

### Harpalus rubripes

(Duftschmid, 1812)

Carabus rubripes Duftschmid, 1812: 77. Type locality: «um Linz [Austria]» (original citation). Syntype(s) probably lost.
Harpalus rufipes Motschulsky, 1844: 212 [secondary homonym of *Harpalus rufipes* (DeGeer, 1774)]. Type locality: «Alpes du Caucase» (original citation). Lectotype (♀), designated by Kataev (1989: 200), in ZMMU. Synonymy established by Kataev (1989: 199).
Harpalus hyperboreus Motschulsky, 1844: 214. Type locality: «bords du fleuve Selenga à Tchertovkino [Siberia, Russia]» (original citation). Lectotype, designated by Kataev (1989: 200), in ZMMU. Synonymy established by Kataev (1989: 199).

#### Distribution.

This Palaearctic species is adventive in North America where it is known from Nova Scotia (Majka et al. 2006: 606) and Prince Edward Island (Majka et al. 2008: 132) to eastern Ontario (near Reids Mills, CNC), including southern Quebec (Chantal 1994: 29), south to eastern Pennsylvania (Davidson et al. 2011: 517). The first inventoried specimen collected on this continent was found in New Hampshire in 1981 (Bell and Davidson 1987: 56).

#### Records.

**CAN**: NB, NS, ON, PE, QC **USA**: CT, MA, ME, NH, NY, PA, RI, VT – **Adventive**

### [amputatus group]

### Harpalus amputatus amputatus

Say, 1830

Harpalus amputatus Say, 1830c: 19. Type locality: «La Junta, Bent Co[unty], Col[orado]» (neotype label), not «San Luis Valley, Colo[rado]» as specified by Lindroth and Freitag (1969: 352). Neotype (♂), designated by Lindroth and Freitag (1969: 352), in MCZ [# 32971]. Note. The type locality of *Harpalus amputatus* was discussed by Noonan (1991). «N[orth] W[est] Territory» was the area originally cited by Say (1830c: 19).
Harpalus rotundicollis Kirby, 1837: 44. Type locality: northern parts of British America (inferred from title of the book). Lectotype (♀), designated by Noonan (1991: 174), in BMNH. Synonymy established by Horn (1876e: 130), confirmed by Lindroth (1953b: 174).
Harpalus stephensii Kirby, 1837: 45. Type locality: «Lat. 54° [= along North Saskatchewan River]» (original citation). Holotype [by monotypy] (♂) in BMNH (Noonan 1991: 174). Synonymy established by LeConte (1847: 397), confirmed by Noonan (1991: 175).
Harpalus transversus Casey, 1914: 77. Type locality: near Santa Fé, Santa Fe County, New Mexico (lectotype label according to Lindroth 1975: 138). Lectotype (♀), designated by Lindroth (1975: 138), in USNM [# 47751]. Synonymy established by Lindroth (1968: 769).
Harpalus papagonalis Casey, 1914: 77. Type locality: «Arizona » (original citation). Holotype [by monotypy] (♀) in USNM [# 47753]. Synonymy established by Lindroth (1968: 769).
Harpalus bracatus Casey, 1924: 95. Type locality: «Miner’s Peak [Iron County], Utah» (original citation). Lectotype (♀), designated by Lindroth (1975: 138), in USNM [# 47752]. Synonymy established by Lindroth (1968: 769).
Harpalus ancillaris Casey, 1924: 95. Type locality: «Columbia River [Clatsop County], Oregon» (original citation). Lectotype (♂), designated by Lindroth (1975: 138), in USNM [# 47754]. Synonymy established by Hatch (1953: 170), confirmed by Noonan (1991: 175).
Harpalus cupreolatus Casey, 1924: 96. Type locality: «Pullman [Whitman County], Washington» (original citation). Lectotype (♂), designated by Lindroth (1975: 138), in USNM [# 47756]. Synonymy established by Hatch (1953: 170), confirmed by Noonan (1991: 175).
Harpalus cuculus Casey, 1924: 96. Type locality: «Las Vegas [San Miguel County], New Mexico» (original citation). Holotype [by monotypy] (♂) in USNM [# 47755]. Synonymy established by Lindroth (1968: 769).

#### Distribution.

This species is found west of the Mississippi River from northeastern Alaska to northwestern Minnesota, south to the highlands of central Mexico (Noonan 1991: 179) and southern California [see Noonan 1991: Fig. 289].

#### Records.

**CAN**: AB, BC, MB, NT, SK, YT **USA**: AK, AZ, CA, CO, ID, KS, MN, MT, ND, NE, NM, NV, OK, OR, SD, TX, UT, WA, WY – Mexico

#### Note.

Three subspecies of *Harpalus amputatus* are known from Asia (see Kataev et al. 2003: 372).

### [atrichatus group]

### Harpalus atrichatus

Hatch, 1949

Harpalus atrichatus Hatch, 1949a: 86. Type locality: «Ocean Park [Pacific County], Wash[ington] (original citation). Holotype (♂) in USNM.

#### Distribution.

This species is known only from a few scattered localities from southwestern British Columbia, including Vancouver Island, south to west-central Oregon, east to northern Idaho [see Noonan 1991: Fig. 291].

#### Records.

**CAN**: BC (VCI) **USA**: ID, OR, WA

### [cautus group]

### Harpalus balli

Noonan, 1991

Harpalus balli Noonan, 1991: 151. Type locality: «Mantoloking, Ocean County, New Jersey» (original citation). Holotype (♂) in MCZ [# 35204].

#### Distribution.

This species is known from only six specimens collected near the coast in Massachusetts and New Jersey [see Noonan 1991: Fig. 283]. The collection date of the last known specimen is 1932 and Noonan (1991: 153) believes that it “might be extinct because of destruction of ocean side habitats.”

#### Records.

**USA**: MA, NJ

### Harpalus cautus

Dejean, 1829

Harpalus cautus Dejean, 1829: 367. Type locality: «Californie» (original citation), herein restricted to Point Reyes National Seashore, Marin County (see Noonan 1991: 142). Lectotype (♀), designated by Noonan (1991: 138), in MHNP.
Harpalus albionicus Mannerheim, 1843: 213 [*nomen dubium*]. Type locality: «California ad Ross [farming community about 75 miles north of San Francisco along the coast]» (original citation). Syntype(s) presumably lost (Lindroth 1968: 802). Synonymy established with doubt by LeConte (1865b: 102).
Harpalus advena LeConte, 1852a: 185. Type locality not stated; «Oregon» selected by Noonan (1991: 138). Holotype [by monotypy] (♂) in MCZ [# 95]. Synonymy established by Casey (1914: 95), confirmed by Lindroth (1968: 802).
Harpalus defixus Walker, 1866: 316 [*nomen dubium*]. Type locality: British Columbia (inferred from title of the book). Syntype(s) lost (Lindroth 1968: 802; Noonan 1991: 138). Synonymy established with doubt by LeConte (1870: 400).
Harpalus crenatellus Casey, 1914: 94. Type locality: «California» (original citation). Holotype [by monotypy] (♂) in USNM [# 47788]. Synonymy established by Lindroth (1968: 802).
Harpalus oregonensis Casey, 1914: 94. Type locality: «Oregon» (original citation). Lectotype (♂), designated by Lindroth (1975: 140), in USNM [# 47789]. Synonymy established by Hatch (1953: 168), confirmed by Lindroth (1968: 802).
Harpalus columbianus Casey, 1924: 104. Type locality: «Goldstream, British Columbia» (original citation). Lectotype (♂), designated by Lindroth (1975: 140), in USNM [# 47809]. Synonymy established by Hatch (1953: 168), confirmed by Lindroth (1968: 802).

#### Distribution.

This western species occurs from central Alaska south to southern California, southeastern Arizona, and south-central Colorado, east to western Nebraska [see Noonan 1991: Fig. 286]. The records from northeastern Kansas (Popenoe 1877: 24) and Beaver Island in Michigan (Hatch 1925: 554) are probably in error. According to Noonan (1991: 143), this species is one of the most abundant *Harpalus* along the west coast.

#### Records.

**CAN**: AB, BC (VCI) **USA**: AK, AZ, CA, CO, ID, MT, NE, NV, OR, UT, WA, WY

### Harpalus ellipsis

LeConte, 1847

Harpalus ellipsis LeConte, 1847: 400. Type locality: «Territorio Missouriensi» (original citation), herein restricted to Halsey, Thomas County, Nebraska (see Noonan 1991: 148). Lectotype (♂), designated by Noonan (1991: 143), in MCZ [# 5893].
Harpalus vespertinus Casey, 1884b: 10. Type locality: «Arizona» (original citation). Lectotype [as holotype] (♂), designated by Noonan (1991: 143), in USNM [# 47826]. Synonymy established by Noonan (1991: 143).
Harpalus fractus Casey, 1924: 107. Type locality: «Akron [Washington County], Colorado» (original citation). Lectotype (♂), designated by Noonan (1991: 143), in USNM [# 47838]. Synonymy established by Noonan (1991: 143).
Harpalus provensis Casey, 1924: 113. Type locality: «North Fork, Provo Cañon [Utah County], Utah» (original citation). Lectotype (♂), designated by Noonan (1991: 143), in USNM [# 47834]. Synonymy established by Noonan (1991: 143).

#### Distribution.

The range of this species extends from central Alberta and southern Saskatchewan south to southwestern Texas and southeastern Arizona; seemingly isolated in Minnesota (Gandhi et al. 2005: 930) and northwestern Oregon [see Noonan 1991: Fig. 287]; also recorded from northern Sonora in Mexico (Bates 1884: 271).

#### Records.

**CAN**: AB SK **USA**: AZ, CO, ID, KS, MN, MT, NE, NM, OR, SD, TX, UT, WY – Mexico

### Harpalus innocuus

LeConte, 1863

Harpalus innocuus LeConte, 1863c: 17. Type locality: «Marquette [Marquette County, Michigan], Lake Superior» (original citation). Holotype [by monotypy] (♀) in MCZ [# 5894].
Harpalus persolus Casey, 1914: 96. Type locality: «Oregon» (original citation). Lectotype (♀), designated by Lindroth (1975: 139), in USNM [# 47787]. Synonymy established by Lindroth (1968: 800).
Harpalus macilentus Casey, 1914: 96. Type locality: «Boulder Co[unty], Colorado» (original citation). Lectotype (♂), designated by Noonan (1991: 155), in USNM [# 47822]. Synonymy established by Noonan (1991: 155).
Harpalus fugitans Casey, 1914: 98. Type locality: «Frazier Valley, British Columbia» (original citation). Lectotype (♂), designated by Lindroth (1975: 140), in USNM [# 47796]. Synonymy established by Lindroth (1968: 800).
Harpalus paululus Casey, 1914: 110. Type locality: «Eldora [Boulder County], Colorado» (original citation). Lectotype (♂), designated by Noonan (1991: 155), in USNM [# 47821]. Synonymy established by Noonan (1991: 155).
Harpalus intactus Casey, 1924: 112 [primary homonym of *Harpalus intactus* Casey, 1914]. Type locality: «Marquette [Marquette County], Michigan» (original citation). Holotype [by monotypy] (♂) in USNM [# 47843]. Synonymy established by Lindroth (1968: 800).

#### Distribution.

This species ranges from western Quebec to Vancouver Island, north to southern Northwest Territories, south to the Sierra Nevada in California, southern Colorado in the Rocky Mountains, southwestern South Dakota (Custer County, Peter W. Messer pers. comm. 2011), the upper peninsula of Michigan, and southwestern Pennsylvania [see Noonan 1991: Fig. 288]. Several specimens simply labeled from “New Mexico” are known (Noonan 1991: 159). East of the Rocky Mountains, the species is known only from a few scattered localities.

#### Records.

**CAN**: AB, BC (VCI), NT, ON, QC, SK **USA**: CA, CO, ID, MI, MN, MT, OR, PA, SD, WA, WI, WY [NM]

### Harpalus ochropus

Kirby, 1837

Harpalus ochropus Kirby, 1837: 42. Type locality: northern parts of British America (inferred from title of the book), restricted to «Nipigon, Ont[ario]» by Lindroth (1968: 808)]. Lectotype [as holotype] (♂), designated by Noonan (1991: 149), in BMNH.

#### Distribution.

The range of this species extends from Alaska (Lindroth 1968: 808) east to Anticosti Island in Quebec, south to northeastern Minnesota (Purrington and Maxey 2007: 219) and the southern part of the Prairie Provinces [see Noonan 1991: Fig. 287]. The records from Colorado (Wickham 1902: 242) and New Mexico (Fall and Cockerell 1907: 161) are likely in error.

#### Records.

**CAN**: AB, BC, MB, ON, QC, SK, YT **USA**: AK, MN

### Harpalus vittatus alaskensis

Lindroth, 1968

Harpalus alaskensis Lindroth, 1968: 801. Type locality: «7-15 mi[les] N[orth] of New Rampart House, Alaska» (original citation). Holotype (♂) in USNM [# 75705]. Note. New Rampart House was a trading post on the Porcupine River in Yukon Territory near the border with Alaska.

#### Distribution.

This Holarctic subspecies ranges from eastern Siberia (Kataev et al. 2003: 384) to the Alaska-Yukon border along the Porcupine River. On this continent, the subspecies is known from extant specimens only from the type locality. It was cited as a dominant taxon in a late Pleistocene site in the Klondike region of Yukon Territory (Zazula et al. 2006: 265).

#### Records.

**USA**: AK – **Holarctic**

#### Note.

This taxon was originally described as a distinct species but Kataev (1990: 396) listed it as a subspecies of *Harpalus vittatus* Gebler. The nominotypical subspecies and *Harpalus vittatus kiselevi* Kataev and Shilenkov are found in Asia (Kataevet al. 2003: 384).

### [obnixus group]

### Harpalus obnixus

Casey, 1924

Harpalus obnixus Casey, 1924: 105. Type locality: «Provo Cañon [Utah County], Utah» (original citation). Lectotype (♂), designated by Lindroth (1975: 140), in USNM [# 47837].
Harpalus antiphon Casey, 1924: 106. Type locality: «Jemez Springs [Sandoval County], New Mexico» (original citation). Lectotype (♀), designated by Noonan (1991: 160), in USNM [# 47839]. Synonymy established by Noonan (1991: 160).

#### Distribution.

This species ranges from southwestern Alberta and British Columbia (Noonan 1991: 162-163) south to west-central Oregon, northeastern Nevada, and northwestern New Mexico (Casey 1924: 106, as *Harpalus antiphon*) [see Noonan 1991: Fig. 290]. One old specimen simply labeled from Nebraska is known (Noonan 1991: 163).

#### Records.

**CAN**: AB, BC **USA**: CO, ID, MT, NM, NV, OR, UT, WA, WY [NE]

### Harpalus plenalis

Casey, 1914

Harpalus plenalis Casey, 1914: 108. Type locality: «New Brunswick» (original citation), herein restricted to Pennfield, Charlotte County (CNC). Lectotype (♀), designated by Lindroth (1975: 140), in USNM [# 47819].
Harpalus latebricola Casey, 1914: 109. Type locality: «Las Vegas [San Miguel County], New Mexico» (original citation). Lectotype (♀), designated by Lindroth (1975: 140), in USNM [# 47845]. Synonymy established by Lindroth (1968: 807).
Harpalus modulatus Casey, 1924: 112. Type locality: «F[or]t Coulonge, Quebec» (original citation). Holotype [by monotypy] (♀) in USNM [# 47844]. Synonymy established by Lindroth (1954b: 142).

#### Distribution.

This species is known from scattered localities from Newfoundland (Lindroth 1955a: 143) to southern British Columbia, north to southwestern Northwest Territories, south to central Arizona, north-central New Mexico, eastern Texas, and southwestern North Carolina [see Noonan 1991: Fig. 292].

#### Records.

**CAN**: BC, MB, NB, NF, NS (CBI), NT, ON, PE, QC, SK **USA**: AR, AZ, CO, ID, IL, KS, MA, ME, MI, NC, NH, NJ, NM, NY, TX, VA, VT, WI

### [opacipennis group]

### Harpalus opacipennis

(Haldeman, 1843)

Ophonus opacipennis Haldeman, 1843b: 301. Type locality: southeastern Pennsylvania (Haldeman 1843a: 297). Lectotype (♀), designated by Noonan (1991: 169), in MCZ [# 28698].
Harpalus oodioides Chaudoir, 1868b: 168. Type locality: «terre de Rupert [former name for the entire drainage basin of Hudson’s Bay, thus including most of northern Quebec, Ontario, Manitoba, etc. (Lamb 1971)]» (original citation). Holotype [by monotypy] (♂) in MHNP. Synonymy established by Lindroth (1968: 804).
Harpalus lacustris Casey, 1914: 111. Type locality: «Bayfield [Bayfield County], Wisconsin» (original citation). Lectotype (♂), designated by Lindroth (1975: 140), in USNM [# 47823]. Synonymy established by Lindroth (1968: 805).
Harpalus coloradensis Casey, 1914: 111. Type locality: «Boulder Co[unty], Colorado» (original citation). Lectotype (♂), designated by Noonan (1991: 169), in USNM [# 47824]. Synonymy established by Noonan (1991: 169).
Harpalus mobilis Casey, 1914: 112. Type locality: «Boulder Co[unty], Colorado» (original citation). Lectotype (♂), designated by Noonan (1991: 169), in USNM [# 47827]. Synonymy established by Noonan (1991: 169).
Harpalus leviceps Casey, 1924: 112. Type locality: «Marquette [Marquette County], Michigan» (original citation). Lectotype (♀), designated by Lindroth (1975: 140), in USNM [# 47846]. Synonymy established by Lindroth (1968: 805).

#### Distribution.

This species ranges from southern New Brunswick (Webster and DeMerchant 2012: 6) to south-central Alaska (Lindroth 1968: 806), south to the Sierra Nevada in California, central Arizona and central New Mexico along the Rocky Mountains, southern Indiana, and Maryland (Erwin 1981b: 175) [see Noonan 1991: Fig. 296].

#### Records.

**CAN**: AB, BC (VCI), MB, NB, NT, ON, QC, SK, YT **USA**: AK, AZ, CA, CO, ID, IN, MA, MD, ME, MI, MN, MT, ND, NM, NV, NY, OH, OR, PA, SD, UT, VT, WA, WI, WY

### [somnulentus group]

### Harpalus herbivagus

Say, 1823

Harpalus herbivagus Say, 1823a: 29. Type locality: «Rumney [Grafton County], N[ew] H[ampshire]» (neotype label). Neotype (♂), designated by Lindroth and Freitag (1969: 353), in MCZ [# 32769].
Ophonus mutabilis Haldeman, 1843b: 301. Type locality: southeastern Pennsylvania (Haldeman 1843a: 297). Lectotype (♂), designated by Noonan (1991: 181), in MCZ [# 34542]. Synonymy established by LeConte (1863b: 13), confirmed by Lindroth (1968: 794).
Harpalus foveicollis LeConte, 1847: 399. Type locality: «fines Aquilones, provinciae Maine» (original citation). Lectotype (♂), designated by Noonan (1991: 181), in MCZ [# 5890]. Synonymy established by Lindroth (1954b: 141).
Harpalus assensus Casey, 1924: 107. Type locality: «Boulder Co[unty], Colorado» (original citation). Holotype [by monotypy] (♂) in USNM [# 47828]. Synonymy established by Lindroth (1968: 794).

#### Distribution.

The range of this species extends from Nova Scotia to south-central British Columbia, north to central Alberta, south to southern Oregon, east-central Arizona and central New Mexico along the Rocky Mountains, the Texas Panhandle (Michels et al. 2010: 743), northern Missouri, and southern Georgia [see Noonan 1991: Fig. 293]. The record from “Florida” (Leng 1920: ﻿71) needs confirmation.

#### Records.

**CAN**: AB, BC, MB, NB, NS, ON, PE, QC, SK **USA**: AL, AR, AZ, CO, CT, DC, DE, GA, IA, ID, IL, IN, KS, KY, MA, MD, ME, MI, MN, MO, MT, NC, ND, NE, NH, NJ, NM, NY, OH, OK, OR, PA, RI, SC, SD, TN, TX, UT, VA, VT, WA, WI, WV, WY [FL]

### Harpalus martini

Van Dyke, 1926

Harpalus martini Van Dyke, 1926a: 124. Type locality: «Bear Lake [= Baldwin Lake, San Bernardino County (see Noonan 1991: 190)], San Bernardino Mountains, California» (original citation). Holotype (♂) in CAS [# 1867]. Etymology. The specific name was proposed for James Otis Martin [1870-1951], a coleopterist by avocation. Martin served as a preparator of insects at the California Academy of Sciences from 1924 to 1932. His collection of 12,000 well-prepared beetles, most from western North America, was presented to the California Academy of Sciences in 1928.

#### Distribution.

This species is known from only 16 specimens (including 15 ♂) from mountains in southern California [see Noonan 1991: Fig. 287] and could be either endangered or extinct according to Noonan (1991: 189). The last known specimen found was collected in 1919.

#### Records.

**USA**: CA

### Harpalus solitaris

Dejean, 1829

Carabus fuliginosus Duftschmid, 1812: 83 [primary homonym of *Carabus fuliginosus* Panzer, 1809]. Type locality: «um Linz [Austria]» (original citation). Syntype(s) probably lost (Lindroth 1968: 798).
Harpalus lapponicus Zetterstedt, 1828: 26 [primary homonym of *Harpalus lapponicus* Sahlberg, 1827]. Type locality: «Lapponia Tornensi; Juckasjervi [Sweden]» (original citation). Five syntypes in ZMLS (Lindroth 1938: 18). Synonymy established by Schaum (1860: 596). Note. This name was listed as *nomen oblitum* by Kataev et al. (2003: 382) and *Harpalus solitaris* Dejean, 1829 as *nomen protectum*. Since *Harpalus lapponicus* Zetterstedt, 1828 is a junior primary homonym, there is no need for such qualifiers.
Harpalus solitaris Dejean, 1829: 337. Type locality: «Kamtschatka [Russia]; nord de la Laponie» (original citation). Syntype(s) in MHNP. Synonymy established, under the name *Harpalus lapponicus* Zetterstedt, by Mäklin (1857: 179).

#### Distribution.

The range of this Holarctic species extends in northern North America from Newfoundland to south-central Alaska (Lindroth 1968: 799), south to east-central British Columbia, “Minnesota” (Lindroth 1968: 799), northeastern Wisconsin (Messer 2010: 41), “Michigan” (Lindroth 1968: 799), and Maine (Noonan 1991: 187) [see Noonan 1991: Fig. 294]. The records from the Sierra Nevada and Colorado (Elias 1987: 633) are probably in error; that from Prince Edward Island (Bousquet and Larochelle 1993: 237) needs confirmation (see Majka et al. 2008: 132).

#### Records.

**FRA**: PM **CAN**: AB, BC, LB, MB, NB, NF, NS, NT, ON, QC, SK, YT **USA**: AK, ME, MI, MN, NH, WI [PE] – **Holarctic**

### Harpalus somnulentus

Dejean, 1829

Harpalus somnulentus Dejean, 1829: 333. Type locality: «détroit de Norfolk [= Norfolk Sound, Baranof Island, Alaska] sur la côte nord-ouest de l’Amérique septentrionale» (original citation). Lectotype (♂), designated by Noonan (1991: 190), in MHNP.
Harpalus pleuriticus Kirby, 1837: 41. Type locality: «Lat. 54° [= along North Saskatchewan River]» (original citation), restricted to «Edmonton, Al[ber]ta» by Lindroth (1968: 792). Lectotype (♂), designated by Noonan (1991: 190), in BMNH. Synonymy established by Noonan (1991: 190).
Harpalus fallax LeConte, 1859c: 2. Type locality: «Santa Fé [Santa Fe County, New Mexico]» (original citation). Lectotype (♂), designated by Noonan (1991: 190), in MCZ [# 5889]. Synonymy established by Noonan (1991: 190).
Harpalus stupidus LeConte, 1859c: 3. Type locality: «route to Fort Bridger» (original citation). Holotype [by monotypy] (♀) in MCZ [# 5906]. Synonymy established by Noonan (1991: 191).
Harpalus viduus LeConte, 1859c: 3. Type locality: «New Jersey» (original citation). Syntype(s) presumably lost. Synonymy established, under the name *Harpalus fallax* LeConte, by Lindroth (1968: 794).
Harpalus carbonatus LeConte, 1860: 319. Type locality: «Saskatchewan» (original citation). Lectotype (♀), designated by Noonan (1991: 191), in MCZ [# 5896]. Synonymy established by Noonan (1991: 191).
Harpalus placidus Casey, 1884b: 10 [primary homonym of *Harpalus placidus* Gyllenhal, 1827]. Type locality: «N[ew] J[ersey]» (holotype label). Holotype [by monotypy] (♂) in USNM [# 47807]. Synonymy established, under the name *Harpalus fallax* LeConte, by Horn (1885b: 109), confirmed by Noonan (1991: 191). Note. «Willets Point, Long Island» was the locality cited in the original description.
Harpalus recisus Casey, 1914: 93. Type locality: «New Jersey» (original citation). Lectotype (♂), designated by Lindroth (1975: 139), in USNM [# 47791]. Synonymy established, under the name *Harpalus fallax* LeConte, by Lindroth (1968: 794).
Harpalus futilis Casey, 1914: 97. Type locality: «Lake Tahoe [Placer County], California» (original citation). Holotype [by monotypy] (♂) in USNM [# 47803]. Synonymy established by Noonan (1991: 191).
Harpalus intactus Casey, 1914: 97. Type locality: «California» (original citation). Holotype [by monotypy] (♀) in USNM [# 47795]. Synonymy established by Noonan (1991: 192).
Harpalus aequabilis Casey, 1914: 100. Type locality: «Buena Vista [Chaffee County], Colorado» (original citation). Lectotype (♀), designated by Lindroth (1975: 139), in USNM [# 47779]. Synonymy established, under the name *Harpalus pleuriticus* Kirby, by Lindroth (1954b: 142).
Harpalus lascivus Casey, 1914: 100. Type locality: «British Columbia» (original citation). Lectotype (♂), designated by Lindroth (1975: 139), in USNM [# 47800]. Synonymy established, under the name *Harpalus pleuriticus* Kirby, by Hatch (1953: 169), confirmed by Noonan (1991: 192).
Harpalus pumilio Casey, 1914: 100 [primary homonym of *Harpalus pumilio* Dejean, 1829]. Type locality: «Bayfield [Bayfield County], Wisconsin» (original citation). Lectotype (♀), designated by Lindroth (1975: 139), in USNM [# 47801]. Synonymy established, under the name *Harpalus pleuriticus* Kirby, by Lindroth (1954b: 142).
Harpalus perspicuus Casey, 1914: 101. Type locality: «Boulder Co[unty], Colorado» (original citation). Lectotype (♂), designated by Lindroth (1975: 139), in USNM [# 47797]. Synonymy established, under the name *Harpalus pleuriticus* Kirby, by Lindroth (1954b: 142).
Harpalus lividulus Casey, 1914: 101. Type locality: «Bayfield [Bayfield County], Wisconsin» (original citation). Lectotype (♂), designated by Lindroth (1975: 139), in USNM [# 47804]. Synonymy established, under the name *Harpalus pleuriticus* Kirby, by Lindroth (1954b: 142).
Harpalus pellax Casey, 1914: 105. Type locality: «Yellowstone National Park» (original citation). Lectotype (♂), designated by Noonan (1991: 190), in USNM [# 47812]. Synonymy established by Noonan (1991: 192).
Harpalus peritus Casey, 1914: 107. Type locality: «Reno [Washoe County], Nevada» (original citation). Lectotype (♂), designated by Noonan (1991: 190), in USNM [# 47814]. Synonymy established by Noonan (1991: 192).
Harpalus celox Casey, 1914: 107 (as *celax*, typographic error, see Casey 1914: 383). Type locality: «Clackamas Co[unty], Oregon» (original citation). Lectotype (♂), designated by Lindroth (1975: 139), in USNM [# 47816]. Synonymy established by Noonan (1991: 191).
Harpalus uteanus Casey, 1914: 118. Type locality: «Provo [Utah County], Utah» (original citation). Lectotype (♂), designated by Lindroth (1975: 139), in USNM [# 47835]. Synonymy established by Noonan (1991: 191).
Harpalus oppositus Casey, 1914: 125. Type locality: «Siskiyou Co[unty], California» (original citation). Lectotype (♂), designated by Lindroth (1975: 139), in USNM [# 47860]. Synonymy established, under the name *Harpalus celox* Casey, by Hatch (1953: 169), confirmed by Lindroth (1968: 789).
Harpalus amiculus Casey, 1924: 102. Type locality: «Lake George [Warren County], New York» (original citation). Lectotype (♂), designated by Lindroth (1975: 139), in USNM [# 47792]. Synonymy established, under the name *Harpalus fallax* LeConte, by Lindroth (1968: 794).
Harpalus contactus Casey, 1924: 106. Type locality: «Cedar City [Iron County], Utah» (original citation). Lectotype (♂), designated by Lindroth (1975: 139), in USNM [# 47818]. Synonymy established by Lindroth (1968: 789).
Harpalus uintanus Casey, 1924: 106. Type locality: «Cedar City [Iron County], Utah» (original citation). Lectotype (♂), designated by Lindroth (1975: 139), in USNM [# 47820]. Synonymy established, under the name *Harpalus intactus* Casey, by Lindroth (1968: 789).
Harpalus blanditus Casey, 1924: 108. Type locality: «Terrace, British Columbia» (original citation). Lectotype (♂), designated by Lindroth (1975: 139), in USNM [# 47802]. Synonymy established, under the name *Harpalus pleuriticus* Kirby, by Hatch (1953: 169), confirmed by Noonan (1991: 193).
Harpalus nivalis Casey, 1924: 109. Type locality: «Redvers, Sask[atchewan]» (holotype label). Holotype [by monotypy] (♂) in USNM [# 47805]. Synonymy established, under the name *Harpalus pleuriticus* Kirby, by Lindroth (1954b: 142).
Harpalus pacificus Fall, 1926a: 136. Type locality: «Ketchikan, Alaska» (original citation). Holotype (♂) in MCZ [# 23876]. Synonymy established by Lindroth (1968: 789).
Harpalus rewolinskii Noonan, 1991: 214. Type locality: «2.3 mi[les] S[outh] Oyen, Alberta» (original citation). Holotype (♂) in CNC [# 23531]. Synonymy established by Ball and Bousquet (2000: 99).

#### Distribution.

This species is transcontinental in the north ranging from Newfoundland to the Kenai Peninsula in Alaska, south to southern California, central Arizona and New Mexico in the Rocky Mountains, Nebraska, and northern Virginia [see Noonan 1991: Figs 297-309]. The records from central Alabama (see Noonan 1991: Figs 302 and 304), “Arkansas” (Bousquet and Larochelle 1993: 237) and southern Kansas (Snow 1903: 194, as *Harpalus fallax*) need confirmation.

#### Records.

**FRA**: PM **CAN**: AB, BC (QCI, VCI), LB, MB, NB, NF, NS (CBI), NT, ON, PE, QC, SK, YT **USA**: AK, AZ, CA, CO, CT, DE, IA, ID, IL, IN, MA, MD, ME, MI, MN, MO, MT, ND, NE, NH, NJ, NM, NV, NY, OH, OR, PA, RI, SD, UT, VA, VT, WA, WI, WV, WY [AL, AR, KS]

#### Note.

Noonan (1991: 196-207) recognized six morphs within this species, the *pleuriticus*, *fallax*, “dark,” *uteanus*, *somnulentus*, and *carbonatus* morphs, which Lindroth (1968) treated as distinct species, except for the “dark” one. Noonan went on to report numerous intergradation zones between all morphs.

### Subgenus Glanodes

Casey, 1914

Glanodes Casey, 1914: 50, 60. Type species: *Harpalus obliquus* Horn, 1880 by original designation. Etymology. Uncertain, possibly from the Greek *glanos* (hyena) and the suffix -*odes* (likeness) [masculine].

#### Diversity.

Six species in southwestern North America.

#### Identification.

Ball (1972) revised the species and provided a key for the identification of the males.

### Harpalus cohni

Ball, 1972

Harpalus cohni Ball, 1972: 195. Type locality: «Marfa [Presidio County], Texas» (original citation). Holotype (♂) in CAS [# 11640].

#### Distribution.

This species is known only from western Texas (Ball 1972: Fig. 13) and southeastern New Mexico (Perrault 1974: 120).

#### Records.

**USA**: NM, TX

### Harpalus corpulentus

(Casey, 1914)

Glanodes corpulentus Casey, 1914: 62. Type locality: «Virgin River, Utah» (original citation), restricted to «S[ain]t George [Washington County]» by Ball (1972: 191). Lectotype [as holotype] (♀), designated by Ball (1972: 191), in USNM [# 47736].

#### Distribution.

This species is known for sure only from Summit and Washington Counties in Utah (Ball 1972: 192). The record from “Arizona” (Bousquet and Larochelle 1993: 237) needs confirmation.

#### Records.

**USA**: UT [AZ]

### Harpalus huachuca

Ball, 1972

Harpalus huachuca Ball, 1972: 193. Type locality: «Huachuca M[oun]t[ain]s, Ariz[ona]» (original citation). Holotype (♂) in USNM [# 72116].

#### Distribution.

This species is known only from the holotype collected in southeastern Arizona [see Ball 1972: Fig. 13].

#### Records.

**USA**: AZ

### Harpalus obliquus

Horn, 1880

Harpalus obliquus G.H. Horn, 1880a: 140. Type locality: «Fort Bayard [Grant County], New Mexico» (original citation). Lectotype [as type] (♂), designated by Ball (1972: 194), in MCZ [# 34601].
Glanodes regressus Casey, 1914: 62. Type locality: «near Benson [Cochise County], Arizona» (original citation). Lectotype [as holotype] (♀), designated by Ball (1972: 196), in USNM [# 47737]. **New synonymy**. Note. The lectotype of *Harpalus regressus* (Casey) is a female that could not be identified with certainty. However, Ball (1972: 196) wrote that the specimen is “probably ... a member of *Harpalus obliquus* Horn.” Based on that statement, I believe it is best to treat Casey’s name as a junior synonym of *Harpalus obliquus*.

#### Distribution.

This species is found in southern (Perrault 1974: 120) and western New Mexico and southeastern Arizona [see Ball 1972: Fig. 13].

#### Records.

**USA**: AZ, NM

### Harpalus puncticeps

(Casey, 1914)

Glanodes puncticeps Casey, 1914: 61. Type locality: «Peach Spring[s] [Mohave County], Arizona» (original citation). Lectotype [as holotype] (♀), designated by Ball (1972: 192), in USNM [# 47735].
Harpalus cunctipeps Ball, 1973: 74. Replacement name for *Harpalus puncticeps* (Casey, 1914).

#### Distribution.

This species is known only from northwestern Arizona [see Ball 1972: Fig. 13; Perrault 1982b: 269].

#### Records.

**USA**: AZ

#### Note.

Ball (1973: 74) proposed *Harpalus cunctipeps* to replace *Harpalus puncticeps* (Casey, 1914), a junior secondary homonym of *Harpalus puncticeps* (Stephens, 1828), both species being then placed in the genus *Harpalus*. Since Stephens’ species is currently included in the genus *Ophonus* and the replacement name was proposed after 1960, the original species-group name is to be reinstated (ICZN 1999: Article 59.4).

### Harpalus stephani

Ball, 1972

Harpalus stephani Ball, 1972: 194. Type locality: «Arivaca, Pima County, Arizona» (original citation). Holotype (♂) in USNM [# 72117]. Etymology. This species was named after Karl Heinz Stephan [1931-2005], an accomplished beetle collector and naturalist. Stephan spent 30 years collecting Coleoptera in Latimer County in Oklahoma. Most of his specimens are deposited in the Florida State Collection of Arthropods, in Texas A&M Insect Collection, and in the Field Museum of Natural History (Staphylinidae and Histeridae).

#### Distribution.

This species is known only from Culberson County in western Texas and Pima County in southern Arizona [Ball 1972: 194, Fig. 13].

#### Records.

**USA**: AZ, TX

### Subgenus Harpalobius

Reitter, 1900

Harpalobius Reitter, 1900: 76. Type species: *Harpalus fuscipalpis* Sturm, 1818 designated by Habu (1973a: 68). Etymology. From the generic name *Harpalus* [*q.v*.] and the Greek *bios* (life) [masculine].
Harpalellus Lindroth, 1968: 815. Type species: *Harpalus basilaris* Kirby, 1837 (= *Harpalus fuscipalpis* Sturm, 1818) by original designation. Synonymy established by Kataev (1989: 219). Etymology. From the generic name *Harpalus* [*q.v*.] and the suffix -*ellus* (small, little) [masculine].

#### Diversity.

Four Palaearctic species, of which one is Holarctic.

#### Identification.

The North American species was covered in Lindroth (1968: 815-817) monograph under the name *Harpalellus basilaris*.

### Harpalus fuscipalpis

Sturm, 1818

Carabus contristatus Duftschmid, 1812: 102 [potential *nomen oblitum*]. Type locality: «Wien [Austria]» (original citation). Syntype(s) probably lost (see Ledoux and Roux 2005: 682).
Harpalus fuscipalpis Sturm, 1818: 66 [potential *nomen protectum*]. Type locality: «Oessterreich [= Austria]» (original citation). Syntype(s) location unknown. Synonymy established by Schaum (1860: 610).
Harpalus basilaris Kirby, 1837: 41. Type locality: «Lat. 54° [= along North Saskatchewan River]» (original citation), restricted to «Edmonton, Al[ber]ta» by Lindroth (1968: 816). Two syntypes in BMNH (Lindroth 1953b: 173). Synonymy established by Kataev (1989: 219).
Harpalus taphrioides Motschulsky, 1844: 201. Type locality: «step[pes] Kislar» (lectotype label). Lectotype (♂), designated by Kataev (1989: 221), in ZMMU. Synonymy established by Kataev (1989: 219).
Harpalus fulvipennis Motschulsky, 1844: 221. Type locality: «Kulscha Bäd[er], Transbaic[alia] [Russia]» (lectotype label). Lectotype (♂), designated by Kataev (1989: 221), in ZMMU. Synonymy established by Kataev (1989: 219).
Harpalus varicornis LeConte, 1847: 401. Type locality: «Lacum Superiorem» (original citation). One syntype in MCZ [# 5909]. Synonymy established, under the name *Harpalellus basilaris* Kirby, by Lindroth (1968: 816).
Harpalus celioïdes Ménétriés, 1848: 39. Type locality: «steppes des Kirghises?» (original citation). Lectotype (♂), designated by Kataev (1989: 221), in ZILR. Synonymy established by Kataev (1989: 219).
Harpalus obesulus LeConte, 1852a: 185. Type locality: «Oregon» (original citation). Holotype [by monotypy] (♀) in MCZ [# 94]. Synonymy established, under the name *Harpalus basilaris* Kirby, by LeConte (1870: 397), confirmed by Lindroth (1968: 816).
Amara extensa Walker, 1866: 314. Type locality: British Columbia (inferred from title of the book). Syntype(s) in BMNH. Synonymy established, under the name *Harpalus basilaris* Kirby, by LeConte (1873b: 325), confirmed by Lindroth (1954b: 142).
Harpalus turculus Bates, 1878b: 714. Type locality: «probably near Yarkand [Kashgaria, China]» (original citation). Lectotype (♂), designated by Kataev (2002: 193), in MHNP. Synonymy established by Kataev (2002: 193).
Harpalus oblongus Casey, 1914: 126. Type locality: «Utah» (original citation). Lectotype (♀), designated by Lindroth (1975: 140), in USNM [# 47836]. Synonymy established, under the name *Harpalus basilaris* Kirby, by Hatch (1953: 171), confirmed by Lindroth (1968: 816).
Harpalus sejunctus Casey, 1914: 126. Type locality: «Eldora [Boulder County], Colorado» (original citation). Lectotype (♂), designated by Lindroth (1975: 140), in USNM [# 47858]. Synonymy established, under the name *Harpalellus basilaris* Kirby, by Lindroth (1968: 816).
Harpalus renoicus Casey, 1914: 127. Type locality: «Reno [Washoe County], Nevada» (original citation). Lectotype (♂), designated by Lindroth (1975: 140), in USNM [# 47857]. Synonymy established, under the name *Harpalellus basilaris* Kirby, by Lindroth (1968: 816).
Harpalus furviculus Casey, 1924: 110. Type locality: «Wawawai [Whitman County], Washington» (original citation for the lectotype). Lectotype (♂), designated by Lindroth (1975: 140), in USNM [# 47840]. Synonymy established, under the name *Harpalus basilaris* Kirby, by Hatch (1953: 171), confirmed by Lindroth (1968: 816).
Harpalus stocktonensis Casey, 1924: 111. Type locality: «Stockton [Tooele County], Utah» (original citation). Holotype [by monotypy] (♀) in USNM [# 47832]. Synonymy established, under the name *Harpalellus basilaris* Kirby, by Lindroth (1968: 816).
Harpalus ventricosus Casey, 1924: 111. Type locality: «Spencer, British Columbia» (original citation). Holotype [by monotypy] (♀) in USNM [# 47841]. Synonymy established, under the name *Harpalus basilaris* Kirby, by Hatch (1953: 171), confirmed by Lindroth (1968: 816).
Harpalus durescans Casey, 1924: 111. Type locality: «F[or]t Coulonge, Quebec» (original citation). Lectotype (♀), designated by Lindroth (1975: 140), in USNM [# 47842]. Synonymy established, under the name *Harpalellus basilaris* Kirby, by Lindroth (1968: 816).
Harpalus subenormis Casey, 1924: 113. Type locality: «Callao [Juab County], Utah» (original citation). Lectotype (♀), designated by Lindroth (1975: 140), in USNM [# 47859]. Synonymy established, under the name *Harpalellus basilaris* Kirby, by Lindroth (1968: 816).

#### Distribution.

This Holarctic species ranges from Germany to eastern Siberia (Kataev et al. 2003: 376) and from Alaska to Nova Scotia (Lindroth 1968: 817, as *Harpalellus basilaris*), south to northwestern Ohio (Lucas County, Harry J. Lee, Jr. pers. comm. 2008), southeastern Wisconsin (Messer 2010: 41), northern Iowa (Wickham 1911b: 8, as *Harpalus basilaris*), northwestern New Mexico (Lindroth 1968: 816), and northern California (Siskiyou County, CNC).

#### Records.

**CAN**: AB, BC (VCI), MB, NS, NT, ON, QC, SK, YT **USA**: AK, AZ, CA, CO, IA, ID, MI, MN, MT, ND, NH, NM, NV, OH, OR, SD, UT, WA, WI, WY – **Holarctic**

### Genus Harpalobrachys

Tschitschérine, 1899

Harpalobrachys Tschitschérine, 1899c: 601. Type species: *Harpalus leiroides* Motschulsky, 1844 by monotypy. Etymology. From the generic name *Harpalus* [*q.v*.] and the Greek *brachys* (short) [masculine].

#### Diversity.

One Holarctic species.

#### Identification.

The species was treated by Lindroth (1968: 748-749) and Noonan (1991: 212-213).

#### Taxonomic Note.

Noonan (1991: 211) treated this taxon as a group within the genus *Harpalus*. Lindroth (1968: 748), Kryzhanovskij et al. (1995: 140), and Kataev et al. (2003: 370) regarded *Harpalobrachys* as a distinct genus.

### Harpalobrachys leiroides

(Motschulsky, 1844)

Harpalus leiroïdes Motschulsky, 1844: 217. Type locality: «environs de Koul [near Nerchinsk, eastern Siberia, Russia] sur la grande route de Nertchinsk» (original citation). Five syntypes in ZMMU (Keleinikova 1976: 203).

#### Distribution.

This Holarctic species ranges from the European part of Russia to eastern Siberia (Kataev et al. 2003: 370) and in the Nearctic Region from east-central Alaska to southern Northwest Territories (Lindroth 1968: 749), south to central Alberta (Bousquet 1987a: 130). The species is rarely collected on this continent.

#### Records.

**CAN**: AB, NT, YT **USA**: AK – **Holarctic**

### Genus Hartonymus

Casey, 1914

Hartonymus Casey, 1914: 165. Type species: *Hartonymus hoodi* Casey, 1914 by original designation. Etymology. From the surname of Charles Arthur Hart [1859-1918], an entomologist at the Illinois State Natural History Survey who first found adults of the type species [masculine].

#### Diversity.

Two allopatric species in temperate areas of North America.

#### Identification.

Ball (1976b) revised the species and provided a key for their recognition.

### Hartonymus alternatus

(LeConte, 1863)

Cratognathus alternatus LeConte, 1863c: 13. Type locality: «Arkansas» (original citation). Lectotype (♂), designated by Ball (1976b: 425), in CMNH (collection Ulke).

#### Distribution.

This species is known from “Arkansas” to eastern New Mexico [see Ball 1976b: Fig. 7], including southeastern Colorado (Michels et al. 2008).

#### Records.

**USA**: AR, CO, NM, OK, TX

### Hartonymus hoodi

Casey, 1914

Hartonymus hoodi Casey, 1914: 167. Type locality: «Topeka [Mason County], Illinois» (original citation). Lectotype (♀), designated by Ball (1976b: 423), in USNM [# 47906]. Etymology. The specific name honors Joseph Douglas Hood [1889-1966], professor at the University of Rochester and later at Cornell University. Hood (1952) recounted that while he already had the description of this species ready for publication and a drawing of the habitus of the adult, for which he paid $6.00 in 1911, he visited Casey in Washington to “ask him to check my determination.” Casey looked at the specimens, closed the box which he carefully placed at the near corner of his desk and said to Hood “I am an old man. This is the finest carabid that I have ever seen or will see, and I hope that you will allow me to describe it.”

#### Distribution.

This species in found along the Mississippi Basin from west-central Wisconsin (Messer 2010: 41) to eastern Nebraska (Colfax County, Foster F. Purrington pers. comm. 2010; Greeley County, R. Michael Brattain collection), south to Oklahoma (Ball and Bousquet 2000: 99) and Missouri [see Ball 1976b: Fig. 7].

#### Records.

**USA**: IA, IL, MO, NE, OK, WI

### Genus Amblygnathus

Dejean, 1829

Amblygnathus Dejean, 1829: 62. Type species: *Amblygnathus cephalotes* Dejean, 1829 designated by Brullé (1835b: 10). Etymology (original). From the Greek *amblys* (blunt, obtuse) and *gnathos* (jaw, by extension mandible), alluding to the obtuse mandibles (“*mandibules … obtuses*”) of adults of the four species Dejean had before him [masculine].

#### Diversity.

Twenty-four species in temperate, subtropical, and tropical areas of the Nearctic (four species, only one endemic) and Neotropical (23 species) Regions, including the West Indies (three species, one of them endemic).

#### Identification.

Ball and Maddison (1987) revised the species and provided a key for their identification.

### Amblygnathus evansi

Ball and Maddison, 1987

Amblygnathus evansi Ball and Maddison, 1987: 214. Type locality: «Mazatlan, State of Sinaloa, México» (original citation). Holotype (♂) in USNM. Etymology. The specific name was proposed in honor of Howard Ensign Evans [1919-2002], an internationally recognized authority on the systematics and behavior of Hymenoptera, especially the Sphecidae, Pompilidae, and Bethylidae. During his academic career, Evans worked at the Kansas State University (1949-1952), Cornell University (1952-1960), the Museum of Comparative Zoology (1960-1973), and the Colorado State University (1973-1986). He described one new wasp family (Scolebythidae), 31 new genera, and almost 800 new species of wasps.

#### Distribution.

This species ranges from southeastern California and southwestern Arizona, south along the Pacific Coast to El Salvador [see Ball and Maddison 1987: Fig. 29].

#### Records.

**USA**: AZ, CA – El Salvador, Guatemala, Mexico

### Amblygnathus iripennis

(Say, 1823)

Harpalus iripennis Say, 1823a: 30. Type locality: «Ent[er]prise [Volusia County], Fl[orid]a» (neotype label). Neotype (♂), designated by Lindroth and Freitag (1969: 353), in MCZ [# 32989].
Selenophorus varicolor LeConte, 1847: 392. Type locality: «Pennsylvania et Georgia» (original citation). Lectotype (♀), designated by Ball and Maddison (1987: 206), in MCZ [# 5920]. Synonymy established by LeConte (1863b: 13), confirmed by Ball and Maddison (1987: 206).

#### Distribution.

This species is found along and near the Atlantic Coastal Plain from New Jersey to the Florida Keys [see Ball and Maddison 1987: Fig. 28] and along the Gulf of Mexico in southwestern Alabama (Löding 1945: 25; Robert L. Davidson pers. comm. 2012). The record from “Pennsylvania” (LeConte 1847: 392), “Illinois,” and “Texas” (Horn 1880e: 182) are probably in error.

#### Records.

**USA**: AL, FL, GA, MD, NC, NJ, SC, VA

### Amblygnathus mexicanus

Bates, 1882

Amblygnathus mexicanus Bates, 1882a: 66. Type locality: «Cordova [Veracruz], Mexico» (original citation). Lectotype (♀), designated by Ball and Maddison (1987: 208), in BMNH.
Hemisopalus delumbis Casey, 1914: 140. Type locality: «Lake Worth [Palm Beach County], Florida» (original citation). Lectotype (♀), designated by Ball and Maddison (1987: 208), in USNM [# 47872]. Synonymy established by Ball and Maddison (1987: 208).

#### Distribution.

This species ranges along the Coastal Plain from New Jersey to southern Florida, west to southeastern Texas, north along the Mississippi Basin to southern Arkansas (Ouachita County, CMNH) and northeastern Mississippi; south of the Rio Grande it occurs through eastern Mexico and Central America to the Canal Zone in Panama [see Ball and Maddison 1987: Fig. 29]. The record from “Pennsylvania” (Ball and Maddison 1987: 210) needs confirmation.

#### Records.

**USA**: AL, AR, FL, GA, LA, MS, NC, NJ, SC, TX, VA [PA] – Guatemala, Honduras, Mexico, Nicaragua, Panama

### Amblygnathus subtinctus

(LeConte, 1867)

Selenophorus subtinctus LeConte, 1867b: 365. Type locality: «Louisiana» (original citation). Holotype [by monotypy] (♀) in MCZ [# 5919].
Hemisopalus angulatus Casey, 1914: 139. Type locality: «Brownsville [Cameron County], Texas» (original citation). Lectotype [as holotype] (♀), designated by Ball and Maddison (1987: 226), in USNM [# 47871]. Synonymy established by Ball and Maddison (1987: 226).

#### Distribution.

The range of this species extends along the Gulf Coast from the Florida Panhandle (Jefferson County, CMNH) to central Veracruz in Mexico [see Ball and Maddison 1987: Fig. 31].

#### Records.

**USA**: FL, LA, MS, TX – Mexico

### Genus Athrostictus

Bates, 1878

Athrostictus Bates, 1878a: 592. Type species: *Athrostictus sericatus* Bates, 1878 designated by Noonan (1976: 41). Etymology. From the Greek *athroos* (assembled, crowded) and *stictos* (punctured), alluding to the dense punctuation on the dorsum of the body (“*corpus *... *supra (praecipue elytris) dense minute punctatum*”) of the adult [masculine]. Note. *Arthrostictus* is an incorrect subsequent spelling, introduced by Rye (1880: 33), not in prevailing usage.

#### Diversity.

Eighteen species in the Neotropical Region, of which one extends into southern Texas.

#### Identification.

There is no modern key for the identification of the species. The genus is in need of a revision.

### Athrostictus punctatulus

(Putzeys, 1878)

Selenophorus punctatulus Putzeys, 1878: 65. Type species: «Yucatan» (original citation). Syntype(s) [8 originally cited] in MHNP (collection Chaudoir) and probably IRSN (collection Putzeys).
Selenophorus perpolitus Casey, 1884c: 76. Type locality: «Texas» (original citation). Three syntypes [6 originally cited] in USNM [# 47866]. **New synonymy** (based on a communication of George E. Ball posted on http://bugguide.net/node/view/204062).

#### Distribution.

This species is known from northeastern Arkansas (Kraim 1983: 137, as *Selenophorus perpolitus*), Amite County in southwestern Mississippi (Peter W. Messer pers. comm. 2012), East Baton Rouge Parish in southern Louisiana (Igor Sokolov pers. comm. 2009), and from southern Texas (Johnson 1978: 68; Zapata, Live Oak, and Uvalde Counties, CMNH) to Yucatán (Putzeys 1878: 65).

#### Records.

**USA**: AR, LA, MS, TX – Mexico

#### Note.

The systematic position of this species is not quite settled. It was transferred to the genus *Athrostictus* by Erwin [in Reichardt 1977: 428, under the spelling *Athrostictus punculatus* (Putzeys, 1878)]. It was retained in the genus *Selenophorus* by Noonan (1985: 41) although he did not study specimens. Bates (1891a: 243) wrote “the species [i.e., *Selenophorus punctatulus*] belongs to a group of the genus in which the thorax (except on the disk) and the elytra are closely punctured, a group which connects *Selenophorus* with *Arthrostictus* [sic].”

### Genus Selenophorus

Dejean, 1829

Selenophorus Dejean, 1829: 80. Type species: *Carabus palliatus* Fabricius, 1798 designated by Hope (1838: 84). Etymology (original). From the Greek *selenis* (half-moon, lunule) and *phero* (to bear, carry), possibly alluding to the shape of the mentum of the adult which, in the absence of a median tooth, is in the shape of a half-moon (“*menton échancré en arc de cercle*”) [masculine].

#### Diversity.

Western Hemisphere, with about 190 species arrayed in two subgenera: *Celiamorphus* and *Selenophorus *s.str., both represented in North America.

#### Identification.

Putzeys (1878) reviewed the genus but his work is outdated. About one-third of the currently recognized valid North American species were described by Casey and there is little doubt that several of them will turn out to be synonyms. A revision of the genus is much needed.

### Subgenus Celiamorphus

Casey, 1914

Celiamorphus Casey, 1914: 141. Type species: *Selenophorus ellipticus* Dejean, 1829 designated by Lindroth (1968: 828). Etymology. From the generic name *Celia* [*q.v*.] and the Greek *morphe* (form), probably alluding to a vague resemblance of adults of the species Casey had before him to those of *Celia* [masculine].

#### Diversity.

Fourteen species in temperate, subtropical, and tropical areas of the Nearctic (nine species) and Neotropical (eight species) Regions.

### Selenophorus adjunctus

(Casey, 1914)

Celiamorphus adjunctus Casey, 1914: 144. Type locality: «Galveston [Galveston County], Texas» (original citation). Four syntypes [5 originally cited] in USNM [# 47875].

#### Distribution.

This species is known only from the type locality in southeastern Texas.

#### Records.

**USA**: TX

### Selenophorus contractus

(Casey, 1914)

Celiamorphus contractus Casey, 1914: 144. Type locality: «Southern Pines [Moore County], North Carolina» (original citation). One syntype in USNM [# 47876].

#### Distribution.

This species is known from North Carolina (Casey 1914: 144) and northeastern Georgia (Fattig 1949: 48).

#### Records.

**USA**: GA, NC

### Selenophorus discopunctatus

Dejean, 1829

Selenophorus discopunctatus Dejean, 1829: 92. Type locality: «île Saint-Barthélemy [Guadeloupe]; Amérique septentrionale» (original citation). Syntype(s) location unknown (not found in MHNP, George E. Ball pers. comm. 2008).
Selenophorus cuprinus Dejean, 1829: 96. Type locality: «île Saint-Barthélemy [Guadeloupe]» (original citation). Syntype(s) in MHNP. Synonymy established by Putzeys (1878: 25).
Selenophorus harpaloides Reiche, 1843b: 142. Type locality: «Caracas, Venezuela» (original citation). Syntype(s) in MHNP (George E. Ball pers. comm. 2008). Synonymy established by Putzeys (1878: 25).
Selenophorus aeratus Reiche, 1843b: 142. Type locality: «Colombia» (original citation). Syntype(s) in MHNP (George E. Ball pers. comm. 2008). Synonymy established by Putzeys (1878: 25).
Selenophorus chokoloskei Leng, 1915: 596. Type locality: «Chokoloskee; Everglade [Florida]» (original citation). Syntype(s) location unknown. Synonymy established by Darlington (1935a: 161).

#### Distribution.

This species is found in Mississippi (Harrison, Jackson, and Stone Counties, Paul K. Lago pers. comm. 2009), throughout the Florida Peninsula (Peck and Thomas 1998: 22), on several islands of the West Indies (Peck 2005: 32; Peck 2011: 13), and in Venezuela (Reiche 1843b: 142, as *Selenophorus harpaloides*) and Colombia (Reiche 1843b: 142, as *Selenophorus aeratus*).

#### Records.

**USA**: FL, MS – Antigua, Bahamas, British Virgin Islands, Cayman Islands, Colombia, Cuba, Dominica, Guana Island, Hispaniola, Jamaica, Montserrat, Navassa, Puerto Rico, Venezuela

### Selenophorus ellipticus

Dejean, 1829

Selenophorus ellipticus Dejean, 1829: 108. Type locality: «Amérique septentrionale» (original citation), restricted to «Atlantic City [Atlantic County], N[ew] J[ersey]» by Lindroth (1968: 829). One syntype in MHNP (Lindroth 1955b: 28).
Selenophorus ovalis Dejean, 1829: 106. Type locality: «Amérique septentrionale» (original citation). Holotype [by monotypy] (♂) in MHNP (Lindroth 1955b: 27). Synonymy established by Lindroth (1968: 829).
Celiamorphus opaculus Casey, 1914: 143. Type locality: «Atlantic City [Atlantic County], New Jersey» (original citation). Lectotype (♂), designated by Lindroth (1975: 141), in USNM [# 47873]. Synonymy established by Lindroth (1968: 829).
Celiamorphus currens Casey, 1914: 143. Type locality: «Catskill M[oun]t[ain]s, New York» (original citation for the lectotype). Lectotype (♂), designated by Lindroth (1975: 141), in USNM [# 47874]. Synonymy established by Lindroth (1968: 829).

#### Distribution.

The range of this species extends from west-central Maine (Majka et al. 2011: 46) to eastern Minnesota (Gandhi et al. 2005: 931), south to “Texas” (Horn 1880e: 183) and southern Florida (Peck and Thomas 1998: 22); also cited from “northern Mexico” (Casey 1914: 143).

#### Records.

**CAN**: ON **USA**: AL, AR, CT, DC, FL, GA, IA, IL, IN, KS, LA, MA, MD, ME, MI, MN, MO, MS, NC, NE, NH, NJ, NY, OH, OK, PA, RI, SC, TN, TX, VA, WI – Mexico

### Selenophorus fossulatus

Dejean, 1829

Selenophorus fossulatus Dejean, 1829: 88. Type locality: «Amérique septentrionale» (original citation). One syntype in MHNP (Lindroth 1955b: 28).

#### Distribution.

This species is known from North Carolina (Brimley 1938: 128), Georgia (Horn 1880e: 182; Fattig 1949: 48), throughout Florida (Peck and Thomas 1998: 22), Alabama (Löding 1945: 25), and southeastern coastal Mississippi (Drew A. Hildebrandt pers. comm. 2007).

#### Records.

**USA**: AL, FL, GA, MS, NC

### Selenophorus granarius

Dejean, 1829

Selenophorus granarius Dejean, 1829: 109. Type locality: «Amérique septentrionale» (original citation), restricted to «Mass[achusetts]» by Lindroth (1968: 830). One syntype in MHNP (Lindroth 1955b: 28).
Selenophorus pulicarius Dejean, 1829: 108. Type locality: «Amérique septentrionale» (original citation). Holotype [by monotypy] (♀) in MHNP (Lindroth 1955b: 28). Synonymy established by Putzeys (1878: 21), confirmed by Lindroth (1955b: 28).

#### Distribution.

This species ranges from “Massachusetts” (Lindroth 1968: 830) to southern Florida (Vince Golia pers. comm. 2007), west to southern Texas (Johnson 1978: 67), and north along the Mississippi Basin to eastern Iowa (Linn County, Doug A. Veal pers. comm. 2009), “Illinois” (Bousquet and Larochelle 1993: 239), and “Indiana” (Schrock 1985: 355).

#### Records.

**USA**: AL, CT, FL, GA, IA, IL, IN, MA, MD, MS, NC, NJ, NY, RI, TX, VA

### Selenophorus municeps

(Casey, 1924)

Celiamorphus municeps Casey, 1924: 117. Type locality: «Southern Pines [Moore County], North Carolina» (original citation). Two syntypes [2 originally cited] in USNM [# 47878].

#### Distribution.

This species is known only from the type locality in central North Carolina.

#### Records.

**USA**: NC

### Selenophorus nanulus

(Casey, 1924)

Celiamorphus nanulus Casey, 1924: 118. Type locality: «Southern Pines [Moore County], North Carolina» (original citation). Two syntypes [2 ♂ originally cited] in USNM [# 47877].

#### Distribution.

This species is known only from the type locality in central North Carolina.

#### Records.

**USA**: NC

### Selenophorus subtropicus

(Casey, 1924)

Celiamorphus subtropicus Casey, 1924: 118. Type locality: «Texas and northern Mexico» (original citation). Five syntypes [5 originally cited] in USNM [# 47879].

#### Distribution.

This species is known only from the type series.

#### Records.

**USA**: TX – Mexico

### Subgenus Selenophorus

Dejean, 1829

Selenophorus Dejean, 1829: 80. Type species: *Carabus palliatus* Fabricius, 1798 designated by Hope (1838: 84).
Gynandropus Dejean, 1831: 810, 817. Type species: *Gynandropus americanus* Dejean, 1831 (= *Harpalus hylacis* Say, 1823) by monotypy. Synonymy established by Noonan (1985: 39). Etymology (original). From the Greek *gyne* (female), *andros* (male), and *pous* (foot), alluding to the expanded first protarsomere of the male and female (“*les quatre premiers articles des *... *tarses antérieurs dilatés dans les mâles *... *le premier des tarses antérieurs des femelles fortement dilaté*”) [masculine].
Hemisopalus Casey, 1914: 135. Type species: *Harpalus opalinus* LeConte, 1863 by original designation. Synonymy established by Noonan (1976: 41).

#### Diversity.

About 175 species in temperate, subtropical, and tropical areas of the Nearctic (29 species) and Neotropical (about 150 species) Regions.

### Selenophorus aeneopiceus

Casey, 1884

Selenophorus aeneopiceus Casey, 1884b: 13. Type locality: «Arizona» (original citation). Six syntypes in USNM [# 47888].

#### Distribution.

This species is known from northern Colorado (Denver and Boulder Counties, UASM) and southern Utah (Kane and Garfield Counties, CMNH) south to the Isthmus of Tehuantepec in Mexico (UASM).

#### Records.

**USA**: AZ, CO, NM, TX, UT – Mexico

### Selenophorus blanchardi

Manee, 1915

Selenophorus blanchardi Manee, 1915: 175. Type locality: «Southern Pines [Moore County], North Carolina» (original citation). One syntype [4 originally cited] in ANSP [# 8223].

#### Distribution.

This species is known from North Carolina (Manee 1915: 175), northeastern Georgia (Fattig 1949: 49), and southern South Carolina (Ciegler 2000: 103).

#### Records.

**USA**: GA, NC, SC

### Selenophorus chaparralus

Purrington, 2000

Selenophorus chaparralus Purrington, 2000: 9. Type locality: «Amistad Reservoir, Val Verde Co[unty], Tex[as]» (original citation). Holotype (♂) in USNM.

#### Distribution.

This species is known from a few localities on the lower Rio Grande of southern Texas (Purrington 2000: 9).

#### Records.

**USA**: TX

### Selenophorus concinnus

Schaeffer, 1910

Selenophorus concinnus Schaeffer, 1910: 403. Type locality: «Huachuca Mountains, Arizona» (original citation). Lectotype (♀), designated by Erwin and House (1978: 248), in USNM [# 42511].

#### Distribution.

This species is known yet only from the Huachuca Mountains in southeastern Arizona.

#### Records.

**USA**: AZ

### Selenophorus cupreolus

Casey, 1914

Selenophorus cupreolus Casey, 1914: 149. Type locality: «Texas» (original citation). One syntype in USNM [# 47883].

#### Distribution.

This species is known only from the type series.

#### Records.

**USA**: TX

### Selenophorus discoderoides

Schaeffer, 1910

Selenophorus discoderoides Schaeffer, 1910: 404. Type locality: «Esperanza Ranch, near Brownsville [Cameron County], Texas» (original citation for the lectotype). Lectotype (♂), designated by Erwin and House (1978: 248), in USNM [# 42512].

#### Distribution.

This species is known only from southeastern Texas.

#### Records.

**USA**: TX

### Selenophorus elongatus

(LeConte, 1847)

Gynandropus elongatus LeConte, 1847: 408. Type locality: «Georgia» (original citation). One syntype in MCZ [# 5883].

#### Distribution.

This species ranges from “Georgia” (LeConte 1847: 408) to central Florida (Peck and Thomas 1998: 22), west to southwestern Alabama (Löding 1945: 25).

#### Records.

**USA**: AL, FL, GA

### Selenophorus famulus

Casey, 1914

Selenophorus famulus Casey, 1914: 146. Type locality: «Arizona (probably southern)» (original citation). Five syntypes [5 originally cited] in USNM [# 47880].

#### Distribution.

This species is known from southern Arizona (Cochise, Graham, Maricopa, Pima and Santa Cruz Counties, UASM), northern Sonora (UASM), Baja California Norte (UASM), and “California” (CMNH).

#### Records.

**USA**: AZ, CA – Mexico

### Selenophorus fatuus

LeConte, 1863

Harpalus fatuus LeConte, 1863c: 17. Type locality: «South Carolina to Texas» (original citation). Syntype(s) in MCZ [# 5917].

#### Distribution.

This species ranges from North Carolina (Brimley 1938: 128) and northern Tennessee (Montgomery County, Foster F. Purrington pers. comm. 2011) to southern Florida (Peck and Thomas 1998: 22), west to eastern Texas (Johnson 1978: 68) and south to eastern Mexico (George E. Ball pers. comm. 2008).

#### Records.

**USA**: AL, FL, GA, LA, MS, NC, SC, TN, TX – Mexico

### Selenophorus gagatinus

Dejean, 1829

Selenophorus gagatinus Dejean, 1829: 112. Type locality: «Amérique septentrionale» (original citation), restricted to «Arlington [Middlesex County], Mass[achusetts]» by Lindroth (1968: 824). One syntype in MHNP (Lindroth 1955b: 27).
Selenophorus maurus Haldeman, 1843b: 301. Type locality: southeastern Pennsylvania (Haldeman 1843a: 297). One possible syntype, a ♀ labeled “[pink disc] / 243. [handwritten] / H. (S.) gagatinus (Dej.) maurus Hald. [handwritten],” in MCZ (collection LeConte). Synonymy established by LeConte (1847: 390).
Selenophorus viridescens LeConte, 1847: 392. Type locality: «insula Longa NovEboraci [= Long Island, New York]» (original citation). Syntype(s) in MCZ [# 5921]. Synonymy established by Horn (1880e: 183).

#### Distribution.

The range of this species extends from Nova Scotia (Kings County, NSNH) to central Wisconsin (Purrington et al. 2002: 201), south to central Texas (Travis County, UASM) and southern Florida (Peck and Thomas 1998: 22). The records from southern Arizona (Horn 1880e: 181; Wickham 1898: 301) need confirmation.

#### Records.

**CAN**: NB, NS, ON, QC **USA**: AL, AR, CT, DC, FL, GA, IL, IN, MA, MD, ME, MI, MO, NC, NH, NJ, NY, OH, OK, PA, RI, SC, TX, VA, VT, WI, WV [AZ]

### Selenophorus houstoni

Casey, 1914

Selenophorus houstoni Casey, 1914: 151. Type locality: «Austin [Travis County], Texas» (original citation). Six syntypes in USNM [# 47886].

#### Distribution.

This species is known only from the type locality in central Texas.

#### Records.

**USA**: TX

### Selenophorus hylacis

(Say, 1823)

Harpalus hylacis Say, 1823a: 31. Type locality: «Dorchester [Suffolk County], Mass[achusetts]» (neotype label). Neotype (♂), designated by Lindroth and Freitag (1969: 343), in MCZ [# 32988].
Gynandropus americanus Dejean, 1831: 818. Type locality: «Amérique septentrionale» (original citation). One syntype [2 originally cited] in MHNP (Lindroth 1955b: 29). Synonymy established by LeConte (1847: 408), confirmed by Lindroth (1955b: 29).

#### Distribution.

This species is found from southwestern Maine (Majka et al. 2011: 46) to eastern Minnesota (Gandhi et al. 2005: 931), including southernmost Ontario (possibly only as strays) (Lindroth 1968: 821), south to eastern Texas (Nacogdoches County, CMNH) and central Florida (Manatee County, CNC).

#### Records.

**CAN**: ON **USA**: AL, AR, CT, DC, DE, FL, GA, IA, IL, IN, KS, KY, LA, MA, MD, ME, MI, MN, MO, MS, NC, NE, NH, NJ, NY, OH, OK, PA, RI, SC, TN, TX, VA, VT, WI, WV

### Selenophorus implicans

Casey, 1914

Selenophorus implicans Casey, 1914: 151. Type locality: «Alexandria [Rapides Parish], Louisiana» (original citation). Three syntypes [3 originally cited] in USNM [# 47887].

#### Distribution.

This species is known from central Louisiana (Casey 1914: 151) and Texas (Leng 1920: 72; McCulloch County, USNM). One specimen determined by Casey (USNM) from Kansas is known.

#### Records.

**USA**: LA, TX [KS]

### Selenophorus integer

(Fabricius, 1798)

Carabus integer Fabricius, 1798: 58 (as *nteger*). Type locality: «Americae Insulis» (original citation). One syntype in ZMUC (Zimsen 1964: 57). Note. The spelling *integer*, used by all subsequent authors, is an incorrect subsequent spelling in prevailing usage and attributed to the publication of the original spelling; therefore it is deemed to be the correct original spelling (ICZN 1999: Article 33.3.1).

#### Distribution.

This species is known from southern Florida and several islands of the West Indies (Peck and Thomas 1998: 22).

#### Records.

**USA**: FL – Bahamas, Cuba, Hispaniola, Jamaica, Puerto Rico

### Selenophorus laesus

(LeConte, 1858)

Harpalus laesus LeConte, 1858b: 59. Type locality: «Fort Gates, Texas and Tampico [Tamaulipas]» (original citation). Syntype(s) in MCZ [# 5914].

#### Distribution.

This species ranges from central Texas south at least to southern Tamaulipas in Mexico (LeConte 1858b: 59). The record from “Arizona” (Bousquet and Larochelle 1993: 241) needs confirmation.

#### Records.

**USA**: TX [AZ] – Mexico

#### Note.

Horn (1880e: 180) regarded *Selenophorus palliatus* (Fabricius) as synonym of this species.

### Selenophorus maritimus

Casey, 1914

Selenophorus maritimus Casey, 1914: 148. Type locality: «Galveston [Galveston County], Texas» (original citation). Two syntypes in USNM [# 47882].

#### Distribution.

This species is known from central and southern Florida (Vince Golia pers. comm. 2007; Highland and Palm Beach Counties, CMNH, UASM), southern Mississippi (Hancock and Jackson Counties, Drew A. Hildebrandt pers. comm. 2008; Lago et al. 2002: 202), and southern Texas (Casey 1914: 148; Zapata and San Patricio Counties, CMNH, UASM).

#### Records.

**USA**: FL, MS, TX

### Selenophorus opalinus

(LeConte, 1863)

Selenophorus iripennis LeConte, 1847: 389 [secondary homonym of *Selenophorus iripennis* (Say, 1823)]. Type locality: «Carolina, et NovEboraci [= New York]» (original citation), restricted to «Carolina» by Lindroth (1968: 824). Syntype(s) in MCZ [# 32989].
Harpalus opalinus LeConte, 1863b: 13. Replacement name for *Harpalus iripennis* (LeConte, 1847).

#### Distribution.

The range of this species extends from western New Brunswick (Webster and Bousquet 2008: 19) to Minnesota (Epstein and Kulman 1990: 215) and South Dakota (Kirk and Balsbaugh 1975: 30), including southern Quebec and Ontario (Lindroth 1968: 824), north to southeastern Manitoba (Roughley et al. 2010: 230), south to southern Texas (Johnson 1978: 67), the Florida Keys (Peck and Thomas 1998: 22), and South Bimini Island in the Bahamas (Ball and Shpeley 1992b: 96).

#### Records.

**CAN**: MB, NB, ON, QC **USA**: AL, AR, CT, DC, DE, FL, GA, IA, IL, IN, KS, KY, MA, MD, ME, MI, MN, MO, MS, NC, NE, NH, NJ, NY, OH, OK, PA, RI, SC, SD, TN, TX, VA, VT, WI, WV – Bahamas

### Selenophorus otiosus

Casey, 1914

Selenophorus otiosus Casey, 1914: 148. Type locality: «Arizona (southern)» (original citation). One syntype in USNM [# 47881].

#### Distribution.

This species is known only from the type series.

#### Records.

**USA**: AZ

### Selenophorus palliatus

(Fabricius, 1798)

Carabus palliatus Fabricius, 1798: 58. Type locality: «America boreali» (original citation). One syntype in ZMUC (Zimsen 1964: 57).
Harpalus stigmosus Germar, 1824: 25. Type locality: «Georgia» (original citation). Syntype(s) probably lost. Synonymy established by Brullé (1835c: 290).
Selenophorus impressus Dejean, 1829: 82. Type locality: «Amérique septentrionale» (original citation). One syntype in MHNP (Lindroth 1955b: 28). Synonymy established with the name *Selenophorus stigmosus* Germar by Dejean (1829: 82), confirmed by Lindroth (1955b: 28).

#### Distribution.

This species is found throughout much of southern United States from North Carolina (Brimley 1938: 128) to the Florida Keys and the Bahamas (Peck and Thomas 1998: 22), west to southeastern California (Andrews et al. 1979: 28) and the Baja California Peninsula (Horn 1894: 312), including southern Illinois (Union and Pope Counties, UASM), south to southeastern Texas (Wickham 1897: 113; Johnson 1978: 68; Cameron and San Patricio Counties, UASM) and San Luis Potosí (UASM). The record from “Virginia” (Bousquet and Larochelle 1993: 241) needs confirmation.

#### Records.

**USA**: AL, AR, AZ, CA, FL, GA, IL, LA, MS, NC, NM, SC, TX [VA] – Bahamas, Mexico

### Selenophorus parumpunctatus

Dejean, 1829

Carabus sinuatus Gyllenhal [in Schönherr], 1806: 203 [primary homonym of *Carabus sinuatus* Gmelin, 1790]. Type locality: «Americae insulis» (original citation). Syntype(s) location unknown (possibly in UZIU).
Selenophorus parumpunctatus Dejean, 1829: 104. Type locality: «je crois qu’il vient d’Amérique et probablement des Antilles, mais je n’en suis pas certain [I believe it came from America and probably from the West Indies but I am not certain]» (original citation). Syntype(s) in MHNP (George E. Ball pers. comm. 2008). **New synonymy** (George E. Ball pers. comm. 2008).
Selenophorus excisus LeConte, 1878b: 377 [primary homonym of *Selenophorus excisus* Putzeys, 1878]. Type locality: «southern Florida» (original citation). Syntype(s) [3 originally cited] in MCZ [# 5918]. **New synonymy** (George E. Ball pers. comm. 2008).
Selenophorus mustus Casey, 1914: 152. Type locality: «Biscayne Bay [Dade County], Florida» (original citation). Lectotype (♀), designated by Lindroth (1975: 141), in USNM [# 47889]. Synonymy established, under the name *Selenophorus excisus* LeConte, by Casey (1918: 413).

#### Distribution.

This species is known from southern Florida (Peck and Thomas 1998: 22, as *Selenophorus sinuatus*) and several islands of the West Indies (Peck 2011: 13). The record from South Carolina (Ciegler 2000: 103, as *Selenophorus mustus*) is probably in error.

#### Records.

**USA**: FL – Antigua, Bahamas, Barbados, Cayman Islands, Cuba, Dominica, Guadeloupe, Guana Island, Hispaniola, Jamaica, Montserrat, Navassa, Puerto Rico, Virgin Islands.

### Selenophorus pedicularius

Dejean, 1829

Selenophorus pedicularius Dejean, 1829: 100. Type locality: «Amérique septentrionale» (original citation), restricted to «Fort Worth [Tarrant County], Texas» by Lindroth (1968: 825). Holotype [by monotypy] (♂) in MHNP (Lindroth 1955b: 29).
Selenophorus troglodytes Dejean, 1829: 101. Type locality: «Amérique septentrionale» (original citation). Holotype [by monotypy] (♂) in MHNP (Lindroth 1955b: 28). Synonymy established by Horn (1880e: 183), confirmed by Lindroth (1955b: 28).
Selenophorus aereus LeConte, 1847: 393. Type locality: «territorio Missouriensi» (original citation). Syntype(s) in MCZ [# 5915]. Synonymy established by Horn (1880e: 183), confirmed by Lindroth (1968: 825).
Selenophorus puellus Putzeys, 1878: 40. Type locality: «Etats-unis» (original citation). Syntype(s) [9 originally cited] in MHNP (collection Chaudoir). Synonymy established by Horn (1880e: 180), confirmed by Lindroth (1955b: 29).

#### Distribution.

This species is found from Connecticut (Krinsky and Oliver 2001: 218) to southern Montana (Hatch 1933a: 10), south to southern New Mexico (Fall and Cockerell 1907: 162), southern Texas (Johnson 1978: 67), and central Florida (Peck and Thomas 1998: 22), west along the south to southern Arizona (Griffith 1900: 566; Snow 1906b: 163; Snow 1907: 142; Greenlee and Pima Counties, CMNH) and Baja California (Horn 1894: 311).

#### Records.

**CAN**: ON **USA**: AL, AR, AZ, CO, CT, DC, FL, GA, IA, IL, IN, KS, LA, MD, MO, MS, MT, NC, NJ, NM, NY, OH, OK, PA, SC, SD, TN, TX, VA, WI, WV, WY – Mexico

### Selenophorus planipennis

LeConte, 1847

Selenophorus planipennis LeConte, 1847: 394. Type locality: «prope Long’s Peak [Boulder County, Colorado], Rocky Mountains» (original citation). Holotype [by monotypy] (♂) in MCZ [# 5916].

#### Distribution.

This species ranges from southernmost Ontario (possibly only as strays) to south-central British Columbia (Lindroth 1968: 828), south to northeastern Oregon (Westcott et al. 2006: 9), Durango in Mexico (Ball and Shpeley 1992a: 59), northwestern Mississippi (Bolivar County, Drew A. Hildebrandt pers. comm. 2008), and northwestern Ohio (Lucas County, Harry J. Lee, Jr. pers. comm. 2008).

#### Records.

**CAN**: AB, BC, MB, ON, SK **USA**: AZ, CO, IA, IL, KS, MI, MN, MS, MT, ND, NE, NM, OH, OK, OR, SD, TX, UT, WI, WY – Mexico

### Selenophorus riparius

Casey, 1914

Selenophorus riparius Casey, 1914: 150. Type locality: «Vicksburg [Warren County], Mississippi» (original citation). One syntype in USNM [# 47885].

#### Distribution.

This species is known only from the type locality in western Mississippi.

#### Records.

**USA**: MS

### Selenophorus schaefferi

Csiki, 1932

Selenophorus schaefferi Csiki, 1932a: 1201. Type locality: «Huachuca Mountains [Cochise County] and Yuma County, Arizona» (original citation for *Selenophorus semirufus* Bates *sensu* Schaeffer, 1910). Syntype(s) [2 originally cited] probably in USNM. Note. This name was proposed for *Selenophorus semirufus* Bates, 1882 *sensu* Schaeffer (1910: 403) and the name is available by indication (bibliographic reference to a previously published description).

#### Distribution.

This species is known from the Huachuca Mountains and Yuma County in southern Arizona (Schaeffer 1910: 403) and from Riverside County in southeastern California (Andrews et al. 1979: 28, as *Selenophorus semirufus*). The record from “Texas” (Csiki 1932a: 1201) is probably in error.

#### Records.

**USA**: AZ, CA

### Selenophorus scolopaceus

Casey, 1914

Selenophorus scolopaceus Casey, 1914: 150. Type locality: «Colorado» (original citation). One syntype in USNM [# 47884].

#### Distribution.

This species is known only from the type series.

#### Records.

**USA**: CO

### Selenophorus sinuaticollis

Notman, 1922

Selenophorus sinuaticollis Notman, 1922b: 102. Type locality: «Tucson [Pima County], Ariz[ona]» (original citation). Holotype (♀) in USNM [# 26590].

#### Distribution.

This species is known from the type locality in southern Arizona, from southern New Mexico (Dona Ana County, CMNH), and from southern Sonora in Mexico (UASM).

#### Records.

**USA**: AZ, NM – Mexico

### Selenophorus striatopunctatus

Putzeys, 1878

Selenophorus striatopunctatus Putzeys, 1878: 33. Type locality: «Antilles; Chiapas, Mexique» (original citation). Syntype(s) in MHNP (collection Chaudoir) and probably IRSN (collection Putzeys).
Hemisopalus vigilans Casey, 1914: 137. Type locality: «Florida» (original citation). One syntype in USNM [# 47869]. Synonymy established by Peck and Thomas (1998: 22).
Hemisopalus depressulus Casey, 1914: 137. Type locality: «Lake Worth [Palm Beach County], Florida» (original citation). Lectotype (♂), designated by Lindroth (1975: 141), in USNM [# 47867]. Synonymy established by Peck and Thomas (1998: 22).

#### Distribution.

This species is known so far from Florida and several islands of the West Indies (Peck and Thomas 1998: 22; Peck 2009a: 13), from south-central Louisiana (Saint Landry Parish, Igor M. Sokolov pers. comm. 2009), as well as from southern Texas (Johnson 1978: 67; San Patricio and Cameron Counties, UASM), southern Mexico (Putzeys 1878: 33), and Colombia (Martínez 2003: 9). The record from Georgia (Fattig 1949: 48, as *Selenophorus depressulus*) needs confirmation. The species is adventive on several islands of Hawaii (Liebherr 2009: 403).

#### Records.

**USA**: FL, LA, TX [GA] – Bahamas, Barbados, Cayman Islands, Cuba, Colombia, Dominican Republic, Jamaica, Leeward Islands, Mexico, Puerto Rico, Windward Islands

### Selenophorus trepidus

(Casey, 1924)

Hemisopalus trepidus Casey, 1924: 117. Type locality: «Cape Sable [Monroe County], Florida» (original citation). Holotype [by monotypy] (♀) in USNM [# 47868].

#### Distribution.

This species is known only from the type locality in southern Florida.

#### Records.

**USA**: FL

### [incertae sedis]

### Selenophorus breviusculus

Horn, 1880

Selenophorus breviusculus G.H. Horn, 1880e: 181. Type locality: «Fort Cobb [Caddo County, Oklahoma], Indian Territory» (original citation). One syntype [2 originally cited] in MCZ [# 34543] and one in CMNH (collection Ulke).

#### Distribution.

This peculiar species is known from southern Kansas (Barber County, Robert L. Davidson pers. comm. 2012), west-central Oklahoma (Horn 1880e: 181), and northeastern Texas (Hunt County, CNC).

#### Records.

**USA**: KS, OK, TX

#### Note.

The systematic position of this species is not settled. It was not listed by Noonan (1985) in his classification and names of the Selenophori group. The species is markedly distinctive in its structural characters.

### Genus Discoderus

LeConte, 1853

Discoderus LeConte, 1853c: 381. Type species: *Selenophorus parallelus* Haldeman, 1843 designated by Lindroth (1968: 830). Etymology. From the Greek *discos* (circular plate) and *dere* (neck, by extension pronotum), alluding to the rounded lateral edges of the pronota (“*thorace *... *lateribus rotundatis*” for *Discoderus parallelus* and “*magis rotundatis*” for *Discoderus tenebrosus*) of adults of the two species LeConte had before him [masculine].
Selenalius Casey, 1914: 153. Type species: *Discoderus cordicollis* Horn, 1891 by original designation. Synonymy established by Noonan (1985: 48).

#### Diversity.

Twenty-eight species in North America (19 species) and Middle America (11 species).

#### Identification.

This genus has never been revised and such study is much needed. More than 60% of the currently valid North American species have been described by Casey and some of them will certainly fall in synonymy.

### Discoderus aequalis

Casey, 1914

Discoderus aequalis Casey, 1914: 161. Type locality: «Texas» (original citation). Two syntypes in USNM [# 47899].

#### Distribution.

This species is known from western Missouri (Vernon County, UASM), Texas (several Counties, UASM), southeastern New Mexico (Chaves County, UASM), and southeastern Arizona (Cochise County, UASM).

#### Records.

**USA**: AZ, MO, NM, TX

### Discoderus amoenus

LeConte, 1863

Discoderus amoenus LeConte, 1863c: 14. Type locality: «New Mexico» (original citation). Syntype(s) location unknown. Note. The single specimen in the LeConte collection, labeled “Utah” and “Type 5881,” is not a syntype.

#### Distribution.

This species ranges from eastern Wyoming (Lavigne 1977: 45) to southeastern Oregon (Westcott et al. 2006: 8), south to southern California (Fall 1901a: 50) and south-central New Mexico (Fall and Cockerell 1907: 161).

#### Records.

**USA**: AZ, CA, CO, ID, NM, NV, OR, UT, WY

### Discoderus congruens

Casey, 1914

Discoderus congruens Casey, 1914: 162. Type locality: «Arizona (probably southern)» (original citation). One syntype in USNM [# 47893].

#### Distribution.

This species is known from southeastern Colorado (Bent County, CNC), central New Mexico (Bernalillo County, CNC), and southern Arizona (Cochise and Santa Cruz Counties, UASM).

#### Records.

**USA**: AZ, CO, NM

### Discoderus cordicollis

Horn, 1891

Discoderus cordicollis G.H. Horn, 1891: 34. Type locality: «from Fort Yuma eastward to Tucson, Ariz[ona]» (original citation). Ten syntypes in MCZ [# 34541].

#### Distribution.

This species is known from southern Arizona (Horn 1891: 34; Griffith 1900: 565; Cochise and Maricopa Counties, UASM), New Mexico (Hidalgo and Quay Counties, CMNH; Luna County, UASM; Fall and Cockerell 1907: 161), western Texas (Brewster County, UASM), and Coahuila, Sonora, and Baja California Norte in Mexico.

#### Records.

**USA**: AZ, NM, TX – Mexico

### Discoderus crassicollis

Horn, 1891

Discoderus crassicollis G.H. Horn, 1891: 35. Type locality: «southern Arizona» (original citation). Five syntypes in MCZ [# 34540].

#### Distribution.

This species is known, besides the type series, from a few specimens collected in Cochise County in southeastern Arizona (Robert L. Davidson, pers. comm. 2012).

#### Records.

**USA**: AZ

### Discoderus dallasensis

Casey, 1924

Discoderus dallasensis Casey, 1924: 120. Type locality: «Dallas and Amarillo, Texas» (original citation). Eight syntypes [8 originally cited] in USNM [# 47901].

#### Distribution.

This species is known from northern Texas only (Casey 1924: 120).

#### Records.

**USA**: TX

### Discoderus impotens

(LeConte, 1858)

Harpalus impotens LeConte, 1858a: 14. Type locality: «El Paso [El Paso County, Texas]» (original citation). Holotype [by monotypy] (♀) in MCZ [# 5879].

#### Distribution.

This species ranges from Kansas (Knaus 1907: 233; Trego, Scott, and Barber Counties, CMNH, CNC) to southeastern Utah (San Juan County, UASM), south to northern Sonora (Bates 1884: 276), Coahuila (UASM), and southeastern Texas (San Patricio County, UASM).

#### Records.

**USA**: AZ, CO, KS, NM, OK, TX, UT – Mexico

### Discoderus longicollis

Casey, 1914

Discoderus longicollis Casey, 1914: 162. Type locality: «Austin [Travis County], Texas» (original citation). Holotype [by monotypy] (♂) in USNM [# 47894].

#### Distribution.

This species is known only from the type locality in central Texas.

#### Records.

**USA**: TX

### Discoderus obsidianus

Casey, 1914

Discoderus obsidianus Casey, 1914: 158. Type locality: «Arizona (probably southern)» (original citation). One syntype in USNM [# 47892].

#### Distribution.

This species is known only from Cochise County in southeastern Arizona (UASM).

#### Records.

**USA**: AZ

### Discoderus papagonis

Casey, 1924

Discoderus papagonis Casey, 1924: 119. Type locality: «Arizona; Alamogordo [Otero County], New Mexico» (original citation). Two syntypes in USNM [# 47891].

#### Distribution.

This species is known from southeastern Arizona (Cochise County, UASM) and southern New Mexico (Casey 1924: 119).

#### Records.

**USA**: AZ, NM

### Discoderus parallelus

(Haldeman, 1843)

Selenophorus parallelus Haldeman, 1843b: 301. Type locality: southeastern Pennsylvania (Haldeman 1843a: 297). Syntype(s) presumably lost.
Pangus americanus Motschulsky, 1859a: 137 [*nomen dubium*]. Type locality: «Californie» (original citation). Syntype(s) location unknown (possibly in ZMMU though not listed in Keleinikova 1976). Synonymy established by Casey (1914: 163). Note. Horn (1883c: iv) stated that this species is probably identical with *Discoderus impotens* (LeConte) and that Motschulsky’s specimen(s) came from the Piccolomini collection. If exact, then the type locality is incorrect and the specimen(s) has probably been collected in Texas or northern Mexico.
Discoderus hesperus Casey, 1914: 163. Type locality: «Boulder [Boulder County], Colorado» (original citation for the lectotype). Lectotype (♀), designated by Lindroth (1975: 141), in USNM [# 47905]. Synonymy established by Lindroth (1968: 831).
Discoderus parvuliceps Casey, 1924: 121. Type locality: «Wray [Yuma County], Colorado» (original citation). Lectotype (♂), designated by Lindroth (1975: 141), in USNM [# 47903]. Synonymy established by Lindroth (1968: 831).
Discoderus gener Casey, 1924: 121. Type locality: «Wawawai [Whitman County], Washington» (original citation). Lectotype (♂), designated by Lindroth (1975: 141), in USNM [# 47904]. Synonymy established by Hatch (1953: 178), confirmed by Lindroth (1968: 831).

#### Distribution.

This species ranges from southeastern New Hampshire (Rockingham County, Ross T. Bell pers. comm. 1992) to the Okanagan Valley in south-central British Columbia (Lindroth 1968: 831), south to northern Oregon (Union, Wallowa, and Wasco Counties, James R. LaBonte pers. comm. 2009), northern Utah (Salt Lake County, Foster F. Purrington pers. comm. 2009), New Mexico (Fall and Cockerell 1907: 161), western and central Texas (Lee and Brewster Counties, MCZ; Lindroth 1968: 831), southwestern Alabama (Clarke County, CMNH), central Georgia (Fattig 1949: 49), and eastern South Carolina (Ciegler 2000: 104).

#### Records.

**CAN**: BC, ON **USA**: AL, AR, AZ, CO, DC, GA, IA, ID, IL, IN, KS, KY, LA, MD, MI, MN, MO, MS, MT, NC, NE, NH, NJ, NM, OH, OK, OR, PA, SC, SD, TN, TX, UT, VA, WA, WI, WY

### Discoderus parilis

(Casey, 1914)

Selenalius parilis Casey, 1914: 155. Type locality: «El Paso [El Paso County], Texas» (original citation). Two syntypes in USNM [# 47890].

#### Distribution.

This species is known only from the type locality in western Texas.

#### Records.

**USA**: TX

### Discoderus peregrinus

Casey, 1924

Discoderus peregrinus Casey, 1924: 120. Type locality: «El Paso [El Paso County], Texas» (original citation). One syntype in USNM [# 47895].

#### Distribution.

This species is known only from the type locality in western Texas.

#### Records.

**USA**: TX

### Discoderus pinguis

Casey, 1884

Discoderus pinguis Casey, 1884c: 75. Type locality: «Arizona» (original citation). One syntype [6 originally cited] in USNM [# 47897].

#### Distribution.

This species is known from southern Arizona (Cochise County, UASM).

#### Records.

**USA**: AZ

### Discoderus robustus piceus

Casey, 1914

Discoderus robustus piceus Casey, 1914: 159. Type locality: «near Benson [Cochise County], Arizona» (original citation). Twelve syntypes [12 originally cited] in USNM [# 47896].

#### Distribution.

This subspecies is known only from the type locality in southeastern Arizona.

#### Records.

**USA**: AZ

### Discoderus robustus robustus

Horn, 1883

Discoderus robustus G.H. Horn, 1883a: 52. Type locality: «Arizona» (original citation). Syntype(s) in MCZ [# 34539].

#### Distribution.

This subspecies ranges from southern Arizona (Wickham 1898: 301; Griffith 1900: 565; Snow 1906b: 162; Snow 1907: 142) and northern Sonora in Mexico (Bates 1884: 276, as *Discoderus* ?, see Horn 1886a: ix) to western Texas (Brewster and Jeff Davis Counties, CMNH, UASM).

#### Records.

**USA**: AZ, NM, TX – Mexico

### Discoderus subviolaceus

Casey, 1914

Discoderus subviolaceus Casey, 1914: 160. Type locality: «near Benson [Cochise County], Arizona» (original citation). One syntype in USNM [# 47898].

#### Distribution.

This species is known from Arizona (Cochise, Gila, Graham, Pima, Santa Cruz, and Yavapai Counties, UASM; Casey 1914: 160) and western Texas (Terrell and Jeff Davis Counties, UASM).

#### Records.

**USA**: AZ, TX

### Discoderus symbolicus

Casey, 1914

Discoderus symbolicus Casey, 1914: 161. Type locality: «Arizona (probably southern)» (original citation). One syntype in USNM [# 47902].

#### Distribution.

This species is known only from the type series.

#### Records.

**USA**: AZ

### Discoderus tenebrosus

(LeConte, 1847)

Sel ﻿*enophorus tenebrosus* LeConte, 1847: 391. Type locality: «ad Rocky Mountains» (original citation). Three syntypes in MCZ [# 5880].

#### Distribution.

This species has been recorded only from Santa Fe in New Mexico (Fall and Cockerell 1907: 161).

#### Records.

**USA**: NM

### Discoderus texanus

Casey, 1924

Discoderus texanus Casey, 1924: 119. Type locality: «Dallas [Dallas County], Texas» (original citation). Two syntypes [2 originally cited] in USNM [# 47900].

#### Distribution.

This species is known only from the type locality in northeastern Texas.

#### Records.

**USA**: TX

### Genus Stenomorphus

Dejean, 1831

Stenomorphus Dejean, 1831: 696. Type species: *Stenomorphus angustatus* Dejean, 1831 by monotypy. Etymology (original). From the Greek *stenos* (slender) and *morphe* (form), alluding to the slender form (“*corselet très-allongé *... *élytres allongées*”) of the species of this genus [masculine].
Agaosoma Ménétriés, 1843: 63. Type species: *Agaosoma californicum* Ménétriés, 1843 by monotypy. Synonymy established by Chaudoir (1844: 478). Etymology (original). From the Greek *agaios* (admirable) and *soma* (body), alluding to the unusual and agreeable body form of adults of this genus [neuter].

#### Diversity.

Six species in North America (three species), Middle and South America (five species), and the West Indies (two species, one of them endemic).

#### Identification.

Ball et al. (1991) revised the species.

#### Taxonomic Note.

Ball et al. (1991: 942) postulated that the Neotropical genus *Trichopselaphus* Chaudoir (eight species) is the sister-group to this genus and that *Anisocnemus* Chaudoir (two Neotropical species) is the sister-group to {*Trichopselaphus* + *Stenomorphus*}.

### [angustatus group]

### Stenomorphus californicus californicus

(Ménétriés, 1843)

Agaosoma californicum Ménétriés, 1843: 63. Type locality: «Californie» (original citation), which is very unlikely (see Ball et al. 1991: 960); Douglas, Cochise County, Arizona (see Ball et al. 1991: 961) herein selected. Lectotype (♂), designated by Ball et al. (1991: 960), in MCZ [# 8235].
Stenomorphus rossi Van Dyke, 1943: 29. Type locality: «Chiricahua [Cochise County], Arizona» (original citation). Holotype (♂) in CAS [# 5313]. Synonymy established by Ball et al. (1991: 960).

#### Distribution.

This subspecies is known from a small area in southeastern Arizona and southwestern New Mexico [see Ball et al. 1991: Fig. 24].

#### Records.

**USA**: AZ, NM

#### Note.

Besides the two subspecies found in North America, two other subspecies are known, one (*Stenomorphus californicus manni* Darlington) from Haiti and the other (*Stenomorphus californicus darlingtoni* Ball and Shpeley) from Central America.

### Stenomorphus californicus rufipes

LeConte, 1858

Stenomorphus rufipes LeConte, 1858b: 59. Type locality not stated; «Brownsville, Cameron County, Texas» selected by Ball et al. (1991: 958). Lectotype [as holotype] (♀), designated by Ball et al. (1991: 958), in MCZ [# 5882].
Stenomorphus batesi Casey, 1914: 168. Type locality: «Guanajuato, Mexico» (holotype label). Holotype [by monotypy] (♂) in BMNH (Ball et al. 1991: 958). Synonymy established by Ball et al. (1991: 958). Etymology. The specific name was proposed in honor of the English naturalist Henry Walter Bates [1825-1892], one of the most remarkable and progressive systematists of his time. After collecting for 11 years in different areas of Amazonia, Bates returned to England and eventually took the position of Assistant Secretary at the Royal Geographical Society of London. Bates published mainly on the systematics of Lepidoptera and Coleoptera. Note. Casey (1914: 168) described this taxon from the illustration published in Bates (1882a: plate III, figure 22). The specimen upon which the illustration was drawn is in the British Museum (Natural History) and is *de facto* the holotype (ICZN 1999: Article 72.5.6).
Stenomorphus scolopax Casey, 1914: 169. Type locality: «Fort Worth [Tarrant County], Texas» (original citation). Lectotype (♂), designated by Ball et al. (1991: 958), in USNM [# 47907]. Synonymy established by Ball et al. (1991: 959).
Stenomorphus parallelus Casey, 1924: 122. Type locality: «McPherson [McPherson County], Kansas» (original citation). Lectotype [as holotype] (♀), designated by Ball et al. (1991: 958), in USNM [# 47908]. Synonymy established by Ball et al. (1991: 959).
Stenomorphus arcuatus Casey, 1924: 122. Type locality: «Dallas [Dallas County], Texas» (original citation). Holotype [by monotypy] (♀) in USNM [# 47909]. Synonymy established by Ball et al. (1991: 959).

#### Distribution.

This subspecies ranges from northern Missouri to southern Nebraska (Adams County, Foster F. Purrington pers. comm. 2009), south to the Yucatán Peninsula, west to the southern portion of the Baja California Peninsula, east to central Alabama [see Ball et al. 1991: Fig. 24]. One specimen is known from Clemson, South Carolina (Ciegler 2000: 104).

#### Records.

**USA**: AL, AR, AZ, CA, KS, LA, MO, MS, NE, OK, TX [SC] – Mexico

### [convexior group]

### Stenomorphus convexior

Notman, 1922

Stenomorphus convexior Notman, 1922b: 103. Type locality: «Tucson [Pima County], Ariz[ona]» (original citation). Holotype (♀) in USNM [# 26593].

#### Distribution.

This species ranges from southern Arizona southwards along the Pacific Versant of Mexico to central Jalisco [see Ball et al. 1991: Fig. 21].

#### Records.

**USA**: AZ – Mexico

### [sinaloae group]

### Stenomorphus sinaloae

Darlington, 1936

Stenomorphus sinaloae Darlington, 1936a: 37. Type locality: «Sinaloa, Mex[ico]» (original citation). Holotype (♂) in USNM [# 75661].

#### Distribution.

This species ranges from southeastern Arizona and southwestern New Mexico south along the Pacific Versant of Mexico to Guerrero; it is also known from the southern parts of the Baja California Peninsula [see Ball et al. 1991: Fig. 23]. As pointed out by Ball et al. (1991: 951) the record from central Colorado based on a male is questionable but not impossible.

#### Records.

**USA**: AZ, NM [CO] – Mexico

### Genus Trichotichnus

Morawitz, 1863

Trichotichnus Morawitz, 1863: 63 [*nomen protectum*, see Bousquet (2008b: 328)]. Type species: *Trichotichnus longitarsis* Morawitz, 1863 by monotypy. Etymology. From the Greek *trichotos* (hairy) and *ichnos* (footprint, track), probably alluding to the long setae present on both side of the tarsomeres (“*Die Füsse sind unten zu beiden Seiten mit langen Seidenhaaren besetzt*”) of the adult [masculine].

#### Diversity.

About 250 species (Lorenz 2005: 380-382), more than 85% of them inhabiting Asia, arrayed in six subgenera: *Amaroschesis* Tschitschérine (about 55 species), *Bellogenus* Clarke (about 45 species), *Harpaloxenus* Schauberger (12 species), *Iridessus* (six species), *Lampetes* Andrewes (nine species), and *Trichotichnus* s.str. (about 120 species). The Western Hemisphere has only four species, all endemic to eastern North America.

#### Identification.

Lindroth (1968) reviewed all four North American species, one of them (*Trichotichnus fulgens*) under the genus *Harpalus*.

### Subgenus Trichotichnus

Morawitz, 1863

Trichotichnus Morawitz, 1863: 63. Type species: *Trichotichnus longitarsis* Morawitz, 1863 by monotypy.
Asmerinx Tschitschérine, 1898b: 183. Type species: *Carabus laevicollis* Duftschmid, 1812 designated by Tschitschérine (1900a: 363). Synonymy established by Reitter (1908: 174).
Pteropalus Casey, 1914: 64, 131. Type species: *Harpalus vulpeculus* Say, 1823 designated by Habu (1954a: 245). Synonymy established by Csiki (1932a: 1217).
Carbanus Andrewes, 1937: 27. Type species: *Carbanus flavipes* Andrewes, 1937 (= *Trichotichnus claripes* Lorenz, 1998) by monotypy. Synonymy established by Noonan (1976: 43).
Velimius Jedlička, 1952: 51. Type species: *Velimius edai* Jedlička, 1952 by monotypy. Synonymy established by Habu (1973a: 224).
Lyter Darlington, 1968: 40, 63. Type species: *Lyter glaber* Darlington, 1968 by original designation. Synonymy established by Noonan (1985: 67).

#### Diversity.

About 120 species in the Nearctic (two eastern species), Australian (six species), Oriental (about 25 species), and Palaearctic (about 90 species, the vast majority in its eastern part) Regions.

### Trichotichnus dichrous

(Dejean, 1829)

Harpalus dichrous Dejean, 1829: 258. Type locality: «Amérique septentrionale» (original citation), restricted to «S[ain]t Charles [Saint Charles County], Missouri» by Lindroth (1968: 819). Holotype [by monotypy] (♀) in MHNP (Lindroth 1955b: 27).
Pteropalus fluvialis Casey, 1914: 133. Type locality: «S[ain]t Louis, Missouri» (original citation). Lectotype (♀), designated by Lindroth (1975: 141), in USNM [# 47864]. Synonymy established by Lindroth (1968: 819).
Pteropalus versutulus Casey, 1924: 116. Type locality: «Southern Pines [Moore County], North Carolina» (original citation). Lectotype (♂), designated by Lindroth (1975: 141), in USNM [# 47865]. Synonymy established by Lindroth (1968: 819).

#### Distribution.

The range of this species extends from southern Quebec (Serge Laplante pers. comm. 2002) to southeastern South Dakota (Kirk and Balsbaugh 1975: 30), south to “Texas” (Lindroth 1968: 819) and central South Carolina (Ciegler 2000: 105).

#### Records.

**CAN**: ON, QC **USA**: AL, AR, CT, DC, DE, GA, IA, IL, IN, KS, KY, LA, MA, MD, MI, MO, MS, NC, NH, NJ, NY, OH, OK, PA, SC, SD, TN, TX, VA, VT, WI, WV

#### Note.

Say (1830c: 19) noted that “*H[arpalus] iricolor*, Say, has been recently described by Dejean under the name of *dichrous*.” I have not found any species described by Say under the name *Harpalus iricolor*. Say’s name has been listed as a junior synonym of *Trichotichnus dichrous* (Dejean) in several checklists but it is a *nomen nudum* as pointed out by Lindroth and Freitag (1969: 353).

**Figure 34.**
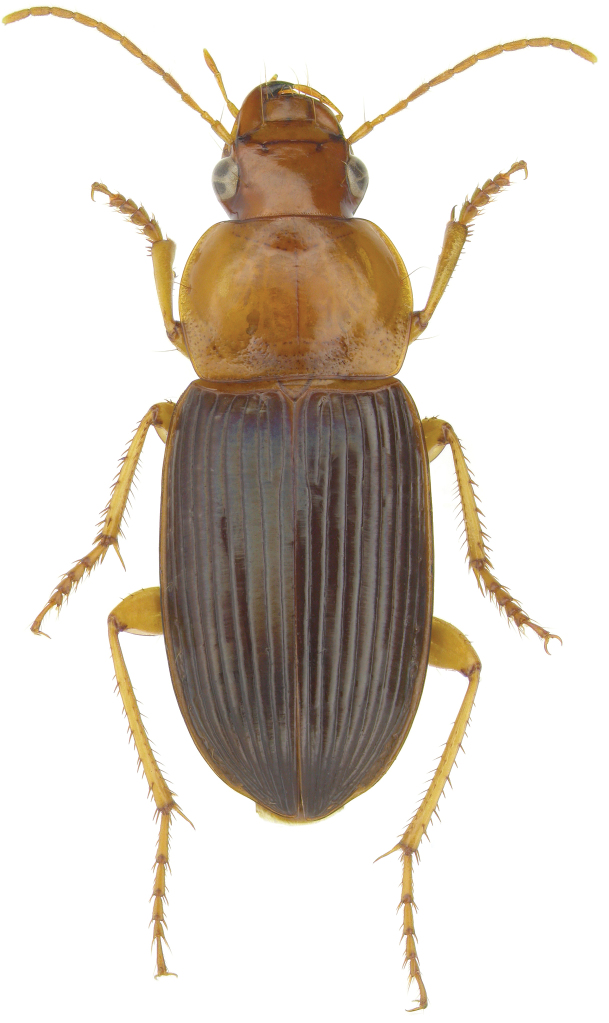
*Trichotichnus dichrous* (Dejean). This eastern species is color-dimorphic, a phenomenon rarely seen in North American carabids. While most adults have the head and pronotum reddish-yellow and the elytra much darker, a small number of adults are known to be uniformly piceous. When he described the species, Dejean had a single specimen of the common morph. The strong color contrast between the forebody and elytra prompt him to propose the name *dichrous*, from the Greek prefix *di*- (two) and *chroma* (color of the skin).

### Trichotichnus vulpeculus

(Say, 1823)

Harpalus vulpeculus Say, 1823a: 30. Type locality: «Washington, D[istrict of] C[olumbia]» (neotype label). Neotype (♂), designated by Lindroth and Freitag (1969: 353), in MCZ [# 32990].
Harpalus nigripennis Dejean, 1829: 260. Type locality: «Amérique septentrionale» (original citation). One syntype in MHNP (Lindroth 1955b: 27). Synonymy established by Say (1830c: 19), confirmed by Lindroth (1955b: 27).

#### Distribution.

The range of this species extends from western New Brunswick (Webster and Bousquet 2008: 19) to southwestern Wisconsin (Grant County, Peter W. Messer pers. comm. 2008), south to northern Arkansas (Boone County, UASM) and eastern Georgia (Fattig 1949: 47). The records from “Florida” and “Minnesota” (Bousquet and Larochelle 1993: 244) need confirmation.

#### Records.

**CAN**: NB, ON, QC **USA**: AL, AR, CT, DC, DE, GA, IA, IL, IN, KY, MA, MD, ME, MI, MO, NC, NH, NJ, NY, OH, PA, RI, TN, VA, VT, WI, WV [FL, MN]

### Subgenus Iridessus

Bates, 1883

Argestes J.E. LeConte, 1849: 26 [*nomen oblitum*, see Bousquet (2008b: 328)]. Type species: *Harpalus nitidulus* Chaudoir, 1843 (= *Trichotichnus fulgens* Csiki, 1932) by monotypy. Etymology. Name of the north-west wind in classical antiquity. Note. Hardy et al. (1986: 472) argued that the paper in which this name was proposed was written by J.E. LeConte and not his son, J.L. LeConte.
Iridessus Bates, 1883b: 240. Type species: *Harpalus lucidus* Morawitz, 1863 designated by Habu (1954a: 245). Etymology. Uncertain, possibly from the Latin *iris* (rainbow) and the French noun *dessus* (above), alluding to the iridescence on the elytra of adults of the species included by Bates in this taxon [masculine].
Episcopellus Casey, 1914: 235. Type species: *Feronia autumnalis* Say, 1823 by original designation. Synonymy established by Ball and Bousquet (2000: 96). Etymology. Uncertain, possibly from the Greek *episcopos* (overseer) and the suffix -*ellus* (small, little) [masculine].

#### Diversity.

Six species in the Nearctic (two species) and east Palaearctic (four species) Regions.

#### Taxonomic Note.

Kataev (in Ball and Bousquet 2000: 96) revalidated *Iridessus* Bates as a subgenus, considered by most authors as a synonym of *Trichotichnus*.

### Trichotichnus autumnalis

(Say, 1823)

Feronia autumnalis Say, 1823a: 48. Type locality: «Nahant [Essex County], Mass[achusetts]» (neotype label). Neotype (♂), designated by Lindroth and Freitag (1969: 355), in MCZ [# 32980].
Episcopellus nitescens Casey, 1914: 236. Type locality: «District of Columbia» (original citation). Lectotype (♀), designated by Lindroth (1975: 140), in USNM [# 47993]. Synonymy established by Lindroth (1968: 813).

#### Distribution.

This species ranges from west-central Maine (Majka et al. 2011: 46) and southern Quebec (CNC) to east-central Minnesota (Gandhi et al. 2011: 673), north to Kapuskasing in central Ontario (CNC), south at least to northeastern Kansas (Popenoe 1877: 24), central Arkansas (Pulaski County, Robert L. Davidson pers. comm. 2008), southwestern Mississippi (Hinds County, CMNH), northern Georgia (Fattig 1949: 52), and eastern South Carolina (Ciegler 2000: 105).

#### Records.

**CAN**: ON, QC **USA**: AR, CT, DC, GA, IA, IL, IN, KS, KY, MA, MD, ME, MI, MN, MO, MS, NC, NH, NJ, NY, OH, PA, RI, SC, VA, VT, WI, WV

### Trichotichnus fulgens

(Csiki, 1932)

Harpalus nitidulus Chaudoir, 1843b: 788 [primary homonym of *Harpalus nitidulus* Stephens, 1828]. Type locality: «Nouvelle Orléans [Orleans Parish, Louisiana]» (original citation). Lectotype (♂), designated by Lindroth (1968: 811), in MHNP.
Harpalus fulgens Csiki, 1932a: 1182 [primary homonym of *Harpalus fulgens* Dejean, 1829]. Replacement name for *Harpalus nitidulus* Chaudoir, 1843. Note. This name is a junior primary homonym of *Harpalus fulgens* Dejean, 1829 (= *Notiobia chalcitis* (Germar, 1824)). Since both names apply to taxa not considered congeneric since 1899, the case is to be referred to the Commission and meanwhile prevailing usage of both names must be maintained (ICZN 1999: Article 23.9.5).

#### Distribution.

This eastern species extends from Massachusetts (Middlesex County, Peter W. Messer pers. comm. 2008) to northeastern Kansas, including southernmost Ontario (Bousquet 1987a: 130) and southeastern Iowa, south to southeastern Texas and southern Florida, west to western Texas [see Noonan 1991: Fig. 286]. The record from southern Wisconsin (Rauterberg 1885: 21, as *Harpalus nitidulus*) needs confirmation.

#### Records.

**CAN**: ON **USA**: AL, AR, CT, DC, FL, GA, IA, IL, IN, KS, KY, LA, MA, MD, MO, MS, NC, NJ, NY, OH, OK, PA, SC, TN, TX, VA, WV [WI]

#### Note.

This species was included in the genus *Harpalus* Latreille by Lindroth (1968: 811) and Noonan (1991: 136) but placed in the genus *Trichotichnus* Morawitz by Ball and Bousquet (2000: 96) on information provided *in litteris* by Boris Kataev.

### Genus Aztecarpalus

Ball, 1970

Aztecarpalus Ball, 1970: 102. Type species: *Harpalus hebescens* Bates, 1882 by original designation. Etymology (original). From Aztec (name of the most important group of Indians living in Mexico) and the generic name *Harpalus* [*q.v*.], alluding to the homeland and superficial affinities of the species to those of the genus *Harpalus* [masculine].

#### Diversity.

Nine species in eastern Mexico, one of them extends into southeastern Texas.

#### Identification.

Ball (1970, 1976a) revised the species and provided a key (Ball 1976a: 62-63) for their identification.

### Aztecarpalus schaefferi

Ball, 1970

Harpalus iripennis Schaeffer, 1910: 402 [primary homonym of *Harpalus iripennis* Say, 1823]. Type locality: «Brownsville [Cameron County], Texas» (original citation). Lectotype (♂), designated by Erwin and House (1978: 238), in USNM [# 42510].
Aztecarpalus schaefferi Ball, 1970: 119. Replacement name for *Aztecarpalus iripennis* (Schaeffer, 1910).

#### Distribution.

This species is known only from southeastern Texas and northeastern Mexico (Ball 1970: 120).

#### Records.

**USA**: TX – Mexico

### Genus Cratacanthus

Dejean, 1829

Cratacanthus Dejean, 1829: 40. Type species: *Cratacanthus pensylvanicus* Dejean, 1829 (= *Harpalus dubius* Palisot de Beauvois, 1811) by monotypy. Etymology (original). From the Greek *cratos* (strong) and *acanthos* (spine), alluding to the strong labial tooth (“*menton *... *au milieu de son échancrure une forte dent aigüe, presque en épine*”) of the adult [masculine].

#### Diversity.

One North American species in the temperate regions.

#### Identification.

The species is covered in Lindroth’s (1968: 744-745) monograph.

### Cratacanthus dubius

(Palisot de Beauvois, 1811)

Harpalus dubius Palisot de Beauvois, 1811: 108. Type locality: «Pensylvanie» (original citation), herein restricted to Philadelphia, Philadelphia County (see Casey 1884c: 75, as *Cratacanthus bisectus*). Syntype(s) probably lost (Lindroth 1968: 744).
Cratacanthus pensylvanicus Dejean, 1829: 41. Type locality: «Amérique septentrionale» (original citation). One syntype in MHNP (Lindroth 1955b: 26). Synonymy established by Duponchel (1843: 327), confirmed by Lindroth (1955b: 26).
Cratacanthus litoreus Casey, 1884c: 74. Type locality: «Atlantic City [Atlantic County], New Jersey» (original citation). Lectotype (♀), designated by Lindroth (1975: 137), in USNM [# 47731]. Synonymy established by Horn (1885b: 108).
Cratacanthus bisectus Casey, 1884c: 75. Type locality: «Fairmont Park, Philadelphia [Philadelphia County, Pennsylvania]» (original citation). Lectotype (♂), designated by Lindroth (1975: 137), in USNM [# 47730]. Synonymy established by Horn (1885b: 108).
Cratacanthus texanus Casey, 1884c: 75. Type locality: «Texas» (original citation). Lectotype (♀), designated by Bousquet and Larochelle (1993: 13), in USNM [# 47732]. Synonymy established by Horn (1885b: 108), confirmed by Bousquet and Larochelle (1993: 13).
Cratacanthus subovalis Casey, 1914: 59. Type locality: «southern Atlantic seaboard» (original citation). Lectotype (♀), designated by Bousquet and Larochelle (1993: 13), in USNM [# 47733]. Synonymy established by Bousquet and Larochelle (1993: 13).
Cratacanthus cephalotes Casey, 1914: 59. Type locality: «S[ain]t Louis, Missouri» (original citation). Lectotype (♂), designated by Bousquet and Larochelle (1993: 13), in USNM [# 47734]. Synonymy established by Bousquet and Larochelle (1993: 13).

#### Distribution.

The range of this species extends from Long Island, New York (Notman 1928: 244), to southeastern Alberta (Lindroth 1968: 745), south to southern Arizona (Cochise, Greenlee and Pima Counties, CNC, UASM), Durango (Bates 1891a: 241) and southern Coahuila (UASM) in Mexico, western Alabama (Pickens and Tuscaloosa Counties, UASM), and “Florida” (Leng 1920: 70, as *Cratacanthus subovalis*).

#### Records.

**CAN**: AB, SK **USA**: AL, AR, AZ, CO, DC, DE, FL, GA, IA, ID, IL, IN, KS, KY, LA, MD, MI, MN, MO, MS, MT, NC, ND, NE, NJ, NM, NY, OH, OK, PA, SC, SD, TN, TX, UT, VA, WI, WY – Mexico

**Figure 35.**
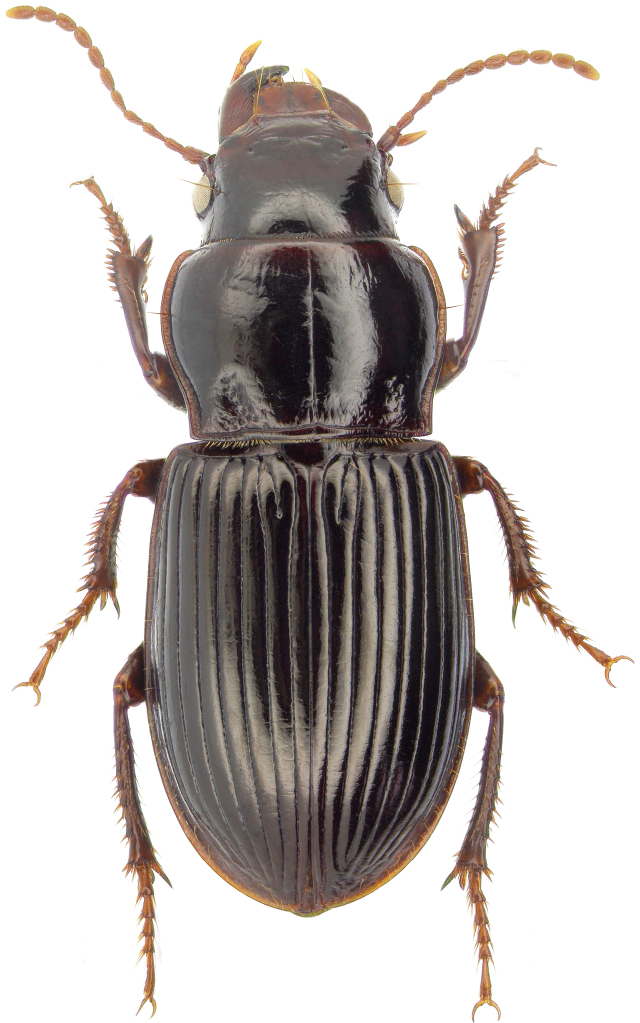
*Cratacanthus dubius* (Palisot de Beauvois). This widely distributed and morphologically variable species was described by the French naturalist traveler Ambroise Marie Joseph Palisot, Baron de Beauvois, one of the first entomologists to collect and describe American insects. On North American soil, Palisot de Beauvois collected from the Ohio River in the west to Savannah in Georgia. Unfortunately most of his collection was lost in a shipwreck off Nova Scotia in 1798 and his subsequent published descriptions were based on his notes and drawings.

### Tribe Sphodrini

Laporte, 1834

Sphodridae Laporte, 1834: 78. Type genus: *Sphodrus* Clairville, 1806.

#### Diversity.

About 825 species in the Nearctic (13 species), Neotropical (16 species in Mexico), Oriental (about eight species), Palaearctic (about 775 species), and Afrotropical (17 species in Ethiopia) Regions, arrayed in six subtribes: Atranopsina (about 100 species), Calathina (about 185 species), Dolichina (17 species), Pristosiina (about 65 Asian species), Synuchina (about 100 species), and Sphodrina (about 360 species).

#### Taxonomic Note.

Several authors, including Lindroth (1966) and Ball and Bousquet (2000), placed the sphodrines within the tribe Platynini.

### Subtribe Atranopsina

Baehr, 1982

Atranopsina Baehr, 1982: 265. Type genus: *Atranopsis* Baehr, 1982.

#### Diversity.

About 100 species in Europe (about 60 species), northern Africa (about 20 species), western Asia, including India (about 25 species), and eastern North America (one species); most species occur in the Mediterranean region.

#### Taxonomic Note.

Lindroth (1968: 653) listed the genus *Pseudamara* in the tribe Amarini (= Zabrini) and pointed out that the sole species of the genus was “not very closely related to *Amara*”. Recently Hieke (2010) concluded that *Pseudamara* belongs to the subtribe Atranopsina, of the Sphodrini, and was probably closely related to the genus *Amaroschema* Jeannel, which includes a single species in the Canary Islands.

### Genus Pseudamara

Lindroth, 1968

Pseudamara Lindroth, 1968: 653. Type species: *Geobaenus arenarius* LeConte, 1847 by original designation. Etymology. From the Greek *pseudos* (fallacy, lie) and the generic name *Amara* [*q.v*.] [feminine].
Disamara Lindroth, 1976b: 132. Unnecessary replacement name for *Pseudamara* Lindroth, 1968. Note. Lindroth (1976b: 132) incorrectly assumed that his name *Pseudamara* was a junior homonym of *Pseudoamara* Baliani, 1934.

#### Diversity.

One species in the boreal and temperate regions of eastern North America.

#### Identification.

Lindroth (1968: 653-654) treated the species in his monograph.

### Pseudamara arenaria

(LeConte, 1847)

Geobaenus arenarius LeConte, 1847: 403. Type locality: «provinciis orientalibus» (original citation), restricted to «M[oun]t Washington [Coos County], N[ew] H[ampshire]» by Lindroth (1968: 654). One syntype in MCZ [# 5673].

#### Distribution.

This species is found from Cape Breton Island (Bousquet 1987a: 128) to northern Minnesota (Gandhi et al. 2005: 929), south to southwestern North Carolina (Macon County, MCZ).

#### Records.

**CAN**: NB, NS (CBI), ON, PE, QC **USA**: IL, MA, ME, MI, MN, NC, NH, NY, OH, PA, RI, VA, VT, WI, WV

### Subtribe Calathina

Laporte, 1834

Calathidae Laporte, 1834: 71. Type genus: *Calathus* Bonelli, 1810.

#### Diversity.

About 185 species (Lorenz 2005: 396-399) placed in the genus *Calathus*. Based on recent molecular sequence analyses (Ruiz et al. 2009), *Synuchidius* Apfelbeck and *Thermoscelis* Putzeys, often ranked as distinct genera, are considered subgenera of *Calathus*.

### Genus Calathus

Bonelli, 1810

Calathus Bonelli, 1810: Tabula Synoptica. Type species: *Carabus cisteloides* Panzer, 1793 (= *Carabus fuscipes* Goeze, 1777) designated by Curtis (1827: plate 184). Etymology. Possibly from the Greek *calathos* (basket) [masculine].

#### Diversity.

About 185 species (Lorenz 2005: 396-399) in North America (eight species, one of them adventive), Mexico (14 species), Ethiopia (17 species), and the Palaearctic Region (about 145 species) arrayed in 11 subgenera: *Calathus* s.str. (50 species), *Lauricalathus* Machado (19 species endemic to the Canary Islands), *Trichocalathus* Bolívar y Pieltain (three species endemic to the Canary Islands), *Neocalathus* (45 species), *Amphyginus* Haliday (two European species), *Tachalus* Ball and Nègre (one Mexican species), *Bedelinus* Ragusa (one species in Europe and northern Africa), *Lindrothius* Kurnakov (13 species restricted to Caucasia), *Acalathus* (11 species), *Synuchidius* Apfelbeck (one southeast European species), and *Thermoscelis* Putzeys (one Caucasian species). About 40 species, including all those from Ethiopia, are currently unplaced (Lorenz 2005: 398-399).

#### Identification.

Ball and Nègre (1972) revised the Western Hemisphere species and provided a key for their identification.

### Subgenus Calathus

Bonelli, 1810

Calathus Bonelli, 1810: Tabula Synoptica. Type species: *Carabus cisteloides* Panzer, 1793 (= *Carabus fuscipes* Goeze, 1777) designated by Curtis (1827: plate 184).

#### Diversity.

Fifty species (Lorenz 2005: 396-397) in Europe, the Middle East, and Nepal. Most of the species are endemic to the Mediterranean region. One species is adventive in western North America.

#### Taxonomic Note.

*Fuscocalathus* Nègre (1969: 7) is usually cited as a junior synonym of this subgenus following Ball and Nègre (1972: 510). However, the name is a *nomen nudum* since Nègre (1969) failed to designate a type species for his new taxon.

### Calathus fuscipes

(Goeze, 1777)

Carabus fuscipes Goeze, 1777: 666. Type locality not stated; «n[ea]r Paris, France» selected by Lindroth (1966: 542). Syntype(s) possibly in MHNP. Note. This taxon was first described by Geoffroy (1762: 161) under the name “*Bupreste noir à pattes brunes*” as mentioned by Goeze (1777: 666). Goeze (1777: 666) reproduced Geoffroy’s original description in Latin and provided a scientific name. I consider that Goeze’s name was made available by a bibliographic reference to a description and as such the type series consists of Geoffroy’s specimens (ICZN 1999: Article 72.4.4).
Carabus cisteloides Panzer, 1793: no 12. Type locality: «Brunsvigiae [= Brunswick, Germany]» (original citation). Syntype(s) location unknown (possibly in ZMHB). Synonymy established by Schönherr (1806: 195).

#### Distribution.

This Palaearctic species is adventive in North America where it is known from southwestern British Columbia (Lindroth 1966: 543) to northwestern Oregon (Westcott et al. 2006: 7). The first inventoried specimen collected on this continent was found in the vicinity of Vancouver, British Columbia, in 1928 (Hatch 1949c: 151).

#### Records.

**CAN**: BC **USA**: OR, WA – **Adventive**

### Subgenus Neocalathus

Ball and Nègre, 1972

Neocalathus Ball and Nègre, 1972: 426. Type species: *Carabus melanocephalus* Linnaeus, 1758 by original designation. Etymology. From the Greek prefix *neo*- (new) and the generic name *Calathus* [*q.v*.] [masculine].

#### Diversity.

Forty-five species in North America (six species), Mexico (13 species), and the Palaearctic Region (27 species, no endemic species in eastern Asia).

#### Taxonomic Note.

Gañán and Novoa (2006) considered *Neocalathus* as a synonym of *Amphyginus* Haliday, 1841 (type species: *Carabus piceus* Marsham, 1802 (= *Calathus rotundicollis* Dejean, 1828)). If this approach is followed, then *Amphyginus* is the valid name for this subgenus (see Alonso-Zarazaga 2006).

#### Faunistic Note.

The record of *Calathus ambigens* Bates from Huachuca Mountains in southeastern Arizona (Schaeffer 1910: 394) is probably in error since the species is recorded only from Durango and Chihuahua in Mexico by Ball and Nègre (1972: 461-462).

### Calathus calceus

Ball and Nègre, 1972

Calathus calceus Ball and Nègre, 1972: 489. Type locality: «Sprague R[iver] Canyon, 5 mi[les] E[ast] Bly, Klamath County, Oregon» (original citation). Holotype (♂) in CAS [# 11313].

#### Distribution.

This species ranges from the southern Gulf Islands of southwestern British Columbia (James C. Bergdahl pers. comm. 1993) to southern Idaho, south to northern Utah and Mono County in the Sierra Nevada of California [see Ball and Nègre 1972: Fig. 53].

#### Records.

**CAN**: BC **USA**: CA, ID, NV, OR, UT, WA

### Calathus gregarius

(Say, 1823)

Feronia gregaria Say, 1823a: 47. Type locality: «Phila[delphia] [Philadelphia County], P[ennsylvani]a» (neotype label). Neotype (♀), designated by Lindroth and Freitag (1969: 345), in MCZ [# 33022].
Calathus piceus T.W. Harris, 1828c: 123 (as *piceous*). Type locality not stated. Syntype(s) presumably lost. Synonymy established with doubt by Harris (1833: 567).
Calathus distinguendus LeConte, 1844: 53 (as *distinguenidus*). Type locality: «Georgia» (original citation). Lectotype [as holotype] (♂), designated by Ball and Nègre (1972: 486), in MCZ [# 5728]. Synonymy established by LeConte (1854b: 36), confirmed by Lindroth (1966: 543).

#### Distribution.

This species is found from Cape Breton Island in Nova Scotia (Lindroth 1966: 544) to western North Dakota (Tinerella 2003: 636), south to Nebraska, northern Alabama, and northeastern Georgia (Fattig 1949: 32) [see Ball and Nègre 1972: Fig. 51]. The records from Prince Edward Island (Bousquet and Larochelle 1993: 245, see Majka et al. 2008: 133) and eastern Kansas (Popenoe 1877: 23) need confirmation; those from “Florida,” Texas (Wickham 1896c: 133; Knaus 1905b: 348), and New Mexico (Fall and Cockerell 1907: 159) are likely in error (see Ball and Nègre 1972: 486).

#### Records.

**CAN**: NB, NS (CBI), ON, QC **USA**: AL, CT, DC, DE, GA, IA, IL, IN, KY, MA, MD, ME, MI, MN, MO, NC, ND, NE, NH, NJ, NY, OH, PA, RI, SC, SD, TN, VA, VT, WI, WV [KS, PE]

### Calathus ingratus

Dejean, 1828

Calathus ingratus Dejean, 1828: 77. Type locality: «île d’Ounalaschka, l’une des îles Aleutiennes [Alaska]» (original citation). One possible syntype (♂) in MHNP (Lindroth 1955b: 18).
Calathus incommodus Mannerheim, 1853: 139. Type locality: «ad ostia fl[umen] Kaktnu [= Kenai River] peninsulae Kenai [Alaska]» (original citation for the lectotype). Lectotype (♂), designated by Lindroth (1966: 544), in ZMH. Synonymy established by LeConte (1860: 317), confirmed by Lindroth (1954b: 137).
Calathus confusus LeConte, 1854b: 36. Type locality: «Lake Superior» (original citation). Syntype(s) in MCZ [# 5729]. Synonymy established by LeConte (1860: 317), confirmed by Lindroth (1954b: 136).
Calathus coloradensis Casey, 1913: 157. Type locality: «Boulder Co[unty], Colorado» (original citation). Lectotype (♀), designated by Lindroth (1975: 125), in USNM [# 47548]. Synonymy established by Lindroth (1954b: 137).
Calathus reductus Casey, 1913: 158. Type locality: «Colorado» (original citation). Five syntypes [5 originally cited] in USNM [# 47546]. Synonymy established by Erwin et al. (1977: 4.39).
Calathus acomanus Casey, 1913: 158. Type locality: «New Mexico» (original citation). Two syntypes in USNM [# 47536]. Synonymy established by Erwin et al. (1977: 4.39).
Calathus labradorinus Casey, 1913: 158. Type locality: «West S[ain]t Modest[e], Labrador» (original citation). Lectotype (♀), designated by Lindroth (1975: 125), in USNM [# 47547]. Synonymy established by Lindroth (1954b: 137).
Calathus planifer Casey, 1920: 217. Type locality: «S[ain]t Paul Island, Alaska» (original citation), which is incorrect (Lindroth 1975: 125). Holotype [by monotypy] (♀) in USNM [# 47545]. Synonymy established by Lindroth (1954b: 137).
Calathus beringi Casey, 1920: 218. Type locality: «S[ain]t Paul Island, Alaska» (original citation), which is no doubt incorrect (Lindroth 1975: 125). Lectotype (♂), designated by Lindroth (1975: 125), in USNM [# 47543]. Synonymy established by Lindroth (1954b: 137).
Calathus nanulus Casey, 1920: 218. Type locality: «S[ain]t Paul Island, Alaska» (original citation), which is incorrect (Lindroth 1975: 125). Lectotype (♂), designated by Lindroth (1975: 125), in USNM [# 47544]. Synonymy established by Lindroth (1954b: 137).
Calathus calator Casey, 1920: 220. Type locality: «Peaceful Valley [Boulder County], Colorado» (original citation). Lectotype (♀), designated by Lindroth (1975: 125), in USNM [# 47549]. Synonymy established by Lindroth (1966: 544).
Calathus aquilus Casey, 1920: 220. Type locality: «Ouray [Ouray County], Colorado» (original citation). Lectotype (♂), designated by Lindroth (1975: 125), in USNM [# 47550]. Synonymy established by Lindroth (1954b: 137).

#### Distribution.

The range of this transamerican species extends from Newfoundland (Lindroth 1955a: 112-113) to Alaska, including the Aleutian Islands (Lindroth 1966: 545), south to Washington (Hatch 1953: 131), central Arizona, southern New Mexico, the Black Hills in southwestern South Dakota (Kirk and Balsbaugh 1975: 23), and the Adirondack Mountains in northeastern New York (Notman 1928: 230) [see Ball and Nègre 1972: Fig. 53].

#### Records.

**FRA**: PM **CAN**: AB, BC, LB, MB, NB, NF, NS (CBI), NT, ON, PE, QC, SK, YT **USA**: AK, AZ, CO, ID, ME, MI, MN, MT, ND, NH, NM, NY, SD, UT, VT, WA, WI, WY

### Calathus opaculus

LeConte, 1854

Calathus opaculus LeConte, 1854b: 37. Type locality: «middle, southern and western states» (original citation), restricted to «Lawrence [Douglas County], Kansas» by Lindroth (1966: 543). Lectotype (♀), designated by Ball and Nègre (1972: 487), in MCZ [# 5730].
Calathus sonoricus Casey, 1913: 156. Type locality: «Arizona» (original citation). Lectotype [as holotype] (♂), designated by Ball and Nègre (1972: 487), in USNM [# 47541]. Synonymy established by Lindroth (1966: 543).
Calathus alutaceus Casey, 1913: 157. Type locality: «S[ain]t Louis, Missouri» (original citation). Lectotype [as holotype] (♂), designated by Ball and Nègre (1972: 488), in USNM [# 47537]. Synonymy established by Ball and Nègre (1972: 488).
Calathus appalachius Casey, 1913: 157. Type locality: «Asheville [Buncombe County], North Carolina» (original citation for the lectotype). Lectotype [as holotype] (♀), designated by Ball and Nègre (1972: 488), in USNM [# 47538]. Synonymy established by Ball and Nègre (1972: 488).
Calathus obesulus Casey, 1913: 157. Type locality: «M[oun]t Hope [Sedgwick County], Kansas» (original citation for the lectotype). Lectotype [as holotype], designated by Ball and Nègre (1972: 488), in USNM [# 47539]. Synonymy established by Ball and Nègre (1972: 488).
Calathus ventricosus Casey, 1920: 219. Type locality: «Vicksburg [Warren County], Mississippi» (original citation). Lectotype [as holotype] (♀), designated by Ball and Nègre (1972: 488), in USNM [# 47542]. Synonymy established by Ball and Nègre (1972: 488).

#### Distribution.

The range of this species extends from southern Quebec (Larochelle 1975: 69) to southeastern Utah, south to southeastern Arizona, southern Texas, and northern Florida [see Ball and Nègre 1972: Fig. 52]. The record from New Brunswick (see Majka et al. 2007: 11) is in error (Christopher G. Majka pers. comm. 2009).

#### Records.

**CAN**: ON, QC **USA**: AL, AR, AZ, CO, CT, DC, DE, FL, GA, IA, IL, IN, KS, KY, LA, MA, MD, ME, MI, MN, MO, MS, NC, NE, NH, NJ, NM, NY, OH, OK, PA, RI, SC, TN, TX, UT, VA, VT, WI, WV, WY

### Calathus peropacus

Casey, 1920

Calathus peropacus Casey, 1920: 219. Type locality: «Palmerlee [= Garcés, Cochise County], Arizona» (original citation). Lectotype (♀), designated by Ball and Nègre (1972: 492), in USNM [# 47540].

#### Distribution.

This species is confined, as far as known, to mountains in southeastern Arizona (Ball and Nègre 1972: 505).

#### Records.

**USA**: AZ

### Calathus ruficollis grandicollis

Casey, 1920

Calathus grandicollis Casey, 1920: 215. Type locality: «Hydesville, Valley of Eel River, Humboldt Co[unty], California» (original citation). Lectotype (♂), designated by Ball and Nègre (1972: 468), in USNM [# 47558].

#### Distribution.

The range of this subspecies extends from southwestern British Columbia, including Vancouver Island, south to Humboldt County in northern California [see Ball and Nègre 1972: Fig. 40].

#### Records.

**CAN**: BC (VCI) **USA**: CA, OR, WA

### Calathus ruficollis ignicollis

Casey, 1920

Calathus ignicollis Casey, 1920: 213. Type locality: «Mokelumne Hill, Calaveras Co[unty], California» (original citation). Lectotype [as holotype] (♀), designated by Ball and Nègre (1972: 468), in USNM [# 47556].

#### Distribution.

This subspecies occurs from eastern Washington and western Idaho south to central California [see Ball and Nègre 1972: Fig. 40].

#### Records.

**USA**: CA, ID, OR, WA

### Calathus ruficollis ruficollis

Dejean, 1828

Calathus ruficollis Dejean, 1828: 78. Type locality: «Californie» (original citation), restricted to «Los Angeles [Los Angeles County]» by Lindroth (1966: 545). One syntype in MHNP (Lindroth 1955b: 18).
Calathus behrensii Mannerheim, 1843: 195. Type locality: «California ad Ross [farming community about 75 miles north of San Francisco along the coast]» (original citation). Lectotype [as holotype] (♀), designated by Ball and Nègre (1972: 466), in ZMH. Synonymy established by Casey (1920: 216), confirmed by Ball and Nègre (1972: 466).
Calathus quadricollis LeConte, 1854b: 37. Type locality: «San Francisco [San Francisco County], California» (original citation). Lectotype (♂), designated by Ball and Nègre (1972: 466), in MCZ [# 5731]. Synonymy established by Lindroth (1966: 546).
Calathus obscurus LeConte, 1854b: 37. Type locality: «southern part of California» (original citation), restricted to «San Diego [San Diego County]» by Ball and Nègre (1972: 466). Holotype [by monotypy] (♀) in MCZ [# 5732]. Synonymy established by Ball and Nègre (1972: 466).
Calathus longulus Casey, 1913: 158. Type locality: «Los Angeles Co[unty], California» (original citation). Lectotype [as holotype] (♀), designated by Ball and Nègre (1972: 466), in USNM [# 47552]. Synonymy established by Ball and Nègre (1972: 466).
Calathus montereyanus Casey, 1920: 213. Type locality: «Monterey [Monterey County], California» (original citation). Lectotype [as holotype] (♀), designated by Ball and Nègre (1972: 466), in USNM [# 47554]. Synonymy established by Ball and Nègre (1972: 466).
Calathus tenuistriatus Casey, 1920: 214. Type locality: «San Diego [San Diego County], California» (original citation). Holotype [by monotypy] (♂) in USNM [# 47557]. Synonymy established by Ball and Nègre (1972: 468).
Calathus minuens Casey, 1920: 216. Type locality: «S[an]ta Clara Co[unty], California» (original citation). Lectotype (♂), designated by Ball and Nègre (1972: 468), in USNM [# 47551]. Synonymy established by Ball and Nègre (1972: 468).
Calathus piceolus Casey, 1920: 217. Type locality: «San Diego [San Diego County], California» (original citation). Lectotype (♀), designated by Ball and Nègre (1972: 468), in USNM [# 47556]. Synonymy established by Ball and Nègre (1972: 468).

#### Distribution.

This subspecies ranges from Mendocino County in California south to the northern parts of the Baja California Peninsula and southern Arizona (Ball and Nègre 1972: 482, Fig. 40); it also occurs on the Channel Islands. The record from “Nevada” (Ball and Nègre 1972: 482) is doubtful. The subspecies is adventive in Hawaii (Liebherr and Zimmerman 2000: 465).

#### Records.

**USA**: AZ, CA (CHI) [NV] – Mexico

#### Note.

According to Ball and Nègre (1972: 482), this form intergrades with the *ignicollis* form along the Great Central Valley of California.

### Subgenus Acalathus

Semenov, 1889

Acalathus Semenov, 1889a: 365. Type species: *Acalathus semirufescens* Semenov, 1889 by monotypy. Etymology. Probably from the Greek prefix *a*- (with, sameness) and the generic name *Calathus* [*q.v*.], possibly alluding to the similarity of the adults with those of *Calathus* [masculine]. Note. This taxon was also described as new by Semenov (1889b: 691).
Procalathus Jedlička, 1953: 106. Type species: *Calathus fallax* Semenov, 1889 by original designation. Synonymy established by Lindroth (1966: 547). Etymology. From the Latin *pro*- (before, in front of) and the generic name *Calathus* [*q.v*.] [masculine].

#### Diversity.

Eleven species in North America (one species) and the Chinese provinces of Kansu, Szechwan, and Tsinghai, and the Autonomous Region of Tibet (ten species).

#### Taxonomic Note.

This taxon has been listed as a distinct genus in the subtribe Dolichina by Hovorka and Sciaky (2003: 529). However, Ruiz et al. (2009) concluded from molecular sequence analyses that the taxon is closely related to *Calathus* and should be ranked as a subgenus of *Calathus*.

### Calathus advena

(LeConte, 1846)

Agonum molle Eschscholtz, 1823: 102 [secondary homonym of *Calathus mollis* (Marsham, 1802)]. Type locality: «Unalaschka [Alaska]» (original citation). Lectotype (♀), designated by Lindroth (1966: 548), in ZMH.
Pristodactyla advena LeConte, 1846b: 217. Type locality: «prope fines Aquilones [apparently along northeastern boundary of Maine, see LeConte (1854b: 38)]» (original citation). Two syntypes in MCZ [# 35336]. Synonymy established by Schaupp (1883c: 49), confirmed by Lindroth (1954b: 137).
Anchomenus brunnescens Mannerheim, 1852: 294. Type locality: «insula Atkha [= Atka Island, Aleutian Islands, Alaska]» (original citation). Lectotype (♂), designated by Lindroth (1966: 548), in ZMH. Synonymy established by Lindroth (1954b: 138).
Anchomenus breviusculus Mannerheim, 1852: 294. Type locality: «insula Atkha [= Atka Island, Aleutian Islands, Alaska]» (original citation). Lectotype (♂), designated by Lindroth (1966: 548), in ZMH. Synonymy established by Lindroth (1954b: 138).
Anchomenus lenis Mannerheim, 1853: 140. Type locality: «insula Kadjak [Alaska]» (original citation for the lectotype). Lectotype (♂), designated by Lindroth (1966: 548), in ZMH. Synonymy established by Van Dyke (1924a: 11), confirmed by Lindroth (1955a: 114).
Anchomenus dulcis Mannerheim, 1853: 141. Type locality: «regionibus interioribus peninsulae Kenai [Alaska]» (original citation). Lectotype (♂), designated by Lindroth (1966: 548), in ZMH. Synonymy established by Harold (1868: 90), confirmed by Lindroth (1954b: 137).
Pristodactyla binaria Casey, 1920: 222. Type locality: «S[outh] Arkansas» (original citation), which is highly doubtful (Lindroth 1975: 125). Lectotype (♀), designated by Lindroth (1975: 125), in USNM [# 47567]. Synonymy established by Lindroth (1954b: 137).
Pristodactyla scolopax Casey, 1920: 223. Type locality: «Colorado» (original citation). Lectotype (♂), designated by Lindroth (1975: 125), in USNM [# 47568]. Synonymy established by Lindroth (1954b: 138).

#### Distribution.

This species ranges in the east from eastern Newfoundland to Lake Superior, south to southwestern New York, and in the west from Kodiak Island in Alaska south to northern Oregon, east-central Nevada, and southern New Mexico along the Rocky Mountains, east to the Black Hills in southwestern South Dakota (Pennington County, CNC) [see Ball and Nègre 1972: Fig. 1]. The records from “California” (Lindroth 1955a: 114) and “Vermont” (Hamilton 1894a: 11) need confirmation.

#### Records.

**FRA**: PM **CAN**: AB, BC, LB, NB, NF, ON, QC, SK **USA**: AK, AZ, CO, ID, ME, MI, MN, MT, ND, NH, NM, NV, NY, OR, SD, UT, WA, WY [CA, VT]

### Subtribe Synuchina

Lindroth, 1956

Synuchi Lindroth, 1956b: 489. Type genus: *Synuchus* Gyllenhal, 1810.

#### Diversity.

About 100 species (Lorenz 2005: 400-401) in North America and Mexico (three species) and the Palaearctic Region (about 95 species), arrayed in four genera: *Parabroscus* Lindroth (two species from Japan and Taiwan), *Nipponosynuchus* Morita (one Japanese species), *Synuchus* (about 80 species), and *Trephionus* Bates (14 Japanese species).

### Genus Synuchus

Gyllenhal, 1810

Synuchus Gyllenhal, 1810: 77. Type species: *Carabus nivalis* Panzer, 1796 (= *Carabus vivalis* Illiger, 1798) by monotypy. Etymology. Probably from the Greek *synochos* (join, continued) rather than from the Greek prefix *syn-* (together, with) and *onyx*, -*ychos* (claw) [masculine].
Taphria Dejean, 1821: 10. Type species: *Carabus vivalis* Illiger, 1798 by monotypy. Etymology. From the Greek *taphros* (ditch, trench, by extension stria, dimple) [feminine]. This name was proposed by Franco Andrea Bonelli and made available by Dejean.
Pristodactyla Dejean, 1828: 82. Type species: *Pristodactyla americana* Dejean, 1828 (= *Feronia impunctata* Say, 1823) by monotypy. Synonymy established by Lindroth (1955a: 115). Etymology (original). From the Greek *pristes* (sawyer) and *dactylos* (finger), alluding to the denticulate claws (“*crochets des tarses dentelés en-dessous*”) of the adult [feminine].
Synochus Agassiz, 1846: 359. Unjustified emendation of *Synuchus* Gyllenhal, 1810.

#### Diversity.

About 80 species in the Nearctic (two species), Neotropical (one species from northwestern Mexico), and Palaearctic (77 species, of which one is Eurasian, nine are from the Himalayas, and the remaining from eastern Asia) Regions.

#### Identification.

Lindroth (1966: 550-552) treated both North American species.

### Synuchus dubius

(LeConte, 1854)

Pristodactyla dubia LeConte, 1854b: 38. Type locality: «New Mexico» (original citation), herein restricted to Cloudcroft, Otero County (UASM). Holotype [by monotypy] possibly in MCZ [# 5734]. Note. The specimen of *Synuchus dubius* in the LeConte collection with the type label is also labeled “Col.” and so is not the holotype. Two other specimens in LeConte’s collection are labeled “N.M.” and one of them could be the holotype.
Pristodactyla arizonica Casey, 1913: 160. Type locality: «Arizona» (original citation). Lectotype (♂), designated by Lindroth (1975: 125), in USNM [# 47563]. Synonymy established by Lindroth (1954b: 137).
Pristodactyla zuniana Casey, 1913: 161. Type locality: «New Mexico» (original citation). Lectotype (♂), designated by Lindroth (1975: 125), in USNM [# 47565]. Synonymy established by Lindroth (1954b: 137).
Pristodactyla neomexicana Casey, 1920: 221. Type locality: «Cloudcroft [Otero County], New Mexico» (original citation). Lectotype (♂), designated by Lindroth (1975: 125), in USNM [# 47566]. Synonymy established by Lindroth (1954b: 137).
Pristodactyla juabitica Casey, 1924: 87. Type locality: «Trout Creek, Juab Co[unty], Utah» (original citation). Lectotype (♀), designated by Lindroth (1975: 126), in USNM [# 47562]. Synonymy established by Lindroth (1954b: 138).

#### Distribution.

This species is found in the southern part of the Rocky Mountains in western Utah (Casey 1924: 87, as *Pristodactyla juabitica*), Colorado (Wickham 1902: 238; Lindroth 1956b: 523), New Mexico (LeConte 1854b: 38; Fall and Cockerell 1907: 159; Lindroth 1956b: 523), and Arizona (Wickham 1898: 300; Casey 1913: 160, as *Pristodactyla arizonica*; UASM).

#### Records.

**USA**: AZ, CO, NM, UT

### Synuchus impunctatus

(Say, 1823)

Feronia impunctata Say, 1823a: 45. Type locality: «Tyngs[boro] [Middlesex County], Mass[achusetts]» (neotype label). Neotype (♂), designated by Lindroth and Freitag (1969: 346), in MCZ [# 33023]. Note. «Germantown [Pennsylvania]» was the area originally cited by Say (1823a: 43).
Pristodactyla americana Dejean, 1828: 83. Type locality: «Amérique septentrionale» (original citation). Holotype [by monotypy] (♂) in MHNP (Lindroth 1955b: 18). Synonymy established by LeConte (1846b: 217), confirmed by Lindroth (1955b: 18).
Pristonychus americanus LeConte, 1844: 52 [secondary homonym of *Pristonychus americanus* (Dejean, 1828)]. Type locality: «Georgia» (original citation). Syntype(s) location unknown. Synonymy established by Lindroth (1956b: 521, as *Pristodactyla corvina*). Note. There is no specimen in LeConte’s collection under the name *Calathus impunctatus* with an orange disc (= southern states which included Georgia). The specimen with the type label is labeled “[pink disc] / Type 5733 / [blank white square label] / P. corvinusLec. Priston. amer. Lec. var. praec. [handwritten] / impunctatus 11 [handwritten].”
Pristodactyla corvina LeConte, 1846b: 217. Replacement name for *Pristodactyla americana* (LeConte, 1844).
Pristodactyla impunctata convexa Casey, 1913: 160. Type locality: «Lake Champlain, New York» (original citation). Lectotype (♀), designated by Lindroth (1975: 125), in USNM [# 47560]. Synonymy established by Lindroth (1954b: 137).

#### Distribution.

This species ranges from Newfoundland (Lindroth 1955a: 115-116) to southwestern British Columbia (Lindroth 1966: 551), south to western Washington (Hatch 1953: 132), northern Idaho (Hatten et al. 2011: 325), eastern Kansas (Cook and Holt 2006: 2313), and northern Georgia (Fattig 1949: 32). The record from southwestern Colorado (Wickham 1902: 238) needs confirmation.

#### Records.

**FRA**: PM **CAN**: AB, BC, MB, NB, NF, NS (CBI), ON, PE, QC, SK **USA**: CT, DC, DE, GA, IA, ID, IL, IN, KS, KY, MA, MD, ME, MI, MN, MO, ND, NE, NH, NJ, NY, OH, PA, RI, SD, VA, VT, WA, WI, WV [CO]

### Subtribe Sphodrina

Laporte, 1834

Sphodridae Laporte, 1834: 78. Type genus: *Sphodrus* Clairville, 1806.
Pristonychidae Gistel, 1848b: [2]. Type genus: *Pristonychus* Dejean, 1828.

#### Diversity.

About 360 species (Lorenz 2005: 401-407), all but one (*Miquihuana rhadiniformis* Barr from Mexico) native to the Palaearctic Region. Two species are adventive in several regions of the world.

#### Identification.

Casale (1988) revised all species then known.

### Genus Laemostenus

Bonelli, 1810

Laemostenus Bonelli, 1810: Tabula Synoptica. Type species: *Carabus janthinus* Duftschmid, 1812 designated by Madge (1975: 583). Etymology. From the Greek *laimos* (throat, gullet, by extension neck) and *stenos* (narrow, tight) (see Bedel 1878: 250) [masculine].

#### Diversity.

About 195 species (Lorenz 2005: 403-406) in Europe, northern Africa, western and central Palaearctic Asia, and the Himalayas arrayed in 12 subgenera. Two species are adventive in several parts of the world.

#### Identification.

Lindroth (1966: 549-550) treated both species found in North America.

### Subgenus Laemostenus

Bonelli, 1810

Laemostenus Bonelli, 1810: Tabula Synoptica. Type species: *Carabus janthinus* Duftschmid, 1812 designated by Madge (1975: 583). Note. *Laemosthenes* is an incorrect subsequent spelling of *Laemostenus* Bonelli, introduced by Agassiz (1846: 199), not in prevailing usage.
Ctenipus Latreille, 1829: 400. Type species: *Carabus janthinus* Duftschmid, 1812 designated by Desmarest (1851: 136). Etymology. From the Greek *ctenos* (comb) and *pous* (foot) [masculine].
Ctenopus Agassiz, 1846: 107. Unjustified emendation of *Ctenipus* Latreille, 1829.

#### Diversity.

Twenty species in Europe (eight species), northern Africa (four species), and Asia (13 species), one of them has been dispersed by trade to several continents and islands.

### Laemostenus complanatus

(Dejean, 1828)

Pristonychus complanatus Dejean, 1828: 58. Type locality: «Portugal, Espagne, midi de la France, Italie, Sicile, Égypte, côte de Barbarie» (original citation), restricted to «Francia meridionale» by Casale (1988: 458). Lectotype (♂), designated by Casale (1988: 458), in MHNP.

#### Distribution.

This species is now subcosmopolitan (Casale 1988: 460). It is adventive in North America and known from southern British Columbia, including Vancouver Island (Lindroth 1966: 550), to southwestern California, east across the Central Valley to the western foothills of the Sierra Nevada (David H. Kavanaugh pers. comm. 2008). The first inventoried specimen collected on this continent was found prior to 1874 since the species is reported by Crotch (1874: 12).

#### Records.

**CAN**: BC (VCI) **USA**: CA, OR, WA – **Adventive**

### Subgenus Pristonychus

Dejean, 1828

Pristonychus Dejean, 1828: 43. Type species: *Carabus terricola* Herbst, 1784 designated by Westwood (1838: 2). Etymology (original). From the Greek *pristes* (saw) and *onychos* (claw), alluding to the denticulate claws (“*crochets des tarses dentelés en-dessous*”) of the adult [masculine].
Eutrichomerus Carret, 1907: 95, 99, 106. Type species: *Carabus terricola* Herbst, 1784 designated by Jeannel (1914: 237). Etymology. From the Greek *eu* (well), *trichos* (hair), and *meros* (femur), alluding to the presence of long setae on the profemur (“*fémurs antérieurs *... *garnis de longs poils dressés et plus ou moins groupés sur la tranche postérieure de l’arête externe*”) of the adult [masculine].

#### Diversity.

About 55 species in Europe, northern Africa, western Asia, and the Himalayas. One European species is adventive in North America and India (Casale 1988: 786-787).

### Laemostenus terricola terricola

(Herbst, 1784)

Carabus terricola Herbst, 1784: 140. Type locality: «Berlin [Germany]» (original citation). Syntype(s) in ZMHB (Casale 1988: 781).
Carabus inaequalis Panzer, 1795: 60. Type locality: Germany (inferred from title of the book). Syntype(s) location unknown (possibly in ZMHB). Synonymy established, under the name *Sphodrus subcyaneus* (Illiger), by Sturm (1824: 152).
Carabus subcyaneus Illiger, 1801: 57. Type locality: Prussia, Germany (inferred from title of Illiger’s 1798 book). Syntype(s) location unknown. Synonymy established by Schönherr (1806: 183). Note. This name was proposed for *Carabus terricola* Herbst, 1784 *sensu* Illiger (1798: 184).
Harpalus episcopus Drapiez, 1819: 130. Type locality: «Mons, en Hainaut [Belgium]» (original citation). One possible syntype in MHNP (see Casale 1988: 781). Synonymy established by Casale (1988: 781).
Sphodrus reichenbachi Schaufuss, 1861: 15. Type locality: «prov[incia] Alaba [= Álava], Hispan[ia] occ[identalis]» (original citation). Lectotype (♂), designated by Casale (1988: 782), in ZMHB. Synonymy established by Casale (1988: 782).

#### Distribution.

This European subspecies is adventive in North America where it is known from Newfoundland (Lindroth 1966: 550; Larson and Langor 1982: 593) and the Maritimes (Lindroth 1966: 550) to the Saint Lawrence Estuary (Larochelle 1975: 98), from three specimens collected in 2005 and 2008 in Boston Harbor, Massachusetts (Davidson et al. 2011: 518), and from one specimen collected in 1980 in southeastern British Columbia (Bousquet 1987a: 125). The first inventoried specimen collected on this continent was found in Nova Scotia prior to 1894 (Bousquet 1992a: 507).

#### Records.

**FRA**: PM **CAN**: BC, NB, NF, NS, PE, QC **USA**: MA – **Adventive**

#### Note.

The subspecies *Laemostenus terricola punctatus* Dejean is restricted to eastern Europe.

### Tribe Platynini

Bonelli, 1810

Platynii Bonelli, 1810: Tabula Synoptica. Type genus: *Platynus* Bonelli, 1810.
Anchomenii Bonelli, 1810: Tabula Synoptica. Type genus: *Anchomenus* Bonelli, 1810.
Sericodiadae Kirby, 1837: 14. Type genus: *Sericoda* Kirby, 1837.
Agonidae Kirby, 1837: 23. Type genus: *Agonum* Bonelli, 1810. Note. The Commission (ICZN 1996: 223) ruled that the stem of the genus *Agonum* is *Agonum-*, not *Agon*- as it should be, to remove homonymy with Agonidae Swainson, 1839 (Osteichthyes, Scorpaeniformes).
Colpodidas Chaudoir, 1872c: 285. Type genus: *Colpodes* Macleay, 1825.
Agelaeina Jacobson, 1907: 334. Type genus: *Agelaea* Gené, 1839.

#### Diversity.

Worldwide, with about 2,670 species arrayed in 170 genera (Lorenz 2005: 407-437, excluding Enoicina and *Atranus*). The Northern Hemisphere has about 750 species (28% of the world fauna) and North America alone about 160 (6%).

### Genus Olisthopus

Dejean, 1828

Odontonyx Stephens, 1827: 67. Type species: *Carabus rotundicollis* Marsham, 1802 (= *Carabus rotundatus* Paykull, 1790) designated by Westwood (1838: 2). Etymology (original, see page 96). From the Greek *odontos* (tooth) and *onyx* (claw), alluding to the denticulate claws (“claws denticulated”) of the adult [masculine]. Note. Lindroth (1966: 553) pointed out that *Carabus rotundicollis sensu* Stephens (1828a: 96), based on Stephens’ key and description as well as his collection, consisted of two species, *Olisthopus rotundicollis* Marsham (= *Olisthopus rotundatus* Paykull) and *Synuchus vivalis* Illiger. There is no provision in the Code to deal with such cases and therefore correct identification of the species is assumed (ICZN 1999: Article 70.1).
Olisthopus Dejean, 1828: 176. Type species: *Carabus rotundatus* Paykull, 1790 designated by Westwood (1838: 3). Etymology (original). From the Greek *olisthos* (slippery, escape easily) and *pous* (foot), alluding to the agility of the species known to Dejean to escape on feet (“*petits carabiques vifs et agiles*”) [masculine]. Note. This name has been in prevailing usage for several decades now. However, reversal of precedence (ICZN 1999: Article 23.9) cannot be applied in this case because *Odontonyx* has been used as a valid name after 1899 by several authors. A submission should be made to the Commission to preserve *Olisthopus* as the valid name for this taxon.

#### Diversity.

Twenty-one species in temperate areas of the Nearctic (seven species) and Palaearctic (14 species, only one extending to eastern Asia) Regions.

#### Identification.

Casey (1913: 169-171) published a key to all North American species except *Olisthopus micans* but the genus is in need of a taxonomic revision.

### [micans group]

### Olisthopus filicornis

Casey, 1913

Olisthopus filicornis Casey, 1913: 171. Type locality: «Rhode Island» (original citation). One syntype in USNM [# 47575].

#### Distribution.

This species has been recorded from Rhode Island (Casey 1913: 171) and from Richland County in South Carolina (Ciegler 2000: 106).

#### Records.

**USA**: RI, SC

### Olisthopus micans

LeConte, 1846

Olisthopus micans LeConte, 1846b: 230. Type locality: «Georgia» (original citation). Two syntypes in MCZ [# 5790].

#### Distribution.

The range of this species extends from southern Quebec (Larochelle 1975: 96) to “Iowa” (Jaques and Redlinger 1946: 295), south to southern Louisiana (Calcasieu Parish, CNC) and northern Florida (Baker County, CMNH). The record from “Wisconsin” (Bousquet and Larochelle 1993: 248) needs confirmation.

#### Records.

**CAN**: ON, QC **USA**: AL, AR, CT, DC, FL, GA, IA, IL, IN, LA, MA, MD, MI, MO, MS, NH, NJ, NY, OH, PA, RI, SC, VA, VT [WI]

### [parmatus group]

### Olisthopus brevicornis

Casey, 1913

Olisthopus brevicornis Casey, 1913: 171. Type locality: «Illinois» (original citation). One syntype in USNM [# 47573].

#### Distribution.

This species is known only from the type series.

#### Records.

**USA**: IL

### Olisthopus innuens

Casey, 1913

Olisthopus innuens Casey, 1913: 170. Type locality: «Boston Neck [Washington County], Rhode Island» (original citation). Holotype [by monotypy] (♂) in USNM [# 47572].

#### Distribution.

This species is known only from the holotype.

#### Records.

**USA**: RI

### Olisthopus iterans

Casey, 1913

Olisthopus parmatus iterans Casey, 1913: 170. Type locality: «Indiana» (original citation). Lectotype (♂), designated by Lindroth (1975: 126), in USNM [# 47570].

#### Distribution.

This species is known only from the original two specimens.

#### Records.

**USA**: IN

#### Note.

Lindroth (1966: 553) listed this form in synonymy with *Olisthopus parmatus* Say but Bousquet and Larochelle (1993: 18) treated it as a distinct species.

### Olisthopus parmatus

(Say, 1823)

Feronia parmata Say, 1823a: 49. Type locality: «Wis[s]ahick[o]n Cr[eek] [Philadelphia County], P[ennsylvani]a» (neotype label). Neotype (♀), designated by Lindroth and Freitag (1969: 348), in MCZ [# 33008].
Olisthopus cinctus Say, 1830b: (5) [3]. Type locality: «Pennsylvania» (original citation). Syntype(s) lost. Synonymy established by LeConte (1854b: 58).
Olisthopus pictus Casey, 1913: 169. Type locality: «Bayfield [Bayfield County], Wisconsin» (original citation). Lectotype (♂), designated by Lindroth (1975: 126), in USNM [# 47571]. Synonymy established by Lindroth (1966: 553).

#### Distribution.

This species ranges from “Nova Scotia” (Larochelle and Larivière 1990a: 30) to eastern Minnesota (Epstein and Kulman 1990: 215; Wickham 1896c: 134), south to northern Oklahoma (French et al. 2001: 229), Arkansas (Logan County, CNC), the Florida Panhandle (Leng 1915: 582), and southwestern Georgia (Fattig 1949: 35). The record from northern Colorado (Armin 1963: 158) needs confirmation.

#### Records.

**CAN**: NB, NS, ON, QC **USA**: AL, AR, CT, DC, DE, FL, GA, IA, IL, IN, KS, KY, MA, MD, ME, MI, MN, MO, MS, NC, NH, NJ, NY, OH, OK, PA, RI, SC, TN, VA, VT, WI, WV [CO]

### Olisthopus pusio

Casey, 1913

Olisthopus pusio Casey, 1913: 171. Type locality: «Alexandria [Rapides Parish], Louisiana» (original citation). Holotype [by monotypy] (♂) in USNM [# 47574].

#### Distribution.

This species is known only from the holotype collected in central Louisiana.

#### Records.

**USA**: LA

### Genus Elliptoleus

Bates, 1882

Elliptoleus Bates, 1882a: 97. Type species: *Anchomenus vixstriatus* Bates, 1878 designated by Casey (1920: 5). Etymology. Unknown [masculine].

#### Diversity.

Eleven Mexican species, of which one extends into southern United States.

#### Identification.

Liebherr (1991b) revised the species.

### Elliptoleus acutesculptus

Bates, 1882

Elliptoleus acutesculptus Bates, 1882a: 98. Type locality: Refugio, Durango, Mexico (lectotype label). Lectotype (♀), designated by Liebherr (1991b: 95), in MHNP. Note. In the original description, the locality listed by Bates (1882a: 98) is “Mexico, near the city.” Liebherr (1991b: 95) pointed out that Bates probably made a mistake in listing this locality since no specimen of *Elliptoleus acutesculptus* in Bates’ collection bears a Mexico City locality label.

#### Distribution.

This species extends from east-central Arizona and southwestern Texas south to the state of San Luis Potosí in central Mexico [see Liebherr 1991b: Fig. 269]. Only two localities have been reported for the United States.

#### Records.

**USA**: AZ, TX – Mexico

### Genus Sericoda

Kirby, 1837

Sericoda Kirby, 1837: 14. Type species: *Sericoda bembidioides* Kirby, 1837 by monotypy. Etymology. From the Greek *sericos* (silk), alluding to the silky appearance of adults (“black underneath, above black-bronze rather silky”) of *Sericoda bembidioides* in the eyes of Kirby [feminine].
Rhytiderus Chaudoir, 1844: 470. Type species: *Dromius decempunctatus* Reiche, 1842 (= *Sericoda bembidioides* Kirby, 1837) by original designation. Etymology (original). From the Greek *rhytis* (wrinkle) and *dere* (neck, by extension pronotum), alluding to the irregular, granulate, transversely strigulate microsculpture on the pronotum, which Chaudoir believed were wrinkles (“*thorax *... *disco toto transverse rugis irregularibus tecto*”), of the adult [masculine].
Rhytidoderus Agassiz, 1846: 327. Unjustified emendation of *Rhytiderus* Chaudoir, 1844.
Agonodromius Reitter, 1908: 139 (as *Agnonodromius*), 239 (index). Type species: *Carabus quadripunctatus* DeGeer, 1774 designated by Jeannel (1942: 873). Synonymy established by Gray and Hatch (1941: 19). Etymology. From the generic names *Agonum* [*q.v*.] and *Dromius* [*q.v*.], alluding to the resemblance of these *Agonum* species to those of *Dromius* (“*Dromius-ähnich*”) [masculine].

#### Diversity.

Eight species in North America (four species, only one of them endemic), Middle America (two species, no endemism), the West Indies (one endemic Cuban species), South America (one species, no endemism), Asia (four species, three of them endemic), and Europe (two species, no endemism).

#### Identification.

Liebherr (1991b) revised the species. One species, *Sericoda balli* Schmidt, was subsequently describedfrom Pakistan.

### [bogemannii group]

### Sericoda bembidioides

Kirby, 1837

Sericoda bembidioides Kirby, 1837: 15. Type locality: northern parts of British America (inferred from title of the paper), restricted to «Edmonton, Al[ber]ta» by Lindroth (1966: 569). Holotype [by monotypy] (♀) in BMNH (Lindroth 1953b: 169).
Dromius 10 punctatus Reiche, 1842: 310. Type locality: «Santa-Fe de Bogota [Colombia]» (original citation). Syntype(s) in MHNP. Synonymy established by Horn (1886b: xii).
Sericoda cicatricosa Mannerheim, 1853: 144. Type locality: «sur les rives du fl[euve] Kwytch-Pack [= apparently Kwikpak, Yukon River Delta (Lindroth 1966: 569)] sur le continent Américain russe» (see Motschulsky 1864: 233). Three syntypes in ZMMU (Keleinikova 1976: 191). Synonymy established by Mannerheim (1853: 144). Note. Motschulsky (1864: 233) first described this species and the name has been attributed to him since. However, Mannerheim (1853: 144) first published the name as a junior synonym of *Anchomenus bembidioides* and therefore the name is available from its first publication as a synonym (ICZN 1999: Article 11.6.1). The fact that the name was previously listed, but not described, by Motschulsky (1850a: 11) in a list is irrelevant. Because no specimen(s) was cited with this name by Mannerheim (1853: 144), the type series consists of the specimens cited when the species was described (ICZN 1999: Article 72.4.3).
Sericoda monticola Casey, 1920: 94. Type locality: «Colorado» (original citation). Lectotype (♀), designated by Lindroth (1975: 126), in USNM [# 47518]. Synonymy established by Lindroth (1966: 569).
Sericoda colonica Casey, 1920: 94. Type locality: «Colonia Garcia, Sierra Madre M[oun]t[ain]s, Chihuahua, Mexico» (original citation). Lectotype (♀), designated by Lindroth (1975: 126), in USNM [# 47519]. Synonymy established by Lindroth (1966: 569).

#### Distribution.

This species is widely distributed in the Western Hemisphere ranging from southeastern Manitoba to central Alaska, south to northern Oregon and through the Rocky Mountains, Transverse Volcanic, Central America, and the Andes to southern Ecuador, east in South America to western Venezuela [see Liebherr 1991b: Fig. 240].

#### Records.

**CAN**: AB, BC (VCI), MB, NT, SK, YT **USA**: AK, AZ, CO, ID, KS, MT, NM, OR, WA – Colombia, Costa Rica, Ecuador, El Salvador, Guatemala, Mexico, Panama, Venezuela

### Sericoda bogemannii

(Gyllenhal, 1813)

Harpalus bogemannii Gyllenhal, 1813: 697. Type locality: «Smolandia [= Småland province, Sweden]» (original citation for the lectotype). Lectotype (♂), designated by Lindroth (1966: 567), in UZIU.
Anchomenus strigicollis Mannerheim, 1852: 294. Type locality: «ad fontes Kaknu [Kenai Peninsula, Alaska] in continenti Americes borealis» (original citation). Lectotype (♂), designated by Lindroth (1966: 567), in ZMH. Synonymy established by Hamilton (1889b: 97), confirmed by Lindroth (1954b: 138).
Batenus costulatus Motschulsky, 1865: 319. Type locality: «Sibérie orientale [Russia]» (original citation). At least one syntype in ZMMU (Schmidt and Liebherr 2009: 254). Synonymy established by Shilenkov (in Kryzhanovskij et al. 1995: 114).
Sericoda invidiosa Casey, 1920: 97. Type locality: «Colorado» (original citation). Lectotype (♂), designated by Lindroth (1975: 126), in USNM [# 47524]. Synonymy established by Lindroth (1954b: 139).
Sericoda tacomae Casey, 1920: 98. Type locality: «Washington State» (original citation). Holotype [by monotypy] (♀) in USNM [# 47523]. Synonymy established by Gray (1937: 311), confirmed by Lindroth (1954b: 139).

#### Distribution.

This Holarctic species is found in the New World from west-central Alaska south to central California and to southern New Mexico along the Rocky Mountains. Disjunct populations are also known from Chiapas in Mexico and Guatemala [see Liebherr 1991b: Fig. 230]. In the Old World, the species is known from disjunct populations in Lapland, the Austrian Alps, and Burma [see Liebherr 1991b: Fig. 231].

#### Records.

**CAN**: AB, BC (VCI), YT **USA**: AK, CA, CO, ID, MT, NM, OR, WA – Guatemala, Mexico – **Holarctic**

### Sericoda obsoleta

(Say, 1823)

Feronia obsoleta Say, 1823a: 57. Type locality: «Bayf[ie]ld [Bayfield County], Wis[consin]» (neotype label). Neotype (♂), designated by Lindroth and Freitag (1969: 348), in MCZ [# 33011].
Agonum luctuosum Dejean, 1828: 172. Type locality: «Amérique septentrionale» (original citation). One syntype in MHNP (Lindroth 1955b: 21). Synonymy established by Say (1830b: (5) [3]), confirmed by Lindroth (1966: 565).
Platynus consimilis LeConte, 1854b: 57 [secondary homonym of *Platynus consimilis* (Gyllenhal, 1810)]. Type locality: «Michipicoton [Ontario], Lake Superior» (original citation). Holotype [by monotypy] (♀) in MCZ [# 5780]. Synonymy established by Lindroth (1966: 565).
Platynus vicinus Gemminger and Harold, 1868a: 377. Replacement name for *Platynus consimilis* LeConte, 1854.
Sericoda insulina Casey, 1920: 97. Type locality: «Victoria, Vancouver Island [British Columbia]» (original citation). Lectotype (♂), designated by Lindroth (1975: 126), in USNM [# 47522]. Synonymy established by Lindroth (1966: 566).

#### Distribution.

This Nearctic species ranges from Newfoundland to Vancouver Island (Casey 1920: 97, as *Sericoda insulina*), north to southern Northwest Territories, south to central Oregon, central Colorado (Wickham 1902: 239), eastern Texas, and coastal Georgia [see Liebherr 1991b: Fig. 229]. One specimen simply labeled from California is known (Liebherr 1991b: 74). There is also one specimen recorded from near Mexico City (Liebherr 1991b: 72) which in my opinion is probably mislabeled.

#### Records.

**CAN**: AB, BC (VCI), LB, MB, NB, NF, NS (CBI), NT, ON, QC, SK **USA**: AL, AR, CO, CT, DC, GA, IA, ID, IL, IN, KS, LA, MA, MD, ME, MI, MN, MO, MS, NC, ND, NH, NJ, NY, OH, OR, PA, RI, SC, TN, TX, VA, VT, WA, WI, WV [CA]

### [quadripunctata group]

### Sericoda quadripunctata

(DeGeer, 1774)

Carabus 4-punctatus DeGeer, 1774: 102. Type locality not stated; «Uppsala, Sweden» selected by Lindroth (1966: 568). Lectotype (♂), designated by Lindroth (1966: 568), in NRSS.
Carabus foveolatus Illiger, 1801: 61. Type locality: Prussia (inferred from title of the paper). Holotype [by monotypy] probably lost. Synonymy established by Gyllenhal (1810: 160).
Anchomenus octocolus Mannerheim, 1853: 144. Type locality: «ad rivulos fl[umen] Tschunuktnu peninsulae Kenai [Alaska]» (original citation). Holotype [by monotypy] probably in ZMMU. Synonymy established by LeConte (1879b: 57). Note. Regarding the type locality, see “Note” section for *Cryobius subcaudatus* Mannerheim.
Platynus stigmosus LeConte, 1854b: 58. Type locality: «Lake Superior, Maine and New Hampshire» (original citation), restricted to «L[ake] Superior» by Lindroth (1966: 568). Three syntypes in MCZ [# 5785]. Synonymy established with doubt, under the name *Sericoda octocola* (Mannerheim), by LeConte (1857c: 8), confirmed by Lindroth (1966: 568).
Anchomenus ambiguus Mäklin, 1855: 36. Type locality: «Ajan [Khabarovsk Kray, Russia]» (original citation). Syntype(s) location unknown (possibly in ZMH). Synonymy established by Jacobson (1907: 330).

#### Distribution.

This Holarctic species ranges in North America from central Alaska to Newfoundland (Lindroth 1955a: 117), south to eastern North Carolina and western Tennessee along the Appalachian Mountains, to northern New Mexico along the Rocky Mountains, and to central Washington [see Liebherr 1991b: Fig. 221]. In the Palaearctic Region, the range of the species extends from Great Britain to the Kamchatka Peninsula, south to the Philippines Islands, the Himalayan Mountains, and Italy in Europe [see Liebherr 1991b: Fig. 222].

#### Records.

**CAN**: AB, BC (VCI), LB, MB, NB, NF, NS (CBI), NT, ON, QC, SK, YT **USA**: AK, ID, IL, IN, MA, ME, MI, MN, MT, NC, NH, NJ, NM, NY, PA, SD, TN, VT, WA, WI – **Holarctic**

**Figure 36.**
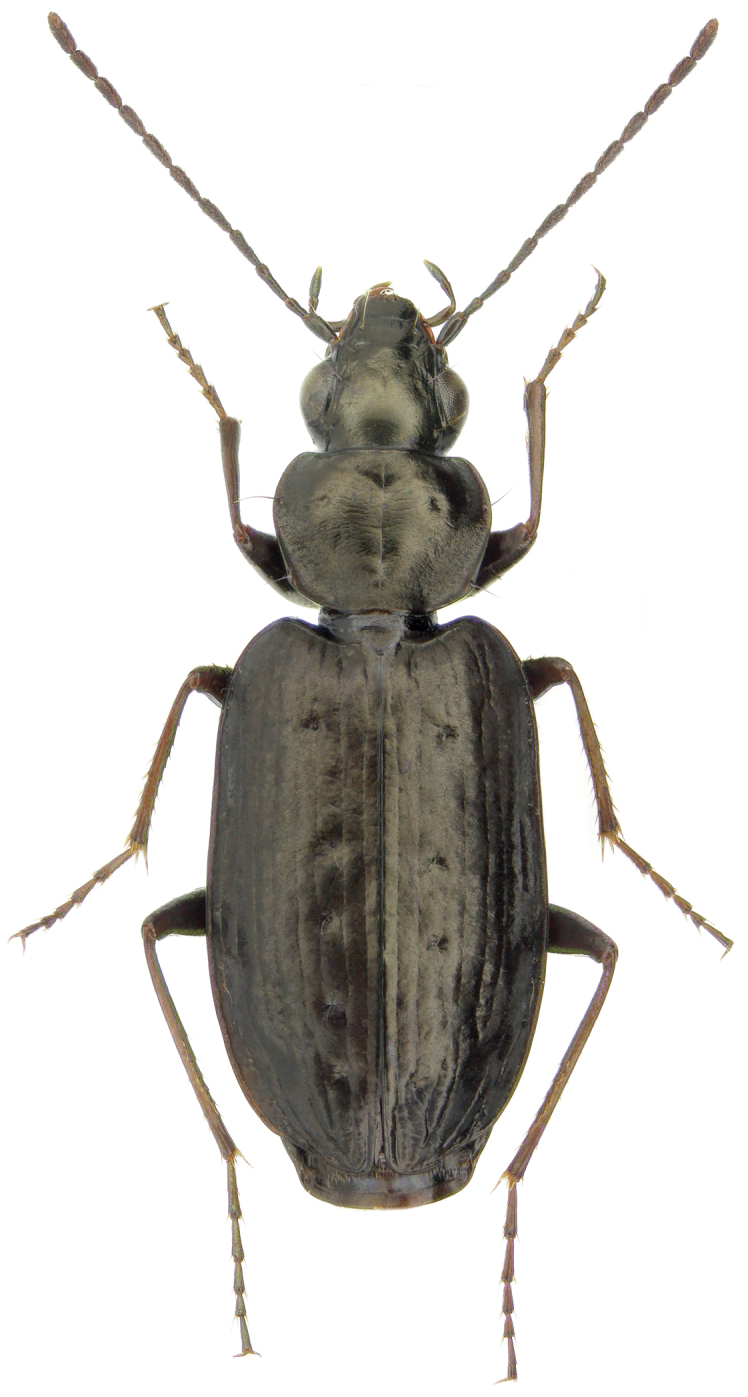
*Sericoda quadripunctata* (DeGeer). This widely distributed Holarctic species is attracted to fire. The adults are often found in great numbers just after forest fires when the ashes are still hot. This behavior is shared by the other species in the genus and a few other carabids, such as *Harpalus laticeps* and *Harpalus laevipes*. The presence of charcoal is probably a stimulus for oviposition. Henri Goulet was unsuccessful in obtaining eggs from *Sericoda obsoleta* in the laboratory until he added charcoal to his rearing containers.

### Genus Tetraleucus

Casey, 1920

Tetraleucus Casey, 1920: 88. Type species: *Anchomenus picticornis* Newman, 1844 by monotypy. Etymology. From the Greek *tetra* (four) and *leukos* (white), probably alluding to the fact that the antennomeres 8-11 of the adults are white (“the last four joints [of the antenna] being very abruptly of a creamy white”) [masculine].

#### Diversity.

One eastern North American species in the temperate regions.

#### Identification.

Liebherr (1991b) redescribed the species and illustrated some of its structural character states.

### Tetraleucus picticornis

(Newman, 1844)

Anchomenus picticornis Newman, 1844: 414. Type locality: «near S[ain]t John’s Bluff [Duval County], in east Florida» (original citation). Holotype [by monotypy] (♀) in BMNH (Lindroth 1966: 615).

#### Distribution.

This species is known from scattered localities in eastern North America ranging from western Vermont to “Minnesota,” south to east-central Texas (Riley 2011), southern Louisiana and central Florida [see Liebherr 1991b: Fig. 187]. The record from Sainte-Rose (= Laval) in Quebec (Hausen 1891b: 159) needs confirmation.

#### Records.

**CAN**: ON **USA**: AL, AR, DC, FL, IA, IL, IN, KY, LA, MD, MN, MS, NC, NY, OH, PA, SC, TX, VA, VT, WI, WV [QC]

### Genus Anchomenus

Bonelli, 1810

Anchomenus Bonelli, 1810: Tabula Synoptica. Type species: *Carabus prasinus* Thunberg, 1784 (= *Carabus dorsalis* Pontoppidan, 1763) designated by Westwood (1838: 3). Etymology. From the Greek *anchomenos* (strangled), possibly alluding to the cordate pronotum (“*th*[*orax*] *obcordatus*,” character of the stripe Anchomenii of Bonelli) of the adult [masculine].

#### Diversity.

Fourteen species in the Nearctic (three species) and Palaearctic (ten species) Regions and one species (*Anchomenus capensis* Liebherr) endemic to Baja California. These species are arrayed in two subgenera: *Anchodemus* Motschulsky (three Palaearctic species) and *Anchomenus* s.str. (11 species).

#### Identification.

Liebherr (1991b) revised the species except for three Palaearctic species of the subgenus *Anchomenus *s.str.

### Subgenus Anchomenus

Bonelli, 1810

Anchomenus Bonelli, 1810: Tabula Synoptica. Type species: *Carabus prasinus* Thunberg, 1784 (= *Carabus dorsalis* Pontoppidan, 1763) designated by Westwood (1838: 3).
Ectenes Billberg, 1820: 29. Type species: *Carabus prasinus* Thunberg, 1784 (= *Carabus dorsalis* Pontoppidan, 1763) designated by Bousquet (2002b: 19).
Clibanarius des Gozis, 1882: 295 [junior homonym of *Clibanarius* Dana, 1852]. Type species: *Carabus dorsalis* Pontoppidan, 1763 by original designation. Etymology. From the Latin *clibanarius* (cuirassier) [masculine].
Chlaeniomimus Semenov, 1889b: 687. Type species: *Chlaenius gracilicollis* Jakowleff, 1887 (= *Atranus virescens* Motschulsky, 1864) by original designation. Synonymy established by Liebherr (1991b: 33). Etymology. From the generic name *Chlaenius* [*q.v*.] and the Latin *mimus* (actor), probably alluding to the resemblance of the adults in the eyes of Semenov to those of some *Chlaenius* [masculine].
Idiochroma Bedel, 1902: 216. Replacement name for *Clibanarius* des Gozis, 1882. Etymology. From the Greek *idios* (personal, individual) and *chroma* (color) [neuter].
Pseudanchus Casey, 1920: 45. Type species: *Platynus funebris* LeConte, 1854 designated by Lindroth (1966: 632). Synonymy established by Liebherr (1991b: 33). Etymology. From the Greek *pseudos* (fallacy, lie) and the generic name *Anchus* [*q.v*.] [masculine].
Nipponachus Habu, 1978: 36. Type species: *Anchomenus leucopus* Bates, 1873 by original designation. Synonymy established by Liebherr (1991b: 33). Etymology. From the English *nippon* (a Japanese name for Japan) and the generic name *Anchus* [*q.v*.] [masculine].

#### Diversity.

Eleven species in North America (three endemic species), Baja California (one endemic species), Asia (seven species, four of them endemic), Europe (three species), and northern Africa (one species).

### Anchomenus aeneolus

(LeConte, 1854)

Platynus aeneolus LeConte, 1854b: 45. Type locality: «Oregon» (original citation), herein restricted to Hilgard Junction State Park, Union County (see Liebherr 1991b: 52). Holotype [by monotypy] (♀) in MCZ [# 5755].
Agonum bjorkmanae Gray and Hatch, 1941: 16. Type locality: «Upper Deschutes River, Thurston Co[unty], Wash[ington]» (original citation). Holotype (♀) in USNM. Synonymy established by Hatch (1953: 136). Note. Hatch (1953: 136) pointed out that the holotype is a composite specimen, the forebody being of *Anchomenus aeneolus* LeConte, the hindbody of *Anchomenus subsericeum* LeConte.

#### Distribution.

This taxon is found from the Rocky Mountains in southwestern Alberta to south-central British Columbia, south to central Oregon and south-central Idaho [see Liebherr 1991b: Fig. 200].

#### Records.

**CAN**: AB, BC **USA**: ID, MT, OR, WA

### Anchomenus funebris

(LeConte, 1854)

Anchomenus micans Ménétriés, 1843: 57 [primary homonym of *Anchomenus micans* Nicolai, 1822]. Type locality: «Californie» (original citation). One syntype in ZMH (collection Mannerheim) (Silfverberg 1987: 20).
Platynus funebris LeConte, 1854b: 45. Type locality: «San Diego [San Diego County], California» (original citation). Lectotype (♀), designated by Liebherr (1991b: 53), in MCZ [# 5757]. Synonymy established by LeConte (1879b: 47), confirmed by Liebherr (1991b: 53).
Scaphiodactylus opacus Motschulsky, 1859a: 160. Type locality: «California» (lectotype label). Lectotype (probably ♀), designated by Bousquet (1997b: 335), in ZMMU. Synonymy established by LeConte (1863b: 7), confirmed by Bousquet (1997b: 335).
Anchomenus morbillosus Casey, 1920: 48. Type locality: «Redwood Creek, Humboldt Co[unty], California» (original citation). Lectotype (♀), designated by Liebherr (1991a: 117), in USNM [# 47419]. Synonymy established by Liebherr (1991a: 117).
Anchomenus nevadensis Casey, 1920: 48. Type locality: «Reno [Washoe County], Nevada» (original citation). Lectotype (♀), designated by Liebherr (1991a: 117), in USNM [# 47420]. Synonymy established by Liebherr (1991a: 117).
Anchomenus concurrens Casey, 1920: 49. Type locality: «S[an]ta Rosa [Sonoma County], California» (original citation for the lectotype). Lectotype (♀), designated by Liebherr (1991a: 116), in USNM [# 47421]. Synonymy established by Hatch (1953: 136), confirmed by Liebherr (1991a: 117).
Anchomenus opacellus Casey, 1920: 50. Type locality: «S[an]ta Rosa [Sonoma County], California» (original citation). Lectotype (♀), designated by Liebherr (1991a: 117), in USNM [# 47422]. Synonymy established by Liebherr (1991a: 117).
Anchomenus renoanus Casey, 1920: 51. Type locality: «Reno [Washoe County], Nevada» (original citation). Lectotype (♂), designated by Liebherr (1991a: 117), in USNM [# 47425]. Synonymy established by Liebherr (1991a: 117).
Anchomenus parvus Casey, 1920: 51. Type locality: «Reno [Washoe County], Nevada» (original citation). Holotype [by monotypy] (♀) in USNM [# 47424]. Synonymy established by Liebherr (1991a: 117).
Agonum funebre var. *micantulum* Csiki, 1931: 861. Replacement name for *Agonum funebre* var. *micans* (Ménétriés, 1843).

#### Distribution.

This western species ranges from central Oregon to Baja California Norte, including west-central Nevada [see Liebherr 1991b: Fig. 200].

#### Records.

**USA**: CA (CHI), NV, OR – Mexico

### Anchomenus quadratus

(LeConte, 1854)

Platynus quadratus LeConte, 1854b: 50. Type locality: «Oregon» (original citation), herein restricted to Dilley, Washington County (see Casey 1920: 52, as *Anchomenus dilleyanus*). Holotype [by monotypy] (♀) in MCZ [# 5756].
Anchomenus cornicula Casey, 1920: 51. Type locality: «Hoopa Valley, Humboldt Co[unty], California» (original citation for the lectotype). Lectotype (♂), designated by Liebherr (1991a: 116), in USNM [# 47423]. Synonymy established by Liebherr (1991a: 116).
Anchomenus metuens Casey, 1920: 52. Type locality: «Newport [Lincoln County], Oregon» (original citation). Lectotype (♂), designated by Lindroth (1975: 128), in USNM [# 47426]. Synonymy established by Hatch (1953: 137), confirmed by Lindroth (1966: 617).
Anchomenus dilleyanus Casey, 1920: 52. Type locality: «Dilley [Washington County], Oregon» (original citation). Lectotype (♂), designated by Lindroth (1975: 128), in USNM [# 47427]. Synonymy established by Hatch (1953: 137), confirmed by Lindroth (1966: 617).

#### Distribution.

This western species is found from west-central British Columbia south to northern California [see Liebherr 1991b: Fig. 187].

#### Records.

**CAN**: BC **USA**: CA, OR, WA

### Genus Rhadine

LeConte, 1846

Rhadine LeConte, 1846b: 218. Type species: *Rhadine larvalis* LeConte, 1846 by monotypy. Etymology. From the Greek *rhadinos* (slender), probably alluding to the slenderness of adults (“*corpus gracillimum*”) of the sole species LeConte had before him [feminine].
Comstockia Van Dyke, 1919a: 179. Type species: *Comstockia subterranea* Van Dyke, 1919 by original designation. Synonymy established implicitly by Jeannel (1931: 407), explicitly by Barr and Lawrence (1960: 138). Etymology. The generic name honors John Henry Comstock [1849-1931], professor of entomology at Cornell University. Comstock established the Department of Entomology at Cornell, the first such department in the United States.
Spelaeorhadine Bolívar y Pieltain, 1944: 27. Type species: *Spelaeorhadine araizai* Bolívar y Pieltain, 1944 by original designation. Synonymy established implicitly by Bolívar y Pieltain and Hendrichs (1964: 6). Etymology. From the Greek *spelaion* (cave, cavern) and the generic name *Rhadine* [*q.v*.], alluding to the place where the adults live [feminine].

#### Diversity.

Forty-nine species in North America (40 species) and Mexico (11 species) arrayed in six species groups.

#### Identification.

Barr (1960b) revised the cavernicolous species and provided a key for their identification, but several new species have been described since. Barr (1974b) published a key to the species groups and a revision of the species of the *subterranea* group. Five new species in this group have been described subsequently by Reddell and Cokendolpher (2001, 2004) and Reddell and Dupérré (2009). Barr (1982) also revised the Mexican species of *Rhadine*. A taxonomic revision of the other five species groups is needed.

### [dissecta group]

### Rhadine anthicoides

Casey, 1913

Rhadine anthicoides Casey, 1913: 167. Type locality: «Jemez Springs [Sandoval County], New Mexico» (original citation). One syntype in USNM [# 35021].

#### Distribution.

This species is known only from the type series collected in northwestern New Mexico.

#### Records.

**USA**: NM

### Rhadine balesi

(Gray, 1937)

Agonum balesi Gray, 1937: 310. Type locality: «Pasco [Franklin County], Wash[ington]» (as cited by Gray and Hatch 1941: 27). Holotype (♂) in USNM.

#### Distribution.

This species is known from a few localities in southeastern Oregon (Harney County, James R. LaBonte pers. comm. 1992), southeastern Washington, and southern Idaho (Hatch 1953: 146; Stafford et al. 1986: 288). The record from northern Colorado (Armin 1963: 147) needs confirmation.

#### Records.

**USA**: ID, OR, WA [CO]

### Rhadine constricta

Casey, 1913

Rhadine constricta Casey, 1913: 165. Type locality: «San Francisco M[oun]t[ain]s [Greenlee County], Arizona» (original citation). One syntype in USNM [# 35014].

#### Distribution.

This species is known only from the type series collected in southeastern Arizona.

#### Records.

**USA**: AZ

### Rhadine dissecta

(LeConte, 1863)

Platynus dissectus LeConte, 1863c: 8. Type locality: «Nebraska; Texas» (original citation). Two syntypes in MCZ [# 5737].

#### Distribution.

This species is known from “Nebraska” (LeConte 1863c: 8) to southwestern Colorado (LeConte 1879b: 54; Wickham 1902: 238), south to northern Arizona (Coconino County, CMNH; Snow 1906b: 162), the Sacramento Mountains in south-central New Mexico (Fall and Cockerell 1907: 159), and “Texas” (LeConte 1863c: 8).

#### Records.

**USA**: AZ, CO, NE, NM, TX

### Rhadine lindrothi

Barr, 1965

Rhadine lindrothi Barr, 1965b: 140. Type locality: «Medicine Hat, Alberta» (original citation). Holotype (♂) in MCZ.

#### Distribution.

This species is found from southern Saskatchewan and southern Alberta (Lindroth 1966: 647) south to Utah (Barr 1965b: 141), northeastern Colorado (Bell 1971: 52), and South Dakota (Kirk and Balsbaugh 1975: 25). The record from “Nebraska” (Bousquet and Larochelle 1993: 251) needs confirmation.

#### Records.

**CAN**: AB, SK **USA**: CO, ID, ND, SD, UT, WY [NE]

**Figure 37.**
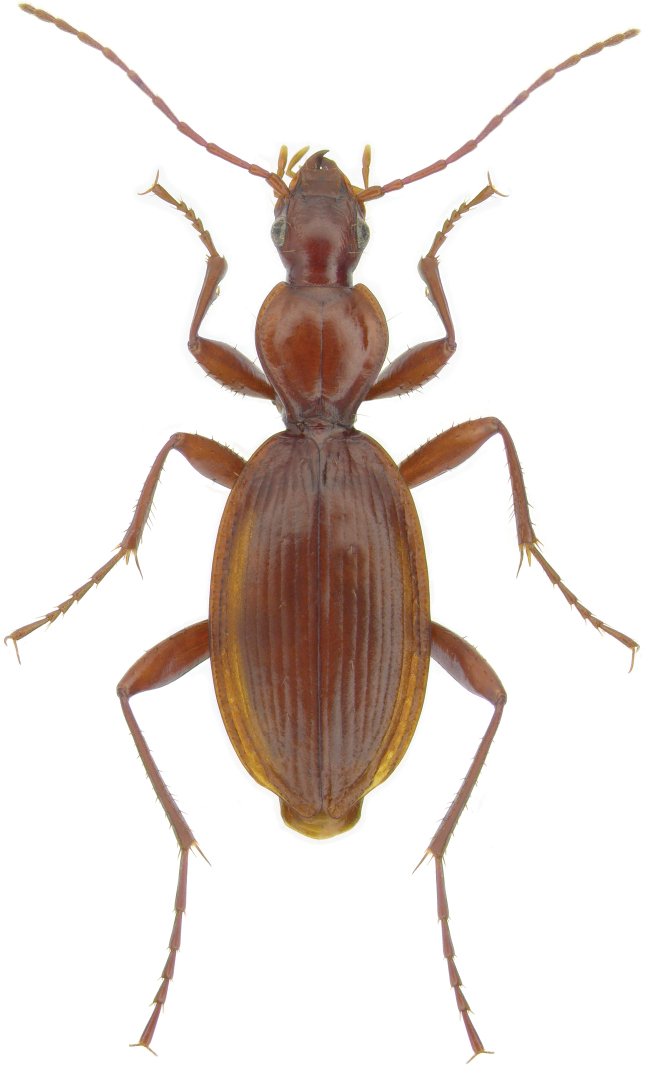
*Rhadine lindrothi* Barr. This species, named after Carl H. Lindroth, is the only one of the genus *Rhadine* that reaches as far north as southern Canada. Ecologically, members of the genus can be divided into two groups, one that includes cavernicolous species and the other that includes surface dwelling species like *Rhadine lindrothi*. Most species are more or less depigmented and with relatively long legs.

### Rhadine longipes

Casey, 1913

Rhadine longipes Casey, 1913: 167. Type locality: «Las Vegas [San Miguel County], New Mexico» (original citation). One syntype in USNM [# 35022].

#### Distribution.

This species is known only from the type locality in northeastern New Mexico.

#### Records.

**USA**: NM

### Rhadine rossi

Van Dyke, 1949

Rhadine rossi Van Dyke, 1949a: 51. Type locality: «near Somerset [Bexar County], Texas» (original citation for the holotype). Holotype (♂) in CAS [# 6011]. Etymology. The specific name was proposed for Edward Shearman Ross [1915-], curator of insects at the California Academy of Sciences. Ross was at one time a specialist on Histeridae and later became interested in the taxonomy, biology, and evolution of Embioptera.

#### Distribution.

This species is known from many specimens collected in south-central Texas.

#### Records.

**USA**: TX

### Rhadine rubra

(Barr, 1960)

Agonum rubrum Barr, 1960b: 49. Type locality: «Big Mouth Cave, 3 mi[les] N[orth] of Shamrock on left bank Red River, Wheeler Co[unty], Texas» (original citation). Holotype (♂) in USNM [# 75389].

#### Distribution.

This species is known from a few specimens collected in southeastern Colorado and northwestern Texas (Barr 1960b: 50).

#### Records.

**USA**: CO, TX

### Rhadine testacea

Casey, 1920

Rhadine testacea Casey, 1920: 12. Type locality: «Cripple Creek [Teller County], Colorado» (original citation). One syntype in USNM [# 35015].

#### Distribution.

This species is known only from the syntype collected in central Colorado.

#### Records.

**USA**: CO

### [jejuna group]

### Rhadine jejuna

(LeConte, 1878)

Platynus jejunus LeConte, 1878a: 449. Type locality: «mountains of California, Oregon, Nevada, and Idaho» (original citation). Three syntypes in MCZ [# 5738].
Rhadine gracilenta Casey, 1913: 166. Type locality: «California» (original citation). One syntype in USNM [# 35019]. **New synonymy**.
Rhadine plumasensis Casey, 1920: 12. Type locality: «Plumas Co[unty], California» (original citation). One syntype in USNM [# 35016]. **New synonymy**.
Rhadine pugetana Casey, 1920: 13. Type locality: «Wawawai [Whitman County], Washington» (original citation). One syntype in USNM [# 35017]. Synonymy established by Gray (1937: 310).
Rhadine tenuipes Casey, 1920: 13. Type locality: «California» (original citation). One syntype in USNM [# 35018]. **New synonymy**.

#### Distribution.

This species ranges from eastern Washington to southwestern Wyoming (Parmenter and MacMahon 1984: 26), south to northern Arizona (Wichkam 1896a: 157) and the Sierra Nevada in California (Casey 1913: 166). The records from Colorado (Cockerell 1893: 72; Wickham 1902: 238) need confirmation.

#### Records.

**USA**: AZ, CA, ID, NV, OR, WA, WY [CO]

#### Note.

A preliminary study of the type specimens of *Rhadine gracilenta* Casey, *Rhadine plumasensis* Casey, and *Rhadine tenuipes* Casey did not show any significant external differences from members of *Rhadine jejuna* (LeConte).

### Rhadine lanei

(Gray, 1937)

Agonum lanei Gray, 1937: 310. Type locality: «Wilma [Whitman County], Wash[ington]» (as cited by Gray and Hatch 1941: 27). Holotype (♂) in USNM.

#### Distribution.

This species is known only from the type locality in southeastern Washington (Hatch 1953: 146).

#### Records.

**USA**: WA

### [larvalis group]

### Rhadine caudata

(LeConte, 1863)

Platynus caudatus LeConte, 1863c: 7. Type locality: «western states» (original citation), herein restricted to Cumberland Caverns, Warren County, Tennessee (see Barr 1960b: 47). Holotype [by monotypy] (♂) in MCZ [# 5736].

#### Distribution.

This species ranges from southwestern Pennsylvania (Hamilton 1895: 350, 379; Ehrman 1900: 499) to southeastern Wisconsin (Rauterberg 1885: 14), south to northern Alabama (Jeannel 1949b: 95; Barr 1960b: 47) and northern Georgia (Fattig 1949: 32). The record from southwestern Colorado (Wickham 1902: 238) must be in error; that from “Arkansas” (Bousquet and Larochelle 1993: 250) needs confirmation.

#### Records.

**USA**: AL, DC, GA, IL, IN, KY, MD, NC, OH, PA, TN, VA, WI, WV [AR]

### Rhadine larvalis

LeConte, 1846

Rhadine larvalis LeConte, 1846b: 219. Type locality: «in vicinia urbis S[ain]t Louis» (original citation). One syntype in MCZ [# 5735].
Agonum jonesi Barr, 1960b: 48. Type locality: «Turk’s Cave, near Brooklyn, Conecuh Co[unty], Alabama» (original citation). Holotype (♂) in ALM. Synonymy established by Barr (1982a: 178).

#### Distribution.

This species is found from the Florida Panhandle (Choate and Rogers 1976: 364) to central Arkansas (Barr 1982a: 178), north to east-central Missouri (LeConte 1846b: 219). The records from northern Colorado (Wickham 1902: 238), northern Arizona (Wickham 1896a: 157), southern Montana (Hatch 1933a: 9), and southwestern Pennsylvania (Ehrman 1900: 500) are probably in error.

#### Records.

**USA**: AL, AR, FL, IL, MO, MS, TN

### Rhadine ozarkensis

Sanderson and Miller, 1941

Rhadine ozarkensis Sanderson and Miller, 1941: 39. Type locality: «Fincher’s Cave, Washington Co[unty], Arkansas» (original citation). Holotype (♂) in INHS (Webb 1980: 78).

#### Distribution.

This species is known only from the original specimens collected in Fincher’s Cave, near Fayetteville, Arkansas (Barr 1960b: 49).

#### Records.

**USA**: AR

### [nivalis group]

### Rhadine nivalis

(Horn, 1881)

Platynus nivalis G.H. Horn [in LeConte and Horn], 1881: 74. Type locality: «Santa Fe canon (7,000 feet), New Mexico» (original citation). Holotype [by monotypy] (♀) in MCZ [# 34453].

#### Distribution.

This species has been recorded from several localities in New Mexico (Fall and Cockerell 1907: 159; Casey 1913: 163).

#### Records.

**USA**: NM

### Rhadine sublustris

Casey, 1913

Rhadine sublustris Casey, 1913: 164. Type locality: «San Francisco M[oun]t[ain]s [Greenlee County], Arizona» (original citation). One syntype in USNM [# 35013].

#### Distribution.

This species is known from Greenlee (Casey 1913: 164) and Coconino Counties (CMNH) in southeastern and northern Arizona.

#### Records.

**USA**: AZ

### Rhadine umbra

Casey, 1913

Rhadine umbra Casey, 1913: 163. Type locality: «Jemez Springs [Sandoval County], New Mexico» (original citation). One syntype in USNM [# 35012].

#### Distribution.

This species is known only from the type series collected in northwestern New Mexico.

#### Records.

**USA**: NM

### [perlevis group]

### Rhadine albamontana

Dajoz, 1998

Rhadine albamontana Dajoz, 1998: 90. Type locality: «White Mountains, Mono County, Californie» (original citation). Holotype in Dajoz’s collection (Paris, France).

#### Distribution.

This species is known only from the original specimens collected in the White Mountains, eastern California.

#### Records.

**USA**: CA

### Rhadine babcocki

(Barr, 1960)

Agonum babcocki Barr, 1960b: 52. Type locality: «Mayfield’s Cave, Sutton Co[unty], Texas» (original citation). Holotype (♂) in USNM [# 75388]. Etymology. The specific name was proposed in honor of Orville Gorman Babcock [1885-1973] who collected some of the paratypes. Babcock was an entomologist who worked with the USDA on animal insect parasites.

#### Distribution.

This species is known from a number of caves in Sutton, Edwards, and Pecos Counties, western Texas (Barr 1960b: 52).

#### Records.

**USA**: TX

#### Note.

This taxon has been considered a subspecies of *Rhadine araizi* by Bolívar y Pieltain and Hendricks (1964: 6) but treated as a distinct species by Barr (1982a: 183-184).

### Rhadine howdeni

(Barr and Lawrence, 1960)

Agonum howdeni Barr and Lawrence, 1960: 143. Type locality: «Wilson’s Cave, 20 miles southwest of Hunt, Kerr County, Texas» (original citation). Holotype (♂) in CNC [# 7117]. Etymology. The specific name was proposed for Henry Fuller Howden [1925-], one of the leading taxonomists on the Scarabaeoidea.

#### Distribution.

This species is known from a number of caves in Kerr, Edwards, Uvalde, and Bexar Counties, southern Texas (Barr and Lawrence 1960: 143; Reddell and Cokendolpher, 2004: 157).

#### Records.

**USA**: TX

### Rhadine longiceps

Van Dyke, 1949

Rhadine longiceps Van Dyke, 1949a: 52. Type locality: «10 miles west of Alpine [Brewster County], Texas» (original citation). Holotype (♀) in CAS [# 6013].

#### Distribution.

This species is known only from the type locality in western Texas.

#### Records.

**USA**: TX

### Rhadine longicollis

Benedict, 1927

Rhadine longicollis Benedict, 1927: 44. Type locality: «Bat Cave [Eddy County, New Mexico]» (original citation). Holotype (♂) in SMEK [# 1079] (Byers and Karren 1968: 3).

#### Distribution.

This species is known from numerous caves in Eddy County, southeastern New Mexico, and Culberson County, western Texas (Cokendolpher and Polyak 2004: 192).

#### Records.

**USA**: NM, TX

### Rhadine myrmecodes

(Horn, 1892)

Platynus myrmecodes G.H. Horn, 1892c: 42. Type locality: «Arizona» (original citation). Holotype [by monotypy] (♀) in MCZ [# 34497].

#### Distribution.

This species has been recorded from southwestern Utah (Tanner 1928: 270), west-central (Bechtel et al. 1983: 474) and southern (Tanner and Tanner 1974: 218) Nevada, “Arizona” (Horn 1892c: 42), and New Mexico as far south as the Sierra Blanca Range (Fall and Cockerell 1907: 159).

#### Records.

**USA**: AZ, NM, NV, UT

### Rhadine perlevis

Casey, 1913

Rhadine perlevis Casey, 1913: 168. Type locality: «Colonia Garcia, Sierra Madre M[oun]t[ain]s, Chihuahua, Mexico» (original citation). One syntype in USNM [# 35023].

#### Distribution.

This species is known from southern Arizona and western Chihuahua in northern Mexico (Barr 1982a: 182).

#### Records.

**USA**: AZ – Mexico

### [subterranea group]

### Rhadine austinica

Barr, 1974

Rhadine austinica Barr, 1974b: 11. Type locality: «Bandit Cave, in the Rollingwood subdivision on the south side of Austin, Travis County, Texas» (original citation). Holotype (♂) in AMNH [# 1301].

#### Distribution.

This species is found in several caves in central Travis County, Texas (Barr 1974b: 12).

#### Records.

**USA**: TX

### Rhadine bullis

Reddell and Cokendolpher, 2004

Rhadine bullis Reddell and Cokendolpher, 2004: 154. Type locality: «Stahl Cave, Camp Bullis, Bexar County, Texas» (original citation). Holotype (♂) in AMNH.

#### Distribution.

This species is known from several caves on Camp Bullis, a U.S. Army installation in Bexar and Comal Counties, south-central Texas (Reddell and Cokendolpher, 2004: 154-155).

#### Records.

**USA**: TX

### Rhadine exilis

(Barr and Lawrence, 1960)

Agonum exile Barr and Lawrence, 1960: 141. Type locality: «Marnock Cave [= John Wagner Ranch Cave No. 3], 1 mile north of Helotes, Bexar County, Texas» (original citation). Holotype (♂) in CAS [# 8154].

#### Distribution.

This species is known only from caves near Helotes and Camp Bullis in Bexar County, south-central Texas (Barr 1974b: 16; Reddell and Cokendolpher, 2004: 156).

#### Records.

**USA**: TX

#### Note.

This species has been listed as endangered by the U.S. Fish and Wildlife Service in December 2000.

### Rhadine grubbsi

Reddell and Dupérré, 2009

Rhadine grubbsi Reddell and Dupérré, 2009: 111. Type locality: «Lime Kiln Quarry Cave, Hays County, Texas» (original citation). Holotype (♂) in AMNH.

#### Distribution.

This species is known from three caves in or near San Marcos, Hays County, in south-central Texas (Reddell and Dupérré 2009: 112).

#### Records.

**USA**: TX

### Rhadine infernalis ewersi

(Barr, 1960)

Agonum infernale ewersi Barr, 1960b: 55. Type locality: «Headquarters Cave, Camp Bullis, Bexar Co[unty], Texas» (original citation). Holotype (♂) in CMC (Vulinec and Davis 1984: 233).

#### Distribution.

This subspecies is known from three caves in southern Bexar County, south-central Texas (Reddell and Cokendolpher, 2004: 157).

#### Records.

**USA**: TX

### Rhadine infernalis infernalis

(Barr and Lawrence, 1960)

Agonum infernale Barr and Lawrence, 1960: 139. Type locality: «Madla Cave, 3 miles north of Helotes, Bexar County, Texas» (original citation). Holotype (♂) in CAS [# 8155].

#### Distribution.

This subspecies is known from a few small caves, near Helotes, in the highlands north of San Antonio, southern Texas (Barr 1974b: 23).

#### Records.

**USA**: TX

#### Note.

A series of eight specimens collected at Bat Cave in Government Canyon, Bexar County, Texas, are reported as hybrids *infernalis* x *ewersi* by Barr (1974b: 24). The species *Rhadine infernalis* has been listed as endangered by the U.S. Fish and Wildlife Service in December 2000.

### Rhadine insolita

Barr, 1974

Rhadine insolita Barr, 1974b: 20. Type locality: «Fischer Cave, near Fischers Store, Comal County, Texas» (original citation). Holotype (♂) in AMNH [# 1307].

#### Distribution.

This species is known only from the type-locality cave in south-central Texas.

#### Records.

**USA**: TX

### Rhadine ivyi

Reddell and Cokendolpher, 2004

Rhadine ivyi Reddell and Cokendolpher, 2004: 159. Type locality: «Vera Cruz Shaft, Camp Bullis, Bexar County, Texas» (original citation). Holotype (♂) in AMNH.

#### Distribution.

This species is known only from the type-locality cave in south-central Texas (Reddell and Cokendolpher, 2004: 161).

#### Records.

**USA**: TX

### Rhadine koepkei koepkei

(Barr, 1960)

Agonum koepkei Barr, 1960b: 56. Type locality: «Schneider’s Ranch Cave, 14 mi[les] N[orth]E[ast] of Boerne, Kendall Co[unty], Texas» (original citation). Holotype (♂) in CAS [# 8156].

#### Distribution.

This subspecies is known from two caves in south-central Texas (Barr 1974b: 24).

#### Records.

**USA**: TX

### Rhadine koepkei privata

Barr, 1974

Rhadine koepkei privata Barr, 1974b: 25. Type locality: «Skunk-X Water Cave, near Boerne, Kendall County, Texas» (original citation). Holotype (♂) in AMNH [# 1311].

#### Distribution.

This subspecies is known from a few caves in south-central Texas (Barr 1974b: 25).

#### Records.

**USA**: TX

### Rhadine noctivaga

Barr, 1974

Rhadine noctivaga Barr, 1974b: 10. Type locality: «Cobb Cavern, 12 miles north of Georgetown, Williamson County, Texas» (original citation). Holotype (♀) in AMNH [# 1300].

#### Distribution.

This species is known only from Cobb Cavern and Cricket Cave in northern Williamson County, central Texas (Barr 1974b: 11).

#### Records.

**USA**: TX

### Rhadine persephone

Barr, 1974

Rhadine persephone Barr, 1974b: 17. Type locality: «Tooth Cave, 15 miles northwest of Austin on the Kretschmarr Ranch, Travis County, Texas» (original citation). Holotype (♂) in AMNH [# 1305].

#### Distribution.

This species has been collected in caves in northern Travis and southern Williamson Counties, central Texas (Reddell and Cokendolpher 2001: 110).

#### Records.

**USA**: TX

#### Note.

This species, also known under the vernacular name “Tooth Cave Ground Beetle”, is listed as an endangered species by the World Wildlife Fund.

### Rhadine reyesi

Reddell and Cokendolpher, 2001

Rhadine reyesi Reddell and Cokendolpher, 2001: 110. Type locality: «Tippit Cave, Fort Hood, Coryell County, Texas» (original citation). Holotype (♂) in AMNH.

#### Distribution.

This species is known from a number of caves on the Fort Hood Military Reservation in Bell and Coryell Counties, central Texas (Reddell and Cokendolpher 2001: 110).

#### Records.

**USA**: TX

### Rhadine russelli

Barr, 1974

Rhadine russelli Barr, 1974b: 9. Type locality: «Lunsford Cave, 7 miles west of Leander on the George Lunsford Ranch, Travis County, Texas» (original citation). Holotype (♂) in AMNH [# 1299].

#### Distribution.

This species is known only from the type-locality cave on the Jollyville plateau in central Texas.

#### Records.

**USA**: TX

### Rhadine specum crinicollis

Barr, 1974

Rhadine speca crinicollis Barr, 1974b: 15. Type locality: «Kappelmans Salamander Cave, 4 miles east of Bulverde, Comal County, Texas» (original citation). Holotype (♂) in AMNH [# 1303].

#### Distribution.

This subspecies has been found yet only in two caves along Cibolo Creek in southern Texas (Barr 1974b: 16).

#### Records.

**USA**: TX

### Rhadine specum gentilis

Barr, 1974

Rhadine speca gentilis Barr, 1974b: 14. Type locality: «Little Gem Cave, on the R.A. Mittman Ranch 4 miles west of New Braunfels, Comal County, Texas» (original citation). Holotype (♂) in AMNH [# 1302].

#### Distribution.

This subspecies is known only from two caves in south-central Texas (Barr 1974b: 15).

#### Records.

**USA**: TX

### Rhadine specum specum

(Barr, 1960)

Agonum specum Barr, 1960b: 58. Type locality: «Cave-Without-A-Name, Kendall Co[unty], Texas» (original citation). Holotype (♂) in CAS [# 8157]. Note. Barr (1960b) did not give the etymology of the specific name but it certainly derives from the Latin noun *specus*, -*us* (cave). Barr believed the word could be used as an adjective since he described later (Barr 1974b: 14) a *Rhadine speca gentilis*. However, the word is a noun (Theil 1883: 297) and the species should have been named *Agonum specus* (nominative singular and genitive singular) or *Agonum specuum* (genitive plural). I consider that Barr’s name is not a Latin or latinized word; as such it does not need to agree in gender with the generic name with which it is combined (ICZN 1999: Article 31.2.3). Therefore, *Rhadine specum*, not *Rhadine speca*, is the correct spelling for this name.

#### Distribution.

This subspecies is known from a few caves in Kendall and Comal Counties, southern Texas (Barr 1974b: 13; Reddell and Cokendolpher, 2004: 158).

#### Records.

**USA**: TX

### Rhadine sprousei

Reddell and Cokendolpher, 2004

Rhadine sprousei Reddell and Cokendolpher, 2004: 158. Type locality: «Cannonball Cave, Camp Bullis, Bexar County, Texas» (original citation). Holotype (♂) in AMNH.

#### Distribution.

This species is known yet only from the type-locality cave in south-central Texas (Reddell and Cokendolpher, 2004: 159).

#### Records.

**USA**: TX

### Rhadine subterranea mitchelli

Barr, 1974

Rhadine subterranea mitchelli Barr, 1974b: 7. Type locality: «Steam Cave, 2 miles south of Georgetown, Williamson County, Texas» (original citation). Holotype (♂) in AMNH [# 1298].

#### Distribution.

This subspecies is known from several caves in central Texas (Barr 1974b: 8).

#### Records.

**USA**: TX

### Rhadine subterranea subterranea

(Van Dyke, 1919)

Comstockia subterranea Van Dyke, 1919a: 182. Type locality: «a cave near Austin, Texas» (original citation); according to Barr (1974b: 7), the holotype is from Sam Bass (= McNeil Quarry) Cave, near McNeil, Williamson County. Holotype (♀) in CUIC [# 259].

#### Distribution.

This subspecies is known from several caves in southern Williamson and northern Travis Counties, central Texas (Barr 1974b: 7).

#### Records.

**USA**: TX

### Rhadine tenebrosa mckenziei

Barr, 1974

Rhadine tenebrosa mckenziei Barr, 1974b: 19. Type locality: «Skeleton Cave, 5 miles north of Leakey on the Orell Ranch, Real County, Texas» (original citation). Holotype (♂) in AMNH [# 1306].

#### Distribution.

This subspecies is found in a few caves in Real and Uvalde Counties, southwestern Texas (Barr 1974b: 20).

#### Records.

**USA**: TX

### Rhadine tenebrosa tenebrosa

(Barr, 1960)

Agonum tenebrosum Barr, 1960b: 57. Type locality: «Wilson’s Cave, 25 mi[les] S[outh]W[est] of Hunt, Kerr Co[unty], Texas» (original citation). Holotype (♂) in CNC [# 7118].

#### Distribution.

This subspecies is known from a few caves in Kerr, Real, and Bandera Counties, southwestern Texas (Barr 1974b: 19).

#### Records.

**USA**: TX

### [incertae sedis]

### Rhadine pertenuis

Casey, 1920

Rhadine pertenuis Casey, 1920: 14. Type locality: «Esmeralda Co[unty], Nevada» (original citation). One syntype in USNM [# 35020].

#### Distribution.

This species is known for sure only from the type locality in southwestern Nevada. The record from “California” (Bousquet and Larochelle 1993: 251) needs confirmation.

#### Records.

**USA**: NV [CA]

### Genus Mexisphodrus

Barr, 1965

Mexisphodrus Barr, 1965c: 65. Type species: *Mexisphodrus veraecrucis* Barr, 1965 by original designation. Etymology. From the English adjective *mexican* (of Mexico) and the generic name *Sphodrus*, alluding to the country where these *Sphodrus*-like species live [masculine].

#### Diversity.

Eleven species in Texas (one species) and Mexico (ten species).

#### Identification.

Barr (1982a) revised the species and provided a key for their identification.

#### Taxonomic Note.

Valentine (1987) believed this genus is closely related to the genera *Bryanites* Valentine of Western Samoa and *Prosphodrus* Britton of New Zealand. He proposed the family-group name Prosphodrini to include the three genera.

### Mexisphodrus valverdensis

Barr, 1982

Mexisphodrus valverdensis Barr, 1982a: 177. Type locality: «Ladder Cave, 25 km N[orth]W[est] Del Rio, Val Verde Co[unty], Texas» (original citation). Holotype (♂) in AMNH.

#### Distribution.

This species is known from two nearby caves in southwestern Texas.

#### Records.

**USA**: TX

#### Note.

This species belongs to the *boneti* species group which includes only the polymorphic *Mexisphodrus boneti* (Bolívar y Pieltain and Hendrichs) from Nuevo León and *Mexisphodrus valverdensis* Barr.

### Genus Tanystoma

Motschulsky, 1845

Tanystoma Motschulsky, 1845b: 341. Type species: *Anchomenus striatus* Dejean, 1828 by original designation. Etymology. From the Greek *tanyo* (long) and *stoma* (mouth) [neuter].
Tanystola Motschulsky, 1850a: 69. Unnecessary replacement name for *Tanystoma* Motschulsky, 1845.
Leucagonum Casey, 1920: 99. Type species: *Agonum maculicolle* Dejean, 1828 designated by Bousquet and Larochelle (1993: 252). Synonymy established by Liebherr (1985: 1184). Etymology. From the Greek *leucos* (white) and the generic name *Agonum* [*q.v*.], probably alluding to the color (“color pale flavo-testaceous”) of the adults [neuter].

#### Diversity.

Five species in western North America (four species) and the Baja California Peninsula and Guadalupe Island in Mexico (two species, one of them endemic).

#### Identification.

Liebherr (1985) revised the species. Subsequently he described a new species, the endemic Baja Californian *Tanystoma diabolicum*, and provided a key to all species (Liebherr 1989).

#### Taxonomic Note.

According to Liebherr (1989: 184), the genus *Rhadine* is the sister-group of *Tanystoma*.

### Tanystoma cuyama

Liebherr, 1985

Tanystoma cuyama Liebherr, 1985: 1194. Type locality: «L[a]k[e] Cachuma (800’), 18 mi[les] S[outh]E[ast] Hwy. 101, Santa Barbara Co[unty], Ca[lifornia]» (original citation). Holotype (♂) in CUIC [# 5860].

#### Distribution.

This species ranges through the Coast Ranges from central California south to the Mexican border [see Liebherr 1985: Fig. 27].

#### Records.

**USA**: CA

### Tanystoma maculicolle

(Dejean, 1828)

Agonum maculicolle Dejean, 1828: 175. Type locality: «Californie» (original citation), restricted to «Santa Barbara [Santa Barbara County]» by Liebherr (1985: 1194). Lectotype (♂), designated by Liebherr (1985: 1194), in MHNP.
Agonum maculicolle guadalupense Casey, 1920: 100. Type locality: «Guadalupe Island, [Baja] California» (original citation). Lectotype (♂), designated by Liebherr (1991a: 117), in USNM [# 47471]. Synonymy established by Liebherr (1985: 1190).
Agonum angustior Casey, 1920: 101. Type locality: «Hoopa Valley, Humboldt Co[unty], California» (original citation). Lectotype (♂), designated by Liebherr (1991a: 116), in USNM [# 47472]. Synonymy established by Liebherr (1985: 1190).

#### Distribution.

This species, also known under the vernacular name “tule Beetle”, ranges from southwestern Oregon south through California, including the Channel Islands, to Baja California Norte and Guadalupe Island [see Liebherr 1985: Fig. 25]. The species is apparently adventive and possibly established at Seattle, Washington (Liebherr 1985: 1194). The record from “Utah” (Bousquet and Larochelle 1993: 252) is likely in error.

#### Records.

**USA**: CA (CHI), OR, WA – Mexico

### Tanystoma striatum

(Dejean, 1828)

Anchomenus striatus Dejean, 1828: 132. Type locality: «Californie» (original citation), restricted to San Bruno M[oun]t[ai]n, San Mateo Co[unty]» by Liebherr (1985: 1198). Lectotype (♂), designated by Liebherr (1985: 1198), in MHNP.

#### Distribution.

This species occurs along the Coast Ranges of California from Siskiyou County to Santa Cruz and Alameda Counties and in the Sierra Nevada between Tulare and Shasta Counties [see Liebherr 1985: Fig. 27].

#### Records.

**USA**: CA

### Tanystoma sulcatum

(Dejean, 1828)

Anchomenus sulcatus Dejean, 1828: 131. Type locality: «Californie» (original citation), herein restricted to Hayfork, Trinity County (see Liebherr 1985: 1202). Lectotype (♀), designated by Liebherr (1985: 1200), in MHNP. Note. Liebherr (1985: 1200) selected «Coos Bay, Oregon» as type locality of *Tanystoma sulcatum* but it should be rejected since Dejean (1828: 131) specified that his specimen(s) came from California.
Agonum charactum Hatch, 1951: 117. Type locality: «Marshfield [= Coos Bay], Coos Co[unty], Ore[gon]» (original citation). Holotype (♂) in USNM. Synonymy established by Liebherr (1985: 1200).

#### Distribution.

This species ranges from northwestern Oregon to northern California [see Liebherr 1985: Fig. 27]. An old specimen labeled from western Washington is known.

#### Records.

**USA**: CA, OR [WA]

### Genus Paranchus

Lindroth, 1974

Paranchus Lindroth, 1974: 81. Type species: *Carabus albipes* Fabricius, 1794 by original designation. Etymology. From the Greek *para* (near, next to) and the generic name *Anchus* [*q.v*.] [masculine].

#### Diversity.

Two species endemic to the Canary Islands and one European species, *Paranchus albipes*, which is adventive in northeastern North America.

#### Identification.

The North American species is covered in Lindroth’s (1966: 630-631, as *Agonum albipes*) monograph.

### Paranchus albipes

(Fabricius, 1794)

Carabus oblongus Fabricius, 1792: 140 [primary homonym of *Carabus oblongus* Fabricius, 1792]. Type locality: «Germania» (original citation). Syntype(s) location unknown. Note. There is one specimen under the name *Carabus pallipes* Fabricius in ZMUC (Zimsen 1964: 55) but, according to Lindroth (1966: 630), the specimen is not a syntype.
Carabus albipes Fabricius, 1794a: 468. Replacement name for *Carabus oblongus* Fabricius, 1792.
Carabus pavidus Panzer, 1799: no 7. Type locality: «Dresdae [Germany]» (original citation). Syntype(s) location unknown (possibly in ZMHB). Synonymy established with doubt by Illiger (1801: 54).
Carabus pallipes Fabricius, 1801: 187 [primary homonym of *Carabus pallipes* Fabricius, 1787]. Replacement name for *Carabus oblongus* Fabricius, 1792.
Carabus circulatus Marsham, 1802: 450. Type locality «fluvium Usk prope Crickhowell et prope Ealing [Great Britain]» (original citation). Syntype(s) location unknown. Synonymy established by Schönherr (1806: 190).
Carabus sordidus Marsham, 1802: 457. Type locality: Great Britain (inferred from title of the book). Two syntypes in BMNH (collection Stephens). Synonymy established implicitely by Stephens (1828a: 82). Note. Stephens (1828a: 82) listed this taxon as a valid species but also said “I do not think them [the two specimens of *sordidus* from Marsham’s collection in his hands] sufficiently distinct from the preceding [*Anchomenus albipes* Illiger].”
Platynus clemens LeConte, 1863c: 8. Type locality: «Nova Scotia» (original citation). Two syntypes in MCZ [# 5758]. Synonymy established by Lindroth (1954b: 138).

#### Distribution.

This Palaearctic species is adventive in North America where it is known from southern Newfoundland, Nova Scotia, New Brunswick, and Maine (Lindroth 1966: 631). The first inventoried specimen collected on this continent was found in Newfoundland before 1835. The Canadian Museum of Nature in Gatineau holds color drawings made by Philip Henry Gosse executed prior to 1834 of insects from Newfoundland and one drawing represents this species; the plates are bound together under the title *Entomologia Terrae Novae - P.H. Gosse - 1833*.

#### Records.

**CAN**: NB, NF, NS (CBI) **USA**: ME – **Adventive**

#### Note.

*Carabus ruficornis* Goeze, 1777 is often listed as a synonym of this species (e.g., Lindroth 1966: 630; Bousquet 2003c: 462). However, Goeze (1777: 663) did not propose a new species under such name since he referred to *Carabus ruficornis* DeGeer, 1774 which is considered a doubtful synonym of *Amara aulica* (Panzer, 1796) following Schönherr (1806: 181). Goeze (1777: 663) apparently misidentified DeGeer’s *Carabus ruficornis*.

### Genus Oxypselaphus

Chaudoir, 1843

Oxypselaphus Chaudoir, 1843a: 415. Type species: *Oxypselaphus pallidulus* Chaudoir, 1843 (= *Carabus obscurus* Herbst, 1784) by monotypy. Etymology (original). From the Greek *oxys* (acute, sharp) and *pselaphos* (palpus), alluding to the acuminate last maxillary palpomere (“*palpi maxillares *... *4o acuminato*”) of the adult [masculine].
Anchus LeConte, 1854b: 38. Type species: *Anchus pusillus* LeConte, 1854 by monotypy. Etymology. From the Greek *ancho* (strangle), probably alluding to the basally narrow pronotum (“thorax ... cordate, almost turned into a pedicel at the base”) of the adult [masculine].

#### Diversity.

Four species, two endemic to northern Africa, one to Europe and western Asia, and one to North America.

#### Identification.

The North American species is covered in Lindroth’s (1966: 633-634, as *Agonum puncticeps*) monograph.

#### Taxonomic Note.

Jeanne (1988: 77-78) pointed out the morphological differences between the type species of *Oxypselaphus* and *Anchus* and suggested that the two should be treated as generically distinct.

### Oxypselaphus pusillus

(LeConte, 1854)

Anchus pusillus LeConte, 1854b: 39. Type locality: «Massachusetts; Illinois» (original citation), restricted to «Mass[achusetts]» by Lindroth (1966: 633). Three syntypes in MCZ [# 5759].
Anchus puncticeps Casey, 1920: 2. Type locality: «Ontario» (original citation). Lectotype (♀), designated by Lindroth (1975: 130), in USNM [# 47452]. Synonymy established by Lindroth (1966: 633).

#### Distribution.

This species ranges from Cape Breton Island (Bousquet 1987a: 126) to the Okanagan river in south-central British Columbia (Lindroth 1966: 634), south to western Oregon (Hatch 1953: 138), northeastern Kansas (Popenoe 1878: 78), and northwestern South Carolina (Anderson County, CNC). The record from “Georgia” (Bousquet and Larochelle 1993: 253) needs confirmation.

#### Records.

**CAN**: AB, BC, MB, NB, NS (CBI), ON, PE, QC, SK **USA**: CT, IA, ID, IL, IN, KS, MA, ME, MI, MN, MO, MT, NC, ND, NE, NH, NJ, NY, OH, OR, PA, RI, SC, SD, VA, VT, WA, WI [GA]

### Genus Agonum

Bonelli, 1810

Agonum Bonelli, 1810: Tabula Synoptica. Type species: *Carabus marginatus* Linnaeus, 1758 designated by Curtis (1827: plate 183). Etymology. Uncertain, possibly from the Greek *a* (without) and *gonia* (angle), alluding to the rounded posterior angles of the pronotum (“*th*[*orax*] ... *angulis baseos rotundatis*”) of the adult or from the Greek *agonos* (barren), or from the Greek *agon*, -*os* (contest, struggle) [neuter].
Amolyntus Gistel, 1848a: viii. Unnecessary replacement name for *Agonum* Bonelli, 1810.
Agonothorax Motschulsky, 1850a: 67. Unnecessary replacement name for *Agonum* Bonelli, 1810.

#### Diversity.

About 130 species (Liebherr and Schmidt 2004: 202-203) in the Nearctic (72 species, one adventive), Neotropical (15 species in Middle America, all but two shared with North America), Palaearctic (63 species, of which seven are Holarctic), and Afrotropical (three endemic species) Regions. The species are arrayed in four subgenera, following Liebherr and Schmidt (2004): *Europhilus*, *Platynomicrus*, *Agonum *s.str., and *Olisares*, all represented in North America. All other subgenera of *Agonum* listed in Lorenz (2005: 411-412) have been excluded by Liebherr and Schmidt (2004: 152) as most of them “are most likely evolutionary affiliates of the highly diverse and taxonomically confused *Platynus*-“*Colpodes*” group of genera.” About 50 species are left unplaced in Lorenz (2005: 412) and some of them may belong to *Agonum*.

#### Identification.

Liebherr (1994) published a key to all species found in the Western Hemisphere as well as a review of the Middle American species. Lindroth (1966, 1969a) covered all North American species except *Agonum anthracinum* Dejean, *Agonum cyanopis* Bates, *Agonum elongatulum* Dejean, *Agonum extimum* Liebherr, *Agonum muiri* Liebherr, *Agonum pacificum* Casey, and *Agonum parextimum* Liebherr.

### Subgenus Platynomicrus

Casey, 1920

Platynomicrus Casey, 1920: 90. Type species: *Agonum nigriceps* LeConte, 1846 by original designation. Etymology. From the generic name *Platynus* [*q.v*.] and the Greek *micros* (small) [masculine].

#### Diversity.

Two species in North America, one of them is Holarctic.

### Agonum ferruginosum

(Dejean, 1828)

Anchomenus ferruginosus Dejean, 1828: 128. Type locality: «Californie» (original citation). One syntype in MHNP (Lindroth 1955b: 20), one specimen [labeled “type”] in MCZ (collection LeConte) (Lindroth 1966: 588).
Platynus erasus LeConte, 1879b: 52. Type locality: «Vancouver Island [British Columbia]» (original citation). Two syntypes [2 originally cited] in MCZ [# 5754]. Synonymy established by Lindroth (1966: 588).
Anchomenus wadei Casey, 1920: 62. Type locality: «Wilbur [Lincoln County], Washington» (original citation). Lectotype (♀), designated by Lindroth (1975: 127), in USNM [# 47442]. Synonymy established, under the name *Agonum erasum* (LeConte), by Gray (1937: 313), confirmed by Lindroth (1966: 588).
Anchomenus marcidus Casey, 1920: 65. Type locality: «Ouray [Ouray County], Colorado» (original citation). Lectotype (♂), designated by Lindroth (1975: 127), in USNM [# 47447]. Synonymy established by Lindroth (1966: 588).

#### Distribution.

The range of this species extends from Saskatchewan to Vancouver Island (Lindroth 1966: 589), north to southeastern Alaska (Lindroth 1966: 589), south to southeastern California (San Bernardino County, CNC; Casey 1920: 65) and southwestern Colorado (Casey 1920: 65, as *Anchomenus marcidus*).

#### Records.

**CAN**: AB, BC (QCI, VCI), SK **USA**: AK, CA, CO, ID, MT, NV, OR, UT, WA, WY

### Agonum nigriceps

LeConte, 1846

Agonum nigriceps LeConte, 1846b: 229. Type locality: «Lacum Superiorem» (original citation), herein restricted to Eagle Harbor, Keweenaw County, Michigan (see LeConte 1854b: 54). One syntype in MCZ [# 5789].
Platynomicrus fragilissimus Casey, 1920: 92. Type locality: «Toronto [Ontario]» (original citation). Lectotype (♂), designated by Lindroth (1975: 127), in USNM [# 47517]. Synonymy established by Lindroth (1954b: 139).

#### Distribution.

This Holarctic species is known from the Kamchatka Peninsula (Lindroth 1966: 584) and from Alaska (Lindroth 1966: 584) to Newfoundland (Lindroth 1955a: 129), south to northern New Hampshire (Coos County, Donald S. Chandler pers. comm. 2008), southeastern Michigan (Oakland County, UMAA), “Iowa” (Lindroth 1966: 584), east-central South Dakota (Brookings County, MCZ), east-central Colorado (Kiowa County, Foster F. Purrington pers. comm. 2010), “Idaho” (Lindroth 1966: 584), and northern Washington (Okanogan County, UASM). The record from “Pennsylvania” (Bousquet and Larochelle 1993: 255) needs confirmation.

#### Records.

**CAN**: AB, BC, MB, NB, NF, NT, ON, PE, QC, SK, YT **USA**: AK, CO, IA, ID, IL, ME, MI, MN, MT, ND, NH, NY, SD, WA, WI, WY [PA] – **Holarctic**

### Subgenus Europhilus

Chaudoir, 1859

Europhilus Chaudoir, 1859: 124. Type species: *Anchomenus micans* Nicolai, 1822 designated by Motschulsky (1865: 317). Etymology. Probably from the Greek *euros* (mold, decay), rather than *euros* (east wind), and *philos* (beloved) [masculine].

#### Diversity.

Twenty-nine species in the Nearctic (16 species) and Palaearctic (17 species, six of them endemic to eastern Asia) Regions. Four species are Holarctic.

### [limbatum group]

### Agonum limbatum

Motschulsky, 1845

Agonum limbatum Motschulsky, 1845a: 21. Type locality: «Californie» (original citation), herein restricted to San Francisco, San Francisco County (see LeConte 1851: 178, as *Platynus variolatus*). Lectotype, designated by Bousquet and Larochelle (1993: 11), in ZMMU.
Platynus variolatus LeConte, 1851: 178. Type locality: «San Francisco [San Francisco County, California]» (original citation). Three syntypes in MCZ [# 69]. Synonymy established by Motschulsky (1855b: 79, as *Platynus variolaris*), confirmed by Bousquet and Larochelle (1993: 11).
Sericoda variolata coronadina Casey, 1920: 96. Type locality: «San Diego [San Diego County], California» (original citation). Lectotype (♀), designated by Lindroth (1975: 127), in USNM [# 47521]. Synonymy established by Lindroth (1966: 589).
Sericoda shastanica Casey, 1920: 96. Type locality: «Siskiyou Co[unty], California» (original citation). Lectotype (♀), designated by Lindroth (1975: 127), in USNM [# 47520]. Synonymy established by Lindroth (1966: 589).

#### Distribution.

This species ranges along the west coast from southern British Columbia, including Vancouver Island (Lindroth 1966: 589, as *Agonum variolatum*), to southern California (Fall 1901a: 46, as *Agonum variolatum*).

#### Records.

**CAN**: BC (VCI) **USA**: CA (CHI), OR, WA

### [sordens group]

### Agonum anchomenoides

Randall, 1838

Agonum anchomenoides Randall, 1838a: 2. Type locality: «Augusta [Kennebec County, Maine]» (original citation). Syntype(s) lost.
Agonum lanellum Gray, 1937: 311. «Type locality: Orient [Ferry County], Washington» (original citation). Holotype (♂) in USNM. Synonymy established by Hatch (1953: 144), confirmed by Lindroth (1966: 589).

#### Distribution.

This species ranges from western Newfoundland (Lindroth 1955a: 125) to eastern Alaska (Lindroth 1966: 590), south to northeastern Washington (Gray 1937: 310, as *Agonum lanellum*), western Montana (Russell 1968: 63), east-central South Dakota (Kirk and Balsbaugh 1975: 24), and southern Pennsylvania (Hamilton 1895: 322; Allegheny, Lancaster, and Westmoreland Counties, Robert L. Davidson pers. comm. 2008). The records from “Wyoming,” “Colorado,” “Iowa,” “Nebraska,” and “Kansas” (Bousquet and Larochelle 1993: 253) are probably in error.

#### Records.

**CAN**: AB, BC, MB, NB, NF, NS, NT, ON, PE, QC, SK, YT **USA**: AK, CT, IL, IN, MA, ME, MI, MN, MT, ND, NH, NJ, NY, OH, PA, SD, VT, WA, WI

### Agonum canadense

Goulet, 1969

Agonum elongatulum Haldeman, 1843b: 300 [secondary homonym of *Agonum elongatulum* (Dejean, 1828)]. Type locality: southeastern Pennsylvania (Haldeman 1843a: 297). One possible syntype, a ♂ labeled “[pink disc] / elongatulum Hald. [handwritten] / picipennis 4 [handwritten],” in MCZ (Lindroth 1966: 581).
Agonum canadense Goulet, 1969: 279. Type locality: «Bala, N[orth]W[est] Gravenhurst, Ontario» (original citation). Holotype (♂) in CNC [# 11574]. **New synonymy**. Note. The specimen probably belonging to *Agonum elongatulum* Haldeman in LeConte’s collection is conspecific with specimens of *Agonum canadense* Goulet. Goulet’s name is retained as valid since Haldeman’s name is a junior homonym of *Agonum elongatulum* (Dejean).

#### Distribution.

This species is found from Cape Breton Island (Bousquet 1987d: 106) to southern Manitoba (Goulet 1969: 280), south to east-central South Dakota (Larsen and Purrington 2010: 570), northern Illinois (Goulet 1969: 280), and northern West Virginia (Tucker and Preston Counties, CMNH). The record from “Nebraska” (Bousquet and Larochelle 1993: 253) needs confirmation.

#### Records.

**CAN**: MB, NB, NS (CBI), ON, PE, QC **USA**: CT, IL, IN, MA, ME, MI, NH, NJ, NY, PA, SD, VT, WI, WV [NE]

### Agonum consimile

(Gyllenhal, 1810)

Harpalus consimilis Gyllenhal, 1810: 161. Type locality: «Lapponia [Sweden]» (original citation), restricted to «Abisko, Torne Lappmark» by Lindroth (1966: 572). Lectotype (♂), designated by Lindroth (1966: 572), in UZIU.
Anchomenus fragilis Mannerheim, 1853: 142. Type locality: «insula Sitkha [= Baranof Island, Alaska]» (original citation). One syntype in MHNP (Lindroth 1966: 572). Synonymy established by Lindroth (1955a: 126).
Batenus borealis Motschulsky, 1865: 319. Type locality: «nord de la Sibérie orientale» (original citation). Two syntypes in ZMMU (Keleinikova 1976: 189). Synonymy established by Shilenkov (in Liebherr 1991b: 78).
Agonum invalidum Casey, 1924: 84. Type locality: «Edmonton, Alberta» (original citation). Holotype [by monotypy] (♂) in USNM [# 47493]. Synonymy established by Lindroth (1953b: 171).

#### Distribution.

A Holarctic species occurring in the Palaearctic Region from Norway to the Far East (Bousquet 2003c: 452) and in North America from Alaska (Lindroth 1966: 573) to Newfoundland (Lindroth 1955a: 126), south to Cape Breton Island (Lindroth 1954c: 307), Vancouver Island (Lindroth 1966: 573), and northern Washington (Glesne et al. 2000: 89). Fossil remnants, dated between 14,000 and 15,400 years B.P., have been unearthed in central Iowa (Schwert 1992: 78).

#### Records.

**FRA**: PM **CAN**: AB, BC (VCI), LB, MB, NB, NF, NS (CBI), NT, ON, QC, YT **USA**: AK, WA – **Holarctic**

### Agonum darlingtoni

Lindroth, 1954

Agonum darlingtoni Lindroth, 1954b: 159. Type locality: «Lexington [Middlesex County], Mass[achusetts]» (holotype label). Holotype (♂) in MCZ [# 29072]. Etymology. The specific name honors Philip Jackson Darlington, Jr. [1904-1983], field naturalist, biogeographer, and systematist, specialist of the family Carabidae. Darlington was curator of entomology at the Museum of Comparative Zoology. Note. Although Lindroth’s description is very short, I believe he met the requirements of availability for a species-group name published after 1930 (ICZN 1999: Article 13.1). Lindroth (1955c: 12) redescribed the species in length.

#### Distribution.

This species is restricted to a small area from Nova Scotia to the Ontario Peninsula (Lindroth 1966: 583), south to central Pennsylvania (Clinton County, CMNH) and New Jersey (Lindroth 1966: 583).

#### Records.

**CAN**: NB, NS, ON, QC **USA**: CT, MA, ME, NH, NJ, NY, PA, RI, VT

### Agonum exaratum

(Mannerheim, 1853)

Anchomenus exaratus Mannerheim, 1853: 143. Type locality: «ora orientali insulae Kadjak [Alaska]» (original citation). Lectotype (♀), designated by Lindroth (1966: 573), in ZMH.
Batenus aeneolus Motschulsky, 1865: 320. Type locality: «Kadiak [Alaska]» (original citation). Lectotype (♀), designated by Bousquet and Larochelle (1993: 12), in ZMMU. Synonymy established by Bousquet and Larochelle (1993: 12).
Platynus aldanicus Poppius, 1906a: 36. Type locality: «Aldan-Mündung, Lena-Ufern [Yakutia, Siberia, Russia]» (original citation). Holotype (♀) in ZMH. Synonymy established by Lindroth (1954b: 139).

#### Distribution.

A Holarctic species ranging from the Kola Peninsula to eastern Siberia and in North America from Alaska to the Hudson Bay in Nunavut and northern Manitoba (Lindroth 1966: 574). Fossil remnants, dated between 14,000 and 20,700 years B.P., have been unearthed in east-central Iowa and central Illinois (Schwert 1992: 78).

#### Records.

**CAN**: BC, MB, NT, NU, YT **USA**: AK – **Holarctic**

### Agonum galvestonicum

(Casey, 1920)

Europhilus galvestonicus Casey, 1920: 126. Type locality: «Galveston [Galveston County], Texas» (original citation). Holotype [by monotypy] (♂) in USNM [# 47532].

#### Distribution.

This rarely collected species is known from Pennsylvania (Carbon, Chester and Greene Counties, Robert L. Davidson pers. comm. 2012), southernmost Ontario (Lindroth 1966: 583) and southern Michigan (Ingham County, CMNH) south to southeastern Texas (Casey 1920: 126) and eastern South Carolina (Kirk 1969: 12; Ciegler 2000: 112).

#### Records.

**CAN**: ON **USA**: MI, MO, MS, OH, PA, SC, TN, TX, VA, WV

### Agonum gratiosum

(Mannerheim, 1853)

Platynus ruficornis LeConte, 1850: 205. Type locality: Lake Superior (inferred from title of the paper), herein restricted to Marquette, Marquette County, Michigan (see Hubbard and Schwarz 1878: 628). Three syntypes in MCZ [# 5784]. Note. This name has been considered a junior secondary homonym of *Agonum ruficorne* (Goeze, 1777). As stated before (see “Note” section for *Paranchus albipes* (Fabricius)), Goeze’s name is based on a misidentification and has no nomenclatural status. Therefore, *Platynus ruficornis* LeConte is not a homonym and should be used as the valid name for this taxon. However, the name *Agonum gratiosum* is well established for this common species since the 1960s and I believe it should be preserved. The case should be submitted to the Commission for a ruling. Reversal of precedence (ICZN 1999: 23.9) cannot be used here since LeConte’s name has been used as valid after 1899 (e.g., Blatchley 1910: 135).
Anchomenus gratiosus Mannerheim, 1853: 142. Type locality: «insula Kadjak [Alaska]» (original citation). Lectotype (♀), designated by Lindroth (1955c: 7), in ZMH. Synonymy established by LeConte (1879b: 58), confirmed by Lindroth (1954b: 139).
Europhilus symmetricus Casey, 1920: 129. Type locality: «Devil’s Lake [Ramsey County], N[orth] Dakota» (original citation for the lectotype). Lectotype (♀), designated by Lindroth (1975: 126), in USNM [# 47528]. Synonymy established by Hatch (1953: 145), confirmed by Lindroth (1954b: 140).
Europhilus properans Casey, 1920: 129. Type locality: «Durham [Strafford County], New Hampshire» (original citation). Lectotype (♀), designated by Lindroth (1975: 126), in USNM [# 47530]. Synonymy established by Lindroth (1954b: 140).
Europhilus antiquus Notman, 1922b: 102. Type locality: «Lyme [New London County], C[onnecticu]t» (original citation). Holotype (♂) in USNM [# 26591]. Synonymy established by Lindroth (1954b: 140).
Europhilus carri Casey, 1924: 85. Type locality: «Edmonton [Alberta]» (original citation). Lectotype (♂), designated by Lindroth (1966: 577), in USNM [# 47529]. Synonymy established with doubt by Lindroth (1955c: 7), confirmed by Lindroth (1966: 577). Etymology. The specific name was proposed in honor of Frederick Stephens Carr [1881-1934], teacher and inspector in the educational system in Alberta and amateur coleopterist. His collection, which consisted at the time of his death of about 6,500 North American species, was deposited at the University of Alberta.

#### Distribution.

This widely distributed species ranges from western Newfoundland (Lindroth 1955a: 128) to Kodiak Island, north to the Anderson River delta in northern Northwest Territories (Lindroth 1966: 578), south to the Sierra Nevada in California (El Dorado County, CAS), San Miguel County in New Mexico (Fall and Cockerell 1907: 159, as *Platynus ruficornis*), northeastern Kansas (Popenoe 1878: 78, as *Platynus ruficornis*), northern Illinois (Lake County, CNC), and northern West Virginia (Tucker and Randolph Counties, CMNH). The species is also known from the Far East (Bousquet 2003c: 453).

#### Records.

**CAN**: AB, BC, LB, MB, NB, NF, NS (CBI), NT, ON, PE, QC, SK, YT **USA**: AK, CA, CO, CT, IA, IL, IN, KS, MA, ME, MI, MN, MT, ND, NE, NH, NJ, NM, NY, OH, OR, PA, RI, SD, UT, VT, WA, WI, WV, WY – **Holarctic**

### Agonum lutulentum

(LeConte, 1854)

Platynus lutulentus LeConte, 1854b: 54. Type locality: «Maine and Lake Superior» (original citation), restricted to «Nipigon, Ont[ario]» by Lindroth (1966: 580). Three syntypes in MCZ [# 5788].
Europhilus lutulentus elegantulus Casey, 1920: 127. Type locality: «New Jersey» (original citation). Lectotype (♂), designated by Lindroth (1975: 126), in USNM [# 47525]. Synonymy established by Casey (1924: 85), confirmed by Lindroth (1966: 580).
Europhilus lutulentus adustus Casey, 1920: 127. Type locality: «Indiana» (original citation). Lectotype (♂), designated by Lindroth (1975: 126), in USNM [# 47526]. Synonymy established by Lindroth (1954b: 140).
Europhilus atriceps Casey, 1920: 128. Type locality: «Massachusetts» (original citation). Lectotype (♀), designated by Lindroth (1975: 126), in USNM [# 47527]. Synonymy established by Lindroth (1955c: 9).

#### Distribution.

The range of this species extends from Cape Breton Island (Lindroth 1954c: 307) to Vancouver Island (Lindroth 1966: 581), north to southern Northwest Territories (Bousquet 1987a: 125), south to eastern Washington (Hatch 1953: 145), central Kansas (Trego and Scott Counties, CNC, CMNH), and northwestern Maryland (Garrett County, CMNH).

#### Records.

**CAN**: AB, BC (VCI), MB, NB, NS (CBI), NT, ON, PE, QC, SK **USA**: CT, DC, DE, IA, IL, IN, KS, MA, MD, ME, MI, MN, MO, MT, ND, NE, NH, NJ, NY, OH, PA, RI, SD, VT, WA, WI, WV

### Agonum palustre

Goulet, 1969

Agonum palustre Goulet, 1969: 280. Type locality: «Amberley, S[outh] Kincardine, Ontario» (original citation). Holotype (♂) in CNC [# 11575].

#### Distribution.

This species is found from southwestern New Brunswick (Webster and Bousquet 2008: 22) to Minnesota (Epstein and Kulman 1990: 214; Sherburne County, CNC), south to southwestern Nebraska (Lincoln County, Foster F. Purrington pers. comm. 2010) and along the Appalachian Mountains to central Alabama (Lee County, CNC).

#### Records.

**CAN**: NB, ON, QC **USA**: AL, CT, IA, IL, IN, KY, MA, MD, ME, MI, MN, NC, NE, NH, NJ, NY, OH, PA, RI, TN, VA, VT, WI, WV

### Agonum picicornoides

Lindroth, 1966

Agonum picicornoides Lindroth, 1966: 574. Type locality: «Nipigon, L[ake] Sup[erior], Ont[ario]» (original citation). Holotype (♂) in CNC [# 9228].

#### Distribution.

This species ranges from the Nova Scotia Peninsula to south-central British Columbia, north to northern Alberta and Anticosti Island in Quebec, south to southeastern Wisconsin (Messer 2010: 42), the Saginaw Bay area in northern Michigan, and southern Vermont (Lindroth 1966: 574). Fossil remnants, dated about 10,800 years B.P., have been unearthed in central North Dakota (Ashworth and Schwert 1992: 260).

#### Records.

**CAN**: AB, BC, MB, NB, NS, ON, PE, QC, SK **USA**: ME, MI, NH, VT, WI

### Agonum retractum

LeConte, 1846

Agonum retractum LeConte, 1846b: 228. Type locality: «Lacum Superiorem» (original citation), restricted to «Nipigon, Ont[ario]» by Lindroth (1966: 576). Syntype(s) in MCZ [# 5786].
Europhilus dilutipennis Motschulsky, 1865: 322. Type locality: «nouveau Mexique [= New Mexico]» (original citation), which is probably incorrect. One syntype in ZMMU (Lindroth 1969a: 1119). Synonymy established by Lindroth (1969a: 1119).
Europhilus collusor Casey, 1920: 129. Type locality: «Montana» (original citation). Lectotype (♂), designated by Lindroth (1975: 126), in USNM [# 47531]. Synonymy established by Lindroth (1954b: 140).
Europhilus facilis Casey, 1920: 130. Type locality: «Boston Neck [Washington County], Rhode Island» (original citation). Lectotype (♀), designated by Lindroth (1975: 126), in USNM [# 47533]. Synonymy established by Lindroth (1954b: 140).
Europhilus serenus Casey, 1920: 131. Type locality: «Bayfield [Bayfield County, Wisconsin], Lake Superior» (original citation for the lectotype). Lectotype (♂), designated by Lindroth (1975: 126), in USNM [# 47534]. Synonymy established by Lindroth (1954b: 140).

#### Distribution.

This species ranges from Newfoundland (Lindroth 1955a: 127) to western British Columbia (Lindroth 1966: 576), north to southern Northwest Territories (CNC), south to “Montana” (Casey 1920: 129, as *Europhilus collusor*), South Dakota (Kirk and Balsbaugh 1975: 25), central West Virginia (Pocahontas County, CMNH), and northern Virginia (Madison and Rappahannock Counties, CNC). The record from New Mexico (type locality of *Agonum dilutipenne* Motschulsky) is probably in error; those from “Illinois” and “Indiana” (Bousquet and Larochelle 1993: 254) need confirmation.

#### Records.

**FRA**: PM **CAN**: AB, BC, MB, NB, NF, NS (CBI), NT, ON, PE, QC, SK **USA**: CT, MA, MD, ME, MI, MN, MT, NH, NY, PA, RI, SD, VA, VT, WI, WV [IL, IN]

### Agonum simile

Kirby, 1837

Agonum simile Kirby, 1837: 27. Type locality: northern parts of British America (inferred from title of the book), restricted to «Pikwitonei, Manit[oba]» by Lindroth (1966: 571). Two syntypes in BMNH (Lindroth 1953b: 171).

#### Distribution.

This rarely collected species is known from scattered localities from north-central Manitoba to the Gulf Coast of Alaska (Lindroth 1966: 571).

#### Records.

**CAN**: AB, BC, MB, SK, YT **USA**: AK

### Agonum sordens

Kirby, 1837

Agonum sordens Kirby, 1837: 25. Type locality: «Lat. 54° [= along North Saskatchewan River]» (original citation), restricted to «Edmonton, Al[ber]ta» by Lindroth (1966: 575). One syntype [2 originally cited] in BMNH (Lindroth 1953b: 170).
Platynus picicornis LeConte, 1860: 319. Type locality: «Jasper House [= Jasper, Alberta]» (original citation). One syntype in MCZ [# 5783]. Synonymy established by Hatch (1953: 144) and Lindroth (1953b: 170).
Anchomenus ineptus Casey, 1920: 63. Type locality: «Coeur d’Alene [Kootenai County], Idaho» (original citation). Lectotype (♂), designated by Lindroth (1975: 126), in USNM [# 47441]. Synonymy established by Lindroth (1966: 575).
Europhilus frosti Casey, 1924: 86. Type locality: «Eastbrook [Hancock County], Maine» (original citation). Lectotype (♂), designated by Lindroth (1975: 126), in USNM [# 47535]. Synonymy established by Lindroth (1954b: 140).

#### Distribution.

This species ranges from Newfoundland (Lindroth 1955a: 127) to central Alaska (Lindroth 1966: 576), south to “Oregon” (Hatch 1953: 144), southern Colorado (Wickham 1902: 239; LeConte 1879d: 500), northern Illinois (Lake County, CNC), and Maryland (Erwin 1981b: 154). The records from “Iowa” (Jaques and Redlinger 1946: 295), northeastern Kansas (Popenoe 1878: 78), and “North Carolina” (Bousquet and Larochelle 1993: 254) need confirmation.

#### Records.

**CAN**: AB, BC, LB, MB, NB, NF, NS (CBI), NT, ON, PE, QC, SK, YT **USA**: AK, CO, ID, IL, MA, MD, ME, MI, MN, MT, ND, NH, NJ, NY, OR, PA, SD, UT, VT, WA, WI, WV [IA, KS, NC]

### Agonum superioris

Lindroth, 1966

Agonum superioris Lindroth, 1966: 578. Type locality: «Nipigon, W[estern] Ont[ario]» (original citation). Holotype (♂) in CNC [# 9229].

#### Distribution.

This species ranges from New Brunswick (Bousquet 1987a: 125) to the Rocky Mountains in Alberta and eastern Alaska (Lindroth 1966: 579), south to northwestern Wisconsin (Messer 2010: 42), Beaver Islands, Lake Michigan, in northern Michigan (Dunn 1987: 11), and New England (Lindroth 1966: 579).

#### Records.

**CAN**: AB, MB, NB, NT, ON, PE, QC, SK, YT **USA**: AK, ME, MI, NH, VT, WI

### Agonum thoreyi

Dejean, 1828

Agonum affine Stephens, 1828a [1 March]: 94 [potential *nomen oblitum*]. Type locality: «near London» (original citation). Holotype [by monotypy] probably in BMNH (collection Stephens, see Dawson 1854: 94). Note. This taxon was first placed in synonymy with *Anchomenus pelidnus* (Herbst) *sensu* Paykull (1798: 134) (= *Agonum thoreyi thoreyi* (Dejean, 1828)), by Dawson (1854: 93).
Agonum thoreyi Dejean, 1828 [29 November]: 165 [potential *nomen protectum*]. Type locality: «environs de Hambourg, nord de l’Allemagne» (original citation). Syntype(s) in MHNP.
Anchomenus melanocephalus Dejean, 1828 [29 November]: 118. Type locality: «Espagne [= Spain]» (original citation). Syntype(s) probably in MHNP. Synonymy established by Bedel (1900b: 248).
Agonum puellum Dejean, 1828 [29 November]: 166. Type locality: «environs de Berlin [Germany]» (original citation). Syntype(s) probably in MHNP. Synonymy established, under the name *Anchomenus pelidnus* (Herbst) *sensu* Paykull (1798: 134) (= *Agonum thoreyi thoreyi* (Dejean, 1828)), by Schiødte (1841: 254).
Agonum lenum Dejean, 1828 [29 November]: 166 [*nomen dubium*]. Type locality: «Amérique septentrionale» (original citation). Syntype(s) presumably lost (Lindroth 1955b: 21). Synonymy established by Lindroth (1954b: 138), subsequently considered a *nomen dubium* by the same author (Lindroth 1955b: 21).
Agonum longicolle Lacordaire [in Boisduval and Lacordaire], 1835: 215. Type locality: «Bondy [northeastern suburbs of Paris, France]» (original citation). Syntype(s) location unknown. Synonymy established, under the name *Anchomenus pelidnus* (Herbst) *sensu* Paykull (1798: 134) (= *Agonum thoreyi thoreyi* (Dejean, 1828)), by Erichson (1837: 117).
Agonum picipenne Kirby, 1837: 24. Type locality: «Lat. 54° [= along North Saskatchewan River]» (original citation). Three syntypes in BMNH (Lindroth 1953b: 170). Synonymy established, under the name *Agonum lenum* Dejean, by Melsheimer (1853: 17), confirmed by Lindroth (1953b: 170).
Agonum lehmanni Chaudoir, 1837b: 25. Type locality: «Livonie [currently in Latvia and Estonia]» (original citation). Syntype(s) probably in MHNP. Synonymy established, under the name *Anchomenus pelidnus* (Herbst) *sensu* Paykull (1798: 134) (= *Agonum thoreyi thoreyi* (Dejean, 1828)), by Chaudoir (1850b: 107).
Agonum convexiusculum Chaudoir, 1843b: 765. Type locality: «Smyrne [= Izmir, Turkey]» (original citation). One syntype in MHNP (Schmidt and Liebherr 2009: 250). Synonymy established by Schmidt and Liebherr (2009: 250).
Agonum longulum Motschulsky, 1844: 133. Type locality: «fl[euve] Ichim [Russia]» (lectotype label). Lectotype (♂), designated by Schmidt and Liebherr (2009: 250), in ZMMU. Synonymy established, under the name *Anchomenus puellus* (Dejean), by Chaudoir (1850b: 108).
Platynus gemellus LeConte, 1879b: 54. Type locality: «Vancouver Island [British Columbia]» (original citation). Two syntypes [2 originally cited] in MCZ [# 5787]. Synonymy established by Hatch (1953: 145) and Lindroth (1953b: 170).

#### Distribution.

A Holarctic species widely distributed in the Palaearctic Region from Ireland to the Far East, south to Spain and Italy (Bousquet 2003c: 453). In North America, the taxon ranges from Alaska (Lindroth 1966: 580) to Newfoundland (Lindroth 1955a: 129), south to the District of Columbia (Ulke 1902: 7, as *Platynus picipennis*), central Kansas (Trego County, CNC), east-central Colorado (Kiowa County, Foster F. Purrington pers. comm. 2010), and “California” (Lindroth 1955a: 129).

#### Records.

**CAN**: AB, BC (VCI), MB, NB, NF, NS (CBI), NT, ON, PE, QC, SK, YT **USA**: AK, CA, CO, CT, DC, IA, ID, IL, IN, KS, MA, ME, MI, MN, MT, ND, NE, NH, NJ, NY, OH, OR, PA, RI, SD, VT, WA, WI – **Holarctic**

### Subgenus Agonum

Bonelli, 1810

Agonum Bonelli, 1810: Tabula Synoptica. Type species: *Carabus marginatus* Linnaeus, 1758 designated by Curtis (1827: plate 183).
Megalonychus Chaudoir, 1843a: 418. Type species: *Megalonychus madagascariensis* Chaudoir, 1843 by monotypy. Synonymy established by Liebherr and Schmidt (2004: 202). Etymology (original). From the Greek prefix *megalo*- (large) and *onychos* (claws), alluding to the size of the last tarsomeres (“*tarsi sublineares; articulis *... *ultimo longissimo*”) of the adult [masculine].
Agonocyrthes Motschulsky, 1865: 317. Type species: *Agonocyrthes orbicollis* Motschulsky, 1865 (= *Agonum sinense* Csiki, 1931) by original designation. Synonymy established by Liebherr and Schmidt (2004: 202).
Agonopsis Semenov, 1889a: 359 [junior homonym of *Agonopsis* Gill, 1861]. Type species: *Anchomenus humerosus* Semenov, 1889 by monotypy. Synonymy established by Liebherr and Schmidt (2004: 202). Etymology. From the generic name *Agonum* [*q.v*.] and the Greek suffix -*opsis* (having the appearance of) [feminine].
Platynopsis Lutshnik, 1915d: 186. Type species: *Agonum semenowi* Lutshnik, 1915 (= *Agonum rugicolle* Chaudoir, 1846) by original designation. Synonymy established by Schmidt andLiebherr (2009: 228). Etymology. From the generic name *Platynus* [*q.v*.] and the Greek suffix -*opsis* (having the appearance of) [feminine].
Paragonum Casey, 1920: 123. Type species: *Feronia placida* Say, 1823 designated by Lindroth (1966: 613). Synonymy established by Ball (1960b: 133). Etymology. From the Greek *para* (near) and the generic name *Agonum* [*q.v*.] [neuter].
Nagonium Habu, 1956c: 166. Type species: *Agonum kitanoi* Habu, 1956 by original designation. Synonymy established by Liebherr and Schmidt (2004: 202).
Liebherrius Shilenkov [in Kryzhanovskij et al.], 1995: 115. Type species: *Anchomenus bicolor* Dejean, 1828 by original designation. Synonymy established by Liebherr and Schmidt (2004: 202).
Agonops Bousquet, 2002b: 5. Replacement name for *Agonopsis* Semenov, 1889. Etymology. From the generic name *Agonum* [*q.v*.] and the Greek suffix -*ops* (having the appearance of) [masculine].

#### Diversity.

Twenty-nine species in the Nearctic (four species, of which one extends into southern Mexico and one is adventive), Palaearctic (24 species, of which one is Holarctic), and Afrotropical (three species in eastern Africa including Madagascar) Regions.

### [bicolor group]

### Agonum bicolor

(Dejean, 1828)

Anchomenus bicolor Dejean, 1828: 126. Type locality: «Kamtschatka [Russia]» (original citation). Three syntypes in MHNP (Lindroth 1955b: 20) and ZMHB (Schmidt 1995: 162).
Agonum alpinum Motschulsky, 1844: 139. Type locality: «sommités des Alpes du Hamar-Daban [Irkutsk Oblast, Russia]» (original citation). Lectotype (♀), designated by Schmidt (1995: 162), in ZMMU. Synonymy established by Schmidt (1995: 162).
Agonum sibiricum Gebler, 1847: 330. Type locality: «Alpe des kusnezkischen Gebirges [= south Kuznetskiy Alatau, Siberia, Russia]» (original citation). Holotype [by monotypy] location unknown. Synonymy established doubtfully with *Agonum alpinum* Motschulsky by Chaudoir (1850b: 105).
Platynus marginellus LeConte, 1860: 315. Type locality: «Fort Simpson [Northwest Territories]» (original citation). Syntype(s) in MCZ [# 5752]. Synonymy established by LeConte (1863b: 7), confirmed by Lindroth (1966: 585).
Agonum jemelianovi Lafer, 1992: 612. Type locality: «r. Samarga, ust’e r. Odui, Primorsk[iy] Kr[ay] [Russia]» (original citation). Holotype location unknown. Synonymy established by Schmidt (1995: 162).

#### Distribution.

A Holarctic species ranging in the Palaearctic Region across Siberia up to the Ob River (Lindroth 1966: 586), south to Kazakhstan and Mongolia (Bousquet 2003c: 454). In North America, the species ranges from Alaska and northern British Columbia to northeastern Alberta (Lindroth 1966: 586). The records from Colorado (Wickham 1902: 238) and New Mexico (Fall and Cockerell 1907: 159) probably refer to *Agonum piceolum*.

#### Records.

**CAN**: AB, BC, NT, YT **USA**: AK – **Holarctic**

### Agonum piceolum

(LeConte, 1879)

Platynus piceolus LeConte, 1879b: 52. Type locality: «Oregon and British Columbia» (original citation), restricted to «Oregon» by Lindroth (1966: 587). Two syntypes [3 originally cited] in MCZ [# 5753].
Anchomenus tritus Casey, 1920: 63. Type locality: «Bayfield [Bayfield County], Wisc[onsin]» (original citation). Lectotype (♂), designated by Lindroth (1975: 127), in USNM [# 47443]. Synonymy established by Lindroth (1966: 587).
Anchomenus tritus fractus Casey, 1920: 63. Type locality: «Provo [Utah County], Utah» (original citation). Lectotype (♂), designated by Lindroth (1975: 127), in USNM [# 47444]. Synonymy established by Lindroth (1966: 587).
Anchomenus lascivus Casey, 1920: 66. Type locality: «Stikine River, British Columbia» (original citation). Lectotype (♂), designated by Lindroth (1975: 127), in USNM [# 47446]. Synonymy established by Hatch (1953: 138), confirmed by Lindroth (1966: 587).
Anchomenus frigidulus Casey, 1920: 66. Type locality: «Stikine River, British Columbia» (original citation). Lectotype (♂), designated by Lindroth (1975: 127), in USNM [# 47445]. Synonymy established by Lindroth (1966: 587).
Anchomenus dissensus Casey, 1920: 67. Type locality: «Priest River [Bonner County], Idaho» (original citation). Lectotype (♀), designated by Lindroth (1975: 127), in USNM [# 47448]. Synonymy established by Hatch (1953: 138), confirmed by Lindroth (1975: 127).

#### Distribution.

This species ranges from Newfoundland (Lindroth 1955a: 125) to Vancouver Island (Lindroth 1966: 588), north to southern Yukon Territory (Hyland River, UASM), south to west-central California (Lindroth 1966: 587), southwestern Arizona (Maricopa County, UASM), New Mexico (Fall and Cockerell 1907: 159; UASM), and mountains of New England (Lindroth 1966: 587). The record from “Connecticut” (Bousquet and Larochelle 1993: 255) was based on a misidentified specimen of *Agonum extensicolle* (Krinsky and Oliver 2001: 5); that from “Massachusetts” (Bousquet and Larochelle 1993: 255) needs confirmation.

#### Records.

**CAN**: AB, BC (VCI), MB, NB, NF, ON, QC, SK, YT **USA**: AZ, CA, CO, ID, ME, MI, MT, NH, NM, NV, OR, UT, WA, WI, WY [MA]

### [muelleri group]

### Agonum muelleri

(Herbst, 1784)

Carabus VI-punctatus O.F. Müller, 1776: 78 [primary homonym of *Carabus sexpunctatus* Linnaeus, 1758]. Type locality: Denmark and Norway (inferred from title of the book). Syntype(s) lost.
Carabus mülleri Herbst, 1784: 139. Replacement name for *Carabus sexpunctatus* Müller, 1776. Etymology. The specific name was proposed in honor of Otto Friedrich Müller [1730-1784], a Danish naturalist, theologian, linguist, and trumpet player. Following his marriage in 1773, Müller became financially independent and devoted himself exclusively to science.
Carabus parumpunctatus Fabricius, 1792: 157. Type locality: «Germania» (original citation). Two syntypes in ZMUC (Zimsen 1964: 57). Synonymy established by Illiger (1798: 195).
Platynus hardyi LeConte, 1879b: 53. Type locality: «Newfoundland» (original citation). Three syntypes [3 originally cited] in MCZ [# 28696]. Synonymy established by Hatch (1953: 141), confirmed by Lindroth (1954b: 138).
Platynus hornii Hausen, 1890: 235. Type locality: «S[ain]te-Rose [= Laval] and Ile Perrot, P[rovince of] Q[uebec]» (original citation). Syntypes presumably lost. **New synonymy**. Note. Lindroth (1966: 629) listed this name as synonym of *Agonum decorum* (Say, 1823) on the basis of an “authentic ♂,” labeled “Montreal. Hausen” in the CNC. Unfortunately the specimen could not be located. However, there is serious doubt that the specimen was really a syntype. Hausen (1890: 236) stated that “on being shown a specimen, Dr. Horn declared he doubted the American origin of this species.” It would have been surprising that Horn did not recognize *Agonum decorum*. On the other hand, *Agonum muelleri* was little known in North America, being first noticed by LeConte in 1879 when he described it under the name *Platynus hardyi*. The description is poor but seems to fit better *Agonum muelleri* than *Agonum decorum*, particularly the size (0.375 in = 9.5 mm).

#### Distribution.

This Palaearctic species is adventive in North America where it is known from west-central Alberta (Emerald Lake, Gerald J. Hilchie pers. comm. 2009) and southern British Columbia, including Vancouver Island (Lindroth 1966: 593), to northwestern California (Humboldt County, James R. LaBonte pers. comm. 1992) in the west and from Newfoundland (Lindroth 1955a: 121) to northeastern Minnesota (Kamal J.K. Gandhi pers. comm. 2008), south to northeastern Iowa (Larsen and Purrington 2010: 570) and northeastern West Virginia (Tucker County, CMNH) in the east. The first inventoried specimen collected in the east was found in Newfoundland around 1840 (Lindroth 1955a: 122) and in the west in 1933 (Lindroth 1966: 593; Leech 1935: 122, as *Platynus hardyi*). The species is also adventive in Hawaii (Liebherr et al. 2009: 98).

#### Records.

**FRA**: PM **CAN**: AB, BC (VCI), NB, NF, NS (CBI), ON, PE, QC **USA**: CA, CT, IA, MA, ME, MI, MN, NH, NY, OH, OR, PA, RI, VA, VT, WA, WI, WV – **Adventive**

### Agonum placidum

(Say, 1823)

Feronia placida Say, 1823a: 43. Type locality: «Dorchester [Suffolk County], Mass[achusetts]» (neotype label). Neotype (♀), designated by Lindroth and Freitag (1969: 348), in MCZ [# 33012].
Agonum morosum Dejean, 1828: 145. Type locality: «Amérique septentrionale» (original citation). One syntype in MHNP (Lindroth 1955b: 21). Synonymy established by LeConte (1854b: 55), confirmed by Lindroth (1966: 613).
Agonum alcyoneum Chaudoir, 1837b: 24. Type locality: «Mexique» (original citation), restricted to «Amecameca, Mexico state» by Liebherr (1991a: 119). Lectotype (♂), designated by Liebherr (1991a: 119), in MHNP. Synonymy established by Liebherr (1991a: 119).
Anchomenus transpunctatus Bates, 1878a: 593. Type locality: «near the capital, Mexico» (original citation). Syntype(s) probably in BMNH. Synonymy established by Bates (1882a: 94).
Agonum placidum amplior Casey, 1920: 124. Type locality: «Boulder Co[unty], Colorado» (original citation for the lectotype). Lectotype (♀), designated by Lindroth (1975: 128), in USNM [# 47491]. Synonymy established by Lindroth (1966: 613).
Agonum placidum aztecanum Casey, 1920: 124. Type locality: «Amecameca [México], Mexico» (original citation). Lectotype (♀), designated by Lindroth (1975: 128), in USNM [# 47492]. Synonymy established by Lindroth (1966: 613).
Agonum placidum citatum Casey, 1920: 124. Type locality: «New Hampshire» (original citation). Lectotype (♂), designated by Lindroth (1975: 128), in USNM [# 47494]. Synonymy established by Lindroth (1966: 613).
Agonum rhodeanum Casey, 1924: 84. Type locality: «Rhode Island» (original citation). Lectotype (♂), designated by Lindroth (1975: 128), in USNM [# 47490]. Synonymy established by Lindroth (1966: 613).

#### Distribution.

This widely distributed species ranges from Newfoundland (Lindroth 1955a: 120; Larson and Langor 1982: 594) to western British Columbia (Lindroth 1966: 614), south to Inyo County in California, the Sierra de Miahuatlán in Oaxaca (Liebherr 1994: 32), and northern Georgia (Fattig 1949: 34).

#### Records.

**CAN**: AB, BC, LB, MB, NB, NF, NS (CBI), ON, PE, QC, SK **USA**: AR, AZ, CA, CO, CT, DC, DE, GA, IA, ID, IL, IN, KS, KY, MA, MD, ME, MI, MN, MO, MS, MT, NC, ND, NE, NH, NJ, NM, NY, OH, OR, PA, RI, SD, TN, TX, UT, VA, VT, WA, WI, WV, WY – Mexico

### Subgenus Olisares

Motschulsky, 1865

Olisares Motschulsky, 1865: 326. Type species: *Olisares flavolimbatus* Motschulsky, 1865 (= *Carabus pallipes* Fabricius, 1792) designated by Liebherr (1991a: 120). Etymology. Probably an arbitrary combination of letters [masculine].
Taphranchus Casey, 1920: 52. Type species: *Agonum excavatum* Dejean, 1828 designated by Lindroth (1966: 623). Synonymy established by Liebherr and Schmidt (2004: 203). Etymology. From the Greek *taphros* (ditch, trench, by extension stria) and the generic name *Anchus* [*q.v*.] [masculine].
Stictanchus Casey, 1920: 54. Type species: *Feronia extensicollis* Say, 1823 designated by Lindroth (1966: 625). Synonymy established by Liebherr and Schmidt (2004: 203). Etymology. From the Greek *stictos* (punctured) and the generic name *Anchus* [*q.v*.] [masculine].
Deratanchus Casey, 1920: 70. Type species: *Platynus quadrimaculatus* Horn, 1885 by monotypy. Synonymy established by Liebherr and Schmidt (2004: 202).
Circinalia Casey, 1920: 72. Type species: *Feronia punctiformis* Say, 1823 by original designation. Synonymy established by Liebherr and Schmidt (2004: 203).
Circinalidia Casey, 1920: 78. Type species: *Agonum aeruginosum* Dejean, 1828 designated by Bousquet and Larochelle (1993: 258). Synonymy established by Liebherr and Schmidt (2004: 203).
Micragonum Casey, 1920: 80. Type species: *Feronia nutans* Say, 1823 by original designation. Synonymy established by Liebherr and Schmidt (2004: 203). Etymology. From the Greek *micros* (small, little) and the generic name *Agonum* [*q.v*.] [neuter].
Stereagonum Casey, 1920: 80. Type species: *Agonum ferreum* Haldeman, 1843 designated by Lindroth (1966: 624). Synonymy established by Liebherr and Schmidt (2004: 203). Etymology. From the Greek *stereos* (solid, firm, hard) and the generic name *Agonum* [*q.v*.] [neuter].
Melanagonum Casey, 1920: 111. Type species: *Agonum melanarium* Dejean, 1828 designated by Lindroth (1966: 598). Synonymy established by Liebherr and Schmidt (2004: 202). Etymology. From the Greek *melanos* (black) and the generic name *Agonum* [*q.v*.] [neuter].
Punctagonum Gray, 1937: 311. Type species: *Platynus belleri* Hatch, 1933 by monotypy. Synonymy established by Liebherr and Schmidt (2004: 203). Etymology. From the Latin *punctum* (small hole, by extension puncture) and the generic name *Agonum* [*q.v*.] [neuter].

#### Diversity.

Seventy-two species in the Nearctic (50 species), Neotropical (14 species in Middle America but only two endemic), and Palaearctic (21 species, one Holarctic) Regions.

### [albicrus group]

### Agonum albicrus

Dejean, 1828

Agonum albicrus Dejean, 1828: 158. Type locality: «Amérique septentrionale» (original citation), restricted to «M[ount] Vernon, Alab[ama]» by Lindroth (1966: 615). One syntype in MHNP (Lindroth 1955b: 21).

#### Distribution.

The range of this species extends from southeastern New Hampshire (Rockingham County, Ross T. Bell pers. comm. 2008) to eastern Kansas (Douglas County, Robert L. Davidson pers. comm. 2009), including southernmost Ontario (Lindroth 1966: 615), south to eastern Oklahoma (Latimer County, UASM), northwestern Louisiana (Bossier Parish, CMNH) and the Florida Panhandle (Jackson County, CNC). The record from “Iowa” (Bousquet and Larochelle 1993: 255) needs confirmation.

#### Records.

**CAN**: ON **USA**: AL, AR, CT, FL, GA, IL, IN, KS, LA, MA, MD, MS, NC, NH, NJ, OH, OK, PA, RI, SC, TN, VA [IA]

### [cupripenne group]

### Agonum belleri

(Hatch, 1933)

Platynus belleri Hatch, 1933c: 120. Type locality: «Chase Lake, Snohomish County, Washington» (original citation). Holotype (♂) in USNM. Etymology. The specific name was proposed for Samuel Beller who did a Master Thesis, under the direction of Melville H. Hatch, on the Chrysomelidae of Washington at the University of Washington in 1931.

#### Distribution.

This species is known from a few localities in the Pacific Northwest from the Queen Charlotte Islands (Kavanaugh 1992: 74) to northwestern Oregon (Clackamas County, James R. LaBonte pers. comm. 1992).

#### Records.

**CAN**: BC (QCI) **USA**: OR, WA

### Agonum cupreum

Dejean, 1831

Agonum cupreum Dejean, 1831: 735. Type locality: «Amérique septentrionale» (original citation), restricted to «Duluth [Saint Louis County], Minnes[ota]» by Lindroth (1966: 596). Holotype [by monotypy] (♀) in MHNP (Lindroth 1955b: 21).
Agonum seminitidum Kirby, 1837: 26. Type locality: «Lat. 54° [= along North Saskatchewan River]» (original citation), restricted to «Edmonton, Al[ber]ta» by Lindroth (1966: 596). Three syntypes in BMNH (Lindroth 1953b: 170). Synonymy established by LeConte (1870: 396), confirmed by Lindroth (1953b: 170).
Agonum chalceum LeConte, 1846b: 224. Type locality: «Lacum Superiorem» (original citation); cited from «Sault Ste Marie, Michigan» by LeConte (1854b: 55). Three syntypes in MCZ [# 5777]. Synonymy established with doubt by LeConte (1863b: 7), confirmed by LeConte (1869b: 248) and Lindroth (1966: 596).
Platynus protractus LeConte, 1854b: 55. Type locality: «Lake Superior and Sandy Lake, Minnesota» (original citation). Four syntypes in MCZ [# 5778]. Synonymy established by LeConte (1879b: 57), confirmed by Lindroth (1966: 596).
Platynus crassicollis LeConte, 1860: 319. Type locality: «Jasper House [= Jasper, Alberta], Rocky Mountains» (original citation). One syntype in MCZ [# 5779]. Synonymy established by Henshaw (1882: 209), confirmed by Lindroth (1966: 596).
Agonum longulum Casey, 1920: 107. Type locality: «[Fort] Douglas [Salt Lake County], Utah» (original citation). Lectotype (♂), designated by Lindroth (1975: 127), in USNM [# 47463]. Synonymy established by Lindroth (1966: 596).
Agonum parallelum Casey, 1920: 108. Type locality: «Magnolia, Boulder Co[unty], Colorado» (original citation). Lectotype (♀), designated by Lindroth (1975: 127), in USNM [# 47464]. Synonymy established by Lindroth (1966: 596).
Agonum marquettense Casey, 1920: 108. Type locality: «Marquette [Marquette County, Michigan], Lake Superior» (original citation). Lectotype (♀), designated by Lindroth (1975: 127), in USNM [# 47465]. Synonymy established by Lindroth (1966: 596).
Agonum seminitidum borealinum Casey, 1920: 109. Type locality: «Duluth [Saint Louis County], Minnesota» (original citation). Lectotype (♂), designated by Lindroth (1975: 127), in USNM [# 47467]. Synonymy established by Lindroth (1966: 596).
Agonum ovalicauda Casey, 1920: 109. Type locality: «Magnolia, Boulder Co[unty], Colorado» (original citation). Lectotype (♂), designated by Lindroth (1975: 128), in USNM [# 47468]. Synonymy established by Lindroth (1966: 596).
Agonum esuriale Casey, 1920: 109. Type locality: «Colorado» (original citation). Lectotype (♂), designated by Lindroth (1975: 128), in USNM [# 47470]. Synonymy established by Lindroth (1966: 596).
Agonum cupreolucens Casey, 1924: 83. Type locality: «Winnipeg [Manitoba]» (original citation for the lectotype). Lectotype (♂), designated by Lindroth (1975: 128), in USNM [# 47466]. Synonymy established by Lindroth (1966: 596).
Agonum uintanum Casey, 1924: 83. Type locality: «Mammoth (10000 ft.) [Juab County], Parowan M[oun]t[ain]s, Utah» (original citation). Lectotype (♂), designated by Lindroth (1975: 128), in USNM [# 47469]. Synonymy established by Liebherr (1991a: 118).

#### Distribution.

This species ranges from central Prince Edward Island (CNC) and New Brunswick (Kouchibouguac National Park, CNC) to central Alaska (Lindroth 1966: 598), south at least to east-central California (Mono County, CAS), southeastern Arizona (Dajoz 2007: 21; UASM), northeastern New Mexico (San Miguel County, UASM), South Dakota including the Black Hills (Kirk and Balsbaugh 1975: 25), and northern Michigan (Dunn 1985a: 8; Casey 1920: 108, as *Agonum marquettense*). The record from “Kansas” (Horn 1872c: 385, as *Platynus chalceus*) needs confirmation.

#### Records.

**CAN**: AB, BC, MB, NB, NT, ON, PE, QC, SK, YT **USA**: AK, AZ, CA, CO, ID, MI, MN, MT, ND, NM, NV, OR, SD, UT, WA, WI, WY [KS]

#### Note.

Lindroth (1966: 597) treated *Agonum uintanum* Casey as a subspecies of *Agonum cupreum* Dejean.

### Agonum cupripenne

(Say, 1823)

Feronia cupripennis Say, 1823a: 50. Type locality: «Fall River [Bristol County], Mass[achusetts]» (neotype label). Neotype (♀), designated by Lindroth and Freitag (1969: 347), in MCZ [# 33014]. Note. Lindroth and Freitag (1969: 347) reported that the neotype was from “W[est] Roxbury [Suffolk County], Mass[achusetts]” but the label on the specimen indicates that it was collected at Fall River, Massachusetts.
Agonum nitidulum Dejean, 1828: 143. Type locality: «Amérique septentrionale» (original citation). One syntype in MHNP (Lindroth 1955b: 20). Synonymy established by LeConte (1869b: 248), confirmed by Lindroth (1955b: 20).
Agonum gemmeum Casey, 1920: 103. Type locality: «Kansas» (original citation). Lectotype (♂), designated by Lindroth (1975: 127), in USNM [# 47457]. Synonymy established by Lindroth (1966: 591).
Agonum tahoense Casey, 1920: 106. Type locality: «Truckee [Nevada County], California» (original citation). Lectotype (♀), designated by Liebherr (1991a: 118), in USNM [# 47460]. Synonymy established by Liebherr (1991a: 118).

#### Distribution.

The range of this species extends from Cape Breton Island (Lindroth 1954c: 305) to Vancouver Island (LeConte 1869c: 370), north to west-central Northwest Territories (Bousquet 1987a: 125), south to the Sierra Nevada in California (Casey, 1920: 106, as *Agonum tahoense*; Dajoz 2007: 16), southeastern Arizona (Greenlee County, UASM), New Mexico (Wickham 1896c: 133; Fall and Cockerell 1907: 159; Cibola County, CMNH), “Kansas” (Lindroth 1966: 591), Tennessee (Sevier County, CNC), and northern Georgia (Fattig 1949: 34). The record from “Louisiana” (Bousquet and Larochelle 1993: 256) needs confirmation.

#### Records.

**CAN**: AB, BC (VCI), MB, NB, NS (CBI), NT, ON, PE, QC, SK **USA**: AZ, CA, CO, CT, DC, DE, GA, IA, ID, IL, IN, KS, MA, ME, MD, MI, MN, MO, MT, NC, ND, NE, NH, NJ, NM, NV, NY, OH, OR, PA, RI, SD, TN, VA, VT, WA, WI, WV, WY [LA]

### Agonum deplanatum

Ménétriés, 1843

Agonum deplanatum Ménétriés, 1843: 57. Type locality: «Californie» (original citation), herein restricted to Berkeley, Alameda County (see Casey 1924: 82, as *Anchomenus amplicollis*). Lectotype (♀), designated by Lindroth (1966: 598), in ZMH.
Platynus fallianus Leng, 1919b: 203. Unnecessary replacement name for *Platynus deplanatus* (Ménétriés, 1843).
Anchomenus amplicollis Casey, 1924: 82. Type locality: «Berkeley [Alameda County], California» (original citation). Lectotype (♂), designated by Liebherr (1991a: 116), in USNM [# 47436]. Synonymy established by Liebherr (1991a: 116).

#### Distribution.

This species is known from southwestern Oregon (Jackson County, CNC) to southern California (Fall 1901a: 46), including the Sierra Nevada (Papp 1978: 163). The record from “Idaho” (Horn 1872c: 385) is probably in error.

#### Records.

**USA**: CA, OR

#### Note.

*Agonum fallianum* Leng has been retained as the valid name for this taxon by most authors since 1919 on the account that *Agonum deplanatum* Ménétriés was a junior homonym of *Agonum deplanatum* (Chaudoir). Leng (1919b: 203) believed the date of publication for Ménétriés’ name was 1844 and Chaudoir’s name 1843. However, both names were published in 1843. Ménétriés’ publication was issued on 29 July 1843 as indicated on page 64 of the journal and Chaudoir’s publication was issued after 7 October 1843, the date of permission for publication as indicated on the recto of the title page. The reversal of precedence (ICZN 1999: Article 23.9) cannot be applied in this case because *Agonum deplanatum* Ménétriés has been used as a valid name after 1899 (e.g., Csiki 1931: 841).

### Agonum fossiger

Dejean, 1828

Agonum fossiger Dejean, 1828: 160. Type locality: «Californie» (original citation), herein restricted to Redwood Creek, Humboldt County (see Casey 1920: 121, as *Agonum tumidulum*). Holotype [by monotypy] in MHNP (Lindroth 1955b: 21). Note. Because Dejean (1828: 160) used the spelling *fossiger* in combination with the genus name *Agonum*, he obviously regarded the specific name as a noun in apposition, not as an adjective. In such case, the noun need not agree in gender with the generic name with which it is combined (ICZN 1999: Articles 31.2.1 and 34.2.1). *Agonum fossiger*, not *Agonum fossigerum*, is the correct spelling for this species.
Agonum famelicum Ménétriés, 1843: 58. Type locality: «Californie» (original citation). Lectotype (♀), designated by Lindroth (1966: 596), in ZMH. Synonymy established by LeConte (1863b: 7), confirmed by Lindroth (1966: 596).
Agonothorax robustus Motschulsky, 1859a: 158. Type locality: California (inferred from title of the paper). Two syntypes in ZMMU (Keleinikova 1976: 214) and one in MCZ (collection LeConte) (Lindroth 1966: 596). Synonymy established by LeConte (1863b: 7), confirmed by Lindroth (1966: 596).
Platynus foveiceps Notman, 1919b: 233. Type locality: «Franktown [Washoe County], Nevada» (original citation). Holotype [by monotypy] (♀) in USNM. Synonymy established by Liebherr (1991a: 121).
Agonum breviusculum Casey, 1920: 119. Type locality: «Lake Tahoe [Placer County], California» (original citation). Lectotype (♀), designated by Lindroth (1975: 127), in USNM [# 47485]. Synonymy established by Lindroth (1966: 596).
Agonum pertinax Casey, 1920: 119. Type locality: «Reno [Washoe County], Nevada» (original citation). Holotype [by monotypy] (♂) in USNM [# 47479]. Synonymy established by Lindroth (1966: 596).
Agonum atromicans Casey, 1920: 120. Type locality: «probably Colorado» (original citation). Holotype [by monotypy] (♂) in USNM [# 47484]. Synonymy established by Lindroth (1966: 596).
Agonum vegetum Casey, 1920: 121. Type locality: «S[an]ta Cruz [Santa Cruz County], California» (original citation). Lectotype (♀), designated by Lindroth (1975: 127), in USNM [# 47487]. Synonymy established by Lindroth (1966: 596).
Agonum columbicum Casey, 1920: 121. Type locality: «The Dalles [Wasco County], Oregon» (original citation). Lectotype (♂), designated by Lindroth (1975: 127), in USNM [# 47488]. Synonymy established by Hatch (1953: 143), confirmed by Lindroth (1966: 596).
Agonum tumidulum Casey, 1920: 121. Type locality: «Redwood Creek, Humboldt Co[unty], California» (original citation for the lectotype). Lectotype (♀), designated by Lindroth (1975: 127), in USNM [# 47489]. Synonymy established by Lindroth (1966: 596).

#### Distribution.

This species is found west of the Rocky Mountains from “Washington” (Hatch 1953: 143) and western Idaho (Nez Perce County, CNC) to southern California (Fall 1901a: 46). The record from Baja California (Horn 1894: 309) needs confirmation since the species is not listed in Liebherr’s (1994) review of the Mexican *Agonum*; those from southwestern British Columbia (Hatch 1953: 143) and Colorado (LeConte 1858a: 28) are probably in error.

#### Records.

**USA**: CA, ID, NV, OR, WA

### Agonum muiri

Liebherr, 1984

Agonum muiri Liebherr, 1984b: 374. Type locality: «M[oun]t Lyell (11,000 ft.) [Madera County], Cal[ifornia]» (original citation). Holotype (♂) in CAS [# 15255]. Etymology. The specific name was proposed in honor of John Muir [1838-1914], naturalist, author, and conservationist. Born in Scotland, Muir came to United States as a boy and in 1868 settled in California where his activism helped save several wilderness areas. The Sierra Club, which he founded, is now one of the most important conservation organizations in the United States.

#### Distribution.

This species is endemic to the Sierra Nevada [see Liebherr 1984b: Fig. 6], ranging from Lassen County to Tulare County.

#### Records.

**USA**: CA

### Agonum pacificum

Casey, 1920

Agonum pacificum Casey, 1920: 102. Type locality: «California» (original citation), restricted to «Julian, San Diego Co[unty]» by Liebherr (1984b: 381). Lectotype (♂), designated by Liebherr (1984b: 381), in USNM [# 47461].

#### Distribution.

This species ranges from west-central California south through the Coast Ranges to Baja California Norte [see Liebherr 1984b: Fig. 6].

#### Records.

**USA**: CA – Mexico

### Agonum quinquepunctatum

Motschulsky, 1844

Agonum 5-punctatum Motschulsky, 1844: 137. Type locality: «Koul [Lake Baikal district, Siberia, Russia] au dela du Baïcal» (original citation). Lectotype (♂), designated by Lindroth (1966: 594), in ZMH.
Platynus perforatus LeConte, 1863c: 9. Type locality: «Methy [Portage] [= Portage La Loche, northern Saskatchewan], Hudson’s Bay Territory» (original citation). Two syntypes in MCZ [# 5776]. Synonymy established by Lindroth (1966: 594). Note. Concerning the type locality, see “Note” under *Pelophila rudis*.

#### Distribution.

This Holarctic species is found in eastern Siberia (Bousquet 2003c: 451) and in North America from Alaska (Lindroth 1966: 595) to Labrador (Goose Bay, CNC). The record from “Newfoundland” (Bousquet and Larochelle 1993: 258) refers to Labrador. Fossil remnants of this species, dated between about 14,000 and 18,100 years B.P., have been unearthed in central and southeastern Iowa (Baker et al. 1986: 96; Schwert 1992: 78).

#### Records.

**CAN**: AB, BC, LB, MB, NT, ON, QC, SK, YT **USA**: AK – **Holarctic**

### Agonum suturale

Say, 1830

Agonum suturale Say, 1830b: (4) [3]. Type locality: «6.8 km N[orth] Tlaxco, Tlaxcala, Mexico» (neotype label). Neotype (♂), designated by Liebherr (1994: 34), in MCZ [# 35413]. Note. «Mexico» was the area originally cited by Say (1830b: (4) [3]).
Platynus subsericeus LeConte, 1863c: 8. Type locality: «Kansas» (original citation). Lectotype [as type] (♀), designated by Lindroth (1966: 592), in CMNH (collection Ulke). Synonymy established by Liebherr (in Ball and Shpeley 1992a: 49).
Agonum viridissimum Casey, 1920: 103. Type locality: «Stockton [Tooele County], Utah» (original citation for the lectotype). Lectotype (♂), designated by Lindroth (1975: 127), in USNM [# 47456]. Synonymy established, under the name *Agonum subsericeum* (LeConte), by Hatch (1953: 141), confirmed by Lindroth (1966: 591).
Agonum suffusum Casey, 1920: 104. Type locality: «Agassiz, British Columbia» (original citation for the lectotype). Lectotype (♀), designated by Lindroth (1975: 127), in USNM [# 47453]. Synonymy established, under the name *Agonum subsericeum* (LeConte), by Hatch (1953: 141), confirmed by Lindroth (1966: 591).
Agonum suffusum latiusculum Casey, 1920: 104. Type locality: «California» (original citation). Lectotype (♀), designated by Lindroth (1975: 127), in USNM [# 47454]. Synonymy established, under the name *Agonum subsericeum* (LeConte), by Lindroth (1966: 591).
Agonum suffusum uteanum Casey, 1920: 104. Type locality: «Ogden [Weber County], Utah» (original citation). Lectotype (♀), designated by Lindroth (1975: 127), in USNM [# 47455]. Synonymy established, under the name *Agonum subsericeum* (LeConte), by Lindroth (1966: 591).
Agonum sierranum Casey, 1920: 105. Type locality: «Truckee [Nevada County], California» (original citation). Lectotype (♀), designated by Liebherr (1991a: 118), in USNM [# 47458]. Synonymy established by Liebherr (1991a: 118).
Agonum sierranum sequoiarum Casey, 1920: 105. Type locality: «Redwood Creek, Humboldt Co[unty], California» (original citation). Holotype [by monotypy] (♀) in USNM [# 47459]. Synonymy established by Liebherr (1991a: 118).
Agonum sybariticum Casey, 1920: 107. Type locality: «California (southern)» (original citation), restricted to «Lake Henshaw, San Diego Co[unty]» by Liebherr (1991a: 118). Lectotype (♀), designated by Liebherr (1991a: 118), in USNM [# 47462]. Synonymy established by Liebherr (1991a: 118).

#### Distribution.

The range of this species extends from southern Saskatchewan to Vancouver Island (Lindroth 1966: 592, as *Agonum subsericeum*), south to southern California (Casey 1920: 107, as *Agonum sybariticum*) and Tlaxcala in Mexico (Liebherr 1994: 34).

#### Records.

**CAN**: AB, BC (VCI), SK **USA**: AZ, CA, CO, ID, KS, MT, NM, NV, OR, UT, WA, WY – Mexico

### [cyclifer group]

### Agonum anthracinum

Dejean, 1831

Agonum anthracinum Dejean, 1831: 739. Type locality: «Mexique» (original citation), herein restricted to Durango, Durango (see Liebherr 1994: 24). Holotype [by monotypy, designated lectotype by Liebherr (1994: 23)] (♂) in MHNP (Liebherr 1994: 23).

#### Distribution.

This species ranges from southeastern Arizona to the Isthmus of Tehuantepec [see Liebherr 1994: Fig. 29].

#### Records.

**USA**: AZ – Mexico

### Agonum cyclifer

(Bates, 1884)

Anchomenus cyclifer Bates, 1884: 281. Type locality: «near the city, Mexico» (original citation). One syntype in MHNP (Liebherr 1994: 22). Note. This species-group name may be either a noun in apposition or an adjective in the masculine gender (ICZN 1999: Example for Article 31.2.2). It was treated as an adjective (e.g., *Agonum cycliferum*) by Csiki (1931: 847), Blackwelder (1944: 41) and Liebherr (1991a: 120) and as a noun in apposition (e.g., *Agonum cyclifer*) by Bousquet and Larochelle (1993: 256), Liebherr (1994: 13) and Lorenz (2005: 410). When the evidence of usage is not decisive as in this case, the name is to be treated as a noun in apposition (Article 31.2.2).
Platynus arizonensis G.H. Horn, 1892c: 42. Type locality: «Camp Grant [Pinal County], Arizona» (original citation). Lectotype (♂), designated by Liebherr (1991a: 120), in MCZ [# 34498]. Synonymy established by Liebherr (1991a: 120). Note. The type locality probably refers to the “old” Camp Grant located in eastern Pinal County near Winkelman, where Horn collected while serving as a surgeon with the California Volunteers, rather than the “new” Camp Grant located in Graham County. The “old” Camp Grant was in operation from 1859 to 1872 and the scene of the infamous Camp Grant Massacre of 1871.

#### Distribution.

The range of this species extends from southeastern Arizona to the Rio Grande drainage in south-central Texas, north to southeastern Colorado (Michels et al. 2008; Las Animas County, Robert L. Davidson pers. comm. 2008), south to the Federal District﻿ of Mexico [see Liebherr 1994: Fig. 28].

#### Records.

**USA**: AZ, CO, NM, TX – Mexico

### [errans group]

### Agonum errans

(Say, 1823)

Feronia errans Say, 1823b: 147. Type locality: «Buena Vista [Chaffee County], Col[orado]» (neotype label). Neotype (♀), designated by Lindroth and Freitag (1969: 347), in MCZ [# 33016].
Agonum erythropum Kirby, 1837: 28 [secondary homonym of *Agonum erythropum* (Dejean, 1828)]. Type locality: «Canada» (original citation). One syntype in BMNH (Lindroth 1953b: 171). Synonymy established by LeConte (1879b: 56), confirmed by Lindroth (1953b: 171).
Platynus subcordatus LeConte, 1850: 205. Replacement name for *Platynus erythropus* (Kirby, 1837). Note. Although not explicitly indicated, I believe LeConte (1850: 205) proposed *Platynus subcordatus* as a replacement name for *Platynus erythropus* (Kirby) but that he forgot to add the parallels (‖) after Kirby’s name as usual; he did so subsequently (LeConte 1854b: 52). Lindroth (1966: 616) made the same assumption. Because LeConte’s name was proposed as a replacement name, the specimen labeled as type [# 5760] of *Platynus subcordatus* in MCZ has no status (see ICZN 1999: Article 72.7).

#### Distribution.

This species ranges from the Saguenay River in Quebec (Larochelle 1975: 35) to southeastern British Columbia (Lindroth 1966: 616), south to “Oregon” (Hatch 1953: 143), New Mexico (LeConte 1879b: 56; UASM), “Texas” (LeConte 1858a: 28, as *Platynus subcordatus*), and southeastern South Carolina (Ciegler 2000: 111).

#### Records.

**CAN**: AB, BC, MB, ON, QC, SK **USA**: AL, AR, CO, GA, IA, ID, IN, KS, MA, MI, MN, MS, MT, NC, ND, NE, NH, NM, NY, OR, PA, SC, SD, TX, UT, VA, VT, WA, WI, WY

### Agonum ferreum

Haldeman, 1843

Agonum ferreum Haldeman, 1843b: 299. Type locality: southeastern Pennsylvania (Haldeman 1843a: 295). One possible syntype, a ♀ labeled “[pink disc] / P. ferreus (Hald.) Lec. A. ocreatum Hald. [handwritten],” in MCZ (collection LeConte).
Agonum ocreatum Haldeman, 1843b: 299. Type locality: «Alleghany M[oun]t[ain]s» (original citation). Syntype(s) presumably lost. Synonymy established by LeConte (1854b: 51).
Micragonum quadrulum Casey, 1920: 81. Type locality: «Illinois» (original citation). Lectotype (♀), designated by Lindroth (1975: 129), in USNM [# 47506]. Synonymy established by Lindroth (1966: 624).
Micragonum solidulum Casey, 1920: 82. Type locality: «Highland Park [Lake County], northern Illinois» (original citation for the lectotype). Lectotype (♀), designated by Lindroth (1975: 129), in USNM [# 47505]. Synonymy established by Lindroth (1966: 624).

#### Distribution.

This species ranges from western New Hampshire (Grafton County, Ross T. Bell pers. comm. 2008) to eastern Oklahoma (Foster F. Purrington pers. comm. 2012), including southernmost Ontario (Lindroth 1966: 624), south to eastern Mississippi (Noxubee County, CMNH), southern Alabama (Löding 1945: 19), and central South Carolina (Ciegler 2000: 112). The record from northern Wisconsin along Lake Superior (Wickham 1896c: 134) is likely in error.

#### Records.

**CAN**: ON **USA**: AL, AR, CT, DC, DE, GA, IA, IL, IN, KS, KY, MA, MD, MI, MO, MS, NC, NH, NJ, NY, OH, OK, PA, RI, SC, TN, VA, VT, WV

### Agonum sulcipenne

(Horn, 1881)

Platynus sulcipennis G.H. Horn [in LeConte and Horn], 1881: 75. Type locality: «Florida» (original citation). Syntype(s) in MCZ [# 34474].

#### Distribution.

This species is known from a few localities along the Coastal Plain from southern Georgia (Fattig 1949: 33) and the Florida Panhandle (Peck and Thomas 1998: 23) west to northeastern Mississippi (Snodgrass and Cross 1983: 13), north along the Mississippi Basin to southern Illinois (Gallatin, Alexander, and Jackson Counties, CMNH, CNC). The record from “North Carolina” (Bousquet and Larochelle 1993: 260) needs confirmation.

#### Records.

**USA**: AL, FL, GA, IL, MS, TN [NC]

### [excavatum group]

### Agonum excavatum

Dejean, 1828

Agonum excavatum Dejean, 1828: 169. Type locality: «Amérique septentrionale» (original citation), restricted to «Dorchester [Suffolk County], Mass[achusetts]» by Lindroth (1966: 623). One syntype in MHNP (Lindroth 1955b: 20).
Anchomenus ontarionis Casey, 1920: 54. Type locality: «Toronto, Ontario» (original citation). Lectotype (♀), designated by Lindroth (1975: 129), in USNM [# 47428]. Synonymy established by Lindroth (1966: 623).
Anchomenus trinarius Casey, 1920: 54. Type locality: «probably Indiana» (original citation). Holotype [by monotypy] (♂) in USNM [# 47429]. Synonymy established by Lindroth (1966: 623).

#### Distribution.

This species ranges from New Brunswick (Lindroth 1966: 623) to southern Wisconsin (Messer 2010: 42), south to eastern Oklahoma (Latimer County, UASM), west-central Arkansas (Logan County, CNC), northern Georgia (Fattig 1949: 34), and eastern South Carolina (Ciegler 2000: 111). The records from “Minnesota” and “Louisiana” (Bousquet and Larochelle 1993: 256) need confirmation.

#### Records.

**USA**: NB, ON, QC **USA**: AL, AR, CT, DC, DE, GA, IA, IL, IN, KY, MA, MD, ME, MI, MO, MS, NC, NH, NJ, NY, OH, OK, PA, RI, SC, TN, VA, VT, WI, WV [LA, MN]

### [extensicolle group]

### Agonum cyanopis

(Bates, 1882)

Anchomenus cyanopis Bates, 1882a: 94. Type locality: «Guanajuato, Mexico» (original citation for the lectotype). Lectotype (♂), designated by Liebherr (1982: 153), in BMNH. Note. The specific name probably derives from the Latin adjective *cyaneus*, -*a*, -*um* (dark blue) and the Latin noun *ops*, -*opis* (riches, wealth) and refers to the dark blue coloration of the adult. The ending of the name remains unchanged whether the name is combined with a masculine generic name such as *Anchomenus* or a neutral generic name such as *Agonum*. The spelling *Agonum cyanope*, used by modern authors since the 1980s, is incorrect.

#### Distribution.

This species is found from eastern Arizona and western New Mexico south through the Sierra Madre Occidental to the Mexican states of Guerrero, Puebla, and Vera Cruz [see Liebherr 1986: Fig. 37].

#### Records.

**USA**: AZ, NM – Mexico

### Agonum decorum

(Say, 1823)

Feronia decora Say, 1823a: 53. Type locality: «Arlington [Middlesex County], Mass[achusetts]» (neotype label). Neotype (♂), designated by Lindroth and Freitag (1969: 343), in MCZ [# 33017].
Anchomenus thoracicus Dejean, 1828: 114. Type locality: «Amérique septentrionale» (original citation), restricted to «Utah» by Lindroth (1966: 629). Holotype [by monotypy] in MHNP (Lindroth 1955b: 19). Synonymy established by LeConte (1846b: 223), confirmed by Liebherr (1986: 124).
Anchomenus californicus Dejean, 1828: 127. Type locality: «Californie» (original citation). One syntype in MHNP (Lindroth 1955b: 20). Synonymy established by Liebherr (1986: 124).
Anchomenus obscurus LeConte, 1846b: 223. Type locality: «Lacum Onondaga [Onondaga County, New York]» (original citation). Six syntypes in MCZ [# 5749]. Synonymy established by LeConte (1854b: 46), confirmed by Lindroth (1966: 629).
Platynus simplex LeConte, 1854b: 46. Type locality: «Colorado River, California» (original citation). Four syntypes in MCZ [# 5748]. Synonymy established by Liebherr (1986: 124).
Anchomenus charmis Bates, 1884: 280. Type locality: «near the city, Mexico» (original citation). Holotype [by monotypy] (♀) in MHNP. Synonymy established by Liebherr (1986: 124).
Platynus testaceonotus Hausen, 1891b: 162. Type locality: «S[ain]te-Rose [= Laval], P[rovince of] Q[uebec]» (original citation). Holotype [by monotypy] presumably lost. Synonymy established by Lindroth (1966: 629).
Anchomenus solutus Casey, 1920: 60. Type locality: «Reno [Washoe County], Nevada» (original citation). Holotype [by monotypy] (♀) in USNM [# 47437]. Synonymy established by Liebherr (1986: 125).
Anchomenus impictus Casey, 1920: 60. Type locality: «San Joaquin Co[unty], California» (original citation). Lectotype (♂), designated by Liebherr (1991a: 117), in USNM [# 47438]. Synonymy established by Liebherr (1986: 125).
Anchomenus irruptus Casey, 1920: 60. Type locality: «Priest River [Bonner County], Idaho» (original citation). Lectotype (♂), designated by Lindroth (1975: 129), in USNM [# 47439]. Synonymy established, under the name *Anchomenus californicum* (Dejean), by Hatch (1953: 137), confirmed by Lindroth (1966: 628).
Anchomenus vinnulus Casey, 1920: 61. Type locality: «Battle M[oun]t[ain]s, Nevada» (original citation). Lectotype (♂), designated by Liebherr (1991a: 118), in USNM [# 47440]. Synonymy established by Liebherr (1986: 125).
Anchomenus luxatus Casey, 1920: 67. Type locality: «Utah» (original citation). Lectotype (♂), designated by Lindroth (1975: 129), in USNM [# 47449]. Synonymy established, under the name *Anchomenus thoracicum* (Dejean), by Lindroth (1955b: 19).
Anchomenus decorus arenarius Casey, 1920: 68. Type locality: «Galveston [Galveston County], Texas» (original citation). Holotype [by monotypy] (♂) in USNM [# 47450]. Synonymy established by Lindroth (1966: 629).
Anchomenus tepidus Casey, 1920: 68. Type locality: «Tuçson [Pima County], Arizona» (original citation). Holotype [by monotypy] (♀) in USNM [# 47451]. Synonymy established by Lindroth (1966: 629).
Anchomenus uteanus Casey, 1924: 81. Type locality: «Callao [Juab County], Utah» (original citation). Lectotype (♂), designated by Liebherr (1991a: 118), in USNM [# 47433]. Synonymy established by Liebherr (1986: 125).
Agonum extensicolle cubanum Darlington, 1934: 97. Type locality: «Soledad (near Cienfuegos), Cuba» (original citation). Holotype (♂) in MCZ [#19517]. Synonymy established by Liebherr (1986: 125).

#### Distribution.

This widely distributed species ranges from southern Quebec to Vancouver Island, south to Baja California Norte, the Isthmus of Tehuantepec, and central Florida; also present on several islands of the West Indies [see Liebherr 1986: Figs 51-54].

#### Records.

**CAN**: BC (VCI), MB, ON, QC, SK **USA**: AL, AR, AZ, CA, CO, CT, DC, DE, FL, GA, IA, ID, IL, IN, KS, KY, LA, MA, MD, ME, MI, MN, MO, MS, MT, NC, ND, NE, NH, NJ, NM, NV, NY, OH, OK, OR, PA, RI, SC, SD, TN, TX, UT, VA, VT, WA, WI, WY – Cayman Islands, Cuba, Hispaniola, Jamaica, Mexico

### Agonum elongatulum

(Dejean, 1828)

Anchomenus elongatulus Dejean, 1828: 112. Type locality: «Amérique septentrionale» (original citation), restricted to «Winter Park [Orange County], Flor[ida]» by Lindroth (1966: 627). Two syntypes in MHNP (Lindroth 1955b: 19).
Platynus floridanus LeConte, 1878b: 374. Type locality: «[Fort] Capron and Lake Harney [Florida]» (original citation). Six syntypes in MCZ [# 5750]. Synonymy established by Lindroth (1955b: 19), confirmed by Liebherr (1986: 137).
Anchomenus gravidulus Casey, 1920: 59. Type locality: «Indian River Haulover, Florida» (original citation). Lectotype (♀), designated by Liebherr (1991a: 117), in USNM [# 47435]. Synonymy established by Liebherr (1986: 137).
Anchomenus collisus Casey, 1920: 59. Type locality: «Marion Co[unty], Florida» (original citation). Lectotype (♂), designated by Liebherr (1991a: 116), in USNM [# 47434]. Synonymy established by Liebherr (1986: 137).

#### Distribution.

This species is found only in southern Georgia, throughout Florida including the Keys, and on Bimini [see Liebherr 1986: Fig. 58].

#### Records.

**USA**: FL, GA – Bahamas

#### Note.

Liebherr (1986: 140) reported the presence of a series of putative hybrids *elongatulum* x *decorum* from Okracoke Island, North Carolina.

### Agonum extensicolle

(Say, 1823)

Feronia extensicollis Say, 1823a: 54. Type locality: «Rumney [Grafton County], N[ew] H[ampshire]» (neotype label). Neotype (♀), designated by Lindroth and Freitag (1969: 346), in MCZ [# 33018].
Anchomenus obscuratus Chaudoir, 1843b: 763. Type locality: «Etats unis de l’Amérique septentrionale» (original citation). Syntype(s) in MHNP. Synonymy established by LeConte (1859c: 32).
Anchomenus le contei LeConte, 1844: 53. Type locality: North America (inferred from title of the paper). Syntype(s) in MCZ. Synonymy established by Melsheimer (1853: 16). Note. LeConte (1844: 53) did not specify the origin of his specimen(s) but most of the other species described in the same paper came from Georgia. There is one specimen in LeConte’s collection with an orange disc, labeled “extensicollis 2,” that could be a syntype.
Anchomenus viridis LeConte, 1846b: 222. Type locality: «Indiana ad flumen Ohio» (original citation). One syntype in MCZ [# 5747]. Synonymy established by LeConte (1859c: 32), confirmed by Lindroth (1966: 625).
Anchomenus cyanescens Motschulsky, 1859a: 159. Type locality: California (inferred from title of the paper). One syntype in ZMMU (Keleinikova 1976: 194). Synonymy established by LeConte (1879b: 55). Note. This name was listed as “*Anchomenus cyaneus*” by Motschulsky (1869: 21).
Anchomenus gaudens Casey, 1920: 55. Type locality: «Lake Champlain, New York» (original citation). Lectotype (♂), designated by Lindroth (1975: 129), in USNM [# 47430]. Synonymy established by Lindroth (1966: 625).
Anchomenus gaudens clientulus Casey, 1920: 55. Type locality: «Rutherford [Bergen County], New Jersey» (original citation). Lectotype (♂), designated by Lindroth (1975: 129), in USNM [# 47431]. Synonymy established by Lindroth (1966: 625).
Anchomenus vigilans Casey, 1920: 56. Type locality: «Asheville [Buncombe County], North Carolina» (original citation). Lectotype (♂), designated by Lindroth (1975: 129), in USNM [# 47432]. Synonymy established by Lindroth (1966: 625).

#### Distribution.

This species ranges from Cape Breton Island (Bousquet 1987d: 106) to northwestern Montana, including southern Manitoba and southern Saskatchewan (Ronald R. Hooper pers. comm. 2007), south to south-central Arizona, Jalisco in Mexico, and northern Florida [see Liebherr 1986: Fig. 46].

#### Records.

**CAN**: MB, NB, NS (CBI), ON, QC, SK **USA**: AL, AR, AZ, CO, CT, DC, DE, FL, GA, IA, IL, IN, KS, KY, LA, MA, MD, ME, MI, MN, MO, MS, MT, NC, ND, NE, NH, NJ, NM, NY, OH, OK, PA, RI, SC, SD, TN, TX, UT, VA, VT, WI, WV, WY – Mexico

### Agonum extimum

Liebherr, 1986

Agonum extimum Liebherr, 1986: 100. Type locality: «31.2 km E[ast] San Pedro (900 m), Coahuila, Mexico» (original citation). Holotype (♀) in CAS [# 15040].

#### Distribution.

This species ranges from southern Arizona to southeastern Texas, south to southern Coahuila in Mexico [see Liebherr 1986: Fig. 41].

#### Records.

**USA**: AZ, NM, TX – Mexico

### Agonum parextimum

Liebherr, 1986

Agonum parextimum Liebherr, 1986: 103. Type locality: «Los Mochis, Sinaloa, Mexico» (original citation). Holotype (♀) in CAS [# 15041].

#### Distribution.

This species ranges from south-central Arizona south to the Mexican states of Sonora and southern Sinaloa along the Gulf of California Coast [see Liebherr 1986: Fig. 41].

#### Records.

**USA**: AZ – Mexico

### Agonum texanum

(LeConte, 1878)

Platynus texanus LeConte, 1878b: 374. Type locality: «Texas» (original citation), restricted to «Clifton, Bosque Co[unty]» by Lindroth (1966: 629). Six syntypes in MCZ [# 5751].
Anchomenus megillus Bates, 1891a: 252. Type locality: «Villa Lerdo, in Durango» (original citation). Lectotype (♂), designated by Liebherr (1982: 153), in BMNH. Synonymy established by Liebherr (1986: 107).

#### Distribution.

This taxon ranges from central Kansas south to Chiapas in Mexico, west to central Arizona [see Liebherr 1986: Fig. 43].

#### Records.

**USA**: AZ, KS, NM, OK, TX – Mexico

### [melanarium group]

### Agonum affine

Kirby, 1837

Agonum affine Kirby, 1837: 27 [primary homonym of *Agonum affine* Stephens, 1828]. Type locality: northern parts of British America (inferred from title of the book), restricted to «Edmonton, Al[ber]ta» by Lindroth (1966: 603). Holotype [by monotypy] (♂) in BMNH (Lindroth 1953b: 171). Note. *Agonum affine* Kirby, 1837 is a primary homonym of *Agonum affine* Stephens, 1828 (a junior synonym of *Agonum thoreyi thoreyi* Dejean, 1828). Although *Agonum affine* Stephens has not been used as a valid name after 1899, I am unable to meet the condition of Article 23.9.1.2 of the ICZN (1999) in order to qualify *Agonum affine* Stephens of *nomen oblitum* and *Agonum affine* Kirby of *nomen protectum*. Nevertheless, I believe that the name *Agonum affine* Kirby should be maintained for this species until the case is submitted to the International Commission on Zoological Nomenclature.
Platynus carbo LeConte, 1850: 205. Type locality: Lake Superior (inferred from title of the paper), cited from the «northern shore of Lake Superior» by LeConte (1854b: 49). Holotype [by monotypy] (♀) in MCZ [# 5767]. Synonymy established by Lindroth (1953b: 171).
Agonothorax planipennis Motschulsky, 1850a: 68 [*nomen dubium*]. Type locality: «Sitka [Alaska]» (original citation). Syntype(s) location unknown (possibly in ZMMU though not listed in Keleinikova 1976). Synonymy established with doubt by Bousquet and Larochelle (1993: 11). Note. This name was listed as “*Agonothorax flavipennis*” by Motschulsky (1869: 21). Horn (in Wickham 1902: 239) stated that this name “is probably a variety of *P[latynus] fossiger* Dej.” If this is correct, then the type locality reported by Motschulsky is in error.

#### Distribution.

This species ranges from Newfoundland (Lindroth 1955a: 123, as *Agonum carbo*) to central Alaska (Lindroth 1966: 605), south to southern Oregon (Westcott et al. 2006: 6), the Sangre de Cristo Mountains in New Mexico (Fall and Cockerell 1907: 159), northern Indiana (Blatchley 1910: 130; Wolcott and Montgomery 1933: 127), southwestern Pennsylvania (Westmoreland County, William L. Krinsky pers. comm. 2012) and the District of Columbia (Ulke 1902: 7, as *Platynus carbo*).

#### Records.

**FRA**: PM **CAN**: AB, BC (VCI), LB, MB, NB, NF, NS (CBI), NT, ON, PE, QC, SK, YT **USA**: AK, CO, CT, DC, IA, ID, IL, IN, MA, ME, MI, MN, MT, NH, NJ, NM, NY, OH, OR, PA, RI, VT, WA, WI, WY

### Agonum brevicolle

Dejean, 1828

Agonum brevicolle Dejean, 1828: 159. Type locality: «Californie» (original citation), herein restricted to San Francisco, San Francisco County (see LeConte 1854b: 49, as *Platynus frater*). Holotype [by monotypy] in MHNP (Lindroth 1955b: 20).
Platynus frater LeConte, 1854b: 49. Type locality: «San Francisco and San Diego, California» (original citation), restricted to «San Francisco [San Francisco County]» by Lindroth (1966: 608). Three syntypes in MCZ [# 5769]. Synonymy established by LeConte (1869b: 248), confirmed by Lindroth (1966: 608).

#### Distribution.

This western species ranges from western British Columbia (Lindroth 1966: 609), as far north as the Skeena river area and Queen Charlotte Islands (Kavanaugh 1992: 75), south to southern California (Fall 1901a: 46, as *Platynus frater*), including eastern Nevada (Liebherr 1986: 130).

#### Records.

**CAN**: BC (QCI, VCI) **USA**: CA, NV, OR, WA

### Agonum collare

(Say, 1830)

Anchomenus collaris Say, 1830b: (4) [3]. Type locality: «Woodbury [Gloucester County], N[ew] J[ersey]» (neotype label). Neotype (♂), designated by Lindroth and Freitag (1969: 347), in MCZ [# 33015]. Note. «Indiana» was the area originally cited by Say (1830b: (4)[3]).

#### Distribution.

This species ranges from Rhode Island (Sikes 2003: 8; CMNH) to northeastern Illinois (Cook County, CMNH), south to eastern Texas along the Gulf Coast (Snow 1906a: 141) and central Florida (Brevard and Highlands Counties, CNC). The records from “Wisconsin” and “Pennsylvania” (Bousquet and Larochelle 1993: 258) need confirmation.

#### Records.

**USA**: AL, CT, FL, GA, IL, IN, KY, LA, MD, MI, MS, NC, NJ, NY, OH, PA, RI, SC, VA, TX [PA, WI]

### Agonum corvus

(LeConte, 1860)

Platynus corvus LeConte, 1860: 319. Type locality: «Black Hills [South Dakota]; Saskatchewan» (original citation), restricted to «Black Hills» by Lindroth (1966: 603). One syntype in MCZ [# 5768].
Agonum hyslopi Casey, 1920: 113. Type locality: «Wilbur [Lincoln County], Washington» (original citation). Lectotype (♂), designated by Lindroth (1975: 128), in USNM [# 47477]. Synonymy established by Lindroth (1966: 603). Etymology. The specific name was proposed for James Augustus Hyslop [1884-1953], entomologist for the Bureau of Entomology in Washington DC. Hyslop worked mainly on elaterids.
Agonum debiliceps Casey, 1920: 118. Type locality: «Duluth [Saint Louis County], Minnesota» (original citation). Holotype [by monotypy] (♀) in USNM [# 47481]. Synonymy established by Lindroth (1966: 603).

#### Distribution.

This species ranges from the Saint Lawrence Valley in southern Quebec (Larochelle 1975: 33) to the Fraser Valley in British Columbia (Lindroth 1966: 603), south to northern California (Siskiyou County, CNC), east-central California (Mono County, Foster F. Purrington pers. comm. 2012), northern Arizona (Coconino and Apache Counties, CNC), southern Colorado (Alamosa County, CNC), and southern Nebraska (Chase County, Foster F. Purrington pers. comm. 2010). The record from “Michigan” (Bousquet and Larochelle 1993: 256) needs confirmation.

#### Records.

**CAN**: AB, BC, MB, ON, QC, SK **USA**: AZ, CA, CO, ID, MN, MT, ND, NE, OR, SD, UT, VT, WA, WV, WY [MI]

### Agonum deceptivum

(LeConte, 1879)

Platynus deceptivus LeConte, 1879b: 53. Type locality: «Nova Scotia and Lake Superior» (original citation), restricted to «Nova Scotia» by Lindroth (1966: 611). Syntype(s) in MCZ [# 5771].

#### Distribution.

This rarely collected species is found along the provinces and states bordering the Atlantic Coast from Cape Breton Island (Lindroth 1966: 611) to Connecticut (New London County, William L. Krinsky pers. comm. 2008).

#### Records.

**CAN**: NS (CBI) **USA**: CT, MA, ME, NH

### Agonum fidele

Casey, 1920

Platynus laevis LeConte, 1854b: 48 [secondary homonym of *Agonum laeve* (Gyllenhal, 1827)]. Type locality: «middle and western states» (original citation). Syntype(s) in MCZ [# 5763].
Platynus molestus LeConte, 1863b: 7 [secondary homonym of *Agonum molestum* Motschulsky, 1844]. Replacement name for *Platynus laevis* LeConte, 1854.
Agonum fidele Casey, 1920: 116. Type locality: «Boston Neck [Washington County], Rhode Island» (original citation). Lectotype (♂), designated by Lindroth (1975: 128), in USNM [# 47480]. Synonymy established by Hatch (1953: 142), confirmed by Lindroth (1954b: 139).
Agonum subinflatum Casey, 1920: 117. Type locality: «Marquette [Marquette County, Michigan], Lake Superior» (original citation for the lectotype). Lectotype (♂), designated by Lindroth (1975: 128), in USNM [# 47478]. Synonymy established by Lindroth (1954b: 140).

#### Distribution.

This species ranges from Cape Breton Island (Lindroth 1954c: 306) to western Wisconsin (Kleintjes et al. 2003: 81; Messer 2010: 42), south to northeastern Mississippi (Snodgrass and Cross 1983: 13) and west-central South Carolina (Ciegler 2000: 112). The record from “Prince Edward Island” (Bousquet and Larochelle 1993: 256) is based on misidentified small specimens of *Agonum melanarium* Dejean (CNC).

#### Records.

**CAN**: NB, NS (CBI), ON, QC **USA**: CT, DC, IA, IL, KY, MA, MD, ME, MI, MS, NC, NH, NJ, NY, OH, PA, RI, SC, TN, VA, VT, WI, WV

### Agonum harrisii

LeConte, 1846

Agonum harrisii LeConte, 1846b: 225. Type locality: «Massachusetts» (original citation). One syntype in MCZ [# 5766]. Etymology. The specific name honors Thaddeus William Harris [1795-1856], American entomologist and botanist. Harris first made his living as a physician but soon became librarian at Harvard University. He also taught natural history at Harvard but never achieved full-time professorship. Harris is best known for his work on economic entomology and is often viewed as the founder of applied entomology in the United States.
Agonum mordax Casey, 1920: 113. Type locality: «Beaver Dam [Dodge County], Wisconsin» (original citation). Lectotype (♂), designated by Lindroth (1975: 128), in USNM [# 47474]. Synonymy established by Lindroth (1966: 609).
Agonum aethiops Casey, 1920: 116. Type locality: «probably British America» (original citation). Holotype [by monotypy] (♀) in USNM [# 47475]. Synonymy established by Lindroth (1966: 609).

#### Distribution.

This species ranges from Cape Breton Island (Lindroth 1954c: 306) to Vancouver Island (Lindroth 1966: 609), south to “Oregon” (CAS), north-central Idaho (Idaho County, CNC), southeastern Wyoming (Lavigne 1977: 43), southern Minnesota (Hennepin County, CNC), northeastern Illinois (Purrington et al. 2002: 200), and southeastern Virginia (Surry County, CMNH). The record from “Nebraska” (Bousquet and Larochelle 1993: 257) is probably in error.

#### Records.

**CAN**: AB, BC (VCI), MB, NB, NS (CBI), ON, PE, QC, SK **USA**: CT, ID, IL, IN, MA, ME, MI, MN, MT, NH, NJ, NY, OH, OR, PA, RI, VA, VT, WA, WI, WV, WY

### Agonum imitans

(Notman, 1919)

Platynus imitans Notman, 1919b: 232. Type locality: «North America» (original citation). Holotype [by monotypy] (♂) in USNM [# 104255].

#### Distribution.

This species is known only from the holotype which has no locality or state label (see Liebherr 1991a: 121-122).

#### Records.

None.

### Agonum melanarium

Dejean, 1828

Feronia scutellaris Say, 1823b: 146 [*nomen dubium*]. Type locality not stated. Syntype(s) lost. Note. LeConte (1879b: 56) remarked that this species was based on “a distorted specimen [of *Agonum melanarium*], the name is therefore rejected.”
Agonum melanarium Dejean, 1828: 152. Type locality: «Amérique septentrionale» (original citation), restricted to «Arlington [Middlesex County], Mass[achusetts]» by Lindroth (1966: 599). One syntype in MHNP (Lindroth 1955b: 20). Synonymy established by LeConte (1879b: 56).
Agonum maurum Haldeman, 1843b: 300. Type locality: southeastern Pennsylvania (Haldeman 1843a: 297). One possible syntype, a ♂ labeled “[pink disc] / P. melanarius (Dej.) Lec.A. maurum Hald. Fer. scutellaris Say [handwritten],” in MCZ (collection LeConte). Synonymy established by LeConte (1846b: 225).
Agonum nitidum T.W. Harris [in Scudder], 1869: 230 [*nomen dubium*]. Type locality not stated. Syntype(s) lost. Synonymy established with doubt by Bousquet and Larochelle (1993: 12).
Agonum politulum T.W. Harris [in Scudder], 1869: 230. Type locality not stated. Syntype(s) lost. Synonymy established by LeConte (in Scudder 1869: 230).
Agonum militare Casey, 1920: 114. Type locality: «West Point [Orange County], New York» (original citation). Lectotype (♀), designated by Lindroth (1975: 128), in USNM [# 47473]. Synonymy established by Lindroth (1966: 600).

#### Distribution.

The range of this species extends from Cape Breton Island (Lindroth 1954c: 306) to southwestern British Columbia (Lindroth 1966: 600), south to northwestern California (Trinity County, CNC), northern Colorado (Haubold 1951: 705; Armin 1963: 150), west-central Kansas (Trego County, CNC), and North Carolina (Haywood County, CNC).

#### Records.

**CAN**: AB, BC, MB, NB, NS (CBI), ON, PE, QC, SK **USA**: CA, CO, CT, DC, DE, IA, ID, IL, IN, KS, MA, MD, ME, MI, MN, MT, NC, ND, NE, NH, NJ, NY, OH, OR, PA, RI, SD, UT, VA, VT, WA, WI, WV, WY

### Agonum metallescens

(LeConte, 1854)

Platynus metallescens LeConte, 1854b: 48. Type locality: «Sault S[ain]te Marie, and northern shore of Lake Superior» (original citation), restricted to «Sault S[ain]te Marie, Ont[ario]» by Lindroth (1966: 605). Syntype(s) in MCZ [# 5770].
Agonum lacustre Casey, 1920: 114. Unnecessary replacement name for *Agonum metallescens* (LeConte, 1854).
Agonum terracense Casey, 1924: 85. Type locality: «Terrace, British Columbia» (original citation). Lectotype (♂), designated by Lindroth (1975: 128), in USNM [# 47476]. Synonymy established by Lindroth (1954b: 139).

#### Distribution.

This species ranges from Newfoundland (Lindroth 1955a: 122) to the Queen Charlotte Islands (Kavanaugh 1992: 74), north to southern Northwest Territories (Bousquet 1987a: 125), south to northern Idaho (Peter W. Messer pers. comm. 2008), northwestern Montana (Glacier County, CNC), northern Wisconsin (Wickham 1896c: 133; Casey 1920: 114, as *Agonum lacustre*), the upper peninsula of Michigan along Lake Superior (Casey 1920: 114, as *Agonum lacustre*), and New Jersey (Smith 1890: 85; Smith 1910: 208).

#### Records.

**FRA**: PM **CAN**: AB, BC (QCI, VCI), MB, NB, NF, NS (CBI), NT, ON, PE, QC, SK **USA**: ID, MA, ME, MI, MN, MT, ND, NH, NJ, NY, VT, WI

### Agonum moerens

Dejean, 1828

Agonum moerens Dejean, 1828: 152. Type locality: «Amérique septentrionale» (original citation), restricted to «Elkhart [Elkhart County], Indiana» by Lindroth (1966: 610). One syntype in MHNP (Lindroth 1955b: 20).

#### Distribution.

This species is known from western Maine (Franklin, Kennebec, and York Counties, Ross T. Bell pers. comm. 2008), southwestern Quebec, the Ontario Peninsula, and southern Manitoba (Lindroth 1966: 611) south to southern Louisiana (Assumption and East Baton Rouge Parishes, LSAM) and northern Florida (Alachua County, CNC).

#### Records.

**CAN**: MB, ON, QC **USA**: AL, AR, CT, DC, FL, GA, IA, IL, IN, LA, MA, MD, ME, MI, MN, MS, NC, NJ, NY, OH, PA, RI, SC, TN, VA, VT, WI

### Agonum mutatum

(Gemminger and Harold, 1868)

Platynus atratus LeConte, 1850: 205 [secondary homonym of *Platynus atratus* (Duftschmid, 1812)]. Type locality: Lake Superior (inferred from title of the paper), cited from the «northern shore of Lake Superior» by LeConte (1854b: 49), herein restricted to Marquette, Marquette County, Michigan (see Hubbard and Schwarz 1878: 628, as *Platynus mutatus*). Two syntypes in MCZ [# 5762].
Platynus mutatus Gemminger and Harold, 1868a: 374. Replacement name for *Platynus atratus* LeConte, 1850.

#### Distribution.

This species is found from Newfoundland (Lindroth 1955a: 124) to British Columbia, north to east-central Alaska (Lindroth 1966: 601), south to west-central Washington (Lindroth 1966: 601), northwestern Montana (Russell 1968: 64), central Indiana (Downie 1957: 116; Schrock 1985: 343), and eastern South Carolina (Kirk 1969: 11; Ciegler 2000: 112).

#### Records.

**CAN**: AB, BC, LB, MB, NB, NF, NS (CBI), NT, ON, PE, QC, SK **USA**: AK, CT, DE, IA, IN, MA, ME, MI, MN, MT, NC, NH, NJ, NY, OH, PA, RI, SC, VT, WA, WI, WV

### Agonum propinquum

(Gemminger and Harold, 1868)

Agonum piceum LeConte, 1846b: 226 [secondary homonym of *Agonum piceum* (Linnaeus, 1758)]. Type locality: «Massachusetts» (original citation). Syntype(s) in MCZ [# 5764]. Note. The specimen in MCZ labeled as “Type” is also labeled “Can” and is not a syntype.
Platynus propinquus Gemminger and Harold, 1868a: 375. Replacement name for *Platynus piceus* (LeConte, 1846).
Platynus fraterculus LeConte, 1869c: 373. Type locality: Vancouver Island, British Columbia (inferred from title of the paper). Holotype [by monotypy] (♀) in MCZ [# 5765]. Synonymy established by Lindroth (1966: 611).
Anchomenus xanthocnemis Bates, 1884: 281. Type locality: «Mexico» (original citation). Holotype [by monotypy] (♀) in BMNH. Synonymy established by Liebherr (1991a: 119).
Agonum humile Casey, 1920: 117. Type locality: «Kalispell [Flathead County], Montana» (original citation). Lectotype (♂), designated by Lindroth (1975: 128), in USNM [# 47482]. Synonymy established by Lindroth (1954b: 140).
Agonum insuetum Casey, 1920: 118. Type locality: «Wilbur [Lincoln County], Washington» (original citation). Lectotype (♀), designated by Lindroth (1975: 128), in USNM [# 47483]. Synonymy established by Hatch (1953: 142), confirmed by Lindroth (1954b: 140).
Agonum amens Casey, 1924: 83. Type locality: «Edmonton, Alberta» (original citation). Lectotype (♂), designated by Lindroth (1975: 128), in USNM [# 47486]. Synonymy established by Lindroth (1954b: 140).

#### Distribution.

This widely distributed species extends from Newfoundland (Lindroth 1955a: 124) to Alaska (Lindroth 1966: 612), south to the Sierra Nevada in northeastern California (Liebherr 1991a: 119), southwestern Colorado (Wickham 1902: 239; Ouray County, CNC), northwestern Nebraska (Sheridan County, CNC), northwestern Pennsylvania (Erie County, CMNH), and “Maryland” (Liebherr 1994: 30). One specimen is also known from the city of Mexico (Liebherr 1994: 30) but in my opinion it is likely mislabeled.

#### Records.

**CAN**: AB, BC (VCI), MB, NB, NF, NS (CBI), NT, ON, PE, QC, SK, YT **USA**: AK, CA, CO, CT, ID, IN, MA, MD, ME, MI, MN, MT, ND, NE, NH, NJ, NY, OR, PA, SD, VT, WA, WI, WY

### Agonum tenue

(LeConte, 1854)

Platynus tenuis LeConte, 1854b: 48. Type locality: «middle and eastern states» (original citation), restricted to «Cambridge [Middlesex County], Mass[achusetts]» by Lindroth (1966: 610). Three syntypes in MCZ [# 5761].

#### Distribution.

This species ranges from Cape Breton Island (Lindroth 1954c: 306) to northeastern Minnesota (Kamal J.K. Gandhi pers. comm. 2008), south to southern Georgia (Fattig 1949: 34). The record from “Nebraska” (Bousquet and Larochelle 1993: 258) needs confirmation.

#### Records.

**CAN**: NB, NS (CBI), ON, PE, QC **USA**: CT, DC, DE, GA, IA, IL, IN, MA, MD, ME, MI, MN, NC, NH, NJ, NY, OH, PA, RI, SC, VA, VT, WI, WV [NE]

### Agonum trigeminum

Lindroth, 1954

Agonum trigeminum Lindroth, 1954b: 156. Type locality: «Sackville [near] Halifax, Nova Scotia» (original citation). Holotype (♂) in CNC [# 6573].

#### Distribution.

This species is known from Cape Breton Island (CNC) to southern Manitoba (Lindroth 1966: 601), south to southwestern North Carolina (Macon County, CNC).

#### Records.

**CAN**: MB, NB, NS (CBI), ON, PE, QC **USA**: CT, MA, MD, ME, MI, MN, NC, NH, NJ, NY, OH, PA, RI, VT, WI, WV

### [nutans group]

### Agonum aeruginosum

Dejean, 1828

Agonum aeruginosum Dejean, 1828: 168. Type locality: «Amérique septentrionale» (original citation), restricted to «Rivervale [Bergen County], N[ew] J[ersey]» by Lindroth (1966: 623). One syntype in MHNP (Lindroth 1955b: 21).
Circinalia undulata Casey, 1920: 79. Type locality: «Southern Pines [Moore County], North Carolina» (original citation). Lectotype (♂), designated by Lindroth (1975: 129), in USNM [# 47516]. Synonymy established by Lindroth (1966: 623).

#### Distribution.

This species ranges from Nova Scotia (Lindroth 1954c: 306) to southeastern South Dakota (Kirk and Balsbaugh 1975: 25), south at least to northeastern Kansas (Popenoe 1877: 23), central Louisiana (East Feliciana Parish, CMNH, CNC), and the Florida Panhandle (Peck and Thomas 1998: 22).

#### Records.

**CAN**: NB, NS, ON, QC **USA**: AL, AR, CT, DC, DE, FL, GA, IA, IL, IN, KS, KY, LA, MA, MD, ME, MI, MN, MO, MS, NC, NH, NJ, NY, OH, PA, RI, SC, SD, TN, VA, VT, WI, WV

### Agonum basale

LeConte, 1846

Agonum basale LeConte, 1846b: 227. Type locality: «ad Rocky Mountains» (original citation); cited from «Nebraska [Territory], near the mountains [probably in present day Colorado]» by LeConte (1854b: 52). One syntype in MCZ [# 5772]. Note. Prior to 1854, Nebraska included the area between the Missouri river and the Rockies from 40°N to the Canadian border.
Platynus vagans LeConte, 1854b: 52. Type locality: «New York» (original citation). Holotype [by monotypy] (♀) in MCZ [# 5773]. Synonymy established by LeConte (1879b: 56), confirmed by Lindroth (1966: 620).
Micragonum concretum Casey, 1920: 83. Type locality: «Illinois» (original citation). Lectotype (♀), designated by Lindroth (1975: 129), in USNM [# 47503]. Synonymy established by Lindroth (1966: 620).
Micragonum concretum amicum Casey, 1920: 83. Type locality: «northern Illinois» (original citation). Lectotype (♀), designated by Lindroth (1975: 129), in USNM [# 47504]. Synonymy established by Lindroth (1966: 620).

#### Distribution.

This species ranges from “New York” (LeConte 1854b: 52, as *Platynus vagans*) and New Jersey (Smith 1890: 85; Smith 1910: 208) to northeastern Colorado (LeConte 1854b: 52), south to northwestern South Carolina (Kirk 1970: 12; Ciegler 2000: 110). The record from “Florida” (Leng and Beutenmüller 1893: 140) is probably in error; that from “Wisconsin” (Bousquet and Larochelle 1993: 258) needs confirmation.

#### Records.

**USA**: CO, IA, IL, IN, KS, NE, NJ, NY, PA, SC [WI]

### Agonum crenulatum

(LeConte, 1854)

Platynus crenulatus LeConte, 1854b: 53. Type locality: «Georgia; Louisiana» (original citation). Syntype(s) in MCZ [# 5775].
Micragonum maritimum Casey, 1920: 85. Type locality: «Galveston [Galveston County], Texas» (original citation). Lectotype (♀), designated by Liebherr (1991a: 117), in USNM [# 47496]. Synonymy established by Liebherr (1991a: 117).
Micragonum semiviride Casey, 1920: 85. Type locality: «Fruitdale [Washington County], Alabama» (original citation). Lectotype (♀), designated by Lindroth (1975: 128), in USNM [# 47498]. Synonymy established by Lindroth (1966: 619).
Micragonum ovalipenne Casey, 1920: 86. Type locality: «Southern Pines [Moore County], North Carolina» (original citation for the lectotype). Lectotype (♀), designated by Lindroth (1975: 129), in USNM [# 47499]. Synonymy established by Lindroth (1966: 619).
Micragonum pinorum Casey, 1920: 86. Type locality: «Southern Pines [Moore County], North Carolina» (original citation). Lectotype (♂), designated by Lindroth (1975: 129), in USNM [# 47500]. Synonymy established by Lindroth (1975: 129).
Micragonum houstoni Casey, 1920: 87. Type locality: «Galveston [Galveston County], Texas» (original citation). Holotype [by monotypy] (♂) in USNM [# 47497]. Synonymy established by Lindroth (1966: 619).

#### Distribution.

This species ranges from central North Carolina (Casey 1920: 86, as *Micragonum ovalipenne* and *Micragonum pinorum*) and South Carolina (Ciegler 2000: 111) west to southeastern Texas (Casey 1920: 85, 87, as *Micragonum maritimum* and *Micragonum houstoni*). The records from Missouri (Summers 1873: 134) and “Florida” (Bousquet and Larochelle 1993: 258) need confirmation.

#### Records.

**USA**: AL, GA, LA, MS, NC, SC, TN, TX [FL, MO]

### Agonum nutans

(Say, 1823)

Feronia nutans Say, 1823a: 52. Type locality: «Phila[delphia] Neck, P[ennsylvani]a» (neotype label). Neotype (♂), designated by Lindroth and Freitag (1969: 347), in MCZ [# 33013].
Agonum femoratum Dejean, 1828: 145. Type locality: «Amérique septentrionale» (original citation). Two syntypes in MHNP (Lindroth 1955b: 20). Synonymy established by LeConte (1846b: 227), confirmed by Lindroth (1955b: 20).

#### Distribution.

This eastern species is found from southern Quebec (Larochelle 1975: 38) to eastern South Dakota (Kirk and Balsbaugh 1975: 24), south to Oklahoma (French et al. 2001: 228; Latimer County, UASM) and the District of Columbia (Ulke 1902: 7). The records from central Arizona (Griffith 1900: 565), northern Colorado (Wickham 1902: 239; Armin 1963: 149), southeastern Texas (Snow 1906a: 141), “Mississippi,” “Alabama” (Bousquet and Larochelle 1993: 259), and “Florida” (LeConte 1879b: 56) are probably in error; those from “Maryland,” “Delaware” (Bousquet and Larochelle 1993: 259), and South Carolina (Kirk 1970: 12, as *Agonum* possibly *nutans*) need confirmation.

#### Records.

**CAN**: ON, QC **USA**: AR, CT, DC, IA, IL, IN, KS, MA, ME, MI, MN, MO, NE, NH, NJ, NY, OH, OK, PA, RI, SD, VT, WI [DE, MD, SC]

### Agonum striatopunctatum

Dejean, 1828

Agonum striatopunctatum Dejean, 1828: 167. Type locality: «Amérique septentrionale» (original citation), restricted to «Mobile [Mobile County], Alab[ama]» by Lindroth (1966: 618). One syntype in MHNP (Lindroth 1955b: 20).
Agonum decipiens LeConte, 1846b: 229. Type locality: «Georgia» (original citation). Syntype(s) in MCZ [# 5774]. Synonymy established by LeConte (1854b: 53), confirmed by Lindroth (1966: 618).
Micragonum breviceps Casey, 1920: 87. Type locality: «Vicksburg [Warren County], Mississippi» (original citation). Lectotype (♂), designated by Lindroth (1975: 128), in USNM [# 47502]. Synonymy established by Lindroth (1966: 618).
Micragonum luculentum Casey, 1920: 88. Type locality: «Indiana» (original citation). Lectotype (♀), designated by Lindroth (1975: 128), in USNM [# 47501]. Synonymy established by Lindroth (1966: 618).

#### Distribution.

This species ranges from New Jersey (Essex County, CMNH) to east-central Iowa (Muscatine County, Doug A. Veal pers. comm. 2010), including southernmost Ontario (Lindroth 1966: 619), south to southeastern Texas (Casey 1920: 88) and northern Florida (Peck and Thomas 1998: 23). The record from “Wisconsin” (Bousquet and Larochelle 1993: 259) needs confirmation.

#### Records.

**CAN**: ON **USA**: AL, AR, DC, FL, GA, IA, IL, IN, KS, KY, LA, MD, MI, MO, MS, NC, NJ, OH, OK, PA, SC, TN, TX, VA [WI]

### [octopunctatum group]

### Agonum octopunctatum

(Fabricius, 1798)

Carabus 8punctatus Fabricius, 1798: 55. Type locality: «America boreali» (original citation), restricted to «Milton [Strafford County], N[ew] H[ampshire]» by Lindroth (1966: 593). Lectotype (♂), designated by Lindroth (1966: 593), in ZMUC.

#### Distribution.

This species ranges from Nova Scotia (Lindroth 1966: 594) to southeastern Manitoba (UASM), south to eastern Texas (Orange and Sabine Counties, CNC; Dajoz 2007: 23) and central Florida (Peck and Thomas 1998: 23).

#### Records.

**CAN**: MB, NB, NS, ON, PE, QC **USA**: AL, AR, CT, DC, DE, FL, GA, IA, IL, IN, KS, KY, LA, MA, MD, ME, MI, MN, MO, MS, NC, NH, NJ, NY, OH, OK, PA, RI, SC, TN, TX, VA, VT, WI, WV

### [pallipes group]

### Agonum crenistriatum

(LeConte, 1863)

Platynus crenistriatus LeConte, 1863c: 9. Type locality: «Illinois» (original citation). One syntype in MCZ [# 5781].
Circinalia politissima Casey, 1920: 76. Type locality: «Duluth [Saint Louis County], Minnesota» (original citation). Lectotype (♀), designated by Lindroth (1975: 129), in USNM [# 47513]. Synonymy established by Lindroth (1966: 621).
Circinalia politissima statenensis Casey, 1920: 77. Type locality: «Staten Island, New York» (original citation for the lectotype). Lectotype (♀), designated by Lindroth (1975: 129), in USNM [# 47514]. Synonymy established by Lindroth (1966: 621).
Circinalia liticola Casey, 1920: 77. Type locality: «Iowa City [Johnson County], Iowa» (original citation). Lectotype (♂), designated by Lindroth (1975: 129), in USNM [# 47512]. Synonymy established by Lindroth (1966: 621).
Circinalia roticollis Casey, 1920: 78. Type locality: «New Jersey» (original citation). Lectotype (♂), designated by Lindroth (1975: 129), in USNM [# 47515]. Synonymy established by Lindroth (1966: 621).

#### Distribution.

The range of this species extends from Cape Breton Island (Bousquet 1987a: 125) to southeastern South Dakota (Kirk and Balsbaugh 1975: 25), south to southeastern Nebraska (Hall County, Foster F. Purrington pers. comm. 2009), east-central Missouri (Saint Louis, CNC), and northwestern South Carolina (Kirk 1970: 12; Ciegler 2000: 111). The records from southern Louisiana (Summers 1874a: 80), “Kansas,” and “Arkansas” (Bousquet and Larochelle 1993: 259) need confirmation.

#### Records.

**CAN**: NB, NS (CBI), ON, PE, QC **USA**: CT, DC, IA, IL, IN, KY, MA, MD, ME, MI, MN, MO, NE, NH, NJ, NY, OH, PA, RI, SC, SD, VA, VT, WI, WV [AR, KS, LA]

### Agonum pallipes

(Fabricius, 1787)

Carabus pallipes Fabricius, 1787: 202. Type locality: «America» (original citation), restricted to «Hope [Hempstead County], Arkans[as]» by Lindroth (1966: 620). Lectotype [as type], designated by Staig (1931: 33), in HMUG.
Feronia limbata Say, 1823a: 49. Type locality: «Camden [Kershaw County], S[outh] C[arolina]» (neotype label). Neotype (♂), designated by Lindroth and Freitag (1969: 343), in MCZ [# 33010]. Synonymy established by Fall (1933: 103).
Agonum palliatum Dejean, 1828: 174. Type locality: «Amérique septentrionale» (original citation). One syntype in MHNP (Lindroth 1955b: 21). Synonymy established, under the name of *Agonum limbatum* (Say), by Say (1830b: (5) [3]), confirmed by Lindroth (1955b: 21).
Olisares flavolimbatus Motschulsky, 1865: 327. Type locality: «environs de Mobile [Mobile County, Alabama]» (original citation). Lectotype (♂), designated by Liebherr (1991a: 120), in ZMMU. Synonymy established by Liebherr (1991a: 120).

#### Distribution.

This species ranges from “Maryland” (CMNH) to north-central Oklahoma (Kay County, CNC), south at least to central Texas (Travis and Blanco Counties, CMNH; Casey 1920: 73), eastern Louisiana (Washington Parish, LSAM), and the Florida Panhandle (Peck and Thomas 1998: 23). The record from “Kansas” (Bousquet and Larochelle 1993: 259) needs confirmation.

#### Records.

**USA**: AL, AR, DC, FL, GA, IL, IN, KY, LA, MD, MO, MS, NC, OK, SC, TN, TX, VA [KS]

### Agonum punctiforme

(Say, 1823)

Feronia punctiformis Say, 1823a: 58. Type locality: «Phila[delphia] Neck, P[ennsylvani]a» (neotype label). Neotype (♂), designated by Lindroth and Freitag (1969: 348), in MCZ [# 33009].
Agonum orbicollis Say, 1830b: (4) [3]. Type locality: «35 km NE Zimapan, Hidalgo, Mexico» (neotype label). Neotype (♀), designated by Liebherr (1994: 17), in MCZ [# 35412]. Synonymy established by Bates (1882a: 95). Note. «Mexico» was the area originally cited by Say (1830b: (4) [3]).
Agonum foveicolle Chaudoir, 1843b: 764. Type locality: «Nouvelle Orléans [Orleans Parish, Louisiana]» (original citation). Lectotype (♀), designated by Lindroth (1966: 622), in MHNP. Synonymy established by LeConte (1854b: 50), confirmed by Lindroth (1966: 622).
Olisares picipes Motschulsky, 1865: 326. Type locality: «Caraccas [Venezuela]» (original citation), which is likely incorrect. Lectotype (♂), designated by Liebherr (1991a: 120), in ZMMU. Synonymy established by Liebherr (1991a: 120).
Circinalia ludoviciana Casey, 1920: 76. Type locality: «Alexandria [Rapides Parish], Louisiana» (original citation). Lectotype (♂), designated by Lindroth (1975: 129), in USNM [# 47511]. Synonymy established by Lindroth (1966: 622).

#### Distribution.

This species ranges from southeastern New Hampshire (Rockingham County, Ross T. Bell pers. comm. 2008) to southeastern Colorado (Michels et al. 2008), including southern Ontario (CNC), south to southern Mexico (Liebherr 1994: 20) and southern Florida (Peck and Thomas 1998: 23), west along the southwest to southern California (Liebherr 1994: 19-20); also recorded from the Bahamas and Bermuda where it is probably adventive (Hilburn and Gordon 1989: 677; Liebherr 1994: 19). The record from Quebec (Lindroth 1966: 622) refers to *Agonum rufipes* (Lindroth 1969a: 1120); that from northern Wisconsin along Lake Superior (Wickham 1896c: 134) is probably in error.

#### Records.

**CAN**: ON **USA**: AL, AR, AZ, CA, CO, CT, DC, DE, FL, GA, IA, IL, IN, KS, KY, LA, MA, MD, MI, MO, MS, NC, NE, NH, NJ, NY, OH, OK, PA, RI, SC, TN, TX, VA, VT, WV – Bahamas, Bermuda, Mexico

### Agonum rigidulum

(Casey, 1920)

Circinalia rigidula Casey, 1920: 75. Type locality: «Southern Pines [Moore County], North Carolina» (original citation for the lectotype). Lectotype (♂), designated by Lindroth (1975: 129), in USNM [# 47507].
Circinalia rigidula semipunctata Casey, 1920: 75. Type locality: «Norfolk, Virginia» (original citation). Lectotype (♀), designated by Lindroth (1975: 129), in USNM [# 47508]. Synonymy established by Lindroth (1966: 621).
Circinalia rigidula putata Casey, 1920: 75. Type locality: «Southern Pines [Moore County], North Carolina» (original citation). Lectotype (♀), designated by Lindroth (1975: 129), in USNM [# 47510]. Synonymy established by Lindroth (1966: 621).
Circinalia ventricula Casey, 1920: 75. Type locality: «Mobile [Mobile County], Alabama» (original citation). Lectotype (♀), designated by Lindroth (1975: 129), in USNM [# 47509]. Synonymy established by Lindroth (1966: 621).

#### Distribution.

This species ranges from New Jersey (Ocean County, CMNH) south to northwestern Louisiana (Bossier Parish, LSAM), southwestern Mississippi (Hancock and Hinds Counties, CMNH), southwestern Alabama (Casey 1920: 75, as *Circinalia ventricula*), and southwestern Georgia (Fattig 1949: 33, as *Platynus ventricula*).

#### Records.

**USA**: AL, GA, LA, MD, MS, NC, NJ, TN, VA

### Agonum rufipes

Dejean, 1828

Agonum rufipes Dejean, 1828: 173. Type locality: «Amérique septentrionale» (original citation), restricted to «Galesburg [Knox County], Illin[ois]» by Lindroth (1966: 621). Two syntypes in MHNP (Lindroth 1955b: 21).
Platynus rubripes Zimmermann [in LeConte], 1869b: 244. Type locality: «M[arylan]d» (syntype label). One syntype in MCZ [# 5782]. Synonymy established by Lindroth (1966: 621).

#### Distribution.

The range of this eastern species extends from southeastern New Hampshire (Rockingham County, Donald S. Chandler pers. comm. 2009) to eastern South Dakota (Kirk and Balsbaugh 1975: 25), including southern Ontario (Lindroth 1969a: 1120), south to southeastern Texas (Casey 1920: 74) and southeastern Georgia (Fattig 1949: 32, as *Platynus rubripes*); also recorded from southern Arizona (Casey 1920: 74). The record from Connecticut (Britton 1920: 215, as *Platynus rubripes*) needs confirmation (see Krinsky and Oliver 2001: 4); that from Quebec (Lindroth 1966: 622, as *Agonum punctiforme*) based on specimens collected by Beaulne are very likely mislabeled.

#### Records.

**CAN**: ON **USA**: AL, AR, AZ, CA, DC, GA, IL, IN, KS, KY, MD, MI, MO, NC, NE, NH, NJ, NY, OH, OK, PA, SD, TN, TX, VA, WI, WV [CT]

### [quadrimaculatum group]

### Agonum quadrimaculatum

(Horn, 1885)

Platynus quadrimaculatus G.H. Horn, 1885a: 130. Type locality: «near Owensburgh [probably Owensboro, Davies County], K[entuck]y (banks of the Ohio, near Louisville)» (original citation). Holotype [by monotypy] (♀) in MCZ [# 34454].

#### Distribution.

This species is known from scattered localities from southwestern Indiana (Blatchley 1910: 123) south to southwestern Georgia (Fattig 1949: 34), southwestern Alabama (Casey 1920: 71; Mobile County, MCZ), southeastern Mississippi (George County, MCZ), north-central Louisiana (Winn Parish, MCZ), and northeastern Texas (Wood County, UASM). The record from Alberta (see Lindroth 1966: 631) must be in error after all; those from northwestern New York (Notman 1928: 235) and “Michigan” (Bousquet and Larochelle 1993: 261) need confirmation.

#### Records.

**USA**: AL, AR, GA, IN, KY, LA, MO, MS, TN, TX [MI, NY]

### Genus Platynus

Bonelli, 1810

Platynus Bonelli, 1810: Tabula Synoptica. Type species: *Carabus angusticollis* Fabricius, 1801 (= *Carabus assimilis* Paykull, 1790) by subsequent monotypy in Germar (1817: 303). Etymology. From the Greek *platyno* (to broaden, widen), possibly alluding to the large abdomen (“*abd*[omen] *latissimum*”) of the adults in the hands of Bonelli [masculine].

#### Diversity.

The number of species of *Platynus* is difficult to assess. Lorenz (2005: 429-434) assigned about 550 species to the genus arrayed in 14 subgenera, with about 95 species left unplaced. However, Liebherr (1998: 997) restricted the concept of *Colpodes* Macleay to three species from Java and suggested assigning the remaining species to “a broader *Platynus*.” Lorenz (2005: 416-418) listed about 270 species in *Colpodes* and if Liebherr’s advice is followed, this brings the total number of *Platynus* species to about 820. The Northern Hemisphere has about 155 species (19% of the world fauna) of the *Platynus*-“*Colpodes*-like” complex and 23 of them, placed in five subgenera, occur in North America.

#### Identification.

Liebherr and Will (1996: 303-307) published a key for the identification of all species found in North America. Bousquet (2012: 77-78) described a new species and issued a key for the species found east of the Mississippi River.

### Subgenus Microplatynus

Barr, 1982

Microplatynus Barr, 1982b: 98. Type species: *Platynus pecki* Barr, 1982 by original designation. Etymology. From the Greek *micros* (small, little) and the generic name *Platynus* [*q.v*.], alluding to the comparatively small size of adults of these *Platynus* species [masculine].

#### Diversity.

Two species in southwestern North America.

### Platynus agilis

LeConte, 1863

Platynus fragilis LeConte, 1854b: 41 [secondary homonym of *Platynus fragilis* (Mannerheim, 1853)]. Type locality: «in the mountains near Santa Isabel [San Bernardino County], in the southern part of California» (original citation). Three syntypes in MCZ [# 5739].
Platynus agilis LeConte, 1863b: 6. Replacement name for *Platynus fragilis* LeConte, 1854.

#### Distribution.

This species is restricted to southern California from the Santa Ynez Mountains to San Diego County (Liebherr and Will 1996: 318).

#### Records.

**USA**: CA

### Platynus pecki

Barr, 1982

Platynus pecki Barr, 1982b: 98. Type locality: «Monjeau Lookout northwest of Ruidoso (9,100 feet), Lincoln County, New Mexico» (original citation). Holotype (♂) location unknown (not in CNC).

#### Distribution.

This species is known only from the Sierra Blanca (Barr 1982b: 98), Capitan, and Sacramento Mountains (Liebherr and Will 1996: 318) in New Mexico.

#### Records.

**USA**: NM

### Subgenus Platynus

Bonelli, 1810

Platynus Bonelli, 1810: Tabula Synoptica. Type species: *Carabus angusticollis* Fabricius, 1801 (= *Carabus assimilis* Paykull, 1790) by subsequent monotypy in Germar (1817: 303).
Lyrophorus Chaudoir [in Chevrolat], 1846: 522. Type species: *Carabus angusticollis* Fabricius, 1801 (= *Carabus assimilis* Paykull, 1790) by monotypy. Etymology (original). From the Greek *lyra* (lyre) and *phoro* (to bear, carry) [masculine]. Note. This name is usually credited to Seidlitz (1872: 15 [Arten]) but it was made available by Chaudoir many years earlier.
Limodromus Motschulsky, 1850a: 70. Type species: *Carabus angusticollis* Fabricius, 1801 (= *Carabus assimilis* Paykull, 1790) designated by Motschulsky (1865: 316). Etymology. Probably from the Greek prefix *limo*- (meadow) and *dromos* (running) [masculine].

#### Diversity.

Sixteen species in the Nearctic (nine species, of which one extends into Mexico) and Palaearctic (seven species) Regions.

### Platynus brunneomarginatus

(Mannerheim, 1843)

Anchomenus brunneomarginatus Mannerheim, 1843 [after 28 March]: 196. Type locality: «California» (original citation), herein restricted to San Diego, San Diego County (see LeConte 1854b: 43, as *Platynus bicolor*). Syntype(s) location unknown.
Anchomenus rugiceps Mannerheim, 1843 [after 28 March]: 196 [*nomen dubium*]. Type locality: «California» (original citation). Syntype(s) location unknown (Lindroth 1966: 637). Synonymy established with doubt by LeConte (1863b: 6).
Anchomenus marginatus Ménétriés, 1843 [29 July]: 56 [secondary homonym of *Platynus marginatus* (Linnaeus, 1758)]. Type locality: «Californie» (original citation). Syntype(s) location unknown (Lindroth 1966: 637). Synonymy established by LeConte (1879b: 55).
Anchomenus ovipennis Motschulsky, 1845b: 339 [primary homonym of *Anchomenus ovipennis* Mannerheim, 1843]. Type locality: «Californie» (original citation). Syntype(s) location unknown (possibly in ZMMU). Synonymy established, under the name *Platynus rugiceps* (Mannerheim), by Motschulsky (1850a: 70).
Platynus bicolor LeConte, 1854b: 43 [secondary homonym of *Platynus bicolor* (Dejean, 1828)]. Type locality: «San Diego [San Diego County], California» (original citation). Syntype(s) in MCZ [# 5746]. Synonymy established by LeConte (1879b: 55).
Platynus cinctellus LeConte, 1854b: 43. Type locality: «San Francisco [San Francisco County], California» (original citation). Syntype(s) in MCZ [# 5745]. Synonymy established by LeConte (1863b: 6), confirmed by Lindroth (1966: 637).
Platynus bicoloratus Gemminger and Harold, 1868a: 368. Replacement name for *Platynus bicolor* LeConte, 1854.
Platynus tenebricosus Gemminger and Harold, 1868a: 377. Replacement name for *Platynus marginatus* (Ménétriés, 1843).

#### Distribution.

This species ranges from southern British Columbia, including Vancouver Island (Lindroth 1966: 639), south to the northern parts of the Baja California Peninsula (Horn 1894: 309), east to western New Mexico (Liebherr and Will 1996: 316) and northern Sonora (Bates 1884: 280).

#### Records.

**CAN**: BC (VCI) **USA**: AZ, CA (CHI), ID, NM, NV, OR, UT, WA – Mexico

### Platynus daviesi

Bousquet, 2012

Platynus daviesi Bousquet, 2012: 70. Type locality: «Powell Gap (2300’), Shenandoah N[ational] P[ark], Virg[inia]» (original citation). Holotype (♂) in CNC [# 23464].

#### Distribution.

This species occurs along the Appalachian Mountains from Connecticut to southern Pennsylvania, south to northwestern Alabama [see Bousquet 2012: Fig. 4]

#### Records.

**USA**: AL, CT, KY, MD, NC, NY, PA, TN, VA, WV

### Platynus decentis

(Say, 1823)

Feronia decentis Say, 1823a: 53. Type locality: «Marion [Plymouth County], Mass[achusetts]» (neotype label). Neotype (♀), designated by Lindroth and Freitag (1969: 346), in MCZ [# 33020]. Note. The specific name derives from the Latin adjective *decens*, -*entis* (decent, proper). All authors seen, starting with LeConte (1850: 205), have used the spelling *decens* (nominative singular) until Lindroth (1966: 636) returned to the spelling *decentis* (genitive singular). Although the ICZN (1999: Article 11.9.1.1) mandates that a Latin adjective is to be proposed in the nominative singular, it could be argued in this case that the adjective is used in a nominal form and so can be formulated in the genitive case (Miguel A. Alonso-Zarazaga pers. comm. 2012).
Anchomenus gagates Dejean, 1828: 107. Type locality: «Amérique septentrionale» (original citation). One syntype in MHNP (Lindroth 1955b: 19). Synonymy established by Dejean (1828: 107), confirmed by Lindroth (1955b: 19).
Anchomenus sinuatus Dejean, 1828: 108. Type locality: «Amérique septentrionale» (original citation), restricted to «Bethel [Oxford County], Maine» by Lindroth (1966: 636). One syntype in MHNP (Lindroth 1955b: 19). Synonymy established by Lindroth (1955b: 118).
Anchomenus depressus Haldeman, 1843b: 299. Type locality: southeastern Pennsylvania (Haldeman 1843a: 296). Syntype(s) presumably lost. Synonymy established, under the name *Platynus sinuatus* (Dejean), by LeConte (1879b: 54).
Anchomenus coracinus LeConte, 1846b: 220. Type locality: «NovEboraci [= New York]» (original citation). Syntype(s) in MCZ [# 5741]. Synonymy established by LeConte (1854b: 44), confirmed by Lindroth (1966: 636). Note. The specimen in the LeConte collection with the type label is also labeled “S.C.” and is probably not a syntype. Two other specimens in LeConte’s collection have pink labels (e.g., middle states, which included New York) and are probably syntypes.
Anchomenus oblongipennis Casey, 1920: 31. Type locality: «Marquette [Marquette County, Michigan], Lake Superior» (original citation). Lectotype (♀), designated by Lindroth (1975: 130), in USNM [# 47402]. Synonymy established by Lindroth (1966: 636).
Anchomenus turbidus Casey, 1920: 32. Type locality: «Bayfield [Bayfield County, Wisconsin], Lake Superior» (original citation for the lectotype). Lectotype (♂), designated by Lindroth (1975: 130), in USNM [47403]. Synonymy established by Lindroth (1966: 636).
Anchomenus iowanus Casey, 1920: 32. Type locality: «Iowa City [Johnson County], Iowa» (original citation). Lectotype (♀), designated by Lindroth (1975: 130), in USNM [# 47401]. Synonymy established by Lindroth (1966: 636).
Anchomenus aleneanus Casey, 1920: 36. Type locality: «Coeur d’Alene [Kootenai County], Idaho» (original citation). Lectotype (♀), designated by Lindroth (1975: 130), in USNM [# 47407]. Synonymy established by Hatch (1953: 135), confirmed by Lindroth (1966: 636).
Anchomenus pacatus Casey, 1920: 43. Type locality: «Peaceful Valley [Boulder County], Colorado» (original citation). Lectotype (♀), designated by Lindroth (1975: 130), in USNM [# 47416]. Synonymy established by Lindroth (1966: 636).
Anchomenus missourianus Casey, 1924: 81. Type locality: «Westminster [= probably Westminster College in Fulton, Callaway County], Missouri» (original citation). Lectotype (♀), designated by Lindroth (1975: 130), in USNM [# 47399]. Synonymy established by Lindroth (1966: 636).
Anchomenus albertanus Casey, 1924: 81. Type locality: «Edmonton, Alberta» (original citation). Lectotype (♀), designated by Lindroth (1975: 130), in USNM [# 47400]. Synonymy established by Lindroth (1966: 636).

#### Distribution.

This species ranges from Newfoundland (Lindroth 1955a: 118, as *Agonum sinuatum*) to south-central British Columbia (Lindroth 1966: 637), north to the coast of southern Alaska (Lindroth 1969a: 1120), south to western Oregon (Hatch 1953: 135), the Sangre de Cristo Mountains in New Mexico (Fall and Cockerell 1907: 159; Lindroth 1955a: 118), northeastern Kansas (Knaus 1901: 110; Ottawa County, CNC), east-central Alabama (Lee County, CNC), and southern Georgia (Torres and Ruberson 2006: 32). The records from “Louisiana” and “Texas” (Bousquet and Larochelle 1993: 262) need confirmation.

#### Records.

**FRA**: PM **CAN**: AB, BC, LB, MB, NB, NF, NS (CBI), NT, ON, PE, QC, SK **USA**: AK, AL, AR, CO, CT, DC, DE, GA, IA, ID, IL, IN, KS, KY, MA, MD, ME, MI, MN, MO, MS, MT, NC, ND, NE, NH, NJ, NM, NY, OH, OR, PA, RI, SC, SD, TN, UT, VA, VT, WA, WI, WV, WY [LA, TX]

### Platynus indecentis

Liebherr and Will, 1996

Platynus indecentis Liebherr and Will, 1996: 307. Type locality: «McLean Bog, Tompkins Co[unty], New York» (original citation). Holotype (♂) in CUIC [# 6964]. Note. The remark made in the “Note” section for *Feronia decentis* (see previous species) also applies in this case.

#### Distribution.

This species ranges from Nova Scotia (Majka et al. 2007: 11) to eastern Ohio, south to West Virginia and Maryland [see Liebherr and Will 1996: Fig. 18].

#### Records.

**CAN**: NB, NS, ON, QC **USA**: CT, MD, ME, NH, NY, OH, PA, RI, VT, WV

### Platynus opaculus

LeConte, 1863

Platynus opaculus LeConte, 1863c: 8. Type locality: «Ohio» (original citation). Syntype(s) in MCZ [# 5742]. Note. The specimen labeled “Type 5742” and “Lectotype” (by Liebherr, 1996; apparently not published) has a pink label and is probably not a syntype. The second specimen in LeConte’s collection has a yellow disc and is probably a syntype.
Anchomenus inquisitor Casey, 1920: 30. Type locality: «Marquette [Marquette County], Michigan» (original citation). Lectotype (♀), designated by Lindroth (1975: 130), in USNM [# 47904]. Synonymy established by Lindroth (1966: 641).
Anchomenus boopis Casey, 1920: 37. Type locality: «S[outh] Meriden [New Haven County], Connecticut» (original citation). Lectotype (♂), designated by Lindroth (1975: 130), in USNM [# 47411]. Synonymy established by Lindroth (1966: 641).

#### Distribution.

This species ranges from southwestern New Brunswick (Webster and Bousquet 2008: 22) to northeastern Iowa (Allamakee County, Doug A. Veal pers. comm. 2009), including the upper peninsula of Michigan (Casey 1920: 30, as *Anchomenus inquisitor*), south to west-central Illinois (Fulton County, Ken Karns pers. comm. 2009), southwestern Indiana (Blatchley 1910: 125), and west-central South Carolina (Ciegler 2000: 115).

#### Records.

**CAN**: NB, ON, QC **USA**: CT, IA, IL, IN, MA, MI, NH, NJ, NY, OH, PA, RI, SC, VT, WI

### Platynus ovipennis

(Mannerheim, 1843)

Anchomenus ovipennis Mannerheim, 1843: 196. Type locality: «California» (original citation), herein restricted to Redwood Creek, Humboldt County (see Casey 1920: 33, as *Anchomenus rugulifer*). Syntype(s) location unknown.
Anchomenus maurus Motschulsky, 1845b: 339. Type locality: «Californie» (original citation). Holotype [by monotypy] location unknown (possibly in ZMMU in collection Eschscholtz). Synonymy established by Hatch (1953: 136).
Anchomenus rotundipennis Motschulsky, 1845b: 340. Unnecessary replacement name for *Anchomenus ovipennis* Mannerheim, 1843.
Anchomenus rugulifer Casey, 1920: 33. Type locality: «Redwood Creek, Humboldt C[ounty], California» (original citation). Holotype [by monotypy] (♀) in USNM [# 47406]. Synonymy established by Liebherr (1991a: 118).
Anchomenus tersus Casey, 1920: 35. Type locality: «Redwood Creek, Humboldt Co[unty], California» (original citation). Lectotype (♂), designated by Liebherr (1991a: 118), in USNM [# 47409]. Synonymy established by Liebherr (1991a: 118).
Anchomenus arachnoides Casey, 1920: 35. Type locality: «Clackamas Co[unty], Oregon» (original citation for the lectotype). Lectotype (♂), designated by Lindroth (1975: 130), in USNM [# 47410]. Synonymy established by Hatch (1953: 136), confirmed by Lindroth (1966: 644).
Anchomenus similatus Casey, 1920: 35. Type locality: «Hydesville, Eel River Valley, Humboldt Co[unty], California» (original citation). Lectotype (♂), designated by Lindroth (1975: 130), in USNM [# 47405]. Synonymy established by Lindroth (1966: 644).

#### Distribution.

This species is found along the west coast from Vancouver Island (Lindroth 1966: 645) to at least west-central California (Marin County, CNC). The record from northern Colorado (Wickham 1902: 238; Armin 1963: 153) is in error.

#### Records.

**CAN**: BC (VCI) **USA**: CA, OR, WA

### Platynus parmarginatus

Hamilton, 1893

Platynus parmarginatus Hamilton, 1893: 305. Type locality: near Allegheny [Pennsylvania] (inferred from title of the paper). Lectotype (♂), designated by Lindroth (1966: 641), in CMNH.

#### Distribution.

This species ranges from southwestern Pennsylvania southwestwards to eastern Oklahoma [see Bousquet 2012: Fig. 4].

#### Records.

**USA**: AR, IL, IN, MO, OK, PA

### Platynus tenuicollis

(LeConte, 1846)

Anchomenus tenuicollis LeConte, 1846b: 222. Type locality: «ad cataractam Sanctae Mariae (Sault de S[ain]te Marie) [= Sault Sainte Marie, Ontario]» (original citation). One syntype in MCZ [# 5743].
Anchomenus marginatus LeConte, 1846b: 221 [primary homonym of *Anchomenus marginatus* Ménétriés, 1843]. Type locality: «NovEboraci [= New York]» (original citation). One syntype in MCZ [# 5744]. Synonymy established by Lindroth (1966: 641).
Limodromus acuticollis Motschulsky, 1865: 319. Type locality: «états méridionaux de l’Amérique du Nord» (original citation), restricted to «Washington, D.C.» by Liebherr (1991a: 119). Lectotype (♂), designated by Liebherr (1991a: 119), in ZMMU. Synonymy established by Liebherr (1991a: 119).
Colpodes approximatus Chaudoir, 1879: 370. Type locality: «du nord du Mexique» (original citation), restricted to «Jefferson County, Colorado» by Whitehead (1973: 212). Holotype [by monotypy] (♀) in MHNP. Synonymy established by Whitehead (1973: 212). Note. The type locality given by Chaudoir (1879: 371) indicates that the holotype was collected in northern Mexico. As far as known, *Platynus tenuicollis* has not been found in Mexico.
Platynus reflexus LeConte, 1879b: 55. Replacement name for *Platynus marginatus* (LeConte, 1846).
Anchomenus pennsylvanicus Casey, 1920: 40. Type locality: «Pennsylvania» (original citation). Lectotype (♀), designated by Lindroth (1975: 130), in USNM [# 47414]. Synonymy established by Lindroth (1966: 641).
Anchomenus distinguendus Casey, 1920: 40. Type locality: «Ridgeway, Ontario» (original citation). Lectotype (♂), designated by Lindroth (1975: 130), in USNM [# 47413]. Synonymy established by Lindroth (1966: 641).

#### Distribution.

The range of this species extends from Cape Breton Island (Lindroth 1966: 643) to the Black Hills in southwestern South Dakota (Kirk and Balsbaugh 1975: 24), south to northern Colorado (Armin 1963: 154; UASM), northwestern Texas (Wheeler County, CMNH), northeastern Oklahoma (Delaware County, CMNH), and southern South Carolina (Ciegler 2000: 115). The record from “Florida” (Bousquet and Larochelle 1993: 262) needs confirmation.

#### Records.

**CAN**: NB, NS (CBI), ON, PE, QC **USA**: AL, AR, CO, CT, DC, GA, IA, IL, IN, KY, LA, MA, MD, ME, MI, MN, MO, MS, NC, NH, NJ, NY, OH, OK, PA, RI, SC, SD, TN, TX, VA, VT, WI, WV [FL]

### Platynus trifoveolatus

Beutenmüller, 1903

Platynus trifoveolatus Beutenmüller, 1903: 516. Type locality: «Pigeon River, Retreat [Haywood County], western North Carolina» (original citation). Syntype(s) [6 originally cited] in USNM, AMNH [# 4], ANSP, and MCZ (collection Horn).

#### Distribution.

This species is known from high mountains along the Tennessee - North Carolina border (Barr 1969: 81); it was also reported from northeastern Georgia (Fattig 1949: 33).

#### Records.

**USA**: GA, NC, TN

### Subgenus Batenus

Motschulsky, 1865

Batenus Motschulsky, 1865: 317. Type species: *Harpalus livens* Gyllenhal, 1810 by original designation. Etymology. Uncertain, possibly from the Greek *bates* (walker) and the Latin suffix -*enus* (pertaining to) [masculine].
Platynidius Casey, 1920: 4. Type species: *Feronia hypolithos* Say, 1823 by original designation. Synonymy established by Liebherr (1990: 434).
Paranchomenus Casey, 1920: 30. Type species: *Platynus stygicus* LeConte, 1854 (= *Anchomenus mannerheimii* Dejean, 1828) designated by Bousquet and Larochelle (1993: 262). Synonymy established by Bousquet and Larochelle (1993: 262). Etymology. From the Greek *para* (near, beside) and the generic name *Anchomenus* [*q.v*.] [masculine].
Pseudoplatynus Habu, 1973b: 11. Type species: *Anchomenus magnus* Bates, 1873 by original designation. Synonymy established by Kryzhanovskij et al. (1995: 117). Etymology. From the Greek *pseudos* (fallacy, lie) and the generic name *Platynus* [*q.v*.] [masculine].
Platynomenus Ádám, 1996: 12. Type species: *Carabus scrobiculatus* Fabricius, 1801 by original designation. Synonymy established by Lorenz (1998: 406). Etymology. From the generic name *Platynus* [*q.v*.] and the Greek *menos* (force, strength) [masculine].

#### Diversity.

Forty-four species are recorded by Lorenz (2005: 429-430), five in the Nearctic Region, with one of them Holarctic, and 40 in the Palaearctic Region. A number of Palaearctic species considered as *incertae sedis* by Lorenz (2005: 433-434) have been listed in this subgenus by Bousquet (2003c: 462-464).

### Platynus angustatus

Dejean, 1828

Platynus angustatus Dejean, 1828: 98. Type locality: «Amérique septentrionale» (original citation), restricted to «Little Switzerland [Mitchell County], Blacks M[oun]t[ain]s (3400 ft), N[orth] Carol[ina]» by Lindroth (1966: 646). One syntype in MHNP (Lindroth 1955b: 18).
Platynus gracilentus Beutenmüller, 1903: 517. Type locality: «summit of the Black Mountains (5000-6717 feet), N[orth] C[arolina]» (original citation). Sixteen syntypes in AMNH [# 2] (Grossbeck 1912: 361), one in ANSP. Synonymy established by Lindroth (1955b: 18).
Platynidius enormis Casey, 1920: 8. Type locality: «Black M[oun]t[ain]s, North Carolina» (original citation). Holotype [by monotypy] (♀) in USNM [# 47385]. Synonymy established by Lindroth (1966: 646).
Platynidius aesopus Casey, 1920: 9. Type locality: «Adirondack M[oun]t[ain]s, New York» (original citation). Lectotype (♀), designated by Lindroth (1975: 130), in USNM [# 47386]. Synonymy established by Lindroth (1966: 646).
Platynidius rhombiceps Casey, 1920: 9. Type locality: «New Jersey» (original citation). Lectotype (♂), designated by Lindroth (1975: 130), in USNM [# 47389]. Synonymy established by Lindroth (1966: 646).
Platynidius cervicalis Casey, 1920: 10. Type locality: «Pennsylvania» (original citation). Lectotype (♂), designated by Lindroth (1975: 130), in USNM [# 47388]. Synonymy established by Lindroth (1966: 646).
Platynidius carolinensis Casey, 1920: 10. Type locality: «Black M[oun]t[ain]s, North Carolina» (original citation). Lectotype (♀), designated by Lindroth (1975: 130), in USNM [# 47387]. Synonymy established by Lindroth (1966: 646).

#### Distribution.

This species is found from the Adirondack Mountains in New York (Casey 1920: 9 as *Platynidius aesopus*; Liebherr and Song 2002: 135) to at least south-central Ohio (Will and Androw 1993), south to southwestern Alabama (Löding 1945: 19) and southeastern South Carolina (Ciegler 2000: 114). The records from “Indiana” (Schrock 1985: 353), “Illinois” (Thomas 1861: 635), and Mount Washington in New Hampshire (Bowditch 1896: 2) need confirmation.

#### Records.

**USA**: AL, GA, KY, MD, NC, NJ, NY, OH, PA, SC, TN, VA, WV [IL, IN, NH]

### Platynus cincticollis

(Say, 1823)

Feronia cincticollis Say, 1823a: 52. Type locality: «Phila[delphia] [Philadelphia County], P[ennsylvani]a» (neotype label). Neotype (♂), designated by Lindroth and Freitag (1969: 343), in MCZ [# 33019].
Feronia maculifrons Say, 1823b: 146 [*nomen dubium*]. Type locality: «Arkansa Territory» (original citation). Syntype(s) lost. Synonymy established with doubt by LeConte (1854b: 43).
Platynus blandus Germar, 1824: 12 [*nomen dubium*]. Type locality: «America septentrionali» (original citation). Syntype(s) probably lost (Lindroth 1966: 640). Synonymy established by Say (1830b: (4) [3]).
Anchomenus corvinus Dejean, 1828: 109. Type locality: «Amérique septentrionale» (original citation). One syntype in MHNP (Lindroth 1955b: 19). Synonymy established by Say (1830b: (4) [3]), confirmed by Lindroth (1955b: 19).
Anchomenus deplanatus Chaudoir, 1843b: 763. Type locality: «Nouvelle Orléans [Orleans Parish, Louisiana]» (original citation). Syntype(s) in MHNP. Synonymy established by LeConte (1854b: 43).
Anchomenus marginalis Haldeman, 1843b: 299. Type locality: southeastern Pennsylvania (Haldeman 1843a: 296). One possible syntype, a ♀ labeled “[pink disc] / marginalis praec. var. Hd. [handwritten] / cincticollis 4 [handwritten],” in MCZ (collection LeConte). Synonymy established, under the name *Platynus deplanatus* (Chaudoir), by LeConte (1846b: 221).

#### Distribution.

This species ranges from Sable Island (CNC) off the Nova Scotia coast to southeastern Minnesota (Gandhi et al. 2005: 932), south to southern Texas (Casey 1920: 40, as *Platynus cincticollis deplanatus*; Dajoz 2007: 23) and southern Florida (Peck and Thomas 1998: 23); also recorded from Bermuda (Hilburn and Gordon 1989: 677).

#### Records.

**CAN**: NS, ON, QC **USA**: AL, AR, CT, DC, DE, FL, GA, IA, IL, IN, KY, LA, MA, MD, MI, MN, MO, MS, NC, NH, NJ, NY, OH, OK, PA, RI, SC, TN, TX, VA, VT, WI, WV – Bermuda

### Platynus hypolithos

(Say, 1823)

Feronia hypolithos Say, 1823a: 59. Type locality: «Cleveland [Cuyahoga County], O[hio]» (neotype label). Neotype (♂), designated by Lindroth and Freitag (1969: 346), in MCZ [# 33021].
Platynus erythropus Dejean, 1828: 97. Type locality: «Amérique septentrionale» (original citation). Holotype [by monotypy] (♀) in MHNP (Lindroth 1955b: 18). Synonymy established by LeConte (1854b: 41), confirmed by Lindroth (1955b: 18).
Platynidius ontariensis Casey, 1920: 8. Type locality: «Ontario» (original citation). Lectotype (♂), designated by Lindroth (1975: 130), in USNM [# 47384]. Synonymy established by Lindroth (1966: 645).

#### Distribution.

This species is found from Vermont (Rutland County, CNC) to the lower peninsula of Michigan (Hubbard and Schwarz 1878: 644), including southern Ontario (Lindroth 1966: 646), south to east-central Kentucky (Powell County, CNC). The record from “Iowa” (Bousquet and Larochelle 1993: 263) needs confirmation; that from “Virginia” (Bousquet and Larochelle 1993: 263) is probably in error (Richard L. Hoffman pers. comm. 2009).

#### Records.

**CAN**: ON **USA**: IN, KY, MA, MD, MI, NY, OH, PA, VT, WV [IA]

#### Note.

Morvan (2000: 76) treated this taxon as a subspecies of *Platynus angustatus* Dejean.

### Platynus mannerheimii

(Dejean, 1828)

Anchomenus mannerheimii Dejean, 1828: 104. Type locality: «Finlande» (original citation). Syntype(s) probably in MHNP.
Anchomenus morio Gebler, 1847: 325. Type locality: «Flusse Ters [= Ters River in the Kuznetsky Alatau Mountain Range, eastern border of Kemerovo Oblast in southwestern Siberia]» (original citation). Syntype(s) [2 originally cited] probably in ZILR. Synonymy established by Chaudoir (1850b: 102).
Anchomenus melas Gebler, 1848: 68. Unnecessary replacement name for *Anchomenus morio* Gebler, 1847.
Platynus stygicus LeConte, 1854b: 42. Type locality: «Sault S[ain]te Marie [Ontario]» (original citation). Holotype [by monotypy] (♂) in MCZ [# 5740]. Synonymy established by Lindroth (1966: 634).
Anchomenus octofoveolatus Mäklin, 1855: 34. Type locality: «Kadjak [Alaska]» (original citation). Lectotype, designated by Silfverberg (1987: 21), in ZMH. Synonymy established, under the name *Platynus mannerheimii stygicus* LeConte, by Lindroth (1954b: 138).
Limodromus interstitialis Motschulsky, 1865: 318. Type locality: «Kadiak [Alaska]» (original citation). Three syntypes in ZMMU (Keleinikova 1976: 201). Synonymy established by Lindroth (1966: 634).

#### Distribution.

This Holarctic species ranges from Newfoundland (Lindroth 1955a: 119) to central British Columbia and Alaska (Lindroth 1966: 636), south to northeastern Minnesota (Casey 1920: 36, as *Platynus stygicus*), central Wisconsin (Messer 2010: 43), and northern West Virginia (Preston County, CMNH). In the Palaearctic Region, the species is found from the Far East to Finland (Bousquet 2003c: 463).

#### Records.

**FRA**: PM **CAN**: AB, BC, LB, MB, NB, NF, NS (CBI), NT, ON, PE, QC, SK, YT **USA**: AK, CT, ME, MI, MN, NH, NY, VT, WI, WV – **Holarctic**

### Platynus prognathus

Van Dyke, 1926

Platynus prognathus Van Dyke, 1926a: 119. Type locality: «S[ain]t Simon[s] Island, Georgia» (original citation). Holotype (♀) in CAS [# 1861]. Note. Van Dyke (1926a: 119) recorded the type locality as «St. Simon Island, Okefinokee Swamp, Georgia». I have not been able to locate such island in the Okefinokee Swamp in Georgia.

#### Distribution.

This characteristic species is known for sure only from the holotype. However, Fattig (1949) recorded it also from Dalton, Georgia but the specimen(s) was apparently destroyed (Liebherr 1990: 432).

#### Records.

**USA**: GA

### Subgenus Glyptolenopsis

Perrault, 1991

Glyptolenopsis Perrault, 1991b: 48. Type species: *Glyptolenopsis degallieri* Perrault, 1991 by original designation. Etymology. From the generic name *Glyptolenus* and the Greek suffix -*opsis* (likeness), alluding to the resemblance of adults of this group to those of *Glyptolenus* [feminine].

#### Diversity.

Thirty-six species in Middle America (28 species, of which one extends into southern North America and two into South America) and the West Indies (eight species).

#### Identification.

Liebherr (1992) revised the species.

### Platynus ovatulus

(Bates, 1884)

Anchomenus ovatulus Bates, 1884: 281. Type locality: «Chihuahua, Mexico» (original citation). Lectotype (♀), designated by Whitehead (1973: 205), in BMNH.
Platynus languidus G.H. Horn, 1892c: 42. Type locality: «southern Arizona» (original citation), restricted to «Cochise Stronghold, Dragoon M[oun]t[ai]ns, Cochise Co[unty]» by Liebherr (1991a: 121). Lectotype (♂), designated by Liebherr (1991a: 121), in MCZ [# 2888]. Synonymy established by Liebherr (1991a: 121).

#### Distribution.

This species is known from southeastern Arizona and the northern Sierra Madre Occidental in Sonora and Chihuahua [see Liebherr 1992: Fig. 160].

#### Records.

**USA**: AZ – Mexico

### Subgenus Trapezodera

Casey, 1920

Trapezodera Casey, 1920: 19. Type species: *Colpodes aeneicauda* Bates, 1891 by original designation. Etymology. From the Greek *trapezion* (an irregular four-shaped figure, by extension trapezoid) and *dere* (neck, by extension pronotum), alluding to the shape of the pronotum (“trapezoidal outline of the prothorax”) of the adult [feminine].

#### Diversity.

Four species are assigned to this subgenus, one from southwestern United States and three from Mexico (Liebherr and Will 1996: 316).

### Platynus cohni

Liebherr and Will, 1996

Platynus cohni Liebherr and Will, 1996: 313. Type locality: «Madera C[an]y[o]n (4400’), Santa Rita M[oun]t[ain]s, Pima Co[unty], Arizona» (original citation). Holotype (♂) in CUIC [# 6965].

#### Distribution.

This species is known from several localities in southeastern Arizona [see Liebherr and Will 1996: Fig. 24].

#### Records.

**USA**: AZ

### Subgenus Dyscolus

Dejean, 1831

Dyscolus Dejean, 1831: 279, 437. Type species: *Dyscolus memnonius* Dejean, 1831 designated by Hope (1838: 105). Etymology (original). From the Greek *dyscolos* (difficult), probably alluding to the difficulty Dejean had to find the systematic place of the three species he had before him (“*formé sur des insectes qui *... *semblent *... *former le passage entre les Féroniens et les Troncatipennes, et que je n’ai de même placé dans cette tribu que faute de pouvoir les mettre convenablement ailleurs*”) [masculine].
Ophryodactylus Chaudoir, 1842: 832. Type species: *Ophryodactylus subviolaceus* Chaudoir, 1842 by monotypy. Synonymy established by Moret (1989: 143). Etymology (original). From the Greek *ophrys* (brow, by extension turned-up margin) and *dactylos* (finger), alluding to the furrowed sides of the protarsomeres (“*tarses antérieurs *... *tous fortement sillonnés sur les côtés, ce qui fait paraître ceux-ci relevés en bourrelet*”) of the adults [masculine].
Paranomus Chaudoir, 1842: 835. Type species: *Paranomus lherminieri* Chaudoir, 1842 by monotypy. Synonymy established by Moret (1989: 143). Etymology (original). From the Greek *para* (beside, near, outside) and *nomos* (usage, law) [masculine].
Pleurasoma Guérin-Méneville, 1844b: 9. Type species: *Pleurasoma sulcatum* Guérin-Méneville, 1844 by monotypy. Synonymy established by Moret (1989: 143). Etymology (original). From the Greek *pleura* (side) and *soma* (body) [neuter]. Note. This genus was described again the same year under the spelling *Pleurosoma* by Guérin-Méneville (1844d: 1).
Metallosomus Motschulsky, 1865: 304. Type species: *Metallosomus virescens* Motschulsky, 1865 designated by Jeannel (1948a: 515). Synonymy established by Moret (1989: 143). Etymology. Probably from the Greek *metallon* (metal) and *soma* (body) and alluding to the metallic coloration of the adults in the hands of Motschulsky [masculine].
Omiastus Motschulsky, 1865: 306. Type species: *Omiastus rutilans* Motschulsky, 1865 designated by Lorenz (1998: 129). Synonymy established by Perrault (1992: 276).

#### Diversity.

About 320 species are listed in this subgenus by Lorenz (2005: 430-433). Of these only five unrelated species are found in southern United States, two of them being endemic (*Platynus falli* and *Platynus rufiventris*). The other species of the subgenus occur in the Neotropical Region, including the West Indies and the Galapagos Islands.

### [cazieri group]

### Platynus cazieri

Liebherr and Will, 1996

Platynus cazieri Liebherr and Will, 1996: 312. Type locality: «El Mirador Ranch, 4 mi[les] N[orth]W[est] Sasabe, Baboquivari M[oun]t[ain]s, Pima Co[unty], Arizona» (original citation). Holotype (♂) in AMNH [# 1610].

#### Distribution.

This species is known only from Pima and Santa Cruz Counties in southeastern Arizona and from one specimen without precise locality collected in Mexico [see Liebherr and Will 1996: Fig. 24].

#### Records.

**USA**: AZ – Mexico

### [falli group]

### Platynus falli

(Darlington, 1936)

Colpodes falli Darlington, 1936e: 152. Type locality: «Baboquivari M[oun]t[ain]s, Arizona» (original citation). Holotype (♂) in MCZ [# 34577].

#### Distribution.

This species is known from Pima, Cochise, and Santa Cruz Counties in southern Arizona (Liebherr and Will 1996: 318).

#### Records.

**USA**: AZ

#### Note.

Whitehead (1973: 196) stated that adults of this form are possibly conspecific with those of *Platynus segregatus* (Bates), a species known from the states of Michoacán, Colima, Morelos, Guerrero, and Oaxaca.

### [lyratus group]

### Platynus lyratus

(Chaudoir, 1879)

Colpodes lyratus Chaudoir, 1879: 347. Type locality: «Capulalpam, Mexique» (original citation for the lectotype). Lectotype (♂), designated by Whitehead (1973: 200), in MHNP.
Anchomenus pinalicus Casey, 1920: 42. Type locality: «Pinal M[oun]t[ain]s [Gila County], Arizona» (original citation). Holotype [by monotypy] (♂) in USNM [# 47415]. Synonymy established by Whitehead (1973: 200).

#### Distribution.

This species ranges from southern Utah, eastern Arizona, and western New Mexico south to Oaxaca in Mexico; it also occurs throughout the Baja California Peninsula (Liebherr and Will 1996: 318).

#### Records.

**USA**: AZ, NM, UT – Mexico

### [megalops group]

### Platynus megalops

(Bates, 1882)

Colpodes megalops Bates, 1882a: 116. Type locality: «Guanajuato, Mexico» (original citation). Lectotype (♂), designated by Whitehead (1973: 200), in BMNH.
Platynus longiceps Schaeffer, 1910: 394. Type locality: «Huachuca M[oun]t[ain]s, Arizona» (original citation). Lectotype (♀), designated by Whitehead (1973: 200), in USNM [# 42502]. Synonymy established by Whitehead (1973: 200).

#### Distribution.

The range of this species extends from southern Arizona and western Texas (Jeff Davis County, CMNH) south to the state of Oaxaca (Whitehead 1973: 201).

#### Records.

**USA**: AZ, TX – Mexico

### [rufiventris group]

### Platynus rufiventris

(Van Dyke, 1926)

Colpodes rufiventris Van Dyke, 1926a: 120. Type locality: «Mount Washington (6000 feet), near Nogales [Santa Cruz County], Arizona» (original citation). Holotype (♂) in CAS [# 1862].

#### Distribution.

This species is yet known only from Cochise, Pima, and Santa Cruz Counties in southern Arizona (Liebherr and Will 1996: 318). The record from southwestern Utah (Tanner 1928: 270) is probably in error.

#### Records.

**USA**: AZ

### Genus Metacolpodes

Jeannel, 1948

Metacolpodes Jeannel, 1948a: 516. Type species: *Colpodes buchanani* Hope, 1831 by original designation. Etymology. From the Greek *meta* (between, among, beyond) and the generic name *Colpodes* [masculine].

#### Diversity.

Twenty-three species in the Palaearctic (11 Asian species) and Oriental (15 species) Regions are listed in this genus by Lorenz (2005: 423). Liebherr (2005), on the other hand, redefined cladistically the genus to include seven Pacific and Asian species. One of these species is adventive in western North America.

#### Identification.

The sole species present in North America is included in Hatch’s (1953: 132) monograph on the beetles of the Pacific Northwest. It is also described in length by Habu (1978: 126-128).

### Metacolpodes buchanani

(Hope, 1831)

Colpodes buchanani Hope, 1831: 21 (as *buchannani*). Type locality: Nepal (inferred from title of the paper). Syntype(s) probably in BMNH. Note. The spelling *buchanani*, used by most authors, is an incorrect subsequent spelling, introduced by Lacordaire (1854: 361), in prevailing usage and attributed to the publication of the original spelling; therefore it is deemed to be the correct original spelling (ICZN 1999: Article 33.3.1).
Agonum sargentorum Malkin and Hatch, 1952: 107. Type locality: «Eugene [Lane County], Oregon» (original citation). Holotype (♂) in USNM. Synonymy established by Malkin and Hatch (1953: 134).

#### Distribution.

This adventive species is known in North America from a few specimens collected in southern British Columbia (Jarrett and Scudder 2001: 382), southwestern Washington (Pacific County, Peter W. Messer pers. comm. 2011), western Oregon (Hatch 1953: 132), and northern Idaho (Liebherr and Will 1996: 303). The first specimen collected on this continent was found in 1931 in Portland, Oregon (Hatch 1953: 132). The species is also adventive in Hawaii (Liebherr and Zimmerman 2000: 466).

#### Records.

**CAN**: BC **USA**: ID, OR, WA – **Adventive**

### Tribe Perigonini

Horn, 1881

Trechichinae Bates, 1873: 224, 281 [*nomen oblitum*, see Bouchard et al. (2011: 140, 836-837)]. Type genus: *Trechicus* LeConte, 1853.
Perigonae G.H. Horn, 1881: 143 [*nomen protectum*]. Type genus: *Perigona* Laporte, 1835.

#### Diversity.

About 120 species (Lorenz 2005: 438-439), mainly in the tropical regions, arrayed in five genera. Only one endemic species (*Perigona pallipennis*) is found in North America and one in Europe (*Galiciotyphlotes weberi* Assmann from northwestern Spain).

### Genus Perigona

Laporte, 1835

Perigona Laporte, 1835: 151. Type species: *Perigona pallida* Laporte, 1835 by monotypy. Etymology. Uncertain, either from the Greek *peri* (around, near, very) and *gone* (offspring) or from Perigone, daughter of the brigand Sinis. According to Desmarest (1851: 104), the name derives from the Greek *peri* and *gonia* (angle) [feminine].

#### Diversity.

Excluding the subcosmopolitan *Perigona nigriceps*, about 105 species (Lorenz 2005: 438-439) in the temperate, subtropical, and tropical areas of the Nearctic (one species), Neotropical (twelve species), Australian (about 17 species), Oriental (about 30 species), eastern Palaearctic (five species), and Afrotropical (about 40 species) Regions. These species are arrayed in 12 subgenera: *Cryptoperigona* Perrault (one Neotropical species), *Euripogena* Basilewsky (four Afrotropical species), *Euryperigona* Jeannel (three species in the Philippines, Indonesia, and New Guinea), *Hirtoperigona* Perrault^9^ (two Oriental species), *Neoperigona* Perrault (three Neotropical species), *Perigona* s.str. (57 species in the Old World as well as the Neotropical and Australian Regions), *Perigonillus* Jeannel (three Afrotropical species), *Ripogena* Jeannel (ten Afrotropical species), *Trechicus* (18 species), *Typhlonestra* Jeannel (one Oriental and one Afrotropical species), *Xenogona* Jeannel (one Afrotropical species), and *Xenogonilla* Basilewsky (one Afrotropical species).

### Subgenus Trechicus

LeConte, 1853

Trechicus LeConte, 1853c: 386. Type species: *Trechicus umbripennis* LeConte, 1853 (= *Bembidium nigriceps* Dejean, 1831) designated by Jeannel (1926: 247). Etymology. From the generic name *Trechus* [*q.v*.] and the Greek *eicon* (image, figure), probably alluding to the resemblance of the adults to those of *Trechus* (“have very much the appearance of minute Trechus”) [masculine].
Extromus Péringuey, 1896: 584, 586. Type species: *Extromus pusillus* Péringuey, 1896 (= *Bembidium nigriceps* Dejean, 1831) by monotypy.

#### Diversity.

Eighteen species in the Nearctic (one endemic species), Australian (seven endemic species), and Afrotropical (ten endemic species) Regions and one (*Perigona nigriceps*) subcosmopolitan species.

#### Identification.

Bousquet (1987a: 128) commented on the structural differences between the two species found in North America.

#### Taxonomic Note.

*Hirtoperigona* Perrault is placed in synonymy with *Trechicus* in Lorenz (2005: 438). However, the position of the median group of setae of the umbilical series in the type species (*Perigona tronqueti* Perrault from Sri Lanka) and in *Perigona pubescens* Jeannel (from Vietnam) suggests that members of *Hirtoperigona* are more closely related to *Perigona* s.str. than to *Trechicus*. Perrault (1988) placed a third species (*Perigona hirtella* Basilewsky from Africa) in *Hirtoperigona* on the basis of the original description alone; according to Basilewsky (1989: 447), the species belongs to *Trechicus*.

The relationship of the endemic North American species (*Perigona pallipennis* LeConte) has not been studied and, in my opinion, it may be more closely related to members of *Cryptoperigona* than to those of *Trechicus*.

### Perigona nigriceps

(Dejean, 1831)

Bembidium nigriceps Dejean, 1831: 44. Type locality: «Amérique septentrionale» (original citation), restricted to «Georgia» by Lindroth (1968: 651). Holotype [by monotypy] in MHNP (Lindroth 1955b: 22; Erwin 1974a: 126).
Trechicus umbripennis LeConte, 1853c: 386. Type locality: «Georgia; Carolina» (original citation). Three syntypes in MCZ [# 5791]. Synonymy established by Horn (1875: 126), confirmed by Bousquet (1987a: 128).
Polyderis testaceolimbata Motschulsky, 1862b: 33. Type locality: «environs de Mobile [Mobile County, Alabama]» (original citation). Lectotype, designated by Erwin (1974a: 127), in ZMMU. Synonymy established by Erwin (1974a: 127).
Polyderis glabrella Motschulsky, 1862b: 34. Type locality: «Mobile [Mobile County, Alabama]» (original citation). Lectotype, designated by Erwin (1974a: 127), in ZMMU. Synonymy established by Erwin (1974a: 127).

#### Distribution.

This species is subcosmopolitan. It is adventive in North America where it is known from Nova Scotia (Pictou County, CNC) to eastern Iowa (Muscatine County, Doug A. Veal pers. comm. 2009), south to east-central Texas (Riley 2011), central Louisiana (Grant Parish, CNC) and southern Florida (Peck and Thomas 1998: 23) in the east, and from Oregon (Hatch 1953: 147) to southern California (Fall 1901a: 47) in the west. The first inventoried specimen collected on this continent was found prior to 1853 (LeConte 1853c: 386, as *Trechicus umbripennis*). Basilewsky (1953a: 108) believed the species originated along the coast of the Indian Ocean where it is very abundant in rubbish and groundnut.

#### Records.

**CAN**: NB, NS, ON, QC **USA**: AL, AR, CA, CT, DC, FL, GA, IA, IL, IN, KY, LA, MA, MD, ME, MI, MS, NC, NE, NH, NJ, NY, OH, OR, PA, RI, SC, TX, VA, VT, WI, WV – **Adventive**

**Figure 38.**
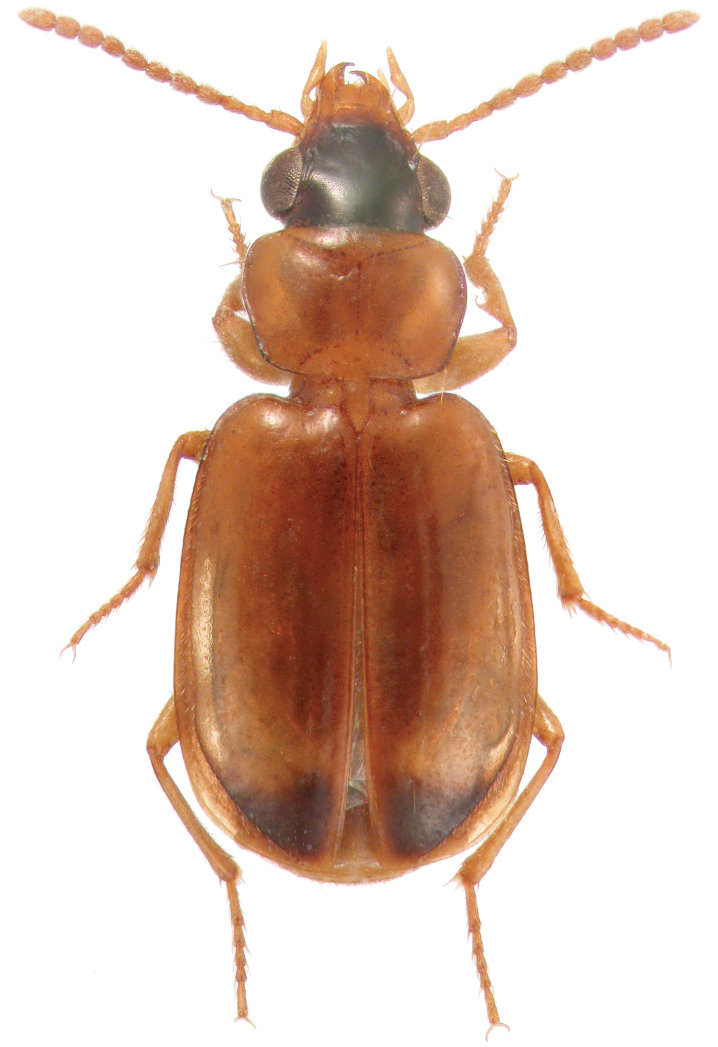
*Perigona nigriceps* (Dejean). While most adventive carabid species have been introduced from Europe, this species, which is now subcosmopolitan, is probably native from southern Asia where it is common around the Indian Ocean. Contrary to European adventive species which have been introduced to North America through ballast and nursery stocks, this species was probably transported around the world among stored products on ships.

### Perigona pallipennis

(LeConte, 1853)

Trechicus pallipennis LeConte, 1853c: 386. Type locality: «Carolina» (original citation). One syntype in MCZ [# 5792].
Perigona pallidipennis Csiki, 1931: 898. Unjustified emendation of *Perigona pallipennis* (LeConte, 1853).

#### Distribution.

The range of this species extends from southwestern Maine (Nelson 1991: 284) to eastern Iowa (Wickham 1911b: 7), including southernmost Ontario, south to “Missouri,” “Alabama” (Bousquet 1987a: 127-128), and northern Florida (Hamilton, Okaloosa, and Taylor Counties, CNC).

#### Records.

**CAN**: ON **USA**: AL, DC, FL, GA, IA, IL, IN, MD, ME, MI, MO, MS, NC, NJ, NY, OH, PA, SC, VA

### Tribe Atranini

Horn, 1881

Atrani G.H. Horn, 1881: 145. Type genus: *Atranus* LeConte, 1847.

#### Diversity.

One genus with two species in the Nearctic and Palaearctic Regions.

### Genus Atranus

LeConte, 1847

Atranus LeConte, 1847: 438. Type species: *Anchomenus pubescens* Dejean, 1828 by monotypy. Etymology (original). From the Greek *a* (privative) and *tranos* (clear, distinct), possibly alluding to the obscure relationships of the genus to the eyes of LeConte [masculine].

#### Diversity.

Two species in the temperate regions of eastern North America (*Atranus pubescens*) and Europe (*Atranus ruficollis* Gautier des Cottes).

#### Identification.

Lindroth (1968: 649) commented on the structural differences between the two species.

### Atranus pubescens

(Dejean, 1828)

Anchomenus pubescens Dejean, 1828: 122. Type locality: «Amérique septentrionale» (original citation), restricted to «White Sulphur Springs [Greenbrier County], W[est] V[irgini]a» by Lindroth (1966: 648). Holotype [by monotypy] in MHNP (Lindroth 1955b: 22).
Anchomenus obconicus Haldeman, 1843b: 299. Type locality: southeastern Pennsylvania (Haldeman 1843a: 296). Syntype(s) presumably lost. Synonymy established by LeConte (1847: 439).
Atranus pallescens Casey, 1913: 172. Type locality: «Fairmont Park, Philadelphia [Philadelphia County, Pennsylvania]» (original citation). Lectotype (♂), designated by Lindroth (1975: 130), in USNM [# 47616]. Synonymy established (as aberration) by Csiki (1931: 740), confirmed by Lindroth (1966: 648).

#### Distribution.

The range of this species extends from central Nova Scotia (Hants County, Christopher G. Majka pers. comm. 2008) to southwestern Nebraska (Foster F. Purrington pers. comm. 2010), south to “Texas” (Barr 1964: 3; Lindroth 1968: 649) and west-central South Carolina (Ciegler 2000: 108).

#### Records.

**CAN**: NB, NS, ON, QC **USA**: AL, AR, CT, DC, GA, IA, IL, IN, KS, KY, LA, MA, MD, ME, MI, MO, MS, NC, NE, NH, NJ, NY, OH, OK, PA, SC, TN, TX, VA, VT, WI, WV

**Figure 39.**
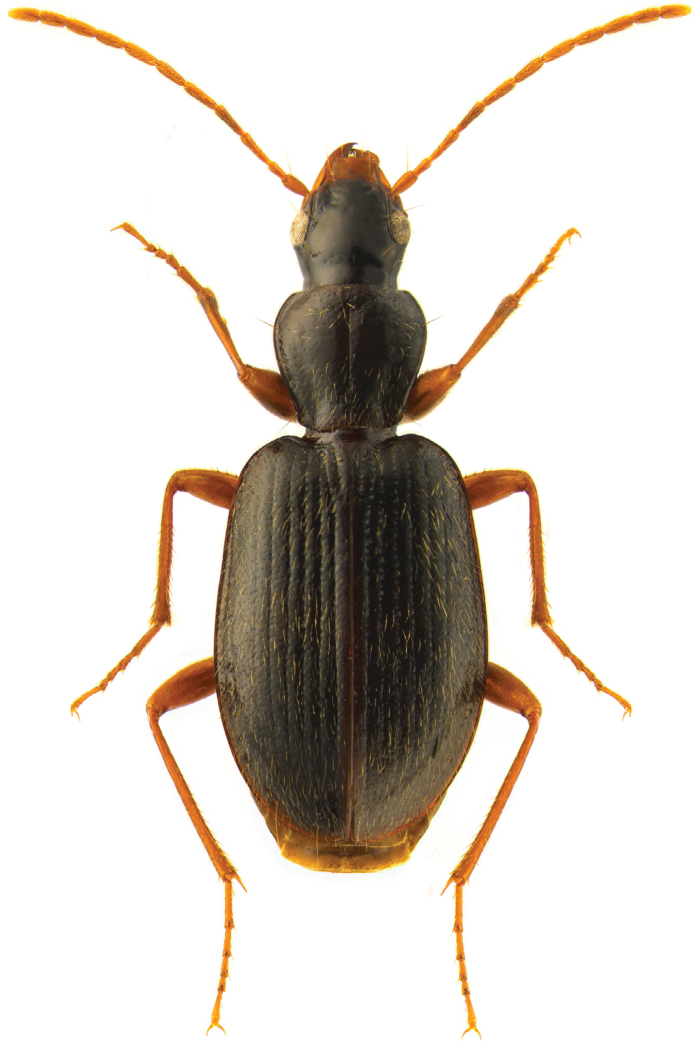
*Atranus pubescens* (Dejean). This species is found over eastern North America and the only other species in the genus, *Atranus ruficollis*, occurs in southern Europe and the Middle East. The only other carabid genus with a similar vicariant distribution is *Olisthopus* although one European species, *Olisthopus sturmii*, extends to the Far East. Most amphiatlantic vicariant carabid groups, such as *Pseudanophthalmus*-*Duvaliopsis*, are at least generically distinct.

### Tribe Lachnophorini

LeConte, 1853

Lachnophori LeConte, 1853c: 370, 375. Type genus: *Lachnophorus* Dejean, 1831.
Anchonodérides Lacordaire, 1854: 373. Type genus: *Anchonoderus* Reiche, 1843.
Eucaeri LeConte, 1861a: 21. Type genus: *Eucaerus* LeConte, 1853.
Egini G.H. Horn, 1881: 125, 152. Type genus: *Ega* Laporte, 1834.
Selinini Jeannel, 1948a: 743. Type genus: *Selina* Motschulsky, 1858.

#### Diversity.

About 115 species (Lorenz 2005: 440-441, as Lachnophorina) in the Nearctic and Neotropical Regions, arrayed in 11 genera, and one species (*Selina westermanni* Motschulsky) in the Oriental and Afrotropical Regions.

#### Taxonomic Note.

Ball and Bousquet (2000: 108) recognized two groups of genera within this tribe. The eucaerine complex comprising *Eucaerus*, *Aporesthus* Bates, *Amphitasus* Bates, and *Asklepia* Liebke and the lachnophorine complex encompassing *Lachnophorus*, *Euphorticus*, *Calybe*, *Anchonoderus*, and *Selina*. Subsequently to their investigation, three new genera have been proposed: *Pseudophorticus* Erwin (four species), *Guatemalteca* Erwin (one species), and *Quammenis* Erwin (one species).

### Genus Anchonoderus

Reiche, 1843

Anchonoderus Reiche, 1843a: 38. Type species: *Platynus elegans* Brullé, 1838 by original designation. Etymology. From the Greek *anchone* (strangling) and *dere* (neck, by extension pronotum), alluding to the cordate shape of the pronotum (“*thorax transversus valde cordatus*”) of the adult [masculine].
Axylosius Liebke, 1936: 461. Type species: *Lachnophorus humeralis* Bates, 1883 by original designation. Synonymy established by Liebherr (1988: 24).

#### Diversity.

Western Hemisphere, with 26 species (Lorenz 2005: 440) in the Nearctic (two species) and Neotropical (24 species) Regions.

#### Identification.

Schaeffer (1910: 395) commented on the structural differences between the two species found in North America.

### Anchonoderus quadrinotatus

Horn, 1878

Anchonoderus quadrinotatus G.H. Horn, 1878b: 53. Type locality: «Texas» (original citation). Two syntypes [2 originally cited] in MCZ [# 8182].

#### Distribution.

This species is known only from Texas (Val Verde County, CNC).

#### Records.

**USA**: TX

### Anchonoderus schaefferi

Liebke, 1928

Anchonoderus unicolor Schaeffer, 1910: 395 [primary homonym of *Anchonoderus unicolor* Chaudoir, 1850]. Type locality: «New Braunfels [Comal County], Tex[as]» (original citation). Holotype (♂) in USNM [# 56139].
Anchonoderus schaefferi Liebke, 1928: 128. Replacement name for *Anchonoderus unicolor* Schaeffer, 1910.

#### Distribution.

This species is known from southeastern Arizona (Graham County, CNC) and southern Texas (Schaeffer 1910: 395).

#### Records.

**USA**: AZ, TX

### Genus Lachnophorus

Dejean, 1831

Lachnophorus Dejean, 1831: 28. Type species: *Lachnophorus pilosus* Dejean, 1831 designated by Desmarest (1851: 189). Etymology (original). From the Greek *lachnos* (soft hair, down) and *phoro* (to bear, carry), alluding to the pubescence covering the dorsal surface (“*corps pubescent*”) of the adults [masculine].
Aretaonus Liebke, 1936: 461, 463. Type species: *Lachnophorus elegantulus* Mannerheim, 1843 by original designation. Synonymy established by Liebherr (1988: 34).

#### Diversity.

Western Hemisphere, with about 35 species in temperate, subtropical, and tropical areas of the Nearctic (one species only) and Neotropical Regions.

#### Taxonomic Note.

According to Liebherr (1988: 34), *Stigmaphorus* Motschulsky, 1855 (type species: *Stigmaphorus tessellatus* Motschulsky, 1855), considered a junior synonym of *Lachnophorus* by several authors, “should not be considered the same as *Lachnophorus*.”

### Lachnophorus elegantulus

Mannerheim, 1843

Lachnophorus elegantulus Mannerheim, 1843 [after 28 March]: 215. Type locality: «California» (original citation). Syntype(s) location unknown (possibly in ZMH).
Bembidium mediosignatum Ménétriés, 1843 [29 July]: 62. Type locality: «Californie» (original citation). Syntype(s) location unknown (possibly in ZMH). Synonymy established by Motschulsky (1845a: 29). Note. This name may be older than *Lachnophorus elegantulus* but is not in “prevailing usage” (see *Principle of priority* under “Nomenclature” section).
Lachnophorus sculptifrons Bates, 1878a: 604. Type locality: «Chinautla (4100 feet) [and] Chontales, Guatemala» (original citation). Syntype(s) probably in BMNH. Synonymy established by Horn (1886b: xii). Note. Liebke (1936: 463) retained this form as a valid species.
Lachnophorus elegantulus ocularis Casey, 1920: 225. Type locality: «Texas» (original citation). Holotype [by monotypy] in USNM [# 47615]. Synonymy established by Erwin et al. (1977: 4.32).

#### Distribution.

This species ranges from Kansas (LeConte 1858a: 28; Horn 1872c: 385) to southwestern Oregon (Hatch 1953: 146; Jackson County, MCZ, UASM), south to southern California (Moore 1937: 11; San Diego County, CNC, MCZ) and Costa Rica (Liebke 1936: 463).

#### Records.

**USA**: AZ, CA, KS, NM, OK, OR, TX, UT – Costa Rica, Guatemala, Mexico, Nicaragua

### Genus Euphorticus

Horn, 1881

Euphorticus G.H. Horn, 1881: 144. Type species: *Lachnophorus pubescens* Dejean, 1831 by monotypy. Etymology. From the Greek *eu* (good, beautiful) and *phorticos* (of bearing) [masculine].

#### Diversity.

Four species in temperate, subtropical, and tropical areas of the Nearctic (two species) and Neotropical (three species) Regions.

#### Identification.

Horn (1891: 38) commented on the structural differences between the two species found in North America.

### Euphorticus occidentalis

Horn, 1891

Euphorticus occidentalis G.H. Horn, 1891: 38. Type locality: «near Los Angeles [Los Angeles County], Cal[ifornia]» (original citation). One syntype in MCZ [# 35339].

#### Distribution.

This species is known so far only from southwestern California (Horn 1891: 38; Fall 1901a: 47; Moore 1937: 11).

#### Records.

**USA**: CA

### Euphorticus pubescens

(Dejean, 1831)

Lachnophorus pubescens Dejean, 1831: 30. Type locality: «Amérique septentrionale» (original citation). One syntype in MHNP (Lindroth 1955b: 22).
Lachnophorus niger Gory, 1833: 245. Type locality: «Cayenne [French Guiana]» (original citation). Syntype(s) probably in MHNP (collection Chaudoir). Synonymy established by Bates (1883a: 156).
Lachnophorus laevicollis Reiche, 1843c: 180. Type locality: «provincia Novae Granatae [= present day Panama and Colombia]» (original citation). Syntype(s) probably in MHNP (collection Chaudoir). Synonymy established by Bates (1883a: 156).
Euphorticus pubescens var. *aeneolus* Bates, 1883a: 156. Type locality: «Jalapa, Oaxaca [in] Mexico; near the city, San Gerónimo, Paso Antonio [in] Guatemala; Amazons to Santa Catharina, S. Brazil» (original citation). Syntype(s) in BMNH. Synonymy established by Liebherr (1988: 28).

#### Distribution.

This species is found along the Coastal Plain from southeastern North Carolina to central Florida, west to northeastern Texas (Dallas County, MCZ) and central Oklahoma (Grady County, Robert L. Davidson pers. comm. 2012), including west-central Louisiana (Allen 1965: 67), along the Mississippi Basin in southwestern Ohio (Blatchley 1910: 137) and central Indiana (Downie and White 1967: 308), in the West Indies from the Bahamas (Turnbow and Thomas 2008: 12) to Trinidad, in tropical mainland from central Mexico to Santa Catarina in Brazil [see Liebherr 1988: Fig. 17], and along southwestern United States from New Mexico (Snow 1885: 66; Fall and Cockerell 1907: 159) to southern Arizona (Pima County, MCZ); also recorded from the southern part of the Baja California Peninsula (Horn 1895: 226). The record from southeastern Pennsylvania (Rathvon 1869: 523) must be in error.

#### Records.

**USA**: AL, AZ, FL, GA, IN, LA, MS, NC, NM, OH, OK, SC, TX – Bahamas, Brazil, Colombia, Cuba, French Guiana, Guatemala, Honduras, Jamaica, Mexico, Venezuela

### Genus Calybe

Laporte, 1834

Calybe Laporte, 1834: 92. Type species: *Calybe leprieuri* Laporte, 1834 by monotypy. Etymology. Mythological name designating a Trojan Nymph loved by King Laomedon of Troy. Literately, *kalybe* or *calybe* designates a rustic cabin or hut [feminine]. Note. *Chalybe* is an incorrect subsequent spelling, introduced by Dejean (1836: 56), not in prevailing usage.

#### Diversity.

Twenty-five species (Lorenz 2005: 440-441) in the Western Hemisphere arrayed in two subgenera: *Calybe* s.str. (eight species) represented in the Neotropical Region only and *Ega* (17 species).

### Subgenus Ega

Laporte, 1834

Ega Laporte, 1834: 93. Type species: *Ega formicaria* Laporte, 1834 by monotypy. Etymology. Uncertain, either from Ega (also spelled Aega), the mayor of the palace and regent of Neustria and Burgundy or from Ega the old name for the city and river port of Tefé in northwestern Brazil [feminine].
Aega Agassiz, 1846: 8, 134. Unjustified emendation of *Ega* Laporte, 1834.

#### Diversity.

Seventeen species in the temperate, subtropical, and tropical areas of the Nearctic (two species) and Neotropical (17 species) Regions.

#### Identification.

Horn (1881: 153) commented on the structural differences between the two species found in North America.

### Calybe laetula

(LeConte, 1851)

Ega laetula LeConte, 1851: 173. Type locality: «ad flumina Colorado et Gila» (original citation). Three syntypes in MCZ [# 59].

#### Distribution.

This species ranges from southernmost Nevada (Clark County, CNC) south to Guatemala (Bates 1883a: 157), including southeastern California (Riverside County, MCZ, UASM) and southern Arizona (Fall 1901a: 48; Maricopa and Pima Counties, MCZ, UASM). The record from “Colorado” (LeConte 1858a: 28) probably refers to the Colorado River.

#### Records.

**USA**: AZ, CA, NV – Guatemala, Mexico

### Calybe sallei

(Chevrolat, 1839)

Ega sallei Chevrolat, 1839: 308. Type locality: «environs de la Nouvelle-Orléans [Orleans Parish, Louisiana]» (original citation). Syntype(s) location unknown (possibly in UMO).
Ega sallaei Bates, 1883a: 157. Unjustified emendation of *Ega sallei* Chevrolat, 1839.

#### Distribution.

This species is known along the Coastal Plain from South Carolina (Kirk 1969: 12; Ciegler 2000: 116; Clarendon County, MCZ) to southern Florida (Peck and Thomas 1998: 23), west to central and southeastern Texas (Lee and Cameron Counties, MCZ; Wickham 1897: 107), and south to Nicaragua (Bates 1883a: 157). The record from “Arizona” (Ball and Bousquet 2000: 109) refers to *Calybe laetula*. Old specimens simply labeled “Ill.” and “Ks” are known (MCZ).

#### Records.

**USA**: AL, AR, FL, GA, LA, MS, SC, TX [IL, KS] – Guatemala, Mexico, Nicaragua

### Genus Eucaerus

LeConte, 1853

Eucaerus LeConte, 1853c: 386. Type species: *Eucaerus varicornis* LeConte, 1853 by monotypy. Etymology. From the Greek *eu* (well) and *caeros* (fit, opportune) [masculine].

#### Diversity.

Fourteen species in the Western Hemisphere arrayed in two subgenera: *Eucarus* s.str. (11 species) and *Lachnaces* Bates (three Amazonian species).

### Subgenus Eucaerus

LeConte, 1853

Eucaerus LeConte, 1853c: 386. Type species: *Eucaerus varicornis* LeConte, 1853 by monotypy.

#### Diversity.

Eleven species in temperate, subtropical, and tropical areas of the Nearctic (one species) and Neotropical (ten species) Regions arrayed in two species groups (Ball and Hilchie 1983: 107-108).

### Eucaerus varicornis

LeConte, 1853

Eucaerus varicornis LeConte, 1853c: 387. Type locality: «New Orleans [Orleans Parish, Louisiana]» (original citation). Holotype [by monotypy] in MCZ [# 5834].

#### Distribution.

This species inhabits the Coastal Plain from central Maryland (Hoffman et al. 2006: 28) to southern Florida (Peck and Thomas 1998: 23), west to eastern Texas (Hardin County, UASM), including southeastern Mississippi (Jackson County, Drew A. Hildebrandt pers. comm. 2008).

#### Records.

**USA**: AL, FL, GA, LA, MD, MS, NC, SC, TX, VA

### Tribe Pentagonicini

Bates, 1873

Pentagonicinae Bates, 1873: 225, 320. Type genus: *Pentagonica* Schmidt-Göbel, 1846.
Scopodinae Bates, 1874: 275. Type genus: *Scopodes* Erichson, 1842.

#### Diversity.

Worldwide but excluding Europe and northern Africa, with about 165 species (Lorenz 2005: 445-447) arrayed in five genera: *Aeolodermus* Andrewes (one Oriental species), *Homethes* Newman (ten australo-oriental species), *Parascopodes* Darlington (one Australian species), *Pentagonica* (about 85 species), and *Scopodes* Erichson (68 australo-oriental species). The Northern Hemisphere is underrepresented with only 17 species (about 10% of the world fauna).

### Genus Pentagonica

Schmidt-Göbel, 1846

Rhombodera Reiche, 1842: 313 [junior homonym of *Rhombodera* Burmeister, 1838]. Type species: *Rhombodera virgata* Reiche, 1842 (= *Lebia trivittata* Dejean, 1831) designated by Bousquet and Larochelle (1993: 245). Etymology. From the Greek *rhombos* (lozenge) and *dere* (neck, by extension pronotum), alluding to the shape of the pronotum (“*thorax sub rhomboideus*”) of the adult [feminine].
Pentagonica Schmidt-Göbel, 1846: 47. Type species: *Pentagonica ruficollis* Schmidt-Göbel, 1846 designated by Andrewes (1938: 137). Synonymy established by Chaudoir (1877: 212). Etymology (original). From the Greek *pente* (five) and *goniakos* (angular), not *gonichos* as originally cited by Schmidt-Göbel, alluding to the shape of the pronotum (“*thorax pentagonus*”) of the adult [feminine].
Didetus LeConte, 1853c: 377. Type species: *Didetus flavipes* LeConte, 1853 by monotypy. Synonymy established by LeConte (1861a: 25). Etymology. From the Greek prefix *di*- (two) and *detos* (bound) [masculine].

#### Diversity.

About 85 species in the temperate, subtropical, and tropical areas of the Nearctic (six species, only one endemic), Neotropical (about 25 species), Australian (10 species), Oriental (about 20 species), Palaearctic (11 Asian species), and Afrotropical (25 species) Regions.

#### Identification.

Lindroth’s (1969a: 1013) key covered all North American known at the time. Two new species have been described subsequently by Bell (1987) and Mateu (1995).

### Pentagonica bicolor

(LeConte, 1863)

Rhombodera bicolor LeConte, 1863c: 7. Type locality: «western states» (original citation), herein restricted to Columbus, Colorado County, Texas (see Reichardt 1968: 150). One syntype in MCZ [# 5836].
Pentagonica semifulva Bates, 1883a: 217. Type locality: «Cordova, Jalapa [in] Mexico; Cerro Zunil, Pantaleon [in] Guatemala» (original citation). Syntype(s) in BMNH. Synonymy established by Reichardt (1968: 150).

#### Distribution.

This species ranges from Colorado County in southeastern Texas to Guatemala (Reichardt 1968: 150).

#### Records.

**USA**: TX – Guatemala, Mexico

### Pentagonica felix

Bell, 1987

Pentagonica felix R.T. Bell, 1987: 373. Type locality: «Rustler Park, Cochise Co[unty], Ariz[ona]» (original citation). Holotype (♀) in CNC [# 19859].

#### Distribution.

This species is known from a few localities from southeastern Arizona and southwestern New Mexico (Bell 1987: 373) south to Chiapas in southern Mexico (Bell 1989a: 156).

#### Records.

**USA**: AZ, NM – Mexico

### Pentagonica flavipes flavipes

(LeConte, 1853)

Didetus flavipes LeConte, 1853c: 377. Type locality: «Louisiana» (original citation). Two syntypes in MCZ [# 5835].
Pentagonica americana Motschulsky, 1864: 224. Type locality: «environs de Mobile [Mobile County, Alabama]» (original citation). Lectotype, designated by Bousquet (1997b: 339), in ZMMU. Synonymy established by Horn (1882: 163), confirmed by Bousquet (1997b: 339).
Pentagonica albipes Bates, 1883a: 218. Type locality: «Mirandilla, Guatemala; Bugaba, Panama» (original citation). Syntype(s) in BMNH. Synonymy established by Reichardt (1968: 153).
Rhombodera picea Fleutiaux and Sallé, 1890: 362. Type locality: «Cascade Vauchelet, Camp- Jacob; Trois-Rivières [Guadeloupe]» (original citation). Syntype(s) probably in MHNP. Synonymy established by Reichardt (1968: 153).

#### Distribution.

This subspecies ranges from southwestern New Jersey (Smith 1910: 212) and northern Virginia (Hoffman and Roble 2000: 39) to northeastern Kansas (Popenoe 1877: 23, as *Rhombodera pallipes*), south to eastern Texas (Sabine County, CNC) and the Florida Keys (Peck and Thomas 1998: 22). It is also known from Central America, Colombia, Brazil, and the West Indies (Reichardt 1968: 153; Bell 1985a: 326). The record from “Arizona” (Leng 1915: 589) needs confirmation.

#### Records.

**USA**: AL, AR, FL, GA, IA, IN, KS, LA, MS, NC, NJ, OH, OK, SC, TX, VA [AZ] – Antigua, Bahamas, Belize, Brazil, Cayman Islands, Colombia, Costa Rica, Cuba, Dominica, Dominican Republic, Guadeloupe, Guana Island, Guatemala, Monserrat, Panama, Trinidad and Tobago

#### Note.

The subspecies *Pentagonica flavipes picipes* Darlington is restricted to Jamaica, Hispaniola, Puerto Rico, and the Virgin Islands (Reichardt 1968: 154; Bell 1985a: 326).

### Pentagonica marshalli

Mateu, 1995

Pentagonica marshalli Mateu, 1995: 141. Type locality: «3 mill. N[orth]W[est] Alligator P[oin]t, Franklin Co[unty], Florida» (original citation). Holotype (♂) in Mateu’s collection (Almería, Spain).

#### Distribution.

This species is known from southeastern Georgia (Camden and Glynn Counties, CMNH), central Florida (Pinellas County, CMNH), north-central Mississippi (Grenada County, Drew A. Hildebrandt pers. comm. 2010), and east-central (Riley 2011) and south-central Texas (Bastrop County, Peter W. Messer pers. comm. 2010).

#### Records.

**USA**: FL, GA, MS, TX

### Pentagonica nigricornis

Darlington, 1934

Pentagonica nigricornis Darlington, 1934: 121. Type locality: «Soledad (near Cienfuegos), Cuba» (original citation). Holotype in MCZ [# 19537].

#### Distribution.

This species is known only from the Florida Peninsula as far north as Alachua County (Peck and Thomas 1998: 22), the Bahamas (Turnbow and Thomas 2008: 14), Cuba (Bell 1985a: 323), and the Cayman Islands (Darlington 1947: 211).

#### Records.

**USA**: FL – Bahamas, Cuba, Cayman Islands

### Pentagonica picticornis

Bates, 1883

Pentagonica picticornis Bates, 1883a: 217. Type locality: «El Jicaro, Guatemala» (original citation). Holotype [by monotypy] in BMNH.

#### Distribution.

This species ranges from southern Quebec (Larochelle 1975: 98) to western Wisconsin (Messer 2010: 42), south to Guatemala (Bates 1883a: 217), southeastern Louisiana (Saint Tammany Parish, Igor M. Sokolov pers. comm. 2009), and central Florida (Vince Golia pers. comm. 2007), west to southern Arizona and the Baja California Peninsula (Horn 1894: 311).

#### Records.

**CAN**: ON, QC **USA**: AR, AZ, FL, GA, IA, IL, IN, KS, LA, MD, MI, MO, MS, NE, NH, NJ, NM, OH, OK, PA, TN, TX, VA, VT, WI, WV – Guatemala, Mexico

### Tribe Odacanthini

Laporte, 1834

Odacanthidae Laporte, 1834: 40. Type genus: *Odacantha* Paykull, 1798.
Casnoniae LeConte, 1861a: 21. Type genus: *Cosnania* Dejean, 1821. Note. This family-group name is based on *Casnonia*, an incorrect subsequent spelling of *Cosnania* Dejean, not in prevailing usage.
Colliurini Bedel, 1910: 72. Type genus: *Colliuris* DeGeer, 1774.
Lasiocerini Jeannel, 1948a: 757. Type genus: *Lasiocera* Dejean, 1831.

#### Diversity.

Worldwide, with about 325 species (Lorenz 2005: 441-445, as Odacanthina) in the Nearctic (five species), Neotropical, Australian, Oriental, Palaearctic (21 species, several shared with the Oriental Region), and Afrotropical Regions.

### Genus Colliuris

DeGeer, 1774

Colliuris DeGeer, 1774: 79. Type species: *Attelabus surinamensis* Linnaeus, 1758 by monotypy. Etymology. From the Latin *collum* (neck, by extension pronotum), alluding to the unusually long pronotum (“*à cause de la grande étendue de leur corcelet qui leur forme comme un très long col*”) of the adult [feminine].
Collyris Agassiz, 1846: 94 [junior homonym of *Collyris* Fabricius, 1801]. Unjustified emendation of *Colliuris* DeGeer, 1774.

#### Diversity.

Western Hemisphere, with about 100 species (Lorenz 2005: 442-444, as *Cosnania* and *Colliuris*) in temperate, subtropical, and tropical areas of the Nearctic (five species) and Neotropical Regions. The species are arrayed in 20 subgenera.

#### Identification.

Bousquet (2010b) reviewed the Nearctic, Mexican and West Indian (Greater Antilles) species and provided a key for their identification.

#### Taxonomic Note.

Liebke (1930, 1938) recognized an array of subgenera and several appear to be polyphyletic assemblages. A taxonomic revision of the genus is much needed. Lorenz (2005: 442) recognized *Cosnania* as generically distinct from *Colliuris*. I see no reason to support this approach particularly since *Cosnania*, as currently recognized, is likely polyphyletic (see below).

### Subgenus Mimocasnonia

Liebke, 1938

Mimocasnonia Liebke, 1938: 51, 57. Type species: *Casnonia pilatei* Chaudoir, 1848 by original designation. Etymology. From the Greek *mimos* (imitator, actor) and *Casnonia*, an incorrect subsequent spelling of the generic name *Cosnania* [*q.v*.] [feminine].

#### Diversity.

Three species in the Neotropical Region (Liebke 1938: 57), one of them extending into southern Arizona.

### Colliuris pilatei

(Chaudoir, 1848)

Casnonia pilatei Chaudoir, 1848: 47 (as *pilati*). Type locality: «Yucatan [Mexico]» (original citation). Syntype(s) [2 originally cited] probably in MHNP. Note. The incorrect subsequent spelling *pilatei*, first used by Bates (1883a: 161), is in prevailing usage and attributed to the publication of the original spelling; therefore it is deemed to be the correct original spelling (ICZN 1999: Article 33.3.1).

#### Distribution.

This species ranges from southern Arizona (Bousquet 2010b: 7) to Panama (FSCA).

#### Records.

**USA**: AZ – Costa Rica, Guatemala, Honduras, Mexico, Panama

### Subgenus Cosnania

Dejean, 1821

Macrotrachelus Latreille, 1818a: 16 [potential *nomen oblitum*, see Bousquet (2002b: 30)]. Type species: *Attelabus pensylvanicus* Linnaeus, 1758 by monotypy. Etymology. From the Greek *macros* (long) and *trachelos* (neck, by extension pronotum), alluding to the elongate pronotum of the adult [masculine].
Cosnania Dejean, 1821: 2 [potential *nomen protectum*]. Type species: *Attelabus pensylvanicus* Linnaeus, 1758 by monotypy. Etymology. Unknown; this is also the case for the spelling *Casnonia* [feminine]. Note. *Casnonia* is an incorrect subsequent spelling, first used by Dejean (1825: 170), not currently in prevailing usage. The spelling was in prevailing usage until the 1990s but not attributed to the publication of the original spelling. Therefore *Casnonia* cannot be considered as the correct original spelling (see ICZN 1999: Article 33.3.1).
Odacanthella Liebke, 1930: 658. Type species: *Attelabus pensylvanicus* Linnaeus, 1758 by original designation. Etymology. From the generic name *Odacantha* and the Greek suffix -*ella* (small, little) [feminine].

#### Diversity.

Fourteen species are listed in this subgenus by Lorenz (2005: 442) but some (e.g., *Cosnania lioptera*, *Cosnania tetrastigma*, and *Cosnania emdeni* Liebke) are not closely related to the type species, *Cosnania pensylvanica*. Two North American species belong to this subgenus.

### Colliuris lengi

(Schaeffer, 1910)

Casnonia lengi Schaeffer, 1910: 395. Type locality: «Nogales [Santa Cruz County], Arizona» (original citation). Holotype [by monotypy] (♀) in USNM [# 56140]. Etymology. The species name honors Charles William Leng [1859-1941], coleopterist and historian. Born on Staten Island, Leng was early in his adult life a business partner with his father in importing iron and steel, with bicycles as a new and promising sideline. He eventually became director of the Staten Island Institute of Arts and Sciences. He is of course well known for the catalogue of North American beetles he published in 1920 at the request of John D. Sherman, Jr.

#### Distribution.

This species has a very restricted distribution; it is known from southeastern Arizona and adjacent northern Mexico [see Bousquet 2010b: Fig. 19].

#### Records.

**USA**: AZ – Mexico

### Colliuris pensylvanica

(Linnaeus, 1758)

Attelabus pensylvanicus Linnaeus, 1758: 387. Type locality: «Philadelphia [Philadelphia County, Pennsylvania]» (original citation). Syntype(s) probably lost (see Lindroth 1957b: 327).
Casnonia picta Chaudoir, 1843b: 697. Type locality: «Californie» (original citation), which is likely incorrect. Syntype(s) in MHNP. Synonymy established by Bousquet (2010b: 9).
Casnonia suturalis Chaudoir, 1872d: 405. Type locality: «midi des Etats-Unis» (original citation). Syntype(s) [3 originally cited] in MHNP. Synonymy established by LeConte (1880a: 85).
Casnonia limbata C.O. Waterhouse, 1879: 304. Type locality: «Jamaica» (lectotype label). Lectotype (♀), designated by Bousquet (2010b: 9), in BMNH. Synonymy established by Bousquet (2010b: 9).
Colliuris picta var. *extrema* Liebke, 1930: 689. Type locality: «Mexico» (original citation). Lectotype (♂), designated by Bousquet (2010b: 10), in ZMHB. Synonymy established by Bousquet (2010b: 9).
Colliuris picta var. *concluda* Liebke, 1930: 689. Type locality: «Durango city [Mexico]» (lectotype label). Lectotype (♂), designated by Bousquet (2010b: 9), in ZMHB. Synonymy established by Bousquet (2010b: 9).
Colliuris yucatana Liebke, 1930: 700. Type locality: «Temax, N[orth] Yucatan [Mexico]» (original citation). Holotype in BMNH. Synonymy established by Bousquet (2010b: 9).

#### Distribution.

This species ranges from Maine (Dearborn and Donahue 1993: 8; Foss 2001: 14) to eastern South Dakota (Kirk and Balsbaugh 1975: 37), including southern Quebec (Larochelle 1975: 78) and the Ontario Peninsula (Lindroth 1969a: 1008), south to the Yucatán Peninsula and southern Florida, west to southern Arizona and Baja California Sur [see Bousquet 2010b: Fig. 18]; also known from Cuba and Jamaica (Bousquet 2010b: 10).

#### Records.

**CAN**: ON, QC **USA**: AL, AR, AZ, CT, DC, DE, FL, GA, IA, IL, IN, KS, KY, LA, MA, MD, ME, MI, MN, MO, MS, NC, ND, NE, NH, NJ, NY, OH, OK, PA, RI, SC, SD, TN, TX, VA, VT, WI, WV – Cuba, Jamaica, Mexico

### Subgenus Calocolliuris

Liebke, 1938

Calocolliuris Liebke, 1938: 55. Type species: *Casnonia ludoviciana* Sallé, 1849 by original designation. Etymology. From the Greek prefix *calo*- (beautiful) and the generic name *Colliuris* [*q.v*.] [feminine].

#### Diversity.

Five Nearctic and Neotropical species were included in this subgenus by Liebke (1938: 55-56). However, a number of species (e.g., *Colliuris liptera* Bates, *Colliuris tetrastigma* Chaudoir, *Colliuris caymanensis* Darlington, *Colliuris ellipticeps* Liebke, and *Colliuris gundlachi* Darlington), placed in other subgenera by Liebke (1938), are closely related to members of *Calocolliuris* (personal observation).

### Colliuris caymanensis

Darlington, 1947

Colliuris tetrastigma caymanensis Darlington, 1947: 211. Type locality: «South Sound, Grand Cayman» (original citation). Holotype (♂) in BMNH.

#### Distribution.

This species is known from southern Florida, Cuba, the Cayman Islands, Jamaica, and Haiti (Bousquet 2010b: 23).

#### Records.

**USA**: FL – Cayman Islands, Cuba, Haiti, Jamaica

### Colliuris lioptera

(Bates, 1891)

Casnonia lioptera Bates, 1891a: 265. Type locality: «Atoyac, in Vera Cruz [Mexico]» (original citation). Syntype(s) in BMNH.

#### Distribution.

This species ranges from southeastern Arizona (Bousquet and Larochelle 1993: 334) south at least to Honduras (USNM).

#### Records.

**USA**: AZ – Honduras, Mexico

### Colliuris ludoviciana

(Sallé, 1849)

Casnonia ludoviciana Sallé, 1849: 297. Type locality: «Nouvelle-Orléans [Orleans Parish], Louisiane» (original citation). One syntype [2 originally cited] in MHNP (collection Chaudoir) and one in MCZ (collection LeConte).

#### Distribution.

This species inhabits the Coastal Plain and ranges from southeastern New York to southern Florida, west to eastern Louisiana [see Bousquet 2010b: Fig. 20]. The species was also recorded from “N. Yucatan” by Liebke (1938: 56) and from “Pennsylvania” by Leng (1920: 65); both records are suspect.

#### Records.

**USA**: AL, DC, FL, LA, MD, NC, NJ, NY, VA [PA]

### Tribe Ctenodactylini

Laporte, 1834

Ctenodactylidae Laporte, 1834: 45. Type genus: *Ctenodactyla* Dejean, 1825.

#### Diversity.

Western Hemisphere, with about 65 species (Lorenz 2005: 393) arrayed in 16 genera.

### Genus Leptotrachelus

Latreille, 1829

Leptotrachelus Latreille, 1829: 371. Type species: *Odacantha dorsalis* Fabricius, 1801 by monotypy. Etymology. From Greek *leptos* (thin, slender) and *trachelos* (neck, by extension pronotum), alluding to the narrow pronotum (“*où cette partie du corps* [pronotum] *est à peu près cylindrique*”) of the adult [masculine].
Spheracra Say, 1830a: 133. Type species: *Odacantha dorsalis* Fabricius, 1801 by monotypy.

#### Diversity.

Thirty-four species (Lorenz 2005: 393) in the temperate, subtropical, and tropical areas of the Nearctic (three species) and Neotropical (32 species) Regions.

#### Identification.

Bousquet (1997b: 336) commented on the structural differences between *Leptotrachelus dorsalis* and *Leptotrachelus pallidulus*. The third species (*Leptotrachelus depressus*) is known only from the holotype which is very similar to members of *Leptotrachelus pallidulus* and may eventually prove to be conspecific with them.

### Leptotrachelus depressus

Blatchley, 1923

Leptotrachelus depressus Blatchley, 1923: 15. Type locality: «Lake Wales [Polk County], Fl[orid]a» (original citation). Holotype [by monotypy] (♂) in PURC.

#### Distribution.

This species is known only from the Florida Peninsula (Peck and Thomas 1998: 23).

#### Records.

**USA**: FL

### Leptotrachelus dorsalis

(Fabricius, 1801)

Odacantha dorsalis Fabricius, 1801: 229. Type locality: «Carolina» (original citation), herein restricted to Yemassee, Hampton County, South Carolina (see Ciegler 2000: 117). Syntype(s) location unknown (Lindroth 1969a: 1006).

#### Distribution.

This species ranges from Rhode Island (William L. Krinsky pers. comm. 2009) and Connecticut (Krinsky and Oliver 2001: 232) to southeastern South Dakota (Kirk and Balsbaugh 1975: 36), including the Ontario Peninsula (Lindroth 1969a: 1006), south to southeastern Texas (Wharton County, CNC) and southern Florida (Peck and Thomas 1998: 23); also found in Cuba (Gundlach 1891: 19), Barbados, and Hispaniola (Peck and Thomas 1998: 23).

#### Records.

**CAN**: ON **USA**: AL, AR, CT, DC, DE, FL, GA, IA, IL, IN, KS, KY, LA, MD, MI, MN, MO, MS, NC, NE, NJ, NY, OH, PA, RI, SC, SD, TN, TX, VA, WI, WV – Barbados, Cuba, Hispaniola

### Leptotrachelus pallidulus

Motschulsky, 1864

Leptotrachelus pallidulus Motschulsky, 1864: 218. Type locality: «Nouv[elle] Orléans [Orleans Parish, Louisiana]» (original citation). Lectotype, designated by Bousquet (1997b: 335), in ZMMU.

#### Distribution.

This species is known from a few scattered localities in Tennessee, Louisiana (Bousquet 1997b: 338), and Texas (Chaudoir 1872d: 412).

#### Records.

**USA**: LA, TN, TX

### Tribe Cyclosomini

Laporte, 1834

Cyclosomidae Laporte, 1834: 69. Type genus: *Cyclosomus* Latreille, 1829.
Tétragonodérides Chaudoir, 1871a: 111. Type genus: *Tetragonoderus* Dejean, 1829.
Sarothrocrepidae Chaudoir, 1876d: 2, 83. Type genus: *Sarothrocrepis* Chaudoir, 1850. Note. The stem of *Sarothrocrepis* is *Sarothrocrepid*- (Madge 1989: 467).

#### Diversity.

About 120 species in the Nearctic (four species), Neotropical (about 40 species of *Tetragonoderus*), Australian (one adventive *Tetragonoderus*), Oriental, Palaearctic (20 species), and Afrotropical Regions. These species are arrayed in four genera: *Cyclosomus* Latreille (13 species), *Cyclicus* Jeannel (22 afro-oriental species), *Mnuphorus* Chaudoir (11 species) and *Tetragonoderus* (about 75 species). The Northern Hemisphere is represented by about 25 species (approximately 21% of the world fauna).

#### Taxonomic Note.

This tribe is treated here in a restricted sense following Ball and Bousquet (2000: 109) and excluded the graphipterines, corsyrines, masoreines, somoplatines, and nemotarsines.

### Genus Tetragonoderus

Dejean, 1829

Tereus Billberg, 1820: 26 [potential *nomen oblitum*, see Bousquet (2002b: 50)]. Type species: *Carabus quadrinotatus* Fabricius, 1798 designated by Bousquet (2002b: 50). Etymology. Unknown [masculine].
Tetragonoderus Dejean, 1829: 485 [potential *nomen protectum*]. Type species: *Carabus quadrum* Fabricius, 1792 designated by Hope (1838: 89). Synonymy established by Bousquet (2002b: 50). Etymology (original). From the Greek *tetragonos* (square) and *dere* (neck, by extension pronotum), alluding to the shape of the pronotum (“*corselet *... *plus ou moins carré*”) of adults of the 16 species Dejean had before him [masculine].

#### Diversity.

About 75 species (Lorenz 2005: 453) arrayed in two subgenera, *Tetragonoderus* s.str. with about 35 species in the Old World and *Crossonychus* for the Western Hemisphere species.

#### Identification.

Lindroth (1969a: 1010) included a key to four of the five species found in North America.

### Subgenus Crossonychus

Chaudoir, 1848

Crossonychus Chaudoir, 1848: 98. Type species: *Dromius viridis* Dejean, 1831 by monotypy. Etymology. From the Greek *crossos* (fringe) and *onyx*, -*ychos* (claw) [masculine].
Lobius Motschulsky, 1864: 230. Type species: *Dromius viridis* Dejean, 1831 designated by Lorenz (1998: 108).
Peronoscelis Chaudoir, 1876d: 56. Type species: *Tetragonoderus figuratus* Dejean, 1831 designated by Bousquet and Larochelle (1993: 266). Synonymy established by Ball (2000: 190). Etymology. From the Greek *perone* (pin, something pointed) and *scelos* (leg) [feminine].

#### Diversity.

Western Hemisphere, with about 40 species in the Nearctic (five species, one of them adventive) and Neotropical (about 40 species) Regions. One South American species (*Tetragonoderus undatus* Dejean) is adventive in Australia (Moore 1976). The species have been arrayed in two species groups by Ball (2000: 190), the *figuratus* and *intersectus* groups; only the last one is represented in North America.

### Tetragonoderus fasciatus

(Haldeman, 1843)

Coptodera fasciata Haldeman, 1843b: 298. Type locality: North America (inferred from title of the paper), «Fairfax Co[unty], V[irgini]a» selected by Lindroth (1969a: 1010). One possible syntype, a ♀ labeled “[pink disc] / T. fasciatus (Hald.) Lec. [handwritten],” in MCZ (collection LeConte).
Tetragonoderus undulatus LeConte, 1863c: 6. Type locality: «Cape San Lucas, Lower California» (original citation). Two syntypes in MCZ [# 5797]. Synonymy established by Horn (1882: 160).
Tetragonoderus distigma Motschulsky, 1864: 222. Type locality: «Etat de Tennessée» (original citation). Lectotype (♀), designated by Bousquet (1997b: 338), in ZMMU. Synonymy established by Bousquet (1997b: 339).

#### Distribution.

This species ranges from southwestern Maine (Majka et al. 2011: 47) and southern Quebec (CNC) to southwestern Minnesota (Gandhi et al. 2005: 932), south to southern Texas (Wickham 1897: 107; Zapata County, CMNH) and central Florida (Peck and Thomas 1998: 23), west along the south to Riverside County in California (Dajoz 2007: 19) and the Baja California Peninsula (Horn 1894: 310; Horn 1895: 226).

#### Records.

**CAN**: ON, QC **USA**: AL, AR, AZ, CA, CT, DC, DE, FL, GA, IA, IL, IN, KS, LA, MA, MD, ME, MI, MN, MO, MS, NC, NE, NH, NJ, NY, OH, OK, PA, SC, TN, TX, VA, VT, WI – Mexico

### Tetragonoderus intersectus

(Germar, 1824)

Bembidion intersectum Germar, 1824: 28. Type locality: «Kentucky» (original citation). Lectotype (♀), designated by Lindroth (1969a: 1012), in ZMHB.
Tetragonoderus lecontei Dejean, 1829: 499. Type locality: «Amérique septentrionale» (original citation). Two syntypes in MHNP (Lindroth 1955b: 22). Synonymy established by LeConte (1853c: 378), confirmed by Lindroth (1955b: 22).

#### Distribution.

This species ranges from North Carolina (Moore County, CNC) to southern Arizona (Pima County, CMNH), south to Jalisco in Mexico (CMNH), southern Texas (Johnson 1978: 68), and southern Florida (Peck and Thomas 1998: 23); also known from the Bahamas (Darlington 1953: 16). The record from “Virginia” (Bousquet and Larochelle 1993: 266) needs confirmation; that from southern Ontario (Belaoussoff et al. 2003: 878) probably refers to *Tetragonoderus fasciatus*.

#### Records.

**USA**: AL, AR, AZ, FL, GA, KY, LA, MS, NC, SC, TN, TX [VA] – Bahamas, Mexico

### Tetragonoderus laevigatus

Chaudoir, 1876

Tetragonoderus laevigatus Chaudoir, 1876d: 46. Type locality: «près de Montevideo, Uruguay» (original citation). One syntype (♂) in MHNP (Shpeley and Ball 2008: 7).
Tetragonoderus unicolor Chaudoir, 1876d: 47 [primary homonym of *Tetragonoderus unicolor* Gemminger and Harold, 1868]. Type locality: «province de Rio-Janeiro [Brazil]» (original citation). Lectotype (♂), designated by Shpeley and Ball (2008: 7), in MHNP. Synonymy established by Shpeley and Ball (2008: 7).
Tetragonoderus chaudoiri Liebke, 1928: 129. Replacement name for *Tetragonoderus unicolor* Chaudoir, 1876.

#### Distribution.

This South American species is adventive in southeastern Florida (Shpeley and Ball 2008: 9). The first inventoried specimen collected on this continent was found in March 2007 (Halbert 2007: 7; Shpeley and Ball 2008: 9). In South America, the species ranges from the Baía de Marajó along the Atlantic Coast in northern Brazil to southern Uruguay and central Argentina (Shpeley and Ball 2008: Fig. 6).

#### Records.

**USA**: FL – **Adventive**

### Tetragonoderus latipennis

LeConte, 1874

Tetragonoderus latipennis LeConte, 1874b: 44. Type locality: «Texas» (original citation). Four syntypes in MCZ [# 5798].

#### Distribution.

This species occurs from southwestern Alabama (Baldwin and Clarke Counties, CMNH) to central Arizona (Yavapai County, CNC), south to southern Texas (Wickham 1897: 107; Johnson 1978: 68); also recorded from Mexico (Blackwelder 1944: 52).

#### Records.

**USA**: AL, AR, AZ, LA, MS, TX – Mexico

### Tetragonoderus pallidus

Horn, 1869

Tetragonoderus pallidus G.H. Horn, 1869b: 130. Type locality: «Temescal, southern California and Arizona» (original citation). Syntype(s) in MCZ [# 34501].

#### Distribution.

This species is known from southwestern California (Fall 1901a: 48; Moore 1937: 11) to the western tip of Texas (El Paso County, CMNH), including western Nevada (Bechtel et al. 1983: 474); also recorded from the Baja California Peninsula (Horn 1895: 226).

#### Records.

**USA**: AZ, CA, NV, TX – Mexico

### Tribe Lebiini

Bonelli, 1810

Lebiotae Bonelli, 1810: Tabula Synoptica. Type genus: *Lebia* Latreille, 1802.

#### Diversity.

Worldwide, with about 4,260 species arrayed in 237 genera (Lorenz 2005: 453-502, as Nemotarsina and Lebiini). The North American fauna is underrepresented both in term of species (about 155 species or 3.6% of the world fauna) and genera (25 genera or 10.5%). The lebiine genera are classified into the following 17 subtribes: Actenonycina (one New Zealand species), Agrina (about 585 species), Apenina (about 115 species), Calleidina (about 660 species), Celaenephina (two species), Cymindidina (about 315 species), Demetriadina (14 species), Dromiusina (about 735 species), Gallerucidiina (ten species), Lebiina (about 800 species), Metallicina (about 70 species), Nemotarsina (nine species), Peliocypadina (about 70 species), Pericalina (about 825 species), Physoderina (45 species), Sugimotoina (one species), and Trichina (five species).

### Subtribe Pericalina

Hope, 1838

Pericalidae Hope, 1838: 105 (as Pericallidae). Type genus: *Pericalus* Macleay, 1825.
Coptodérides Chaudoir, 1848: 116 (as Costodérides). Type genus: *Coptodera* Dejean, 1825.
Mormolycites Blanchard, 1845: 390. Type genus: *Mormolyce* Hagenbach, 1825. Synonymy established by Ball (1975: 149).
Thyréoptérides Chaudoir, 1870a: 113. Type genus: *Thyreopterus* Dejean, 1831.
Eucheilinae Bates, 1883a: 168. Type genus: *Eucheila* Dejean, 1829.
Miscelini Sloane, 1907: 473. Type genus: *Miscelus* Klug, 1834.
Thysanotini Jeannel, 1949a: 947, 975. Type genus: *Thysanotus* Chaudoir, 1848.
Lobodontini Jeannel, 1949a: 1007. Type genus: *Lobodontus* Chaudoir, 1842.
Somotrichini Mateu, 1963: 122, 131. Type genus: *Somotrichus* Seidlitz, 1887. Synonymy established by Ball (1975: 147).

#### Diversity.

Worldwide, with about 825 species arrayed in 77 genera (Lorenz 2005: 454-464). The subtribe is underrepresented in the Northern Hemisphere with about 70 species (8.5% of the world fauna), of which nine (about 1%) only occur in North America.

#### Identification.

Shpeley and Ball (2001) reviewed all 111 native species-group taxa found in the Western Hemisphere and provided keys for their identification. Their work is to be used in conjunction with a previous study (Ball and Shpeley 1983).

#### Taxonomic Note.

In a cladistic analysis based on characters of the adults performed by Ball et al. (1995: Fig. 7), this subtribe is positioned as the sister-group to {Sugimotoina + Actenonycina}. In Casale’s (1998: Fig. 91) cladistic analysis, the pericalines showed up as the sister-group to the remaining Lebiini.

### Genus Mochtherus

Schmidt-Göbel, 1846

Mochtherus Schmidt-Göbel, 1846: 76. Type species: *Mochtherus angulatus* Schmidt-Göbel, 1846 (= *Dromius tetraspilotus* Macleay, 1825) designated by Andrewes (1938: 136). Etymology (original). From the Greek *mochtheros* (toiling, suffering, wretched, by extension cheaply, stunted) [masculine].
Cyrtopterus Motschulsky, 1861: 106. Type species: *Cyrtopterus quadrinotatus* Motschulsky, 1861 (= *Dromius tetraspilotus* Macleay, 1825) designated by Habu (1967: 103). Etymology. From the Greek *cyrtos* (curved) and *pteron* (wing, by extension elytron) [masculine].

#### Diversity.

Nine species (Lorenz 2005: 460) in the Palaearctic (eastern Asia only), Oriental, and Australian Regions, with one of them adventive in Florida.

#### Identification.

The character states of the sole species found in North America are described in detail by Habu (1967: 104-105) under the name *Dolichoctis tetraspilotus*.

#### Taxonomic Note.

Habu (1967: 100) treated this taxon as a subgenus of *Dolichoctis* Schmidt-Göbel.

### Mochtherus tetraspilotus

(Macleay, 1825)

Dromius tetraspilotus W.S. Macleay, 1825: 25. Type locality: Java (inferred from title of the book). Syntype(s) location unknown (possibly in USS).

#### Distribution.

This species is widely distributed in Asia from India to Japan, south through the Malay Archipelago to New Guinea; it is also known from Christmas Island and Samoa. The species is adventive in southeastern United States and currently known from Florida, where the first specimens were detected in 1992 in Palm Beach County (Choate 2001), southern Mississippi (Pearl River County, Peter W. Messer pers. comm. 2010), and east-central Louisiana (West Feliciana Parish, Igor M. Sokolov pers. comm. 2009).

#### Records.

**USA**: FL, LA, MS – **Adventive**

### Genus Phloeoxena

Chaudoir, 1870

Phloeoxena Chaudoir, 1870a: 145. Type species: *Phloeoxena picta* Chaudoir, 1870 designated by Ball (1975: 178). Etymology. From the Greek *phloios* (bark) and *xenos* (stranger, guest) [feminine].

#### Diversity.

Thirty-four species in the temperate, subtropical, and tropical areas of the Nearctic (one eastern species) and Neotropical (34 species) Regions. These species are arrayed in five subgenera: *Oenaphelox* Ball (nine species), *Tacana* Ball (one species), *Phloeoxena* s.str. (15 species), *Oxephloena* Shpeley and Ball (one species), and *Ochropisus* Bates (eight species).

#### Identification.

Shpeley and Ball (2001: 78-80) published a key for the identification of all known species.

### Subgenus Oenaphelox

Ball, 1975

Oenaphelox Ball, 1975: 205. Type species: *Coptodera signata* Dejean, 1825 by original designation. Etymology. Anagram of the generic name *Phloeoxena* [*q.v*.] [masculine].

#### Diversity.

Nine species in North America and Middle America.

### Phloeoxena signata

(Dejean, 1825)

Coptodera signata Dejean, 1825: 275. Type locality: «Géorgie» (original citation), herein restricted to Billy’s Island, Okefinokee Swamp, Charlton County (see Ball 1975: 222). Lectotype [as holotype] (♂), designated by Ball (1975: 213), in MHNP.
Coptodera collaris LeConte, 1846b: 197. Type locality: «Georgia» (original citation). Lectotype (♀), designated by Ball (1975: 215), in MCZ [#5810]. Synonymy established by Horn (1882: 160), confirmed by Ball (1975: 215).
Phloeoxena maculicollis Chaudoir, 1870a: 151. Type locality: «Nouvelle-Grenade» (original citation). Holotype [by monotypy] (♀) in MHNP. Synonymy established by Ball (1975: 215).
Phloeoxena högei Bates, 1883a: 178. Type locality: «Mirador [Veracruz], Mexico» (original citation for the lectotype). Lectotype (♀), designated by Ball (1975: 215), in BMNH. Synonymy established by Ball (1975: 215).
Phloeoxena signata var. *nigripennis* Leng, 1915: 587. Type locality: «Enterprise [Volusia County, Florida]» (original citation). Syntype(s) location unknown. Synonymy established (as aberration) by Csiki (1932b: 1359), accepted by Ball (1975: 215).

#### Distribution.

This species extends from Maryland (Steiner et al. 2007: 224) to central Florida (Peck and Thomas 1998: 24) and southeastern Louisiana, and from the states of Tamaulipas and Jalisco in Mexico to Panama [see Ball 1975: Fig. 112].

#### Records.

**USA**: AL, FL, GA, LA, MD, MS, NC, SC, TN, VA – Costa Rica, Guatemala, Honduras, Mexico, Nicaragua, Panama

### Genus Eucheila

Dejean, 1829

Eucheila Dejean [in Dejean and Boisduval], 1829: 60 (as *Eucheyla*). Type species: *Eucheyla flavilabris* Dejean, 1829 by monotypy. Etymology (original). From the Greek *eu* (well, large) and *cheilos* (lip), alluding to the large size of the labrum (“*lèvre supérieure très-grande*”) of the adult [feminine]. Note. *Eucheila* is an incorrect subsequent spelling of *Eucheyla* Dejean, 1829 introduced by Dejean (1831: 455). The spelling *Eucheila* is in prevailing usage and attributed to the original publication by recent authors (e.g., Lorenz 2005: 463; Anichtchenko 2010: 189); therefore, it is deemed to be the correct original spelling (ICZN 1999: Article 33.3.1).
Euchila Agassiz, 1846: 146 [junior homonym of *Euchila* Billberg, 1820]. Unjustified emendation of *Eucheila* Dejean, 1829.

#### Diversity.

Twenty-four species (Lorenz 2005: 463-464) in the Neotropical Region, one of them reaching southeastern Texas. The species are arrayed in five subgenera: *Inna* Putzeys (ten species), *Eucheila* s.str. (six species), *Pseudoinna* Mateu (four species), *Bordoniella* Mateu (two species), and *Hansus* Ball and Shpeley (two species).

### Subgenus Inna

Putzeys, 1861

Inna Putzeys, 1861: 71. Type species: *Inna punctata* Putzeys, 1861 (= *Polistichus boyeri* Solier, 1835) by monotypy. Etymology. Unknown [feminine].
Periglossium Liebke, 1929: 246. Type species: *Periglossium nevermanni* Liebke, 1929 by original designation. Synonymy established by Reichardt (1966: 14), confirmed by Ball and Shpeley (1983: 756).

#### Diversity.

Ten species in the Neotropical Region, one of them reaching southeastern Texas.

### Eucheila boyeri

(Solier, 1835)

Polistichus boyeri Solier, 1835: 111. Type locality: «Colombie» (original citation). Lectotype [as holotype] (♂), designated by Ball and Shpeley (1983: 775), in MHNP (collection Oberthür). Etymology. The specific name was proposed for Etienne Laurent Joseph Hippolyte Boyer de Fonscolombe [1772-1853], pharmacist and entomologist in Aix-en-Provence in France. Boyer published mainly on economic insects and Hymenoptera and had a large entomological collection.
Inna punctata Putzeys, 1861: 72. Type locality: «Aragua [Venezuela]» (original citation). Holotype [by monotypy] (♂) in ZMHB. Synonymy established by Ball and Shpeley (1983: 775).
Inna texana Schaeffer, 1910: 400. Type locality: «Esperanza Ranch, Brownsville [Cameron County], Texas» (original citation). Lectotype (♂), designated by Erwin and House (1978: 239), in USNM [# 42508]. Synonymy established by Ball and Shpeley (1983: 775).

#### Distribution.

This species ranges from southern Texas (Shpeley and Ball 2001: 162) south to northeastern Brazil [see Ball and Shpeley 1983: Fig. 62].

#### Records.

**USA**: TX – Brazil, Colombia, Costa Rica, Guatemala, Honduras, Mexico, Netherlands Antilles, Nicaragua, Panama, Venezuela

### Genus Somotrichus

Seidlitz, 1887

Somotrichus Seidlitz, 1887: 7 [Gattung]. Type species: *Carabus elevatus* Fabricius, 1792 (= *Lebia unifasciata* Dejean, 1831) by monotypy. Etymology. From the Greek *soma* (body) and *trichos* (hair), probably alluding to the pubescence on the elytra (“*Fl*[*ü*]*g*[*el*]*d*[*ecken*]... *dicht behaart*”) of the adult [masculine].

#### Diversity.

Two species from the Afrotropical Region, one restricted to Madagascar, the other subcosmopolitan, having been dispersed through commerce.

#### Identification.

The character states of the species found in North America are described in detail by Habu (1967: 75-77).

#### Taxonomic Note.

This genus was listed in the tribe Singilini (currently considered a synonym of Dromiusina) by Jeannel (1949a: 915-916) along with the genera *Singilis* Rambur (eight species in the Mediterranean region and Middle East), *Phloeozetus* Peyron (about 55 species in Africa, Mediterranean region, and Middle East), *Velindopsis* Burgeon (three Afrotropical species), *Pephrica* Alluaud (six Afrotropical species), and *Paulianites* Jeannel (one Madagascan species).

### Somotrichus unifasciatus

(Dejean, 1831)

Carabus elevatus Fabricius, 1792: 162 [primary homonym of *Carabus elevatus* Fabricius, 1787]. Type locality: «Parisiis [France]» (original citation). One syntype in ZMUC (Zimsen 1964: 59).
Lebia unifasciata Dejean, 1831: 389. Type locality: «Ile-de-France» (original citation). Holotype [by monotypy] in MHNP. Synonymy established by Brullé (1834a: 108).
Coptodera 2-cincta Hope, 1845: 15. Type locality: Canton (= Guangzhou in Guangdong province), China (inferred from title on page 13). Syntype(s) location unknown (possibly in UMO). Synonymy established by Andrewes (1919: 178).
Coptodera massiliensis Fairmaire, 1850: 419. Type locality: «Marseille [France]» (original citation). Syntype(s) location unknown (possibly in MHNP). Synonymy established by Chaudoir (1854: 133).

#### Distribution.

This species is adventive in North America where it is known from Randolph County in North Carolina (Peter W. Messer pers. comm. 2012), Aiken County in South Carolina (Peter W. Messer pers. comm. 2011), Telfair and Jeff Davis Counties in southern Georgia (Harry J. Lee, Jr. pers. comm. 2010), Broward County in Florida (Peck and Thomas 1998: 25), Polk and Garland Counties in Arkansas (CMNH), and Santa Cruz County in southern Arizona (Ober and Maddison 2008: 30, as *Somotrichus elevatus*). The record from Seattle, Washington (Hatch 1953: 153) is based on specimens intercepted in nuts from Brazil; that from “Texas” (Bousquet and Larochelle 1993: 270) needs confirmation.

#### Records.

**USA**: AR, AZ, FL, GA, NC, SC [TX] – **Adventive**

### Genus Coptodera

Dejean, 1825

Coptodera Dejean, 1825: 273. Type species: *Coptodera festiva* Dejean, 1825 designated by Hope (1838: 85). Etymology (original). From the Greek *copto* (to cut, chop) and *dere* (neck, by extension pronotum), alluding to the straight (i.e., without lobe as in *Lebia*) basal edge of the pronotum (“*corselet *... *coupé carrément postérieurement*”) of the adult [feminine].

#### Diversity.

About 105 species (Lorenz 2005: 457-458) in the Nearctic (five species), Neotropical (43 species), Australian, Afrotropical, Oriental, and Palaearctic (13 species in eastern Asia only) Regions. These species are arrayed in four subgenera: *Coptodera* s.str. (about 45 species), *Haplocrepis* Jeannel (six Afrotropical species), *Coptoderina* Jeannel (53 species), and *Coptoderinella* Hansen (one Afrotropical species).

### Subgenus Coptodera

Dejean, 1825

Coptodera Dejean, 1825: 273. Type species: *Coptodera festiva* Dejean, 1825 designated by Hope (1838: 85).
Stenoglossa Chaudoir, 1848: 116. Type species: *Stenoglossa variegata* Chaudoir, 1848 (= *Coptodera sallei* Shpeley and Ball, 1994) by monotypy. Synonymy established by Shpeley and Ball (1994: 18). Etymology. From the Greek *stenos* (narrow) and *glossa* (tongue), alluding to the narrow ligula (“*ligula longissima angustissimaque*”) of the adult [feminine].

#### Diversity.

About 45 species in temperate, subtropical, and tropical areas of the Western Hemisphere arrayed in 11 species groups. Five species are found in North America.

#### Identification.

Shpeley and Ball (1994) revised the species and provided a key for their identification.

### [aerata group]

### Coptodera aerata

Dejean, 1825

Coptodera aerata Dejean, 1825: 277. Type locality: «Amérique septentrionale» (original citation), restricted to «Jone’s Creek, Lee Co[unty], V[irgini]a» by Lindroth (1969a: 1039). Lectotype (♂), designated by Shpeley and Ball (1994: 118), in MHNP.
Coptodera viridipennis Gory, 1833: 194. Type locality: «Java» (original citation), which is incorrect (Shpeley and Ball 1994: 118). Lectotype [as holotype] (♂), designated by Shpeley and Ball (1994: 118), in MHNP (collection Chaudoir). Synonymy established by Chaudoir (1850a: 357), confirmed by Shpeley and Ball (1994: 118).
Coptodera viridipennis LeConte, 1846b: 196 [primary homonym of *Coptodera viridipennis* Gory, 1833]. Type locality: «Alabama» (original citation). Lectotype (♂), designated by Shpeley and Ball (1994: 118), in MCZ [# 34513]. Synonymy established by Melsheimer (1853: 6), confirmed by Shpeley and Ball (1994: 118).
Coptodera ruficornis Chaudoir, 1870b: 179. Type locality: «états méridionaux de l’Union américaine» (original citation). Holotype [by monotypy; designated lectotype by Shpeley and Ball (1994: 119)] (♀) in MHNP. Synonymy established by Horn (1882: 160), confirmed by Shpeley and Ball (1994: 119).

#### Distribution.

The range of this species extends from Connecticut (New London County, William L. Krinsky pers. comm. 2012) to southeastern Nebraska, south to eastern Texas and west-central Florida [see Shpeley and Ball 1994: map 19]. According to Shpeley and Ball (1994: 119), the specimens labeled from Gallatin County in Montana (CAS) and Santa Cruz County in Arizona (AMNH) are probably mislabeled.

#### Records.

**USA**: AL, AR, CT, DC, DE, FL, GA, IA, IL, IN, KS, KY, LA, MD, MI, MO, MS, NC, NE, NJ, NY, OH, OK, PA, SC, TN, TX, VA, WV

### Coptodera brunnea

Shpeley and Ball, 1994

Coptodera brunnea Shpeley and Ball, 1994: 116. Type locality: «Guadalupe Canyon, Cochise County, Arizona» (original citation). Holotype (♂) in USNM.

#### Distribution.

This species is found from southeastern Arizona and southeastern New Mexico south to Honduras and Belize [see Shpeley and Ball 1994: map 18].

#### Records.

**USA**: AZ, NM – Belize, Honduras, Mexico

### Coptodera nitidula

(Buquet, 1835)

Lebia nitidula Buquet, 1835: 677. Type locality: «Guyane centrale» (original citation). Lectotype [as holotype] (♂), designated by Shpeley and Ball (1994: 112), in MHNP. Note. The specimen selected as lectotype is labeled “Brézil” (Shpeley and Ball 1994: 112) and therefore may not be a syntype.
Lebia triangularis Buquet, 1835: 678. Type locality: «non loin des sources du Jari [Brazil]» (original citation). Lectotype [as holotype] (♂), designated by Shpeley and Ball (1994: 112), in MHNP. Synonymy established by Shpeley and Ball (1994: 112).
Coptodera luculenta Erichson, 1847: 69. Type locality: Peru (inferred from title of the paper). Lectotype [as holotype] (♂), designated by Shpeley and Ball (1994: 112), in MHNP. Synonymy established, under the name *Coptodera triangularis* (Buquet), by Chaudoir (1870b: 185), confirmed by Shpeley and Ball (1994: 112).
Coptodera debilis Bates, 1869: 76. Type locality: «St. Paulo [= possibly Sāo Paulo de Oliviença, Brazil], Upper Amazons» (original citation). Lectotype (♀), designated by Shpeley and Ball (1994: 112), in MHNP. Synonymy established by Bates (1871: xvi), confirmed by Shpeley and Ball (1994: 112).
Coptodera nubiculosa Chaudoir, 1870b: 186. Type locality: «Paramaribo [Surinam]» (lectotype label). Lectotype (♂), designated by Shpeley and Ball (1994: 112), in MHNP. Synonymy established by Shpeley and Ball (1994: 112).
Coptodera flavodisca Chaudoir, 1870b: 187. Type locality: «Ega (Haut-Amazone) [Brazil]» (original citation). Holotype [by monotypy] (♀) in MHNP. Synonymy established by Shpeley and Ball (1994: 112).
Coptodera flavodisca var. *immaculipennis* Bates, 1883a: 182. Type locality: «San Gerónimo, Guatemala» (original citation for the lectotype). Lectotype, designated by Shpeley and Ball (1994: 112), in BMNH. Synonymy established by Shpeley and Ball (1994: 112).

#### Distribution.

The range of this species extends from southern Arizona and southwestern New Mexico south to southeastern Brazil and northern Argentina [see Shpeley and Ball 1994: map 17].

#### Records.

**USA**: AZ, NM – Argentina, Bolivia, Brazil, Colombia, Costa Rica, Ecuador, Guatemala, Guyana, Mexico, Nicaragua, Panama, Paraguay, Peru, Surinam, Trinidad and Tobago, Venezuela

### [festiva group]

### Coptodera festiva

Dejean, 1825

Coptodera festiva Dejean, 1825: 274. Type locality: «île de Cuba» (original citation). Holotype [by monotypy; designated lectotype by Shpeley and Ball (1994: 82)] (♂) in MHNP.
Coptodera chloris Bates, 1883a: 182. Type locality: «Mirador [Veracruz], Mexico» (original citation). Lectotype (♂), designated by Shpeley and Ball (1994: 82), in BMNH. Synonymy established by Shpeley and Ball (1994: 82).

#### Distribution.

This species occurs in southern Florida, the West Indies, and from Mexico (as far north as San Luis Potosí) to Brazil [see Shpeley and Ball 1994: map 12].

#### Records.

**USA**: FL – Brazil, Colombia, Costa Rica, Cuba, Ecuador, Guatemala, Haiti, Jamaica, Mexico, Panama, Puerto Rico, Venezuela

### [picea group]

### Coptodera picea

Dejean, 1826

Coptodera picea Dejean, 1826: 458. Type locality: «Brésil» (original citation). Lectotype (♂), designated by Shpeley and Ball (1994: 108), in MHNP.
Coptodera velox Gory, 1833: 195. Type locality: «Cayenne [French Guiana]» (original citation). Lectotype [as holotype] (♂), designated by Shpeley and Ball (1994: 108), in MHNP. Synonymy established by Chaudoir (1850a: 357), confirmed by Shpeley and Ball (1994: 108).
Coptodera unicolor Chevrolat, 1834: [no. 40]. Type locality: «Orixaba [Mexico]» (original citation). Lectotype (♀), designated by Shpeley and Ball (1994: 108), in UMO. Synonymy established by Shpeley and Ball (1994: 108).
Coptodera obscura Laporte, 1834: 51. Type locality: «Orizaba [Veracruz], Mexique» (original citation). Syntype(s) location unknown (possibly in MHNP). Synonymy established, under the name *Coptodera unicolor* Chevrolat, by Chaudoir (1850a: 358).
Lebia rufula Buquet, 1835: 680. Type locality: «environs de Cayenne [French Guiana]» (original citation). Lectotype [as holotype] (♂), designated by Shpeley and Ball (1994: 108), in MHNP. Synonymy established by Chaudoir (1870b: 178), confirmed by Shpeley and Ball (1994: 108).

#### Distribution.

This species is known from the tip of Florida, the West Indies, including the Bahamas (Turnbow and Thomas 2008: 12), Cuba, and the Dominican Republic (Shpeley and Ball 2001: 148), and from the state of San Luis Potosí in Mexico to southeastern Brazil [see Shpeley and Ball 1994: map 16].

#### Records.

**USA**: FL – Bahamas, Belize, Bolivia, Brazil, Colombia, Cuba, Dominican Republic, Ecuador, French Guiana, Guatemala, Guyana, Honduras, Mexico, Nicaragua, Panama, Paraguay, Peru, Surinam, Trinidad and Tobago, Venezuela

### Subtribe Cymindidina

Laporte, 1834

Cymindidae Laporte, 1834: 46. Type genus: *Cymindis* Latreille, 1805. Note. The stem of *Cymindis* is *Cymindid*- (Madge 1989: 462).
Taridae Gistel, 1848b: [2]. Type genus: *Tarus* Clairville, 1806 (= *Cymindis* Latreille, 1805).
Pseudomasoreini Jeannel, 1942: 1039. Type genus: *Pseudomasoreus* Desbrochers des Loges, 1904. Synonymy established by Ball and Hilchie (1983: 132).

#### Diversity.

About 315 species (Lorenz 2005: 465-470, excluding *Metaxymorphus*) in the Nearctic (27 species), Neotropical (12 species), Oriental (six species), Palaearctic (about 170 species), and Afrotropical (about 115 species) Regions. These species are arrayed in nine genera: *Taridius* Chaudoir (four Oriental species), *Afrotarus* Jeannel (seven species in Africa, Arabia, and India), *Cymindis* (about 200 species), *Petrimagnia* Kryzhanovskij and Mikhailov (one species from Tadzhikistan), *Leptosarcus* Péringuey (two Afrotropical species), *Pseudomasoreus* Desbrochers des Loges (20 species in Africa, one of them extending into southwestern Europe), *Assadecma* Basilewsky (one Madagascan species), *Hystrichopus* Boheman (about 70 species in Africa and one in Yemen), and *Plagiopyga* Boheman (13 Afrotropical species). According to Ball and Hilchie (1983: 197), the Afrotropical genus *Metaxymorphus* Chaudoir belongs to the subtribe Dromiusina. On the other hand, Basilewsky (1984: 551), followed by Lorenz (2005: 470), listed it, but with strong doubt, with the Cymindidina.

### Genus Cymindis

Latreille, 1805

Cymindis Latreille, 1805: 190. Type species: *Buprestis humeralis* Geoffroy, 1785 by monotypy. Etymology. From the Greek *cymindis* (kind of hawk in Pliny the Elder) [feminine]. Note. Bousquet and Larochelle (1993: 267-269) treated *Cymindis* as masculine but the name is feminine (Theil 1882: 709).
Tarus Clairville, 1806: 94, 95. Type species: *Buprestis humeralis* Geoffroy, 1785 designated by Curtis (1828: plate 235).

#### Diversity.

About 200 species (Lorenz 2005: 465-469, as *Pinacodera* and *Cymindis*) in the Nearctic (27 species), Neotropical (13 species), and Palaearctic (167 species) Regions arrayed in 15 subgenera.

#### Taxonomic Note.

Ball and Hilchie (1983) regarded *Afrotarus*, *Pinacodera*, and *Taridius* as subgenera of *Cymindis*, implicitly considering all 14 subgenera of *Cymindis* currently recognized in Lorenz (2005) as synonyms of *Cymindis* s.str. For practical reasons this approach is not followed here except that *Pinacodera* is retained as a subgenus of *Cymindis*.

### Subgenus Tarulus

Bedel, 1906

Tarulus Bedel, 1906: 253. Type species: *Tarus zargoides* Wollaston, 1863 by monotypy. Etymology. From the generic name *Tarus* [*q.v*.] and the Latin suffix -*ulus* (small, little) [masculine].

#### Diversity.

Twenty-one species in North America (16 species, one of them Holarctic), Mexico (two species shared with North America), Eurasia (two species, one endemic to eastern Asia), and North Africa (four endemic species).

#### Identification.

Lindroth (1969a) reviewed all North American species. Since then, one Palaearctic species (*Coptodera vaporariorum*) has been found to occur naturally also in North America. Shpeley and Ball (1999: 420-421) published an emendation to Lindroth’s (1969a: 1072-1073) key of *Cymindis* to incorporate *Coptodera vaporariorum* and commented on the structural differences to separate that species from *Coptodera unicolor* which is most similar externally.

#### Taxonomic Note.

Lorenz (2005) listed all Nearctic species in the subgenus *Cymindis* s.str. However, Lindroth (1969a: 1070) noted that the North American species belong to the subgenus *Tarulus*, to which *Coptodera vaporariorum* is included.

### Cymindis americana

Dejean, 1826

Cymindis americana Dejean, 1826: 446. Type locality: «Amérique septentrionale» (original citation), restricted to «Concord [Middlesex County], Mass[achusetts]» by Lindroth (1969a: 1079). One syntype in MHNP (Lindroth 1955b: 25).
Cymindis venator Dejean, 1831: 311. Type locality: «Amérique septentrionale» (original citation). One syntype in MHNP (Lindroth 1955b: 25). Synonymy established by Horn (1882: 162), confirmed by Lindroth (1955b: 25).
Cymindis continens Casey, 1920: 287. Type locality: «Colorado» (original citation), which is probably incorrect (Lindroth 1969a: 1082). Lectotype (♀), designated by Lindroth (1975: 146), in USNM [# 47607]. Synonymy established by Lindroth (1969a: 1079).

#### Distribution.

This species ranges from southern Quebec (Larochelle 1975: 78) to eastern South Dakota (Kirk and Balsbaugh 1975: 39), south to northeastern Texas (Lamar County, Brian Raber pers. comm. 2010), southern Louisiana (Chaudoir 1873c: 103), northern Alabama (Madison County, CMNH), and southeastern South Carolina (Ciegler 2000: 119). The record from “Florida” (Bousquet and Larochelle 1993: 267) needs confirmation.

#### Records.

**CAN**: ON, QC **USA**: AL, AR, CT, DC, GA, KS, IA, IL, IN, KY, LA, MA, MD, MI, MN, MO, MS, NC, NE, NH, NJ, NY, OH, PA, RI, SC, SD, TN, TX, VA, VT, WI, WV [FL]

### Cymindis arizonensis

Schaeffer, 1910

Cymindis arizonensis Schaeffer, 1910: 400. Type locality: «Huachuca M[oun]t[ain]s, Arizona» (original citation for the lectotype). Lectotype (♀), designated by Lindroth (1969a: 1074), in USNM.
Cymindis zuniana Casey, 1913: 181. Type locality: «Benson [Cochise County], Arizona» (original citation for the lectotype). Lectotype (♂), designated by Lindroth (1975: 145), in USNM [# 47591]. Synonymy established by Lindroth (1969a: 1075).

#### Distribution.

This species ranges from southwestern California to “New Mexico” (Lindroth 1969a: 1075).

#### Records.

**USA**: AZ, CA, NM

### Cymindis borealis

LeConte, 1863

Cymindis borealis LeConte, 1863c: 7. Type locality: «North Red River; Nova Scotia» (original citation), restricted to «North Red River [either in southern Manitoba or on Minnesota-Dakota line]» by Lindroth (1969a: 1082). Five syntypes in MCZ [# 5830].

#### Distribution.

The range of this species extends from Newfoundland (Lindroth 1955a: 132) to southern British Columbia, south to “New Mexico” (Lindroth 1969a: 1082), northern Wisconsin along Lake Superior (Wickham 1896c: 135; MCZ), northwestern Pennsylvania (Erie County, CMNH), and Connecticut (Krinsky and Oliver 2001: 272). The record from “South Dakota” (Bousquet and Larochelle 1993: 267) is probably in error.

#### Records.

**CAN**: AB, BC, MB, NB, NF, NS (CBI), ON, QC, SK **USA**: CO, CT, ME, MI, MN, ND, NH, NM, NY, PA, UT, VT, WI, WY

### Cymindis californica

Horn, 1895

Cymindis californica G.H. Horn, 1895: 231. Type locality: «San Luis Obispo [San Luis Obispo County, California]» (original citation). Holotype [by monotypy] (♂) in MCZ [# 34519].

#### Distribution.

This species is known so far only from the holotype (Lindroth 1969a: 1086) collected in southwestern California.

#### Records.

**USA**: CA

### Cymindis cribricollis

Dejean, 1831

Cymindis cribricollis Dejean, 1831: 311. Type locality: «Amérique septentrionale» (original citation), restricted to «Rumney [Grafton County], N[ew] H[ampshire]» by Lindroth (1969a: 1075). One syntype in MHNP (Lindroth 1955b: 24).
Cymindis marginatus Kirby, 1837: 13. Type locality: «from New York to Cumberland-house; Lat. 65° [= apparently region of Great Bear Lake, Northwest Territories]» (original citation), restricted to «Fort Wrigley, N[orth] W[est] T[erritories]» by Lindroth (1969a: 1075). Two syntypes [2 originally cited] in BMNH (Lindroth 1953b: 169). Synonymy established by LeConte (1846b: 186), confirmed by Lindroth (1953b: 169).
Cymindis reflexa LeConte, 1850: 203. Type locality: Lake Superior (inferred from title of the paper). Four syntypes in MCZ [# 5824]. Synonymy established by LeConte (1869b: 244), confirmed by Lindroth (1955a: 131).
Cymindis abstrusa LeConte, 1859a: 82. Type locality: «Washington Territory» (original citation). Syntype(s) in MCZ [# 5825]. Synonymy established by Henshaw (1882: 209), confirmed by Lindroth (1969a: 1075).
Cymindis acomana Casey, 1913: 181. Type locality: «New Mexico» (original citation). Lectotype (♂), designated by Lindroth (1975: 145), in USNM [# 47597]. Synonymy established by Lindroth (1954b: 140).
Cymindis rupimontis Casey, 1913: 183. Type locality: «Boulder Co[unty], Colorado» (original citation). Lectotype (♀), designated by Lindroth (1975: 145), in USNM [# 47601]. Synonymy established by Lindroth (1954b: 141).
Cymindis alticola Casey, 1913: 183. Type locality: «White M[oun]t[ain]s, New Hampshire» (original citation). Lectotype (♂), designated by Lindroth (1975: 145), in USNM [# 47603]. Synonymy established by Lindroth (1954b: 141).
Cymindis kirbyi Casey, 1924: 88. Type locality: «Caribou District, British Columbia» (original citation). Holotype [by monotypy] (♂) in USNM [# 47602]. Synonymy established by Hatch (1953: 158), confirmed by Lindroth (1954b: 141).
Cymindis planifera Casey, 1924: 89. Type locality: «probably Colorado» (original citation). Holotype [by monotypy] (♂) in USNM [# 47598]. Synonymy established by Lindroth (1954b: 141).
Cymindis obliqua Casey, 1924: 89. Type locality: «Edmonton, Alberta» (original citation). Lectotype (♀), designated by Lindroth (1975: 145), in USNM [# 47598]. Synonymy established by Lindroth (1954b: 141).
Cymindis sinuata Casey, 1924: 90 [primary homonym of *Cymindis sinuata* Reiche and Saulcy, 1855]. Type locality: «New Mexico» (original citation). Lectotype (♀), designated by Lindroth (1975: 145), in USNM [# 47595]. Synonymy established by Lindroth (1954b: 141).
Cymindis alternans Casey, 1924: 90 [primary homonym of *Cymindis alternans* Rambur, 1837]. Type locality: «probably Colorado» (original citation). Holotype [by monotypy] (♀) in USNM [# 47596]. Synonymy established by Lindroth (1954b: 141).
Cymindis tarda Liebke, 1927: 104. Replacement name for *Cymindis sinuata* Casey, 1924.
Cymindis caseyi Liebke, 1927: 104. Replacement name for *Cymindis alternans* Casey, 1924.

#### Distribution.

This widely distributed species ranges from Newfoundland (Lindroth 1955a: 131) to Vancouver Island, north to Yukon Territory (Lindroth 1969a: 1076), south to “Oregon” (Horn 1882: 152), southeastern Arizona (Snow 1906b: 162), southern New Mexico (Otero County, CMNH; Lindroth 1969a: 1076), northeastern South Dakota (Kirk and Balsbaugh 1975: 38), and western Maryland (Bailey et al. 1994: 320). The records from “Prince Edward Island” (Bousquet and Larochelle 1993: 267, see Majka et al. 2008: 133), “Kansas” (Horn 1872c: 385), and “Nebraska” (Wickham 1896c: 135) need confirmation; that from southern California (Moore 1937: 12) is probably in error though one old specimen labeled “Cal.” is known (MCZ).

#### Records.

**FRA**: PM **CAN**: AB, BC (VCI), LB, MB, NB, NF, NS (CBI), NT, ON, QC, SK, YT **USA**: AZ, CO, CT, MA, MD, ME, MI, MN, MT, ND, NH, NJ, NM, NY, OH, OR, PA, RI, SD, UT, VT, WA, WI, WY [CA, KS, NE, PE]

### Cymindis elegans

LeConte, 1846

Cymindis elegans LeConte, 1846b: 186. Type locality: «provinciis australibus» (original citation). Syntype(s) in MCZ [# 5827].
Cymindis elegans mobilensis Casey, 1920: 288. Type locality: «Mobile [Mobile County], Alabama» (original citation). Two syntypes in USNM [# 47606]. **New synonymy**.

#### Distribution.

This species is found from Massachusetts (Darlington 1936b: 148; Hampden County, MCZ) south to the Florida Panhandle (Peck and Thomas 1998: 24) and southwestern Alabama (Casey 1920: 288).

#### Records.

**USA**: AL, DC, FL, GA, MA, NC, NJ, NY, RI, SC

### Cymindis evanescens

Casey, 1913

Cymindis evanescens Casey, 1913: 179. Type locality: «Marysvale [Piute County], Utah» (original citation). Lectotype (♂), designated by Lindroth (1975: 146), in USNM [# 47594].

#### Distribution.

This species is known from a few localities in southern Oregon (Westcott et al. 2006: 8; Harney County, CMNH; Lake County, Foster F. Purrington pers. comm. 2009), west-central Idaho (Valley County, USNM), southwestern Wyoming (Parmenter and MacMahon 1984: 26), south-central Utah (Casey 1913: 179; Knowlton 1939: 2; Piute County, MCZ), “Nevada” (CMNH, MCZ), and east-central California (Lindroth 1969a: 1079). The record from Seattle, Washington (Hatch 1953: 158) needs confirmation.

#### Records.

**USA**: CA, ID, NV, OR, UT, WY [WA]

### Cymindis interior

Lindroth, 1969

Cymindis interior Lindroth, 1969a: 1074. Type locality: «Albuquerque [Bernalillo County], New Mex[ico]» (original citation). Holotype (♂) in MCZ [# 34676].

#### Distribution.

This species is found from the southern part of the Prairie Provinces south to southeastern Arizona (Graham County, UASM), central New Mexico (Lindroth 1969a: 1074), western Texas (Ward County, Ken Karns pers. comm. 2009), and northwestern Oklahoma (Woods County, CMNH).

#### Records.

**CAN**: AB, MB, SK **USA**: AZ, CO, KS, MN, ND, NE, NM, OK, SD, TX, WY

### Cymindis laticollis

Say, 1830

Cymindis laticollis Say, 1830a: 134. Type locality: «Col[orado]» (neotype label). Neotype (♂), designated by Lindroth and Freitag (1969: 350), in MCZ [# 33002]. Note. «near the Rocky Mountains» was the area originally cited by Say (1830a: 134).
Cymindis villigera Chaudoir, 1873c: 96. Type locality: «Texas» (original citation). Holotype [by monotypy] (♀) in MHNP. Synonymy established by Horn (1882: 162), confirmed by Lindroth (1969a: 1073).

#### Distribution.

This species ranges from northwestern South Dakota (Kirk and Balsbaugh 1975: 38) to western Montana (Hatch 1933a: 9), south to northern Sonora (Bates 1884: 296) and southern Texas (Sutton, Val Verde, Edwards, and Colorado Counties, CMNH). The record from “Arkansas” (Bousquet and Larochelle 1993: 268) needs confirmation; those from southern Wisconsin (Rauterberg 1885: 14) and southwestern California (Moore 1937: 12) are probably in error.

#### Records.

**USA**: AZ, CO, KS, MT, NM, OK, SD, TX, WY [AR] – Mexico

### Cymindis neglecta

Haldeman, 1843

Cymindis neglecta Haldeman, 1843b: 298. Type locality: southeastern Pennsylvania (Haldeman 1843a: 295). One possible syntype, a ♀ labeled “[pink disc] / C. neglecta Hald. [handwritten],” in MCZ (collection LeConte).

#### Distribution.

The range of this species extends from Sable Island (Christopher G. Majka pers. comm. 2007) off the coast of Nova Scotia to southern Alberta (Lindroth 1969a: 1086), south to northwestern South Dakota (Kirk and Balsbaugh 1975: 39) and southern South Carolina (Ciegler 2000: 119).

#### Records.

**CAN**: AB, MB, NB, NS, ON, QC, SK **USA**: CT, DC, DE, IA, IL, IN, MA, ME, MI, MN, NC, ND, NH, NJ, NY, OH, PA, SC, SD, VA, VT, WI, WV

### Cymindis pilosa

Say, 1823

Cymindis pilosus Say, 1823a: 10. Type locality: «Dorchester [Suffolk County], Mass[achusetts]» (neotype label). Neotype (♂), designated by Lindroth and Freitag (1969: 350), in MCZ [# 33000].
Cymindis pubescens Dejean, 1825: 215. Type locality: «Amérique septentrionale» (original citation). Two syntypes in MHNP (Lindroth 1955b: 24). Synonymy established by Dejean (1826: 446), confirmed by Lindroth (1955b: 24).
Cymindis cribrata LeConte, 1859c: 2. Type locality: «Nebraska» (original citation). Holotype [by monotypy] (♀) in MCZ [# 5829]. Synonymy established by Lindroth (1969a: 1083).

#### Distribution.

This species ranges from southern Quebec (Larochelle 1975: 79) to southeastern North Dakota (Ransom County, CNC), north to southwestern Manitoba (Stjernberg 2011: 71), south to “Texas” (Lindroth 1969a: 1083) and northeastern Florida (Duval County, USNM). The record from the state of Puebla in Mexico (Bates 1891a: 270) needs confirmation.

#### Records.

**CAN**: MB, ON, QC **USA**: AR, CT, DC, DE, FL, GA, IA, IL, IN, KS, KY, MA, MD, ME, MI, MN, MO, NC, ND, NE, NH, NJ, NY, OH, OK, PA, RI, SC, SD, TN, TX, VA, VT, WI, WV

### Cymindis planipennis

LeConte, 1863

Cymindis planipennis LeConte, 1863c: 6. Type locality: «New Mexico» (original citation). Four syntypes in MCZ [# 5826].
Cymindis brevipennis Zimmermann [in LeConte], 1869b: 243. Type locality: «Kansas» (original citation). Syntype(s) probably lost. Synonymy established by Horn (1882: 162), confirmed by Lindroth (1969a: 1077).
Cymindis bipartita Casey, 1913: 185. Type locality: «Green River [Sweetwater County], Wyoming» (original citation). Lectotype (♂), designated by Lindroth (1975: 145), in USNM [# 47604]. Synonymy established by Lindroth (1969a: 1077).
Cymindis directa Casey, 1920: 286. Type locality: «Akron [Washington County], Colorado» (original citation). Lectotype (♀), designated by Lindroth (1975: 145), in USNM [# 47605]. Synonymy established by Lindroth (1969a: 1077).
Cymindis govanica Casey, 1924: 89. Type locality: «Govan [Lincoln County], Washington» (original citation). Lectotype (♀), designated by Lindroth (1975: 146), in USNM [# 47599]. Synonymy established, under the name *Cymindis brevipennis* Zimmermann, by Hatch (1953: 159), confirmed by Lindroth (1969a: 1077).

#### Distribution.

The range of this species extends from southern Manitoba to the Okanagan Valley in south-central British Columbia, north to southern Northwest Territories (Lindroth 1969a: 1078), south to northern Oregon (Baker and Wasco Counties, CMNH, USNM; Hatch 1953: 158), northern Arizona (Coconino County, USNM; Snow 1906b: 162), northwestern Texas (Potter County, USNM), and Wisconsin (Purrington and Maxwell 1998: 190); also known from one specimen collected in southern Quebec (Lindroth 1969a: 1078).

#### Records.

**CAN**: AB, BC, MB, NT, QC, SK **USA**: AZ, CO, IA, ID, KS, MN, MT, ND, NE, NM, NV, OR, SD, TX, UT, WA, WI, WY

### Cymindis seriata

Hatch, 1953

Cymindis seriata Hatch, 1953: 158. Type locality: «Steilacoom [Pierce County], Wash[ington]» (original citation). Holotype (♂) in USNM.

#### Distribution.

This species is known from a few localities in Washington (Hatch 1953: 158) and western Oregon (Westcott et al. 2006: 8).

#### Records.

**USA**: OR, WA

### Cymindis unicolor

Kirby, 1837

Cymindis unicolor Kirby, 1837: 14. Type locality: northern parts of British America (inferred from title of the paper), restricted to «Fort Smith, N[orth] W[est] Terr[itories]» by Lindroth (1969a: 1083). Holotype [by monotypy] in BMNH (Lindroth 1953b: 169).
Cymindis hudsonica LeConte, 1863c: 6. Type locality: «Methy Lake [Saskatchewan] and Labrador» (original citation). One syntype in MCZ [# 5828]. Synonymy established by LeConte (1873b: 322), confirmed by Lindroth (1955a: 130).
Cymindis parowana Casey, 1924: 88. Type locality: «Parowan M[oun]t[ain]s (10000 ft.) [Juab County] Utah» (original citation for the lectotype). Lectotype (♂), designated by Lindroth (1975: 146), in USNM [# 47592]. Synonymy established by Lindroth (1955a: 130).

#### Distribution.

This species occurs from Newfoundland (Lindroth 1955a: 131) to southwestern Yukon Territory (Shpeley and Ball 1999: 422), south to the Sierra Nevada in east-central California (Papp 1978: 165; Nelson 1988a: Fig. 1), Santa Catalina Mountains in southern Arizona (Nelson 1988a: Fig. 1), and southern Colorado (Wickham 1902: 240; Lindroth 1969a: 1085); isolated on some mountains of northern New England. The records from “Vermont,” “Massachusetts,” “Connecticut,” and “Rhode Island” (Erwin et al. 1977: 4.58) are likely in error; that from Alaska (Lindroth 1969a: 1085) probably refers to *Cymindis vaporariorum* Linnaeus (Shpeley and Ball 1999: 423). Fossil remnants of this species, dated between 11,200 and 20,530 years old, have been found in northeastern Wisconsin (Schwert 1992: 78), south-central Minnesota (Ashworth et al. 1981: 77), and southeastern and northeastern Iowa (Baker et al. 1986: 96; Schwert 1992: 78; Woodman et al. 1996: 17).

#### Records.

**CAN**: AB, BC, LB, MB, NF, NT, NU, ON, QC, SK, YT **USA**: AZ, CA, CO, ME, MT, NH, OR, UT, WA, WY

### Cymindis uniseriata

Bates, 1884

Cymindis uniseriata Bates, 1884: 296. Type locality: «Pinos Altos in Chihuahua, Mexico» (original citation). Syntype(s) probably in BMNH.
Cymindis agitata Casey, 1920: 285. Type locality: «Colonia Garcia, Chihuahua, Mexico» (original citation). Lectotype (♀), designated by Lindroth (1975: 146), in USNM [# 47593]. Synonymy established by Lindroth (1969a: 1085).

#### Distribution.

This species is known from southeastern Arizona (Bousquet and Larochelle 1993: 334) and central New Mexico (Torrance County, Ken Karns pers. comm. 2009) south to the state of Durango along the Sierra Madre Occidental (Ball and Shpeley 1992a: 61).

#### Records.

**USA**: AZ, NM – Mexico

### Cymindis vaporariorum

(Linnaeus, 1758)

Carabus vaporariorum Linnaeus, 1758: 415. Type locality: «Europa» (original citation), restricted to «Uppsala, Suecia» by Lindroth (1957b: 339). One possible syntype in LSL (Lindroth 1957b: 335).
Carabus humeralis Paykull, 1790: 40 [secondary homonym of *Cymindis humeralis* (Geoffroy, 1785)]. Type locality: Sweden (inferred from title of the book). Syntype(s) probably in NRSS. Synonymy established by Chaudoir (1850b: 86).
Cymidis basalis Gyllenhal, 1810: 174. Type locality: Sweden (inferred from title of the book). Syntype(s) probably in UZIU. Synonymy established by Germar (1840b: 442). Note. Gyllenhal (1810: 172-175) used the spelling *Cymidis*, an incorrect subsequent spelling of *Cymindis*.
Cymindis punctata Dejean, 1825: 214. Type locality: «Suède; Alpes de la Haute Styrie; dans les Pyrénées orientales au Canigou et Carlitte; Alpes du Piémont» (original citation). Syntype(s) in MHNP. Synonymy established with the name *Cymindis basalis* Gyllenhal by Dejean (1825: 214).
Cymindis immaculata Dejean [in Dejean and Boisduval], 1829: 93. Type locality: «Kamtschatka [Russia]» (original citation). Syntype(s) in MHNP. Synonymy established (as aberration) by Csiki (1932b: 1482).
Cymindis intricata Motschulsky, 1844: 46. Type locality: «Alpes du Hamar-Daban au sud du Baïcal et auprès de Tourkinsk au N[ord] O[uest] du Baïcal, Sibérie orientale [Irkutsk Oblast, Russia]» (original citation). Three syntypes in ZMMU (Keleinikova 1976: 201). Synonymy established, under the name *Cymindis immaculata* Dejean, by Chaudoir (1850b: 87).
Tarus gebleri Motschulsky, 1850a: 40. Type locality: «Altaïi» (original citation). One syntype, listed as “corruptum,” in ZMMU (Keleinikova 1976: 199). Synonymy established, under the name *Cymindis vaporariorum immaculata* Dejean, by Emetz (1976: 227). Note. According to Emetz (1976: 227), the syntype is labeled “Alp. Altai, Ajan [= Ayan, a city along the coast of the Sea of Okhotsk, in northern Khabarovskii Kray Province, Russia].”
Tarus dilatipennis Motschulsky, 1865: 300. Type locality: «Ajan [Khabarovsk Kray], Sib[érie] or[ientale]» (original citation). One syntype in ZMMU (Keleinikova 1976: 195). Synonymy established by Chaudoir (1873c: 99).
Cymindis subarcticus Kano, 1933: 96. Type locality: «Oboedomari near Ohtomari, S. Saghalien [Russia]» (original citation). Holotype (♀) in USMT (Habu 1967: 69). Synonymy established, under the name *Cymindis vaporariorum immaculata* Dejean, by Habu (1982: 122).
Cymindis subarcticus asahiensis Habu and Baba, 1962: 13. Type locality: «M[oun]t Asahidake (2,300 m), Niigata Prefecture, Honshu, Japan» (original citation). Holotype (♂) in NIAS. Synonymy established, under the name *Cymindis vaporariorum immaculata* Dejean, by Habu (1982: 122).

#### Distribution.

This Holarctic species ranges from Ireland to the Far East, south to Spain, Portugal, Greece, Kazakhstan, Mongolia, northern China (Kabak 2003: 418), and in the Nearctic Region from Alaska to northwestern Northwest Territories [see Shpeley and Ball 1999: Fig. 6].

#### Records.

**CAN**: NT, YT **USA**: AK – **Holarctic**

### Subgenus Pinacodera

Schaum, 1857

Pinacodera Schaum, 1857a: 294. Type species: *Cymindis limbata* Dejean, 1831 designated by Lindroth (1969a: 1067). Etymology. From the Greek *pinacos* (board, tablet, chart) and *dere* (neck, by extension pronotum) [feminine].
Planesus Motschulsky, 1864: 240. Type species: *Cymindis fuscata* Dejean, 1831 (= *Lebia platicollis* Say, 1823) by original designation. Synonymy established by Chaudoir (1875: 2).

#### Diversity.

Nineteen species in the temperate, subtropical, and tropical regions of North America (11 species) and Middle America (11 species).

#### Identification.

Casey (1920) published a key to all but three (*Pinacodera complanata*, *Pinacodera punctigera*, and *Pinacodera punctifera*) of the currently valid North American species. As usual his key is difficult to use. A taxonomic revision of the subgenus is needed.

#### Taxonomic Note.

Lorenz (2005: 465) listed *Pinacodera* as a valid genus. However, Ball and Hilchie (1983: 149) presented arguments to treat this group as a subgenus of *Cymindis*.

### Cymindis abbreviata

(Casey, 1920)

Pinacodera abbreviata Casey, 1920: 283. Type locality: «Colorado» (original citation). One syntype in USNM [# 47614].

#### Distribution.

This species is known only from the type series.

#### Records.

**USA**: CO

### Cymindis ampliata

(Casey, 1920)

Pinacodera ampliata Casey, 1920: 282. Type locality: «Colorado» (original citation). One syntype in USNM [# 47611].

#### Distribution.

This species is known only from the type series.

#### Records.

**USA**: CO

### Cymindis atripennis

(Casey, 1920)

Pinacodera atripennis Casey, 1920: 284. Type locality: «Florida» (original citation). Holotype [by monotypy] in USNM [# 47613].

#### Distribution.

This species ranges from east-central Georgia (Emanuel County, UASM) to southern Florida (Collier County, CMNH). The record from “South Carolina” (Bousquet and Larochelle 1993: 268) needs confirmation.

#### Records.

**USA**: FL, GA [SC]

### Cymindis blanda

Casey, 1913

Cymindis blanda Casey, 1913: 184. Type locality: «Douglas, Cochise Co[unty], Arizona» (original citation). Three syntypes [3 originally cited] in USNM [# 47608].

#### Distribution.

This species is known only from the type locality in southeastern Arizona.

#### Records.

**USA**: AZ

### Cymindis complanata

Dejean, 1826

Cymindis complanata Dejean, 1826: 448. Type locality: «Amérique septentrionale» (original citation), restricted to «S[ain]t John’s Bluff [Duval County], Flor[ida]» by Lindroth (1969a: 1070). Two syntypes in MHNP (Lindroth 1955b: 24).
Lebia russata Newman, 1840: 31. Type locality: «S[ain]t John’s Bluff [Duval County], East Florida» (original citation). One syntype in BMNH (Lindroth 1969a: 1070). Synonymy established by Lindroth (1969a: 1070).

#### Distribution.

This species ranges from Maryland (Prince Georges County, UASM) and northeastern Virginia (UASM) south to central Florida (Peck and Thomas 1998: 24) and southwestern Alabama (Mobile and Washington Counties, UASM). All other state records listed in Bousquet and Larochelle (1993: 269) as well as that from southeastern Pennsylvania (Rathvon 1869: 524, as *Lebia russata*) need confirmation.

#### Records.

**USA**: AL, FL, GA, MD, NC, SC, VA [LA, NJ, PA, TX]

### Cymindis limbata

Dejean, 1831

Cymindis limbata Dejean, 1831: 320. Type locality: «Amérique septentrionale» (original citation), restricted to «Marion [Plymouth County], Mass[achusetts]» by Lindroth (1969a: 1067). One syntype in MHNP (Lindroth 1955b: 24).
Cymindis comma T.W. Harris [in Scudder], 1869: 82 [*nomen dubium*]. Type locality not stated. Syntype(s) presumably lost. Synonymy established with doubt by Bousquet and Larochelle (1993: 13).

#### Distribution.

This species ranges from Nova Scotia (Lindroth 1954c: 307) to northwestern Minnesota (Becker County, UASM), south to southeastern Texas (Brazoria County, CMNH) and central Florida (Peck and Thomas 1998: 24). The record from “North Dakota” (Bousquet and Larochelle 1993: 269) needs confirmation.

#### Records.

**CAN**: NB, NS, ON, QC **USA**: AL, AR, CT, DC, DE, FL, GA, IA, IL, IN, KS, KY, LA, MA, MD, ME, MI, MN, MO, MS, NC, NE, NH, NJ, NY, OH, OK, PA, RI, SC, TN, TX, VA, VT, WI, WV [ND]

### Cymindis obscura

(Casey, 1920)

Pinacodera obscura Casey, 1920: 284. Type locality: «Southern Pines [Moore County], North Carolina» (original citation). One syntype in USNM [# 47612].

#### Distribution.

This species is known only from the type locality in central North Carolina and one locality in northern Georgia (Fattig 1949: 40).

#### Records.

**USA**: GA, NC

### Cymindis platicollis

(Say, 1823)

Lebia platicollis Say, 1823a: 14. Type locality: «Allegheny, P[ennsylvani]a» (neotype label). Neotype (♂), designated by Lindroth and Freitag (1969: 349), in MCZ [# 33001].
Cymindis fuscata Dejean, 1831: 321. Type locality: «Amérique septentrionale» (original citation). One syntype in MHNP (Lindroth 1955b: 24). Synonymy established by Lindroth (1969a: 1068).
Planesus laevigatus Motschulsky, 1865: 297. Type locality: «environs de Mobile [Mobile County, Alabama]» (original citation). Lectotype, designated by Bousquet and Larochelle (1993: 17), in ZMMU. Synonymy established by Bousquet and Larochelle (1993: 17).
Planesus fuscicollis Motschulsky, 1865: 298. Type locality: «états méridionaux de l’Amérique du Nord» (original citation). Lectotype (♀), designated by Bousquet and Larochelle (1993: 17), in ZMMU. Synonymy established by Bousquet and Larochelle (1993: 17).
Pinacodera virescens Dury, 1911: 275. Type locality: «Plott Balsam M[oun]t[ain]s, North Carolina» (original citation). Holotype [by monotypy] (♂) in CMC (Vulinec and Davis 1984: 233). Synonymy established by Ball (in Vulinec and Davis 1984: 233).

#### Distribution.

This species ranges from southwestern Maine (Majka et al. 2011: 47) and southern Quebec (Bousquet 1987a: 133) to eastern North Dakota (Tinerella 2003: 636), south to western Texas, Nuevo León in Mexico, and southern South Carolina [see Hunting 2009: Fig. 4.12]. The record from northern Sonora (Bates 1884: 296) needs confirmation.

#### Records.

**CAN**: ON, QC **USA**: AL, AR, CT, DC, GA, IA, IL, IN, KS, LA, MA, MD, ME, MI, MN, MS, NC, ND, NE, NH, NJ, NM, NY, OH, OK, PA, RI, SC, TN, TX, VA, VT, WI, WV – Mexico

**Figure 40.**
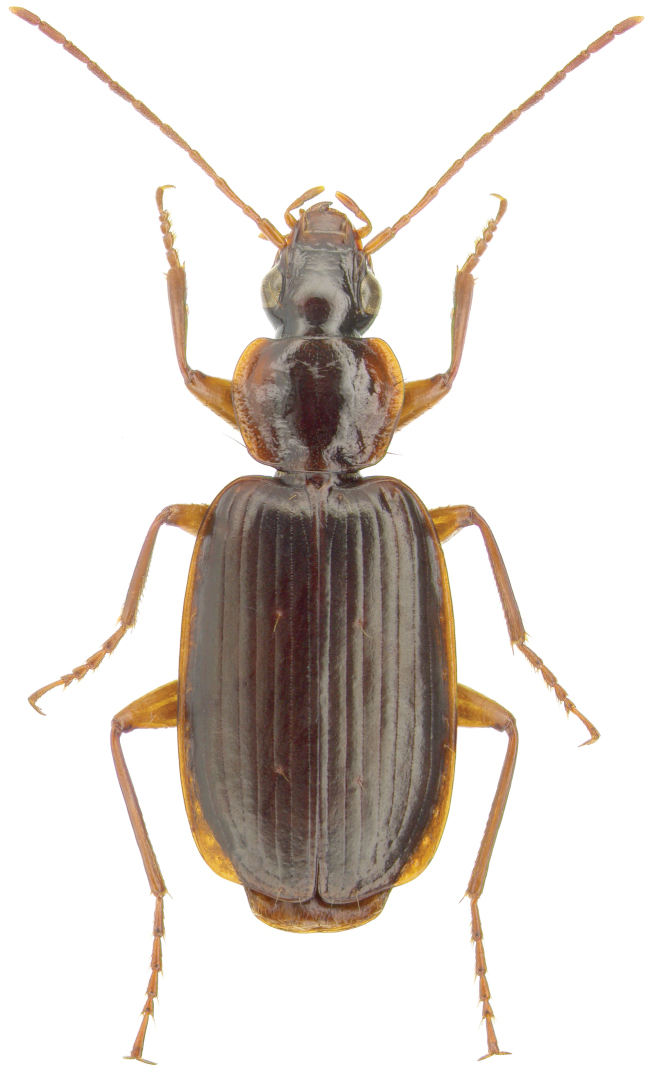
*Cymindis platicollis* (Say). This *Cymindis* is a typical element of the temperate forest of eastern North America. Unlike most carabids inhabiting the temperate and boreal regions, this species lives above the surface of the ground in trees and is usually collected by beating branches. An arboreal way of life is a common feature among tropical carabids.

### Cymindis punctifera

(LeConte, 1884)

Lebia punctifera LeConte, 1884: 2. Type locality: «Arizona» (original citation). Holotype [by monotypy] (♀) in MCZ [# 5800].

#### Distribution.

This species is known from western Arizona to western Texas, south to Nuevo León, Coahuila, Durango, and Sonora in Mexico (Ball and Shpeley 1992a: 62).

#### Records.

**USA**: AZ, NM, TX – Mexico

### Cymindis punctigera

LeConte, 1851

Cymindis punctigera LeConte, 1851: 178. Type locality: «ad flumina Colorado et Gila» (original citation). Three syntypes in MCZ [# 66].

#### Distribution.

This species ranges from northeastern California to north-central Colorado, south to eastern Michoacán in Mexico and southern California [see Hunting 2009: Fig. 4.23].

#### Records.

**USA**: AZ, CA, CO, NM, NV, TX, UT – Mexico

### Cymindis subcarinata

(Casey, 1920)

Pinacodera subcarinata Casey, 1920: 281. Type locality: «Tuçson [Pima County], Arizona» (original citation). One syntype in USNM [# 47609].

#### Distribution.

This species is known only from the type locality in southern Arizona.

#### Records.

**USA**: AZ

### Subtribe Apenina

Ball, 1983

Apenina Ball, 1983: 516. Type genus: *Apenes* LeConte, 1851.

#### Diversity.

About 115 species (Lorenz 2005: 464-465) in the Nearctic (eight species of *Apenes*), Neotropical (72 species of *Apenes*), Australian (one species in New Guinea), Oriental (nine species), Palaearctic (22 species), and Afrotropical (14 species) Regions. These species are arrayed in five genera: *Apenes* (75 species), *Habutarus* Ball and Hilchie (one species from New Guinea), *Cymindoidea* Laporte (15 species), *Platytarus* Fairmaire (12 species), and *Trymosternus* Chaudoir (13 species from the Iberian Peninsula and northern Africa).

#### Taxonomic Note.

1. The family-group names Platytarini and Trymosternini were proposed by Zaballos and Jeanne (1994: 116) and recorded as synonyms of Apenina by Lorenz (2005: 464). However, both names are unavailable since they were not accompanied by a description or a reference to a published description (ICZN 1999: Article 13.1). 2. Apenina came out as the sister-group to the remaining lebiine subtribes, with the exclusion of sugimotoinines, actenocynines, and pericalines, in Ball et al. (1995) and Casale (1998: Fig. 91) cladistic analyses.

### Genus Apenes

LeConte, 1851

Sphenopalpus Blanchard, 1842: plate1 [*nomen oblitum*, see Ball and Shpeley (2009: 95)]. Type species: *Sphenopalpus parallelus* Blanchard, 1842 (= *Cymindis aenea* Dejean, 1831) by monotypy. Etymology. From the Greek *sphenos* (wedge) and the Latin *palpus* (feeler, by extension palp), alluding to the shape of the last maxillary and labial palpomeres (“*palpes cylindriques, ayant leur dernier article ovalaire terminé en pointe*,” see Blanchard 1853: 32) of the adult [masculine]. Note. Since Chaudoir (1875: 38) listed *Sphenopalpus parallelus* Blanchard as a junior synonym of *Apenes aenea* (Dejean), *Sphenopalpus* has been considered a junior synonym of *Apenes* on the account that it was made available with the publication of the text in 1853. However, the name was first made available by the publication of the plate in 1842 (see Emberson 1992).
Apenes LeConte, 1851: 174 [*nomen protectum*]. Type species: *Cymindis lucidula* Dejean, 1831 designated by Lacordaire (1854: 110). Synonymy established by Chaudoir (1875: 38). Etymology. From the Greek *apenes* (rough, harsh, cruel, ferocious) [feminine]. Note. *Apenes* have been treated as masculine (e.g., Bousquet and Larochelle 1993: 269-270; Lorenz 2005: 464-465) or feminine (e.g., Lindroth 1969a: 1087-1088; Ball and Shpeley 1992b). It comes from a transliteration of the Greek adjective Aπηνής, ές and so could be masculine or feminine. In such case, the name is to be treated as masculine unless its author, when establishing the name, stated that it is feminine or treated it as feminine in combination with an adjectival species-group name (ICZN 1999: Article 30.1.4.2). LeConte (1851) did not specify the gender but described the species “*Apenes opaca*”, so treating the generic name *Apenes* as feminine. As such *Apenes* is feminine.
Sphenopselaphus Gemminger and Harold, 1868a: 299. Unjustified emendation of *Sphenopalpus* Blanchard, 1842.

#### Diversity.

Seventy-five species in the Western Hemisphere arrayed in two subgenera: *Apenes* s.str. (67 species) and *Didymochaeta* Chaudoir (eight Neotropical species).

### Subgenus Apenes

LeConte, 1851

Apenes LeConte, 1851: 174. Type species: *Cymindis lucidula* Dejean, 1831 designated by Lacordaire (1854: 110).
Nominus Motschulsky, 1864: 240. Type species: *Cymindis pustulata* Dejean, 1831 (= *Cymindis sinuatus* Say, 1823) by original designation. Synonymy established by Gemminger and Harold (1868a: 124).
Malisus Motschulsky, 1864: 240. Type species: *Cymindis variegata* Dejean, 1825 (= *Carabus pallipes* Fabricius, 1792) by original designation. Synonymy established by Bates (1883a: 188).

#### Diversity.

Sixty-seven species in the temperate, subtropical, and tropical areas of the Nearctic (nine species) and Neotropical (64 species) Regions.

#### Identification.

Horn (1882: 156) wrote a key to four of the species found in North America. A taxonomic revision of the subgenus is needed.

### Apenes angustata

Schwarz, 1878

Apenes angustata Schwarz, 1878: 354. Type locality: «Enterprise [Volusia County, Florida]» (original citation). Lectotype (♂), designated by Erwin and House (1978: 234), in USNM [# 4495].

#### Distribution.

This species is known from Alabama (Löding 1945: 22) and northwestern Georgia (Fattig 1949: 41) south to southern Florida (Peck and Thomas 1998: 23).

#### Records.

**USA**: AL, FL, GA

### Apenes coriacea

(Chevrolat, 1863)

Cymindis coriacea Chevrolat, 1863: 188. Type locality: Cuba (inferred from title of the paper). Lectotype (♀), designated by Ball and Shpeley (2009: 109), in UMO.
Apenes laevis Liebke, 1939: 473. Type locality: «Amer[ica] mer[idionalis]» (lectotype label according to Ball and Shpeley 2009: 111). Lectotype (♀), designated by Ball and Shpeley (2009: 111), in IZWP. Synonymy established by Ball and Shpeley (2009: 111). Note. Liebke (1939: 473) originally cited the type locality as “Nord Amerika.”

#### Distribution.

This species is known from Monroe and Dade Counties in southern Florida (Thomas 2011: 308), Cuba, Jamaica, and the Grand Cayman Island [see Ball and Shpeley 2009: Fig. 9].

#### Records.

**USA**: FL – Cayman Islands, Cuba, Jamaica

### Apenes hilariola

Bates, 1891

Apenes hilariola Bates, 1891a: 271. Type locality: «Cuernavaca [Morelos], Colima City [Colima]» (original citation). Syntype(s) [2 originally mentioned] in BMNH.

#### Distribution.

This species is known from the Mexican states of Morelos, Colima (Bates 1891a: 271) and Baja California Norte (Notman 1919b: 234) and from southern Arizona (Ober and Maddison 2008: 30).

#### Records.

**USA**: AZ – Mexico

### Apenes lucidula lucidula

(Dejean, 1831)

Cymindis lucidula Dejean, 1831: 320. Type locality: «Amérique septentrionale» (original citation), restricted to «Baltimore, M[arylan]d» by Lindroth (1969a: 1087). One syntype in MHNP (Lindroth 1955b: 25).

#### Distribution.

This subspecies ranges from Massachusetts (Davidson et al. 2011: 514) to southeastern Minnesota (Gandhi et al. 2005: 932), south to southern Texas (Live Oak County, Brian Raber pers. comm. 2010), northwestern Louisiana (Natchitoches Parish, USNM), and central Florida (Highlands and Pasco Counties, CMNH; Horn 1882: 157); also known from southern Arizona (Pima and Santa Cruz Counties, UASM, CMNH) and the Baja California Peninsula (Horn 1895: 226).

#### Records.

**USA**: AL, AZ, CT, DC, DE, FL, GA, IA, IL, IN, KS, KY, LA, MA, MD, MI, MN, NC, NJ, NY, OH, PA, RI, SC, TN, TX, VA, WV – Mexico

#### Note.

The subspecies *Apenes lucidulus dulculia* Ball and Shpeley and *Apenes lucidulus michelii* Ball and Shpeley are found in the West Indies.

### Apenes nebulosa

LeConte, 1867

Apenes nebulosa LeConte, 1867b: 364. Type locality: «Cape San Lucas, Lower California [= Baja California Sur]» (original citation). Two syntypes in MCZ [# 5833].

#### Distribution.

This species ranges from southern and western Texas (Duval, Val Verde, and Jeff Davis Counties, CMNH) to southeastern California (Dajoz 2007: 20) and the Baja California Peninsula (LeConte 1867b: 364).

#### Records.

**USA**: AZ, CA, NM, TX – Mexico

### Apenes opaca

LeConte, 1851

Apenes opaca LeConte, 1851: 175. Type locality: «Georgia» (original citation). Holotype [by monotypy] (♂) in MCZ [# 5832].

#### Distribution.

This species is found along the Coastal Plain from South Carolina (Ciegler 2000: 120) to southern Florida (Peck and Thomas 1998: 23), west to southern Alabama (Löding 1945: 22); also recorded from the Bahamas (Turnbow and Thomas 2008: 11).

#### Records.

**USA**: AL, FL, GA, SC – Bahamas

### Apenes pallidipes

(Chevrolat, 1836)

Cymindis pallidipes Chevrolat, 1836a: [no. 153]. Type locality: «environs de Tuspan [Mexico]» (original citation). Holotype [by monotypy] location unknown (possibly in UMO).
Apenes marginipennis Chaudoir, 1875: 24. Type locality: «Yucatan [Mexico]» (original citation). Holotype [by monotypy] (♂) in MHNP. Synonymy established by Bates (1883a: 189).
Apenes mexicana Chaudoir, 1875: 25. Type locality: «Mexique» (original citation). Holotype [by monotypy] (♀) in IZWP (Mroczkowski 1960: 372). Synonymy established by Chaudoir (1875: 25).

#### Distribution.

This species ranges from southern Arizona (Bousquet and Larochelle 1993: 334) to Panama (Bates 1883a: 189).

#### Records.

**USA**: AZ – Guatemala, Mexico, Panama

### Apenes parallela parallela

(Dejean, 1825)

Cymindis parallela Dejean, 1825: 218. Type locality: «île de Cuba» (original citation). One syntype in MHNP (Ball and Shpeley 2009: 115). Note. Ball and Shpeley (2009: 115) mentioned that the specimen is MHNP is the holotype. However, Dejean (1825: 218) did not specify that he had a single specimen and so the specimen is a syntype.

#### Distribution.

This subspecies is known from Dade County in southeastern Florida (Thomas 2011: 307), the Bahamas, Cuba, and the Cayman Islands [see Ball and Shpeley 2009: Fig. 13].

#### Records.

**USA**: FL – Bahamas, Cayman Islands, Cuba

#### Note.

Two other subspecies, *Apenes parallela inaguae* Darlington and *Apenes parallela sublaevis* Darlington, are known from the West Indies.

### Apenes sinuata

(Say, 1823)

Cymindis sinuatus Say, 1823a: 8. Type locality: «M[arylan]d» (neotype label). Neotype (♀), designated by Lindroth and Freitag (1969: 350), in MCZ [# 32999]. Note. «Maryland» was the area originally cited by Say (1823a: 8).
Cymindis pustulata Dejean, 1831: 316. Type locality: «Amérique septentrionale» (original citation). Two syntypes [2 originally cited] in MHNP (Lindroth 1955b: 25). Synonymy established by LeConte (1846b: 189), confirmed by Lindroth (1955b: 25).

#### Distribution.

The range of this species extends from Rhode Island (USNM) to northeastern Kansas (Popenoe 1877: 23), including southernmost Ontario (Lindroth 1969a: 1088), south to southern Texas (Wickham 1897: 112; Galveston and San Patricio Counties, UASM) and the Florida Keys (Peck and Thomas 1998: 23).

#### Records.

**CAN**: ON **USA**: AL, AR, CT, DC, DE, FL, GA, IA, IL, IN, KS, KY, LA, MA, MD, MI, MO, MS, NC, NE, NJ, NY, OH, OK, PA, RI, SC, TN, TX, VA, WV

### Subtribe Dromiusina

Bonelli, 1810

Dromiei Bonelli, 1810: Tabula Synoptica. Type genus: *Dromius* Bonelli, 1810. Note. The spelling of the family-group name based on *Dromius* was emended to Dromiusidae by the International Commission on Zoological Nomenclature (ICZN 2006).
Lichnasthenitae J. Thomson, 1858: 35 (as Lichnastenitae), 458. Type genus: *Lichnasthenus* Thomson, 1858.
Lionychidae Jeannel, 1948a: 378, 380. Type genus: *Lionychus* Wissmann, 1846.
Singilini Jeannel, 1949a: 881, 915. Type genus: *Singilis* Rambur, 1837.
Metadromiina Basilewsky, 1984: 543. Type genus: *Metadromius* Bedel, 1907.
Metaxymorphina Basilewsky, 1984: 549. Type genus: *Metaxymorphus* Chaudoir, 1850.
Singiliomimina Basilewsky, 1984: 552. Type genus: *Singiliomimus* Péringuey, 1896.

#### Diversity.

Worldwide, with about 735 species arrayed in 48 genera (Lorenz 2005: 470-478, 480, as Lionychina, Pseudotrechina, Dromiina, Lichnasthenina, Singilina, plus *Metaxymorphus*). The North American fauna is represented by 33 species (about 4.5 % of the world fauna), of which two are adventive.

#### Taxonomic Note.

Jeannel (1949a: 991) grouped this subtribe and the demetriadines under the subfamily Dromiitae and stated that this complex is characterized by having the basal bulb of the median lobe reduced. The peliocypidines were included in this subtribe by Ball and Bousquet (2000: 112).

### Genus Dromius

Bonelli, 1810

Dromius Bonelli, 1810: Tabula Synoptica. Type species: *Carabus quadrimaculatus* Linnaeus, 1758 by subsequent monotypy in Samouelle (1819: 155). Etymology. Probably from the Greek *dromeus* (runner) and alluding to the agility of the adults in the field [masculine]. Note. Madge (1975: 582) pointed out that the type species of *Dromius* Bonelli, 1810 should be *Carabus truncatellus* Linnaeus, 1760 by subsequent monotypy in Panzer (1813: 73). That species is now included in the genus *Syntomus* Hope, 1838. Acceptance of this species as type species of *Dromius* would require nomenclatural changes for both *Dromius* and *Syntomus*. A request should be addressed to the International Commission on Zoological Nomenclature to set aside *Carabus truncatellus* Linnaeus, 1760 as type species of *Dromius* Bonelli, 1810.
Dromaeus Billberg, 1820: 26. Unjustified emendation of *Dromius* Bonelli, 1810.
Ocaeus Gistel, 1848a: ix. Unnecessary replacement name for *Dromius* Bonelli, 1810.

#### Diversity.

About 105 species (Lorenz 2005: 475-477) in the Nearctic (two species), Neotropical (20 species), Australian (one adventive species in New Zealand), Oriental, Palaearctic (53 species), and Afrotropical Regions. These species are placed in three subgenera: *Dromius* s.str. (about 50 species), *Obodromius* Jedlička (one species), and *Klepterus* Péringuey (47 species), with six species unplaced.

### Subgenus Dromius

Bonelli, 1810

Dromius Bonelli, 1810: Tabula Synoptica. Type species: *Carabus quadrimaculatus* Linnaeus, 1758 by subsequent monotypy in Samouelle (1819: 155).
Eudromius Acloque, 1896: 49 [junior homonym of *Eudromius* Gould, 1841]. Type species: *Dromius testaceus* Erichson, 1837 (= *Dromius angustus* Brullé, 1834) designated by Bousquet (2002b: 20). Synonymy established by Bousquet (2002b: 20).
Dinodromius Casey, 1920: 277. Type species: *Dromius piceus* Dejean, 1831 designated by Lindroth (1969a: 1041). Synonymy established by Lindroth (1969a: 1041). Etymology. Probably from the Greek prefix *dino*- (terrible, fearful) and the generic name *Dromius* [*q.v*.] [masculine].

#### Diversity.

About 50 species in the Nearctic (two species, one of them adventive), Neotropical (20 species), Australian (one adventive species in New Zealand), Oriental (four species), and Palaearctic (28 species) Regions.

#### Identification.

Larson (1998: 126) discussed the structural differences between the two species found in North America.

### Dromius fenestratus

(Fabricius, 1794)

Carabus fenestratus Fabricius, 1794a: 443 [primary homonym of *Carabus fenestratus* Müller, 1776]. Type locality: «Germania» (original citation). One syntype, without head and pronotum, in ZMUC (Zimsen 1964: 59). Note. Fabricius’ name should be permanently invalid because it is a primary homonym. However, *Carabus fenestratus* Müller has never been interpreted since its original description to my knowledge and the name is a *nomen dubium*.

#### Distribution.

This European species is adventive in North America where it is known only from three specimens collected in Newfoundland (Larson 1998: 126) and Nova Scotia (Majka and Klimaszewski 2004: 9; Halifax County, UASM). The first inventoried specimen collected on this continent was found in 1952 in Armdale, near Halifax, Nova Scotia (UASM).

#### Records.

**CAN**: NF, NS – **Adventive**

### Dromius piceus

Dejean, 1831

Dromius piceus Dejean, 1831: 353. Type locality: «Amérique septentrionale» (original citation), restricted to «Bethel [Oxford County], Maine» by Lindroth (1969a: 1041). Holotype [by monotypy] in MHNP (Lindroth 1955b: 24).
Dromius quadricollis LeConte, 1859a: 82. Type locality: «Puget Sound [Washington]» (original citation). One syntype in MCZ [# 5812]. Synonymy established by Horn (1882: 160).

#### Distribution.

The range of this species extends from Cape Breton Island (Bousquet 1987d: 107) to Vancouver Island (Lindroth 1969a: 1043), south to southern California (Fall 1901a: 48; MCZ), the states of Sonora (CNC) and Durango (CNC) in Mexico, and the Florida Panhandle (Peck and Thomas 1998: 24). Fossil remnants, undated but believed to be younger than 3 million years B.P., have been unearthed in northwestern Yukon Territory (Elias and Matthews 2002: 914).

#### Records.

**CAN**: AB, BC (VCI), MB, NB, NS (CBI), ON, PE, QC, SK **USA**: AL, AR, AZ, CA, CO, CT, DC, FL, GA, IA, IL, IN, KY, LA, MA, MD, ME, MI, MN, MS, MT, NC, NE, NH, NJ, NM, NY, OH, OR, PA, RI, SC, SD, TN, TX, VA, VT, WA, WI, WV, WY – Mexico

#### Note.

Lindroth (1969a: 1042-1043) studied superficially the structural variation in this species and concluded there was a single species but that the specimens west of the Rockies “may very well be treated” as a subspecies for which the name *Dromius quadricollis* LeConte, 1859 is available. Until a detailed study of the variation is done, I prefer not to recognize subspecies for this taxon.

### Genus Philorhizus

Hope, 1838

Philorhizus Hope, 1838: 63. Type species: *Carabus fasciatus* Paykull, 1790 (= *Carabus sigma* Rossi, 1790) by original designation. Etymology (original). Anagram of the generic name *Risophilus* (as *Rizophilus*), derived from the Greek *philos* (beloved) and *rhiza* (root) [masculine].
Dromiolus Reitter, 1905: 230, 235. Type species: *Dromius nigriventris* Thomson, 1857 (= *Dromius notatus* Stephens, 1827) by original designation. Etymology. From the generic name *Dromius* [*q.v*.] and the Latin suffix -*olus* (diminutive) [masculine].
Similidromius Mateu, 1953: 140. Type species: *Dromius elliptipennis* Wollaston, 1864 by monotypy. Synonymy established by Machado (1992: 519). Etymology. From the Latin adjective *similis*, -*e* (like) and the generic name *Dromius* [*q.v*.], alluding to the resemblance of the species in the hands of Mateu to those of *Dromius* [masculine].

#### Diversity.

About 50 species (Lorenz 2005: 477) in the Nearctic (two species, one of them adventive), Oriental (two species), Palaearctic (about 35 species, 15 of them endemic to the Canary and Madeira Islands), and Afrotropical (11 species) Regions.

#### Identification.

Bousquet (2004b: 49-51) commented on the structural differences between the two species found in North America.

### Philorhizus atriceps

(LeConte, 1880)

Dromius atriceps LeConte, 1880b: 163. Type locality: «Bayou Sara [West Feliciana Parish], L[ouisian]a» (original citation). One syntype in MCZ [# 5813].

#### Distribution.

This Coastal Plain species is found from “Massachusetts” (Lindroth 1969a: 1043) to southern Florida (Peck and Thomas 1998: 24), west to Louisiana (LeConte 1880b: 163) and north to western Tennessee (Tipton County, CMNH).

#### Records.

**USA**: AL, CT, FL, GA, LA, MA, MS, NJ, NY, RI, SC, TN, VA

### Philorhizus melanocephalus

(Dejean, 1825)

Dromius melanocephalus Dejean, 1825: 234. Type locality: «environs de Paris et de Lyon, France; Allemagne; Angleterre» (original citation). Syntype(s) probably in MHNP.

#### Distribution.

This Palaearctic species is adventive in North America where it known from four specimens (CNC) collected in 1996 on Vancouver Island, at Saanich Peninsula, Island View Beach (Bousquet 2004b: 49).

#### Records.

**CAN**: BC (VCI) – **Adventive**

### Genus Microlestes

Schmidt-Göbel, 1846

Microlestes Schmidt-Göbel, 1846: 41. Type species: *Microlestes inconspicuus* Schmidt-Göbel, 1846 designated by Andrewes (1934: 201). Etymology (original). From the Greek *micros* (small, little) and *lestes* (robber, mugger) [masculine].
Blechrus Motschulsky, 1847: 219. Type species: *Lebia glabrata* Duftschmid, 1812 (= *Carabus minutulus* Goeze, 1777) designated by Desmarest (1851: 70). Synonymy established by Chaudoir (1848: 95). Etymology. From the Greek *blechros* (faint, gentle, slight) [masculine].
Bomius LeConte, 1851: 177. Type species: *Bomius linearis* LeConte, 1851 designated by Lindroth (1969a: 1048). Synonymy established by Zimmermann (in LeConte 1869b: 243). Etymology. From the Greek *bomos* (base, plinth, pedestal), alluding to the lobate posterior edge of the pronotum (“*basi pedunculatus*”) of the adult [masculine].

#### Diversity.

About 130 species (Lorenz 2005: 473-474) in the Nearctic (eight species), Neotropical (one species endemic to Cuba and one species in northern Mexico), Oriental, Palaearctic (about 55 species), and Afrotropical Regions.

#### Identification.

Lindroth (1969a: 1047-1054) reviewed the Nearctic species. Since then, one new North American species (*Microlestes lindrothi*) has been described by Mateu (1995).

### Microlestes brevilobus brevilobus

Lindroth, 1969

Microlestes brevilobus Lindroth, 1969a: 1054. Type locality: «Long Point, Ont[ario]» (original citation). Holotype (♂) in CNC [# 11577].

#### Distribution.

This subspecies is known from scattered localities from southernmost Ontario (Lindroth 1969a: 1054) to southern Arizona (Pima County, CMNH), southwestern Texas (Val Verde County, CNC), northwestern Alabama (Walker County, CNC), and southwestern North Carolina (Macon County, CMNH). One specimen simply labeled from California is also known (Lindroth 1969a: 1054).

#### Records.

**CAN**: ON **USA**: AL, AR, AZ, CO, IA, KS, MO, MS, NC, OH, OK, TX, WV [CA]

#### Note.

The subspecies *Microlestes brevilobus mexicanus* Mateu is known from Michoacán in Mexico.

### Microlestes curtipennis

(Casey, 1920)

Blechrus curtipennis Casey, 1920: 270. Type locality: «San Francisco [San Francisco County], California» (original citation). Lectotype (♀), designated by Lindroth (1975: 145), in USNM [# 47685].

#### Distribution.

This species is known from scattered localities from central Saskatchewan (Lindroth 1969a: 1053) to the Okanagan Valley in south-central British Columbia (Blades and Maier 1996: 66), south to the San Francisco area (Casey 1920: 270), northern Utah (Lindroth 1969a: 1053), northeastern Colorado (Bell 1971: 47), and southwestern South Dakota (Kirk and Balsbaugh 1975: 38).

#### Records.

**CAN**: AB, BC, SK **USA**: CA, CO, OR, SD, UT, WY

### Microlestes lindrothi

Mateu, 1995

Microlestes lindrothi Mateu, 1995: 144. Type locality: «Berkeley [Alameda County], California» (original citation). Holotype (♂) in Mateu’s collection (Almería, Spain).

#### Distribution.

This species is known only from the original two specimens collected at Berkeley, on the San Francisco Bay in western California.

#### Records.

**USA**: CA

### Microlestes linearis

(LeConte, 1851)

Dromius angustus LeConte, 1846b: 191 [primary homonym of *Dromius angustus* Brullé, 1834]. Type locality: «ad Rocky Mountains» (original citation). Two syntypes in MCZ [# 5815].
Bomius linearis LeConte, 1851: 177. Replacement name for *Bomius angustus* (LeConte, 1846).
Blechrus prominulus Casey, 1920: 269. Type locality: «Reno [Washoe County], Nevada» (original citation for the lectotype). Lectotype (♂), designated by Lindroth (1975: 145), in USNM [# 47686]. Synonymy established by Lindroth (1969a: 1050).

#### Distribution.

The range of this species extends from Nova Scotia to southwestern British Columbia, though not quite reaching the coast (Lindroth 1969a: 1051), south to northern California (Lindroth 1969a: 1051; Casey 1920: 269, as *Blechrus prominulus*), northern Arizona (Lindroth 1969a: 1051), southern Oklahoma (Elliott et al. 2006: 126), and “Virginia” (CMNH, collection Ulke). The record from “Mississippi” (Bousquet and Larochelle 1993: 274) needs confirmation.

#### Records.

**CAN**: AB, BC, MB, NS, ON, QC, SK **USA**: AR, AZ, CA, CO, CT, DC, IA, ID, IL, IN, KS, MA, MD, ME, MI, MN, MO, MT, ND, NE, NH, NJ, NV, NY, OH, OK, OR, PA, UT, VA, VT, WA, WI, WY [MS]

#### Note.

This species has passed under the name of *Blechrus glabratus* (Duftschmid, 1812) until the 1960s.

### Microlestes lucidus lucidus

(LeConte, 1851)

Bomius lucidus LeConte, 1851: 177. Type locality: «Colorado [River, California]» (original citation). Four syntypes in MCZ [# 65].

#### Distribution.

This subspecies is known from southern California (Fall 1901a: 48; Mateu 1974: 264), “Nevada” (Horn 1882: 134), southeastern Colorado (Bent County, CNC), and central Texas (Casey 1920: 270). The records from northeastern Kansas (Popenoe 1877: 23) and “Oregon” (Horn 1882: 134) need confirmation. The previous records from “Colorado” (Wickham 1902: 239; Mateu 1974: 264) were probably based on a misinterpretation of the type locality.

#### Records.

**USA**: CA, CO, NV, TX [KS, OR]

#### Note.

The subspecies *Microlestes lucidus subdeserticus* Mateu is known from the states of Durango and Querétaro in Mexico.

### Microlestes major

Lindroth, 1969

Microlestes major Lindroth, 1969a: 1049. Type locality: «W[ashington] T[erritory]» (holotype label), herein restricted to Seattle, King County, Washington (see Lindroth 1969a: 1049). Holotype (♂) in MCZ [# 34512].

#### Distribution.

This species is known from a few specimens collected along the coast in southwestern British Columbia, west-central Washington (Lindroth 1969a: 1049), and Oregon (Lane County, CNC).

#### Records.

**CAN**: BC **USA**: OR, WA

### Microlestes nigrinus

(Mannerheim, 1843)

Dromius nigrinus Mannerheim, 1843: 184. Type locality: «California» (original citation), herein restricted to Port Harford, San Luis Obispo County (see Casey 1920: 269, as *Blechrus nigrinus obispinus*). Lectotype (♂), designated by Lindroth (1969a: 1051), in ZMH.
Blechrus nigrinus fretus Casey, 1920: 269. Type locality: «San Francisco [San Francisco County], California» (original citation). Lectotype (♀), designated by Lindroth (1975: 145), in USNM [# 47683]. Synonymy established by Lindroth (1969a: 1051).
Blechrus nigrinus obispinus Casey, 1920: 269. Type locality: «Port Harford, S[an] L[uis] Obispo Co[unty], California» (original citation). Lectotype (♀), designated by Lindroth (1975: 145), in USNM [# 47684]. Synonymy established by Lindroth (1969a: 1051).

#### Distribution.

The range of this species extends from Vancouver Island to eastern South Dakota (Kirk and Balsbaugh 1975: 38; French et al. 2004: 557), south to southern Colorado (Wickham 1902: 239; Boulder County, CMNH), northern Arizona (Coconino County, CMNH), and southern California (Lindroth 1969a: 1053). The record from “New Mexico” (Wickham 1896c: 135) needs confirmation.

#### Records.

**CAN**: BC (VCI) **USA**: AZ, CA, CO, ID, OR, SD, UT, WA, WY [NM]

### Microlestes pusio

(LeConte, 1863)

Blechrus pusio LeConte, 1863c: 6. Type locality: «Louisiana» (original citation). Syntype(s) location unknown. Note. All four specimens under this name in the LeConte collection (MCZ # 5816) are labeled either “Tex” or “Dallas, Texas” suggesting that none of these specimens are syntypes.

#### Distribution.

This species is found from Long Island, New York (Notman 1928: 238) to eastern South Dakota (Kirk and Balsbaugh 1975: 38), including southernmost Ontario (Lindroth 1969a: 1054), south to southeastern Texas (Horn 1882: 135; Snow 1906a: 141; Casey 1920: 270), east-central Mississippi (Snodgrass and Cross 1983: 17), and northeastern Tennessee (Hylton 1980: 25). The record from the District of Columbia (Ulke 1902: 7) needs confirmation.

#### Records.

**CAN**: ON **USA**: IL, IN, KS, LA, MO, MS, NY, OH, OK, PA, SD, TN, TX, WI [DC]

### Genus Apristus

Chaudoir, 1846

Apristus Chaudoir [in Chaudoir and Hochhuth], 1846: 62. Type species: *Apristus subaeneus* Chaudoir, 1846 by monotypy. Etymology (original). From the Greek *a* (absence) and *prizos* (saw-like), alluding to the smooth (i.e., not denticulate) tarsal claws (“*crochets des tarses non dentelés*”) of the adults [masculine].

#### Diversity.

About 60 species (Lorenz 2005: 472) in the Nearctic (13 species), Neotropical (five species in Middle America and the West Indies), Australian, Oriental, Palaearctic (27 species), and Afrotropical Regions.

#### Identification.

Casey (1920: 272-276) wrote a key to all but one (*Apristus latens*) North American species. As usual his key is difficult to use. Ten of the current 13 species in North America have been described by Casey (1920) and only two (*Apristus constrictus* and *Apristus pugetanus*) have been study subsequently (Lindroth 1969a). A taxonomic revision of the genus is needed.

### Apristus actuosus

Casey, 1920

Apristus actuosus Casey, 1920: 274. Type locality: «Southern Pines [Moore County], North Carolina» (original citation). Holotype [by monotypy] (♂) in USNM [# 47687].

#### Distribution.

This species is known only from the type locality in central North Carolina and two localities in northern Georgia (Fattig 1949: 39).

#### Records.

**USA**: GA, NC

### Apristus agitatus

Casey, 1920

Apristus liratus agitatus Casey, 1920: 276. Type locality: «San Diego [San Diego County], Ca[l]ifornia» (original citation). Holotype [by monotypy] (♂) in USNM [# 47691].

#### Distribution.

This species is known only from the holotype collected in southwestern California.

#### Records.

**USA**: CA

### Apristus cephalus

Casey, 1920

Apristus cephalus Casey, 1920: 272. Type locality: «Cloverdale, Sonoma Co[unty], California» (original citation). Holotype [by monotypy] (♀) in USNM [# 47692].

#### Distribution.

This species is known only from the holotype collected along the coast of California.

#### Records.

**USA**: CA

### Apristus constrictus

Casey, 1920

Apristus constrictus Casey, 1920: 276. Type locality: «S[an]ta Rosa [Sonoma County], California» (original citation). Lectotype, designated by Lindroth (1975: 145), in USNM [# 47696].

#### Distribution.

The range of this species extends from southwestern Alberta to Vancouver Island, south to west-central California (Lindroth 1969a: 1046) and southwestern Colorado (Elias 1987: 634).

#### Records.

**CAN**: AB, BC (VCI) **USA**: CA, CO, MT, OR, WA

### Apristus latens

(LeConte, 1846)

Dromius latens LeConte, 1846b: 191. Type locality: «ad Mississippi scaturigines [according to Lindroth (1969a: 1046) apparently in present day Minnesota]» (original citation). One syntype in MCZ [# 5814].
Apristus fuscipennis Motschulsky, 1864: 233. Type locality: «nouveau Mexique [= New Mexico]» (original citation). Lectotype, designated by Bousquet and Larochelle (1993: 12), in ZMMU. Synonymy established by Horn (1882: 160), confirmed by Bousquet and Larochelle (1993: 12).

#### Distribution.

The range of this species extends from Cape Breton Island (Lindroth 1969a: 1046) to southern Saskatchewan (Ronald R. Hooper pers. comm. 2007), south to southern Arizona (Pima County, CMNH), central Texas (Casey 1920: 274, as *Apristus fuscipennis*; Terrell County, CMNH), and southern Georgia (Torres and Ruberson 2006: 31). The record from “Montana” (Bousquet and Larochelle 1993: 273) needs confirmation.

#### Records.

**CAN**: MB, NB, NS (CBI), ON, QC, SK **USA**: AL, AR, AZ, CO, GA, IA, IL, MA, MD, ME, MN, MS, NE, NH, NJ, NM, OH, OK, PA, SD, TN, TX, VA, VT, WV [MT]

#### Note.

This species has passed under the name *Apristus subsulcatus* (Dejean, 1826) in the literature until Lindroth (1969a: 1045-1046).

### Apristus laticollis

LeConte, 1851

Apristus laticollis LeConte, 1851: 176. Type locality: «San Diego [San Diego County, California]» (original citation). Four syntypes in MCZ [# 64].
Apristus subcyaneus G.E. Horn, 1894: 360. Type locality: «San José del Cabo [Baja California Sur, Mexico]» (original citation). One syntype in CAS [# 3]. Synonymy established by Leng (1920: 66).

#### Distribution.

This species is known from east-central Oregon (Grant County, UASM; Horn 1882: 134; Lindroth 1969a: 1044) to northern Colorado (Armin 1963: 179), including southern Idaho (Horning and Barr 1970: 25), south to central New Mexico (Bernalillo County, CMNH), southern Arizona (Pima County, CMNH; Horn 1882: 134), and the Baja California Peninsula (Horn 1894: 310).

#### Records.

**USA**: AZ, CA, CO, ID, NM, NV, OR, UT – Mexico

### Apristus liratus

Casey, 1920

Apristus liratus Casey, 1920: 276. Type locality: «Humboldt Co[unty] and S[an]ta Rosa [Sonoma County], California» (original citation). Two syntypes [2 originally cited] in USNM [# 47690].

#### Distribution.

This species is known only from the two syntypes collected along coastal California.

#### Records.

**USA**: CA

### Apristus nevadensis

Casey, 1920

Apristus nevadensis Casey, 1920: 273. Type locality: «Reno [Washoe County], Nevada» (original citation). Holotype [by monotypy] in USNM [# 47695].

#### Distribution.

This species is known only from the holotype collected in northwestern Nevada.

#### Records.

**USA**: NV

### Apristus pugetanus

Casey, 1920

Apristus pugetanus Casey, 1920: 275. Type locality: «Washington State» (original citation). Four syntypes [4 originally cited] in USNM [# 47689].

#### Distribution.

This species ranges from Vancouver Island (Lindroth 1969a: 1047) to northwestern Montana (Flathead County, Ken Karns pers. comm. 2009), south to southwestern New Mexico (Hidalgo County, CMNH), southern Arizona (Graham, Pinal, Cochise, Pima, and Greenlee Counties, CMNH), and northern California (Shasta and Yolo Counties, CMNH).

#### Records.

**CAN**: BC (VCI) **USA**: AZ, CA, CO, ID, MT, NM, OR, UT, WA

### Apristus subdeletus

Casey, 1920

Apristus subdeletus Casey, 1920: 272. Type locality: «M[oun]t Diablo [Contra Costa County], California» (original citation). Holotype [by monotypy] (♂) in USNM [# 47694].

#### Distribution.

This species is known only from the holotype collected in western California.

#### Records.

**USA**: CA

### Apristus subsulcatus

(Dejean, 1826)

Dromius subsulcatus Dejean, 1826: 451. Type locality: «Amérique septentrionale» (original citation), restricted to «Northfield [Franklin County], Mass[achusetts]» by Lindroth (1969a: 1044). One syntype in MHNP (Lindroth 1955b: 24).
Dromius cordicollis LeConte, 1846b: 190. Type locality: «NovEboraci [= New York]» (original citation). Three syntypes in MCZ [# 35338]. Synonymy established by Lindroth (1955b: 24).

#### Distribution.

The range of this species extends from Cape Breton Island (Lindroth 1954c: 307, as *Apristus cordicollis*) to Minnesota (Gandhi et al. 2005: 932), south to Mississippi (Casey 1920: 275, as *Apristus cordicollis*), southeastern Alabama (Kharboutli and Mack 1991: 1017), and southern South Carolina (Ciegler 2000: 122). The records from Colorado (LeConte 1879d: 500; Armin 1963: 179), “New Mexico,” and Texas (Horn 1882: 133; Wickham 1897: 109) need confirmation since they could refer to *Apristus latens* (LeConte).

#### Records.

**CAN**: NB, NS (CBI), ON, QC **USA**: AL, AR, CT, DC, GA, IA, IL, IN, MA, MD, ME, MI, MN, MO, MS, NH, NJ, NY, OH, PA, RI, SC, VA, VT, WI, WV [CO, NM, TX]

### Apristus thoracicus

Casey, 1920

Apristus thoracicus Casey, 1920: 275. Type locality: «Jemez Springs [Sandoval County], New Mexico» (original citation). Three syntypes [3 originally cited] in USNM [# 47688].

#### Distribution.

This species is known only from the syntypes collected in northwestern New Mexico.

#### Records.

**USA**: NM

### Apristus tuckeri

Casey, 1920

Apristus tuckeri Casey, 1920: 273. Type locality: «Tuçson [Pima County], Arizona» (original citation). One syntype in USNM [# 47693].

#### Distribution.

This species is known only from the type series collected in southern Arizona.

#### Records.

**USA**: AZ

### Genus Syntomus

Hope, 1838

Syntomus Hope, 1838: 64. Type species: *Carabus truncatellus* Linnaeus, 1760 by monotypy. Etymology (original). From the Greek *syntomos* (shortened), alluding to the small size (“body short”) of the adults [masculine].
Metabletus Schmidt-Göbel, 1846: 38. Type species: *Lebia obscuroguttata* Duftschmid, 1812 designated by Andrewes (1934: 201). Synonymy established by Lacordaire (1854: 122). Etymology (original). From the Greek *metabletos* (variable) [masculine].
Charopterus Motschulsky, 1858: 155. Type species: *Carabus truncatellus* Linnaeus, 1760 designated by Habu (1967: 238). Etymology. From the Greek *charops* (glad-eyed, joyous) and *pteron* (wing, by extension elytron) [masculine].
Apristomorphus Motschulsky, 1861: 104. Type species: *Apristomorphus sexpunctatus* Motschulsky, 1861 (= *Metabletus quadripunctatus* Schmidt-Göbel, 1846) by monotypy. Synonymy established, under the name *Metabletus* Schmidt-Göbel, by Andrewes (1928: 7). Etymology. From the generic name *Apristus* [*q.v*.] and the Greek *morphe* (form), alluding to the resemblance of the adults to those of *Apristus* (“*dromiide de la forme parallèle des Apristus*”) [masculine].

#### Diversity.

About 50 species (Lorenz 2005: 470-471) in the boreal, temperate, and tropical areas of the Nearctic (one species), Australian (one widely distributed Old World species), Oriental, Palaearctic (about 35 species), and Afrotropical Regions.

#### Identification.

The North American species is included in Lindroth’s (1969a: 1056) monograph on the Canadian and Alaskan Carabidae.

### Syntomus americanus

(Dejean, 1831)

Dromius americanus Dejean, 1831: 361. Type locality: «Amérique septentrionale» (original citation), restricted to «Rumney [Grafton County], N[ew] H[ampshire]» by Lindroth (1969a: 1056). Holotype [by monotypy] in MHNP (Lindroth 1955b: 24).
Metabletus borealis Zimmermann [in LeConte], 1869b: 243. Type locality: «Lake Superior» (original citation). Syntype(s) probably lost (Lindroth 1969a: 1056). Synonymy established by Horn (1882: 161).

#### Distribution.

The range of this species extends from Newfoundland (Lindroth 1955a: 130) to east-central Alaska (Lindroth 1969a: 1056), south to the Sierra Nevada in California (Dajoz 2007: 16), southeastern Arizona (Graham and Greenlee Counties, UASM), central New Mexico (Fall and Cockerell 1907: 160), south-central South Dakota (Kirk and Balsbaugh 1975: 38), central Illinois (Wolcott 1895: 309), and western North Carolina (Yancey County, USNM) along the Appalachians. The records from “Nebraska” and “Texas” (Bousquet and Larochelle 1993: 274) need confirmation.

#### Records.

**CAN**: AB, BC (VCI), LB, MB, NB, NF, NS (CBI), NT, ON, PE, QC, SK, YT **USA**: AK, AZ, CA, CO, CT, DC, IA, ID, IL, IN, MA, MD, ME, MI, MN, MT, NC, ND, NH, NJ, NM, NY, OH, OR, PA, RI, SD, TN, UT, VA, VT, WA, WI, WV, WY [NE, TX]

### Genus Axinopalpus

LeConte, 1846

Axinopalpus LeConte, 1846b: 190. Type species: *Dromius biplagiatus* Dejean, 1825 by monotypy. Etymology. From the Greek *axine* (ax, wedge) and the Latin *palpus* (feeler, by extension palp), alluding to the shape of the terminal labial palpomere (“*palpi labiales *... *articulo ultimo magno, obconico, subsecuriformi*”) of the adult [masculine].
Variopalpis Solier, 1849: 148. Type species: *Variopalpis humeralis* Solier, 1849 by monotypy. Synonymy established by Reed (1874: 69-70). Etymology. From the Latin *vario* (change, different) and *palpus* (feeler, by extension palp) [masculine]. Note. *Variopalpus* is an incorrect subsequent spelling of *Variopalpis* Solier used by Reed (1874: 69).
Axinopselaphus Gemminger and Harold, 1868a: 128. Unjustified emendation of *Axinopalpus* LeConte, 1846.

#### Diversity.

Fifteen species in the temperate, subtropical, and tropical areas of the Nearctic (seven species) and Neotropical (nine species) Regions.

#### Identification.

There is no modern taxonomic revision of the species of *Axinopalpus* and such study is much needed. A preliminary study of the type material of the species described by Casey and Hatch led to the three new synonyms proposed herein.

### Axinopalpus biplagiatus

(Dejean, 1825)

Dromius biplagiatus Dejean, 1825: 243. Type locality: «Amérique septentrionale» (original citation), restricted to «Hays [Ellis County], Kansas» by Lindroth (1969a: 1058). Syntype(s) presumably lost (Lindroth 1969a: 1058).
Dromius californicus Motschulsky, 1845b: 336 [*nomen dubium*]. Type locality: «Californie» (original citation). Syntype(s) location unknown (possibly in ZMMU though not listed in Keleinikova 1976). Synonymy established by LeConte (1880b: 164).
Axinopalpus coloradensis Casey, 1920: 266. Type locality: «Boulder Co[unty], Colorado» (original citation). One syntype in USNM [# 47680]. **New synonymy**.
Axinopalpus habilis Casey, 1920: 266. Type locality: «Austin [Travis County], Texas» (original citation). Holotype [by monotypy] in USNM [# 47679]. **New synonymy**.
Axinopalpus demissus Casey, 1920: 267. Type locality: «San Francisco to Los Angeles, California» (original citation). Two syntypes in USNM [# 47681]. **New synonymy**.

#### Distribution.

This species ranges from “Maine” (Larochelle and Larivière 1990a: 32) to “Washington” (Hatch 1953: 156), north to the southern part of the Prairie Provinces (Bousquet 1987a: 133), south to southwestern California (Casey 1920: 266, as *Axinopalpus demissus*; Moore 1937: 12), southern Arizona (Wickham 1898: 300; Ober and Maddison 2008: 31), southern Texas (Wickham 1897: 109; Kerr and Hidalgo Counties, CNC, USNM), and west-central Georgia (Fattig 1949: 39). The records from “Pennsylvania,” “Florida” (Bousquet and Larochelle 1993: 272), Vancouver Island (see Lindroth 1969a: 1058), and Mexico (Blackwelder 1944: 58) need confirmation.

#### Records.

**CAN**: AB, MB, ON, QC, SK **USA**: AL, AZ, CA, CO, CT, DE, GA, IA, ID, IL, IN, KS, MA, ME, MI, MN, MO, ND, NE, NH, NJ, NM, NY, OK, OR, RI, SD, TX, UT, VT, WA, WI, WY [BC, FL, PA]

### Axinopalpus denticulatus

Hatch, 1949

Axinopalpus denticulatus Hatch, 1949a: 84. Type locality: «Grand Coulee [Grant County], Wash[ington]» (original citation). Holotype (♀) in USNM.

#### Distribution.

This species is known only from the holotype collected in central Washington.

#### Records.

**USA**: WA

### Axinopalpus fusciceps

LeConte, 1851

Axinopalpus fusciceps LeConte, 1851: 175. Type locality: «San Jose [Santa Clara County, California]» (original citation). Three syntypes in MCZ [# 61].
Axinopalpus nigriceps LeConte, 1880b: 164. Type locality: «Tex[as]» (syntype labels). Three syntypes in MCZ [# 5817]. Synonymy established by Henshaw (1882: 209).

#### Distribution.

This species ranges from western Idaho (Washington County, CNC) to northern Oklahoma (Alfalfa County, CMNH), south to Guatemala (Bates 1883a: 193) and southwestern California (LeConte 1851: 175; Fall 1901a: 48).

#### Records.

**USA**: AZ, CA, CO, ID, NM, OK, TX – Guatemala, Mexico

### Axinopalpus illectus

Casey, 1920

Axinopalpus illectus Casey, 1920: 265. Type locality: «Jountville, Napa Co[unty], California» (original citation). One syntype in USNM [# 47682].

#### Distribution.

This species is known only from the type series collected in west-central California.

#### Records.

**USA**: CA

### Axinopalpus pratti

Hatch, 1949

Axinopalpus pratti Hatch, 1949a: 83. Type locality: «Coupeville [Island County], Wash[ington]» (original citation). Holotype (♀) in USNM. Etymology. The specific name honors Robert Y. Pratt [1915-1999], a resident of Coupeville, on Whidbey Island, Washington. Pratt collected insects in the Pacific Northwest and left his collection of several thousand specimens, mostly beetles, to Au Sable-Pacific Rim. He also bequeathed 147 acres of his land to the Nature Conservancy. The reserve now safeguards a unique grassland ecosystem, including rare populations of golden paintbrush and brittle cactus, the only cactus native to western Washington.

#### Distribution.

This species is known only from the holotype collected in northwestern Washington.

#### Records.

**USA**: WA

### Axinopalpus utahensis

Tanner, 1928

Axinopalpus utahensis Tanner, 1928: 270. Type locality: «S[ain]t George [Washington County], Utah» (holotype label). Holotype (♂) in BYUC (Shawn M. Clark pers. comm. 2007). Note. The type locality is incorrectly spelled “St. Geroge” on the holotype label (Shawn M. Clark pers. comm. 2007).

#### Distribution.

This species is known only from Utah and Washington Counties in Utah (Tanner 1928: 270).

#### Records.

**USA**: UT

### Axinopalpus vittatus

Hatch, 1949

Axinopalpus vittatus Hatch, 1949a: 84. Type locality: «Seattle [King County], Wash[ington]» (original citation). Holotype (♀) in USNM.

#### Distribution.

This species is known from the type locality in west-central Washington (Hatch 1953: 156) and from southeastern Oregon (Westcott et al. 2006: 6).

#### Records.

**USA**: OR, WA

### Subtribe Lebiina

Bonelli, 1810

Lebiotae Bonelli, 1810: Tabula Synoptica. Type genus: *Lebia* Latreille, 1802.
Encratidae Gistel, 1856: 355. Type genus: *Encrates* Gistel, 1848 (= *Lebia* Latreille, 1802).
Lampriadae Chaudoir, 1871a: 115. Type genus: *Lamprias* Bonelli, 1810.

#### Diversity.

Worldwide, with about 800 species arrayed in 25 genera (Lorenz 2005: 481-489). The North American fauna has 49 species (about 6 % of the world fauna) placed in two genera.

#### Taxonomic Note.

Casale’s (1998: Fig. 91) cladistic analysis suggests that this subtribe is possibly the sister-group to {cymindidines + dromiusines + physoderines + agrines + metallicines + calleidines + demetriadines + peliocypadines}.

### Genus Lebia

Latreille, 1802

Lebia Latreille, 1802: 85. Type species: *Carabus haemorrhoidalis* Fabricius, 1792 (= *Buprestis marginatus* Geoffroy, 1785) designated by Desmarest (1851: 76). Etymology (see Bedel 1878: 247). From the Greek *lebias* (kind of fish) [feminine]. Note. As stated by Andrewes (1935: 24), the first valid type species designation for *Lebia* Latreille, 1802 is that of *Carabus quadrimaculatus* Linnaeus, 1758 by Latreille (1810: 426). That species is also the type species of *Dromius* Bonelli, 1810. Acceptance of Latreille’s designation would require nomenclatural changes for the taxa *Lebia* Latreille, 1802 and *Dromius* Bonelli, 1810. A request should be addressed to the International Commission on Zoological Nomenclature to suppress Latreille’s designation. A first request was postponed (ICZN 1950).
Encrates Gistel, 1848a: ix. Unnecessary replacement name for *Lebia* Latreille, 1802.

#### Diversity.

Worldwide, with about 740 species (Lorenz 2005: 481-488) arrayed in 17 subgenera. The North American fauna includes 48 species (about 6.5 % of the world fauna) in the boreal, temperate, and subtropical regions. These species are currently placed in four subgenera.

#### Identification.

Madge (1967) revised the North American species. Since the publication of his work, one of his species (*Lebia viridis*) has been shown to be a complex of two species (*Lebia viridis* and *Lebia moesta*) by Lindroth (1969a).

### Subgenus Loxopeza

Chaudoir, 1871

Loxopeza Chaudoir, 1871a: 117, 138. Type species: *Lebia grandis* Hentz, 1830 designated by Madge (1967: 153). Etymology (original). From Greek *loxos* (slanting, by extension oblique) and *peza* (foot), alluding to the oblique anterior edge of the male first three protarsomeres (“*tarsi antici maris articulis tribus apice valde oblique truncatis*”) [feminine].

#### Diversity.

Twenty-five species in the Nearctic (eight species) and Neotropical (18 species) Regions.

### [atriventris group]

### Lebia atriceps

LeConte, 1863

Lebia atriceps LeConte, 1863c: 5. Type locality: «Nebraska» (original citation), herein restricted to Mitchell, Scotts Bluff County (see Madge 1967: 156). One syntype in MCZ [# 5803].
Loxopeza nanulina Casey, 1920: 238. Type locality: «Boulder Co[unty], Colorado» (original citation for the lectotype). Lectotype (♀), designated by Lindroth (1975: 144), in USNM [# 47622]. Synonymy established by Madge (1967: 155).

#### Distribution.

This species ranges from southern Manitoba to eastern Oregon (Baker County, James R. LaBonte pers. comm. 1992), as far north as central Alberta, south to northern Sonora (Bates 1884: 298) and southwestern Texas [see Madge 1967: Fig. 141]. On specimen is known from Strafford County, northeastern Virginia (Hoffman 2010: 23), probably resulting from anthropogenic transport.

#### Records.

**CAN**: AB, MB, SK **USA**: AZ, CO, ID, KS, MT, ND, NE, NM, NV, OK, OR, SD, TX, UT, WY – Mexico

### Lebia atriventris

Say, 1823

Lebia atriventris Say, 1823a: 13. Type locality: «Arlington [Middlesex County], Mass[achusetts]» (neotype label). Neotype (♀), designated by Lindroth and Freitag (1969: 349), in MCZ [# 33007].
Loxopeza enormis Casey, 1920: 237. Type locality: «near the city [of New York], New York» (original citation). Lectotype (♀), designated by Lindroth (1975: 144), in USNM [# 47620]. Synonymy established by Madge (1967: 154).

#### Distribution.

This species ranges from “Maine” (Larochelle and Larivière 1990a: 32) to southwestern Montana, north to northern Alberta (Fort McMurray area, Gerald J. Hilchie pers. comm. 2009), Saskatchewan and southern Manitoba, south to central New Mexico (Fall and Cockerell 1907: 159), central Texas, and northeastern Florida [see Madge 1967: Fig. 117]. The records from “California” (Erwin et al. 1977: 4.61; Bousquet and Larochelle 1993: 274) and Mexico (Chaudoir 1871a: 143) are likely in error.

#### Records.

**CAN**: AB, MB, ON, QC, SK **USA**: AL, AR, CT, DC, DE, FL, GA, IA, IL, IN, KS, KY, LA, MA, MD, ME, MI, MN, MO, MS, MT, NC, ND, NE, NH, NJ, NM, NY, OH, OK, PA, RI, SC, SD, TN, TX, VA, VT, WI, WV

### [tricolor group]

### Lebia deceptrix

Madge, 1967

Lebia deceptrix Madge, 1967: 158. Type locality: «Pena Blanca (4000’), Santa Cruz Co[unty], Ariz[ona]» (original citation). Holotype (♂) in CNC [# 9559].

#### Distribution.

This species is known from southern Arizona and western Texas (Madge 1967: 159).

#### Records.

**USA**: AZ, TX

### Lebia grandis

Hentz, 1830

Lebia grandis Hentz, 1830: 255. Type locality: «North Carolina» (original citation), herein restricted to Asheville, Buncombe County (see Madge 1967: 163). Syntype(s) [2 originally cited] lost.
Loxopeza majuscula Chaudoir, 1871a: 141. Type locality: «Texas» (original citation). Syntype(s) [2 originally cited] in MHNP. Synonymy established by Madge (1967: 161).
Loxopeza grandis rivularis Casey, 1920: 235. Type locality: «Brownsville [Cameron County], Texas» (original citation). One syntype in USNM [# 47618]. Synonymy established by Madge (1967: 161).
Loxopeza magister Casey, 1920: 236. Type locality: «Marquette [Marquette County, Michigan], Lake Superior» (original citation). Lectotype (♀), designated by Lindroth (1975: 144), in USNM [# 47617]. Synonymy established by Madge (1967: 161).

#### Distribution.

This species is found east of the Rocky Mountains from southern Quebec (Larochelle 1975: 91) to northeastern North Dakota (Tinerella 2003: 637), south to southeastern Texas along the Rio Grande and southern South Carolina, west to western Texas and western Oklahoma [see Madge 1967: Fig. 137]. The records from Baja California (Horn 1894: 310), Arizona (Wickham 1898: 300; Snow 1907: 142, as *Lebia majuscula*), New Mexico (Fall and Cockerell 1907: 159, 160 as *Lebia majuscula*), and “Colorado” (Snow 1877: 17; Wickham 1902: 239) need confirmation.

#### Records.

**CAN**: ON, QC **USA**: AL, AR, CT, DC, DE, GA, IA, IL, IN, KS, KY, LA, MA, MD, MI, MN, MO, MS, NC, ND, NE, NH, NJ, NY, OH, OK, PA, RI, SC, SD, TN, TX, VA, VT, WI, WV [AZ, CO, NM]

### Lebia pimalis

(Casey, 1920)

Loxopeza pimalis Casey, 1920: 237. Type locality: «Arizona» (original citation), herein restricted to Brown’s Canyon, Baboquivari Mountains, Pima County (see Madge 1967: 159). One syntype in USNM [# 47619].

#### Distribution.

This species is known so far only from southern Arizona (Madge 1967: 159).

#### Records.

**USA**: AZ

### Lebia subdola

Madge, 1967

Lebia subdola Madge, 1967: 157. Type locality: «Madera C[a]n[yon] Sta[tion] (5000’-5800’), Rita M[oun]t[ain]s, S[an]ta Cruz Co[unty], Ariz[ona]» (original citation). Holotype (♂) in CNC [# 9558].

#### Distribution.

This species is known from southern Arizona and western Texas (Madge 1967: 158), including southwestern New Mexico (Luna County, Ken Karns pers. comm. 2009).

#### Records.

**USA**: AZ, NM, TX

### Lebia subgrandis

Madge, 1967

Lebia subgrandis Madge, 1967: 160. Type locality: «Pena Blanca (4000’), Santa Cruz Co[unty], Ariz[ona]» (original citation). Holotype (♂) in CNC [# 9557].

#### Distribution.

This species ranges from southern Arizona to western Texas (Madge 1967: 161).

#### Records.

**USA**: AZ, NM, TX

### Lebia tricolor

Say, 1823

Lebia tricolor Say, 1823a: 11. Type locality: «Pennsylvania; on the Missouri» (original citation), restricted to «Pennsylvania» by Lindroth and Freitag (1969: 349), herein to Milford, Pike County (see Madge 1967: 157). Lectotype (♀), designated by Lindroth and Freitag (1969: 349), in MHNP.

#### Distribution.

This species occurs from Nova Scotia (Halifax and Colchester Counties, CNC) to eastern Minnesota, north to southern Manitoba (CNC), south to northern Oklahoma (Alfalfa County, Foster F. Purrington pers. comm. 2010), southern Louisiana, and central Florida [see Madge 1967: Fig. 126]. Old specimens simply labeled from “Texas” (Madge 1967: 157) are known.

#### Records.

**CAN**: MB, NB, NS, ON, QC **USA**: AL, AR, CT, DC, DE, FL, GA, IA, IL, IN, KS, KY, LA, MA, MD, ME, MI, MN, MS, NC, NH, NJ, NY, OH, OK, PA, RI, SC, TN, VA, VT, WI, WV [TX]

### Subgenus Polycheloma

Madge, 1967

Polycheloma Madge, 1967: 163. Type species: *Lebia lecontei* Madge, 1967 by original designation. Etymology (original). From the Greek *polys* (many) and *cheloma* (notch), alluding to the presence of several preapical notches on the mesotibia of the male [neuter].

#### Diversity.

One species in southern United States and northeastern Mexico.

### Lebia lecontei

Madge, 1967

Lebia lecontei Madge, 1967: 164. Type locality: «Texas» (original citation), herein restricted to 2.5 miles east of Nickle Creek Station, Culberson County (see Madge 1967: 164). Syntype(s) in MCZ [# 5802]. Note. Madge (1967: 164) proposed the name *Lebia lecontei* as a replacement name for *Lebia testacea* (LeConte, 1880). However, LeConte never provided a description for *Lebia testacea*. The first description of the species was done by Madge (1967: 164).

#### Distribution.

This species is known from a few specimens collected in western Texas (Madge 1967: 164), southeastern Arizona (Cochise County, CNC, UASM), and “Tamaulipas” (MCZ, collection LeConte) and Nuevo León (UASM) in Mexico. The record from Baja California (Horn 1894: 310, as *Lebia testacea*) needs confirmation.

#### Records.

**USA**: AZ, TX – Mexico

### Subgenus Lamprias

Bonelli, 1810

Lamprias Bonelli, 1810: Tabula Synoptica. Type species: *Carabus cyanocephalus* Linnaeus, 1758 by subsequent monotypy in Panzer (1813: 71). Etymology. From the Greek *lampros* (bright, brilliant) and the suffix -*ios* (pertaining to), possibly alluding to the vivid coloration of the adults in the hands of Bonelli [masculine].
Echimuthus Leach, 1815: 81. Type species: *Carabus cyanocephalus* Linnaeus, 1758 by monotypy.
Lamprus Billberg, 1820: 26. Unjustified emendation of *Lamprias* Bonelli, 1810.
Omalomorpha Motschulsky, 1844: 13[table], 42 [junior homonym of *Omalomorpha* Brullé, 1835]. Type species: *Lebia punctata* Gebler, 1843 designated by Lorenz (1998: 129). Synonymy established by Chaudoir (1871a: 129). Etymology. From the Greek *homalos* (even, equal) and *morphe* (form) [feminine].
Homalops Motschulsky, 1850a: viii, 42. Replacement name for *Omalomorpha* Motschulsky, 1844.
Rhopalostyla Chaudoir, 1850b: 96. Replacement name for *Omalomorpha* Motschulsky, 1844. Etymology (original). From the Greek *rhopalon* (club) and *stylos* (pillar, base) [feminine].
Lebida Motschulsky, 1862b: 51. Type species: *Lebida pilosella* Motschulsky, 1862 (= *Carabus cyanocephalus* Linnaeus, 1758) designated by Lorenz (1998: 104).

#### Diversity.

Fourteen species in the Nearctic (one species) and Palaearctic (13 species) Regions.

### Lebia divisa

LeConte, 1850

Lebia concinna LeConte, 1846b: 192 [primary homonym of *Lebia concinna* Brullé, 1838]. Type locality: «Lacum Superiorem» (original citation), herein restricted to Eagle Harbor, Keweenaw County, Michigan (see Hubbard and Schwarz 1878: 627). One syntype in MCZ [# 5801].
Lebia divisa LeConte, 1850: 203. Replacement name for *Lebia concinna* LeConte, 1846.

#### Distribution.

This species ranges from the Prairie Provinces, as far north as central Alberta, south to northern Colorado (Wickham 1902: 239) and eastern Kansas (Popenoe 1877: 23), east to west-central Indiana (Downie 1957: 116; Schrock 1985: 351), west to northwestern Idaho [see Madge 1967: Fig. 120]. The record from northeastern New Mexico (Fall and Cockerell 1907: 159) needs confirmation.

#### Records.

**CAN**: AB, MB, SK **USA**: CO, ID, IL, IN, KS, MN, MT, SD, WI, WY [NM]

### Subgenus Lebia

Latreille, 1802

Lebia Latreille, 1802: 85. Type species: *Carabus haemorrhoidalis* Fabricius, 1792 (= *Buprestis marginatus* Geoffroy, 1785) designated by Desmarest (1851: 76).
Metabola Chaudoir, 1871a: 160 [junior homonym of *Metabola* Mayer, 1867]. Type species: *Metabola rufopyga* Chaudoir, 1871 (= *Lebia pulchella* Dejean, 1826) by monotypy. Synonymy established by Madge (1967: 166).
Aphelogenia Chaudoir, 1871b: 25. Type species: *Carabus vittatus* Fabricius, 1777 designated by Madge (1967: 166). Synonymy established by Madge (1967: 166).
Dianchomena Chaudoir, 1871b: 45. Type species: *Lebia abdominalis* Chaudoir, 1843 designated by Casey (1920: 239). Synonymy established by Madge (1967: 166). Etymology. From the Greek *dis* (twice, double) and *anchomenos* (strangled), possibly alluding to the constriction of the vertex (“*caput basi valde strangulatum*”) of the adult [feminine].

#### Diversity.

Worldwide, with about 525 species (Lorenz 2005: 481-488) of which 38 (about 7 % of the world fauna) occur in North America.

### [analis group]

### Lebia analis

Dejean, 1825

Lebia analis Dejean, 1825: 265. Type locality: «Amérique septentrionale» (original citation), restricted to «Newark [Essex County], N[ew] J[ersey]» by Lindroth (1969a: 1030). One syntype in MHNP (Lindroth 1955b: 23).
Lebia anchora Chevrolat, 1835c: [no. 132]. Type locality: «environs d’Orixaba [Veracruz, Mexico]» (original citation). Holotype [by monotypy] location unknown (Lindroth 1969a: 1030). Synonymy established by Madge (1967: 184).
Lebia bonellii Putzeys, 1845a: 39. Type locality: «Mexique» (original citation). Holotype [by monotypy] location unknown (Lindroth 1969a: 1030). Synonymy established, under the name *Lebia anchora* Chevrolat, by Chaudoir (1871a: 212). Etymology. The specific name honors Franco Andrea Bonelli [1784-1830], professional entomologist and professor of zoology at the University of Turin. Bonelli proposed the first classification of the family Carabidae when he classified the species into 22 “stirpes” in his “Tabula Synoptica” published in 1810.
Lebia subfigurata Motschulsky, 1864: 227. Type locality: «Amér[ique] bor[éale]» (original citation). Lectotype, designated by Bousquet and Larochelle (1993: 14), in ZMMU. Synonymy established with doubt with the name *Lebia analis* var. *appendiculata* Chaudoir by Horn (1872a: 142), confirmed by Bousquet and Larochelle (1993: 14).
Lebia sublimbata Motschulsky, 1864: 226. Type locality: «états septentrionaux de l’Amérique du Nord» (original citation). Lectotype, designated by Bousquet and Larochelle (1993: 15), in ZMMU. Synonymy established by Bousquet and Larochelle (1993: 15).
Lebia appendiculata Chaudoir, 1871a: 212. Type locality: «Louisiane» (original citation). Syntype(s) [4 originally cited] in MHNP. Synonymy established by LeConte (1880a: 88).

#### Distribution.

This species ranges from northwestern Vermont to eastern South Dakota (Kirk and Balsbaugh 1975: 37), including southernmost Ontario (Bousquet 1987a: 133), south to Guatemala (Bates 1883a: 229, as *Lebia anchora*) and the Florida Keys, west along the southwest to southeastern Arizona [see Madge 1967: Fig. 130] and the Baja California Peninsula (Blackwelder 1944: 52); also recorded from the Bahamas (Turnbow and Thomas 2008: 13). The record from “Maine” (Bousquet and Larochelle 1993: 276) needs confirmation.

#### Records.

**CAN**: ON **USA**: AL, AR, AZ, CO, CT, DC, DE, FL, GA, IA, IL, IN, KS, KY, LA, MA, MD, MI, MN, MO, MS, NC, NE, NJ, NM, NY, OH, OK, PA, RI, SC, SD, TN, TX, VA, VT, WI, WV [ME] – Bahamas, Guatemala, Mexico

### [bitaeniata group]

### Lebia bitaeniata

Chevrolat, 1834

Lebia bitaeniata Chevrolat, 1834 [6 August]: [no. 37]. Type locality: «Orixaba [Veracruz, Mexico]» (original citation). Syntype(s) location unknown (possibly in UMO).
Lebia bicincta Laporte, 1834 [30 June]: 47. Type locality: «Orizaba [Veracruz], Mexique» (original citation). Syntype(s) location unknown (possibly in MHNP). Synonymy established by Chaudoir (1871a: 208). Note. Based on bibliographic research I made, this name may be older than Chevrolat’s name but is not in “prevailing usage” (see *Principle of priority* under “Nomenclature” section).
Lia femorata Motschulsky, 1864: 228. Type locality: «Am[érique] centr[ale]» (original citation). One syntype in ZMMU (Keleinikova 1976: 197). Synonymy established with doubt by Chaudoir (1871a: 208).
Lebia callizona Bates, 1878a: 607. Type locality: «Guatemala» (original citation). Syntype(s) probably in BMNH. Synonymy established (as aberration) by Csiki (1932b: 1332).

#### Distribution.

This species ranges from southeastern Texas (Madge 1967: 172) to Colombia (Martínez 2003: 14); also recorded from the Bahamas (Turnbow and Thomas 2008: 13), Cuba (Gundlach 1891: 16), Jamaica (Darlington 1941a: 14), Puerto Rico (Wolcott 1936: 188), and Dominica (Peck 2006: 176).

#### Records.

**USA**: TX – Bahamas, Colombia, Costa Rica, Cuba, Dominica, Guatemala, Jamaica, Mexico, Nicaragua, Puerto Rico

### [bivittata group]

### Lebia abdominalis

Chaudoir, 1843

Lebia abdominalis Chaudoir, 1843b: 704. Type locality: «près de la Nouvelle Orléans [Orleans Parish, Louisiana]» (original citation). Syntype(s) in MHNP.
Dianchomena convictor Casey, 1920: 262. Type locality: «Cairo [Alexander County], Illinois» (original citation). One syntype in USNM [# 47665]. Synonymy established by Madge (1967: 198).

#### Distribution.

This species ranges from New Jersey to northeastern Kansas (Popenoe 1877: 23), south to Nicaragua (Bates 1883a: 240) and southern Florida, west to northwestern Texas [see Madge 1967: Fig. 119]; also recorded from Cuba (Gundlach 1891: 16; Darlington 1934: 113) and Jamaica (Darlington 1941a: 14). The record from “Wisconsin” (Bousquet and Larochelle 1993: 276) needs confirmation.

#### Records.

**USA**: AL, AR, DC, DE, FL, GA, IL, IN, KS, LA, MD, MO, NC, NJ, OH, PA, SC, TN, TX, VA, WV [WI] – Belize, Cuba, Guatemala, Jamaica, Mexico, Nicaragua

### Lebia bilineata

Motschulsky, 1859

Lebia bilineata Motschulsky, 1859a: 145. Type locality: «Col[onie] Ross [farming community about 75 miles north of San Francisco along the coast, California]» (original citation). Syntype(s) location unknown (possibly in ZMMU though not listed in Keleinikova 1976).

#### Distribution.

This species ranges from central Idaho and northern Oregon south to southern California along the Mexican border [see Madge 1967: Fig. 133].

#### Records.

**USA**: CA, ID, NV, OR

### Lebia bivittata

(Fabricius, 1798)

Carabus 2vittatus Fabricius, 1798: 59. Type locality: «America» (original citation), restricted to «Galesburg [Knox County], Illin[ois]» by Lindroth (1969a: 1034). Syntype(s) apparently lost (Lindroth 1969a: 1034).
Lebia quadrivittata Dejean, 1825: 268. Type locality: «Amérique septentrionale» (original citation). One syntype in MHNP (Lindroth 1955b: 23). Synonymy established by LeConte (1863b: 5), confirmed by Lindroth (1955b: 23).
Dianchomena aemula Casey, 1920: 263. Type locality: «Kansas» (original citation). Two syntypes in USNM [# 47666]. Synonymy established by Madge (1967: 196).
Dianchomena devincta Casey, 1920: 264. Type locality: «Boulder Co[unty], Colorado» (original citation). One syntype in USNM [# 47667]. Synonymy established by Madge (1967: 196).

#### Distribution.

The range of this species extends from northern New Jersey to north-central Colorado, north to southeastern Michigan, south at least to southern Arizona, Guanajuato and the Federal District in Mexico (Bates 1883a: 241), and central Georgia [see Madge 1967: Fig. 124]. At least one old specimen simply labeled from Wisconsin is known (Madge 1967: Fig. 124). The record from “Massachusetts” (Leng and Beutenmüller 1893: 142) needs confirmation.

#### Records.

**USA**: AL, AR, AZ, CO, DC, DE, GA, IA, IL, IN, KS, KY, LA, MD, MI, MO, MS, NE, NJ, NM, NY, OH, PA, TX, VA [MA, WI] – Mexico

### [collaris group]

### Lebia collaris

Dejean, 1826

Lebia collaris Dejean, 1826: 456. Type locality: «Amérique septentrionale» (original citation), herein restricted to Southern Pines, Moore County, North Carolina (see Madge 1967: 215). One syntype in MHNP (Chaudoir 1871a: 200).

#### Distribution.

This rarely collected species is known from southern Ohio (Purrington et al. 1999: 48) and southern Indiana, from North Carolina to southern Florida [see Madge 1967: Fig. 136], from southeastern Louisiana (Saint Tammany Parish, Igor M. Sokolov pers. comm. 2009), from east-central Texas (Riley 2011), and from western Arkansas (Polk and Garland Counties, Robert L. Davidson pers. comm. 2012). The records from New York (LeConte 1846b: 195), Alabama (Löding 1945: 21), and Cuba (Darlington 1934: 113) need confirmation.

#### Records.

**USA**: AR, FL, GA, IN, LA, NC, OH, TX [AL, NY]

### [fuscata group]

### Lebia abdita

Madge, 1967

Lebia abdita Madge, 1967: 201. Type locality: «Pena Blanca (4000’), S[an]ta Cruz Co[unty], Ariz[ona]» (original citation). Holotype (♂) in CNC [# 9560].

#### Distribution.

This species is known from southern Arizona and the Baja California Peninsula (Madge 1967: 202).

#### Records.

**USA**: AZ – Mexico

### Lebia calliope

Bates, 1883

Lebia calliope Bates, 1883a: 231. Type locality: «Mirador [and] Cerro de Plumas, Mexico; San Gerónimo, Guatemala» (original citation). Syntype(s) probably in BMNH.
Lebia serpentina Casey, 1920: 256. Type locality: «Brownsville [Cameron County], Texas» (original citation). Seven syntypes [7 originally cited] in USNM [# 47655]. Synonymy established by Madge (1967: 212).

#### Distribution.

This species ranges from southeastern Texas (Madge 1967: 212) along the Rio Grande south at least to Guatemala (Bates 1883a: 231).

#### Records.

**USA**: TX – Guatemala, Mexico

### Lebia esurialis

Casey, 1920

Lebia esurialis Casey, 1920: 257. Type locality: «Brownsville [Cameron County], Texas» (original citation). Two syntypes [2 originally cited] in USNM [# 47656].

#### Distribution.

This species is known from northwestern and south-central Louisiana (Caddo and Saint Landry Parishes, Igor M. Sokolov pers. comm. 2009), eastern Texas (Madge 1967: 212; Riley 2011), and “Mexico” (Erwin et al. 1977: 4.62); also recorded from the Bahamas (Turnbow and Thomas 2008: 13) and Cuba (Mateu 1977: 378). The record from South Dakota (Kirk and Balsbaugh 1975: 38) needs confirmation.

#### Records.

**USA**: LA, TX [SD] – Bahamas, Cuba, Mexico

### Lebia fuscata

Dejean, 1825

Lebia fuscata Dejean, 1825: 270. Type locality: «Amérique septentrionale» (original citation), restricted to «Rumney [Grafton County], N[ew] H[ampshire]» by Lindroth (1969a: 1035). One syntype in MHNP (Lindroth 1955b: 23).
Lebia canonica Casey, 1920: 257. Type locality: «Marquette [Marquette County, Michigan], Lake Superior» (original citation for the lectotype). Lectotype (♀), designated by Lindroth (1975: 144), in USNM [# 47657]. Synonymy established by Madge (1967: 203).

#### Distribution.

This species is found from Nova Scotia to western Washington, as far north as southern Manitoba and southwestern British Columbia (Lindroth 1969a: 1036), south to west-central California, east-central Texas (Riley 2011), and southern Florida [see Madge 1967: Fig. 127]. The species has been rarely collected in the Great Plains.

#### Records.

**CAN**: BC (VCI), MB, NB, NS, ON, PE, QC **USA**: AL, AR, CA, CT, DC, DE, FL, GA, IA, ID, IL, IN, KS, LA, MA, MD, ME, MI, MN, MO, MS, MT, NC, ND, NE, NH, NJ, NY, OH, OR, PA, RI, SC, SD, TN, TX, VA, VT, WA, WI, WV

### Lebia guttula

LeConte, 1851

Lebia guttula LeConte, 1851: 178. Type locality: «ad Colorado [River, California]» (original citation). Holotype [by monotypy] (♀) in MCZ [# 67].
Lebia metuens Casey, 1920: 258. Type locality: «California» (original citation). Two syntypes in USNM [# 47658]. Synonymy established by Madge (1967: 199).
Lebia pacifica Casey, 1920: 259. Type locality: «Lake Co[unty], California» (original citation). Lectotype (♀), designated by Lindroth (1975: 144), in USNM [# 47659]. Synonymy established by Madge (1967: 200).

#### Distribution.

The range of this species extends from southern Alberta (Drumheller, CNC) and southern British Columbia south to southern California, southern New Mexico [see Madge 1967: Fig. 134], and western Texas (Jeff Davis County, Ken Karns pers. comm. 2009). One old specimen simply labeled from Kansas is known (Madge 1967: 201).

#### Records.

**CAN**: AB, BC **USA**: AZ, CA, CO, ID, MT, ND, NM, NV, OR, TX, UT, WA, WY [KS]

### Lebia insulata

Madge, 1967

Lebia insulata Madge, 1967: 202. Type locality: «Esperanza Ranch, Brownsville [Cameron County], Tex[as]» (original citation). Holotype (♂) in MCZ [# 34669].

#### Distribution.

This species is known only from southeastern Texas (Madge 1967: 203).

#### Records.

**USA**: TX

### Lebia lobulata

LeConte, 1863

Lebia lobulata LeConte, 1863c: 5. Type locality: «Ohio and Louisiana» (original citation). Syntypes location unknown. Note. The specimen in LeConte’s collection [Type 5807] is labeled “V[irgini]a” and so is not a syntype.
Lebia brunnicollis Motschulsky, 1864: 227. Type locality: «Atlanta [Georgia]» (lectotype label). Lectotype, designated by Bousquet and Larochelle (1993: 14), in ZMMU. Synonymy established by Horn (1872a: 142), confirmed by Bousquet and Larochelle (1993: 14).

#### Distribution.

This species ranges from southeastern Maine (Majka et al. 2011: 47) to eastern North Dakota (Tinerella 2003: 637), south to eastern Texas and central Florida [see Madge 1967: Fig. 135].

#### Records.

**CAN**: ON **USA**: AL, AR, CT, DC, FL, GA, IA, IL, IN, KS, LA, MD, ME, MO, MS, NC, ND, NJ, NY, OH, OK, PA, SC, SD, TN, TX, VA, WI, WV

### Lebia ornata

Say, 1823

Lebia ornata Say, 1823a: 13. Type locality: «Wis[s]ahick[o]n Cr[eek] [Philadelphia County], P[ennsylvani]a» (neotype label). Neotype (♀), designated by Lindroth and Freitag (1969: 349), in MCZ [# 33005].
Lebia axillaris Dejean, 1831: 372. Type locality: «Amérique septentrionale» (original citation). Holotype [by monotypy] in MHNP (Lindroth 1955b: 23). Synonymy established by LeConte (1869b: 248), confirmed by Lindroth (1969a: 1036).
Lebia marginella Dejean, 1831: 373. Type locality: «Amérique septentrionale» (original citation). One syntype in MHNP (Lindroth 1955b: 23). Synonymy established, under the name *Lebia axillaris* Dejean, by LeConte (1869b: 248), confirmed by Lindroth (1969a: 1036).
Lebia nigripennis Dejean, 1831: 373. Type locality: «Amérique septentrionale» (original citation). Two syntypes in MHNP (Lindroth 1955b: 23). Synonymy established by Madge (1967: 208).
Lebia nigripennis var. *erythrocephala* Dejean, 1831: 373. Type locality: «Amérique septentrionale» (original citation). Syntype(s) in MHNP. Synonymy established by Chaudoir (1871a: 201).
Dromius apicalis Haldeman, 1843b: 298. Type locality: southeastern Pennsylvania (Haldeman 1843a: 296). Syntype(s) presumably lost. Synonymy established, under the name *Lebia axillaris* Dejean, by LeConte (1846b: 194).
Lebia brunnea Haldeman, 1843b: 298. Type locality: southeastern Pennsylvania (Haldeman 1843a: 296). Syntype(s) presumably lost. Synonymy established, under the name *Lebia axillaris* Dejean, by Melsheimer (1853: 5).
Lebia flaviventris Motschulsky, 1864: 227 [*nomen dubium*]. Type locality: «Am[érique] bor[éale]» (original citation). One syntype, represented only by parts of legs (Bousquet 1997b: 339), in ZMMU. Synonymy established with doubt by Horn (1872a: 142).
Lebia frigida Chaudoir, 1871a: 242. Type locality: «Boston? [Massachusetts]» (original citation). Lectotype (♂), designated by Lindroth (1969a: 1036), in MHNP. Synonymy established by Casey (1920: 260), confirmed by Lindroth (1969a: 1036).
Lebia reperta Casey, 1920: 255. Type locality: «New York» (original citation). Lectotype (♀), designated by Lindroth (1975: 144), in USNM [# 47651]. Synonymy established by Madge (1967: 208).
Lebia virginica Casey, 1920: 255. Type locality: «Va [with a black dot below the “a”] [= Norfolk, Virginia]» (lectotype label). Lectotype (♀), designated by Lindroth (1975: 144), in USNM [# 47652]. Synonymy established, under the name *Lebia axillaris* Dejean, by Lindroth (1955b: 23).
Lebia virginica ashevillensis Casey, 1920: 256. Type locality: «Asheville [Buncombe County], North Carolina» (original citation). Holotype [by monotypy] (♀) in USNM [# 47653]. Synonymy established, under the name *Lebia axillaris* Dejean, by Lindroth (1955b: 23).
Lebia fluviatilis Casey, 1920: 256. Type locality: «Vicksburg [Warren County], Mississippi» (original citation for the lectotype). Lectotype (♀), designated by Lindroth (1975: 145), in USNM [# 47654]. Synonymy established by Madge (1967: 208).

#### Distribution.

This species ranges from Cape Breton Island, Nova Scotia (Christopher G. Majka pers. comm. 2007) to northwestern South Dakota (Kirk and Balsbaugh 1975: 37), including northern Minnesota, south to eastern Texas and central Florida (Peck and Thomas 1998: 24) [see Madge 1967: Fig. 128].

#### Records.

**CAN**: NB, NS (CBI), ON, QC **USA**: AL, AR, CT, DC, FL, GA, IA, IL, IN, KS, KY, LA, MA, MD, ME, MI, MN, MO, MS, NC, NE, NH, NJ, NY, OH, OK, PA, RI, SC, SD, TN, TX, VA, VT, WI, WV

### Lebia perpallida

Madge, 1967

Lebia perpallida Madge, 1967: 206. Type locality: «Pena Blanca (4000’), S[an]ta Cruz Co[unty], Ariz[ona]» (original citation). Holotype (♂) in CNC [# 9561].

#### Distribution.

This species is known only from a few specimens collected in southern Arizona (Madge 1967: 207).

#### Records.

**USA**: AZ

### Lebia subrugosa

Chaudoir, 1871

Lebia subrugosa Chaudoir, 1871a: 227. Type locality: «Mexique» (original citation). Syntype(s) in MHNP.

#### Distribution.

This species is known from southern Arizona to western Texas (Madge 1967: 206), south to Guatemala (Bates 1883a: 230).

#### Records.

**USA**: AZ, NM, TX – Guatemala, Mexico

### [lecta group]

### Lebia bumeliae

Schaeffer, 1910

Lebia bumeliae Schaeffer, 1910: 399. Type locality: «Brownsville [Cameron County], Texas» (original citation). Lectotype (♀), designated by Erwin and House (1978: 239), in USNM [# 42507].

#### Distribution.

This species is known only from a few specimens collected in southeastern Texas (Madge 1967: 213).

#### Records.

**USA**: TX

### Lebia lecta

Horn, 1885

Lebia lecta G.H. Horn, 1885a: 131. Type locality: «Florida» (original citation), herein restricted to Miami, Dade County (see Madge 1967: 214). Holotype [by monotypy] (♀) in MCZ [# 34502].

#### Distribution.

This species is known only from Dade, Monroe, and Walton Counties in Florida (Peck and Thomas 1998: 24).

#### Records.

**USA**: FL

### [pulchella group]

### Lebia pulchella

Dejean, 1826

Lebia pulchella Dejean, 1826: 457. Type locality: «Amérique septentrionale» (original citation), restricted to «Texas» by Lindroth (1969a: 1023), herein to Brownsville, Cameron County (see Madge 1967: 169). One syntype in MHNP (Lindroth 1955b: 22).
Metabola rufopyga Chaudoir, 1871a: 160 [*nomen dubium*]. Type locality: «Mexique» (original citation). Holotype [by monotypy] (♂) in MHNP. Synonymy established with doubt by Lindroth (1969a: 1023). Note. Lindroth (1969a: 1023) stated that Madge (1967) synonymized *Lebia rufopyga* (Chaudoir) with *Lebia pulchella* though with a question mark. In fact Madge (1967: 167) simply wrote that “at least one [*Metabola vivida* Bates] and probably both species of *Metabola* are variants of *Lebia pulchella*.” However, he did not list *Metabola rufopyga* Chaudoir in synonymy with *Lebia pulchella*.
Metabola vivida Bates, 1884: 298. Type locality: «Arizona; northern Sonora, Mexico» (original citation). Syntype(s) in BMNH and ANSP. Synonymy established by Madge (1967: 167).
Lebia tahoensis Casey, 1920: 252. Type locality: «Lake Tahoe [Placer County], California» (original citation). Three syntypes [3 originally cited] in USNM [# 47623]. Synonymy established by Madge (1967: 167).

#### Distribution.

This species is found from northern New Hampshire to central Alberta, south to central California, the state of Hidalgo in Mexico (Ball and Shpeley 1992a: 64), and southern Florida [see Madge 1967: Fig. 123].

#### Records.

**CAN**: AB, MB, ON, SK **USA**: AL, AR, AZ, CA, CO, CT, DC, DE, FL, GA, IL, IN, KS, LA, MA, MD, MI, MN, MO, MS, NC, ND, NE, NH, NJ, NM, NV, NY, OH, OK, PA, RI, SC, TN, TX, VA, WI, WV, WY – Mexico

### [pumila group]

### Lebia pumila

Dejean, 1831

Lebia pumila Dejean, 1831: 388. Type locality: «Amérique septentrionale» (original citation), restricted to «S[outh] McAlester [Pittsburg County, Oklahoma], Ind[ian Territory]» by Lindroth (1969a: 1038). One syntype in MHNP (Lindroth 1955b: 23).
Lebia maculicornis LeConte, 1846b: 195. Type locality: «Georgia» (original citation). Syntype(s) in MCZ [# 5805]. Synonymy established by LeConte (1880a: 88), confirmed by Lindroth (1969a: 1038).
Lebia rhodopus Schwarz, 1878: 354. Type locality: «Tampa [Hillsborough County, Florida]» (original citation). Lectotype (♂), designated by Erwin and House (1978: 239), in USNM [# 4494]. Synonymy established by Madge (1967: 215).
Lebia tertiaria Casey, 1920: 248. Type locality: «District of Columbia» (original citation). Lectotype (♀), designated by Lindroth (1975: 145), in USNM [# 47650]. Synonymy established by Madge (1967: 215).
Lebia ludoviciana Casey, 1920: 248. Type locality: «Alexandria [Rapides Parish], Louisiana» (original citation). Lectotype (♂), designated by Lindroth (1975: 145), in USNM [# 47646]. Synonymy established by Madge (1967: 215).
Lebia quadrata Casey, 1920: 249. Type locality: «Southern Pines [Moore County], North Carolina» (original citation). Lectotype, designated by Lindroth (1975: 145), in USNM [# 47647]. Synonymy established by Lindroth (1955b: 23).
Lebia illini Casey, 1920: 249. Type locality: «Illinois» (original citation). Lectotype (♀), designated by Lindroth (1975: 145), in USNM [# 47648]. Synonymy established by Madge (1967: 215).
Lebia frugalis Casey, 1920: 250. Type locality: «Bayfield [Bayfield County], Wisconsin» (original citation). Lectotype (♀), designated by Lindroth (1975: 145), in USNM [# 47649]. Synonymy established by Madge (1967: 215).

#### Distribution.

This species ranges from Nova Scotia (Lindroth 1954c: 307) and Prince Edward Island (Majka et al. 2008: 133) to southeastern Alberta, south to southeastern Texas along the Rio Grande and southern Florida. The species is also known from one locality in southwestern British Columbia and one in western Washington [see Madge 1967: Fig. 132].

#### Records.

**CAN**: AB, BC, MB, NB, NS, ON, PE, QC, SK **USA**: AL, AR, CT, DC, FL, GA, IA, IL, IN, KS, KY, LA, MA, MD, ME, MI, MN, MO, MS, NC, ND, NE, NH, NJ, NY, OH, OK, PA, RI, SC, SD, TN, TX, VA, VT, WA, WI, WV

#### Note.

*Lebia floricola*, credited to Harris, has been cited as a synonym of this species, first by LeConte (1846b: 195), on the basis that the name was published in the “N[ew] E[ngland] Farmer.” I have not found any publication of Harris citing such a name. The name is a *nomen nudum* as mentioned by Madge (1967: 216).

### [scalpta group]

### Lebia scalpta

Bates, 1883

Lebia scalpta Bates, 1883a: 230. Type locality: «Jalapa [and] Yucatan, Mexico» (original citation). Syntype(s) probably in BMNH.

#### Distribution.

This species ranges from southern Arizona and southern Texas (Madge 1967: 186) south at least to the Yucatán Peninsula (Bates 1883a: 230) in Mexico.

#### Records.

**USA**: AZ, TX – Mexico

### [scapula group]

### Lebia scapula

Horn, 1885

Lebia scapula G.H. Horn, 1885a: 132. Type locality: «Arizona» (original citation), herein restricted to Nogales, Santa Cruz County (see Madge 1967: 184). Holotype [by monotypy] (♀) in MCZ [# 34504].

#### Distribution.

This species is found from Arizona (Madge 1967: 184) to western Texas (Graves and Suter 1979: 6).

#### Records.

**USA**: AZ, NM, TX

### [viridipennis group]

### Lebia viridipennis

Dejean, 1826

Lebia viridipennis Dejean, 1826: 452. Type locality: «Amérique septentrionale» (original citation), restricted to «Brookline [Norfolk County], Mass[achusetts]» by Lindroth (1969a: 1025). One syntype in MHNP (Lindroth 1955b: 23).
Lebia borea Hentz, 1830: 255. Type locality: «Massachusetts» (original citation). Syntype(s) lost. Synonymy established by LeConte (1846b: 193).
Lebia abrupta Casey, 1920: 250. Type locality: «Indiana» (original citation). One syntype in USNM [# 47624]. Synonymy established by Madge (1967: 170).
Lebia viridipennis frontalis Casey, 1920: 251. Type locality: «Keokuk [Lee County], Iowa» (original citation). One syntype in USNM [# 47625]. Synonymy established by Madge (1967: 170).
Lebia rhodeana Casey, 1920: 251. Type locality: «Boston Neck [Washington County], Rhode Island» (original citation). One syntype in USNM [# 47626]. Synonymy established by Madge (1967: 170).

#### Distribution.

The range of this species extends from southwestern Maine (Majka et al. 2011: 47) and southwestern Quebec (LeSage 1996: 22) to southeastern South Dakota (Kirk and Balsbaugh 1975: 37), including southernmost Ontario (Pettit 1869: 107; CNC), south to southeastern Texas along the Rio Grande and southern Florida [see Madge 1967: Fig. 122]. The record from “North Dakota” (Bousquet and Larochelle 1993: 278) needs confirmation.

#### Records.

**CAN**: ON, QC **USA**: AL, AR, CT, DC, DE, FL, GA, IA, IL, IN, KS, LA, MA, MD, ME, MI, MN, MO, MS, NC, NE, NH, NJ, NY, OH, OK, PA, RI, SC, SD, TN, TX, VA, VT, WI, WV [ND]

### [viridis group]

### Lebia arizonica

Schaeffer, 1910

Lebia arizonica Schaeffer, 1910: 398. Type locality: «Huachuca M[oun]t[ain]s [Cochise County], Arizona» (original citation). Lectotype (♂), designated by Erwin and House (1978: 239), in USNM [# 42506].

#### Distribution.

The range of this species extends from southern Arizona to western Texas (Madge 1967: 175). The record from “Nevada” (Bousquet and Larochelle 1993: 276) needs confirmation.

#### Records.

**USA**: AZ, NM, TX [NV]

### Lebia cyanipennis

Dejean, 1831

Lebia cyanipennis Dejean, 1831: 385. Type locality: «Californie» (original citation), restricted to «San Diego [San Diego County]» by Lindroth (1969a: 1026). Holotype [by monotypy] in MHNP (Lindroth 1955b: 22).
Lebia ruficollis LeConte, 1851: 178. Type locality: «San Diego [San Diego County, California]» (original citation). Two syntypes in MCZ [# 68] and at least one in MHNP (collection Chaudoir). Synonymy established by Casey (1920: 251), confirmed by Madge (1967: 170).
Lebia montana G.H. Horn, 1885a: 131. Type locality: «Montana» (original citation). Two syntypes in MCZ [# 34503]. Synonymy established by Madge (1967: 176).
Lebia barbarae Casey, 1920: 242. Type locality: «S[an]ta Barbara [Santa Barbara County], California» (original citation). One syntype in USNM [# 47637]. Synonymy established by Madge (1967: 176).
Lebia melaena Hatch, 1953: 152. Type locality: «Pullman [Whitman County], Wash[ington]» (original citation). Holotype (♂) in USNM. Synonymy established by Madge (1967: 176).

#### Distribution.

This species ranges from southern Saskatchewan to south-central British Columbia, south to southern California along the Mexican border and northern New Mexico [see Madge 1967: Fig. 129]. At least one specimen is known from “Texas” (Madge 1967: 177). The record from “North Dakota” (Bousquet and Larochelle 1993: 276) needs confirmation.

#### Records.

**CAN**: AB, BC, SK **USA**: AZ, CA, CO, ID, MT, NM, OR, UT, WA, WY [ND, TX]

### Lebia marginicollis

Dejean, 1825

Lebia marginicollis Dejean, 1825: 271. Type locality: «Géorgie» (original citation), herein restricted to Tifton, Tift County (see Madge 1967: 181). One syntype in MHNP (Lindroth 1955b: 22).
Lebia cyanea Dejean, 1831: 386. Type locality: «île de Cuba; Cayenne [French Guiana]» (original citation), restricted to «Cuba» by Madge (1967: 180). Syntype(s) apparently lost (Lindroth 1969a: 1030). Synonymy established by Madge (1967: 180). Note. Lindroth (1969a: 1030) stated that the sole specimen in Chaudoir’s collection under this name cannot be a syntype since its structural features do not match those reported in the original description. He agreed with Madge (1967: 180) that the syntypes were probably conspecific with members of *Lebia marginicollis* Dejean.
Lebia affinis Dejean, 1831: 387. Type locality: «Amérique septentrionale» (original citation). Holotype [by monotypy] in MHNP (Lindroth 1955b: 22). Synonymy established by LeConte (1869b: 248), confirmed by Lindroth (1955b: 22).
Lamprias limbicollis Motschulsky, 1859a: 145. Type locality: «Canada» (original citation), which is likely incorrect. Lectotype (probably ♀), designated by Bousquet (1997b: 334), in ZMMU. Synonymy established, under the name *Lebia affinis* Dejean, by LeConte (1863b: 5), confirmed by Bousquet (1997b: 334).

#### Distribution.

The range of this species extends from New Jersey (Smith 1890: 87; Smith 1910: 210) to Arizona, north to northwestern Ohio (Hancock County, Foster F. Purrington pers. comm. 2009), southern Michigan, and northern Illinois, south to Brazil (Chaudoir 1871a: 185) and southern Florida [see Madge 1967: Fig. 125]; also found “on all the Greater Antilles” (Darlington 1953: 11, as *Lebia cyanea*) and Dominica (Peck 2006: 176). The record from Montana (Hatch 1933a: 9) is probably in error.

#### Records.

**USA**: AL, AR, AZ, DC, FL, GA, IL, IN, LA, MI, MO, MS, NC, NJ, NM, OH, OK, SC, TN, TX, VA – Bahamas, Brazil, Cuba, Dominica, French Guiana, Guatemala, Jamaica, Mexico, Nicaragua, Puerto Rico

### Lebia moesta

LeConte, 1850

Lebia moesta LeConte, 1850: 203. Type locality: «Michipicotin [Ontario]» (original citation). Syntype(s) in MCZ [# 5811].
Lebia cynica Casey, 1920: 241. Type locality: «Boston Neck [Washington County], Rhode Island» (original citation). Lectotype (♂), designated by Lindroth (1975: 144), in USNM [# 47634]. Synonymy established by Lindroth (1969a: 1028).

#### Distribution.

This species ranges from Newfoundland (Larson and Langor 1982: 594) and southern Labrador to Vancouver Island (Lindroth 1969a: 1028-1029), south at least to northern Oregon (Tillamook and Umatilla Counties, CNC), southern Wyoming (Lavigne 1977: 46; Lincoln County, CNC), eastern Nebraska (Cuming County, Foster F. Purrington pers. comm. 2009), east-central Ohio (Usis and MacLean 1998: 67), and the District of Columbia (Ulke 1902: 7). The record from east-central Missouri (Summers 1873: 134) needs confirmation.

#### Records.

**CAN**: AB, BC (VCI), LB, MB, NB, NF, NS, ON, PE, QC, SK **USA**: DC, ID, IL, MA, ME, MI, MN, MT, NE, NH, NJ, NY, OH, OR, RI, SD, VT, WA, WI, WY [MO]

#### Note.

Madge (1967: 177) listed this form as synonym of *Lebia viridis* Say.

### Lebia perita

Casey, 1920

Lebia perita Casey, 1920: 241. Type locality: «Hoopa Valley, Humboldt Co[unty], California» (original citation). One syntype in USNM [# 47636].

#### Distribution.

This species occurs from southern British Columbia, including Vancouver Island, south to southern California along the Mexican border, east to southwestern Idaho [see Madge 1967: Fig. 140].

#### Records.

**CAN**: BC (VCI) **USA**: CA, ID, OR, WA

### Lebia pleuritica

LeConte, 1846

Lebia pleuritica LeConte, 1846b: 193. Type locality: «Lacum Superiorem» (original citation), herein restricted to Eagle Harbor, Keweenaw County, Michigan (see Hubbard and Schwarz 1878: 627). Two syntypes in MCZ [# 5806].

#### Distribution.

This species extends from southwestern Quebec (LeSage 1996: 22) to south-central Saskatchewan (Hooper 1977: 51), south to northeastern Kansas (Madge 1967: 174), central Missouri (Cooper County, CMNH), and northwestern North Carolina (Watauga County, CMNH). The records from the Organ Mountains in southern New Mexico (Fall and Cockerell 1907: 160) and “Mexico” (Blackwelder 1944: 55) are probably in error.

#### Records.

**CAN**: ON, QC, SK **USA**: DC, IA, IL, IN, KS, MA, MI, MN, MO, NC, NE, NJ, NY, PA, SD, VT, WI

### Lebia rufopleura

Schaeffer, 1910

Lebia rufopleura Schaeffer, 1910: 398. Type locality: «Brownsville [Cameron County], Texas» (original citation). Lectotype (♂), designated by Erwin and House (1978: 239), in USNM [# 42505].

#### Distribution.

This species is known only from a few localities in southeastern Texas (Madge 1967: 173).

#### Records.

**USA**: TX

### Lebia tuckeri

(Casey, 1920)

Loxopeza tuckeri Casey, 1920: 237. Type locality: «Tuçson [Pima County], Arizona» (original citation). Five syntypes [5 originally cited] in USNM [# 47621].

#### Distribution.

This species is found from southern California to western Texas, north to Mesa County in Colorado (Madge 1967: 175).

#### Records.

**USA**: AZ, CA, CO, NM, TX

### Lebia viridis

Say, 1823

Lebia viridis Say, 1823a: 14. Type locality: «Camp Hill [Cumberland County], P[ennsylvani]a» (neotype label). Neotype (♂), designated by Lindroth and Freitag (1969: 349), in MCZ [# 33006].
Lebia viridis Dejean, 1825: 271 [primary homonym of *Lebia viridis* Say, 1823]. Type locality: «Kentucki» (syntype label). One syntype in MHNP (Lindroth 1955b: 22). Synonymy established with doubt by Say (1830a: 135), confirmed by Lindroth (1955b: 22).
Lebia smaragdula Dejean, 1831: 387. Type locality: «Amérique septentrionale» (original citation). One syntype in MHNP (Lindroth 1955b: 23). Synonymy established by LeConte (1880a: 88).
Lamprias cyanellus Motschulsky, 1850a: 42. Type locality: «California» (original citation), cited from «St. Francisco [San Francisco County]» by Motschulsky (1859a: 145). Lectotype, designated by Bousquet (1997b: 331), in ZMMU. Synonymy established, under the name *Lebia smaragdula* Dejean, by LeConte (1869b: 248), confirmed by Bousquet (1997b: 331).
Lebia viridis var. *violaceipennis* Chaudoir, 1871a: 193. Type locality: «Guatemala» (original citation). Syntype(s) in MHNP. Synonymy established by Bates (1883a: 223).
Lebia viridis var. *subopaca* Schaeffer, 1910: 397. Type locality: «Huachuca M[oun]t[ain]s [Cochise County], Arizona» (original citation). Lectotype (♀), designated by Erwin and House (1978: 239), in USNM [# 42504]. Synonymy established by Madge (1967: 177).
Lebia truckeensis Casey, 1920: 241. Type locality: «Reno [Washoe County], Nevada» (original citation). Holotype [by monotypy] in USNM [# 47635]. Synonymy established by Madge (1967: 178).
Lebia castigata Casey, 1920: 242. Type locality: «Placer Co[unty], California» (original citation). One syntype in USNM [# 47638]. Synonymy established by Madge (1967: 178).
Lebia adolescens Casey, 1920: 242. Type locality: «Boston Neck, Rhode Island; Southern Pines, North Carolina» (original citation), restricted to «Boston Neck [Washington County]» by Madge (1967: 178). Four syntypes in USNM [# 47639]. Synonymy established by Madge (1967: 178).
Lebia evoluta Casey, 1920: 243. Type locality: «Las Vegas, New Mexico; Kansas» (original citation), restricted to «Las Vegas [San Miguel County]» by Madge (1967: 178). Two syntypes in USNM [# 47640]. Synonymy established by Madge (1967: 178).
Lebia histrica Casey, 1920: 243. Type locality: «Boston Neck [Washington County], Rhode Island» (original citation). One syntype in USNM [# 47641]. Synonymy established by Madge (1967: 178).
Lebia bracata Casey, 1920: 243. Type locality: «probably Indiana» (original citation). Holotype [by monotypy] (♀) in USNM [# 47642]. Synonymy established by Madge (1967: 178).
Lebia magica Casey, 1920: 244. Type locality: «S[ain]t Louis, Missouri» (original citation). One syntype in USNM [# 47643]. Synonymy established by Madge (1967: 178).
Lebia incitata Casey, 1920: 244. Type locality: «Hoopa Valley, Humboldt Co[unty], California» (original citation). One syntype in USNM [# 47644]. Synonymy established by Madge (1967: 178).
Lebia subaffinis Casey, 1920: 244. Type locality: «Fort Wingate [McKinley County], New Mexico» (original citation). Two syntypes in USNM [# 47645]. Synonymy established by Madge (1967: 178).
Lebia vermiculina Casey, 1920: 245. Type locality: «Boston Neck [Washington County], Rhode Island» (original citation). One syntype in USNM [# 47628]. Synonymy established by Madge (1967: 178).
Lebia prominens Casey, 1920: 245. Type locality: «probably Indiana» (original citation). Holotype [by monotypy] in USNM [# 47627]. Synonymy established by Madge (1967: 178).
Lebia planifera Casey, 1920: 246. Type locality: «Tuçson [Pima County], Arizona» (original citation). Six syntypes in USNM [# 47629]. Synonymy established by Madge (1967: 178).
Lebia cobaltina Casey, 1920: 246. Type locality: «Colonia Garcia, Sierra Madre M[oun]t[ain]s, Chihuahua, Mexico» (original citation). One syntype in USNM [# 47630]. Synonymy established by Madge (1967: 178).
Lebia papago Casey, 1920: 247. Type locality: «Tuçson [Pima County], Arizona» (original citation). One syntype in USNM [# 47631]. Synonymy established by Madge (1967: 178).
Lebia papago trajecta Casey, 1920: 247. Type locality: «Arizona» (original citation). One syntype in USNM [# 47632]. Synonymy established by Madge (1967: 178).
Lebia duluthiana Casey, 1920: 247. Type locality: «Duluth [Saint Louis County], Minnesota» (original citation). One syntype in USNM [# 47633]. Synonymy established by Madge (1967: 178).

#### Distribution.

This widely distributed species ranges from Nova Scotia to northwestern Yukon Territory, south to southern California along the Mexican border, Guatemala (Chaudoir 1871a: 192), and southern Florida [see Madge 1967: Fig. 139]; also reported from some islands of the Greater Antilles (Blackwelder 1944: 56). According to Shimonoya (2004), this species has been recently found in Fukui Prefecture, Japan.

#### Records.

**CAN**: AB, BC (VCI), MB, NB, NS (CBI), NT, ON, PE, QC, SK, YT **USA**: AL, AR, AZ, CA, CO, CT, DC, DE, FL, GA, IA, ID, IL, IN, KS, KY, LA, MA, MD, ME, MI, MN, MO, MS, MT, NC, ND, NE, NH, NJ, NM, NV, NY, OH, OK, OR, PA, RI, SC, SD, TN, TX, UT, VA, VT, WA, WI, WV, WY – Cuba, Dominican Republic, Guatemala, Mexico, Puerto Rico

### [vittata group]

### Lebia histrionica

Bates, 1883

Lebia histrionica Bates, 1883a: 240. Type locality: «Mexico; Guatemala» (original citation). Syntype(s) probably in BMNH.
Lebia histrionica var. *scutellata* Bates, 1883a: 241. Type locality: «Playa Vicente [Veracruz], Mexico» (original citation). Syntype(s) probably in BMNH. Synonymy established by Madge (1967: 193).
Lebia histrionica var. *nigrosignata* Bates, 1883a: 241. Type locality: «Guanajuato, Mexico» (original citation). Syntype(s) probably in BMNH. Synonymy established by Madge (1967: 193).

#### Distribution.

This species ranges from southern Arizona (Madge 1967: 194) to Guatemala (Bates 1883a: 240).

#### Records.

**USA**: AZ – Guatemala, Mexico

### Lebia miranda

(Horn, 1872)

Dianchomena miranda G.H. Horn, 1872a: 139. Type locality: «Camp Grant [Pinal County], Arizona» (original citation). Syntype(s) in MCZ [# 8325].

#### Distribution.

This species is found in southern Arizona (Madge 1967: 189). At least one specimen labeled from “Texas” is known.

#### Records.

**USA**: AZ [TX]

#### Note.

Madge (1967: 189) retained this name only for the Arizona (and possibly Texas) populations but pointed out that populations, probably representing the same species but with different color patterns, were seen from Mexico and Colombia.

### Lebia nigricapitata

Madge, 1967

Lebia nigricapitata Madge, 1967: 195. Type locality: «Oak C[ree]k Canyon [Coconino County], Ariz[ona]» (original citation). Holotype (♂) in CAS [# 8162].

#### Distribution.

This species is known only from a few localities in Arizona (Madge 1967: 196).

#### Records.

**USA**: AZ

### Lebia pectita

Horn, 1885

Lebia pectita G.H. Horn, 1885a: 133. Type locality: «Pennsylvania to Texas» (original citation for *Lebia vittata* Fabricius *sensu* Horn, 1872), herein restricted to Jacksonville, Duval County, Florida (see Madge 1967: 195). Syntype(s) in MCZ [# 34507]. Note. This name was proposed for *Lebia vittata* (Fabricius, 1777) *sensu* Horn (1872a: 140). Horn’s collection in MCZ contains three specimens under this name: two are labeled “N.M.” (one of which has a type label) and the other one “Fla.”

#### Distribution.

This species is found from New Hampshire to northeastern Kansas (Knaus 1903: 188), south to eastern Texas and northern Florida [see Madge 1967: Fig. 121]. The state record of “New Mexico” (Bousquet and Larochelle 1993: 277), based on two specimens in Horn collection (MCZ) labeled “N.M.,” needs confirmation.

#### Records.

**USA**: AL, AR, CT, DC, FL, GA, IL, IN, KS, KY, LA, MA, MD, MI, MS, NC, NH, NJ, NY, OH, OK, PA, RI, SC, TN, TX, VA [NM]

### Lebia solea

Hentz, 1830

Lebia solea Hentz, 1830: 256. Type locality: «Massachusetts» (original citation), herein restricted to Framingham, Middlesex County (see Madge 1967: 188). Syntype(s) lost.
Lebia scapularis Dejean, 1831: 377. Type locality: «Amérique septentrionale» (original citation). One syntype in MHNP (Lindroth 1955b: 23). Synonymy established by LeConte (1859c: 31), confirmed by Lindroth (1955b: 23).
Lebia conjungens LeConte, 1846b: 194. Type locality: «NovEboraci [= New York]» (original citation). Two syntypes in MCZ [# 5809]. Synonymy established, under the name *Lebia scapularis* Dejean, by Horn (1872a: 138). Note. Madge (1967: 190) listed this name in synonymy with *Lebia vittata* Fabricius. Horn (1872a: 138) referred it to this species and the original description clearly suggests that his interpretation is correct. The specimen labeled as type in MCZ is not a syntype since it bears the label “N.J.”
Lebia flavolineata Motschulsky, 1864: 227. Type locality: «Am[érique] bor[éale]» (original citation). Lectotype (♀), designated by Bousquet (1997b: 339), in ZMMU. Synonymy established with doubt by Horn (1872a: 142), confirmed by Bousquet (1997b: 339).
Lebia scapularis var. *limbigera* Chaudoir, 1871b: 54. Type locality: «Louisiane» (original citation). Holotype [by monotypy] probably in MHNP. **New synonymy**. Note. This name has been forgotten in the literature. The elytral color of the sole specimen seen by Chaudoir (1871b: 54), which was the reason for proposing the new taxon, falls within the variability of *Lebia solea* as expressed by Madge (1967: 187).
Lebia websteri Casey, 1920: 260. Type locality: «Indiana» (original citation). Lectotype (♀), designated by Lindroth (1975: 144), in USNM [# 47664]. Synonymy established by Madge (1967: 187). Etymology. The specific name honors Francis Marion Webster [1849-1916], a self-made entomologist who worked as a special field agent to the United States Department of Agriculture under Charles Valentine Riley before joining the U.S. Bureau of Entomology.

#### Distribution.

This species is found east of the Rocky Mountains from southern Nova Scotia to southwestern Saskatchewan, south to southeastern Texas along the Rio Grande and southern Florida [see Madge 1967: Fig. 131]; also recorded from Cuba (Darlington 1934: 113) and “Mexico” (Lindroth 1969a: 1031). The record from southwestern New Mexico (Fall and Cockerell 1907: 160, as *Lebia scapularis*) needs confirmation.

#### Records.

**CAN**: MB, NS, ON, QC, SK **USA**: AL, AR, CO, CT, DC, DE, FL, GA, IA, IL, IN, KS, KY, LA, MA, MD, ME, MI, MN, MO, MS, NC, ND, NE, NH, NJ, NY, OH, OK, PA, RI, SC, SD, TN, TX, VA, VT, WI, WV [NM] – Cuba, Mexico

### Lebia vittata

(Fabricius, 1777)

Carabus vittatus Fabricius, 1777: 240. Type locality: «America boreali» (original citation), restricted to «Elkhart [Elkhart County], Ind[iana]» by Lindroth (1969a: 1031). Syntype(s) apparently lost (Lindroth 1969a: 1031).
Lebia flavovittata Chevrolat, 1836a: [no. 161]. Type locality: «environs de Mexico» (original citation). Syntype(s) [2 originally cited] location unknown (possibly in UMO). Synonymy established by Chaudoir (1871b: 40).
Lebia furcata LeConte, 1846b: 193. Type locality: «ad flumen Platte, et ad Lacum Superiorem» (original citation), restricted to «Eagle Harbor [Keweenaw County], Mich[igan]» by Lindroth (1969a: 1031). Syntype(s) in MCZ [# 5808]. Synonymy established by Madge (1967: 189).
Aphelogenia vittata var. *connecta* Chaudoir, 1871b: 40. Type locality: Etats de l’Union américaine (inferred from text on page 41). Syntype(s) in MHNP. Synonymy established by Madge (1967: 190).
Aphelogenia spraguei G.H. Horn, 1872a: 139. Type locality: «Texas» (original citation). Holotype [by monotypy] (♀) in MCZ [# 34506]. Synonymy established by Horn (1885a: 133).
Lebia depicta G.H. Horn, 1885a: 133. Type locality: «Montana» (original citation). Two syntypes in MCZ [# 34505]. Synonymy established by Madge (1967: 190).
Lebia sonomae Casey, 1913: 191. Type locality: «Mendocino Co[unty], California» (original citation). Two syntypes in USNM [# 47662]. Synonymy established by Madge (1967: 190).
Lebia debiliceps Casey, 1913: 192. Type locality: «Indiana» (original citation). Lectotype (♂), designated by Lindroth (1975: 144), in USNM [# 47660]. Synonymy established, under the name *Lebia furcata* LeConte, by Hatch (1953: 153), confirmed by Lindroth (1969a: 1032).
Lebia amnicola Casey, 1913: 192. Type locality: «Brownsville [Cameron County], Texas» (original citation). One syntype in USNM [# 47661]. Synonymy established by Madge (1967: 190).
Lebia tempeana Casey, 1924: 92. Type locality: «Tempe [Maricopa County], Arizona» (original citation). One syntype in USNM [# 47663]. Synonymy established by Madge (1967: 190).

#### Distribution.

This species is found from Nova Scotia to southeastern British Columbia (Lindroth 1969a: 1033), south to southern California, southwestern New Mexico (Fall and Cockerell 1907: 160), southeastern Texas, and southern Florida [see Madge 1967: Fig. 118]; also recorded from “Mexico” (Bates 1883a: 240) and the state of Tabasco (Bates 1891a: 274, as *Lebia furcata*).

#### Records.

**CAN**: AB, BC, MB, NS, ON, QC, SK **USA**: AL, AR, AZ, CA, CO, CT, DC, DE, FL, GA, IA, ID, IL, IN, KS, KY, LA, MA, MD, ME, MI, MN, MO, MS, MT, NC, ND, NE, NH, NJ, NM, NV, NY, OH, OK, OR, PA, RI, SC, SD, TN, TX, UT, VA, VT, WA, WI, WY – Mexico

### Genus Hyboptera

Chaudoir, 1873

Hyboptera Chaudoir, 1873b: 161. Type species: *Hyboptera angulicollis* Chaudoir, 1873 designated by Reichardt (1973: 48). Etymology (original). From Greek *hybos* (hump, tubercle) and *pteron* (wing, by extension elytron), alluding to the presence of linear series of tubercles on the elytra (“*elytra *... *supra seriatim tuberculata*”) of the adult [feminine].

#### Diversity.

Seven species are known (Erwin 2004: 33) in the Neotropical Region, one of which extends into southern Texas.

#### Identification.

Reichardt (1973) reviewed the species then known and published a key for their identification. Subsequently, two new species were described by Erwin (2004).

### Hyboptera auxiliadora

Erwin, 2004

Hyboptera auxiliadora Erwin, 2004: 35. Type locality: «Bentsen State Park, Mission, Hidalgo County, Texas» (original citation). Holotype (♂) in USNM.

#### Distribution.

This species ranges from southern Texas to Costa Rica (Erwin 2004: 37).

#### Records.

**USA**: TX – Costa Rica, Mexico

### Subtribe Calleidina

Chaudoir, 1873

Callidides Chaudoir, 1873b: 97. Type genus: *Callida* Agassiz, 1846 (unjustified emendation of *Calleida* Latreille, 1824, not in prevailing usage) (= *Calleida* Latreille, 1824).
Plochionidae des Gozis, 1875: 3. Type genus: *Plochionus* Dejean, 1821.
Anomotarina Habu, 1967: 117. Type genus: *Anomotarus* Chaudoir, 1875. Synonymy established by Ball and Hilchie (1983: 173).

#### Diversity.

Worldwide, with about 660 species arrayed in 46 genera (Lorenz 2005: 491-498). The North American fauna is represented by 24 species (about 3.5 % of the world fauna).

#### Taxonomic Note.

Erwin (2004: 6) advocated combining calleidines with agrines into one subtribe (Agrina) but did not provide any justification. In a cladistic analysis of lebiine exemplars performed by Casale (1998: Fig. 91), calleidines turned out as the sister-group to metallicines and agrines as the sister-group to physoderines. In this work, agrines are retained as a distinct subtribe. In the analysis of Ball et al. (1995), calleidines showed up as the sister-group to gallerucidiines. Jeannel (1949a: 946) listed the gallerucidiines and thysanotines (currently considered as pericalines) with the calleidines (including trichines).

### Genus Plochionus

Dejean, 1821

Plochionus Dejean, 1821: 5. Type species: *Lebia bonfilsii* Audinet-Serville, 1821 (= *Carabus pallens* Fabricius, 1775) by monotypy. Etymology (see Dejean 1825: 250). From the Greek *plochion* (chain), alluding to the moniliform antennomeres 4-11 of the adult which reminded Dejean of a pearl necklace (“*antennes *... *dont les sept derniers articles sont *... *arrondis comme des perles formant un collier*”) [masculine].
Plocionus Agassiz, 1846: 299. Unjustified emendation of *Plochionus* Dejean, 1821.

#### Diversity.

Seventeen species (Lorenz 2005: 495) in the Nearctic (four species), Neotropical (14 species), and Australian (one species from New Caledonia) Regions. One species, *Plochionus pallens*, has become subcosmopolitan through commerce.

#### Identification.

Horn (1882: 145) provided a key for the identification of the North American species. Since then, one new species (*Plochionus bicolor*) has been described by Notman (1919b: 234). A taxonomic revision of the species would be useful.

### Subgenus Menidius

Chaudoir, 1872

Menidius Chaudoir, 1872b: 170. Type species: *Plochionus timidus* Haldeman, 1843 designated by Lindroth (1969a: 1066). Etymology (original). From the Greek *mene* (moon) and *eidos* (form), alluding to the semicircular (i.e., half-moon) shape of the pronotum (“*thorax *... *fere semicircularis*”) of the adult [masculine].

#### Diversity.

Fourteen species in the temperate, subtropical, and tropical areas of the Nearctic (four species, all of them extending into the West Indies or Mexico) and Neotropical Regions.

### Plochionus amandus

Newman, 1840

Plochionus amandus Newman, 1840: 32. Type locality: «S[ain]t John’s Bluff [Duval County], East Florida» (original citation). Syntype(s) probably in BMNH.
Plochionus vittatus LeConte, 1844: 48. Type locality: «Florida» (original citation). One syntype in MCZ. Synonymy established by LeConte (1863b: 5).

#### Distribution.

This species ranges from southeastern Georgia (Fattig 1949: 40; McIntosh County, CNC) to southern Florida (Peck and Thomas 1998: 24), west to southwestern Alabama (Leng 1915: 588; Mobile County, MCZ, USNM); also recorded from the Bahamas (Turnbow and Thomas 2008: 13). The record from Guana Island (Valentine and Ivie 2005: 275) in the British Virgin Islands needs confirmation.

#### Records.

**USA**: AL, FL, GA – Bahamas

### Plochionus bicolor

Notman, 1919

Plochionus bicolor Notman, 1919b: 234. Type locality: «Key Largo [Monroe County], Fl[orid]a» (original citation). One syntype [2 ♂ originally cited] in SIM (Hennessey 1990: 466).

#### Distribution.

This species is known only from southern Florida (Peck and Thomas 1998: 24), Cuba (Darlington 1934: 117), and Navassa (Steiner 2008: 132).

#### Records.

**USA**: FL – Cuba, Navassa

#### Note.

According to Ball (in Peck 2005: 39), *Plochionus rubrofasciatus* Zayas, 1988, described from Cuba, is probably a synonym of this species.

### Plochionus discoideus

LeConte, 1880

Plochionus amandus var. *discoideus* LeConte, 1880a: 86. Type locality: «Fl[orid]a» (original citation). Five syntypes in MCZ.
Plochionus dorsalis G.H. Horn, 1882: 146. Type locality: «Florida» (original citation). Six syntypes in MCZ [# 8186]. Synonymy established by Leng (1915: 588).

#### Distribution.

This species is known from northern Georgia (Fattig 1949: 40), the Florida Peninsula, as far south as Dade County (Peck and Thomas 1998: 24), and the Bahamas (Darlington 1953: 13).

#### Records.

**USA**: FL, GA – Bahamas

### Plochionus timidus

Haldeman, 1843

Plochionus timidus Haldeman, 1843b: 298. Type locality: «Alabama» (original citation). One possible syntype, a ♀ labeled “[orange disc] / Plochionus timidus Hald. [handwritten],” in MCZ (collection LeConte).

#### Distribution.

The range of this species extends from southeastern New Hampshire (Strafford County, Ross T. Bell pers. comm. 2008) to southern Wisconsin (Messer 2010: 44) and northern Iowa (Wickham 1911b: 7), including southern Ontario (Pettit 1869: 107), south to central Texas (Haldeman 1852: 373; Blanco County, CMNH), southern Louisiana (East Baton Rouge, Iberville, Saint Martin, and Saint Tammany Parishes, Igor M. Sokolov pers. comm. 2009), and central Florida (Pinellas County, CMNH; Schwarz 1878: 435), west along southwestern United States to southern California (Moore 1937: 12; Kern and Fresno Counties, CNC) and the Baja California Peninsula (Horn 1894: 310).

#### Records.

**CAN**: ON **USA**: AL, AR, AZ, CA, CT, DC, DE, FL, GA, IA, IL, IN, KS, LA, MA, MD, MI, MO, MS, NC, NH, NJ, NM, NV, NY, OH, PA, RI, SC, TN, TX, VA, WI, WV – Mexico

### Subgenus Plochionus

Dejean, 1821

Plochionus Dejean, 1821: 5. Type species: *Lebia bonfilsii* Audinet-Serville, 1821 (= *Carabus pallens* Fabricius, 1775) by monotypy.

#### Diversity.

Two Neotropical species, one of them subcosmopolitan, and one New Caledonian species (*Plochionus niger* Fauvel).

### Plochionus pallens

(Fabricius, 1775)

Carabus pallens Fabricius, 1775: 244. Type locality: «Dresdae [Germany]» (original citation). Syntype(s) apparently lost (Lindroth 1969a: 1066).
Lebia bonfilsii Audinet-Serville, 1821: 11. Type locality: «Bordeaux [France]» (original citation). Syntype(s) probably lost. Synonymy established by Chevrolat (1863: 189). Note. LeConte’s collection holds a specimen labeled “P. Bonfilsii Dej. type! [handwritten] / pallens 2 [handwritten].”
Plochionus valens LeConte, 1863c: 5. Type locality: «Pennsylvania; Tampico, Mexico» (original citation), restricted to «Penns[ylvania]» by Lindroth (1969a: 1066). Two syntypes in MCZ [# 5823]. Synonymy established, under the name *Plochionus bonfilsi* (Audinet-Serville), by LeConte (1880a: 86).

#### Distribution.

Lindroth (1969a: 1066) stated that this species is almost cosmopolitan and probably of South American origin. In North America, it is known from “Massachusetts” (Harris 1833: 566, as *Plochionus bonfilsii*?), “Pennsylvania” (LeConte 1846b: 192; Lindroth 1969a: 1066), Maryland (Charles County, USNM), District of Columbia (USNM), northern Georgia (Rabun County, USNM), eastern Florida (Leng 1915: 588), Missouri (Summers 1873: 133, as *Plochionus bonfilsii*), Kansas (Douglas County, USNM), and Indiana (Alien County, USNM). The record from “California” (Csiki 1932b: 1451) needs confirmation.

#### Records.

**USA**: DC, FL, GA, IN, KS, MA, MD, MO, PA [CA] – **Adventive**

### Genus Tecnophilus

Chaudoir, 1877

Tecnophilus Chaudoir, 1877: 239. Type species: *Calleida croceicollis* Ménétriés, 1843 designated by Larson (1969: 44). Etymology. Anagram of the generic name *Philotecnus*, derived from the Greek *tecnon* (child, young) and *philos* (beloved), although Chaudoir was aware that there was no data suggesting that adults of these species care for their young (“*quoique l’amour de ces insectes pour leur progéniture ne soit rien moins que prouvé*”) [masculine].

#### Diversity.

Two Nearctic species, one of them extending into northern Mexico.

#### Identification.

Larson (1969) revised the species.

### Tecnophilus croceicollis croceicollis

(Ménétriés, 1843)

Calleida croceicollis Ménétriés, 1843: 53. Type locality: «Californie» (original citation), herein restricted to Bay Farm Island, Alameda County (see Larson 1969: 61). Syntype(s) location unknown (Lindroth 1969a: 1064).
Callida chloridipennis Motschulsky, 1850a: 39. Type locality: «California» (original citation), cited from «Col[onie] Ross [farming community about 75 miles north of San Francisco along the coast]» by Motschulsky (1859a: 140). One syntype in ZMMU (Keleinikova 1976: 191). Synonymy established by Horn (1882: 161).
Philotecnus nigricollis LeConte, 1851: 176. Type locality: «San Jose [Santa Clara County, California]» (original citation). Syntype(s) in MCZ [# 63] and MHNP (collection Chaudoir). Synonymy established by Horn (1882: 161).
Philotecnus ruficollis LeConte, 1851: 176. Type locality: «San Diego [San Diego County, California]» (original citation). Holotype [by monotypy] (♀) in MCZ [# 62]. Synonymy established by LeConte (1853c: 379).
Tecnophilus glabripennis Chaudoir, 1877: 242. Type locality: «Nevada» (original citation). Holotype [by monotypy] in MHNP. Synonymy established by Horn (1882: 161).

#### Distribution.

This subspecies occurs from northern California to eastern Colorado, south to southeastern Texas, Durango and Sinaloa in Mexico, and southern California [see Larson 1969: Fig. 63]. The record from “Oregon” (Bousquet and Larochelle 1993: 280) needs confirmation.

#### Records.

**USA**: AZ, CA, CO, NM, NV, TX, UT [OR] – Mexico

### Tecnophilus croceicollis peigani

Larson, 1969

Tecnophilus croceicollis peigani Larson, 1969: 61. Type locality: «Milk River near junction with Lost River, Lost River Ranch, Alberta» (original citation). Holotype (♂) in CNC [# 10540].

#### Distribution.

The range of this subspecies extends from southern Saskatchewan (several localities, CNC) and southern Alberta south to southwestern Idaho, northern Utah, and “Colorado” [Larson 1969: 62, Fig. 63].

#### Records.

**CAN**: AB, SK **USA**: CO, ID, MT, UT, WY

**Figure 41.**
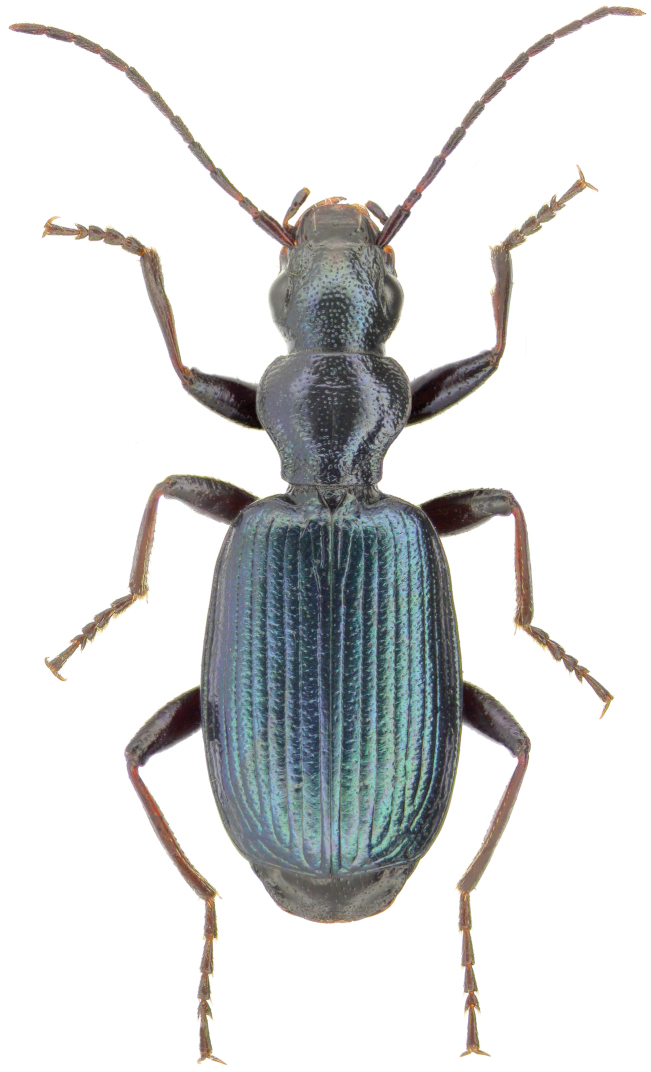
*Tecnophilus croceicollis peigani* Larson. Adults of this handsome subspecies are usually found during the day on clay-sand soil on the ground or climbing on very low vegetation along the bases of south facing coulees. The subspecific name derives from the name of an Indian tribe of the Blackfoot Confederation, Peigan, which inhabited the prairies of southern Alberta where the taxon is found in Canada.

### Tecnophilus pilatei

Chaudoir, 1877

Tecnophilus pilatei Chaudoir, 1877: 239. Type locality: «Texas» (original citation), herein restricted to Goose Island State Park, Aransas County (see Larson 1969: 46). Holotype [by monotypy] (♀) in MHNP.

#### Distribution.

This species is restricted to the Gulf Coast of Texas [see Larson 1969: Fig. 62].

#### Records.

**USA**: TX

### Genus Calleida

Latreille, 1824

Calleida Latreille [in Latreille and Dejean], 1824: 132. Type species: *Carabus decorus* Fabricius, 1801 designated by Desmarest (1851: 72). Etymology. From the Greek *callos* (beauty) and *eidos* (form), alluding to the elegant form of adults of the two species known to Latreille [feminine].
Callida Agassiz, 1846: 58. Unjustified emendation of *Calleida* Latreille, 1824.
Caloidea Maindron, 1905: 331. Unjustified emendation of *Calleida* Latreille, 1824.
Caloida Bedel, 1907: 265. Unjustified emendation of *Calleida* Latreille, 1824.

#### Diversity.

About 275 species primarily in tropical areas of the Neotropical, Oriental, and Afrotropical Regions (over 95 % of the world fauna) with a few elements found in the Nearctic (11 species) and Palaearctic (ten species in Asia) Regions. The species are arrayed in three subgenera: *Calleida* s.str. (about 175 species), *Callidiola* Jeannel (60 species), and *Stenocallida* Jeannel (40 species), though some authors (e.g., Casale 2008) treat *Callidiola* and *Stenocallida* as distinct genera.

### Subgenus Calleida

Latreille, 1824

Calleida Latreille [in Latreille and Dejean], 1824: 132. Type species: *Carabus decorus* Fabricius, 1801 designated by Desmarest (1851: 72).
Spongoloba Chaudoir, 1873b: 152. Type species: *Calleida fulgida* Dejean, 1831 designated by Habu (1960: 157). Synonymy established by Horn (1882: 139). Etymology (original). From the Greek *spongos* (sponge) and *lobos* (lobe), alluding to the bilobed, with spongy pubescence underneath, of the male fourth protarsomere (“*tarsi *... *anteriores articulo *... *quarto fortiter bilobo, lobis ovatis latis, subtus dense spongiosis*”) [feminine].
Lecalida Casey, 1920: 288. Type species: *Lecalida pimalis* Casey, 1920 (= *Callida platynoides* Horn, 1882) by original designation. Synonymy established by Bousquet and Larochelle (1993: 280). Etymology. Anagram of the generic name *Calleida* [*q.v*.] [feminine].

#### Diversity.

About 175 species confined to the Western Hemisphere. Only 11 species are endemic (five species) or extends (six species) into the Nearctic Region.

#### Identification.

There is no key for the identification of the North American species.

### Calleida circumcincta

Bates, 1883

Calleida circumcincta Bates, 1883a: 212. Type locality: «Jalapa [Veracruz], Mexico» (original citation). Syntype(s) location unknown (possibly in BMNH). Note. The type series was collected by Höge. According to Bates (1884: 256), Höge, on his return to Europe, mislabeled many specimens collected in the city of Oaxaca as “Jalapa” and vice versa.

#### Distribution.

This species is known from southeastern Texas (Schaeffer 1905: 142; MCZ) south at least to Veracruz, Mexico (Bates 1883a: 212); it may occur much further south, as far as Venezuela (Schaeffer 1910: 397).

#### Records.

**USA**: TX – Mexico

### Calleida decora

(Fabricius, 1801)

Carabus decorus Fabricius, 1801: 181 [primary homonym of *Carabus decorus* Panzer, 1799]. Type locality: «Carolina» (original citation). Syntype(s) location unknown (Zimsen 1964: 53), though according to Motschulsky (1855a: 51) in ZMUC. Note. This name is a junior primary homonym of *Carabus decorus* Panzer, 1799 (= *Bembidion decorum* (Panzer, 1799)). Since both names apply to taxa not considered congeneric since 1899, the case is to be referred to the Commission and meanwhile prevailing usage of both names must be maintained (ICZN 1999: Article 23.9.5).
Calleida cordicollis Putzeys, 1845a: 21. Type locality: «Oaxaca, Mexique» (original citation). Holotype [by monotypy] location unknown. Synonymy established by Horn (1882: 161).
Calleida cyanoptera LeConte, 1858b: 59 [primary homonym of *Calleida cyanoptera* Solier, 1849]. Type locality not stated. Three syntype(s) in MCZ [# 5819]. Synonymy established by Horn (1882: 161).
Callida coeruleipennis Gemminger and Harold, 1868a: 115. Replacement name for *Calleida cyanoptera* LeConte, 1858.

#### Distribution.

This species ranges from south-central North Dakota (Burleigh County, Donald P. Schwert pers. comm. 1989) to southeastern Virginia (Hoffman and Roble 2000: 40), south to southern Florida (Peck and Thomas 1998: 24), the Bahamas (Darlington 1953: 11), and Nicaragua (CMNH), west to southern Arizona (Santa Cruz and Pima Counties, CMNH, UASM).

#### Records.

**USA**: AL, AR, AZ, FL, GA, IA, IL, IN, KS, LA, MS, NC, ND, NE, OK, SC, SD, TN, TX, VA – Bahamas, Belize, Mexico, Nicaragua

### Calleida fimbriata

Bates, 1883

Calleida fimbriata Bates, 1883a: 212. Type locality: «Jalapa [Veracruz], Mexico» (original citation). Syntype(s) location unknown (possibly in BMNH).

#### Distribution.

This species is found in southern Texas (Bousquet and Larochelle 1993: 334) south at least to Veracruz (Bates 1883a: 212) in Mexico.

#### Records.

**USA**: TX – Mexico

### Calleida fulgida

Dejean, 1831

Calleida fulgida Dejean, 1831: 330. Type locality: «Amérique septentrionale» (original citation). One syntype in MHNP (Lindroth 1955b: 24).
Calleida striata Casey, 1913: 177. Type locality: «Florida» (original citation). Two syntypes in USNM [# 47675]. **New synonymy**.

#### Distribution.

This species ranges from southern South Carolina (Ciegler 2000: 126) to southern Florida (Peck and Thomas 1998: 24), west to “Texas” (Leng 1915: 587) and central Oklahoma (Hatch and Ortenburger 1930: 11). The records from Colorado (Wickham 1902: 240) and northeastern Kansas (Knaus 1901: 110) need confirmation.

#### Records.

**USA**: AL, FL, GA, OK, SC, TX [CO, KS]

### Calleida obrieni

Mateu, 1995

Calleida obrieni Mateu, 1995: 146. Type locality: «Myakka R[iver] St[ate] Park, Sarasota Co[unty], Florida» (original citation). Holotype (♀) in Mateu’s collection (Almería, Spain).

#### Distribution.

This species is known only from the holotype collected in central Florida.

#### Records.

**USA**: FL

### Calleida planulata

LeConte, 1858

Calleida planulata LeConte, 1858b: 59. Type locality not stated. Holotype [by monotypy] (♂) in MCZ [# 34515]. Note. Horn (1882: 140) stated that the specimen upon which LeConte described this species “may have been taken in Mexico or Texas.” It was described by LeConte in a paper on new species chiefly collected by the United States and Mexican Boundary Commission.
Callida metallescens Chaudoir, 1873b: 120. Type locality: «Vera-Cruz, Mexique» (original citation. Holotype [by monotypy] (♀) probably in MHNP. Synonymy established with doubt by Horn (1882: 140).

#### Distribution.

This species ranges from the Rio Grande Valley in southern Texas (Wickham 1897: 111) south to Guatemala and Belize (Bates 1883a: 208, as *Calleida metallescens*).

#### Records.

**USA**: TX – Belize, Guatemala, Mexico

### Calleida platynoides

Horn, 1882

Callida platynoides G.H. Horn, 1882: 139. Type locality: «mountains east of Visalia, California; southwestern Utah» (original citation). Two syntypes in MCZ [# 8185].
Lecalida pimalis Casey, 1920: 288. Type locality: «Tuçson [Pima County], Arizona» (original citation). Seven syntypes [7 originally cited] in USNM [# 47676]. Synonymy established by Ball (in Bousquet and Larochelle 1993: 281).
Lecalida nigritula Casey, 1920: 289. Type locality: «Tuçson [Pima County], Arizona» (original citation). Holotype [by monotypy] (♀) in USNM [# 47677]. Synonymy established by Ball (in Bousquet and Larochelle 1993: 281).

#### Distribution.

This species ranges from southwestern California (Moore 1937: 12) to southeastern Texas (Cameron County, CNC), including northern Sonora (Bates 1884: 298); also known from Baja California Sur (Santa Rosa, MCZ).

#### Records.

**USA**: AZ, CA, NM, NV, TX, UT – Mexico

### Calleida punctata

LeConte, 1846

Calleida punctata LeConte, 1846b: 189. Type locality: «ad flumen Kansas [Kansas]» (original citation). Two syntypes in MCZ [# 5820].

#### Distribution.

The range of this species extends from Maine (Dearborn and Donahue 1993: 8; Foss 2001: 14) to southern Manitoba (Bousquet 1987a: 133), south to “Kansas” (Horn 1882: 141) and northwestern South Carolina (Anderson County, Robert L. Davidson pers. comm. 2012). The records from “Texas,” “Florida” (Erwin et al. 1977: 4.59), “Louisiana” (Chaudoir 1873b: 153), “Arkansas” (Bousquet and Larochelle 1993: 281), northern Alabama (Löding 1945: 22), and southwestern Georgia (Fattig 1949: 40) need confirmation.

#### Records.

**CAN**: MB, ON, QC **USA**: CT, IA, IL, IN, KS, KY, MA, MD, ME, MI, MN, MO, NC, ND, NE, NH, NJ, NY, OH, PA, SC, SD, TN, VA, VT, WI, WV [AL, AR, FL, GA, LA, TX]

### Calleida punctulata

Chaudoir, 1848

Calleida punctulata Chaudoir, 1848: 86. Type locality: Mexique (inferred from text on page 87). Syntype(s) in MHNP.
Callida rugicollis G.H. Horn, 1894: 361. Type locality: «Coral de Piedra, Sierra El Taste, Pescadero and San José del Cabo [Mexico]» (original citation). Two syntypes in CAS [# 4, 5]. Synonymy established by Schaeffer (1910: 396).

#### Distribution.

This species is known from the Baja California Peninsula (Horn 1894: 361, as *Calleida rugicollis*) and southern Texas (Wickham 1897: 111; Cameron County, CNC), south at least to Nicaragua (CMNH). The record from “California” (Bousquet and Larochelle 1993: 281) is probably in error.

#### Records.

**USA**: TX – Guatemala, Mexico, Nicaragua

### Calleida purpurea

(Say, 1823)

Cymindis purpureus Say, 1823a: 10. Type locality: «Neb[raska]» (neotype label). Neotype (♂), designated by Lindroth and Freitag (1969: 349), in MCZ [# 33003]. Note. «the Missouri» was the area originally cited by Say (1823a: 10).
Calleida smaragdina Dejean, 1825: 225. Type locality: «Géorgie» (original citation). Two syntypes in MHNP (Lindroth 1955b: 24). Synonymy established by Horn (1882: 161), confirmed by Lindroth (1955b: 24)**.**
Calleida cyanipennis Chaudoir, 1844: 467. Type locality: «Amérique» (original citation). Syntype(s) probably in MHNP. Synonymy established, under the name *Calleida smaragdina* Dejean, by LeConte (1869b: 248).

#### Distribution.

This species ranges from “New Hampshire” (Lindroth 1969a: 1062) to northeastern South Dakota (Kirk and Balsbaugh 1975: 38), north to southern Manitoba (Lindroth 1969a: 1062; Bousquet 1987a: 133), south to eastern Kansas (Popenoe 1877: 23), central Louisiana (Rapides Parish, Igor M. Sokolov pers. comm. 2009), and northern Florida (Peck and Thomas 1998: 24). The records from Colorado (LeConte 1846b: 188; Snow 1877: 17) need confirmation.

#### Records.

**CAN**: MB **USA**: AL, CT, FL, GA, IA, IL, KS, LA, MA, MI, MO, MN, NC, NE, NH, NJ, NY, OH, PA, RI, SC, SD, WI [CO]

### Calleida viridipennis

(Say, 1823)

Cymindis viridipennis Say, 1823a: 9. Type locality: «Wash[ington] Co[unty], P[ennsylvani]a» (neotype label). Neotype (♀), designated by Lindroth and Freitag (1969: 349), in MCZ [# 33004].
Calleida marginata Dejean, 1825: 222. Type locality: «Géorgie» (original citation). One syntype in MHNP (Lindroth 1955b: 24). Synonymy established by Dejean (1826: 450), confirmed by Lindroth (1955b: 24).

#### Distribution.

This species ranges from southwestern New York (Notman 1928: 239) to eastern New Mexico (Chaves and De Baca Counties, CMNH), including southwestern Iowa (Fremont County, Doug A. Veal pers. comm. 2009), south to southeastern Texas (Wickham 1897: 111), southern Louisiana (East Baton Rouge, Iberville, Pointe Coupee, Saint Martin, and Saint Tammany Parishes, Igor M. Sokolov pers. comm. 2009), and southern Florida (Peck and Thomas 1998: 24). The records from “Utah,” “Arizona,” and “California” (Csiki 1932b: 1449) are likely in error.

#### Records.

**USA**: AL, AR, DC, DE, FL, GA, IA, IL, KY, LA, MD, MO, MS, NM, NY, OH, OK, PA, SC, TN, TX, VA, WV

### Genus Philophuga

Motschulsky, 1859

Philophuga Motschulsky, 1859a: 140. Type species: *Calleida cyanea* Motschulsky, 1850 (= *Cymindis viridis* Dejean, 1831) designated by Larson (1969: 29). Etymology. From the Greek *philos* (beloved) and *phyge* or *pheuge* (escape) [feminine].
Philophyga Gemminger and Harold, 1868a: 118. Unjustified emendation of *Philophuga* Motschulsky, 1859.
Philopheuga Bates, 1883a: 202. Unjustified emendation of *Philophuga* Motschulsky, 1859.

#### Diversity.

Four species comprised this genus: one (*Philophuga brachinoides* Bates) is found in Mexico only, another one (*Anchomenus viridis*) in Canada and United States, and the other two in United States and Mexico.

#### Identification.

Larson (1969) revised the species.

#### Taxonomic Note.

This taxon is ranked as a subgenus of *Calleida* by some authors.

### Philophuga caerulea

Casey, 1913

Calleida viridis Chevrolat, 1836a: [no. 155] [secondary homonym of *Calleida viridis* (Dejean, 1831)]. Type locality: «Las Vigas [Veracruz, Mexico]» (original citation). Syntype(s) [2 originally cited] location unknown (possibly in UMO).
Philophuga caerulea Casey, 1913: 174. Type locality: «Arizona» (original citation). Lectotype [as holotype] (♀), designated by Larson (1969: 33), in USNM [# 47668]. Synonymy established by Larson (1969: 33).

#### Distribution.

This species ranges from Arizona south to the Isthmus of Tehuantepec [see Larson 1969: Fig. 58].

#### Records.

**USA**: AZ – Mexico

### Philophuga viridicollis

(LeConte, 1846)

Cymindis viridicollis LeConte, 1846b: 188. Type locality: «Long’s Peak [Boulder County, Colorado], Rocky Mountains» (original citation). Lectotype (♀), designated by Larson (1969: 31), in MCZ [# 5821].
Philopheuga subcordata Chaudoir, 1877: 246. Type locality: «Mexique» (original citation). Syntype(s) [2 originally cited] in MHNP. Synonymy established by Horn (1882: 161).

#### Distribution.

This species ranges from eastern Colorado and Kansas south to northeastern Mexico and southwestern Texas [see Larson 1969: Fig. 58]. The record from South Dakota (Kirk and Balsbaugh 1975: 38) needs confirmation. One specimen labeled from “Alabama” and two specimens from California seen by Larson (1969: 32) are probably mislabeled.

#### Records.

**USA**: CO, KS, NM, OK, TX [SD] – Mexico

### Philophuga viridis amoena

(LeConte, 1846)

Cymindis amoena LeConte, 1846b: 188. Type locality: «Long’s Peak [Boulder County, Colorado], Rocky Mountains» (original citation). Lectotype (♀), designated by Larson (1969: 42), in MCZ [# 5822].
Philophuga canora Casey, 1913: 174. Type locality: «Texas» (original citation). Lectotype [as holotype] (♀), designated by Larson (1969: 42), in USNM [# 47669]. Synonymy established by Larson (1969: 42).
Philophuga puella Casey, 1913: 176. Type locality: «Boulder Co[unty], Colorado» (original citation). Lectotype [as holotype] (♂), designated by Larson (1969: 42), in USNM [# 47671]. Synonymy established by Larson (1969: 42).
Philophuga obscura Casey, 1924: 91. Type locality: «Lake George, New York» (original citation), which is incorrect. Lectotype [as holotype] (♀), designated by Larson (1969: 42), in USNM [# 47674]. Synonymy established by Larson (1969: 42).

#### Distribution.

The range of this subspecies extends from southern Manitoba to south-central British Columbia, south to northern Oregon, Colorado (Larson 1969: 42-43), and east-central Kansas (Knaus 1907: 233). The records from “New Mexico” and “Utah” (Bousquet and Larochelle 1993: 281), based on misplaced dots on Larson’s (1969) figure 59, are in error; those from “Oklahoma” (Arnold 2008) and “Texas” (Casey 1913: 174, as *Philophuga canora*) need confirmation.

#### Records.

**CAN**: AB, BC, MB, SK **USA**: CO, ID, KS, MT, ND, NE, OR, WA, WY [OK, TX]

### Philophuga viridis horni

Chaudoir, 1877

Philopheuga horni Chaudoir, 1877: 245. Type locality: «Nevada» (original citation), herein restricted to Winnemucca, Humboldt County (see Larson 1969: 41). Syntype(s) [2 originally cited] in MHNP.
Philophuga cobaltina Casey, 1924: 91. Type locality: «Trout Creek, Juab Co[unty], Utah» (original citation). Lectotype [as holotype] (♂), designated by Larson (1969: 41), in USNM [# 47672]. Synonymy established by Larson (1969: 41).
Philophuga uteana Casey, 1924: 92. Type locality: «Stockton [Tooele County], Utah» (original citation for the lectotype). Lectotype [as holotype] (♂), designated by Larson (1969: 41), in USNM [# 47673]. Synonymy established by Larson (1969: 41).

#### Distribution.

This subspecies ranges from northern Washington to eastern Wyoming (Lavigne 1977: 45, as *Calleida viridis viridis*), south to northeastern New Mexico, south-central Arizona, and northeastern California (Larson 1969: 41, Fig. 59).

#### Records.

**USA**: AZ, CA, CO, ID, NM, NV, OR, UT, WA, WY

### Philophuga viridis klamathea

Larson, 1969

Philophuga viridis klamathea Larson, 1969: 40. Type locality: «Klamath Falls [Klamath County], Oregon» (original citation). Holotype (♂) in CAS [# 9812].

#### Distribution.

This subspecies is known only from south-central Oregon and northern California [see Larson 1969: Fig. 59].

#### Records.

**USA**: CA, OR

### Philophuga viridis viridis

(Dejean, 1831)

Cymindis viridis Dejean, 1831: 325. Type locality: «Californie» (original citation), herein restricted to Lake Merced, San Francisco County (see Larson 1969: 38). Syntype(s) presumably lost (Lindroth 1955b: 24).
Calleida cyanea Motschulsky, 1850a: 39. Type locality: «California» (original citation), cited from «St. Francisco [San Francisco County]» by Motschulsky (1859a: 144). One syntype, described as “destructum,” in ZMMU (Keleinikova 1976: 194). Synonymy established by Horn (1882: 161).
Philophuga lauta Casey, 1913: 175. Type locality: «California» (original citation). Lectotype [as holotype] (♂), designated by Larson (1969: 38), in USNM [# 47670]. Synonymy established by Larson (1969: 38).

#### Distribution.

This subspecies is known only from the San Francisco Bay area in California [see Larson 1969: Fig. 59].

#### Records.

**USA**: CA

### Genus Infernophilus

Larson, 1969

Infernophilus Larson, 1969: 43. Type species: *Philophuga castanea* Horn, 1882 by original designation. Etymology (original). From the Latin *infernus* (hell) and the Greek *philos* (beloved), alluding to the hot desert region where the sole species is found [masculine].

#### Diversity.

One Nearctic species extending into northern Mexico.

#### Identification.

The character states of the sole species are described in detail by Larson (1969: 43-44).

### Infernophilus castaneus

(Horn, 1882)

Philophuga castanea G.H. Horn, 1882: 144. Type locality: «high mountains in Kern Co[unty], California» (original citation). Two syntypes [2 originally cited] in MCZ [# 34516].

#### Distribution.

This species is known from “Nevada” and the Lake Tahoe region in the Sierra Nevada south to the Mexican border in California [see Larson 1969: Fig. 61]. According to Ball and Bousquet (2000: 114), the species is also found in northwestern Mexico.

#### Records.

**USA**: CA, NV – Mexico

### Genus Onota

Chaudoir, 1873

Onota Chaudoir, 1873b: 165. Type species: *Onota bicolor* Chaudoir, 1873 designated by Bousquet and Larochelle (1993: 280). Etymology (original). From the Greek *onos* (donkey, ass) and *otos* (ear), alluding to the shape of the paraglossae (*paraglossae membranaceae, parallelae, glabrae, utroque angulo antico late subproducto-rotundato (inde nom. gen.)*) of the adults [feminine].

#### Diversity.

Ten species in Central America and South America, of which two reach southern United States.

#### Identification.

This genus has not been revised in modern times and such work is needed.

### Onota angulicollis

(Reiche, 1842)

Lebia angulicollis Reiche, 1842: 312. Type locality: «Provincia novae Granatae [= present day Panama and Colombia]» (original citation). On syntype in MHNP (see Erwin 2004: 38).

#### Distribution.

This species ranges from southeastern Texas (Cameron County, Edward G. Riley pers. comm. 2009) south to the Amazonia (Chaudoir 1873b: 166). This is a **new record** for America north of Mexico.

#### Records.

**USA**: TX – Brazil, Colombia, Costa Rica, Guatemala, Nicaragua, Panama, Peru, Surinam.

### Onota floridana

Horn, 1881

Onota floridana G.H. Horn, 1881: 159. Type locality: «near Lake Poinsett [Brevard County], Florida» (original citation). Syntype(s) [3 originally cited] in MCZ [# 8187].

#### Distribution.

This species is known only from Brevard County in central Florida (Peck and Thomas 1998: 24).

#### Records.

**USA**: FL

### Genus Cylindronotum

Putzeys, 1845

Cylindronotum Putzeys, 1845a: 22. Type species: *Cylindronotum aeneum* Putzeys, 1845 by monotypy. Etymology. From the Greek *cylindros* (roller, cylinder) and *notos* (back, upper surface), probably alluding to the cylindrical pronotum (“*corselet *... *presque cylindrique*”) of the adult [neuter].
Stenonotum Lacordaire, 1854: 107. Unnecessary replacement name for *Cylindronotum* Putzeys, 1845. Etymology. From the Greek *stenos* (narrow) and *notos* (back, dorsum) [neuter].
Micragra Chaudoir, 1873b: 155. Unnecessary replacement name for *Cylindronotum* Putzeys, 1845. Etymology (original). From the Greek *micros* (small) and the generic name *Agra* [*q.v*.], alluding to the resemblance of adults of these small insects to those of *Agra* (“*ces petits insectes, qui ont le facies des Agra*”) [feminine].
Pseudometabletus Liebke, 1930: 722. Type species: *Pseudometabletus nevermanni* Liebke, 1930 by original designation. Synonymy established by Erwin (2004: 22). Etymology. From the Greek *pseudos* (fallacy, lie) and the generic name *Metabletus* [*q.v*.] [masculine].

#### Diversity.

Seven Neotropical species, of which one reaches southeastern Texas.

#### Identification.

This genus has not been revised in modern times and such work would be useful.

### Cylindronotum aeneum

Putzeys, 1845

Cylindronotum aeneum Putzeys, 1845a: 22. Type locality: «Cayenne [French Guiana]» (original citation). Lectotype, designated by Erwin (2004: 22), in MHNP.

#### Distribution.

This species is found from southeastern Texas (Wickham 1897: 108; Snow 1906a: 141 as “*Micragra brunnea* Putzeys”) south to Panama and French Guiana (Bates 1883a: 199).

#### Records.

**USA**: TX – Belize, French Guiana, Guatemala, Mexico, Nicaragua, Panama.

### Subtribe Agrina

Kirby, 1837

Agridae Kirby, 1837: 13. Type genus: *Agra* Fabricius, 1801.

#### Diversity.

About 585 species (Lorenz 2005: 498-502) belonging to the Western Hemisphere genus *Agra*.

### Genus Agra

Fabricius, 1801

Agra Fabricius, 1801: 224. Type species: *Agra aenea* Fabricius, 1801 designated by Latreille (1810: 426). Etymology. From the Greek *agra* (catch, booty, prey, seizure) [feminine].
Agrana Rafinesque, 1815: 109. Unjustified emendation of *Agra* Fabricius, 1801.
Agridia Chaudoir, 1861d: 109. Type species: *Agridia platyscelis* Chaudoir, 1861 designated by Erwin (1982a: 45). Synonymy established by Erwin (1982a: 45).

#### Diversity.

About 585 species in the Neotropical Region, one of them extending into southeastern Texas.

#### Taxonomic Note.

1. Erwin (1982a: 45) postulated that *Agra* is the putative sister-group of *Callidiola* Jeannel of Africa and Asia, currently recognized as a subgenus of *Calleida*. Casale (1998: 401) challenged this hypothesis and postulated that *Agra* could be the sister-group to physoderines which are found in Australia, some Pacific islands, eastern Asia, and central and south Africa. 2. According to Erwin (in Ball and Bousquet 2000: 115), two species, both undescribed, reach southeastern Texas. Therefore, the species currently recorded from United States is probably not a resident of North America.

### Agra oblongopunctata oblongopunctata

Chevrolat, 1836

Agra oblongopunctata Chevrolat, 1836b: [no. 183]. Type locality: «environs de la Véra-Cruz [Mexico]» (original citation). Syntype(s) location unknown (possibly in UMO).

#### Distribution.

This subspecies ranges from southeastern Texas (Wickham 1897: 106) to Guatemala (Bates 1883a: 247). As mentioned previously, the Texas specimens actually belong to an undescribed species.

#### Records.

**USA**: TX – Guatemala, Mexico

#### Note.

The subspecies *Agra oblongopunctata hypsophila* Straneo is found in Costa Rica.

### Subtribe Metallicina

Basilewsky, 1984

Metallicini Basilewsky, 1984: 534, 542. Type genus: *Metallica* Chaudoir, 1873.

#### Diversity.

About 70 species (Lorenz 2005: 490) in the Nearctic (three species), Neotropical (16 species of *Euproctinus*), Oriental, Palaearctic (31 species), and Afrotropical Regions. These species are arrayed in four genera: *Parena* Motschulsky (47 species), *Metallica* Chaudoir (six species), *Pachycallida* Jeannel (three species), and *Euproctinus* (16 species).

#### Taxonomic Note.

This subtribe came out as the sister-group to {Calleidina + Gallerucidiina} in the phylogenetic analysis of Ball et al. (1995).

### Genus Euproctinus

Leng and Mutchler, 1927

Euproctus Solier, 1849: 131 [junior homonym of *Euproctus* Gené, 1839]. Type species: *Euproctus fasciatus* Solier, 1849 by monotypy. Etymology. Uncertain, possibly from the Greek *eu* (well, by extension large) and *proctos* (anus, tail) [masculine].
Euproctinus Leng and Mutchler, 1927: 14. Replacement name for *Euproctus* Solier, 1849. Etymology. From the generic name *Euproctus* [*q.v*.] and the Latin suffix -*inus* (pertaining to) [masculine].
Andrewesella Csiki, 1932b: 1456. Replacement name for *Euproctus* Solier, 1849. Etymology. Although not specified, this name probably originates from the surname of Herbert Edward Andrewes [1863-1950] who devoted a large part of his life to the study of Carabidae of the Oriental Region.

#### Diversity.

Sixteen species restricted to the Western Hemisphere, currently arrayed in two subgenera: *Euproctinus* s.str. (one species, *Euproctinus fasciatus* Solier from Chile and Argentina) and *Neoeuproctus* (15 species).

#### Identification.

Shpeley (1986) revised the species and provided a key for their identification.

### Subgenus Neoeuproctus

Shpeley, 1986

Neoeuproctus Shpeley, 1986: 284. Type species: *Euproctus sigillatus* Bates, 1883 by original designation. Etymology. From the Greek prefix *neo*- (new) and the generic name *Euproctus* [*q.v*.] [masculine].

#### Diversity.

Fifteen species in the Neotropical Region, of which three extend into southern United States.

### [abjectus group]

### Euproctinus abjectus

(Bates, 1883)

Euproctus abjectus Bates, 1883a: 196. Type locality: «Almolonga [Veracruz], Mexico» (original citation for the lectotype). Lectotype (♂), designated by Shpeley (1986: 291), in BMNH.
Euproctus texanus Wickham, 1897: 109. Type locality: «Brownsville [Cameron County] Texas» (lectotype label). Lectotype (♀), designated by Shpeley (1986: 292), in USNM. Synonymy established by Shpeley (1986: 292).

#### Distribution.

This species is found from southern California and southeastern Texas south to Guatemala and southern Baja California [see Shpeley 1986: map 4].

#### Records.

**USA**: CA, TX – Guatemala, Mexico

### Euproctinus balli

Shpeley, 1986

Euproctinus balli Shpeley, 1986: 286. Type locality: «21.8 miles north of Juchatengo (7100’), Oaxaca, México» (original citation). Holotype (♂) in USNM.

#### Distribution.

This species ranges from southeastern Arizona south to southern Mexico [see Shpeley 1986: map 3].

#### Records.

**USA**: AZ – Mexico

### [trivittatus group]

### Euproctinus trivittatus

(LeConte, 1878)

Onota trivittata LeConte, 1878b: 373. Type locality: «Florida» (original citation), restricted to «F[or]t Capron [Saint Lucie County]» by Shpeley (1986: 295). Lectotype (♀), designated by Shpeley (1986: 295), in MCZ [# 5818].

#### Distribution.

This species is found along the east coast of Florida, including the Keys, and in Cuba [see Shpeley 1986: map 6].

#### Records.

**USA**: FL – Cuba

### Subtribe Nemotarsina

Bates, 1883

Nemotarsinae Bates, 1883a: 173. Type genus: *Nemotarsus* LeConte, 1853.

#### Diversity.

One genus endemic to the Western Hemisphere.

#### Taxonomic Note.

Jeannel (1949a: 860) and Basilewsky (1984: 527) considered this taxon as a masoreimorph (i.e., masoreines and cyclosomines). Ball and Bousquet (2000: 113-114) retained it as a lebiines, as previously done by Ball (1960b: 158) and Lindroth (1969a: 1014), but recognized that its position remains to be determined.

### Genus Nemotarsus

LeConte, 1853

Nemotarsus LeConte, 1853c: 377. Type species: *Nemotarsus elegans* LeConte, 1853 by monotypy. Etymology. From the Greek *nema* (thread) and *tarsos* (tarsus), alluding to the filiform tarsi (“*tarsi filiformes*”) of the adult [masculine].
Nematotarsus Gemminger and Harold, 1868a: 145. Unjustified emendation of *Nemotarsus* LeConte, 1853.

#### Diversity.

Nine species in the temperate and tropical areas of the Nearctic (two species) and Neotropical (eight species) Regions.

#### Identification.

This genus has not been revised in modern times and such work is needed.

### Nemotarsus elegans

LeConte, 1853

Nemotarsus elegans LeConte, 1853c: 378. Type locality: «Illinois; Upper Georgia» (original citation), restricted to «Illin[ois]» by Lindroth (1969a: 1014). Two syntypes [2 originally cited] in MCZ [# 5799].

#### Distribution.

The range of this species extends from the District of Columbia (CMNH) to “Illinois” (LeConte 1853c: 378), including southwestern Pennsylvania (Westmoreland County, Robert L. Davidson pers. comm. 2008), south to southern Texas (Cameron, Colorado, and Sabine Counties, USNM, Brian Raber pers. comm. 2010) and northern Florida (Peck and Thomas 1998: 24). The record from “Iowa” (Bousquet and Larochelle 1993: 279) needs confirmation.

#### Records.

**USA**: AL, AR, DC, FL, GA, IL, LA, MD, MS, NC, OK, PA, SC, TX, VA [IA]

### Nemotarsus rhombifer

Bates, 1883

Nemotarsus rhombifer Bates, 1883a: 173. Type locality: «Jalapa, Mexico; Dueñas, Guatemala» (original citation). Syntype(s) probably in BMNH.

#### Distribution.

This species ranges from southern Texas (Cameron and Hidalgo Counties, Edward G. Riley pers. comm. 2009; Brian Raber pers. comm. 2010) south to Guatemala (Bates 1883a: 173). This is a **new record** for America north of Mexico.

#### Records.

**USA**: TX – Guatemala, Mexico

### Tribe Zuphiini

Bonelli, 1810

Zuphietae Bonelli, 1810: Tabula Synoptica. Type genus: *Zuphium* Latreille, 1805.

#### Diversity.

Worldwide, with about 290 species (Lorenz 2005: 504-507, excluding Planetina) arrayed in 20 genera. The tribe is represented in the Northern Hemisphere by about 65 species (22% of the world fauna). Three subtribes are recognized: Leleupidiina (about 90 species in the Old World and the Australian Region), Zuphiina (about 200 species), and Dicrodontina (three species endemic to the Canary Islands).

#### Taxonomic Note.

The planetines are included in this tribe by Lorenz (2005: 507). However Basilewsky (1963b), Reichardt (1967), Ball (1985), and Baehr (1986) presented arguments suggesting that planetines are probably more closely related to galeritines than to zuphiines.

### Subtribe Zuphiina

Bonelli, 1810

Zuphietae Bonelli, 1810: Tabula Synoptica. Type genus: *Zuphium* Latreille, 1805.
Patriziini Basilewsky, 1953b: 266. Type genus: *Patrizia* Alluaud, 1931.

#### Diversity.

Worldwide, with about 200 species placed in 14 genera. The North American fauna is represented by 18 species (about 9% of the world fauna).

### Genus Zuphium

Latreille, 1805

Zuphium Latreille, 1805: 198. Type species: *Carabus olens* Rossi, 1790 designated by Latreille (1810: 426). Etymology. Uncertain, possibly from the Greek *zoyphion* (small animal) or *zophos* (obscure, dark) [neuter].
Zophium Gistel, 1840: 112. Unjustified emendation of *Zuphium* Latreille, 1805.
Zoyphium Agassiz, 1846: 393. Unjustified emendation of *Zuphium* Latreille, 1805.

#### Diversity.

Worldwide, with about 75 species of which six are found in the temperate and subtropical areas of North America.

#### Identification.

Mateu (1981) reviewed the North American species and provided a key for their identification.

### Zuphium americanum

Dejean, 1831

Zuphium americanum Dejean, 1831: 298. Type locality: «Amérique septentrionale» (original citation), restricted to «Brownsville [Cameron County], Tex[as]» by Lindroth (1969a: 1090). Holotype [by monotypy] (♂) in MHNP (Lindroth 1955b: 22).

#### Distribution.

This species ranges from New Jersey (Morris County, USNM) to southern South Dakota (Kirk and Balsbaugh 1975: 39), including southernmost Ontario (Lindroth 1969a: 1090), south to southeastern Texas (Snow 1906a: 141; Cameron, Kleberg, and Gonzales Counties, CMNH, MCZ) and the Florida Keys (Peck and Thomas 1998: 25), west to southern Arizona (Mateu 1981: 118); seemingly isolated in western Oregon (Malkin 1943: 52).

#### Records.

**CAN**: ON **USA**: AL, AR, AZ, FL, GA, IA, IL, IN, KS, LA, MD, MI, MO, MS, NC, NE, NJ, NM, OH, OK, OR, PA, SC, SD, TN, TX, VA, WV

### Zuphium delectum

Liebke, 1933

Zuphium delectum Liebke, 1933: 469. Type locality: «Framingham [Middlesex County], Massachusetts» (original citation). Syntype(s) [2 originally cited] in DEI (Döbler 1975: 110) and IZWP [# 1704] (Mroczkowski 1960: 399).

#### Distribution.

This species is known only from the original two specimens.

#### Records.

**USA**: MA

### Zuphium longicolle

LeConte, 1879

Zuphium longicolle LeConte, 1879c: 62. Type locality: «San Joaquin Co[unty], Cal[ifornia]; Texas» (original citation), restricted to «San Joaquín Co[unty]» by Mateu (1981: 123). Two syntypes in MCZ [# 5795].

#### Distribution.

This species is known from western Arkansas (Polk and Hempstead Counties, CMNH, MCZ), Texas, as far south as the Rio Grande in the southeast (Mateu 1981: 125; Dajoz 2007: 18-19), southern Arizona (Pima County, MCZ), and central California (LeConte 1879c: 62). The record from “Oklahoma” (Arnold 2008) needs confirmation.

#### Records.

**USA**: AR, AZ, CA, TX [OK]

### Zuphium magnum

Schaeffer, 1910

Zuphium magnum Schaeffer, 1910: 396. Type locality: «Brownsville [Cameron County], Texas» (original citation). Holotype (♀) in USNM [# 42503].

#### Distribution.

This species is known from “Louisiana” (USNM) and from Cameron (Schaeffer 1910: 396) and Zapata Counties (CMNH) in southern Texas.

#### Records.

**USA**: LA, TX

### Zuphium mexicanum

Chaudoir, 1863

Zuphium mexicanum Chaudoir, 1863: 314. Type locality: «Veracruz?, Mexique» (original citation). Lectotype (♀), designated by Mateu (1985: 330), in MHNP.
Zuphium vicinum Liebke, 1933: 471. Type locality: «Mexiko» (original citation). Holotype [by monotypy] in IZWP [# 1711] (Mroczkowski 1960: 400). Synonymy established by Mateu (1985: 330).

#### Distribution.

This species ranges from southwestern Arizona to southeastern Texas (Mateu 1981: 127). It was also reported from “Mexico” (Chaudoir 1863: 314; Liebke 1933: 471).

#### Records.

**USA**: AZ, NM, TX – Mexico

### Zuphium pseudamericanum

Mateu, 1981

Zuphium pseudamericanum Mateu, 1981: 118. Type locality: «Fort Yuma [Imperial County, California], Arizona» (original citation). Holotype (♂) in USNM. Note. Mateu (1981) used two different original spellings for this species: *pseudoamericanum* (page 115) and *pseudamericanum* (page 118). I am acting as First Reviser (see ICZN 1999: Article 24.2.3) and select *pseudamericanum* to have precedence over *pseudoamericanum*.

#### Distribution.

This species is known from a few scattered localities from southern California to southeastern Texas (Mateu 1981: 119).

#### Records.

**USA**: AZ, CA, TX

### Genus Pseudaptinus

Laporte, 1834

Diaphorus Dejean, 1831: 300 [junior homonym of *Diaphorus* Meigen, 1824]. Type species: *Diaphorus lecontei* Dejean, 1831 by monotypy. Etymology (original). From the Greek *diaphoros* (different) [masculine].
Pseudaptinus Laporte, 1834: 56. Type species: *Polistichus albicornis* Klug, 1834 by monotypy. Synonymy established by Laporte (1840: 33). Etymology. From the Greek *pseudos* (fallacy, lie) and the generic name *Aptinus* [*q.v*.], alluding to the resemblance of the adults to those of *Aptinus pigmaeus* (“*le petit insecte sur lequel je forme ce genre a le faciès de l’Aptinus Pigmaeus*”) [masculine].
Tiphys Gistel, 1848a: ix [junior homonym of *Tiphys* Koch, 1837]. Replacement name for *Diaphorus* Dejean, 1831.

#### Diversity.

About 55 species in temperate, subtropical, and tropical areas of the Nearctic (12 species but only four endemic), Neotropical (about 40 species), and Australian (nine species in Australia) Regions.

### Subgenus Pseudaptinus

Laporte, 1834

Pseudaptinus Laporte, 1834: 56. Type species: *Polistichus albicornis* Klug, 1834 by monotypy.

#### Diversity.

Eighteen species (Lorenz 2005: 505, as *Pseudaptinus *s.str.) in the Nearctic (three species) and Neotropical (16 species) Regions.

#### Identification.

Two of the North American species are included in Liebke’s (1934: 372-373) key to the species of this genus. A taxonomic revision is needed.

### Pseudaptinus lecontei

(Dejean, 1831)

Diaphorus lecontei Dejean, 1831: 301. Type locality: «Amérique septentrionale» (original citation), herein restricted to Florence, Florence County, South Carolina (see Ciegler 2000: 127). Holotype [by monotypy] (♀) in MHNP (Lindroth 1955b: 22). Etymology. The specific name was proposed for Major John Eatton LeConte [1784-1860] who devoted most of his time after his early retirement from the army to the education of his only child, John Lawrence LeConte [1825–1883]. Himself a naturalist, the Major was interested in botany, Lepidoptera, and Coleoptera. In 1845 he published the first taxonomic revision of the North American Histeridae for which some of the nice plates were drew by his son. To my knowledge, this is the first revision of a North American beetle family ever published. The LeContes came from a wealthy and prominent family in France of Huguenot ancestry. They fled to the United States to escape the intolerance of Louis XIV and the revocation of the Edict of Nantes (Mallis 1971: 243).

#### Distribution.

This Coastal Plain species ranges from southeastern Virginia (Davidson 1995: 18) to central Florida (Peck and Thomas 1998: 25), west to southeastern Texas (Cameron County, USNM).

#### Records.

**USA**: AL, AR, FL, GA, LA, MS, NC, SC, TX, VA

### Pseudaptinus oviceps

Van Dyke, 1926

Pseudaptinus oviceps Van Dyke, 1926a: 121. Type locality: «Griffith Park [Los Angeles, Los Angeles County], California» (original citation). Holotype (♀) in CAS [# 1863].

#### Distribution.

This species is known only from the type locality in southwestern California.

#### Records.

**USA**: CA

### Pseudaptinus tenuicollis

(LeConte, 1851)

Diaphorus tenuicollis LeConte, 1851: 173. Type locality: «San Jose [Santa Clara County, California]» (original citation). Holotype [by monotypy] in MCZ [# 665].
Diaphorus tenuicornis Chaudoir, 1872b: 106. Type locality: «intérieur du Mexique» (original citation). Syntype(s) in MHNP. Synonymy established by LeConte (1880a: 85).

#### Distribution.

The range of this species extends from southern Washington (Zack et al. 2003) to southern California (Fall 1901a: 47), east along the southwest to western Arkansas (Polk County, Robert L. Davidson pers. comm. 2008) and southern Texas (Zapata County, CMNH; Liebke 1934: 373), north to central Kansas (Knaus 1905a: 218), south at least to Oaxaca (Bates 1883a: 166).

#### Records.

**USA**: AR, AZ, CA, KS, NM, OK, OR, TX, WA – Mexico

### Subgenus Thalpius

LeConte, 1851

Thalpius LeConte, 1851: 174. Type species: *Helluo pygmaeus* Dejean, 1826 by monotypy. Etymology. Probably from the Greek *thalpos* (warmth, heat), possibly alluding to the climatic region where the sole species then known lived [masculine].
Enaphorus LeConte, 1851: 174. Type species: *Enaphorus rufulus* LeConte, 1851 by monotypy. Synonymy established by LeConte (1853c: 373).
Zuphiosoma Laporte, 1867: 103. Type species: *Zuphiosoma fulva* Laporte, 1867 by monotypy. Synonymy established by Csiki (1932b: 1561). Etymology. From the generic name *Zuphium* [*q.v*.] and the Greek *soma* (body) [neuter].

#### Diversity.

Thirty-four species (Lorenz 2005: 505) in the Nearctic (nine species but only two endemic), Neotropical (23 species), and Australian (nine species in Australia) Regions.

#### Identification.

Messer (2011: 422-423) published a key to all species found in the United States and Mexico.

#### Taxonomic Note.

This taxon is considered as a distinct genus by some authors (e.g., Ball and Bousquet 2000: 115) but Baehr (1985: 36) found out that the character states between *Thalpius* and *Pseudaptinus* were “very few and rather weak.” I am following him and treat *Thalpius* as a subgenus of *Pseudaptinus*.

### Pseudaptinus cubanus

(Chaudoir, 1877)

Diaphorus cubanus Chaudoir, 1877: 252. Type locality: «Cuba» (original citation). Holotype [by monotypy] in MHNP.

#### Distribution.

This species is found in central and southern Florida (Darlington 1935a: 161; Peck and Thomas 1998: 25), the Bahamas (Turnbow and Thomas 2008: 15), Cuba (Chaudoir 1877: 252; Peck 2005: 39), and the Dominican Republic (CMNH).

#### Records.

**USA**: FL – Bahamas, Cuba, Dominican Republic

### Pseudaptinus deceptor

Darlington, 1934

Pseudaptinus deceptor Darlington, 1934: 128. Type locality: «Soledad (near Cienfuegos), Cuba» (original citation). Holotype in MCZ [# 19545].

#### Distribution.

This species is known from Hidalgo and Cameron Counties in southern Texas (Peter W. Messer pers. comm. 2011), from southern Florida (Bousquet and Larochelle 1993: 334), and from Cuba (Darlington 1934: 128), the Cayman Islands, and Dominican Republic (Messer 2011: 422).

#### Records.

**USA**: FL, TX – Cayman Islands, Cuba, Dominican Republic

### Pseudaptinus dorsalis

(Brullé, 1834)

Diaphorus dorsalis Brullé, 1834b: 181. Type locality: «Amérique septentrionale» (original citation). Syntype(s) location unknown (possibly in MHNP).
Pseudaptinus bierigi Liebke, 1934: 387. Type locality: «Habana, Kuba» (original citation). Holotype [by monotypy] in IZWP [# 1704]. Synonymy established by Darlington (1937: 117). Etymology. The specific name was proposed for Alexander Bierig [1884-1963]. Born in Germany, Bierig moved with his family after World War I to Cuba and finally in 1939 to Costa Rica where he became professor of entomology at the University of Costa Rica in San José. Although he published quite extensively on Staphylinidae, most of his publications as professional dealt with the biology and control of insects injurious to tropical crops. His collection of about 26,000 specimens was purchased in 1966 by the Field Museum of Natural History.

#### Distribution.

The range of this species extends from the District of Columbia (Ulke 1902: 7) to western South Dakota (Kirk and Balsbaugh 1975: 39), south to southern Texas (Johnson 1978: 68) and southern Florida (Peck and Thomas 1998: 25); also recorded from the Bahamas (Turnbow and Thomas 2008: 15), Cuba (Darlington 1934: 128), Jamaica (Darlington 1941a: 14), Guana Island in the British Virgin Islands (Valentine and Ivie 2005: 275), Dominican Republic, and Mexico (Messer 2011: 423). The record from Arizona (Snow 1906b: 162) is probably in error.

#### Records.

**USA**: AL, AR, DC, FL, GA, KY, LA, MS, OH, OK, SC, SD, TN, TX, VA – Bahamas, British Virgin Islands, Cuba, Dominican Republic, Jamaica, Mexico

### Pseudaptinus hoegei

(Bates, 1883)

Diaphorus högei Bates, 1883a: 166. Type locality: «Maltrata [Veracruz], Mexico» (original citation). Syntype(s) in MCZ [# 1668]. Etymology. The specific name was proposed for Carl Friedrich [Frederik] Höge [1834-1908], an insect dealer in Hamburg. Högue travelled several times to Mexico between 1878 and 1889 for collecting. He left his Mexican collection of tiger beetles, *Calosoma*, and *Pasimachus* to the Zoological Museum in Hamburg.

#### Distribution.

This species is known from Horn Island in southeastern Mississippi (Drew A. Hildebrandt pers. comm. 2010), “Texas” (Liebke 1934: 374), and Veracruz in Mexico (Bates 1883a: 166).

#### Records.

**USA**: MS, TX – Mexico

### Pseudaptinus horni

(Chaudoir, 1872)

Diaphorus horni Chaudoir, 1872b: 107. Type locality: «Californie» (original citation). Syntype(s) location unknown (possibly in MHNP).

#### Distribution.

This species ranges from southeastern California (Fall 1901a: 47; Andrews et al. 1979: 28) and west-central Nevada (Storey County, David H. Kavanaugh pers. comm. 2008) east to southeastern Texas (Cameron County, CMNH, MCZ; LeConte 1880a: 85; Snow 1906a: 141), south to the states of Veracruz and Nayarit in Mexico (Peter W. Messer pers. comm. 2011); also recorded from “Oklahoma” (Messer 2011: 423).

#### Records.

**USA**: AZ, CA, NM, NV, OK, TX – Mexico

### Pseudaptinus microcephalus

(Van Dyke, 1926)

Thalpius microcephalus Van Dyke, 1926a: 122. Type locality: «Griffith Park, Los Angeles [Los Angeles County], California» (original citation). Holotype (♀) in CAS [# 1864].

#### Distribution.

This species is known only from the type locality in southwestern California.

#### Records.

**USA**: CA

### Pseudaptinus nobilis

Liebke, 1934

Pseudaptinus nobilis Liebke, 1934: 380. Type locality: «Mexiko» (original citation). Holotype [by monotypy] in IZWP [# 1655] (Mroczkowski 1960: 397).

#### Distribution.

This species is known from Veracruz in Mexico and from two specimens collected in Live Oak County in southern Texas (Messer 2011: 420).

#### Records.

**USA**: TX – Mexico

### Pseudaptinus pygmaeus

(Dejean, 1826)

Helluo pygmaeus Dejean, 1826: 460. Type locality: «Amérique septentrionale» (original citation). One syntype in MHNP (Lindroth 1955b: 22).

#### Distribution.

This species ranges from southeastern Virginia (Davidson 1995: 18) to eastern Missouri (Anonymous 2007), south to southern Texas (Johnson 1978: 68) and central Florida (Peck and Thomas 1998: 25); also recorded from Cuba (Liebke 1934: 373; Peck 2005: 39).

#### Records.

**USA**: AL, AR, FL, GA, LA, MO, MS, NC, OK, SC, TN, TX, VA – Cuba

### Pseudaptinus rufulus

(LeConte, 1851)

Enaphorus rufulus LeConte, 1851: 174. Type locality: «San Jose [Santa Clara County, California]» (original citation). Two syntypes in MCZ [# 60].

#### Distribution.

This species ranges from southeastern Oregon (LaBonte 1996: 357) to the Baja California Peninsula (Horn 1897: 367) and southern Arizona (Maricopa and Pima Counties, MCZ); its is also known from western Texas (El Paso and Brewster Counties, Robert L. Davidson pers. comm. 2012).

#### Records.

**USA**: AZ, CA, OR, TX – Mexico

### Tribe Galeritini

Kirby, 1825

Galeritae Kirby, 1825: 564. Type genus: *Galerita* Fabricius, 1801. Note. This family-group name is a homonym of Galeritidae Gray, 1825 (based on *Galerites* Lamark, 1801), a valid family of fossil Echinodermata.

#### Diversity.

Worldwide (though not represented in Europe), with about 130 species (Lorenz 2005: 507-509, as Planetina and Galeritini). The group is underrepresented in the Northern Hemisphere with only 17 species (about 12.5% of the world fauna). Two subtribes are recognized: Galeritina (about 105 species) and Planetina (27 species in the Eastern Hemisphere).

### Subtribe Galeritina

Kirby, 1825

Galeritidae Kirby, 1825: 564. Type genus: *Galerita* Fabricius, 1801.
Galeritinini Jeannel, 1949a: 1057. Type genus: *Galeritina* Jeannel, 1949 (= *Galerita* Fabricius, 1801). Note. This name has been placed on the Official Index of Rejected and Invalid Family-Group Names in Zoology (ICZN 1968: 98).
Galeritulini Jedlička, 1963: 279. Type genus: *Galeritula* Strand, 1936 (= *Galerita* Fabricius, 1801). Note. This name has been placed on the Official Index of Rejected and Invalid Family-Group Names in Zoology (ICZN 1968: 98).

#### Diversity.

About 105 species in the temperate, subtropical, and tropical areas of the Nearctic (eight species of *Galerita*), Neotropical (64 species), Oriental (seven species of *Galerita*), Palaearctic (five species of *Galerita*), and Afrotropical (31 species) Regions. The species are placed in four genera: *Ancystroglossus* Chaudoir (six Neotropical species), *Eunostus* Laporte (14 Afrotropical species), *Galerita* (82 species), and *Trichognathus* Latreille (one Neotropical species).

### Genus Galerita

Fabricius, 1801

Galerita Fabricius, 1801: 214. Type species: *Carabus americanus* Linnaeus, 1758 designated by Latreille (1810: 426). Etymology. Uncertain, most likely from the Latin adjective *galeritus*, -*a*, *um* (wearing a hood) but possibly also from the Greek *galeros* (cheerful) [feminine]. According to Latreille (1804: 260), the name *galerite* was used formerly to designate a bird or a fish with some kind of crest. Note. The name *Galerita* Gouan, 1770 has been suppressed for the purposes of the Principles of Priority and Homonymy and the name *Galerita* Fabricius, 1801 placed on the Official List of Generic Names in Zoology (ICZN 1968: 98).
Galeritula Strand, 1936: 168. Replacement name for *Galerita* Fabricius, 1801.
Galeritina Jeannel, 1949a: 1057. Replacement name for *Galerita* Fabricius, 1801.

#### Diversity.

Eighty-two species in the Western Hemisphere (58 species) and the Old World (24 species) currently arrayed in two subgenera, both represented in the Nearctic Region. Only eight species are found in North America and a single one (*Galerita bicolor*) is endemic.

#### Identification.

Reichardt (1967) revised the Western Hemisphere species and provided keys for their identification.

### Subgenus Progaleritina

Jeannel, 1949

Progaleritina Jeannel, 1949a: 1058. Type species: *Carabus janus* Fabricius, 1792 by original designation. Etymology. From the Latin prefix *pro*- (before) and the generic name *Galeritina* [*q.v*.] [feminine].

#### Diversity.

Seven species in North America (seven species), Middle America (six species), and the West Indies (one species).

#### Identification.

Subsequent to Reichardt’s (1967) revision, Ball and Nimmo (1983) published a synopsis of the species of *Progaleritina* leading to two new species-group taxa (*Galerita reichardti* and *Galerita lecontei veracrucis*) and subspecific status for two of Reichardt’s species (*Galerita tenebricosa* Klug and *Galerita bicoloripes* Reichardt).

### Galerita atripes

LeConte, 1858

Galerita atripes LeConte, 1858b: 59. Type locality: «Fort Riley [junction of Republican and Smoky Hill Rivers], Kansas» (original citation). Lectotype (♂), designated by Reichardt (1967: 28), in MCZ [# 5793].
Galerita decipiens G.H. Horn, 1885a: 131. Type locality: «Arizona» (original citation). Lectotype (♀), designated by Reichardt (1967: 28), in MCZ [# 34500]. Synonymy established by Reichardt (1967: 28).

#### Distribution.

This species is known from southeastern Louisiana (Saint Tammany Parish, Igor M. Sokolov pers. comm. 2009), Missouri, southeastern Nebraska (Nemaha County, Foster F. Purrington pers. comm. 2011), eastern Kansas (Popenoe 1877: 23; Horn 1872c: 385), eastern Texas, several mountain ranges in southern Arizona, and from the Sierra Huachinera of eastern Sonora and western Chihuahua in northern Mexico [see Ball and Nimmo 1983: Fig. 10]; it is also listed from “Oklahoma” by Arnold (2008). The record from eastern Iowa (Wickham 1911b: 7) needs confirmation.

#### Records.

**USA**: AZ, KS, LA, MO, NE, OK, TX [IA] – Mexico

### Galerita bicolor

(Drury, 1773)

Carabus bicolor Drury, 1773: [index]. Type locality: «Virginie» (original citation, see Drury 1770: 95). Syntype(s) lost (Reichardt 1967: 41).
Galerita longicollis Chaudoir, 1843b: 700. Type locality: «environs de la Nouvelle Orléans [Orleans Parish, Louisiana]» (original citation). Lectotype (♂), designated by Reichardt (1967: 41), in MHNP. Synonymy established by LeConte (1863b: 5), confirmed by Reichardt (1967: 41).
Galerita dubia LeConte, 1844: 48. Type locality: «Georgia» (original citation). Lectotype [as type] (♂), designated by Reichardt (1967: 41), in MCZ [# 5794]. Synonymy established by LeConte (1863b: 5), confirmed by Reichardt (1967: 41).
Galerita bicolor obliqua Casey, 1897: 350. Type locality: «Lake Worth [Palm Beach County], Florida» (original citation). Two syntypes in USNM [# 47582]. Synonymy established by Reichardt (1967: 41).
Galerita bicolor iowensis Casey, 1920: 230. Type locality: «Iowa» (original citation). One syntype in USNM [# 47580]. Synonymy established by Reichardt (1967: 41).
Galerita bicolor rhombiceps Casey, 1920: 230. Type locality: «Indiana» (original citation). One syntype in USNM [# 47579]. Synonymy established by Reichardt (1967: 41).

#### Distribution.

The range of this eastern species extends from Rhode Island (Sikes and Webster 2005: 317) to southeastern South Dakota (Kirk and Balsbaugh 1975: 39), south to southeastern Texas and southern Florida [see Ball and Nimmo 1983: Fig. 14].

#### Records.

**USA**: AL, AR, CT, DC, DE, FL, GA, IA, IL, IN, KS, KY, LA, MD, MI, MN, MO, MS, NC, NJ, NY, OH, OK, PA, RI, SC, SD, TN, TX, VA, WI, WV

### Galerita forreri

Bates, 1883

Galerita forreri Bates, 1883a: 165. Type locality: «Presidio [either the Río Presidio or the village on the river, Sinaloa], Mexico» (original citation). Lectotype (♀), designated by Reichardt (1967: 37), in BMNH.

#### Distribution.

This species ranges from southern Arizona southwards along the Pacific Coast to Guerrero [see Ball and Nimmo 1983: Fig. 7].

#### Records.

**USA**: AZ – Mexico

### Galerita janus

(Fabricius, 1792)

Carabus janus Fabricius, 1792: 136. Type locality: «Carolina» (original citation). Lectotype (♂), designated by Lindroth (1969a: 1093), in ZMUC.
Galerita cyanipennis Dejean, 1831: 293. Type locality: «Amérique septentrionale» (original citation). Lectotype (♂), designated by Reichardt (1967: 24), in MHNP. Synonymy established by Mannerheim (1843: 183), confirmed by Lindroth (1955b: 22).
Galerita borealis Laporte, 1840: 35. Type locality: «Amérique boréale» (original citation). Syntype(s) probably in MHNP (collection Oberthür). Synonymy established by Reichardt (1967: 24).
Galerita cordicollis Chaudoir, 1843b: 699. Type locality: environs de Nouvelle Orleans [Orleans Parish, Louisiana] (lectotype label according to Reichardt 1967: 24). Lectotype (♂), designated by Reichardt (1967: 24), in MHNP. Synonymy established by Melsheimer (1853: 3), confirmed by Reichardt (1967: 24).
Galerita thoracica Casey, 1897: 350 [primary homonym of *Galerita thoracica* Chevrolat, 1834]. Type locality: «Florida» (original citation). One syntype in USNM [# 47578]. Synonymy established by Reichardt (1967: 24).
Galerita bicolor intermedia Casey, 1897: 351 [primary homonym of *Galerita intermedia* Fairmaire, 1887]. Type locality: «Iowa» (original citation). Holotype [by monotypy] (♂) in USNM [# 47577]. Synonymy established by Reichardt (1967: 24).
Galerita caseyi Leng, 1919b: 203. Replacement name for *Galerita thoracica* Casey, 1897.
Galerita angusticeps Casey, 1920: 227. Type locality: «S[ain]t Louis, Missouri» (original citation). Lectotype (♂), designated by Bousquet and Larochelle (1993: 14), in USNM [# 47586]. Synonymy established by Bousquet and Larochelle (1993: 14).
Galerita occipitalis Casey, 1920: 228. Type locality: «Boston Neck [Washington County], Rhode Island» (original citation). One syntype in USNM [# 47576]. Synonymy established by Reichardt (1967: 24).
Galerita caseyi Liebke, 1928: 129 [primary homonym of *Galerita caseyi* Leng, 1919]. Replacement name for *Galerita intermedia* Casey, 1897.
Galerita ahasverus Liebke, 1929: 297. Replacement name for *Galerita caseyi* Liebke, 1928.

#### Distribution.

This species is found from southern Quebec to southeastern South Dakota, north to southern Manitoba (Bousquet 1987a: 134), south to east-central Texas and central Florida; isolated populations are known from southwestern New Mexico (Grant County, Robert L. Davidson pers. comm. 2008), southeastern Arizona, and Chihuahua in Mexico [see Ball and Nimmo 1983: Fig. 10].

#### Records.

**CAN**: MB, ON, QC **USA**: AL, AR, AZ, CO, CT, DC, DE, FL, GA, IA, IL, IN, KS, KY, LA, MA, MD, MI, MN, MO, MS, NC, ND, NE, NH, NJ, NM, NY, OH, OK, PA, RI, SC, SD, TN, TX, VA, VT, WI, WV – Mexico

### Galerita lecontei lecontei

Dejean, 1831

Galerita lecontei Dejean, 1831: 294. Type locality: «Amérique septentrionale» (original citation), herein restricted to Port Arthur, Jefferson County, Texas (see Reichardt 1967: 32). Lectotype (♂), designated by Reichardt (1967: 31), in MHNP.
Galerita californica Mannerheim, 1843: 183. Type locality: «California» (original citation). Syntype(s) location unknown (possibly in ZMH). Synonymy established by LeConte (1879c: 61).
Galerita infirma Casey, 1897: 350. Type locality: «Yuma [Yuma County], Arizona» (original citation). One syntype in USNM [# 47585]. Synonymy established by Reichardt (1967: 31).
Galerita crinicornis Casey, 1920: 229. Type locality: «near El Paso [El Paso County], Texas» (original citation). One syntype in USNM [# 47581]. Synonymy established by Reichardt (1967: 31).
Galerita californica arizonica Casey, 1920: 231. Type locality: «Arizona and Texas» (original citation). Three syntypes in USNM [# 47583]. Synonymy established by Reichardt (1967: 31).

#### Distribution.

The range of this subspecies is disjunct: there is an eastern component ranging from Virginia to southwestern Kentucky (Trigg County, Foster F. Purrington pers. comm. 2011) southwards to southern Florida and South Bimini Island in the Bahamas, westwards to eastern Texas; the western population ranges from central California southeastwards to the state of Durango in Mexico [see Ball and Nimmo 1983: Fig. 17]. The record from east-central Missouri (Summers 1873: 133) needs confirmation.

#### Records.

**USA**: AL, AR, AZ, CA, FL, GA, KY, LA, MS, NC, NM, SC, TN, TX, VA [MO] – Bahamas, Mexico

#### Note.

The subspecies *Galerita lecontei bicoloripes* Reichardt is found in central Mexico, that of *Galerita lecontei veracrucis* Ball and Nimmo along the Gulf states of Mexico, and that of *Galerita lecontei tenebricosa* Klug in several islands of the Greater Antilles.

### Galerita mexicana

Chaudoir, 1872

Galerita mexicana Chaudoir, 1872b: 103. Type locality: «Mexique» (original citation), herein restricted to Ciudad Victoria, Tamaulipas (see Reichardt 1967: 29). Lectotype (♀), designated by Reichardt (1967: 29), in MHNP.
Galerita melanopus Casey, 1920: 232. Type locality: «Amarillo [Potter County], Texas» (original citation). One syntype in USNM [# 47584]. Synonymy established by Reichardt (1967: 29).

#### Distribution.

This species ranges from southeastern Nebraska (Lancaster County, Foster F. Purrington pers. comm. 2011) to south-central Arizona, south to Costa Rica [see Ball and Nimmo 1983: Fig. 12].

#### Records.

**USA**: AZ, KS, NE, NM, OK, TX – Costa Rica, El Salvador, Guatemala, Honduras, Mexico

### Galerita reichardti

Ball and Nimmo, 1983

Galerita reichardti Ball and Nimmo, 1983: 304. Type locality: «3.5 km e[ast] Jaltipan, Veracruz, Mexico» (original citation). Holotype (♂) in USNM. Etymology. The specific name was proposed for Hans Reichardt [1939-1976], Brazilian taxonomist with an interest in the South American carabid fauna. Reichardt tragically died in a car accident.

#### Distribution.

This species is known from a few localities from eastern Texas southwards along the Gulf and Caribbean Coasts to Honduras [see Ball and Nimmo 1983: Fig. 7].

#### Records.

**USA**: TX – Honduras, Mexico

### Subgenus Galerita

Fabricius, 1801

Galerita Fabricius, 1801: 214. Type species: *Carabus americanus* Linnaeus, 1758 designated by Latreille (1810: 426).

#### Diversity.

Seventy-five species in the Western Hemisphere (51 species) and the Old World (24 species). One Neotropical species extends into southern Texas.

### Galerita aequinoctialis

Chaudoir, 1852

Galerita aequinoctialis Chaudoir, 1852: 37 (as *aeguinoctialis*). Type locality: «Mexique» (original citation), herein restricted to Jalapa Enríquez, Veracruz (see Reichardt 1967: 90). Syntype(s) probably in MHNP. Note. The fact that Chaudoir’s name is spelled *aequinoctialis* in “Table des Matières” of the original publication (page 103) should be regarded as a clear indication of an inadvertent error in the original spelling *aeguinoctialis* (page 37).

#### Distribution.

This species ranges from the Rio Grande in southeastern Texas south to Colombia (Martínez 2003: 7) [see Reichardt 1967: Fig. 76]. The record from “Arkansas” (Bousquet and Larochelle 1993: 285) is in error.

#### Records.

**USA**: TX – Colombia, Mexico, Panama

### Tribe Helluonini

Hope, 1838

Helluonidae Hope, 1838: 110. Type genus: *Helluo* Bonelli, 1813.

#### Diversity.

Worldwide (though absent from Europe) with about 180 species (Lorenz 2005: 510-512) arrayed in 26 genera: five of these genera (about 60 species) are represented in the Western Hemisphere and 21 (about 120 species) in the Eastern Hemisphere. The group is underrepresented in the Northern Hemisphere with 14 species (about 8% of the world fauna), only five of them being endemic (about 3%). Two subtribes are recognized: Helluonina, with 40 species in the Australian Region, and Omphrina with about 140 species.

### Subtribe Omphrina

Jedlička, 1941

Omphrini Jedlička, 1941: 6, 26. Type genus: *Omphra* Dejean, 1825.
Helluomorphina Reichardt, 1974: 224, 226. Type genus: *Helluomorpha* Laporte, 1834.

#### Diversity.

About 140 species in the Nearctic (seven species of *Helluomorphoides*), Neotropical (about 55 species), Australian (two species of *Creagris* Nietner, one of them endemic), Oriental (27 species), Palaearctic (seven Asian species but only two endemic, both in the Middle East), and Afrotropical (about 50 species) Regions.

### Genus Helluomorphoides

Ball, 1951

Helluomorphoides Ball, 1951: 136. Type species: *Helluomorpha texana* LeConte, 1853 by original designation. Etymology. From the generic name *Helluomorpha* and the Greek suffix -*oides* (resembling, having the form of), alluding to the resemblance of adults to those of *Helluomorpha* [masculine].

#### Diversity.

Twenty-three species in temperate, subtropical, and tropical areas of the Nearctic (seven species) and Neotropical (20 species) Regions.

#### Identification.

Ball (1956a) revised the North American species. The taxonomy and nomenclature of these species have not changed since.

### Helluomorphoides clairvillei

(Dejean, 1831)

Helluo clairvillei Dejean, 1831: 406. Type locality: «Amérique septentrionale» (original citation), herein restricted to Lucedale, George County, Mississippi (see Lindroth 1955b: 25). One syntype in MHNP.

#### Distribution.

This species is found along the Coastal Plain and Piedmont from southern New Jersey to southern Florida (Peck and Thomas 1998: 25), west to southeastern Mississippi (Ball 1956a: 85, Fig. 34). The record from south-central Kansas (Knaus 1901: 110) is probably in error.

#### Records.

**USA**: AL, FL, GA, MS, NC, NJ, SC

### Helluomorphoides ferrugineus

(LeConte, 1853)

Helluomorpha ferruginea LeConte, 1853c: 373. Type locality: «Texas» (original citation), herein restricted to Brownsville, Cameron County (see Ball 1956a: 77). Lectotype [as type], designated by Ball (1956a: 78), in MCZ [# 5838].
Helluomorpha languida Casey, 1913: 190. Type locality: «Brownsville [Cameron County], Texas» (original citation). Four syntypes [4 originally cited] in USNM [# 47589]. Synonymy established by Ball (1956a: 75).

#### Distribution.

This species ranges from New Jersey (Smith 1890: 90; Smith 1910: 212) to northwestern South Carolina (Ciegler 2000: 130), west to the Baboquivari Mountains in southern Arizona, north to southwestern Utah (Washington County, CMNH) and northeastern Kansas, south to Sinaloa in Mexico (Ball 1956a: 78). The records from New York City and vicinity (Leng and Beutenmüller 1893: 143) and “Florida” (Leng 1920: 67) need confirmation.

#### Records.

**USA**: AL, AR, AZ, GA, KS, NC, NJ, NM, OK, SC, TX, UT, VA [FL, NY] – Mexico

### Helluomorphoides latitarsis

(Casey, 1913)

Helluomorpha latitarsis Casey, 1913: 189. Type locality: «Arizona» (original citation), herein restricted to Baboquivari Mountains, Pima County (see Ball 1956a: 83). Lectotype [as type] (♂), designated by Ball (1956a: 84), in USNM [# 47590].

#### Distribution.

This species is found from western Texas to south-central Arizona, south at least to Durango in Mexico (Ball 1956a: 84, Fig. 34). The record from “Oklahoma” (Arnold 2008) needs confirmation.

#### Records.

**USA**: AZ, NM, TX [OK] – Mexico

### Helluomorphoides nigripennis

(Dejean, 1831)

Helluo nigripennis Dejean, 1831: 408. Type locality: «Amérique septentrionale» (original citation), herein restricted to Raleigh, Wake County, North Carolina (CNC). Two possible syntypes in MHNP (Lindroth 1955b: 25).

#### Distribution.

This species is found from Massachusetts to west-central Georgia (Fattig 1949: 41), west to eastern Texas (Ball 1956a: 75), and north to central Illinois (Purrington et al. 2002: 200) and western Michigan (Newaygo County, CMNH).

#### Records.

**USA**: AL, AR, DC, GA, IL, LA, MA, MD, MI, MS, NC, NJ, NY, OK, SC, TX, VA

### Helluomorphoides papago

(Casey, 1913)

Helluomorpha papago Casey, 1913: 190. Type locality: «Arizona» (original citation), herein restricted to Ramsey Canyon, 15 miles south Sierra Vista, Cochise County (CNC). Lectotype [as type] (♂), designated by Ball (1956a: 86), in USNM [# 47588].

#### Distribution.

This species ranges from the mountains of southern Arizona to southwestern Texas, south at least to Durango (Ball 1956a: 86, Fig. 34).

#### Records.

**USA**: AZ, TX – Mexico

### Helluomorphoides praeustus bicolor

(Harris, 1828)

Zuphium bicolor T.W. Harris, 1828b: 117. Type locality: «vicinity of Salem [Essex County, Massachusetts]» (original citation). Holotype [by monotypy] (♂) in MCZ [# 28191].
Helluo laticornis Dejean, 1831: 407. Type locality: «Amérique septentrionale» (original citation). Holotype [by monotypy] presumably lost (Lindroth 1955b: 25). Synonymy established by LeConte (1863b: 5).
Helluo pubescens Klug, 1834: 77. Type locality not stated. Syntype(s) location unknown. Synonymy established by LeConte (1879c: 61).

#### Distribution.

This subspecies occurs from southeastern New Hampshire (Cooper 1976: 165) to southeastern Wyoming (Platte County, CMNH) and northern Colorado (Ball 1956a: Fig. 33), south to Oklahoma (French et al. 2001: 228; Grady County, CMNH), west-central Arkansas (Garland County, Robert L. Davidson pers. comm. 2012), and northeastern Georgia (Fattig 1949: 42). The record from southwestern Alabama (Löding 1945: 23) needs confirmation.

#### Records.

**USA**: AR, CO, CT, DC, DE, GA, IA, IL, IN, KS, MA, MD, MI, MN, MO, ND, NE, NH, NJ, NY, OH, OK, PA, SD, TN, VA, WI, WV, WY [AL]

**Figure 42.**
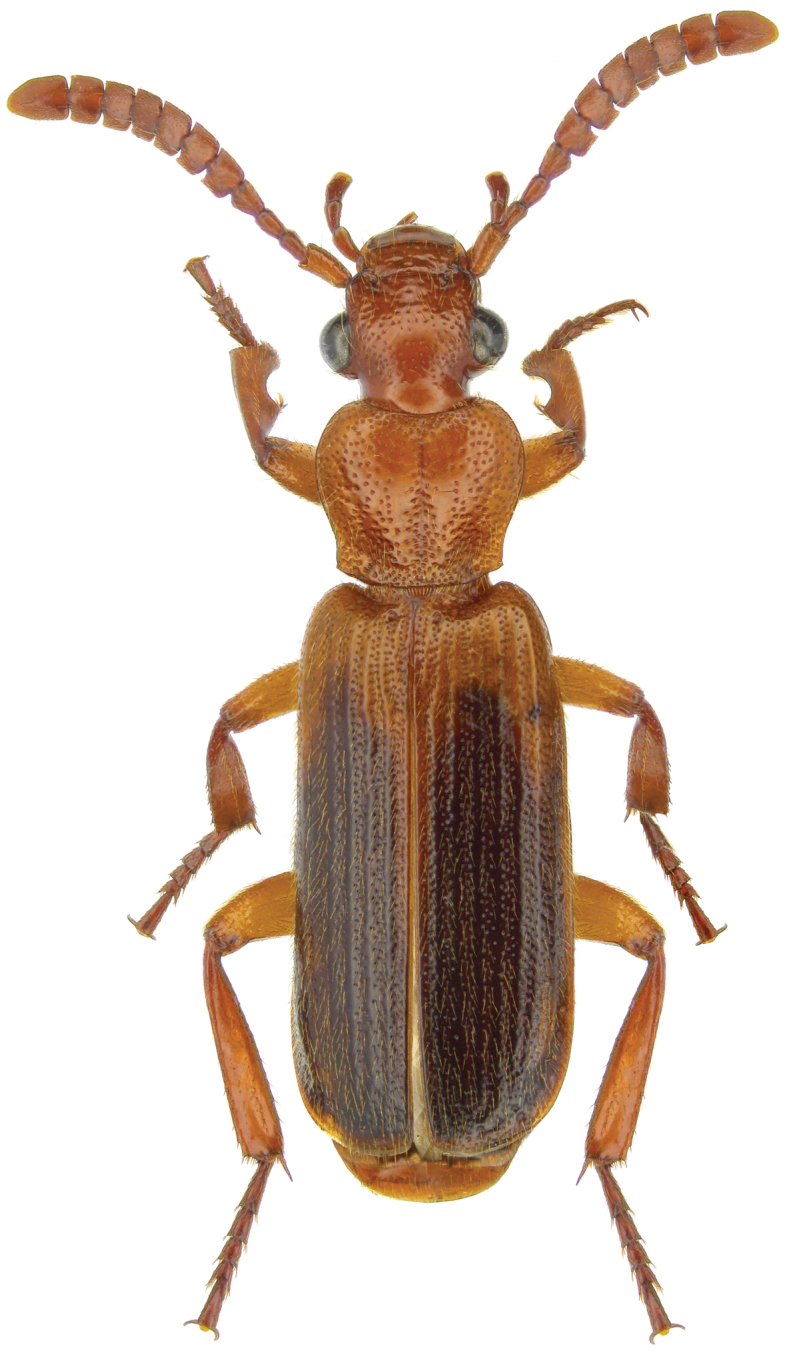
*Helluomorphoides praeustus bicolor* (Harris). Adults of most carabids are opportunist predators or scavengers but those of *Helluomorphoides* seem to have adopted a different, more dangerous life-style. Adults of two North American species have been observed to plunder foraging and migrating columns of army ant species of the genus *Neivamyrmex* and run away with their prey and broods. The beetles do not inhabit the ant nest or bivouac but can detect and follow the chemical trails of the ants at night. Whether this habit is common to all species of *Helluomorphoides* remains to be investigated.

### Helluomorphoides praeustus floridanus

Ball, 1956

Helluomorphoides praeustus floridanus Ball, 1956a: 80. Type locality: «Ormond, Volusia County, Florida» (original citation). Holotype (♂) in USNM [# 75438].

#### Distribution.

This subspecies is endemic to the Florida Peninsula (Peck and Thomas 1998: 25).

#### Records.

**USA**: FL

### Helluomorphoides praeustus praeustus

(Dejean, 1825)

Helluo praeustus Dejean, 1825: 289. Type locality: «Amérique septentrionale» (original citation). One syntype in MHNP (Lindroth 1955b: 25).

#### Distribution.

This subspecies is known for sure only from Mississippi (Harrison and Lafayette Counties, Drew A. Hildebrandt pers. comm. 2008), “New Jersey,” and “Alabama” (Ball 1956a: 79) where Löding (1945: 23) recorded it from Colbert and Mobile Counties. The record from southwestern Ohio (Blatchley 1910: 155) is probably in error; those from Sand Point and Tampa in Florida (Leng 1915: 589) need confirmation.

#### Records.

**USA**: AL, NJ, MS [FL]

### Helluomorphoides texanus

(LeConte, 1853)

Helluomorpha texana LeConte, 1853c: 374. Type locality: «Texas» (original citation), herein restricted to Tyler, Smith County (CNC). Holotype [by monotypy] (♂) in MCZ [# 5837].

#### Distribution.

This species ranges from southwestern Kentucky (Trigg County, Foster F. Purrington pers. comm. 2010) and central Tennessee to southeastern New Mexico (Lea and Chaves Counties, CMNH), north to southwestern Iowa (Fremont County, Doug A. Veal pers. comm. 2009) and southeastern Nebraska (Jefferson County, Foster F. Purrington pers. comm. 2010), south to northeastern Mexico (Ball 1956a: 82-83, Fig. 34) and southwestern Georgia (Fattig 1949: 42). The records from southern Arizona (Snow 1907: 142) and Indiana (Blatchley 1910: 156) need confirmation.

#### Records.

**USA**: AL, AR, CO, GA, IA, KS, KY, MO, MS, NE, NM, OK, TN, TX [AZ, IN] – Mexico

### Tribe Pseudomorphini

Hope, 1838

Pseudomorphidae Hope, 1838: 108 (as Heteromorphidae). Type genus: *Pseudomorpha* Kirby, 1823 (as *Heteromorpha* Kirby, 1823). Note. This family-group name is based on an incorrect original spelling of the name of its type genus (*Heteromorpha*) and must be corrected (ICZN 1999: Article 35.4.1). Therefore Heteromorphidae is corrected to Pseudomorphidae Hope, 1838.

#### Diversity.

About 325 species (Lorenz 2005: 388-390) in the Western Hemisphere (35 species) and the Australian (285 species, close to 90% of the world fauna), Oriental (four species of *Cryptocephalomorpha*), and Afrotropical (one species of *Cryptocephalomorpha* in South Africa) Regions. These species are arrayed in 12 genera: *Adelotopus* Hope (123 Australian species), *Cainogenion* Notman (11 Australian species), *Cryptocephalomorpha* Ritsema (seven species in the Eastern Hemisphere), *Manumorpha* Erwin and Geraci (one South American species), *Notopseudomorpha* Baehr (two species in South America), *Paussotropus* Waterhouse (one Australian species), *Procainogenion* Baehr (one Australian species), *Pseudomorpha* (30 species), *Samiriamorpha* Erwin and Geraci (one species in Peru), *Sphallomorpha* Westwood (145 Australian species), *Tuxtlamorpha* Erwin and Geraci (one species in Middle America), and *Yasunimorpha* Erwin and Geraci (one species from Ecuador).

### Genus Pseudomorpha

Kirby, 1823

Pseudomorpha Kirby, 1823a: 98. Type species: *Pseudomorpha excrucians* Kirby, 1823 by monotypy. Etymology. From the Greek *pseudos* (fallacy, lie) and *morphe* (form), probably alluding to the fact that the shape of the adult is completely different from that of other Carabidae (“though its exhibits the characters, has not the aspect, of that tribe [i.e., Carabi]”) [feminine]. Note. Kirby (1823a) originally used two different names for this taxon, *Pseudomorpha* (in the description on page 98) and *Heteromorpha* (in the explanation of the figures on page 109). Both names are considered different original spellings of the same name. Kirby reissued the paper later the same year in another journal, without the plate, and so only *Pseudomorpha* was used. As such, Kirby (1823b) is the “First Reviser” and *Pseudomorpha* is the correct original spelling (ICZN 1999: Article 24.2.4).
Axinophorus Dejean [in Dejean and Boisduval], 1829: 60. Type species: *Axinophorus lecontei* Dejean, 1829 (= *Pseudomorpha excrucians* Kirby, 1823) designated by Notman (1925: 30). Etymology (original). From the Greek *axine* (ax) and *phero* (to carry), alluding to the markedly securiform last labial palpomere (“*dernier article *... *des labiaux très fortement sécuriforme*”) of the adult [masculine].
Drepanus Dejean, 1831: 434. Type species: *Axinophorus lecontei* Dejean, 1829 (= *Pseudomorpha excrucians* Kirby, 1823) designated by Hope (1838: 109). Etymology. Uncertain, possibly from the Greek *drepane* (sickle, scimitar), or from Drepanum, the old name of the city of Trapani, in Sicily, or from Drepanus the surname of Saturne [masculine]. Note. This name was first used, but not made available, by Illiger (1807: 344).

#### Diversity.

Thirty species in temperate, subtropical, and tropical areas of the Western Hemisphere (30 species) and southern Australia (three species) arrayed in two subgenera: *Pseudomorpha* (27 species) and *Austropseudomorpha* Baehr (three Australian species). The North American fauna has 19 known species (57.5% of the world fauna).

#### Taxonomic Note.

According to Baehr (1994: Fig. 1), *Pseudomorpha* is the sister-group to a clade including *Adelotopus* Hope, *Cainogenion* Notman, *Paussotropus* Waterhouse, and *Cryptocephalomorpha* Ritsema.

**Figure 43.**
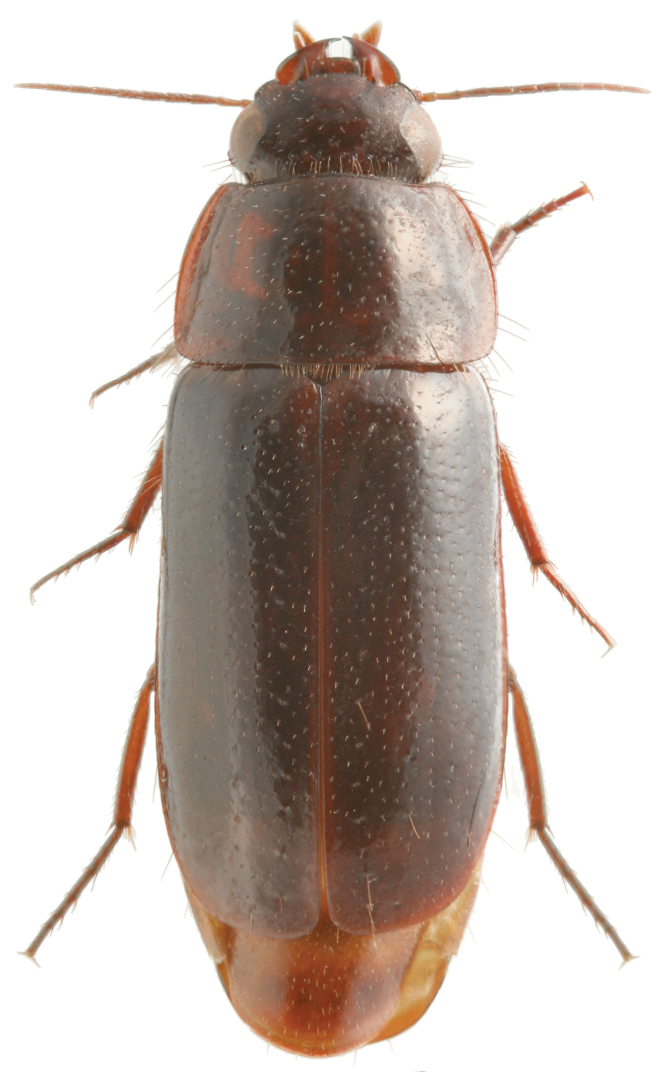
*Pseudomorpha* sp. Members of *Pseudomorpha* are among the most unusual North American carabids. The body shape of the adults is very characteristic. In addition, contrary to other Nearctic carabids that lay eggs, they are ovoviviparous. The females carry hatched larvae in their bursa copulatrix, vagina, and lateral oviducts. Such behavior probably greatly reduces egg mortality.

### Subgenus Pseudomorpha

Kirby, 1823

Pseudomorpha Kirby, 1823a: 98. Type species: *Pseudomorpha excrucians* Kirby, 1823 by monotypy.

#### Diversity.

Twenty-seven species in the Western Hemisphere.

#### Identification.

Notman (1925) published a key to all species known at the time except *Pseudomorpha ruficollis* described the previous year. Since then, two new North American species have been described by Van Dyke (1943, 1953a). Species identification of the Nearctic fauna is almost impossible without recourse to type material. A thorough revision is needed.

### Pseudomorpha alleni

Van Dyke, 1953

Pseudomorpha alleni Van Dyke, 1953a: 99. Type locality: «Tuba City [Coconino County], Arizona» (original citation). Holotype (♂) in CAS [# 8166].

#### Distribution.

This species is known only from the type locality in northern Arizona.

#### Records.

**USA**: AZ

### Pseudomorpha alutacea

Notman, 1925

Pseudomorpha alutacea Notman, 1925: 17. Type locality: «Mesilla [Dona Ana County], New Mexico» (original citation). Holotype (♀) in USNM [# 26171].

#### Distribution.

This species is known only from the type locality in southern New Mexico.

#### Records.

**USA**: NM

### Pseudomorpha augustata

Horn, 1883

Pseudomorpha augustata G.H. Horn, 1883b: 274. Type locality: «Arizona» (original citation). Syntype(s) in MCZ [# 7383].

#### Distribution.

This species is known from central and southern Arizona (Griffith 1900: 566; Notman 1925: 22) and southwestern New Mexico (Fall and Cockerell 1907: 162; Notman 1925: 22).

#### Records.

**USA**: AZ, NM

### Pseudomorpha behrensi

Horn, 1870

Pseudomorpha behrensi G.H. Horn, 1870a: 76. Type locality: United States (inferred from title of the paper). Holotype [by monotypy] in MCZ [# 7382]. Etymology. The specific name honors James H. Behrens [1824-1898], an insect collector in San Francisco who sent many beetles from the western part of the continent to LeConte and Horn. Born in Germany, Behrens was elected resident member of the California Academy of Sciences. A few years prior to his death, he sent his collection to the Naturhistorisches Museum in Lübeck.

#### Distribution.

This species has been cited from Butte County in southeastern Idaho (Stafford et al. 1986: 289), Churchill County in western Nevada (Bechtel et al. 1983: 474), Contra Costa County (Notman 1925: 21) and the San Joaquin Valley (Fall 1901a: 51) in California, and the state of Durango (Bates 1883a: 255) in Mexico.

#### Records.

**USA**: CA, ID, NV – Mexico

### Pseudomorpha castanea

Casey, 1909

Pseudomorpha castanea Casey, 1909: 278. Type locality: «Stockton [Tooele County], Utah» (original citation). Syntype(s) in USNM.

#### Distribution.

This species is known from southeastern Oregon (Hatch 1953: 187), Sacramento County in California, and northwestern Utah (Notman 1925: 22).

#### Records.

**USA**: CA, OR, UT

### Pseudomorpha champlaini

Notman, 1925

Pseudomorpha champlaini Notman, 1925: 20. Type locality: «Paradise [Cochise County], Arizona» (original citation). Holotype (♂) in USNM [# 26173]. Etymology. The specific name was proposed for Alfred B. Champlain [1882-1957], entomologist for the U.S. Bureau of Entomology.

#### Distribution.

This species is known from southeastern Arizona (Notman 1925: 20). The record from “California” (Csiki 1933a: 1638) needs confirmation.

#### Records.

**USA**: AZ [CA]

### Pseudomorpha consanguinea

Notman, 1925

Pseudomorpha consanguinea Notman, 1925: 18. Type locality: «Morena Dam, San Diego County, California» (original citation). Holotype (♂) in MCZ [# 27143].

#### Distribution.

This species is known from San Diego County in southwestern California and Yavapai County in central Arizona (Notman 1925: 19).

#### Records.

**USA**: AZ, CA

### Pseudomorpha cronkhitei

Horn, 1867

Pseudomorpha cronkhitei G.H. Horn, 1867b: 151. Type locality: «Owen’s Valley [Inyo County], California» (original citation). Syntype(s) [3 originally cited] in MCZ [# 7381].

#### Distribution.

This species is known from Inyo (Horn 1867b: 151) and Tulare (Notman 1925: 21) Counties in California.

#### Records.

**USA**: CA

### Pseudomorpha cylindrica

Casey, 1889

Pseudomorpha cylindrica Casey, 1889: 40. Type locality: «El Paso [El Paso County], Texas» (original citation). Holotype [by monotypy] (♂) in USNM.

#### Distribution.

This species is known only from the type locality in westernmost Texas.

#### Records.

**USA**: TX

### Pseudomorpha excrucians

Kirby, 1823

Pseudomorpha excrucians Kirby, 1823a: 101. Type locality: «in Georgiae forsan aquaticis?» (original citation). One syntype in BMNH (Erwin and Geraci 2008: 84).
Axinophorus lecontei Dejean [in Dejean and Boisduval], 1830: 176. Type locality: «Amérique septentrionale» (original citation). One syntype in MHNP (Erwin and Geraci 2008: 85). Synonymy established with doubt by Dejean (1831: 435), confirmed by Erwin and Geraci (2008: 85).
Pseudomorpha ruficollis Casey, 1924: 148. Type locality: «Covington [Saint Tammany Parish], Louisiana» (original citation). One syntype in USNM [# 48078]. Synonymy established by Erwin and Geraci (2008: 84).

#### Distribution.

This Coastal Plain species is found from northern South Carolina (Ciegler 2000: 130) to Louisiana (Notman 1925: 22; Erwin and Geraci 2008: 85).

#### Records.

**USA**: AL, GA, LA, MS, SC

### Pseudomorpha falli

Notman, 1925

Pseudomorpha falli Notman, 1925: 15. Type locality: «Jacumba, San Diego County, California» (original citation). Holotype (♂) in MCZ [# 27142].

#### Distribution.

This species is known only from the holotype collected in southwestern California.

#### Records.

**USA**: CA

### Pseudomorpha hubbardi

Notman, 1925

Pseudomorpha hubbardi Notman, 1925: 15. Type locality: «Rincon Mountains [Pima County], Arizona» (original citation). Holotype (♂) in SIM (Hennessey 1990: 466).

#### Distribution.

This species is known from Pima, Pinal (Notman 1925: 15), and Cochise (MCZ) Counties in southern Arizona.

#### Records.

**USA**: AZ

### Pseudomorpha parallela

Van Dyke, 1943

Pseudomorpha parallela Van Dyke, 1943: 30. Type locality: «Warners, San Diego County, California» (original citation). Holotype (♂) in CAS [# 5315].

#### Distribution.

This species is known from southern California (Van Dyke 1943: 30; Andrews et al. 1979: 28).

#### Records.

**USA**: CA

### Pseudomorpha schwarzi

Notman, 1925

Pseudomorpha schwarzi Notman, 1925: 21. Type locality: «Santa Rita Mountains, Arizona» (original citation). Holotype (♂) in USNM [# 26174]. Etymology. The specific name was proposed for Eugene Amandus Schwarz [1844-1928], entomologist at the U.S. Department of Agriculture with a specialization in the taxonomy of Coleoptera, especially the more obscure groups.

#### Distribution.

This species is known only from the holotype collected in southern Arizona.

#### Records.

**USA**: AZ

### Pseudomorpha tenebroides

Notman, 1925

Pseudomorpha tenebroides Notman, 1925: 16. Type locality: «Tucson [Pima County], Arizona» (original citation). Holotype (♂) in USNM [# 26170].

#### Distribution.

This species is known only from the holotype collected in southern Arizona.

#### Records.

**USA**: AZ

### Pseudomorpha vandykei

Notman, 1925

Pseudomorpha van dykei Notman, 1925: 18. Type locality: «Santa Cruz Village (about 3,100 feet), Cobabi Mountains [= Ko Vaya Hills, Pima County], Arizona» (original citation). Holotype (♀) in AMNH [# 334].

#### Distribution.

This species is known only from the holotype collected in southern Arizona.

#### Records.

**USA**: AZ

### Pseudomorpha vicina

Notman, 1925

Pseudomorpha vicina Notman, 1925: 17. Type locality: «Jacumba, San Diego County, California» (original citation). Holotype (♂) in MCZ [# 27141].

#### Distribution.

This species has been reported from San Diego County in southwestern California and Lyon County in west-central Nevada (Notman 1925: 18).

#### Records.

**USA**: CA, NV

### Pseudomorpha vindicata

Notman, 1925

Pseudomorpha vindicata Notman, 1925: 19. Type locality: «Stockton [Tooele County], Utah» (original citation). Holotype (♂) in SIM (Hennessey 1990: 466).

#### Distribution.

This species is known only from the holotype collected in northwestern Utah.

#### Records.

**USA**: UT

## Appendix 1. List of North American fossil Geadephaga

### Nebriini

*Nebria abstracta* Scudder, 1900b: 70. Type locality: «Reservoir Park, Toronto [Ontario]» (original citation), from the Pleistocene period. Holotype [by monotypy] location unknown. Note. According to Kavanaugh (1978: 276), the holotype probably belongs to the genus *Agonum*.

*Nebria occlusa* Scudder, 1900a: 17. Type locality: «Florissant [Teller County], Colorado» (original citation), from the early Oligocene period. Holotype [by monotypy] in MCZ [# 1755]. Note. 1. Although noted originally as from the Miocene period, most authors agree that the shales of the Florissant Basin are from the Early Oligocene (Wilson 1978: 17). 2. According to Kavanaugh (1978: 275), the holotype probably belongs to the genus *Amara*.

*Nebria paleomelas* Scudder, 1879: 178B. Type locality: «Nicola River, below main coal seam [British Columbia]» (original citation), from the Miocene period. Holotype [by monotypy] location unknown. Note. According to Kavanaugh (1978: 275), the holotype probably belongs to the genus *Amara*.

### Cychrini

*Cychrus minor* Horn, 1876b: 243. Type locality: «Port Kennedy, Penn[sylvani]a» (original citation), from the Pleistocene period. Holotype location unknown. Note. According to Horn (1876b: 243), this species is “almost exactly that of *andrewsii*” but Roeschke (1907a: 270) believed it belongs to the *elevatus* group rather than to be related to * Scaphinotus andrewsii*.

*Cychrus wheatleyi* Horn, 1876b: 242. Type locality: «Port Kennedy, Penn[sylvani]a» (original citation), from the Pleistocene period. Holotype location unknown. Etymology. The specific name was proposed for Charles M. Wheatley [1822-1882], mine operator, mineralogist, palaeontologist, naturalist, conchologist, and copper metallurgist. Born in England, Wheatley emigrated to the United States with his parents as a young boy. Note. Horn (1876b: 242) believed this species is related to *Scaphinotus viduus*.

*Nomaretus serus* Scudder, 1900a: 13. Type locality: «Florissant [Teller County], Colorado» (original citation), from the early Oligocene period. Holotype [by monotypy] in MCZ [# 1762]. Note. Roeschke (1907a: 271) stated that this species is not a member of the tribe Cychrini.

### Carabini

*Calosoma calvini* Wickham, 1909: 126. Type locality: «Florissant [Teller County, Colorado]» (original citation), from the early Oligocene period. Holotype in PMNH [# 4]. Note. According to Jeannel (1940a: 34), the only difference between this species and *Calosoma emmonsii* is the slightly longer size of the elytra and the specimen could be conspecific with those of *Calosoma scrutator*.

*Calosoma cockerelli* Wickham, 1910: 47. Type locality: Florissant [Teller County], Colorado (inferred from title of the book), from the early Oligocene period. Holotype in PMNH [# 10]. Note. Jeannel (1940a: 34) had no opinion on the specimen.

*Calosoma emmonsii* Scudder, 1900a: 16. Type locality: «Florissant [Teller County], Colorado» (original citation), from the early Oligocene period. Holotype [by monotypy] in MCZ [# 1756, 1757]. Etymology. The specific name honors the American geologist, educator, and physician Ebenezer Emmons [1799-1863] who is credited for having named the Adirondack Mountains in 1838 and the Taconic Mountains in 1844. Note. Lapouge (1930: 159) stated that this species does not differ from *Calosoma externum* and Jeannel (1940a: 34) believed it could be identical to *Calosoma scrutator*.

*Calosoma fernquisti* Cockerell, 1924: 14. Type locality: «Deep Creek Canon, near Spokane, Washington» (original citation), from the Upper Eocene period. Holotype in USNM [# 69218]. Note. 1. Jeannel (1940a: 34) believed the species is very likely *Calosoma externum*. 2. According to Nel (1989: 265), fossil specimens described as *Calosoma* by Wickham, Scudder, and Cockerell could all be conspecific.

*Carabus jeffersoni* Scudder, 1900a: 15. Type locality: «Florissant [Teller County], Colorado» (original citation), from the early Oligocene period. Two syntypes [2 specimens originally cited] in MCZ [# 1758, 1759]. Etymology. The specific name honors Thomas Jefferson [1743-1826], third president of the United States and one of the earliest writers on American palaeontology. Jefferson is also famous for writing the Declaration of Independence in 1776. Note. According to Lapouge (1930: 159), this species does not belong to the subfamily Carabinae.

*Carabus maeander sangamon* Wickham, 1917b: 139. Type locality: «north bank of the Sangamon River near Mahomet, Champaign County, Illinois» (original citation), from the Pleistocene period. Holotype [by monotypy] in UICU.

*Neothanes testeus* (Scudder, 1878: 758) (as *Cychrus testeus*). Type locality: «immediate vicinity of Green River Station on the Union Pacific Railroad in Wyoming» (original citation), from the Eocene period. Holotype [by monotypy] location unknown. Note. The genus *Neothanes* was proposed by Scudder (1890: 535) for *Cychrus testeus* Scudder, 1878 and, according to him, is “allied to *Carabus*.”

### Cicindelini

*Cicindelopsis eophilus* Cockerell, 1920: 254. Type locality: «White River, Colorado» (original citation), from the “Eocene rock of Green River Age.” Holotype in USNM [# 66576]. Note. 1. According to Wilson (1978: 14), the “White River” collections made by Scudder, some of which were described by Cockerell, came from the Green River Formation localities along the White River near the Colorado-Utah border. 2. According to Cockerell (1920: 254), the single elytron representing this species “suggests a cicindelid” but is narrower, without any distinct inner apical angle, and the surface is neither punctured nor striate. He also stated that “the Collyrinae have narrow elytra shaped essentially as in *Cicindelopsis*.” The species was subsequently transferred to the Melandryidae (Cockerell 1924: 12) based on Herbert S. Barber’s opinion. The holotype was also studied by Walther Horn who believed it did not belong to the Cicindelinae (Cockerell 1924: 12).

### Elaphrini

*Diacheila matthewsi* Böcher, 1995: 21. Type locality: «Peary Land, Greenland» (original citation), from the Plio-Pleistocene period (2-2.5 million years B.P.). Holotype in ZMUC. Note. Böcher (1995: 21, 22) noted that fossil remnants of *Diacheila* reported from Alaska (about 2.9 million B.P.), Yukon Territory, Banks, Prince Patrick and Meighen Islands (Pliocene), and Ellesmere Island probably belong to this species.

*Elaphrus clairvillei lynni* Pierce, 1948a: 52. Type locality: «Lynn Creek, British Columbia» (original citation), from the Pleistocene period. Holotype in LACM. Note. Goulet (1983: 271) listed this name in synonymy with *Elaphrus clairvillei* Kirby, 1837.

*Elaphrus irregularis* Scudder, 1890: 534. Type locality: «Scarboro[ugh], Ontario» (original citation), from the Pleistocene period. Holotype [by monotypy] in MCZ. Note. Goulet (1983: 321) concluded from a study of the fossil elytron that several character states present in the holotype suggest “a match with *Elaphrus parviceps* [= *Elaphrus trossulus* Semenov].”

*Elaphrus ruscarius foveatus* Pierce, 1948b: 54. Type locality: «McKittrick [Kern County, California]» (original citation), from the Pleistocene period. Holotype in LACM. Note. Goulet (1983: 303) listed this name in synonymy with *Elaphrus finitimus* Casey, 1920.

### Loricerini

*Loricera exita* Scudder, 1900b: 70. Type locality: «Scarborough [Ontario]» (original citation), from the Pleistocene period. Holotype [by monotypy] location unknown. Note. Ball and Erwin (1969: 894) stated that the single fragment of an elytron representing this species probably belongs “to a carabid, possibly an agonine.”

*Loricera glacialis* Scudder, 1877: 763. Type locality: Scarborough, Ontario (inferred from title of the paper), from the Pleistocene period. Holotype [by monotypy] location unknown. Note. Ball and Erwin (1969: 894) indicated that the elytra of two specimens assigned to this species “are probably those of a weevil.”

*Loricera* ? *lutosa* Scudder, 1890: 533. Type locality: «Scarboro[ugh], Ontario» (original citation), from the Pleistocene period. Holotype [by monotypy] location unknown. Note. Ball and Erwin (1969: 894) stated that the single elytron representing this species has characteristics “of a brachyrhinine weevil, not of a carabid, and certainly not of *Loricera*.” Morgan and Morgan (1980: 23) stated that the species is “definitely a member of the Scarabaeidae, almost certainly of the genus *Aegialia* and probably *Aegialia lacustris* LeC.”

### Trechini

*Trechus fractus* Wickham, 1912: 6. Type locality: Florissant [Teller County], Colorado (inferred from title of the paper), from the early Oligocene period. Holotype in UCM [# 8272]. Note. Based on the illustration provided, this species, if really of a carabid, could belong to any groups and not necessary to the genus *Trechus*.

### Bembidiini

*Bembidium damnosum* Scudder, 1900b: 73. Type locality: «Scarborough [Ontario]» (original citation), from the Pleistocene period. Holotype [by monotypy] location unknown.

*Bembidion davidae* Pierce, 1944: 7. Type locality: «Cuyama River outcrop section on the west bank of the river, near its junction with Huasna Creek, in San Luis Obispo County near center of section 27, Township 11 N, R. 33 W. San Bernardino Base Line and Meridian [California]» (original citation), from the “Upper Lower Miocene.” Holotype in LACM. Etymology. The specific name was proposed for the American palaeontologist Lore Rose David [1905-1985]. Note. This species is represented by a single elytron and was assigned to the subgenus *Ochthedromus* by Pierce (1944: 7).

*Bembidion everestae* Pierce, 1944: 11. Type locality: «Bellevue District, near town of Rosedale, 6 miles due west of Bakersfield, Kern Co[unty], California» (original citation), from the “Upper Lower Miocene, Saucesian Age.” Holotype in LACM. Note. This species, represented by a fragment of an elytron and its impression in oil shale core, was tentatively assigned to the subgenus *Odontium* LeConte by Pierce (1944: 12).

*Bembidium exoletum* Scudder, 1876: 77. Type locality: «Green River group, near White River, in one of the two localities called ... Chagrin Valley and Fossil Cañon» (original citation), from the Eocene period. Holotype [by monotypy] location unknown.

*Bembidium expletum* Scudder, 1900b: 72. Type locality: «Reservoir Park, Toronto [Ontario]» (original citation), from the Pleistocene period. Holotype [by monotypy] location unknown.

*Bembidium florissantensis* Wickham, 1913: 6. Type locality: Florissant [Teller County], Colorado (inferred from title of the paper), from the early Oligocene period. Holotype in USNM [# 90451].

*Bembidion fragmentum* Scudder, 1890: 531. Type locality: «shores of Lake Erie, near Cleveland, Ohio» (Scudder 1895: 54), from the Pleistocene period. Holotype [by monotypy] location unknown. Note. The type locality was originally cited as “Scarboro[ugh], Ontario” but corrected to the present one subsequently. Scudder (1890: 531) indicated that this species “is represented by a single elytron with the tip broken off” and agrees “better among modern types with *Bembidion constrictum*.”

*Bembidion glaciatum* Scudder, 1890: 531. Type locality: «Scarboro[ugh], Ontario» (original citation), from the Pleistocene period. Syntype(s) [2 originally cited] location unknown. Note. According to Scudder (1890: 531), this species is represented by a couple of elytra and is “nearly allied to the scarcely smaller *Bembidion longulum* LeC. [= *Bembidion concolor* (Kirby)].”

*Bembidium haywardi* Scudder, 1900b: 70. Type locality: «Logan’s brickyard, Toronto [Ontario]» (original citation), from the Pleistocene period. Holotype [by monotypy] location unknown.

*Bembidium obductum* Scudder, 1900a: 18. Type locality: «Florissant [Teller County], Colorado» (original citation), from the early Oligocene period. Holotype in MCZ [# 1768].

*Bembidium praeteritum* Scudder, 1900b: 72. Type locality: «Scarborough [Ontario]» (original citation), from the Pleistocene period. Holotype [by monotypy] location unknown.

*Bembidium tumulorum* Scudder, 1900a: 19. Type locality: «Florissant [Teller County], Colorado» (original citation), from the early Oligocene period. Holotype in USNM.

*Bembidium vanum* Scudder, 1900b: 71. Type locality: «Scarborough; Reservoir Park, Toronto [Ontario]» (original citation), from the Pleistocene period. Syntypes [2 originally cited] location unknown.

*Bembidium vestigium* Scudder, 1900b: 71. Type locality: «Scarborough [Ontario]» (original citation), from the Pleistocene period. Holotype [by monotypy] location unknown.

*Tachys haywardi* Wickham, 1913: 7. Type locality: Florissant [Teller County], Colorado (inferred from title of the paper), from the early Oligocene period. Holotype in USNM [# 90582B].

### Patrobini

*Patrobus decessus* Scudder, 1900b: 73. Type locality: «Reservoir Park, Toronto; Scarborough [Ontario]» (original citation), from the Pleistocene period. Lectotype [as type], designated by Darlington (1938: 155), in MCZ. Note. Darlington (1938: 155) listed this name in synonymy with *Patrobus stygicus* Chaudoir, 1871.

*Patrobus frigidus* Scudder, 1900b: 74. Type locality: «Reservoir Park, Toronto [Ontario]» (original citation), from the Pleistocene period. Holotype [by monotypy] in MCZ. Note. Darlington (1938: 155) listed this name in synonymy with *Patrobus stygicus* Chaudoir, 1871.

*Patrobus gelatus* Scudder, 1890: 530. Type locality: «Scarboro[ugh], Ontario» (original citation), from the Pleistocene period. Holotype [by monotypy] in MCZ. Note. Darlington (1938: 155) listed this name in synonymy with *Patrobus stygicus* Chaudoir, 1871.

*Patrobus henshawi* Wickham, 1917b: 140. Type locality: «north bank of the Sangamon River near Mahomet, Champaign County, Illinois» (original citation), from the Pleistocene period. Holotype [by monotypy] in UICU.

### Brachinini

*Brachinus newberryi* Scudder, 1900a: 32 (as *Brachynus newberryi*). Type locality: «Florissant [Teller County], Colorado» (original citation), from the early Oligocene period. Four syntypes [4 originally cited] in MCZ [# 1850-1853]. Etymology. The specific name was proposed in honor of the American geologist, palaeontologist, physician, explorer, and author John Strong Newberry [1822-1892]. Note. Erwin (1970a: 164) noted that the specimens assigned to this species “should be placed in the genus *Lebia*.”

*Brachinus repressus* Scudder, 1900a: 33 (as *Brachynus repressus*). Type locality: «Florissant [Teller County], Colorado» (original citation), from the early Oligocene period. Holotype in MCZ [# 1848]. Note. According to Erwin (1970a: 164), the single specimen of this species “is too fragmentary to make definitive comparisons, but is similar in some respects to members of *Cymindis*.”

### Paussini

*Paussopsis nearctica* Cockerell, 1911: 71. Type locality: «Florissant [Teller County, Colorado]» (original citation), from the early Oligocene period. Holotype in UCM [# 4508]. Note. The genus *Paussopsis* Cockerell, 1911 was proposed for this species.

*Paussopsis secunda* Wickham, 1912: 10. Type locality: «Florissant [Teller County], Colorado» (original citation), from the early Oligocene period. Holotype in UCM [# 8200]. Note. According to Wasmann (1926: 25), members of *Paussopsis* do not belong to paussines.

### Pterostichini

*Cyclotrachelus tenebricus* (Scudder, 1900a: 24) (as *Evarthrus tenebricus*). Type locality: «Florissant [Teller County], Colorado» (original citation), from the early Oligocene period. Holotype [by monotypy] in MCZ [# 1786]. Note. This species is represented only by a head. Based on the original description, Freitag (1969: 168) stated that the specimen “may or may not be a member of the genus *Evarthrus* [= *Cyclotrachelus*].”

*Loxandrus gelidus* Scudder, 1877: 763. Type locality: Scarborough, Ontario (inferred from title of the paper), from the Pleistocene period. Syntype(s) [5 originally cited] location unknown. Note. According to Scudder (1890: 528), this species closely resembles *Pterostichus hudsonicus* LeConte.

*Myas rigefactus* Scudder, 1900a: 20. Type locality: «Florissant [Teller County], Colorado» (original citation), from the early Oligocene period. Holotype [by monotypy] in MCZ [# 1766].

*Myas umbrarum* Scudder, 1900a: 21. Type locality: «Florissant [Teller County], Colorado» (original citation), from the early Oligocene period. Syntype(s) [3 originally cited] in MCZ [# 1763, 1764, 1767].

*Pterostichus abrogatus* Scudder, 1890: 525. Type locality: «Scarboro[ugh], Ontario» (original citation), from the Pleistocene period. Holotype [by monotypy] location unknown. Note. According to Scudder (1890: 525), this species is represented by a “fragment of an elytron” and its features suggest “a species closely allied to *Pterostichus herculaneus* Mann.”

*Pterostichus depletus* Scudder, 1900b: 75. Type locality: «Scarborough [Ontario]» (original citation), from the Pleistocene period. Holotype [by monotypy] location unknown.

*Pterostichus destitutus* Scudder, 1890: 526. Type locality: «Scarboro[ugh], Ontario» (original citation), from the Pleistocene period. Holotype [by monotypy] location unknown. Note. According to Scudder (1890: 526), this species is “represented by a single elytron of a mahogany color, which seems to be nearly related to *Pterostichus sayi* Brullé [= *Poecilus chalcites* (Say)].”

*Pterostichus destructus* Scudder, 1890: 527. Type locality: «Scarboro[ugh], Ontario» (original citation), from the Pleistocene period. Syntype(s) [2 originally cited] location unknown. Note. According to Scudder (1890: 527), this species, represented by a couple of elytra, “closely resembles *Pterostichus patruelis* Dej.”

*Pterostichus dormitans* Scudder, 1890: 526. Type locality: «shores of Lake Erie, near Cleveland, Ohio» (Scudder 1895: 51), from the Pleistocene period. Syntype(s) [2 originally cited] location unknown. Note. The type locality was originally listed as “Scarboro[ugh], Ontario” but corrected to the present one subsequently. According to Scudder (1890: 526), this species, “represented by two opposite ends of elytra, is very close indeed in size and general character to *Pterostichus laetulus* LeC. [= *Poecilus laetulus* (LeConte)].”

*Pterostichus fernquisti* Wickham, 1931: 318. Type locality: «Deep Creek Canyon [near Spokane, Washington]» (original citation), from the Miocene period. Holotype in MCZ. Note. According to Wickham (1931: 318), this species, represented by three elytra, is perhaps “most reminiscent of *Pterostichus gracilior*.”

*Pterostichus fractus* Scudder, 1890: 527. Type locality: «Scarboro[ugh], Ontario» (original citation), from the Pleistocene period. Holotype [by monotypy] location unknown. Note. This species is represented by a single elytron with the extreme apex broken. According to Scudder (1890: 527), the species is “closely allied to *Pterostichus destitutus*.”

*Pterostichs laevigatus* Scudder, 1890: 528. Type locality: «Bone caves of Port Kennedy, Pennsylvania» (original citation), from the Pleistocene period. Syntype(s) location unknown.

*Pterostichus pumpellyi* Scudder, 1900a: 23. Type locality: «Florissant [Teller County], Colorado» (original citation), from the early Oligocene period. Two syntypes [2 originally cited] in MCZ [# 1775, 1776]. Etymology. The specific name was proposed for the American geologist and explorer Raphael Pumpelly [1837-1923].

*Pterostichus walcotti* Scudder, 1900a: 23. Type locality: «Florissant [Teller County], Colorado» (original citation), from the early Oligocene period. Syntype(s) [6 originally cited] in MCZ [# 1769-1772, 1774] and USNM. Etymology. The specific name honors the American geologist and palaeontologist Charles Doolittle Walcott [1850-1927] who for some time was director of the United States Geological Survey.

### Zabrini

*Amara cockerelli* Wickham, 1912: 7. Type locality: Florissant [Teller County], Colorado (inferred from title of the paper), from the early Oligocene period. Holotype in UCM [# 8236].

*Amara danae* Scudder, 1900a: 27. Type locality: «Florissant [Teller County], Colorado» (original citation), from the early Oligocene period. Syntype(s) [15 originally cited] in MCZ [# 1787, 1788, 1801, 1802, 1804-1806, 1808, 1809, 1811, 1812, 4167] and USNM. Etymology. The specific name honors the American geologist, mineralogist, and zoologist James Dwight Dana [1813-1895].

*Amara powellii* Scudder, 1900a: 26. Type locality: «Florissant [Teller County], Colorado» (original citation), from the early Oligocene period. Syntype(s) [12 originally cited] in MCZ [# 1777, 1790-1794, 1796-1800] and USNM [# 59646]. Etymology. This species was named after the American geologist and explorer Major John Wesley Powell [1834-1902] who led a three-month expedition in 1867 down the Green and Colorado rivers that included the first passage through the Grand Canyon. Powell served as the second director of the United States Geological Survey from 1881 to 1894.

*Amara revocata* Scudder, 1900a: 25. Type locality: «Florissant [Teller County], Colorado» (original citation), from the early Oligocene period. Holotype [by monotypy] in MCZ [# 1785].

*Amara sterilis* Scudder, 1900a: 25. Type locality: «Florissant [Teller County], Colorado» (original citation), from the early Oligocene period. Syntype(s) [4 originally cited] in MCZ [# 1782-1784] and USNM.

*Amara veterata* Scudder, 1900a: 26. Type locality: «Florissant [Teller County], Colorado» (original citation), from the early Oligocene period. Syntype(s) [5 originally cited] in MCZ [# 1778-1781, 1813].

### Chlaeniini

*Chlaenius plicatipennis* Wickham, 1917b: 143. Type locality: «north bank of the Sangamon River near Mahomet, Champaign County, Illinois» (original citation), from the Pleistocene period. Holotype [by monotypy] in UICU.

*Chlaenius punctulatus* Horn, 1876b: 244 [homonym of *Chlaenius punctulatus* Chaudoir, 1876]. Type locality: «Port Kennedy, Penn[sylvani]a» (original citation), from the Pleistocene period. Holotype [by monotypy] location unknown. Note. This species is represented by one elytron “of the size and very similar to that of *Chlaenius laticollis*” (Horn 1876b: 244).

### Licinini

*Badister antecursor* Scudder, 1900b: 75. Type locality: «Logan’s brickyard, Toronto [Ontario]» (original citation), from the Pleistocene period. Holotype [by monotypy] location unknown.

*Dicaelus alutaceus* Horn, 1876b: 244. Type locality: «Port Kennedy, Penn[sylvani]a» (original citation), from the Pleistocene period. Holotype [by monotypy] location unknown.

*Diplocheila* ? *henshawi* Scudder, 1890: 523. Type locality: «Florissant [Teller County, Colorado]» (original citation), from the early Oligocene period. Holotype [by monotypy] location unknown.

### Harpalini

*Cratacanthus florissantensis* Wickham, 1917a: 464. Type locality: Florissant [Teller County], Colorado (inferred from title of the paper), from the early Oligocene period. Holotype in USNM [# 63442]. Note. Based on the illustration provided by Wickham (1917a: plate 37, figure 2), the specimen is not necessarily a *Cratacanthus*, not even a carabid.

*Euryderus kingii* (Scudder, 1900a: 34) (as *Nothopus kingii*). Type locality: «Florissant [Teller County], Colorado» (original citation), from the early Oligocene period. Holotype [by monotypy] in MCZ [# 1847]. Etymology. The species was named for the American geologist and mountaineer Clarence King [1842-1901] who served as first director of the United States Geological Survey from 1879 to 1881.

*Harpalus conditus* Scudder, 1900b: 77. Type locality: «Scarborough [Ontario]» (original citation), from the Pleistocene period. Holotype [by monotypy] location unknown.

*Harpalus maceratus* Wickham, 1911a: 54. Type locality: «Florissant [Teller County, Colorado]» (original citation), from the early Oligocene period. Holotype in UCM [# 8246].

*Harpalus nuperus* Scudder, 1900a: 34. Type locality: «Florissant [Teller County], Colorado» (original citation), from the early Oligocene period. Holotype in MCZ [# 1846]

*Harpalus redivivus* Wickham, 1917a: 465. Type locality: Florissant [Teller County], Colorado (inferred from title of the paper), from the early Oligocene period. Holotype in USNM [# 63444].

*Harpalus ulomaeformis* Wickham, 1917a: 464. Type locality: Florissant [Teller County], Colorado (inferred from title of the paper), from the early Oligocene period. Holotype in USNM [# 63443].

*Harpalus veterum* Cockerell, 1920: 256. Type locality: «Roan Mountain, Colorado» (original citation), from the Eocene period. Holotype in USNM [# 66578].

*Harpalus whitfieldi* Scudder, 1900a: 35. Type locality: «Florissant [Teller County], Colorado» (original citation), from the early Oligocene period. One syntype [3 originally cited] in MCZ [# 1845] and two in USNM. Etymology. The species was named after the American palaeontologist, teacher, and curator Robert Parr Whitfield [1828-1910].

*Stenolophus religatus* Scudder, 1900a: 35. Type locality: «Florissant [Teller County], Colorado» (original citation), from the early Oligocene period. Holotype [by monotypy] in MCZ [# 1858].

### Platynini

*Platynus caesus* Scudder, 1890: 522. Type locality: «Green River, Wyoming» (original citation), from the Eocene period. Syntype(s) [2 originally cited] location unknown.

*Platynus calvini* Wickham, 1917b: 142. Type locality: «north bank of the Sangamon River near Mahomet, Champaign County, Illinois» (original citation), from the Pleistocene period. Syntypes [2 originally cited] in UICU. Etymology. The specific name was proposed for Samuel Calvin [1840-1911], professor of geology at the State University of Iowa.

*Platynus casus* Scudder, 1890: 519. Type locality: «Scarboro[ugh], Ontario» (original citation), from the Pleistocene period. Holotype [by monotypy] location unknown. Note. This species is represented by a single elytron. According to Scudder (1890: 519), it is best comparable to *Platynus rubripes* Zimmermann (= *Agonum rufipes* Dejean) among extant species.

*Platynus desuetus* Scudder, 1890: 521. Type locality: «Scarboro[ugh], Ontario» (original citation), from the Pleistocene period. Syntype(s) [6 originally cited] location unknown. Note. According to Scudder (1890: 521), this species “is more nearly allied to *Platynus crenistriatus* LeC. than to any other living species.”

*Platynus dilapidatus* Scudder, 1895: 49. Type locality: «Scarboro[ugh], Ontario» (original citation), from the Pleistocene period. Holotype [by monotypy] location unknown. Note. According to Scudder (1895: 49), this species, represented by a fragment of a single elytron, “belongs rather in the near vicinity of *Platynus maculicollis* Dej. [= *Tanystoma maculicolle* (Dejean)].”

*Platynus dissipatus* Scudder, 1890: 521. Type locality: «Scarboro[ugh], Ontario» (original citation), from the Pleistocene period. Syntype(s) [2 originally cited] location unknown. Note. According to Scudder (1890: 521), this species agrees with *Platynus halli* “in its general features and in the minute texture of the surface.”

*Platynus exterminatus* Scudder, 1900b: 75. Type locality: «Scarborough [Ontario]» (original citation), from the Pleistocene period. Holotype [by monotypy] location unknown.

*Platynus florissantensis* Wickham, 1913: 7. Type locality: Florissant [Teller County], Colorado (inferred from title of the paper), from the early Oligocene period. Holotype in USNM [# 63441]. Note. Wickham (1913: 7) noted that this species “compared with recent species ... remind one of *Platynus placidus* by the fine striae and of *Platynus melanarius* by the size and form.”

*Platynus halli* Scudder, 1890: 520. Type locality: «Scarboro[ugh], Ontario» (original citation), from the Pleistocene period. Syntype(s) [3 originally cited] location unknown. Etymology. The specific name honors the American geologist and palaeontologist James Hall [1811-1898]. Note. According to Scudder (1890: 520), this species is “allied to *Platynus crenistriatum* LeC. ... [but] more nearly related to *Platynus hindei*.”

*Platynus harttii* Scudder, 1890: 522. Type locality: «Scarboro[ugh], Ontario» (original citation), from the Pleistocene period. Syntype(s) [2 originally cited] location unknown. Etymology. The specific name was proposed in honor of the American geologist and teacher Charles Frederic Hartt [1840-1878] who led, in his last years, the Brazilian Geological Commission. Note. According to Scudder (1890: 522), this small species “is most nearly allied to the largest [= *Platynus desuetus* Scudder].”

*Platynus hindei* Scudder, 1890: 520. Type locality: «Scarboro[ugh], Ontario» (original citation), from the “clay beds of interglacial deposits” [Pleistocene period]. Syntype(s) [9 originally cited] location unknown. Etymology. This species was named for the geologist and palaeontologist George Jennings Hinde [1839-1918]. Note. The species is represented by “a number of fragments” and, according to Scudder (1890: 520), “seems to be allied to *Platynus rubripes* Zimm. [= *Agonum rufipes* Dejean].”

*Platynus interglacialis* Scudder, 1900b: 76. Type locality: «Scarborough [Ontario]» (original citation), from the Pleistocene period. Holotype [by monotypy] location unknown.

*Platynus interitus* Scudder, 1900b: 76. Type locality: «Reservoir Park, Toronto [Ontario]» (original citation), from the Pleistocene period. Holotype [by monotypy] location unknown.

*Platynus insculptipennis* Wickham, 1917a: 463. Type locality: Florissant [Teller County], Colorado (inferred from title of the paper), from the early Oligocene period. Holotype in USNM [# 63441].

*Platynus longaevus* Scudder, 1900b: 77. Type locality: «Scarborough [Ontario]» (original citation), from the Pleistocene period. Holotype [by monotypy] location unknown.

*Platynus pleistocenicus* Wickham, 1917b: 141. Type locality: «north bank of the Sangamon River near Mahomet, Champaign County, Illinois» (original citation), from the Pleistocene period. Holotype [by monotypy] in UICU.

*Platynus senex* Scudder, 1878: 759. Type locality: «immediate vicinity of Green River Station on the Union Pacific Railroad in Wyoming» (original citation), from Eocene period. Holotype [by monotypy] location unknown.

*Platynus subgelidus* Wickham, 1917b: 142. Type locality: «north bank of the Sangamon River near Mahomet, Champaign County, Illinois» (original citation), from the Pleistocene period. Holotype [by monotypy] in UICU.

*Platynus tartareus* Scudder, 1900a: 30. Type locality: «Florissant [Teller County], Colorado» (original citation), from the early Oligocene period. Syntype(s) [4 originally cited] in MCZ [# 1838, 1840, 1841, 1857].

### Lebiini

*Cymindis aurora* Horn, 1876b: 243. Type locality: «Port Kennedy, Penn[sylvani]a» (original citation), from the Pleistocene period. Syntype(s) location unknown. Note. According to Horn (1876b: 243), the species “is closely related to *Cymindis americana*, but somewhat larger.”

*Cymindis extorpescens* Scudder, 1898: 743. Type locality: «Fort River in Hadley, Massachusetts, near its entrance into the Connecticut» (original citation), from the Pleistocene period. Holotype [by monotypy] location unknown.

*Lebia protospiloptera* Cockerell, 1921: 35. Type locality: «back of house at Smith’s ranch, shale of Green River formation, Colorado» (original citation), from the Eocene period. Holotype in USNM [# 66928].

*Plochionus lesquereuxi* Scudder, 1900a: 31. Type locality: «Florissant [Teller County], Colorado» (original citation), from the early Oligocene period. Holotype [by monotypy] in MCZ [# 1855]. Etymology. The species was named after the bryologist and paleobotanist Leo Lesquereux [1806-1889]. Born in Switzerland, Lesquereux progressively lost his hearing, consequent to a near-fatal fall from the top of a cliff at the age of seven, which had considerable ramifications in his professional life. He moved with his wife and five children to the United States in 1847. Note. According to Scudder (1900a: 31), this species could rather belong to the genus *Cymindis* of the subgenus *Pinacodera*.

### Galeritini

*Galerita marshii* Scudder, 1900a: 31. Type locality: «Green River, Wyoming» (original citation), from the Eocene period. Holotype [by monotypy] location unknown. Etymology. The specific name was proposed for the American palaeontologist Othniel Charles Marsh [1831-1899] who uncovered the first pterosaur fossil in America. Marsh is also known for his heated rivalry with Edward Drinker Cope [1840-1897] which led the two palaeontologists to use underhanded methods to out-compete the other in the field, resorting to bribery, theft, and destruction of bones (the Bone Wars). They attack each other in scientific publications, attempting to ruin the other’s credibility. Marsh is credited to have persuaded his uncle, George Peabody [1795-1869], to establish the Peabody Museum of Natural History at Yale.

### Incertae Sedis

*Carabites* (?) *arapahoensis* Cockerell, 1918: 307. Type locality: «one mile west of Spicer, Arapahoe Pass Road, North Park, Colorado, 2½ miles south of fork of road» (original citation), from the Eocene period. Holotype [by monotypy] in UCM [# 5822].

*Carabites eocenicus* Cockerell, 1920: 255. Type locality: «White River, Colorado» (original citation), from the Eocene period. Holotype in USNM [# 66577].

*Carabites exanimus* Scudder, 1892: 17. Type locality: «White River, Utah, about 5 miles from the Colorado line» (original citation), from the Eocene period. Holotype [by monotypy] location unknown. Note. Based on Scudder’s description, this species, which is represented only by the elytra, is that of a large species with the elytral epipleura crossed. Scudder (1892: 17) stated that the species “seems to agree best with the Pterostichini.”

*Carabites feildenianus* Heer, 1878: 38. Type locality: Grinnell-Landes [= central section of Ellesmere Island, Nunavut] (inferred from title of the paper), from the Miocene period. Syntype(s) location unknown. Etymology. The specific name was proposed for Henry Wemyss Feilden [1838-1921], naturalist of the British north-polar expedition of Captain George Nares in 1875-76.

*Carabites kincaidi* Cockerell, 1908: 51. Type locality: «Green River, Wyoming» (original citation), from the Eocene period. Holotype [by monotypy] in PMNH. Note. This species was described from a larva. Cockerell (1908: 52) noted that the larva may be that of “the genus *Neothanes* Scudder, described from the Green River beds; but as this cannot be demonstrated, I leave it in the blanket genus *Carabites*.”

*Carabites russelli* Cockerell, 1928: 37. Type locality: Fox Hills, South Dakota (inferred from title of the paper), from the Cretaceous period. Holotype probably in PMNH.

## Appendix 2. List of North American Geadephaga larvae described or illustrated

Along with the reference, the following information is provided: pages where the character states of the species are indicated, the figures illustrating character states, the stage described, and the provenance of the material, either reared (RR) or field collected (FC). A question mark in front of a reference indicates that the identification of the taxon is uncertain according to the author(s). A dagger after the name of a species indicates that the taxon is adventive in North America. The abbreviations used are:

**FC** field collected

**LIT** Literature

**L_1_** first instar

**L_2_** second instar

**L_3_** third instar

**L_1-3_** instar not determined

**L_2-3_** second and/or third instar

**RR** reared

### Trachypachini

*Trachypachus gibbsii* LeConte^10^: Lindroth (1960a: 33; Figs 2, 4a-h) [L_2_; RR]; Thompson (1979a: Figs 26a-e); Luff (1993: Figs 25-32); Luna de Carvalho (1989: Figs 27c-d).

*Trachypachus inermis* Motschulsky: Bousquet and Goulet (1984: Figs 25, 37, 42, 51, as *Trachypachus holmbergi*); Beutel (1993: Figs 2, 8, 13, 16, 21, 29, as *Trachypachus holmbergi*); Arndt and Beutel (1995: 442; Figs 4, 6, 12, as *Trachypachus holmbergi*) [L_1_; RR].

### Clinidiini

*Clinidium (Arctoclinidium) sculptile* (Newman): Böving and Craighead (1931: Pl. 3); Hůrka (1978: Fig. H-10); Thompson (1979a: Figs 10a-c).

### Pelophilini

*Pelophila borealis* (Paykull): Johnson and Carpenter (1898: 136-139; Figs 1-12) [L_3_; RR]; Sharova (1958: 37; Figs 31, 81, 82) [L_1-3_; ?]; Sharova (1964: 143, 144; Figs 99-4, 128, 129-1) [L_1-3_; ?]; Haberman (1968: Fig. 57-1); Andersen (1970: 92, 93; Figs 2a-i, 3a-c) [L_1-3_; FC]; Luff (1972: 176, 177; Figs 51-62) [L_1-3_; RR+FC]; Thompson (1979a: Figs 34a-c); Luff (1993: 58, 59; Figs 160-163) [L_1_, L_2-3_; ?]; Makarov (1994: Fig. 84); Bousquet (2010a: Figs 373-376).

### Opisthiini

*Opisthius richardsoni* Kirby: Garner (1954: 160, 161; Figs a-c [Pl. 9]) [L_1_, L_2-3_; RR+FC]; Lindroth (1960a: 38, 40, 41; Figs 3, 6a-g) [L_1_, L_2_, L_3_; FC]; Thompson (1979a: Figs 37a-c); Bousquet and Smetana (1991: 110; Figs 3, 9a) [L_1_, L_2_, L_3_; RR].

### Nebriini

*Leistus (Leistus) ferrugineus* (Linnaeus)**†**: Larsson (1941: 280) [L_1-3_; ?]; Verhoeff (1921: Fig. 22); Larsson (1968: 324) [L_1-3_; ?]; Luff (1972: 162, 165; Fig. 12) [L_1-3_; FC]; Arndt (1991b: 72) [L_1-3_; LIT]; Luff (1993: 48; Figs 122, 123) [L_1-3_; ?]; Bousquet (2010a: Figs 378, 379).

*Nebria (Nebria) brevicollis* (Fabricius)**†**: Blisson (1848: 73-76; Figs 1-6 [Pl. 1]) [L_2-3_; FC]; Schiødte (1867: 461-465; Figs 1-7 [Pl. 14]) [L_1-3_; ?]; Znojko (1929: Fig. 4b); Raynaud (1935: 12, 13, 16) [L_3_?; FC]; Larsson (1941: 278; Figs 5, 6, 17a, 18a) [L_1-3_; ?]; Jeannel (1941b: Figs 74a-h); Sharova (1958: 38; Figs 6, 18, 85a) [L_1-3_; ?]; Habu (1958: Figs 12, 14); Sharova (1964: 145; Figs 88, 93-2, 130-1) [L_1-3_; ?]; Larsson (1968: 321, 322; Figs 5, 6, 17a, 18a) [L_1-3_; ?]; Haberman (1968: Fis 57-2, 57-4, 58-2); Andersen (1970: 91) [L_3_; LIT]; Luff (1972: 171, 173; Figs 32-41) [L_1_, L_2-3_; FC]; Arndt (1991b: 71, 72; Figs 2, 62) [L_1_, L_2-3_; ?]; Luff (1993: 49, 54, 57; Figs 2, 3, 7, 8, 143-155) [L_1_, L_2-3_; ?]; Sustek (1993: 522) [L_3_; LIT].

*Nebria (Boreonebria) gyllenhali* Schönherr: Larsson and Gígja (1959: 15, as *Nebria gyllenhali munsteri*) [L_1-3_; FC]; Andersen (1970: 91-93; Figs 4a-i, 5a-c) [L_1-3_; FC]; Luff (1972: 171, 174, 176, as *Nebria rufescens*; Figs 45-49) [L_1_, L_2-3_; RR+FC]; Böcher (1988: Fig. 1, as *Nebria rufescens*); Arndt (1991b: 71, 72) [L_1_, L_2-3_; ?]; Luff (1993: 50-52, as *Nebria rufescens*; Figs 131-136) [L_1_, L_2-3_; ?]; Sustek (1993: 522) [L_3_; LIT]; Makarov (1994: Figs 85, 86).

*Nebria (Boreonebria) lacustris* Casey: Spence (1974: 13, 17; Fig. 4) [L_1_, L_2_, L_3_; RR]; Spence et al. (1976: 81; Figs 2a-c, 3a) [L_1_, L_2_, L_3_; RR]; Spence and Sutcliffe (1982: Figs 1, 2, 6-19).

*Nebria (Boreonebria) nivalis* (Paykull): Andersen (1970: 91-93; Figs 6a-d) [L_1-3_; FC]; Luff (1993: 50, 52; Figs 137-142) [L_1-3_; FC].

*Nebria (Reductonebria) pallipes* Say: Spence (1974: 13, 17; Fig. 4) [L_1_, L_2_, L_3_; RR]; Spence et al. (1976: 81; Figs 1, 3b) [L_1_, L_2_, L_3_; RR].

*Nebria* prob. *schwarzi* (Alberta): Garner (1954: 186, 202, 203) [L_2-3_; FC].

*Nebria* prob. *trifaria* (Oregon): Garner (1954: 189, 204, 205) [L_2-3_; FC].

*Nebria* *sp.* A (Washington, D.C.): Garner (1954: 189-191) [L_2-3_; FC].

*Nebria* *sp.* B (California): Garner (1954: 189, 191, 192) [L_2-3_; FC].

*Nebria* *sp.* C (Utah): Garner (1954: 189, 192-194) [L_2-3_; FC].

*Nebria* *sp.* D (Oregon): Garner (1954: 189, 194, 195) [L_2-3_; FC].

*Nebria* *sp.* E (Oregon): Garner (1954: 189, 196, 197) [L_2-3_; FC].

*Nebria* *sp.* F (Tennessee): Garner (1954: 189, 197-199) [L_2-3_; FC].

*Nebria* *sp.* G (Montana): Garner (1954: 189, 199, 200) [L_2-3_; FC].

*Nebria* *sp.* H (Illinois): Garner (1954: 189, 200-202) [L_2-3_; FC].

*Nebria* *sp.* (Colorado): Thompson (1979a: Figs 36a-c).

*Nebria* *sp.*: Bousquet and Goulet (1984: Figs 27, 48); Bousquet (1991a: Figs 34.77, 34.101); Bousquet (2010a: Figs 377, 380, 382-385).

### Notiophilini

*Notiophilus aquaticus* (Linnaeus): Schiødte (1867: 456; Fig. 19 [Pl. 13]) [L_1-3_; ?]; Larsson (1941: 283) [L_1-3_; ?]; Garner (1954: 174, 175) [L_2-3_; FC]; Sharova (1958: 38) [L_1-3_; ?]; Davies (1964: 208) [L_1-3_; LIT]; Sharova (1964: 145) [L_1-3_; ?]; Larsson (1968: 326-328) [L_1-3_; ?]; Haberman (1968: 135) [L_2-3_; ?]; Luff (1976: 52, 59; Figs 17-19) [L_1_, L_3_; FC]; Arndt (1991b: 74) [L_1-3_; ?]; Luff (1993: 61, 62; Figs 168-170) [L_1_, L_2-3_; ?].

*Notiophilus biguttatus* (Fabricius)**†**: Schiødte (1867: 456; Figs 12-17 [Pl. 13]) [L_1-3_; ?]; Saalas (1923: 667) [L_1-3_; ?]; Larsson (1941: 283, 284; Figs 20, 22a) [L_1-3_; ?]; Jeannel (1941b: Figs 75a-d); van Emden (1942: Fig. 4); Garner (1954: 176, 177) [L_2-3_; FC]; Sharova (1958: 39; Fig. 86a) [L_1-3_; ?]; Davies (1964: 207, 208; Fig. 1) [L_1_, L_2-3_; FC]; Sharova (1964: 146; Fig. 130-3) [L_1-3_; ?]; Larsson (1968: 327, 328; Figs 20, 22a) [L_1-3_; ?]; Haberman (1968: 135; Fig. 68-3) [L_2-3_; ?]; Luff (1976: 52, 53, 55; Figs 1-10) [L_1_, L_2-3_; FC]; Arndt (1991b: 74) [L_1-3_; ?]; Luff (1993: 61, 64; Figs 178-183) [L_1_, L_2-3_; ?].

*Notiophilus novemstriatus* LeConte: Thompson and Allen (1974: 186-189; Figs 1a-c) [L_2-3_; FC]; Thompson (1979a: Figs 38a-b).

*Notiophilus palustris* (Duftschmid)**†**: Larsson (1941: 283, 284) [L_1-3_; ?]; Sharova (1958: 39; Fig. 80) [L_1-3_; ?]; Davies (1964: 207, 208) [L_1_, L_2-3_; FC]; Sharova (1964: 146; Fig. 127) [L_1-3_; ?]; Larsson (1968: 327-329) [L_1-3_; ?]; Haberman (1968: 136) [L_2-3_; ?]; Luff (1976: 52, 56; Figs 12, 13) [L_2_; FC]; Arndt (1991b: 74; Fig. 64) [L_1-3_; ?]; Luff (1993: 61-63) [L_1-3_; ?].

*Notiophilus* *sp.* A (California): Garner (1954: 169, 170; Figs a-c [Pl. 10]) [L_2-3_; FC].

*Notiophilus* *sp.* B (Illinois): Garner (1954: 170-172) [L_2-3_; FC].

*Notiophilus* *sp.* C (Illinois): Garner (1954: 172-174) [L_1_, L_2-3_; FC].

*Notiophilus* *sp.* D (Oregon): Garner (1954: 177, 178) [L_2-3_; FC].

*Notiophilus* *sp.* E (Texas): Garner (1954: 179, 180) [L_2-3_; FC].

*Notiophilus* *sp.*: Bousquet (2010a: Fig. 381).

### Cychrini

*Scaphinotus (Nomaretus) bilobus* (Say): Bousquet (1977: 4.9; Figs 12, 13) [L_3_; FC]; Bousquet (2010a: 396; Fig. 396) [L_3_; FC].

*Scaphinotus (Scaphinotus) elevatus* (Fabricius): Wickham (1895b: 763; Fig. 1) [L_3_; FC].

*Scaphinotus (Maronetus) hubbardi* (Schwarz): Lapouge (1929a: 60) [L_2-3_; ?].

*Scaphinotus (Pseudonomaretus) mannii* Wickham: Greene (1975: Figs 2-3).

*Scaphinotus (Brennus) marginatus* (Fischer von Waldheim): Garner (1954: 58, 67, 68) [L_2-3_; FC].

*Scaphinotus (Irichroa) viduus* (Dejean): Bousquet (1977: 4.4-4.8; Figs 1-11) [L_2-3_; FC]; Bousquet (2010a: 396; Figs 390, 394) [L_2-3_; FC].

*Scaphinotus (Brennus)* *sp.* A (California): Garner (1954: 58, 59) [L_2-3_; FC].

*Scaphinotus (Brennus)* *sp.* B (California): Garner (1954: 55, 60, 61) [L_2-3_; FC].

*Scaphinotus (Brennus)* *sp.* C (California): Garner (1954: 58, 61, 62; Figs a-c [Pl. 2]) [L_2-3_; FC].

*Scaphinotus (Brennus)* *sp.* D (California): Garner (1954: 55, 63, 64) [L_2-3_; FC].

*Scaphinotus* (*Nomaretus*?) *sp*. E (Pennsylvania, North Carolina): Garner (1954: 55, 64, 65) [L_2-3_; FC].

*Scaphinotus* *sp.* F (Massachusetts): Garner (1954: 58, 66, 67) [L_2-3_; FC].

*Scaphinotus* (*Nomaretus* ?) *sp*. (Pennsylvania): van Emden (1942: 56) [L_2-3_; FC].

*Scaphinotus* *sp.* (Arkansas): Thompson (1979a: Figs 32a-b, as *Sphaeroderus* *sp.*).

*Sphaeroderus canadensis* Chaudoir: Bousquet (1977: 1.9; Figs 2, 3, 8, 16) [L_1_, L_2_, L_3_; RR+FC]; Bousquet (2010a: 398) [L_2-3_; RR+FC].

*Sphaeroderus nitidicollis* Guérin-Méneville: Lindroth (1955a: Fig. 7d); Bousquet (1977: 1.9, 1.10; Figs 5, 7, 17, as *Sphaeroderus nitidicollis brevoorti*) [L_1_, L_2_, L_3_; RR+FC]; Bousquet (2010a: 398) [L_2-3_; RR+FC].

*Sphaeroderus stenostomus lecontei* Dejean: Lindroth (1955a: Fig. 7c); Bousquet (1977: 1.9, 1.10; Figs 4, 6, 9-15, 18-20) [L_1_, L_2_, L_3_; RR+FC]; Bousquet (1991a: Figs 34.75, 34.79, 34.87, 34.93, 34.96, 34.99, 34.102); Bousquet (2010a: 398; Figs 391-393, 395, 397) [L_2-3_; RR+FC].

### Carabini

*Calosoma (Chrysostigma) affine* Chaudoir: Garner (1954: 91, 108, 109) [L_2-3_; FC].

*Calosoma (Chrysostigma) calidum* (Fabricius): Schaupp (1882d: 33-34) [L_3_; FC]; Burgess (1897: 426-428; Figs 10 [Pl. 3], 3, 5-6 [Pl. 4]) [L_1_, L_2_, L_3_; RR]; Lapouge (1905: 172) [L_3_; ?RR]; Lapouge (1908b: 164) [L_1-3_; LIT]; Burgess and Collins (1917: 19, 101; Figs a-d [Pl. 18], a-b [Pl. 19]) [L_1_, L_2_, L_3_; RR]; Garner (1954: 91, 100-102) [L_2-3_; FC]; Kirk (1972: 1355; Figs 7 [Pl. 1], 3-7) [L_1-3_; FC]; Bousquet (2010a: 399; Fig. 403) [L_2-3_; RR+FC].

*Calosoma (Chrysostigma) cancellatum* Eschscholtz: Burgess and Collins (1917: 19, 112, 113; Fig. g [Pl. 17]) [L_1_, L_2_, L_3_; RR]; Garner (1954: 91, 99, 100) [L_2-3_; FC].

*Calosoma (Callitropa) externum* (Say): Burgess and Collins (1917: 20, 23, 24) [L_1_, L_2_, L_3_; RR]; Lapouge (1927: 438) [L_2-3_; ?]; Lapouge (1929a: 56) [L_2-3_; ?]; Garner (1954: 90, 113, 114) [L_2-3_; FC].

*Calosoma (Calosoma) frigidum* Kirby: Burgess (1897: 420-425; Figs 3-9 [Pl. 3], 1, 4, 7 [PL. 4]) [L_1_, L_2_, L_3_; RR]; Lapouge (1905: 172) [L_3_; ?RR]; Lapouge (1908b: 163) [L_1-3_; LIT]; Burgess and Collins (1917: 19, 48-50) [L_1_, L_2_, L_3_; RR]; Lapouge (1927: 439) [L_2-3_; ?]; Lapouge (1929a: 57) [L_2-3_; ?]; Garner (1954: 90, 93, 94) [L_2-3_; FC]; Bousquet and Goulet (1984: Figs 40, 45, 47); Bousquet (1991a: Fig. 34.86); Bousquet (2010a: 399; Figs 398-402, 404, 405) [L_2-3_; RR+FC].

*Calosoma (Blaptosoma) haydeni* Horn: Burgess and Collins (1917: 20, 96; Fig. e [Pl. 17]) [L_1_, L_2_, L_3_; RR]; Garner (1954: 91, 106, 107) [L_2-3_; FC].

*Calosoma (Chrysostigma) lepidum* LeConte: Lapouge (1927: 439) [L_2-3_; ?]; Lapouge (1929a: 58) [L_2-3_; ?].

*Calosoma (Camegonia) marginale* Casey: Burgess and Collins (1917: 20, 83, as *Calosoma lugubre*) [L_1_, L_2_, L_3_; RR]; Lapouge (1927: 439, as *Calosoma lugubris*) [L_2-3_; ?]; Lapouge (1929a: 57, as *Calosoma lugubris*) [L_2-3_; ?]; Garner (1954: 90, 109-111, as *Calosoma lugubre*) [L_2-3_; FC].

*Calosoma (Chrysostigma) obsoletum* Say: Burgess and Collins (1917: 19, 89; Fig. d [Pl. 17]) [L_1_, L_2_, L_3_; RR]; Garner (1954: 91, 104-106) [L_2-3_; FC].

*Calosoma (Carabosoma) peregrinator* Guérin-Méneville: Burgess and Collins (1917: 20, 77) [L_1_, L_3_; RR]; Lapouge (1927: 438) [L_2-3_; ?]; Lapouge (1929a: 56) [L_2-3_; ?]; Garner (1954: 90, 111, 112) [L_2-3_; FC].

*Calosoma (Castrida) sayi* Dejean: Lapouge (1927: 437, 438) [L_2-3_; ?]; Lapouge (1929a: 56) [L_2-3_; ?]; Thompson (1979a: Figs a-d).

*Calosoma (Calodrepa) scrutator* (Fabricius): Chapuis and Candèze (1853: 372, 373; Fig. 4 [Pl. 1]) [L_1-3_; FC]; Schaupp (1879: 14) [L_2-3_; LIT]; Burgess and Collins (1917: 19, 31; Figs a-d [Pl. 8], a,b [Pl. 9]) [L_1_, L_2_, L_3_; RR]; Lapouge (1927: 440) [L_2-3_; ?]; Lapouge (1929a: 59) [L_2-3_; ?]; Garner (1954: 90, 95, 96; Figs a-c [Pl. 4]) [L_2-3_; FC].

*Calosoma (Chrysostigma) semilaeve* LeConte: Burgess and Collins (1917: 19, 92; Fig. f [Pl. 17]) [L_1_, L_2_, L_3_; RR]; Garner (1954: 91, 102-104) [L_2-3_; FC].

*Calosoma (Calosoma) sycophanta* (Linnaeus)**†**: Burmeister (1837: 235, 236; Figs 1-5 [Pl. 13]) [L_1-3_; FC]; Lapouge (1905: 172) [L_3_; ?RR]; Lapouge (1908b: 159, 160) [L_1-3_; FC]; Burgess (1911: 26, 27) [L_1_, L_2_, L_3_; RR]; Burgess and Collins (1915: 7, 8) [L_1-3_; RR]; Burgess and Collins (1917: 18, 19, 64, 65; Figs a-d [Pl. 10], Figs a,b [Pl. 11]) [L_1_, L_2_, L_3_; RR]; Lapouge (1927: 440) [L_2-3_; ?]; Lapouge (1929a: 59) [L_2-3_; ?]; Larsson (1941: 263-265; Figs 9b, 10b) [L_1-3_; ?]; Jeannel (1941b: Fig. 61k); Garner (1954: 90, 97, 98) [L_2-3_; FC]; Sharova (1957: 882; Figs 1a, 2a, 3a) [L_1-3_; LIT]; Sharova (1958: 28; Figs 69a, 71a) [L_1-3_; ?]; Sturani (1962: Fig. 38); Sharova (1964: 135; Figs 118-1, 120-1) [L_1-3_; ?]; Larsson (1968: 306, 308; Figs 9b, 10b) [L_1-3_; ?]; Haberman (1968: 114, 115; Figs 55-1, 55-6) [L_2-3_; ?]; Thompson (1979a: Figs 13a-c); Casale et al. (1982: 82-83; Fig. 26b) [L_1-3_; ?]; Arndt (1991b: 62; Fig. 29) [L_1-3_; LIT]; Luff (1993: 33, 34; Figs 58-62) [L_1_, L_2-3_; ?].

*Calosoma (Calodrepa) wilcoxi* LeConte: Burgess and Collins (1917: 19, 41, 42) [L_1_, L_2_, L_3_; RR]; Lapouge (1927: 439, 440) [L_2-3_; ?]; Lapouge (1929a: 58) [L_2-3_; ?]; Garner (1954: 90-93) [L_2-3_; FC].

*Carabus (Tachypus) auratus* Linnaeus**†**: Laboulbène (1862: 562, 563, as *Calathus gallicus*, see Raynaud 1940a; Figs 8-15 [Pl. 13]) [L_3_; FC]; Lapouge (1905: 172) [L_3_; ?RR]; Lapouge (1907: 52, 53) [L_1_; RR]; Lengerken (1921: 82-111; Figs 1-3, 7-21, 23-31, 33-51, 57-70) [L_1_, L_2_, L_3_; RR]; Verhoeff (1921: 185, 188) [L_2-3_; ?]; van Emden (1925; Fig. 1); Bengtsson (1927: 37, 43-46; Figs 14, 15, 22-26) [L_1_, L_3_; ?FC]; Lapouge (1927: 447) [L_2-3_; ?]; Lapouge (1929a: 53) [L_2-3_; ?]; Sturani (1962: Figs 37, 38); Hůrka (1971: 23, 24, 44, 46, 47; Figs 13a-o) [L_1_, L_2_, L_3_; FC]; Raynaud (1976a: 38; Pl. 71) [L_1-3_; ?]; Arndt (1985: 50; Figs 13, 64, 66, 70, 104) [L_1-3_; ?]; Arndt (1991b: 64) [L_1-3_; LIT]; Luff (1993: 38) [L_1-3_; ?].

*Carabus (Diocarabus) chamissonis* Fischer von Waldheim: **?**Garner (1954: 75, 81, 82) [L_2-3_; FC]; Lindroth (1955a: Figs 8b,e); Bousquet (2010a: 401; Figs 406, 413) [L_2-3_; FC].

*Carabus (Carabus) goryi* Dejean: Garner (1954: 75, 79, 80, as *Carabus limbatus*) [L_2-3_; FC].

*Carabus (Carabus) granulatus* Linnaeus**†**: Schiødte (1867: 493-494; Figs 13-15 [Pl. 17]) [L_1-3_; ?]; Lapouge (1905: 172) [L_3_; ?RR]; Lapouge (1908b: 152, 153) [L_1-3_; RR]; Verhoeff (1917: 42) [L_1-3_; ?]; Verhoeff (1921: 175, 176, 184, 187; Fig. 7) [L_2_, L_3_; RR]; Oertel (1924: 326-356; Figs h, j-z, a^1^-h^1^, j^1^) [L_1_, L_2_, L_3_; RR]; Bengtsson (1927: 38, 57-59; Figs 46-50) [L_1_, L_2-3_; FC]; Lapouge (1927: 448) [L_2-3_; ?]; Lapouge (1929a: 54) [L_2-3_; ?]; Larsson (1941: 267, 269, 270; Fig. 12a) [L_1-3_; ?]; Garner (1954: 75, 85, 86) [L_2-3_; FC]; Sharova (1958: 32; Figs 7, 72h, 73d, 76g, 79e) [L_1-3_; ?]; Sturani (1962: Fig. 37); Sharova (1964: 139; Figs 89-1, 121-8, 122-4, 124-7, 126-5) [L_1-3_; ?]; Larsson (1968: 310, 313; Fig. 12a) [L_1-3_; ?]; Haberman (1968: 95; Figs 35-5, 37-a, 38-1, 41-4) [L_2-3_; ?]; Luff (1969: 251, 255; Figs 40-46) [L_1-3_; FC]; Hůrka (1971: 23, 24, 54-56; Figs 16a-u) [L_1_, L_2_, L_3_; FC]; Raynaud (1976a: 107; Pls 90, 91, 107) [L_1-3_; ?]; Casale et al. (1982: 285; Fig. 103) [L_1-3_; ?]; Arndt (1985: 52; Figs 14, 68b, 105) [L_1-3_; ?]; Arndt (1991b: 66) [L_1-3_; LIT]; Luff (1993: 38, 42, 43; Figs 101-103) [L_1-3_; ?]; Turin et al. (1993; Figs 4a,c,e); Huk and Kühne (2000: Fig. 1); Bousquet (2010a: 401; Fig. 407) [L_2-3_; FC].

*Carabus (Homoeocarabus) maeander* Fischer von Waldheim: Lindroth (1955a: Figs 8a,d); Raynaud (1976a: 107) [L_1-3_; ?]; Bousquet (2010a: 401; Figs 408-410) [L_2-3_; FC].

*Carabus (Archicarabus) nemoralis* Müller**†**: Schiødte (1867: 490-491; Figs 9-11 [Pl. 16]) [L_1-3_; ?]; Zang (1901: 274-275; Figs 2-5) [L_1_; RR]; Lapouge (1905: 172) [L_3_; ?RR]; Lapouge (1908b: 150, 151) [L_1-3_; RR]; Verhoeff (1917: 42, 43) [L_1-3_; ?]; Verhoeff (1921: 186-188; Figs j-1, q [Pl. 9]) [L_2-3_; ?]; van Emden (1925; Fig. 2); Bengtsson (1927: 38, 51-54; Figs 1, 2, 37-41) [L_1_, L_2-3_; RR]; Lapouge (1927: 448) [L_2-3_; ?]; Lapouge (1929a: 53) [L_2-3_; ?]; Delkeskamp (1930: 30-54; Figs 12-40) [L_1_, L_2_, L_3_; RR]; Larsson (1941: 267, 269; Figs 7, 11c) [L_1-3_; ?]; Garner (1954: 75-77) [L_2-3_; FC]; Sharova (1958: 31; Figs 72a, 74a, 76a, 79a) [L_1-3_; ?]; Sturani (1962: Fig. 44b); Sharova (1964: 137, 138; Figs 121-1, 123-1, 124-1, 126-1) [L_1-3_; ?]; Larsson (1968: 310, 312; Figs 7, 11c) [L_1-3_; ?]; Haberman (1968: 93, 95; Figs 35-1, 38-7, 39-1, 41-1) [L_2-3_; ?]; Luff (1969: 251, 255, 257; Figs 47-52) [L_1-3_; FC]; Raynaud (1969: 24) [L_3_; ?]; Sturani (1971: Fig. 2); Hůrka (1971: 22, 25, 62, 64, 65; Figs 19a-o) [L_1_, L_2_, L_3_; FC]; Raynaud (1976a: 66; Pl. 76) [L_1-3_; ?]; Hůrka (1978: Fig. H-2); Casale et al. (1982: 177; Fig. 71b) [L_1_; FC]; Arndt (1985: 52; Figs 1, 18, 68a, 107, 110) [L_1-3_; ?]; Arndt (1991b: 66; Figs 26, 39, 51) [L_1-3_; LIT]; Makarov (1992: Fig. 9e); Luff (1993: 38, 42; Figs 6, 98-100) [L_1-3_; ?]; Deuve (1994a: Fig. 36); Bousquet (2010a: 401) [L_2-3_; FC].

*Carabus (Hemicarabus) serratus* Say: Lapouge (1927: 446) [L_2-3_; FC]; Lapouge (1929a: 50) [L_2-3_; FC]; Lindroth (1955a: Figs 8c,f); Raynaud (1975: 308) [L_1-3_; ?]; Bousquet (2010a: 401; Fig. 411) [L_2-3_; FC].

*Carabus (Tanaocarabus) sylvosus* Say: Lapouge (1927: 446) [L_2-3_; FC]; Lapouge (1929a: 50) [L_2-3_; FC]; Garner (1954: 75, 77-79; Figs a-c [Pl. 3]) [L_2-3_; FC]; Raynaud (1975: 264; Pl. 14) [L_1-3_; ?]; Thompson (1979a: Figs 29a-b); Bousquet (2010a: 402; Fig. 412) [L_2-3_; FC].

*Carabus (Tanaocarabus) taedatus* Fabricius: **?**Lapouge (1927: 446) [L_2-3_; FC]; Lapouge (1929a: 50) [L_2-3_; FC]; **?**Garner (1954: 75, 83, 84) [L_2-3_; FC]; Sturani (1971: Fig. 3t); Raynaud (1975: 222; Pl. 5) [L_1-3_; ?].

*Carabus (Aulonocarabus) truncaticollis* Eschscholtz: Kincaid (1900: 368, 369; Figs 1-6 [Pl. 22]) [L_2-3_; FC]; Lapouge (1907: 34) [L_3_; LIT]; Lapouge (1927: 445) [L_2-3_; ?]; Lapouge (1929a: 50) [L_2-3_; ?]; Garner (1954: 75) [L_2-3_; LIT]; Raynaud (1975: 260; Pl. 12) [L_1-3_; ?]; Makarov (1992: Figs 10b,g).

*Carabus (Megodontus) vietinghoffii* Adams: Lapouge (1927: 443) [L_2-3_; ?]; Raynaud (1976a: 14; Pl. 57) [L_1-3_; ?].

*Carabus* *sp.* N^o^ 19 (Alabama): Lapouge (1908b: 164) [L_1_; FC].

### Amblycheilini

*Amblycheila baroni* Rivers: Knisley and Pearson (1984: 502; Figs 25-31) (L_3_; FC].

*Amblycheila cylindriformis* (Say): Horn (1878a: 29-31; Figs 1a-g [Pl. II]) [L_2-3_; FC]; Riley et al. (1878: Figs 53a-h); Schaupp (1879: 3) [L_2-3_; LIT]; Hamilton (1925: 70; Figs 4, 19, 11, 16, 20, 26, 31, 36, 41, 81, 123, 148) [L_2-3_; RR]; Böving and Craighead (1931: Figs b,d [Pl. 4]).

*Amblycheila schwarzi* Horn**?**: Horn (1908a: 285, 286; Figs 1-5) [L_2-3_; FC]; Putchkov and Arndt (1994: Figs 6, 13, 23, 44, 46, 50).

*Omus audouini* Reiche: Hamilton (1925: 66-68, as *Omus ambiguus* [see Leffler 1986b: 54]); Figs 47, 86, 121) [L_2-3_; RR]; Leffler (1979a: 70-71; Fig. 7a) [L_3_; FC]; Leffler (1986b: 55, 62; Fig. 2) [L_3_; FC].

*Omus californicus* Eschscholtz: Hamilton (1925: 66-67; Figs 3, 12, 13, 19, 25, 30, 35, 44, 46, 82-84, 118, 119) [L_2-3_; RR]; Hamilton (1925: 69, as *Omus edwardsii* [see Leffler 1986b: 54]) [_L2-3_; FC]; Hamilton (1925: 66, 68, 69, as *Omus sequoiarum* [see Leffler 1986b: 54]; Figs 45, 85, 120) [L_2-3_; RR]; Böving and Craighead (1931: Fig. e [Pl. 4]); van Emden (1935: Fig. 1); Leffler (1986b: 55-56, 62) [L_3_; FC]; Leffler (1979a: Fig. 4a).

*Omus dejeanii* Reiche: Horn (1878a: 31-33; Figs 2a-e [Pl. II]) [L_2-3_; FC]; Schaupp (1879: 3) [L_2-3_; LIT]; Leffler (1979a: 68-69) [L_1_, L_2_, L_3_; FC]; Leffler (1986b: 56-57, 62; Fig. 1) [L_3_; FC].

*Omus submetallicus* Horn: Leffler (1986b: 57-58, 62) [L_3_; FC].

*Omus* *sp.*: Putchkov and Arndt (1994: Fig. 24).

### Megacephalini

*Tetracha (Tetracha) carolina* (Linnaeus): Horn (1878a: 34-35; Figs 3a-e [Pl. II]) [L_2-3_; FC]; Schaupp (1879: 3) [L_2-3_; LIT]; Hamilton (1925: 59-60; Figs 2, 9, 15, 18, 24, 29, 34, 43, 80, 116) [L_2-3_; RR]; Putchkov and Arndt (1997: 61) [L_3_; LIT]; Leonard and Bell (1999: 27, 153; Figs 35-37, 148, 149) [L_3_; LIT].

*Tetracha (Tetracha) virginica* (Linnaeus): Hamilton (1925: 60-61; Figs 79, 117) [L_2-3_; RR]; Putchkov and Arndt (1997: 61) [L_3_; LIT].

### Cicindelini

*Cicindela (Cicindelidia) abdominalis* Fabricius: Hamilton (1925: 19, 44-45; Figs 72, 109, 139) [L_2-3_; FC]; Spangler (1956: 84) [L_2-3_; LIT]; Willis (1967: 173) [L_2-3_; LIT].

*Cicindela (Cicindelidia) amargosae* Dahl: Leffler (1979a: 86-87) [L_2-3_; FC].

*Cicindela (Cicindela) ancocisconensis* Harris: Leonard and Bell (1999: 33, 63; Figs 76-78) [L_3_; FC]; Bousquet (2010a: 405) [L_2-3_; LIT].

*Cicindela (Cicindela) arenicola* Rumpp: Rumpp (1967: 133; Figs 1[a-b]) [L_3_; FC]; Shook and Clark (1988: Fig. 2).

*Cicindela (Cicindela) bellissima* Leng: Leffler (1979a: 76-77; Fig. 5e) [L_1-2_, L_3_; FC].

*Cicindela (Cicindela) decemnotata* Say: Leffler (1979a: 80-81) [L_3_; FC].

*Cicindela (Cicindela) denikei* Brown: Kaulbars and Freitag (1993b: 45, 46, 48; Figs 1-7) [L_3_; FC].

*Cicindela (Cicindela) depressula* Casey: Leffler (1979a: 73-74) [L_1-2_, L_3_; FC].

*Cicindela (Cicindela) duodecimguttata* Dejean: Shelford (1908: 170, 172, 173; Figs 37-39) [L_1_, L_2_, L_3_; RR]; Hamilton (1925: 19, 39-40; Figs 68, 105, 135) [L_2-3_; RR]; Leonard and Bell (1999: 33, 97; Figs 51, 104, 105) [L_3_; LIT]; Bousquet (2010a: 405) [L_2-3_; LIT].

*Cicindela (Cicindela) formosa* Say: Shelford (1908: 170, 172, 173; Figs 40-42) [L_1_, L_2_, L_3_; RR]; Hamilton (1925: 19, 46-48; Figs 49-51, 74, 88-90, 111, 141, 142) [L_2-3_; FC]; Gaumer^11^ (1977: 182, 190-193, 205, 206, 211-213, 217-219, 223-225, 230, 231; Figs 45d, 46d, 47d, 48d, 49d, 50c) [L_1_, L_2_, L_3_; FC]; Leonard and Bell (1999: 34, 58; Figs 41, 53, 72, 73) [L_3_; LIT]; Bousquet (2010a: 404) [L_2-3_; LIT].

*Cicindela (Cicindela) fulgida* Say: Willis (1967: 165-168, 173; Figs 41-46) [L_3_; FC].

*Cicindela (Cicindelidia) hemorrhagica* LeConte: Leffler (1979a: 88-89) [L_3_; FC]; Knisley and Pearson (1984: 497-499, 533-534; Figs 86-91) [L_1_, L_2_, L_3_; RR+FC].

*Cicindela (Cicindela) hirticollis* Say: Shelford (1908: 172, 173) [L_1_, L_2_, L_3_; RR]; Hamilton (1925: 20, 55; Figs 55-57, 92, 93, 146) [L_2-3_; RR]; Leffler (1979a: 75; Fig. 5f) [L_2_, L_3_; FC]; Thompson (1979a: Figs 12a-e); Leonard and Bell (1999: 36, 80, 81; Figs 57, 92, 93) [L_3_; LIT]; Bousquet (2010a: 404; Fig. 418) [L_2-3_; LIT].

*Cicindela (Cicindelidia) hornii* Schaupp: Knisley and Pearson (1984: 496-498, 511-514; Figs 44-49) [L_1_,L_2_, L_3_; RR+FC].

*Cicindela (Cicindela) latesignata* LeConte: Hamilton (1925: 18, 29; Figs 61, 98, 128) [L_2-3_; FC].

*Cicindela (Cicindela) lengi* Horn: Leffler (1979a: 89-90) [L_3_; FC].

*Cicindela (Cicindela) limbalis* Klug: Shelford (1908: 172, 173; Figs 49-51) [L_1_, L_2_, L_3_; RR]; Hamilton (1925: 18, 25-26; Figs 1, 5-8, 14, 17, 21-23, 27, 28, 32, 33, 37-40, 42, 52-54, 96, 126) [L_2-3_; RR]; Böving and Craighead (1931: Fig. c [Pl. 4]); **?**Bousquet and Goulet (1984: Fig. 28); Leonard and Bell (1999: 30, 119; Figs 44, 122-124) [L_3_; LIT]; Bousquet (2010a: 405; Figs 421, 423) [L_2-3_; LIT].

*Cicindela (Cicindela) limbata* Say: Hamilton (1925: 20, 56-57; Figs 77, 114, 145) [L_2-3_; FC]; Leonard and Bell (1999: 36, 89; Figs 101, 102) [L_3_; LIT].

*Cicindela (Cicindela) longilabris* Say: Leffler (1979a: 81-82) [L_2_; FC]; Spanton (1988: 99; Figs 28-32) [L_1-3_; RR]; Leonard and Bell (1999: 32, 107, 108; Figs 113, 114) [L_2_, L_3_; LIT]; Bousquet (2010a: 405) [L_2-3_; LIT].

*Cicindela (Cicindela) nebraskana* Casey: Leffler (1979a: 83-84) [L_3_; FC]; Brust and Hoback (2010: 345; Figs 3-8) [L_1_, L_2_, L_3_; RR].

*Cicindela (Cicindelidia) nigrocoerulea* LeConte: Knisley and Pearson (1984: 497-499, 528-530; Figs 11, 74-79) [L_1_, L_2_, L_3_; RR].

*Cicindela (Cicindelidia) obsoleta* Say: Hamilton (1925: 18, 34-35, as *Cicindela* species D [see Knisley and Pearson (1984: 507)]; Figs 152, 164, 178) [L_2-3_; FC]; Knisley and Pearson (1984: 496-498, 505, 507; Figs 32-37) [L_1_, L_2_, L_3_; RR+FC].

*Cicindela (Cicindelidia) ocellata* Klug: Hamilton (1925: 19, 42-43, as *Cicindela flavopunctata rectilatera*; Figs 70, 107, 137) [L_2-3_; RR]; Knisley and Pearson (1984: 497, 499, 536-537; Figs 18, 21, 92-97) [L_1_, L_2_, L_3_; RR+FC].

*Cicindela (Cicindela) oregona* LeConte: Hamilton (1925: 18, 38; Figs 67, 104, 134) [L_2-3_; FC]; Leffler (1979a: 72; Figs 5b, 7b-c) [L_2_, L_3_; FC].

*Cicindela (Cicindela) parowana* Wickham: Leffler (1979a: 84-85) [L_3_; FC].

*Cicindela (Cicindela) patruela* Dejean: Willis (1982: 49, 56; Figs 1-6) [L_3_; RR]; Leonard and Bell (1999: 33, 46, 47; Figs 63-65) [L_3_; LIT].

*Cicindela (Cicindela) pimeriana* LeConte: Knisley and Pearson (1984: 496, 498, 519-520; Figs 13, 56-61) [L_1_, L_2_, L_3_; RR].

*Cicindela (Cicindela) pugetana* Casey: Leffler (1979a: 78-79) [L_3_; FC].

*Cicindela (Cicindela) pulchra* Say: Hamilton (1925: 19, 51; Figs 76, 113, 144) [L_2-3_; FC]; Hamilton (1925: 19, 51-52, as *Cicindela obsoleta* [see Knisley and Pearson (1984: 507)]; Figs 158, 169, 179) [L_2-3_; FC]; Knisley and Pearson (1984: 496-498, 508-509; Figs 1-9, 22-24, 38-43) [L_1_, L_2_, L_3_; RR+FC].

*Cicindela (Cicindelidia) punctulata* Olivier: Shelford (1908: 172, 173) [L_1_, L_2_, L_3_; RR]; Hamilton (1925: 19, 41; Figs 69, 106, 136) [L_2-3_; RR]; Knisley and Pearson (1984: 497-499, 525-526; Figs 68-73) [L_1_, L_2_, L_3_; RR]; Leonard and Bell (1999: 34, 112; Figs 52, 117, 118) [L_3_; LIT]; Bousquet (2010a: 405) [L_2-3_; LIT].

*Cicindela (Cicindela) purpurea* Olivier: Shelford (1908: 170, 172-173; Figs 7-11, 25-27) [L_1_, L_2_, L_3_; RR]; Hamilton (1925: 18, 27; Figs 60, 97, 127) [L_2-3_; RR]; Leffler (1979a: 77-78; Fig. 5c) [L_3_; FC]; Leonard and Bell (1999: 31, 124; Figs 47, 128, 129) [L_3_; LIT]; Bousquet (2010a: 405) [L_2-3_; LIT].

*Cicindela (Cicindela) repanda* Dejean: Horn (1878a: 35-37; Figs a-d [Pl. II]) [L_2-3_; FC]; Schaupp (1879: 13) [L_2-3_; LIT]; Schaupp (1884b: 123; Fig. 124) [L_2-3_; FC]; Shelford (1908: 170, 172, 173; Figs 34-36) [L_1_, L_2_, L_3_; RR]; Hamilton (1925: 18, 31; Figs 63, 100, 130) [L_2-3_; RR]; Leffler (1979a: 71; Fig. 5a) [L_2_, L_3_; FC]; Leonard and Bell (1999: 32, 53, 54; Figs 49, 68, 69) [L_3_; LIT]; Bousquet (2010a: 405; Figs 420, 422) [L_2-3_; LIT].

*Cicindela (Cicindelidia) rufiventris* Dejean: Beatty and Knisley (1984: 4-8; Figs 1-11) [L_1_, L_2_, L_3_; RR]; Leonard and Bell (1999: 32, 103, 104; Figs 46, 48, 109, 110) [L_3_; LIT].

*Cicindela (Cicindela) scutellaris* Say: Shelford (1908: 170-173; Figs 46-48) [L_1_, L_2_, L_3_; RR]; Hamilton (1925: 19, 48-49; Figs 75, 112, 143) [L_2-3_; RR]; Leonard and Bell (1999: 35, 131, 132; Figs 55, 133, 134) [L_3_; LIT]; Bousquet (2010a: 404; Fig. 417) [L_2-3_; LIT].

*Cicindela (Cicindelidia) sedecimpunctata* Klug: Knisley and Pearson (1984: 497, 539; Figs 116-119) [L_3_; RR].

*Cicindela (Cicindela) sexguttata* Fabricius: Shelford (1908: 170, 172, 173; Figs 28-30) [L_1_, L_2_, L_3_; RR]; Hamilton (1925: 17, 23-24; Figs 58, 94, 124) [L_2-3_; RR]; Willis (1967: 172) [L_2-3_; LIT]; Kaulbars and Freitag (1993b: 48) [L_3_; LIT]; Leonard and Bell (1999: 29, 41, 42; Figs 59, 60) [L_3_; LIT]; Bousquet (2010a: 405) [L_2-3_; LIT].

*Cicindela (Cicindelidia) tenuisignata* LeConte: Knisley and Pearson (1984: 497-499, 530-531; Figs 80-85) [L_1_, L_2_, L_3_; RR].

*Cicindela (Cicindela) theatina* Rotger: Pineda and Kondratieff (2002: 166, 170, 171; Figs 1-8) [L_1_, L_2_, L_3_; RR+FC].

*Cicindela (Cicindela) tranquebarica* Herbst: Shelford (1908: 172-173; Figs 43-45) [L_1_, L_2_, L_3_; RR]; Hamilton (1925: 18, 36; Figs 66, 103, 133) [L_2-3_; RR]; Leffler (1979a: 91) [L_3_; FC]; Leonard and Bell (1999: 33, 69; Figs 82, 83) [L_3_; LIT]; Bousquet (2010a: 405; Fig. 424) [L_2-3_; LIT].

*Cicindela (Cicindelidia) willistoni* LeConte: Willis (1967: 172, 173; Figs 59-64) [L_3_; FC]; Leffler (1979a: 87) [L_3_; FC]; Knisley and Pearson (1984: 497, 498, 514, 515; Figs 120-121) [L_1_, L_2_; FC].

*Cicindela* *sp.* A (California; probably *Cicindela trifasciatus sigmoidea*): Hamilton (1925: 17, 22-23; Figs 59, 95, 125) [L_2-3_; FC].

*Cicindela* *sp.* B (Colorado; possibly *Cicindela cimarrona* or *Cicindela obsoleta*): Hamilton (1925: 18, 28; Figs 62, 99, 129; according to Leffler (1979a: 78) and Knisley and Pearson (1984: 507) Hamilton’s species is neither *Cicindela cimarrona* or *Cicindela obsoleta* respectively) [L_2-3_; FC].

*Cicindela* *sp.* C (Indiana): Hamilton (1925: 18, 30; Figs 151, 163, 175) [L_2-3_; FC].

*Cicindela* *sp.* D (Colorado): Hamilton (1925: 18, 34-35; Figs 152, 164, 178) [L_2-3_; FC].

*Cicindela* *sp.*: Bousquet (1991a: Figs 34.76, 34.78, 34.92); Bousquet (2010a: Figs 414-416, 425).

*Cylindera (Cylindera) cursitans* (LeConte): Brust et al. (2005: 383-385; Figs 2-7) [L_1_, L_2_, L_3_; RR].

*Cylindera (Cylindera) debilis* (Bates): Knisley and Pearson (1984: 540, 542; Figs 16-17, 98-103) [L_1_, L_2_, L_3_; RR+FC].

*Cylindera (Cylindera) lemniscata* (LeConte): Knisley and Pearson (1984: 496, 498, 499, 542-545; Figs 104-109) [L_1_,L_2_, L_3_; RR+FC].

*Cylindera (Cylindera) terricola* Say: Leffler (1979a: 91-92, as *Cylindera cinctipennis*) [L_3_; FC].

*Cylindera (Cylindera) unipunctata* (Fabricius): Hamilton (1925: 19, 43-44; Figs 71, 108, 138) [L_2-3_; FC].

*Cylindera (Cylindera) viridisticta* (Bates): Knisley and Pearson (1984: 496, 498, 499, 545-546; Figs 110-115) [L_1_, L_2_, L_3_; RR+FC].

*Dromochorus pruininus* Casey: Spomer et al. (2008b: 38-40; Figs 1-6) [L_1_, L_2_, L_3_; RR].

*Ellipsoptera gratiosa* (Guérin-Méneville): Hamilton (1925: 18, 34; Figs 65, 102, 132) [L_2-3_; RR].

*Ellipsoptera lepida* (Dejean): Shelford (1908: 171-173; Figs 49-51) [L_1_, L_2_, L_3_; RR]; Hamilton (1925: 18, 32-33; Figs 64, 101, 131) [L_2-3_; RR]; Leonard and Bell (1999: 32, 146, 147; Figs 50, 144, 145) [L_3_; LIT]; Bousquet (2010a: 405; Fig. 419) [L_2-3_; LIT].

*Ellipsoptera macra* (LeConte): Shelford (1908: 171-173, as *Ellipsoptera cuprascens*; Figs 55, 56) [L_1_, L_2_; RR]; Hamilton (1925: 19, 49-50; Figs 153, 165, 172) [L_2-3_; RR+FC].

*Ellipsoptera marginata* (Fabricius): Hamilton (1925: 19, 45-46; Figs 73, 110, 140) [L_2-3_; RR]; Spangler (1956: 84) [L_2-3_; LIT]; Willis (1967: 173) [L_2-3_; LIT]; Leonard and Bell (1999: 29, 86; Figs 40, 98, 99) [L_3_; LIT]; Bousquet (2010a: 404) [L_2-3_; LIT].

*Ellipsoptera nevadica* (LeConte): Willis (1967: 168, 173; Figs 47-52) [L_3_; RR].

*Ellipsoptera puritana* (Horn): Leonard and Bell (1999: 33, 74, 75; Figs 87-90) [L_3_; LIT (unpublished)]; Bousquet (2010a: 405) [L_2-3_; LIT].

*Ellipsoptera rubicunda* (Harris): Knisley and Pearson (1984: 496-498, 516-517, as *Ellipsoptera marutha*; Figs 12, 50-55) [L_1_, L_2_, L_3_; RR+FC].

*Eunota togata* (LaFerté-Sénectère): Willis (1967: 170, 173; Figs 53-58) [L_3_; RR].

*Habroscelimorpha circumpicta* (LaFerté-Sénectère): Spangler (1956: 82, 84; 14 figs) [L_2-3_; FC]; Willis (1967: 163-165, 173; Figs 35-40) [L_3_; RR].

*Habroscelimorpha dorsalis* (Say): Hamilton (1925: 17, 20-21; Figs 78, 115, 147) [L_2-3_; RR]; Leonard and Bell (1999: 29, 142; Figs 141, 142) [L_3_; LIT].

*Habroscelimorpha fulgoris* (Casey): Knisley and Pearson (1984: 496-498, 522-523; Figs 14-15, 19-20, 62-67) [L_1_, L_2_, L_3_; RR+FC].

### Loricerini

*Loricera (Loricera) decempunctata* Eschscholtz: Ball and Erwin (1969: 897; Fig. 49) [L_3_; FC].

*Loricera (Loricera) pilicornis* (Fabricius): Schiødte (1867: 465-469; Figs 8-16 [Pl. 14]) [L_1-3_; ?]; Gernet (1867: 10-12; Figs 2a-f [Pl. 1]) [L_1-3_; FC]; Larsson (1941: 290, as *Loricera coerulescens*; Figs 27, 28a-c) [L_1-3_; ?]; Jeannel (1941b: Figs 92a-f); van Emden (1942: Fig. 48); Garner (1954: 149, 150) [L_2-3_; FC]; Sharova (1958: 40; Figs 25a-e) [L_1-3_; ?]; Sharova (1964: 146; Figs 97-1,2,3,4,5,6) [L_1-3_; ?]; Larsson (1968: 335; Figs 27, 28a-c) [L_1-3_; ?]; Haberman (1968: 155, 156; Figs 85.1-6) [L_2-3_; ?]; Ball and Erwin (1969: 894, 896; Figs 50, 53, 56, 57) [L_1_, L_3_; RR+FC]; Raynaud (1976d: 167, 168; Figs 1-7 [Pl. 16B]) [L_3_; FC]; Luff (1978: 269, 270; Figs 9-17) [L_1_, L_2-3_; FC]; Thompson (1979a: Figs 39a-c, as *Loricera* *sp.*); Bousquet and Goulet (1984: Figs 30, 49); Bousquet (1991a: Figs 34.85, 34.91); Arndt (1991b: Figs 67, 68); Luff (1993: 69, 70; Figs 1, 206-213) [L_1_, L_2-3_; ?]; Makarov (1994: Fig. 102); Makarov (1996; Fig. 39); Bousquet (2010a: Figs 426-430).

*Loricera* *sp.* A (California): Garner (1954: 149, 151, 152; Figs a-c [Pl. 8]) [L_2-3_; FC].

*Loricera* *sp.* B (California): Garner (1954: 149, 153, 154) [L_2-3_; FC].

### Elaphrini

*Blethisa julii* LeConte: Lindroth (1954a: 23, 24; Figs 9b, 10b, 11b, 12b, 13c, 15b) [L_2-3_; FC]; Sharova (1958: Fig. 90b); Sharova (1964: Figs 133-1); Thompson (1979a: Figs 41a-e).

*Blethisa multipunctata* (Linnaeus): Bøving (1910: 357-363; Figs 8, 12, 15) [L_2-3_; FC]; Znojko (1929: Fig. 4e); Larsson (1941: 287; Fig. 25) [L_1-3_; ?]; Jeannel (1941b: Figs 87a-e); van Emden (1942: Fig. 74); Lindroth (1954a: 24, Figs 11c, 13b, 14b, 15a) [L_2-3_; FC]; Garner (1954: 121, 122; Figs a-c [Pl. 6]) [L_2-3_; FC]; Sharova (1958: Figs 89a-e); Sharova (1964: Figs 132-1,2,3,4,5); Larsson (1968: 332; Fig. 25) [L_1-3_; ?]; Haberman (1968: Figs 83.1-5); Luff (1976: 60, 65, 67; Figs 39-47) [L_3_; FC]; Goulet (1983: Figs 96a-b); Arndt (1991b: 75; Fig. 66) [L_1-3_; ?]; Luff (1993: 65; Figs 184-189) [L_2-3_; ?]; Makarov (1994: Fig. 51); Makarov (1996: Fig. 26); Bousquet (2010a: 411; Figs 432, 435) [L_2-3_; RR+FC].

*Blethisa quadricollis* Haldeman: Lindroth (1954a: 23; Figs 9c, 11d, 13d, 14c) [L_2-3_; FC]; Goulet (1983: Figs 88a-b, 95); Bousquet (2010a: 411; Fig. 436) [L_2-3_; FC].

*Diacheila arctica* (Gyllenhal): Lindroth (1954a: Figs 9a, 10a, 11a, 12a, 13a, 14a); Sharova (1958: 41; Figs 27b, 90a, 92a) [L_1-3_; ?]; Sharova (1964: 149; Figs 98-2,3, 133-1, 135-1) [L_1-3_; ?]; Goulet (1983: Figs 78, 84, 94); Luff (1993: 66; Figs 190-192) [L_1-3_; ?]; Makarov (1994: Fig. 50).

*Diacheila polita* (Faldermann): Sharova (1958: 41; Figs 26, 27a, 88, 92b) [L_1-3_; ?]; Sharova (1964: 149; Figs 98-1, 131-2, 135-2) [L_1-3_; ?]; Goulet (1983: Figs 79, 83a-c, 87a-b, 93a-b); Luff (1993: 66) [L_1-3_; LIT]; Bousquet (2010a: Figs 431, 434).

*Elaphrus (Elaphrus) americanus americanus* Dejean: Goulet (1983: 287, 288, 309; Figs 100a-b) [L_1_, L_2_, L_3_; RR]; Bousquet (2010a: 413; Figs 433, 437) [L_2-3_; RR].

*Elaphrus (Elaphrus) americanus sylvanus* Goulet: Goulet (1983: 311) [L_1_, L_3_; FC].

*Elaphrus (Elaphroterus) angusticollis* Sahlberg: Goulet (1983: 325, 326, 335-336; Figs 151, 152, 156) [L_1_, L_2_, L_3_; RR].

*Elaphrus (Elaphrus) californicus* Mannerheim: Goulet (1983: 286-288, 302; Figs 154, 155) [L_1_, L_2_, L_3_; RR]; Bousquet (2010a: 413) [L_2-3_; RR].

*Elaphrus (Neoelaphrus) cicatricosus* LeConte: Goulet (1983: 250, 251, 253, 263-264; Figs 99a-b) [L_1_, L_2_, L_3_; RR]; Bousquet (2010a: 412; Fig. 439) [L_2-3_; RR].

*Elaphrus (Neoelaphrus) clairvillei* Kirby: Lindroth (1954a: 26; Figs 12c, 14d, 16c, 17c) [L_2-3_; FC]; Thompson (1979a: Fig. 40b); Goulet (1983: 251-253, 275; Figs 76a-g, 80, 82a-b, 85a-c, 90a-b, 98a-c) [L_1_, L_2_, L_3_; RR]; Beutel (1993: Figs 17, 25); Bousquet (2010a: 412; Fig. 438) [L_2-3_; RR].

*Elaphrus (Elaphrus) finitimus* Casey: Goulet (1983: 288, 305-306) [L_3_; FC].

*Elaphrus (Neoelaphrus) fuliginosus* Say: Goulet (1983: 250, 251, 253, 261-262) [L_1_, L_2_, L_3_; RR]; Bousquet (2010a: 412) [L_2-3_; RR].

*Elaphrus (Neoelaphrus) laevigatus* LeConte: Goulet (1983: 251-253, 281-282) [L_1_, L_2_, L_3_; RR].

*Elaphrus (Arctelaphrus) lapponicus* Gyllenhal: Lindroth (1954a: 25; Figs 13e, 16a, 17a) [L_2-3_; FC]; Sharova (1958: Fig. 91); Sharova (1964: Fig. 134); Luff (1976: 62, 63, 65; Figs 34-38) [L_1_, L_2-3_; FC]; Thompson (1979a: Fig. 40a); Luff (1993: 67, 68; Figs 193-194) [L_1_, L_2-3_; ?]; Goulet (1983: 243; Figs 89a-b, 97) [L_3_; FC]; Bousquet (2010a: 412) [L_2-3_; FC].

*Elaphrus (Elaphrus) lecontei* Crotch: Garner (1954: 127, 128) [L_3_; FC]; Goulet (1983: 286-288, 297-298; Figs 81, 91a-b) [L_1_, L_2_, L_3_; RR].

*Elaphrus (Neoelaphrus) lindrothi* Goulet: Goulet (1983: 250-252, 265-266) [L_1_, L_2_, L_3_; RR].

*Elaphrus (Neoelaphrus) olivaceus* LeConte: Lindroth (1954a: 25; Figs 16b, 17b) [L_2-3_; FC]; Goulet (1983: 251-253, 279-280) [L_1_, L_2_, L_3_; RR]; Bousquet (2010a: 413) [L_2-3_; RR].

*Elaphrus (Elaphroterus) purpurans* Hausen: Goulet (1983: 324-326, 332-333; Figs 86, 92a-b, 102a-b) [L_1_, L_2_, L_3_; RR].

*Elaphrus (Elaphrus) ruscarius* Say: Goulet (1983: 287, 288, 295) [L_1_, L_2_, L_3_; RR].

*Elaphrus (Elaphrus) tuberculatus* Mäklin: Goulet (1983: 287, 288, 318) [L_1_, L_2_, L_3_; RR].

*Elaphrus (Elaphrus) viridis* Horn: Goulet and Smetana (1997: 215) [L_3_; FC].

### Omophronini

*Omophron (Omophron) americanum* Dejean: Silvey (1936: 691-692; Figs 17-19) [L_3_; FC].

*Omophron (Omophron) nitidum* LeConte: **?**Böving and Craighead (1931: Figs a-e [Pl. 5]).

*Omophron (Omophron) tessellatum* Say: Silvey (1936: 688-690; Figs 13, 15, 16) [L_3_; FC]; Landry and Bousquet (1984: Figs 1-21); Bousquet and Goulet (1984: Figs 29, 50, 52, 56); Bousquet (1991a: Figs 34.73, 34.88, 34.94, 34.97); Bousquet (2010a: Figs 440-447).

*Omophron* *sp.* A (California): Garner (1954: 134-136) [L_2-3_; FC].

*Omophron* *sp.* B (California): Garner (1954: 134, 136, 137) [L_2-3_; FC].

*Omophron* *sp.* C (Nebraska): Garner (1954: 134, 138, 139) [L_2-3_; FC].

*Omophron* *sp.* D (Nebraska): Garner (1954: 134, 140, 141; Pl. 7, Figs a-c) [L_2-3_; FC].

*Omophron* *sp.* (Ontario): Thompson (1979a: Figs 33a-e).

### Pasimachini

*Pasimachus (Pasimachus) elongatus* LeConte: Garner (1954: 234, 237, 238) [L_2-3_; FC]; Kirk (1972: 1355; Figs 22 [Pl. 2], 3-22) [L_1-3_; FC]; Thompson and Allen (1974: 189-192; Figs 2a-c) [L_3_; FC]; Thompson (1979a: Figs 45a-d).

*Pasimachus* *sp.* A (North Carolina): Garner (1954: 234, 238, 239; Pl. 16, Figs a-c) [L_2-3_; FC].

*Pasimachus* *sp.* B (Texas): Garner (1954: 234, 240, 241) [L_2-3_; FC].

*Pasimachus* *sp.* C (Florida): Garner (1954: 234, 241, 242) [L_2-3_; FC].

*Pasimachus* *sp.* D (Montana): Garner (1954: 234, 242, 243) [L_2-3_; FC].

### Scaritini

*Scarites (Scarites) quadriceps* Chaudoir: van Emden (1942: Fig. 51); Garner (1954: 227, 231, 232, as *Scarites* prob. *substriatus*) [L_2-3_; FC]; Kirk (1972: 1355; Figs 23 [Pl. 2], 3-23, as *Scarites substriatus*) [L_2-3_; FC]; Thompson (1979a: Figs 46a-c, as *Scarites substriatus*).

*Scarites (Scarites) subterraneus* Fabricius: Wickham (1893b: 332, 333; Figs 3-4 [Pl. 9]) [L_3_; FC]; Garner (1954: 227, 230; Pl. 15, Figs a-c) [L_2-3_; FC]; Bousquet (2010a: Figs 448-450).

### Clivinini

*Clivina (Clivina) collaris* (Herbst)**†**: Luff (1978: 272, 281; Fig. 57) [L_1_, L_2_; RR+FC]; Vaněk (1984: 100-103, 111; Figs 1-47) [L_1_, L_2_, L_3_; RR]; Arndt (1991b: 78, Figs 78, 80) [L_1-3_; LIT]; Luff (1993: 71, 72; Fig. 222) [L_1_, L_2-3_; ?]; Makarov (1994: Fig. 24).

*Clivina (Semiclivina) dentipes* Dejean: Thompson (1979a: Figs 47a-c).

*Clivina (Clivina) fossor* (Linnaeus)**†**: Bøving (1911: 148-153, 172-178; Pl. 6; Figs 4, 10, 15, 20, 23) [L_2-3_; FC]; Larsson (1941: 296) [L_1-3_; ?]; Jeannel (1941b: Figs 100a-g); van Emden (1942: Fig. 76); Garner (1954: 258, 262, 263) [L_2-3_; FC]; Sharova (1958: 44; Figs 32, 34, 97, 99a) [L_1-3_; ?]; Sharova (1964: 153; Figs 100-1, 101-1, 138, 139-2) [L_1-3_; ?]; Larsson (1968: 342; Fig. 32) [L_1-3_; ?]; Haberman (1968: Figs 87.1, 88.1,3,4); Luff (1978: 272, 280, 281; Figs 49-56) [L_1_, L_2-3_; RR+FC]; Vaněk (1984: 108, 109, 111; Figs 48-53) [L_1_, L_2_, L_3_; RR]; Arndt (1991b: 78) [L_1-3_; LIT]; Luff (1993: 71, 72; Figs 214-221) [L_1_, L_2-3_; ?]; Bousquet (2010a: Figs 451, 458).

*Clivina (Clivina) impressefrons* LeConte: Phillips (1909: 14-16; Figs 8b,e,f) [L_3_; FC]; Kirk (1972: 1355; Figs 16 [Pl. 2], 3-16) [L_1-3_; FC].

*Clivina* *sp.* A (Nebraska): Garner (1954: 258, 261, 262; Pl. 18, Figs a-c) [L_2-3_; FC].

*Clivina* *sp.* B (Louisiana): Garner (1954: 258, 264, 265) [L_2-3_; FC].

*Clivina* *sp.* C (Illinois): Garner (1954: 258, 265, 266) [L_2-3_; FC].

*Schizogenius (Schizogenius) lineolatus* (Say): Bousquet (1996b: 348-351; Figs 1-11) [L_1_, L_2-3_; RR+FC]; Bousquet (2010a: Figs 452, 454, 456, 459, 461).

*Semiardistomis viridis* (Say): Bousquet (2006c: 5; Figs 36-39) [L_1_; RR].

Genus A sp. A (Florida): Garner (1954: 270, 271; Pl. 19, Figs a-c) [L_2-3_; FC].

### Dyschiriini

*Akephorus marinus* LeConte: Garner (1954: 248, 251; Pl. 17, Figs a-c) [L_2-3_; FC].

*Dyschirius dejeanii* Putzeys: Kirk (1972: 1355; Figs 18 [Pl. 2], 3-18, as *Dyschirius integer*) [L_1-3_; FC].

*Dyschirius globosus* (Herbst)**†**: Larsson (1941: 292, 295) [L_1-3_; ?]; Sharova (1958: 44) [L_1-3_; ?]; Sharova (1964: 154) [L_1-3_; ?]; Larsson (1968: 338, 341) [L_1-3_; ?]; Haberman (1968: 167) [L_1-3_; ?]; Raynaud (1976e: 276, 277; Figs 4-8) [F_3_; FC]; Luff (1978: 271, 277; Figs 41-47) [L_3_; FC]; Arndt (1991b: 78) [L_1-3_; ?]; Luff (1993: 73, 76; Figs 243-245) [L_1-3_; ?].

*Dyschirius globulosus* (Say): Garner (1954: 248, 254) [L_3_; FC].

*Dyschirius politus* (Dejean): Larsson (1941: 293, 294; Figs 30d, 31e) [L_1-3_; ?]; Sharova (1958: 45; Fig. 101e) [L_1-3_; ?]; Sharova (1964: 154; Fig. 139-7) [L_1-3_; ?]; Larsson (1968: 338 340; Figs 30d, 31e) [L_1-3_; ?]; Haberman (1968: 168; Fig. 95.7) [L_1-3_; ?]; Luff (1978: 271, 277; Figs 38-40) [L_3_; FC]; Arndt (1991b: 78) [L_1-3_; ?]; Luff (1993: 73, 75, 76; Figs 231, 232) [L_1-3_; ?].

*Dyschirius sphaericollis* (Say): Silvey (1936: 685-687; Figs 9, 11, 12) [L_3_; FC].

*Dyschirius* *sp.* A (Louisiana): Garner (1954: 248, 252, 253) [L_2-3_; FC].

*Dyschirius* *sp.* (Quebec): Bousquet (1988a: Figs 31-34); Bousquet (2010a: Figs 453, 455, 457, 460).

### Promecognathini

*Promecognathus laevissimus* (Dejean)^12^: Bousquet and Smetana (1986: 27-30; Figs 1-12) [L_1_; RR]; Bousquet (1991a: Fig. 34.80).

### Broscini

*Broscus cephalotes* (Linnaeus)**†**: Schiødte (1867: 504-507; Figs 1-8 [Pl. 19]) [L_1-3_; ?]; Znojko (1929: Fig. 8a); Larsson (1941: 297; Figs 32a-b) [L_1-3_; ?]; Jeannel (1941b: Figs 110a-e); van Emden (1942: Fig. 39); Sharova (1958: 45; Figs 36, 37) [L_1-3_; ?]; Sharova (1964: 154, 155; Figs 102-1,2) [L_1-3_; ?]; Larsson (1968: 343; Figs 33a-b) [L_1-3_; ?]; Andersen (1968: 74) [L_3_; ?]; Haberman (1968: Figs 112d1-d2); Luff (1978: 282-285; Figs 58-65) [L_1_, L_2-3_; FC]; Sharova and Makarov (1984: 742, 744, 746; Figs 1-2) [L_1_, L_2_, L_3_; FC]; Arndt (1991b: Figs 69-71); Luff (1993: 77, 78; Figs 246-251) [L_1_, L_2-3_; ?]; Beutel (1993: Figs 4, 10, 18); Makarov (1994: Fig. 26); Bousquet (2010a: Figs 464, 468).

*Miscodera arctica* (Paykull): Andersen (1968: 71-74; Fig. 1, 2a-c, 3a-b) [L_3_; FC]; Larsson (1968: 343) [L_1-3_; ?]; Luff (1978: 282, 285, 286; Figs 66-75) [L_1_, L_2-3_; FC]; Thompson (1979a: Figs 43a-f); Luff (1993: 79; Figs 252, 253) [L_1_, L_2-3_; FC]; Makarov (1994: Fig. 25); Bousquet (2010a: Figs 462, 463, 465-467).

### Gehringiini

*Gehringia olympica* Darlington: Lindroth (1960a: 37; Figs 5a-f) [L_1_; RR]; Thompson (1979a: Figs 27a-f); Luna de Carvalho (1989: 55; Fig. 27b) [L_1-3_; LIT].

### Trechini

*Blemus discus* (Fabricius)**†**: **?**Bousquet (2010a: Figs 469-473).

*Neaphaenops tellkampfii* (Erichson): Packard (1874: 563, as *Pseudanophthalmus* *sp.*) [L_3_; FC]; Hubbard (1888: 77-78; Figs 18a-b, as *Pseudanophthalmus* *sp.*) [L_3_; FC]; Jeannel (1920a: 538, 539; Fig. 62) [L_3_; FC]; Jeannel (1926: 396) [L_3_; FC].

*Pseudanophthalmus* *sp.* A (Higginbotham’s Cave, West Virginia): Boldori (1951: 143, 144; Figs 39, 42) [L_2-3_; FC].

*Pseudanophthalmus* *sp.* B (Higginbotham’s Cave, West Virginia): Boldori (1951: 145) [L_2-3_; FC].

*Pseudanophthalmus* *sp.* C (Coffman’s Cave, West Virginia): Boldori (1951: 145; Fig. 40) [L_2-3_; FC].

*Pseudanophthalmus* *sp.* D (Shelta Cave, Alabama): Boldori (1951: 145; Fig. 41) [L_2-3_; FC].

*Pseudanophthalmus* *sp.* E (Johnson’s Cave, Tennessee): Boldori (1951: 145) [L_2-3_; FC].

*Trechus (Trechus) apicalis* Motschulsky: Thompson (1979a: Figs a-d); **?**Bousquet (2010a: Fig. 474).

*Trechus (Trechus) obtusus* Erichson**†**^13^: Boldori (1931a: 6) [L_3_; FC]; Boldori (1931b: 157; Figs 1-3, 2-3) [L_3_; FC]; Larsson (1941: 303, 304; Fig. 34c) [L_1-3_; ?]; Boldori (1951: Fig. 3); Sharova (1958: 47; Fig. 105c) [L_1-3_; ?]; Sharova (1964: 157; Fig. 143-3) [L_1-3_; ?]; Larsson (1968: 352, 353; Fig. 36c) [L_1-3_; ?]; Haberman (1968: 274; Fig. 122.3) [L_1-3_; ?]; Luff (1985: 302, 304; Fig. 11) [L_2-3_; FC]; Arndt (1991b: 82) [L_1-3_; ?]; Luff (1993: 85, 86; Figs 284-286) [L_1-3_; ?].

*Trechus (Trechus) quadristriatus* (Schrank)**†**: Bøving (1911: 141-147, 172-178; Figs 3, 9, 14, 19, 22 [Pl. 5]) [L_2-3_; FC]; Jeannel (1920a: 523, 539; Figs 13-17) [L_1-3_; FC]; Jeannel (1926: 387) [L_2-3_; FC]; Beier and Strouhal (1928: 1-4; Figs 1a-b, 2a-e, 3 [Pl. 1]) [L_3_; FC]; Boldori (1931a: 6) [L_3_; FC]; Boldori (1931b: 158; Figs 1-2, 2-2) [L_3_; FC]; Larsson (1941: 303; Fig. 34b) [L_1-3_; ?]; Jeannel (1941b: Figs 113a-f); Boldori (1951: Fig. 2); Sharova (1958: 47; Fig. 105d) [L_1-3_; ?]; Sharova (1964: 157; Fig. 143-4) [L_1-3_; ?]; Larsson (1968: 352, 353; Fig. 36b) [L_1-3_; ?]; Haberman (1968: 273; Fig. 122.3) [L_1-3_; ?]; Luff (1985: 302, 304; Figs 1-10) [L_1_, L_2-3_; FC]; Arndt (1991b: 82; Figs 72, 73) [L_1-3_; ?]; Luff (1993: 84-86; Figs 281-283) [L_1_, L_2-3_; ?]; Makarov (1994: Fig. 32).

*Trechus (Trechus) rubens* (Fabricius)**†**: Larsson (1941: 303, 304; Fig. 34a) [L_1-3_; ?]; Sharova (1958: 47; Fig. 105b) [L_1-3_; ?]; Larsson and Gígja (1959: 27) [L_1-3_; FC]; Sharova (1964: 157; Fig. 143-2) [L_1-3_; ?]; Larsson (1968: 352, 353; Fig. 36a) [L_1-3_; ?]; Haberman (1968: 273; Fig. 122.2) [L_2-3_; ?]; Luff (1985: 302, 304, 306; Figs 12-16) [L_3_; FC]; Bousquet (1991a: Figs 34.95, 34.98); Arndt (1991b: 82) [L_1-3_; ?]; Luff (1993: 85; Figs 278-280) [L_1-3_; ?].

### Bembidiini

*Asaphidion alaskanum* Wickham: Maddison (1993: 153, 271; Figs 195, 225) [L_1_; RR].

*Asaphidion yukonense* Wickham: Thompson (1979a: Figs a-c).

*Bembidion (Odontium) aenulum* Hayward: Maddison (1993: 172; Fig. 223) [L_1_; RR].

*Bembidion (Bracteon) alaskense* Lindroth: Maddison (1993: 172, 178; Figs 212, 227, 233, 236) [L_1_, L_2_; RR].

*Bembidion (Pseudoperyphus) antiquum* Dejean: Maddison (1993: Figs 222, 247).

*Bembidion (Bracteon) balli* Lindroth: Maddison (1993: 173-174; Figs 191, 196, 210, 240, 242, 245, 248, 252, 255, 257, 258) [L_1_, L_2_; RR].

*Bembidion (Odontium) bowditchii* LeConte: Maddison (1993: 173) [L_1_; RR].

*Bembidion (Peryphus) bruxellense* Wesmael**†**: Larsson (1941: 299, 300, as *Bembidion rupestre*; Fig. 33g) [L_1-3_; ?]; Sharova (1958: 49, as *Bembidion rupestre*; Fig. 108f) [L_1-3_; ?]; Sharova (1964: 159, as *Bembidion rupestre*; Fig. 143-10) [L_1-3_; ?]; Larsson (1968: 347, 349; Fig. 35g) [L_1-3_; ?]; Luff (1993: 91, 98, 99; Figs 327, 328) [L_1-3_; ?].

*Bembidion (Bracteon) carinula* Chaudoir: Maddison (1993: 172, 181; Figs 197, 198, 213, 228, 234, 243, 246, 250, 253, 256, 260) [L_1_, L_2_; RR].

*Bembidion (Pseudoperyphus) chalceum* Dejean: Maddison (1993: Fig. 221).

*Bembidion (Ochthedromus) cheyennense* Casey: Maddison (1993: 172; Fig. 220) [L_1_; RR].

*Bembidion (Odontium) confusum* Hayward: Silvey (1936: 682-684; Figs 5, 7, 8) [L_3_; FC]; Maddison (1993: 173) [L_1_; RR].

*Bembidion (Notaphus) contractum* Say: Lindroth (1955a: Fig. 15b).

*Bembidion (Odontium) coxendix* Say: Maddison (1993: 173; Figs 192, 200, 219) [L_1_; RR].

*Bembidion (Peryphus) femoratum* Sturm**†**: Netolitzky (1926: 118; Fig. 3) [L_1_; RR]; Arndt (1991b: Fig. 75).

*Bembidion (Bracteon) foveum* Motschulsky: Maddison (1993: 173, 175; Figs 199, 201-203, 205, 211, 226, 232, 238, 249) [L_1_, RR].

*Bembidion (Peryphanes) grapii* Gyllenhal: Larsson and Gígja (1959: 23) [L_1-3_; FC]; Böcher (1988: Fig. 4); Luff (1993: 92, 99; Figs 329, 330) [L_1-3_; ?].

*Bembidion (Bracteon) hesperium* Fall: Maddison (1993: 173, 186) [L_1_, L_2_; RR].

*Bembidion (Bracteon) inaequale* Say: Maddison (1993: 192; Fig. 193) [L_1_, L_2_; RR].

*Bembidion (Pseudoperyphus) integrum* Casey: Maddison (1993: Fig. 244).

*Bembidion (Leja) lampros* (Herbst)**†**: Larsson (1941: 299; Fig. 33b) [L_1-3_; ?]; Larsson (1968: 346, 348; Fig. 35b) [L_1-3_; ?]; Desender and Crappé (1983: 39, 46; Figs 4a-i, 5a-h, 6a-h) [L_1-3_; RR+FC]; Luff (1993: 91, 93; Figs 304, 305) [L_1_, L_2-3_; ?].

*Bembidion (Bracteon) lapponicum* Zetterstedt: Andersen (1966: 446, 448, 449; Figs 1, 17-26) [L_1_, L_2_, L_3_; RR+FC]; Maddison (1993: 172, 184; Figs 206-209, 214, 229, 235, 259) [L_1_, L_2_; RR]; Luff (1993: 91-93; Figs 299-301) [L_1_, L_2-3_; LIT].

*Bembidion (Bracteon) levettei* Casey: Maddison (1993: 190; Figs 218, 231) [L_1_, L_2_; RR].

*Bembidion (Bracteon) lorquinii* Chaudoir: Maddison (1993: 173, 187-188; Fig. 216) [L_1_, L_2_; RR].

*Bembidion (Eurytrachelus) nitidum* (Kirby): Kirk (1972: 1355; Figs 26 [Pl. 3], 3-26) [L_2-3_; FC].

*Bembidion (Phyla) obtusum* Audinet-Serville**†**: Maddison (1993: Fig. 224).

*Bembidion (Peryphus) petrosum* Gebler: Andersen (1966: Fig. 43); Luff (1993: 91,98; Figs 325, 326) [L_1-3_; ?].

*Bembidion (Leja) properans* (Stephens)**†**: Desender and Crappé (1983: 39, 46; Figs 1a-h, 2a-h, 3a-h) [L_1-3_; RR+FC].

*Bembidion (Bracteon) punctatostriatum* Say: Maddison (1993: 172, 185; Figs 194, 204, 215, 230, 237, 239, 241, 251, 254) [L_1_, L_2_; RR].

*Bembidion (Bembidion) quadrimaculatum* (Linnaeus): Kirk (1972: 1355; Figs 25 [Pl. 3], 3-25, as *Bembidion quadrimaculatum*) [L_1-3_; FC].

*Bembidion (Notaphus) semipunctatum* (Donovan): Makarov (1994: Fig. 37).

*Bembidion (Plataphus) sulcipenne prasinoides* Lindroth: Lindroth (1955a: Fig. 15a, as *Bembidion lenense*).

*Bembidion (Peryphus) tetracolum* Say**†**; Larsson (1941: 299, 300, as *Bembidion ustulatum*; Fig. 33f) [L_1-3_; ?]; Sharova (1958: 49, as *Bembidion ustulatum*; Fig. 108e) [L_1-3_; ?]; Sharova (1964: 159, as *Bembidion ustulatum*; Fig. 143-9) [L_1-3_; ?]; Larsson (1968: 347, 349; Fig. 35f) [L_1-3_; ?]; Kirk (1972: 1355; Figs 27 [Pl. 3], 3-27) [L_1-3_; FC]; Smrž (1979: 247, 249; Figs 1, 4, 6, 7, 10, 12, 14, 15, 17, 19, 21, 24, 26, 27, 29, 31, 33, 35, 38, 39, 42, 44, 46, 47, 50, 52, 54, 56) [L_1_, L_2_, L_3_; RR]; Luff (1993: 91, 97; Figs 320-324) [L_1_, L_2-3_; ?].

*Bembidion (Bracteon) zephyrum* Fall: Maddison (1993: 189; Fig. 217) [L_1_, L_2_; RR].

*Bembidion* *sp.* (Arkansas): Thompson (1979a: Figs a-b).

*Bembidion* *sp.*: Bousquet (2010a: Figs 475, 478, 480, 482).

*Elaphropus (Barytachys) anceps* (LeConte): Kirk (1972: 1355; Figs 24 [Pl. 2], 3-24) [L_1-3_; FC].

*Elaphropus (Tachyura) parvulus* (Dejean)**†**: Cerruti (1939: 121-123; Figs 1-3) [L_1_; RR]; van Emden (1942 : 31) [L_1_; LIT]; Luff (1993: 102; Figs 337-339) [L_1_; FC].

*Elaphropus (Barytachys) tripunctatus* (Say): Grebennikov and Maddison (2000: 237; Figs 8, 33) [L_1_, L_2_; RR].

*Elaphropus (Barytachys)* *sp.*: Bousquet (2010a: Figs 477, 484).

*Mioptachys flavicauda* (Say): Thompson (1979a: Figs 48); Grebennikov and Maddison (2000: 230; Figs 3, 12, 22, 40) [L_1_; RR]; Bousquet (2010a: Figs 476, 479, 481, 483).

*Phrypeus rickseckeri* (Hayward): Grebennikov and Maddison (2005: 43, 44; Figs 5-10) [L_1_; RR].

*Polyderis laeva* (Say): Grebennikov and Maddison (2000: 236; Figs 20, 30, 52) [L_2_; RR].

*Polyderis rufotestacea* (Hayward): **?**Grebennikov and Maddison (2000: 236; Figs 6, 29) [L_1_; RR].

*Porotachys bisulcatus* (Nicolai)**†**: Grebennikov and Maddison (2000: 238; Figs 11, 17, 38, 39, 48, 56, 60) [L_1_, L_2_, L_3_; RR].

*Tachys (Tachys) halophilus* Lindroth: Grebennikov and Maddison (2000: 235; Fig. 19) [L_1_; RR].

*Tachyta (Tachyta) inornata* (Say): Erwin (1975: Figs 158, 160, 162, 164, 169, 171-173); Thompson (1979a: Figs 49a-d).

### Pogonini

*Thalassotrechus barbarae* (Horn): Moore (1956: 215; Figs 1-7) [L_2-3_; FC]; Thompson (1979a: Figs a-g); Grebennikov and Bousquet (1999: 438; Figs 6, 13, 16, 20) [L_1_; RR].

### Patrobini

*Diplous (Platidius) aterrimus* (Dejean): Thompson (1979a: Figs a-c).

*Diplous (Platidius) rugicollis* (Randall): Bousquet (2010a: Figs 487, 490, 492).

*Patrobus foveocollis* (Eschscholtz): Bousquet and Goulet (1984: Figs 31, 53, 57).

*Patrobus longicornis* (Say): Schaupp (1882a: 56) [L_2-3_; FC]; Thompson (1979a: Figs 55a-d); Bousquet (2010a: Figs 485, 488, 489).

*Patrobus septentrionis* Dejean: Larsson (1941: 305, 306; Fig. 35b) [L_1-3_; ?]; Larsson and Gígja (1959: 30) [L_1-3_; FC]; Larsson (1968: 356, 357; Fig. 38b) [L_1-3_; ?]; Houston and Luff (1975: 60, 64; Fig. 16) [L_1_, L_2-3_; FC]; Böcher (1988: Fig. 2); Arndt (1991b: 84, 85) [L_1_, L_2-3_; LIT]; Luff (1993: 79, 80; Figs 254-257) [L_1_, L_2-3_; ?].

*Platypatrobus lacustris* Darlington: Bousquet and Grebennikov (1999: 6, 8, 10; Figs 7-15) [L_1_, L_2_, L_3_; RR+FC].

### Psydrini

*Nomius pygmaeus* (Dejean): Jeannel (1948b: 74, 75; Figs 1a-c) [L_3_; FC]; Hůrka (1978: 54) [L_1-3_; LIT].

*Psydrus piceus* LeConte: Dajoz (1997b: 133-134; Figs 1-11) [L_3_; FC].

### Metriini

*Metrius contractus* Eschscholtz^14^: Bousquet and Goulet (1984: Figs 39, 44); Bousquet (1986: 375, 376; Figs 1-20) [L_1_; RR]; Luna de Carvalho (1989: 993; Figs 597a-j) [L_1_; LIT]; Luna de Carvalho (1992: 287, 288; Figs 1-9 [Pl. 1]) [L_1_; LIT]; Moore and Di Giulio (2006: 15) [L_1-3_; ?].

### Ozaenini

*Goniotropis kuntzeni* Bänninger: Moore and Di Giulio (2006: 10-14, 16; Figs 9-36) [L_1_, L_2_; RR].

### Brachinini

*Brachinus (Neobrachinus) americanus* (LeConte): Thompson (1979a: Figs 86a-b).

*Brachinus (Neobrachinus) cyanipennis* Say: Clausen (1940: Figs 226a-c).

*Brachinus (Neobrachinus) janthinipennis* (Dejean): Wickham (1893b: 330-332; Figs 1-2 [Pl. 9]) [L_1-3_; FC]; Dimmock and Knab (1904: 31-34; Fig. 4; Figs 1-2 [Pl. 3]) [L_1_, L_2-5_; FC].

*Brachinus (Neobrachinus) pallidus* Erwin: Erwin (1967: 44-47; Figs 1-3 [Pl. 1]; Figs 1-6 [Pl. 2]; Figs 1-6 [Pl. 3]) [L_1_, L_2_, L_3_; L_4-5_; FC]; Thompson (1979a: Figs 85a-b).

*Brachinus (Neobrachinus)* *sp.*: Bousquet and Goulet (1984: Figs 26, 38, 43, 55); Bousquet (1991a: Figs 34.90, 34.100); Bousquet (2010a: Figs 493-497).

### Morionini

*Morion monilicornis* (Latreille): van Emden (1953a: 51, as *Morion georgiae*) [L_2-3_; FC]; Thompson (1979b: 85, 86; Figs 48a-g) [L_2-3_; FC].

### Abacetini

*Loxandrus velocipes* Casey: Thompson (1979a: Figs a-d); Thompson (1979b: 53, 54; Figs 28a-d) [L_1_, L_2-3_; RR]; Bousquet (1985c: 200, 203, 207-208; Figs 14, 26) [L_1_, L_2_, L_3_; RR]; Bousquet (1989: 30, 33) [L_1_, L_2_, L_3_; RR].

### Pterostichini

*Abax (Abax) parallelepipedus* (Piller and Mitterpacher)**†**: Schiødte (1872: 179-180, as *Pterostichus striola*; Figs 3-8 [Pl. 1]) [L_1-3_; ?]; Larsson (1941: 330, as *Abax ater*; Fig. 49g) [L_1-3_; ?]; van Emden (1942: Fig. 99, as *Abax ater*); Jeannel (1942: Figs 251a-f); Sharova (1958: Figs 50a-e, *Abax ater*); Sharova (1964: 168, as *Abax ater*; Figs 108-1,2,3,4,5) [L_1-3_; ?]; Larsson (1968: 390, *Abax ater*; Fig. 53g) [L_1-3_; ?]; Raynaud (1976d: 171, 172, 174, as *Abax ater*; Figs 1-7 [Pl. 17B]) [L_3_; FC]; Bousquet (1985c: 200, 203, 221; Figs 17, 58, 65, 73, 84, 96) [L_1_, L_2_, L_3_; RR]; Bousquet (1989: 30, 33; Figs 33, 38) [L_1_, L_2_, L_3_; RR]; Arndt (1989: 264-268; Figs 16-21) [L_1_, L_2_, L_3_; RR+FC]; Bousquet (1991a: Fig. 34.89); Arndt (1991b: 94; Fig. 14) [L_1_, L_2-3_; LIT]; Luff (1993: 104, 115; Figs 379-384) [L_1_, L_2-3_; ?]; Makarov (1994: Fig. 72); Bousquet (1999: Fig. 126); Bousquet (2010a: Figs 500, 526, 531).

*Cyclotrachelus (Evarthrus) alternans* (Casey): Kirk (1972: 1355; Figs 20 [Pl. 2], 3-20) [L_1-3_; FC]; Bousquet (2010a: Fig. 499).

*Cyclotrachelus (Evarthrus) parasodalis* (Freitag): Thompson (1979a: Figs 62a-c); Thompson (1979b: 52; Figs 26a-h) [L_3_; FC].

*Cyclotrachelus (Evarthrus) seximpressus* (LeConte): Thompson (1979b: 52; Figs 27a-g) [L_1_, L_2-3_; RR]; Bousquet (1985c: 200, 202, 222; Figs 18, 32, 66, 69, 74) [L_1_, L_2_; RR]; Bousquet (1989: 30, 33) [L_1_, L_2_; RR]; Bousquet (1999: Fig. 122).

*Cyclotrachelus (Evarthrus) torvus* (LeConte): Kirk (1972: 1355; Figs 21 [Pl. 2], 3-21) [L_1_; FC].

*Cyclotrachelus* *sp.*: Bousquet (2010a: Figs 509, 542, 546).

*Gastrellarius blanchardi* (Horn): Bousquet (1989: 29, 32, 33; Figs 5-7) [L_1_, L_2_, L_3_; RR].

*Gastrellarius honestus* (Say): Bousquet (1985c: 200, 203, 224-225; Figs 19, 33, 34, 59, 75, 97) [L_1_, L_2_, L_3_; RR]; Bousquet (1989: 30, 33; Figs 15, 34) [L_1_, L_2_, L_3_; RR]; Bousquet (2010a: Figs 510, 533, 549).

*Lophoglossus haldemanni* (LeConte): Thompson (1979b: 57, 68, 69; Figs 37a-e) [L_1_, L_2_; RR].

*Lophoglossus scrutator* (LeConte): Bousquet (1985c: 200, 204, 211; Figs 15, 27, 28, 63, 71, 88) [L_1_, L_2_, L_3_; RR]; Bousquet (1989: 30, 34; Figs 8, 13, 32) [L_1_, L_2_, L_3_; RR]; Bousquet (1999: Fig. 120); Bousquet (2010a: Figs 505, 506, 539).

*Myas (Trigonognatha) cyanescens* Dejean: Thompson (1979a: Figs a-b); Thompson (1979b: 54, 55; Figs 29a-g) [L_3_; FC]; Bousquet (1985c: 203, 219-220; Figs 31, 77, 95) [L_3_; FC]; Bousquet (1989: 33; Figs 14, 36) [L_3_; FC]; Makarov (1994: Figs 73, 76); Bousquet (1999: Fig. 125); Bousquet (2010a: Figs 501, 508, 550).

*Piesmus submarginatus* (Say): Bousquet (1999: 47, 48, 71, 72) [L_1_, L_2_, L_3_; RR].

*Poecilus (Poecilus) chalcites* (Say): Kirk (1972: 1355; Figs 29 [Pl. 3], 3-29) [L_1-3_; FC]; Thompson (1979b: 55, 62, 63; Figs 33a-f) [L_1_, L_2-3_; RR]; Bousquet (1985c: 201, 204, 213-214; Figs 16, 57, 76, 93, 100) [L_1_, L_2_, L_3_; RR]; Bousquet (1989: 31, 34; Figs 9-10, 30) [L_1_, L_2_, L_3_; RR]; Bousquet (1999: Figs 119, 123); Bousquet (2010a: 441; Figs 532, 547) [L_2_, L_3_; RR].

*Poecilus (Poecilus) corvus* (LeConte): Thompson (1979b: 55, 65, 66; Figs 35a-h) [L_1_, L_2_; RR].

*Poecilus (Poecilus) lucublandus* (Say): Schaupp (1881a: 88, 89; Figs Di-v) [L_3_; FC]; van Emden (1942: Fig. 23); Larsson (1968: Fig. 54c); Kirk (1972: 1355; Figs 28 [Pl. 3], 3-28) [L_1-3_; FC]; Thompson (1979b: 55, 74, 75; Figs 41a-g) [L_1_, L_2-3_; RR]; Bousquet (1985c: 201, 204, 212-213; Fig. 29) [L_1_, L_2_, L_3_; RR]; Bousquet (1989: 31, 34) [L_1_, L_2_, L_3_; RR]; Bousquet (2010a: 441; Fig. 498) [L_2_, L_3_; RR].

*Poecilus (Derus) nearcticus* (Lindroth): Bousquet (1999: 65) [L_2_, L_3_; FC].

*Poecilus (Poecilus)* *sp.*: Dogger and Olson (1966: 93, 94, as *Micromaseus femoralis*; Figs 1-2) [L_3_; FC].

*Pterostichus (Hypherpes) adoxus* (Say): Bousquet (1985c: 200, 203, 246; Figs 55, 99) [L_1_, L_2_, L_3_; RR]; Bousquet (1989: 30, 34) [L_1_, L_2_, L_3_; RR]; Bousquet (2010a: 444; Fig. 523) [L_2_, L_3_; RR].

*Pterostichus (Bothriopterus) adstrictus* Eschscholtz: Larsson and Gígja (1959: 37) [L_1-3_; FC]; Goulet (1974a: 21, 22) [L_1_, L_2_, L_3_; RR]; Thompson (1979b: 56, 59; Figs 30a-c) [L_1_, L_2-3_; RR]; Bousquet (1985c: 202, 205, 234) [L_1_, L_2_, L_3_; RR]; Bousquet (1989: 32, 35) [L_1_, L_2_, L_3_; RR]; Bousquet (1991a: Fig. 34.103); Luff (1993: 107, 109) [L_1-3_; ?]; Bousquet (2010a: 445) [L_2_, L_3_; RR].

*Pterostichus (Hypherpes) algidus* LeConte: Thompson (1979b: 56, 59, 60; Figs 31a-h) [L_1_, L_2-3_; RR].

*Pterostichus (Hypherpes) amethystinus* Mannerheim: Thompson (1979b: 57, 61, 62; Figs 32a-e) [L_1_, L_2-3_; RR].

*Pterostichus (Cryobius) arcticola* (Chaudoir): Bousquet (1985c: 202, 206, 249) [L_1_, L_2_, L_3_; RR]; Bousquet (1989: 32, 37) [L_1_, L_2_, L_3_; RR]; Bousquet (2010a: 447) [L_2_, L_3_; RR].

*Pterostichus (Cryobius) brevicornis* (Kirby): Bousquet (1985c: 202, 203, 248; Fig. 98) [L_1_, L_2_, L_3_; RR]; Bousquet (1989: 32, 33) [L_1_, L_2_, L_3_; RR]; Bousquet (2010a: 444; Fig. 553) [L_2_, L_3_; RR].

*Pterostichus (Melanius) castor* Goulet and Bousquet: Bousquet (1985c: 202, 206, 237) [L_1_, L_2_, L_3_; RR]; Bousquet (1989: 33, 36) [L_1_, L_2_, L_3_; RR]; Bousquet (2010a: 446) [L_2_, L_3_; RR].

*Pterostichus (Lamenius) caudicalis* (Say): Bousquet (1985c: 201, 206, 235; Figs 24, 42, 91) [L_1_, L_2_, L_3_; RR]; Bousquet (1989: 31, 36; Fig. 12) [L_1_, L_2_, L_3_; RR]; Bousquet (2010a: 446; Figs 515, 529) [L_2_, L_3_; RR].

*Pterostichus (Argutor) commutabilis* (Motschulsky): Bousquet (1985c: 202, 206, 225-226, as *Pterostichus leconteianus*; Fig. 35) [L_1_, L_2_, L_3_; RR]; Bousquet (1989: 32, 36, as *Pterostichus leconteianus*; Fig. 16) [L_1_, L_2_, L_3_; RR]; Bousquet (2010a: 446, 447; Fig. 511) [L_2_, L_3_; RR].

*Pterostichus (Euferonia) coracinus* (Newman): Bousquet (1985c: 201, 205, 242-243; Figs 62, 81) [L_1_, L_2_, L_3_; RR]; Bousquet (1989: 31, 35; Fig. 20) [L_1_, L_2_, L_3_; RR]; Bousquet (2010a: 446; Fig. 534) [L_2_, L_3_; RR].

*Pterostichus (Melanius) corvinus* (Dejean): Thompson (1979b: 56, 65; Figs 34a-g) [L_1_, L_2-3_; RR+FC]; Bousquet (1985c: 202, 206, 236; Figs 43, 44, 86, 92) [L_1_, L_2_, L_3_; RR]; Bousquet (1989: 33, 36; Fig. 28) [L_1_, L_2_, L_3_; RR]; Bousquet (2010a: 446; Figs 516, 530, 538) [L_2_, L_3_; RR].

*Pterostichus (Monoferonia) diligendus* (Chaudoir): Bousquet (1985c: 202, 206, 239; Figs 1-13, 48, 70, 82, 87) [L_1_, L_2_, L_3_; RR]; Bousquet (1989: 32, 36; Figs 21, 29, 31) [L_1_, L_2_, L_3_; RR]; Bousquet (1991a: Figs 34.74, 34.81); Bousquet (2010a: 446; Figs 502, 503, 537, 545, 551) [L_2_, L_3_; RR].

*Pterostichus (Phonias) femoralis* (Kirby): Bousquet (1985c: 201, 204, 227-228) [L_1_, L_2_, L_3_; RR]; Bousquet (1989: 31, 34) [L_1_, L_2_, L_3_; RR]; Bousquet (2010a: 444, 445) [L_2_, L_3_; RR].

*Pterostichus (Euferonia) lachrymosus* (Newman): Thompson (1979b: 56, 71; Figs 38a-g) [L_1_, L_3_; RR]; Bousquet (1985c: 201, 205, 241-242; Figs 51, 61) [L_1_, L_2_, L_3_; RR]; Bousquet (1989: 31, 35; Fig. 19) [L_1_, L_2_, L_3_; RR]; Bousquet (2010a: 446; Figs 520, 535) [L_2_, L_3_; RR].

*Pterostichus (Hypherpes) lama* (Ménétriés): Thompson (1979b: 56, 71, 72; Figs 39a-f) [L_3_; FC].

*Pterostichus (Pseudomaseus) luctuosus* (Dejean): Thompson (1979b: 56, 74; Figs 40a-g) [L_1_, L_2-3_; RR]; Bousquet (1985c: 201, 204, 238; Figs 45-47, 68) [L_1_, L_2_, L_3_; RR]; Bousquet (1989: 31, 34; Fig. 27) [L_1_, L_2_, L_3_; RR]; Bousquet (2010a: 444; Figs 517, 518, 544) [L_2_, L_3_; RR].

*Pterostichus (Morphnosoma) melanarius* (Illiger)**†**: Schiødte (1867: 511) [L_1-3_; ?]; Rupertsberger (1872: 573-575) [L_3_; FC]; van Emden (1936b: Fig. 1, as *Pterostichus vulgaris*); Larsson (1941: 326-328, as *Pterostichus vulgaris*; Fig. 49d) [L_1-3_; ?]; Sharova (1958: 64, as *Pterostichus vulgaris*; Figs 120aa, 123f) [L_1-3_; ?]; Sharova (1964: 175, as *Pterostichus vulgaris*; Figs 150-24, 154-6) [L_1-3_; ?]; Larsson (1968: 384, 387, as *Pterostichus vulgaris*; Fig. 53d) [L_1-3_; ?]; Haberman (1968: 461; Fig. 275.11, 276.9) [L_2-3_; ?]; Thompson (1979b: 56, 77; Figs 42a-f) [L_2-3_; FC]; Bousquet (1985c: 201, 205, 241; Figs 50, 80, 85) [L_1_, L_2_, L_3_; RR]; Bousquet (1989: 31, 35) [L_1_, L_2_, L_3_; RR]; Arndt (1991b: 89, 93) [L_1_, L_2-3_; ?]; Arndt and Hůrka (1992a: 106; Figs 7, 10) [L_1_, L_2_, L_3_; RR]; Luff (1993: 108, 111; Figs 364-366) [L_1_, L_2-3_; ?]; Arndt and Hůrka (1993: 45, 48) [L_1_, L_2-3_; ?]; Bousquet (2010a: 445; Figs 519, 525) [L_2_, L_3_; RR].

*Pterostichus (Eosteropus) moestus* (Say): Bousquet (1984b: 1616; Figs 7-12) [L_1_, L_2_, L_3_; RR]; Bousquet (1989: 31, 35, 36; Figs 22, 35) [L_1_, L_2_, L_3_; RR].

*Pterostichus (Bothriopterus) mutus* (Say): Schaupp (1881a: 89; Figs Ei-v) [L_3_; FC]; Bousquet (1985c: 201, 204, 232; Figs 22, 40, 78) [L_1_, L_2_, L_3_; RR]; Bousquet (1989: 32, 35) [L_1_, L_2_, L_3_; RR]; Bousquet (2010a: 445) [L_2_, L_3_; RR].

*Pterostichus (Phonias) patruelis* (Dejean): Thompson (1979b: 56, 77, 78; Figs 43a-f) [L_1_, L_2-3_; RR]; Bousquet (1985c: 201, 204, 227; Figs 21, 38, 39, 67, 89) [L_1_, L_2_, L_3_; RR]; Bousquet (1989: 31, 34; Figs 11, 25-26) [L_1_, L_2_, L_3_; RR]; Bousquet (1999: Fig. 121); Bousquet (2010a: 444, 445; Figs 513, 514, 528, 543) [L_2_, L_3_; RR].

*Pterostichus (Bothriopterus) pensylvanicus* LeConte: Goulet (1974a: 19) [L_1_, L_2_, L_3_; RR]; Thompson (1979b: 55, 80; Figs 44a-d) [L_1_, L_2-3_; RR+FC]; Bousquet (1985c: 202, 205, 233; Figs 23, 41, 79, 90) [L_1_, L_2_, L_3_; RR]; Bousquet (1989: 32, 35; Fig. 24) [L_1_, L_2_, L_3_; RR]; Bousquet (2010a: 445; Fig. 527) [L_2_, L_3_; RR].

*Pterostichus (Abacidus) permundus* (Say): Kirk (1972: 1355; Figs 17 [Pl. 2], 3-17) [L_1_; FC]; Thompson (1979a: Fig. 61); Thompson (1979b: 49, 50; Figs 25a-g) [L_1_, L_3_; RR]; Bousquet (1985c: 200, 240; Figs 25, 49, 60) [L_1_; RR]; Bousquet (1989: 30; Fig. 18) [L_1_; RR]; Bousquet (2010a: Fig. 524).

*Pterostichus (Cryobius) pinguedineus* (Eschscholtz): Bousquet (1985c: 202, 205, 206, 248-249; Figs 56, 83) [L_1_, L_2_, L_3_; RR]; Bousquet (1989: 32, 37) [L_1_, L_2_, L_3_; RR]; Bousquet (2010a: 446, 447; Fig. 504) [L_2_, L_3_; RR].

*Pterostichus (Lenapterus) punctatissimus* (Randall): Thompson (1979b: 56, 80, 81; Figs 45a-i) [L_1_, L_2-3_; RR]; Bousquet (1985c: 202, 205, 244; Figs 52, 53) [L_1_, L_2_, L_3_; RR]; Bousquet (1989: 33, 35) [L_1_, L_2_, L_3_; RR]; Bousquet (2010a: 445; Fig. 521) [L_2_, L_3_; RR].

*Pterostichus (Cylindrocharis) rostratus* (Newman): Bousquet (1985c: 202, 206, 245; Fig. 54) [L_1_, L_2_, L_3_; RR]; Bousquet (1989: 32, 36) [L_1_, L_2_, L_3_; RR]; Bousquet (2010a: 446; Fig. 522) [L_2_, L_3_; RR].

*Pterostichus (Leptoferonia) sphodrinus* LeConte: Thompson (1979b: 57, 83, 84; Figs 47a-h) [L_1_, L_2-3_; RR].

*Pterostichus (Phonias) strenuus* (Panzer)**†**: Larsson (1941: 325, 329; Fig. 48b) [L_1-3_; ?]; Sharova (1958: 62; Fig. 120s) [L_1-3_; ?]; Sharova (1964: 173; Fig. 150-16) [L_1-3_; ?]; Larsson (1968: 383, 388, 389) [L_1-3_; ?]; Haberman (1968: 460; Fig. 276.2) [L_2-3_; ?]; Desender and Panné (1983: 139, 141-143; Figs 1, 2a-g, 3a-g, 4a-h) [L_1_, L_2-3_; RR]; Bousquet (1985c: 202, 206, 226-227; Figs 20, 36, 37) [L_1_, L_2_, L_3_; RR]; Bousquet (1989: 32, 36; Fig. 17) [L_1_, L_2_, L_3_; RR]; Arndt (1991b: 90, 92; Fig. 85) [L_1_, L_2-3_; ?]; Arndt and Hůrka (1992b: 264; Figs 26, 27) [L_1_, L_2-3_; RR]; Luff (1993: 108, 113; Figs 373, 374) [L_1-3_; ?]; Arndt and Hůrka (1993: 46, 48) [L_1_, L_2-3_; RR]; Makarov (1996: Figs 27, 53); Bousquet (2010a: 446, 447; Fig. 512) [L_2_, L_3_; RR].

*Pterostichus (Euferonia) stygicus* (Say): Dimmock and Knab (1904: 41-43; Fig. 3; Figs 1, 4 [Pl. 4]); Thompson (1979a: Fig. 60); Bousquet (1985c: 201, 205, 243) [L_1_, L_2_, L_3_; RR]; Bousquet (1989: 31, 35) [L_1_, L_2_, L_3_; RR]; Bousquet (2010a: 446) [L_2_, L_3_; RR].

*Pterostichus (Eosteropus) superciliosus* (Say): Bousquet (1984b: 1616) [L_1_, L_2_, L_3_; RR+FC]; Bousquet (1989: 31, 36; Fig. 23) [L_1_, L_2_, L_3_; RR+FC].

*Pterostichus (Pseudomaseus) tenuis* (Casey): Bousquet (1985c: 201, 204, 238) [L_1_, L_2_, L_3_; RR]; Bousquet (1989: 31, 34) [L_1_, L_2_, L_3_; RR]; Bousquet (2010a: 444) [L_2_, L_3_; RR].

*Pterostichus (Bothriopterus) trinarius* (Casey, 1918): Bousquet (1989: 27-28, 32, 35, as *Pterostichus ohionis*; Figs 1-4) [L_1_, L_2_, L_3_; RR].

*Pterostichus (Hypherpes) tristis* (Dejean): Dimmock and Knab (1904: 46-47, as *Pterostichus adoxus*; Fig. 6 [Pl.4]); Bousquet (1985c: 200, 203, 246-247) [L_1_, L_2_, L_3_; RR]; Bousquet (1989: 30, 34) [L_1_, L_2_, L_3_; RR]; Bousquet (2010a: 444) [L_2_, L_3_; RR].

*Pterostichus (Argutor) vernalis* (Panzer)**†**: Larsson (1941: 326, 327; Figs 48d, 49c) [L_1-3_; ?]; Sharova (1958: 64; Fig. 120ab) [L_1-3_; ?]; Sharova (1964: 175; Fig. 150-25) [L_1-3_; ?]; Larsson (1968: 383, 386; Figs 52b, 53c) [L_1-3_; ?]; Haberman (1968: 460; Fig. 276.10) [L_2-3_; ?]; Desender and Panné (1983: 139, 141-143; Figs 5a-g, 6a-g, 7a-h, 8) [L_1_, L_2-3_; RR]; Arndt (1991b: 90, 93) [L_1_, L_2-3_; ?]; Arndt and Hůrka (1992b: 263) [L_1_, L_2-3_; RR]; Luff (1993: 108, 109; Figs 357, 358) [L_1_, L_2-3_; ?]; Arndt and Hůrka (1993: 46, 48) [L_1_, L_2-3_; RR].

*Stereocerus haematopus* (Dejean): Thompson (1979b: 56, 68; Figs 36a-h) [L_1_, L_2-3_; RR+FC]; Bousquet and Goulet (1984: Fig. 58); Bousquet (1985c: 201, 203, 214-219; Figs 30, 64, 72, 94) [L_1_, L_2_, L_3_; RR]; Bousquet (1989: 31, 33; Fig. 37) [L_1_, L_2_, L_3_; RR]; Bousquet (1999: Fig. 124); Bousquet (2010a: Figs 507, 540, 548).

*Stomis (Stomis) pumicatus* (Panzer)**†**: Luff (1993: 106; Figs 350-352) [L_2-3_; RR+FC]; Zetto Brandmayr and Marano (1994: 30-32; Figs 1-6 [Pl. 4], Figs 1-10 [Pl. 5]) [L_1_, L_2_, L_3_; FC].

### Zabrini

*Amara (Amara) aenea* (DeGeer)**†**: Boldori (1935: 150-151; Figs 1, 6) [L_1_; RR]; Scatizzi Branchini (1938: 216-220; Fig. 1) [L_1_; RR]; Larsson (1941: 333, 335) [L_1-3_; ?]; Sharova (1958: 68) [L_1-3_; ?]; Sharova (1964: 180) [L_1-3_; ?]; Larsson (1968: 395, 397) [L_1-3_; ?]; Haberman (1968: 394) [L_2-3_; ?]; Raynaud (1976b: 24; Figs 17-20 [Pl. 8]) [L_3_; FC]; Desender (1988: 154, 158; Figs 1-7 [Pl. 1], 1-7 [Pl. 2], 1-7 [Pl. 3]) [L_1_, L_2_, L_3_; RR+FC]; Arndt (1991b: 109) [L_1_, L_2-3_; ?]; Luff (1993: 133, 134, 136; Fig. 440) [L_1_, L_2-3_; ?]; Saska (2004: 198, 200, 232, 233; Figs 1, 11, 12, 15, 16, 26, 33, 39, 40, 42, 44, 46, 49-51, 57-61, 71, 73, 78, 83-84, 89, 93, 102, 111, 113, 115-117, 124-126, 128) [L_1_, L_2_, L_3_; RR].

*Amara (Curtonotus) alpina* (Paykull): Forsskåhl (1966: 29, 31; Figs 1-7) [L_2-3_; FC]; Luff (1993: 133, 143) [L_1-3_; LIT].

*Amara (Amara) anthobia* Villa and Villa**†**: Saska (2004: 200, 202, 232, 233; Figs 18, 27, 52, 62, 91) [L_1_, L_2_, L_3_; RR].

*Amara (Bradytus) apricaria* (Paykull)**†**: Schiødte (1867: 530) [L_1-3_; ?]; Xambeu (1892: 13, 14) [L_1-3_; FC]; Larsson (1941: 333, 336; Fig. 52i) [L_1-3_; ?]; Sharova (1958: 69; Figs 54, 58, 126b) [L_2-3_; ?]; Sharova (1964: 181; Figs 110-2, 112, 158-9) [L_1-3_; ?]; Larsson (1968: 393, 400; Fig. 57i) [L_1-3_; ?]; Haberman (1968: 426; Figs 114.4, 114.6, 240.2, 240.10, 240.16) [L_2-3_; ?]; Hůrka and Ducháč (1980a: 167, 169, 181; Figs 1-19) [L_1_, L_2_, L_3_; RR]; Arndt (1991b: 106, 107; Fig. 103) [L_1_, L_2-3_; LIT]; Luff (1993: 132, 141; Figs 448-451) [L_1_, L_2-3_; ?].

*Amara (Curtonotus) aulica* (Panzer)**†**: Larsson (1941: 333, 334; Fig. 52a) [L_1-3_; ?]; Sharova (1958: 66; Figs 125, 127c) [L_1-3_; ?]; Sharova (1964: 178; Figs 157, 158-3) [L_1-3_; ?]; Larsson (1968: 394, 395; Fig. 57a) [L_1-3_; ?]; Haberman (1968: 432; Figs 240.1, 240.6) [L_2-3_; ?]; Bílý (1975: Fig. 12-4); Hůrka and Ducháč (1980b: 259-261, 263, 269, 352; Figs 1-18, 29) [L_1_, L_2_, L_3_; RR]; Arndt (1991b: 108, 109) [L_1_, L_2-3_; LIT]; Luff (1993: 133, 142, 143; Figs 456-461) [L_1_, L_2-3_; ?]; Zetto Brandmayr et al. (1995: 196) [L_1-3_; ?]; Makarov and Brinev (2001: Fig. 75).

*Amara (Bradytus) avida* (Say): Bousquet (2010a: Figs 555, 558, 561, 563).

*Amara (Celia) bifrons* (Gyllenhal)**†**: Schiødte (1867: 530, as *Amara livida*) [L_1-3_; ?]; Larsson (1941: 333, 336; Fig. 52h) [L_1-3_; ?]; Sharova (1958: 67; Figs 127e, 129b) [L_1-3_; ?]; Sharova (1964: 179; Figs 158-5, 159-2) [L_1-3_; ?]; Larsson (1968: 393, 399; Fig. 57h) [L_1-3_; ?]; Haberman (1968: 414; Figs 240.8, 240.11) [L_2-3_; ?]; Bílý (1975: 35-36, 62; Fig. 5) [L_2_, L_3_; RR]; Raynaud (1976b: 23, 24; Figs 14-16 [Pl. 8]) [L_3_; FC]; Thompson (1979b: 87-89; Figs 49a-f) [L_2_; FC]; Arndt (1991b: 107, 108) [L_1_, L_2-3_; LIT]; Luff (1993: 133, 140; Fig. 445) [L_1_, L_2-3_; ?]; Makarov (1994: Fig. 54).

*Amara (Celia) brunnea* (Gyllenhal): Larsson (1941: 332, 336) [L_1-3_; ?]; Sharova (1958: 67) [L_1-3_; ?]; Sharova (1964: 179) [L_1-3_; ?]; Larsson (1968: 394, 399) [L_1-3_; ?]; Bílý (1975: Fig. 12-3); Arndt (1991b: 106) [L_1-3_; ?]; Luff (1993: 132, 141; Figs 446, 447) [L_1-3_; ?]; Makarov (1996: Fig. 30); Makarov and Brinev (2001: Fig. 68).

*Amara (Curtonotus) carinata* (LeConte): Kirk (1972: 1355; Figs 14 [Pl. 2], 3-14) [L_1-3_; FC]; Thompson (1979b: 87, 89; Figs 50a-h) [L_1_, L_2-3_; RR].

*Amara (Amara) communis* (Panzer)**†**: Larsson (1941: 333, 335; Fig. 52c) [L_1-3_; ?]; Sharova (1958: 68) [L_1-3_; ?]; Sharova (1964: 180) [L_1-3_; ?]; Larsson (1968: 394, 396; Fig. 57c) [L_1-3_; ?]; Haberman (1968: 394) [L_2-3_; ?]; Arndt (1991b: 109, 110) [L_1_, L_2-3_; ?]; Luff (1993: 134, 135; Figs 435, 436) [L_1_, L_2-3_; ?]; Hůrka and Růžičková (1999: 452, 457, 461; Figs 9-19) [L_1_, L_2_, L_3_; RR].

*Amara (Amara) cupreolata* Putzeys: Dogger and Olson (1966: 94, 95; Figs 3-4) [L_3_; FC].

*Amara (Amarocelia) erratica* (Duftschmid): Xambeu (1903: 8-9) [L_1-3_; FC]; Sharova (1958: 68) [L_1-3_; ?]; Sharova (1964: 180) [L_1-3_; ?]; Haberman (1968: 414) [L_2-3_; ?]; Bílý (1971: 89-93; Figs 1-15) [L_1_, L_2_, L_3_; RR]; Bílý (1975: 48-49, 60, 62; Figs 11, 12-1) [L_1_, L_2_, L_3_; RR]; Arndt (1991b: 107, 108,110) [L_1_, L_2-3_; LIT]; Luff (1993: 134, 140) [L_1-3_; LIT]; Zetto Brandmayr et al. (1995: 196) [L_1-3_; ?].

*Amara (Amara) eurynota* (Panzer)**†**: Larsson (1941: 334; Fig. 52b) [L_1-3_; ?]; Sharova (1958: 68; Fig. 129d) [L_1-3_; ?]; Sharova (1964: 179; Fig. 159-4) [L_1-3_; ?]; Larsson (1968: 393, 394, 396; Fig. 57b) [L_1-3_; ?]; Haberman (1968: 394; Fig. 240.12) [L_2-3_; ?]; Bílý (1972: 324-328; Figs 1-19) [L_1_, L_2_, L_3_; RR]; Raynaud (1976b: 22, 23; Figs 7-13 [Pl. 8]) [L_3_; FC]; Arndt (1991b: 109, 110; Figs 102, 144) [L_1_, L_2-3_; ?]; Luff (1993: 133, 136) [L_1_, L_2-3_; LIT].

*Amara (Bradytus) exarata* Dejean: Kirk (1972: 1355; Figs 12 [Pl. 1], 3-12) [L_1-3_; FC]; Thompson (1979b: 87, 91; Figs 51a-f) [L_1_, L_2-3_; RR+FC].

*Amara (Amara) familiaris* (Duftschmid)**†**: Schiødte (1867: 531) [L_1-3_; ?]; Beling (1877: 45-47) [L_1-3_; ?]; Larsson (1941: 333, 335; Fig. 52e) [L_1-3_; ?]; Sharova (1958: 67; Fig. 129a) [L_1-3_; ?]; Sharova (1964: 178; Fig. 159-1) [L_1-3_; ?]; Larsson (1968: 393, 397; Fig. 57e) [L_1-3_; ?]; Haberman (1968: 394; Fig. 240.14) [L_2-3_; ?]; Desender (1988: 159; Figs 1-7 [Pl. 4], 1-7 [Pl. 5], 1-7 [Pl. 6]) [L_1_, L_2_, L_3_; RR+FC]; Arndt (1991b: 109; Fig. 104) [L_1_, L_2-3_; ?]; Luff (1993: 132, 137; Fig. 441) [L_1_, L_2-3_; ?]; Saska (2004: 202, 204, 232, 233; Figs 5, 17, 35, 63, 72, 90, 103, 122, 129, 137) [L_1_, L_2_, L_3_; RR].

*Amara (Amarocelia) farcta* LeConte: Kirk (1972: 1355; Figs 13 [Pl. 2], 3-13) [L_2-3_; FC]; Thompson (1979b: 87, 91, 92; Figs 52a-f) [L_1_, L_2-3_; RR].

*Amara (Bradytus) fulva* (Müller)**†**: Boldori (1935: 151; Figs 3, 5) [L_1_; RR]; Larsson (1941: 333, 336) [L_1-3_; ?]; van Emden (1942: Fig. 10); Sharova (1958: 70; Fig. 130) [L_1-3_; ?]; Sharova (1964: 180; Fig. 160) [L_2-3_; ?]; Larsson (1968: 393, 400, 401) [L_2-3_; ?]; Haberman (1968: 426; Fig. 240.3) [L_2-3_; ?]; Raynaud (1976b: 20, 22; Figs 1-6 [Pl. 8]) [L_3_; FC]; Hůrka and Ducháč (1980a: 173, 174, 176, 181; Figs 20-33, 670) [L_1_, L_2_, L_3_; RR]; Arndt (1991b: 106, 107) [L_1_, L_2-3_; LIT]; Luff (1993: 132, 141, 142; Figs 452, 453) [L_1_, L_2-3_; ?].

*Amara (Amara) impuncticollis* (Say): Thompson (1979a: Figs 66a-c); Thompson (1979b: 87, 93, 94; Figs 53a-c) [L_3_; RR].

*Amara (Amara) littoralis* Dejean: Thompson (1979b: 87, 94; Figs 54a-f) [L_1_, L_2_; RR].

*Amara (Amara) lunicollis* Schiødte: Larsson (1941: 333, 335; Figs 52d, 53) [L_1-3_; ?]; Sharova (1958: 66; Fig. 128) [L_1-3_; ?]; Sharova (1964: 178; Fig. 158-7) [L_1-3_; ?]; Larsson (1968: 394, 396, 397; Figs 57d, 58) [L_1-3_; ?]; Haberman (1968: 394; Fig. 240.9) [L_2-3_; ?]; Luff (1993: 132, 135; Figs 437-439) [L_1-3_; ?]; Saska (2004: 206, 208, 232, 233; Figs 2, 19, 32, 34, 48, 55, 64, 76, 79, 104, 114, 119, 130, 135) [L_1_, L_2_, L_3_; RR].

*Amara (Celia) musculis* (Say): Bousquet (2010a: Figs 557, 560, 565).

*Amara (Percosia) obesa* (Say): Riley et al. (1878: 291, 292; Figs 25a,d-g) [L_2-3_; FC]; Lugger (1899: Figs 20a,d-g); Kirk (1972: 1355; Figs 11 [Pl. 1], 3-11) [L_1-3_; FC]; **?**Bousquet (2010a: Figs 556, 559, 564).

*Amara (Amara) ovata* (Fabricius)**†**: Arndt (1991b: 110) [L_2-3_; ?]; Makarov and Brinev (2001: Fig. 74).

*Amara (Paracelia) quenseli* (Schönherr): Sharova (1958: 67; Fig. 127d) [L_1-3_; ?]; Larsson and Gígja (1959: 42, 43) [L_1-3_; FC]; Sharova (1964: 179; Fig. 158-4) [L_1-3_; ?]; Larsson (1968: 393, 399) [L_1-3_; ?]; Haberman (1968: 414; Fig. 240.7) [L_2-3_; ?]; Bílý (1975: 51-54, 60, 61; Figs 8-10) [L_1_, L_2_, L_3_; RR]; Arndt (1991b: 106) [L_1-3_; ?]; Luff (1993: 132, 139; Figs 46, 443, 444) [L_1_, L_2-3_; ?]; Zetto Brandmayr et al. (1995: 193) [L_1-3_; ?].

*Amara (Curtonotus)* *sp.*: Bousquet (2010a: Figs 554, 562).

### Oodini

*Lachnocrepis parallela* (Say): Bousquet (2010a: Figs 566, 569).

*Oodes americanus* Dejean: Thompson (1979a: Figs 75a-c).

*Oodes fluvialis* LeConte: Bousquet (2010a: Figs 567, 568).

*Oodes* *sp.*: Chu (1945: 29, 30; Figs 6, 75, 79) [L_2-3_; FC].

### Chlaeniini

*Chlaenius (Lithochlaenius) cordicollis* Kirby: Schaupp (1880b: 26; Figs Bi-viii, as *Chlaenius leucoscelis*) [L_3_; FC]; Bousquet and Goulet (1984: Figs 34, 41); Bousquet (2010a: Fig. 574).

*Chlaenius (Chlaeniellus) impunctifrons* Say: Claassen (1919: 97-99, Figs 3, 4, 7 [Pl. 6]) [L_1_, L_2_, L_3_; RR].

*Chlaenius (Chlaenius) laticollis* Say: Schaupp (1880a: 17-18) [L_2-3_; FC].

*Chlaenius (Agostenus) niger* Randall: Makarova (2001: 302-305) [L_2_; RR].

*Chlaenius (Chlaeniellus) pennsylvanicus* Say: Chu (1945: 21, 24, 25; Figs 5, 8, 14, 17, 18) [L_3_; FC].

*Chlaenius (Chlaenius) platyderus* Chaudoir: Kirk (1972: 1355; Figs 8 [Pl. 1], 3-8) [L_2-3_; FC].

*Chlaenius (Lithochlaenius) prasinus* Dejean: Thompson and Allen (1974: 196-199; Figs 4a-c) [L_3_; FC]; Thompson (1979a: Fig. 74).

*Chlaenius (Chlaenius) sericeus* (Forster): Wickham (1895b: 763, 764) [L_3_; FC]; Boldori (1940: 285) [L_1-3_; LIT]; **?**van Emden (1942: Figs 62, 79); Chu (1945: 22, 28, 29; Figs 2, 15, 16, 47) [L_2-3_; FC]; Kirk (1972: 1355; Figs 9 [Pl. 1], 3-9) [L_2-3_; FC]; Bousquet (2010a: Fig. 571).

*Chlaenius (Eurydactylus) tomentosus* (Say): Kirk (1972: 1355; Figs 10 [Pl. 1], 3-10) [L_1-3_; FC]; Thompson (1979a: Figs 73a-d); Bousquet (2010a: Figs 570, 575).

*Chlaenius (Chlaeniellus) tricolor* Dejean: Chu (1945: 21, 25, 26; Figs 4, 11, 13) [L_3_; FC]; Bousquet (2010a: Figs 572, 573, 576).

### Licinini

*Badister* *sp.*: Bousquet (2010a: Figs 579-581, 583, 585).

*Dicaelus (Paradicaelus) dilatatus* Say: Schaupp (1878a: 3; Fig. 1) [L_3_; FC]; Schaupp (1879: 14) [L_3_; LIT].

*Dicaelus (Paradicaelus) elongatus* Bonelli: Schaupp (1878b: 43) [L_3_; FC]; Schaupp (1879: 14) [L_3_; LIT].

*Dicaelus (Paradicaelus) politus* Dejean: Schaupp (1878c: 44) [L_3_; FC]; Bousquet (2010a: Figs 577, 582, 584).

*Dicaelus (Dicaelus) purpuratus* Bonelli: Dimmock and Knab (1904: 21-26; Figs 1-2 [Pl. 1], Figs 1-10 [Pl. 2]) [L_3_; FC].

*Dicaelus (Dicaelus) purpuratus splendidus* Say: Wickham (1893a: 195-197; Figs 1, 1c-e) [L_3_; FC].

*Dicaelus (Paradicaelus) sculptilis* Say: Kirk (1972: 1355; Figs 19 [Pl. 2], 3-19) [L_2-3_; FC]; Thompson (1979a: Figs a-c).

*Dicaelus* *sp.*: Horn (1878a: 37-39; Figs 5a-e [Pl. II]) [L_2-3_; FC]; Schaupp (1879: 14) [L_3_; LIT]; Beutel (1992c: Figs 9, 10).

*Diplocheila (Isorembus) impressicollis* (Dejean): van Emden (1942: Fig. 15, as *Rembus laticollis*); **?**Bousquet (2010a: 465) [L_2_, L_3_; FC].

*Diplocheila (Isorembus) obtusa* (LeConte): Bousquet (2010a: 465; Figs 578, 586) [L_3_; FC].

*Diplocheila (Isorembus) striatopunctata* (LeConte): Bousquet and Goulet (1984: Fig. 33); Bousquet (1991a: Fig. 34.83); Makarov (1994: Fig. 62); Bousquet (2010a: 465) [L_2_, L_3_; FC].

### Harpalini

*Acupalpus (Acupalpus) meridianus* (Linnaeus)**†**: Larsson (1941: 350; Figs 60d, 62c) [L_1-3_; ?]; Sharova (1958: Figs 133f, 134b); Sharova (1964; Figs 161-6, 162-1); Larsson (1968: 420; Figs 66d, 68c) [L_1-3_; ?]; Haberman (1968: Figs 206.15, 207.2); Arndt (1991a: 50; Fig. 5.9) [L_2_; RR]; Arndt (1991b: 121; Fig. 120) [L_1-3_; ?]; Luff (1993: 159; Fig. 510) [L_1-3_; RR].

*Agonoleptus conjunctus* (Say): Chu (1945: 22, 49, 50; Figs 54, 63, 64) [L_3_; FC]; Bousquet (2010a: Figs 596, 621).

*Amphasia (Amphasia) interstitialis* (Say): van Emden (1942: Fig. 12); Chu (1945: 46-47; Figs 36, 67, 68) [L_2-3_; FC]; Bousquet and Tchang (1992: Figs 4, 13, 21); Bousquet (2010a: Fig. 599).

*Anisodactylus (Anisodactylus) binotatus* (Fabricius)**†**: Rupertsberger (1872: 576) [L_3_; FC]; Xambeu (1911: 70, 71) [L_1-3_; FC]; Bøving (1911: 163-169, 172-178; Figs 6, 12, 17, 21 [Pl. 6]) [L_2-3_; FC]; Larsson (1941: 342; Fig. 57a) [L_1-3_; ?]; Jeannel (1942: Figs 217a-h); Sharova (1958: 76; Fig. 133n) [L_1-3_; ?]; Sharova (1964: 187; Fig. 161-12) [L_1-3_; ?]; Larsson (1968: 414, 415; Fig. 65a) [L_1-3_; ?]; Haberman (1968: Fig. 206.19); Arndt (1991b: 112) [L_1_, L_2-3_; ?]; Luff (1993: 152, 153; Figs 493-496) [L_1_, L_2-3_; ?].

*Anisodactylus (Anisodactylus) californicus* Dejean: Chu (1945: 22, 44; Figs 39, 65, 66) [L_2-3_; FC].

*Anadaptus (Anadaptus) discoideus* Dejean: Dogger and Olson (1966: 95, 96; Figs 5-6) [L_3_; FC].

*Anisodactylus (Anisodactylus) harrisii* LeConte: Bousquet and Tchang (1992: Figs 23, 30).

*Anisodactylus (Anisodactylus) kirbyi* Lindroth: Bousquet and Tchang (1992: Figs 16, 40); Bousquet (2010a: Figs 602, 619).

*Anisodactylus (Gynandrotarsus) merula* (Germar): Bousquet and Tchang (1992: Figs 9, 24); Bousquet (2010a: Figs 592, 612, 618).

*Anisodactylus (Anisodactylus) nigrita* Dejean: Bousquet and Goulet (1984: Fig. 32); Bousquet (1991a: Fig. 34.82); Bousquet and Tchang (1992: Fig. 7).

*Anisodactylus (Gynandrotarsus) rusticus* (Say): Bousquet and Tchang (1992: Figs 18, 27, 39); Bousquet (2010a: Figs 604, 606).

*Anisodactylus (Anadaptus) sanctaecrucis* (Fabricius): Kirk (1976: 354; Fig. 1) [L_1-3_; RR]; Thompson (1979a: Figs 69a-c); Bousquet and Tchang (1992: Figs 8, 17, 33, 35); Bousquet (2010a: Figs 593, 603, 622).

*Anisodactylus (Spongopus) verticalis* (LeConte): Bousquet and Tchang (1992: Figs 6, 15, 34); Bousquet (2010a: Figs 601, 624).

*Anisodactylus* (or close) *sp*.: Chu (1945: 22, 45, 46; Figs 40, 69, 70) [L_2-3_; FC].

*Bradycellus (Bradycellus) harpalinus* (Audinet-Serville)**†**: van Emden (1945: 27) [L_1-3_; ?]; **?**Larsson (1968: 418) [L_1-3_; ?]; Luff (1993: 156, 157; Figs 507, 508) [L_1_, L_2-3_; FC].

*Bradycellus (Stenocellus) rupestris* (Say): Chu (1945: 48, 49; Figs 56, 58, 60) [L_3_; FC]; Matalin (1996: 290) [L_1-3_; LIT]; Hůrka (1997: 197) [L_1-3_; LIT]; Matalin (2001: 317) [L_1-3_; LIT].

*Cratacanthus dubius* (Palisot de Beauvois): van Emden (1942: Fig. 77); Chu (1945: 22, 32, 33; Figs 37, 48, 50) [L_3_; FC]; Chu (1945: 22, 33, 34, as *Cratacanthus* *sp.*; Figs 38, 43, 45, 46) [L_2-3_; FC]; Thompson (1979a: Figs a-f).

*Dicheirotrichus (Trichocellus) cognatus* (Gyllenhal): Larsson (1941: 347, 348; Fig. 61b) [L_1-3_; ?]; Larsson and Gígja (1959: 46) [L_1-3_; FC]; Larsson (1968: 417; Fig. 67b) [L_1-3_; ?]; Böcher (1988: Fig. 5); Luff (1993: 154, 155; Figs 503, 504) [L_1_, L_2-3_; ?]; Matalin (2001: 314, 320; Figs 15, 22, 30, 31, 51, 59, 60) [L_2_; RR].

*Euryderus grossus* (Say): van Emden (1942: 73, as *Euryderus zabroides*) [L_3_; FC]; Chu (1945: 30, 31, as *Nothopus zabroides*; Figs 35, 41, 42) [L_3_; FC].

*Geopinus incrassatus* (Dejean): Kirk (1972: 1354, 1355; Figs 6 [Pl. 1], 3-6) [L_1-3_; FC]; Bousquet and Tchang (1992: Figs 10, 11, 19, 26, 36, 41); Bousquet (2010a: Figs 594, 605, 610, 620, 623).

*Harpalus (Harpalus) affinis* (Schrank)**†**: Schiødte (1867: 531-535, as *Harpalus aeneus*; Figs 1-3 [Pl. 22]) [L_1-3_; ?]; Larsson (1941: 344, 345, as *Harpalus aeneus*; Figs 58b, 59b) [L_1-3_; ?]; Chu (1945: 23, 36, 37, as *Harpalus viridiaeneus*; Figs 25, 80, 83) [L_3_; FC]; Sharova (1958: Figs 133q, 134a, as *Harpalus aeneus*); Sharova (1964: 187, as *Harpalus aeneus*; Figs 163-1, 164-1) [L_1-3_; ?]; Sharova (1967: Figs 8.6, as *Harpalus aeneus*); Larsson (1968: 408, 410, 411, as *Harpalus aeneus*; Figs 63b, 64b) [L_1-3_; ?]; Haberman (1968: Fig. 206.4, 207.5, as *Harpalus aeneus*); Arndt (1991b: 115, as *Harpalus aeneus*; Figs 107, 145) [L_1-3_; ?]; Putchkov (1992: 73) [L_1_; ?]; Luff (1993: 145, 149; Figs 481-483) [L_1_, L_2-3_; ?].

*Harpalus (Megapangus) caliginosus* (Fabricius): **?**Chu (1945: 23, 34, 35; Figs 26, 89, 90) [L_2-3_; FC]; Kirk (1972: 1354; Figs 1 [Pl. 1], 3-1) [L_2-3_; FC]; Thompson (1979a: Figs 71a-e).

*Harpalus (Pseudoophonus) compar* LeConte: Chu (1945: 24, 40, 41; Figs 22, 73, 74) [F_3_; FC].

*Harpalus (Plectralidus) erraticus* Say: Kirk (1972: 1354; Figs 4 [Pl. 1], 1, 3-4) [L_1-3_; FC].

*Harpalus (Pseudoophonus) erythropus* Dejean: Chu (1945: 23, 39, 40; Figs 23, 27, 77, 78) [L_3_; FC].

*Harpalus (Harpalus) herbivagus* Say: Lugger (1899: Figs 21a-c); Kirk (1972: 1354; Figs 2 [Pl. 1], 2, 3-2) [L_1-3_; FC].

*Harpalus (Opadius) laevipes* Zetterstedt: Sharova (1958: Fig. 51, as *Harpalus quadripunctatus*); Sharova (1964: 188, 189, as *Harpalus quadripunctatus*; Figs 109, 163-7, 164-7) [L_1-3_; ?]; Sharova (1967: Fig. 8.3, as *Harpalus quadripunctatus*); Larsson (1968: 407, 412, as *Harpalus quadripunctatus*) [L_1-3_; ?]; Haberman (1968: Figs 203, 206.8, 207.10, as *Harpalus quadripunctatus*); Arndt (1991b: 116, as *Harpalus quadripunctatus*) [L_1-3_; ?]; Luff (1993: 145, 150, 151, as *Harpalus quadripunctatus*) [L_1-3_; ?].

*Harpalus (Pseudoophonus) pensylvanicus* (DeGeer): Quaintance and Jenne (1912: Figs 32b-d); Chu (1945: 23, 41, 42; Figs 21, 29-31, 33, 84) [L_3_; FC]; Kirk (1972: 1354; Figs 3 [Pl. 1], 3-3) [L_1-3_; FC]; Bousquet (2010a: Figs 588, 611).

*Harpalus (Harpalus) rubripes* (Duftschmid)**†**: Xambeu (1894: 119-121) [L_1-3_; FC]; Larsson (1941: 344-346; Figs 58d, 59d) [L_1-3_; ?]; Sharova (1958: Figs 133s, 134g); Sharova (1964: 189; Figs 163-8, 164-8) [L_1-3_; ?]; Sharova (1967: Figs 1.4, 2.4); Larsson (1968: 407, 412; Fig. 64d) [L_1-3_; ?]; Haberman (1968: Figs 206.9, 207.11); Arndt (1991b: 116; Figs 108, 113) [L_1-3_; ?]; Luff (1993: 145, 151; Figs 490, 491) [L_1_, L_2-3_; ?].

*Harpalus (Pseudoophonus) rufipes* (DeGeer)**†**: Schiødte (1867: 535, as *Harpalus ruficornis*; Figs 4-11 [Pl. 22]) [L_1-3_; ?]; Znojko (1929: 351, 352, as *Harpalus pubescens*; Figs 19, 20a-b, 21a-e, 22, 23, 24) [L_1-3_; ?]; Larsson (1941: 343, as *Pseudophonus pubescens*; Figs 58a, 59a) [L_1-3_; ?]; van Emden (1942: Fig. 78); van Emden (1945: 27; Figs 19, 26) [L_1-3_; ?]; Sharova (1958: 74; Figs 52, 133p) [L_1-3_; ?]; Sharova (1964: 186; Figs 110-1, 161-14) [L_1-3_; ?]; Sharova (1967: 892; Figs 1.2, 2.3, 8.1) [L_1-3_; ?]; Larsson (1968: 406, 409, 410, as *Harpalus pubescens*; Figs 60, 63a, 64a) [L_1-3_; ?]; Brandmayr et al. (1980; Fig. 1d); Brandmayr and Zetto Brandmayr (1982: Fig. 2); Arndt (1991b: Figs 115, 116); Luff (1993: 145, 147; Figs 476-480) [L_1_, L_2-3_; ?]; Makarov (1994: Fig. 97).

*Harpalus (Harpalus) solitaris* Dejean: Hůrka and Papoušek (2002: 112; Figs 54-61) [L_3_; RR].

*Harpalus (Pseudoophonus) vagans* LeConte: Chu (1945: 24, 37, 38; Figs 20, 49, 85, 86) [L_3_; FC].

*Harpalus* *sp.* [probably *Harpalus herbivagus*]: Riley et al. (1878: 290; Figs 24a-c) [L_2-3_; FC].

*Harpalus* *sp.* [might be a young instar of *Harpalus caliginosus*]: Chu (1945: 23, 35, 36; Figs 19, 28, 76) [L_1-3_; FC].

*Notiobia (Anisotarsus) nitidipennis* (LeConte): Bousquet and Tchang (1992: 756; Figs 2, 12, 20, 25, 29, 31) [L_1_, L_2-3_; RR]; Bousquet (2010a: 469; Figs 590, 598, 608, 613) [L_2_, L_3_; RR].

*Notiobia (Anisotarsus) sayi* (Blatchley): van Emden (1942: Fig. 11); Chu (1945: 47-48; Figs 32, 44, 51, 71, 72) [L_2-3_; FC].

*Notiobia (Anisotarsus) terminata* (Say): Thompson (1979a: 68a-b); Bousquet and Tchang (1992: 756; Figs 1, 3, 28) [L_1_, L_2-3_; RR]; Bousquet (2010a: 469; Fig. 607) [L_2_, L_3_; RR].

*Ophonus (Metaphonus) puncticeps* Stephens**†**: Larsson (1941: 340, 341, as *Ophonus angusticollis*; Figs 55c, 56c) [L_1-3_; ?]; Larsson (1968: 406, 409, as *Harpalus angusticollis*; Figs 61c, 62c) [L_1-3_; ?]; Brandmayr and Zetto Brandmayr (1982: 87, 89, 98; Figs 69-75, 102, 103, 115) [L_1_, L_3_; RR]; Arndt (1991b: 117) [L_1-3_; LIT]; Luff (1993: 145, 146; Figs 472, 473) [L_1_, L_2-3_; ?]; Bousquet (2010a: Figs 587, 597, 609, 616).

*Ophonus (Metaphonus) rufibarbis* (Fabricius)**†**: Larsson (1941: 340, 341, as *Ophonus seladon*; Figs 55d, 56d) [L_1-3_; ?]; Larsson (1968: 406, 409, as *Harpalus seladon*; Figs 61d, 62d) [L_1-3_; ?]; **?**Brandmayr and Zetto Brandmayr (1982: 95, 98; Figs 91-95, 106) [L_3_; FC]; Arndt (1991b: 118) [L_1-3_; LIT].

*Polpochila (Phymatocephalus) capitata* (Chaudoir): Chu (1945: 53, 54; Figs 34, 57, 59) [L_2-3_; FC].

*Stenolophus (Agonoderus) comma* (Fabricius): **?**Chu (1945: 24, 52, 53, as *Agonoderus pallipes*; Figs 53, 87, 88) [L_2-3_; FC]; Kirk (1972: 1354; Figs 5 [Pl. 1], 3-5) [L_1-3_; FC]; Bousquet (2010a: Figs 595, 615).

*Stenolophus (Stenolophus) dissimilis* Dejean: Thompson (1979a: a-b).

*Stenolophus (Agonoderus) lecontei* (Chaudoir): Bryson and Dillon (1941: 50; Fig. 2, as *Agonoderus pallipes* Fabricius) [L_3_; FC].

*Stenolophus (Agonoderus) lineola* (Fabricius): van Emden (1942: Fig. 61); Chu (1945: 24, 51, 52; Figs 52, 91, 92) [L_2-3_; FC].

*Stenolophus* (*Stenolophus*) sp.: Chu (1945: 22, 50, 51) [L_2-3_; FC]; Sharova (1958: Fig. 133e); Sharova (1964; Fig. 161-5).

*Trichotichnus (Trichotichnus) dichrous* (Dejean): Chu (1945: 21, 43; Figs 24, 81, 82) [L_3_; FC].

*Trichotichnus (Trichotichnus) vulpeculus* (Say): Bousquet (2010a: Figs 589, 614).

*Xestonotus lugubris* (Dejean): Bousquet and Tchang (1992: Figs 5, 14, 22, 32, 37, 38); Bousquet (2010a: Figs 591, 600, 617).

### Sphodrini

*Calathus (Acalathus) advena* (LeConte): Lindroth (1956b: Fig. 35d, 36-Id, 36-IId, 36-IIId, 36-Ivd). Bousquet (2010a: Figs 627, 639).

*Calathus (Calathus) fuscipes* (Goeze)**†**: Bøving (1910: 335-341; Figs 5, 9, 13) [L_2-3_; FC]; Raynaud (1940b: 83, 84, 86, 87) [L_1_, L_2_, L_3_; FC]; Larsson (1941: 317; Fig. 41b) [L_1-3_; ?]; van Emden (1942: Fig. 47); Raynaud (1944: 89) [L_3_; FC]; van Emden (1945: Fig. 24); Sharova (1958: 59, 60; Fig. 120b) [L_1-3_; ?]; Sharova (1964: 171; Fig. 152-1) [L_1-3_; ?]; Larsson (1968: 370; Fig. 44b) [L_1-3_; ?]; Haberman (1968: 481; Fig. 297.1) [L_2-3_; ?]; Kůrka (1971: 247, 248, 260, 261; Figs 1-4, 6-7, 8a, 9, 11a-e, 13a-c, 14a) [L_1_, L_2_, L_3_; RR]; Arndt (1991b: 104; Fig. 97) [L_1_, L_2-3_; LIT]; Hovorka (1991: Fig. 18); Luff (1993: 117, 118; Figs 385-388) [L_1_, L_2-3_; ?].

*Calathus (Neocalathus) gregarius* (Say): Thompson (1979b: 35-37; Figs 17a-d) [L_1_, L_2-3_; RR+FC]; Bousquet (2010a: 482) [L_2_; RR].

*Calathus (Neocalathus) ingratus* Dejean: Thompson (1979a: Figs 65a-b); Thompson (1979b: 35, 39; Figs 18a-i) [L_1_, L_2-3_; RR+FC]; Bousquet (2010a: 482) [L_2_; RR].

*Calathus (Neocalathus) opaculus* LeConte: Thompson (1979b: 35, 40, 41; Figs 19a-c) [L_3_; FC]; Bousquet (2010a: 482) [L_3_; LIT].

*Laemostenus (Laemostenus) complanatus* (Dejean)**†**: van Emden (1945: 24) [L_1-3_; ?]; Thompson (1979b: 46, 47) [L_2-3_; FC]; Vereshchagina and Makarov (1986: 372) [L_1-3_; ?]; Casale (1988: 90) [L_1-3_; ?]; Arndt (1991b: 103) [L_1-3_; ?]; Luff (1993: 121) [L_1-3_; ?].

*Laemostenus (Pristonychus) terricola terricola* (Herbst)**†**: Chapuis and Candèze (1853: 376-378; Fig. 3 [Pl. 1]) [L_1-3_; FC]; Böving and Craighead (1931; Figs f-h [Pl. 4]); Larsson (1941: 315; Fig. 41a) [L_1-3_; ?]; van Emden (1945: 24; Figs 17, 18) [L_1-3_; ?]; Sharova (1958: 57; Fig. 120a) [L_1-3_; ?]; Sharova (1964: 167; Fig. 150-1) [L_1-3_; ?]; Larsson (1968: 368; Fig. 44a) [L_1-3_; ?]; Haberman (1968: 445; Fig. 306.1) [L_2-3_; ?]; Raynaud (1976c: 256, 257, as *Pristonichus terricola subcyanescens*; Figs 1-4) [L_3_; RR]; Vereshchagina and Makarov (1986: 372) [L_1-3_; ?]; Casale (1988: 90; Fig. 114) [L_1-3_; ?]; Arndt (1991b: 103) [L_1-3_; ?]; Hovorka (1991: 102, 103; Figs 8-12) [L_1_, L_2_, L_3_; RR]; Luff (1993: 120; Figs 399-401) [L_1_, L_2-3_; ?]; Bousquet (2010a: Figs 629, 638).

*Synuchus impunctatus* (Say): Lindroth (1956b: 564; Figs 35b, 36-Ib, 36-IIbg, 36-IIIb, 36-IVb) [L_1_; RR]; Thompson (1979a: Figs 64a-d); Thompson (1979b: 48; Figs 24a-f) [L_1_, L_2-3_; RR+FC]; Bousquet (2010a: Figs 626, 637).

### Platynini

*Agonum (Europhilus) anchomenoides* Randall: Thompson (1979b: 7, 10; Figs 1a-e) [L_3_; FC].

*Agonum (Europhilus) consimile* Gyllenhal: Lindroth (1955c: Fig. 1b).

*Agonum (Olisares) cupripenne* (Say): Bousquet (2010a: Fig. 634).

*Agonum (Olisares) errans* (Say): Bousquet (2010a: Figs 635, 640).

*Agonum (Europhilus) exaratum* (Mannerheim): Makarov (1996: Fig. 29).

*Agonum (Olisares) extensicolle* (Say): Schaupp (1881b: 91-92; Figs Ai-v) [L_3_; FC]; Thompson (1979b: 9, 11, 12; Figs 2a-e) [L_3_; RR].

*Agonum (Europhilus) gratiosum* (Mannerheim): Thompson (1979b: 7, 16; Figs 4a-g) [L_1_, L_3_; RR+FC]; Bousquet (2010a: Fig. 631).

*Agonum (Olisares) melanarium* Dejean: Thompson (1979b: 8, 18; Figs 5a-d) [L_3_; FC]; Bousquet (2010a: Fig. 633).

*Agonum (Agonum) muelleri* (Herbst)**†**: Raynaud (1940b: 77-79) [L_3_; FC]; Larsson (1941: 321; Figs 43b, 44a) [L_1-3_; ?]; van Emden (1942: Fig. 52); Sharova (1958: 61; Fig. 120k) [L_1-3_; ?]; Sharova (1964: 172; Fig. 150-8) [L_1-3_; ?]; Larsson (1968: 376, 378, 379; Figs 47f, 48a-b) [L_1-3_; ?]; Haberman (1968: 501; Fig. 306.8) [L_2-3_; ?]; Arndt (1991b: 98, 100; Figs 1, 6-11, 16, 92, 96) [L_1_; ?]; Luff (1993: 124, 128, 130; Figs 417-419) [L_1_, L_2-3_; ?]; Makarov (1996: Fig. 23).

*Agonum (Platynomicrus) nigriceps* LeConte: Bousquet (2010a: Fig. 630).

*Agonum (Olisares) octopunctatum* (Fabricius): Thompson (1979b: 8, 21; Figs 7a-e) [L_2-3_; RR].

*Agonum (Olisares) pallipes* (Fabricius): Thompson (1979b: 8, 21, 22; Figs 8a-b) [L_3_; FC].

*Agonum (Agonum) placidum* (Say): Kirk (1972: 1355; Figs 15 [Pl. 2], 3-15) [L_1-3_; FC].

*Agonum (Olisares) propinquum* (Gemminger and Harold): Thompson (1979b: 8, 24, 25; Figs 10a-g) [L_1_, L_2-3_; RR+FC].

*Agonum (Olisares) punctiforme* (Say): Thompson and Allen (1974: 192-195; Figs 3a-d) [L_3_; FC]; Thompson (1979a: Figs a-b); Thompson (1979b: 8, 26, 27; Figs 11a-e) [L_3_; FC].

*Agonum (Europhilus) retractum* LeConte: Thompson (1979b: 7, 29; Figs 12a-f) [L_1_, L_2-3_; RR+FC].

*Agonum (Olisares) striatopunctatum* Dejean: Thompson (1979a: Figs a-d); Thompson (1979b: 8, 31; Figs 13a-e) [L_2_; RR].

*Agonum (Olisares) sulcipenne* (Horn): Thompson (1979b: 8, 31, 32; Figs 14a-f) [L_2-3_; FC].

*Agonum (Olisares) tenue* (LeConte): Thompson (1979b: 8, 34; Figs 15a-h) [L_1_, L_2-3_; RR].

*Agonum (Europhilus) thoreyi* Dejean: Larsson (1941: 323; Figs 45c, 46d-e) [L_1-3_; ?]; Lindroth (1955c: Fig. 1a); Sharova (1958: 60; Fig. 120g) [L_1-3_; ?]; Sharova (1964: 172; Fig. 150-4) [L_1-3_; ?]; Larsson (1968: 376, 380; Figs 49c, 50d-e) [L_1-3_; ?]; Haberman (1968: 525; Fig. 306.4) [L_2-3_; ?]; Thompson (1979b: 7, 34, 35; Figs 16a-f) [L_1_, L_2_; RR+FC]; Arndt (1991b: 100; Fig. 94) [L_2-3_; ?]; Luff (1993: 123, 126; Figs 411, 412) [L_1_, L_2-3_; ?].

*Anchomenus (Anchomenus) funebris* (LeConte): Thompson (1979b: 9, 13, 14; Figs 3a-f) [L_1_, L_2_; RR]; Liebherr (1991b: 128-130; Figs 317-327) [L_1_; RR].

*Metacolpodes buchanani* (Hope): Liebherr (2000: Fig. 3g).

*Olisthopus parmatus* (Say): Bousquet (2010a: 483; Fig. 625) [L_1_; RR].

*Oxypselaphus pusillus* (LeConte): Bousquet (2010a: Fig. 628).

*Paranchus albipes* (Fabricius)**†**: Xambeu (1898: 55-57, as *Anchomenus pallipes*) [L_1-3_; ?FC]; Kemner (1913: 21, 22, as *Platynus ruficornis*; Figs 12, 3-4 [Pl. 2]) [L_3_; FC]; Raynaud (1940b: 76-78, as *Platynus ruficornis*) [L_3_; FC]; Larsson (1941: 319, 320, as *Platynus ruficornis*; Fig. 42b) [L_1-3_; ?]; Sharova (1958: 61, as *Agonum ruficorne*; Fig. 120o) [L_1-3_; ?]; Sharova (1964: 172, 173, as *Agonum ruficorne*; Fig. 150-12) [L_1-3_; ?]; Larsson (1968: 376, 377, as *Agonum ruficorne*; Fig. 47b) [L_1-3_; ?]; Haberman (1968: 517, as *Agonum ruficorne*; Fig. 306.12) [L_2-3_; ?]; Arndt (1991b: 98, 99, as *Platynus ruficornis*) [L_1_, L_2-3_; ?]; Luff (1993: 125, 130, 131; Figs 422-424) [L_1_, L_2-3_; ?].

*Platynus (Platynus) decentis* (Say): Thompson (1979a: Figs a-d); Thompson (1979b: 41, 42; Figs 20a-j) [L_1_, L_2-3_; RR+FC]; Bousquet (2010a: Figs 632, 636, 641).

*Platynus (Batenus) hypolithos* (Say): Thompson (1979b: 41, 44; Figs 21a-i) [L_3_; FC].

*Platynus (Batenus) mannerheimii* (Dejean): **?**Saalas (1917: 286; Figs 1-2) [L_2-3_; FC]; **?**Saalas (1923: 667) [L_2-3_; FC]; Thompson (1979b: 41, 45, 46; Figs 22a-f) [L_2-3_; FC].

*Sericoda bembidioides* Kirby: Liebherr (1991b: 130, 132; Figs 328-338) [L_1_; RR].

*Sericoda obsoleta* (Say): Thompson (1979b: 8, 18, 19; Figs 6a-f) [L_1_, L_2-3_; RR].

*Tanystoma maculicolle* (Dejean): van Emden (1945: 24) [L_1-3_; ?]; Liebherr (1984a: 531, 532, 534, 536; Figs 1, 2a-g, 3a-i) [L_1_, L_2_, L_3_; RR].

*Tetraleucus picticornis* (Newman): Thompson (1979b: 8, 24; Figs 9a-f) [L_1_, L_2-3_; RR]; Liebherr (1991b: 125; Figs 293-304) [L_1_; RR].

### Atranini

*Atranus pubescens* (Dejean): Bousquet (1985b: 330-331; Figs 1-6) [L_1_, L_2_, L_3_; FC]; Makarov (1994: Fig. 81); Bousquet (2010a: Figs 642-646).

### Lachnophorini

*Calybe (Ega) sallei* (Chevrolat): Liebherr (1983: 255; Figs 1-7) [L_2_, L_3_; FC].

### Odacanthini

*Colliuris (Cosnania) pensylvanica* (Linnaeus): Thompson (1979a: Figs 77a-c); Bousquet (2010a: 491; Figs 647-650) [L_1_, L_2-3_; FC].

### Ctenodactylini

*Leptotrachelus dorsalis* (Fabricius): Erwin and White (2012: 22, 24, 25, 27, 28 ; Figs 3-12) [L_1,3_; RR].

### Lebiini

*Apristus subsulcatus* (Dejean): Bousquet (2010a: 496-498; Figs 660, 669, 671, 673) [L_1_, L_2_, L_3_; RR+FC].

*Axinopalpus biplagiatus* (Dejean): Bousquet (2010a: 496; Fig. 659) [L_2-3_; FC].

*Calleida (Calleida) decora* (Fabricius): Thompson (1979a: Figs 78a-d); Zhou and Goyer (1993: Fig. 3a).

*Calleida (Calleida) punctata* LeConte: Bousquet and Goulet (1984: Fig. 36); Bousquet (1991a: Fig. 34.84); Zhou and Goyer (1993: Fig. 3b); Bousquet (2010a: Figs 663, 664, 676).

*Calleida (Calleida) viridipennis* (Say): Zhou and Goyer (1993: 234-236; Figs 1a-d) [L_1_, L_2_, L_3_; RR].

*Cymindis (Tarulus) vaporariorum* (Linnaeus): Hůrka (1969: 105-107; Figs 3a-m) [L_1_, L_2_, L_3_; FC]; Hůrka (1986: 47; Figs 85-99) [L_1_, L_2_, L_3_; RR]; Hůrka (1986: 48, as *Cymindis vaporariorum immaculata*; Figs 100-103) [L_1_, L_2_; RR]; Arndt (1991b: 133) [L1, L_1_, L_2-3_; LIT]; Luff (1993: 176, 177; Figs 581, 582) [L_1_, L_2-3_; ?]; Makarov (1996: Fig. 31).

*Cymindis* *sp.*: Bousquet and Goulet (1984: Figs 35, 46, 54, 59); Bousquet (1991a: Fig. 34.104); Bousquet (2010a: Figs 665, 666, 677, 678).

*Dromius (Dromius) piceus* Dejean: Mahar et al. (1983: 24-26; Figs 1-7) [L_1_, L_2_, L_3_; FC].

*Lebia (Lebia) analis* Dejean: Thompson (1979a: Figs 80a-b).

*Lebia (Loxopeza) grandis* Hentz: Chaboussou (1939: 414-419; Figs 18-20, 22-34) [L_1_, L_2_; RR].

*Lebia (Lebia) viridis* Say: Capogreco (1989a: 187-191; Figs 2-5, 7-16) [L_1_, L_2_; RR]; Makarov (1994: Figs 123, 125); Bousquet (2010a: Figs 656-658).

*Microlestes* *sp.*: Bousquet (2010a: 499; Figs 662, 670, 672, 674) [L_2-3_; FC].

*Mochtherus tetraspilotus* (Macleay)**†**: Gardner (1936: 193, 194; Figs 47-50) [L_3_; FC]; van Emden (1942: 78) [L_3_; FC].

*Onota floridana* Horn: van Emden (1942: 79) [L_2_; FC].

*Philophuga viridicollis* (LeConte): Larson (1969: 26; Figs 9, 11) [L_3_; FC].

*Philophuga viridis amoena* (LeConte): Mahar et al. (1983: Fig. 9).

*Philorhizus melanocephalus* (Dejean)**†**: Xambeu (1904: 88, 89) [L_1-3_; FC]; Larsson (1941: 357, 358; Fig. 67f) [L_1-3_; ?]; Larsson (1968: 429, 431; Fig. 73f) [L_1-3_; ?]; Luff (1993: 169, 172, 173; Figs 564-568) [L_1_, L_2-3_; FC].

*Plochionus (Plochionus) pallens* (Fabricius): van Emden (1945: 25) [L_1-3_; ?]; Arndt (1991b: Fig. 143).

*Plochionus (Menidius) timidus* Haldeman: Duffey (1892: 538; Figs a-e [Pl. 10]) [L_1_, L_3_; RR]; Larson (1969: 25; Figs 7, 8) [L_2-3_; FC]; Thompson (1979a: Figs 79a-b); Zhou and Goyer (1993: 237-240; Figs 2a-d) [L_1_, L_2_, L_3_; RR].

*Somotrichus unifasciatus* (Dejean): van Emden (1945: 27) [L_1-3_; ?].

*Syntomus americanus* (Dejean): Bousquet (2010a: Figs 661, 667, 668, 675).

*Tecnophilus croceicollis croceicollis* (Ménétriés): Larson (1969: 24; Figs 6, 10, 12) [L_1-3_; RR]; Mahar et al. (1983: Fig. 8).

*Tecnophilus croceicollis peigani* Larson: Larson (1969: 24; Figs 13, 14) [L_1-3_; RR].

### Galeritini

*Galerita (Progaleritina) janus* (Fabricius): Packard (1871: 29, 30; Figs 13a-b) [L_3_; FC]; Hubbard (1875: 49-51) [L_3_; FC]; Schaupp (1879: 14) [L_2-3_; LIT]; Kirk (1981: 368; Fig. 1) [L_2-3_; FC]; Arndt and Drechsel (1998: Fig. 6); Bousquet (2010a: Figs 679-681).

*Galerita (Progaleritina) lecontei* Dejean: Sallé (1849: 298-299; Figs 2c-d [Pl. 8]) [L_3_; FC]; Chapuis and Candèze (1853: 367-369) [L_3_; FC]; Schaupp (1879: 14) [L_2-3_; LIT].

*Galerita* *sp.* (Arkansas): Thompson (1979a: Figs 82a-c).

### Helluonini

*Helluomorphoides praeustus bicolor* (Harris): Bousquet (1987c: 922, 925-927; Figs 1-7) [L_1_, L_2_; FC]; Bousquet (2010a: Figs 682-686).

*Helluomorphoides* *sp.* (Georgia): Thompson (1979a: Figs 83a-c).

### Pseudomorphini

*Pseudomorpha (Pseudomorpha) augustata* Horn: Liebherr and Kavanaugh (1985: 1081) [L_1_; RR].

*Pseudomorpha (Pseudomorpha) hubbardi* Notman: Liebherr and Kavanaugh (1985: 1081; Figs 1a-b) [L_1_; RR].

*Pseudomorpha* *sp.* [Arizona]: Erwin (1981a: 56, 58; Fig. 24) [?L_2_; FC].

*Pseudomorpha* *sp.* [Nevada]: Thompson (1979a: Figs 84a-f); Erwin (1981a: 54-56; Figs 1-23) [L_1_, L_2-3_; FC].

## Appendix 3. List of Geadephaga species described from specimens mislabeled as from North America

A number of available species were listed originally as from North America and were subsequently found not to belong to the region. The following is an annotated alphabetical list of species described from specimens mislabeled or incorrectly assumed to be from North America.

*Agonum splendidulum* Motschulsky, 1844: 138. Type locality listed as “Sitka et du Kamtchatka.” Chaudoir (1850b: 123) stated that the specimens he identified as *Agonum splendidulum* from Kamchatka seemed to represent a very minor variety of *Agonum impressum* (Panzer). *Agonum splendidulum* is currently considered a junior synonym of *Agonum impressum*, a Palaearctic species (Bousquet 2003c: 450). The original description is very short and uninterpretable (see Lindroth 1966: 594). According to Keleinikova (1976: 218), three syntypes from Kamchatka are present in ZMMU.

*Aretharea helluonis* Say, 1830a: 68. Type locality listed as “Pennsylv[ania]” although Say (1830a: 68) added “I am not sure whether I obtained it on our journey to the Rocky Mountains, to St. Peter’s river, or in Pennsylvania, but I think the latter.” LeConte (1859e: 524) wrote the following about this species: “No insect has yet been seen by other entomologists, which at all approaches the description here given ... In fact, there is nothing in the description of the head, thorax and anterior legs, which forbids a reference to *Cryptobium bicolor* or *Cryptobium melanocephalum* [Staphylinidae]. With regard to the elytra and posterior legs, I must suppose them to have been derived from some foreign *Carabide*, and that the union was effected unintentionally, before the specimen came under Say’s notice.”

*Bembidium glabriusculum* Motschulsky, 1844: 272. Type locality given as “Sitka [Baranof Island, Alaska].” Maddison (1993: 176) remarked that the holotype of this species is conspecific with members of *Bembidion argenteolum* Ahrens, a species found only west of the Lena River and Ussuri River areas of Russia. The original locality given by Motschulsky (1844: 272) is in error.

*Calleida rubricollis* Dejean, 1825: 225. Type locality given as “Amérique septentrionale.” Dejean (1831: 328) acknowledged receiving specimens of this species from Cuba, including some from Klug under the name *elegans*, which did not differ from his specimens of *Calleida rubricollis*. Chaudoir (1844: 469) described *Calleida elegans* (which he credited to Klug) by pointing out structural differences between his sole specimen and those of *Calleida rubricollis*. Later Chaudoir (1873b: 144) concluded that the differences reported in 1844 between the specimens from the United States and Cuba were only “accidental” and that all the specimens belonged to the same species. I have seen a specimen from Chaudoir’s collection in MHNP labeled “rubricollis mihi elegans [illegible letters] in Ins. Cuba D. Klug [handwritten] / Lectotype ♂ Calleida rubricollis Dej. by Erwin ‘76 [partly handwritten].” The specimen is probably one received from Klug and noticed in 1831 by Dejean and therefore is likely not a syntype. Based on the original description by Dejean (1825: 225) and this specimen, I believe that *Calleida rubricollis* is structurally most similar to *Calleida decora* (Fabricius) among the North American species but specifically distinct. I have not seen a conspecific specimen from North America and believe the type locality listed by Dejean (i.e., Amérique septentrionale) is in error. The statement by Chaudoir (1873b: 144) that the specimens from United States were identical with those from Cuba may be based on a comparison of his Cuban specimens with those of Dejean, which he believed came from the United States. At the time, Chaudoir owned Dejean’s carabid collection. Already in his first catalogue, LeConte (1863b: 6) stated that “*Calleida rubricollis* Dej. is not found within the present limits of the United States, but is confined to Cuba.”

*Carabus beauvoisi* Dejean, 1826: 67. Type locality listed as “Amérique septentrionale.” Motschulsky (1850a: 81) listed this name as a “var.?” of *Carabus californicus*, a species he described only in 1866 [see next entry]. LeConte (1863b: 3) reported that this species is identical to “*Carabus catenulatus* Fabricius.” Motschulsky (1866: 287, as *Carabus bauvoisii* Dej.) stated that Haldeman gave him specimen(s) of this species during his trip to America. Kraatz (1878: 158) agreed with LeConte and stated that Dejean’s specimen(s) was conspecific with *Carabus catenulatus* and that the locality given was in error. Lapouge (1931: 570) considered this name to be a questionable junior synonym of *Carabus taedatus agassii* LeConte. Csiki (1927: 81) listed it as a variety of *Carabus problematicus* Herbst, and Breuning (1933b: 810) as a junior synonym of *Carabus problematicus problematicus* natio *gallicus* Géhin. There is no indication that either Csiki or Breuning saw the name-bearing type specimen(s) and therefore, if their conclusions are correct, the type locality was in error. Jeannel (1941b: 132) listed also the name as a junior synonym of *Carabus problematicus*, adding that the type locality of “Amér. du Nord” is in error. In the latest catalogue of the genus *Carabus* (Deuve 2004: 240), this name is listed as a junior synonym of *Carabus problematicus harcyniae* Sturm. Dejean’s original specimens came from Palisot de Beauvois’ collection which, according to Dejean (1825: 101), was not in order.

*Carabus bifasciatus* Olivier, 1790b: 347. Type locality listed as “Amérique septentrionale.” This species was transferred to the genus *Calophaena* by Klug (1821: 297) which is confirmed by the illustration provided by Olivier (1795: Fig. 80 [plate 7]). The genus is not found in North America, north of Mexico, and the type locality is obviously in error.

*Carabus californicus* Motschulsky, 1866: 288. This name was listed as a variety of the Palaearctic *Carabus problematicus* Herbst by Csiki (1927: 81), as a junior synonym of *Carabus problematicus problematicus* natio *gallicus* Géhin by Breuning (1933b: 810), and as a junior synonym of *Carabus problematicus gallicus* by Kryzhanovskij (1968: 175). In the latest catalogue of the genus *Carabus* (Deuve 2004: 240), this name is listed as a questionable junior synonym of *Carabus problematicus solutus* Oberthür. Obviously the provenance, implied by the specific name, is incorrect.

*Carabus carolinus* Fabricius, 1792: 126. Type locality given as “Carolina meridionali.” LeConte (1863b: 3) reported that Fabricius’ taxon was identical to *Carabus splendens* Olivier. The name is still listed in synonymy with *Carabus splendens* in Deuve’s (2004) latest catalogue of the genus *Carabus*. Since this species is found only in southwestern France, the provenance given by Fabricius is obviously incorrect.

*Carabus latus* Linnaeus, 1758: 415. Type locality listed as “Europa; America septentrionali.” Lindroth (1957b: 332) discussed the status of this species and came to the conclusion that the name should be retained for *Harpalus latus* auctorum, one of the commonest species in Sweden. However, none of the six “Linnean” specimens in LSL belong to this species. Motschulsky (1855a: 44) interpreted Linnaeus’ species as the North American *Harpalus herbivagus* Say.

*Casnonia rufipes* Dejean, 1825: 172. Type locality listed as “Amérique septentrionale.” The holotype came from Palisot de Beauvois’ collection. Chaudoir (1863: 278) indicated that the species is not found in North America but occurs from Cayenne (in French Guiana) to Brazil.

*Chlaenius nimrod* Horn, 1897: 372. Type locality given as “Custer, South Dakota.” Bell (1960: 116) stated that the original specimens were obviously mislabeled and probably belonged to an Old World species.

*Cicindela baltimorensis* Herbst, 1806: 181. Type locality given as “Baltimore.” Schaum (see LeConte 1863b: 1) reported that this taxon is the East Indian *Cylindera minuta* (Olivier) with an erroneous locality. Acciavatti and Pearson (1989: 291) found syntypes of *Cicindela baltimorensis* in ZMHB and confirmed Schaum’s identification.

*Cicindela californica californica* Ménétriés, 1843: 52. Type locality listed as “Californie.” The nominotypical subspecies of *Habroscelimorpha californica* is known from the coast of central and southern Baja California (Pearson et al. 2006: 138) suggesting that the type locality is in error. However, all the other species described from California by Ménétriés (1843), except maybe *Agaosoma californicum*, are currently found in California. Two other possibilities are conceivable. The subspecies had a wider range in the xix Century and did occur in California or our concept of *Habroscelimorpha californica californica* is in error and the name actually applies to one of the two subspecies of *Habroscelimorpha californica* found in California. As far as I know, nobody has reported studying syntype(s) of the nominotypical subspecies.

*Cicindela coerulea* Herbst, 1806: 182. Type locality given as “Nordamerica.” LeConte (1863b: 2) listed this name as a synonym of the Palaearctic *Cylindera germanica* (Linnaeus). The type locality is obviously in error.

*Cicindela magdalenae* LeConte, 1873b: 321. Type locality listed as “found in turpentine barrels brought to London, supposed to be from North Carolina.” Horn (1880f: xix) noted the similarity between the elytral markings of LeConte’s species and those of some South African species and later (1883b: 269-270) reported that the species was identical to *Lophyra senegalensis* (Dejean).

*Cicindela tortuosa* Dejean, 1825: 87. Type locality: “Amérique septentrionale et dans les Antilles.” Gistel (1837: 49) listed this name in synonymy with *Cicindela trifasciata* Fabricius. LeConte’s (1851: 172) record of this species in “Georgia et Louisiana, in oryzaceis” refers to *Cicindela trifasciata ascendens* LeConte (Horn 1926: 294). Currently *Cicindela tortuosa* is listed in catalogues (e.g., Wiesner 1992: 146; Lorenz 2005: 49) as a junior synonym of *Cicindela trifasciata trifasciata* Fabricius, which occurs commonly over the West Indies. However, Dejean’s type series likely includes specimens of *Cicindela trifasciata trifasciata* and *Cicindela trifasciata ascendens* LeConte. As far as I know, a lectotype has not been designated for *Cicindela tortuosa* Dejean.

*Cicindela triguttata* Herbst, 1806: 182. Type locality given as “Nordamerica.” LeConte (1863b: 2) stated that Herbst’s species was “not North American.” Acciavatti and Pearson (1989: 253), upon an examination of the syntypes in ZMHB, concluded that *Cicindela triguttata* Herbst is a synonym of *Cylindera viduata* (Fabricius), a species found in Asia. The type locality originally reported is in error.

*Clivina biguttata* Putzeys, 1867b: 157. Type locality given as “Cuba. Louisiane.” Nichols (1988a: 129) reported this species from Cuba, Isla de Pinos, and Jamaica. As far as I know, the species has not been reported subsequently from North America.

*Clivina picipes* Bonelli, 1813: 481. Bonelli originally stated that the country of origin was unknown. Dejean (1825: 416) reported that his specimen(s) of this species originated from “Amérique” and Putzeys (1846: 105) redescribed the sole specimen in Dejean’s collection and listed “Amérique boréale” as the provenance. Subsequently Putzeys (1867b: 110) listed this name as a junior synonym of *Clivina attenuata* (Herbst) and pointed out that the species was from India.

*Curtonotus transversicollis* Putzeys, 1866b: 236. Type locality given as “Amér[ique] Russe. (Akina).” According to Lindroth (1968: 665), the lectotype is conspecific with members of the Palaearctic *Amara brevicollis* (Chaudoir) and probably originated from Akima in Transbaikalia.

*Cymindis morio* Dejean, 1825: 219. Type locality listed as “Amérique septentrionale.” The specimen(s) was given to Dejean by Palisot de Beauvois. Schaum (1857a: 293) stated that this species belongs to the genus *Apenes* LeConte and occurs in Haiti. LeConte (1863b: 6) said that it “does not occur in the United States.” According to Ball (1992b: Fig. 5.6), the species is found in the Bahamas and on Hispaniola.

*Dinodes rotundicollis* Dejean, 1826: 373. Type locality given as “Amérique septentrionale.” The specimen(s) was given to Dejean by Palisot de Beauvois. According to LeConte (1863b: 11), this species “is probably not North American.” Chaudoir (1876b: 58), who had access to Dejean’s types, stated that *Dinodes rotundicollis* “n’est qu’un individu de l’*azureus* [= *Chlaenius decipiens* (Dufour)]” and that the locality originally given was in error.

*Dromius geminatus* Haldeman, 1843b: 298. Type locality indicated as “south-eastern Pennsylvania” by Haldeman (1843a: 296). LeConte (1863b: 5) stated that this species is identical to *Dromius quadrinotatus* (Panzer) [= *Calodromius spilotus* (Illiger)] of Europe, suggesting that the type locality given was in error.

*Feronia quadricollis* LeConte, 1846b: 343. Type locality given as “Pennsylvania.” LeConte (1853a: 235) stated that his species is identical to *Abax parallelus* Duftschmid of Europe, suggesting that the type locality was in error.

*Lebia angulata* Boheman, 1858: 7. Type locality given as “California.” As pointed out by Horn (1872a: 141), the California record is unreliable and the specimen(s) probably originated from South America (see also LeConte 1869b: 248). Chaudoir (1871a: 113) transferred the species to the genus *Rhombodera* (= *Pentagonica*). Reichardt (1968: 154) listed *Lebia angulata* as a questionable synonym of *Pentagonica picea* Chaudoir. *Lebia goniodera* Gemminger and Harold, 1868 is a replacement name for *Lebia angulata* Boheman, 1858.

*Lebia cupripennis* Boheman, 1858: 7. Type locality given as “California.” LeConte (1869b: 248) stated that *Lebia cupripennis* Boheman is identical to *Lebia cupripennis* Chaudoir and that Boheman’s specimens did not originate from California but from Peru and Chile. *Lebia chalcoptera* Gemminger and Harold, 1868 is a replacement name for *Lebia cupripennis* Boheman, 1858.

*Myosodus femoratus* Motschulsky, 1845a: 22. Type locality given as “Sitka.” Motschulsky (1850a: 47) listed this species from “Sitka? Irkutsk?” Tschitschérine (1902b: 105) suspected this taxon to be a form of *Pterostichus kamtschaticus* (Motschulsky). Csiki (1930: 681) listed the name as a valid species in section *Lyperopherus* Motschulsky and reported it from Siberia and Alaska. Lindroth (1966: 526) believed the type locality was “almost certainly false.” Finally, Kryzhanovskij et al. (1995: 105), followed by Bousquet (2003d: 497), listed the name as a questionable junior synonym of *Pterostichus costatus* (Ménétriés). According to Keleinikova (1976: 197), there is one “corruptum” syntype in Motschulsky’s collection (ZMMU). *Pterostichus cruralis* (Tschitschérine, 1902) is a replacement name for *Pterostichus femoratus* (Motschulsky, 1845).

*Nebria macrocephala* Motschulsky, 1844: 128. Type locality listed as “probablement de Sitka ou d’Ounalachka [Alaska].” Mannerheim (1853: 111), who apparently saw syntypes of this taxon, noted that they were conspecific with specimens of *Nebria stigmula* Dejean, 1826 (= *Nebria hellwigii* Panzer, 1803). Farkač and Janata (2003: 95) listed this form as a valid species of the subgenus *Tetungonebria* Shilenkov from “Siberia.” The provenance given by Motschulsky is likely incorrect.

*Oodes schaumi* Chaudoir, 1882: 346. Type locality given as “trouvée probablement dans la Louisiane.” The holotype in ZMHB belongs to the genus *Coptocarpus* Chaudoir (Bousquet 1996a: 471) which is endemic to the Australian Region. The provenance originally given is obviously in error.

*Pogonus minutus* Dejean, 1828: 15. Type locality listed as “Amérique septentrionale.” The sole specimen came from Palisot de Beauvois’ collection. Chaudoir (1872a: 27), who owned Dejean’s collection at the time, stated that the type of this species was conspecific with that of *Pogonus senegalensis* Dejean [= *Pogonus minutus* Dejean] from western Africa. The locality as originally given is in error.

*Scarites attenuatus* Herbst, 1806: 255. Type locality given as “Nordamerica.” Putzeys (1867b: 110) stated that this species, which belongs to the genus *Clivina* Latreille, occurs in India and it is by error that Herbst listed it from North America.

*Scarites emarginatus* Herbst, 1806: 255. Type locality listed as “Nordamerica.” Bänninger (1933: 116) studied the “type” of Herbst’s species in ZMHB and concluded that it was conspecific with *Scarites dentatus* Andrewes of India. The locality as originally given is in error.

*Scarites gagates* Bonelli, 1813: 475. Type locality given as “Amérique septentrionale.” Bänninger (1938: 112) stated that Bonelli’s specimen(s) is conspecific with those of *Scarites indus* Olivier of the Oriental Region and that the provenance originally given was obviously incorrect.

*Scarites polyphemus* Herbst, 1806: 254. Type locality given as “Nordamerica.” Dejean (1825: 372) listed Herbst’s name in synonymy with a new species, *Scarites herbstii* Dejean, from Cape of Good Hope although Dejean’s description indicates that he considered the two as distinct species. Bänninger (1938: 176), who saw Herbst’s type specimen(s), listed *Scarites polyphemus* as a synonym of *Scarites herbstii* Dejean and noted that the species does not occur in North America but in South Africa.

*Selenophorus beauvoisii* Dejean, 1829: 98. Type locality listed as “Amérique septentrionale.” Dejean’s specimen(s) came from Palisot de Beauvois’ collection. LeConte (1870: 403) saw the name-bearing type(s) in Paris and reported that the species was “abundant in the Antilles, but does not occur in the United States.” In Blackwelder (1944: 49) the species is listed as valid and from Jamaica, with *Selenophorus aeneocupreus* Dejean as a junior synonym.

*Steropus rugulosus* Motschulsky, 1845b: 342. Type locality given as “Ounalaschka.” Motschulsky’s original description was based on a single specimen in ZMMU. Csiki (1930: 658) listed the taxon as a valid species in the subgenus *Cryobius* and reported it from Alaska. Based on a study of the holotype, Bousquet and Larochelle (1993: 333) concluded that it belongs to an undetermined species of *Abacetus* Dejean. The provenance originally given by Motschulsky is obviously incorrect. *Pterostichus rugatinus* Csiki, 1930 is a replacement name for *Pterostichus rugulosus* (Motschulsky, 1845).

*Tachypus elongatus* Motschulsky, 1850a: 16. Type locality given as “Sitka?” According to Lindroth (1963b: 204), this *Asaphidion* is “almost certainly not from N. America.” A syntype of Motschulsky’s species is present in ZMMU (Keleinikova 1976: 196) and a study of the specimen is needed to determine its identity.

*Trechus ruficollis* Van Dyke, 1926a: 66. Type locality given as “Lawrence, Massachusetts.” Barr (1962a: 76-77) stated that the four original specimens of this species were likely mislabeled and Donabauer (2010b: 45) pointed out that they are conspecific with members of *Trechus striatulus* Putzeys, a species endemic to the western Carpathians. Because of homonymy, Van Dyke’s name was first changed to *Trechus vandykei* Jeannel, 1928 (also a homonym) and subsequently to *Trechus dietrichi* Barr, 1962. The holotype (♂) is in CUIC [# 1635].

## Appendix 4. List of unavailable Geadephaga names listed from North America

Many species- or genus-group names have been reported from North America but are unavailable (*nomina nuda*) because they were either not described [**DES**], or proposed as infrasubspecific entities [**INF**], or first proposed as junior synonyms and not treated as valid names or senior homonyms before 1961 [**SYN**], or proposed as generic entities after 1930 without type species designations [**TYP**], or not published [**PUB**]. All these names have no taxonomic status and are not listed in the catalogue itself. This list does not pretend to be complete.

*Acupalpus minuatus* Rathvon, 1869: 527. **DES**

*Agonothorax anchomenoides* Motschulsky, 1850a: 68. **DES**

*Agonum aeneicolle* Sturm, 1843: 23. **DES**

*Agonum coracinum* Sturm, 1843: 22. **DES**

*Agonum formosum* Sturm, 1826: 89. **DES**

*Agonum foveolatum* Sturm, 1826: 89. **DES**

*Agonum rotundicolle* Sturm, 1843: 23. **DES**

*Agonum saltuum* Sturm, 1826: 89. **DES**

*Agonum sulcatum* T.W. Harris [in Scudder], 1869: 230. **SYN**

*Amara aerea* Sturm, 1843: 29. **DES**

*Amara cylindrica* Motschulsky, 1850a: 60. **DES**

*Amara elongatula* Motschulsky, 1850a: 60. **DES**

*Amara fulvipes* Sturm, 1843: 29. **DES**

*Amara misera* Dejean, 1828: 462. **SYN**

*Amara ovalis* Sturm, 1826: 91. **DES**

*Amara quadrifoveolata* Motschulsky, 1850a: 61. **DES**

*Anchomenus aeruginosus* Sturm, 1826: 91. **DES**

*Anchomenus decorus* ab. *syracusensis* Hatch, 1926: 247. **INF**

*Anchomenus laticollis* Sturm, 1826: 91. **DES**

*Anchomenus proximus* T.W. Harris, 1828d: 132. **SYN**

*Anchomenus viridanus* Dejean, 1821: 10. **DES**

*Angoleus picipes* Sturm, 1843: 24. **DES**

*Anisodactylus brevis* Sturm, 1843: 32. **DES**

*Argutor ornatus* Sturm, 1826: 97. **DES**

*Argutor rufipes* Dejean, 1821: 11. **DES**

*Argutor rufitarsis* Sturm, 1843: 24. **DES**

*Bembidium apicale* Sturm, 1843: 36. **DES**

*Bembidium axillare* Sturm, 1843: 37. **DES**

*Bembidium bisbiguttatum* Sturm, 1843: 38. **DES**

*Bembidion browni* Lindroth, 1954b: 158. **DES**

*Bembidium elegans* Sturm, 1843: 36. **DES**

*Bembidium maeklini* LeConte, 1863b: 14. **DES**

*Bembidion planiusculum* ab. *pallidum* Hatch, 1950: 99. **INF**

*Bembidium unistriatum* Sturm, 1843: 36. **DES**

*Brachinus glabripennis* LeConte, 1858a: 28. **DES**

*Brachinus puncticollis* LeConte, 1858a: 28. **DES**

*Brachinus similis* Sturm, 1843: 2. **DES**

*Brachystylus megas* LeConte, 1853a: 250. **DES**

*Bradytus planiusculus* Sturm, 1843: 29. **DES**

*Calathus flavipes* Sturm, 1843: 21. **DES**

*Calosoma luxatum zimmermanni* ab. *striata* Breuning, 1928b: 86. **INF** + **DES**

*Carabus catena* Gosse, 1840: 107, 150, 185, 307. **DES**

*Carabus fimbriatus* Sturm, 1826: 110. **DES**

*Carabus maeander* monstr. *excatenatus* Kraatz, 1880: 337. **INF**

*Celia compacta* Motschulsky, 1850a: 59. **DES**

*Chlaenius aeratus* Dejean, 1821: 8. **DES**

*Chlaenius angusticollis* Dejean, 1821: 8. **DES**

*Chlaenius aureliensis* LaFerté-Sénectère, 1851: 248. **DES**

*Chlaenius dimidiatus* Motschulsky, 1858: 157. **DES**

*Chlaenius discolor* Strum, 1826: 115. **DES**

*Chlaenius exaratus* LaFerté-Sénectère, 1851: 249. **DES**

*Chlaenius feisthamelii* LaFerté-Sénectère, 1851: 248. **DES**

*Chlaenius indutus* LaFerté-Sénectère, 1851: 247. **DES**

*Chlaenius lugubris* Motschulsky, 1850a: 64. **DES**

*Chlaenius nigrocoeruleus* Sturm, 1843: 18. **DES**

*Chlaenius nigrovirens* Sturm, 1843: 18. **DES**

*Chlaenius opacus* Sturm, 1843: 18. **DES**

*Chlaenius thalassinus* Sturm, 1843: 18. **DES**

*Chlaenius viridiceps* LaFerté-Sénectère, 1851: 248. **DES**

*Cicindela carthagena haemorrhagica* var. *nigroides* Hatch, 1938: 236. **INF**

*Cicindela collaris* LaFerté-Sénectère, 1841a: 40, 41, 42. **DES**

*Cicindela continua* Sturm, 1843: 2. **DES**

*Cicindela deflexa* Sturm, 1843: 2. **DES**

*Cicindela deflua* Sturm, 1843: 2. **DES**

*Cicindela dejeanii* Dejean, 1831: 215. **SYN**

*Cicindela deleta* Sturm, 1843: 2. **DES**

*Cicindela erythrogaster* T.W. Harris [in Scudder], 1869: 20. **DES**

*Cicindela excisa* Sturm, 1843: 2. **DES**

*Cicindela formosa mescalero* Gaumer, 1977: 225. **PUB**

*Cicindela formosa yampa* Gaumer, 1977: 219. **PUB**

*Cicindela hamata* Sturm, 1843: 2. **DES**

*Cicindela humeralis* Dejean, 1831: 215. **SYN**

*Cicindela hydropica* Dejean, 1836: 3. **SYN**

*Cicindela latecincta* Gould, 1834: 42. **SYN**

*Cicindela lemnisticta* Smyth, 1908: 188. **SYN**

*Cicindela maculosa* Sturm, 1843: 2. **DES**

*Cicindela purpurea* ab. *nigerrimoides* Hatch, 1938: 233. **INF**

*Cicindela raiana* Frost, 1920: 229. **DES**

*Cicindela scripta* Sturm, 1843: 2. **DES**

*Cicindela thalassina* Dejean, 1821: 1. **DES**

*Clivina brunnipes* Sturm, 1843: 2. **DES**

*Clivina trisulcata* Sturm, 1843: 2. **DES**

*Clivina unicolor* Sturm, 1843: 2. **DES**

*Cratacanthus pallidus* Sturm, 1843: 30. **DES**

*Dicaelus ambiguus* Dejean, 1836: 31. **DES**

*Dicaelus lecontei* Dejean, 1836: 30. **DES**

*Dicaelus quadratus* Dejean, 1836: 31. **DES**

*Dicaelus tristis* Sturm, 1843: 19. **DES**

*Dromius auratus* Sturm, 1826: 134. **DES**

*Dromius collaris* Sturm, 1826: 134. **DES**

*Dromius festinans* Dejean, 1821: 3. **DES**

*Dromius picipes* Sturm, 1826: 134. **DES**

*Dyschirius californicus* Motschulsky, 1850a: 18. **DES**

*Emphanes flavopictus* Motschulsky, 1850a: 13. **DES**

*Galerita elegans* Chaudoir, 1861b: 553. **SYN**

*Harpalus brevis* Sturm, 1826: 148. **DES**

*Harpalus brunnipennis* Dejean, 1821: 15. **DES**

*Harpalus cuprescens* Sturm, 1843: 33. **DES**

*Harpalus laevigatus* Sturm, 1843: 33. **DES**

*Harpalus moestus* Dejean, 1821: 14. **DES**

*Hirmoplataphus* Netolitzky, 1942: 46. **TYP**

*Lebia albomarginata* Sturm, 1826: 161. **DES**

*Lebia marginella* Sturm, 1826: 161. **DES**

*Limodromus parallelus* Motschulsky, 1850a: 70. **DES**

*Lorocera flavipes* Motschulsky, 1850a: 66. **DES**

*Loxopeza testacea* LeConte, 1880b: 164. **DES**

*Nebria fusiformis* Van Dyke, 1926b: 11. **DES**

*Nebria strawberriensis* Kavanaugh, 1978: 413. **PUB**

*Neonebria* Hatch, 1939a: 117. **TYP**

*Notiophilus intermedius* Lindroth, 1954b: 157. **DES**

*Notiophilus plumbeus* Sturm, 1843: 17. **DES**

*Ochthedromus compar* LeConte, 1857a: 5. **DES**

*Olisthopus dorsalis* Sturm, 1843: 23. **DES**

*Omaseus cephalotes* Sturm, 1843: 24. **DES**

*Omaseus punctatostriatus* Sturm, 1843: 24. **DES**

*Omaseus sulcatus* Sturm, 1843: 24. **DES**

*Pangus melanarius* Sturm, 1843: 31. **DES**

*Pasimachus laterisulcatus* Sturm, 1826: 182. **DES**

*Pasimachus obtusus* Sturm, 1826: 182. **DES**

*Pasimachus sulcatus* Dejean, 1821: 4. **DES**

*Platynus dixianus* Zimmermann [in LeConte], 1869b: 246. **DES**

*Platysma alternata* Motschulsky, 1850a: 53. **DES**

*Platysma breviuscula* Motschulsky, 1850a: 54. **DES**

*Platysma punctatosulcata* Sturm, 1826: 185. **DES**

*Poecilus albionicus* Motschulsky, 1850a: 53. **DES**

*Poecilus cupreonitens* Sturm, 1843: 23. **DES**

*Poecilus similis* Dejean, 1821: 11. **DES**

*Pogonus californicus* Motschulsky, 1850a: 6. **DES**

*Pseudomaseus dilutus* Motschulsky, 1850a: 51. **DES**

*Pterostichus maeklini* LeConte, 1863b: 9. **DES**

*Pterostichus muticus* LeConte, 1863b: 8. **DES**

*Rhombodera pallipes* LeConte, 1863b: 6. **DES**

*Scarites laevistriatus* Sturm, 1843: 2. **DES**

*Scarites picicornis* Sturm, 1843: 2. **DES**

*Selenophorus pedicularius* var. *semiopacus* Snow, 1903: 194. **DES**

*Stenolophus americanus* Dejean, 1821: 15. **DES**

*Stenolophus marginellus* Dejean, 1821: 15. **DES**

*Stenolophus palliatus* Motschulsky, 1850a: 21. **DES**

*Steropus picipes* Sturm, 1843: 25. **DES**

*Steropus politus* Sturm, 1843: 25. **DES**

*Tachys carolinus* Schwarz, 1878: 438. **DES**

*Tachys columbiensis* Schwarz, 1878: 438. **DES**

*Tachys truncorum* Crotch, 1874a 21. **DES**

*Tennessarius* Valentine, 1952: 15. **TYP**

*Trechus laevigatus* LeConte, 1863b: 14. **DES**

## Appendix 5. List of nomenclatural acts included in this catalogue

**New taxon**

*Randallius* Bousquet, new subgenus. Type species: *Chlaenius purpuricollis* Randall, 1838.

**New replacement name**

*Pterostichus amadeus* Bousquet for *Pterostichus vexatus* Bousquet, 1985.

**New synonymies**

*Agonum canadense* Goulet, 1969 = *Agonum elongatulum* Haldeman, 1843.

*Anisodactylus lecontei* Chaudoir, 1868 = *Anisodactylus nigrita* Dejean, 1829.

*Axinopalpus coloradensis* Casey, 1920 = *Axinopalpus biplagiatus* (Dejean, 1825).

*Axinopalpus demissus* Casey, 1920 = *Axinopalpus biplagiatus* (Dejean, 1825).

*Axinopalpus habilis* Casey, 1920 = *Axinopalpus biplagiatus* (Dejean, 1825).

*Badister laticeps* Blatchley, 1910 = *Badister flavipes flavipes* LeConte, 1853.

*Bembidion arizonae* Lindroth, 1963 = *Bembidion durangoense* Bates, 1891.

*Bembidium lapponicum* C.G. Thomson, 1857 = *Bembidion lapponicum* Zetterstedt, 1828.

*Brachylobus lithophilus indigaceus* Casey, 1914 = *Chlaenius lithophilus* Say, 1823.

*Calleida striata* Casey, 1913 = *Calleida fulgida* Dejean, 1831.

*Carabus franciscanus* Casey, 1913 = *Carabus taedatus agassii* LeConte, 1850.

*Celia impunctata* Putzeys, 1867 = *Amara chalcea* Dejean, 1828.

*Chlaenius frostii* Carr, 1920 = *Chlaenius purpuricollis* Randall, 1838.

*Chlaenius purpuricollis* Randall, 1838 = *Chlaenius purpuratus* Harris, 1836.

*Chlaenius sierricola* Casey, 1914 = *Chlaenius sericeus* (Forster, 1771).

*Chlaenius texanellus* Casey, 1914 = *Chlaenius brevilabris* LeConte, 1847.

*Chlaenius viridifrons* Eschscholtz, 1833 = *Chlaenius sericeus* (Forster, 1771).

*Clivina cordata* Putzeys, 1846 = *Clivina acuducta* Haldeman, 1843.

*Cryobius delicatus* Casey, 1918 = *Pterostichus brevicornis brevicornis* (Kirby, 1837).

*Cymindis elegans mobilensis* Casey, 1920 = *Cymindis elegans* LeConte, 1846.

*Darlingtonea kentuckensis lexingtoni* Valentine, 1952 = *Darlingtonea kentuckensis* Valentine, 1952.

*Dicaelus darlingtoni* Fall, 1932 = *Dicaelus quadratus* LeConte, 1847.

*Euharpalops* Casey, 1924 = *Opadius* Casey, 1914.

*Glanodes regressus* Casey, 1914 = *Harpalus obliquus* Horn, 1880.

*Goniolophus lucens* Casey, 1914 = *Philodes flavilimbus* (LeConte, 1869).

*Haploharpalus* Schauberger, 1926 = *Opadius* Casey, 1914.

*Harpalus cyrtonotoides* Notman, 1919 = *Harpalus desertus* LeConte, 1859.

*Harpalus subaeneus* Mannerheim, 1853 = *Harpalus fulvilabris* Mannerheim, 1853.

*Hypherpes alamedae* Casey, 1918 = *Pterostichus vicinus* Mannerheim, 1843.

*Hypherpes intectus* Casey, 1918 = *Pterostichus protractus* LeConte, 1860.

*Hypherpes kansanus* Casey, 1918 = *Pterostichus algidus* LeConte, 1853.

*Hypherpes placerensis* Casey, 1918 = *Pterostichus laborans* Casey, 1913.

*Hypherpes protensipennis* Casey, 1918 = *Pterostichus mercedianus* (Casey, 1918).

*Lebia scapularis* var. *limbigera* Chaudoir, 1871 = *Lebia solea* Hentz, 1830.

*Micranillodes* Jeannel, 1963 = *Anillinus* Casey, 1918.

*Moritapterus* O. Berlov, 2000 = *Euferonia* Casey, 1918.

*Neomyas lindrothi* Allen, 1980 = *Myas cyanescens* Dejean, 1828.

*Pharalus* Casey, 1914 = *Opadius* Casey, 1914.

*Platynus hornii* Hausen, 1891 = *Agonum muelleri* (Herbst, 1784).

*Poecilus lucublandus acomanus* Casey, 1924 = *Poecilus lucublandus* (Say, 1823).

*Poecilus lucublandus louisinus* Casey, 1924 = *Poecilus lucublandus* (Say, 1823).

*Pterostichus breviusculus* Casey, 1913 = *Pterostichus congestus* (Ménétriés, 1843).

*Pterostichus breviusculus mimus* Casey, 1913 = *Pterostichus congestus* (Ménétriés, 1843).

*Pterostichus bucolicus* Casey, 1913 = *Pterostichus algidus* LeConte, 1853.

*Pterostichus cuneatulus* Casey, 1913 = *Pterostichus suffusus* Casey, 1913.

*Pterostichus diabolus* Casey, 1913 = *Pterostichus californicus* (Dejean, 1828).

*Pterostichus gregalis* Casey, 1913 = *Pterostichus castanipes* (Ménétriés, 1843).

*Pterostichus humboldti* Casey, 1913 = *Pterostichus algidus* LeConte, 1853.

*Pterostichus plutonicus* Casey, 1913 = *Pterostichus congestus* (Ménétriés, 1843).

*Pterostichus tahoensis* Casey, 1913 = *Pterostichus laborans* Casey, 1913.

*Pterostichus zunianus* Casey, 1913 = *Pterostichus protractus* LeConte, 1860.

*Rhadine gracilenta* Casey, 1913 = *Rhadine jejuna* (LeConte, 1878).

*Rhadine plumasensis* Casey, 1920 = *Rhadine jejuna* (LeConte, 1878).

*Rhadine tenuipes* Casey, 1920 = *Rhadine jejuna* (LeConte, 1878).

*Selenophorus excisus* LeConte, 1878 = *Selenophorus parumpunctatus* (Dejean, 1829).

*Selenophorus parumpunctatus* Dejean, 1829 = *Selenophorus sinuatus* (Gyllenhal, 1806).

*Selenophorus perpolitus* Casey, 1884 = *Athrostictus punctatulus* Putzeys, 1878.

*Steniridia andrewsi barksdalei* Valentine, 1936 = *Scaphinotus andrewsi darlingtoni* (Valentine, 1935).

*Steniridia andrewsi montana* Valentine, 1935 = *Scaphinotus andrewsii amplicollis* (Casey, 1920).

*Steniridia andrewsi nantahalae* Valentine, 1936 = *Scaphinotus andrewsi darlingtoni* (Valentine, 1935).

*Steniridia andrewsi saludae* Valentine, 1936 = *Scaphinotus andrewsi darlingtoni* (Valentine, 1935).

*Steniridia ridingsi intermedia* Valentine, 1935 = *Scaphinotus ridingsii ridingsii* (Bland, 1863).

*Steniridia violacea carolinae* Valentine, 1935 = *Scaphinotus violaceus* (LeConte, 1863).

*Stenolophus abstinens* Casey, 1914 = *Stenolophus incultus* Casey, 1914.

*Stenolophus extensicollis* Casey, 1924 = *Stenolophus cincticollis* LeConte, 1858.

*Stenolophus lamprotus* Bates, 1891 = *Stenolophus cincticollis* LeConte, 1858.

*Stenolophus peregrinus* Casey, 1914 = *Stenolophus anceps* LeConte, 1857.

*Stenolophus quadripunctatus* Mannerheim, 1853 = *Dicheirotrichus cognatus* (Gyllenhal, 1827).

*Stenolophus remissus* Casey, 1914 = *Stenolophus incultus* Casey, 1914.

*Stenolophus semitinctus* Casey, 1914 = *Stenolophus cincticollis* LeConte, 1858.

**New status**

*Agonoleptus dolosus* Casey, 1914 from *Agonoleptus unicolor dolosus*.

**New combinations**

*Agonoleptus conjunctus* (Say, 1823) from *Stenolophus*.

*Agonoleptus dolosus* Casey, 1914 from *Stenolophus*.

*Agonoleptus rotundatus* (LeConte, 1863) from *Stenolophus*.

*Agonoleptus rotundicollis* (Haldeman, 1843) from *Stenolophus*.

*Agonoleptus thoracicus* (Casey, 1914) from *Stenolophus*.

*Agonoleptus unicolor* (Dejean, 1829) from *Stenolophus*.

*Anillinus affabilis* (Brues, 1902) from *Anillodes*.

*Anillinus depressus* (Jeannel, 1963) from *Micranillodes*.

*Anillinus sinuatus* (Jeannel, 1963) from *Anillodes*.

*Calosoma dawsoni* (Dajoz, 1997) from *Callisthenes*.

*Scaphinotus hoffmani* (Barr, 2009) from *Maronetus*.

*Scaphinotus reichlei* (Barr, 2009) from *Maronetus*.

**Changes in precedence**

*Agonum deplanatum* Ménétriés, 1843 for *Agonum fallianum* (Leng, 1919).

*Badister micans* LeConte, 1844 for *Badister ocularis* Casey, 1920.

*Ellipsoptera rubicunda* Harris, 1911 for *Ellipsoptera marutha* Dow, 1911.

## Footnotes

These asterisks were dropped in the publication of Say’s entomological works by LeConte.

These names have been credited to Eschscholtz by almost all authors I have seen although there are proper citations of Fischer von Waldheim’s plates in Eschscholtz’s work. This suggests that the plates were available before the publication of Eschscholtz’s work. However because Fischer von Waldheim’s validation of the names is through illustrations, it is always possible that Fischer von Waldheim simply gave Eschscholtz the position of these species on his forthcoming plates.

By 1825, Major LeConte had sent Dejean more than 600 species of beetles (Dejean 1825: xxv). He also visited Paris in 1828 and gave Dejean a huge [“une immense”] collection of insects from the United States (Dejean 1828: vi). All species named “*lecontei*” by Dejean were in the honour of the Major and not his son.

A copy of the letter was published in *The Coleopterists Bulletin* in December 1961.

Following Maddison et al. (1999: 104), the expression ‘basal grade carabids’ is restricted to lineages branching off early along the evolutionary path of the family, ‘middle grade carabids’ to the lineages placed by Jeannel (1941b) in his ‘Stylifera’ and ‘higher carabids’ to the numerous lineages currently included in the subfamily Harpalinae.

† = adventive species in North America; ‡ = Holarctic species. The species list is alphabetic within the subgenera and does not necessary follow the catalogue listing.

All vernacular names of tiger beetles are taken from Pearson et al. (2006) and Erwin and Pearson (2008).

Several new synonyms are proposed in this subgenus. All are based on a manuscript written by Serge Laplante in the 1990s originating from a study of the type specimens of all species then considered valid and the specimens in the CNC. The new synonymies should be credited to him.

This subgenus is herein removed from synonymy with *Trechicus*, see “Taxonomic Note” under *Trechicus*.

The field-collected larva described as *Trachypachus* sp. by Garner (1954: 49, 50; Figs a-c [Pl. 1]) is misidentified.

Gaumer (1977) described larvae of five subspecies of *Cicindela formosa*.

The field-collected and reared larvae described by Garner (1954: 220, 221; Pl. 14, Figs a-c) as *Promecognathus laevissimus* are misidentified.

As pointed out by Jeannel (1920a: 510), the field-collected larva described by Xambeu (1901: 58, 59) as *Trechus obtusus* has been incorrectly associated.

The field-collected larvae described by Garner (1954: 211, 212; Pl. 13, Figs a-c) as *Metrius contractus* are obviously misidentified.
